# Projecting the global impact of fossil fuel production from the Former Soviet Union

**DOI:** 10.1007/s40789-021-00449-x

**Published:** 2021-08-09

**Authors:** Steve Mohr, Jianliang Wang, James Ward, Damien Giurco

**Affiliations:** 1 The University of Newcastle, University Drive, Callaghan, NSW 2308 Australia; 2 School of Business Administration, China University of Petroleum, Beijing, China; 3 Engineering and the Environment, University of South Australia, Mawson Lakes Campus, Mawson Lakes Boulevard, Mawson Lakes, SA 5095 Australia; 4 Institute for Sustainable Futures, University of Technology Sydney, UTS Building 10, 235 Jones St., Ultimo, NSW 2007 Australia

**Keywords:** Former Soviet Union, Fossil Fuel Production, Fossil Fuel Projection

## Abstract

Detailed projections of the Former Soviet Union (FSU) fossil fuel production has been created. Russian production has been modelled at the region (oblast) level where possible. The projections were made using the Geologic Resource Supply-Demand Model (GeRS-DeMo). Low, Best Guess and High scenarios were created. FSU fossil fuels are projected to peak between 2027 and 2087 with the range due to spread of Ultimately Recoverable Resources (URR) values used. The Best Guess (BG) scenario anticipates FSU will peak in 2087 with production over 170 EJ per year. The FSU projections were combined with rest of the world projections (Mohr et al. 2015b), the emissions from the High scenario for the world are similar to the IPCC A1 AIM scenario.

**Supplementary Information:**

The online version contains supplementary material available at 10.1007/s40789-021-00449-x.

## Introduction

The Former Soviet Union (FSU) region^1^ is a major contributor to the world’s fossil fuel production. The region accounts for over 7% (coal), 15% (oil) and 21% (gas) of the world’s production in 2018 BP (2019). The large contribution of the FSU is matched by its resources which are over 18% (coal), 12% (oil) and 28% (gas) of the world’s total BGR (2016). The fate of the FSU’s fossil fuel future production therefore will have a major influence on the world.

Despite the importance of the FSU region, the literature has limited detailed projections for this region compared to comparable regions such as China and USA. For example, Mohr et al. (2015b) projected both China and USA by province/state for fossil fuels and Höök and Aleklett (2009) examined USA coal production by state. A literature review highlights the limited current fossil fuel production modelling for the FSU region. The literature can be divided into three categories:

The first is to model the world fossil fuel production as a whole and differences of regions are excluded in these analyses. For example, Cavallo (2004) modelled the whole world oil production. Brecha (2008) analysed the whole world fossil fuel production in different scenarios. Kharecha and Hansen (2008) analysed the whole fossil fuels production for the world and their impacts on CO_2_ and climate. Nel and Cooper (2009) forecast the whole world fossil fuels production and their implications on economic growth and global warming. Maggio and Cacciola (2009) projected the world oil production as a whole by using a variant of the Hubbert curve. Wang et al. (2011) analysed the whole world conventional oil production by using two different multi-cycle curve-fitting models. Maggio and Cacciola (2012) modelled the peak of world oil, gas and coal by using the multi-cycle Hubert method. Similarly Nehring (2009) projected fossil fuels for the world. Ward et al. (2012) presented a high estimate for the whole world fossil fuels production. In these studies, the contribution of FSU is unknown.

The second category includes world fossil fuel production estimates by geographic/political regions. For example, Al-Fattah and Startzman (2000) and Imam et al. (2004) forecast the gas production of Eastern Europe and FSU as a whole in their world natural gas production modelling. Mohr and Evans (2011, 2009) have projected natural gas and coal production at the FSU region level. Mohr et al. (2015b) projected fossil fuel scenarios at the country level for most countries, however the FSU region was mostly projected as a whole. Höök et al. (2010) analysed Russian coal production and total Euroasian coal in their forecast of global coal production. Nashawi et al. (2010) analysed the crude oil production of Russia and Kazakhstan when they forecast world crude oil production. Rutledge (2011) analysed the coal production of Russia when they estimated long-term coal production. Reynolds and Kolodziej (2008) forecast FSU oil production as a whole by using a modified multi-cycle Hubbert model. Wang and Bentley (2020) modelled CIS gas production as a whole when they forecast world natural gas production. In these analyses, FSU is primarily treated as a whole.

The third category is to model the fossil fuel production for specific countries in FSU. Henderson (2019) projected Russian oil production in high detail to 2030, and Kapustin and Grushevenko (2019) projected Russian oil production to 2040. In terms of gas projections, Anon (2020) modelled Russian gas production by region to 2030.

Based on the above analysis, we note that the number of studies for FSU fossil fuels production is limited, despite the importance of the FSU region. Furthermore, several studies on FSU fossil fuels generally treated the region as a whole in their modelling. This appears to be due to the paucity of disaggregated production data during the Soviet Union years. The importance of the region necessitates the need for more detailed and disaggregated projections of this region.

The purpose of this paper is to examine by region the Former Soviet Union fossil fuel production in an attempt to reduce the uncertainty in global fossil fuel projection models and the associated greenhouse gas emissions. This study will continue to use the three URR scenarios of Mohr et al. (2015b) for all other regions of the world. The GeRs-DeMo approach assumes no global action to reduce global greenhouse gas emissions and no significant breakthroughs in alternative (non fossil fuel) energy technologies. The resultant models are therefore not intended as a prediction of future fossil fuel energy use, but instead estimate an informative, geographical and mineralogical picture of the upper limits to business as usual growth in fossil fuel use and its associated greenhouse gas emissions (Mohr et al. 2015b).

Due to the border disputes in what was until recently Eastern Ukraine, the Donetsk, Luhansk and Crimea regions have been modelled individually. This has been done to ensure that data is as granular as possible and to remain as neutral as possible to the politics surrounding these regions. The GeRS-DeMo model has the term ‘country’ and these regions will be modelled as such. This labelling by the authors is for modelling purposes only and is not an indication of support for or against any separatist movements in these regions or for any particular nations claims to these regions.

## Modelling methodology

**Fig. 1:**
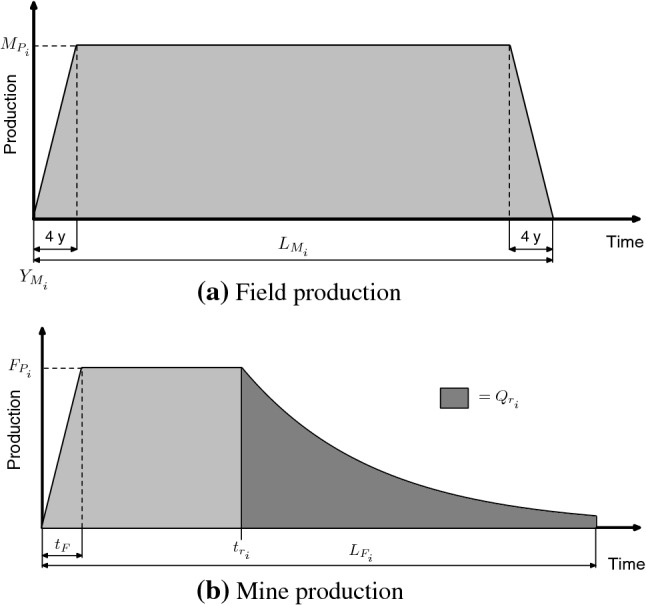
Idealised production from fields and mines

The model used to create the projections is the Geologic Resources Supply-Demand Model (GeRS-DeMo). GeRS-DeMo incorporates a supply and demand components with interact, so that if demand is high, supply is increased and vice versa. The model has been used to model a wide variety of resources such as fossil fuels, lithium, copper, lead, zinc, and iron ore (Mohr et al. 2012, 2015a; Northey et al. 2014; Mohr et al. 2018). The model was selected due to its ease of use and capability to model supply and demand interaction and handle supply disruptions (e.g. global conflicts). The model was developed previously (Mohr 2010), and has been briefly described elsewhere^2^. The model has two methods of supplying resources either from mines or from oil/gas fields as indicated in Fig. 1.

### Supply–Oil and gas fields

The production for a region is determined from the production of all idealised fields. The production of an individual idealised field has a one year ramp up to a plateau period, followed by an exponential decline in production, as shown in Fig. 1. Two key variables to calculate are the number of fields on-line over time, and the URR of the individual fields. The number of fields on-line *n*(*t*) is determined by Eq. 1.

1 $$\begin{aligned} n(t)= & {} \left\lceil r_Fn_{T}\frac{Q(t)}{Q_T} \right\rceil \end{aligned}$$

where, $n_T$ is the total number of fields to be placed on-line, $r_F$ is a rate constant, $Q_T$ is the URR of the region, and *Q*(*t*) is the cumulative production. The URR of the individual field, is calculated through the exploitable URR. The exploitable URR, is the sum of the URR in fields (or mines) that have already been brought on-line. The exploitable URR $Q_e(t)$ is estimated via Eq. (2).

2 $$\begin{aligned} Q_e(t)= & {} Q_T\left( \frac{n(t)}{n_T}\right) ^{r_Q} \end{aligned}$$

where, $r_Q$ is a rate constant. The URR of an individual field brought on-line in year *t*, $Q_F(t)$ is determined as:

3 $$\begin{aligned} Q_F(t)= & {} \frac{Q_e(t)-Q_e(t-1)}{N(t)-N(t-1)} \end{aligned}$$

### Supply–Coal, natural bitumen, extra heavy and kerogen mines

The production from mines is determined from the sum of the individual idealised mines’ production. The idealised mines have a four year ramp up and ramp down period, with a steady production rate in between, as shown in Fig. 1.

The life of an individual mine and its production rate is dependent on the year the mine is brought on-line as described in Eqs. (4) and (5).

4 $$\begin{aligned} M_P(t)= & {} \frac{M_H+M_L}{2}+\frac{M_H-M_L}{2}\tanh (r_t(t-t_t)) \end{aligned}$$

5 $$\begin{aligned} L_M(t)= & {} \left\{ \begin{array}{ll} L_H +(L_L - L_H)\frac{\log _{10}\left( M_P(t)/M_H\right) }{\log _{10}\left( M_L/M_H\right) } &{} \text{; } \text{ if } M_L \ne M_H\\ \frac{(L_L + L_H)}{2} &{} \text{; } \text{ otherwise } \end{array}\right. \end{aligned}$$

where, $r_t$ and $t_t$ are rate and time constants, $M_L$, $M_H$ is the minimum and maximum mine production rates, and $L_L$, $L_H$ are the minimum and maximum mine lives. The rate and time constants used are the same as those from Mohr (2010). Finally, the number of mines brought on-line in year *t* is calculated via the estimated exploitable URR $Q_E(t)$ as:

6 $$\begin{aligned} Q_E(t)= & {} \frac{Q_T - Q_{T1}e^{-r_T}}{1-e^{-r_T}} - \frac{Q_T-Q_{T1}}{1-e^{-r}}e^{-r_T\frac{Q(t)}{Q_T}} \end{aligned}$$

where, $Q_{T1}$ is the URR of the first mine brought on-line in the region and $r_T$ is a rate constant. The number of mines brought on-line is determined by increasing the number of mines on-line until the actual exploitable URR is larger than the estimated exploitable URR.

### Demand

The demand used is identical to Mohr et al. (2015b). Specifically, the global population *p*(*t*) (in billions) is estimated to level off at 11 billion U.N. (2013) based on the following equation:

7 $$\begin{aligned} p(t)= & {} \frac{11-0.82}{\left[ 1+1.5\exp (-0.023\times 2(t-2014))\right] ^{1/2}}+0.82 \end{aligned}$$

The per-capita demand, *D*(*t*) is calculated as:

8 $$\begin{aligned} D(t)= & {} \left\{ \begin{array}{ll} 60\exp (0.025(t-1973)) &{} \text{; } \text{ if } t<1973\\ 60 &{} \text{; } \text{ if } t\ge 1973 \end{array} \right. \end{aligned}$$

## Data source

**Fig. 2:**
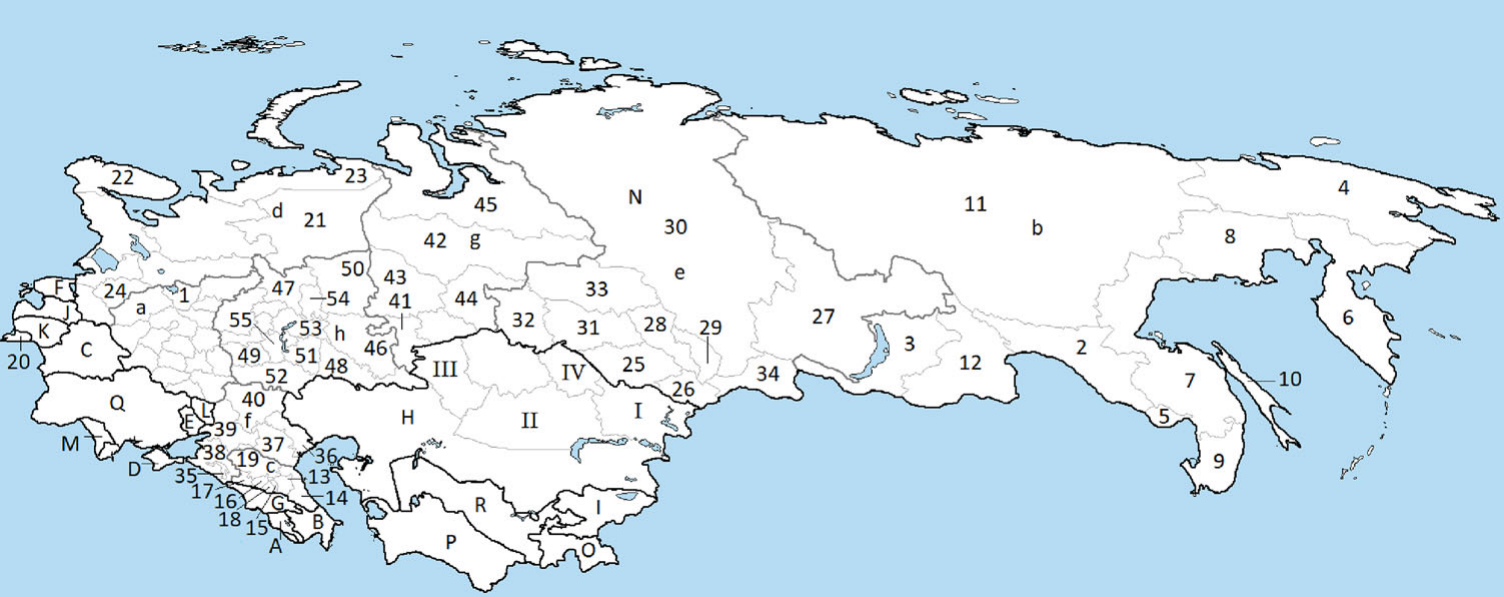
Regions of the Former Soviet Union. A – Armenia, B – Azerbaijan, C – Belarus, D – Crimea, E – Donetsk, F – Estonia, G – Georgia, H – Kazakhstan, I – Kyrgyzstan, J – Lativa, K – Lithuania, L – Luhansk, M – Moldova, N – Russia, O – Tajikistan, P – Turkmenistan, Q – Ukraine, R – Uzbekistan, I – East Kazakhstan, II – Karaganda, III – Kostanay, IV – Pavlodar, a – Central, b – Far Eastern, c – North Caucasian, d – Northwestern, e – Siberian, f – Southern, g – Ural, h – Volga, 1 – Yaroslavl, 2 – Amur, 3 – Buryatia, 4 – Chukotka AO, 5 – Jewish AO, 6 – Kamchatka, 7 – Khabarovsk, 8 – Magadan, 9 – Primorsky, 10 – Sakhalin, 11 – Yakutia, 12 – Zabaykalsky, 13 – Chechnya, 14 – Dagestan, 15 – Ingushetia, 16 – Kabardino-Balkaria, 17 – Karachay-Cherkessia, 18 – North Ossetia-Alania, 19 – Stavropol, 20 – Kaliningrad, 21 – Komi, 22 – Murmansk, 23 – Nenets AO, 24 – Novgorod, 25 – Altai Krai, 26 – Altai Rep, 27 – Irkutsk, 28 – Kemerovo, 29 – Khakassia, 30 – Krasnoyarsk, 31 – Novosibirsk, 32 – Omsk, 33 – Tomsk, 34 – Tuva, 35 – Adygea, 36 – Astrakhan, 37 – Kalmykia, 38 – Krasnodar, 39 – Rostov, 40 – Volgograd, 41 – Chelyabinsk, 42 – Khanty-Mansi AO, 43 – Sverdlovsk, 44 – Tyumen, 45 – Yamalo-Nenets AO, 46 – Bashkortostan, 47 – Kirov, 48 – Orenburg, 49 – Penza, 50 – Perm, 51 – Samara, 52 – Saratov, 53 – Tatarstan, 54 – Udmurtia, 55 – Ulyanovsk

Historic production for the FSU needed to be split into the individual countries. Russia’s production was split into regions (oblast’s/krai’s etc) where possible due to Russia’s importance to world fossil fuel supply. Where this was not possible the production was reported at the Federal Districts level. The regions of the Former Soviet Union are shown in Fig. 2. In general the word krai, oblast or republic is dropped with the exception to distinguish between Altai Republic and Altai Krai. The region Tyumen denotes the Tyumen oblast excluding Khanty-Mansi AO and Yamalo-Nenets AO which are modelled separately. In addition the Donetsk, Luhansk and Crimea regions' production was split out into individual regions. Acquiring production data at this granular level proved to be difficult. To the best of the authors’ knowledge a comprehensive, publicly available dataset does not exist covering the full time period and region, which our current paper seeks to address.

**Fig. 3:**
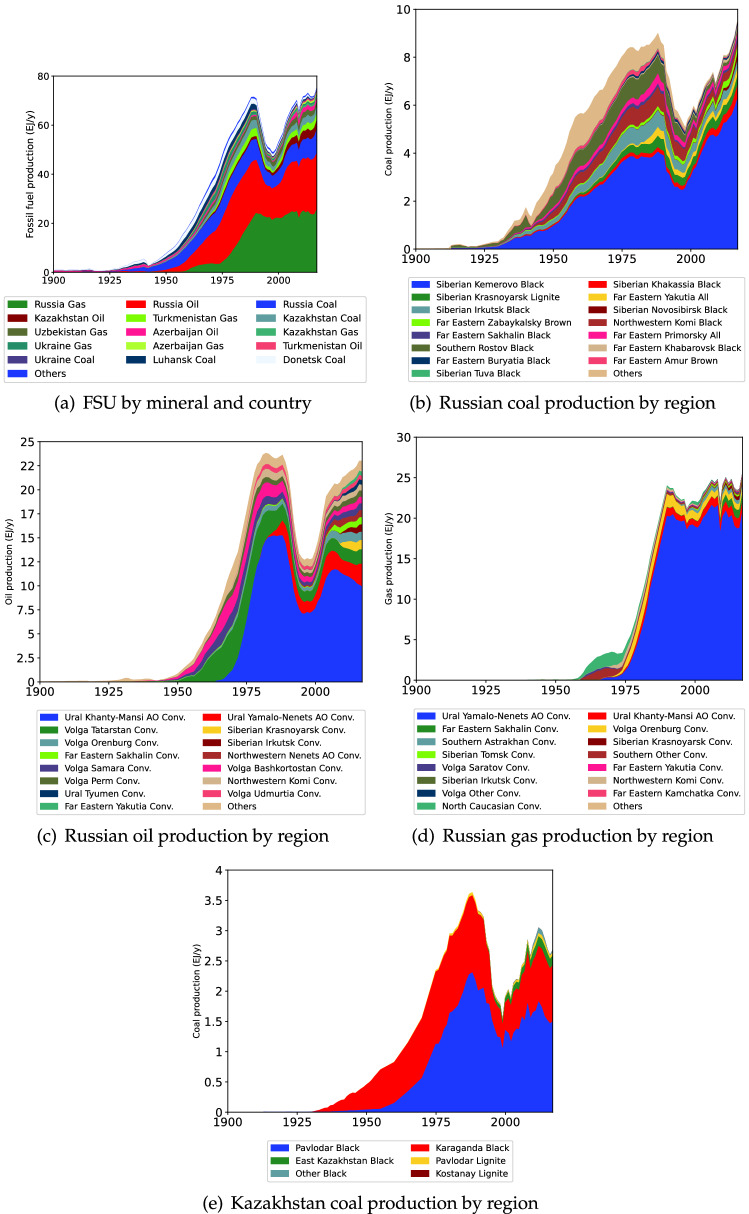
Historic fossil fuel production of the FSU

Recent production data after the end of the Soviet Union is readily available through the various statistical agencies and yearbooks e.g. (Ukrstat 2017; Rosstat 2018) and usual sources such as the BP (2019) and BGS (2017). Declassified documents from the US Central Intelligence Agency contain a wealth of data on Soviet fossil fuel production from both before and during the Cold War. Production data between 1955 and 1980 in particular was challenging to acquire and typically was only reported every 5 years. As a result, production data in between these 5 year intervals had to be estimated. The historical production dataset was constructed by combining the data from the following literature^3^. The historical production data for the FSU is shown in Fig. 3.

The dominance of the Kuznetsk basin (in Kemerovo Oblast), Khanty-Mansi Autonomous Oblast and Yamalo-Nenets Autonomous Oblast to Russia’s coal, oil and gas production respectively is readily observed. Coal production in regions closer to Moscow have historical peaked and declined, such as Central, Northwestern, Ural and Volga regions. To assist future researchers the collated production dataset is available in the electronic supplement.

## Fossil fuel URR

The Ultimately Recoverable Resources (URR) are the total amount of the fossil fuels that can be recovered from the resource in the ground before production starts ASPO (2014). Due to the uncertainty surrounding the URR, three URR values have been used, specifically a Low Estimate, a High estimate and a Best Guess (BG) estimate. The URR estimates for the FSU region have been collated from a wide range of sources (see Table 8). The Low estimate was determined primarily through Hubbert Linearisation, and the High estimate was primarily from BGR (2016). The new URR values for the FSU are compared to Mohr et al. (2015b) results in Table 1 and detailed URR values for FSU are shown in Tables 2–4. As shown the High URR is higher than the previous estimate across each fuel source. Similarly the Low URR is slightly lower than the previous estimate. The main difference is in the BG estimate, with the current URR substantially higher in this study, particularly for coal and gas.

The mass to energy conversions are the same as Mohr et al. (2015b). A small number of regions the coal quality is not known for these regions, the energy density assumed is half way between brown and black coal energy densities (19.5 EJ/Gt). The conversion to greenhouse gas emissions, carbon dioxide equivalents (CO_2_e), assumes the bituminous values for these regions.

**Table 1:** URR in EJ used in this study; Mohr et al. (2015b) (in brackets) for comparison

Projection	Low		BG		High	
Coal	1425.8	(1668.8)	7902.6	(1668.8)	10,592.3	(444.8)
Gas	2605.5	(2670.6)	8454.6	(4102.7)	11,341.0	(10,061.6)
Oil	3036.4	(3556.7)	5059.0	(4046.6)	5764.9	(4599.4)
Total	7067.7	(7896.1)	21,416.2	(9818.1)	27,698.2	(19,105.7)

**Table 2:** Coal URR values used in this study by country and type

Type	Country	Low	BG	High
All	Russia	43.6	402.1	403.6
Bituminous	Moldova	<0	<0	<0
Bituminous	Tajikistan	4.8	10.1	10.1
Bituminous	Turkmenistan	<0	<0	<0
Black	Crimea	<0	<0	<0
Black	Donetsk	169.0	783.0	783.0
Black	Kazakhstan	178.8	702.7	1959.8
Black	Kyrgyzstan	2.1	12.9	30.5
Black	Luhansk	117.0	582.5	582.5
Black	Russia	758.2	4408.3	4911.0
Black	Ukraine	34.8	34.8	243.8
Black	Uzbekistan	0.2	1.3	1.3
Brown	Russia	51.6	144.3	155.6
Lignite	Kazakhstan	3.0	117.0	727.6
Lignite	Kyrgyzstan	1.8	9.3	14.0
Lignite	Russia	50.9	682.0	720.5
Lignite	Ukraine	2.3	2.3	24.5
Lignite	Uzbekistan	5.2	5.2	19.8
Sub Bituminous	Georgia	2.4	4.7	4.7
Sub Bituminous	Tajikistan	<0	<0	<0
Total		1425.8	7902.6	10,592.3

**Table 3:** Oil URR values used in this study by country and type

Type	Country	Low	BG	High
Conventional	Azerbaijan	122.4	176.3	176.3
Conventional	Belarus	8.4	8.4	8.1
Conventional	Crimea	0.6	0.6	0.6
Conventional	Georgia	1.3	1.3	3.6
Conventional	Kazakhstan	184.5	184.5	425.6
Conventional	Kyrgyzstan	0.2	0.2	0.7
Conventional	Lithuania	0.2	0.2	2.8
Conventional	Luhansk	<0	<0	<0
Conventional	Moldova			0.4
Conventional	Russia	1832.6	2054.2	2267.7
Conventional	Tajikistan	0.1	0.1	2.7
Conventional	Turkmenistan	35.5	35.5	99.3
Conventional	Ukraine	17.2	17.2	24.7
Conventional	Uzbekistan	12.1	12.1	30.4
Extra Heavy	Azerbaijan			0.7
Extra Heavy	Russia			0.1
Kerogen	Armenia			1.8
Kerogen	Belarus		40.0	40.0
Kerogen	Estonia	5.7	5.7	94.6
Kerogen	Kazakhstan			16.3
Kerogen	Russia	0.7	1421.1	1421.1
Kerogen	Turkmenistan			22.0
Kerogen	Ukraine			24.0
Kerogen	Uzbekistan		70.1	70.1
Natural Bitumen	Kazakhstan	312.5	312.5	312.5
Natural Bitumen	Russia		219.4	219.4
Tight	Kazakhstan	60.7	60.7	60.5
Tight	Lithuania	4.0		
Tight	Russia	431.5	432.6	432.6
Tight	Ukraine	6.3	6.3	6.3
Total		3036.4	5059.0	5764.9

**Table 4:** Gas URR values used in this study by country and type

Type	Country	Low	BG	High
CBM	Kazakhstan	10.5	10.5	52.0
CBM	Russia	209.9	209.9	466.8
CBM	Ukraine	26.2	26.2	111.2
Conventional	Armenia			0.4
Conventional	Azerbaijan	70.4	70.4	132.4
Conventional	Belarus	0.4	0.4	0.9
Conventional	Crimea	1.1	1.1	1.1
Conventional	Donetsk	<0	<0	<0
Conventional	Georgia	<0	<0	4.1
Conventional	Kazakhstan	131.2	131.2	161.8
Conventional	Kyrgyzstan	0.3	0.3	1.2
Conventional	Lithuania			14.1
Conventional	Luhansk	0.1	0.1	0.1
Conventional	Moldova			0.7
Conventional	Russia	1591.1	5811.5	6971.9
Conventional	Tajikistan	0.3	1.3	1.3
Conventional	Turkmenistan	200.4	200.4	1026.0
Conventional	Ukraine	111.2	128.8	128.8
Conventional	Uzbekistan	126.5	201.6	201.6
Hydrates	Russia		403.8	807.7
Shale	Kazakhstan	2.9	28.9	28.9
Shale	Russia	35.2	352.1	352.1
Shale	Ukraine	13.4	134.6	134.6
Tight	Russia	74.1	741.3	741.3
Total		2605.5	8454.5	11,341.0

## Results and discussion

The results and discussion will examine first detailed projections of Russia’s fossil fuels and Kazakhstan’s coal production. Following this the results for the entire FSU region will be examined. All results shown are the dynamic model where the new FSU model was combined with projections from the rest of the world from Mohr et al. (2015b). The electronic Supplement contains the complete results of the projections.

### Regional results

**Fig. 4:**
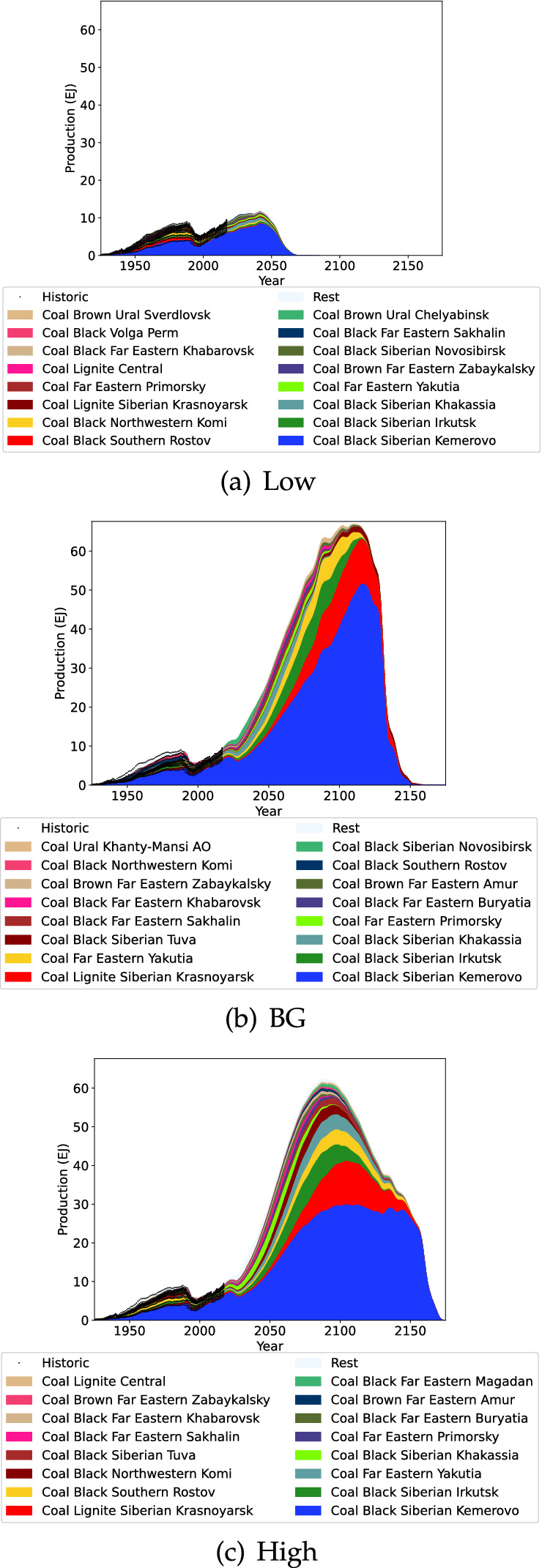
Russian coal projection

The projections of Russian coal are shown in Fig. 4. Coal production for Russia is likely to increase for several more decades with the earliest peak estimated at 2042 in the Low projection. In all projections of Russian coal production we can see the dominance of the Kuznetsk basin (in Kemerovo Oblast) will continue into the future, with the earliest peak estimated 2 decades away in 2042 (Low estimate triggering Russia’s coal peak). The projection in this study is slightly higher than the Russian Government’s estimate for 2035 (This Study 465–734 Mt, Russian Government 429–588 Mt) (Mishustin 2020). More generally the dominance of Siberian and Far Eastern regions is evident. The sharp decline evidenced in the projections is due to the dynamic interactions in the model attempting to keep coal production for the world increasing. Note that this model assumes continuing underlying demand for coal to explore the character of peak estimates arising due to constrained supply. In practice, reduced future demand for coal could alter estimates of peak production to be earlier or later.

**Fig. 5:**
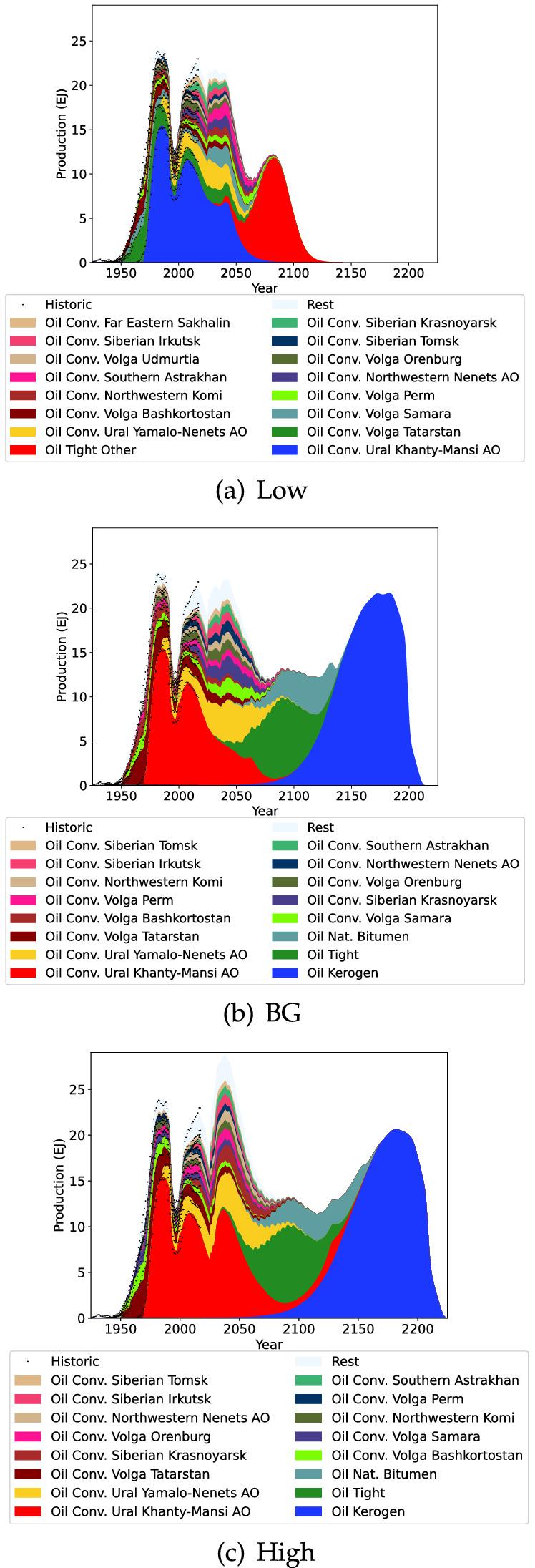
Russian oil projection

Russian oil production is rather disjointed as indicated in Fig. 5. The collapse of the Soviet Union caused oil production to sharply decline, and while it has managed to approximately reach its pre collapse heights there is cause for concern. An important factor is that the dominant Khanty-Mansi AO oil production has been in declining since 2007. All projections indicate that there will be a short term decline in Russian oil production in the near future as a result. The conventional oil decline is in line with other literature projections, however the projections presented here are on the more optimistic end of the literature (Table 5) (Henderson 2019; Kapustin and Grushevenko 2019). These projected declines are partially offset in the short term by Yamalo-Nenets AO production and in the longer term by unconventional oil sources.

**Table 5:** Russia conventional oil production comparison to literature (EJ/yr)

Year	This Study	Henderson (2019)	Kapustin and Grushevenko (2019)
2030	21.5–25.4	19.7	20.4–21.2
2040	20.6–27.8	–	17.1–21.2

**Fig. 6:**
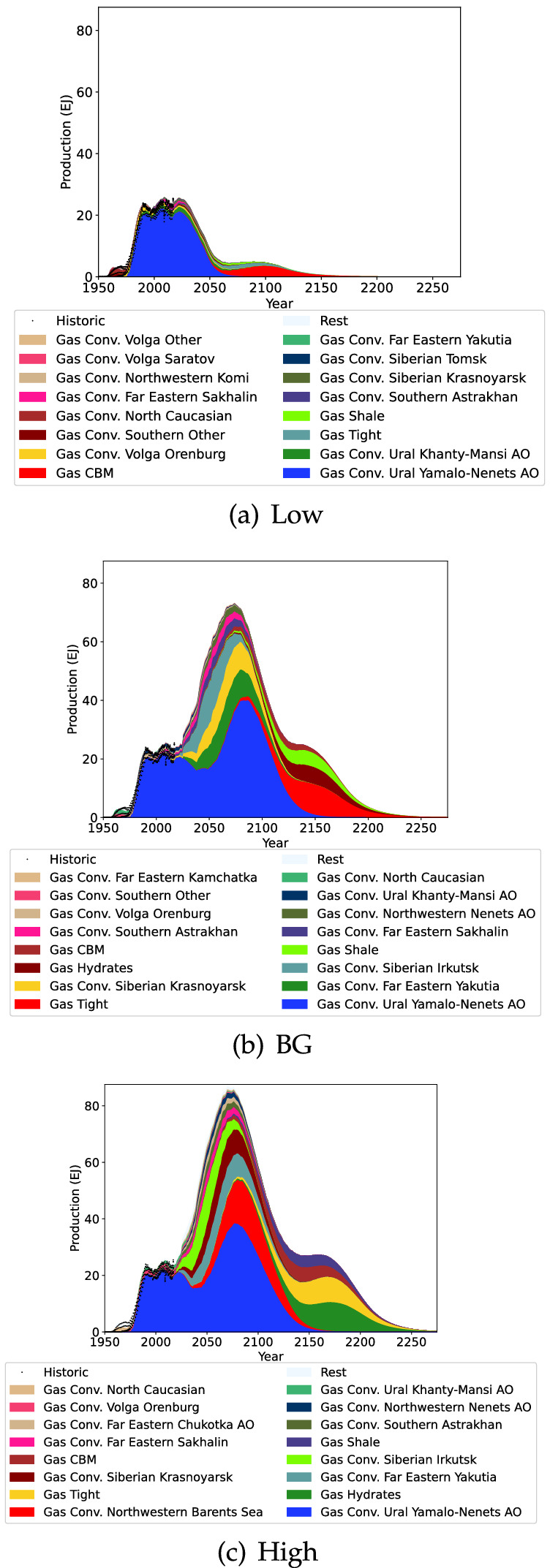
Russian gas projection

Russian gas production is driven almost entirely by Yamalo-Nenets AO production (Fig. 6) and this region has been producing a steady production level for decades. It is difficult to predict what will happen to Russian gas production in the future, but the BG and High scenarios indicate that substantial growth is possible. In contrast, the Low scenario with a substantially smaller URR indicates that Russian gas production would peak in 2022 before sharply declining.

**Fig. 7:**
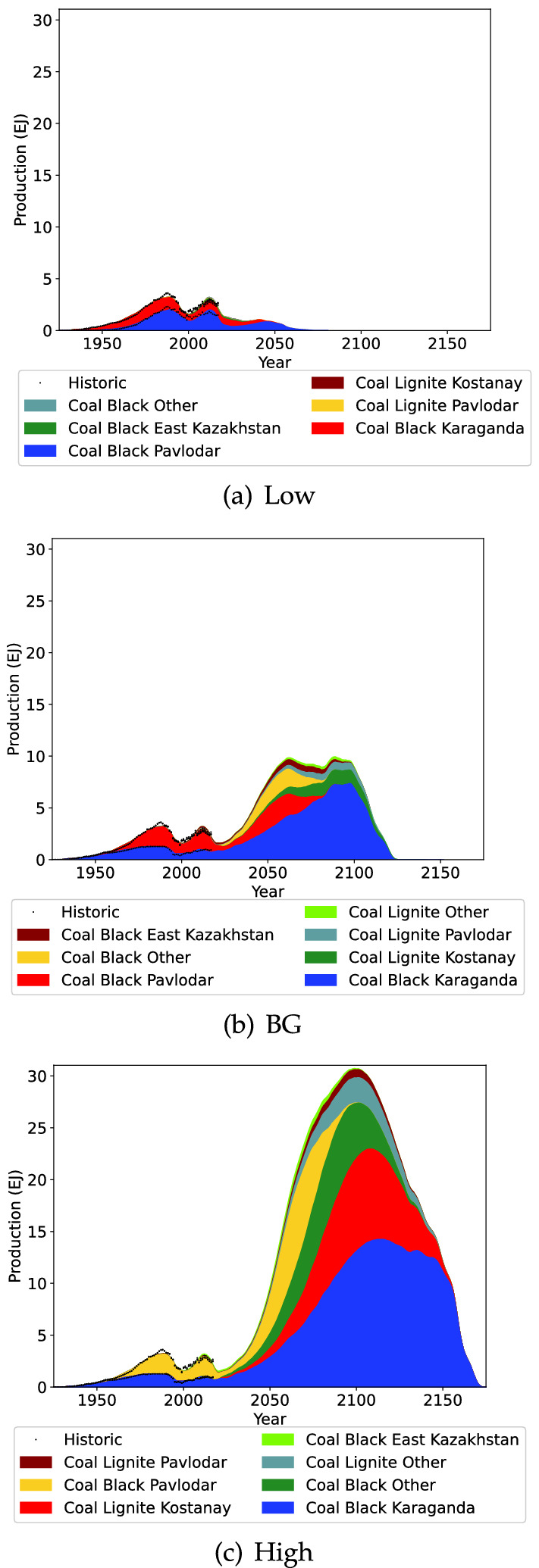
Kazakhstan coal projection

Kazakhstan coal production projection is highlighted in Fig. 7. Coal production in Kazakhstan is currently declining due to stagnant production in Karaganda and declining production in Pavlodar. For the Low scenario this declining production is expected to continue. In the BG and High scenarios however production is projected to start increasing again in the near future, and decline after 2100.

### FSU total results

**Fig. 8:**
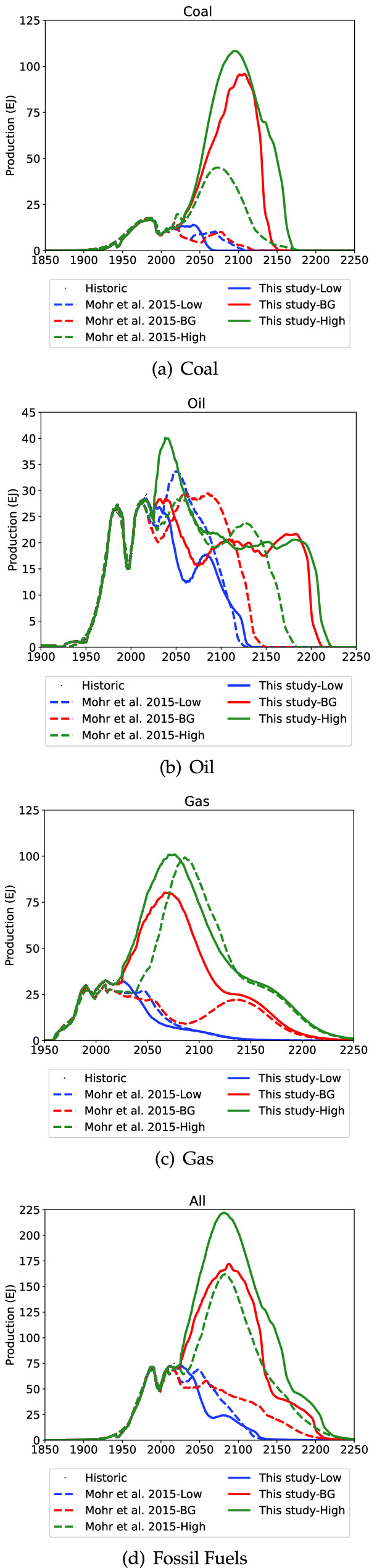
Comparison between this study and Mohr et al. (2015b) for FSU

**Table 6:** Peak year comparison between this study (Mohr et al. 2015b in brackets)

Region		Peak Year						Peak Rate (EJ/yr)					
		Low		BG		High		Low		BG		High	
FSU	Coal	1984	(1985)	2108	(1986)	2095	(2073)	16.9	(17.7)	96.0	(17.7)	108.3	(45.1)
FSU	Gas	2009	(2009)	2067	(2009)	2076	(2086)	32.6	(30.4)	80.4	(30.3)	101.0	(99.3)
FSU	Oil	2017	(2052)	2038	(2059)	2038	(2056)	28.5	(33.7)	28.4	(29.6)	40.1	(28.8)
FSU	Total	2027	(1988)	2087	(1988)	2082	(2083)	72.7	(69.9)	171.9	(69.9)	222.0	(162.0)

The FSU projections are compared to Mohr et al. (2015b) in Fig. 8 and Table 6. FSU coal production in the High scenario is projected to increase faster than Mohr et al. (2015b) and ultimately peak at over 100 EJ/year compared to under 50 EJ/year in Mohr et al. (2015b). The substantial increase in the FSU BG coal URR in this study is evident as the projection shows BG FSU coal production peaking after 2100 instead of choppily continuing to decline. In terms of oil, the current projection is more optimistic than Reynolds and Kolodziej (2008) with a peak year estimate of 2017–2038 at 28.4–40.1 EJ compared to a peak at 26 EJ in 2009. For the fossil fuels overall, compared to Mohr et al. (2015b), there is little difference in the Low scenarios; the High scenario peak year is almost identical (2082–3), however the peak rate is notably higher (222 EJ/yr compared to 162 EJ/yr).

The results shown in Fig. 8 highlight that the specific URR value used has a large impact on the projections. It could be argued that detailed modelling of the FSU region was not necessary, and efforts instead could be restricted to towards more detailed and accurate URR information. Modelling at a granular level does however result in a more nuanced understanding that would otherwise have been missed. For example the rapid increase gas production in the Far Eastern and Siberian regions^4^. Similarly the depletion of coal closer to Russia’s population such as the Central lignite and the increases in more remote locations such as the Kuznetsk basin.

## Global implications

**Fig. 9:**
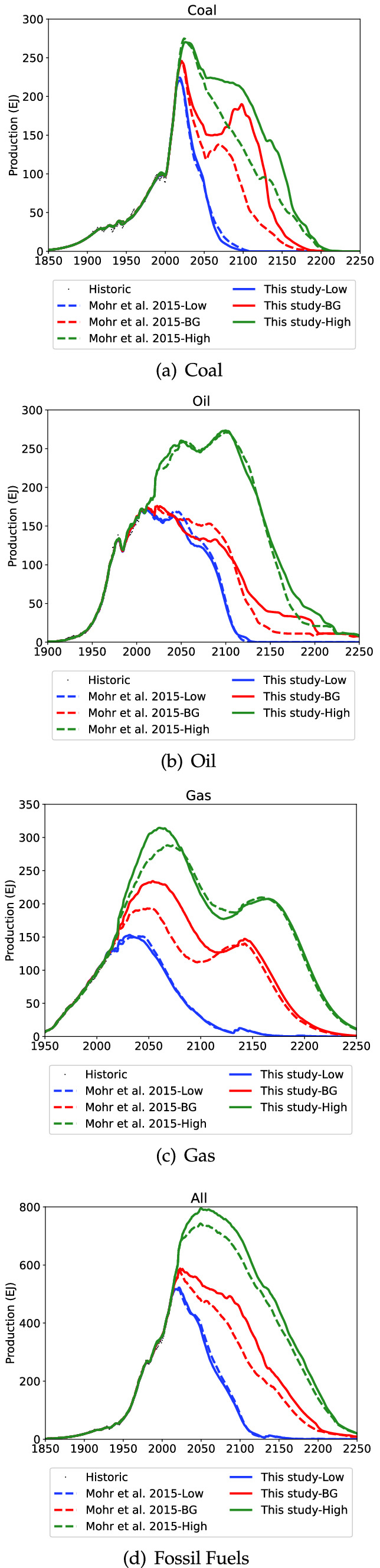
Comparison between this study and Mohr et al. (2015b) for the world

The impact of the new FSU projection for the world fossil fuel production is shown in Fig. 9 and the peak year and rates are shown in Table 7.

**Fig. 10:**
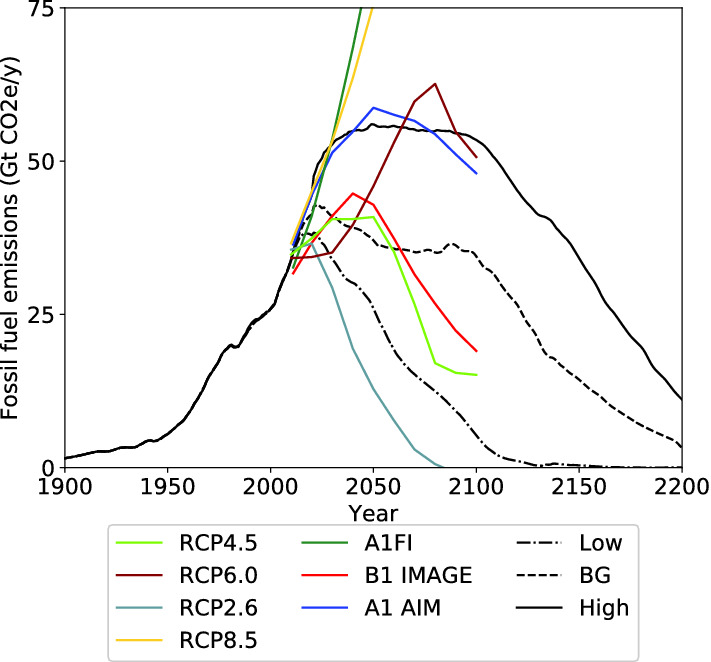
World Emission projections compared to IPCC scenarios (Nakicenovic et al. 2001; IPCC 2013; Meinhausen et al. 2011)

The comparisons for the world between the two FSU models shows little difference to world oil production, with the slight change in the BG scenario of a longer slower decline compared to Mohr et al. (2015b). For gas the new FSU projection causes world production to increase slightly higher and faster in the BG and High cases, with the Low scenario mostly unchanged. World fossil fuel production from the new FSU projection is anticipated to be virtually unchanged in the Low scenario, decline more gradually in the BG scenario and peak at a higher rate in the High scenario. The comparison to selected IPCC projections (Nakicenovic et al. 2001; IPCC 2013; Meinhausen et al. 2011) is shown in Fig. 10. The high scenario now very closely aligns with the A1 Aim, and the BG scenario declines more slowly than the A1Fl or RCP4.5 scenarios. The potential decline in near future could have significant implications on responses to climate change, and accelerate the use of renewable energy.

**Table 7:** Peak year comparison between this study (Mohr et al. 2015b in brackets)

Region		Peak Year						Peak Rate (EJ/yr)					
		Low		BG		High		Low		BG		High	
World	Coal	2019	(2018)	2021	(2021)	2026	(2024)	220.6	(224.5)	244.5	(245.9)	270.3	(274.9)
World	Gas	2032	(2041)	2054	(2052)	2060	(2068)	153.0	(151.2)	234.3	(193.6)	314.6	(288.2)
World	Oil	2011	(2011)	2023	(2011)	2100	(2100)	172.2	(172.6)	176.0	(174.7)	273.5	(271.3)
World	Total	2022	(2021)	2023	(2023)	2050	(2049)	522.2	(516.4)	587.9	(577.5)	795.1	(743.1)

## Conclusions

This paper utilises comprehensive data from the FSU to establish scenarios for future projections of fossil fuel supply from known FSU resources, with comprehensive geographical and mineralogical detail. This additional detail is added to the work of Mohr et al. (2015b) to produce updated global projections of fossil fuel supply from known resources assuming an increasing global demand arising from population growth (with demand per person assumed constant). Comparisons of emissions from the scenarios presented in the paper with IPCC projections representing significant climate change are also given. The most striking finding is the substantial increase in FSU ultimately recoverable resources, particularly for coal but also for gas and oil. At the aggregate global level, the Best Guess and High supply projections increase somewhat whilst Low scenario is broadly similar to the 2015 study. The value of geographically resolved projections for future work, is to more readily be able to visualise both upper bound scenarios – were fossil fuel demand to continue at current per capita rates – as well as the contribution to meeting climate change goals which might be achieved through reducing demand and in turn supply from various regions, or the impact of supply interruptions from various regions. Given that fossil fuel demand has declined in 2020 due to the global impact of the coronavirus, the assumption of constant per capita supply must be qualified. Rather than likely projections of demand, the projections presented in this paper illustrate a time-dependent supply landscape from different countries under low, high and best-guess estimates of ultimately recoverable resources.

### Supplementary Information

Below is the link to the electronic supplementary material.

Supplementary file1 (ZIP 23581 kb)

Supplementary file2 (PDF 21837 kb)

Supplementary file3 (PDF 22162 kb)

Supplementary file4 (PDF 21927 kb)

Supplementary file5 (PDF 22356 kb)

Supplementary file6 (PDF 22451 kb)

Supplementary file7 (PDF 22103 kb)

## Appendix

See Table 8

**Table 8:** The list of all scenarios with the URR value and source

Mineral	Country	Type	Region	Subregion	Low	BG	High
Coal	Crimea	Black	Crimea		<0.0$^{a}$	<0.0$^{a}$	<0.0$^{a}$
Coal	Donetsk	Black	Donetsk		169.0$^{b}$	783.0$^{c}$	783.0$^{c}$
Coal	Georgia	Sub Bituminous			2.4$^{d}$	4.7$^{e}$	4.7$^{e}$
Coal	Kazakhstan	Black	East Kazakhstan		4.5$^{d}$	29.0$^{f}$	33.4$^{g}$
Coal	Kazakhstan	Black	Karaganda		73.6$^{d}$	456.9$^{f}$	1273.3$^{g}$
Coal	Kazakhstan	Black	Other		1.3$^{d}$	62.1$^{f}$	337.8$^{g}$
Coal	Kazakhstan	Black	Pavlodar		99.4$^{d}$	154.7$^{f}$	315.3$^{g}$
Coal	Kazakhstan	Lignite	Kostanay		0.1$^{b}$	67.1$^{f}$	533.5$^{g}$
Coal	Kazakhstan	Lignite	Other			12.6$^{f}$	143.1$^{g}$
Coal	Kazakhstan	Lignite	Pavlodar		2.9$^{d}$	37.3$^{f}$	51.1$^{g}$
Coal	Kyrgyzstan	Black			2.1$^{d}$	12.9$^{h}$	30.5$^{h}$
Coal	Kyrgyzstan	Lignite			1.8$^{d}$	9.3$^{h}$	14.0$^{h}$
Coal	Luhansk	Black	Luhansk		117.0$^{b}$	582.5$^{c}$	582.5$^{c}$
Coal	Moldova	Bituminous			<0.0$^{a}$	<0.0$^{a}$	<0.0$^{a}$
Coal	Russia	All	Far Eastern	Primorsky	20.2$^{d}$	87.6$^{i}$	87.6$^{i}$
Coal	Russia	All	Far Eastern	Yakutia	23.4$^{b}$	288.2$^{i}$	288.2$^{i}$
Coal	Russia	All	Siberian	Altai Rep			1.6$^{i}$
Coal	Russia	All	Ural	Khanty-Mansi AO		26.3$^{i}$	26.3$^{i}$
Coal	Russia	Black	Far Eastern	Buryatia	5.2$^{d}$	71.8$^{i}$	71.8$^{i}$
Coal	Russia	Black	Far Eastern	Chukotka AO	1.0$^{d}$	1.0$^{d}$	19.1$^{i}$
Coal	Russia	Black	Far Eastern	Khabarovsk	13.0$^{a}$	62.9$^{i}$	62.9$^{i}$
Coal	Russia	Black	Far Eastern	Magadan	2.5$^{d}$	2.5$^{d}$	54.0$^{i}$
Coal	Russia	Black	Far Eastern	Sakhalin	13.0$^{a}$	77.1$^{i}$	77.1$^{i}$
Coal	Russia	Black	North Caucasian	Karachay-Cherkessia	0.1$^{d}$	0.1$^{a}$	0.3$^{i}$
Coal	Russia	Black	Northwestern	Komi	42.5$^{d}$	42.5$^{d}$	225.6$^{i}$
Coal	Russia	Black	Northwestern	Murmansk	0.5$^{a}$	0.5$^{a}$	0.5$^{a}$
Coal	Russia	Black	Northwestern	Nenets AO			2.6$^{i}$
Coal	Russia	Black	Siberian	Irkutsk	46.2$^{d}$	412.4$^{i}$	412.4$^{i}$
Coal	Russia	Black	Siberian	Kemerovo	520.0$^{b}$	3378.9$^{i}$	3378.9$^{i}$
Coal	Russia	Black	Siberian	Khakassia	39.0$^{b}$	153.4$^{i}$	153.4$^{i}$
Coal	Russia	Black	Siberian	Novosibirsk	13.0$^{b}$	39.4$^{i}$	39.4$^{i}$
Coal	Russia	Black	Siberian	Tuva	1.6$^{d}$	99.9$^{i}$	99.9$^{i}$
Coal	Russia	Black	Southern	Rostov	48.6$^{d}$	48.6$^{d}$	295.9$^{i}$
Coal	Russia	Black	Volga	Perm	12.1$^{a}$	17.3$^{i}$	17.3$^{i}$
Coal	Russia	Brown	Far Eastern	Amur	9.5$^{d}$	55.7$^{i}$	55.7$^{i}$
Coal	Russia	Brown	Far Eastern	Jewish AO	<0.0$^{a}$	<0.0$^{a}$	0.7$^{i}$
Coal	Russia	Brown	Far Eastern	Kamchatka	<0.0$^{a}$	3.9$^{i}$	3.9$^{i}$
Coal	Russia	Brown	Far Eastern	Zabaykalsky	19.4$^{d}$	55.6$^{i}$	55.6$^{i}$
Coal	Russia	Brown	Northwestern	Novgorod	<0.0$^{a}$	<0.0$^{a}$	<0.0$^{a}$
Coal	Russia	Brown	Siberian	Altai Krai	<0.0$^{a}$	<0.0$^{a}$	0.4$^{i}$
Coal	Russia	Brown	Ural	Chelyabinsk	12.0$^{d}$	18.4$^{i}$	18.4$^{i}$
Coal	Russia	Brown	Ural	Sverdlovsk	10.0$^{d}$	10.0$^{d}$	11.2$^{i}$
Coal	Russia	Brown	Volga	Orenburg	0.6$^{b}$	0.6$^{b}$	9.6$^{i}$
Coal	Russia	Lignite	Central		15.4$^{d}$	15.4$^{d}$	51.5$^{i}$
Coal	Russia	Lignite	Far Eastern	Zabaykalsky	<0.0$^{a}$	<0.0$^{a}$	<0.0$^{a}$
Coal	Russia	Lignite	Siberian	Krasnoyarsk	33.8$^{d}$	664.9$^{i}$	664.9$^{i}$
Coal	Russia	Lignite	Volga	Bashkortostan	1.7$^{a}$	1.7$^{a}$	4.1$^{i}$
Coal	Tajikistan	Bituminous			4.8$^{b}$	10.1$^{e}$	10.1$^{e}$
Coal	Tajikistan	Sub Bituminous			<0.0$^{d}$	<0.0$^{d}$	<0.0$^{d}$
Coal	Turkmenistan	Bituminous			<0.0$^{a}$	<0.0$^{a}$	<0.0$^{e}$
Coal	Ukraine	Black			34.8$^{d}$	34.8$^{d}$	243.8$^{c}$
Coal	Ukraine	Lignite			2.3$^{a}$	2.3$^{a}$	24.5$^{e}$
Coal	Uzbekistan	Black			0.2$^{d}$	1.3$^{j}$	1.3$^{j}$
Coal	Uzbekistan	Lignite			5.2$^{d}$	5.2$^{d}$	19.8$^{j}$
Gas	Armenia	Conventional					0.4$^{k}$
Gas	Azerbaijan	Conventional			70.4$^{d}$	70.4$^{d}$	132.4$^{k}$
Gas	Belarus	Conventional			0.4$^{d}$	0.4$^{d}$	0.9$^{k}$
Gas	Crimea	Conventional	Crimea		1.1$^{d}$	1.1$^{d}$	1.1$^{d}$
Gas	Donetsk	Conventional	Donetsk		<0.0$^{a}$	<0.0$^{a}$	<0.0$^{a}$
Gas	Georgia	Conventional			<0.0$^{d}$	<0.0$^{d}$	4.1$^{k}$
Gas	Kazakhstan	CBM			10.5$^{l}$	10.5$^{l}$	52.0$^{k}$
Gas	Kazakhstan	Conventional			131.2$^{m}$	131.2$^{m}$	161.8$^{k}$
Gas	Kazakhstan	Shale			2.9$^{n}$	28.9$^{k}$	28.9$^{k}$
Gas	Kyrgyzstan	Conventional			0.3$^{d}$	0.3$^{d}$	1.2$^{k}$
Gas	Lithuania	Conventional					14.1$^{k}$
Gas	Luhansk	Conventional	Luhansk		0.1$^{b}$	0.1$^{b}$	0.1$^{b}$
Gas	Moldova	Conventional					0.7$^{k}$
Gas	Russia	CBM			209.9$^{l}$	209.9$^{l}$	466.8$^{k}$
Gas	Russia	Conventional	Far Eastern	Chukotka AO			124.2$^{i}$
Gas	Russia	Conventional	Far Eastern	Kamchatka	0.4$^{b}$	24.2$^{i}$	24.2$^{i}$
Gas	Russia	Conventional	Far Eastern	Primorsky			7.4$^{i}$
Gas	Russia	Conventional	Far Eastern	Sakhalin	19.3$^{d}$	195.4$^{i}$	195.4$^{i}$
Gas	Russia	Conventional	Far Eastern	Yakutia	4.2$^{d}$	574.7$^{i}$	574.7$^{i}$
Gas	Russia	Conventional	North Caucasian		31.5$^{d}$	31.5$^{d}$	79.5$^{i}$
Gas	Russia	Conventional	Northwestern	Barents Sea			937.7$^{i}$
Gas	Russia	Conventional	Northwestern	Komi	17.9$^{d}$	17.9$^{d}$	60.4$^{i}$
Gas	Russia	Conventional	Northwestern	Nenets AO	0.4$^{b}$	122.4$^{i}$	122.4$^{i}$
Gas	Russia	Conventional	Siberian	Irkutsk	0.9$^{b}$	496.3$^{i}$	496.3$^{i}$
Gas	Russia	Conventional	Siberian	Krasnoyarsk	18.0$^{b}$	561.4$^{i}$	561.4$^{i}$
Gas	Russia	Conventional	Siberian	Tomsk	11.0$^{b}$	20.1$^{i}$	20.1$^{i}$
Gas	Russia	Conventional	Southern	Astrakhan	24.6$^{d}$	176.3$^{i}$	176.3$^{i}$
Gas	Russia	Conventional	Southern	Other	47.5$^{d}$	36.5$^{i}$	36.5$^{i}$
Gas	Russia	Conventional	Ural	Khanty-Mansi AO	97.2$^{d}$	79.6$^{i}$	79.6$^{i}$
Gas	Russia	Conventional	Ural	Tyumen			0.6$^{i}$
Gas	Russia	Conventional	Ural	Yamalo-Nenets AO	1232.1$^{d}$	3364.8$^{i}$	3364.8$^{i}$
Gas	Russia	Conventional	Volga	Orenburg	75.0$^{d}$	93.0$^{i}$	93.0$^{i}$
Gas	Russia	Conventional	Volga	Other	1.9$^{b}$	6.3$^{i}$	6.3$^{i}$
Gas	Russia	Conventional	Volga	Saratov	9.3$^{b}$	11.2$^{i}$	11.2$^{i}$
Gas	Russia	Hydrates				403.8$^{o}$	807.7$^{p}$
Gas	Russia	Shale			35.2$^{n}$	352.1$^{k}$	352.1$^{k}$
Gas	Russia	Tight			74.1$^{n}$	741.3$^{k}$	741.3$^{k}$
Gas	Tajikistan	Conventional			0.3$^{d}$	1.3$^{k}$	1.3$^{k}$
Gas	Turkmenistan	Conventional			200.4$^{d}$	200.4$^{d}$	1026.0$^{k}$
Gas	Ukraine	CBM			26.2$^{l}$	26.2$^{l}$	111.2$^{k}$
Gas	Ukraine	Conventional			111.2$^{b}$	128.8$^{k}$	128.8$^{k}$
Gas	Ukraine	Shale			13.4$^{n}$	134.6$^{k}$	134.6$^{k}$
Gas	Uzbekistan	Conventional			126.5$^{d}$	201.6$^{k}$	201.6$^{k}$
Oil	Armenia	Kerogen					1.8$^{q}$
Oil	Azerbaijan	Conventional			122.4$^{d}$	176.3$^{k}$	176.3$^{k}$
Oil	Azerbaijan	Extra Heavy					0.7$^{k}$
Oil	Belarus	Conventional			8.4$^{d}$	8.4$^{d}$	8.1$^{k}$
Oil	Belarus	Kerogen				40.0$^{q}$	40.0$^{q}$
Oil	Crimea	Conventional	Crimea		0.6$^{b}$	0.6$^{b}$	0.6$^{b}$
Oil	Estonia	Kerogen			5.7$^{b}$	5.7$^{b}$	94.6$^{q}$
Oil	Georgia	Conventional			1.3$^{d}$	1.3$^{d}$	3.6$^{k}$
Oil	Kazakhstan	Conventional			184.5$^{d}$	184.5$^{d}$	425.6$^{k}$
Oil	Kazakhstan	Kerogen					16.3$^{q}$
Oil	Kazakhstan	Natural Bitumen			312.5$^{k}$	312.5$^{k}$	312.5$^{k}$
Oil	Kazakhstan	Tight			60.7$^{r}$	60.7$^{r}$	60.5$^{k}$
Oil	Kyrgyzstan	Conventional			0.2$^{d}$	0.2$^{d}$	0.7$^{k}$
Oil	Lithuania	Conventional			0.2$^{d}$	0.2$^{d}$	2.8$^{k}$
Oil	Lithuania	Tight			4.0$^{r}$		
Oil	Luhansk	Conventional	Luhansk		<0.0$^{a}$	<0.0$^{a}$	<0.0$^{a}$
Oil	Moldova	Conventional					0.4$^{k}$
Oil	Russia	Conventional	Central	Yaroslavl	<0.0$^{a}$	<0.0$^{a}$	<0.0$^{a}$
Oil	Russia	Conventional	Far Eastern	Sakhalin	25.5$^{d}$	29.5$^{i}$	29.5$^{i}$
Oil	Russia	Conventional	Far Eastern	Yakutia	17.2$^{b}$	32.4$^{i}$	32.4$^{i}$
Oil	Russia	Conventional	North Caucasian	Chechnya	18.9$^{d}$	18.9$^{d}$	18.9$^{d}$
Oil	Russia	Conventional	North Caucasian	Dagestan	1.8$^{d}$	1.8$^{d}$	1.8$^{d}$
Oil	Russia	Conventional	North Caucasian	Ingushetia	0.1$^{a}$	0.1$^{a}$	0.1$^{a}$
Oil	Russia	Conventional	North Caucasian	Kabardino-Balkaria	<0.0$^{a}$	<0.0$^{a}$	<0.0$^{a}$
Oil	Russia	Conventional	North Caucasian	North Ossetia-Alania	<0.0$^{a}$	<0.0$^{a}$	<0.0$^{a}$
Oil	Russia	Conventional	North Caucasian	Stavropol	5.9$^{d}$	5.9$^{d}$	9.9$^{i}$
Oil	Russia	Conventional	Northwestern	Kaliningrad	2.2$^{d}$	2.2$^{d}$	2.2$^{d}$
Oil	Russia	Conventional	Northwestern	Komi	65.0$^{d}$	65.4$^{i}$	65.4$^{i}$
Oil	Russia	Conventional	Northwestern	Murmansk			16.8$^{i}$
Oil	Russia	Conventional	Northwestern	Nenets AO	57.3$^{b}$	57.0$^{i}$	57.0$^{i}$
Oil	Russia	Conventional	Siberian	Irkutsk	28.6$^{b}$	46.2$^{i}$	46.2$^{i}$
Oil	Russia	Conventional	Siberian	Krasnoyarsk	28.6$^{b}$	86.6$^{i}$	86.6$^{i}$
Oil	Russia	Conventional	Siberian	Novosibirsk	0.7$^{d}$	0.7$^{d}$	0.7$^{d}$
Oil	Russia	Conventional	Siberian	Omsk	0.4$^{d}$	0.4$^{d}$	0.4$^{d}$
Oil	Russia	Conventional	Siberian	Tomsk	31.3$^{d}$	37.2$^{i}$	37.2$^{i}$
Oil	Russia	Conventional	Southern	Adygea	0.1$^{a}$	0.1$^{a}$	0.1$^{a}$
Oil	Russia	Conventional	Southern	Astrakhan	57.3$^{b}$	37.8$^{i}$	37.8$^{i}$
Oil	Russia	Conventional	Southern	Kalmykia	0.7$^{d}$	0.7$^{d}$	0.7$^{d}$
Oil	Russia	Conventional	Southern	Krasnodar	11.3$^{d}$	11.3$^{d}$	11.3$^{d}$
Oil	Russia	Conventional	Southern	Volgograd	13.5$^{d}$	13.5$^{d}$	13.5$^{d}$
Oil	Russia	Conventional	Ural	Khanty-Mansi AO	738.0$^{d}$	738.0$^{d}$	990.0$^{i}$
Oil	Russia	Conventional	Ural	Tyumen	12.1$^{d}$	24.7$^{i}$	24.7$^{i}$
Oil	Russia	Conventional	Ural	Yamalo-Nenets AO	143.2$^{b}$	255.3$^{i}$	255.3$^{i}$
Oil	Russia	Conventional	Volga	Bashkortostan	90.4$^{d}$	90.6$^{i}$	90.6$^{i}$
Oil	Russia	Conventional	Volga	Kirov	<0.0$^{a}$	<0.0$^{a}$	<0.0$^{a}$
Oil	Russia	Conventional	Volga	Orenburg	48.4$^{d}$	72.1$^{i}$	72.1$^{i}$
Oil	Russia	Conventional	Volga	Penza	<0.0$^{a}$	<0.0$^{a}$	<0.0$^{a}$
Oil	Russia	Conventional	Volga	Perm	74.4$^{d}$	74.4$^{d}$	56.5$^{i}$
Oil	Russia	Conventional	Volga	Samara	118.8$^{d}$	118.8$^{d}$	77.5$^{i}$
Oil	Russia	Conventional	Volga	Saratov	5.2$^{d}$	8.5$^{i}$	8.5$^{i}$
Oil	Russia	Conventional	Volga	Tatarstan	200.5$^{b}$	184.7$^{i}$	184.7$^{i}$
Oil	Russia	Conventional	Volga	Udmurtia	33.9$^{d}$	34.7$^{i}$	34.7$^{i}$
Oil	Russia	Conventional	Volga	Ulyanovsk	1.2$^{d}$	4.8$^{i}$	4.8$^{i}$
Oil	Russia	Extra Heavy					0.1$^{k}$
Oil	Russia	Kerogen			0.7$^{a}$	1421.1$^{q}$	1421.1$^{q}$
Oil	Russia	Natural Bitumen				219.4$^{k}$	219.4$^{k}$
Oil	Russia	Tight	Northwestern	Kalingrad	4.0$^{r}$		
Oil	Russia	Tight	Other		427.5$^{r}$		
Oil	Russia	Tight				432.6$^{k}$	432.6$^{k}$
Oil	Tajikistan	Conventional			0.1$^{d}$	0.1$^{d}$	2.7$^{k}$
Oil	Turkmenistan	Conventional			35.5$^{d}$	35.5$^{d}$	99.3$^{k}$
Oil	Turkmenistan	Kerogen					22.0$^{q}$
Oil	Ukraine	Conventional			17.2$^{d}$	17.2$^{d}$	24.7$^{k}$
Oil	Ukraine	Kerogen					24.0$^{q}$
Oil	Ukraine	Tight			6.3$^{r}$	6.3$^{k}$	6.3$^{k}$
Oil	Uzbekistan	Conventional			12.1$^{d}$	12.1$^{d}$	30.4$^{k}$
Oil	Uzbekistan	Kerogen				70.1$^{q}$	70.1$^{q}$
Total					7067.7	21,416.2	27,698.2

$$^{a}$$Cumulative production

$^{b}$Estimated - Hubbert linearisation unstable

$$^{c}$$Fikkers (2013)

$$^{d}$$Hubbert linearisation

$^{e}$World Energy Council (2016)

$$^{f}$$Uvaisova (2013)

$$^{g}$$Oprisan (2013)

$^{h}$US Department of the Interior, USGS (1997)

$^{i}$Vasilkov et al. (2018)

$$^{j}$$Kholikov (2019)

$$^{k}$$BGR (2016)

$^{l}$Kuuskraa and Stevens (2009)

$^{m}$Campbell and Heaps (2009)

$$^{n}$$10% of BGR (2016)

$^{o}$50% of Rogner et al. (2012)

$^{p}$Rogner et al. (2012)

$$^{q}$$Dyni (2006)

$$^{r}$$EIA (2015)

## Supplementry material

The electronic supplement contains the inputs, model and outputs of the models. The associated CO_2_e emission for the models, as is the the collated production statistics.

## Footnotes

1 Comprised of the following countries: Armenia, Azerbaijan, Belarus,Estonia, Georgia, Kazakhstan, Kyrgyzstan, Latvia, Lithuania, Moldova, Russia, Tajikistan, Turkmenistan, Ukraine, Uzbekistan

2 (Mohr and Evans 2013; Mohr et al. 2012; Mohr and Ward 2014; Northey et al. 2014; Mohr et al. 2018, 2015b)

3 (BP 2019; Mohr et al. 2015b; Ukrstat 2017; Rosstat 2018; BGS 2017; CIA 1954, 1985, 1955b, a, 1990, 1978; L 1951; Rosstat 2018; Fedstat 2020; Lydolp and Shabad 1960; Meyerhoff 1983; Stern 1983; Shabad 1983; Bokserman et al. 1998; Surgai and Tolstoy 2006; Mykhnenko 2014; Kazanskyi et al. 2017; Mishina 2018; Ministry of National Economy of the Republic of Kazakhstan Statistics Committee 2017; Olson 1980; Hopkins et al. 1973; Little Earth 2017; Landis et al. 1997; World Bank 1994; Sergeevich and Ivanovna 2016; Chibrik et al. 2018; Kornilkov et al. 2000; Bespalov 2013; Russian Nature 2020; Liuhto et al. 2004; Kontorovich et al. 2018; Kiyaev 2018; Anon 2013; ROSSTANDART 2017; Prishchepa and Orlova 2007; Perkins 2012; OECD 1998; Bogoyavlensky 2016; Korzhubaev and Eder 2011; Eder et al. 2016; EIA 2017; Savosin 2019; Doroshenko et al. 2013; Oil and Limited 2019; Sagers 1986; Rzayeva 2015; EaP CSF 2018; EIA 2019; Stern 1980; Rothwell 1922; Eder et al. 2018b; Vasilkov et al. 2018; Alexandrovich 2017; Eder et al. 2018a; Rep. of Komi Official portal 2020; USGS 1993; Sugimoto 2013; Engerer and Kemfert 2008; Sagers 2006)

4 e.g. Sakhalin Island which has seen a ten fold increase in production in years 2008–2017
