# Supplementary material for: Projecting the global impact of fossil fuel production from the Former Soviet Union
Source: Int J Coal Sci Technol. 2021 Aug 9;8(6):1208–26. doi: 10.1007/s40789-021-00449-x (PMC8351787; doi:10.1007/s40789-021-00449-x)
Supplement: Supplementary file 3 — Supplementary file3 (PDF 22162 kb) [file 40789_2021_449_MOESM3_ESM.pdf]

GeRS-DeMo Post Processor

Compilation ran on scenario: Dynamic High

Date: 7/6/2020

Contents

1 Africa **21**

1.1 Algeria 21

1.1.1 All Projections 21

1.1.2 By Mineral 23

1.2 Angola 25

1.2.1 All Projections 25

1.2.2 By Mineral 26

1.3 Benin 27

1.3.1 All Projections 27

1.3.2 By Mineral 28

1.4 Botswana 29

1.4.1 All Projections 29

1.4.2 By Mineral 30

1.5 Cameroon 31

1.5.1 All Projections 31

1.5.2 By Mineral 32

1.6 Central African Republic 33

1.6.1 All Projections 33

1.6.2 By Mineral 34

1.7 Chad 35

1.7.1 All Projections 35

1.7.2 By Mineral 36

1.8 Congo 37

1.8.1 All Projections 37

1.8.2 By Mineral 38

1.9 Egypt 39

1.9.1 All Projections 39

1.9.2 By Mineral 41

1.10 Equatorial Guinea 43
1.10.1 All Projections 43
1.10.2 By Mineral 44
1.11 Eritrea 45
1.11.1 All Projections 45
1.11.2 By Mineral 46
1.12 Ethiopia 47
1.12.1 All Projections 47
1.12.2 By Mineral 48
1.13 Gabon 49
1.13.1 All Projections 49
1.13.2 By Mineral 50
1.14 Gambia 51
1.14.1 All Projections 51
1.14.2 By Mineral 52
1.15 Ghana 53
1.15.1 All Projections 53
1.15.2 By Mineral 54
1.16 Guinea 55
1.16.1 All Projections 55
1.16.2 By Mineral 56
1.17 Guinea-Bissau 57
1.17.1 All Projections 57
1.17.2 By Mineral 58
1.18 Ivory Coast 59
1.18.1 All Projections 59
1.18.2 By Mineral 60
1.19 Kenya 61
1.19.1 All Projections 61
1.19.2 By Mineral 62
1.20 Liberia 63
1.20.1 All Projections 63
1.20.2 By Mineral 64
1.21 Libya 65
1.21.1 All Projections 65
1.21.2 By Mineral 67

1.22 Madagascar 69
1.22.1 All Projections 69
1.22.2 By Mineral 71
1.23 Malawi 73
1.23.1 All Projections 73
1.23.2 By Mineral 74
1.24 Mauritania 75
1.24.1 All Projections 75
1.24.2 By Mineral 76
1.25 Morocco 77
1.25.1 All Projections 77
1.25.2 By Mineral 79
1.26 Mozambique 81
1.26.1 All Projections 81
1.26.2 By Mineral 83
1.27 Namibia 85
1.27.1 All Projections 85
1.27.2 By Mineral 86
1.28 Niger 87
1.28.1 All Projections 87
1.28.2 By Mineral 88
1.29 Nigeria 89
1.29.1 All Projections 89
1.29.2 By Mineral 91
1.30 Rwanda 93
1.30.1 All Projections 93
1.30.2 By Mineral 94
1.31 Sao Tome and Principe 95
1.31.1 All Projections 95
1.31.2 By Mineral 96
1.32 Senegal 97
1.32.1 All Projections 97
1.32.2 By Mineral 98
1.33 Seychelles 99
1.33.1 All Projections 99
1.33.2 By Mineral 100

1.34 Sierra Leone 101
1.34.1 All Projections 101
1.34.2 By Mineral 102
1.35 Somalia 103
1.35.1 All Projections 103
1.35.2 By Mineral 104
1.36 South Africa 105
1.36.1 All Projections 105
1.36.2 By Mineral 107
1.37 Sudan 109
1.37.1 All Projections 109
1.37.2 By Mineral 110
1.37.3 Regional Projections 110
South Sudan 111
Sudan 113
Sudan Region 115
1.37.4 Projection by region 117
1.38 Swaziland 118
1.38.1 All Projections 118
1.38.2 By Mineral 119
1.39 Tanzania 120
1.39.1 All Projections 120
1.39.2 By Mineral 122
1.40 Togo 124
1.40.1 All Projections 124
1.40.2 By Mineral 125
1.41 Tunisia 126
1.41.1 All Projections 126
1.41.2 By Mineral 128
1.42 Uganda 130
1.42.1 All Projections 130
1.42.2 By Mineral 131
1.43 Western Sahara 132
1.43.1 All Projections 132
1.43.2 By Mineral 133
1.44 Zaire 134

1.44.1 All Projections . . . 134
1.44.2 By Mineral . . . 136
1.45 Zambia . . . 138
1.45.1 All Projections . . . 138
1.45.2 By Mineral . . . 139
1.46 Zimbabwe . . . 140
1.46.1 All Projections . . . 140
1.46.2 By Mineral . . . 141
1.47 Total . . . 142
1.47.1 By country . . . 142
1.47.2 By mineral . . . 144

2 Asia **146**
2.1 Afghanistan . . . 146
2.1.1 All Projections . . . 146
2.1.2 By Mineral . . . 148
2.2 Australia . . . 149
2.2.1 All Projections . . . 149
2.2.2 By Mineral . . . 152
2.2.3 Regional Projections . . . 152
Australia . . . 154
JPDA . . . 156
NSW . . . 158
Northern Territory . . . 161
Queensland . . . 164
South Australia . . . 167
Tasmania . . . 170
Victoria . . . 173
Western Australia . . . 176
2.2.4 Projection by region . . . 179
2.3 Bangladesh . . . 181
2.3.1 All Projections . . . 181
2.3.2 By Mineral . . . 183
2.4 Bhutan . . . 185
2.4.1 All Projections . . . 185
2.4.2 By Mineral . . . 186

2.5 Brunei 187
2.5.1 All Projections 187
2.5.2 By Mineral 189
2.6 Burma 191
2.6.1 All Projections 191
2.6.2 By Mineral 193
2.7 Cambodia 195
2.7.1 All Projections 195
2.7.2 By Mineral 196
2.8 China 197
2.8.1 All Projections 197
2.8.2 By Mineral 200
2.8.3 Regional Projections 200
Anhui 202
Beijing 204
China 206
Chongqing 209
Fujian 211
Gansu 213
Guangdong 215
Guangxi 217
Guizhou 219
Hainan 221
Hebei 223
Heilongjiang 225
Henan 227
Historic 229
Hubei 231
Hunan 233
Inner Mongolia 235
Jiangsu 237
Jiangxi 239
Jilin 241
Liaoning 243
Ningxia 245
Offshore 247

Qinghai 249
Shaanxi 251
Shandong 253
Shanghai 255
Shanxi 257
Sichuan 259
Tianjin 261
Tibet 263
Xinjiang 265
Yunnan 267
Zhejiang 269
2.8.4 Projection by region 271
2.9 East Timor 273
2.9.1 All Projections 273
2.9.2 By Mineral 274
2.10 India 275
2.10.1 All Projections 275
2.10.2 By Mineral 277
2.11 Indonesia 279
2.11.1 All Projections 279
2.11.2 By Mineral 281
2.12 Japan 283
2.12.1 All Projections 283
2.12.2 By Mineral 285
2.13 Laos 287
2.13.1 All Projections 287
2.13.2 By Mineral 288
2.14 Malaysia 289
2.14.1 All Projections 289
2.14.2 By Mineral 290
2.15 Mongolia 291
2.15.1 All Projections 291
2.15.2 By Mineral 293
2.16 Nepal 295
2.16.1 All Projections 295
2.16.2 By Mineral 296

2.17 New Caledonia 297
2.17.1 All Projections 297
2.17.2 By Mineral 298
2.18 New Zealand 299
2.18.1 All Projections 299
2.18.2 By Mineral 301
2.19 North Korea 303
2.19.1 All Projections 303
2.19.2 By Mineral 304
2.20 PNG 305
2.20.1 All Projections 305
2.20.2 By Mineral 306
2.21 Pakistan 307
2.21.1 All Projections 307
2.21.2 By Mineral 309
2.22 Philippines 311
2.22.1 All Projections 311
2.22.2 By Mineral 313
2.23 South Korea 315
2.23.1 All Projections 315
2.23.2 By Mineral 317
2.24 Sri Lanka 319
2.24.1 All Projections 319
2.24.2 By Mineral 320
2.25 Taiwan 321
2.25.1 All Projections 321
2.25.2 By Mineral 322
2.26 Thailand 323
2.26.1 All Projections 323
2.26.2 By Mineral 325
2.27 Vietnam 327
2.27.1 All Projections 327
2.27.2 By Mineral 329
2.28 Total 331
2.28.1 By country 331
2.28.2 By mineral 333

3 Europe **335**
3.1 Albania 335
3.1.1 All Projections 335
3.1.2 By Mineral 337
3.2 Austria 339
3.2.1 All Projections 339
3.2.2 By Mineral 341
3.3 Belgium 343
3.3.1 All Projections 343
3.3.2 By Mineral 344
3.4 Bulgaria 345
3.4.1 All Projections 345
3.4.2 By Mineral 347
3.5 Cyprus 349
3.5.1 All Projections 349
3.5.2 By Mineral 350
3.6 Czech Republic 351
3.6.1 All Projections 351
3.6.2 By Mineral 353
3.7 Denmark 355
3.7.1 All Projections 355
3.7.2 By Mineral 357
3.8 France 359
3.8.1 All Projections 359
3.8.2 By Mineral 361
3.9 Germany 363
3.9.1 All Projections 363
3.9.2 By Mineral 365
3.10 Greece 367
3.10.1 All Projections 367
3.10.2 By Mineral 369
3.11 Greenland 371
3.11.1 All Projections 371
3.11.2 By Mineral 373
3.12 Hungary 375
3.12.1 All Projections 375

3.12.2 By Mineral . . . 377
3.13 Ireland . . . 379
3.13.1 All Projections . . . 379
3.13.2 By Mineral . . . 381
3.14 Italy . . . 383
3.14.1 All Projections . . . 383
3.14.2 By Mineral . . . 385
3.14.3 Regional Projections . . . 385
Italy . . . 387
Mainland . . . 390
Sicily . . . 392
3.14.4 Projection by region . . . 394
3.15 Malta . . . 395
3.15.1 All Projections . . . 395
3.15.2 By Mineral . . . 396
3.16 Netherlands . . . 397
3.16.1 All Projections . . . 397
3.16.2 By Mineral . . . 399
3.17 Norway . . . 401
3.17.1 All Projections . . . 401
3.17.2 By Mineral . . . 403
3.18 Poland . . . 405
3.18.1 All Projections . . . 405
3.18.2 By Mineral . . . 407
3.19 Portugal . . . 409
3.19.1 All Projections . . . 409
3.19.2 By Mineral . . . 411
3.20 Romania . . . 413
3.20.1 All Projections . . . 413
3.20.2 By Mineral . . . 415
3.21 Slovakia . . . 417
3.21.1 All Projections . . . 417
3.21.2 By Mineral . . . 419
3.22 Spain . . . 421
3.22.1 All Projections . . . 421
3.22.2 By Mineral . . . 423

3.23 Sweden 425
3.23.1 All Projections 425
3.23.2 By Mineral 426
3.24 Switzerland 427
3.24.1 All Projections 427
3.24.2 By Mineral 428
3.25 Turkey 429
3.25.1 All Projections 429
3.25.2 By Mineral 431
3.26 UK 433
3.26.1 All Projections 433
3.26.2 By Mineral 435
3.26.3 Regional Projections 435
England and Wales 437
Northern Ireland 440
Scotland 442
UK 444
3.26.4 Projection by region 446
3.27 Yugoslavia 448
3.27.1 All Projections 448
3.27.2 By Mineral 450
3.27.3 Regional Projections 450
Bosnia and Herzegovina 452
Croatia 454
Serbia 456
Slovenia 458
Yugoslavia 460
3.27.4 Projection by region 462
3.28 Total 464
3.28.1 By country 464
3.28.2 By mineral 466

4 FSU 468
4.1 Armenia 468
4.1.1 All Projections 468
4.1.2 By Mineral 470

4.2 Azerbaijan . . . 471
4.2.1 All Projections . . . 471
4.2.2 By Mineral . . . 472
4.3 Belarus . . . 473
4.3.1 All Projections . . . 473
4.3.2 By Mineral . . . 474
4.4 Crimea . . . 475
4.4.1 All Projections . . . 475
4.4.2 By Mineral . . . 476
4.4.3 Regional Projections . . . 476
Crimea . . . 477
4.4.4 Projection by region . . . 479
4.5 Donetsk . . . 480
4.5.1 All Projections . . . 480
4.5.2 By Mineral . . . 481
4.5.3 Regional Projections . . . 481
Donetsk . . . 482
4.5.4 Projection by region . . . 484
4.6 Estonia . . . 485
4.6.1 All Projections . . . 485
4.6.2 By Mineral . . . 486
4.7 Georgia . . . 487
4.7.1 All Projections . . . 487
4.7.2 By Mineral . . . 488
4.8 Kazakhstan . . . 489
4.8.1 All Projections . . . 489
4.8.2 By Mineral . . . 491
4.8.3 Regional Projections . . . 491
All . . . 493
East Kazakhstan . . . 496
Karaganda . . . 498
Kostanay . . . 500
Other . . . 502
Pavlodar . . . 504
4.8.4 Projection by region . . . 506
4.9 Kyrgyzstan . . . 508

4.9.1 All Projections 508
4.9.2 By Mineral 510
4.10 Lithuania 512
4.10.1 All Projections 512
4.10.2 By Mineral 513
4.11 Luhansk 514
4.11.1 All Projections 514
4.11.2 By Mineral 516
4.11.3 Regional Projections 516
Luhansk 518
4.11.4 Projection by region 520
4.12 Moldova 521
4.12.1 All Projections 521
4.12.2 By Mineral 522
4.13 Russia 523
4.13.1 All Projections 523
4.13.2 By Mineral 526
4.13.3 Regional Projections 526
All 528
Central 531
Far Eastern 533
North Caucasian 536
Northwestern 538
Siberian 541
Southern 544
Ural 547
Volga 550
4.13.4 Projection by region 553
4.14 Tajikistan 555
4.14.1 All Projections 555
4.14.2 By Mineral 557
4.15 Turkmenistan 559
4.15.1 All Projections 559
4.15.2 By Mineral 561
4.16 Ukraine 563
4.16.1 All Projections 563

4.16.2 By Mineral 565
4.17 Uzbekistan 567
4.17.1 All Projections 567
4.17.2 By Mineral 569
4.18 Total 571
4.18.1 By country 571
4.18.2 By mineral 572

5 Middle East **575**
5.1 Bahrain 575
5.1.1 All Projections 575
5.1.2 By Mineral 577
5.2 Iran 578
5.2.1 All Projections 578
5.2.2 By Mineral 580
5.3 Iraq 582
5.3.1 All Projections 582
5.3.2 By Mineral 583
5.4 Israel 584
5.4.1 All Projections 584
5.4.2 By Mineral 585
5.5 Jordan 586
5.5.1 All Projections 586
5.5.2 By Mineral 587
5.6 Kuwait 588
5.6.1 All Projections 588
5.6.2 By Mineral 589
5.7 Lebanon 590
5.7.1 All Projections 590
5.7.2 By Mineral 591
5.8 Oman 592
5.8.1 All Projections 592
5.8.2 By Mineral 593
5.9 Palestine 594
5.9.1 All Projections 594
5.9.2 By Mineral 595

5.10 Qatar . . . 596
5.10.1 All Projections . . . 596
5.10.2 By Mineral . . . 597
5.11 Saudi Arabia . . . 598
5.11.1 All Projections . . . 598
5.11.2 By Mineral . . . 599
5.12 Syria . . . 600
5.12.1 All Projections . . . 600
5.12.2 By Mineral . . . 601
5.13 UAE . . . 602
5.13.1 All Projections . . . 602
5.13.2 By Mineral . . . 603
5.14 Yemen . . . 604
5.14.1 All Projections . . . 604
5.14.2 By Mineral . . . 605
5.15 Total . . . 606
5.15.1 By country . . . 606
5.15.2 By mineral . . . 606

6 North America 609
6.1 Canada . . . 609
6.1.1 All Projections . . . 609
6.1.2 By Mineral . . . 613
6.1.3 Regional Projections . . . 613
Alberta . . . 615
British Columbia . . . 618
Canada . . . 621
East Coast Offshore . . . 623
Manitoba . . . 625
New Brunswick . . . 627
Northwest Territories . . . 629
Nova Scotia . . . 631
Ontario . . . 633
Quebec . . . 635
Saskatchewan . . . 637
Yukon . . . 640

6.1.4 Projection by region 642
6.2 USA 644
6.2.1 All Projections 644
6.2.2 By Mineral 649
6.2.3 Regional Projections 649
Alabama 651
Alaska 653
Arizona 655
Arkansas 657
California 660
Colorado 662
Eastern 665
Florida 667
Georgia 669
Illinois 671
Indiana 673
Iowa 675
Kansas 677
Kentucky 679
Louisiana 682
Maryland 685
Michigan 687
Mississippi 689
Missouri 691
Montana 693
Nebraska 696
Nevada 698
New Mexico 700
New York 703
North Dakota 705
Ohio 707
Oklahoma 710
Oregon 713
Other 715
Pennsylvania 717
South Dakota 720

Tennessee 722
Texas 724
USA 727
Utah 729
Virginia 732
Washington 734
West Virginia 736
Wyoming 739
6.2.4 Projection by region 742
6.3 Total 744
6.3.1 By country 744
6.3.2 By mineral 745

7 South America 747
7.1 Argentina 747
7.1.1 All Projections 747
7.1.2 By Mineral 749
7.2 Barbados 751
7.2.1 All Projections 751
7.2.2 By Mineral 752
7.3 Belize 753
7.3.1 All Projections 753
7.3.2 By Mineral 754
7.4 Bolivia 755
7.4.1 All Projections 755
7.4.2 By Mineral 756
7.5 Brazil 757
7.5.1 All Projections 757
7.5.2 By Mineral 759
7.6 Chile 761
7.6.1 All Projections 761
7.6.2 By Mineral 763
7.7 Colombia 765
7.7.1 All Projections 765
7.7.2 By Mineral 767
7.8 Cuba 769

7.8.1 All Projections 769
7.8.2 By Mineral 770
7.9 Dominican Republic 771
7.9.1 All Projections 771
7.9.2 By Mineral 772
7.10 Ecuador 773
7.10.1 All Projections 773
7.10.2 By Mineral 775
7.11 Falkland Islands 777
7.11.1 All Projections 777
7.11.2 By Mineral 778
7.12 French Guiana 779
7.12.1 All Projections 779
7.12.2 By Mineral 780
7.13 Grenada 781
7.13.1 All Projections 781
7.13.2 By Mineral 782
7.14 Guatemala 783
7.14.1 All Projections 783
7.14.2 By Mineral 784
7.15 Guyana 785
7.15.1 All Projections 785
7.15.2 By Mineral 786
7.16 Haiti 787
7.16.1 All Projections 787
7.16.2 By Mineral 788
7.17 Mexico 789
7.17.1 All Projections 789
7.17.2 By Mineral 791
7.18 Paraguay 793
7.18.1 All Projections 793
7.18.2 By Mineral 794
7.19 Peru 795
7.19.1 All Projections 795
7.19.2 By Mineral 797
7.20 Puerto Rico 799

7.20.1 All Projections . . . 799
7.20.2 By Mineral . . . 800
7.21 Suriname . . . 801
7.21.1 All Projections . . . 801
7.21.2 By Mineral . . . 802
7.22 Trinidad and Tobago . . . 803
7.22.1 All Projections . . . 803
7.22.2 By Mineral . . . 804
7.23 Uruguay . . . 805
7.23.1 All Projections . . . 805
7.23.2 By Mineral . . . 806
7.24 Venezuela . . . 807
7.24.1 All Projections . . . 807
7.24.2 By Mineral . . . 809
7.25 Total . . . 811
7.25.1 By country . . . 811
7.25.2 By mineral . . . 813

8 Total 815
8.1 By continent . . . 815
8.2 By mineral . . . 817
8.3 By Country . . . 819

Chapter 1

Africa

1.1 Algeria

1.1.1 All Projections

Table 1.1: Peak years - All

**Name**	**URR**	**Peak Year**	**Peak Rate**
Gas Conv.	293.0	2008	3.31
Oil Conv.	274.69	2023	4.37
Gas Shale	241.54	2135	2.83
Gas Tight	203.94	2134	2.41
Coal Bit.	0.13	1952	0.01
Coal Lignite	–	1942	–
**Total**	**1013.3**	**2007**	**7.23**

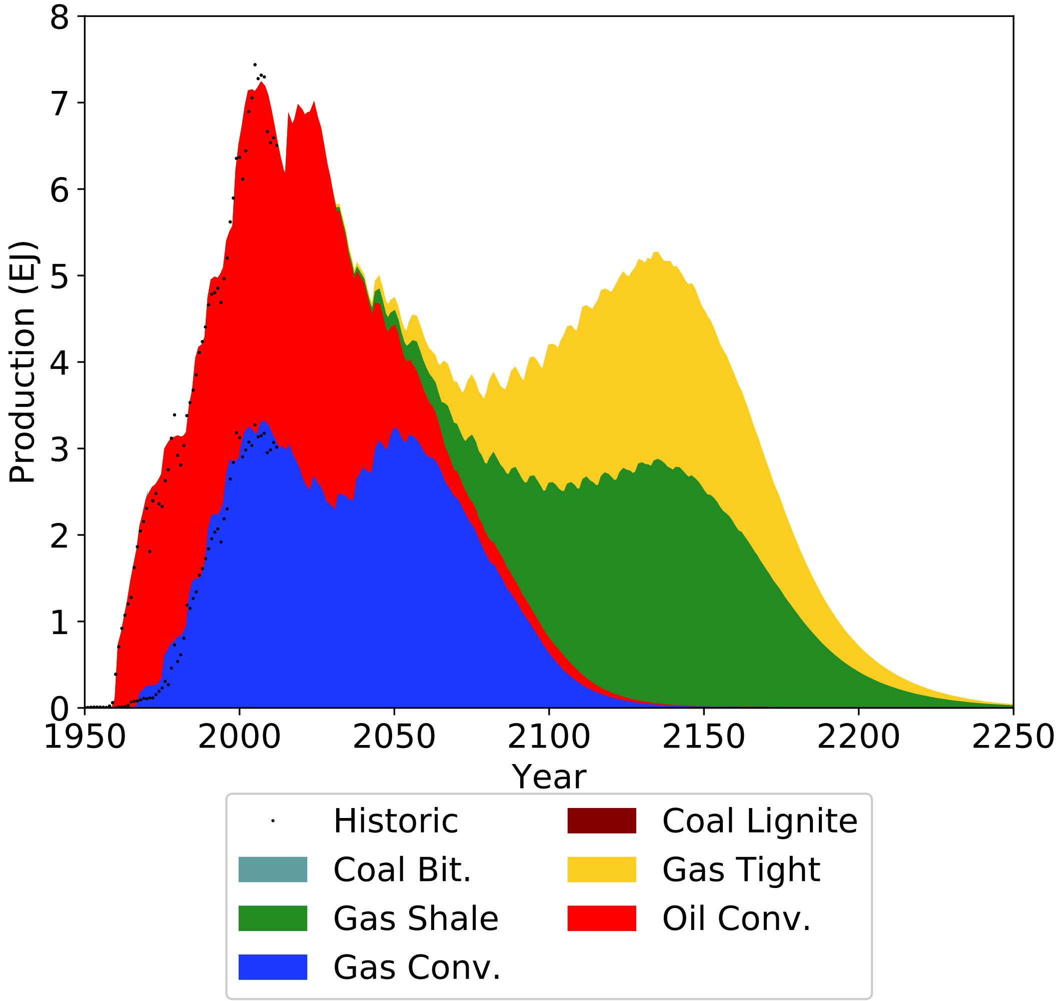

Figure 1.1: Algeria projections capped at 16

1.1.2 By Mineral

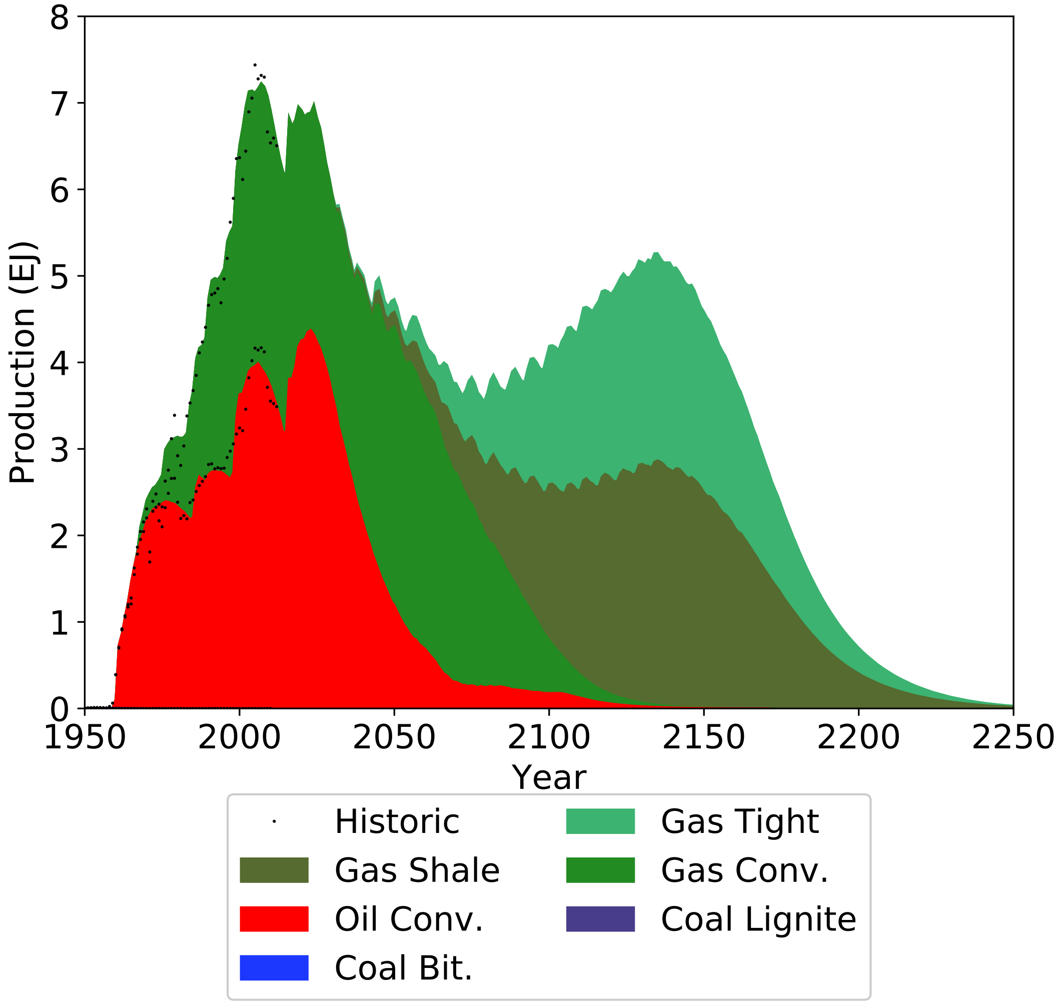

Figure 1.2: Algeria projection by mineral type

Table 1.2: Peak years - Minerals

**Name**	**URR**	**Peak Year**	**Peak Rate**
Coal Bit.	0.13	1952	0.01
Coal Lignite	–	1942	–
Oil Conv.	274.69	2023	4.37
Gas Conv.	293.0	2008	3.31
Gas Shale	241.54	2135	2.83
Gas Tight	203.94	2134	2.41
**Total**	**1013.3**	**2007**	**7.23**

1.2 Angola

1.2.1 All Projections

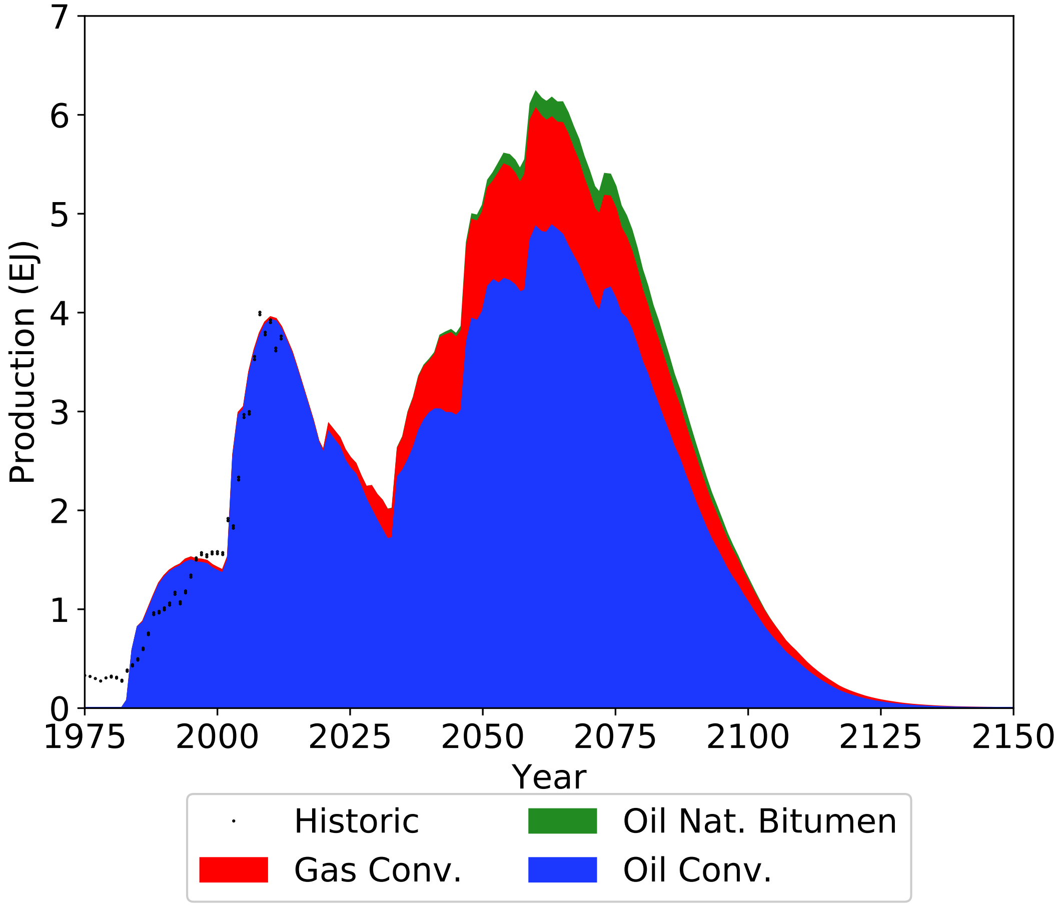

Figure 1.3: Angola projections capped at 16

Table 1.3: Peak years - All

**Name**	**URR**	**Peak Year**	**Peak Rate**
Oil Conv.	347.12	2063	4.88
Gas Conv.	56.67	2059	1.21
Oil Nat. Bitumen	8.42	2073	0.22
**Total**	**412.21**	**2060**	**6.23**

1.2.2 By Mineral

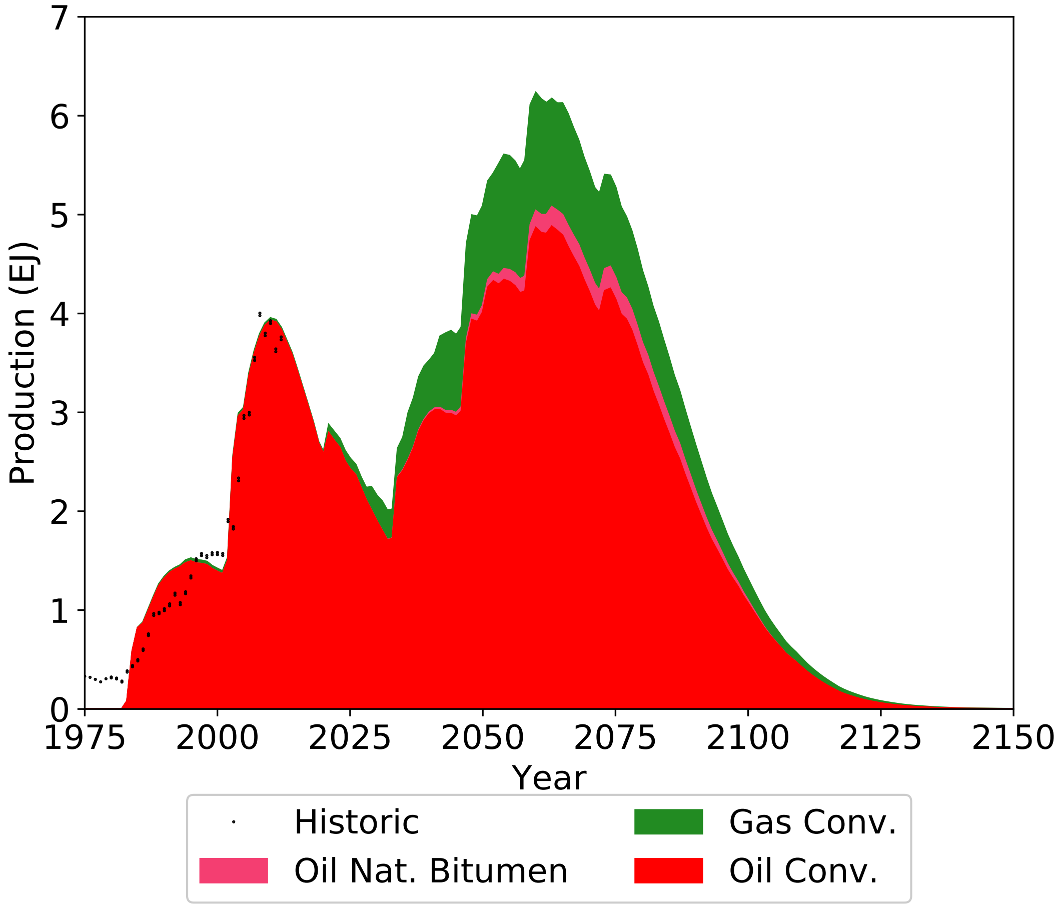

Figure 1.4: Angola projection by mineral type

Table 1.4: Peak years - Minerals

Name	URR	Peak Year	Peak Rate
Oil Conv.	347.12	2063	4.88
Oil Nat. Bitumen	8.42	2073	0.22
Gas Conv.	56.67	2059	1.21
**Total**	**412.21**	**2060**	**6.23**

1.3 Benin

1.3.1 All Projections

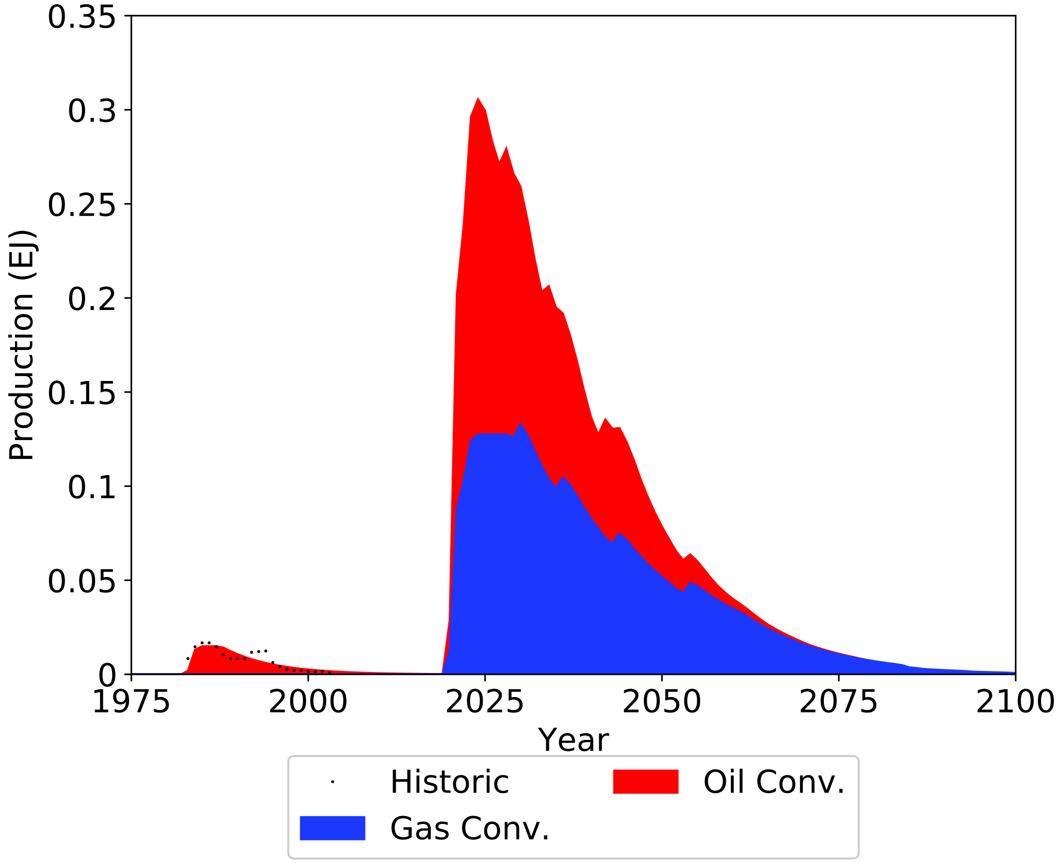

Figure 1.5: Benin projections capped at 16

Table 1.5: Peak years - All

Name	URR	Peak Year	Peak Rate
Gas Conv.	3.75	2030	0.13
Oil Conv.	3.16	2024	0.18
**Total**	**6.9**	**2024**	**0.31**

1.3.2 By Mineral

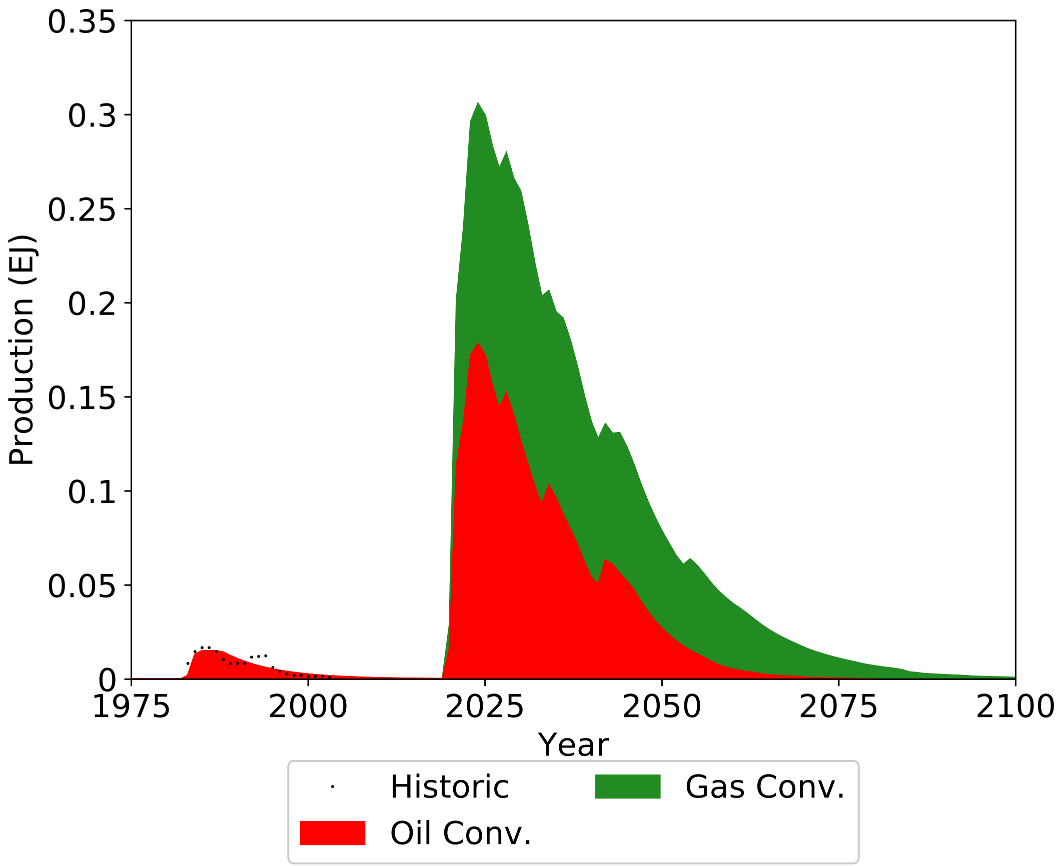

Figure 1.6: Benin projection by mineral type

Table 1.6: Peak years - Minerals

Name	URR	Peak Year	Peak Rate
Oil Conv.	3.16	2024	0.18
Gas Conv.	3.75	2030	0.13
**Total**	**6.9**	**2024**	**0.31**

1.4 Botswana

1.4.1 All Projections

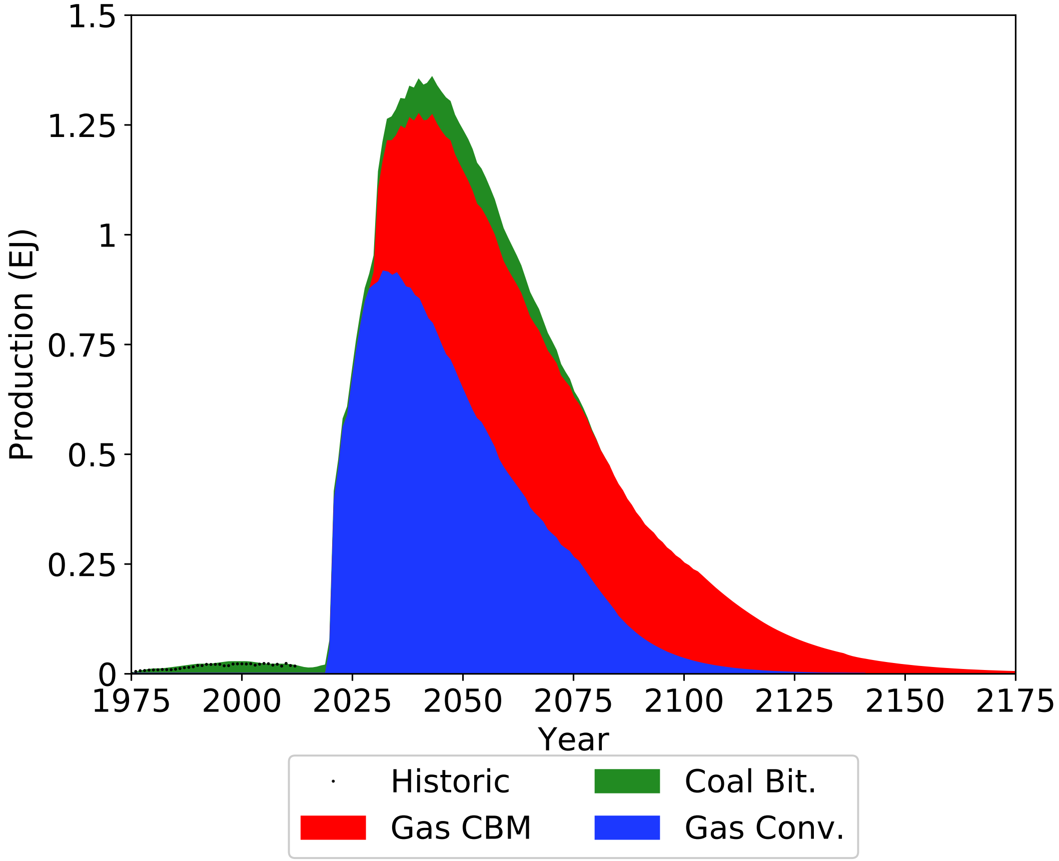

Figure 1.7: Botswana projections capped at 16

Table 1.7: Peak years - All

Name	URR	Peak Year	Peak Rate
Gas Conv.	37.08	2032	0.92
Gas CBM	31.15	2051	0.5
Coal Bit.	4.08	2052	0.09
**Total**	**72.31**	**2043**	**1.36**

1.4.2 By Mineral

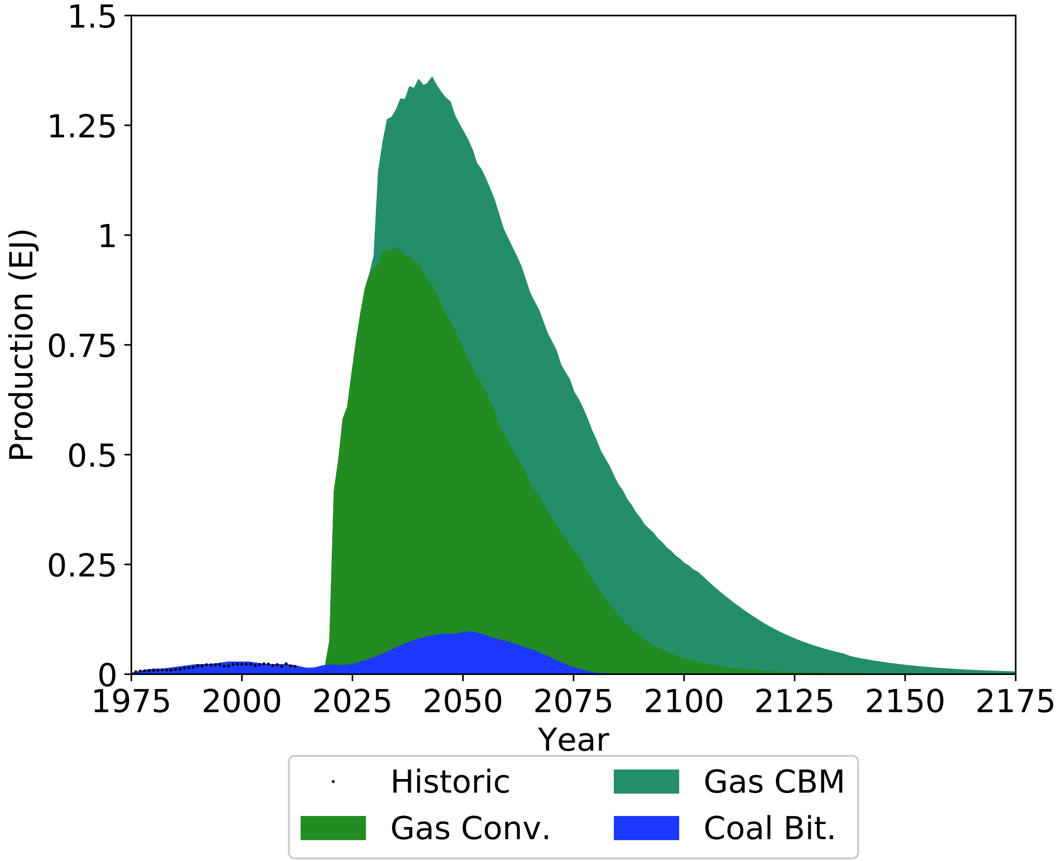

Figure 1.8: Botswana projection by mineral type

Table 1.8: Peak years - Minerals

**Name**	**URR**	**Peak Year**	**Peak Rate**
Coal Bit.	4.08	2052	0.09
Gas Conv.	37.08	2032	0.92
Gas CBM	31.15	2051	0.5
**Total**	**72.31**	**2043**	**1.36**

1.5 Cameroon

1.5.1 All Projections

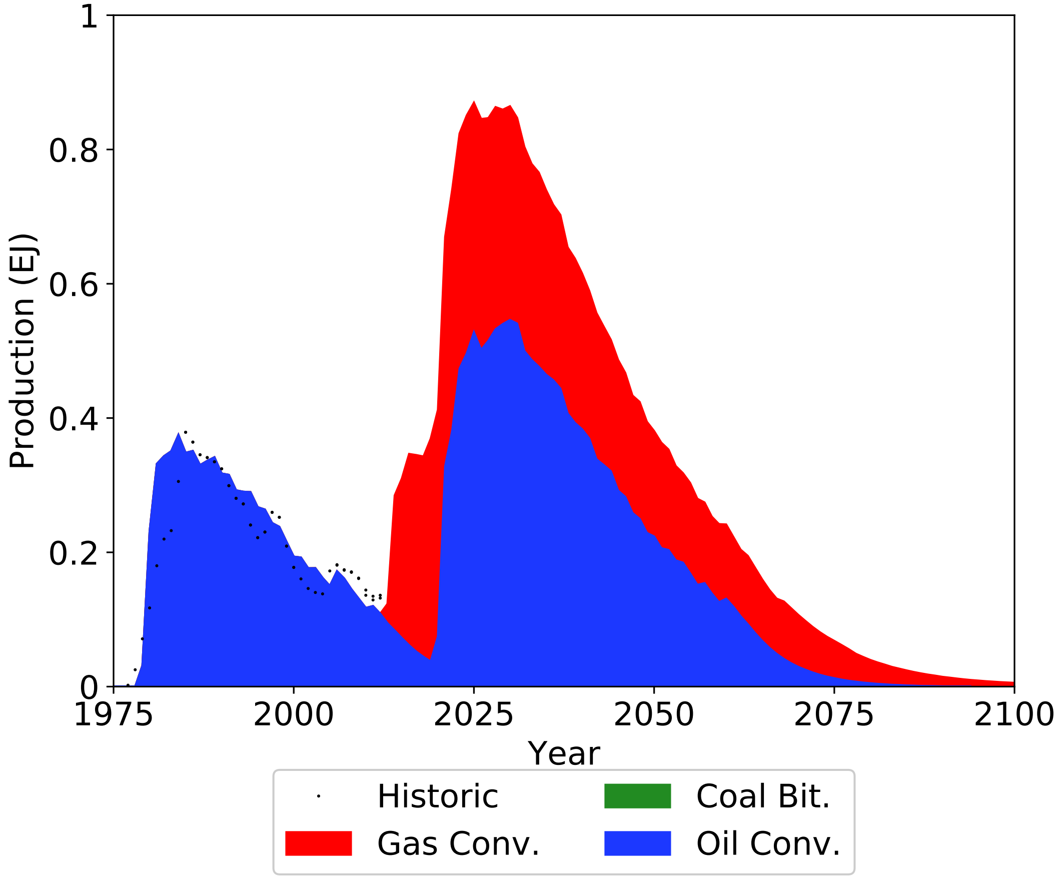

Figure 1.9: Cameroon projections capped at 16

Table 1.9: Peak years - All

Name	URR	Peak Year	Peak Rate
Oil Conv.	23.4	2030	0.55
Gas Conv.	13.11	2022	0.36
Coal Bit.	–	1988	–
**Total**	**36.51**	**2025**	**0.87**

1.5.2 By Mineral

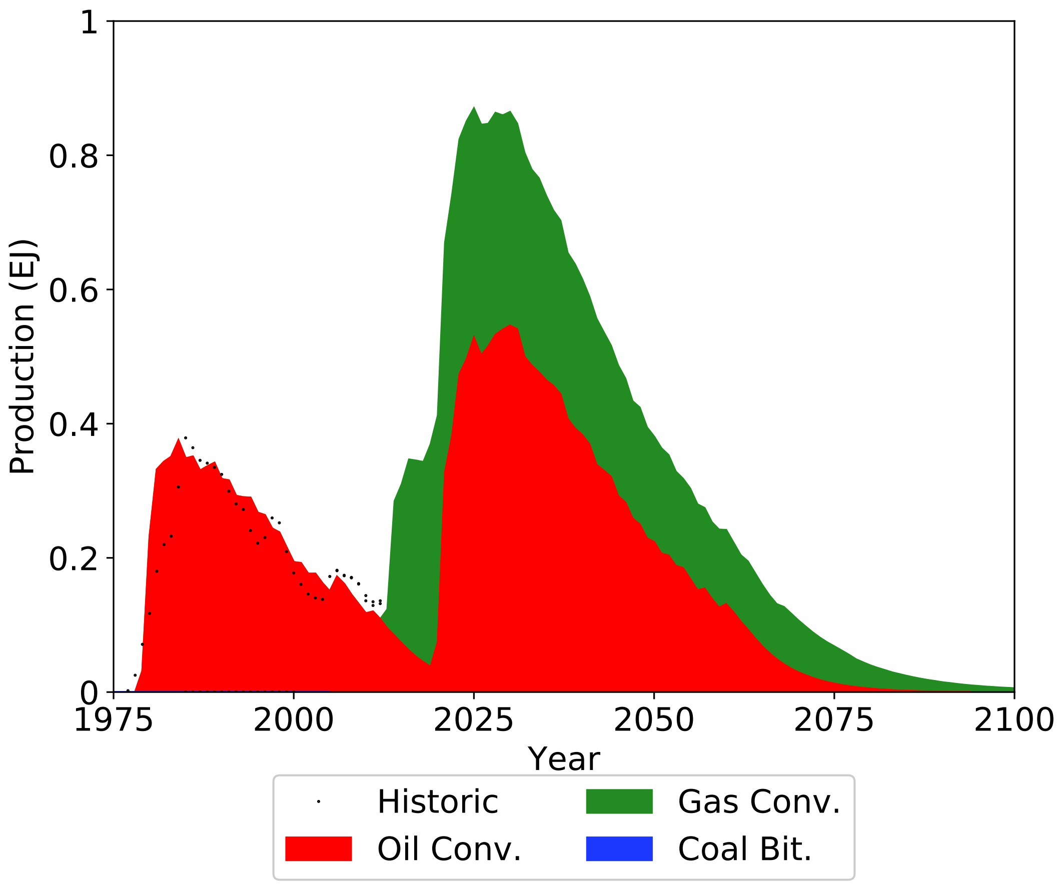

Figure 1.10: Cameroon projection by mineral type

Table 1.10: Peak years - Minerals

**Name**	**URR**	**Peak Year**	**Peak Rate**
Coal Bit.	–	1988	–
Oil Conv.	23.4	2030	0.55
Gas Conv.	13.11	2022	0.36
**Total**	**36.51**	**2025**	**0.87**

1.6 Central African Republic

1.6.1 All Projections

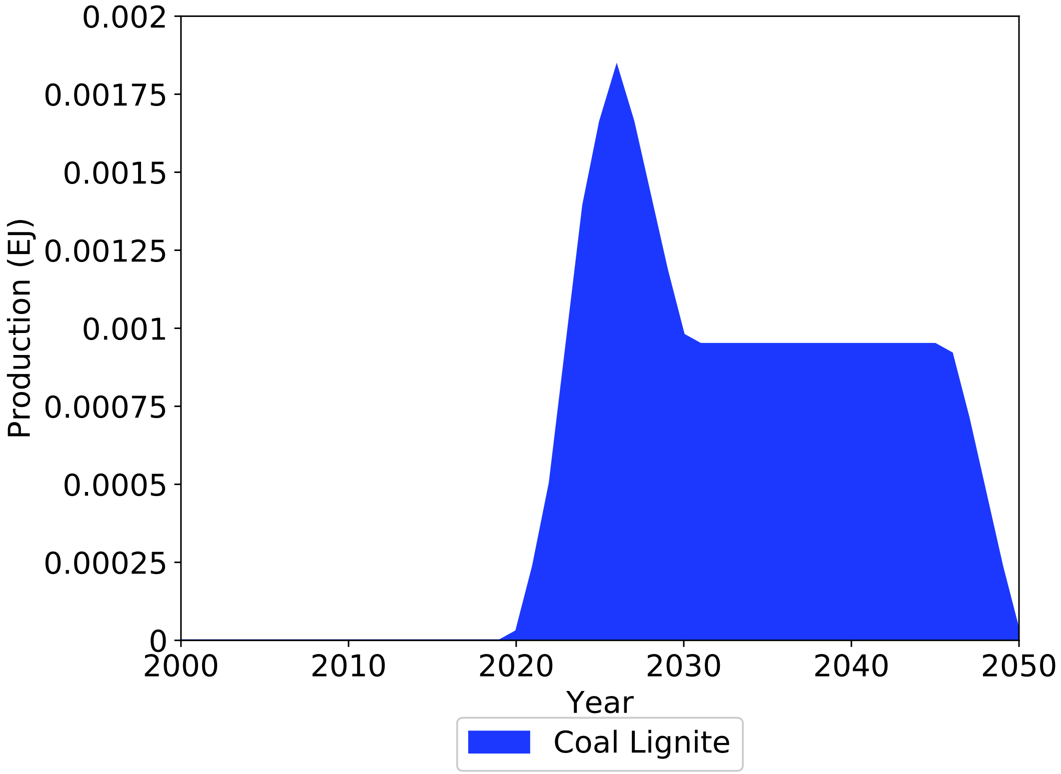

Figure 1.11: Central African Republic projections capped at 16

Table 1.11: Peak years - All

Name	URR	Peak Year	Peak Rate
Coal Lignite	0.03	2026	–
**Total**	**0.03**	**2026**	–

1.6.2 By Mineral

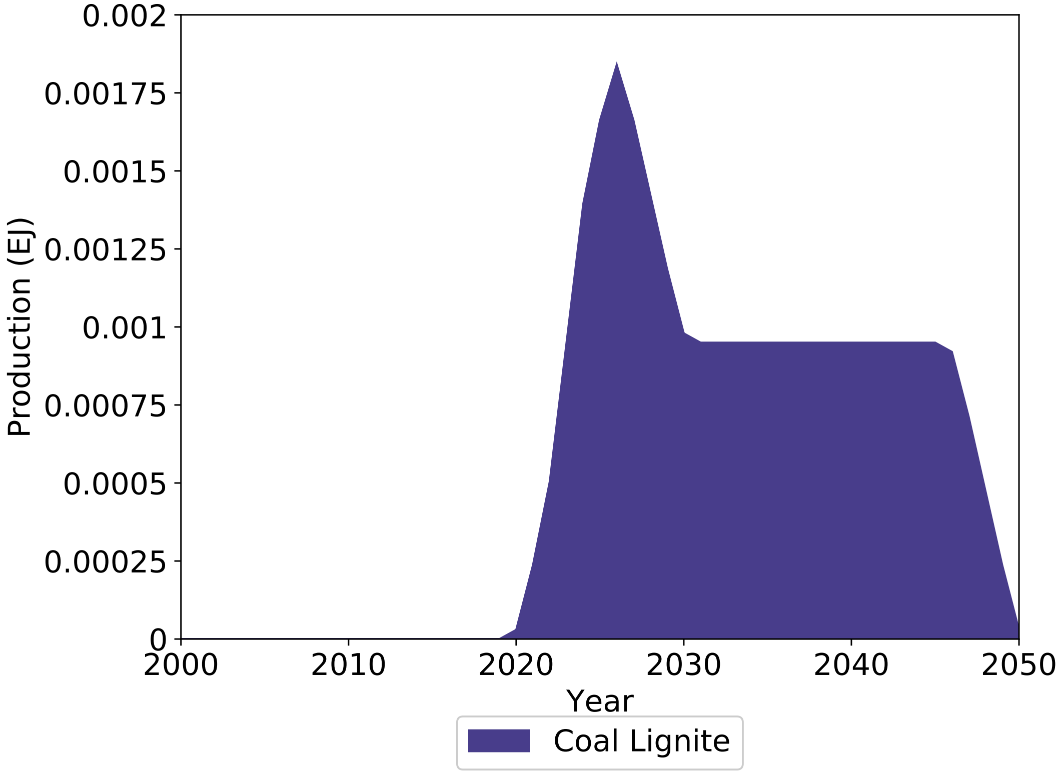

Figure 1.12: Central African Republic projection by mineral type

Table 1.12: Peak years - Minerals

**Name**	**URR**	**Peak Year**	**Peak Rate**
Coal Lignite	0.03	2026	–
**Total**	**0.03**	**2026**	–

1.7 Chad

1.7.1 All Projections

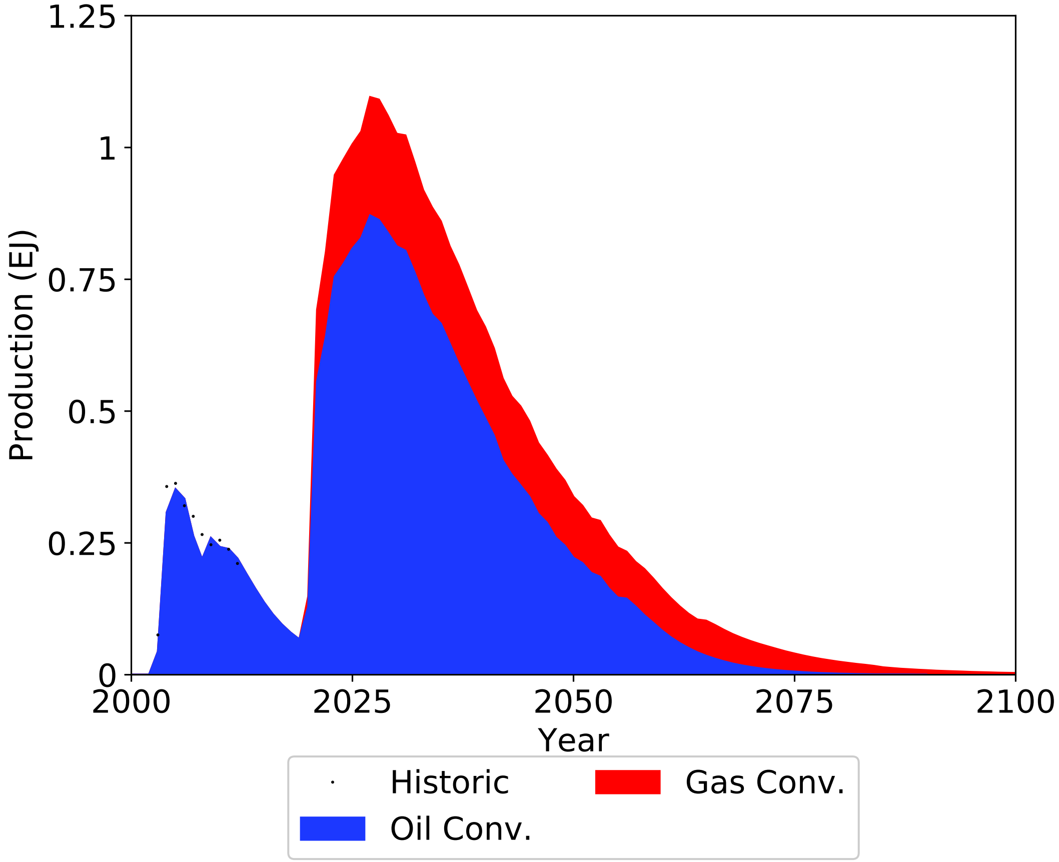

Figure 1.13: Chad projections capped at 16

Table 1.13: Peak years - All

Name	URR	Peak Year	Peak Rate
Oil Conv.	22.8	2027	0.87
Gas Conv.	7.42	2028	0.23
**Total**	**30.22**	**2027**	**1.1**

1.7.2 By Mineral

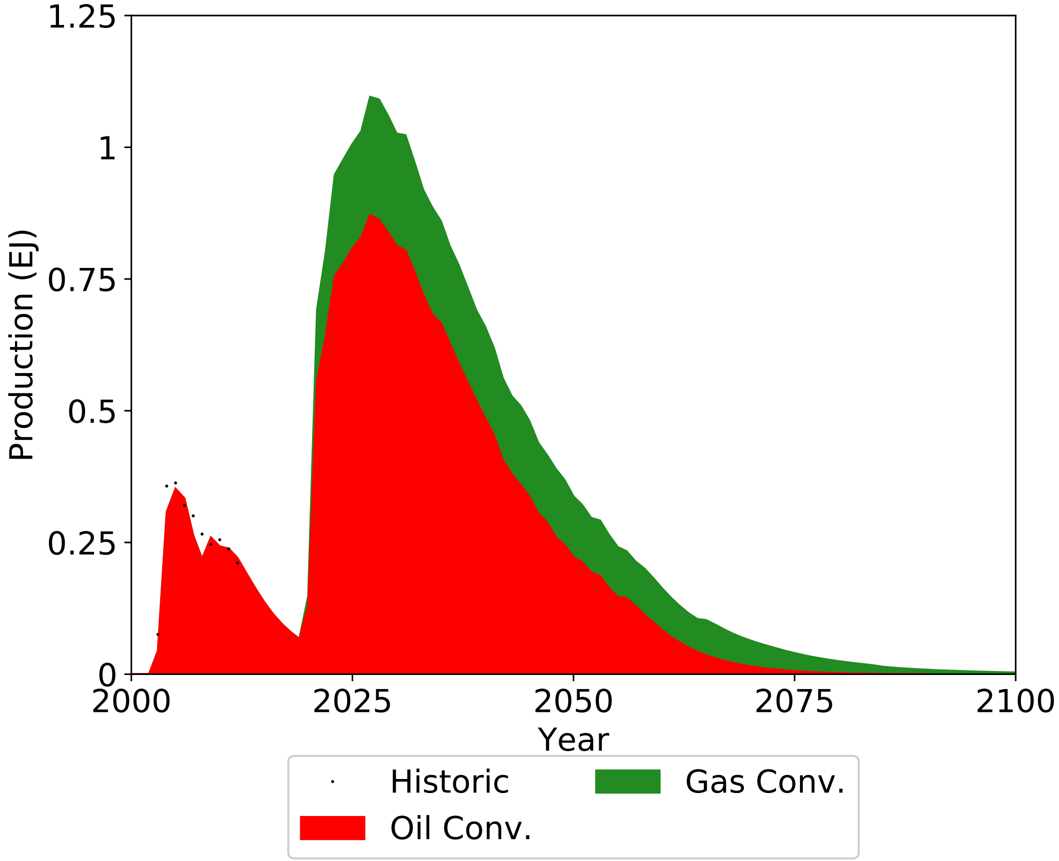

Figure 1.14: Chad projection by mineral type

Table 1.14: Peak years - Minerals

Name	URR	Peak Year	Peak Rate
Oil Conv.	22.8	2027	0.87
Gas Conv.	7.42	2028	0.23
**Total**	**30.22**	**2027**	**1.1**

1.8 Congo

1.8.1 All Projections

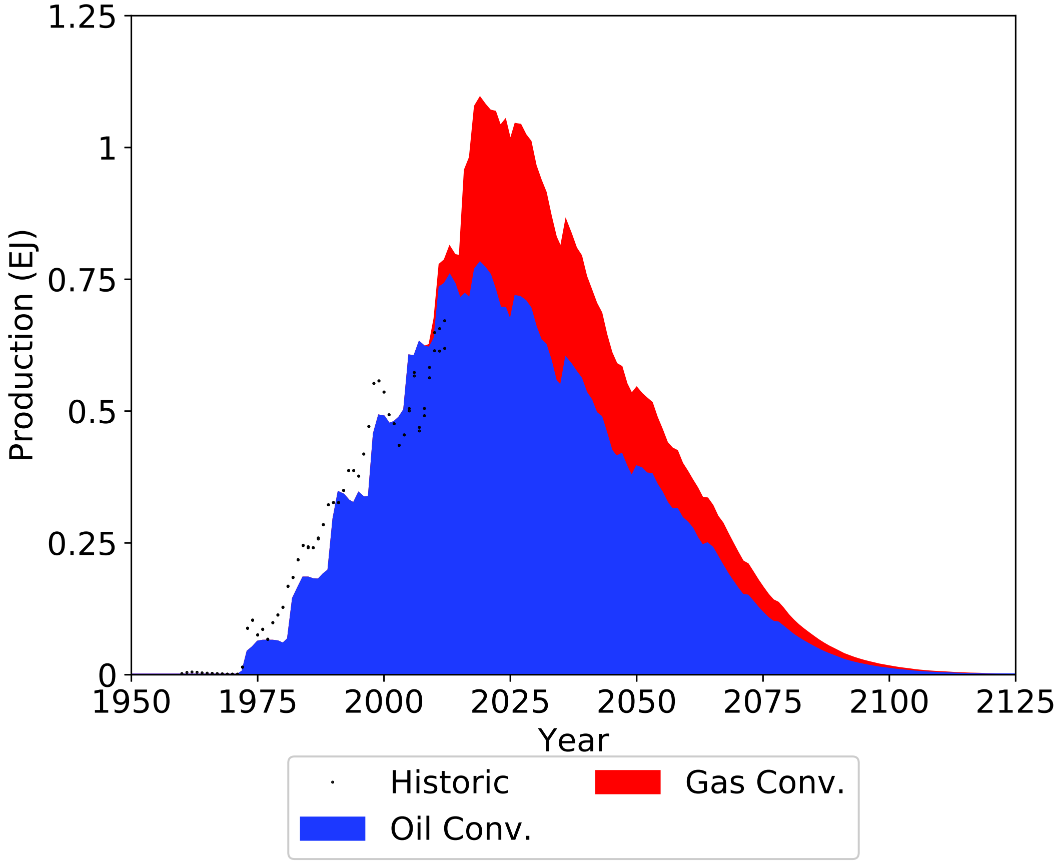

Figure 1.15: Congo projections capped at 16

Table 1.15: Peak years - All

Name	URR	Peak Year	Peak Rate
Oil Conv.	44.05	2019	0.78
Gas Conv.	12.21	2024	0.36
**Total**	**56.26**	**2019**	**1.09**

1.8.2 By Mineral

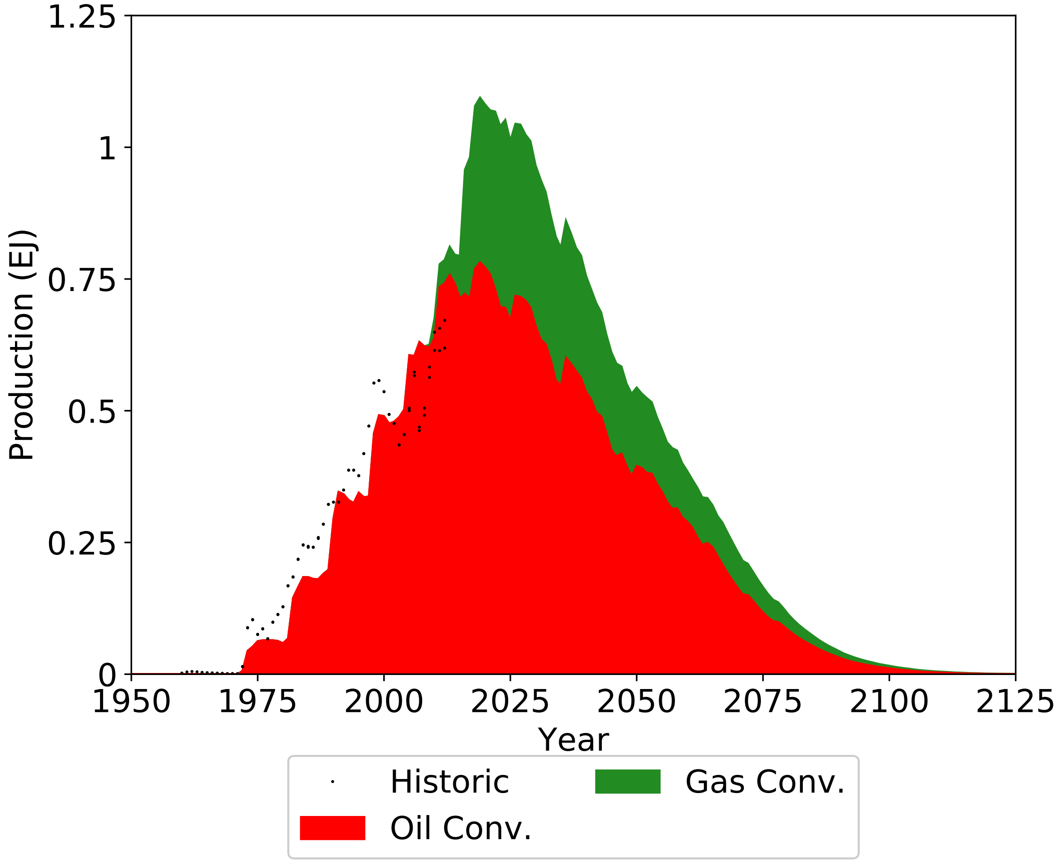

Figure 1.16: Congo projection by mineral type

Table 1.16: Peak years - Minerals

Name	URR	Peak Year	Peak Rate
Oil Conv.	44.05	2019	0.78
Gas Conv.	12.21	2024	0.36
**Total**	**56.26**	**2019**	**1.09**

1.9 Egypt

1.9.1 All Projections

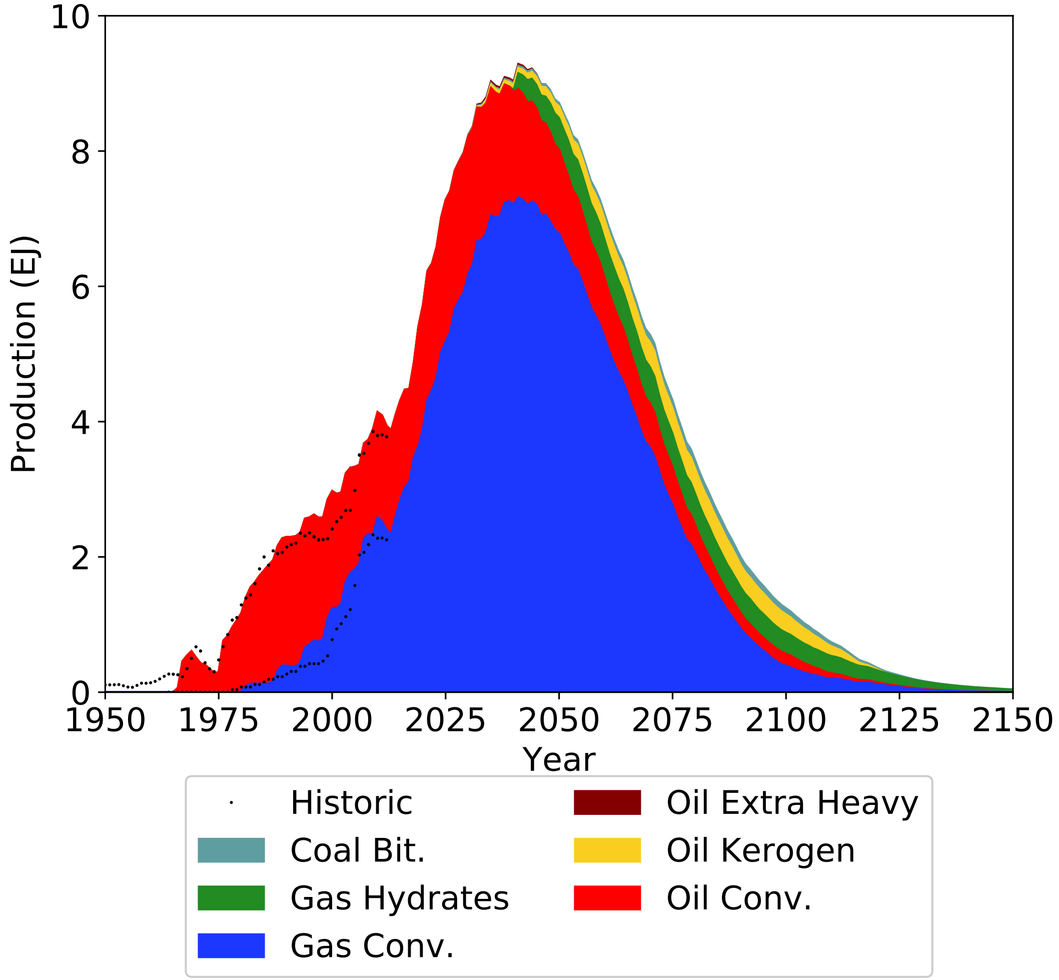

Figure 1.17: Egypt projections capped at 16

Table 1.17: Peak years - All

**Name**	**URR**	**Peak Year**	**Peak Rate**
Gas Conv.	406.11	2041	7.31
Oil Conv.	157.63	2025	2.09
Gas Hydrates	35.5	2058	0.57
Oil Kerogen	20.6	2072	0.37
Coal Bit.	7.26	2081	0.12
Oil Extra Heavy	0.34	2035	0.03
**Total**	**627.43**	**2041**	**9.29**

1.9.2 By Mineral

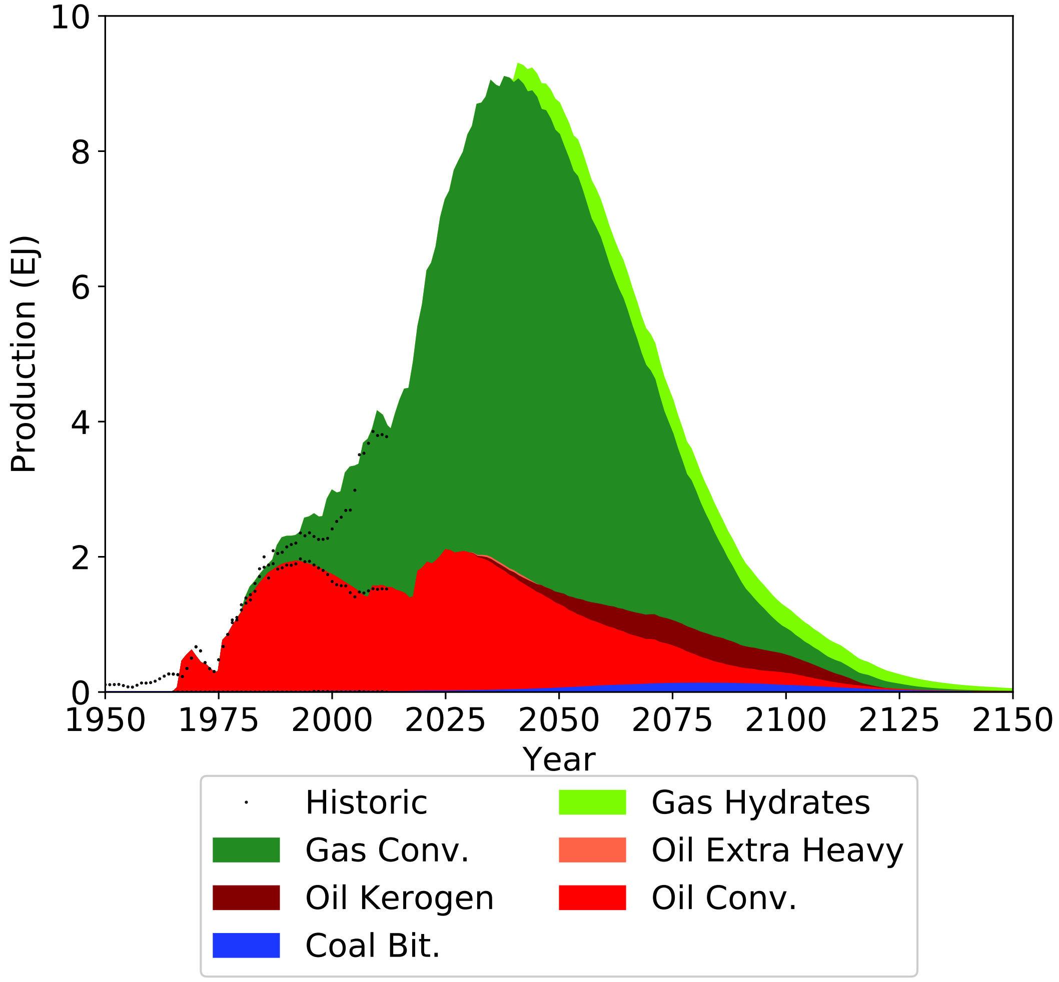

Figure 1.18: Egypt projection by mineral type

Table 1.18: Peak years - Minerals

**Name**	**URR**	**Peak Year**	**Peak Rate**
Coal Bit.	7.26	2081	0.12
Oil Conv.	157.63	2025	2.09
Oil Kerogen	20.6	2072	0.37
Oil Extra Heavy	0.34	2035	0.03
Gas Conv.	406.11	2041	7.31
Gas Hydrates	35.5	2058	0.57
**Total**	**627.43**	**2041**	**9.29**

1.10 Equatorial Guinea

1.10.1 All Projections

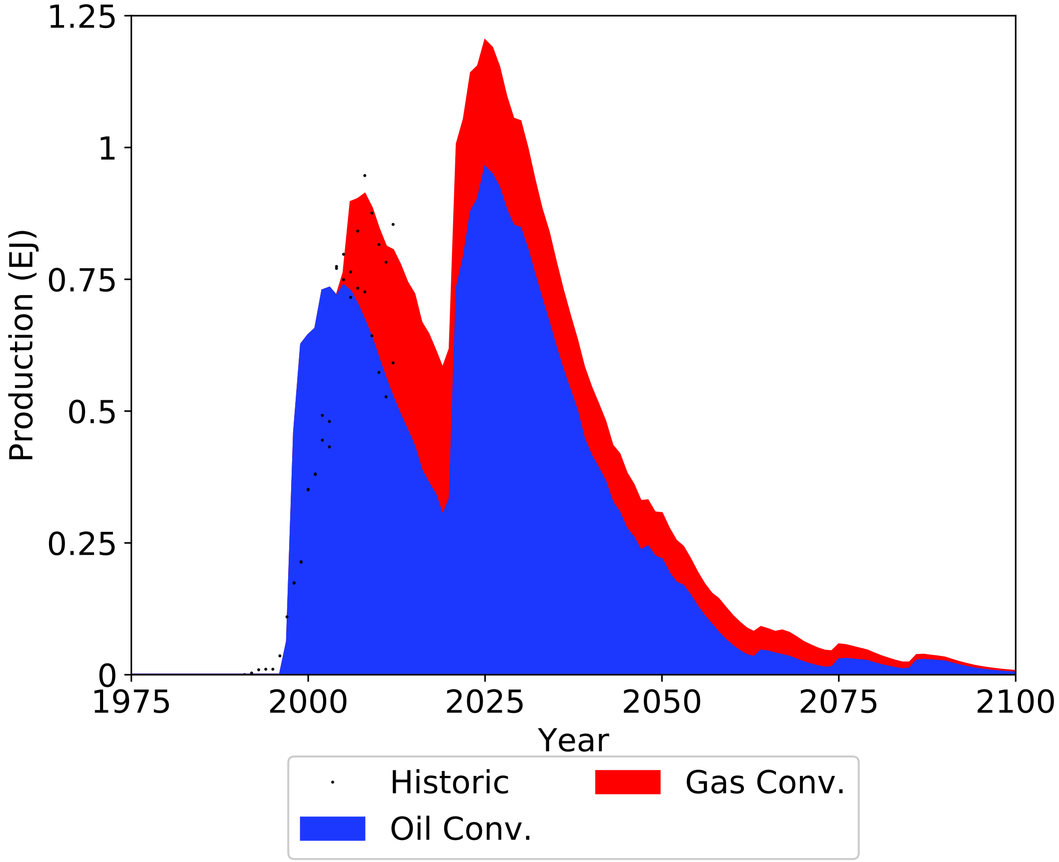

Figure 1.19: Equatorial Guinea projections capped at 16

Table 1.19: Peak years - All

Name	URR	Peak Year	Peak Rate
Oil Conv.	32.71	2025	0.96
Gas Conv.	10.46	2015	0.29
**Total**	**43.17**	**2025**	**1.2**

1.10.2 By Mineral

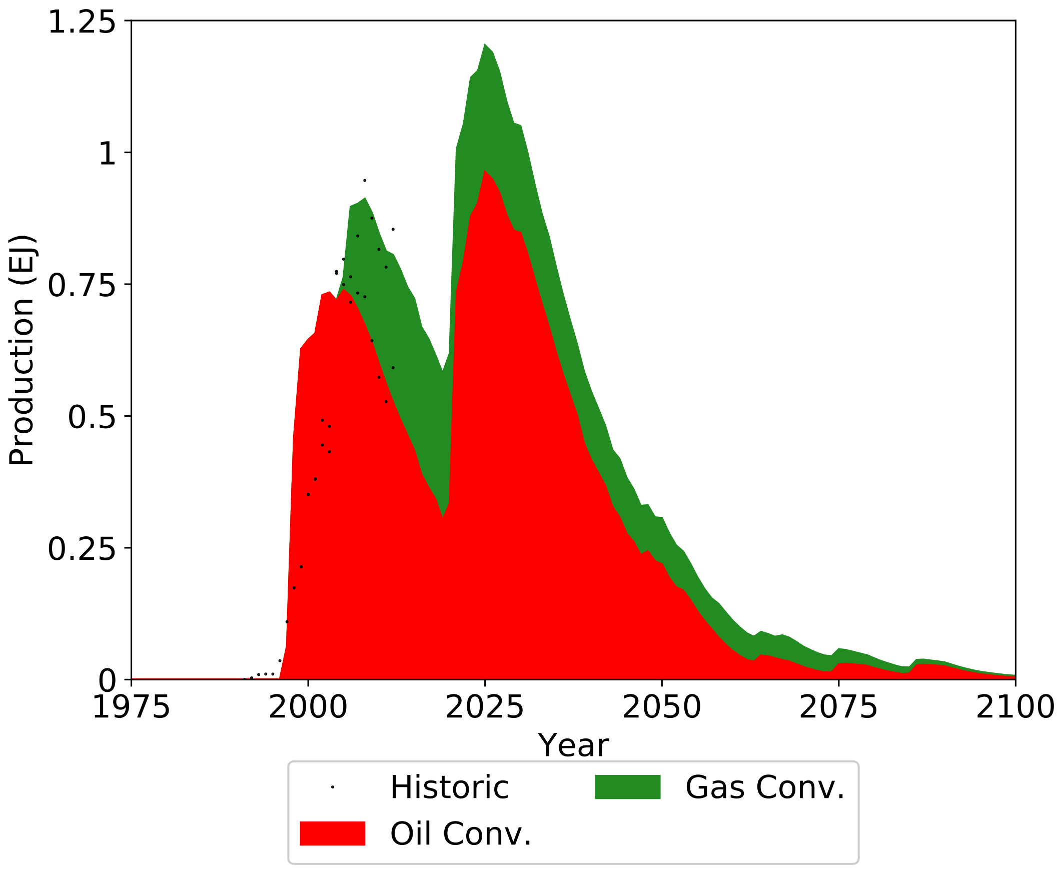

Figure 1.20: Equatorial Guinea projection by mineral type

Table 1.20: Peak years - Minerals

Name	URR	Peak Year	Peak Rate
Oil Conv.	32.71	2025	0.96
Gas Conv.	10.46	2015	0.29
**Total**	**43.17**	**2025**	**1.2**

1.11 Eritrea

1.11.1 All Projections

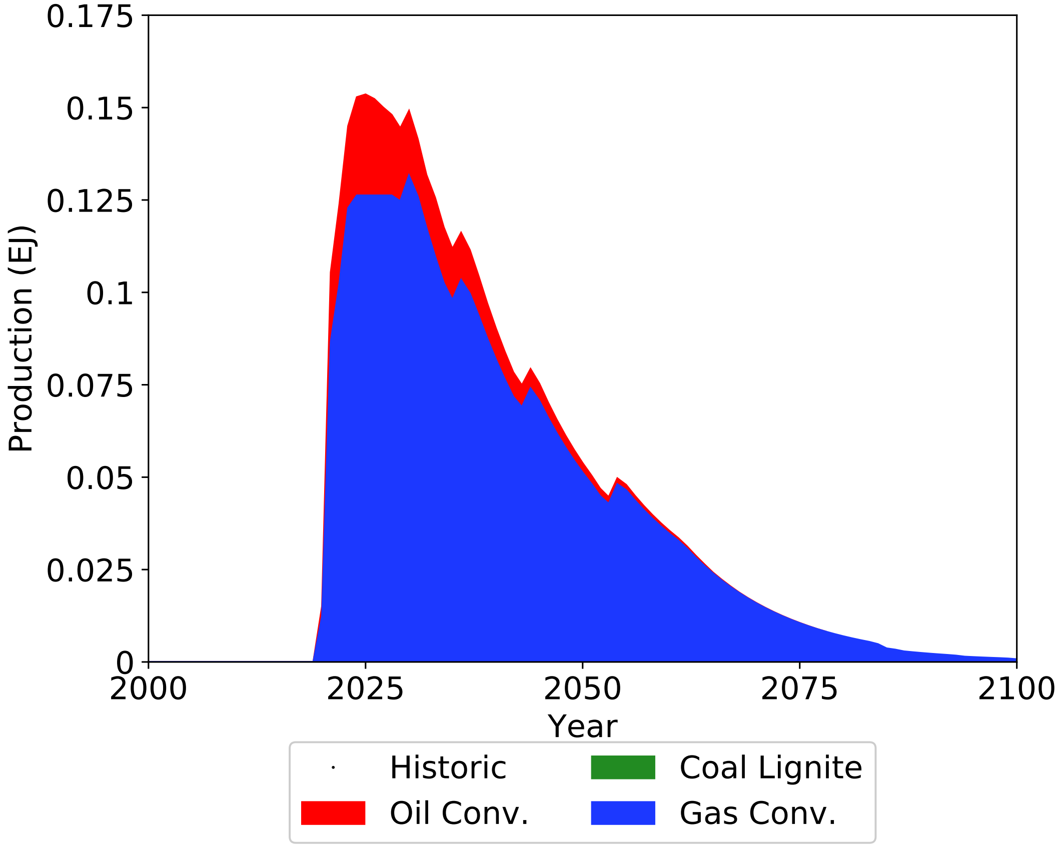

Figure 1.21: Eritrea projections capped at 16

Table 1.21: Peak years - All

Name	URR	Peak Year	Peak Rate
Gas Conv.	3.71	2030	0.13
Oil Conv.	0.42	2025	0.03
Coal Lignite	–	1936	–
**Total**	**4.13**	**2025**	**0.15**

1.11.2 By Mineral

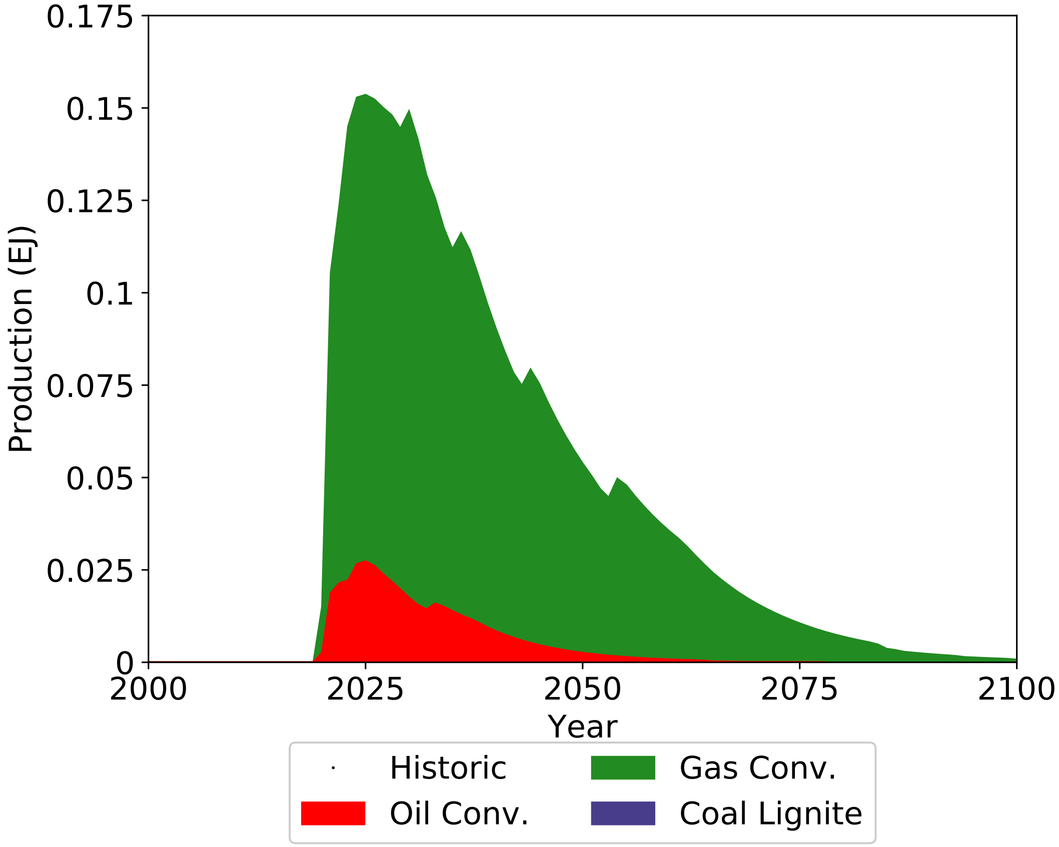

Figure 1.22: Eritrea projection by mineral type

Table 1.22: Peak years - Minerals

Name	URR	Peak Year	Peak Rate
Coal Lignite	–	1936	–
Oil Conv.	0.42	2025	0.03
Gas Conv.	3.71	2030	0.13
**Total**	**4.13**	**2025**	**0.15**

1.12 Ethiopia

1.12.1 All Projections

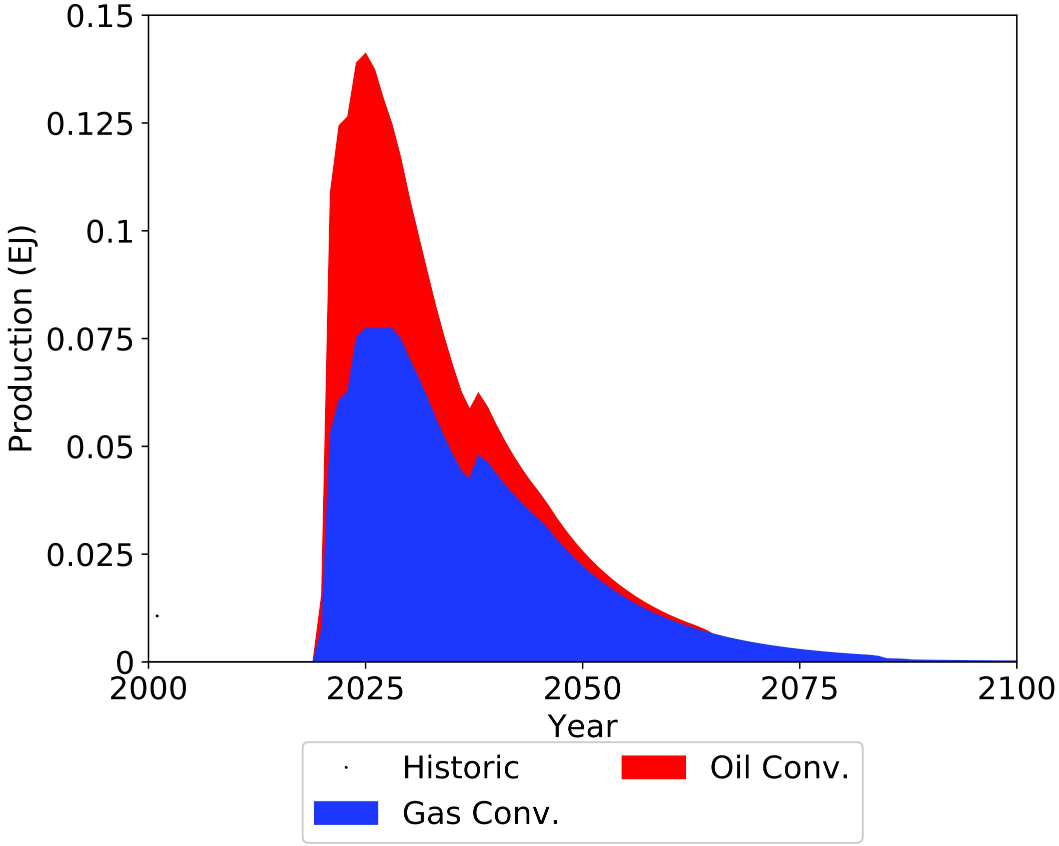

Figure 1.23: Ethiopia projections capped at 16

Table 1.23: Peak years - All

Name	URR	Peak Year	Peak Rate
Gas Conv.	1.78	2025	0.08
Oil Conv.	0.85	2022	0.06
**Total**	**2.63**	**2025**	**0.14**

1.12.2 By Mineral

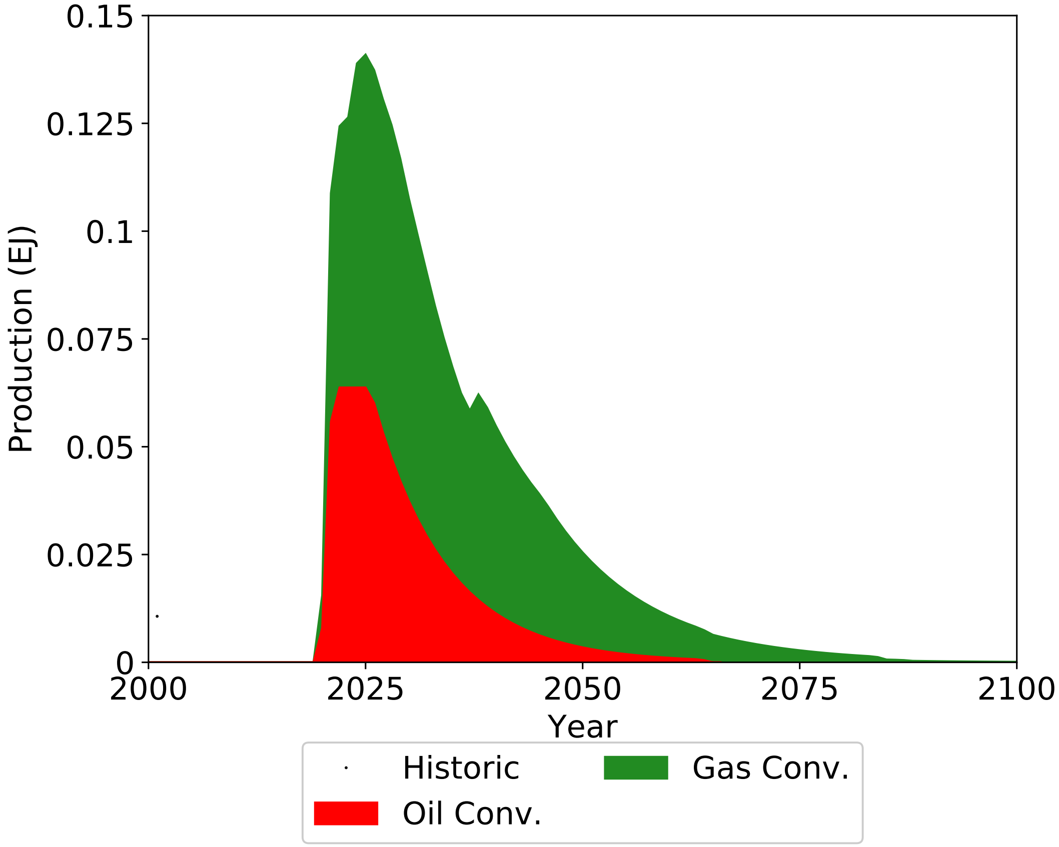

Figure 1.24: Ethiopia projection by mineral type

Table 1.24: Peak years - Minerals

Name	URR	Peak Year	Peak Rate
Oil Conv.	0.85	2022	0.06
Gas Conv.	1.78	2025	0.08
**Total**	**2.63**	**2025**	**0.14**

1.13 Gabon

1.13.1 All Projections

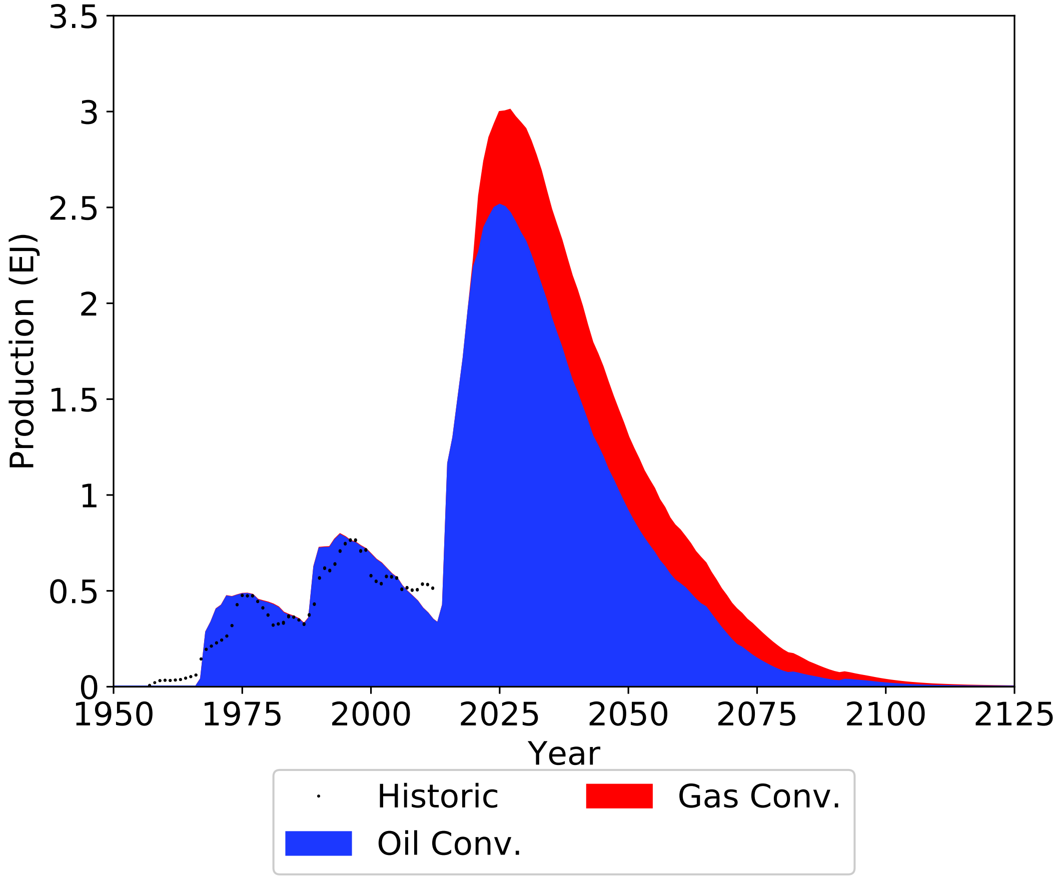

Figure 1.25: Gabon projections capped at 16

Table 1.25: Peak years - All

Name	URR	Peak Year	Peak Rate
Oil Conv.	102.07	2025	2.51
Gas Conv.	23.38	2032	0.6
**Total**	**125.45**	**2027**	**3.01**

1.13.2 By Mineral

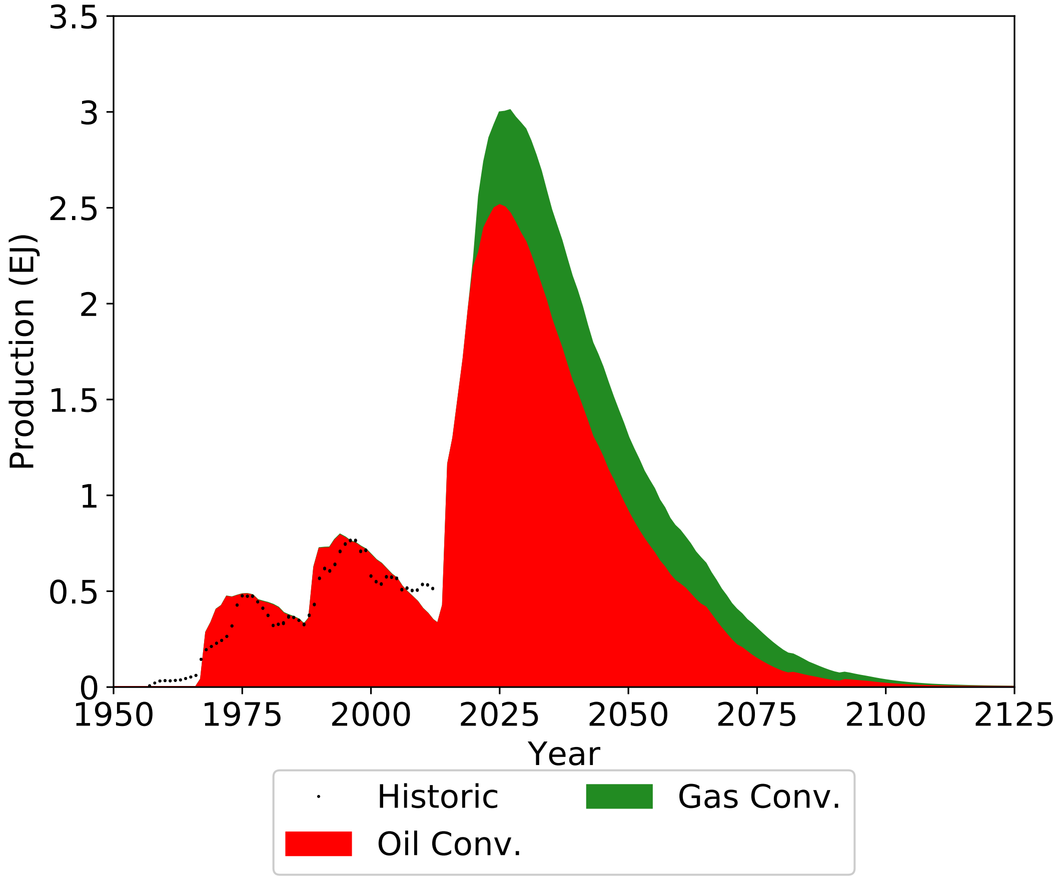

Figure 1.26: Gabon projection by mineral type

Table 1.26: Peak years - Minerals

Name	URR	Peak Year	Peak Rate
Oil Conv.	102.07	2025	2.51
Gas Conv.	23.38	2032	0.6
**Total**	**125.45**	**2027**	**3.01**

1.14 Gambia

1.14.1 All Projections

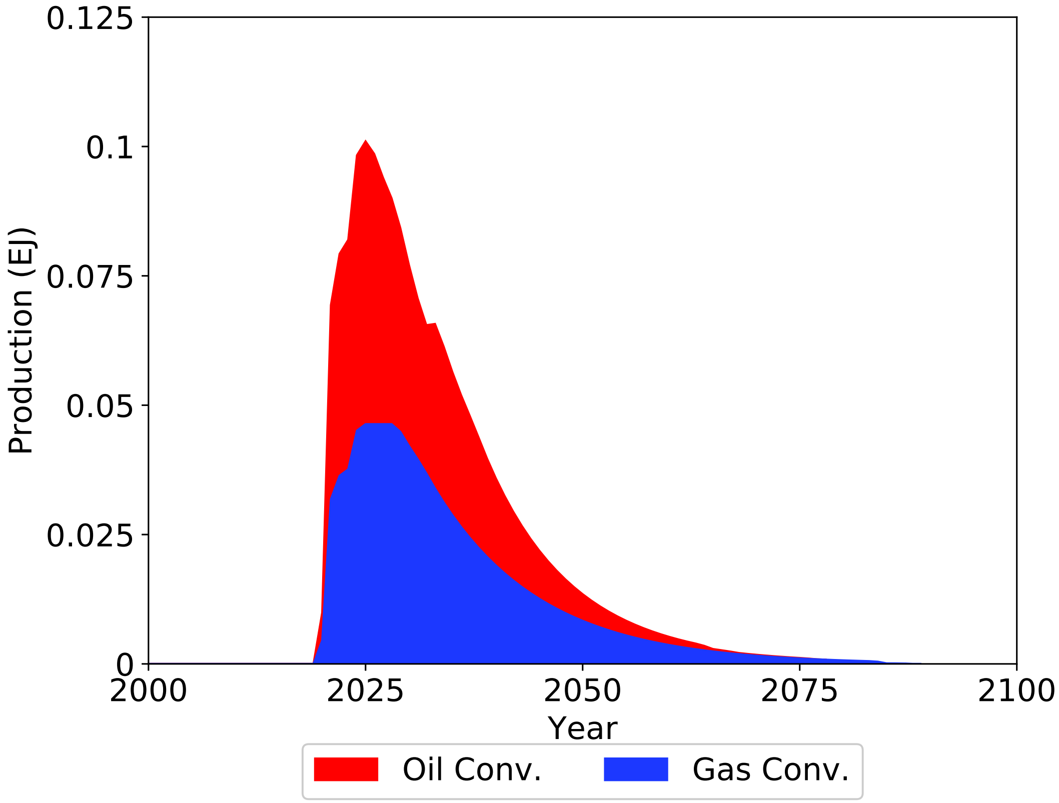

Figure 1.27: Gambia projections capped at 16

Table 1.27: Peak years - All

**Name**	**URR**	**Peak Year**	**Peak Rate**
Gas Conv.	0.93	2025	0.05
Oil Conv.	0.84	2025	0.05
**Total**	**1.77**	**2025**	**0.1**

1.14.2 By Mineral

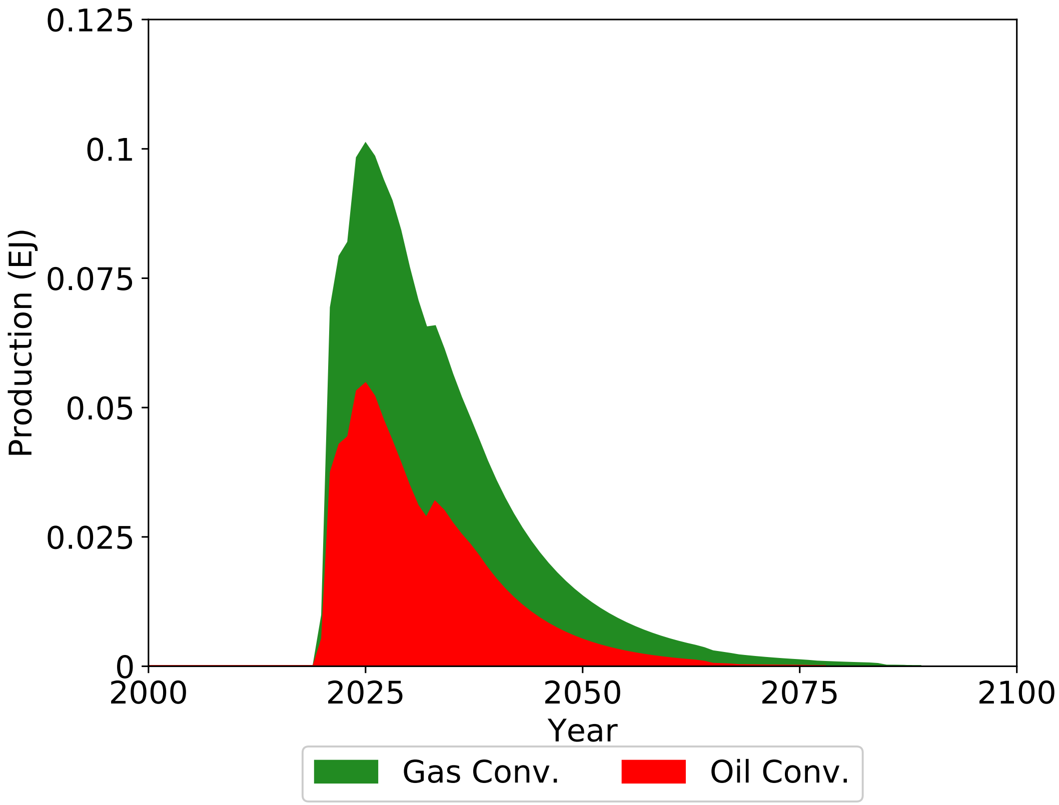

Figure 1.28: Gambia projection by mineral type

Table 1.28: Peak years - Minerals

Name	URR	Peak Year	Peak Rate
Oil Conv.	0.84	2025	0.05
Gas Conv.	0.93	2025	0.05
**Total**	**1.77**	**2025**	**0.1**

1.15 Ghana

1.15.1 All Projections

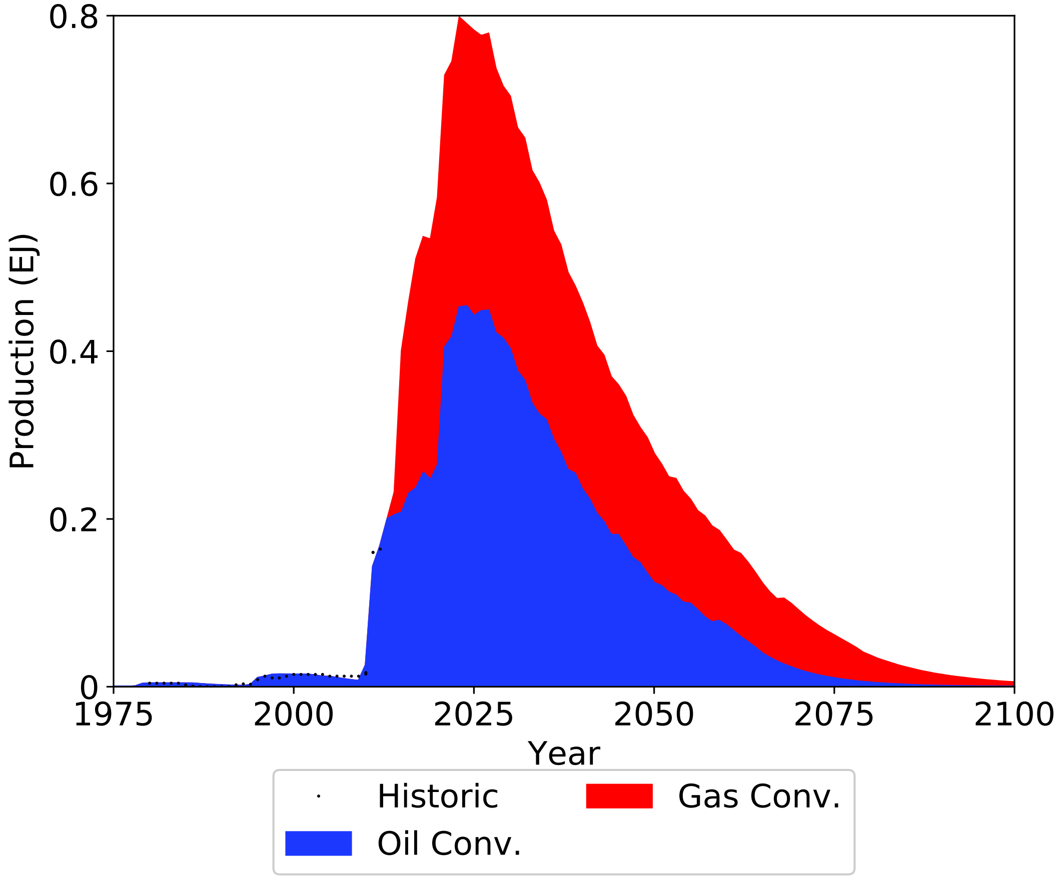

Figure 1.29: Ghana projections capped at 16

Table 1.29: Peak years - All

Name	URR	Peak Year	Peak Rate
Oil Conv.	12.96	2024	0.45
Gas Conv.	12.13	2023	0.35
**Total**	**25.09**	**2023**	**0.8**

1.15.2 By Mineral

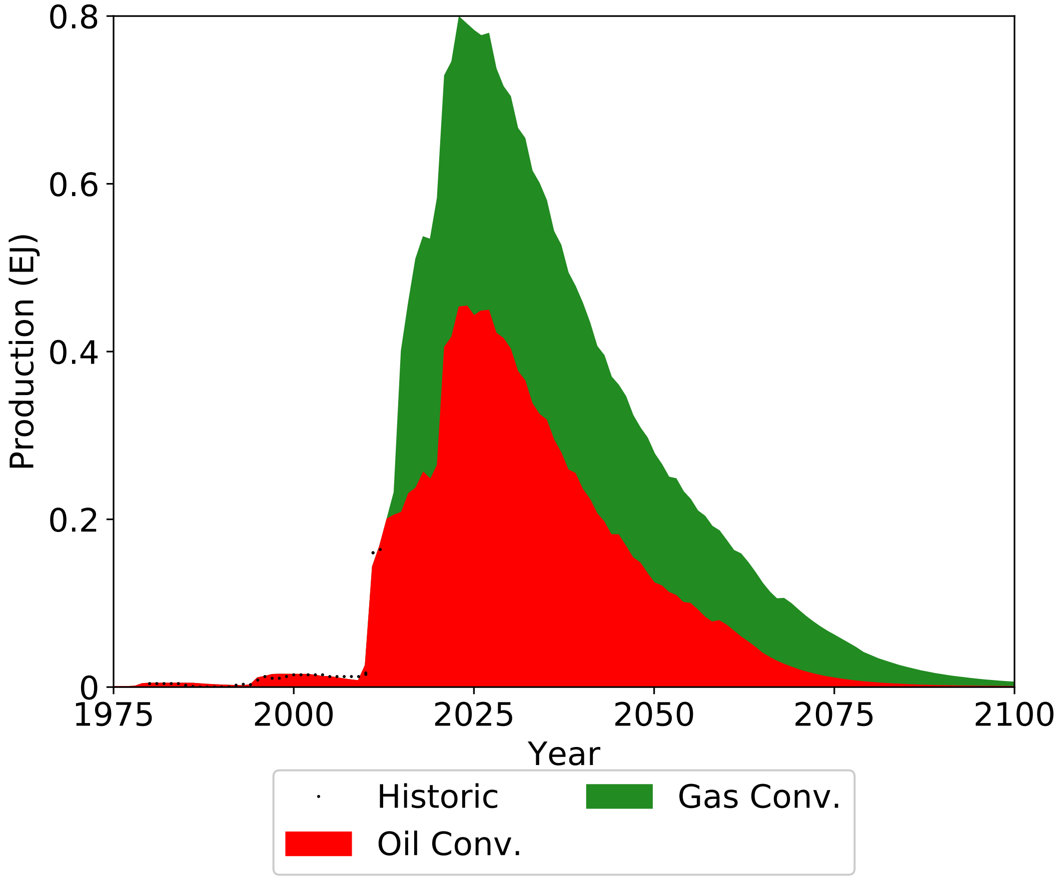

Figure 1.30: Ghana projection by mineral type

Table 1.30: Peak years - Minerals

Name	URR	Peak Year	Peak Rate
Oil Conv.	12.96	2024	0.45
Gas Conv.	12.13	2023	0.35
**Total**	**25.09**	**2023**	**0.8**

1.16 Guinea

1.16.1 All Projections

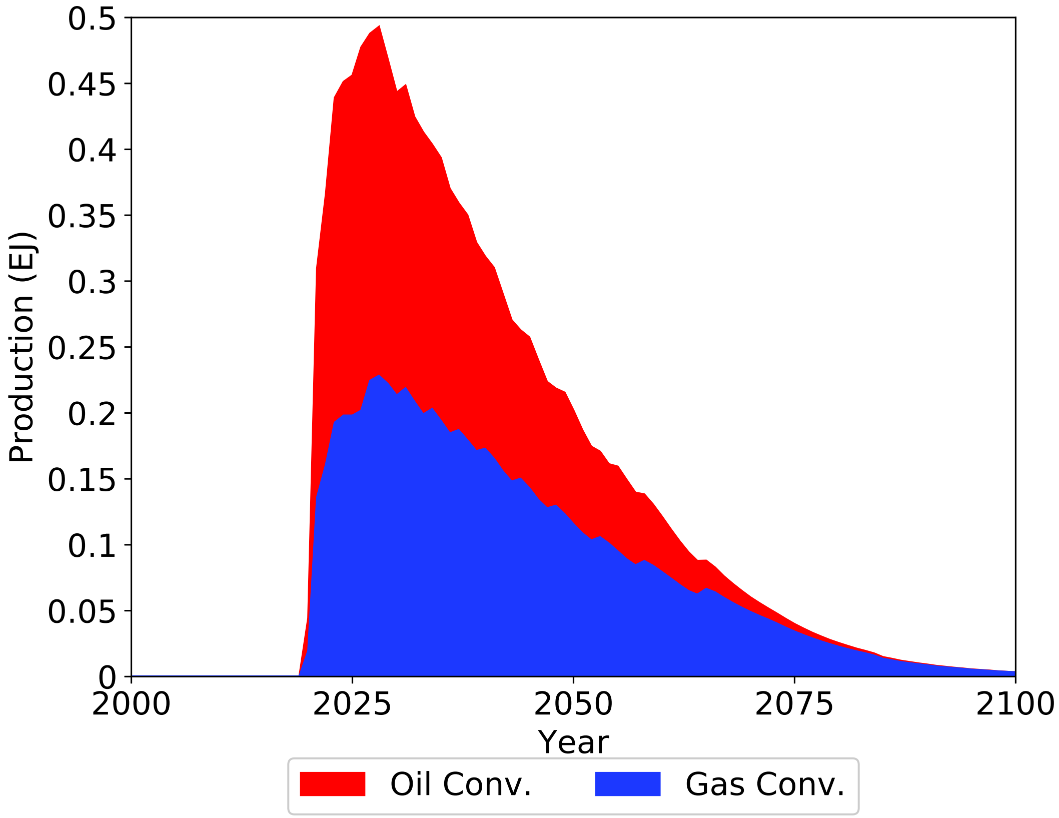

Figure 1.31: Guinea projections capped at 16

Table 1.31: Peak years - All

Name	URR	Peak Year	Peak Rate
Gas Conv.	7.42	2028	0.23
Oil Conv.	6.32	2026	0.28
**Total**	**13.74**	**2028**	**0.49**

1.16.2 By Mineral

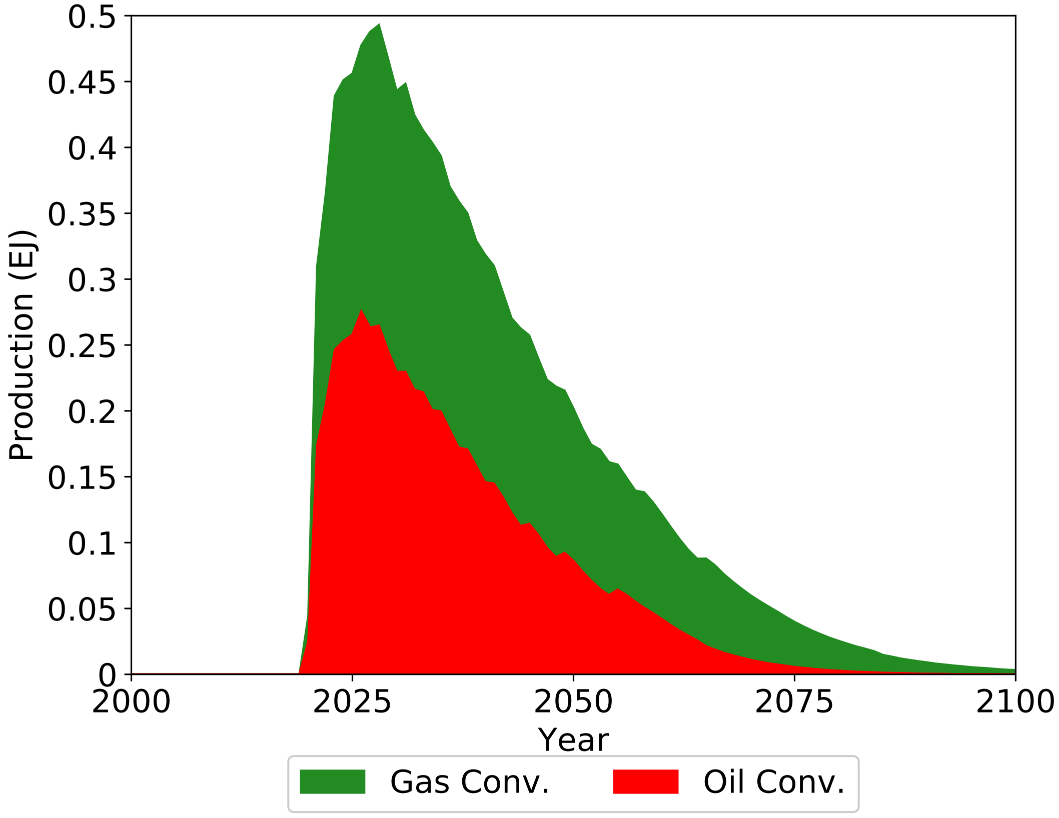

Figure 1.32: Guinea projection by mineral type

Table 1.32: Peak years - Minerals

Name	URR	Peak Year	Peak Rate
Oil Conv.	6.32	2026	0.28
Gas Conv.	7.42	2028	0.23
**Total**	**13.74**	**2028**	**0.49**

1.17 Guinea-Bissau

1.17.1 All Projections

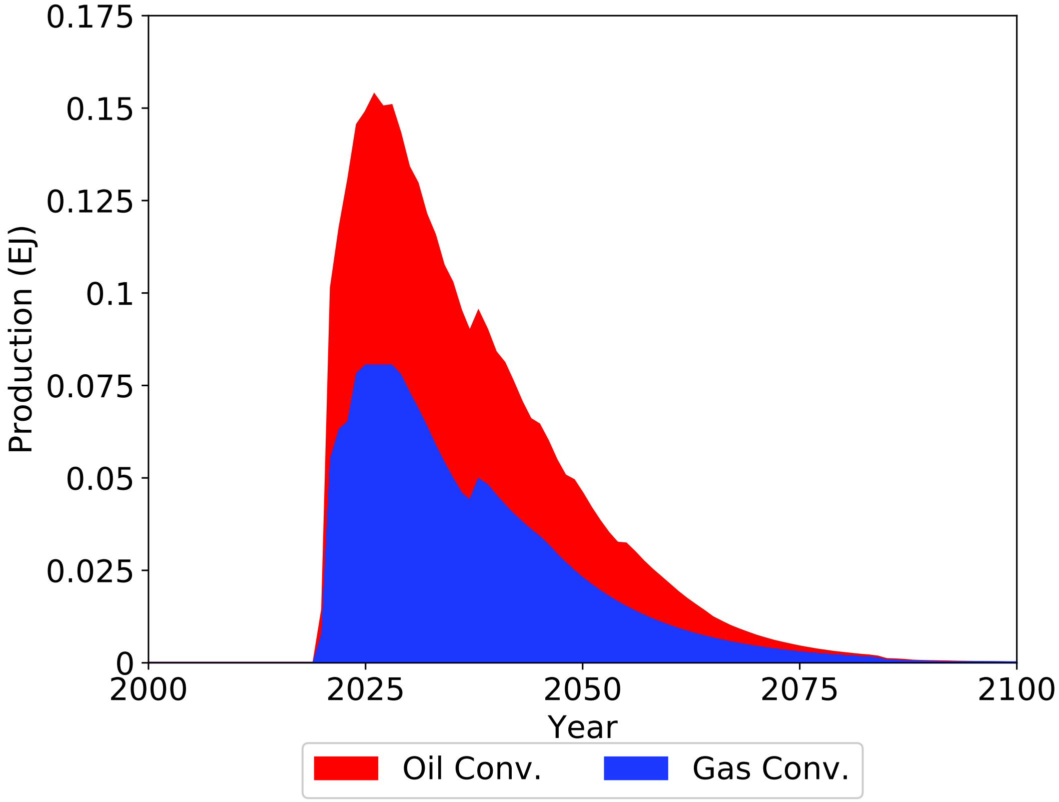

Figure 1.33: Guinea-Bissau projections capped at 16

Table 1.33: Peak years - All

Name	URR	Peak Year	Peak Rate
Gas Conv.	1.85	2025	0.08
Oil Conv.	1.68	2026	0.07
**Total**	**3.53**	**2026**	**0.15**

1.17.2 By Mineral

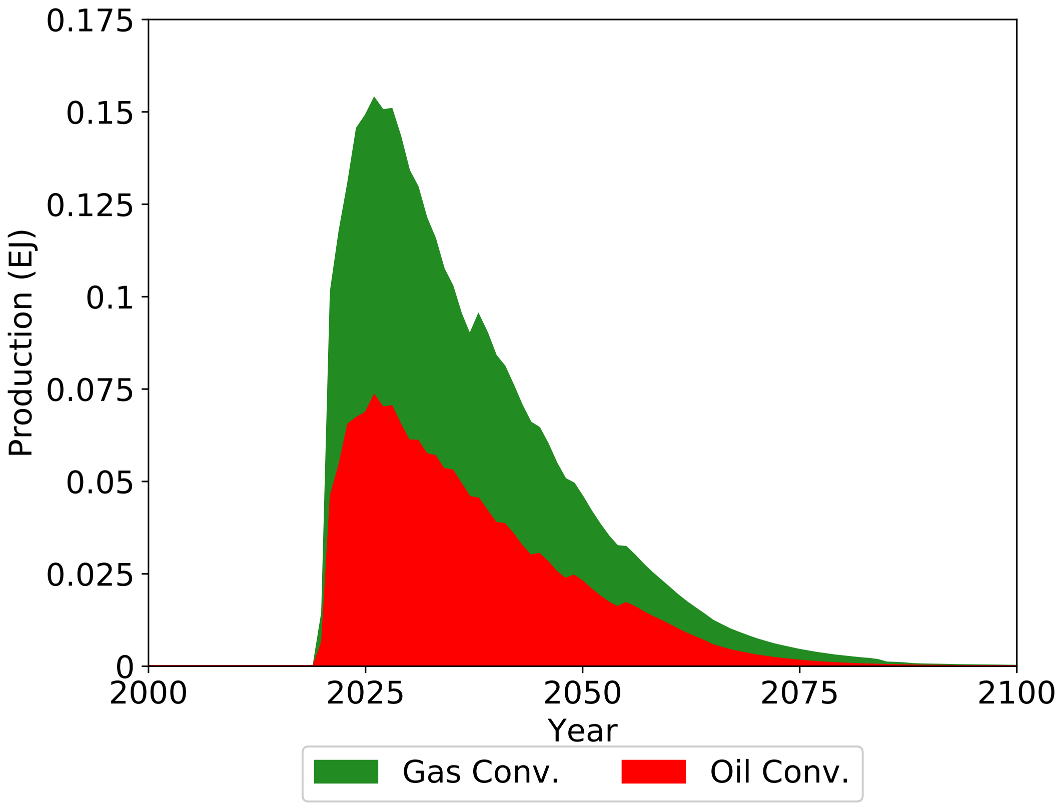

Figure 1.34: Guinea-Bissau projection by mineral type

Table 1.34: Peak years - Minerals

**Name**	**URR**	**Peak Year**	**Peak Rate**
Oil Conv.	1.68	2026	0.07
Gas Conv.	1.85	2025	0.08
**Total**	**3.53**	**2026**	**0.15**

1.18 Ivory Coast

1.18.1 All Projections

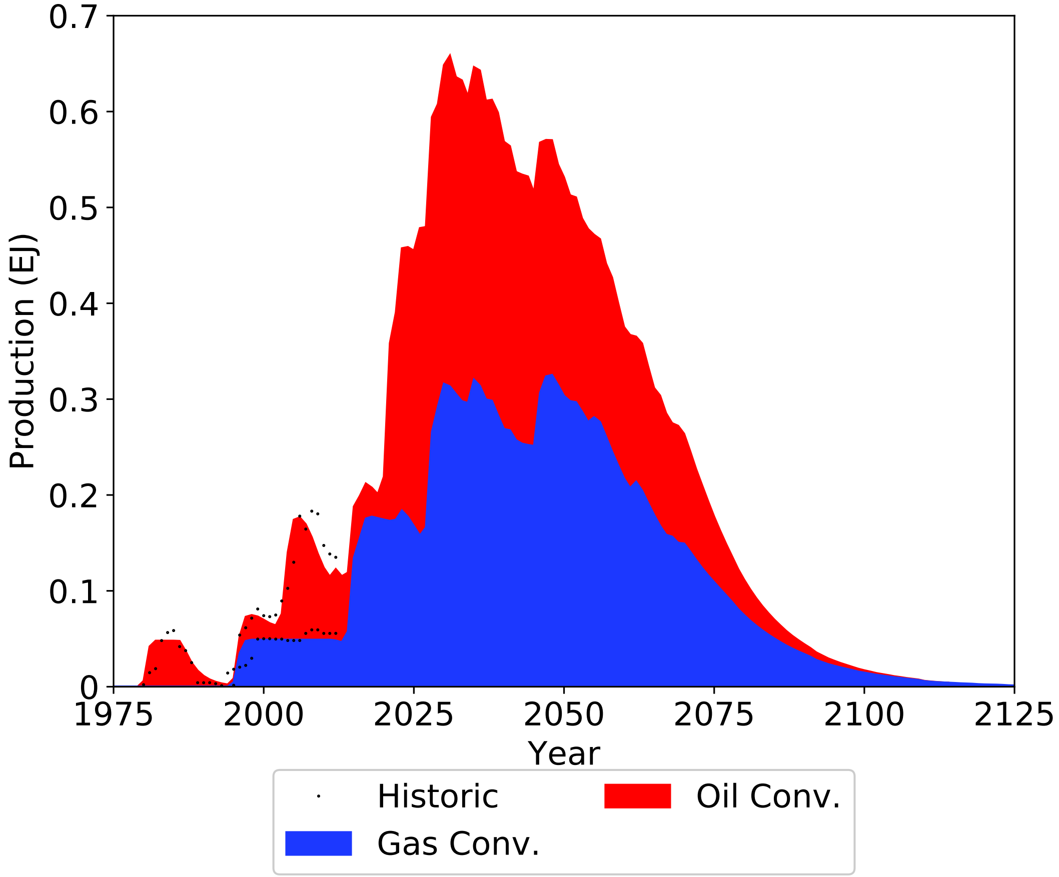

Figure 1.35: Ivory Coast projections capped at 16

Table 1.35: Peak years - All

Name	URR	Peak Year	Peak Rate
Gas Conv.	16.23	2048	0.33
Oil Conv.	14.7	2031	0.35
**Total**	**30.93**	**2031**	**0.66**

1.18.2 By Mineral

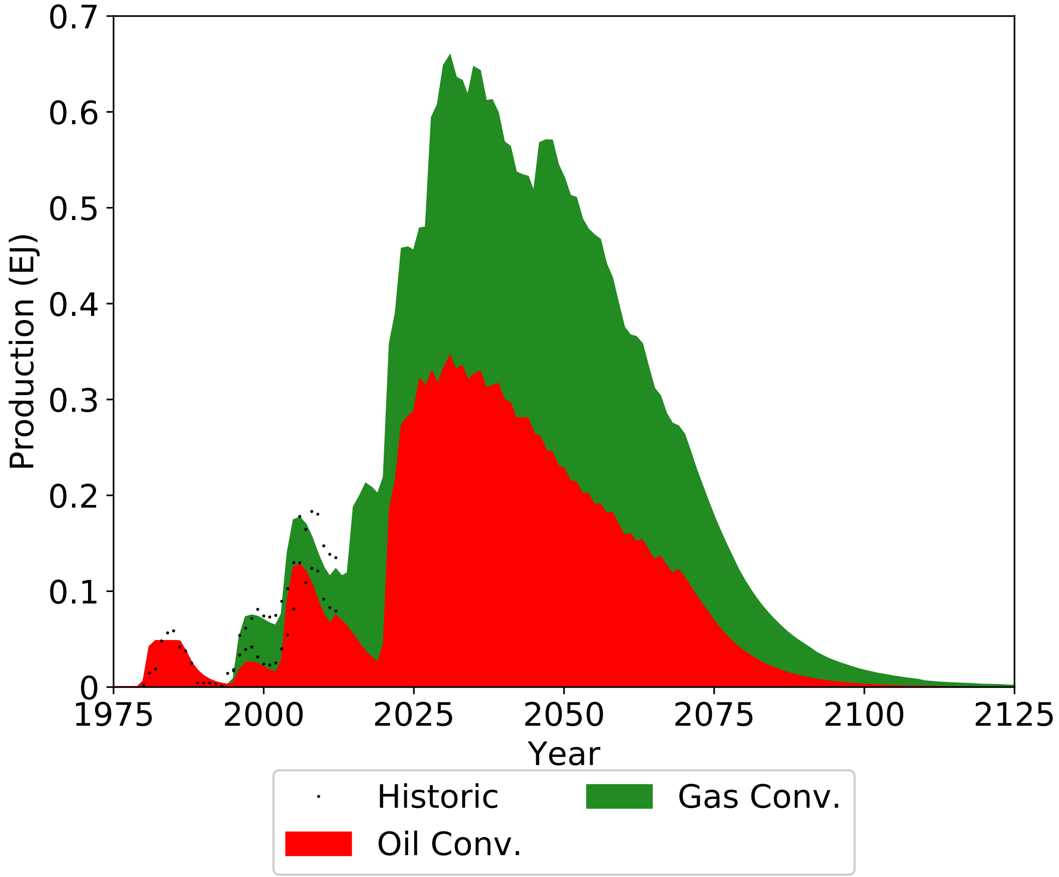

Figure 1.36: Ivory Coast projection by mineral type

Table 1.36: Peak years - Minerals

Name	URR	Peak Year	Peak Rate
Oil Conv.	14.7	2031	0.35
Gas Conv.	16.23	2048	0.33
**Total**	**30.93**	**2031**	**0.66**

1.19 Kenya

1.19.1 All Projections

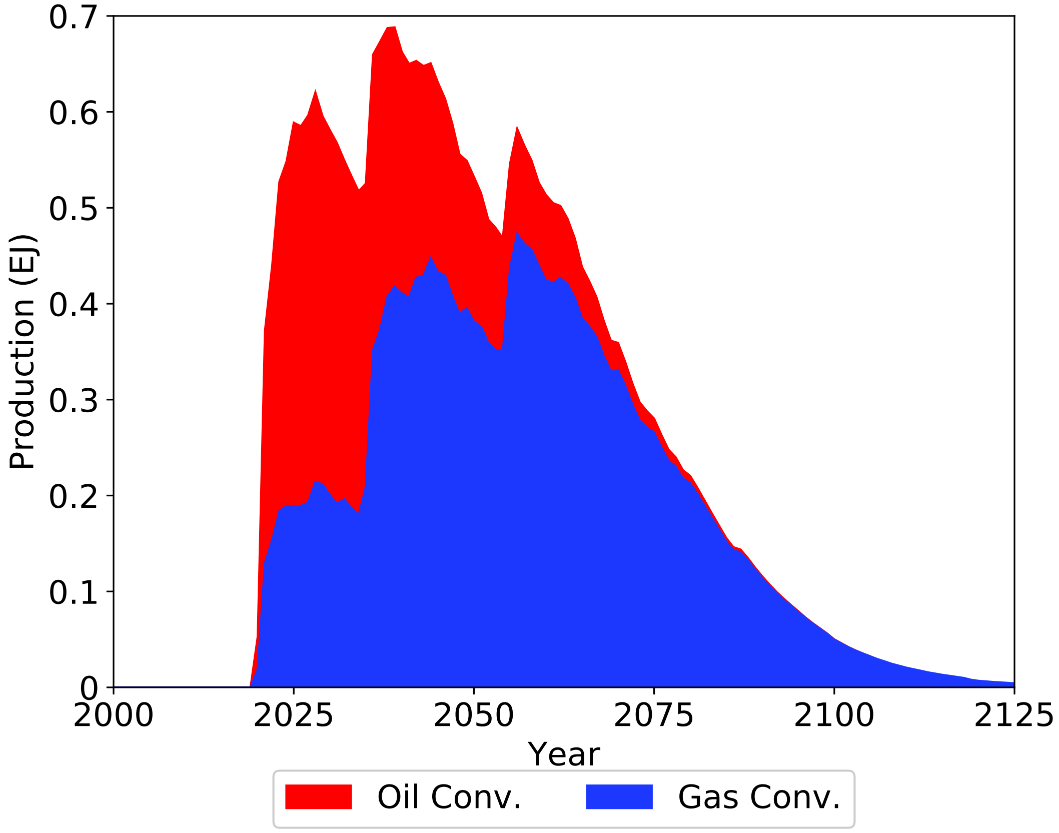

Figure 1.37: Kenya projections capped at 16

Table 1.37: Peak years - All

Name	URR	Peak Year	Peak Rate
Gas Conv.	22.25	2056	0.47
Oil Conv.	10.53	2028	0.41
**Total**	**32.78**	**2039**	**0.69**

1.19.2 By Mineral

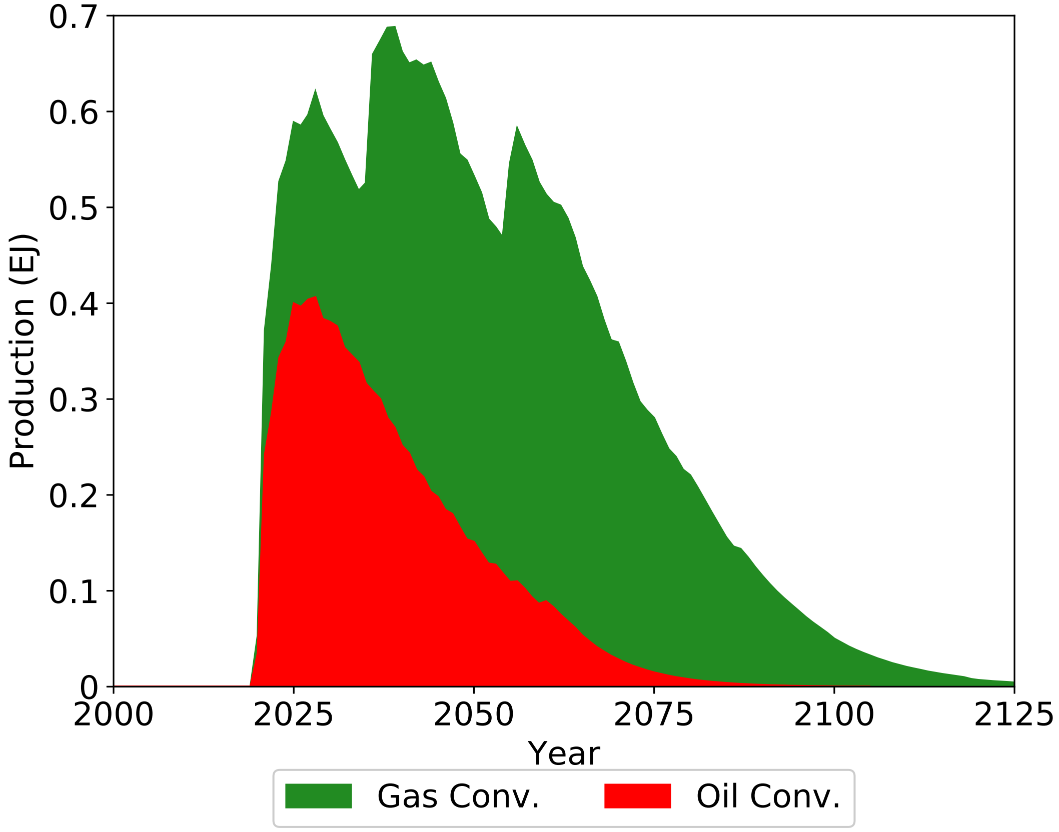

Figure 1.38: Kenya projection by mineral type

Table 1.38: Peak years - Minerals

Name	URR	Peak Year	Peak Rate
Oil Conv.	10.53	2028	0.41
Gas Conv.	22.25	2056	0.47
**Total**	**32.78**	**2039**	**0.69**

1.20 Liberia

1.20.1 All Projections

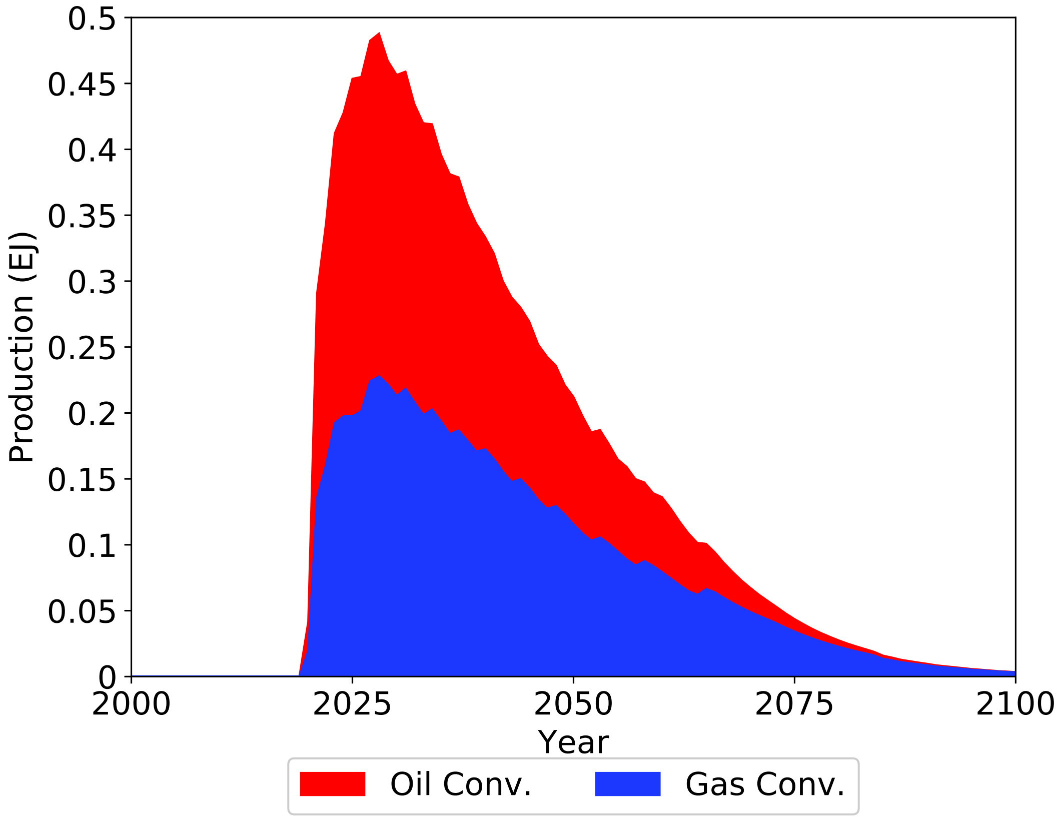

Figure 1.39: Liberia projections capped at 16

Table 1.39: Peak years - All

Name	URR	Peak Year	Peak Rate
Gas Conv.	7.4	2028	0.23
Oil Conv.	6.74	2028	0.26
**Total**	**14.14**	**2028**	**0.49**

1.20.2 By Mineral

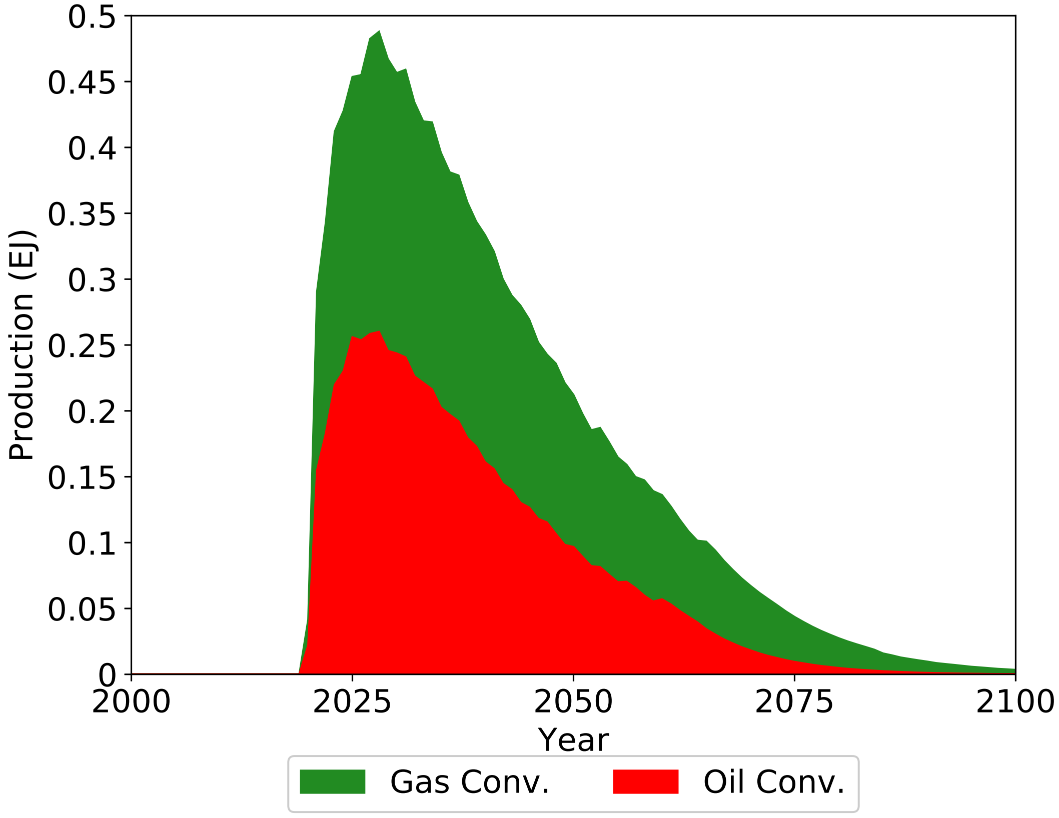

Figure 1.40: Liberia projection by mineral type

Table 1.40: Peak years - Minerals

Name	URR	Peak Year	Peak Rate
Oil Conv.	6.74	2028	0.26
Gas Conv.	7.4	2028	0.23
**Total**	**14.14**	**2028**	**0.49**

1.21 Libya

1.21.1 All Projections

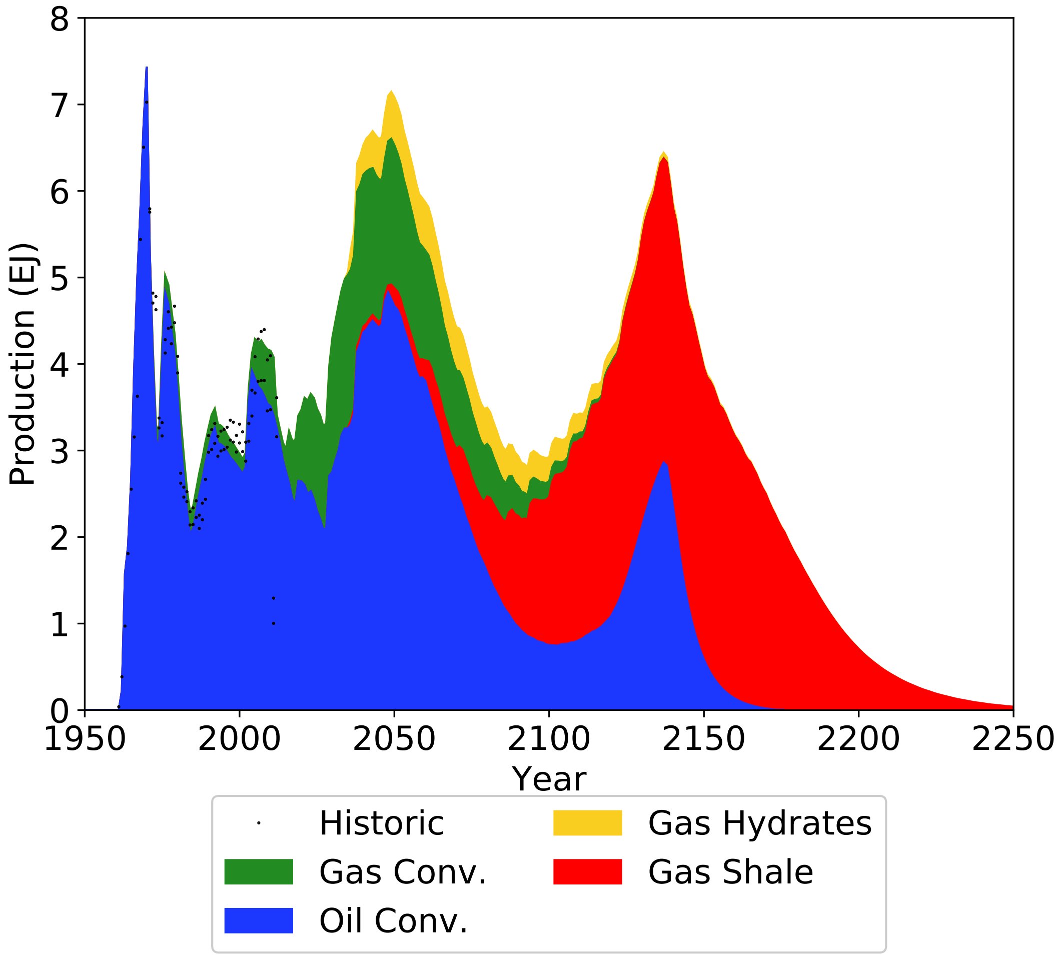

Figure 1.41: Libya projections capped at 16

Table 1.41: Peak years - All

**Name**	**URR**	**Peak Year**	**Peak Rate**
Oil Conv.	487.21	1970	7.44
Gas Shale	304.54	2142	3.56
Gas Conv.	102.88	2038	1.79
Gas Hydrates	35.5	2052	0.57
**Total**	**930.13**	**1970**	**7.44**

1.21.2 By Mineral

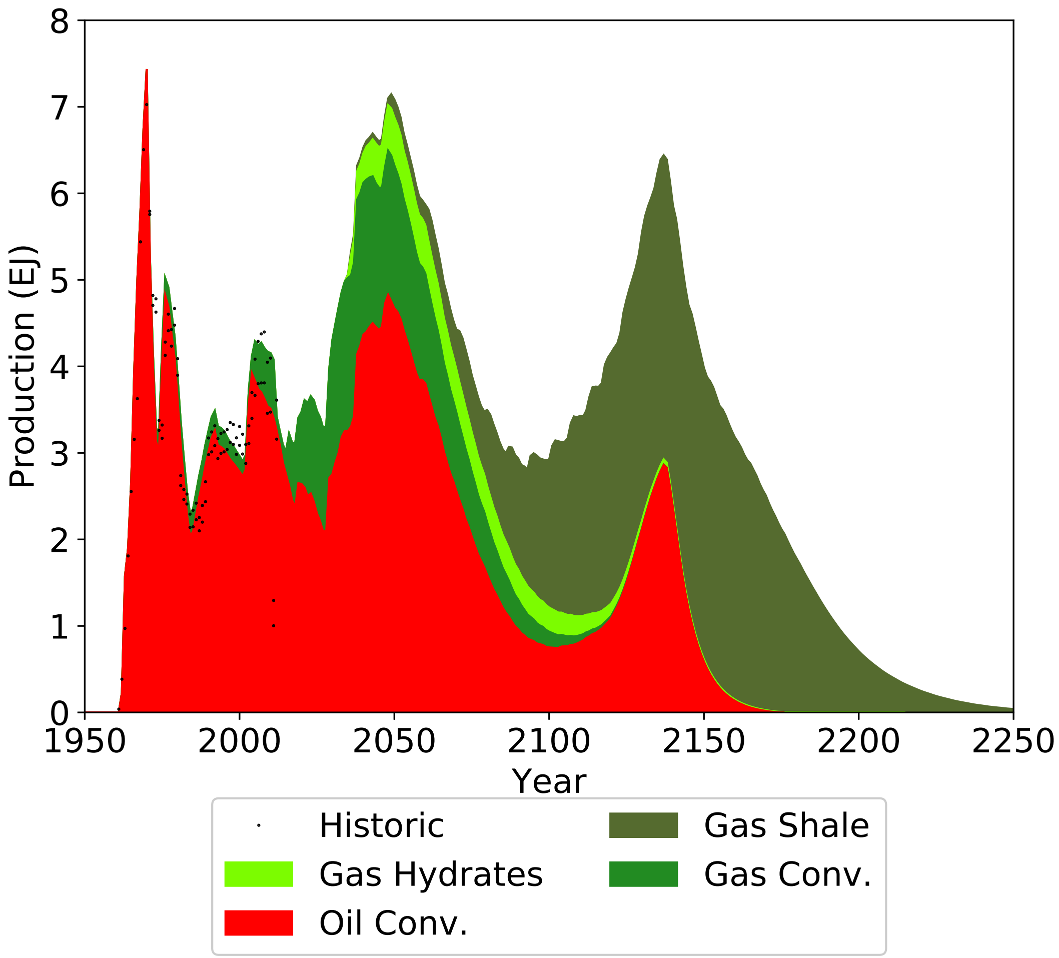

Figure 1.42: Libya projection by mineral type

Table 1.42: Peak years - Minerals

**Name**	**URR**	**Peak Year**	**Peak Rate**
Oil Conv.	487.21	1970	7.44
Gas Conv.	102.88	2038	1.79
Gas Hydrates	35.5	2052	0.57
Gas Shale	304.54	2142	3.56
**Total**	**930.13**	**1970**	**7.44**

1.22 Madagascar

1.22.1 All Projections

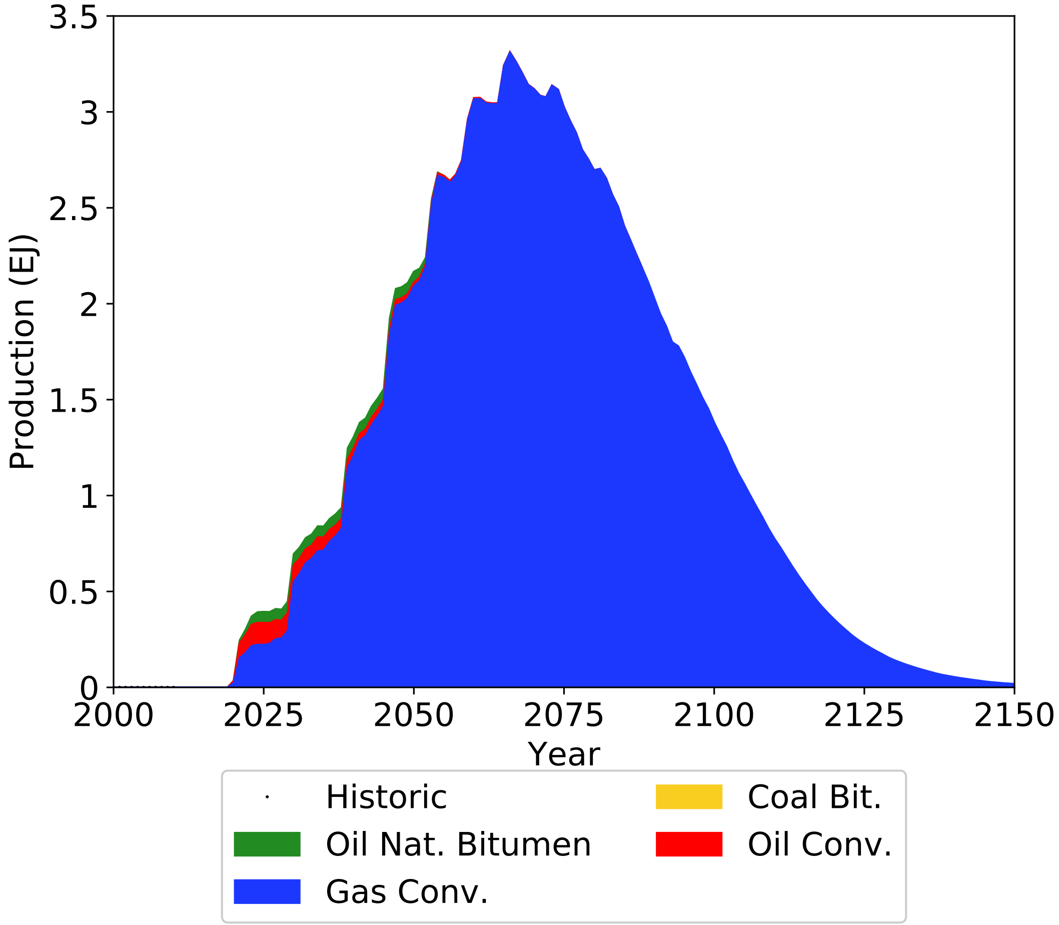

Figure 1.43: Madagascar projections capped at 16

Table 1.43: Peak years - All

**Name**	**URR**	**Peak Year**	**Peak Rate**
Gas Conv.	174.28	2066	3.31
Oil Conv.	2.1	2024	0.11
Oil Nat. Bitumen	1.73	2025	0.06
Coal Bit.	–	1945	–
**Total**	**178.11**	**2066**	**3.31**

1.22.2 By Mineral

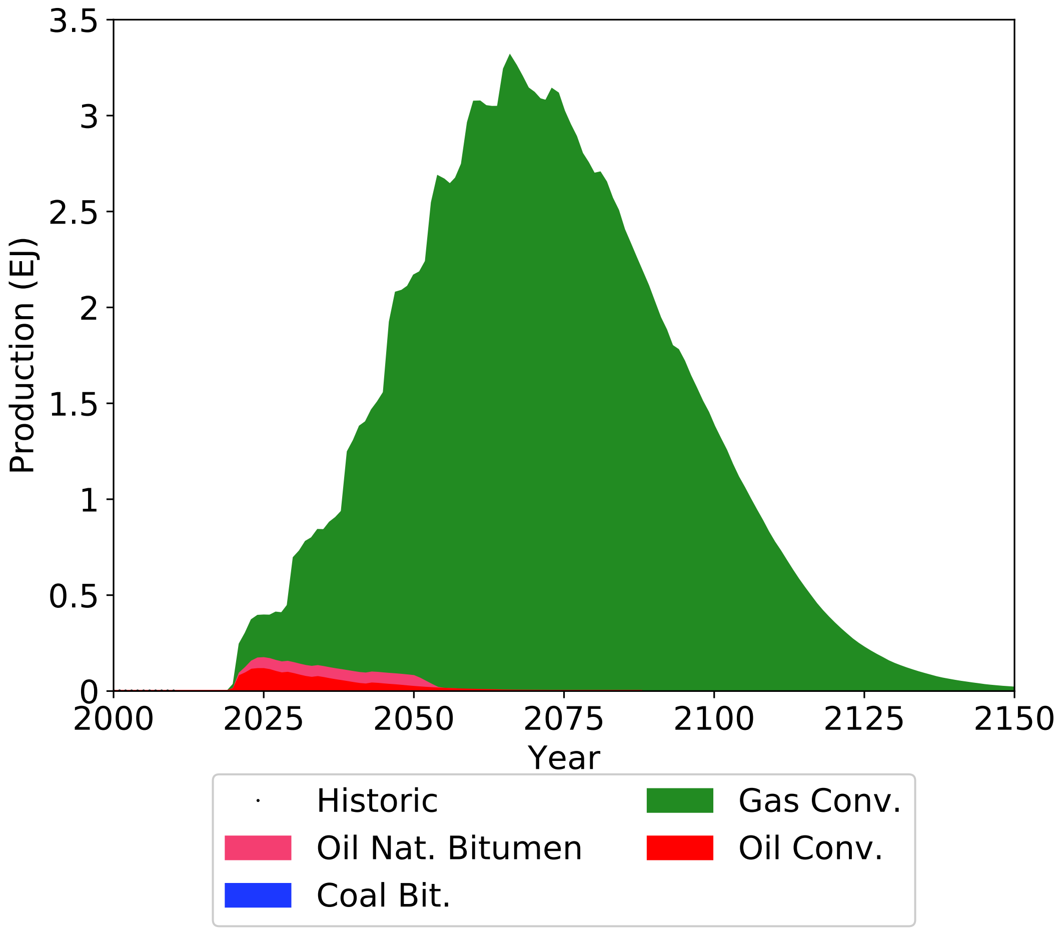

Figure 1.44: Madagascar projection by mineral type

Table 1.44: Peak years - Minerals

**Name**	**URR**	**Peak Year**	**Peak Rate**
Coal Bit.	–	1945	–
Oil Conv.	2.1	2024	0.11
Oil Nat. Bitumen	1.73	2025	0.06
Gas Conv.	174.28	2066	3.31
**Total**	**178.11**	**2066**	**3.31**

1.23 Malawi

1.23.1 All Projections

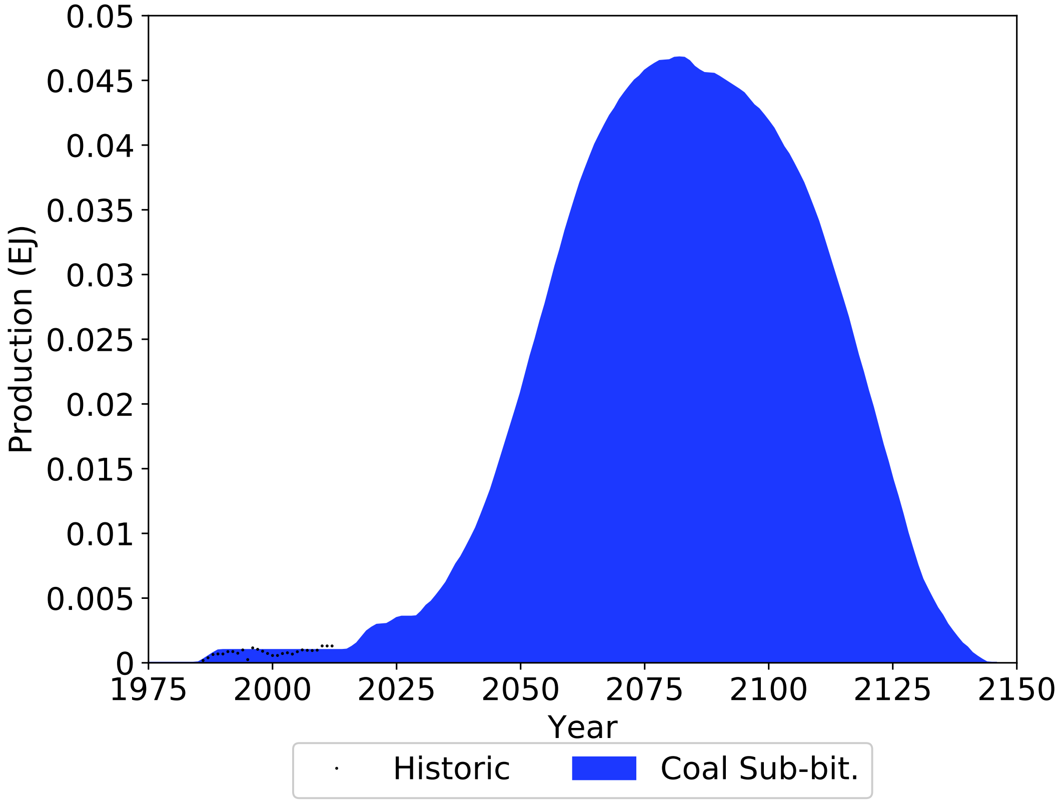

Figure 1.45: Malawi projections capped at 16

Table 1.45: Peak years - All

Name	URR	Peak Year	Peak Rate
Coal Sub-bit.	3.16	2082	0.05
**Total**	**3.16**	**2082**	**0.05**

1.23.2 By Mineral

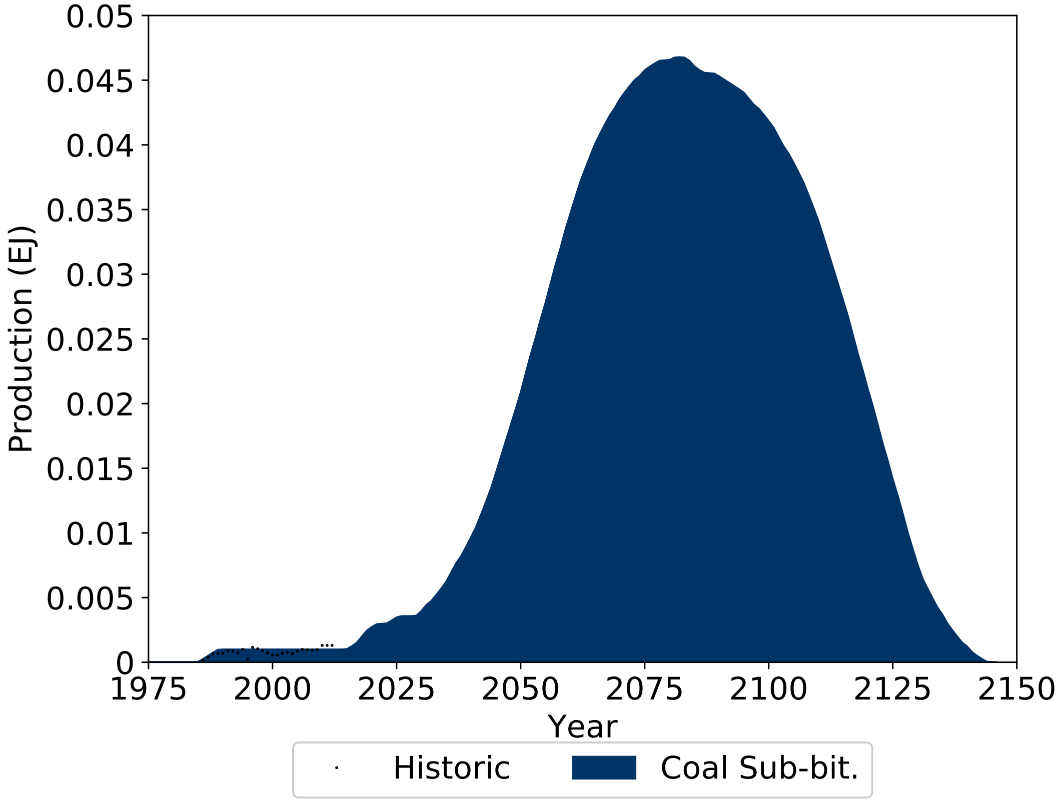

Figure 1.46: Malawi projection by mineral type

Table 1.46: Peak years - Minerals

Name	URR	Peak Year	Peak Rate
Coal Sub-bit.	3.16	2082	0.05
**Total**	**3.16**	**2082**	**0.05**

1.24 Mauritania

1.24.1 All Projections

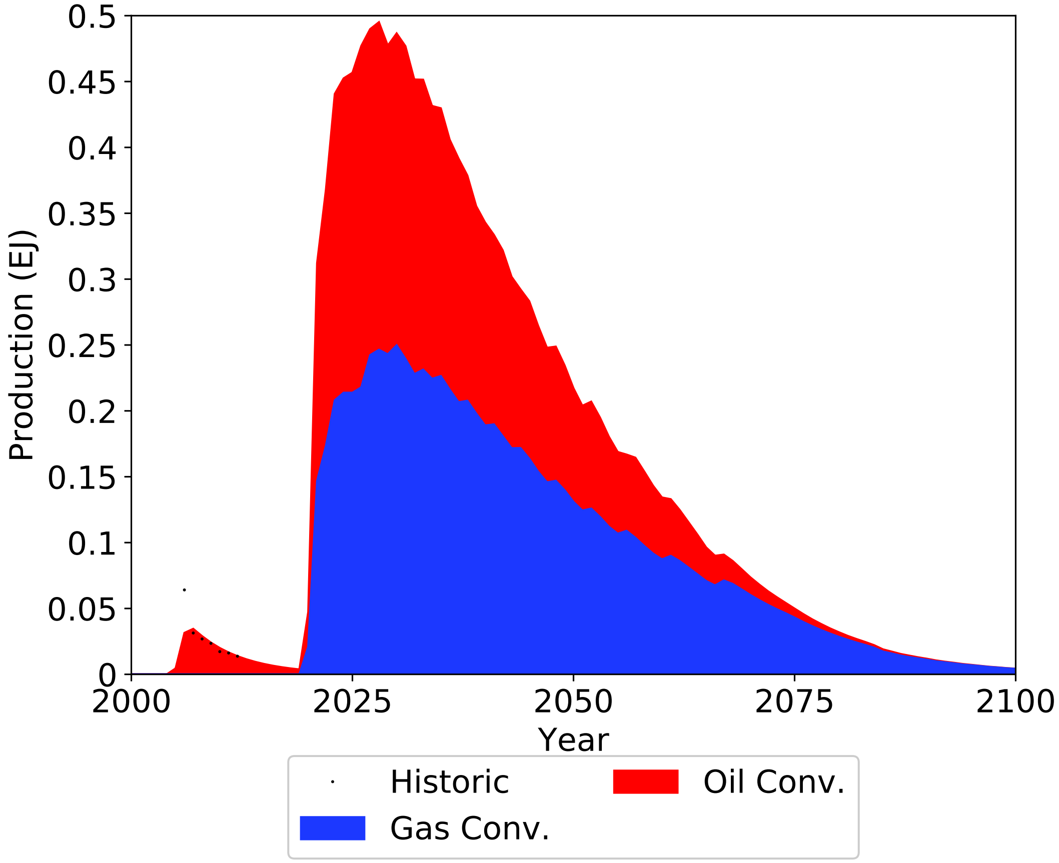

Figure 1.47: Mauritania projections capped at 16

Table 1.47: Peak years - All

Name	URR	Peak Year	Peak Rate
Gas Conv.	8.45	2030	0.25
Oil Conv.	6.64	2026	0.26
**Total**	**15.09**	**2028**	**0.5**

1.24.2 By Mineral

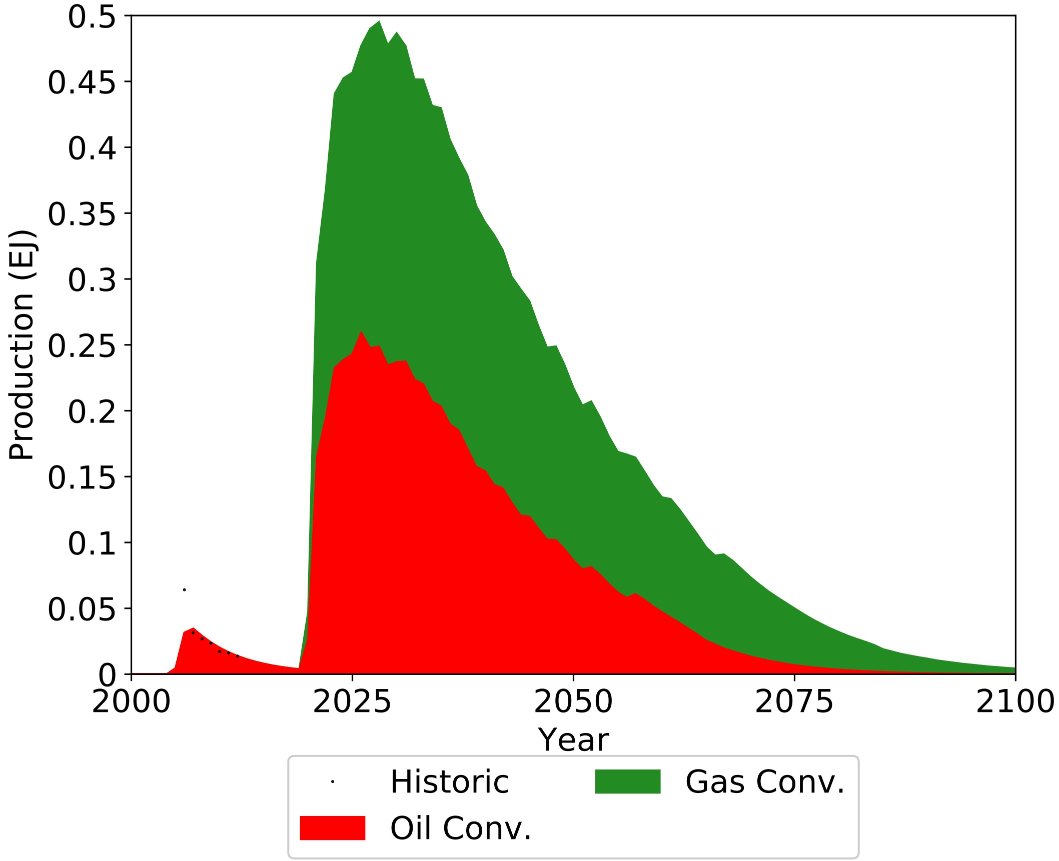

Figure 1.48: Mauritania projection by mineral type

Table 1.48: Peak years - Minerals

Name	URR	Peak Year	Peak Rate
Oil Conv.	6.64	2026	0.26
Gas Conv.	8.45	2030	0.25
**Total**	**15.09**	**2028**	**0.5**

1.25 Morocco

1.25.1 All Projections

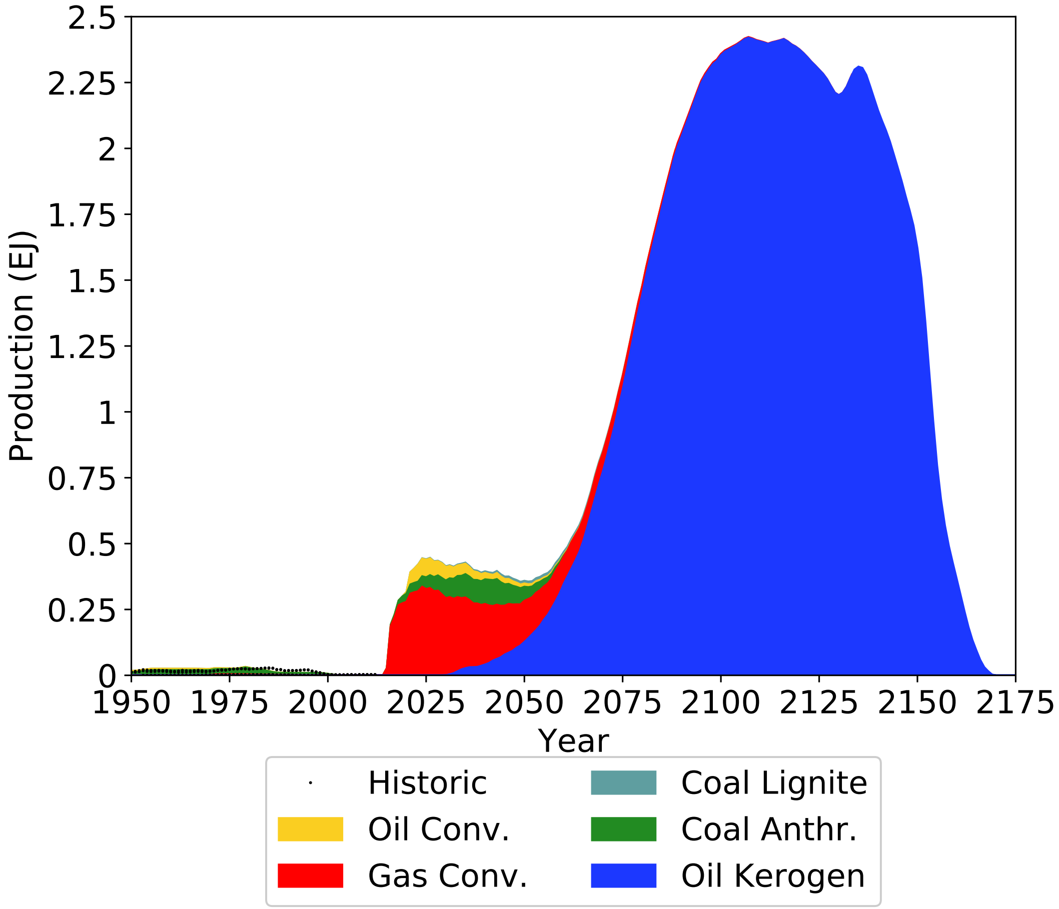

Figure 1.49: Morocco projections capped at 16

Table 1.49: Peak years - All

**Name**	**URR**	**Peak Year**	**Peak Rate**
Oil Kerogen	182.2	2107	2.42
Gas Conv.	11.89	2024	0.34
Coal Anthr.	3.34	2042	0.1
Oil Conv.	1.38	2024	0.07
Coal Lignite	0.38	2055	0.01
**Total**	**199.19**	**2107**	**2.42**

1.25.2 By Mineral

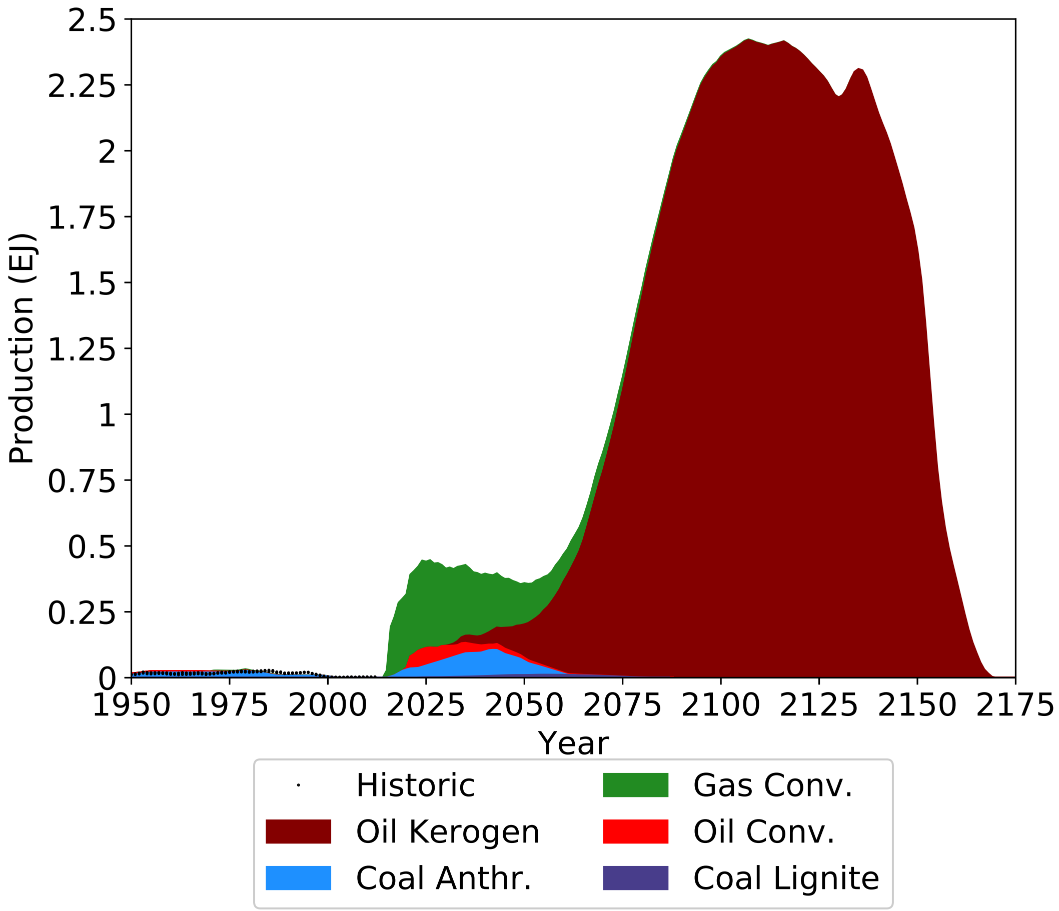

Figure 1.50: Morocco projection by mineral type

Table 1.50: Peak years - Minerals

**Name**	**URR**	**Peak Year**	**Peak Rate**
Coal Lignite	0.38	2055	0.01
Coal Anthr.	3.34	2042	0.1
Oil Conv.	1.38	2024	0.07
Oil Kerogen	182.2	2107	2.42
Gas Conv.	11.89	2024	0.34
**Total**	**199.19**	**2107**	**2.42**

1.26 Mozambique

1.26.1 All Projections

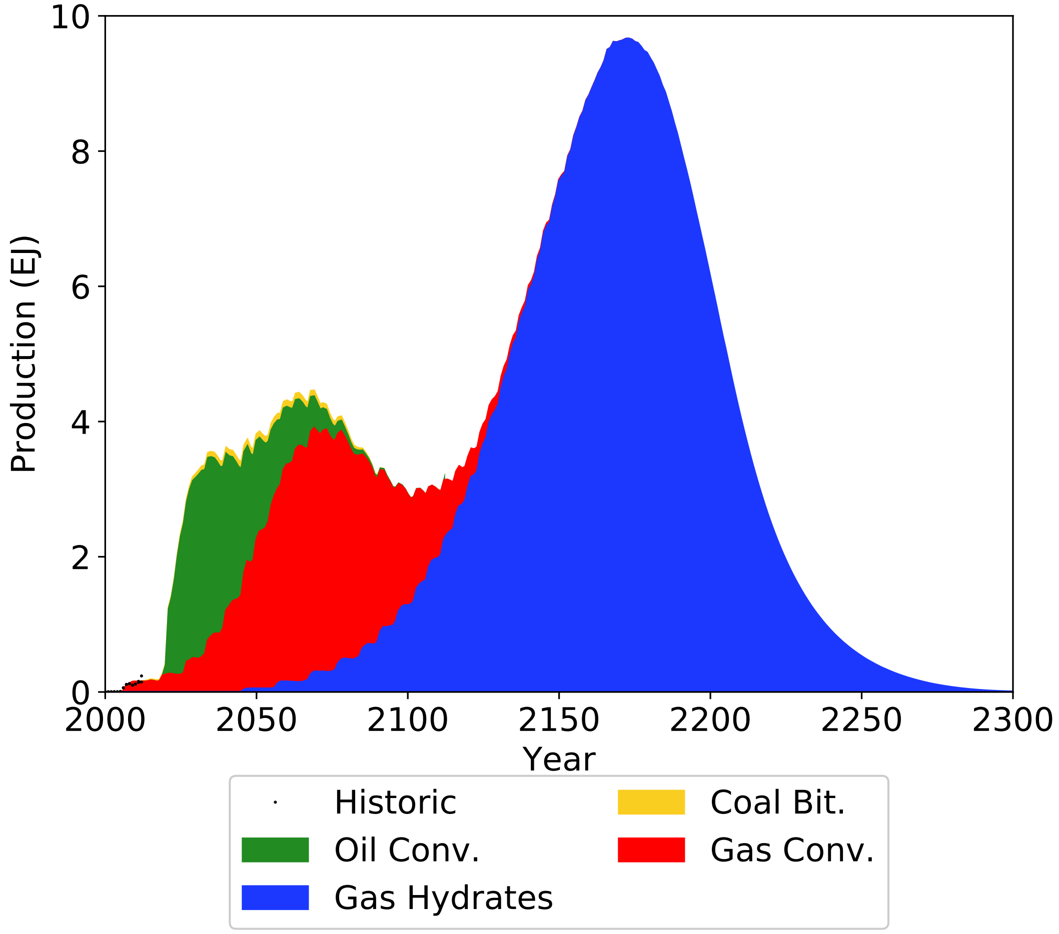

Figure 1.51: Mozambique projections capped at 16

Table 1.51: Peak years - All

Name	URR	Peak Year	Peak Rate
Gas Hydrates	779.0	2173	9.66
Gas Conv.	198.35	2069	3.6
Oil Conv.	84.3	2032	2.75
Coal Bit.	5.44	2048	0.1
**Total**	**1067.09**	**2173**	**9.66**

1.26.2 By Mineral

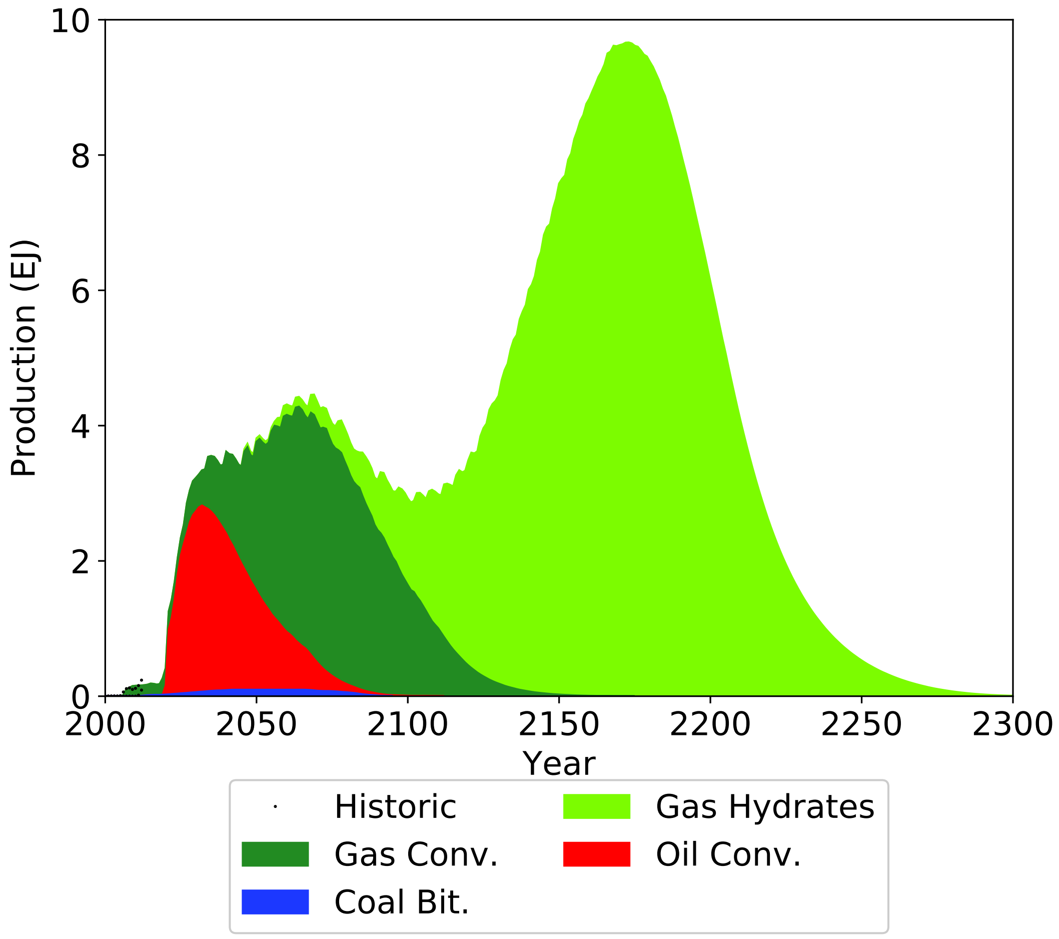

Figure 1.52: Mozambique projection by mineral type

Table 1.52: Peak years - Minerals

Name	URR	Peak Year	Peak Rate
Coal Bit.	5.44	2048	0.1
Oil Conv.	84.3	2032	2.75
Gas Conv.	198.35	2069	3.6
Gas Hydrates	779.0	2173	9.66
**Total**	**1067.09**	**2173**	**9.66**

1.27 Namibia

1.27.1 All Projections

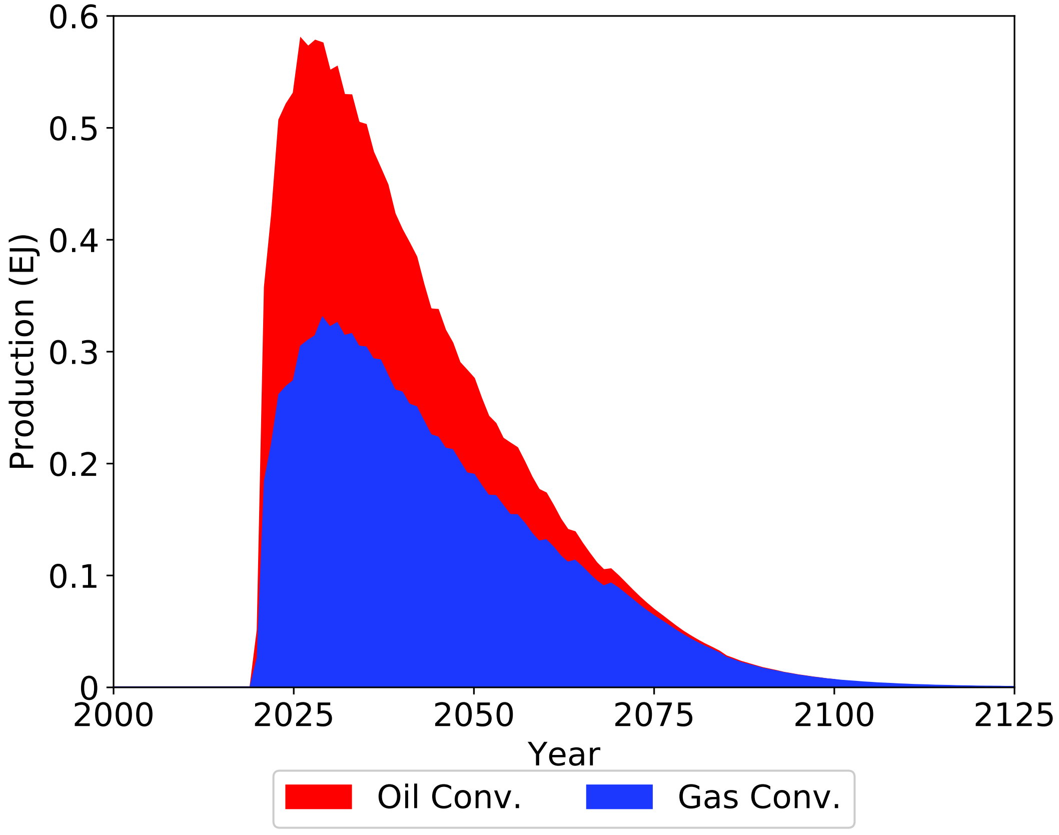

Figure 1.53: Namibia projections capped at 16

Table 1.53: Peak years - All

Name	URR	Peak Year	Peak Rate
Gas Conv.	11.6	2029	0.33
Oil Conv.	6.3	2026	0.27
**Total**	**17.9**	**2026**	**0.58**

1.27.2 By Mineral

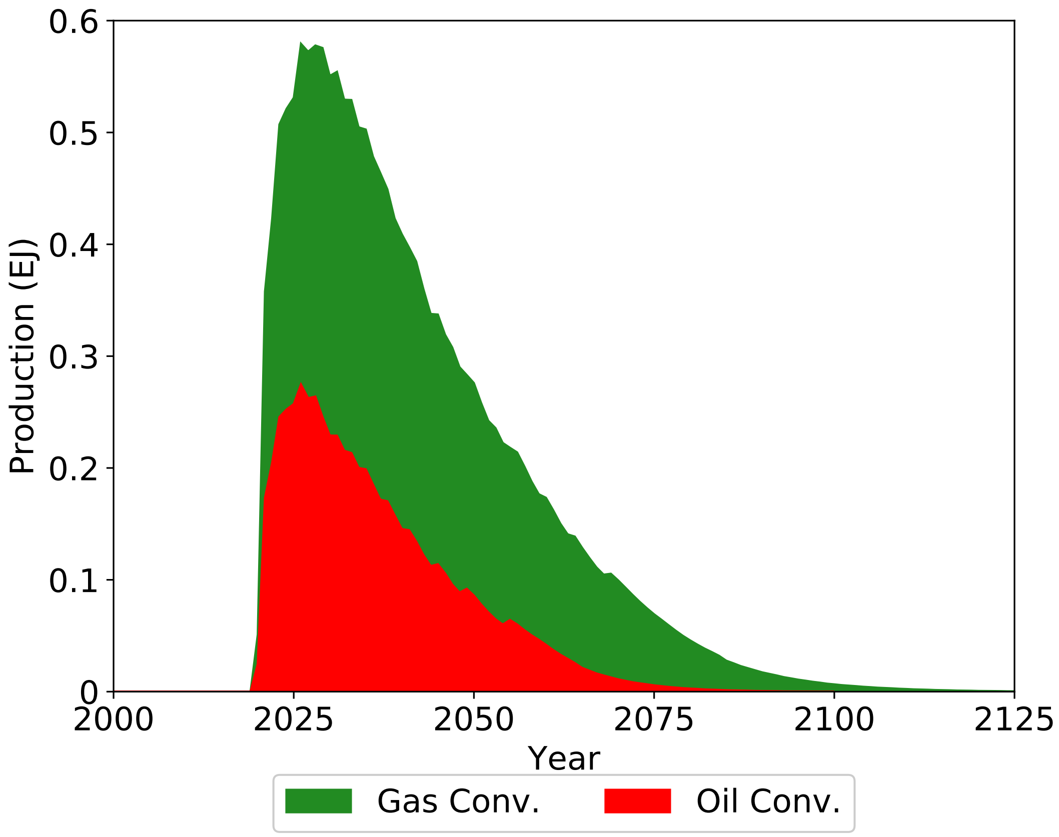

Figure 1.54: Namibia projection by mineral type

Table 1.54: Peak years - Minerals

Name	URR	Peak Year	Peak Rate
Oil Conv.	6.3	2026	0.27
Gas Conv.	11.6	2029	0.33
**Total**	**17.9**	**2026**	**0.58**

1.28 Niger

1.28.1 All Projections

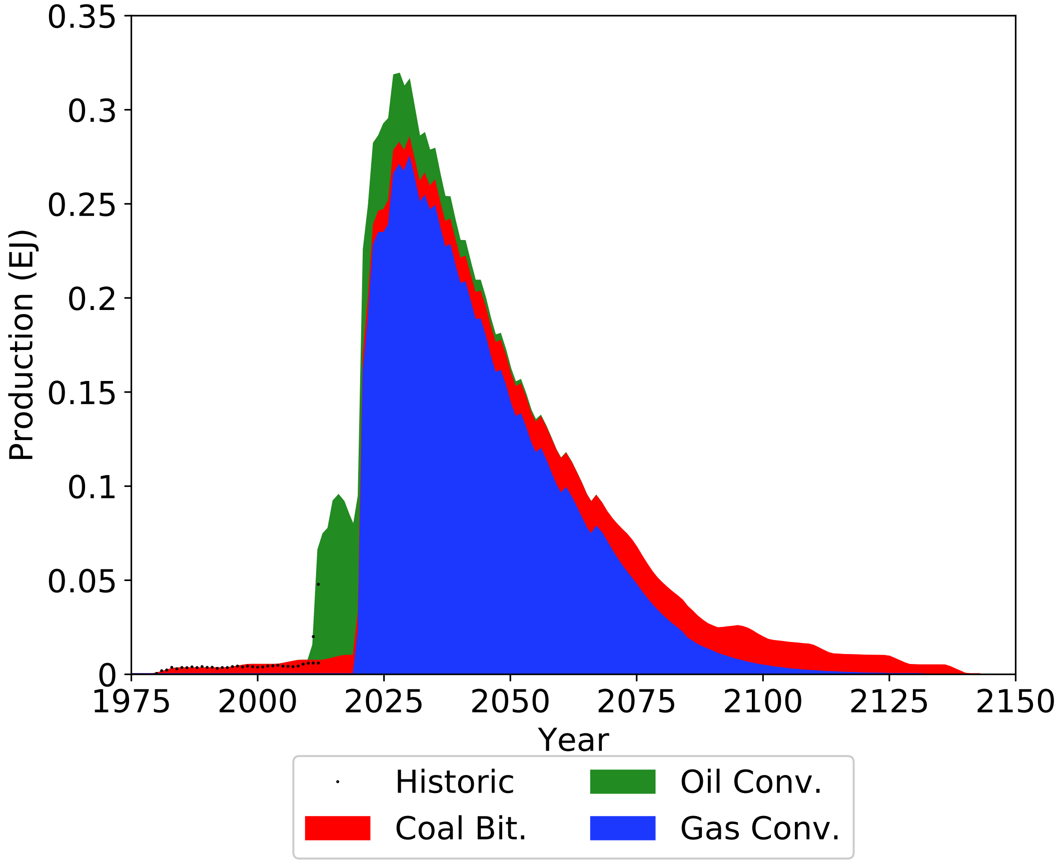

Figure 1.55: Niger projections capped at 16

Table 1.55: Peak years - All

Name	URR	Peak Year	Peak Rate
Gas Conv.	9.27	2030	0.27
Coal Bit.	1.81	2074	0.02
Oil Conv.	1.32	2016	0.09
**Total**	**12.4**	**2028**	**0.32**

1.28.2 By Mineral

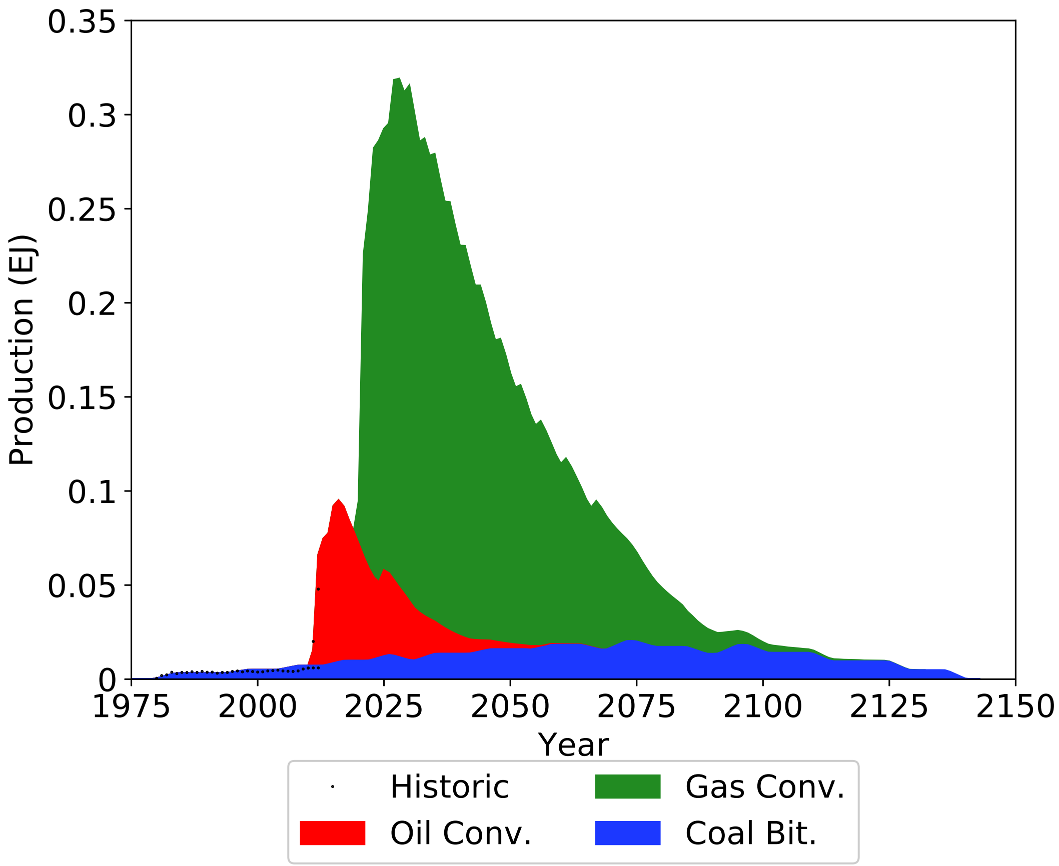

Figure 1.56: Niger projection by mineral type

Table 1.56: Peak years - Minerals

Name	URR	Peak Year	Peak Rate
Coal Bit.	1.81	2074	0.02
Oil Conv.	1.32	2016	0.09
Gas Conv.	9.27	2030	0.27
**Total**	**12.4**	**2028**	**0.32**

1.29 Nigeria

1.29.1 All Projections

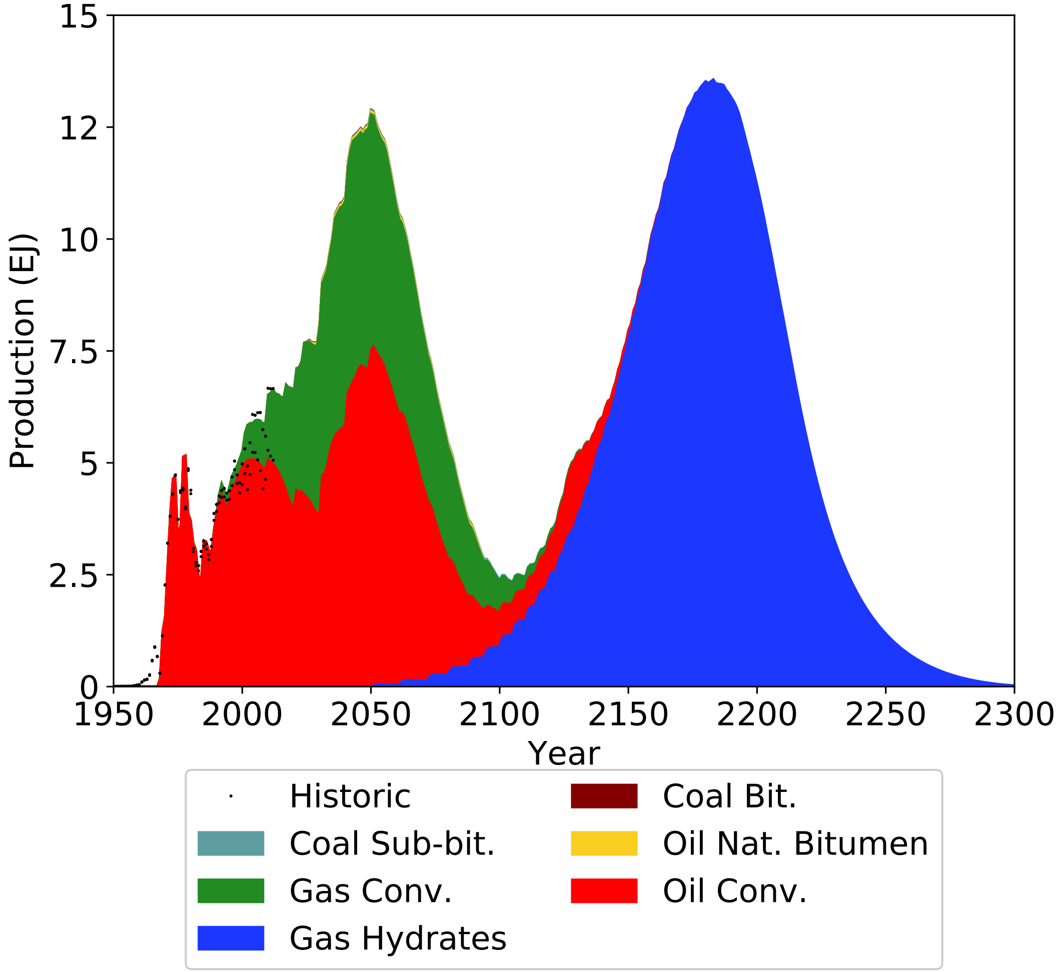

Figure 1.57: Nigeria projections capped at 16

Table 1.57: Peak years - All

Name	URR	Peak Year	Peak Rate
Gas Hydrates	1054.0	2183	13.57
Oil Conv.	605.3	2051	7.57
Gas Conv.	318.52	2043	5.37
Oil Nat. Bitumen	3.79	2036	0.06
Coal Sub-bit.	3.58	2076	0.05
Coal Bit.	0.5	2035	0.02
**Total**	**1985.7**	**2183**	**13.57**

1.29.2 By Mineral

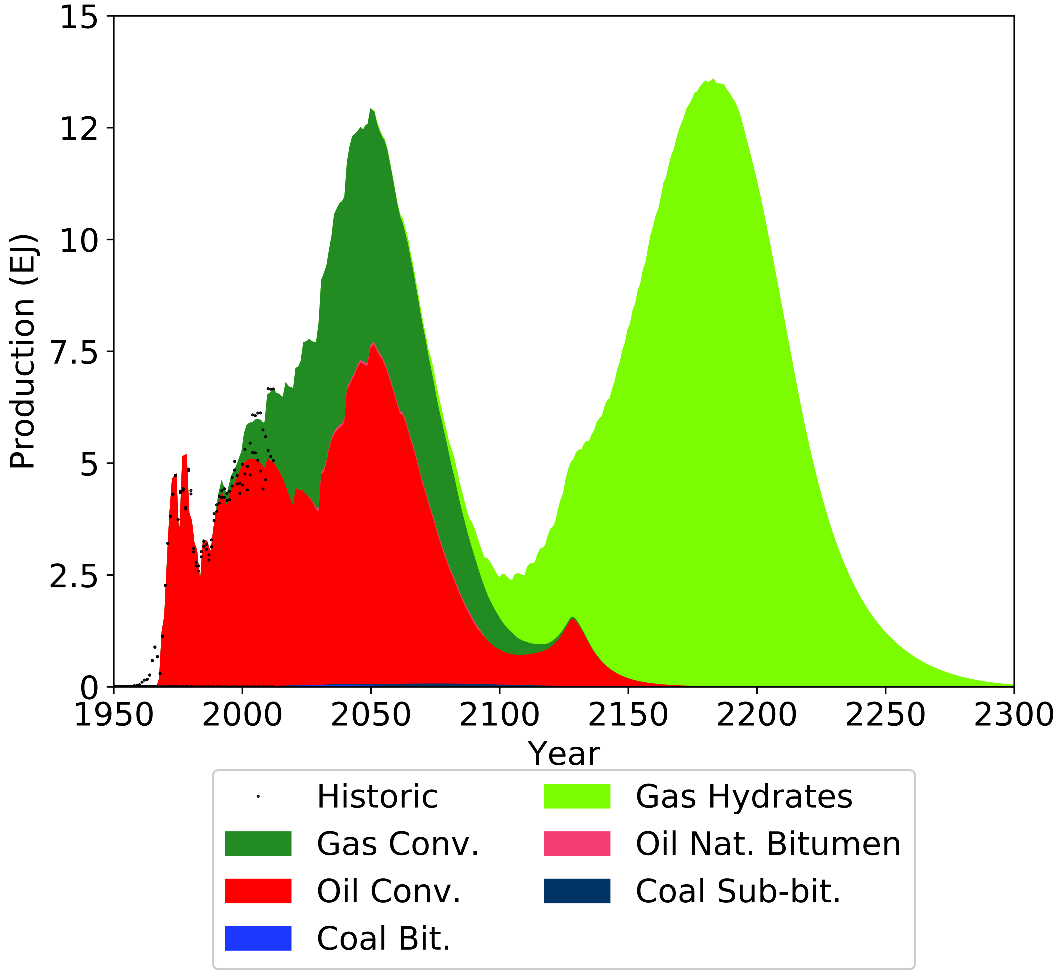

Figure 1.58: Nigeria projection by mineral type

Table 1.58: Peak years - Minerals

**Name**	**URR**	**Peak Year**	**Peak Rate**
Coal Bit.	0.5	2035	0.02
Coal Sub-bit.	3.58	2076	0.05
Oil Conv.	605.3	2051	7.57
Oil Nat. Bitumen	3.79	2036	0.06
Gas Conv.	318.52	2043	5.37
Gas Hydrates	1054.0	2183	13.57
**Total**	**1985.7**	**2183**	**13.57**

1.30 Rwanda

1.30.1 All Projections

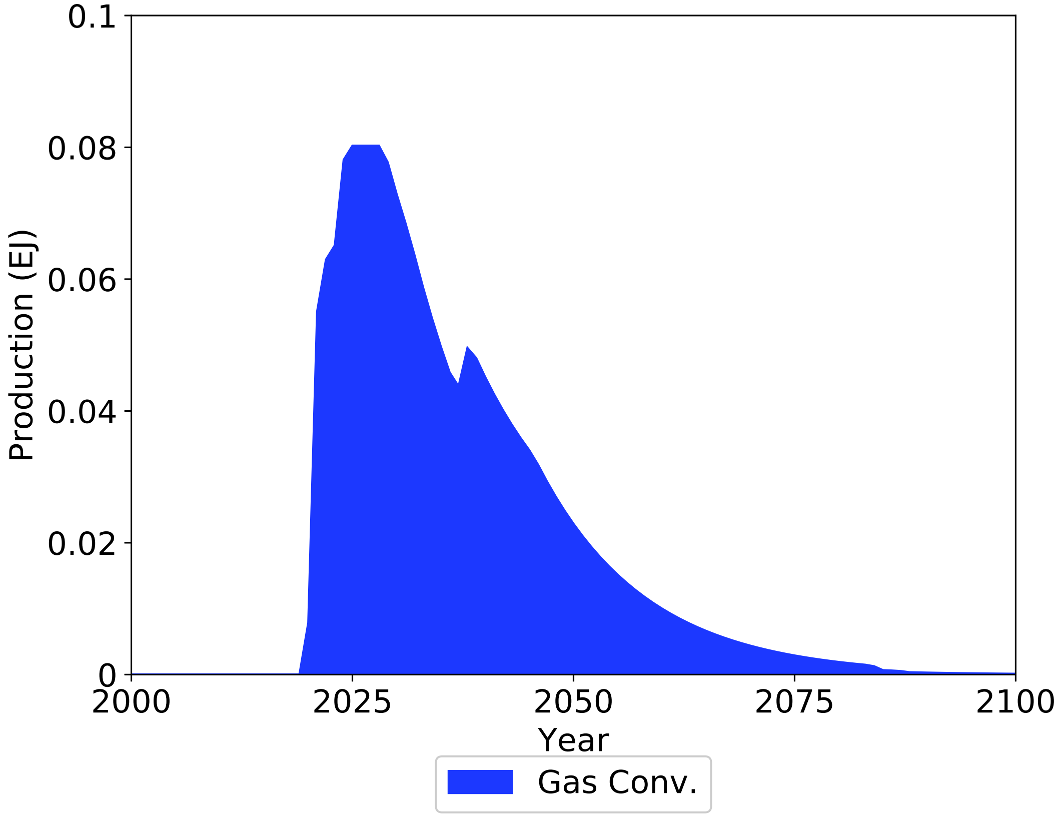

Figure 1.59: Rwanda projections capped at 16

Table 1.59: Peak years - All

Name	URR	Peak Year	Peak Rate
Gas Conv.	1.85	2025	0.08
**Total**	**1.85**	**2025**	**0.08**

1.30.2 By Mineral

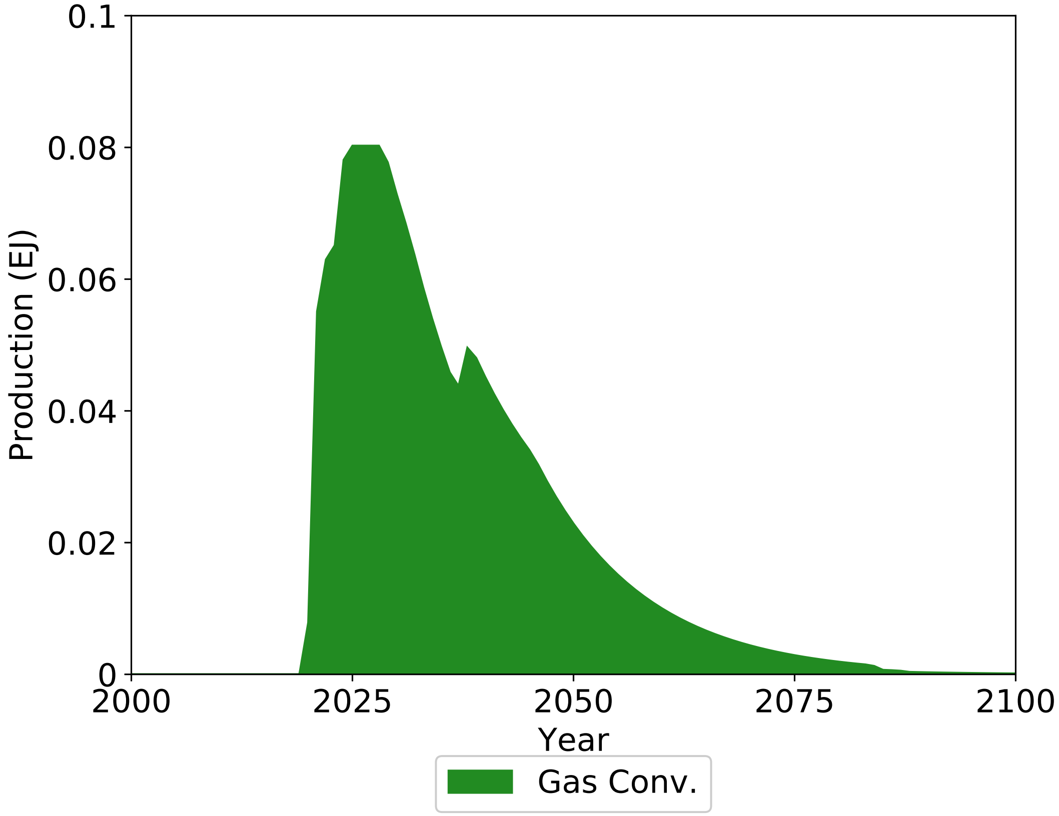

Figure 1.60: Rwanda projection by mineral type

Table 1.60: Peak years - Minerals

Name	URR	Peak Year	Peak Rate
Gas Conv.	1.85	2025	0.08
**Total**	**1.85**	**2025**	**0.08**

1.31 Sao Tome and Principe

1.31.1 All Projections

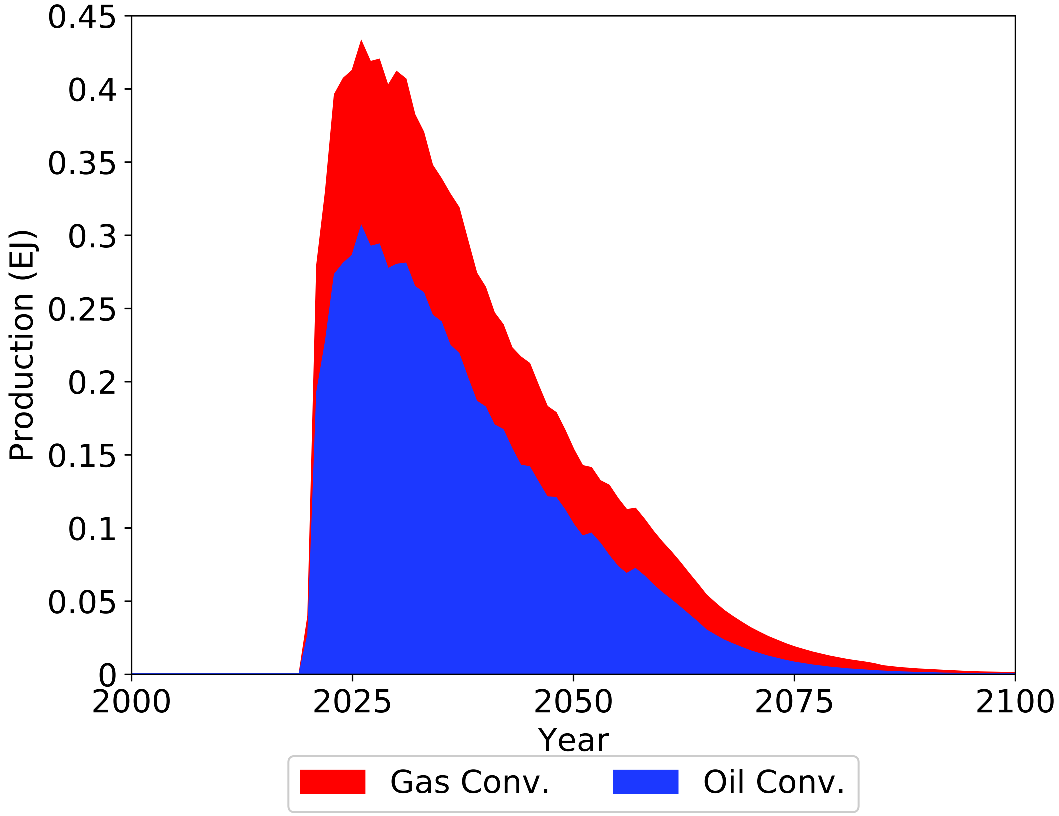

Figure 1.61: Sao Tome and Principe projections capped at 16

Table 1.61: Peak years - All

Name	URR	Peak Year	Peak Rate
Oil Conv.	7.58	2026	0.31
Gas Conv.	3.71	2030	0.13
**Total**	**11.29**	**2026**	**0.43**

1.31.2 By Mineral

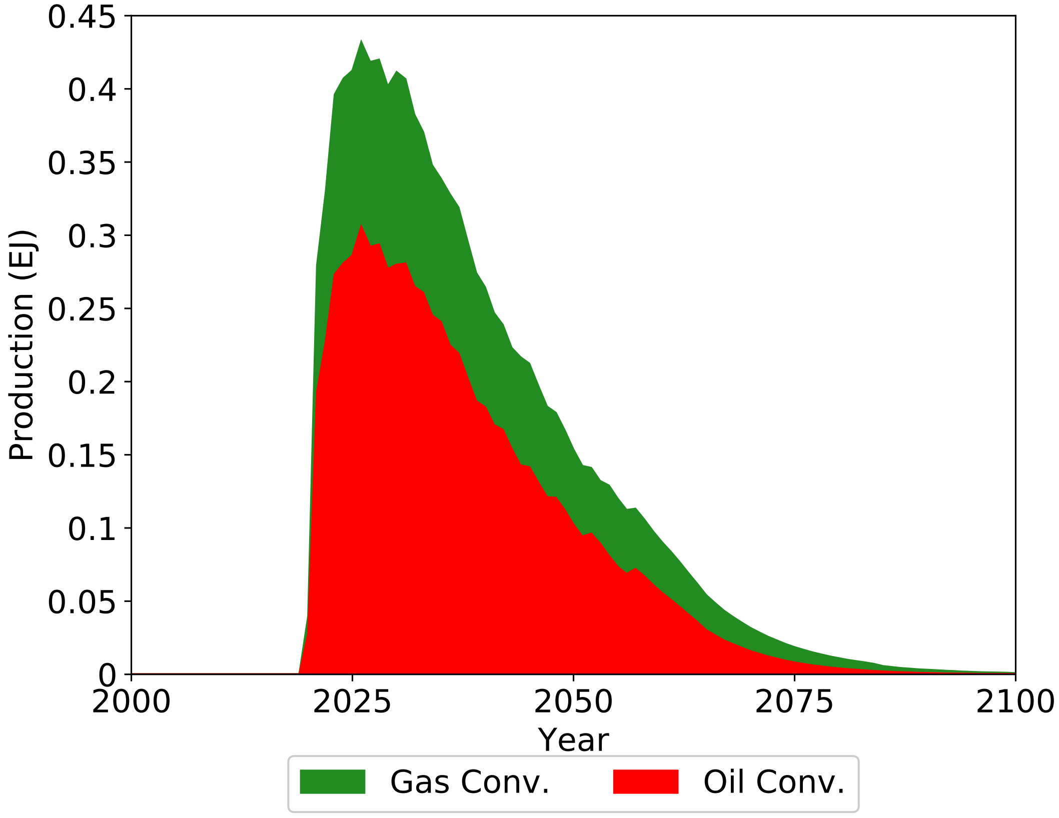

Figure 1.62: Sao Tome and Principe projection by mineral type

Table 1.62: Peak years - Minerals

Name	URR	Peak Year	Peak Rate
Oil Conv.	7.58	2026	0.31
Gas Conv.	3.71	2030	0.13
**Total**	**11.29**	**2026**	**0.43**

1.32 Senegal

1.32.1 All Projections

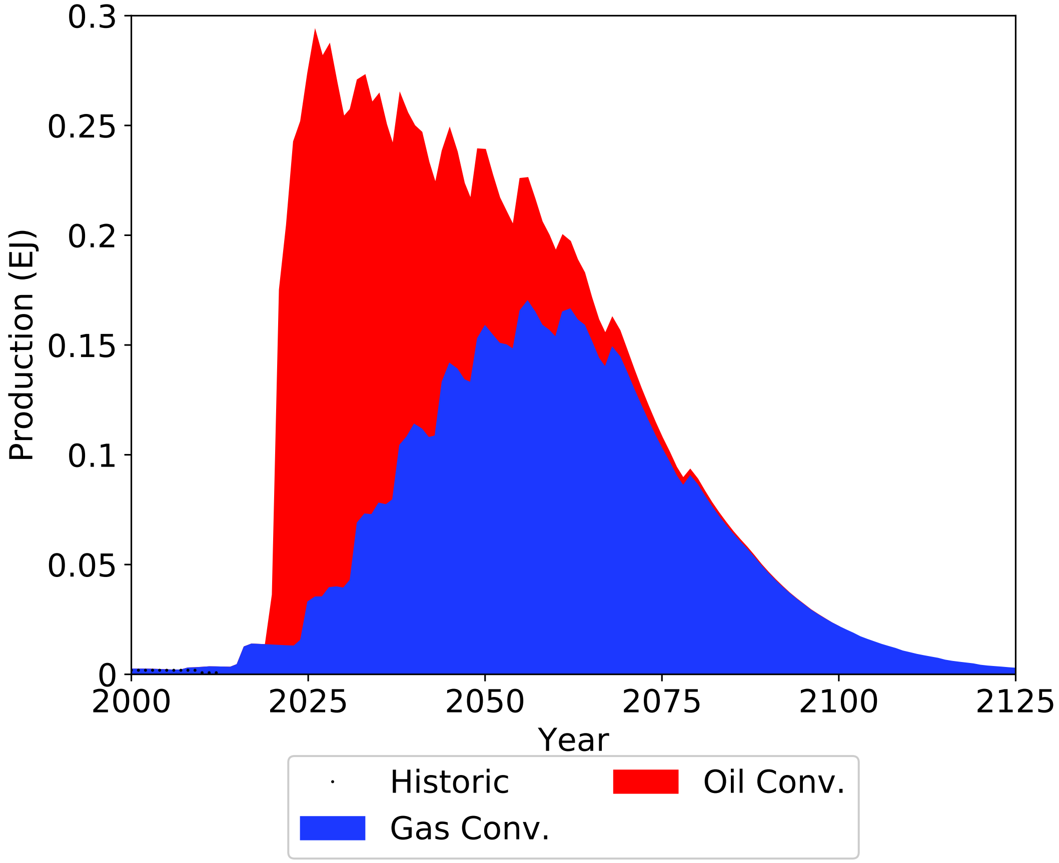

Figure 1.63: Senegal projections capped at 16

Table 1.63: Peak years - All

Name	URR	Peak Year	Peak Rate
Gas Conv.	7.82	2056	0.17
Oil Conv.	5.9	2026	0.26
**Total**	**13.72**	**2026**	**0.29**

1.32.2 By Mineral

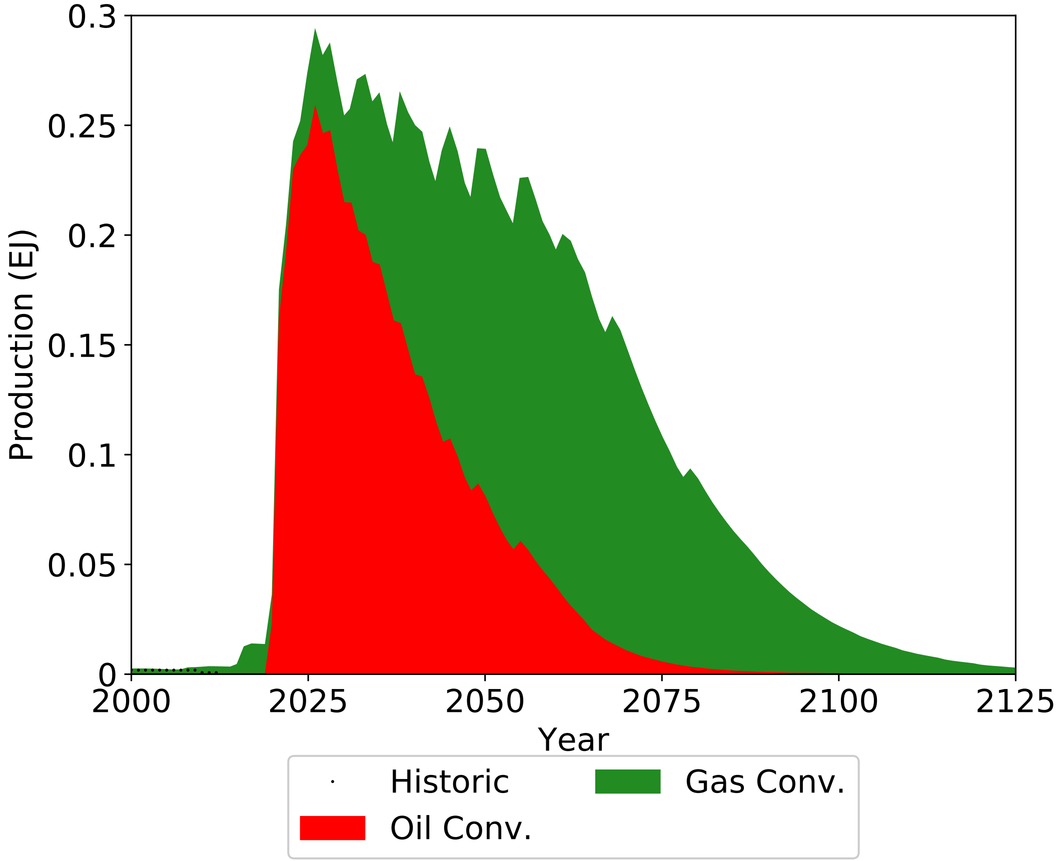

Figure 1.64: Senegal projection by mineral type

Table 1.64: Peak years - Minerals

Name	URR	Peak Year	Peak Rate
Oil Conv.	5.9	2026	0.26
Gas Conv.	7.82	2056	0.17
**Total**	**13.72**	**2026**	**0.29**

1.33 Seychelles

1.33.1 All Projections

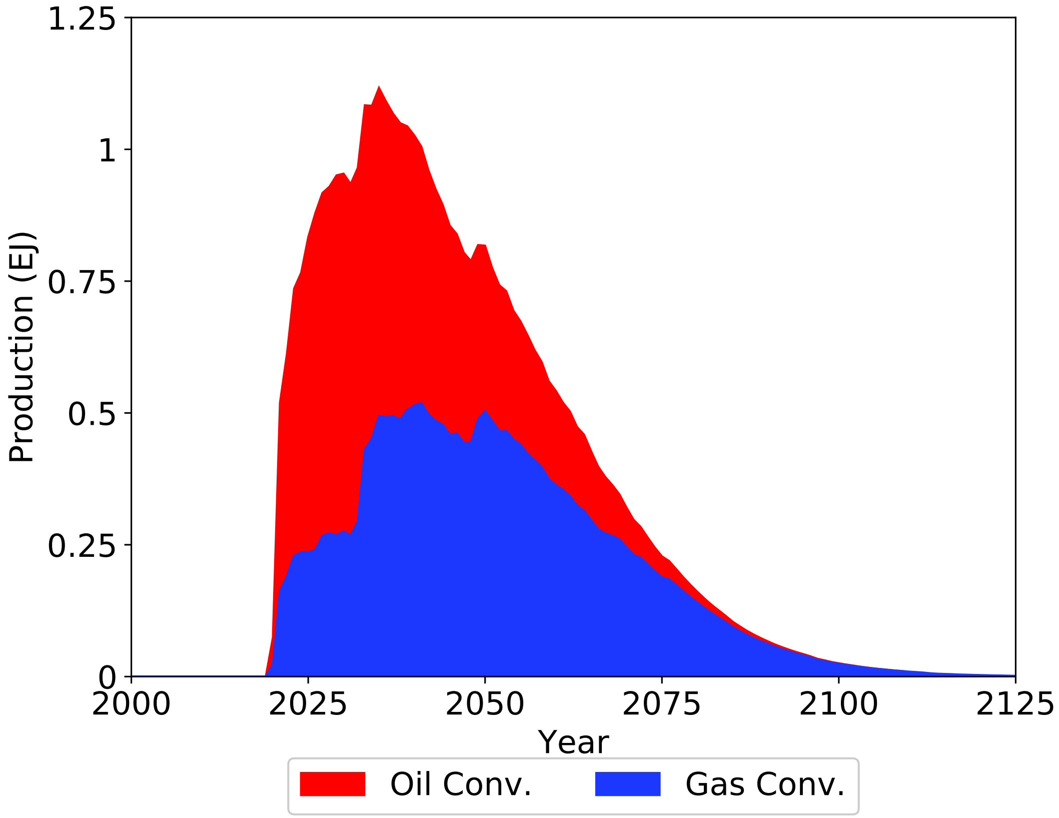

Figure 1.65: Seychelles projections capped at 16

Table 1.65: Peak years - All

Name	URR	Peak Year	Peak Rate
Gas Conv.	22.25	2041	0.52
Oil Conv.	19.8	2029	0.68
**Total**	**42.05**	**2035**	**1.12**

1.33.2 By Mineral

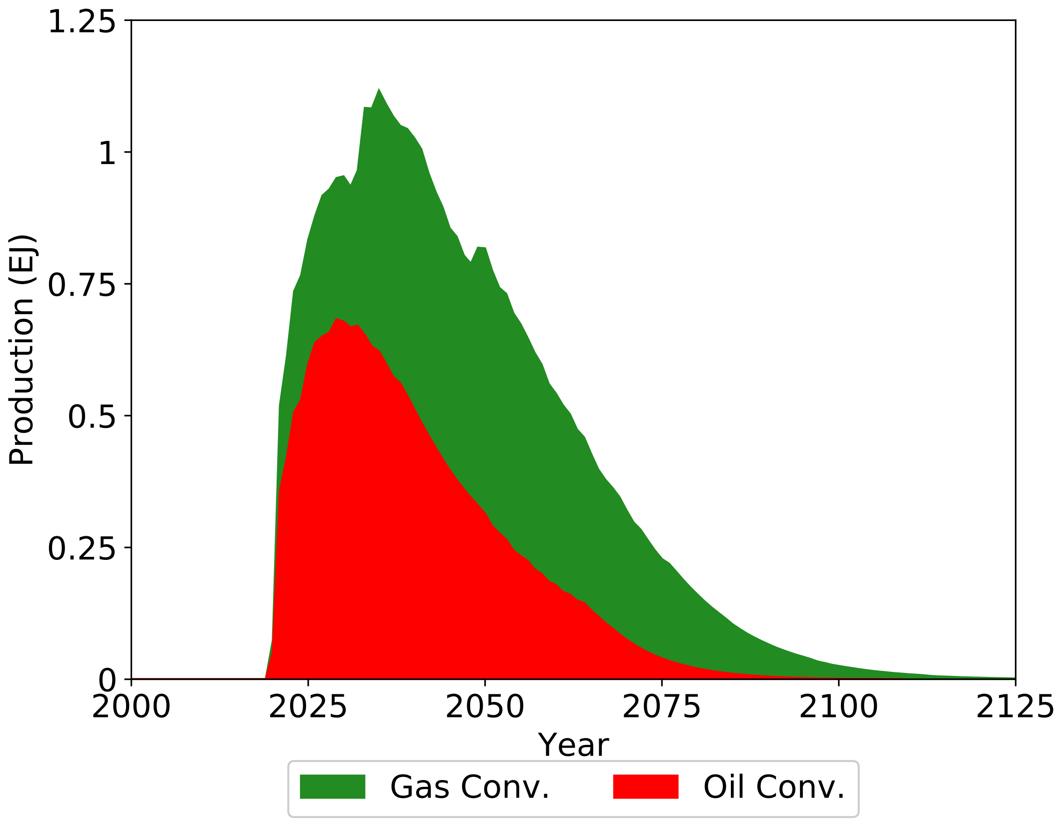

Figure 1.66: Seychelles projection by mineral type

Table 1.66: Peak years - Minerals

**Name**	**URR**	**Peak Year**	**Peak Rate**
Oil Conv.	19.8	2029	0.68
Gas Conv.	22.25	2041	0.52
**Total**	**42.05**	**2035**	**1.12**

1.34 Sierra Leone

1.34.1 All Projections

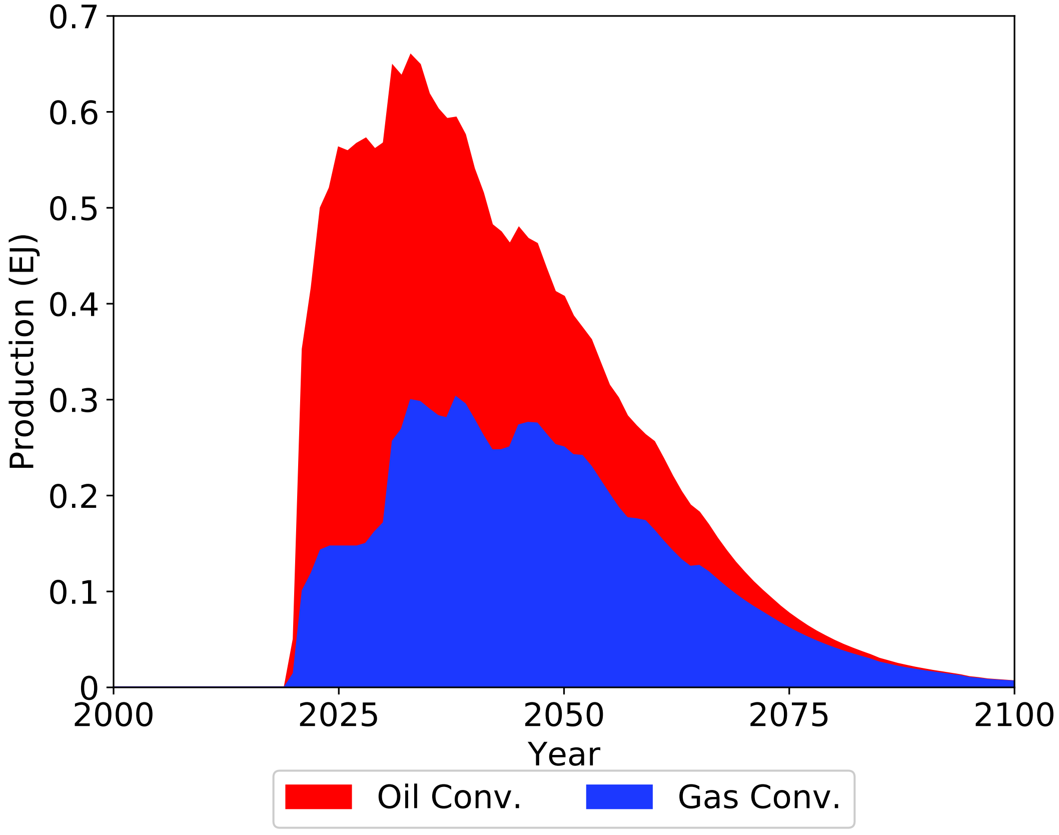

Figure 1.67: Sierra Leone projections capped at 16

Table 1.67: Peak years - All

Name	URR	Peak Year	Peak Rate
Gas Conv.	11.12	2038	0.3
Oil Conv.	10.95	2028	0.42
**Total**	**22.07**	**2033**	**0.66**

1.34.2 By Mineral

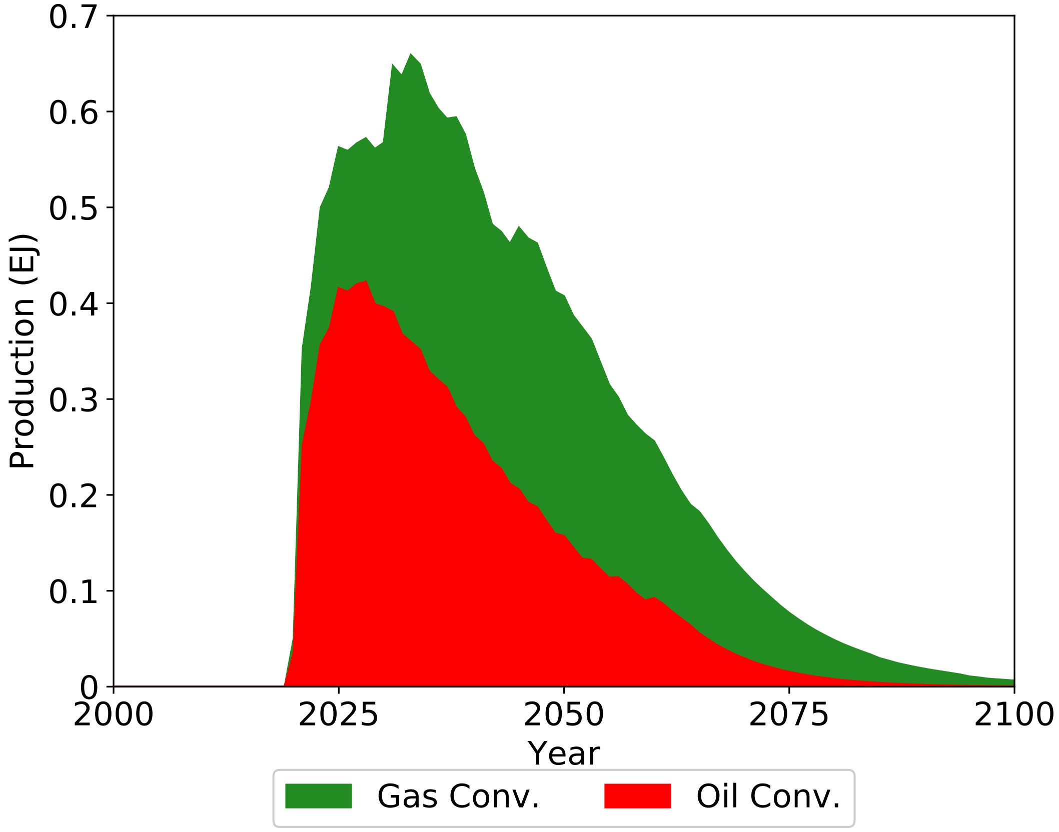

Figure 1.68: Sierra Leone projection by mineral type

Table 1.68: Peak years - Minerals

Name	URR	Peak Year	Peak Rate
Oil Conv.	10.95	2028	0.42
Gas Conv.	11.12	2038	0.3
**Total**	**22.07**	**2033**	**0.66**

1.35 Somalia

1.35.1 All Projections

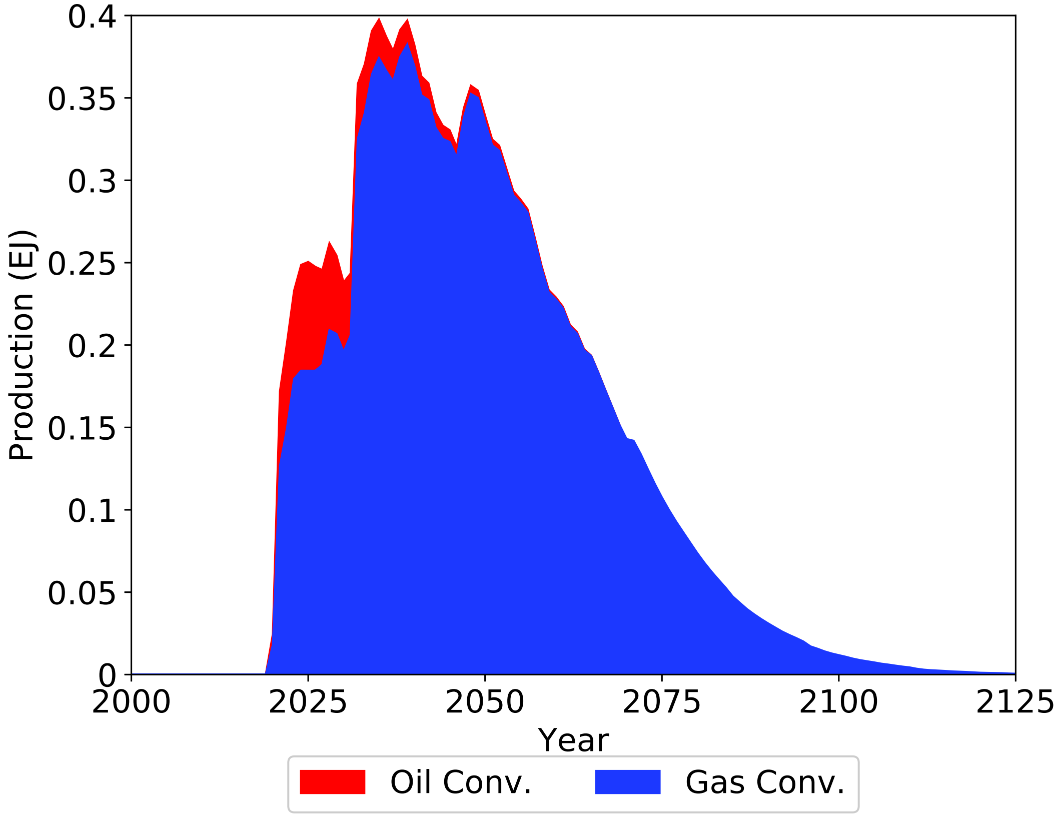

Figure 1.69: Somalia projections capped at 16

Table 1.69: Peak years - All

**Name**	**URR**	**Peak Year**	**Peak Rate**
Gas Conv.	15.1	2039	0.38
Oil Conv.	0.88	2025	0.07
**Total**	**15.98**	**2035**	**0.4**

1.35.2 By Mineral

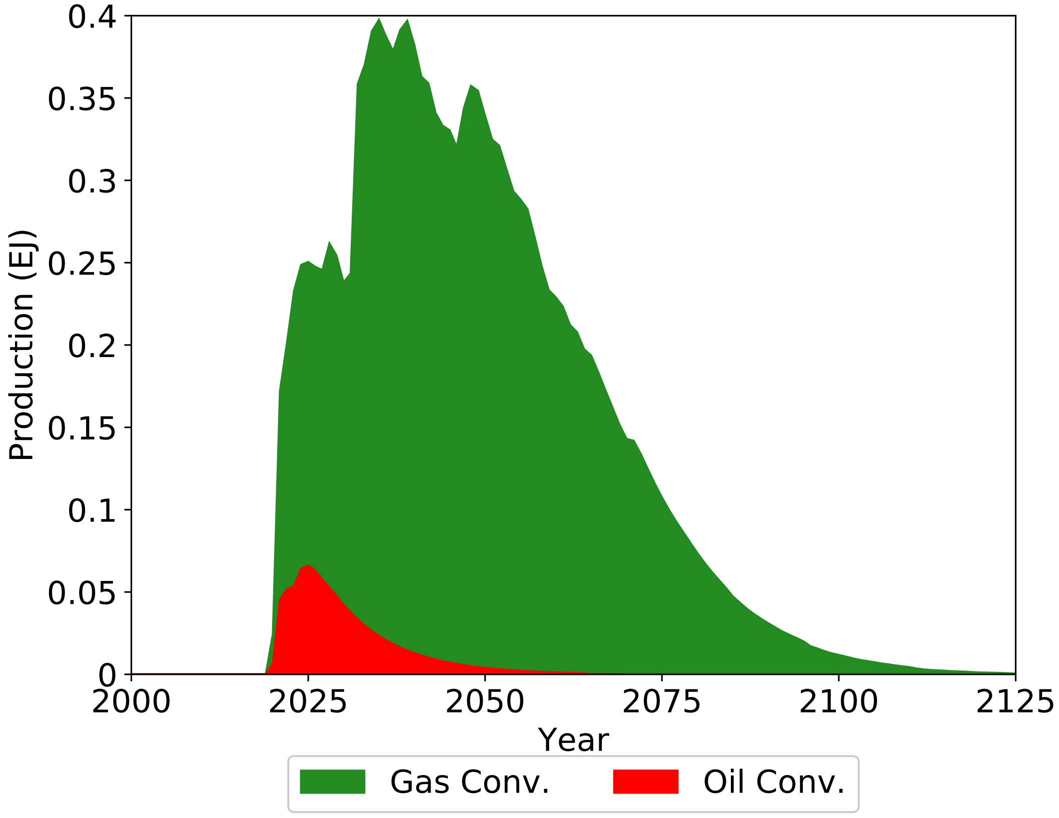

Figure 1.70: Somalia projection by mineral type

Table 1.70: Peak years - Minerals

Name	URR	Peak Year	Peak Rate
Oil Conv.	0.88	2025	0.07
Gas Conv.	15.1	2039	0.38
**Total**	**15.98**	**2035**	**0.4**

1.36 South Africa

1.36.1 All Projections

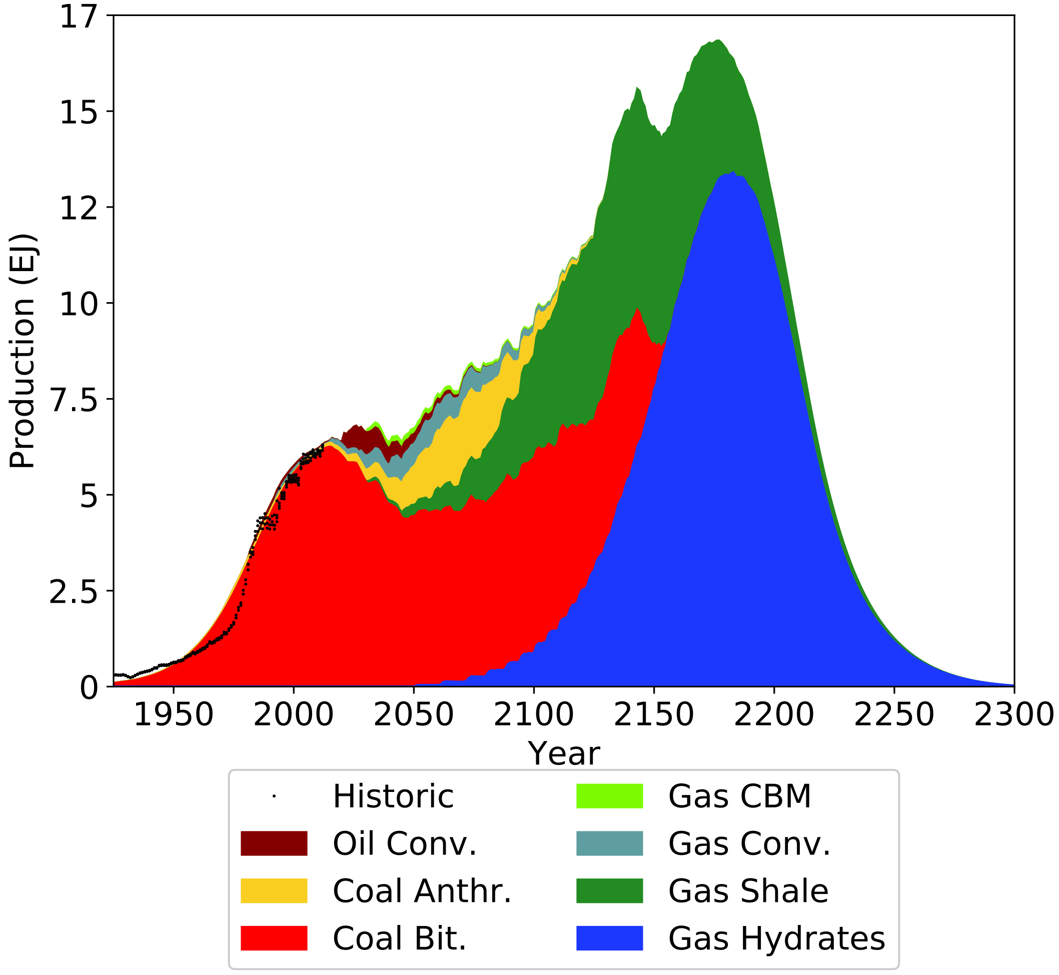

Figure 1.71: South Africa projections capped at 16

Table 1.71: Peak years - All

**Name**	**URR**	**Peak Year**	**Peak Rate**
Gas Hydrates	1043.0	2183	13.41
Coal Bit.	859.57	2015	6.26
Gas Shale	509.34	2144	5.79
Coal Anthr.	95.02	2070	1.8
Gas Conv.	38.9	2055	0.73
Oil Conv.	20.1	2028	0.61
Gas CBM	8.4	2044	0.16
**Total**	**2574.33**	**2177**	**16.84**

1.36.2 By Mineral

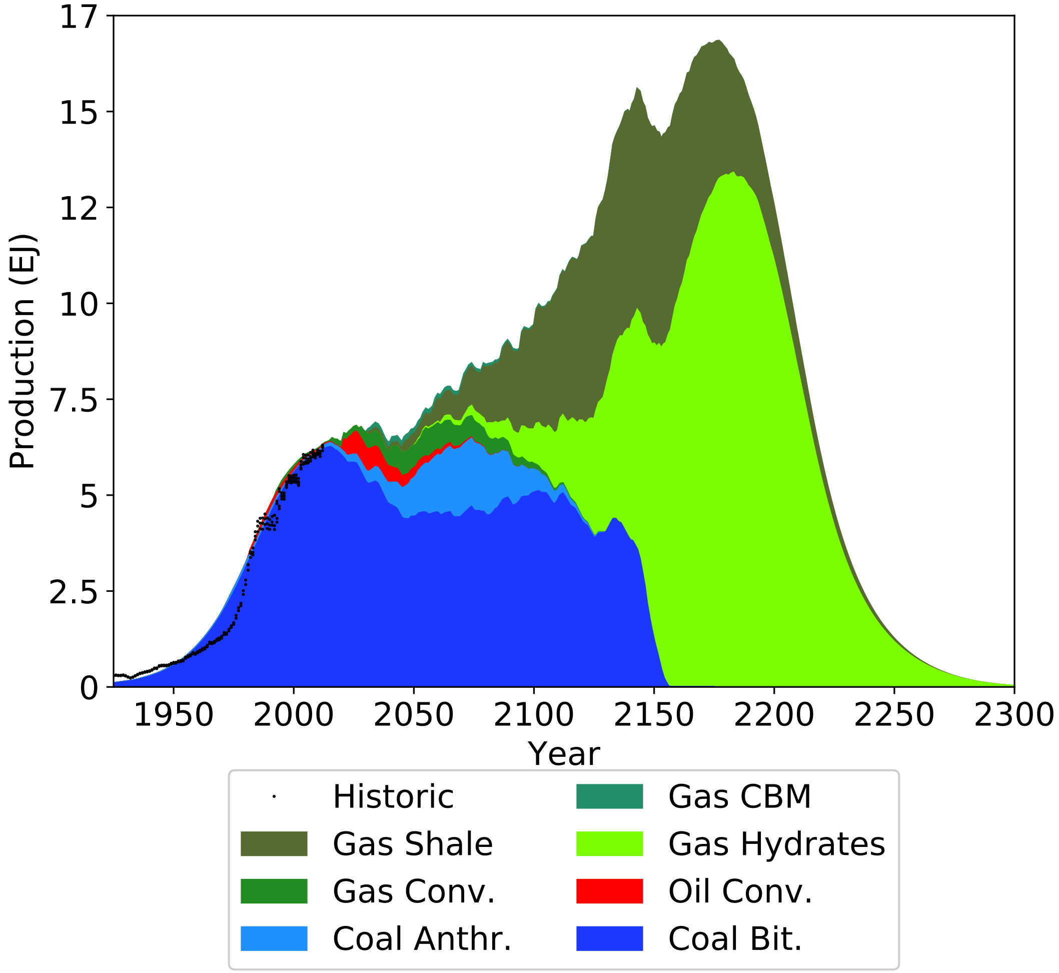

Figure 1.72: South Africa projection by mineral type

Table 1.72: Peak years - Minerals

**Name**	**URR**	**Peak Year**	**Peak Rate**
Coal Bit.	859.57	2015	6.26
Coal Anthr.	95.02	2070	1.8
Oil Conv.	20.1	2028	0.61
Gas Conv.	38.9	2055	0.73
Gas Hydrates	1043.0	2183	13.41
Gas Shale	509.34	2144	5.79
Gas CBM	8.4	2044	0.16
**Total**	**2574.33**	**2177**	**16.84**

1.37 Sudan

1.37.1 All Projections

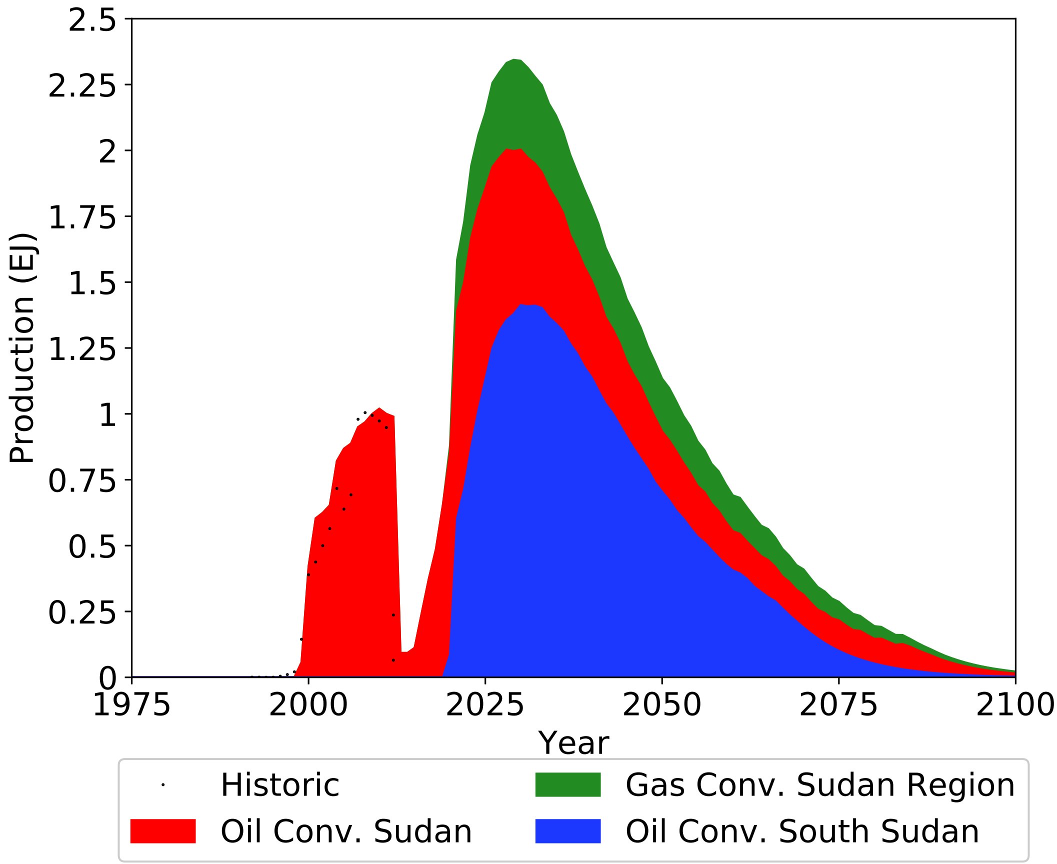

Figure 1.73: Sudan projections capped at 16

Table 1.73: Peak years - All

Name	URR	Peak Year	Peak Rate
Oil Conv. South Sudan	42.69	2030	1.41
Oil Conv. Sudan	33.66	2010	1.02
Gas Conv. Sudan Region	12.4	2029	0.35
**Total**	**88.75**	**2029**	**2.34**

1.37.2 By Mineral

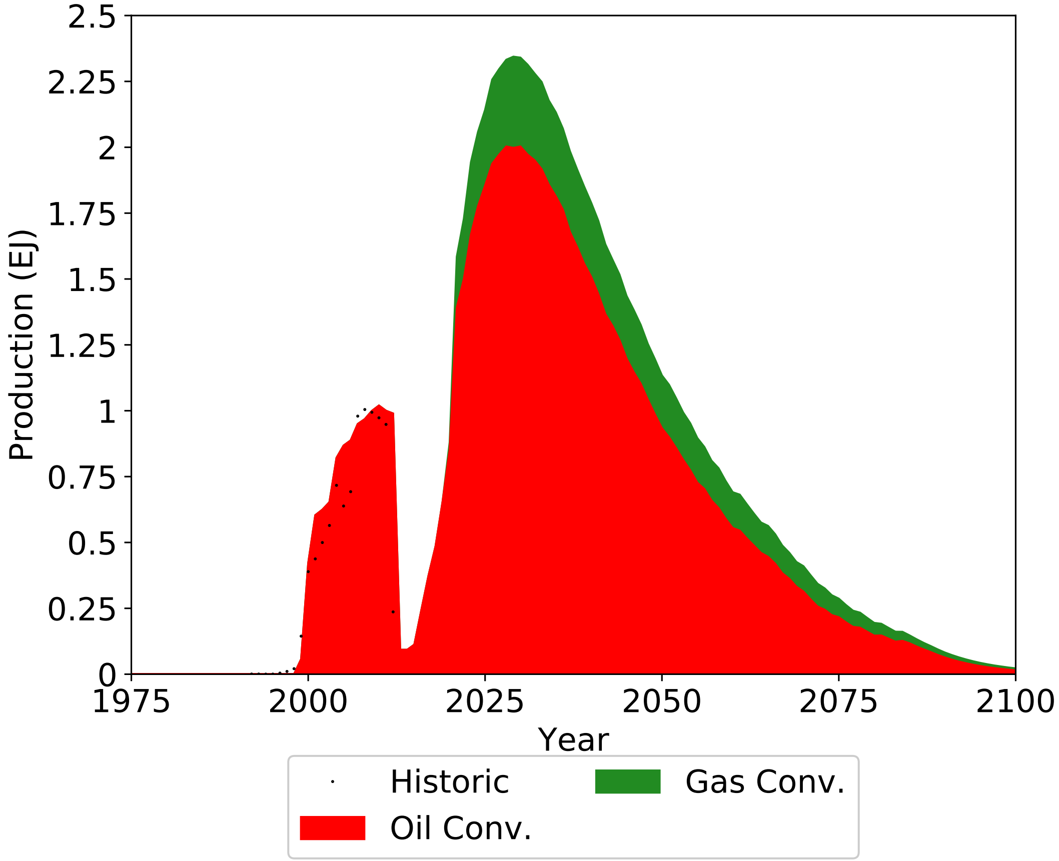

Figure 1.74: Sudan projection by mineral type

Table 1.74: Peak years - Minerals

Name	URR	Peak Year	Peak Rate
Oil Conv.	76.35	2028	2.0
Gas Conv.	12.4	2029	0.35
**Total**	**88.75**	**2029**	**2.34**

1.37.3 Regional Projections

South Sudan

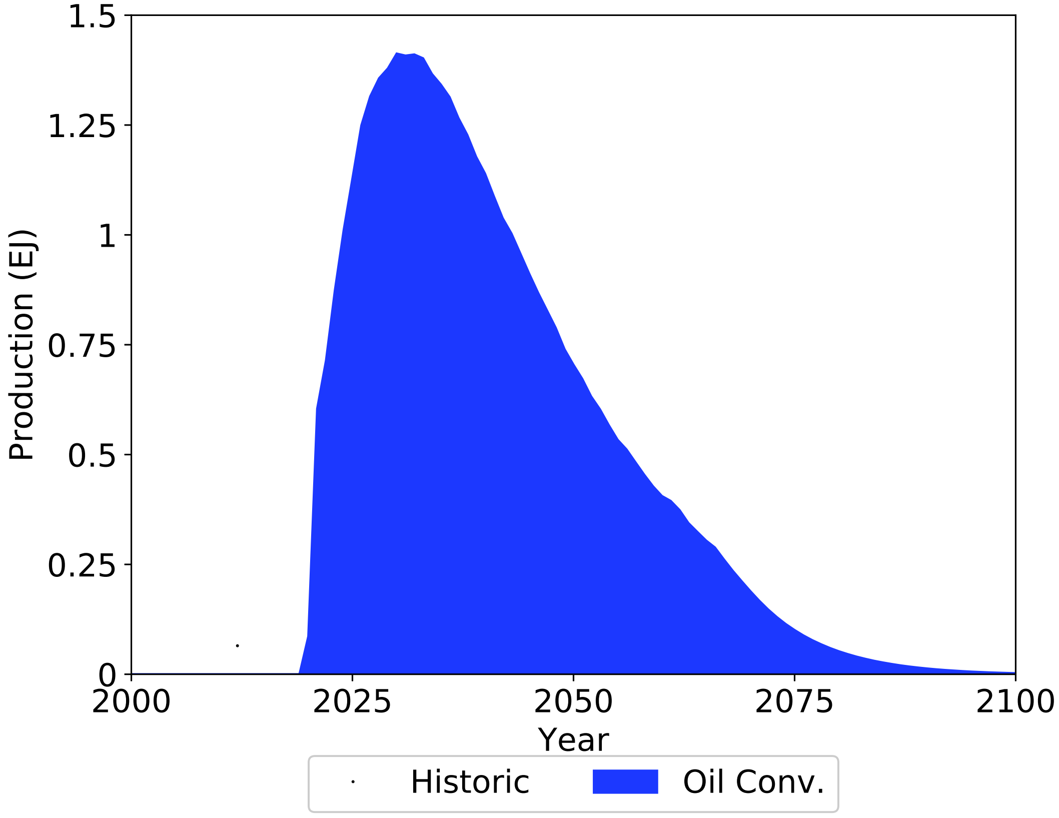

Figure 1.75: Sudan - South Sudan projections capped at 16

Table 1.75: Peak years - All

Name	URR	Peak Year	Peak Rate
Oil Conv. South Sudan	42.69	2030	1.41
**Total**	**42.69**	**2030**	**1.41**

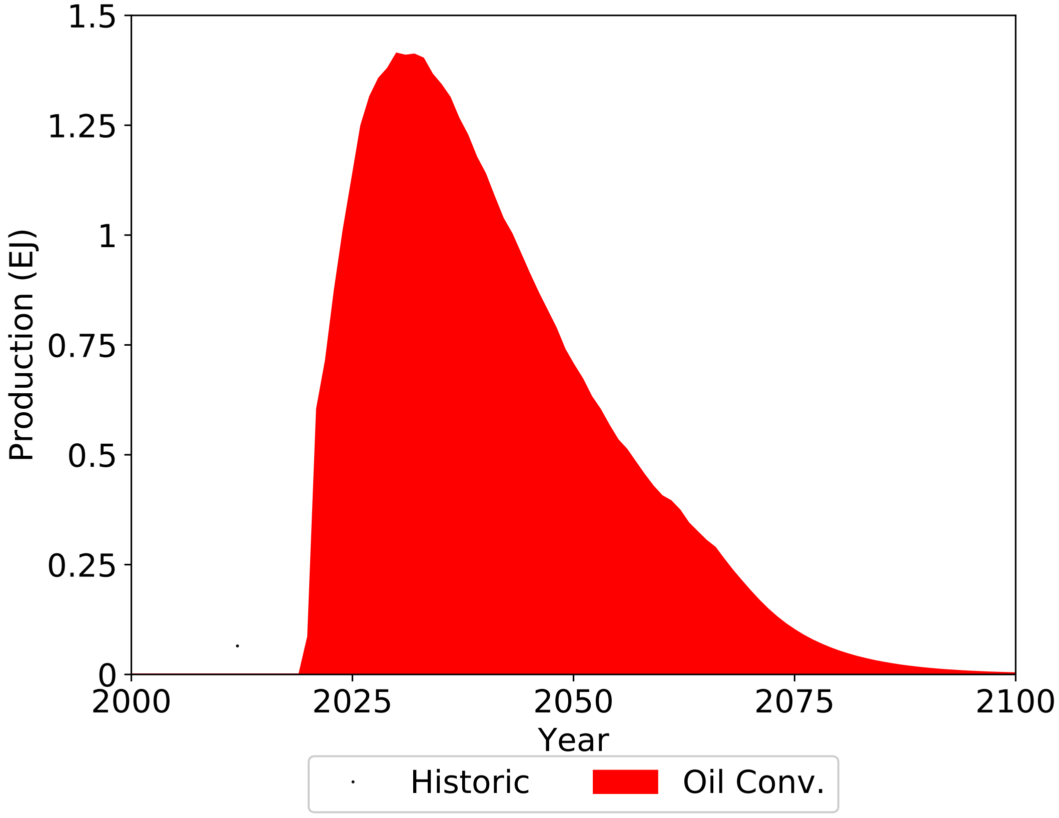

Figure 1.76: Sudan - South Sudan projection by mineral type

Table 1.76: Peak years - Minerals

Name	URR	Peak Year	Peak Rate
Oil Conv.	42.69	2030	1.41
**Total**	**42.69**	**2030**	**1.41**

Sudan

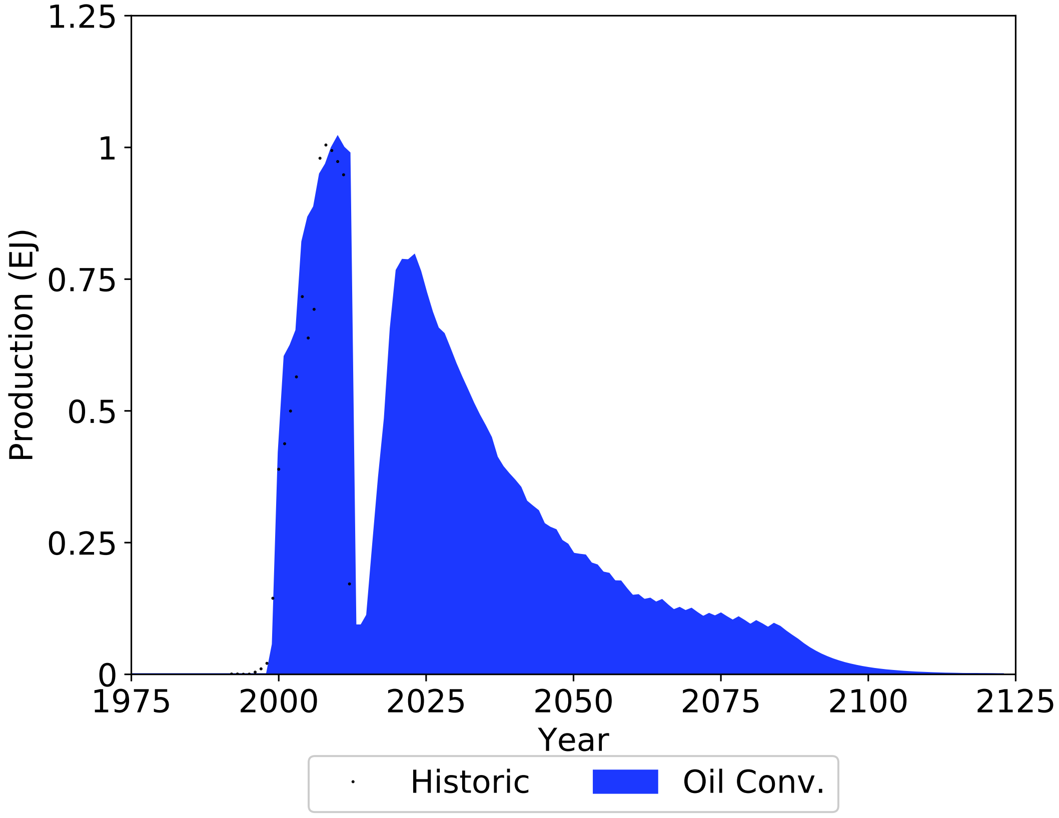

Figure 1.77: Sudan - Sudan projections capped at 16

Table 1.77: Peak years - All

Name	URR	Peak Year	Peak Rate
Oil Conv. Sudan	33.66	2010	1.02
**Total**	**33.66**	**2010**	**1.02**

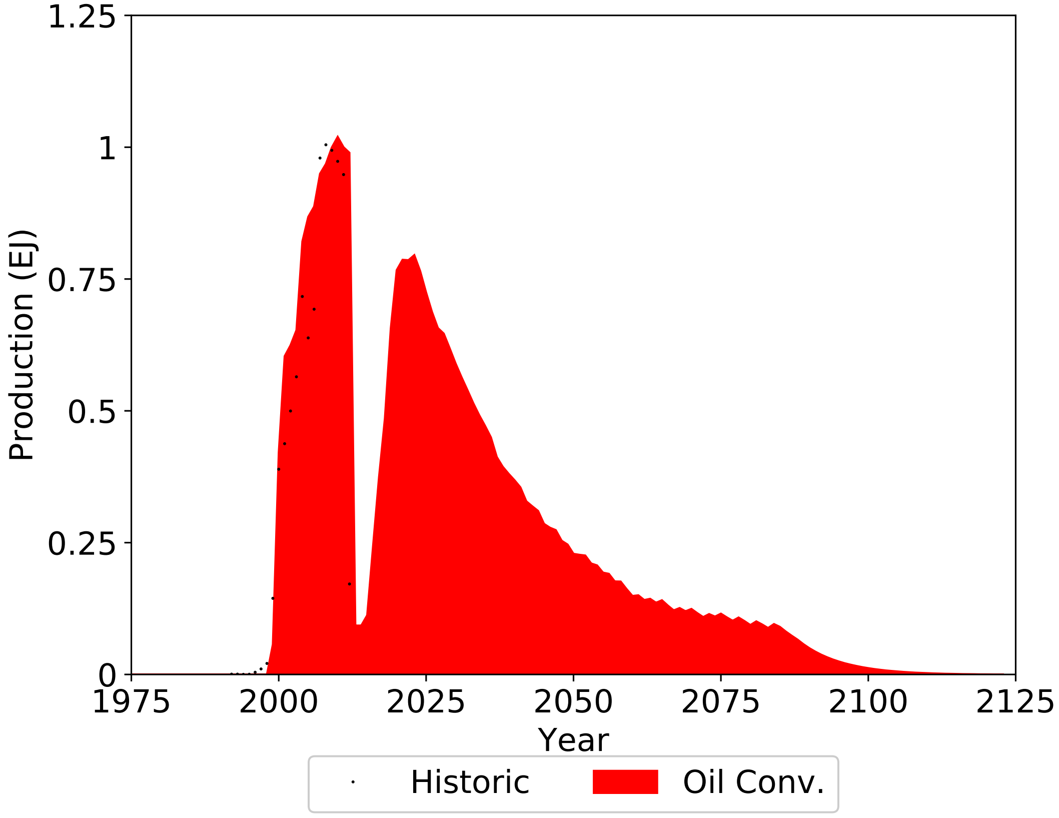

Figure 1.78: Sudan - Sudan projection by mineral type

Table 1.78: Peak years - Minerals

Name	URR	Peak Year	Peak Rate
Oil Conv.	33.66	2010	1.02
**Total**	**33.66**	**2010**	**1.02**

Sudan Region

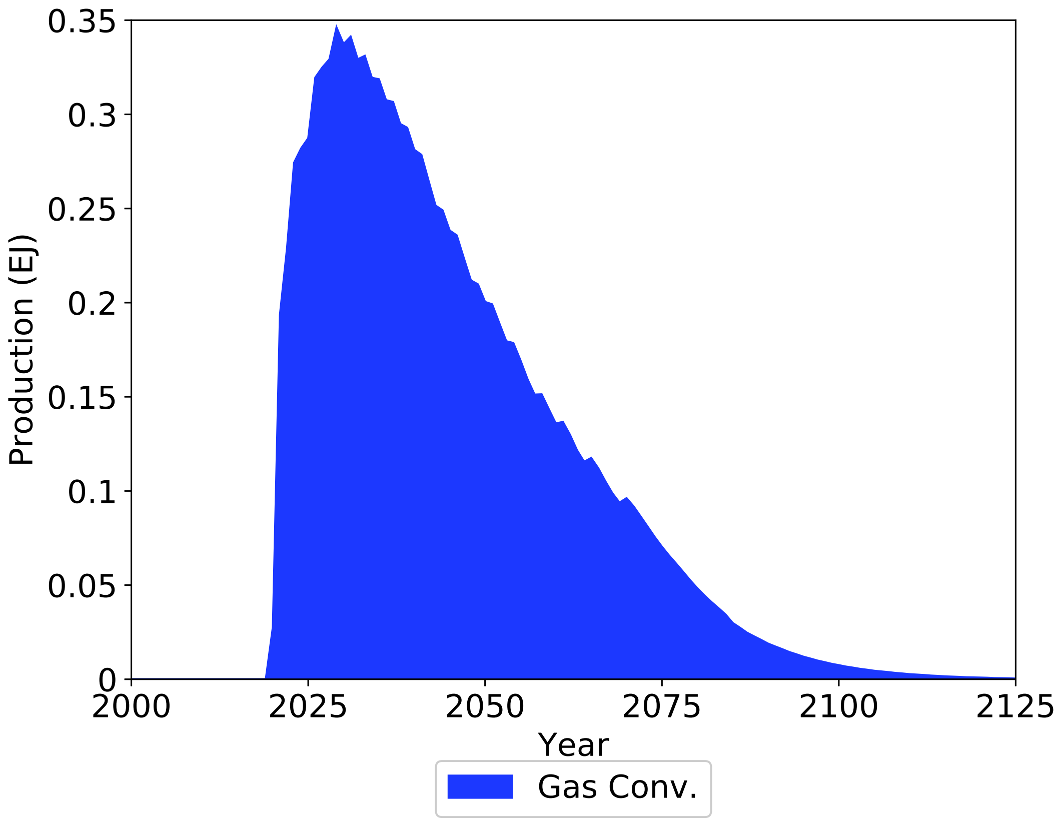

Figure 1.79: Sudan - Sudan Region projections capped at 16

Table 1.79: Peak years - All

Name	URR	Peak Year	Peak Rate
Gas Conv. Sudan Region	12.4	2029	0.35
**Total**	**12.4**	**2029**	**0.35**

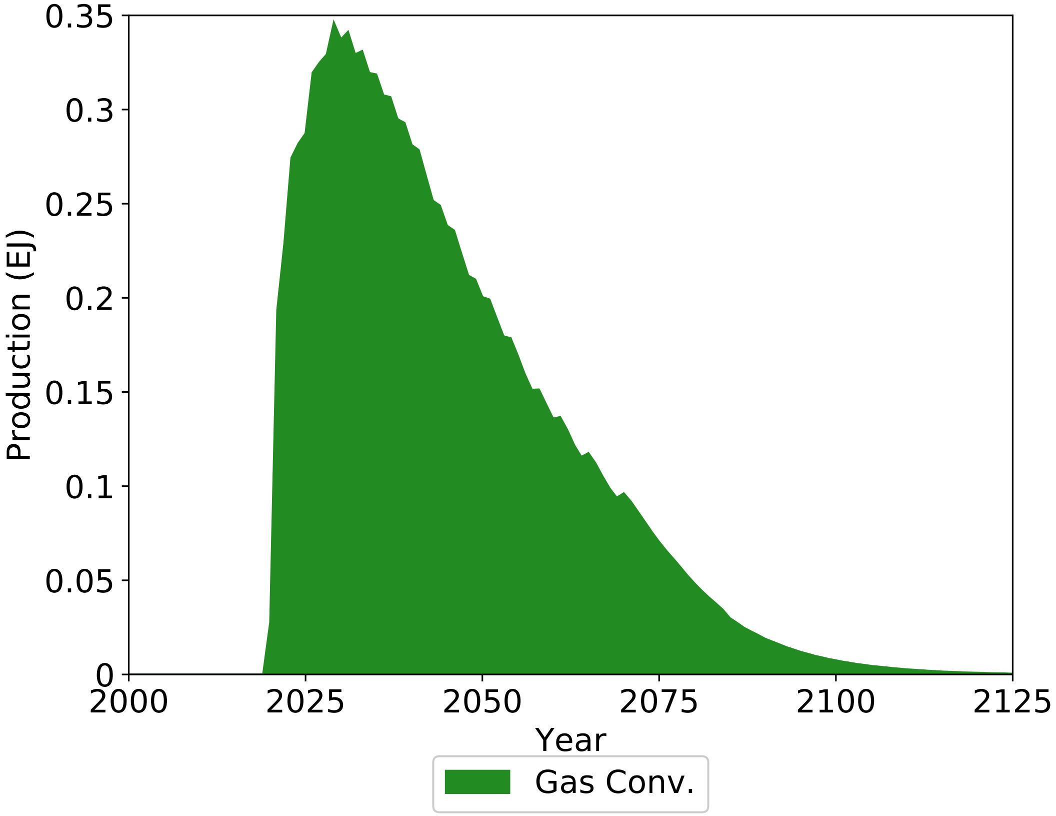

Figure 1.80: Sudan - Sudan Region projection by mineral type

Table 1.80: Peak years - Minerals

Name	URR	Peak Year	Peak Rate
Gas Conv.	12.4	2029	0.35
**Total**	**12.4**	**2029**	**0.35**

1.37.4 Projection by region

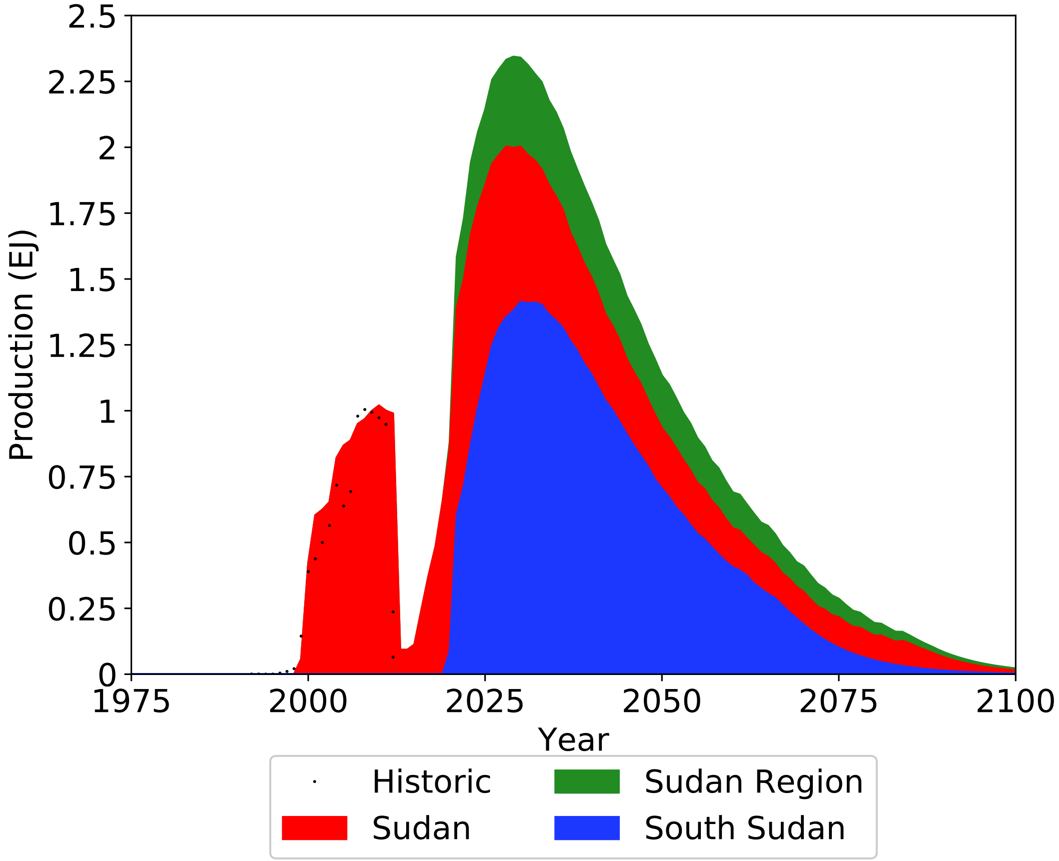

Figure 1.81: Sudan by region projections capped at 16

Table 1.81: Peak years - All

**Name**	**URR**	**Peak Year**	**Peak Rate**
South Sudan	42.69	2030	1.41
Sudan	33.66	2010	1.02
Sudan Region	12.4	2029	0.35
**Total**	**88.75**	**2029**	**2.34**

1.38 Swaziland

1.38.1 All Projections

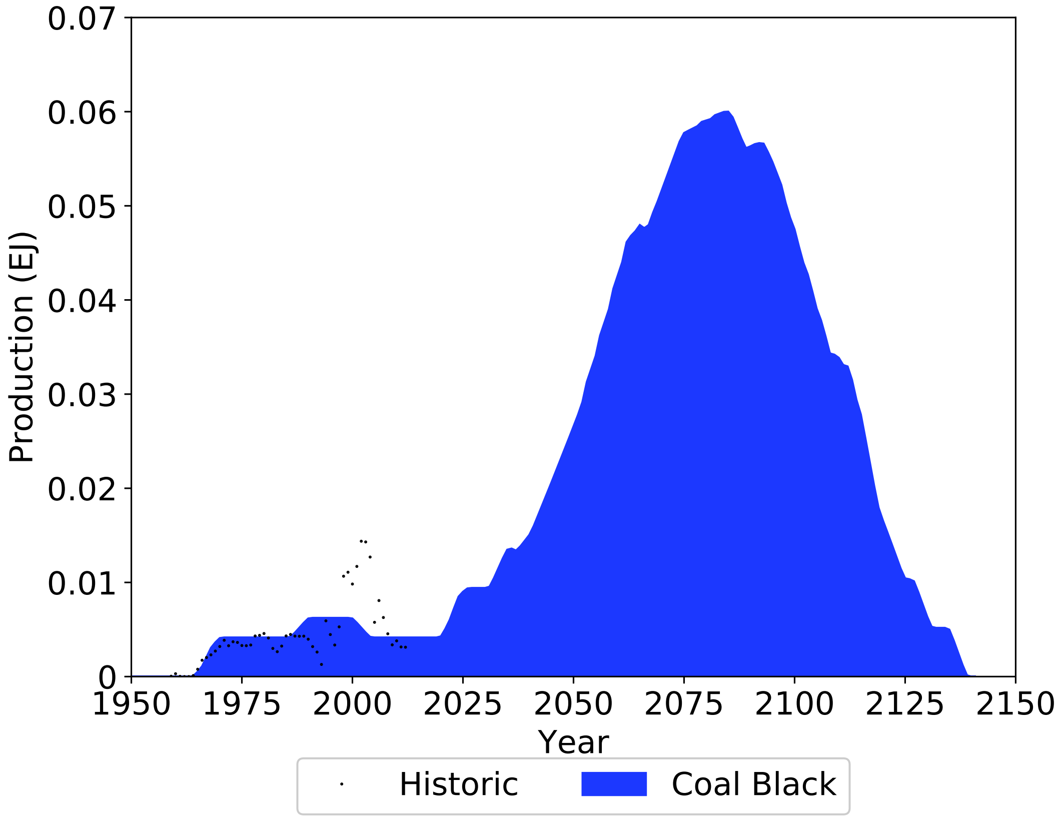

Figure 1.82: Swaziland projections capped at 16

Table 1.82: Peak years - All

**Name**	**URR**	**Peak Year**	**Peak Rate**
Coal Black	3.98	2085	0.06
**Total**	**3.98**	**2085**	**0.06**

1.38.2 By Mineral

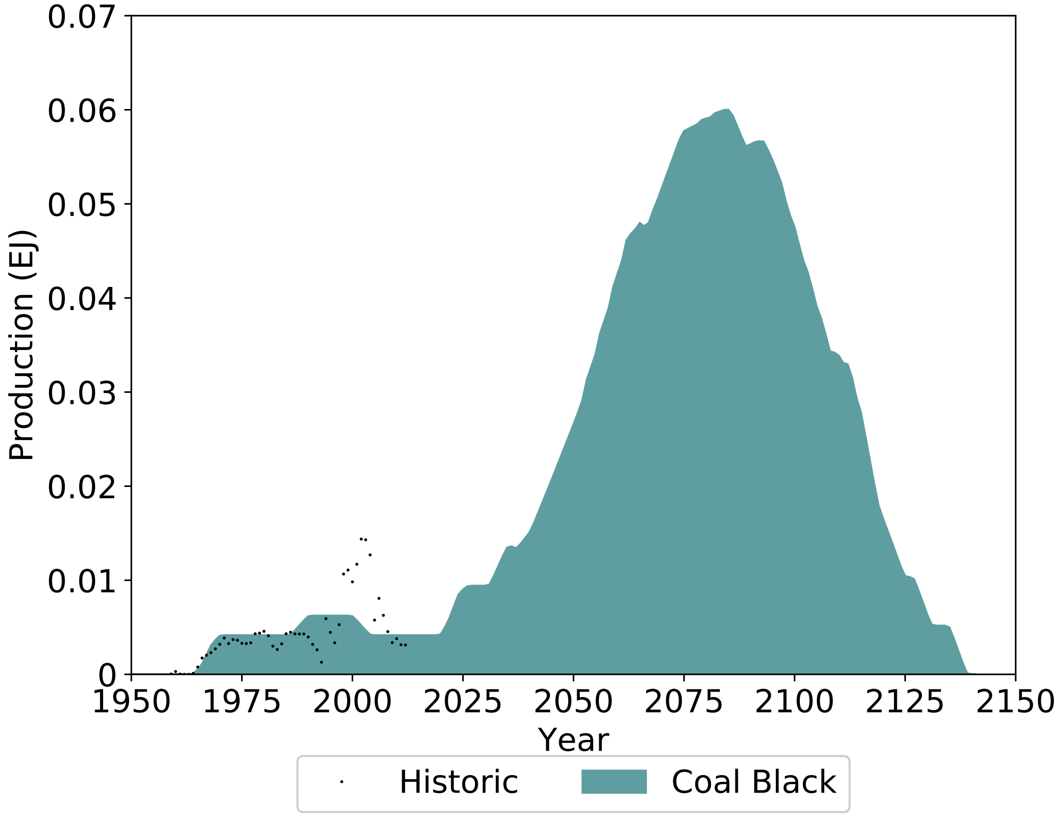

Figure 1.83: Swaziland projection by mineral type

Table 1.83: Peak years - Minerals

Name	URR	Peak Year	Peak Rate
Coal Black	3.98	2085	0.06
**Total**	**3.98**	**2085**	**0.06**

1.39 Tanzania

1.39.1 All Projections

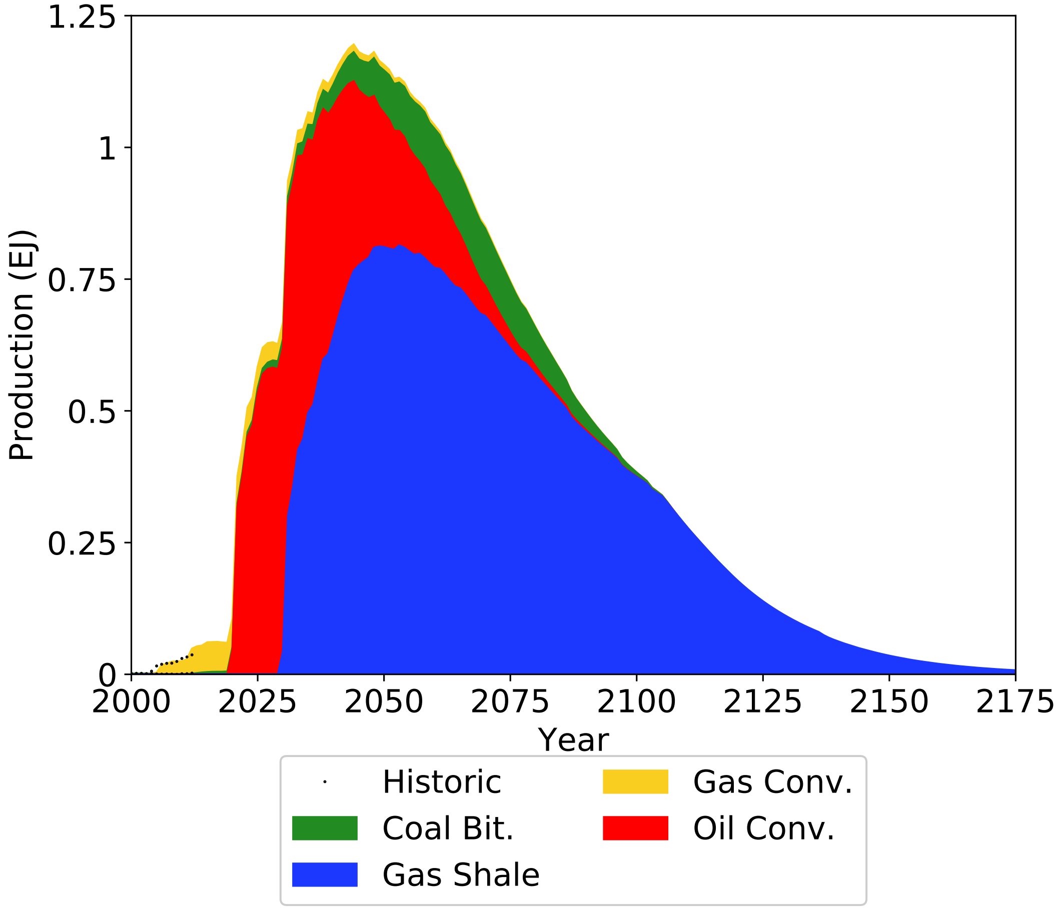

Figure 1.84: Tanzania projections capped at 16

Table 1.84: Peak years - All

**Name**	**URR**	**Peak Year**	**Peak Rate**
Gas Shale	51.91	2053	0.81
Oil Conv.	16.85	2031	0.59
Coal Bit.	4.83	2064	0.12
Gas Conv.	1.57	2017	0.06
**Total**	**75.16**	**2044**	**1.2**

1.39.2 By Mineral

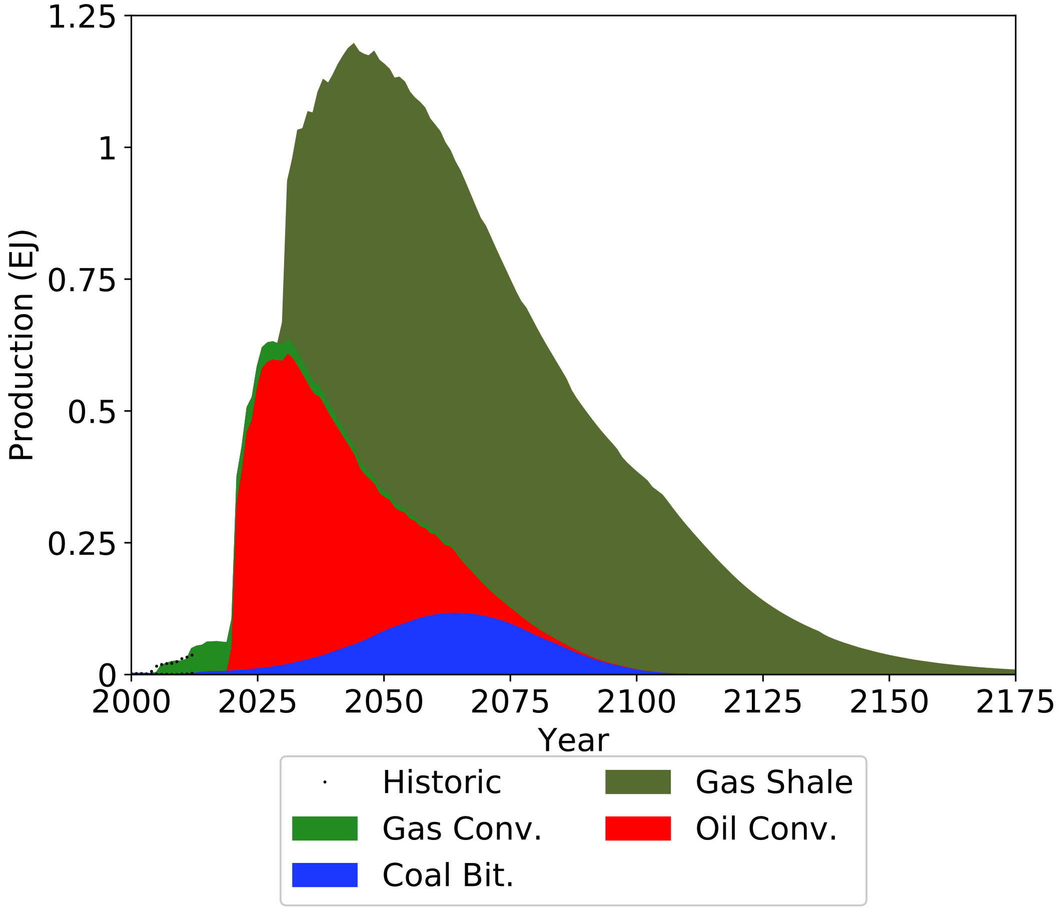

Figure 1.85: Tanzania projection by mineral type

Table 1.85: Peak years - Minerals

Name	URR	Peak Year	Peak Rate
Coal Bit.	4.83	2064	0.12
Oil Conv.	16.85	2031	0.59
Gas Conv.	1.57	2017	0.06
Gas Shale	51.91	2053	0.81
**Total**	**75.16**	**2044**	**1.2**

1.40 Togo

1.40.1 All Projections

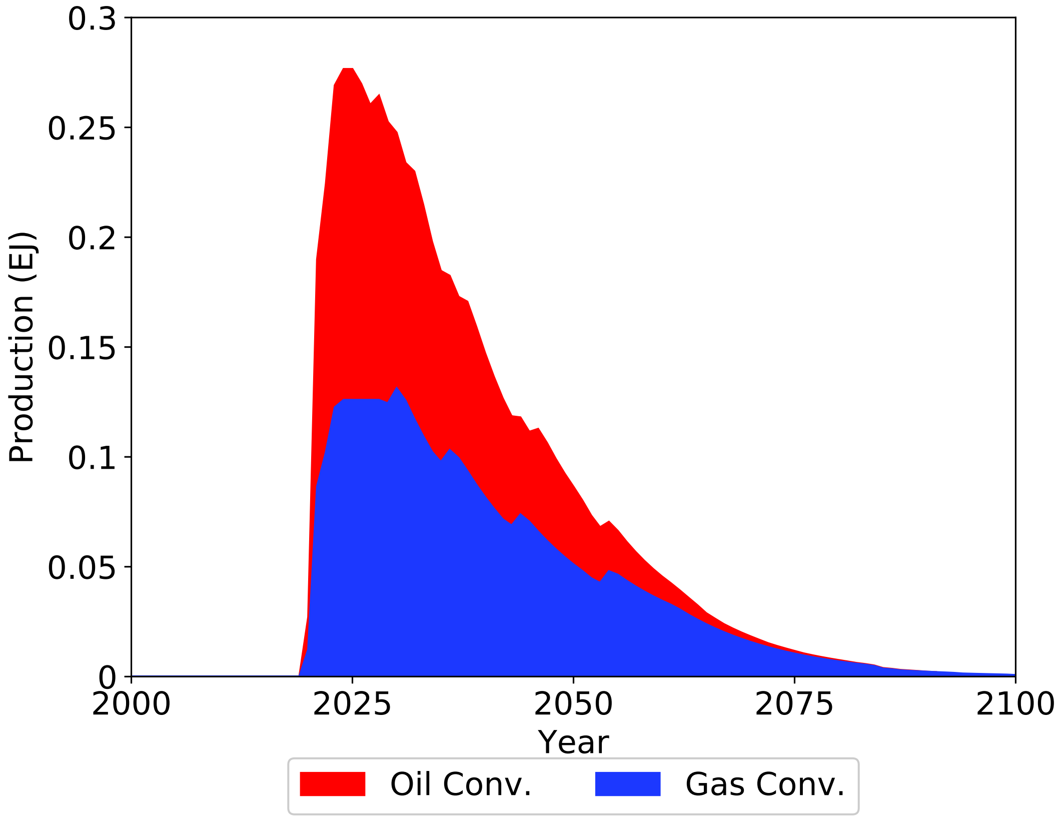

Figure 1.86: Togo projections capped at 16

Table 1.86: Peak years - All

Name	URR	Peak Year	Peak Rate
Gas Conv.	3.7	2030	0.13
Oil Conv.	2.95	2024	0.15
**Total**	**6.65**	**2024**	**0.28**

1.40.2 By Mineral

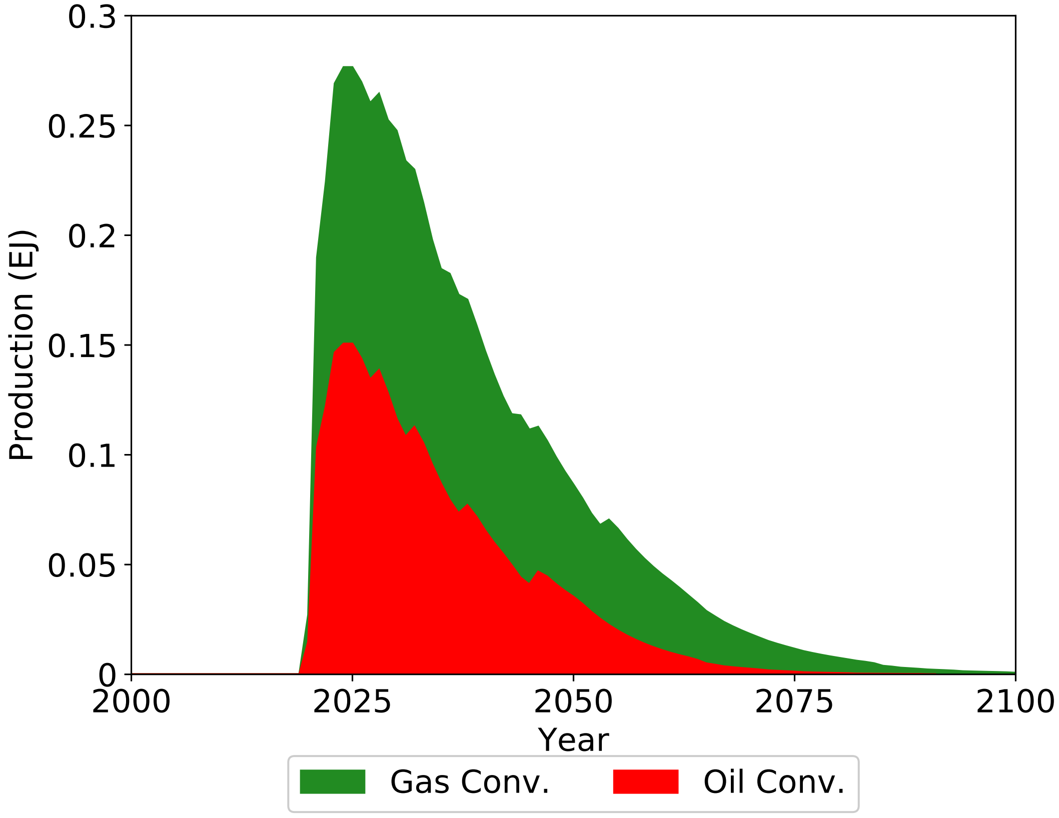

Figure 1.87: Togo projection by mineral type

Table 1.87: Peak years - Minerals

Name	URR	Peak Year	Peak Rate
Oil Conv.	2.95	2024	0.15
Gas Conv.	3.7	2030	0.13
**Total**	**6.65**	**2024**	**0.28**

1.41 Tunisia

1.41.1 All Projections

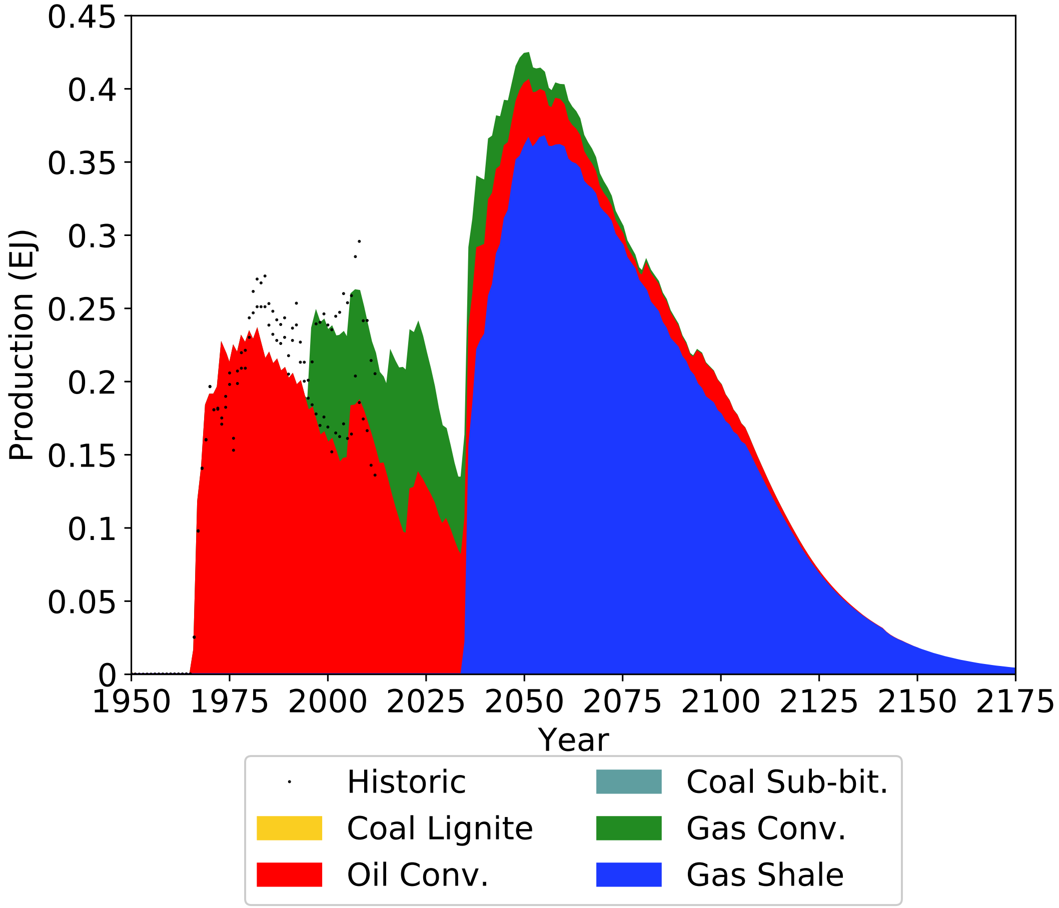

Figure 1.88: Tunisia projections capped at 16

Table 1.88: Peak years - All

**Name**	**URR**	**Peak Year**	**Peak Rate**
Gas Shale	22.62	2055	0.37
Oil Conv.	13.38	1982	0.23
Gas Conv.	4.0	2019	0.11
Coal Lignite	0.01	1943	–
Coal Sub-bit.	–	1919	–
**Total**	**40.01**	**2051**	**0.42**

1.41.2 By Mineral

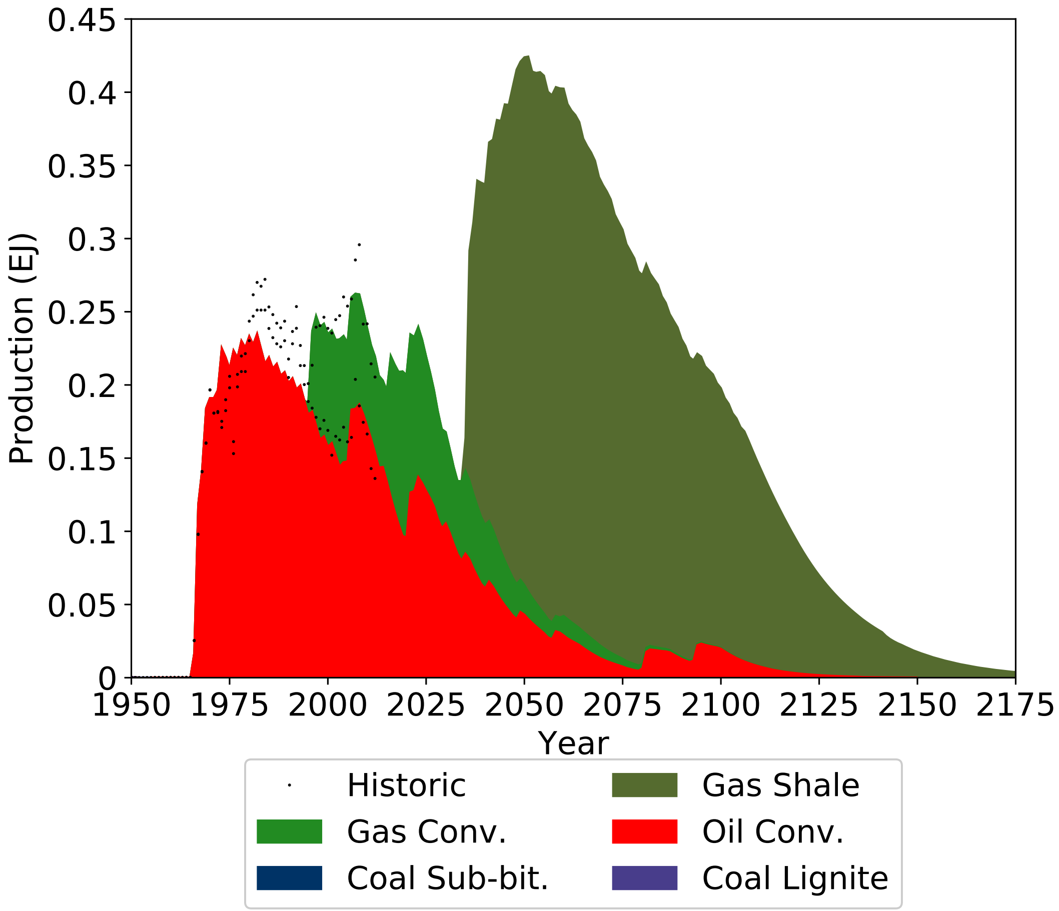

Figure 1.89: Tunisia projection by mineral type

Table 1.89: Peak years - Minerals

**Name**	**URR**	**Peak Year**	**Peak Rate**
Coal Lignite	0.01	1943	–
Coal Sub-bit.	–	1919	–
Oil Conv.	13.38	1982	0.23
Gas Conv.	4.0	2019	0.11
Gas Shale	22.62	2055	0.37
**Total**	**40.01**	**2051**	**0.42**

1.42 Uganda

1.42.1 All Projections

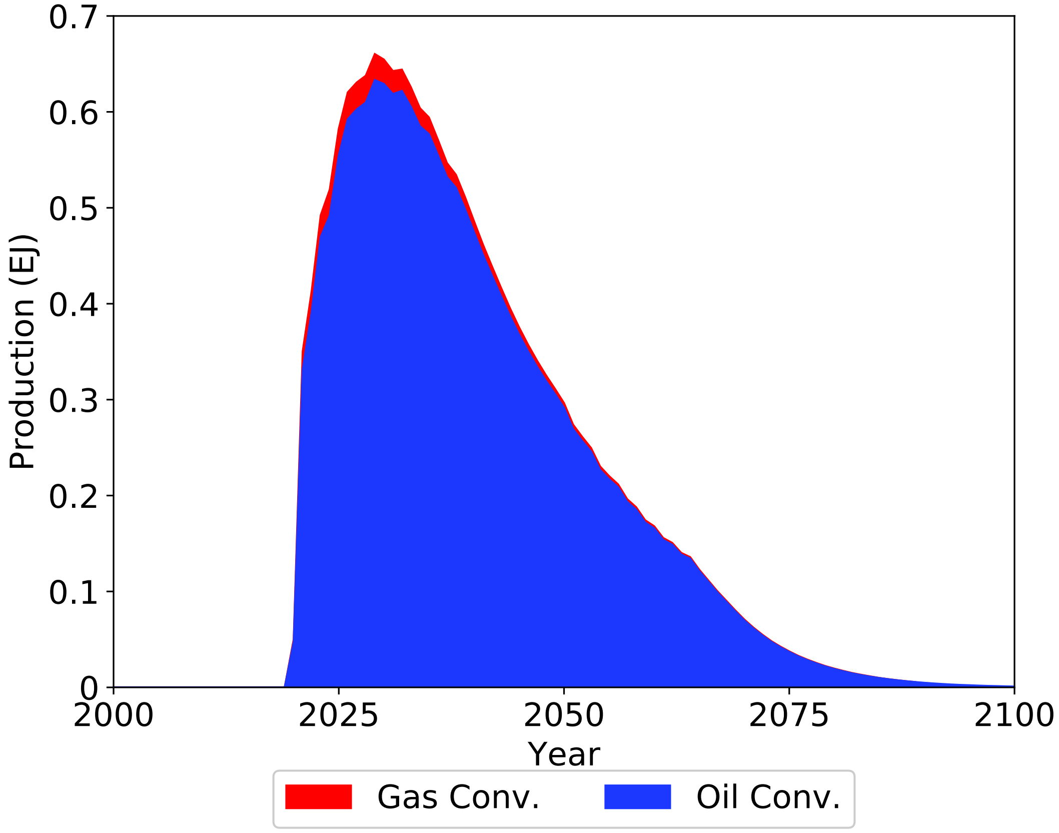

Figure 1.90: Uganda projections capped at 16

Table 1.90: Peak years - All

Name	URR	Peak Year	Peak Rate
Oil Conv.	18.36	2029	0.63
Gas Conv.	0.56	2025	0.03
**Total**	**18.92**	**2029**	**0.66**

1.42.2 By Mineral

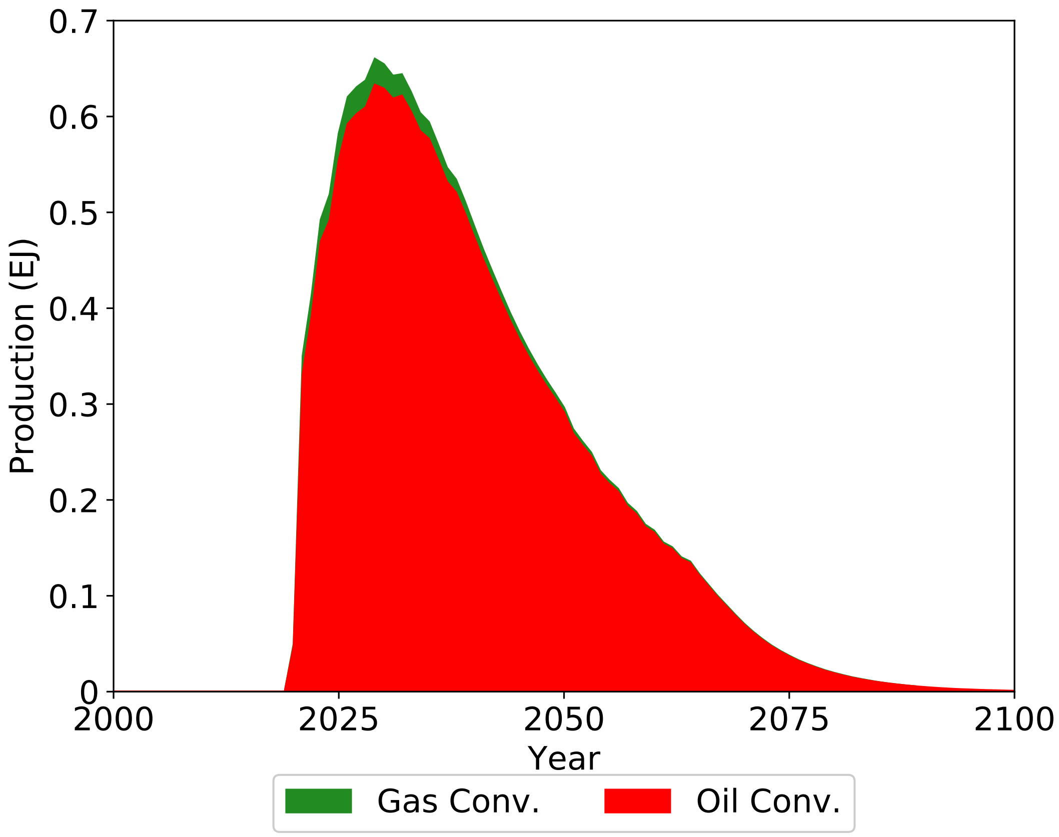

Figure 1.91: Uganda projection by mineral type

Table 1.91: Peak years - Minerals

Name	URR	Peak Year	Peak Rate
Oil Conv.	18.36	2029	0.63
Gas Conv.	0.56	2025	0.03
**Total**	**18.92**	**2029**	**0.66**

1.43 Western Sahara

1.43.1 All Projections

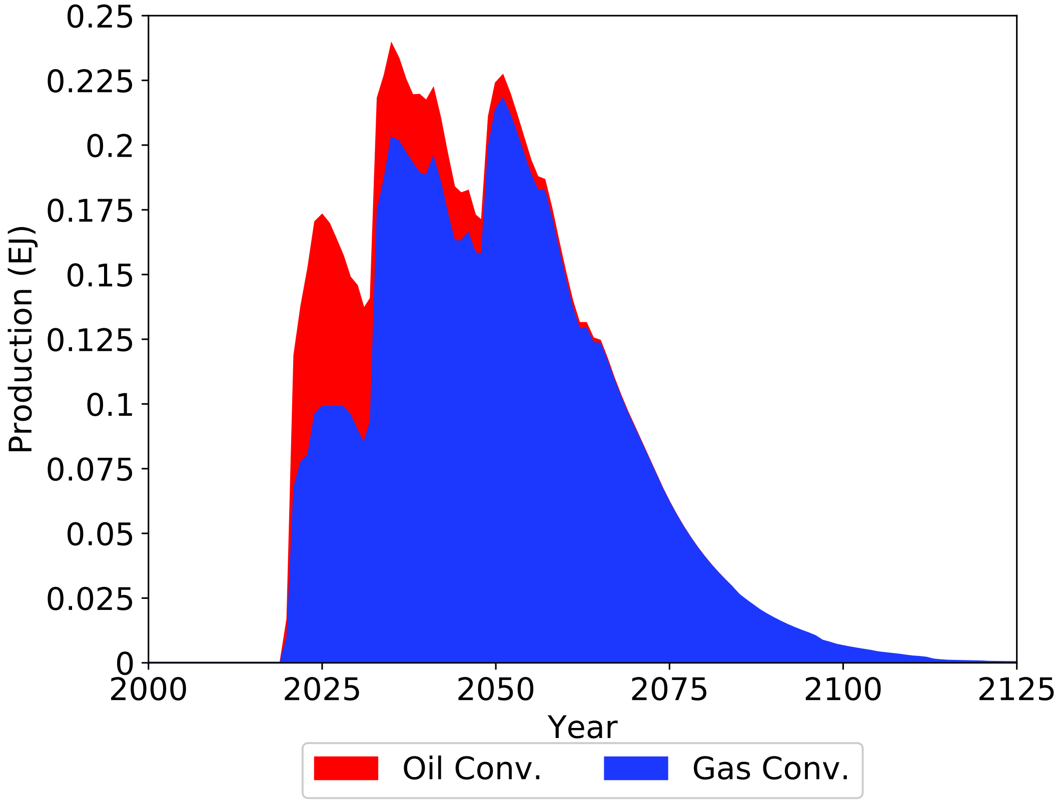

Figure 1.92: Western Sahara projections capped at 16

Table 1.92: Peak years - All

Name	URR	Peak Year	Peak Rate
Gas Conv.	8.45	2051	0.22
Oil Conv.	1.26	2024	0.07
**Total**	**9.71**	**2035**	**0.24**

1.43.2 By Mineral

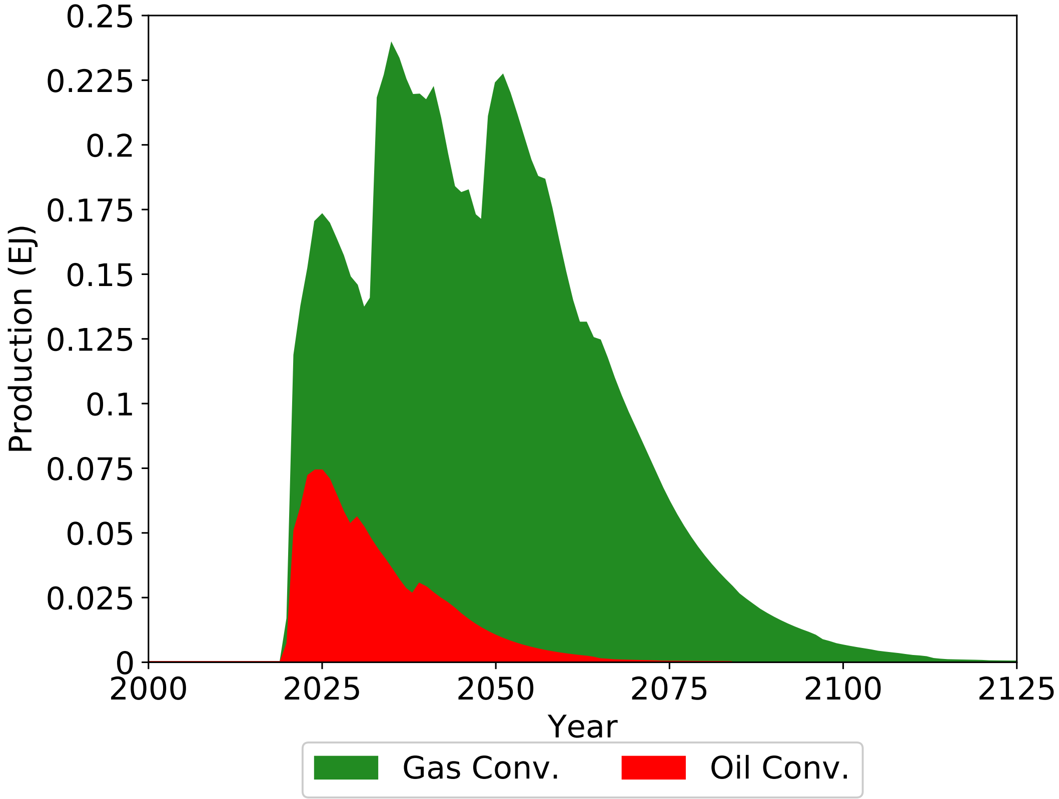

Figure 1.93: Western Sahara projection by mineral type

Table 1.93: Peak years - Minerals

Name	URR	Peak Year	Peak Rate
Oil Conv.	1.26	2024	0.07
Gas Conv.	8.45	2051	0.22
**Total**	**9.71**	**2035**	**0.24**

1.44 Zaire

1.44.1 All Projections

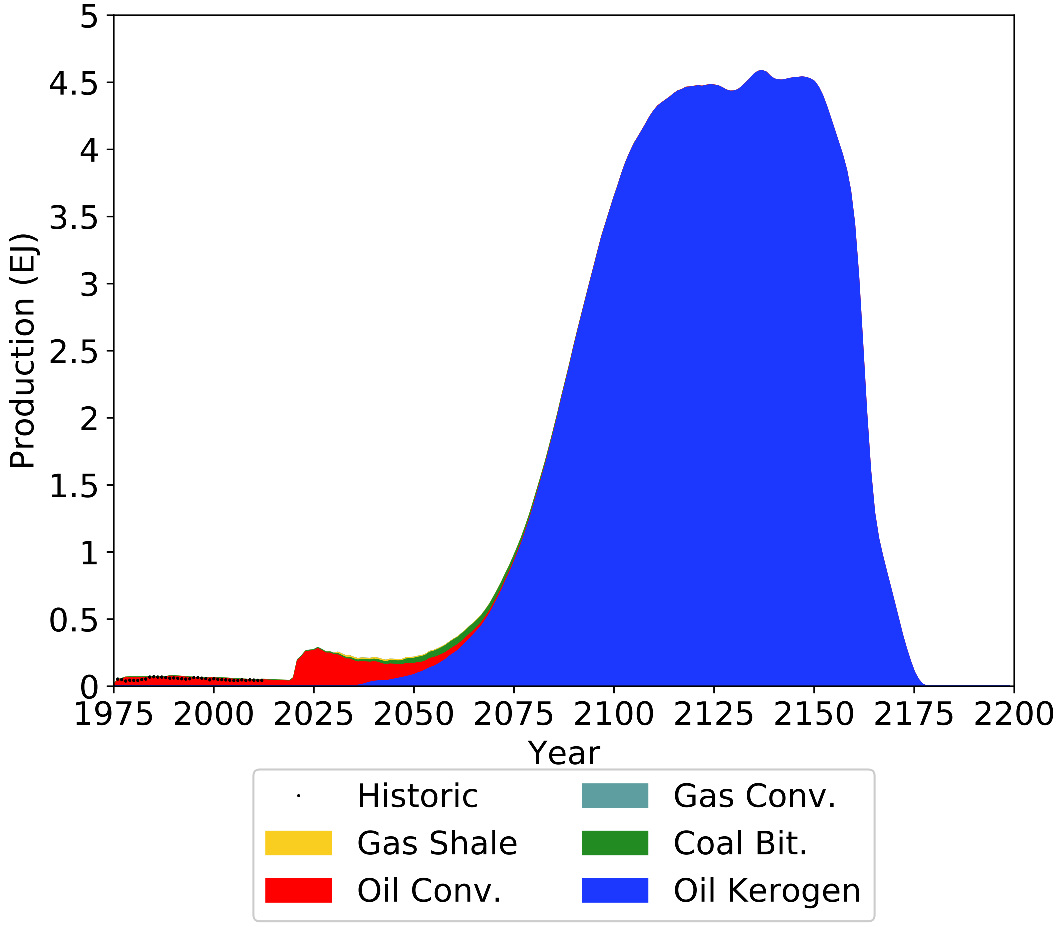

Figure 1.94: Zaire projections capped at 16

Table 1.94: Peak years - All

**Name**	**URR**	**Peak Year**	**Peak Rate**
Oil Kerogen	343.8	2137	4.59
Oil Conv.	8.98	2026	0.28
Coal Bit.	2.37	2062	0.06
Gas Shale	0.37	2032	0.01
Gas Conv.	0.04	2025	–
**Total**	**355.56**	**2137**	**4.59**

1.44.2 By Mineral

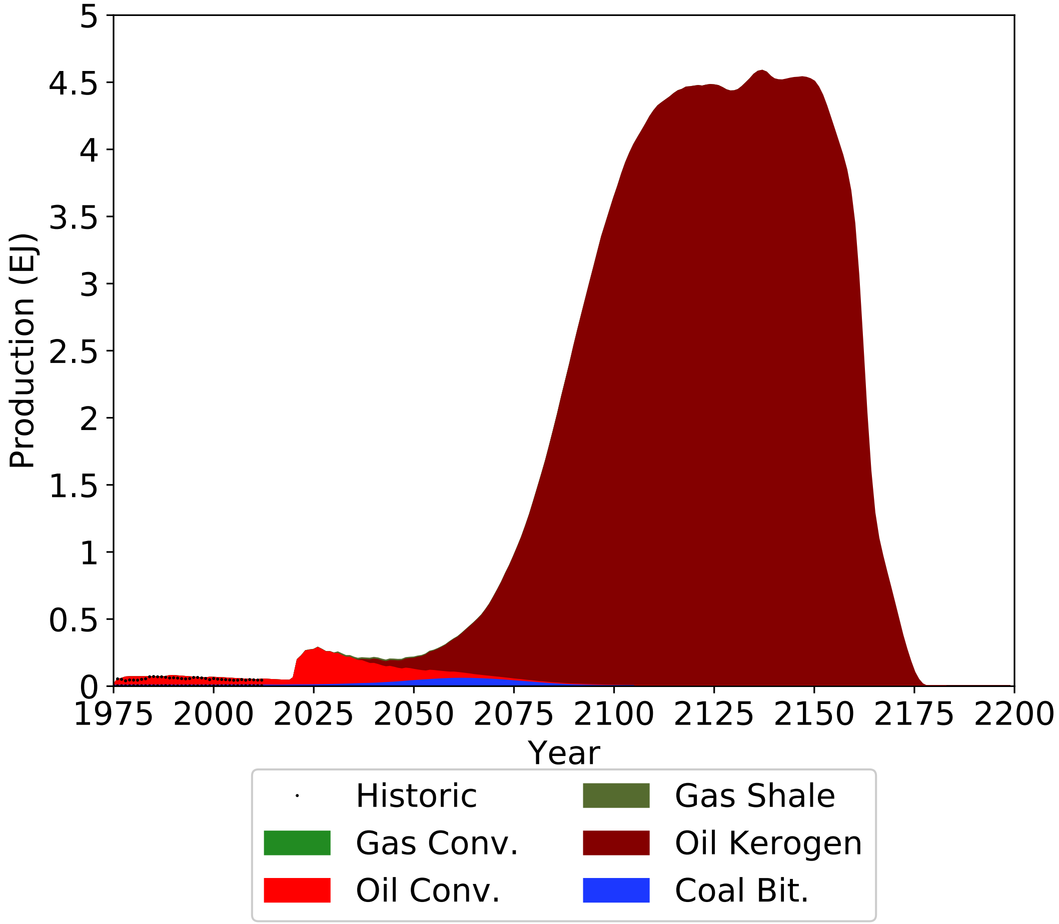

Figure 1.95: Zaire projection by mineral type

Table 1.95: Peak years - Minerals

Name	URR	Peak Year	Peak Rate
Coal Bit.	2.37	2062	0.06
Oil Conv.	8.98	2026	0.28
Oil Kerogen	343.8	2137	4.59
Gas Conv.	0.04	2025	–
Gas Shale	0.37	2032	0.01
**Total**	**355.56**	**2137**	**4.59**

1.45 Zambia

1.45.1 All Projections

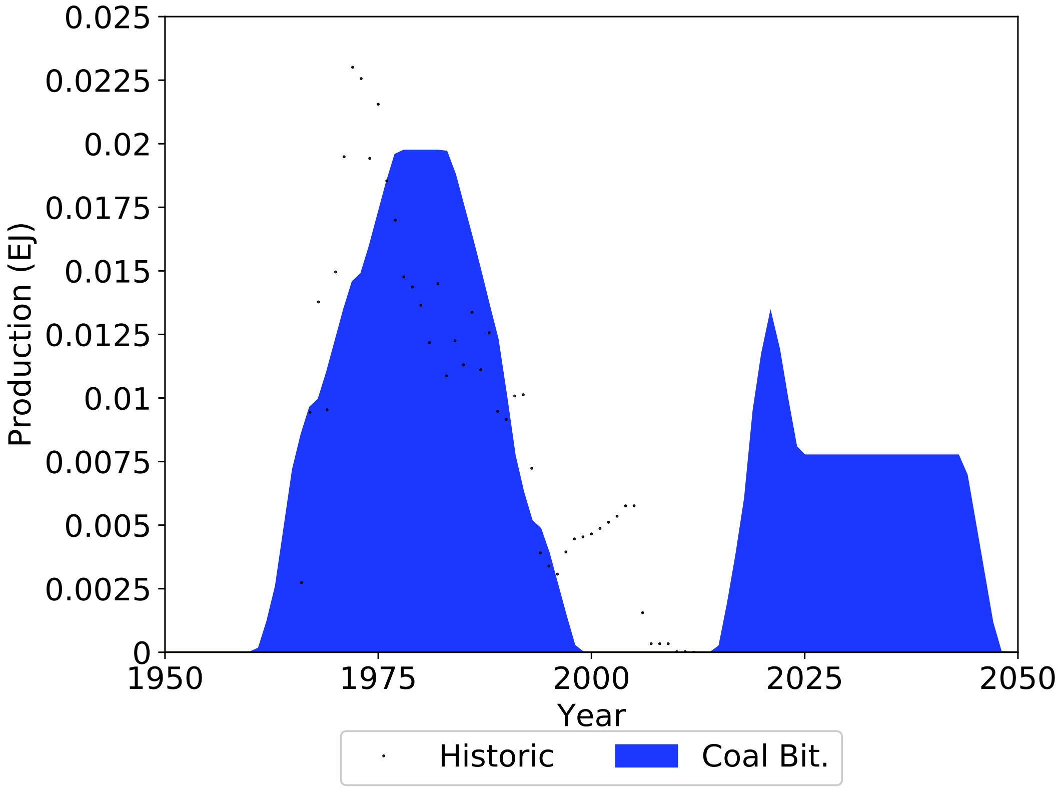

Figure 1.96: Zambia projections capped at 16

Table 1.96: Peak years - All

Name	URR	Peak Year	Peak Rate
Coal Bit.	0.68	1978	0.02
**Total**	**0.68**	**1978**	**0.02**

1.45.2 By Mineral

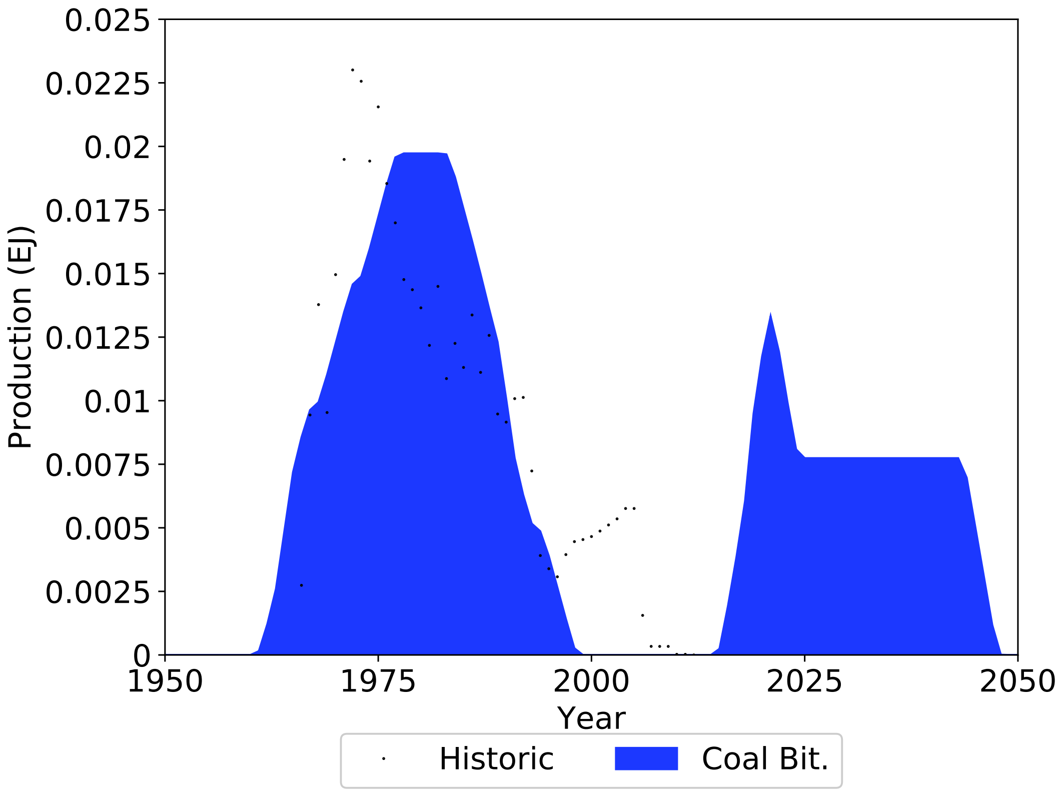

Figure 1.97: Zambia projection by mineral type

Table 1.97: Peak years - Minerals

Name	URR	Peak Year	Peak Rate
Coal Bit.	0.68	1978	0.02
**Total**	**0.68**	**1978**	**0.02**

1.46 Zimbabwe

1.46.1 All Projections

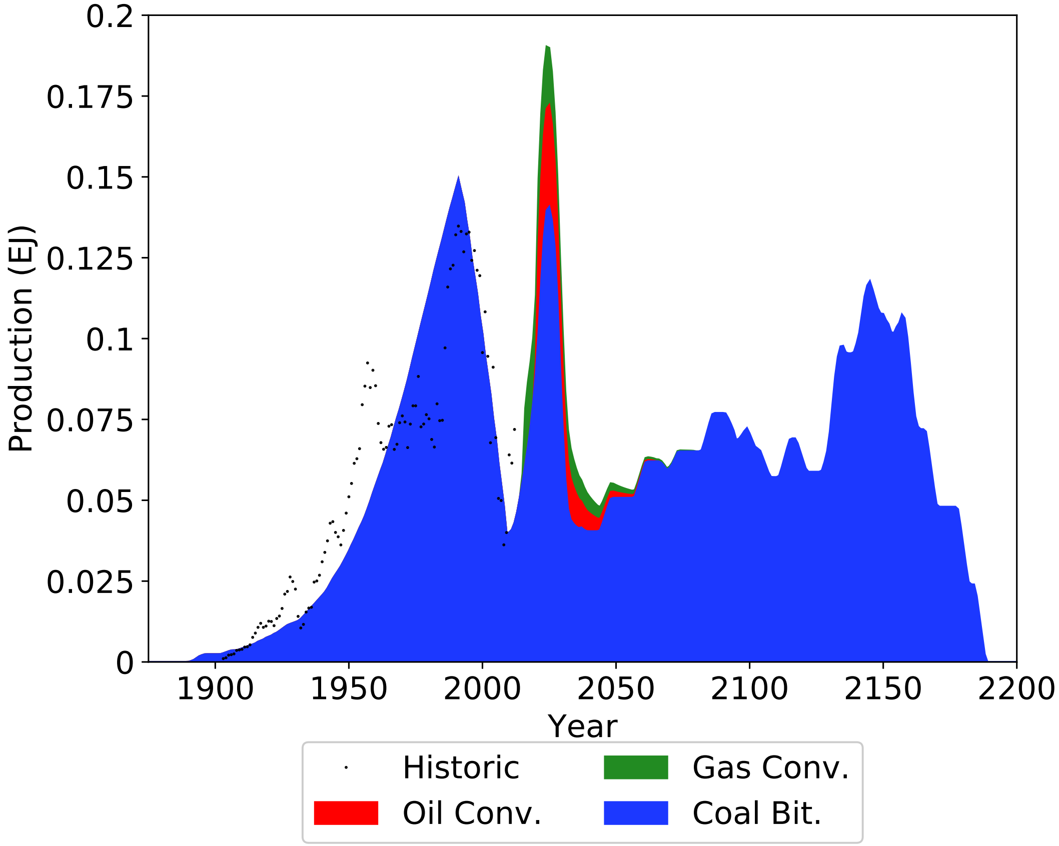

Figure 1.98: Zimbabwe projections capped at 16

Table 1.98: Peak years - All

**Name**	**URR**	**Peak Year**	**Peak Rate**
Coal Bit.	18.18	1991	0.15
Oil Conv.	0.42	2022	0.03
Gas Conv.	0.4	2017	0.02
**Total**	**19.0**	**2024**	**0.19**

1.46.2 By Mineral

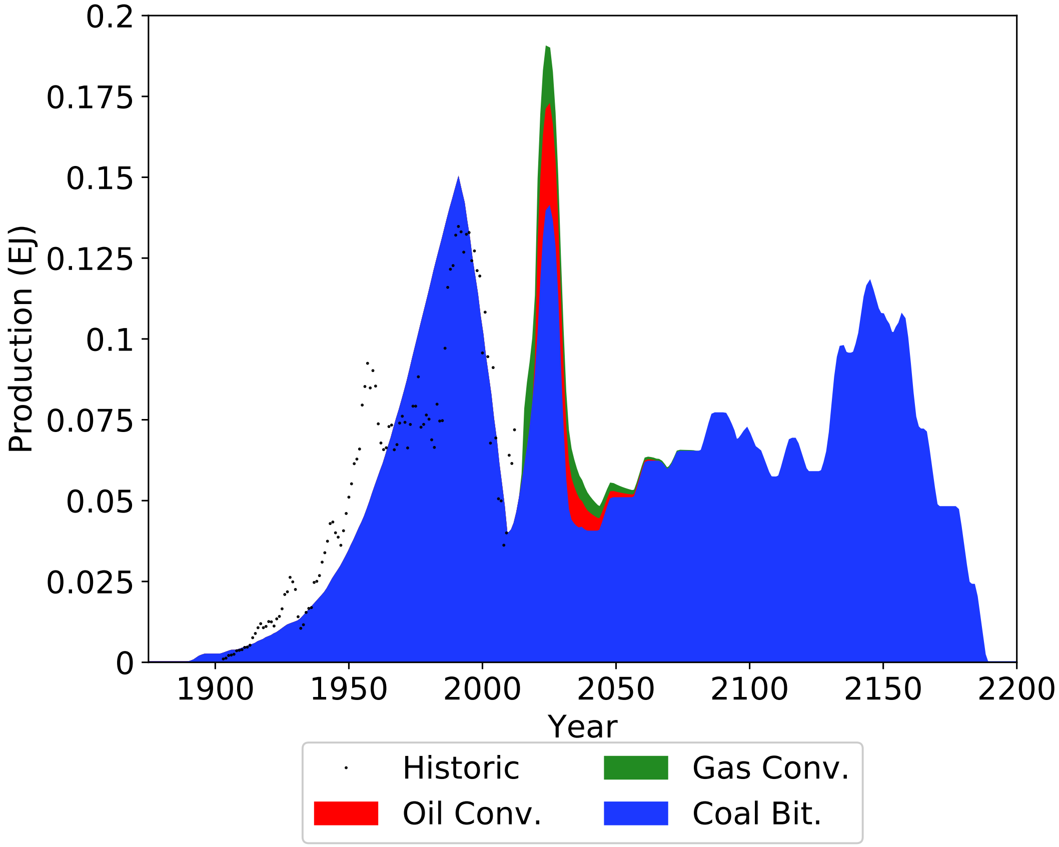

Figure 1.99: Zimbabwe projection by mineral type

Table 1.99: Peak years - Minerals

Name	URR	Peak Year	Peak Rate
Coal Bit.	18.18	1991	0.15
Oil Conv.	0.42	2022	0.03
Gas Conv.	0.4	2017	0.02
**Total**	**19.0**	**2024**	**0.19**

1.47 Total

1.47.1 By country

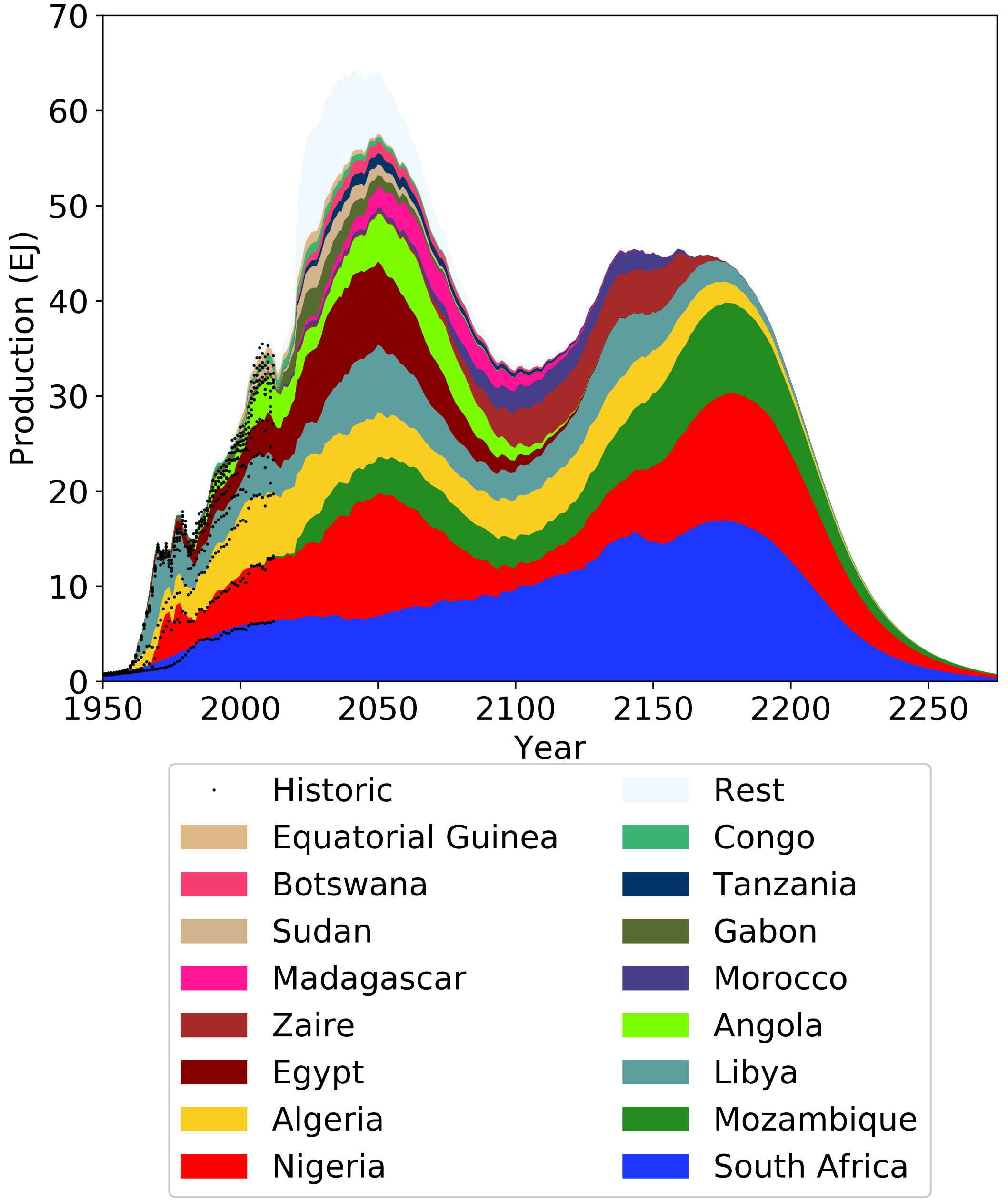

Figure 1.100: Africa projections by country

Table 1.100: Peak years - All

**Name**	**URR**	**Peak Year**	**Peak Rate**
South Africa	2574.33	2177	16.84
Nigeria	1985.7	2183	13.57
Mozambique	1067.09	2173	9.66
Algeria	1013.3	2007	7.23
Libya	930.13	1970	7.44
Egypt	627.43	2041	9.29
Angola	412.21	2060	6.23
Zaire	355.56	2137	4.59
Morocco	199.19	2107	2.42
Madagascar	178.11	2066	3.31
Gabon	125.45	2027	3.01
Sudan	88.75	2029	2.34
Tanzania	75.16	2044	1.2
Botswana	72.31	2043	1.36
Congo	56.26	2019	1.09
Equatorial Guinea	43.17	2025	1.2
Seychelles	42.05	2035	1.12
Tunisia	40.01	2051	0.42
Cameroon	36.51	2025	0.87
Kenya	32.78	2039	0.69
Ivory Coast	30.93	2031	0.66
Chad	30.22	2027	1.1
Ghana	25.09	2023	0.8
Sierra Leone	22.07	2033	0.66
Zimbabwe	19.0	2024	0.19
Uganda	18.92	2029	0.66
Namibia	17.9	2026	0.58
Somalia	15.98	2035	0.4
Mauritania	15.09	2028	0.5
Liberia	14.14	2028	0.49
Guinea	13.74	2028	0.49
Senegal	13.72	2026	0.29
Niger	12.4	2028	0.32
Sao Tome and Principe	11.29	2026	0.43
Western Sahara	9.71	2035	0.24
Benin	6.9	2024	0.31
Togo	6.65	2024	0.28
Eritrea	4.13	2025	0.15
Swaziland	3.98	2085	0.06
Guinea-Bissau	3.53	2026	0.15
Malawi	3.16	2082	0.05
Ethiopia	2.63	2025	0.14
Rwanda	1.85	2025	0.08
Gambia	1.77	2025	0.1
Zambia	0.68	1978	0.02
Central African Republic	0.03	2026	–
**Total**	**10260.99**	**2041**	**64.1**

1.47.2 By mineral

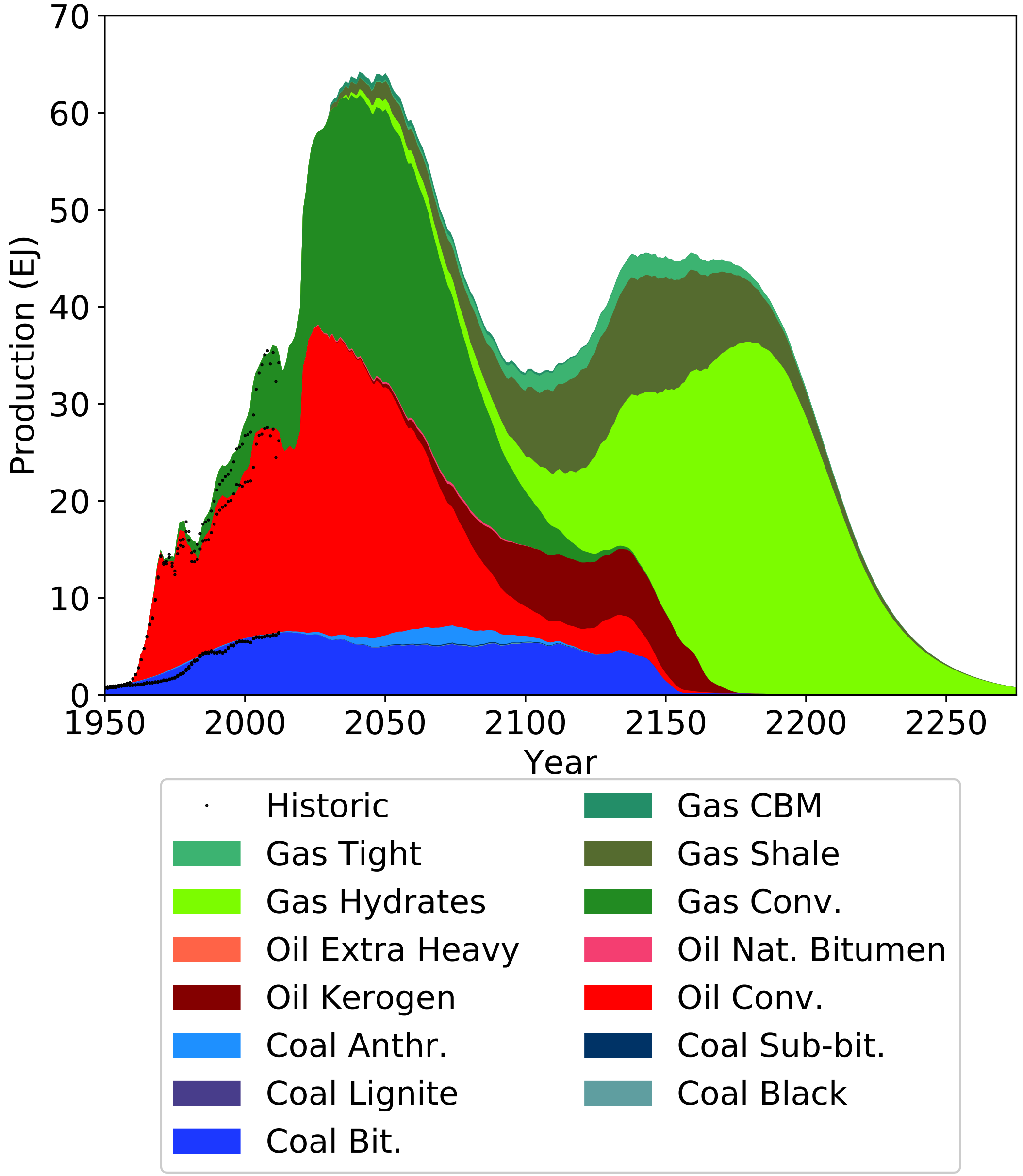

Figure 1.101: Africa projection by mineral type

Table 1.101: Peak years - Minerals

**Name**	**URR**	**Peak Year**	**Peak Rate**
Coal Bit.	904.84	2015	6.36
Coal Black	3.98	2085	0.06
Coal Lignite	0.42	2055	0.01
Coal Sub-bit.	6.74	2077	0.1
Coal Anthr.	98.36	2070	1.8
Oil Conv.	2460.98	2026	31.54
Oil Kerogen	546.6	2116	6.9
Oil Nat. Bitumen	13.94	2073	0.28
Oil Extra Heavy	0.34	2035	0.03
Gas Conv.	1903.98	2050	28.04
Gas Hydrates	2947.0	2180	36.27
Gas Shale	1130.32	2138	12.08
Gas Tight	203.94	2134	2.41
Gas CBM	39.55	2046	0.65
**Total**	**10260.99**	**2041**	**64.1**

Chapter 2

Asia

2.1 Afghanistan

2.1.1 All Projections

Table 2.1: Peak years - All

Name	URR	Peak Year	Peak Rate
Gas Conv.	15.32	2029	0.35
Oil Conv.	12.21	2028	0.45
Coal Bit.	1.79	2029	0.06
**Total**	**29.32**	**2029**	**0.85**

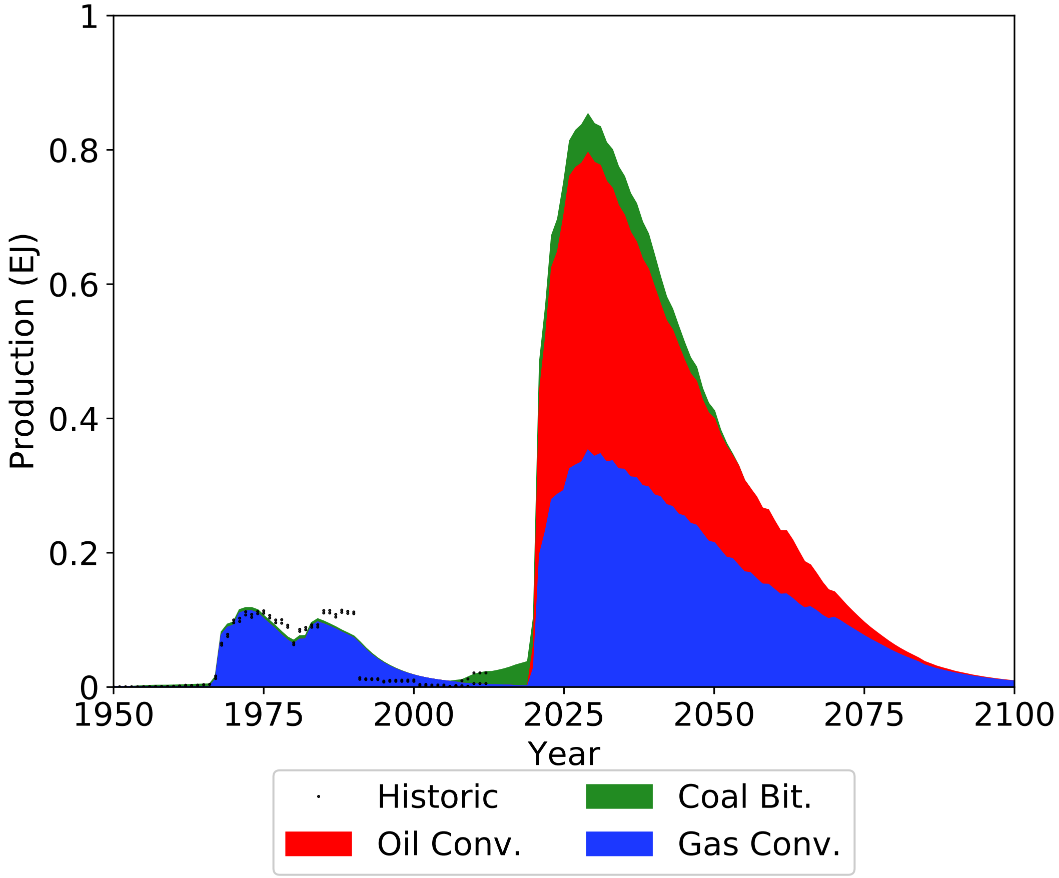

Figure 2.1: Afghanistan projections capped at 16

2.1.2 By Mineral

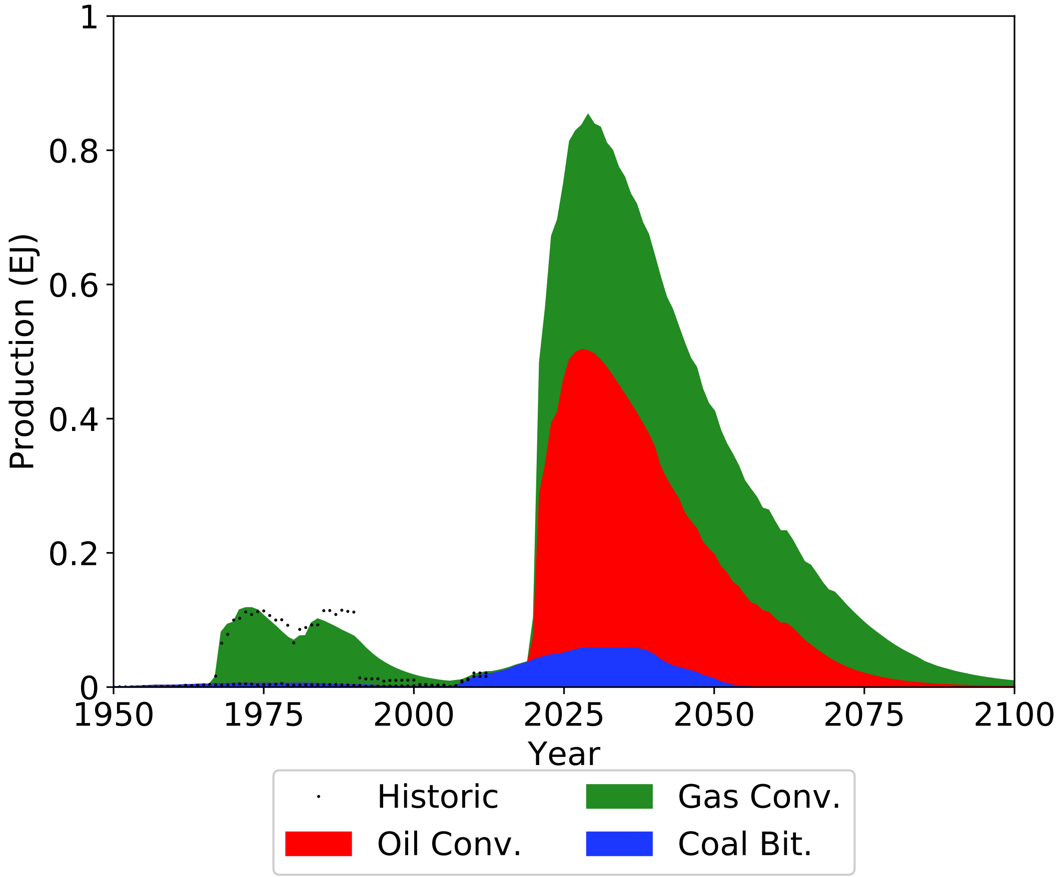

Figure 2.2: Afghanistan projection by mineral type

Table 2.2: Peak years - Minerals

Name	URR	Peak Year	Peak Rate
Coal Bit.	1.79	2029	0.06
Oil Conv.	12.21	2028	0.45
Gas Conv.	15.32	2029	0.35
**Total**	**29.32**	**2029**	**0.85**

2.2 Australia

2.2.1 All Projections

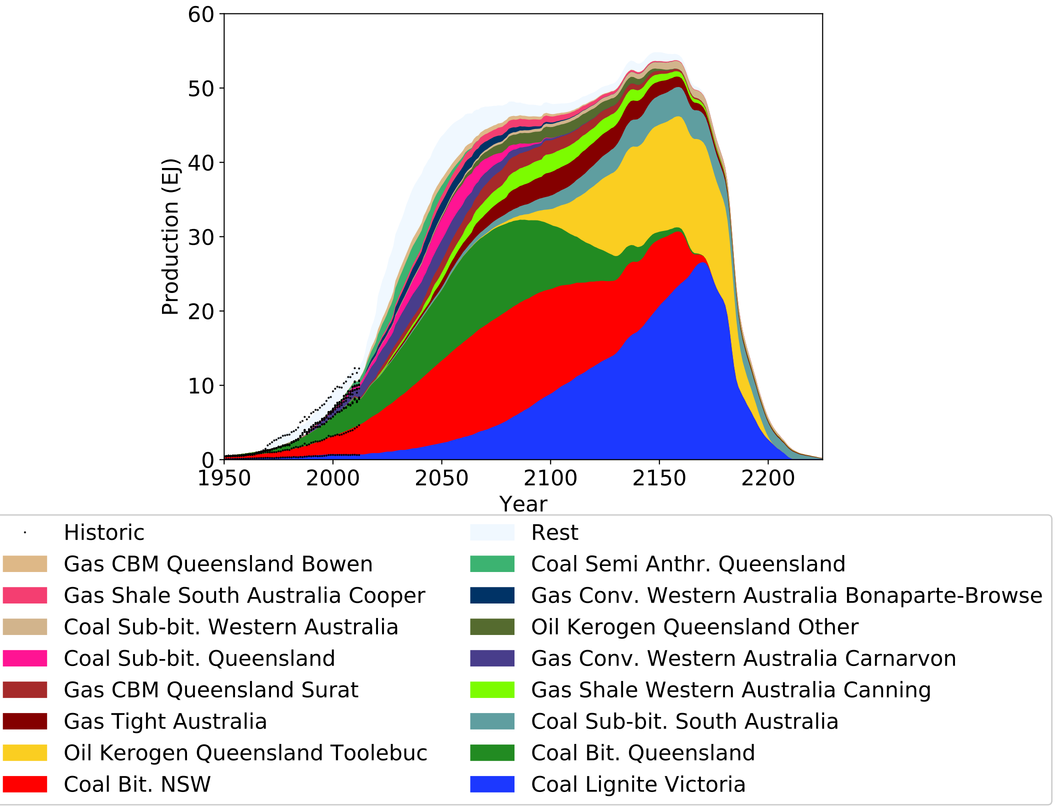

Figure 2.3: Australia projections capped at 16

Table 2.3: Peak years - All

**Name**	**URR**	**Peak Year**	**Peak Rate**
Coal Lignite Victoria	1900.0	2170	26.48
Coal Bit. NSW	1737.24	2086	14.85
Coal Bit. Queensland	1104.24	2074	12.18
Oil Kerogen Queensland Toolebuc	1031.4	2167	15.39
Coal Sub-bit. South Australia	397.9	2161	3.95
Gas Tight Australia	296.65	2115	3.19
Gas Shale Western Australia Canning	223.63	2098	2.51
Gas CBM Queensland Surat	190.57	2080	2.13
Gas Conv. Western Australia Carnarvon	185.66	2037	3.1
Coal Sub-bit. Queensland	136.95	2053	2.98
Oil Kerogen Queensland Other	106.73	2103	1.47
Coal Sub-bit. Western Australia	99.66	2160	1.07
Gas Conv. Western Australia Bonaparte-Browse	89.93	2046	1.81
Gas Shale South Australia Cooper	88.5	2077	1.1
Coal Semi Anthr. Queensland	79.9	2036	2.46
Gas CBM Queensland Bowen	69.37	2037	1.03
Oil Tight Western Australia Canning	57.52	2050	1.51
Coal Lignite South Australia	47.5	2152	0.85
Oil Conv. Victoria Gippsland	43.75	1980	0.96
Gas Shale Northern Territory Beetaloo	41.87	2045	0.66
Oil Conv. Western Australia Carnarvon	41.47	2021	0.92
Gas Shale Western Australia Perth	31.4	2042	0.5
Gas Conv. Victoria Gippsland	28.71	2023	0.47
Oil Tight Northern Territory Beetaloo	27.87	2048	0.79
Oil Conv. Northern Territory Browse-Bonaparte	20.18	2029	0.66
Gas CBM NSW	19.54	2030	0.32
Coal Lignite Western Australia	19.0	2135	0.33
Gas Shale Queensland Maryborough	18.08	2045	0.3
Gas Shale Northern Territory Georgina	12.37	2045	0.22
Gas Conv. South Australia Cooper-Eromanga	10.96	1978	0.2
Gas Conv. JPDA Timor Gap	10.31	2025	0.27
Coal Bit. Tasmania	8.88	2094	0.08
Oil Tight South Australia Cooper	5.93	2024	0.25
Oil Tight Northern Territory Georgina	5.93	2026	0.25
Gas Conv. Victoria Otway	3.77	2018	0.12
Oil Conv. South Australia Cooper-Eromanga	3.7	1986	0.11
Gas Conv. Queensland Cooper-Eromanga	3.66	1997	0.16
Gas CBM Queensland Clarence	3.24	2039	0.07
Oil Tight Western Australia Perth	2.97	2025	0.14
Oil Tight Queensland Cooper	2.97	2022	0.14
Oil Conv. JPDA Timor Gap	2.11	2017	0.09
Oil Kerogen South Australia	2.06	2040	0.04
Gas Conv. Queensland Surat-Bowen	1.83	2023	0.04
Oil Conv. Queensland Cooper-Eromanga	1.7	1985	0.05
Oil Conv. Northern Territory Timor Sea	1.59	2001	0.16
Gas Conv. Western Australia Perth	1.54	2020	0.03
Coal Lignite Tasmania	1.43	2055	0.05
Gas Conv. Tasmania Bass	1.38	2020	0.05
Gas Conv. Northern Territory Amadeus	1.13	2023	0.03

Table 2.3: Peak years - All – Continued

**Name**	**URR**	**Peak Year**	**Peak Rate**
Oil Conv. Tasmania Bass	0.99	2025	0.06
Gas Conv. Queensland Denison	0.67	1992	0.02
Coal Bit. Victoria	0.55	1917	0.02
Oil Conv. Queensland Surat-Bowen	0.51	1964	0.02
Oil Conv. Western Australia Perth	0.3	2005	0.03
Oil Conv. Northern Territory Amadeus	0.24	2025	0.01
Oil Conv. Western Australia Timor Sea	0.17	2002	0.03
Oil Conv. Victoria Otway	0.17	2010	0.01
Oil Kerogen Tasmania	0.17	2030	0.01
Oil Kerogen NSW	0.16	2030	0.01
Gas Conv. South Australia Otway	0.14	2000	0.01
Gas Conv. Queensland Clarence	0.12	2022	0.01
Gas Conv. Queensland Adavale	0.05	2020	–
Oil Conv. Western Australia Canning	0.02	1987	–
Gas Conv. Western Australia Canning	0.02	2022	–
Gas Conv. NSW Gunnedah	0.02	2017	–
Oil Conv. South Australia Otway	–	1994	–
Coal Sub-bit. NSW	–	1944	–
Coal Semi Anthr. Tasmania	–	1953	–
Gas CBM Western Australia	–	2008	–
**Total**	**8228.99**	**2148**	**54.73**

2.2.2 By Mineral

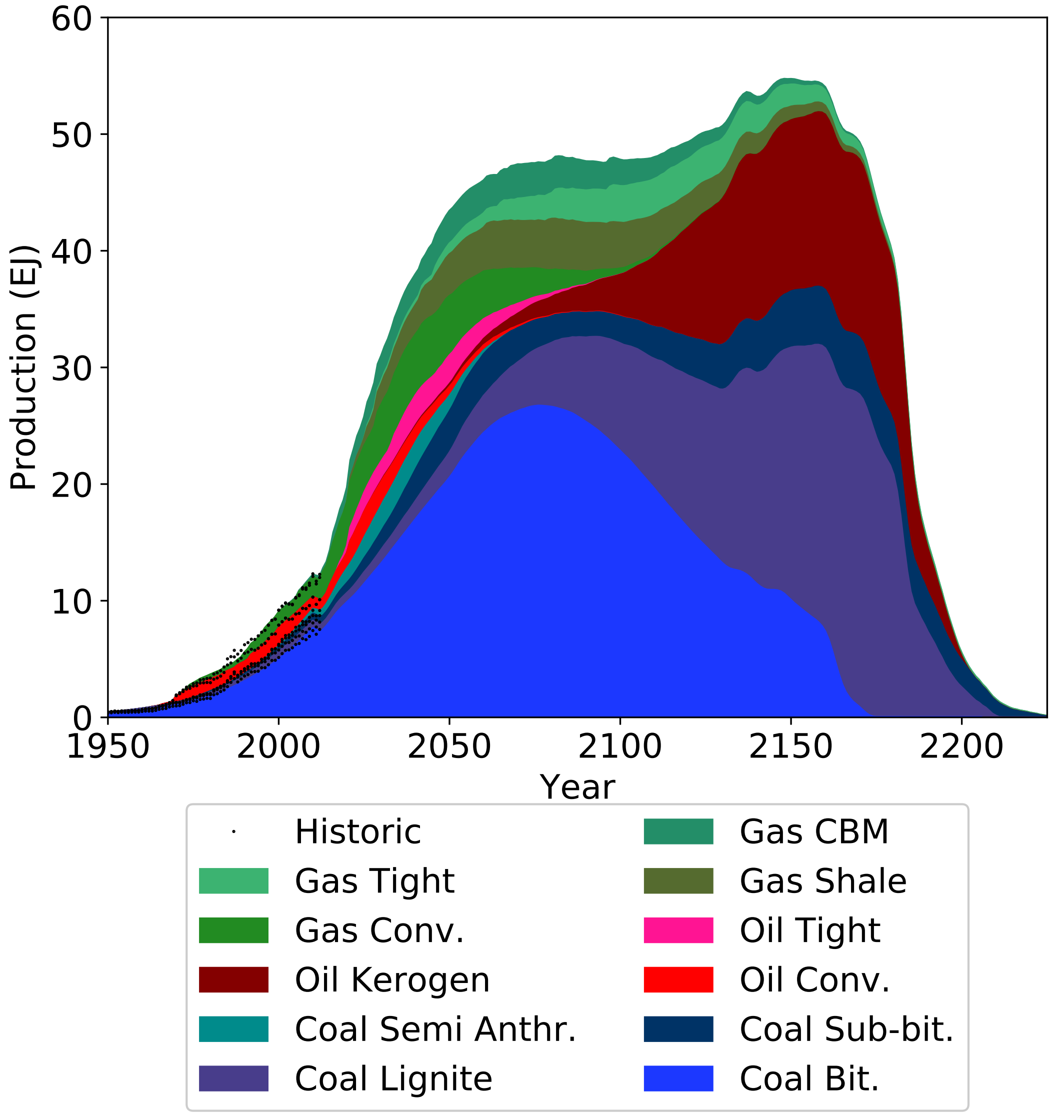

Figure 2.4: Australia projection by mineral type

2.2.3 Regional Projections

Table 2.4: Peak years - Minerals

**Name**	**URR**	**Peak Year**	**Peak Rate**
Coal Bit.	2850.91	2076	26.72
Coal Lignite	1967.93	2170	26.78
Coal Sub-bit.	634.51	2160	5.01
Coal Semi Anthr.	79.9	2036	2.46
Oil Conv.	116.91	2026	2.21
Oil Kerogen	1140.52	2167	15.39
Oil Tight	103.19	2040	2.59
Gas Conv.	339.9	2037	5.35
Gas Shale	415.85	2080	4.39
Gas Tight	296.65	2115	3.19
Gas CBM	282.72	2069	2.95
**Total**	**8228.99**	**2148**	**54.73**

Australia

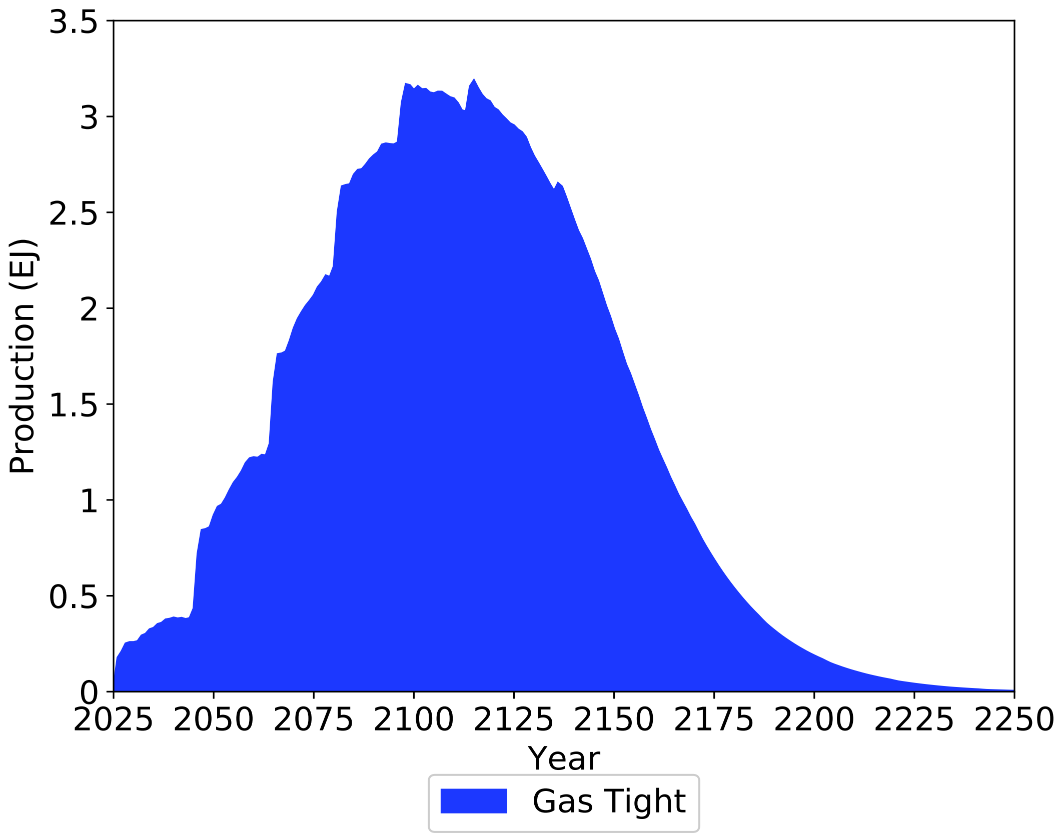

Figure 2.5: Australia - Australia projections capped at 16

Table 2.5: Peak years - All

Name	URR	Peak Year	Peak Rate
Gas Tight Australia	296.65	2115	3.19
**Total**	**296.65**	**2115**	**3.19**

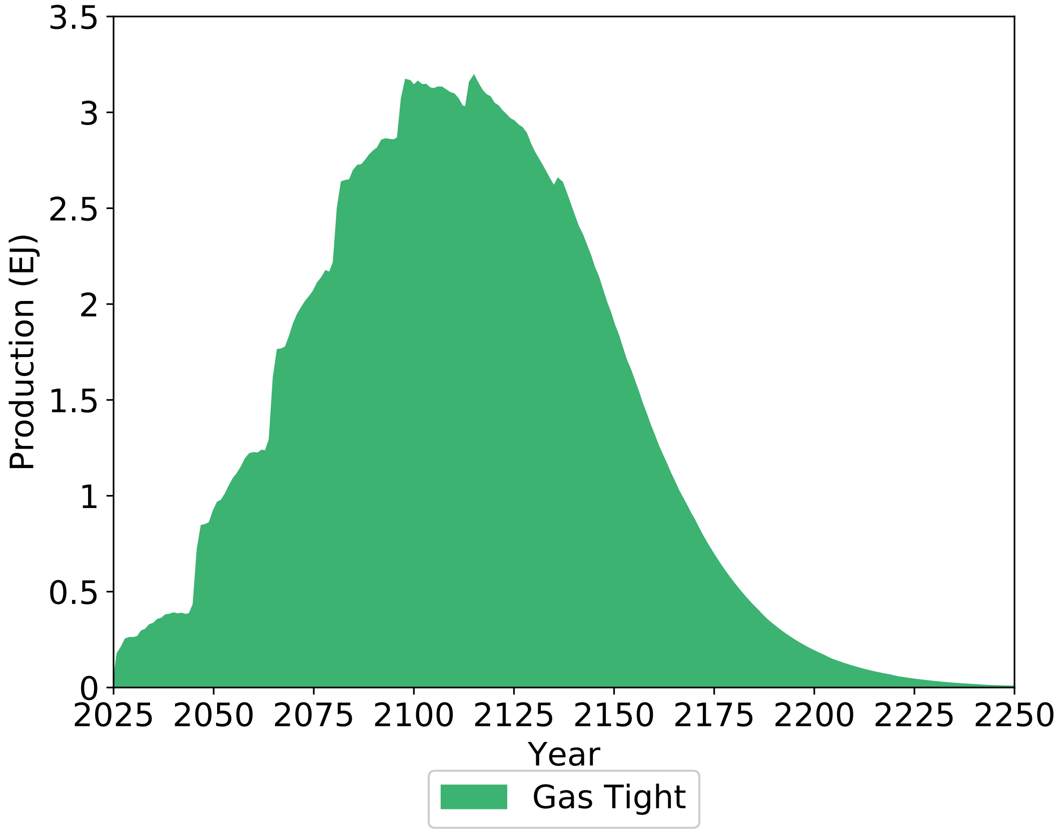

Figure 2.6: Australia - Australia projection by mineral type

Table 2.6: Peak years - Minerals

**Name**	**URR**	**Peak Year**	**Peak Rate**
Gas Tight	296.65	2115	3.19
**Total**	**296.65**	**2115**	**3.19**

JPDA

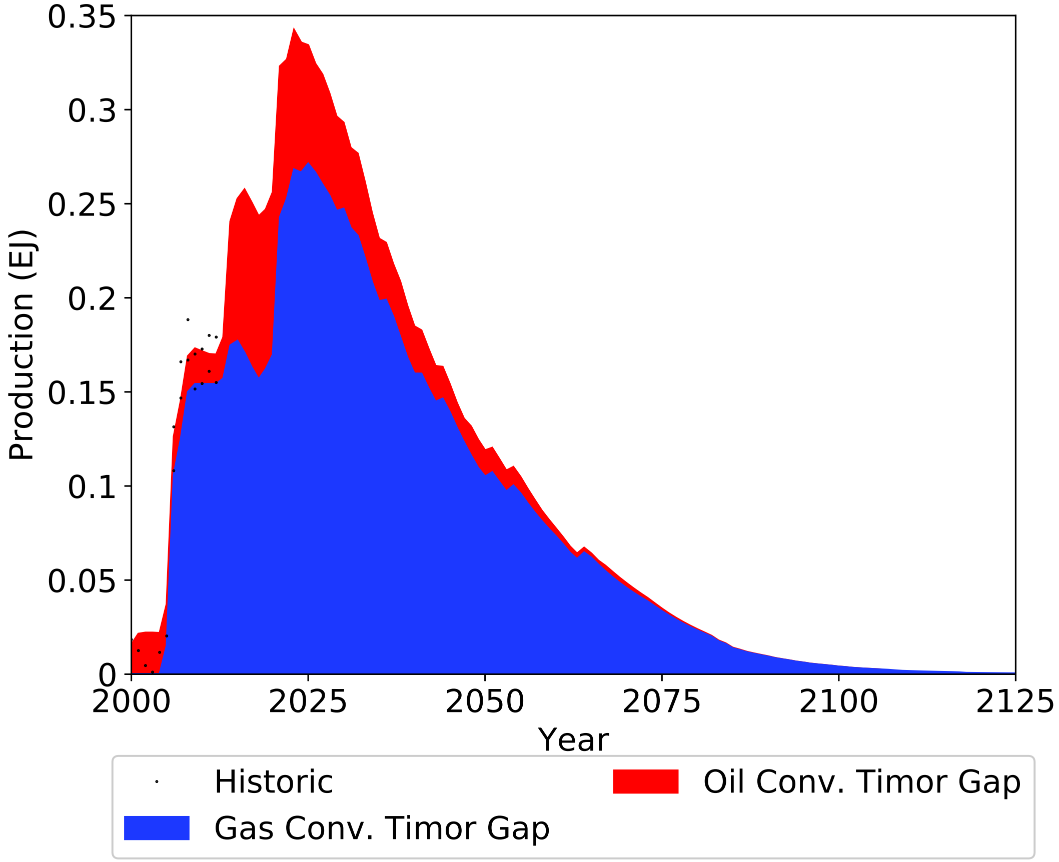

Figure 2.7: Australia - JPDA projections capped at 16

Table 2.7: Peak years - All

**Name**	**URR**	**Peak Year**	**Peak Rate**
Gas Conv. JPDA Timor Gap	10.31	2025	0.27
Oil Conv. JPDA Timor Gap	2.11	2017	0.09
**Total**	**12.42**	**2023**	**0.34**

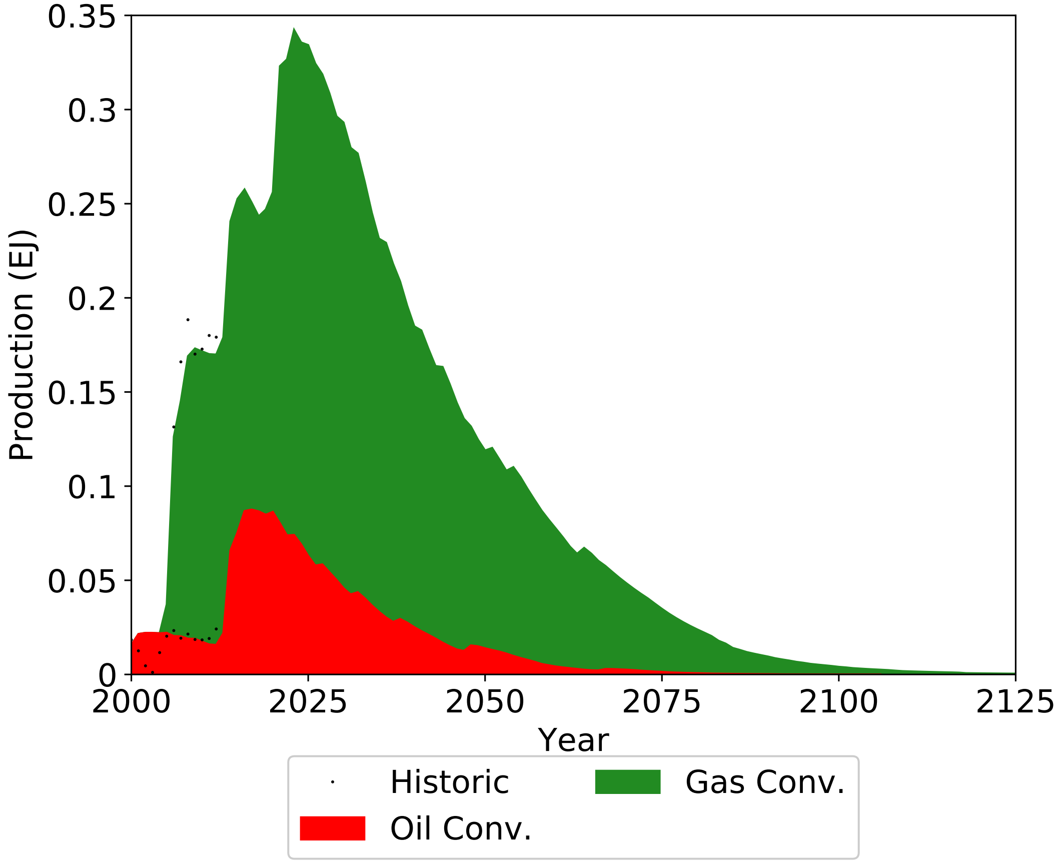

Figure 2.8: Australia - JPDA projection by mineral type

Table 2.8: Peak years - Minerals

Name	URR	Peak Year	Peak Rate
Oil Conv.	2.11	2017	0.09
Gas Conv.	10.31	2025	0.27
**Total**	**12.42**	**2023**	**0.34**

NSW

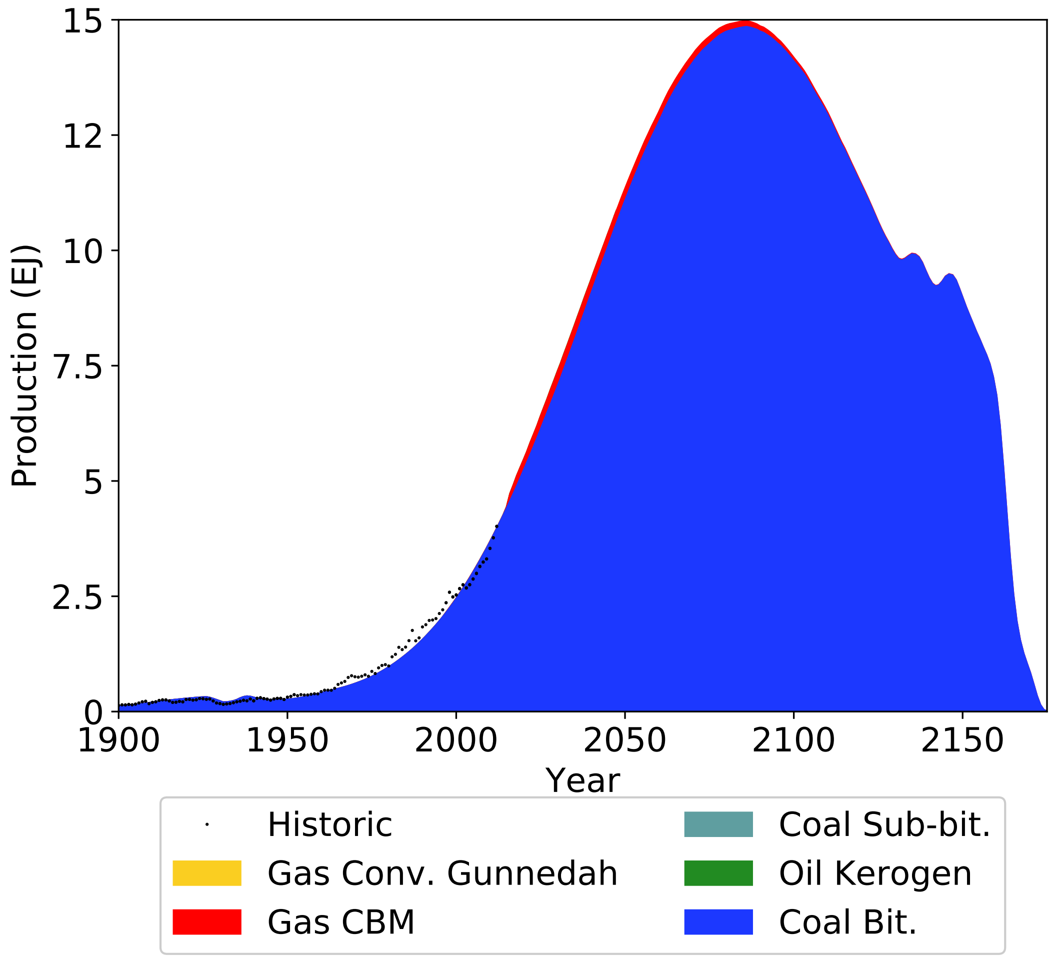

Figure 2.9: Australia - NSW projections capped at 16

Table 2.9: Peak years - All

**Name**	**URR**	**Peak Year**	**Peak Rate**
Coal Bit. NSW	1737.24	2086	14.85
Gas CBM NSW	19.54	2030	0.32
Oil Kerogen NSW	0.16	2030	0.01
Gas Conv. NSW Gunnedah	0.02	2017	–
Coal Sub-bit. NSW	–	1944	–
**Total**	**1756.96**	**2086**	**14.97**

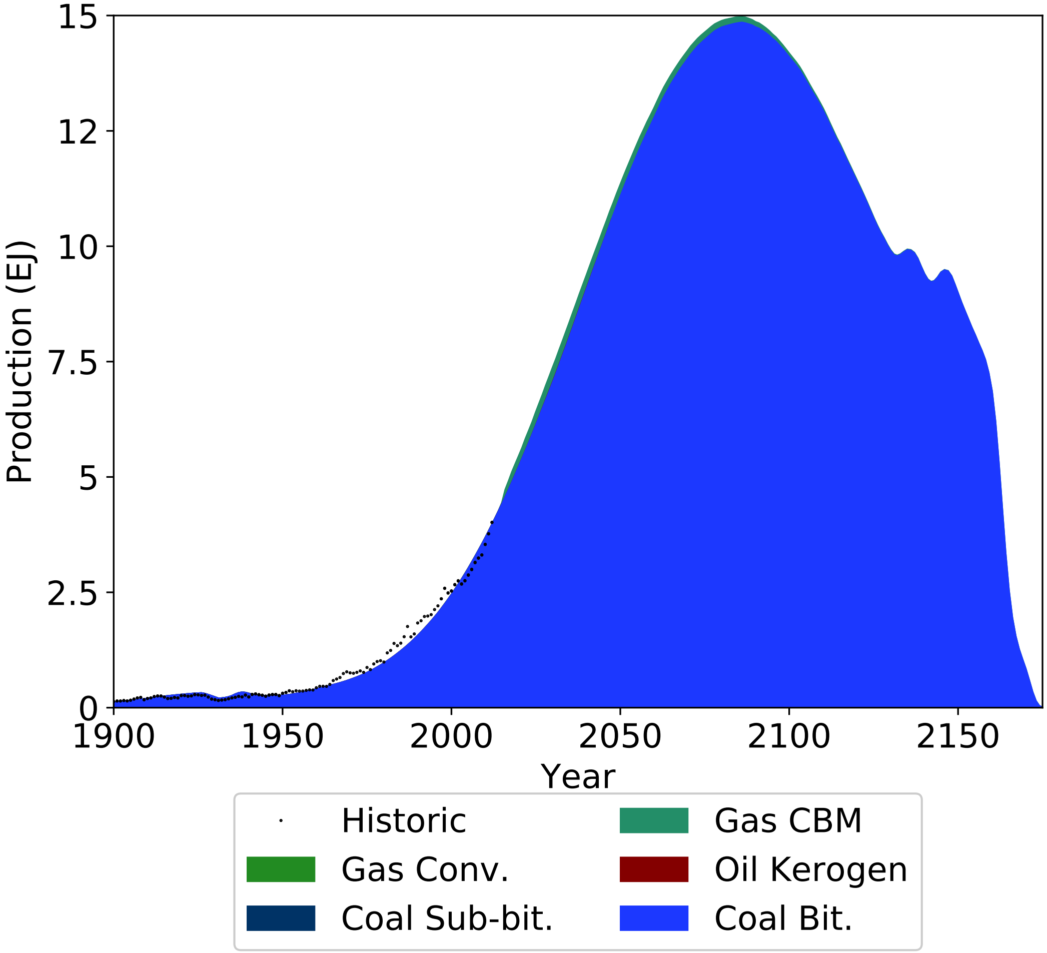

Figure 2.10: Australia - NSW projection by mineral type

Table 2.10: Peak years - Minerals

Name	URR	Peak Year	Peak Rate
Coal Bit.	1737.24	2086	14.85
Coal Sub-bit.	–	1944	–
Oil Kerogen	0.16	2030	0.01
Gas Conv.	0.02	2017	–
Gas CBM	19.54	2030	0.32
**Total**	**1756.96**	**2086**	**14.97**

Northern Territory

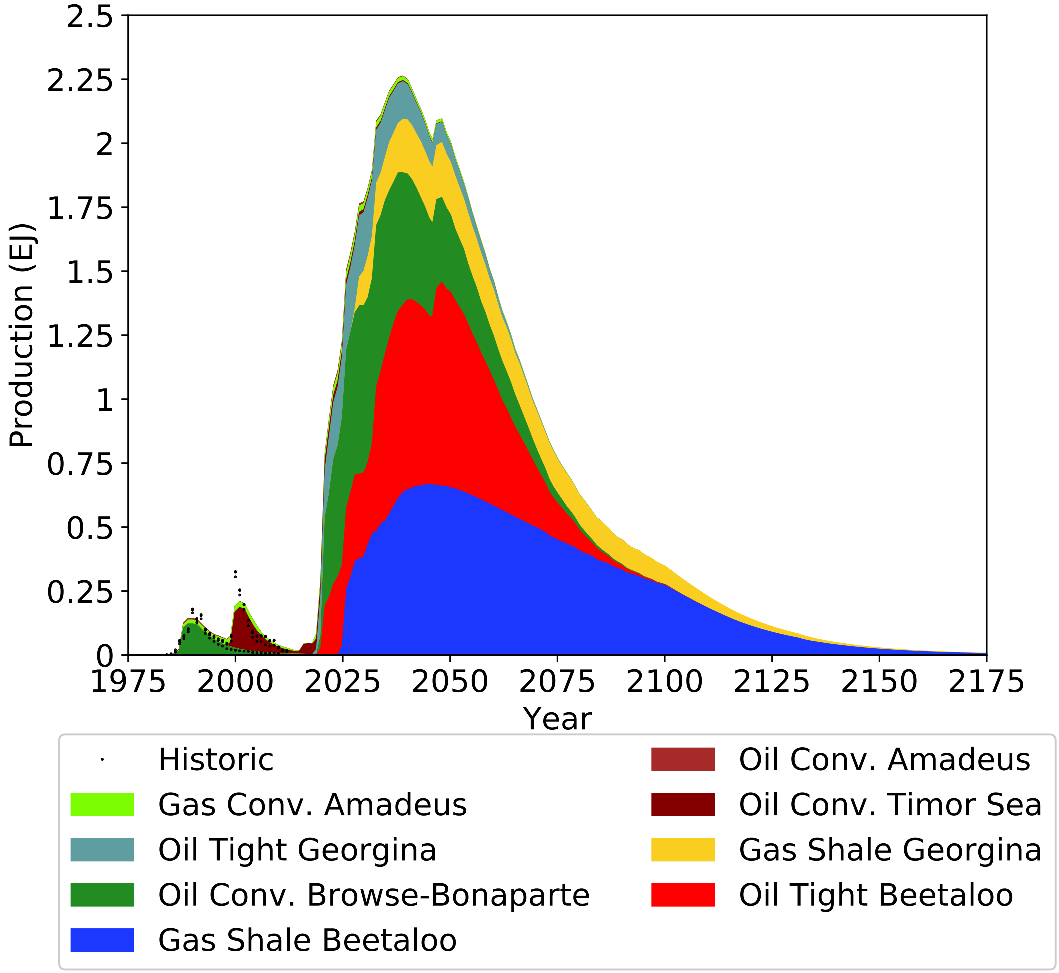

Figure 2.11: Australia - Northern Territory projections capped at 16

Table 2.11: Peak years - All

**Name**	**URR**	**Peak Year**	**Peak Rate**
Gas Shale Northern Territory Beetaloo	41.87	2045	0.66
Oil Tight Northern Territory Beetaloo	27.87	2048	0.79
Oil Conv. Northern Territory Browse-Bonaparte	20.18	2029	0.66
Gas Shale Northern Territory Georgina	12.37	2045	0.22
Oil Tight Northern Territory Georgina	5.93	2026	0.25
Oil Conv. Northern Territory Timor Sea	1.59	2001	0.16
Gas Conv. Northern Territory Amadeus	1.13	2023	0.03
Oil Conv. Northern Territory Amadeus	0.24	2025	0.01
**Total**	**111.18**	**2039**	**2.26**

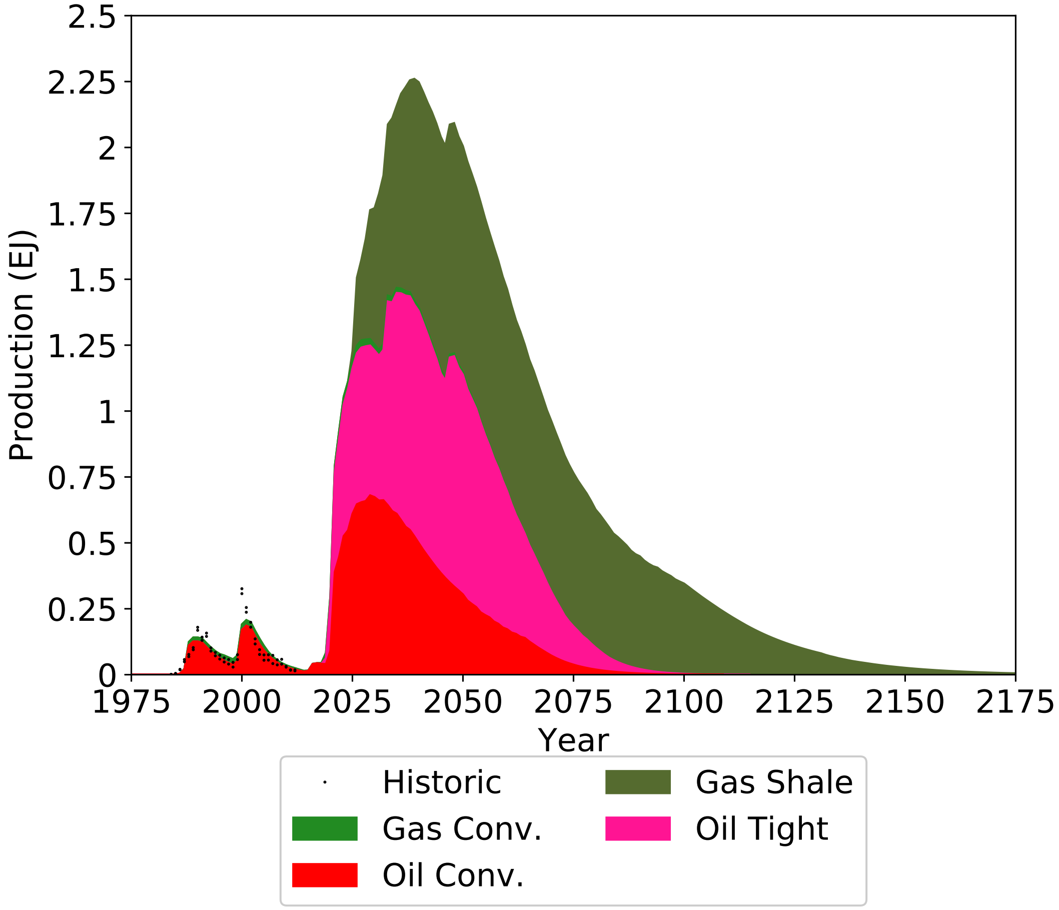

Figure 2.12: Australia - Northern Territory projection by mineral type

Table 2.12: Peak years - Minerals

**Name**	**URR**	**Peak Year**	**Peak Rate**
Oil Conv.	22.01	2029	0.68
Oil Tight	33.8	2038	0.89
Gas Conv.	1.13	2023	0.03
Gas Shale	54.24	2045	0.88
**Total**	**111.18**	**2039**	**2.26**

Queensland

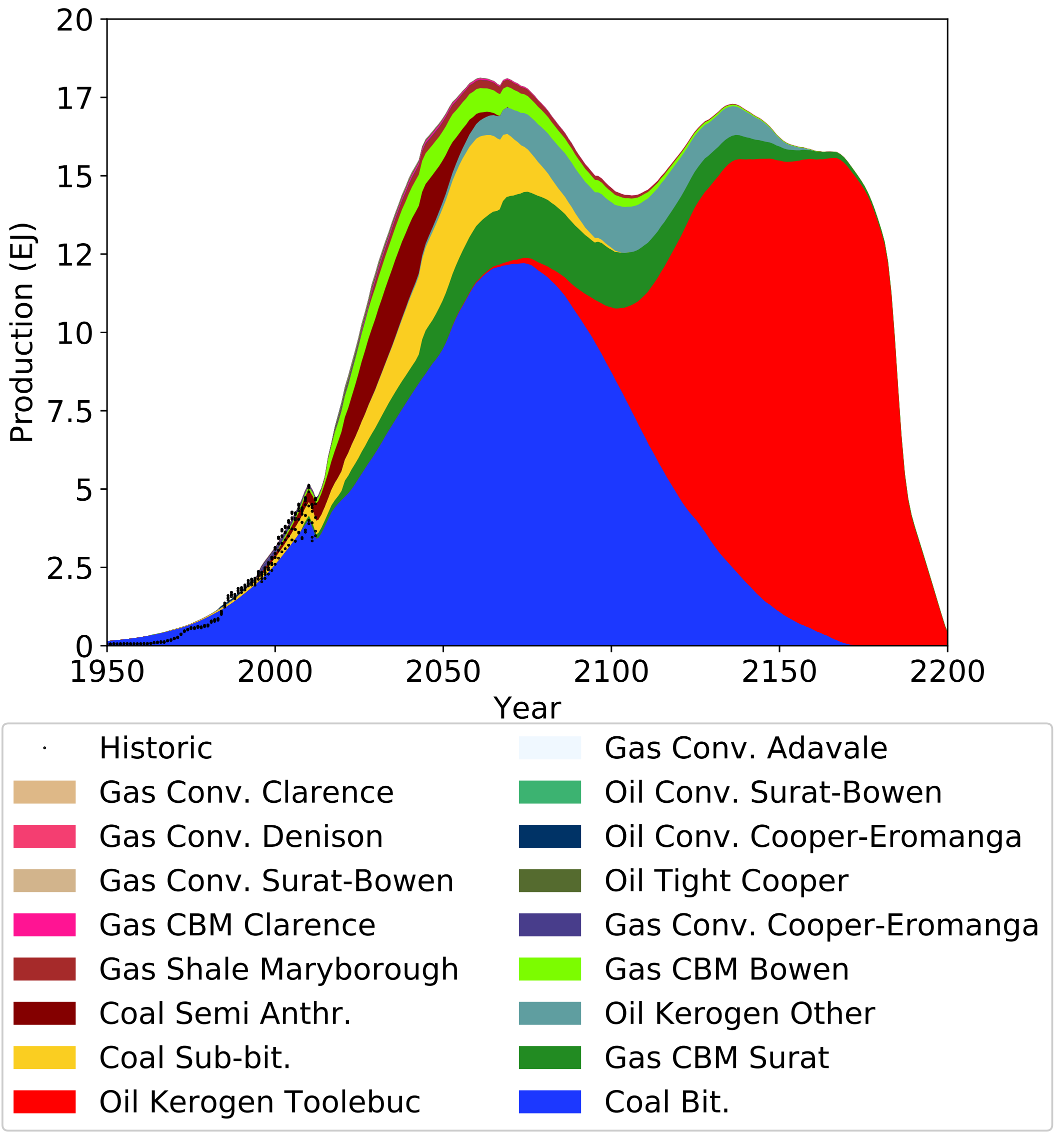

Figure 2.13: Australia - Queensland projections capped at 16

Table 2.13: Peak years - All

Name	URR	Peak Year	Peak Rate
Coal Bit. Queensland	1104.24	2074	12.18
Oil Kerogen Queensland Toolebuc	1031.4	2167	15.39
Gas CBM Queensland Surat	190.57	2080	2.13
Coal Sub-bit. Queensland	136.95	2053	2.98
Oil Kerogen Queensland Other	106.73	2103	1.47
Coal Semi Anthr. Queensland	79.9	2036	2.46
Gas CBM Queensland Bowen	69.37	2037	1.03
Gas Shale Queensland Maryborough	18.08	2045	0.3
Gas Conv. Queensland Cooper-Eromanga	3.66	1997	0.16
Gas CBM Queensland Clarence	3.24	2039	0.07
Oil Tight Queensland Cooper	2.97	2022	0.14
Gas Conv. Queensland Surat-Bowen	1.83	2023	0.04
Oil Conv. Queensland Cooper-Eromanga	1.7	1985	0.05
Gas Conv. Queensland Denison	0.67	1992	0.02
Oil Conv. Queensland Surat-Bowen	0.51	1964	0.02
Gas Conv. Queensland Clarence	0.12	2022	0.01
Gas Conv. Queensland Adavale	0.05	2020	–
**Total**	**2752.0**	**2061**	**18.1**

Table 2.14: Peak years - Minerals

Name	URR	Peak Year	Peak Rate
Coal Bit.	1104.24	2074	12.18
Coal Sub-bit.	136.95	2053	2.98
Coal Semi Anthr.	79.9	2036	2.46
Oil Conv.	2.22	1987	0.06
Oil Kerogen	1138.13	2167	15.39
Oil Tight	2.97	2022	0.14
Gas Conv.	6.33	1997	0.2
Gas Shale	18.08	2045	0.3
Gas CBM	263.18	2069	2.77
**Total**	**2752.0**	**2061**	**18.1**

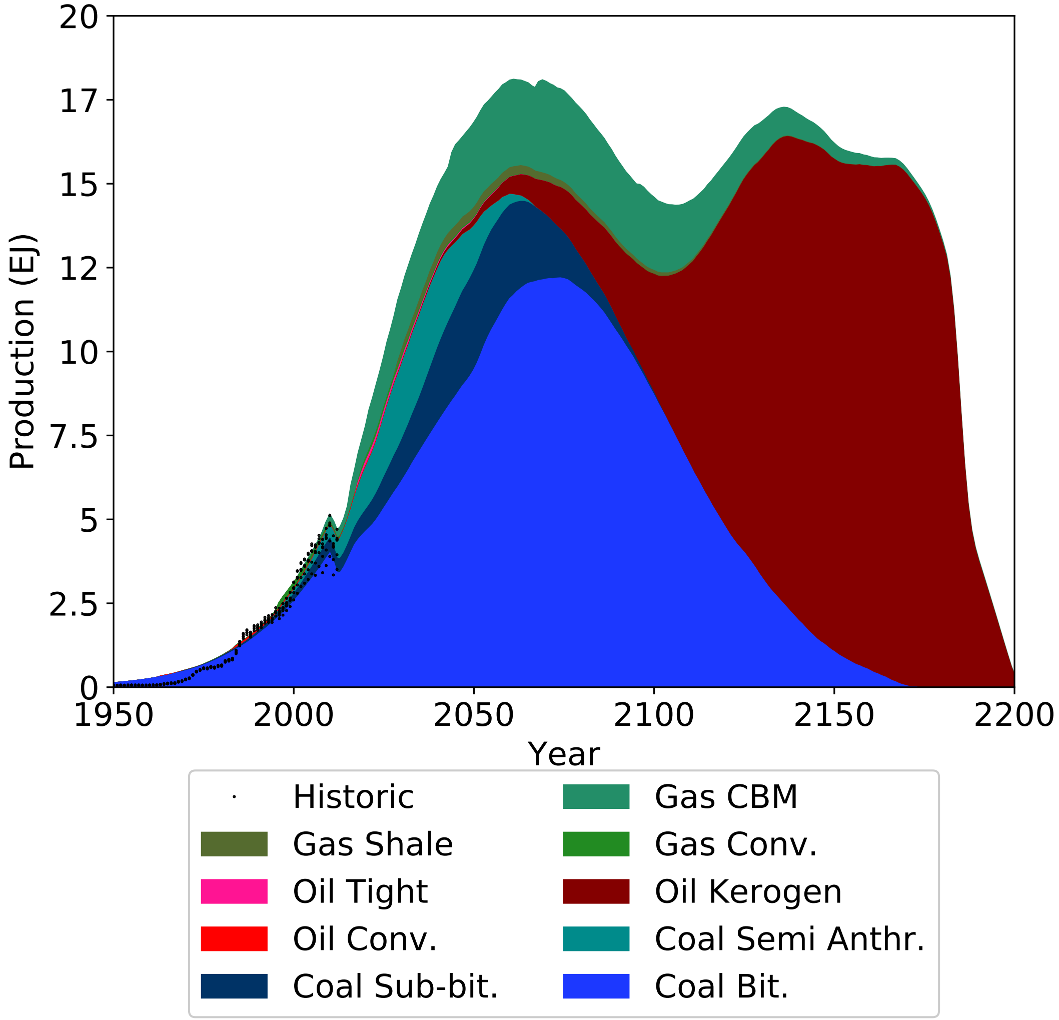

Figure 2.14: Australia - Queensland projection by mineral type

South Australia

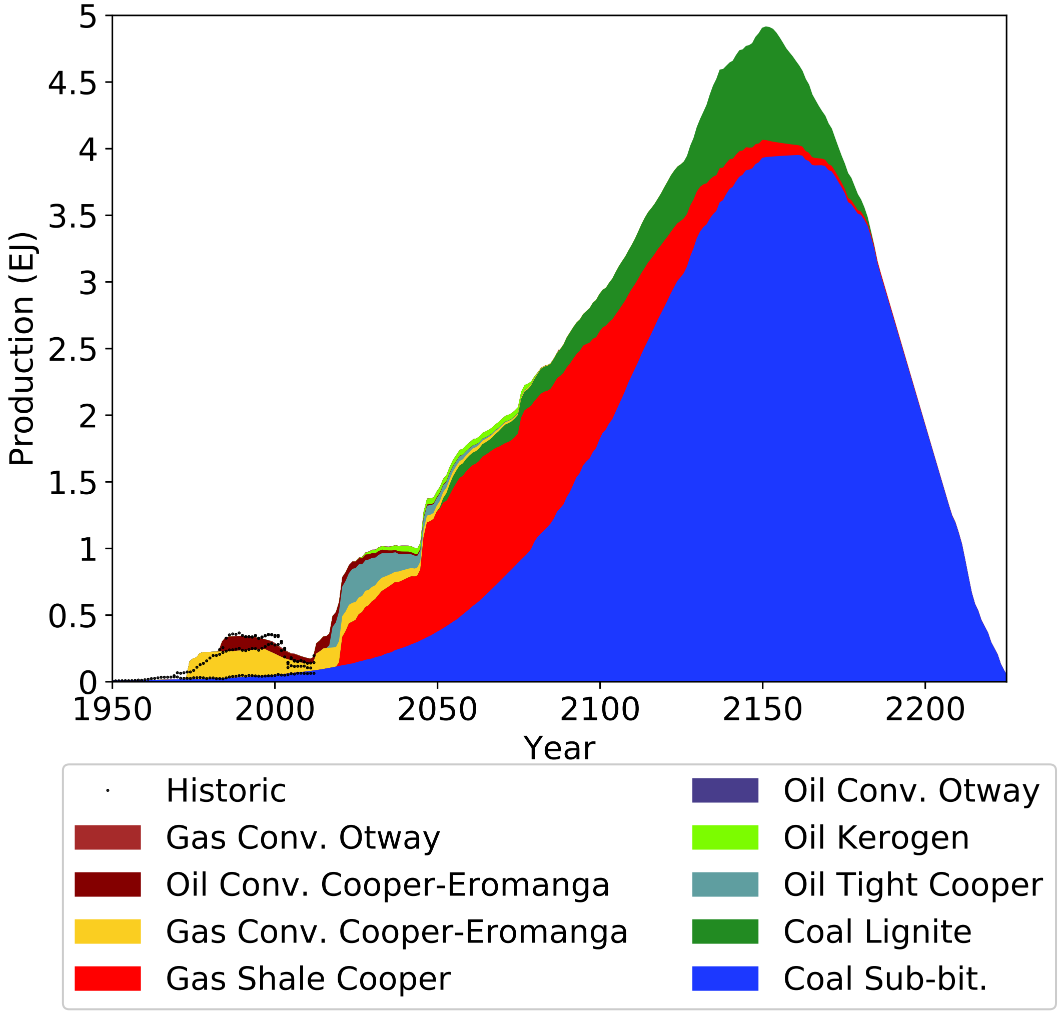

Figure 2.15: Australia - South Australia projections capped at 16

Table 2.15: Peak years - All

**Name**	**URR**	**Peak Year**	**Peak Rate**
Coal Sub-bit. South Australia	397.9	2161	3.95
Gas Shale South Australia Cooper	88.5	2077	1.1
Coal Lignite South Australia	47.5	2152	0.85
Gas Conv. South Australia Cooper-Eromanga	10.96	1978	0.2
Oil Tight South Australia Cooper	5.93	2024	0.25
Oil Conv. South Australia Cooper-Eromanga	3.7	1986	0.11
Oil Kerogen South Australia	2.06	2040	0.04
Gas Conv. South Australia Otway	0.14	2000	0.01
Oil Conv. South Australia Otway	–	1994	–
**Total**	**556.7**	**2151**	**4.91**

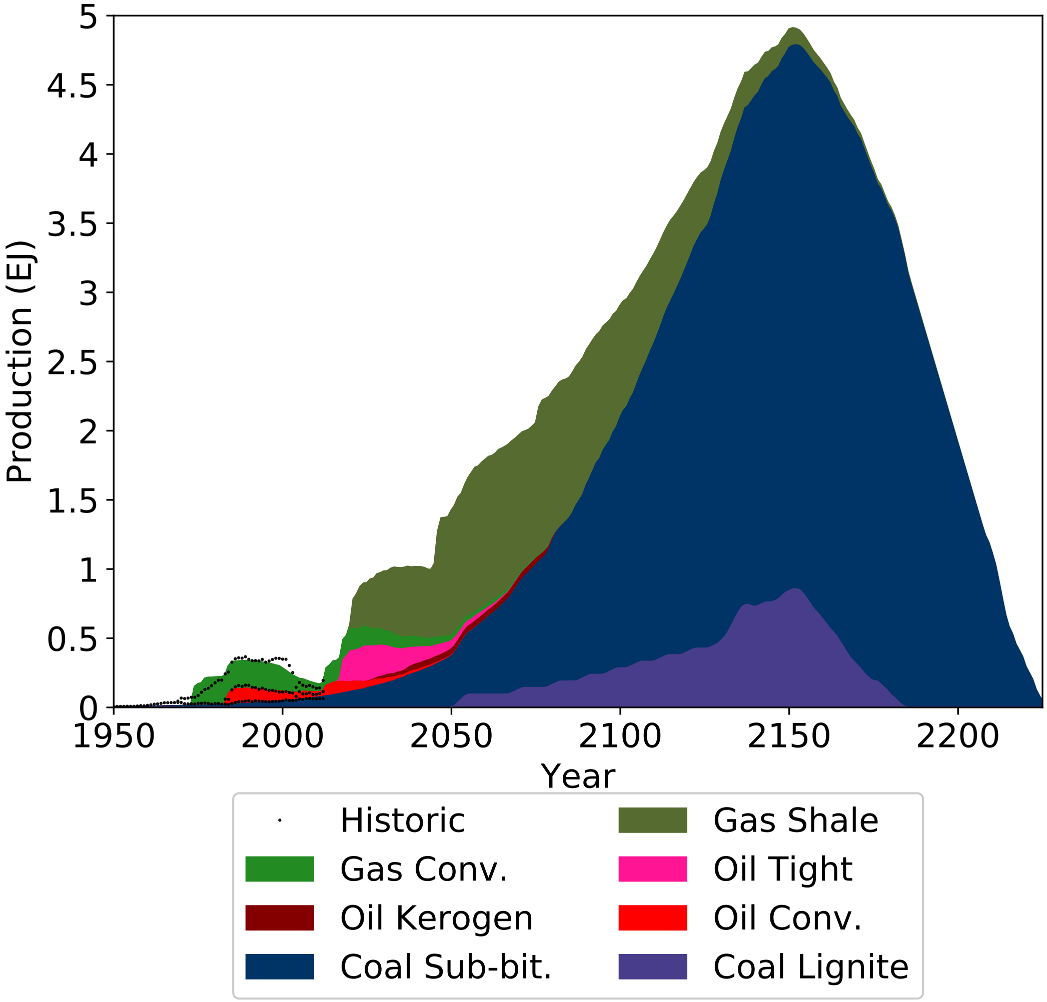

Figure 2.16: Australia - South Australia projection by mineral type

Table 2.16: Peak years - Minerals

**Name**	**URR**	**Peak Year**	**Peak Rate**
Coal Lignite	47.5	2152	0.85
Coal Sub-bit.	397.9	2161	3.95
Oil Conv.	3.7	1986	0.11
Oil Kerogen	2.06	2040	0.04
Oil Tight	5.93	2024	0.25
Gas Conv.	11.1	1978	0.2
Gas Shale	88.5	2077	1.1
**Total**	**556.7**	**2151**	**4.91**

Tasmania

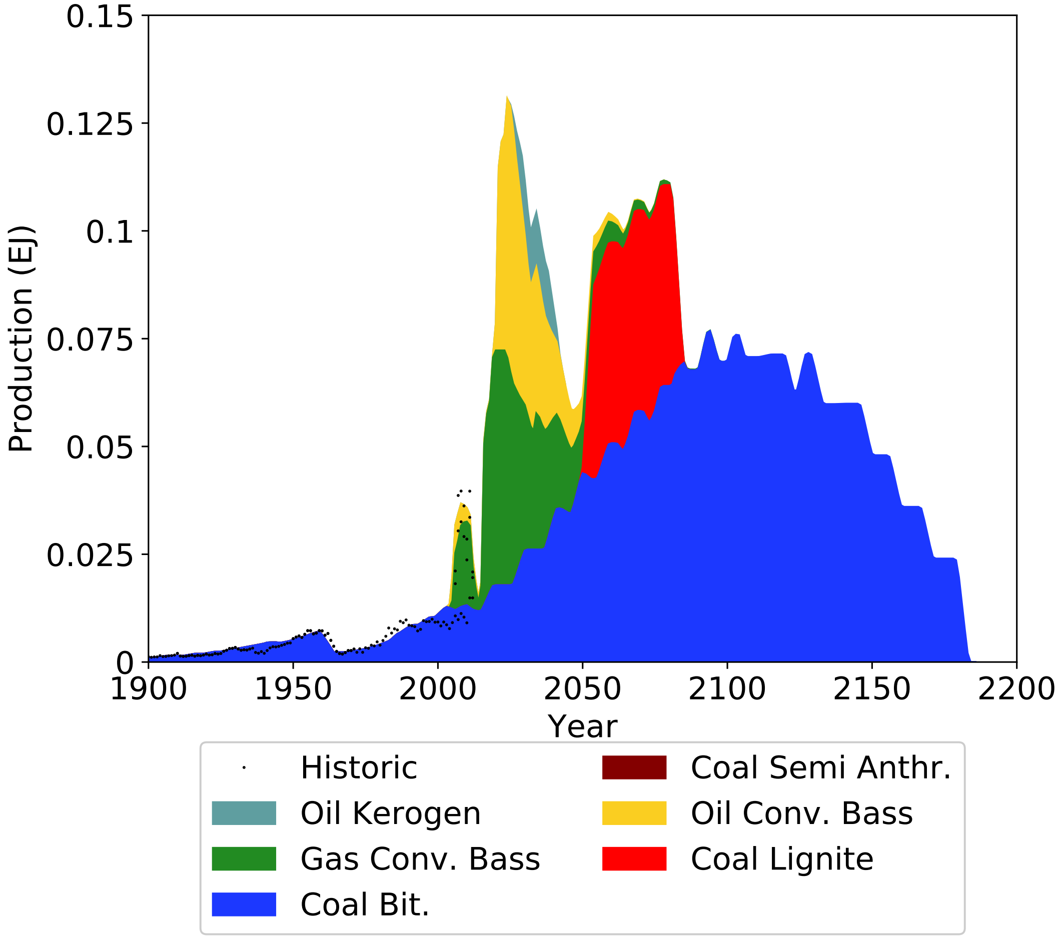

Figure 2.17: Australia - Tasmania projections capped at 16

Table 2.17: Peak years - All

**Name**	**URR**	**Peak Year**	**Peak Rate**
Coal Bit. Tasmania	8.88	2094	0.08
Coal Lignite Tasmania	1.43	2055	0.05
Gas Conv. Tasmania Bass	1.38	2020	0.05
Oil Conv. Tasmania Bass	0.99	2025	0.06
Oil Kerogen Tasmania	0.17	2030	0.01
Coal Semi Anthr. Tasmania	–	1953	–
**Total**	**12.84**	**2024**	**0.13**

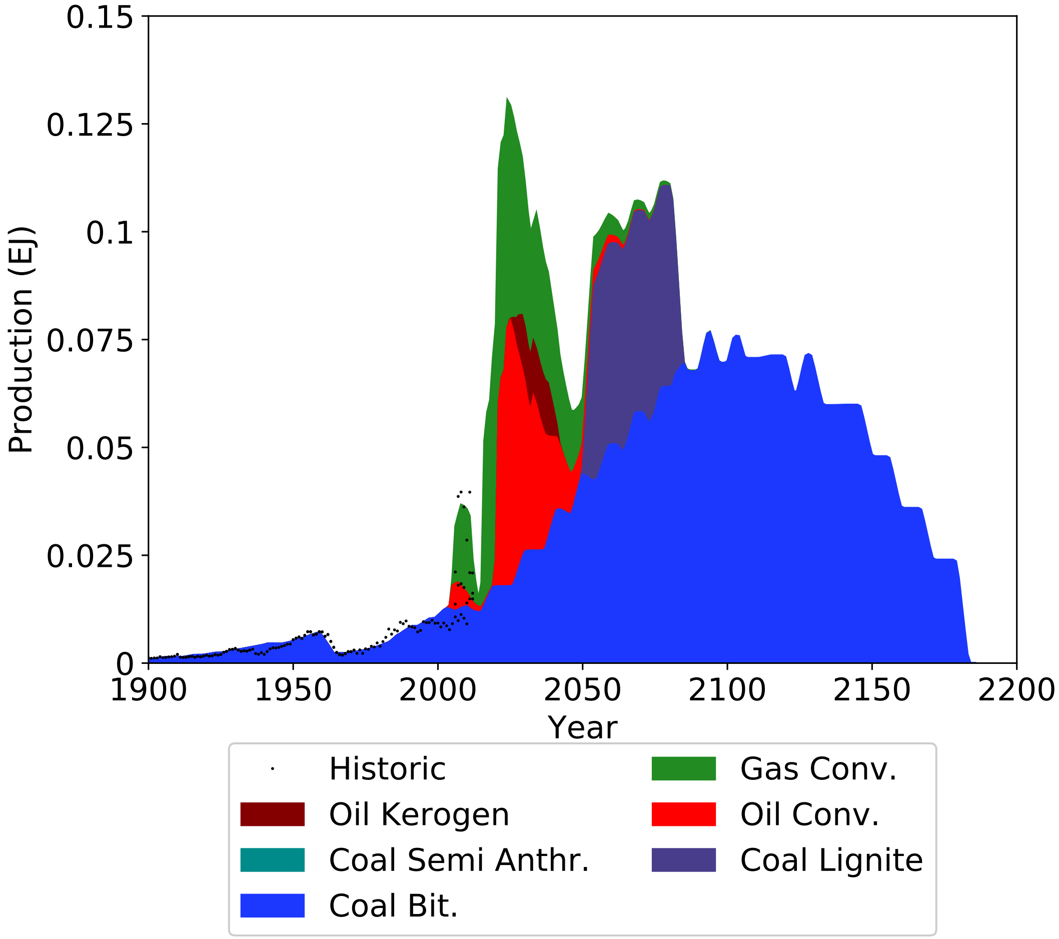

Figure 2.18: Australia - Tasmania projection by mineral type

Table 2.18: Peak years - Minerals

Name	URR	Peak Year	Peak Rate
Coal Bit.	8.88	2094	0.08
Coal Lignite	1.43	2055	0.05
Coal Semi Anthr.	–	1953	–
Oil Conv.	0.99	2025	0.06
Oil Kerogen	0.17	2030	0.01
Gas Conv.	1.38	2020	0.05
**Total**	**12.84**	**2024**	**0.13**

Victoria

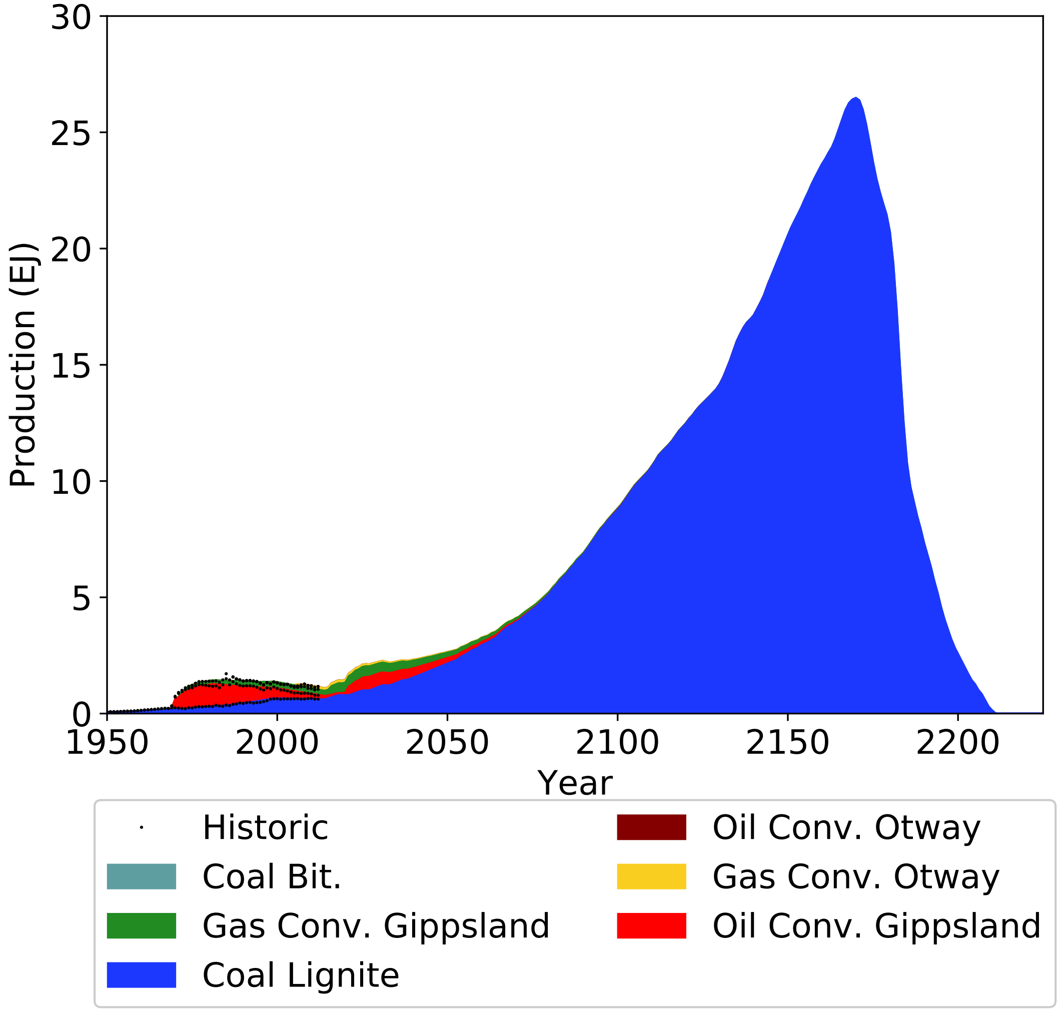

Figure 2.19: Australia - Victoria projections capped at 16

Table 2.19: Peak years - All

**Name**	**URR**	**Peak Year**	**Peak Rate**
Coal Lignite Victoria	1900.0	2170	26.48
Oil Conv. Victoria Gippsland	43.75	1980	0.96
Gas Conv. Victoria Gippsland	28.71	2023	0.47
Gas Conv. Victoria Otway	3.77	2018	0.12
Coal Bit. Victoria	0.55	1917	0.02
Oil Conv. Victoria Otway	0.17	2010	0.01
**Total**	**1976.95**	**2170**	**26.48**

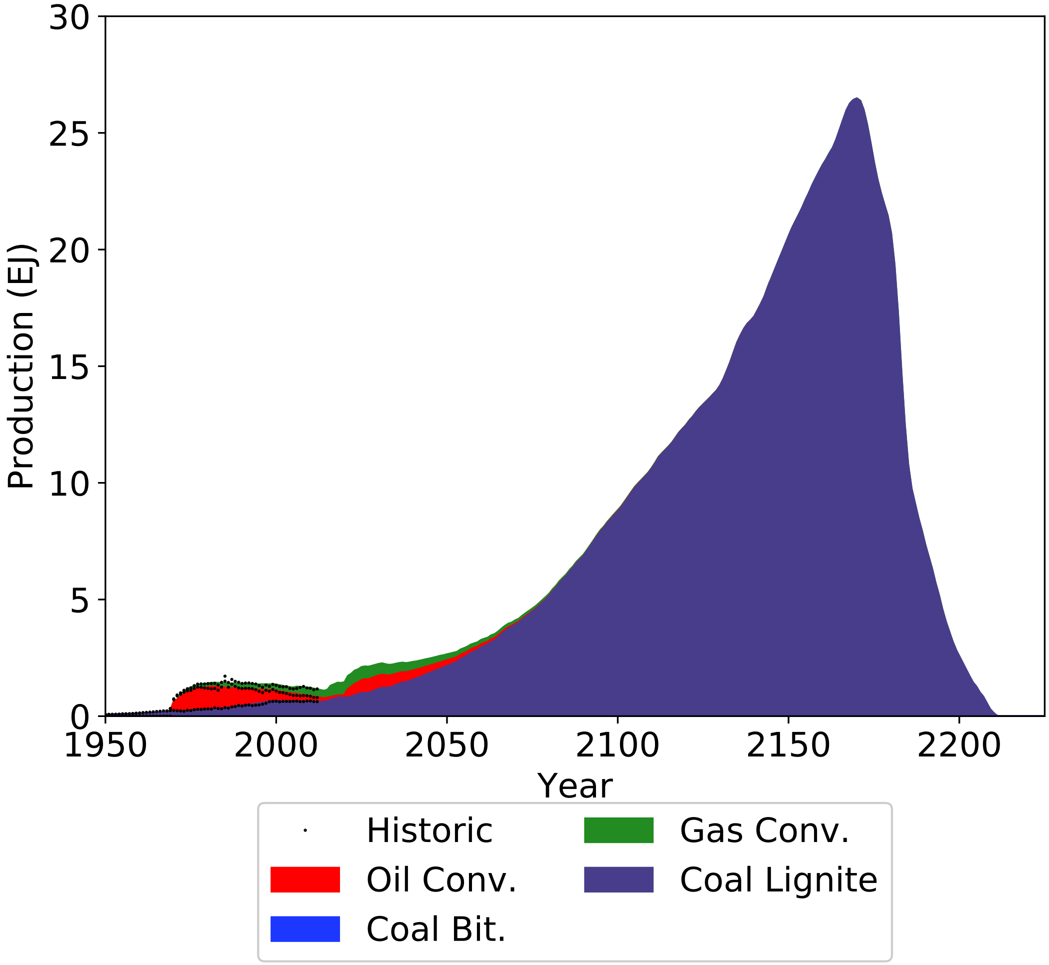

Figure 2.20: Australia - Victoria projection by mineral type

Table 2.20: Peak years - Minerals

**Name**	**URR**	**Peak Year**	**Peak Rate**
Coal Bit.	0.55	1917	0.02
Coal Lignite	1900.0	2170	26.48
Oil Conv.	43.92	1980	0.96
Gas Conv.	32.48	2023	0.57
**Total**	**1976.95**	**2170**	**26.48**

Western Australia

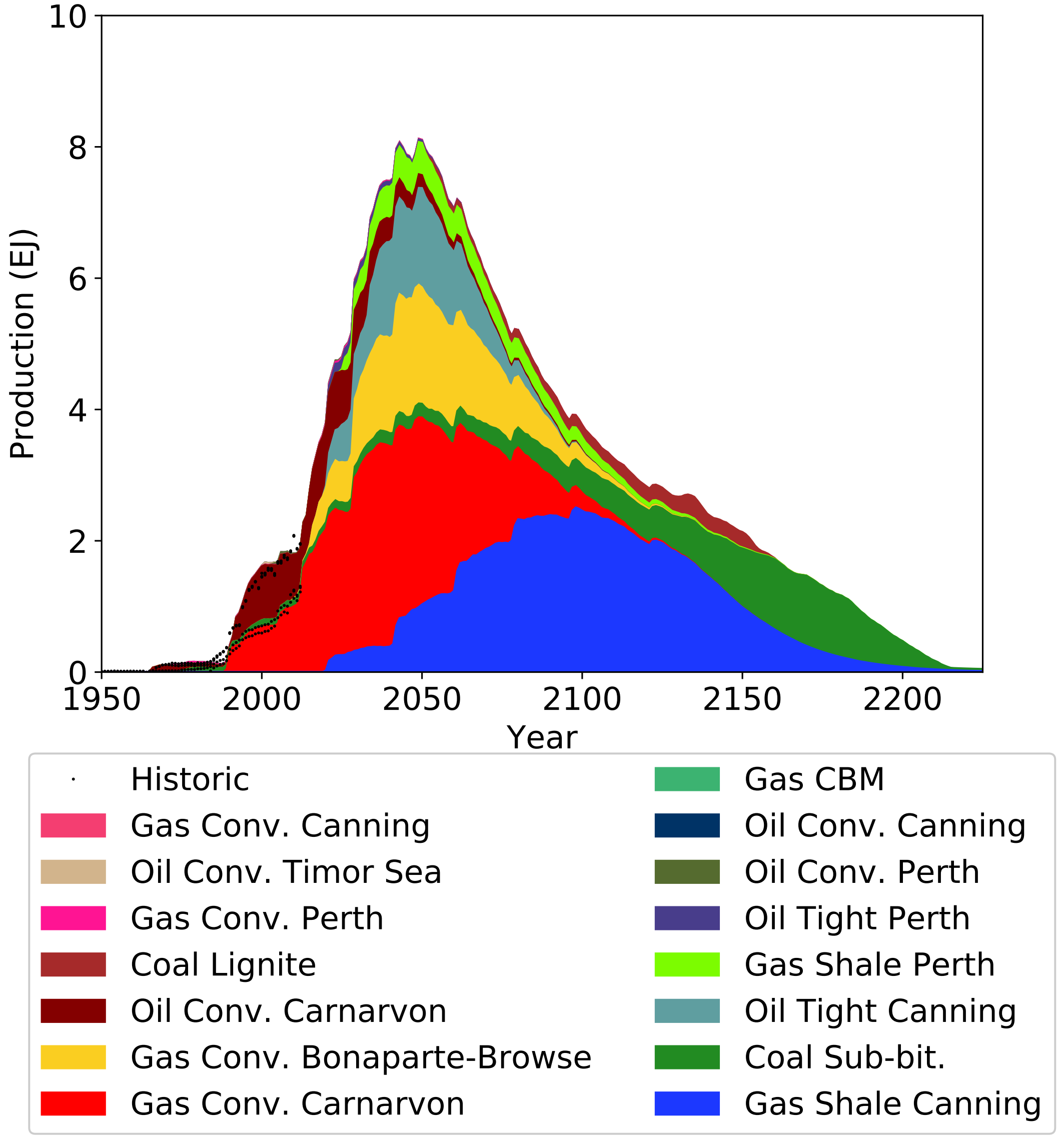

Figure 2.21: Australia - Western Australia projections capped at 16

Table 2.21: Peak years - All

Name	URR	Peak Year	Peak Rate
Gas Shale Western Australia Canning	223.63	2098	2.51
Gas Conv. Western Australia Carnarvon	185.66	2037	3.1
Coal Sub-bit. Western Australia	99.66	2160	1.07
Gas Conv. Western Australia Bonaparte-Browse	89.93	2046	1.81
Oil Tight Western Australia Canning	57.52	2050	1.51
Oil Conv. Western Australia Carnarvon	41.47	2021	0.92
Gas Shale Western Australia Perth	31.4	2042	0.5
Coal Lignite Western Australia	19.0	2135	0.33
Oil Tight Western Australia Perth	2.97	2025	0.14
Gas Conv. Western Australia Perth	1.54	2020	0.03
Oil Conv. Western Australia Perth	0.3	2005	0.03
Oil Conv. Western Australia Timor Sea	0.17	2002	0.03
Oil Conv. Western Australia Canning	0.02	1987	–
Gas Conv. Western Australia Canning	0.02	2022	–
Gas CBM Western Australia	–	2008	–
**Total**	**753.3**	**2049**	**8.12**

Table 2.22: Peak years - Minerals

Name	URR	Peak Year	Peak Rate
Coal Lignite	19.0	2135	0.33
Coal Sub-bit.	99.66	2160	1.07
Oil Conv.	41.97	2021	0.94
Oil Tight	60.49	2050	1.55
Gas Conv.	277.15	2043	4.74
Gas Shale	255.03	2098	2.71
Gas CBM	–	2008	–
**Total**	**753.3**	**2049**	**8.12**

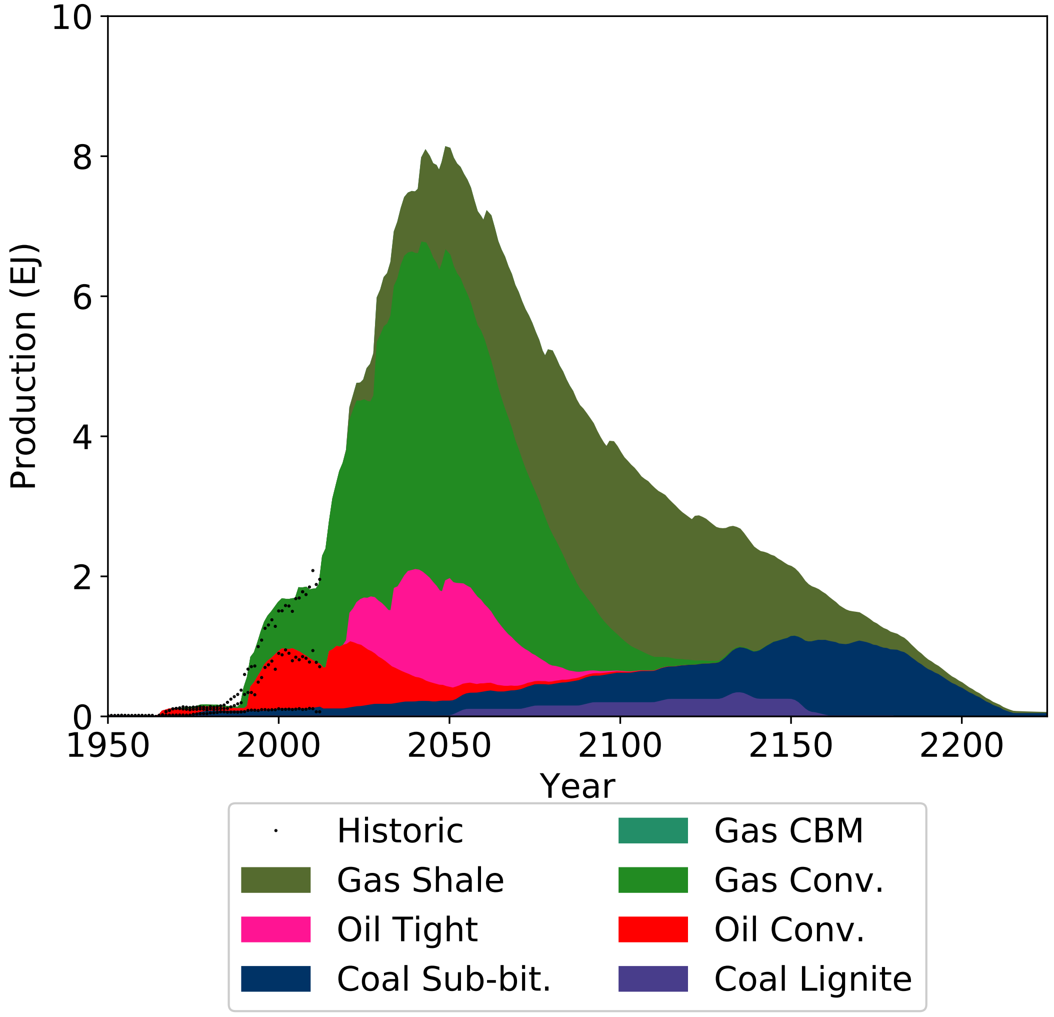

Figure 2.22: Australia - Western Australia projection by mineral type

2.2.4 Projection by region

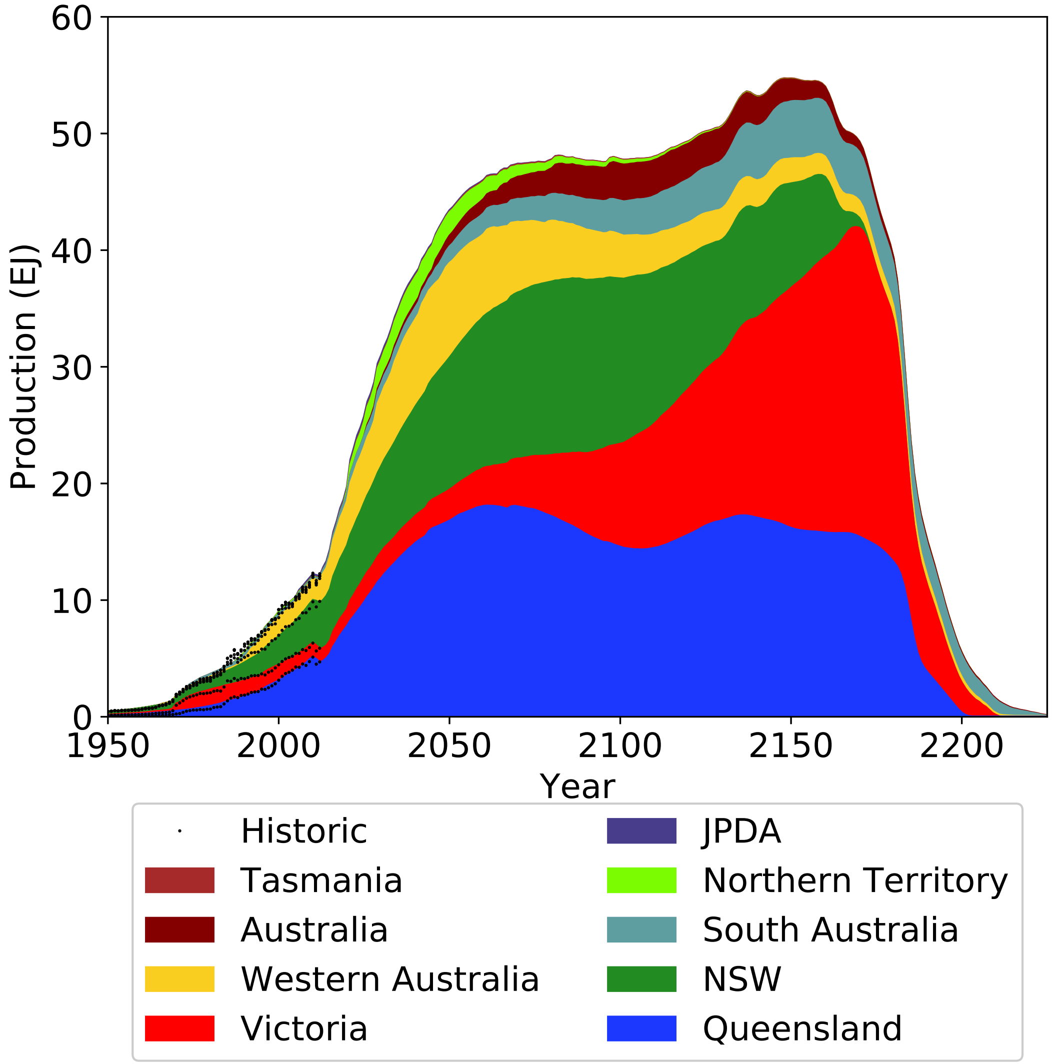

Figure 2.23: Australia by region projections capped at 16

Table 2.23: Peak years - All

Name	URR	Peak Year	Peak Rate
Queensland	2752.0	2061	18.1
Victoria	1976.95	2170	26.48
NSW	1756.96	2086	14.97
Western Australia	753.3	2049	8.12
South Australia	556.7	2151	4.91
Australia	296.65	2115	3.19
Northern Territory	111.18	2039	2.26
Tasmania	12.84	2024	0.13
JPDA	12.42	2023	0.34
**Total**	**8228.99**	**2148**	**54.73**

2.3 Bangladesh

2.3.1 All Projections

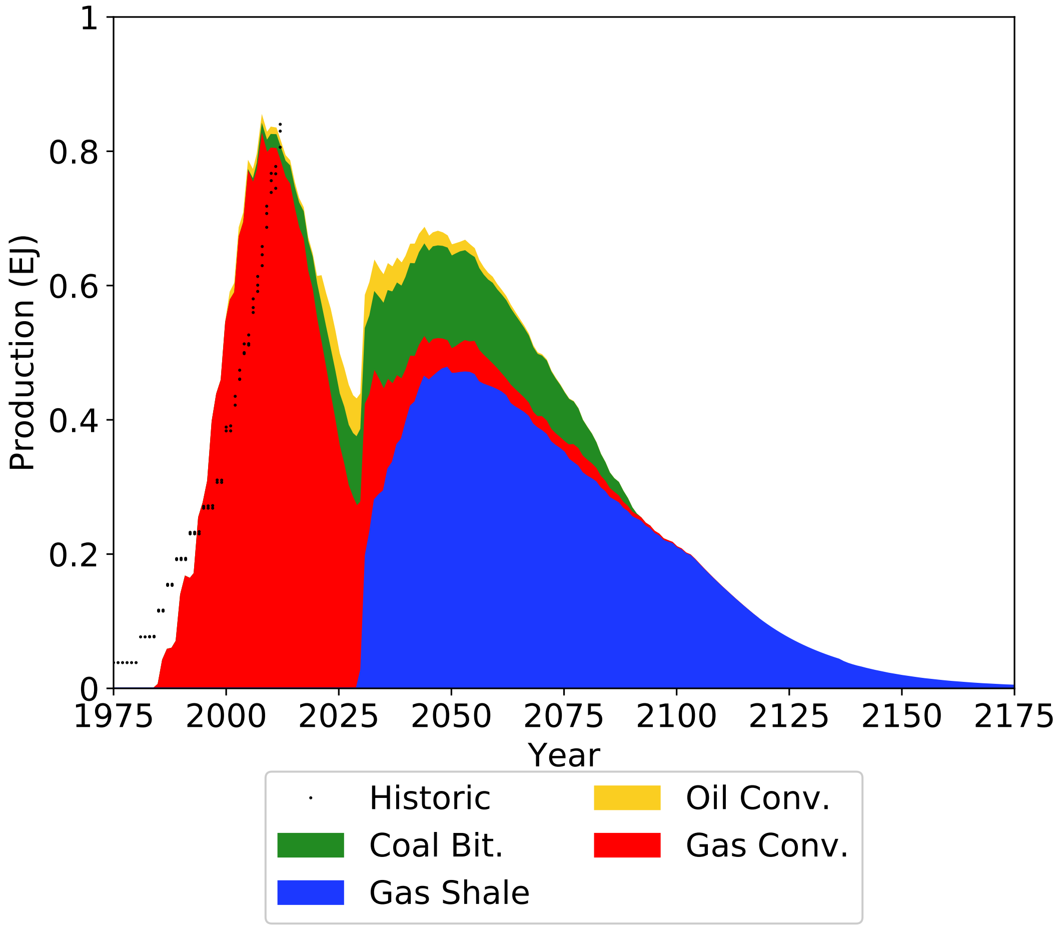

Figure 2.24: Bangladesh projections capped at 16

Table 2.24: Peak years - All

**Name**	**URR**	**Peak Year**	**Peak Rate**
Gas Shale	29.66	2049	0.48
Gas Conv.	24.4	2008	0.82
Coal Bit.	7.15	2038	0.14
Oil Conv.	1.58	2024	0.06
**Total**	**62.79**	**2008**	**0.85**

2.3.2 By Mineral

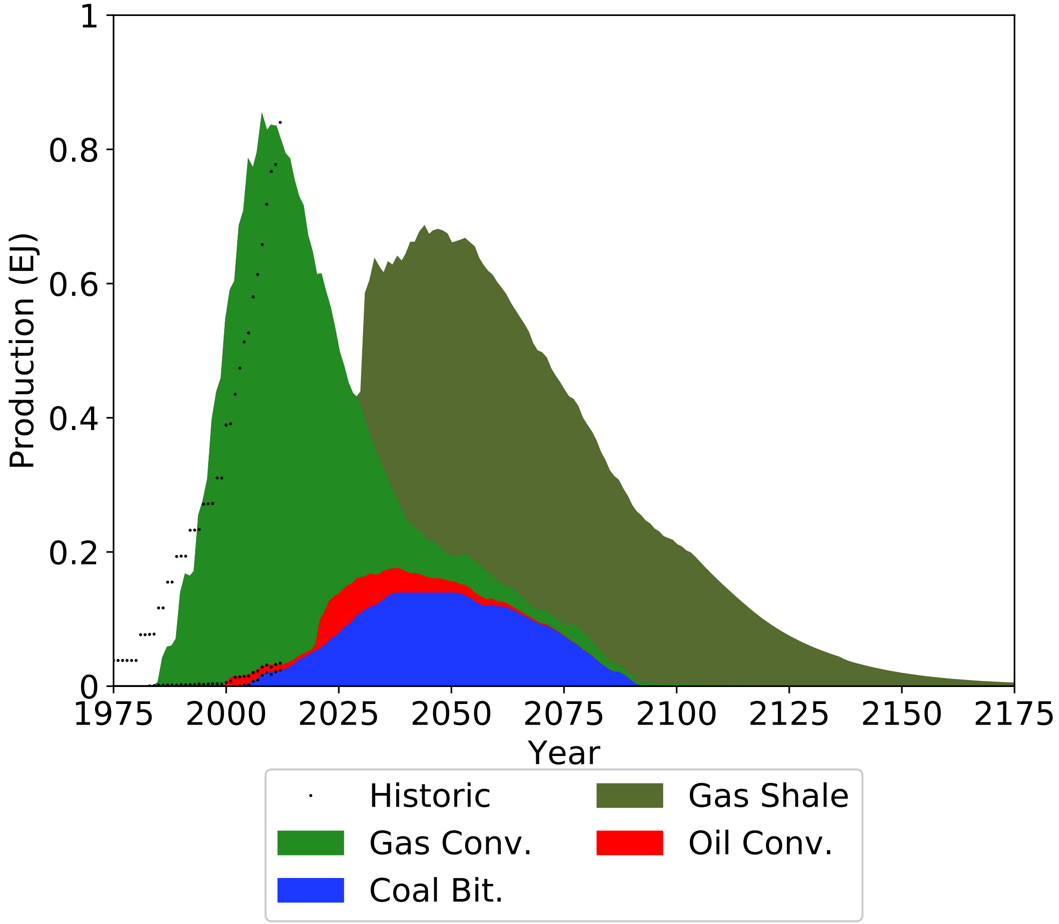

Figure 2.25: Bangladesh projection by mineral type

Table 2.25: Peak years - Minerals

**Name**	**URR**	**Peak Year**	**Peak Rate**
Coal Bit.	7.15	2038	0.14
Oil Conv.	1.58	2024	0.06
Gas Conv.	24.4	2008	0.82
Gas Shale	29.66	2049	0.48
**Total**	**62.79**	**2008**	**0.85**

2.4 Bhutan

2.4.1 All Projections

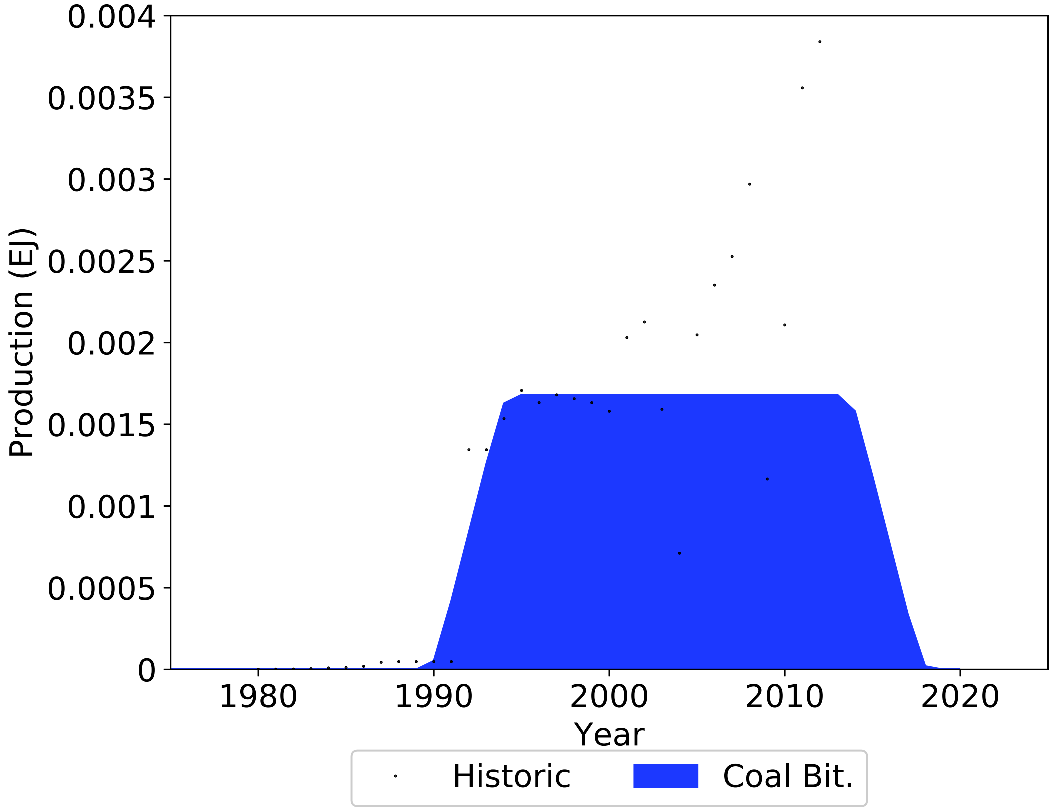

Figure 2.26: Bhutan projections capped at 16

Table 2.26: Peak years - All

Name	URR	Peak Year	Peak Rate
Coal Bit.	0.04	1995	–
**Total**	**0.04**	**1995**	–

2.4.2 By Mineral

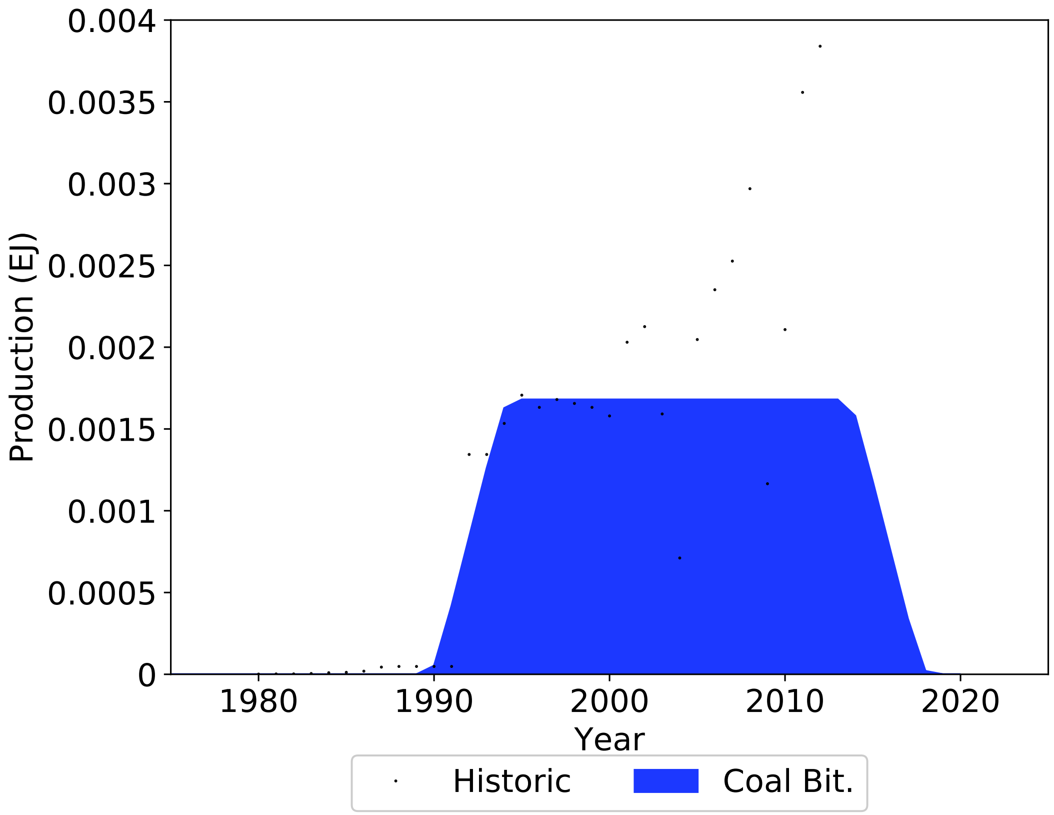

Figure 2.27: Bhutan projection by mineral type

Table 2.27: Peak years - Minerals

Name	URR	Peak Year	Peak Rate
Coal Bit.	0.04	1995	–
**Total**	**0.04**	**1995**	–

2.5 Brunei

2.5.1 All Projections

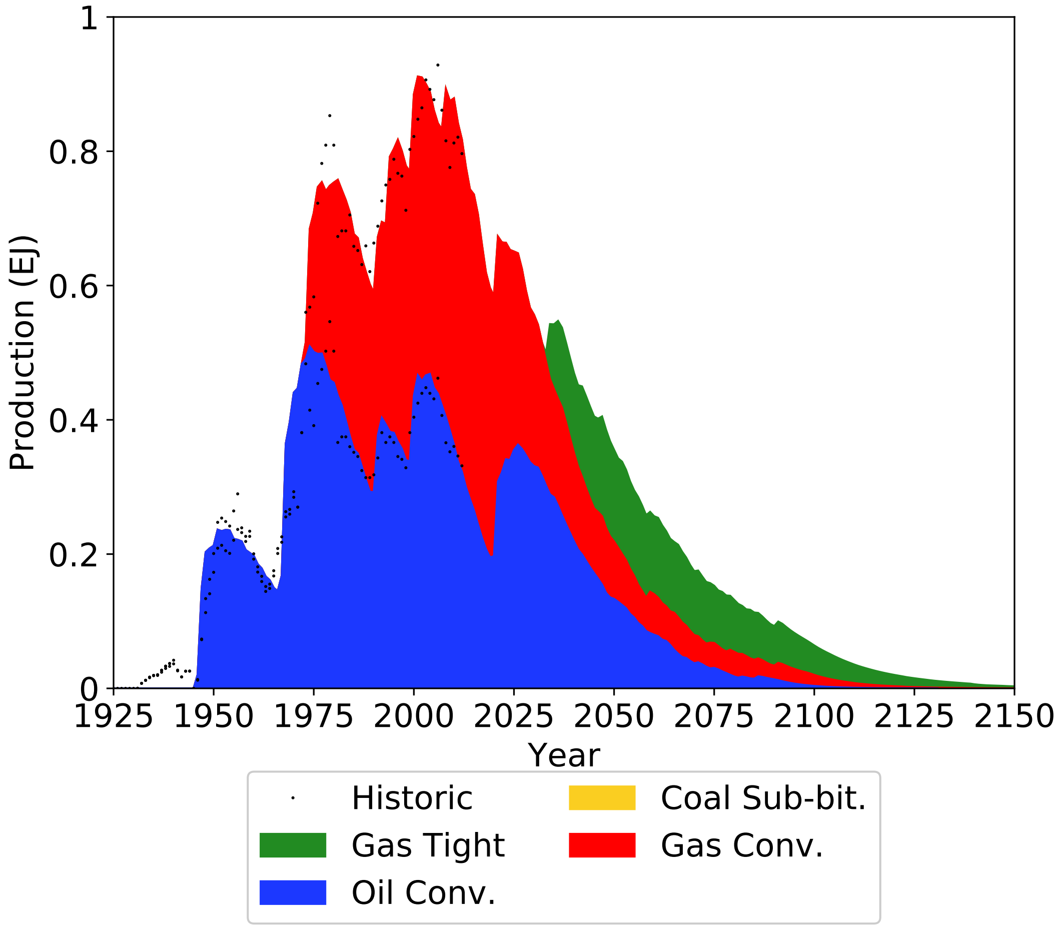

Figure 2.28: Brunei projections capped at 16

Table 2.28: Peak years - All

**Name**	**URR**	**Peak Year**	**Peak Rate**
Oil Conv.	34.37	1974	0.51
Gas Conv.	25.6	2010	0.52
Gas Tight	7.42	2047	0.15
Coal Sub-bit.	0.01	1893	–
**Total**	**67.4**	**2001**	**0.91**

2.5.2 By Mineral

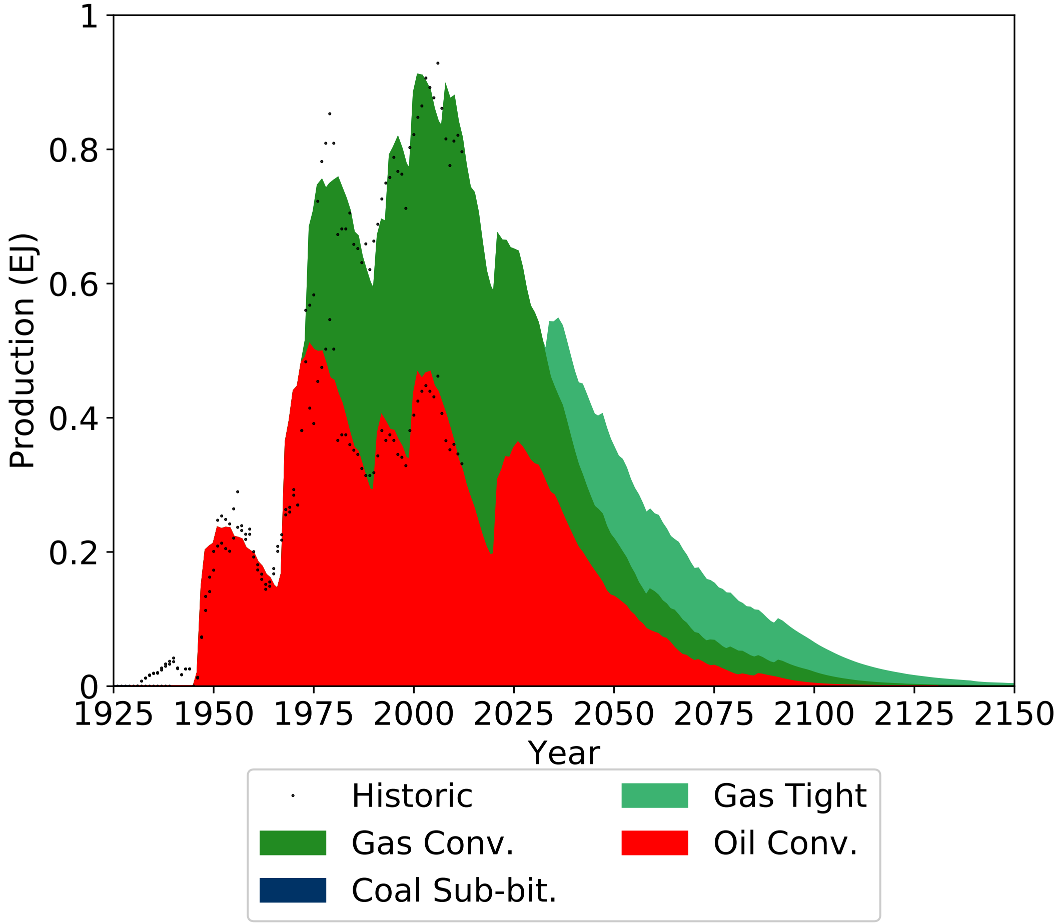

Figure 2.29: Brunei projection by mineral type

Table 2.29: Peak years - Minerals

Name	URR	Peak Year	Peak Rate
Coal Sub-bit.	0.01	1893	–
Oil Conv.	34.37	1974	0.51
Gas Conv.	25.6	2010	0.52
Gas Tight	7.42	2047	0.15
**Total**	**67.4**	**2001**	**0.91**

2.6 Burma

2.6.1 All Projections

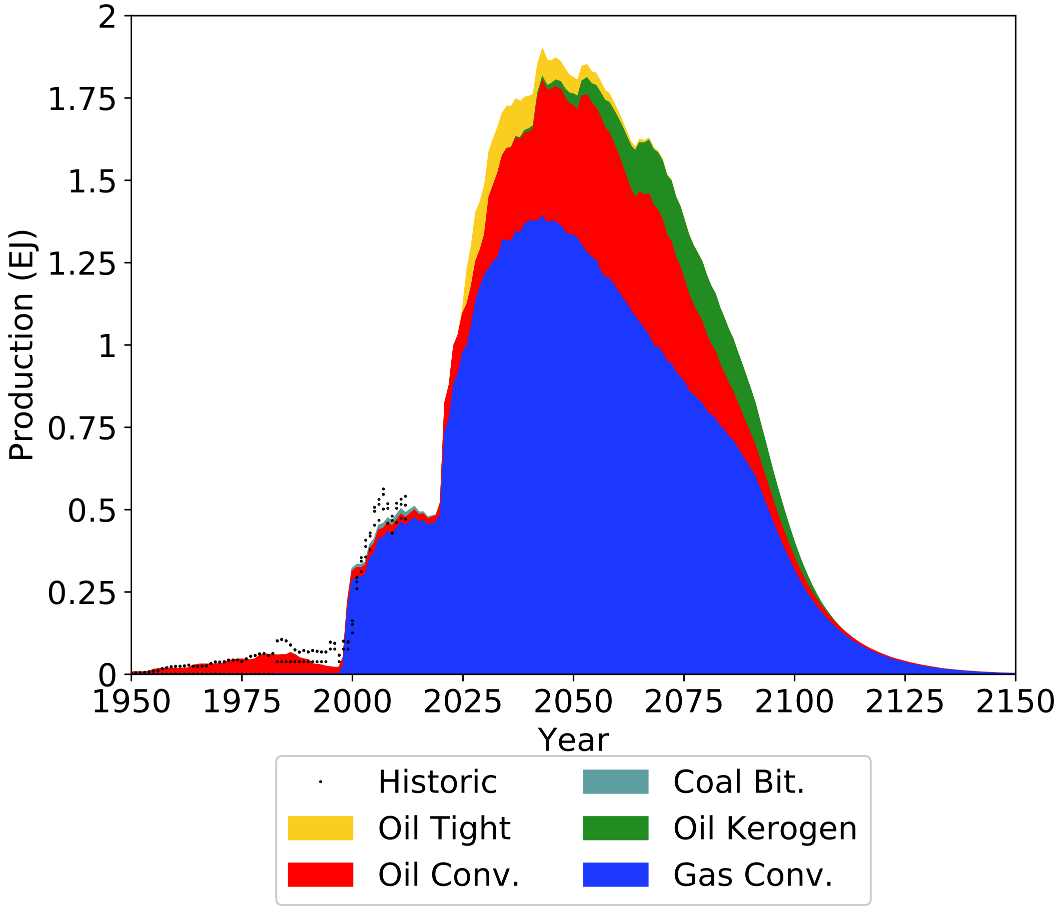

Figure 2.30: Burma projections capped at 16

Table 2.30: Peak years - All

**Name**	**URR**	**Peak Year**	**Peak Rate**
Gas Conv.	92.1	2043	1.39
Oil Conv.	23.95	2053	0.48
Oil Kerogen	6.9	2072	0.18
Oil Tight	3.07	2030	0.15
Coal Bit.	0.26	2010	0.02
**Total**	**126.28**	**2043**	**1.9**

2.6.2 By Mineral

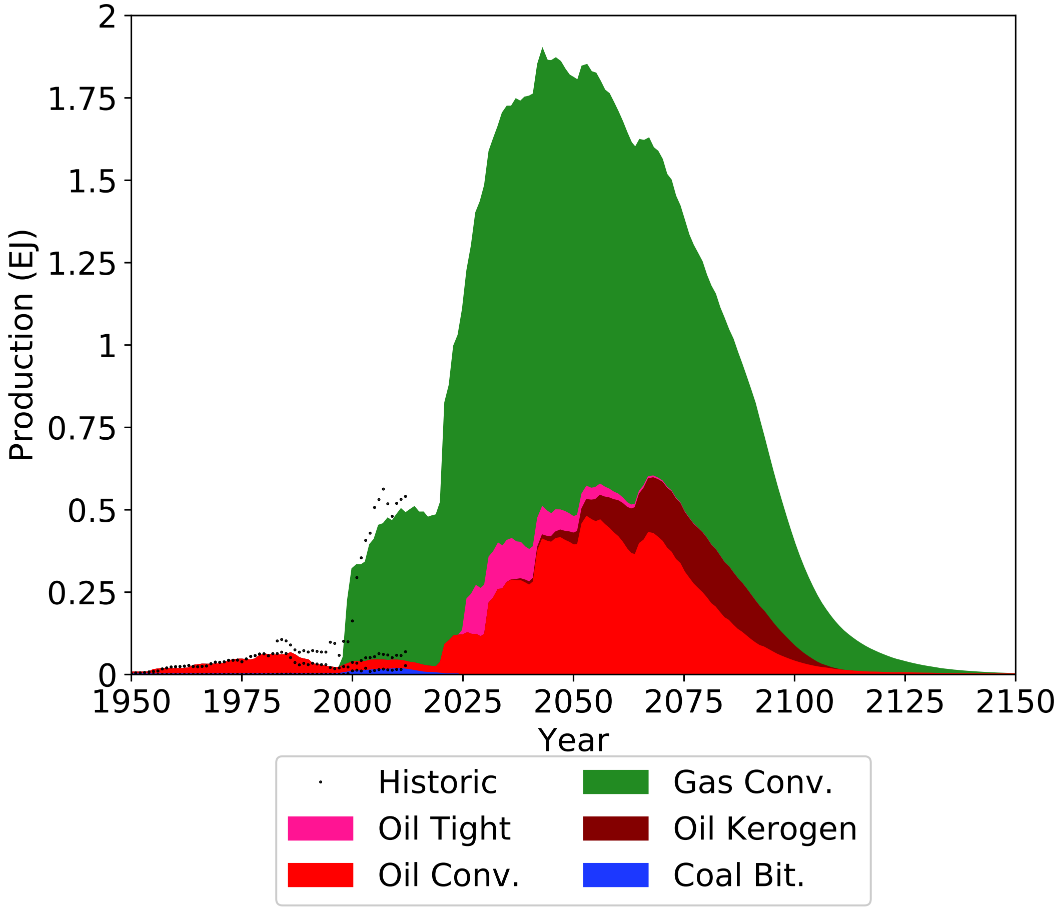

Figure 2.31: Burma projection by mineral type

Table 2.31: Peak years - Minerals

**Name**	**URR**	**Peak Year**	**Peak Rate**
Coal Bit.	0.26	2010	0.02
Oil Conv.	23.95	2053	0.48
Oil Kerogen	6.9	2072	0.18
Oil Tight	3.07	2030	0.15
Gas Conv.	92.1	2043	1.39
**Total**	**126.28**	**2043**	**1.9**

2.7 Cambodia

2.7.1 All Projections

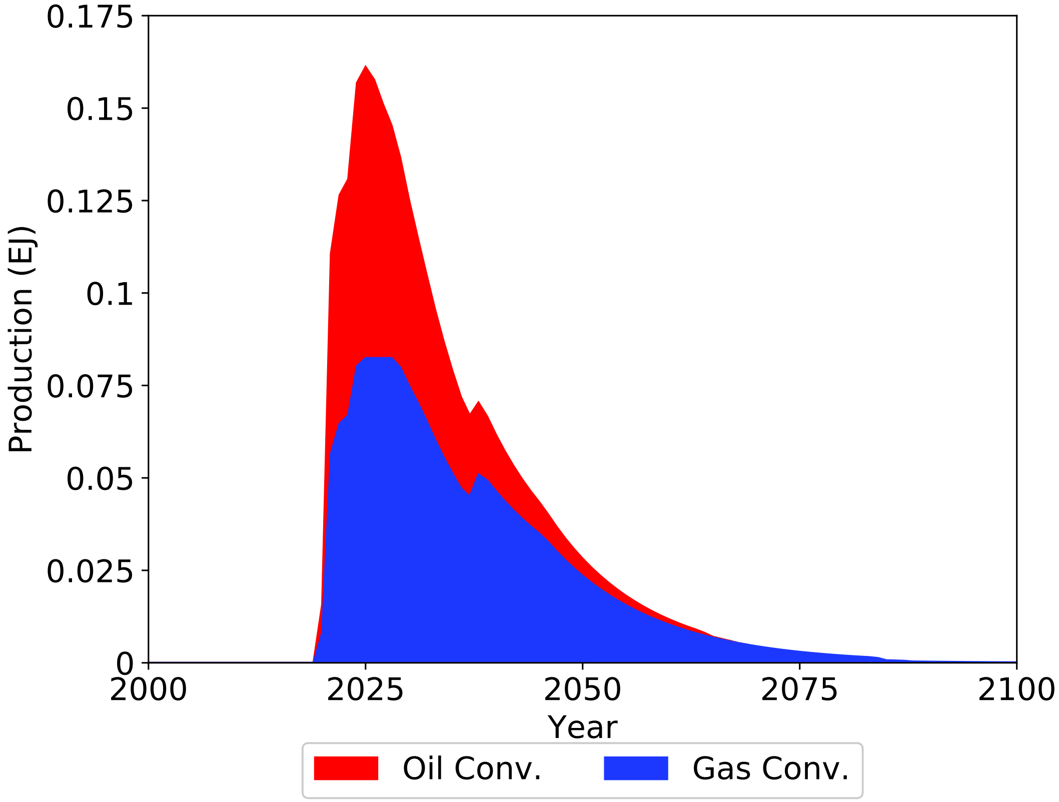

Figure 2.32: Cambodia projections capped at 16

Table 2.32: Peak years - All

Name	URR	Peak Year	Peak Rate
Gas Conv.	1.9	2025	0.08
Oil Conv.	1.05	2025	0.08
**Total**	**2.95**	**2025**	**0.16**

2.7.2 By Mineral

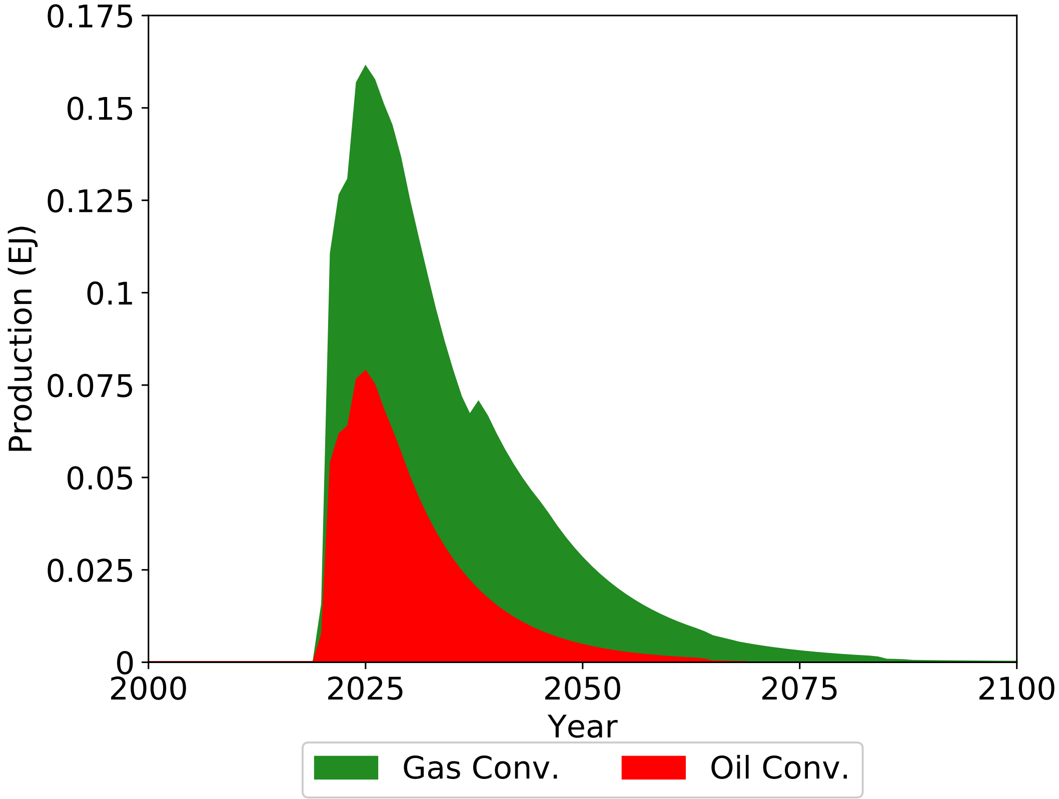

Figure 2.33: Cambodia projection by mineral type

Table 2.33: Peak years - Minerals

Name	URR	Peak Year	Peak Rate
Oil Conv.	1.05	2025	0.08
Gas Conv.	1.9	2025	0.08
**Total**	**2.95**	**2025**	**0.16**

2.8 China

2.8.1 All Projections

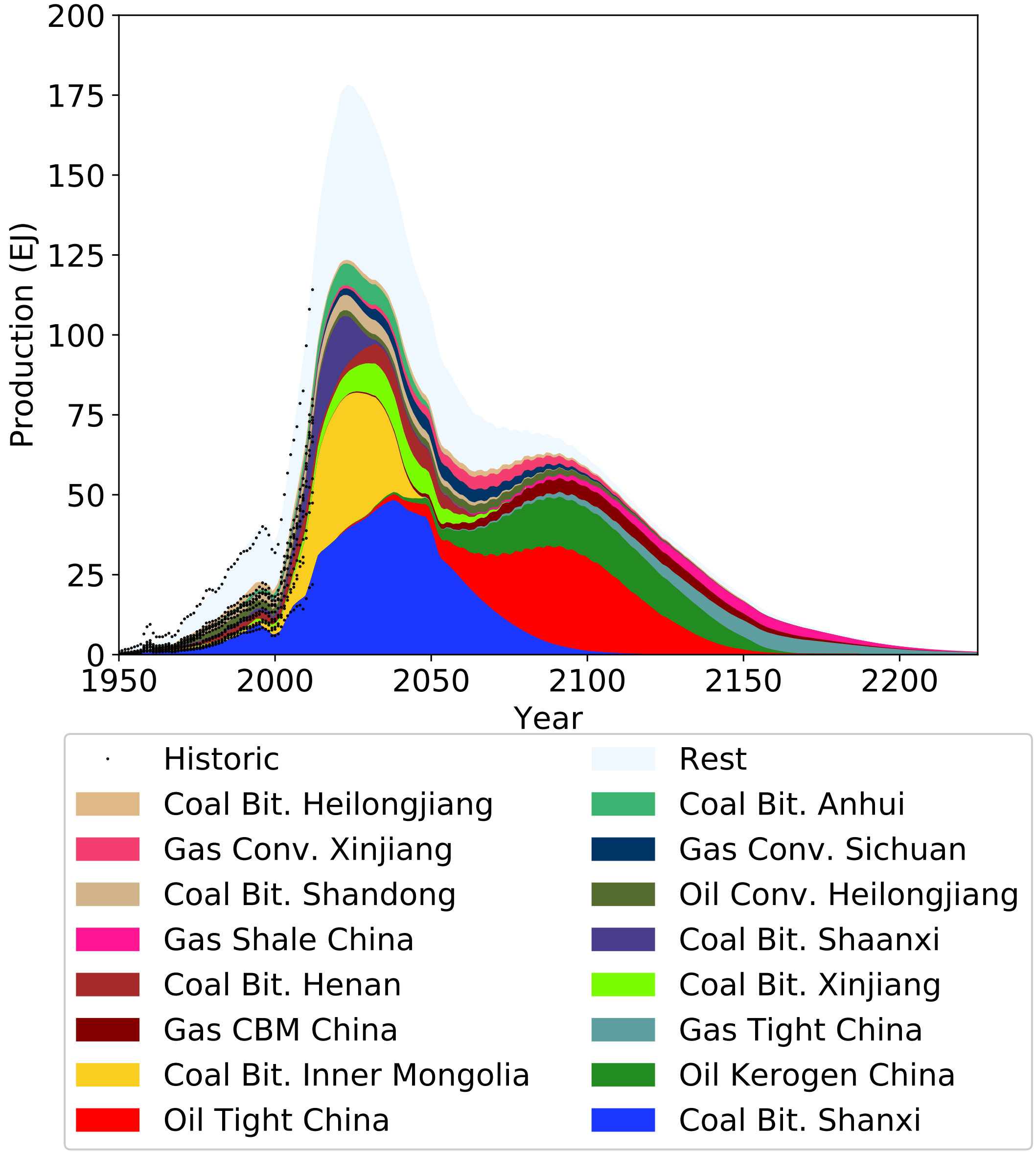

Figure 2.34: China projections capped at 16

Table 2.34: Peak years - All

Name	URR	Peak Year	Peak Rate
Coal Bit. Shanxi	2508.19	2038	48.04
Oil Tight China	1718.31	2091	31.17
Oil Kerogen China	1146.0	2096	15.47
Coal Bit. Inner Mongolia	1122.81	2023	41.31
Gas Tight China	444.97	2150	5.18
Gas CBM China	407.03	2103	4.55
Coal Bit. Xinjiang	397.78	2037	10.89
Coal Bit. Henan	346.51	2042	7.65
Coal Bit. Shaanxi	346.35	2018	19.61
Gas Shale China	318.89	2145	3.83
Oil Conv. Heilongjiang	302.68	2067	2.74
Coal Bit. Shandong	278.09	2023	4.79
Gas Conv. Sichuan	277.77	2047	4.82
Gas Conv. Xinjiang	251.57	2066	4.14
Coal Bit. Anhui	247.75	2027	6.95
Coal Bit. Heilongjiang	222.88	2007	2.66
Coal Bit. Guizhou	221.34	2024	8.4
Gas Conv. Inner Mongolia	208.83	2046	3.58
Coal Bit. Sichuan	184.59	2027	4.61
Coal Bit. Yunnan	176.66	2031	4.01
Gas Conv. Shaanxi	175.87	2050	2.83
Coal Bit. Hebei	170.41	2030	2.84
Oil Conv. Shandong	165.18	2041	2.95
Gas Hydrates China	142.0	2131	1.73
Coal Bit. Liaoning	141.13	2016	1.92
Oil Conv. Xinjiang	125.62	2042	2.18
Coal Bit. Gansu	102.87	2034	1.64
Coal Bit. Ningxia	99.05	2021	5.02
Oil Conv. Hebei	78.91	2042	1.6
Coal Bit. Chongqing	74.71	2020	1.29
Gas Conv. Offshore	70.95	2047	1.47
Oil Conv. Shaanxi	69.46	2019	1.96
Oil Conv. Liaoning	68.87	2050	0.81
Gas Conv. Chongqing	56.59	2042	1.16
Coal Bit. Hunan	56.4	2012	2.15
Gas Conv. Heilongjiang	52.91	2043	0.97
Coal Bit. Jilin	52.66	2017	1.75
Coal Bit. Jiangsu	48.95	2000	0.66
Oil Conv. Tianjin	44.06	2011	1.39
Coal Bit. Qinghai	43.41	2027	1.72
Oil Conv. Jilin	38.88	2026	0.8
Gas Conv. Qinghai	37.32	2033	0.88
Oil Conv. Gansu	31.5	2031	0.95
Coal Bit. Jiangxi	31.21	2007	0.8
Oil Conv. Henan	30.55	2027	0.71
Oil Conv. Guangdong	28.79	2023	0.64
Coal Bit. Historic	25.96	1937	0.96
Coal Bit. Fujian	20.67	2031	0.83
Gas Conv. Jilin	20.22	2021	0.51

Table 2.34: Peak years - All – Continued

**Name**	**URR**	**Peak Year**	**Peak Rate**
Coal Bit. Beijing	19.54	2048	0.27
Gas Conv. Shandong	17.34	2030	0.41
Gas Conv. Guangdong	17.26	2017	0.36
Coal Bit. Hubei	16.89	2009	0.28
Gas Conv. Liaoning	16.56	2027	0.37
Oil Conv. Inner Mongolia	13.32	2021	0.45
Coal Bit. Guangxi	13.25	1993	0.25
Gas Conv. Tianjin	13.15	2026	0.31
Oil Conv. Qinghai	12.64	2027	0.39
Gas Conv. Hebei	10.39	2024	0.28
Gas Conv. Henan	9.64	2029	0.24
Oil Conv. Jiangsu	8.1	2023	0.23
Coal Bit. Guangdong	8.07	1972	0.24
Coal Bit. Tianjin	7.13	2042	0.27
Gas Conv. Ningxia	6.63	2025	0.24
Oil Conv. Hubei	5.97	2024	0.2
Gas Conv. Gansu	5.66	2028	0.17
Oil Extra Heavy China	5.01	2030	0.15
Oil Conv. Ningxia	4.84	2027	0.16
Coal Bit. Hainan	2.86	2059	0.09
Coal Bit. Zhejiang	2.2	1975	0.04
Gas Conv. Hubei	1.73	2023	0.06
Oil Conv. Sichuan	1.3	2025	0.07
Gas Conv. Shanghai	1.19	2025	0.04
Oil Nat. Bitumen China	1.05	2054	0.03
Gas Conv. Jiangsu	0.79	2025	0.03
Oil Conv. Hainan	0.5	2025	0.02
Oil Conv. Shanghai	0.42	2000	0.03
Gas Conv. Guizhou	0.42	2025	0.02
Gas Conv. Hainan	0.34	2015	0.02
Coal Bit. Tibet	0.31	2037	0.01
Oil Conv. Anhui	0.3	2025	0.02
Oil Conv. Guangxi	0.29	2020	0.02
Coal Bit. Shanghai	0.25	1982	0.08
Oil Conv. Chongqing	0.19	2025	0.01
Gas Conv. Yunnan	0.12	2020	0.01
Gas Conv. Guangxi	0.03	2022	–
Gas Conv. Jiangxi	0.02	2007	–
Oil Conv. Yunnan	0.01	2022	–
Gas Conv. Anhui	0.01	2022	–
**Total**	**13459.83**	**2024**	**177.93**

2.8.2 By Mineral

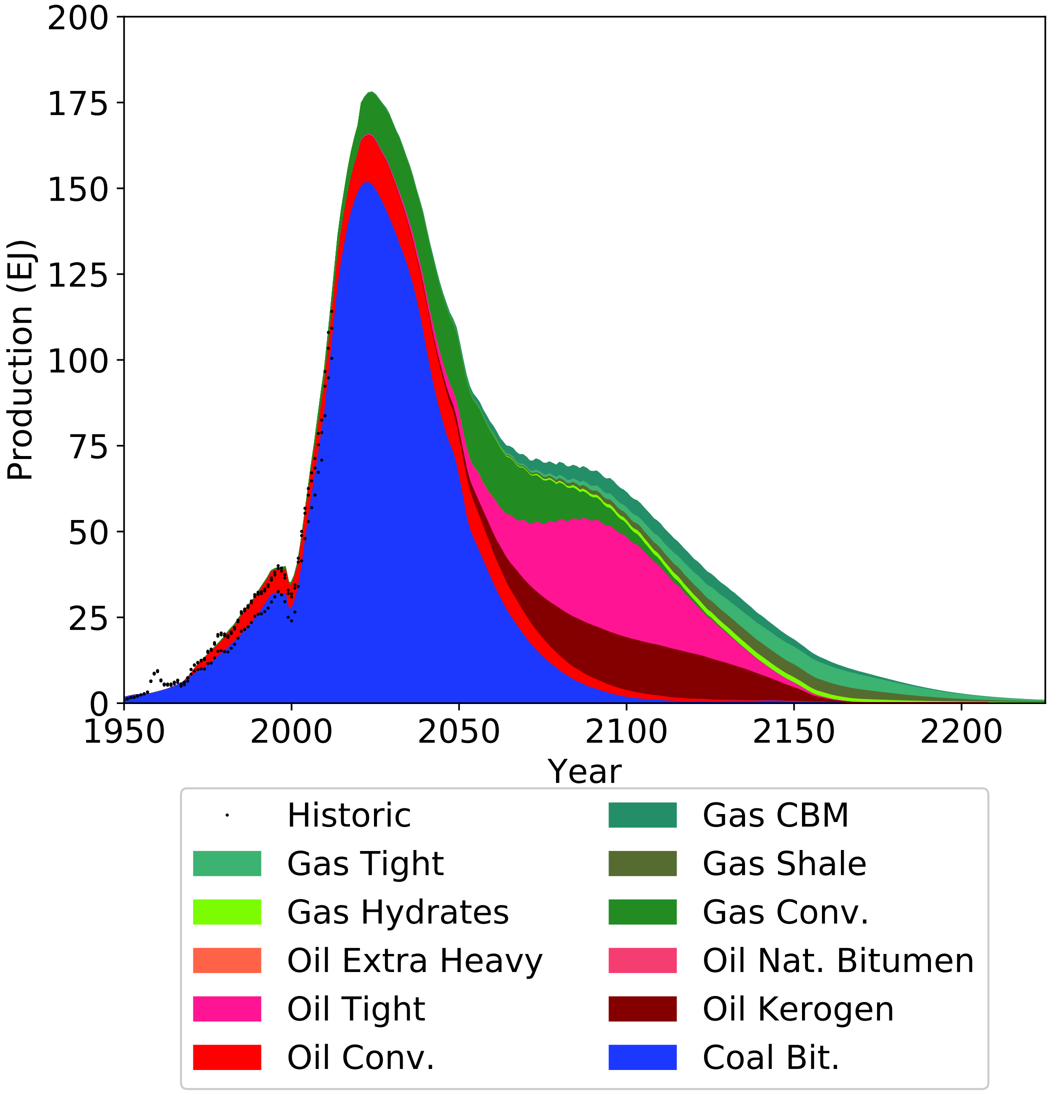

Figure 2.35: China projection by mineral type

2.8.3 Regional Projections

Table 2.35: Peak years - Minerals

Name	URR	Peak Year	Peak Rate
Coal Bit.	6990.88	2023	151.47
Oil Conv.	1032.38	2028	14.24
Oil Kerogen	1146.0	2096	15.47
Oil Tight	1718.31	2091	31.17
Oil Nat. Bitumen	1.05	2054	0.03
Oil Extra Heavy	5.01	2030	0.15
Gas Conv.	1253.31	2049	19.85
Gas Hydrates	142.0	2131	1.73
Gas Shale	318.89	2145	3.83
Gas Tight	444.97	2150	5.18
Gas CBM	407.03	2103	4.55
**Total**	**13459.83**	**2024**	**177.93**

Anhui

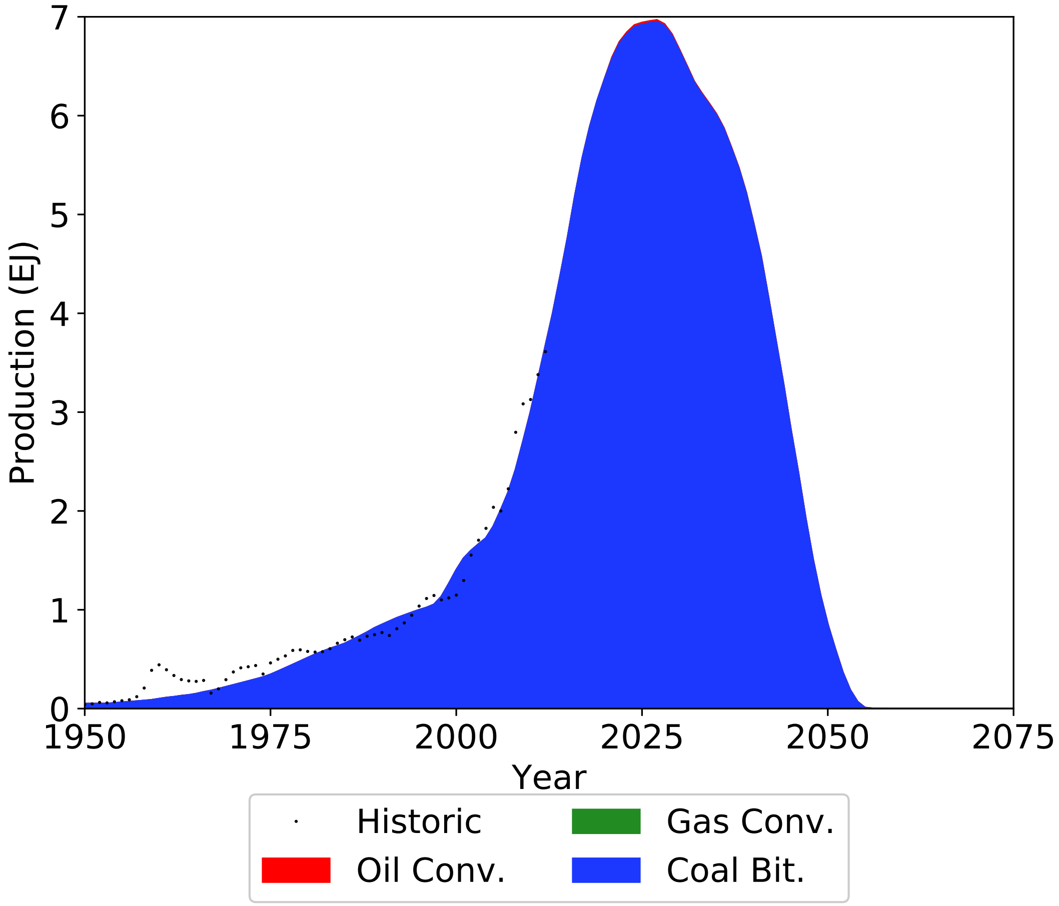

Figure 2.36: China - Anhui projections capped at 16

Table 2.36: Peak years - All

Name	URR	Peak Year	Peak Rate
Coal Bit. Anhui	247.75	2027	6.95
Oil Conv. Anhui	0.3	2025	0.02
Gas Conv. Anhui	0.01	2022	–
**Total**	**248.06**	**2027**	**6.97**

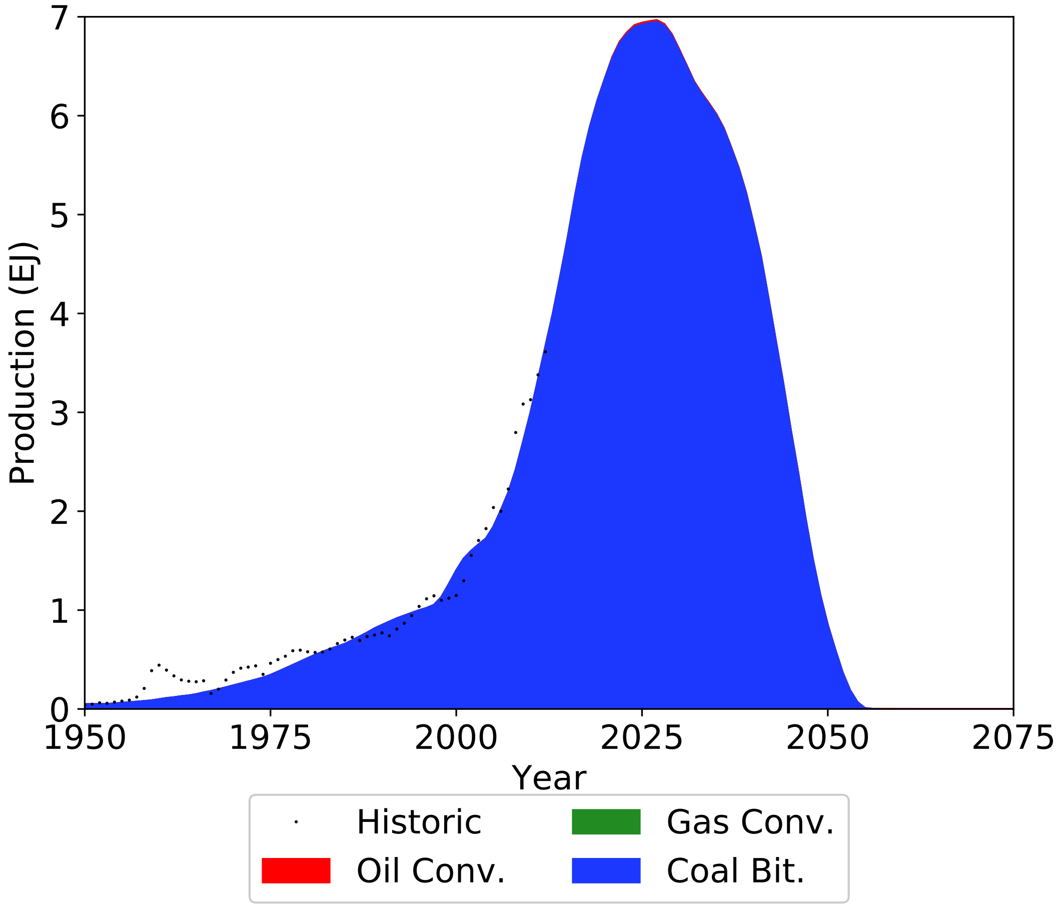

Figure 2.37: China - Anhui projection by mineral type

Table 2.37: Peak years - Minerals

Name	URR	Peak Year	Peak Rate
Coal Bit.	247.75	2027	6.95
Oil Conv.	0.3	2025	0.02
Gas Conv.	0.01	2022	–
**Total**	**248.06**	**2027**	**6.97**

Beijing

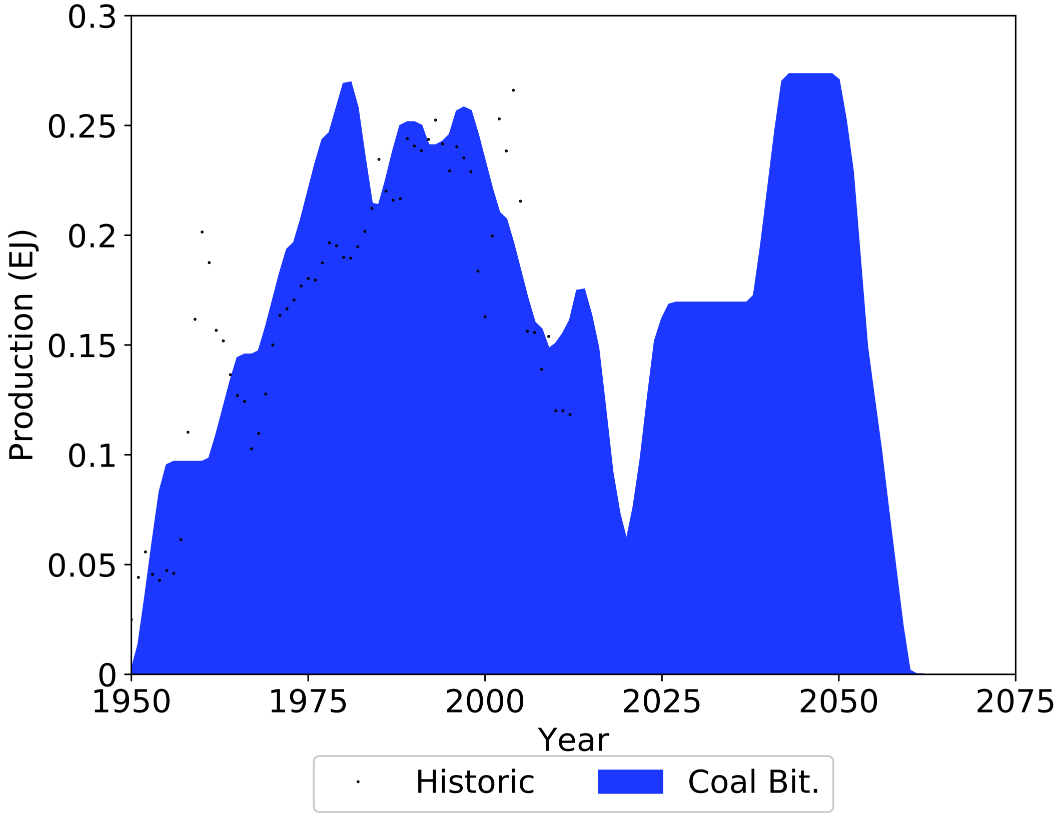

Figure 2.38: China - Beijing projections capped at 16

Table 2.38: Peak years - All

Name	URR	Peak Year	Peak Rate
Coal Bit. Beijing	19.54	2048	0.27
**Total**	**19.54**	**2048**	**0.27**

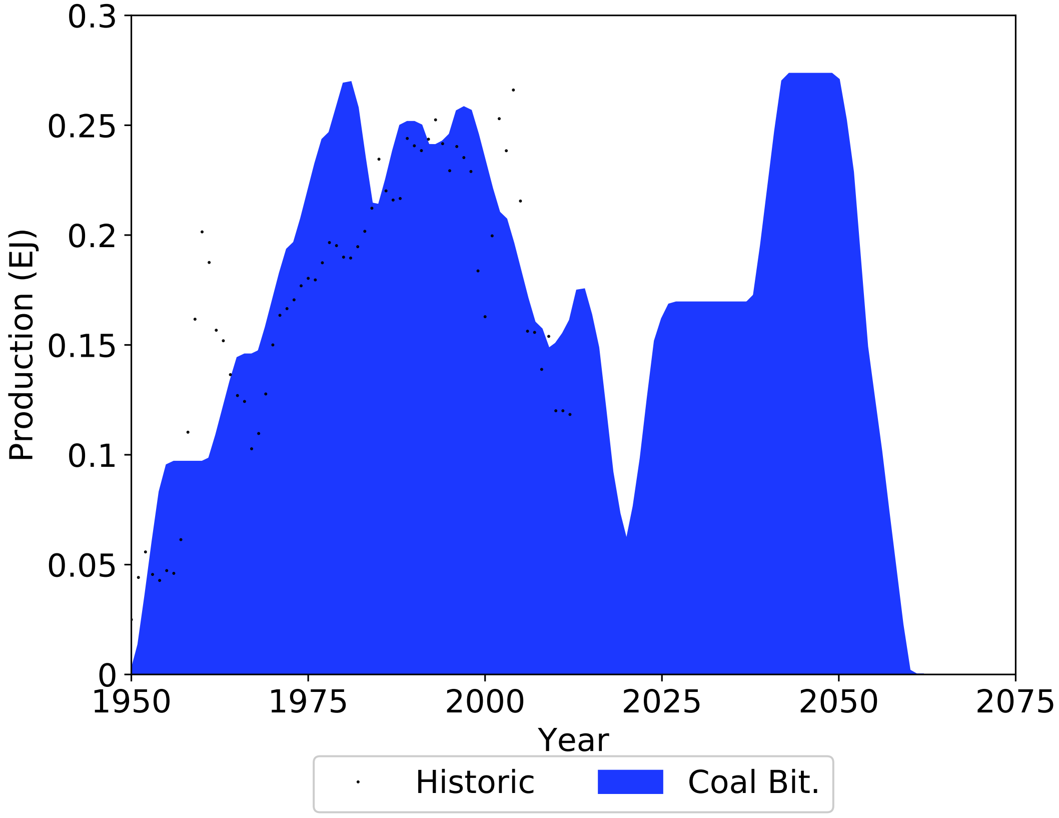

Figure 2.39: China - Beijing projection by mineral type

Table 2.39: Peak years - Minerals

Name	URR	Peak Year	Peak Rate
Coal Bit.	19.54	2048	0.27
**Total**	**19.54**	**2048**	**0.27**

China

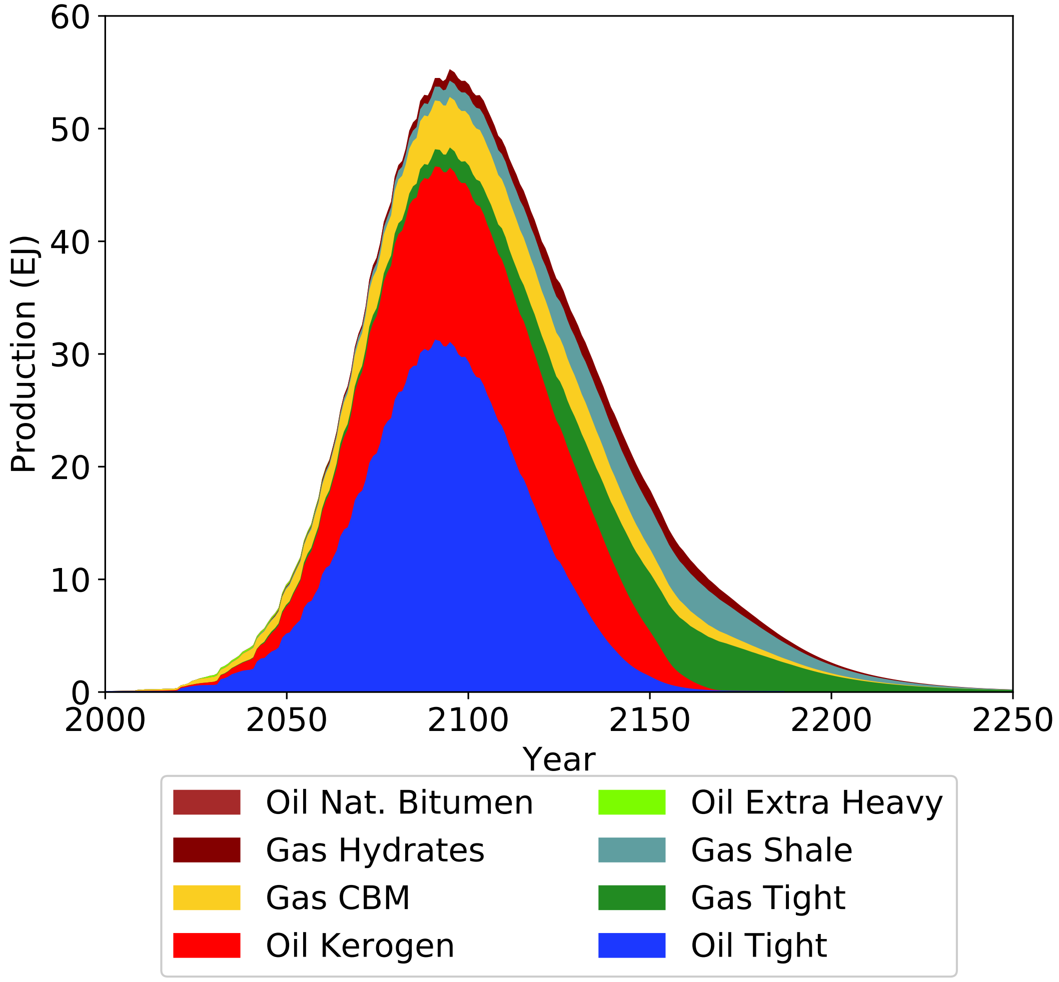

Figure 2.40: China - China projections capped at 16

Table 2.40: Peak years - All

Name	URR	Peak Year	Peak Rate
Oil Tight China	1718.31	2091	31.17
Oil Kerogen China	1146.0	2096	15.47
Gas Tight China	444.97	2150	5.18
Gas CBM China	407.03	2103	4.55
Gas Shale China	318.89	2145	3.83
Gas Hydrates China	142.0	2131	1.73
Oil Extra Heavy China	5.01	2030	0.15
Oil Nat. Bitumen China	1.05	2054	0.03
**Total**	**4183.26**	**2095**	**55.14**

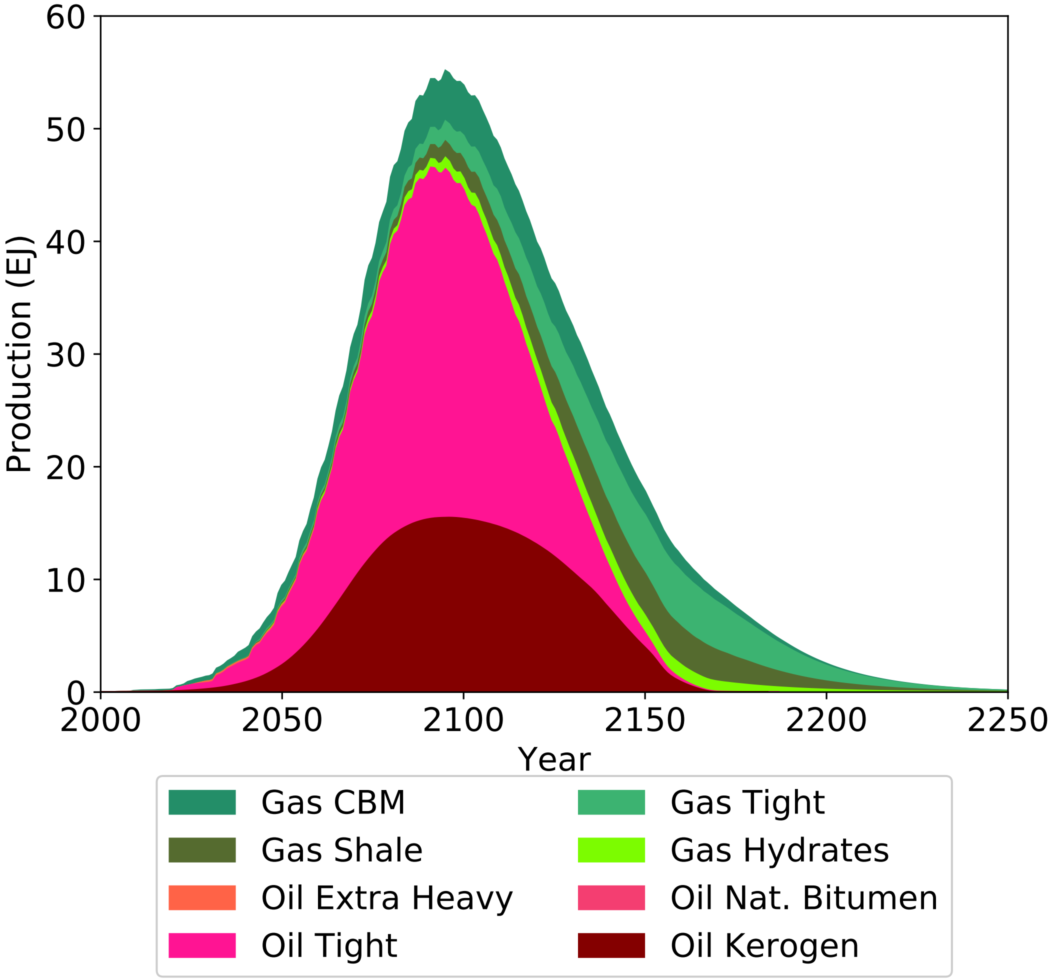

Figure 2.41: China - China projection by mineral type

Table 2.41: Peak years - Minerals

**Name**	**URR**	**Peak Year**	**Peak Rate**
Oil Kerogen	1146.0	2096	15.47
Oil Tight	1718.31	2091	31.17
Oil Nat. Bitumen	1.05	2054	0.03
Oil Extra Heavy	5.01	2030	0.15
Gas Hydrates	142.0	2131	1.73
Gas Shale	318.89	2145	3.83
Gas Tight	444.97	2150	5.18
Gas CBM	407.03	2103	4.55
**Total**	**4183.26**	**2095**	**55.14**

Chongqing

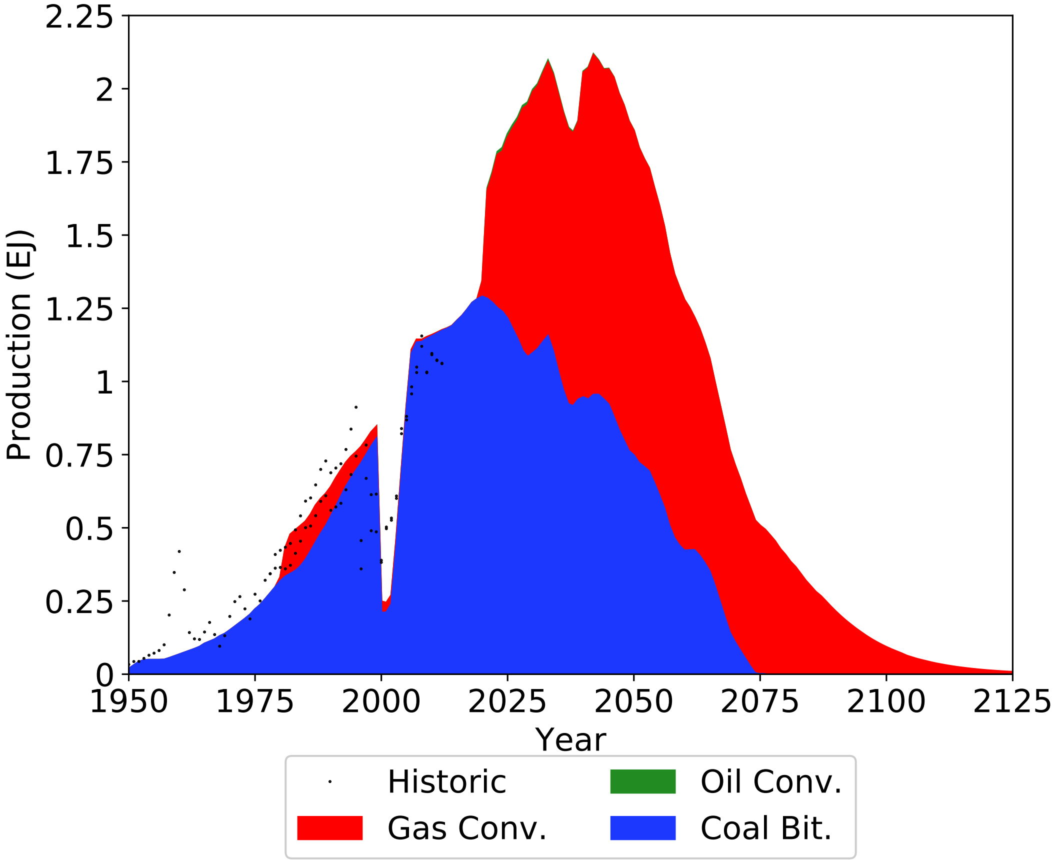

Figure 2.42: China - Chongqing projections capped at 16

Table 2.42: Peak years - All

**Name**	**URR**	**Peak Year**	**Peak Rate**
Coal Bit. Chongqing	74.71	2020	1.29
Gas Conv. Chongqing	56.59	2042	1.16
Oil Conv. Chongqing	0.19	2025	0.01
**Total**	**131.49**	**2042**	**2.12**

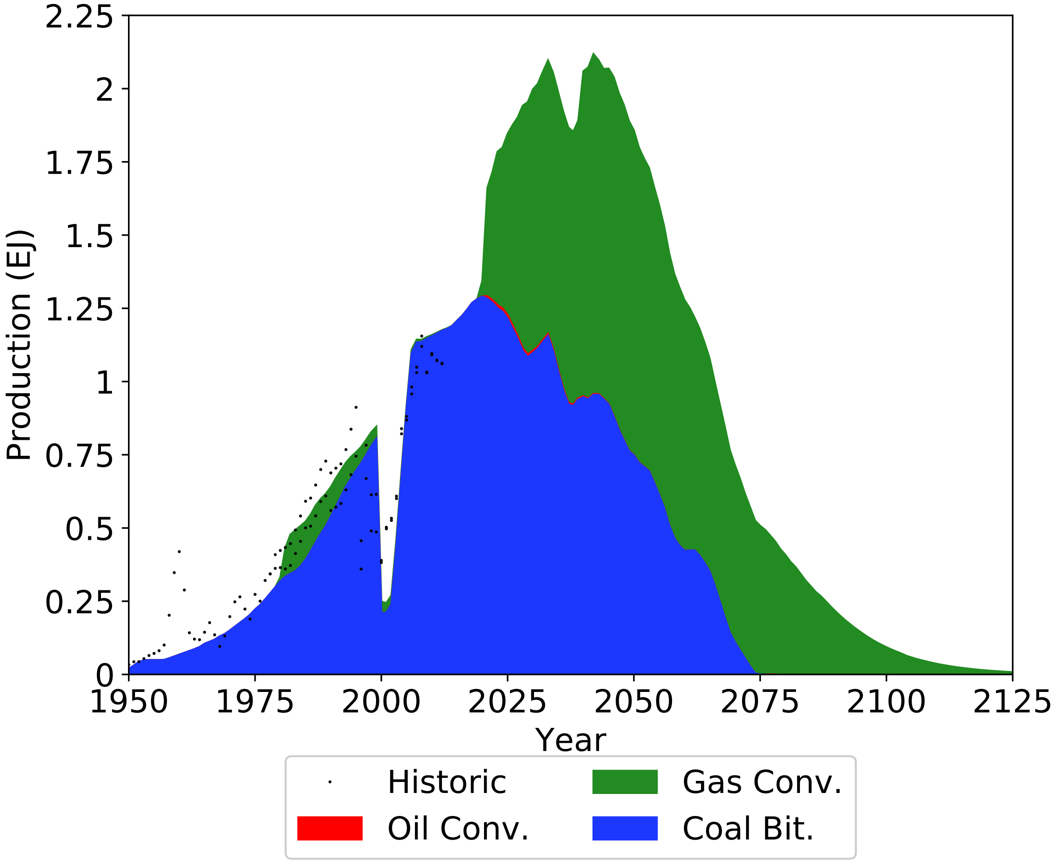

Figure 2.43: China - Chongqing projection by mineral type

Table 2.43: Peak years - Minerals

Name	URR	Peak Year	Peak Rate
Coal Bit.	74.71	2020	1.29
Oil Conv.	0.19	2025	0.01
Gas Conv.	56.59	2042	1.16
**Total**	**131.49**	**2042**	**2.12**

Fujian

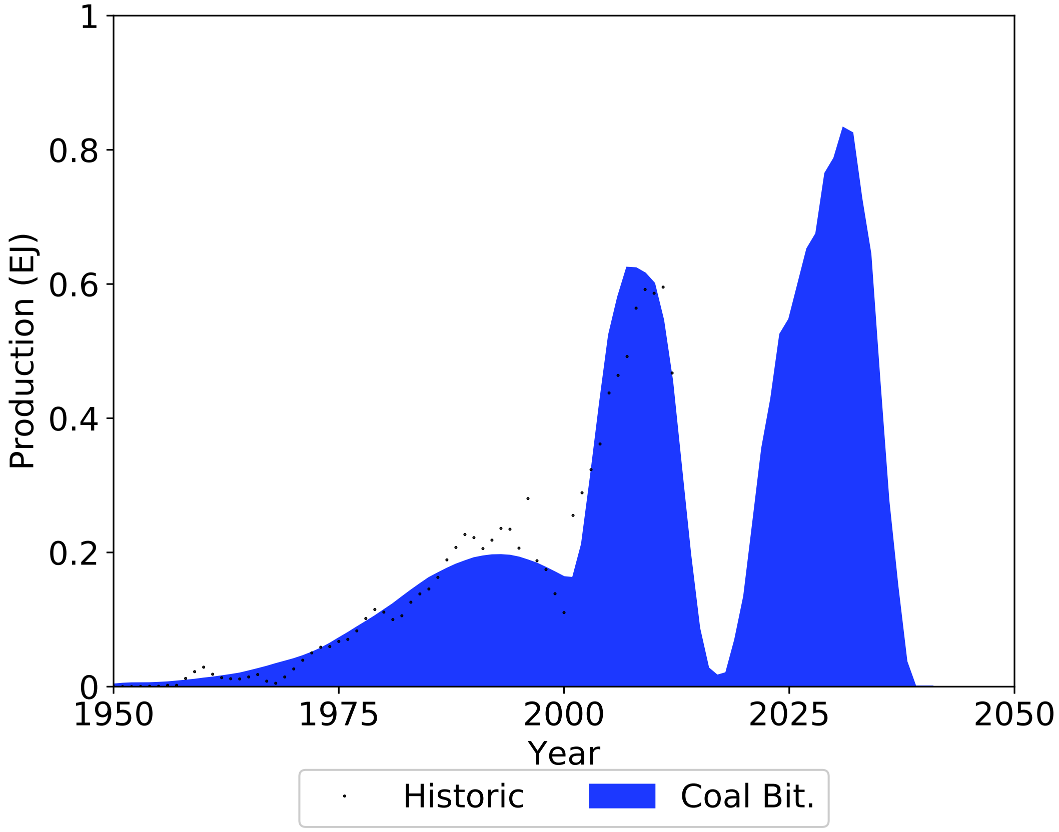

Figure 2.44: China - Fujian projections capped at 16

Table 2.44: Peak years - All

**Name**	**URR**	**Peak Year**	**Peak Rate**
Coal Bit. Fujian	20.67	2031	0.83
**Total**	**20.67**	**2031**	**0.83**

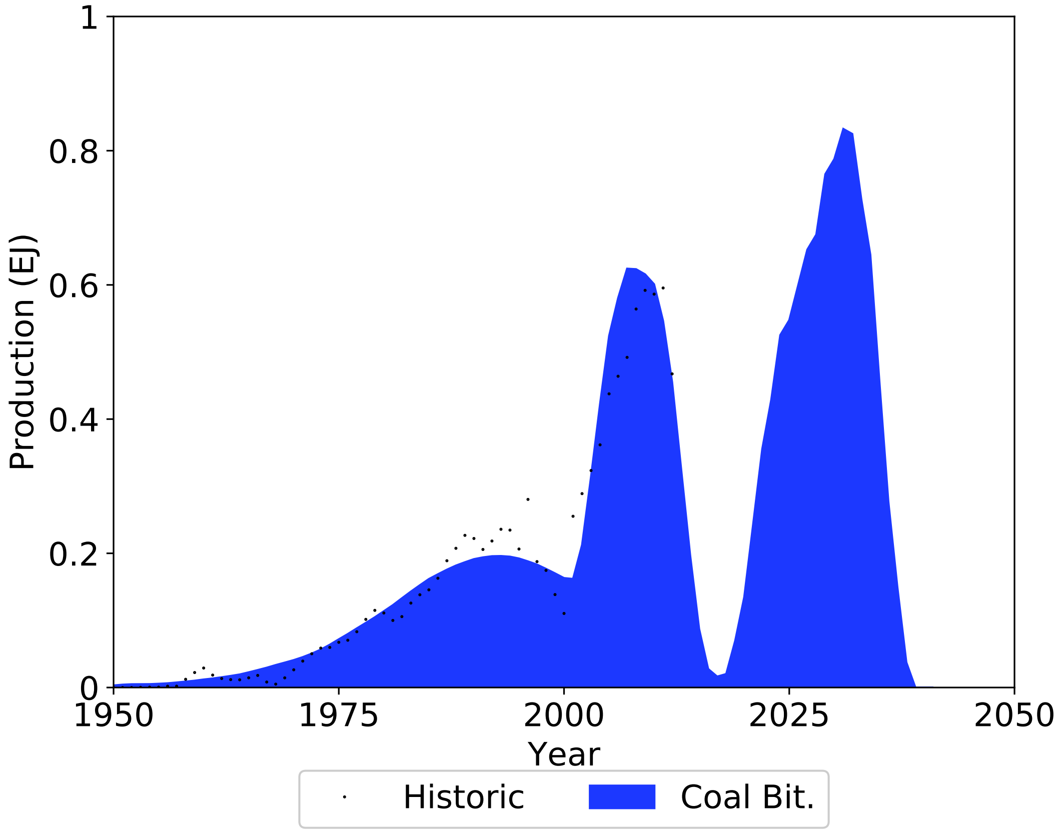

Figure 2.45: China - Fujian projection by mineral type

Table 2.45: Peak years - Minerals

Name	URR	Peak Year	Peak Rate
Coal Bit.	20.67	2031	0.83
**Total**	**20.67**	**2031**	**0.83**

Gansu

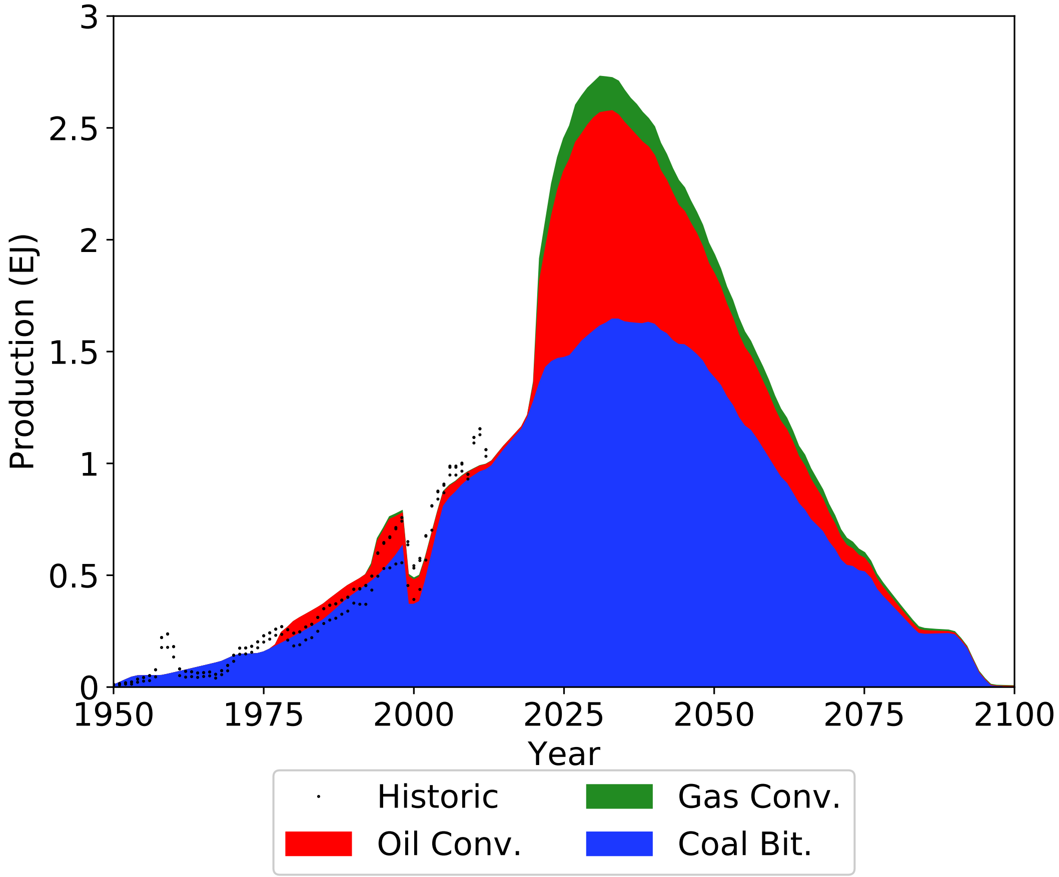

Figure 2.46: China - Gansu projections capped at 16

Table 2.46: Peak years - All

**Name**	**URR**	**Peak Year**	**Peak Rate**
Coal Bit. Gansu	102.87	2034	1.64
Oil Conv. Gansu	31.5	2031	0.95
Gas Conv. Gansu	5.66	2028	0.17
**Total**	**140.03**	**2031**	**2.73**

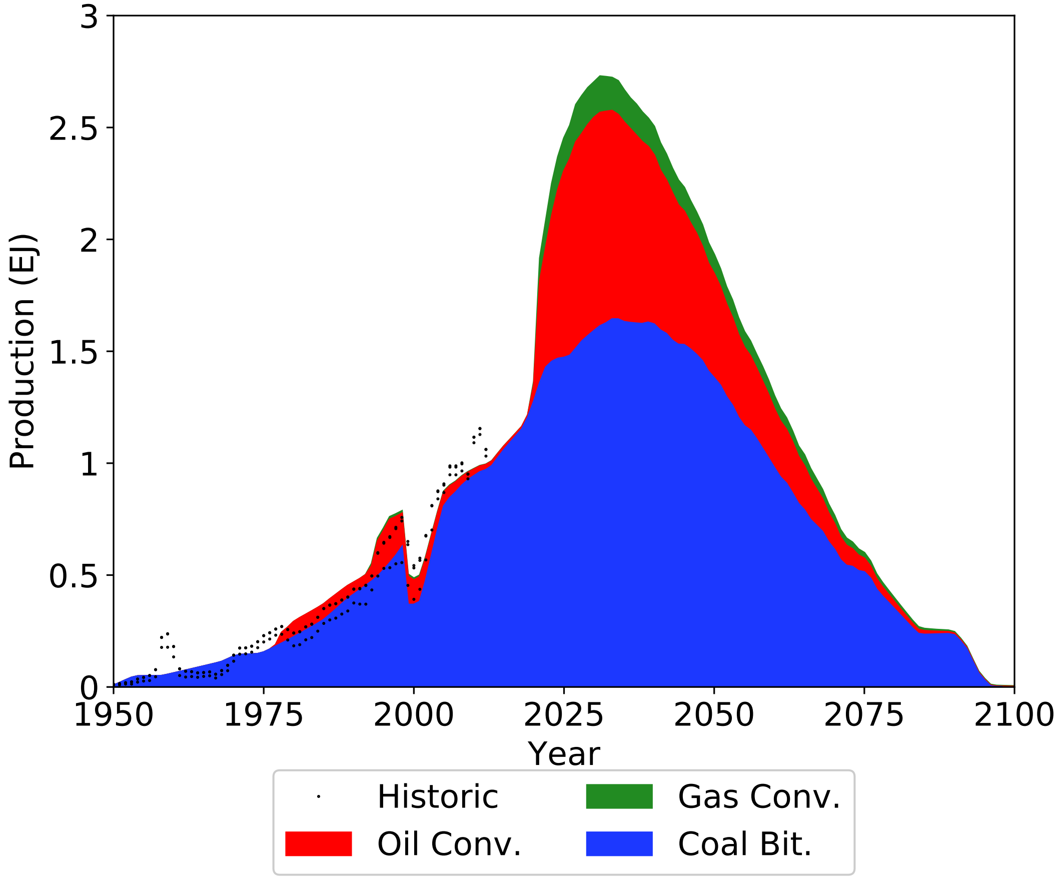

Figure 2.47: China - Gansu projection by mineral type

Table 2.47: Peak years - Minerals

Name	URR	Peak Year	Peak Rate
Coal Bit.	102.87	2034	1.64
Oil Conv.	31.5	2031	0.95
Gas Conv.	5.66	2028	0.17
**Total**	**140.03**	**2031**	**2.73**

Guangdong

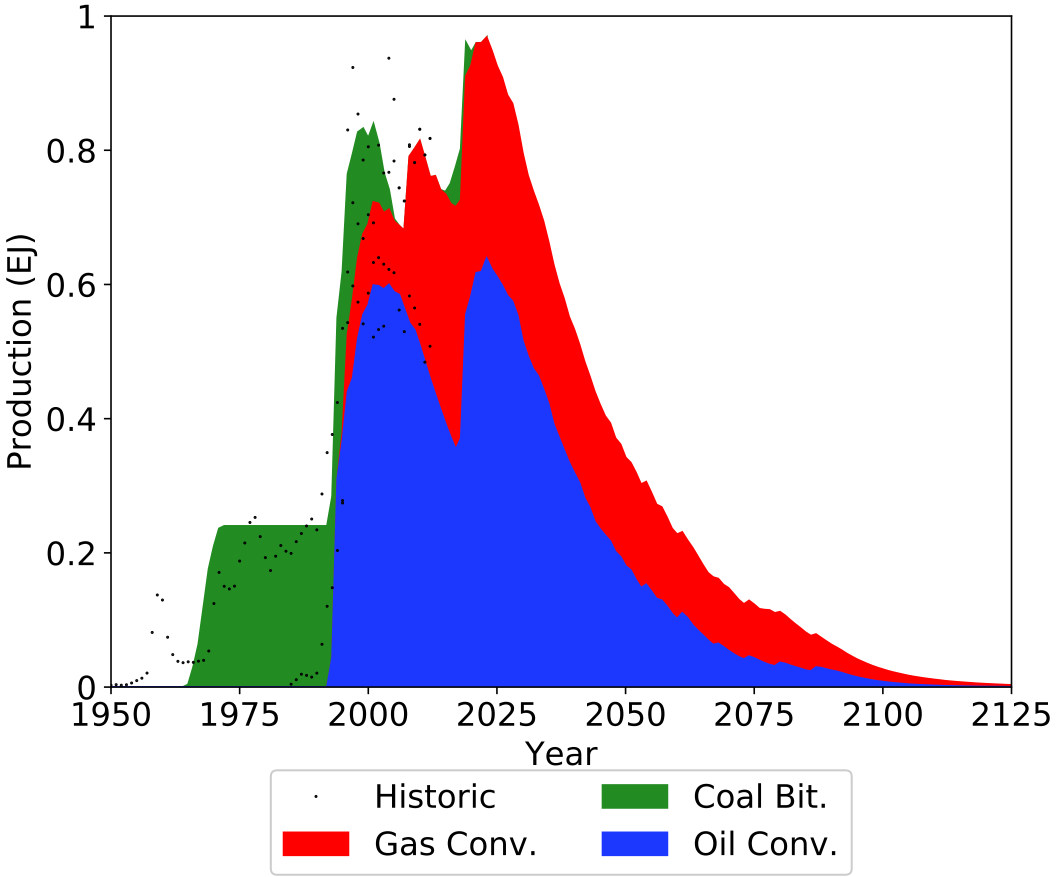

Figure 2.48: China - Guangdong projections capped at 16

Table 2.48: Peak years - All

Name	URR	Peak Year	Peak Rate
Oil Conv. Guangdong	28.79	2023	0.64
Gas Conv. Guangdong	17.26	2017	0.36
Coal Bit. Guangdong	8.07	1972	0.24
**Total**	**54.12**	**2023**	**0.97**

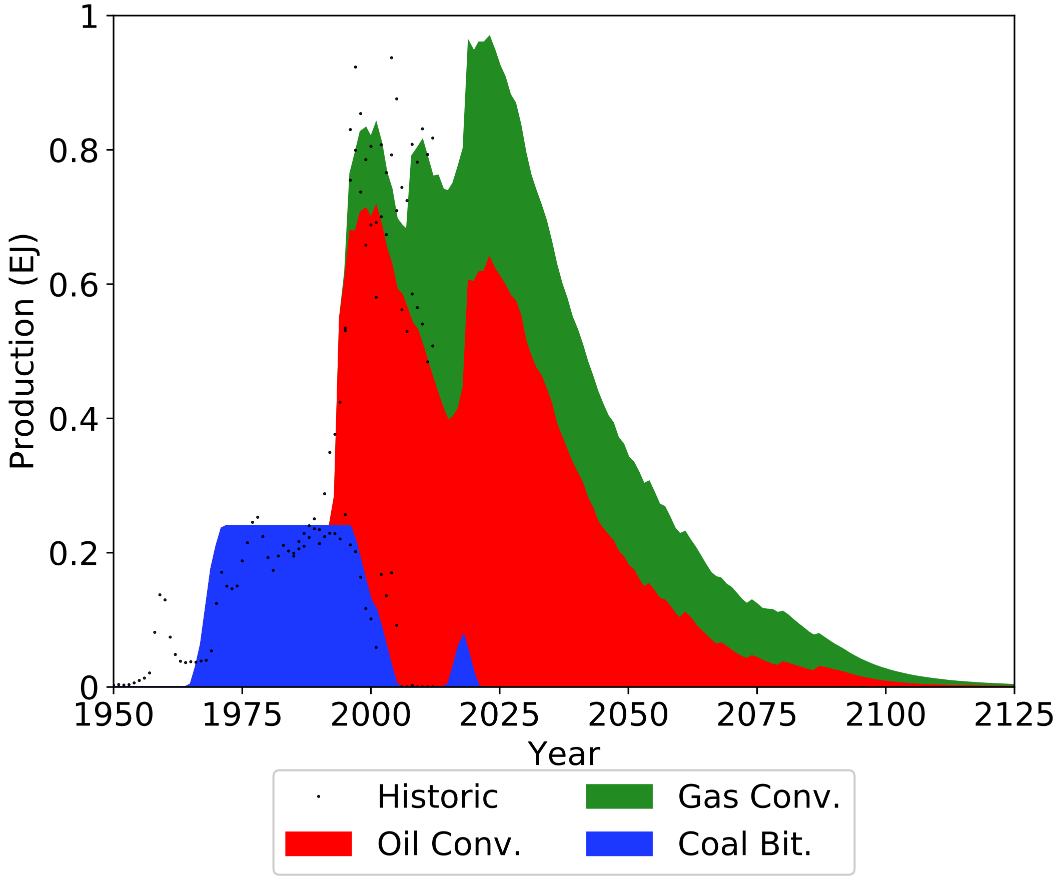

Figure 2.49: China - Guangdong projection by mineral type

Table 2.49: Peak years - Minerals

Name	URR	Peak Year	Peak Rate
Coal Bit.	8.07	1972	0.24
Oil Conv.	28.79	2023	0.64
Gas Conv.	17.26	2017	0.36
**Total**	**54.12**	**2023**	**0.97**

Guangxi

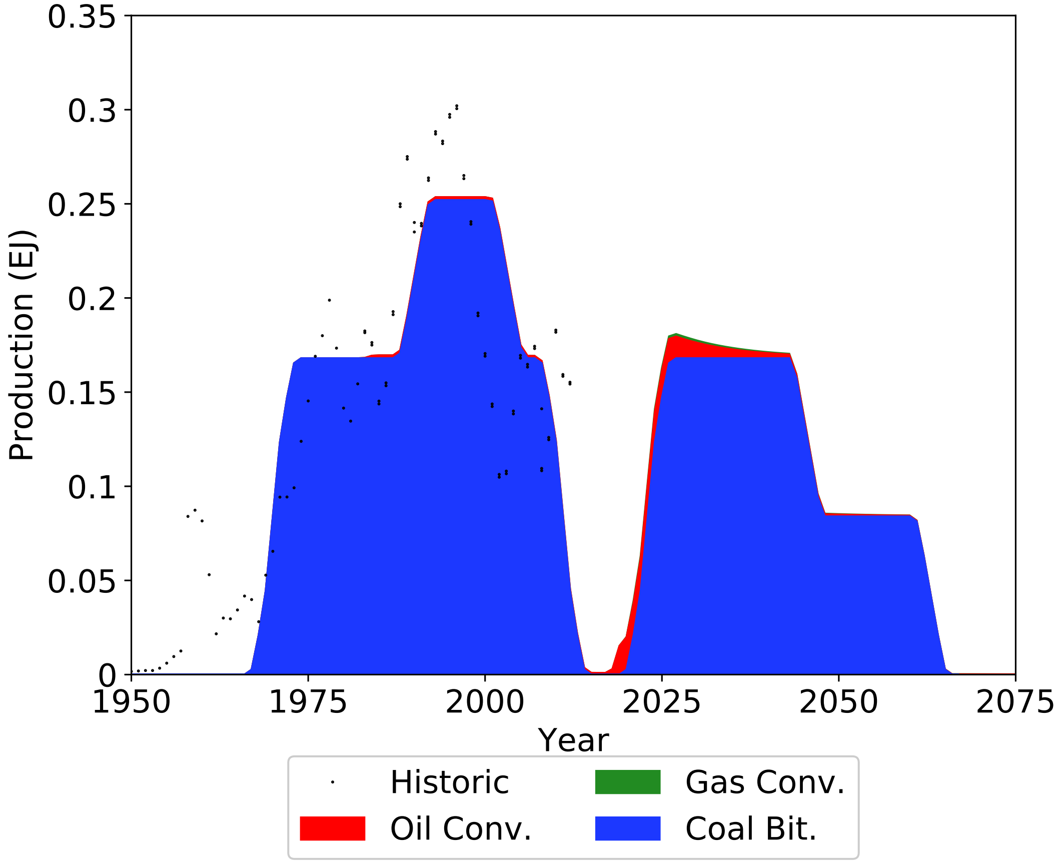

Figure 2.50: China - Guangxi projections capped at 16

Table 2.50: Peak years - All

Name	URR	Peak Year	Peak Rate
Coal Bit. Guangxi	13.25	1993	0.25
Oil Conv. Guangxi	0.29	2020	0.02
Gas Conv. Guangxi	0.03	2022	–
**Total**	**13.57**	**1993**	**0.25**

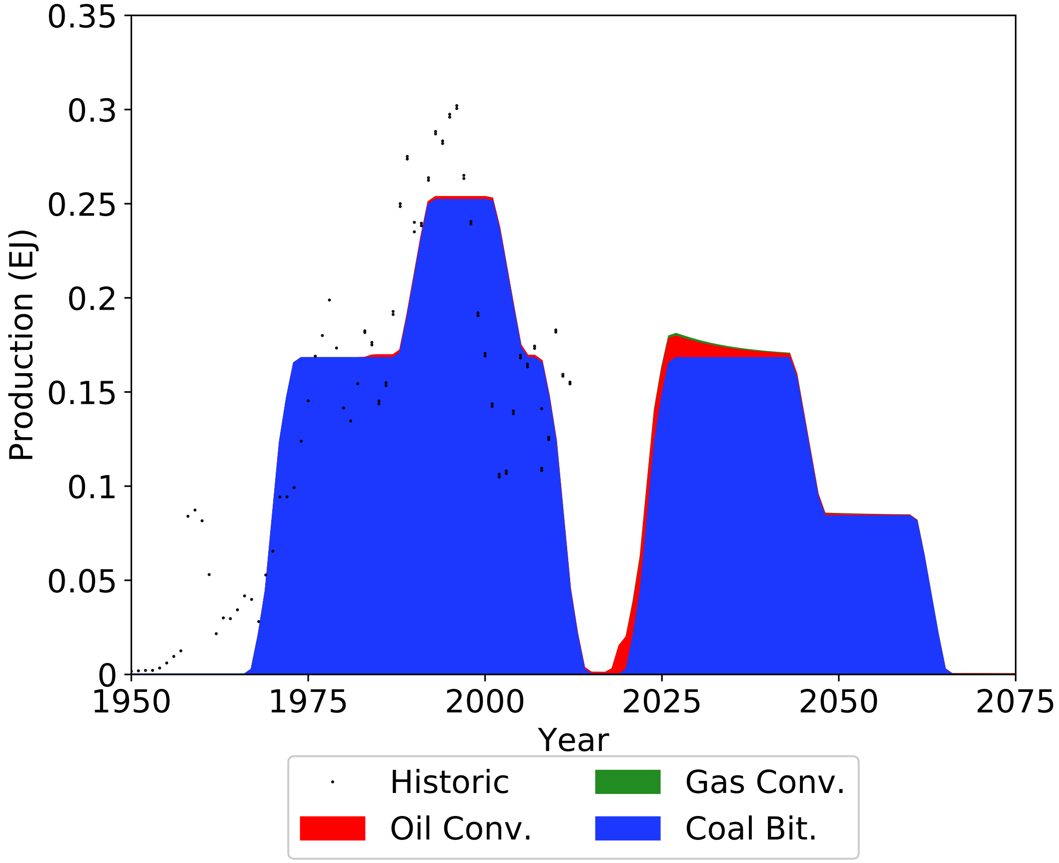

Figure 2.51: China - Guangxi projection by mineral type

Table 2.51: Peak years - Minerals

Name	URR	Peak Year	Peak Rate
Coal Bit.	13.25	1993	0.25
Oil Conv.	0.29	2020	0.02
Gas Conv.	0.03	2022	–
**Total**	**13.57**	**1993**	**0.25**

Guizhou

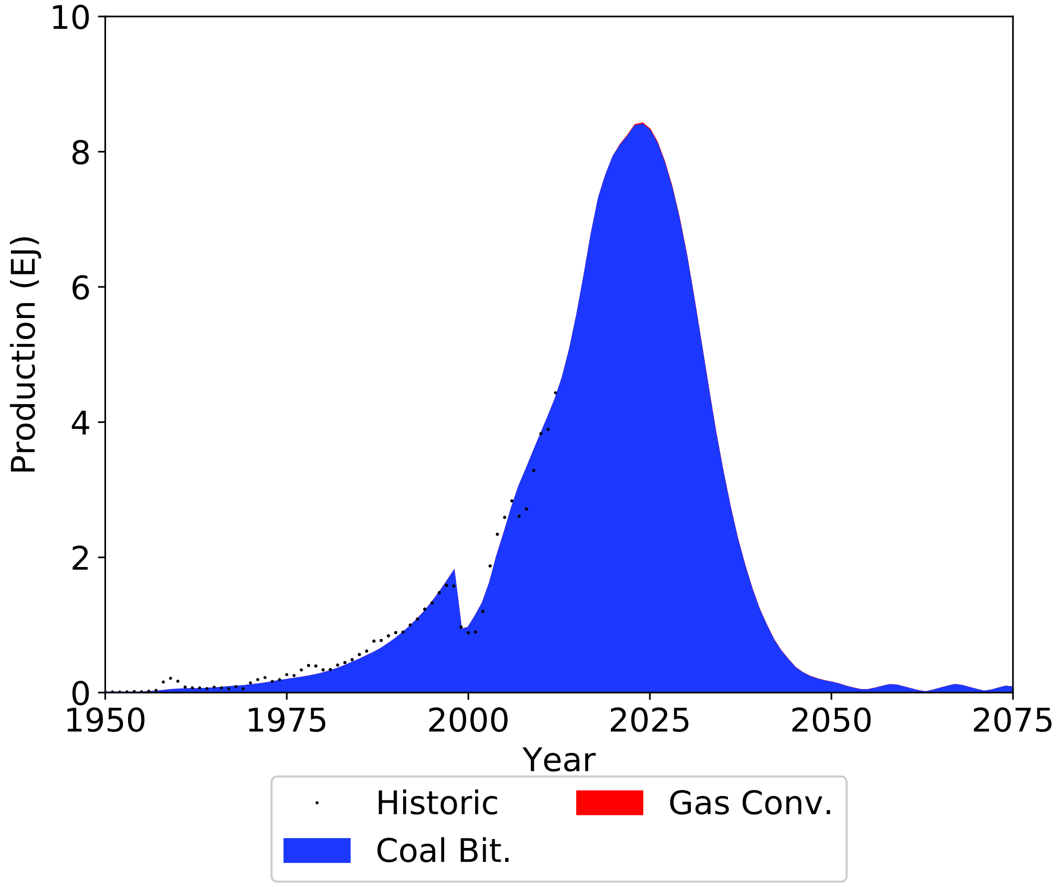

Figure 2.52: China - Guizhou projections capped at 16

Table 2.52: Peak years - All

**Name**	**URR**	**Peak Year**	**Peak Rate**
Coal Bit. Guizhou	221.34	2024	8.4
Gas Conv. Guizhou	0.42	2025	0.02
**Total**	**221.76**	**2024**	**8.42**

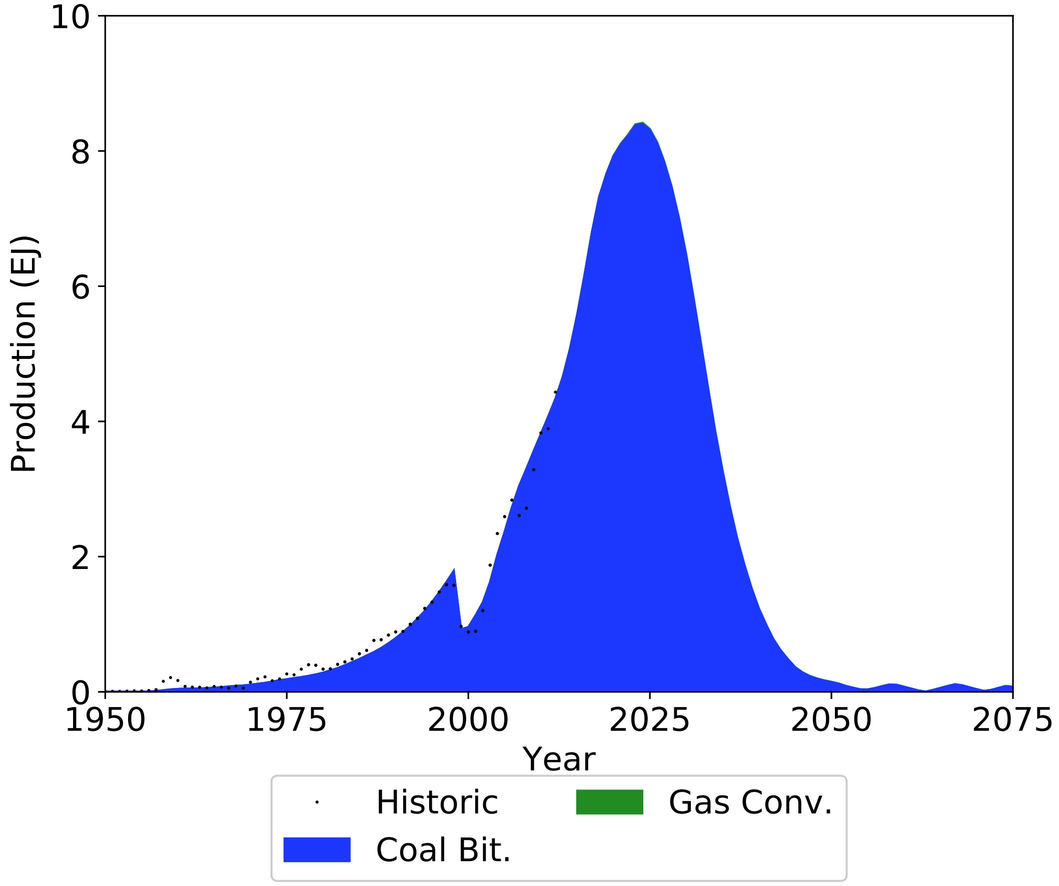

Figure 2.53: China - Guizhou projection by mineral type

Table 2.53: Peak years - Minerals

Name	URR	Peak Year	Peak Rate
Coal Bit.	221.34	2024	8.4
Gas Conv.	0.42	2025	0.02
**Total**	**221.76**	**2024**	**8.42**

Hainan

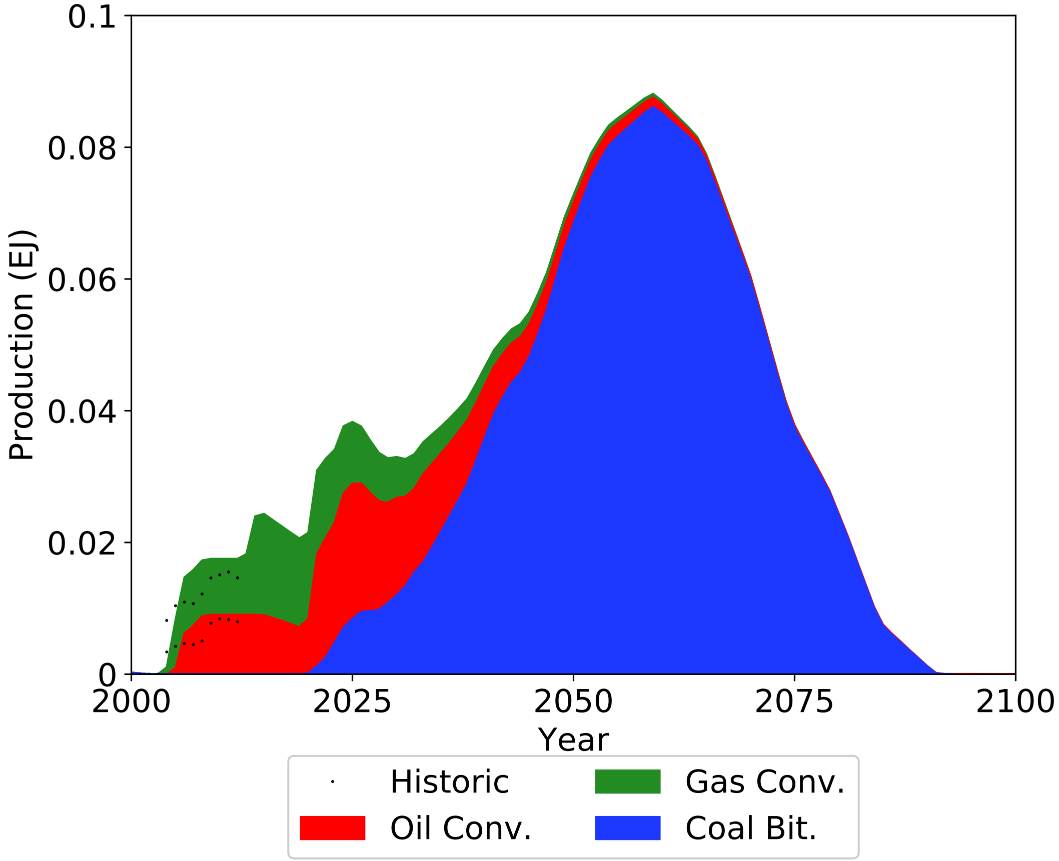

Figure 2.54: China - Hainan projections capped at 16

Table 2.54: Peak years - All

Name	URR	Peak Year	Peak Rate
Coal Bit. Hainan	2.86	2059	0.09
Oil Conv. Hainan	0.5	2025	0.02
Gas Conv. Hainan	0.34	2015	0.02
**Total**	**3.7**	**2059**	**0.09**

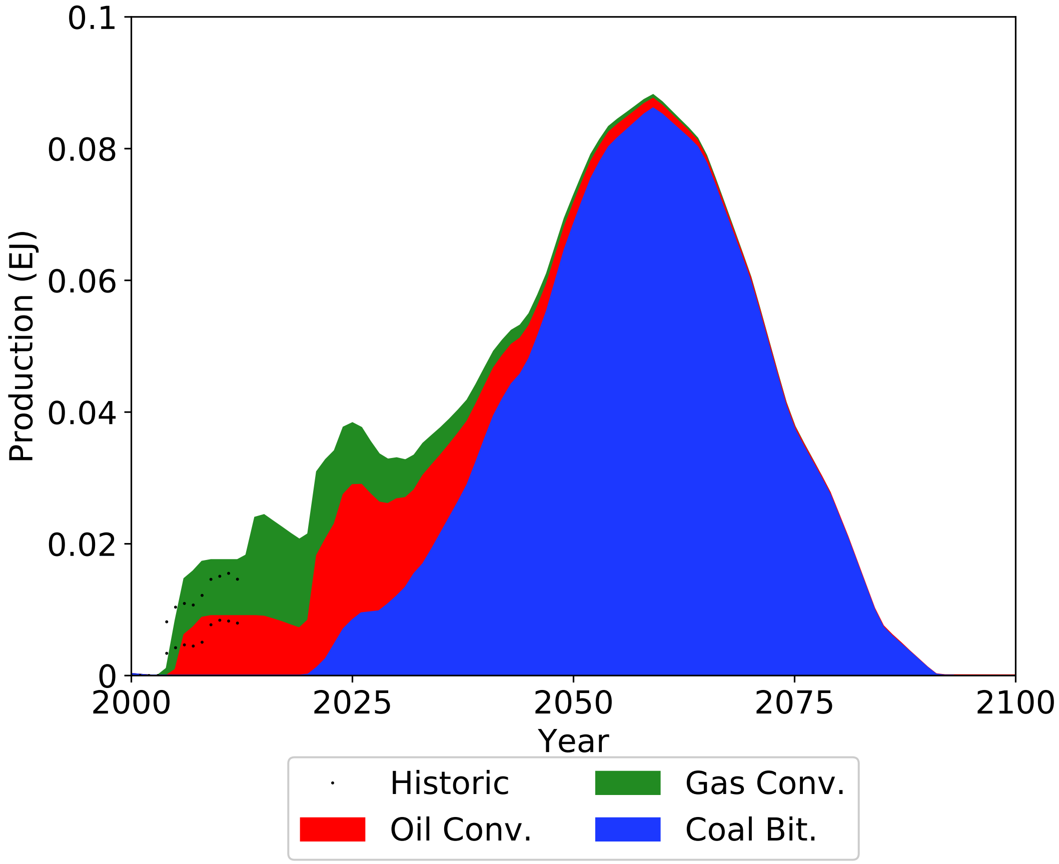

Figure 2.55: China - Hainan projection by mineral type

Table 2.55: Peak years - Minerals

Name	URR	Peak Year	Peak Rate
Coal Bit.	2.86	2059	0.09
Oil Conv.	0.5	2025	0.02
Gas Conv.	0.34	2015	0.02
**Total**	**3.7**	**2059**	**0.09**

Hebei

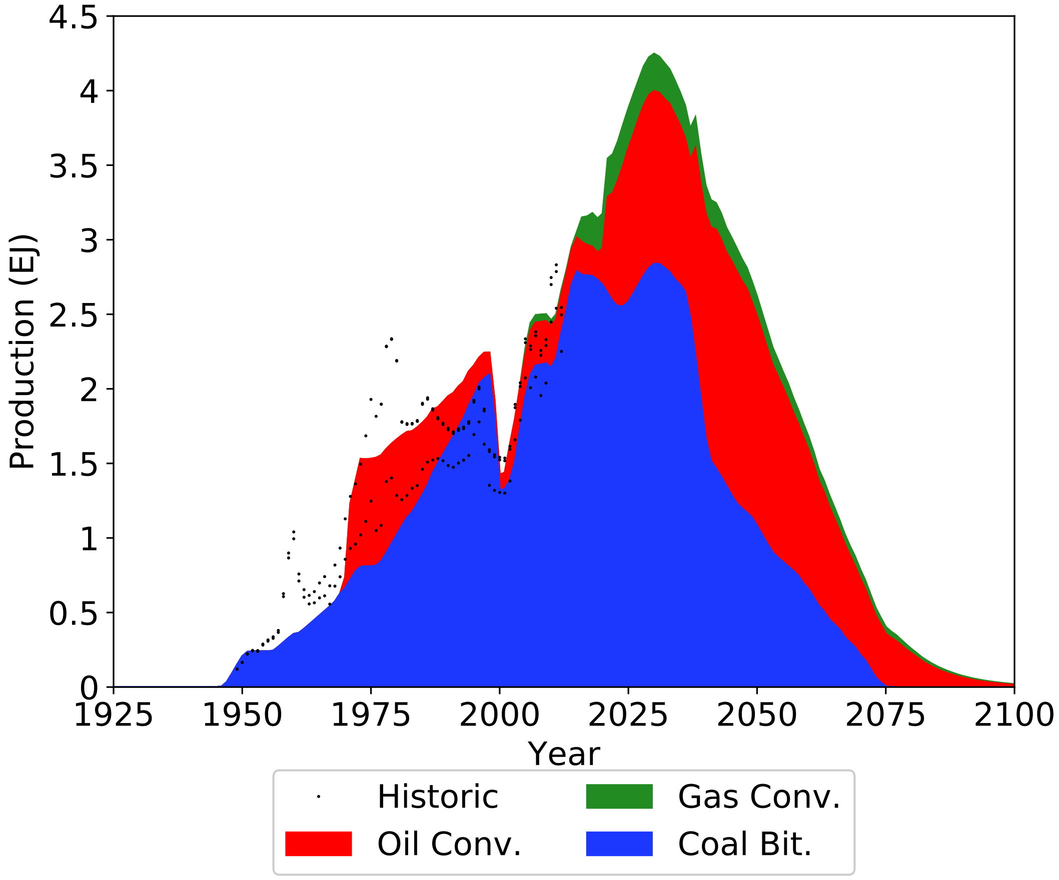

Figure 2.56: China - Hebei projections capped at 16

Table 2.56: Peak years - All

Name	URR	Peak Year	Peak Rate
Coal Bit. Hebei	170.41	2030	2.84
Oil Conv. Hebei	78.91	2042	1.6
Gas Conv. Hebei	10.39	2024	0.28
**Total**	**259.71**	**2030**	**4.25**

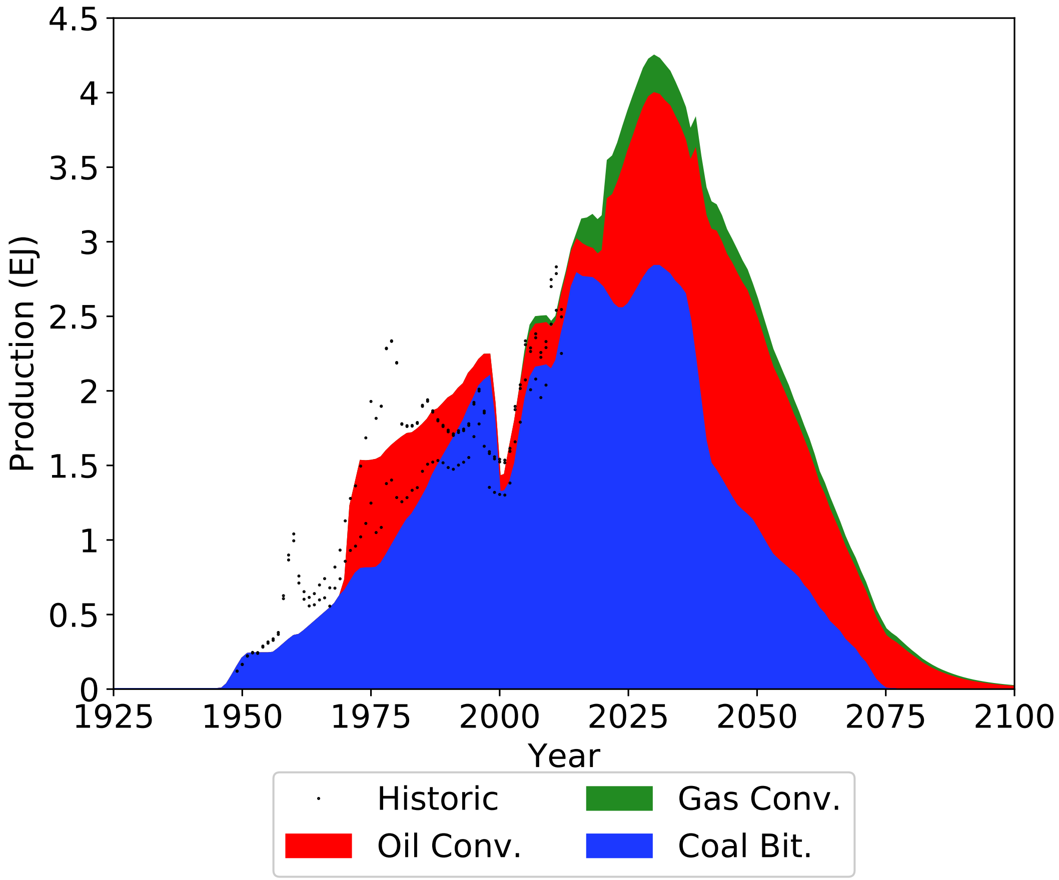

Figure 2.57: China - Hebei projection by mineral type

Table 2.57: Peak years - Minerals

**Name**	**URR**	**Peak Year**	**Peak Rate**
Coal Bit.	170.41	2030	2.84
Oil Conv.	78.91	2042	1.6
Gas Conv.	10.39	2024	0.28
**Total**	**259.71**	**2030**	**4.25**

Heilongjiang

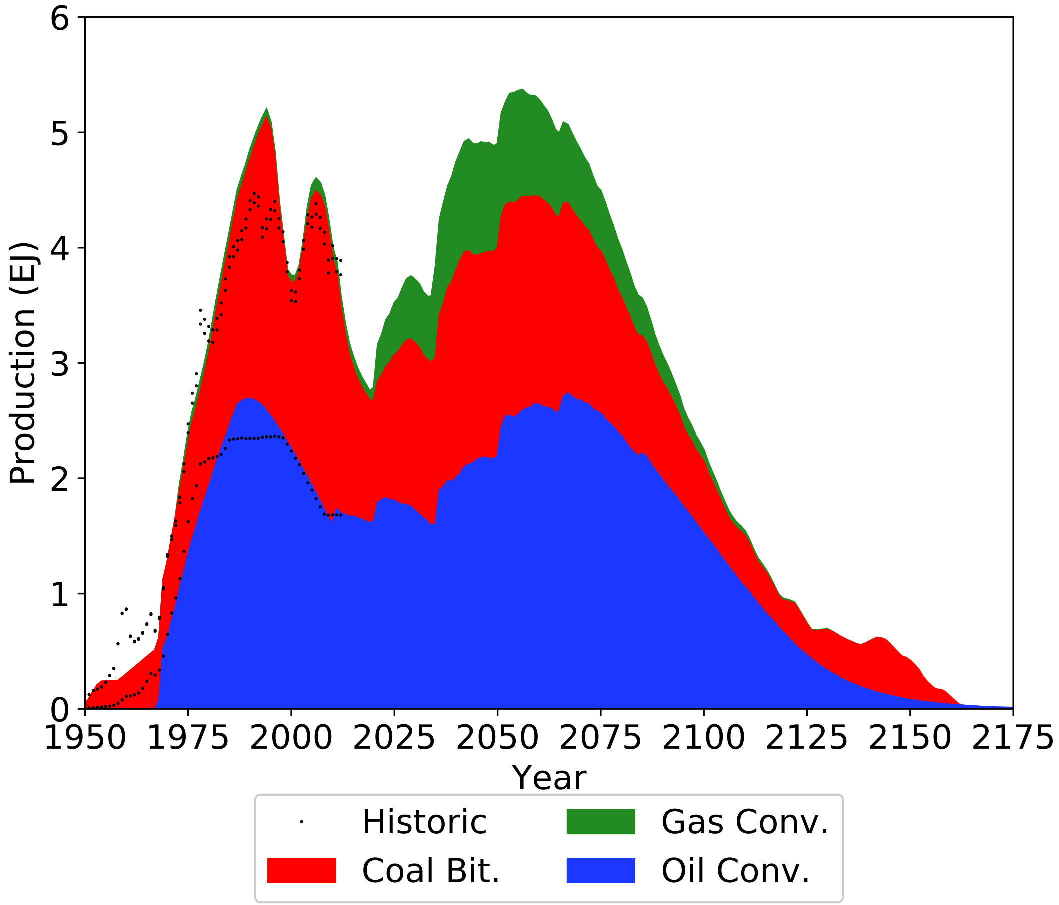

Figure 2.58: China - Heilongjiang projections capped at 16

Table 2.58: Peak years - All

Name	URR	Peak Year	Peak Rate
Oil Conv. Heilongjiang	302.68	2067	2.74
Coal Bit. Heilongjiang	222.88	2007	2.66
Gas Conv. Heilongjiang	52.91	2043	0.97
**Total**	**578.47**	**2056**	**5.37**

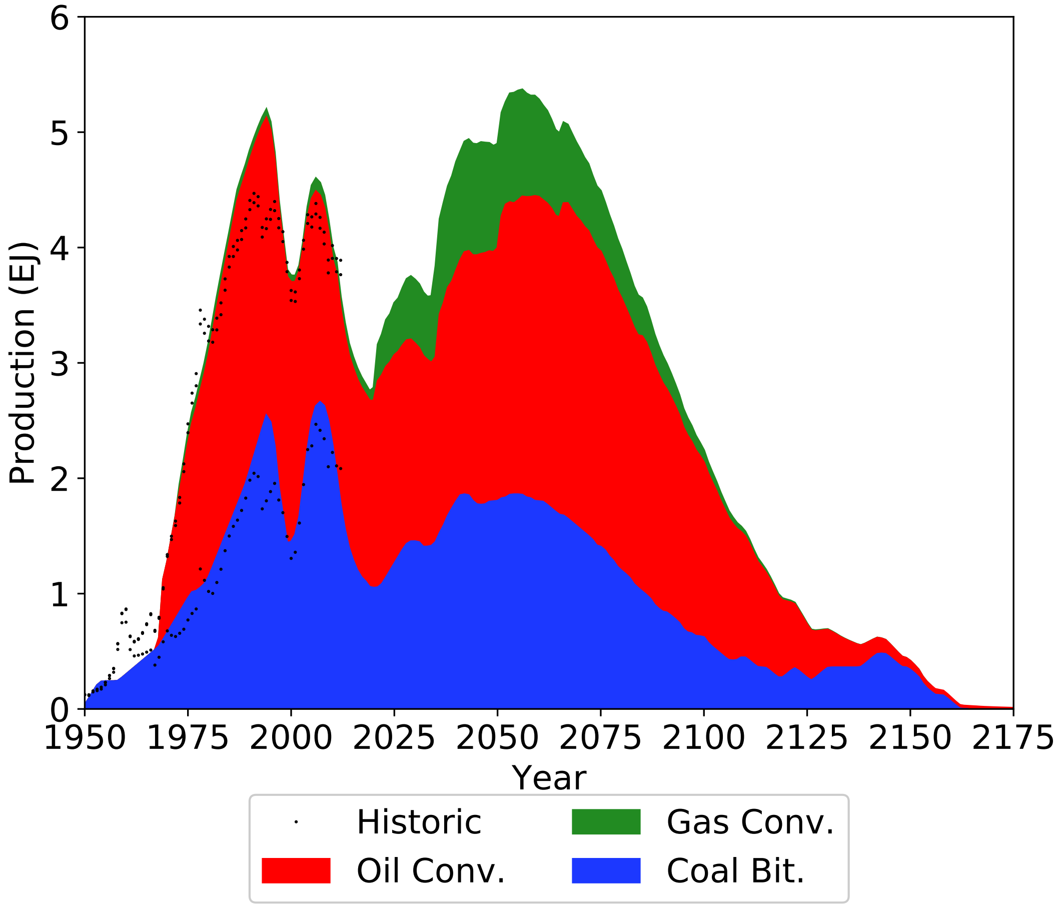

Figure 2.59: China - Heilongjiang projection by mineral type

Table 2.59: Peak years - Minerals

Name	URR	Peak Year	Peak Rate
Coal Bit.	222.88	2007	2.66
Oil Conv.	302.68	2067	2.74
Gas Conv.	52.91	2043	0.97
**Total**	**578.47**	**2056**	**5.37**

Henan

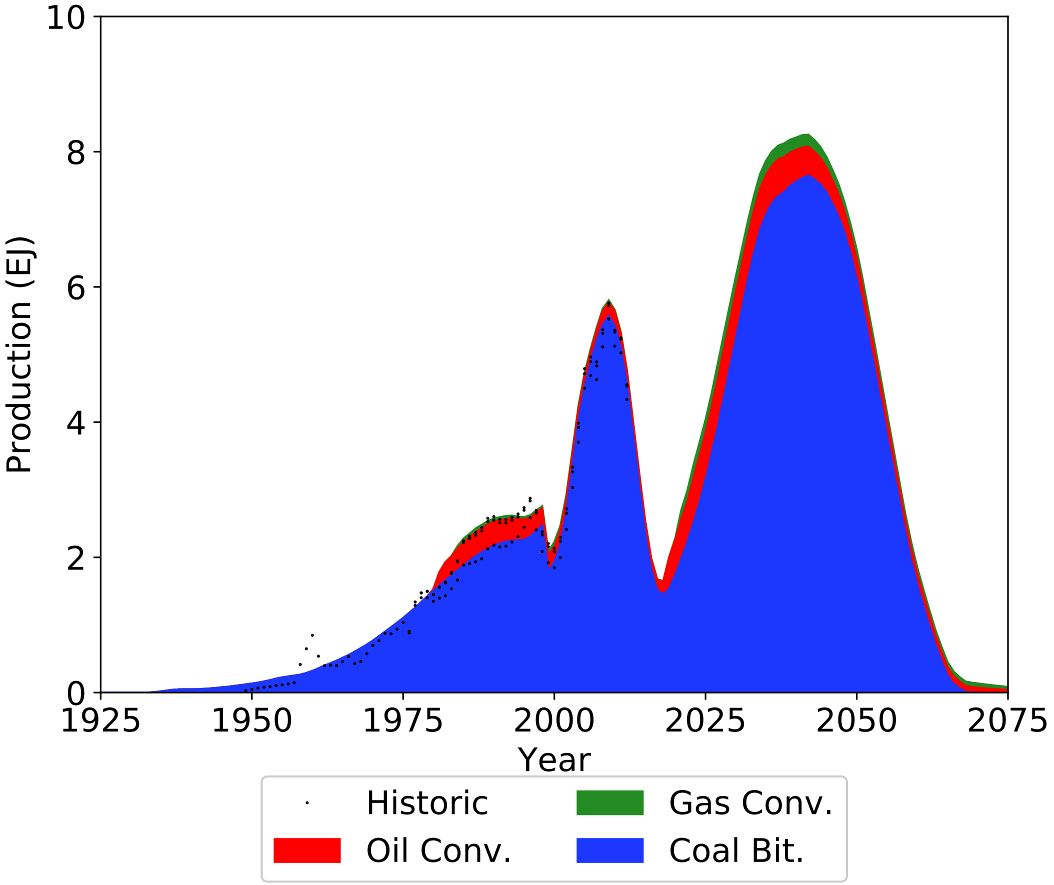

Figure 2.60: China - Henan projections capped at 16

Table 2.60: Peak years - All

Name	URR	Peak Year	Peak Rate
Coal Bit. Henan	346.51	2042	7.65
Oil Conv. Henan	30.55	2027	0.71
Gas Conv. Henan	9.64	2029	0.24
**Total**	**386.7**	**2042**	**8.25**

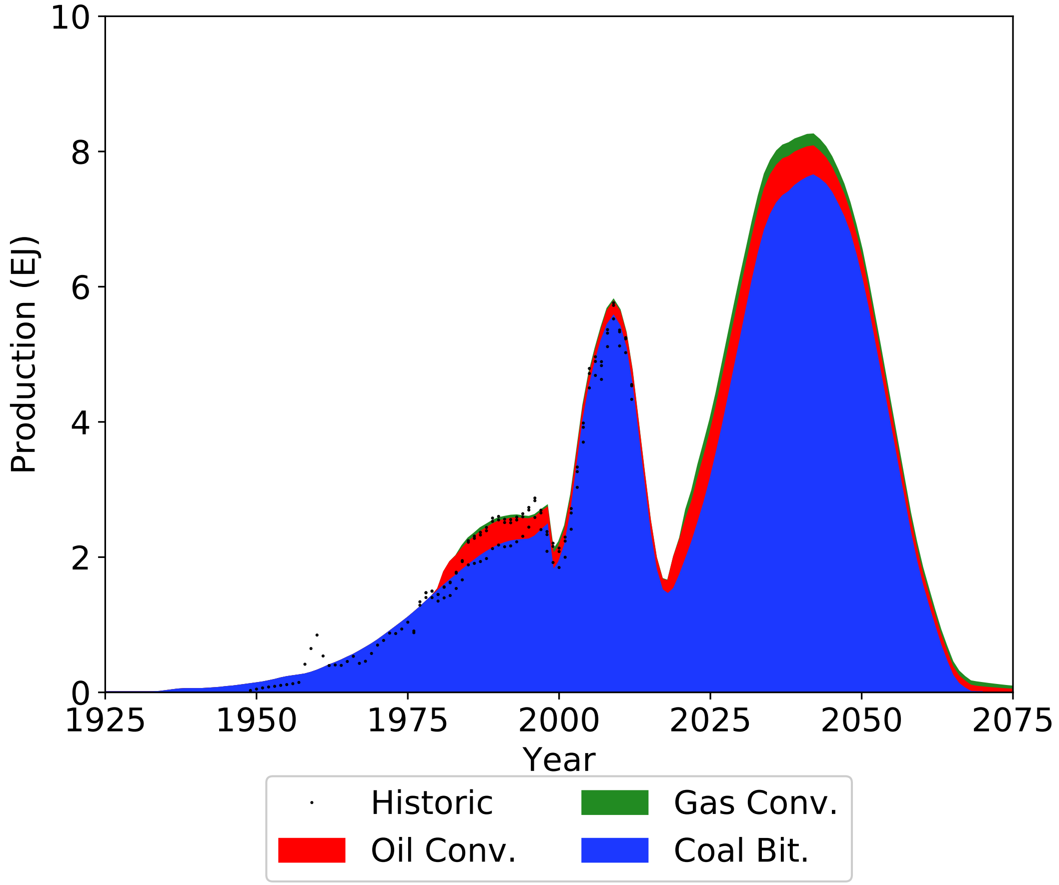

Figure 2.61: China - Henan projection by mineral type

Table 2.61: Peak years - Minerals

Name	URR	Peak Year	Peak Rate
Coal Bit.	346.51	2042	7.65
Oil Conv.	30.55	2027	0.71
Gas Conv.	9.64	2029	0.24
**Total**	**386.7**	**2042**	**8.25**

Historic

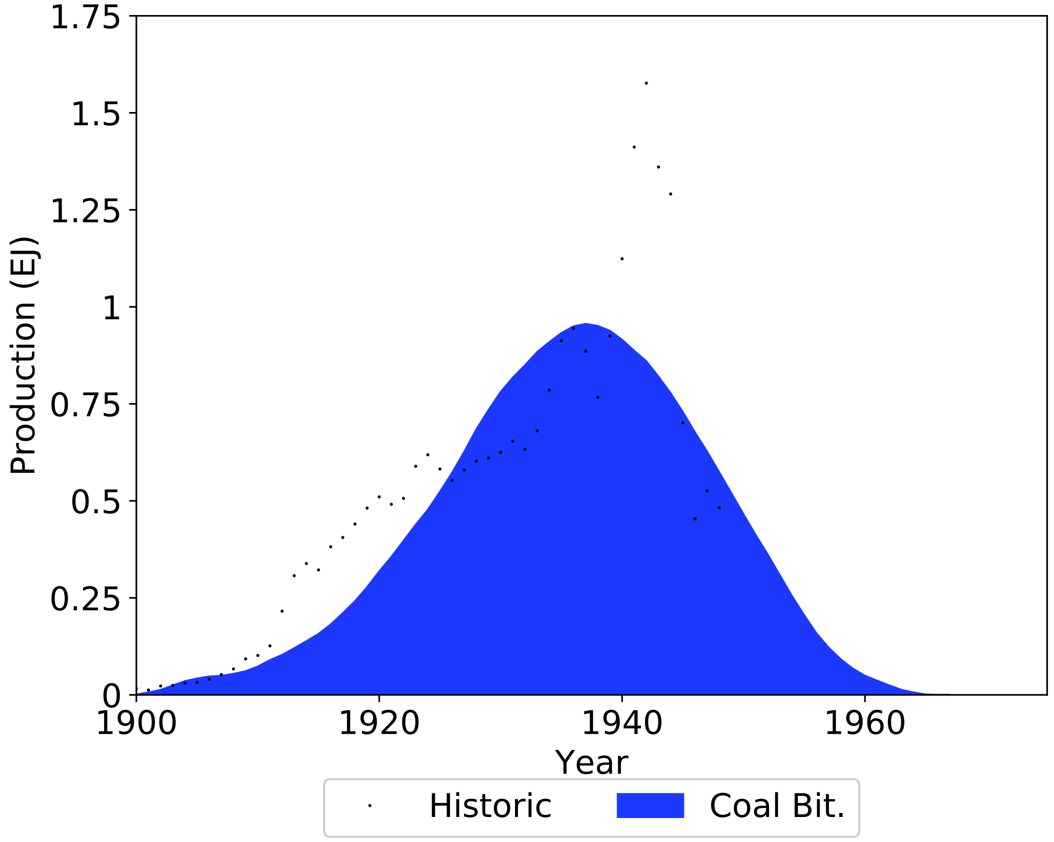

Figure 2.62: China - Historic projections capped at 16

Table 2.62: Peak years - All

Name	URR	Peak Year	Peak Rate
Coal Bit. Historic	25.96	1937	0.96
**Total**	**25.96**	**1937**	**0.96**

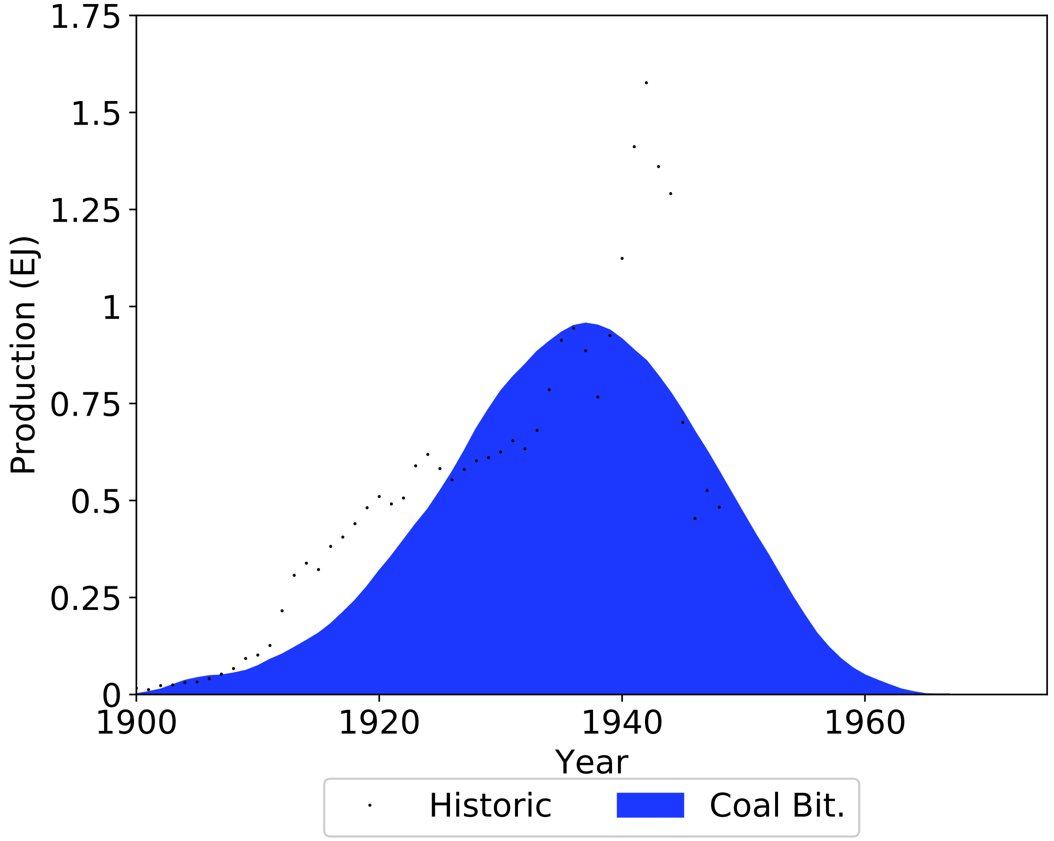

Figure 2.63: China - Historic projection by mineral type

Table 2.63: Peak years - Minerals

Name	URR	Peak Year	Peak Rate
Coal Bit.	25.96	1937	0.96
**Total**	**25.96**	**1937**	**0.96**

Hubei

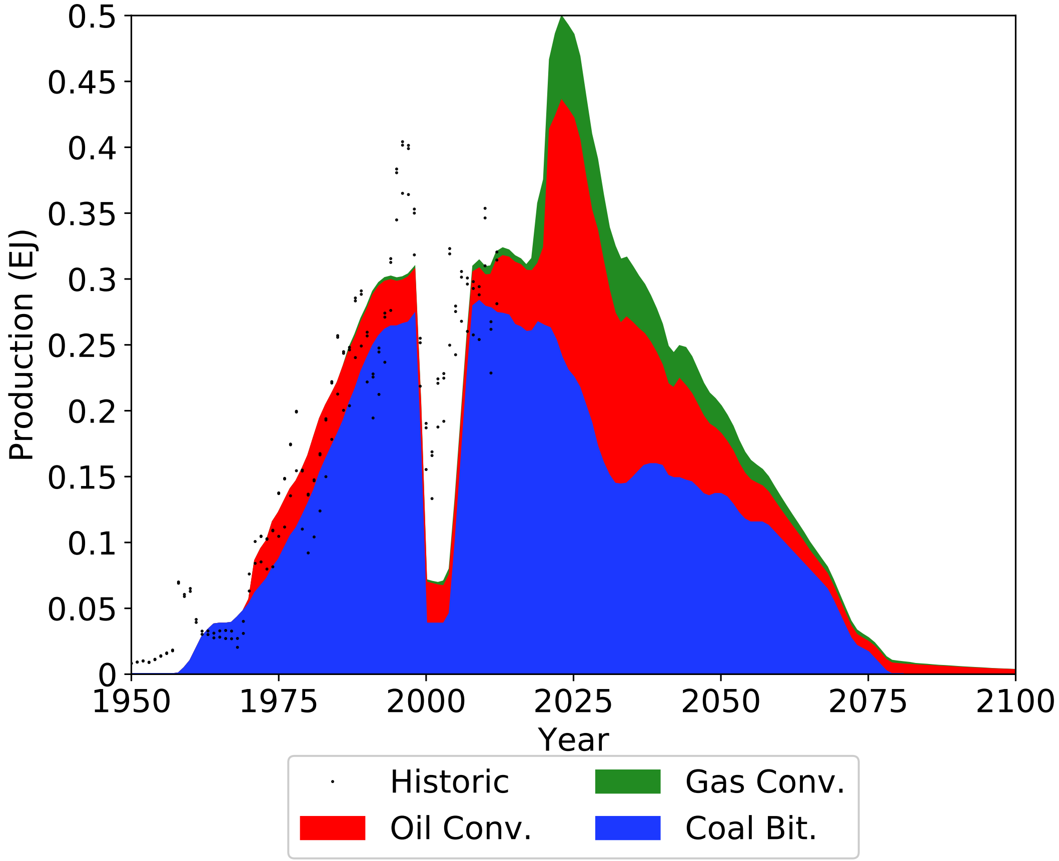

Figure 2.64: China - Hubei projections capped at 16

Table 2.64: Peak years - All

Name	URR	Peak Year	Peak Rate
Coal Bit. Hubei	16.89	2009	0.28
Oil Conv. Hubei	5.97	2024	0.2
Gas Conv. Hubei	1.73	2023	0.06
**Total**	**24.59**	**2023**	**0.5**

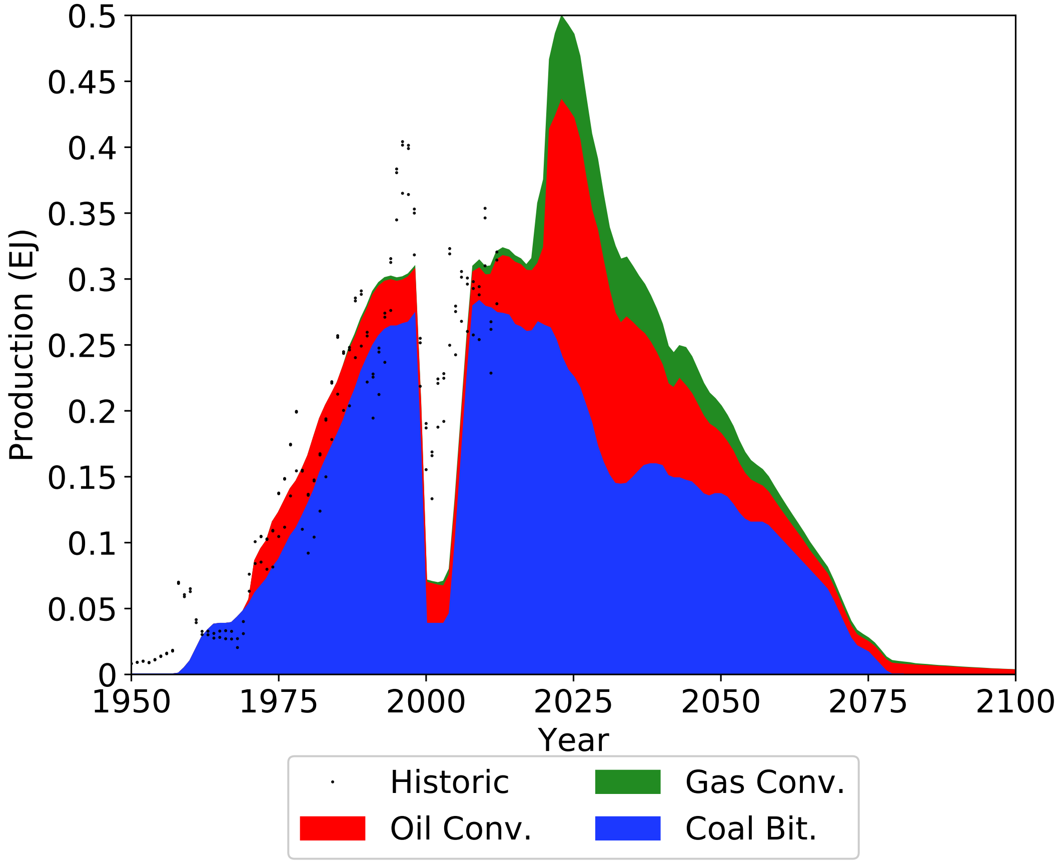

Figure 2.65: China - Hubei projection by mineral type

Table 2.65: Peak years - Minerals

Name	URR	Peak Year	Peak Rate
Coal Bit.	16.89	2009	0.28
Oil Conv.	5.97	2024	0.2
Gas Conv.	1.73	2023	0.06
**Total**	**24.59**	**2023**	**0.5**

Hunan

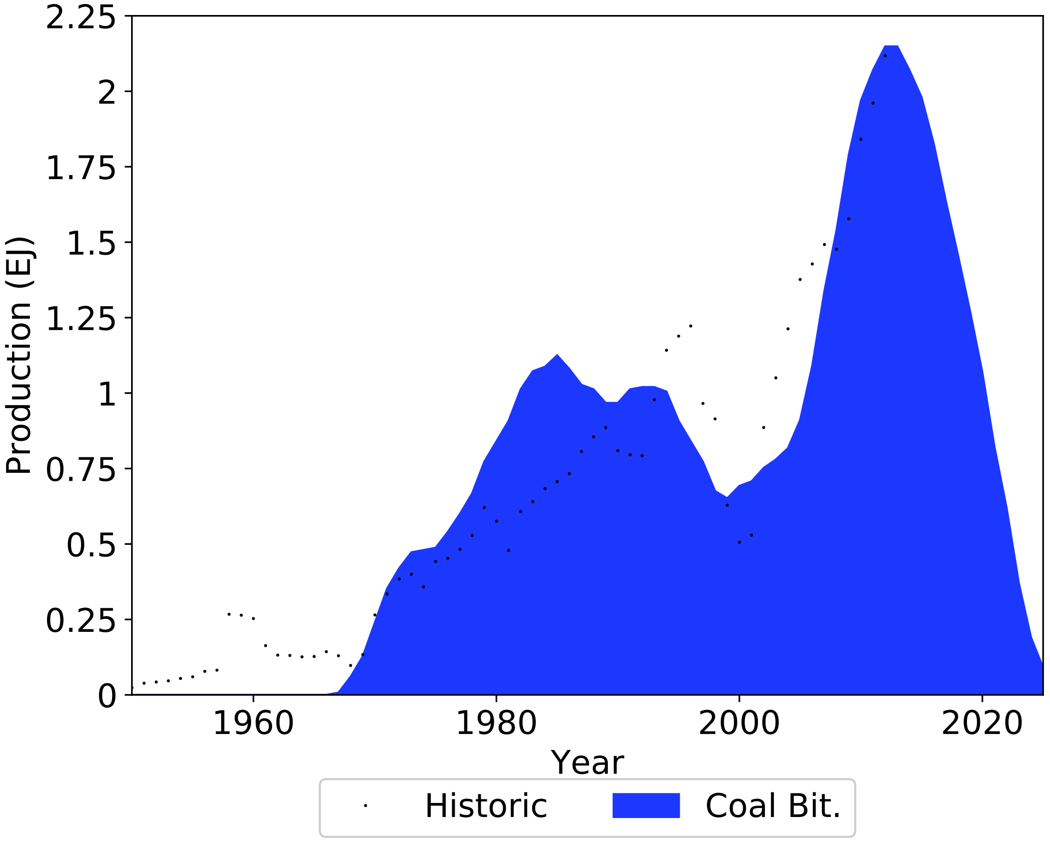

Figure 2.66: China - Hunan projections capped at 16

Table 2.66: Peak years - All

Name	URR	Peak Year	Peak Rate
Coal Bit. Hunan	56.4	2012	2.15
**Total**	**56.4**	**2012**	**2.15**

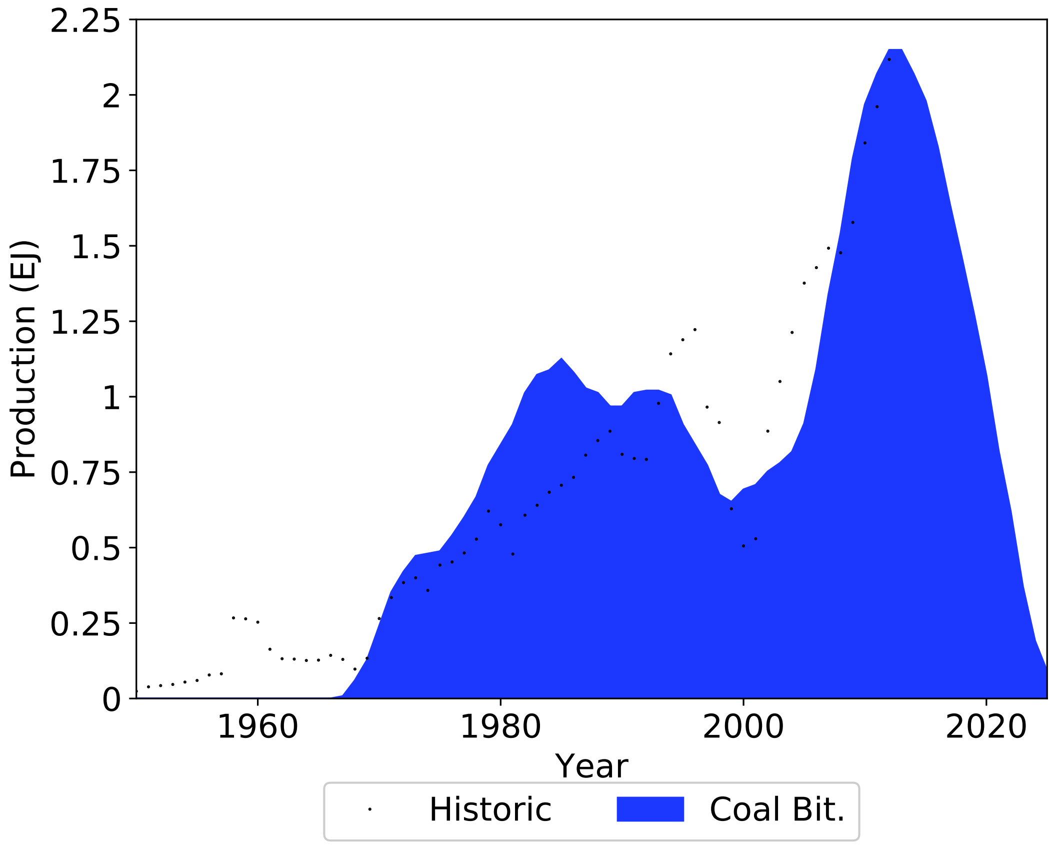

Figure 2.67: China - Hunan projection by mineral type

Table 2.67: Peak years - Minerals

Name	URR	Peak Year	Peak Rate
Coal Bit.	56.4	2012	2.15
**Total**	**56.4**	**2012**	**2.15**

Inner Mongolia

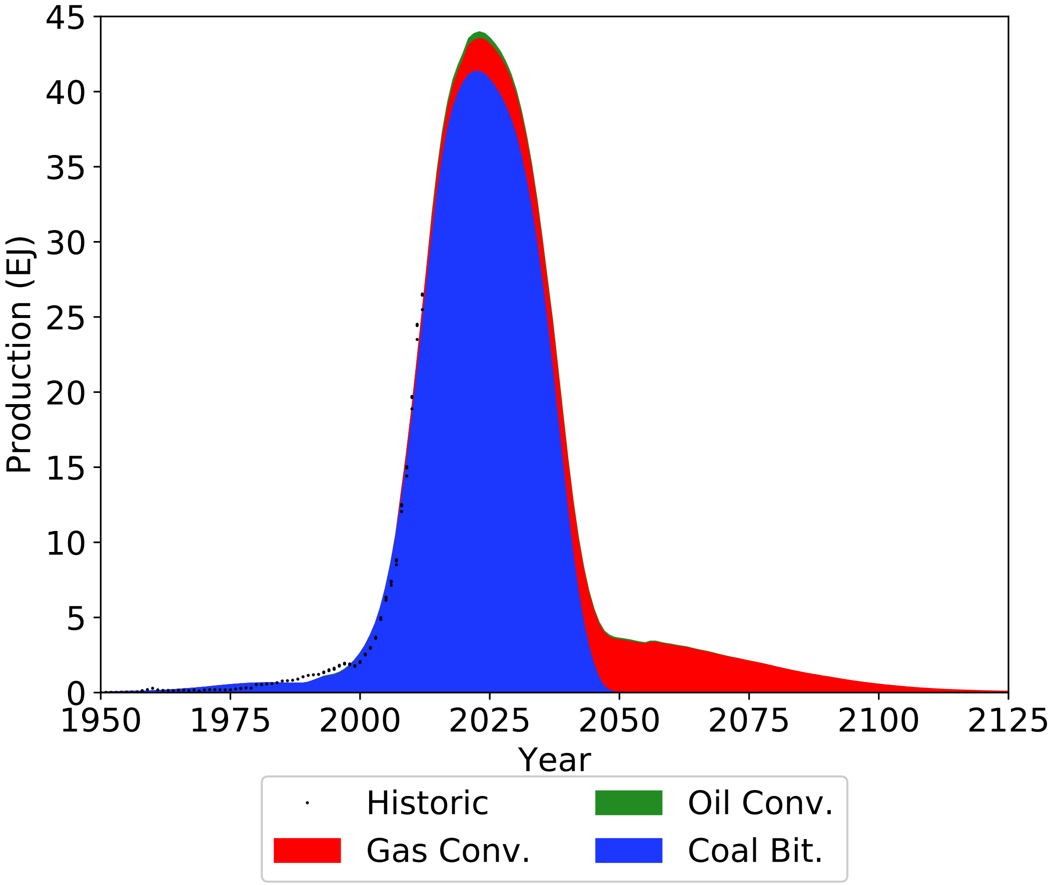

Figure 2.68: China - Inner Mongolia projections capped at 16

Table 2.68: Peak years - All

Name	URR	Peak Year	Peak Rate
Coal Bit. Inner Mongolia	1122.81	2023	41.31
Gas Conv. Inner Mongolia	208.83	2046	3.58
Oil Conv. Inner Mongolia	13.32	2021	0.45
**Total**	**1344.96**	**2023**	**43.95**

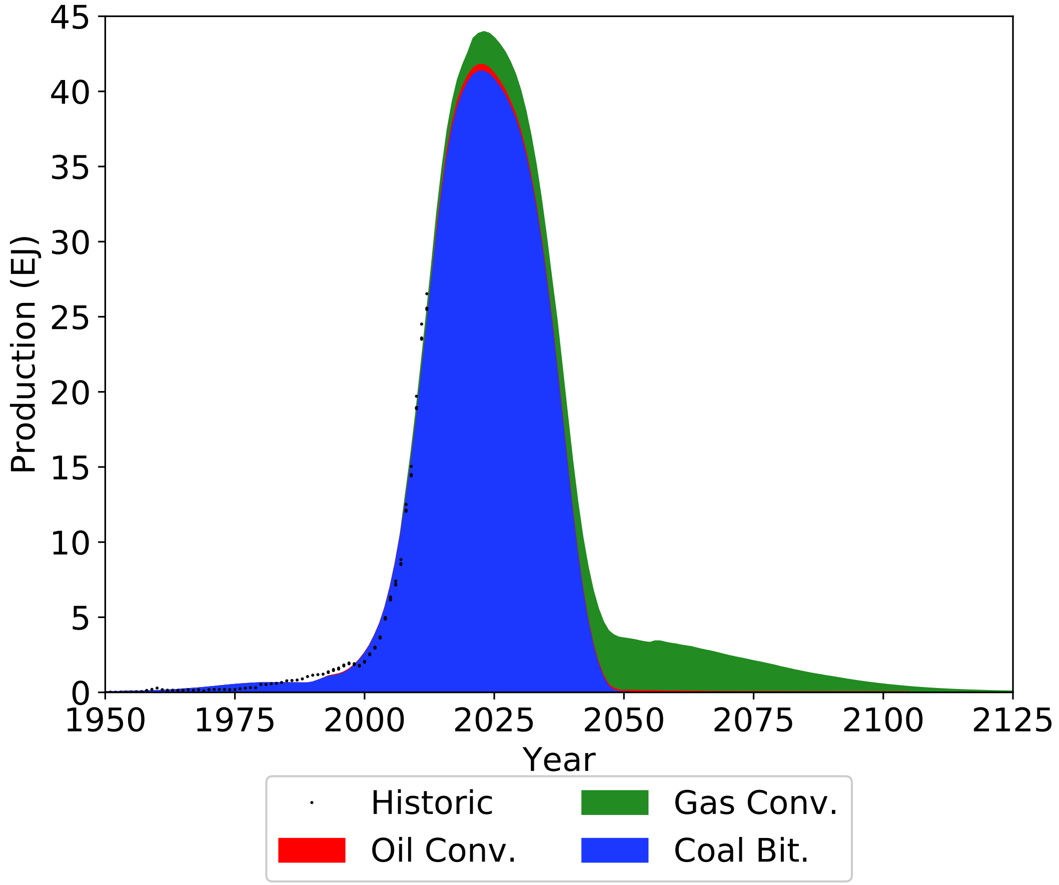

Figure 2.69: China - Inner Mongolia projection by mineral type

Table 2.69: Peak years - Minerals

Name	URR	Peak Year	Peak Rate
Coal Bit.	1122.81	2023	41.31
Oil Conv.	13.32	2021	0.45
Gas Conv.	208.83	2046	3.58
**Total**	**1344.96**	**2023**	**43.95**

Jiangsu

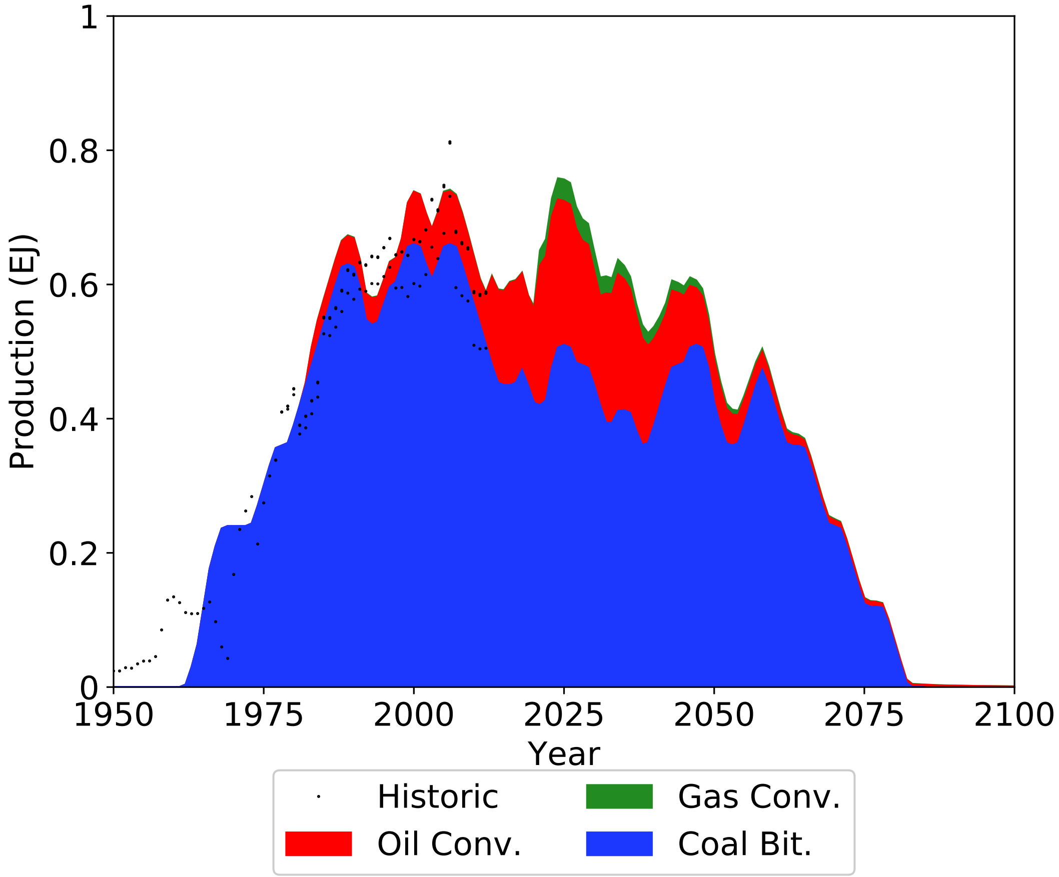

Figure 2.70: China - Jiangsu projections capped at 16

Table 2.70: Peak years - All

Name	URR	Peak Year	Peak Rate
Coal Bit. Jiangsu	48.95	2000	0.66
Oil Conv. Jiangsu	8.1	2023	0.23
Gas Conv. Jiangsu	0.79	2025	0.03
**Total**	**57.84**	**2024**	**0.76**

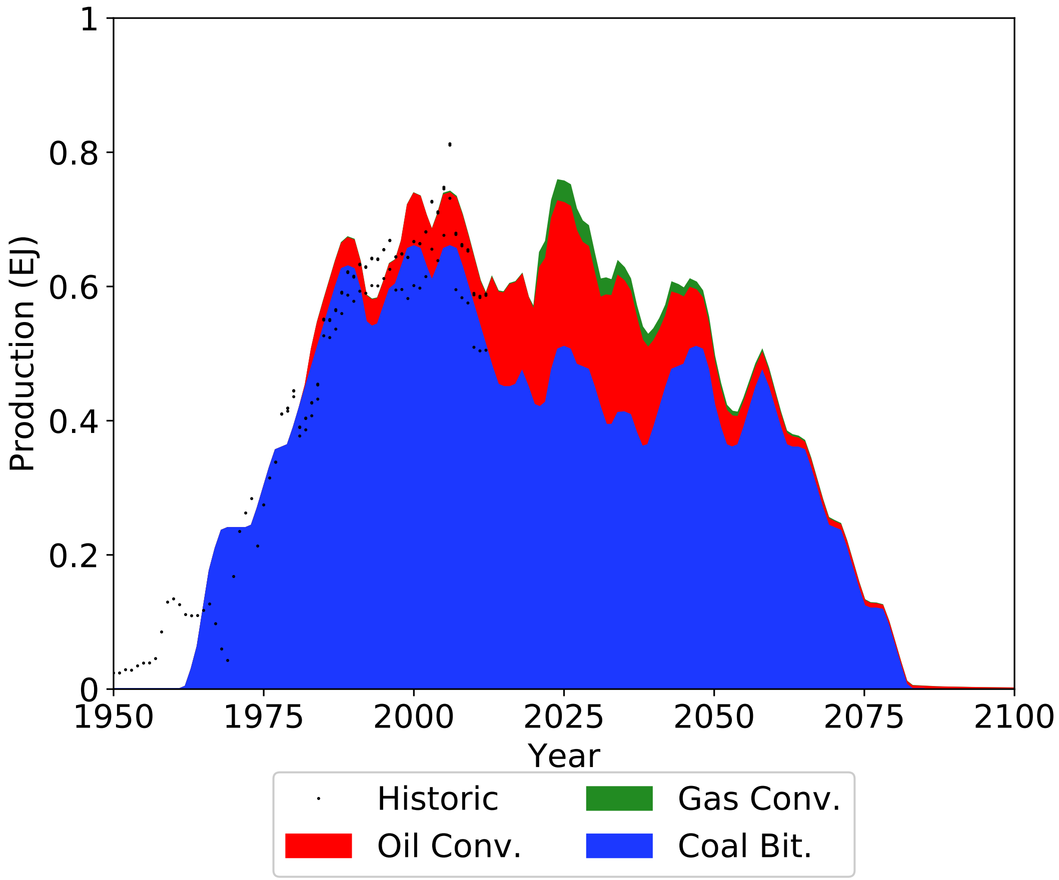

Figure 2.71: China - Jiangsu projection by mineral type

Table 2.71: Peak years - Minerals

Name	URR	Peak Year	Peak Rate
Coal Bit.	48.95	2000	0.66
Oil Conv.	8.1	2023	0.23
Gas Conv.	0.79	2025	0.03
**Total**	**57.84**	**2024**	**0.76**

Jiangxi

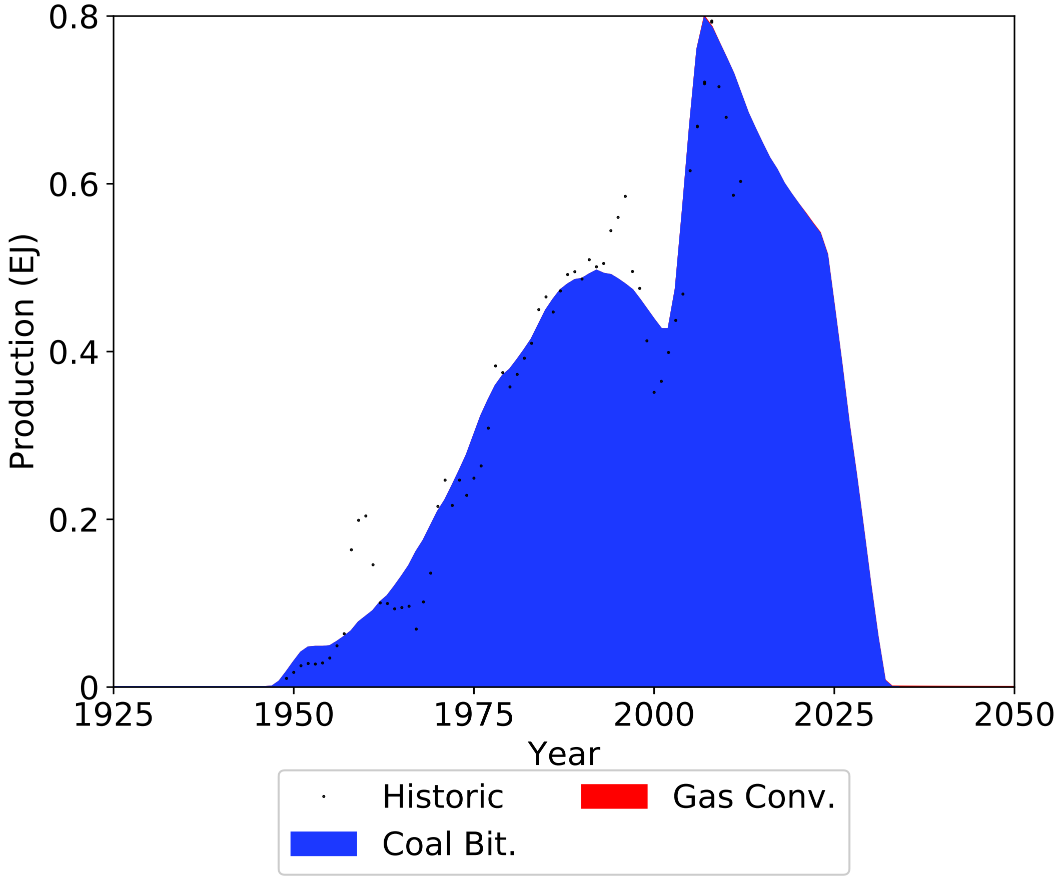

Figure 2.72: China - Jiangxi projections capped at 16

Table 2.72: Peak years - All

**Name**	**URR**	**Peak Year**	**Peak Rate**
Coal Bit. Jiangxi	31.21	2007	0.8
Gas Conv. Jiangxi	0.02	2007	–
**Total**	**31.23**	**2007**	**0.8**

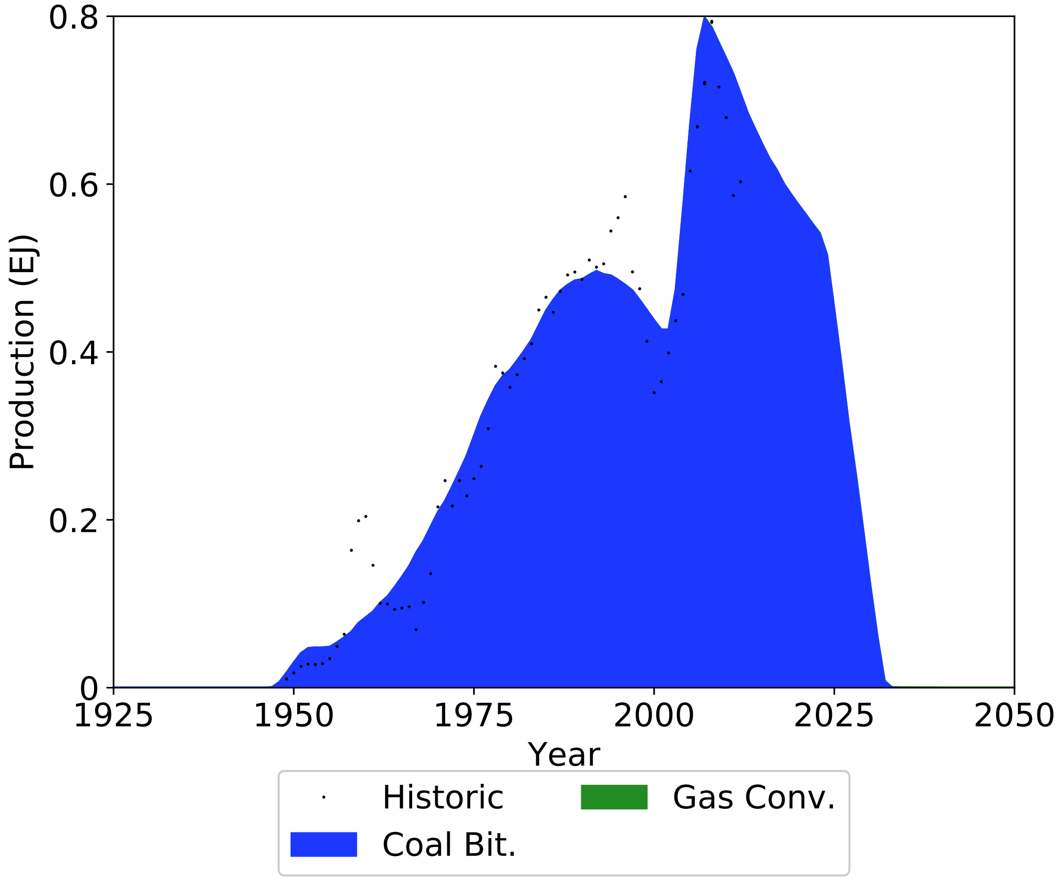

Figure 2.73: China - Jiangxi projection by mineral type

Table 2.73: Peak years - Minerals

Name	URR	Peak Year	Peak Rate
Coal Bit.	31.21	2007	0.8
Gas Conv.	0.02	2007	–
**Total**	**31.23**	**2007**	**0.8**

Jilin

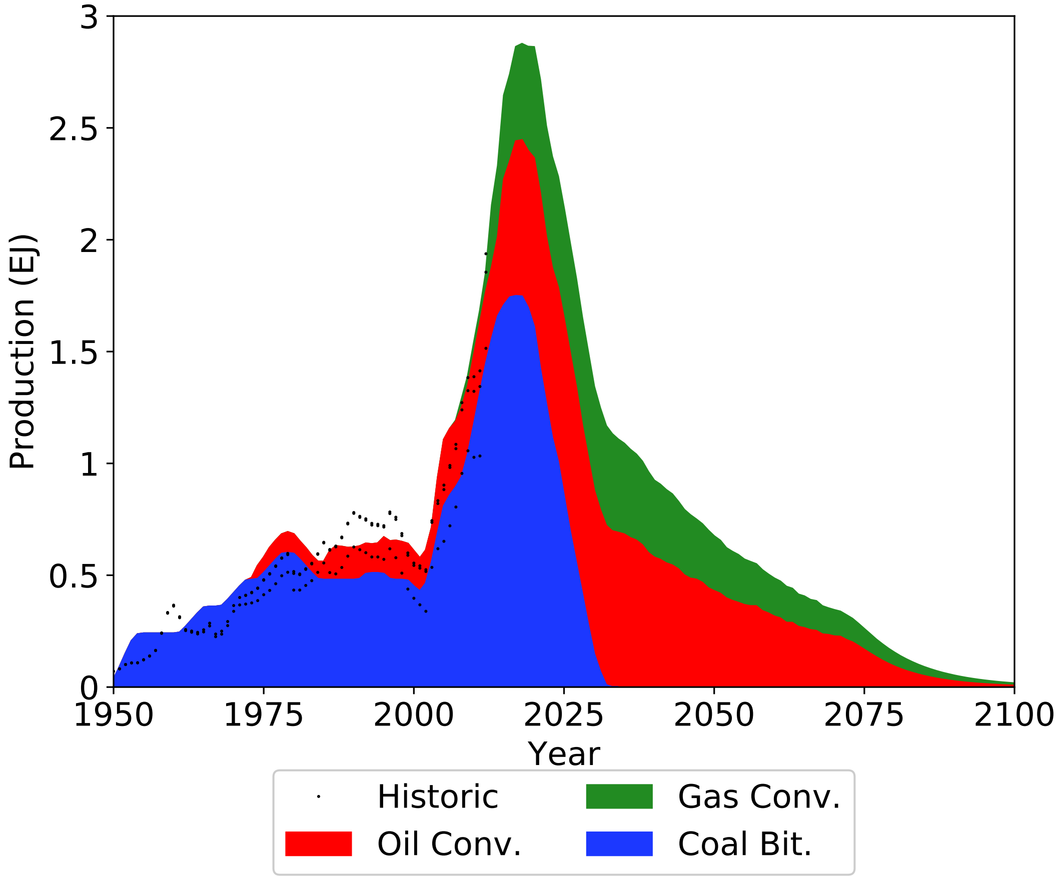

Figure 2.74: China - Jilin projections capped at 16

Table 2.74: Peak years - All

**Name**	**URR**	**Peak Year**	**Peak Rate**
Coal Bit. Jilin	52.66	2017	1.75
Oil Conv. Jilin	38.88	2026	0.8
Gas Conv. Jilin	20.22	2021	0.51
**Total**	**111.76**	**2018**	**2.88**

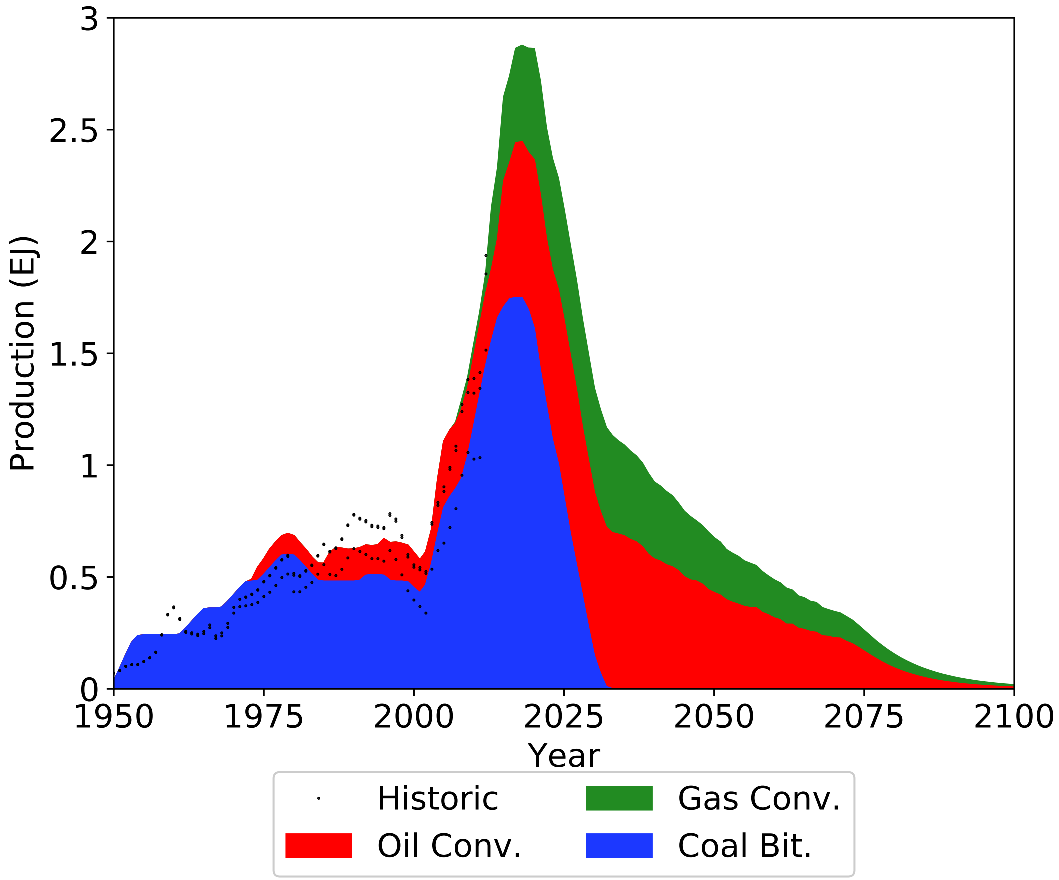

Figure 2.75: China - Jilin projection by mineral type

Table 2.75: Peak years - Minerals

Name	URR	Peak Year	Peak Rate
Coal Bit.	52.66	2017	1.75
Oil Conv.	38.88	2026	0.8
Gas Conv.	20.22	2021	0.51
**Total**	**111.76**	**2018**	**2.88**

Liaoning

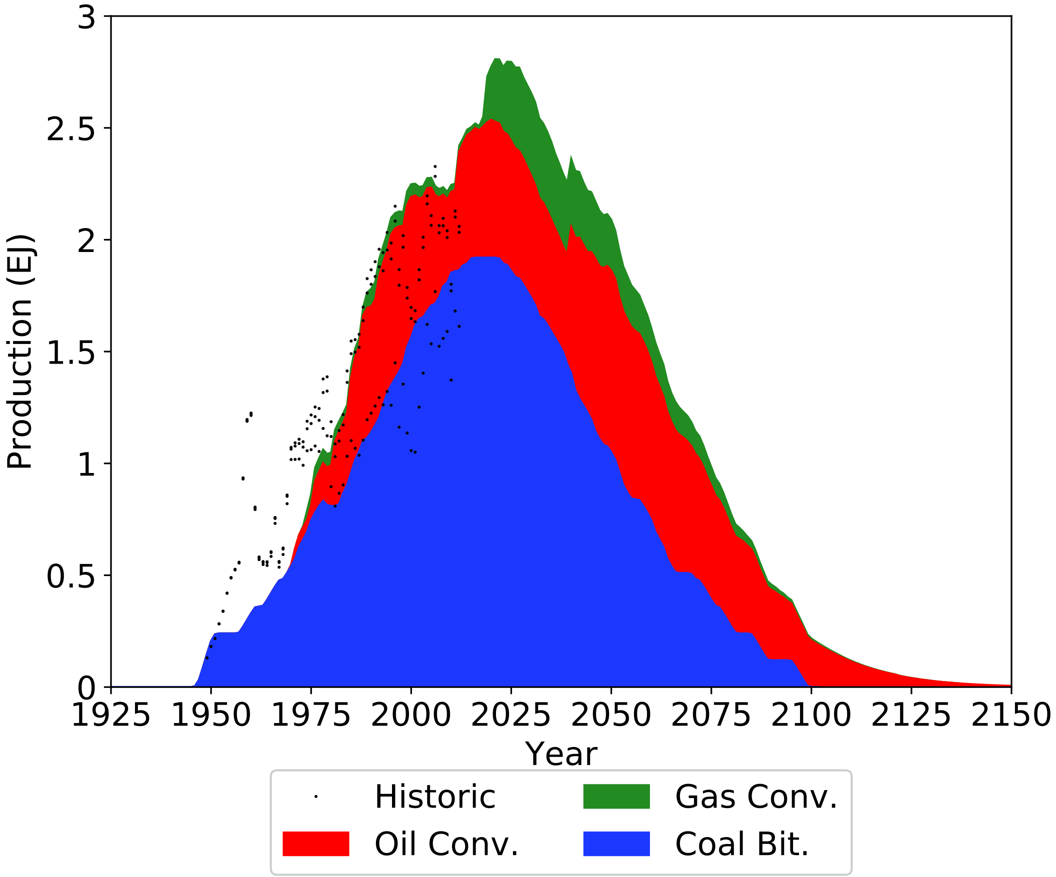

Figure 2.76: China - Liaoning projections capped at 16

Table 2.76: Peak years - All

**Name**	**URR**	**Peak Year**	**Peak Rate**
Coal Bit. Liaoning	141.13	2016	1.92
Oil Conv. Liaoning	68.87	2050	0.81
Gas Conv. Liaoning	16.56	2027	0.37
**Total**	**226.56**	**2021**	**2.81**

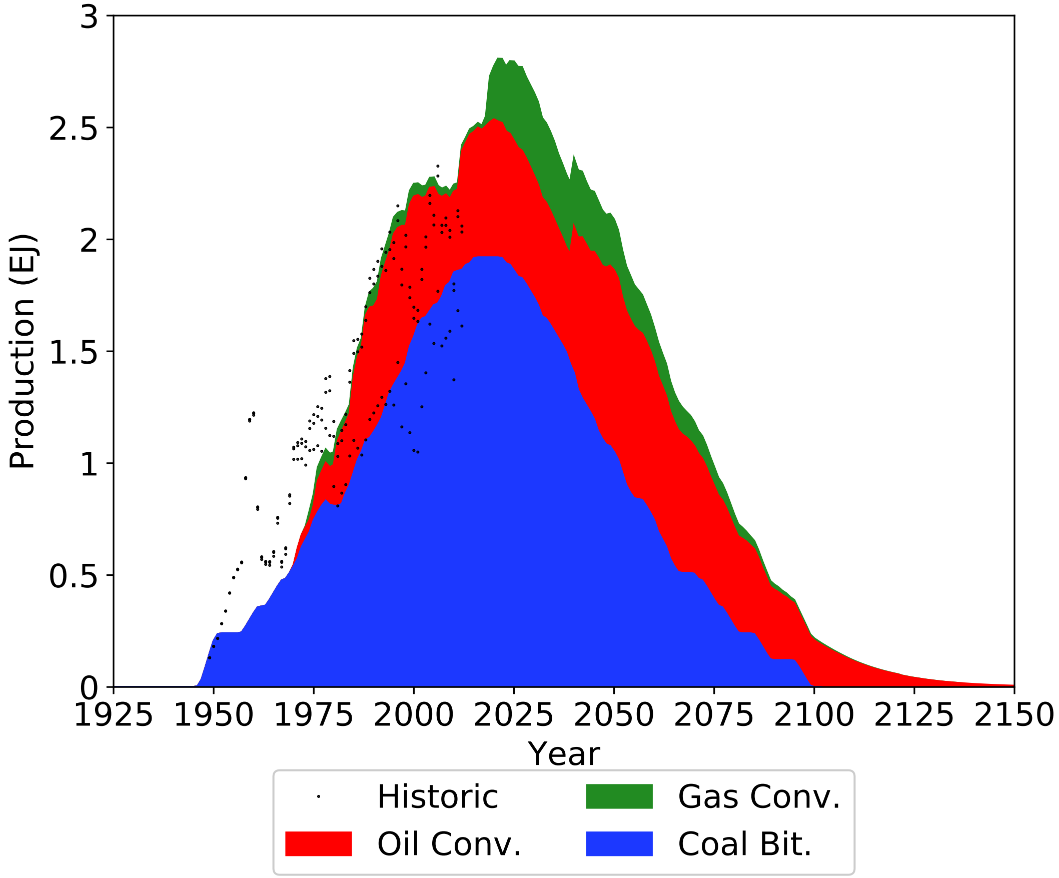

Figure 2.77: China - Liaoning projection by mineral type

Table 2.77: Peak years - Minerals

Name	URR	Peak Year	Peak Rate
Coal Bit.	141.13	2016	1.92
Oil Conv.	68.87	2050	0.81
Gas Conv.	16.56	2027	0.37
**Total**	**226.56**	**2021**	**2.81**

Ningxia

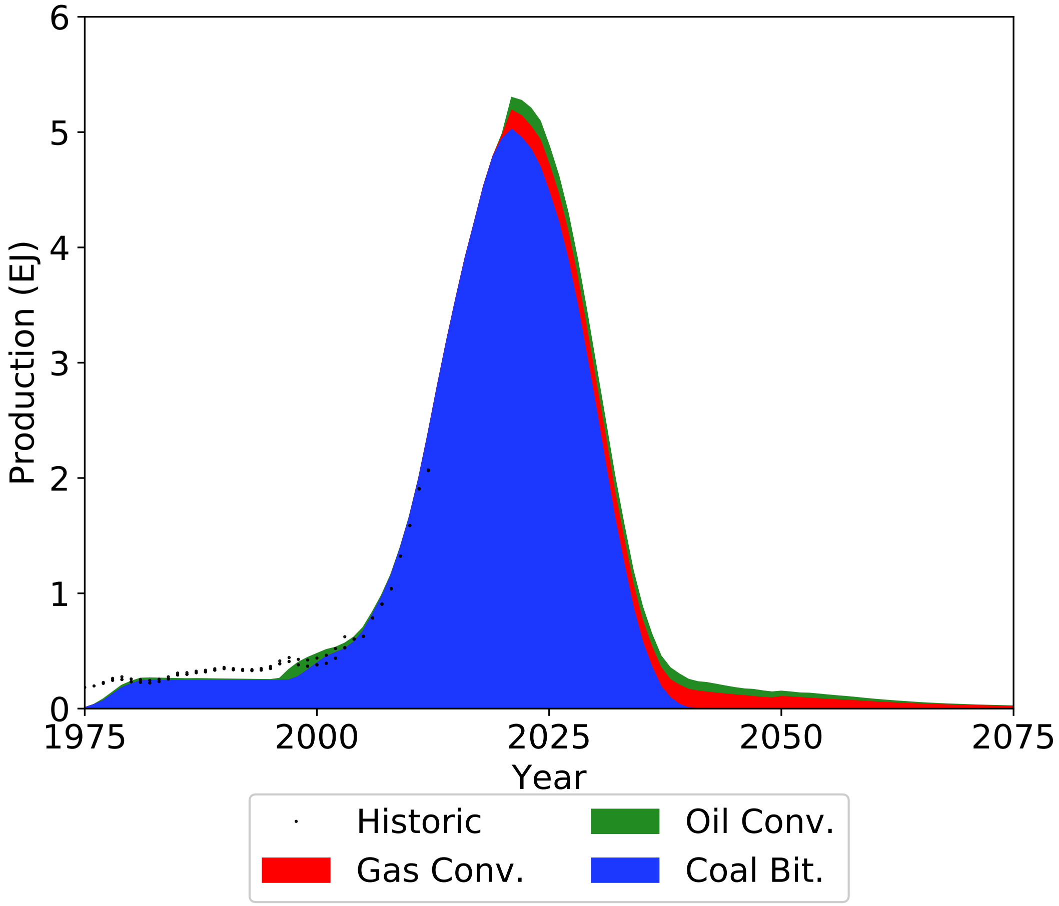

Figure 2.78: China - Ningxia projections capped at 16

Table 2.78: Peak years - All

Name	URR	Peak Year	Peak Rate
Coal Bit. Ningxia	99.05	2021	5.02
Gas Conv. Ningxia	6.63	2025	0.24
Oil Conv. Ningxia	4.84	2027	0.16
**Total**	**110.52**	**2021**	**5.3**

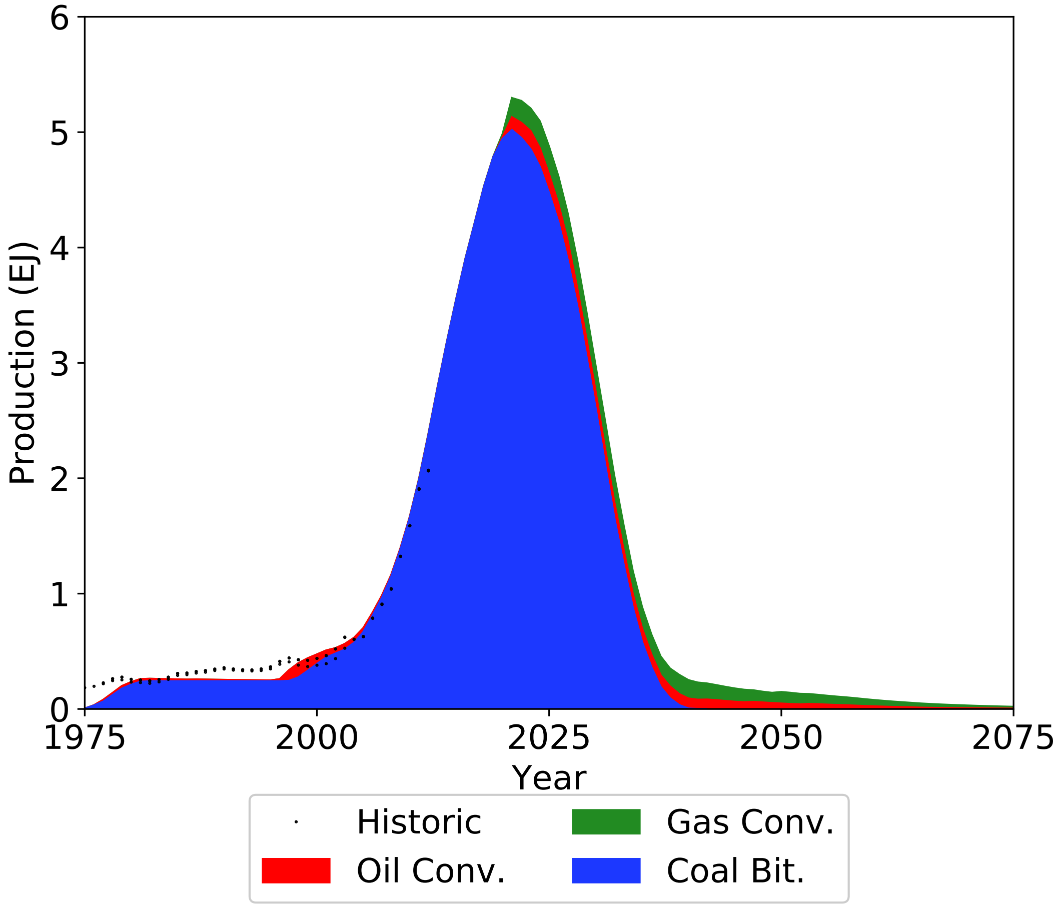

Figure 2.79: China - Ningxia projection by mineral type

Table 2.79: Peak years - Minerals

Name	URR	Peak Year	Peak Rate
Coal Bit.	99.05	2021	5.02
Oil Conv.	4.84	2027	0.16
Gas Conv.	6.63	2025	0.24
**Total**	**110.52**	**2021**	**5.3**

Offshore

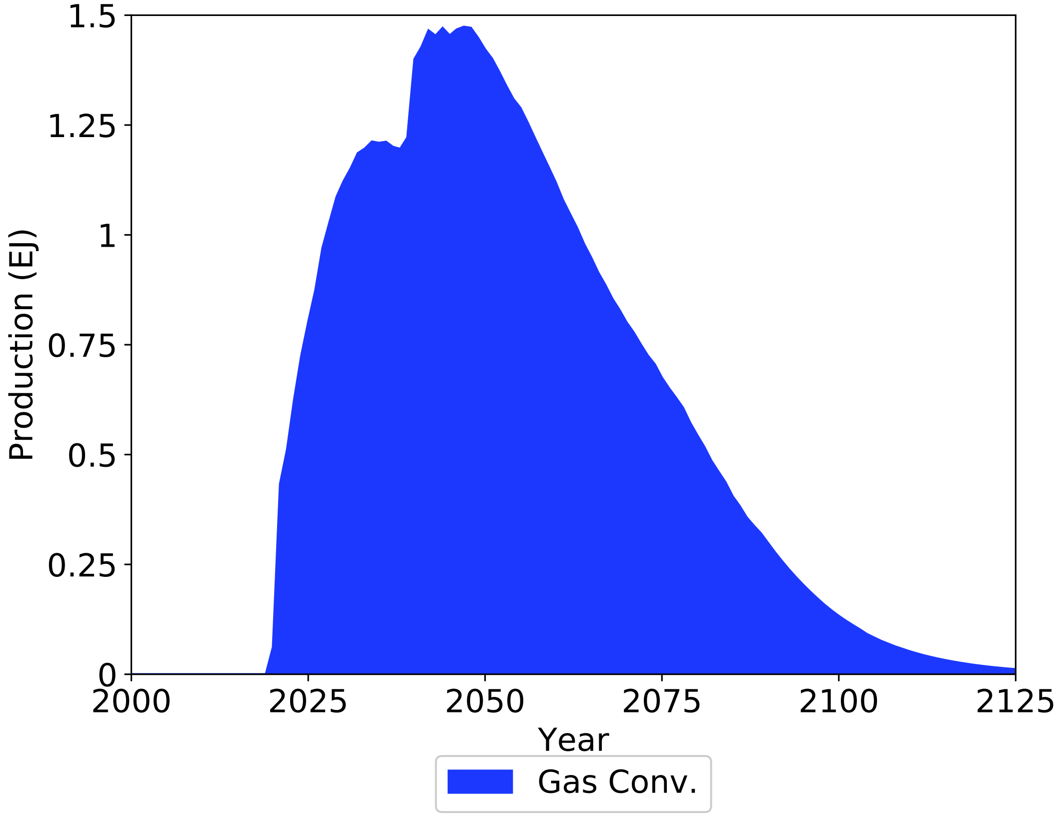

Figure 2.80: China - Offshore projections capped at 16

Table 2.80: Peak years - All

Name	URR	Peak Year	Peak Rate
Gas Conv. Offshore	70.95	2047	1.47
**Total**	**70.95**	**2047**	**1.47**

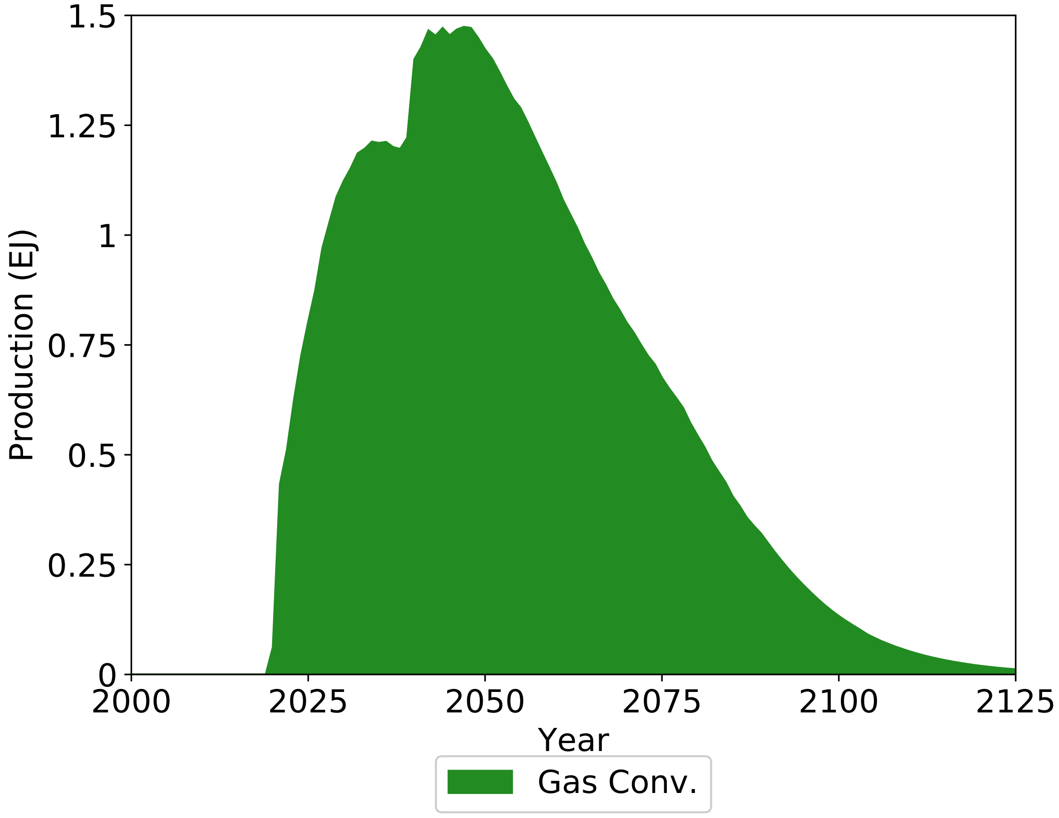

Figure 2.81: China - Offshore projection by mineral type

Table 2.81: Peak years - Minerals

Name	URR	Peak Year	Peak Rate
Gas Conv.	70.95	2047	1.47
**Total**	**70.95**	**2047**	**1.47**

Qinghai

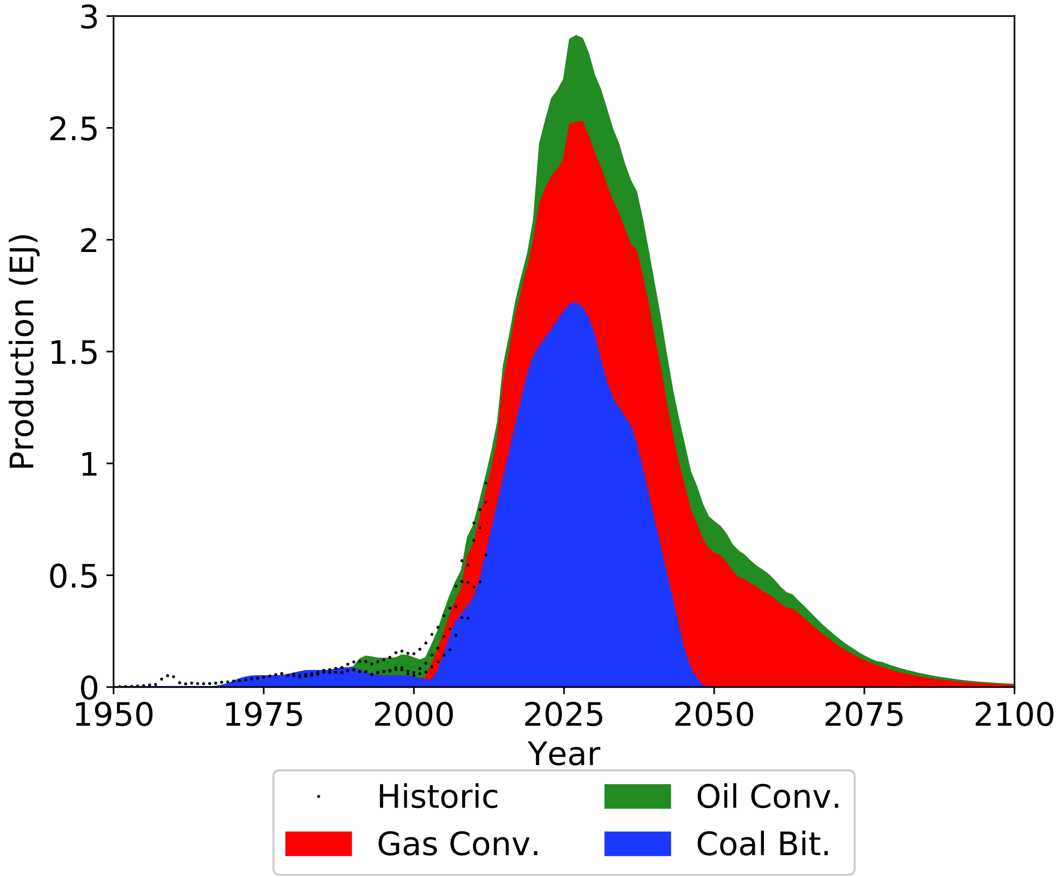

Figure 2.82: China - Qinghai projections capped at 16

Table 2.82: Peak years - All

Name	URR	Peak Year	Peak Rate
Coal Bit. Qinghai	43.41	2027	1.72
Gas Conv. Qinghai	37.32	2033	0.88
Oil Conv. Qinghai	12.64	2027	0.39
**Total**	**93.37**	**2027**	**2.91**

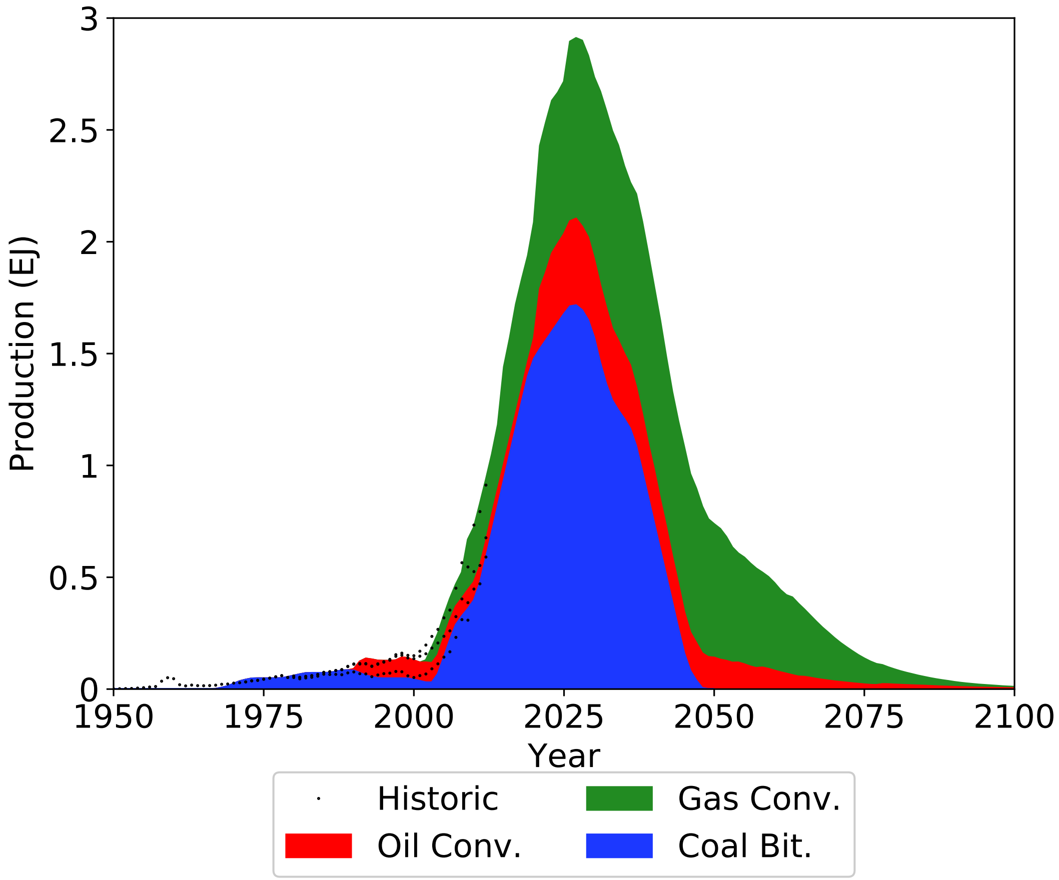

Figure 2.83: China - Qinghai projection by mineral type

Table 2.83: Peak years - Minerals

Name	URR	Peak Year	Peak Rate
Coal Bit.	43.41	2027	1.72
Oil Conv.	12.64	2027	0.39
Gas Conv.	37.32	2033	0.88
**Total**	**93.37**	**2027**	**2.91**

Shaanxi

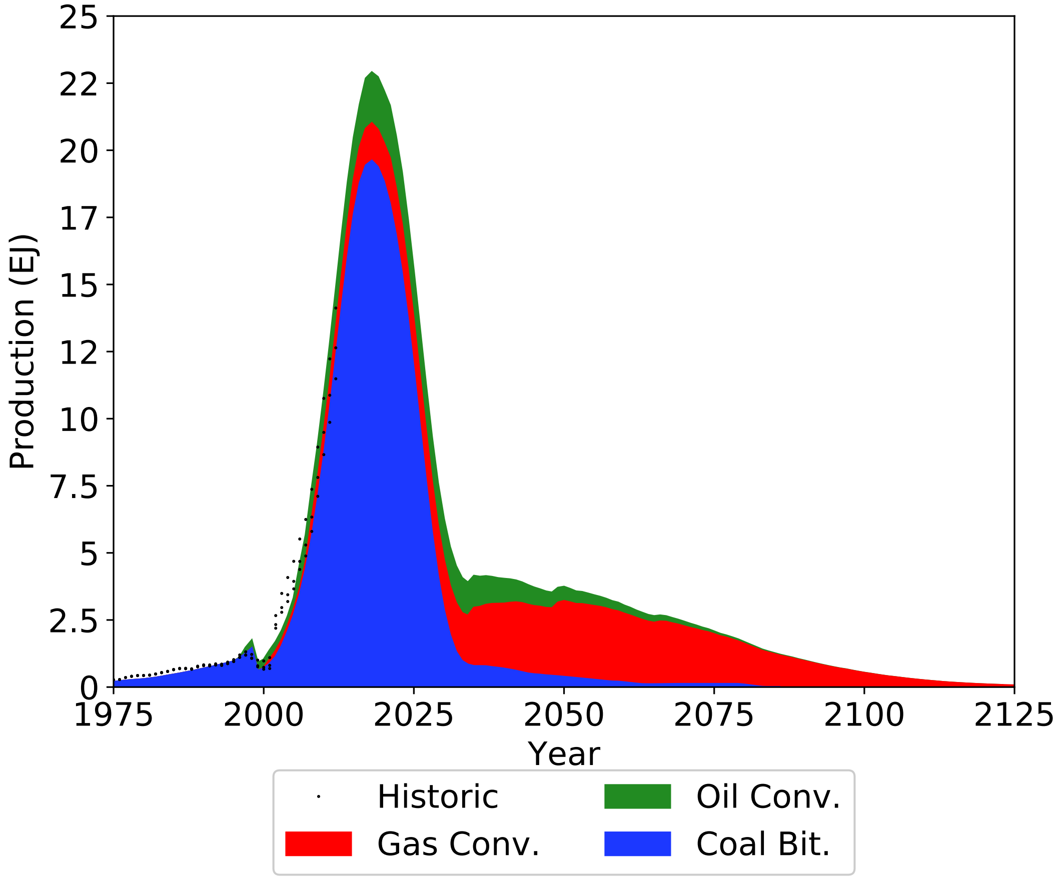

Figure 2.84: China - Shaanxi projections capped at 16

Table 2.84: Peak years - All

Name	URR	Peak Year	Peak Rate
Coal Bit. Shaanxi	346.35	2018	19.61
Gas Conv. Shaanxi	175.87	2050	2.83
Oil Conv. Shaanxi	69.46	2019	1.96
**Total**	**591.68**	**2018**	**22.91**

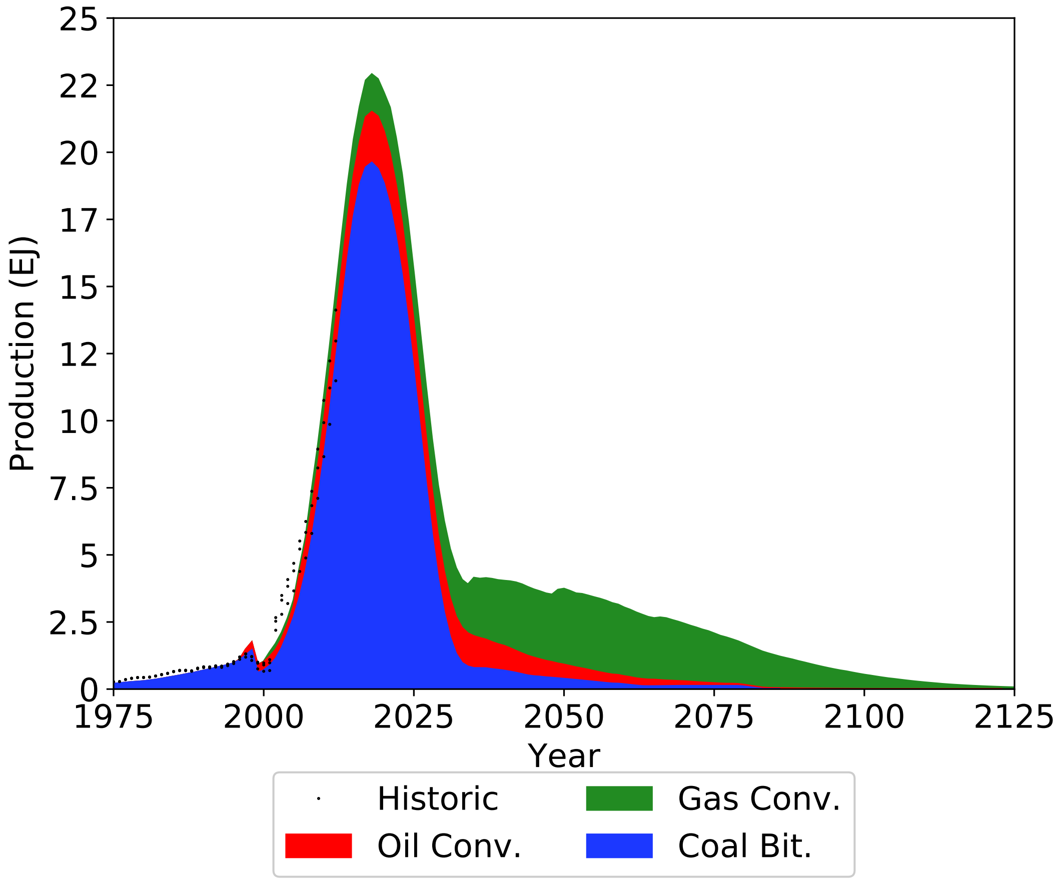

Figure 2.85: China - Shaanxi projection by mineral type

Table 2.85: Peak years - Minerals

Name	URR	Peak Year	Peak Rate
Coal Bit.	346.35	2018	19.61
Oil Conv.	69.46	2019	1.96
Gas Conv.	175.87	2050	2.83
**Total**	**591.68**	**2018**	**22.91**

Shandong

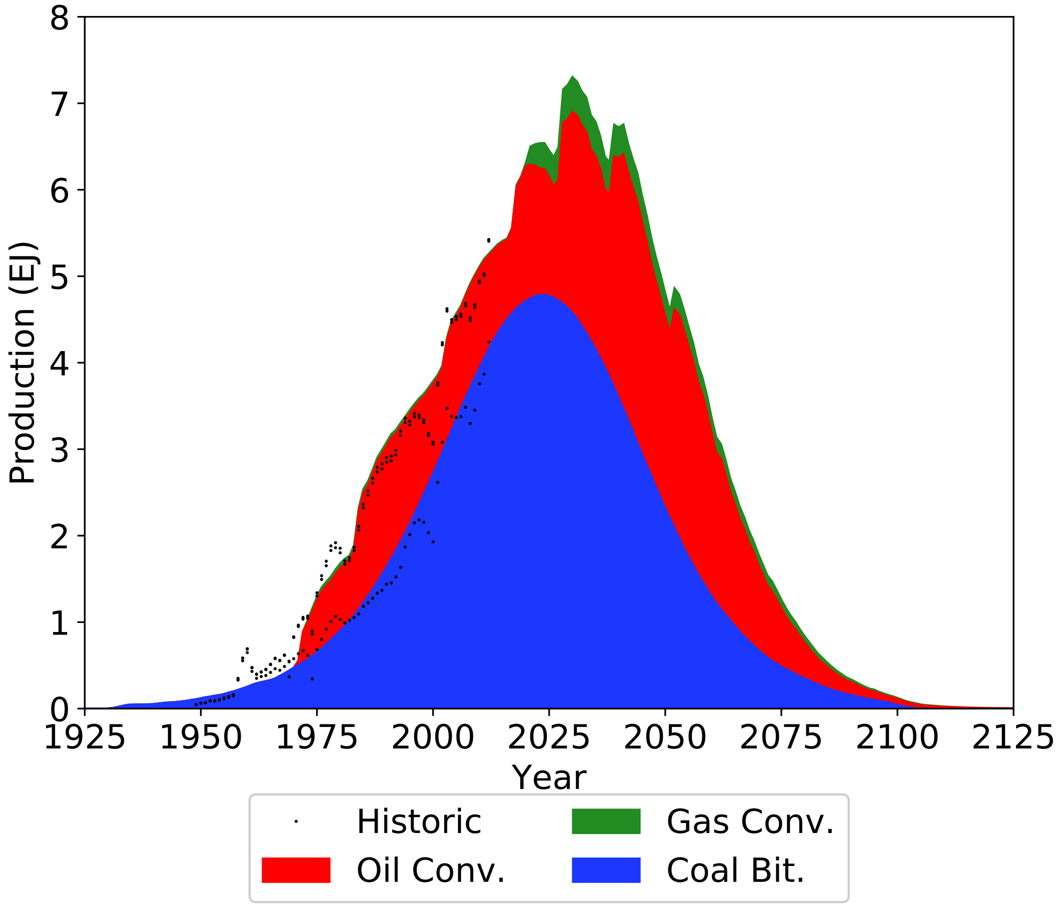

Figure 2.86: China - Shandong projections capped at 16

Table 2.86: Peak years - All

Name	URR	Peak Year	Peak Rate
Coal Bit. Shandong	278.09	2023	4.79
Oil Conv. Shandong	165.18	2041	2.95
Gas Conv. Shandong	17.34	2030	0.41
**Total**	**460.61**	**2030**	**7.3**

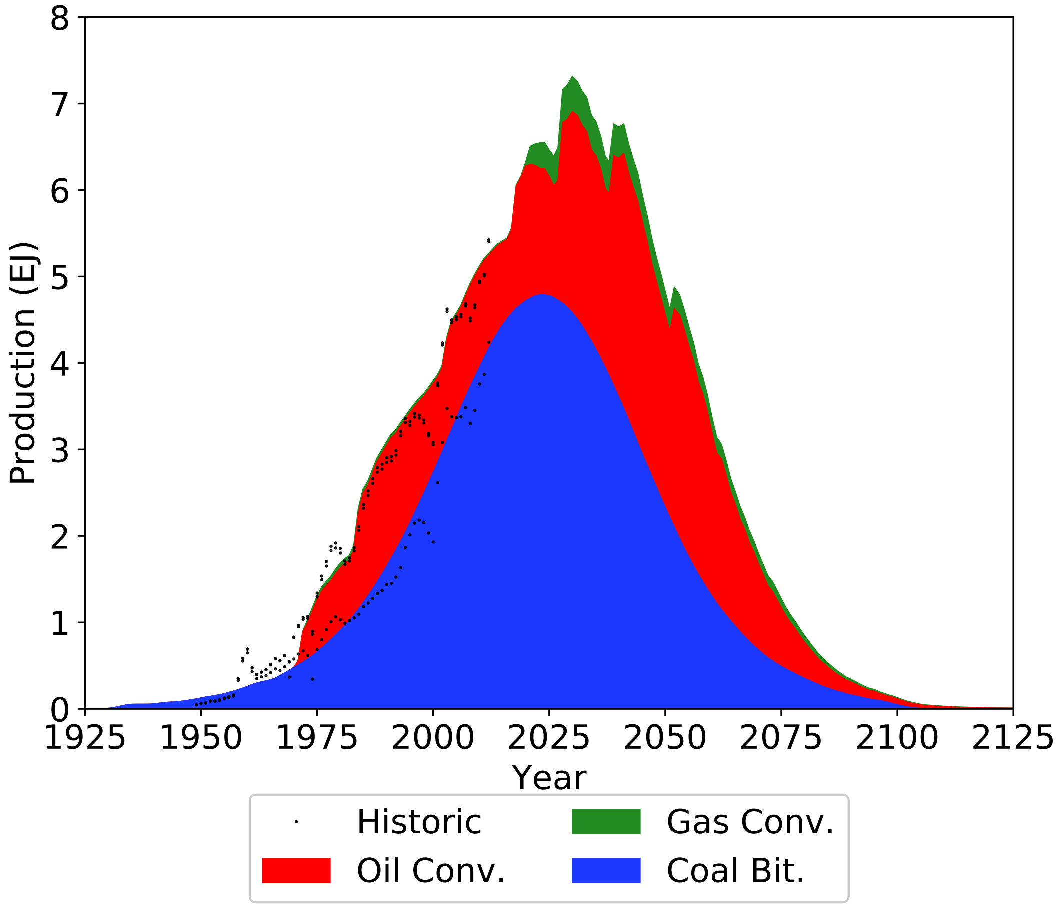

Figure 2.87: China - Shandong projection by mineral type

Table 2.87: Peak years - Minerals

Name	URR	Peak Year	Peak Rate
Coal Bit.	278.09	2023	4.79
Oil Conv.	165.18	2041	2.95
Gas Conv.	17.34	2030	0.41
**Total**	**460.61**	**2030**	**7.3**

Shanghai

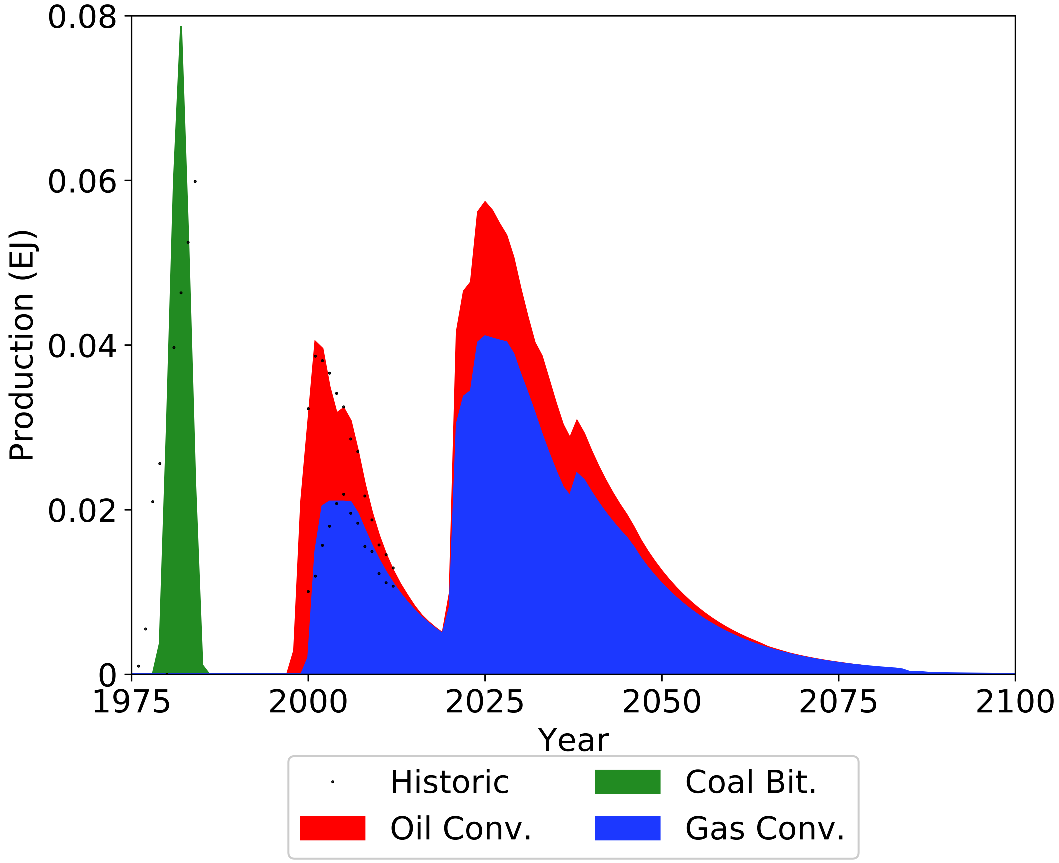

Figure 2.88: China - Shanghai projections capped at 16

Table 2.88: Peak years - All

Name	URR	Peak Year	Peak Rate
Gas Conv. Shanghai	1.19	2025	0.04
Oil Conv. Shanghai	0.42	2000	0.03
Coal Bit. Shanghai	0.25	1982	0.08
**Total**	**1.86**	**1982**	**0.08**

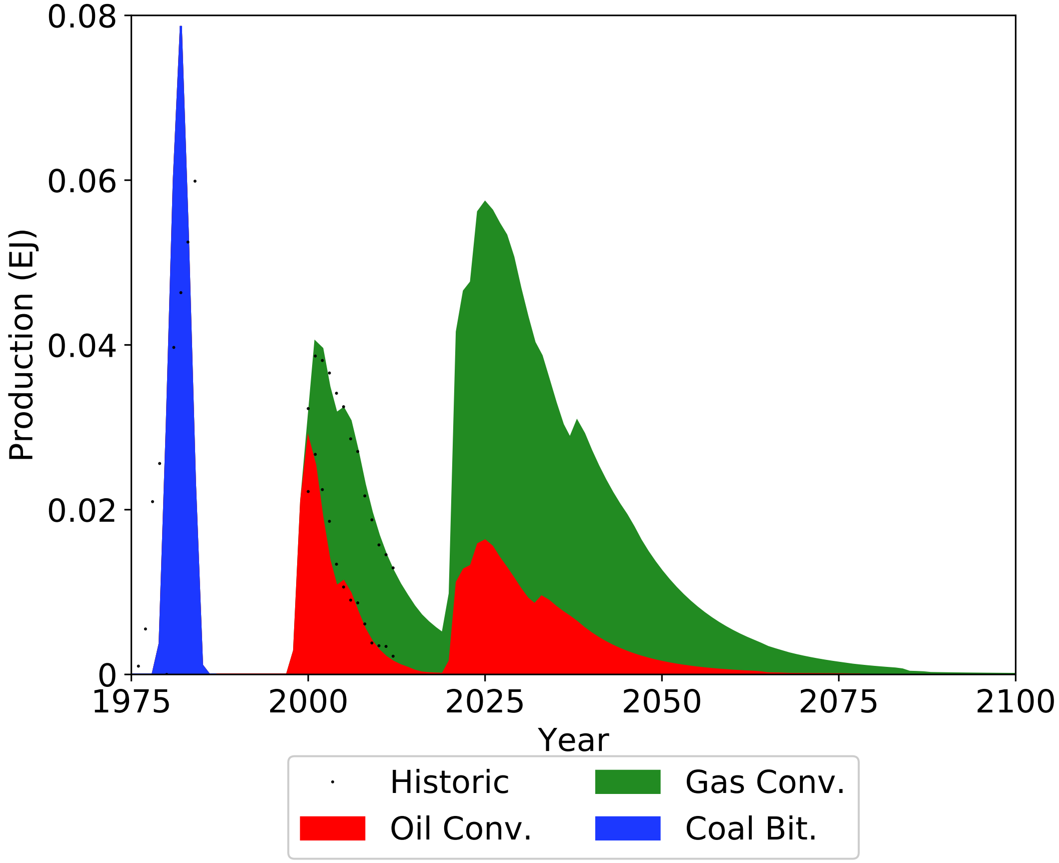

Figure 2.89: China - Shanghai projection by mineral type

Table 2.89: Peak years - Minerals

Name	URR	Peak Year	Peak Rate
Coal Bit.	0.25	1982	0.08
Oil Conv.	0.42	2000	0.03
Gas Conv.	1.19	2025	0.04
**Total**	**1.86**	**1982**	**0.08**

Shanxi

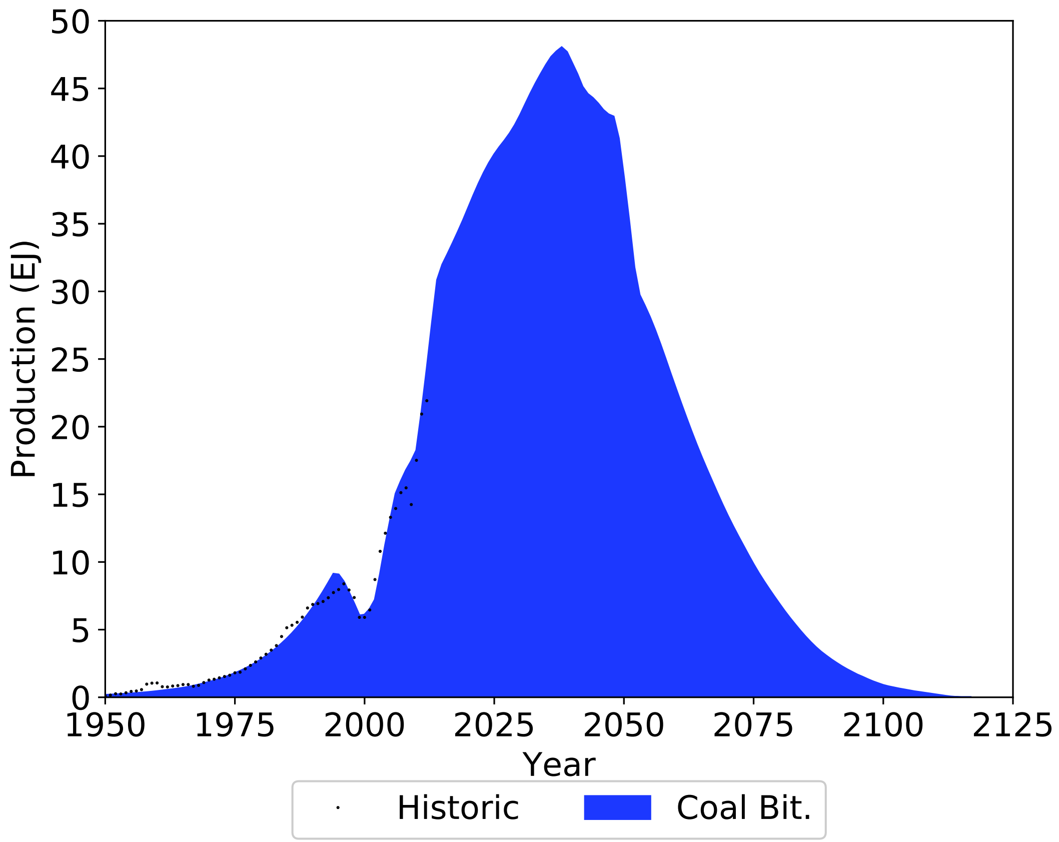

Figure 2.90: China - Shanxi projections capped at 16

Table 2.90: Peak years - All

**Name**	**URR**	**Peak Year**	**Peak Rate**
Coal Bit. Shanxi	2508.19	2038	48.04
**Total**	**2508.19**	**2038**	**48.04**

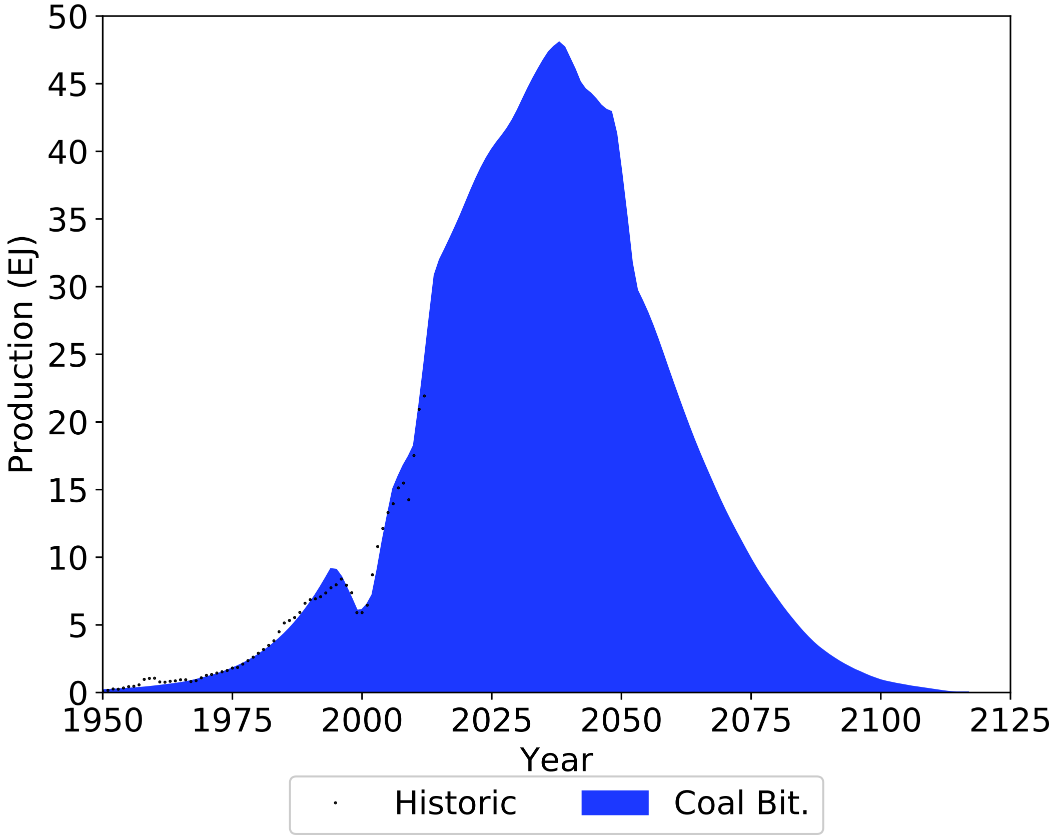

Figure 2.91: China - Shanxi projection by mineral type

Table 2.91: Peak years - Minerals

**Name**	**URR**	**Peak Year**	**Peak Rate**
Coal Bit.	2508.19	2038	48.04
**Total**	**2508.19**	**2038**	**48.04**

Sichuan

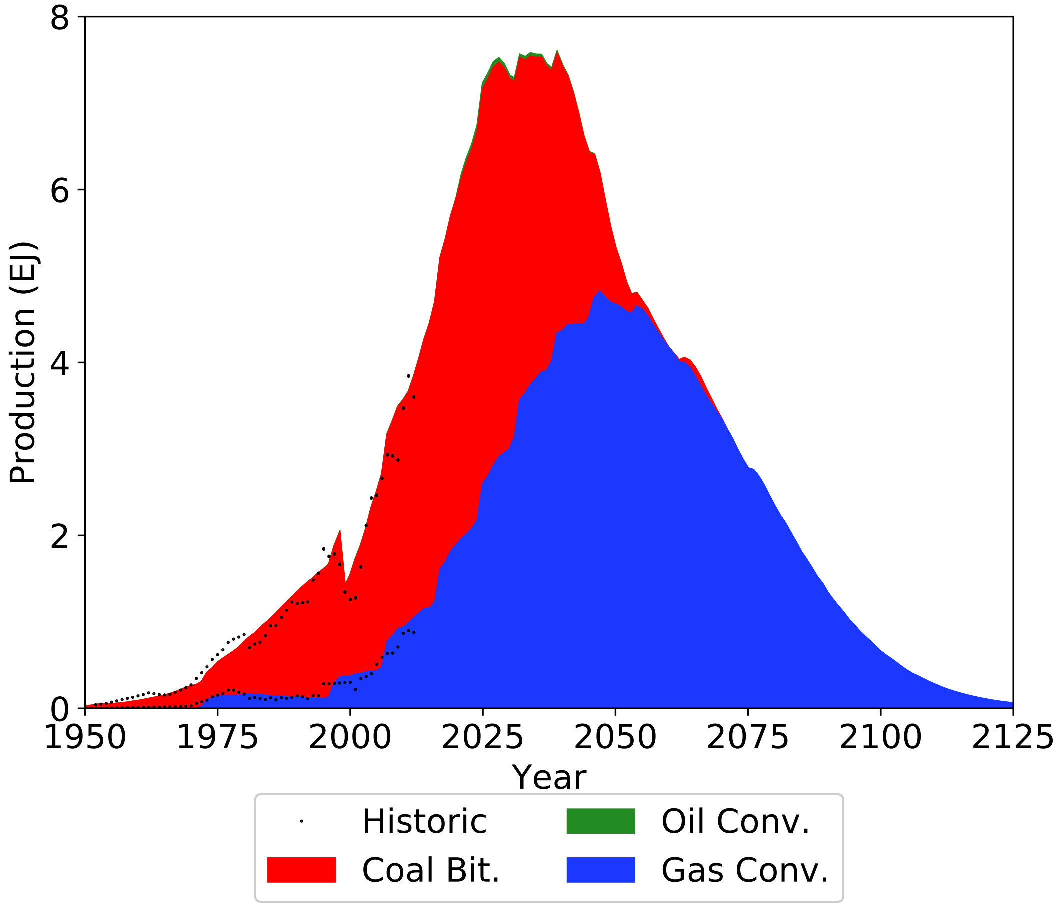

Figure 2.92: China - Sichuan projections capped at 16

Table 2.92: Peak years - All

Name	URR	Peak Year	Peak Rate
Gas Conv. Sichuan	277.77	2047	4.82
Coal Bit. Sichuan	184.59	2027	4.61
Oil Conv. Sichuan	1.3	2025	0.07
**Total**	**463.66**	**2039**	**7.59**

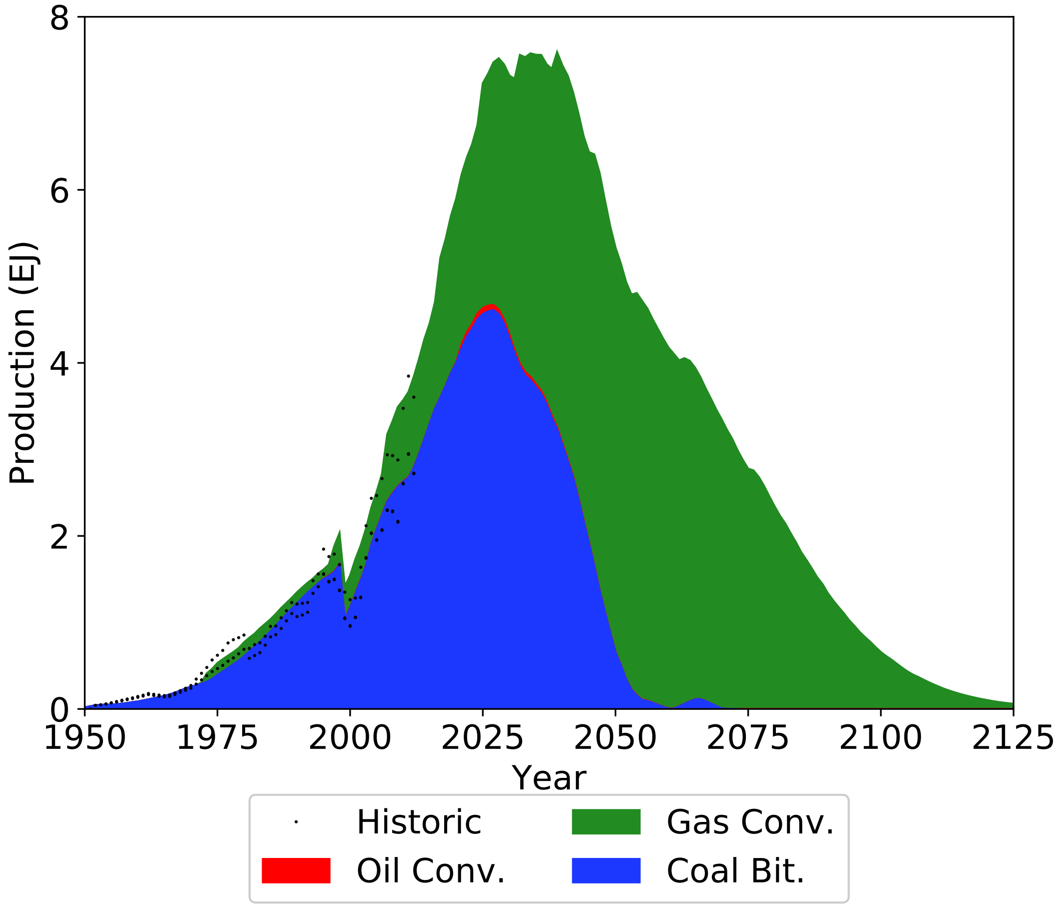

Figure 2.93: China - Sichuan projection by mineral type

Table 2.93: Peak years - Minerals

**Name**	**URR**	**Peak Year**	**Peak Rate**
Coal Bit.	184.59	2027	4.61
Oil Conv.	1.3	2025	0.07
Gas Conv.	277.77	2047	4.82
**Total**	**463.66**	**2039**	**7.59**

Tianjin

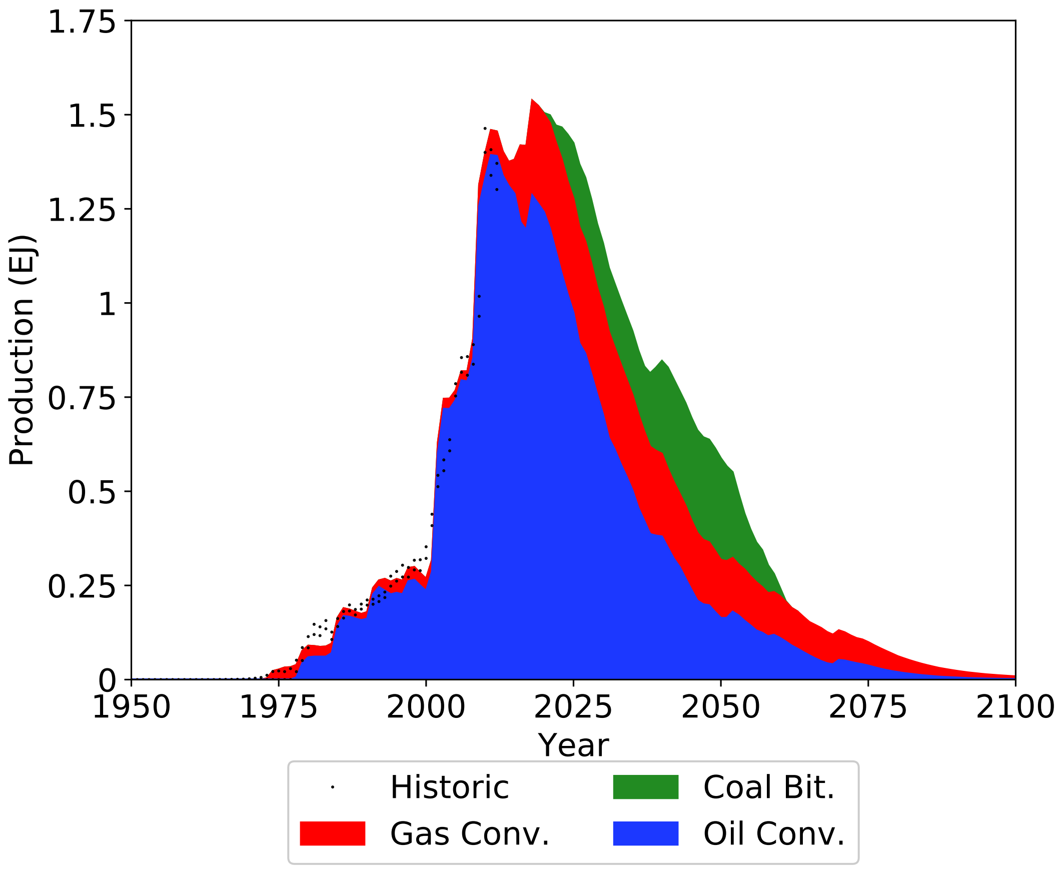

Figure 2.94: China - Tianjin projections capped at 16

Table 2.94: Peak years - All

Name	URR	Peak Year	Peak Rate
Oil Conv. Tianjin	44.06	2011	1.39
Gas Conv. Tianjin	13.15	2026	0.31
Coal Bit. Tianjin	7.13	2042	0.27
**Total**	**64.34**	**2018**	**1.54**

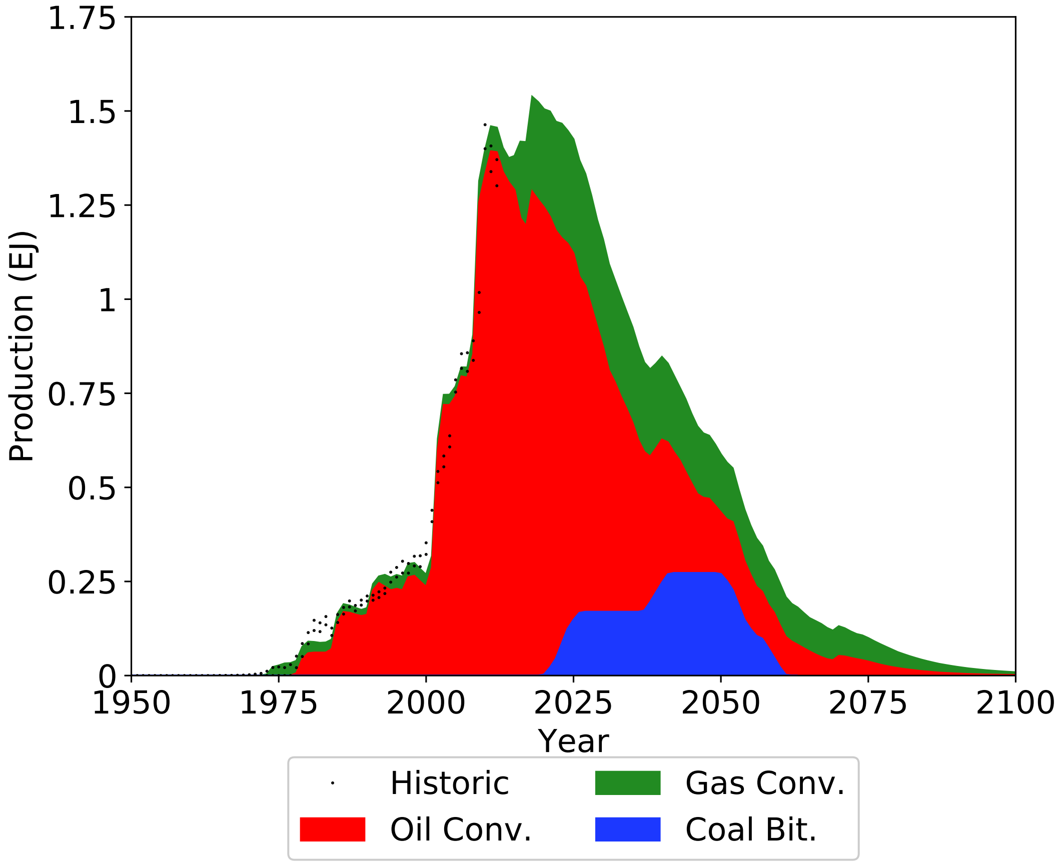

Figure 2.95: China - Tianjin projection by mineral type

Table 2.95: Peak years - Minerals

Name	URR	Peak Year	Peak Rate
Coal Bit.	7.13	2042	0.27
Oil Conv.	44.06	2011	1.39
Gas Conv.	13.15	2026	0.31
**Total**	**64.34**	**2018**	**1.54**

Tibet

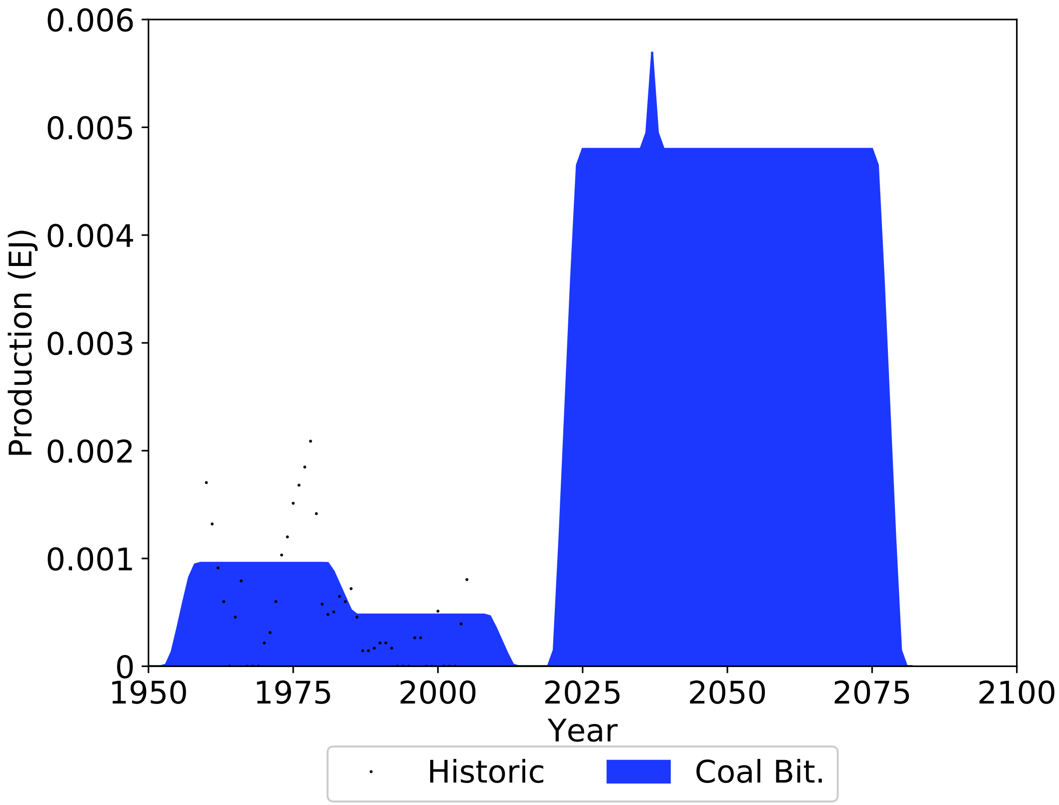

Figure 2.96: China - Tibet projections capped at 16

Table 2.96: Peak years - All

Name	URR	Peak Year	Peak Rate
Coal Bit. Tibet	0.31	2037	0.01
**Total**	**0.31**	**2037**	**0.01**

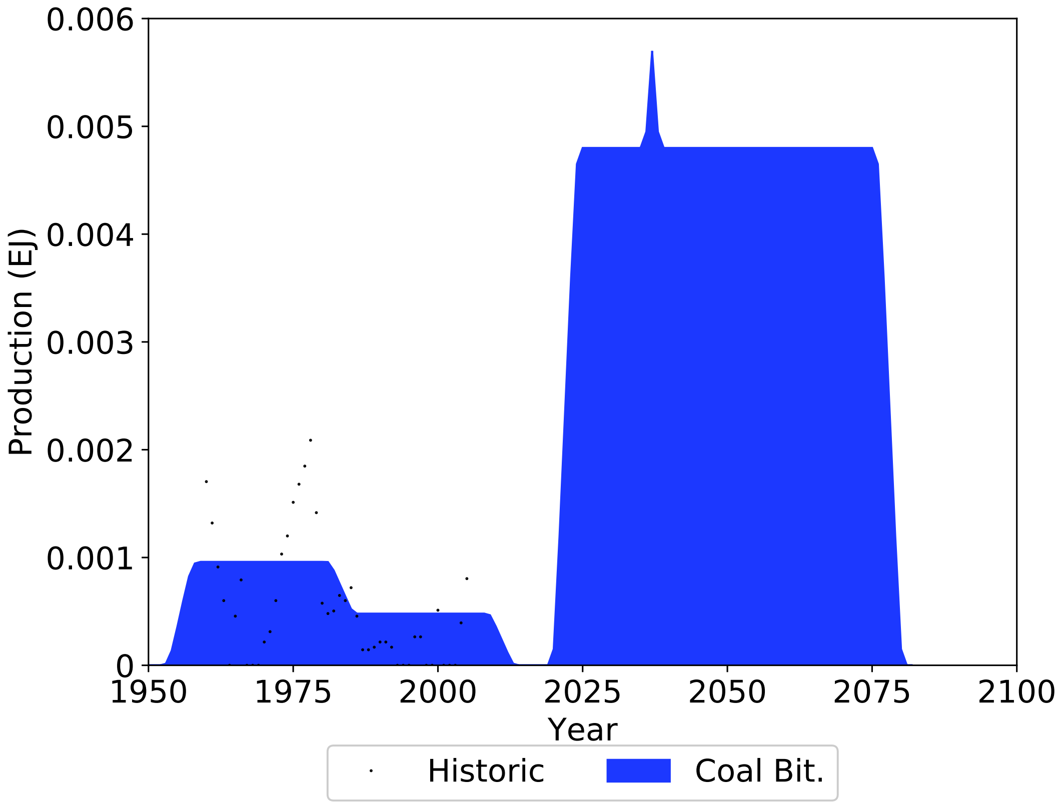

Figure 2.97: China - Tibet projection by mineral type

Table 2.97: Peak years - Minerals

Name	URR	Peak Year	Peak Rate
Coal Bit.	0.31	2037	0.01
**Total**	**0.31**	**2037**	**0.01**

Xinjiang

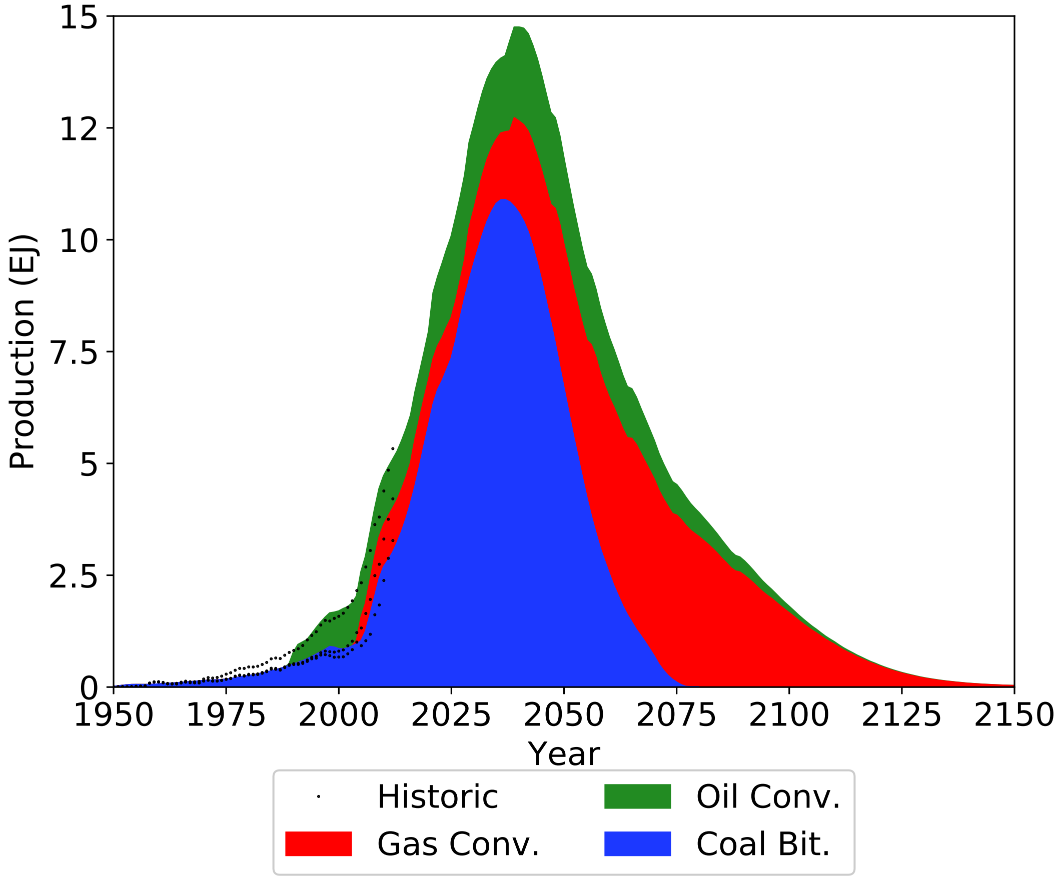

Figure 2.98: China - Xinjiang projections capped at 16

Table 2.98: Peak years - All

**Name**	**URR**	**Peak Year**	**Peak Rate**
Coal Bit. Xinjiang	397.78	2037	10.89
Gas Conv. Xinjiang	251.57	2066	4.14
Oil Conv. Xinjiang	125.62	2042	2.18
**Total**	**774.97**	**2040**	**14.75**

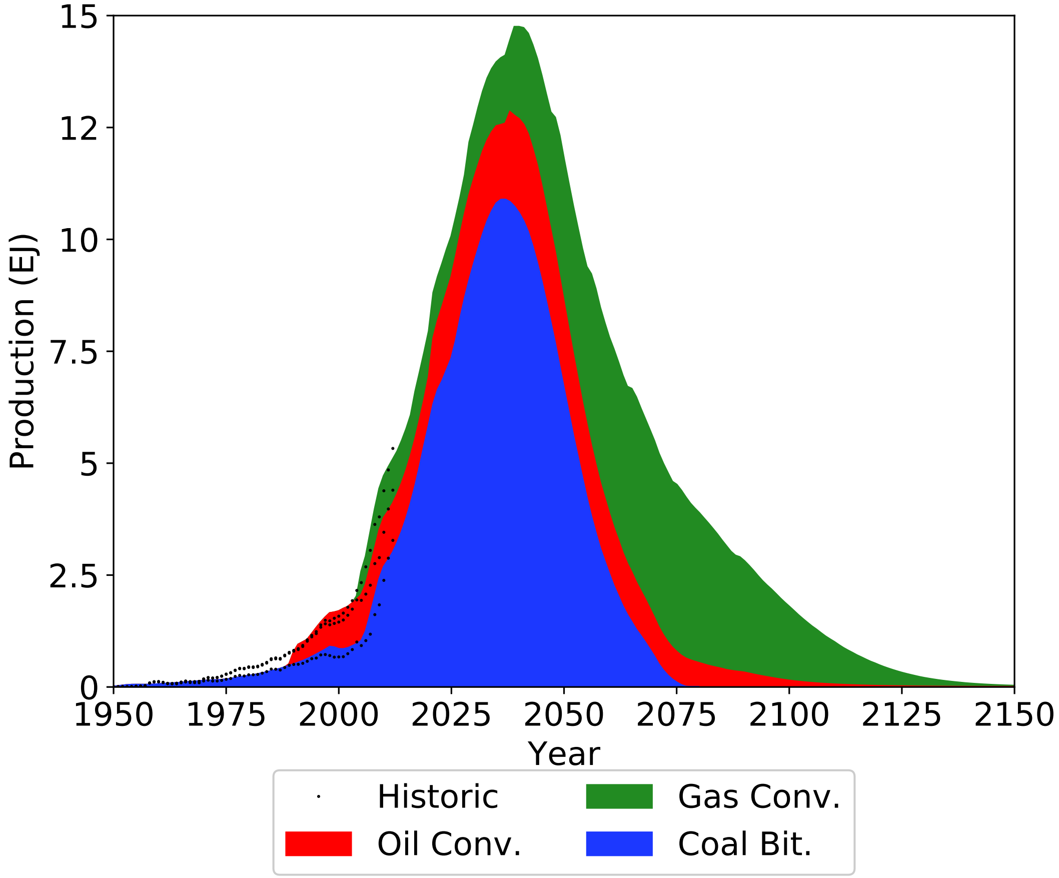

Figure 2.99: China - Xinjiang projection by mineral type

Table 2.99: Peak years - Minerals

Name	URR	Peak Year	Peak Rate
Coal Bit.	397.78	2037	10.89
Oil Conv.	125.62	2042	2.18
Gas Conv.	251.57	2066	4.14
**Total**	**774.97**	**2040**	**14.75**

Yunnan

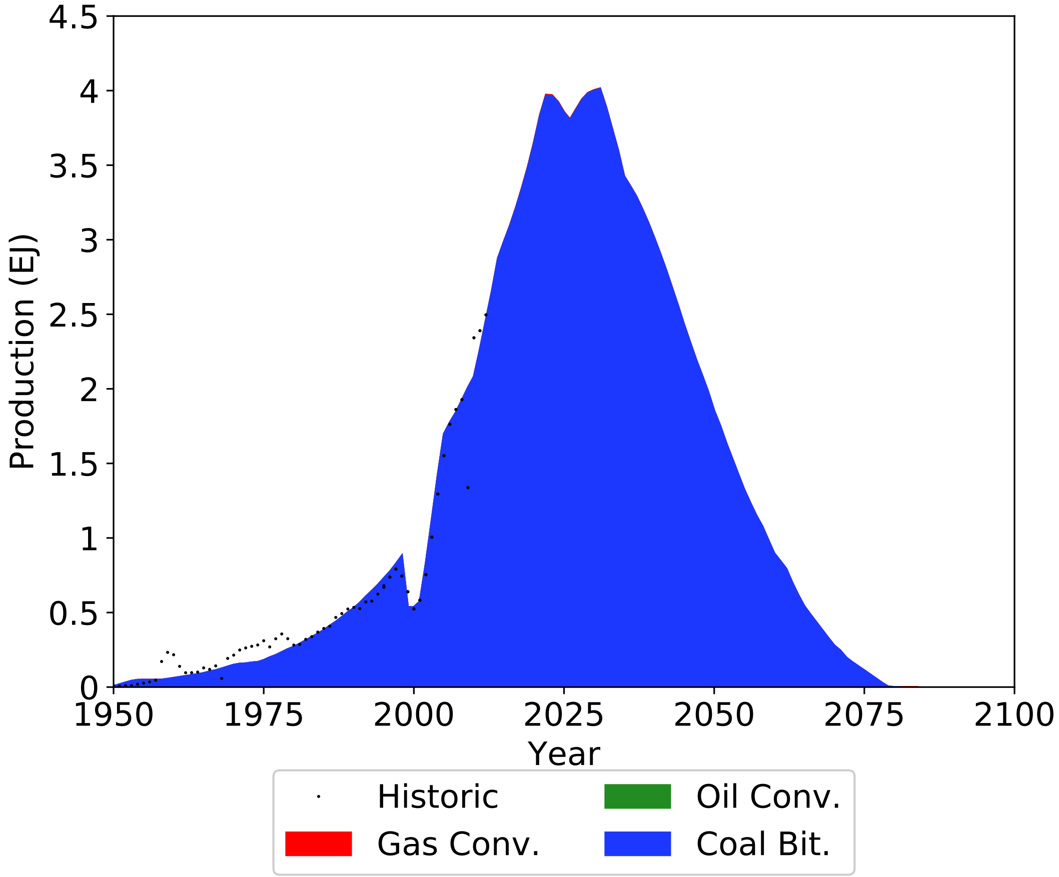

Figure 2.100: China - Yunnan projections capped at 16

Table 2.100: Peak years - All

Name	URR	Peak Year	Peak Rate
Coal Bit. Yunnan	176.66	2031	4.01
Gas Conv. Yunnan	0.12	2020	0.01
Oil Conv. Yunnan	0.01	2022	–
**Total**	**176.79**	**2031**	**4.01**

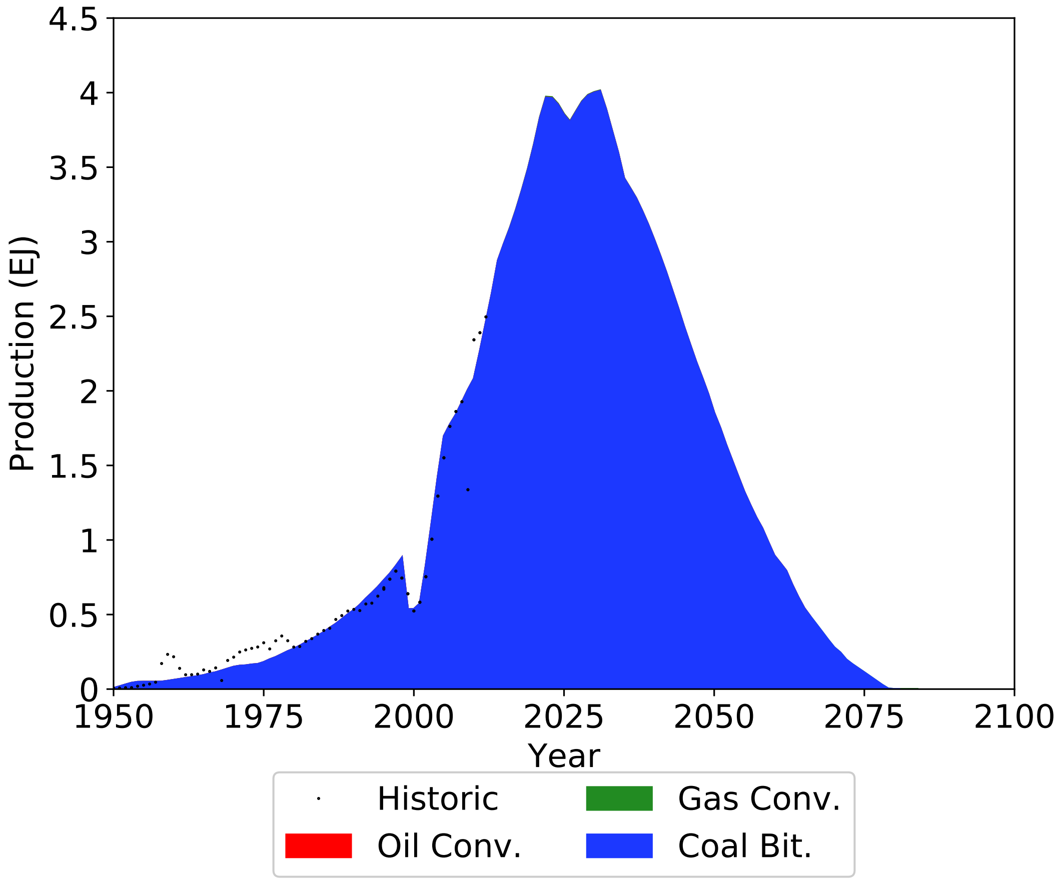

Figure 2.101: China - Yunnan projection by mineral type

Table 2.101: Peak years - Minerals

Name	URR	Peak Year	Peak Rate
Coal Bit.	176.66	2031	4.01
Oil Conv.	0.01	2022	–
Gas Conv.	0.12	2020	0.01
**Total**	**176.79**	**2031**	**4.01**

Zhejiang

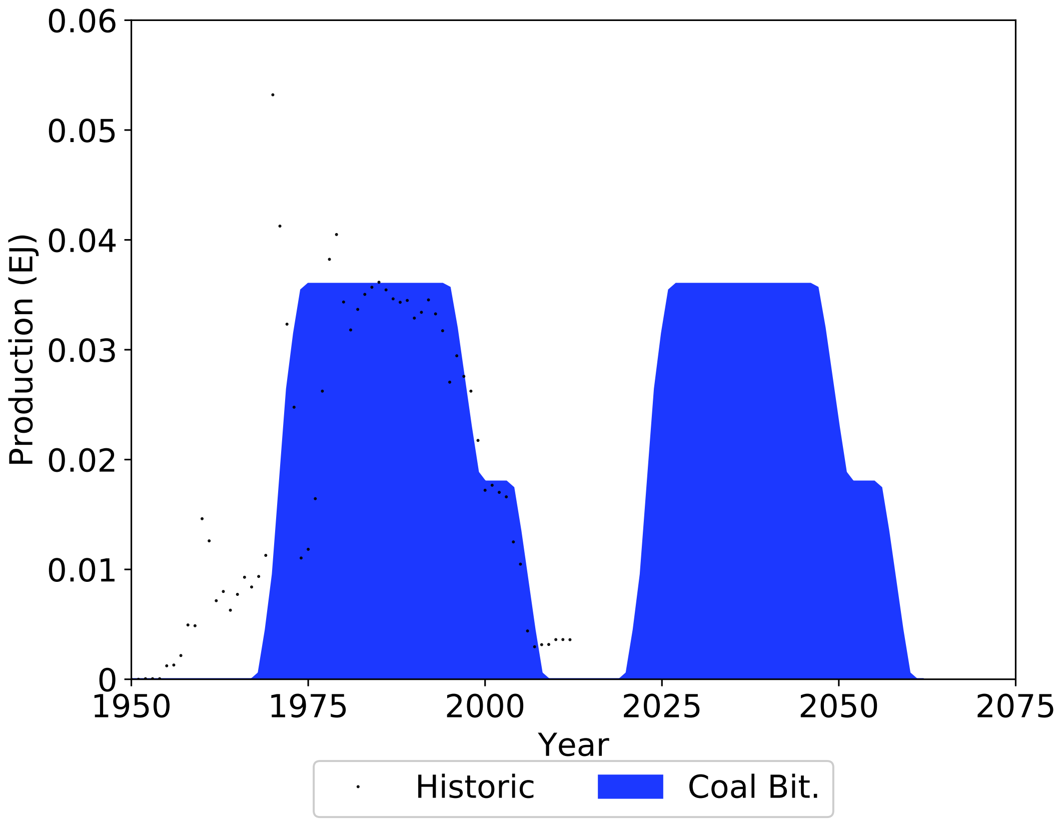

Figure 2.102: China - Zhejiang projections capped at 16

Table 2.102: Peak years - All

Name	URR	Peak Year	Peak Rate
Coal Bit. Zhejiang	2.2	1975	0.04
**Total**	**2.2**	**1975**	**0.04**

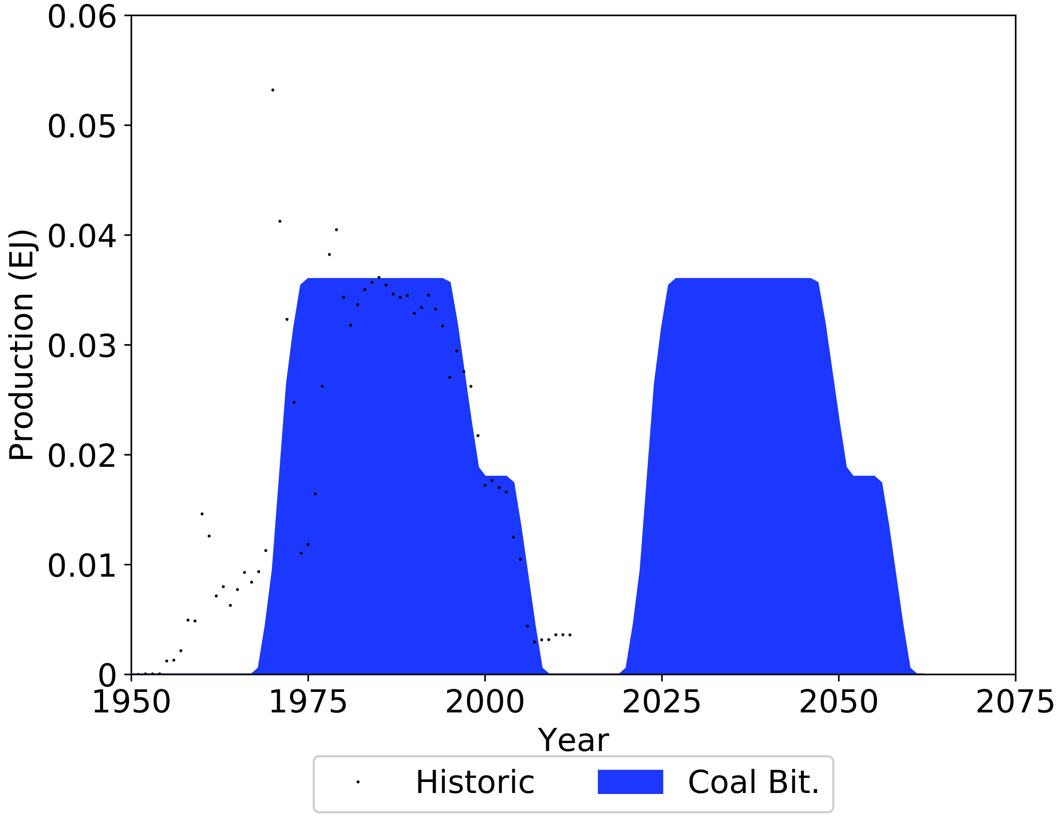

Figure 2.103: China - Zhejiang projection by mineral type

Table 2.103: Peak years - Minerals

Name	URR	Peak Year	Peak Rate
Coal Bit.	2.2	1975	0.04
**Total**	**2.2**	**1975**	**0.04**

2.8.4 Projection by region

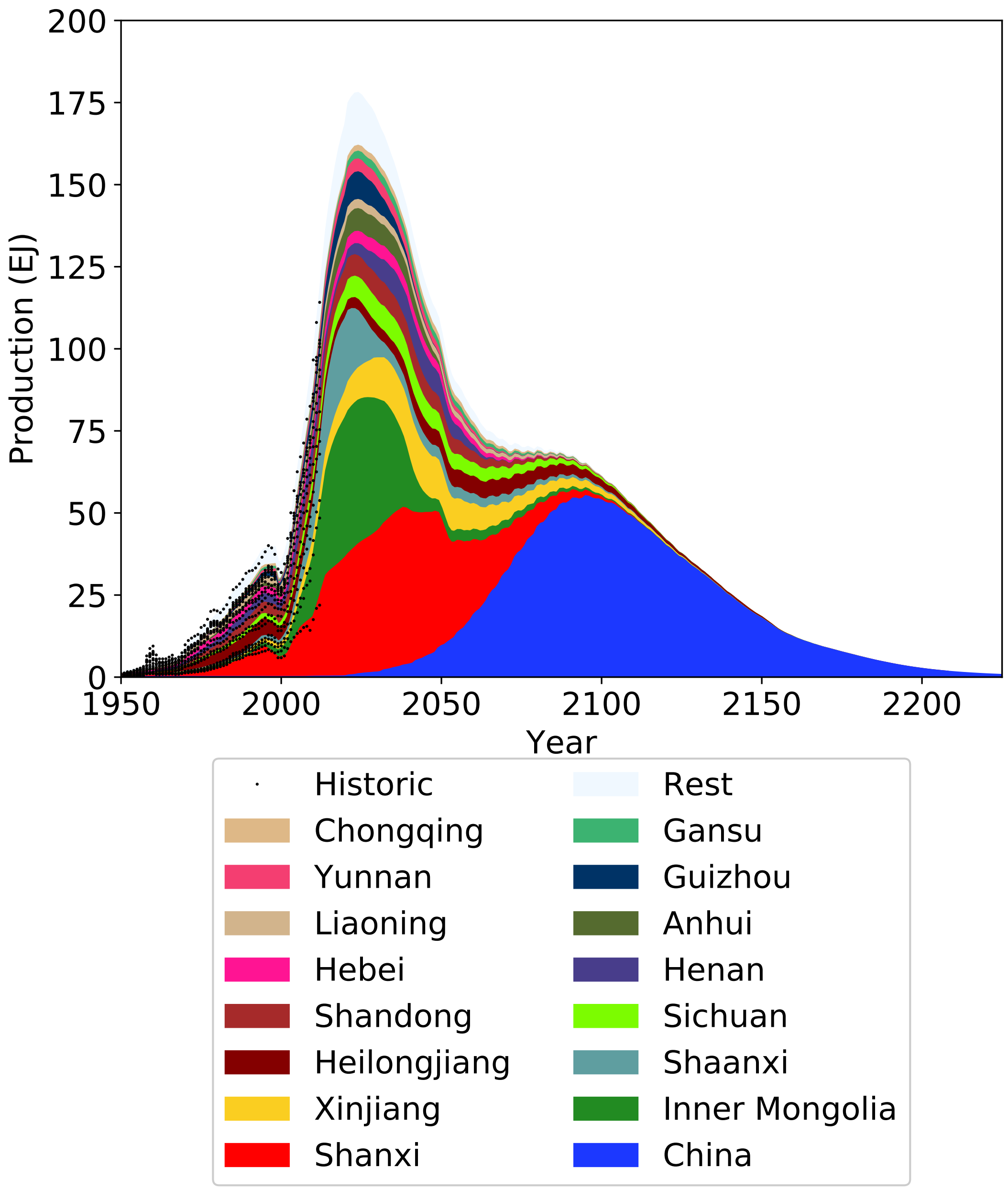

Figure 2.104: China by region projections capped at 16

Table 2.104: Peak years - All

Name	URR	Peak Year	Peak Rate
China	4183.26	2095	55.14
Shanxi	2508.19	2038	48.04
Inner Mongolia	1344.96	2023	43.95
Xinjiang	774.97	2040	14.75
Shaanxi	591.68	2018	22.91
Heilongjiang	578.47	2056	5.37
Sichuan	463.66	2039	7.59
Shandong	460.61	2030	7.3
Henan	386.7	2042	8.25
Hebei	259.71	2030	4.25
Anhui	248.06	2027	6.97
Liaoning	226.56	2021	2.81
Guizhou	221.76	2024	8.42
Yunnan	176.79	2031	4.01
Gansu	140.03	2031	2.73
Chongqing	131.49	2042	2.12
Jilin	111.76	2018	2.88
Ningxia	110.52	2021	5.3
Qinghai	93.37	2027	2.91
Offshore	70.95	2047	1.47
Tianjin	64.34	2018	1.54
Jiangsu	57.84	2024	0.76
Hunan	56.4	2012	2.15
Guangdong	54.12	2023	0.97
Jiangxi	31.23	2007	0.8
Historic	25.96	1937	0.96
Hubei	24.59	2023	0.5
Fujian	20.67	2031	0.83
Beijing	19.54	2048	0.27
Guangxi	13.57	1993	0.25
Hainan	3.7	2059	0.09
Zhejiang	2.2	1975	0.04
Shanghai	1.86	1982	0.08
Tibet	0.31	2037	0.01
**Total**	**13459.83**	**2024**	**177.93**

2.9 East Timor

2.9.1 All Projections

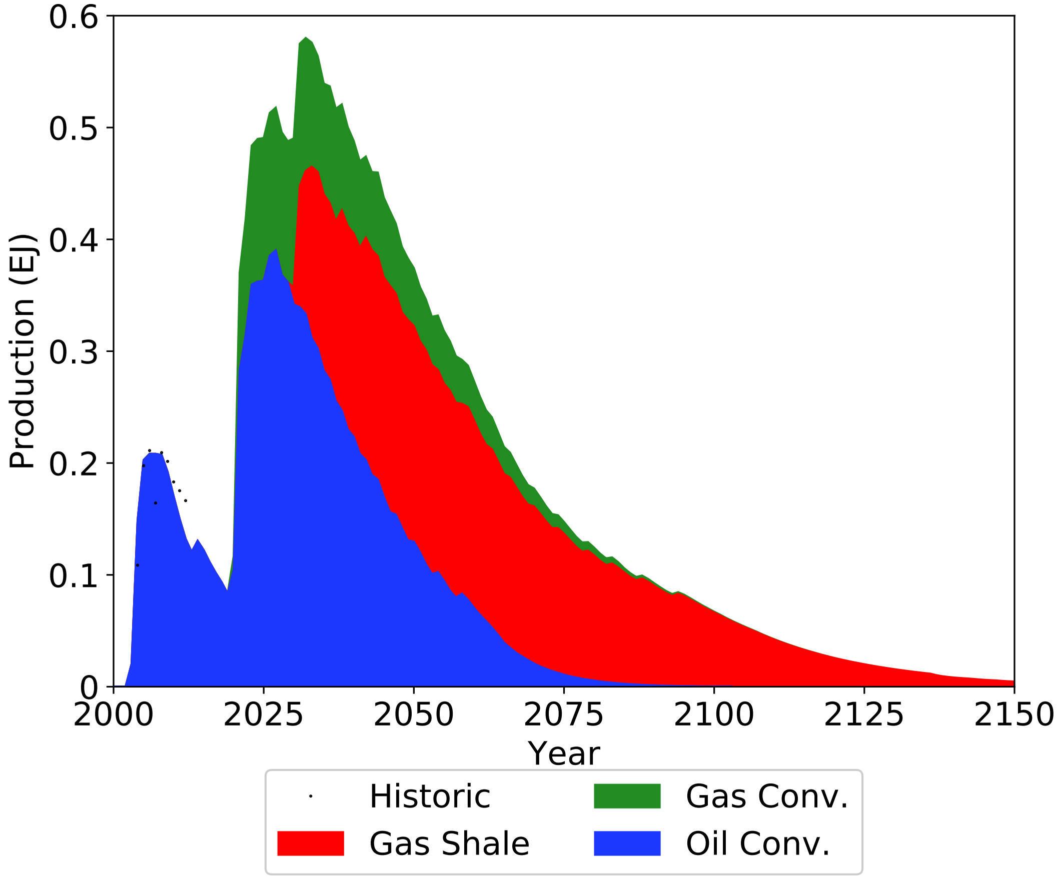

Figure 2.105: East Timor projections capped at 16

Table 2.105: Peak years - All

Name	URR	Peak Year	Peak Rate
Oil Conv.	11.97	2027	0.39
Gas Shale	11.12	2046	0.2
Gas Conv.	3.75	2030	0.13
**Total**	**26.84**	**2032**	**0.58**

2.9.2 By Mineral

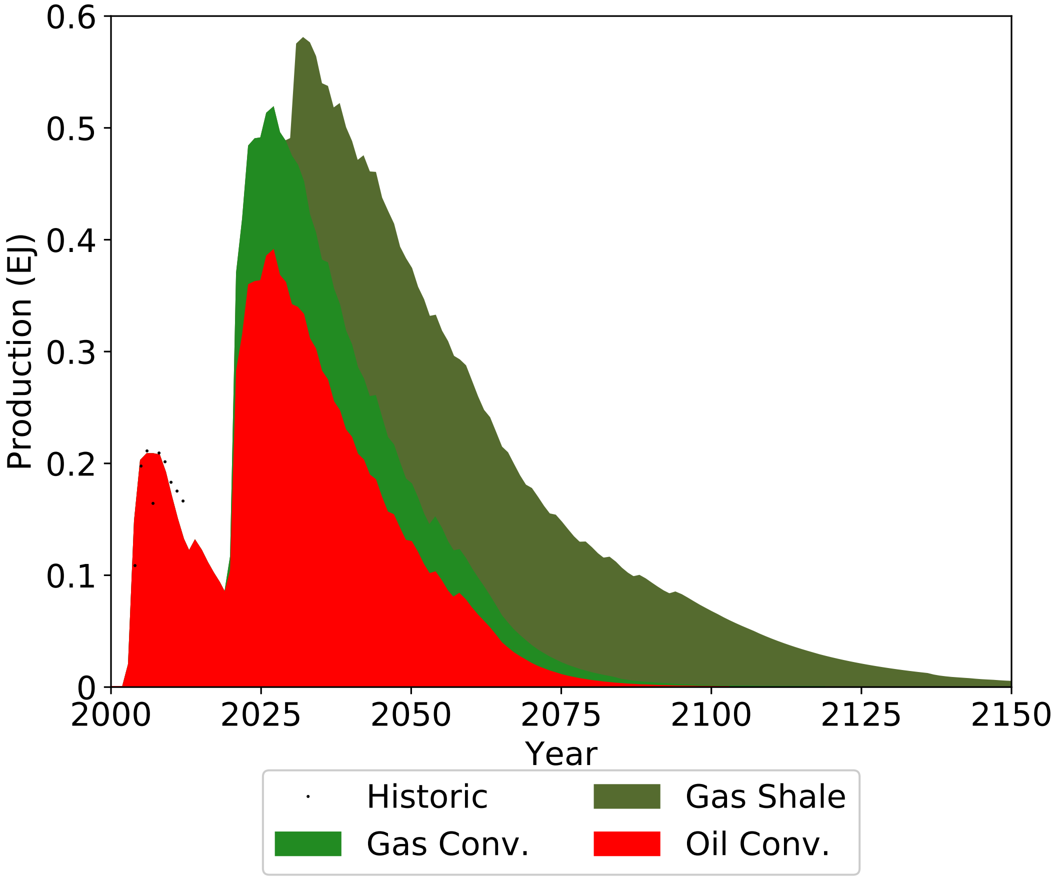

Figure 2.106: East Timor projection by mineral type

Table 2.106: Peak years - Minerals

Name	URR	Peak Year	Peak Rate
Oil Conv.	11.97	2027	0.39
Gas Conv.	3.75	2030	0.13
Gas Shale	11.12	2046	0.2
**Total**	**26.84**	**2032**	**0.58**

2.10 India

2.10.1 All Projections

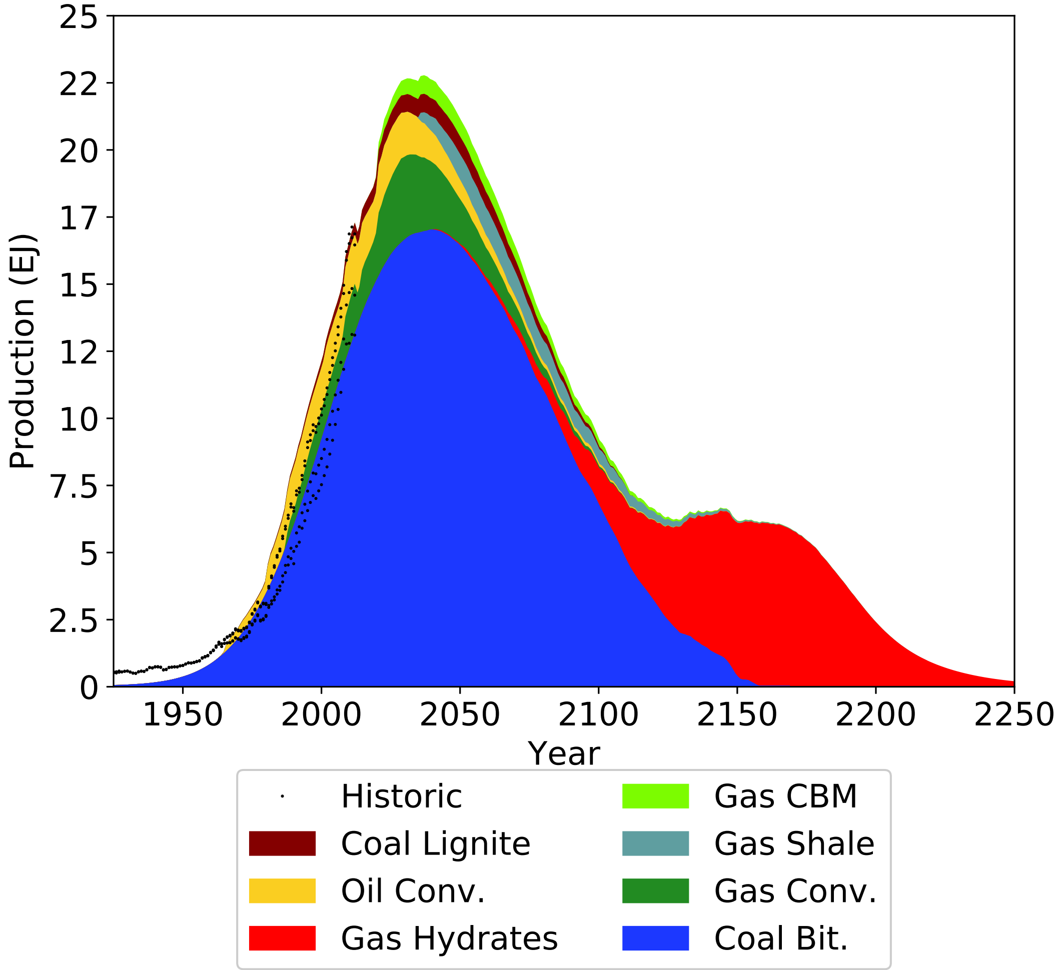

Figure 2.107: India projections capped at 16

Table 2.107: Peak years - All

Name	URR	Peak Year	Peak Rate
Coal Bit.	1652.7	2040	17.0
Gas Hydrates	496.0	2159	6.07
Gas Conv.	145.5	2029	3.13
Oil Conv.	124.57	2011	1.88
Gas Shale	66.15	2059	1.03
Coal Lignite	49.9	2045	0.69
Gas CBM	45.61	2044	0.7
**Total**	**2580.43**	**2037**	**22.74**

2.10.2 By Mineral

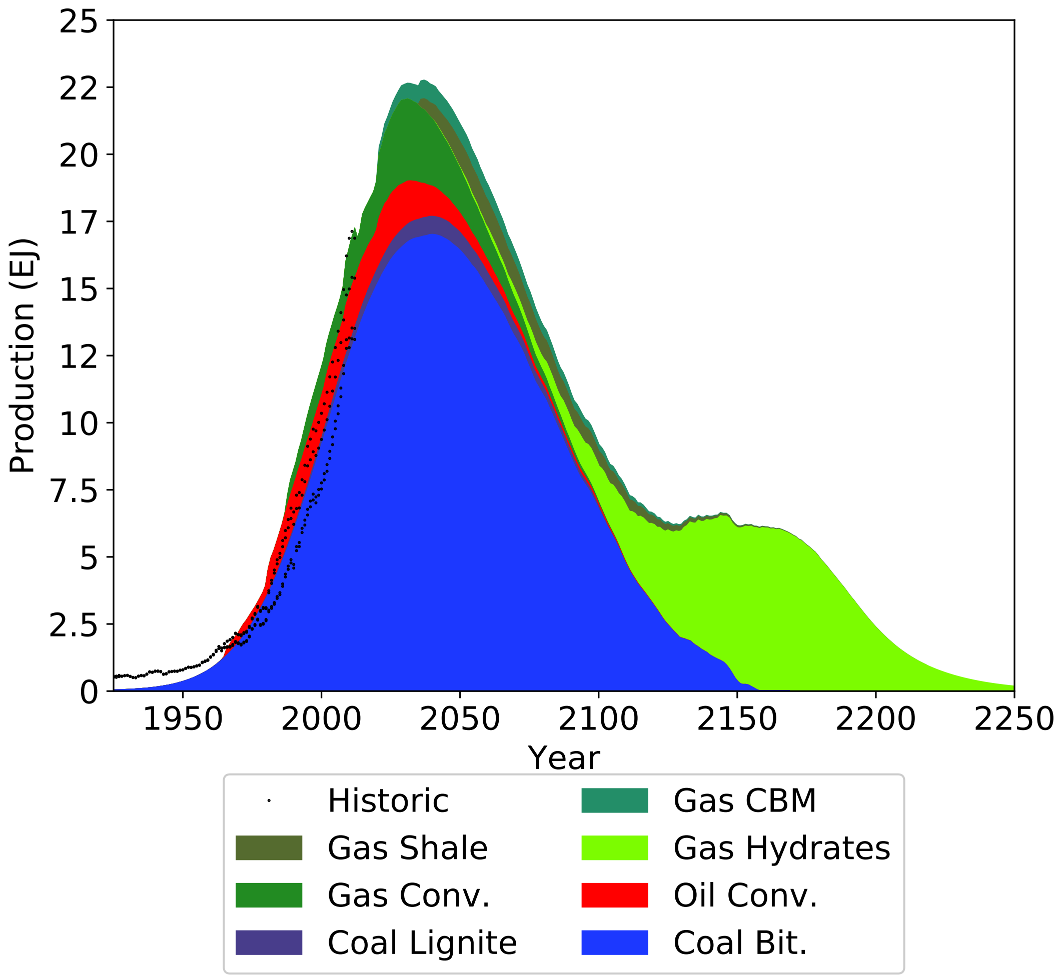

Figure 2.108: India projection by mineral type

Table 2.108: Peak years - Minerals

Name	URR	Peak Year	Peak Rate
Coal Bit.	1652.7	2040	17.0
Coal Lignite	49.9	2045	0.69
Oil Conv.	124.57	2011	1.88
Gas Conv.	145.5	2029	3.13
Gas Hydrates	496.0	2159	6.07
Gas Shale	66.15	2059	1.03
Gas CBM	45.61	2044	0.7
**Total**	**2580.43**	**2037**	**22.74**

2.11 Indonesia

2.11.1 All Projections

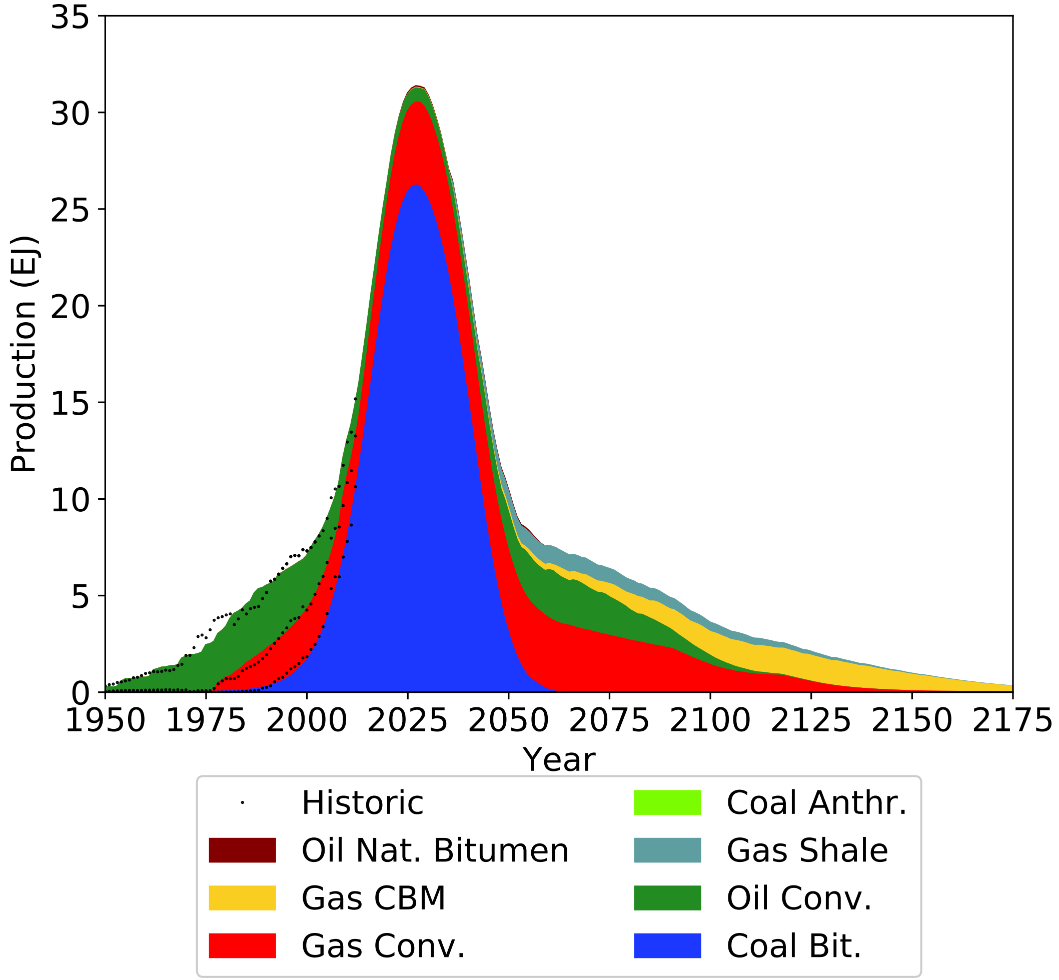

Figure 2.109: Indonesia projections capped at 16

Table 2.109: Peak years - All

**Name**	**URR**	**Peak Year**	**Peak Rate**
Coal Bit.	752.1	2027	26.23
Gas Conv.	403.4	2038	4.57
Oil Conv.	266.85	1988	3.4
Gas CBM	117.92	2112	1.38
Gas Shale	59.7	2057	0.94
Oil Nat. Bitumen	3.12	2048	0.08
Coal Anthr.	0.03	1992	–
**Total**	**1603.11**	**2027**	**31.36**

2.11.2 By Mineral

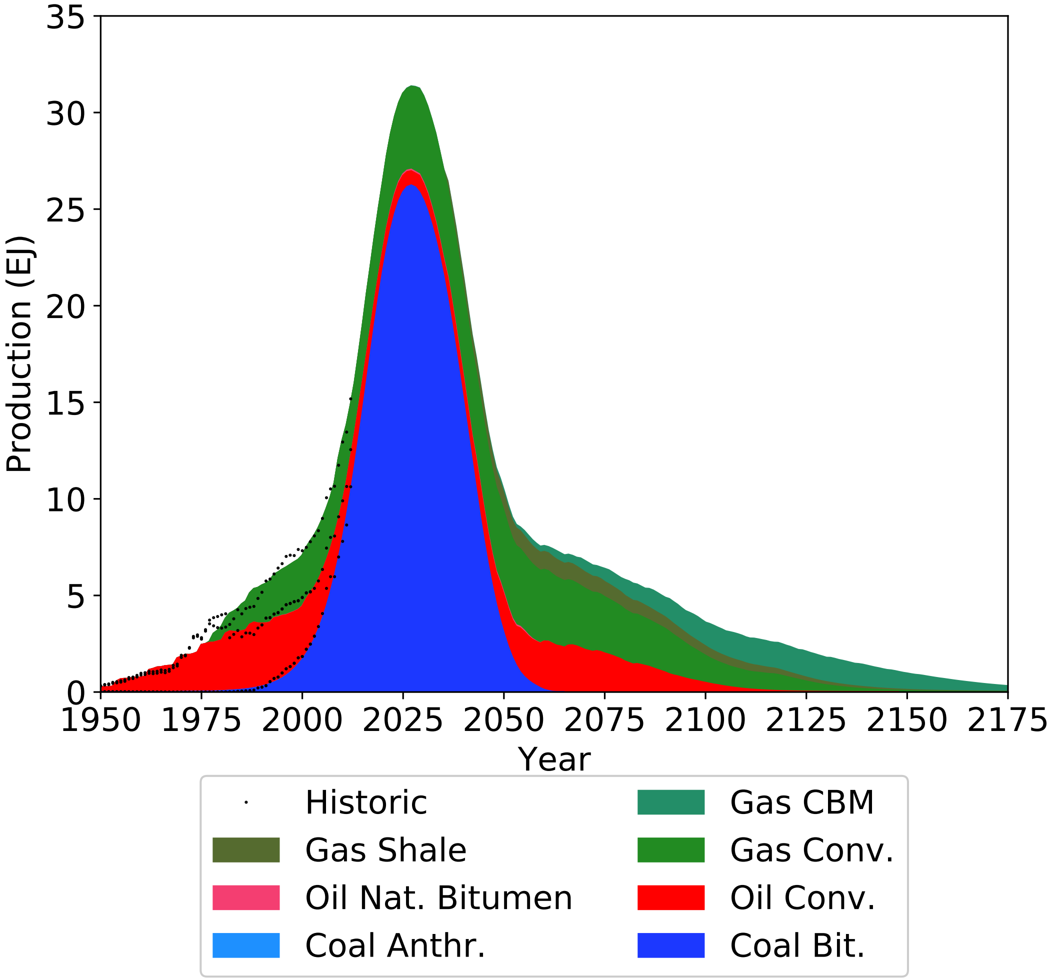

Figure 2.110: Indonesia projection by mineral type

Table 2.110: Peak years - Minerals

**Name**	**URR**	**Peak Year**	**Peak Rate**
Coal Bit.	752.1	2027	26.23
Coal Anthr.	0.03	1992	–
Oil Conv.	266.85	1988	3.4
Oil Nat. Bitumen	3.12	2048	0.08
Gas Conv.	403.4	2038	4.57
Gas Shale	59.7	2057	0.94
Gas CBM	117.92	2112	1.38
**Total**	**1603.11**	**2027**	**31.36**

2.12 Japan

2.12.1 All Projections

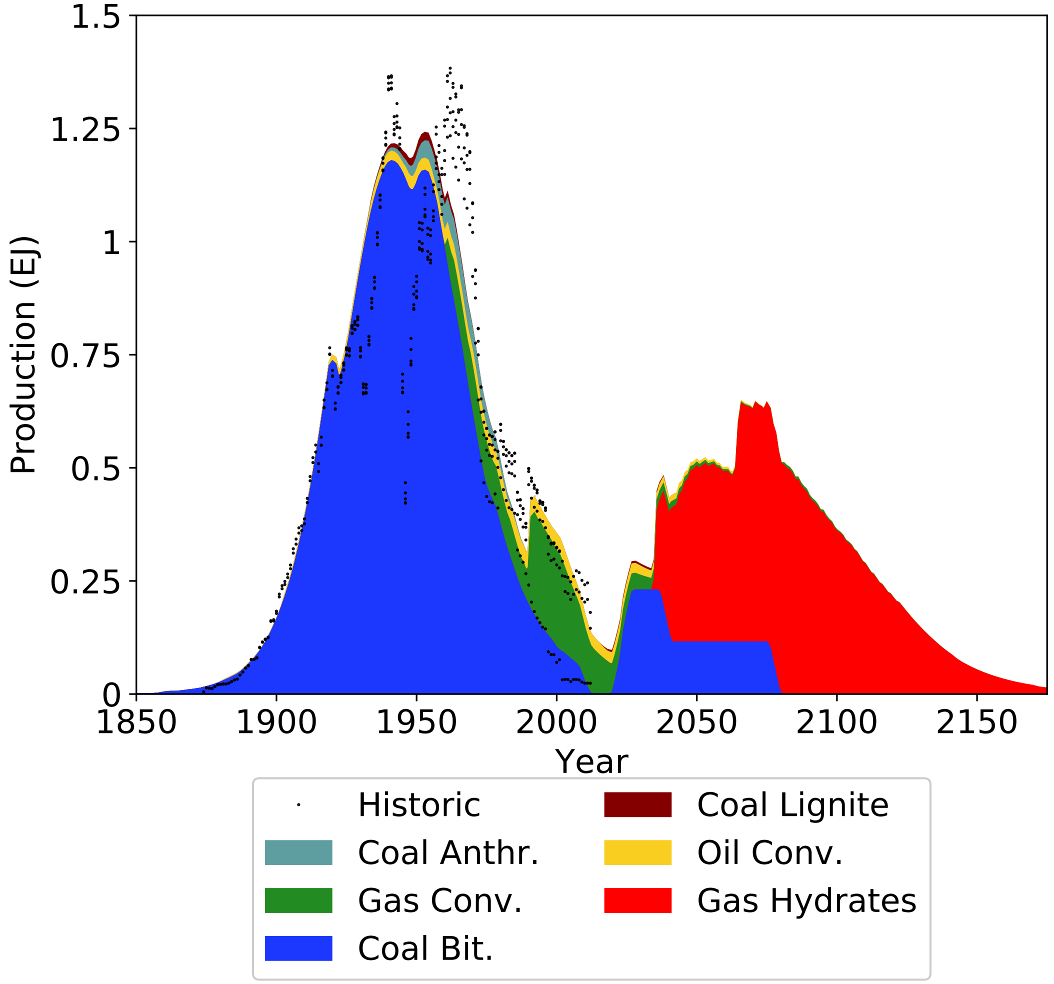

Figure 2.111: Japan projections capped at 16

Table 2.111: Peak years - All

**Name**	**URR**	**Peak Year**	**Peak Rate**
Coal Bit.	76.57	1941	1.18
Gas Hydrates	37.0	2078	0.53
Gas Conv.	8.26	1992	0.23
Oil Conv.	3.6	1985	0.04
Coal Anthr.	1.58	1963	0.06
Coal Lignite	0.52	1952	0.02
**Total**	**127.53**	**1953**	**1.24**

2.12.2 By Mineral

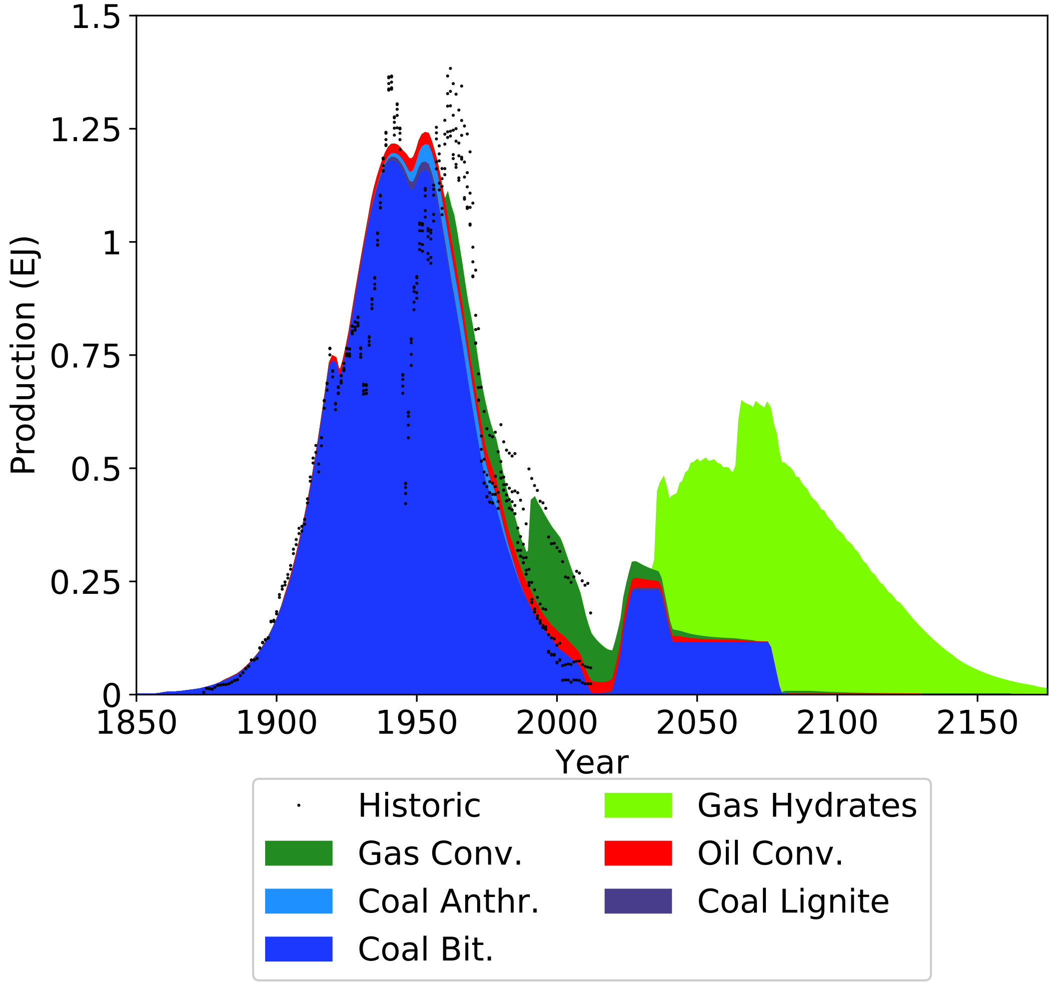

Figure 2.112: Japan projection by mineral type

Table 2.112: Peak years - Minerals

Name	URR	Peak Year	Peak Rate
Coal Bit.	76.57	1941	1.18
Coal Lignite	0.52	1952	0.02
Coal Anthr.	1.58	1963	0.06
Oil Conv.	3.6	1985	0.04
Gas Conv.	8.26	1992	0.23
Gas Hydrates	37.0	2078	0.53
**Total**	**127.53**	**1953**	**1.24**

2.13 Laos

2.13.1 All Projections

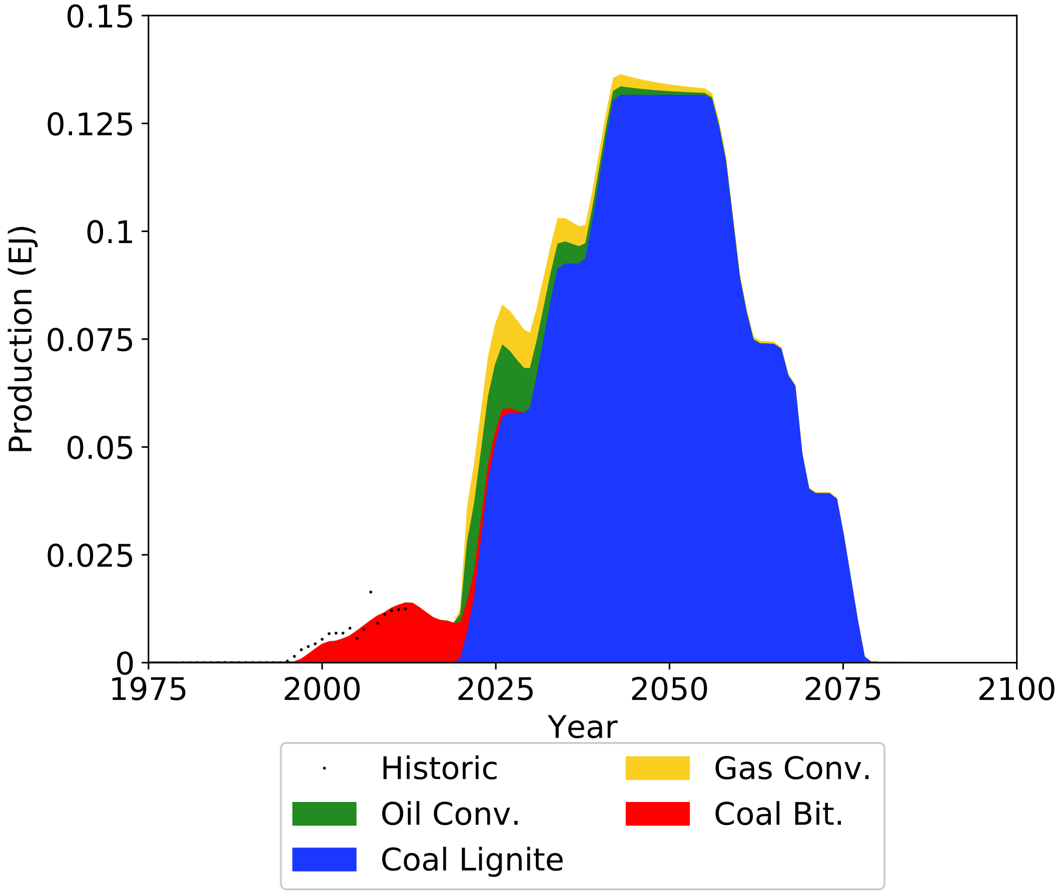

Figure 2.113: Laos projections capped at 16

Table 2.113: Peak years - All

Name	URR	Peak Year	Peak Rate
Coal Lignite	4.74	2043	0.13
Coal Bit.	0.23	2012	0.01
Oil Conv.	0.21	2022	0.02
Gas Conv.	0.19	2022	0.01
**Total**	**5.37**	**2043**	**0.14**

2.13.2 By Mineral

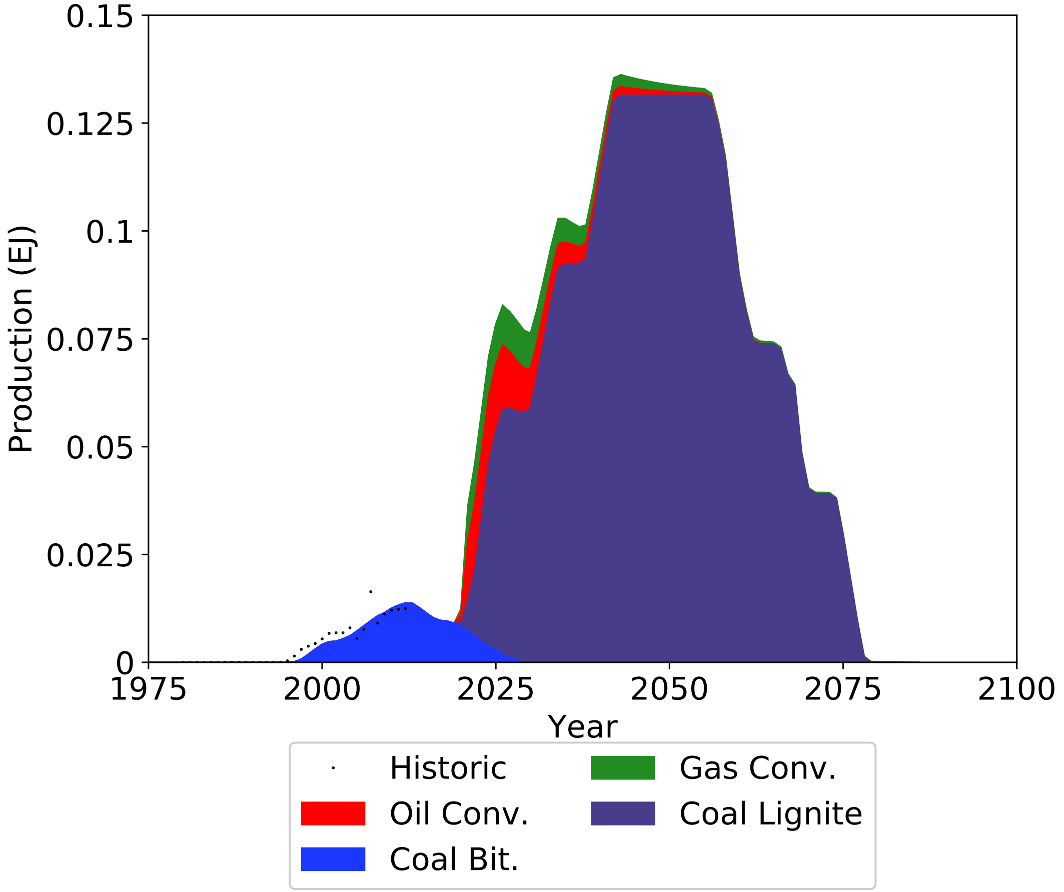

Figure 2.114: Laos projection by mineral type

Table 2.114: Peak years - Minerals

Name	URR	Peak Year	Peak Rate
Coal Bit.	0.23	2012	0.01
Coal Lignite	4.74	2043	0.13
Oil Conv.	0.21	2022	0.02
Gas Conv.	0.19	2022	0.01
**Total**	**5.37**	**2043**	**0.14**

2.14 Malaysia

2.14.1 All Projections

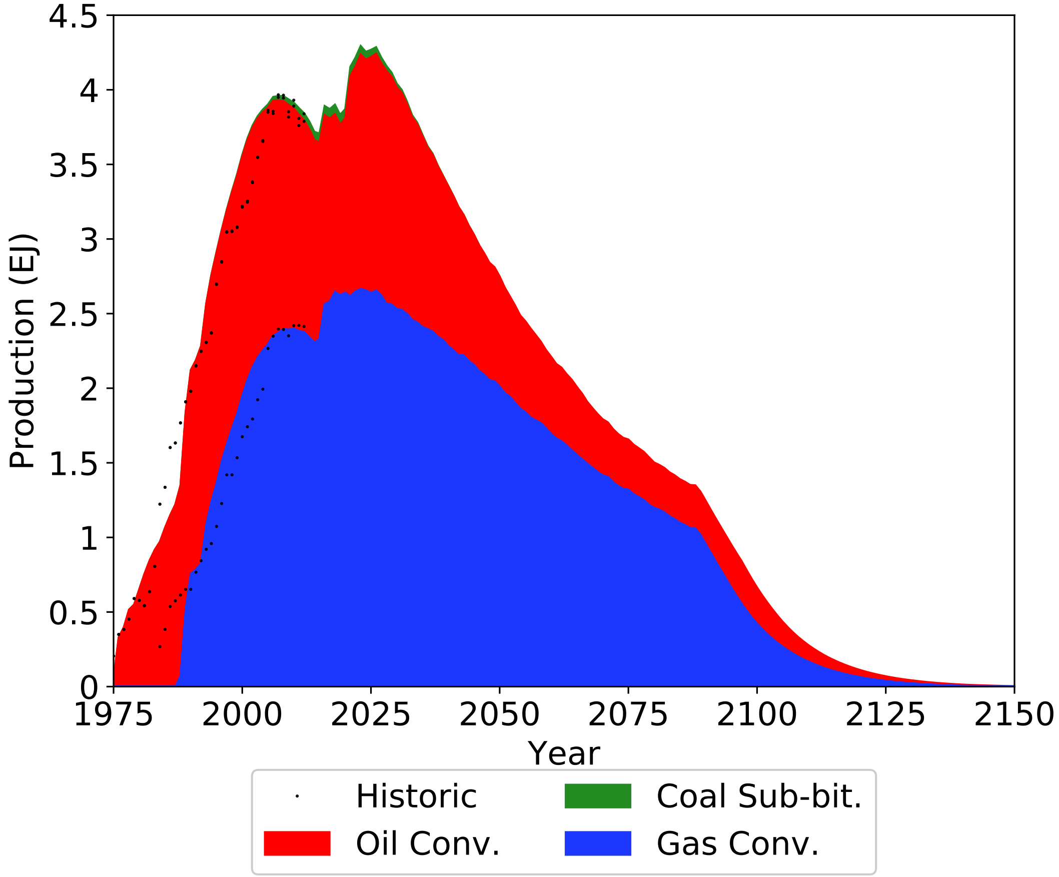

Figure 2.115: Malaysia projections capped at 16

Table 2.115: Peak years - All

Name	URR	Peak Year	Peak Rate
Gas Conv.	202.73	2023	2.66
Oil Conv.	116.24	2001	1.61
Coal Sub-bit.	1.69	2019	0.06
**Total**	**320.66**	**2023**	**4.29**

2.14.2 By Mineral

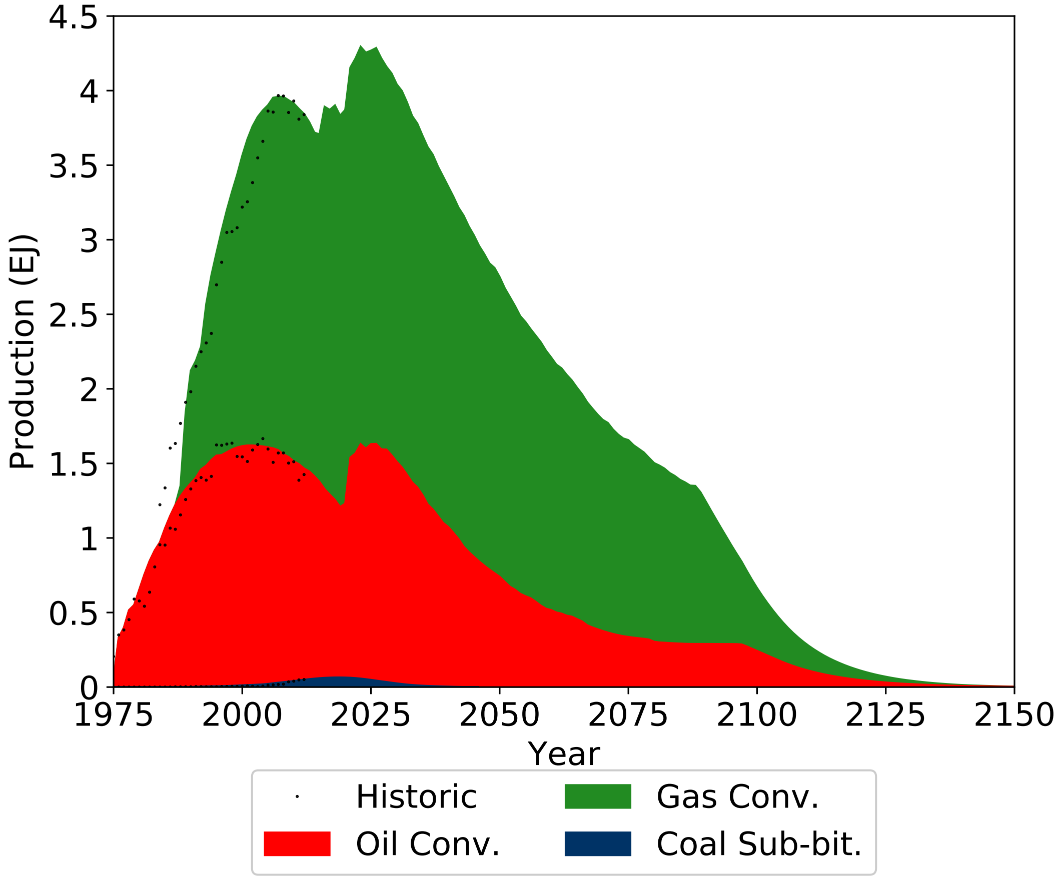

Figure 2.116: Malaysia projection by mineral type

Table 2.116: Peak years - Minerals

Name	URR	Peak Year	Peak Rate
Coal Sub-bit.	1.69	2019	0.06
Oil Conv.	116.24	2001	1.61
Gas Conv.	202.73	2023	2.66
**Total**	**320.66**	**2023**	**4.29**

2.15 Mongolia

2.15.1 All Projections

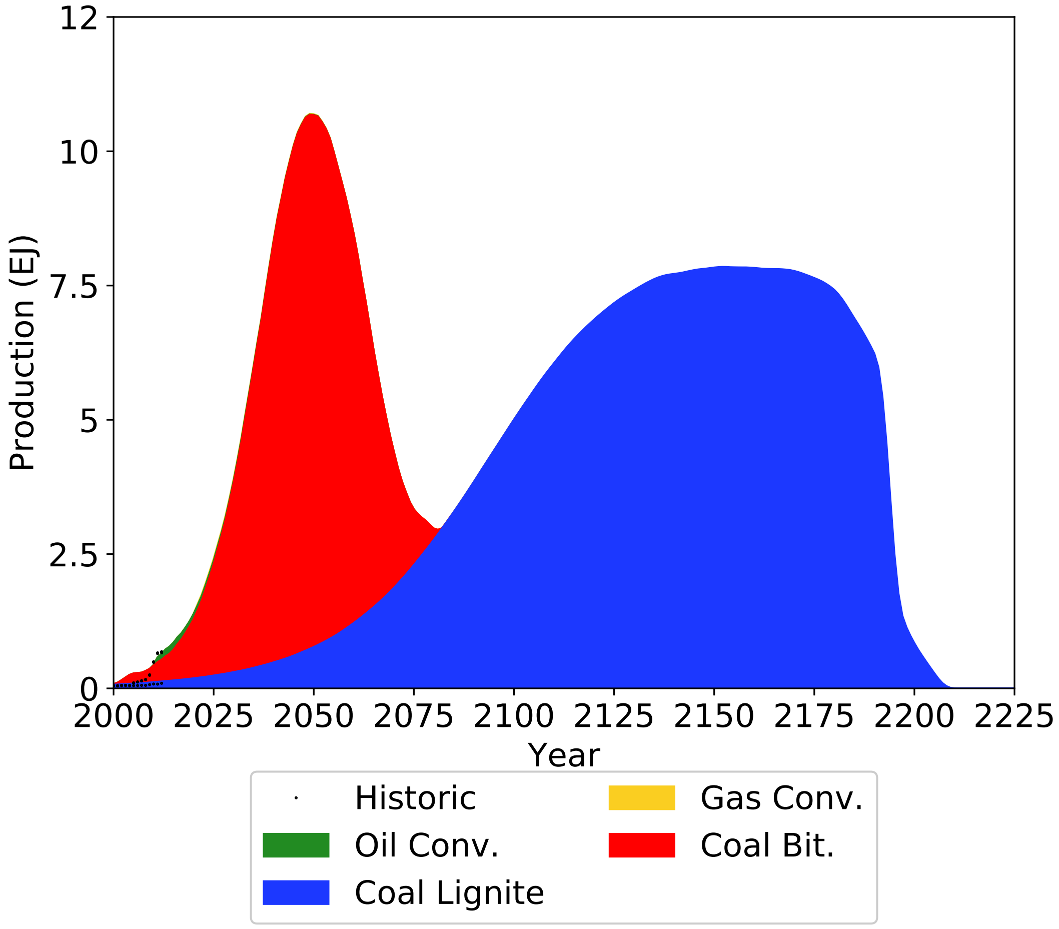

Figure 2.117: Mongolia projections capped at 16

Table 2.117: Peak years - All

Name	URR	Peak Year	Peak Rate
Coal Lignite	822.6	2152	7.85
Coal Bit.	327.1	2049	9.94
Oil Conv.	2.27	2014	0.13
Gas Conv.	0.85	2025	0.04
**Total**	**1152.82**	**2049**	**10.7**

2.15.2 By Mineral

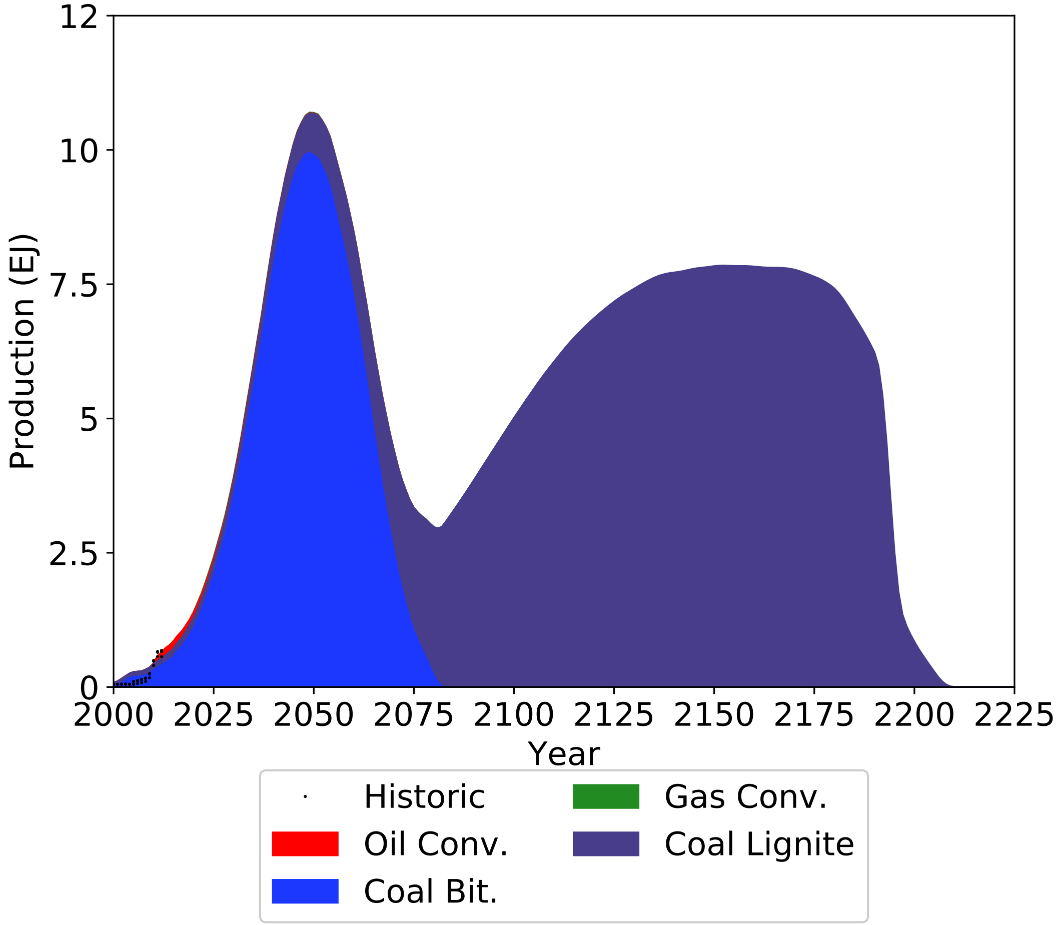

Figure 2.118: Mongolia projection by mineral type

Table 2.118: Peak years - Minerals

**Name**	**URR**	**Peak Year**	**Peak Rate**
Coal Bit.	327.1	2049	9.94
Coal Lignite	822.6	2152	7.85
Oil Conv.	2.27	2014	0.13
Gas Conv.	0.85	2025	0.04
**Total**	**1152.82**	**2049**	**10.7**

2.16 Nepal

2.16.1 All Projections

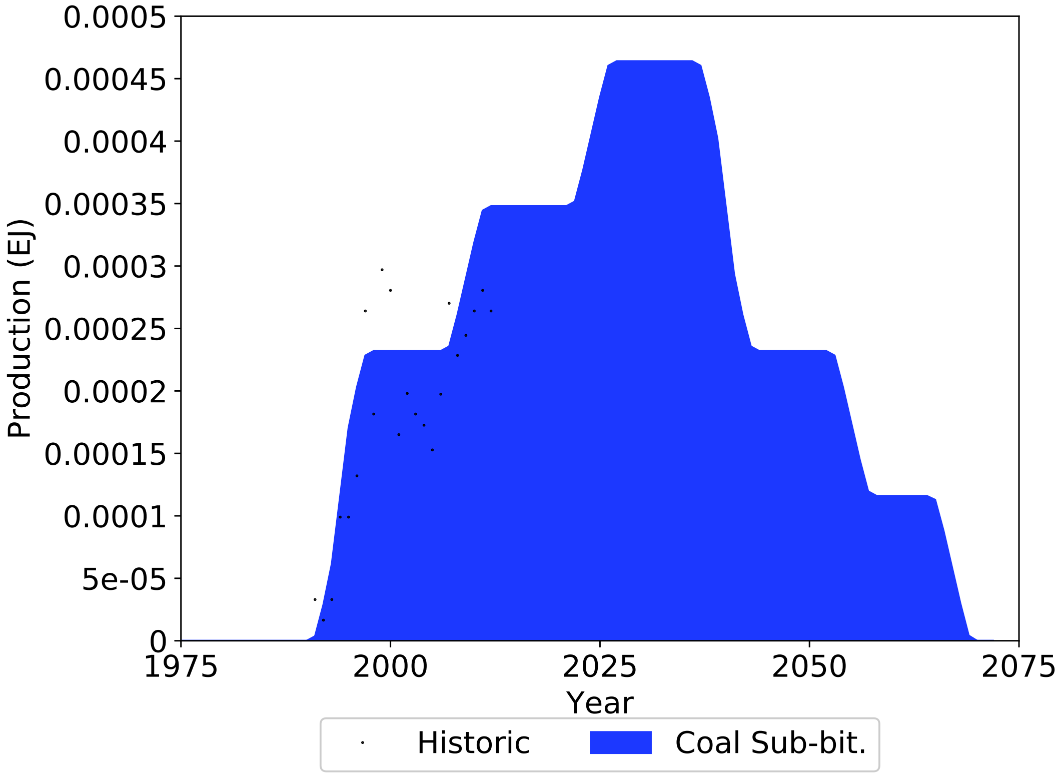

Figure 2.119: Nepal projections capped at 16

Table 2.119: Peak years - All

Name	URR	Peak Year	Peak Rate
Coal Sub-bit.	0.02	2027	–
**Total**	**0.02**	**2027**	–

2.16.2 By Mineral

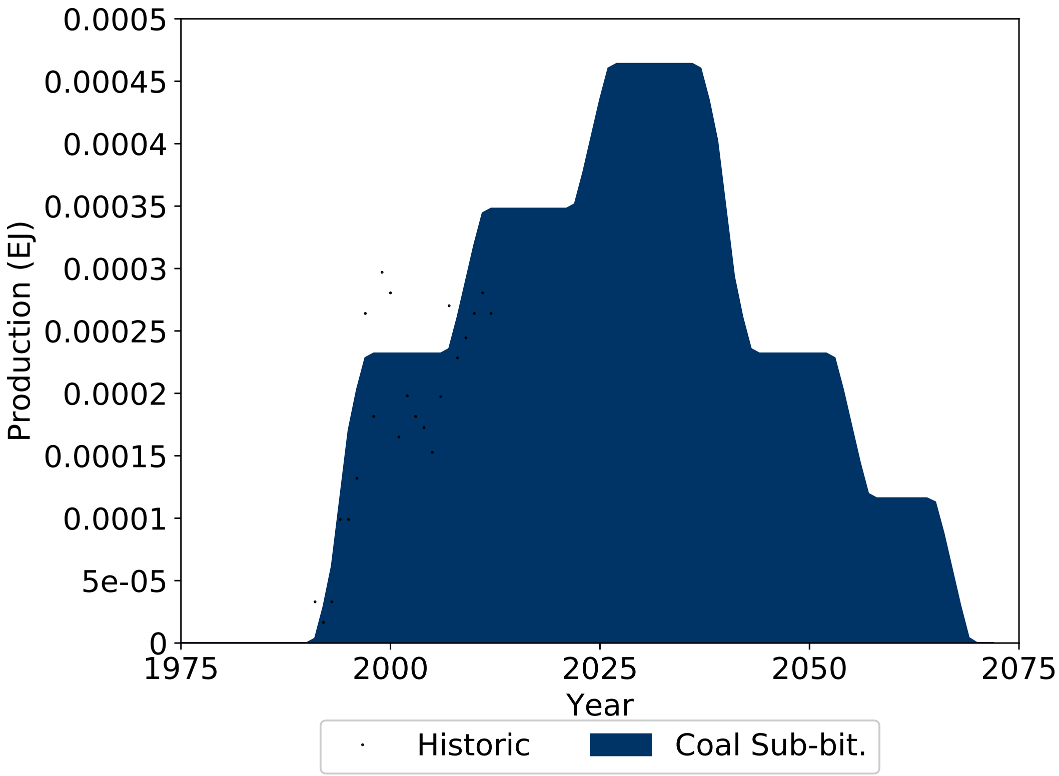

Figure 2.120: Nepal projection by mineral type

Table 2.120: Peak years - Minerals

Name	URR	Peak Year	Peak Rate
Coal Sub-bit.	0.02	2027	–
**Total**	**0.02**	**2027**	–

2.17 New Caledonia

2.17.1 All Projections

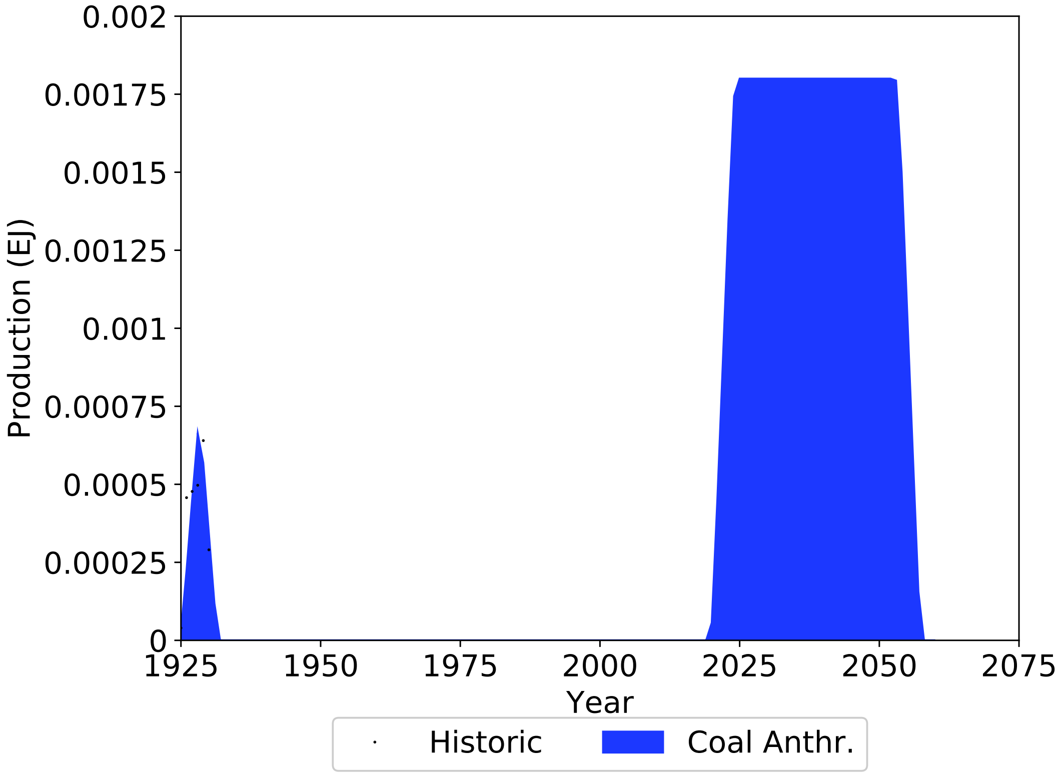

Figure 2.121: New Caledonia projections capped at 16

Table 2.121: Peak years - All

**Name**	**URR**	**Peak Year**	**Peak Rate**
Coal Anthr.	0.06	2048	–
**Total**	**0.06**	**2048**	–

2.17.2 By Mineral

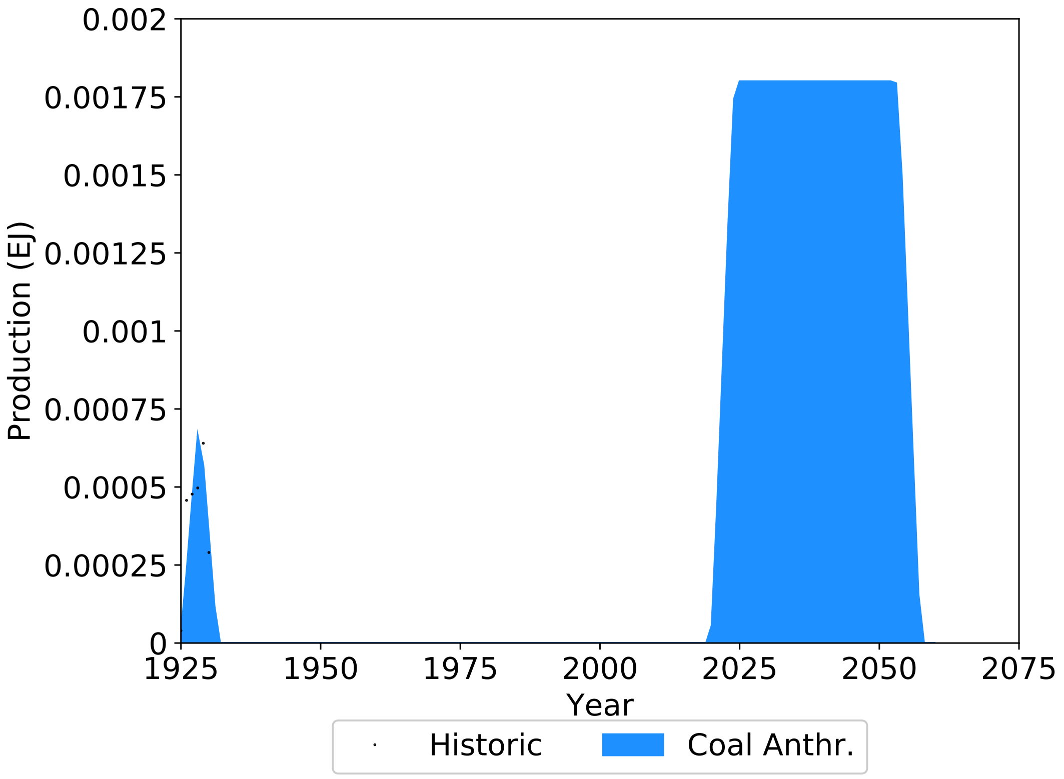

Figure 2.122: New Caledonia projection by mineral type

Table 2.122: Peak years - Minerals

Name	URR	Peak Year	Peak Rate
Coal Anthr.	0.06	2048	–
**Total**	**0.06**	**2048**	–

2.18 New Zealand

2.18.1 All Projections

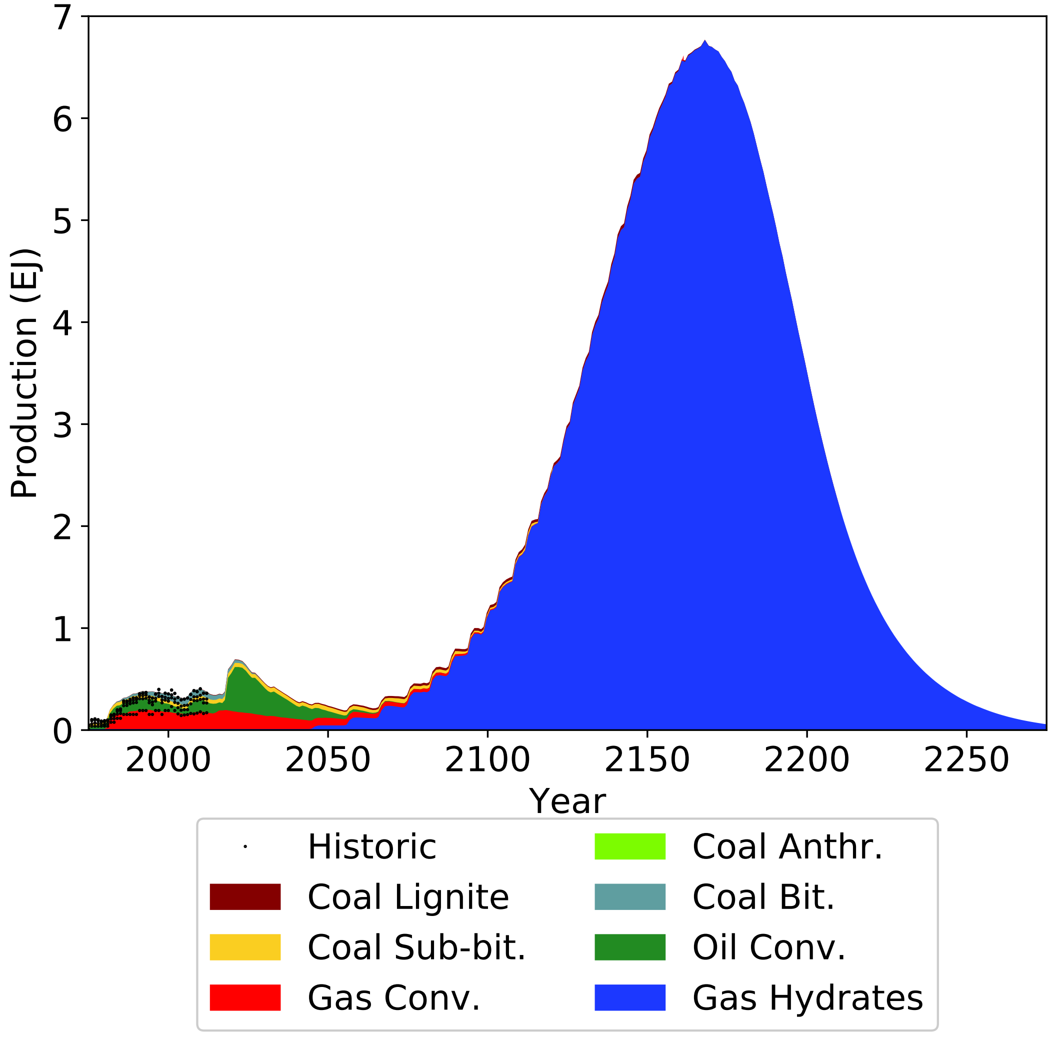

Figure 2.123: New Zealand projections capped at 16

Table 2.123: Peak years - All

**Name**	**URR**	**Peak Year**	**Peak Rate**
Gas Hydrates	533.0	2168	6.75
Gas Conv.	11.9	1991	0.19
Oil Conv.	11.5	2022	0.44
Coal Sub-bit.	6.2	2031	0.04
Coal Bit.	3.7	2007	0.07
Coal Lignite	3.38	2138	0.04
Coal Anthr.	–	1949	–
**Total**	**569.68**	**2168**	**6.75**

2.18.2 By Mineral

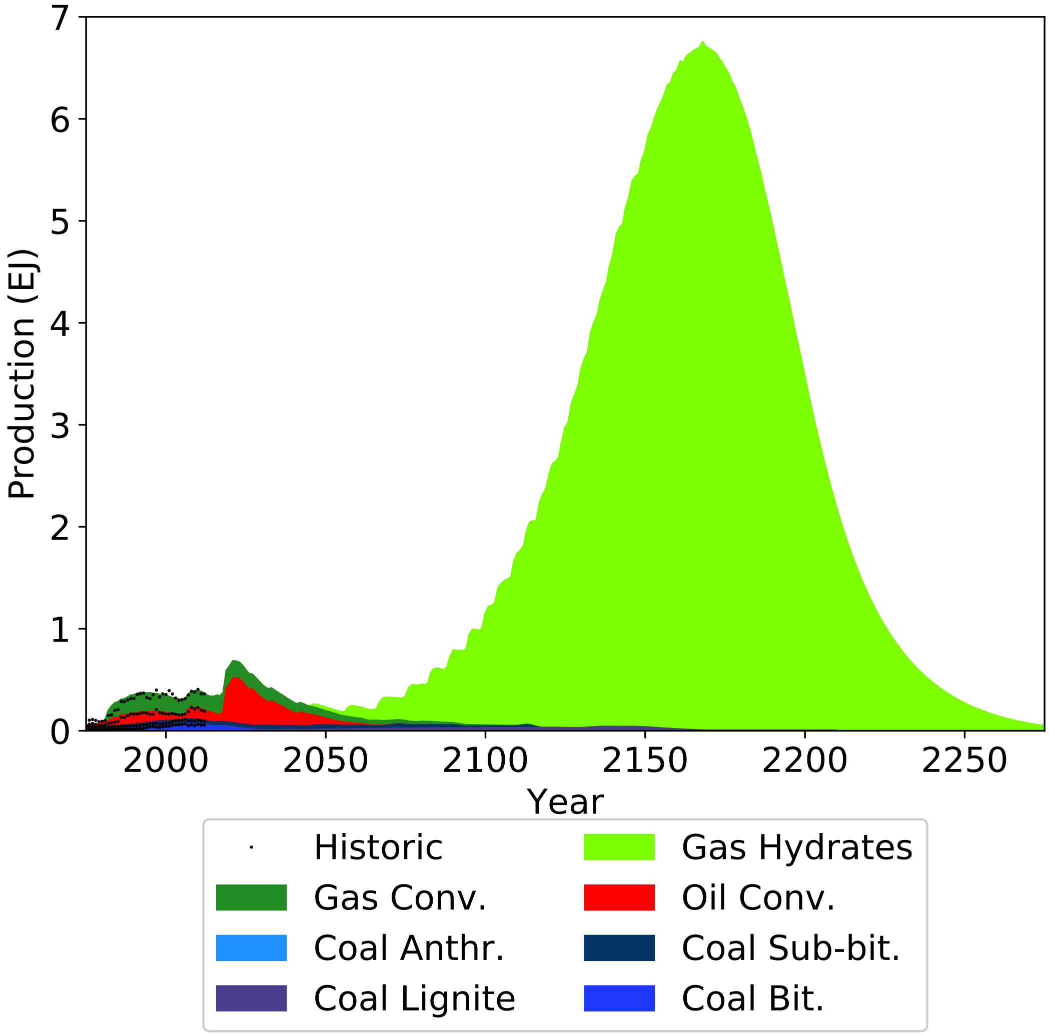

Figure 2.124: New Zealand projection by mineral type

Table 2.124: Peak years - Minerals

Name	URR	Peak Year	Peak Rate
Coal Bit.	3.7	2007	0.07
Coal Lignite	3.38	2138	0.04
Coal Sub-bit.	6.2	2031	0.04
Coal Anthr.	–	1949	–
Oil Conv.	11.5	2022	0.44
Gas Conv.	11.9	1991	0.19
Gas Hydrates	533.0	2168	6.75
**Total**	**569.68**	**2168**	**6.75**

2.19 North Korea

2.19.1 All Projections

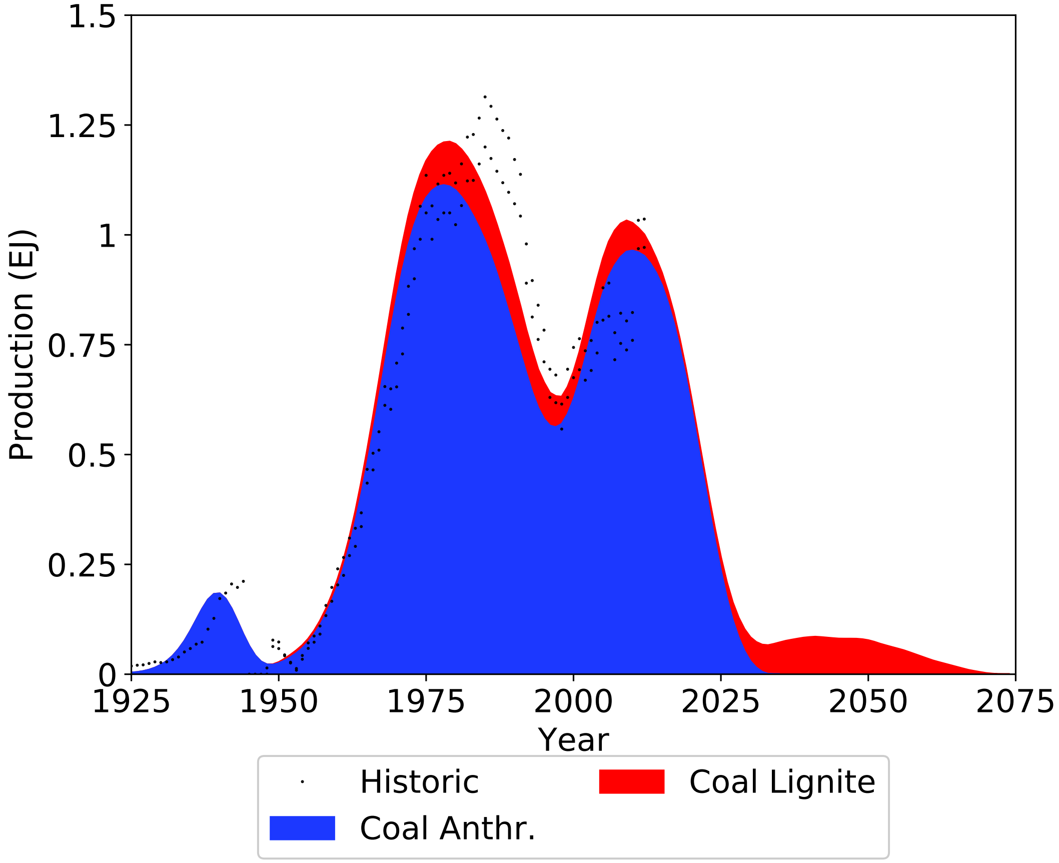

Figure 2.125: North Korea projections capped at 16

Table 2.125: Peak years - All

Name	URR	Peak Year	Peak Rate
Coal Anthr.	53.7	1978	1.11
Coal Lignite	6.88	1989	0.11
**Total**	**60.58**	**1979**	**1.21**

2.19.2 By Mineral

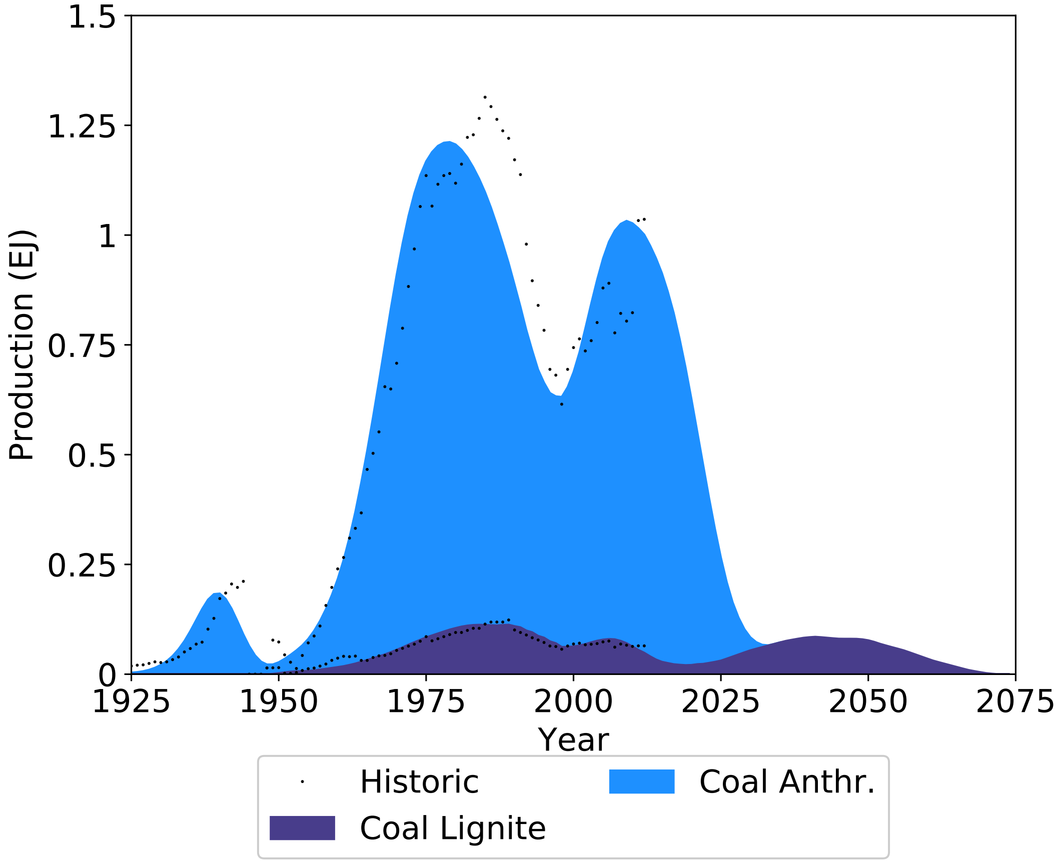

Figure 2.126: North Korea projection by mineral type

Table 2.126: Peak years - Minerals

Name	URR	Peak Year	Peak Rate
Coal Lignite	6.88	1989	0.11
Coal Anthr.	53.7	1978	1.11
**Total**	**60.58**	**1979**	**1.21**

2.20 PNG

2.20.1 All Projections

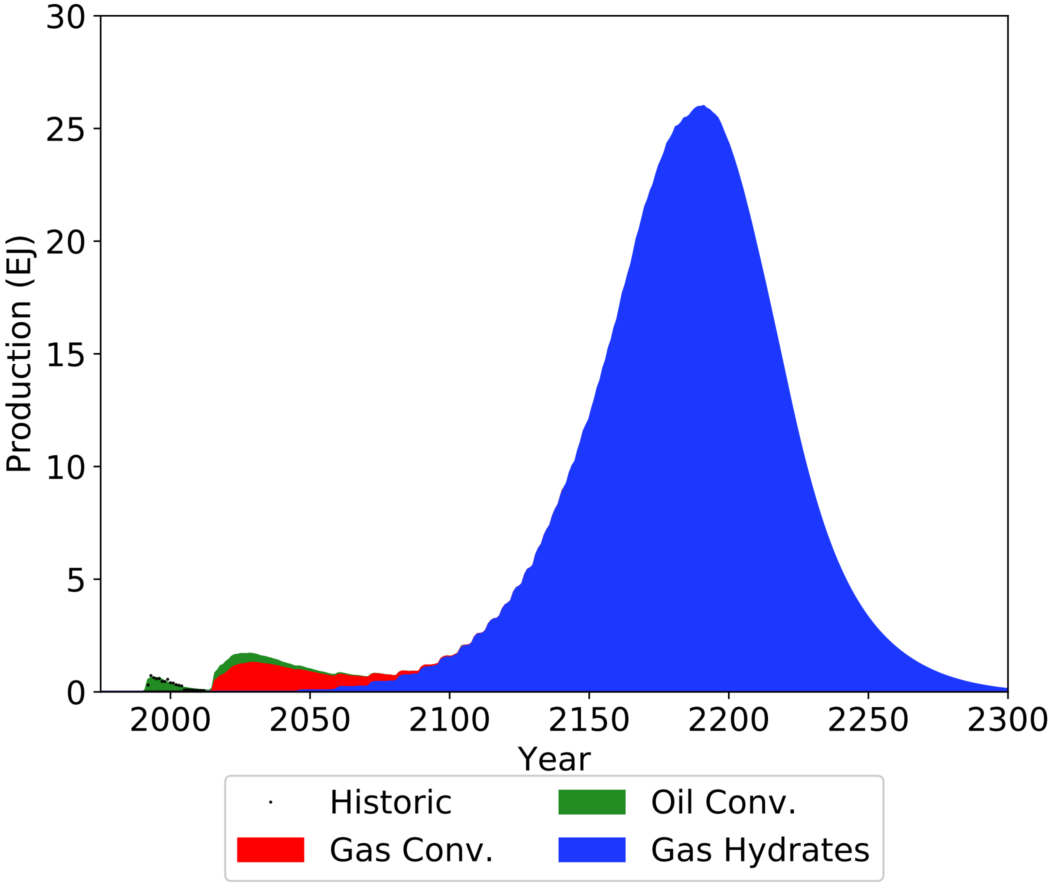

Figure 2.127: PNG projections capped at 16

Table 2.127: Peak years - All

Name	URR	Peak Year	Peak Rate
Gas Hydrates	2053.0	2191	26.0
Gas Conv.	53.55	2030	1.29
Oil Conv.	19.81	1993	0.62
**Total**	**2126.36**	**2191**	**26.0**

2.20.2 By Mineral

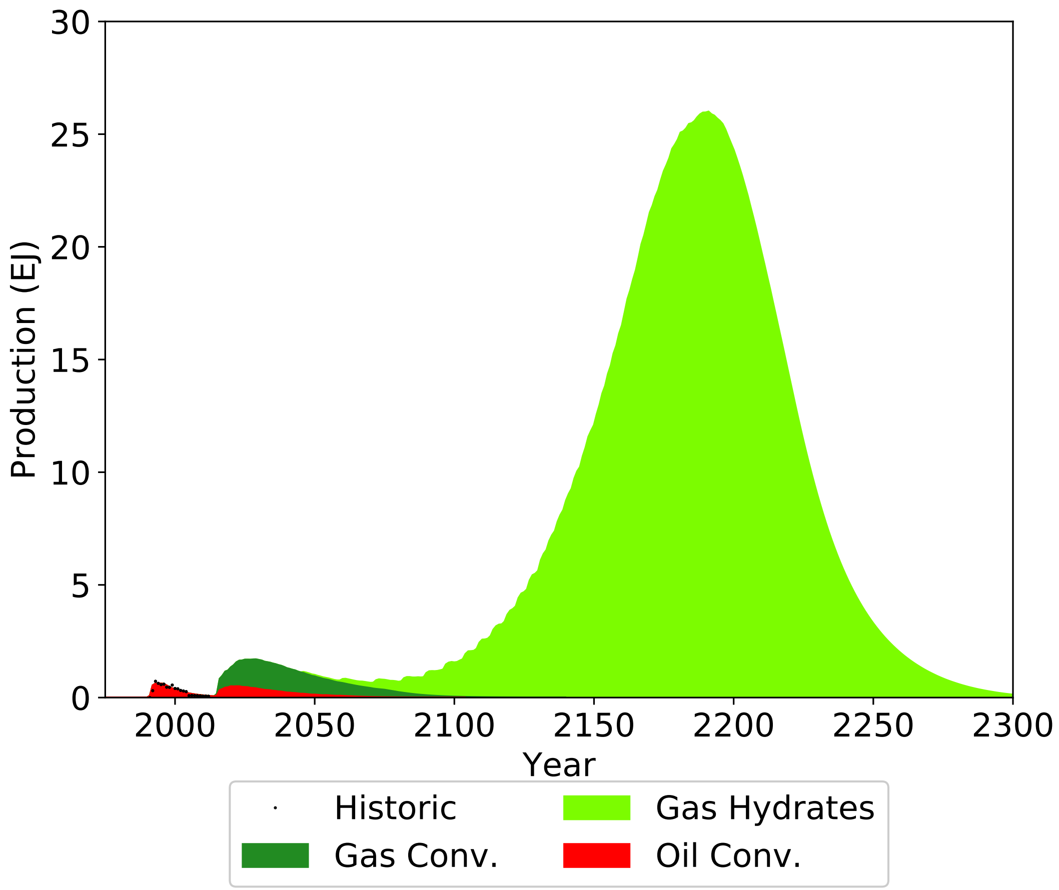

Figure 2.128: PNG projection by mineral type

Table 2.128: Peak years - Minerals

Name	URR	Peak Year	Peak Rate
Oil Conv.	19.81	1993	0.62
Gas Conv.	53.55	2030	1.29
Gas Hydrates	2053.0	2191	26.0
**Total**	**2126.36**	**2191**	**26.0**

2.21 Pakistan

2.21.1 All Projections

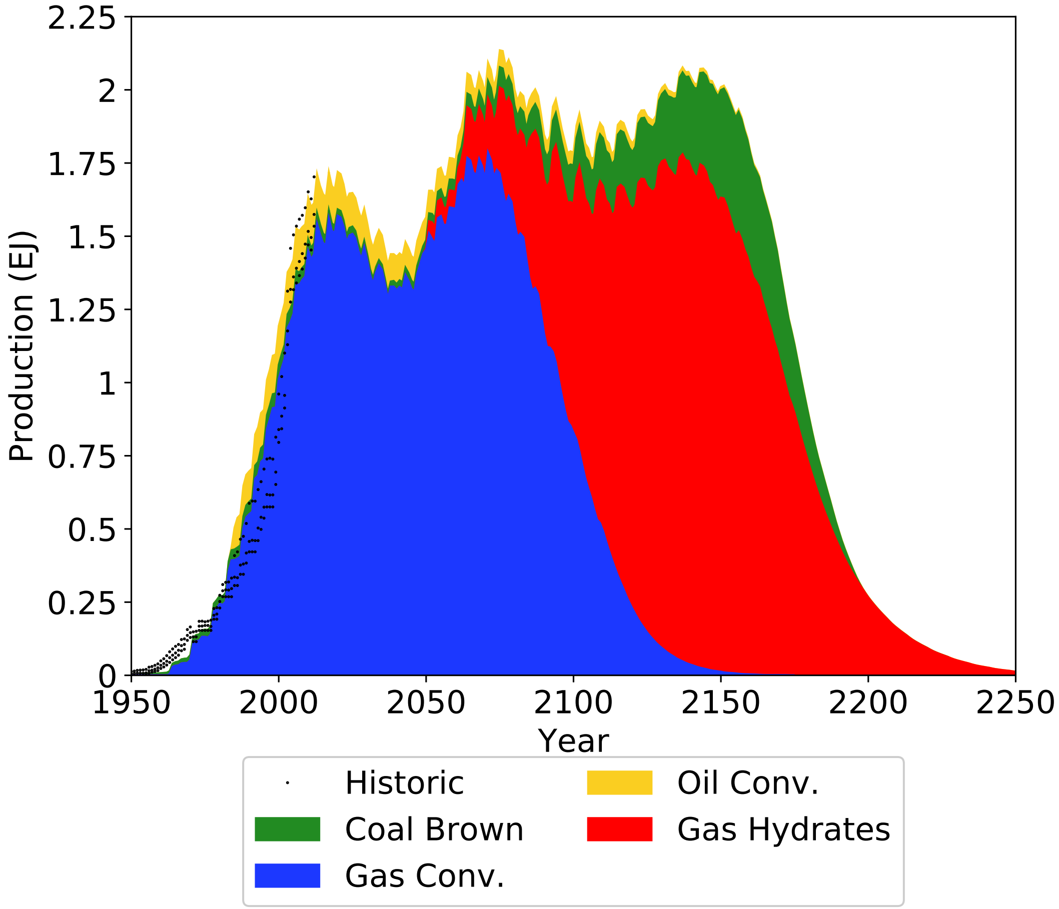

Figure 2.129: Pakistan projections capped at 16

Table 2.129: Peak years - All

**Name**	**URR**	**Peak Year**	**Peak Rate**
Gas Conv.	168.3	2071	1.79
Gas Hydrates	138.0	2137	1.73
Coal Brown	28.6	2157	0.41
Oil Conv.	12.08	2006	0.14
**Total**	**346.98**	**2075**	**2.14**

2.21.2 By Mineral

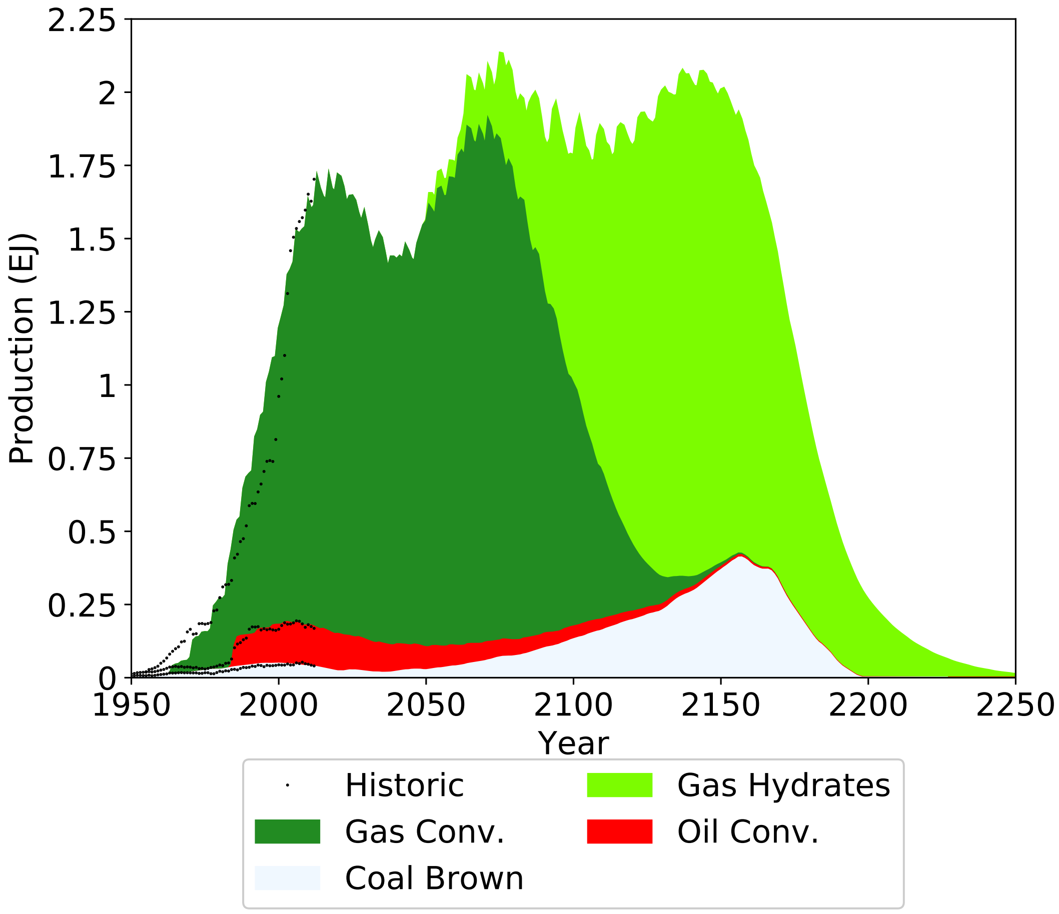

Figure 2.130: Pakistan projection by mineral type

Table 2.130: Peak years - Minerals

**Name**	**URR**	**Peak Year**	**Peak Rate**
Coal Brown	28.6	2157	0.41
Oil Conv.	12.08	2006	0.14
Gas Conv.	168.3	2071	1.79
Gas Hydrates	138.0	2137	1.73
**Total**	**346.98**	**2075**	**2.14**

2.22 Philippines

2.22.1 All Projections

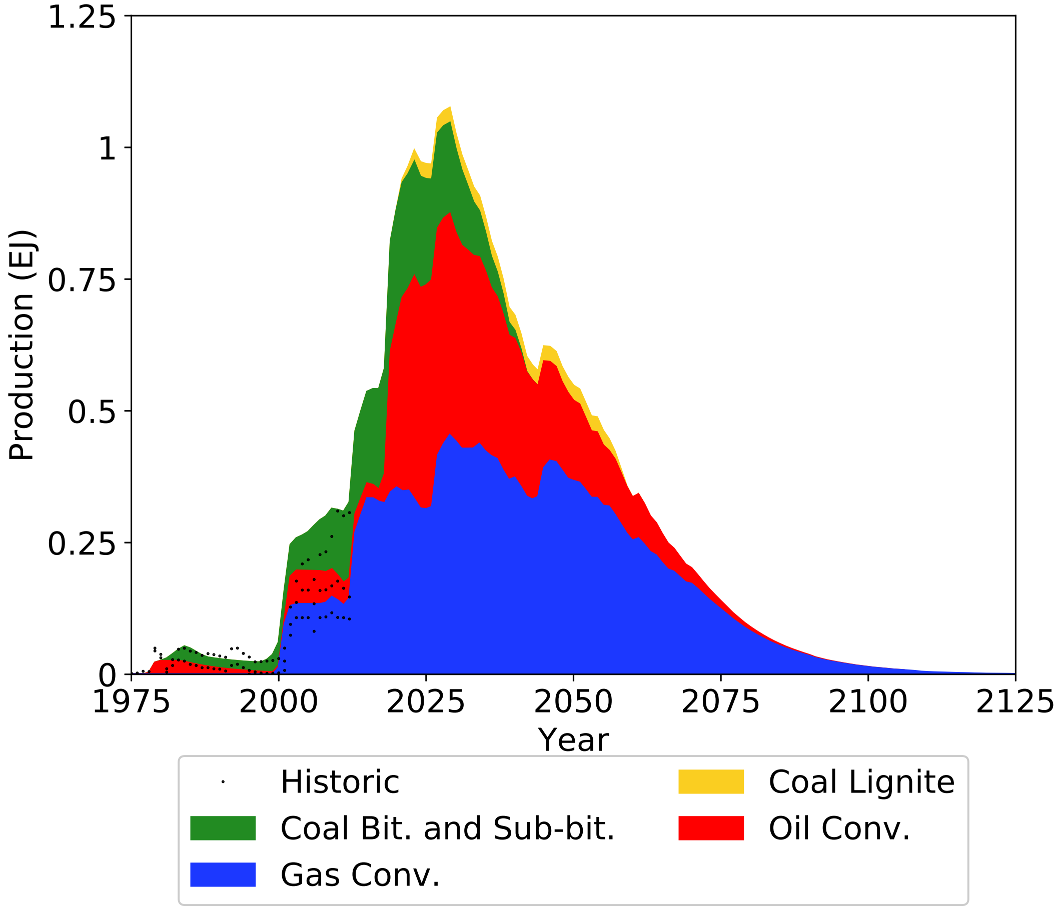

Figure 2.131: Philippines projections capped at 16

Table 2.131: Peak years - All

**Name**	**URR**	**Peak Year**	**Peak Rate**
Gas Conv.	23.0	2029	0.45
Oil Conv.	12.98	2027	0.43
Coal Bit. and Sub-bit.	5.7	2021	0.22
Coal Lignite	1.0	2025	0.03
**Total**	**42.68**	**2029**	**1.08**

2.22.2 By Mineral

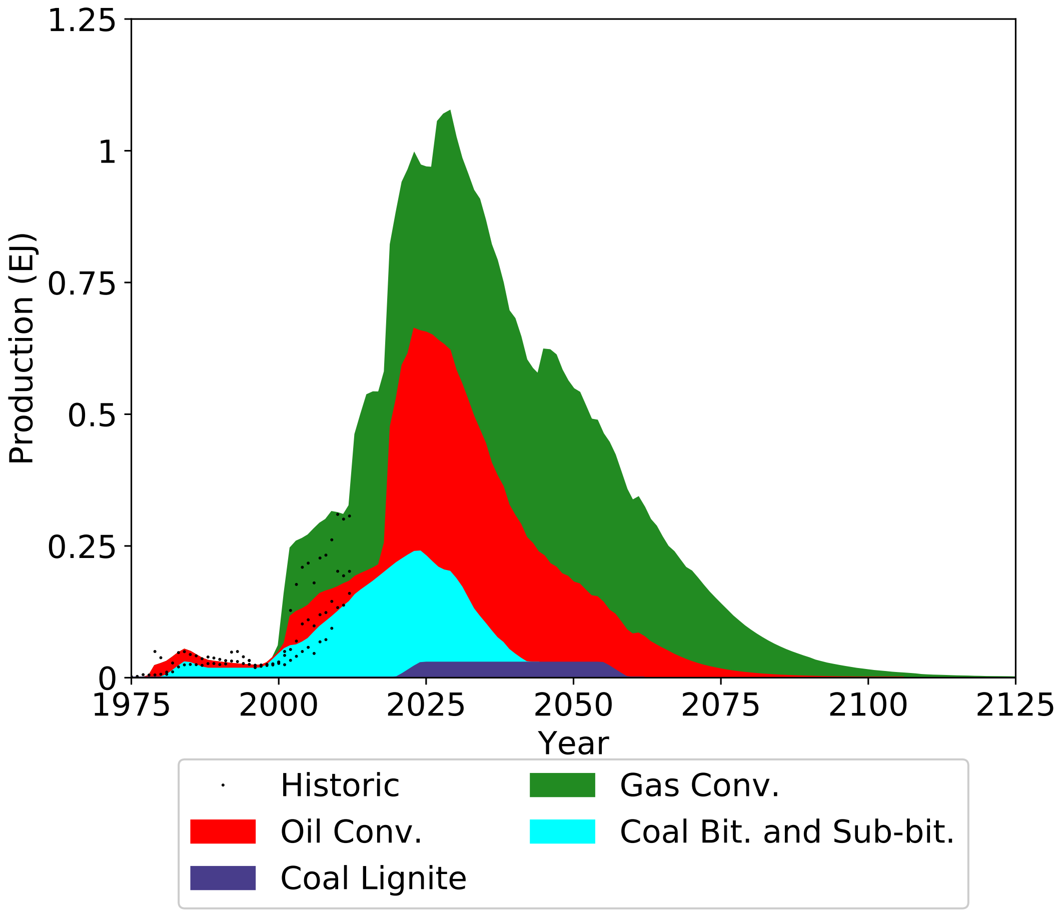

Figure 2.132: Philippines projection by mineral type

Table 2.132: Peak years - Minerals

Name	URR	Peak Year	Peak Rate
Coal Lignite	1.0	2025	0.03
Coal Bit. and Sub-bit.	5.7	2021	0.22
Oil Conv.	12.98	2027	0.43
Gas Conv.	23.0	2029	0.45
**Total**	**42.68**	**2029**	**1.08**

2.23 South Korea

2.23.1 All Projections

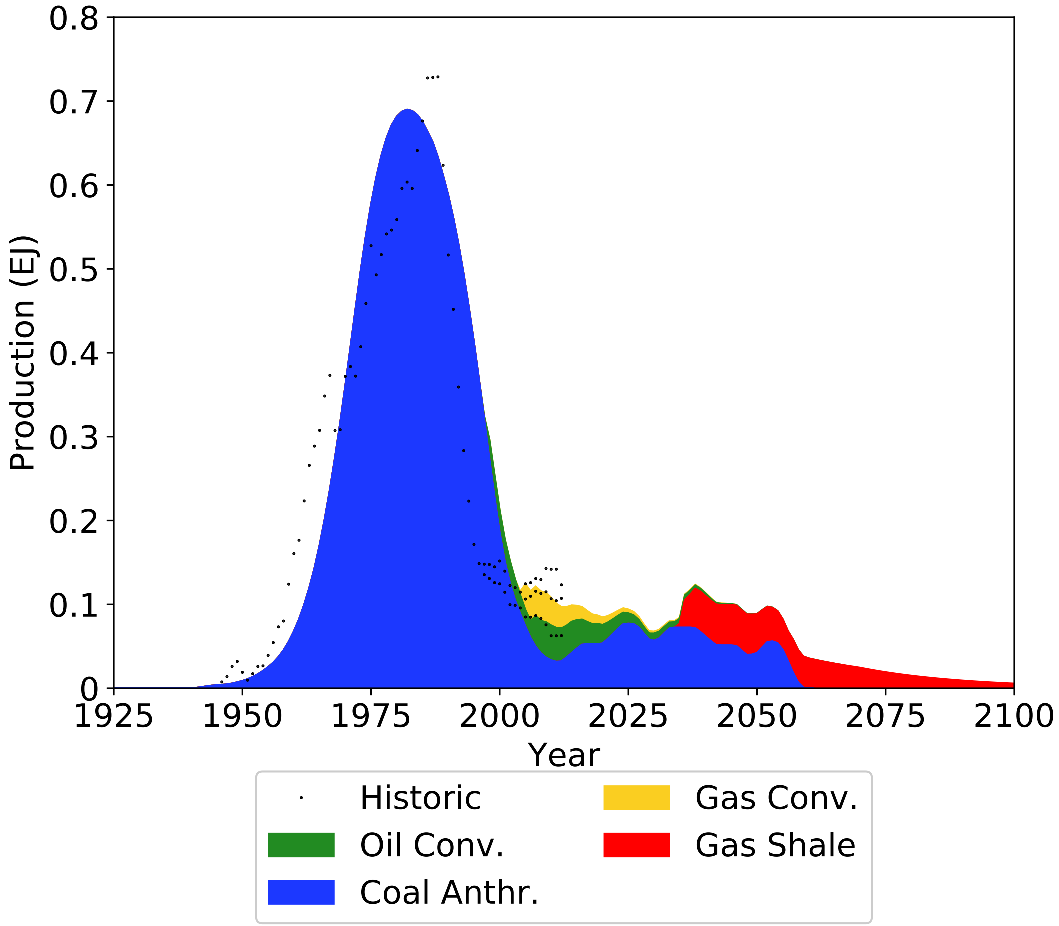

Figure 2.133: South Korea projections capped at 16

Table 2.133: Peak years - All

**Name**	**URR**	**Peak Year**	**Peak Rate**
Coal Anthr.	21.87	1982	0.69
Gas Shale	1.85	2039	0.05
Oil Conv.	0.88	2009	0.04
Gas Conv.	0.44	2006	0.04
**Total**	**25.04**	**1982**	**0.69**

2.23.2 By Mineral

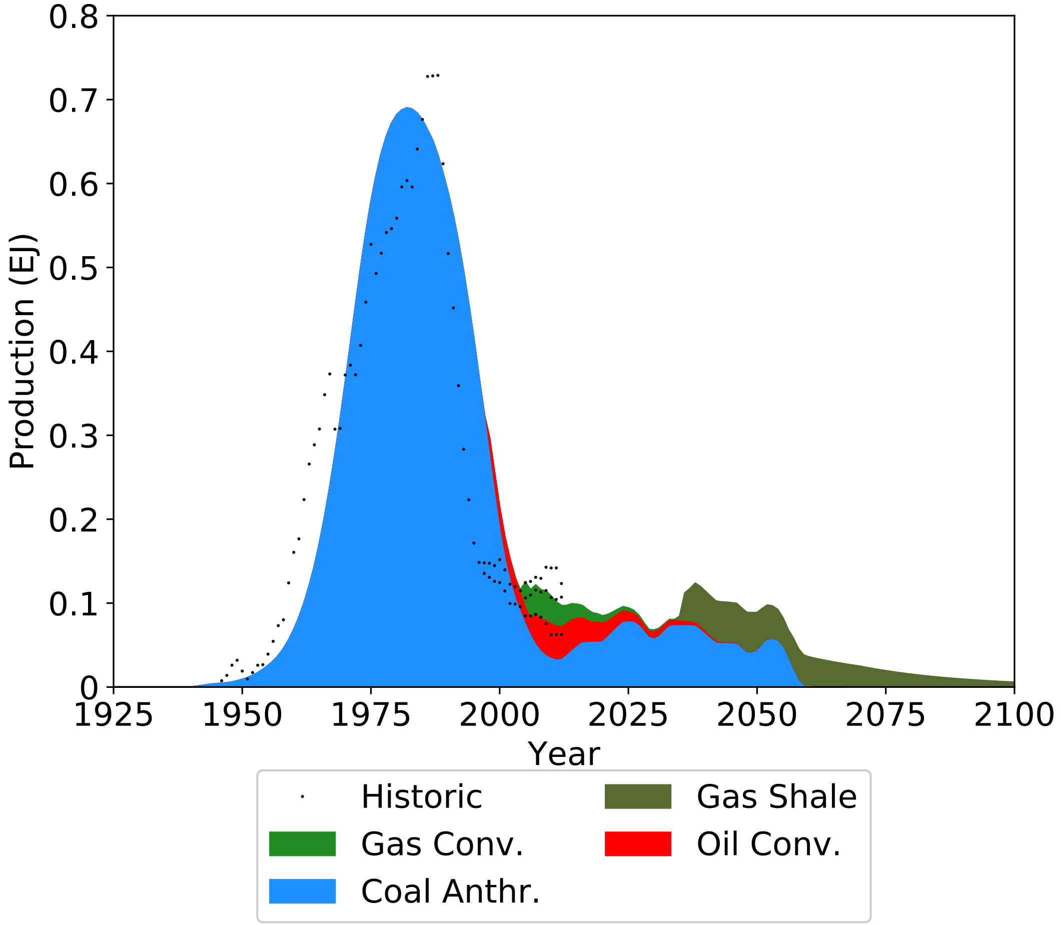

Figure 2.134: South Korea projection by mineral type

Table 2.134: Peak years - Minerals

**Name**	**URR**	**Peak Year**	**Peak Rate**
Coal Anthr.	21.87	1982	0.69
Oil Conv.	0.88	2009	0.04
Gas Conv.	0.44	2006	0.04
Gas Shale	1.85	2039	0.05
**Total**	**25.04**	**1982**	**0.69**

2.24 Sri Lanka

2.24.1 All Projections

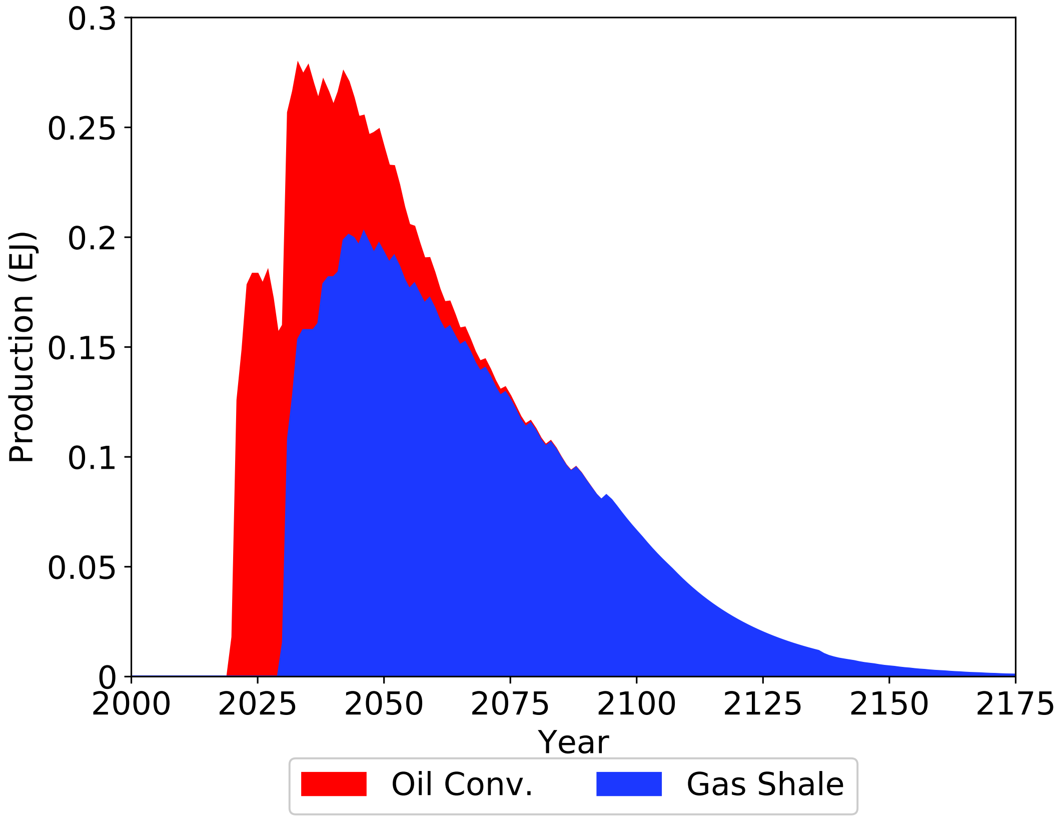

Figure 2.135: Sri Lanka projections capped at 16

Table 2.135: Peak years - All

Name	URR	Peak Year	Peak Rate
Gas Shale	11.12	2046	0.2
Oil Conv.	3.79	2027	0.18
**Total**	**14.91**	**2033**	**0.28**

2.24.2 By Mineral

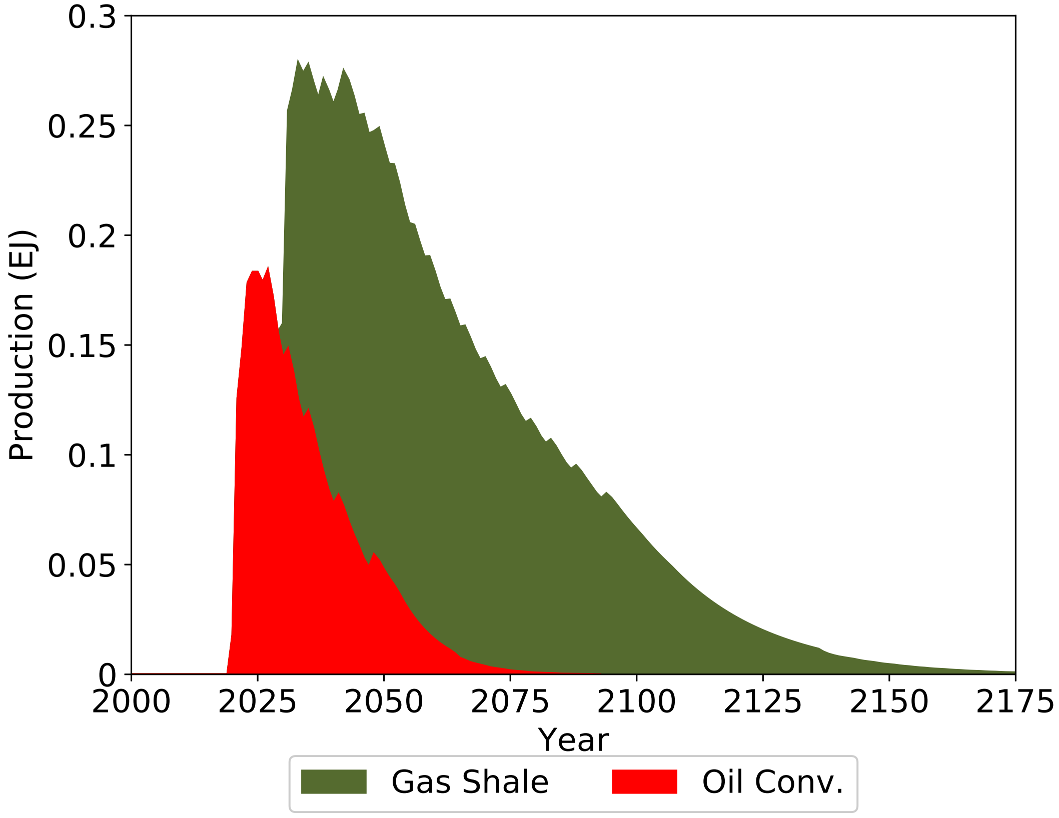

Figure 2.136: Sri Lanka projection by mineral type

Table 2.136: Peak years - Minerals

Name	URR	Peak Year	Peak Rate
Oil Conv.	3.79	2027	0.18
Gas Shale	11.12	2046	0.2
**Total**	**14.91**	**2033**	**0.28**

2.25 Taiwan

2.25.1 All Projections

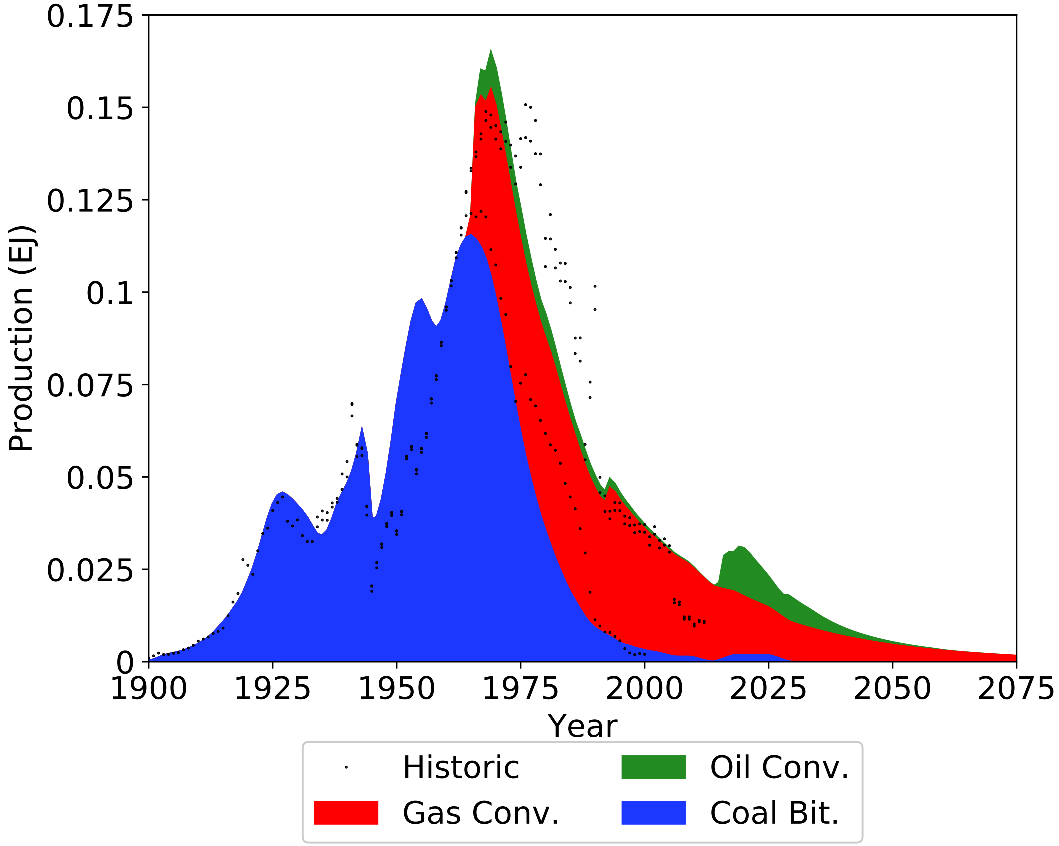

Figure 2.137: Taiwan projections capped at 16

Table 2.137: Peak years - All

Name	URR	Peak Year	Peak Rate
Coal Bit.	4.37	1965	0.12
Gas Conv.	2.4	1970	0.05
Oil Conv.	0.39	2020	0.01
**Total**	**7.16**	**1969**	**0.16**

2.25.2 By Mineral

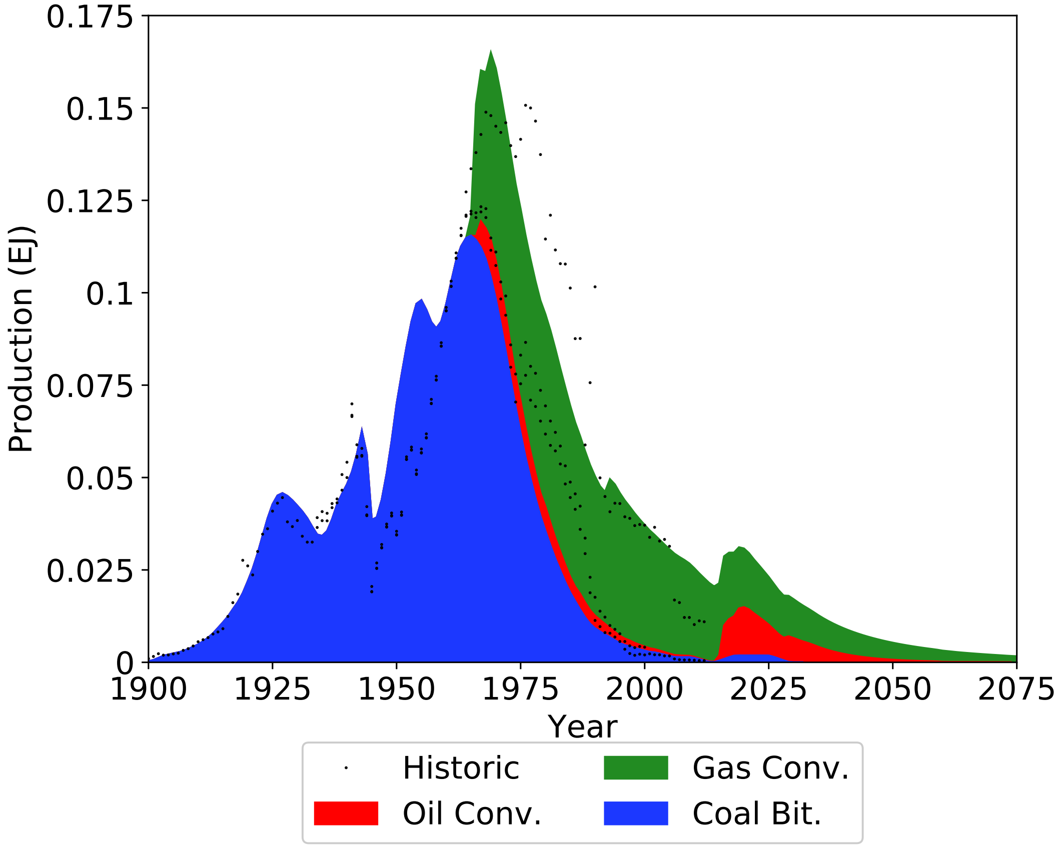

Figure 2.138: Taiwan projection by mineral type

Table 2.138: Peak years - Minerals

Name	URR	Peak Year	Peak Rate
Coal Bit.	4.37	1965	0.12
Oil Conv.	0.39	2020	0.01
Gas Conv.	2.4	1970	0.05
**Total**	**7.16**	**1969**	**0.16**

2.26 Thailand

2.26.1 All Projections

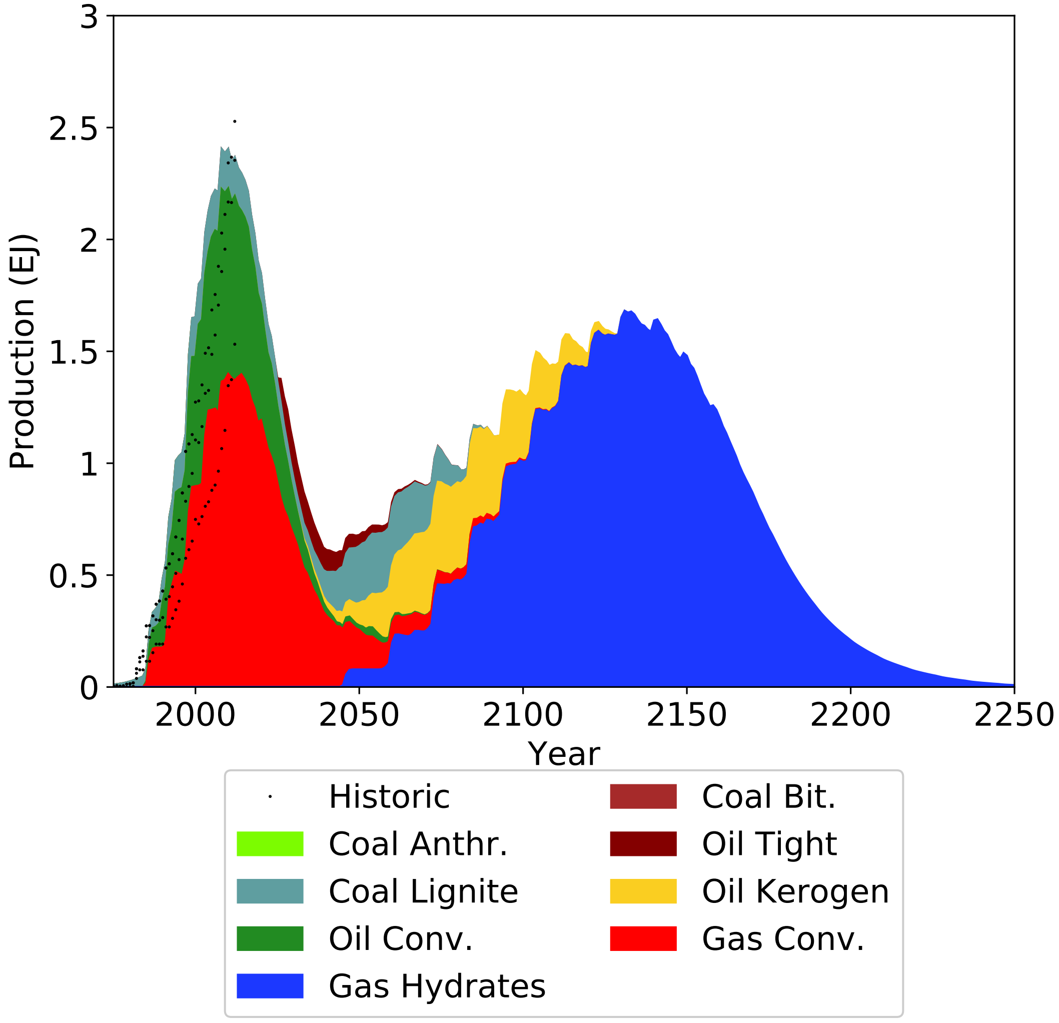

Figure 2.139: Thailand projections capped at 16

Table 2.139: Peak years - All

Name	URR	Peak Year	Peak Rate
Gas Hydrates	138.0	2131	1.68
Gas Conv.	50.8	2010	1.4
Oil Conv.	23.97	2008	0.86
Oil Kerogen	20.6	2085	0.4
Coal Lignite	16.28	2056	0.27
Oil Tight	3.07	2030	0.15
Coal Anthr.	–	1991	–
Coal Bit.	–	1982	–
**Total**	**252.72**	**2010**	**2.4**

2.26.2 By Mineral

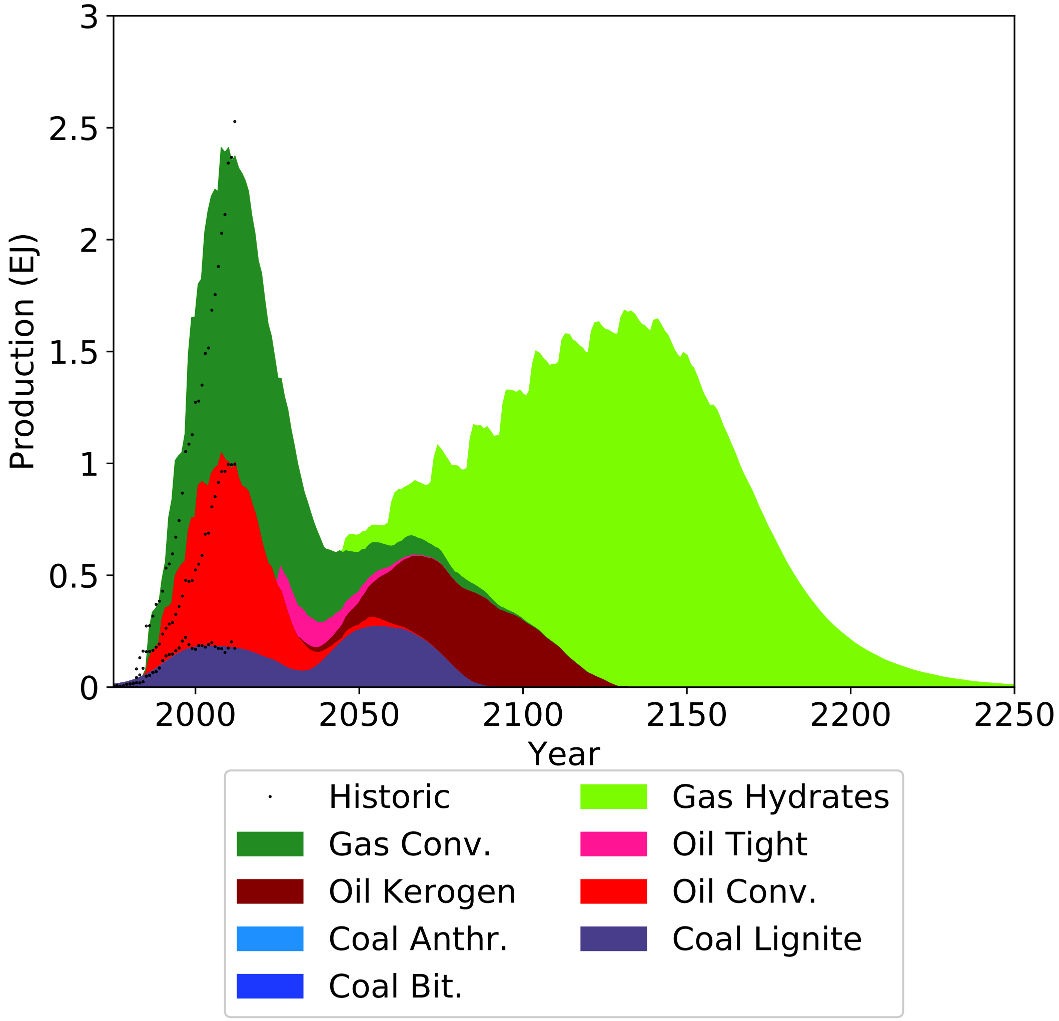

Figure 2.140: Thailand projection by mineral type

Table 2.140: Peak years - Minerals

Name	URR	Peak Year	Peak Rate
Coal Bit.	–	1982	–
Coal Lignite	16.28	2056	0.27
Coal Anthr.	–	1991	–
Oil Conv.	23.97	2008	0.86
Oil Kerogen	20.6	2085	0.4
Oil Tight	3.07	2030	0.15
Gas Conv.	50.8	2010	1.4
Gas Hydrates	138.0	2131	1.68
**Total**	**252.72**	**2010**	**2.4**

2.27 Vietnam

2.27.1 All Projections

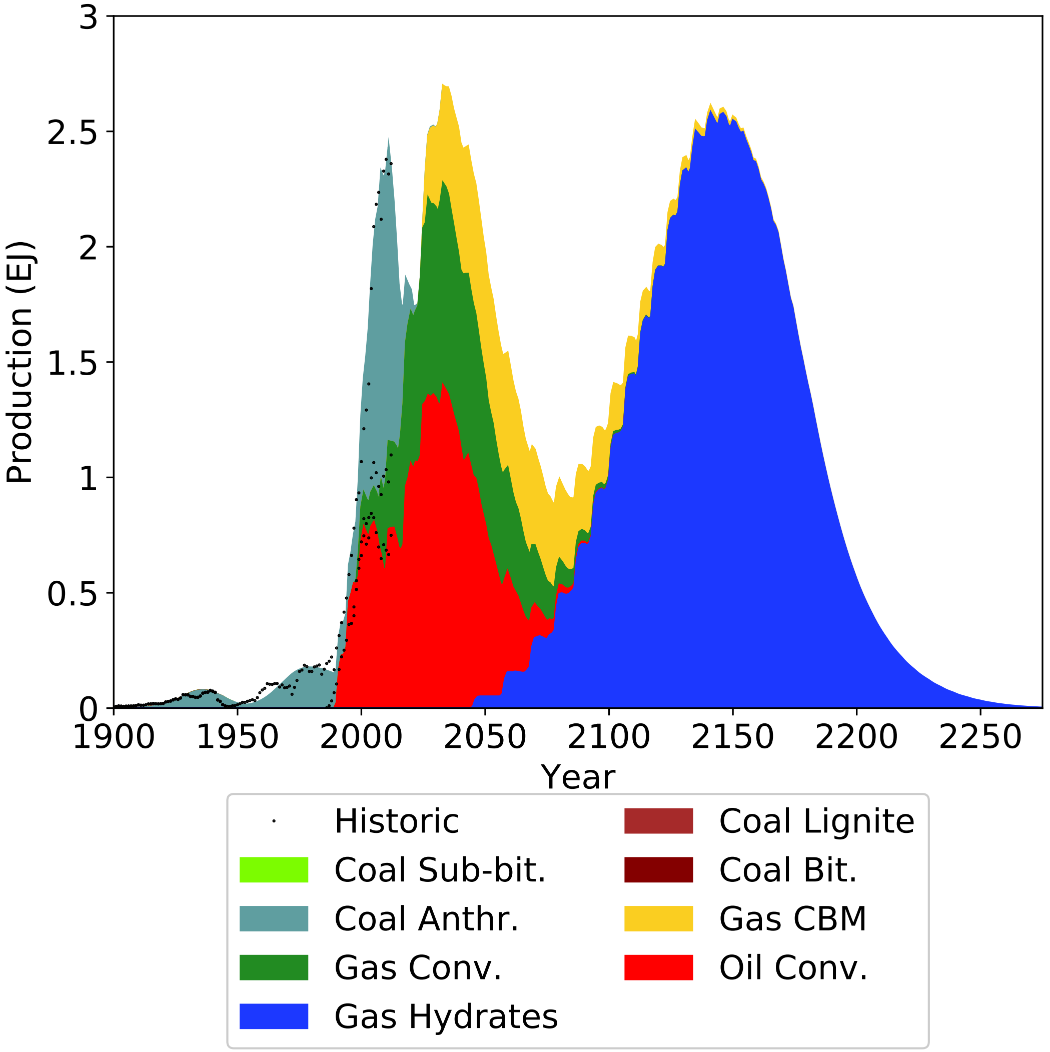

Figure 2.141: Vietnam projections capped at 16

Table 2.141: Peak years - All

**Name**	**URR**	**Peak Year**	**Peak Rate**
Gas Hydrates	212.0	2141	2.58
Oil Conv.	63.34	2033	1.4
Gas Conv.	41.94	2032	0.88
Gas CBM	35.5	2047	0.56
Coal Anthr.	25.1	2009	1.36
Coal Bit.	0.03	1914	–
Coal Sub-bit.	0.01	1931	–
Coal Lignite	–	1941	–
**Total**	**377.92**	**2033**	**2.7**

2.27.2 By Mineral

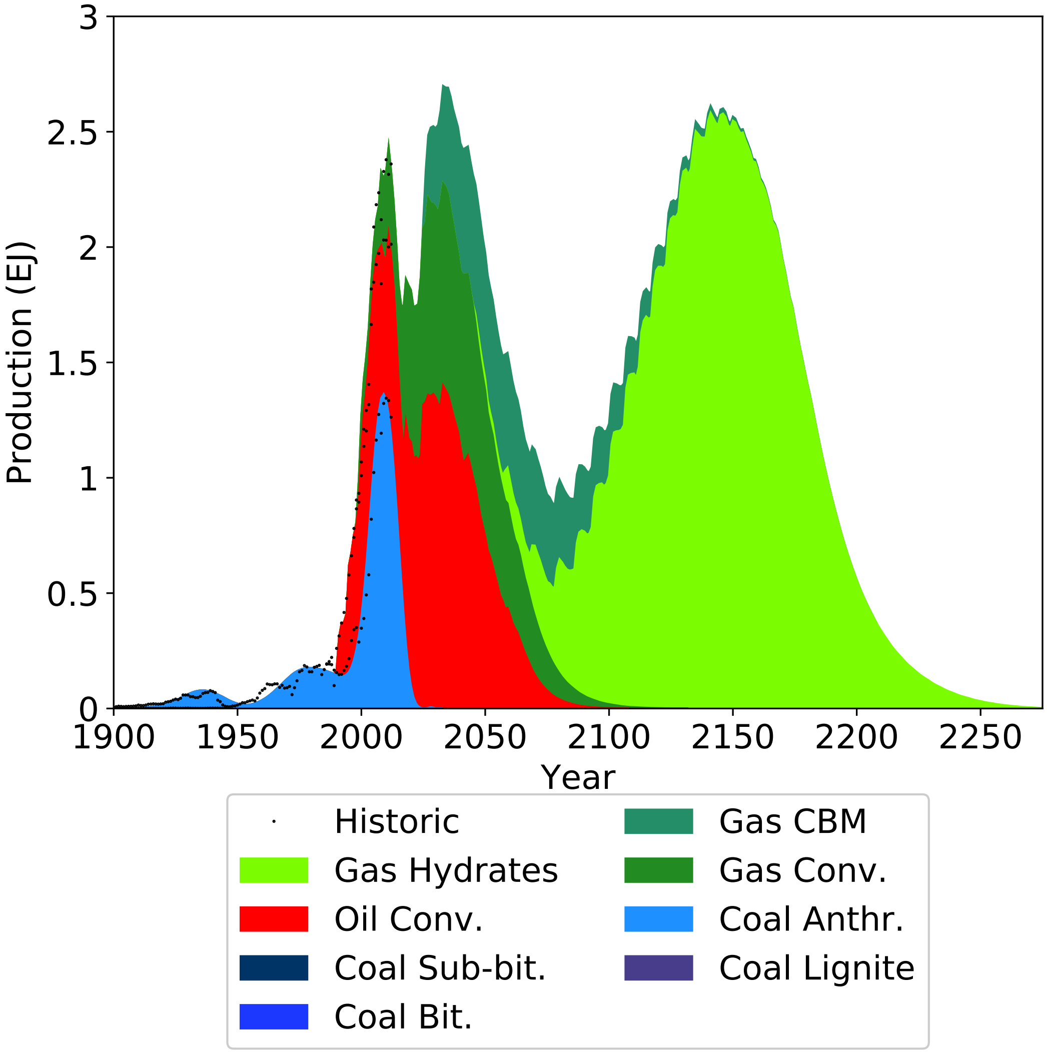

Figure 2.142: Vietnam projection by mineral type

Table 2.142: Peak years - Minerals

**Name**	**URR**	**Peak Year**	**Peak Rate**
Coal Bit.	0.03	1914	–
Coal Lignite	–	1941	–
Coal Sub-bit.	0.01	1931	–
Coal Anthr.	25.1	2009	1.36
Oil Conv.	63.34	2033	1.4
Gas Conv.	41.94	2032	0.88
Gas Hydrates	212.0	2141	2.58
Gas CBM	35.5	2047	0.56
**Total**	**377.92**	**2033**	**2.7**

2.28 Total

2.28.1 By country

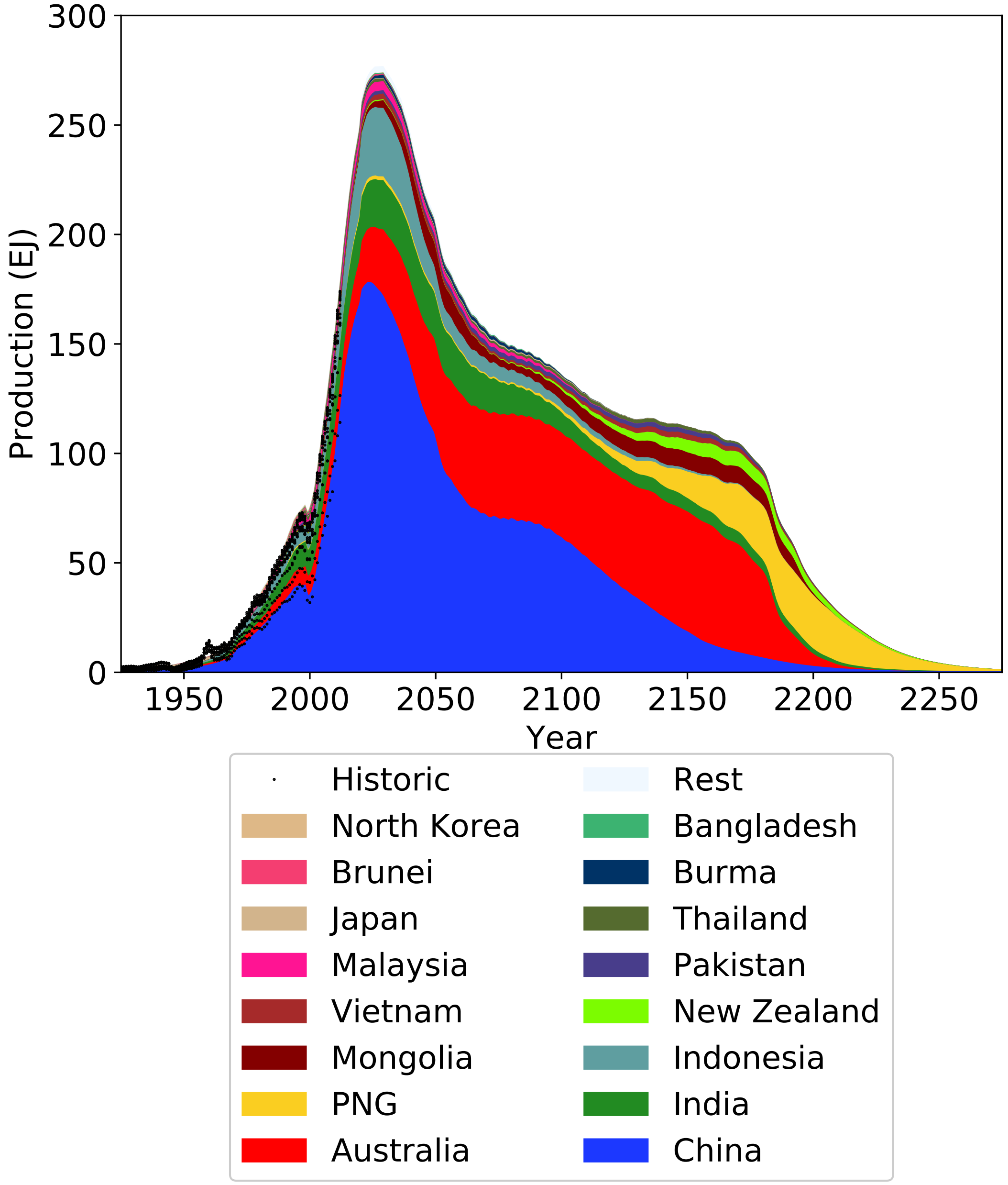

Figure 2.143: Asia projections by country

Table 2.143: Peak years - All

**Name**	**URR**	**Peak Year**	**Peak Rate**
China	13459.83	2024	177.93
Australia	8228.99	2148	54.73
India	2580.43	2037	22.74
PNG	2126.36	2191	26.0
Indonesia	1603.11	2027	31.36
Mongolia	1152.82	2049	10.7
New Zealand	569.68	2168	6.75
Vietnam	377.92	2033	2.7
Pakistan	346.98	2075	2.14
Malaysia	320.66	2023	4.29
Thailand	252.72	2010	2.4
Japan	127.53	1953	1.24
Burma	126.28	2043	1.9
Brunei	67.4	2001	0.91
Bangladesh	62.79	2008	0.85
North Korea	60.58	1979	1.21
Philippines	42.68	2029	1.08
Afghanistan	29.32	2029	0.85
East Timor	26.84	2032	0.58
South Korea	25.04	1982	0.69
Sri Lanka	14.91	2033	0.28
Taiwan	7.16	1969	0.16
Laos	5.37	2043	0.14
Cambodia	2.95	2025	0.16
New Caledonia	0.06	2048	–
Bhutan	0.04	1995	–
Nepal	0.02	2027	–
**Total**	**31618.47**	**2029**	**276.47**

2.28.2 By mineral

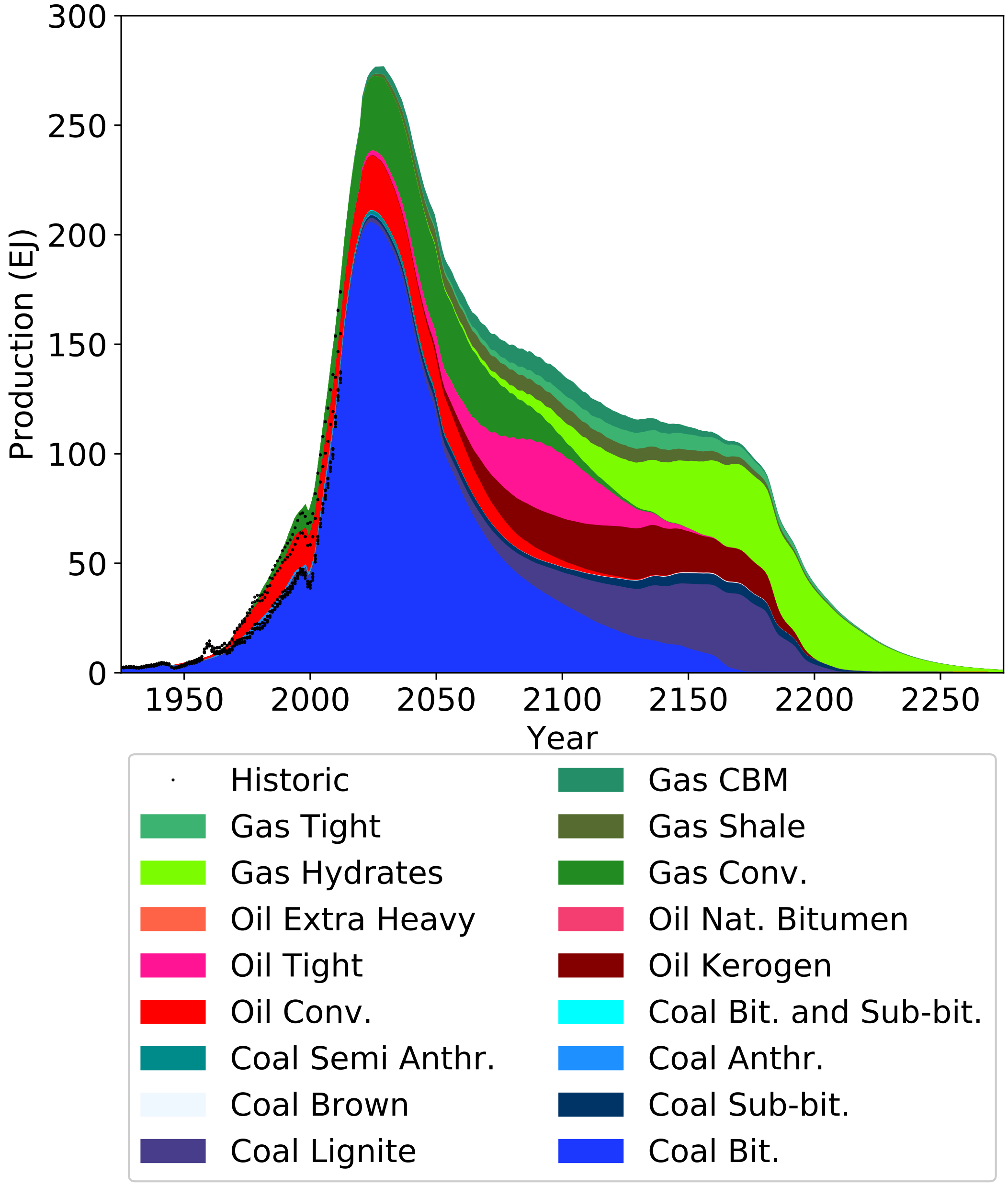

Figure 2.144: Asia projection by mineral type

Table 2.144: Peak years - Minerals

**Name**	**URR**	**Peak Year**	**Peak Rate**
Coal Bit.	12667.83	2024	205.35
Coal Lignite	2873.23	2170	34.56
Coal Sub-bit.	642.44	2160	5.01
Coal Brown	28.6	2157	0.41
Coal Anthr.	102.34	2009	2.36
Coal Semi Anthr.	79.9	2036	2.46
Coal Bit. and Sub-bit.	5.7	2021	0.22
Oil Conv.	1896.9	2025	25.02
Oil Kerogen	2314.02	2127	23.19
Oil Tight	1827.64	2091	31.23
Oil Nat. Bitumen	4.17	2053	0.12
Oil Extra Heavy	5.01	2030	0.15
Gas Conv.	2869.54	2042	39.31
Gas Hydrates	3749.0	2178	39.39
Gas Shale	914.34	2080	7.23
Gas Tight	749.04	2137	7.57
Gas CBM	888.78	2097	8.68
**Total**	**31618.47**	**2029**	**276.47**

Chapter 3

Europe

3.1 Albania

3.1.1 All Projections

Table 3.1: Peak years - All

Name	URR	Peak Year	Peak Rate
Coal Lignite	7.92	2058	0.3
Oil Conv.	5.01	1977	0.09
Gas Shale	0.37	2032	0.01
Gas Conv.	0.26	1981	0.01
Oil Extra Heavy	0.13	2025	0.01
**Total**	**13.69**	**2058**	**0.32**

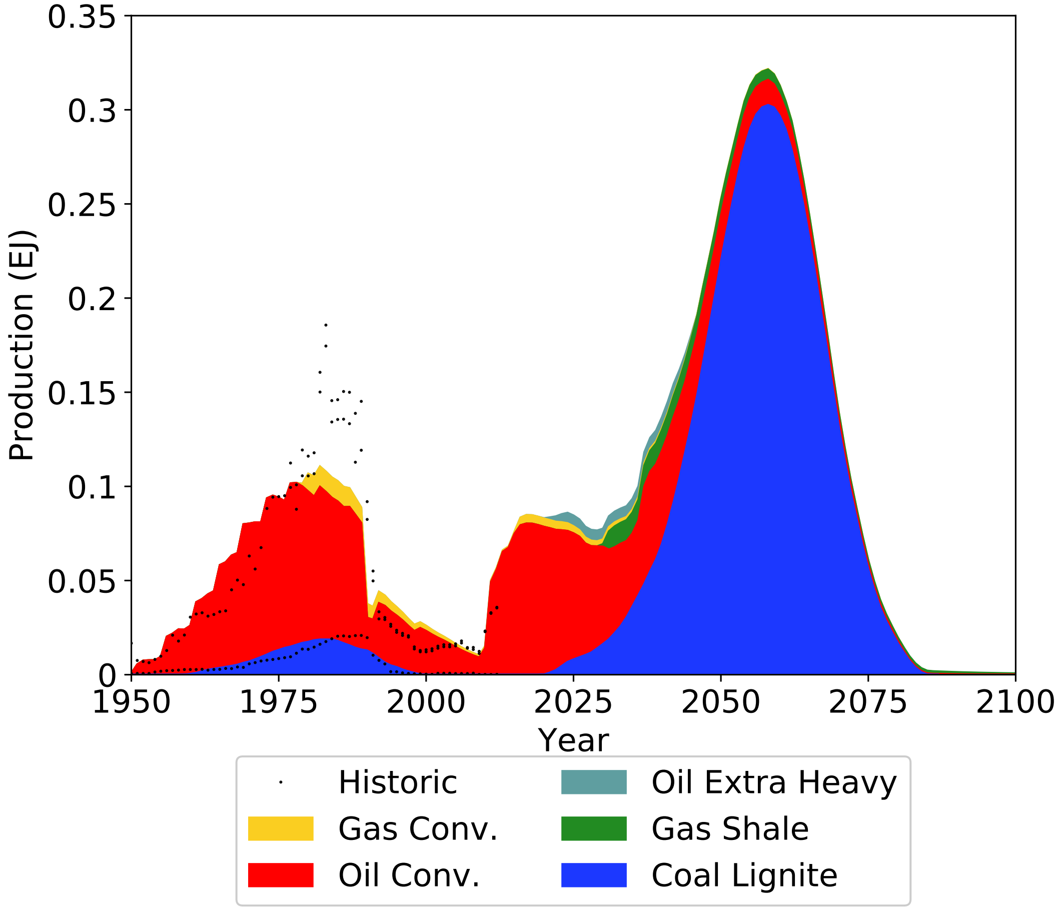

Figure 3.1: Albania projections capped at 16

3.1.2 By Mineral

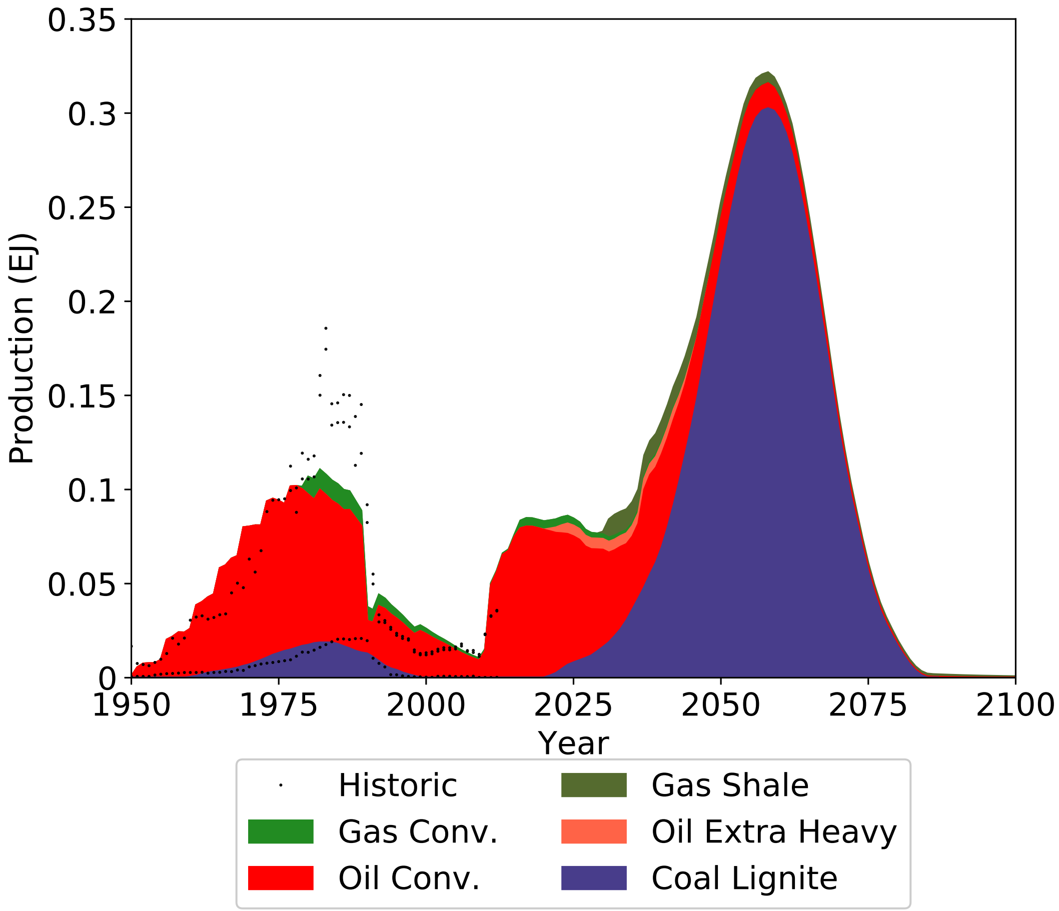

Figure 3.2: Albania projection by mineral type

Table 3.2: Peak years - Minerals

**Name**	**URR**	**Peak Year**	**Peak Rate**
Coal Lignite	7.92	2058	0.3
Oil Conv.	5.01	1977	0.09
Oil Extra Heavy	0.13	2025	0.01
Gas Conv.	0.26	1981	0.01
Gas Shale	0.37	2032	0.01
**Total**	**13.69**	**2058**	**0.32**

3.2 Austria

3.2.1 All Projections

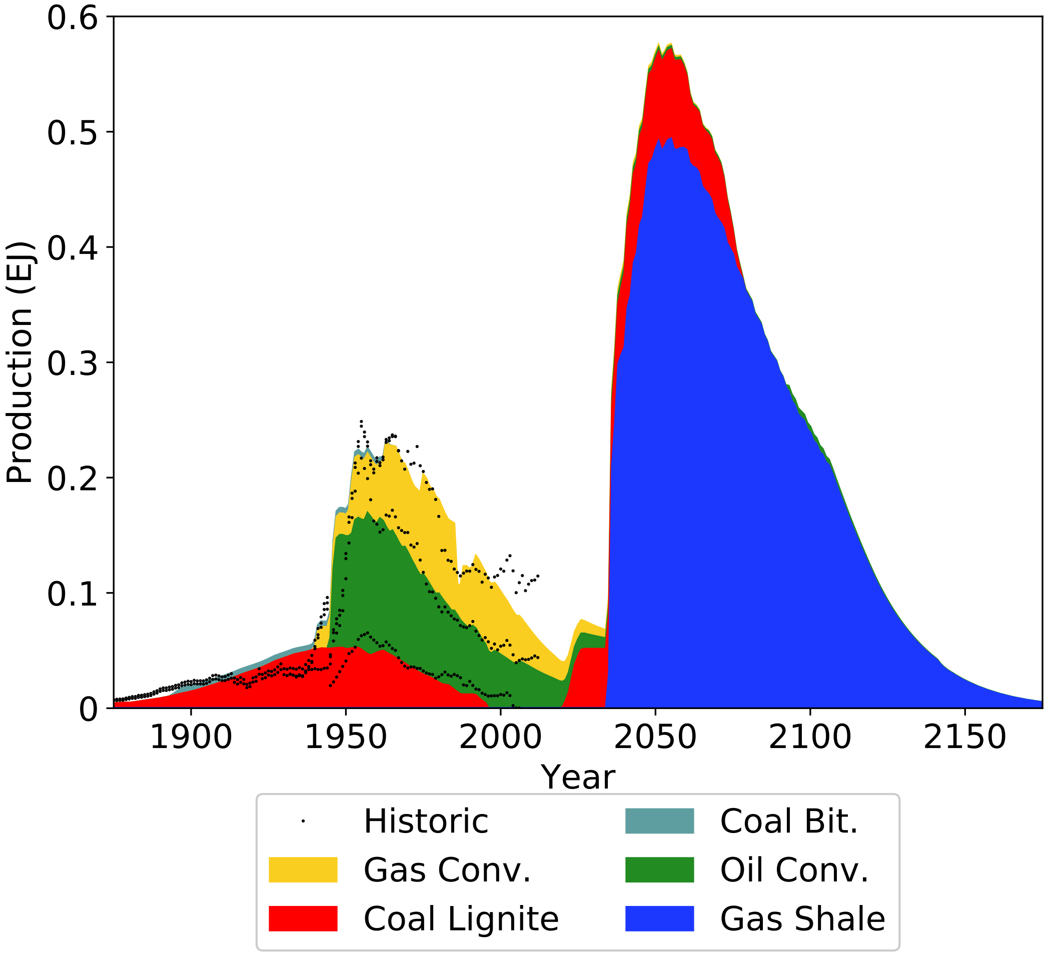

Figure 3.3: Austria projections capped at 16

Table 3.3: Peak years - All

**Name**	**URR**	**Peak Year**	**Peak Rate**
Gas Shale	30.41	2055	0.49
Coal Lignite	6.7	2048	0.08
Oil Conv.	5.86	1957	0.12
Gas Conv.	4.23	1976	0.09
Coal Bit.	0.32	1898	–
**Total**	**47.53**	**2055**	**0.58**

3.2.2 By Mineral

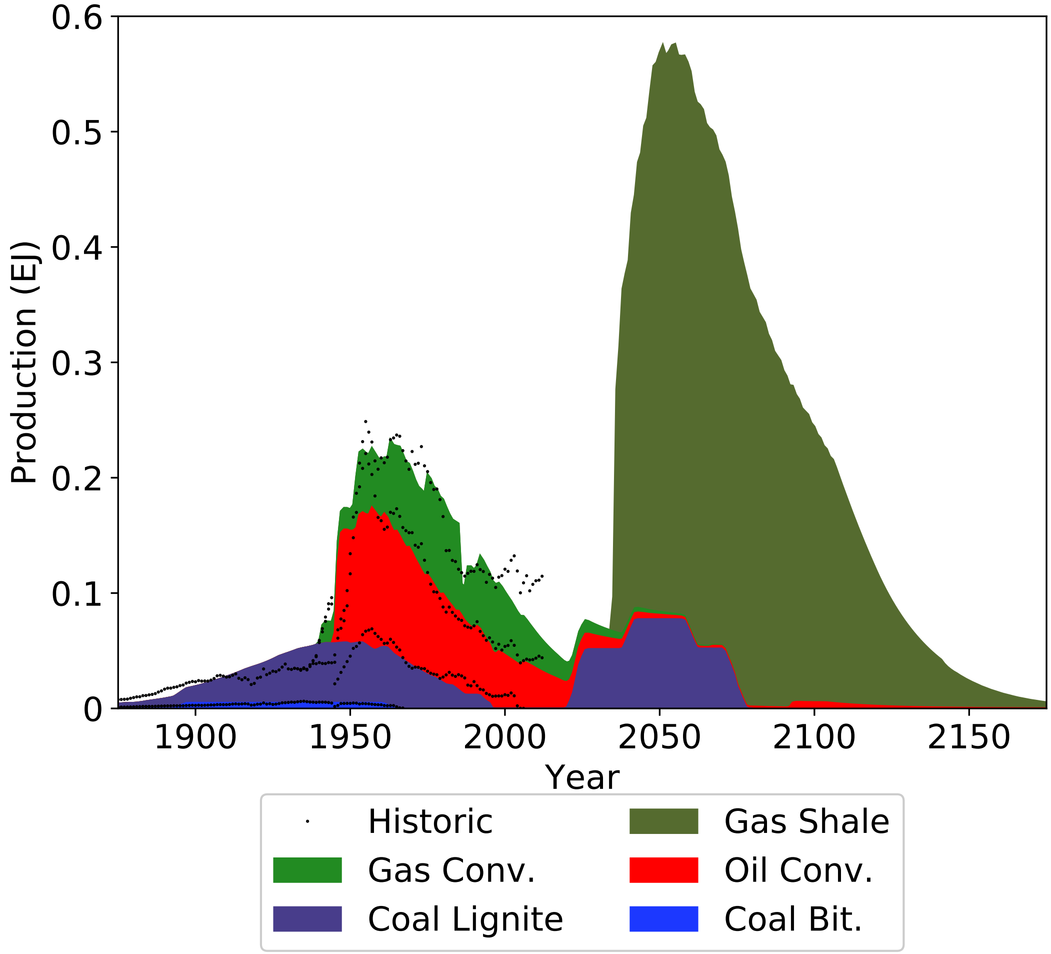

Figure 3.4: Austria projection by mineral type

Table 3.4: Peak years - Minerals

**Name**	**URR**	**Peak Year**	**Peak Rate**
Coal Bit.	0.32	1898	–
Coal Lignite	6.7	2048	0.08
Oil Conv.	5.86	1957	0.12
Gas Conv.	4.23	1976	0.09
Gas Shale	30.41	2055	0.49
**Total**	**47.53**	**2055**	**0.58**

3.3 Belgium

3.3.1 All Projections

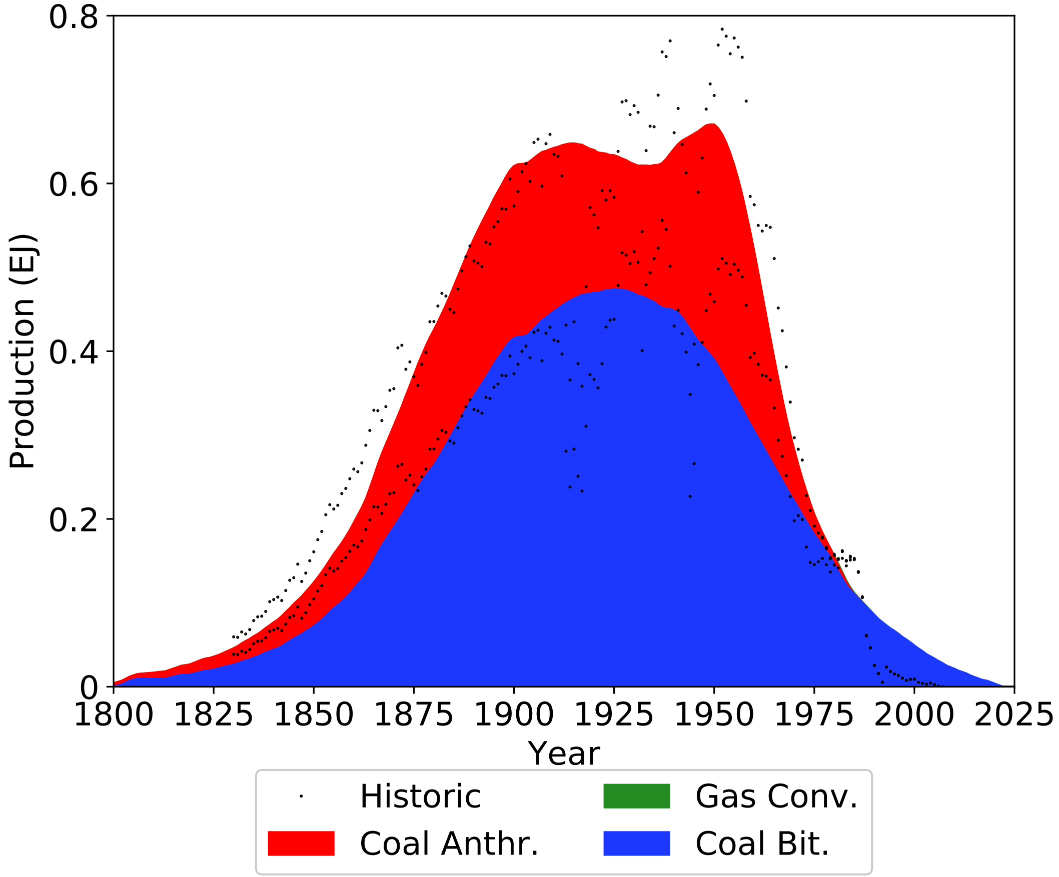

Figure 3.5: Belgium projections capped at 16

Table 3.5: Peak years - All

Name	URR	Peak Year	Peak Rate
Coal Bit.	45.5	1925	0.47
Coal Anthr.	21.6	1952	0.29
Gas Conv.	0.02	1982	–
**Total**	**67.12**	**1949**	**0.67**

3.3.2 By Mineral

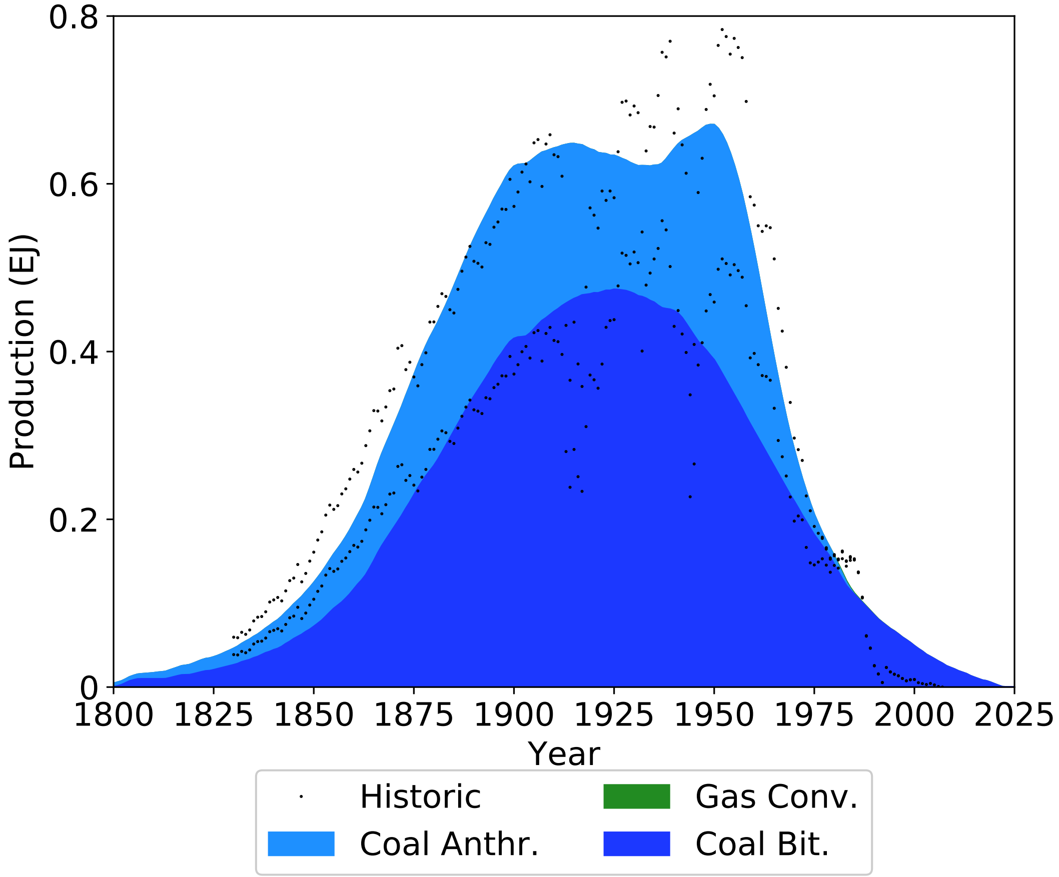

Figure 3.6: Belgium projection by mineral type

Table 3.6: Peak years - Minerals

Name	URR	Peak Year	Peak Rate
Coal Bit.	45.5	1925	0.47
Coal Anthr.	21.6	1952	0.29
Gas Conv.	0.02	1982	–
**Total**	**67.12**	**1949**	**0.67**

3.4 Bulgaria

3.4.1 All Projections

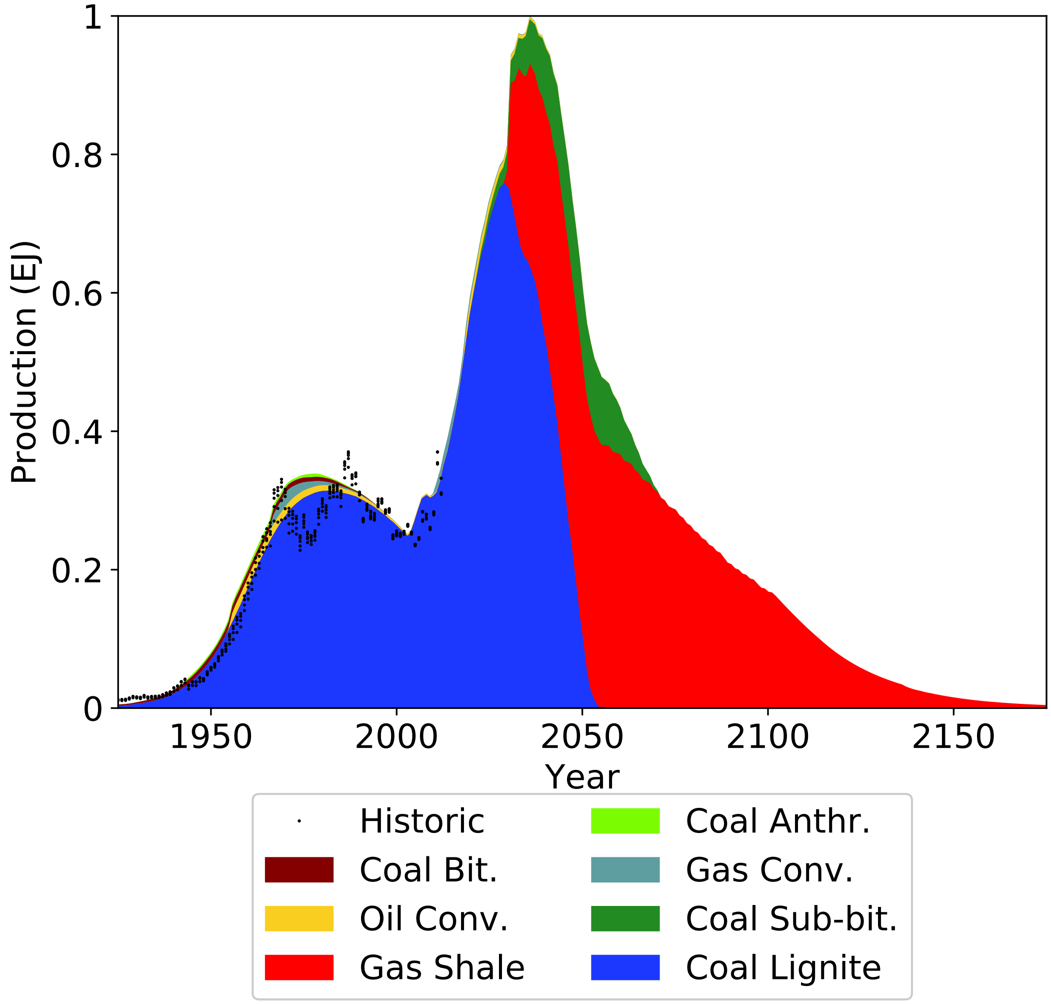

Figure 3.7: Bulgaria projections capped at 16

Table 3.7: Peak years - All

**Name**	**URR**	**Peak Year**	**Peak Rate**
Coal Lignite	36.3	2029	0.76
Gas Shale	24.29	2049	0.4
Coal Sub-bit.	3.1	2047	0.12
Oil Conv.	0.7	2020	0.02
Gas Conv.	0.41	1971	0.02
Coal Bit.	0.39	1957	0.01
Coal Anthr.	0.18	1964	0.01
**Total**	**65.37**	**2036**	**1.0**

3.4.2 By Mineral

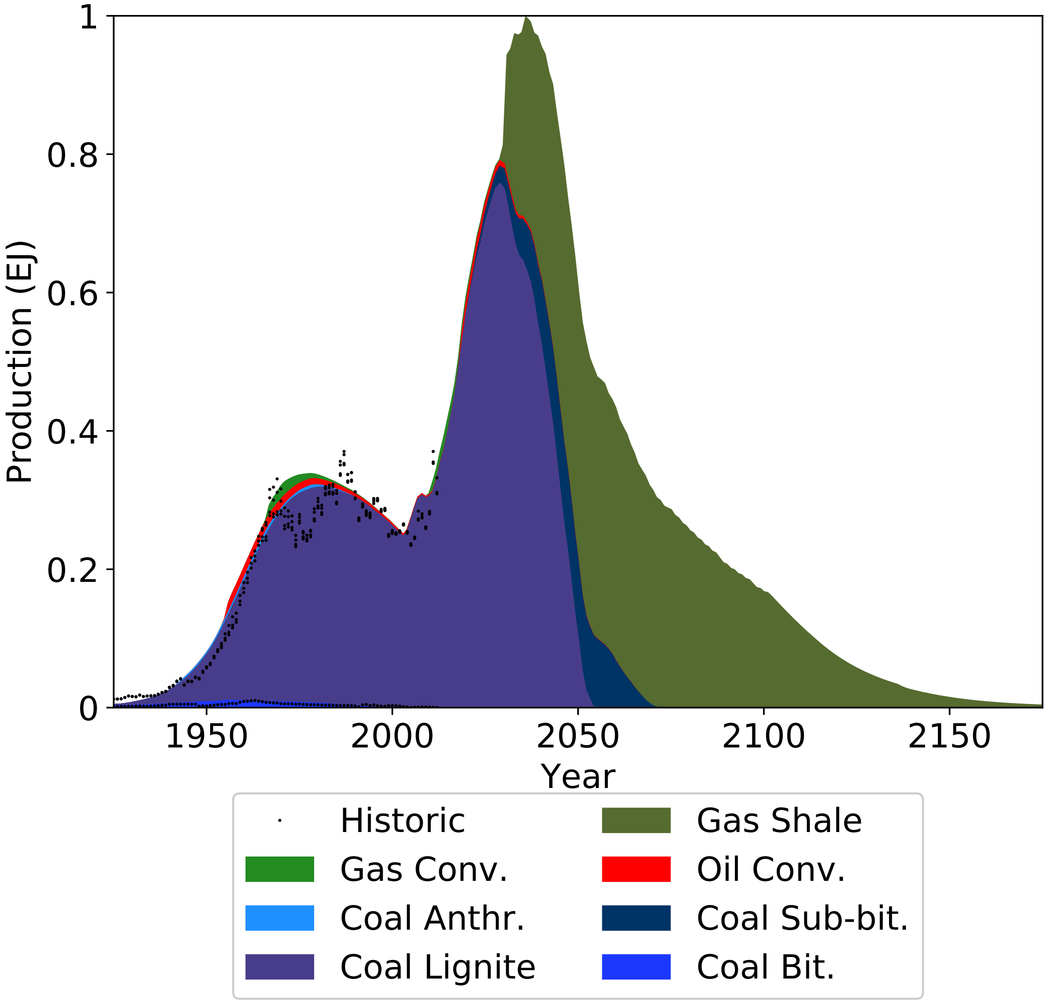

Figure 3.8: Bulgaria projection by mineral type

Table 3.8: Peak years - Minerals

**Name**	**URR**	**Peak Year**	**Peak Rate**
Coal Bit.	0.39	1957	0.01
Coal Lignite	36.3	2029	0.76
Coal Sub-bit.	3.1	2047	0.12
Coal Anthr.	0.18	1964	0.01
Oil Conv.	0.7	2020	0.02
Gas Conv.	0.41	1971	0.02
Gas Shale	24.29	2049	0.4
**Total**	**65.37**	**2036**	**1.0**

3.5 Cyprus

3.5.1 All Projections

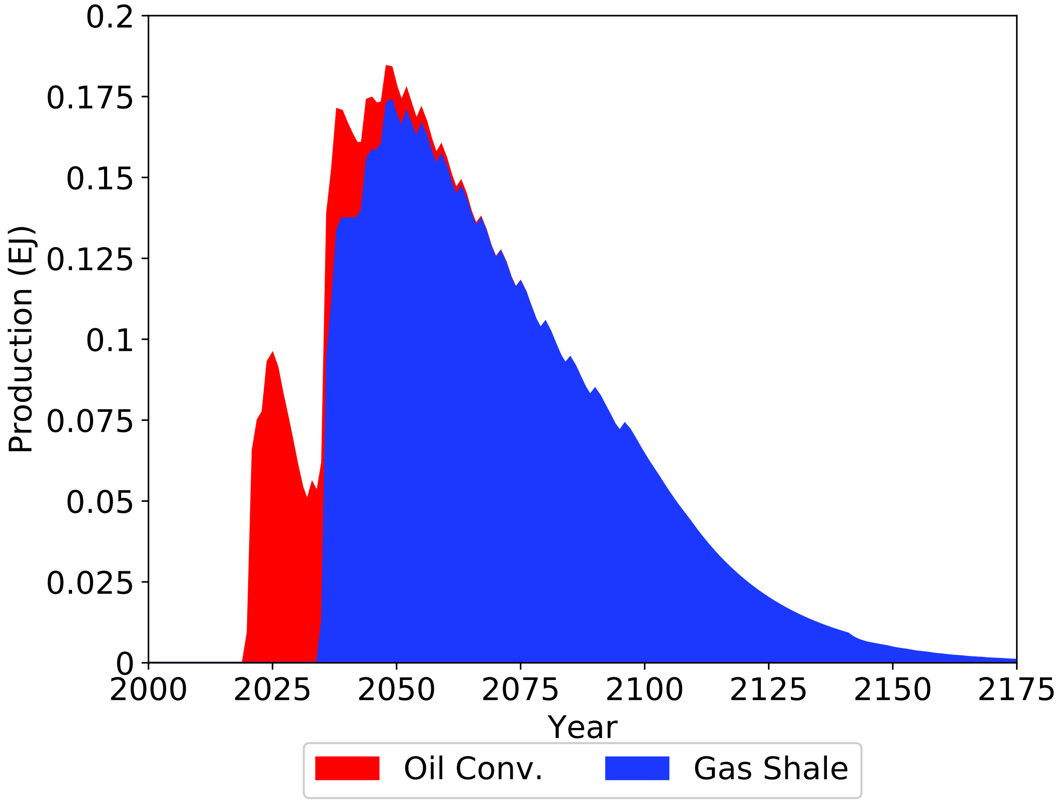

Figure 3.9: Cyprus projections capped at 16

Table 3.9: Peak years - All

Name	URR	Peak Year	Peak Rate
Gas Shale	9.27	2049	0.17
Oil Conv.	1.47	2025	0.1
**Total**	**10.74**	**2048**	**0.18**

3.5.2 By Mineral

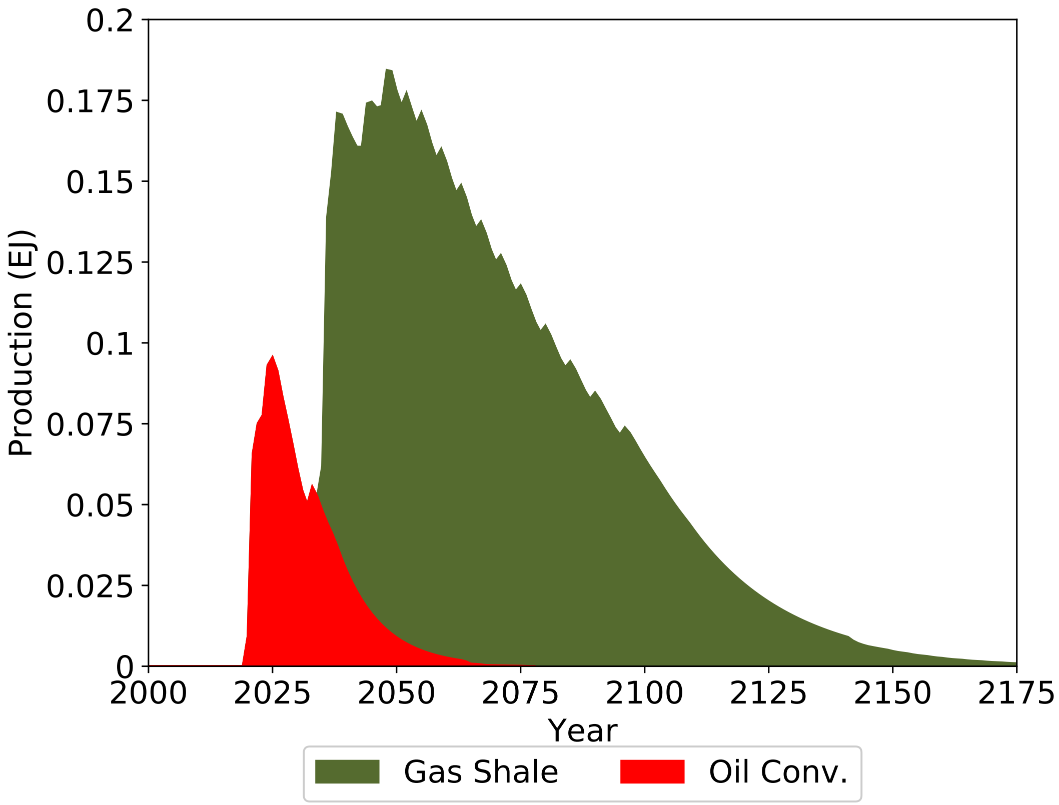

Figure 3.10: Cyprus projection by mineral type

Table 3.10: Peak years - Minerals

Name	URR	Peak Year	Peak Rate
Oil Conv.	1.47	2025	0.1
Gas Shale	9.27	2049	0.17
**Total**	**10.74**	**2048**	**0.18**

3.6 Czech Republic

3.6.1 All Projections

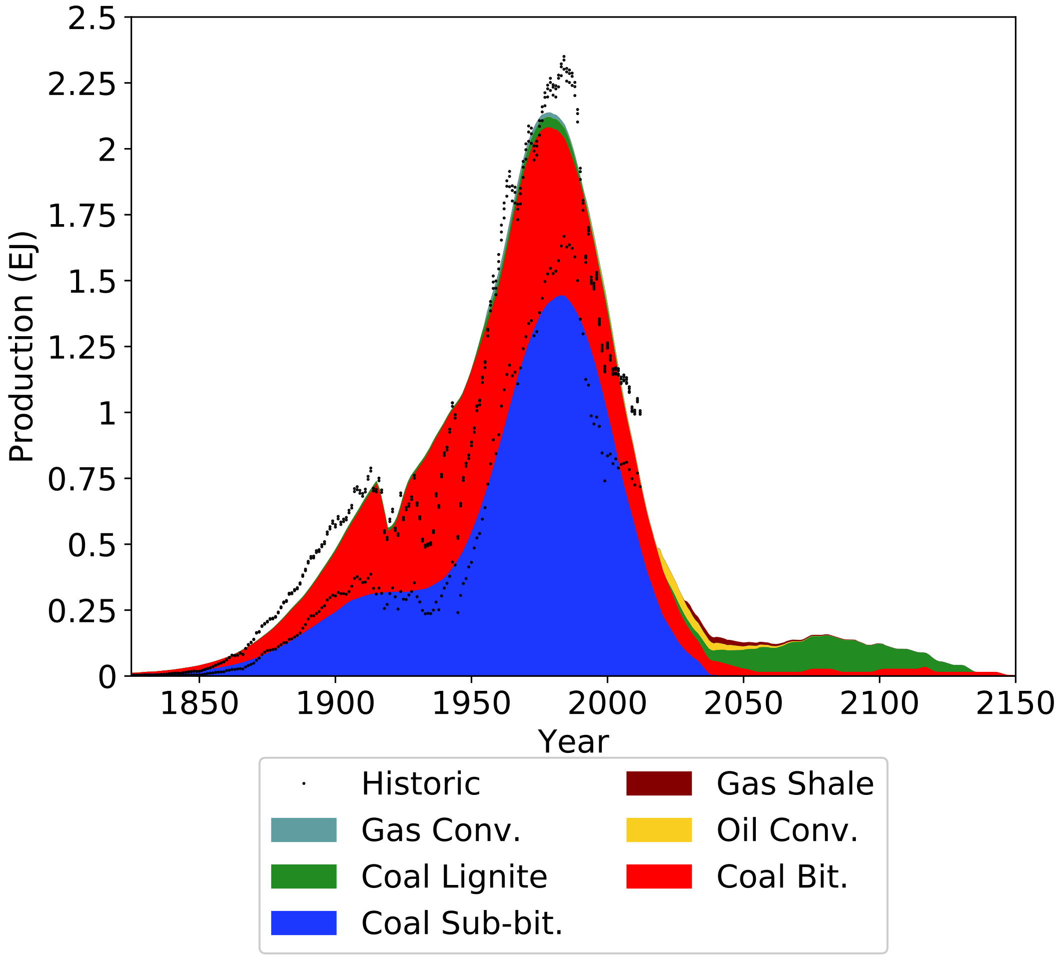

Figure 3.11: Czech Republic projections capped at 16

Table 3.11: Peak years - All

**Name**	**URR**	**Peak Year**	**Peak Rate**
Coal Sub-bit.	91.8	1983	1.44
Coal Bit.	64.86	1972	0.71
Coal Lignite	10.31	2080	0.13
Oil Conv.	1.53	2022	0.06
Gas Conv.	0.91	1968	0.03
Gas Shale	0.74	2030	0.02
**Total**	**170.15**	**1979**	**2.13**

3.6.2 By Mineral

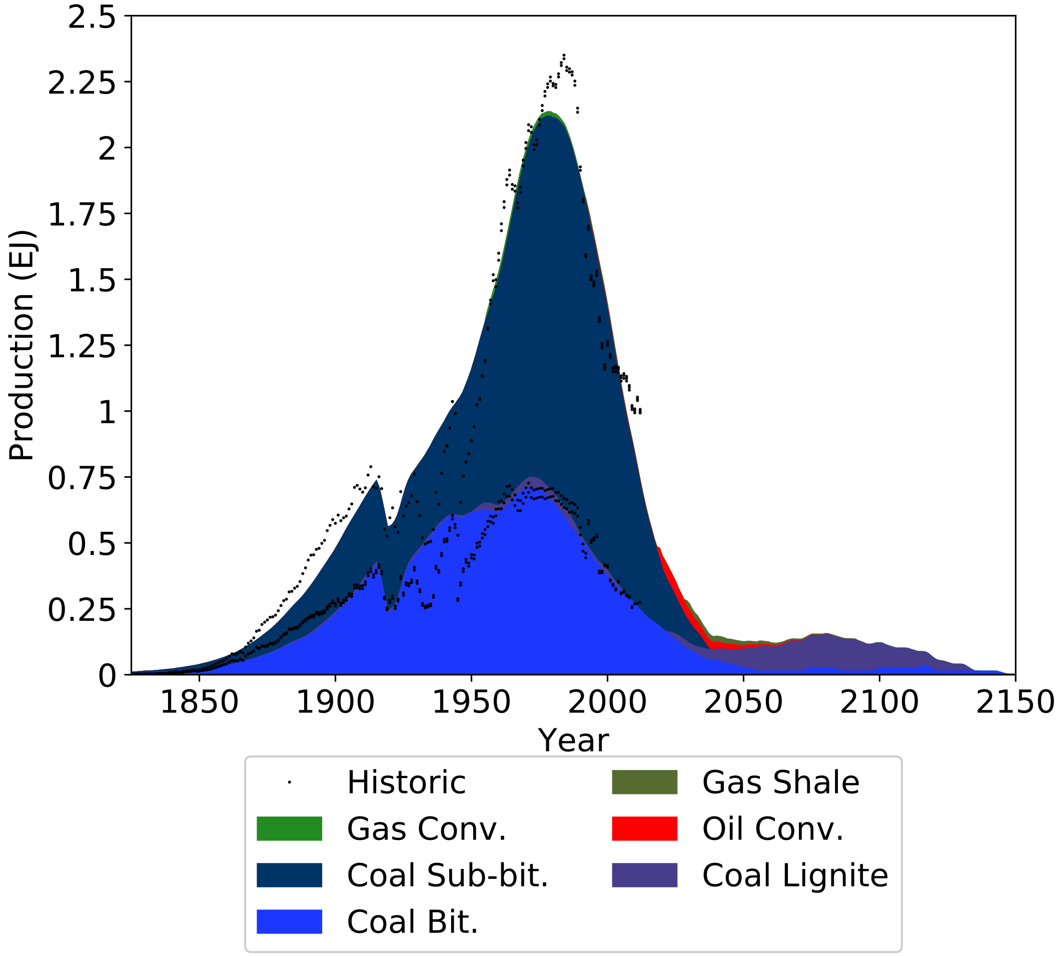

Figure 3.12: Czech Republic projection by mineral type

Table 3.12: Peak years - Minerals

**Name**	**URR**	**Peak Year**	**Peak Rate**
Coal Bit.	64.86	1972	0.71
Coal Lignite	10.31	2080	0.13
Coal Sub-bit.	91.8	1983	1.44
Oil Conv.	1.53	2022	0.06
Gas Conv.	0.91	1968	0.03
Gas Shale	0.74	2030	0.02
**Total**	**170.15**	**1979**	**2.13**

3.7 Denmark

3.7.1 All Projections

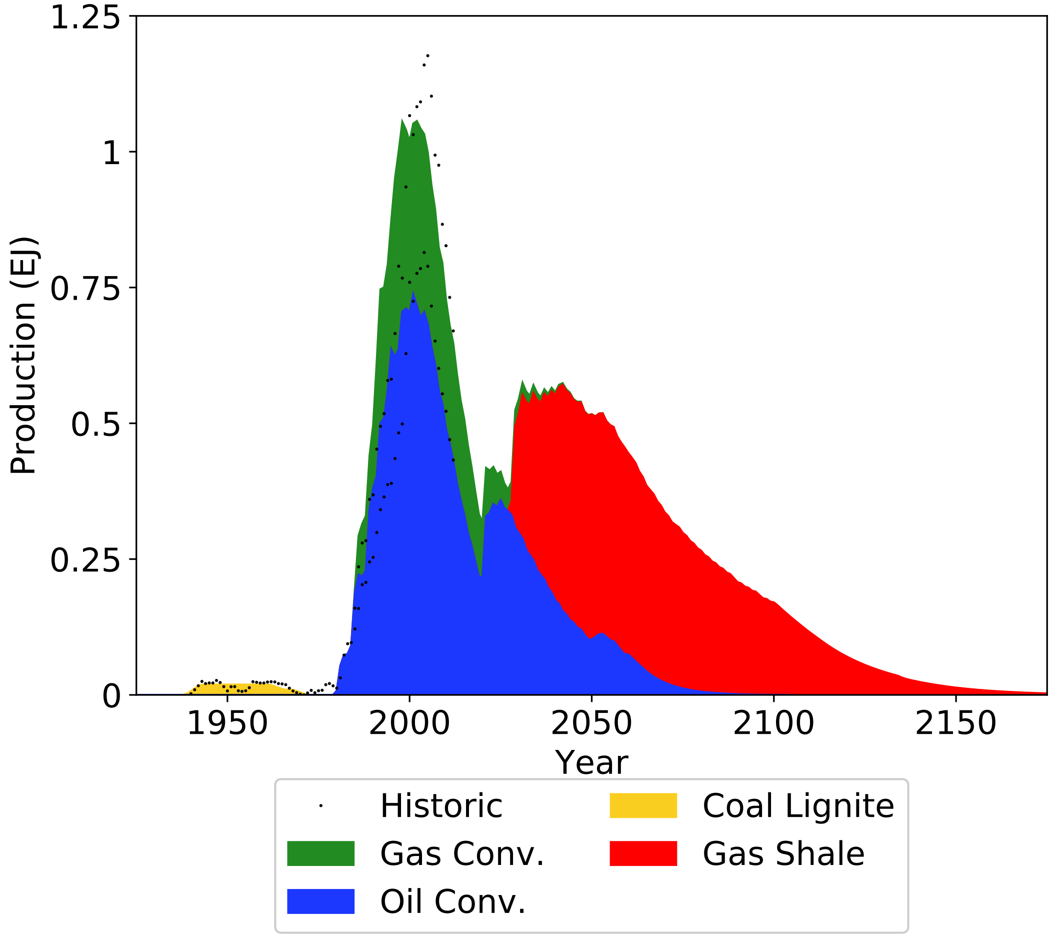

Figure 3.13: Denmark projections capped at 16

Table 3.13: Peak years - All

**Name**	**URR**	**Peak Year**	**Peak Rate**
Oil Conv.	25.92	2001	0.74
Gas Shale	25.62	2047	0.42
Gas Conv.	8.67	1997	0.37
Coal Lignite	0.5	1944	0.02
**Total**	**60.71**	**2002**	**1.06**

3.7.2 By Mineral

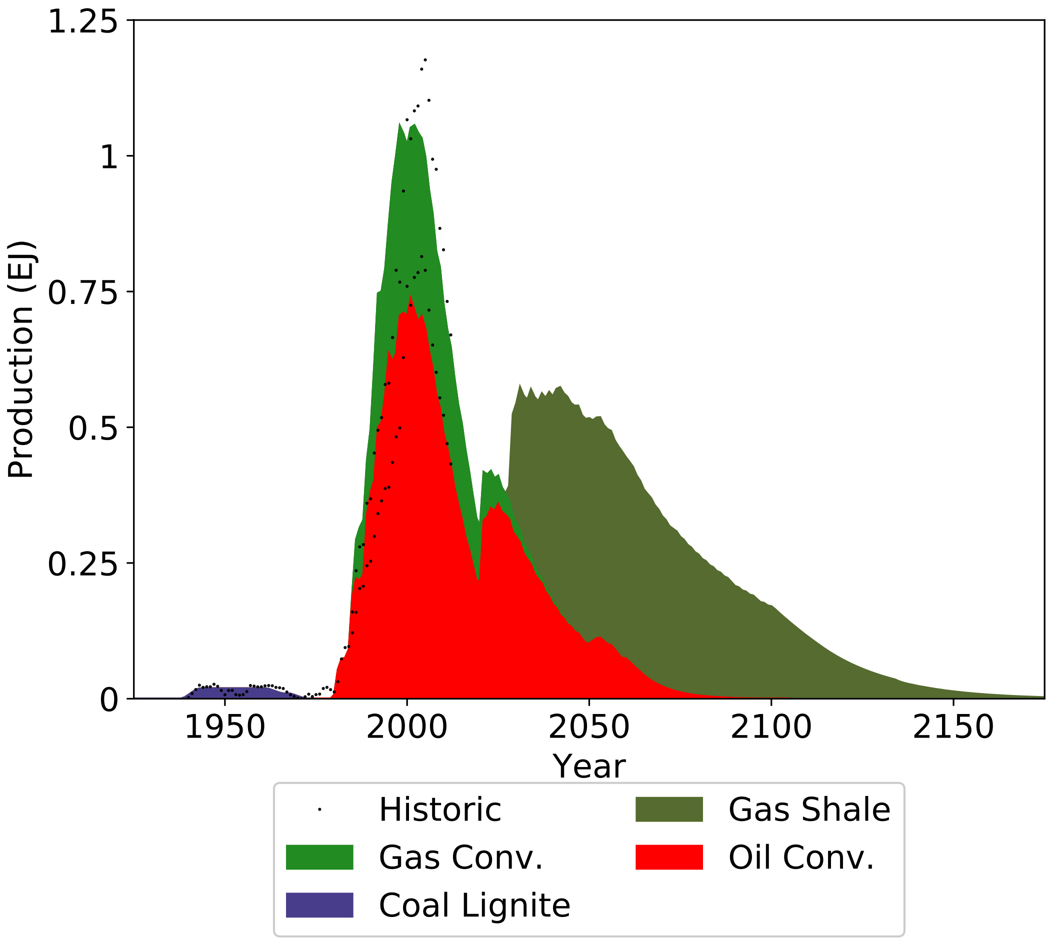

Figure 3.14: Denmark projection by mineral type

Table 3.14: Peak years - Minerals

Name	URR	Peak Year	Peak Rate
Coal Lignite	0.5	1944	0.02
Oil Conv.	25.92	2001	0.74
Gas Conv.	8.67	1997	0.37
Gas Shale	25.62	2047	0.42
**Total**	**60.71**	**2002**	**1.06**

3.8 France

3.8.1 All Projections

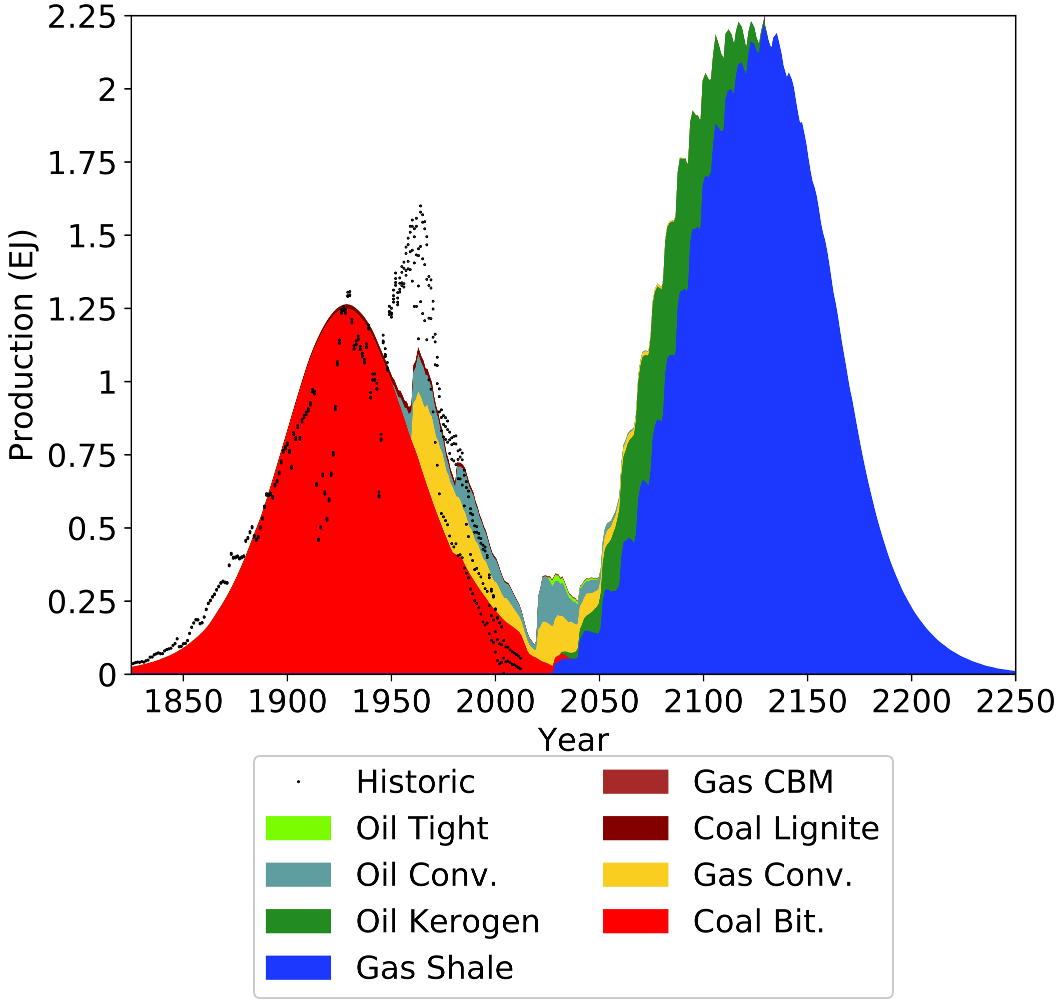

Figure 3.15: France projections capped at 16

Table 3.15: Peak years - All

**Name**	**URR**	**Peak Year**	**Peak Rate**
Gas Shale	189.04	2129	2.21
Coal Bit.	105.4	1928	1.25
Oil Kerogen	24.1	2087	0.46
Gas Conv.	12.7	1969	0.28
Oil Conv.	7.95	2024	0.15
Coal Lignite	1.8	1956	0.02
Oil Tight	0.3	2028	0.02
Gas CBM	0.15	2022	–
**Total**	**341.44**	**2129**	**2.22**

3.8.2 By Mineral

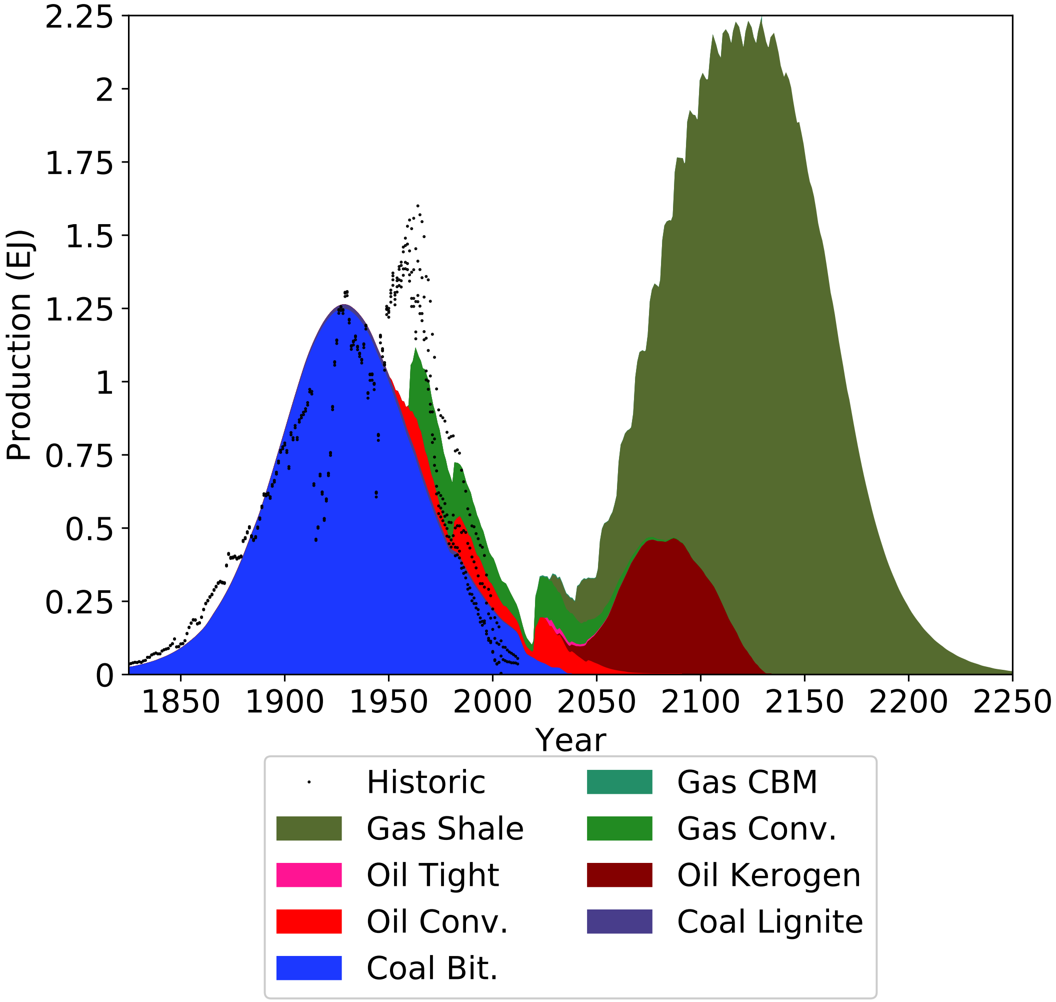

Figure 3.16: France projection by mineral type

Table 3.16: Peak years - Minerals

**Name**	**URR**	**Peak Year**	**Peak Rate**
Coal Bit.	105.4	1928	1.25
Coal Lignite	1.8	1956	0.02
Oil Conv.	7.95	2024	0.15
Oil Kerogen	24.1	2087	0.46
Oil Tight	0.3	2028	0.02
Gas Conv.	12.7	1969	0.28
Gas Shale	189.04	2129	2.21
Gas CBM	0.15	2022	–
**Total**	**341.43**	**2129**	**2.22**

3.9 Germany

3.9.1 All Projections

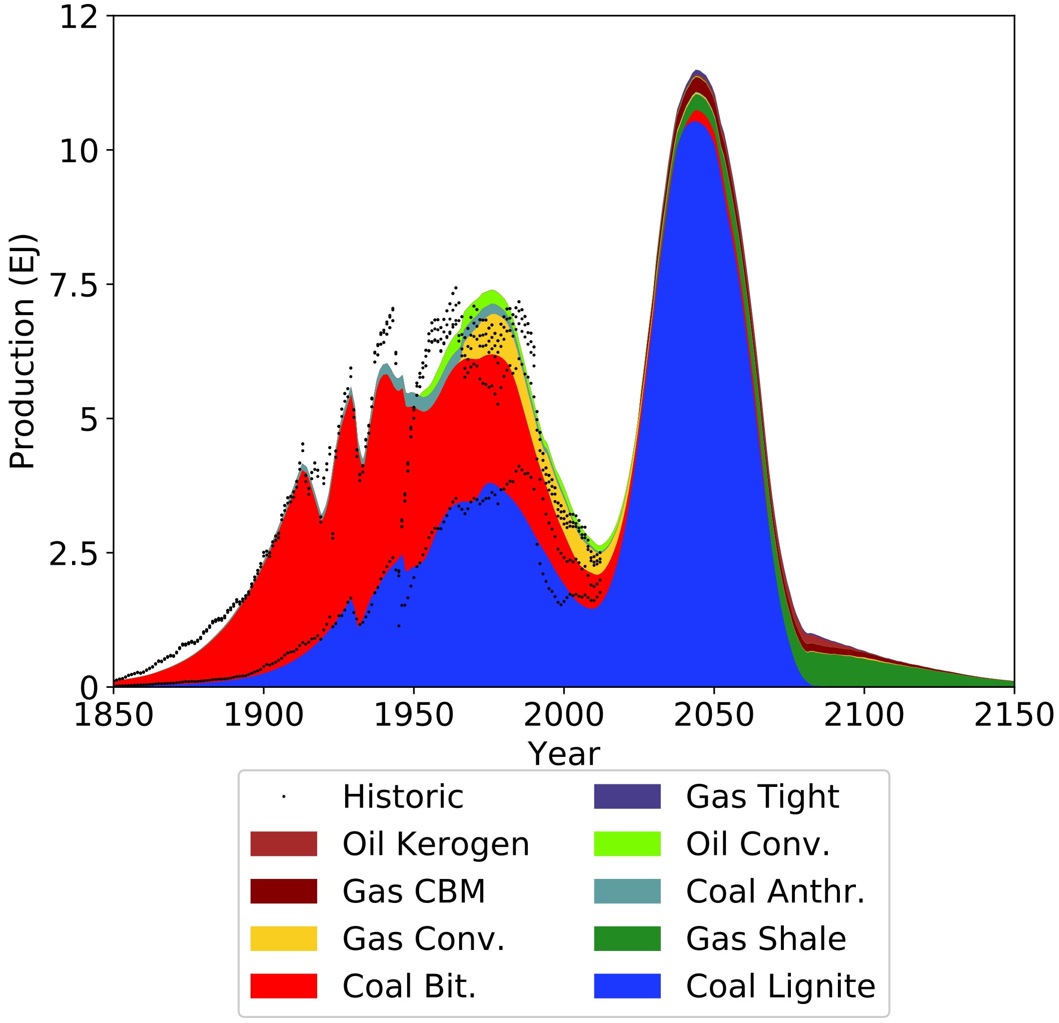

Figure 3.17: Germany projections capped at 16

Table 3.17: Peak years - All

**Name**	**URR**	**Peak Year**	**Peak Rate**
Coal Lignite	627.4	2044	10.52
Coal Bit.	316.3	1939	3.8
Gas Shale	48.2	2083	0.63
Gas Conv.	33.0	1977	0.76
Coal Anthr.	18.9	1955	0.27
Gas CBM	16.7	2040	0.29
Oil Conv.	14.48	1966	0.33
Oil Kerogen	6.97	2067	0.18
Gas Tight	3.71	2044	0.09
**Total**	**1085.66**	**2044**	**11.48**

3.9.2 By Mineral

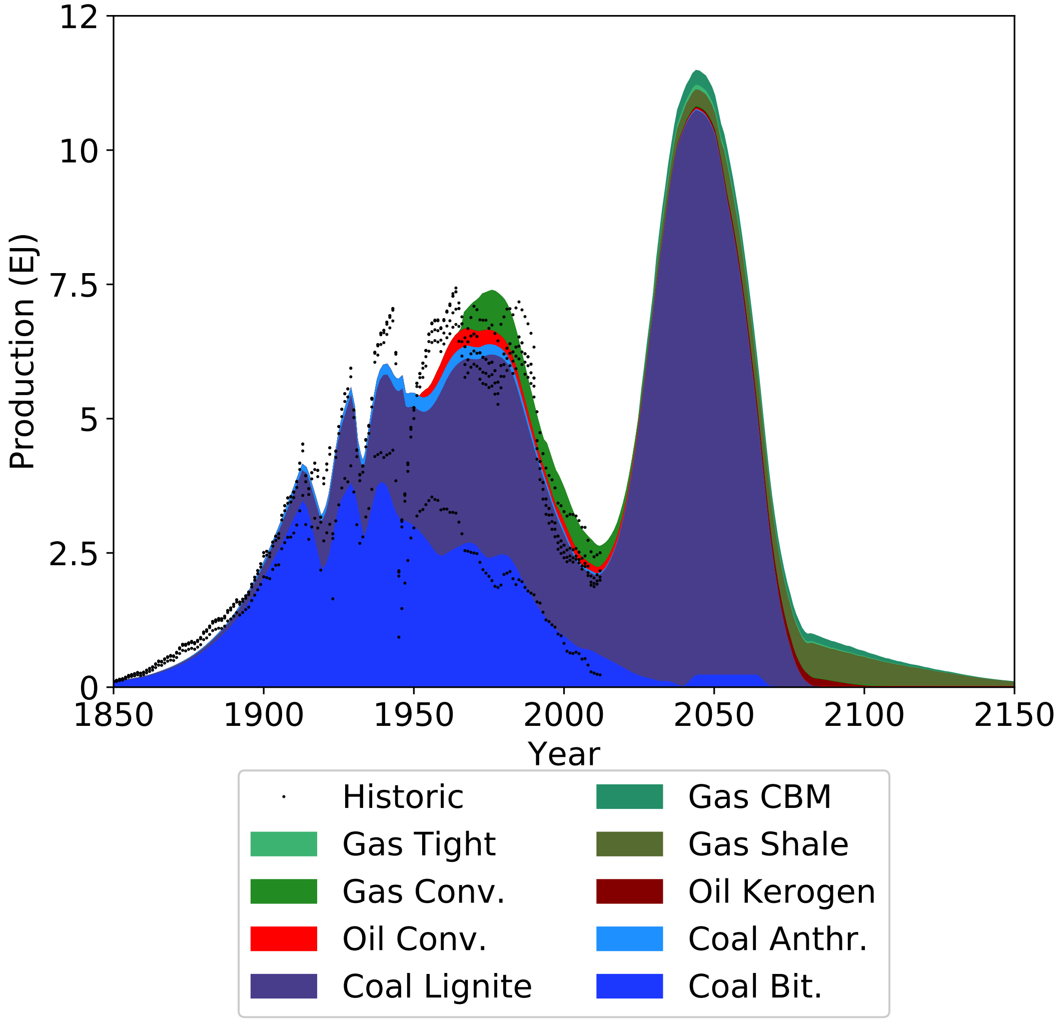

Figure 3.18: Germany projection by mineral type

Table 3.18: Peak years - Minerals

**Name**	**URR**	**Peak Year**	**Peak Rate**
Coal Bit.	316.3	1939	3.8
Coal Lignite	627.4	2044	10.52
Coal Anthr.	18.9	1955	0.27
Oil Conv.	14.48	1966	0.33
Oil Kerogen	6.97	2067	0.18
Gas Conv.	33.0	1977	0.76
Gas Shale	48.2	2083	0.63
Gas Tight	3.71	2044	0.09
Gas CBM	16.7	2040	0.29
**Total**	**1085.66**	**2044**	**11.48**

3.10 Greece

3.10.1 All Projections

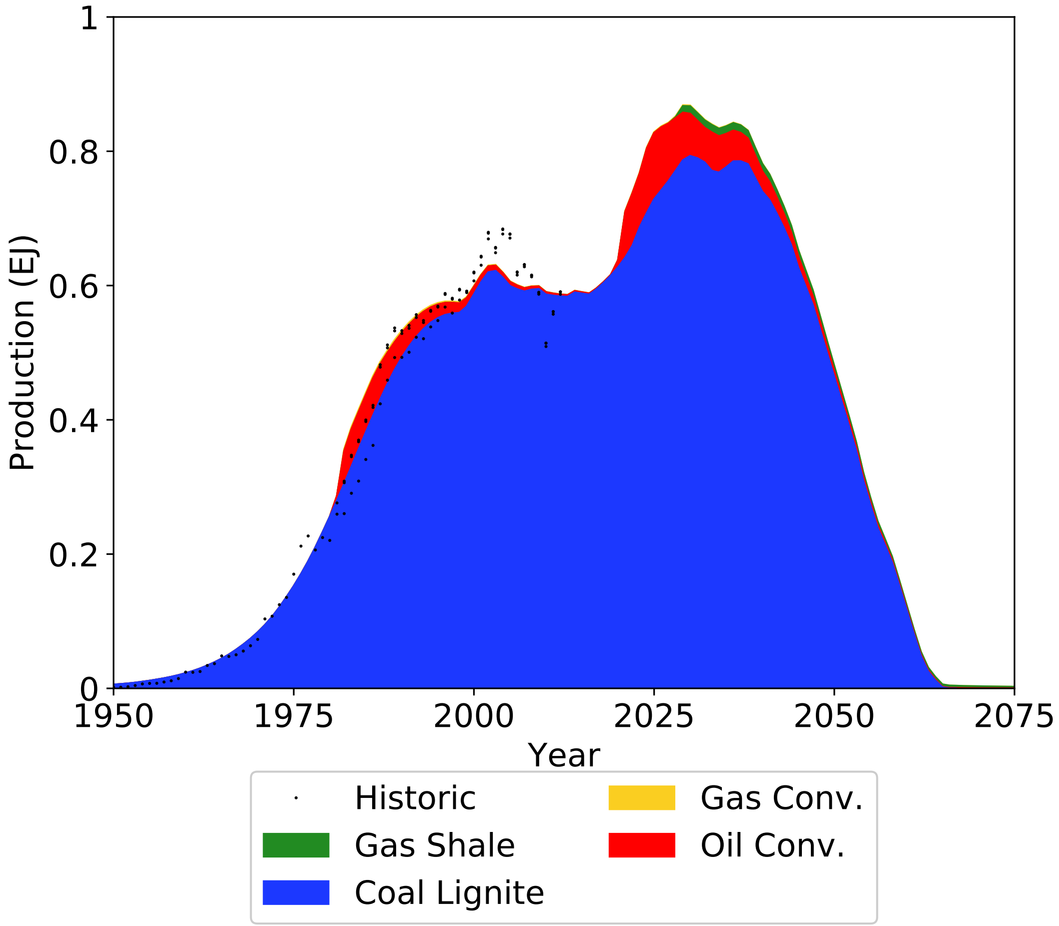

Figure 3.19: Greece projections capped at 16

Table 3.19: Peak years - All

**Name**	**URR**	**Peak Year**	**Peak Rate**
Coal Lignite	47.64	2030	0.79
Oil Conv.	2.25	2025	0.1
Gas Shale	0.37	2030	0.01
Gas Conv.	0.09	1983	–
**Total**	**50.35**	**2029**	**0.87**

3.10.2 By Mineral

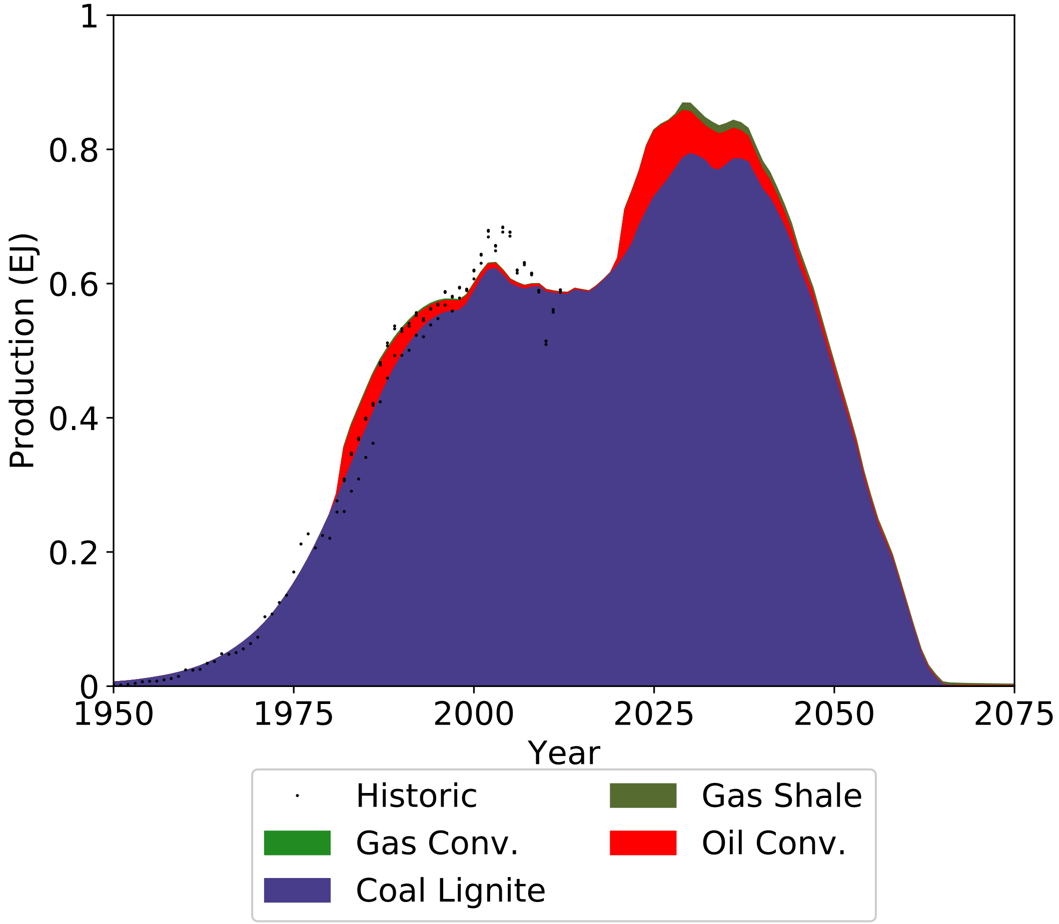

Figure 3.20: Greece projection by mineral type

Table 3.20: Peak years - Minerals

**Name**	**URR**	**Peak Year**	**Peak Rate**
Coal Lignite	47.64	2030	0.79
Oil Conv.	2.25	2025	0.1
Gas Conv.	0.09	1983	–
Gas Shale	0.37	2030	0.01
**Total**	**50.35**	**2029**	**0.87**

3.11 Greenland

3.11.1 All Projections

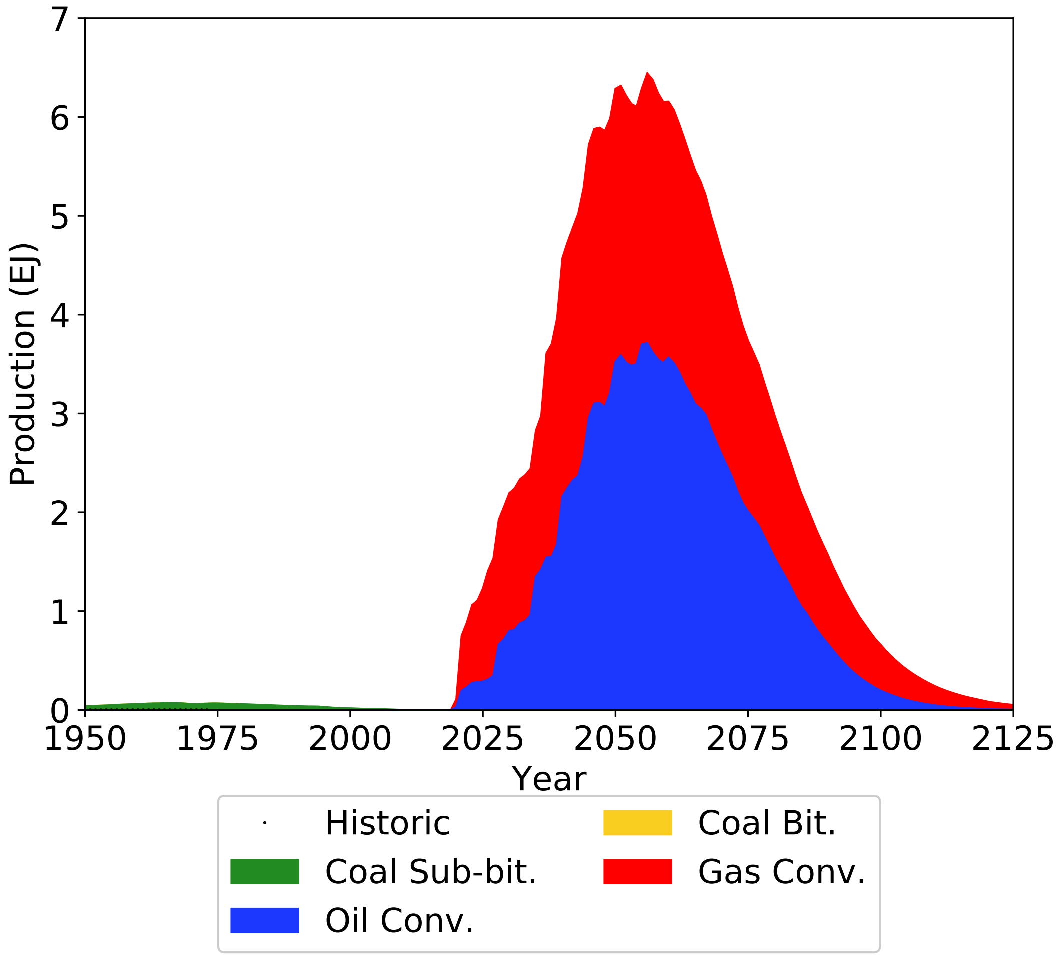

Figure 3.21: Greenland projections capped at 16

Table 3.21: Peak years - All

**Name**	**URR**	**Peak Year**	**Peak Rate**
Oil Conv.	147.4	2056	3.71
Gas Conv.	144.6	2048	2.8
Coal Sub-bit.	3.0	1966	0.07
Coal Bit.	0.02	1952	–
**Total**	**295.02**	**2056**	**6.44**

3.11.2 By Mineral

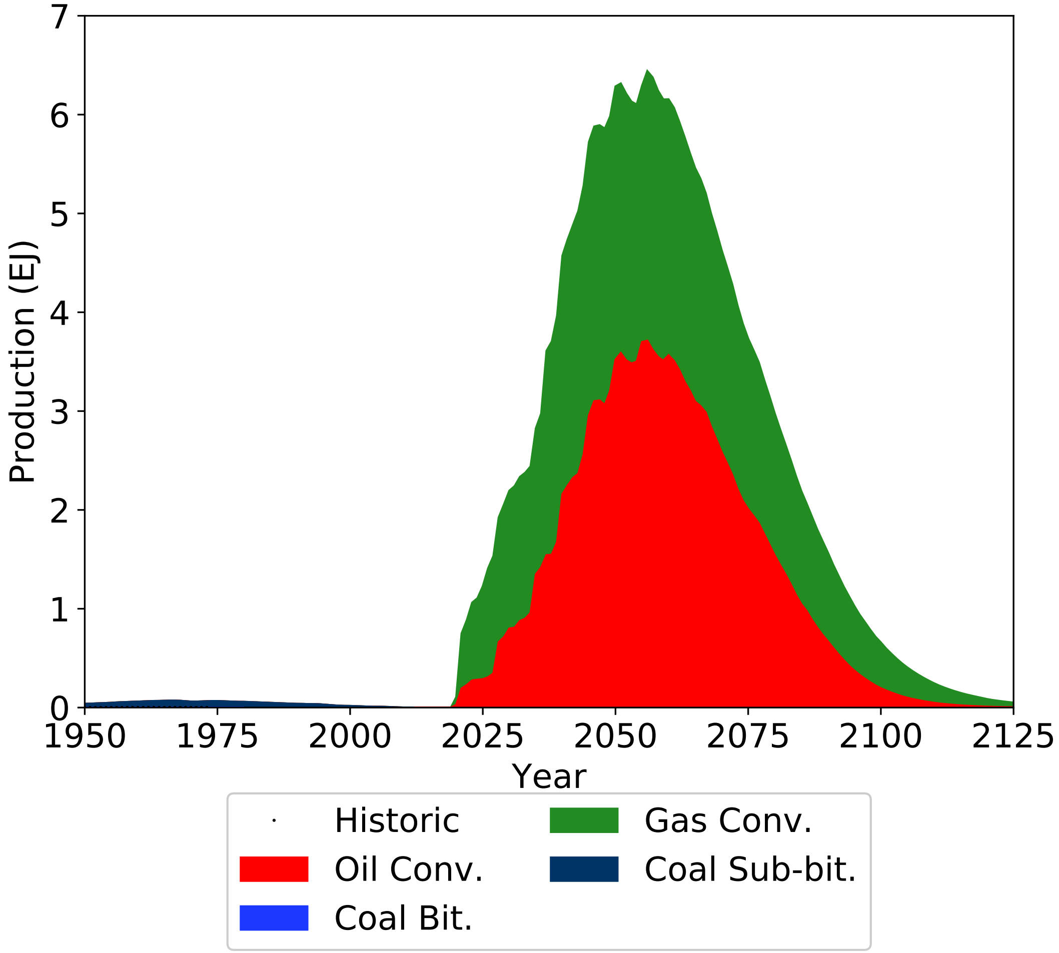

Figure 3.22: Greenland projection by mineral type

Table 3.22: Peak years - Minerals

**Name**	**URR**	**Peak Year**	**Peak Rate**
Coal Bit.	0.02	1952	–
Coal Sub-bit.	3.0	1966	0.07
Oil Conv.	147.4	2056	3.71
Gas Conv.	144.6	2048	2.8
**Total**	**295.02**	**2056**	**6.44**

3.12 Hungary

3.12.1 All Projections

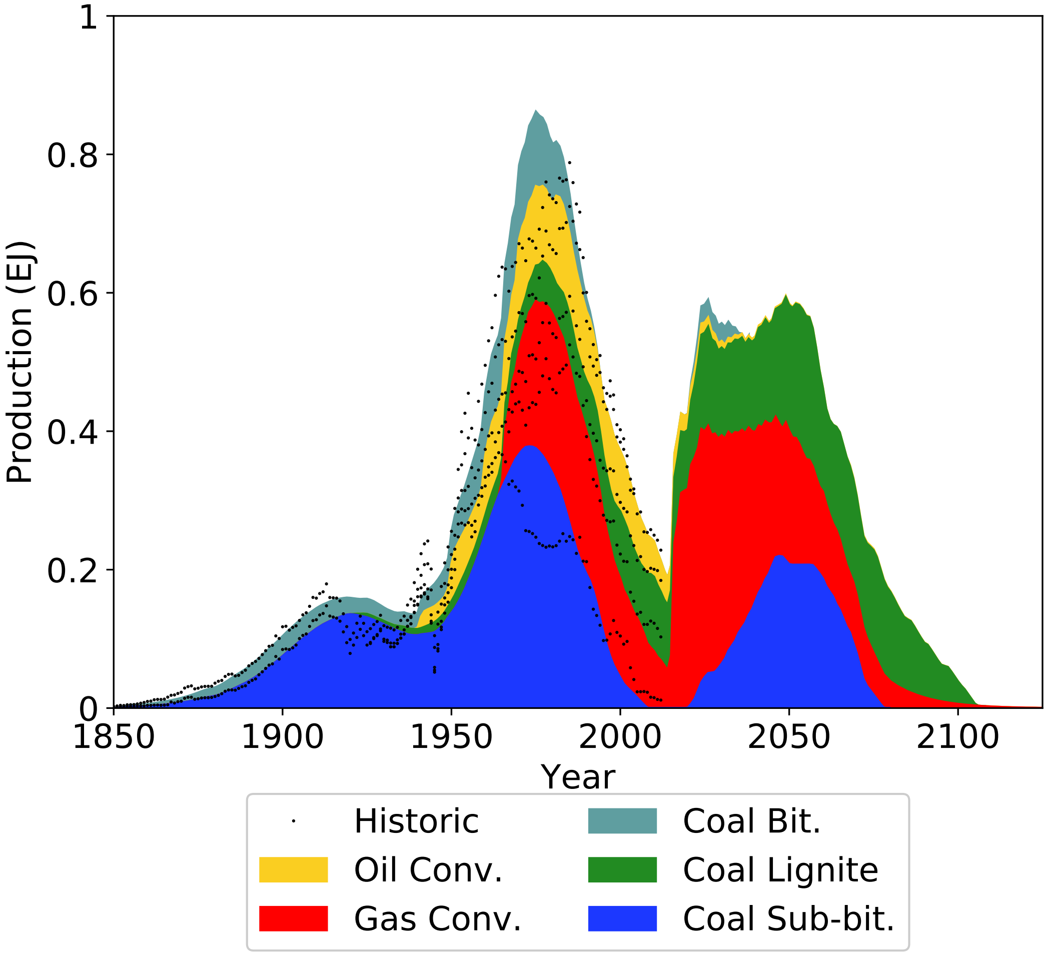

Figure 3.23: Hungary projections capped at 16

Table 3.23: Peak years - All

**Name**	**URR**	**Peak Year**	**Peak Rate**
Coal Sub-bit.	26.6	1973	0.38
Gas Conv.	21.4	2024	0.37
Coal Lignite	15.37	2055	0.21
Oil Conv.	6.36	1982	0.13
Coal Bit.	5.5	1968	0.11
**Total**	**75.23**	**1975**	**0.86**

3.12.2 By Mineral

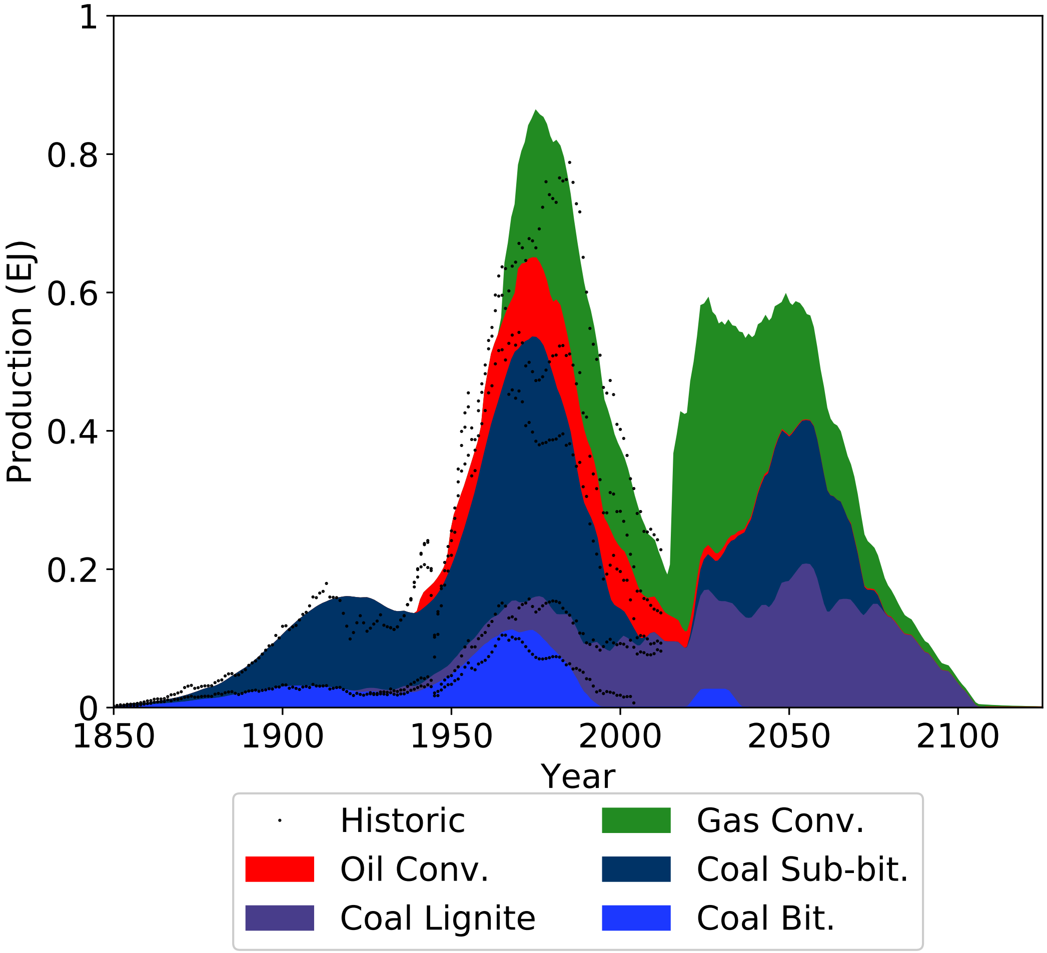

Figure 3.24: Hungary projection by mineral type

Table 3.24: Peak years - Minerals

**Name**	**URR**	**Peak Year**	**Peak Rate**
Coal Bit.	5.5	1968	0.11
Coal Lignite	15.37	2055	0.21
Coal Sub-bit.	26.6	1973	0.38
Oil Conv.	6.36	1982	0.13
Gas Conv.	21.4	2024	0.37
**Total**	**75.23**	**1975**	**0.86**

3.13 Ireland

3.13.1 All Projections

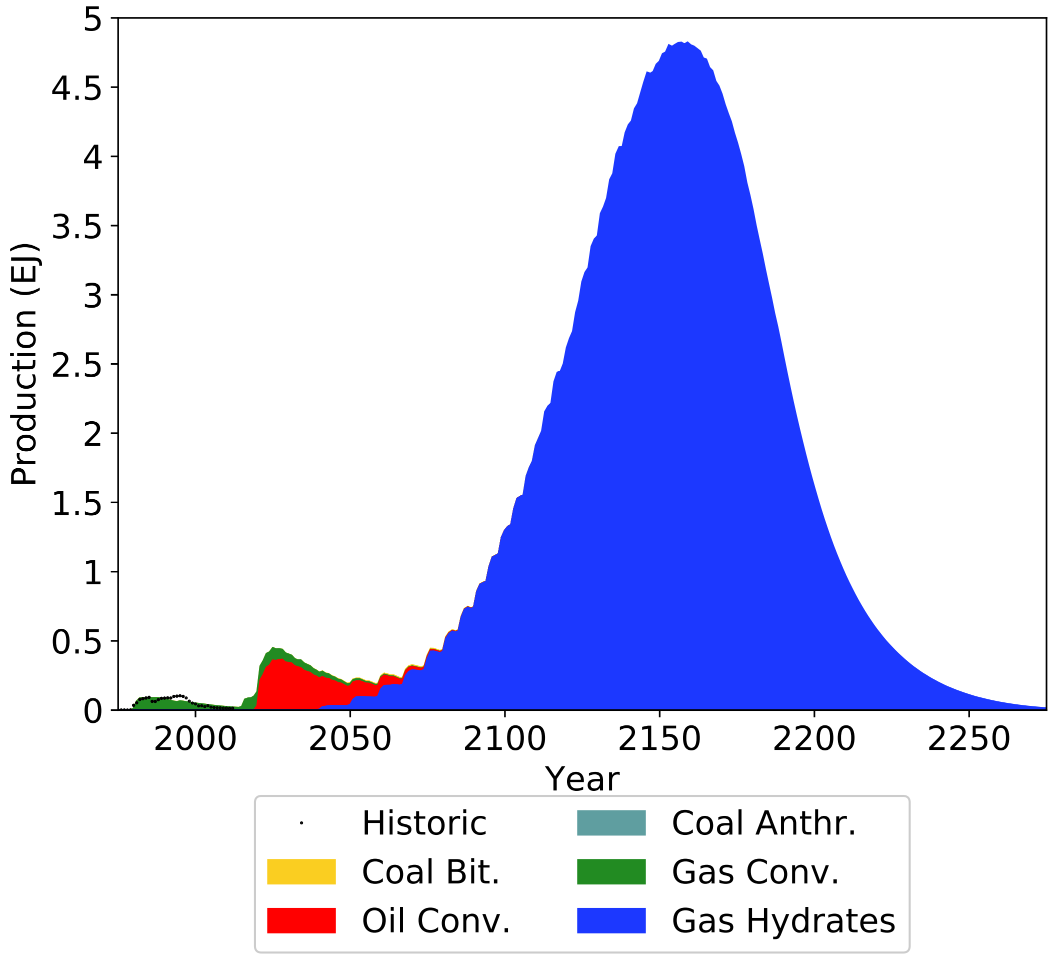

Figure 3.25: Ireland projections capped at 16

Table 3.25: Peak years - All

**Name**	**URR**	**Peak Year**	**Peak Rate**
Gas Hydrates	392.3	2159	4.82
Oil Conv.	9.44	2028	0.36
Gas Conv.	4.1	2020	0.1
Coal Bit.	0.45	2068	0.01
Coal Anthr.	0.27	1882	–
**Total**	**406.56**	**2159**	**4.82**

3.13.2 By Mineral

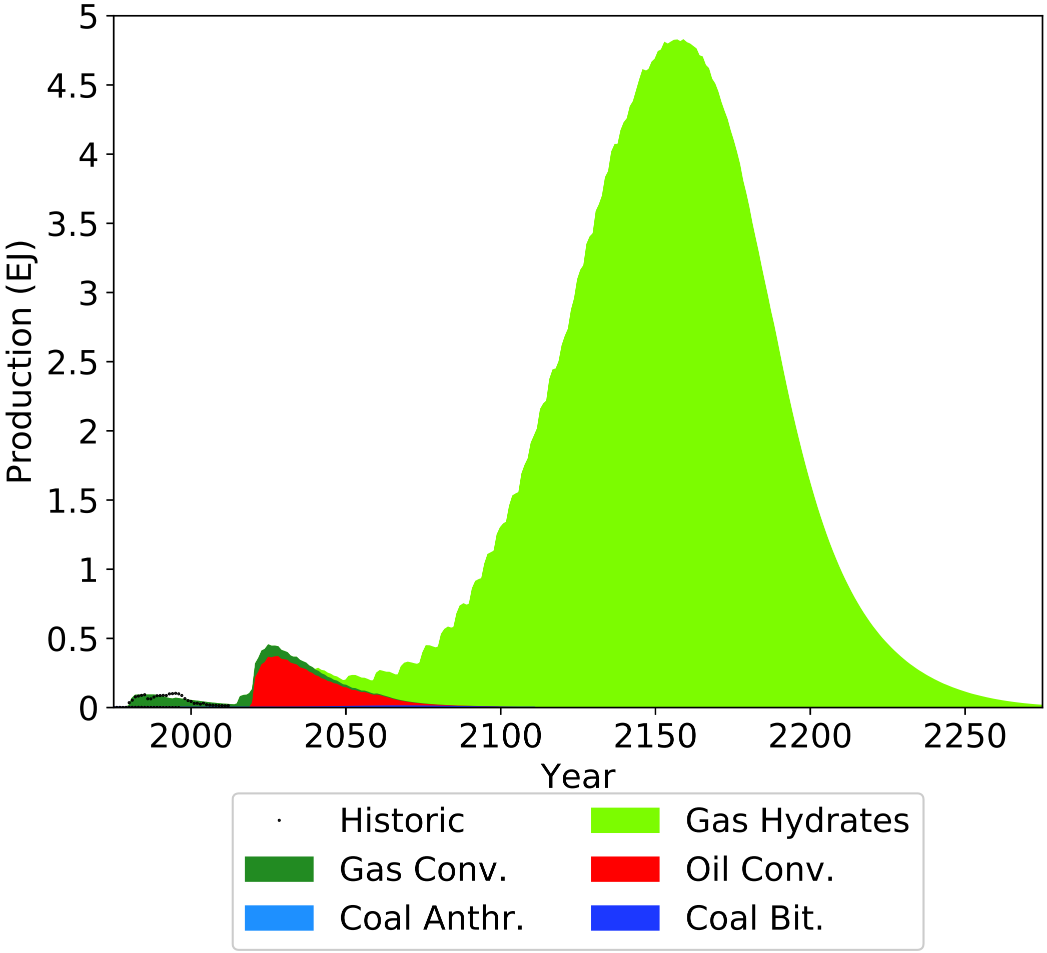

Figure 3.26: Ireland projection by mineral type

Table 3.26: Peak years - Minerals

**Name**	**URR**	**Peak Year**	**Peak Rate**
Coal Bit.	0.45	2068	0.01
Coal Anthr.	0.27	1882	–
Oil Conv.	9.44	2028	0.36
Gas Conv.	4.1	2020	0.1
Gas Hydrates	392.3	2159	4.82
**Total**	**406.56**	**2159**	**4.82**

3.14 Italy

3.14.1 All Projections

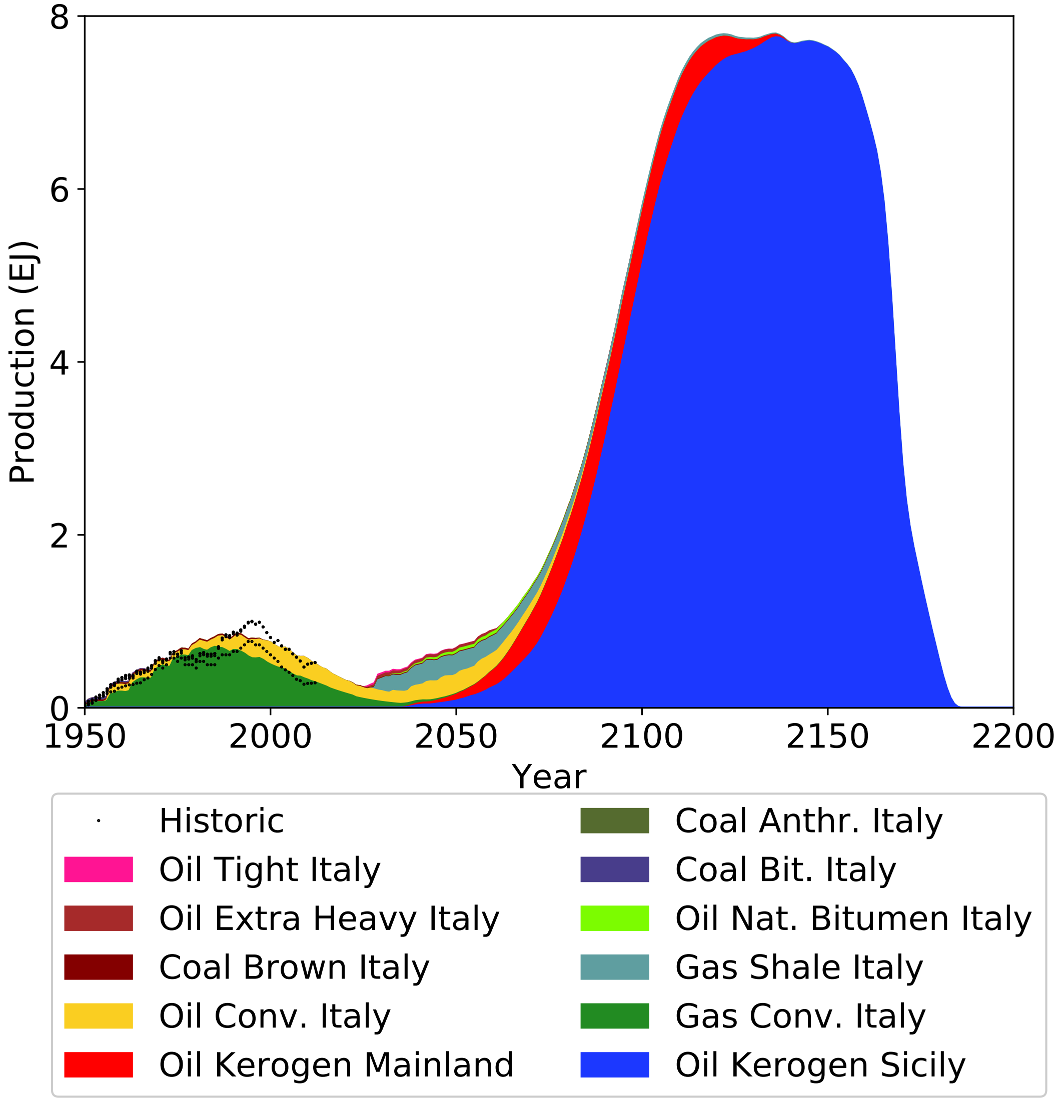

Figure 3.27: Italy projections capped at 16

Table 3.27: Peak years - All

**Name**	**URR**	**Peak Year**	**Peak Rate**
Oil Kerogen Sicily	584.5	2136	7.75
Oil Kerogen Mainland	34.4	2089	0.64
Gas Conv. Italy	31.0	1985	0.71
Oil Conv. Italy	19.09	2004	0.25
Gas Shale Italy	13.57	2042	0.24
Coal Brown Italy	2.17	1917	0.02
Oil Nat. Bitumen Italy	1.26	2060	0.04
Oil Extra Heavy Italy	1.1	2030	0.03
Coal Bit. Italy	0.9	1945	0.03
Oil Tight Italy	0.34	2028	0.02
Coal Anthr. Italy	0.08	1937	–
**Total**	**688.4**	**2136**	**7.8**

3.14.2 By Mineral

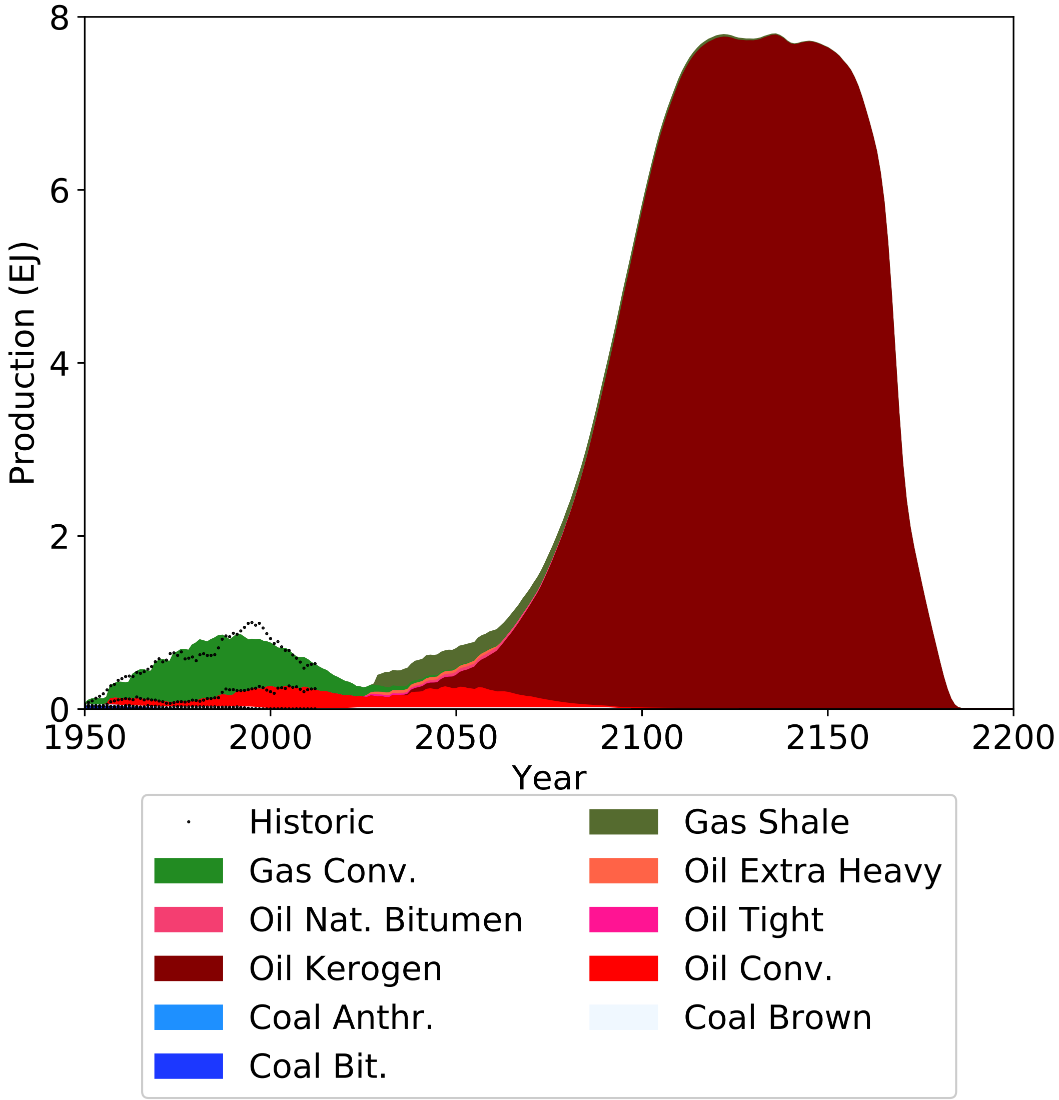

Figure 3.28: Italy projection by mineral type

3.14.3 Regional Projections

Table 3.28: Peak years - Minerals

**Name**	**URR**	**Peak Year**	**Peak Rate**
Coal Bit.	0.9	1945	0.03
Coal Brown	2.17	1917	0.02
Coal Anthr.	0.08	1937	–
Oil Conv.	19.09	2004	0.25
Oil Kerogen	618.9	2136	7.78
Oil Tight	0.34	2028	0.02
Oil Nat. Bitumen	1.26	2060	0.04
Oil Extra Heavy	1.1	2030	0.03
Gas Conv.	31.0	1985	0.71
Gas Shale	13.57	2042	0.24
**Total**	**688.4**	**2136**	**7.8**

Italy

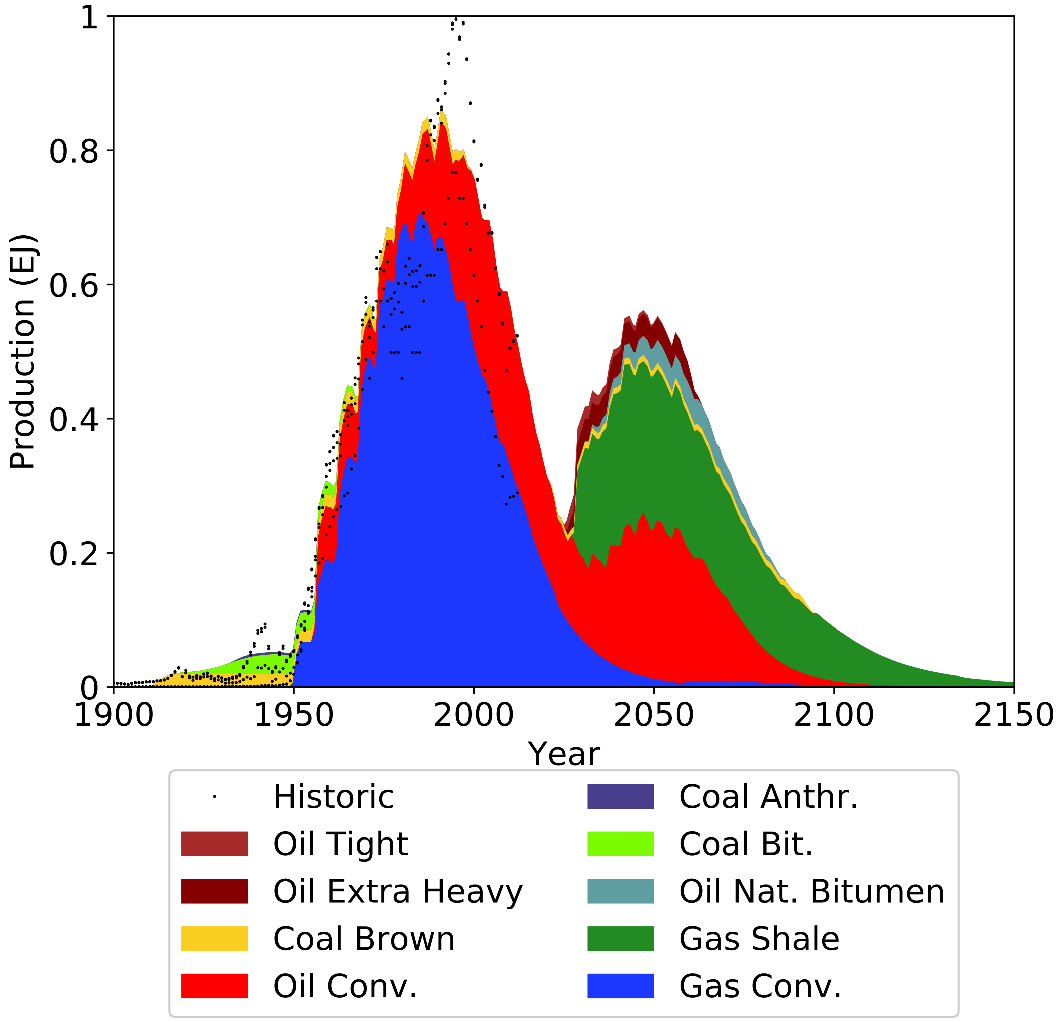

Figure 3.29: Italy - Italy projections capped at 16

Table 3.29: Peak years - All

**Name**	**URR**	**Peak Year**	**Peak Rate**
Gas Conv. Italy	31.0	1985	0.71
Oil Conv. Italy	19.09	2004	0.25
Gas Shale Italy	13.57	2042	0.24
Coal Brown Italy	2.17	1917	0.02
Oil Nat. Bitumen Italy	1.26	2060	0.04
Oil Extra Heavy Italy	1.1	2030	0.03
Coal Bit. Italy	0.9	1945	0.03
Oil Tight Italy	0.34	2028	0.02
Coal Anthr. Italy	0.08	1937	–
**Total**	**69.5**	**1991**	**0.86**

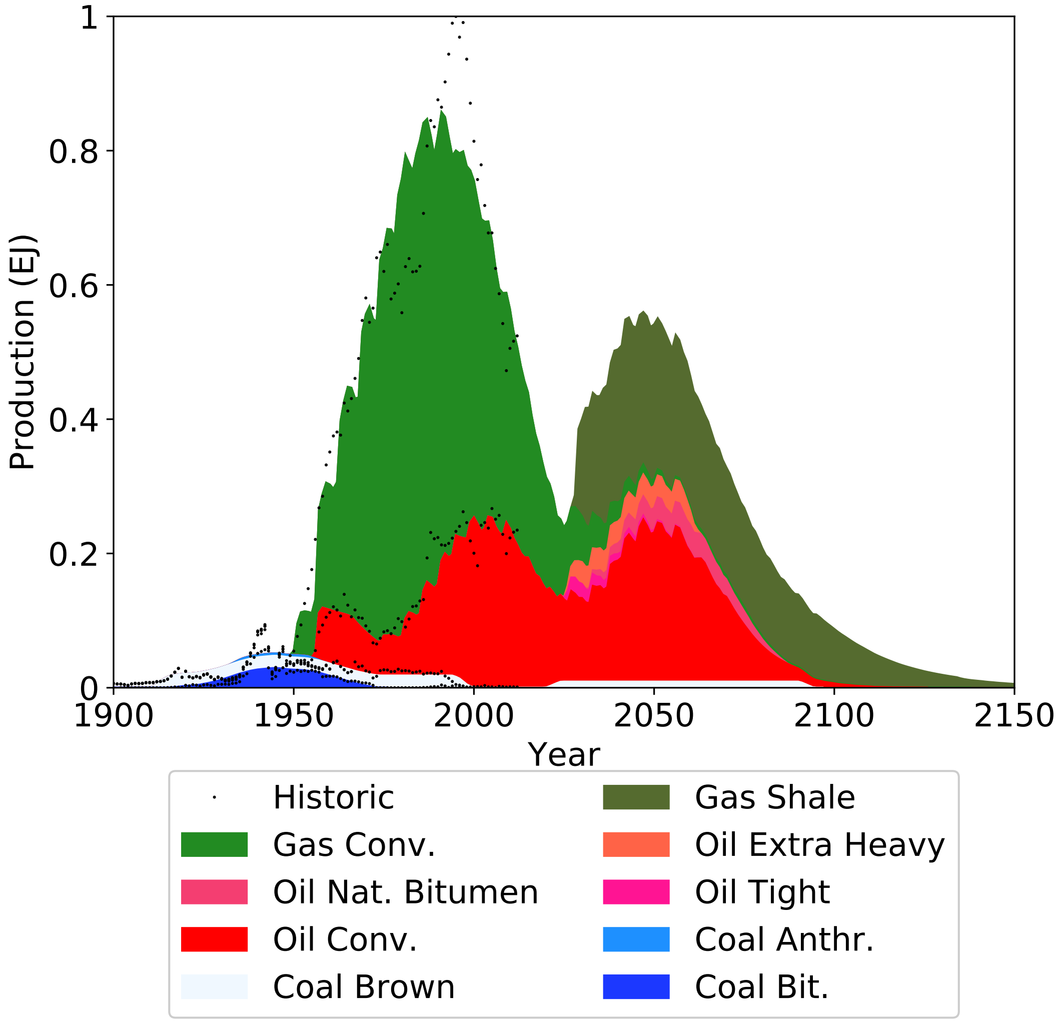

Figure 3.30: Italy - Italy projection by mineral type

Table 3.30: Peak years - Minerals

**Name**	**URR**	**Peak Year**	**Peak Rate**
Coal Bit.	0.9	1945	0.03
Coal Brown	2.17	1917	0.02
Coal Anthr.	0.08	1937	–
Oil Conv.	19.09	2004	0.25
Oil Tight	0.34	2028	0.02
Oil Nat. Bitumen	1.26	2060	0.04
Oil Extra Heavy	1.1	2030	0.03
Gas Conv.	31.0	1985	0.71
Gas Shale	13.57	2042	0.24
**Total**	**69.5**	**1991**	**0.86**

Mainland

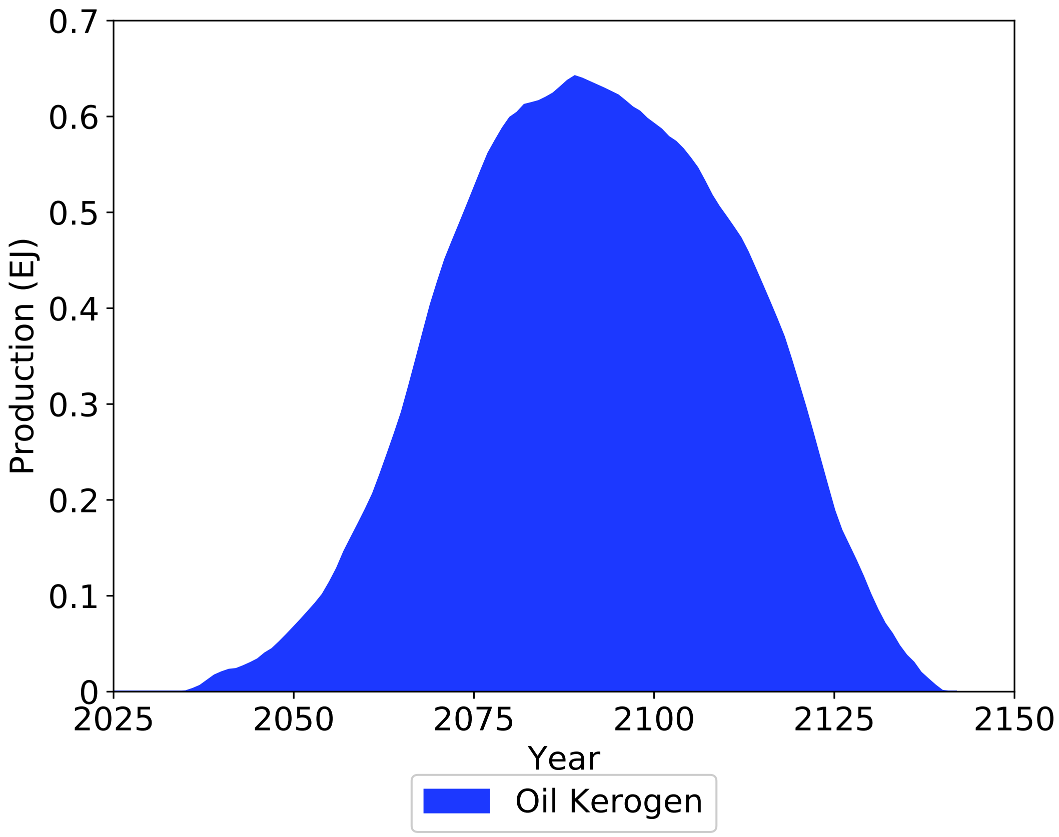

Figure 3.31: Italy - Mainland projections capped at 16

Table 3.31: Peak years - All

Name	URR	Peak Year	Peak Rate
Oil Kerogen Mainland	34.4	2089	0.64
**Total**	**34.4**	**2089**	**0.64**

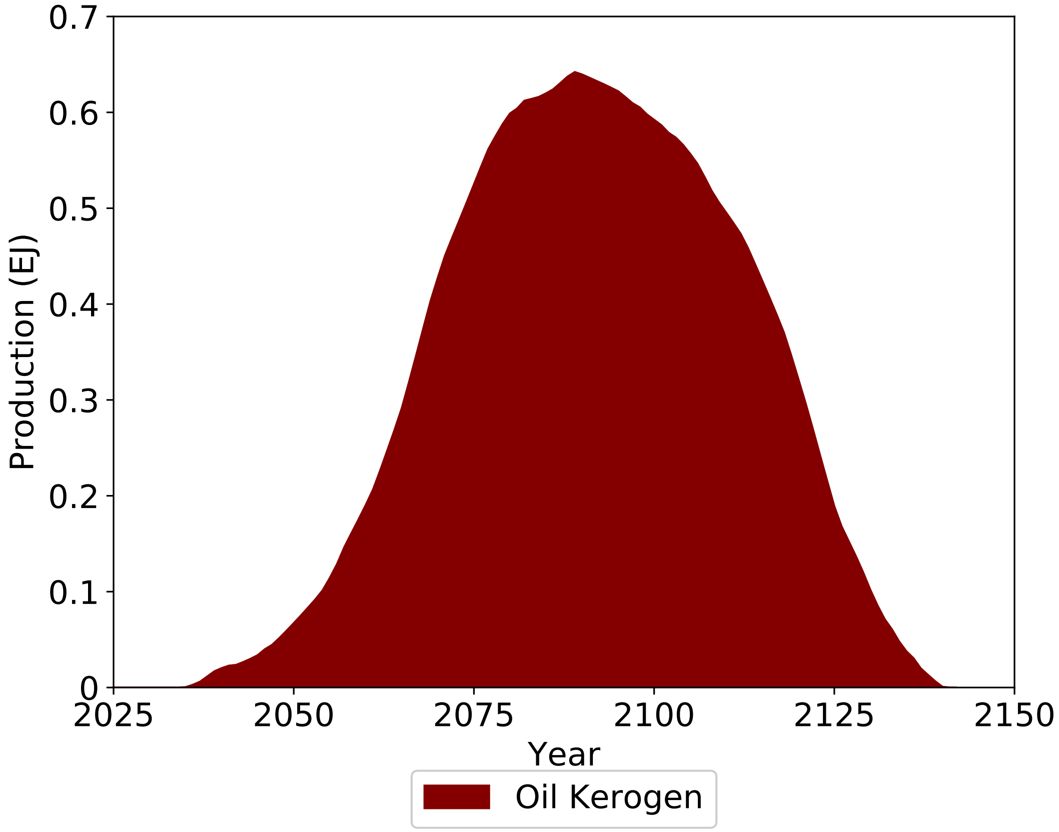

Figure 3.32: Italy - Mainland projection by mineral type

Table 3.32: Peak years - Minerals

Name	URR	Peak Year	Peak Rate
Oil Kerogen	34.4	2089	0.64
**Total**	**34.4**	**2089**	**0.64**

Sicily

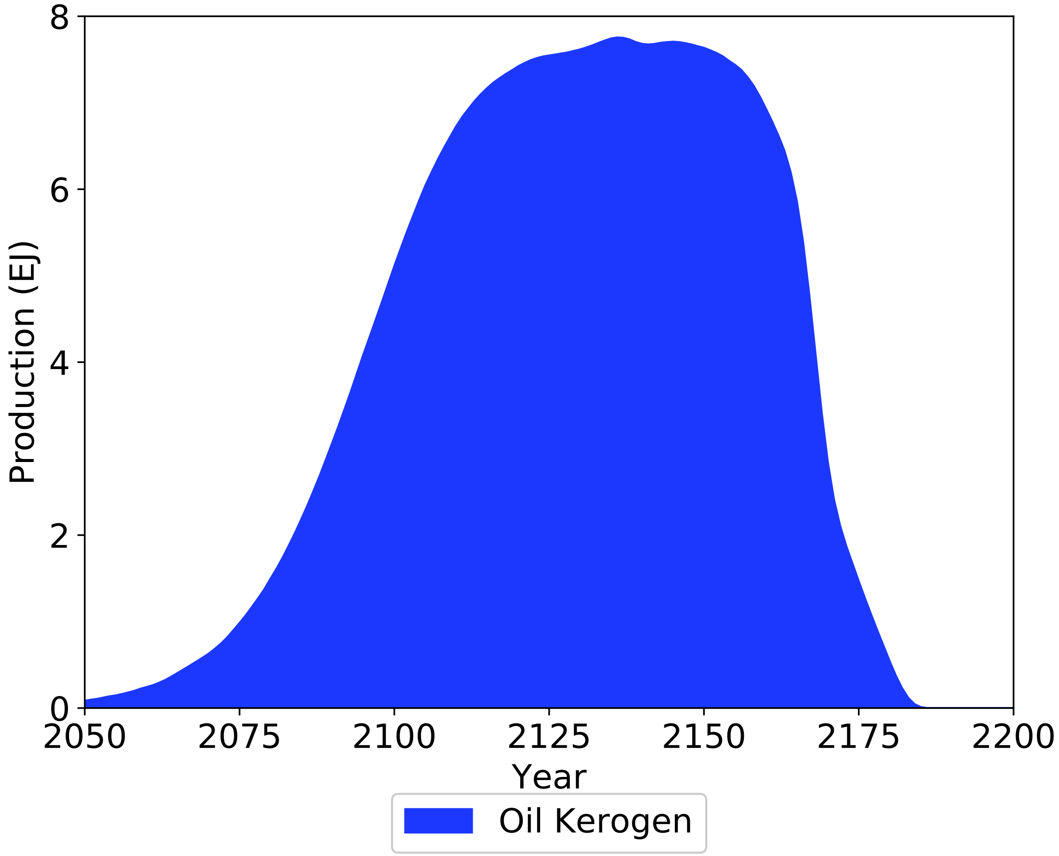

Figure 3.33: Italy - Sicily projections capped at 16

Table 3.33: Peak years - All

Name	URR	Peak Year	Peak Rate
Oil Kerogen Sicily	584.5	2136	7.75
**Total**	**584.5**	**2136**	**7.75**

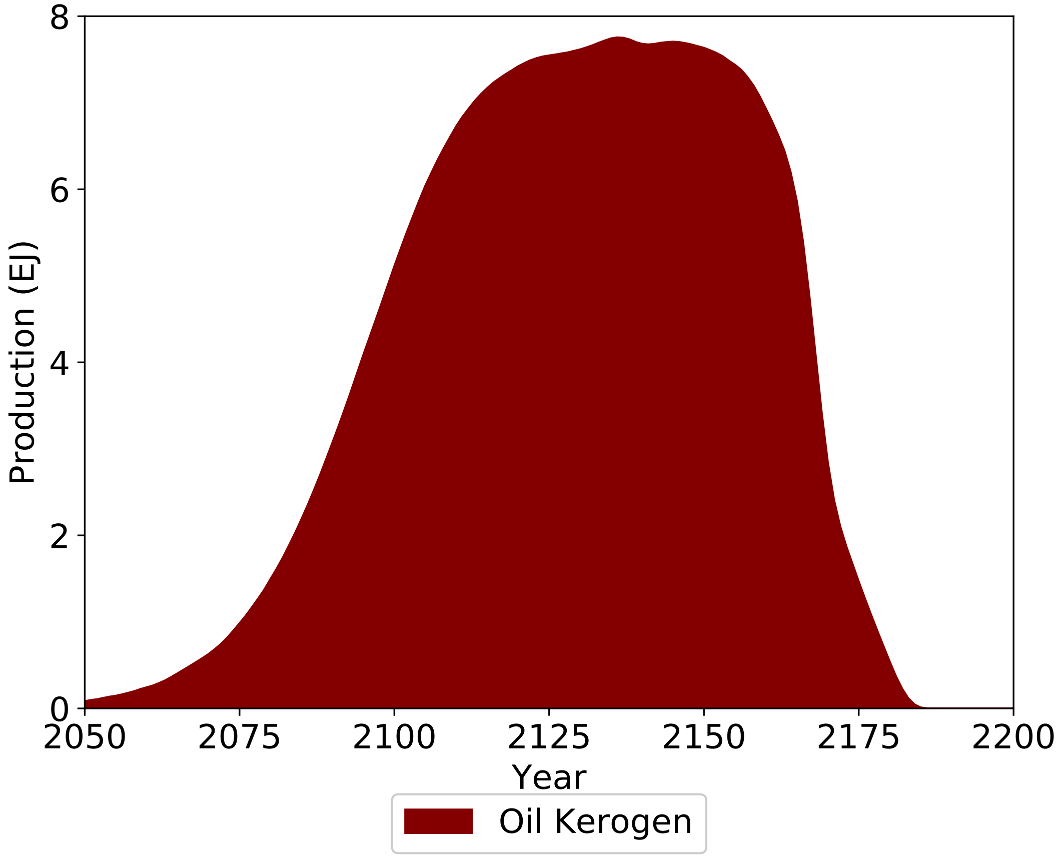

Figure 3.34: Italy - Sicily projection by mineral type

Table 3.34: Peak years - Minerals

Name	URR	Peak Year	Peak Rate
Oil Kerogen	584.5	2136	7.75
**Total**	**584.5**	**2136**	**7.75**

3.14.4 Projection by region

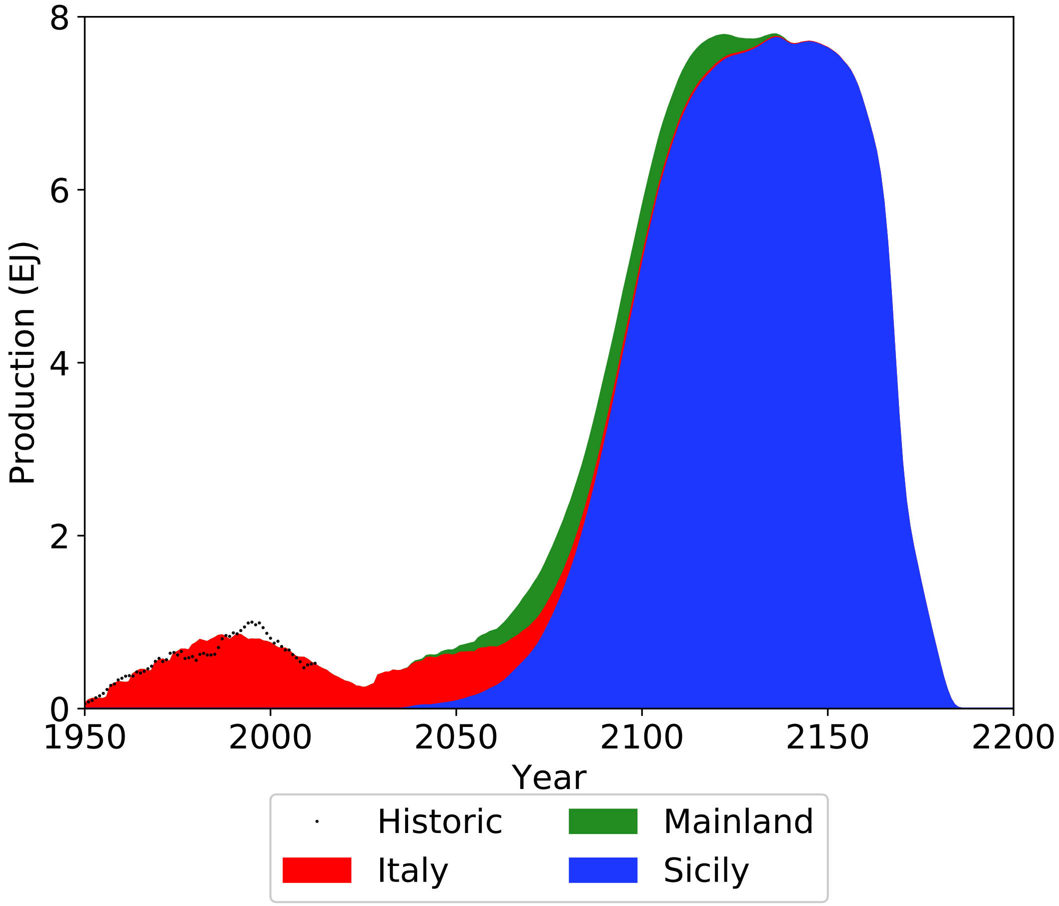

Figure 3.35: Italy by region projections capped at 16

Table 3.35: Peak years - All

Name	URR	Peak Year	Peak Rate
Sicily	584.5	2136	7.75
Italy	69.5	1991	0.86
Mainland	34.4	2089	0.64
**Total**	**688.4**	**2136**	**7.8**

3.15 Malta

3.15.1 All Projections

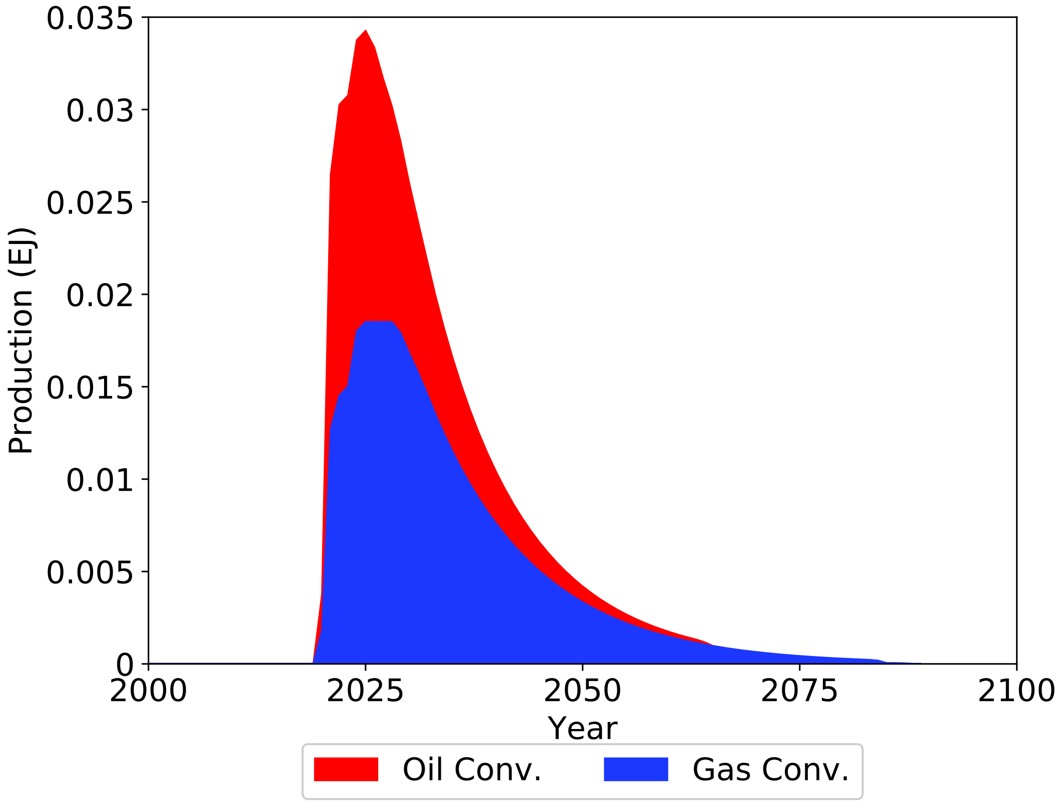

Figure 3.36: Malta projections capped at 16

Table 3.36: Peak years - All

Name	URR	Peak Year	Peak Rate
Gas Conv.	0.37	2025	0.02
Oil Conv.	0.21	2022	0.02
**Total**	**0.58**	**2025**	**0.03**

3.15.2 By Mineral

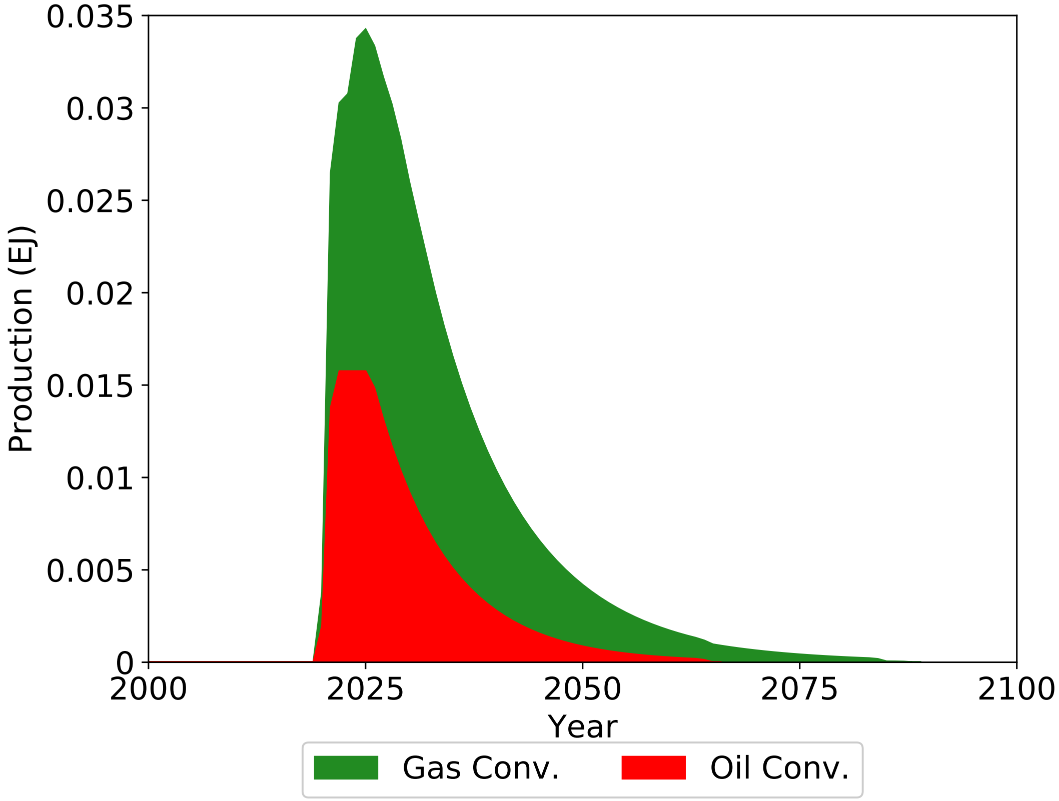

Figure 3.37: Malta projection by mineral type

Table 3.37: Peak years - Minerals

**Name**	**URR**	**Peak Year**	**Peak Rate**
Oil Conv.	0.21	2022	0.02
Gas Conv.	0.37	2025	0.02
**Total**	**0.58**	**2025**	**0.03**

3.16 Netherlands

3.16.1 All Projections

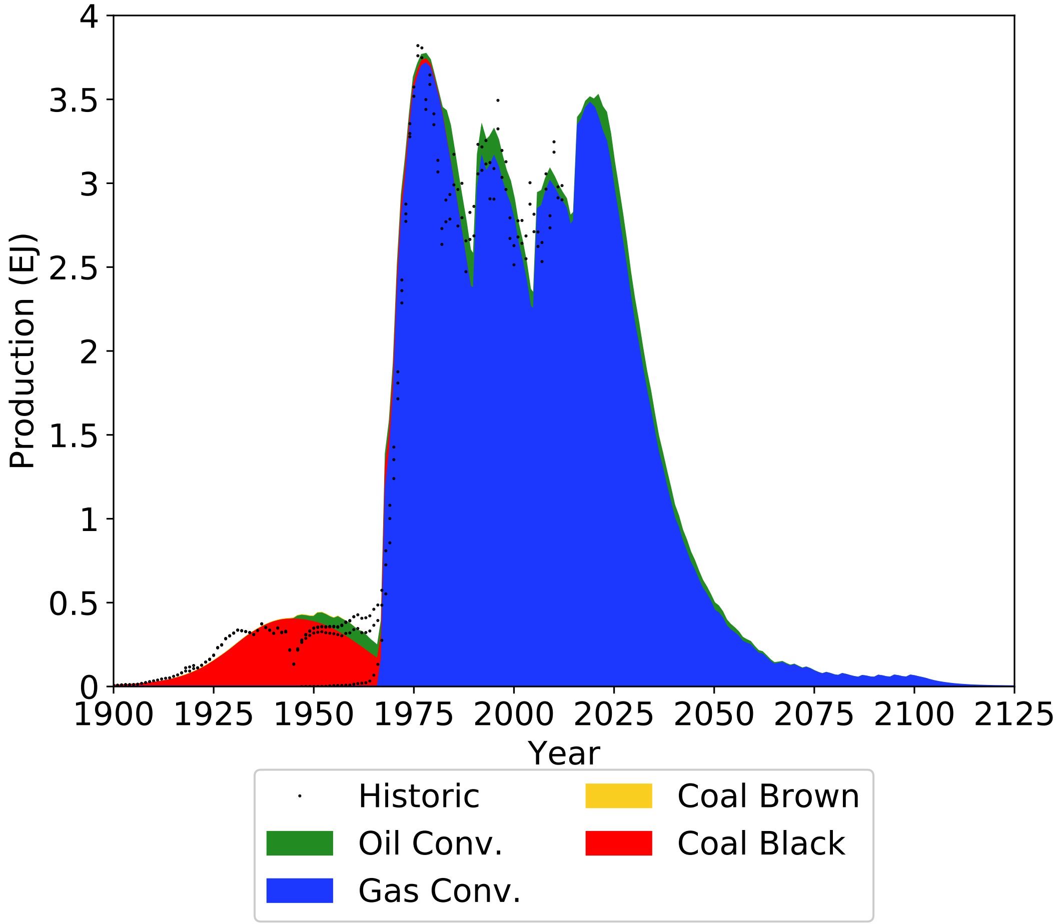

Figure 3.38: Netherlands projections capped at 16

Table 3.38: Peak years - All

**Name**	**URR**	**Peak Year**	**Peak Rate**
Gas Conv.	212.43	1978	3.71
Coal Black	14.9	1945	0.4
Oil Conv.	10.53	1988	0.23
Coal Brown	0.17	1918	–
**Total**	**238.03**	**1978**	**3.77**

3.16.2 By Mineral

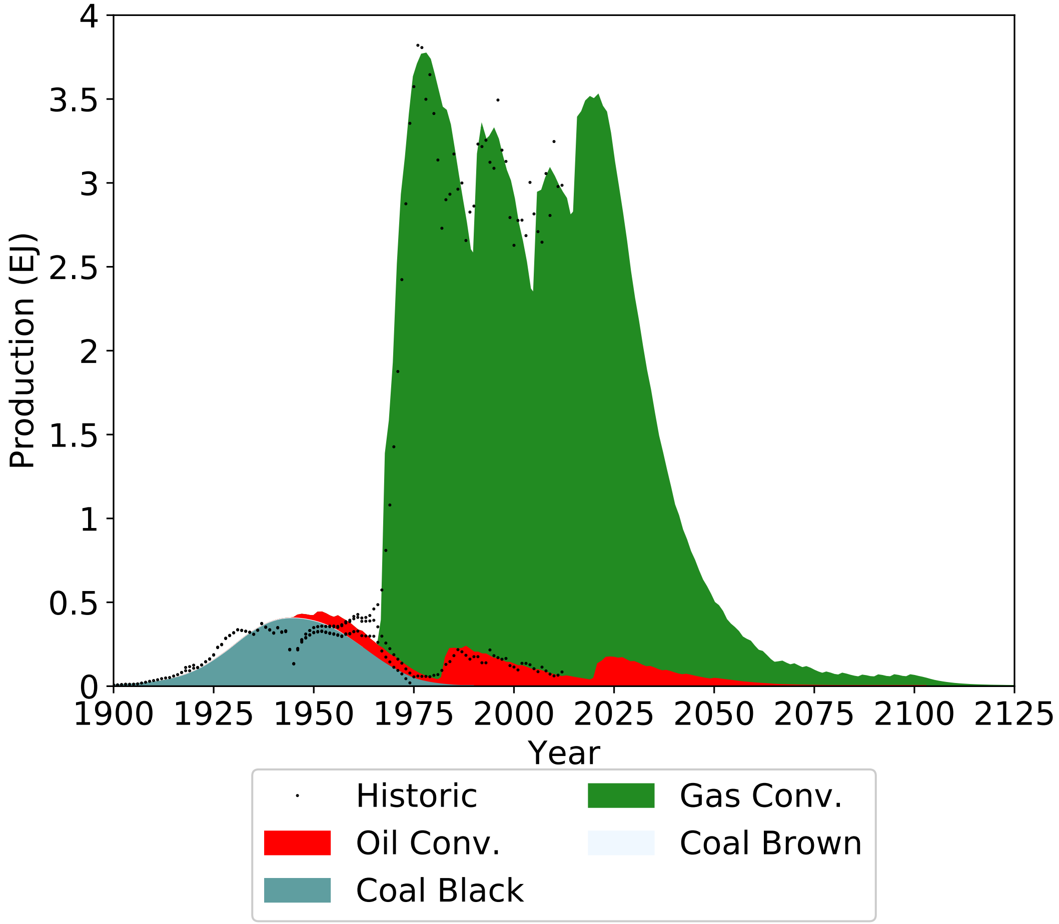

Figure 3.39: Netherlands projection by mineral type

Table 3.39: Peak years - Minerals

**Name**	**URR**	**Peak Year**	**Peak Rate**
Coal Black	14.9	1945	0.4
Coal Brown	0.17	1918	–
Oil Conv.	10.53	1988	0.23
Gas Conv.	212.43	1978	3.71
**Total**	**238.03**	**1978**	**3.77**

3.17 Norway

3.17.1 All Projections

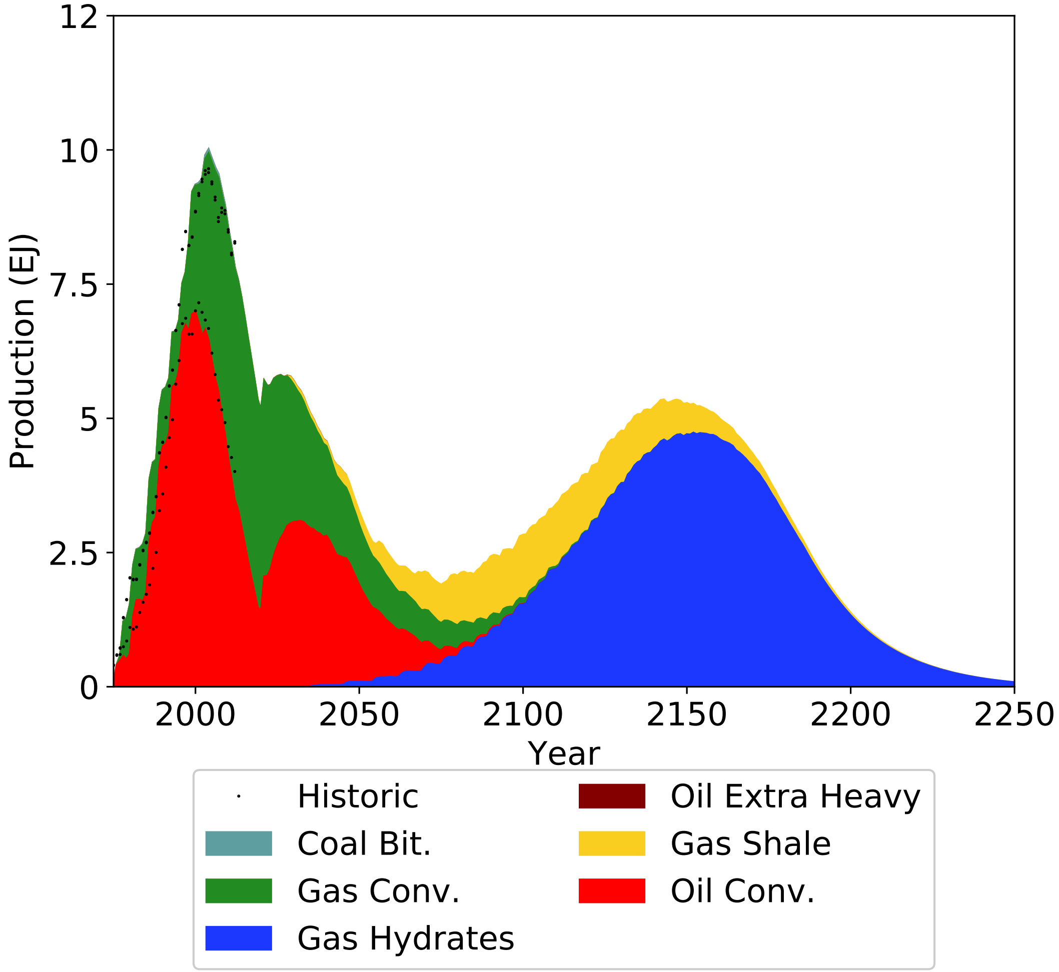

Figure 3.40: Norway projections capped at 16

Table 3.40: Peak years - All

**Name**	**URR**	**Peak Year**	**Peak Rate**
Gas Hydrates	392.3	2152	4.73
Oil Conv.	273.04	2000	6.98
Gas Conv.	195.19	2012	4.3
Gas Shale	100.7	2101	1.19
Coal Bit.	1.44	2004	0.07
Oil Extra Heavy	0.13	2023	0.01
**Total**	**962.8**	**2004**	**10.01**

3.17.2 By Mineral

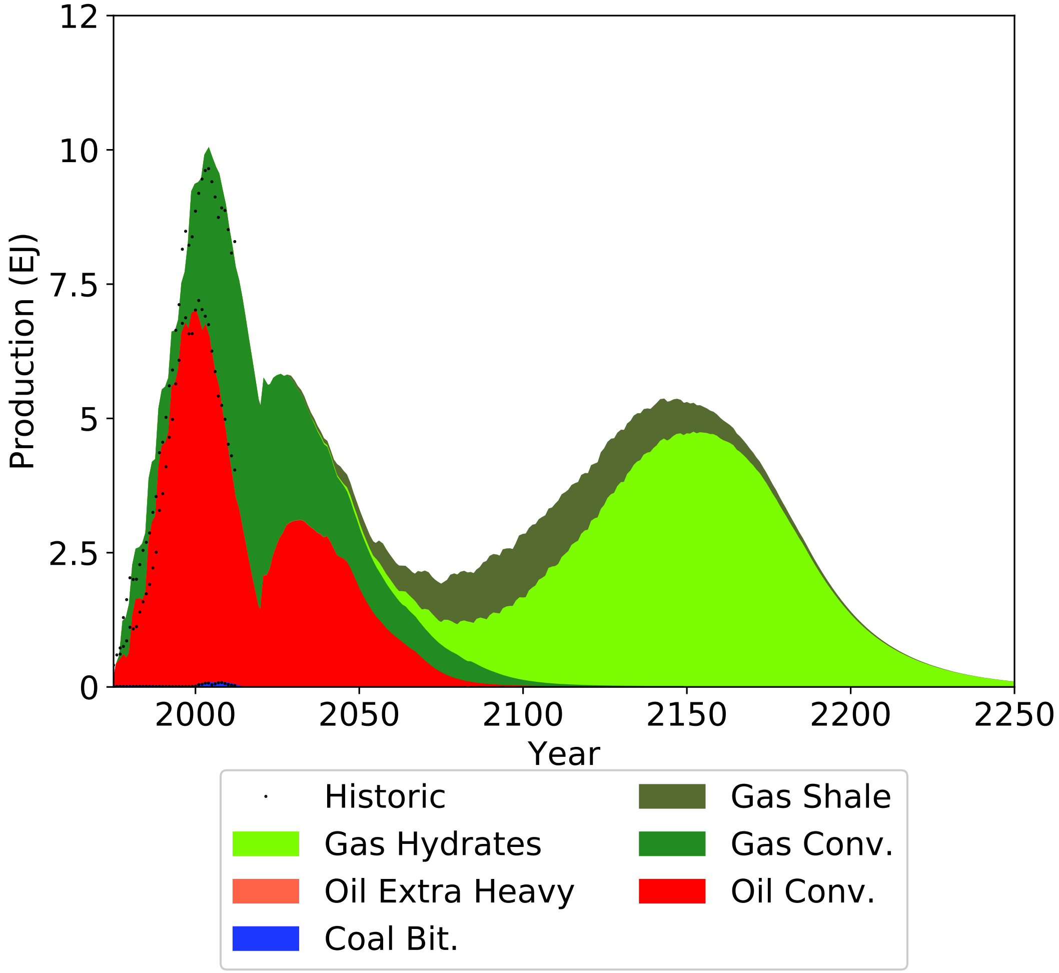

Figure 3.41: Norway projection by mineral type

Table 3.41: Peak years - Minerals

**Name**	**URR**	**Peak Year**	**Peak Rate**
Coal Bit.	1.44	2004	0.07
Oil Conv.	273.04	2000	6.98
Oil Extra Heavy	0.13	2023	0.01
Gas Conv.	195.19	2012	4.3
Gas Hydrates	392.3	2152	4.73
Gas Shale	100.7	2101	1.19
**Total**	**962.8**	**2004**	**10.01**

3.18 Poland

3.18.1 All Projections

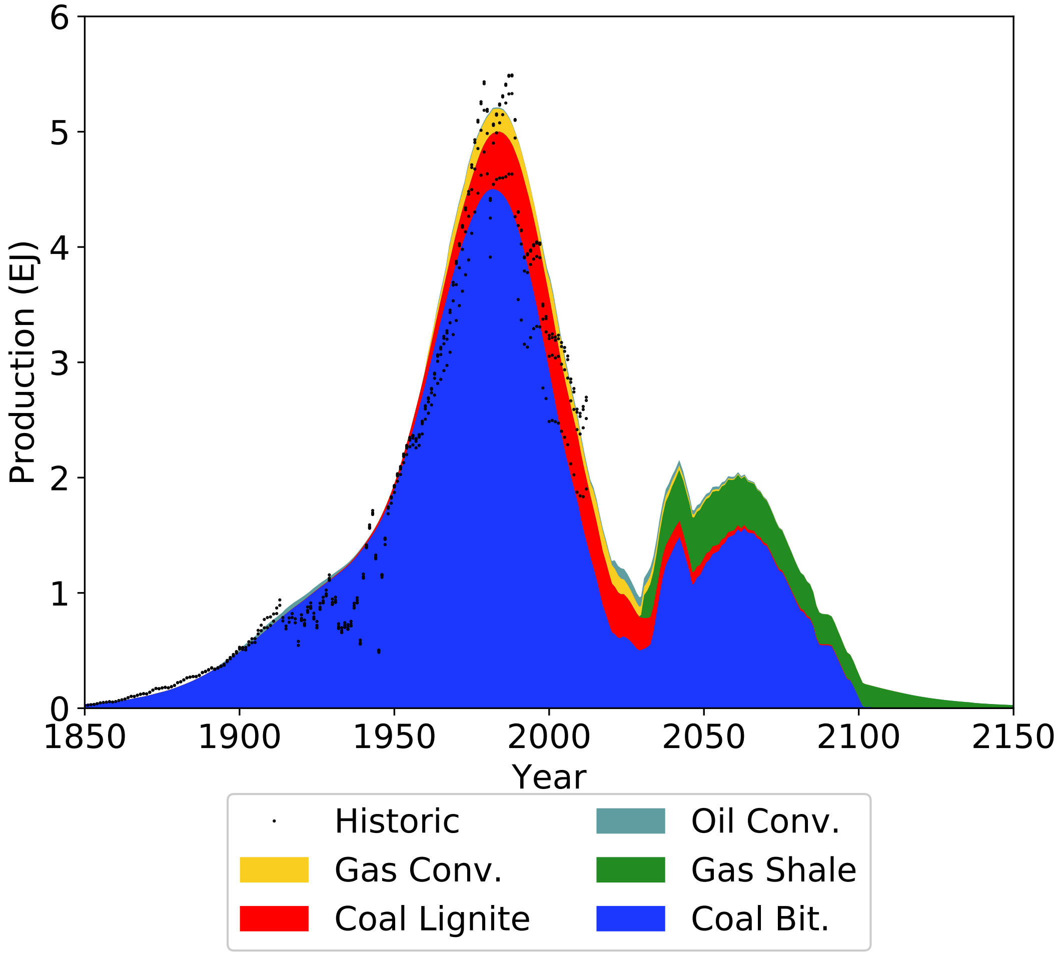

Figure 3.42: Poland projections capped at 16

Table 3.42: Peak years - All

**Name**	**URR**	**Peak Year**	**Peak Rate**
Coal Bit.	349.1	1982	4.49
Coal Lignite	37.3	1998	0.65
Gas Shale	29.55	2050	0.48
Gas Conv.	11.36	1976	0.21
Oil Conv.	4.77	2024	0.09
**Total**	**432.08**	**1983**	**5.2**

3.18.2 By Mineral

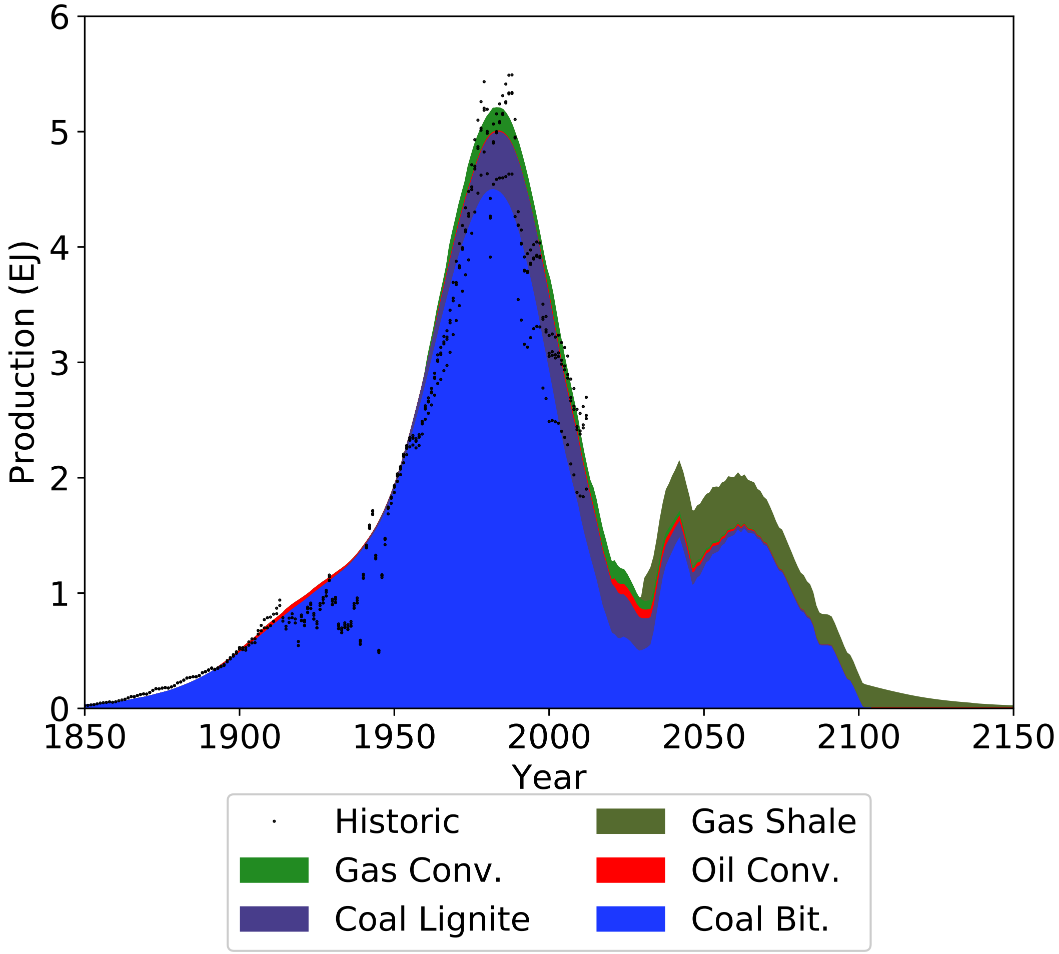

Figure 3.43: Poland projection by mineral type

Table 3.43: Peak years - Minerals

Name	URR	Peak Year	Peak Rate
Coal Bit.	349.1	1982	4.49
Coal Lignite	37.3	1998	0.65
Oil Conv.	4.77	2024	0.09
Gas Conv.	11.36	1976	0.21
Gas Shale	29.55	2050	0.48
**Total**	**432.08**	**1983**	**5.2**

3.19 Portugal

3.19.1 All Projections

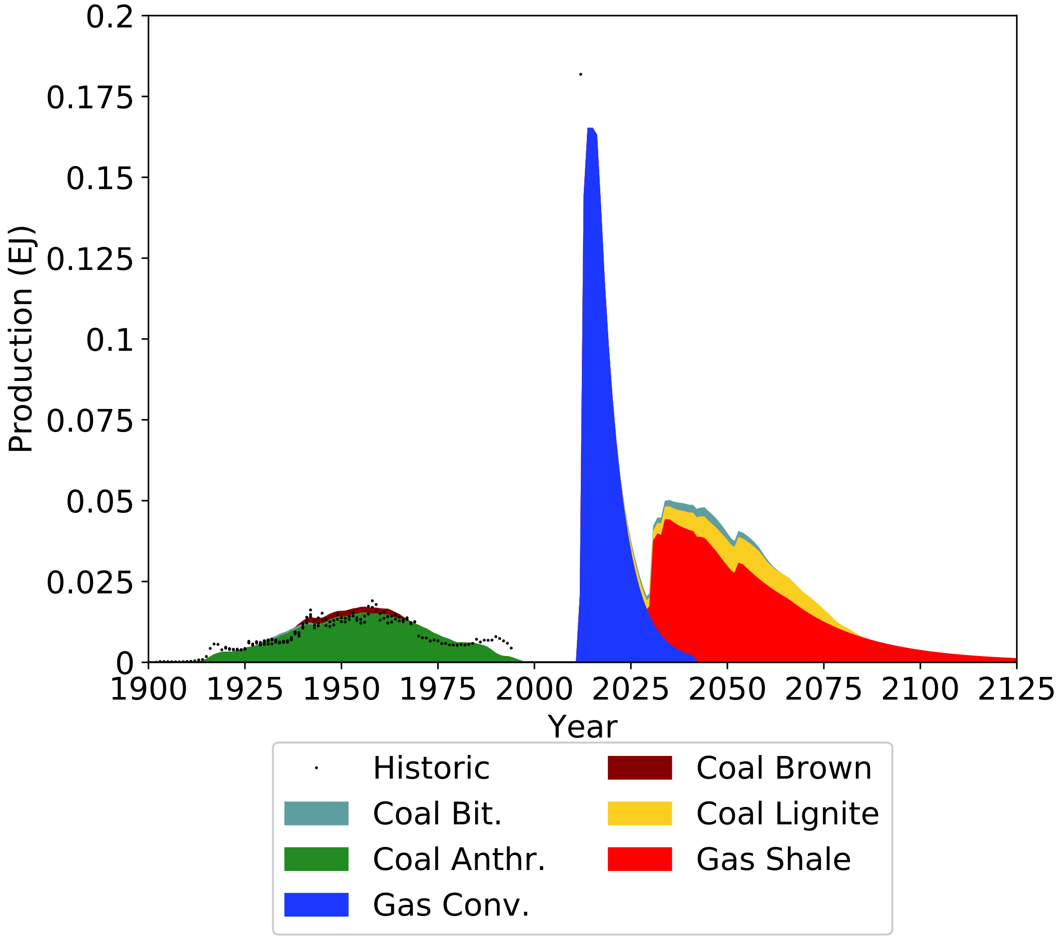

Figure 3.44: Portugal projections capped at 16

Table 3.44: Peak years - All

**Name**	**URR**	**Peak Year**	**Peak Rate**
Gas Conv.	1.5	2014	0.17
Gas Shale	1.48	2035	0.04
Coal Anthr.	0.68	1956	0.02
Coal Lignite	0.31	2057	0.01
Coal Bit.	0.08	2045	–
Coal Brown	0.05	1943	–
**Total**	**4.1**	**2014**	**0.17**

3.19.2 By Mineral

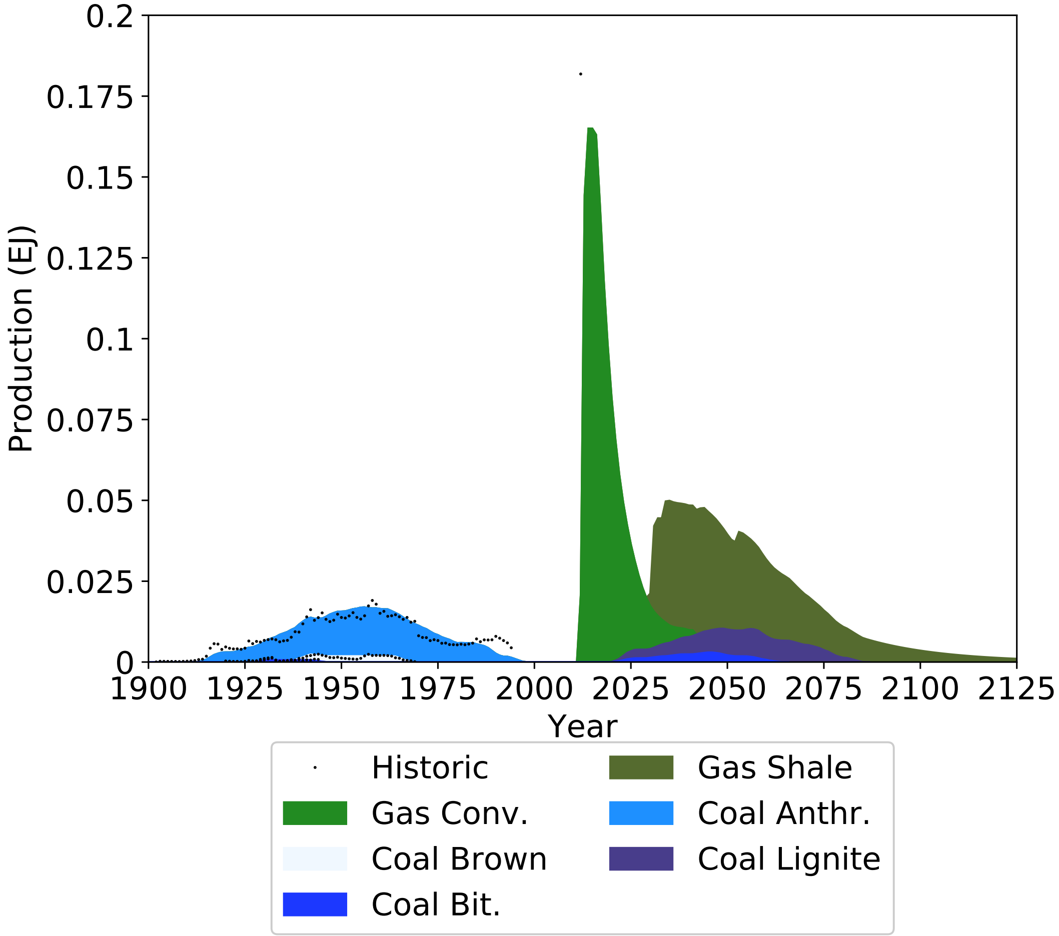

Figure 3.45: Portugal projection by mineral type

Table 3.45: Peak years - Minerals

Name	URR	Peak Year	Peak Rate
Coal Bit.	0.08	2045	–
Coal Lignite	0.31	2057	0.01
Coal Brown	0.05	1943	–
Coal Anthr.	0.68	1956	0.02
Gas Conv.	1.5	2014	0.17
Gas Shale	1.48	2035	0.04
**Total**	**4.1**	**2014**	**0.17**

3.20 Romania

3.20.1 All Projections

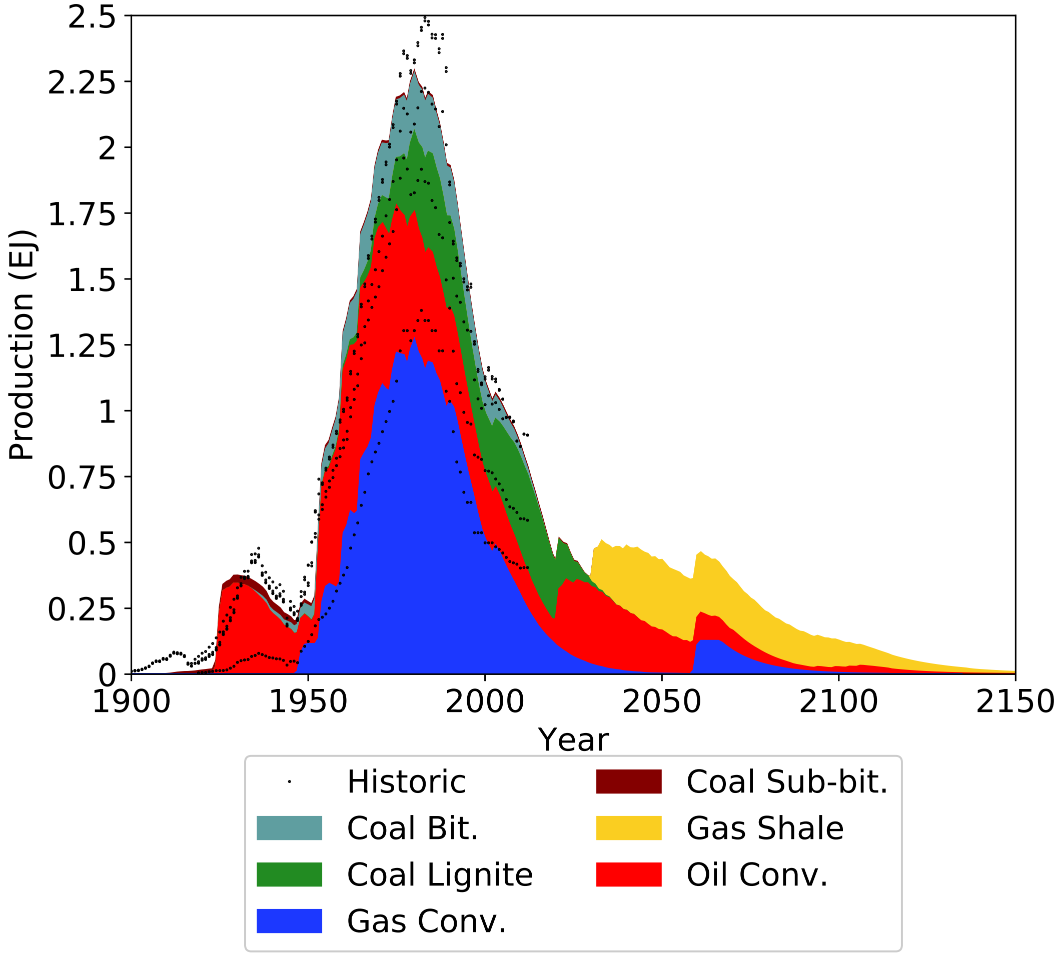

Figure 3.46: Romania projections capped at 16

Table 3.46: Peak years - All

**Name**	**URR**	**Peak Year**	**Peak Rate**
Gas Conv.	50.64	1980	1.27
Oil Conv.	43.64	1965	0.65
Coal Lignite	15.8	1986	0.38
Gas Shale	15.57	2046	0.27
Coal Bit.	9.3	1979	0.22
Coal Sub-bit.	1.6	1936	0.04
**Total**	**136.55**	**1980**	**2.29**

3.20.2 By Mineral

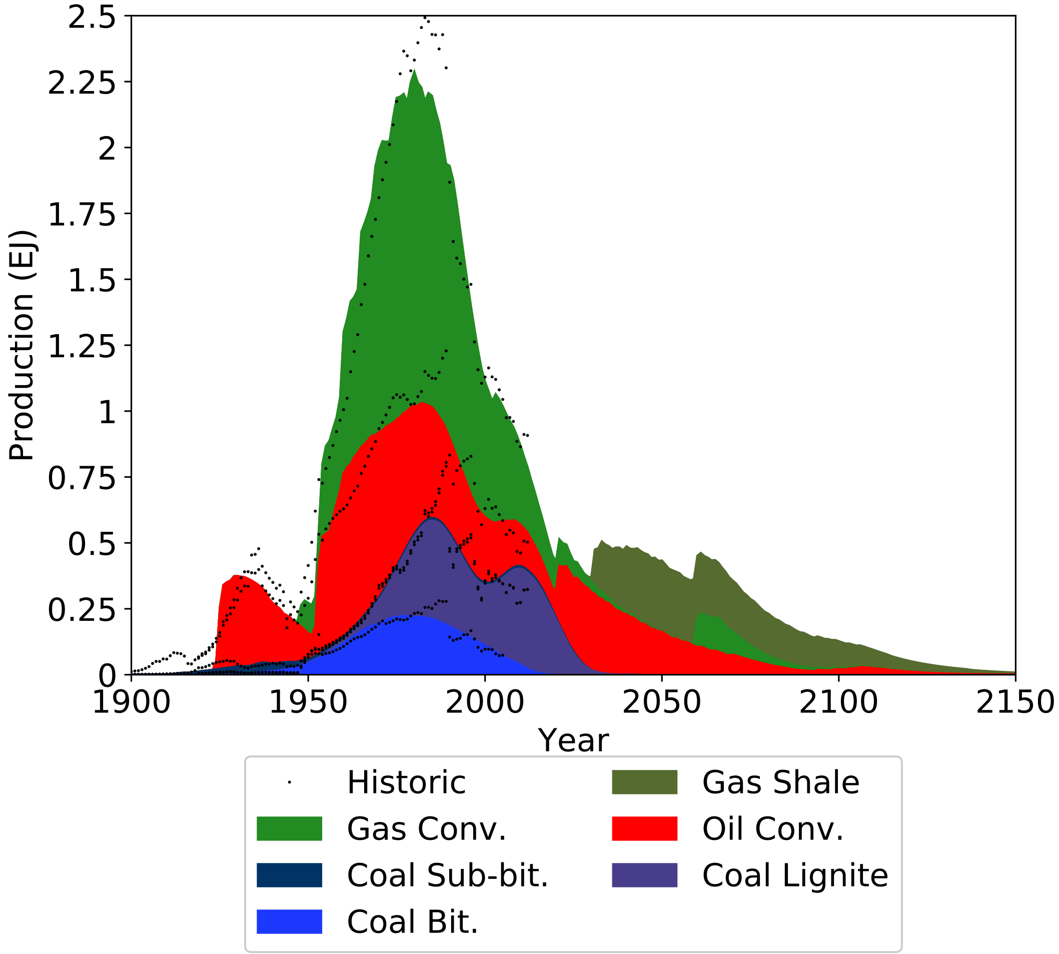

Figure 3.47: Romania projection by mineral type

Table 3.47: Peak years - Minerals

**Name**	**URR**	**Peak Year**	**Peak Rate**
Coal Bit.	9.3	1979	0.22
Coal Lignite	15.8	1986	0.38
Coal Sub-bit.	1.6	1936	0.04
Oil Conv.	43.64	1965	0.65
Gas Conv.	50.64	1980	1.27
Gas Shale	15.57	2046	0.27
**Total**	**136.55**	**1980**	**2.29**

3.21 Slovakia

3.21.1 All Projections

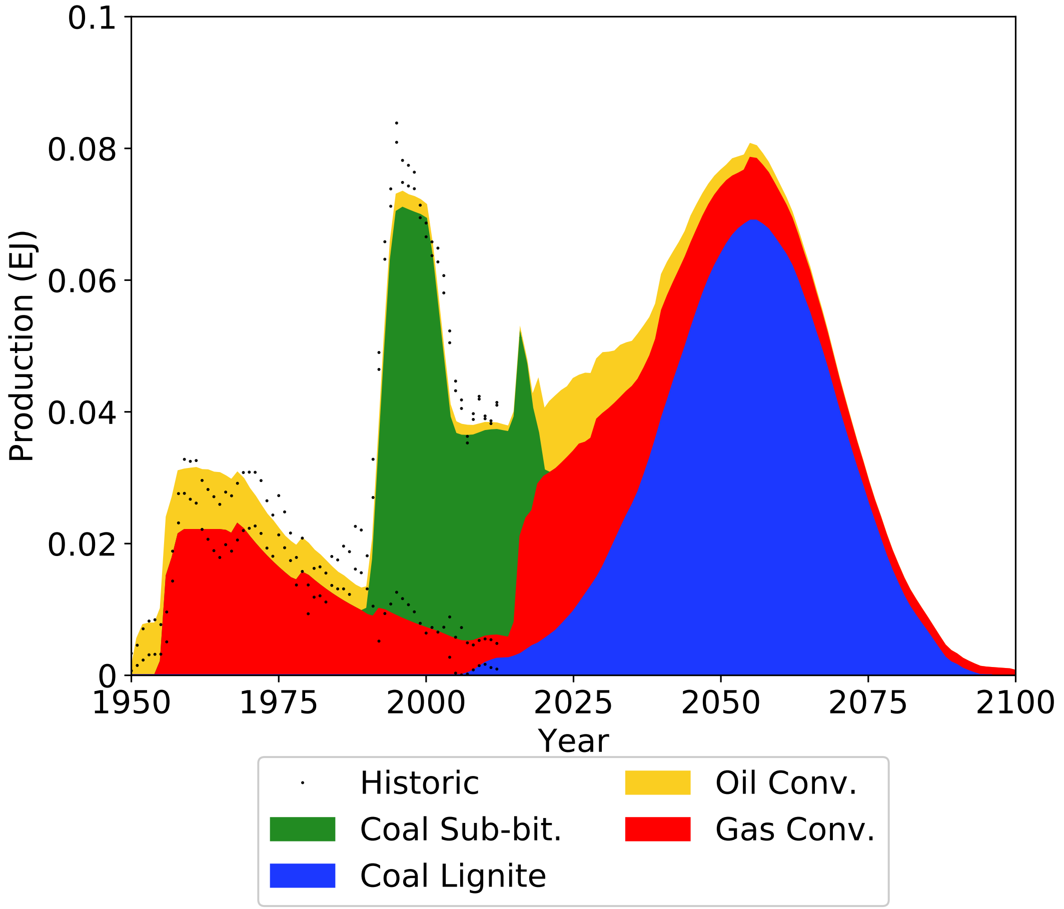

Figure 3.48: Slovakia projections capped at 16

Table 3.48: Peak years - All

**Name**	**URR**	**Peak Year**	**Peak Rate**
Coal Lignite	2.48	2056	0.07
Gas Conv.	1.64	2020	0.02
Coal Sub-bit.	1.1	1996	0.06
Oil Conv.	0.57	2022	0.01
**Total**	**5.79**	**2055**	**0.08**

3.21.2 By Mineral

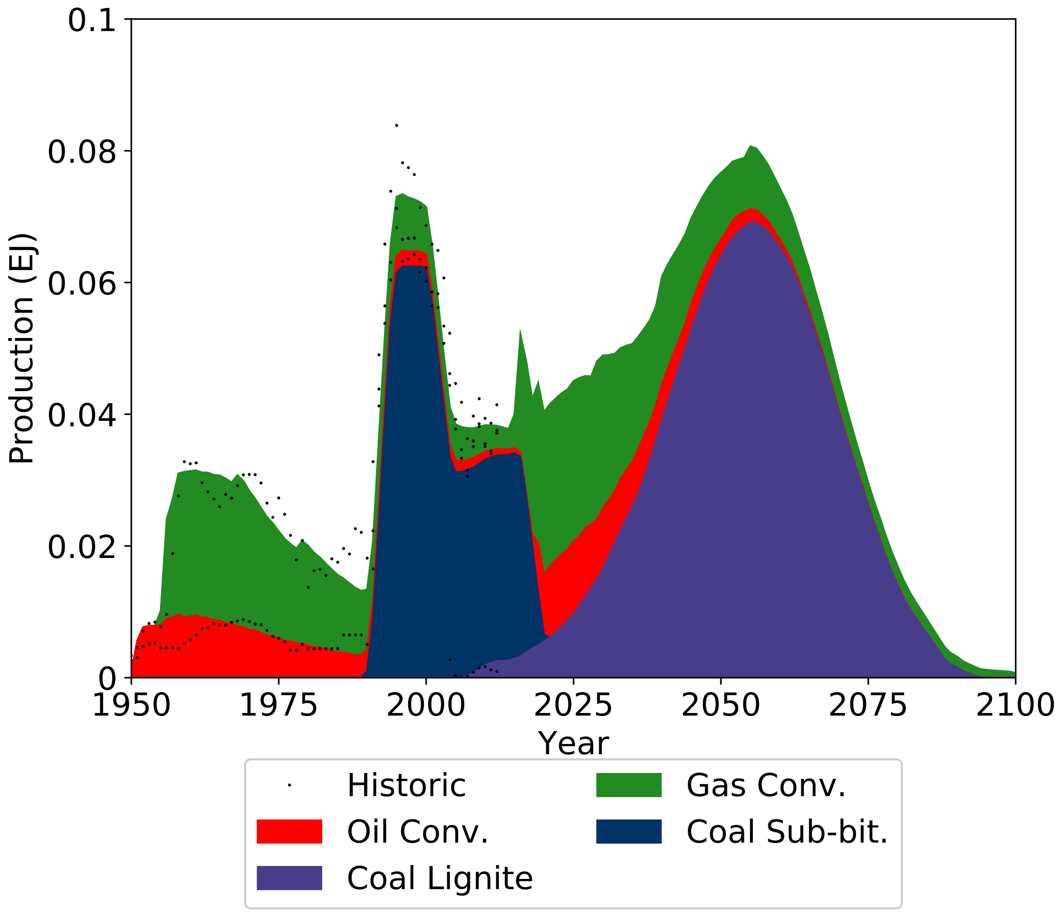

Figure 3.49: Slovakia projection by mineral type

Table 3.49: Peak years - Minerals

Name	URR	Peak Year	Peak Rate
Coal Lignite	2.48	2056	0.07
Coal Sub-bit.	1.1	1996	0.06
Oil Conv.	0.57	2022	0.01
Gas Conv.	1.64	2020	0.02
**Total**	**5.79**	**2055**	**0.08**

3.22 Spain

3.22.1 All Projections

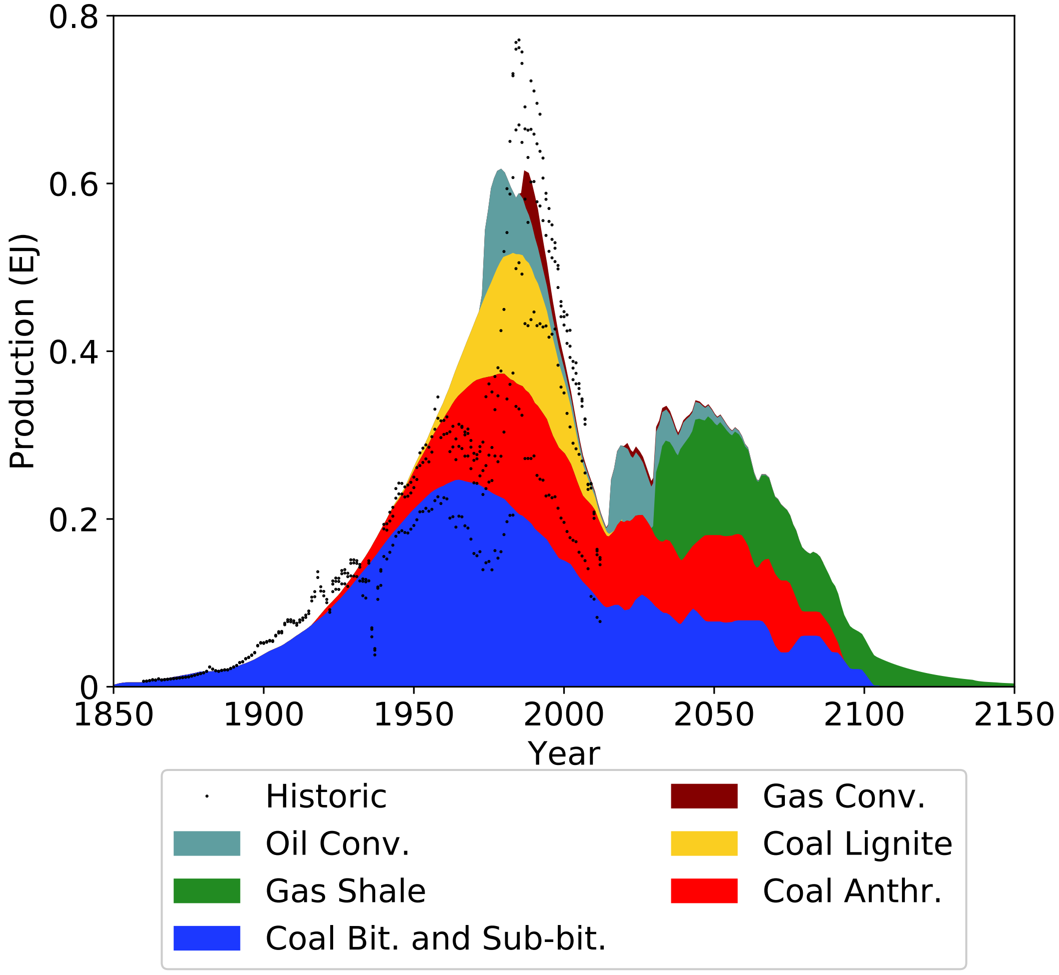

Figure 3.50: Spain projections capped at 16

Table 3.50: Peak years - All

**Name**	**URR**	**Peak Year**	**Peak Rate**
Coal Bit. and Sub-bit.	24.7	1965	0.25
Coal Anthr.	13.95	1985	0.15
Gas Shale	7.56	2044	0.15
Coal Lignite	4.8	1986	0.16
Oil Conv.	3.42	1977	0.12
Gas Conv.	0.64	1988	0.05
**Total**	**55.07**	**1979**	**0.62**

3.22.2 By Mineral

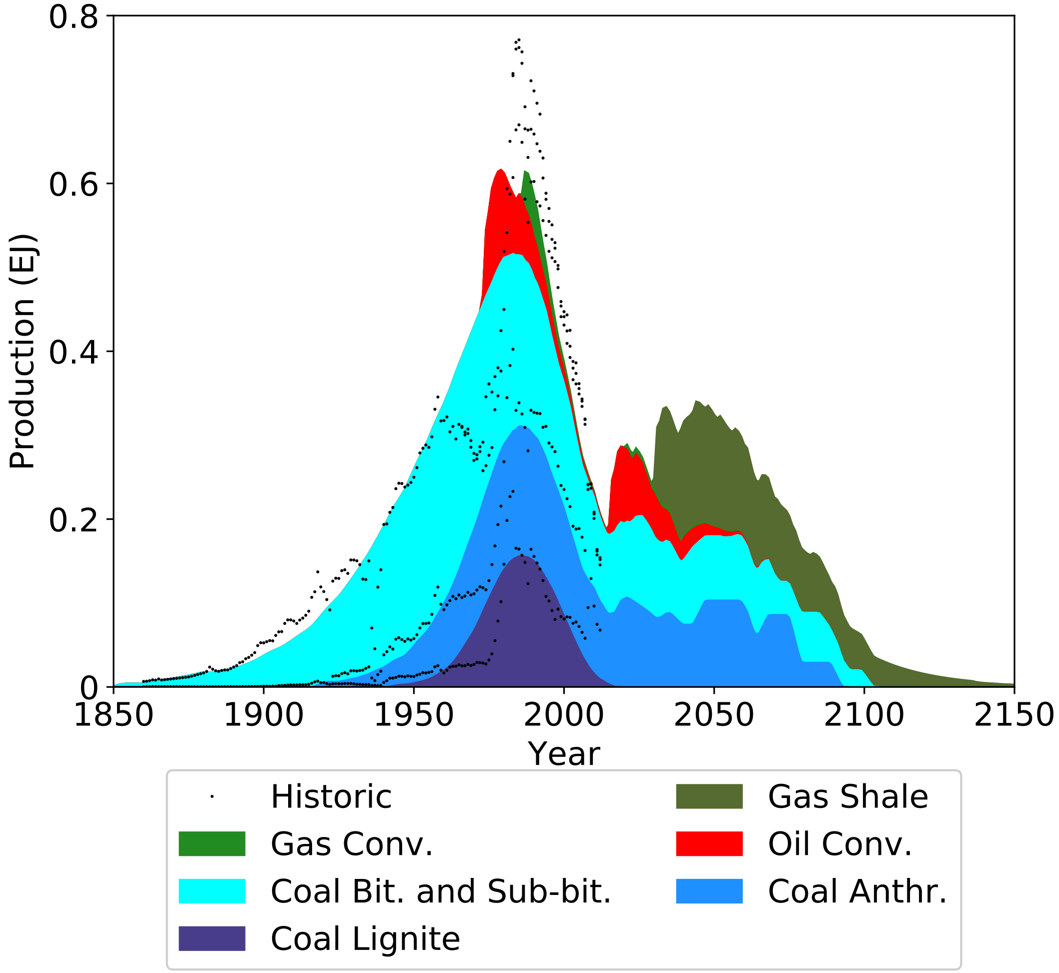

Figure 3.51: Spain projection by mineral type

Table 3.51: Peak years - Minerals

**Name**	**URR**	**Peak Year**	**Peak Rate**
Coal Lignite	4.8	1986	0.16
Coal Anthr.	13.95	1985	0.15
Coal Bit. and Sub-bit.	24.7	1965	0.25
Oil Conv.	3.42	1977	0.12
Gas Conv.	0.64	1988	0.05
Gas Shale	7.56	2044	0.15
**Total**	**55.07**	**1979**	**0.62**

3.23 Sweden

3.23.1 All Projections

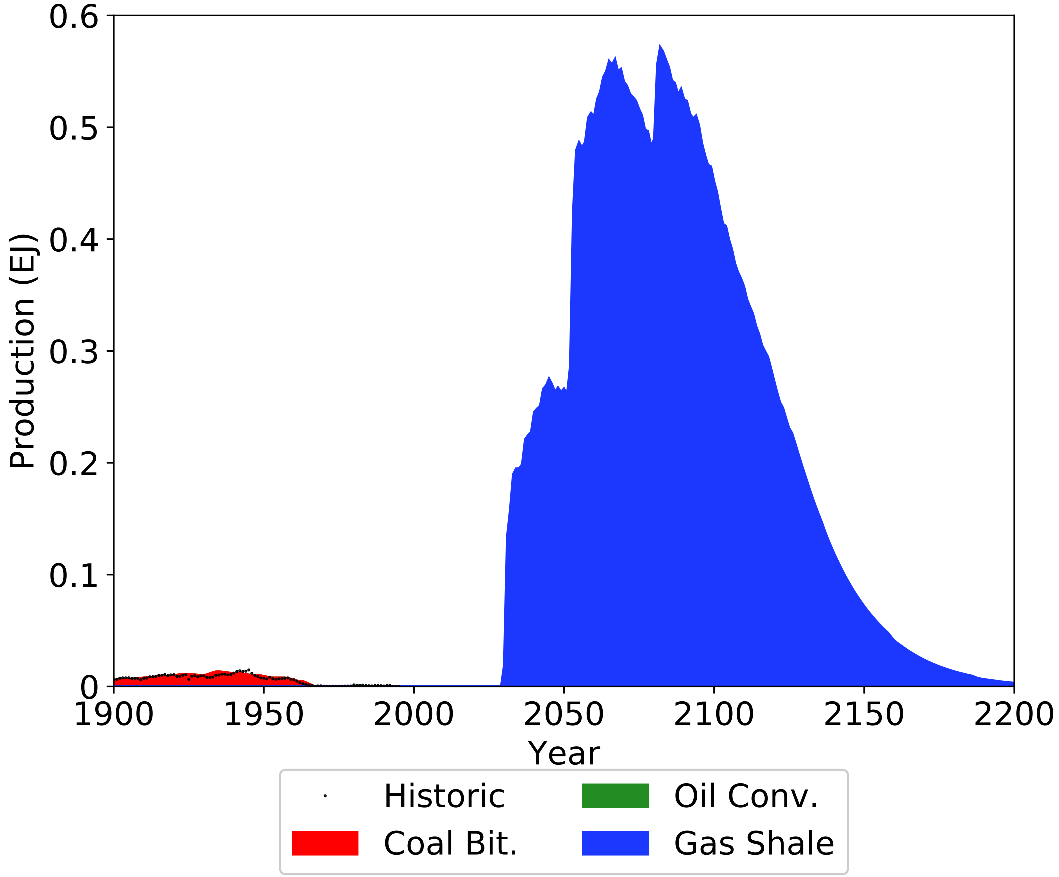

Figure 3.52: Sweden projections capped at 16

Table 3.52: Peak years - All

Name	URR	Peak Year	Peak Rate
Gas Shale	43.05	2082	0.57
Coal Bit.	0.69	1935	0.01
Oil Conv.	–	1982	–
**Total**	**43.74**	**2082**	**0.57**

3.23.2 By Mineral

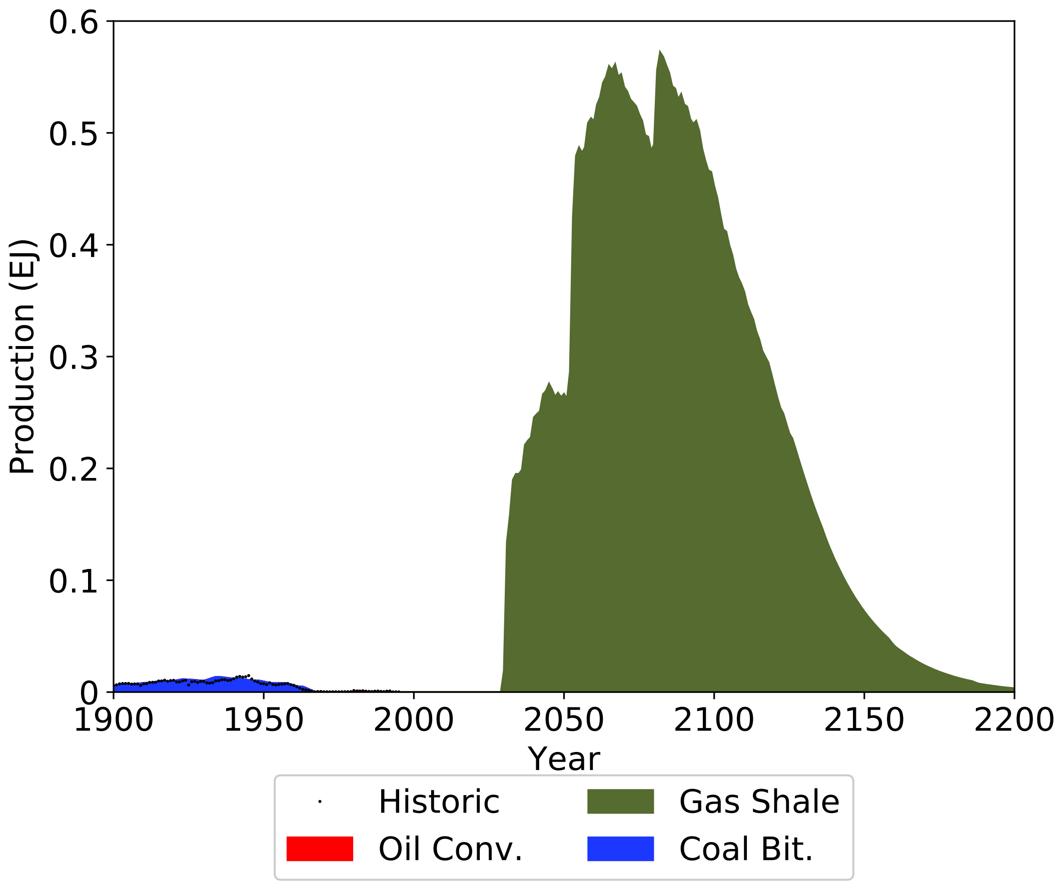

Figure 3.53: Sweden projection by mineral type

Table 3.53: Peak years - Minerals

Name	URR	Peak Year	Peak Rate
Coal Bit.	0.69	1935	0.01
Oil Conv.	–	1982	–
Gas Shale	43.05	2082	0.57
**Total**	**43.74**	**2082**	**0.57**

3.24 Switzerland

3.24.1 All Projections

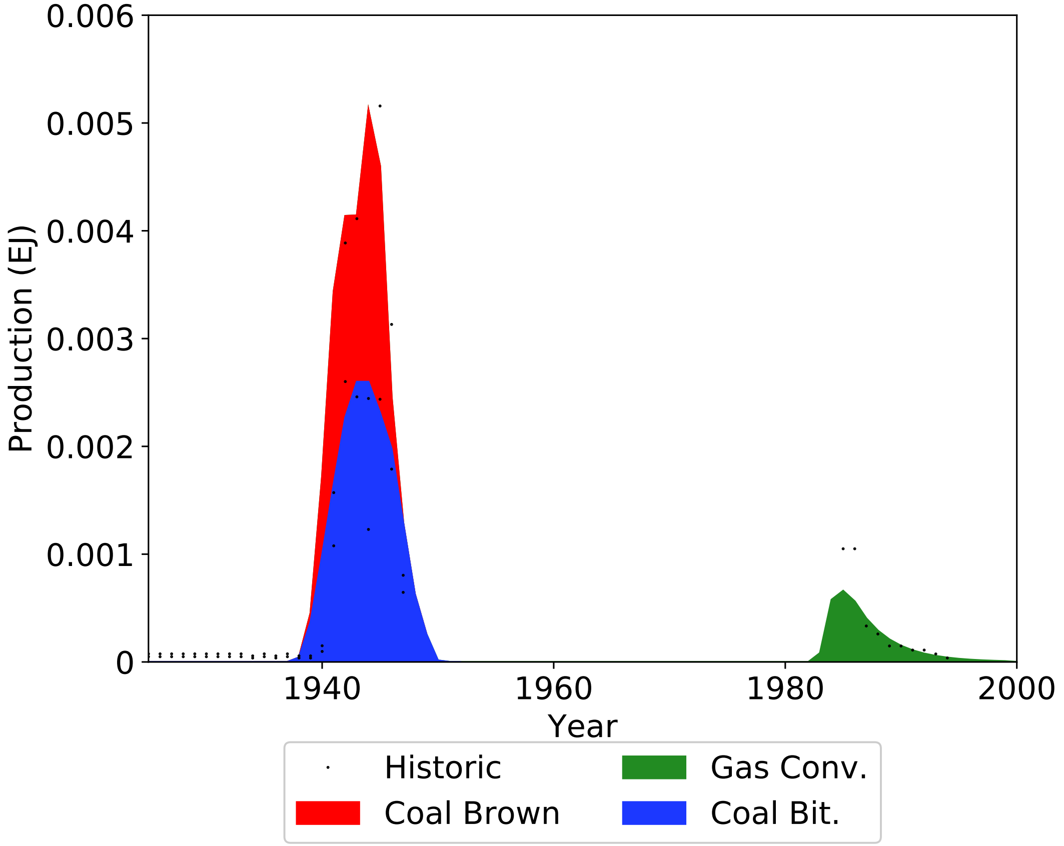

Figure 3.54: Switzerland projections capped at 16

Table 3.54: Peak years - All

Name	URR	Peak Year	Peak Rate
Coal Bit.	0.02	1943	–
Coal Brown	0.01	1944	–
Gas Conv.	–	1985	–
**Total**	**0.03**	**1944**	**0.01**

3.24.2 By Mineral

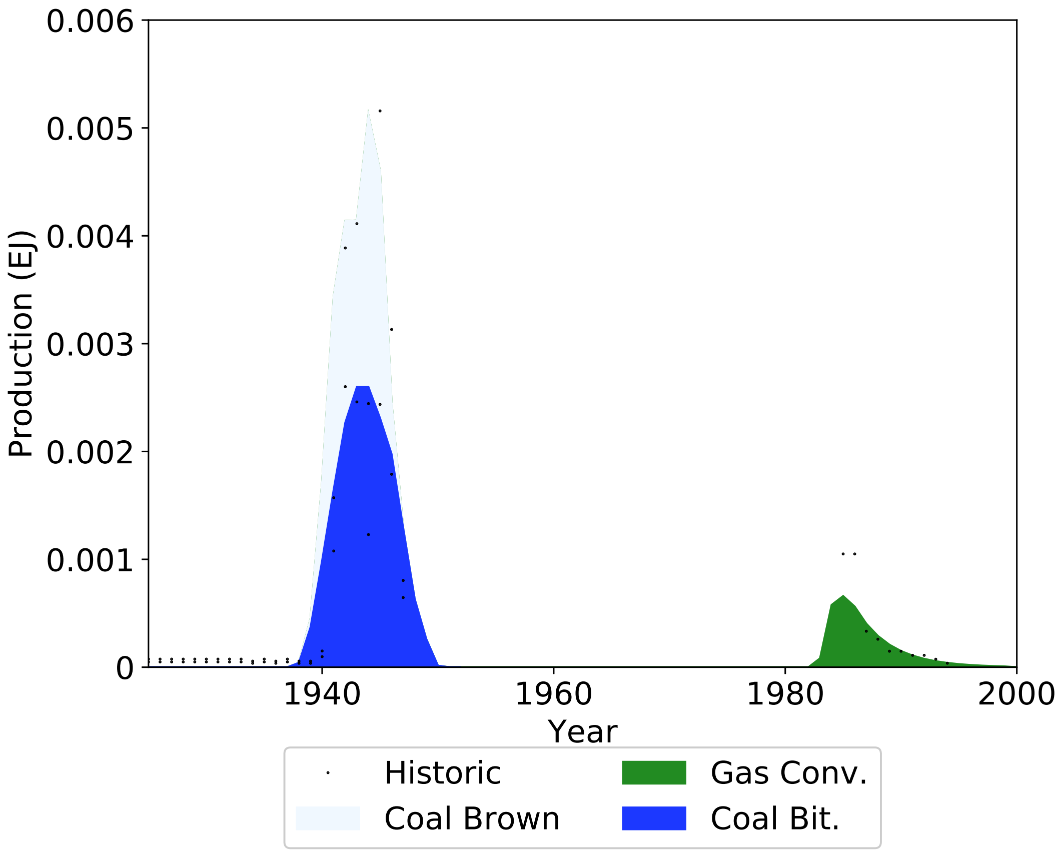

Figure 3.55: Switzerland projection by mineral type

Table 3.55: Peak years - Minerals

Name	URR	Peak Year	Peak Rate
Coal Bit.	0.02	1943	–
Coal Brown	0.01	1944	–
Gas Conv.	–	1985	–
**Total**	**0.03**	**1944**	**0.01**

3.25 Turkey

3.25.1 All Projections

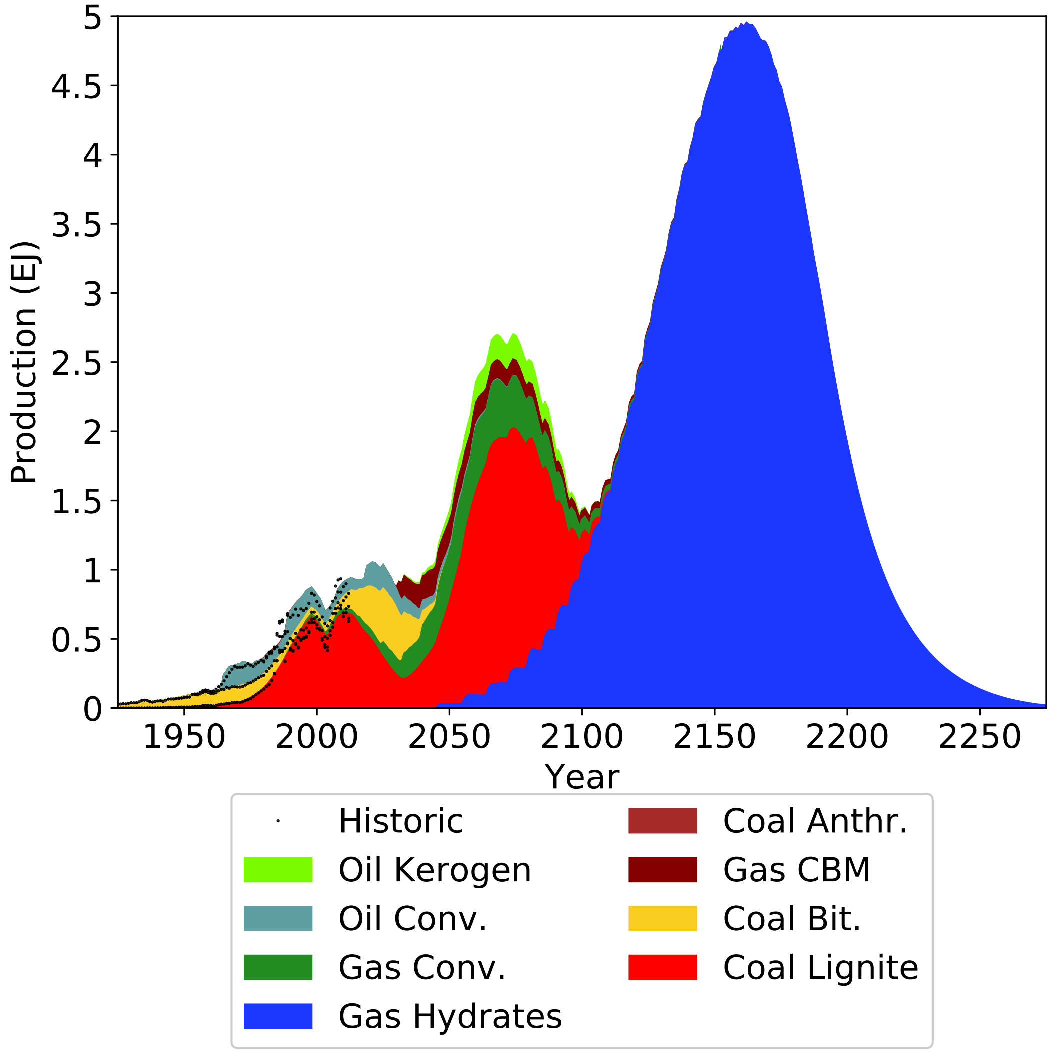

Figure 3.56: Turkey projections capped at 16

Table 3.56: Peak years - All

**Name**	**URR**	**Peak Year**	**Peak Rate**
Gas Hydrates	392.3	2162	4.95
Coal Lignite	96.4	2071	1.77
Gas Conv.	23.89	2060	0.47
Coal Bit.	14.8	2026	0.39
Oil Conv.	10.56	1990	0.2
Gas CBM	10.5	2046	0.2
Oil Kerogen	6.9	2067	0.18
Coal Anthr.	0.13	1983	0.01
**Total**	**555.47**	**2162**	**4.95**

3.25.2 By Mineral

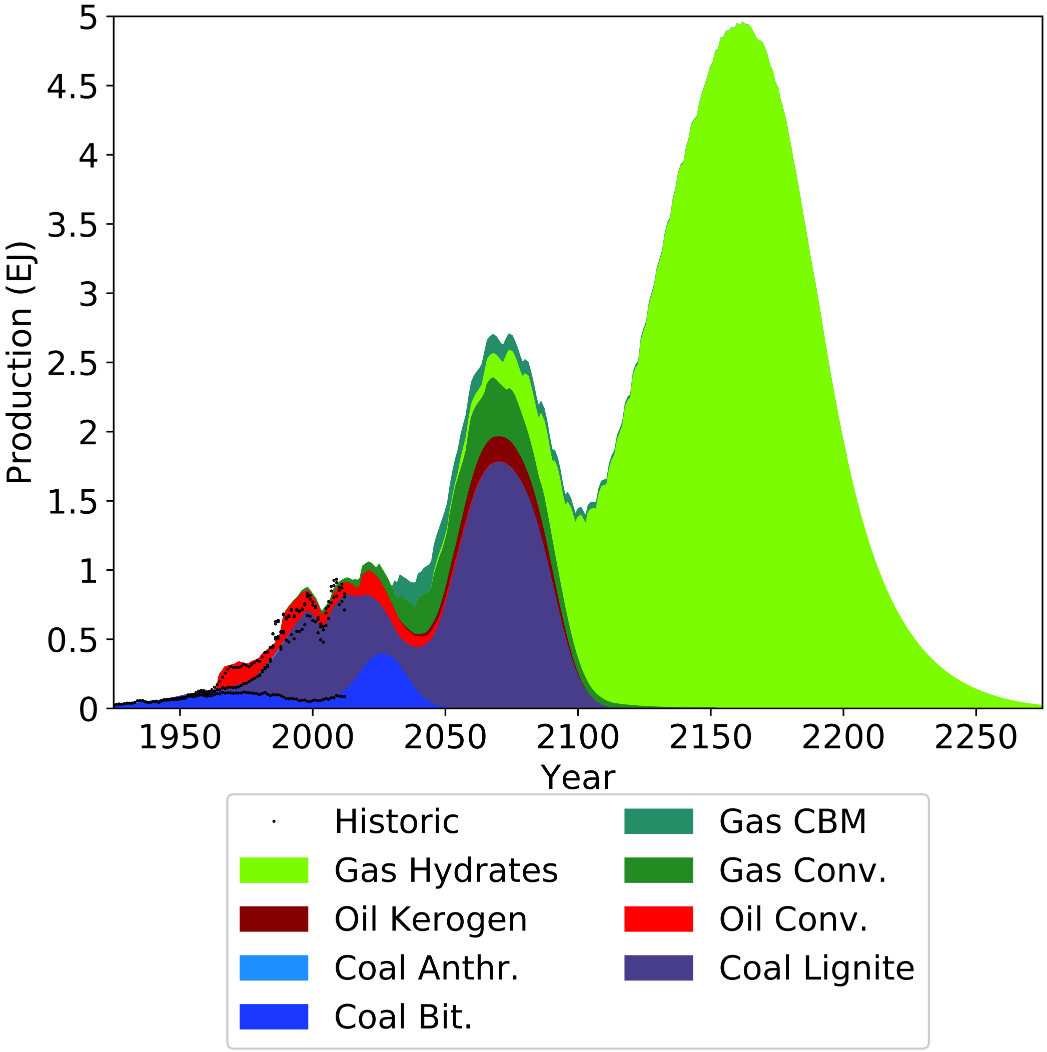

Figure 3.57: Turkey projection by mineral type

Table 3.57: Peak years - Minerals

**Name**	**URR**	**Peak Year**	**Peak Rate**
Coal Bit.	14.8	2026	0.39
Coal Lignite	96.4	2071	1.77
Coal Anthr.	0.13	1983	0.01
Oil Conv.	10.56	1990	0.2
Oil Kerogen	6.9	2067	0.18
Gas Conv.	23.89	2060	0.47
Gas Hydrates	392.3	2162	4.95
Gas CBM	10.5	2046	0.2
**Total**	**555.48**	**2162**	**4.95**

3.26 UK

3.26.1 All Projections

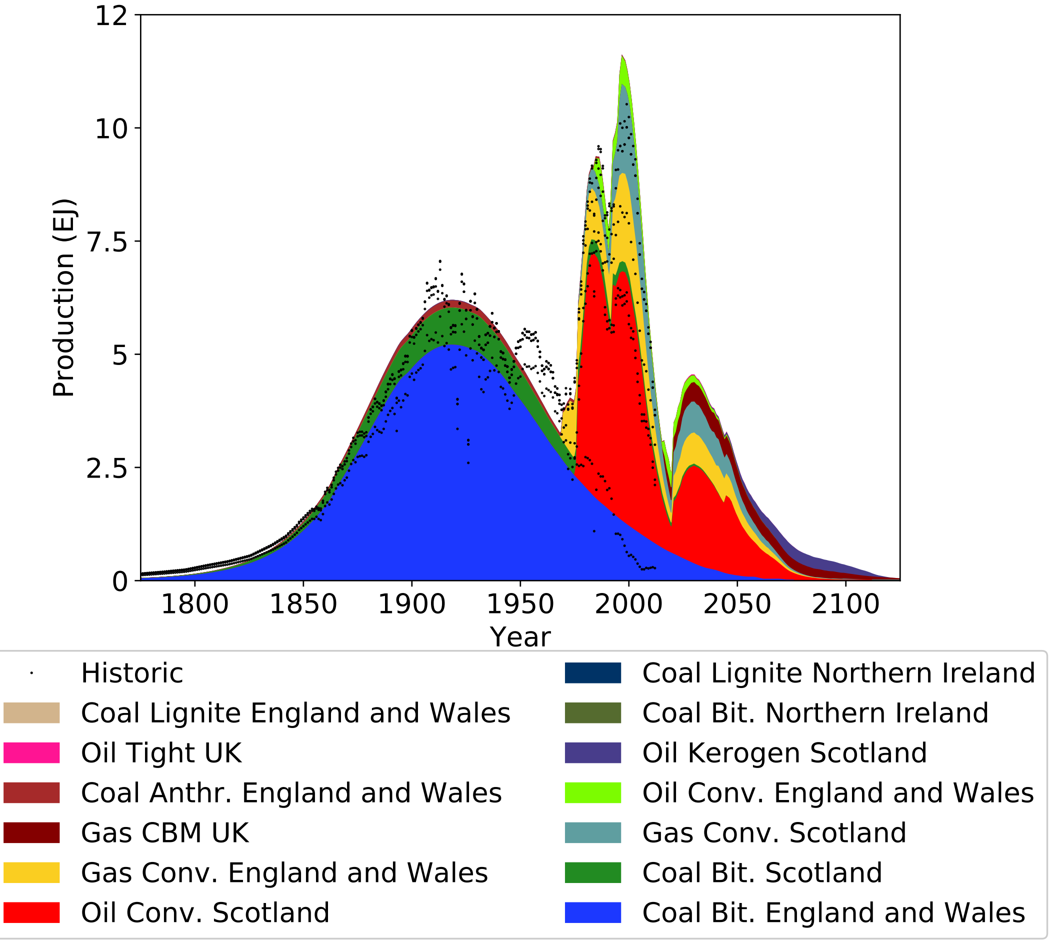

Figure 3.58: UK projections capped at 16

Table 3.58: Peak years - All

Name	URR	Peak Year	Peak Rate
Coal Bit. England and Wales	556.9	1918	5.19
Oil Conv. Scotland	229.54	1998	5.53
Coal Bit. Scotland	87.4	1919	0.82
Gas Conv. England and Wales	77.73	1998	1.96
Gas Conv. Scotland	60.31	1997	1.95
Gas CBM UK	27.5	2034	0.44
Oil Conv. England and Wales	17.19	1997	0.6
Coal Anthr. England and Wales	17.0	1926	0.16
Oil Kerogen Scotland	14.48	2073	0.28
Oil Tight UK	0.3	2028	0.02
Coal Bit. Northern Ireland	–	1932	–
Coal Lignite England and Wales	–	1947	–
Coal Lignite Northern Ireland	–	1944	–
**Total**	**1088.35**	**1997**	**11.56**

3.26.2 By Mineral

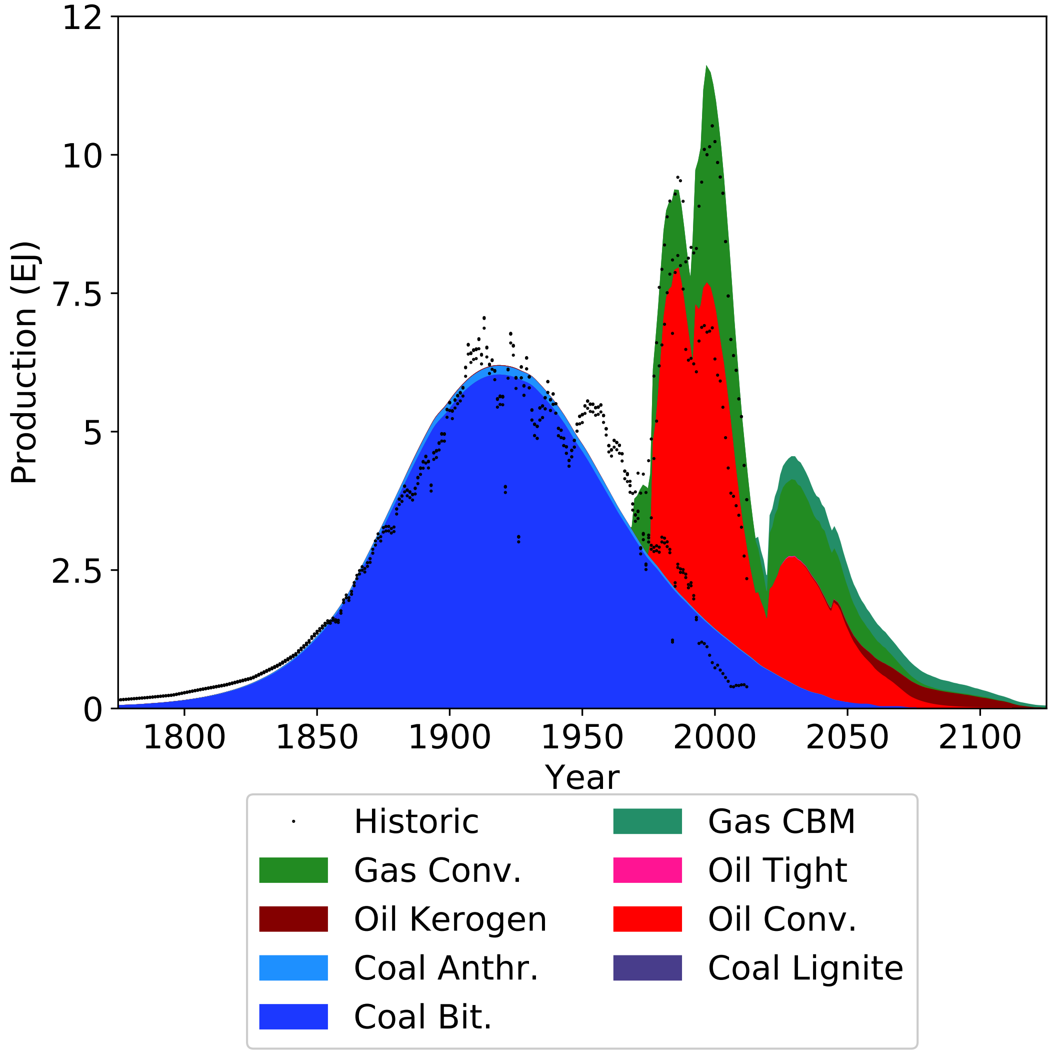

Figure 3.59: UK projection by mineral type

3.26.3 Regional Projections

Table 3.59: Peak years - Minerals

**Name**	**URR**	**Peak Year**	**Peak Rate**
Coal Bit.	644.3	1919	6.01
Coal Lignite	–	1947	–
Coal Anthr.	17.0	1926	0.16
Oil Conv.	246.73	1997	6.12
Oil Kerogen	14.48	2073	0.28
Oil Tight	0.3	2028	0.02
Gas Conv.	138.04	1997	3.9
Gas CBM	27.5	2034	0.44
**Total**	**1088.35**	**1997**	**11.56**

England and Wales

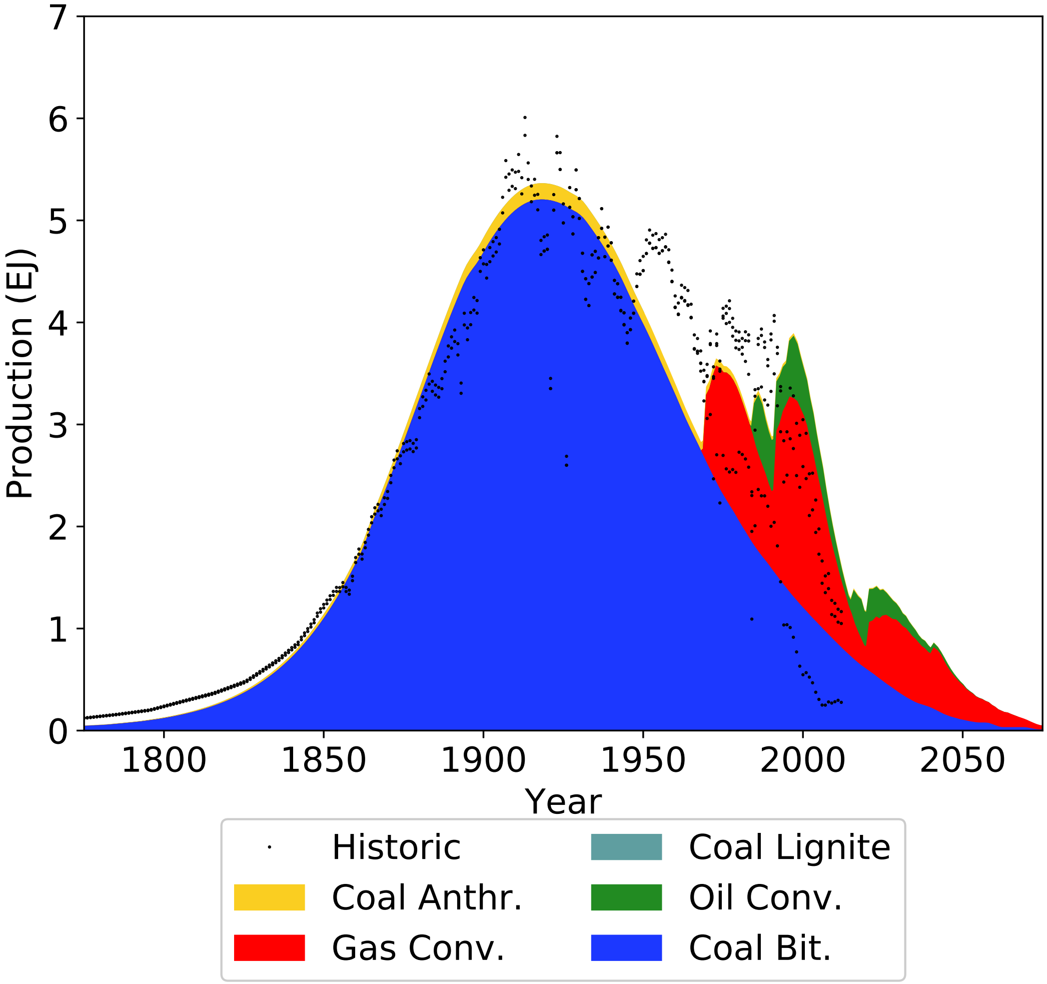

Figure 3.60: UK - England and Wales projections capped at 16

Table 3.60: Peak years - All

Name	URR	Peak Year	Peak Rate
Coal Bit. England and Wales	556.9	1918	5.19
Gas Conv. England and Wales	77.73	1998	1.96
Oil Conv. England and Wales	17.19	1997	0.6
Coal Anthr. England and Wales	17.0	1926	0.16
Coal Lignite England and Wales	–	1947	–
**Total**	**668.82**	**1918**	**5.35**

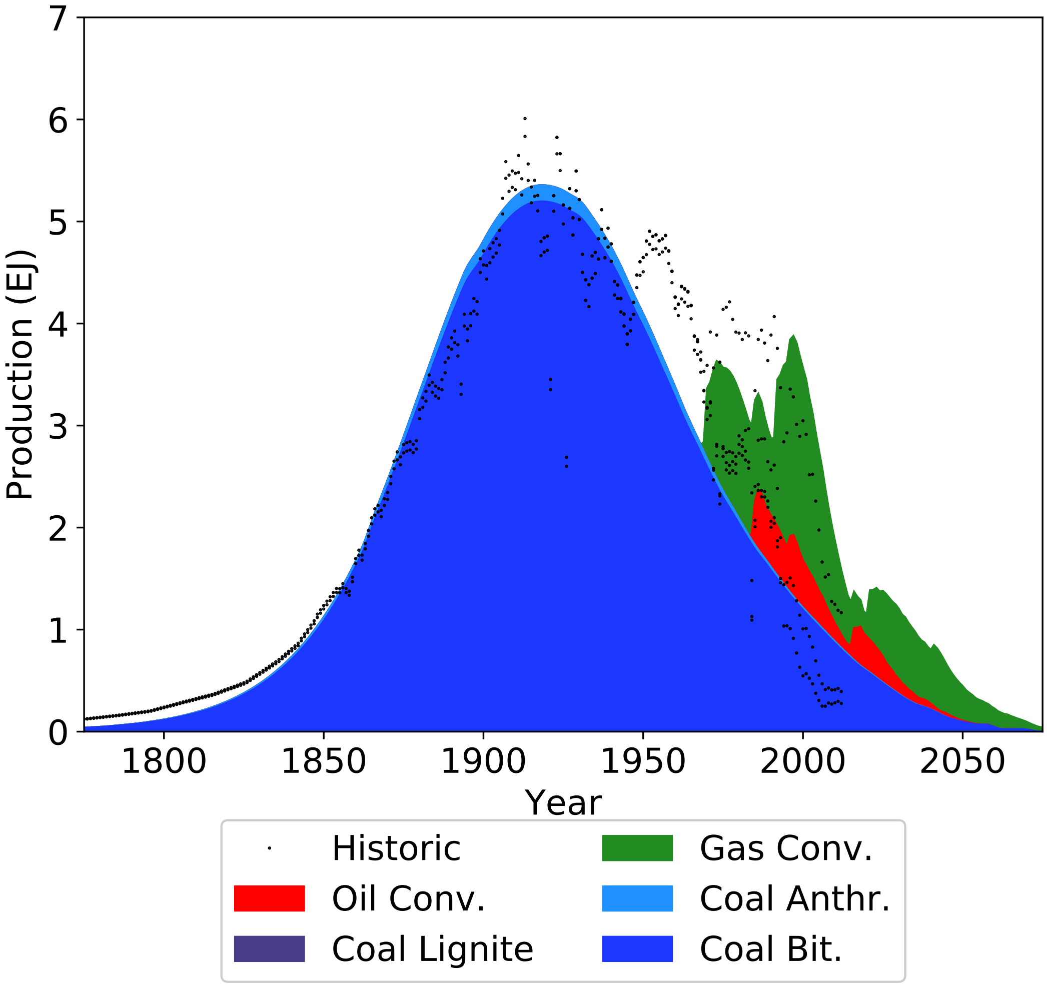

Figure 3.61: UK - England and Wales projection by mineral type

Table 3.61: Peak years - Minerals

**Name**	**URR**	**Peak Year**	**Peak Rate**
Coal Bit.	556.9	1918	5.19
Coal Lignite	–	1947	–
Coal Anthr.	17.0	1926	0.16
Oil Conv.	17.19	1997	0.6
Gas Conv.	77.73	1998	1.96
**Total**	**668.82**	**1918**	**5.35**

Northern Ireland

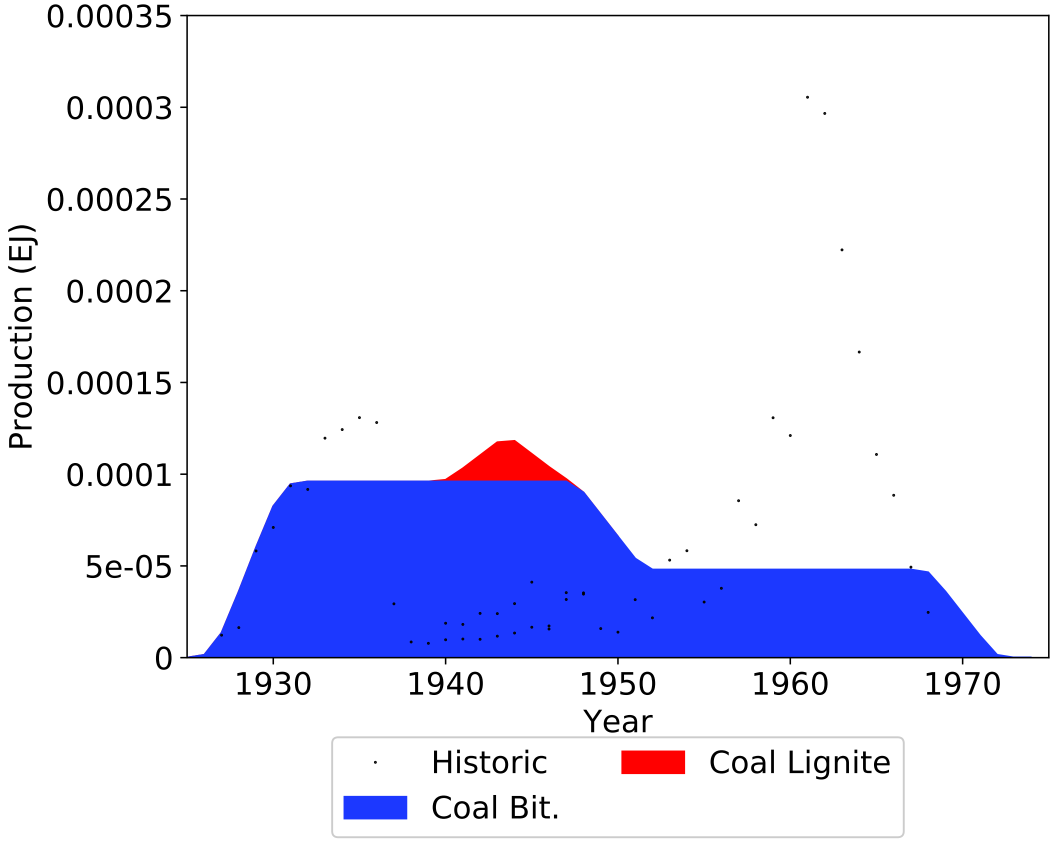

Figure 3.62: UK - Northern Ireland projections capped at 16

Table 3.62: Peak years - All

Name	URR	Peak Year	Peak Rate
Coal Bit. Northern Ireland	–	1932	–
Coal Lignite Northern Ireland	–	1944	–
**Total**	–	**1944**	–

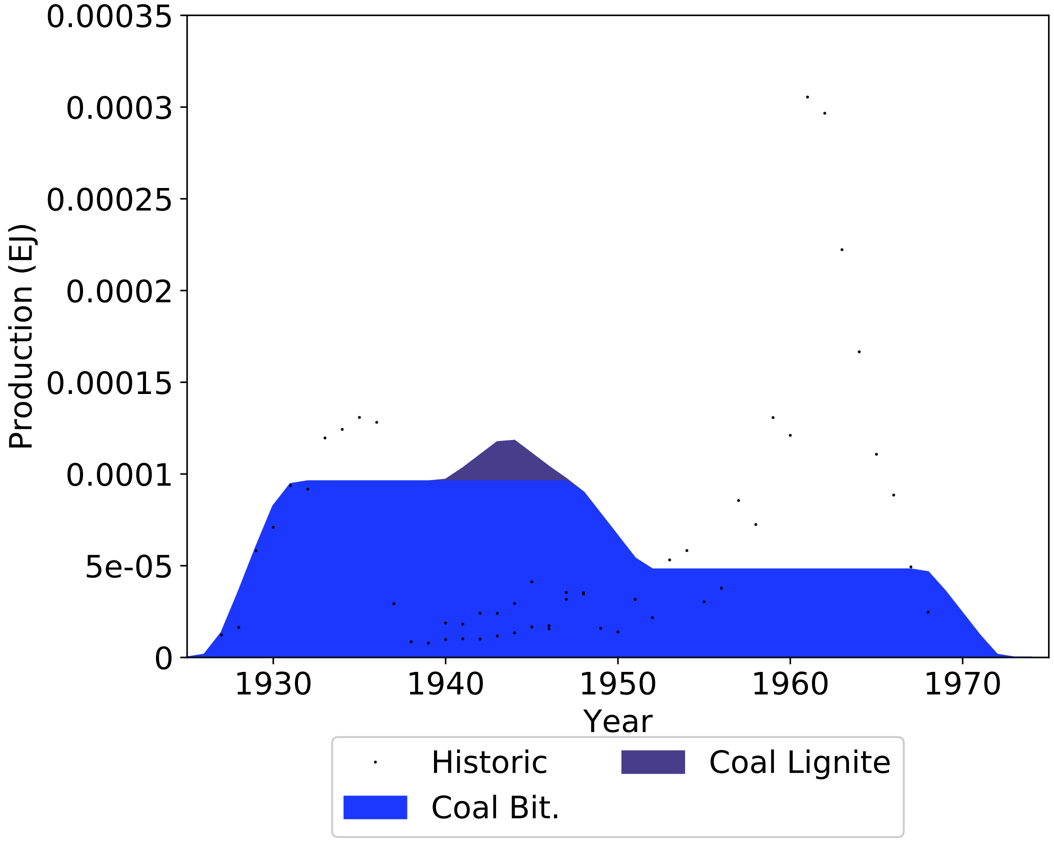

Figure 3.63: UK - Northern Ireland projection by mineral type

Table 3.63: Peak years - Minerals

**Name**	**URR**	**Peak Year**	**Peak Rate**
Coal Bit.	–	1932	–
Coal Lignite	–	1944	–
**Total**	–	**1944**	–

Scotland

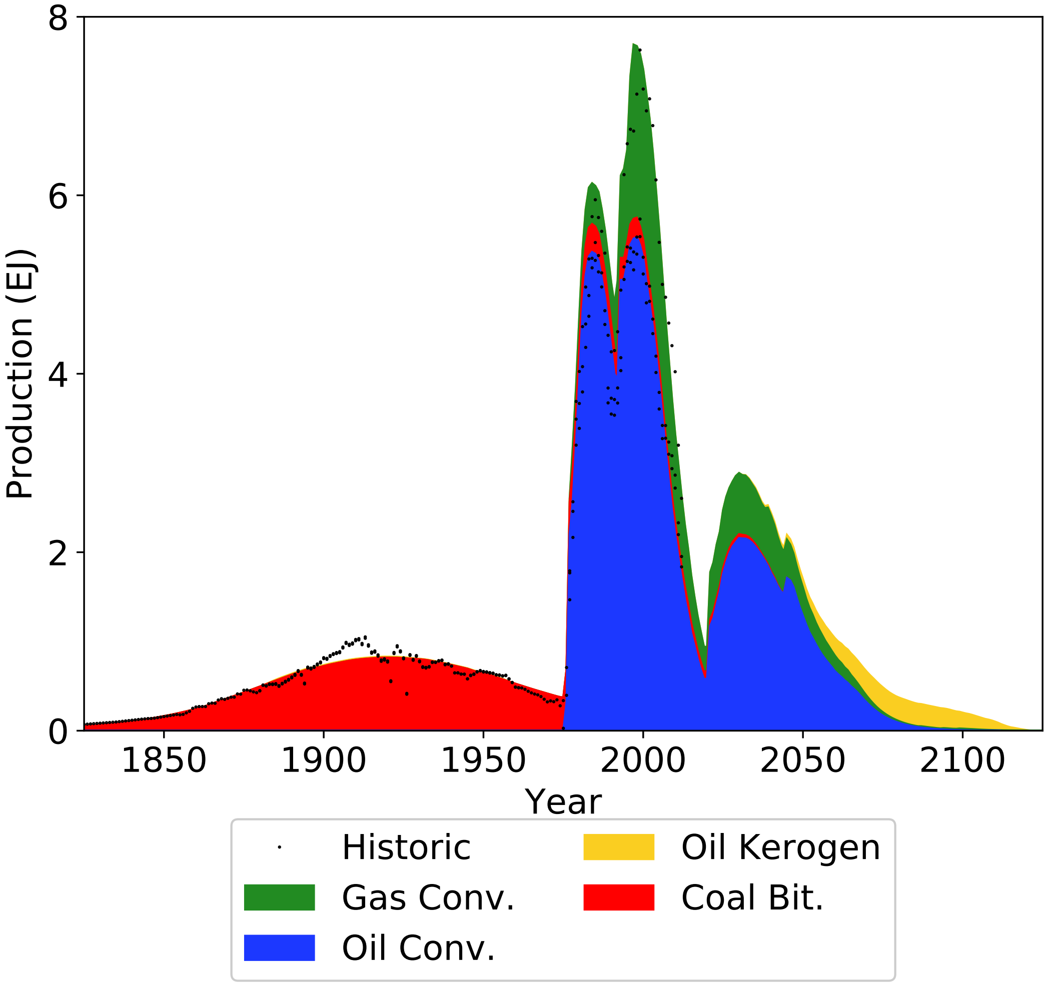

Figure 3.64: UK - Scotland projections capped at 16

Table 3.64: Peak years - All

Name	URR	Peak Year	Peak Rate
Oil Conv. Scotland	229.54	1998	5.53
Coal Bit. Scotland	87.4	1919	0.82
Gas Conv. Scotland	60.31	1997	1.95
Oil Kerogen Scotland	14.48	2073	0.28
**Total**	**391.73**	**1997**	**7.69**

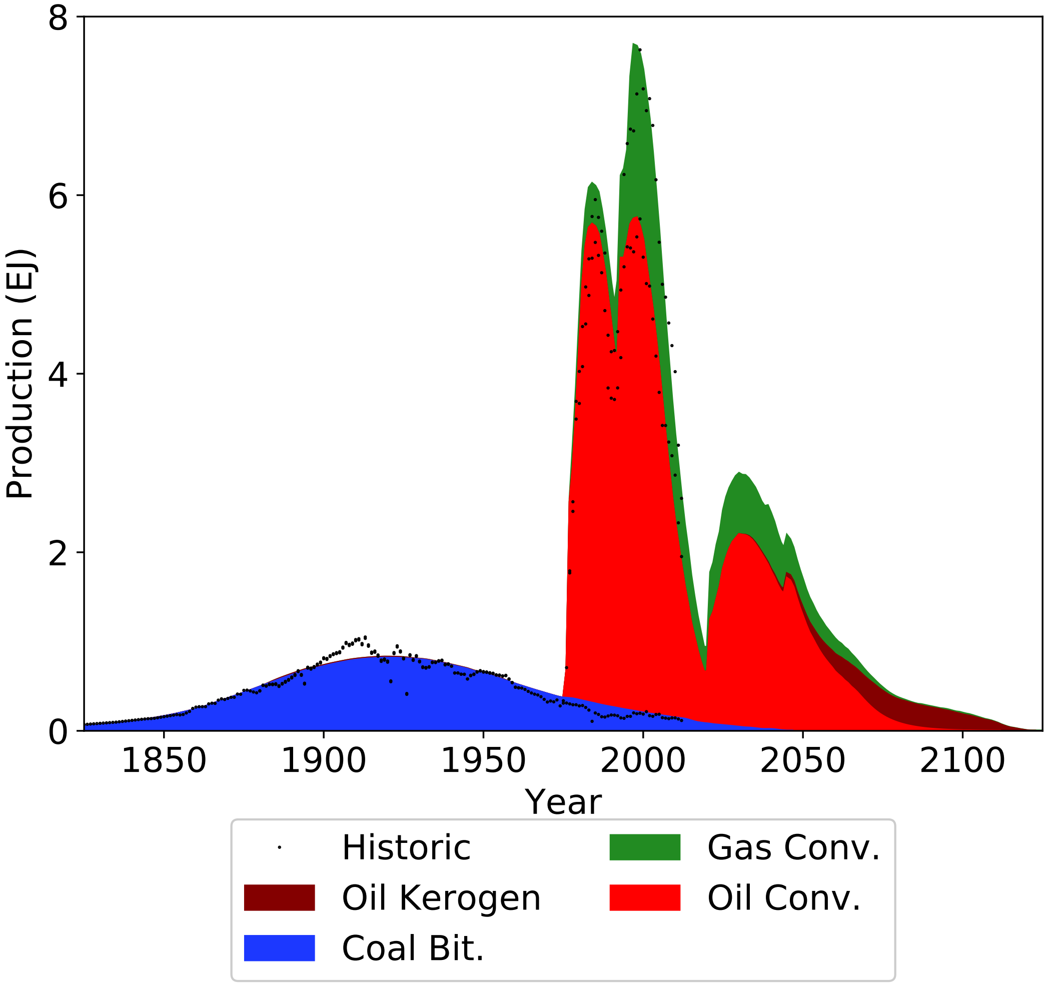

Figure 3.65: UK - Scotland projection by mineral type

Table 3.65: Peak years - Minerals

Name	URR	Peak Year	Peak Rate
Coal Bit.	87.4	1919	0.82
Oil Conv.	229.54	1998	5.53
Oil Kerogen	14.48	2073	0.28
Gas Conv.	60.31	1997	1.95
**Total**	**391.73**	**1997**	**7.69**

UK

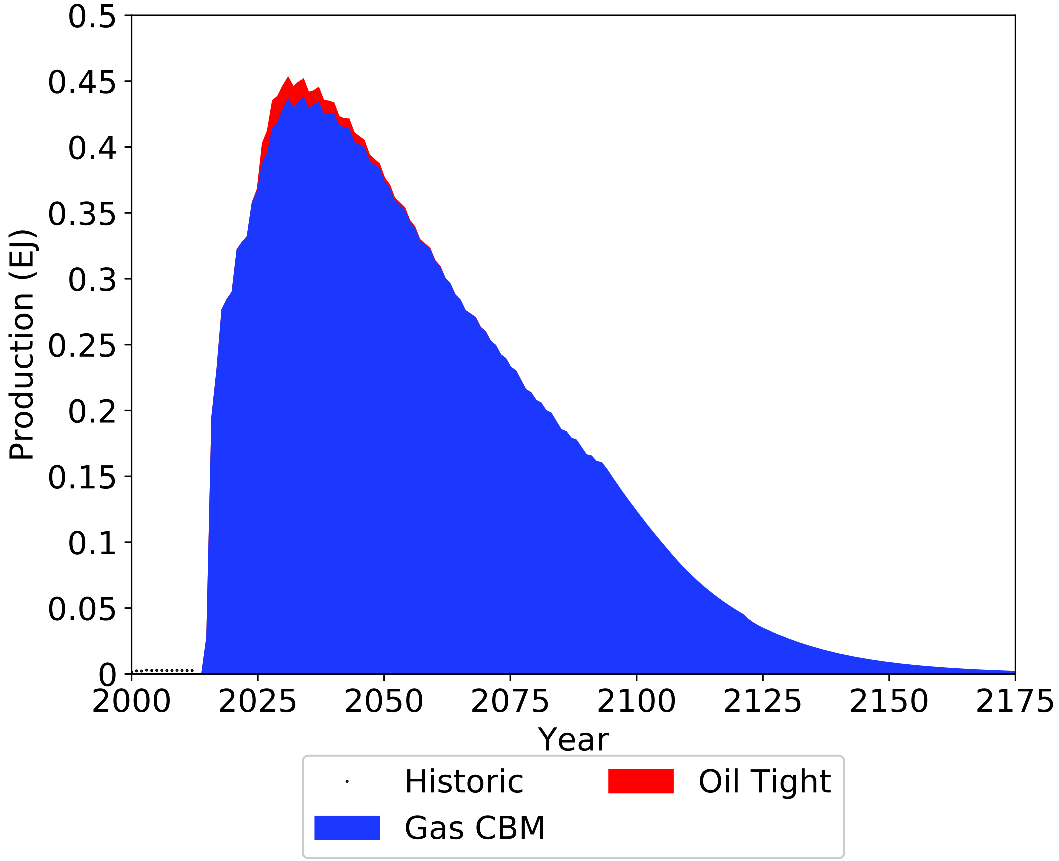

Figure 3.66: UK - UK projections capped at 16

Table 3.66: Peak years - All

Name	URR	Peak Year	Peak Rate
Gas CBM UK	27.5	2034	0.44
Oil Tight UK	0.3	2028	0.02
**Total**	**27.8**	**2031**	**0.45**

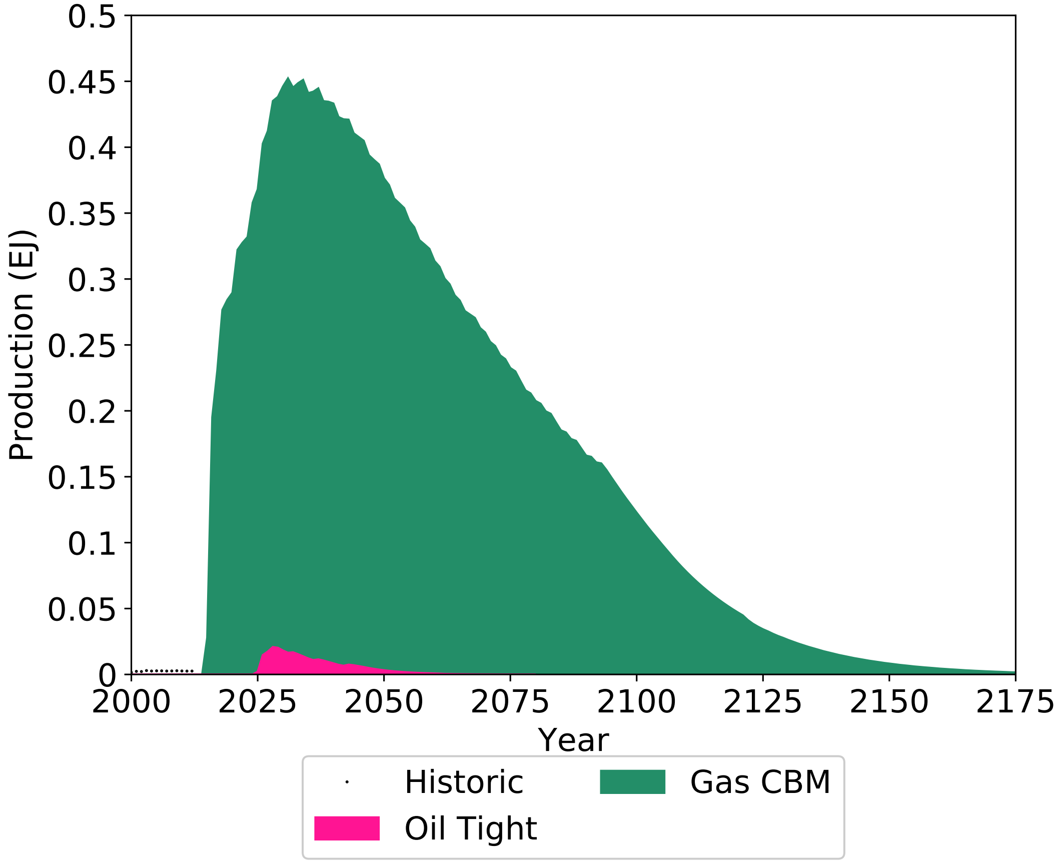

Figure 3.67: UK - UK projection by mineral type

Table 3.67: Peak years - Minerals

Name	URR	Peak Year	Peak Rate
Oil Tight	0.3	2028	0.02
Gas CBM	27.5	2034	0.44
**Total**	**27.8**	**2031**	**0.45**

3.26.4 Projection by region

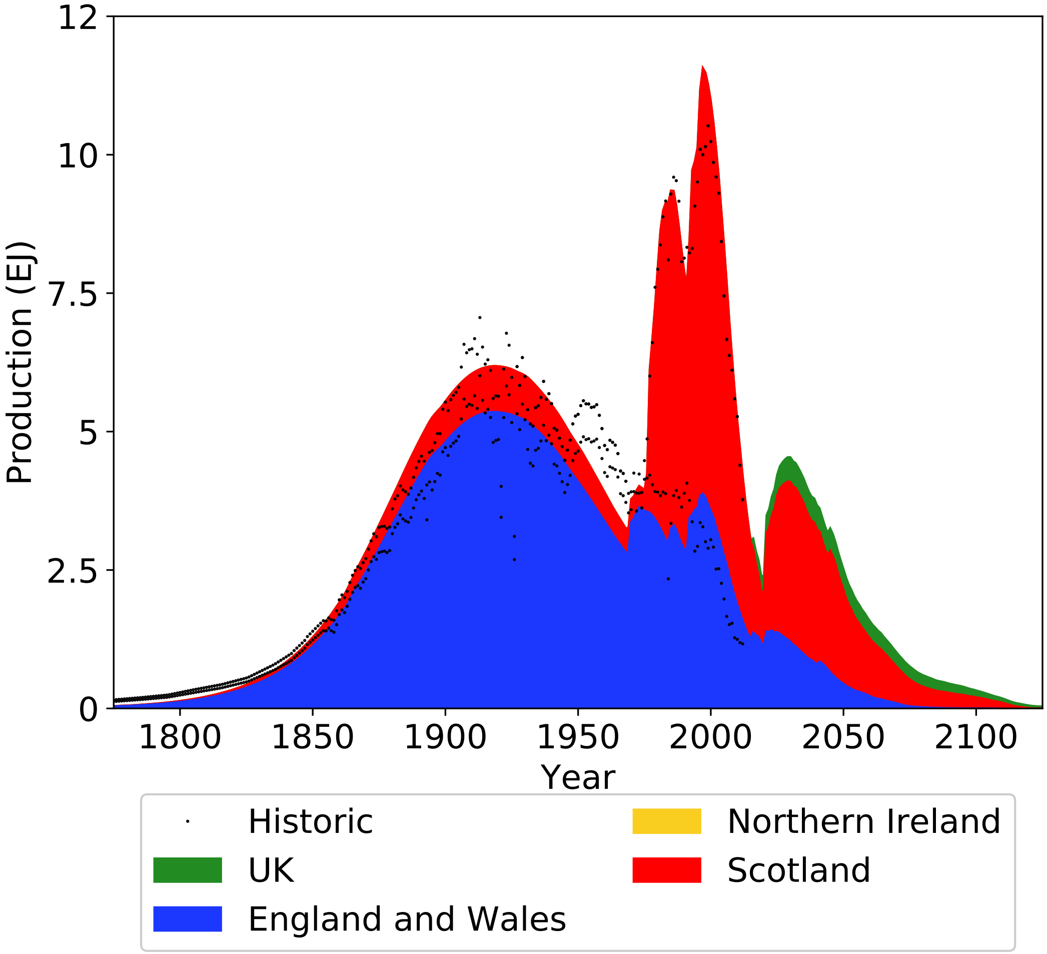

Figure 3.68: UK by region projections capped at 16

Table 3.68: Peak years - All

**Name**	**URR**	**Peak Year**	**Peak Rate**
England and Wales	668.82	1918	5.35
Scotland	391.73	1997	7.69
UK	27.8	2031	0.45
Northern Ireland	–	1944	–
**Total**	**1088.35**	**1997**	**11.56**

3.27 Yugoslavia

3.27.1 All Projections

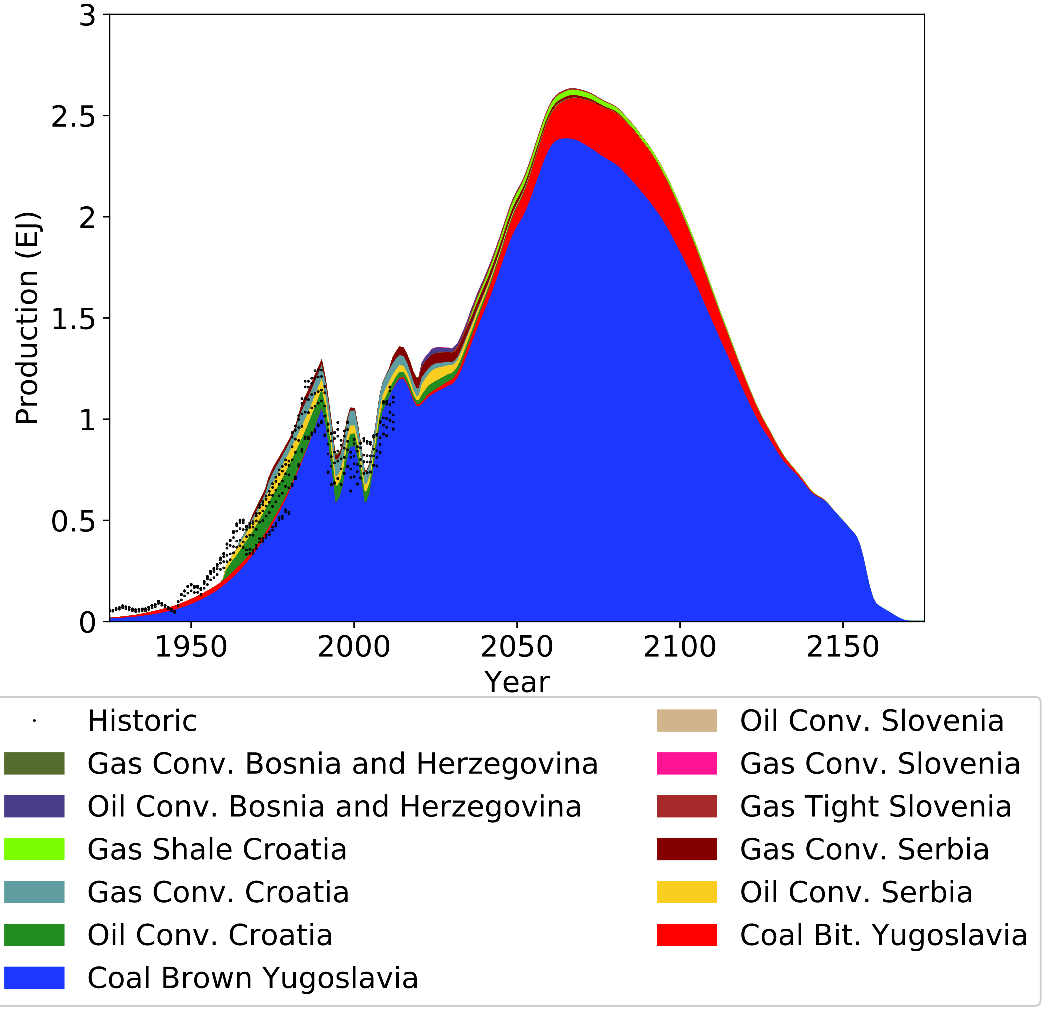

Figure 3.69: Yugoslavia projections capped at 16

Table 3.69: Peak years - All

Name	URR	Peak Year	Peak Rate
Coal Brown Yugoslavia	251.5	2065	2.38
Coal Bit. Yugoslavia	16.29	2085	0.26
Oil Conv. Croatia	5.46	1975	0.13
Oil Conv. Serbia	3.69	1979	0.06
Gas Conv. Croatia	3.43	1993	0.08
Gas Conv. Serbia	2.81	2022	0.05
Gas Shale Croatia	1.85	2054	0.03
Gas Tight Slovenia	0.56	2035	0.01
Oil Conv. Bosnia and Herzegovina	0.42	2025	0.03
Gas Conv. Slovenia	0.04	2022	–
Gas Conv. Bosnia and Herzegovina	0.03	1995	0.01
Oil Conv. Slovenia	–	1996	–
**Total**	**286.08**	**2067**	**2.63**

3.27.2 By Mineral

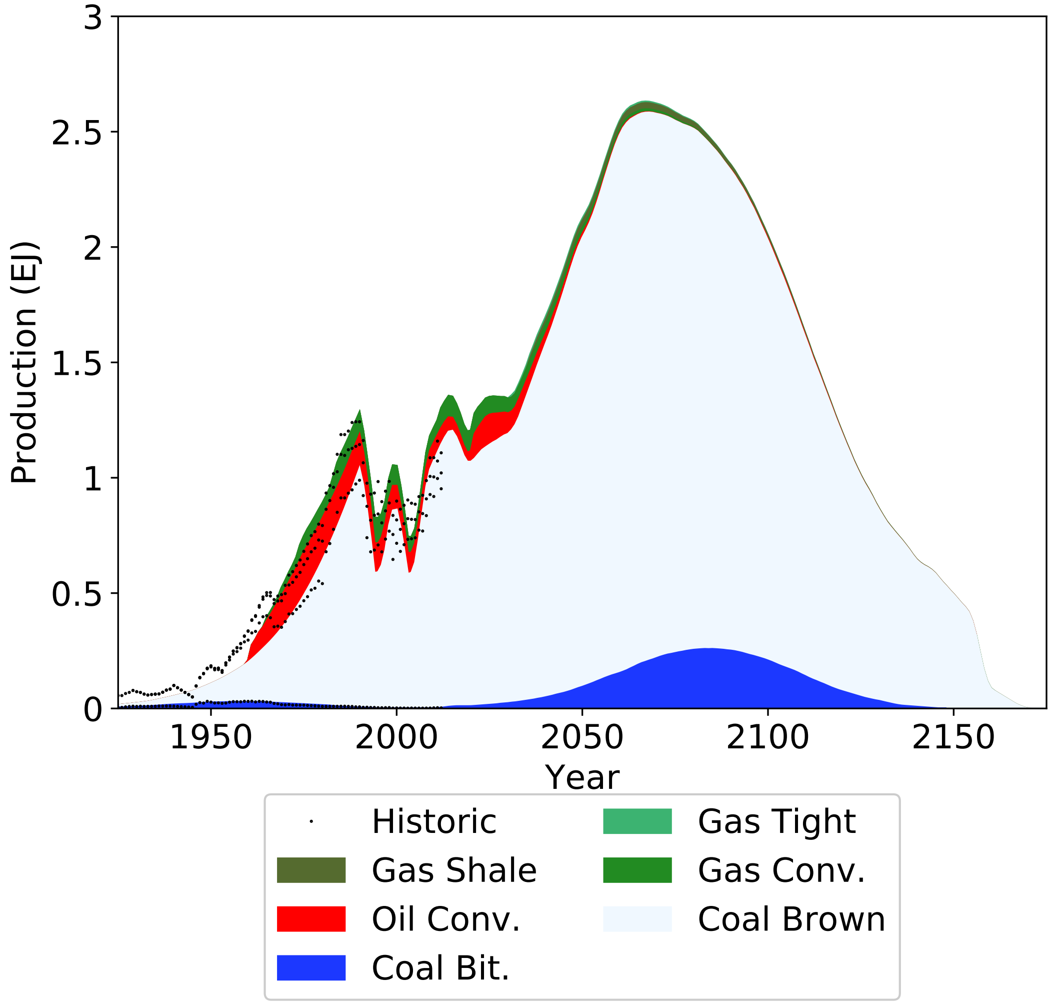

Figure 3.70: Yugoslavia projection by mineral type

3.27.3 Regional Projections

Table 3.70: Peak years - Minerals

**Name**	**URR**	**Peak Year**	**Peak Rate**
Coal Bit.	16.29	2085	0.26
Coal Brown	251.5	2065	2.38
Oil Conv.	9.57	1975	0.19
Gas Conv.	6.32	1985	0.11
Gas Shale	1.85	2054	0.03
Gas Tight	0.56	2035	0.01
**Total**	**286.08**	**2067**	**2.63**

Bosnia and Herzegovina

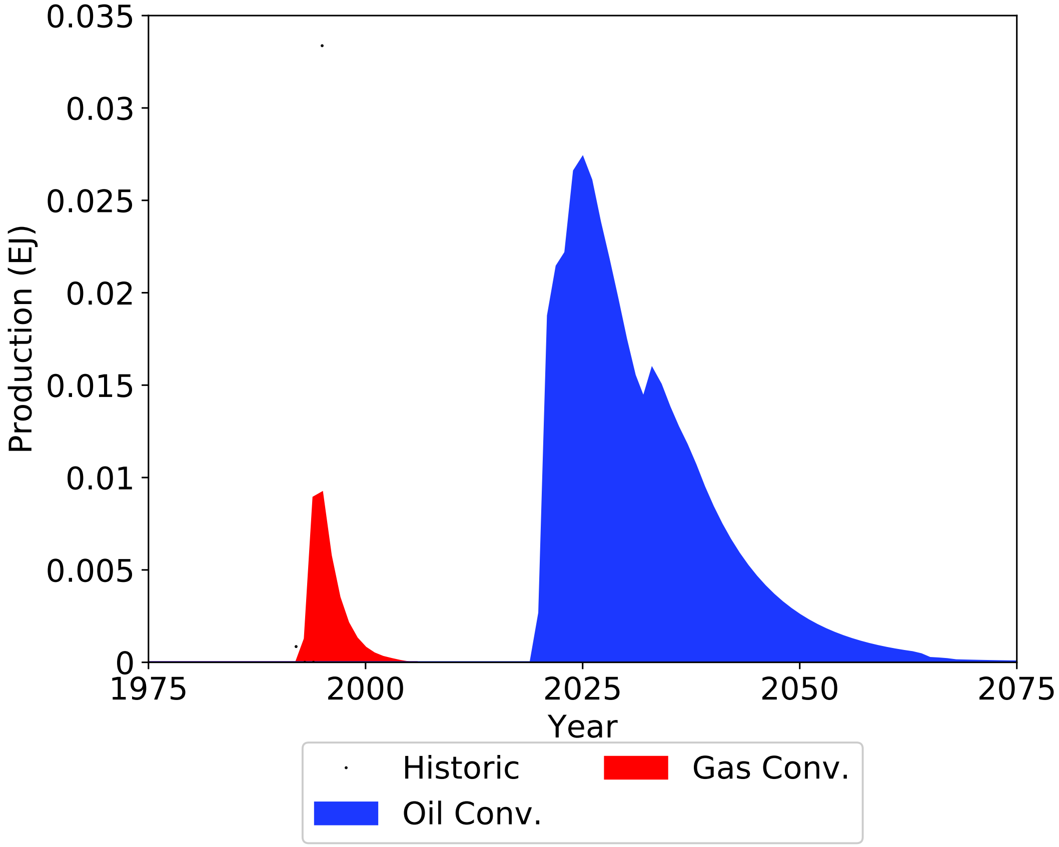

Figure 3.71: Yugoslavia - Bosnia and Herzegovina projections capped at 16

Table 3.71: Peak years - All

**Name**	**URR**	**Peak Year**	**Peak Rate**
Oil Conv. Bosnia and Herzegovina	0.42	2025	0.03
Gas Conv. Bosnia and Herzegovina	0.03	1995	0.01
**Total**	**0.45**	**2025**	**0.03**

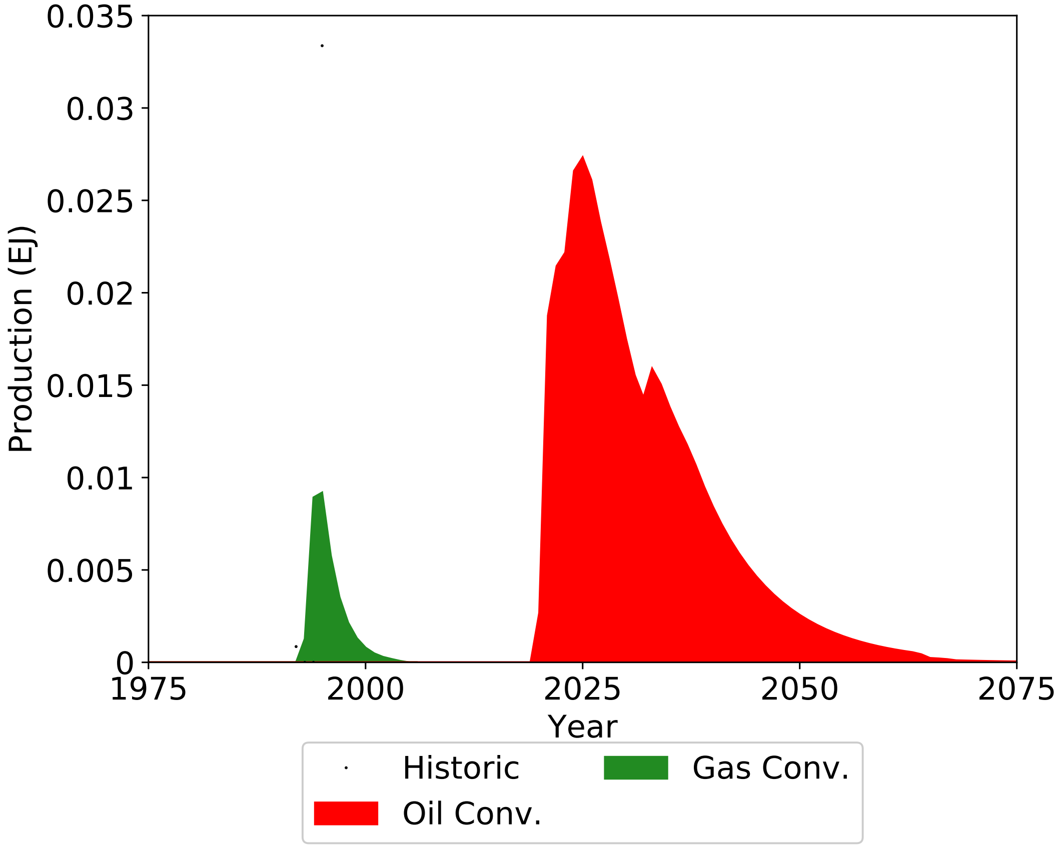

Figure 3.72: Yugoslavia - Bosnia and Herzegovina projection by mineral type

Table 3.72: Peak years - Minerals

Name	URR	Peak Year	Peak Rate
Oil Conv.	0.42	2025	0.03
Gas Conv.	0.03	1995	0.01
**Total**	**0.45**	**2025**	**0.03**

Croatia

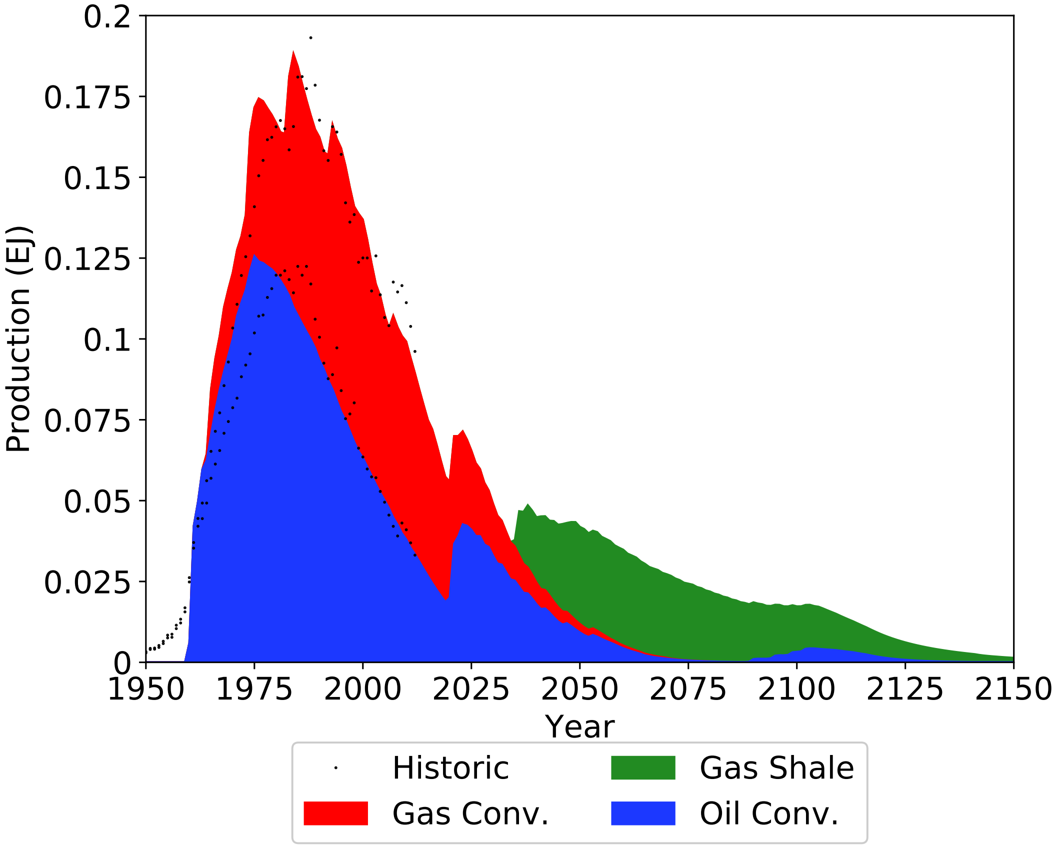

Figure 3.73: Yugoslavia - Croatia projections capped at 16

Table 3.73: Peak years - All

Name	URR	Peak Year	Peak Rate
Oil Conv. Croatia	5.46	1975	0.13
Gas Conv. Croatia	3.43	1993	0.08
Gas Shale Croatia	1.85	2054	0.03
**Total**	**10.74**	**1984**	**0.19**

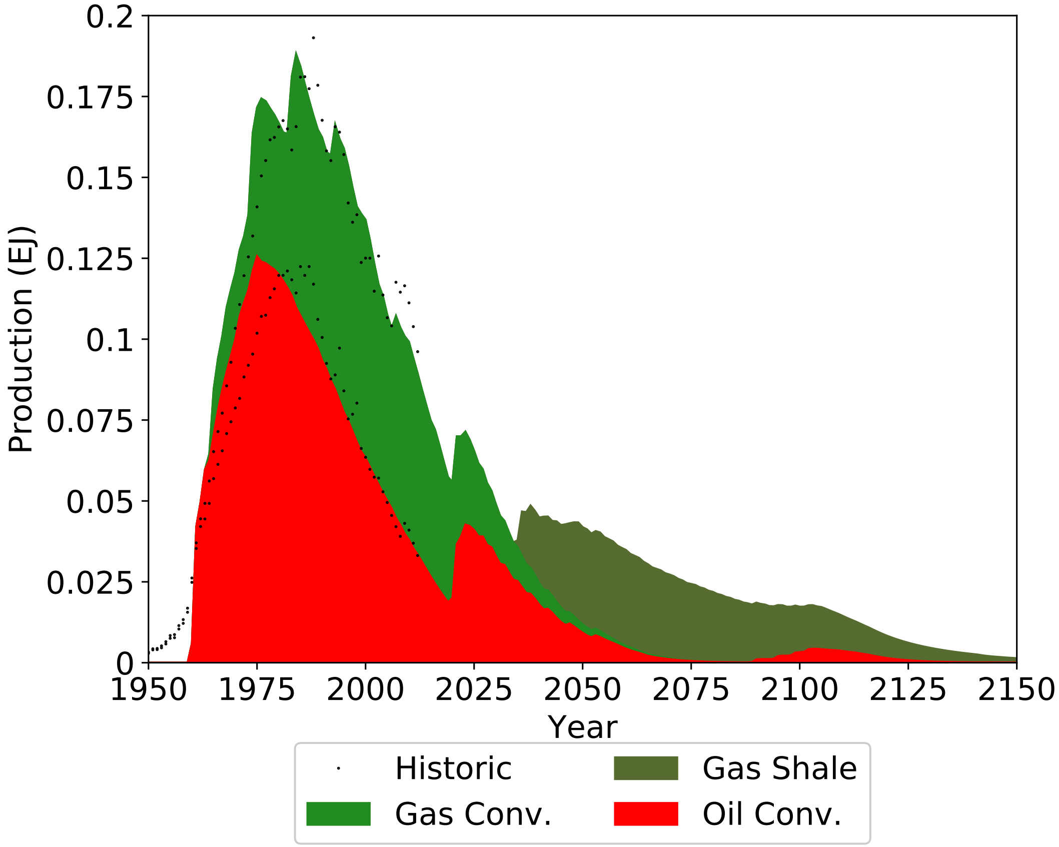

Figure 3.74: Yugoslavia - Croatia projection by mineral type

Table 3.74: Peak years - Minerals

Name	URR	Peak Year	Peak Rate
Oil Conv.	5.46	1975	0.13
Gas Conv.	3.43	1993	0.08
Gas Shale	1.85	2054	0.03
**Total**	**10.74**	**1984**	**0.19**

Serbia

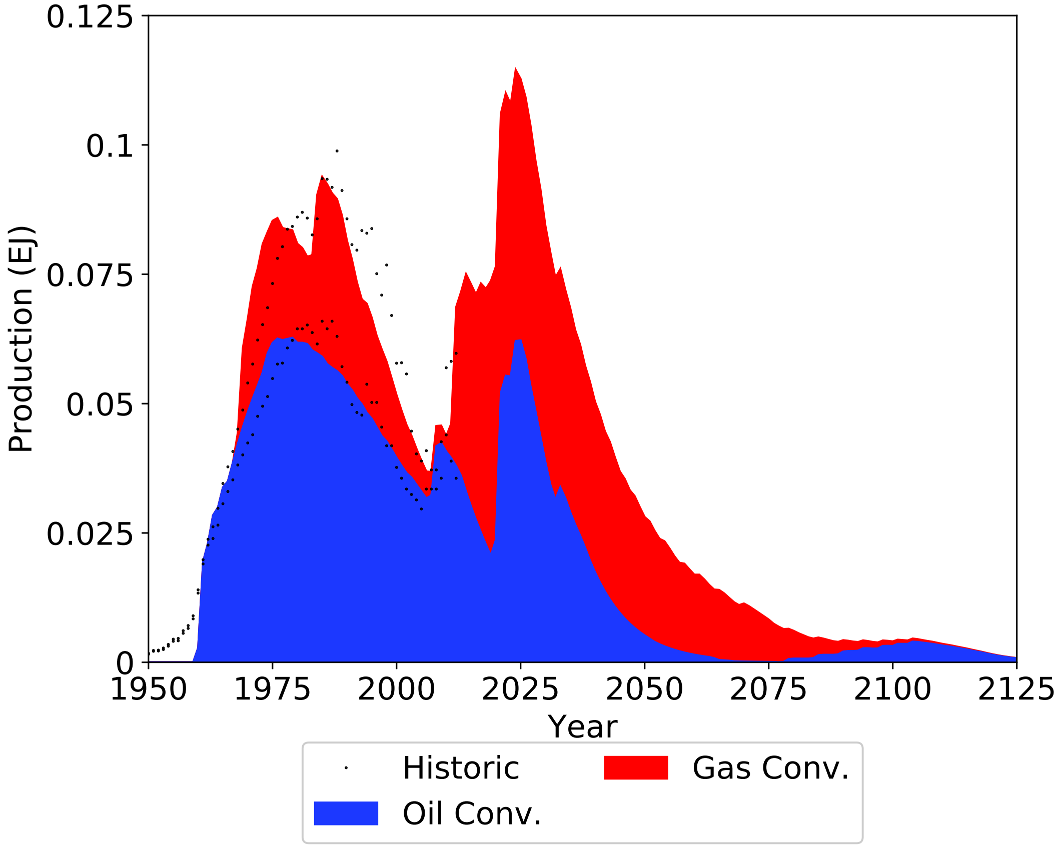

Figure 3.75: Yugoslavia - Serbia projections capped at 16

Table 3.75: Peak years - All

Name	URR	Peak Year	Peak Rate
Oil Conv. Serbia	3.69	1979	0.06
Gas Conv. Serbia	2.81	2022	0.05
**Total**	**6.5**	**2024**	**0.11**

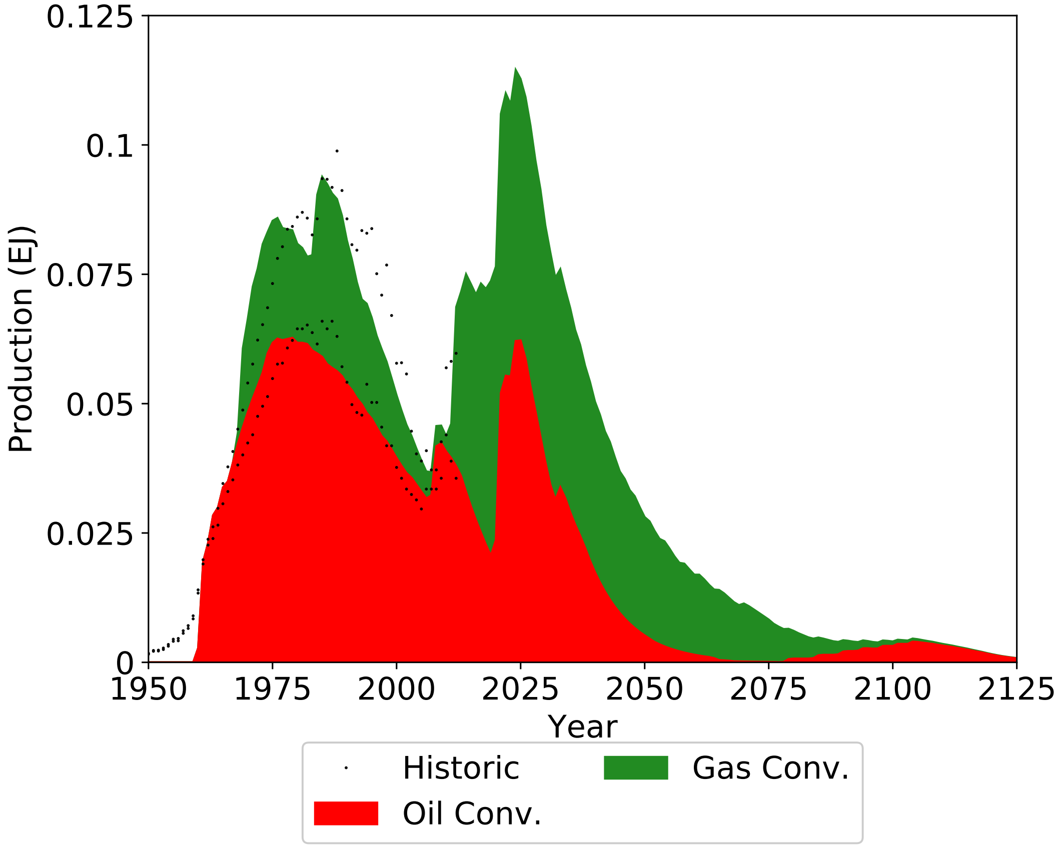

Figure 3.76: Yugoslavia - Serbia projection by mineral type

Table 3.76: Peak years - Minerals

Name	URR	Peak Year	Peak Rate
Oil Conv.	3.69	1979	0.06
Gas Conv.	2.81	2022	0.05
**Total**	**6.5**	**2024**	**0.11**

Slovenia

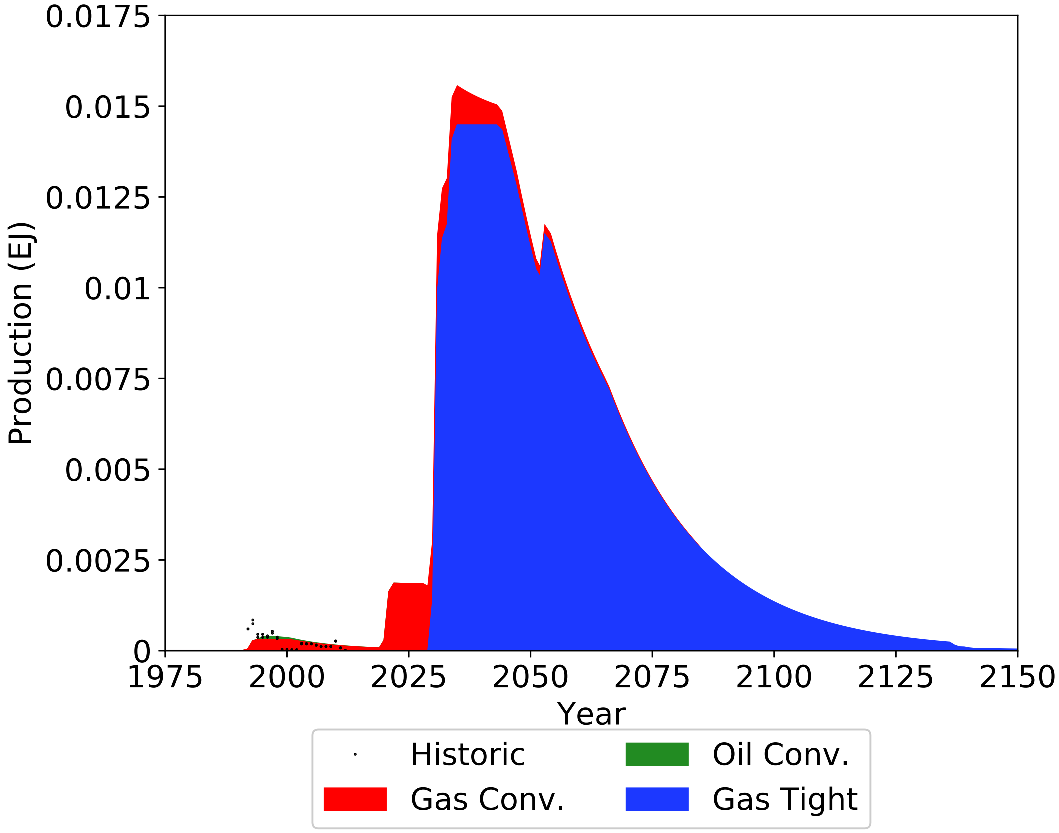

Figure 3.77: Yugoslavia - Slovenia projections capped at 16

Table 3.77: Peak years - All

Name	URR	Peak Year	Peak Rate
Gas Tight Slovenia	0.56	2035	0.01
Gas Conv. Slovenia	0.04	2022	–
Oil Conv. Slovenia	–	1996	–
**Total**	**0.6**	**2035**	**0.02**

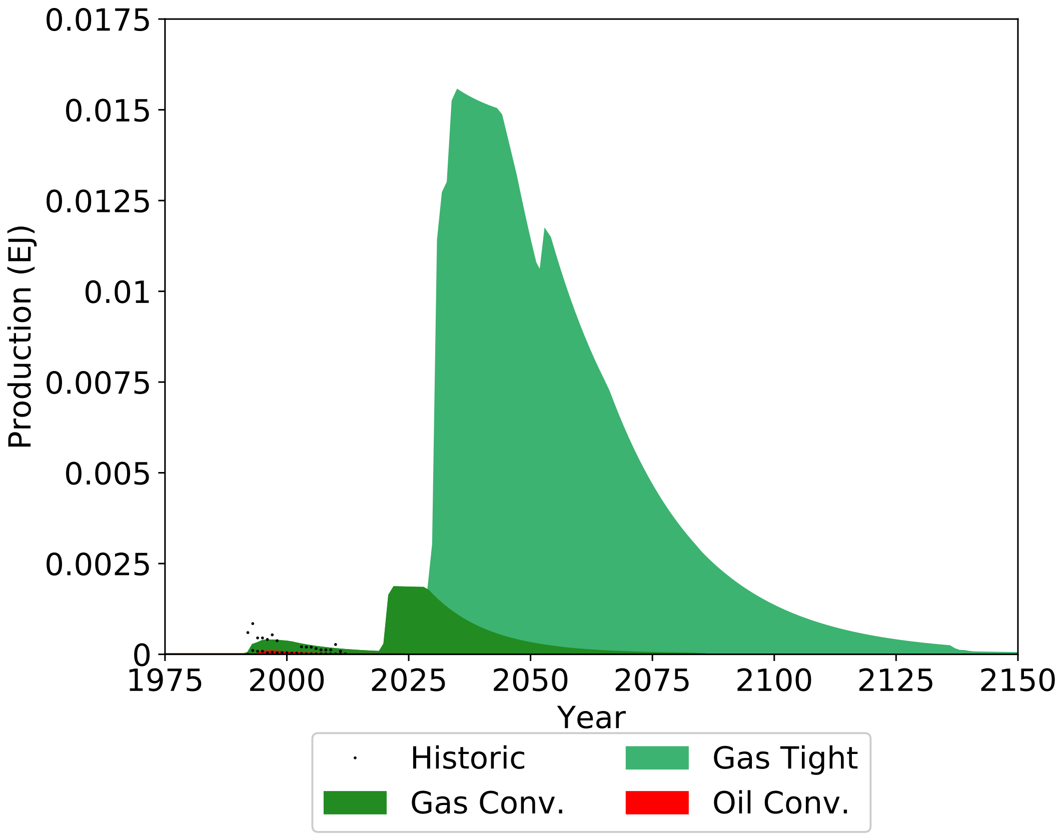

Figure 3.78: Yugoslavia - Slovenia projection by mineral type

Table 3.78: Peak years - Minerals

**Name**	**URR**	**Peak Year**	**Peak Rate**
Oil Conv.	–	1996	–
Gas Conv.	0.04	2022	–
Gas Tight	0.56	2035	0.01
**Total**	**0.6**	**2035**	**0.02**

Yugoslavia

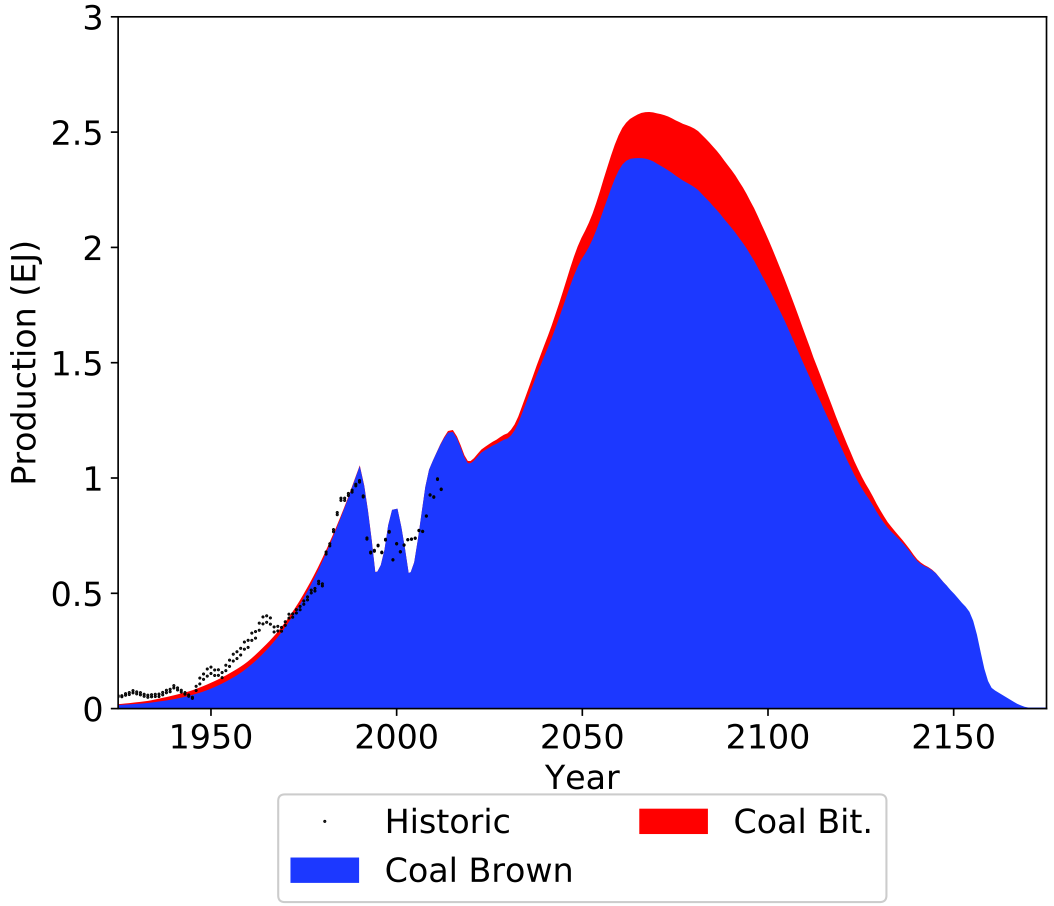

Figure 3.79: Yugoslavia - Yugoslavia projections capped at 16

Table 3.79: Peak years - All

Name	URR	Peak Year	Peak Rate
Coal Brown Yugoslavia	251.5	2065	2.38
Coal Bit. Yugoslavia	16.29	2085	0.26
**Total**	**267.79**	**2068**	**2.58**

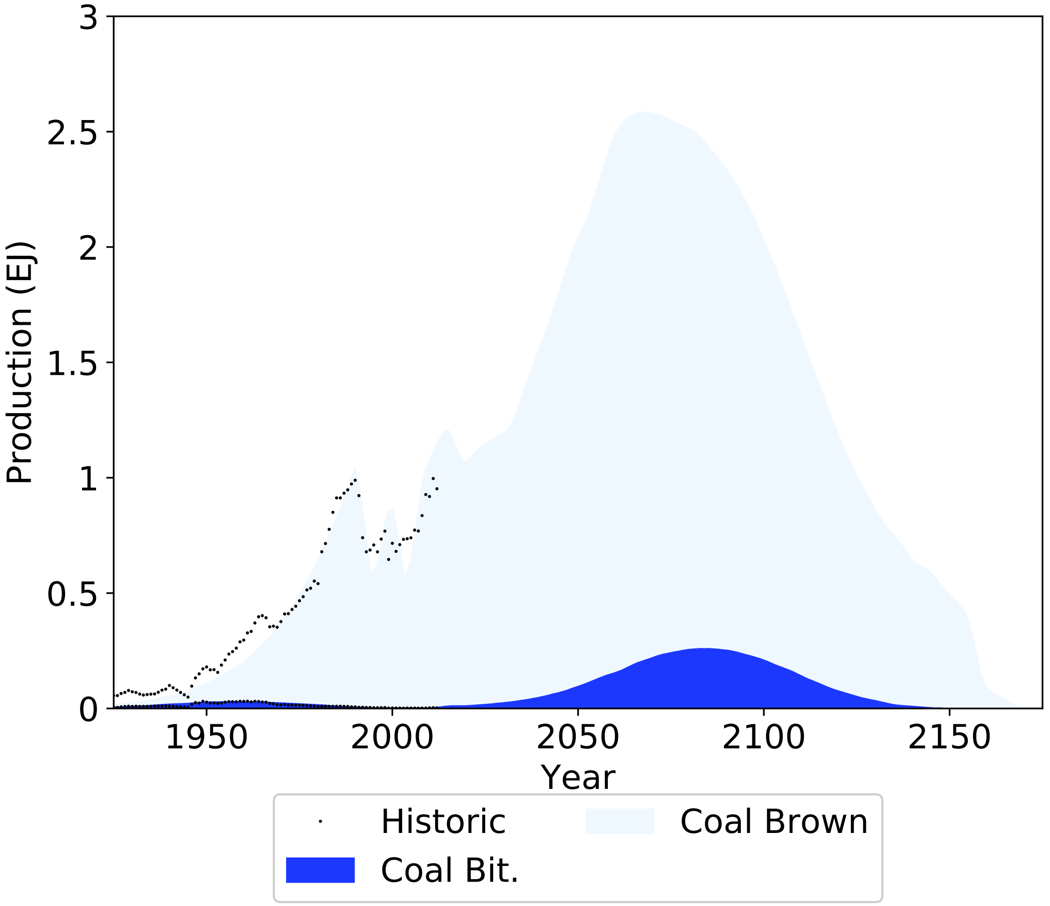

Figure 3.80: Yugoslavia - Yugoslavia projection by mineral type

Table 3.80: Peak years - Minerals

Name	URR	Peak Year	Peak Rate
Coal Bit.	16.29	2085	0.26
Coal Brown	251.5	2065	2.38
**Total**	**267.79**	**2068**	**2.58**

3.27.4 Projection by region

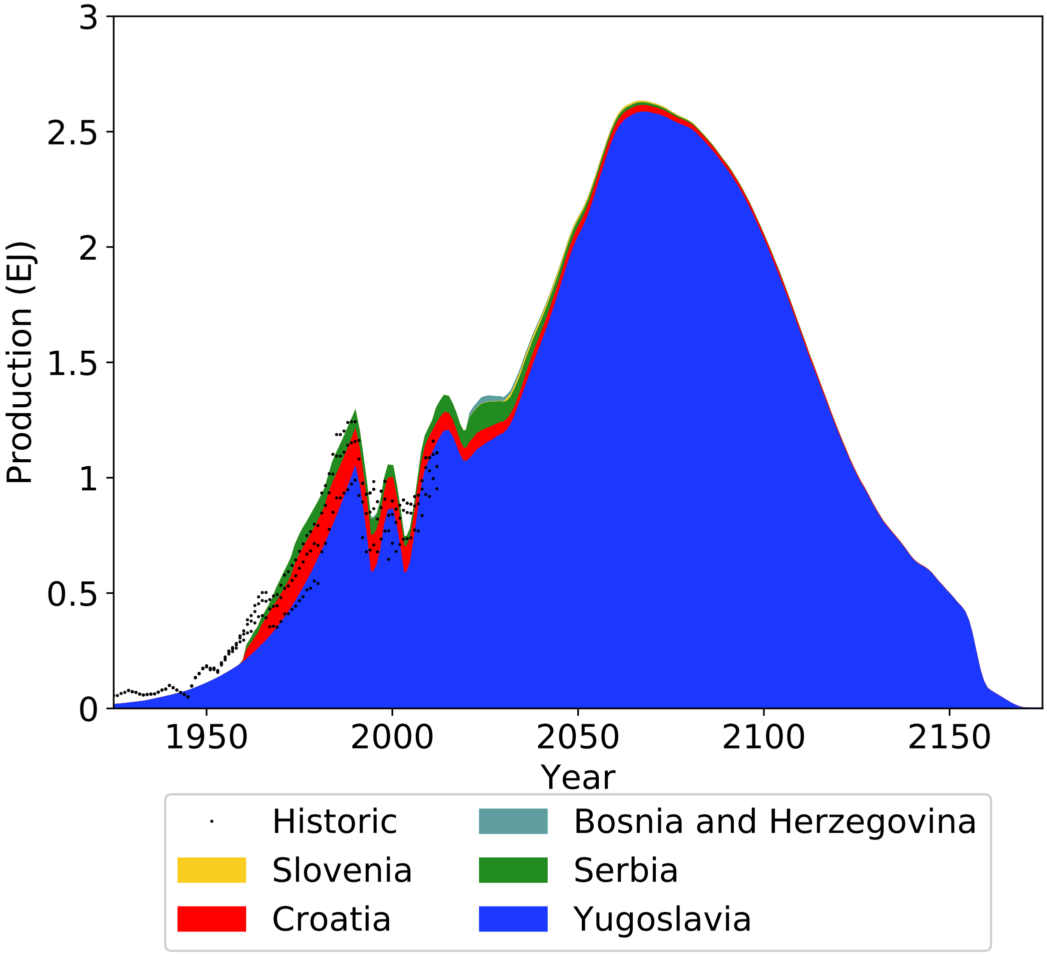

Figure 3.81: Yugoslavia by region projections capped at 16

Table 3.81: Peak years - All

**Name**	**URR**	**Peak Year**	**Peak Rate**
Yugoslavia	267.79	2068	2.58
Croatia	10.74	1984	0.19
Serbia	6.5	2024	0.11
Slovenia	0.6	2035	0.02
Bosnia and Herzegovina	0.45	2025	0.03
**Total**	**286.08**	**2067**	**2.63**

3.28 Total

3.28.1 By country

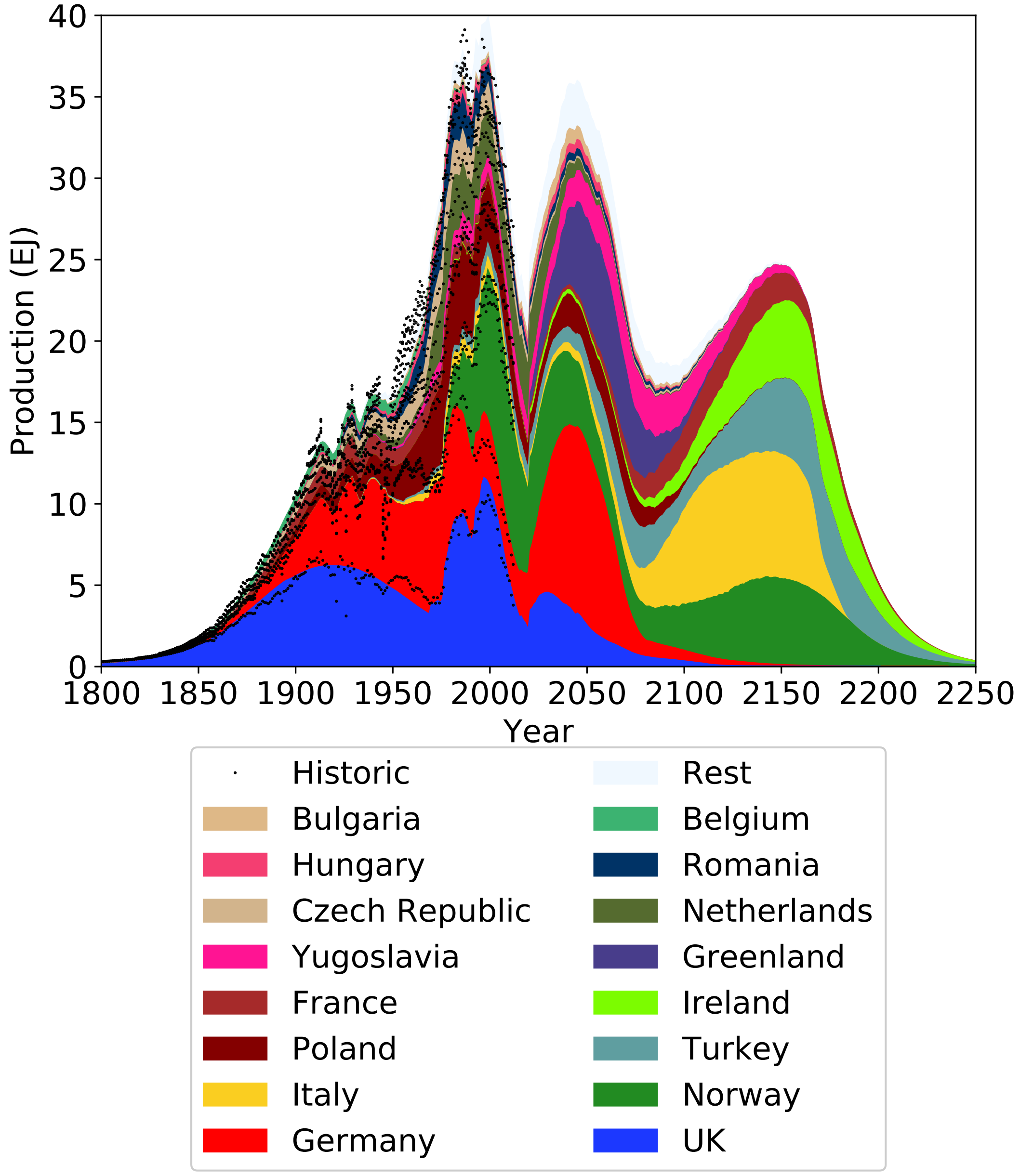

Figure 3.82: Europe projections by country

Table 3.82: Peak years - All

**Name**	**URR**	**Peak Year**	**Peak Rate**
UK	1088.35	1997	11.56
Germany	1085.66	2044	11.48
Norway	962.8	2004	10.01
Italy	688.4	2136	7.8
Turkey	555.48	2162	4.95
Poland	432.08	1983	5.2
Ireland	406.56	2159	4.82
France	341.44	2129	2.22
Greenland	295.02	2056	6.44
Yugoslavia	286.08	2067	2.63
Netherlands	238.03	1978	3.77
Czech Republic	170.15	1979	2.13
Romania	136.55	1980	2.29
Hungary	75.23	1975	0.86
Belgium	67.12	1949	0.67
Bulgaria	65.37	2036	1.0
Denmark	60.71	2002	1.06
Spain	55.07	1979	0.62
Greece	50.35	2029	0.87
Austria	47.53	2055	0.58
Sweden	43.74	2082	0.57
Albania	13.69	2058	0.32
Cyprus	10.74	2048	0.18
Slovakia	5.79	2055	0.08
Portugal	4.1	2014	0.17
Malta	0.58	2025	0.03
Switzerland	0.03	1944	0.01
**Total**	**7186.63**	**1999**	**39.82**

3.28.2 By mineral

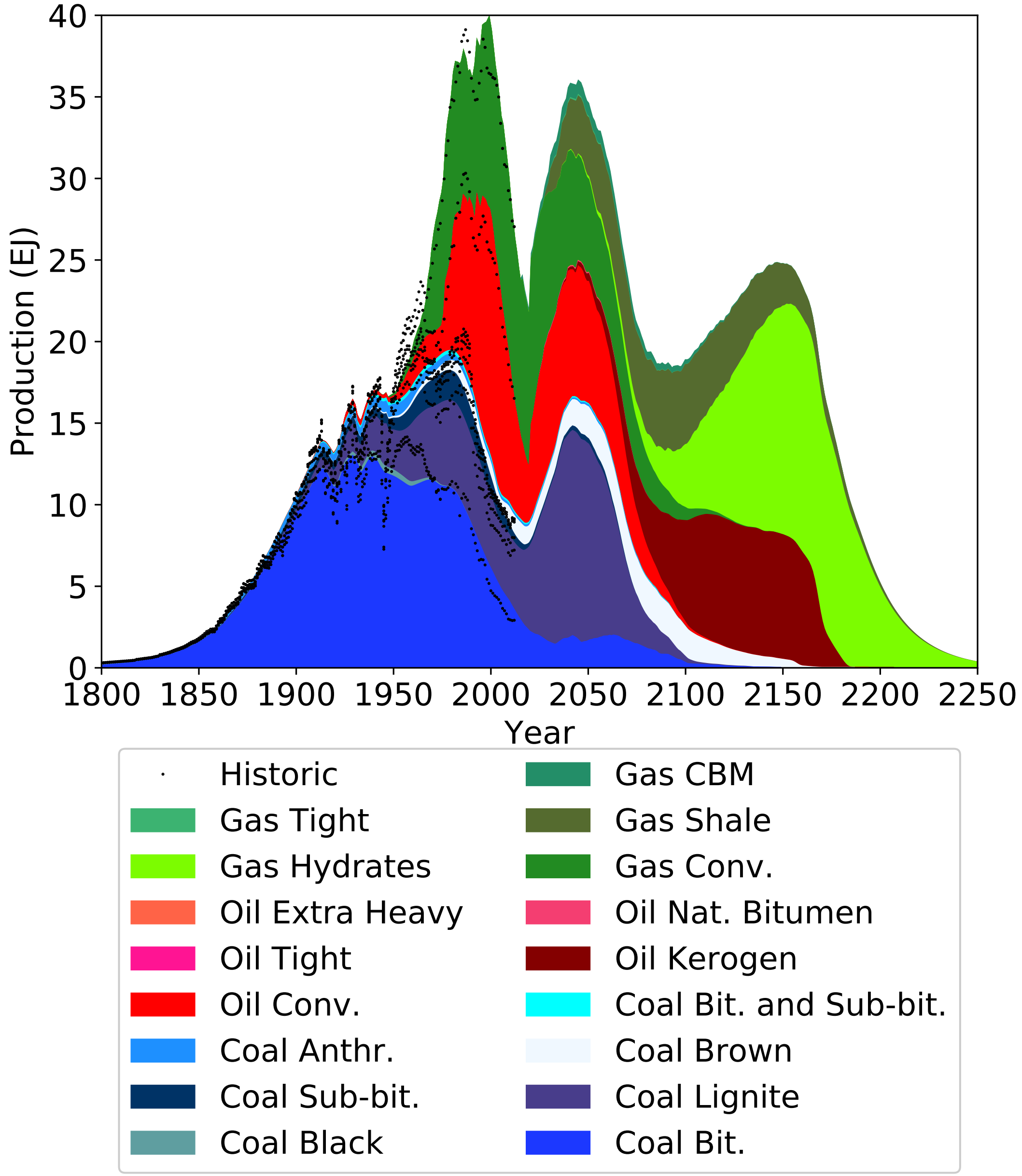

Figure 3.83: Europe projection by mineral type

Table 3.83: Peak years - Minerals

**Name**	**URR**	**Peak Year**	**Peak Rate**
Coal Bit.	1575.66	1929	12.98
Coal Black	14.9	1945	0.4
Coal Lignite	911.04	2043	12.58
Coal Sub-bit.	127.2	1978	1.83
Coal Brown	253.89	2065	2.39
Coal Anthr.	72.78	1953	0.76
Coal Bit. and Sub-bit.	24.7	1965	0.25
Oil Conv.	850.51	1999	14.97
Oil Kerogen	671.35	2120	7.84
Oil Tight	0.93	2028	0.07
Oil Nat. Bitumen	1.26	2060	0.04
Oil Extra Heavy	1.35	2030	0.04
Gas Conv.	903.41	1999	11.66
Gas Hydrates	1176.9	2156	14.42
Gas Shale	541.64	2100	4.91
Gas Tight	4.27	2044	0.1
Gas CBM	54.85	2040	0.89
**Total**	**7186.63**	**1999**	**39.82**

Chapter 4

FSU

4.1 Armenia

4.1.1 All Projections

Table 4.1: Peak years - All

Name	URR	Peak Year	Peak Rate
Oil Kerogen	1.75	2058	0.06
Gas Conv.	0.37	2030	0.02
**Total**	**2.12**	**2058**	**0.06**

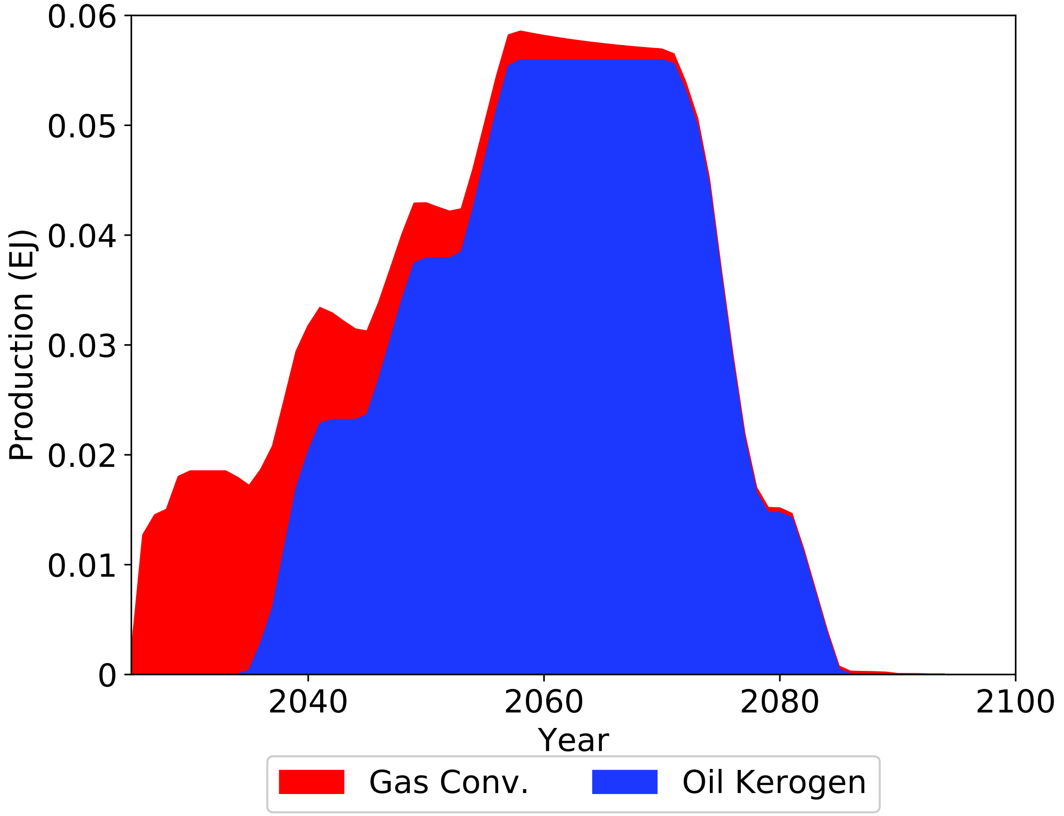

Figure 4.1: Armenia projections capped at 16

4.1.2 By Mineral

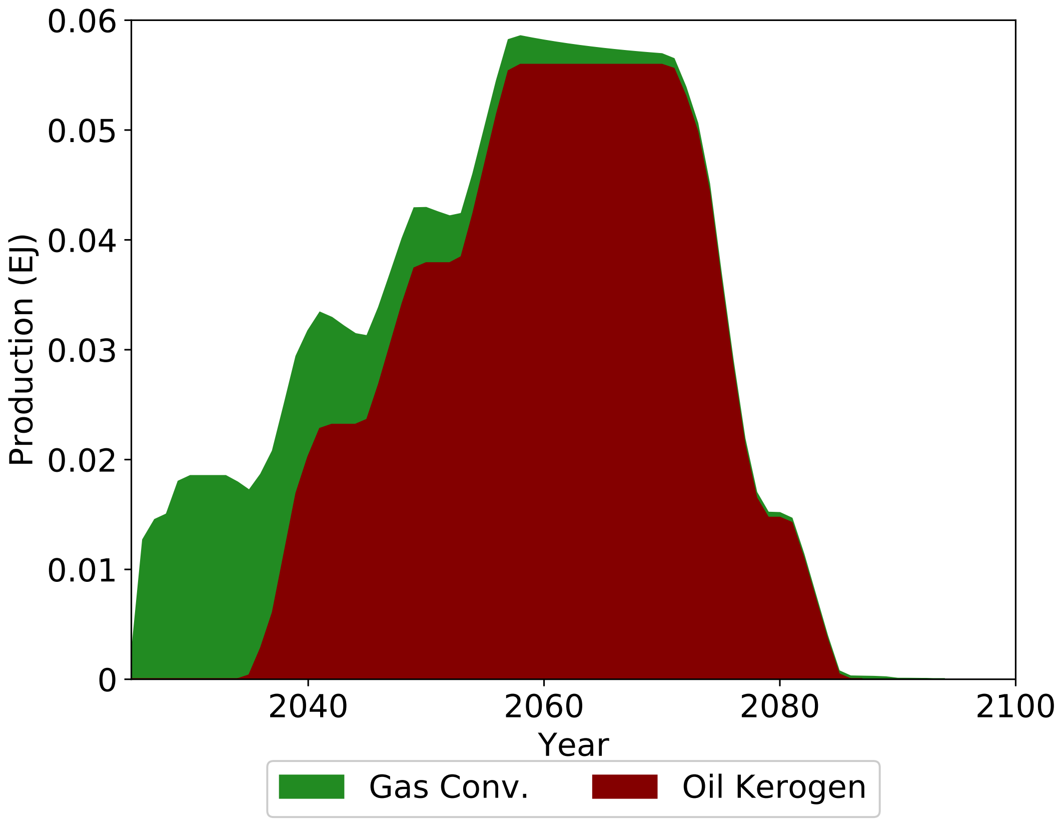

Figure 4.2: Armenia projection by mineral type

Table 4.2: Peak years - Minerals

Name	URR	Peak Year	Peak Rate
Oil Kerogen	1.75	2058	0.06
Gas Conv.	0.37	2030	0.02
**Total**	**2.12**	**2058**	**0.06**

4.2 Azerbaijan

4.2.1 All Projections

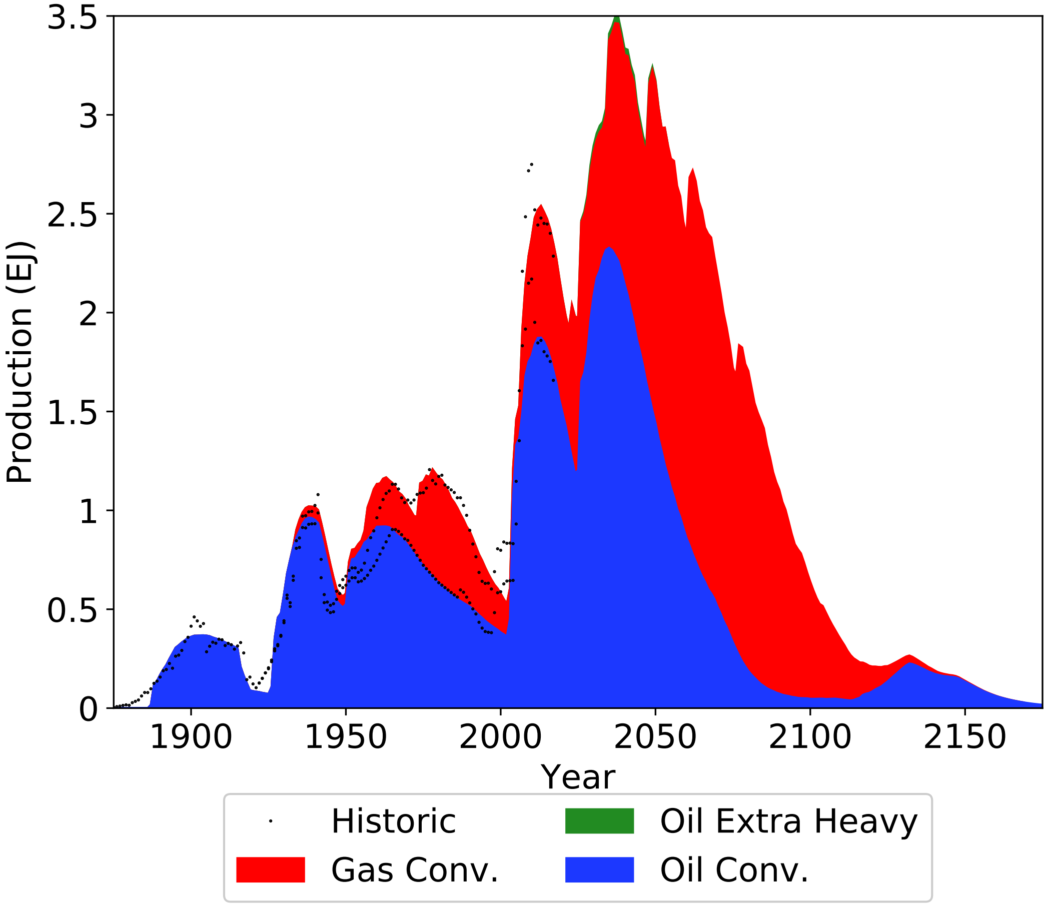

Figure 4.3: Azerbaijan projections capped at 16

Table 4.3: Peak years - All

Name	URR	Peak Year	Peak Rate
Oil Conv.	176.3	2035	2.33
Gas Conv.	132.43	2062	1.94
Oil Extra Heavy	0.72	2030	0.03
**Total**	**309.45**	**2037**	**3.5**

4.2.2 By Mineral

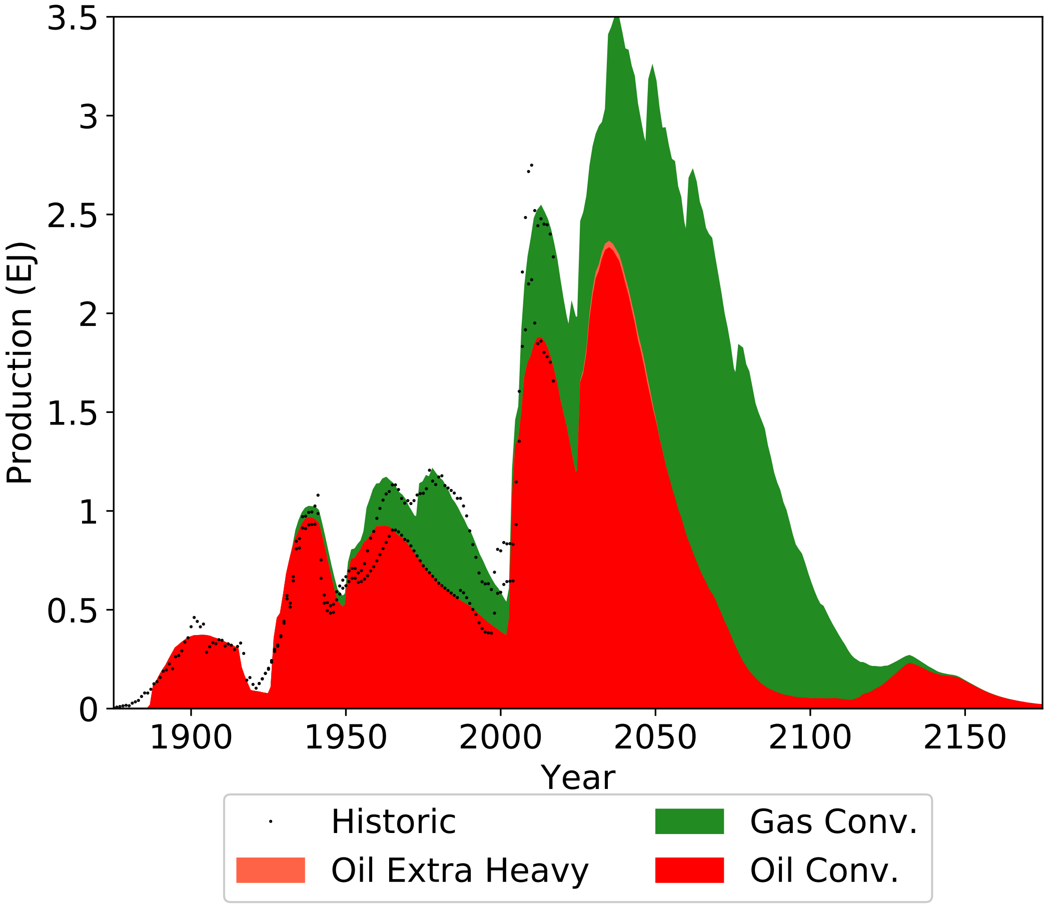

Figure 4.4: Azerbaijan projection by mineral type

Table 4.4: Peak years - Minerals

**Name**	**URR**	**Peak Year**	**Peak Rate**
Oil Conv.	176.3	2035	2.33
Oil Extra Heavy	0.72	2030	0.03
Gas Conv.	132.43	2062	1.94
**Total**	**309.45**	**2037**	**3.5**

4.3 Belarus

4.3.1 All Projections

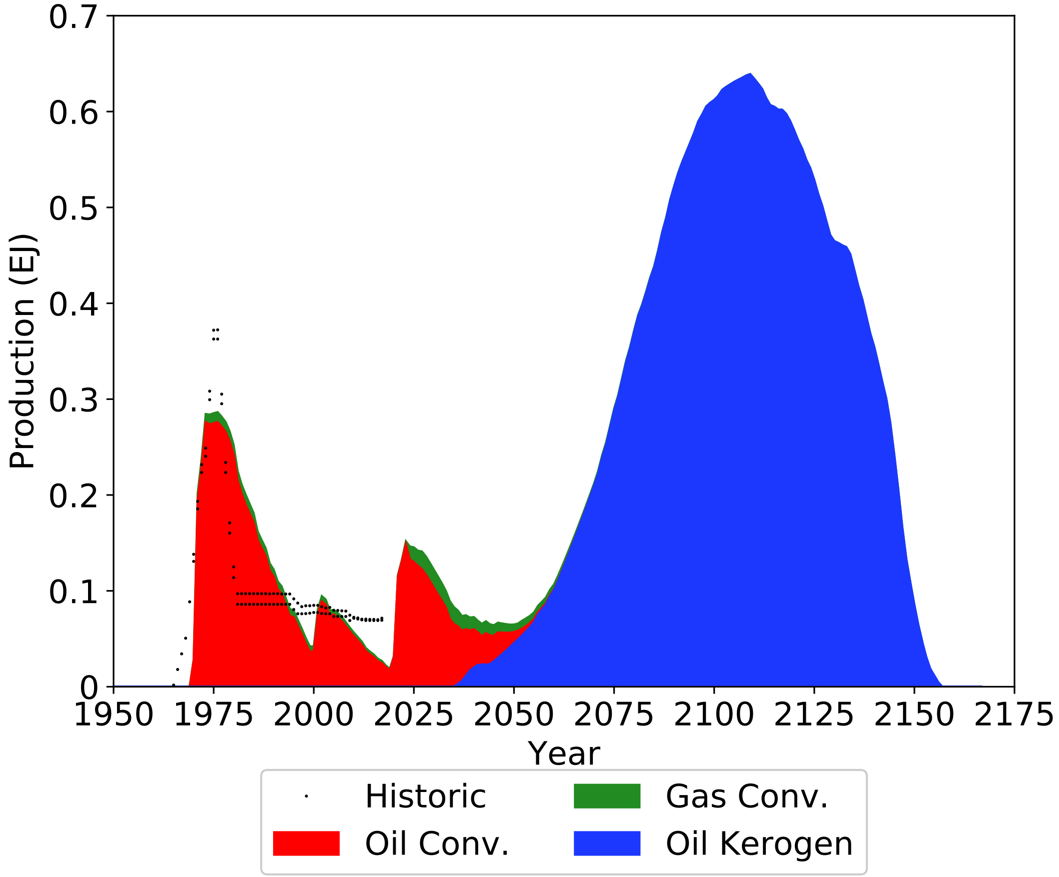

Figure 4.5: Belarus projections capped at 16

Table 4.5: Peak years - All

Name	URR	Peak Year	Peak Rate
Oil Kerogen	40.04	2109	0.64
Oil Conv.	8.08	1976	0.28
Gas Conv.	0.86	2028	0.02
**Total**	**48.98**	**2109**	**0.64**

4.3.2 By Mineral

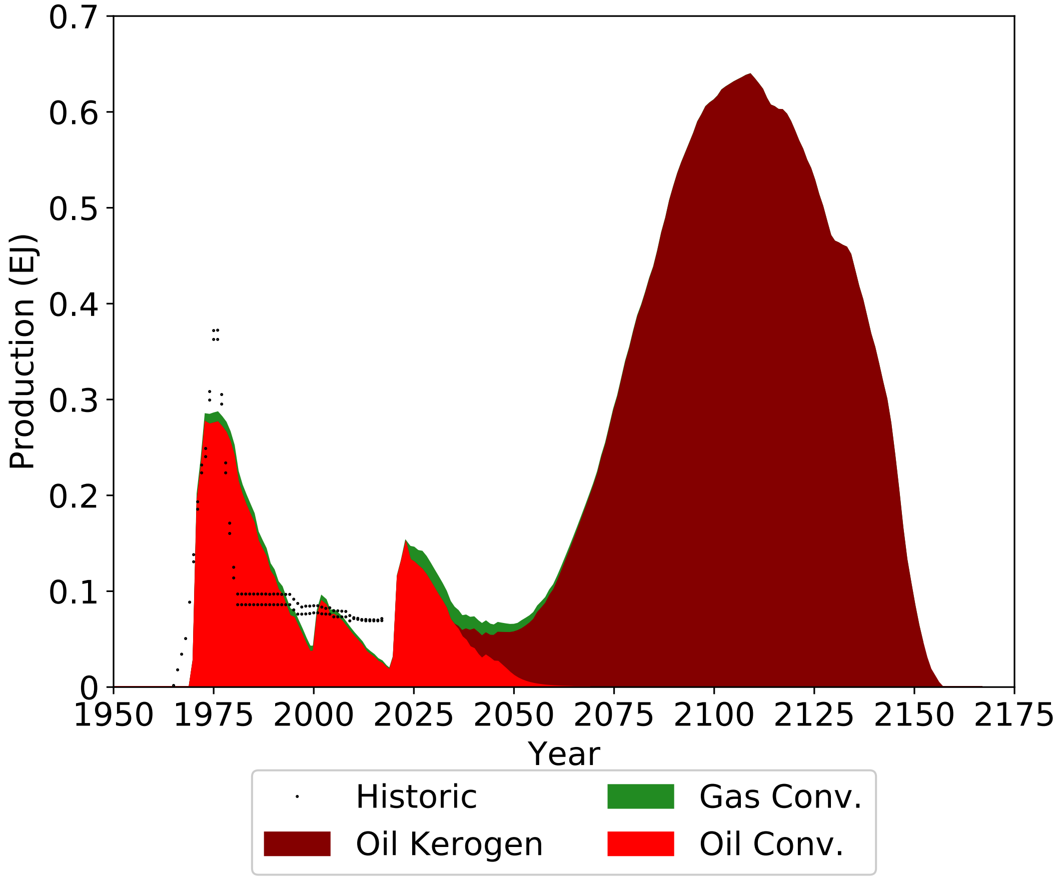

Figure 4.6: Belarus projection by mineral type

Table 4.6: Peak years - Minerals

Name	URR	Peak Year	Peak Rate
Oil Conv.	8.08	1976	0.28
Oil Kerogen	40.04	2109	0.64
Gas Conv.	0.86	2028	0.02
**Total**	**48.98**	**2109**	**0.64**

4.4 Crimea

4.4.1 All Projections

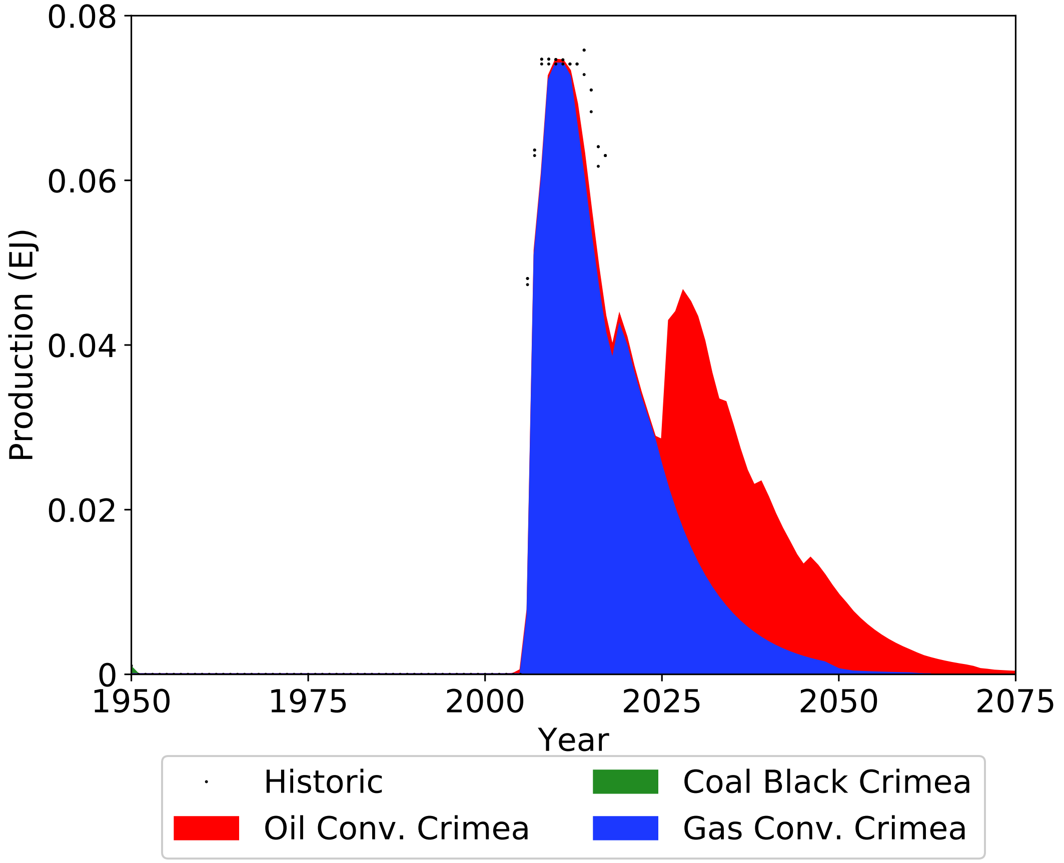

Figure 4.7: Crimea projections capped at 16

Table 4.7: Peak years - All

**Name**	**URR**	**Peak Year**	**Peak Rate**
Gas Conv. Crimea	1.14	2010	0.07
Oil Conv. Crimea	0.57	2029	0.03
Coal Black Crimea	–	1950	–
**Total**	**1.71**	**2010**	**0.07**

4.4.2 By Mineral

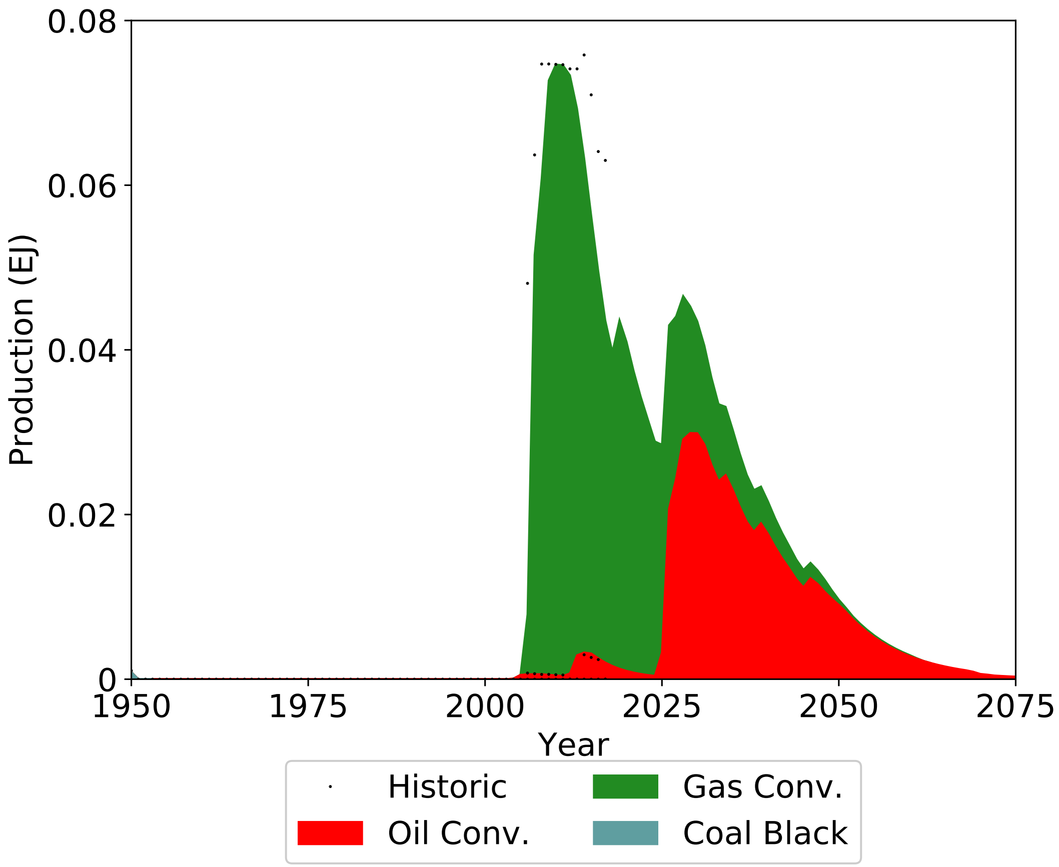

Figure 4.8: Crimea projection by mineral type

Table 4.8: Peak years - Minerals

**Name**	**URR**	**Peak Year**	**Peak Rate**
Coal Black	–	1950	–
Oil Conv.	0.57	2029	0.03
Gas Conv.	1.14	2010	0.07
**Total**	**1.71**	**2010**	**0.07**

4.4.3 Regional Projections

Crimea

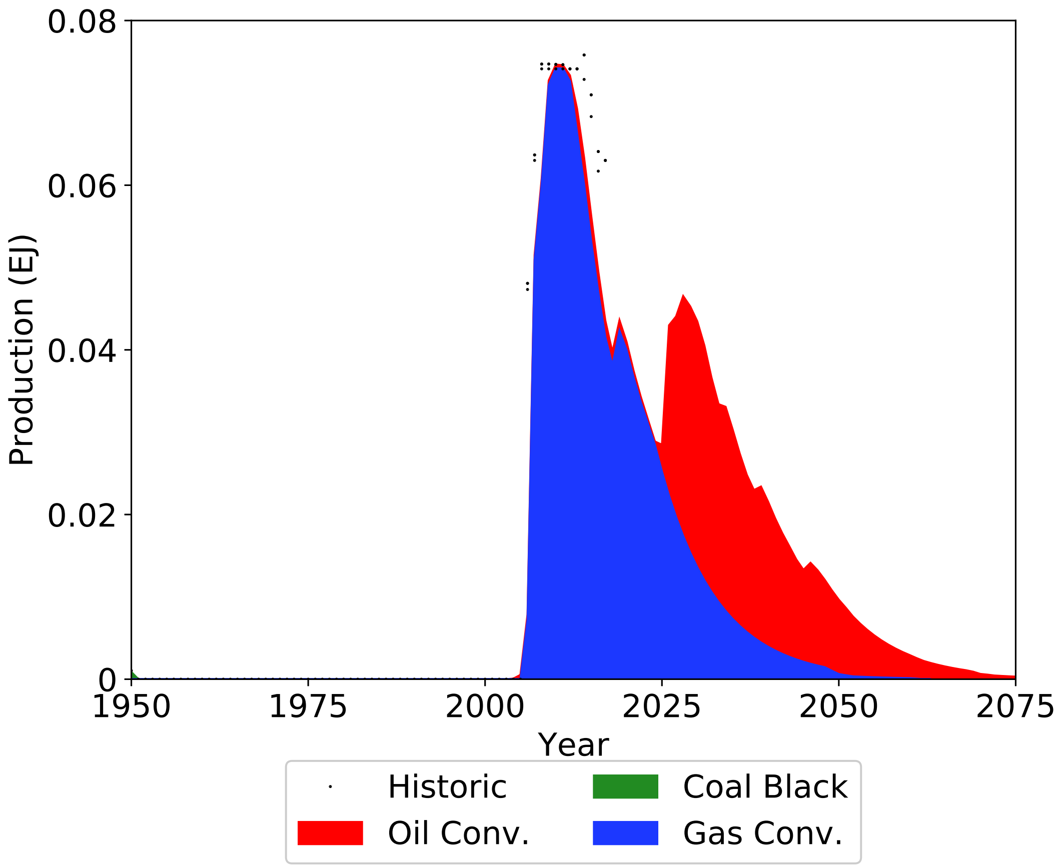

Figure 4.9: Crimea - Crimea projections capped at 16

Table 4.9: Peak years - All

Name	URR	Peak Year	Peak Rate
Gas Conv. Crimea	1.14	2010	0.07
Oil Conv. Crimea	0.57	2029	0.03
Coal Black Crimea	–	1950	–
**Total**	**1.71**	**2010**	**0.07**

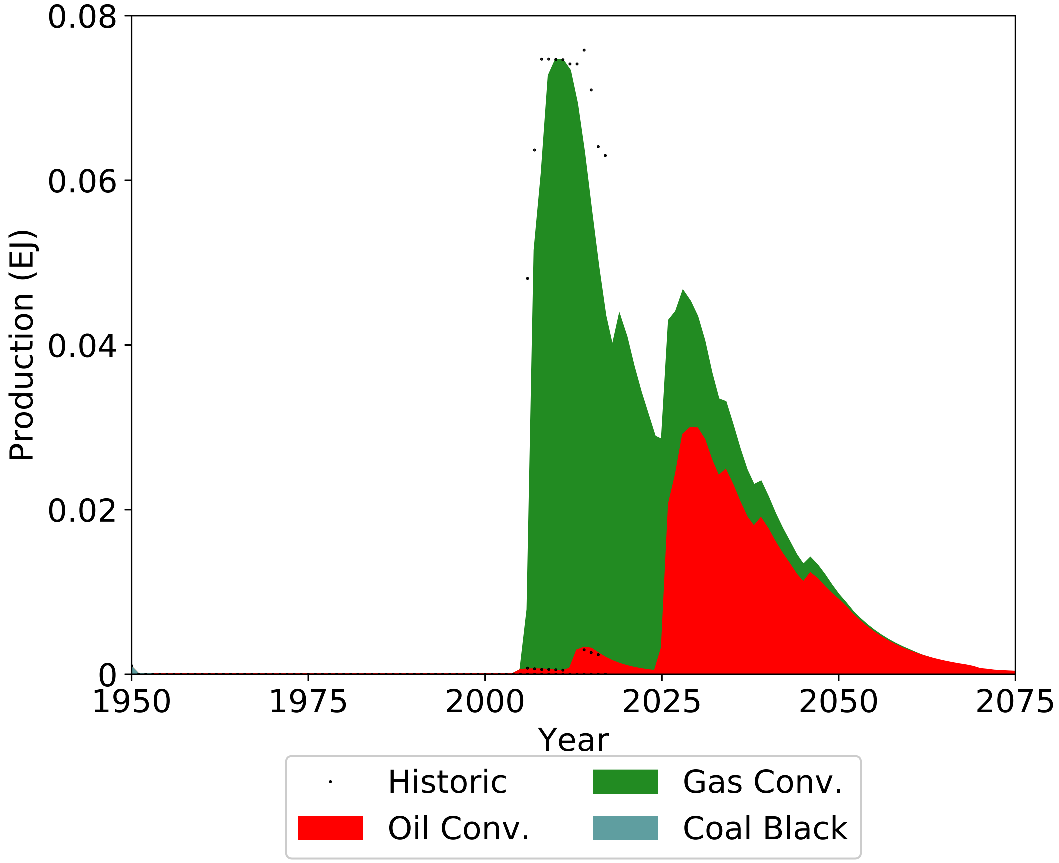

Figure 4.10: Crimea - Crimea projection by mineral type

Table 4.10: Peak years - Minerals

Name	URR	Peak Year	Peak Rate
Coal Black	–	1950	–
Oil Conv.	0.57	2029	0.03
Gas Conv.	1.14	2010	0.07
**Total**	**1.71**	**2010**	**0.07**

4.4.4 Projection by region

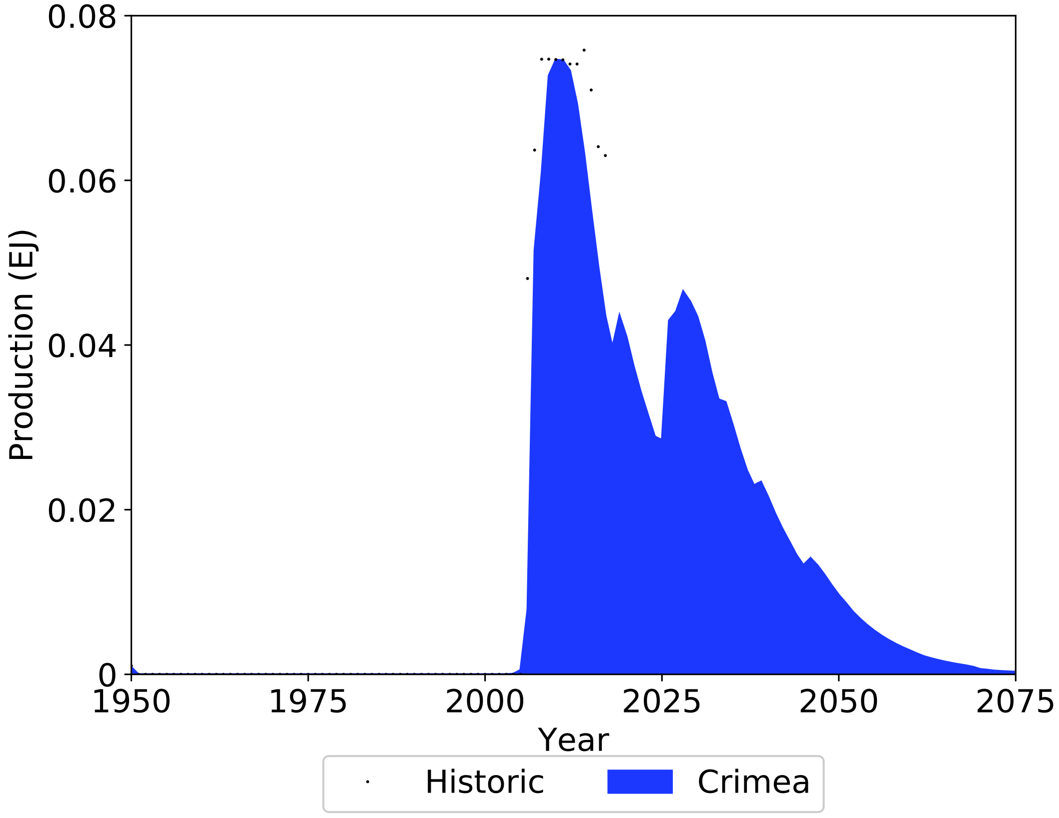

Figure 4.11: Crimea by region projections capped at 16

Table 4.11: Peak years - All

Name	URR	Peak Year	Peak Rate
Crimea	1.71	2010	0.07
**Total**	**1.71**	**2010**	**0.07**

4.5 Donetsk

4.5.1 All Projections

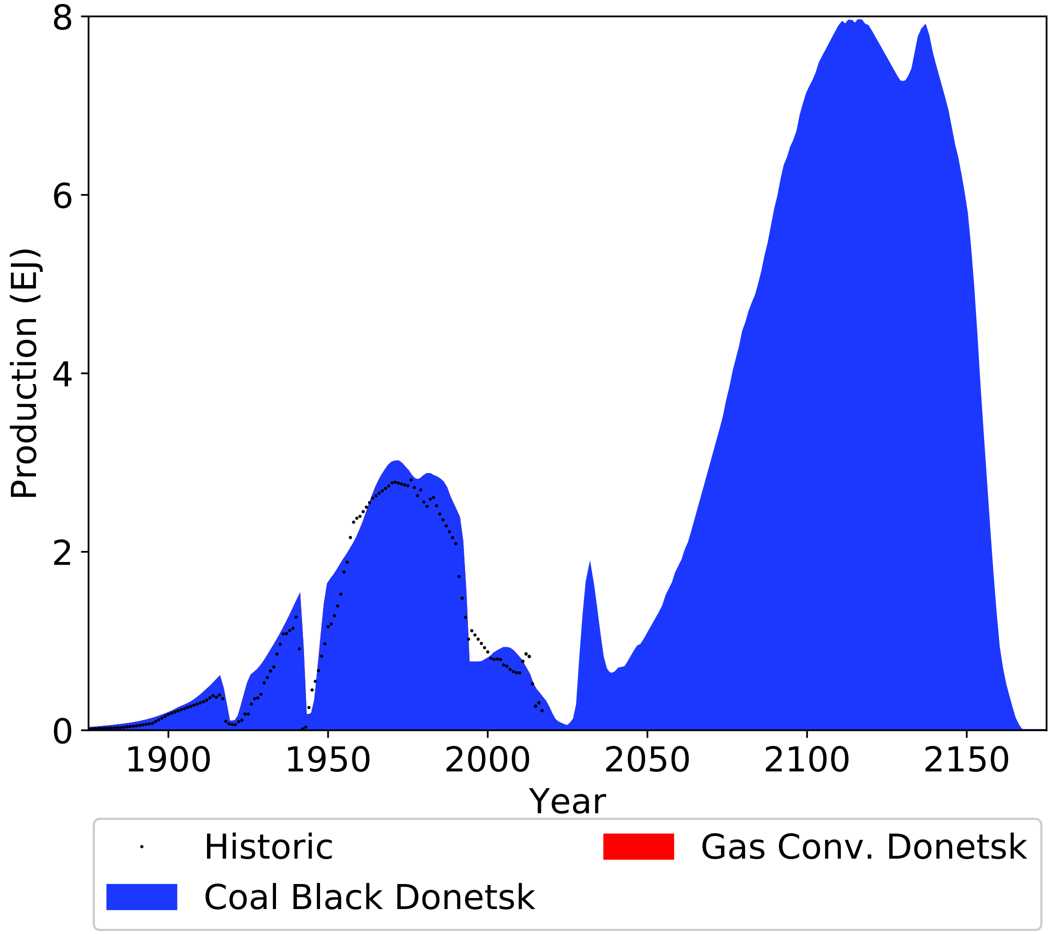

Figure 4.12: Donetsk projections capped at 16

Table 4.12: Peak years - All

Name	URR	Peak Year	Peak Rate
Coal Black Donetsk	783.02	2116	7.96
Gas Conv. Donetsk	0.01	2012	–
**Total**	**783.03**	**2116**	**7.96**

4.5.2 By Mineral

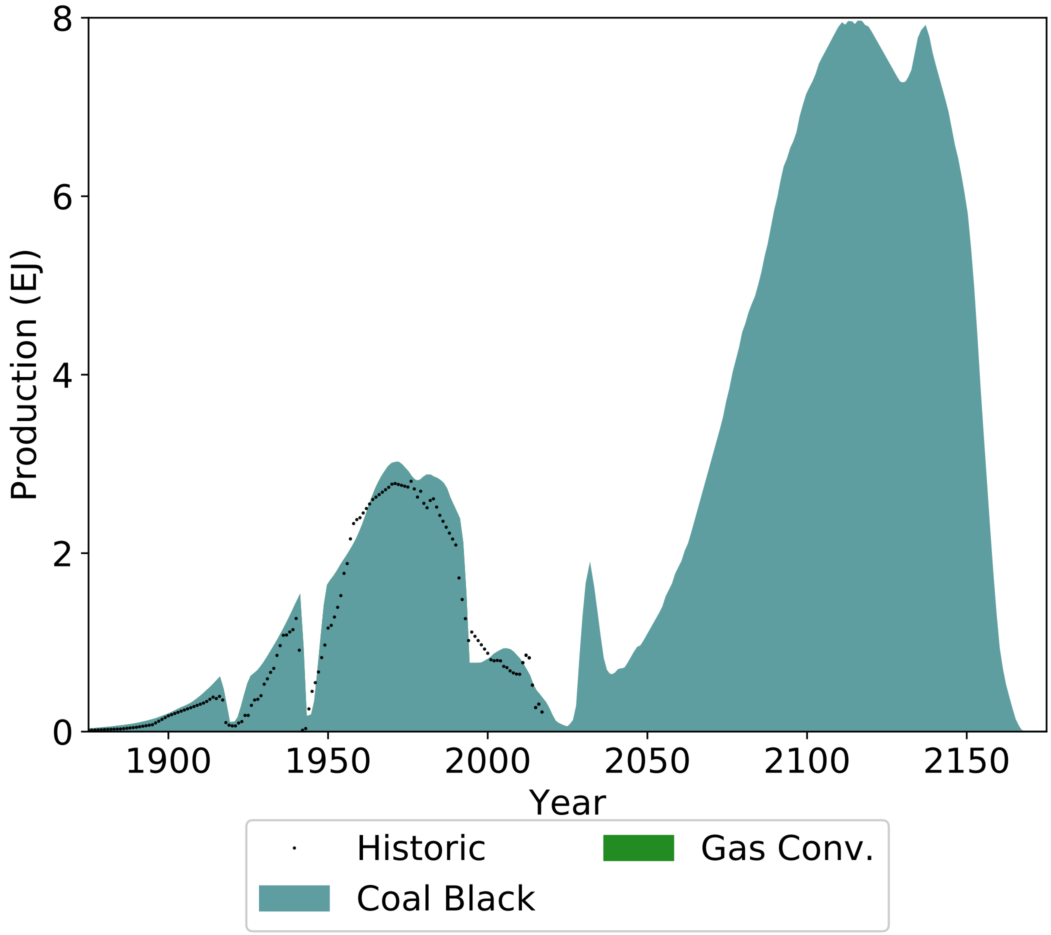

Figure 4.13: Donetsk projection by mineral type

Table 4.13: Peak years - Minerals

Name	URR	Peak Year	Peak Rate
Coal Black	783.02	2116	7.96
Gas Conv.	0.01	2012	–
**Total**	**783.03**	**2116**	**7.96**

4.5.3 Regional Projections

Donetsk

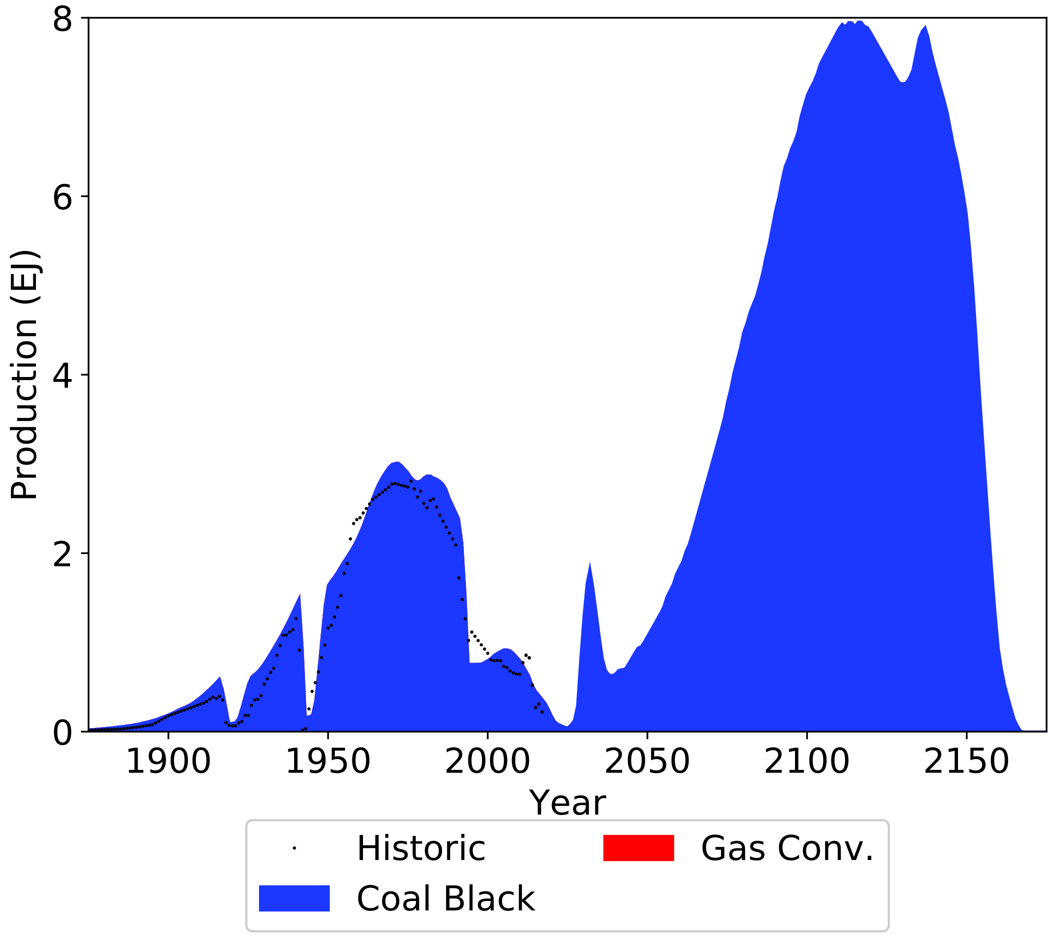

Figure 4.14: Donetsk - Donetsk projections capped at 16

Table 4.14: Peak years - All

**Name**	**URR**	**Peak Year**	**Peak Rate**
Coal Black Donetsk	783.02	2116	7.96
Gas Conv. Donetsk	0.01	2012	–
**Total**	**783.03**	**2116**	**7.96**

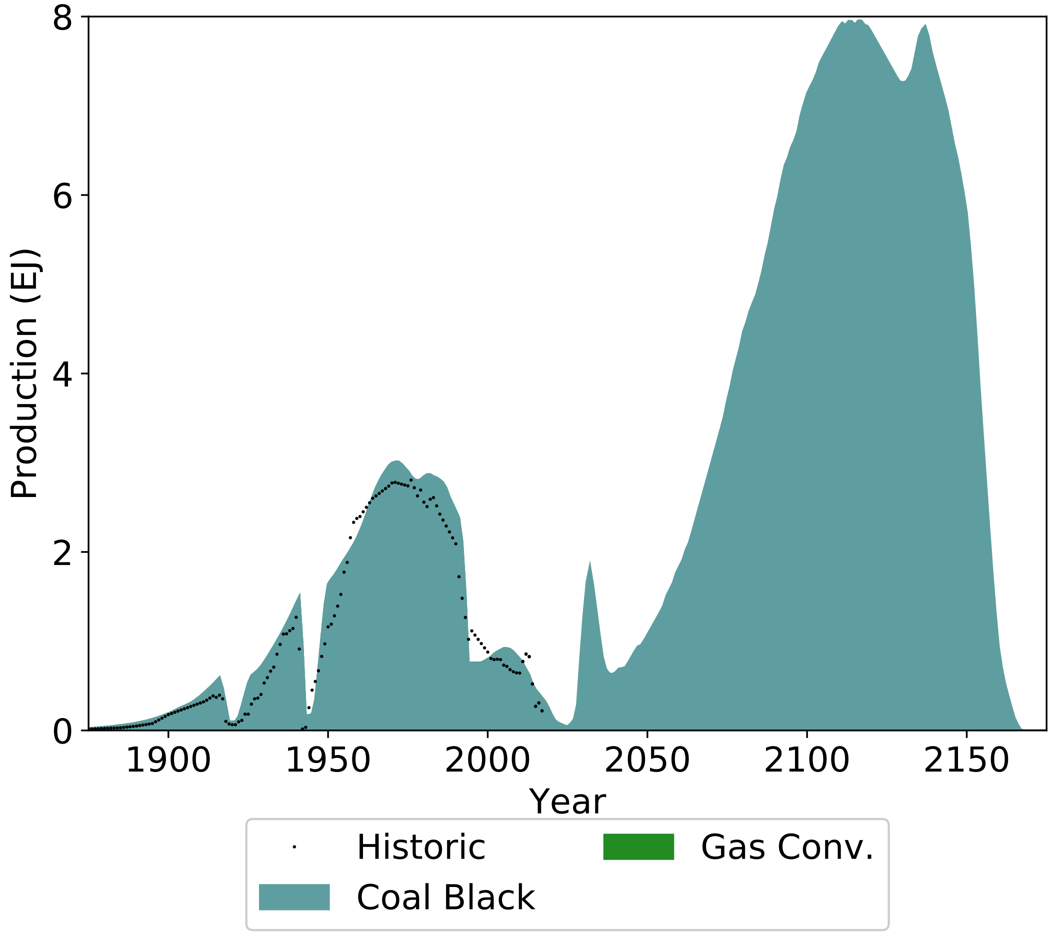

Figure 4.15: Donetsk - Donetsk projection by mineral type

Table 4.15: Peak years - Minerals

Name	URR	Peak Year	Peak Rate
Coal Black	783.02	2116	7.96
Gas Conv.	0.01	2012	–
**Total**	**783.03**	**2116**	**7.96**

4.5.4 Projection by region

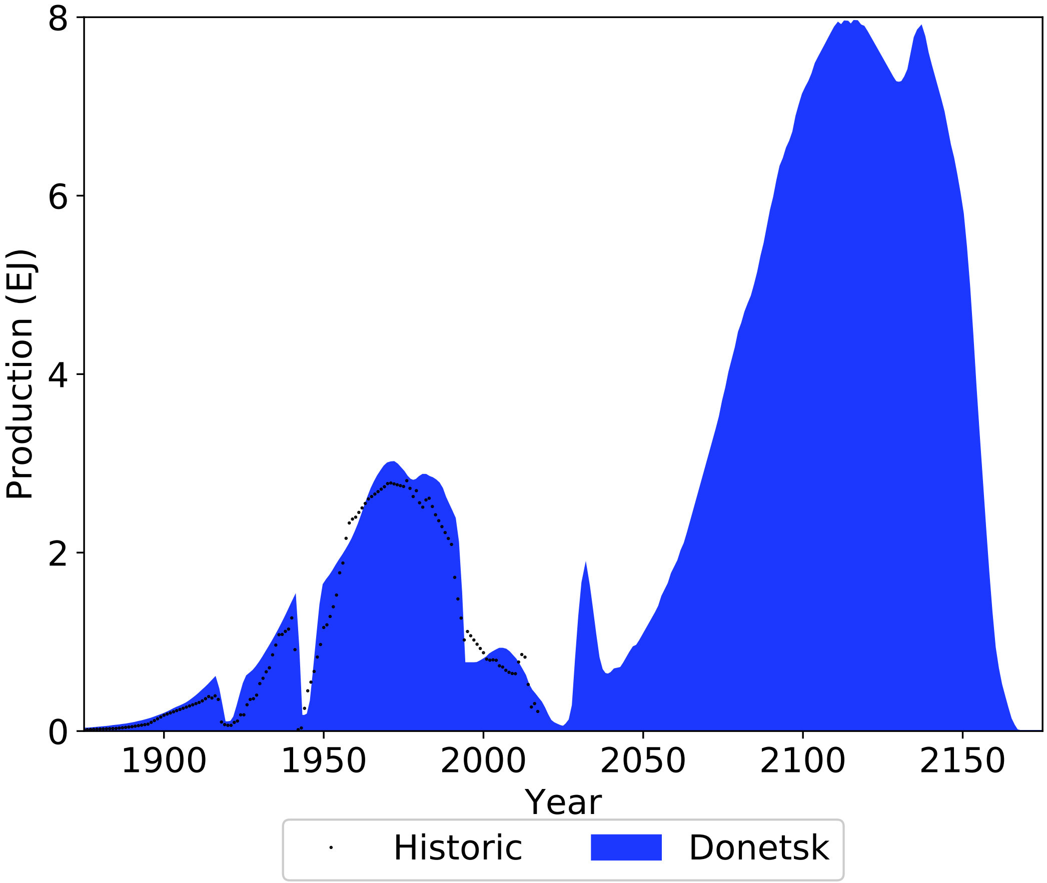

Figure 4.16: Donetsk by region projections capped at 16

Table 4.16: Peak years - All

**Name**	**URR**	**Peak Year**	**Peak Rate**
Donetsk	783.03	2116	7.96
**Total**	**783.03**	**2116**	**7.96**

4.6 Estonia

4.6.1 All Projections

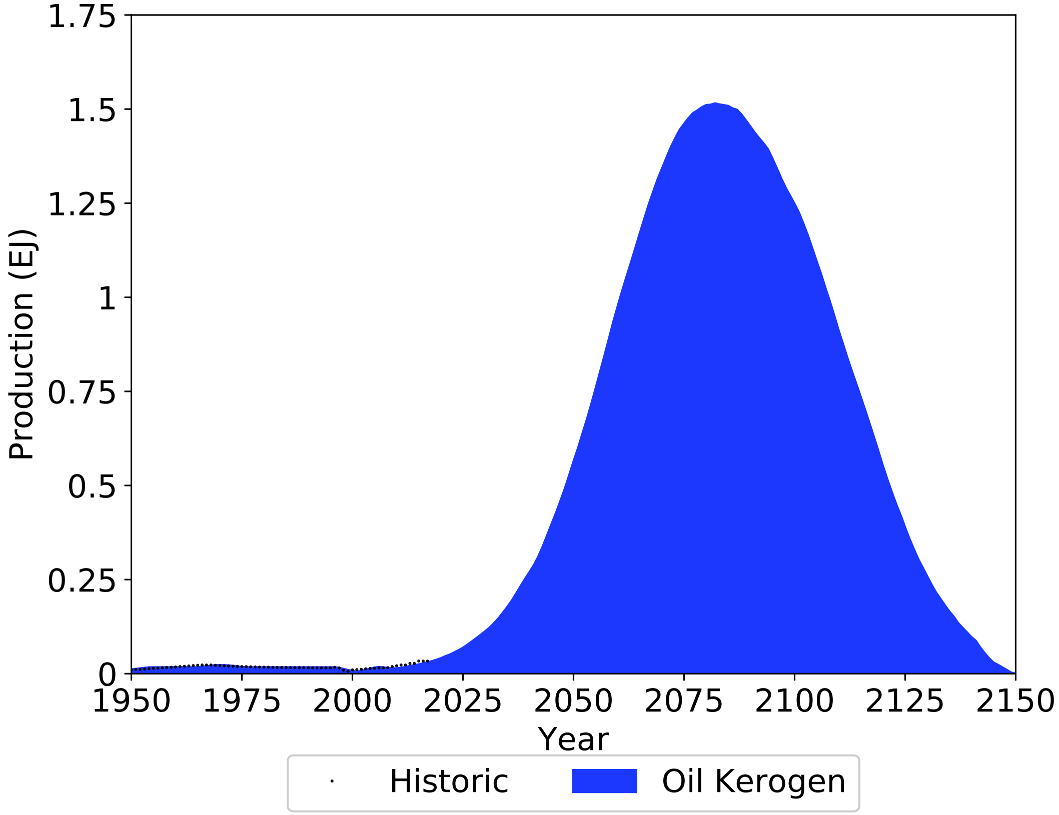

Figure 4.17: Estonia projections capped at 16

Table 4.17: Peak years - All

**Name**	**URR**	**Peak Year**	**Peak Rate**
Oil Kerogen	94.61	2082	1.52
**Total**	**94.61**	**2082**	**1.52**

4.6.2 By Mineral

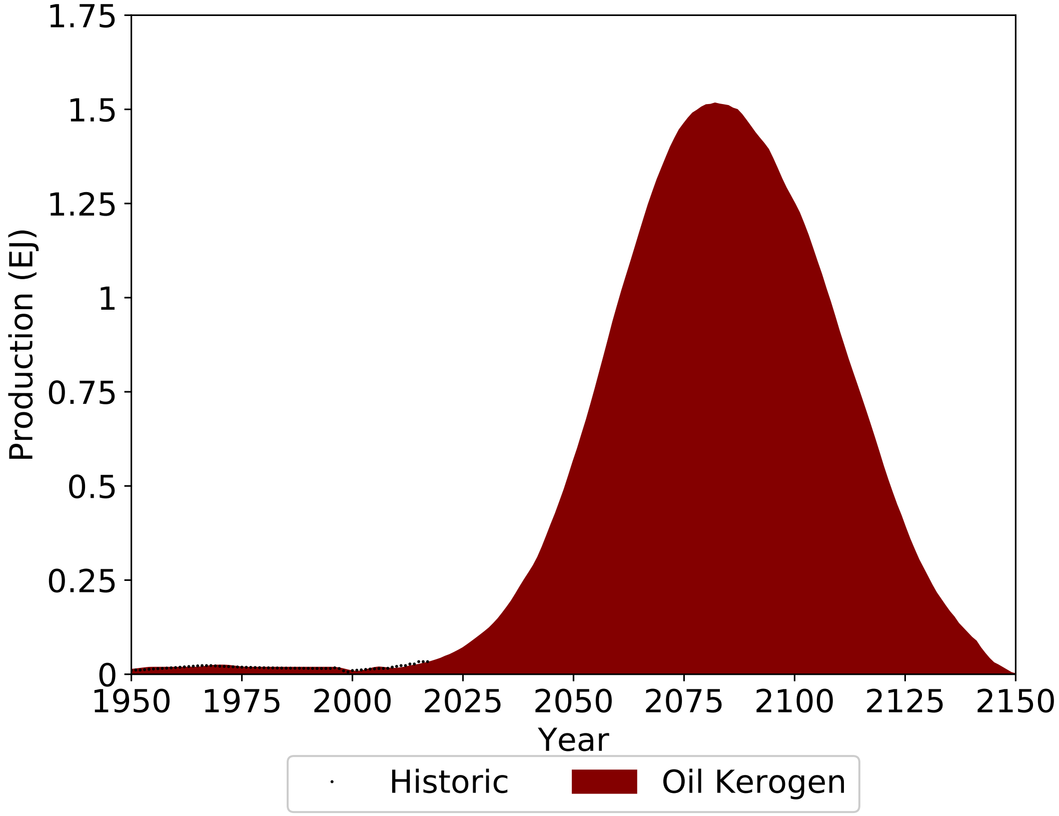

Figure 4.18: Estonia projection by mineral type

Table 4.18: Peak years - Minerals

Name	URR	Peak Year	Peak Rate
Oil Kerogen	94.61	2082	1.52
**Total**	**94.61**	**2082**	**1.52**

4.7 Georgia

4.7.1 All Projections

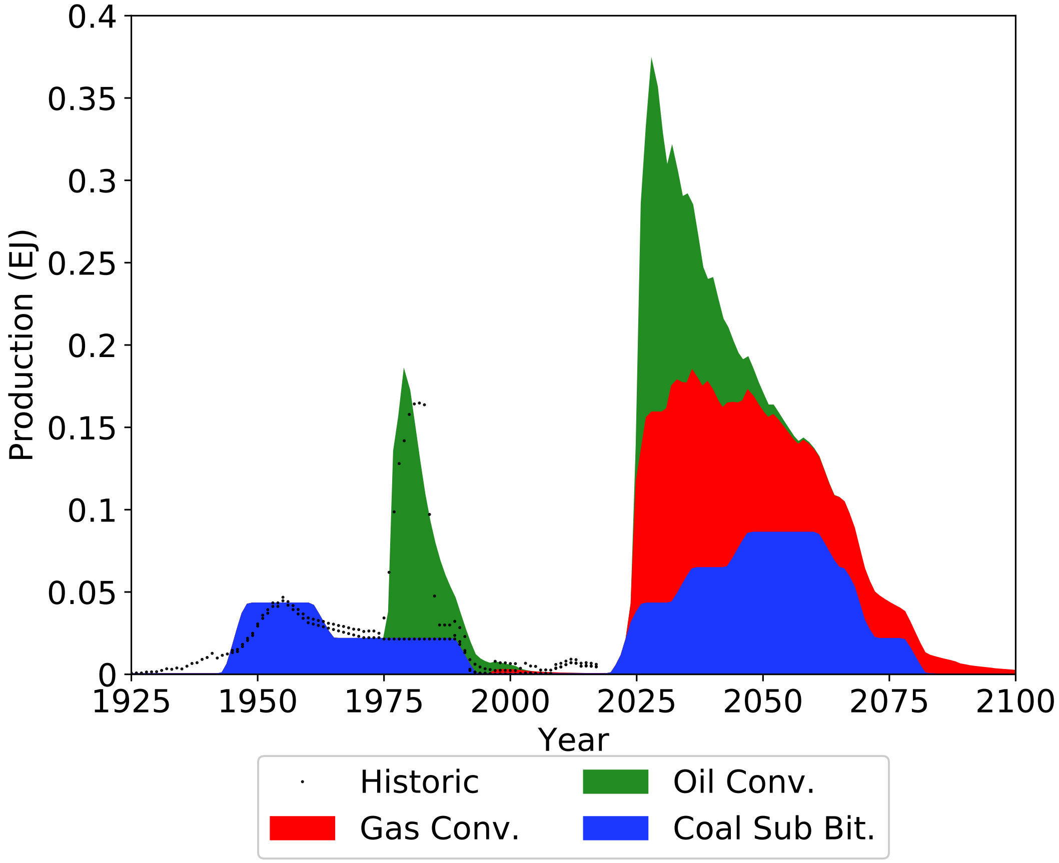

Figure 4.19: Georgia projections capped at 16

Table 4.19: Peak years - All

Name	URR	Peak Year	Peak Rate
Coal Sub Bit.	4.7	2048	0.09
Gas Conv.	4.1	2032	0.13
Oil Conv.	3.59	2028	0.21
**Total**	**12.39**	**2028**	**0.37**

4.7.2 By Mineral

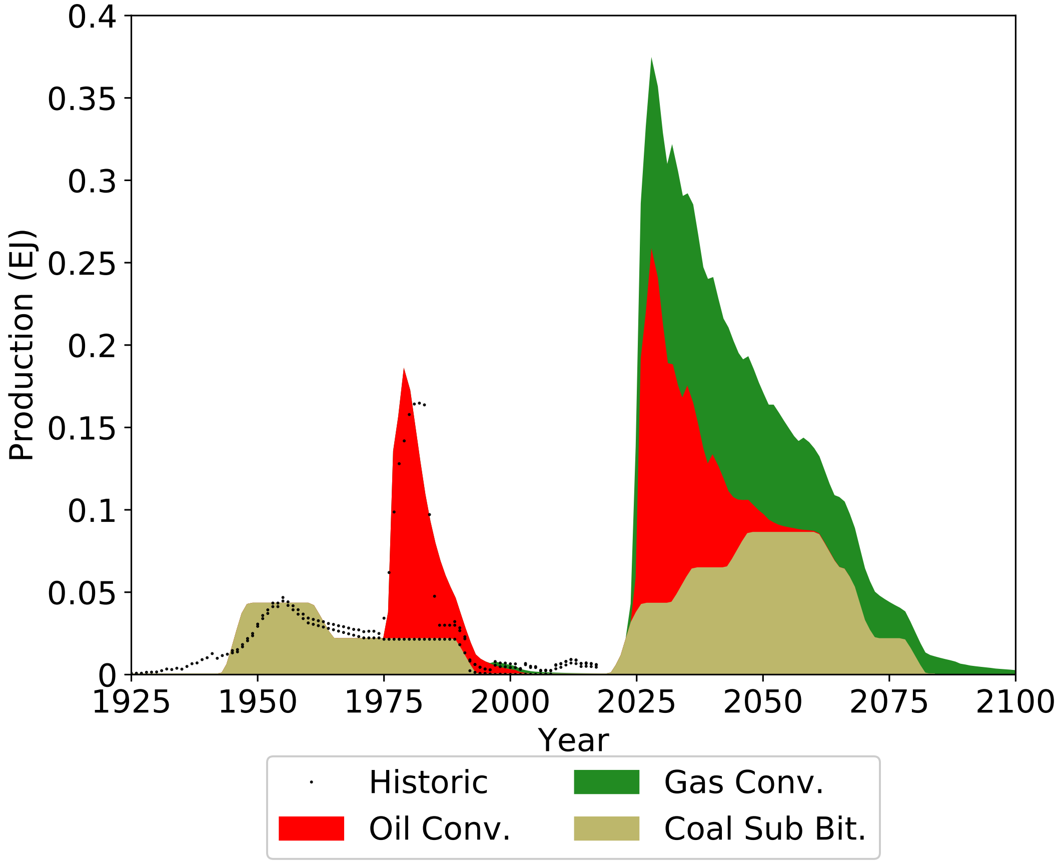

Figure 4.20: Georgia projection by mineral type

Table 4.20: Peak years - Minerals

**Name**	**URR**	**Peak Year**	**Peak Rate**
Coal Sub Bit.	4.7	2048	0.09
Oil Conv.	3.59	2028	0.21
Gas Conv.	4.1	2032	0.13
**Total**	**12.39**	**2028**	**0.37**

4.8 Kazakhstan

4.8.1 All Projections

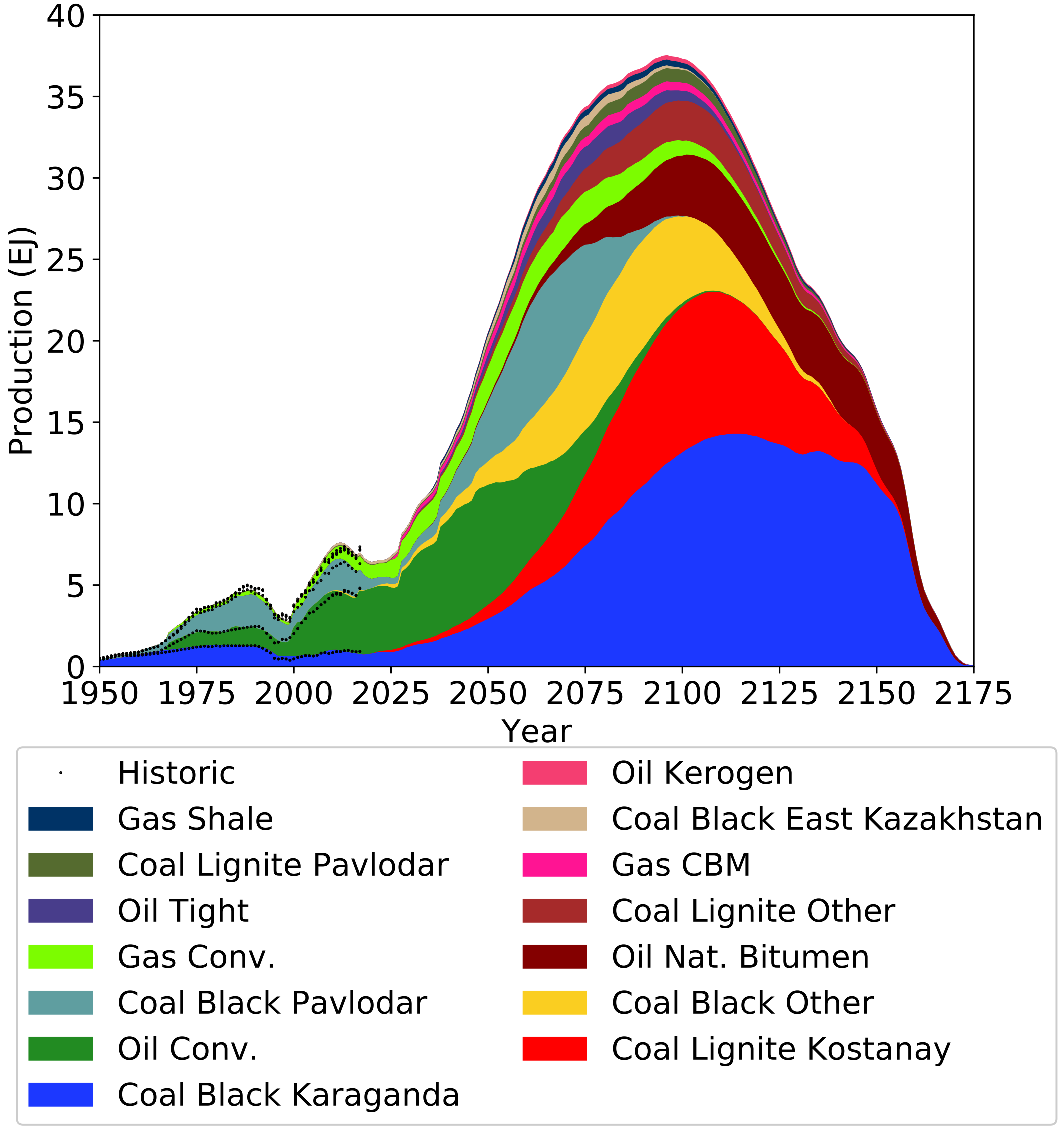

Figure 4.21: Kazakhstan projections capped at 16

Table 4.21: Peak years - All

**Name**	**URR**	**Peak Year**	**Peak Rate**
Coal Black Karaganda	1273.26	2113	14.24
Coal Lignite Kostanay	533.46	2104	9.04
Oil Conv.	425.58	2048	7.55
Coal Black Other	337.75	2087	6.72
Coal Black Pavlodar	315.33	2065	7.41
Oil Nat. Bitumen	312.53	2117	4.1
Gas Conv.	161.77	2053	2.15
Coal Lignite Other	143.07	2102	2.44
Oil Tight	60.48	2074	1.39
Gas CBM	51.97	2080	0.68
Coal Lignite Pavlodar	51.07	2092	0.83
Coal Black East Kazakhstan	33.44	2069	0.67
Gas Shale	28.91	2085	0.4
Oil Kerogen	16.26	2093	0.28
**Total**	**3744.88**	**2096**	**37.48**

4.8.2 By Mineral

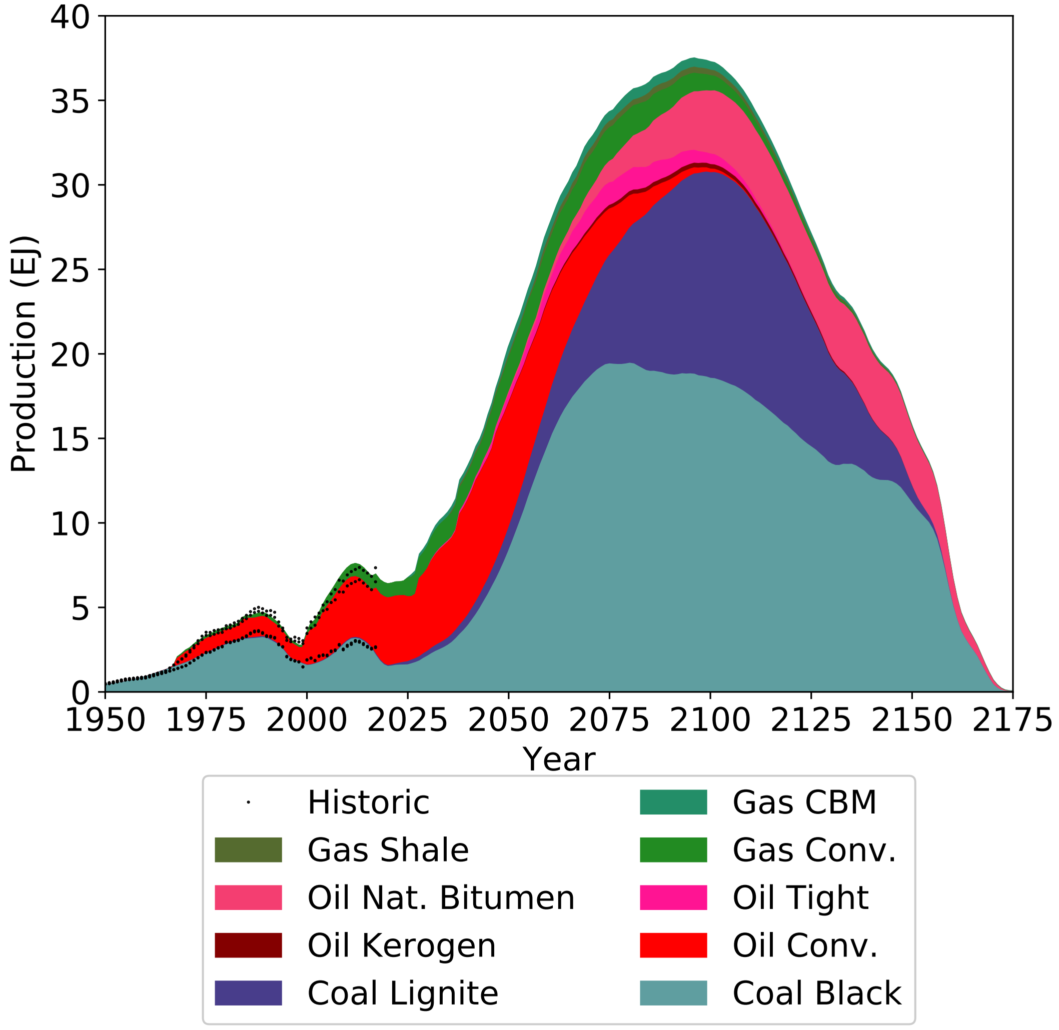

Figure 4.22: Kazakhstan projection by mineral type

4.8.3 Regional Projections

Table 4.22: Peak years - Minerals

**Name**	**URR**	**Peak Year**	**Peak Rate**
Coal Black	1959.78	2080	19.42
Coal Lignite	727.6	2102	12.21
Oil Conv.	425.58	2048	7.55
Oil Kerogen	16.26	2093	0.28
Oil Tight	60.48	2074	1.39
Oil Nat. Bitumen	312.53	2117	4.1
Gas Conv.	161.77	2053	2.15
Gas Shale	28.91	2085	0.4
Gas CBM	51.97	2080	0.68
**Total**	**3744.88**	**2096**	**37.48**

All

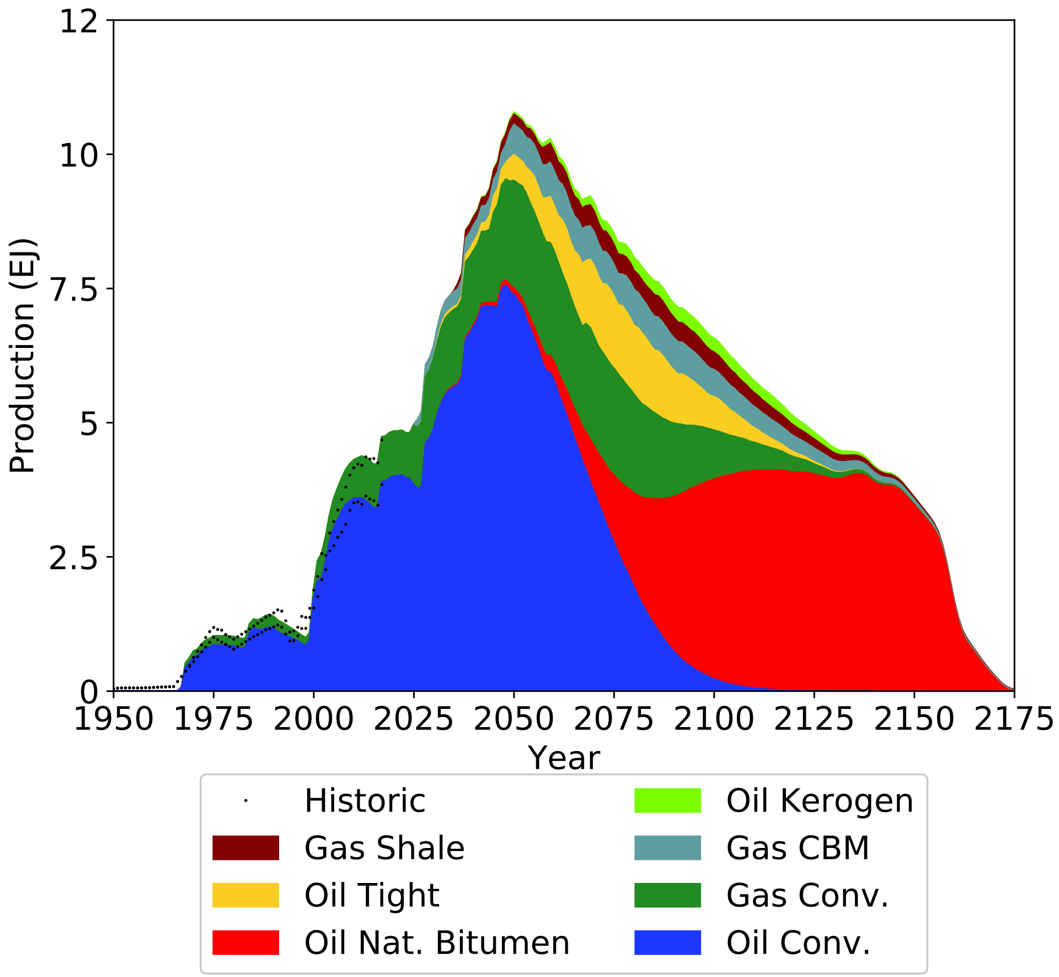

Figure 4.23: Kazakhstan - All projections capped at 16

Table 4.23: Peak years - All

Name	URR	Peak Year	Peak Rate
Oil Conv.	425.58	2048	7.55
Oil Nat. Bitumen	312.53	2117	4.1
Gas Conv.	161.77	2053	2.15
Oil Tight	60.48	2074	1.39
Gas CBM	51.97	2080	0.68
Gas Shale	28.91	2085	0.4
Oil Kerogen	16.26	2093	0.28
**Total**	**1057.5**	**2050**	**10.78**

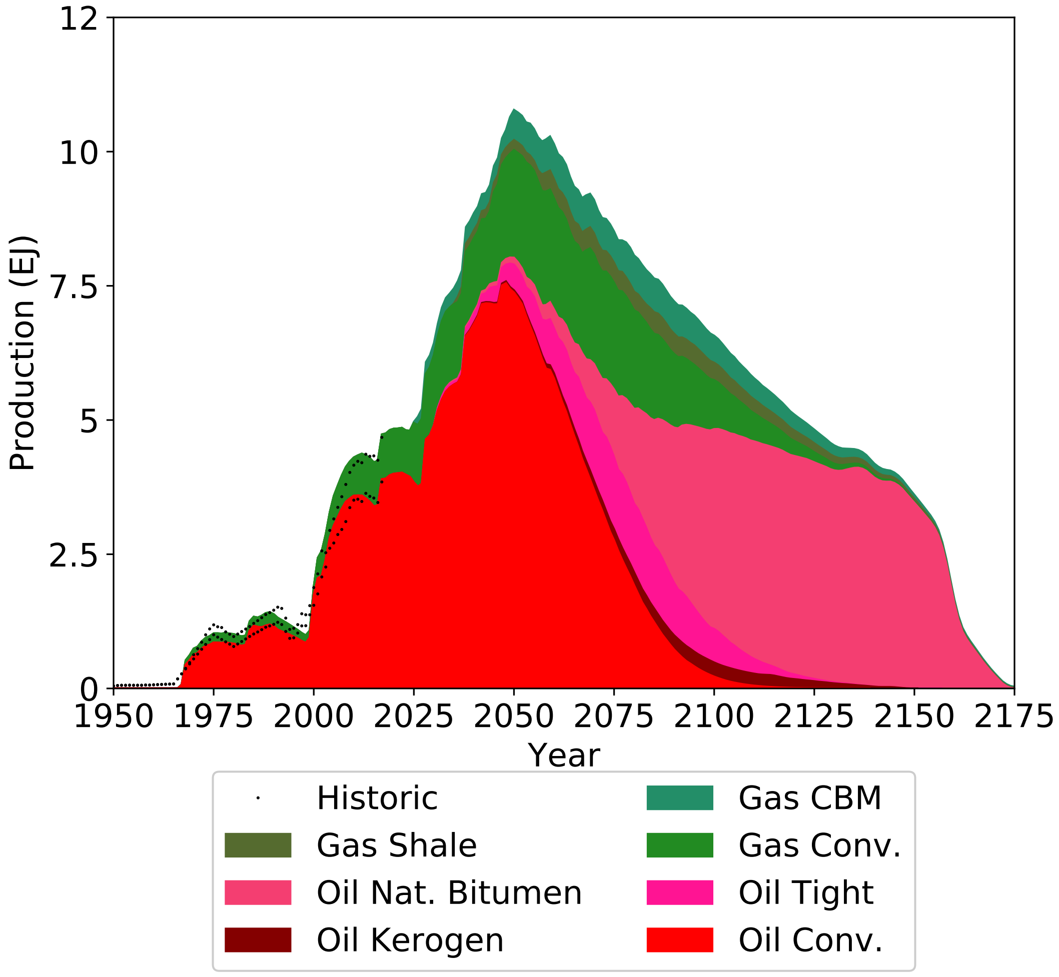

Figure 4.24: Kazakhstan - All projection by mineral type

Table 4.24: Peak years - Minerals

**Name**	**URR**	**Peak Year**	**Peak Rate**
Oil Conv.	425.58	2048	7.55
Oil Kerogen	16.26	2093	0.28
Oil Tight	60.48	2074	1.39
Oil Nat. Bitumen	312.53	2117	4.1
Gas Conv.	161.77	2053	2.15
Gas Shale	28.91	2085	0.4
Gas CBM	51.97	2080	0.68
**Total**	**1057.5**	**2050**	**10.78**

East Kazakhstan

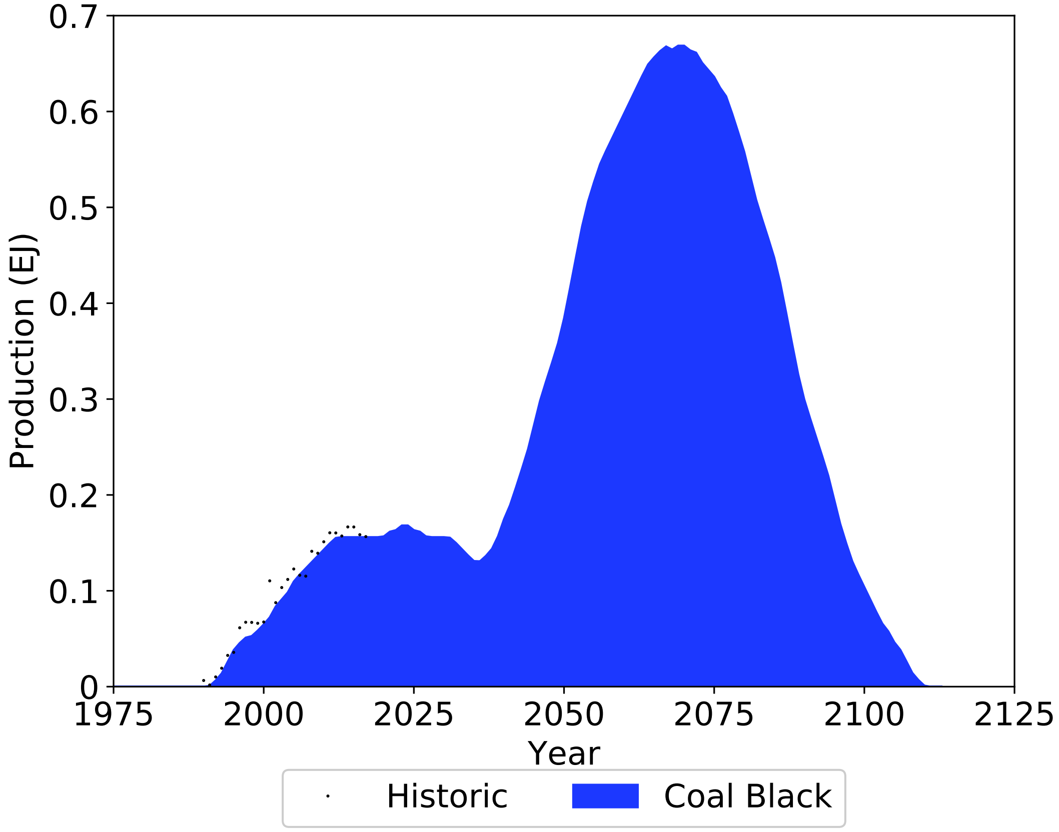

Figure 4.25: Kazakhstan - East Kazakhstan projections capped at 16

Table 4.25: Peak years - All

Name	URR	Peak Year	Peak Rate
Coal Black East Kazakhstan	33.44	2069	0.67
**Total**	**33.44**	**2069**	**0.67**

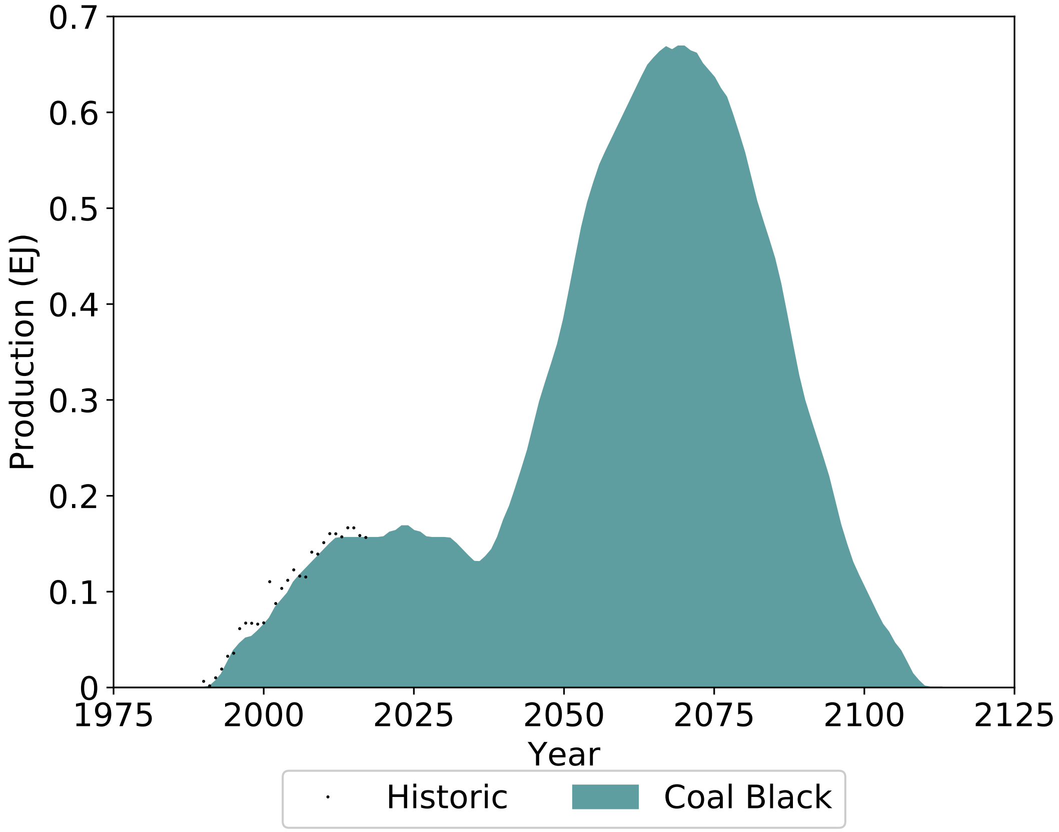

Figure 4.26: Kazakhstan - East Kazakhstan projection by mineral type

Table 4.26: Peak years - Minerals

**Name**	**URR**	**Peak Year**	**Peak Rate**
Coal Black	33.44	2069	0.67
**Total**	**33.44**	**2069**	**0.67**

Karaganda

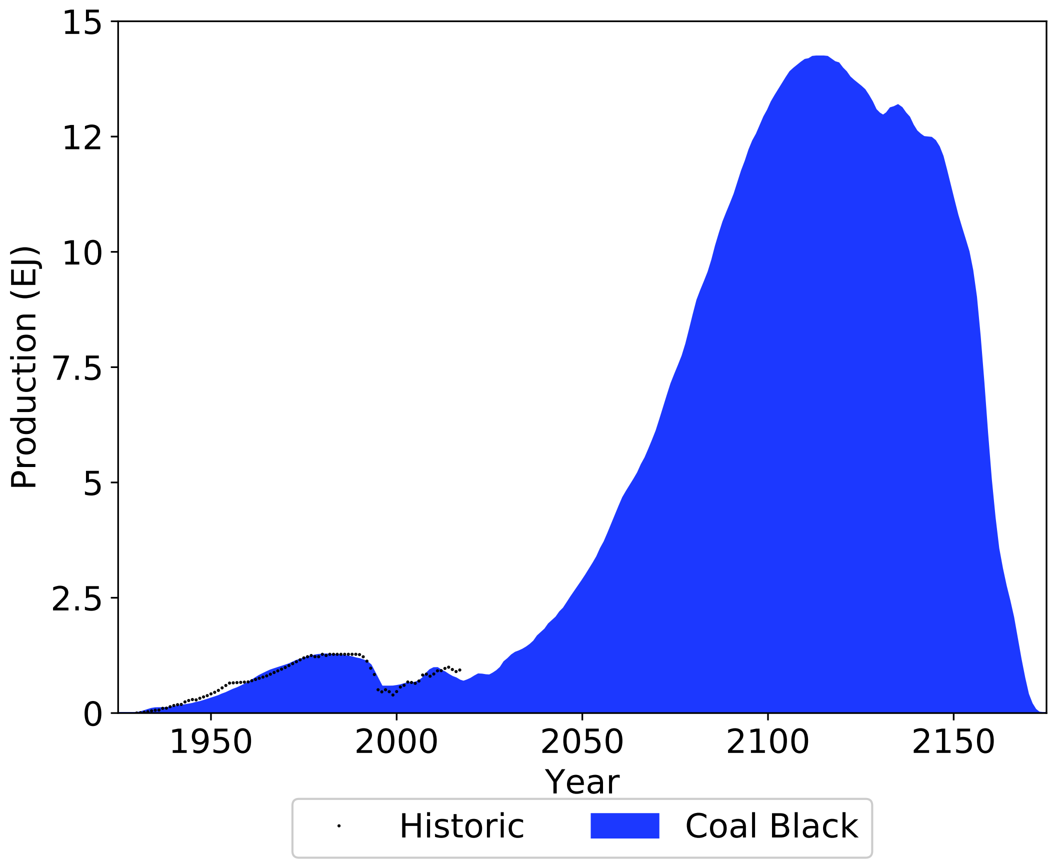

Figure 4.27: Kazakhstan - Karaganda projections capped at 16

Table 4.27: Peak years - All

**Name**	**URR**	**Peak Year**	**Peak Rate**
Coal Black Karaganda	1273.26	2113	14.24
**Total**	**1273.26**	**2113**	**14.24**

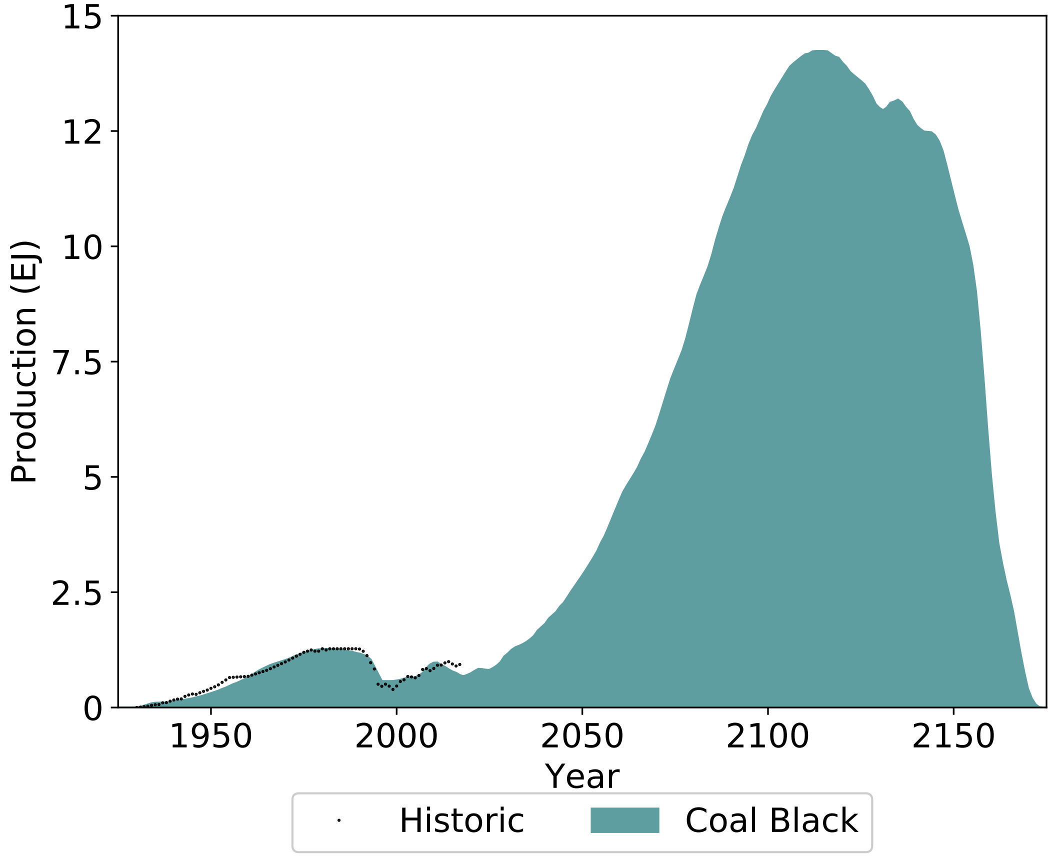

Figure 4.28: Kazakhstan - Karaganda projection by mineral type

Table 4.28: Peak years - Minerals

**Name**	**URR**	**Peak Year**	**Peak Rate**
Coal Black	1273.26	2113	14.24
**Total**	**1273.26**	**2113**	**14.24**

Kostanay

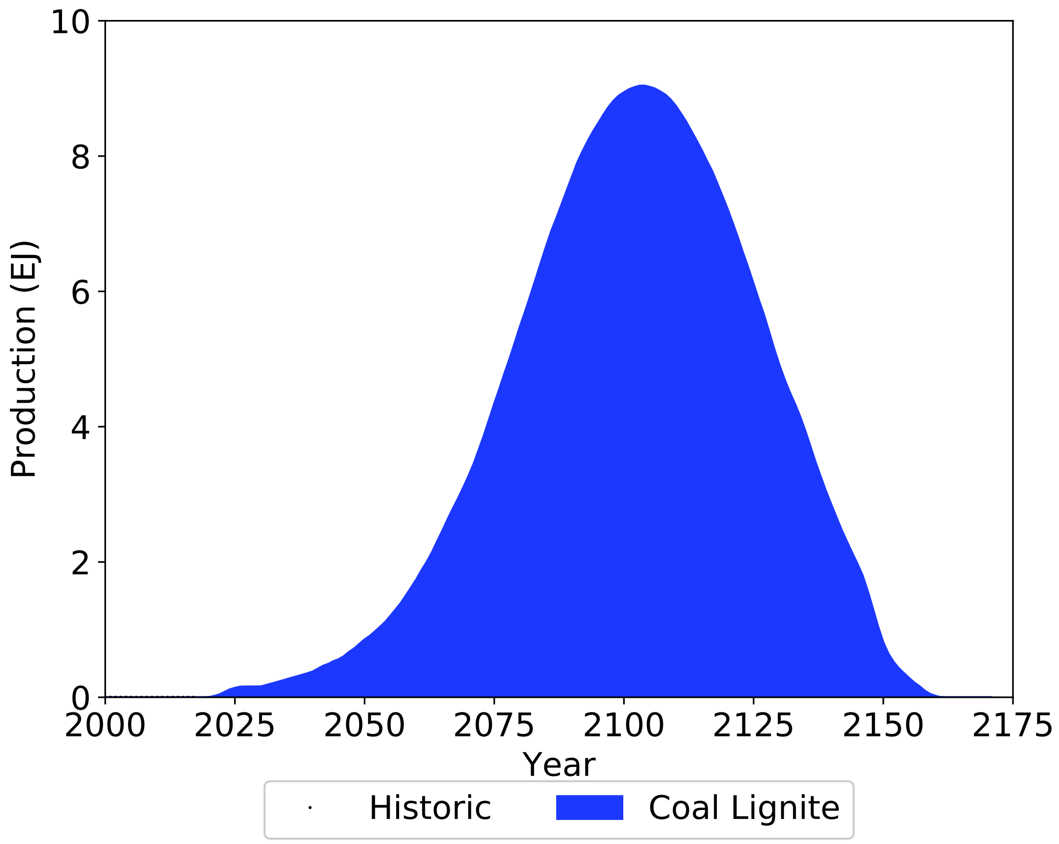

Figure 4.29: Kazakhstan - Kostanay projections capped at 16

Table 4.29: Peak years - All

**Name**	**URR**	**Peak Year**	**Peak Rate**
Coal Lignite Kostanay	533.46	2104	9.04
**Total**	**533.46**	**2104**	**9.04**

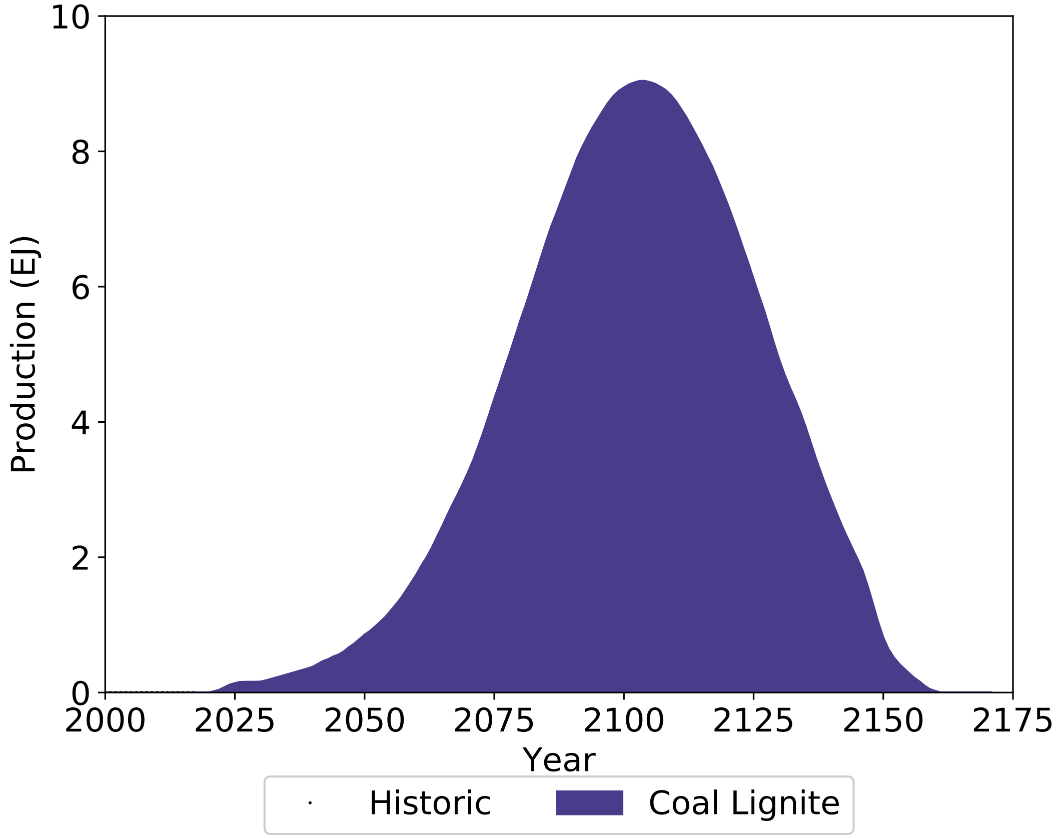

Figure 4.30: Kazakhstan - Kostanay projection by mineral type

Table 4.30: Peak years - Minerals

**Name**	**URR**	**Peak Year**	**Peak Rate**
Coal Lignite	533.46	2104	9.04
**Total**	**533.46**	**2104**	**9.04**

Other

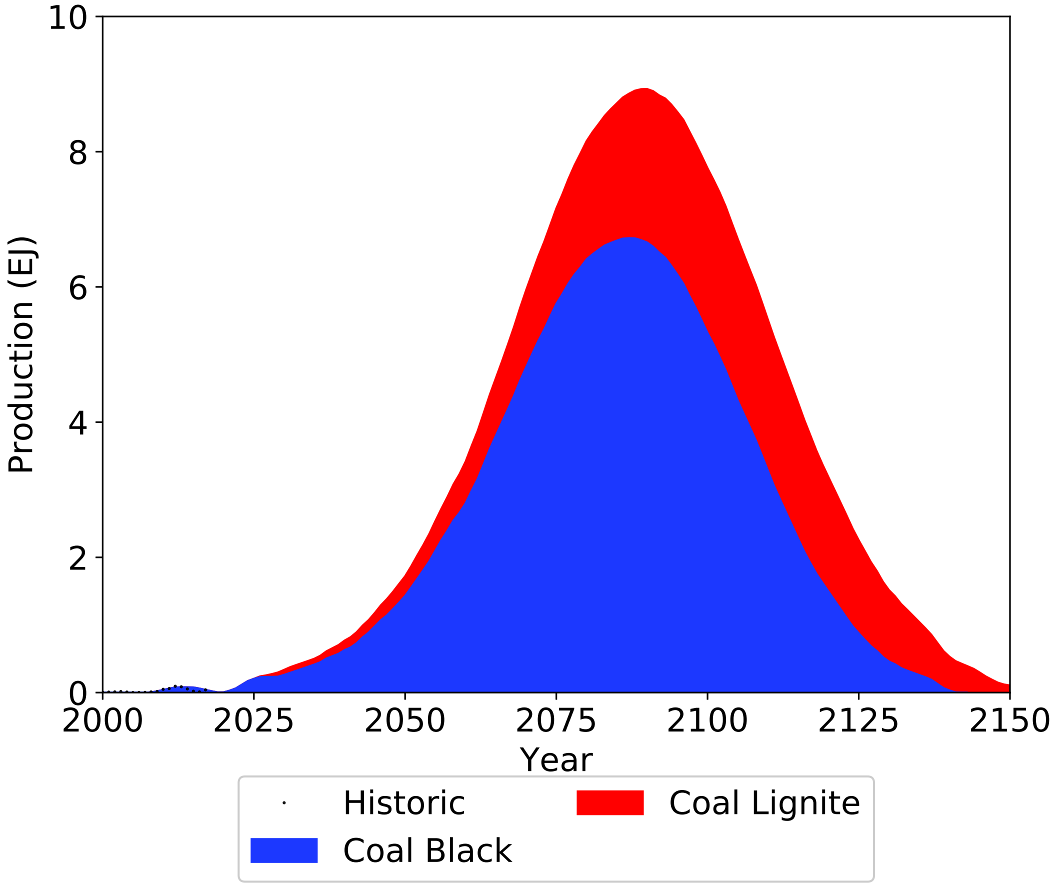

Figure 4.31: Kazakhstan - Other projections capped at 16

Table 4.31: Peak years - All

Name	URR	Peak Year	Peak Rate
Coal Black Other	337.75	2087	6.72
Coal Lignite Other	143.07	2102	2.44
**Total**	**480.82**	**2090**	**8.93**

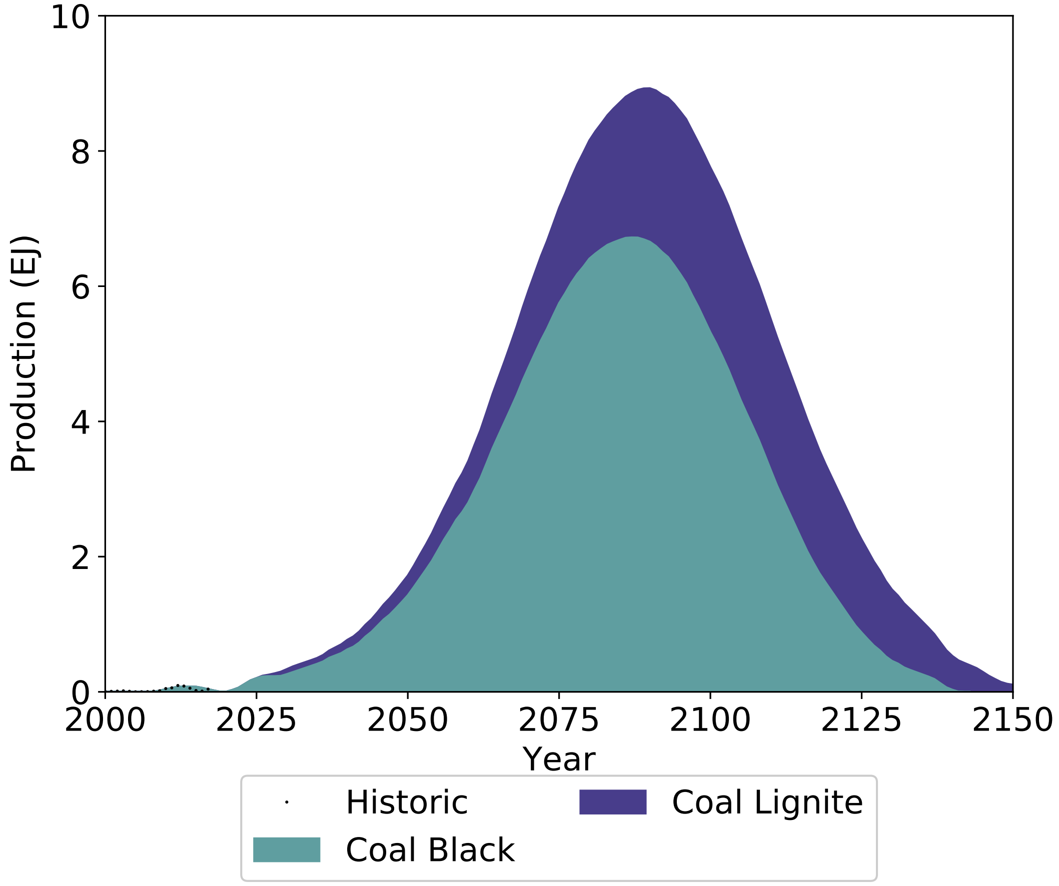

Figure 4.32: Kazakhstan - Other projection by mineral type

Table 4.32: Peak years - Minerals

**Name**	**URR**	**Peak Year**	**Peak Rate**
Coal Black	337.75	2087	6.72
Coal Lignite	143.07	2102	2.44
**Total**	**480.82**	**2090**	**8.93**

Pavlodar

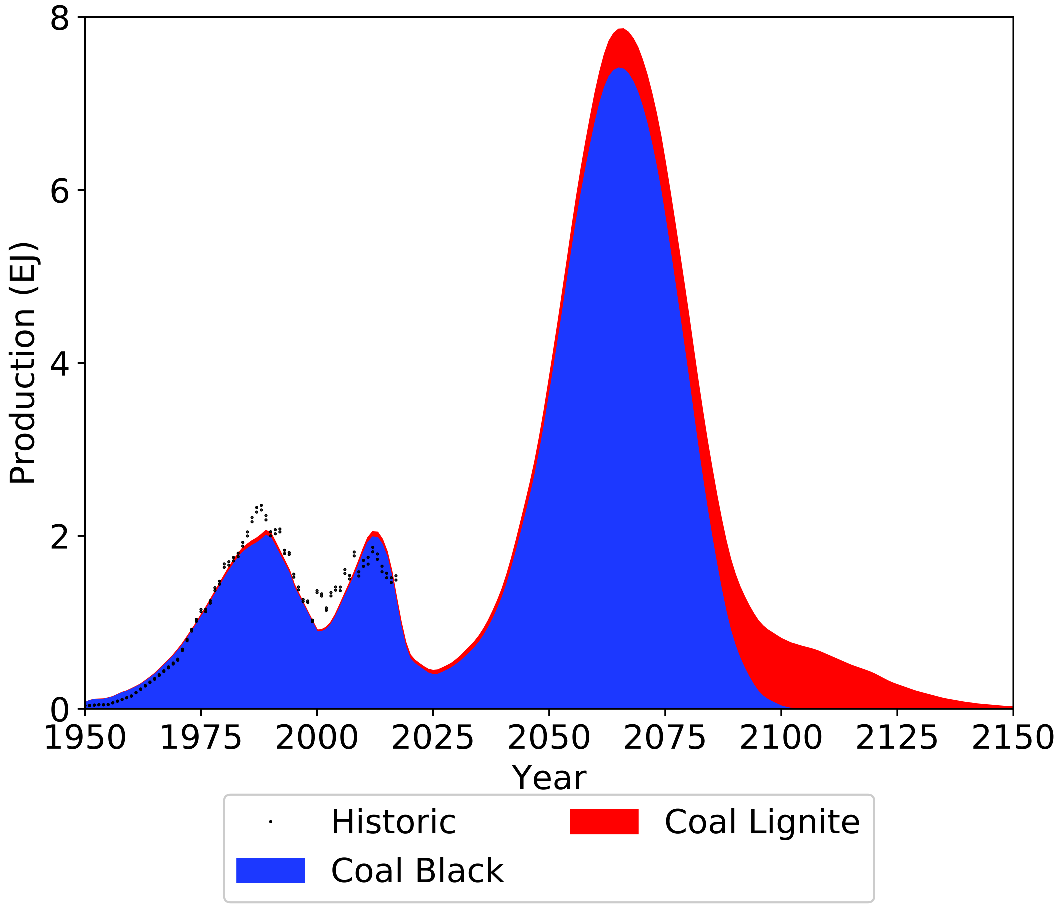

Figure 4.33: Kazakhstan - Pavlodar projections capped at 16

Table 4.33: Peak years - All

Name	URR	Peak Year	Peak Rate
Coal Black Pavlodar	315.33	2065	7.41
Coal Lignite Pavlodar	51.07	2092	0.83
**Total**	**366.4**	**2066**	**7.86**

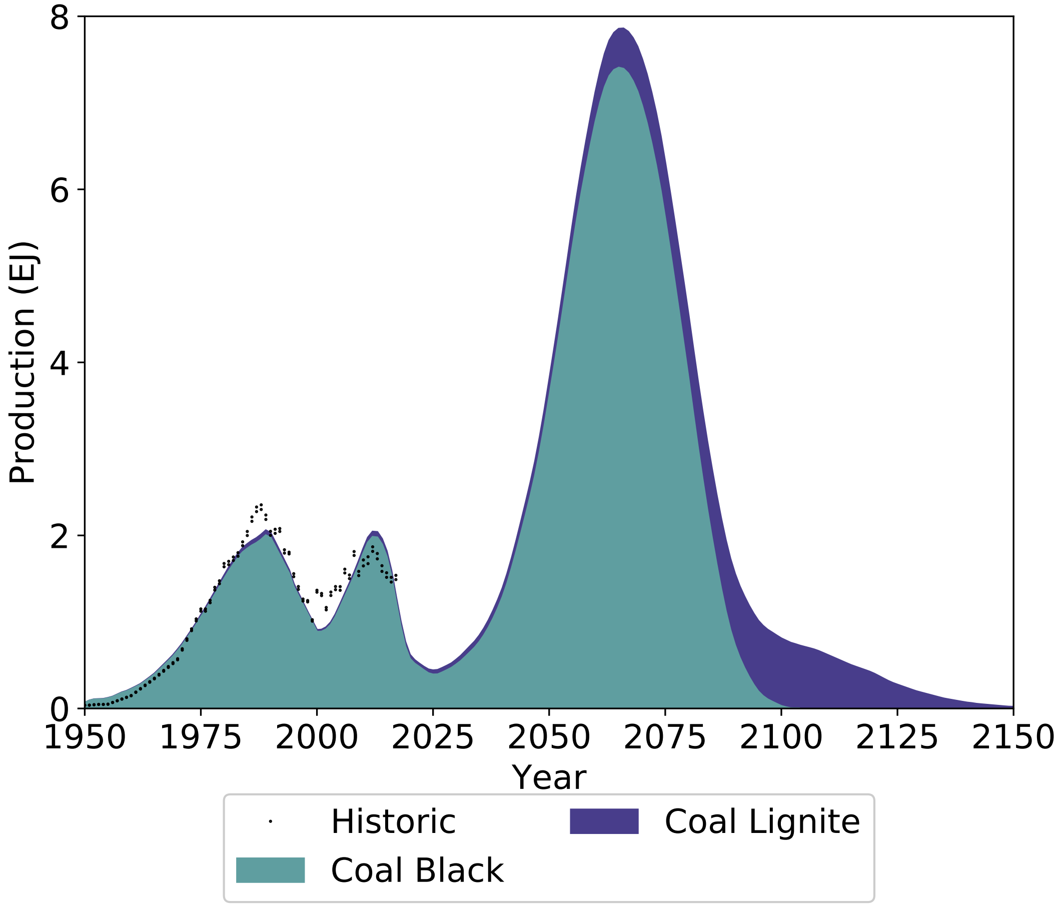

Figure 4.34: Kazakhstan - Pavlodar projection by mineral type

Table 4.34: Peak years - Minerals

**Name**	**URR**	**Peak Year**	**Peak Rate**
Coal Black	315.33	2065	7.41
Coal Lignite	51.07	2092	0.83
**Total**	**366.4**	**2066**	**7.86**

4.8.4 Projection by region

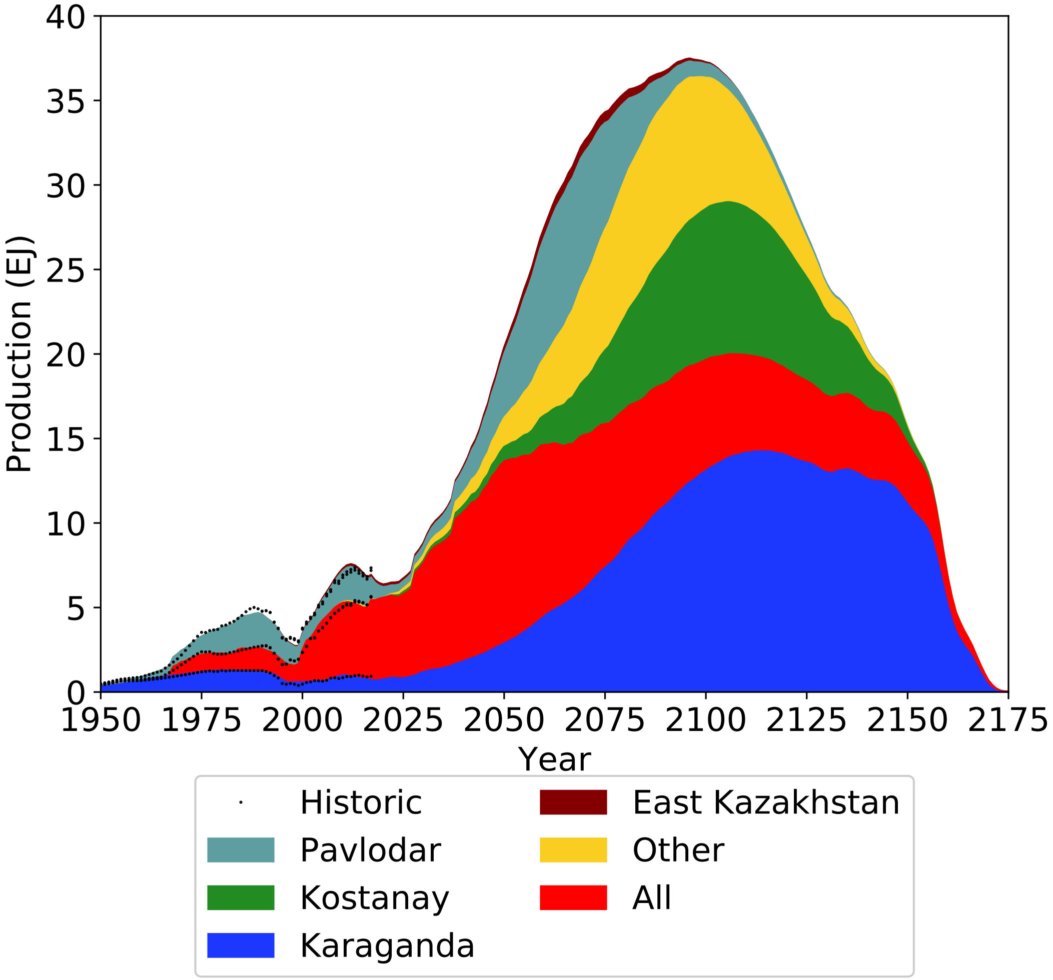

Figure 4.35: Kazakhstan by region projections capped at 16

Table 4.35: Peak years - All

**Name**	**URR**	**Peak Year**	**Peak Rate**
Karaganda	1273.26	2113	14.24
All	1057.5	2050	10.78
Kostanay	533.46	2104	9.04
Other	480.82	2090	8.93
Pavlodar	366.4	2066	7.86
East Kazakhstan	33.44	2069	0.67
**Total**	**3744.88**	**2096**	**37.48**

4.9 Kyrgyzstan

4.9.1 All Projections

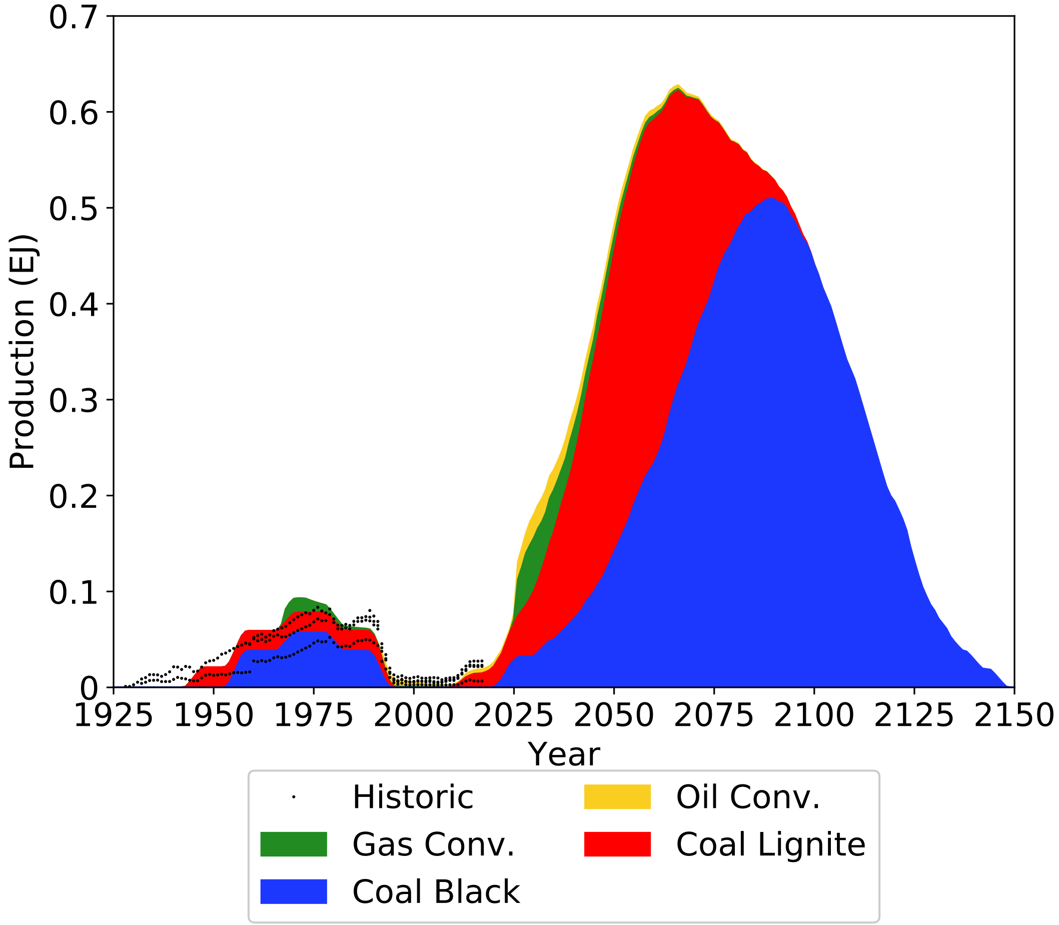

Figure 4.36: Kyrgyzstan projections capped at 16

Table 4.36: Peak years - All

**Name**	**URR**	**Peak Year**	**Peak Rate**
Coal Black	30.52	2089	0.51
Coal Lignite	13.98	2058	0.36
Gas Conv.	1.22	2029	0.06
Oil Conv.	0.74	2030	0.02
**Total**	**46.46**	**2066**	**0.63**

4.9.2 By Mineral

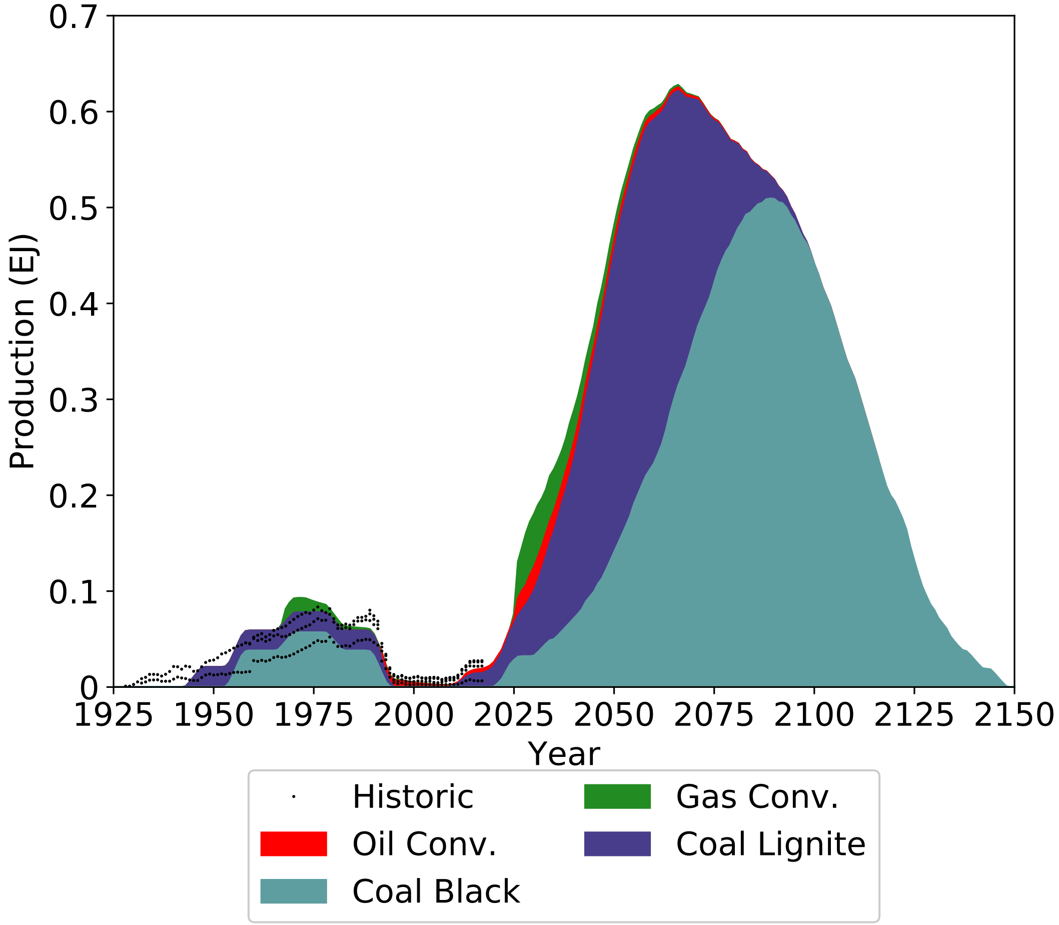

Figure 4.37: Kyrgyzstan projection by mineral type

Table 4.37: Peak years - Minerals

**Name**	**URR**	**Peak Year**	**Peak Rate**
Coal Black	30.52	2089	0.51
Coal Lignite	13.98	2058	0.36
Oil Conv.	0.74	2030	0.02
Gas Conv.	1.22	2029	0.06
**Total**	**46.46**	**2066**	**0.63**

4.10 Lithuania

4.10.1 All Projections

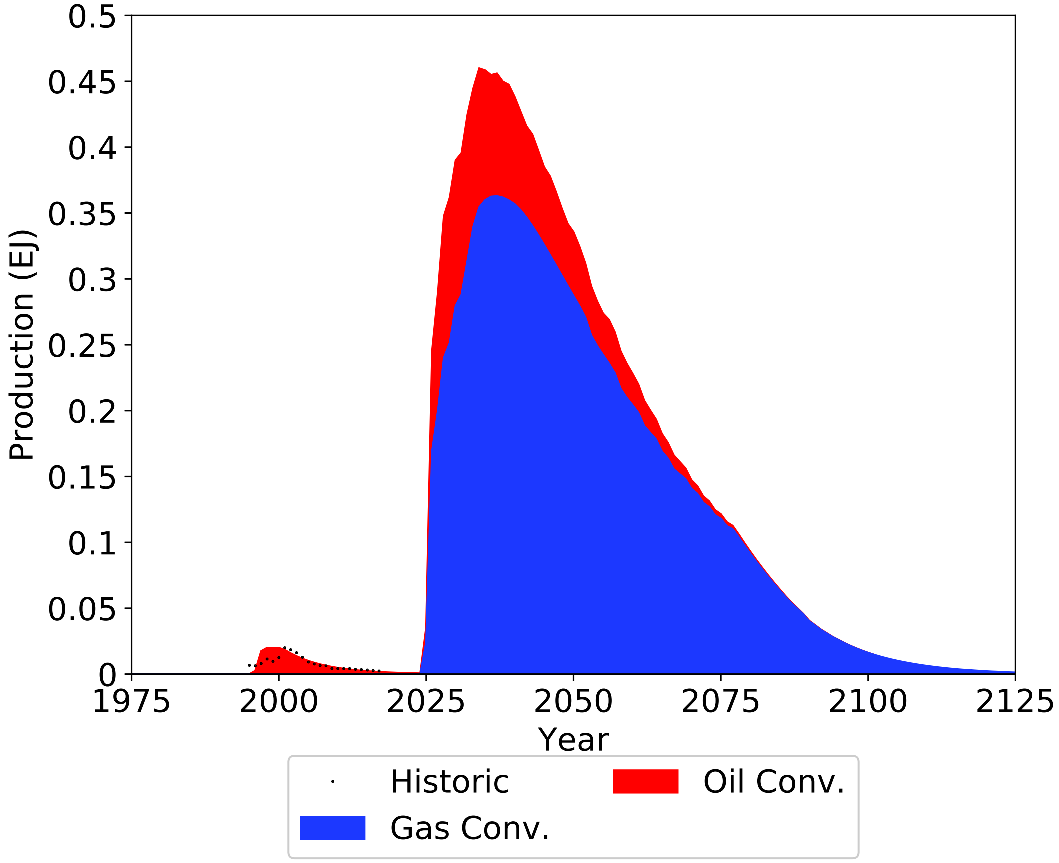

Figure 4.38: Lithuania projections capped at 16

Table 4.38: Peak years - All

Name	URR	Peak Year	Peak Rate
Gas Conv.	14.08	2037	0.36
Oil Conv.	2.79	2032	0.11
**Total**	**16.87**	**2034**	**0.46**

4.10.2 By Mineral

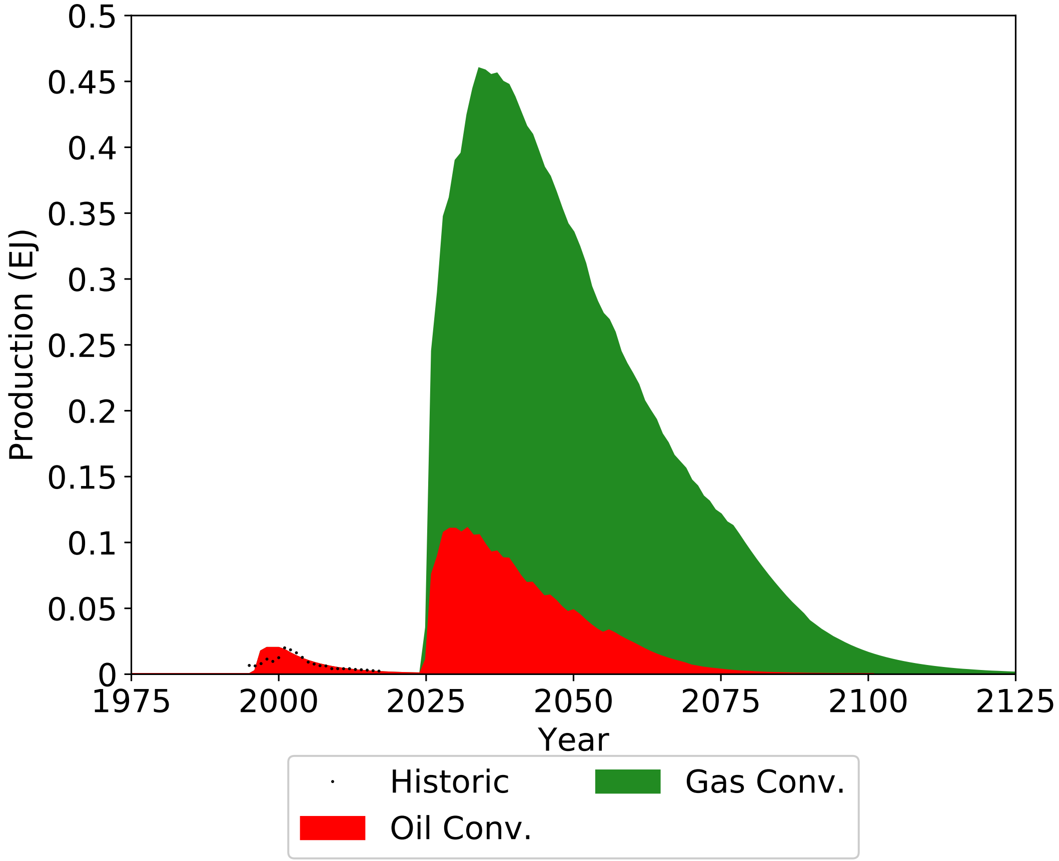

Figure 4.39: Lithuania projection by mineral type

Table 4.39: Peak years - Minerals

Name	URR	Peak Year	Peak Rate
Oil Conv.	2.79	2032	0.11
Gas Conv.	14.08	2037	0.36
**Total**	**16.87**	**2034**	**0.46**

4.11 Luhansk

4.11.1 All Projections

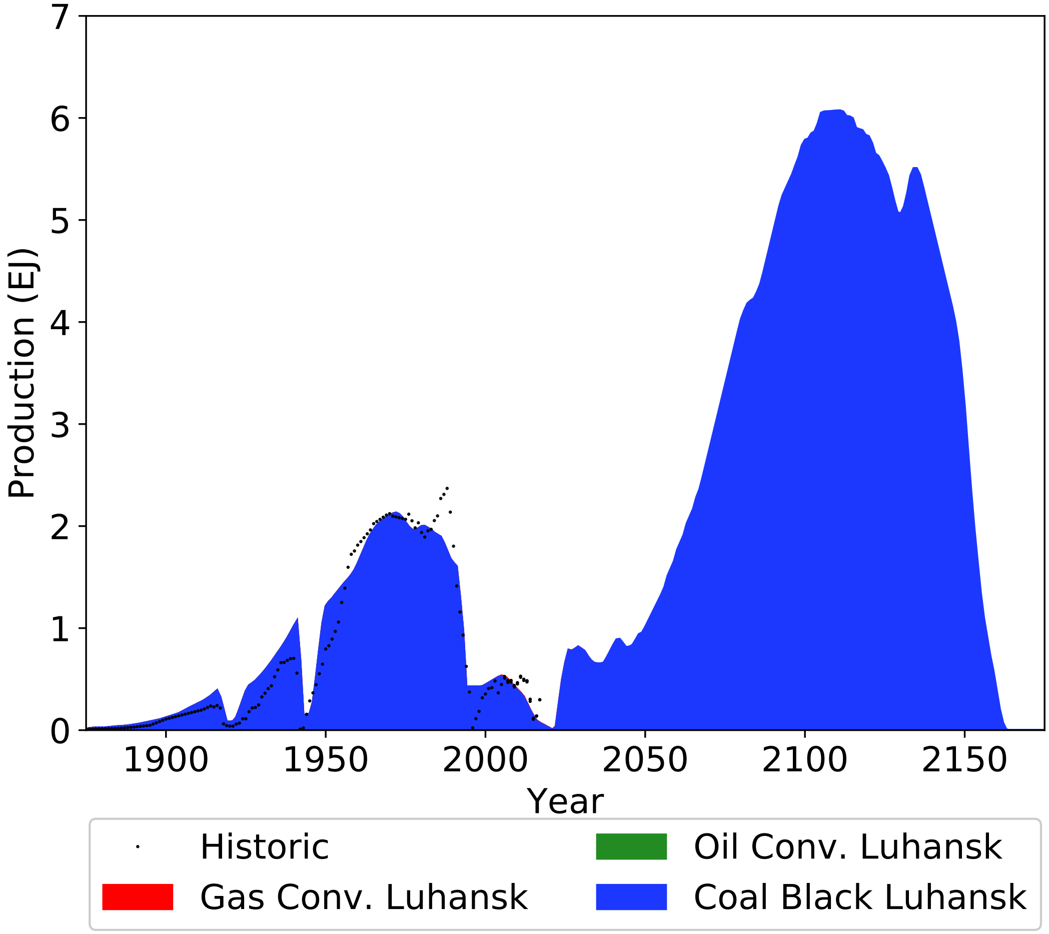

Figure 4.40: Luhansk projections capped at 16

Table 4.40: Peak years - All

Name	URR	Peak Year	Peak Rate
Coal Black Luhansk	582.48	2111	6.07
Gas Conv. Luhansk	0.14	2006	0.01
Oil Conv. Luhansk	–	2006	–
**Total**	**582.62**	**2111**	**6.07**

4.11.2 By Mineral

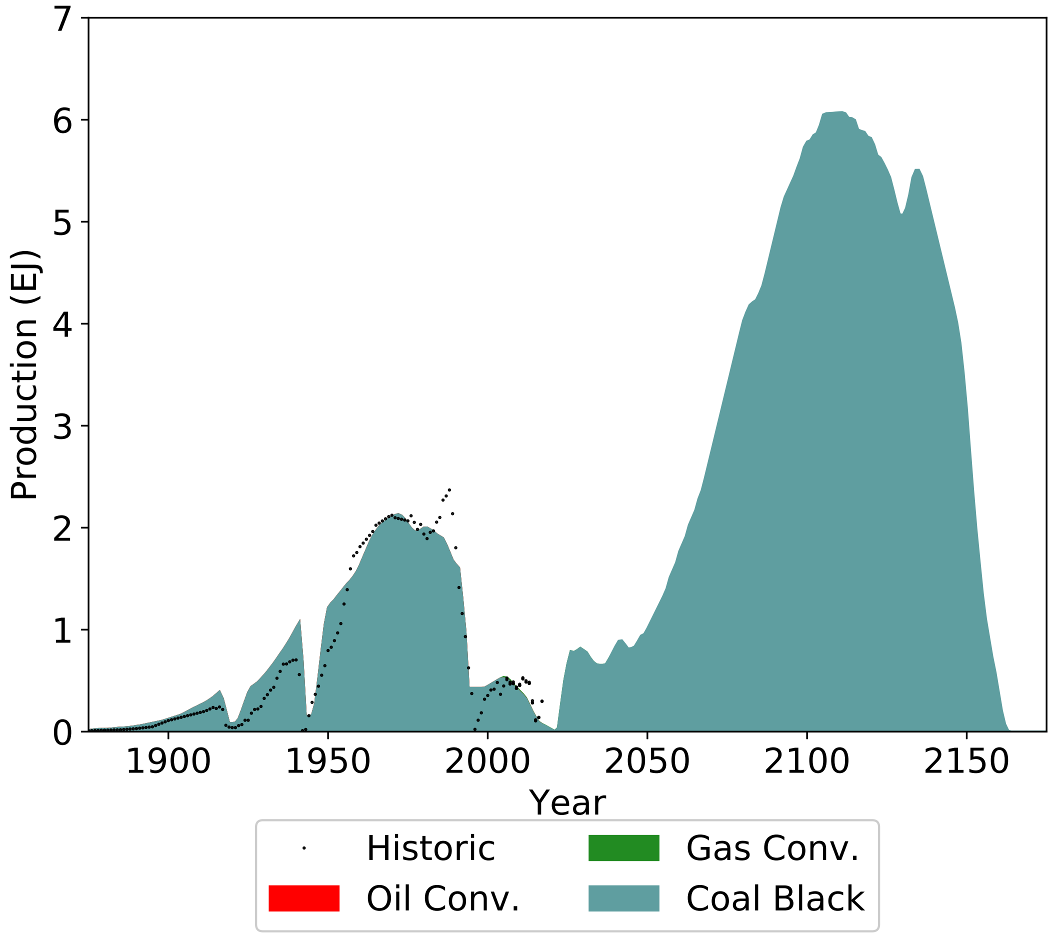

Figure 4.41: Luhansk projection by mineral type

4.11.3 Regional Projections

Table 4.41: Peak years - Minerals

**Name**	**URR**	**Peak Year**	**Peak Rate**
Coal Black	582.48	2111	6.07
Oil Conv.	–	2006	–
Gas Conv.	0.14	2006	0.01
**Total**	**582.62**	**2111**	**6.07**

Luhansk

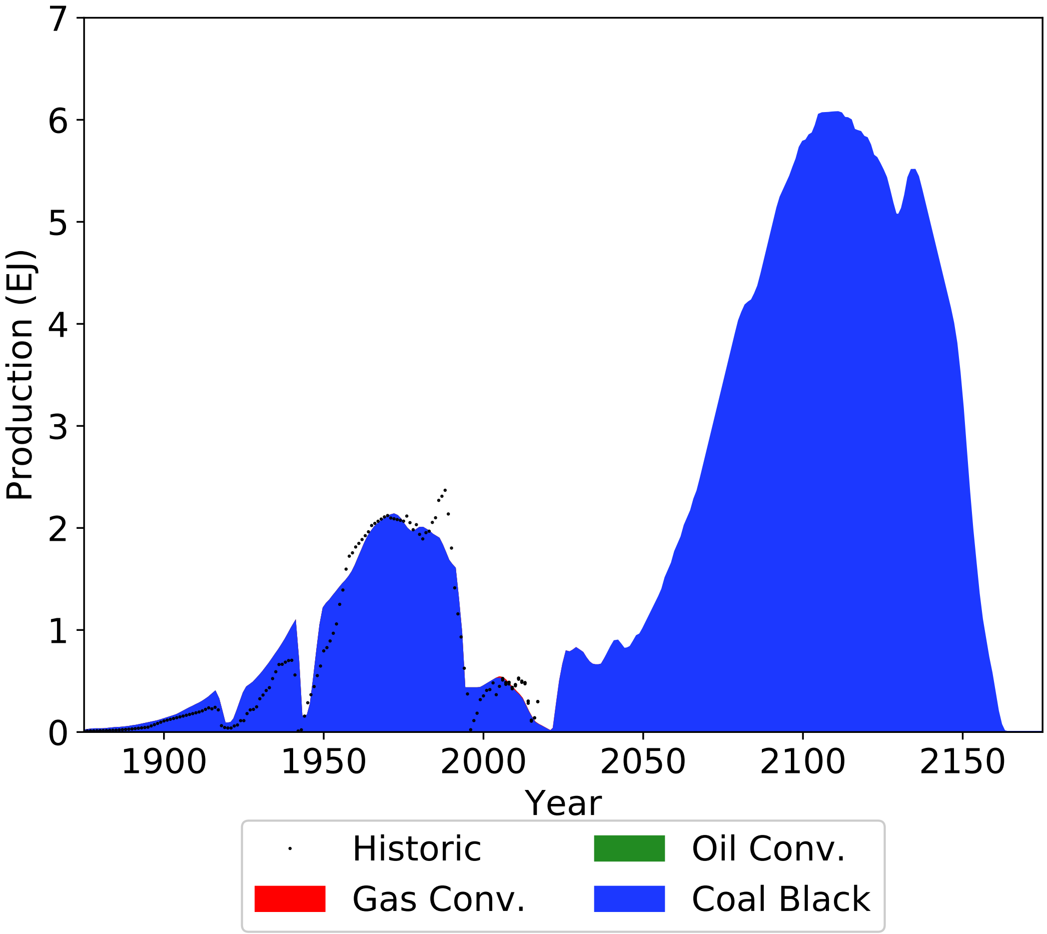

Figure 4.42: Luhansk - Luhansk projections capped at 16

Table 4.42: Peak years - All

**Name**	**URR**	**Peak Year**	**Peak Rate**
Coal Black Luhansk	582.48	2111	6.07
Gas Conv. Luhansk	0.14	2006	0.01
Oil Conv. Luhansk	–	2006	–
**Total**	**582.62**	**2111**	**6.07**

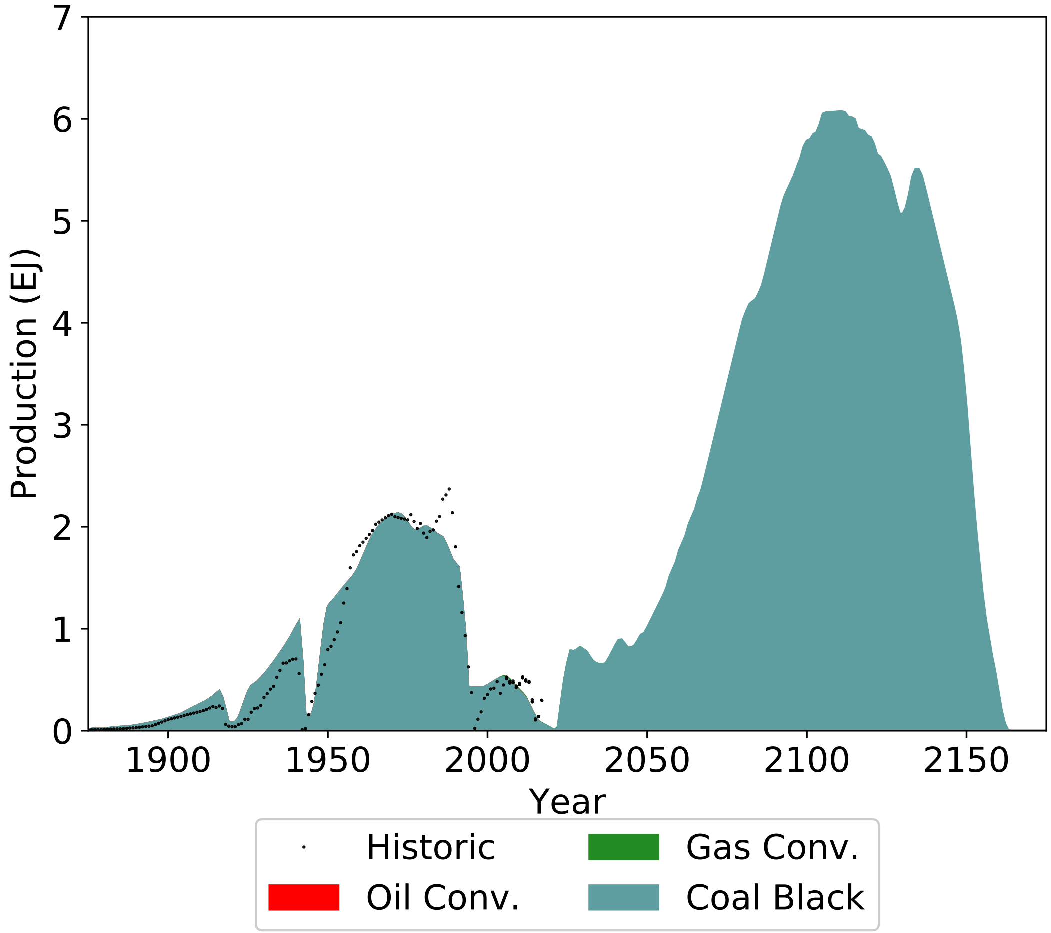

Figure 4.43: Luhansk - Luhansk projection by mineral type

Table 4.43: Peak years - Minerals

**Name**	**URR**	**Peak Year**	**Peak Rate**
Coal Black	582.48	2111	6.07
Oil Conv.	–	2006	–
Gas Conv.	0.14	2006	0.01
**Total**	**582.62**	**2111**	**6.07**

4.11.4 Projection by region

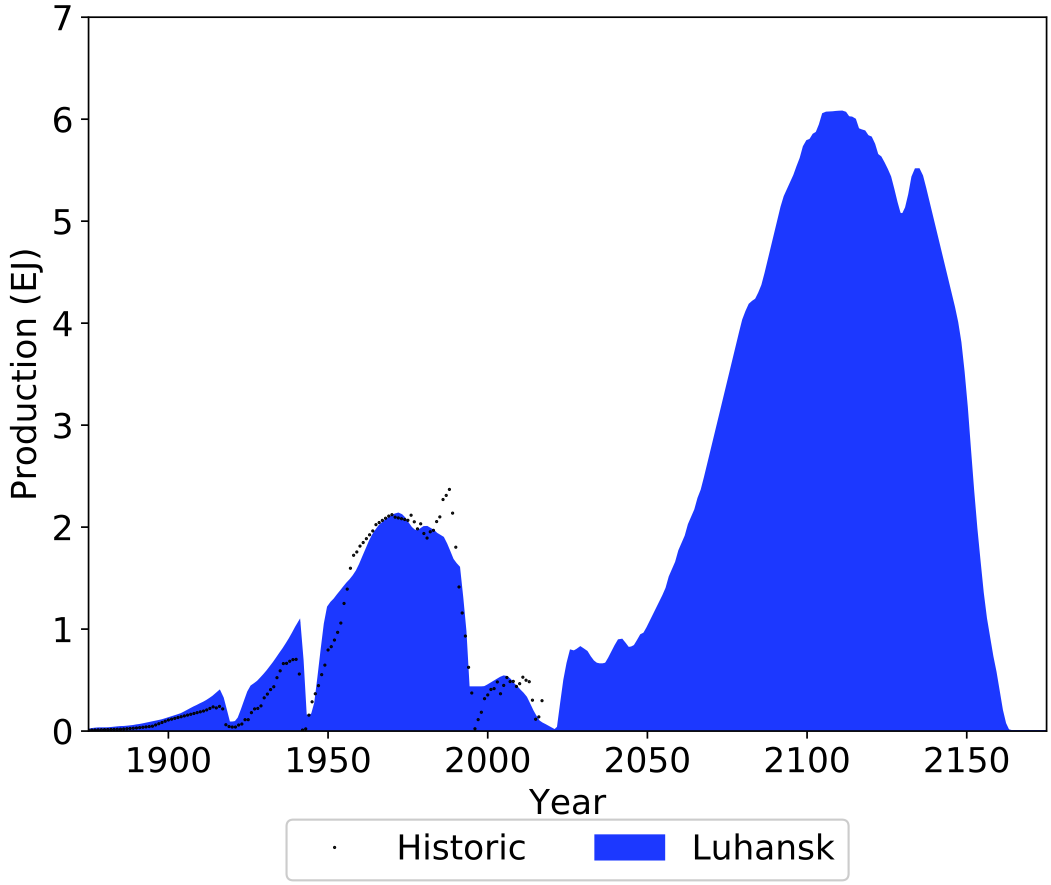

Figure 4.44: Luhansk by region projections capped at 16

Table 4.44: Peak years - All

Name	URR	Peak Year	Peak Rate
Luhansk	582.62	2111	6.07
**Total**	**582.62**	**2111**	**6.07**

4.12 Moldova

4.12.1 All Projections

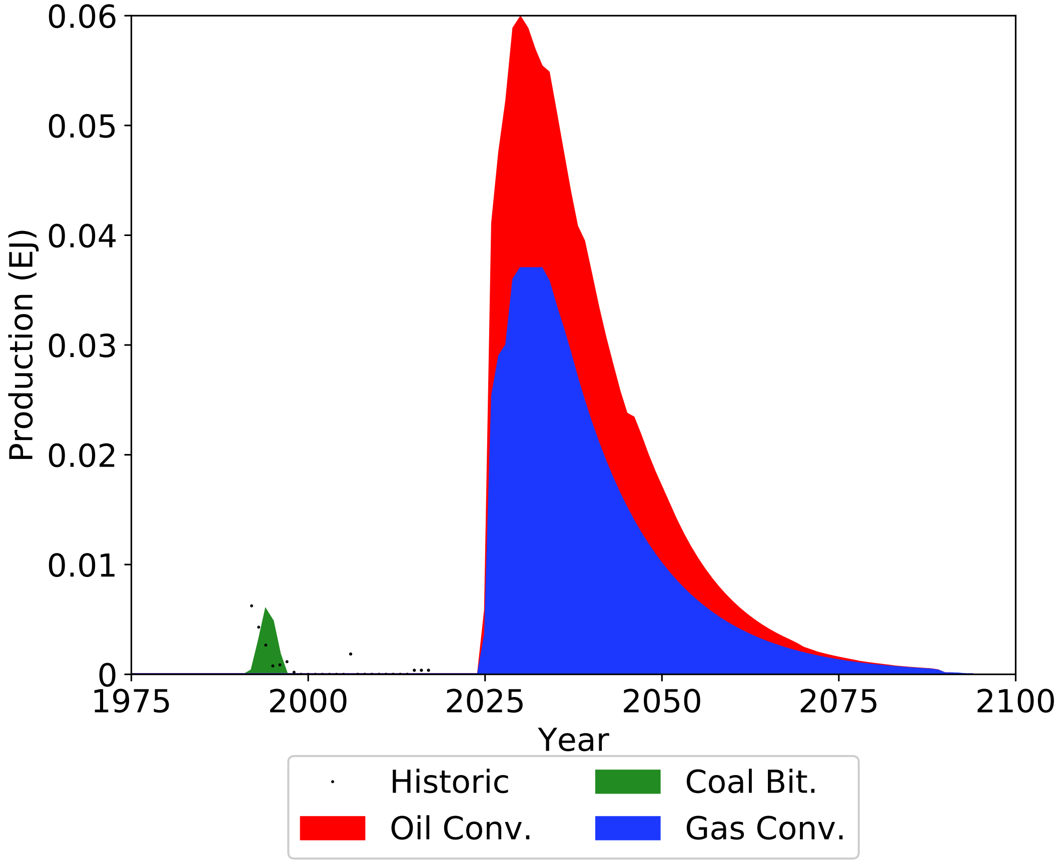

Figure 4.45: Moldova projections capped at 16

Table 4.45: Peak years - All

Name	URR	Peak Year	Peak Rate
Gas Conv.	0.74	2030	0.04
Oil Conv.	0.42	2029	0.02
Coal Bit.	0.02	1994	0.01
**Total**	**1.18**	**2030**	**0.06**

4.12.2 By Mineral

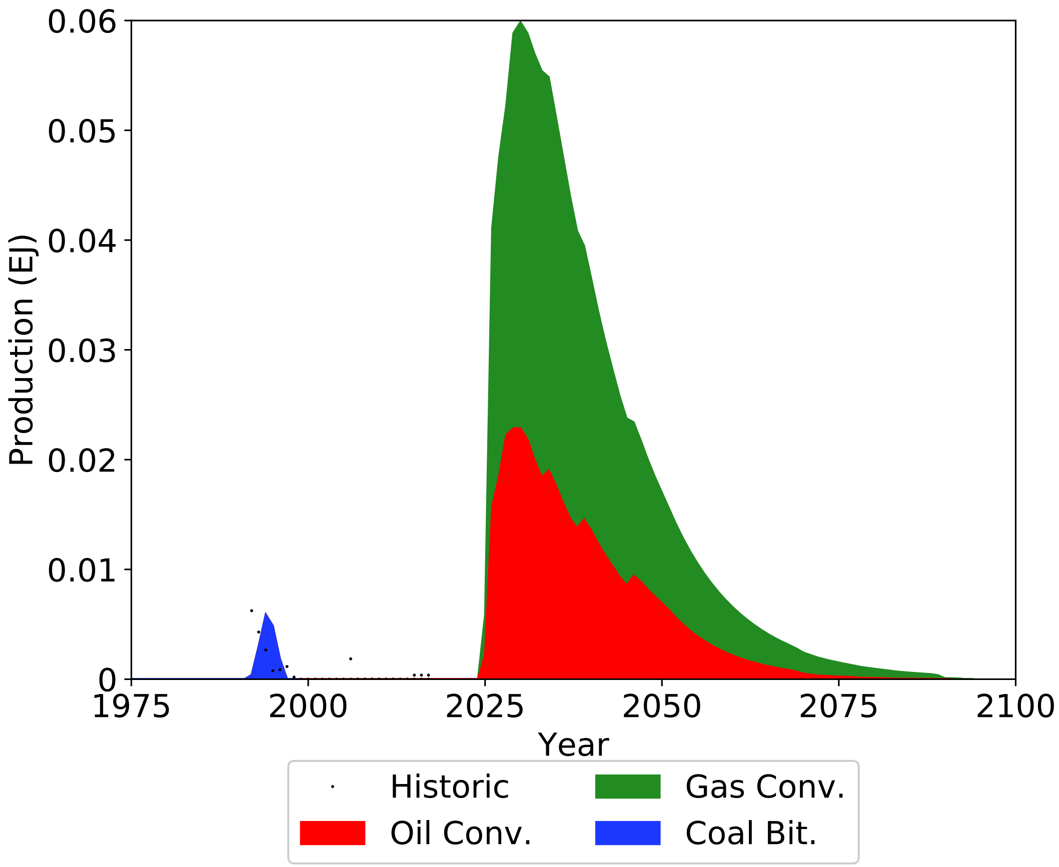

Figure 4.46: Moldova projection by mineral type

Table 4.46: Peak years - Minerals

Name	URR	Peak Year	Peak Rate
Coal Bit.	0.02	1994	0.01
Oil Conv.	0.42	2029	0.02
Gas Conv.	0.74	2030	0.04
**Total**	**1.18**	**2030**	**0.06**

4.13 Russia

4.13.1 All Projections

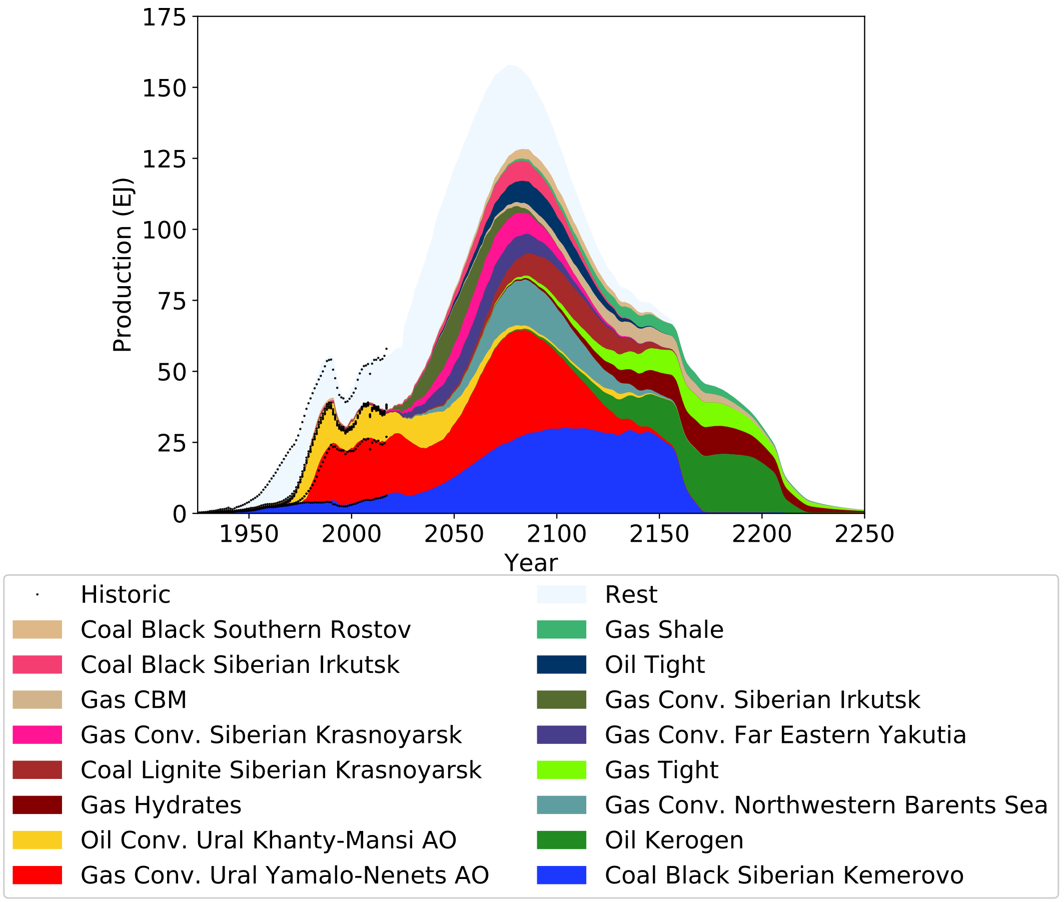

Figure 4.47: Russia projections capped at 16

Table 4.47: Peak years - All

**Name**	**URR**	**Peak Year**	**Peak Rate**
Coal Black Siberian Kemerovo	3378.92	2105	29.84
Gas Conv. Ural Yamalo-Nenets AO	3364.8	2077	38.25
Oil Kerogen	1421.06	2182	20.6
Oil Conv. Ural Khanty-Mansi AO	989.95	1985	15.37
Gas Conv. Northwestern Barents Sea	937.74	2085	16.22
Gas Hydrates	807.66	2171	10.2
Gas Tight	741.3	2164	9.03
Coal Lignite Siberian Krasnoyarsk	664.93	2105	11.25
Gas Conv. Far Eastern Yakutia	574.68	2063	10.73
Gas Conv. Siberian Krasnoyarsk	561.39	2066	10.33
Gas Conv. Siberian Irkutsk	496.29	2049	13.91
Gas CBM	466.8	2140	5.28
Oil Tight	432.61	2091	8.52
Coal Black Siberian Irkutsk	412.45	2081	7.11
Gas Shale	352.12	2146	4.18
Coal Black Southern Rostov	295.92	2096	3.87
Coal Far Eastern Yakutia	288.18	2087	4.34
Oil Conv. Ural Yamalo-Nenets AO	255.27	2045	4.37
Coal Black Northwestern Komi	225.55	2078	3.63
Oil Nat. Bitumen	219.45	2113	2.91
Gas Conv. Far Eastern Sakhalin	195.44	2052	3.54
Oil Conv. Volga Tatarstan	184.67	1972	4.3
Gas Conv. Southern Astrakhan	176.28	2050	3.24
Coal Black Siberian Khakassia	153.37	2057	3.07
Gas Conv. Far Eastern Chukota AO	124.17	2052	2.52
Gas Conv. Northwestern Nenets AO	122.36	2058	2.43
Coal Black Siberian Tuva	99.86	2091	1.67
Gas Conv. Volga Orenburg	93.03	1983	1.76
Oil Conv. Volga Bashkortostan	90.64	1969	1.91
Coal Far Eastern Primorsky	87.56	2067	1.61
Oil Conv. Siberian Krasnoyarsk	86.64	2042	1.94
Gas Conv. Ural Khanty-Mansi AO	79.6	2016	1.23
Gas Conv. North Caucasian	79.49	1963	1.58
Oil Conv. Volga Samara	77.52	1971	1.48
Coal Black Far Eastern Sakhalin	77.12	2058	1.55
Oil Conv. Volga Orenburg	72.11	2035	1.24
Coal Black Far Eastern Buryatia	71.78	2064	1.54
Oil Conv. Northwestern Komi	65.44	2036	0.88
Coal Black Far Eastern Khabarovsk	62.88	2070	1.06
Gas Conv. Northwestern Komi	60.38	2041	1.05
Oil Conv. Northwestern Nenets AO	56.97	2041	1.17
Oil Conv. Volga Perm	56.46	2031	0.85
Coal Brown Far Eastern Amur	55.72	2083	0.76
Coal Brown Far Eastern Zabaykalsky	55.6	2070	0.48
Coal Black Far Eastern Magadan	54.0	2102	0.88
Coal Lignite Central	51.45	2106	0.55
Oil Conv. Siberian Irkutsk	46.16	2031	1.19
Coal Black Siberian Novosibirsk	39.44	2033	1.88
Oil Conv. Southern Astrakhan	37.77	2035	0.88

Table 4.47: Peak years - All – Continued

Name	URR	Peak Year	Peak Rate
Oil Conv. Siberian Tomsk	37.17	2031	0.66
Gas Conv. Southern Other	36.55	1963	1.08
Oil Conv. Volga Udmurtia	34.67	1986	0.51
Oil Conv. Far Eastern Yakutia	32.37	2041	0.73
Oil Conv. Far Eastern Sakhalin	29.53	2018	0.76
Coal Ural Khanty-Mansi AO	26.33	2081	0.48
Oil Conv. Ural Tyumen	24.72	2033	0.65
Gas Conv. Far Eastern Kamchatka	24.15	2037	0.6
Gas Conv. Siberian Tomsk	20.05	2034	0.43
Coal Black Far Eastern Chukotka AO	19.05	2083	0.47
Oil Conv. North Caucasian Chechnya	18.92	1969	0.81
Coal Brown Ural Chelyabinsk	18.45	1966	0.29
Coal Black Volga Perm	17.31	1958	0.34
Oil Conv. Northwestern Murmansk	16.8	2042	0.46
Oil Conv. Southern Volgograd	13.47	1965	0.3
Oil Conv. Southern Krasnodar	11.33	1960	0.25
Gas Conv. Volga Saratov	11.23	1963	0.27
Coal Brown Ural Sverdlovsk	11.22	1960	0.36
Oil Conv. North Caucasian Stavropol	9.9	1971	0.3
Coal Brown Volga Orenburg	9.62	2048	0.39
Oil Conv. Volga Saratov	8.46	2023	0.14
Gas Conv. Far Eastern Primorsky	7.41	2022	0.2
Gas Conv. Volga Other	6.26	2034	0.15
Oil Conv. Volga Ulyanovsk	4.77	2033	0.15
Coal Lignite Volga Bashkortostan	4.09	2045	0.11
Coal Brown Far Eastern Kamchatka	3.93	2060	0.12
Coal Black Northwestern Nenetsk AO	2.6	2030	0.12
Oil Conv. Northwestern Kaliningrad	2.19	1980	0.07
Oil Conv. North Caucasian Dagestan	1.78	1972	0.08
Coal Siberian Altai Rep	1.56	2058	0.06
Oil Conv. Siberian Novosibirsk	0.7	2004	0.09
Oil Conv. Southern Kalmykia	0.68	1977	0.03
Coal Brown Far Eastern Jewish AO	0.66	2056	0.03
Gas Conv. Ural Tyumen	0.62	2027	0.03
Coal Black Northwestern Murmansk	0.47	1970	0.01
Oil Conv. Siberian Omsk	0.43	2005	0.04
Coal Brown Siberian Altai Krai	0.4	2051	0.02
Coal Black North Caucasian Karachay-Cherkessia	0.32	2048	0.01
Oil Conv. Southern Adygea	0.09	1971	0.01
Oil Extra Heavy	0.08	2030	0.01
Oil Conv. North Caucasian Ingushetia	0.07	1996	0.01
Oil Conv. Volga Penza	0.04	2002	0.01
Coal Brown Northwestern Novgorod	0.01	1951	0.01
Oil Conv. North Caucasian North Ossetia-Alania	–	2002	–
Oil Conv. North Caucasian Kabardino-Balkaria	–	1998	–
Oil Conv. Volga Kirov	–	2001	–
Coal Lignite Far Eastern Zabaykalsky	–	1997	–
Oil Conv. Central Yaroslavl	–	2005	–
**Total**	**19871.44**	**2077**	**157.74**

4.13.2 By Mineral

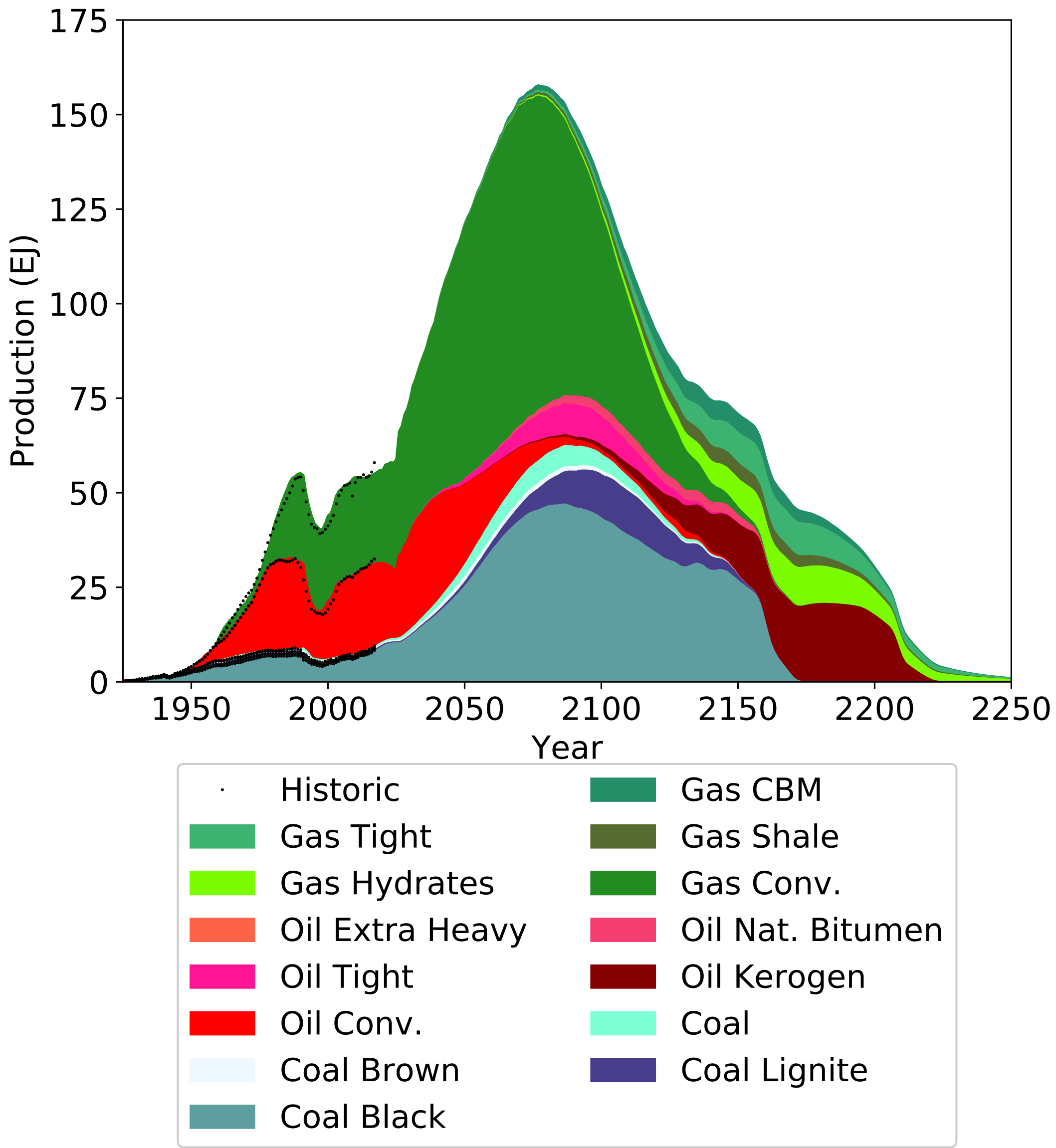

Figure 4.48: Russia projection by mineral type

4.13.3 Regional Projections

Table 4.48: Peak years - Minerals

**Name**	**URR**	**Peak Year**	**Peak Rate**
Coal Black	4911.04	2086	46.91
Coal Lignite	720.47	2105	11.8
Coal Brown	155.6	2058	1.52
Coal	403.63	2078	6.02
Oil Conv.	2267.7	2038	28.45
Oil Kerogen	1421.06	2182	20.6
Oil Tight	432.61	2091	8.52
Oil Nat. Bitumen	219.45	2113	2.91
Oil Extra Heavy	0.08	2030	0.01
Gas Conv.	6971.92	2070	84.69
Gas Hydrates	807.66	2171	10.2
Gas Shale	352.12	2146	4.18
Gas Tight	741.3	2164	9.03
Gas CBM	466.8	2140	5.28
**Total**	**19871.44**	**2077**	**157.74**

All

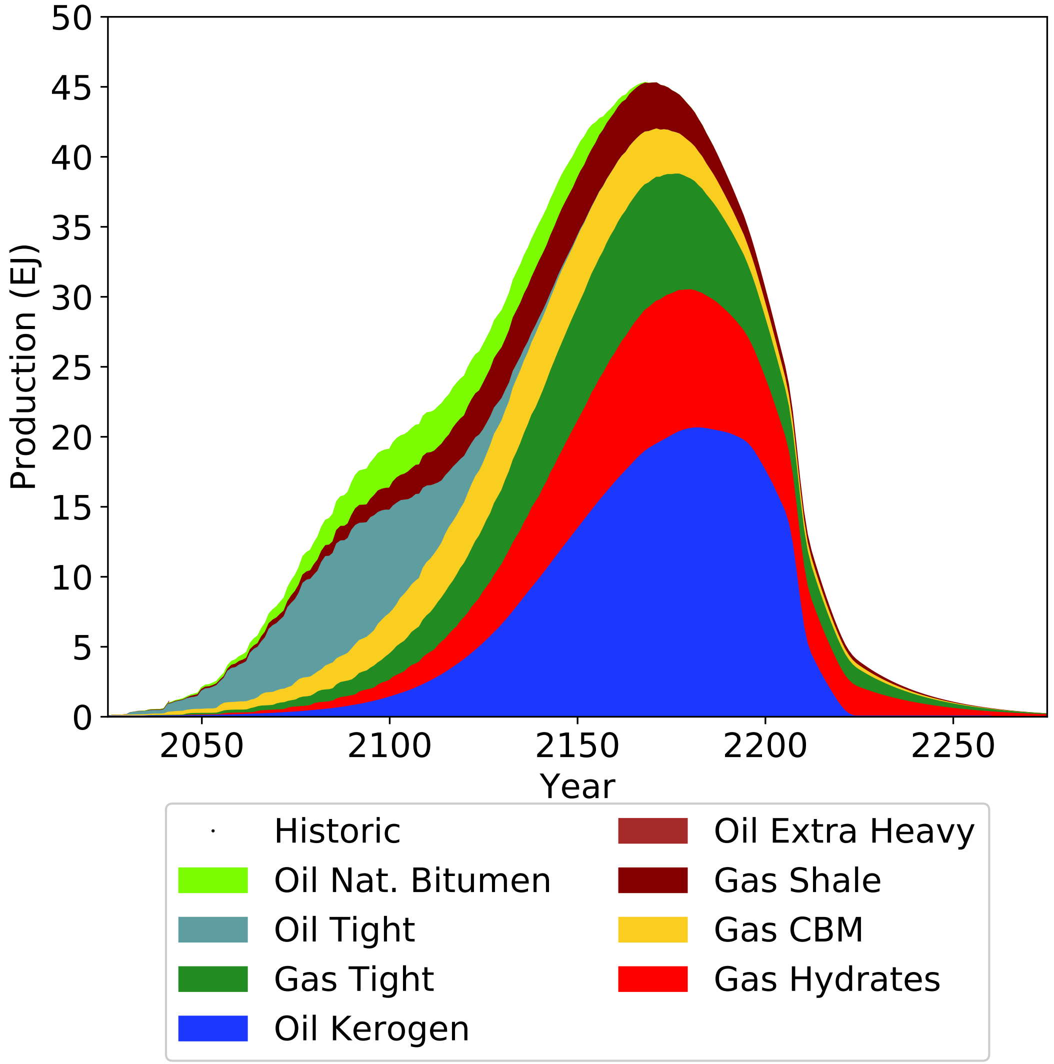

Figure 4.49: Russia - All projections capped at 16

Table 4.49: Peak years - All

Name	URR	Peak Year	Peak Rate
Oil Kerogen	1421.06	2182	20.6
Gas Hydrates	807.66	2171	10.2
Gas Tight	741.3	2164	9.03
Gas CBM	466.8	2140	5.28
Oil Tight	432.61	2091	8.52
Gas Shale	352.12	2146	4.18
Oil Nat. Bitumen	219.45	2113	2.91
Oil Extra Heavy	0.08	2030	0.01
**Total**	**4441.08**	**2168**	**45.27**

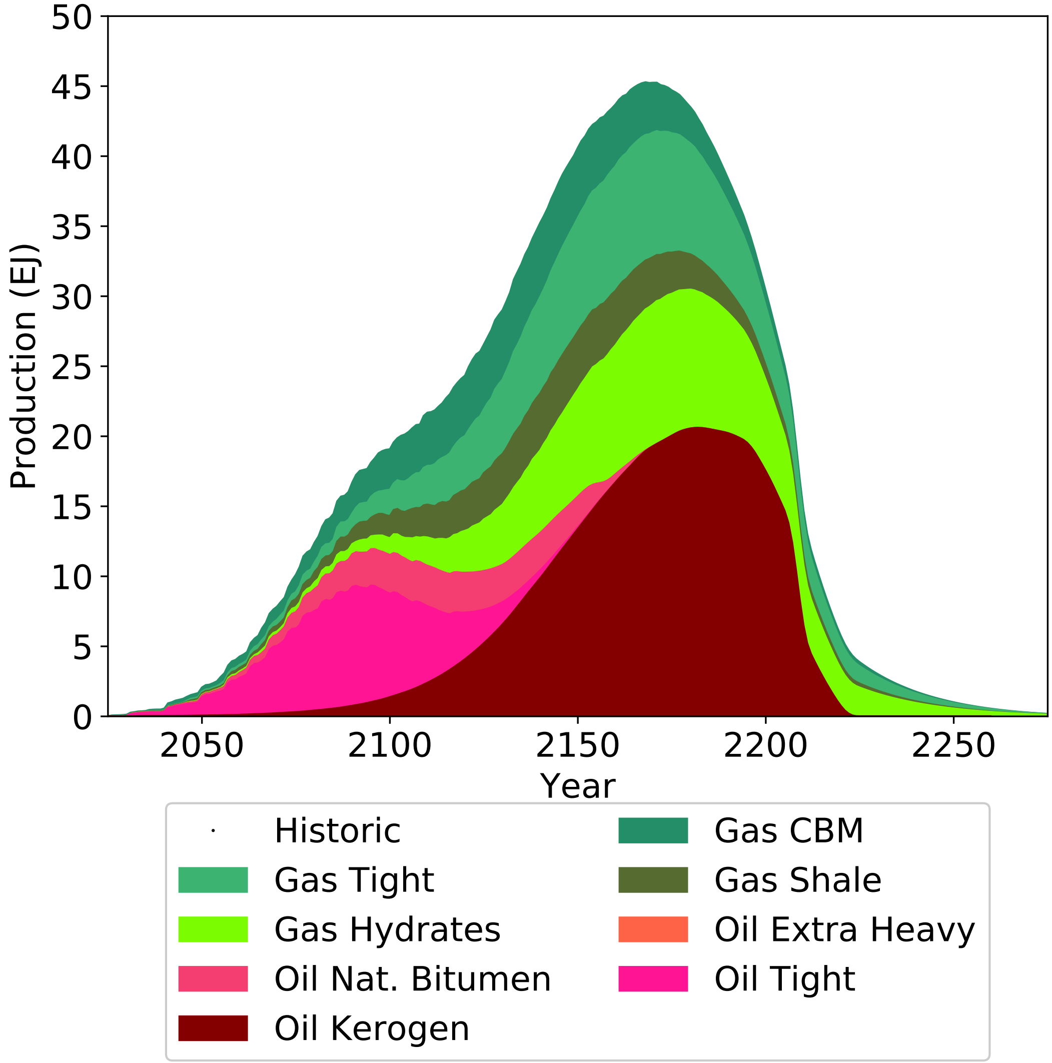

Figure 4.50: Russia - All projection by mineral type

Table 4.50: Peak years - Minerals

**Name**	**URR**	**Peak Year**	**Peak Rate**
Oil Kerogen	1421.06	2182	20.6
Oil Tight	432.61	2091	8.52
Oil Nat. Bitumen	219.45	2113	2.91
Oil Extra Heavy	0.08	2030	0.01
Gas Hydrates	807.66	2171	10.2
Gas Shale	352.12	2146	4.18
Gas Tight	741.3	2164	9.03
Gas CBM	466.8	2140	5.28
**Total**	**4441.08**	**2168**	**45.27**

Central

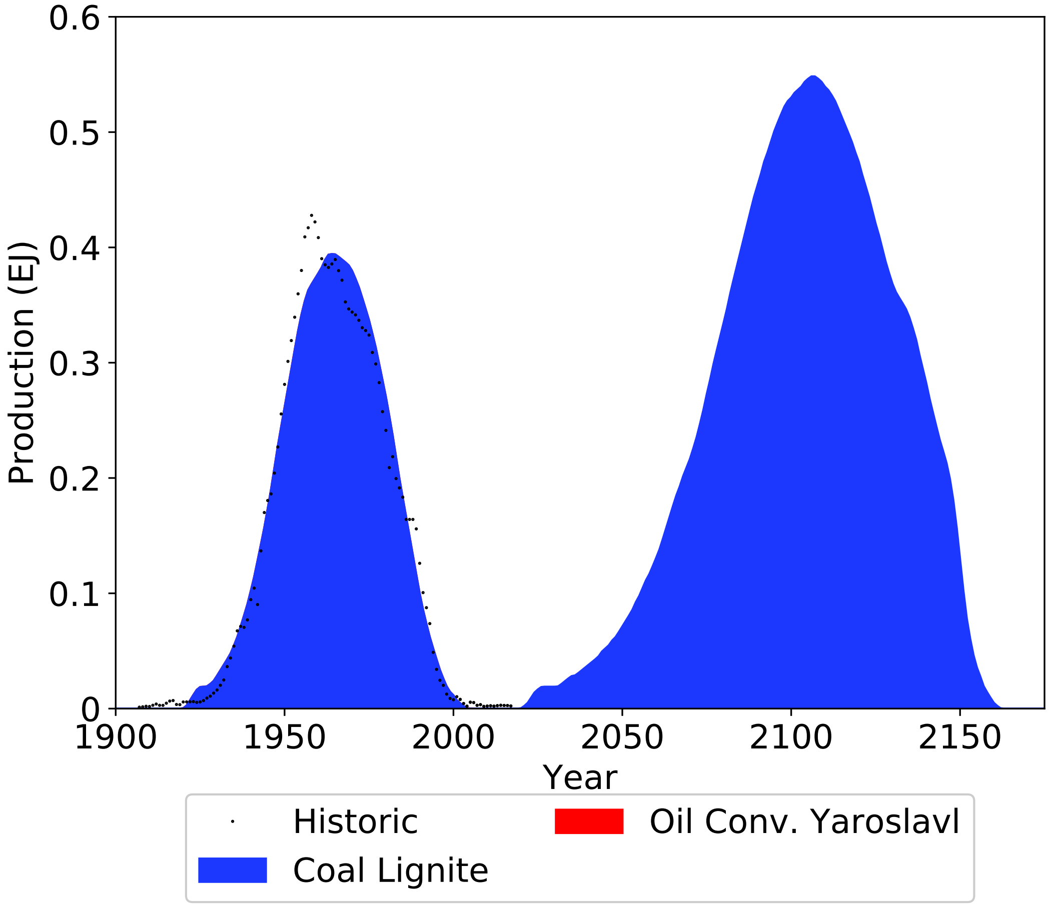

Figure 4.51: Russia - Central projections capped at 16

Table 4.51: Peak years - All

**Name**	**URR**	**Peak Year**	**Peak Rate**
Coal Lignite Central	51.45	2106	0.55
Oil Conv. Central Yaroslavl	–	2005	–
**Total**	**51.45**	**2106**	**0.55**

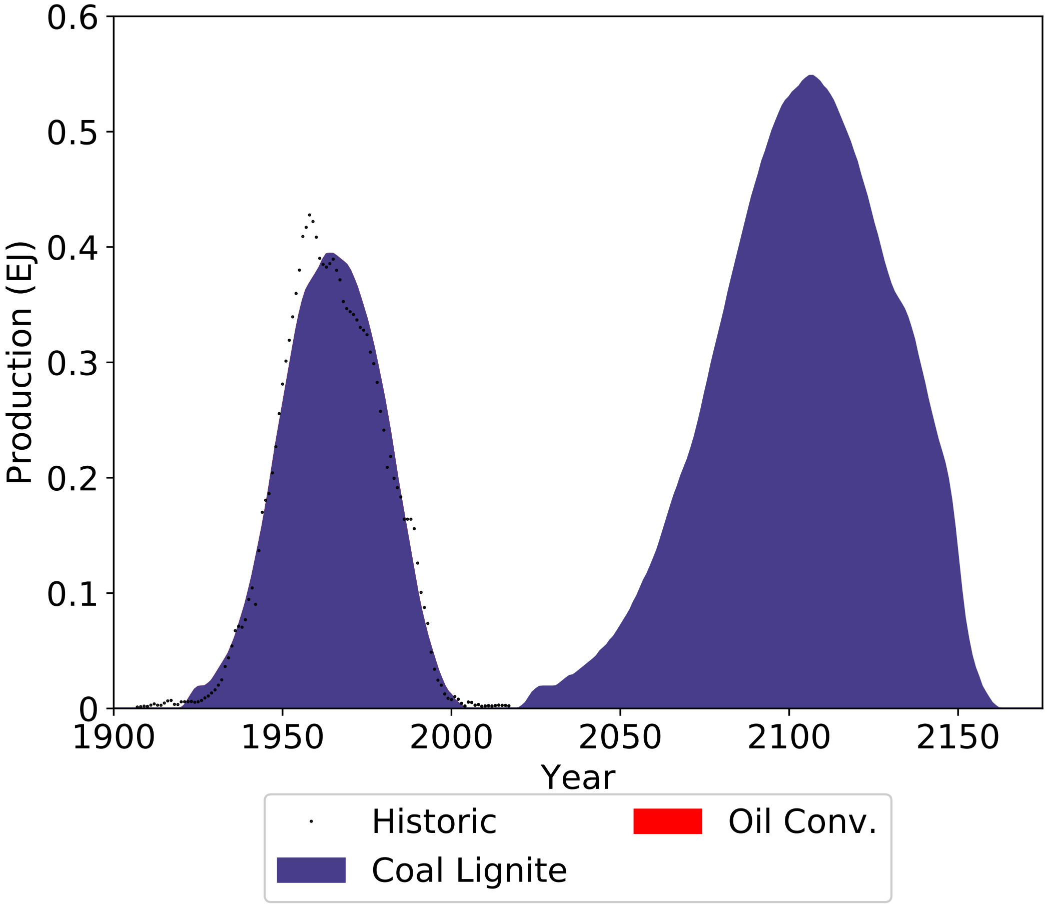

Figure 4.52: Russia - Central projection by mineral type

Table 4.52: Peak years - Minerals

Name	URR	Peak Year	Peak Rate
Coal Lignite	51.45	2106	0.55
Oil Conv.	–	2005	–
**Total**	**51.45**	**2106**	**0.55**

Far Eastern

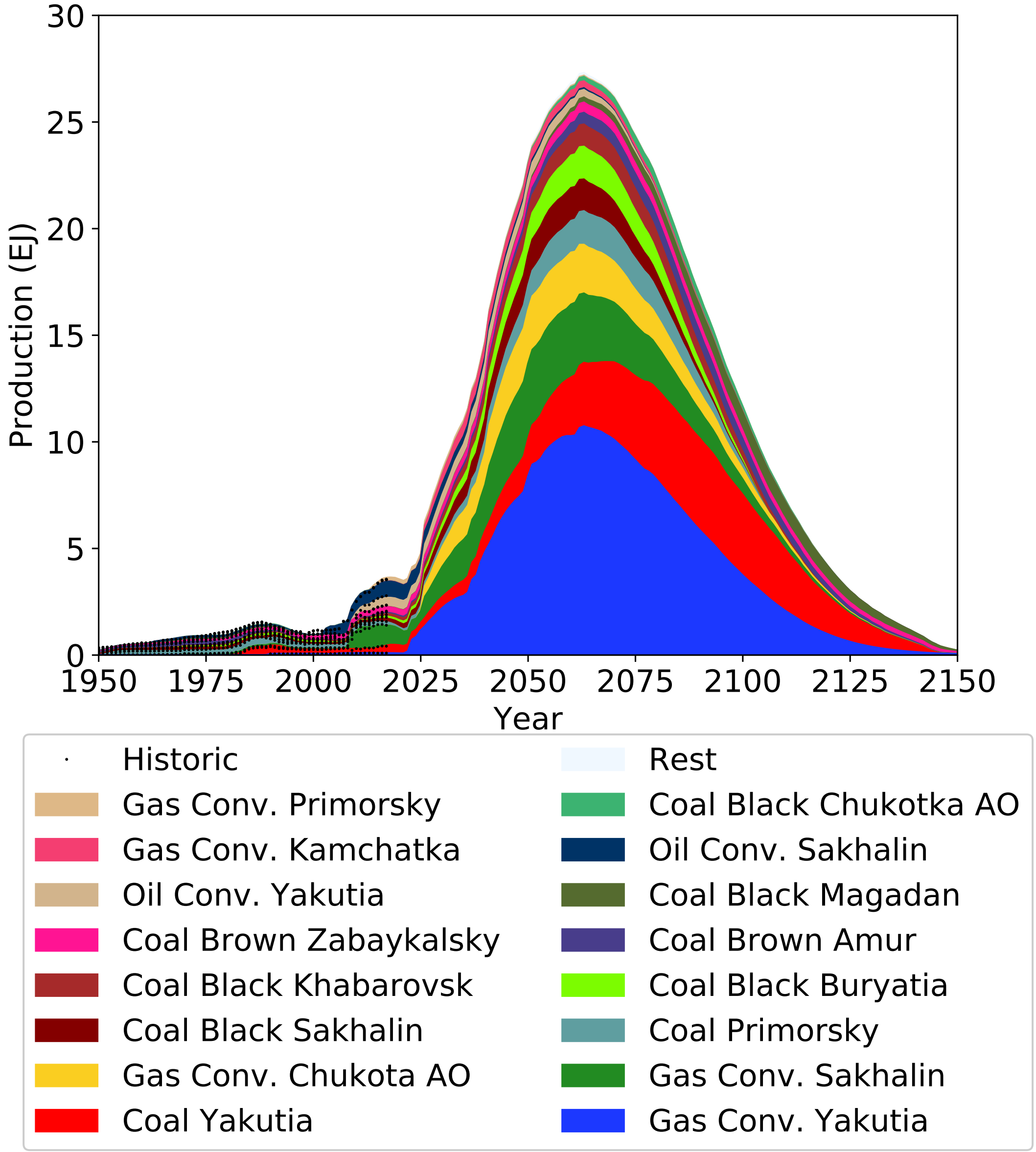

Figure 4.53: Russia - Far Eastern projections capped at 16

Table 4.53: Peak years - All

Name	URR	Peak Year	Peak Rate
Gas Conv. Far Eastern Yakutia	574.68	2063	10.73
Coal Far Eastern Yakutia	288.18	2087	4.34
Gas Conv. Far Eastern Sakhalin	195.44	2052	3.54
Gas Conv. Far Eastern Chukota AO	124.17	2052	2.52
Coal Far Eastern Primorsky	87.56	2067	1.61
Coal Black Far Eastern Sakhalin	77.12	2058	1.55
Coal Black Far Eastern Buryatia	71.78	2064	1.54
Coal Black Far Eastern Khabarovsk	62.88	2070	1.06
Coal Brown Far Eastern Amur	55.72	2083	0.76
Coal Brown Far Eastern Zabaykalsky	55.6	2070	0.48
Coal Black Far Eastern Magadan	54.0	2102	0.88
Oil Conv. Far Eastern Yakutia	32.37	2041	0.73
Oil Conv. Far Eastern Sakhalin	29.53	2018	0.76
Gas Conv. Far Eastern Kamchatka	24.15	2037	0.6
Coal Black Far Eastern Chukotka AO	19.05	2083	0.47
Gas Conv. Far Eastern Primorsky	7.41	2022	0.2
Coal Brown Far Eastern Kamchatka	3.93	2060	0.12
Coal Brown Far Eastern Jewish AO	0.66	2056	0.03
Coal Lignite Far Eastern Zabaykalsky	–	1997	–
**Total**	**1764.23**	**2063**	**27.31**

Table 4.54: Peak years - Minerals

Name	URR	Peak Year	Peak Rate
Coal Black	284.83	2065	4.53
Coal Lignite	–	1997	–
Coal Brown	115.91	2073	1.26
Coal	375.74	2078	5.55
Oil Conv.	61.9	2019	1.2
Gas Conv.	925.85	2062	16.71
**Total**	**1764.23**	**2063**	**27.31**

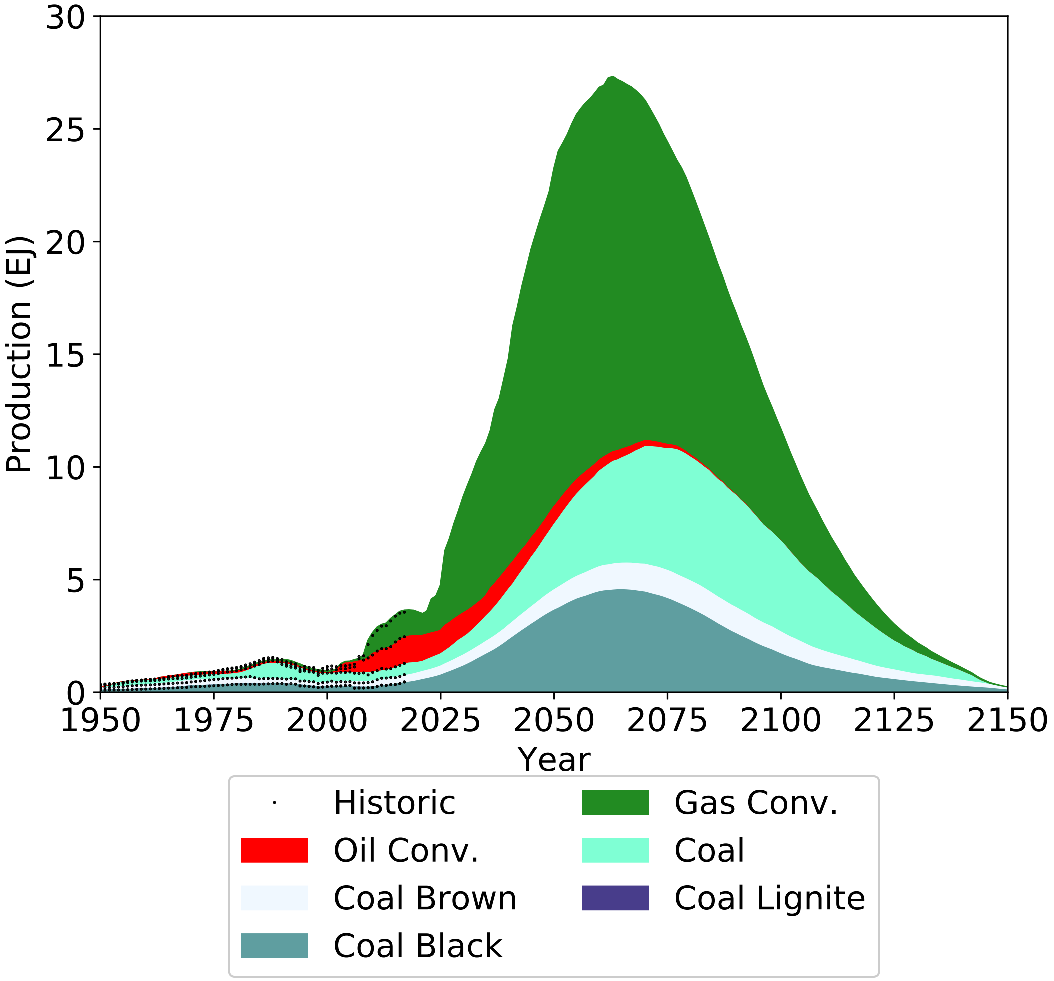

Figure 4.54: Russia - Far Eastern projection by mineral type

North Caucasian

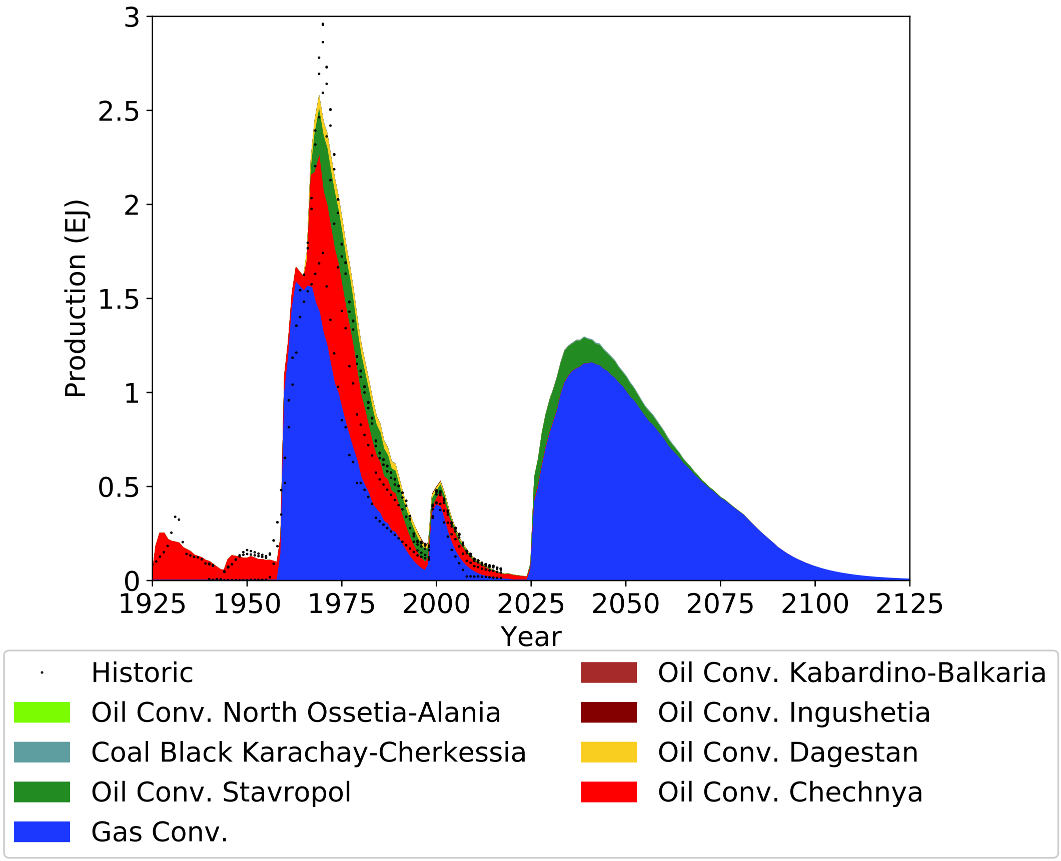

Figure 4.55: Russia - North Caucasian projections capped at 16

Table 4.55: Peak years - All

Name	URR	Peak Year	Peak Rate
Gas Conv. North Caucasian	79.49	1963	1.58
Oil Conv. North Caucasian Chechnya	18.92	1969	0.81
Oil Conv. North Caucasian Stavropol	9.9	1971	0.3
Oil Conv. North Caucasian Dagestan	1.78	1972	0.08
Coal Black North Caucasian Karachay-Cherkessia	0.32	2048	0.01
Oil Conv. North Caucasian Ingushetia	0.07	1996	0.01
Oil Conv. North Caucasian North Ossetia-Alania	–	2002	–
Oil Conv. North Caucasian Kabardino-Balkaria	–	1998	–
**Total**	**110.49**	**1969**	**2.56**

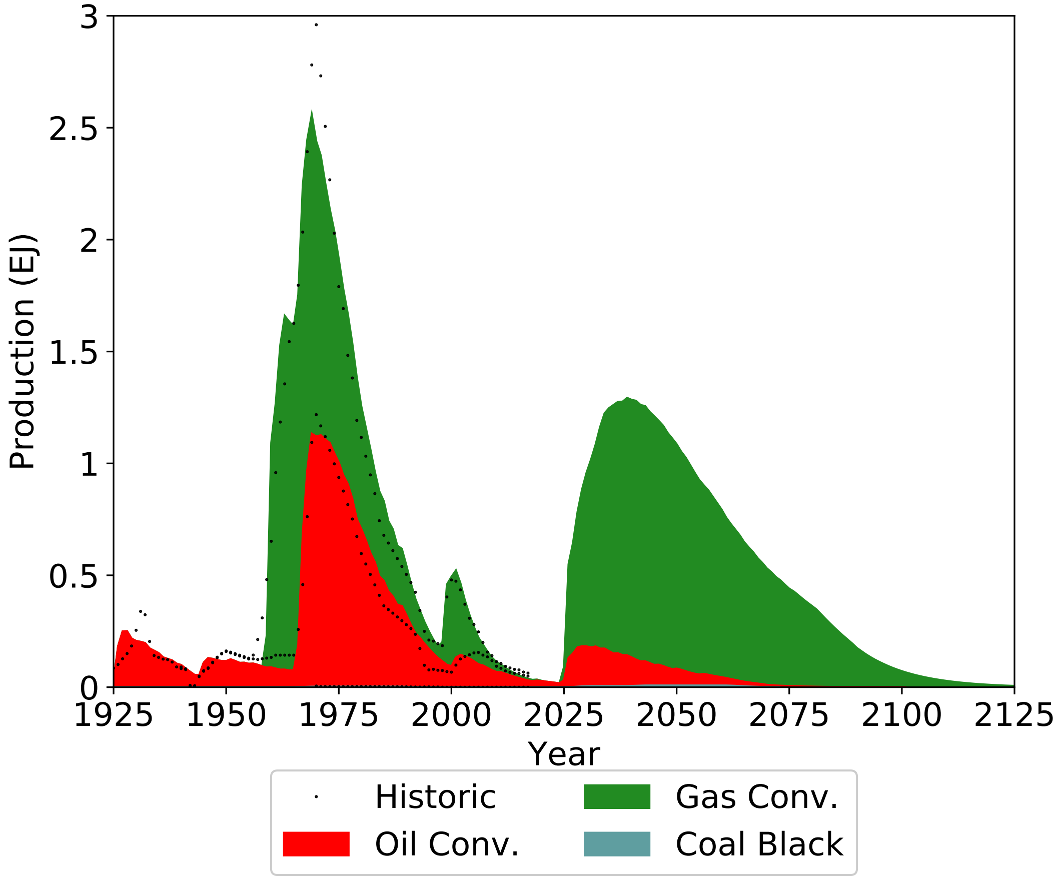

Figure 4.56: Russia - North Caucasian projection by mineral type

Table 4.56: Peak years - Minerals

Name	URR	Peak Year	Peak Rate
Coal Black	0.32	2048	0.01
Oil Conv.	30.68	1969	1.13
Gas Conv.	79.49	1963	1.58
**Total**	**110.49**	**1969**	**2.56**

Northwestern

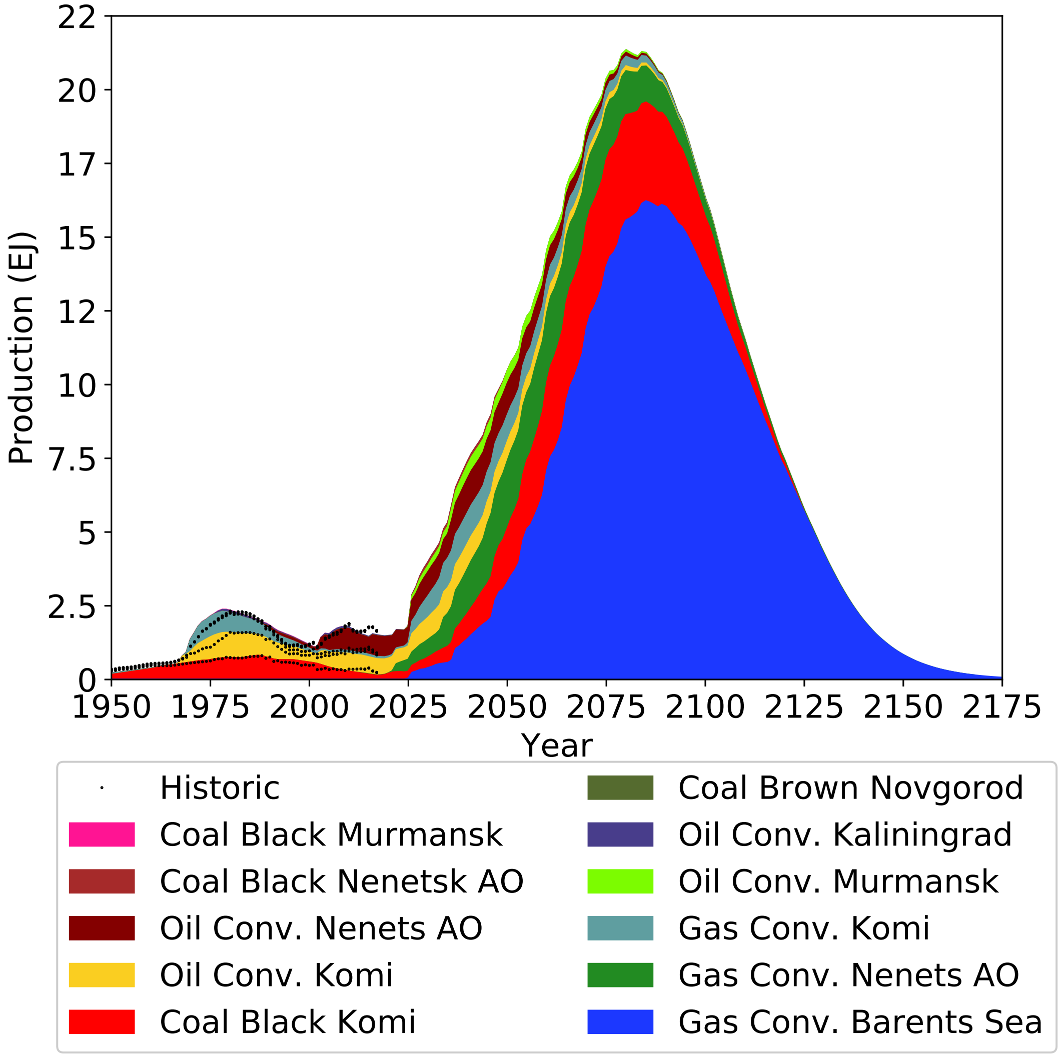

Figure 4.57: Russia - Northwestern projections capped at 16

Table 4.57: Peak years - All

Name	URR	Peak Year	Peak Rate
Gas Conv. Northwestern Barents Sea	937.74	2085	16.22
Coal Black Northwestern Komi	225.55	2078	3.63
Gas Conv. Northwestern Nenets AO	122.36	2058	2.43
Oil Conv. Northwestern Komi	65.44	2036	0.88
Gas Conv. Northwestern Komi	60.38	2041	1.05
Oil Conv. Northwestern Nenets AO	56.97	2041	1.17
Oil Conv. Northwestern Murmansk	16.8	2042	0.46
Coal Black Northwestern Nenetsk AO	2.6	2030	0.12
Oil Conv. Northwestern Kaliningrad	2.19	1980	0.07
Coal Black Northwestern Murmansk	0.47	1970	0.01
Coal Brown Northwestern Novgorod	0.01	1951	0.01
**Total**	**1490.51**	**2080**	**21.34**

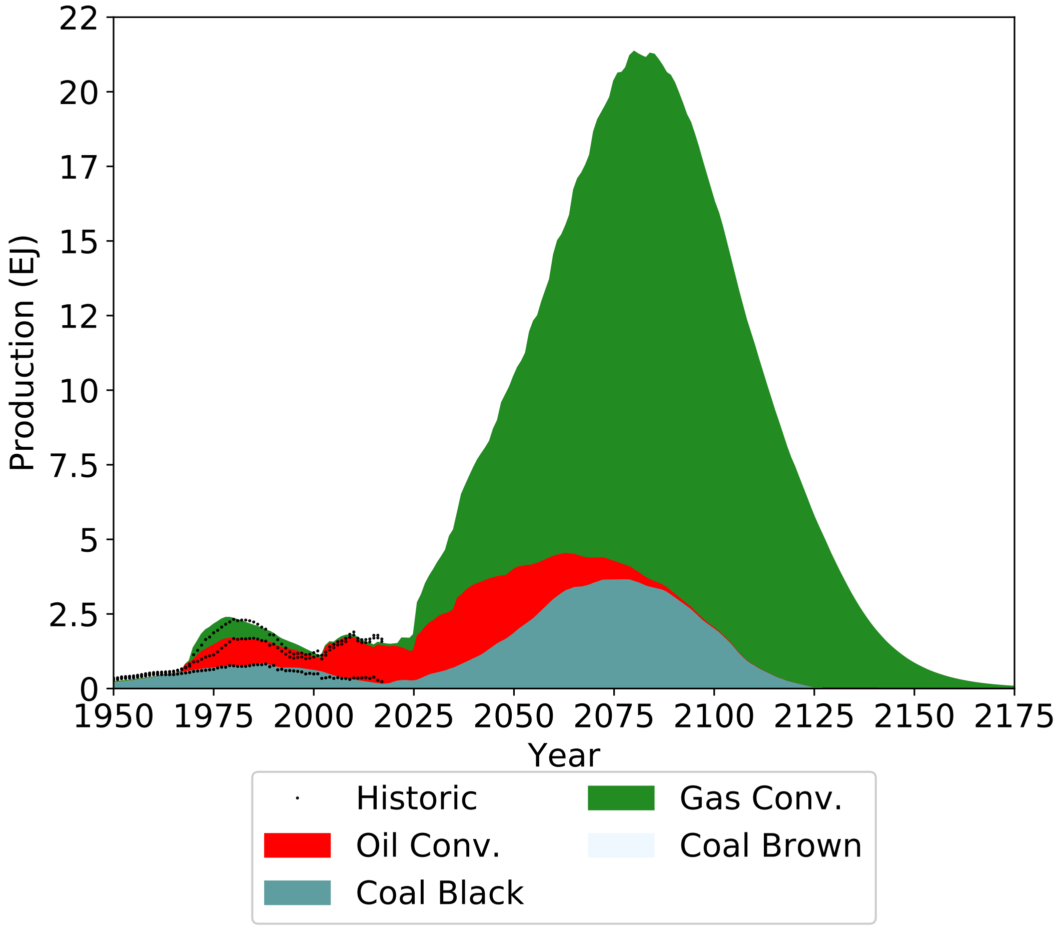

Figure 4.58: Russia - Northwestern projection by mineral type

Table 4.58: Peak years - Minerals

Name	URR	Peak Year	Peak Rate
Coal Black	228.62	2078	3.63
Coal Brown	0.01	1951	0.01
Oil Conv.	141.4	2040	2.47
Gas Conv.	1120.48	2085	17.67
**Total**	**1490.51**	**2080**	**21.34**

Siberian

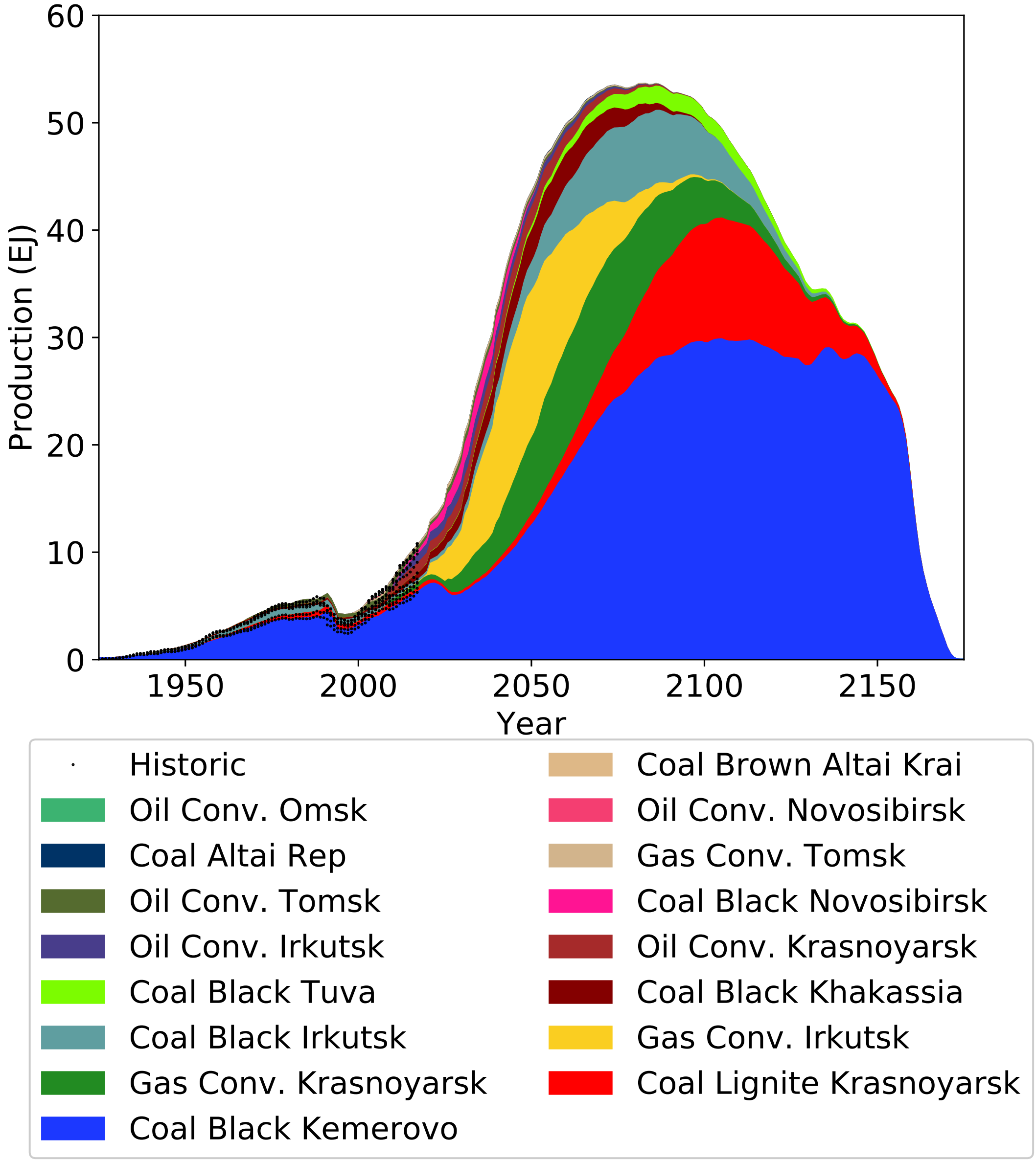

Figure 4.59: Russia - Siberian projections capped at 16

Table 4.59: Peak years - All

Name	URR	Peak Year	Peak Rate
Coal Black Siberian Kemerovo	3378.92	2105	29.84
Coal Lignite Siberian Krasnoyarsk	664.93	2105	11.25
Gas Conv. Siberian Krasnoyarsk	561.39	2066	10.33
Gas Conv. Siberian Irkutsk	496.29	2049	13.91
Coal Black Siberian Irkutsk	412.45	2081	7.11
Coal Black Siberian Khakassia	153.37	2057	3.07
Coal Black Siberian Tuva	99.86	2091	1.67
Oil Conv. Siberian Krasnoyarsk	86.64	2042	1.94
Oil Conv. Siberian Irkutsk	46.16	2031	1.19
Coal Black Siberian Novosibirsk	39.44	2033	1.88
Oil Conv. Siberian Tomsk	37.17	2031	0.66
Gas Conv. Siberian Tomsk	20.05	2034	0.43
Coal Siberian Altai Rep	1.56	2058	0.06
Oil Conv. Siberian Novosibirsk	0.7	2004	0.09
Oil Conv. Siberian Omsk	0.43	2005	0.04
Coal Brown Siberian Altai Krai	0.4	2051	0.02
**Total**	**5999.76**	**2083**	**53.63**

Table 4.60: Peak years - Minerals

Name	URR	Peak Year	Peak Rate
Coal Black	4084.04	2087	37.07
Coal Lignite	664.93	2105	11.25
Coal Brown	0.4	2051	0.02
Coal	1.56	2058	0.06
Oil Conv.	171.1	2037	3.4
Gas Conv.	1077.73	2054	21.72
**Total**	**5999.76**	**2083**	**53.63**

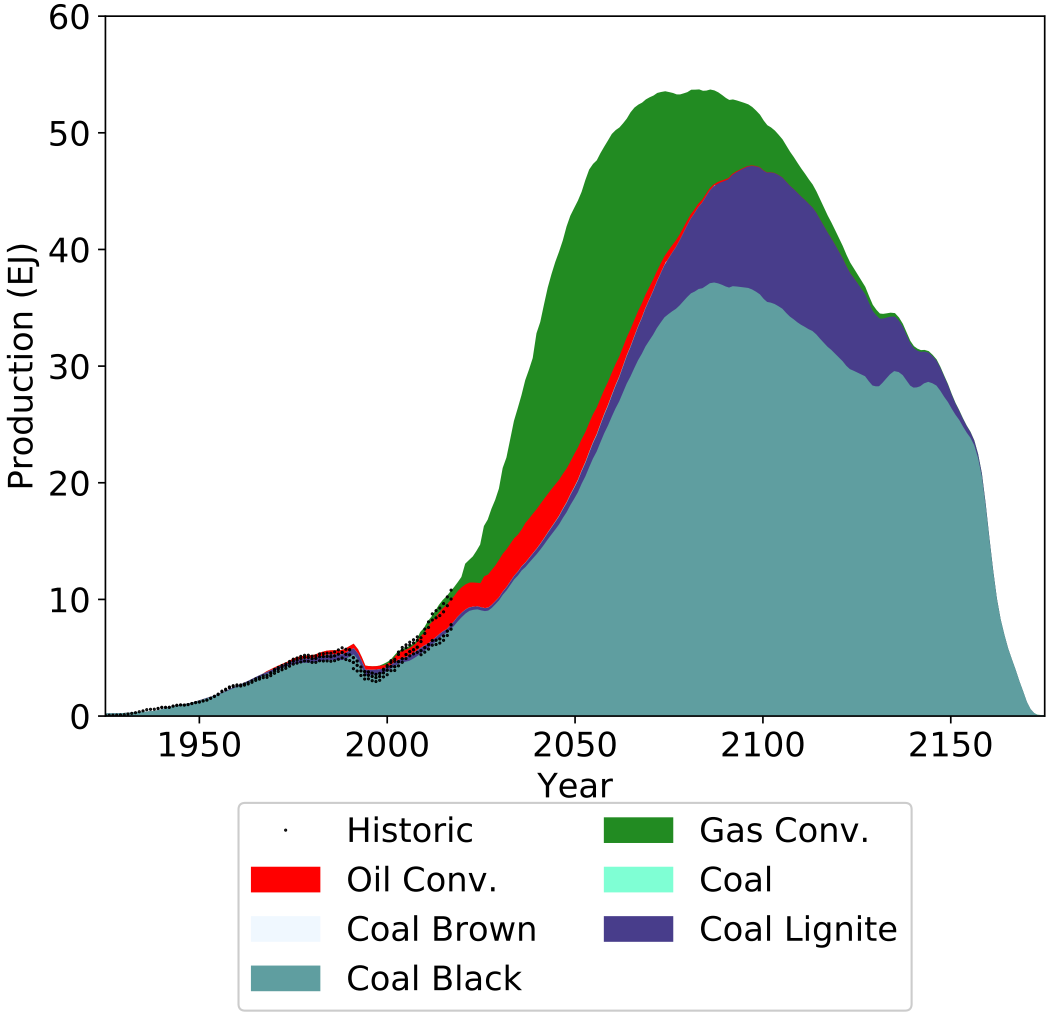

Figure 4.60: Russia - Siberian projection by mineral type

Southern

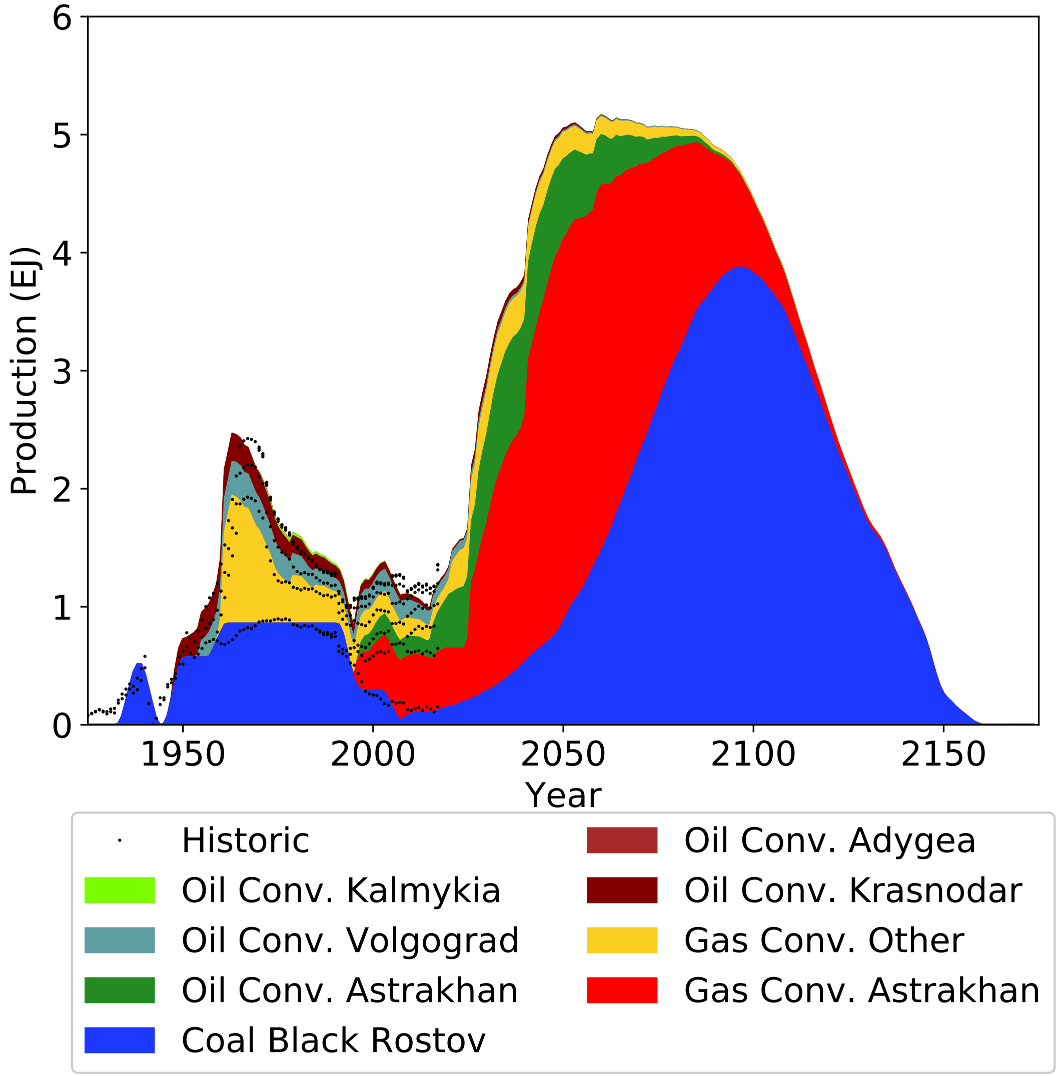

Figure 4.61: Russia - Southern projections capped at 16

Table 4.61: Peak years - All

Name	URR	Peak Year	Peak Rate
Coal Black Southern Rostov	295.92	2096	3.87
Gas Conv. Southern Astrakhan	176.28	2050	3.24
Oil Conv. Southern Astrakhan	37.77	2035	0.88
Gas Conv. Southern Other	36.55	1963	1.08
Oil Conv. Southern Volgograd	13.47	1965	0.3
Oil Conv. Southern Krasnodar	11.33	1960	0.25
Oil Conv. Southern Kalmykia	0.68	1977	0.03
Oil Conv. Southern Adygea	0.09	1971	0.01
**Total**	**572.09**	**2060**	**5.16**

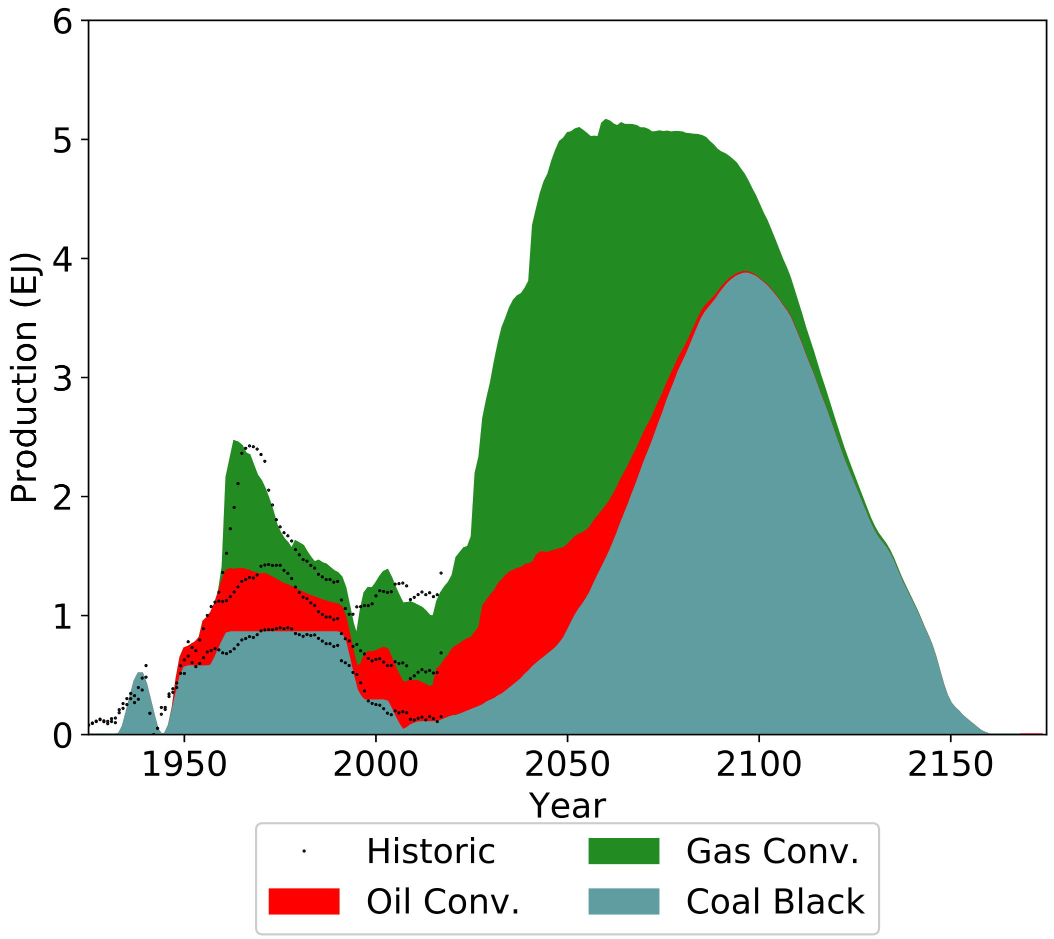

Figure 4.62: Russia - Southern projection by mineral type

Table 4.62: Peak years - Minerals

**Name**	**URR**	**Peak Year**	**Peak Rate**
Coal Black	295.92	2096	3.87
Oil Conv.	63.34	2035	0.97
Gas Conv.	212.83	2050	3.46
**Total**	**572.09**	**2060**	**5.16**

Ural

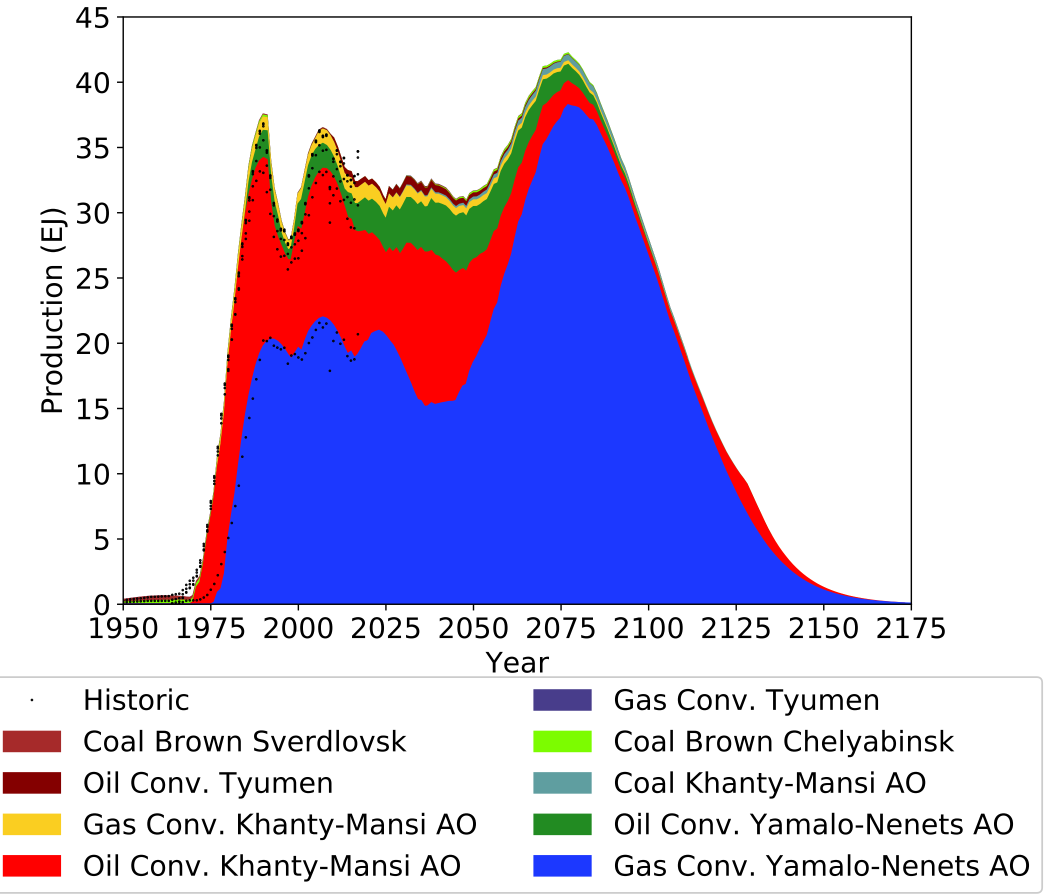

Figure 4.63: Russia - Ural projections capped at 16

Table 4.63: Peak years - All

**Name**	**URR**	**Peak Year**	**Peak Rate**
Gas Conv. Ural Yamalo-Nenets AO	3364.8	2077	38.25
Oil Conv. Ural Khanty-Mansi AO	989.95	1985	15.37
Oil Conv. Ural Yamalo-Nenets AO	255.27	2045	4.37
Gas Conv. Ural Khanty-Mansi AO	79.6	2016	1.23
Coal Ural Khanty-Mansi AO	26.33	2081	0.48
Oil Conv. Ural Tyumen	24.72	2033	0.65
Coal Brown Ural Chelyabinsk	18.45	1966	0.29
Coal Brown Ural Sverdlovsk	11.22	1960	0.36
Gas Conv. Ural Tyumen	0.62	2027	0.03
**Total**	**4770.96**	**2077**	**42.22**

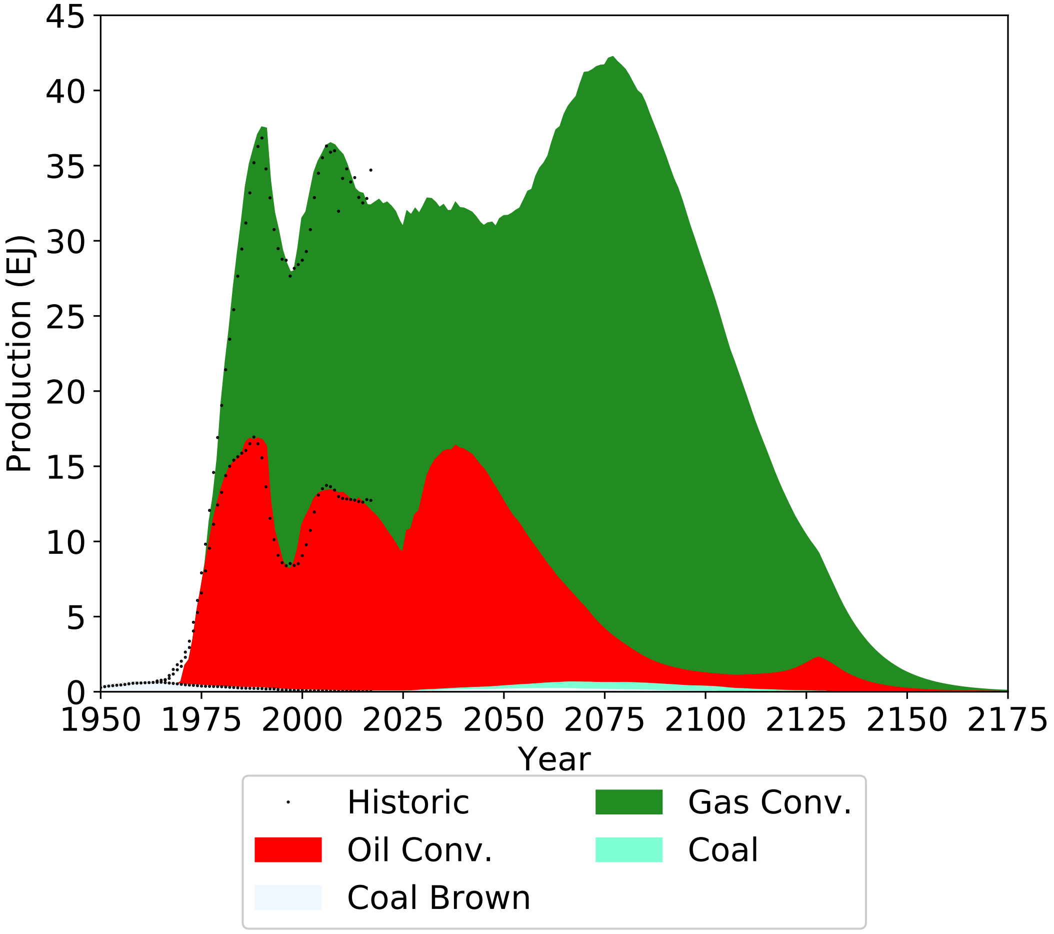

Figure 4.64: Russia - Ural projection by mineral type

Table 4.64: Peak years - Minerals

Name	URR	Peak Year	Peak Rate
Coal Brown	29.67	1961	0.62
Coal	26.33	2081	0.48
Oil Conv.	1269.94	1989	16.61
Gas Conv.	3445.02	2077	38.53
**Total**	**4770.96**	**2077**	**42.22**

Volga

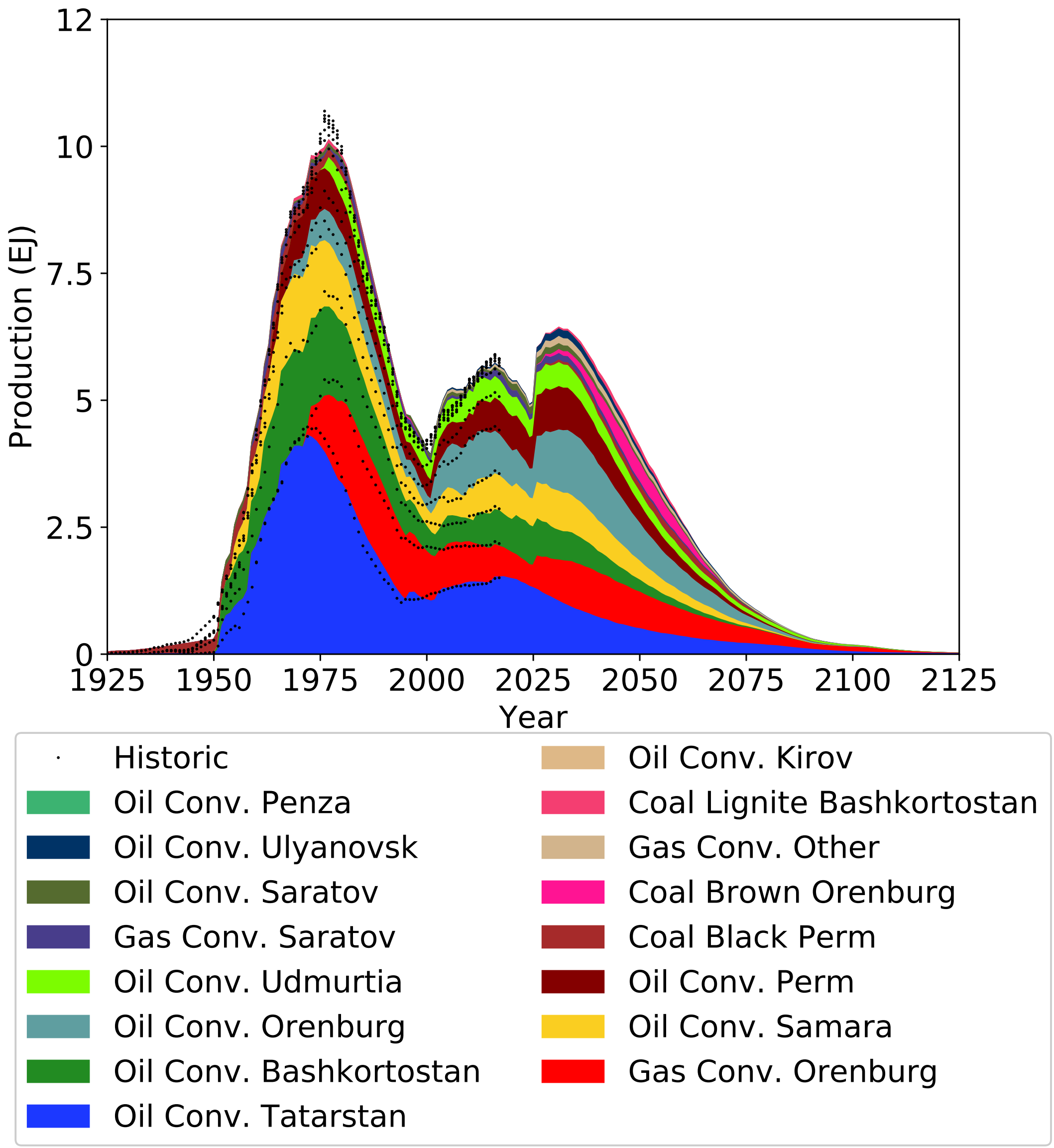

Figure 4.65: Russia - Volga projections capped at 16

Table 4.65: Peak years - All

Name	URR	Peak Year	Peak Rate
Oil Conv. Volga Tatarstan	184.67	1972	4.3
Gas Conv. Volga Orenburg	93.03	1983	1.76
Oil Conv. Volga Bashkortostan	90.64	1969	1.91
Oil Conv. Volga Samara	77.52	1971	1.48
Oil Conv. Volga Orenburg	72.11	2035	1.24
Oil Conv. Volga Perm	56.46	2031	0.85
Oil Conv. Volga Udmurtia	34.67	1986	0.51
Coal Black Volga Perm	17.31	1958	0.34
Gas Conv. Volga Saratov	11.23	1963	0.27
Coal Brown Volga Orenburg	9.62	2048	0.39
Oil Conv. Volga Saratov	8.46	2023	0.14
Gas Conv. Volga Other	6.26	2034	0.15
Oil Conv. Volga Ulyanovsk	4.77	2033	0.15
Coal Lignite Volga Bashkortostan	4.09	2045	0.11
Oil Conv. Volga Penza	0.04	2002	0.01
Oil Conv. Volga Kirov	–	2001	–
**Total**	**670.88**	**1977**	**10.11**

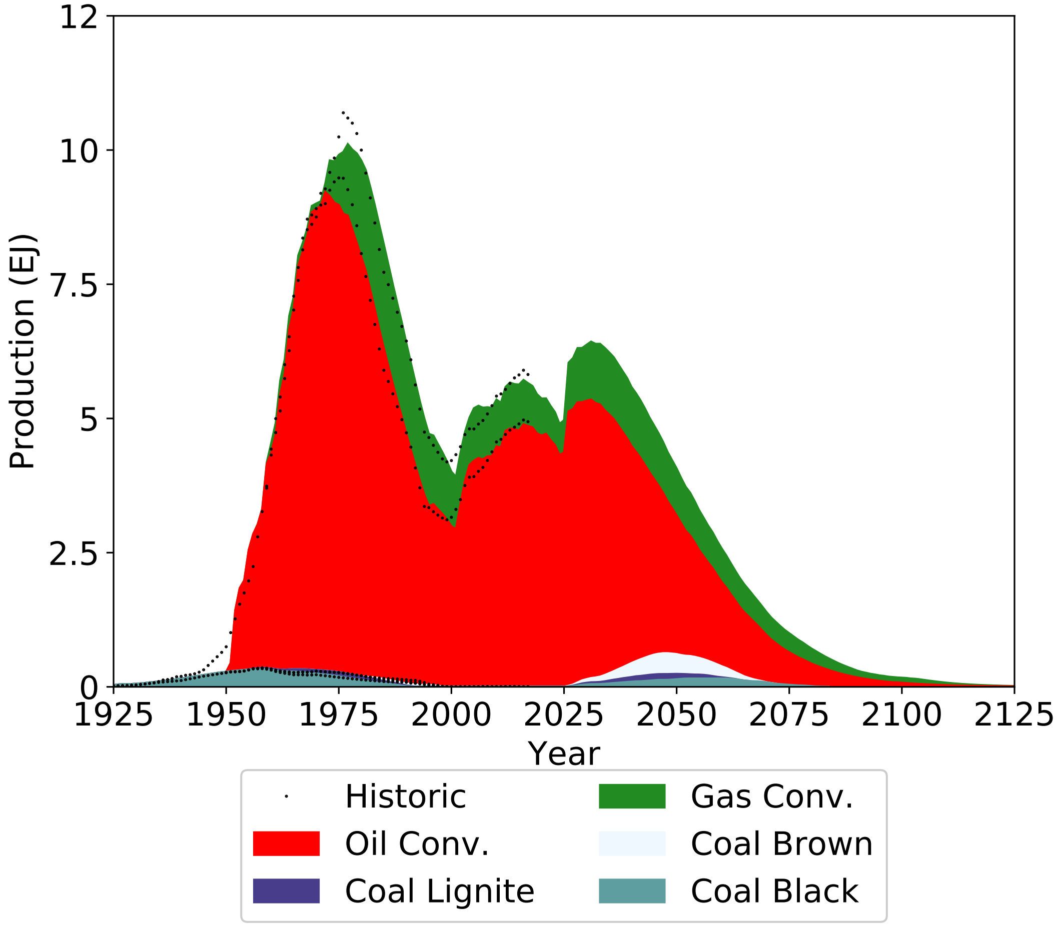

Figure 4.66: Russia - Volga projection by mineral type

Table 4.66: Peak years - Minerals

Name	URR	Peak Year	Peak Rate
Coal Black	17.31	1958	0.34
Coal Lignite	4.09	2045	0.11
Coal Brown	9.62	2048	0.39
Oil Conv.	529.34	1972	8.92
Gas Conv.	110.52	1984	1.95
**Total**	**670.88**	**1977**	**10.11**

4.13.4 Projection by region

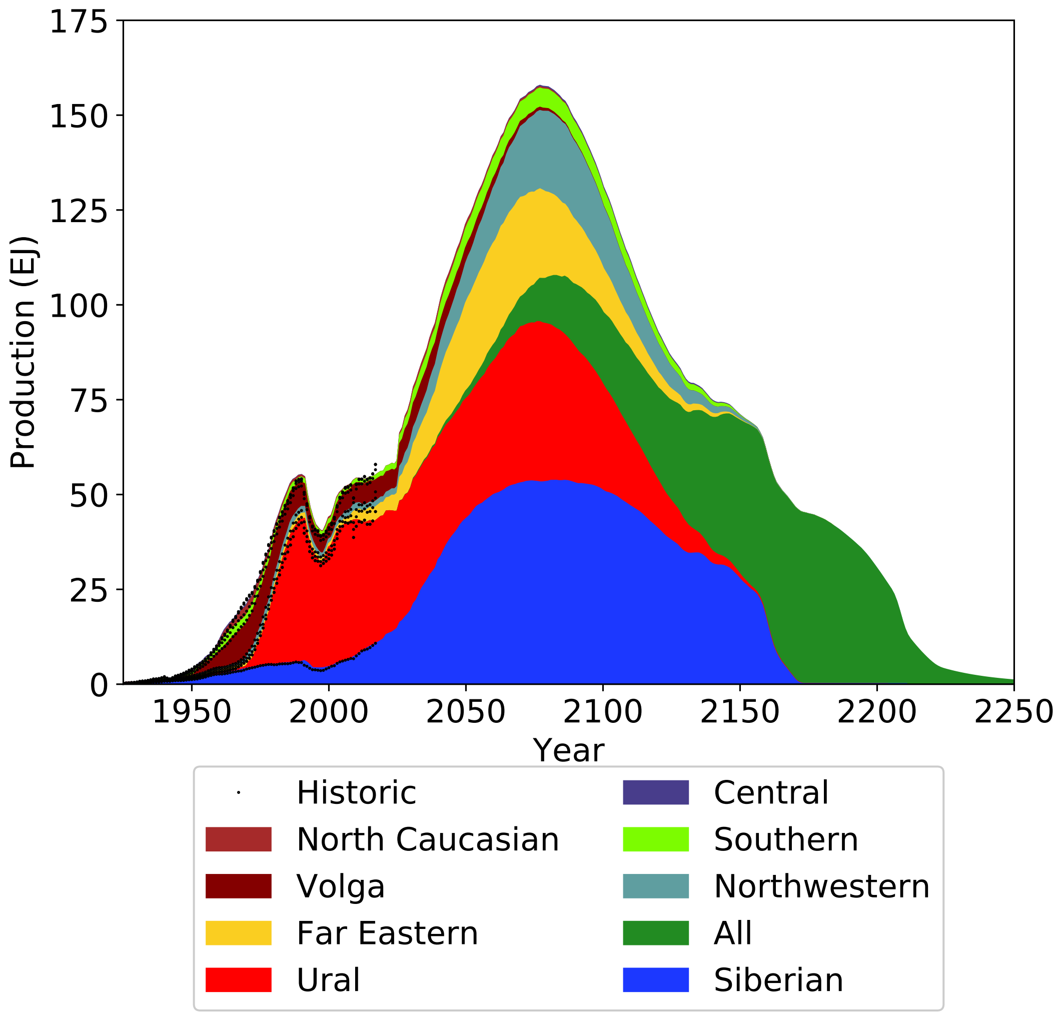

Figure 4.67: Russia by region projections capped at 16

Table 4.67: Peak years - All

**Name**	**URR**	**Peak Year**	**Peak Rate**
Siberian	5999.76	2083	53.63
Ural	4770.96	2077	42.22
All	4441.08	2168	45.27
Far Eastern	1764.23	2063	27.31
Northwestern	1490.51	2080	21.34
Volga	670.88	1977	10.11
Southern	572.09	2060	5.16
North Caucasian	110.49	1969	2.56
Central	51.45	2106	0.55
**Total**	**19871.44**	**2077**	**157.74**

4.14 Tajikistan

4.14.1 All Projections

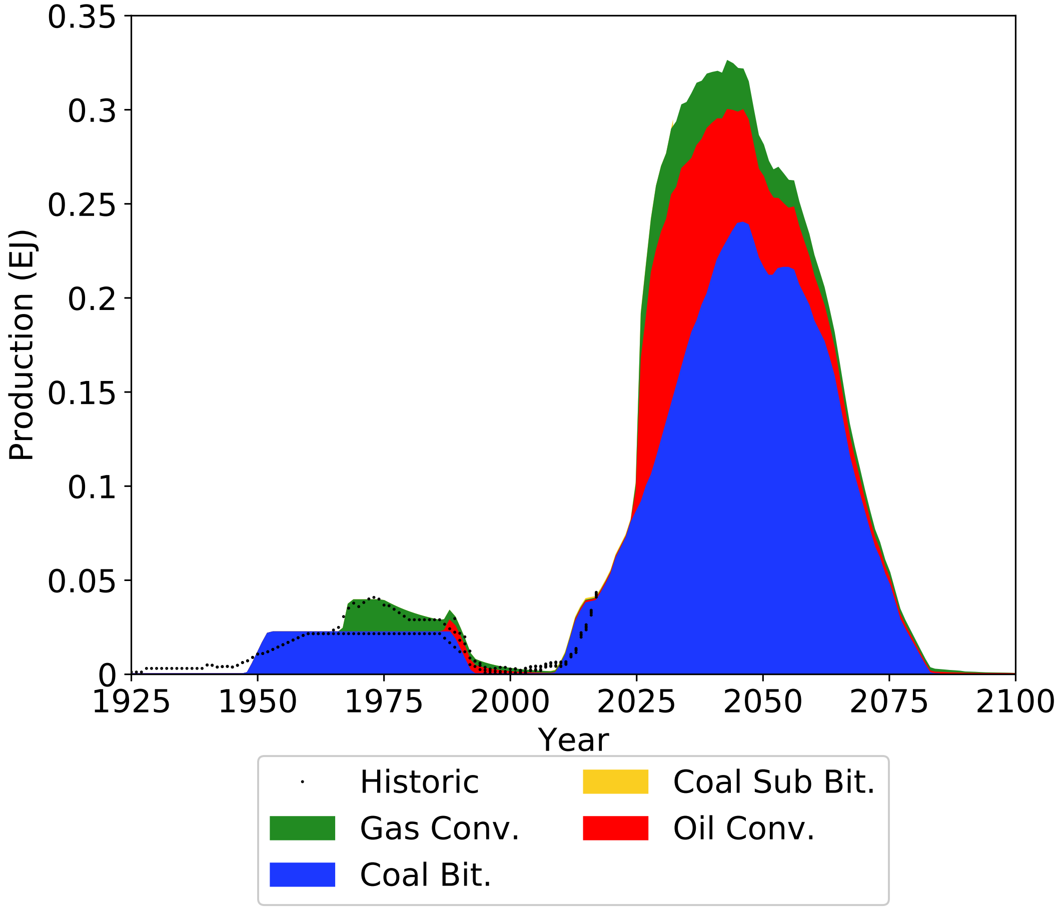

Figure 4.68: Tajikistan projections capped at 16

Table 4.68: Peak years - All

**Name**	**URR**	**Peak Year**	**Peak Rate**
Coal Bit.	10.1	2046	0.24
Oil Conv.	2.65	2032	0.11
Gas Conv.	1.27	2030	0.03
Coal Sub Bit.	0.02	2012	–
**Total**	**14.04**	**2043**	**0.33**

4.14.2 By Mineral

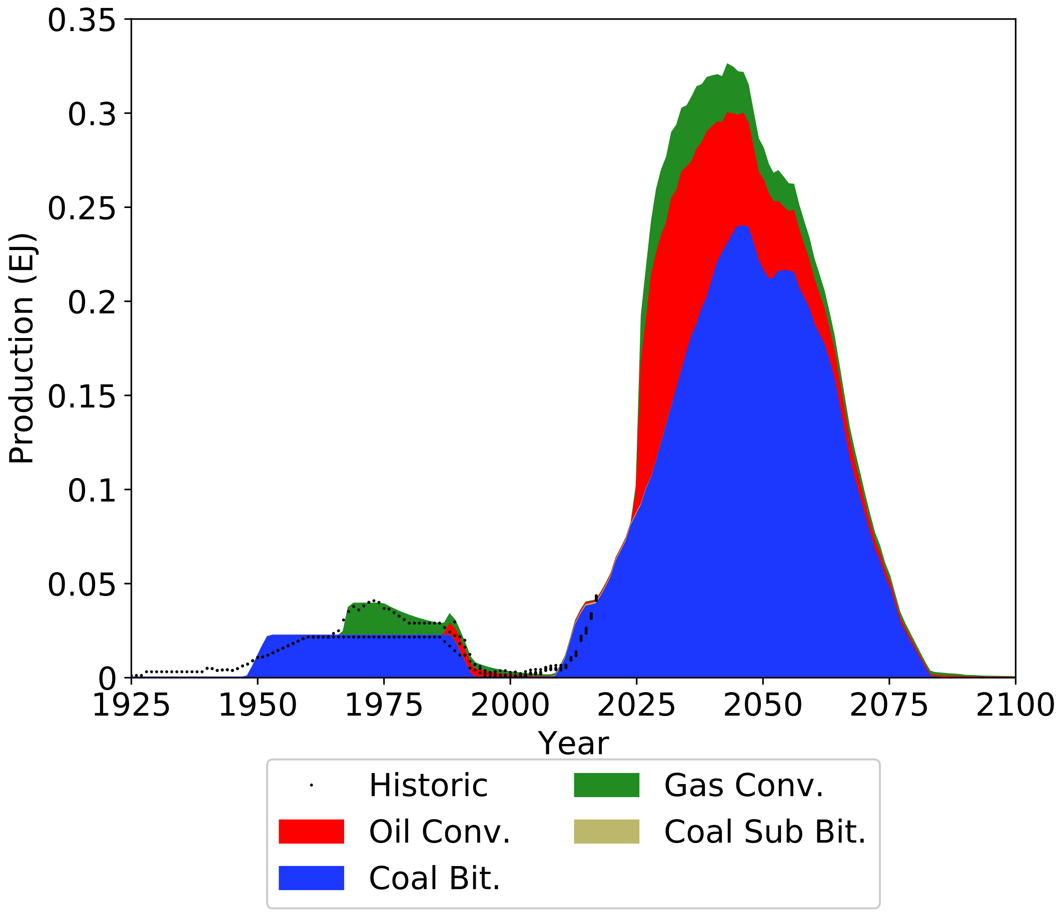

Figure 4.69: Tajikistan projection by mineral type

Table 4.69: Peak years - Minerals

Name	URR	Peak Year	Peak Rate
Coal Bit.	10.1	2046	0.24
Coal Sub Bit.	0.02	2012	–
Oil Conv.	2.65	2032	0.11
Gas Conv.	1.27	2030	0.03
**Total**	**14.04**	**2043**	**0.33**

4.15 Turkmenistan

4.15.1 All Projections

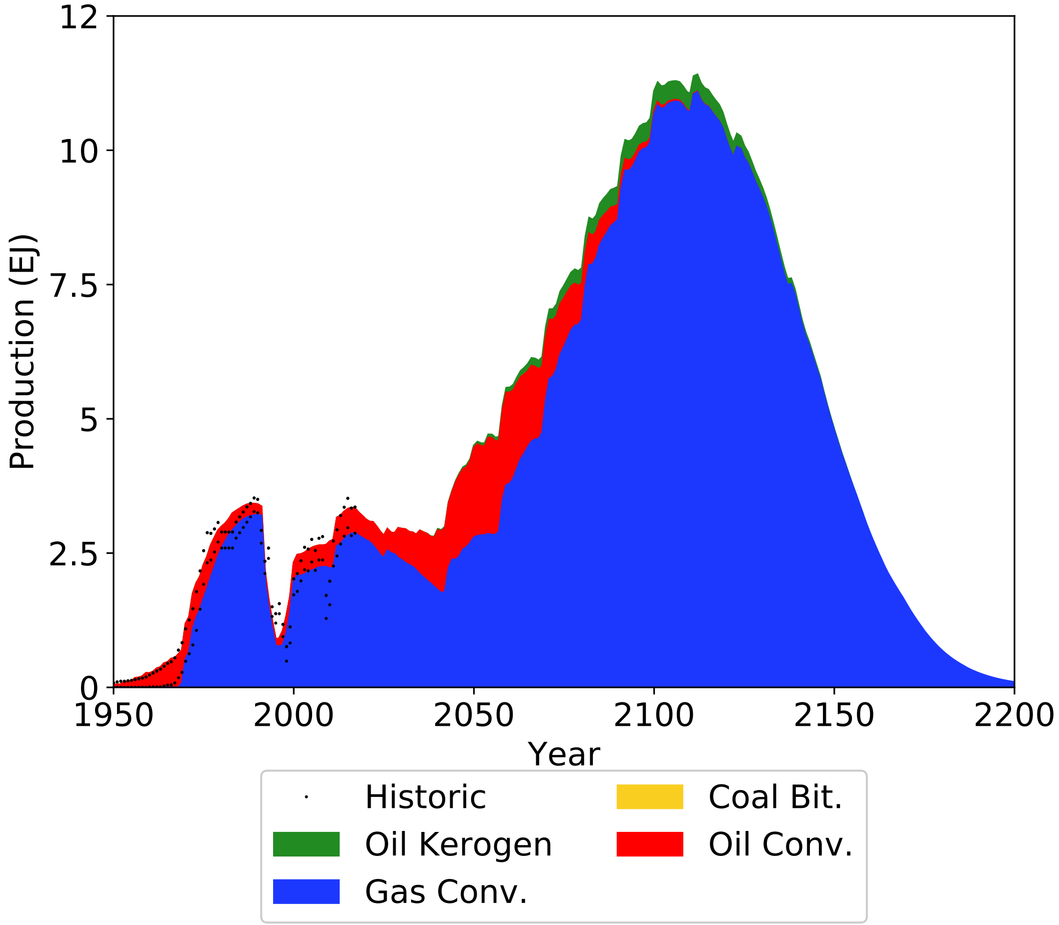

Figure 4.70: Turkmenistan projections capped at 16

Table 4.70: Peak years - All

**Name**	**URR**	**Peak Year**	**Peak Rate**
Gas Conv.	1025.97	2112	11.08
Oil Conv.	99.32	2055	1.8
Oil Kerogen	22.02	2100	0.37
Coal Bit.	0.01	1947	–
**Total**	**1147.32**	**2112**	**11.41**

4.15.2 By Mineral

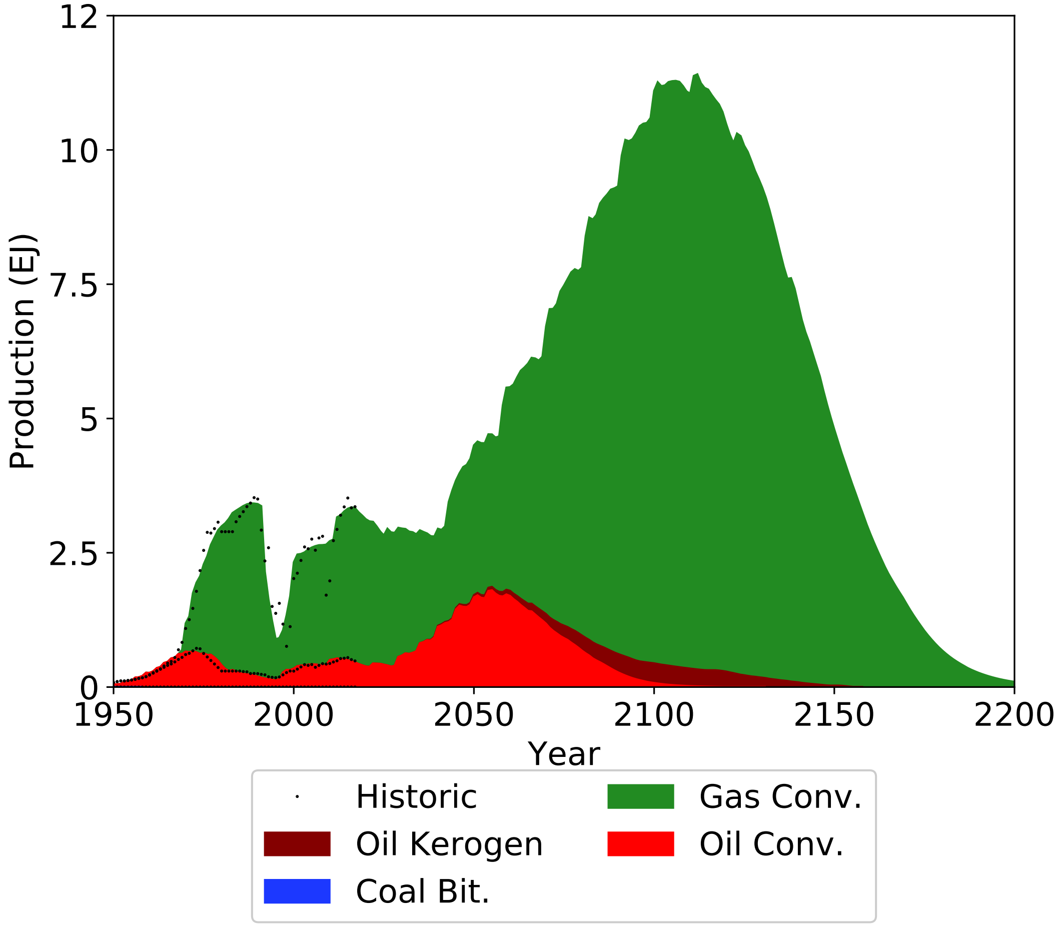

Figure 4.71: Turkmenistan projection by mineral type

Table 4.71: Peak years - Minerals

**Name**	**URR**	**Peak Year**	**Peak Rate**
Coal Bit.	0.01	1947	–
Oil Conv.	99.32	2055	1.8
Oil Kerogen	22.02	2100	0.37
Gas Conv.	1025.97	2112	11.08
**Total**	**1147.32**	**2112**	**11.41**

4.16 Ukraine

4.16.1 All Projections

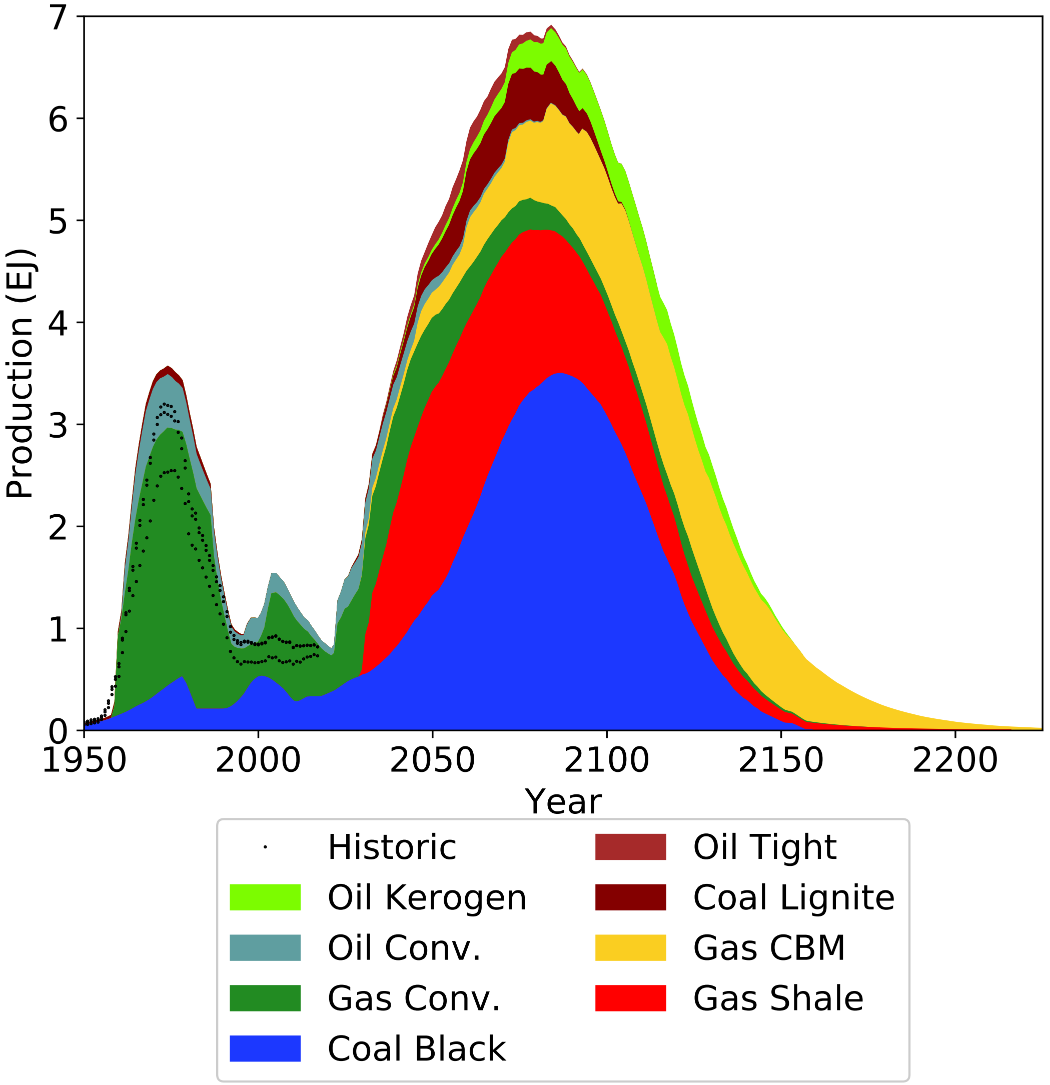

Figure 4.72: Ukraine projections capped at 16

Table 4.72: Peak years - All

**Name**	**URR**	**Peak Year**	**Peak Rate**
Coal Black	243.78	2087	3.5
Gas Shale	134.55	2055	2.05
Gas Conv.	128.82	1974	2.53
Gas CBM	111.2	2105	1.28
Oil Conv.	24.67	1970	0.57
Coal Lignite	24.49	2070	0.55
Oil Kerogen	24.03	2098	0.4
Oil Tight	6.3	2058	0.22
**Total**	**697.84**	**2084**	**6.9**

4.16.2 By Mineral

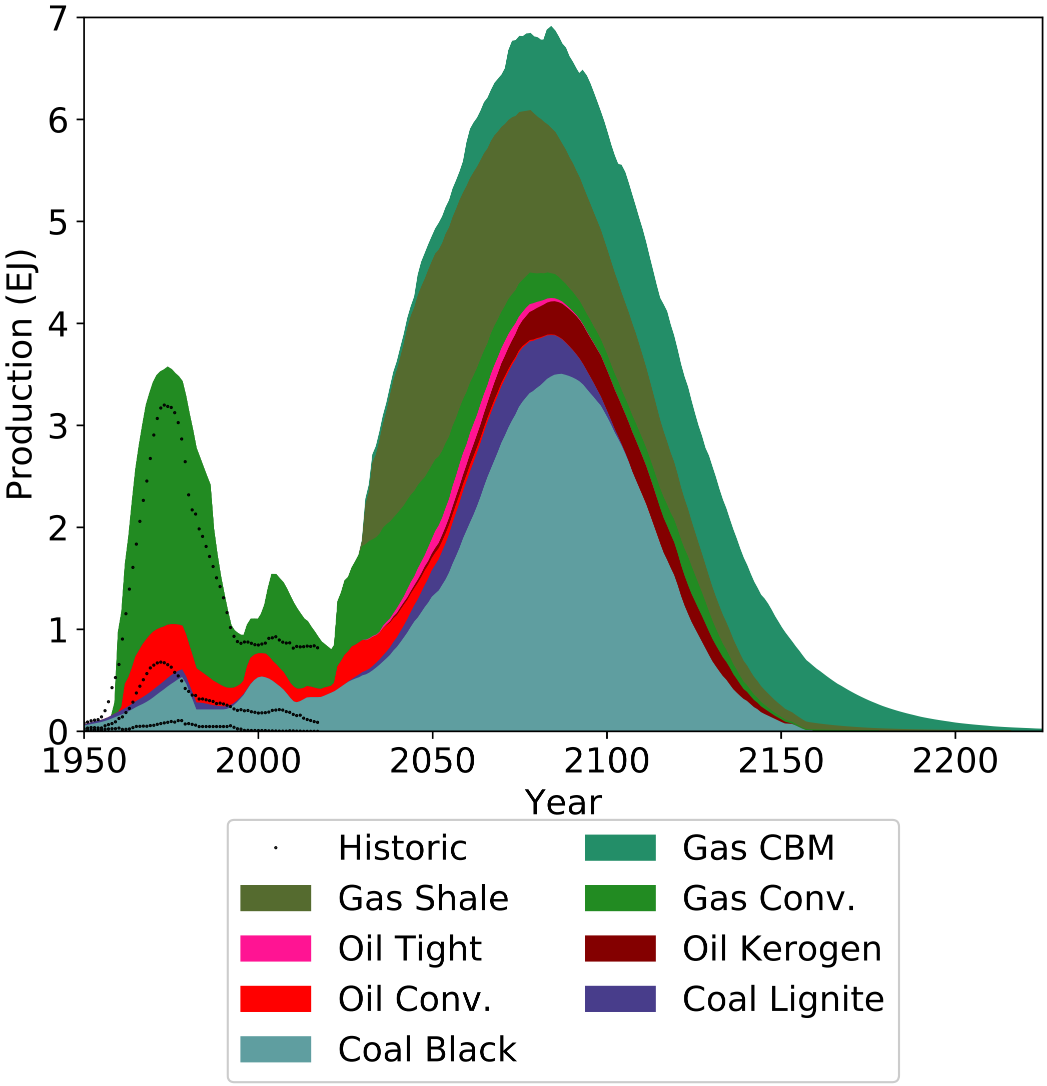

Figure 4.73: Ukraine projection by mineral type

Table 4.73: Peak years - Minerals

**Name**	**URR**	**Peak Year**	**Peak Rate**
Coal Black	243.78	2087	3.5
Coal Lignite	24.49	2070	0.55
Oil Conv.	24.67	1970	0.57
Oil Kerogen	24.03	2098	0.4
Oil Tight	6.3	2058	0.22
Gas Conv.	128.82	1974	2.53
Gas Shale	134.55	2055	2.05
Gas CBM	111.2	2105	1.28
**Total**	**697.84**	**2084**	**6.9**

4.17 Uzbekistan

4.17.1 All Projections

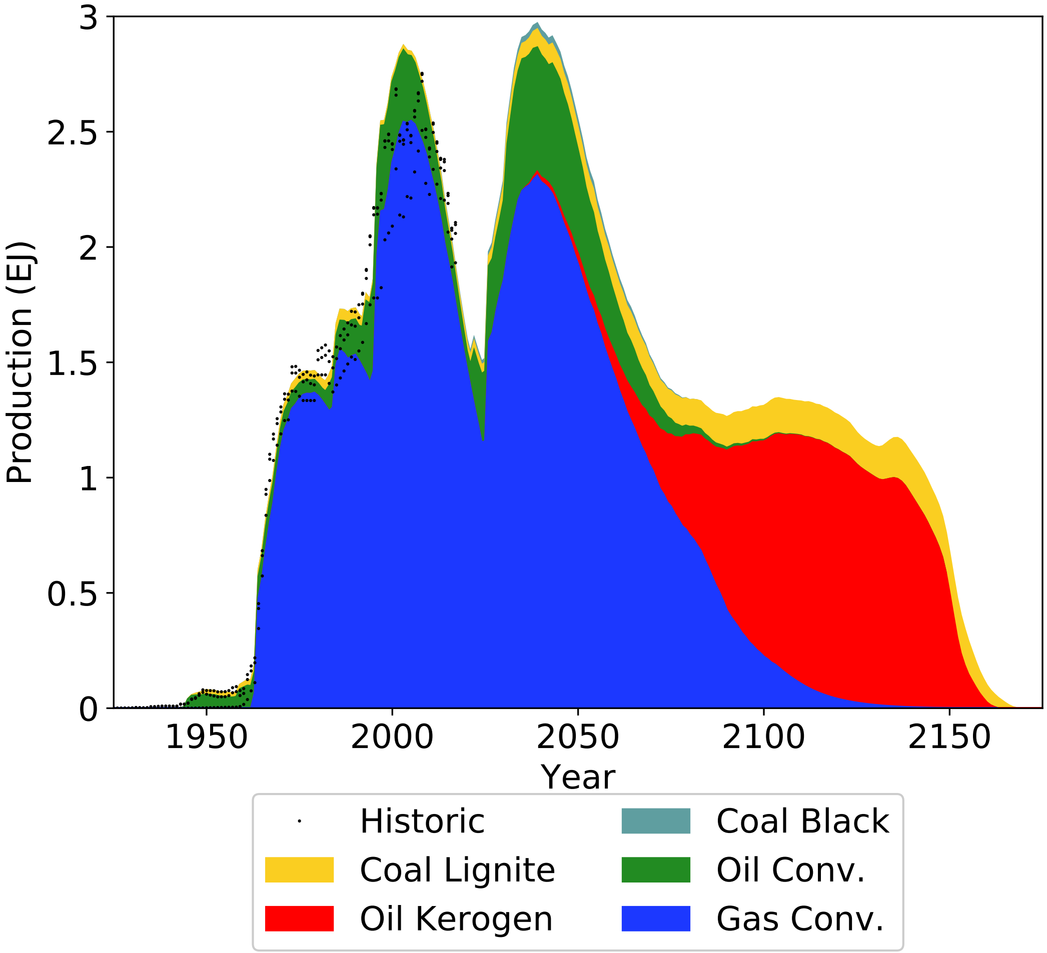

Figure 4.74: Uzbekistan projections capped at 16

Table 4.74: Peak years - All

Name	URR	Peak Year	Peak Rate
Gas Conv.	201.64	2005	2.55
Oil Kerogen	70.08	2115	1.09
Oil Conv.	30.44	2035	0.57
Coal Lignite	19.76	2142	0.19
Coal Black	1.33	2044	0.03
**Total**	**323.25**	**2039**	**2.97**

4.17.2 By Mineral

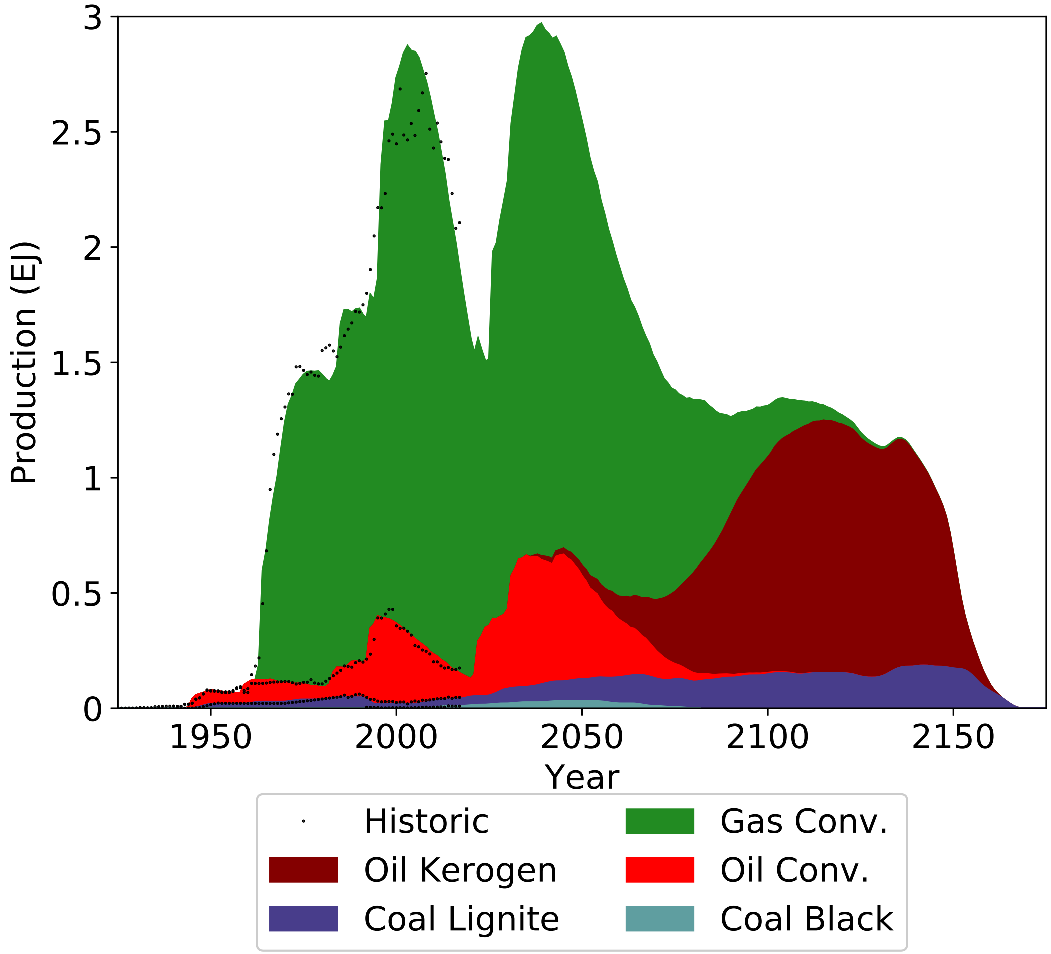

Figure 4.75: Uzbekistan projection by mineral type

Table 4.75: Peak years - Minerals

**Name**	**URR**	**Peak Year**	**Peak Rate**
Coal Black	1.33	2044	0.03
Coal Lignite	19.76	2142	0.19
Oil Conv.	30.44	2035	0.57
Oil Kerogen	70.08	2115	1.09
Gas Conv.	201.64	2005	2.55
**Total**	**323.25**	**2039**	**2.97**

4.18 Total

4.18.1 By country

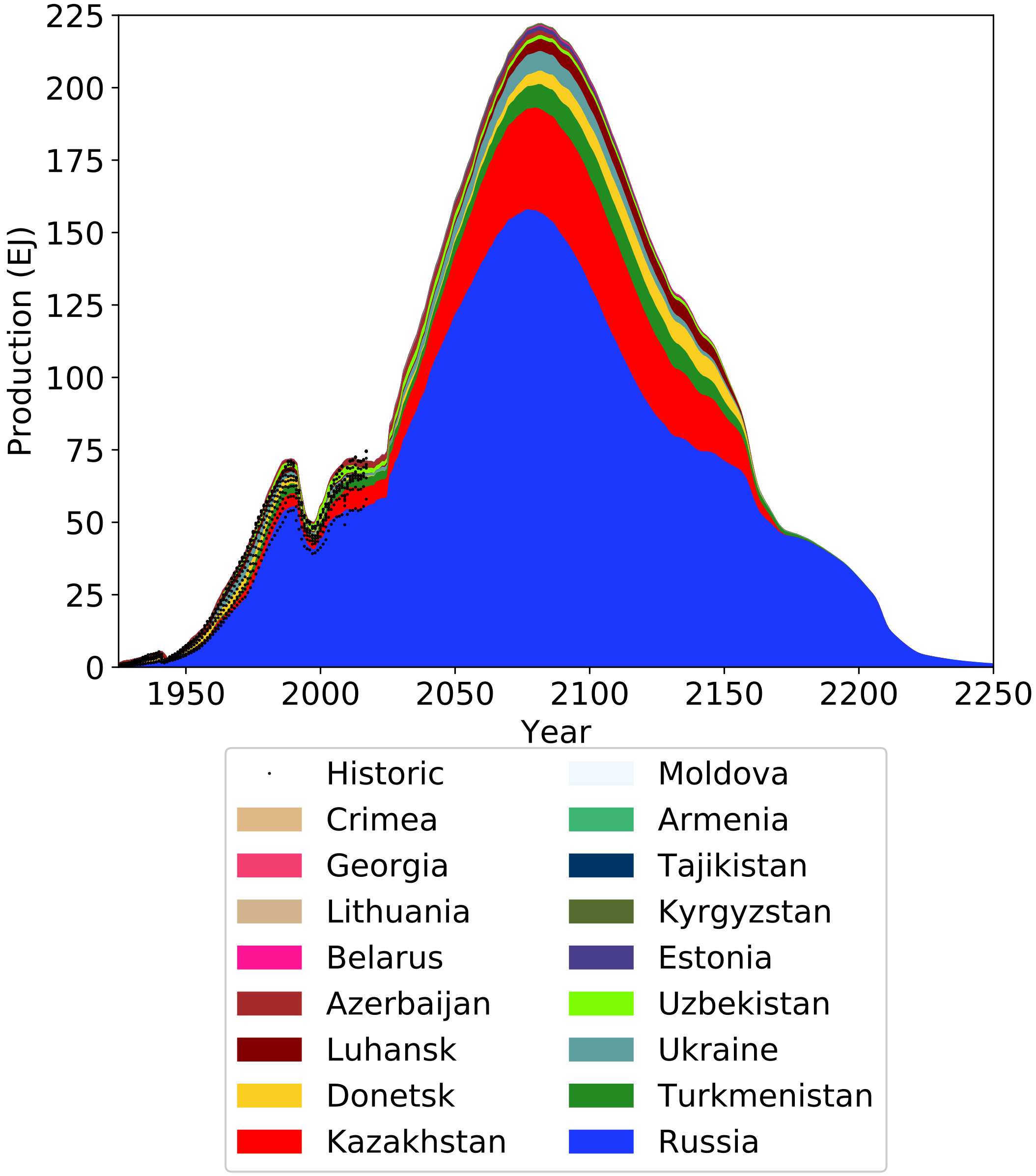

Figure 4.76: FSU projections by country

Table 4.76: Peak years - All

Name	URR	Peak Year	Peak Rate
Russia	19871.44	2077	157.74
Kazakhstan	3744.88	2096	37.48
Turkmenistan	1147.32	2112	11.41
Donetsk	783.03	2116	7.96
Ukraine	697.84	2084	6.9
Luhansk	582.62	2111	6.07
Uzbekistan	323.25	2039	2.97
Azerbaijan	309.45	2037	3.5
Estonia	94.61	2082	1.52
Belarus	48.98	2109	0.64
Kyrgyzstan	46.46	2066	0.63
Lithuania	16.87	2034	0.46
Tajikistan	14.04	2043	0.33
Georgia	12.39	2028	0.37
Armenia	2.12	2058	0.06
Crimea	1.71	2010	0.07
Moldova	1.18	2030	0.06
**Total**	**27698.19**	**2082**	**221.99**

4.18.2 By mineral

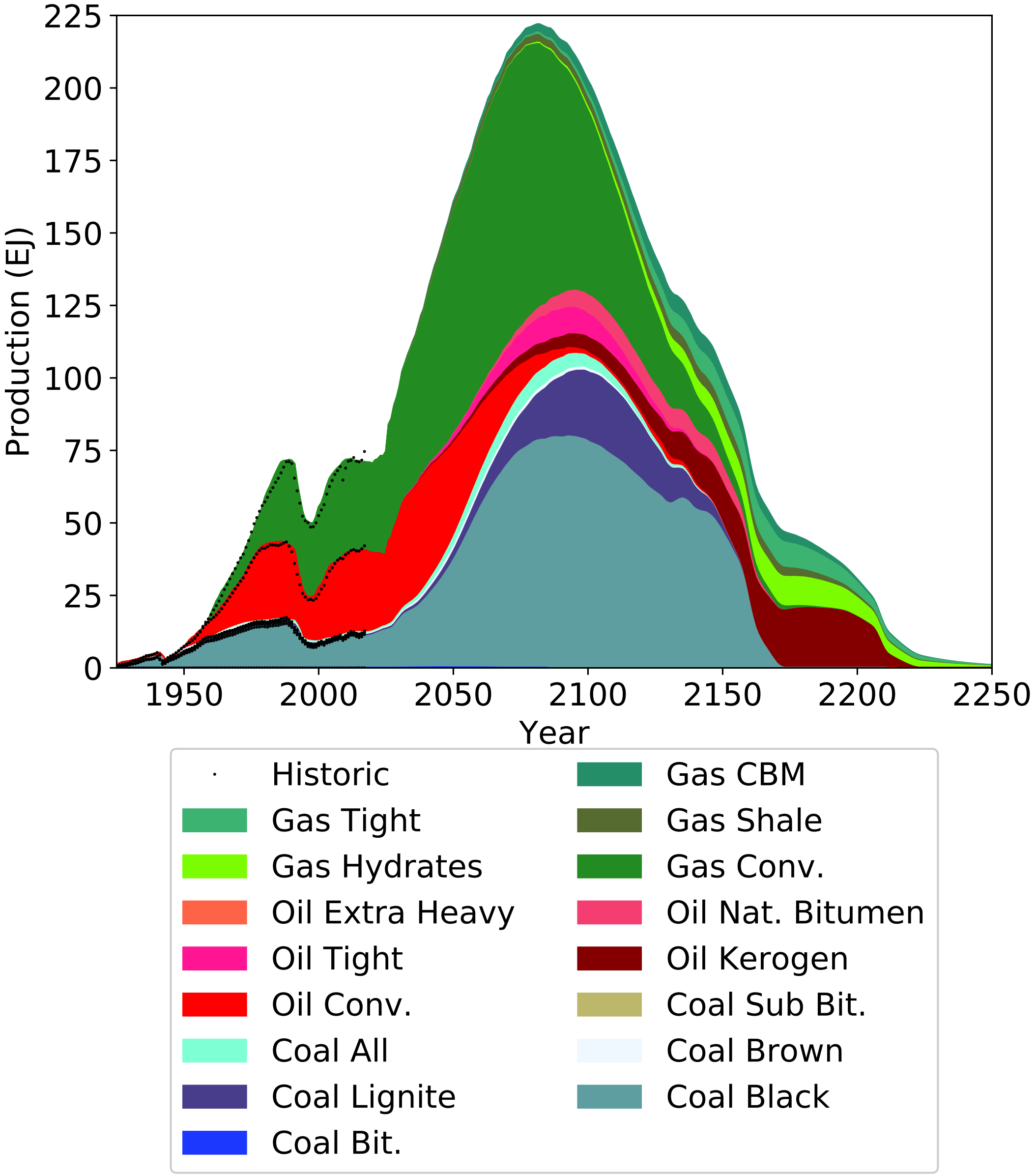

Figure 4.77: FSU projection by mineral type

Table 4.77: Peak years - Minerals

**Name**	**URR**	**Peak Year**	**Peak Rate**
Coal Bit.	10.12	2046	0.24
Coal Black	8511.95	2093	79.86
Coal Lignite	1506.3	2103	24.14
Coal Brown	155.6	2058	1.52
Coal All	403.63	2078	6.02
Coal Sub Bit.	4.72	2048	0.09
Oil Conv.	3042.85	2038	39.29
Oil Kerogen	1689.85	2182	20.6
Oil Tight	499.39	2090	9.49
Oil Nat. Bitumen	531.98	2113	6.99
Oil Extra Heavy	0.8	2030	0.04
Gas Conv.	8646.48	2071	95.41
Gas Hydrates	807.66	2171	10.2
Gas Shale	515.58	2146	4.38
Gas Tight	741.3	2164	9.03
Gas CBM	629.97	2134	6.5
**Total**	**27698.19**	**2082**	**221.99**

Chapter 5

Middle East

5.1 Bahrain

5.1.1 All Projections

Table 5.1: Peak years - All

Name	URR	Peak Year	Peak Rate
Gas Conv.	25.8	2013	0.53
Oil Conv.	16.34	1999	0.1
**Total**	**42.14**	**2013**	**0.63**

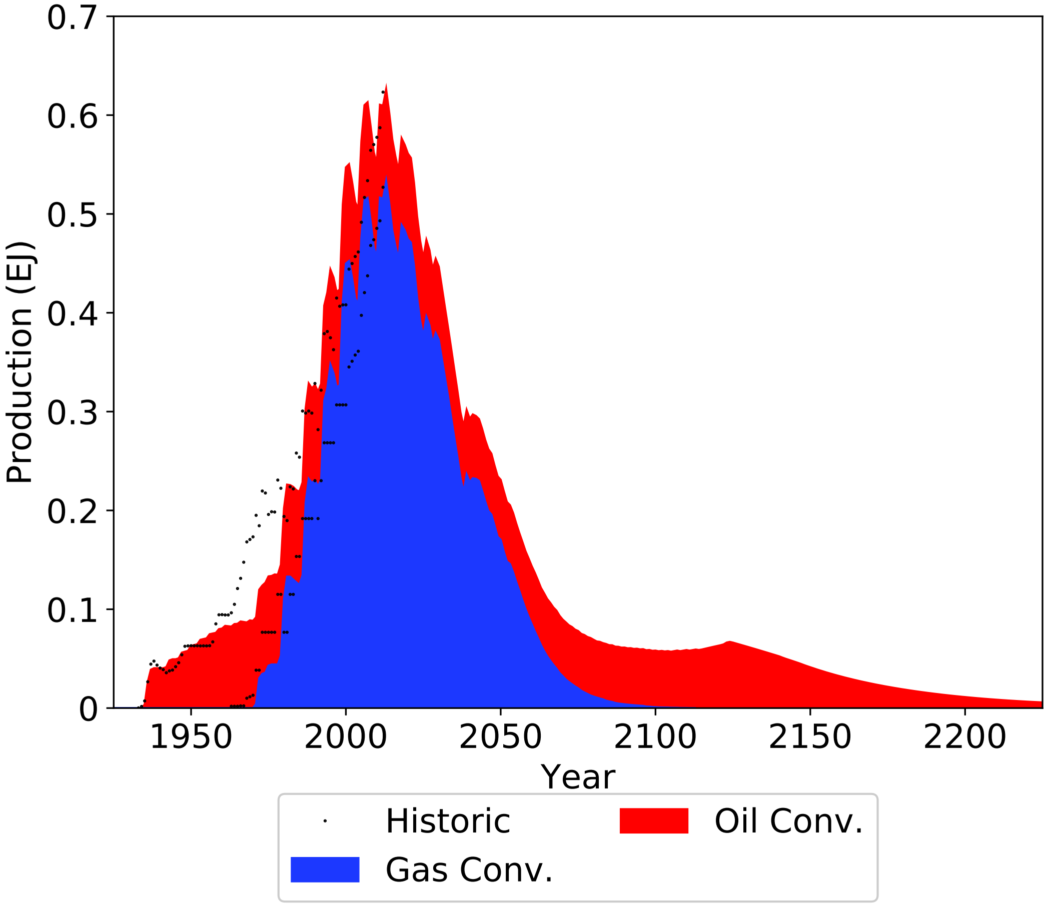

Figure 5.1: Bahrain projections capped at 16

5.1.2 By Mineral

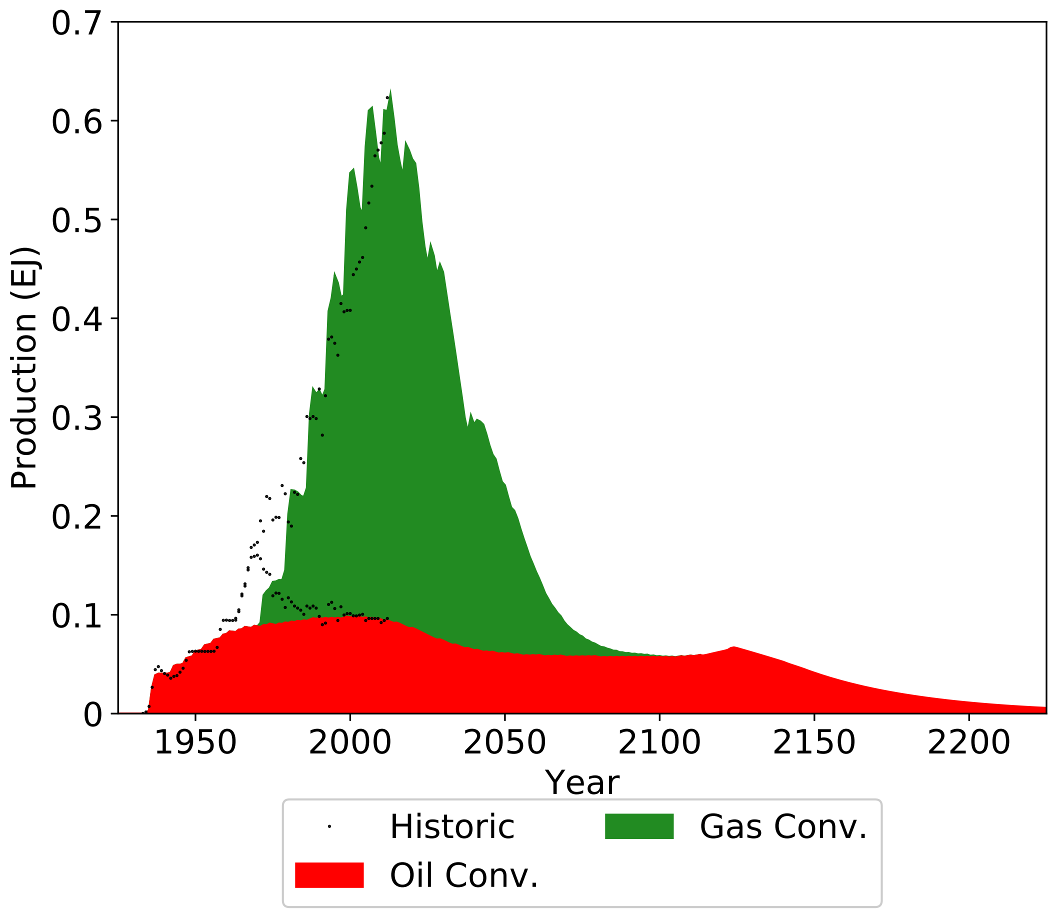

Figure 5.2: Bahrain projection by mineral type

Table 5.2: Peak years - Minerals

Name	URR	Peak Year	Peak Rate
Oil Conv.	16.34	1999	0.1
Gas Conv.	25.8	2013	0.53
**Total**	**42.14**	**2013**	**0.63**

5.2 Iran

5.2.1 All Projections

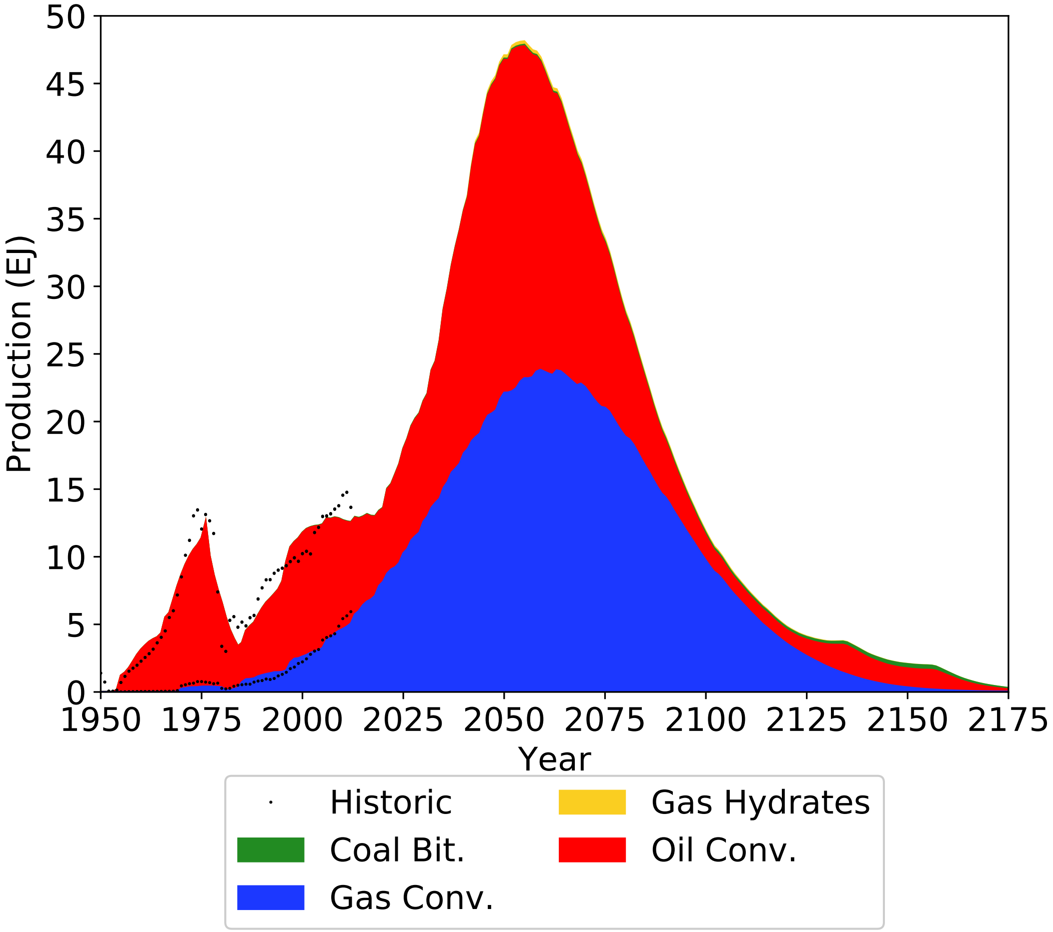

Figure 5.3: Iran projections capped at 16

Table 5.3: Peak years - All

**Name**	**URR**	**Peak Year**	**Peak Rate**
Gas Conv.	1712.1	2059	23.82
Oil Conv.	1593.39	2052	25.2
Coal Bit.	28.2	2153	0.32
Gas Hydrates	15.0	2056	0.26
**Total**	**3348.69**	**2055**	**48.12**

5.2.2 By Mineral

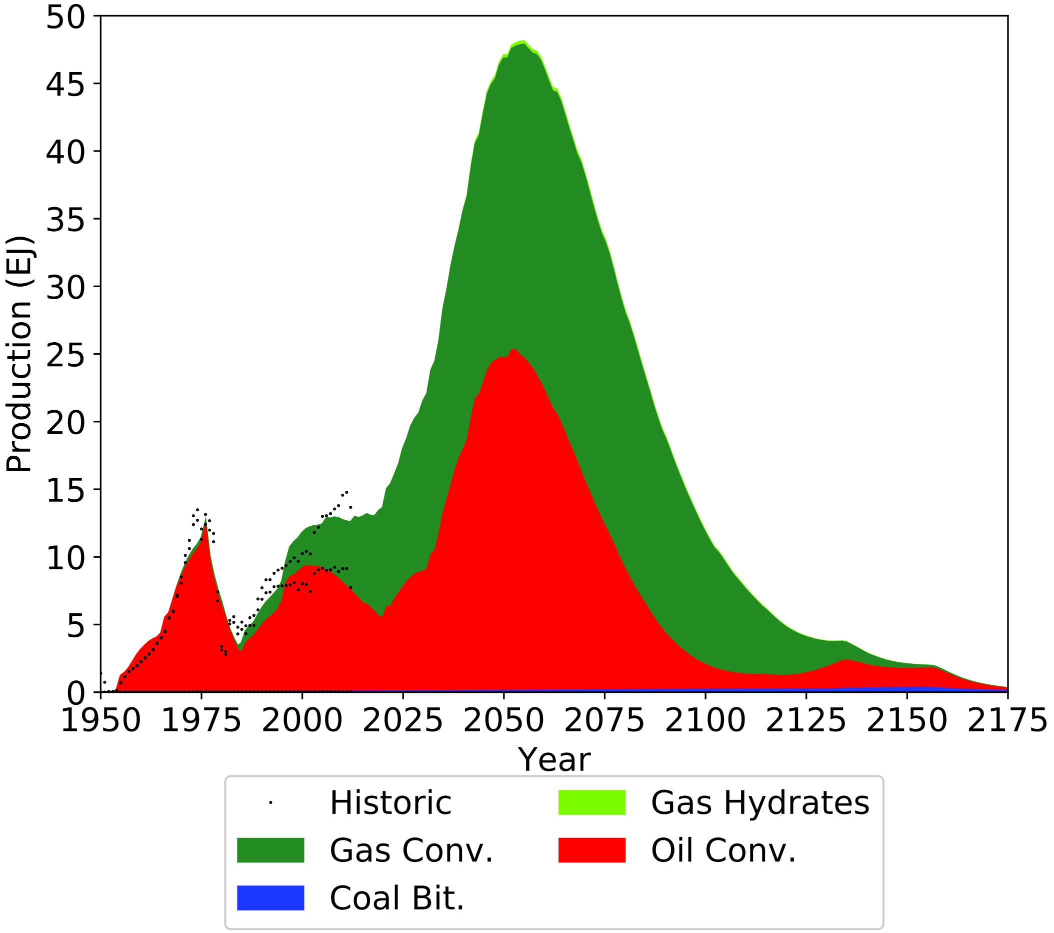

Figure 5.4: Iran projection by mineral type

Table 5.4: Peak years - Minerals

**Name**	**URR**	**Peak Year**	**Peak Rate**
Coal Bit.	28.2	2153	0.32
Oil Conv.	1593.39	2052	25.2
Gas Conv.	1712.1	2059	23.82
Gas Hydrates	15.0	2056	0.26
**Total**	**3348.69**	**2055**	**48.12**

5.3 Iraq

5.3.1 All Projections

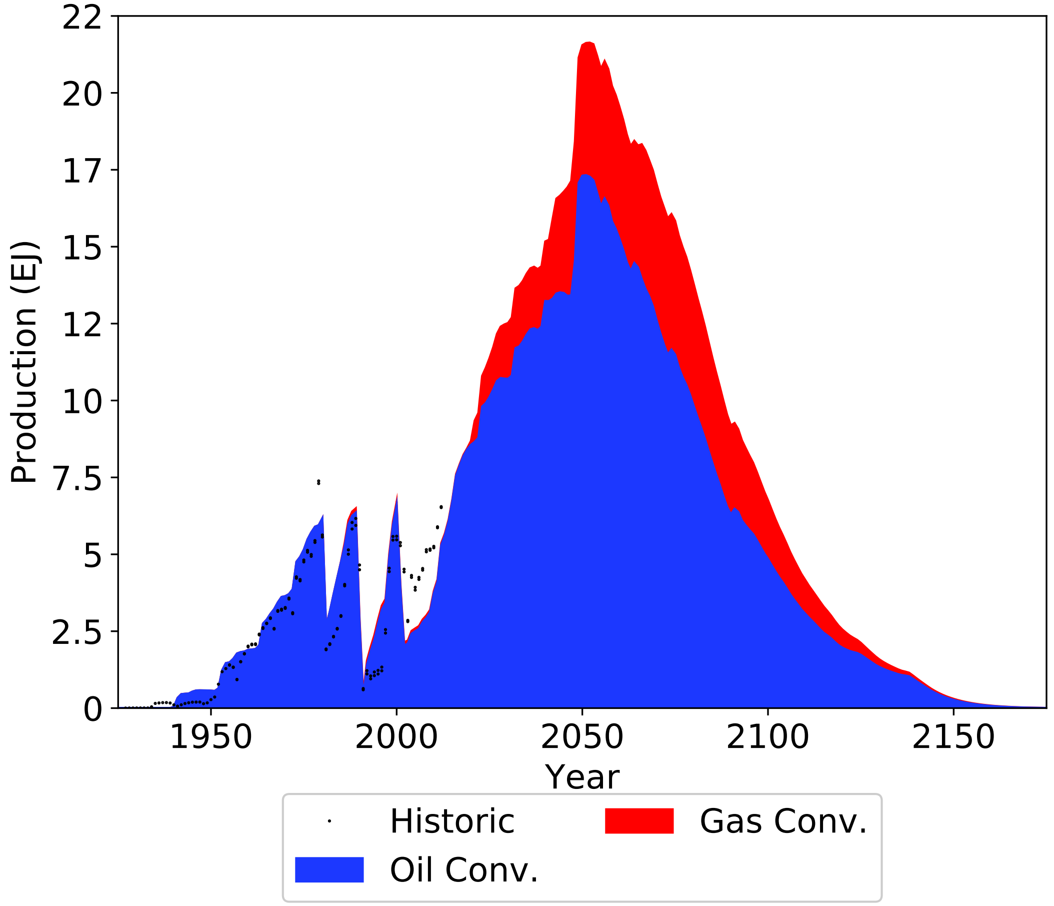

Figure 5.5: Iraq projections capped at 16

Table 5.5: Peak years - All

Name	URR	Peak Year	Peak Rate
Oil Conv.	1284.95	2051	17.33
Gas Conv.	284.9	2067	4.5
**Total**	**1569.85**	**2052**	**21.63**

5.3.2 By Mineral

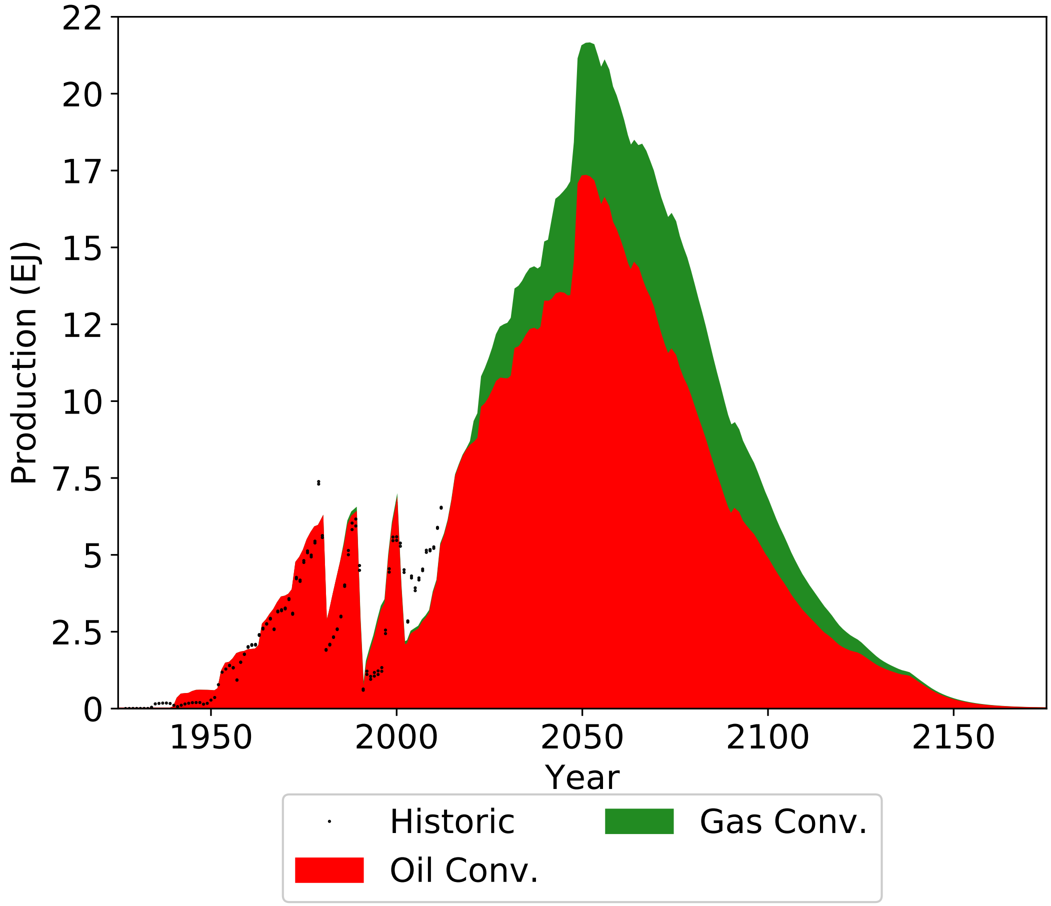

Figure 5.6: Iraq projection by mineral type

Table 5.6: Peak years - Minerals

Name	URR	Peak Year	Peak Rate
Oil Conv.	1284.95	2051	17.33
Gas Conv.	284.9	2067	4.5
**Total**	**1569.85**	**2052**	**21.63**

5.4 Israel

5.4.1 All Projections

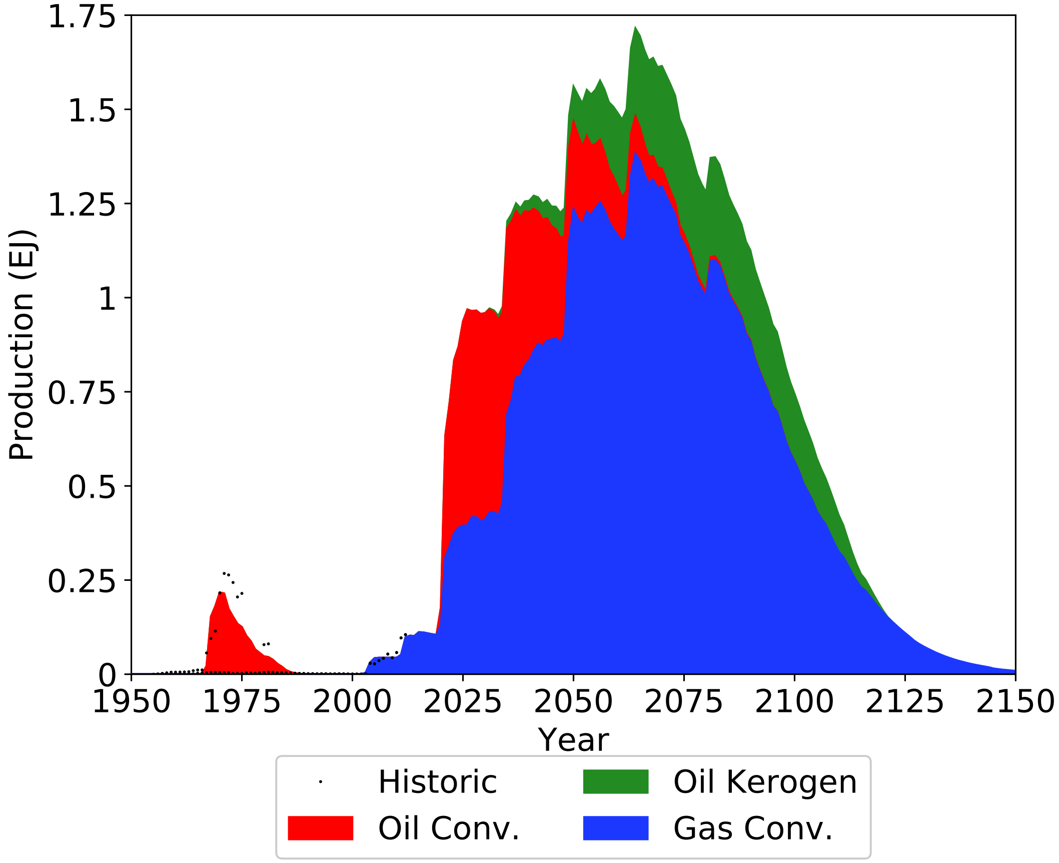

Figure 5.7: Israel projections capped at 16

Table 5.7: Peak years - All

**Name**	**URR**	**Peak Year**	**Peak Rate**
Gas Conv.	82.71	2064	1.38
Oil Conv.	17.54	2026	0.57
Oil Kerogen	13.8	2073	0.28
**Total**	**114.05**	**2064**	**1.72**

5.4.2 By Mineral

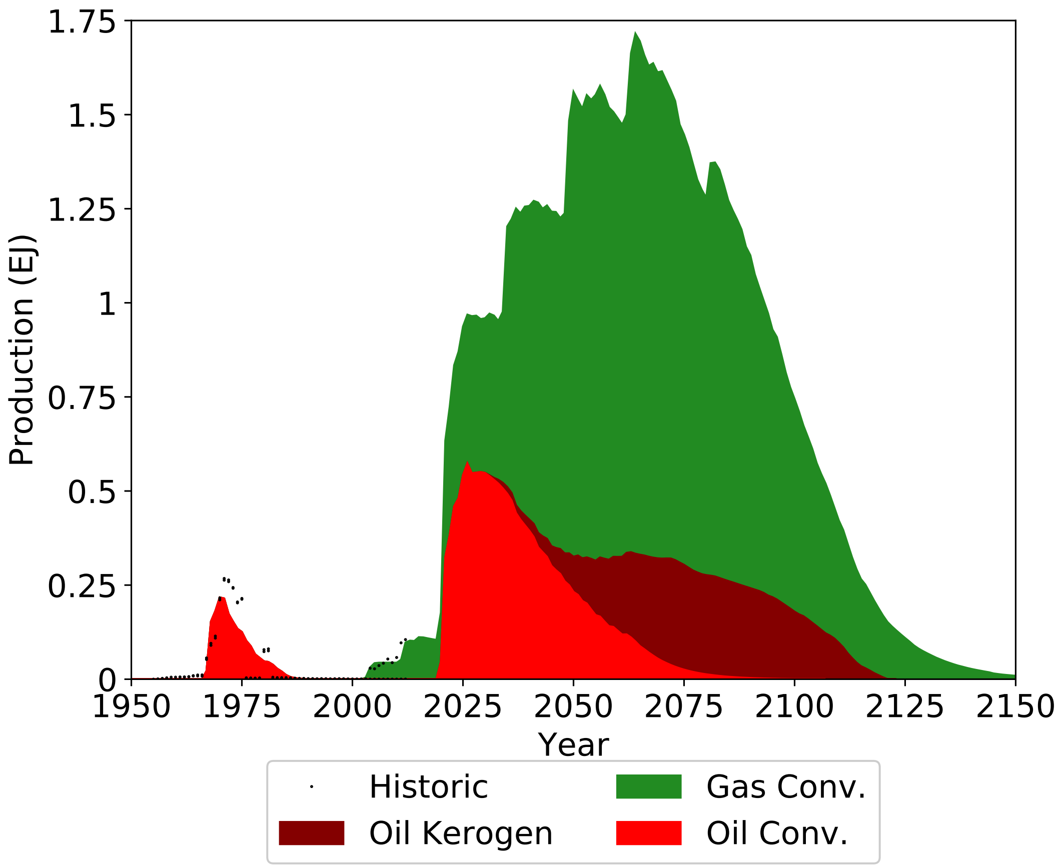

Figure 5.8: Israel projection by mineral type

Table 5.8: Peak years - Minerals

Name	URR	Peak Year	Peak Rate
Oil Conv.	17.54	2026	0.57
Oil Kerogen	13.8	2073	0.28
Gas Conv.	82.71	2064	1.38
**Total**	**114.05**	**2064**	**1.72**

5.5 Jordan

5.5.1 All Projections

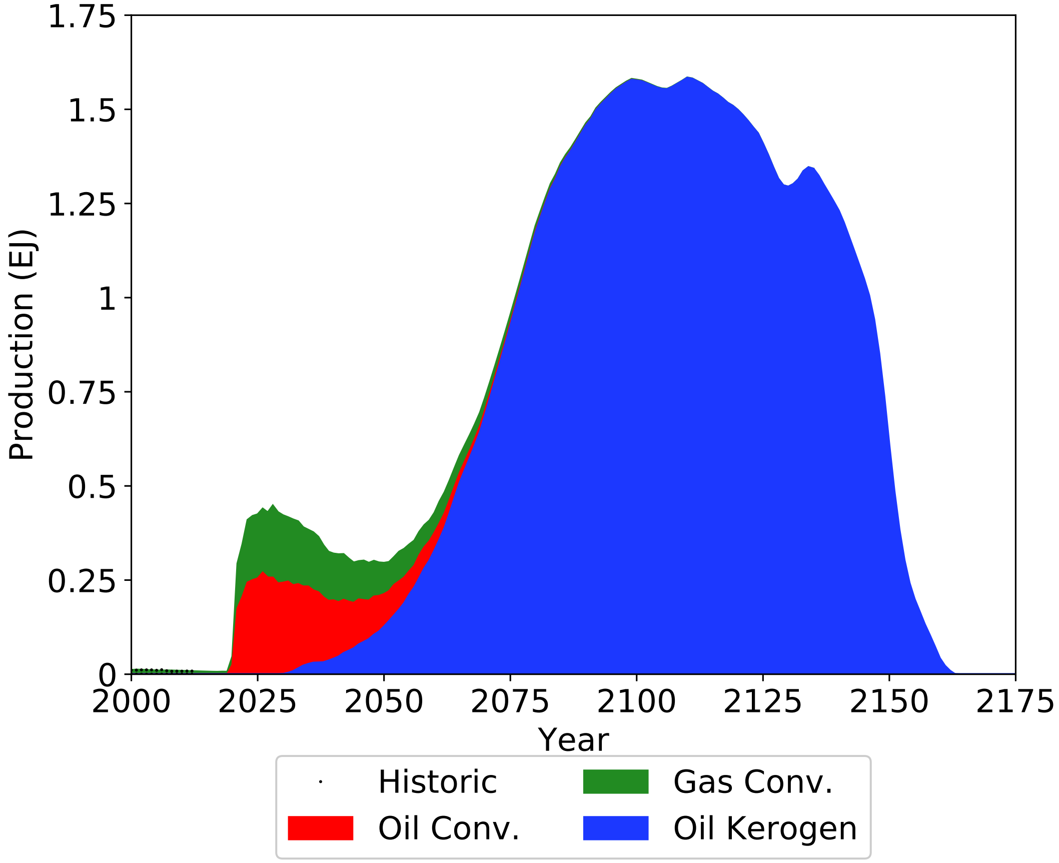

Figure 5.9: Jordan projections capped at 16

Table 5.9: Peak years - All

Name	URR	Peak Year	Peak Rate
Oil Kerogen	116.9	2110	1.58
Oil Conv.	6.54	2026	0.27
Gas Conv.	6.0	2028	0.19
**Total**	**129.44**	**2110**	**1.58**

5.5.2 By Mineral

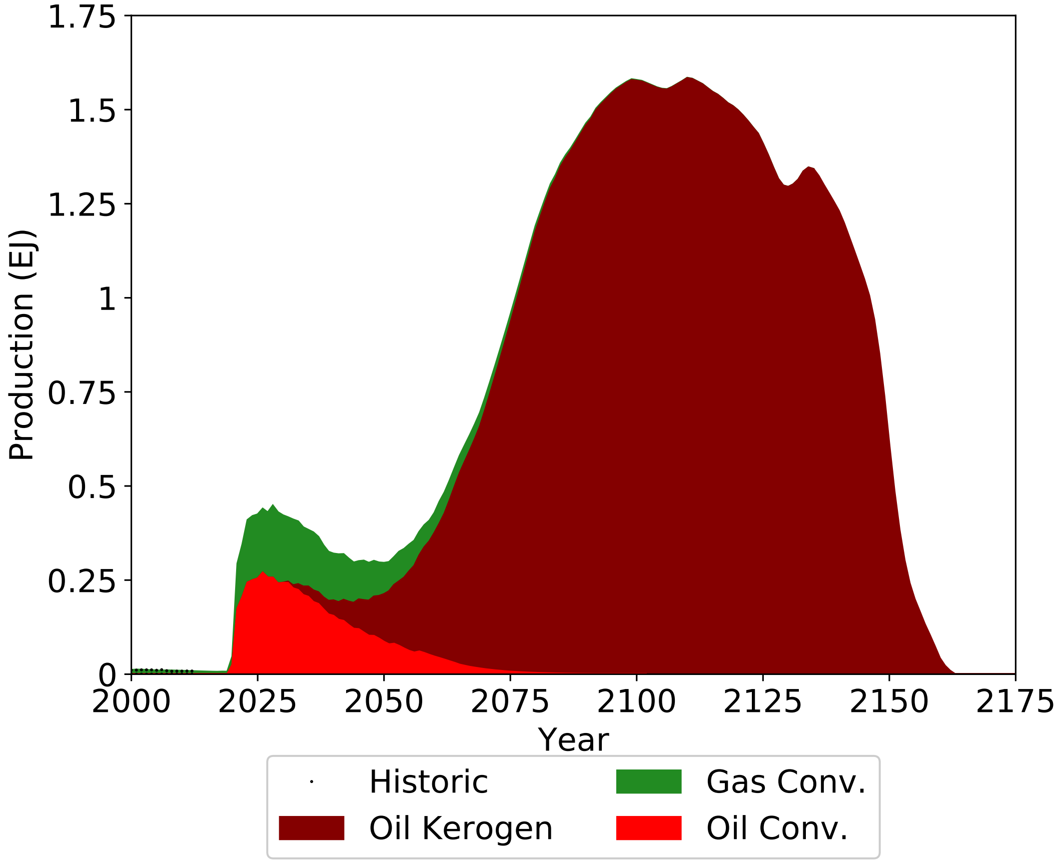

Figure 5.10: Jordan projection by mineral type

Table 5.10: Peak years - Minerals

**Name**	**URR**	**Peak Year**	**Peak Rate**
Oil Conv.	6.54	2026	0.27
Oil Kerogen	116.9	2110	1.58
Gas Conv.	6.0	2028	0.19
**Total**	**129.44**	**2110**	**1.58**

5.6 Kuwait

5.6.1 All Projections

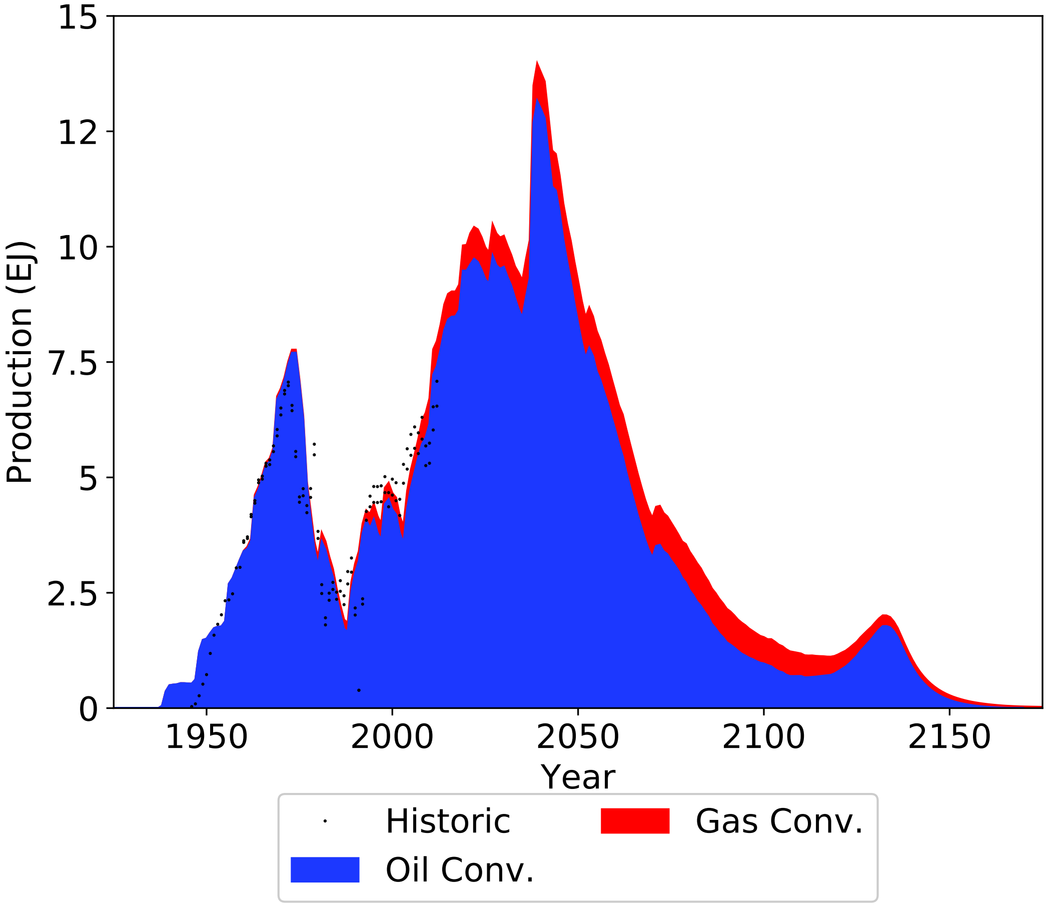

Figure 5.11: Kuwait projections capped at 16

Table 5.11: Peak years - All

Name	URR	Peak Year	Peak Rate
Oil Conv.	865.76	2039	13.16
Gas Conv.	96.8	2063	0.93
**Total**	**962.56**	**2039**	**13.97**

5.6.2 By Mineral

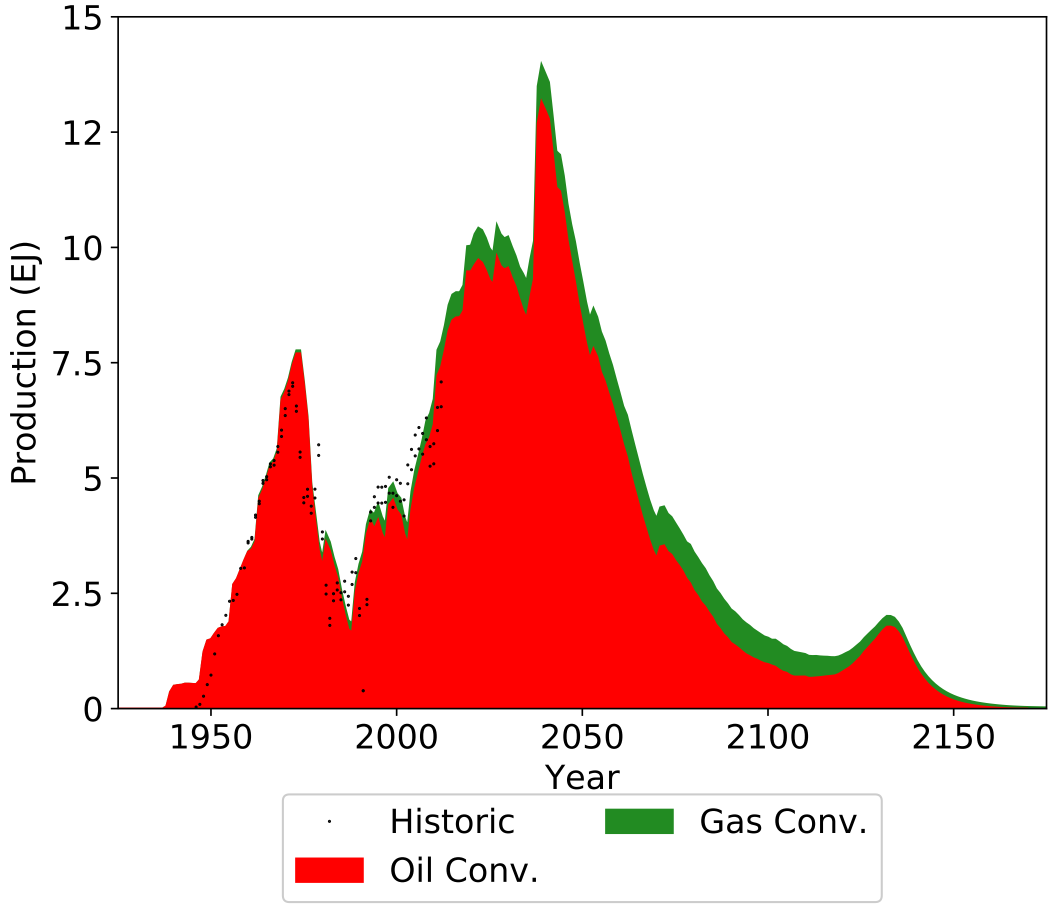

Figure 5.12: Kuwait projection by mineral type

Table 5.12: Peak years - Minerals

Name	URR	Peak Year	Peak Rate
Oil Conv.	865.76	2039	13.16
Gas Conv.	96.8	2063	0.93
**Total**	**962.56**	**2039**	**13.97**

5.7 Lebanon

5.7.1 All Projections

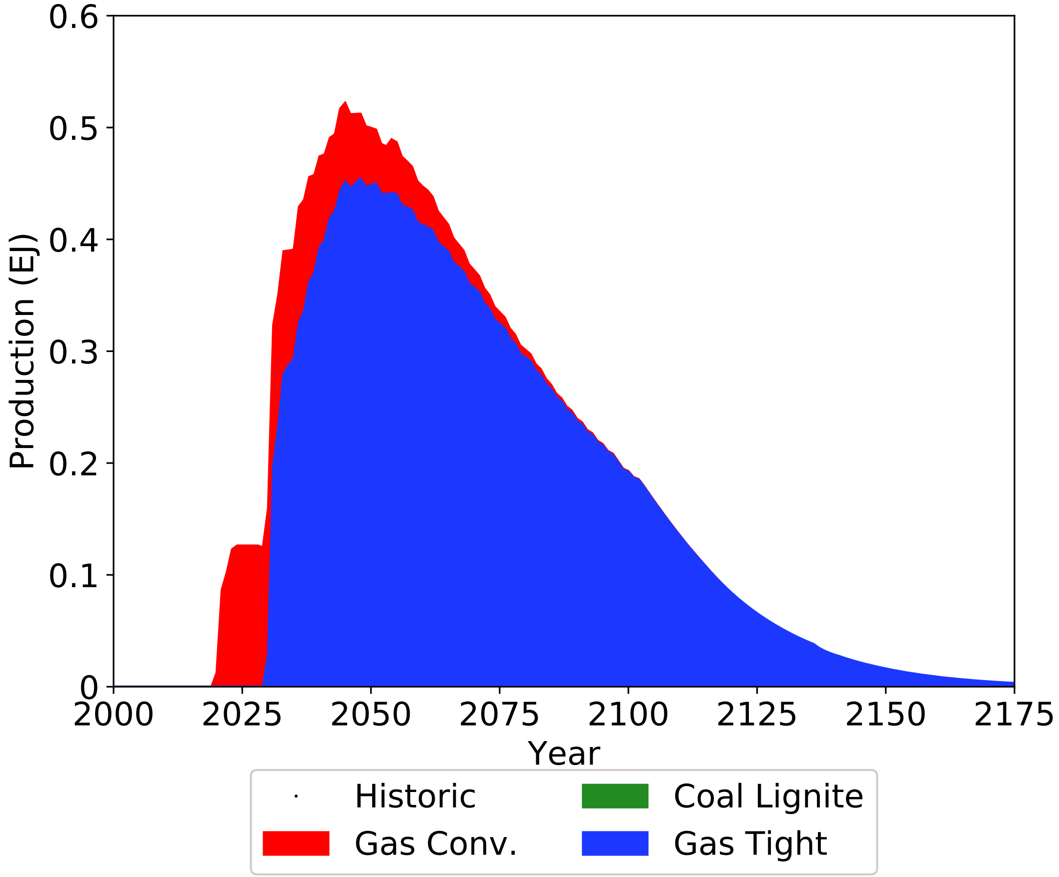

Figure 5.13: Lebanon projections capped at 16

Table 5.13: Peak years - All

**Name**	**URR**	**Peak Year**	**Peak Rate**
Gas Tight	27.81	2048	0.45
Gas Conv.	3.71	2030	0.13
Coal Lignite	–	1942	–
**Total**	**31.52**	**2045**	**0.52**

5.7.2 By Mineral

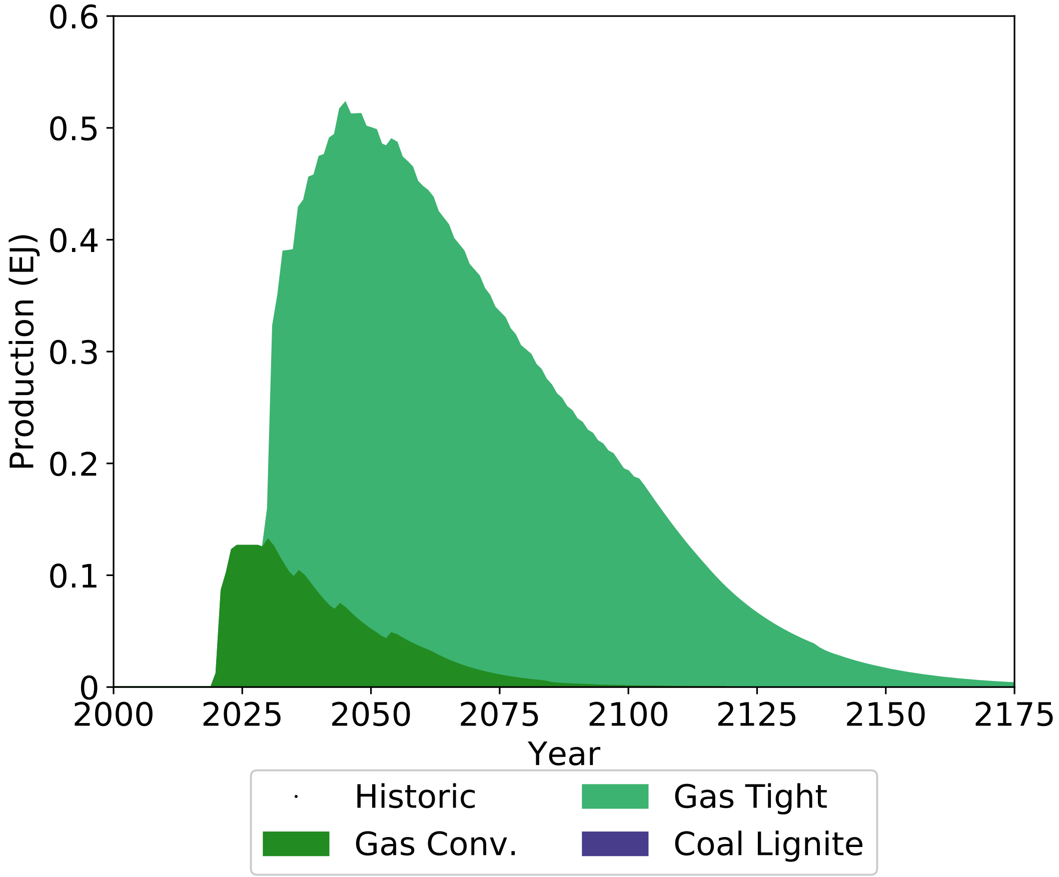

Figure 5.14: Lebanon projection by mineral type

Table 5.14: Peak years - Minerals

**Name**	**URR**	**Peak Year**	**Peak Rate**
Coal Lignite	–	1942	–
Gas Conv.	3.71	2030	0.13
Gas Tight	27.81	2048	0.45
**Total**	**31.52**	**2045**	**0.52**

5.8 Oman

5.8.1 All Projections

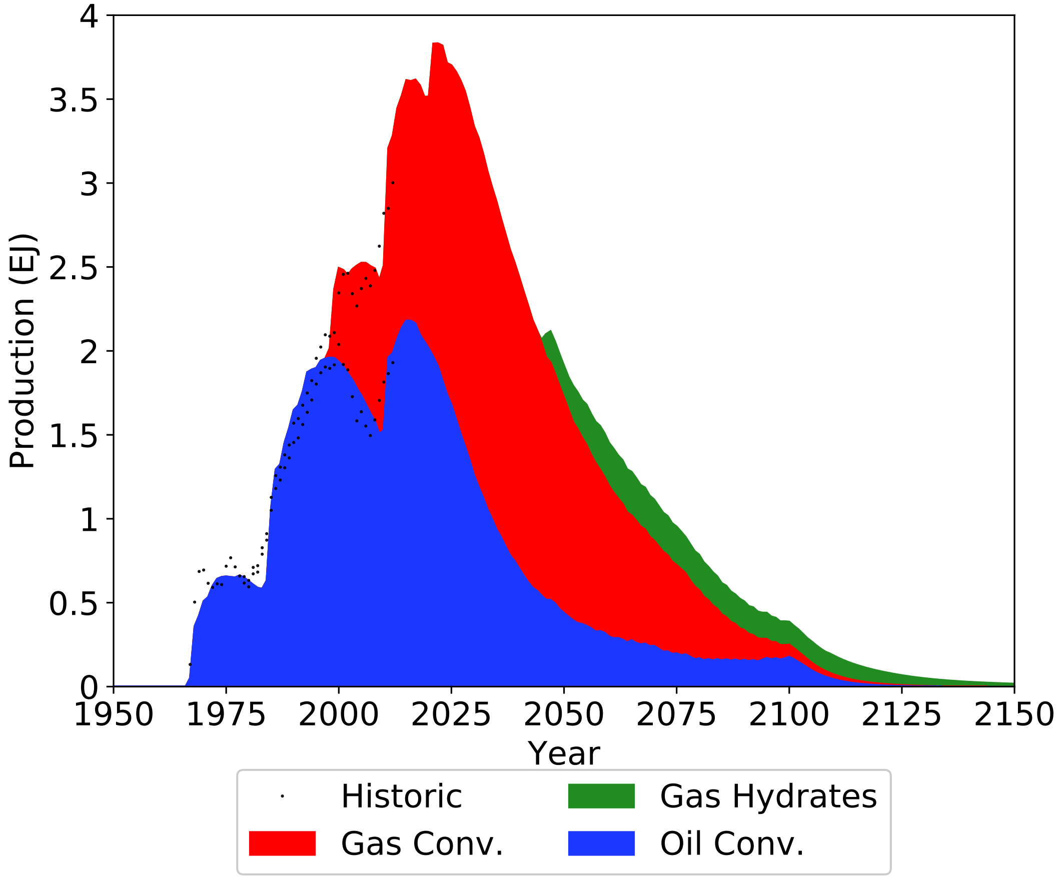

Figure 5.15: Oman projections capped at 16

Table 5.15: Peak years - All

Name	URR	Peak Year	Peak Rate
Oil Conv.	118.61	2015	2.18
Gas Conv.	104.87	2028	2.12
Gas Hydrates	15.0	2059	0.26
**Total**	**238.48**	**2022**	**3.83**

5.8.2 By Mineral

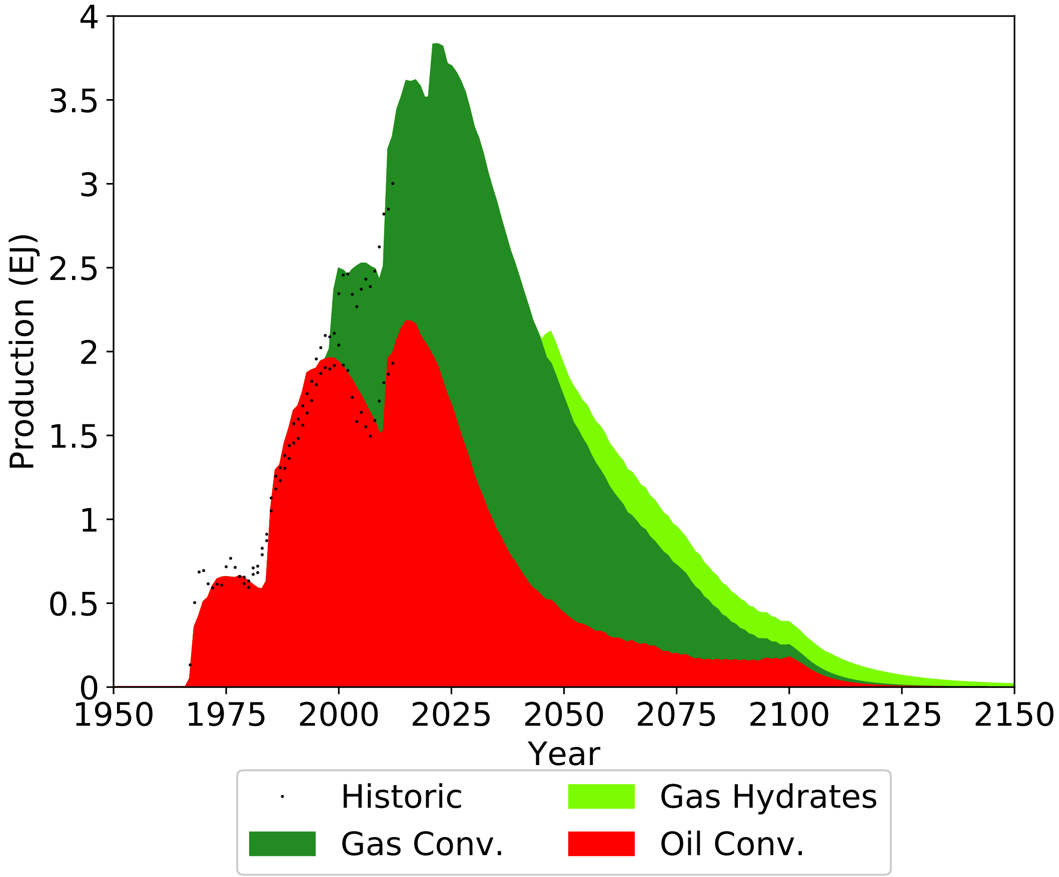

Figure 5.16: Oman projection by mineral type

Table 5.16: Peak years - Minerals

Name	URR	Peak Year	Peak Rate
Oil Conv.	118.61	2015	2.18
Gas Conv.	104.87	2028	2.12
Gas Hydrates	15.0	2059	0.26
**Total**	**238.48**	**2022**	**3.83**

5.9 Palestine

5.9.1 All Projections

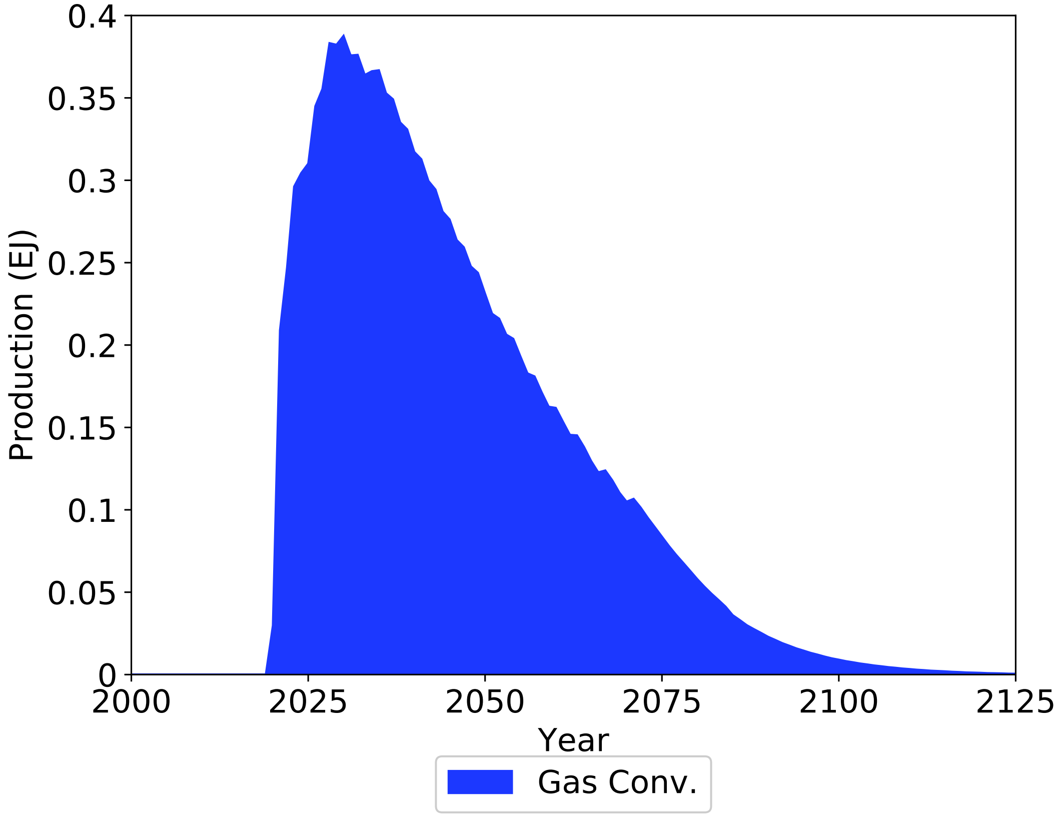

Figure 5.17: Palestine projections capped at 16

Table 5.17: Peak years - All

**Name**	**URR**	**Peak Year**	**Peak Rate**
Gas Conv.	14.09	2030	0.39
**Total**	**14.09**	**2030**	**0.39**

5.9.2 By Mineral

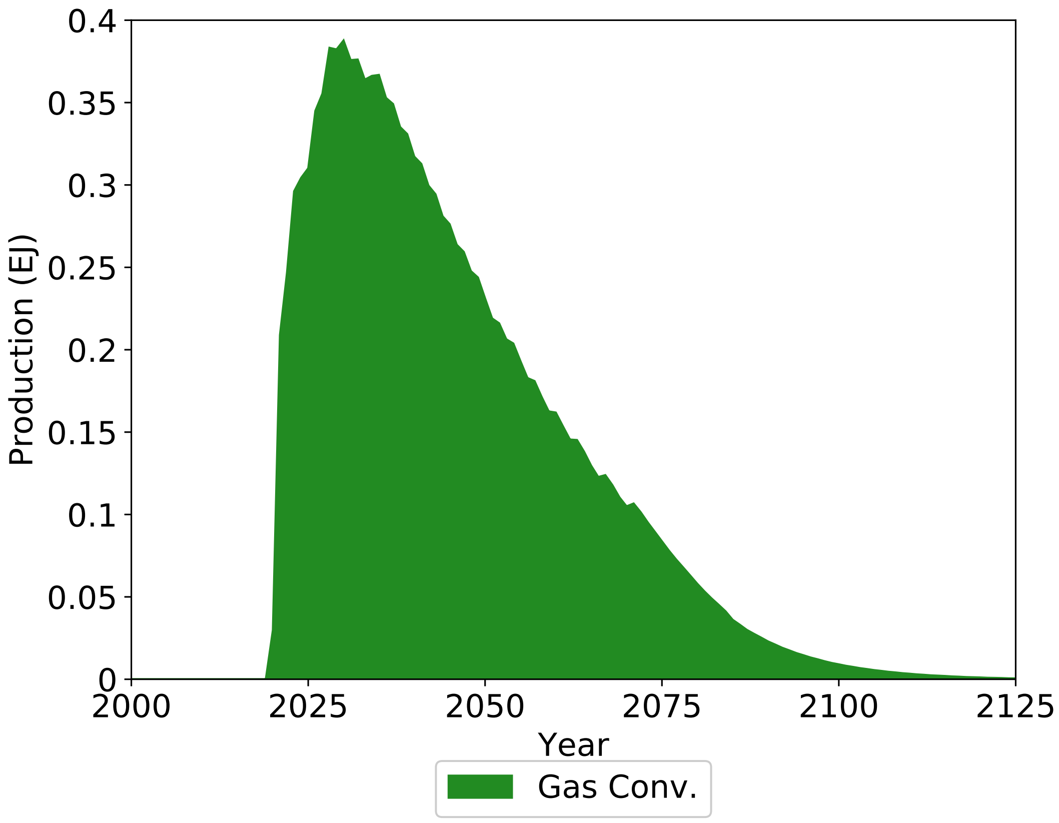

Figure 5.18: Palestine projection by mineral type

Table 5.18: Peak years - Minerals

Name	URR	Peak Year	Peak Rate
Gas Conv.	14.09	2030	0.39
**Total**	**14.09**	**2030**	**0.39**

5.10 Qatar

5.10.1 All Projections

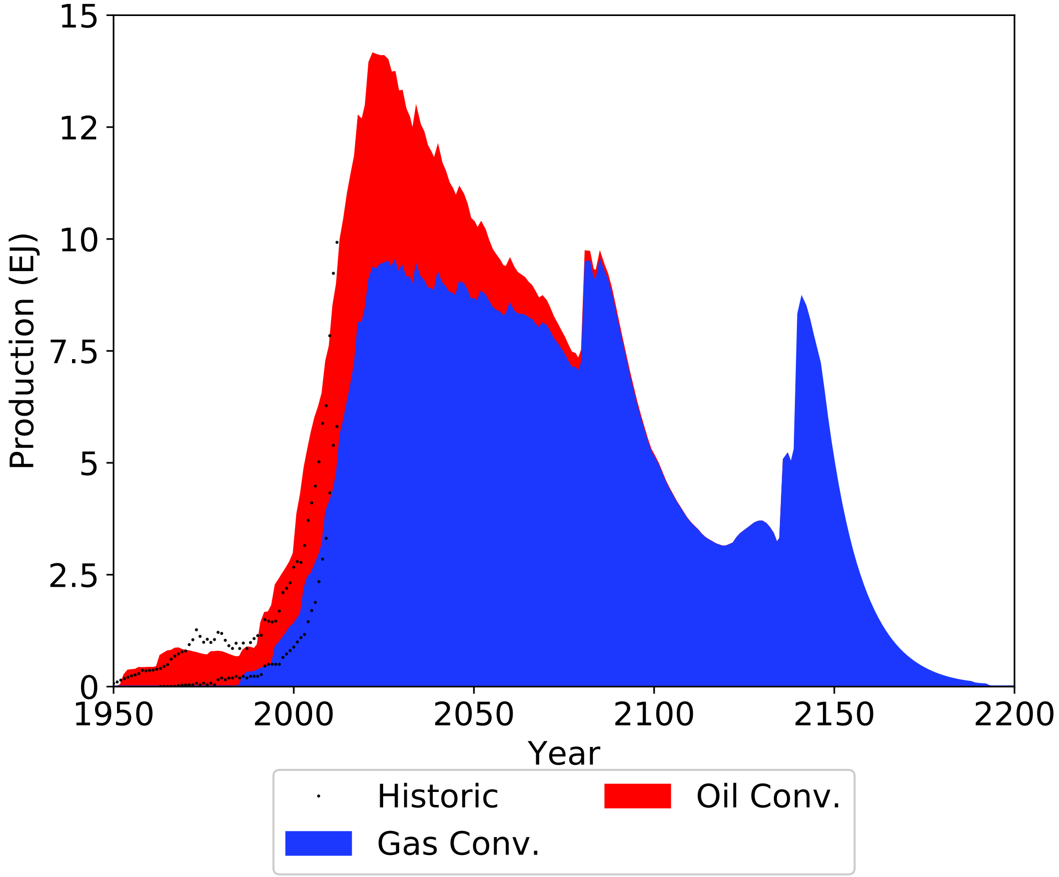

Figure 5.19: Qatar projections capped at 16

Table 5.19: Peak years - All

Name	URR	Peak Year	Peak Rate
Gas Conv.	1043.98	2085	9.5
Oil Conv.	239.78	2021	4.84
**Total**	**1283.76**	**2022**	**14.14**

5.10.2 By Mineral

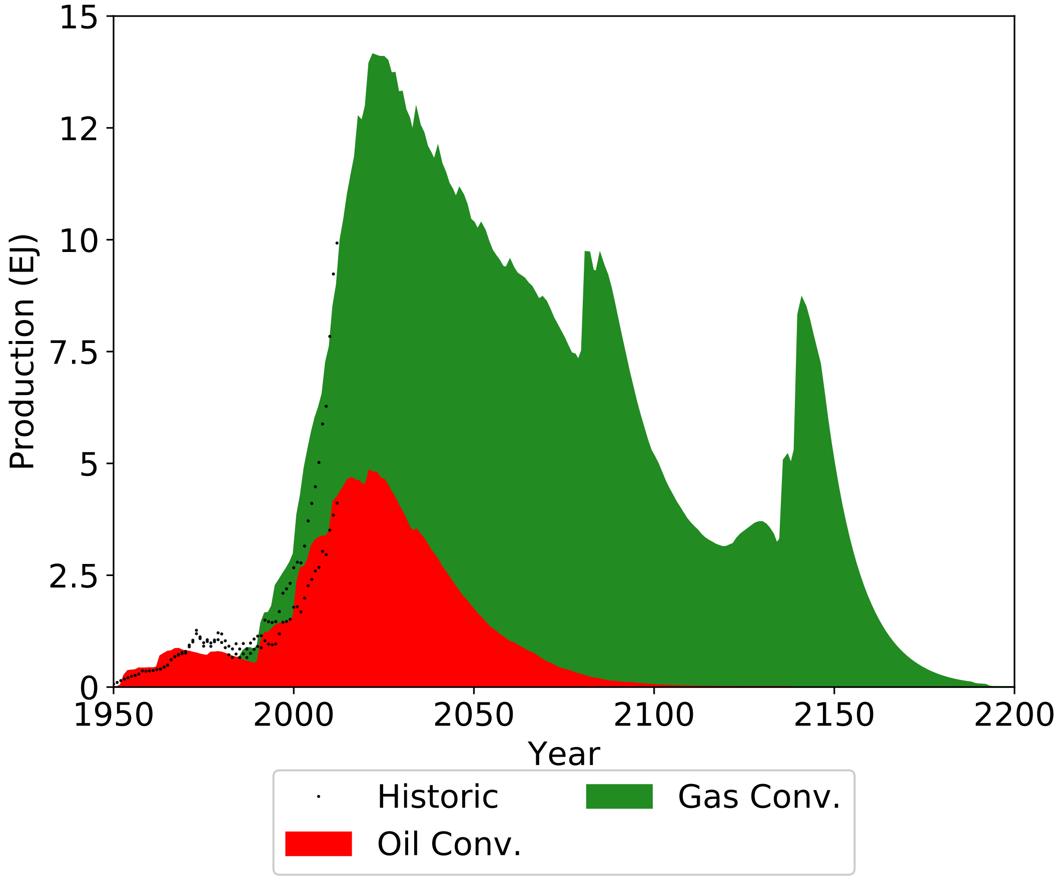

Figure 5.20: Qatar projection by mineral type

Table 5.20: Peak years - Minerals

Name	URR	Peak Year	Peak Rate
Oil Conv.	239.78	2021	4.84
Gas Conv.	1043.98	2085	9.5
**Total**	**1283.76**	**2022**	**14.14**

5.11 Saudi Arabia

5.11.1 All Projections

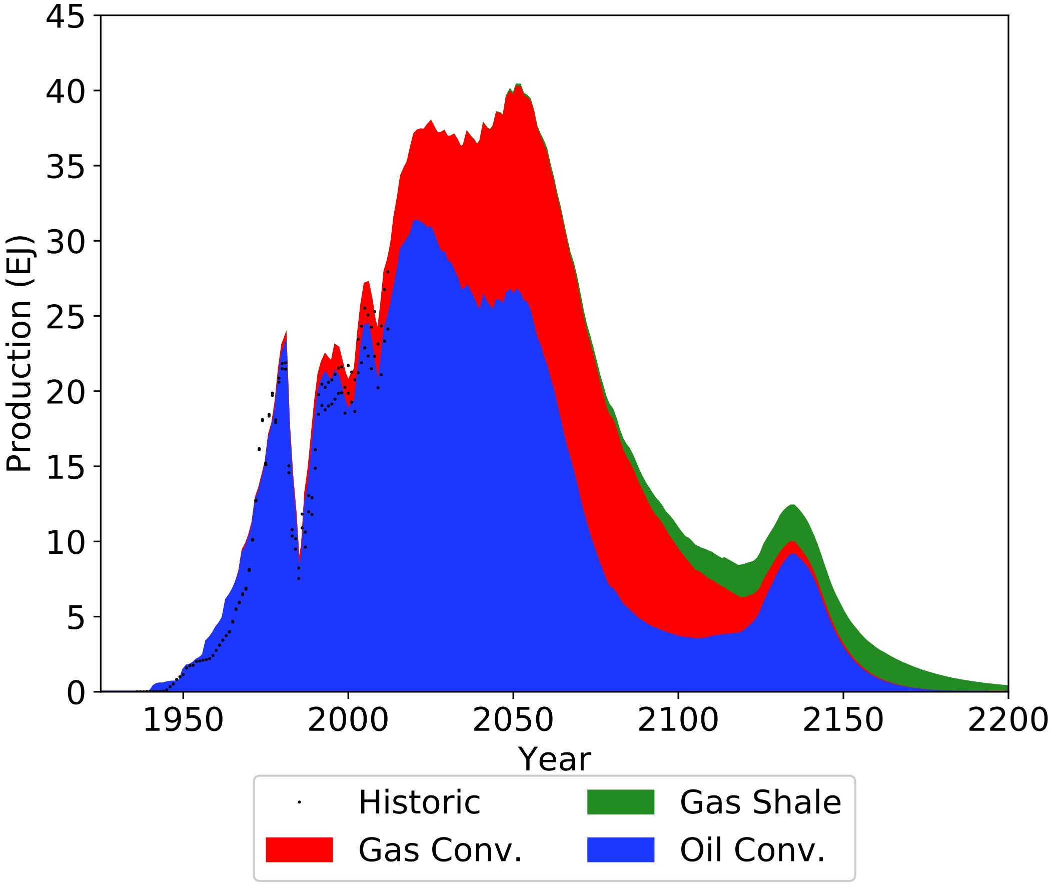

Figure 5.21: Saudi Arabia projections capped at 16

Table 5.21: Peak years - All

Name	URR	Peak Year	Peak Rate
Oil Conv.	2837.7	2021	31.31
Gas Conv.	1060.86	2060	14.14
Gas Shale	210.0	2138	2.5
**Total**	**4108.56**	**2051**	**40.4**

5.11.2 By Mineral

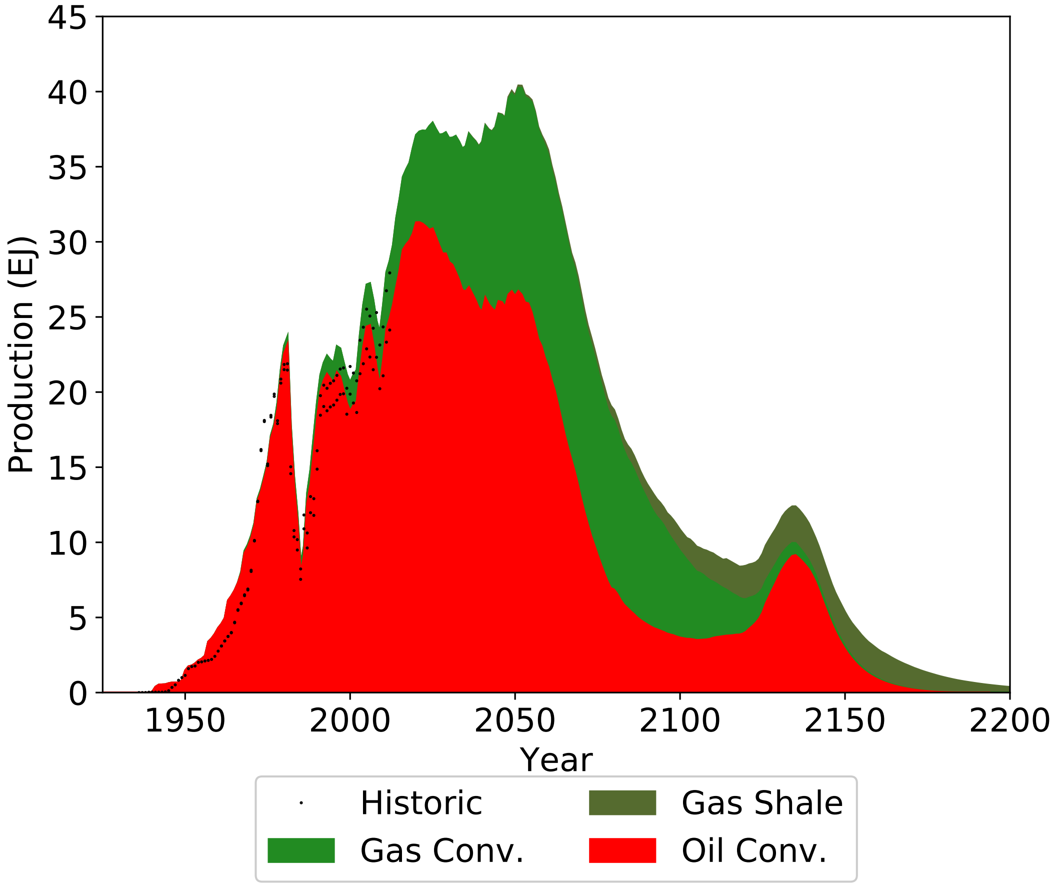

Figure 5.22: Saudi Arabia projection by mineral type

Table 5.22: Peak years - Minerals

Name	URR	Peak Year	Peak Rate
Oil Conv.	2837.7	2021	31.31
Gas Conv.	1060.86	2060	14.14
Gas Shale	210.0	2138	2.5
**Total**	**4108.56**	**2051**	**40.4**

5.12 Syria

5.12.1 All Projections

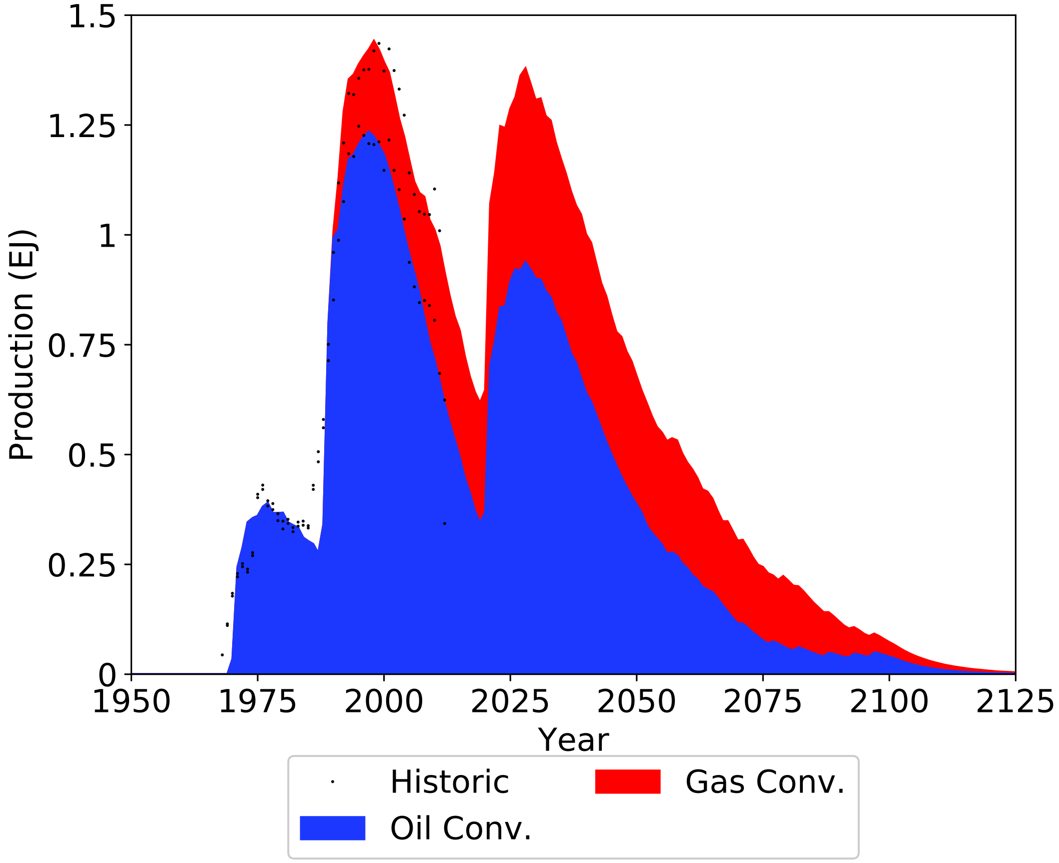

Figure 5.23: Syria projections capped at 16

Table 5.23: Peak years - All

Name	URR	Peak Year	Peak Rate
Oil Conv.	61.8	1997	1.23
Gas Conv.	26.5	2027	0.44
**Total**	**88.3**	**1998**	**1.44**

5.12.2 By Mineral

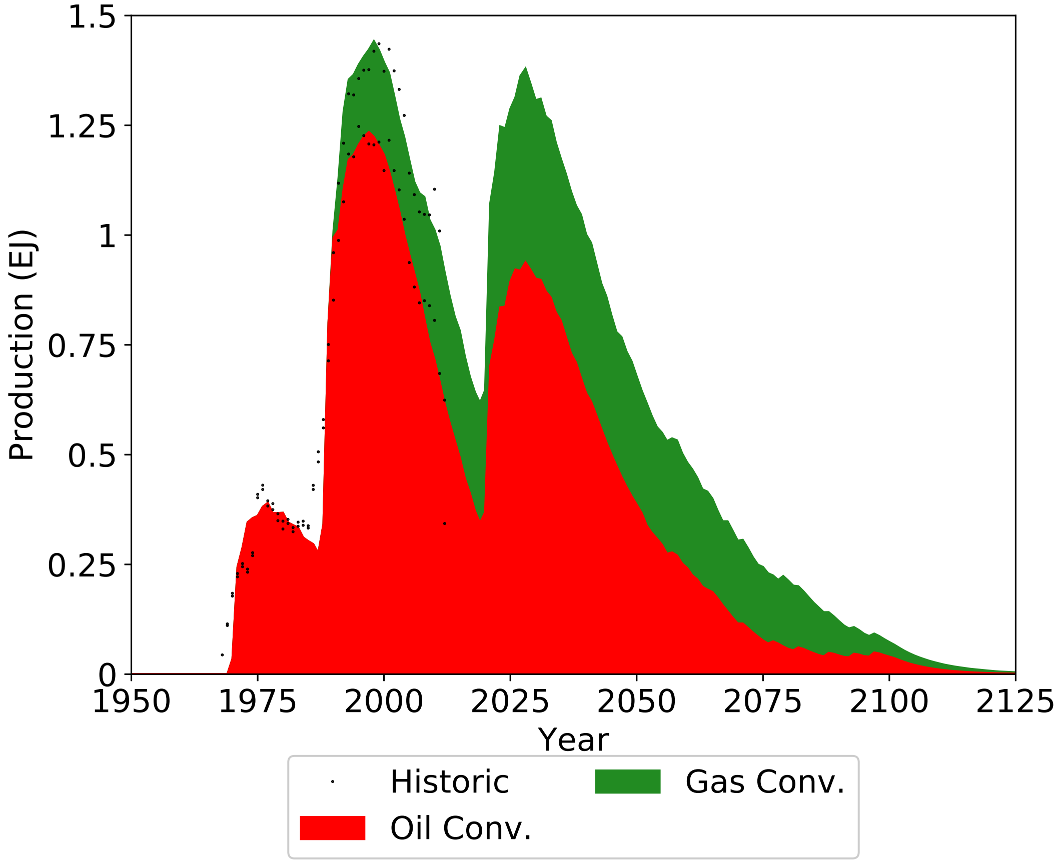

Figure 5.24: Syria projection by mineral type

Table 5.24: Peak years - Minerals

Name	URR	Peak Year	Peak Rate
Oil Conv.	61.8	1997	1.23
Gas Conv.	26.5	2027	0.44
**Total**	**88.3**	**1998**	**1.44**

5.13 UAE

5.13.1 All Projections

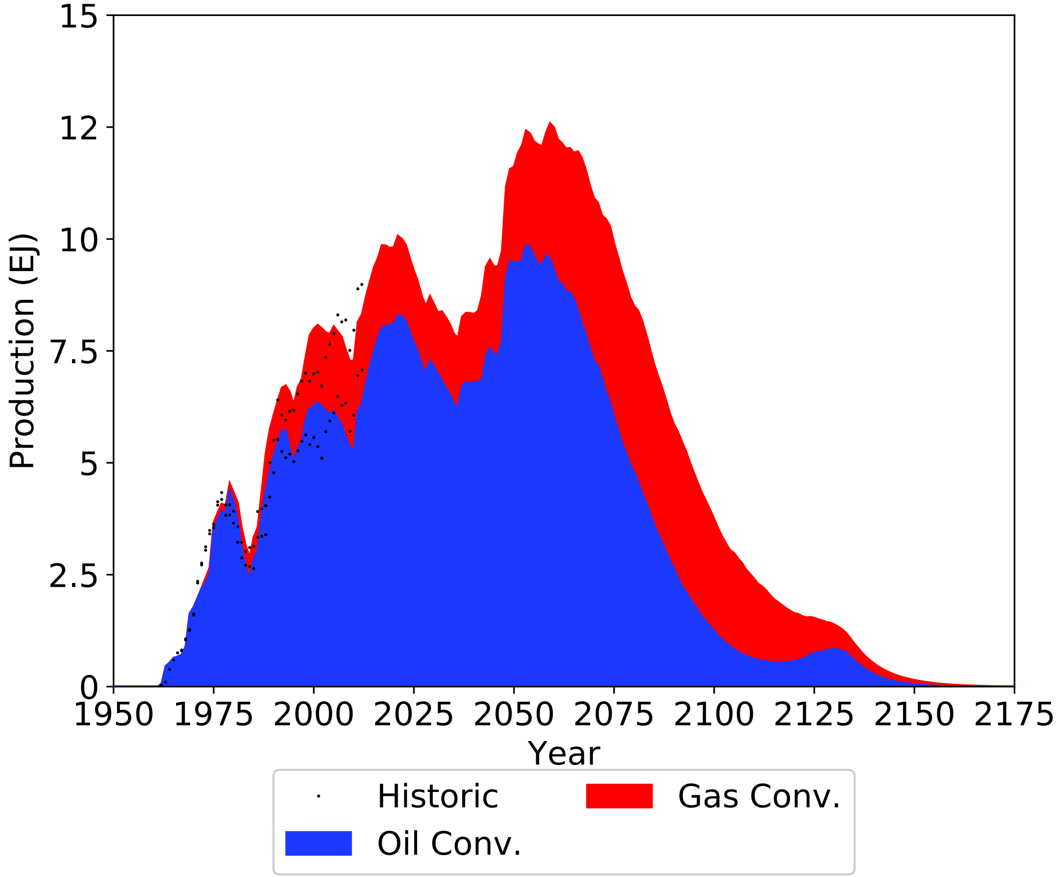

Figure 5.25: UAE projections capped at 16

Table 5.25: Peak years - All

**Name**	**URR**	**Peak Year**	**Peak Rate**
Oil Conv.	807.65	2053	9.86
Gas Conv.	321.1	2074	3.95
**Total**	**1128.75**	**2059**	**12.59**

5.13.2 By Mineral

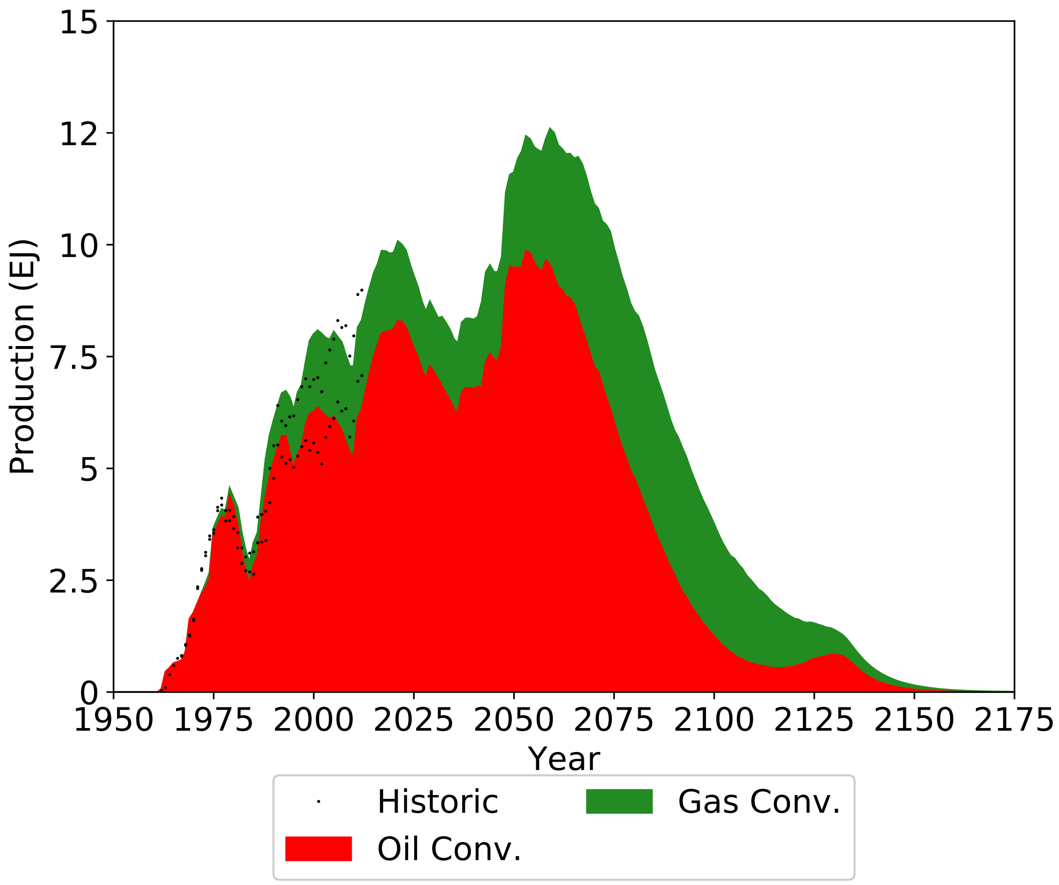

Figure 5.26: UAE projection by mineral type

Table 5.26: Peak years - Minerals

**Name**	**URR**	**Peak Year**	**Peak Rate**
Oil Conv.	807.65	2053	9.86
Gas Conv.	321.1	2074	3.95
**Total**	**1128.75**	**2059**	**12.59**

5.14 Yemen

5.14.1 All Projections

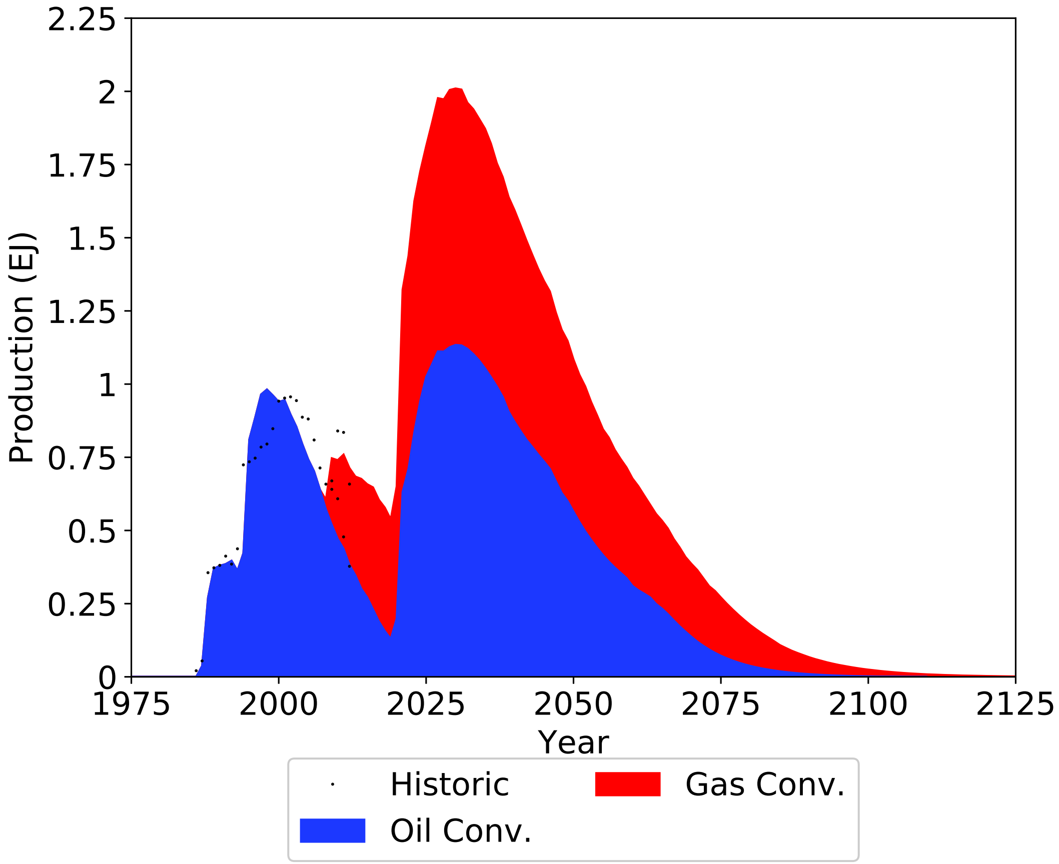

Figure 5.27: Yemen projections capped at 16

Table 5.27: Peak years - All

Name	URR	Peak Year	Peak Rate
Oil Conv.	52.28	2030	1.13
Gas Conv.	37.2	2029	0.88
**Total**	**89.48**	**2030**	**2.01**

5.14.2 By Mineral

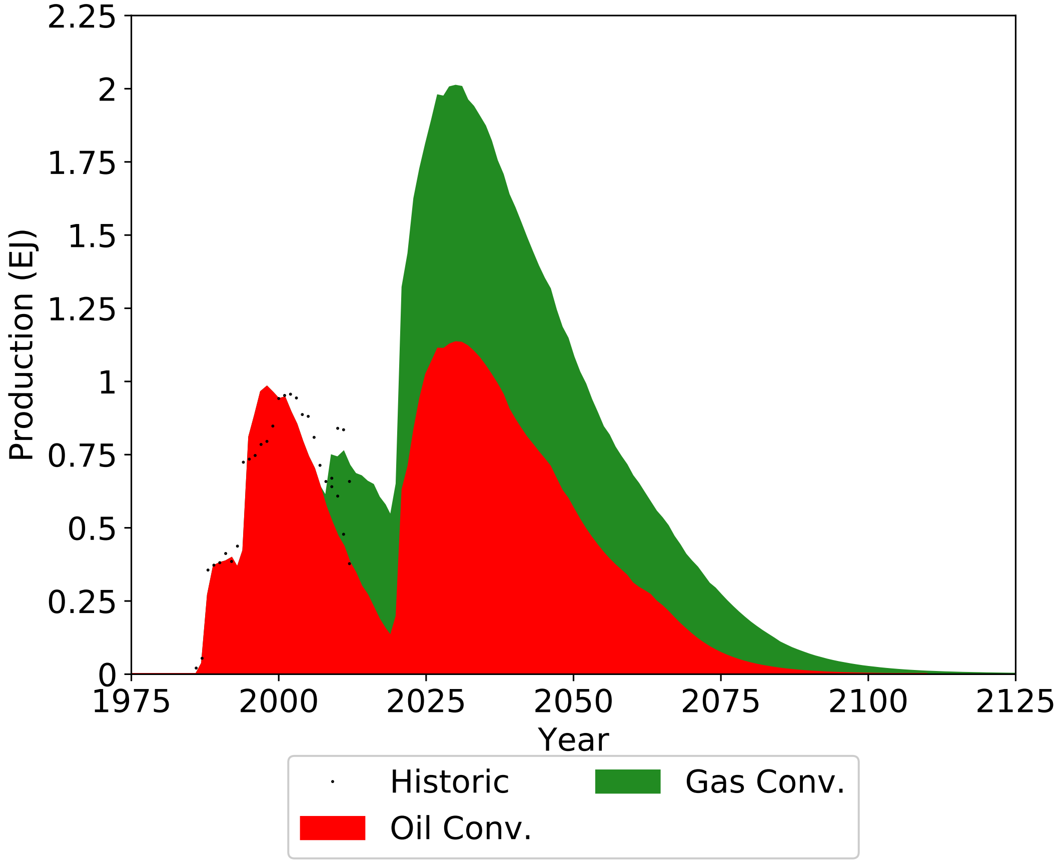

Figure 5.28: Yemen projection by mineral type

Table 5.28: Peak years - Minerals

Name	URR	Peak Year	Peak Rate
Oil Conv.	52.28	2030	1.13
Gas Conv.	37.2	2029	0.88
**Total**	**89.48**	**2030**	**2.01**

5.15 Total

5.15.1 By country

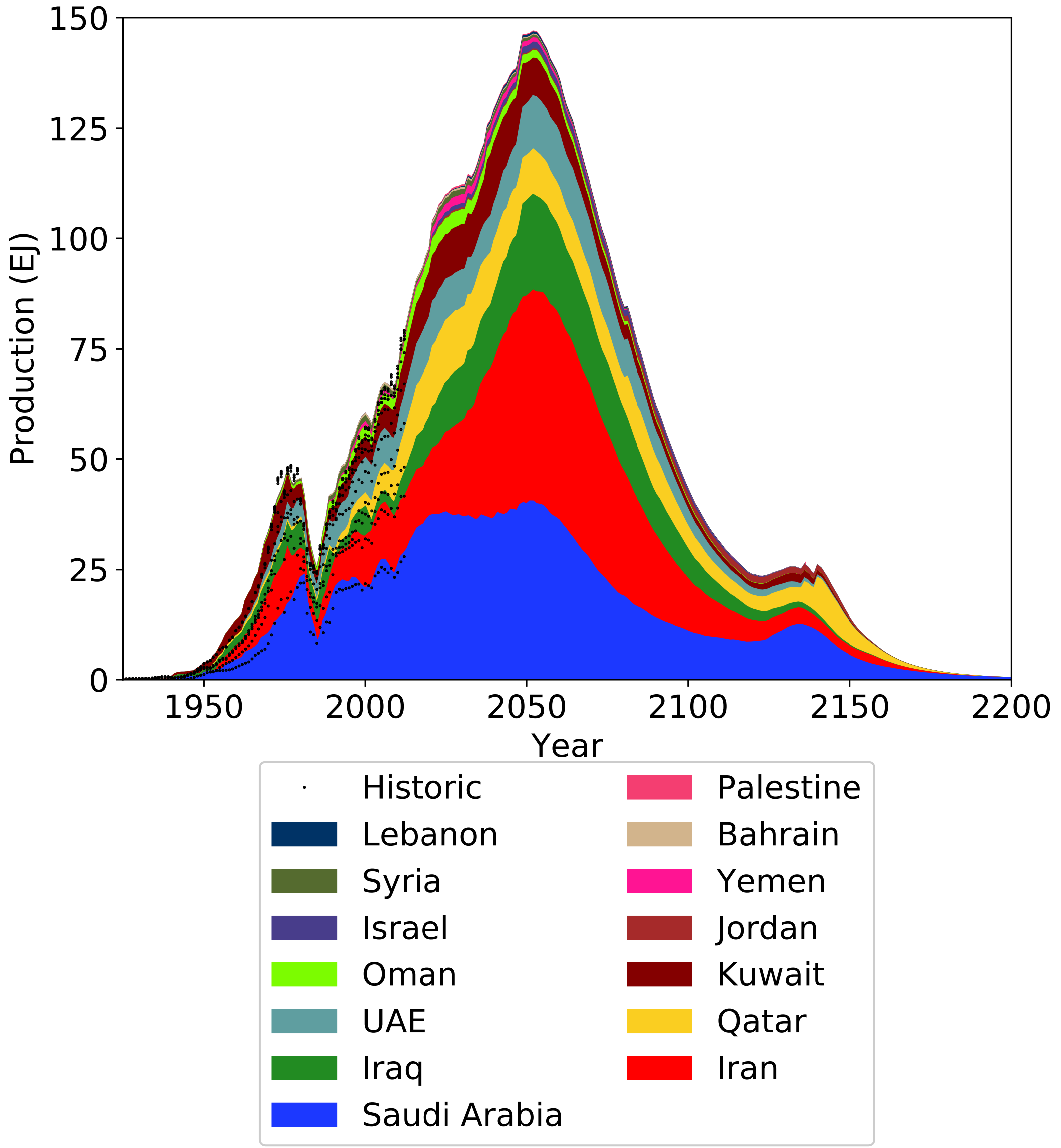

Figure 5.29: Middle East projections by country

5.15.2 By mineral

Table 5.29: Peak years - All

**Name**	**URR**	**Peak Year**	**Peak Rate**
Saudi Arabia	4108.56	2051	40.4
Iran	3348.69	2055	48.12
Iraq	1569.85	2052	21.63
Qatar	1283.76	2022	14.14
UAE	1128.75	2059	12.59
Kuwait	962.56	2039	13.97
Oman	238.48	2022	3.83
Jordan	129.44	2110	1.58
Israel	114.05	2064	1.72
Yemen	89.48	2030	2.01
Syria	88.3	1998	1.44
Bahrain	42.14	2013	0.63
Lebanon	31.52	2045	0.52
Palestine	14.09	2030	0.39
**Total**	**13149.67**	**2052**	**146.88**

Table 5.30: Peak years - Minerals

**Name**	**URR**	**Peak Year**	**Peak Rate**
Coal Bit.	28.2	2153	0.32
Coal Lignite	–	1942	–
Oil Conv.	7902.34	2049	90.36
Oil Kerogen	130.7	2098	1.77
Gas Conv.	4820.62	2060	57.67
Gas Hydrates	30.0	2058	0.52
Gas Shale	210.0	2138	2.5
Gas Tight	27.81	2048	0.45
**Total**	**13149.67**	**2052**	**146.88**

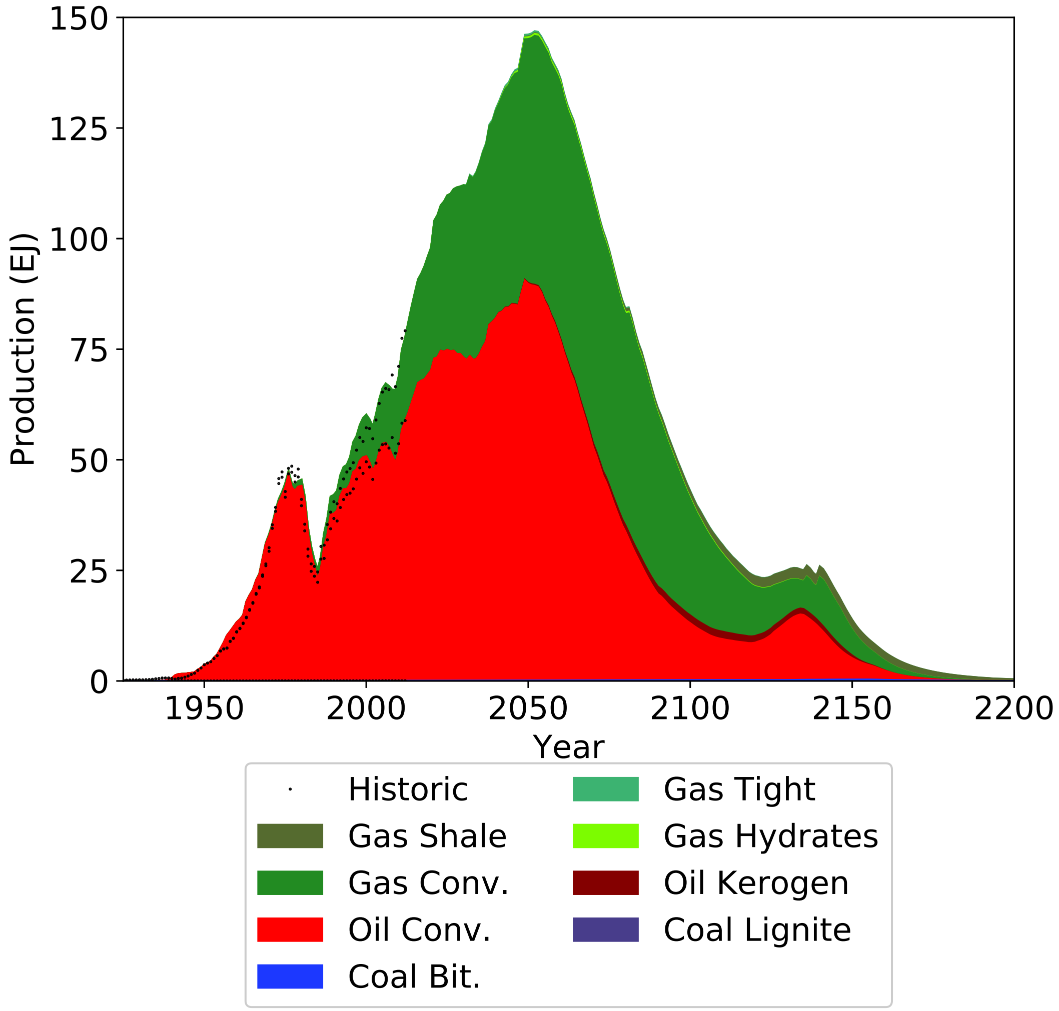

Figure 5.30: Middle East projection by mineral type

Chapter 6

North America

6.1 Canada

6.1.1 All Projections

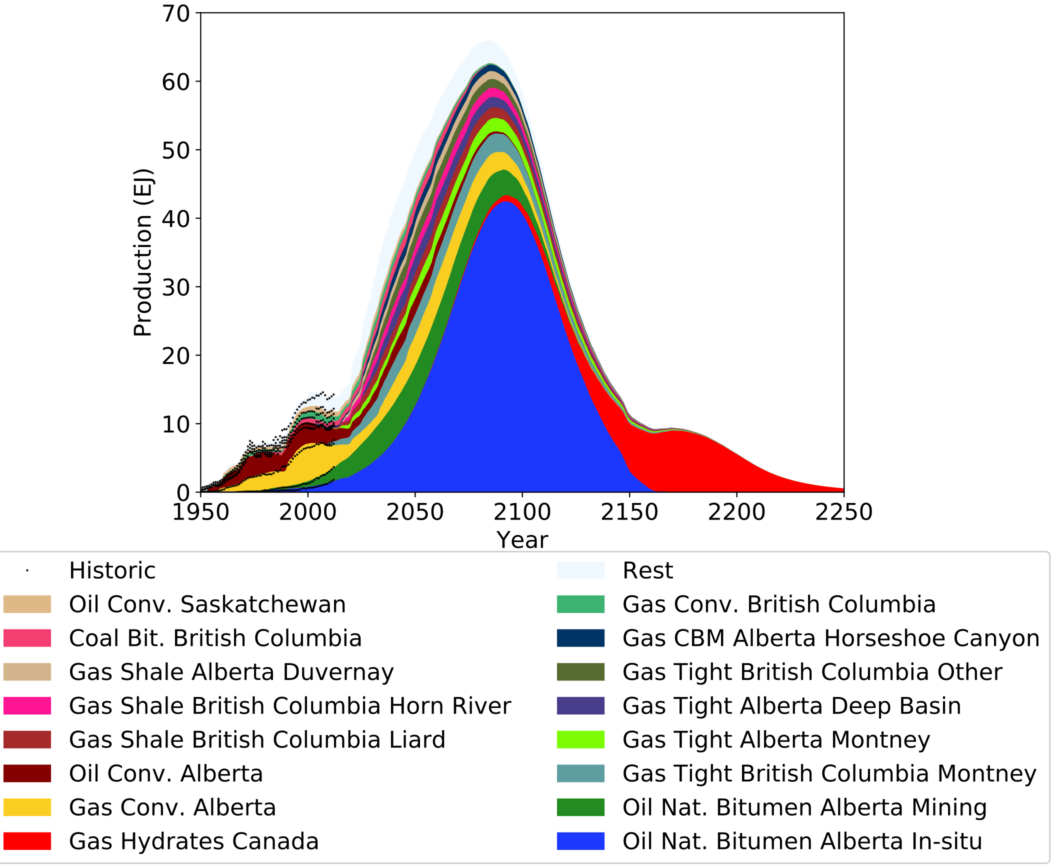

Figure 6.1: Canada projections capped at 16

Table 6.1: Peak years - All

**Name**	**URR**	**Peak Year**	**Peak Rate**
Oil Nat. Bitumen Alberta In-situ	2811.21	2092	42.34
Gas Hydrates Canada	712.0	2170	8.89
Oil Nat. Bitumen Alberta Mining	468.53	2055	5.89
Gas Conv. Alberta	467.07	1999	5.5
Gas Tight British Columbia Montney	285.99	2070	3.21
Oil Conv. Alberta	257.05	1995	3.34
Gas Tight Alberta Montney	187.12	2061	2.14
Gas Shale British Columbia Liard	165.9	2064	2.07
Gas Tight Alberta Deep Basin	162.75	2051	2.46
Gas Shale British Columbia Horn River	140.0	2065	1.74
Gas Tight British Columbia Other	138.6	2051	2.1
Gas Shale Alberta Duvernay	118.65	2063	1.51
Gas CBM Alberta Horseshoe Canyon	108.93	2048	1.58
Coal Bit. British Columbia	94.22	2048	1.57
Gas Conv. British Columbia	77.35	2002	1.1
Oil Conv. Saskatchewan	69.92	2032	0.93
Oil Tight Alberta	48.33	2037	1.26
Gas Shale Alberta Colorado	45.15	2053	0.7
Oil Kerogen Ontario	41.26	2084	0.76
Gas Shale Quebec Utica	32.55	2042	0.51
Gas Shale Alberta Muskwa	32.55	2045	0.51
Coal Sub-bit. Alberta	29.87	2007	0.52
Coal Lignite Saskatchewan	25.43	2070	0.43
Gas Tight British Columbia Jean Marie	24.15	2036	0.41
Gas Shale British Columbia Cordova	21.11	2031	0.33
Gas Conv. Saskatchewan	19.34	2031	0.31
Coal Bit. Nova Scotia	18.66	2048	0.29
Gas CBM Alberta Mannville	18.23	2044	0.29
Oil Conv. East Coast Offshore	16.04	2003	0.66
Oil Conv. British Columbia	14.4	2019	0.27
Coal Bit. Alberta	14.27	1990	0.25
Gas Shale Alberta North Nordegg	13.65	2046	0.23
Gas Shale British Columbia Doig	13.13	2041	0.22
Gas Shale Alberta Doig	13.13	2041	0.22
Oil Tight Saskatchewan	8.68	2016	0.33
Gas CBM British Columbia	8.48	2040	0.15
Oil Kerogen Nova Scotia	5.86	2062	0.13
Gas Conv. East Coast Offshore	5.21	2002	0.18
Gas CBM Nova Scotia	4.24	2037	0.08
Gas Conv. Ontario	3.51	2033	0.06
Gas Shale Nova Scotia Horton Bluff	3.15	2042	0.07
Oil Conv. Northwest Territories	2.85	1987	0.07
Oil Conv. Manitoba	2.7	2007	0.05
Oil Kerogen Manitoba	2.15	2059	0.06
Oil Kerogen Saskatchewan	2.15	2059	0.06
Gas Shale Saskatchewan Bakken	2.1	2037	0.05
Oil Tight Manitoba	2.0	2015	0.1
Gas Conv. Northwest Territories	1.9	2000	0.04
Coal Bit. New Brunswick	1.45	2030	0.02

Table 6.1: Peak years - All – Continued

**Name**	**URR**	**Peak Year**	**Peak Rate**
Gas CBM Saskatchewan	1.06	2030	0.02
Oil Kerogen New Brunswick	0.98	2048	0.03
Oil Conv. Ontario	0.79	2022	0.02
Oil Conv. Nova Scotia Offshore	0.36	2003	0.02
Gas Conv. New Brunswick	0.07	2025	–
Oil Extra Heavy Saskatchewan	0.02	2029	0.01
Oil Extra Heavy Alberta	0.02	2029	0.01
Coal Bit. Yukon	0.02	2025	–
Oil Conv. New Brunswick	0.01	2024	–
Gas Conv. Quebec	0.01	2025	–
Coal Lignite Manitoba	–	2027	–
Gas Conv. Manitoba	–	1928	–
**Total**	**6766.31**	**2084**	**65.92**

6.1.2 By Mineral

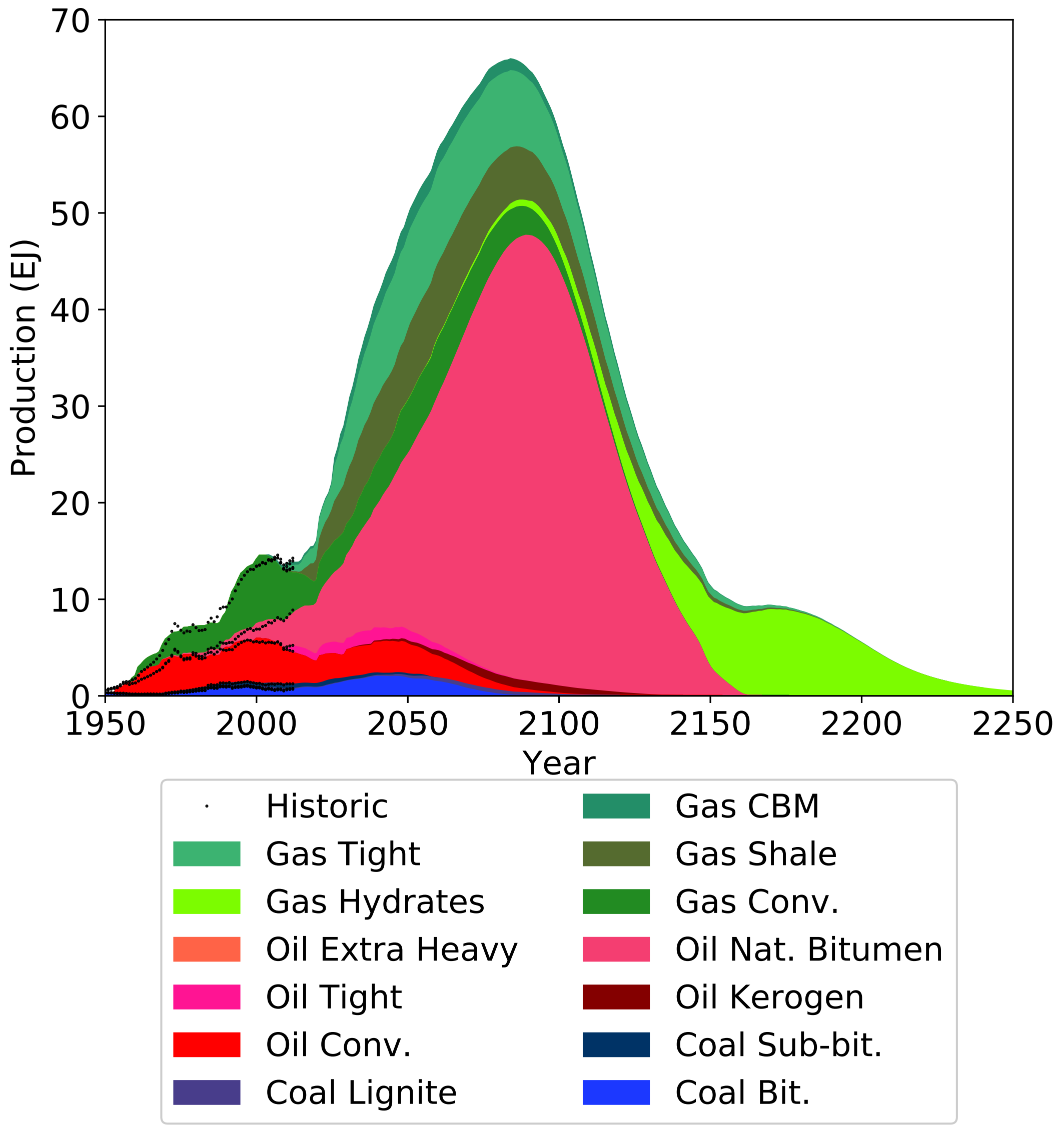

Figure 6.2: Canada projection by mineral type

6.1.3 Regional Projections

Table 6.2: Peak years - Minerals

**Name**	**URR**	**Peak Year**	**Peak Rate**
Coal Bit.	128.62	2040	1.97
Coal Lignite	25.43	2070	0.43
Coal Sub-bit.	29.87	2007	0.52
Oil Conv.	364.12	2001	4.68
Oil Kerogen	52.4	2083	0.92
Oil Tight	59.01	2036	1.41
Oil Nat. Bitumen	3279.74	2091	46.16
Oil Extra Heavy	0.04	2029	0.01
Gas Conv.	574.46	2001	6.95
Gas Hydrates	712.0	2170	8.89
Gas Shale	601.07	2060	7.64
Gas Tight	798.61	2061	9.9
Gas CBM	140.94	2048	2.09
**Total**	**6766.31**	**2084**	**65.92**

Alberta

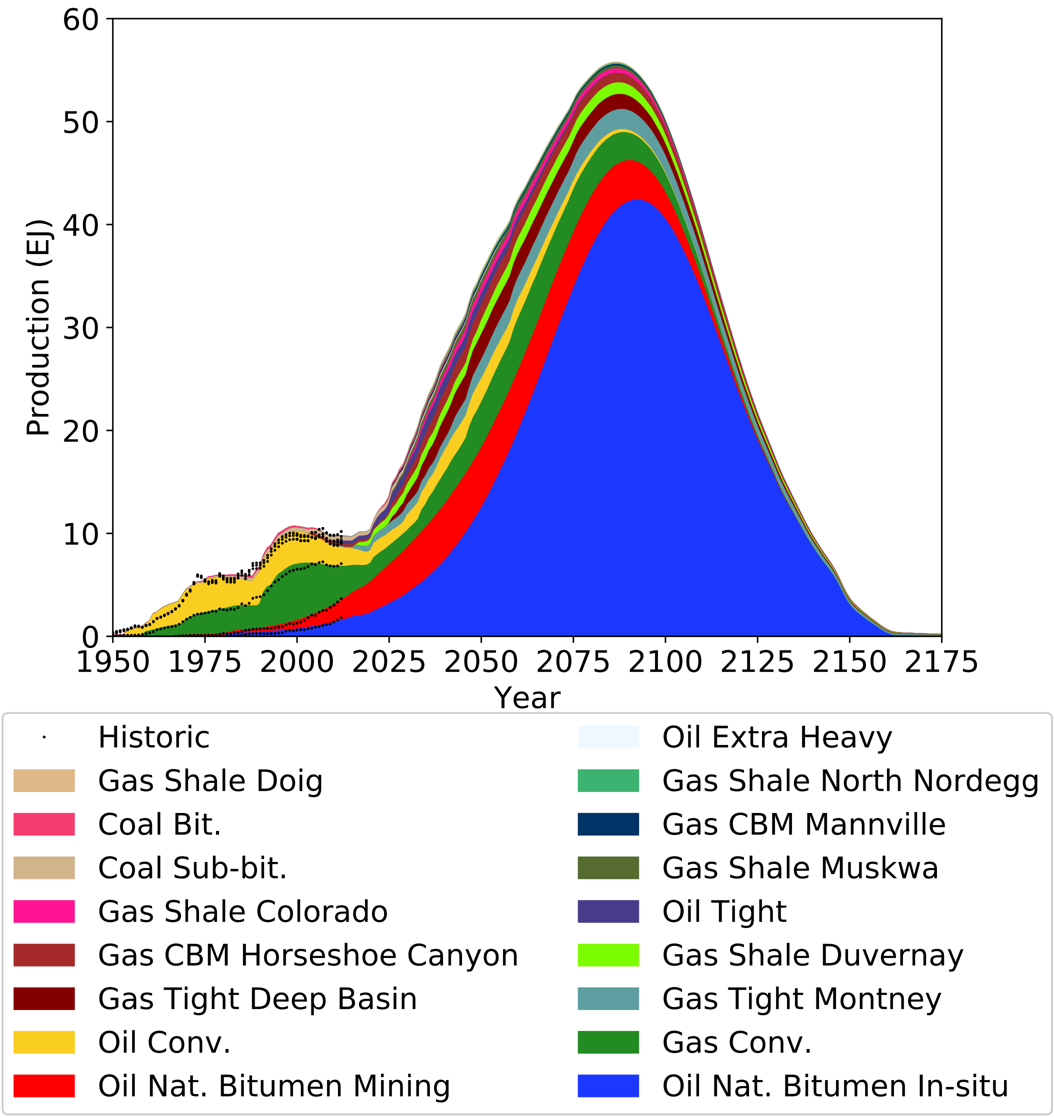

Figure 6.3: Canada - Alberta projections capped at 16

Table 6.3: Peak years - All

Name	URR	Peak Year	Peak Rate
Oil Nat. Bitumen Alberta In-situ	2811.21	2092	42.34
Oil Nat. Bitumen Alberta Mining	468.53	2055	5.89
Gas Conv. Alberta	467.07	1999	5.5
Oil Conv. Alberta	257.05	1995	3.34
Gas Tight Alberta Montney	187.12	2061	2.14
Gas Tight Alberta Deep Basin	162.75	2051	2.46
Gas Shale Alberta Duvernay	118.65	2063	1.51
Gas CBM Alberta Horseshoe Canyon	108.93	2048	1.58
Oil Tight Alberta	48.33	2037	1.26
Gas Shale Alberta Colorado	45.15	2053	0.7
Gas Shale Alberta Muskwa	32.55	2045	0.51
Coal Sub-bit. Alberta	29.87	2007	0.52
Gas CBM Alberta Mannville	18.23	2044	0.29
Coal Bit. Alberta	14.27	1990	0.25
Gas Shale Alberta North Nordegg	13.65	2046	0.23
Gas Shale Alberta Doig	13.13	2041	0.22
Oil Extra Heavy Alberta	0.02	2029	0.01
**Total**	**4796.51**	**2087**	**55.74**

Table 6.4: Peak years - Minerals

Name	URR	Peak Year	Peak Rate
Coal Bit.	14.27	1990	0.25
Coal Sub-bit.	29.87	2007	0.52
Oil Conv.	257.05	1995	3.34
Oil Tight	48.33	2037	1.26
Oil Nat. Bitumen	3279.74	2091	46.16
Oil Extra Heavy	0.02	2029	0.01
Gas Conv.	467.07	1999	5.5
Gas Shale	223.13	2051	3.03
Gas Tight	349.87	2057	4.51
Gas CBM	127.16	2048	1.86
**Total**	**4796.51**	**2087**	**55.74**

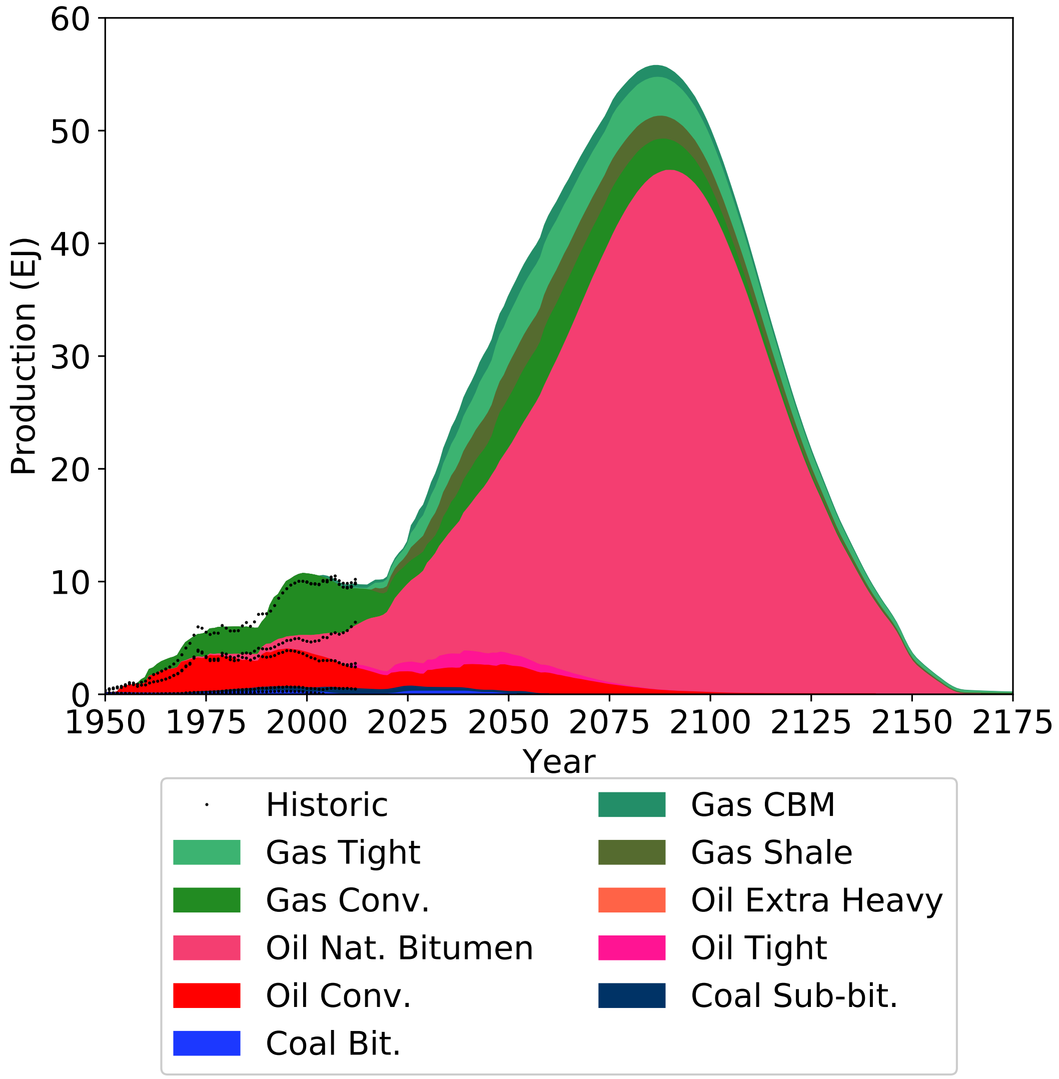

Figure 6.4: Canada - Alberta projection by mineral type

British Columbia

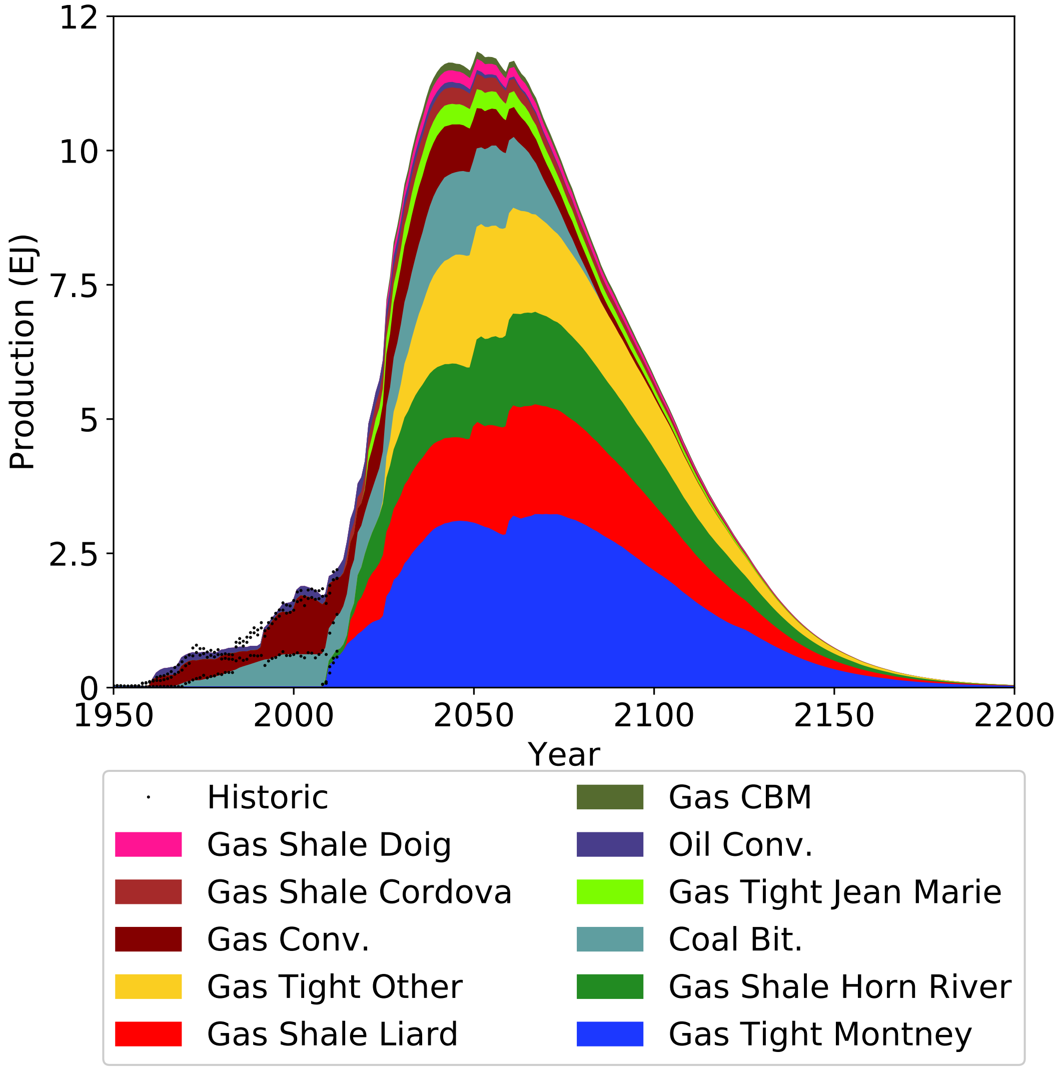

Figure 6.5: Canada - British Columbia projections capped at 16

Table 6.5: Peak years - All

Name	URR	Peak Year	Peak Rate
Gas Tight British Columbia Montney	285.99	2070	3.21
Gas Shale British Columbia Liard	165.9	2064	2.07
Gas Shale British Columbia Horn River	140.0	2065	1.74
Gas Tight British Columbia Other	138.6	2051	2.1
Coal Bit. British Columbia	94.22	2048	1.57
Gas Conv. British Columbia	77.35	2002	1.1
Gas Tight British Columbia Jean Marie	24.15	2036	0.41
Gas Shale British Columbia Cordova	21.11	2031	0.33
Oil Conv. British Columbia	14.4	2019	0.27
Gas Shale British Columbia Doig	13.13	2041	0.22
Gas CBM British Columbia	8.48	2040	0.15
**Total**	**983.33**	**2051**	**11.82**

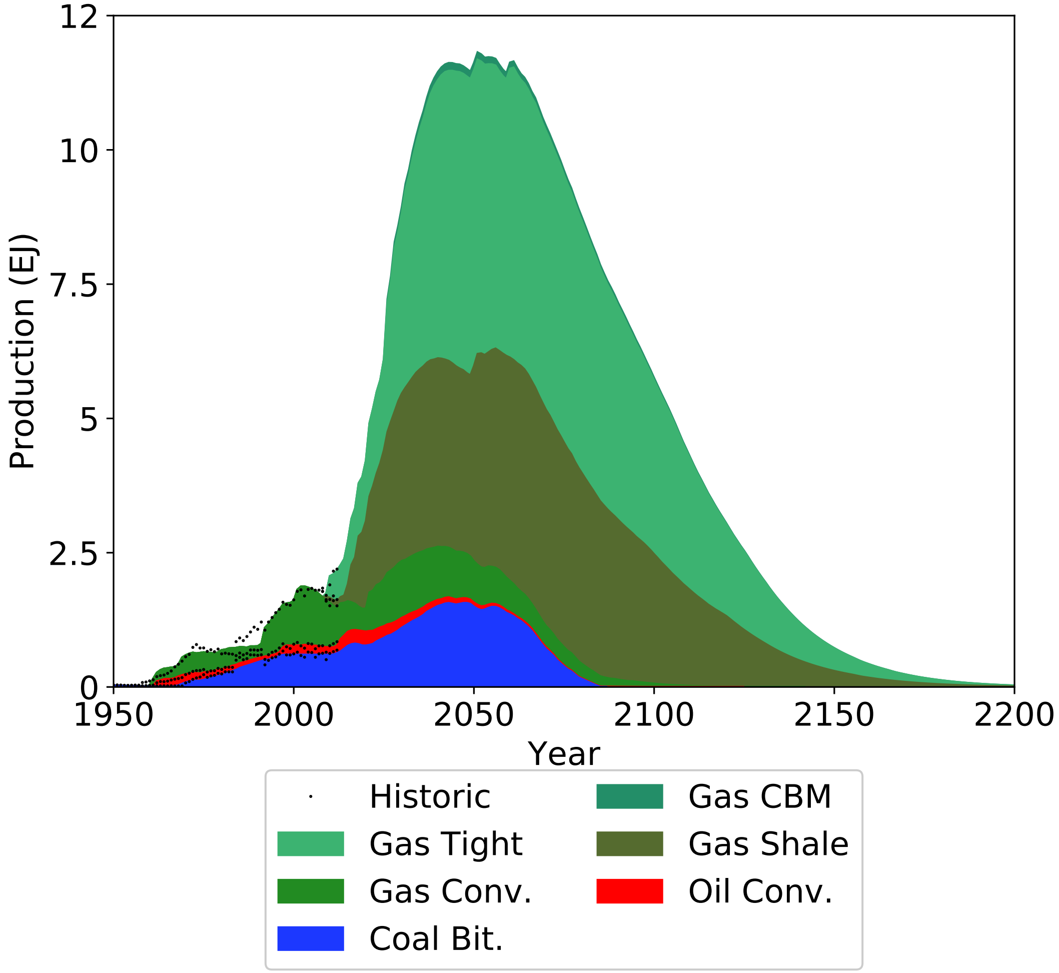

Figure 6.6: Canada - British Columbia projection by mineral type

Table 6.6: Peak years - Minerals

**Name**	**URR**	**Peak Year**	**Peak Rate**
Coal Bit.	94.22	2048	1.57
Oil Conv.	14.4	2019	0.27
Gas Conv.	77.35	2002	1.1
Gas Shale	340.14	2063	4.2
Gas Tight	448.74	2048	5.53
Gas CBM	8.48	2040	0.15
**Total**	**983.33**	**2051**	**11.82**

Canada

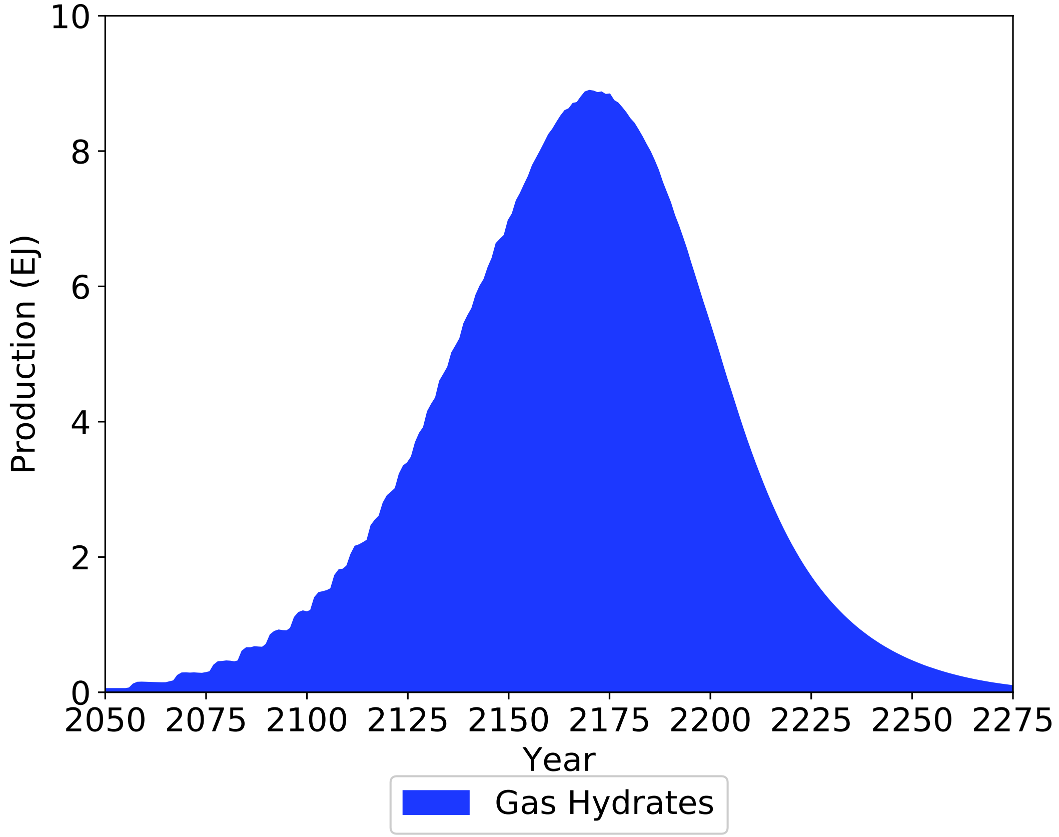

Figure 6.7: Canada - Canada projections capped at 16

Table 6.7: Peak years - All

**Name**	**URR**	**Peak Year**	**Peak Rate**
Gas Hydrates Canada	712.0	2170	8.89
**Total**	**712.0**	**2170**	**8.89**

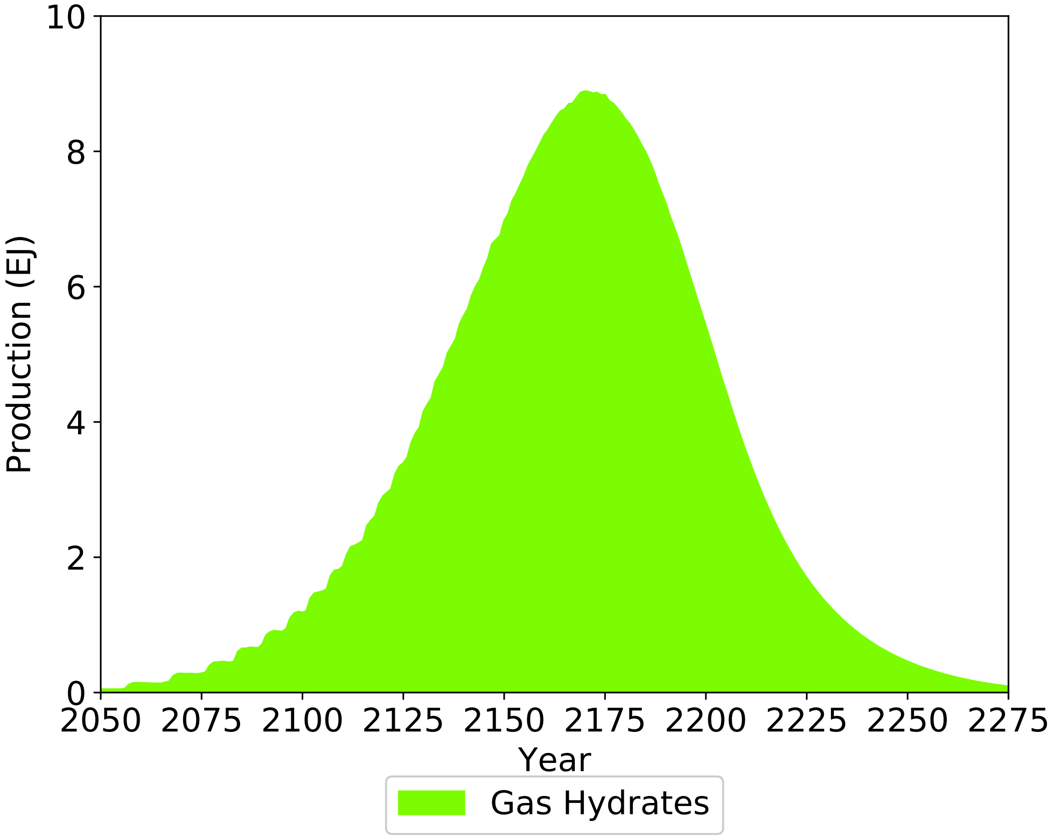

Figure 6.8: Canada - Canada projection by mineral type

Table 6.8: Peak years - Minerals

Name	URR	Peak Year	Peak Rate
Gas Hydrates	712.0	2170	8.89
**Total**	**712.0**	**2170**	**8.89**

East Coast Offshore

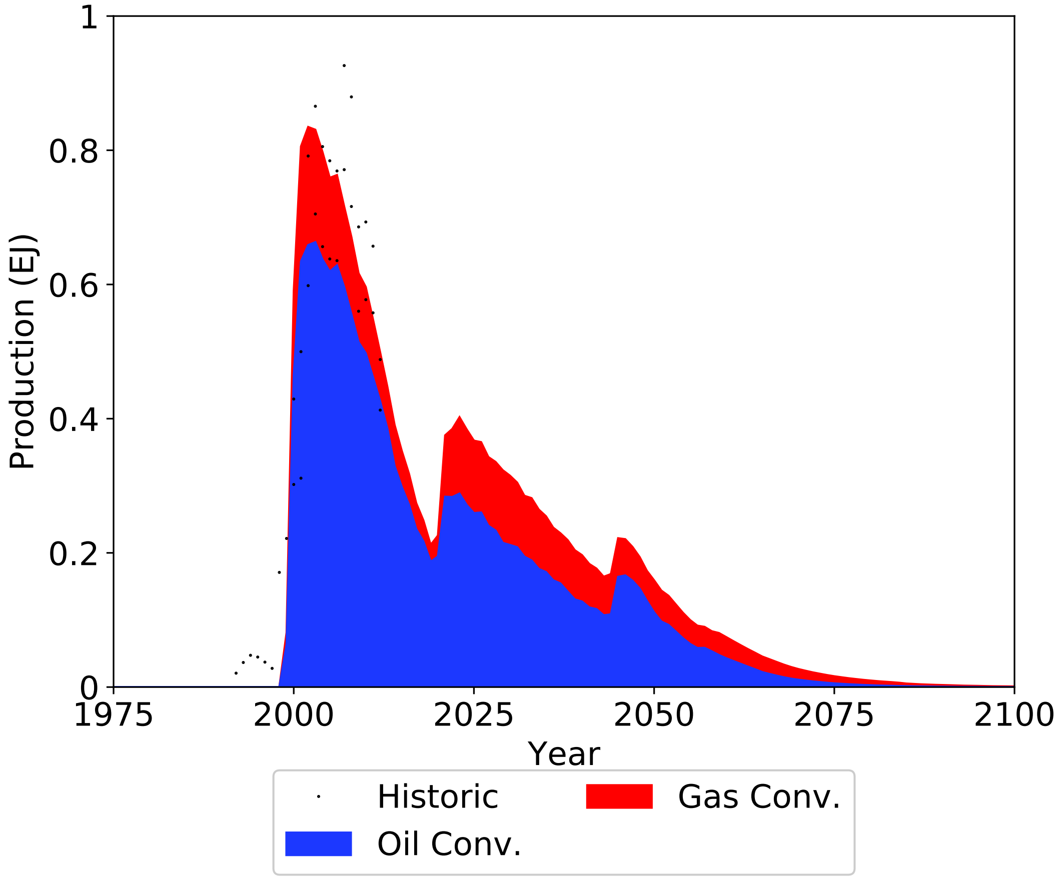

Figure 6.9: Canada - East Coast Offshore projections capped at 16

Table 6.9: Peak years - All

Name	URR	Peak Year	Peak Rate
Oil Conv. East Coast Offshore	16.04	2003	0.66
Gas Conv. East Coast Offshore	5.21	2002	0.18
**Total**	**21.25**	**2002**	**0.83**

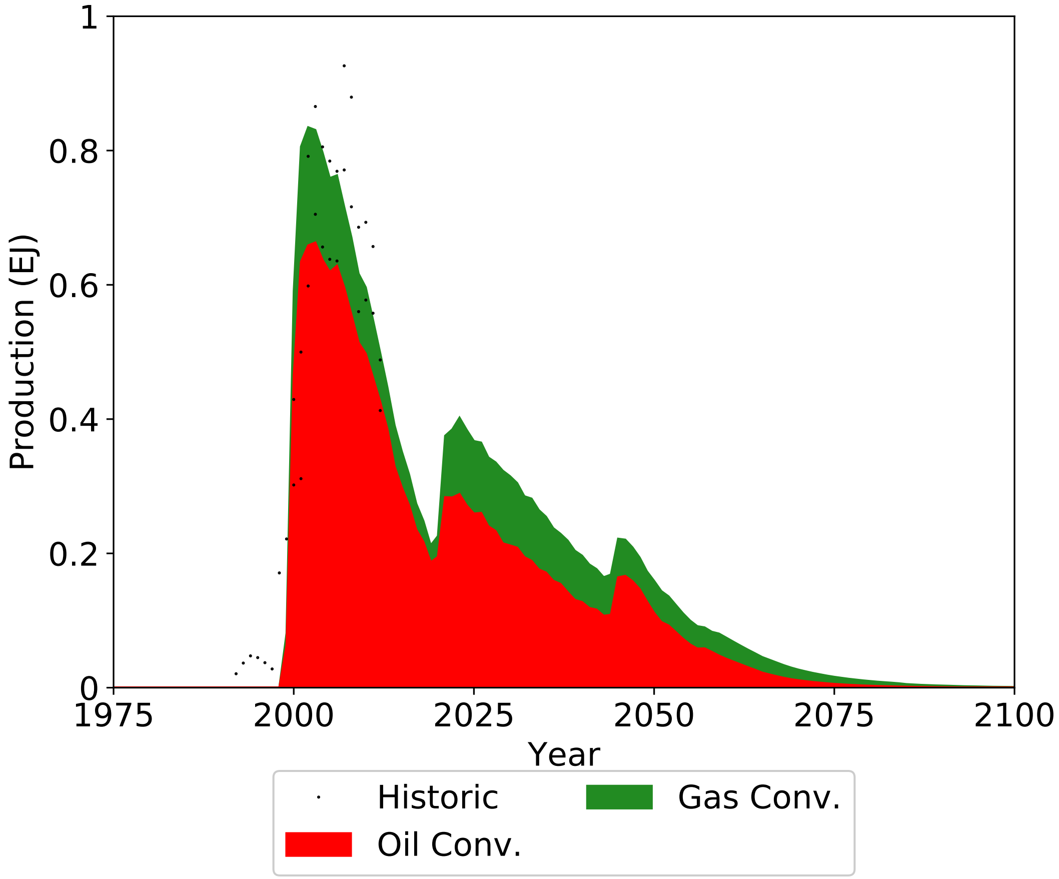

Figure 6.10: Canada - East Coast Offshore projection by mineral type

Table 6.10: Peak years - Minerals

Name	URR	Peak Year	Peak Rate
Oil Conv.	16.04	2003	0.66
Gas Conv.	5.21	2002	0.18
**Total**	**21.25**	**2002**	**0.83**

Manitoba

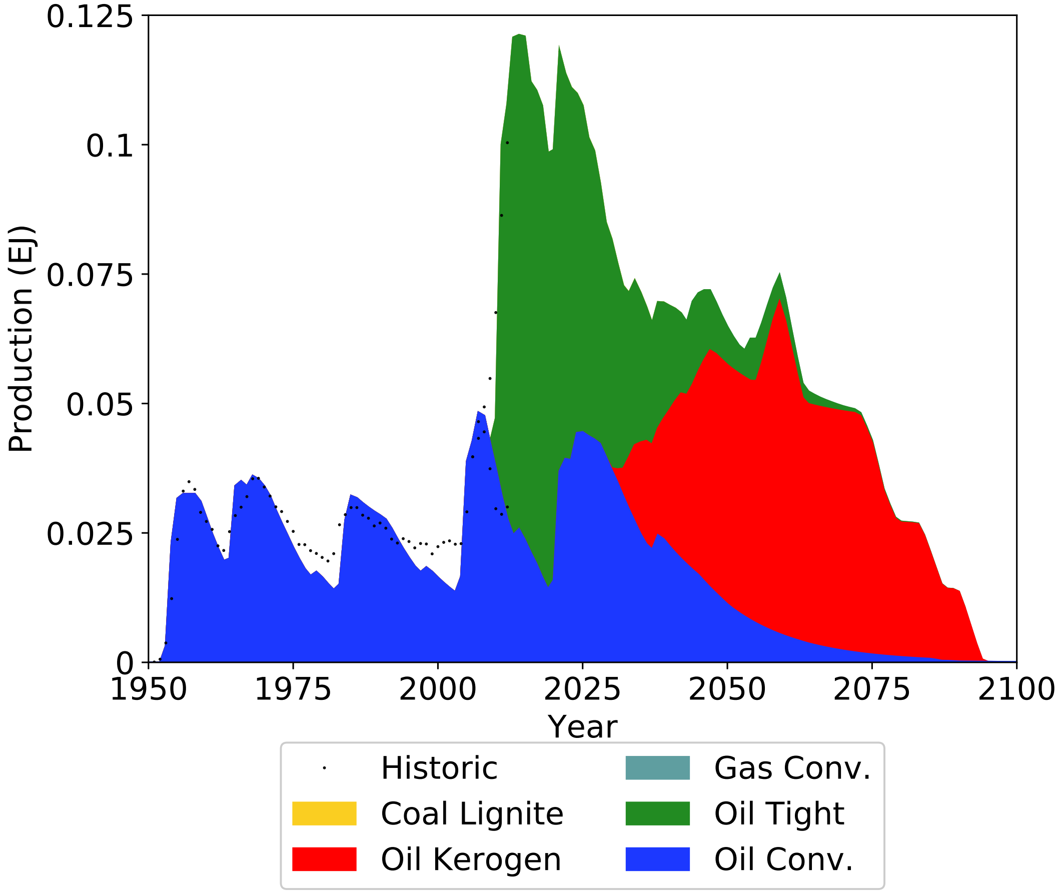

Figure 6.11: Canada - Manitoba projections capped at 16

Table 6.11: Peak years - All

Name	URR	Peak Year	Peak Rate
Oil Conv. Manitoba	2.7	2007	0.05
Oil Kerogen Manitoba	2.15	2059	0.06
Oil Tight Manitoba	2.0	2015	0.1
Coal Lignite Manitoba	–	2027	–
Gas Conv. Manitoba	–	1928	–
**Total**	**6.85**	**2014**	**0.12**

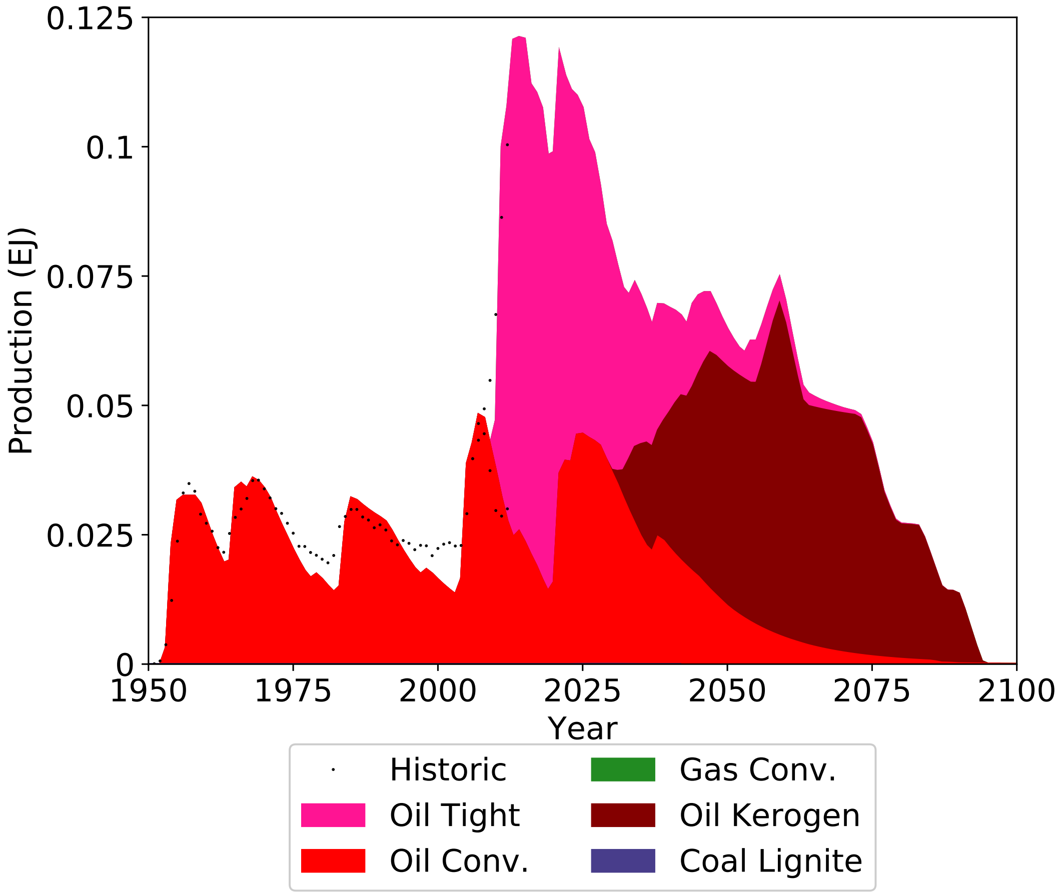

Figure 6.12: Canada - Manitoba projection by mineral type

Table 6.12: Peak years - Minerals

Name	URR	Peak Year	Peak Rate
Coal Lignite	–	2027	–
Oil Conv.	2.7	2007	0.05
Oil Kerogen	2.15	2059	0.06
Oil Tight	2.0	2015	0.1
Gas Conv.	–	1928	–
**Total**	**6.85**	**2014**	**0.12**

New Brunswick

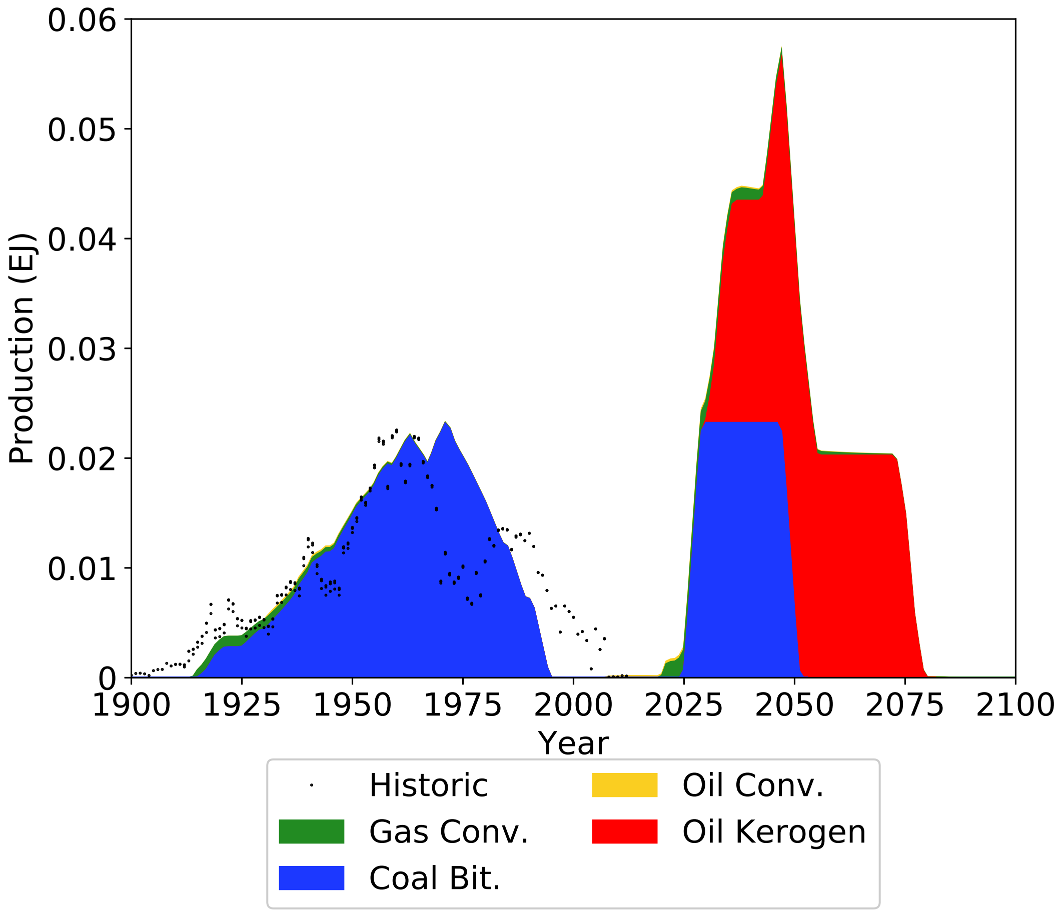

Figure 6.13: Canada - New Brunswick projections capped at 16

Table 6.13: Peak years - All

Name	URR	Peak Year	Peak Rate
Coal Bit. New Brunswick	1.45	2030	0.02
Oil Kerogen New Brunswick	0.98	2048	0.03
Gas Conv. New Brunswick	0.07	2025	–
Oil Conv. New Brunswick	0.01	2024	–
**Total**	**2.51**	**2047**	**0.06**

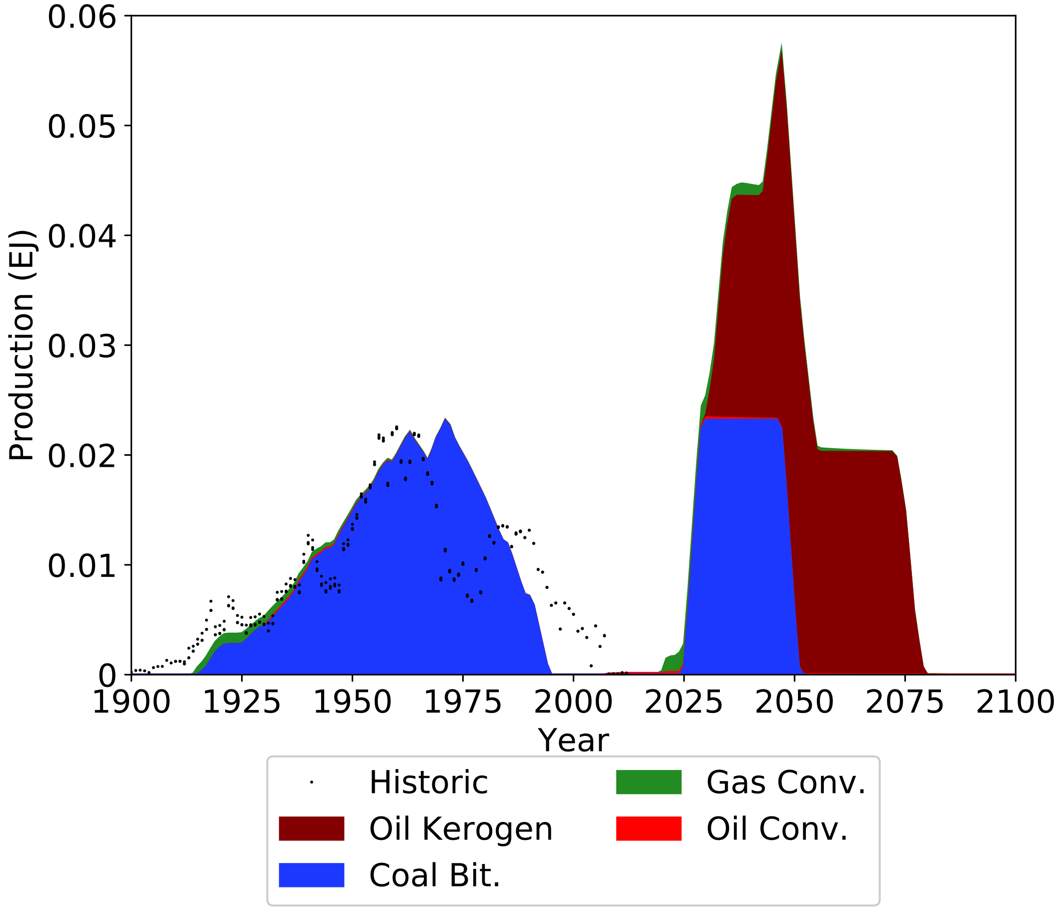

Figure 6.14: Canada - New Brunswick projection by mineral type

Table 6.14: Peak years - Minerals

Name	URR	Peak Year	Peak Rate
Coal Bit.	1.45	2030	0.02
Oil Conv.	0.01	2024	–
Oil Kerogen	0.98	2048	0.03
Gas Conv.	0.07	2025	–
**Total**	**2.51**	**2047**	**0.06**

Northwest Territories

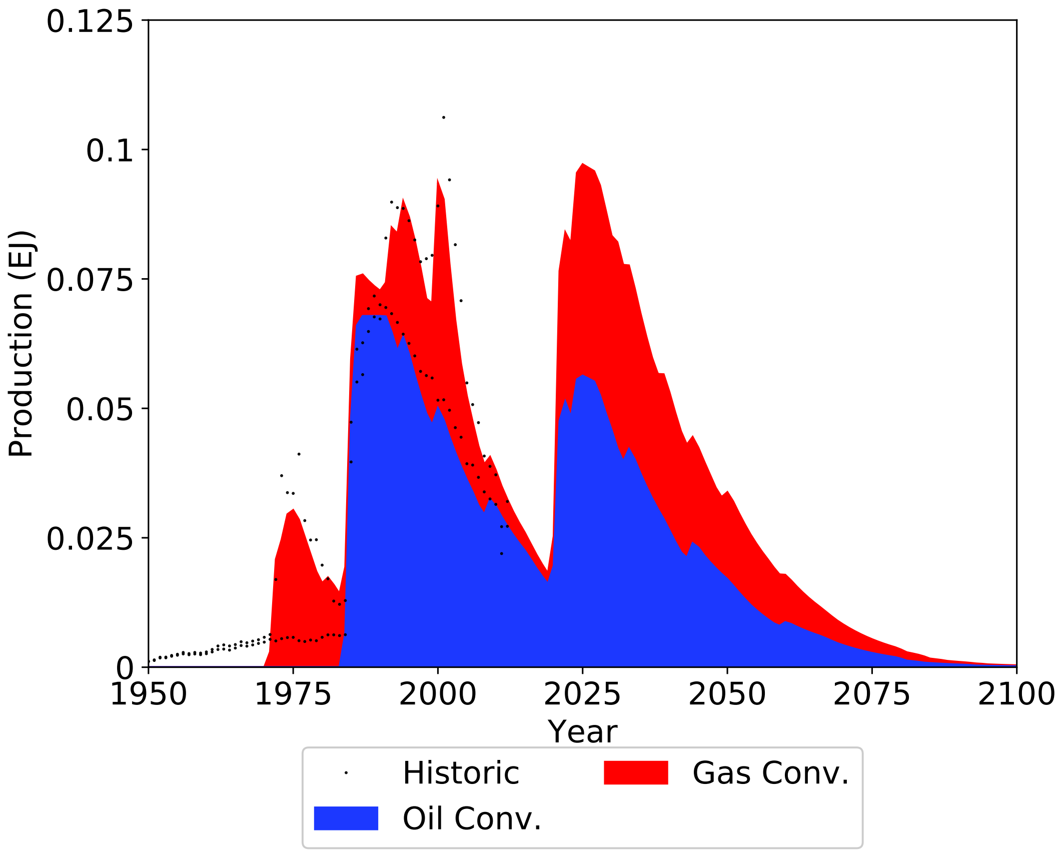

Figure 6.15: Canada - Northwest Territories projections capped at 16

Table 6.15: Peak years - All

Name	URR	Peak Year	Peak Rate
Oil Conv. Northwest Territories	2.85	1987	0.07
Gas Conv. Northwest Territories	1.9	2000	0.04
**Total**	**4.75**	**2025**	**0.1**

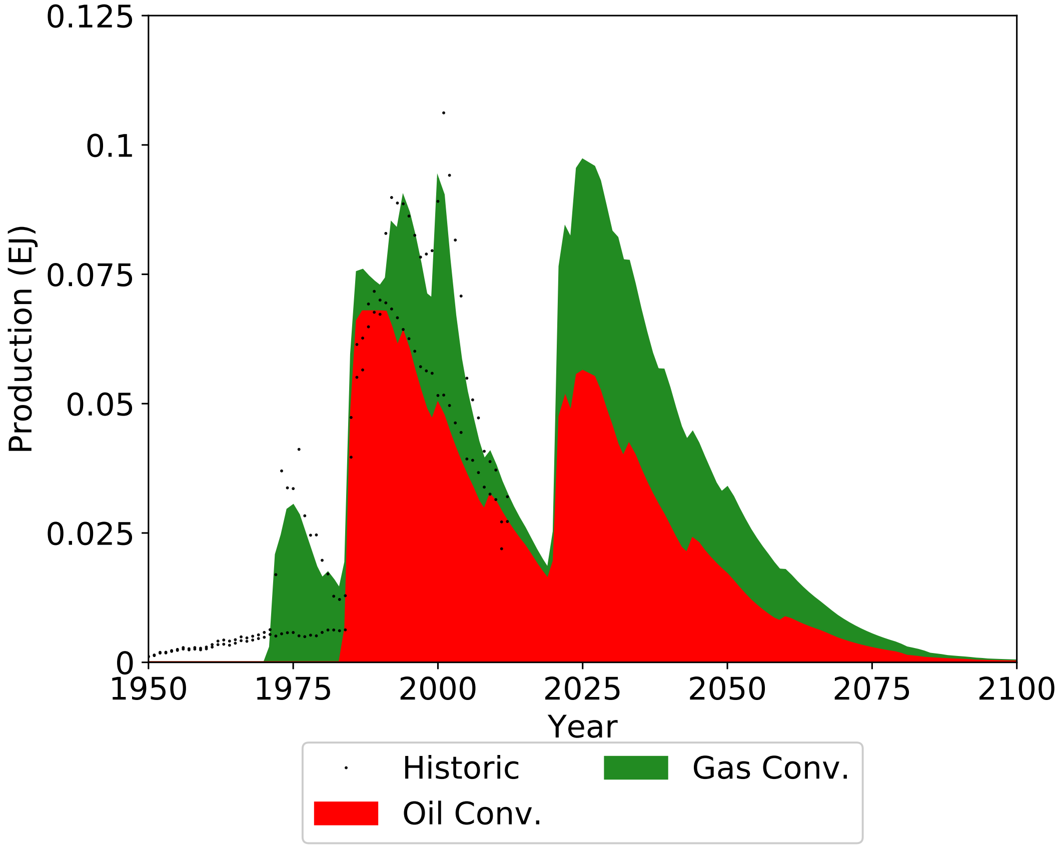

Figure 6.16: Canada - Northwest Territories projection by mineral type

Table 6.16: Peak years - Minerals

**Name**	**URR**	**Peak Year**	**Peak Rate**
Oil Conv.	2.85	1987	0.07
Gas Conv.	1.9	2000	0.04
**Total**	**4.75**	**2025**	**0.1**

Nova Scotia

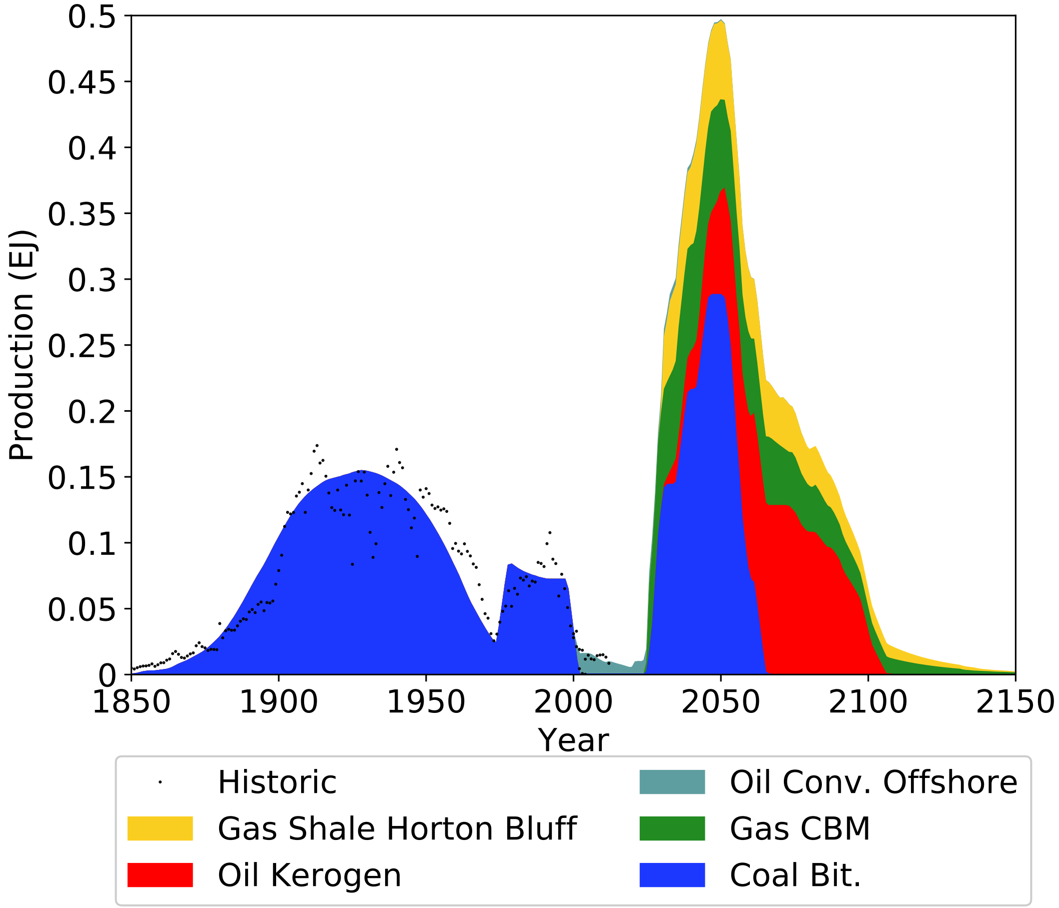

Figure 6.17: Canada - Nova Scotia projections capped at 16

Table 6.17: Peak years - All

Name	URR	Peak Year	Peak Rate
Coal Bit. Nova Scotia	18.66	2048	0.29
Oil Kerogen Nova Scotia	5.86	2062	0.13
Gas CBM Nova Scotia	4.24	2037	0.08
Gas Shale Nova Scotia Horton Bluff	3.15	2042	0.07
Oil Conv. Nova Scotia Offshore	0.36	2003	0.02
**Total**	**32.27**	**2050**	**0.5**

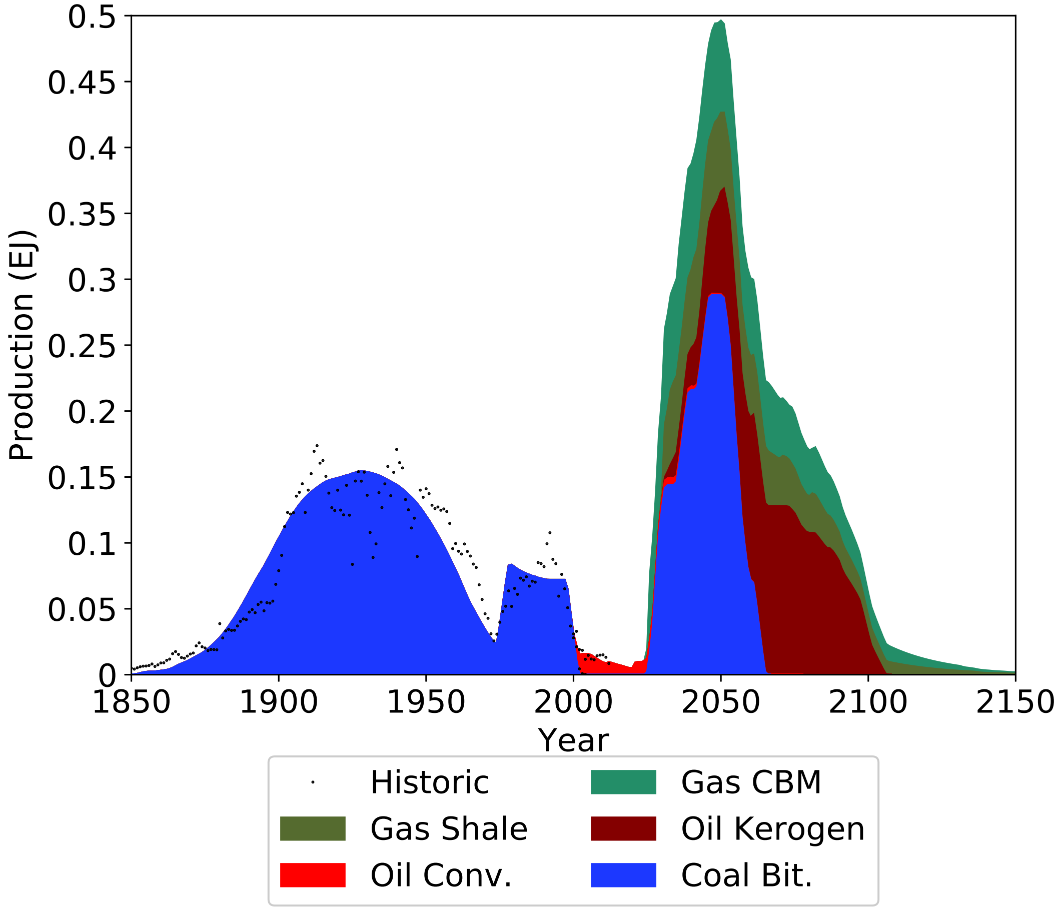

Figure 6.18: Canada - Nova Scotia projection by mineral type

Table 6.18: Peak years - Minerals

Name	URR	Peak Year	Peak Rate
Coal Bit.	18.66	2048	0.29
Oil Conv.	0.36	2003	0.02
Oil Kerogen	5.86	2062	0.13
Gas Shale	3.15	2042	0.07
Gas CBM	4.24	2037	0.08
**Total**	**32.27**	**2050**	**0.5**

Ontario

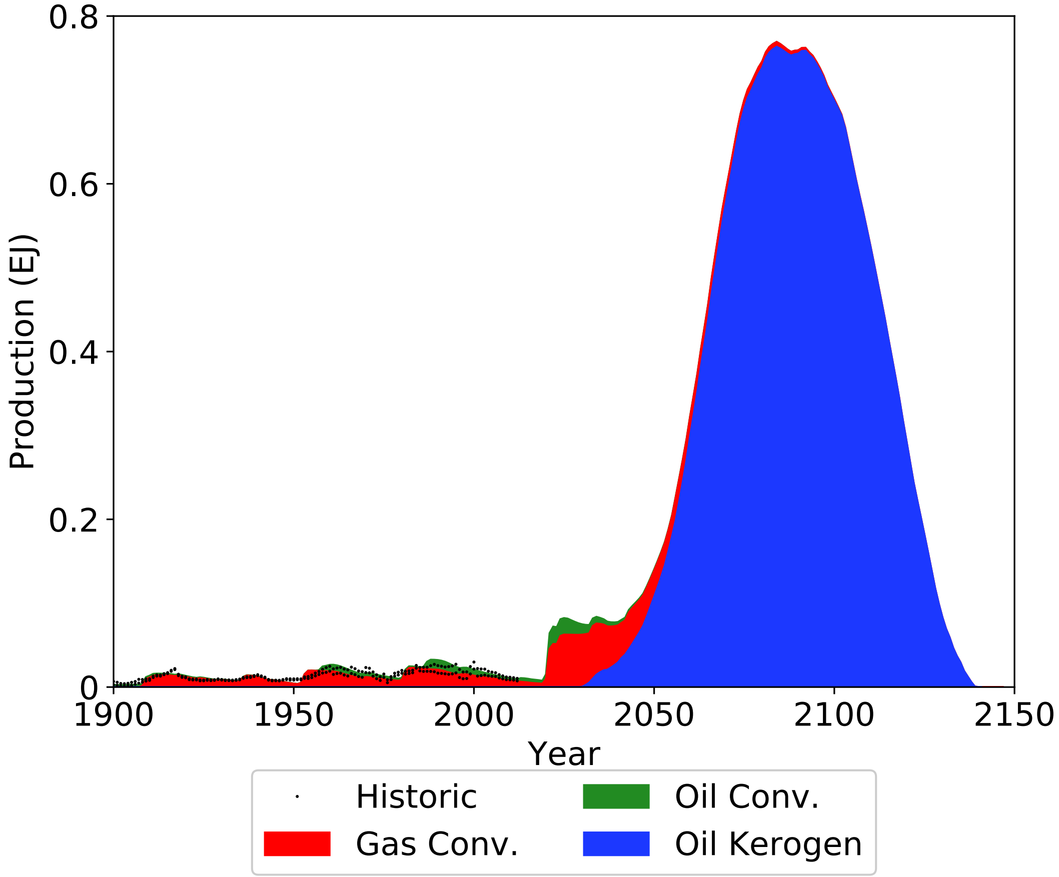

Figure 6.19: Canada - Ontario projections capped at 16

Table 6.19: Peak years - All

**Name**	**URR**	**Peak Year**	**Peak Rate**
Oil Kerogen Ontario	41.26	2084	0.76
Gas Conv. Ontario	3.51	2033	0.06
Oil Conv. Ontario	0.79	2022	0.02
**Total**	**45.56**	**2084**	**0.77**

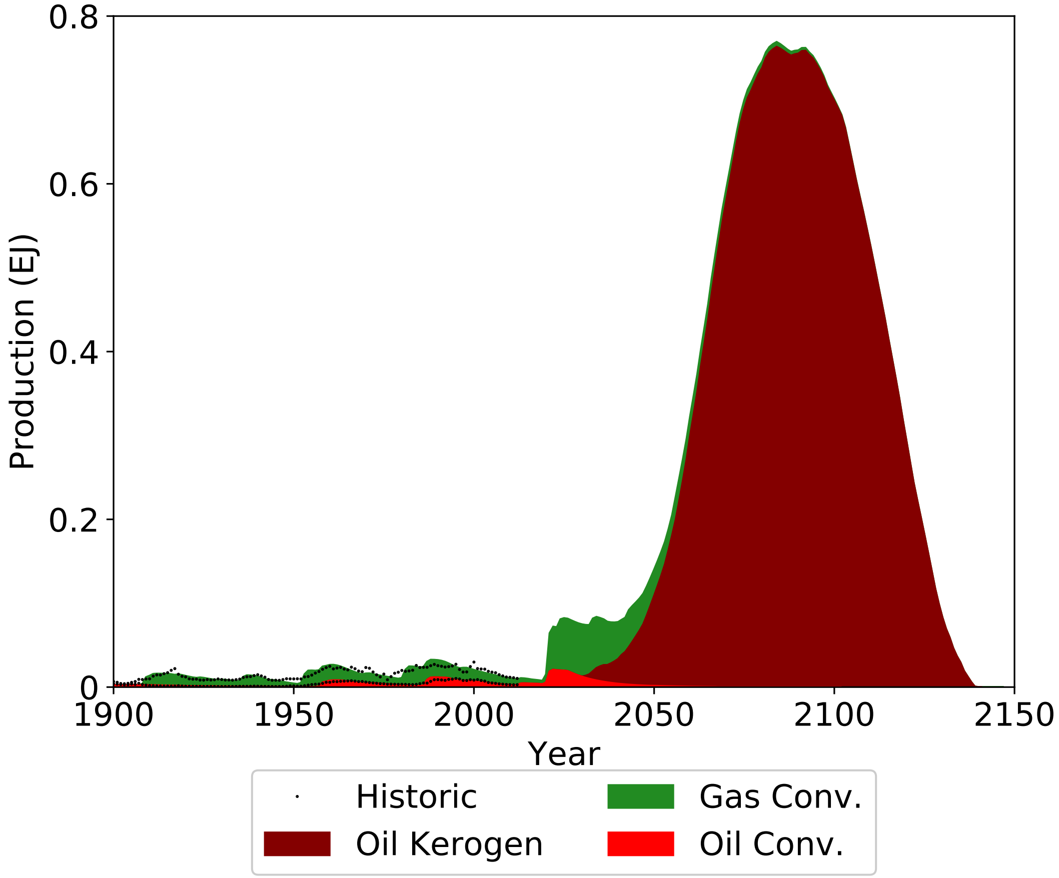

Figure 6.20: Canada - Ontario projection by mineral type

Table 6.20: Peak years - Minerals

Name	URR	Peak Year	Peak Rate
Oil Conv.	0.79	2022	0.02
Oil Kerogen	41.26	2084	0.76
Gas Conv.	3.51	2033	0.06
**Total**	**45.56**	**2084**	**0.77**

Quebec

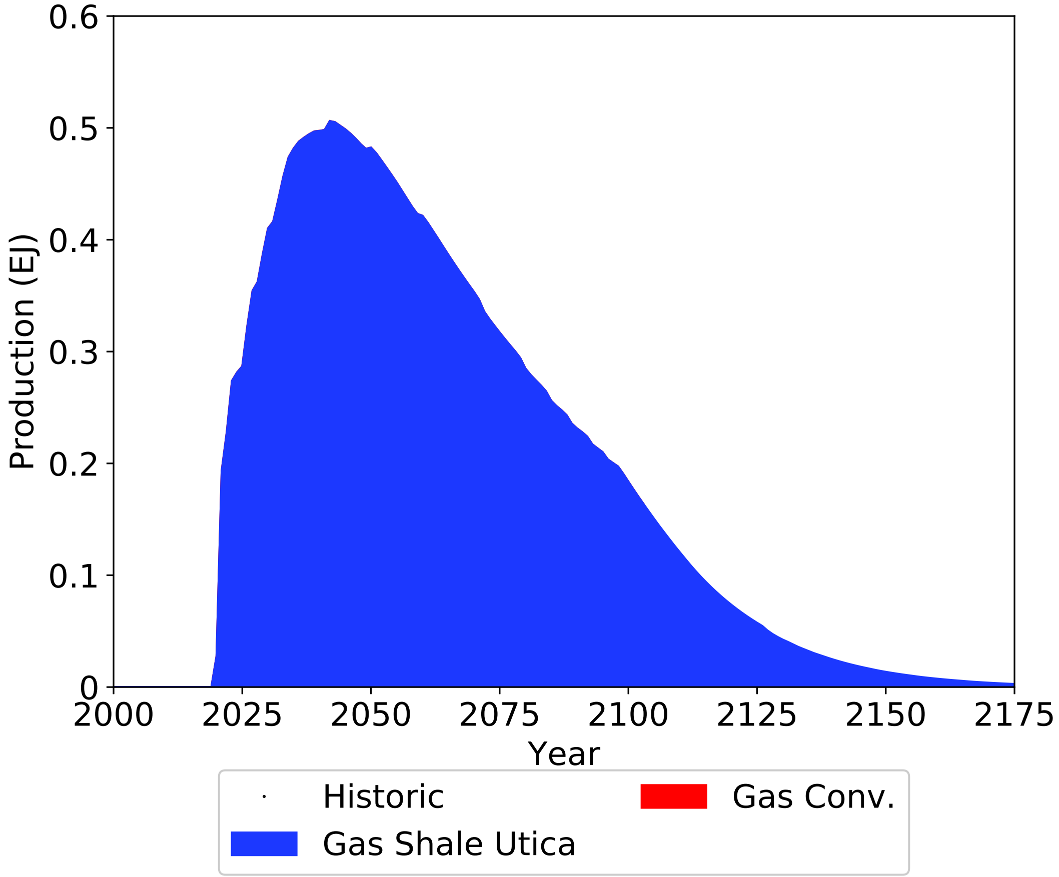

Figure 6.21: Canada - Quebec projections capped at 16

Table 6.21: Peak years - All

**Name**	**URR**	**Peak Year**	**Peak Rate**
Gas Shale Quebec Utica	32.55	2042	0.51
Gas Conv. Quebec	0.01	2025	–
**Total**	**32.56**	**2042**	**0.51**

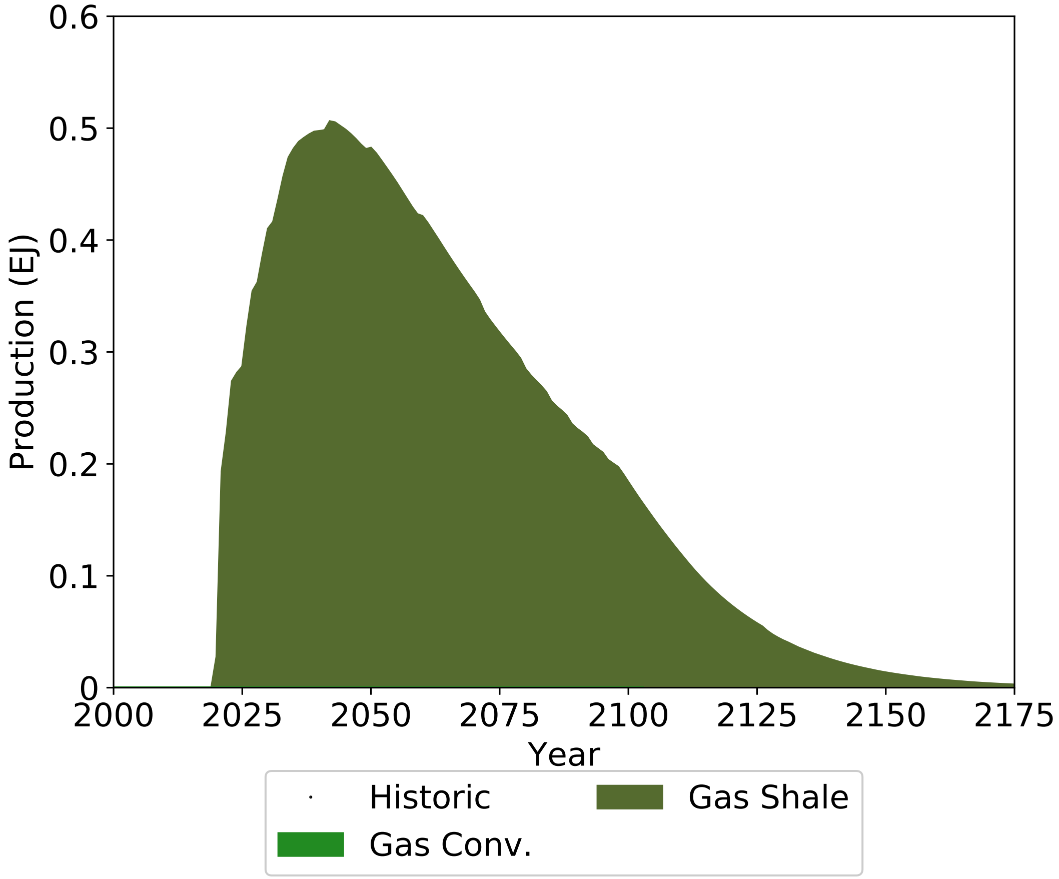

Figure 6.22: Canada - Quebec projection by mineral type

Table 6.22: Peak years - Minerals

Name	URR	Peak Year	Peak Rate
Gas Conv.	0.01	2025	–
Gas Shale	32.55	2042	0.51
**Total**	**32.56**	**2042**	**0.51**

Saskatchewan

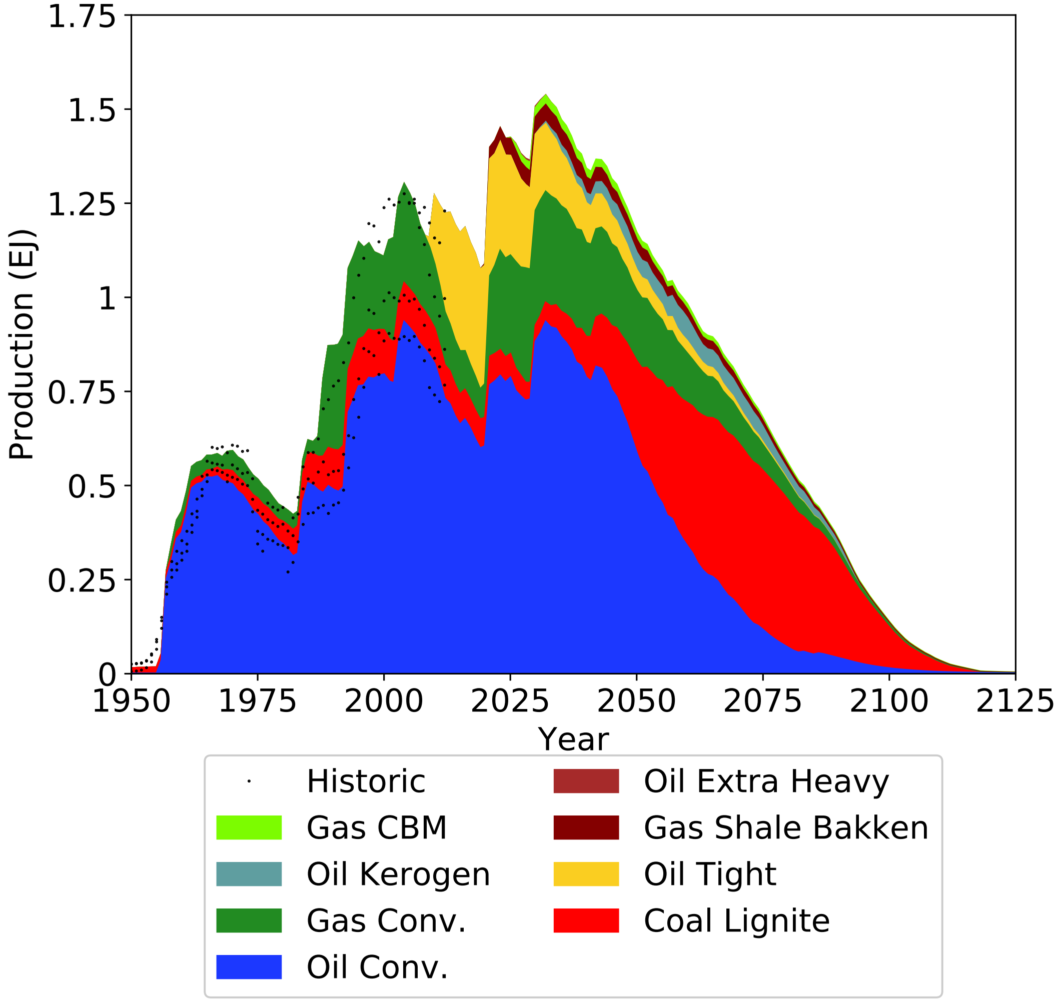

Figure 6.23: Canada - Saskatchewan projections capped at 16

Table 6.23: Peak years - All

**Name**	**URR**	**Peak Year**	**Peak Rate**
Oil Conv. Saskatchewan	69.92	2032	0.93
Coal Lignite Saskatchewan	25.43	2070	0.43
Gas Conv. Saskatchewan	19.34	2031	0.31
Oil Tight Saskatchewan	8.68	2016	0.33
Oil Kerogen Saskatchewan	2.15	2059	0.06
Gas Shale Saskatchewan Bakken	2.1	2037	0.05
Gas CBM Saskatchewan	1.06	2030	0.02
Oil Extra Heavy Saskatchewan	0.02	2029	0.01
**Total**	**128.7**	**2032**	**1.54**

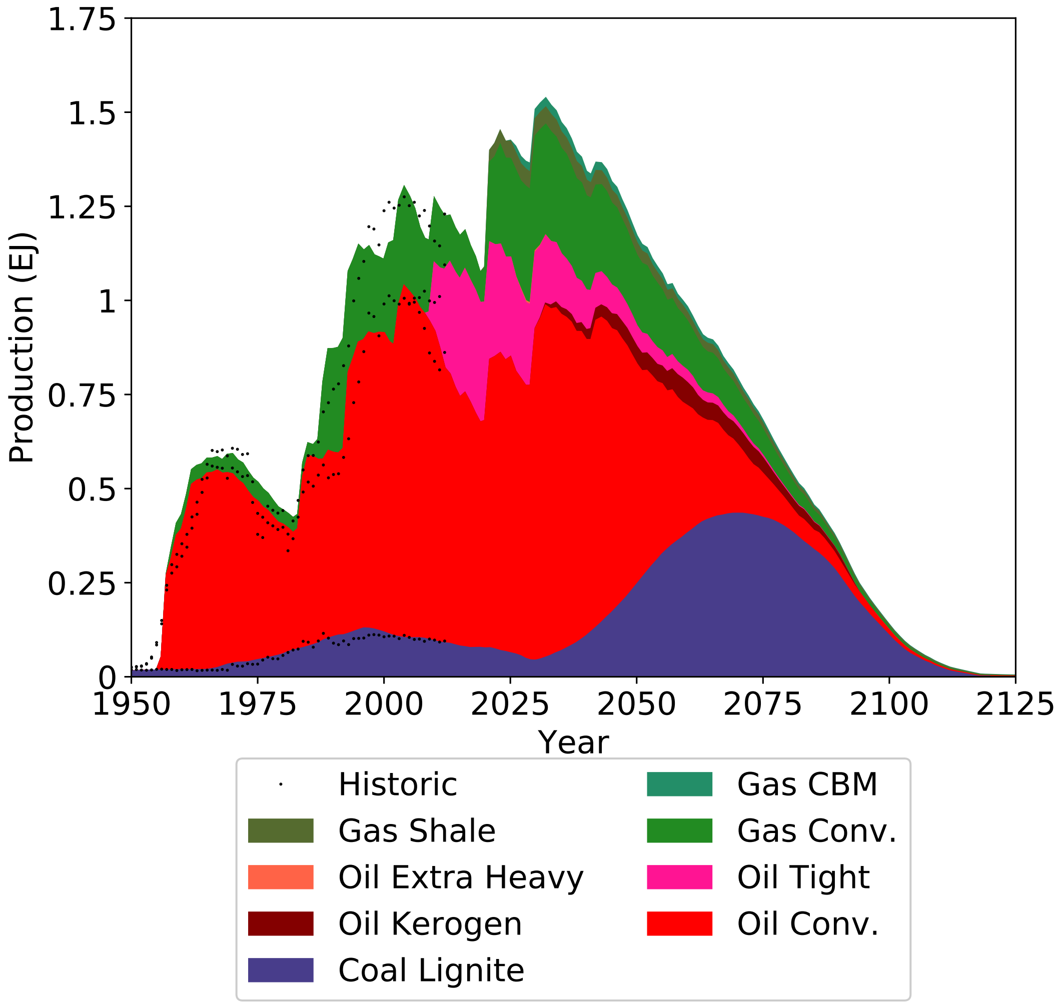

Figure 6.24: Canada - Saskatchewan projection by mineral type

Table 6.24: Peak years - Minerals

**Name**	**URR**	**Peak Year**	**Peak Rate**
Coal Lignite	25.43	2070	0.43
Oil Conv.	69.92	2032	0.93
Oil Kerogen	2.15	2059	0.06
Oil Tight	8.68	2016	0.33
Oil Extra Heavy	0.02	2029	0.01
Gas Conv.	19.34	2031	0.31
Gas Shale	2.1	2037	0.05
Gas CBM	1.06	2030	0.02
**Total**	**128.7**	**2032**	**1.54**

Yukon

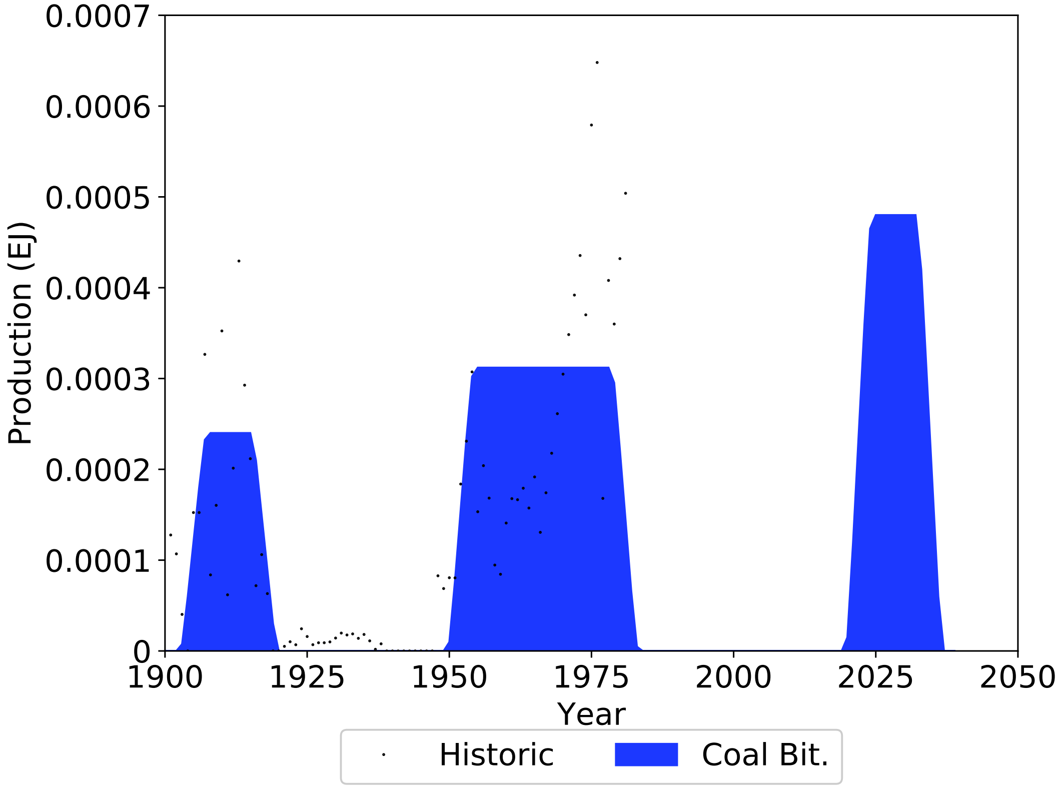

Figure 6.25: Canada - Yukon projections capped at 16

Table 6.25: Peak years - All

Name	URR	Peak Year	Peak Rate
Coal Bit. Yukon	0.02	2025	–
**Total**	**0.02**	**2025**	–

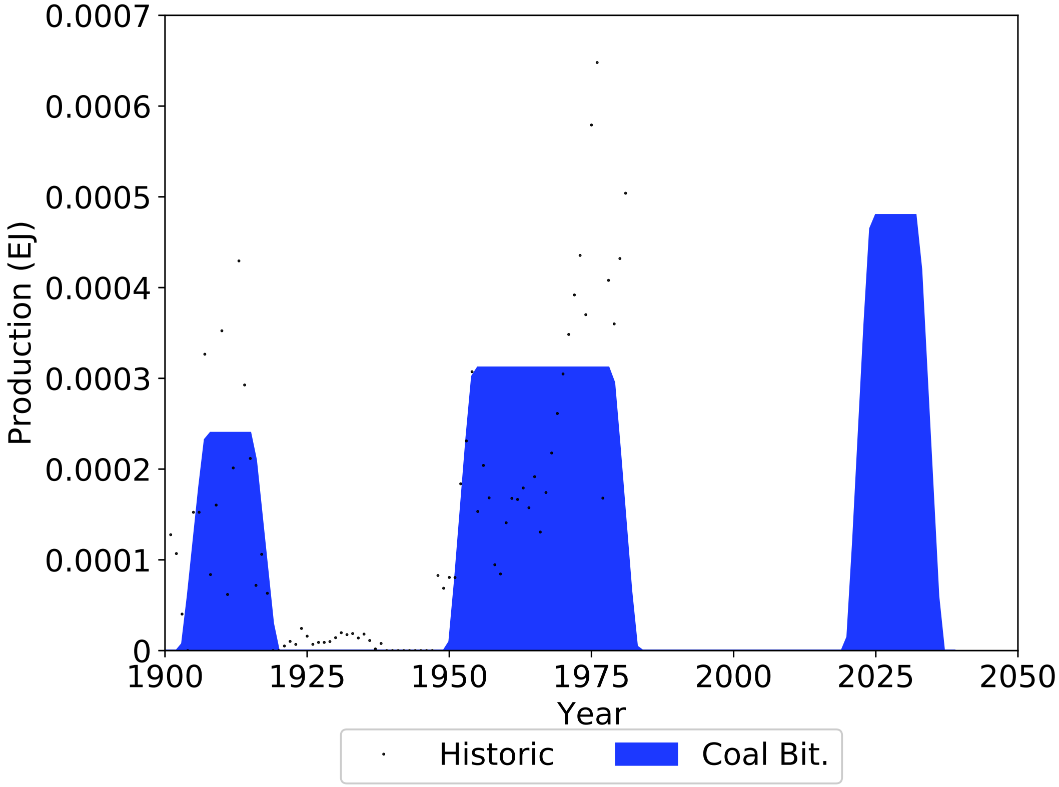

Figure 6.26: Canada - Yukon projection by mineral type

Table 6.26: Peak years - Minerals

Name	URR	Peak Year	Peak Rate
Coal Bit.	0.02	2025	–
**Total**	**0.02**	**2025**	–

6.1.4 Projection by region

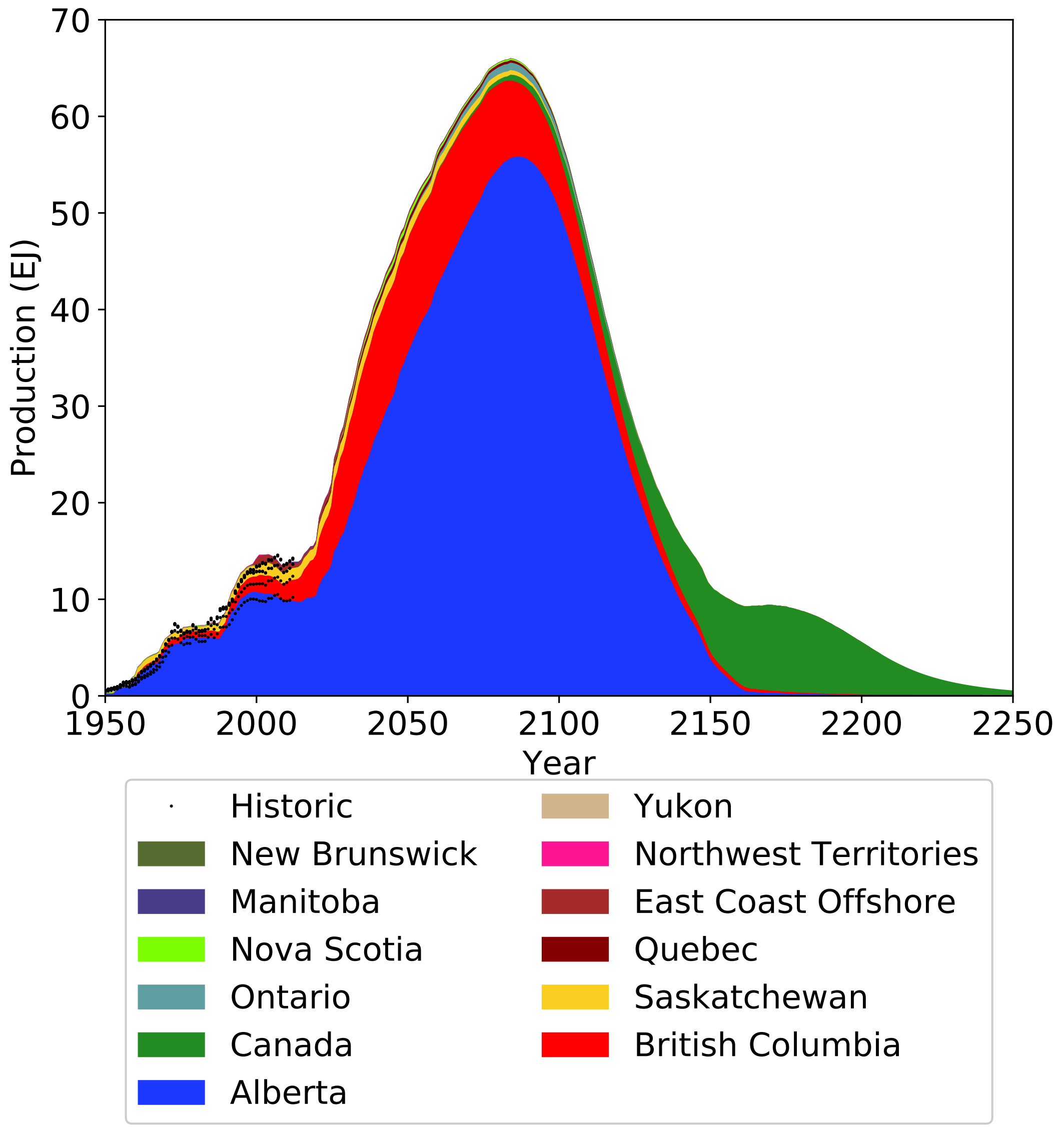

Figure 6.27: Canada by region projections capped at 16

Table 6.27: Peak years - All

**Name**	**URR**	**Peak Year**	**Peak Rate**
Alberta	4796.51	2087	55.74
British Columbia	983.33	2051	11.82
Canada	712.0	2170	8.89
Saskatchewan	128.7	2032	1.54
Ontario	45.56	2084	0.77
Quebec	32.56	2042	0.51
Nova Scotia	32.27	2050	0.5
East Coast Offshore	21.25	2002	0.83
Manitoba	6.85	2014	0.12
Northwest Territories	4.75	2025	0.1
New Brunswick	2.51	2047	0.06
Yukon	0.02	2025	–
**Total**	**6766.31**	**2084**	**65.92**

6.2 USA

6.2.1 All Projections

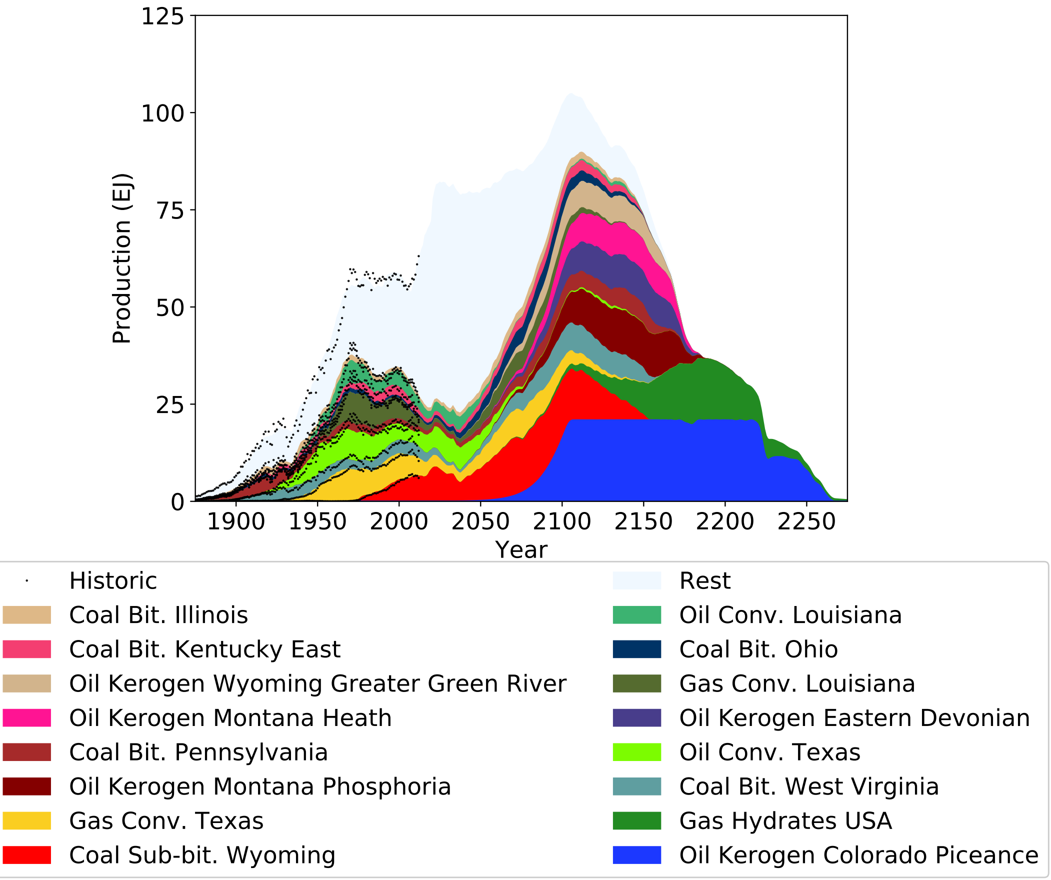

Figure 6.28: USA projections capped at 16

Table 6.28: Peak years - All

Name	URR	Peak Year	Peak Rate
Oil Kerogen Colorado Piceance	3101.7	2155	20.91
Coal Sub-bit. Wyoming	1489.93	2086	15.45
Gas Hydrates USA	1229.0	2185	15.8
Gas Conv. Texas	921.15	1968	8.26
Coal Bit. West Virginia	896.76	2112	7.23
Oil Kerogen Montana Phosphoria	859.5	2136	11.39
Oil Conv. Texas	731.08	1968	7.7
Coal Bit. Pennsylvania	699.56	2137	5.43
Oil Kerogen Eastern Devonian	649.8	2136	8.59
Oil Kerogen Montana Heath	618.8	2136	8.2
Gas Conv. Louisiana	602.93	1974	8.12
Oil Kerogen Wyoming Greater Green River	523.2	2119	6.81
Coal Bit. Ohio	414.76	2082	4.26
Coal Bit. Kentucky East	404.29	2099	2.93
Oil Conv. Louisiana	389.88	1971	5.91
Coal Bit. Illinois	349.04	2066	2.47
Oil Kerogen Utah Unita	336.3	2112	4.42
Coal Bit. Indiana	305.37	2070	2.35
Gas Shale Pennsylvania Marcellus	294.39	2030	5.55
Gas Conv. Oklahoma	251.13	2035	4.31
Oil Conv. California	240.2	1967	2.35
Coal Sub-bit. Montana	193.17	2066	2.38
Coal Bit. Kentucky West	180.45	2045	2.58
Gas Shale Louisiana Haynesville	175.11	2023	4.74
Coal Lignite Texas	170.81	2072	2.79
Oil Conv. Oklahoma	156.34	1965	1.35
Coal Anthr. Pennsylvania	151.2	1919	2.66
Oil Conv. Alaska	144.15	1986	4.1
Coal Bit. Virginia	143.49	2150	1.13
Gas Conv. New Mexico	142.55	2063	1.3
Coal Bit. Alabama	141.58	2077	1.72
Gas Shale Texas Eagle Ford	126.25	2026	2.92
Coal Lignite North Dakota	118.29	2081	1.87
Gas Conv. Colorado	110.24	2021	1.31
Gas Conv. Wyoming	108.86	2011	1.68
Oil Conv. New Mexico	100.05	2028	1.17
Gas Shale West Virginia Marcellus	97.52	2035	1.44
Oil Conv. Wyoming	92.73	1970	0.89
Gas Shale Texas Barnett	88.36	2020	2.24
Gas Shale Oklahoma Woodford	82.9	2034	1.03
Gas Conv. Kansas	82.45	1968	0.87
Gas Conv. California	77.75	2058	0.79
Oil Conv. Kansas	69.8	1953	0.8
Oil Conv. Colorado	69.4	2025	0.91
Coal Sub-bit. New Mexico	68.57	2057	1.54
Coal Bit. Colorado	67.33	2039	1.29
Coal Bit. Utah	62.29	2063	0.83
Gas Shale Texas Haynesville	61.8	2035	0.78
Gas Shale Colorado Niobrara	59.86	2036	0.9

Table 6.28: Peak years - All – Continued

**Name**	**URR**	**Peak Year**	**Peak Rate**
Gas Shale Ohio Utica	58.28	2038	0.88
Gas Shale New York Utica	58.28	2041	0.89
Gas CBM New Mexico	56.28	2038	0.64
Gas CBM Colorado	55.8	2031	0.86
Gas Conv. Pennsylvania	55.12	2040	0.8
Gas Shale Arkansas Fayetteville	54.17	2020	1.49
Gas Conv. Alaska	44.44	1997	0.53
Oil Tight North Dakota Bakken	43.91	2019	1.68
Gas Conv. West Virginia	41.11	2031	0.52
Oil Tight Texas Eagle Ford	39.84	2019	1.57
Gas CBM Virginia	39.4	2041	0.59
Coal Bit. Tennessee	37.06	2088	0.31
Gas Conv. Utah	36.75	2026	0.58
Coal Bit. New Mexico	35.07	2035	0.84
Coal Sub-bit. Colorado	34.6	2073	0.57
Coal Bit. Arizona	33.7	2050	0.59
Gas CBM Wyoming	32.61	2023	0.65
Oil Tight Texas Permian	30.68	2017	1.23
Gas Shale New York Other	30.45	2040	0.48
Gas Shale Texas Permian	28.68	2021	0.73
Gas Conv. Kentucky	27.56	2057	0.3
Oil Conv. Illinois	26.62	1953	0.49
Oil Conv. North Dakota	25.2	2024	0.33
Oil Conv. Mississippi	24.33	1963	0.37
Coal Sub-bit. Washington	22.91	2061	0.41
Coal Bit. Maryland	22.19	2076	0.21
Coal Bit. Missouri	21.97	2048	0.23
Oil Conv. Utah	20.82	2023	0.36
Gas Conv. Ohio	19.23	2038	0.26
Gas Conv. Arkansas	18.98	1992	0.23
Coal Bit. Iowa	18.76	2071	0.22
Coal Lignite Louisiana	18.25	2056	0.49
Oil Nat. Bitumen Utah	17.91	2070	0.28
Gas Conv. Mississippi	17.76	2040	0.21
Coal Bit. Oklahoma	17.56	2048	0.23
Gas Conv. Alabama	16.83	1996	0.48
Coal Bit. Kansas	15.31	2071	0.14
Gas Shale North Dakota Bakken	15.24	2027	0.21
Oil Conv. Montana	15.15	2005	0.23
Gas CBM Alabama	14.4	2032	0.26
Oil Conv. Arkansas	14.07	1923	0.4
Oil Conv. Michigan	13.77	1979	0.28
Oil Conv. Alabama	12.2	2027	0.19
Oil Conv. Kentucky	11.68	2020	0.19
Oil Conv. Pennsylvania	10.19	1883	0.13
Gas Shale Michigan Antrim	9.39	2019	0.28
Gas Conv. Michigan	9.19	1997	0.16
Gas Shale New Mexico Permian	8.96	2027	0.16
Oil Conv. Ohio	8.58	1899	0.12

Table 6.28: Peak years - All – Continued

Name	URR	Peak Year	Peak Rate
Coal Sub-bit. Alaska	8.51	2146	0.07
Oil Nat. Bitumen Texas	8.36	2076	0.17
Oil Conv. West Virginia	8.0	1902	0.08
Gas Conv. Montana	7.37	2028	0.12
Oil Tight Ohio Utica	7.19	2020	0.35
Oil Conv. Florida	6.21	1976	0.35
Oil Tight Wyoming Niobrara	6.15	2019	0.19
Oil Tight Colorado Niobrara	6.15	2019	0.19
Gas CBM Utah	5.86	2023	0.1
Oil Tight California Monterey	5.62	2016	0.22
Oil Tight Oklahoma Woodford	5.5	2016	0.29
Gas Conv. North Dakota	5.41	2028	0.09
Coal Bit. Arkansas	5.36	2069	0.06
Gas Shale Montana Bakken	5.06	2030	0.1
Gas Conv. Virginia	4.48	2023	0.1
Oil Conv. Indiana	4.28	1955	0.07
Coal Lignite Mississippi	4.19	2048	0.06
Oil Conv. Nebraska	3.96	1959	0.15
Gas CBM Oklahoma	3.94	2005	0.07
Gas CBM Kansas	3.94	2024	0.08
Oil Nat. Bitumen Alabama	3.89	2052	0.08
Oil Nat. Bitumen Kentucky	3.43	2053	0.08
Oil Extra Heavy California	3.33	2035	0.15
Gas Conv. New York	3.15	2025	0.06
Coal Bit. Michigan	2.39	2048	0.03
Oil Nat. Bitumen California	2.13	2047	0.05
Gas Shale Kentucky New Albany	2.12	2030	0.05
Gas Conv. Indiana	1.84	2022	0.03
Gas Conv. Florida	1.84	1976	0.04
Oil Tight Texas Austin Chalk	1.75	1999	0.12
Oil Conv. New York	1.5	1940	0.04
Oil Tight Texas Barnett	1.22	2013	0.06
Gas CBM West Virginia	1.21	2018	0.03
Oil Tight West Virginia Marcellus	1.15	2022	0.05
Oil Tight Pennsylvania Marcellus	1.15	2022	0.05
Coal Bit. Wyoming	1.11	1999	0.07
Gas Conv. Illinois	0.98	1942	0.02
Oil Tight Texas Granite Wash	0.89	2011	0.07
Oil Tight Oklahoma Granite Wash	0.89	2011	0.07
Coal Lignite Montana	0.85	2057	0.02
Gas CBM Pennsylvania	0.79	2025	0.02
Oil Conv. South Dakota	0.71	2007	0.01
Coal Bit. Georgia	0.61	2047	0.01
Gas Shale New Mexico Lewis	0.53	2021	0.02
Gas Shale Colorado Lewis	0.53	2021	0.02
Gas CBM Montana	0.44	2004	0.01
Gas CBM Arkansas	0.42	2025	0.01
Oil Conv. Nevada	0.42	1980	0.02
Gas Conv. Oregon	0.37	2020	0.01

Table 6.28: Peak years - All – Continued

**Name**	**URR**	**Peak Year**	**Peak Rate**
Oil Conv. Tennessee	0.27	1973	–
Coal Bit. Montana	0.26	2022	0.07
Gas Conv. Tennessee	0.25	2025	0.01
Gas Conv. Nebraska	0.23	1969	0.01
Gas Conv. South Dakota	0.2	2020	0.01
Oil Conv. Arizona	0.15	1968	0.01
Coal Bit. Texas	0.12	2023	0.02
Oil Conv. Missouri	0.09	2015	–
Oil Nat. Bitumen Wyoming	0.07	2035	0.01
Coal Lignite South Dakota	0.06	2025	–
Gas Conv. Arizona	0.06	2024	–
Coal Bit. Washington	0.05	2023	0.01
Gas Conv. Other	0.04	1887	–
Gas Conv. Maryland	0.01	1969	–
Gas CBM Louisiana	0.01	2008	–
Oil Conv. Virginia	0.01	1982	–
Gas Conv. Missouri	–	2022	–
Coal Anthr. Arkansas	–	1994	–
Gas Conv. Nevada	–	1993	–
Oil Conv. Washington	–	1956	–
Gas Conv. Iowa	–	1919	–
**Total**	**19952.34**	**2106**	**104.83**

6.2.2 By Mineral

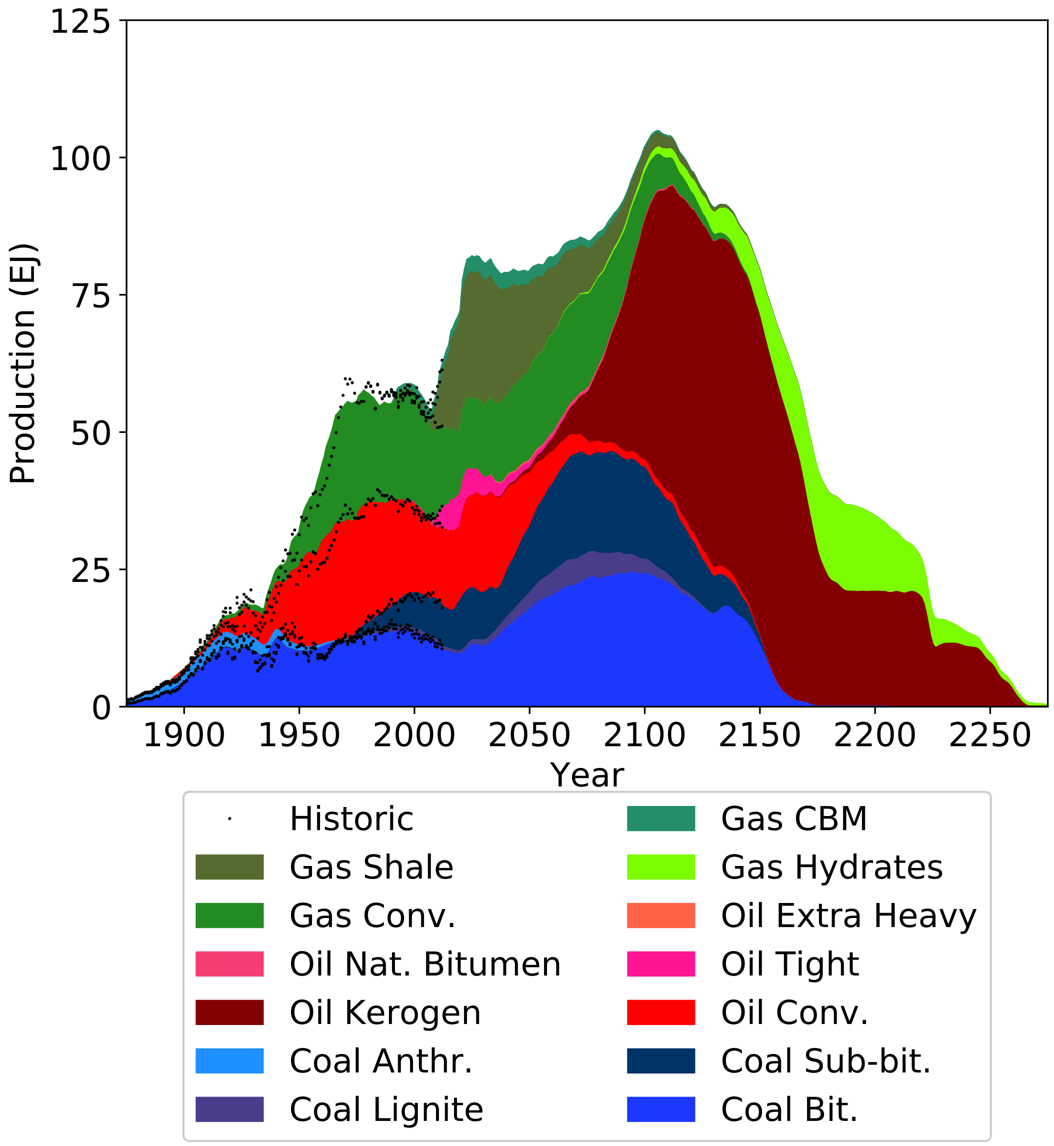

Figure 6.29: USA projection by mineral type

6.2.3 Regional Projections

Table 6.29: Peak years - Minerals

**Name**	**URR**	**Peak Year**	**Peak Rate**
Coal Bit.	3876.43	2095	24.4
Coal Lignite	312.45	2068	4.76
Coal Sub-bit.	1817.69	2071	19.12
Coal Anthr.	151.2	1919	2.66
Oil Conv.	2201.84	1978	21.88
Oil Kerogen	6089.3	2136	59.84
Oil Tight	152.09	2019	5.85
Oil Nat. Bitumen	35.79	2076	0.61
Oil Extra Heavy	3.33	2035	0.15
Gas Conv.	2610.26	1974	21.56
Gas Hydrates	1229.0	2185	15.8
Gas Shale	1257.88	2027	23.17
Gas CBM	215.1	2032	3.11
**Total**	**19952.34**	**2106**	**104.83**

Alabama

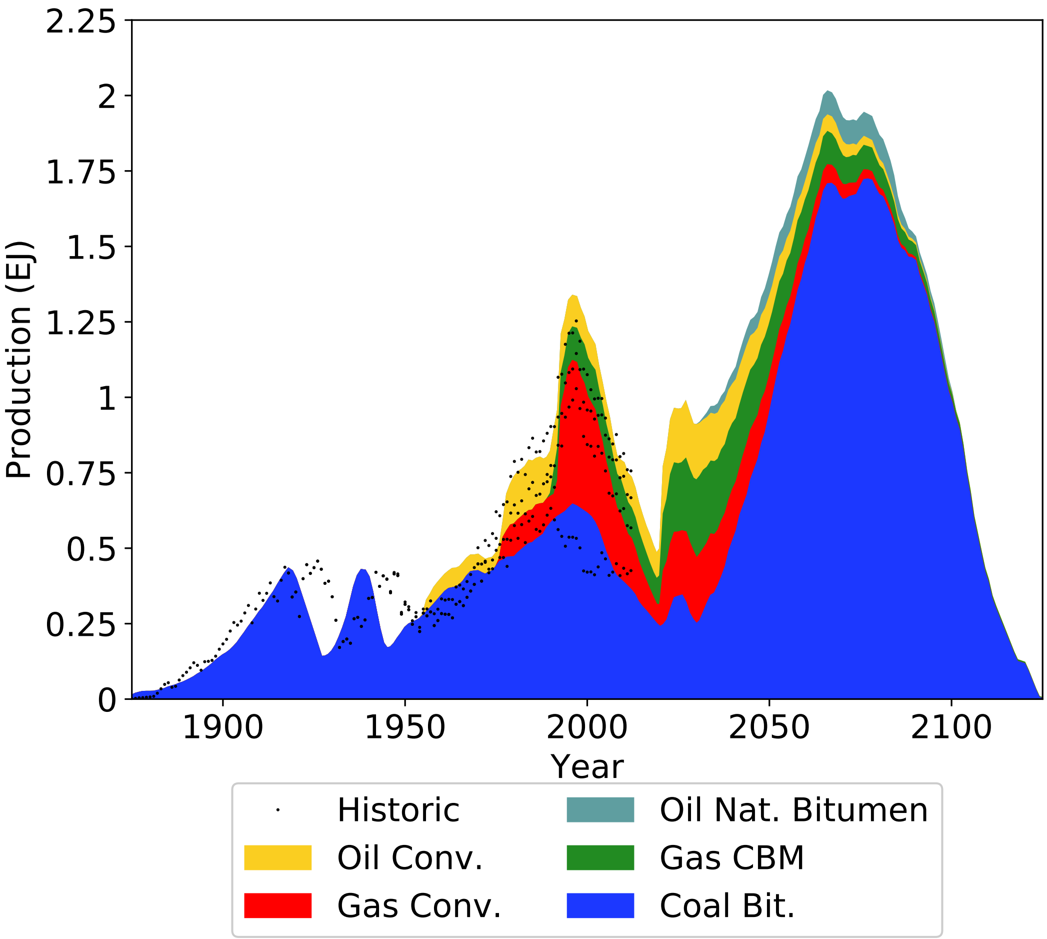

Figure 6.30: USA - Alabama projections capped at 16

Table 6.30: Peak years - All

Name	URR	Peak Year	Peak Rate
Coal Bit. Alabama	141.58	2077	1.72
Gas Conv. Alabama	16.83	1996	0.48
Gas CBM Alabama	14.4	2032	0.26
Oil Conv. Alabama	12.2	2027	0.19
Oil Nat. Bitumen Alabama	3.89	2052	0.08
**Total**	**188.9**	**2066**	**2.01**

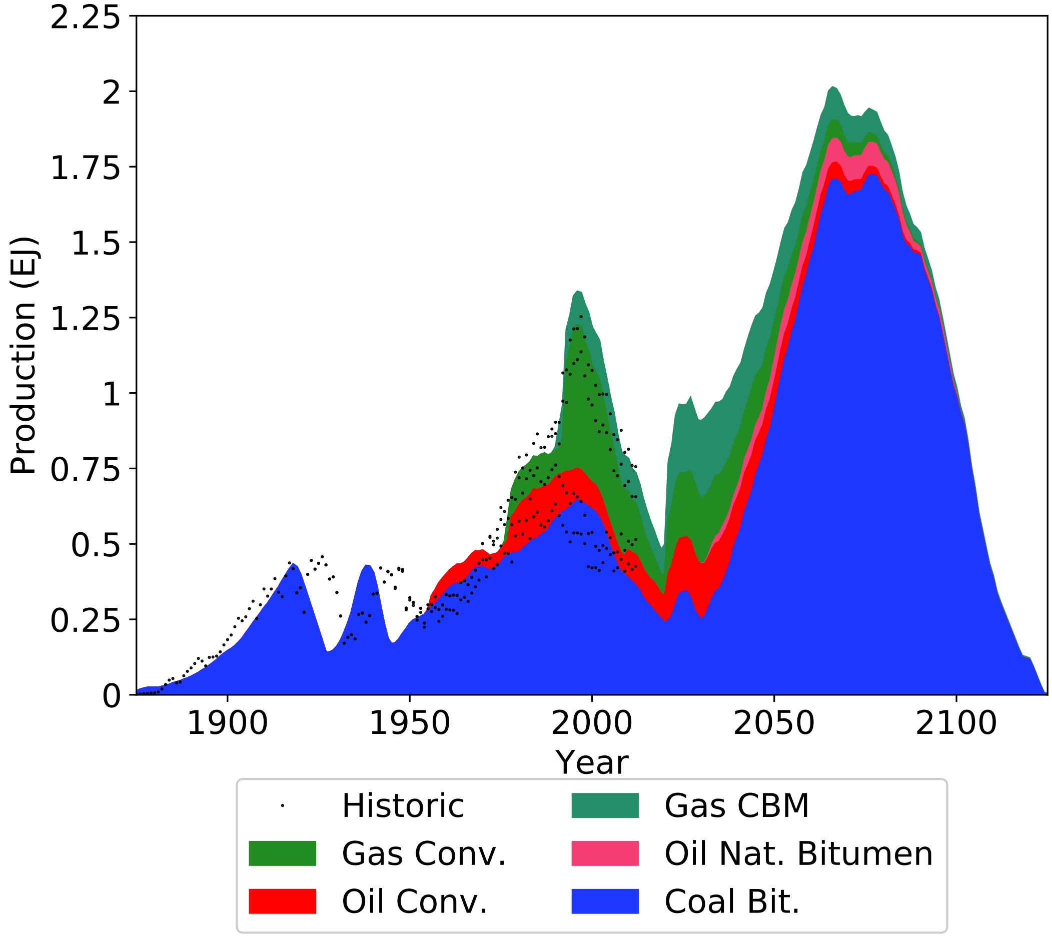

Figure 6.31: USA - Alabama projection by mineral type

Table 6.31: Peak years - Minerals

Name	URR	Peak Year	Peak Rate
Coal Bit.	141.58	2077	1.72
Oil Conv.	12.2	2027	0.19
Oil Nat. Bitumen	3.89	2052	0.08
Gas Conv.	16.83	1996	0.48
Gas CBM	14.4	2032	0.26
**Total**	**188.9**	**2066**	**2.01**

Alaska

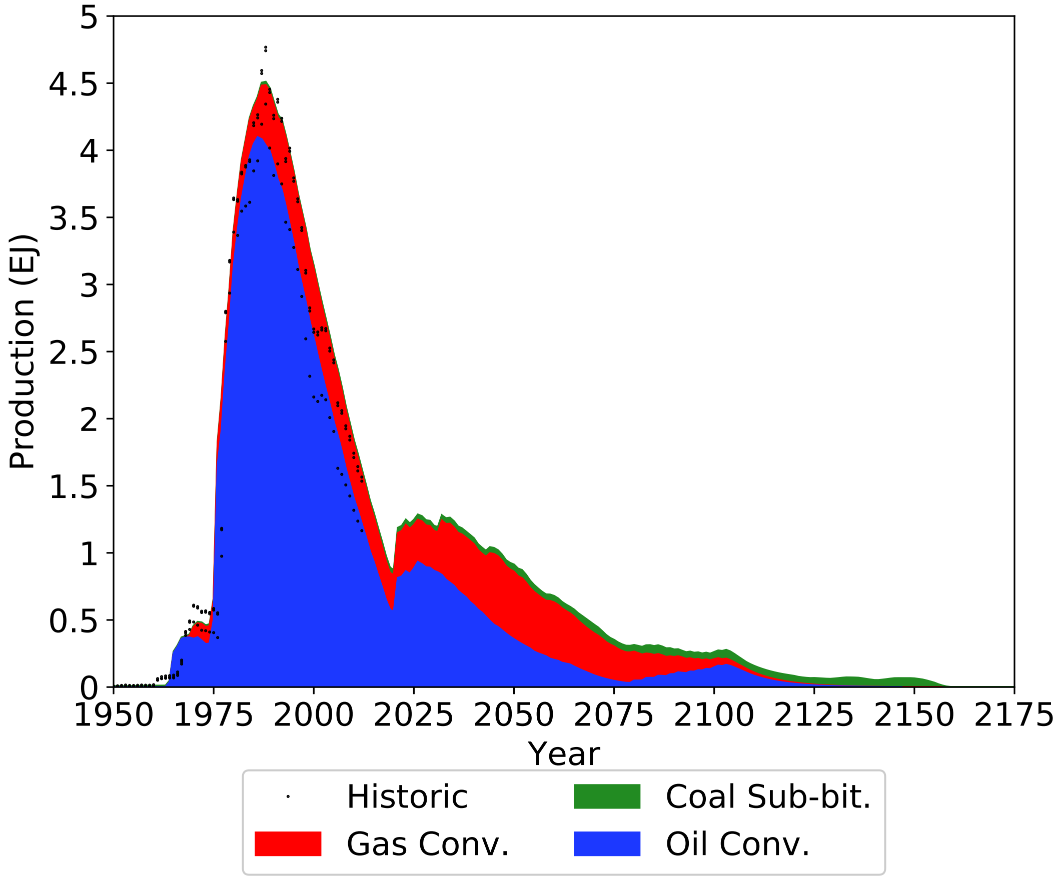

Figure 6.32: USA - Alaska projections capped at 16

Table 6.32: Peak years - All

**Name**	**URR**	**Peak Year**	**Peak Rate**
Oil Conv. Alaska	144.15	1986	4.1
Gas Conv. Alaska	44.44	1997	0.53
Coal Sub-bit. Alaska	8.51	2146	0.07
**Total**	**197.1**	**1988**	**4.51**

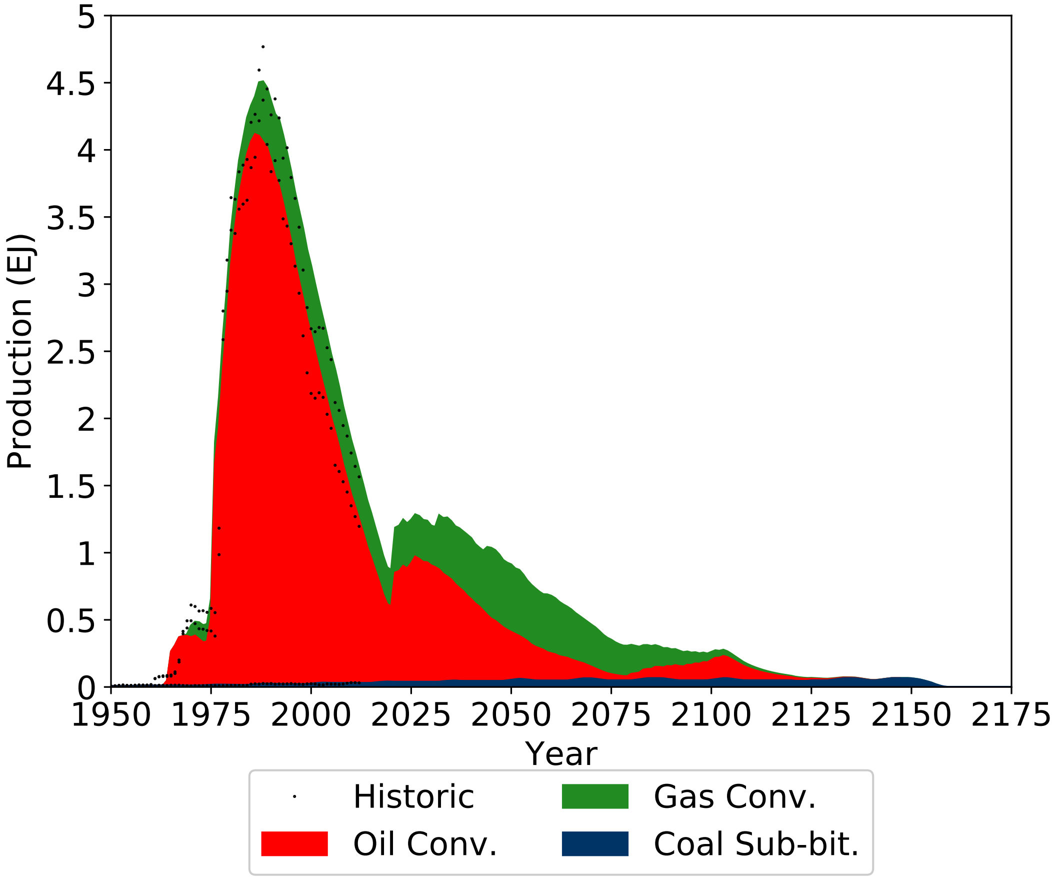

Figure 6.33: USA - Alaska projection by mineral type

Table 6.33: Peak years - Minerals

Name	URR	Peak Year	Peak Rate
Coal Sub-bit.	8.51	2146	0.07
Oil Conv.	144.15	1986	4.1
Gas Conv.	44.44	1997	0.53
**Total**	**197.1**	**1988**	**4.51**

Arizona

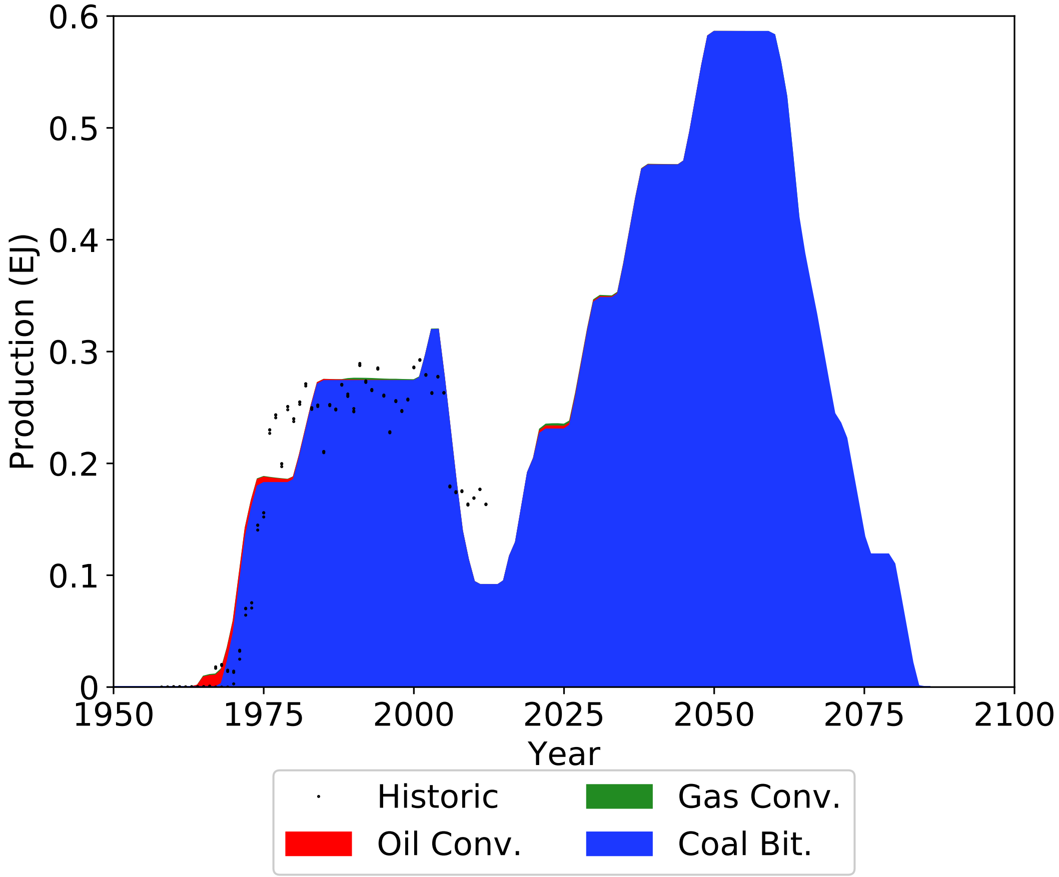

Figure 6.34: USA - Arizona projections capped at 16

Table 6.34: Peak years - All

Name	URR	Peak Year	Peak Rate
Coal Bit. Arizona	33.7	2050	0.59
Oil Conv. Arizona	0.15	1968	0.01
Gas Conv. Arizona	0.06	2024	–
**Total**	**33.91**	**2050**	**0.59**

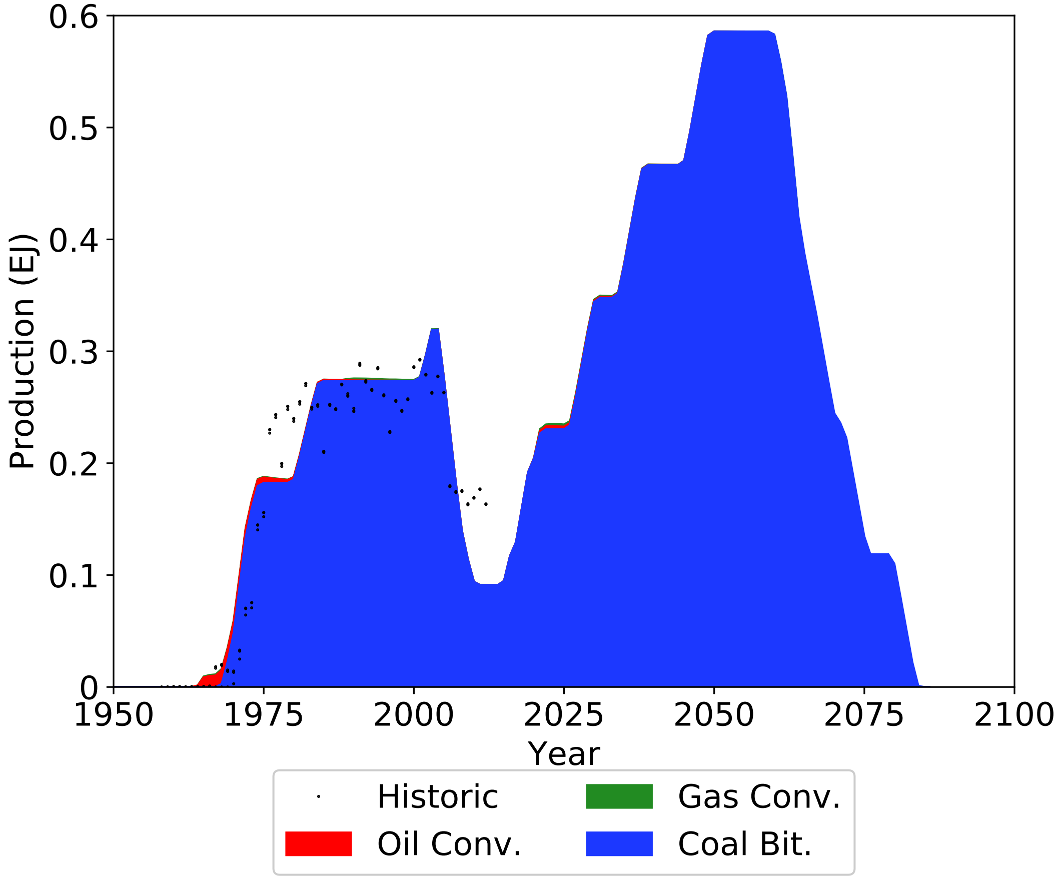

Figure 6.35: USA - Arizona projection by mineral type

Table 6.35: Peak years - Minerals

**Name**	**URR**	**Peak Year**	**Peak Rate**
Coal Bit.	33.7	2050	0.59
Oil Conv.	0.15	1968	0.01
Gas Conv.	0.06	2024	–
**Total**	**33.91**	**2050**	**0.59**

Arkansas

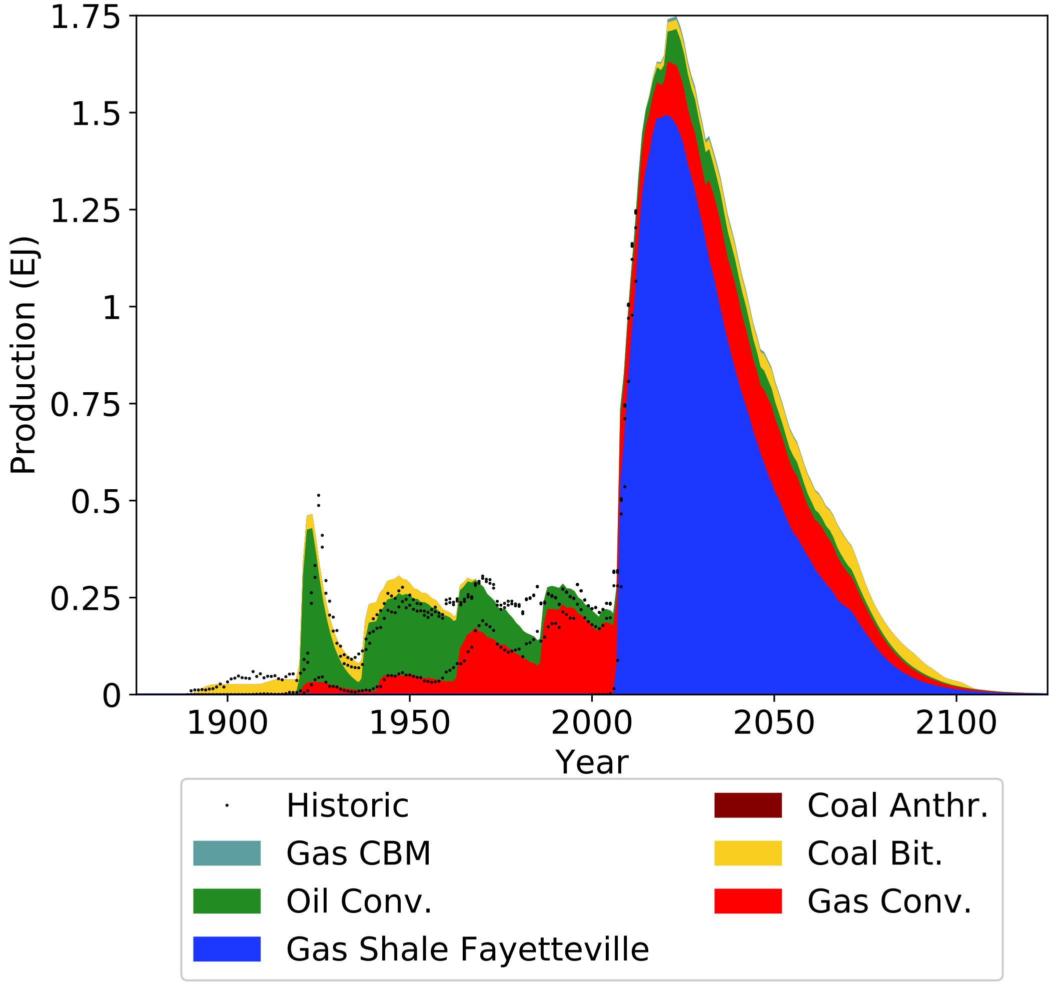

Figure 6.36: USA - Arkansas projections capped at 16

Table 6.36: Peak years - All

Name	URR	Peak Year	Peak Rate
Gas Shale Arkansas Fayetteville	54.17	2020	1.49
Gas Conv. Arkansas	18.98	1992	0.23
Oil Conv. Arkansas	14.07	1923	0.4
Coal Bit. Arkansas	5.36	2069	0.06
Gas CBM Arkansas	0.42	2025	0.01
Coal Anthr. Arkansas	–	1994	–
**Total**	**93.0**	**2023**	**1.74**

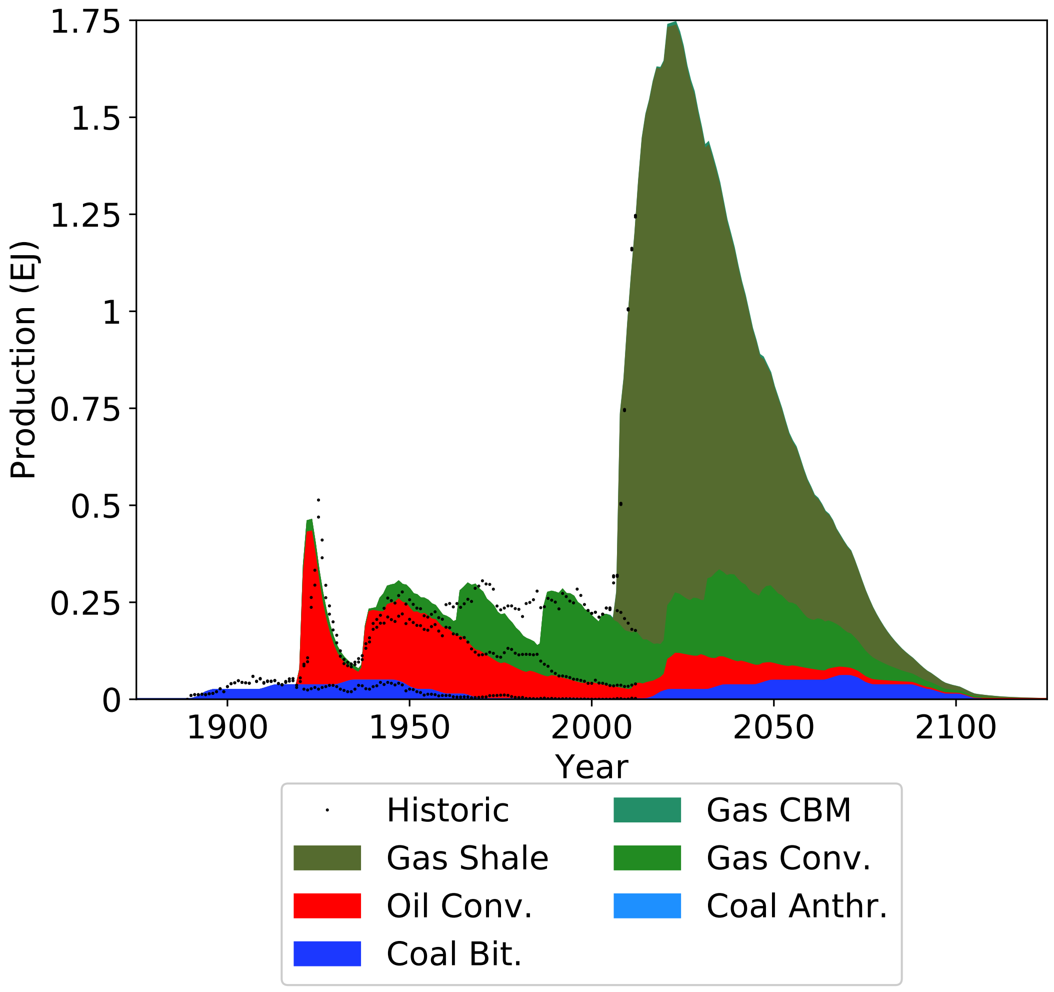

Figure 6.37: USA - Arkansas projection by mineral type

Table 6.37: Peak years - Minerals

**Name**	**URR**	**Peak Year**	**Peak Rate**
Coal Bit.	5.36	2069	0.06
Coal Anthr.	–	1994	–
Oil Conv.	14.07	1923	0.4
Gas Conv.	18.98	1992	0.23
Gas Shale	54.17	2020	1.49
Gas CBM	0.42	2025	0.01
**Total**	**93.0**	**2023**	**1.74**

California

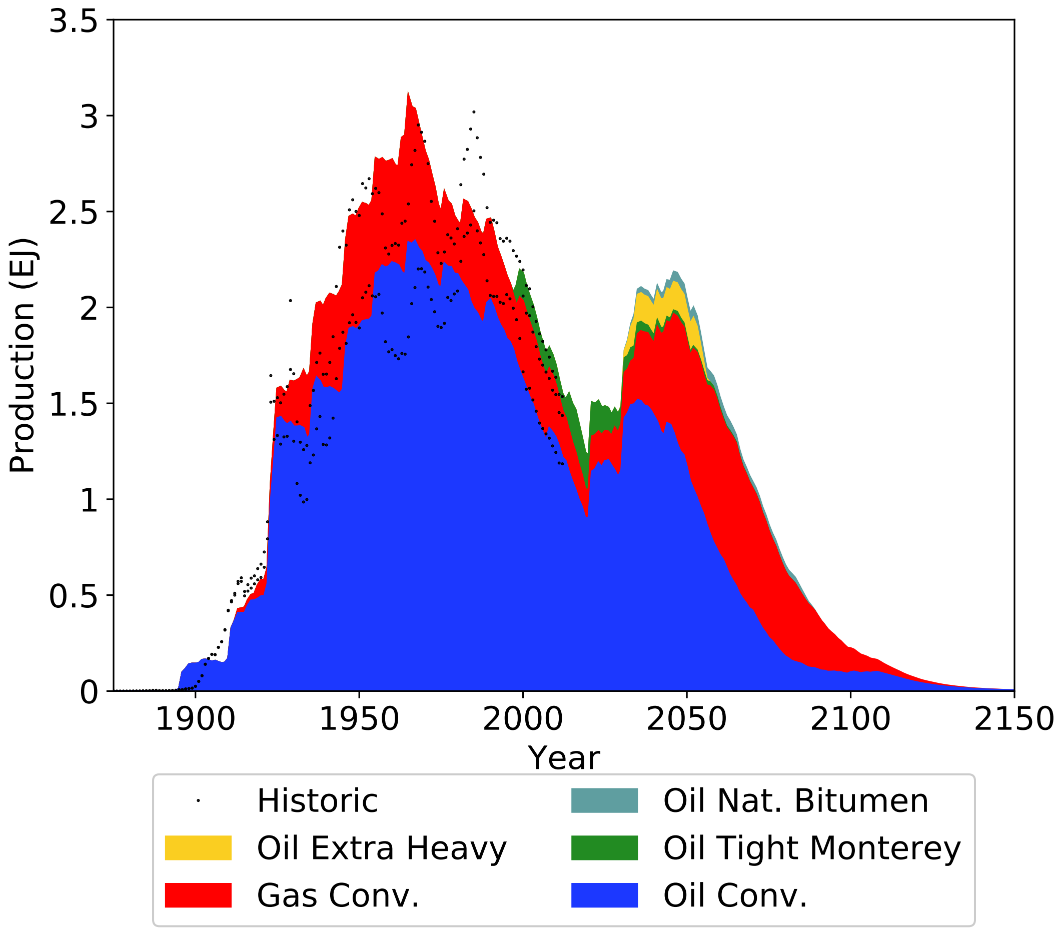

Figure 6.38: USA - California projections capped at 16

Table 6.38: Peak years - All

Name	URR	Peak Year	Peak Rate
Oil Conv. California	240.2	1967	2.35
Gas Conv. California	77.75	2058	0.79
Oil Tight California Monterey	5.62	2016	0.22
Oil Extra Heavy California	3.33	2035	0.15
Oil Nat. Bitumen California	2.13	2047	0.05
**Total**	**329.03**	**1965**	**3.1**

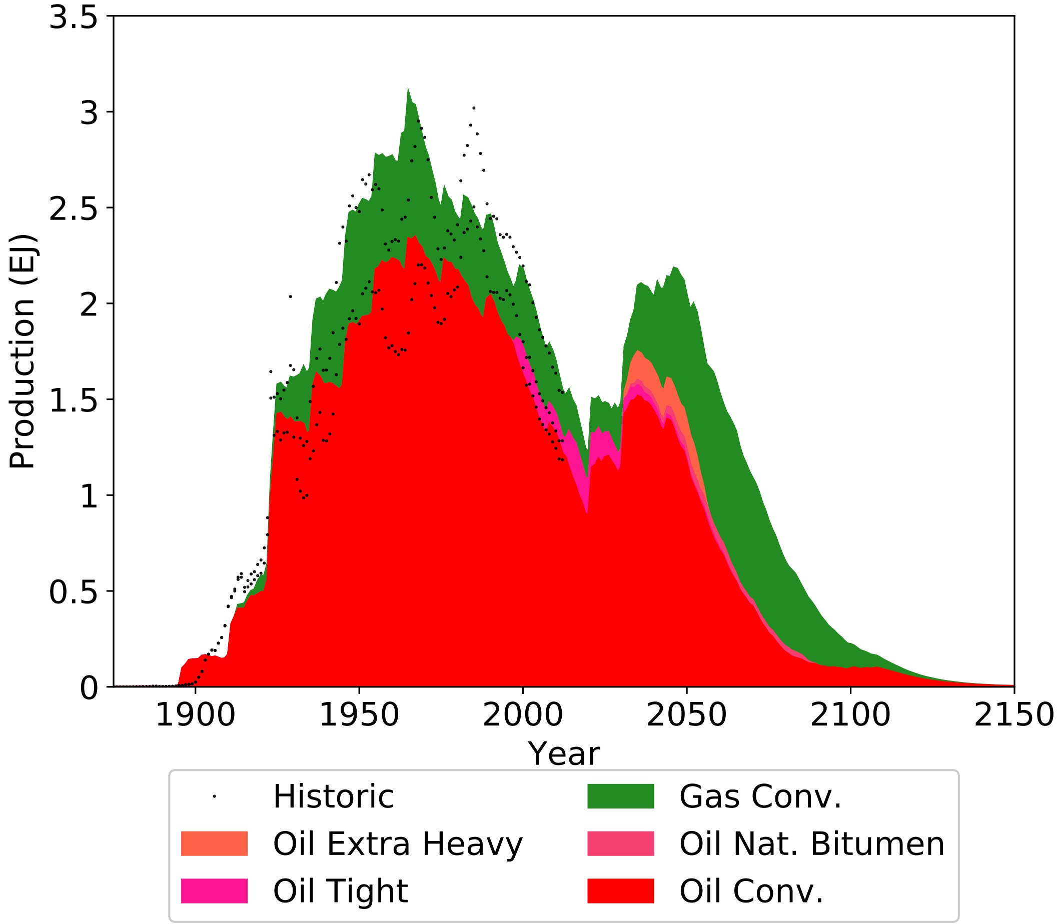

Figure 6.39: USA - California projection by mineral type

Table 6.39: Peak years - Minerals

Name	URR	Peak Year	Peak Rate
Oil Conv.	240.2	1967	2.35
Oil Tight	5.62	2016	0.22
Oil Nat. Bitumen	2.13	2047	0.05
Oil Extra Heavy	3.33	2035	0.15
Gas Conv.	77.75	2058	0.79
**Total**	**329.03**	**1965**	**3.1**

Colorado

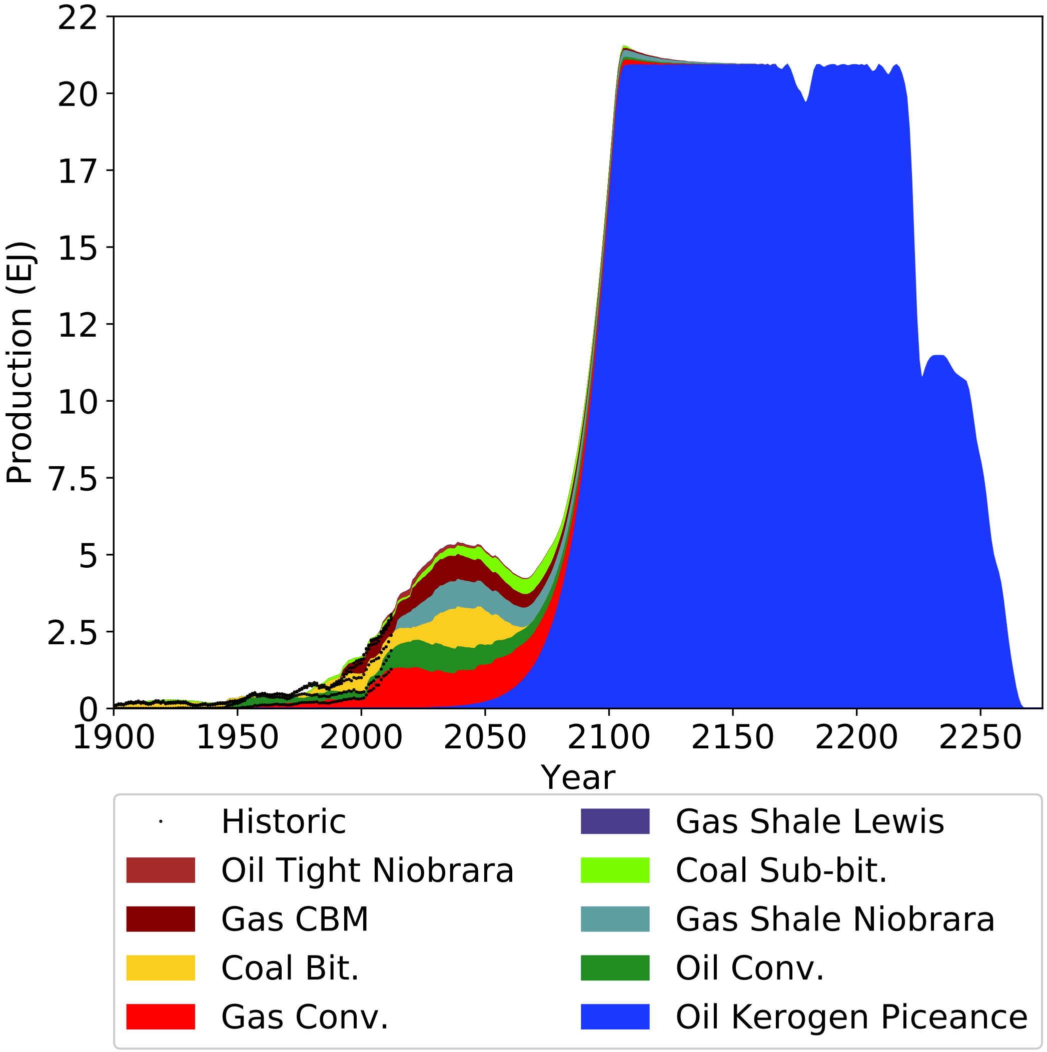

Figure 6.40: USA - Colorado projections capped at 16

Table 6.40: Peak years - All

Name	URR	Peak Year	Peak Rate
Oil Kerogen Colorado Piceance	3101.7	2155	20.91
Gas Conv. Colorado	110.24	2021	1.31
Oil Conv. Colorado	69.4	2025	0.91
Coal Bit. Colorado	67.33	2039	1.29
Gas Shale Colorado Niobrara	59.86	2036	0.9
Gas CBM Colorado	55.8	2031	0.86
Coal Sub-bit. Colorado	34.6	2073	0.57
Oil Tight Colorado Niobrara	6.15	2019	0.19
Gas Shale Colorado Lewis	0.53	2021	0.02
**Total**	**3505.61**	**2106**	**21.52**

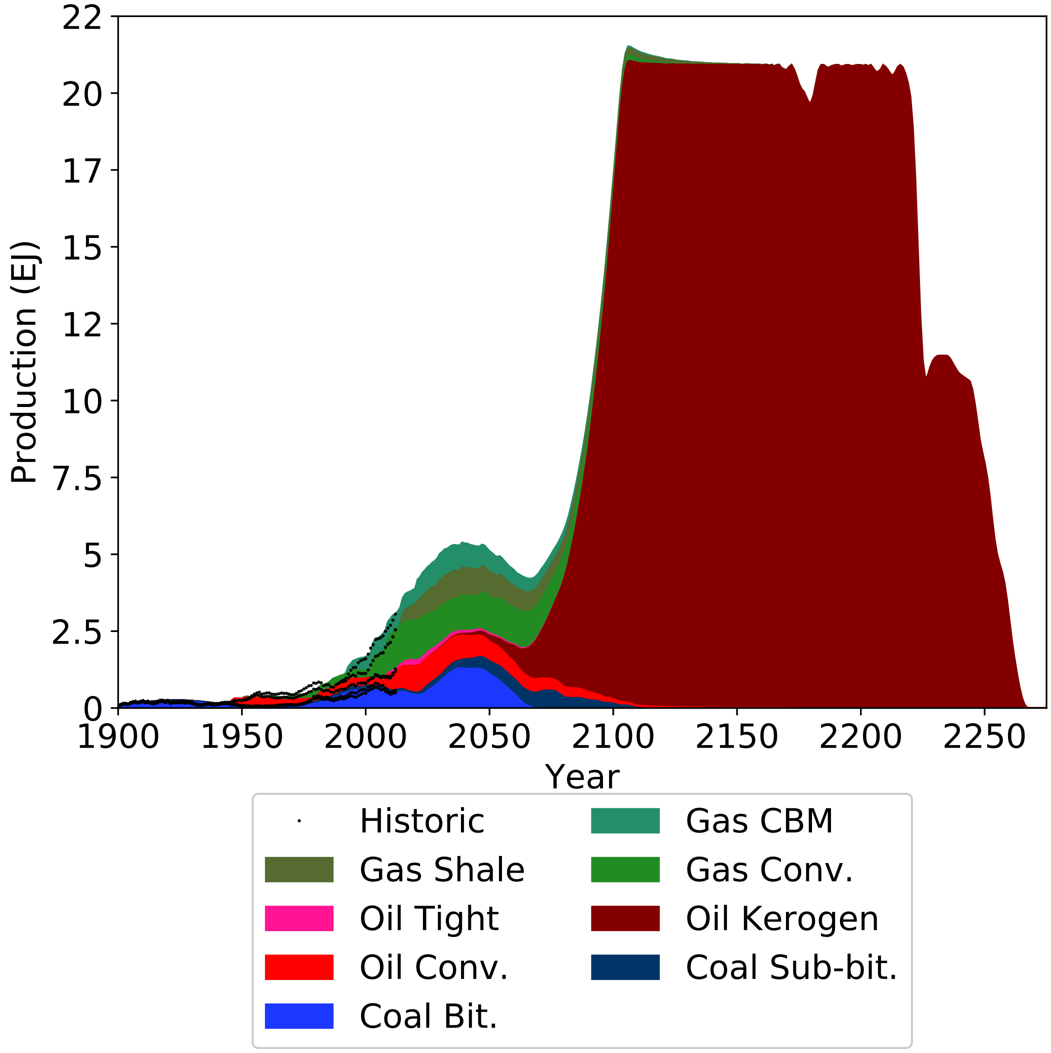

Figure 6.41: USA - Colorado projection by mineral type

Table 6.41: Peak years - Minerals

**Name**	**URR**	**Peak Year**	**Peak Rate**
Coal Bit.	67.33	2039	1.29
Coal Sub-bit.	34.6	2073	0.57
Oil Conv.	69.4	2025	0.91
Oil Kerogen	3101.7	2155	20.91
Oil Tight	6.15	2019	0.19
Gas Conv.	110.24	2021	1.31
Gas Shale	60.39	2036	0.91
Gas CBM	55.8	2031	0.86
**Total**	**3505.61**	**2106**	**21.52**

Eastern

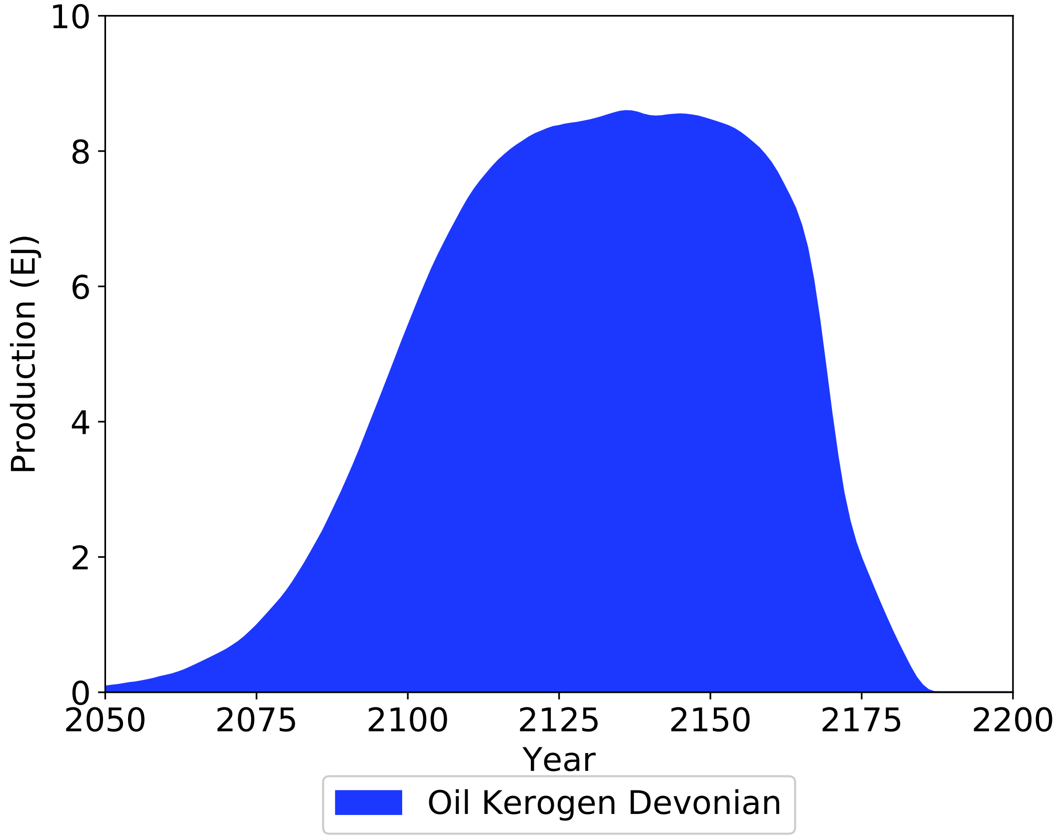

Figure 6.42: USA - Eastern projections capped at 16

Table 6.42: Peak years - All

Name	URR	Peak Year	Peak Rate
Oil Kerogen Eastern Devonian	649.8	2136	8.59
**Total**	**649.8**	**2136**	**8.59**

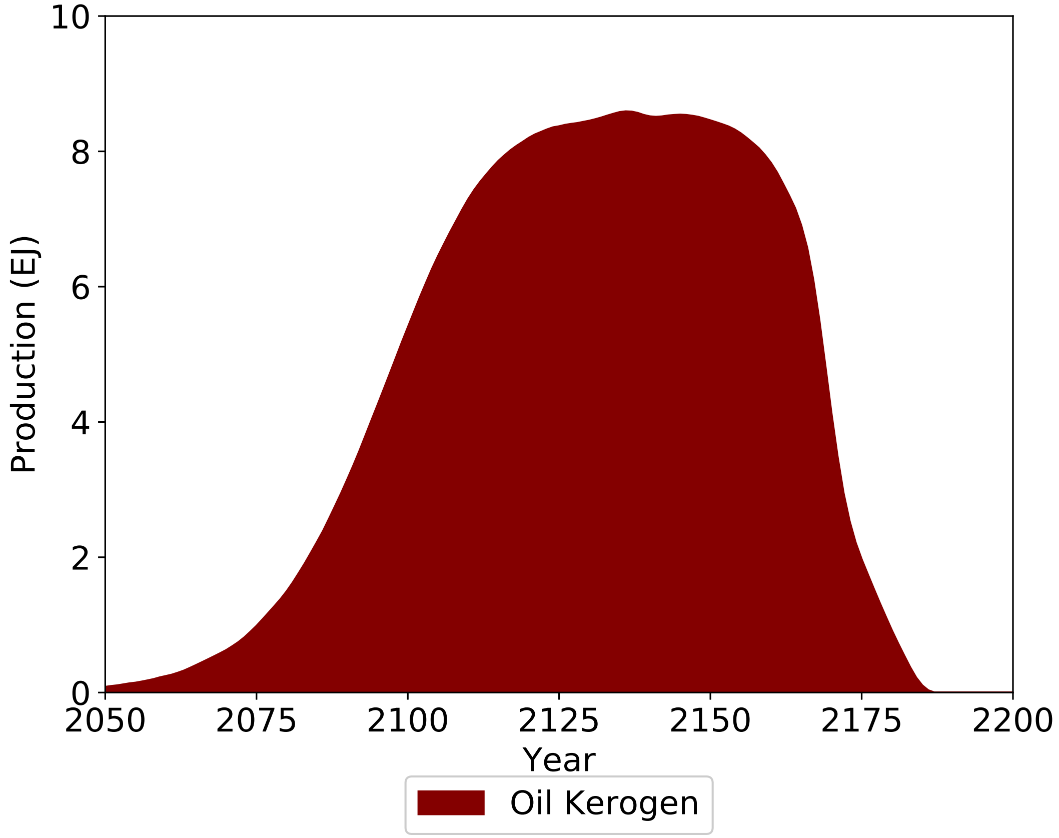

Figure 6.43: USA - Eastern projection by mineral type

Table 6.43: Peak years - Minerals

**Name**	**URR**	**Peak Year**	**Peak Rate**
Oil Kerogen	649.8	2136	8.59
**Total**	**649.8**	**2136**	**8.59**

Florida

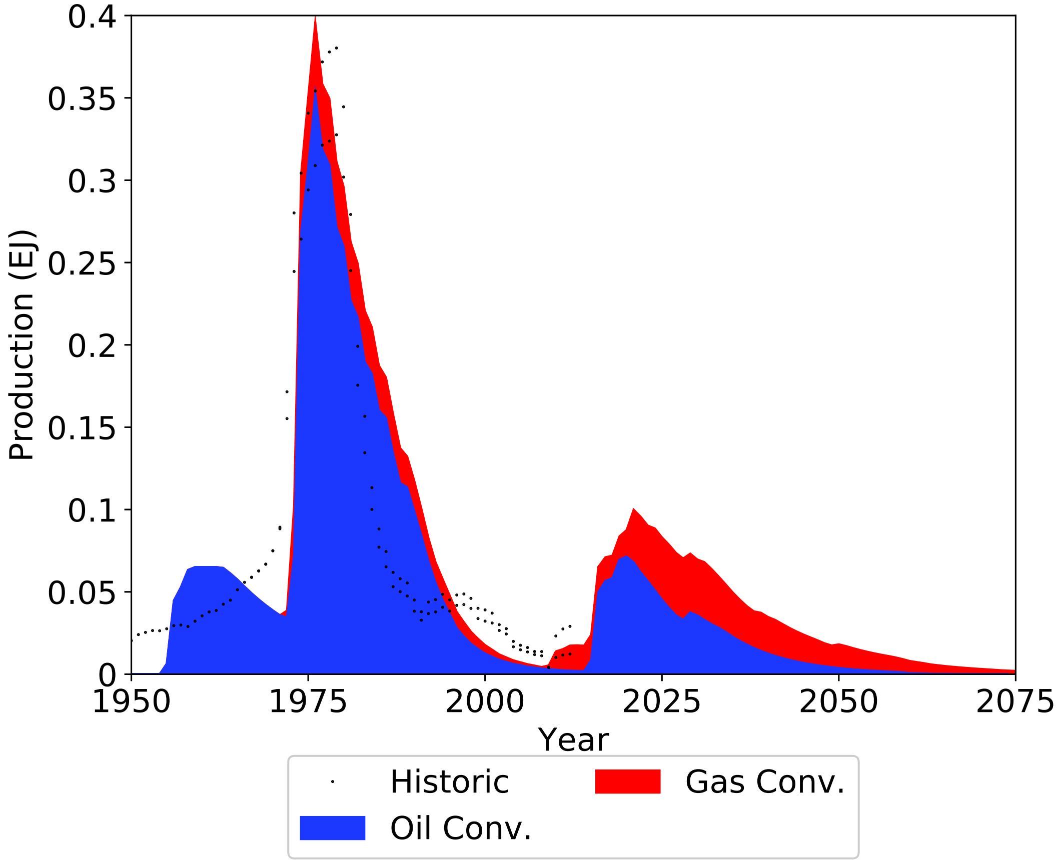

Figure 6.44: USA - Florida projections capped at 16

Table 6.44: Peak years - All

Name	URR	Peak Year	Peak Rate
Oil Conv. Florida	6.21	1976	0.35
Gas Conv. Florida	1.84	1976	0.04
**Total**	**8.05**	**1976**	**0.4**

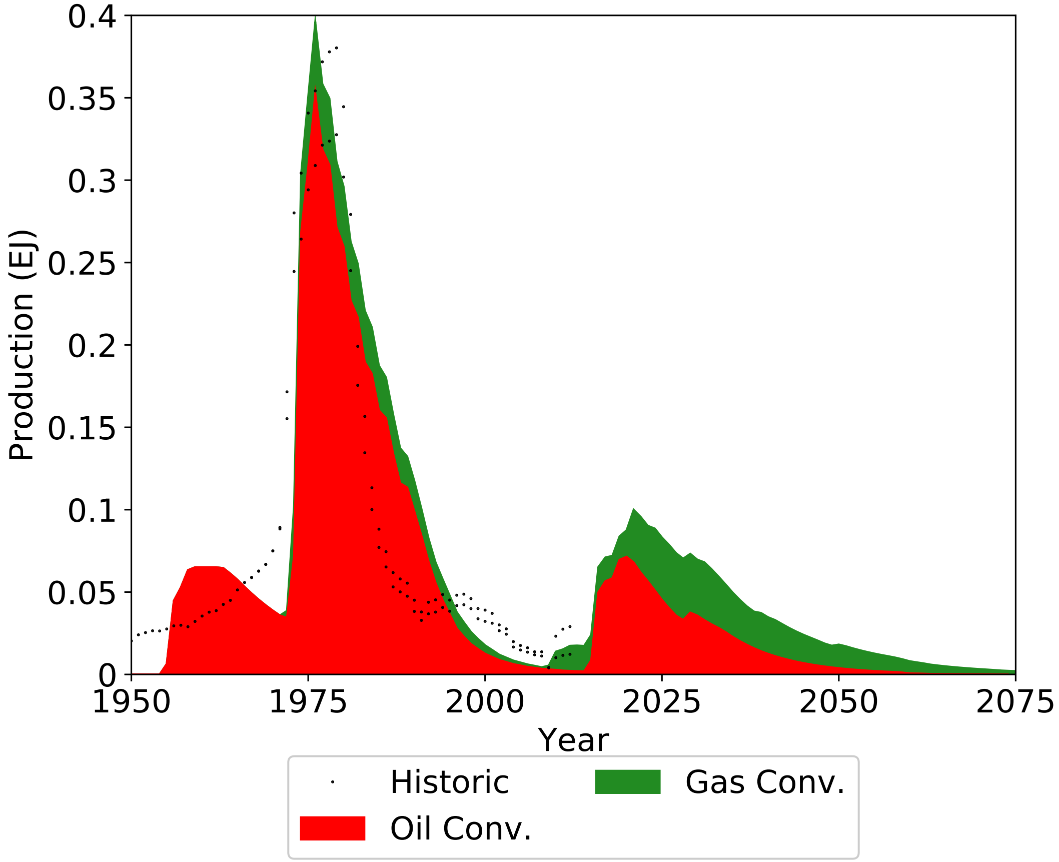

Figure 6.45: USA - Florida projection by mineral type

Table 6.45: Peak years - Minerals

Name	URR	Peak Year	Peak Rate
Oil Conv.	6.21	1976	0.35
Gas Conv.	1.84	1976	0.04
**Total**	**8.05**	**1976**	**0.4**

Georgia

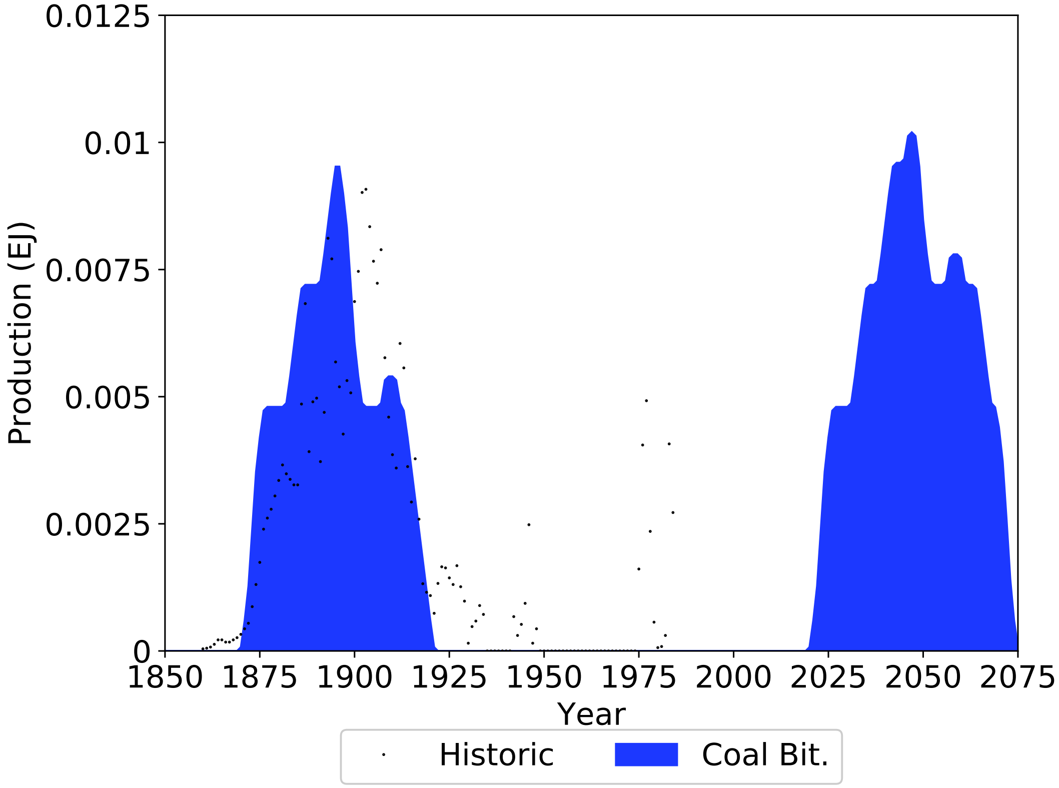

Figure 6.46: USA - Georgia projections capped at 16

Table 6.46: Peak years - All

Name	URR	Peak Year	Peak Rate
Coal Bit. Georgia	0.61	2047	0.01
**Total**	**0.61**	**2047**	**0.01**

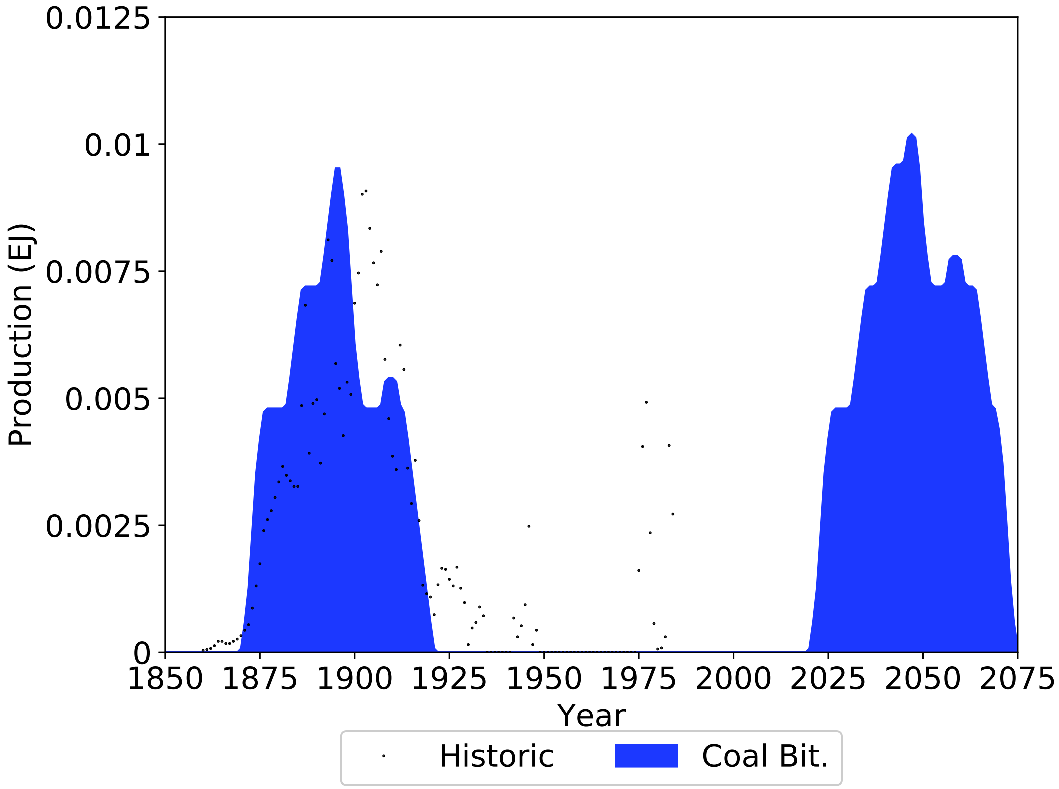

Figure 6.47: USA - Georgia projection by mineral type

Table 6.47: Peak years - Minerals

Name	URR	Peak Year	Peak Rate
Coal Bit.	0.61	2047	0.01
**Total**	**0.61**	**2047**	**0.01**

Illinois

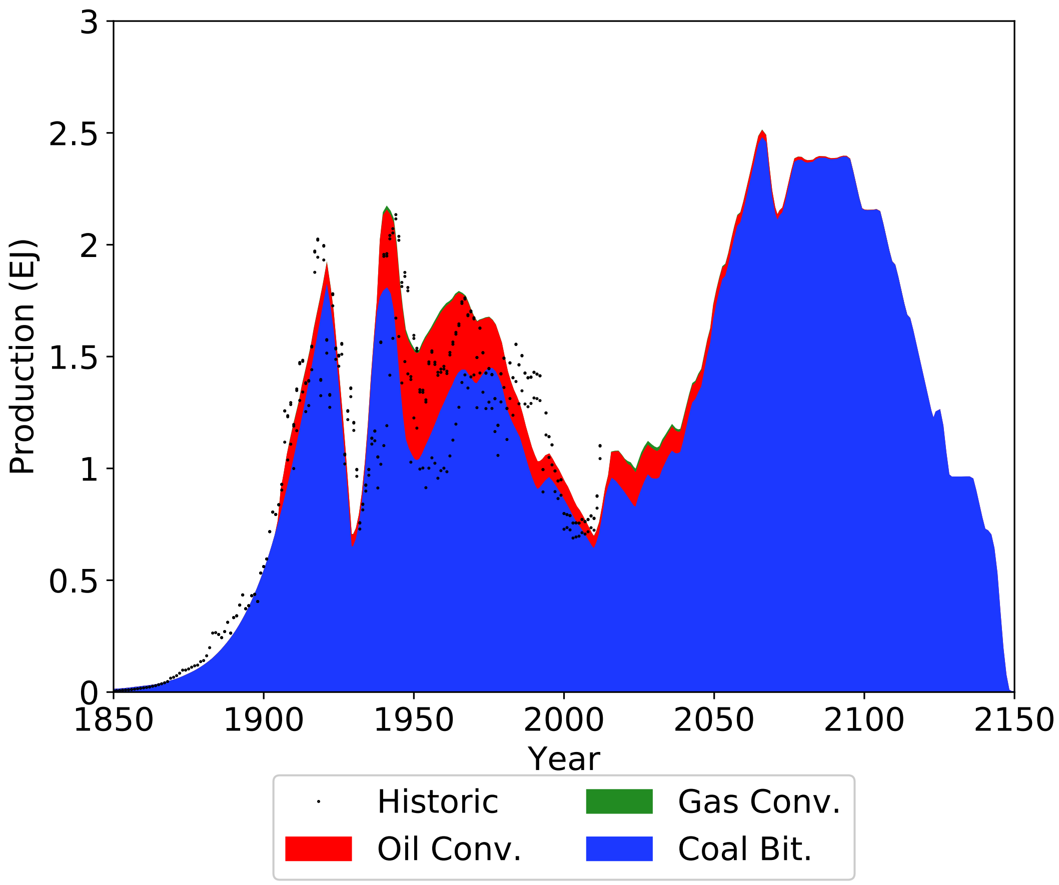

Figure 6.48: USA - Illinois projections capped at 16

Table 6.48: Peak years - All

Name	URR	Peak Year	Peak Rate
Coal Bit. Illinois	349.04	2066	2.47
Oil Conv. Illinois	26.62	1953	0.49
Gas Conv. Illinois	0.98	1942	0.02
**Total**	**376.64**	**2066**	**2.51**

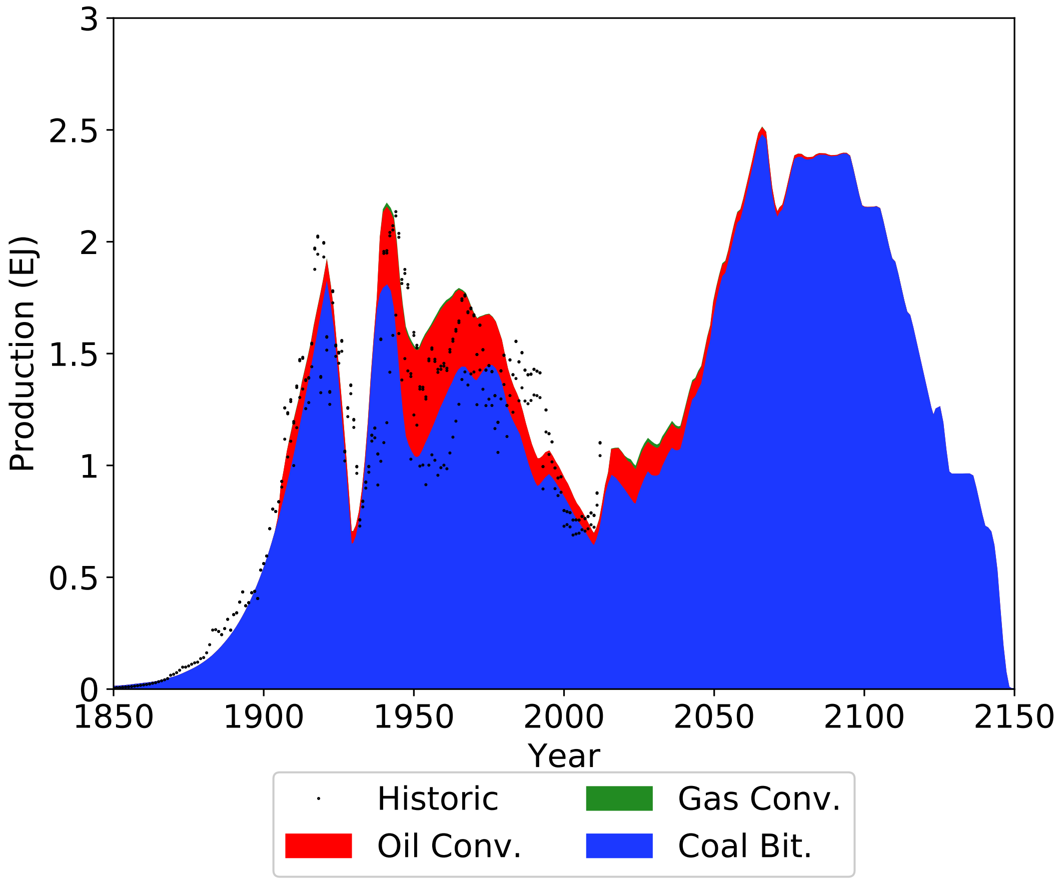

Figure 6.49: USA - Illinois projection by mineral type

Table 6.49: Peak years - Minerals

Name	URR	Peak Year	Peak Rate
Coal Bit.	349.04	2066	2.47
Oil Conv.	26.62	1953	0.49
Gas Conv.	0.98	1942	0.02
**Total**	**376.64**	**2066**	**2.51**

Indiana

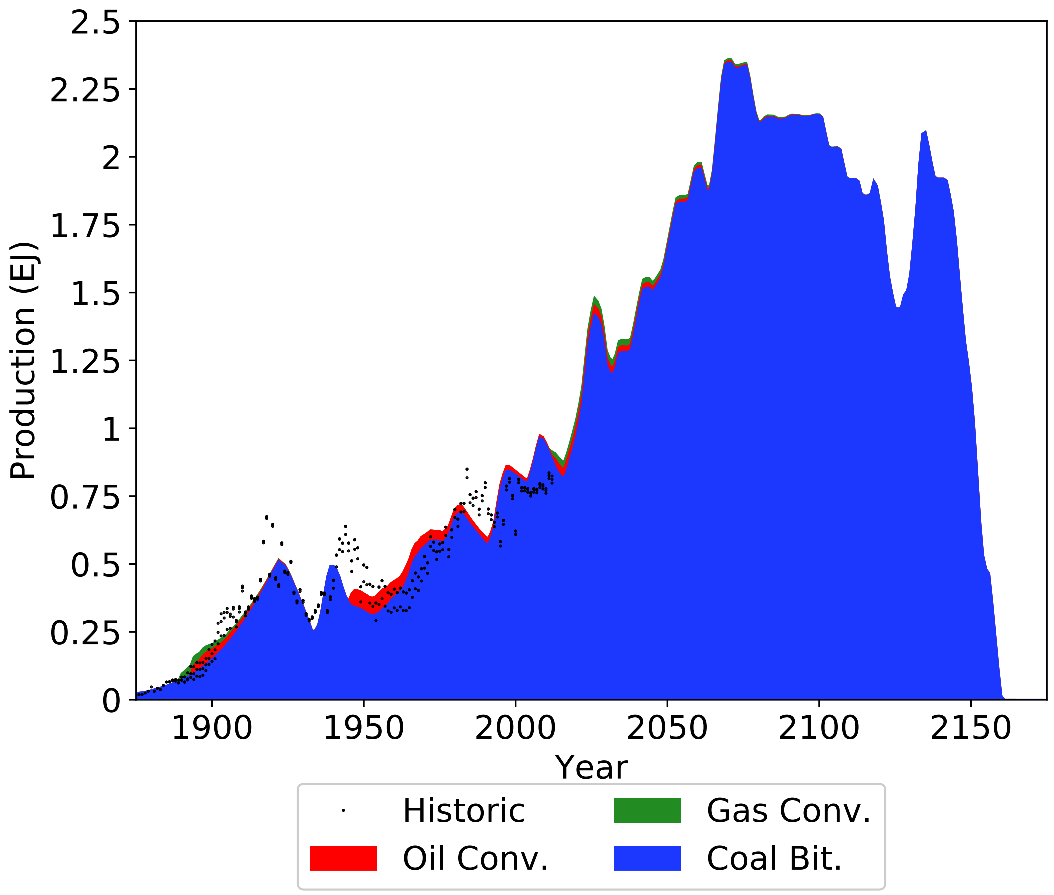

Figure 6.50: USA - Indiana projections capped at 16

Table 6.50: Peak years - All

Name	URR	Peak Year	Peak Rate
Coal Bit. Indiana	305.37	2070	2.35
Oil Conv. Indiana	4.28	1955	0.07
Gas Conv. Indiana	1.84	2022	0.03
**Total**	**311.49**	**2070**	**2.36**

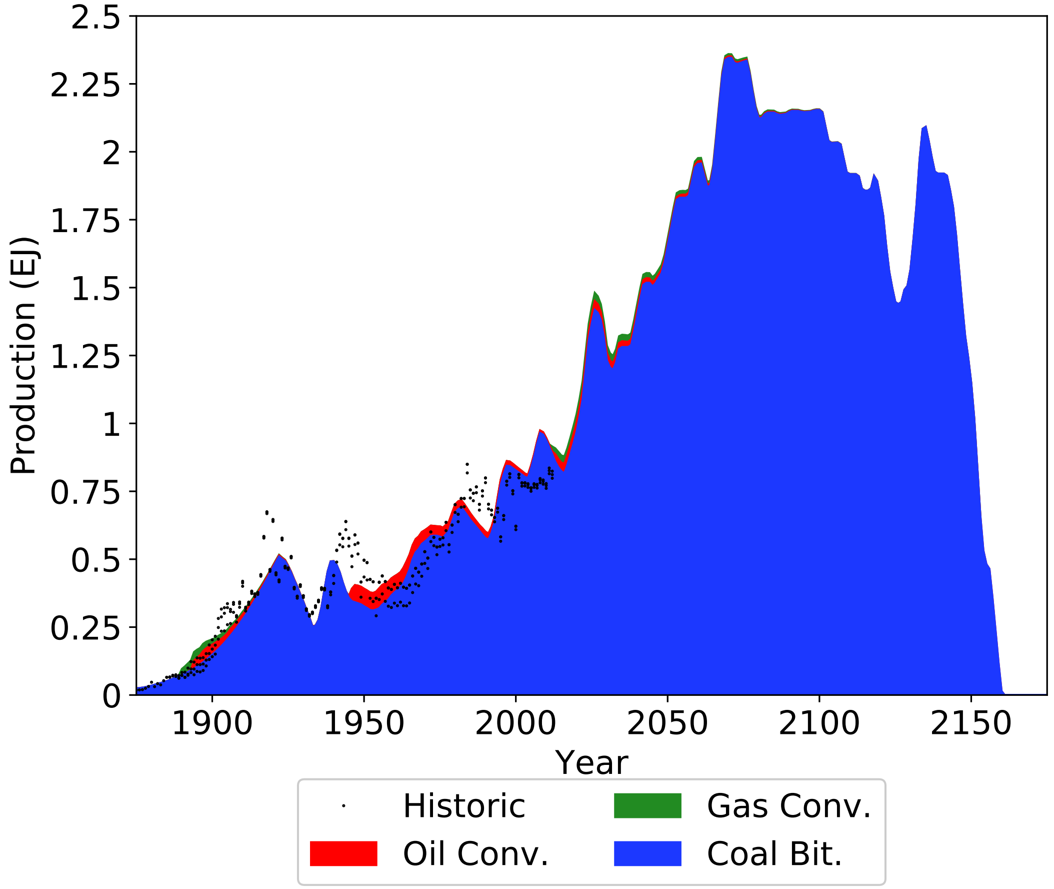

Figure 6.51: USA - Indiana projection by mineral type

Table 6.51: Peak years - Minerals

Name	URR	Peak Year	Peak Rate
Coal Bit.	305.37	2070	2.35
Oil Conv.	4.28	1955	0.07
Gas Conv.	1.84	2022	0.03
**Total**	**311.49**	**2070**	**2.36**

Iowa

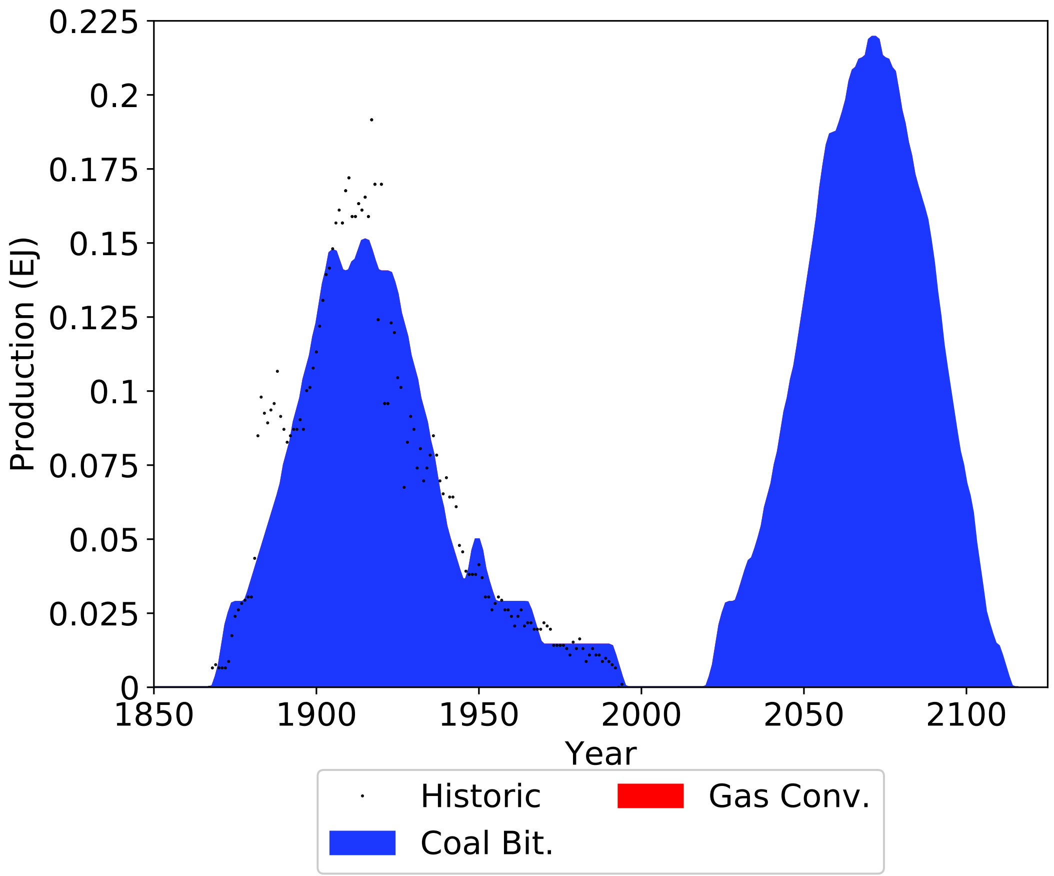

Figure 6.52: USA - Iowa projections capped at 16

Table 6.52: Peak years - All

Name	URR	Peak Year	Peak Rate
Coal Bit. Iowa	18.76	2071	0.22
Gas Conv. Iowa	–	1919	–
**Total**	**18.76**	**2071**	**0.22**

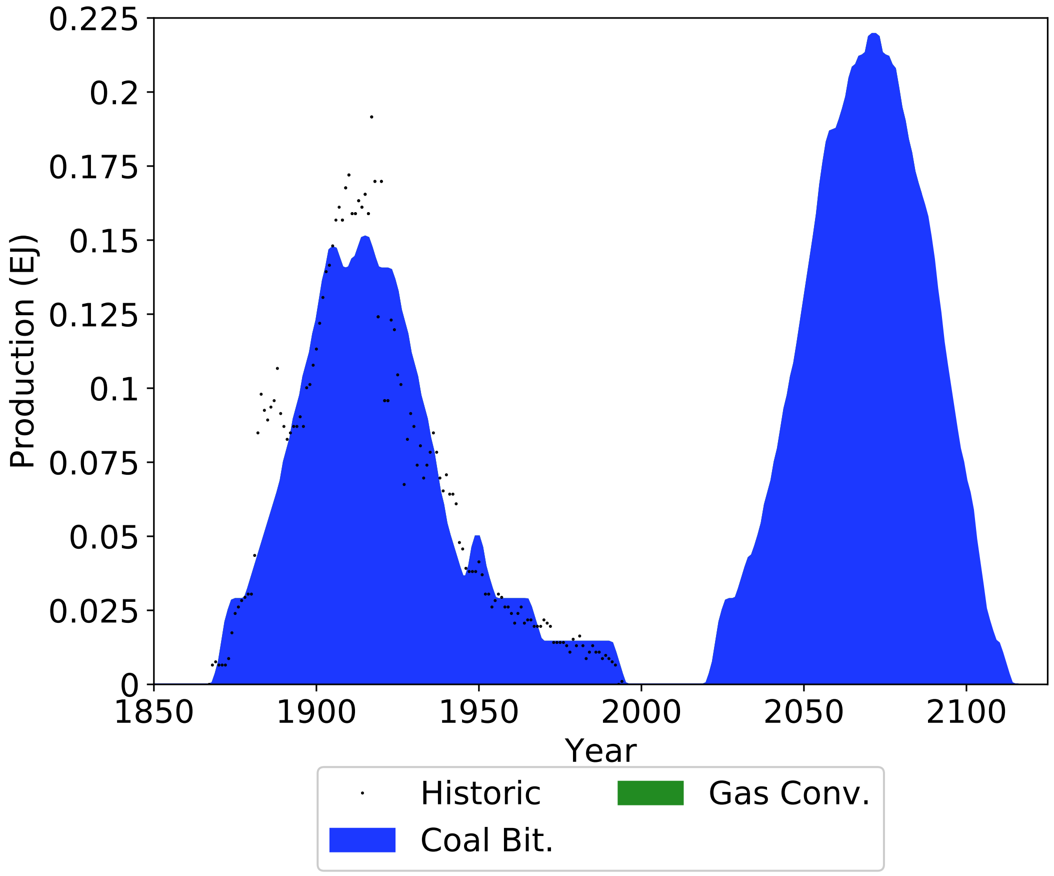

Figure 6.53: USA - Iowa projection by mineral type

Table 6.53: Peak years - Minerals

Name	URR	Peak Year	Peak Rate
Coal Bit.	18.76	2071	0.22
Gas Conv.	–	1919	–
**Total**	**18.76**	**2071**	**0.22**

Kansas

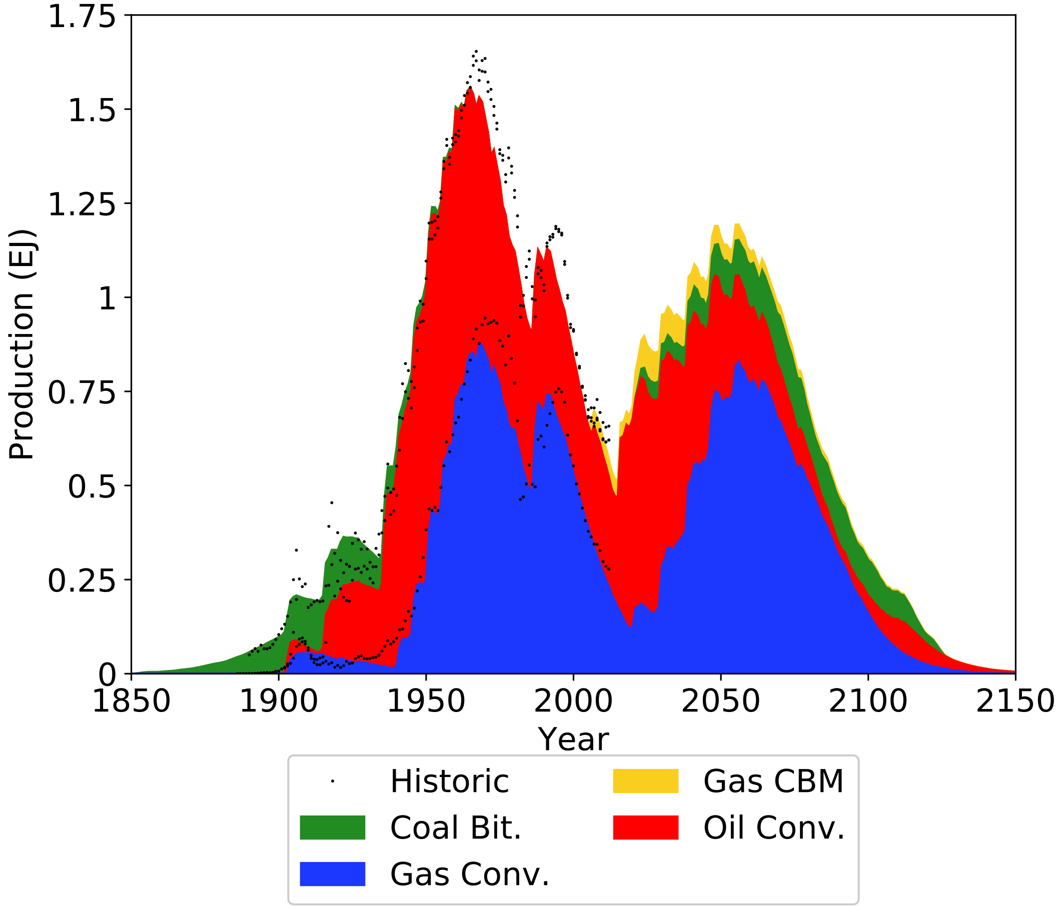

Figure 6.54: USA - Kansas projections capped at 16

Table 6.54: Peak years - All

Name	URR	Peak Year	Peak Rate
Gas Conv. Kansas	82.45	1968	0.87
Oil Conv. Kansas	69.8	1953	0.8
Coal Bit. Kansas	15.31	2071	0.14
Gas CBM Kansas	3.94	2024	0.08
**Total**	**171.5**	**1965**	**1.56**

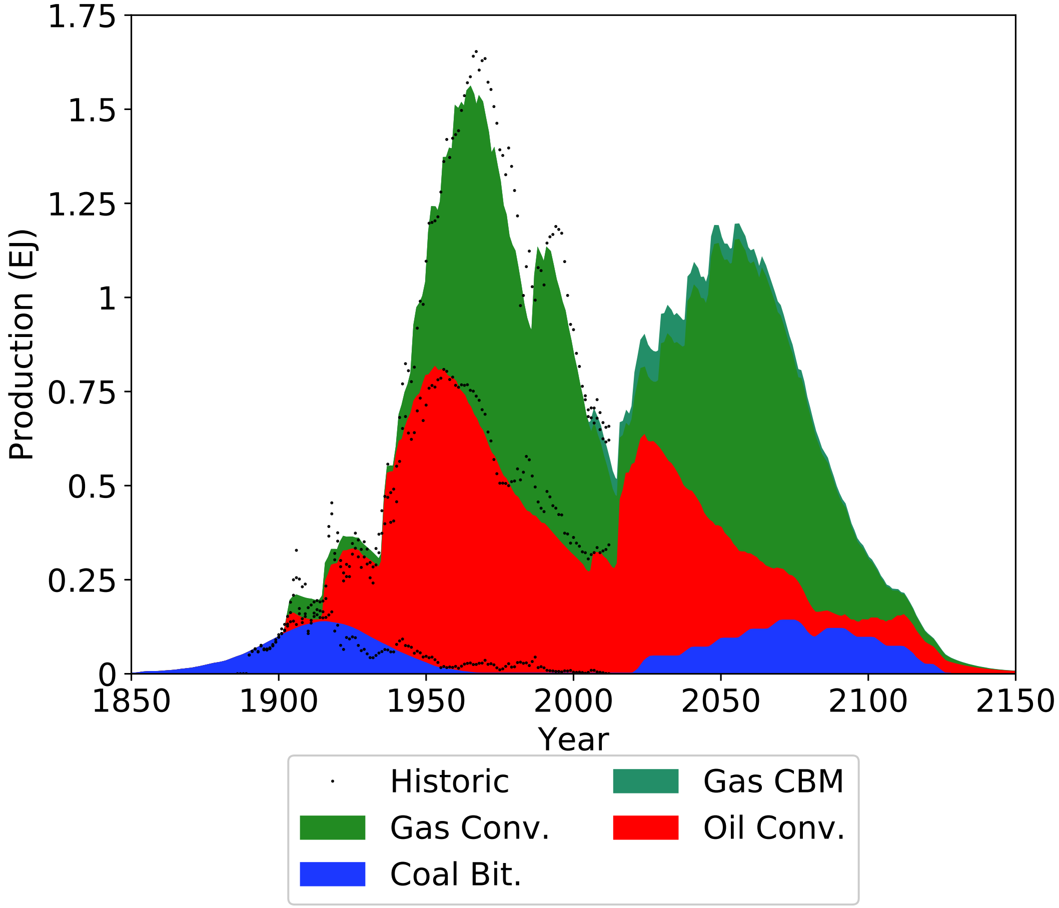

Figure 6.55: USA - Kansas projection by mineral type

Table 6.55: Peak years - Minerals

Name	URR	Peak Year	Peak Rate
Coal Bit.	15.31	2071	0.14
Oil Conv.	69.8	1953	0.8
Gas Conv.	82.45	1968	0.87
Gas CBM	3.94	2024	0.08
**Total**	**171.5**	**1965**	**1.56**

Kentucky

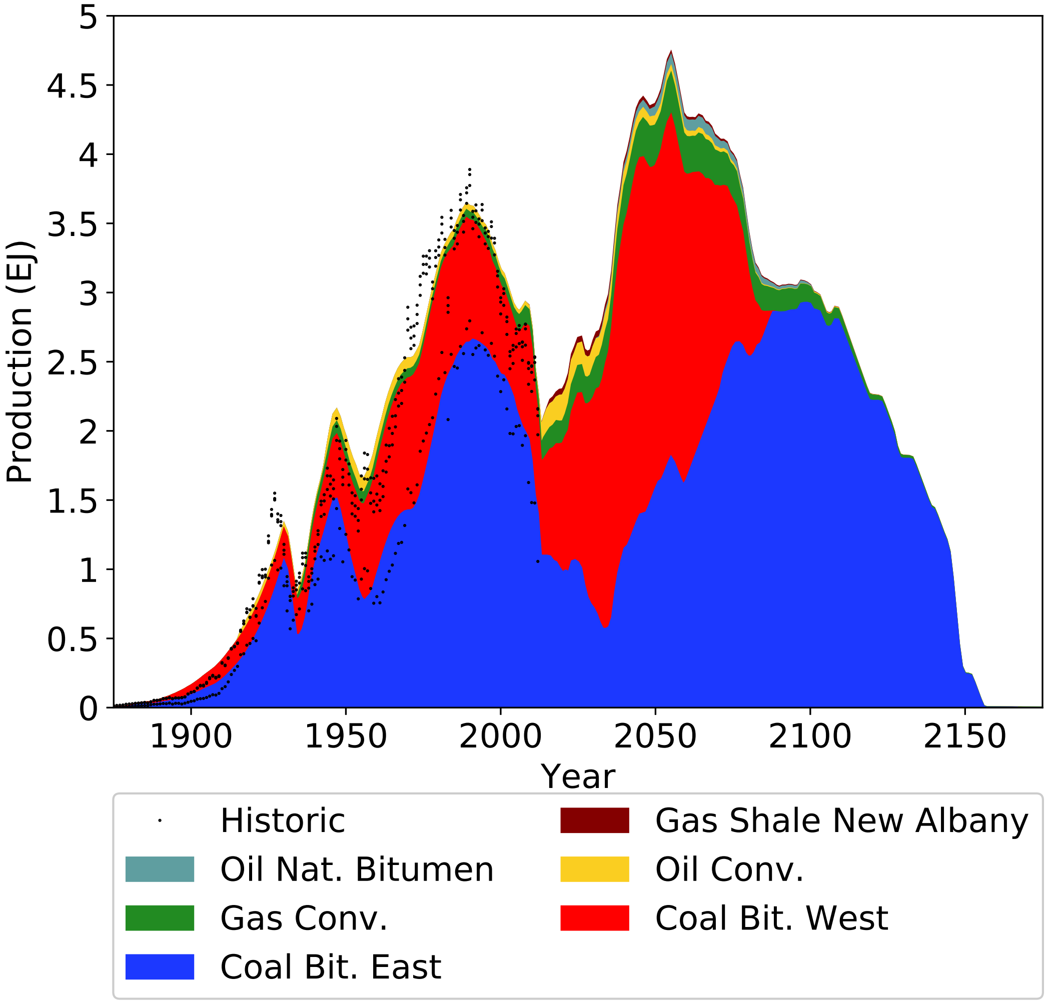

Figure 6.56: USA - Kentucky projections capped at 16

Table 6.56: Peak years - All

Name	URR	Peak Year	Peak Rate
Coal Bit. Kentucky East	404.29	2099	2.93
Coal Bit. Kentucky West	180.45	2045	2.58
Gas Conv. Kentucky	27.56	2057	0.3
Oil Conv. Kentucky	11.68	2020	0.19
Oil Nat. Bitumen Kentucky	3.43	2053	0.08
Gas Shale Kentucky New Albany	2.12	2030	0.05
**Total**	**629.53**	**2055**	**4.73**

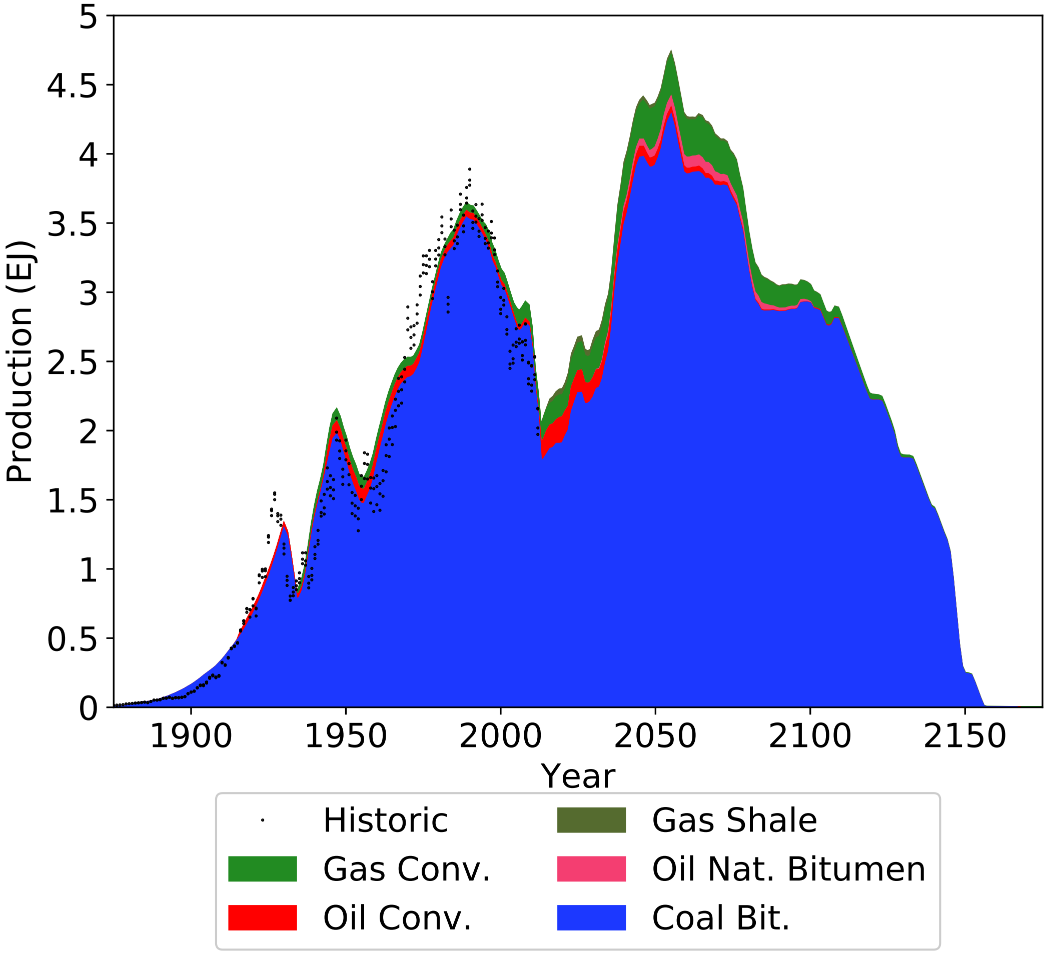

Figure 6.57: USA - Kentucky projection by mineral type

Table 6.57: Peak years - Minerals

**Name**	**URR**	**Peak Year**	**Peak Rate**
Coal Bit.	584.74	2055	4.28
Oil Conv.	11.68	2020	0.19
Oil Nat. Bitumen	3.43	2053	0.08
Gas Conv.	27.56	2057	0.3
Gas Shale	2.12	2030	0.05
**Total**	**629.53**	**2055**	**4.73**

Louisiana

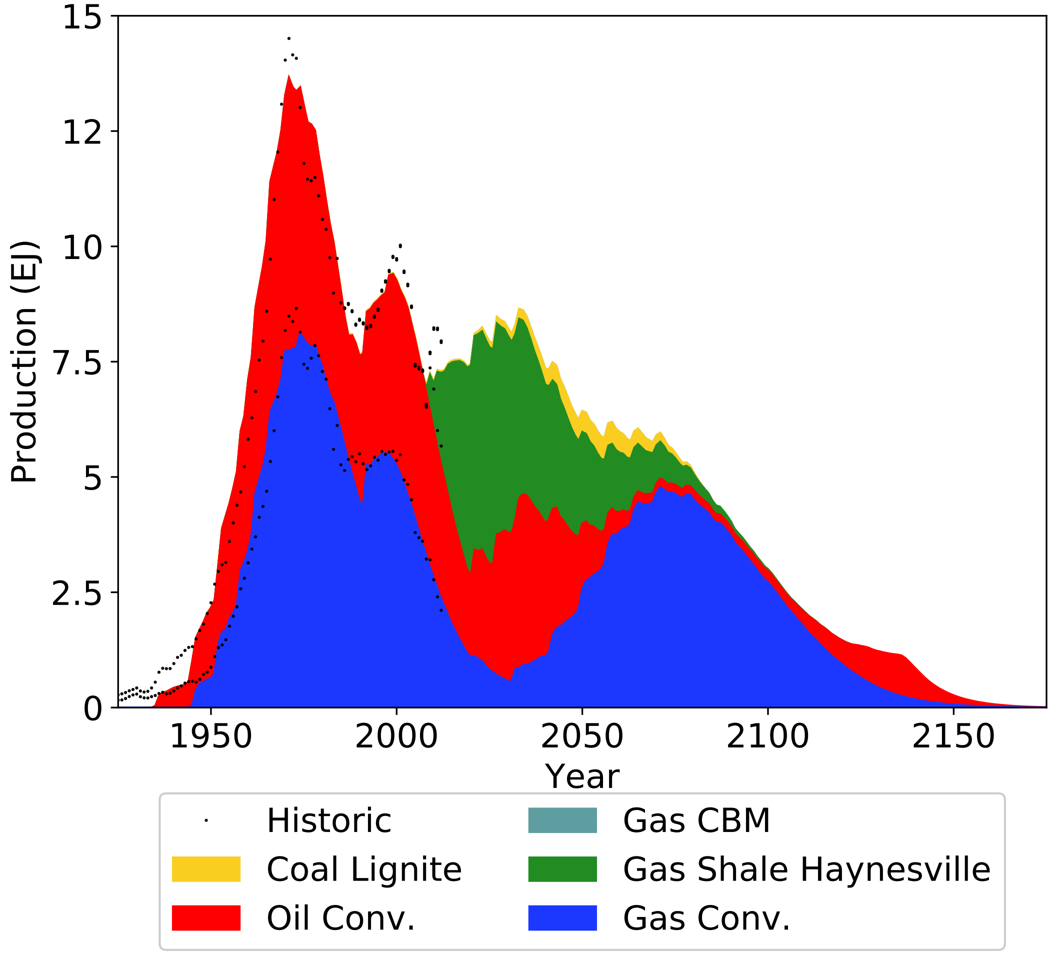

Figure 6.58: USA - Louisiana projections capped at 16

Table 6.58: Peak years - All

Name	URR	Peak Year	Peak Rate
Gas Conv. Louisiana	602.93	1974	8.12
Oil Conv. Louisiana	389.88	1971	5.91
Gas Shale Louisiana Haynesville	175.11	2023	4.74
Coal Lignite Louisiana	18.25	2056	0.49
Gas CBM Louisiana	0.01	2008	–
**Total**	**1186.18**	**1971**	**13.65**

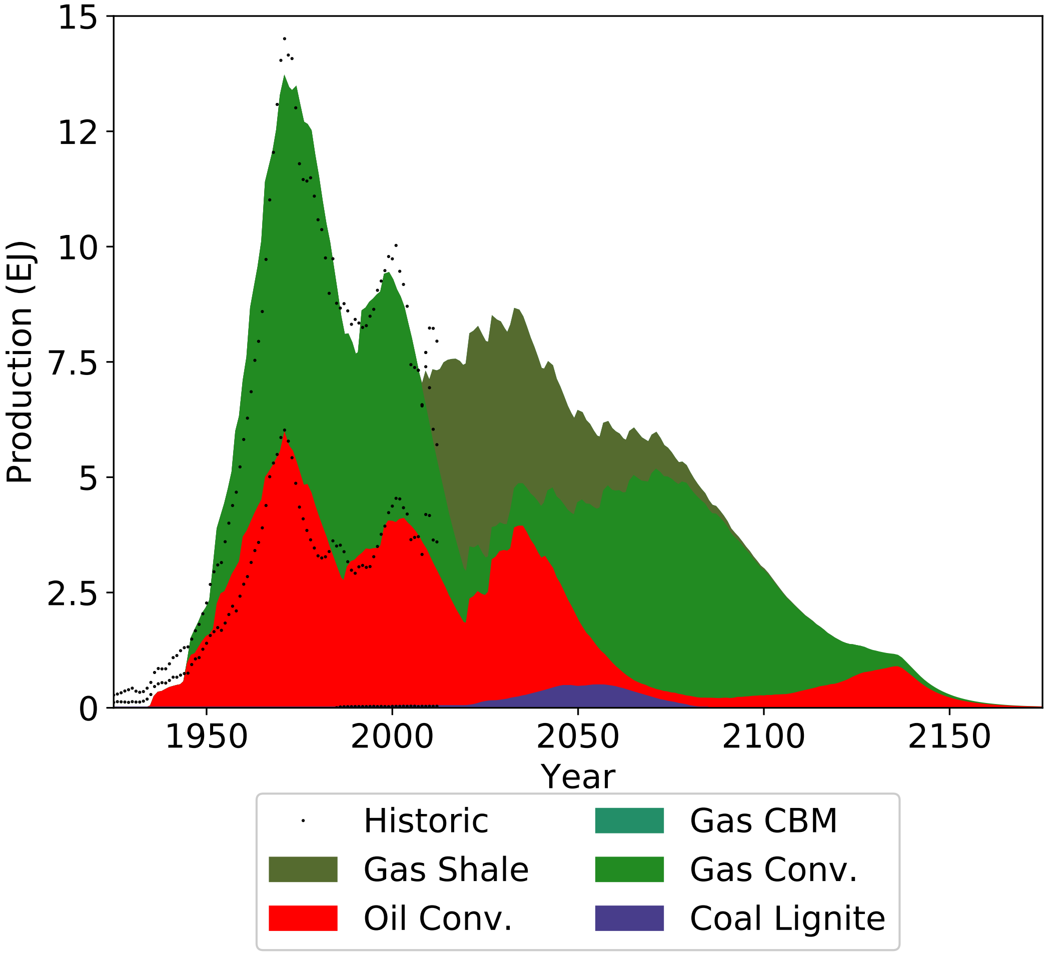

Figure 6.59: USA - Louisiana projection by mineral type

Table 6.59: Peak years - Minerals

**Name**	**URR**	**Peak Year**	**Peak Rate**
Coal Lignite	18.25	2056	0.49
Oil Conv.	389.88	1971	5.91
Gas Conv.	602.93	1974	8.12
Gas Shale	175.11	2023	4.74
Gas CBM	0.01	2008	–
**Total**	**1186.18**	**1971**	**13.65**

Maryland

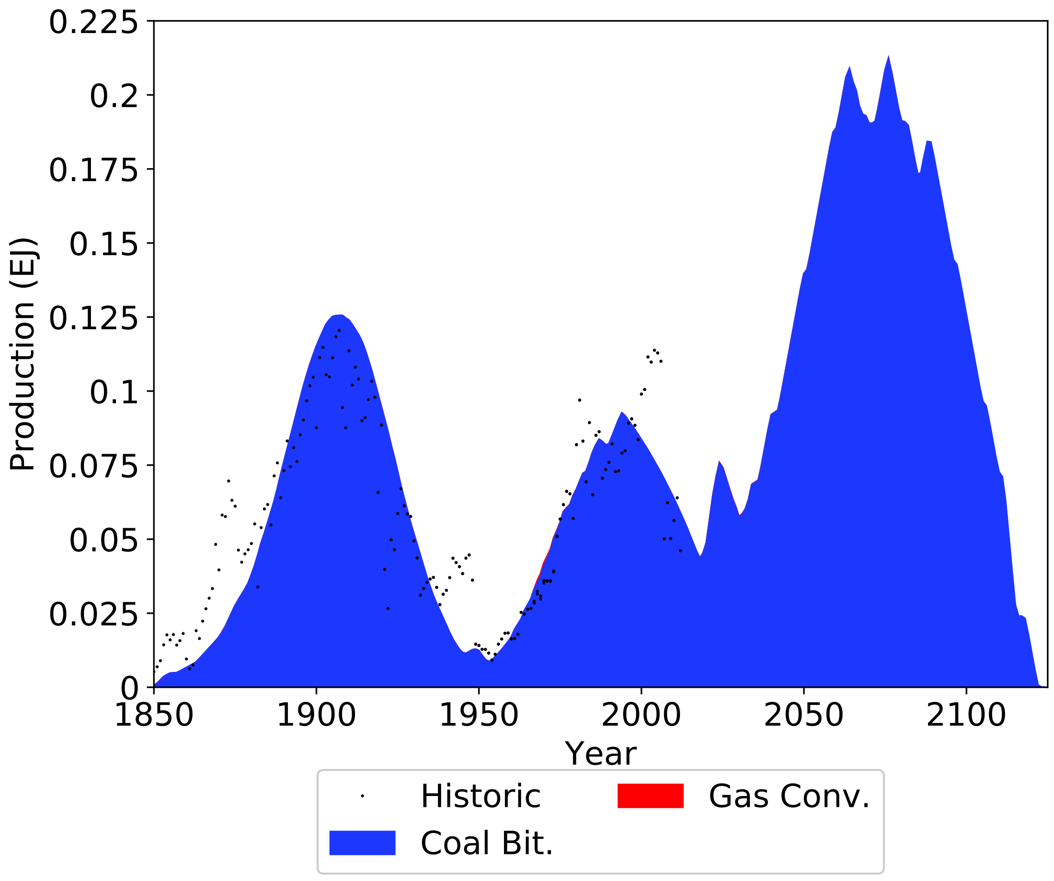

Figure 6.60: USA - Maryland projections capped at 16

Table 6.60: Peak years - All

Name	URR	Peak Year	Peak Rate
Coal Bit. Maryland	22.19	2076	0.21
Gas Conv. Maryland	0.01	1969	–
**Total**	**22.2**	**2076**	**0.21**

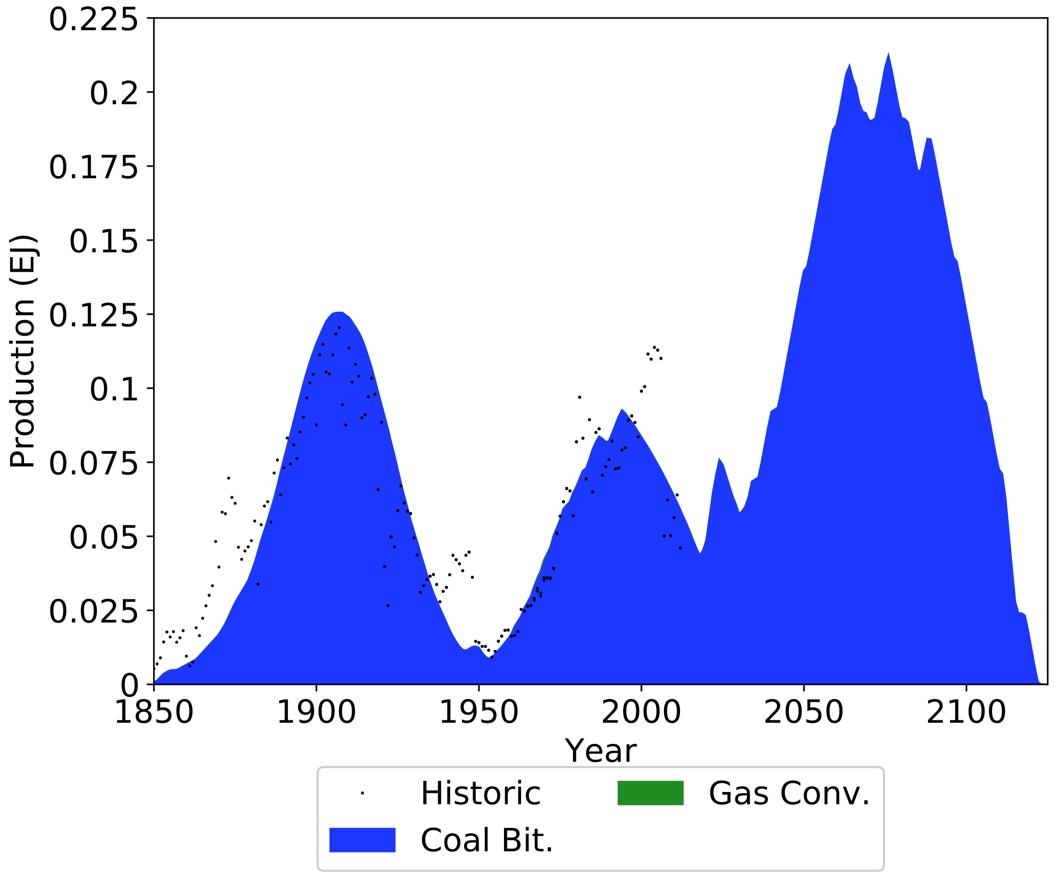

Figure 6.61: USA - Maryland projection by mineral type

Table 6.61: Peak years - Minerals

Name	URR	Peak Year	Peak Rate
Coal Bit.	22.19	2076	0.21
Gas Conv.	0.01	1969	–
**Total**	**22.2**	**2076**	**0.21**

Michigan

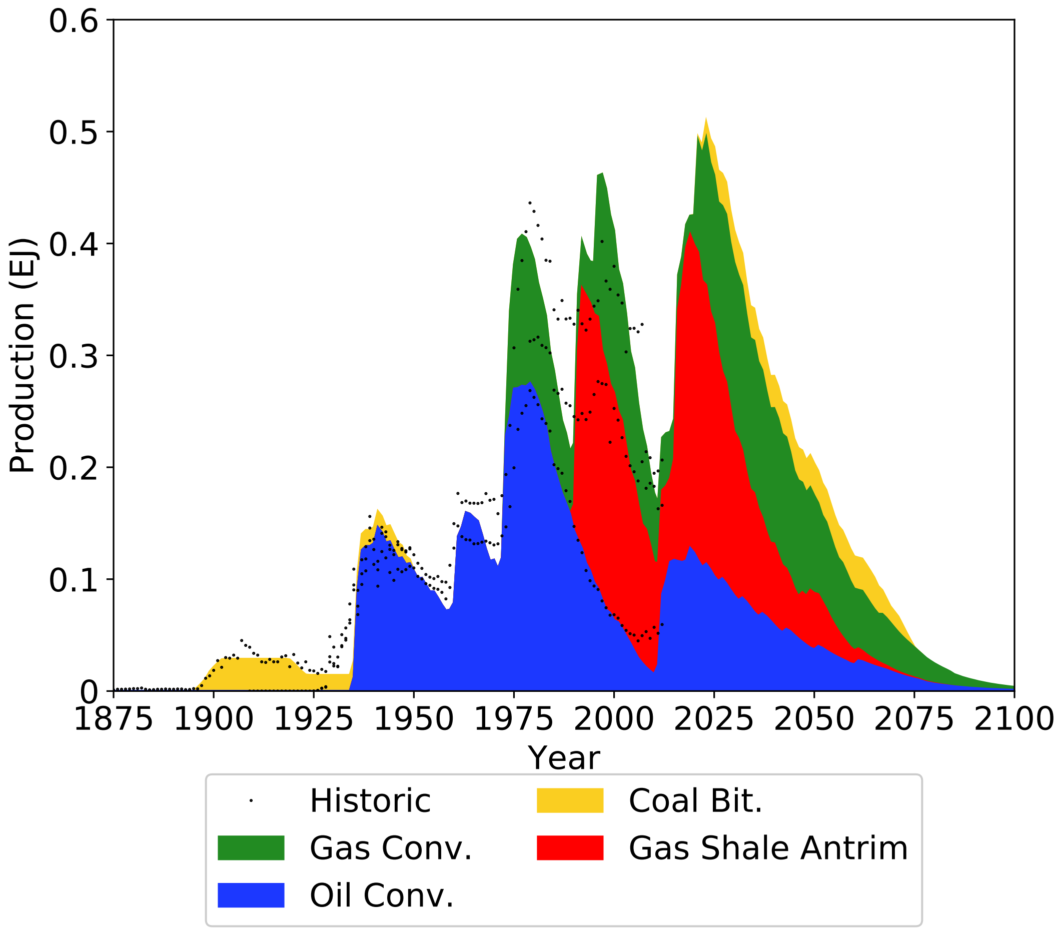

Figure 6.62: USA - Michigan projections capped at 16

Table 6.62: Peak years - All

Name	URR	Peak Year	Peak Rate
Oil Conv. Michigan	13.77	1979	0.28
Gas Shale Michigan Antrim	9.39	2019	0.28
Gas Conv. Michigan	9.19	1997	0.16
Coal Bit. Michigan	2.39	2048	0.03
**Total**	**34.74**	**2023**	**0.51**

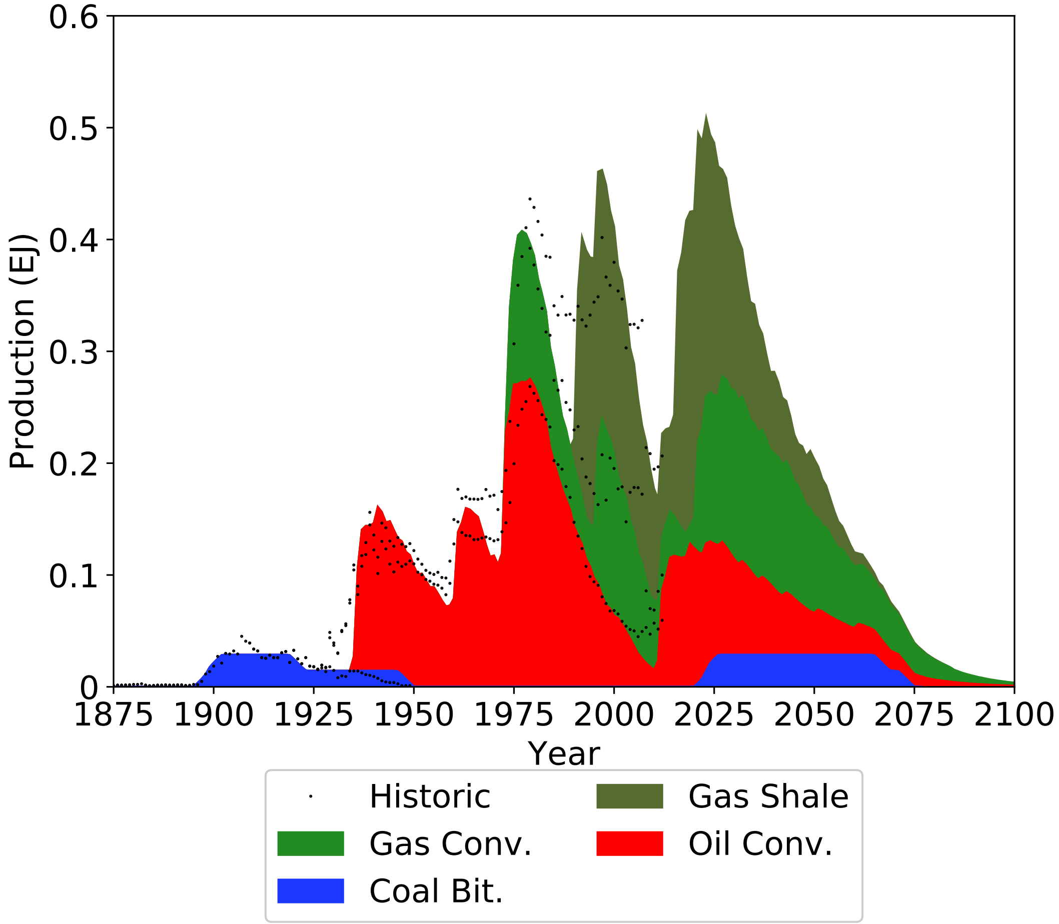

Figure 6.63: USA - Michigan projection by mineral type

Table 6.63: Peak years - Minerals

Name	URR	Peak Year	Peak Rate
Coal Bit.	2.39	2048	0.03
Oil Conv.	13.77	1979	0.28
Gas Conv.	9.19	1997	0.16
Gas Shale	9.39	2019	0.28
**Total**	**34.74**	**2023**	**0.51**

Mississippi

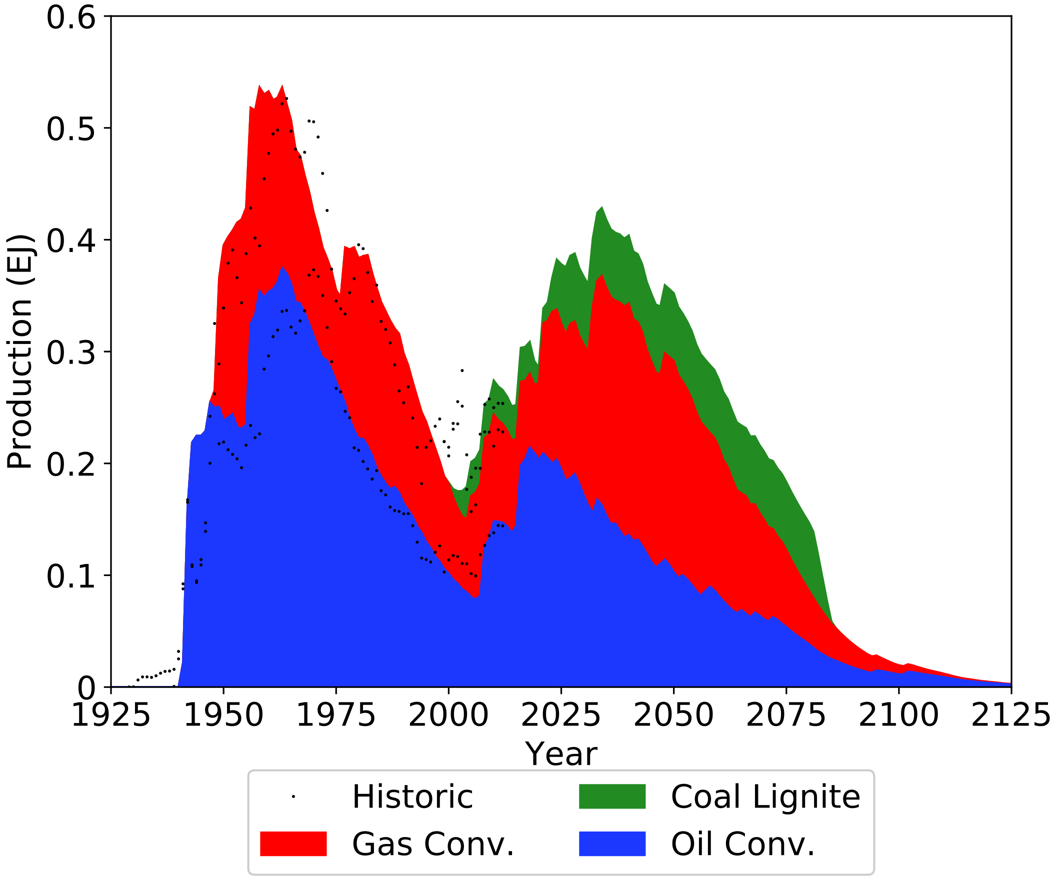

Figure 6.64: USA - Mississippi projections capped at 16

Table 6.64: Peak years - All

Name	URR	Peak Year	Peak Rate
Oil Conv. Mississippi	24.33	1963	0.37
Gas Conv. Mississippi	17.76	2040	0.21
Coal Lignite Mississippi	4.19	2048	0.06
**Total**	**46.28**	**1958**	**0.54**

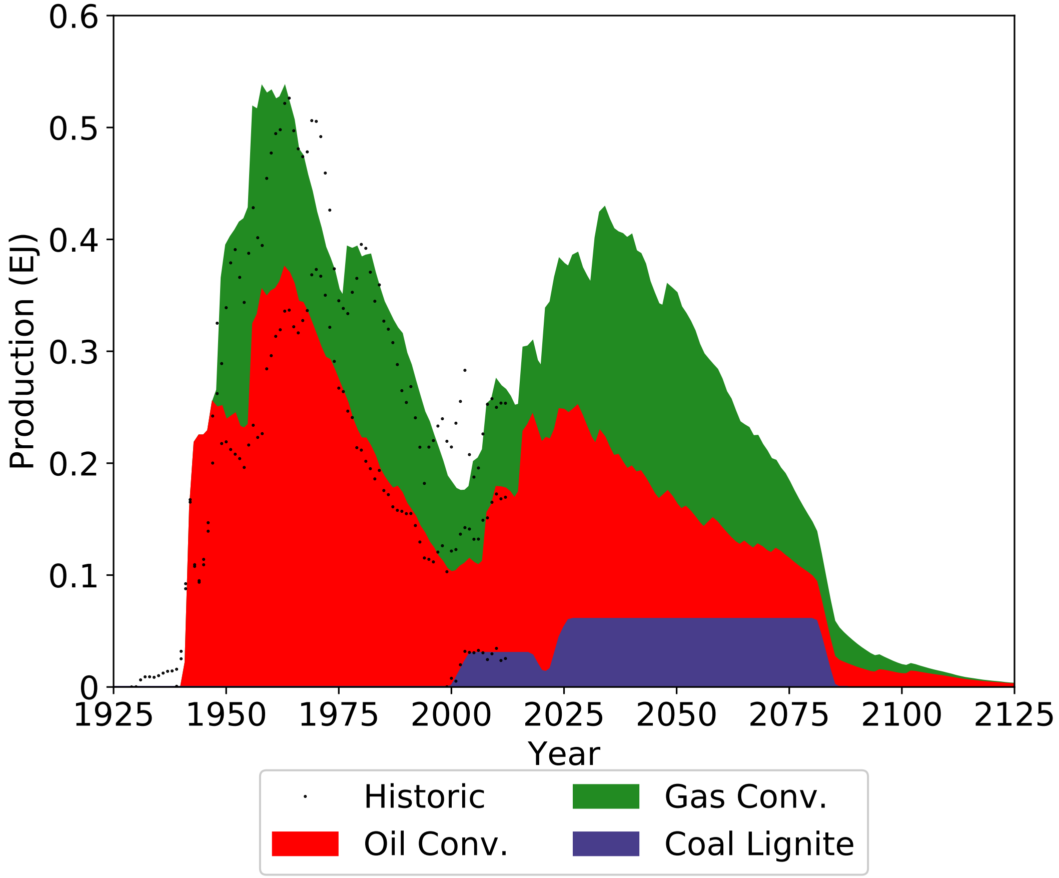

Figure 6.65: USA - Mississippi projection by mineral type

Table 6.65: Peak years - Minerals

Name	URR	Peak Year	Peak Rate
Coal Lignite	4.19	2048	0.06
Oil Conv.	24.33	1963	0.37
Gas Conv.	17.76	2040	0.21
**Total**	**46.28**	**1958**	**0.54**

Missouri

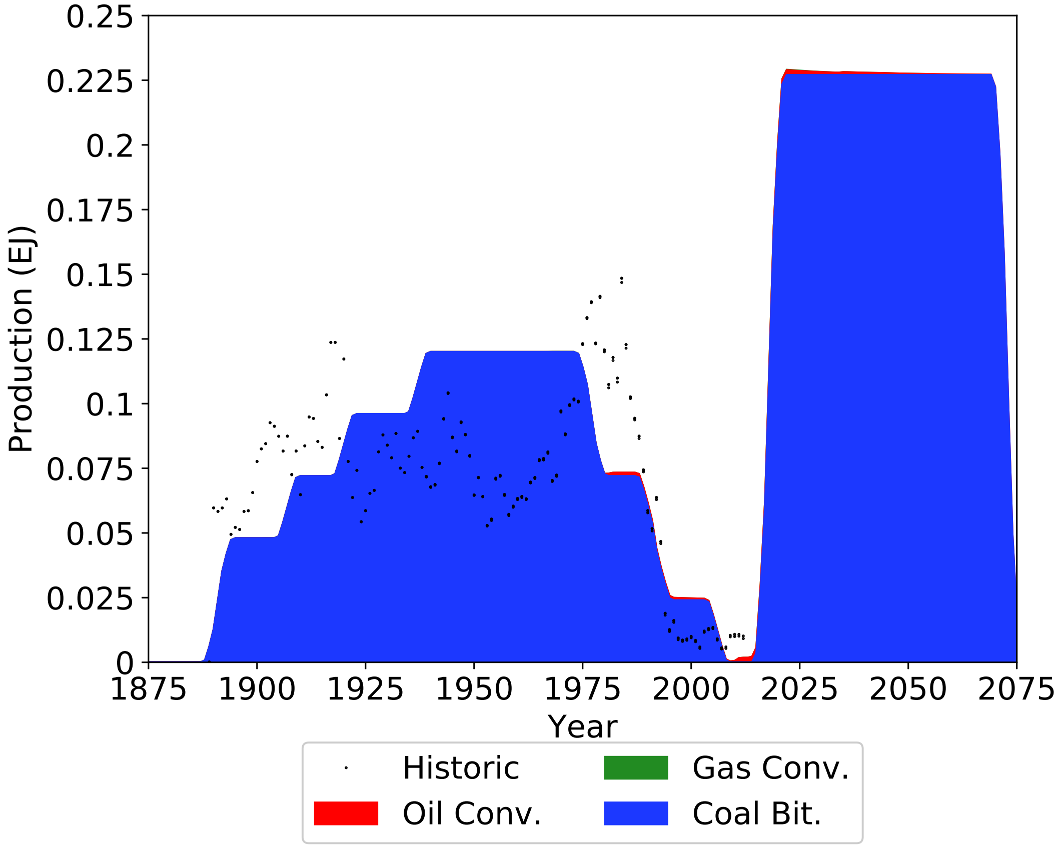

Figure 6.66: USA - Missouri projections capped at 16

Table 6.66: Peak years - All

Name	URR	Peak Year	Peak Rate
Coal Bit. Missouri	21.97	2048	0.23
Oil Conv. Missouri	0.09	2015	–
Gas Conv. Missouri	–	2022	–
**Total**	**22.06**	**2022**	**0.23**

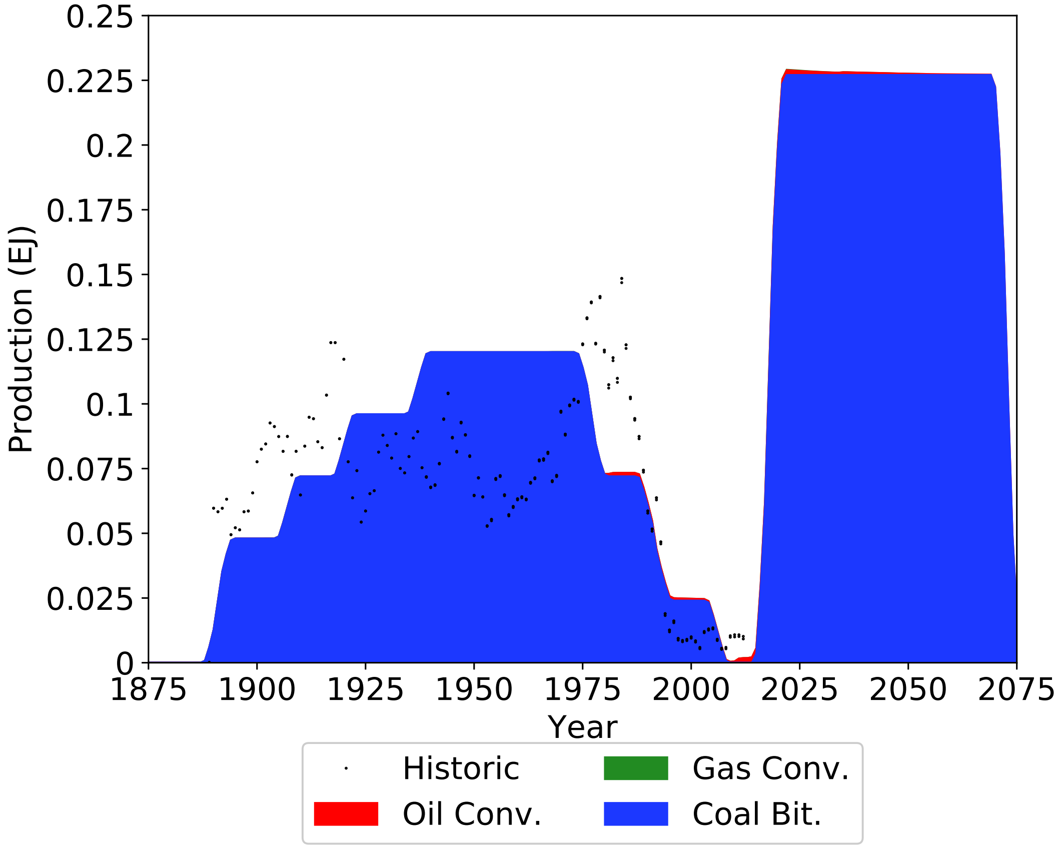

Figure 6.67: USA - Missouri projection by mineral type

Table 6.67: Peak years - Minerals

Name	URR	Peak Year	Peak Rate
Coal Bit.	21.97	2048	0.23
Oil Conv.	0.09	2015	–
Gas Conv.	–	2022	–
**Total**	**22.06**	**2022**	**0.23**

Montana

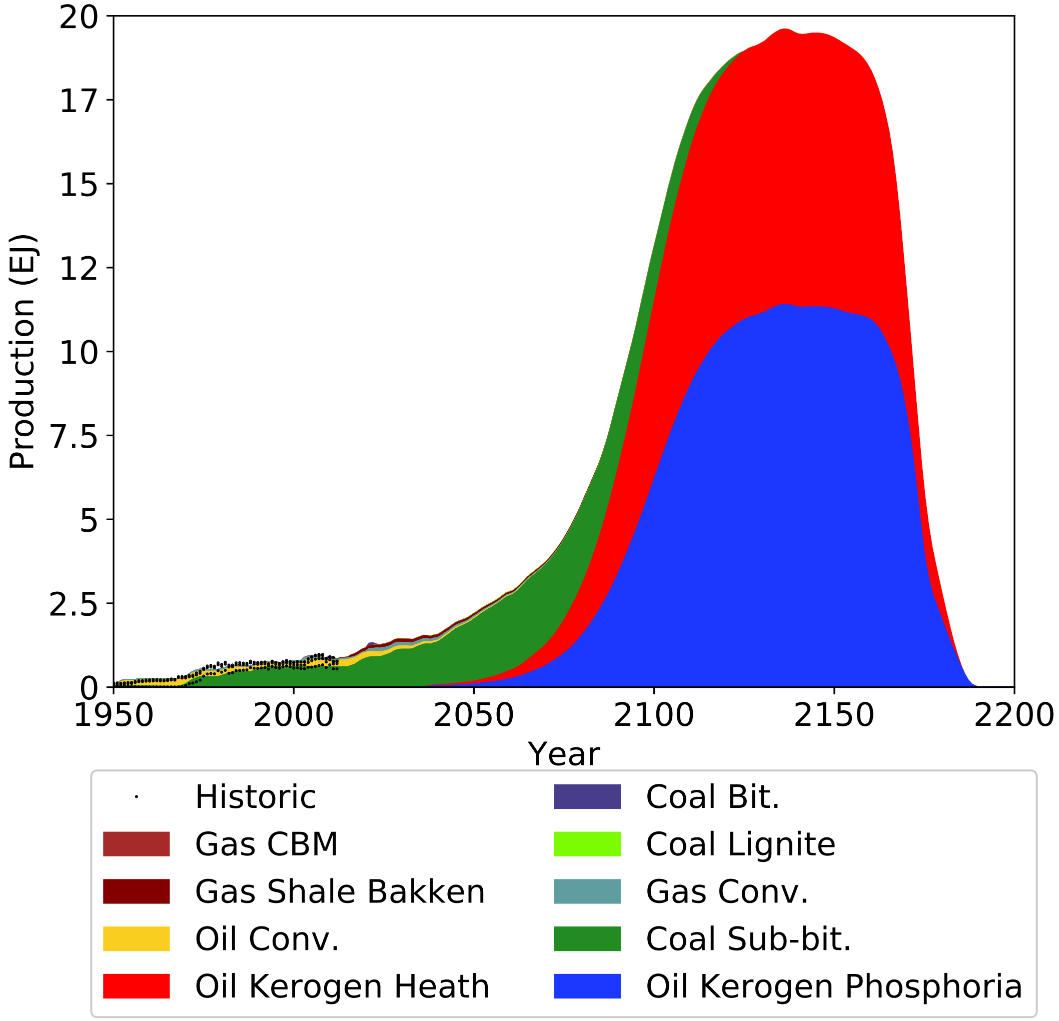

Figure 6.68: USA - Montana projections capped at 16

Table 6.68: Peak years - All

Name	URR	Peak Year	Peak Rate
Oil Kerogen Montana Phosphoria	859.5	2136	11.39
Oil Kerogen Montana Heath	618.8	2136	8.2
Coal Sub-bit. Montana	193.17	2066	2.38
Oil Conv. Montana	15.15	2005	0.23
Gas Conv. Montana	7.37	2028	0.12
Gas Shale Montana Bakken	5.06	2030	0.1
Coal Lignite Montana	0.85	2057	0.02
Gas CBM Montana	0.44	2004	0.01
Coal Bit. Montana	0.26	2022	0.07
**Total**	**1700.6**	**2136**	**19.59**

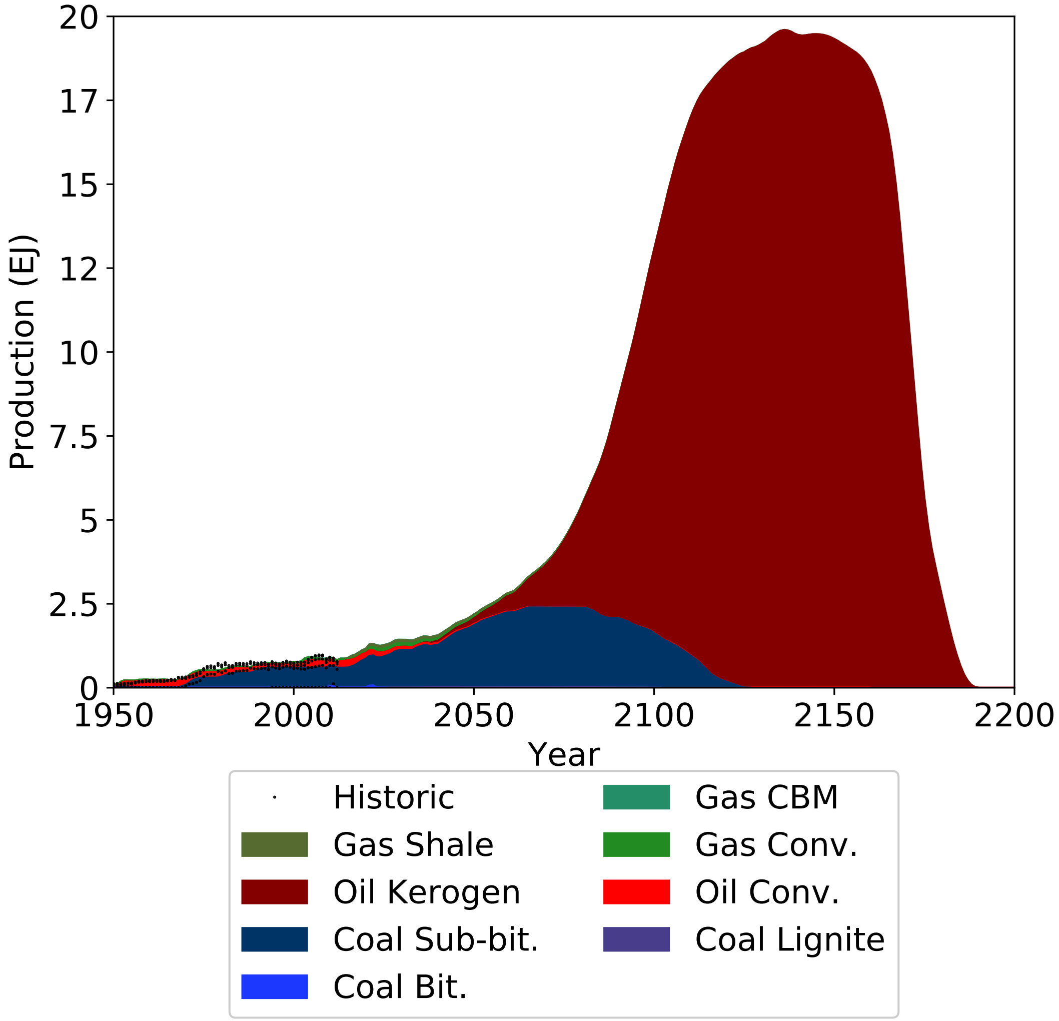

Figure 6.69: USA - Montana projection by mineral type

Table 6.69: Peak years - Minerals

**Name**	**URR**	**Peak Year**	**Peak Rate**
Coal Bit.	0.26	2022	0.07
Coal Lignite	0.85	2057	0.02
Coal Sub-bit.	193.17	2066	2.38
Oil Conv.	15.15	2005	0.23
Oil Kerogen	1478.3	2136	19.59
Gas Conv.	7.37	2028	0.12
Gas Shale	5.06	2030	0.1
Gas CBM	0.44	2004	0.01
**Total**	**1700.6**	**2136**	**19.59**

Nebraska

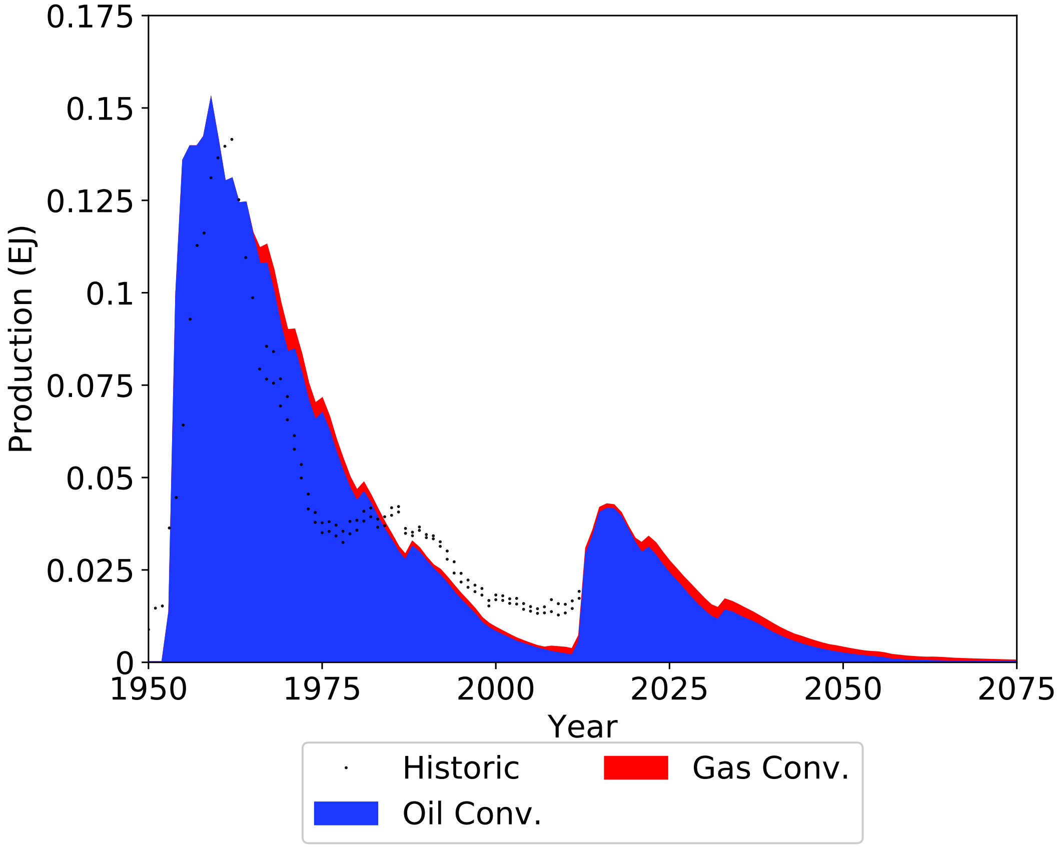

Figure 6.70: USA - Nebraska projections capped at 16

Table 6.70: Peak years - All

Name	URR	Peak Year	Peak Rate
Oil Conv. Nebraska	3.96	1959	0.15
Gas Conv. Nebraska	0.23	1969	0.01
**Total**	**4.19**	**1959**	**0.15**

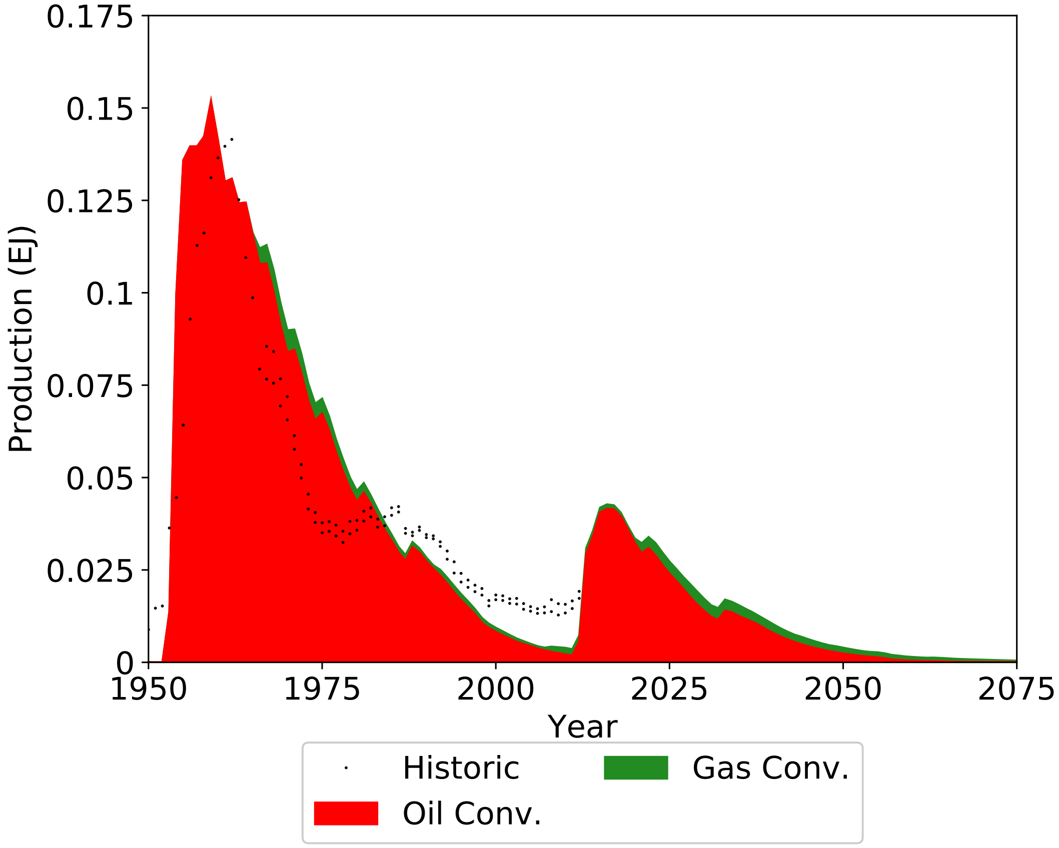

Figure 6.71: USA - Nebraska projection by mineral type

Table 6.71: Peak years - Minerals

**Name**	**URR**	**Peak Year**	**Peak Rate**
Oil Conv.	3.96	1959	0.15
Gas Conv.	0.23	1969	0.01
**Total**	**4.19**	**1959**	**0.15**

Nevada

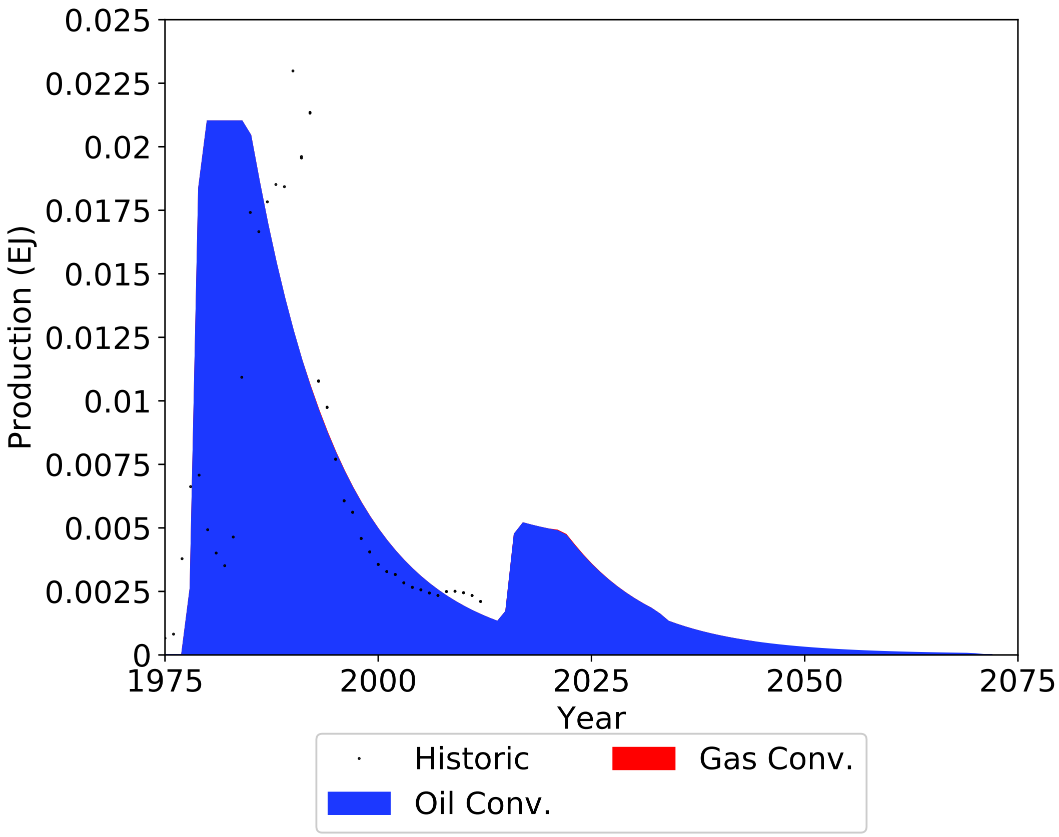

Figure 6.72: USA - Nevada projections capped at 16

Table 6.72: Peak years - All

**Name**	**URR**	**Peak Year**	**Peak Rate**
Oil Conv. Nevada	0.42	1980	0.02
Gas Conv. Nevada	–	1993	–
**Total**	**0.42**	**1980**	**0.02**

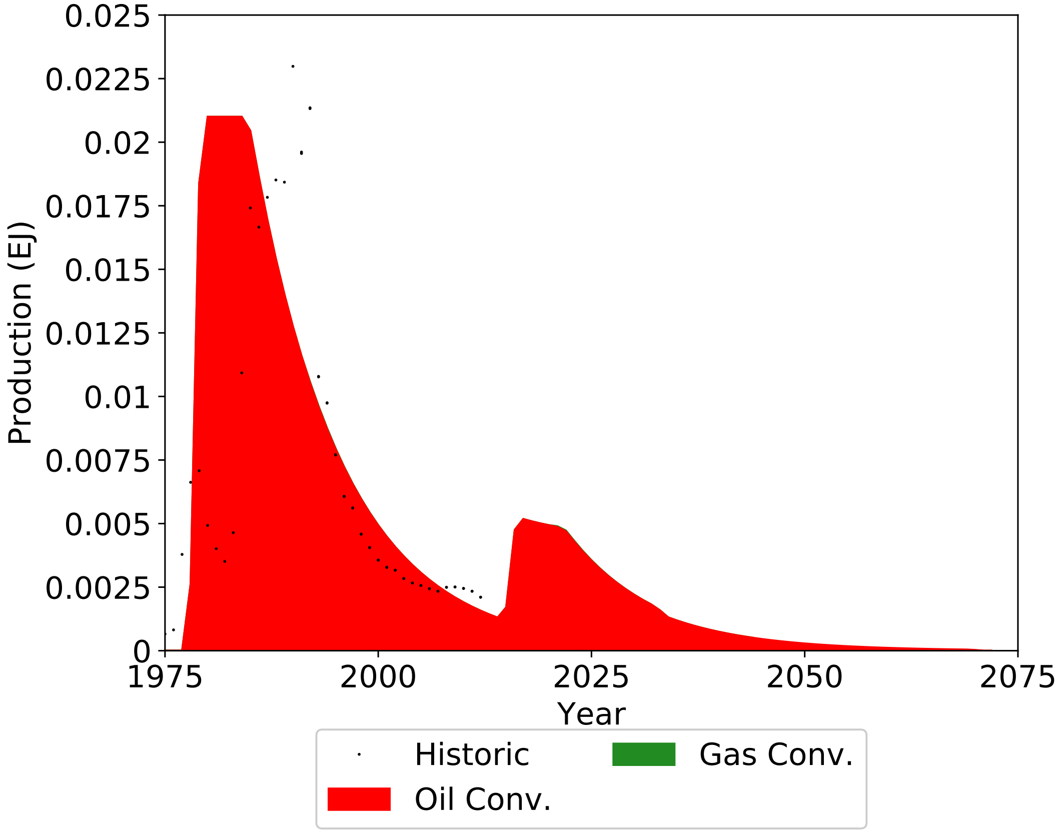

Figure 6.73: USA - Nevada projection by mineral type

Table 6.73: Peak years - Minerals

**Name**	**URR**	**Peak Year**	**Peak Rate**
Oil Conv.	0.42	1980	0.02
Gas Conv.	–	1993	–
**Total**	**0.42**	**1980**	**0.02**

New Mexico

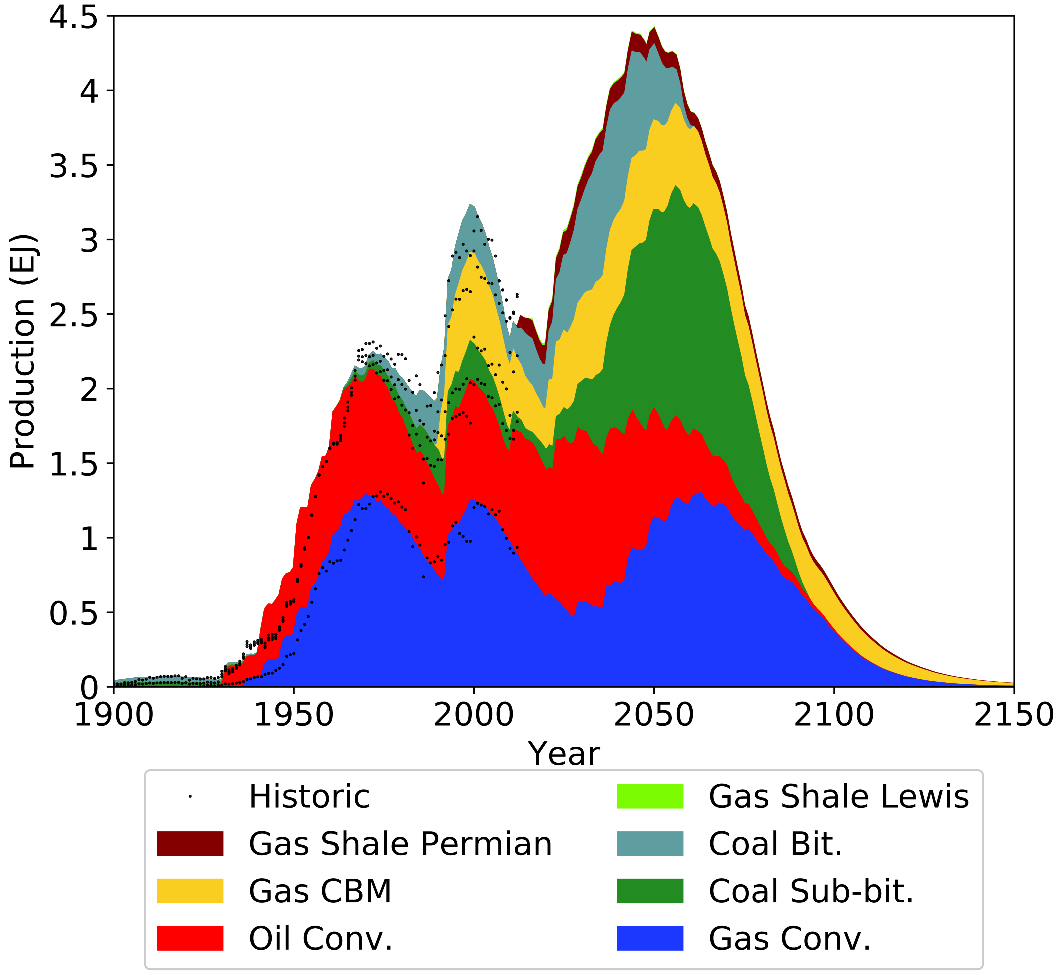

Figure 6.74: USA - New Mexico projections capped at 16

Table 6.74: Peak years - All

Name	URR	Peak Year	Peak Rate
Gas Conv. New Mexico	142.55	2063	1.3
Oil Conv. New Mexico	100.05	2028	1.17
Coal Sub-bit. New Mexico	68.57	2057	1.54
Gas CBM New Mexico	56.28	2038	0.64
Coal Bit. New Mexico	35.07	2035	0.84
Gas Shale New Mexico Permian	8.96	2027	0.16
Gas Shale New Mexico Lewis	0.53	2021	0.02
**Total**	**412.01**	**2050**	**4.42**

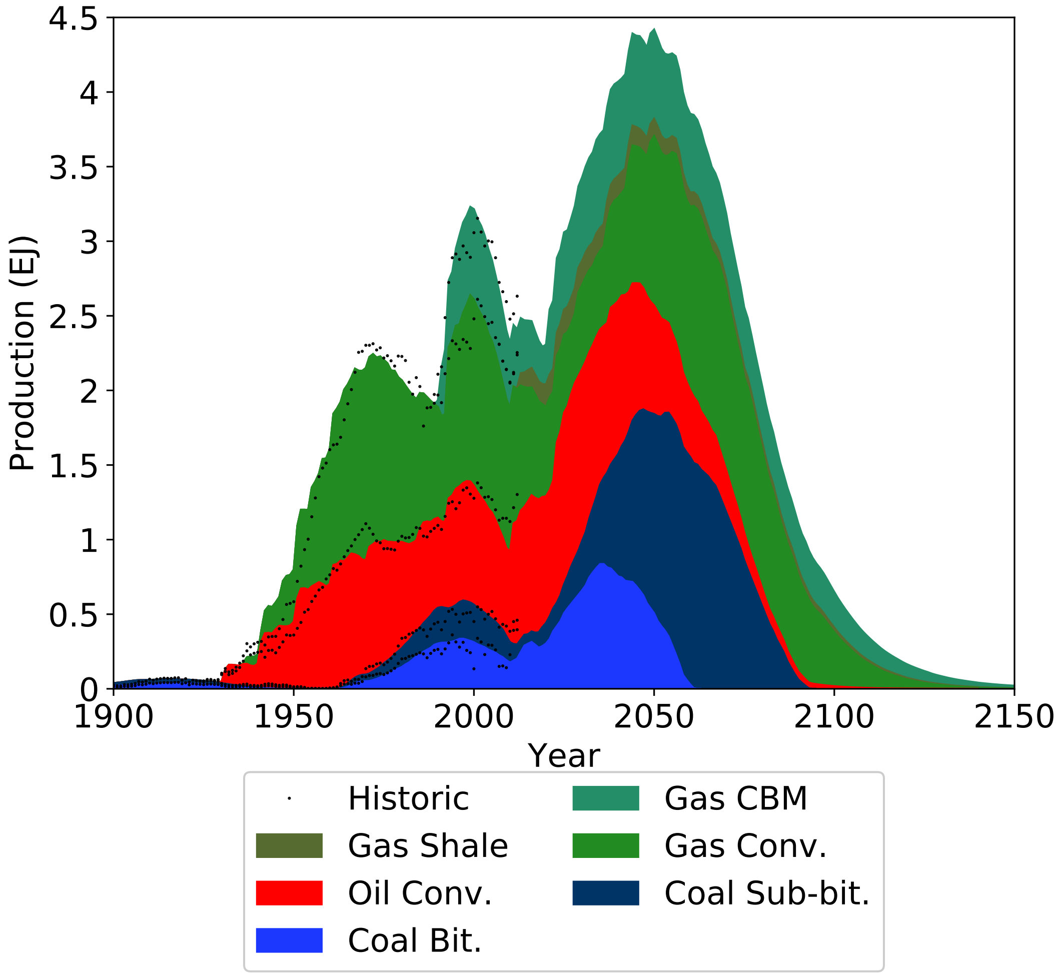

Figure 6.75: USA - New Mexico projection by mineral type

Table 6.75: Peak years - Minerals

Name	URR	Peak Year	Peak Rate
Coal Bit.	35.07	2035	0.84
Coal Sub-bit.	68.57	2057	1.54
Oil Conv.	100.05	2028	1.17
Gas Conv.	142.55	2063	1.3
Gas Shale	9.49	2027	0.17
Gas CBM	56.28	2038	0.64
**Total**	**412.01**	**2050**	**4.42**

New York

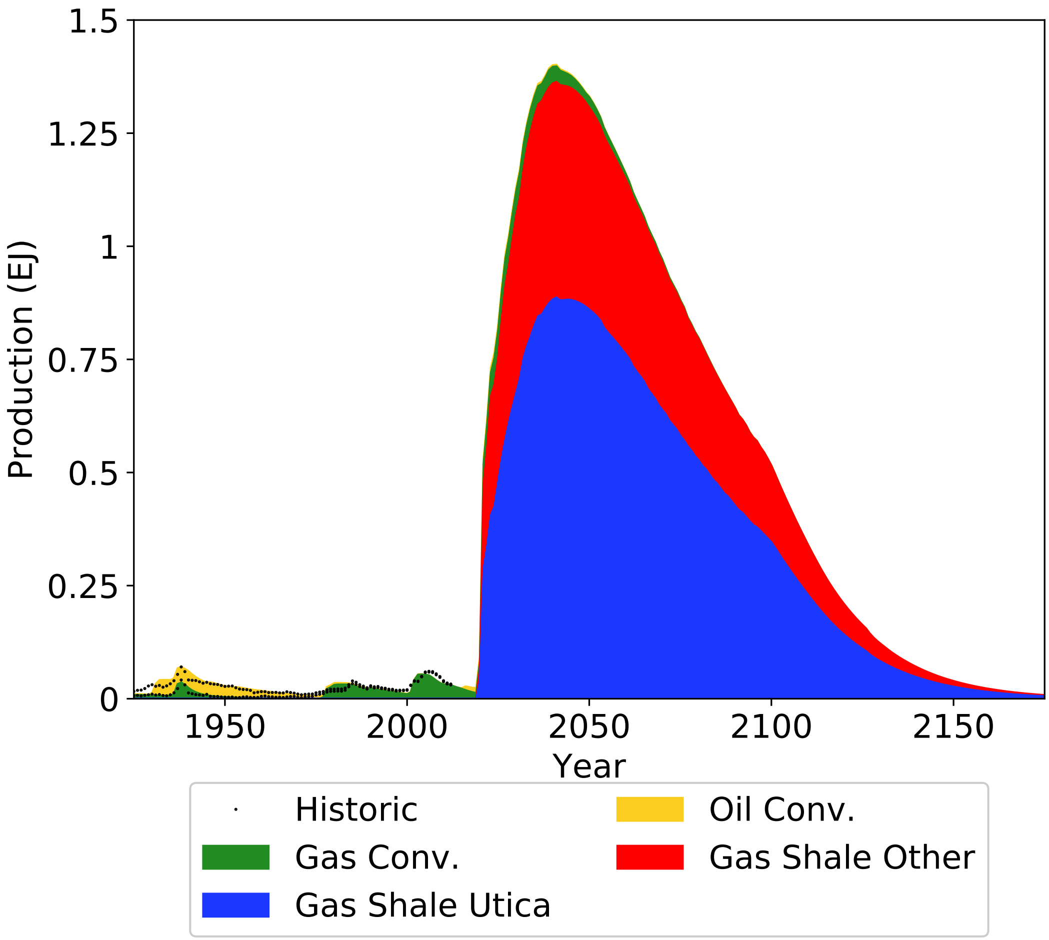

Figure 6.76: USA - New York projections capped at 16

Table 6.76: Peak years - All

Name	URR	Peak Year	Peak Rate
Gas Shale New York Utica	58.28	2041	0.89
Gas Shale New York Other	30.45	2040	0.48
Gas Conv. New York	3.15	2025	0.06
Oil Conv. New York	1.5	1940	0.04
**Total**	**93.38**	**2041**	**1.4**

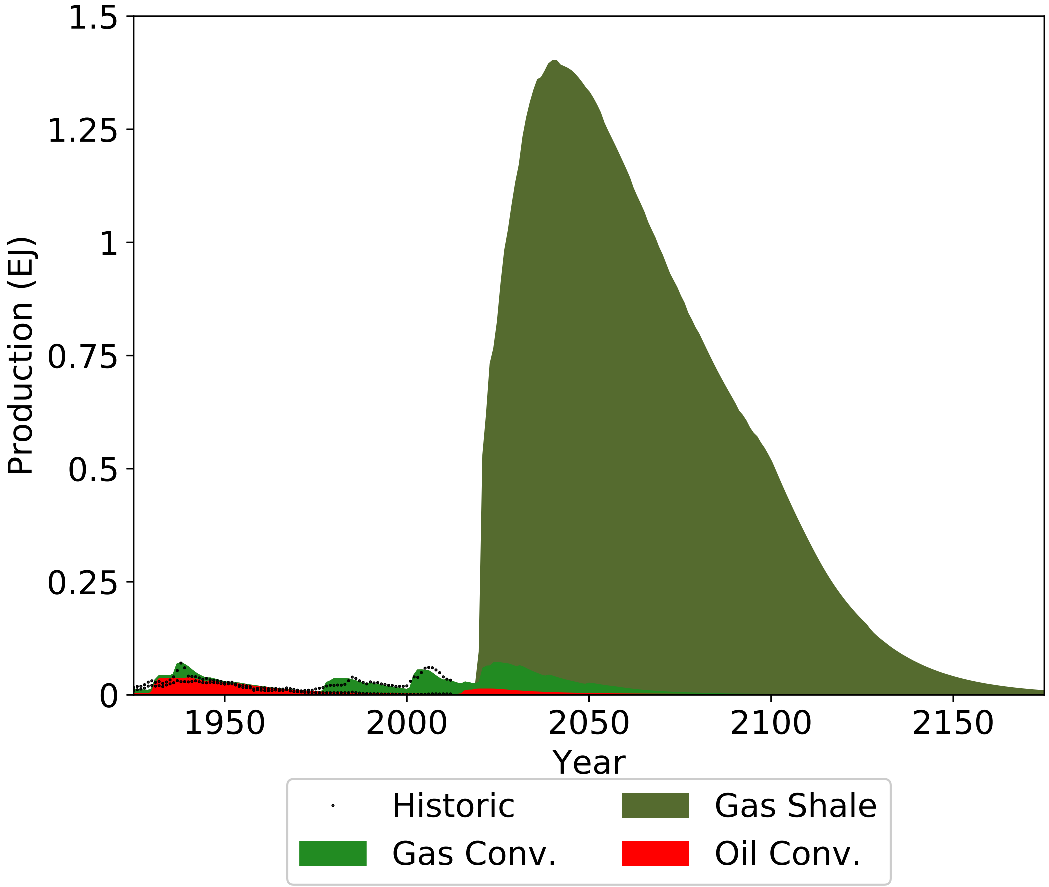

Figure 6.77: USA - New York projection by mineral type

Table 6.77: Peak years - Minerals

Name	URR	Peak Year	Peak Rate
Oil Conv.	1.5	1940	0.04
Gas Conv.	3.15	2025	0.06
Gas Shale	88.73	2041	1.36
**Total**	**93.38**	**2041**	**1.4**

North Dakota

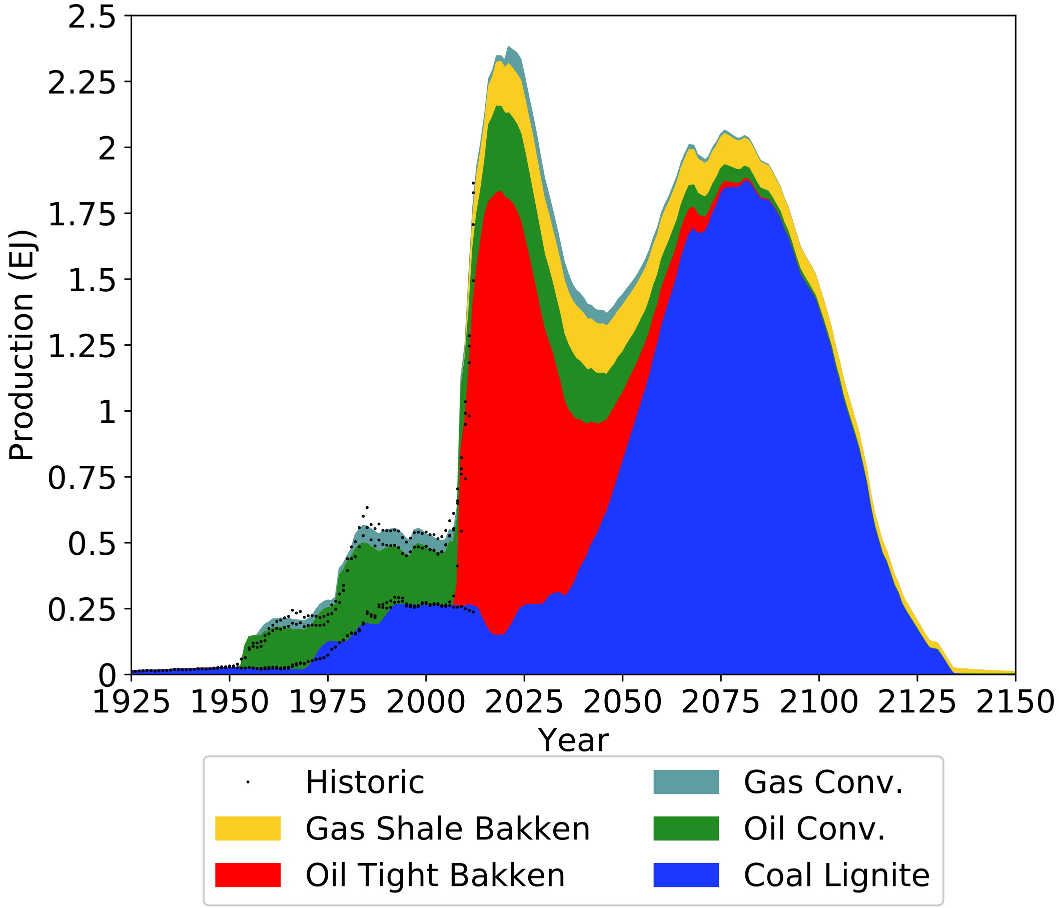

Figure 6.78: USA - North Dakota projections capped at 16

Table 6.78: Peak years - All

Name	URR	Peak Year	Peak Rate
Coal Lignite North Dakota	118.29	2081	1.87
Oil Tight North Dakota Bakken	43.91	2019	1.68
Oil Conv. North Dakota	25.2	2024	0.33
Gas Shale North Dakota Bakken	15.24	2027	0.21
Gas Conv. North Dakota	5.41	2028	0.09
**Total**	**208.05**	**2021**	**2.38**

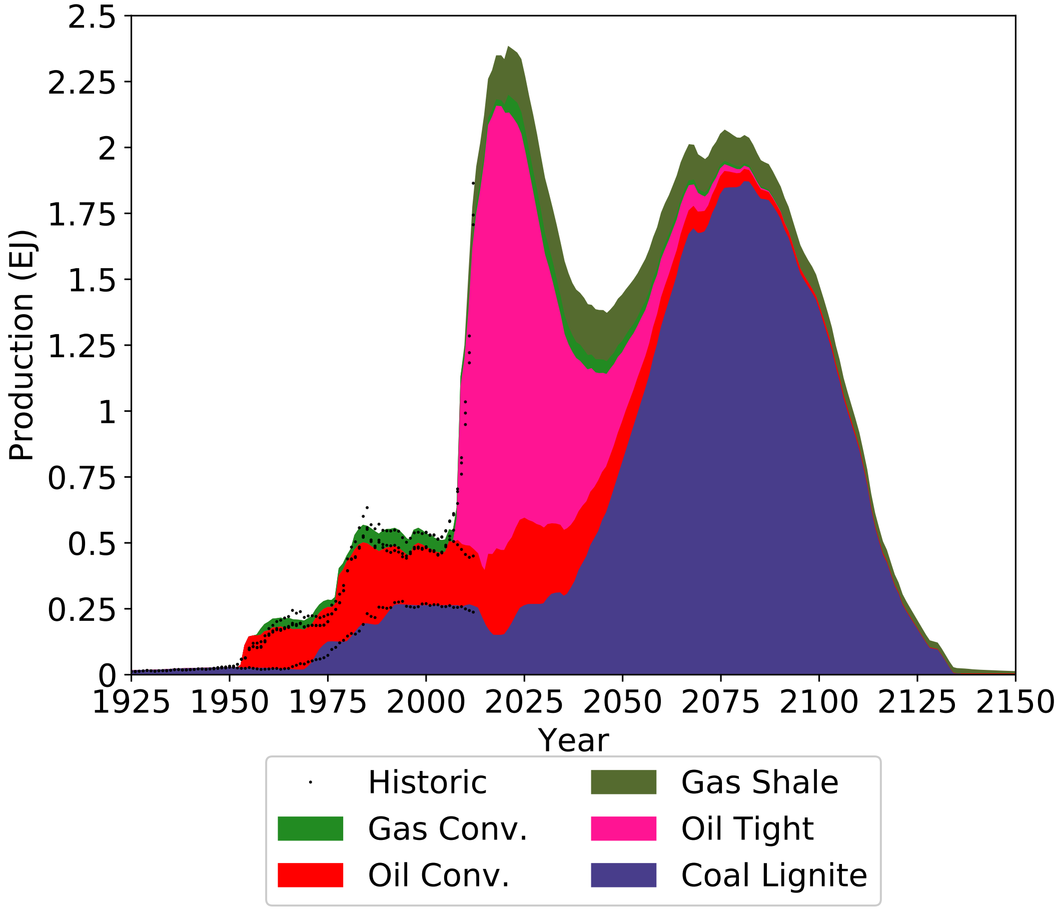

Figure 6.79: USA - North Dakota projection by mineral type

Table 6.79: Peak years - Minerals

Name	URR	Peak Year	Peak Rate
Coal Lignite	118.29	2081	1.87
Oil Conv.	25.2	2024	0.33
Oil Tight	43.91	2019	1.68
Gas Conv.	5.41	2028	0.09
Gas Shale	15.24	2027	0.21
**Total**	**208.05**	**2021**	**2.38**

Ohio

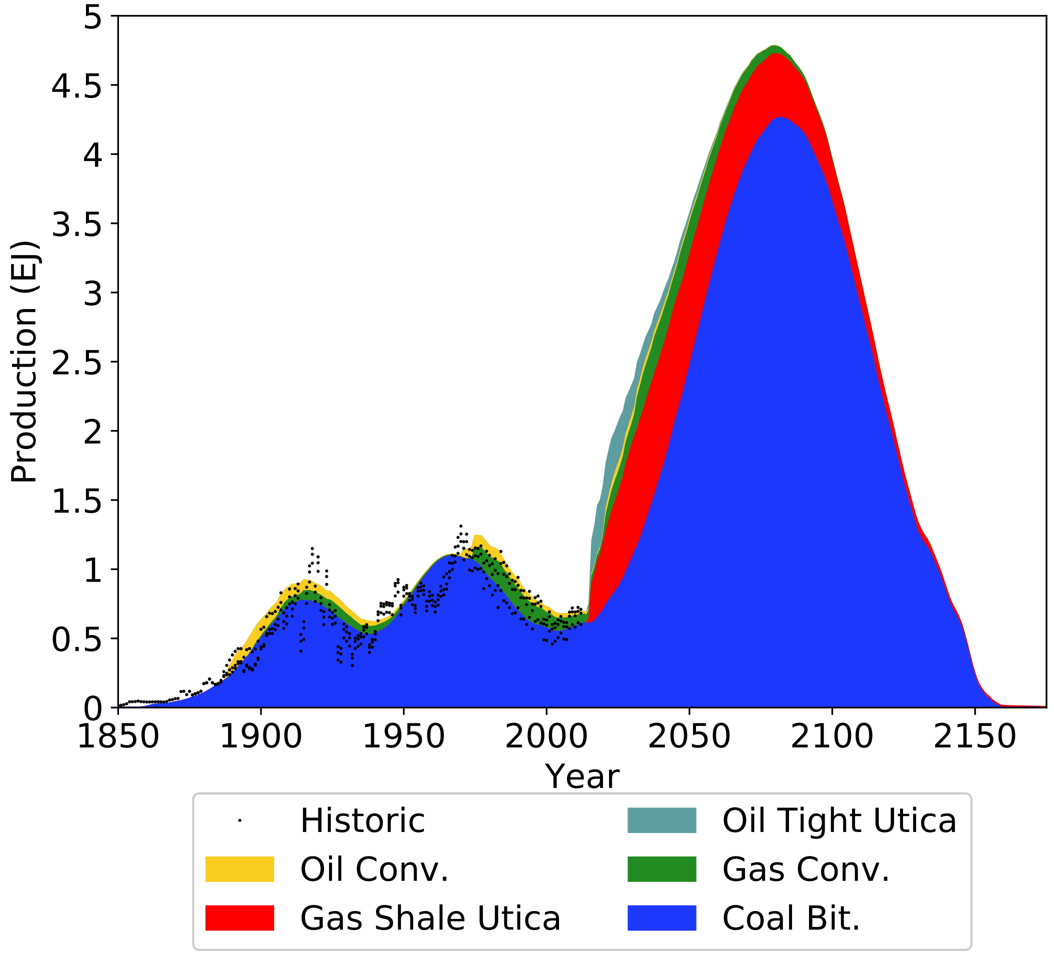

Figure 6.80: USA - Ohio projections capped at 16

Table 6.80: Peak years - All

Name	URR	Peak Year	Peak Rate
Coal Bit. Ohio	414.76	2082	4.26
Gas Shale Ohio Utica	58.28	2038	0.88
Gas Conv. Ohio	19.23	2038	0.26
Oil Conv. Ohio	8.58	1899	0.12
Oil Tight Ohio Utica	7.19	2020	0.35
**Total**	**508.04**	**2080**	**4.78**

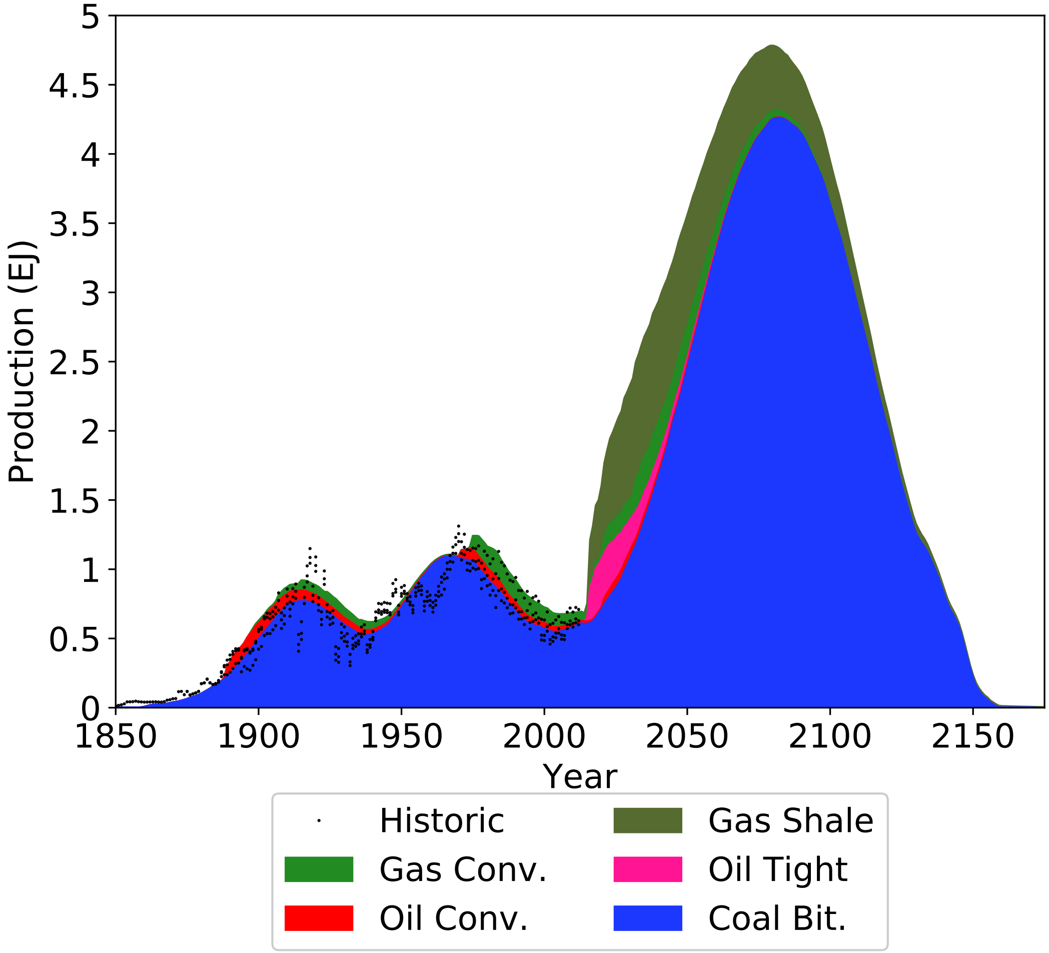

Figure 6.81: USA - Ohio projection by mineral type

Table 6.81: Peak years - Minerals

**Name**	**URR**	**Peak Year**	**Peak Rate**
Coal Bit.	414.76	2082	4.26
Oil Conv.	8.58	1899	0.12
Oil Tight	7.19	2020	0.35
Gas Conv.	19.23	2038	0.26
Gas Shale	58.28	2038	0.88
**Total**	**508.04**	**2080**	**4.78**

Oklahoma

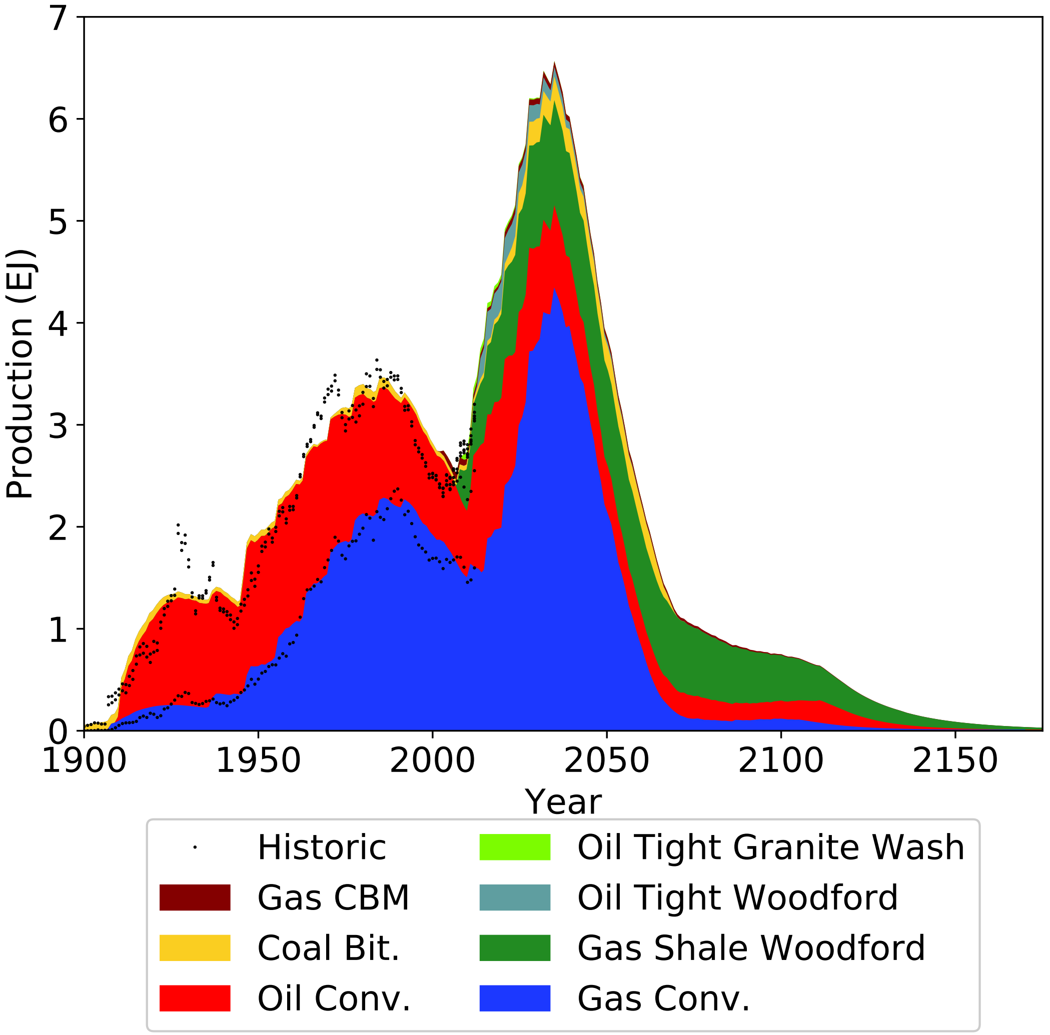

Figure 6.82: USA - Oklahoma projections capped at 16

Table 6.82: Peak years - All

**Name**	**URR**	**Peak Year**	**Peak Rate**
Gas Conv. Oklahoma	251.13	2035	4.31
Oil Conv. Oklahoma	156.34	1965	1.35
Gas Shale Oklahoma Woodford	82.9	2034	1.03
Coal Bit. Oklahoma	17.56	2048	0.23
Oil Tight Oklahoma Woodford	5.5	2016	0.29
Gas CBM Oklahoma	3.94	2005	0.07
Oil Tight Oklahoma Granite Wash	0.89	2011	0.07
**Total**	**518.26**	**2035**	**6.52**

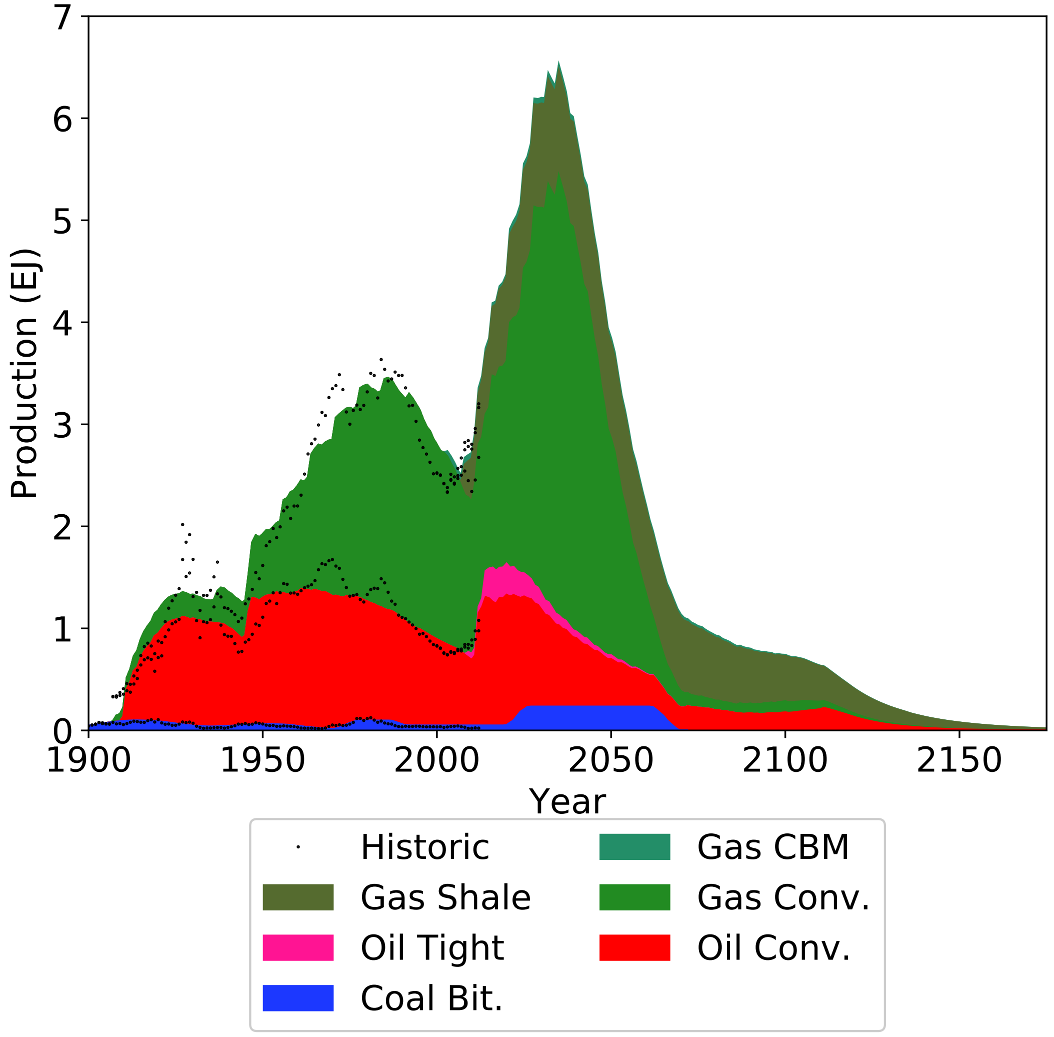

Figure 6.83: USA - Oklahoma projection by mineral type

Table 6.83: Peak years - Minerals

**Name**	**URR**	**Peak Year**	**Peak Rate**
Coal Bit.	17.56	2048	0.23
Oil Conv.	156.34	1965	1.35
Oil Tight	6.39	2016	0.34
Gas Conv.	251.13	2035	4.31
Gas Shale	82.9	2034	1.03
Gas CBM	3.94	2005	0.07
**Total**	**518.26**	**2035**	**6.52**

Oregon

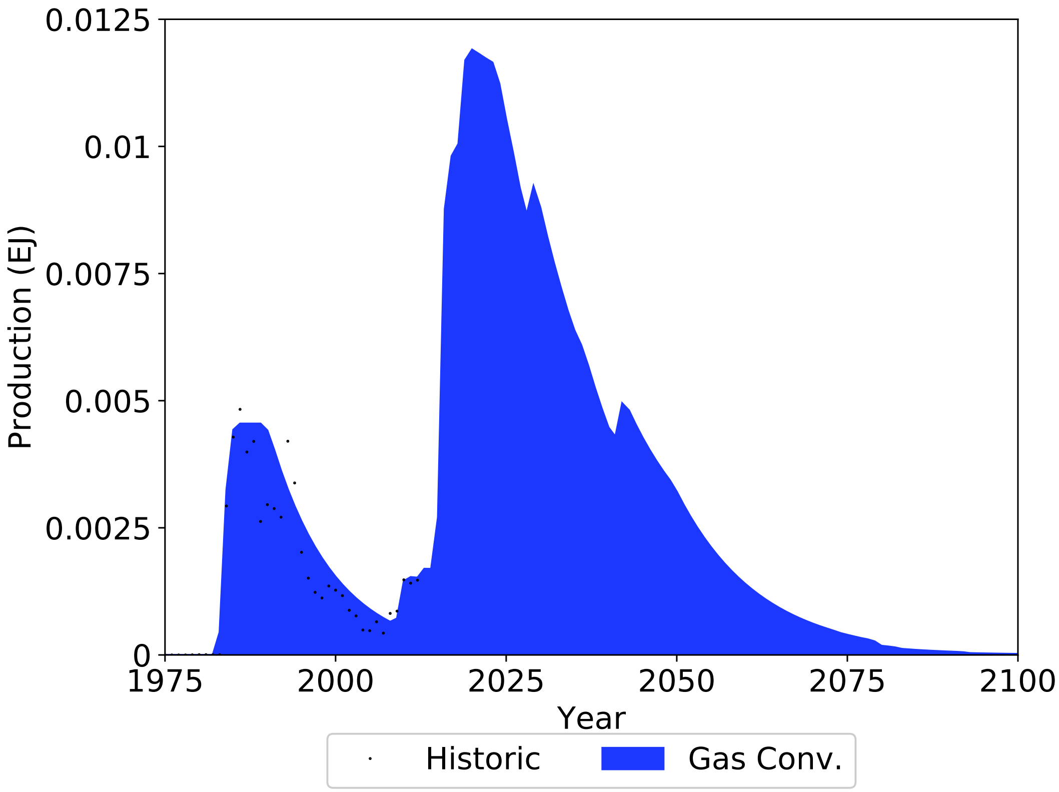

Figure 6.84: USA - Oregon projections capped at 16

Table 6.84: Peak years - All

Name	URR	Peak Year	Peak Rate
Gas Conv. Oregon	0.37	2020	0.01
**Total**	**0.37**	**2020**	**0.01**

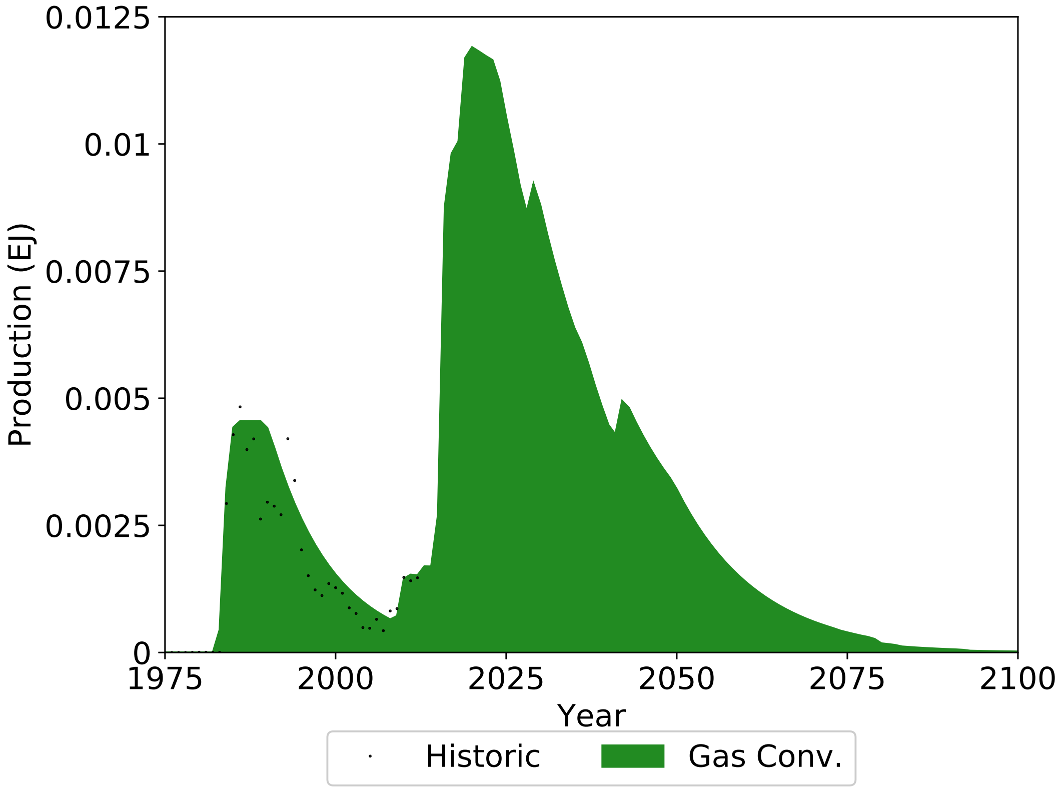

Figure 6.85: USA - Oregon projection by mineral type

Table 6.85: Peak years - Minerals

Name	URR	Peak Year	Peak Rate
Gas Conv.	0.37	2020	0.01
**Total**	**0.37**	**2020**	**0.01**

Other

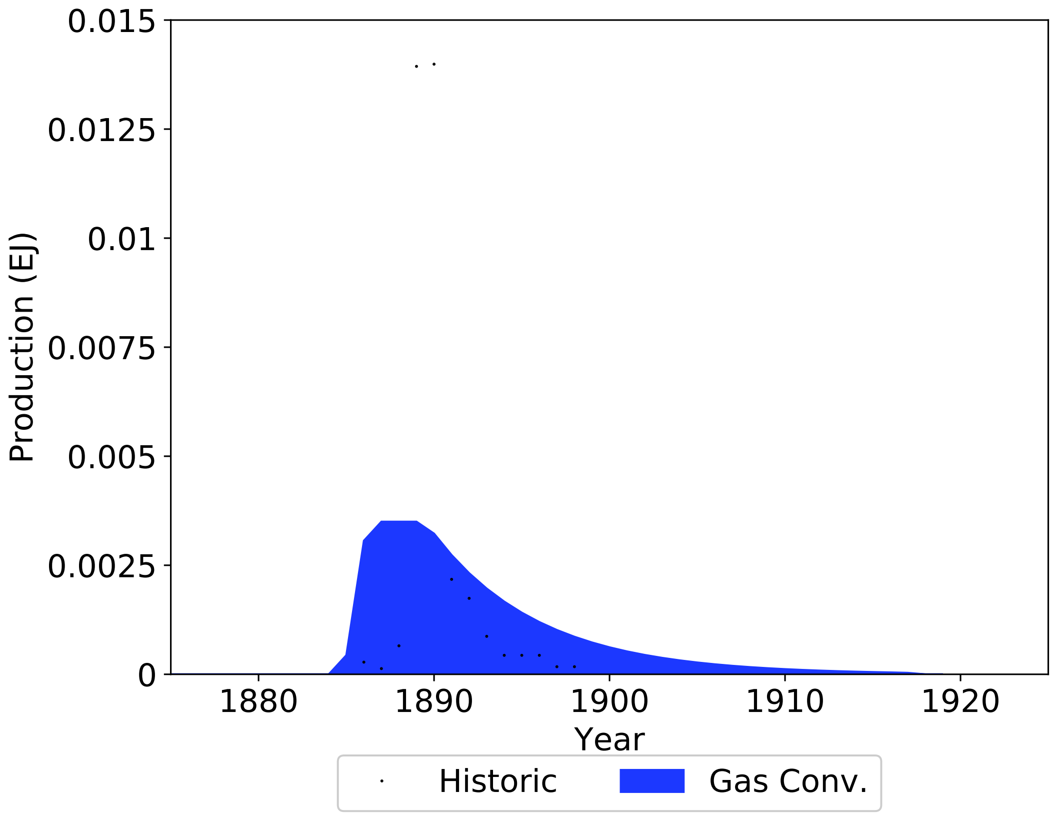

Figure 6.86: USA - Other projections capped at 16

Table 6.86: Peak years - All

**Name**	**URR**	**Peak Year**	**Peak Rate**
Gas Conv. Other	0.04	1887	–
**Total**	**0.04**	**1887**	–

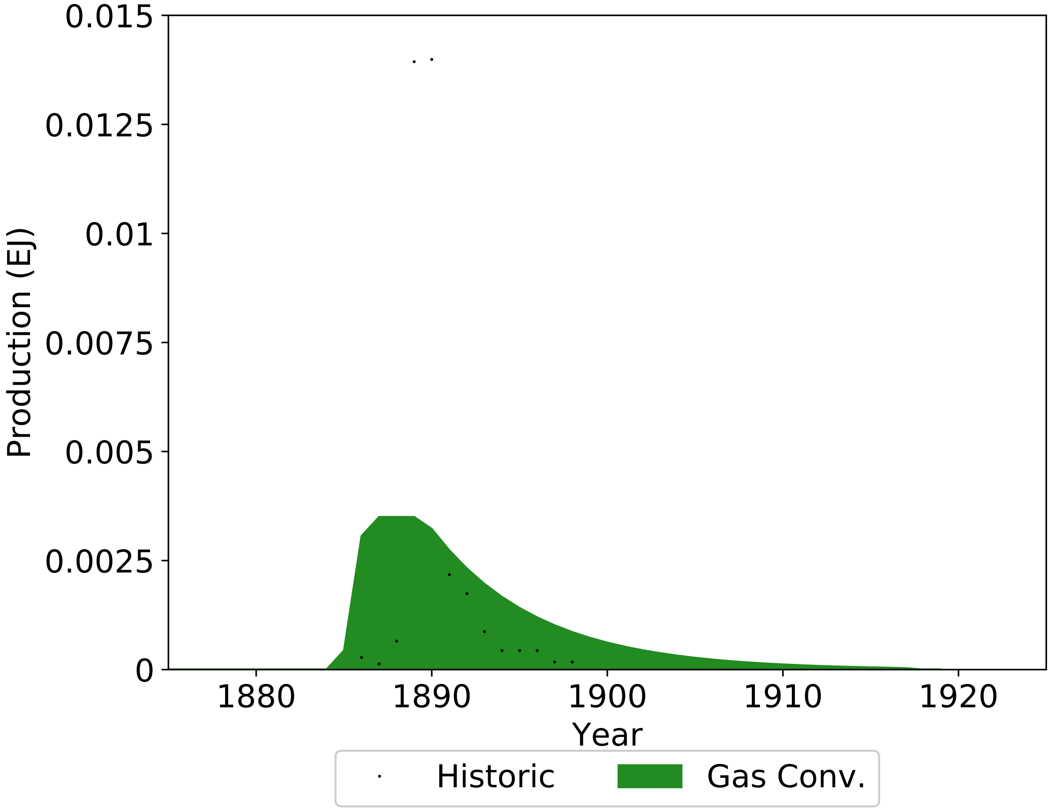

Figure 6.87: USA - Other projection by mineral type

Table 6.87: Peak years - Minerals

Name	URR	Peak Year	Peak Rate
Gas Conv.	0.04	1887	–
**Total**	**0.04**	**1887**	–

Pennsylvania

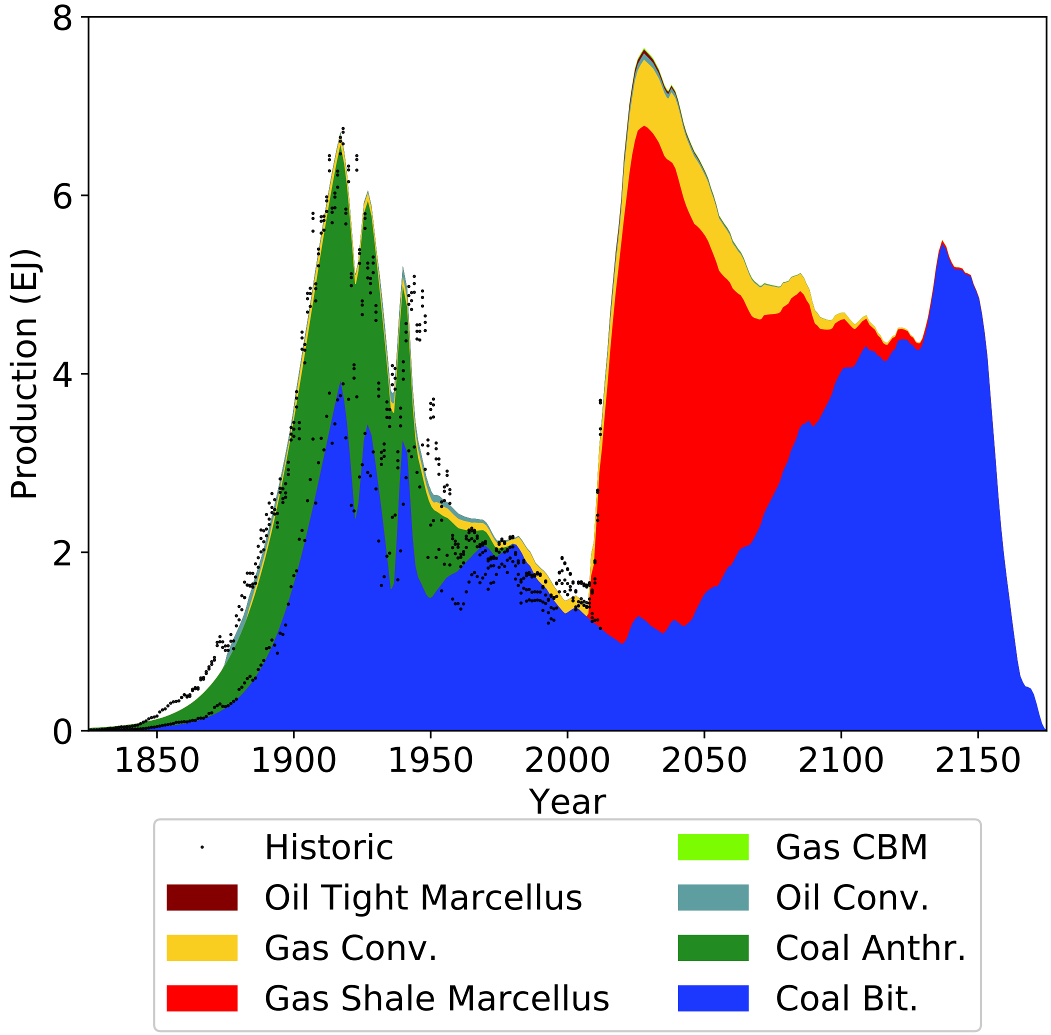

Figure 6.88: USA - Pennsylvania projections capped at 16

Table 6.88: Peak years - All

Name	URR	Peak Year	Peak Rate
Coal Bit. Pennsylvania	699.56	2137	5.43
Gas Shale Pennsylvania Marcellus	294.39	2030	5.55
Coal Anthr. Pennsylvania	151.2	1919	2.66
Gas Conv. Pennsylvania	55.12	2040	0.8
Oil Conv. Pennsylvania	10.19	1883	0.13
Oil Tight Pennsylvania Marcellus	1.15	2022	0.05
Gas CBM Pennsylvania	0.79	2025	0.02
**Total**	**1212.4**	**2028**	**7.63**

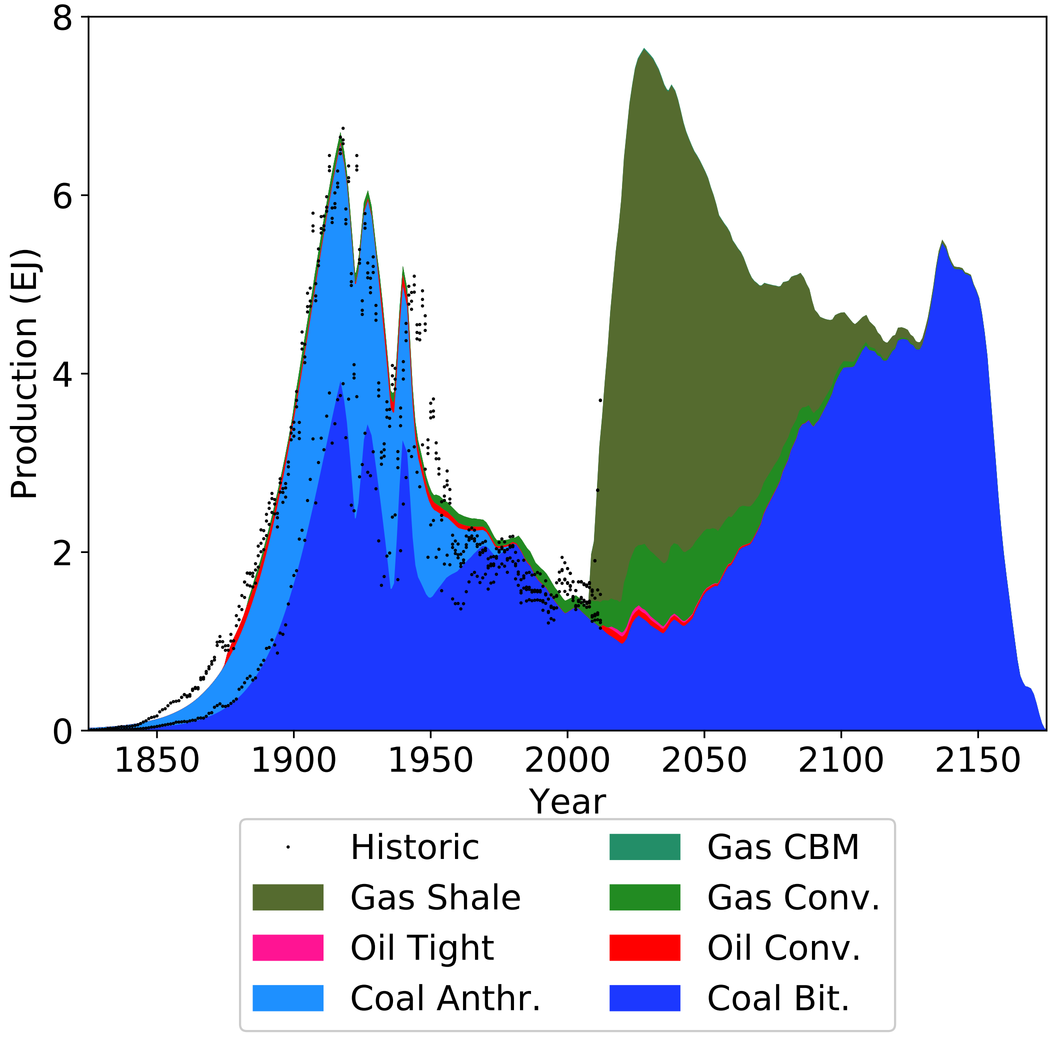

Figure 6.89: USA - Pennsylvania projection by mineral type

Table 6.89: Peak years - Minerals

Name	URR	Peak Year	Peak Rate
Coal Bit.	699.56	2137	5.43
Coal Anthr.	151.2	1919	2.66
Oil Conv.	10.19	1883	0.13
Oil Tight	1.15	2022	0.05
Gas Conv.	55.12	2040	0.8
Gas Shale	294.39	2030	5.55
Gas CBM	0.79	2025	0.02
**Total**	**1212.4**	**2028**	**7.63**

South Dakota

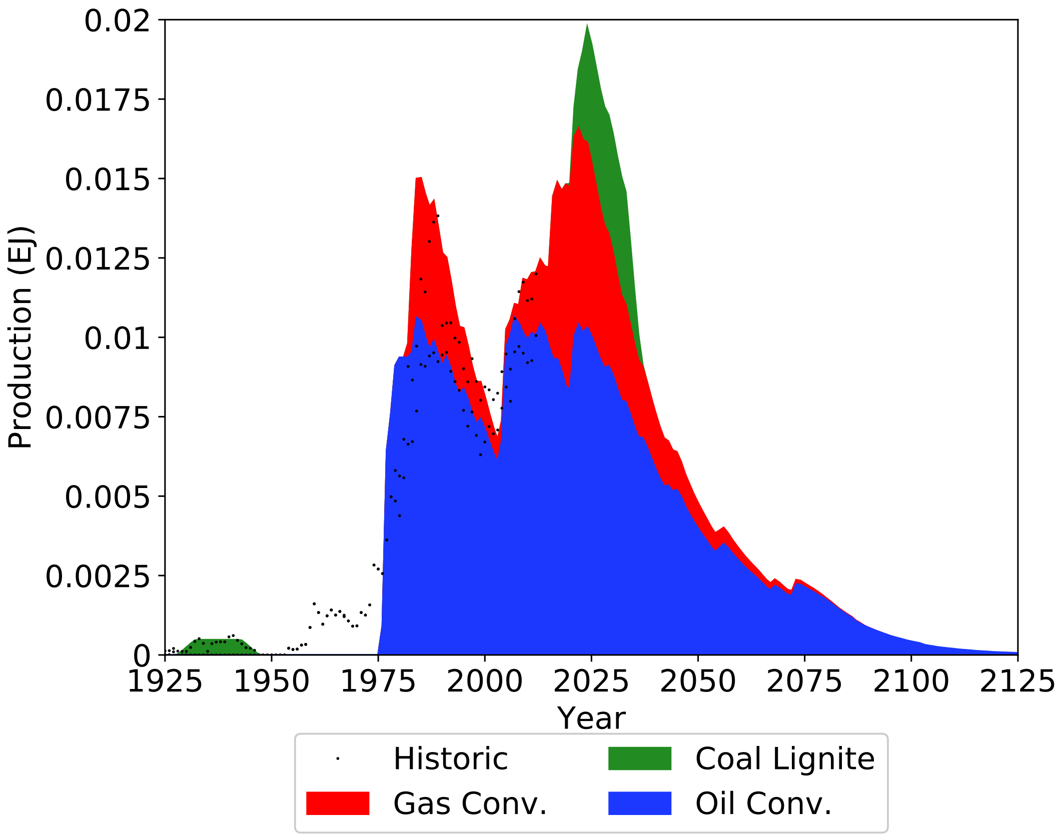

Figure 6.90: USA - South Dakota projections capped at 16

Table 6.90: Peak years - All

Name	URR	Peak Year	Peak Rate
Oil Conv. South Dakota	0.71	2007	0.01
Gas Conv. South Dakota	0.2	2020	0.01
Coal Lignite South Dakota	0.06	2025	–
**Total**	**0.97**	**2024**	**0.02**

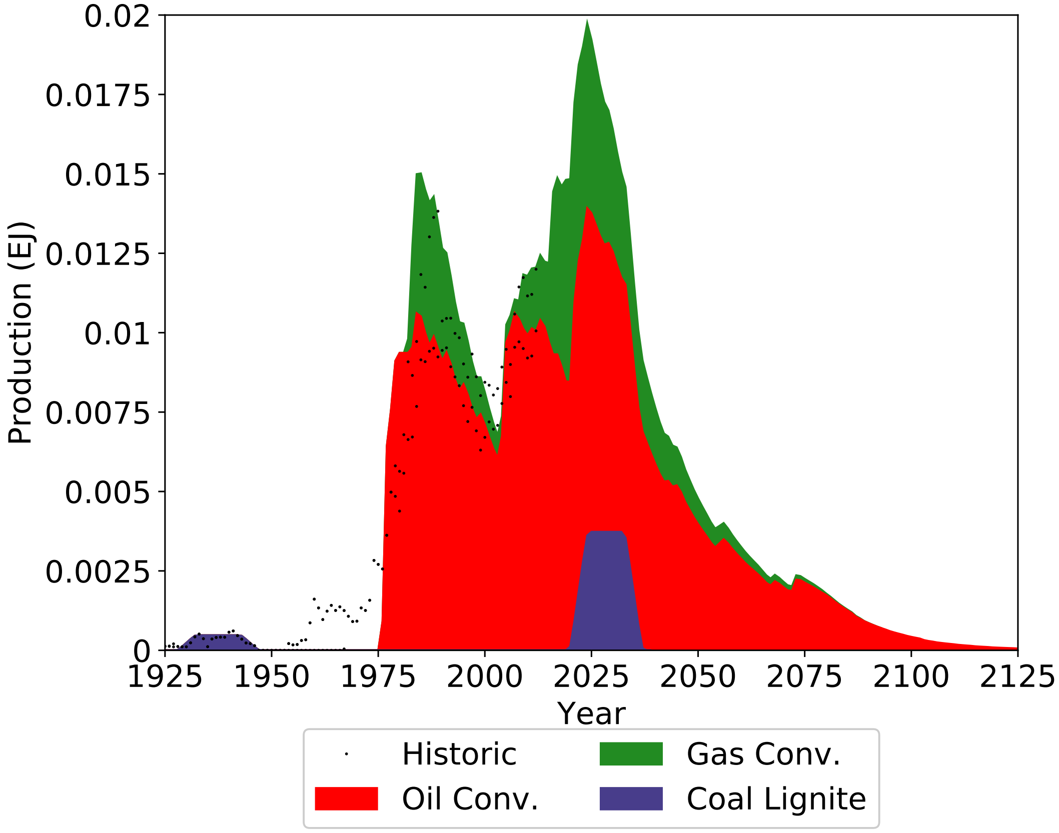

Figure 6.91: USA - South Dakota projection by mineral type

Table 6.91: Peak years - Minerals

Name	URR	Peak Year	Peak Rate
Coal Lignite	0.06	2025	–
Oil Conv.	0.71	2007	0.01
Gas Conv.	0.2	2020	0.01
**Total**	**0.97**	**2024**	**0.02**

Tennessee

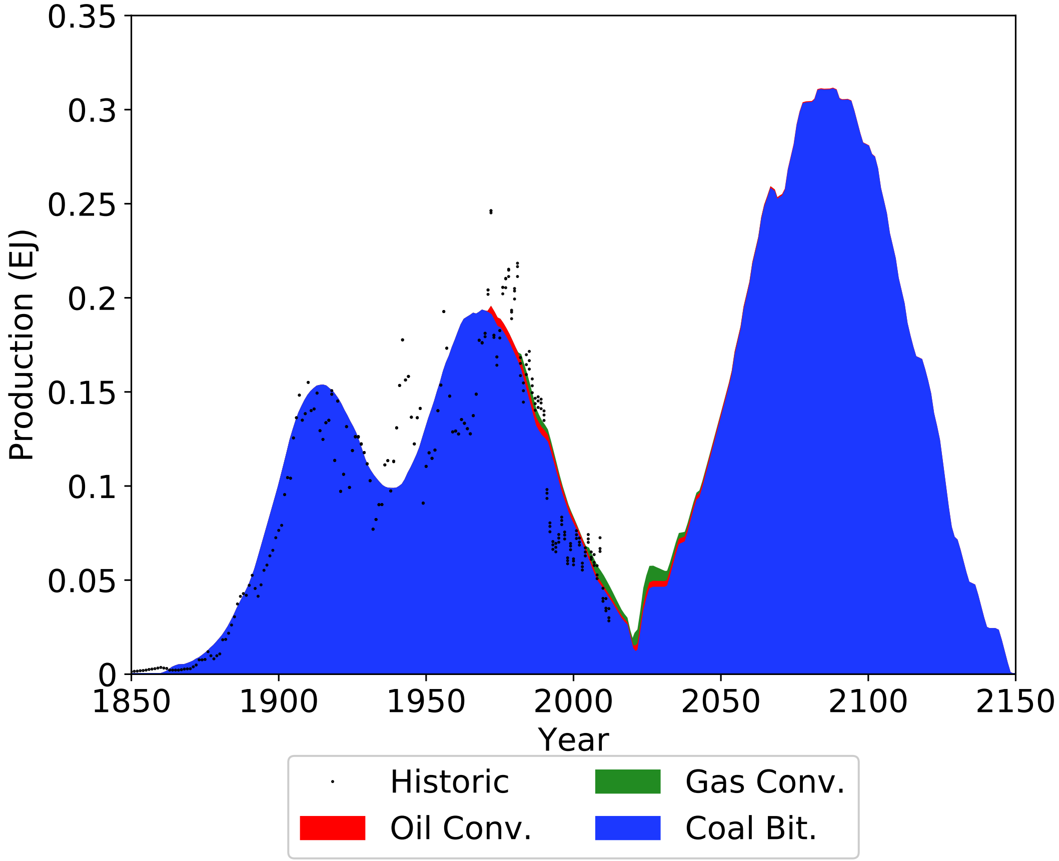

Figure 6.92: USA - Tennessee projections capped at 16

Table 6.92: Peak years - All

Name	URR	Peak Year	Peak Rate
Coal Bit. Tennessee	37.06	2088	0.31
Oil Conv. Tennessee	0.27	1973	–
Gas Conv. Tennessee	0.25	2025	0.01
**Total**	**37.58**	**2088**	**0.31**

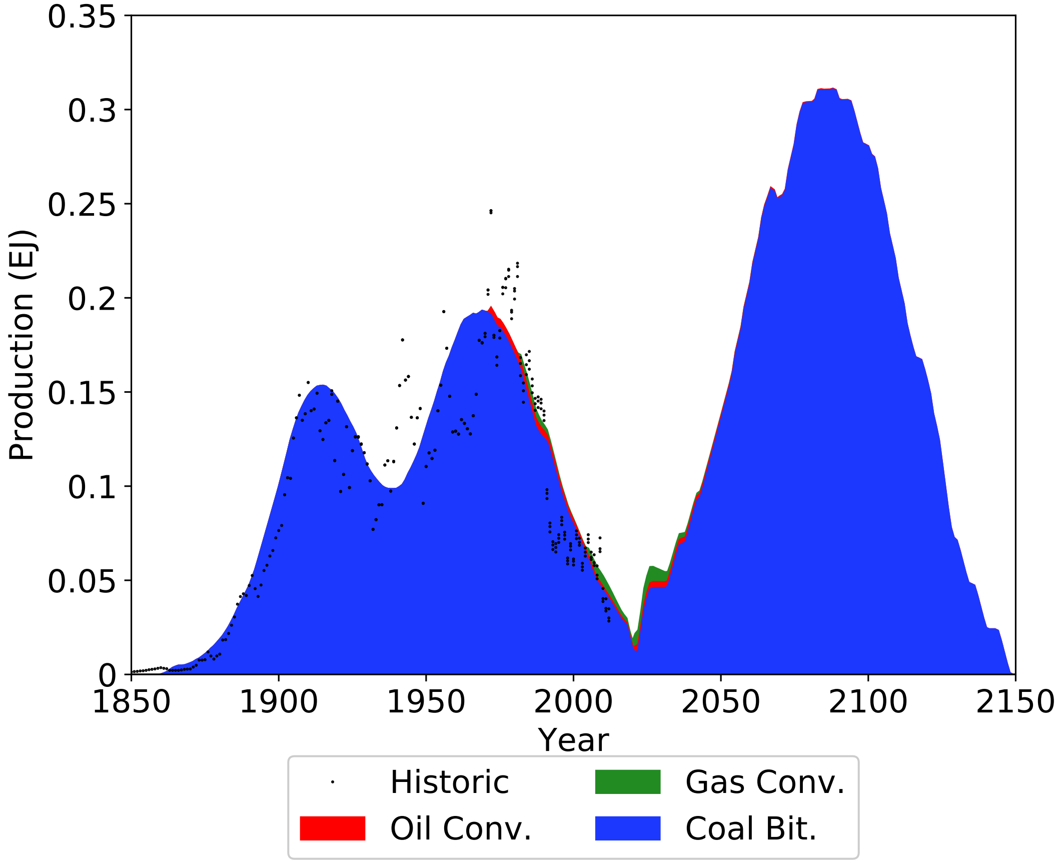

Figure 6.93: USA - Tennessee projection by mineral type

Table 6.93: Peak years - Minerals

Name	URR	Peak Year	Peak Rate
Coal Bit.	37.06	2088	0.31
Oil Conv.	0.27	1973	–
Gas Conv.	0.25	2025	0.01
**Total**	**37.58**	**2088**	**0.31**

Texas

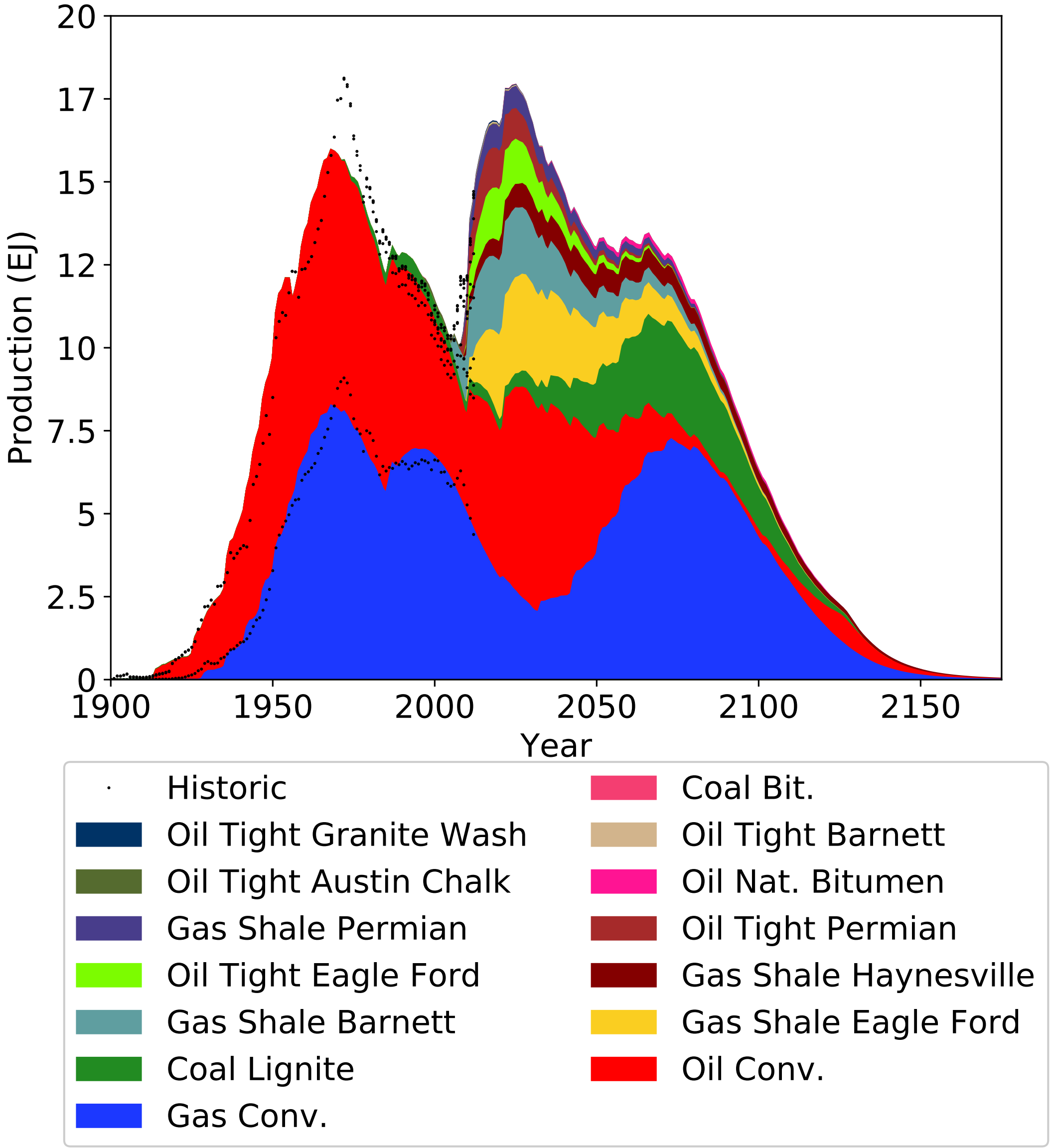

Figure 6.94: USA - Texas projections capped at 16

Table 6.94: Peak years - All

**Name**	**URR**	**Peak Year**	**Peak Rate**
Gas Conv. Texas	921.15	1968	8.26
Oil Conv. Texas	731.08	1968	7.7
Coal Lignite Texas	170.81	2072	2.79
Gas Shale Texas Eagle Ford	126.25	2026	2.92
Gas Shale Texas Barnett	88.36	2020	2.24
Gas Shale Texas Haynesville	61.8	2035	0.78
Oil Tight Texas Eagle Ford	39.84	2019	1.57
Oil Tight Texas Permian	30.68	2017	1.23
Gas Shale Texas Permian	28.68	2021	0.73
Oil Nat. Bitumen Texas	8.36	2076	0.17
Oil Tight Texas Austin Chalk	1.75	1999	0.12
Oil Tight Texas Barnett	1.22	2013	0.06
Oil Tight Texas Granite Wash	0.89	2011	0.07
Coal Bit. Texas	0.12	2023	0.02
**Total**	**2210.99**	**2025**	**17.93**

Table 6.95: Peak years - Minerals

**Name**	**URR**	**Peak Year**	**Peak Rate**
Coal Bit.	0.12	2023	0.02
Coal Lignite	170.81	2072	2.79
Oil Conv.	731.08	1968	7.7
Oil Tight	74.38	2019	2.87
Oil Nat. Bitumen	8.36	2076	0.17
Gas Conv.	921.15	1968	8.26
Gas Shale	305.09	2025	6.37
**Total**	**2210.99**	**2025**	**17.93**

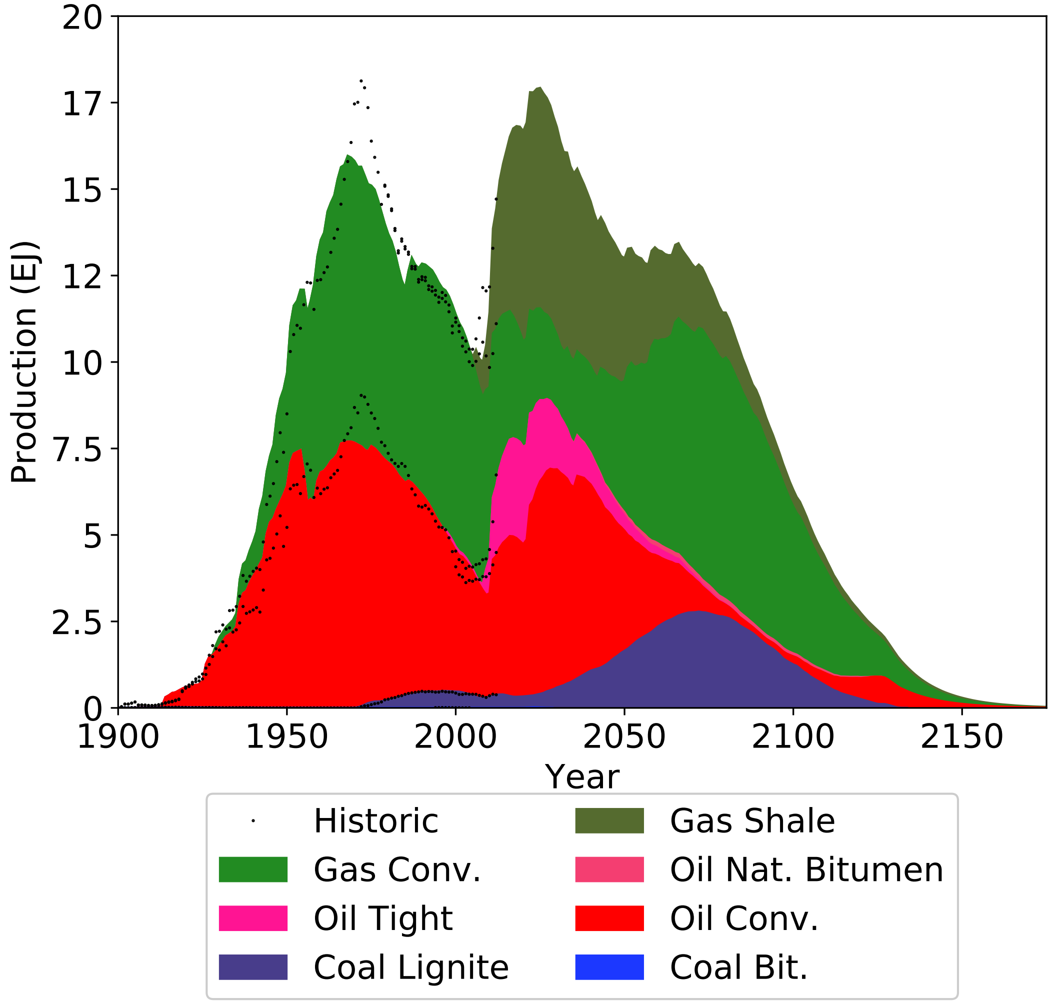

Figure 6.95: USA - Texas projection by mineral type

USA

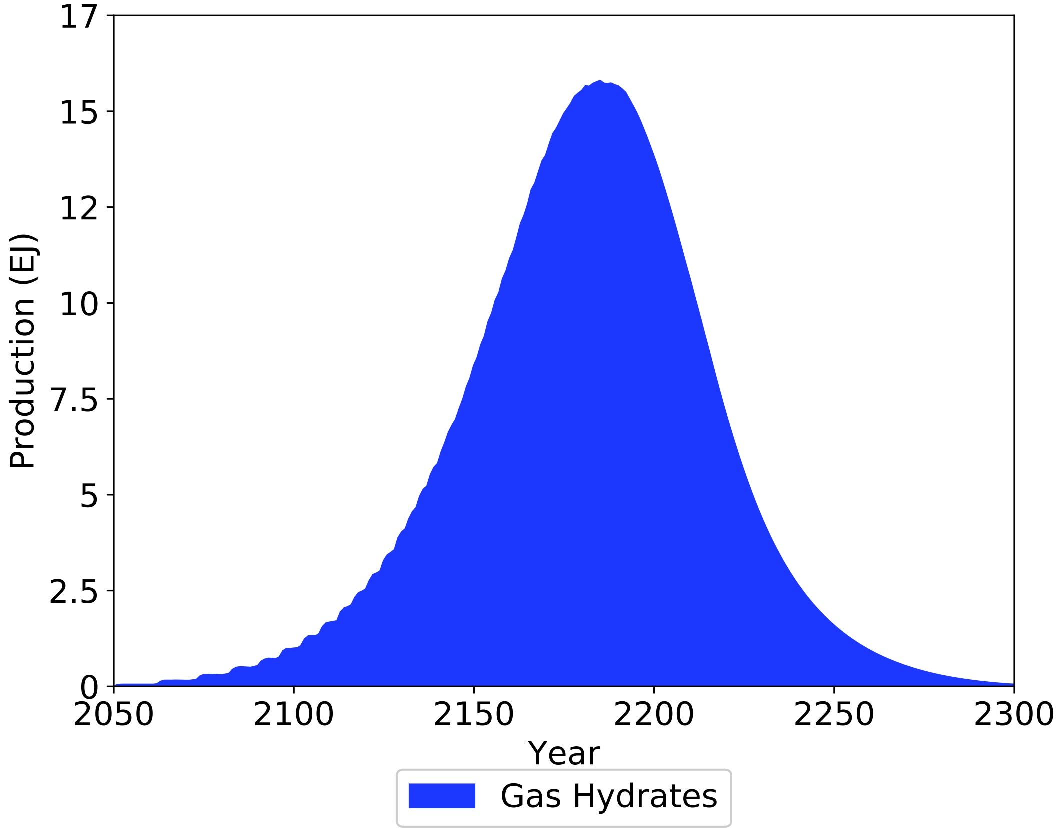

Figure 6.96: USA - USA projections capped at 16

Table 6.96: Peak years - All

Name	URR	Peak Year	Peak Rate
Gas Hydrates USA	1229.0	2185	15.8
**Total**	**1229.0**	**2185**	**15.8**

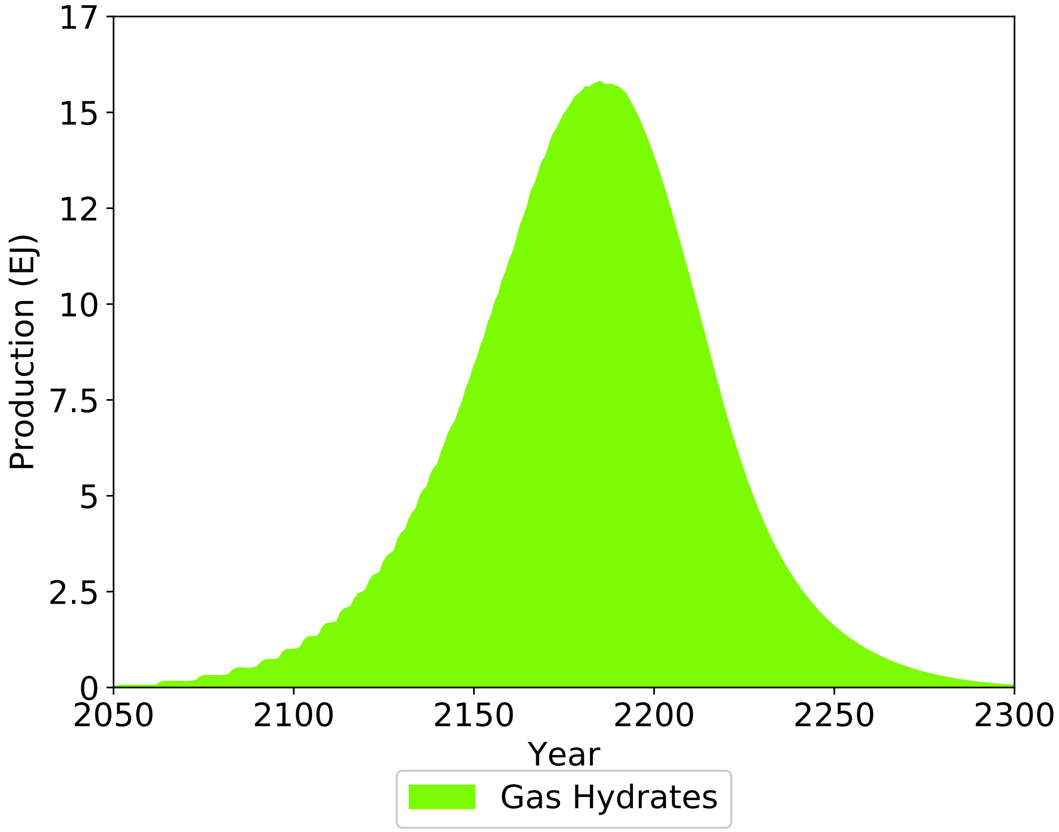

Figure 6.97: USA - USA projection by mineral type

Table 6.97: Peak years - Minerals

Name	URR	Peak Year	Peak Rate
Gas Hydrates	1229.0	2185	15.8
**Total**	**1229.0**	**2185**	**15.8**

Utah

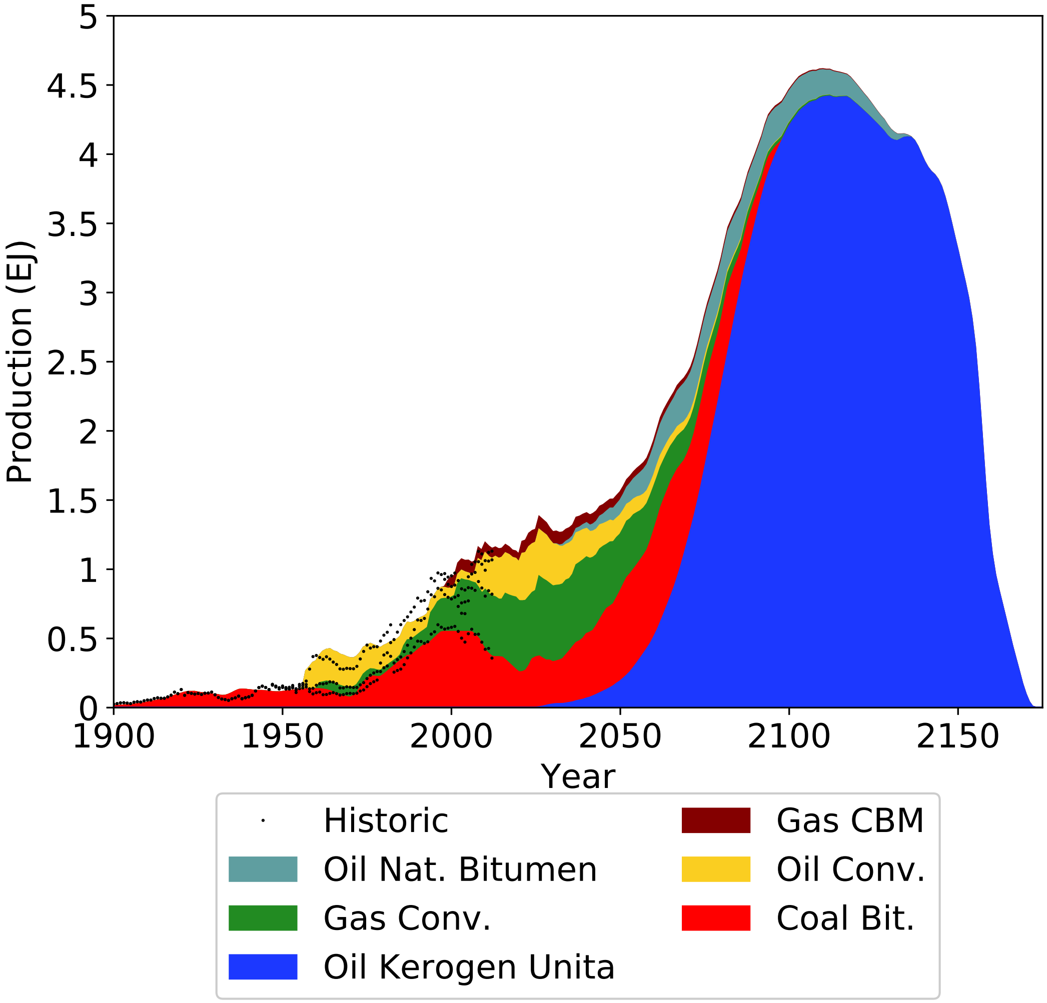

Figure 6.98: USA - Utah projections capped at 16

Table 6.98: Peak years - All

Name	URR	Peak Year	Peak Rate
Oil Kerogen Utah Unita	336.3	2112	4.42
Coal Bit. Utah	62.29	2063	0.83
Gas Conv. Utah	36.75	2026	0.58
Oil Conv. Utah	20.82	2023	0.36
Oil Nat. Bitumen Utah	17.91	2070	0.28
Gas CBM Utah	5.86	2023	0.1
**Total**	**479.93**	**2110**	**4.61**

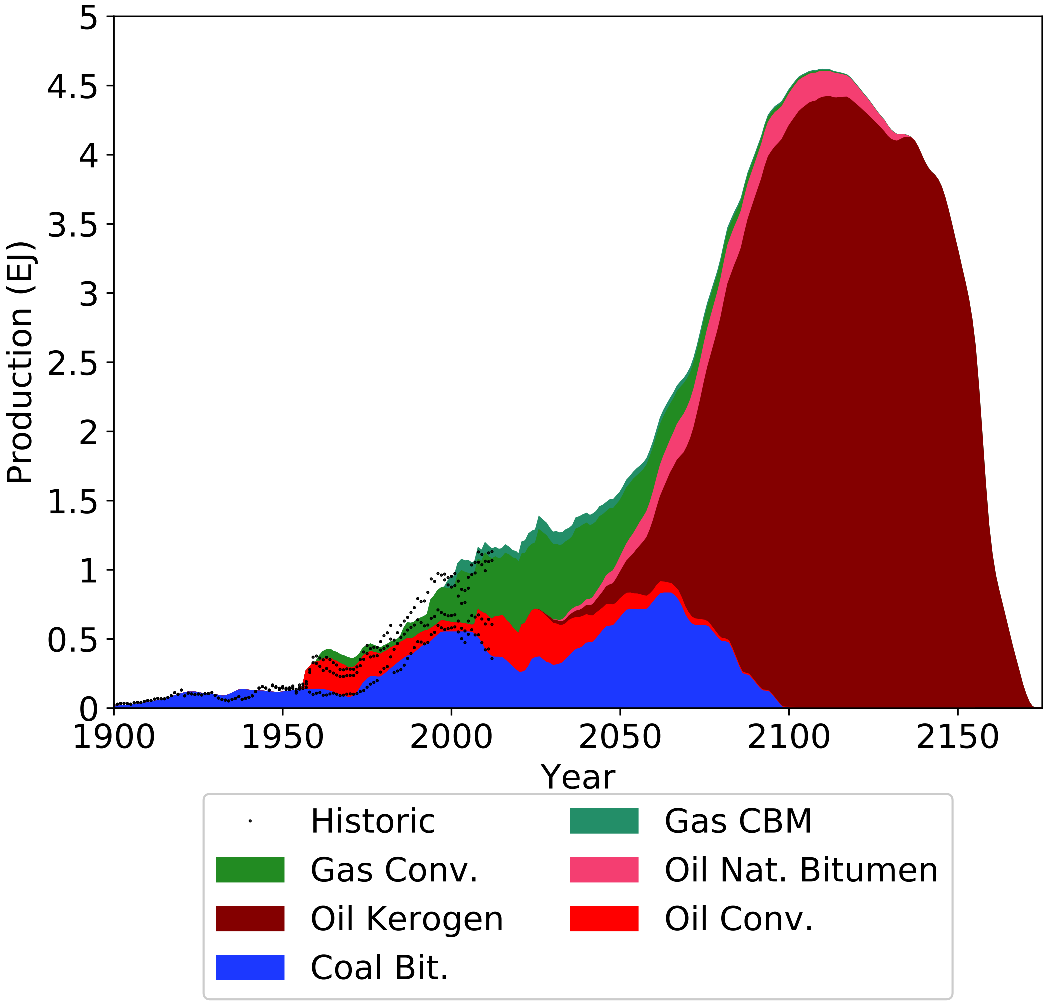

Figure 6.99: USA - Utah projection by mineral type

Table 6.99: Peak years - Minerals

**Name**	**URR**	**Peak Year**	**Peak Rate**
Coal Bit.	62.29	2063	0.83
Oil Conv.	20.82	2023	0.36
Oil Kerogen	336.3	2112	4.42
Oil Nat. Bitumen	17.91	2070	0.28
Gas Conv.	36.75	2026	0.58
Gas CBM	5.86	2023	0.1
**Total**	**479.93**	**2110**	**4.61**

Virginia

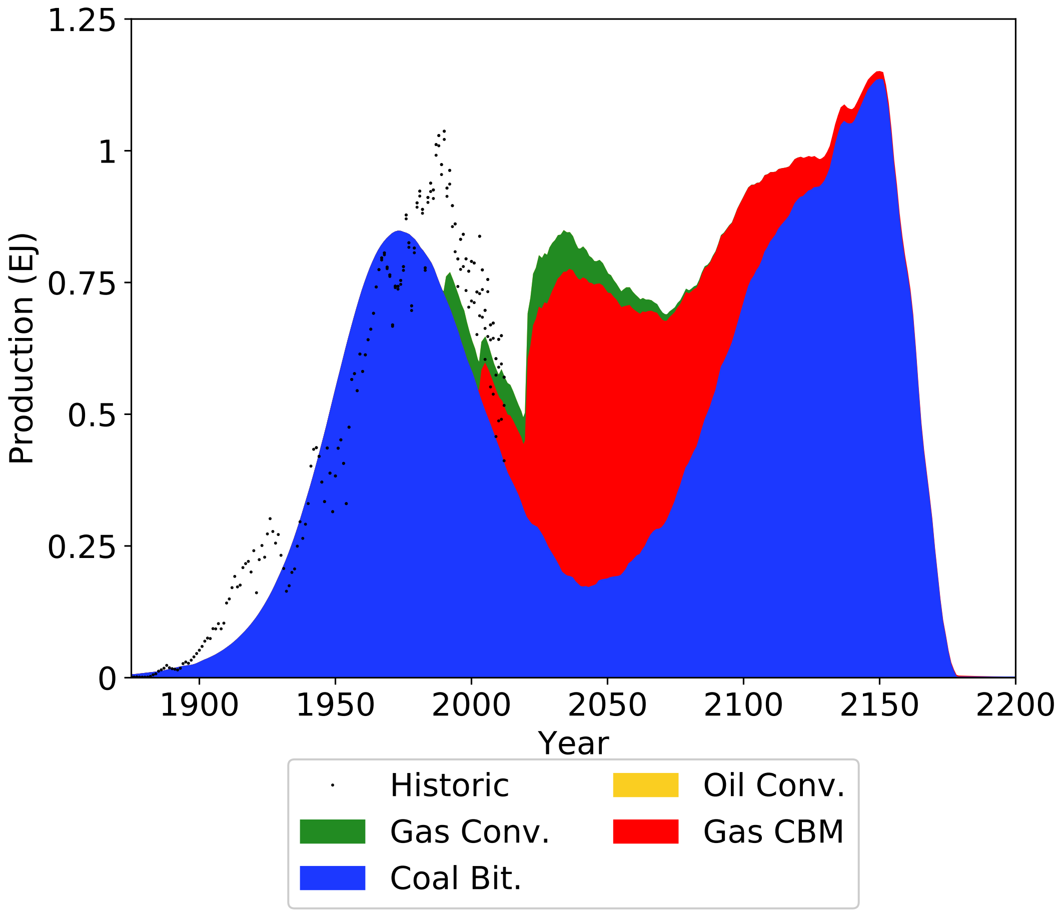

Figure 6.100: USA - Virginia projections capped at 16

Table 6.100: Peak years - All

Name	URR	Peak Year	Peak Rate
Coal Bit. Virginia	143.49	2150	1.13
Gas CBM Virginia	39.4	2041	0.59
Gas Conv. Virginia	4.48	2023	0.1
Oil Conv. Virginia	0.01	1982	–
**Total**	**187.38**	**2150**	**1.15**

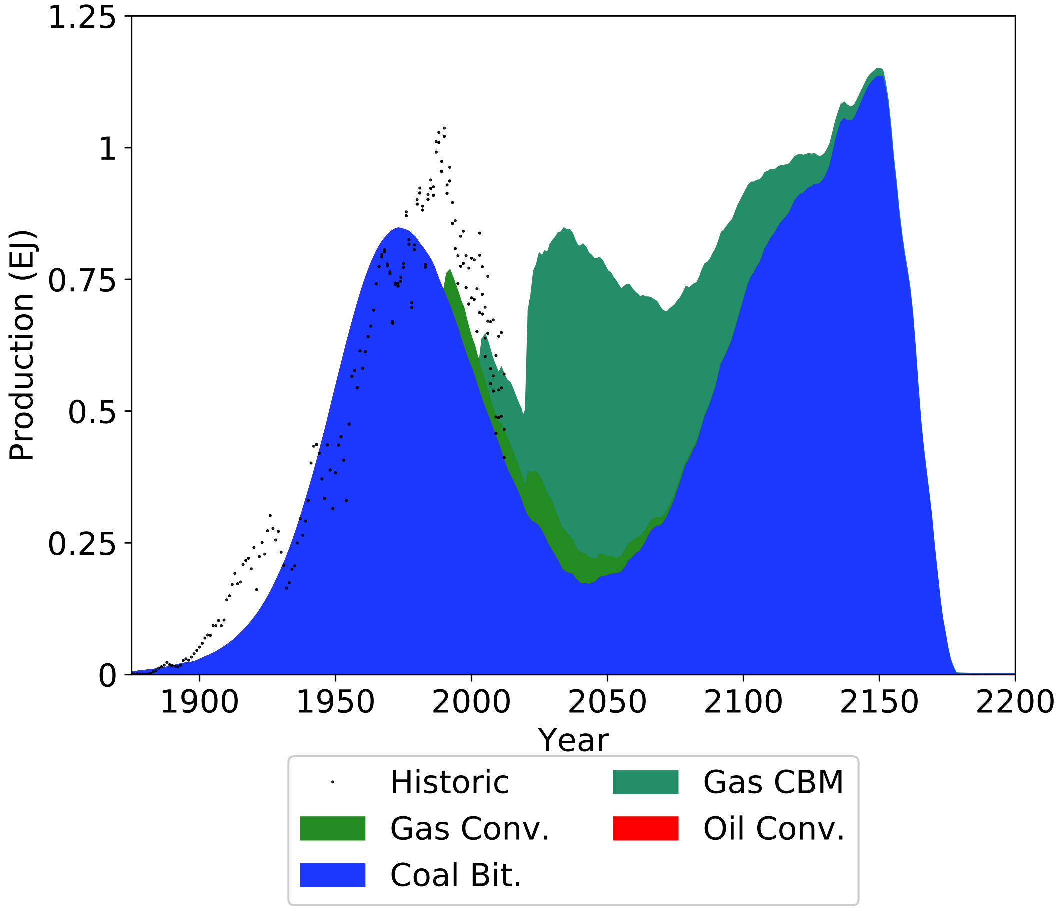

Figure 6.101: USA - Virginia projection by mineral type

Table 6.101: Peak years - Minerals

Name	URR	Peak Year	Peak Rate
Coal Bit.	143.49	2150	1.13
Oil Conv.	0.01	1982	–
Gas Conv.	4.48	2023	0.1
Gas CBM	39.4	2041	0.59
**Total**	**187.38**	**2150**	**1.15**

Washington

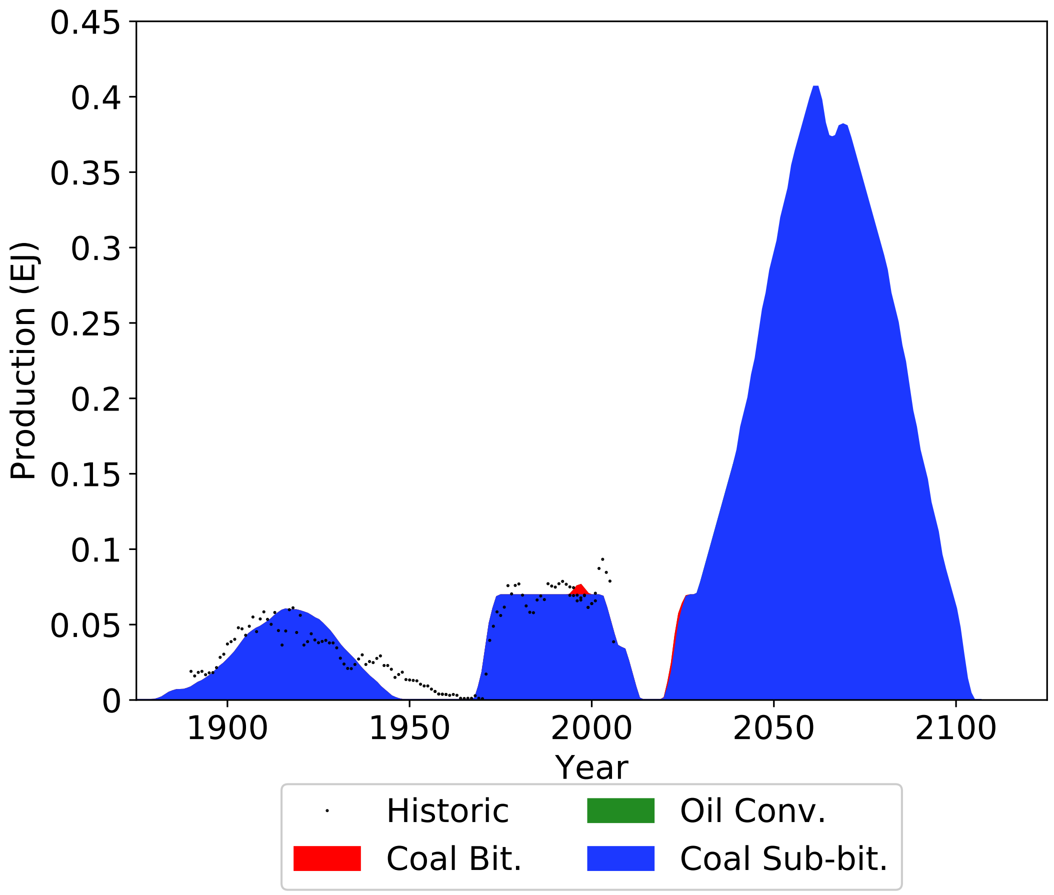

Figure 6.102: USA - Washington projections capped at 16

Table 6.102: Peak years - All

Name	URR	Peak Year	Peak Rate
Coal Sub-bit. Washington	22.91	2061	0.41
Coal Bit. Washington	0.05	2023	0.01
Oil Conv. Washington	–	1956	–
**Total**	**22.96**	**2061**	**0.41**

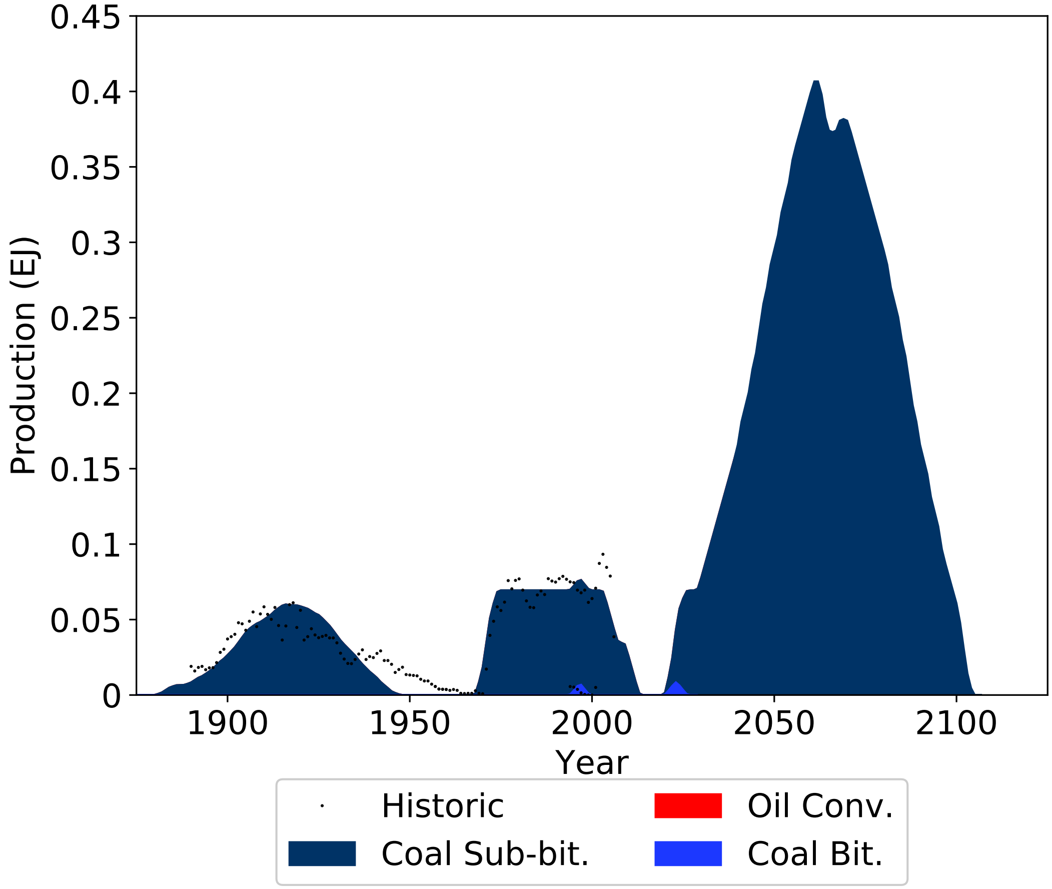

Figure 6.103: USA - Washington projection by mineral type

Table 6.103: Peak years - Minerals

Name	URR	Peak Year	Peak Rate
Coal Bit.	0.05	2023	0.01
Coal Sub-bit.	22.91	2061	0.41
Oil Conv.	–	1956	–
**Total**	**22.96**	**2061**	**0.41**

West Virginia

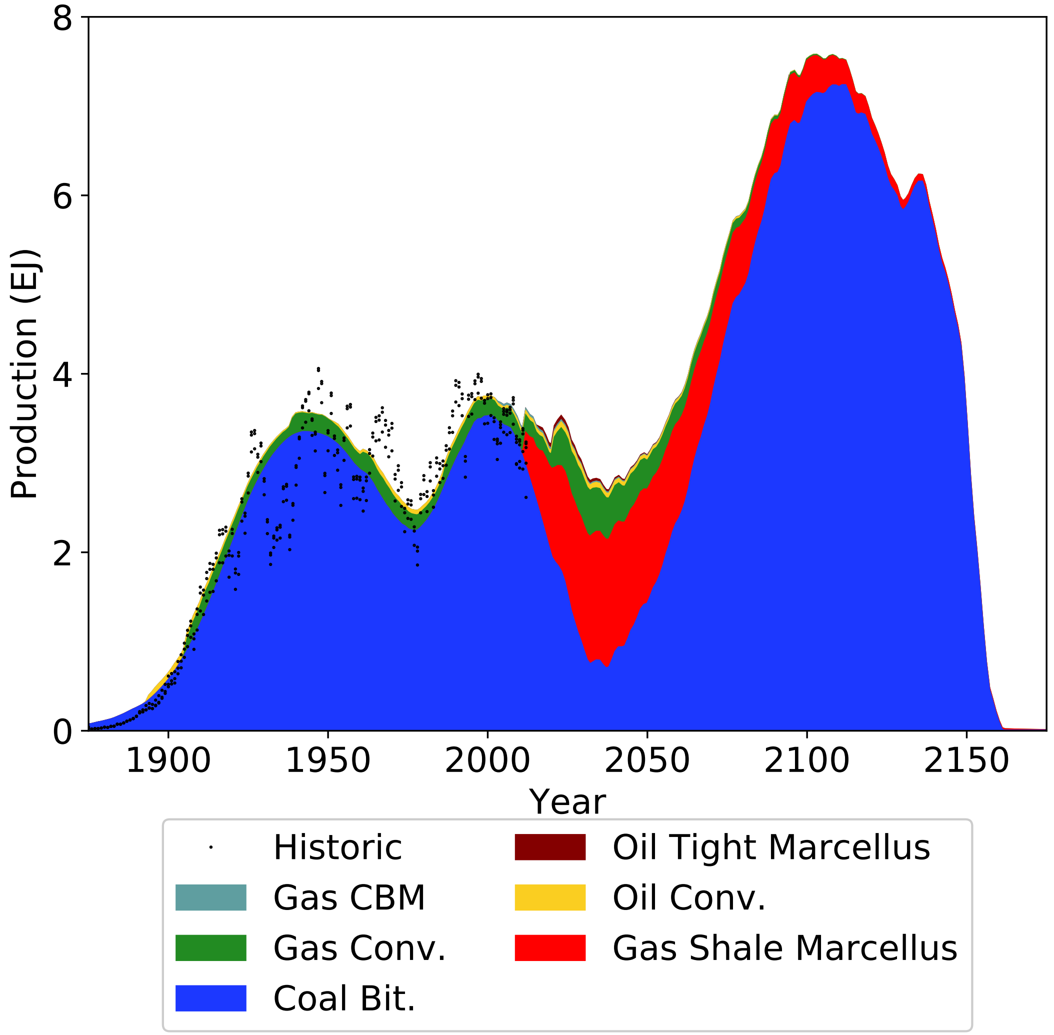

Figure 6.104: USA - West Virginia projections capped at 16

Table 6.104: Peak years - All

**Name**	**URR**	**Peak Year**	**Peak Rate**
Coal Bit. West Virginia	896.76	2112	7.23
Gas Shale West Virginia Marcellus	97.52	2035	1.44
Gas Conv. West Virginia	41.11	2031	0.52
Oil Conv. West Virginia	8.0	1902	0.08
Gas CBM West Virginia	1.21	2018	0.03
Oil Tight West Virginia Marcellus	1.15	2022	0.05
**Total**	**1045.75**	**2103**	**7.58**

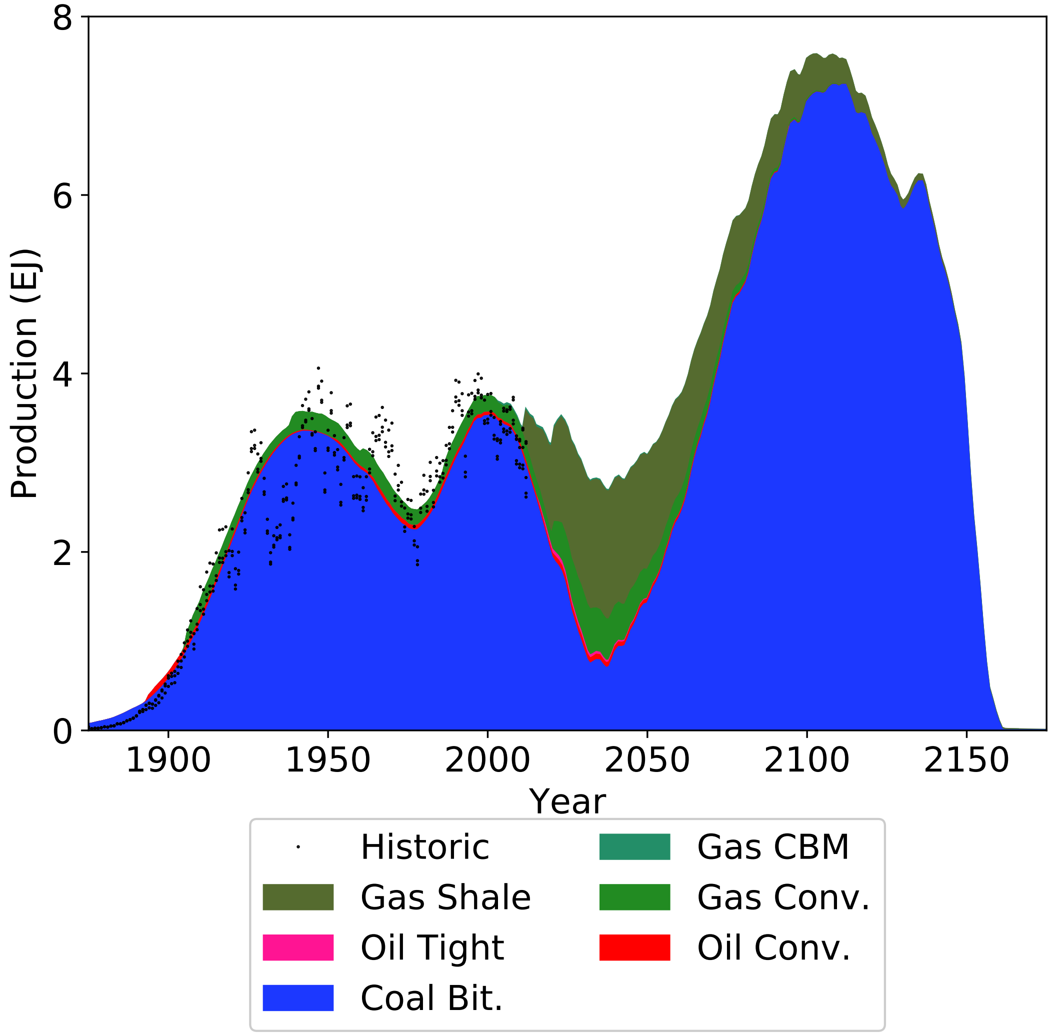

Figure 6.105: USA - West Virginia projection by mineral type

Table 6.105: Peak years - Minerals

**Name**	**URR**	**Peak Year**	**Peak Rate**
Coal Bit.	896.76	2112	7.23
Oil Conv.	8.0	1902	0.08
Oil Tight	1.15	2022	0.05
Gas Conv.	41.11	2031	0.52
Gas Shale	97.52	2035	1.44
Gas CBM	1.21	2018	0.03
**Total**	**1045.75**	**2103**	**7.58**

Wyoming

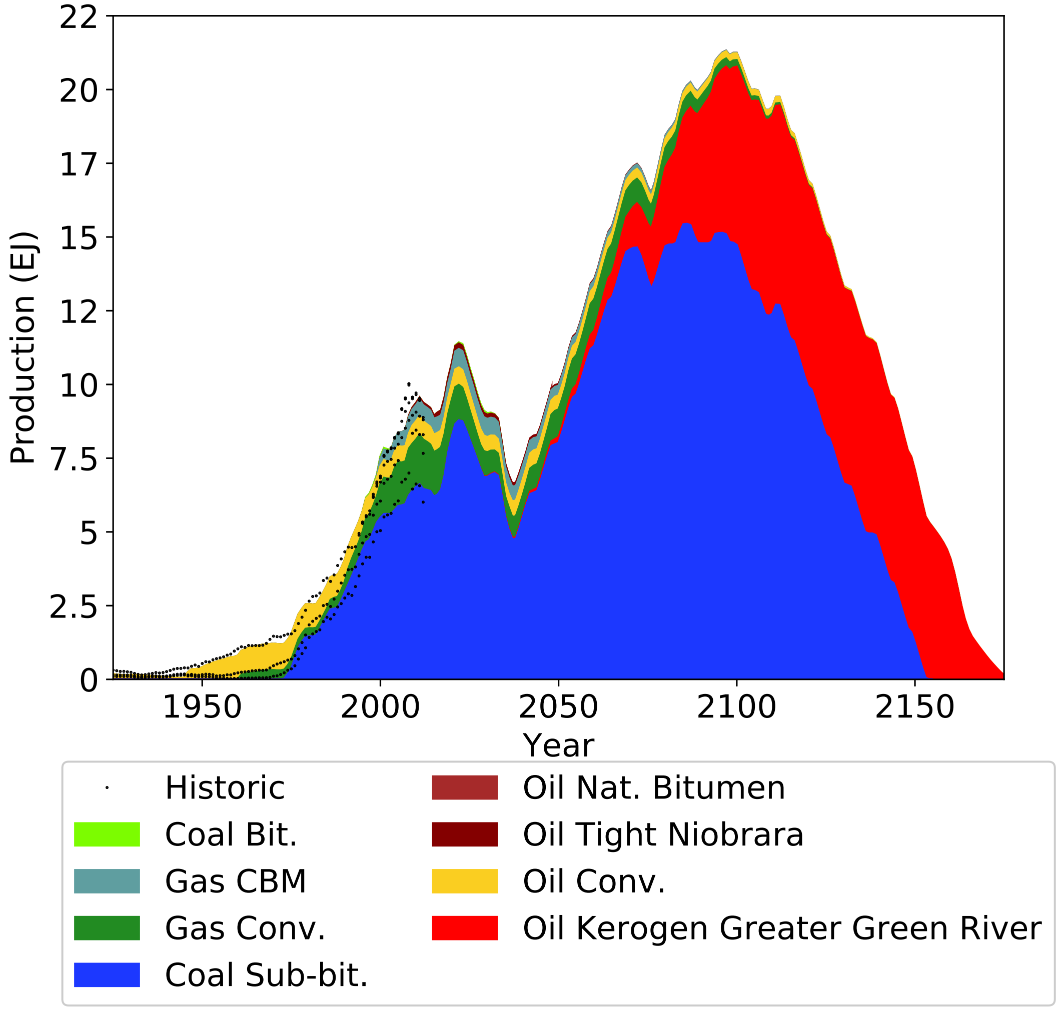

Figure 6.106: USA - Wyoming projections capped at 16

Table 6.106: Peak years - All

Name	URR	Peak Year	Peak Rate
Coal Sub-bit. Wyoming	1489.93	2086	15.45
Oil Kerogen Wyoming Greater Green River	523.2	2119	6.81
Gas Conv. Wyoming	108.86	2011	1.68
Oil Conv. Wyoming	92.73	1970	0.89
Gas CBM Wyoming	32.61	2023	0.65
Oil Tight Wyoming Niobrara	6.15	2019	0.19
Coal Bit. Wyoming	1.11	1999	0.07
Oil Nat. Bitumen Wyoming	0.07	2035	0.01
**Total**	**2254.66**	**2097**	**21.32**

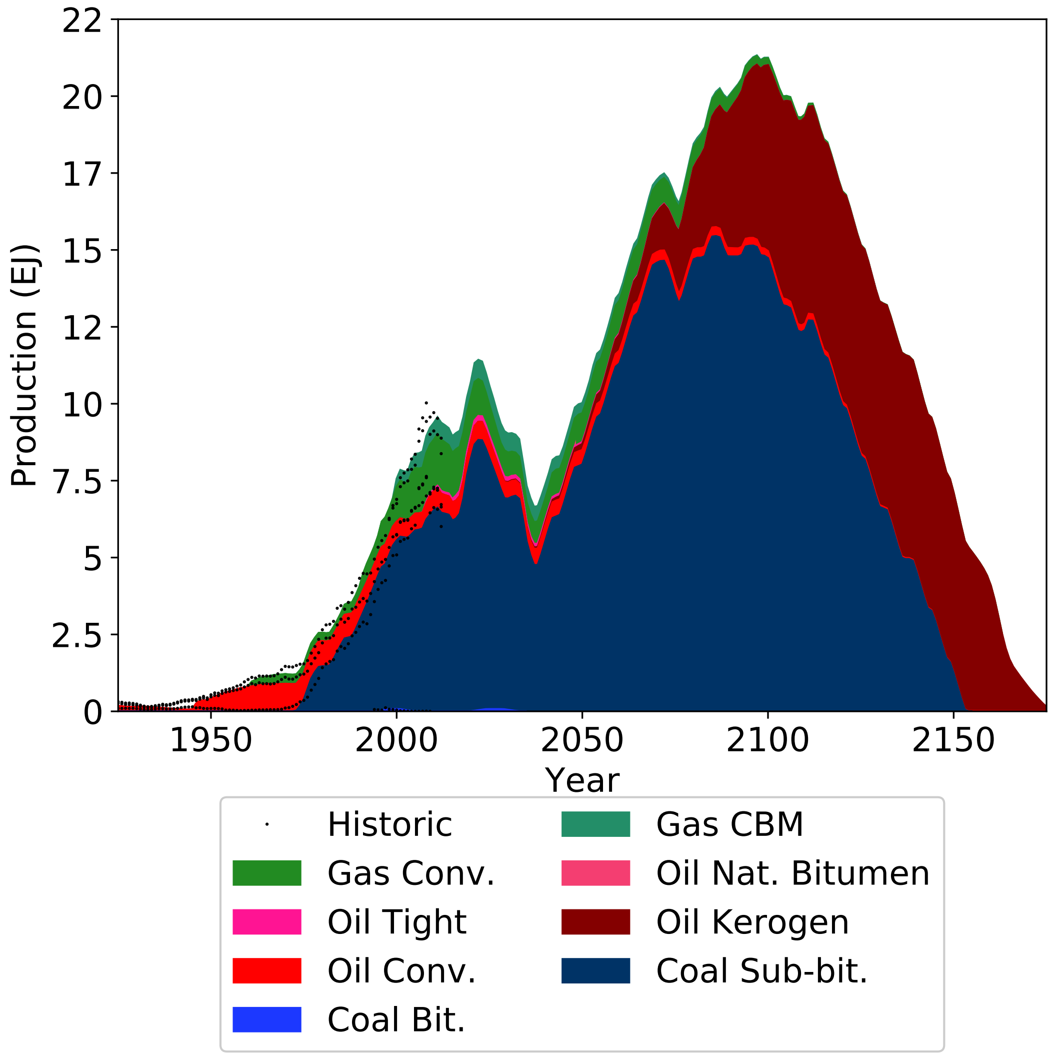

Figure 6.107: USA - Wyoming projection by mineral type

Table 6.107: Peak years - Minerals

**Name**	**URR**	**Peak Year**	**Peak Rate**
Coal Bit.	1.11	1999	0.07
Coal Sub-bit.	1489.93	2086	15.45
Oil Conv.	92.73	1970	0.89
Oil Kerogen	523.2	2119	6.81
Oil Tight	6.15	2019	0.19
Oil Nat. Bitumen	0.07	2035	0.01
Gas Conv.	108.86	2011	1.68
Gas CBM	32.61	2023	0.65
**Total**	**2254.66**	**2097**	**21.32**

6.2.4 Projection by region

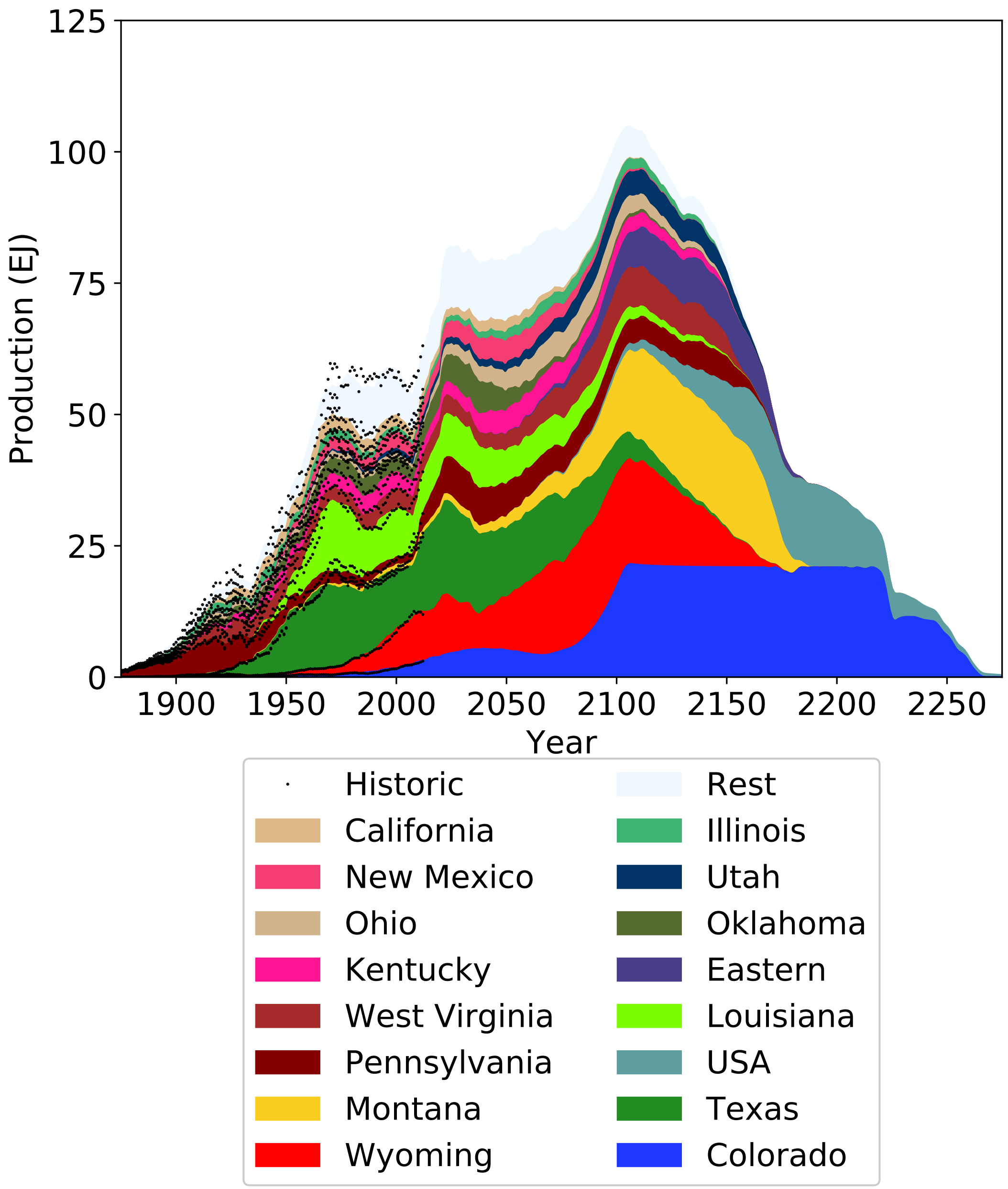

Figure 6.108: USA by region projections capped at 16

Table 6.108: Peak years - All

**Name**	**URR**	**Peak Year**	**Peak Rate**
Colorado	3505.61	2106	21.52
Wyoming	2254.66	2097	21.32
Texas	2210.99	2025	17.93
Montana	1700.6	2136	19.59
USA	1229.0	2185	15.8
Pennsylvania	1212.4	2028	7.63
Louisiana	1186.18	1971	13.65
West Virginia	1045.75	2103	7.58
Eastern	649.8	2136	8.59
Kentucky	629.53	2055	4.73
Oklahoma	518.26	2035	6.52
Ohio	508.04	2080	4.78
Utah	479.93	2110	4.61
New Mexico	412.01	2050	4.42
Illinois	376.64	2066	2.51
California	329.03	1965	3.1
Indiana	311.49	2070	2.36
North Dakota	208.05	2021	2.38
Alaska	197.1	1988	4.51
Alabama	188.9	2066	2.01
Virginia	187.38	2150	1.15
Kansas	171.5	1965	1.56
New York	93.38	2041	1.4
Arkansas	93.0	2023	1.74
Mississippi	46.28	1958	0.54
Tennessee	37.58	2088	0.31
Michigan	34.74	2023	0.51
Arizona	33.91	2050	0.59
Washington	22.96	2061	0.41
Maryland	22.2	2076	0.21
Missouri	22.06	2022	0.23
Iowa	18.76	2071	0.22
Florida	8.05	1976	0.4
Nebraska	4.19	1959	0.15
South Dakota	0.97	2024	0.02
Georgia	0.61	2047	0.01
Nevada	0.42	1980	0.02
Oregon	0.37	2020	0.01
Other	0.04	1887	–
**Total**	**19952.34**	**2106**	**104.83**

6.3 Total

6.3.1 By country

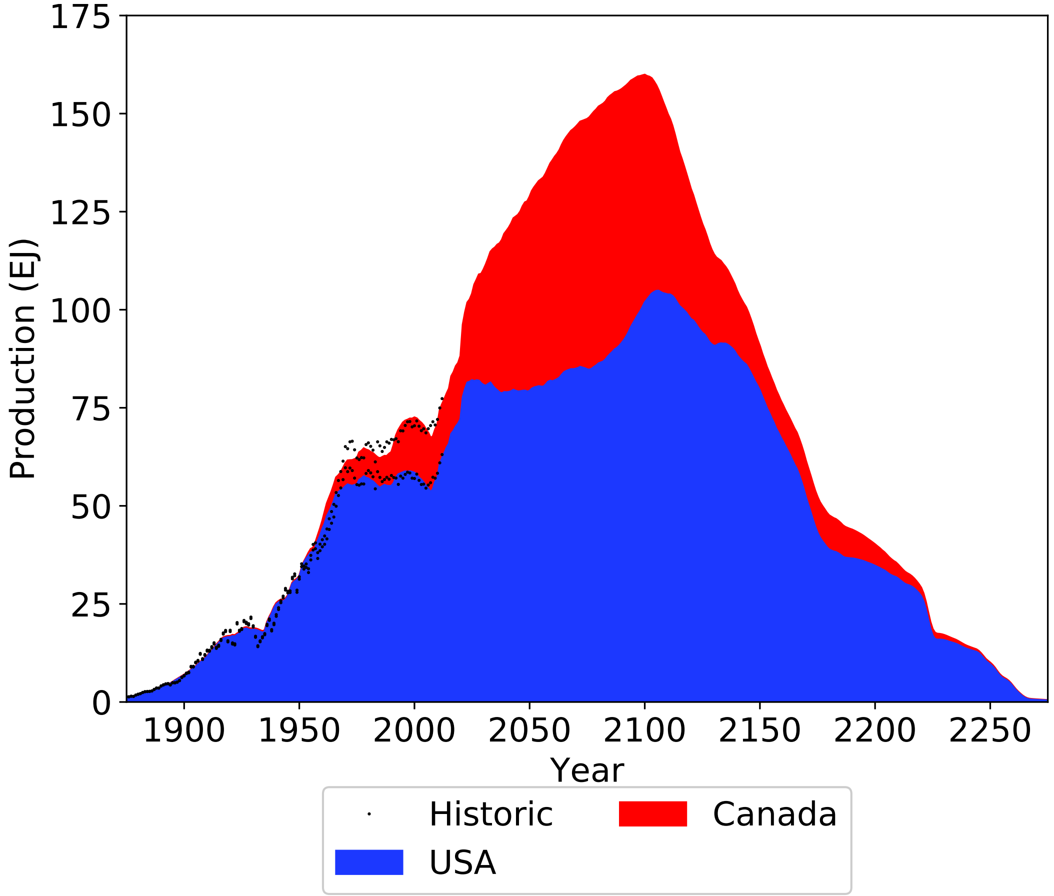

Figure 6.109: North America projections by country

Table 6.109: Peak years - All

Name	URR	Peak Year	Peak Rate
USA	19952.34	2106	104.83
Canada	6766.31	2084	65.92
**Total**	**26718.66**	**2100**	**159.89**

6.3.2 By mineral

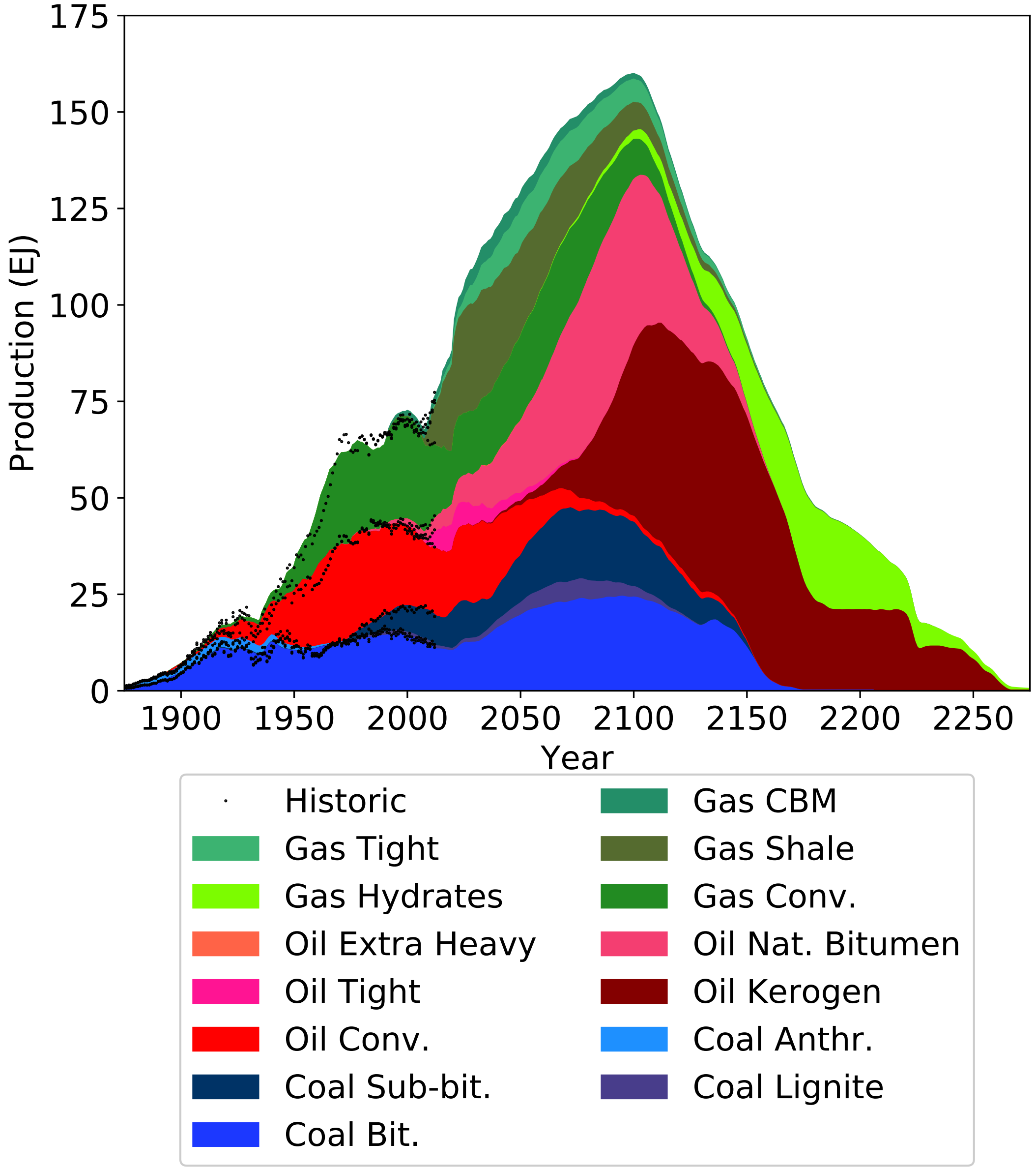

Figure 6.110: North America projection by mineral type

Table 6.110: Peak years - Minerals

**Name**	**URR**	**Peak Year**	**Peak Rate**
Coal Bit.	4005.05	2095	24.4
Coal Lignite	337.88	2068	5.19
Coal Sub-bit.	1847.56	2071	19.12
Coal Anthr.	151.2	1919	2.66
Oil Conv.	2565.96	1978	25.52
Oil Kerogen	6141.7	2136	59.86
Oil Tight	211.1	2019	6.61
Oil Nat. Bitumen	3315.53	2091	46.56
Oil Extra Heavy	3.37	2048	0.15
Gas Conv.	3184.71	1997	26.5
Gas Hydrates	1941.0	2181	24.07
Gas Shale	1858.95	2031	28.25
Gas Tight	798.61	2061	9.9
Gas CBM	356.04	2039	4.9
**Total**	**26718.66**	**2100**	**159.89**

Chapter 7

South America

7.1 Argentina

7.1.1 All Projections

Table 7.1: Peak years - All

Name	URR	Peak Year	Peak Rate
Gas Shale	812.85	2166	9.88
Gas Hydrates	496.5	2164	6.2
Oil Conv.	102.25	1998	2.03
Oil Tight	93.5	2079	2.11
Gas Conv.	88.4	2002	1.71
Coal Bit.	13.7	2069	0.32
**Total**	**1607.2**	**2166**	**16.05**

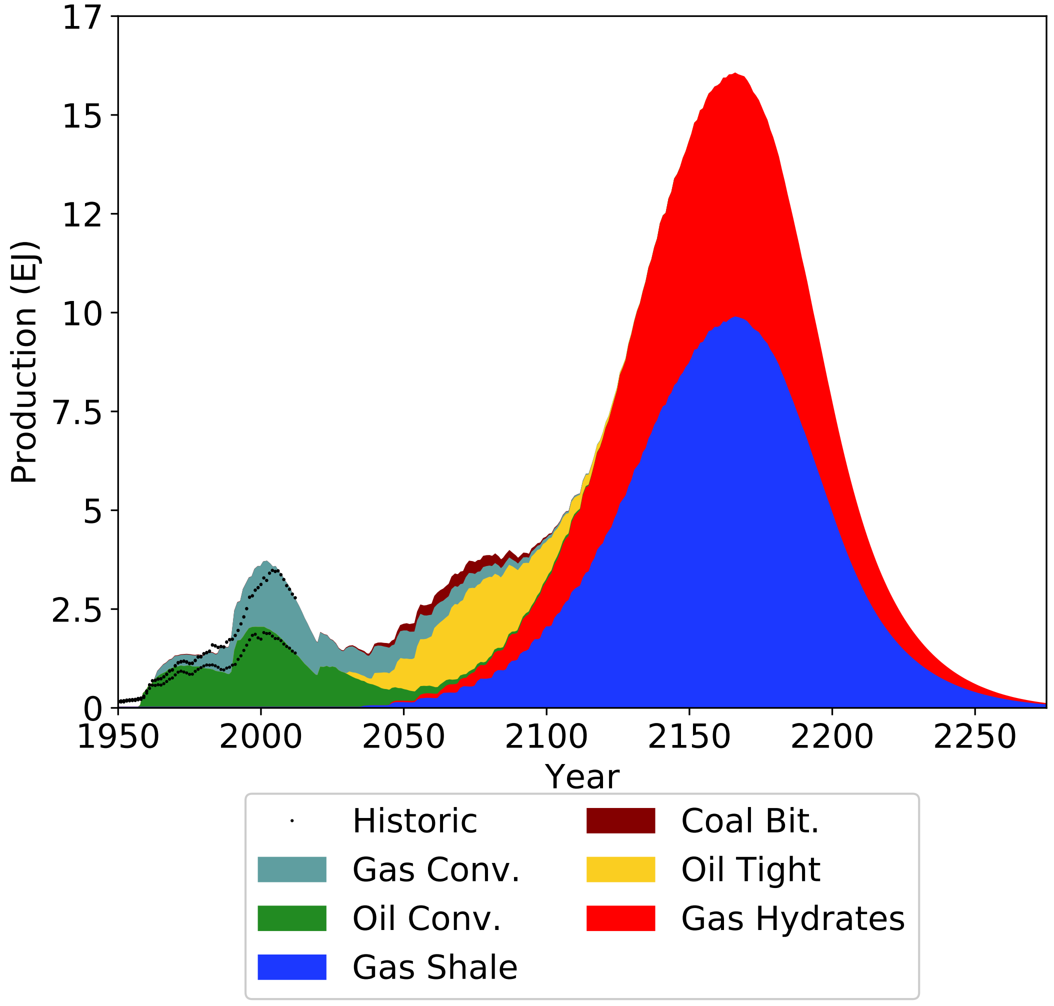

Figure 7.1: Argentina projections capped at 16

7.1.2 By Mineral

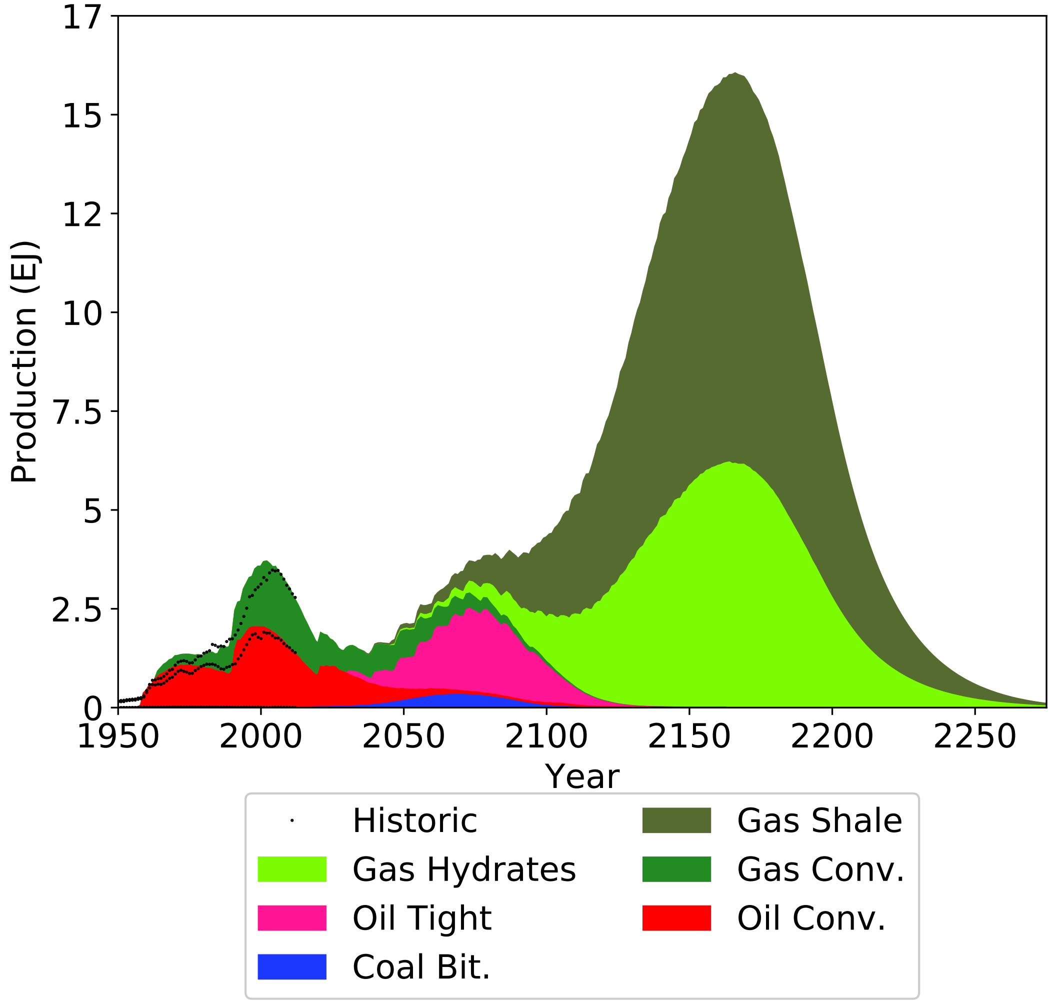

Figure 7.2: Argentina projection by mineral type

Table 7.2: Peak years - Minerals

**Name**	**URR**	**Peak Year**	**Peak Rate**
Coal Bit.	13.7	2069	0.32
Oil Conv.	102.25	1998	2.03
Oil Tight	93.5	2079	2.11
Gas Conv.	88.4	2002	1.71
Gas Hydrates	496.5	2164	6.2
Gas Shale	812.85	2166	9.88
**Total**	**1607.2**	**2166**	**16.05**

7.2 Barbados

7.2.1 All Projections

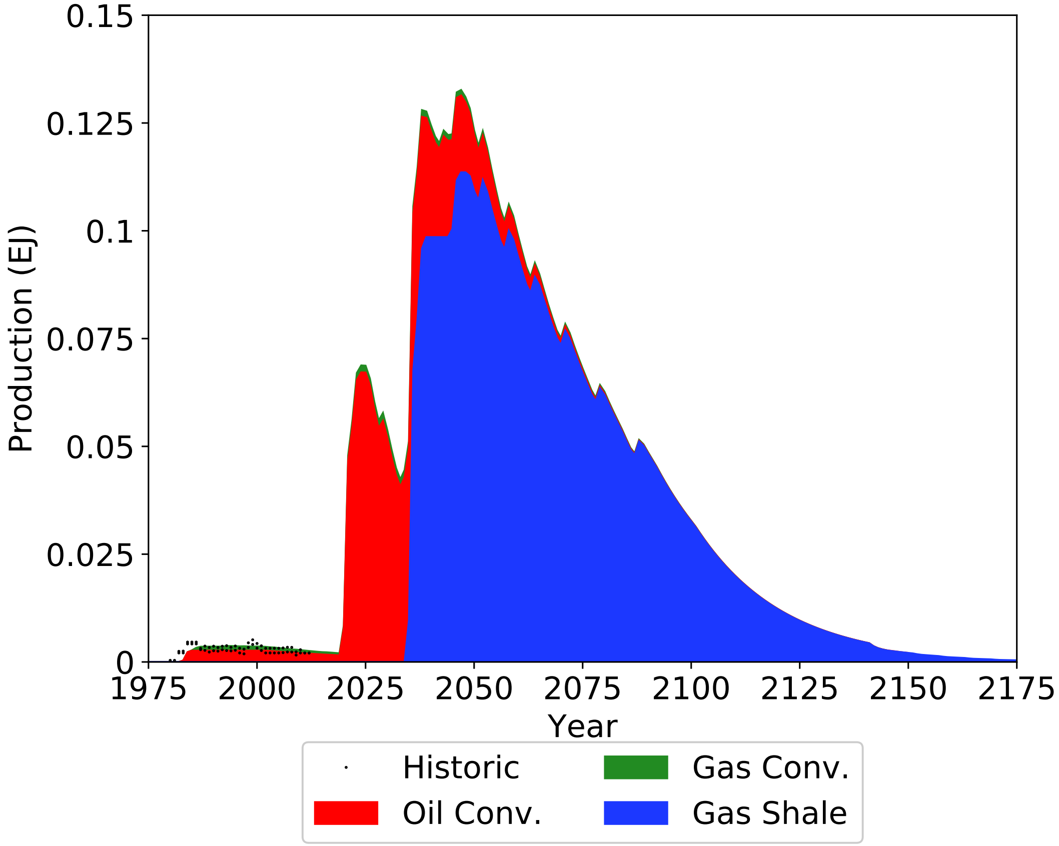

Figure 7.3: Barbados projections capped at 16

Table 7.3: Peak years - All

**Name**	**URR**	**Peak Year**	**Peak Rate**
Gas Shale	5.56	2047	0.11
Oil Conv.	1.34	2024	0.07
Gas Conv.	0.1	2029	–
**Total**	**7.0**	**2047**	**0.13**

7.2.2 By Mineral

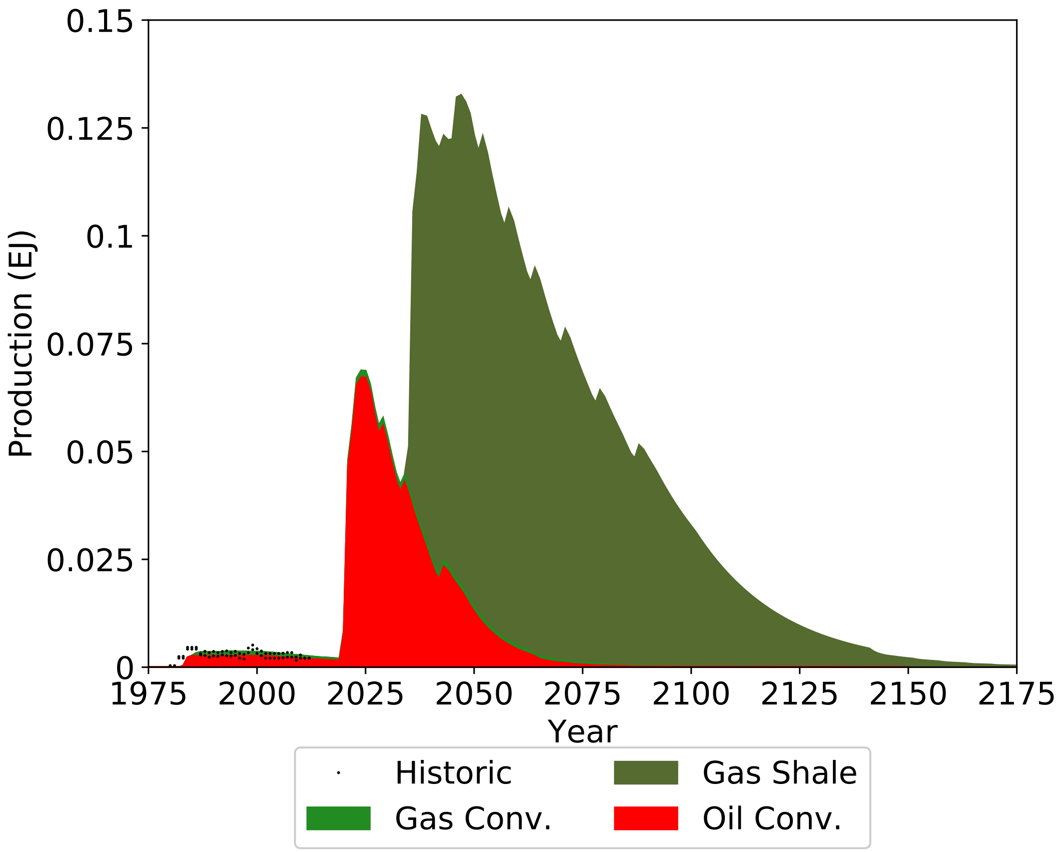

Figure 7.4: Barbados projection by mineral type

Table 7.4: Peak years - Minerals

Name	URR	Peak Year	Peak Rate
Oil Conv.	1.34	2024	0.07
Gas Conv.	0.1	2029	–
Gas Shale	5.56	2047	0.11
**Total**	**7.0**	**2047**	**0.13**

7.3 Belize

7.3.1 All Projections

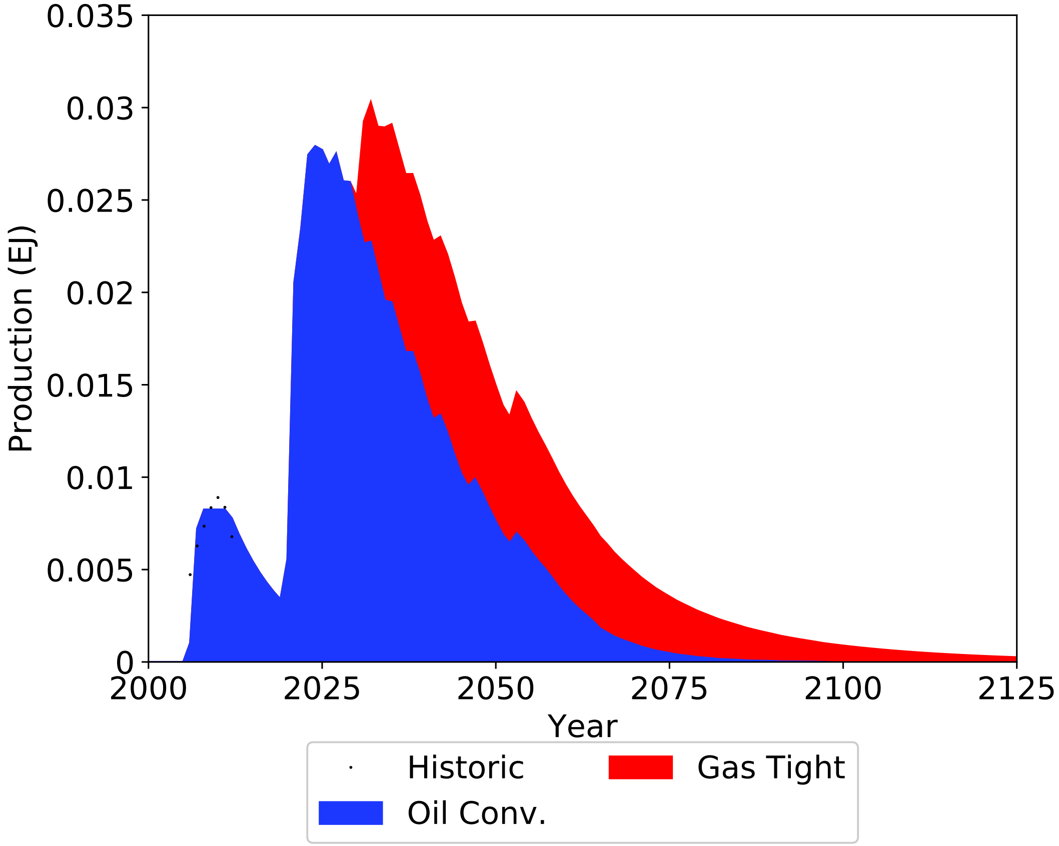

Figure 7.5: Belize projections capped at 16

Table 7.5: Peak years - All

Name	URR	Peak Year	Peak Rate
Oil Conv.	0.72	2024	0.03
Gas Tight	0.37	2035	0.01
**Total**	**1.09**	**2032**	**0.03**

7.3.2 By Mineral

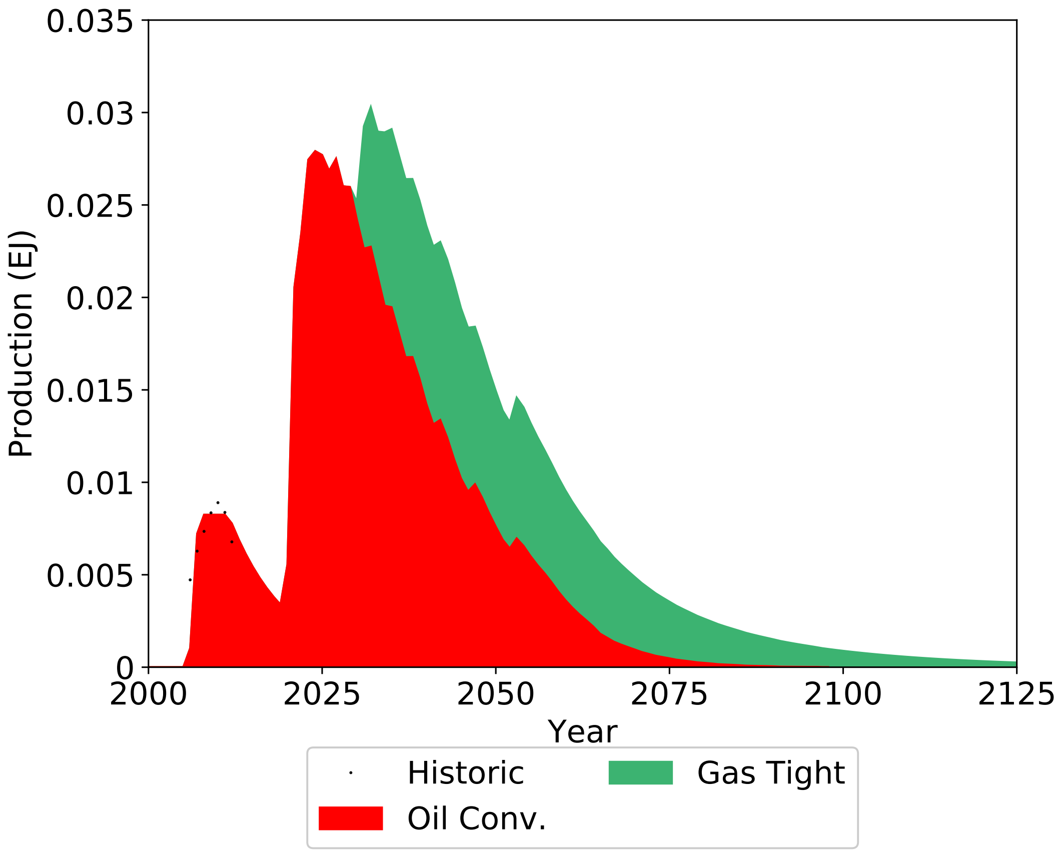

Figure 7.6: Belize projection by mineral type

Table 7.6: Peak years - Minerals

Name	URR	Peak Year	Peak Rate
Oil Conv.	0.72	2024	0.03
Gas Tight	0.37	2035	0.01
**Total**	**1.09**	**2032**	**0.03**

7.4 Bolivia

7.4.1 All Projections

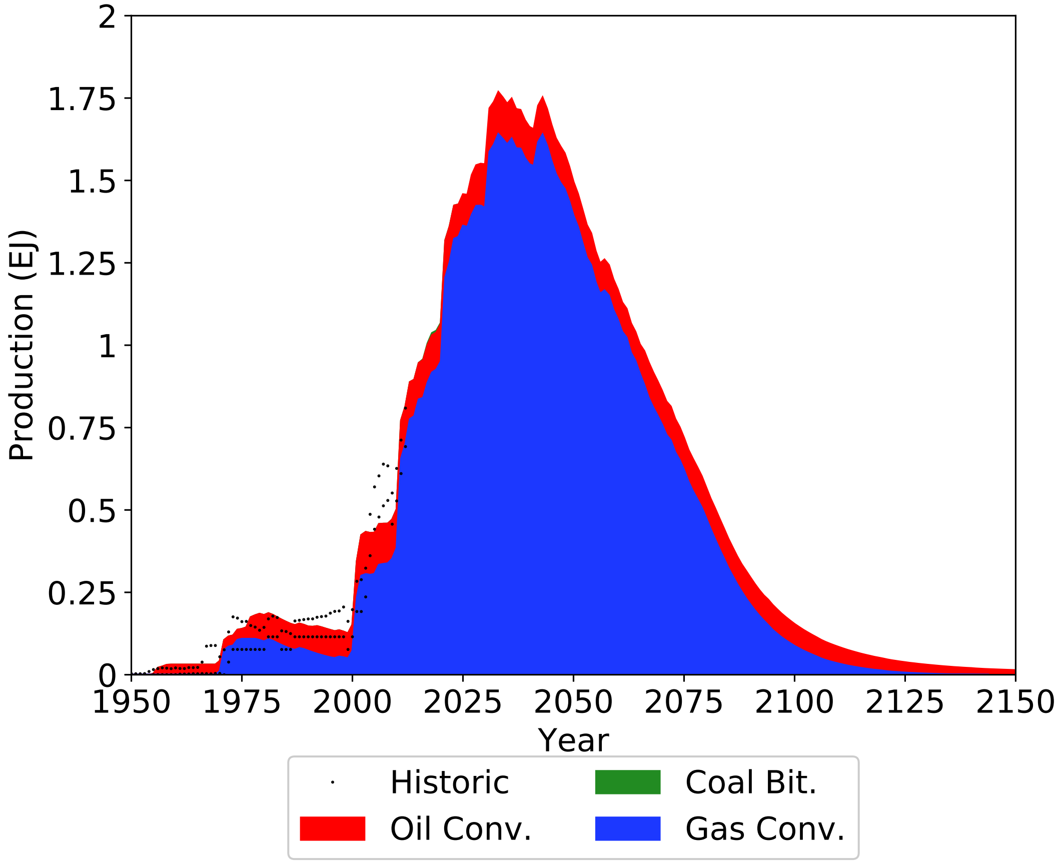

Figure 7.7: Bolivia projections capped at 16

Table 7.7: Peak years - All

**Name**	**URR**	**Peak Year**	**Peak Rate**
Gas Conv.	91.12	2033	1.64
Oil Conv.	14.86	2030	0.13
Coal Bit.	0.02	2018	0.01
**Total**	**106.0**	**2033**	**1.77**

7.4.2 By Mineral

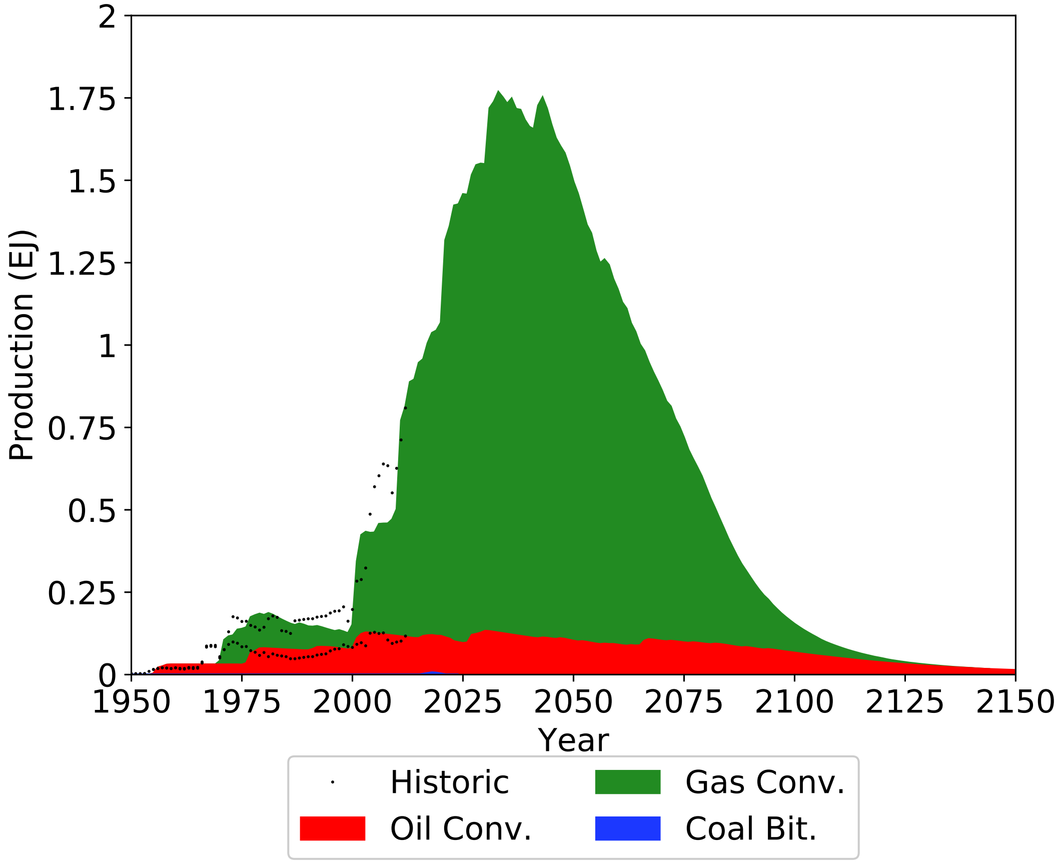

Figure 7.8: Bolivia projection by mineral type

Table 7.8: Peak years - Minerals

Name	URR	Peak Year	Peak Rate
Coal Bit.	0.02	2018	0.01
Oil Conv.	14.86	2030	0.13
Gas Conv.	91.12	2033	1.64
**Total**	**106.0**	**2033**	**1.77**

7.5 Brazil

7.5.1 All Projections

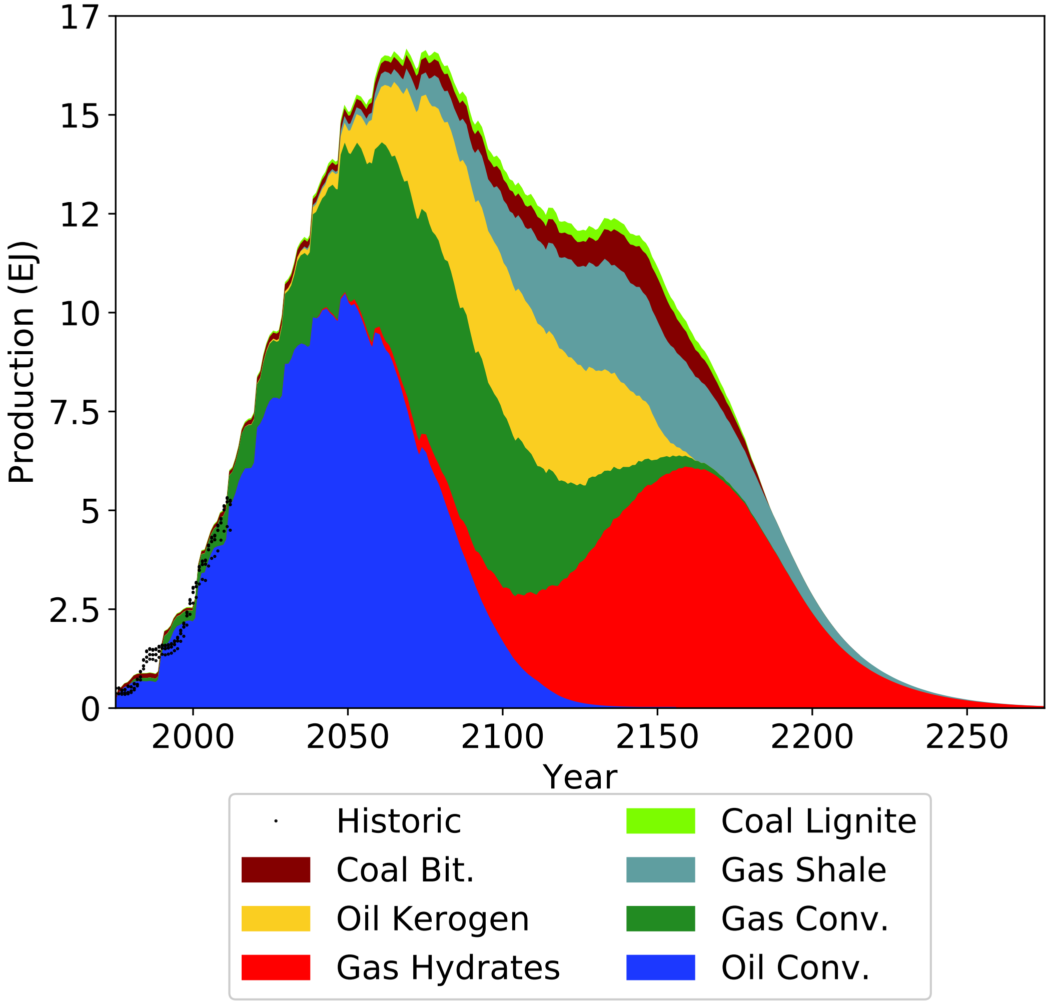

Figure 7.9: Brazil projections capped at 16

Table 7.9: Peak years - All

**Name**	**URR**	**Peak Year**	**Peak Rate**
Oil Conv.	713.83	2049	10.43
Gas Hydrates	496.5	2159	6.08
Gas Conv.	452.15	2073	5.67
Oil Kerogen	282.07	2096	3.84
Gas Shale	237.35	2138	2.83
Coal Bit.	85.2	2150	1.1
Coal Lignite	32.4	2121	0.29
**Total**	**2299.5**	**2069**	**16.61**

7.5.2 By Mineral

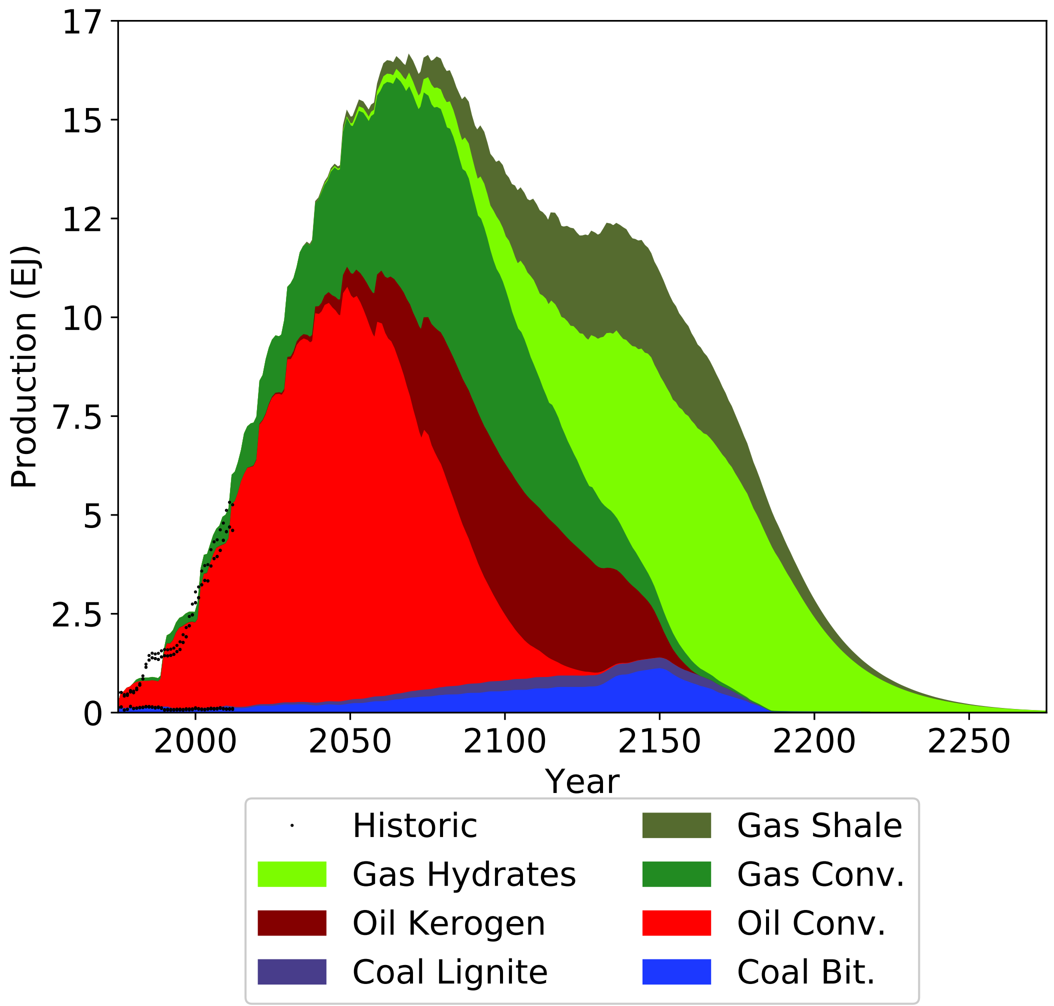

Figure 7.10: Brazil projection by mineral type

Table 7.10: Peak years - Minerals

**Name**	**URR**	**Peak Year**	**Peak Rate**
Coal Bit.	85.2	2150	1.1
Coal Lignite	32.4	2121	0.29
Oil Conv.	713.83	2049	10.43
Oil Kerogen	282.07	2096	3.84
Gas Conv.	452.15	2073	5.67
Gas Hydrates	496.5	2159	6.08
Gas Shale	237.35	2138	2.83
**Total**	**2299.5**	**2069**	**16.61**

7.6 Chile

7.6.1 All Projections

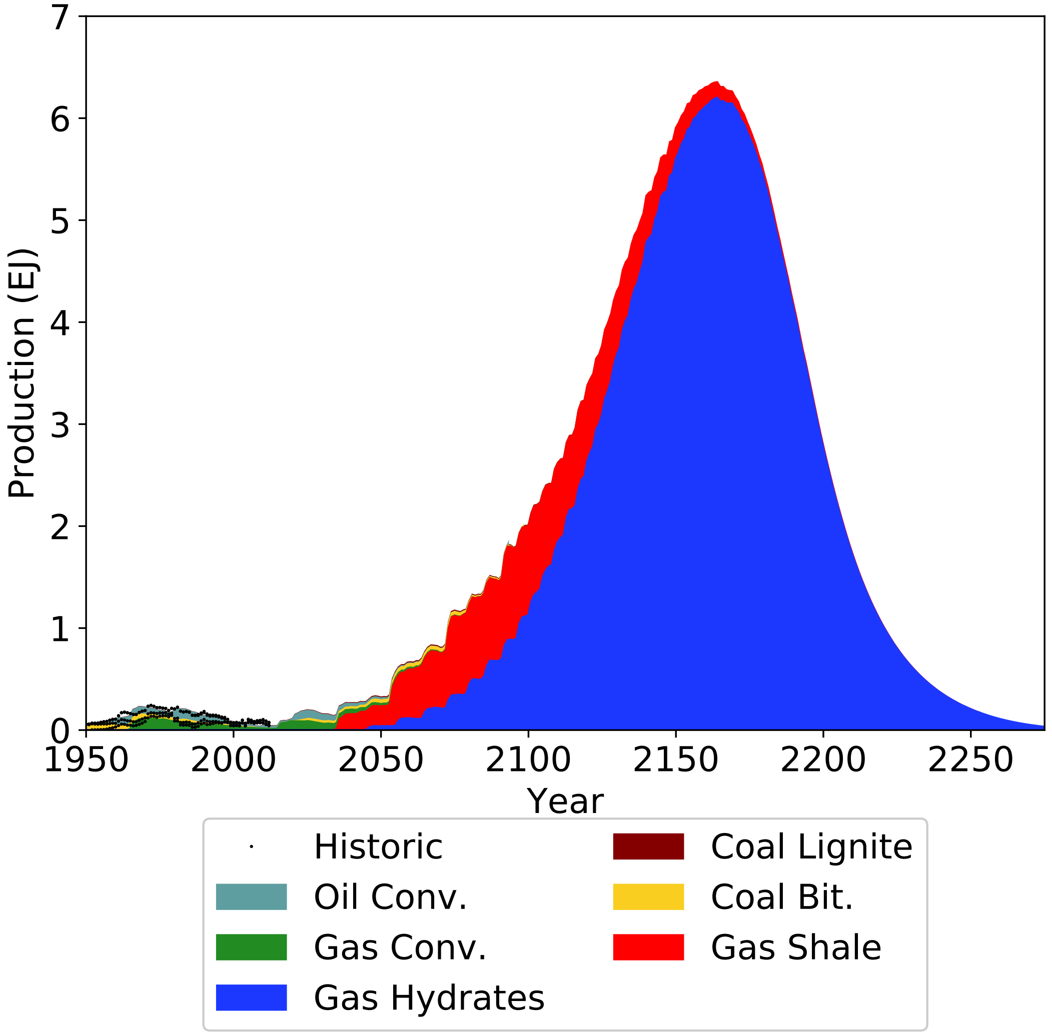

Figure 7.11: Chile projections capped at 16

Table 7.11: Peak years - All

**Name**	**URR**	**Peak Year**	**Peak Rate**
Gas Hydrates	496.5	2164	6.2
Gas Shale	72.79	2093	0.92
Gas Conv.	5.35	1969	0.11
Coal Bit.	5.2	1950	0.06
Oil Conv.	4.76	1981	0.1
Coal Lignite	1.02	2073	0.01
**Total**	**585.62**	**2164**	**6.35**

7.6.2 By Mineral

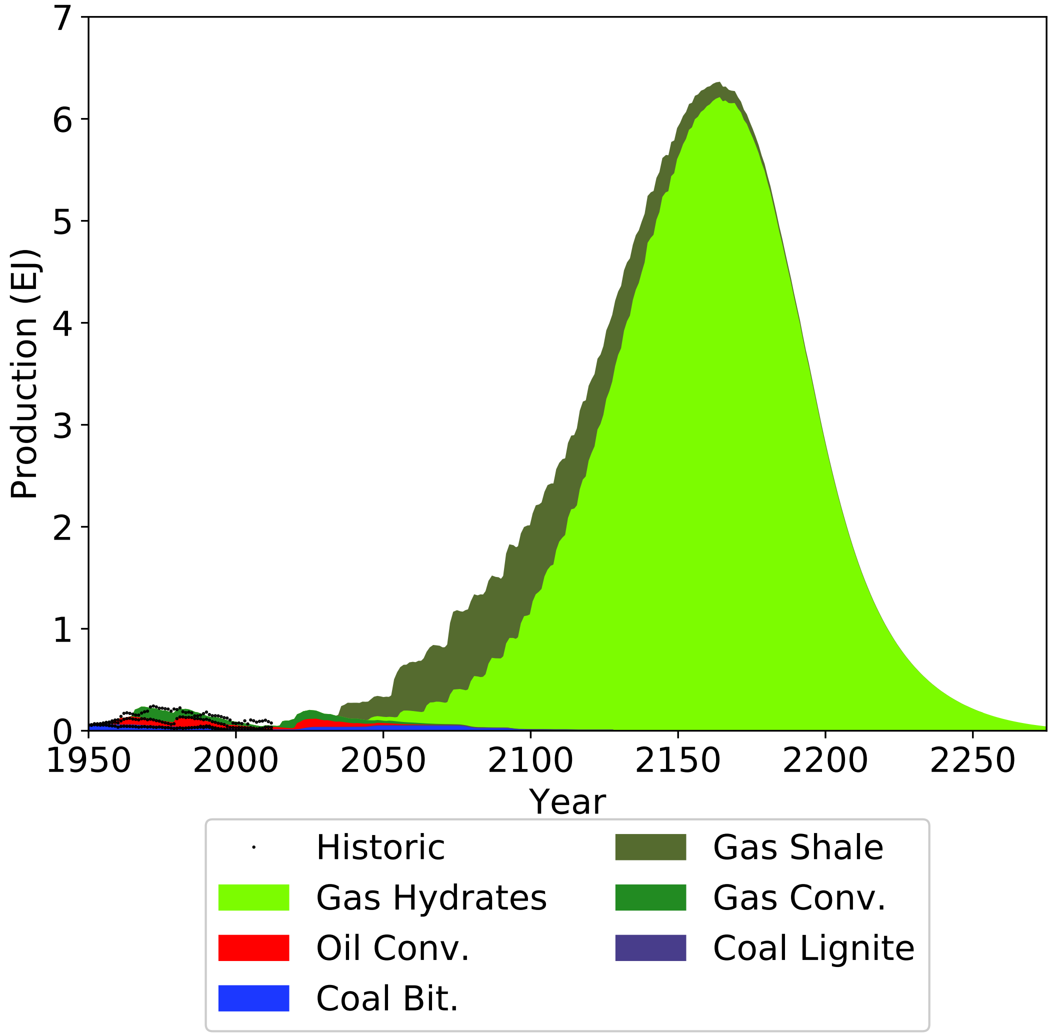

Figure 7.12: Chile projection by mineral type

Table 7.12: Peak years - Minerals

**Name**	**URR**	**Peak Year**	**Peak Rate**
Coal Bit.	5.2	1950	0.06
Coal Lignite	1.02	2073	0.01
Oil Conv.	4.76	1981	0.1
Gas Conv.	5.35	1969	0.11
Gas Hydrates	496.5	2164	6.2
Gas Shale	72.79	2093	0.92
**Total**	**585.62**	**2164**	**6.35**

7.7 Colombia

7.7.1 All Projections

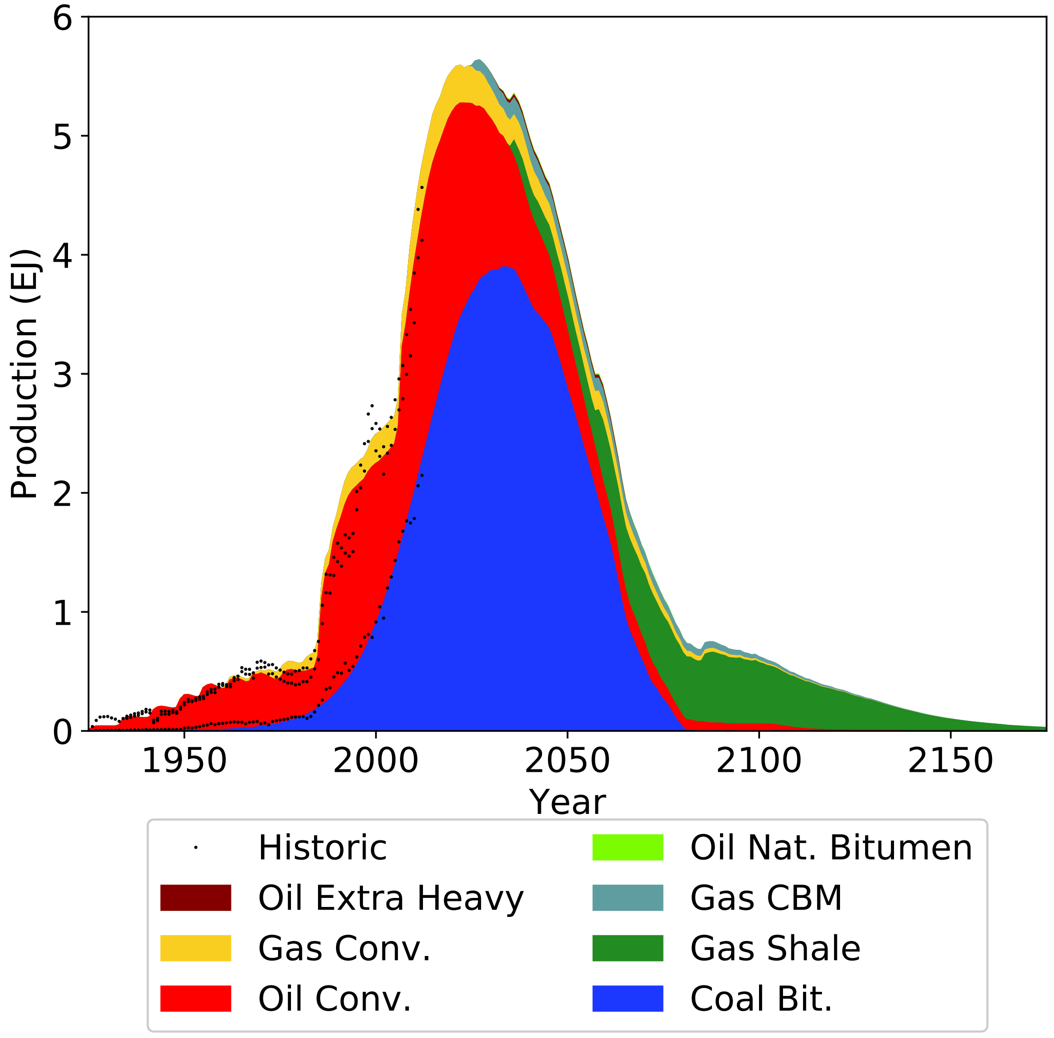

Figure 7.13: Colombia projections capped at 16

Table 7.13: Peak years - All

**Name**	**URR**	**Peak Year**	**Peak Rate**
Coal Bit.	192.3	2033	3.89
Oil Conv.	112.15	2014	2.13
Gas Shale	44.72	2088	0.59
Gas Conv.	21.78	2011	0.43
Gas CBM	7.86	2040	0.15
Oil Extra Heavy	0.8	2035	0.02
Oil Nat. Bitumen	0.42	2037	0.01
**Total**	**380.03**	**2027**	**5.63**

7.7.2 By Mineral

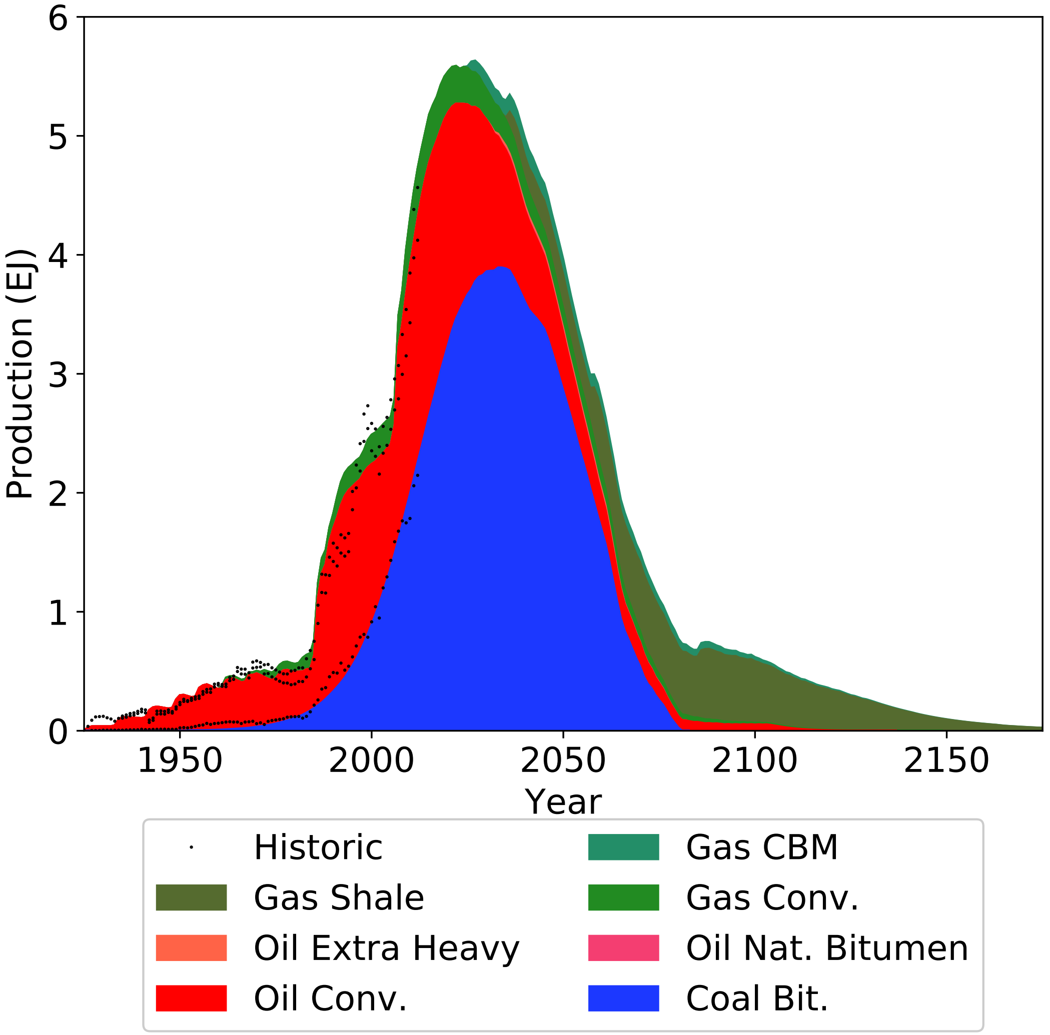

Figure 7.14: Colombia projection by mineral type

Table 7.14: Peak years - Minerals

**Name**	**URR**	**Peak Year**	**Peak Rate**
Coal Bit.	192.3	2033	3.89
Oil Conv.	112.15	2014	2.13
Oil Nat. Bitumen	0.42	2037	0.01
Oil Extra Heavy	0.8	2035	0.02
Gas Conv.	21.78	2011	0.43
Gas Shale	44.72	2088	0.59
Gas CBM	7.86	2040	0.15
**Total**	**380.03**	**2027**	**5.63**

7.8 Cuba

7.8.1 All Projections

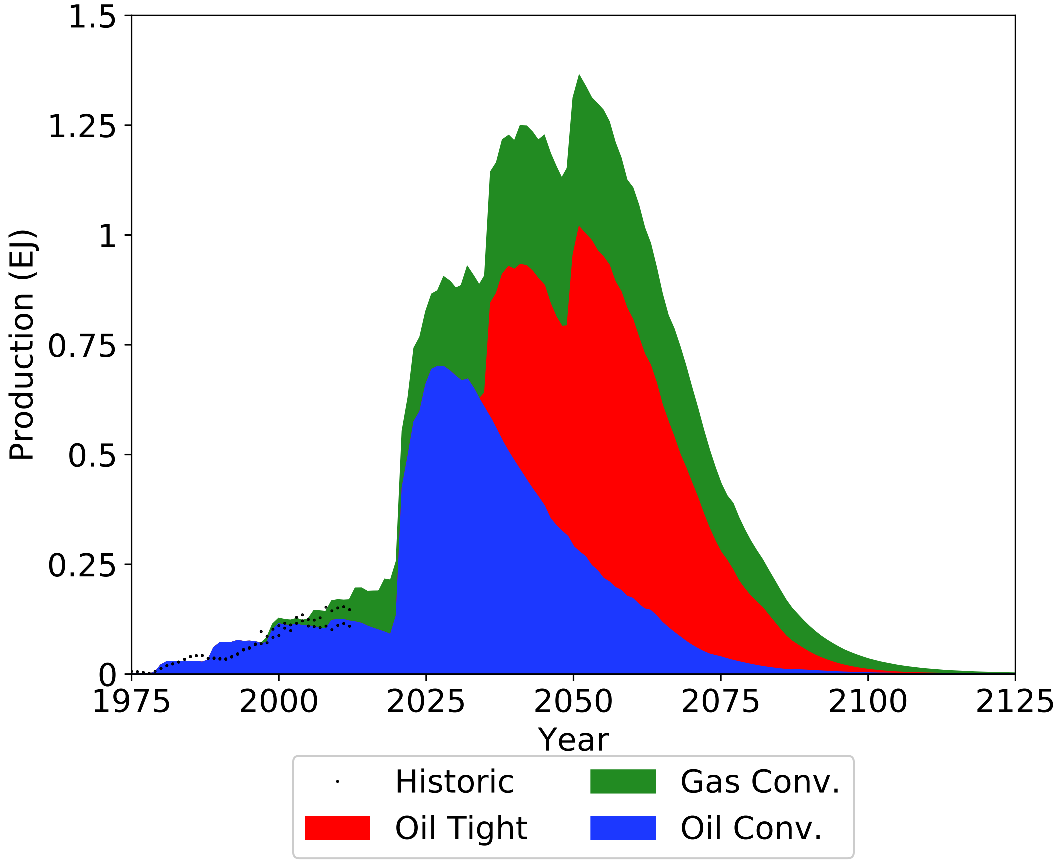

Figure 7.15: Cuba projections capped at 16

Table 7.15: Peak years - All

**Name**	**URR**	**Peak Year**	**Peak Rate**
Oil Conv.	23.04	2027	0.7
Oil Tight	22.32	2053	0.74
Gas Conv.	17.83	2049	0.36
**Total**	**63.19**	**2051**	**1.36**

7.8.2 By Mineral

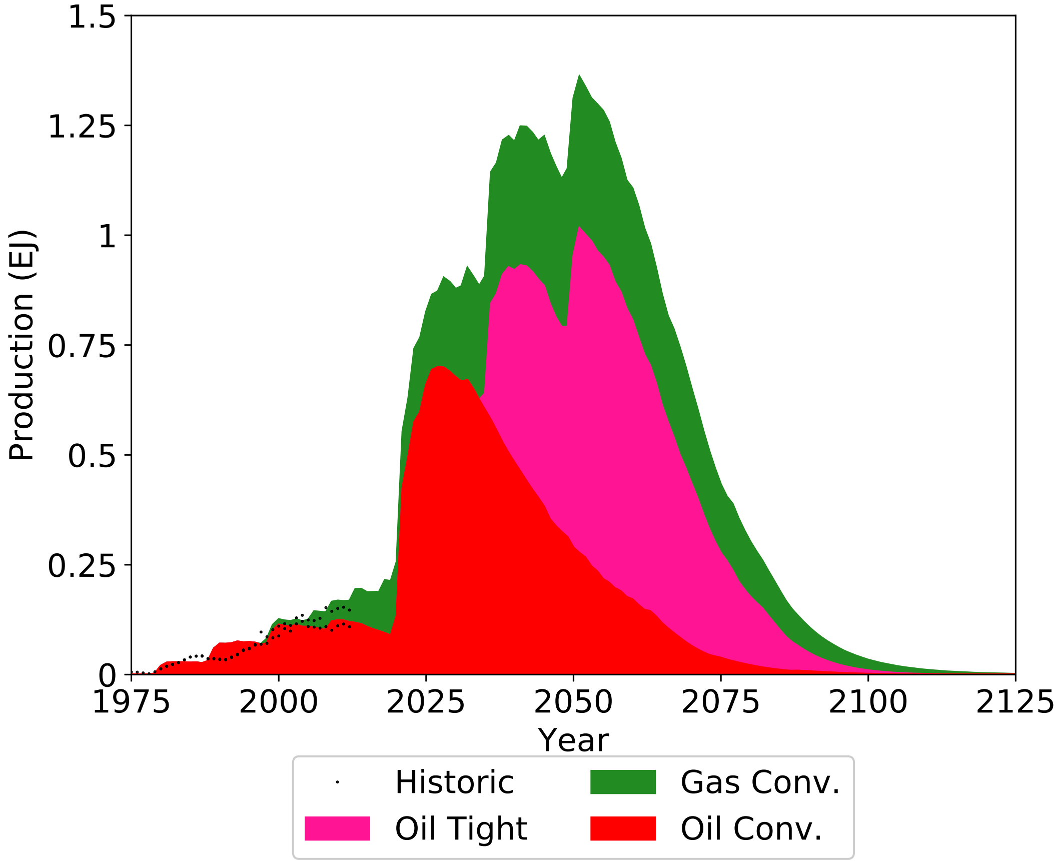

Figure 7.16: Cuba projection by mineral type

Table 7.16: Peak years - Minerals

Name	URR	Peak Year	Peak Rate
Oil Conv.	23.04	2027	0.7
Oil Tight	22.32	2053	0.74
Gas Conv.	17.83	2049	0.36
**Total**	**63.19**	**2051**	**1.36**

7.9 Dominican Republic

7.9.1 All Projections

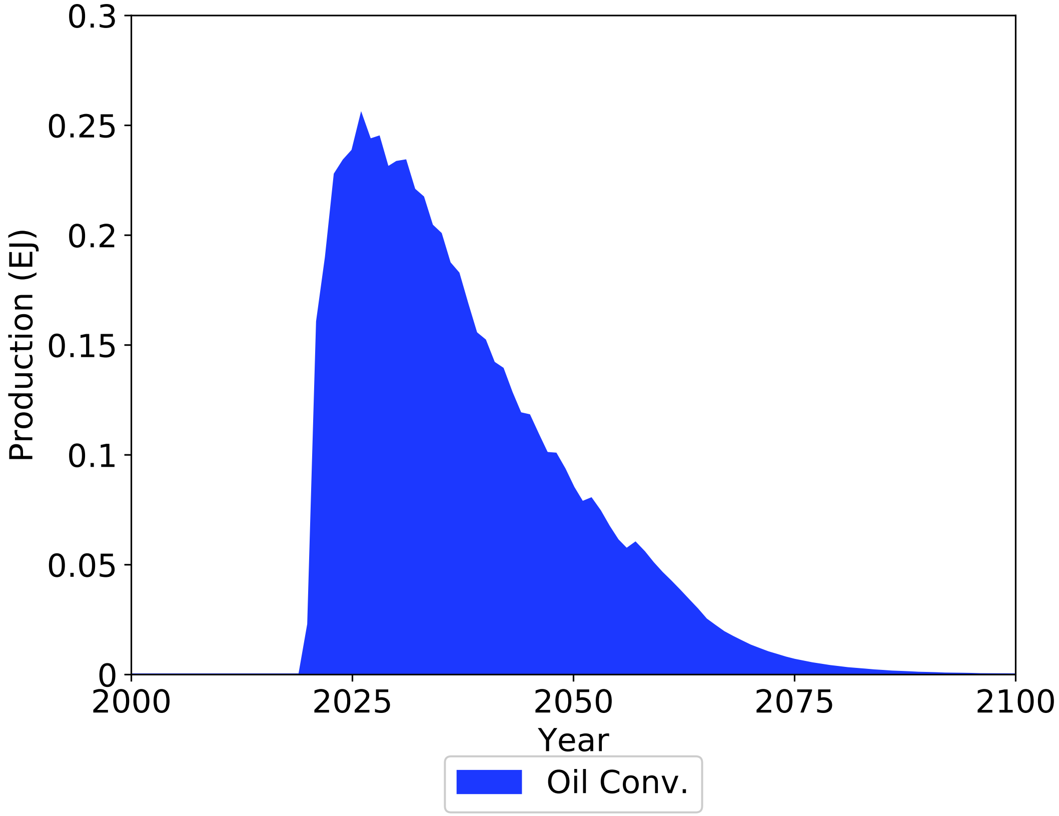

Figure 7.17: Dominican Republic projections capped at 16

Table 7.17: Peak years - All

Name	URR	Peak Year	Peak Rate
Oil Conv.	6.32	2026	0.25
**Total**	**6.32**	**2026**	**0.25**

7.9.2 By Mineral

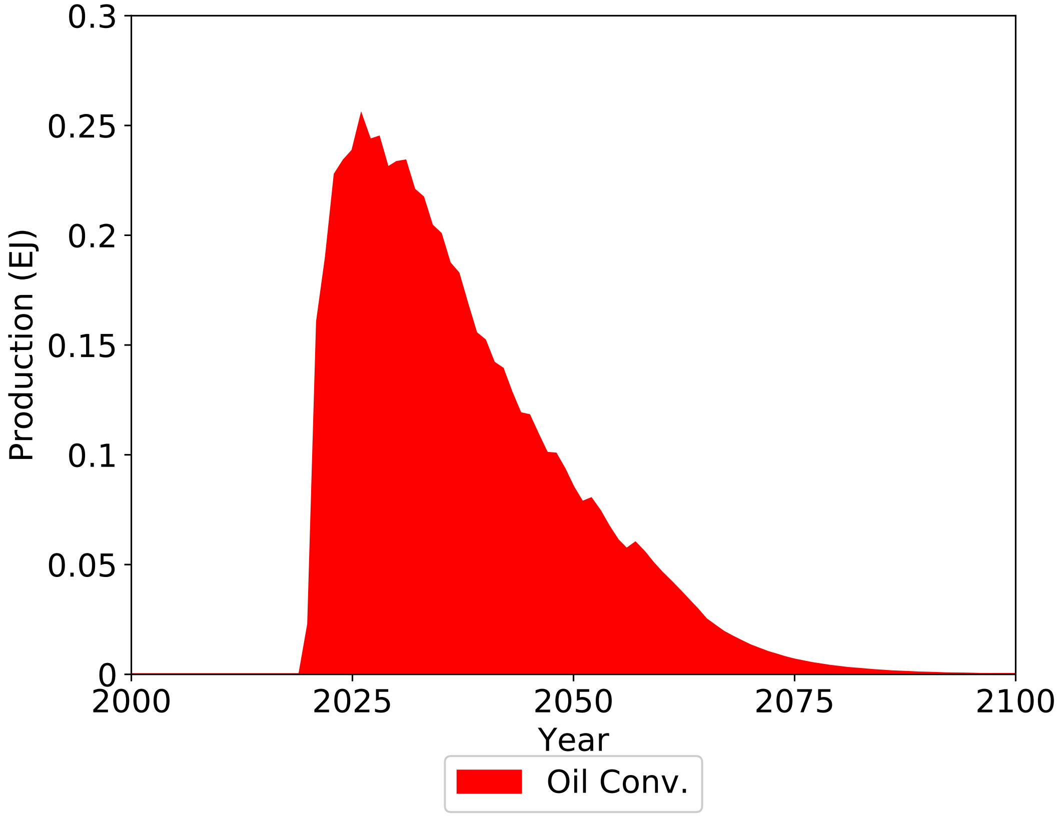

Figure 7.18: Dominican Republic projection by mineral type

Table 7.18: Peak years - Minerals

Name	URR	Peak Year	Peak Rate
Oil Conv.	6.32	2026	0.25
**Total**	**6.32**	**2026**	**0.25**

7.10 Ecuador

7.10.1 All Projections

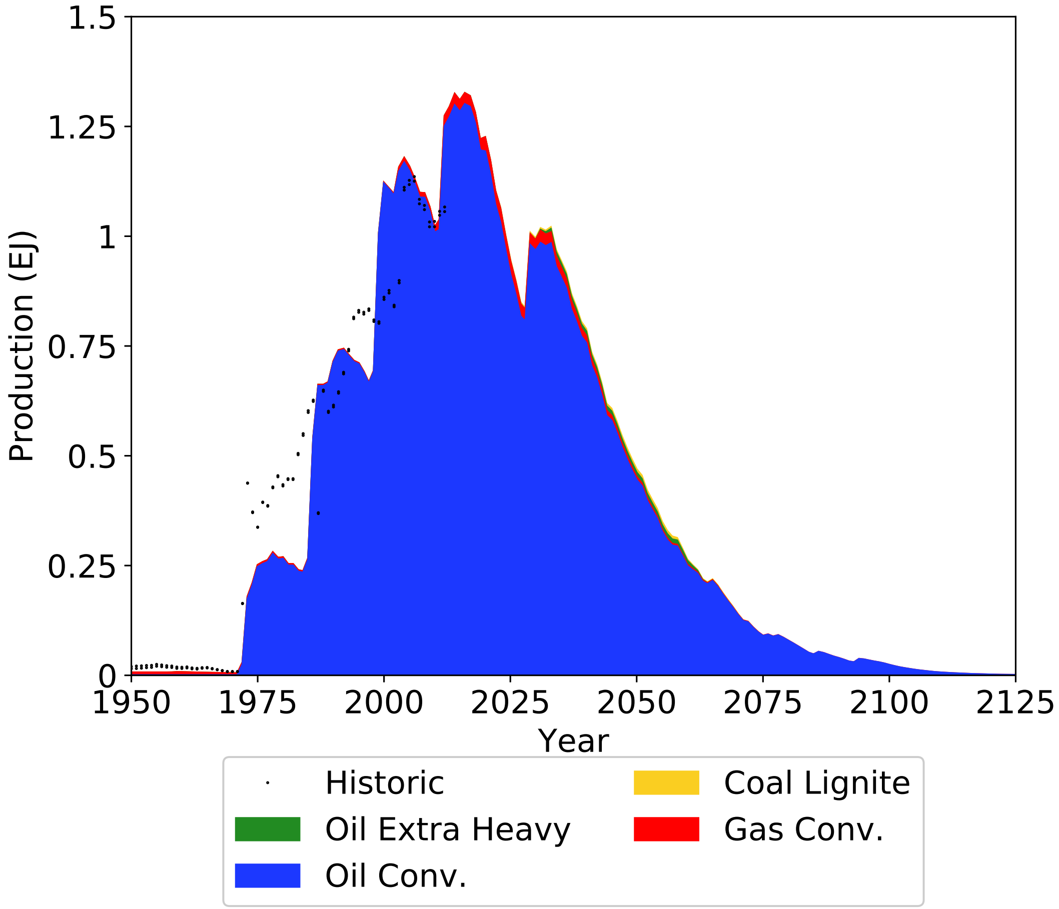

Figure 7.19: Ecuador projections capped at 16

Table 7.19: Peak years - All

**Name**	**URR**	**Peak Year**	**Peak Rate**
Oil Conv.	68.94	2016	1.3
Gas Conv.	1.3	2021	0.03
Oil Extra Heavy	0.29	2035	0.01
Coal Lignite	0.23	2049	0.01
**Total**	**70.76**	**2016**	**1.33**

7.10.2 By Mineral

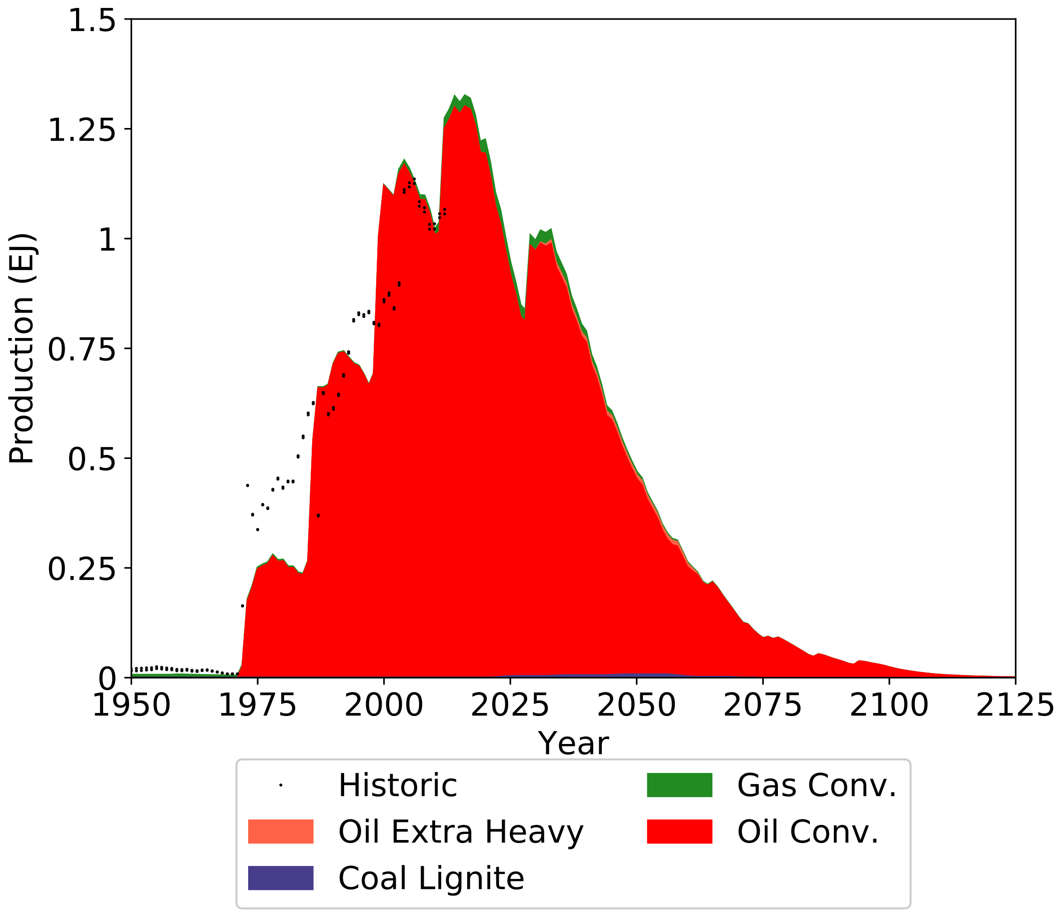

Figure 7.20: Ecuador projection by mineral type

Table 7.20: Peak years - Minerals

Name	URR	Peak Year	Peak Rate
Coal Lignite	0.23	2049	0.01
Oil Conv.	68.94	2016	1.3
Oil Extra Heavy	0.29	2035	0.01
Gas Conv.	1.3	2021	0.03
**Total**	**70.76**	**2016**	**1.33**

7.11 Falkland Islands

7.11.1 All Projections

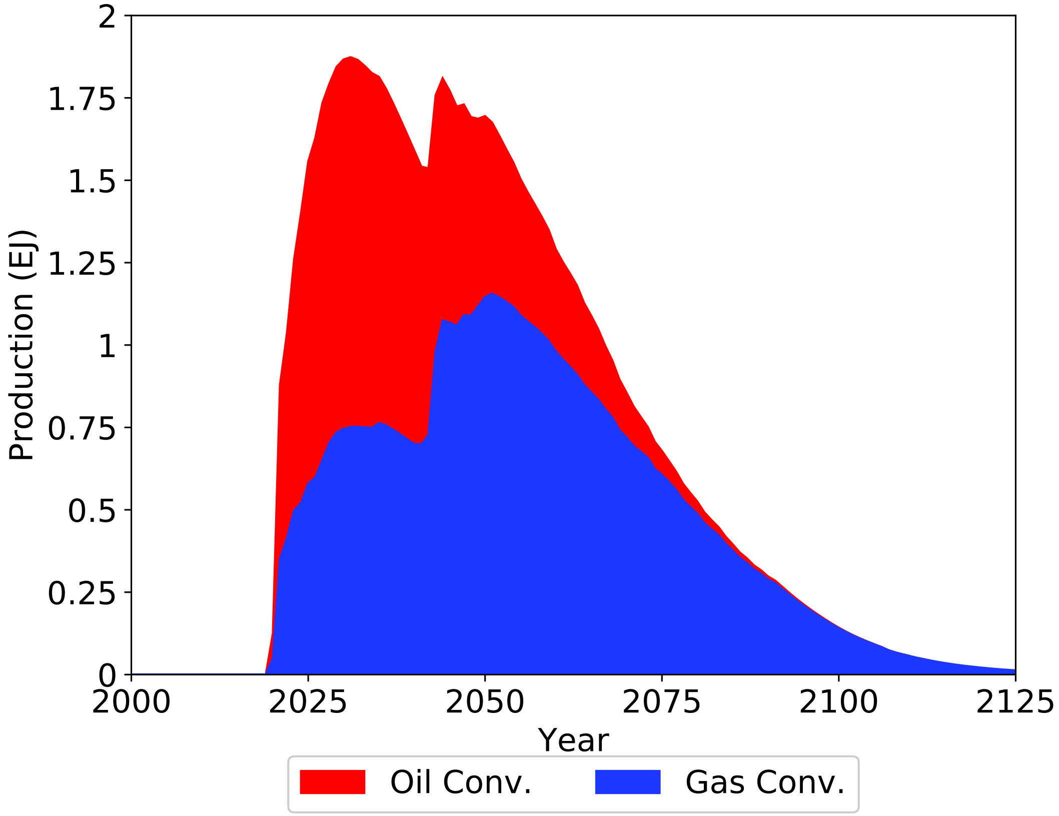

Figure 7.21: Falkland Islands projections capped at 16

Table 7.21: Peak years - All

Name	URR	Peak Year	Peak Rate
Gas Conv.	55.62	2051	1.16
Oil Conv.	33.69	2031	1.12
**Total**	**89.31**	**2031**	**1.87**

7.11.2 By Mineral

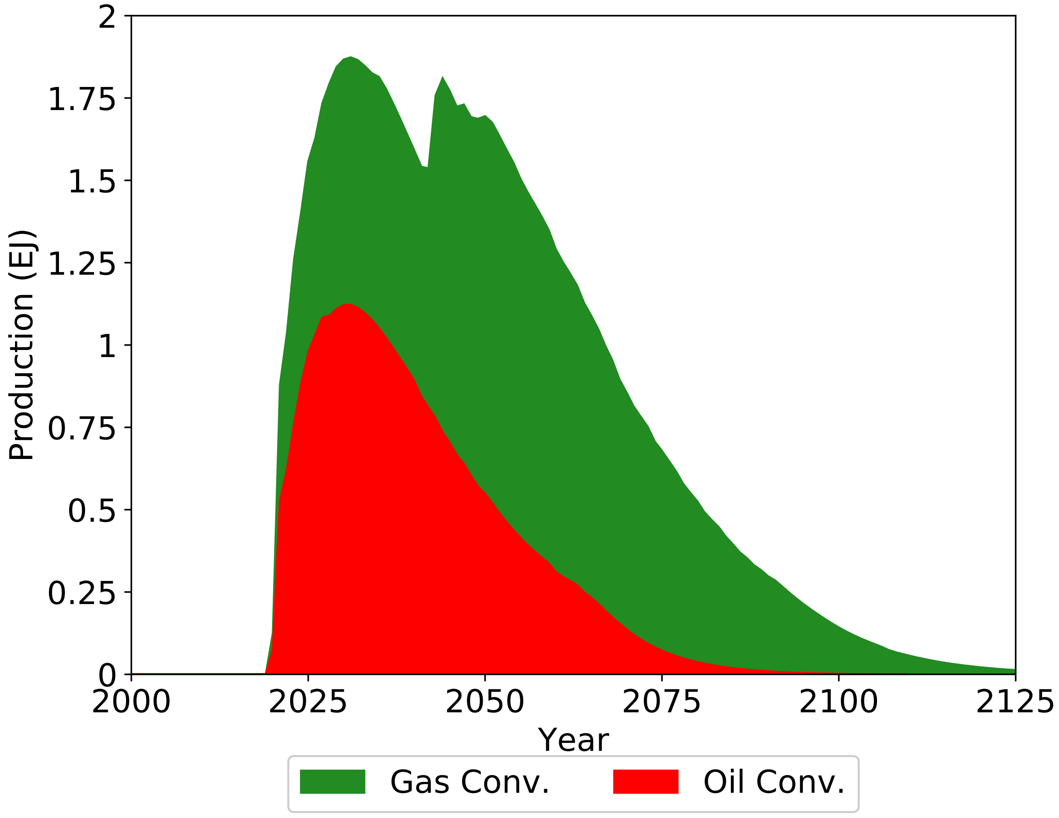

Figure 7.22: Falkland Islands projection by mineral type

Table 7.22: Peak years - Minerals

Name	URR	Peak Year	Peak Rate
Oil Conv.	33.69	2031	1.12
Gas Conv.	55.62	2051	1.16
**Total**	**89.31**	**2031**	**1.87**

7.12 French Guiana

7.12.1 All Projections

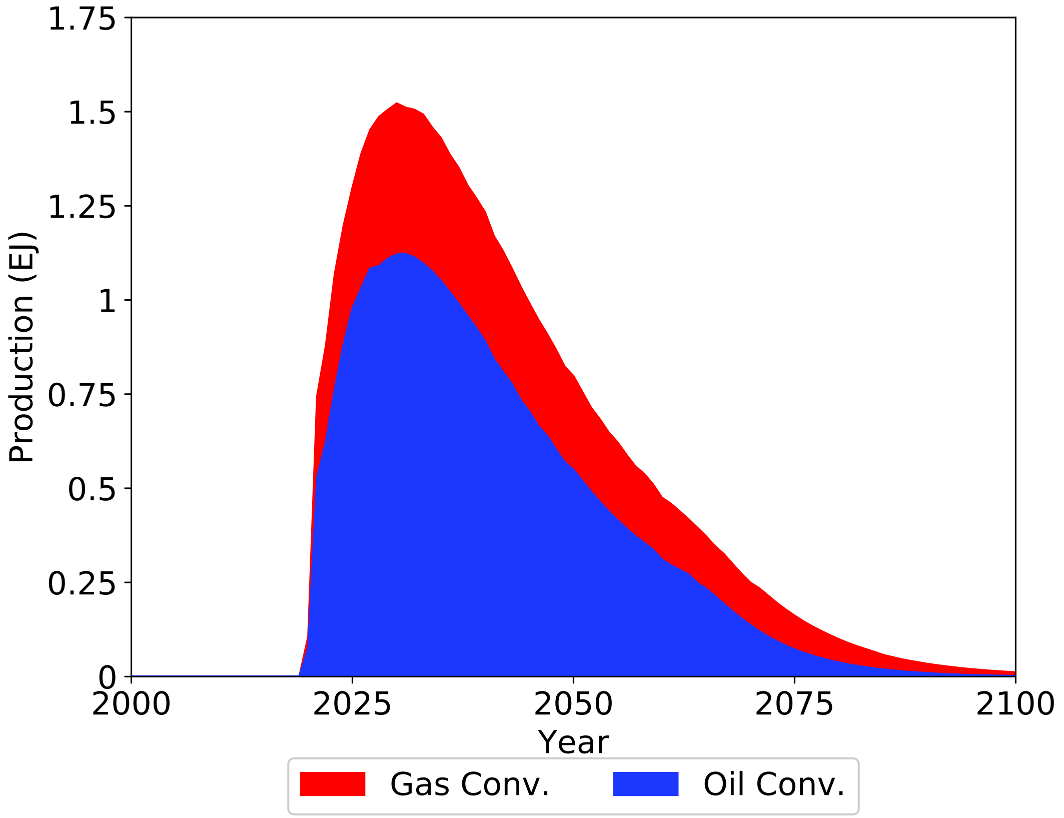

Figure 7.23: French Guiana projections capped at 16

Table 7.23: Peak years - All

Name	URR	Peak Year	Peak Rate
Oil Conv.	33.69	2031	1.12
Gas Conv.	14.8	2030	0.4
**Total**	**48.49**	**2030**	**1.52**

7.12.2 By Mineral

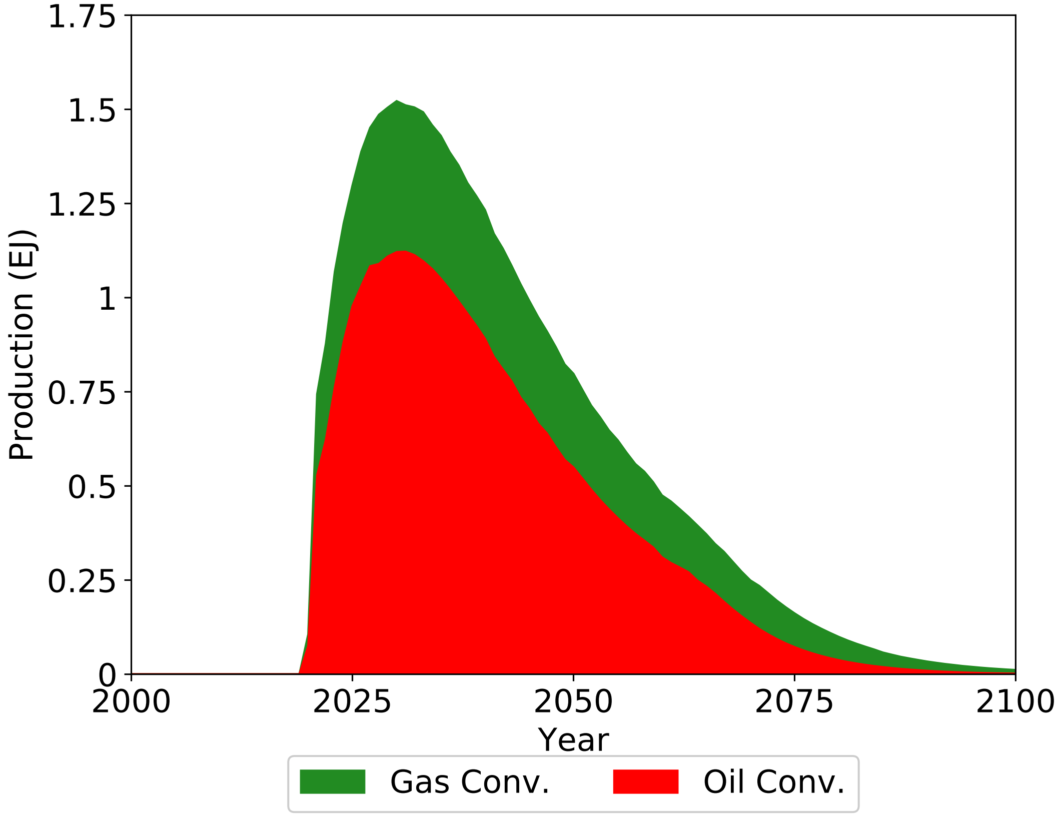

Figure 7.24: French Guiana projection by mineral type

Table 7.24: Peak years - Minerals

Name	URR	Peak Year	Peak Rate
Oil Conv.	33.69	2031	1.12
Gas Conv.	14.8	2030	0.4
**Total**	**48.49**	**2030**	**1.52**

7.13 Grenada

7.13.1 All Projections

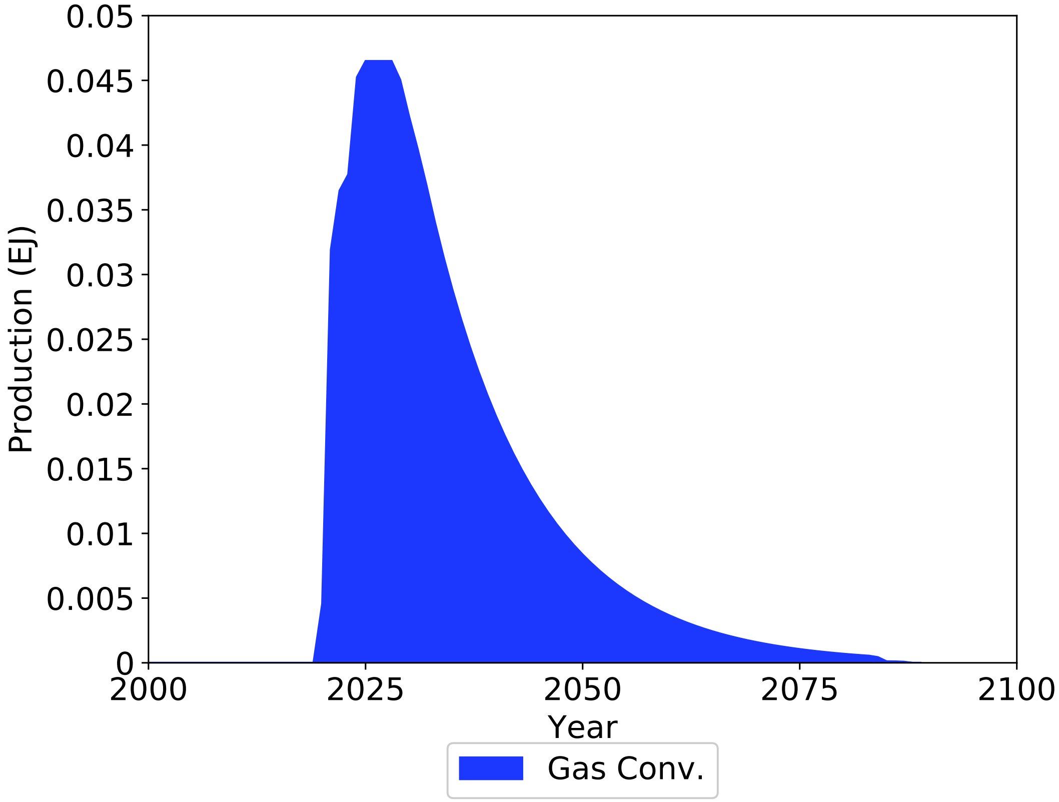

Figure 7.25: Grenada projections capped at 16

Table 7.25: Peak years - All

**Name**	**URR**	**Peak Year**	**Peak Rate**
Gas Conv.	0.93	2025	0.05
**Total**	**0.93**	**2025**	**0.05**

7.13.2 By Mineral

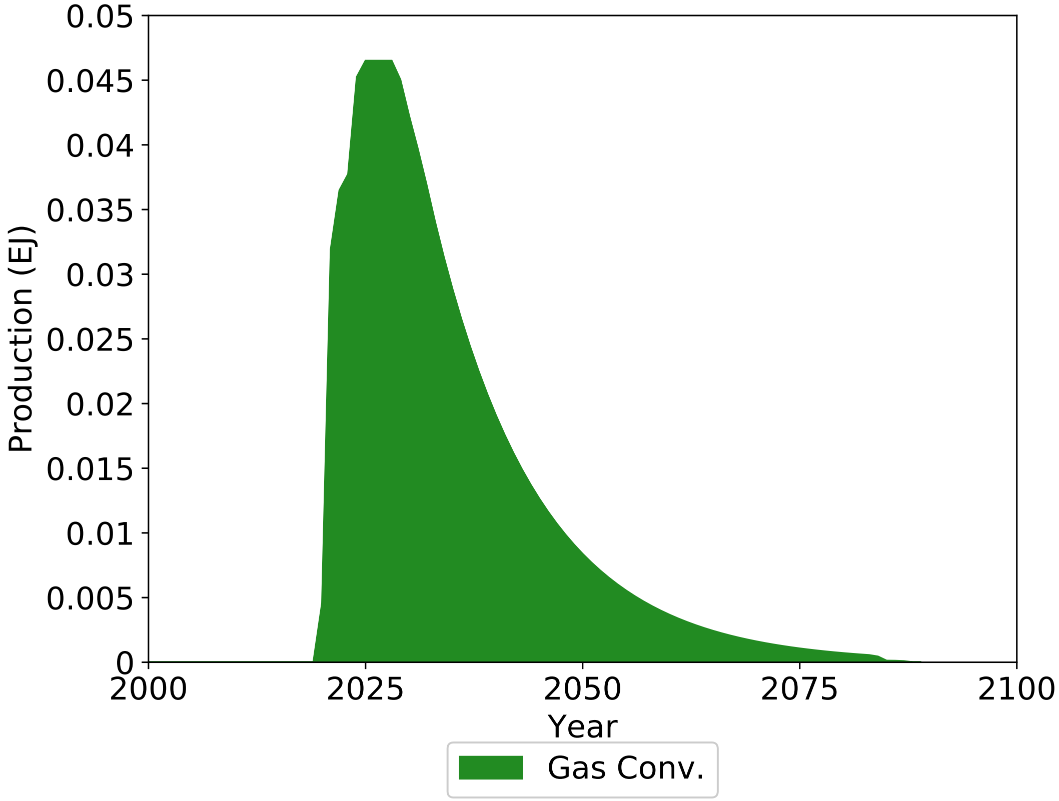

Figure 7.26: Grenada projection by mineral type

Table 7.26: Peak years - Minerals

**Name**	**URR**	**Peak Year**	**Peak Rate**
Gas Conv.	0.93	2025	0.05
**Total**	**0.93**	**2025**	**0.05**

7.14 Guatemala

7.14.1 All Projections

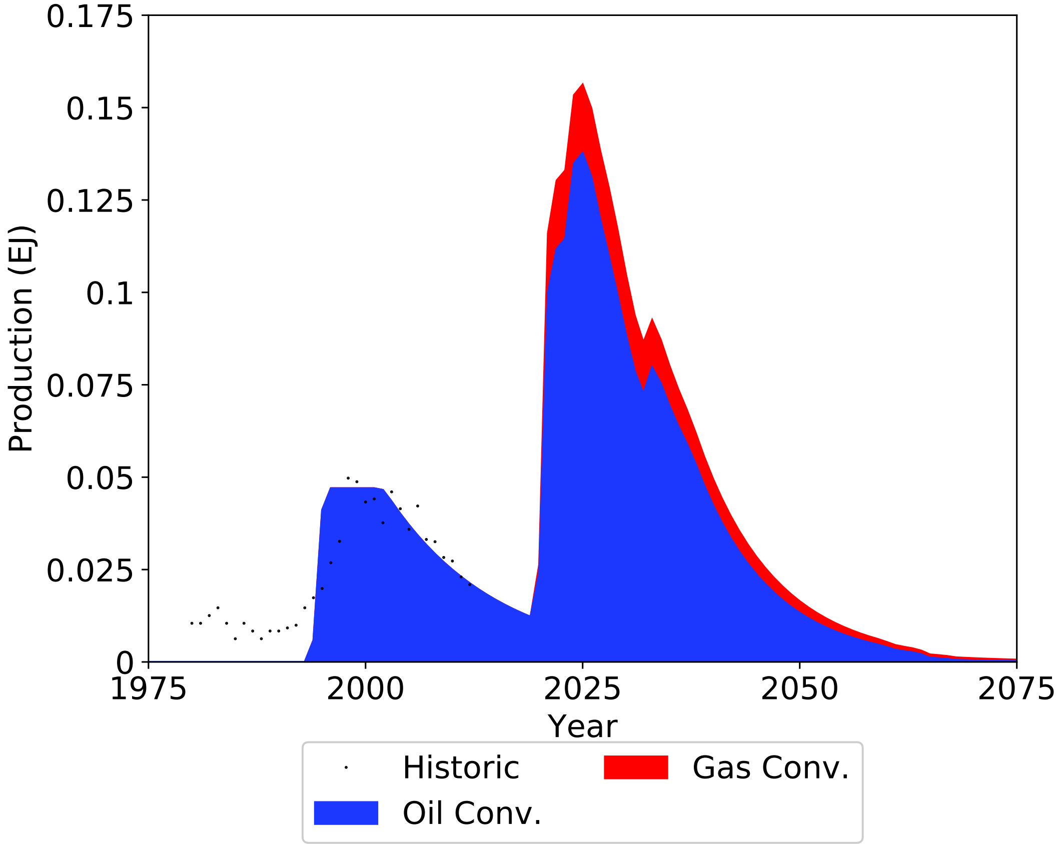

Figure 7.27: Guatemala projections capped at 16

Table 7.27: Peak years - All

**Name**	**URR**	**Peak Year**	**Peak Rate**
Oil Conv.	2.94	2025	0.14
Gas Conv.	0.37	2022	0.02
**Total**	**3.31**	**2025**	**0.16**

7.14.2 By Mineral

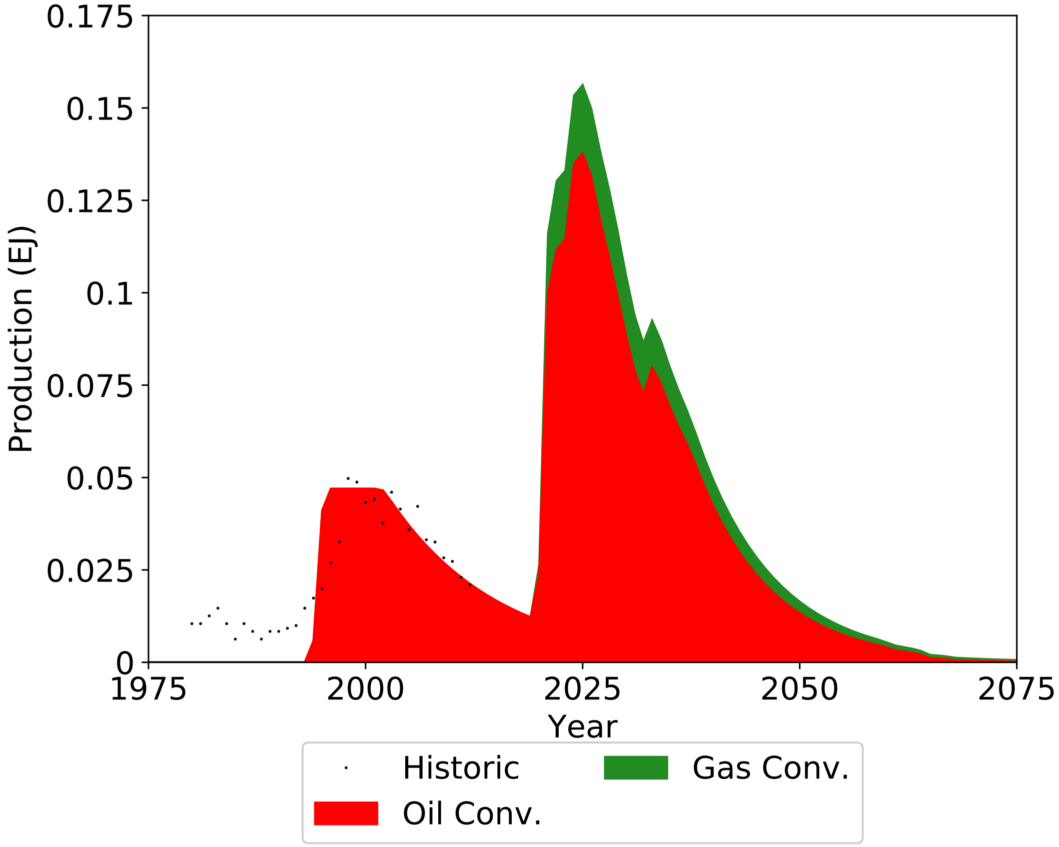

Figure 7.28: Guatemala projection by mineral type

Table 7.28: Peak years - Minerals

Name	URR	Peak Year	Peak Rate
Oil Conv.	2.94	2025	0.14
Gas Conv.	0.37	2022	0.02
**Total**	**3.31**	**2025**	**0.16**

7.15 Guyana

7.15.1 All Projections

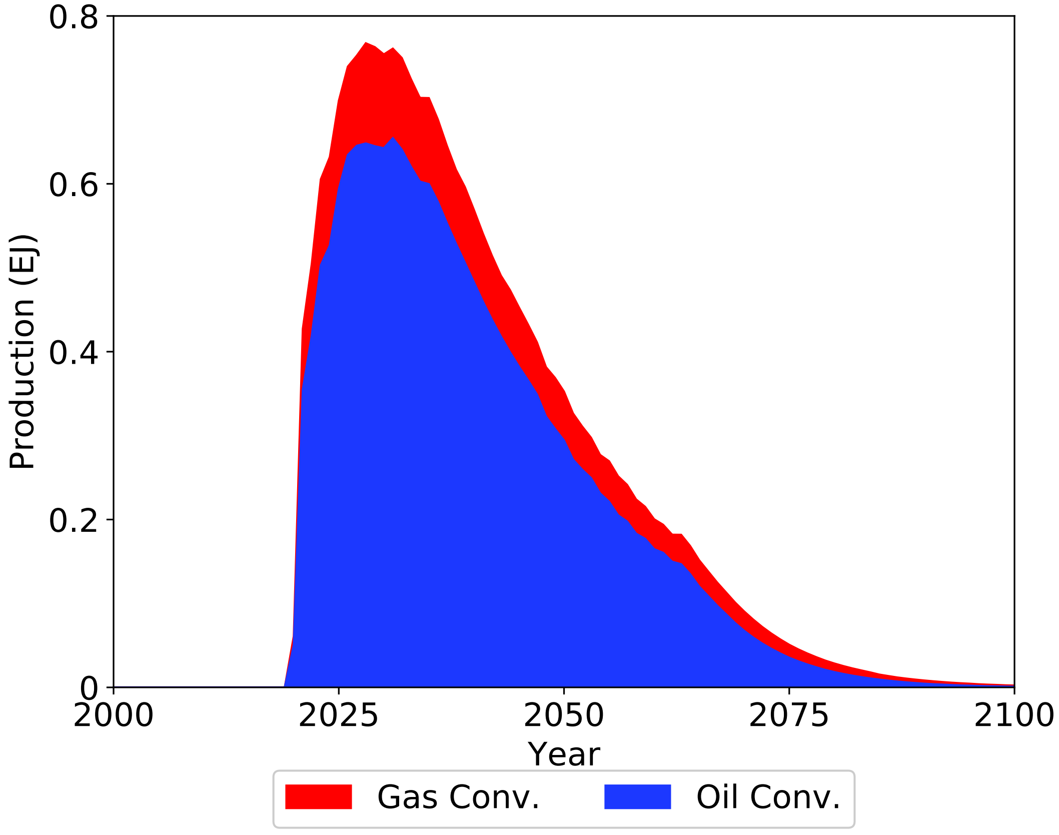

Figure 7.29: Guyana projections capped at 16

Table 7.29: Peak years - All

Name	URR	Peak Year	Peak Rate
Oil Conv.	18.95	2031	0.65
Gas Conv.	3.7	2028	0.12
**Total**	**22.65**	**2028**	**0.77**

7.15.2 By Mineral

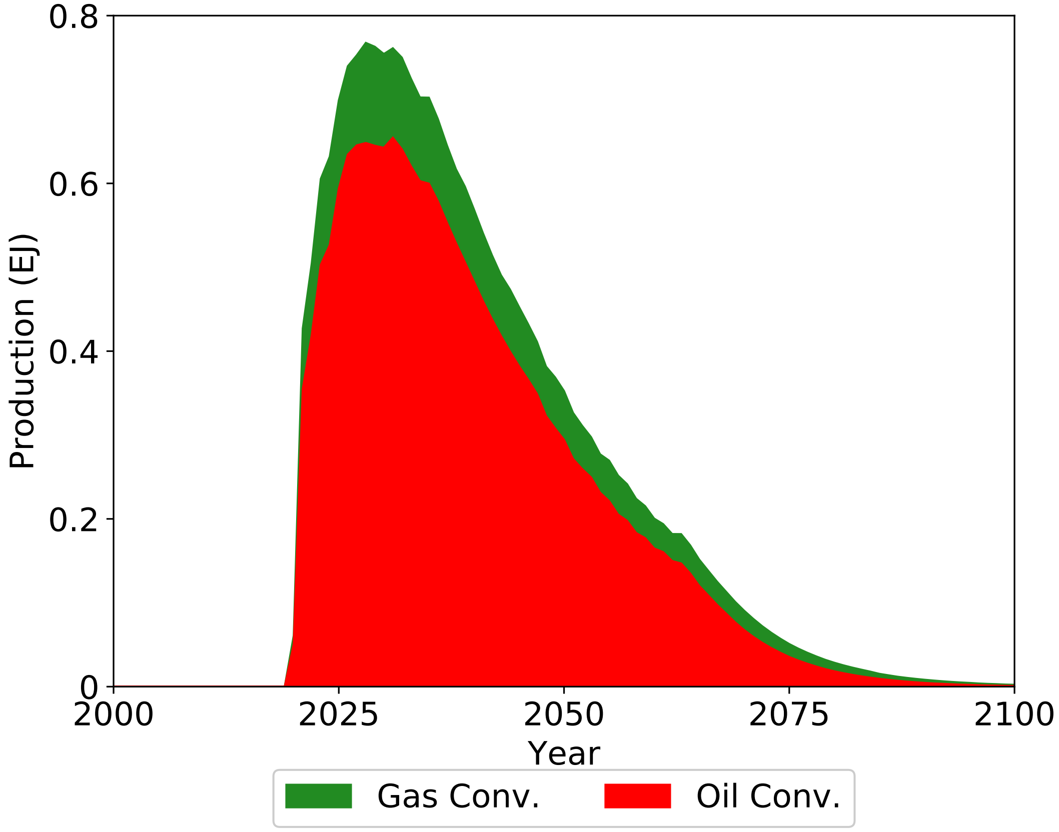

Figure 7.30: Guyana projection by mineral type

Table 7.30: Peak years - Minerals

Name	URR	Peak Year	Peak Rate
Oil Conv.	18.95	2031	0.65
Gas Conv.	3.7	2028	0.12
**Total**	**22.65**	**2028**	**0.77**

7.16 Haiti

7.16.1 All Projections

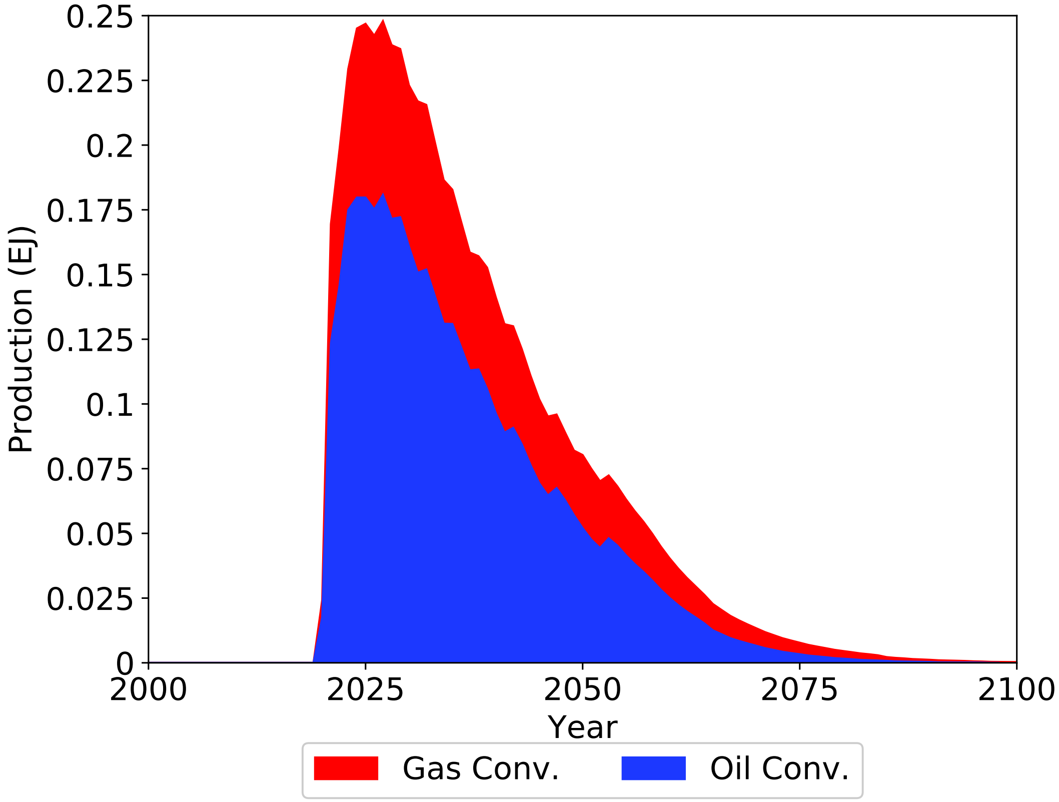

Figure 7.31: Haiti projections capped at 16

Table 7.31: Peak years - All

**Name**	**URR**	**Peak Year**	**Peak Rate**
Oil Conv.	4.21	2027	0.18
Gas Conv.	1.85	2025	0.07
**Total**	**6.06**	**2027**	**0.25**

7.16.2 By Mineral

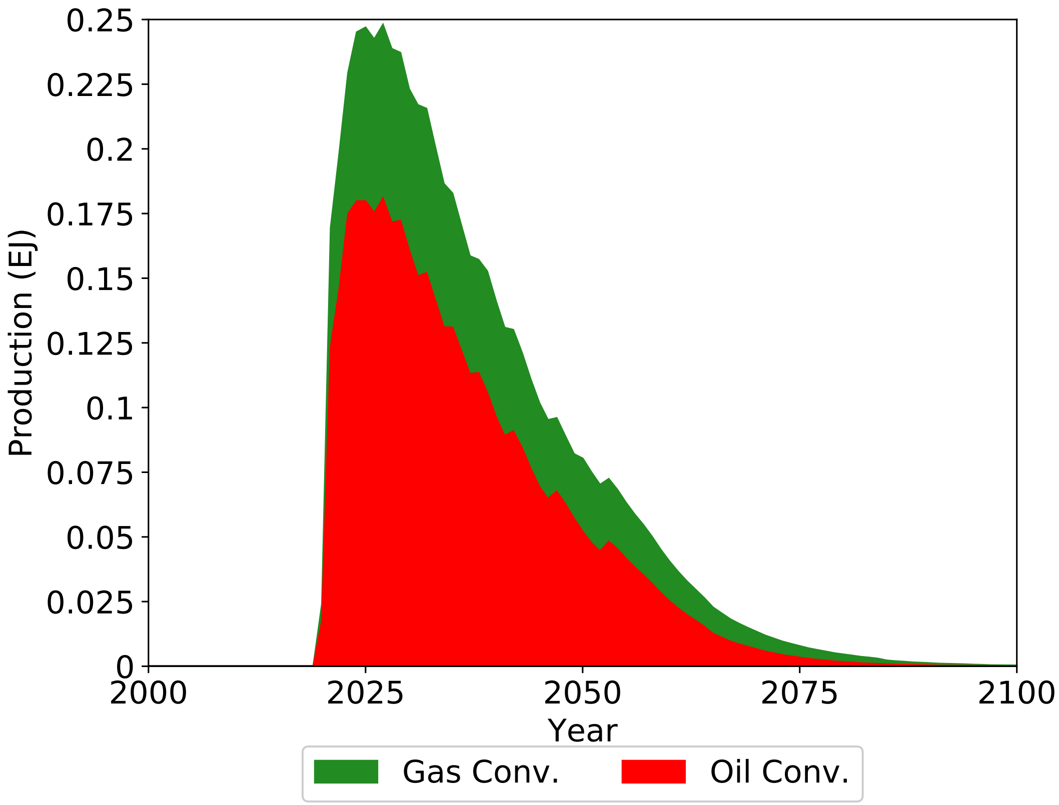

Figure 7.32: Haiti projection by mineral type

Table 7.32: Peak years - Minerals

**Name**	**URR**	**Peak Year**	**Peak Rate**
Oil Conv.	4.21	2027	0.18
Gas Conv.	1.85	2025	0.07
**Total**	**6.06**	**2027**	**0.25**

7.17 Mexico

7.17.1 All Projections

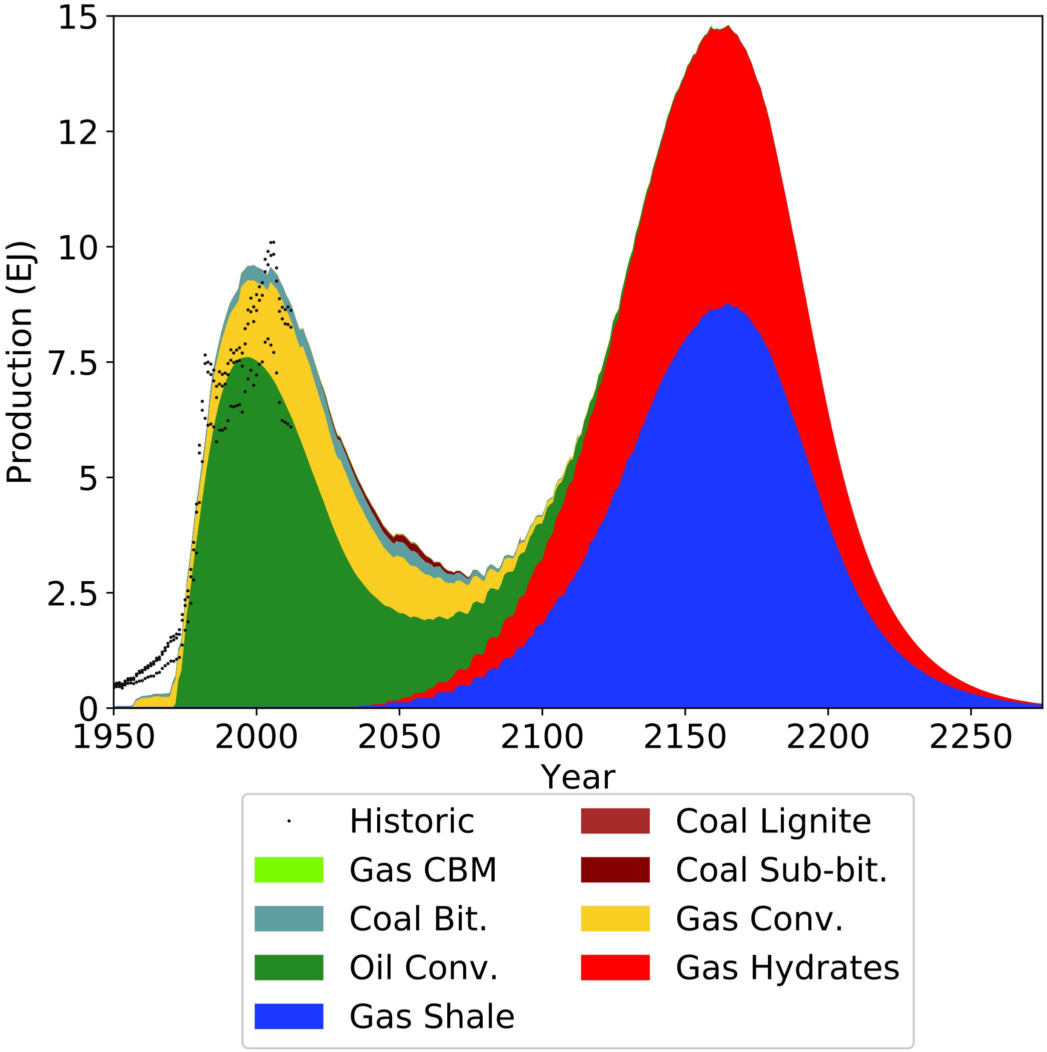

Figure 7.33: Mexico projections capped at 16

Table 7.33: Peak years - All

**Name**	**URR**	**Peak Year**	**Peak Rate**
Gas Shale	715.2	2165	8.75
Gas Hydrates	496.5	2159	6.08
Oil Conv.	445.73	1997	7.59
Gas Conv.	153.2	2016	2.17
Coal Bit.	32.7	2028	0.42
Coal Sub-bit.	4.95	2053	0.16
Gas CBM	1.11	2020	0.03
Coal Lignite	0.48	2055	0.01
**Total**	**1849.87**	**2165**	**14.78**

7.17.2 By Mineral

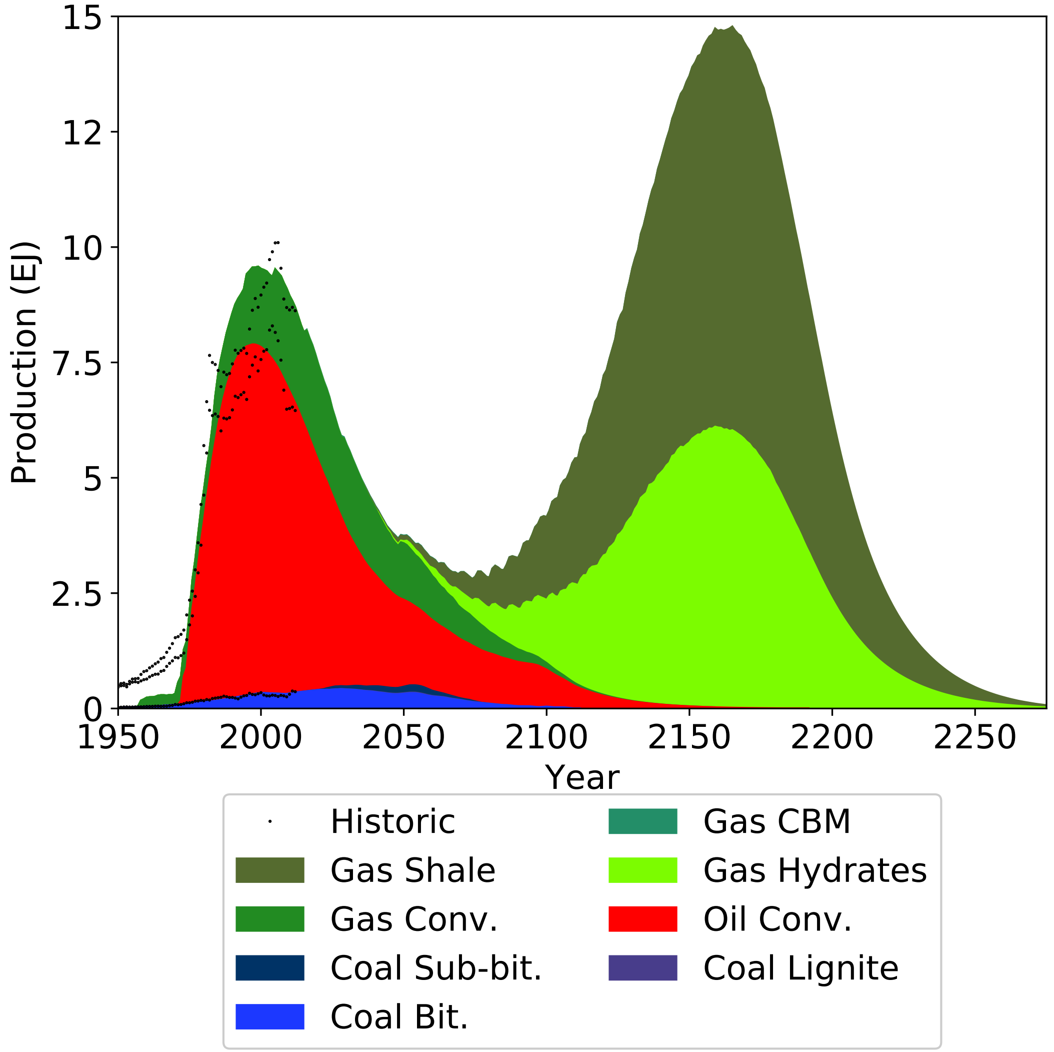

Figure 7.34: Mexico projection by mineral type

Table 7.34: Peak years - Minerals

**Name**	**URR**	**Peak Year**	**Peak Rate**
Coal Bit.	32.7	2028	0.42
Coal Lignite	0.48	2055	0.01
Coal Sub-bit.	4.95	2053	0.16
Oil Conv.	445.73	1997	7.59
Gas Conv.	153.2	2016	2.17
Gas Hydrates	496.5	2159	6.08
Gas Shale	715.2	2165	8.75
Gas CBM	1.11	2020	0.03
**Total**	**1849.87**	**2165**	**14.78**

7.18 Paraguay

7.18.1 All Projections

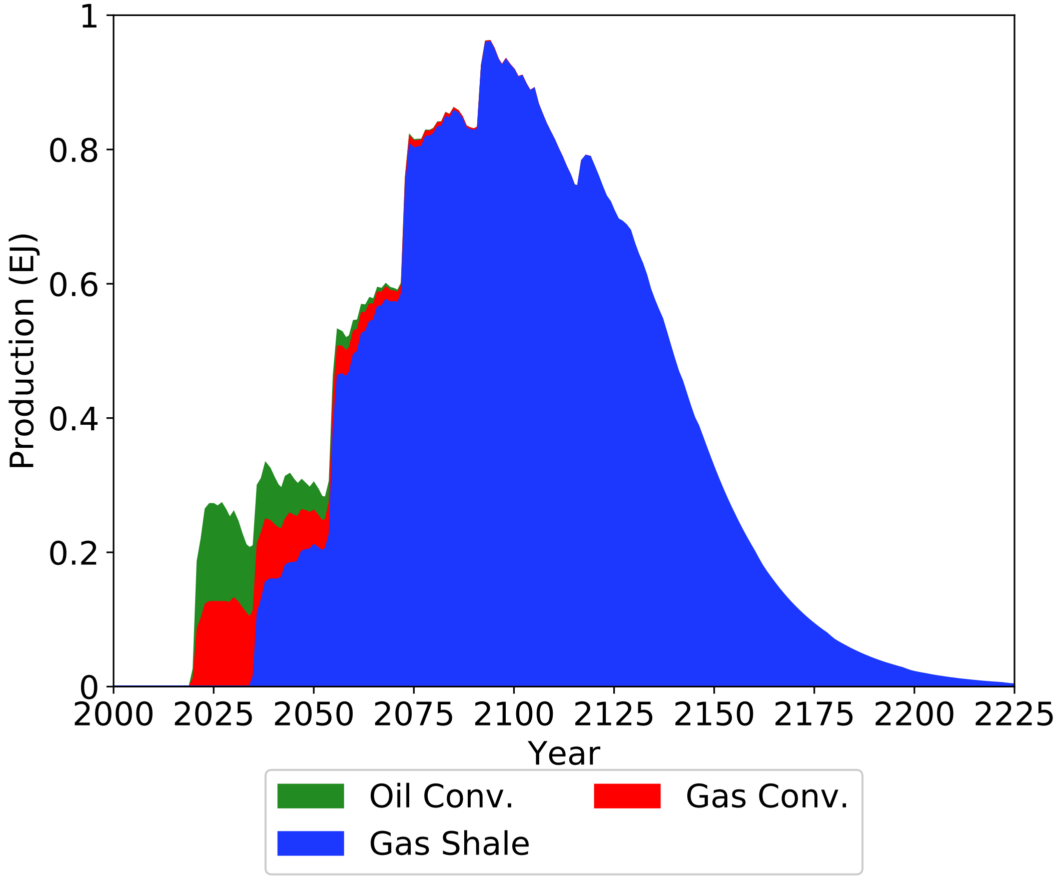

Figure 7.35: Paraguay projections capped at 16

Table 7.35: Peak years - All

Name	URR	Peak Year	Peak Rate
Gas Shale	76.2	2094	0.96
Gas Conv.	3.7	2030	0.13
Oil Conv.	3.16	2027	0.15
**Total**	**83.06**	**2094**	**0.96**

7.18.2 By Mineral

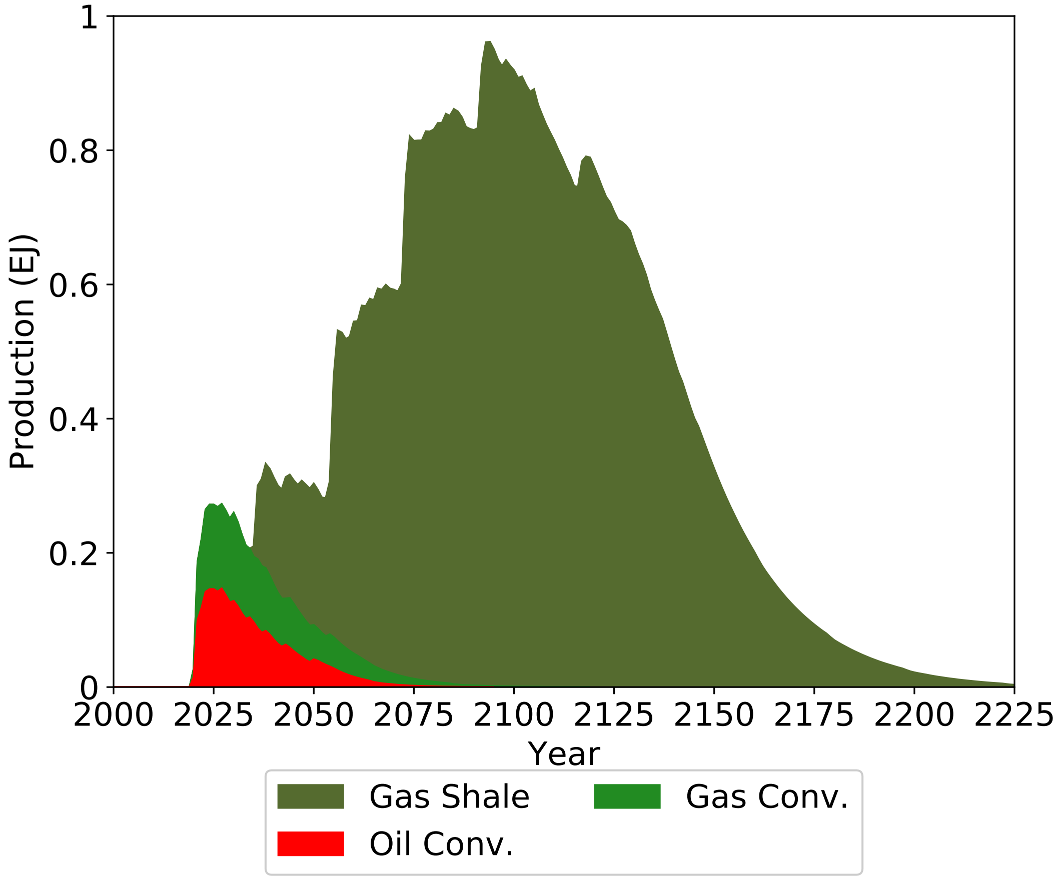

Figure 7.36: Paraguay projection by mineral type

Table 7.36: Peak years - Minerals

Name	URR	Peak Year	Peak Rate
Oil Conv.	3.16	2027	0.15
Gas Conv.	3.7	2030	0.13
Gas Shale	76.2	2094	0.96
**Total**	**83.06**	**2094**	**0.96**

7.19 Peru

7.19.1 All Projections

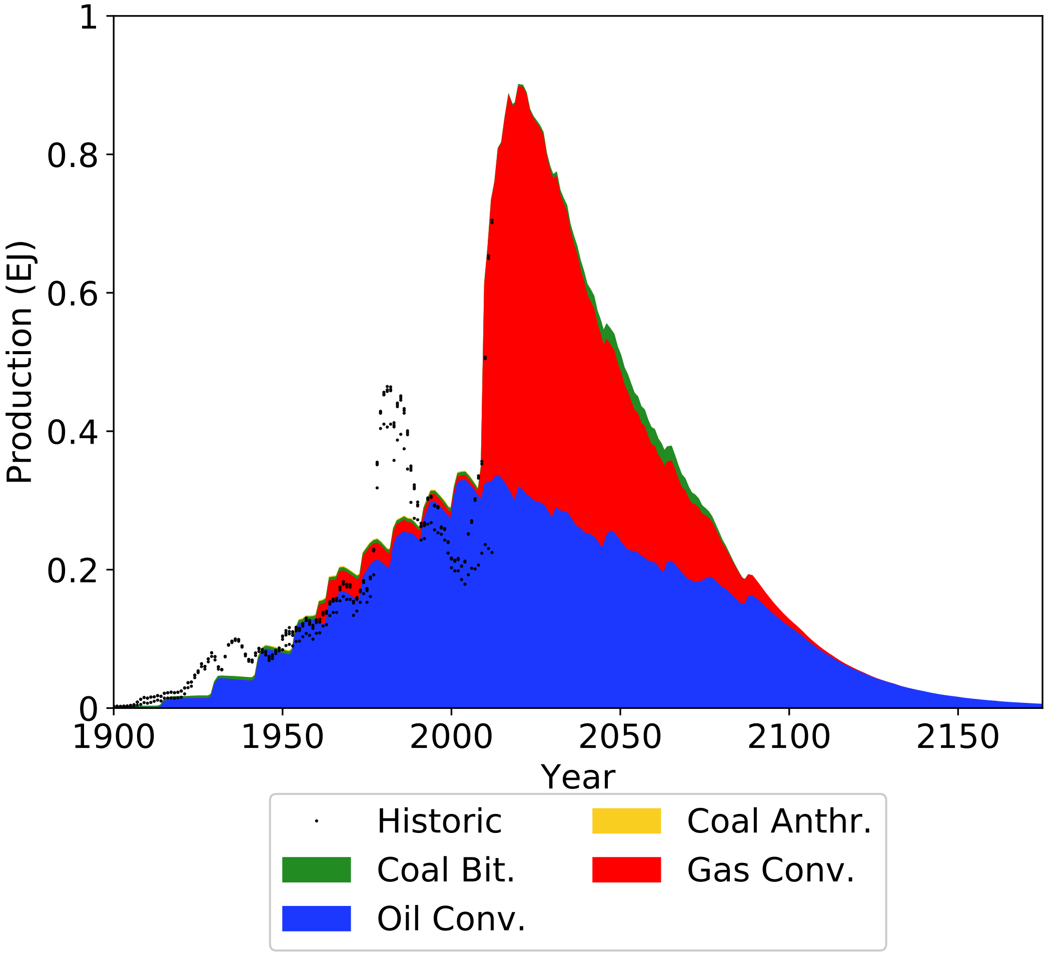

Figure 7.37: Peru projections capped at 16

Table 7.37: Peak years - All

**Name**	**URR**	**Peak Year**	**Peak Rate**
Oil Conv.	37.2	2014	0.33
Gas Conv.	23.5	2021	0.58
Coal Bit.	1.42	2052	0.03
Coal Anthr.	0.1	1943	–
**Total**	**62.22**	**2020**	**0.9**

7.19.2 By Mineral

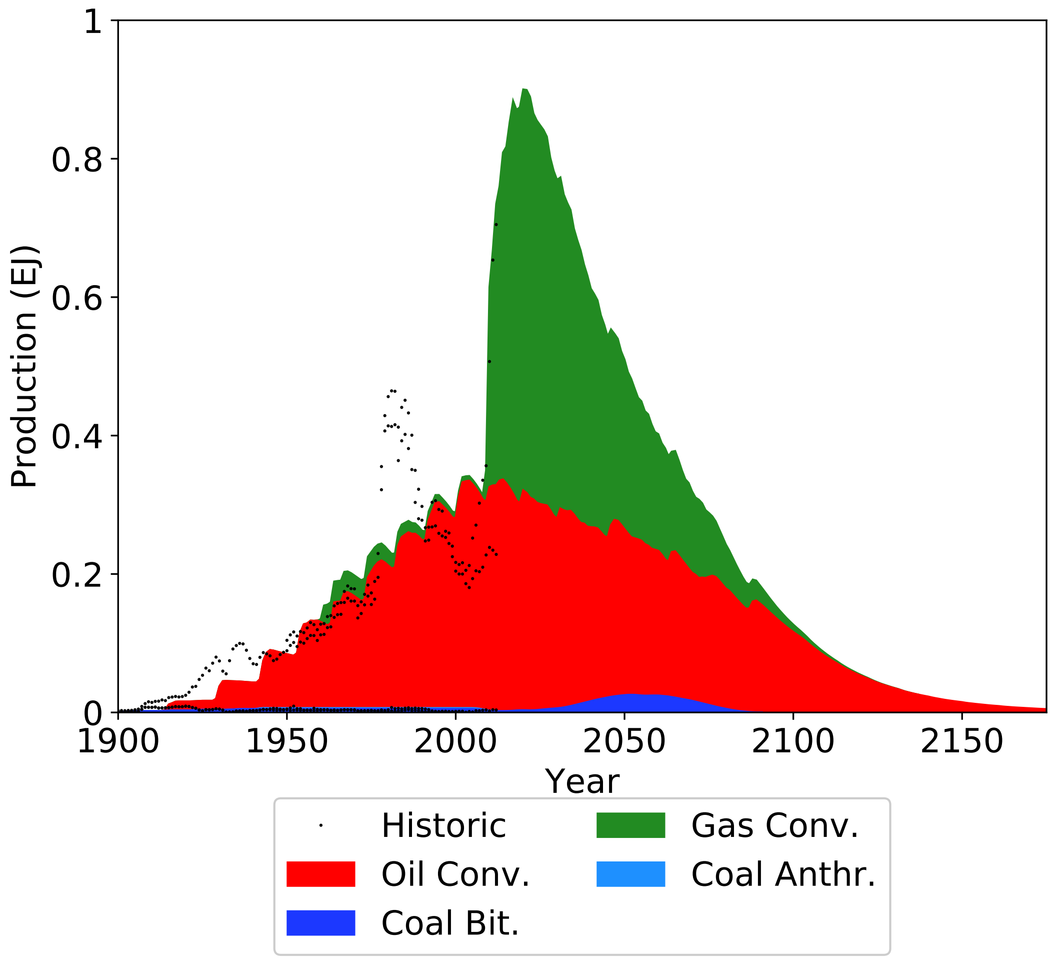

Figure 7.38: Peru projection by mineral type

Table 7.38: Peak years - Minerals

**Name**	**URR**	**Peak Year**	**Peak Rate**
Coal Bit.	1.42	2052	0.03
Coal Anthr.	0.1	1943	–
Oil Conv.	37.2	2014	0.33
Gas Conv.	23.5	2021	0.58
**Total**	**62.22**	**2020**	**0.9**

7.20 Puerto Rico

7.20.1 All Projections

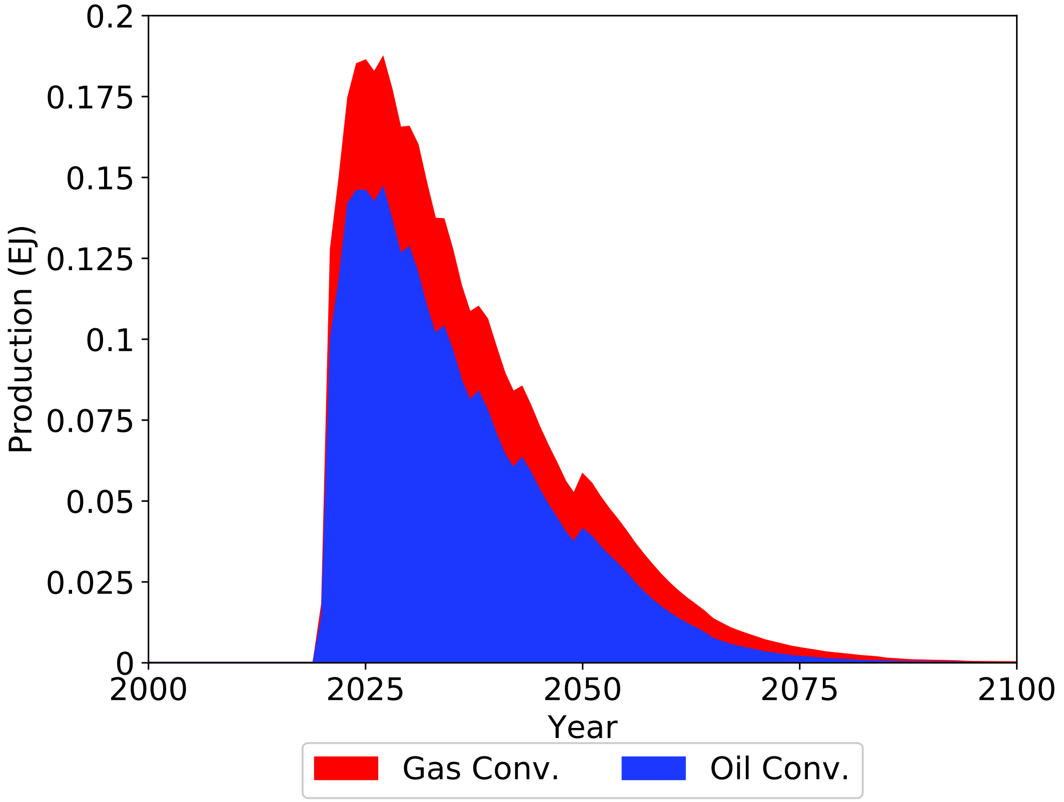

Figure 7.39: Puerto Rico projections capped at 16

Table 7.39: Peak years - All

Name	URR	Peak Year	Peak Rate
Oil Conv.	3.16	2027	0.15
Gas Conv.	1.11	2025	0.04
**Total**	**4.27**	**2027**	**0.19**

7.20.2 By Mineral

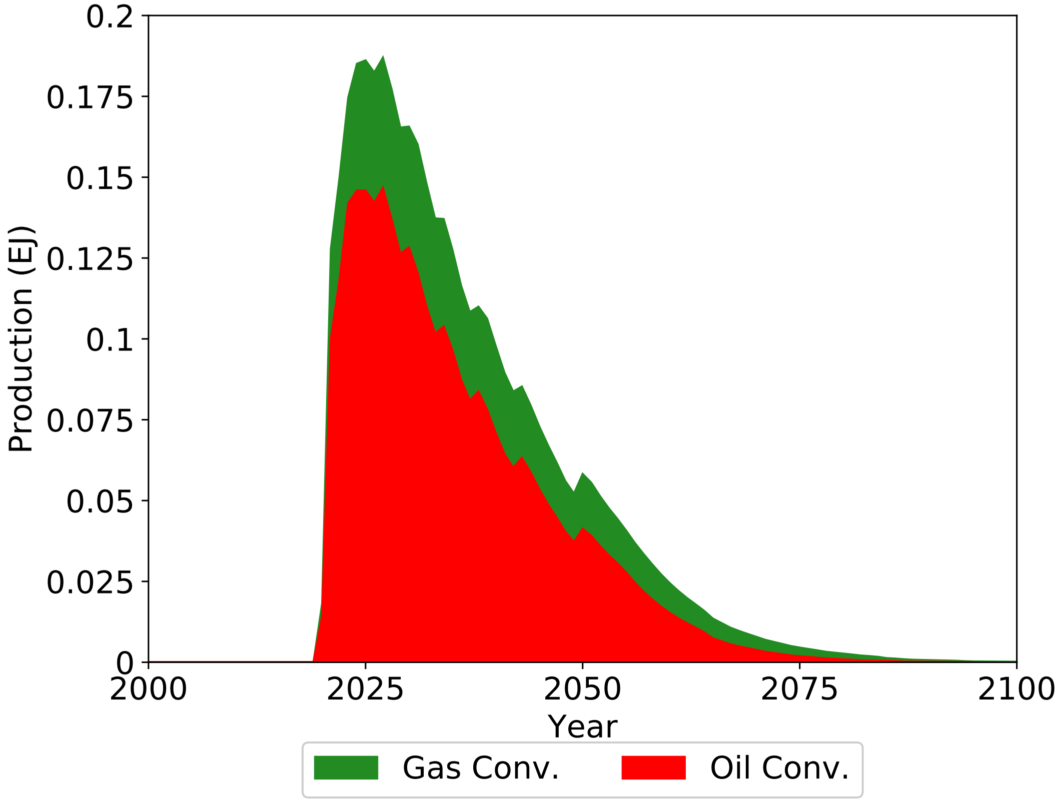

Figure 7.40: Puerto Rico projection by mineral type

Table 7.40: Peak years - Minerals

Name	URR	Peak Year	Peak Rate
Oil Conv.	3.16	2027	0.15
Gas Conv.	1.11	2025	0.04
**Total**	**4.27**	**2027**	**0.19**

7.21 Suriname

7.21.1 All Projections

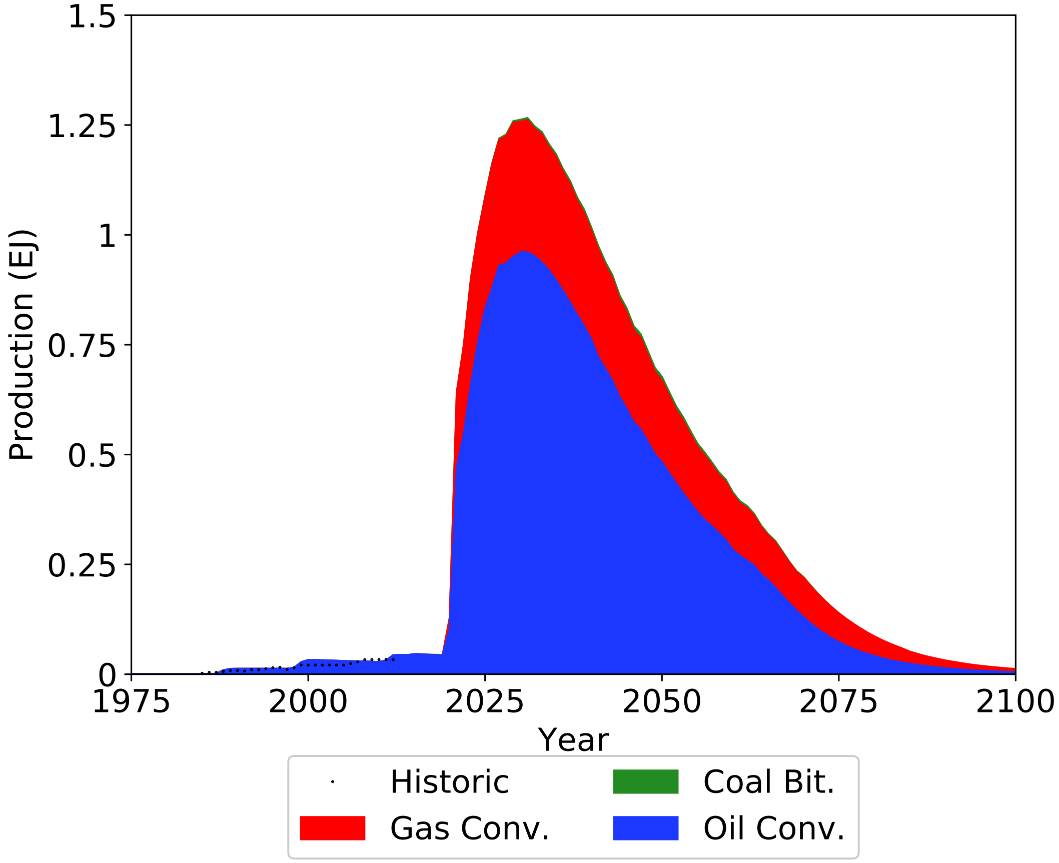

Figure 7.41: Suriname projections capped at 16

Table 7.41: Peak years - All

Name	URR	Peak Year	Peak Rate
Oil Conv.	30.4	2031	0.96
Gas Conv.	11.12	2029	0.3
Coal Bit.	0.29	2046	0.01
**Total**	**41.81**	**2031**	**1.27**

7.21.2 By Mineral

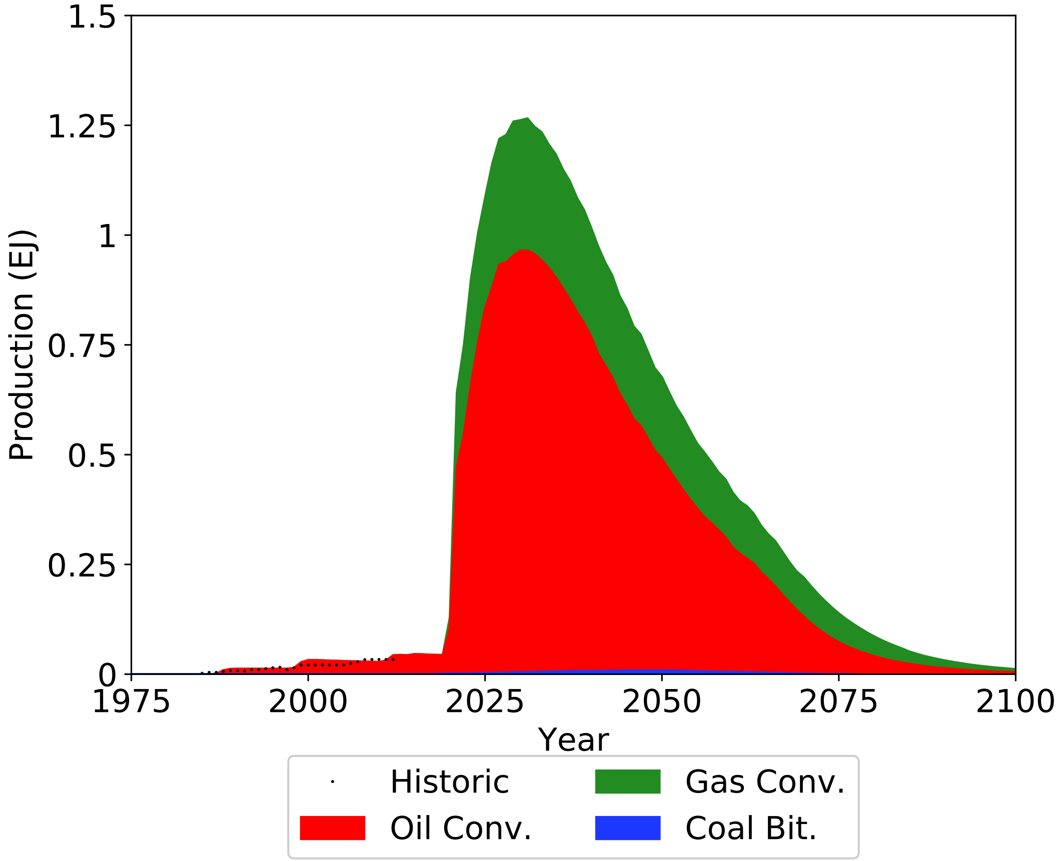

Figure 7.42: Suriname projection by mineral type

Table 7.42: Peak years - Minerals

**Name**	**URR**	**Peak Year**	**Peak Rate**
Coal Bit.	0.29	2046	0.01
Oil Conv.	30.4	2031	0.96
Gas Conv.	11.12	2029	0.3
**Total**	**41.81**	**2031**	**1.27**

7.22 Trinidad and Tobago

7.22.1 All Projections

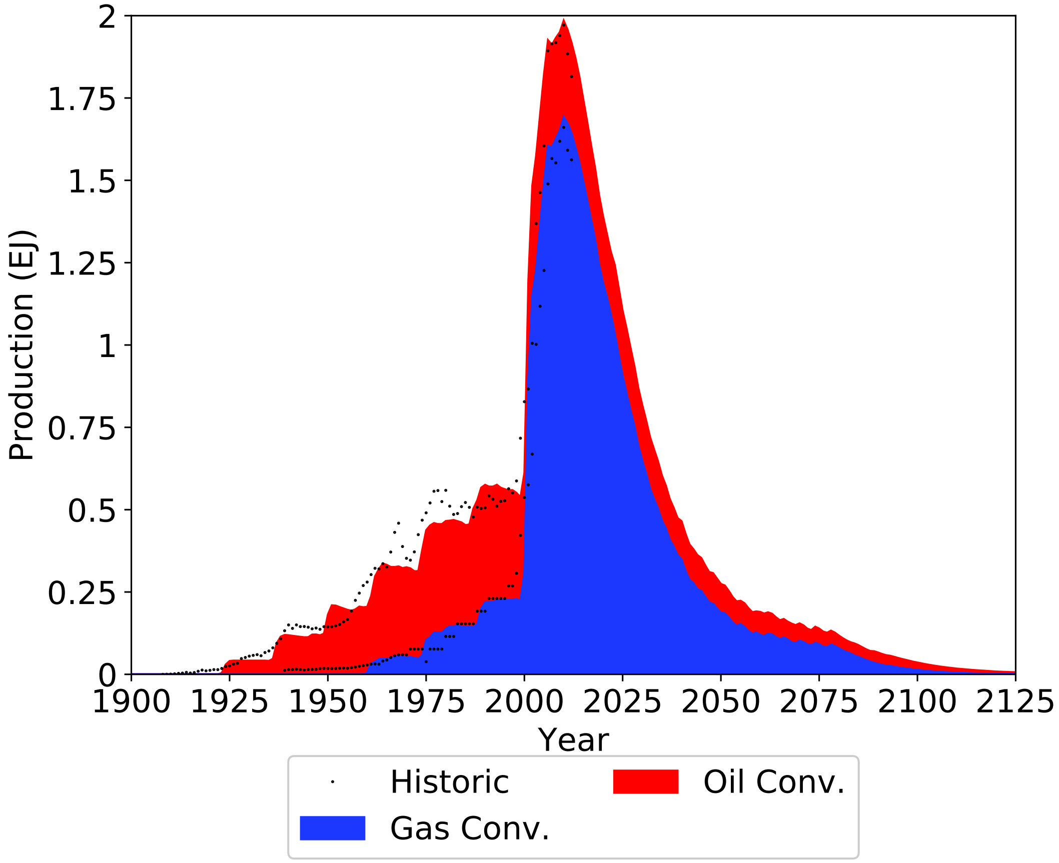

Figure 7.43: Trinidad and Tobago projections capped at 16

Table 7.43: Peak years - All

Name	URR	Peak Year	Peak Rate
Gas Conv.	54.45	2010	1.69
Oil Conv.	29.36	1988	0.38
**Total**	**83.81**	**2010**	**1.99**

7.22.2 By Mineral

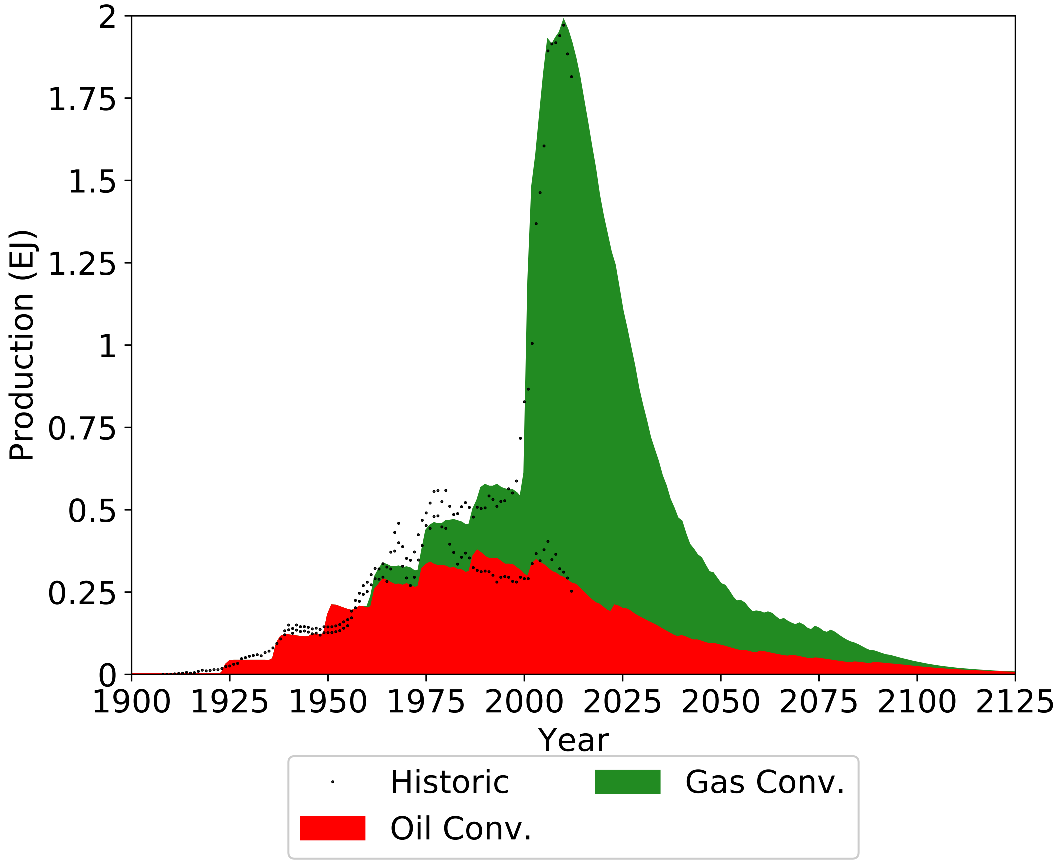

Figure 7.44: Trinidad and Tobago projection by mineral type

Table 7.44: Peak years - Minerals

Name	URR	Peak Year	Peak Rate
Oil Conv.	29.36	1988	0.38
Gas Conv.	54.45	2010	1.69
**Total**	**83.81**	**2010**	**1.99**

7.23 Uruguay

7.23.1 All Projections

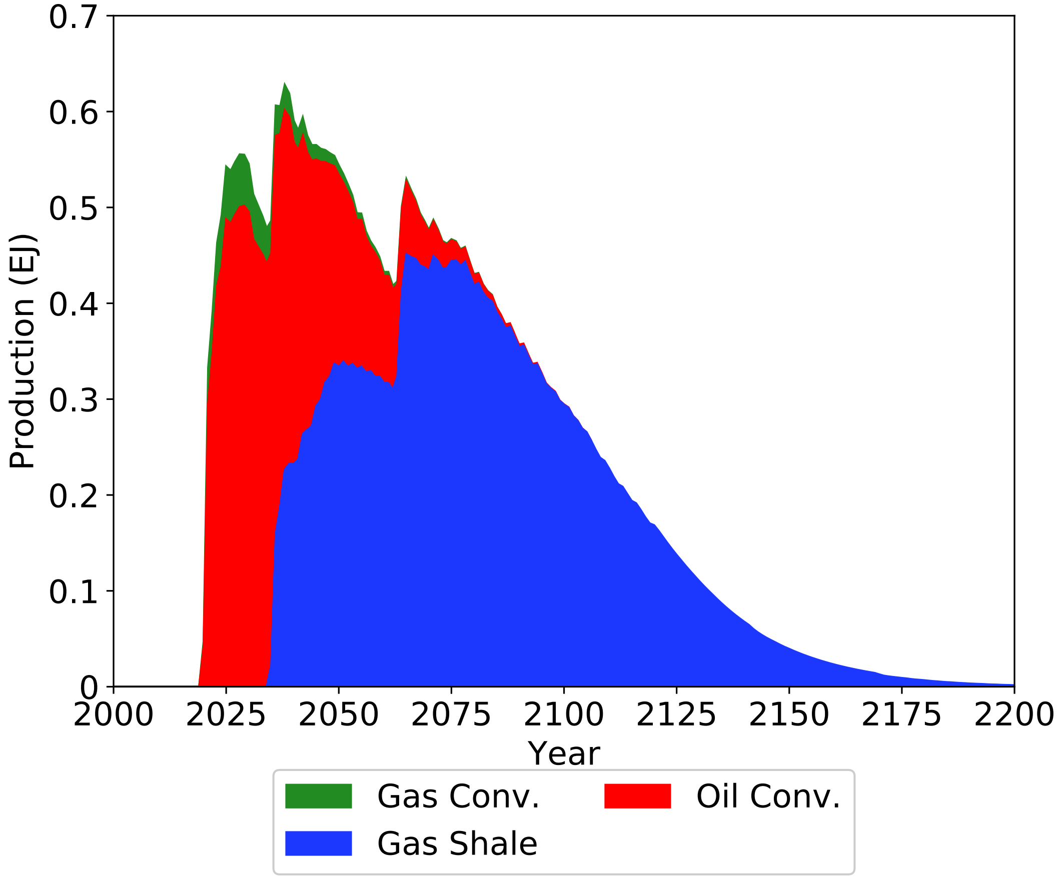

Figure 7.45: Uruguay projections capped at 16

Table 7.45: Peak years - All

Name	URR	Peak Year	Peak Rate
Gas Shale	30.7	2065	0.45
Oil Conv.	13.69	2029	0.5
Gas Conv.	1.1	2025	0.06
**Total**	**45.49**	**2038**	**0.63**

7.23.2 By Mineral

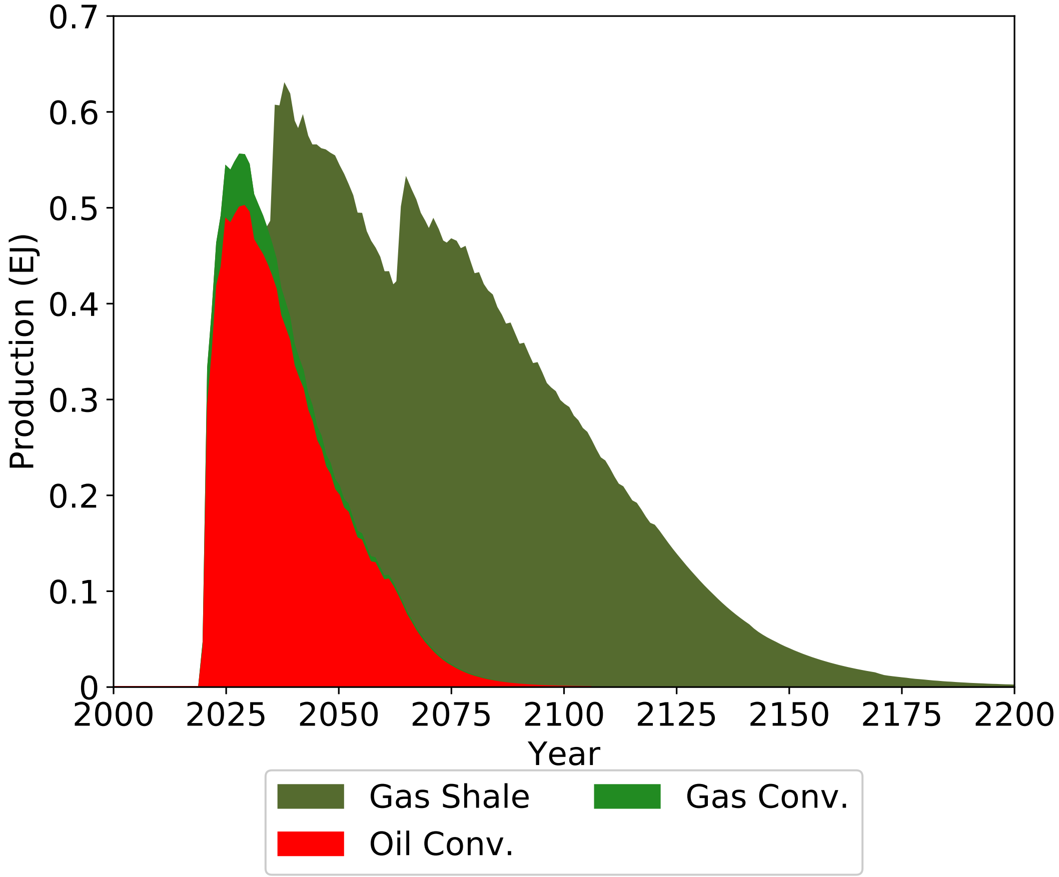

Figure 7.46: Uruguay projection by mineral type

Table 7.46: Peak years - Minerals

Name	URR	Peak Year	Peak Rate
Oil Conv.	13.69	2029	0.5
Gas Conv.	1.1	2025	0.06
Gas Shale	30.7	2065	0.45
**Total**	**45.49**	**2038**	**0.63**

7.24 Venezuela

7.24.1 All Projections

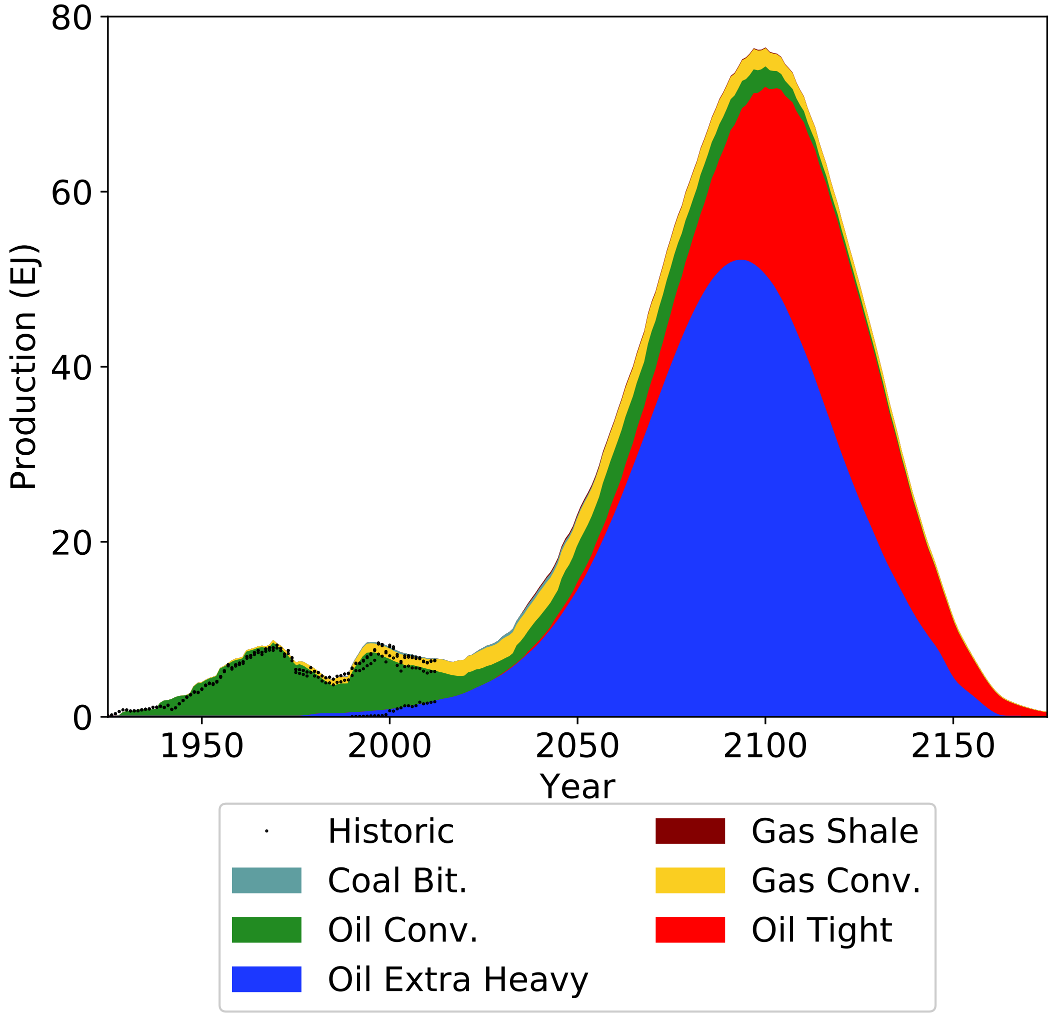

Figure 7.47: Venezuela projections capped at 16

Table 7.47: Peak years - All

Name	URR	Peak Year	Peak Rate
Oil Extra Heavy	3458.16	2093	52.1
Oil Tight	1431.93	2113	26.17
Oil Conv.	728.88	1969	8.33
Gas Conv.	335.2	2062	3.4
Coal Bit.	14.5	2042	0.48
Gas Shale	11.57	2050	0.21
**Total**	**5980.24**	**2100**	**76.33**

7.24.2 By Mineral

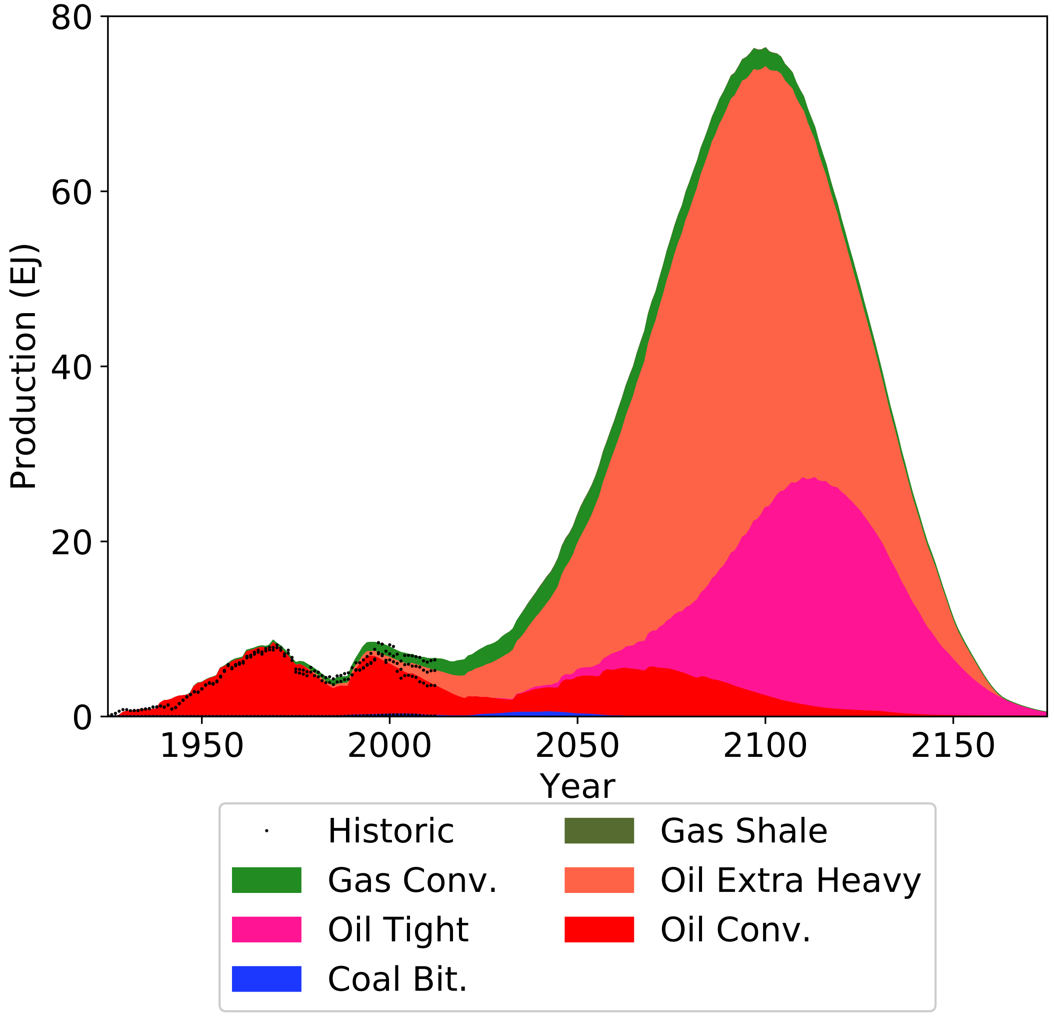

Figure 7.48: Venezuela projection by mineral type

Table 7.48: Peak years - Minerals

**Name**	**URR**	**Peak Year**	**Peak Rate**
Coal Bit.	14.5	2042	0.48
Oil Conv.	728.88	1969	8.33
Oil Tight	1431.93	2113	26.17
Oil Extra Heavy	3458.16	2093	52.1
Gas Conv.	335.2	2062	3.4
Gas Shale	11.57	2050	0.21
**Total**	**5980.24**	**2100**	**76.33**

7.25 Total

7.25.1 By country

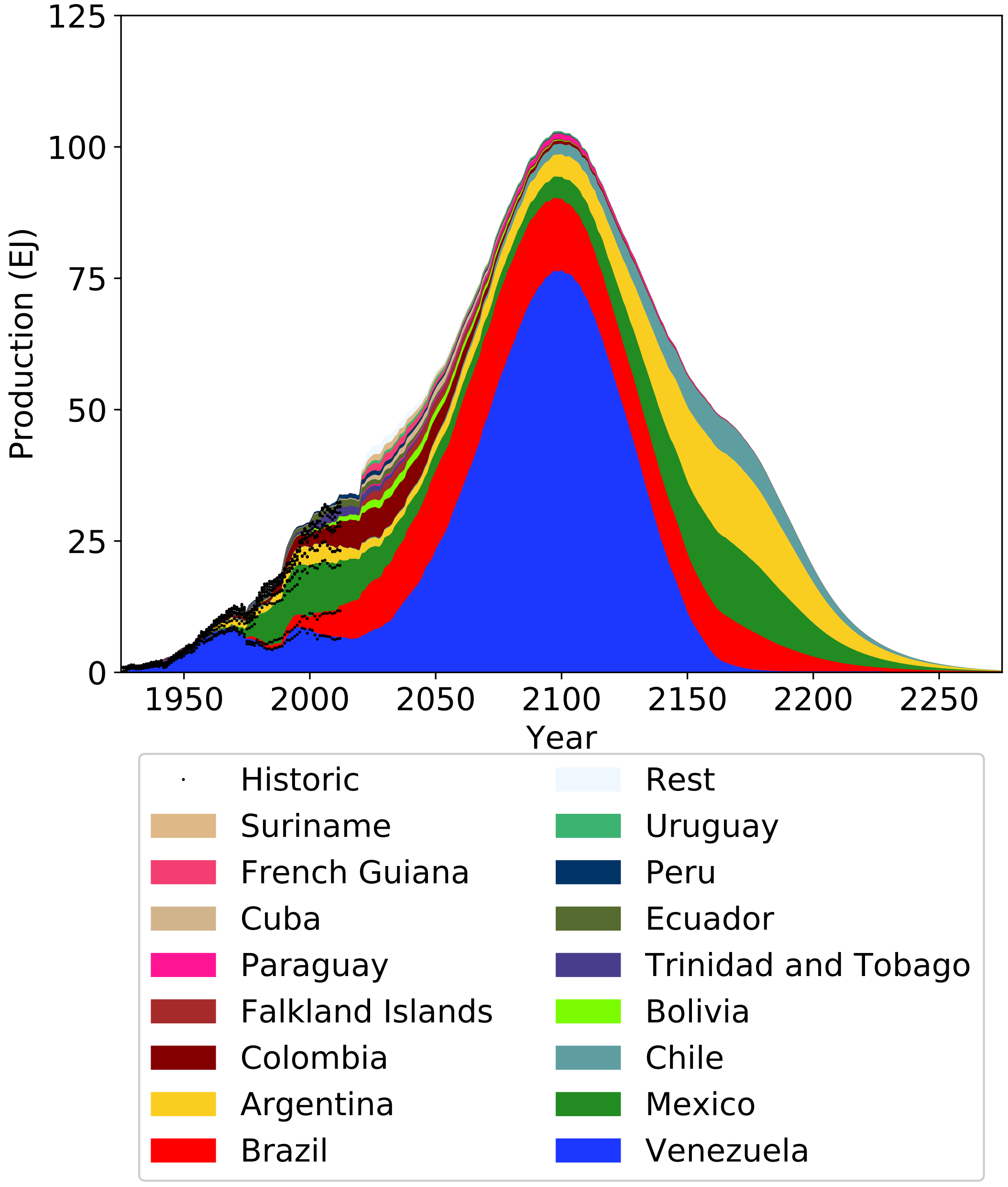

Figure 7.49: South America projections by country

Table 7.49: Peak years - All

Name	URR	Peak Year	Peak Rate
Venezuela	5980.24	2100	76.33
Brazil	2299.5	2069	16.61
Mexico	1849.87	2165	14.78
Argentina	1607.2	2166	16.05
Chile	585.62	2164	6.35
Colombia	380.03	2027	5.63
Bolivia	106.0	2033	1.77
Falkland Islands	89.31	2031	1.87
Trinidad and Tobago	83.81	2010	1.99
Paraguay	83.06	2094	0.96
Ecuador	70.76	2016	1.33
Cuba	63.19	2051	1.36
Peru	62.22	2020	0.9
French Guiana	48.49	2030	1.52
Uruguay	45.49	2038	0.63
Suriname	41.81	2031	1.27
Guyana	22.65	2028	0.77
Barbados	7.0	2047	0.13
Dominican Republic	6.32	2026	0.25
Haiti	6.06	2027	0.25
Puerto Rico	4.27	2027	0.19
Guatemala	3.31	2025	0.16
Belize	1.09	2032	0.03
Grenada	0.93	2025	0.05
**Total**	**13448.24**	**2099**	**102.88**

7.25.2 By mineral

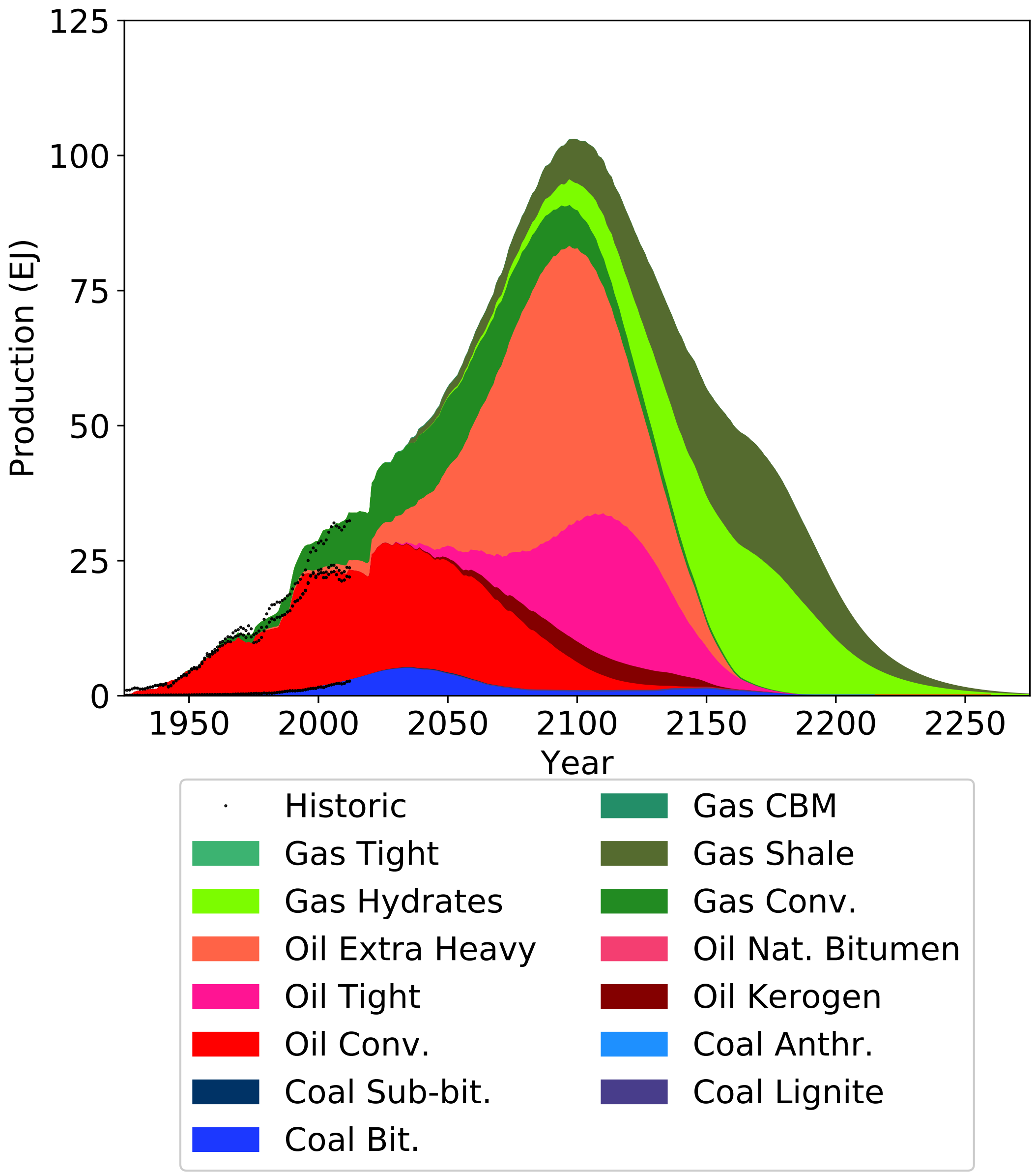

Figure 7.50: South America projection by mineral type

Table 7.50: Peak years - Minerals

**Name**	**URR**	**Peak Year**	**Peak Rate**
Coal Bit.	345.33	2034	4.96
Coal Lignite	34.13	2114	0.29
Coal Sub-bit.	4.95	2053	0.16
Coal Anthr.	0.1	1943	–
Oil Conv.	2433.27	2025	23.5
Oil Kerogen	282.07	2096	3.84
Oil Tight	1547.75	2113	26.48
Oil Nat. Bitumen	0.42	2037	0.01
Oil Extra Heavy	3459.25	2093	52.1
Gas Conv.	1338.68	2053	12.98
Gas Hydrates	1986.0	2163	24.44
Gas Shale	2006.94	2159	21.02
Gas Tight	0.37	2035	0.01
Gas CBM	8.97	2040	0.17
**Total**	**13448.24**	**2099**	**102.88**

Chapter 8

Total

8.1 By continent

Table 8.1: Peak years - All

Name	URR	Peak Year	Peak Rate
Asia	31618.47	2029	276.47
FSU	27698.19	2082	221.99
North America	26718.66	2100	159.89
South America	13448.24	2099	102.88
Middle East	13149.67	2052	146.88
Africa	10260.99	2041	64.1
Europe	7186.63	1999	39.82
**Total**	**130080.84**	**2050**	**795.11**

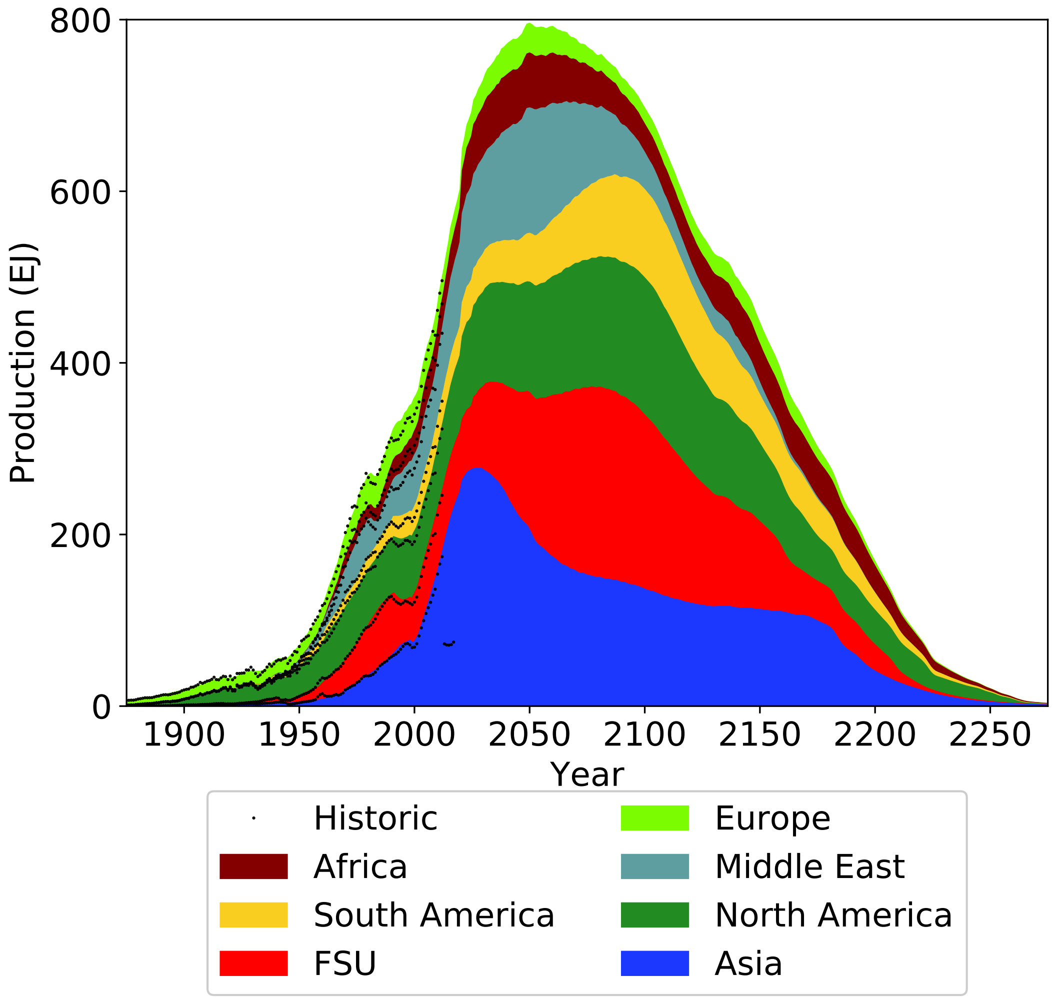

Figure 8.1: Total projections by continent

8.2 By mineral

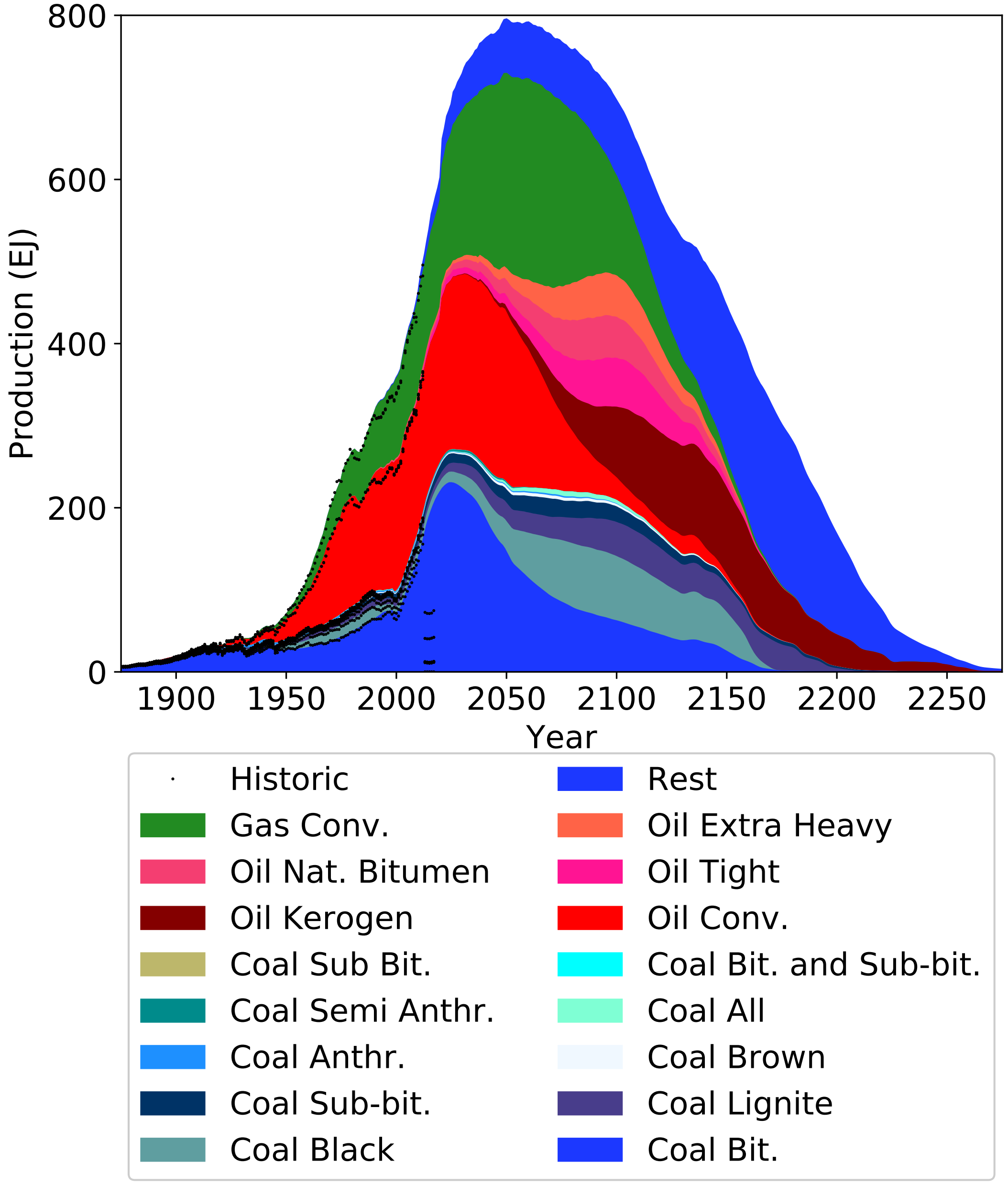

Figure 8.2: Total projection by mineral type

Table 8.2: Peak year by mineral

**Name**	**URR**	**Peak Year**	**Peak Rate**
Gas Conv.	23667.42	2060	245.35
Oil Conv.	21152.8	2039	218.46
Coal Bit.	19537.04	2025	229.94
Gas Hydrates	12637.56	2173	143.78
Oil Kerogen	11776.29	2136	111.48
Coal Black	8530.84	2093	79.91
Gas Shale	7177.77	2143	47.54
Coal Lignite	5662.99	2107	42.54
Oil Tight	4086.81	2100	59.63
Oil Nat. Bitumen	3867.29	2092	52.18
Oil Extra Heavy	3470.12	2093	52.1
Coal Sub-bit.	2628.89	2069	22.35
Gas Tight	2525.34	2144	18.29
Gas CBM	1978.16	2097	15.4
Coal Brown	438.1	2061	3.92
Coal Anthr.	424.78	1918	3.13
Coal All	403.63	2078	6.02
Coal Semi Anthr.	79.9	2036	2.46
Coal Bit. and Sub-bit.	30.4	2024	0.31
Coal Sub Bit.	4.72	2048	0.09
**Total**	**130080.84**	**2050**	**795.11**

8.3 By Country

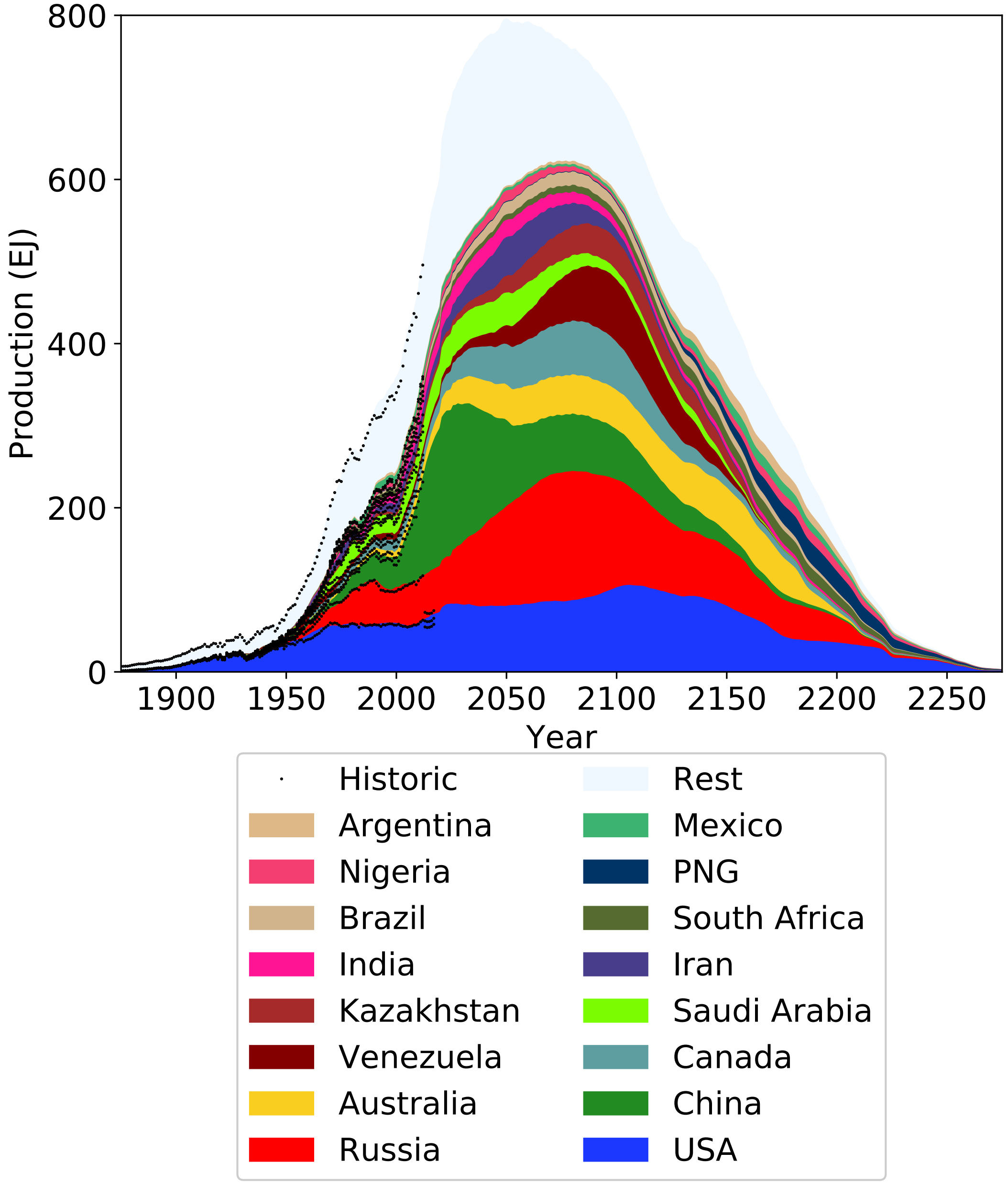

Figure 8.3: Total projection by country

Table 8.3: Peak year - Country

Name	URR	Peak Year	Peak Rate
USA	19952.34	2106	104.83
Russia	19871.44	2077	157.74
China	13459.83	2024	177.93
Australia	8228.99	2148	54.73
Canada	6766.31	2084	65.92
Venezuela	5980.24	2100	76.33
Saudi Arabia	4108.56	2051	40.4
Kazakhstan	3744.88	2096	37.48
Iran	3348.69	2055	48.12
India	2580.43	2037	22.74
South Africa	2574.33	2177	16.84
Brazil	2299.5	2069	16.61
PNG	2126.36	2191	26.0
Nigeria	1985.7	2183	13.57
Mexico	1849.87	2165	14.78
Argentina	1607.2	2166	16.05
Indonesia	1603.11	2027	31.36
Iraq	1569.85	2052	21.63
Qatar	1283.76	2022	14.14
Mongolia	1152.82	2049	10.7
Turkmenistan	1147.32	2112	11.41
UAE	1128.75	2059	12.59
UK	1088.35	1997	11.56
Germany	1085.66	2044	11.48
Mozambique	1067.09	2173	9.66
Algeria	1013.3	2007	7.23
Norway	962.8	2004	10.01
Kuwait	962.56	2039	13.97
Libya	930.13	1970	7.44
Donetsk	783.03	2116	7.96
Ukraine	697.84	2084	6.9
Italy	688.4	2136	7.8
Egypt	627.43	2041	9.29
Chile	585.62	2164	6.35
Luhansk	582.62	2111	6.07
New Zealand	569.68	2168	6.75
Turkey	555.48	2162	4.95
Poland	432.08	1983	5.2
Angola	412.21	2060	6.23
Ireland	406.56	2159	4.82
Colombia	380.03	2027	5.63
Vietnam	377.92	2033	2.7
Zaire	355.56	2137	4.59
Pakistan	346.98	2075	2.14
France	341.44	2129	2.22
Uzbekistan	323.25	2039	2.97
Malaysia	320.66	2023	4.29
Azerbaijan	309.45	2037	3.5
Greenland	295.02	2056	6.44

Table 8.3: Peak year - Country – Continued

Name	URR	Peak Year	Peak Rate
Yugoslavia	286.08	2067	2.63
Thailand	252.72	2010	2.4
Oman	238.48	2022	3.83
Netherlands	238.03	1978	3.77
Morocco	199.19	2107	2.42
Madagascar	178.11	2066	3.31
Czech Republic	170.15	1979	2.13
Romania	136.55	1980	2.29
Jordan	129.44	2110	1.58
Japan	127.53	1953	1.24
Burma	126.28	2043	1.9
Gabon	125.45	2027	3.01
Israel	114.05	2064	1.72
Bolivia	106.0	2033	1.77
Estonia	94.61	2082	1.52
Yemen	89.48	2030	2.01
Falkland Islands	89.31	2031	1.87
Sudan	88.75	2029	2.34
Syria	88.3	1998	1.44
Trinidad and Tobago	83.81	2010	1.99
Paraguay	83.06	2094	0.96
Hungary	75.23	1975	0.86
Tanzania	75.16	2044	1.2
Botswana	72.31	2043	1.36
Ecuador	70.76	2016	1.33
Brunei	67.4	2001	0.91
Belgium	67.12	1949	0.67
Bulgaria	65.37	2036	1.0
Cuba	63.19	2051	1.36
Bangladesh	62.79	2008	0.85
Peru	62.22	2020	0.9
Denmark	60.71	2002	1.06
North Korea	60.58	1979	1.21
Congo	56.26	2019	1.09
Spain	55.07	1979	0.62
Greece	50.35	2029	0.87
Belarus	48.98	2109	0.64
French Guiana	48.49	2030	1.52
Austria	47.53	2055	0.58
Kyrgyzstan	46.46	2066	0.63
Uruguay	45.49	2038	0.63
Sweden	43.74	2082	0.57
Equatorial Guinea	43.17	2025	1.2
Philippines	42.68	2029	1.08
Bahrain	42.14	2013	0.63
Seychelles	42.05	2035	1.12
Suriname	41.81	2031	1.27
Tunisia	40.01	2051	0.42
Cameroon	36.51	2025	0.87

Table 8.3: Peak year - Country – Continued

Name	URR	Peak Year	Peak Rate
Kenya	32.78	2039	0.69
Lebanon	31.52	2045	0.52
Ivory Coast	30.93	2031	0.66
Chad	30.22	2027	1.1
Afghanistan	29.32	2029	0.85
East Timor	26.84	2032	0.58
Ghana	25.09	2023	0.8
South Korea	25.04	1982	0.69
Guyana	22.65	2028	0.77
Sierra Leone	22.07	2033	0.66
Zimbabwe	19.0	2024	0.19
Uganda	18.92	2029	0.66
Namibia	17.9	2026	0.58
Lithuania	16.87	2034	0.46
Somalia	15.98	2035	0.4
Mauritania	15.09	2028	0.5
Sri Lanka	14.91	2033	0.28
Liberia	14.14	2028	0.49
Palestine	14.09	2030	0.39
Tajikistan	14.04	2043	0.33
Guinea	13.74	2028	0.49
Senegal	13.72	2026	0.29
Albania	13.69	2058	0.32
Niger	12.4	2028	0.32
Georgia	12.39	2028	0.37
Sao Tome and Principe	11.29	2026	0.43
Cyprus	10.74	2048	0.18
Western Sahara	9.71	2035	0.24
Taiwan	7.16	1969	0.16
Barbados	7.0	2047	0.13
Benin	6.9	2024	0.31
Togo	6.65	2024	0.28
Dominican Republic	6.32	2026	0.25
Haiti	6.06	2027	0.25
Slovakia	5.79	2055	0.08
Laos	5.37	2043	0.14
Puerto Rico	4.27	2027	0.19
Eritrea	4.13	2025	0.15
Portugal	4.1	2014	0.17
Swaziland	3.98	2085	0.06
Guinea-Bissau	3.53	2026	0.15
Guatemala	3.31	2025	0.16
Malawi	3.16	2082	0.05
Cambodia	2.95	2025	0.16
Ethiopia	2.63	2025	0.14
Armenia	2.12	2058	0.06
Rwanda	1.85	2025	0.08
Gambia	1.77	2025	0.1
Crimea	1.71	2010	0.07

Table 8.3: Peak year - Country – Continued

**Name**	**URR**	**Peak Year**	**Peak Rate**
Moldova	1.18	2030	0.06
Belize	1.09	2032	0.03
Grenada	0.93	2025	0.05
Zambia	0.68	1978	0.02
Malta	0.58	2025	0.03
New Caledonia	0.06	2048	–
Bhutan	0.04	1995	–
Switzerland	0.03	1944	0.01
Central African Republic	0.03	2026	–
Nepal	0.02	2027	–
**Total**	**130080.84**	**2050**	**795.11**
