# Supplementary material for: Projecting the global impact of fossil fuel production from the Former Soviet Union
Source: Int J Coal Sci Technol. 2021 Aug 9;8(6):1208–26. doi: 10.1007/s40789-021-00449-x (PMC8351787; doi:10.1007/s40789-021-00449-x)
Supplement: Supplementary file 5 — Supplementary file5 (PDF 22356 kb) [file 40789_2021_449_MOESM5_ESM.pdf]

GeRS-DeMo Post Processor

Compilation ran on scenario: Static BG

Date: 7/6/2020

Contents

1 Africa **21**

1.1 Algeria . . . 21
1.1.1 All Projections . . . 21
1.1.2 By Mineral . . . 23
1.2 Angola . . . 25
1.2.1 All Projections . . . 25
1.2.2 By Mineral . . . 26
1.3 Benin . . . 27
1.3.1 All Projections . . . 27
1.3.2 By Mineral . . . 28
1.4 Botswana . . . 29
1.4.1 All Projections . . . 29
1.4.2 By Mineral . . . 30
1.5 Cameroon . . . 31
1.5.1 All Projections . . . 31
1.5.2 By Mineral . . . 32
1.6 Central African Republic . . . 33
1.6.1 All Projections . . . 33
1.6.2 By Mineral . . . 34
1.7 Chad . . . 35
1.7.1 All Projections . . . 35
1.7.2 By Mineral . . . 36
1.8 Congo . . . 37
1.8.1 All Projections . . . 37
1.8.2 By Mineral . . . 38
1.9 Egypt . . . 39
1.9.1 All Projections . . . 39
1.9.2 By Mineral . . . 40

1.10 Equatorial Guinea 41
1.10.1 All Projections 41
1.10.2 By Mineral 42
1.11 Eritrea 43
1.11.1 All Projections 43
1.11.2 By Mineral 44
1.12 Ethiopia 45
1.12.1 All Projections 45
1.12.2 By Mineral 46
1.13 Gabon 47
1.13.1 All Projections 47
1.13.2 By Mineral 48
1.14 Gambia 49
1.14.1 All Projections 49
1.14.2 By Mineral 50
1.15 Ghana 51
1.15.1 All Projections 51
1.15.2 By Mineral 52
1.16 Guinea 53
1.16.1 All Projections 53
1.16.2 By Mineral 54
1.17 Guinea-Bissau 55
1.17.1 All Projections 55
1.17.2 By Mineral 56
1.18 Ivory Coast 57
1.18.1 All Projections 57
1.18.2 By Mineral 58
1.19 Kenya 59
1.19.1 All Projections 59
1.19.2 By Mineral 60
1.20 Liberia 61
1.20.1 All Projections 61
1.20.2 By Mineral 62
1.21 Libya 63
1.21.1 All Projections 63
1.21.2 By Mineral 65

1.22 Madagascar 67
1.22.1 All Projections 67
1.22.2 By Mineral 68
1.23 Malawi 69
1.23.1 All Projections 69
1.23.2 By Mineral 70
1.24 Mauritania 71
1.24.1 All Projections 71
1.24.2 By Mineral 72
1.25 Morocco 73
1.25.1 All Projections 73
1.25.2 By Mineral 74
1.26 Mozambique 75
1.26.1 All Projections 75
1.26.2 By Mineral 77
1.27 Namibia 79
1.27.1 All Projections 79
1.27.2 By Mineral 80
1.28 Niger 81
1.28.1 All Projections 81
1.28.2 By Mineral 82
1.29 Nigeria 83
1.29.1 All Projections 83
1.29.2 By Mineral 85
1.30 Rwanda 87
1.30.1 All Projections 87
1.30.2 By Mineral 88
1.31 Sao Tome and Principe 89
1.31.1 All Projections 89
1.31.2 By Mineral 90
1.32 Senegal 91
1.32.1 All Projections 91
1.32.2 By Mineral 92
1.33 Seychelles 93
1.33.1 All Projections 93
1.33.2 By Mineral 94

1.34 Sierra Leone 95
1.34.1 All Projections 95
1.34.2 By Mineral 96
1.35 Somalia 97
1.35.1 All Projections 97
1.35.2 By Mineral 98
1.36 South Africa 99
1.36.1 All Projections 99
1.36.2 By Mineral 101
1.37 Sudan 103
1.37.1 All Projections 103
1.37.2 By Mineral 104
1.37.3 Regional Projections 104
South Sudan 105
Sudan 107
Sudan Region 109
1.37.4 Projection by region 111
1.38 Swaziland 112
1.38.1 All Projections 112
1.38.2 By Mineral 113
1.39 Tanzania 114
1.39.1 All Projections 114
1.39.2 By Mineral 116
1.40 Togo 118
1.40.1 All Projections 118
1.40.2 By Mineral 119
1.41 Tunisia 120
1.41.1 All Projections 120
1.41.2 By Mineral 122
1.42 Uganda 124
1.42.1 All Projections 124
1.42.2 By Mineral 125
1.43 Western Sahara 126
1.43.1 All Projections 126
1.43.2 By Mineral 127
1.44 Zaire 128

1.44.1 All Projections 128
1.44.2 By Mineral 130
1.45 Zambia 132
1.45.1 All Projections 132
1.45.2 By Mineral 133
1.46 Zimbabwe 134
1.46.1 All Projections 134
1.46.2 By Mineral 135
1.47 Total 136
1.47.1 By country 136
1.47.2 By mineral 138

2 Asia **140**
2.1 Afghanistan 140
2.1.1 All Projections 140
2.1.2 By Mineral 142
2.2 Australia 143
2.2.1 All Projections 143
2.2.2 By Mineral 146
2.2.3 Regional Projections 146
Australia 148
JPDA 150
NSW 152
Northern Territory 155
Queensland 158
South Australia 161
Tasmania 164
Victoria 167
Western Australia 169
2.2.4 Projection by region 172
2.3 Bangladesh 174
2.3.1 All Projections 174
2.3.2 By Mineral 176
2.4 Bhutan 178
2.4.1 All Projections 178
2.4.2 By Mineral 179

2.5 Brunei 180
2.5.1 All Projections 180
2.5.2 By Mineral 182
2.6 Burma 184
2.6.1 All Projections 184
2.6.2 By Mineral 185
2.7 Cambodia 186
2.7.1 All Projections 186
2.7.2 By Mineral 187
2.8 China 188
2.8.1 All Projections 188
2.8.2 By Mineral 191
2.8.3 Regional Projections 191
Anhui 193
Beijing 195
China 197
Chongqing 200
Fujian 202
Gansu 204
Guangdong 206
Guangxi 208
Guizhou 210
Hainan 212
Hebei 214
Heilongjiang 216
Henan 218
Historic 220
Hubei 222
Hunan 224
Inner Mongolia 226
Jiangsu 228
Jiangxi 230
Jilin 232
Liaoning 234
Ningxia 236
Offshore 238

Qinghai 240
Shaanxi 242
Shandong 244
Shanghai 246
Shanxi 248
Sichuan 250
Tianjin 252
Tibet 254
Xinjiang 256
Yunnan 258
Zhejiang 260
2.8.4 Projection by region 262
2.9 East Timor 264
2.9.1 All Projections 264
2.9.2 By Mineral 265
2.10 India 266
2.10.1 All Projections 266
2.10.2 By Mineral 268
2.11 Indonesia 270
2.11.1 All Projections 270
2.11.2 By Mineral 272
2.12 Japan 274
2.12.1 All Projections 274
2.12.2 By Mineral 276
2.13 Laos 278
2.13.1 All Projections 278
2.13.2 By Mineral 279
2.14 Malaysia 280
2.14.1 All Projections 280
2.14.2 By Mineral 281
2.15 Mongolia 282
2.15.1 All Projections 282
2.15.2 By Mineral 284
2.16 Nepal 286
2.16.1 All Projections 286
2.16.2 By Mineral 287

2.17 New Caledonia 288
2.17.1 All Projections 288
2.17.2 By Mineral 289
2.18 New Zealand 290
2.18.1 All Projections 290
2.18.2 By Mineral 292
2.19 North Korea 294
2.19.1 All Projections 294
2.19.2 By Mineral 295
2.20 PNG 296
2.20.1 All Projections 296
2.20.2 By Mineral 297
2.21 Pakistan 298
2.21.1 All Projections 298
2.21.2 By Mineral 300
2.22 Philippines 302
2.22.1 All Projections 302
2.22.2 By Mineral 304
2.23 South Korea 306
2.23.1 All Projections 306
2.23.2 By Mineral 308
2.24 Sri Lanka 310
2.24.1 All Projections 310
2.24.2 By Mineral 311
2.25 Taiwan 312
2.25.1 All Projections 312
2.25.2 By Mineral 313
2.26 Thailand 314
2.26.1 All Projections 314
2.26.2 By Mineral 316
2.27 Vietnam 318
2.27.1 All Projections 318
2.27.2 By Mineral 320
2.28 Total 322
2.28.1 By country 322
2.28.2 By mineral 324

3 Europe **326**
3.1 Albania 326
3.1.1 All Projections 326
3.1.2 By Mineral 328
3.2 Austria 329
3.2.1 All Projections 329
3.2.2 By Mineral 331
3.3 Belgium 333
3.3.1 All Projections 333
3.3.2 By Mineral 334
3.4 Bulgaria 335
3.4.1 All Projections 335
3.4.2 By Mineral 337
3.5 Cyprus 339
3.5.1 All Projections 339
3.5.2 By Mineral 340
3.6 Czech Republic 341
3.6.1 All Projections 341
3.6.2 By Mineral 343
3.7 Denmark 345
3.7.1 All Projections 345
3.7.2 By Mineral 347
3.8 France 349
3.8.1 All Projections 349
3.8.2 By Mineral 351
3.9 Germany 353
3.9.1 All Projections 353
3.9.2 By Mineral 355
3.10 Greece 357
3.10.1 All Projections 357
3.10.2 By Mineral 359
3.11 Greenland 361
3.11.1 All Projections 361
3.11.2 By Mineral 362
3.12 Hungary 363
3.12.1 All Projections 363

3.12.2 By Mineral 365
3.13 Ireland 367
3.13.1 All Projections 367
3.13.2 By Mineral 369
3.14 Italy 371
3.14.1 All Projections 371
3.14.2 By Mineral 373
3.14.3 Regional Projections 373
Italy 375
Sicily 378
3.14.4 Projection by region 380
3.15 Malta 381
3.15.1 All Projections 381
3.15.2 By Mineral 382
3.16 Netherlands 383
3.16.1 All Projections 383
3.16.2 By Mineral 385
3.17 Norway 387
3.17.1 All Projections 387
3.17.2 By Mineral 389
3.18 Poland 391
3.18.1 All Projections 391
3.18.2 By Mineral 393
3.19 Portugal 395
3.19.1 All Projections 395
3.19.2 By Mineral 397
3.20 Romania 399
3.20.1 All Projections 399
3.20.2 By Mineral 401
3.21 Slovakia 403
3.21.1 All Projections 403
3.21.2 By Mineral 405
3.22 Spain 407
3.22.1 All Projections 407
3.22.2 By Mineral 409
3.23 Sweden 411

3.23.1 All Projections 411
3.23.2 By Mineral 412
3.24 Switzerland 413
3.24.1 All Projections 413
3.24.2 By Mineral 414
3.25 Turkey 415
3.25.1 All Projections 415
3.25.2 By Mineral 417
3.26 UK 419
3.26.1 All Projections 419
3.26.2 By Mineral 421
3.26.3 Regional Projections 421
England and Wales 423
Northern Ireland 426
Scotland 428
UK 430
3.26.4 Projection by region 432
3.27 Yugoslavia 434
3.27.1 All Projections 434
3.27.2 By Mineral 436
3.27.3 Regional Projections 436
Bosnia and Herzegovina 438
Croatia 440
Serbia 442
Slovenia 444
Yugoslavia 446
3.27.4 Projection by region 448
3.28 Total 450
3.28.1 By country 450
3.28.2 By mineral 452

4 FSU **454**
4.1 Azerbaijan 454
4.1.1 All Projections 454
4.1.2 By Mineral 456
4.2 Belarus 457

4.2.1 All Projections . . . 457
4.2.2 By Mineral . . . 458
4.3 Crimea . . . 459
4.3.1 All Projections . . . 459
4.3.2 By Mineral . . . 460
4.3.3 Regional Projections . . . 460
Crimea . . . 461
4.3.4 Projection by region . . . 463
4.4 Donetsk . . . 464
4.4.1 All Projections . . . 464
4.4.2 By Mineral . . . 465
4.4.3 Regional Projections . . . 465
Donetsk . . . 466
4.4.4 Projection by region . . . 468
4.5 Estonia . . . 469
4.5.1 All Projections . . . 469
4.5.2 By Mineral . . . 470
4.6 Georgia . . . 471
4.6.1 All Projections . . . 471
4.6.2 By Mineral . . . 472
4.7 Kazakhstan . . . 473
4.7.1 All Projections . . . 473
4.7.2 By Mineral . . . 475
4.7.3 Regional Projections . . . 475
All . . . 477
East Kazakhstan . . . 480
Karaganda . . . 482
Kostanay . . . 484
Other . . . 486
Pavlodar . . . 488
4.7.4 Projection by region . . . 490
4.8 Kyrgyzstan . . . 492
4.8.1 All Projections . . . 492
4.8.2 By Mineral . . . 494
4.9 Lithuania . . . 496
4.9.1 All Projections . . . 496

4.9.2 By Mineral 497
4.10 Luhansk 498
4.10.1 All Projections 498
4.10.2 By Mineral 499
4.10.3 Regional Projections 499
Luhansk 500
4.10.4 Projection by region 502
4.11 Moldova 503
4.11.1 All Projections 503
4.11.2 By Mineral 504
4.12 Russia 505
4.12.1 All Projections 505
4.12.2 By Mineral 508
4.12.3 Regional Projections 508
All 510
Central 513
Far Eastern 515
North Caucasian 518
Northwestern 520
Siberian 523
Southern 526
Ural 528
Volga 531
4.12.4 Projection by region 534
4.13 Tajikistan 536
4.13.1 All Projections 536
4.13.2 By Mineral 538
4.14 Turkmenistan 540
4.14.1 All Projections 540
4.14.2 By Mineral 541
4.15 Ukraine 542
4.15.1 All Projections 542
4.15.2 By Mineral 544
4.16 Uzbekistan 546
4.16.1 All Projections 546
4.16.2 By Mineral 548

4.17 Total 550
4.17.1 By country 550
4.17.2 By mineral 551

5 Middle East **554**
5.1 Bahrain 554
5.1.1 All Projections 554
5.1.2 By Mineral 556
5.2 Iran 557
5.2.1 All Projections 557
5.2.2 By Mineral 558
5.3 Iraq 559
5.3.1 All Projections 559
5.3.2 By Mineral 560
5.4 Israel 561
5.4.1 All Projections 561
5.4.2 By Mineral 562
5.5 Jordan 563
5.5.1 All Projections 563
5.5.2 By Mineral 564
5.6 Kuwait 565
5.6.1 All Projections 565
5.6.2 By Mineral 566
5.7 Lebanon 567
5.7.1 All Projections 567
5.7.2 By Mineral 568
5.8 Oman 569
5.8.1 All Projections 569
5.8.2 By Mineral 570
5.9 Qatar 571
5.9.1 All Projections 571
5.9.2 By Mineral 572
5.10 Saudi Arabia 573
5.10.1 All Projections 573
5.10.2 By Mineral 574
5.11 Syria 575

5.11.1 All Projections 575
5.11.2 By Mineral 576
5.12 UAE 577
5.12.1 All Projections 577
5.12.2 By Mineral 578
5.13 Yemen 579
5.13.1 All Projections 579
5.13.2 By Mineral 580
5.14 Total 581
5.14.1 By country 581
5.14.2 By mineral 581

6 North America **584**
6.1 Canada 584
6.1.1 All Projections 584
6.1.2 By Mineral 588
6.1.3 Regional Projections 588
Alberta 590
British Columbia 593
Canada 596
East Coast Offshore 598
Manitoba 600
New Brunswick 602
Northwest Territories 604
Nova Scotia 606
Ontario 609
Quebec 611
Saskatchewan 613
Yukon 616
6.1.4 Projection by region 618
6.2 USA 620
6.2.1 All Projections 620
6.2.2 By Mineral 625
6.2.3 Regional Projections 625
Alabama 627
Alaska 629

Arizona 631
Arkansas 633
California 636
Colorado 638
Eastern 641
Florida 643
Georgia 645
Illinois 647
Indiana 649
Iowa 651
Kansas 653
Kentucky 655
Louisiana 658
Maryland 661
Michigan 663
Mississippi 665
Missouri 667
Montana 669
Nebraska 672
Nevada 674
New Mexico 676
New York 679
North Dakota 681
Ohio 683
Oklahoma 686
Oregon 689
Other 691
Pennsylvania 693
South Dakota 696
Tennessee 698
Texas 700
USA 703
Utah 705
Virginia 708
Washington 710
West Virginia 712

Wyoming . . . 715
6.2.4 Projection by region . . . 718
6.3 Total . . . 720
6.3.1 By country . . . 720
6.3.2 By mineral . . . 721

7 South America 723
7.1 Argentina . . . 723
7.1.1 All Projections . . . 723
7.1.2 By Mineral . . . 725
7.2 Barbados . . . 727
7.2.1 All Projections . . . 727
7.2.2 By Mineral . . . 728
7.3 Belize . . . 729
7.3.1 All Projections . . . 729
7.3.2 By Mineral . . . 730
7.4 Bolivia . . . 731
7.4.1 All Projections . . . 731
7.4.2 By Mineral . . . 732
7.5 Brazil . . . 733
7.5.1 All Projections . . . 733
7.5.2 By Mineral . . . 735
7.6 Chile . . . 737
7.6.1 All Projections . . . 737
7.6.2 By Mineral . . . 739
7.7 Colombia . . . 741
7.7.1 All Projections . . . 741
7.7.2 By Mineral . . . 743
7.8 Cuba . . . 745
7.8.1 All Projections . . . 745
7.8.2 By Mineral . . . 746
7.9 Dominican Republic . . . 747
7.9.1 All Projections . . . 747
7.9.2 By Mineral . . . 748
7.10 Ecuador . . . 749
7.10.1 All Projections . . . 749

7.10.2 By Mineral 750
7.11 Falkland Islands 751
7.11.1 All Projections 751
7.11.2 By Mineral 752
7.12 French Guiana 753
7.12.1 All Projections 753
7.12.2 By Mineral 754
7.13 Grenada 755
7.13.1 All Projections 755
7.13.2 By Mineral 756
7.14 Guatemala 757
7.14.1 All Projections 757
7.14.2 By Mineral 758
7.15 Guyana 759
7.15.1 All Projections 759
7.15.2 By Mineral 760
7.16 Haiti 761
7.16.1 All Projections 761
7.16.2 By Mineral 762
7.17 Mexico 763
7.17.1 All Projections 763
7.17.2 By Mineral 765
7.18 Paraguay 767
7.18.1 All Projections 767
7.18.2 By Mineral 768
7.19 Peru 769
7.19.1 All Projections 769
7.19.2 By Mineral 771
7.20 Puerto Rico 773
7.20.1 All Projections 773
7.20.2 By Mineral 774
7.21 Suriname 775
7.21.1 All Projections 775
7.21.2 By Mineral 776
7.22 Trinidad and Tobago 777
7.22.1 All Projections 777

7.22.2 By Mineral 778
7.23 Uruguay 779
7.23.1 All Projections 779
7.23.2 By Mineral 780
7.24 Venezuela 781
7.24.1 All Projections 781
7.24.2 By Mineral 783
7.25 Total 785
7.25.1 By country 785
7.25.2 By mineral 787

8 Total 789
8.1 By continent 789
8.2 By mineral 791
8.3 By Country 793

Chapter 1

Africa

1.1 Algeria

1.1.1 All Projections

Table 1.1: Peak years - All

**Name**	**URR**	**Peak Year**	**Peak Rate**
Oil Conv.	261.15	2023	4.27
Gas Shale	241.54	2126	2.46
Gas Conv.	231.0	2008	3.26
Gas Tight	203.94	2128	2.16
Coal Bit.	0.13	1952	0.01
Coal Lignite	–	1942	–
**Total**	**937.76**	**2007**	**7.16**

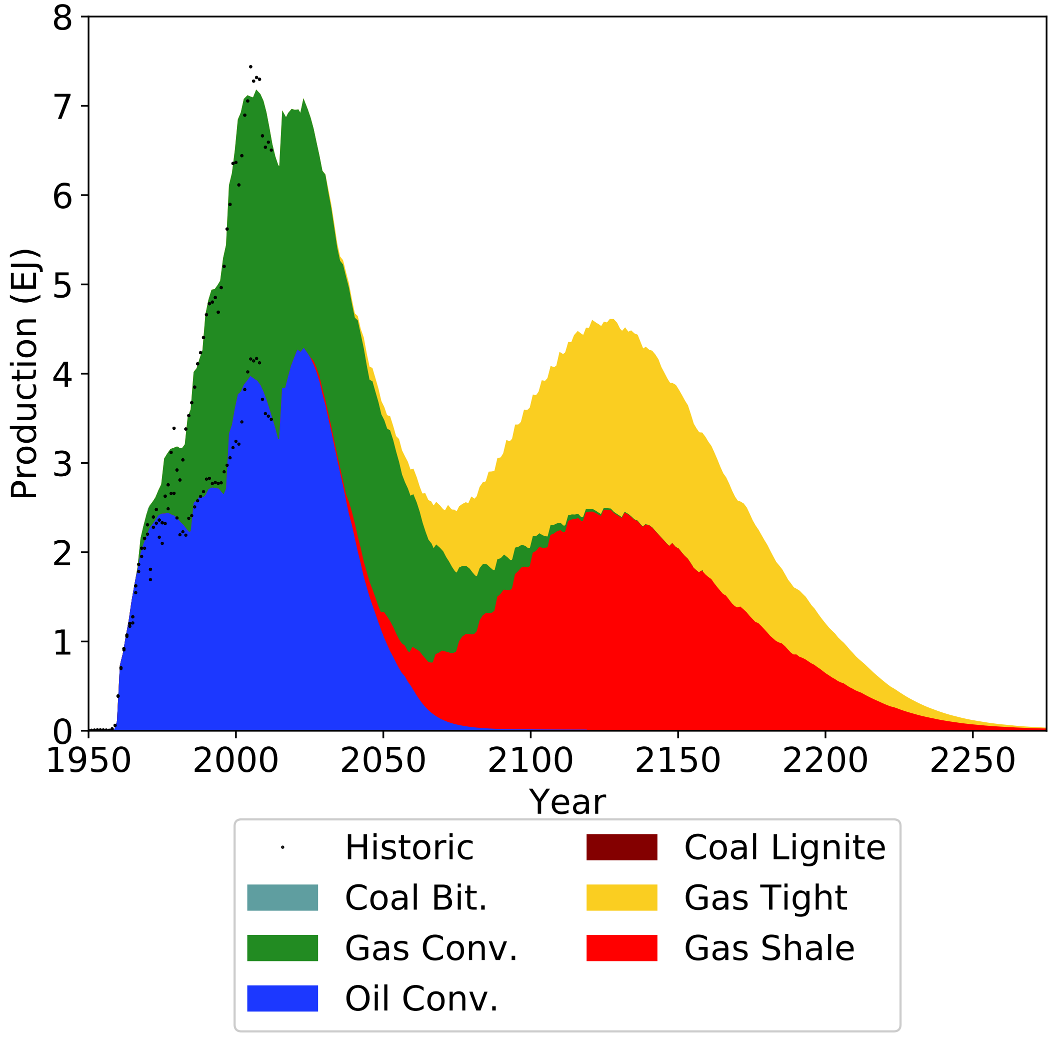

Figure 1.1: Algeria projections capped at 16

1.1.2 By Mineral

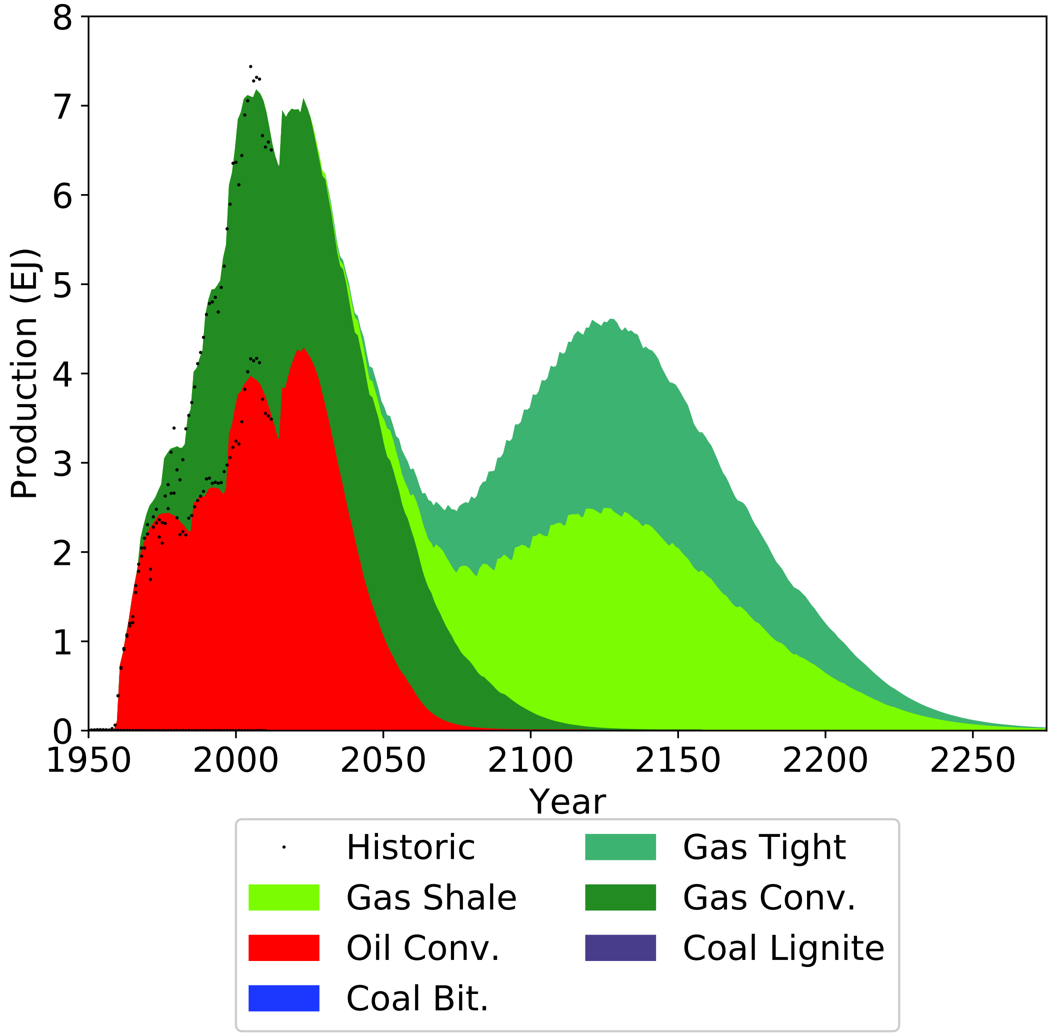

Figure 1.2: Algeria projection by mineral type

Table 1.2: Peak years - Minerals

**Name**	**URR**	**Peak Year**	**Peak Rate**
Coal Bit.	0.13	1952	0.01
Coal Lignite	–	1942	–
Oil Conv.	261.15	2023	4.27
Gas Conv.	231.0	2008	3.26
Gas Shale	241.54	2126	2.46
Gas Tight	203.94	2128	2.16
**Total**	**937.76**	**2007**	**7.16**

1.2 Angola

1.2.1 All Projections

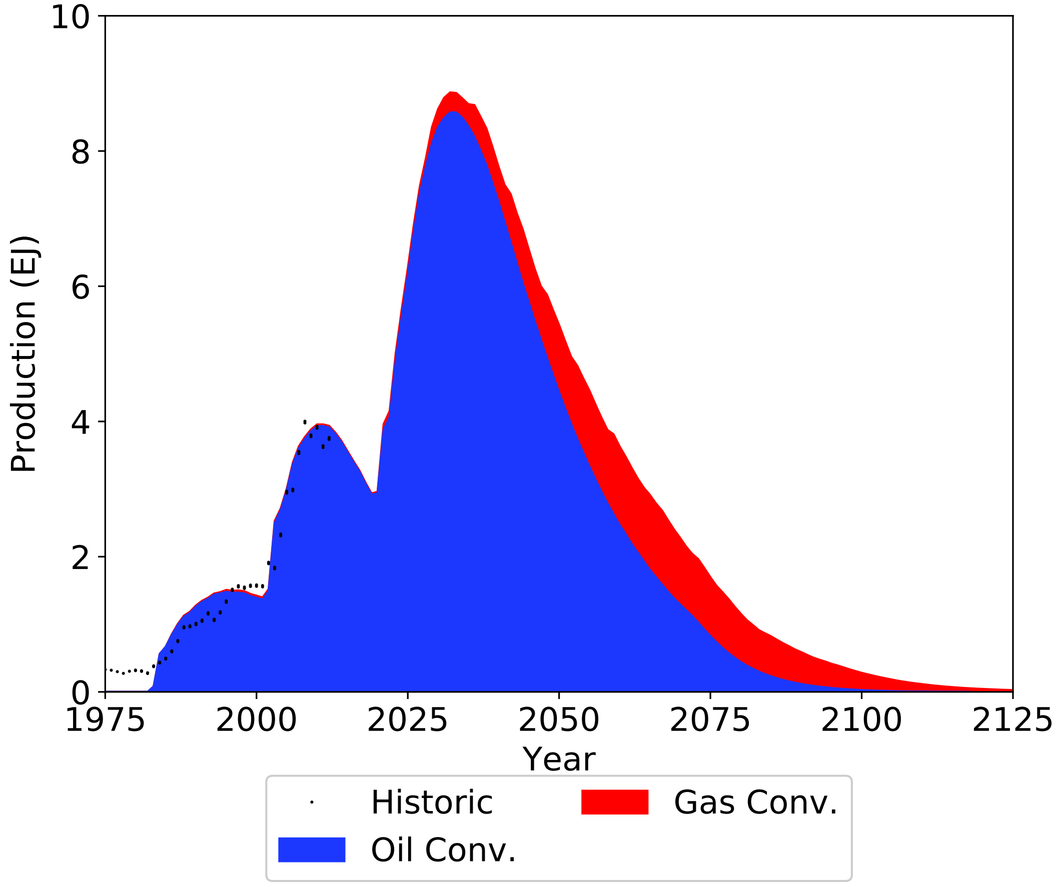

Figure 1.3: Angola projections capped at 16

Table 1.3: Peak years - All

Name	URR	Peak Year	Peak Rate
Oil Conv.	347.12	2032	8.57
Gas Conv.	56.67	2059	1.18
**Total**	**403.79**	**2032**	**8.87**

1.2.2 By Mineral

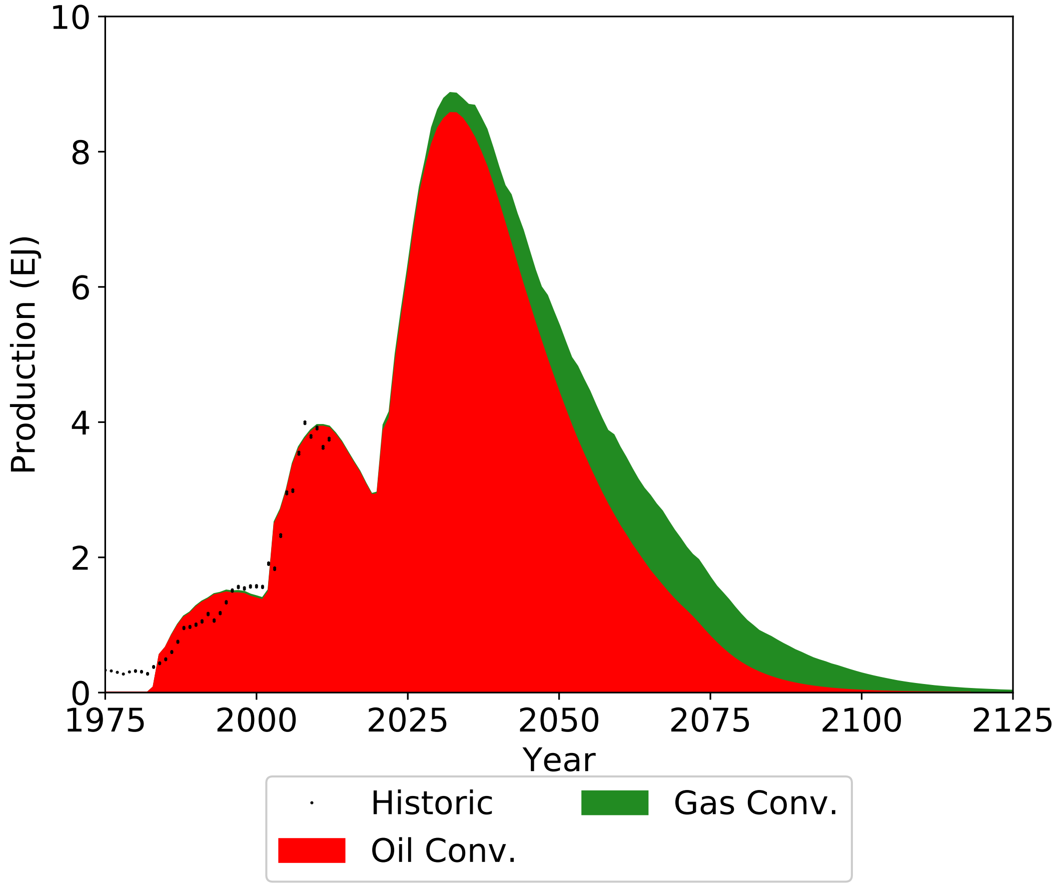

Figure 1.4: Angola projection by mineral type

Table 1.4: Peak years - Minerals

**Name**	**URR**	**Peak Year**	**Peak Rate**
Oil Conv.	347.12	2032	8.57
Gas Conv.	56.67	2059	1.18
**Total**	**403.79**	**2032**	**8.87**

1.3 Benin

1.3.1 All Projections

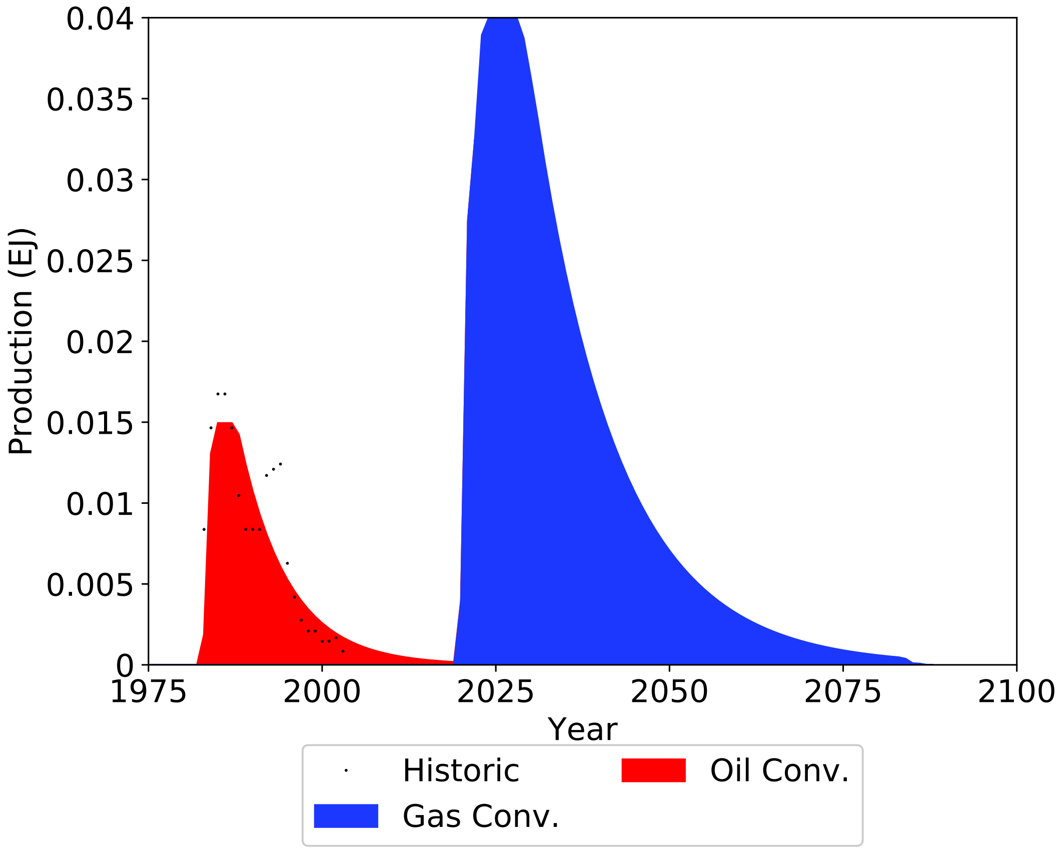

Figure 1.5: Benin projections capped at 16

Table 1.5: Peak years - All

Name	URR	Peak Year	Peak Rate
Gas Conv.	0.8	2024	0.04
Oil Conv.	0.17	1985	0.01
**Total**	**0.97**	**2024**	**0.04**

1.3.2 By Mineral

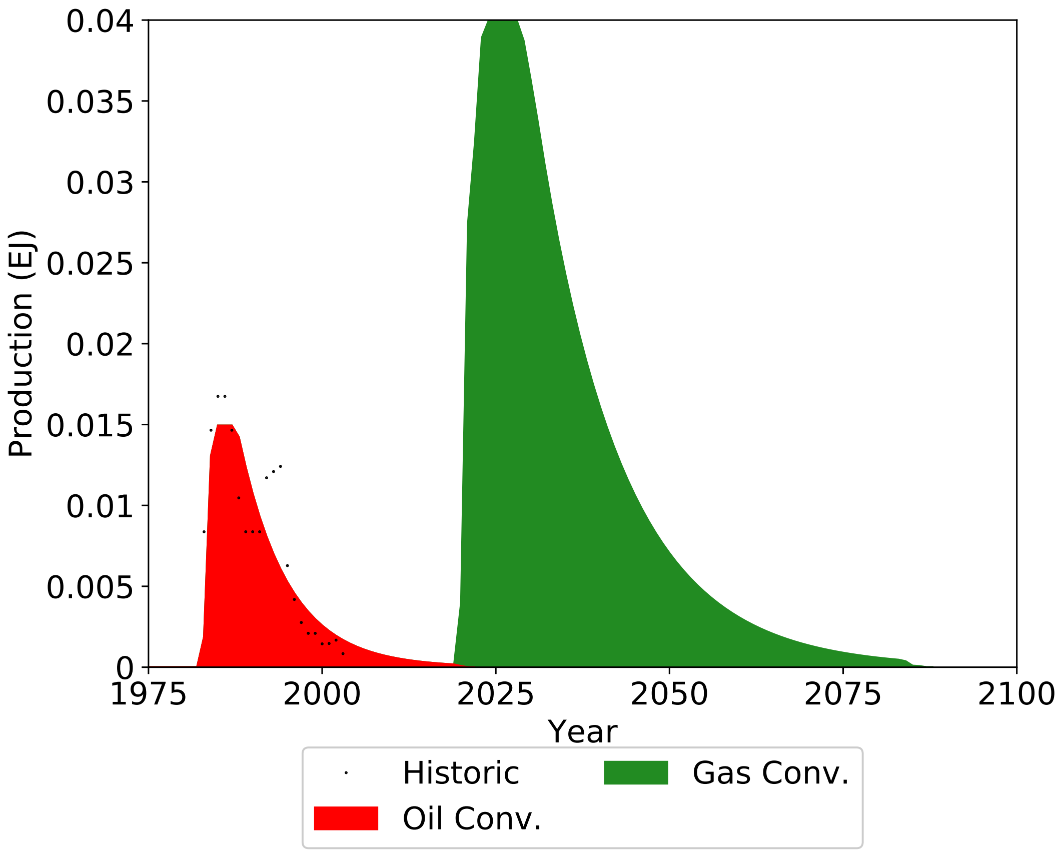

Figure 1.6: Benin projection by mineral type

Table 1.6: Peak years - Minerals

Name	URR	Peak Year	Peak Rate
Oil Conv.	0.17	1985	0.01
Gas Conv.	0.8	2024	0.04
**Total**	**0.97**	**2024**	**0.04**

1.4 Botswana

1.4.1 All Projections

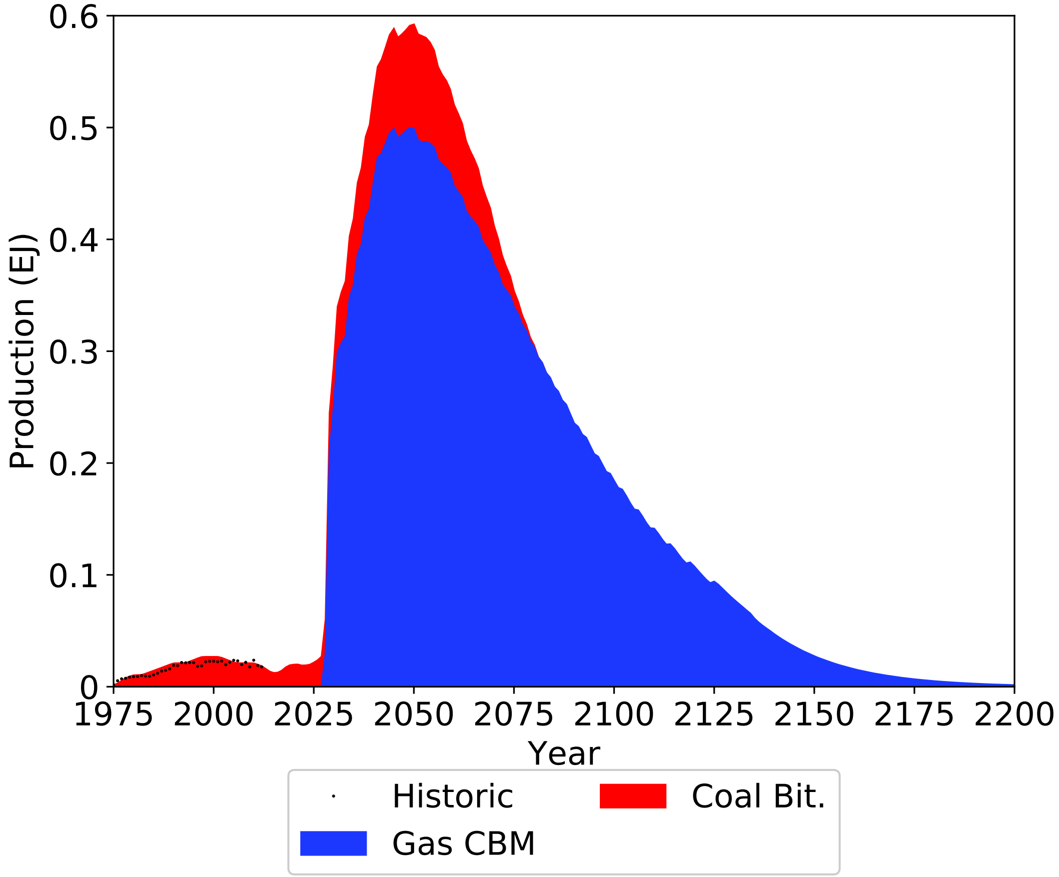

Figure 1.7: Botswana projections capped at 16

Table 1.7: Peak years - All

**Name**	**URR**	**Peak Year**	**Peak Rate**
Gas CBM	31.15	2049	0.5
Coal Bit.	4.08	2052	0.09
**Total**	**35.23**	**2050**	**0.59**

1.4.2 By Mineral

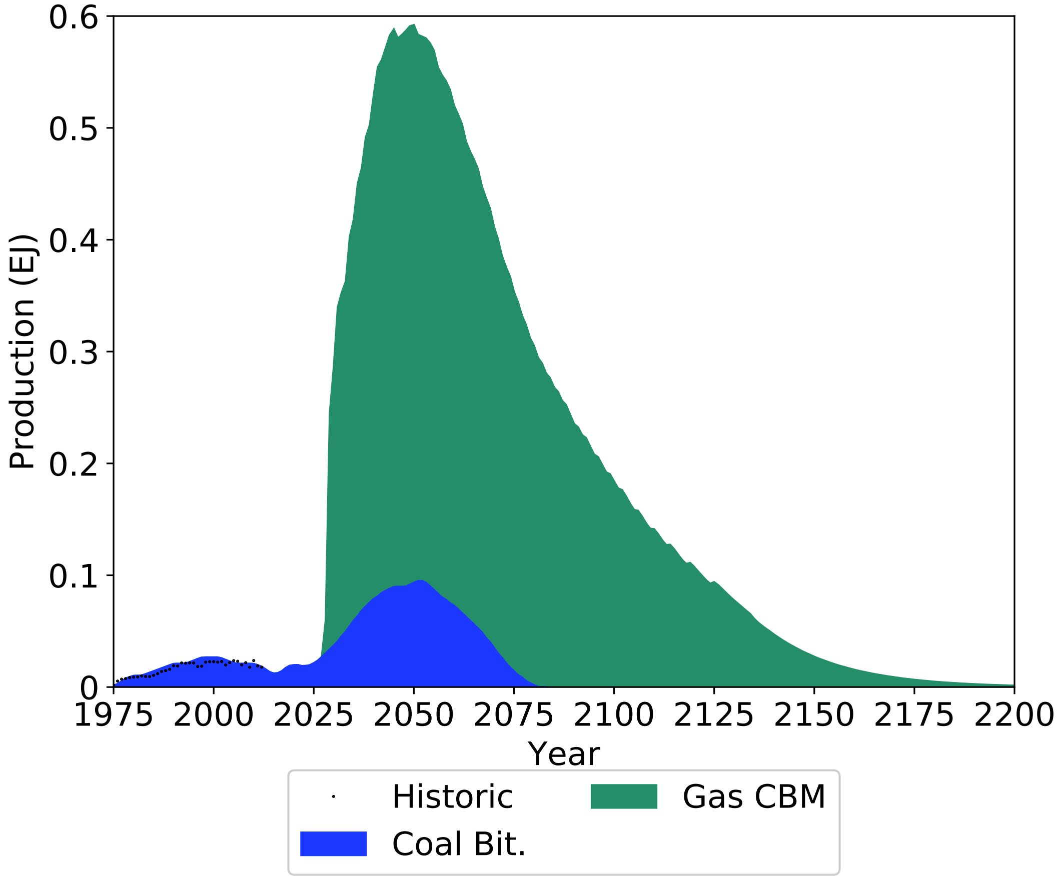

Figure 1.8: Botswana projection by mineral type

Table 1.8: Peak years - Minerals

Name	URR	Peak Year	Peak Rate
Coal Bit.	4.08	2052	0.09
Gas CBM	31.15	2049	0.5
**Total**	**35.23**	**2050**	**0.59**

1.5 Cameroon

1.5.1 All Projections

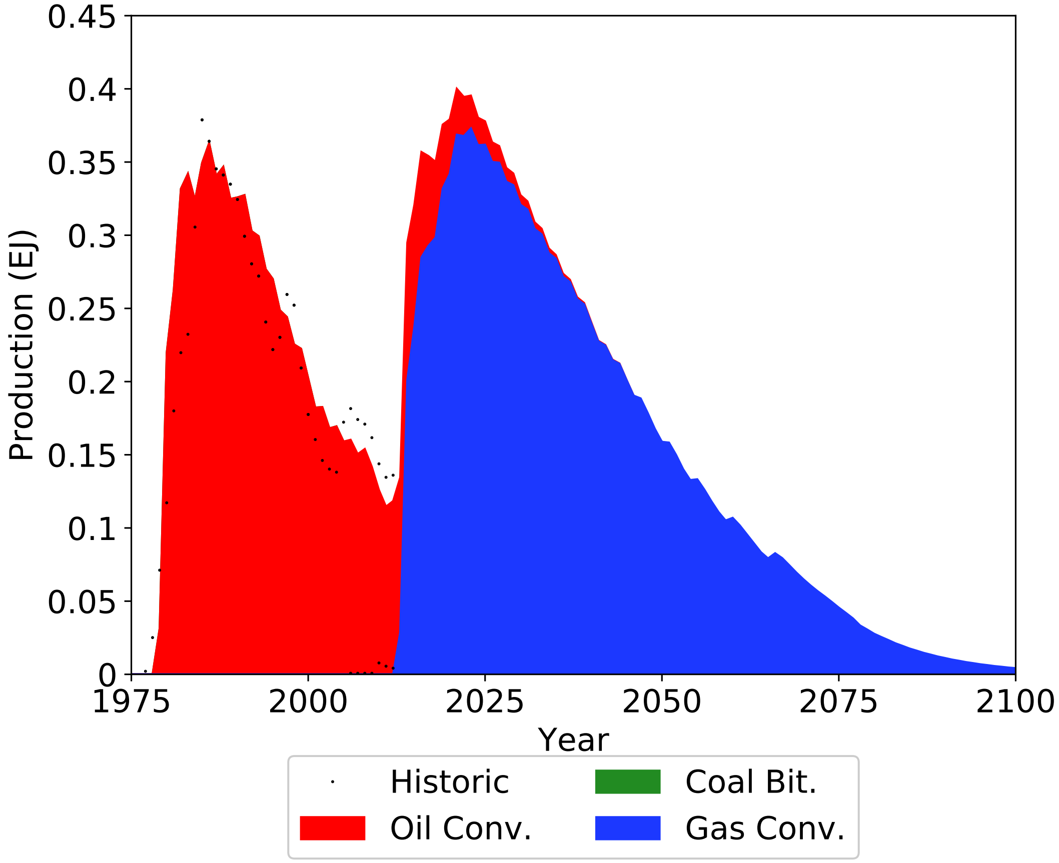

Figure 1.9: Cameroon projections capped at 16

Table 1.9: Peak years - All

**Name**	**URR**	**Peak Year**	**Peak Rate**
Gas Conv.	13.11	2023	0.37
Oil Conv.	8.75	1986	0.36
Coal Bit.	–	1988	–
**Total**	**21.86**	**2021**	**0.4**

1.5.2 By Mineral

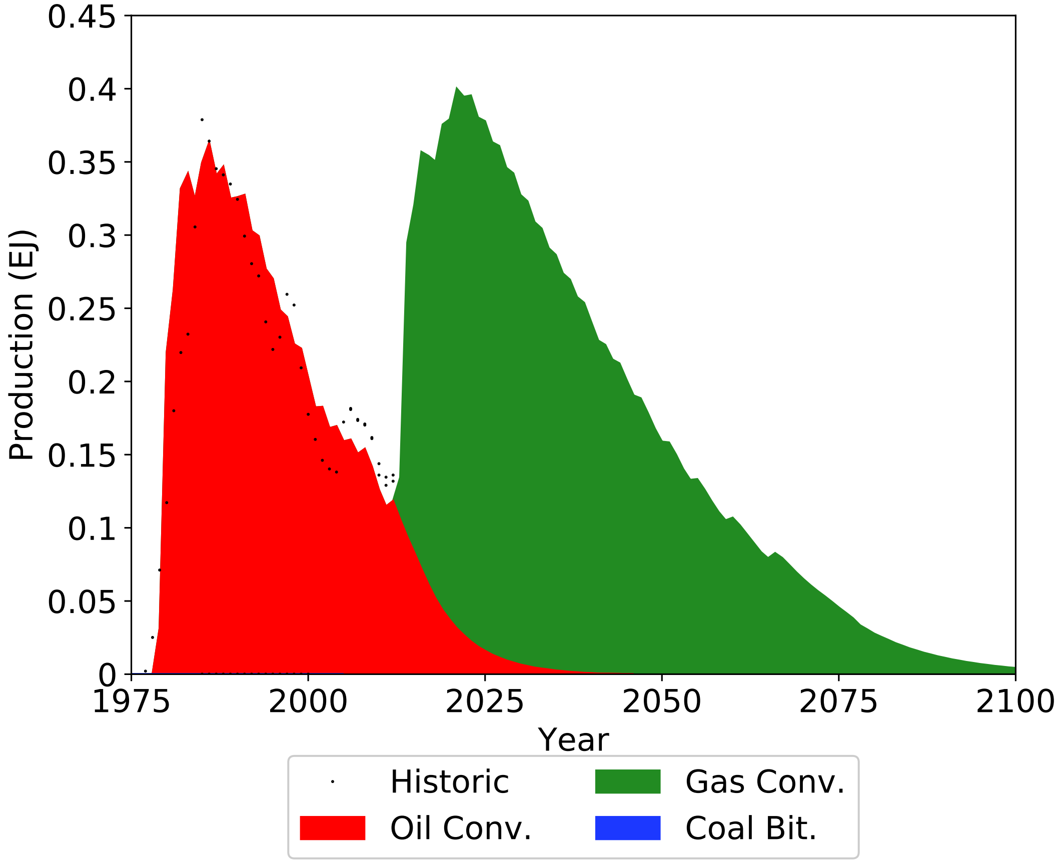

Figure 1.10: Cameroon projection by mineral type

Table 1.10: Peak years - Minerals

Name	URR	Peak Year	Peak Rate
Coal Bit.	–	1988	–
Oil Conv.	8.75	1986	0.36
Gas Conv.	13.11	2023	0.37
**Total**	**21.86**	**2021**	**0.4**

1.6 Central African Republic

1.6.1 All Projections

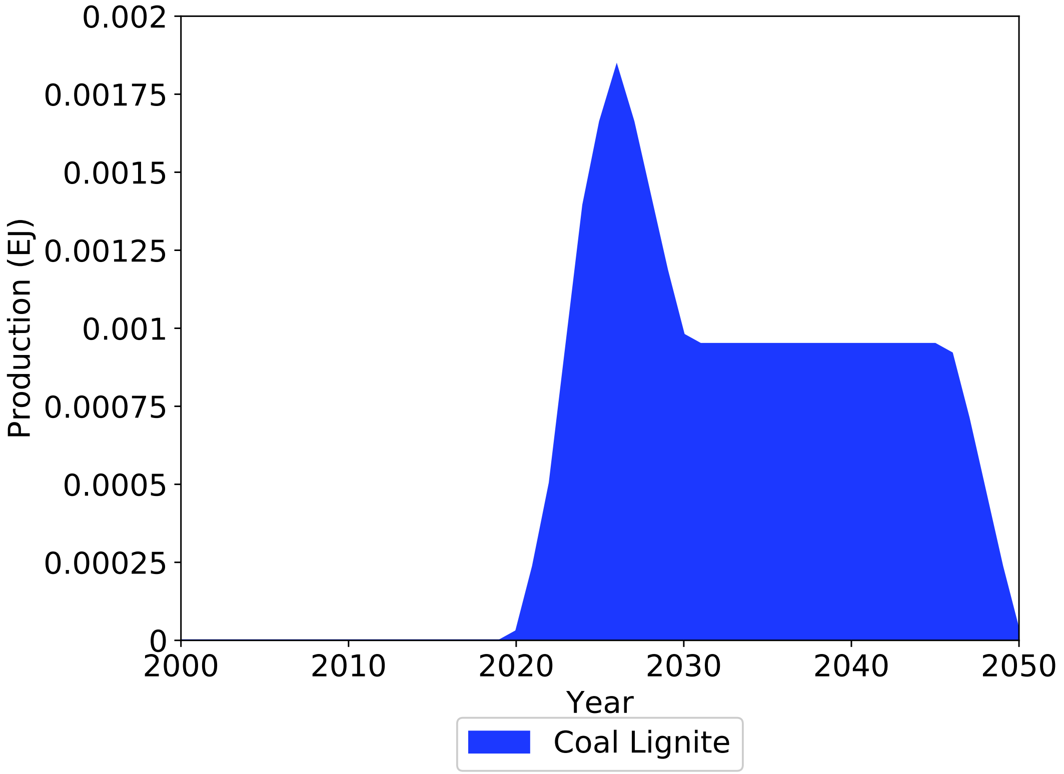

Figure 1.11: Central African Republic projections capped at 16

Table 1.11: Peak years - All

Name	URR	Peak Year	Peak Rate
Coal Lignite	0.03	2026	–
**Total**	**0.03**	**2026**	–

1.6.2 By Mineral

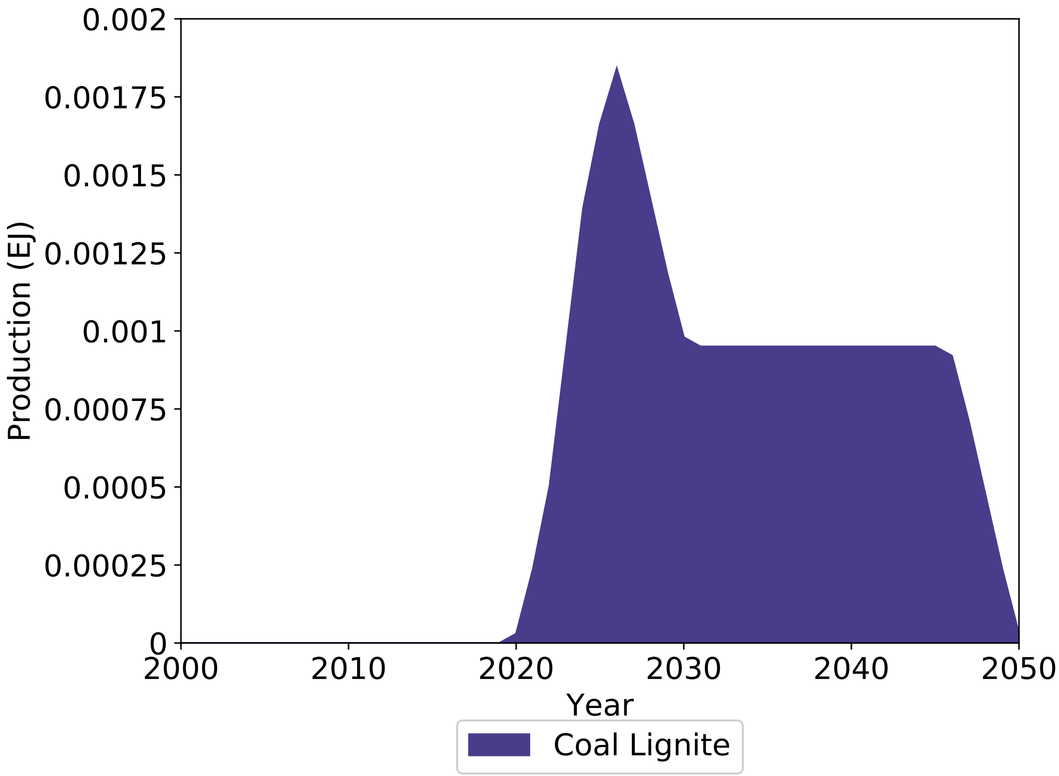

Figure 1.12: Central African Republic projection by mineral type

Table 1.12: Peak years - Minerals

**Name**	**URR**	**Peak Year**	**Peak Rate**
Coal Lignite	0.03	2026	–
**Total**	**0.03**	**2026**	–

1.7 Chad

1.7.1 All Projections

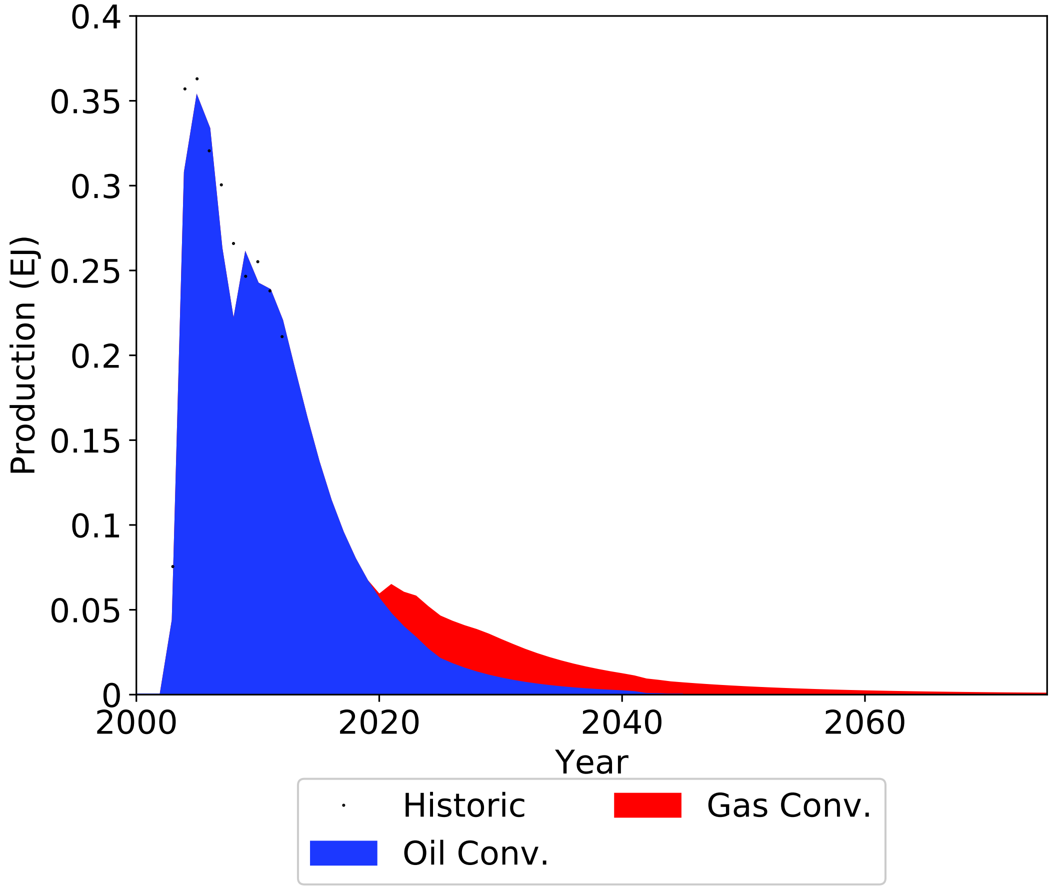

Figure 1.13: Chad projections capped at 16

Table 1.13: Peak years - All

Name	URR	Peak Year	Peak Rate
Oil Conv.	3.67	2005	0.35
Gas Conv.	0.5	2024	0.03
**Total**	**4.17**	**2005**	**0.35**

1.7.2 By Mineral

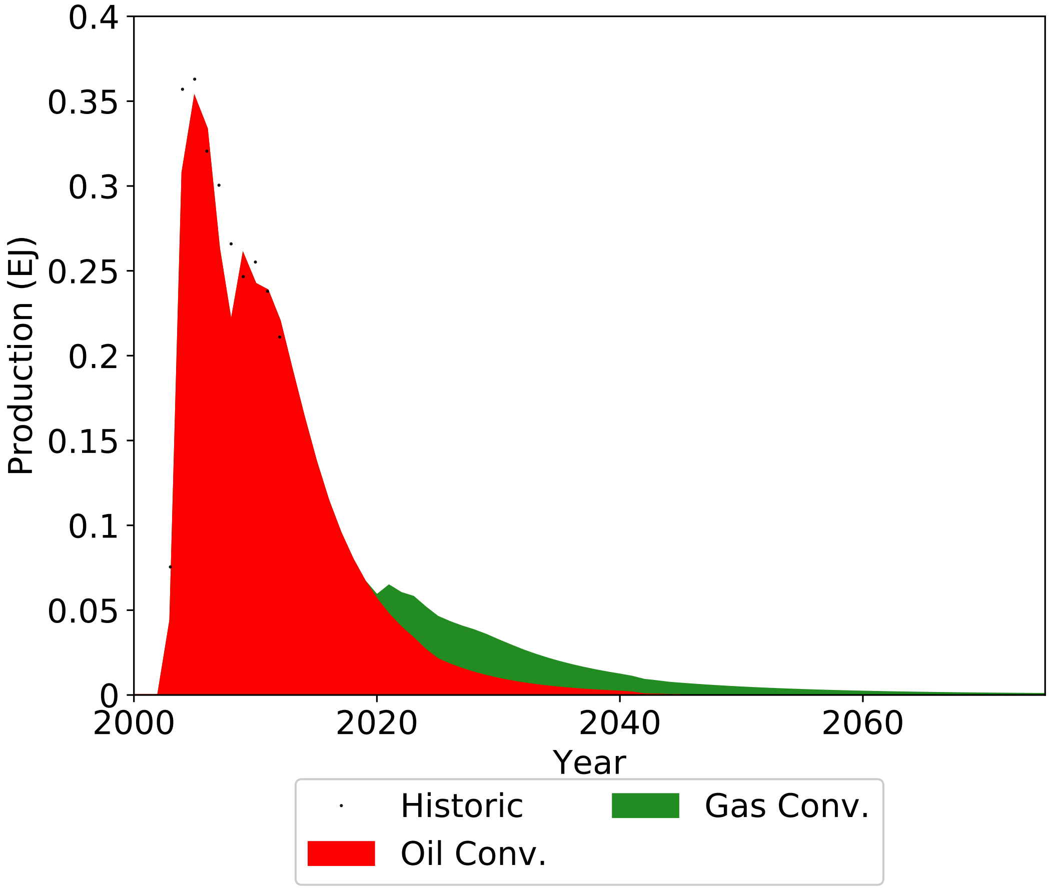

Figure 1.14: Chad projection by mineral type

Table 1.14: Peak years - Minerals

Name	URR	Peak Year	Peak Rate
Oil Conv.	3.67	2005	0.35
Gas Conv.	0.5	2024	0.03
**Total**	**4.17**	**2005**	**0.35**

1.8 Congo

1.8.1 All Projections

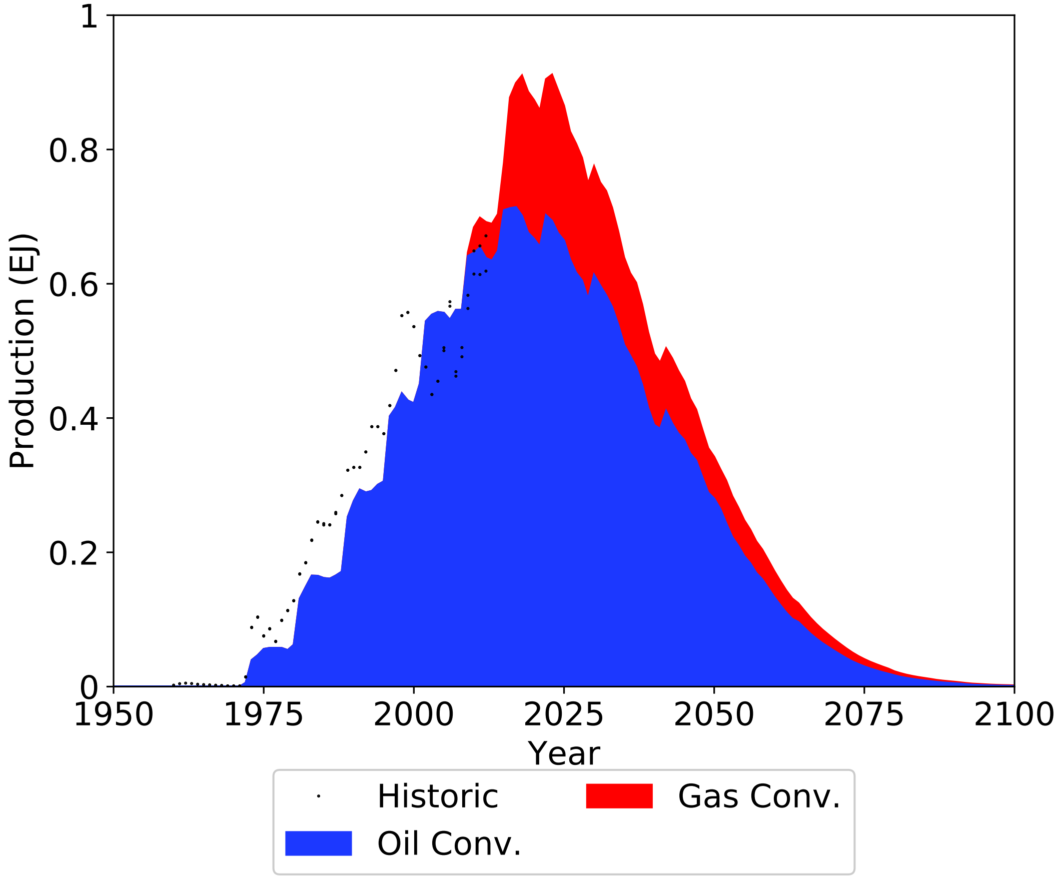

Figure 1.15: Congo projections capped at 16

Table 1.15: Peak years - All

Name	URR	Peak Year	Peak Rate
Oil Conv.	36.34	2017	0.71
Gas Conv.	6.3	2023	0.22
**Total**	**42.64**	**2023**	**0.91**

1.8.2 By Mineral

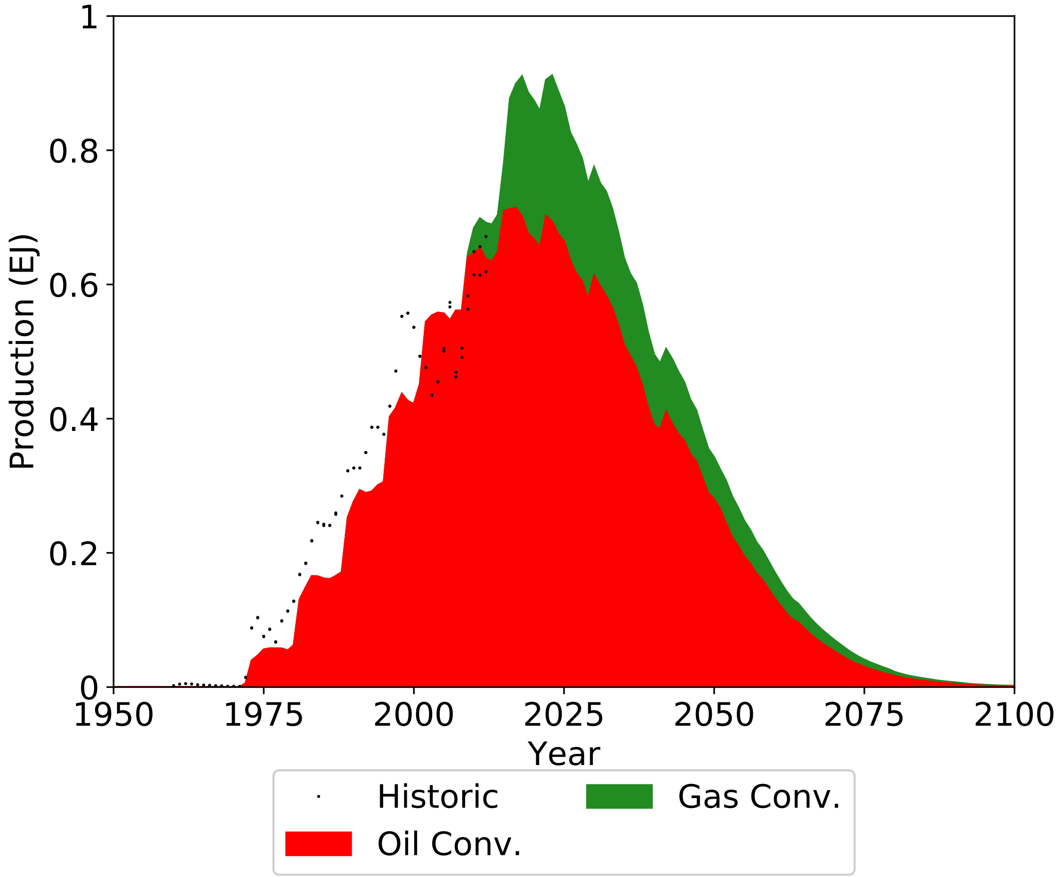

Figure 1.16: Congo projection by mineral type

Table 1.16: Peak years - Minerals

Name	URR	Peak Year	Peak Rate
Oil Conv.	36.34	2017	0.71
Gas Conv.	6.3	2023	0.22
**Total**	**42.64**	**2023**	**0.91**

1.9 Egypt

1.9.1 All Projections

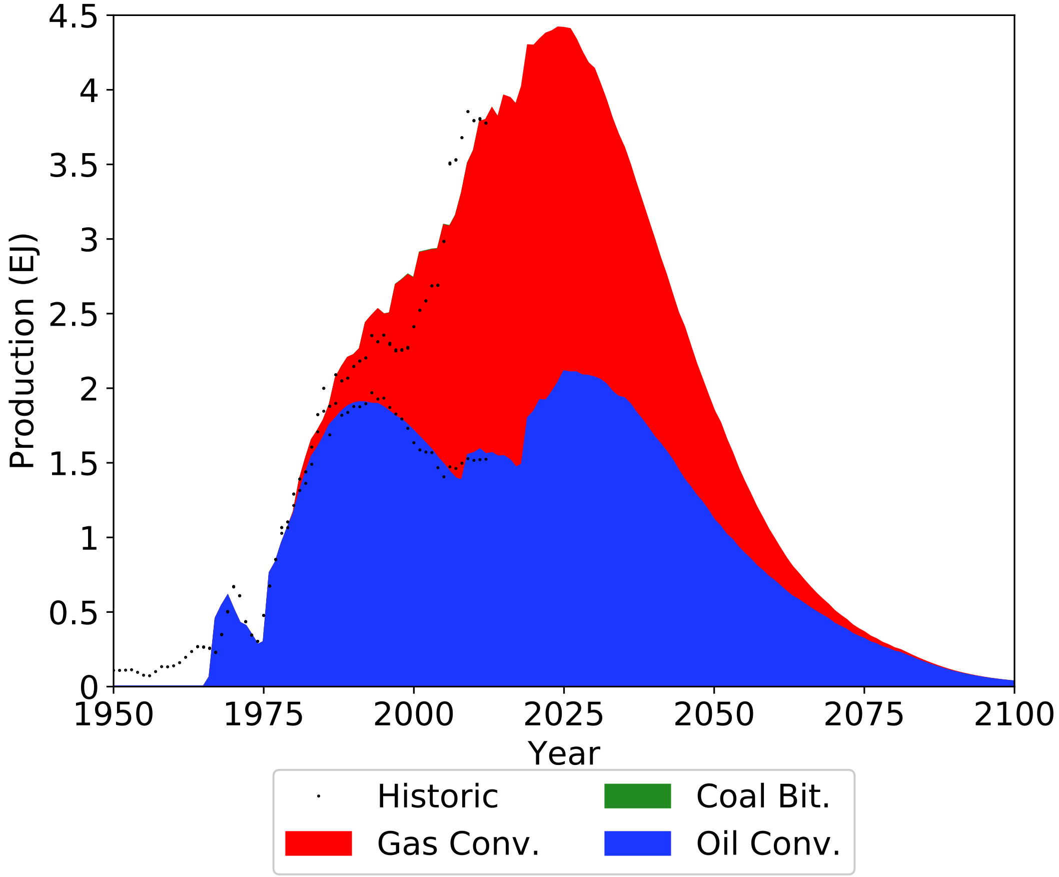

Figure 1.17: Egypt projections capped at 16

Table 1.17: Peak years - All

**Name**	**URR**	**Peak Year**	**Peak Rate**
Oil Conv.	147.78	2025	2.11
Gas Conv.	105.0	2018	2.53
Coal Bit.	0.06	1999	–
**Total**	**252.84**	**2024**	**4.42**

1.9.2 By Mineral

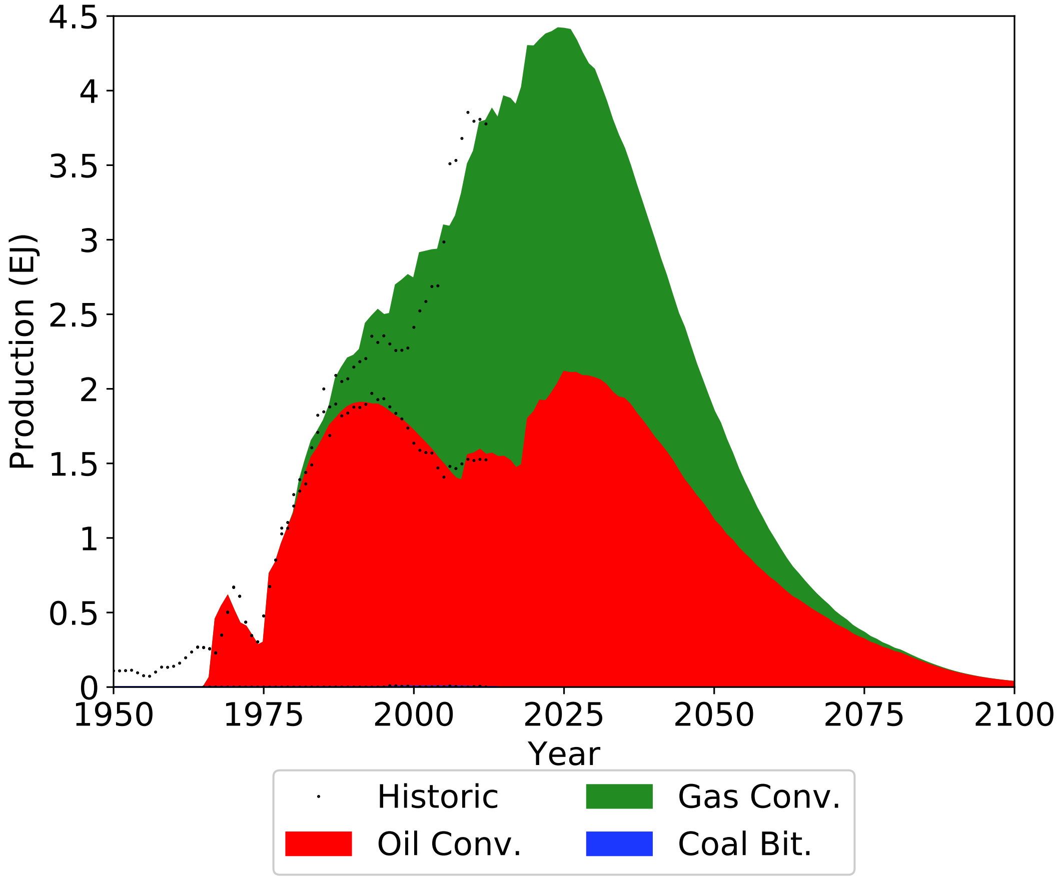

Figure 1.18: Egypt projection by mineral type

Table 1.18: Peak years - Minerals

Name	URR	Peak Year	Peak Rate
Coal Bit.	0.06	1999	–
Oil Conv.	147.78	2025	2.11
Gas Conv.	105.0	2018	2.53
**Total**	**252.84**	**2024**	**4.42**

1.10 Equatorial Guinea

1.10.1 All Projections

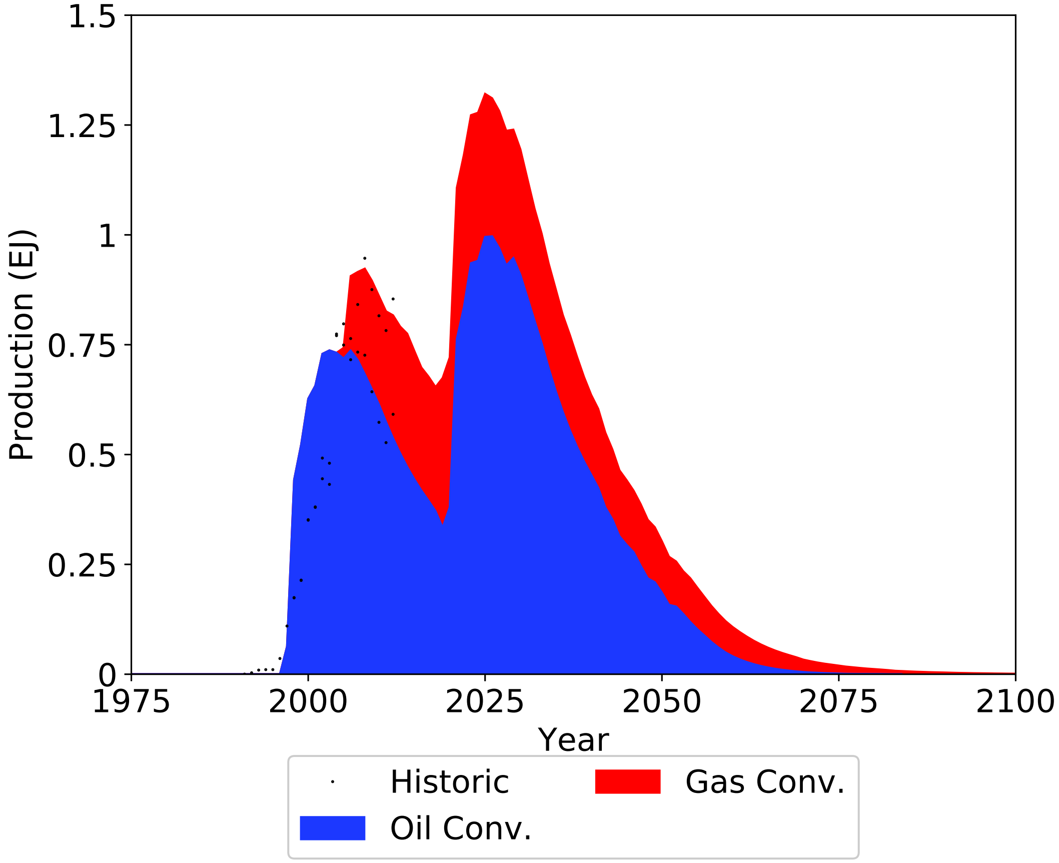

Figure 1.19: Equatorial Guinea projections capped at 16

Table 1.19: Peak years - All

Name	URR	Peak Year	Peak Rate
Oil Conv.	32.71	2026	1.0
Gas Conv.	12.6	2022	0.35
**Total**	**45.31**	**2025**	**1.32**

1.10.2 By Mineral

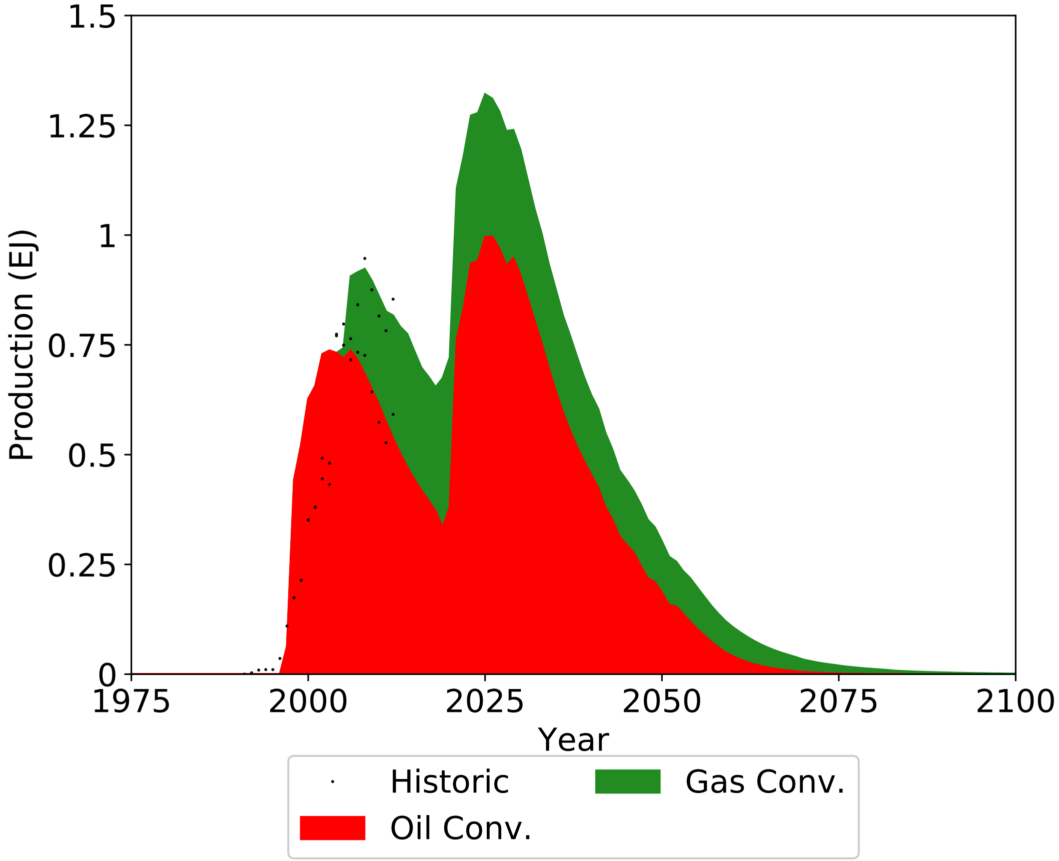

Figure 1.20: Equatorial Guinea projection by mineral type

Table 1.20: Peak years - Minerals

Name	URR	Peak Year	Peak Rate
Oil Conv.	32.71	2026	1.0
Gas Conv.	12.6	2022	0.35
**Total**	**45.31**	**2025**	**1.32**

1.11 Eritrea

1.11.1 All Projections

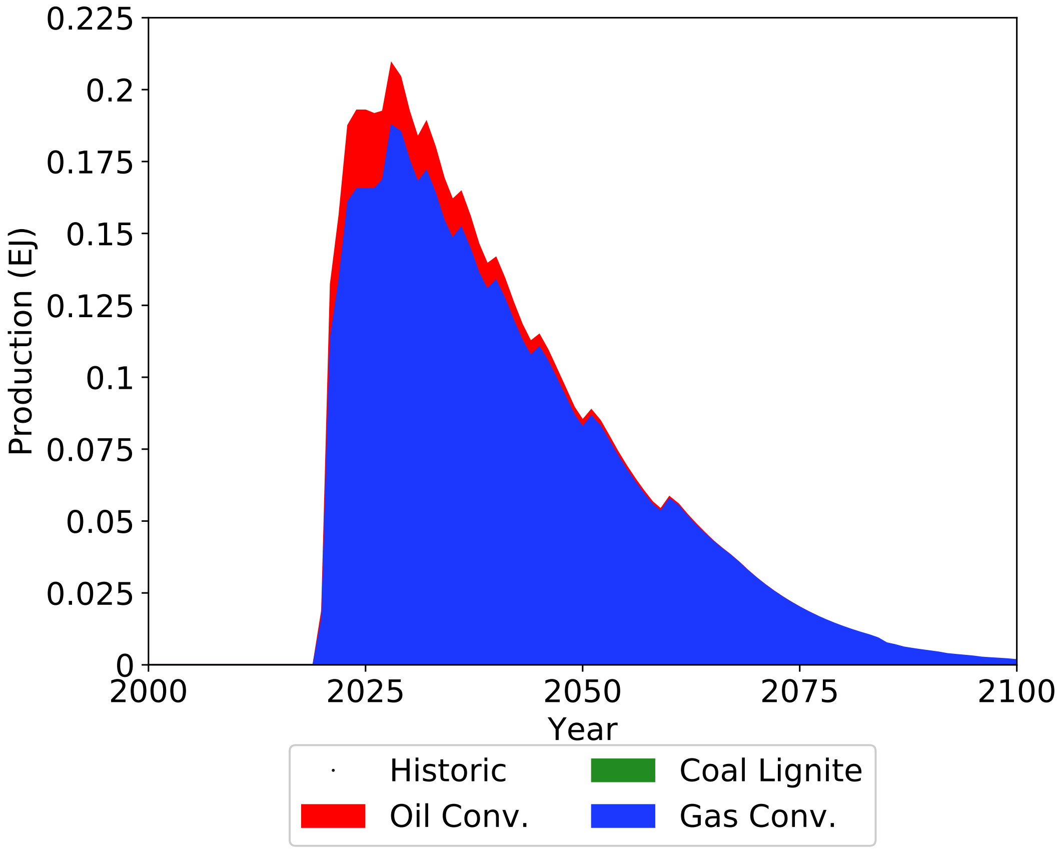

Figure 1.21: Eritrea projections capped at 16

Table 1.21: Peak years - All

Name	URR	Peak Year	Peak Rate
Gas Conv.	5.6	2028	0.19
Oil Conv.	0.42	2024	0.03
Coal Lignite	–	1936	–
**Total**	**6.02**	**2028**	**0.21**

1.11.2 By Mineral

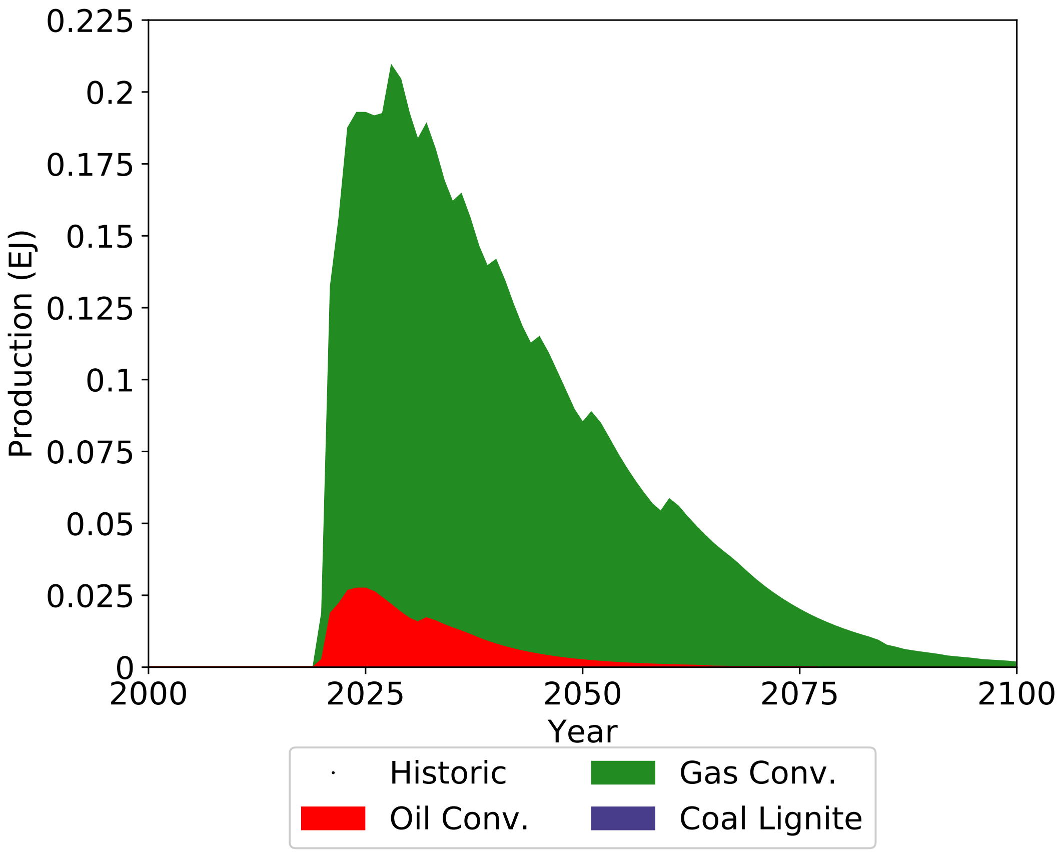

Figure 1.22: Eritrea projection by mineral type

Table 1.22: Peak years - Minerals

**Name**	**URR**	**Peak Year**	**Peak Rate**
Coal Lignite	–	1936	–
Oil Conv.	0.42	2024	0.03
Gas Conv.	5.6	2028	0.19
**Total**	**6.02**	**2028**	**0.21**

1.12 Ethiopia

1.12.1 All Projections

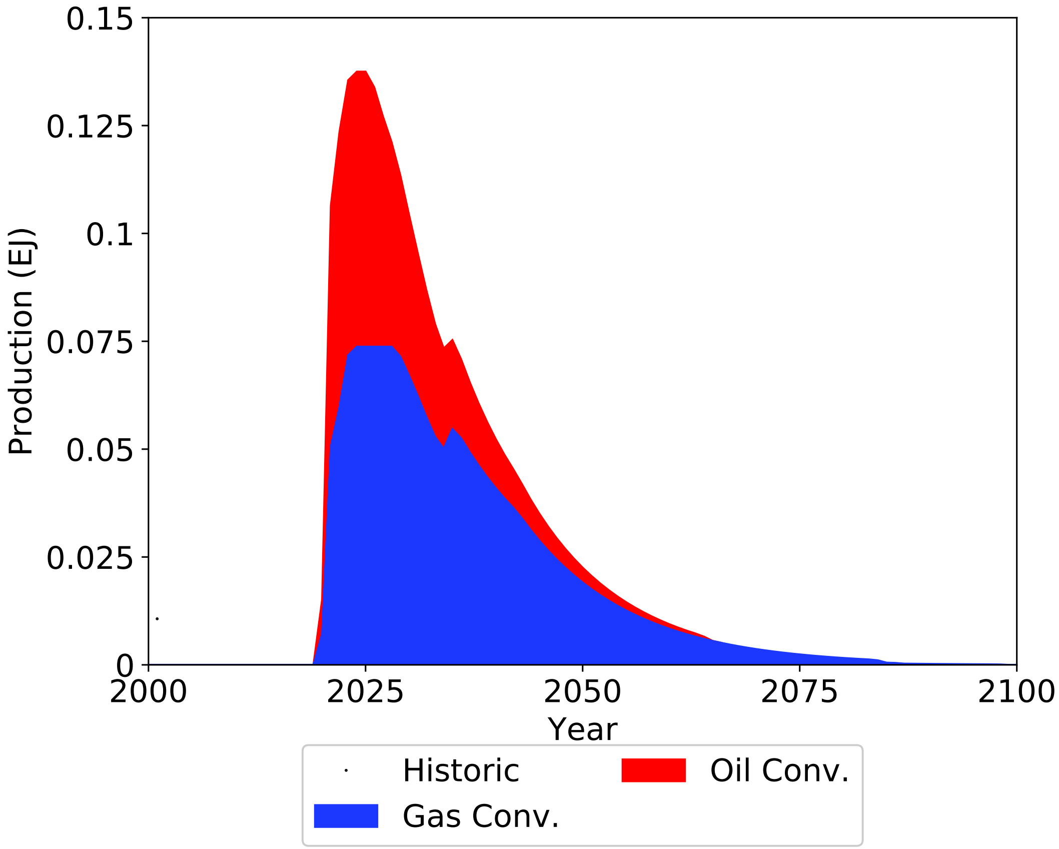

Figure 1.23: Ethiopia projections capped at 16

Table 1.23: Peak years - All

**Name**	**URR**	**Peak Year**	**Peak Rate**
Gas Conv.	1.7	2024	0.07
Oil Conv.	0.85	2022	0.06
**Total**	**2.55**	**2024**	**0.14**

1.12.2 By Mineral

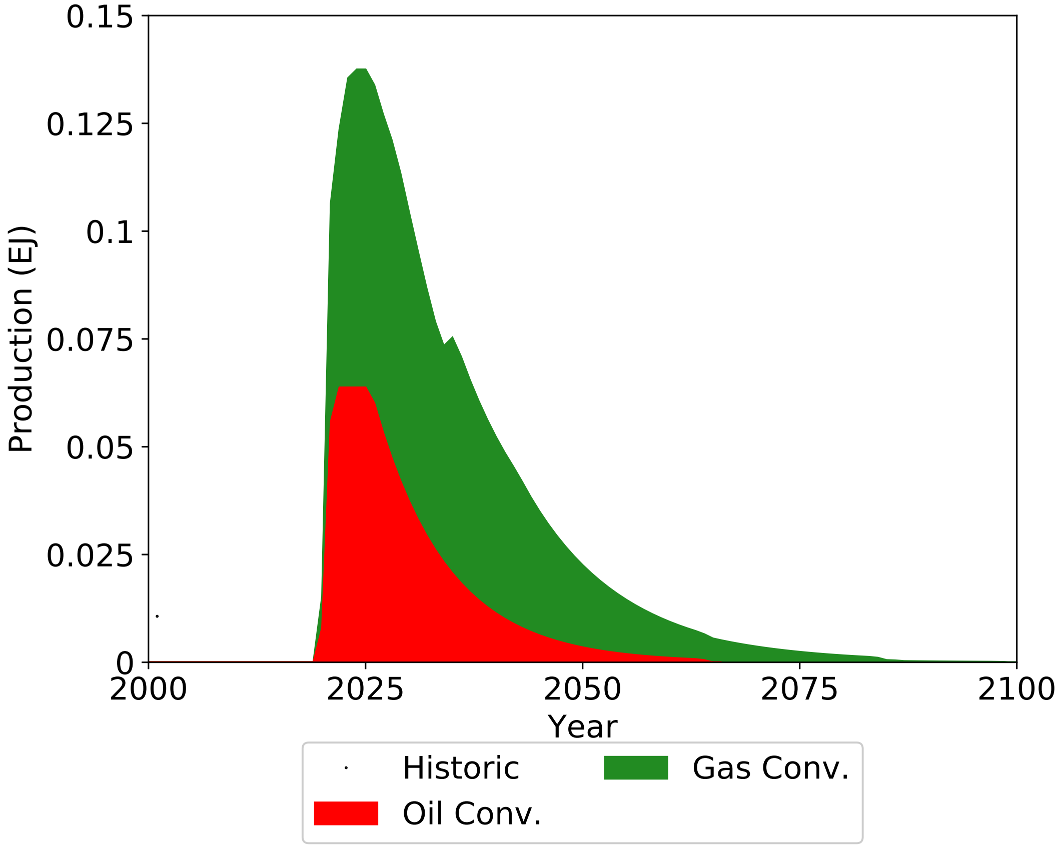

Figure 1.24: Ethiopia projection by mineral type

Table 1.24: Peak years - Minerals

Name	URR	Peak Year	Peak Rate
Oil Conv.	0.85	2022	0.06
Gas Conv.	1.7	2024	0.07
**Total**	**2.55**	**2024**	**0.14**

1.13 Gabon

1.13.1 All Projections

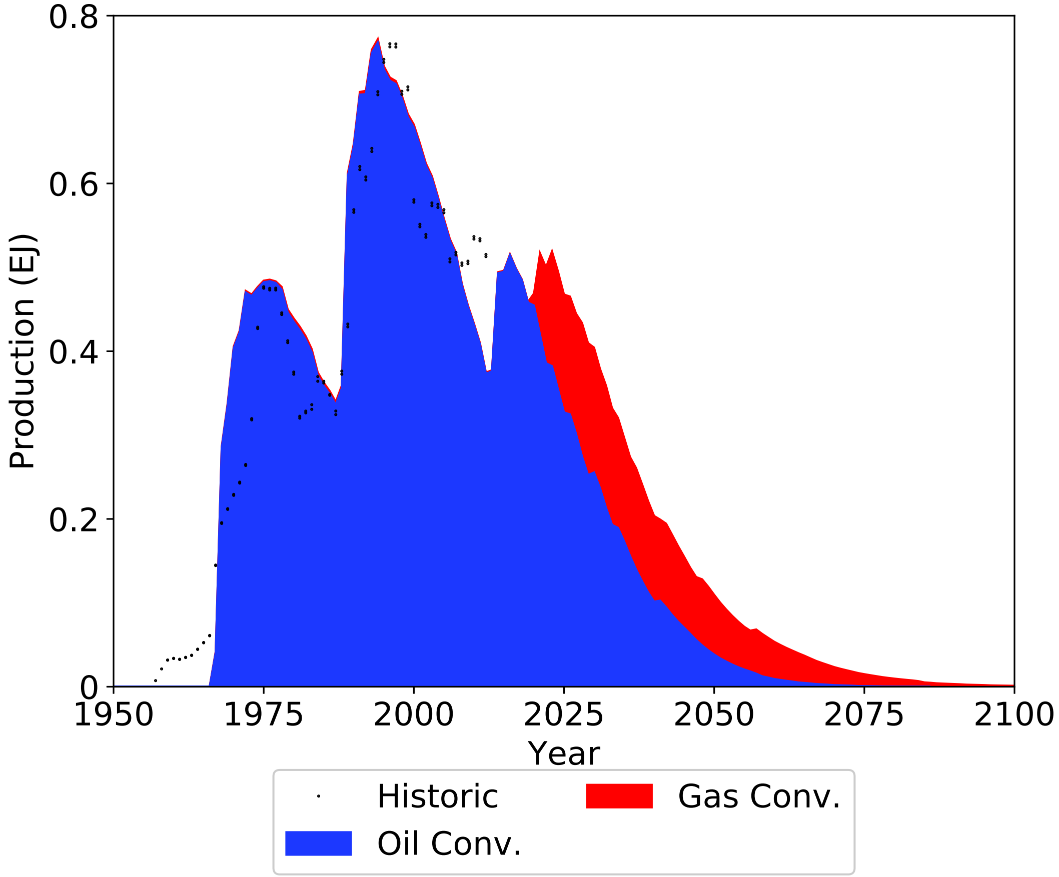

Figure 1.25: Gabon projections capped at 16

Table 1.25: Peak years - All

Name	URR	Peak Year	Peak Rate
Oil Conv.	32.95	1994	0.77
Gas Conv.	4.7	2028	0.16
**Total**	**37.65**	**1994**	**0.77**

1.13.2 By Mineral

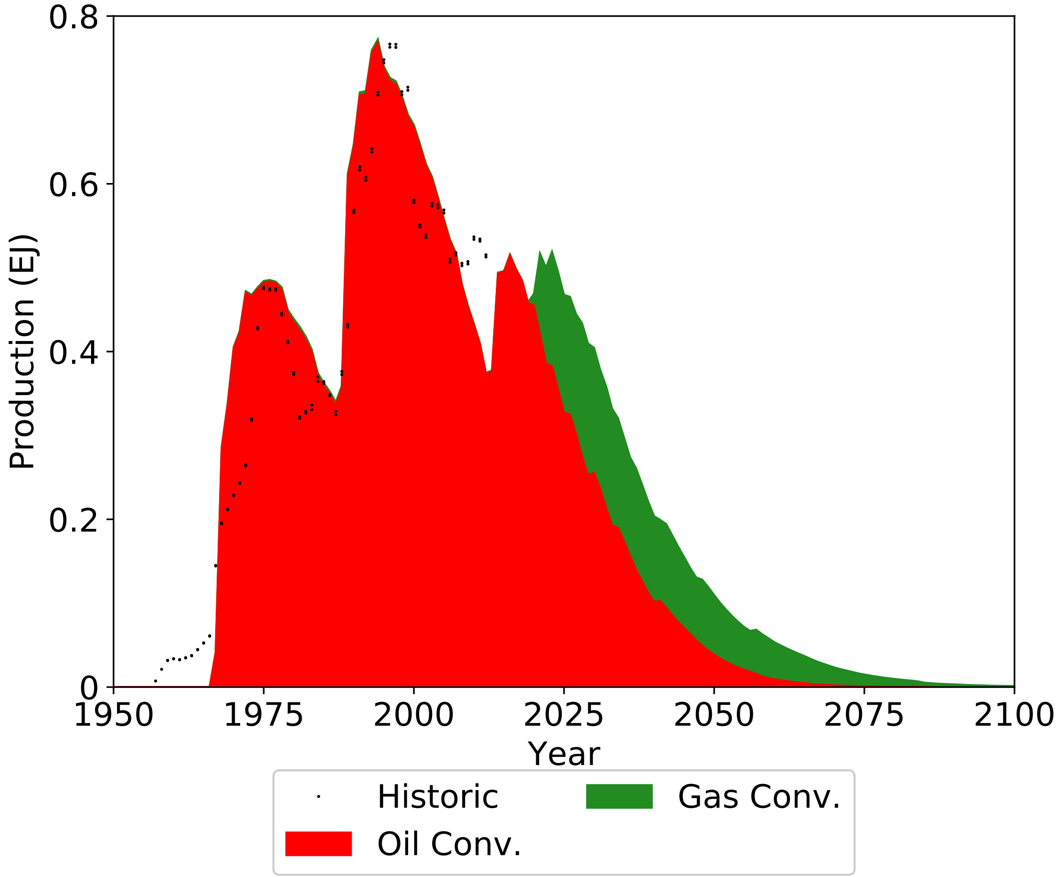

Figure 1.26: Gabon projection by mineral type

Table 1.26: Peak years - Minerals

Name	URR	Peak Year	Peak Rate
Oil Conv.	32.95	1994	0.77
Gas Conv.	4.7	2028	0.16
**Total**	**37.65**	**1994**	**0.77**

1.14 Gambia

1.14.1 All Projections

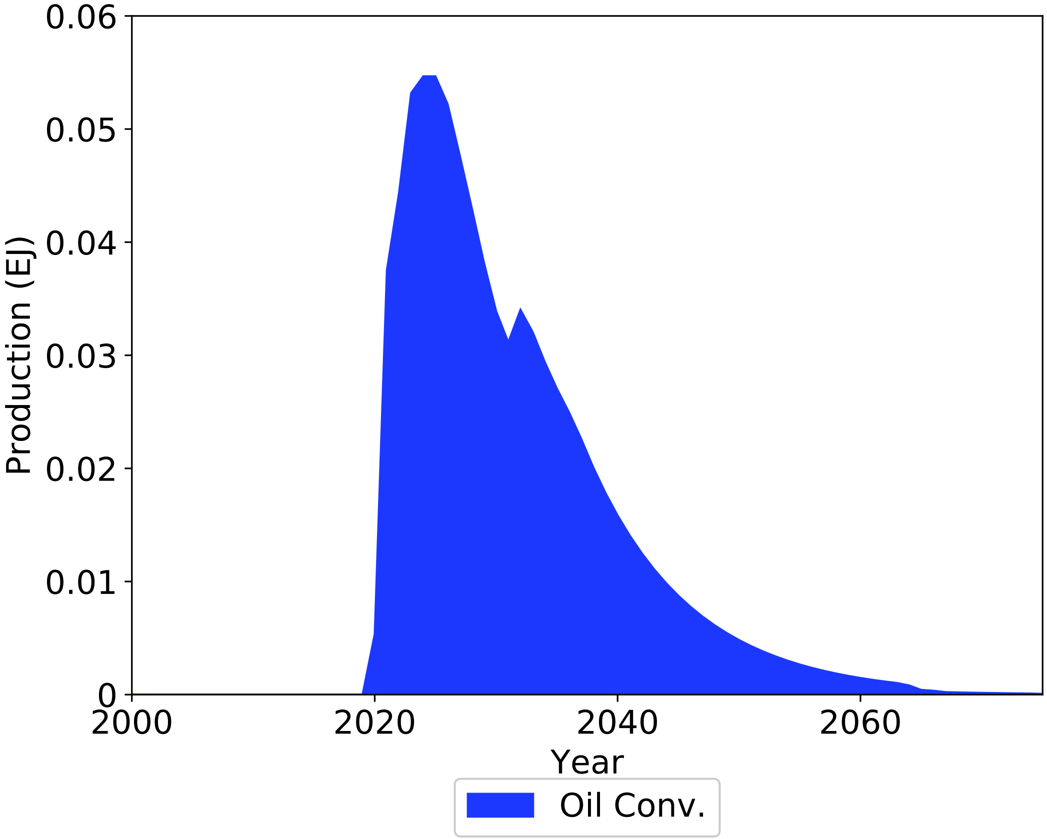

Figure 1.27: Gambia projections capped at 16

Table 1.27: Peak years - All

Name	URR	Peak Year	Peak Rate
Oil Conv.	0.84	2024	0.05
**Total**	**0.84**	**2024**	**0.05**

1.14.2 By Mineral

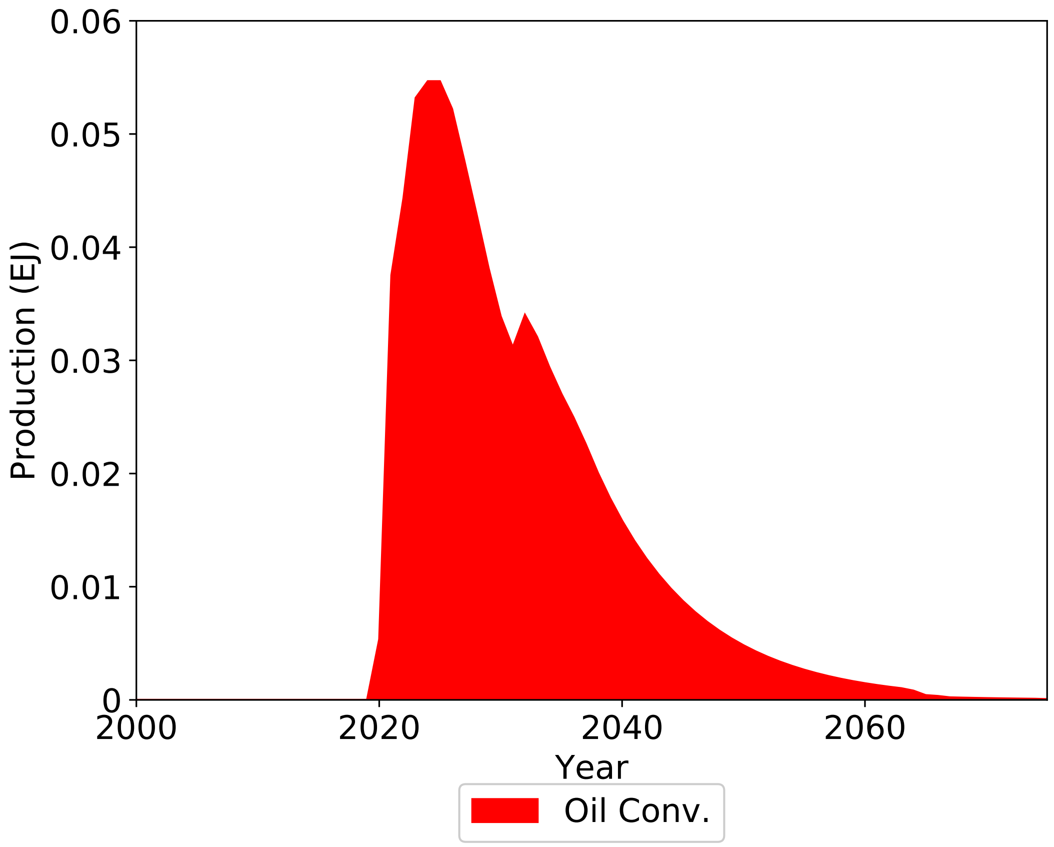

Figure 1.28: Gambia projection by mineral type

Table 1.28: Peak years - Minerals

Name	URR	Peak Year	Peak Rate
Oil Conv.	0.84	2024	0.05
**Total**	**0.84**	**2024**	**0.05**

1.15 Ghana

1.15.1 All Projections

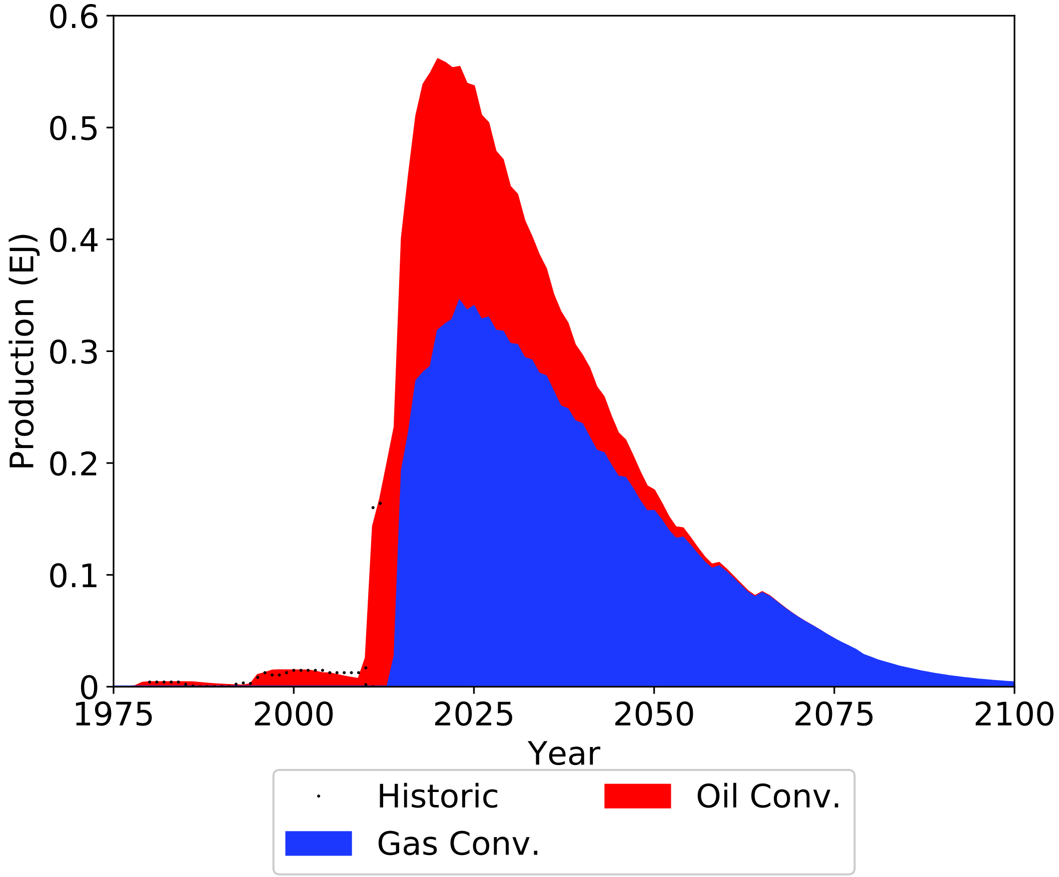

Figure 1.29: Ghana projections capped at 16

Table 1.29: Peak years - All

Name	URR	Peak Year	Peak Rate
Gas Conv.	12.13	2023	0.35
Oil Conv.	5.7	2019	0.26
**Total**	**17.83**	**2020**	**0.56**

1.15.2 By Mineral

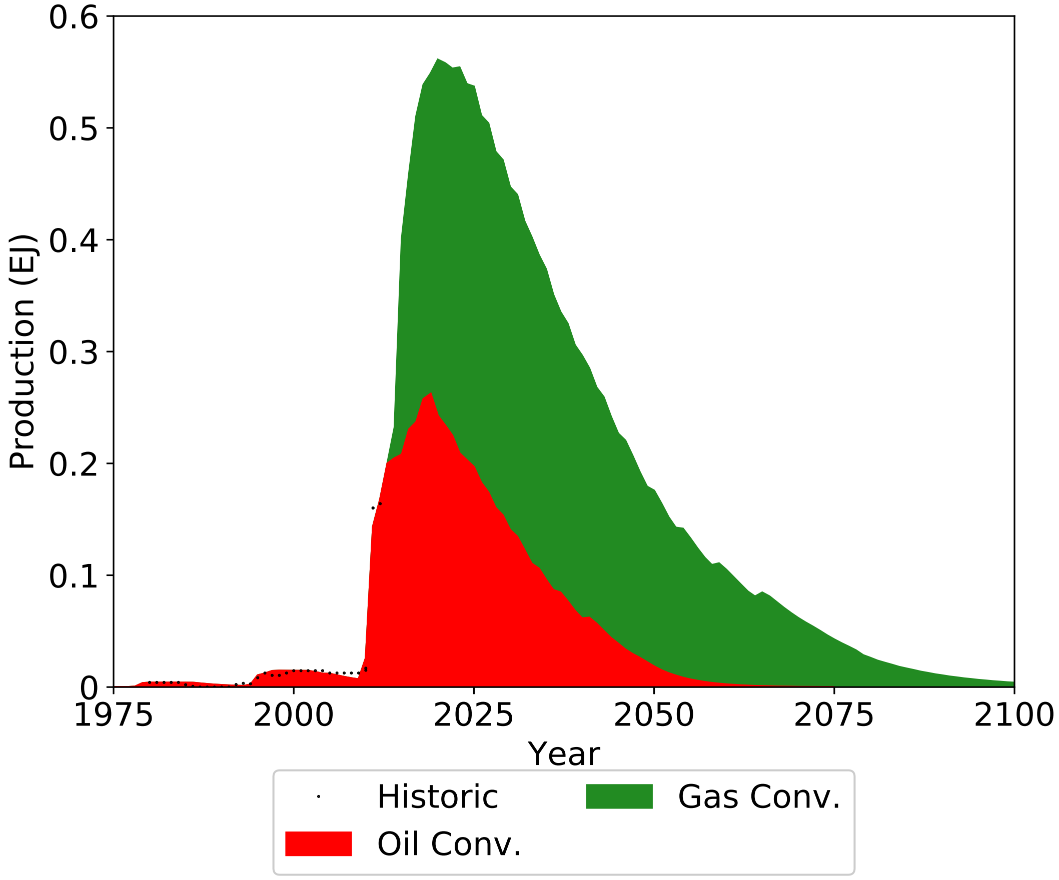

Figure 1.30: Ghana projection by mineral type

Table 1.30: Peak years - Minerals

**Name**	**URR**	**Peak Year**	**Peak Rate**
Oil Conv.	5.7	2019	0.26
Gas Conv.	12.13	2023	0.35
**Total**	**17.83**	**2020**	**0.56**

1.16 Guinea

1.16.1 All Projections

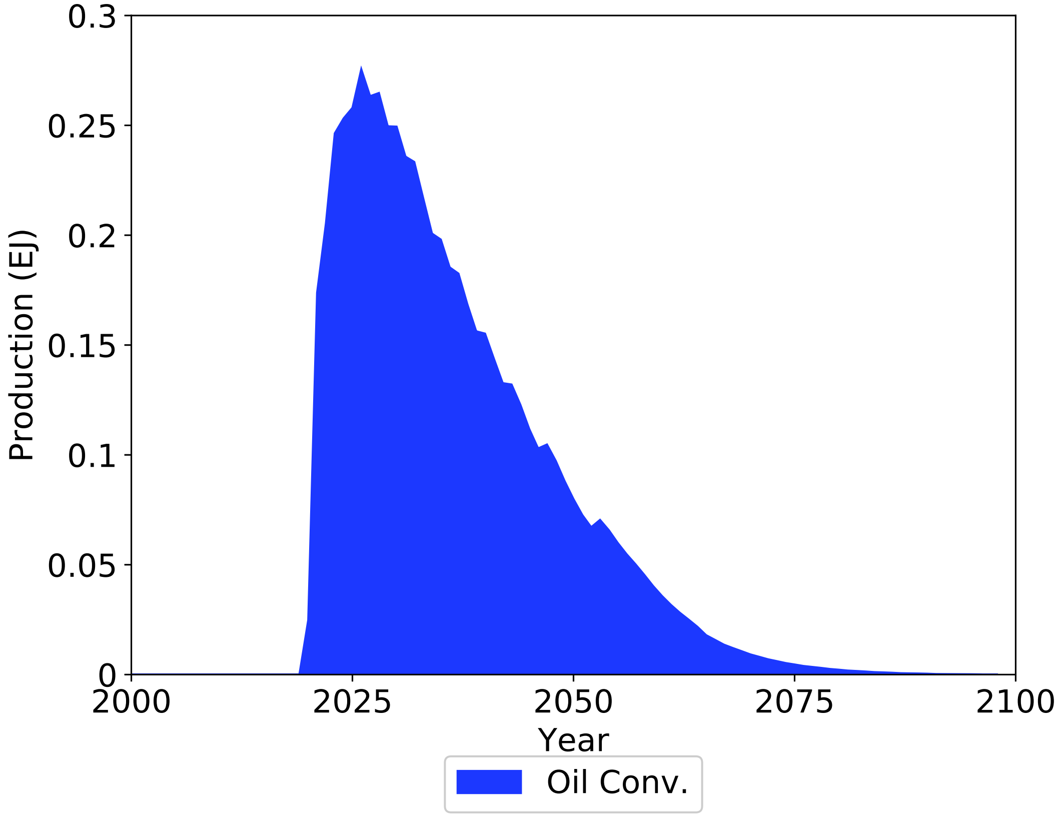

Figure 1.31: Guinea projections capped at 16

Table 1.31: Peak years - All

Name	URR	Peak Year	Peak Rate
Oil Conv.	6.32	2026	0.28
**Total**	**6.32**	**2026**	**0.28**

1.16.2 By Mineral

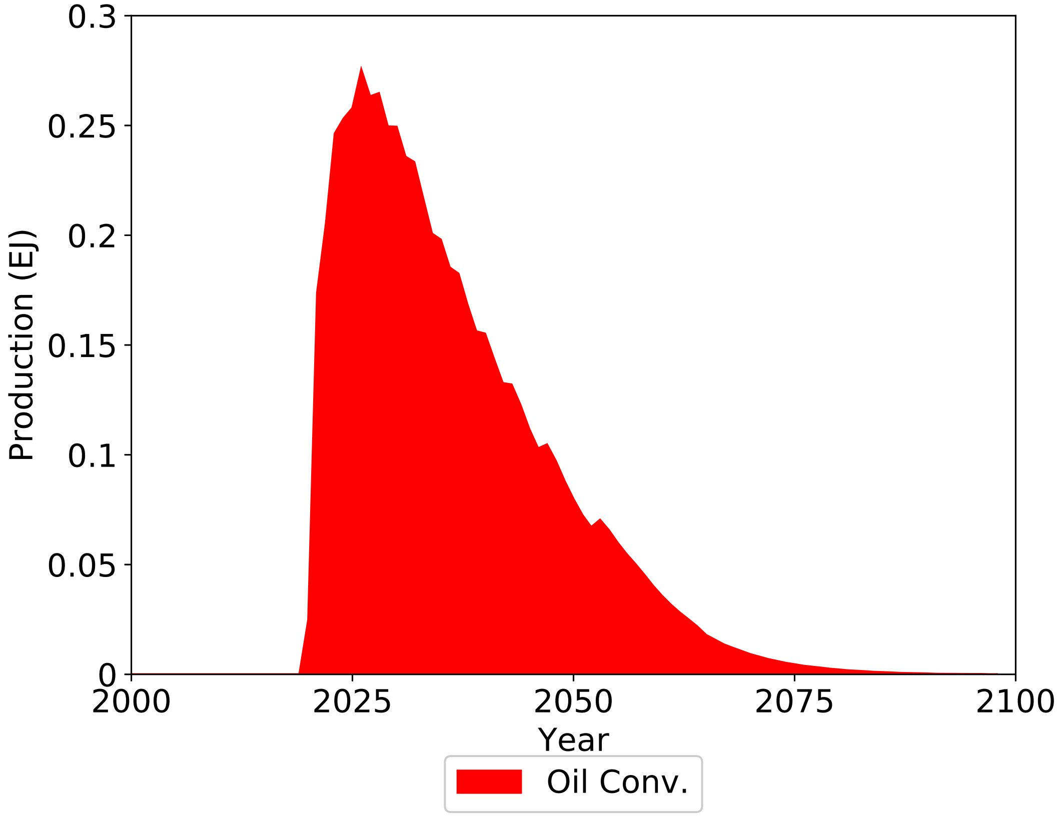

Figure 1.32: Guinea projection by mineral type

Table 1.32: Peak years - Minerals

Name	URR	Peak Year	Peak Rate
Oil Conv.	6.32	2026	0.28
**Total**	**6.32**	**2026**	**0.28**

1.17 Guinea-Bissau

1.17.1 All Projections

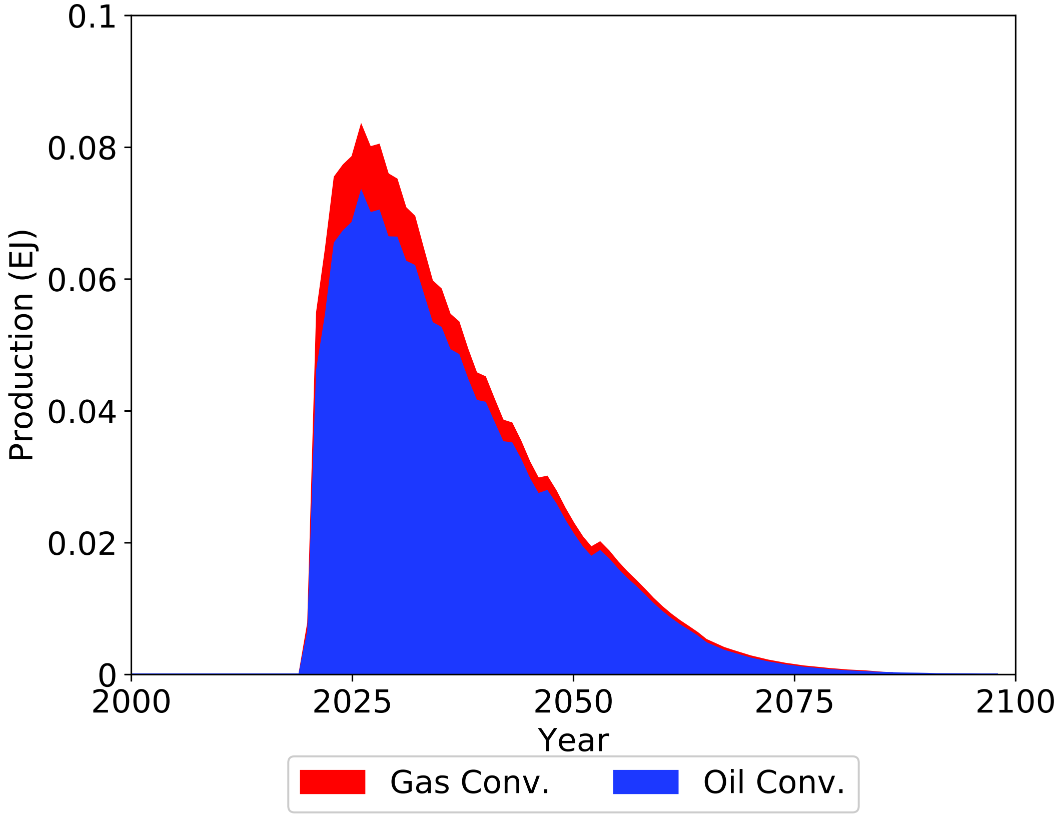

Figure 1.33: Guinea-Bissau projections capped at 16

Table 1.33: Peak years - All

Name	URR	Peak Year	Peak Rate
Oil Conv.	1.68	2026	0.07
Gas Conv.	0.2	2022	0.01
**Total**	**1.88**	**2026**	**0.08**

1.17.2 By Mineral

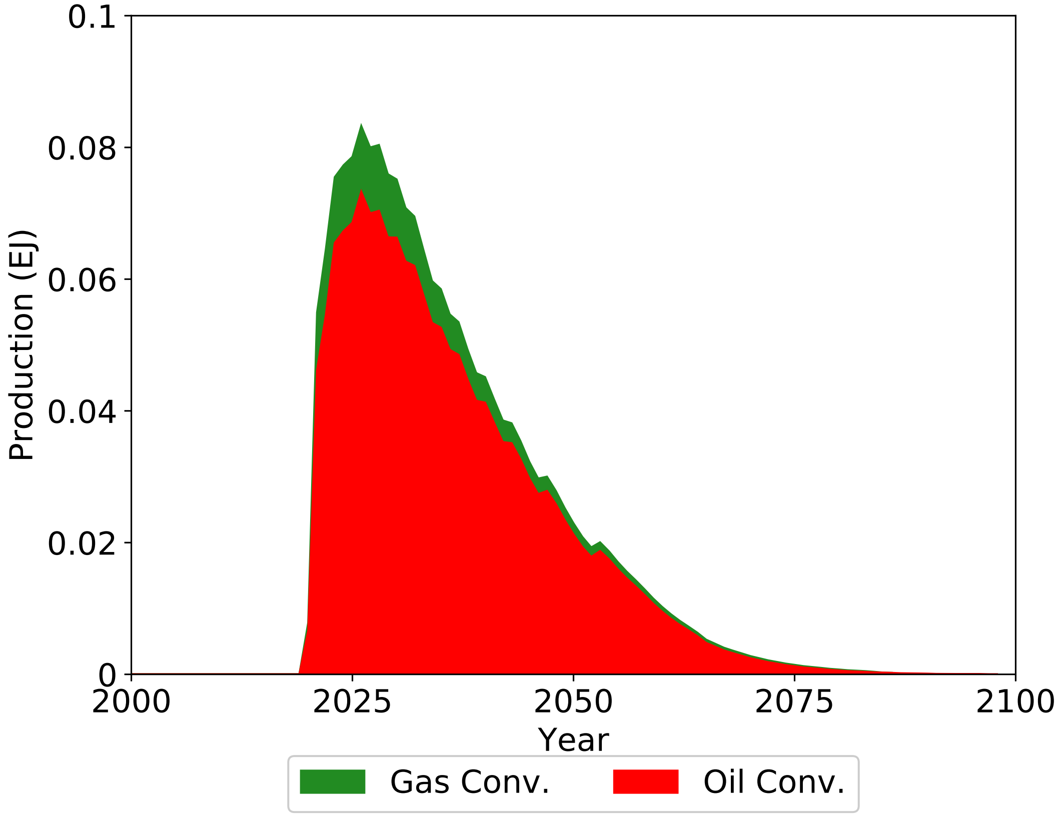

Figure 1.34: Guinea-Bissau projection by mineral type

Table 1.34: Peak years - Minerals

Name	URR	Peak Year	Peak Rate
Oil Conv.	1.68	2026	0.07
Gas Conv.	0.2	2022	0.01
**Total**	**1.88**	**2026**	**0.08**

1.18 Ivory Coast

1.18.1 All Projections

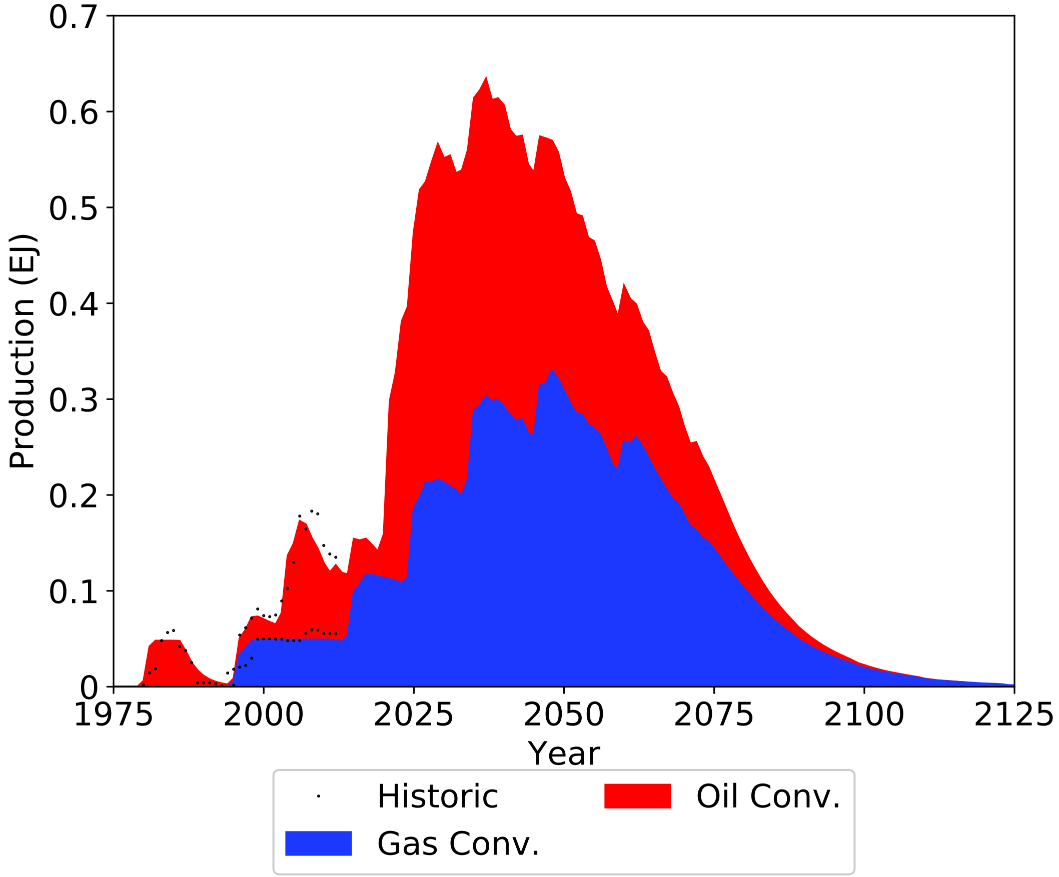

Figure 1.35: Ivory Coast projections capped at 16

Table 1.35: Peak years - All

Name	URR	Peak Year	Peak Rate
Gas Conv.	16.23	2048	0.33
Oil Conv.	14.7	2029	0.35
**Total**	**30.93**	**2037**	**0.63**

1.18.2 By Mineral

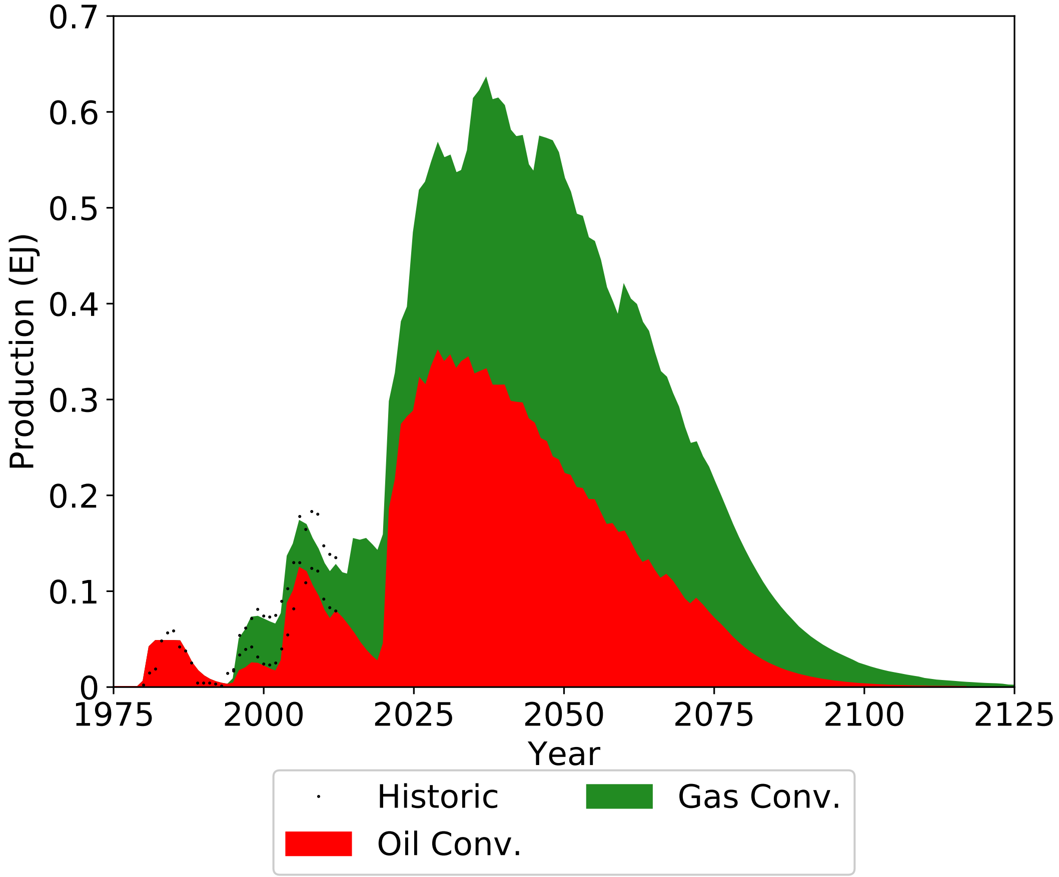

Figure 1.36: Ivory Coast projection by mineral type

Table 1.36: Peak years - Minerals

Name	URR	Peak Year	Peak Rate
Oil Conv.	14.7	2029	0.35
Gas Conv.	16.23	2048	0.33
**Total**	**30.93**	**2037**	**0.63**

1.19 Kenya

1.19.1 All Projections

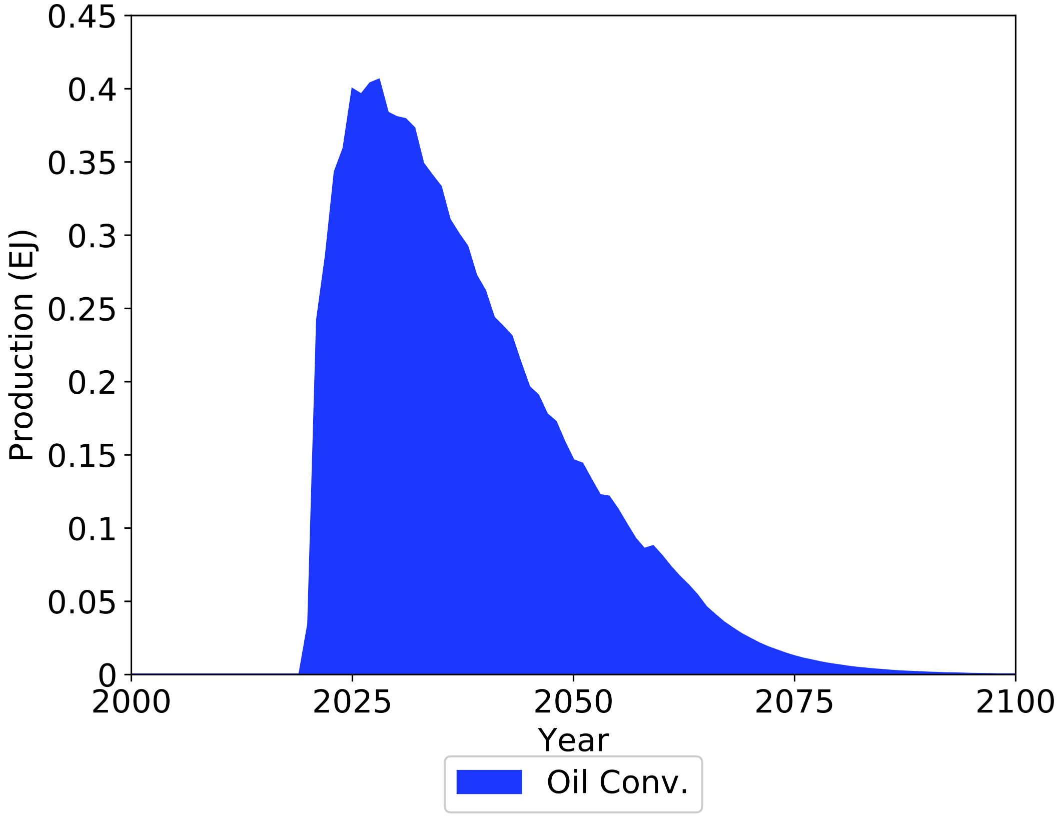

Figure 1.37: Kenya projections capped at 16

Table 1.37: Peak years - All

Name	URR	Peak Year	Peak Rate
Oil Conv.	10.53	2028	0.41
**Total**	**10.53**	**2028**	**0.41**

1.19.2 By Mineral

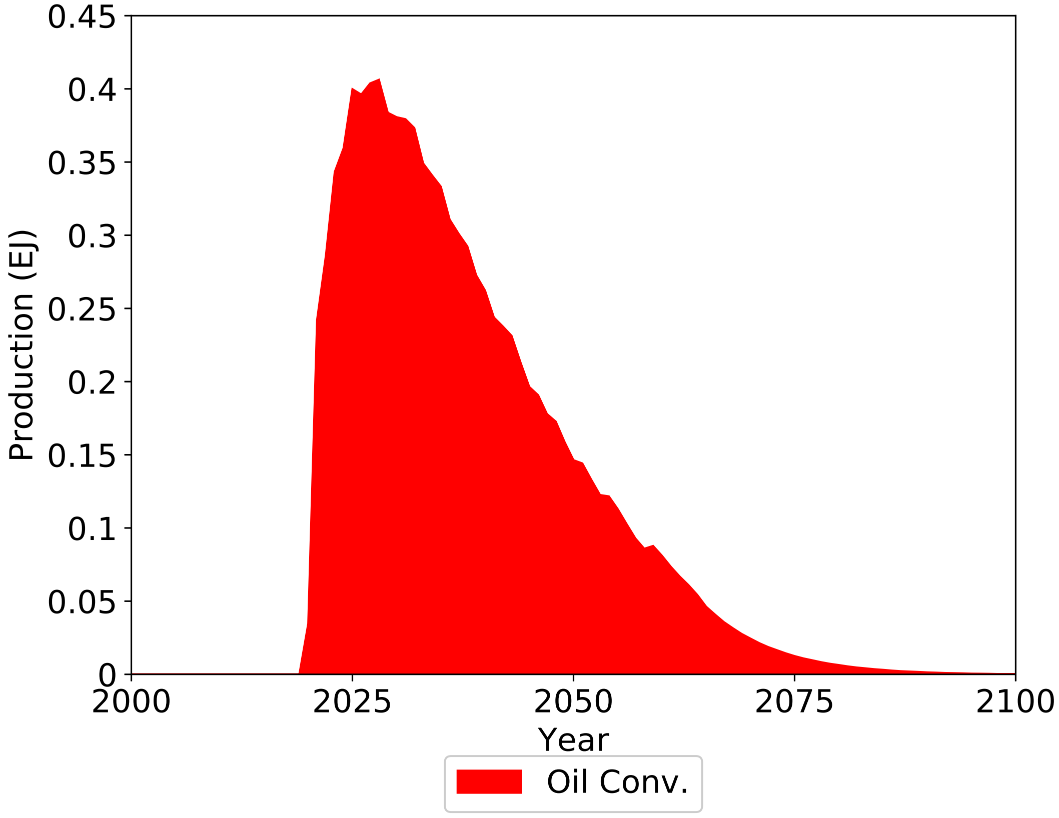

Figure 1.38: Kenya projection by mineral type

Table 1.38: Peak years - Minerals

Name	URR	Peak Year	Peak Rate
Oil Conv.	10.53	2028	0.41
**Total**	**10.53**	**2028**	**0.41**

1.20 Liberia

1.20.1 All Projections

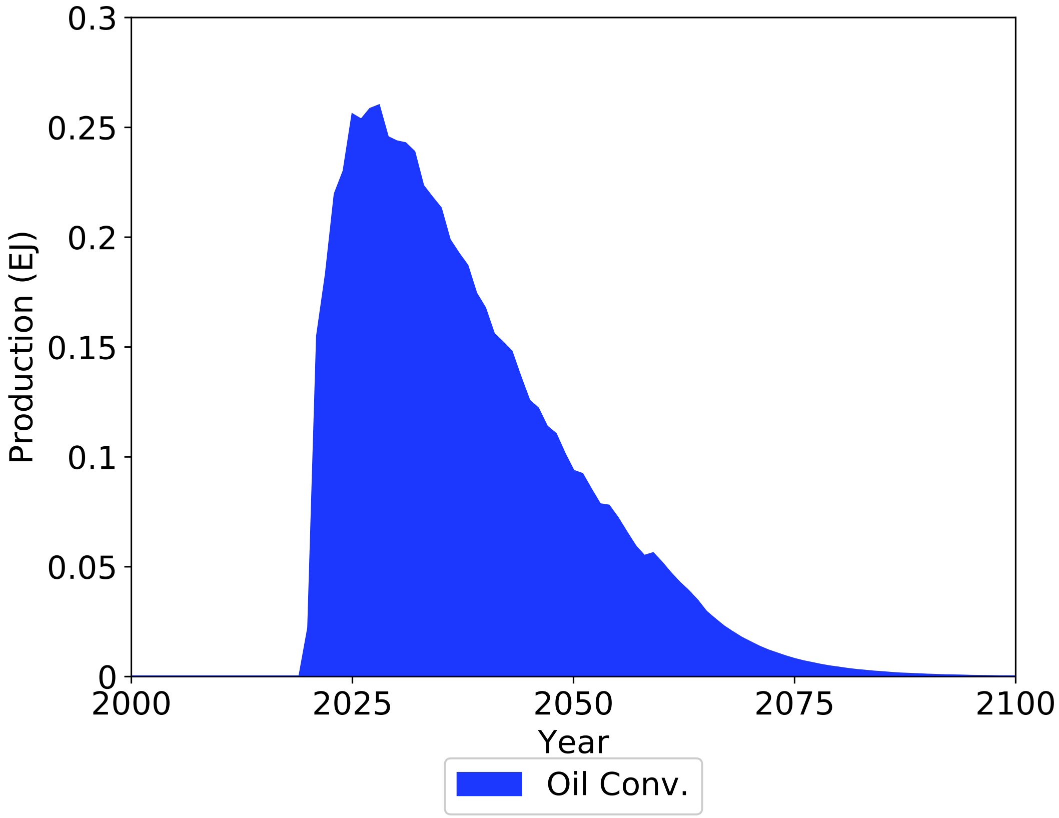

Figure 1.39: Liberia projections capped at 16

Table 1.39: Peak years - All

Name	URR	Peak Year	Peak Rate
Oil Conv.	6.74	2028	0.26
**Total**	**6.74**	**2028**	**0.26**

1.20.2 By Mineral

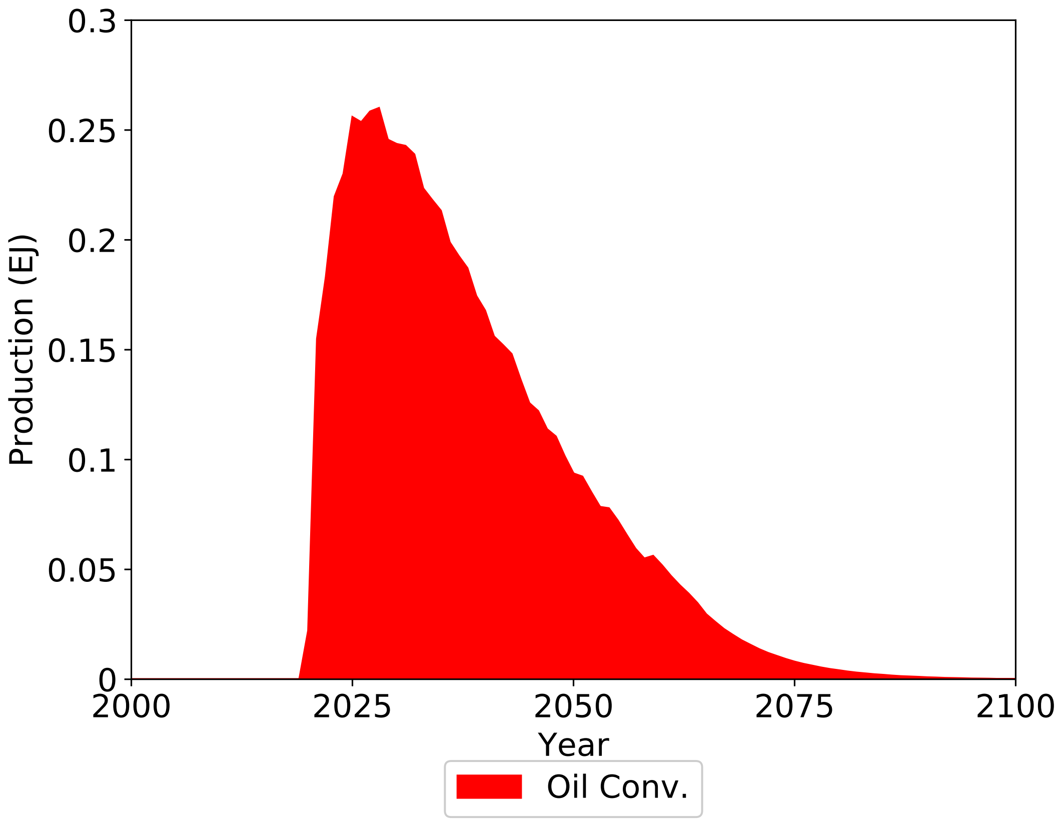

Figure 1.40: Liberia projection by mineral type

Table 1.40: Peak years - Minerals

Name	URR	Peak Year	Peak Rate
Oil Conv.	6.74	2028	0.26
**Total**	**6.74**	**2028**	**0.26**

1.21 Libya

1.21.1 All Projections

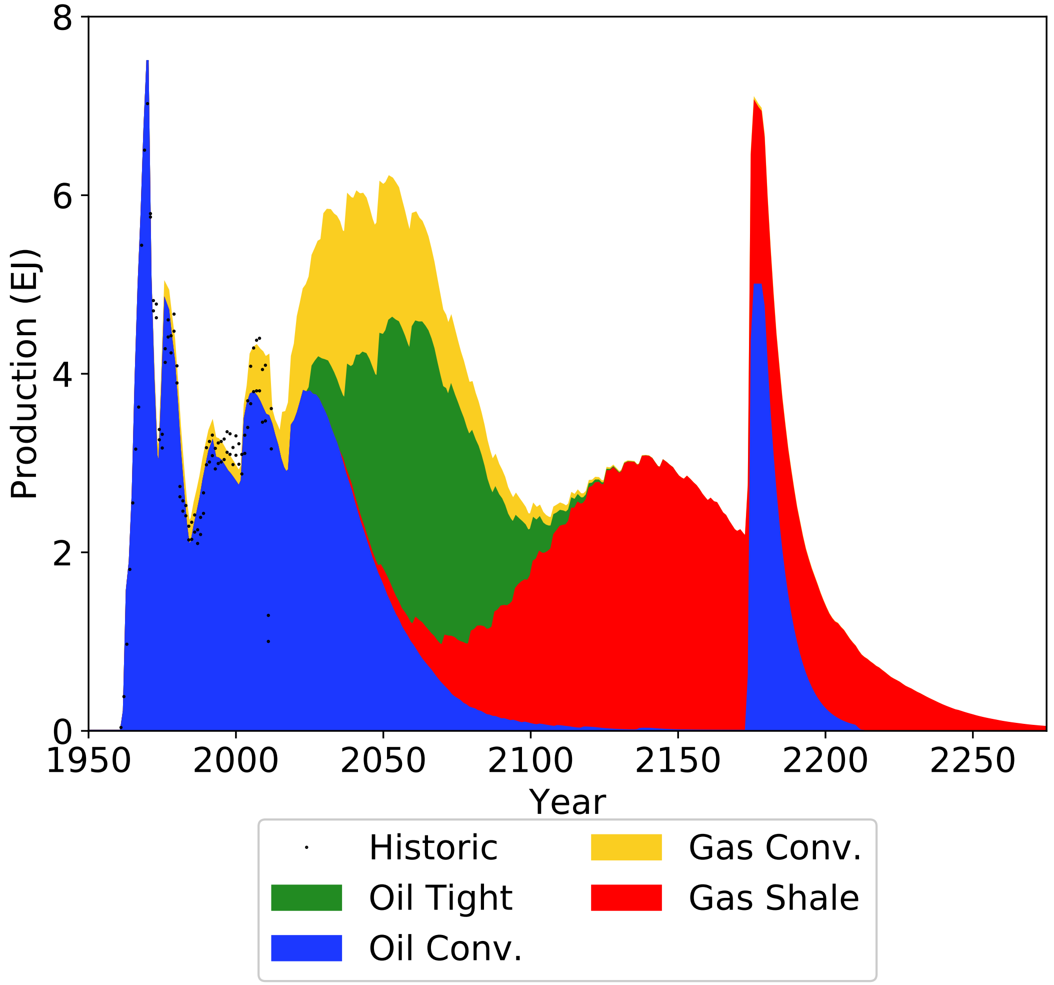

Figure 1.41: Libya projections capped at 16

Table 1.41: Peak years - All

Name	URR	Peak Year	Peak Rate
Oil Conv.	368.66	1970	7.51
Gas Shale	304.54	2139	3.05
Oil Tight	149.55	2064	3.37
Gas Conv.	105.0	2038	1.91
**Total**	**927.75**	**1970**	**7.51**

1.21.2 By Mineral

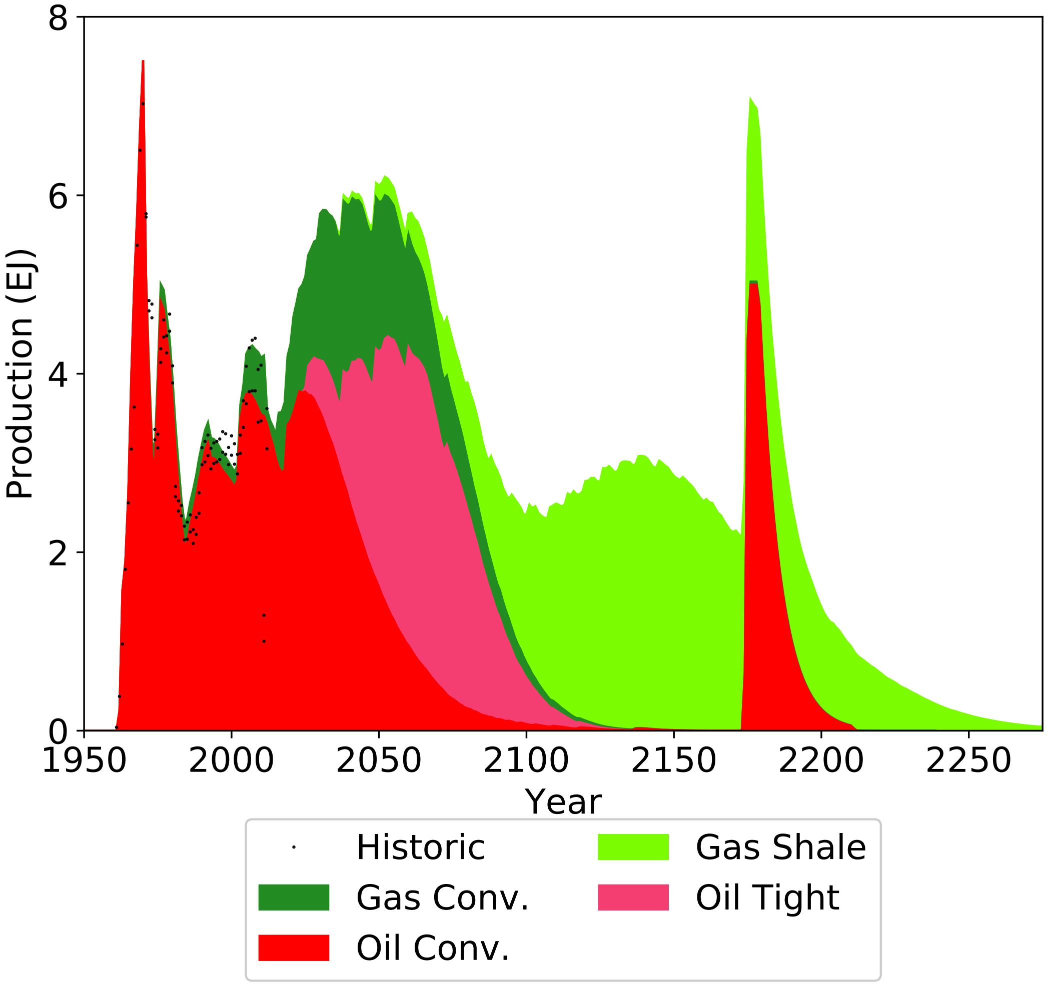

Figure 1.42: Libya projection by mineral type

Table 1.42: Peak years - Minerals

**Name**	**URR**	**Peak Year**	**Peak Rate**
Oil Conv.	368.66	1970	7.51
Oil Tight	149.55	2064	3.37
Gas Conv.	105.0	2038	1.91
Gas Shale	304.54	2139	3.05
**Total**	**927.75**	**1970**	**7.51**

1.22 Madagascar

1.22.1 All Projections

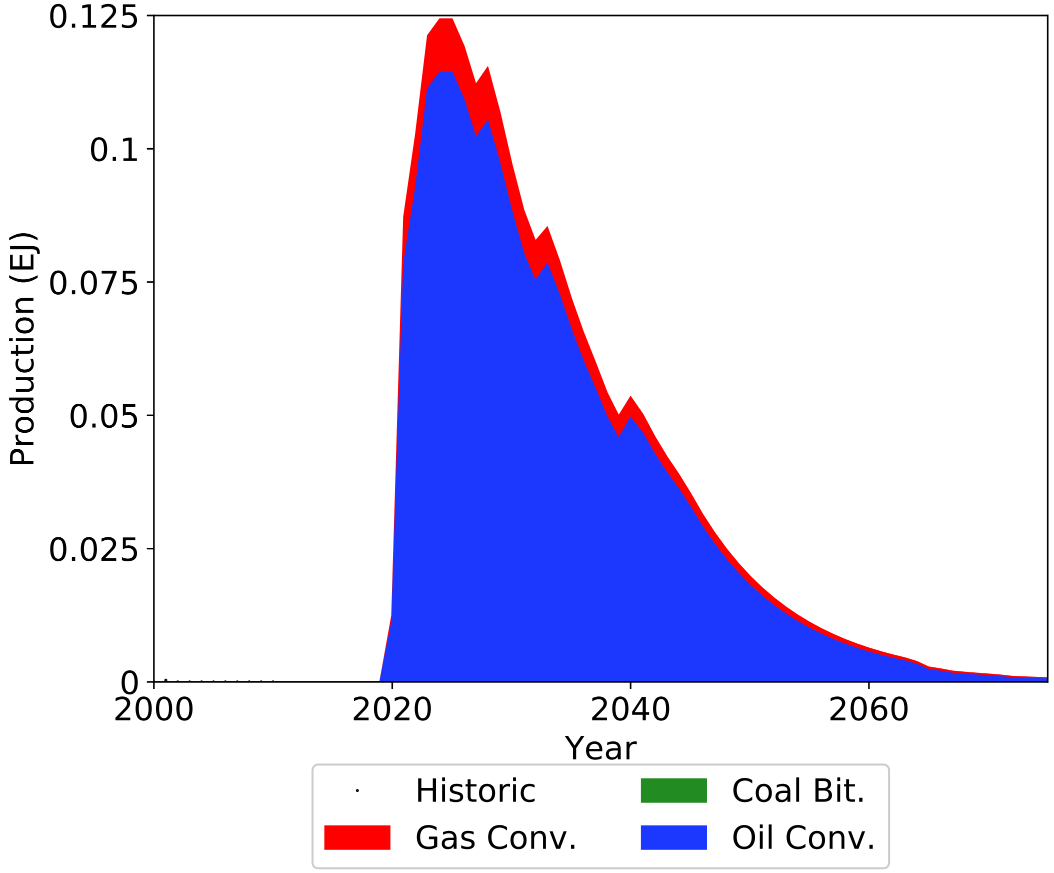

Figure 1.43: Madagascar projections capped at 16

Table 1.43: Peak years - All

Name	URR	Peak Year	Peak Rate
Oil Conv.	2.1	2024	0.11
Gas Conv.	0.2	2022	0.01
Coal Bit.	–	1945	–
**Total**	**2.3**	**2024**	**0.12**

1.22.2 By Mineral

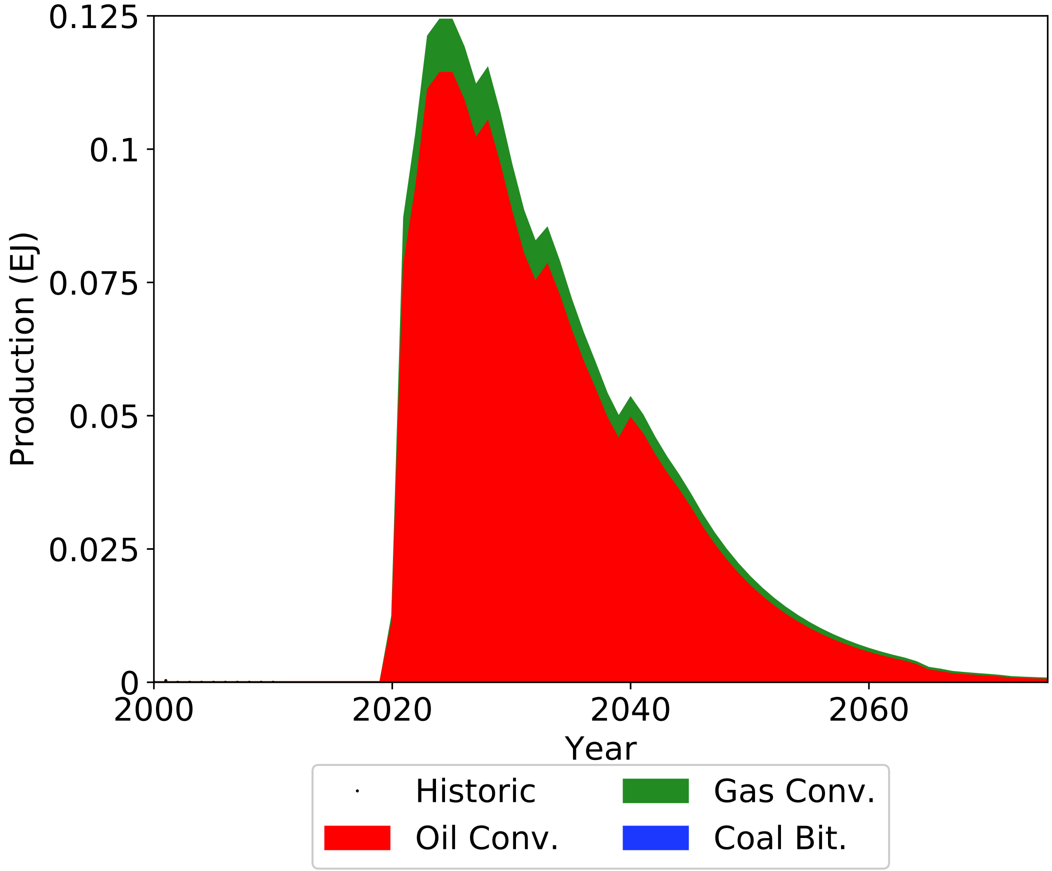

Figure 1.44: Madagascar projection by mineral type

Table 1.44: Peak years - Minerals

Name	URR	Peak Year	Peak Rate
Coal Bit.	–	1945	–
Oil Conv.	2.1	2024	0.11
Gas Conv.	0.2	2022	0.01
**Total**	**2.3**	**2024**	**0.12**

1.23 Malawi

1.23.1 All Projections

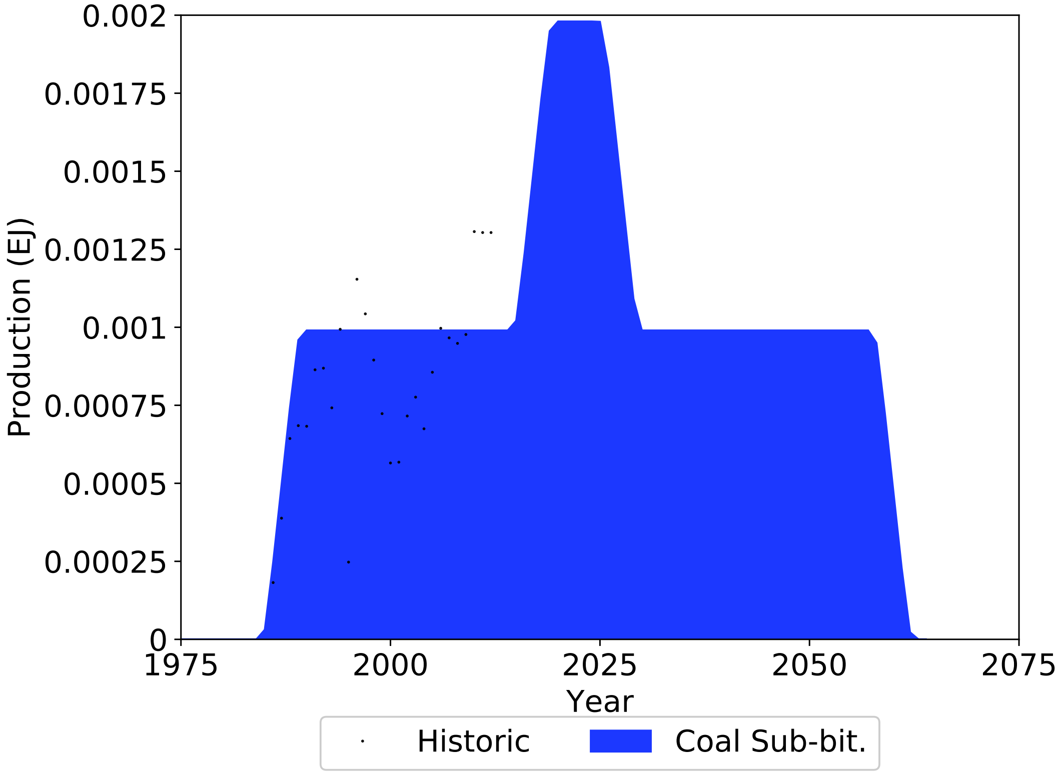

Figure 1.45: Malawi projections capped at 16

Table 1.45: Peak years - All

Name	URR	Peak Year	Peak Rate
Coal Sub-bit.	0.08	2020	–
**Total**	**0.08**	**2020**	–

1.23.2 By Mineral

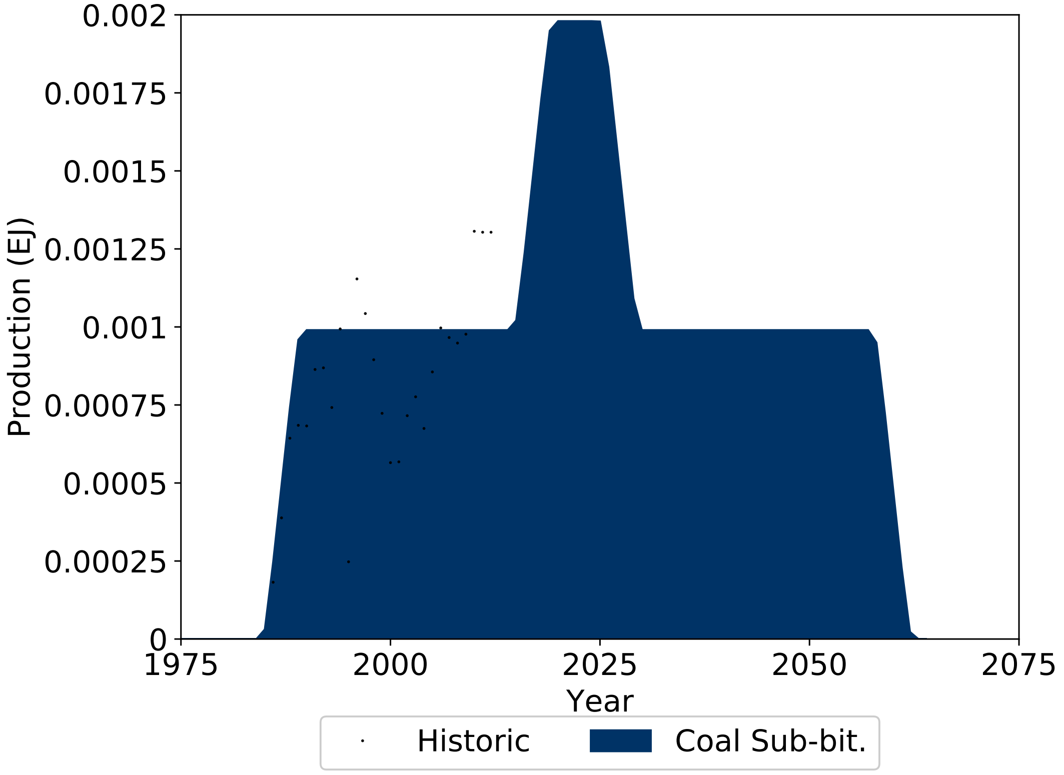

Figure 1.46: Malawi projection by mineral type

Table 1.46: Peak years - Minerals

Name	URR	Peak Year	Peak Rate
Coal Sub-bit.	0.08	2020	–
**Total**	**0.08**	**2020**	–

1.24 Mauritania

1.24.1 All Projections

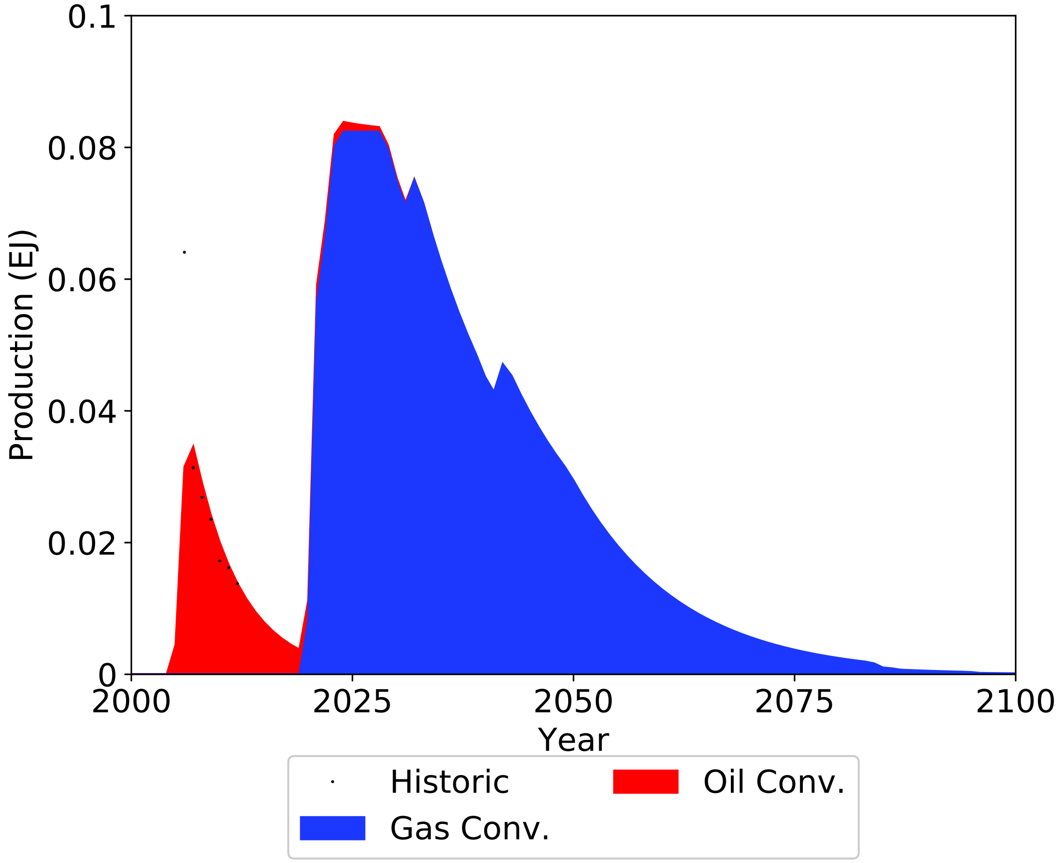

Figure 1.47: Mauritania projections capped at 16

Table 1.47: Peak years - All

Name	URR	Peak Year	Peak Rate
Gas Conv.	2.1	2024	0.08
Oil Conv.	0.24	2007	0.03
**Total**	**2.34**	**2024**	**0.08**

1.24.2 By Mineral

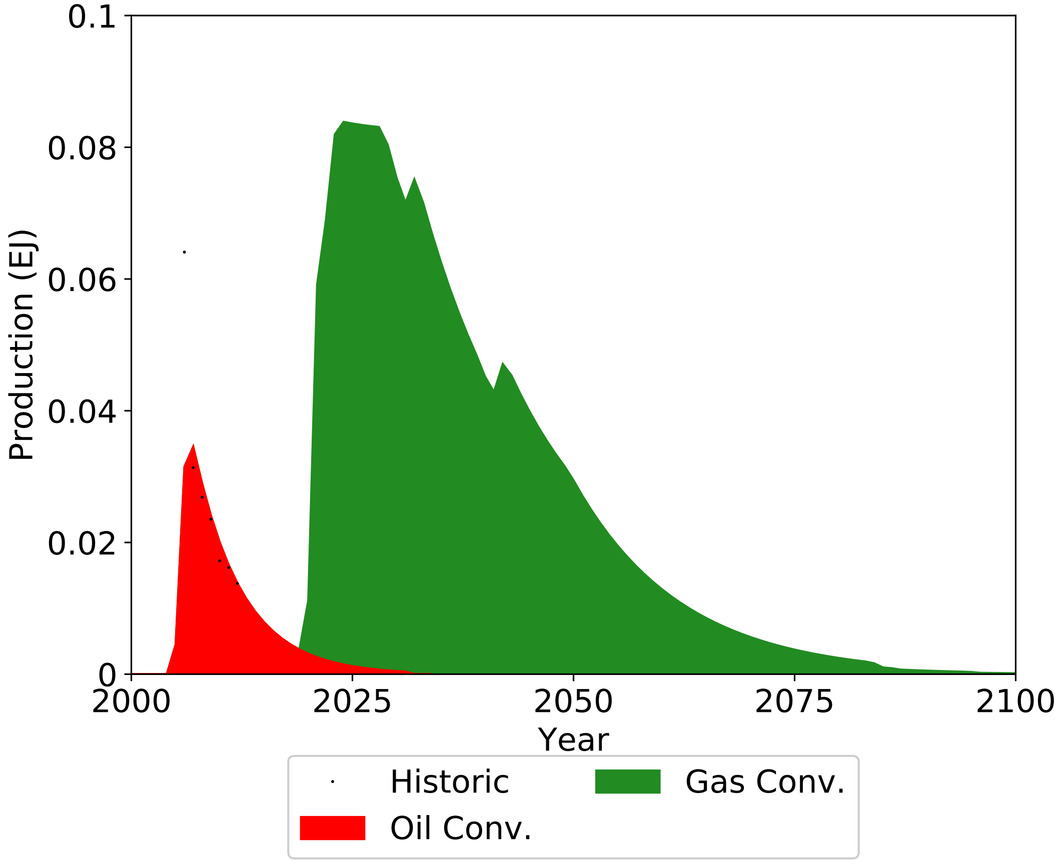

Figure 1.48: Mauritania projection by mineral type

Table 1.48: Peak years - Minerals

Name	URR	Peak Year	Peak Rate
Oil Conv.	0.24	2007	0.03
Gas Conv.	2.1	2024	0.08
**Total**	**2.34**	**2024**	**0.08**

1.25 Morocco

1.25.1 All Projections

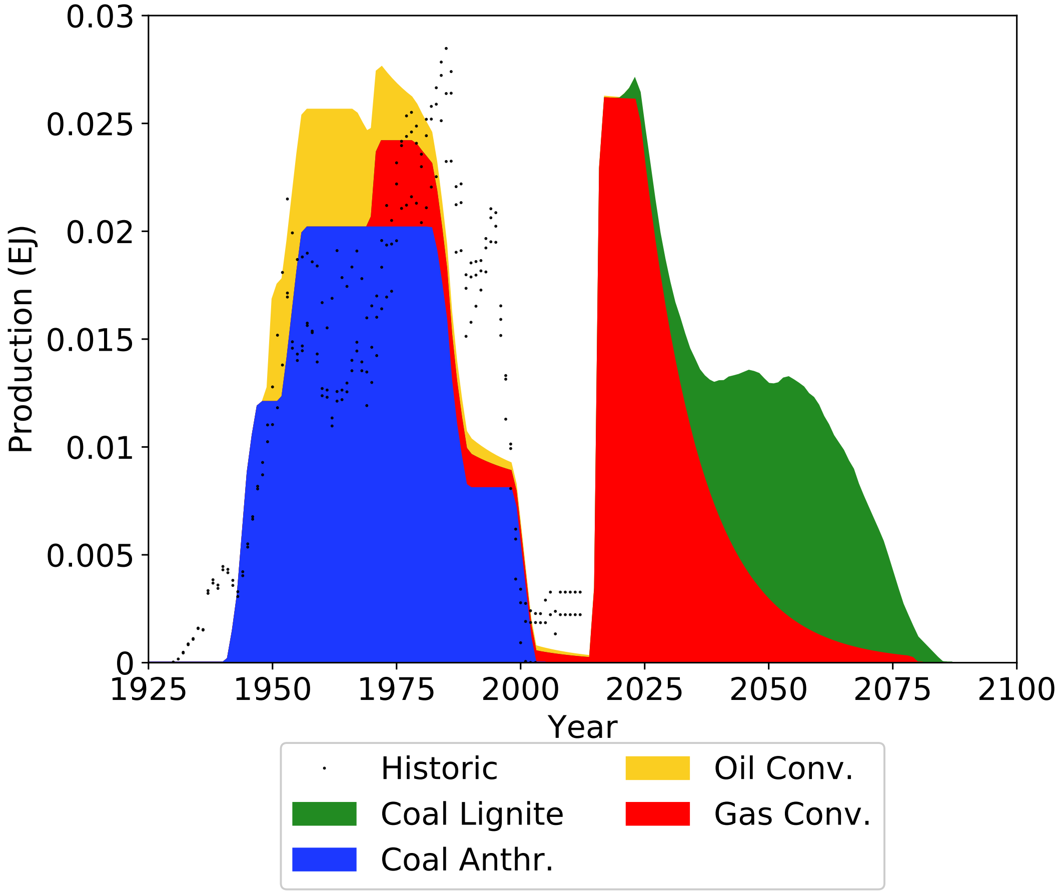

Figure 1.49: Morocco projections capped at 16

Table 1.49: Peak years - All

Name	URR	Peak Year	Peak Rate
Coal Anthr.	0.88	1957	0.02
Gas Conv.	0.6	2017	0.03
Coal Lignite	0.38	2055	0.01
Oil Conv.	0.16	1951	0.01
**Total**	**2.02**	**1972**	**0.03**

1.25.2 By Mineral

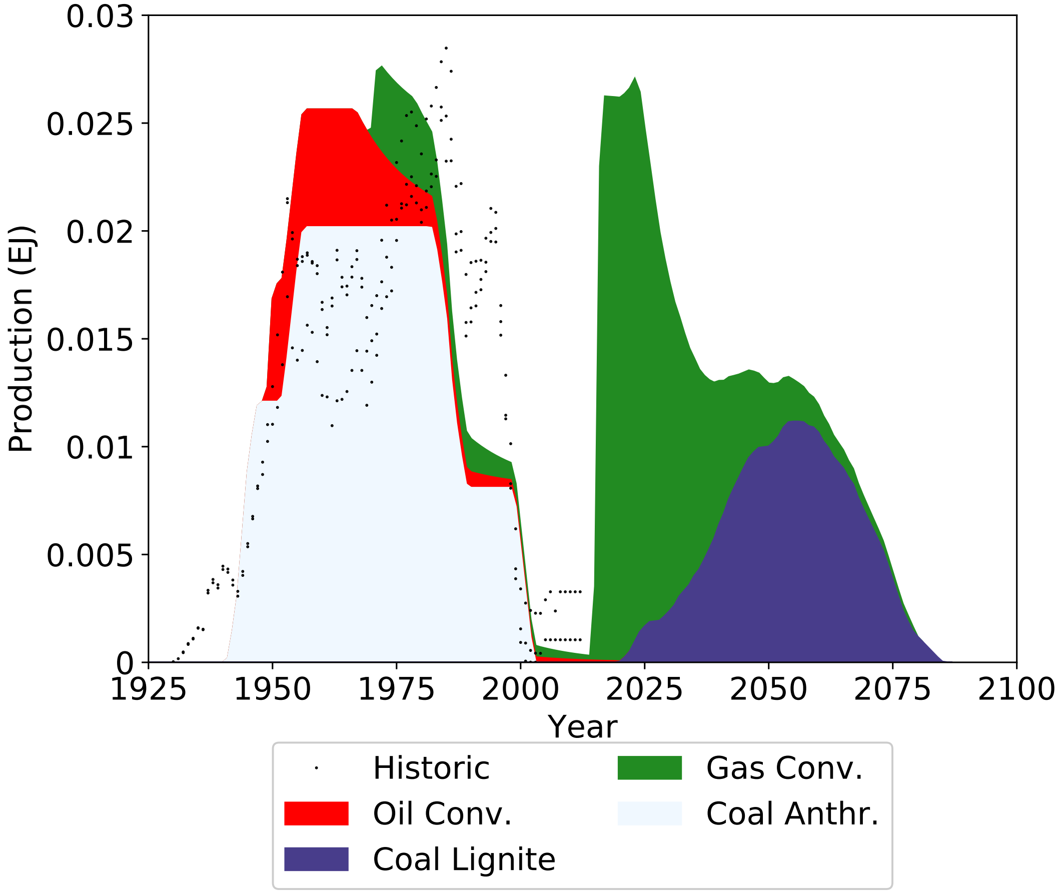

Figure 1.50: Morocco projection by mineral type

Table 1.50: Peak years - Minerals

Name	URR	Peak Year	Peak Rate
Coal Lignite	0.38	2055	0.01
Coal Anthr.	0.88	1957	0.02
Oil Conv.	0.16	1951	0.01
Gas Conv.	0.6	2017	0.03
**Total**	**2.02**	**1972**	**0.03**

1.26 Mozambique

1.26.1 All Projections

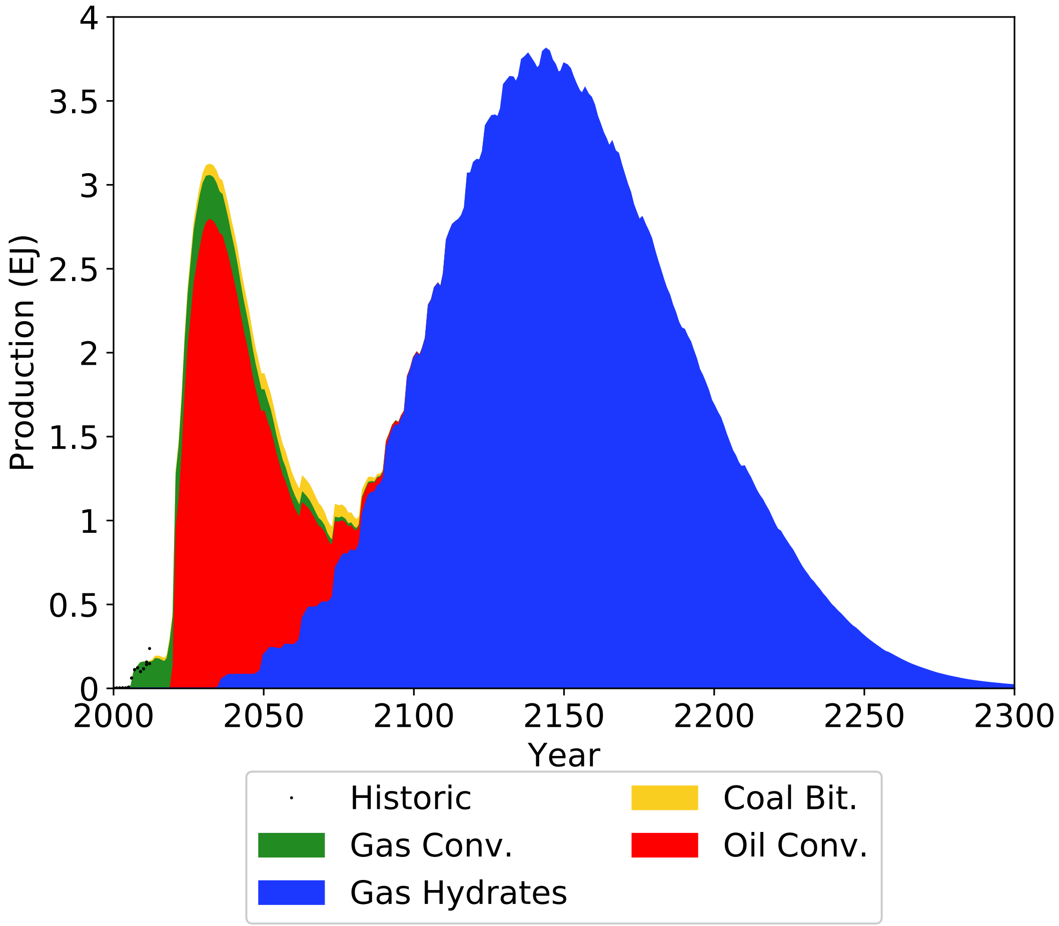

Figure 1.51: Mozambique projections capped at 16

Table 1.51: Peak years - All

Name	URR	Peak Year	Peak Rate
Gas Hydrates	389.5	2144	3.81
Oil Conv.	84.3	2032	2.79
Gas Conv.	11.5	2026	0.32
Coal Bit.	5.44	2048	0.1
**Total**	**490.74**	**2144**	**3.81**

1.26.2 By Mineral

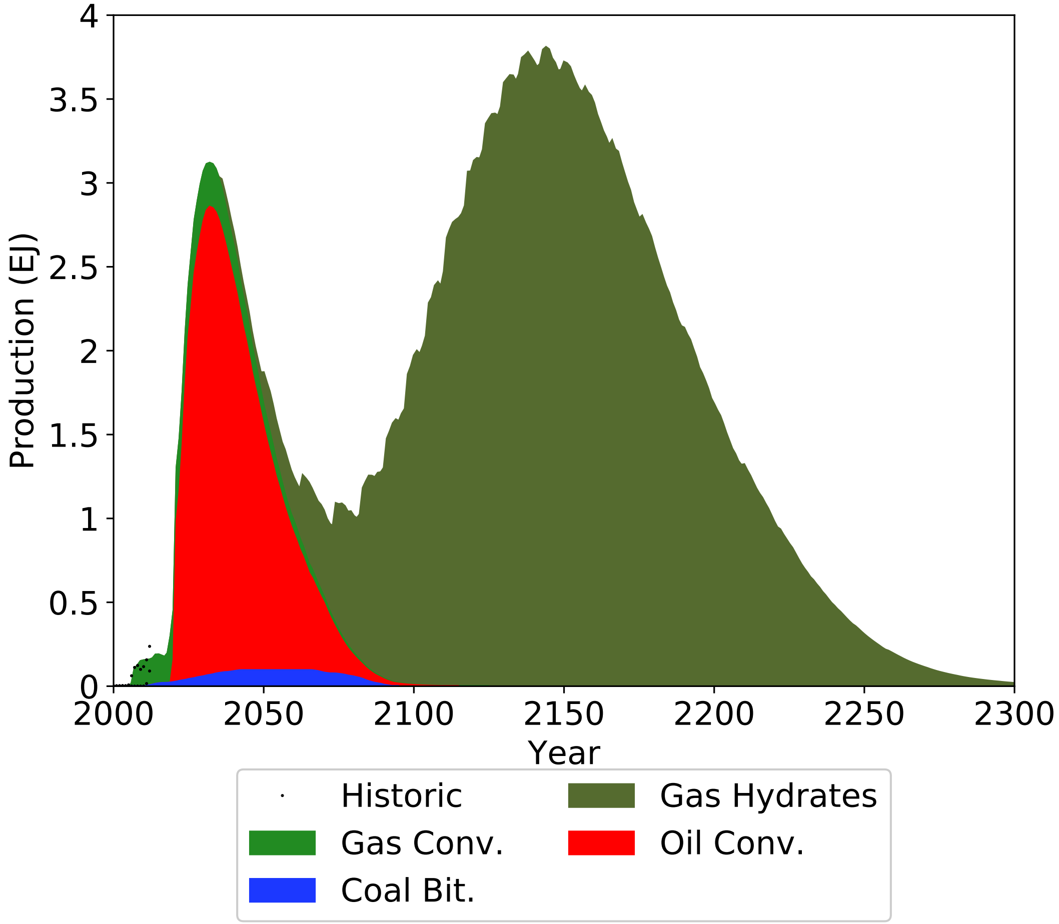

Figure 1.52: Mozambique projection by mineral type

Table 1.52: Peak years - Minerals

Name	URR	Peak Year	Peak Rate
Coal Bit.	5.44	2048	0.1
Oil Conv.	84.3	2032	2.79
Gas Conv.	11.5	2026	0.32
Gas Hydrates	389.5	2144	3.81
**Total**	**490.74**	**2144**	**3.81**

1.27 Namibia

1.27.1 All Projections

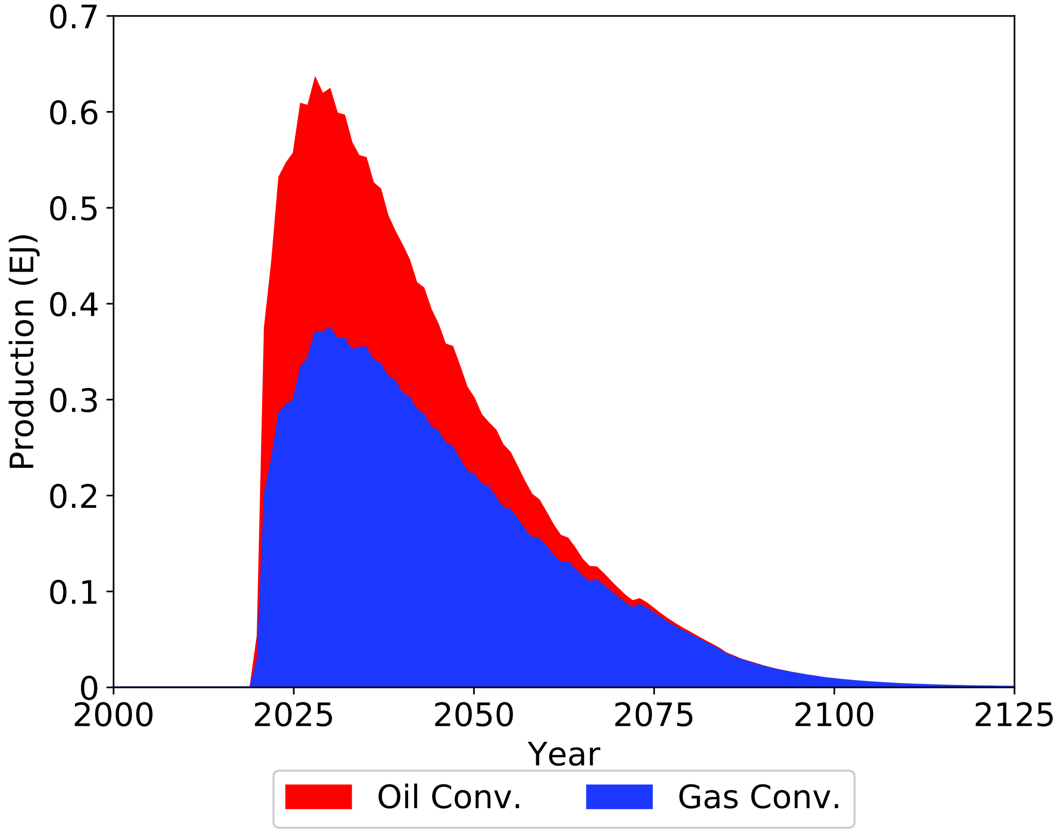

Figure 1.53: Namibia projections capped at 16

Table 1.53: Peak years - All

Name	URR	Peak Year	Peak Rate
Gas Conv.	13.4	2030	0.37
Oil Conv.	6.3	2026	0.27
**Total**	**19.7**	**2028**	**0.63**

1.27.2 By Mineral

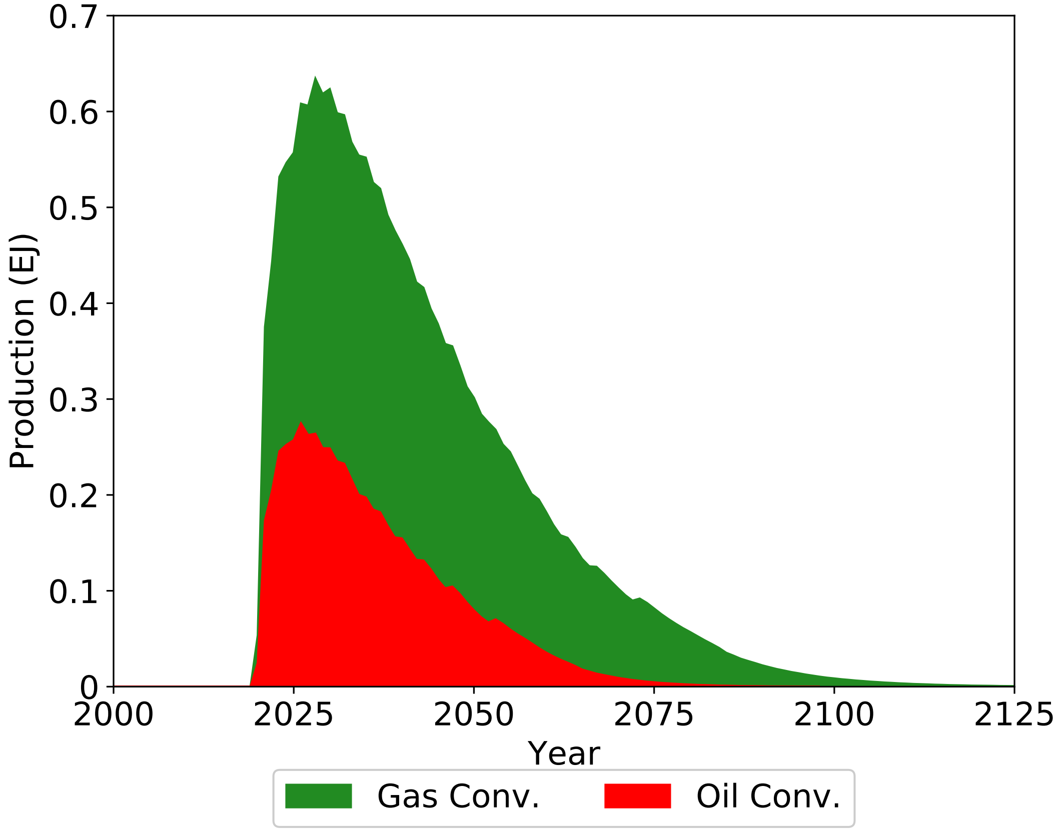

Figure 1.54: Namibia projection by mineral type

Table 1.54: Peak years - Minerals

Name	URR	Peak Year	Peak Rate
Oil Conv.	6.3	2026	0.27
Gas Conv.	13.4	2030	0.37
**Total**	**19.7**	**2028**	**0.63**

1.28 Niger

1.28.1 All Projections

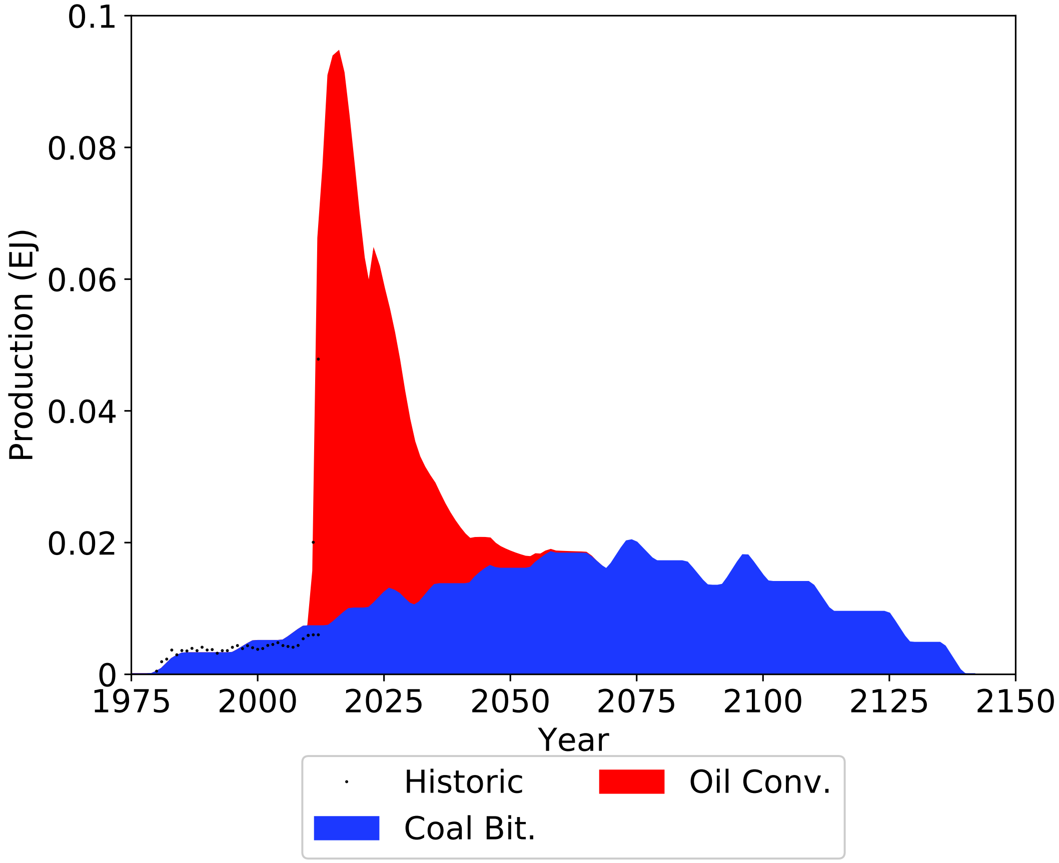

Figure 1.55: Niger projections capped at 16

Table 1.55: Peak years - All

Name	URR	Peak Year	Peak Rate
Coal Bit.	1.81	2074	0.02
Oil Conv.	1.32	2015	0.09
**Total**	**3.13**	**2016**	**0.09**

1.28.2 By Mineral

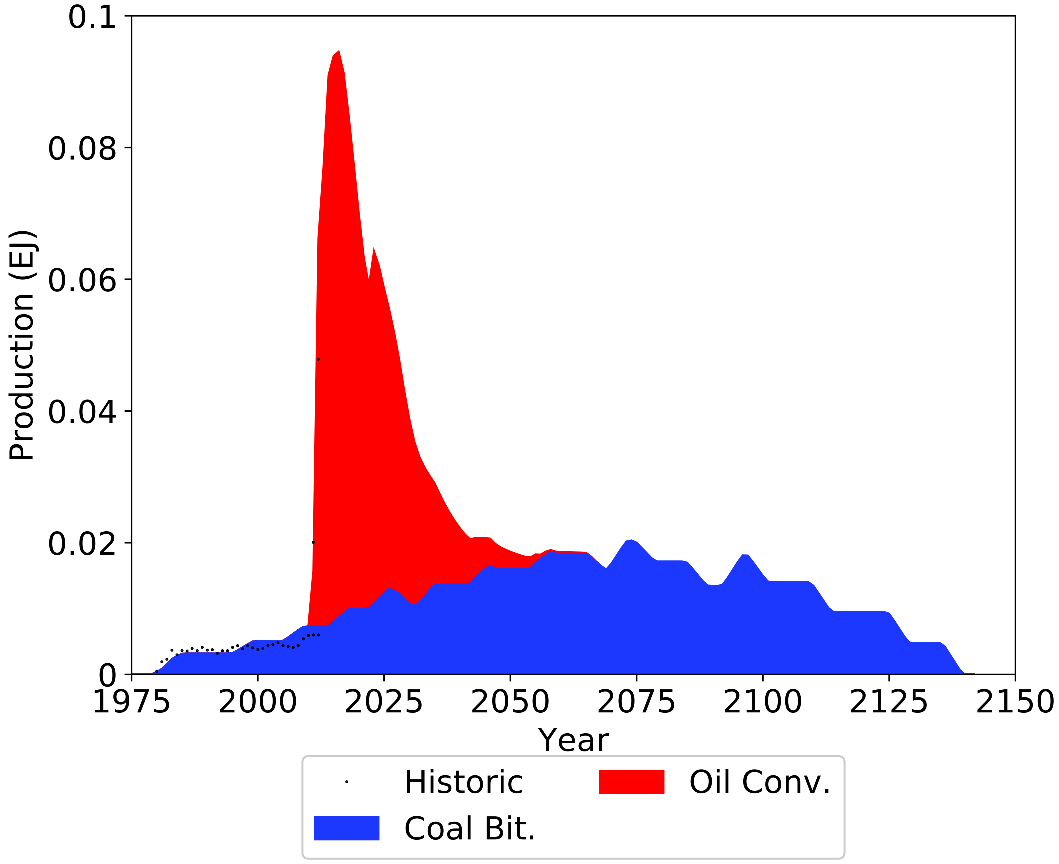

Figure 1.56: Niger projection by mineral type

Table 1.56: Peak years - Minerals

Name	URR	Peak Year	Peak Rate
Coal Bit.	1.81	2074	0.02
Oil Conv.	1.32	2015	0.09
**Total**	**3.13**	**2016**	**0.09**

1.29 Nigeria

1.29.1 All Projections

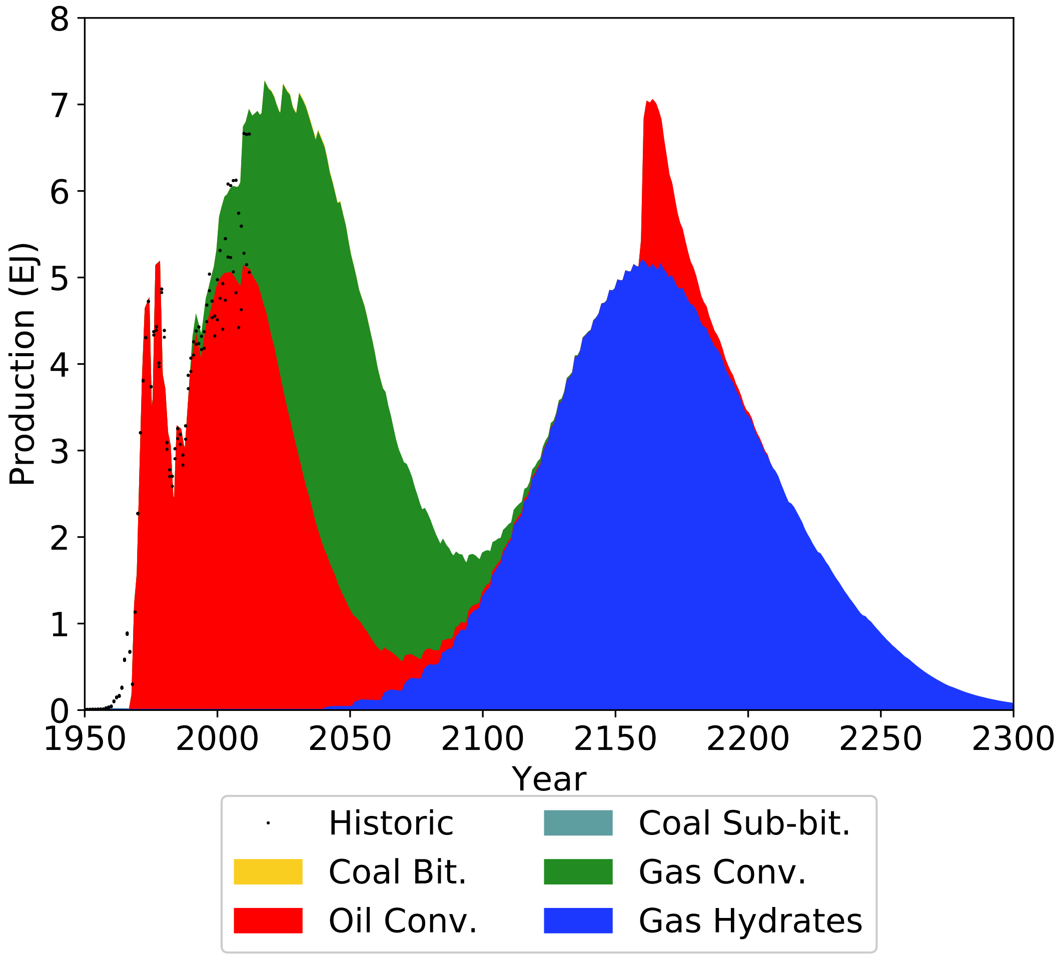

Figure 1.57: Nigeria projections capped at 16

Table 1.57: Peak years - All

**Name**	**URR**	**Peak Year**	**Peak Rate**
Gas Hydrates	527.0	2160	5.19
Oil Conv.	334.9	1978	5.17
Gas Conv.	262.5	2040	4.66
Coal Bit.	0.5	2035	0.02
Coal Sub-bit.	0.45	1961	0.01
**Total**	**1125.36**	**2018**	**7.24**

1.29.2 By Mineral

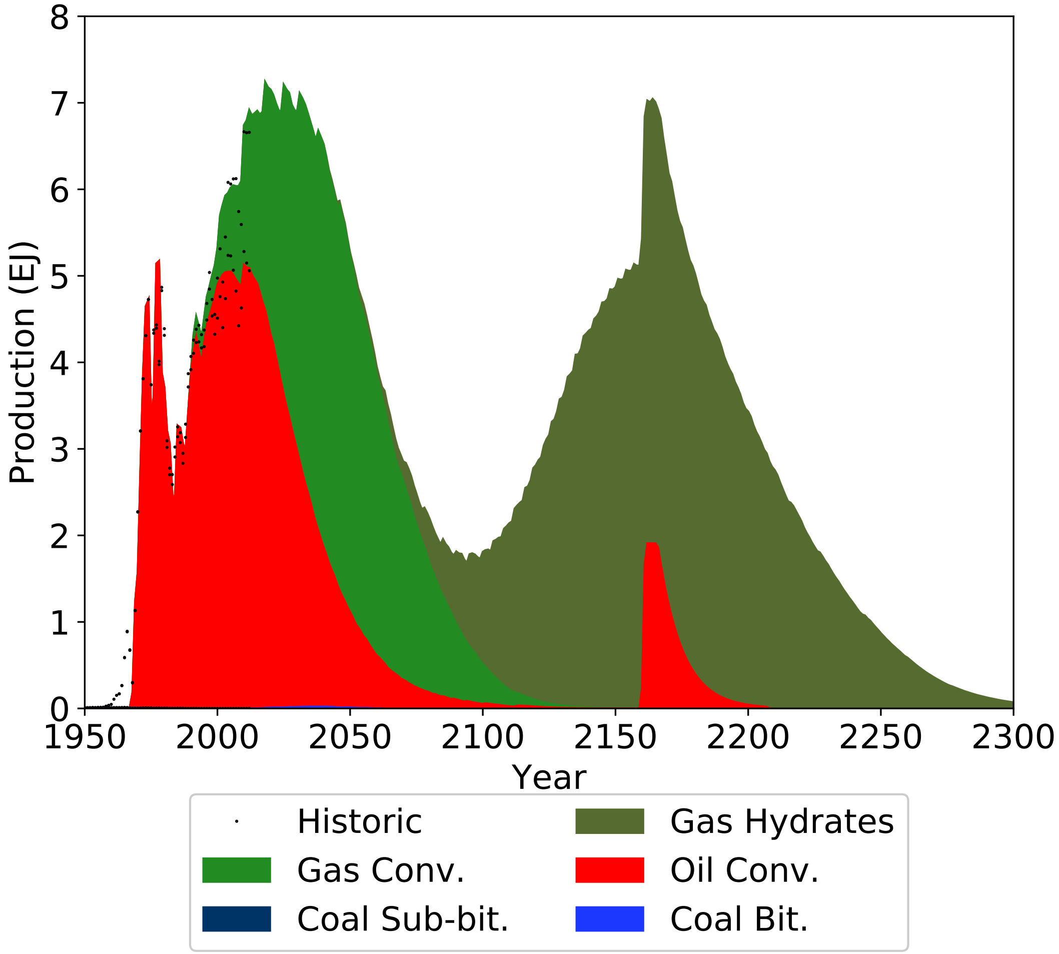

Figure 1.58: Nigeria projection by mineral type

Table 1.58: Peak years - Minerals

**Name**	**URR**	**Peak Year**	**Peak Rate**
Coal Bit.	0.5	2035	0.02
Coal Sub-bit.	0.45	1961	0.01
Oil Conv.	334.9	1978	5.17
Gas Conv.	262.5	2040	4.66
Gas Hydrates	527.0	2160	5.19
**Total**	**1125.36**	**2018**	**7.24**

1.30 Rwanda

1.30.1 All Projections

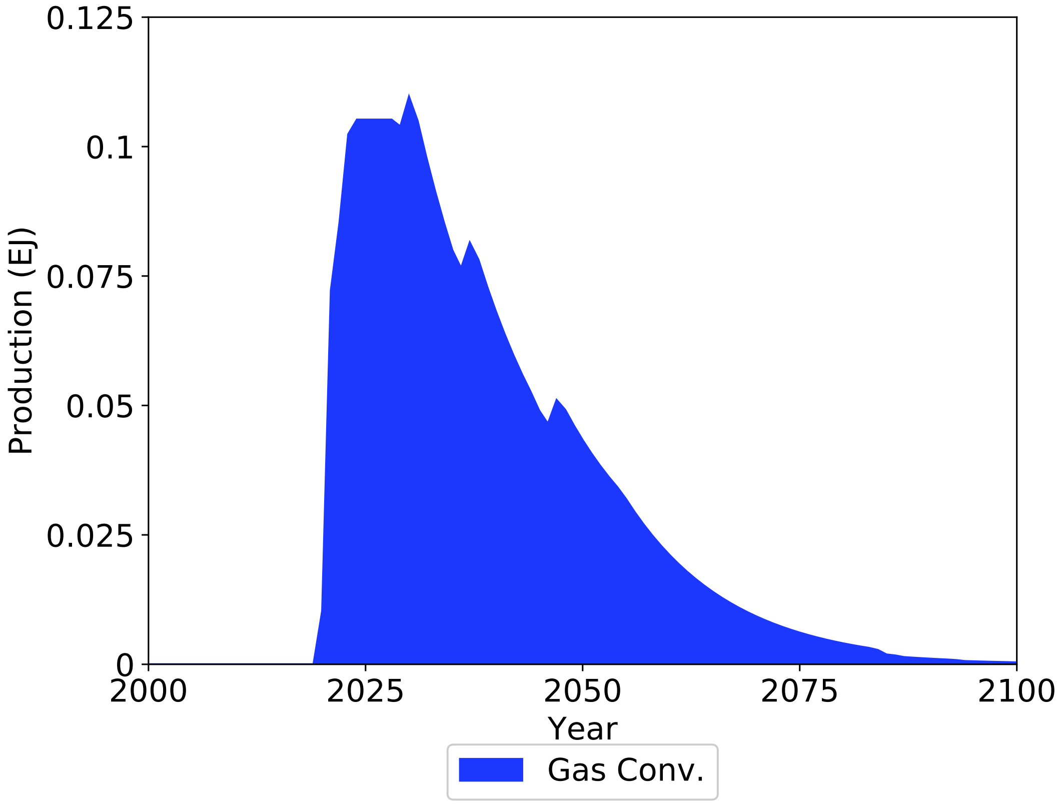

Figure 1.59: Rwanda projections capped at 16

Table 1.59: Peak years - All

Name	URR	Peak Year	Peak Rate
Gas Conv.	2.9	2030	0.11
**Total**	**2.9**	**2030**	**0.11**

1.30.2 By Mineral

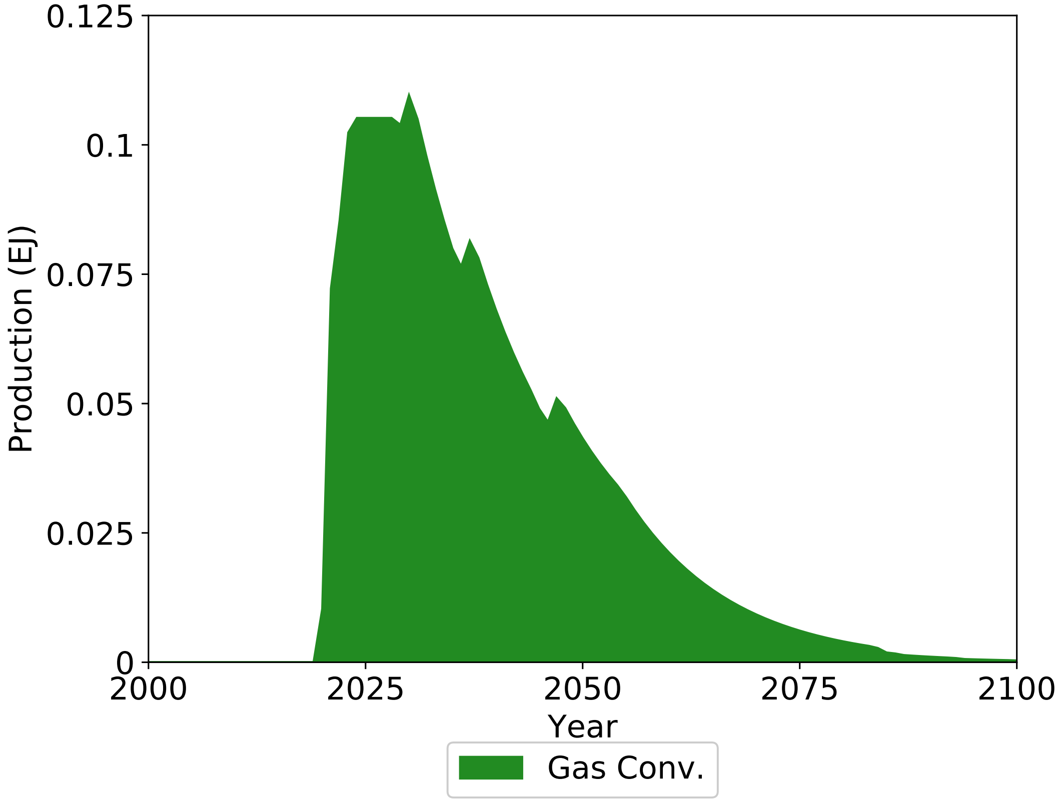

Figure 1.60: Rwanda projection by mineral type

Table 1.60: Peak years - Minerals

**Name**	**URR**	**Peak Year**	**Peak Rate**
Gas Conv.	2.9	2030	0.11
**Total**	**2.9**	**2030**	**0.11**

1.31 Sao Tome and Principe

1.31.1 All Projections

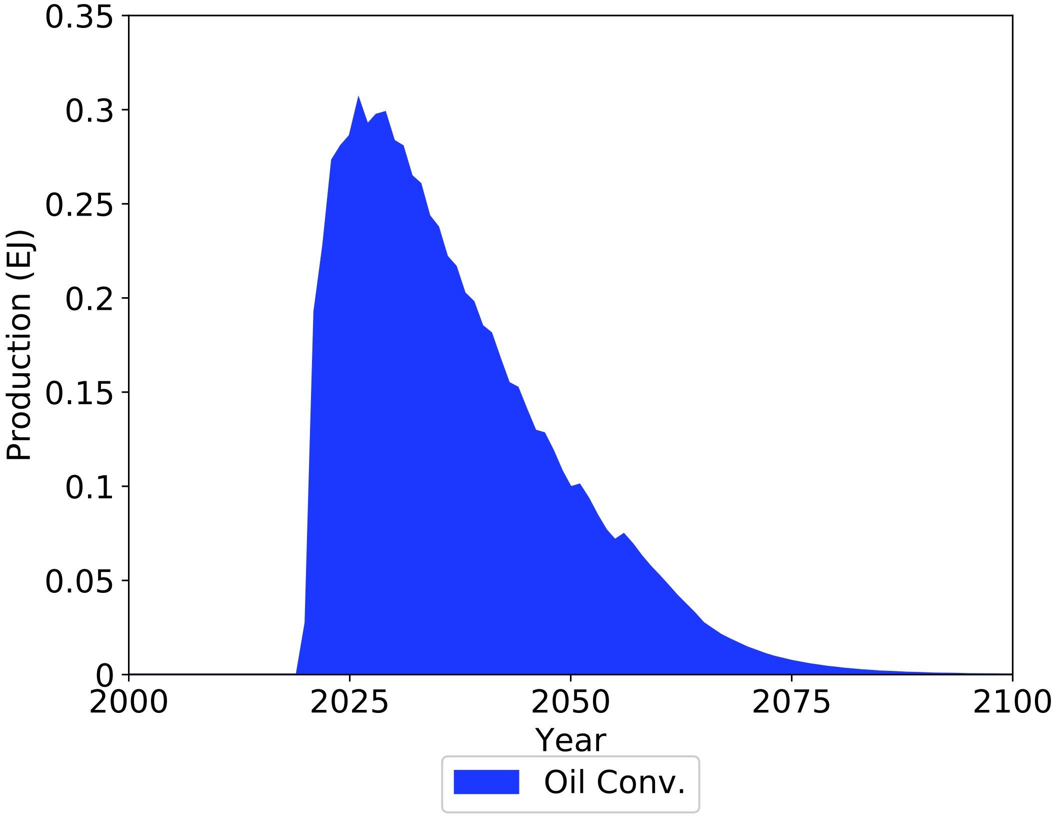

Figure 1.61: Sao Tome and Principe projections capped at 16

Table 1.61: Peak years - All

Name	URR	Peak Year	Peak Rate
Oil Conv.	7.58	2026	0.31
**Total**	**7.58**	**2026**	**0.31**

1.31.2 By Mineral

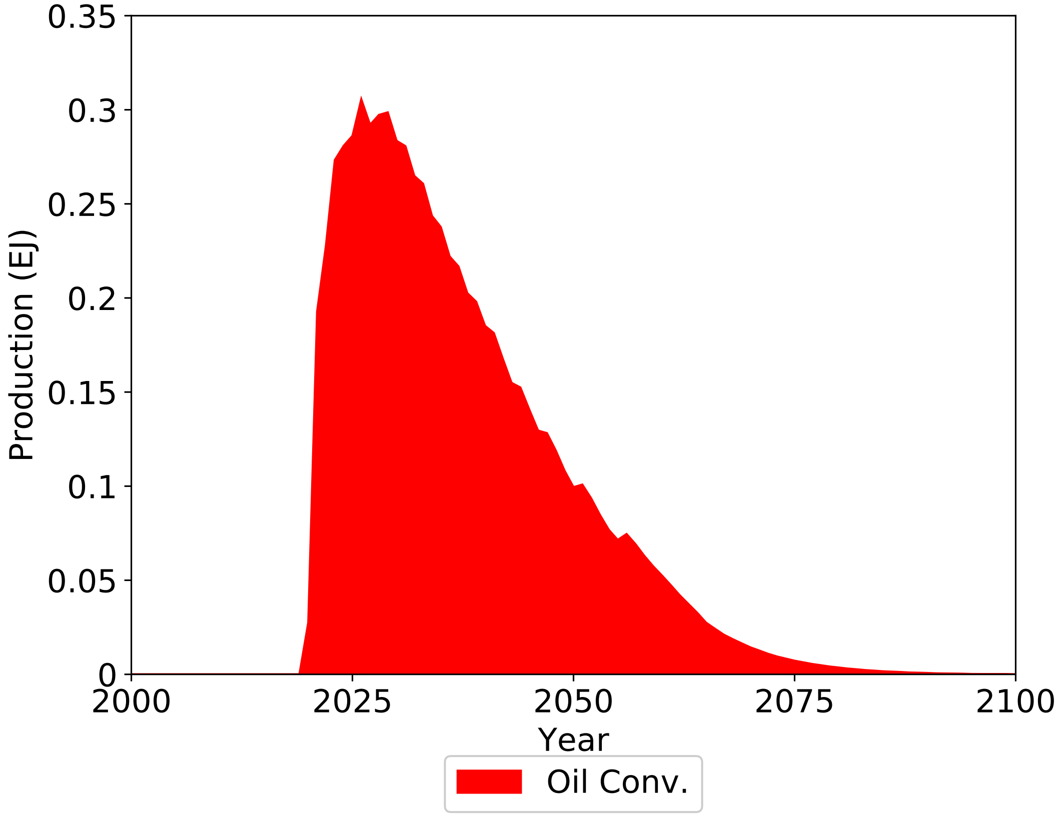

Figure 1.62: Sao Tome and Principe projection by mineral type

Table 1.62: Peak years - Minerals

**Name**	**URR**	**Peak Year**	**Peak Rate**
Oil Conv.	7.58	2026	0.31
**Total**	**7.58**	**2026**	**0.31**

1.32 Senegal

1.32.1 All Projections

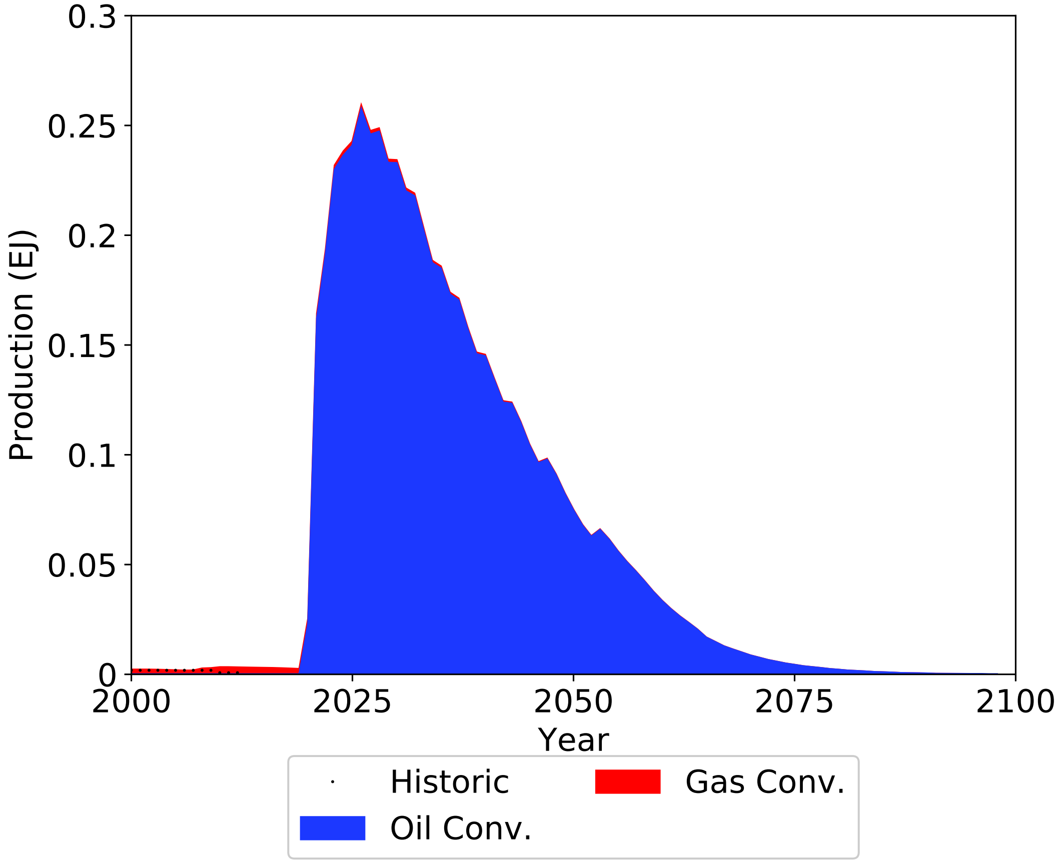

Figure 1.63: Senegal projections capped at 16

Table 1.63: Peak years - All

**Name**	**URR**	**Peak Year**	**Peak Rate**
Oil Conv.	5.9	2026	0.26
Gas Conv.	0.1	2010	–
**Total**	**6.0**	**2026**	**0.26**

1.32.2 By Mineral

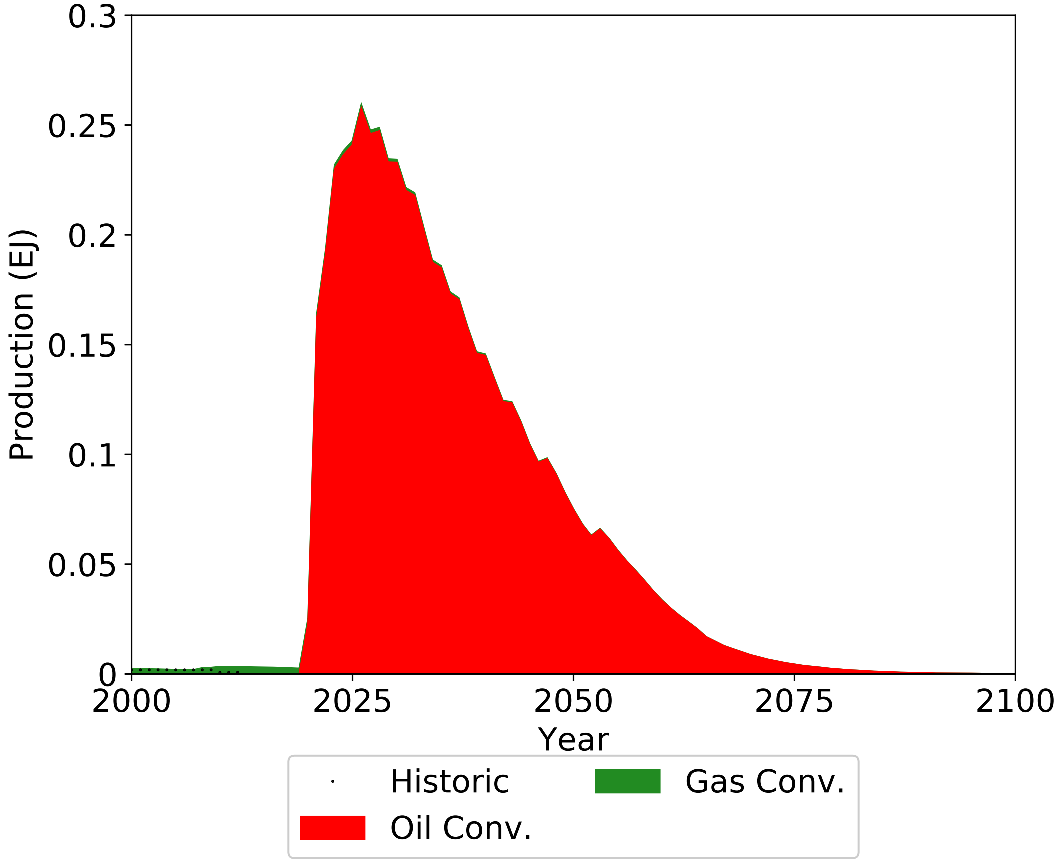

Figure 1.64: Senegal projection by mineral type

Table 1.64: Peak years - Minerals

**Name**	**URR**	**Peak Year**	**Peak Rate**
Oil Conv.	5.9	2026	0.26
Gas Conv.	0.1	2010	–
**Total**	**6.0**	**2026**	**0.26**

1.33 Seychelles

1.33.1 All Projections

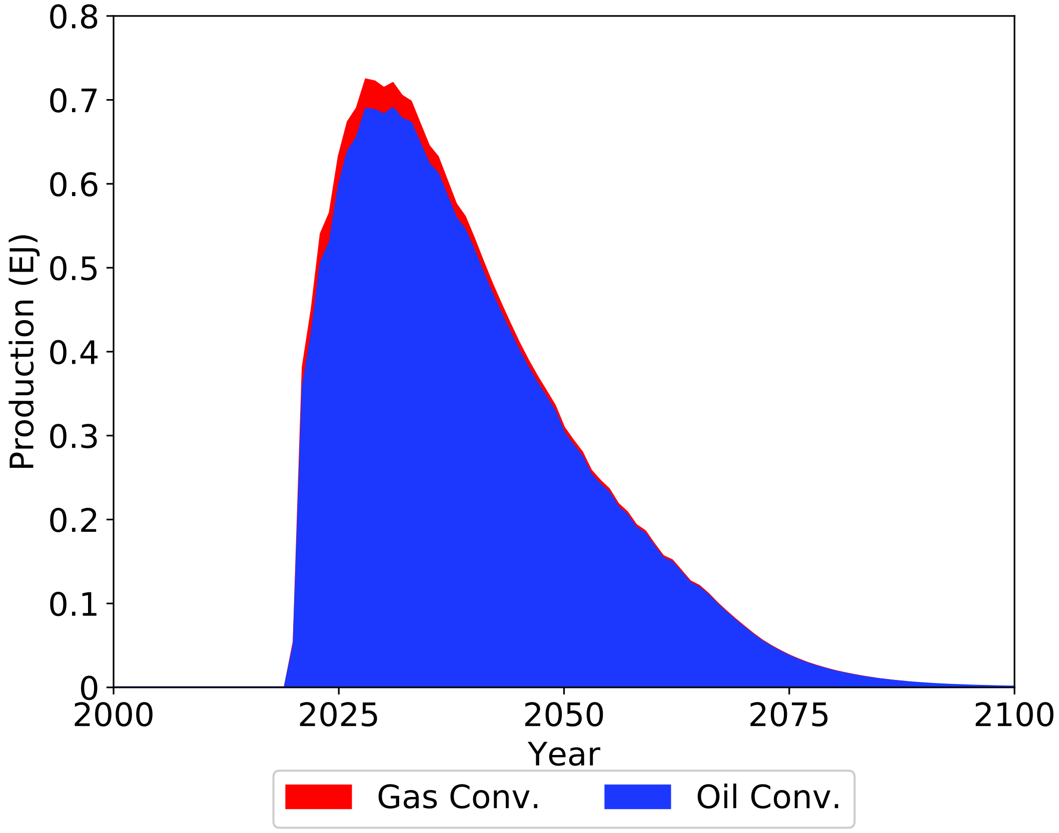

Figure 1.65: Seychelles projections capped at 16

Table 1.65: Peak years - All

Name	URR	Peak Year	Peak Rate
Oil Conv.	19.8	2031	0.69
Gas Conv.	0.7	2024	0.04
**Total**	**20.5**	**2028**	**0.72**

1.33.2 By Mineral

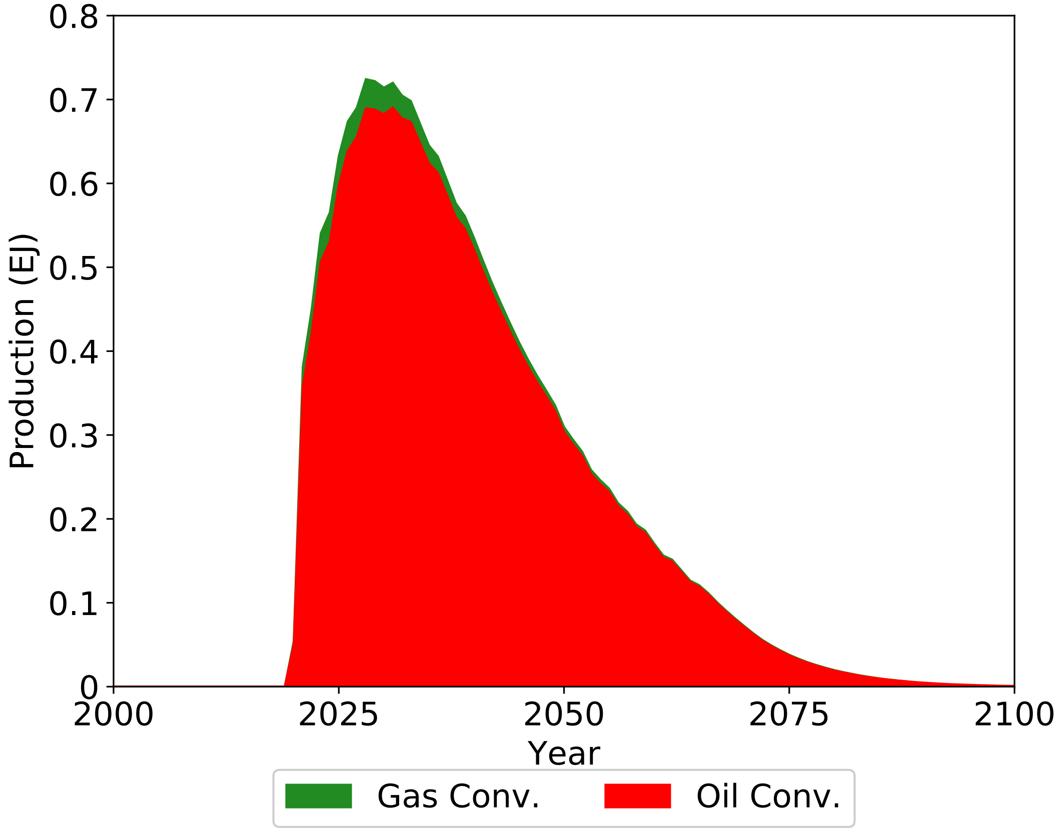

Figure 1.66: Seychelles projection by mineral type

Table 1.66: Peak years - Minerals

**Name**	**URR**	**Peak Year**	**Peak Rate**
Oil Conv.	19.8	2031	0.69
Gas Conv.	0.7	2024	0.04
**Total**	**20.5**	**2028**	**0.72**

1.34 Sierra Leone

1.34.1 All Projections

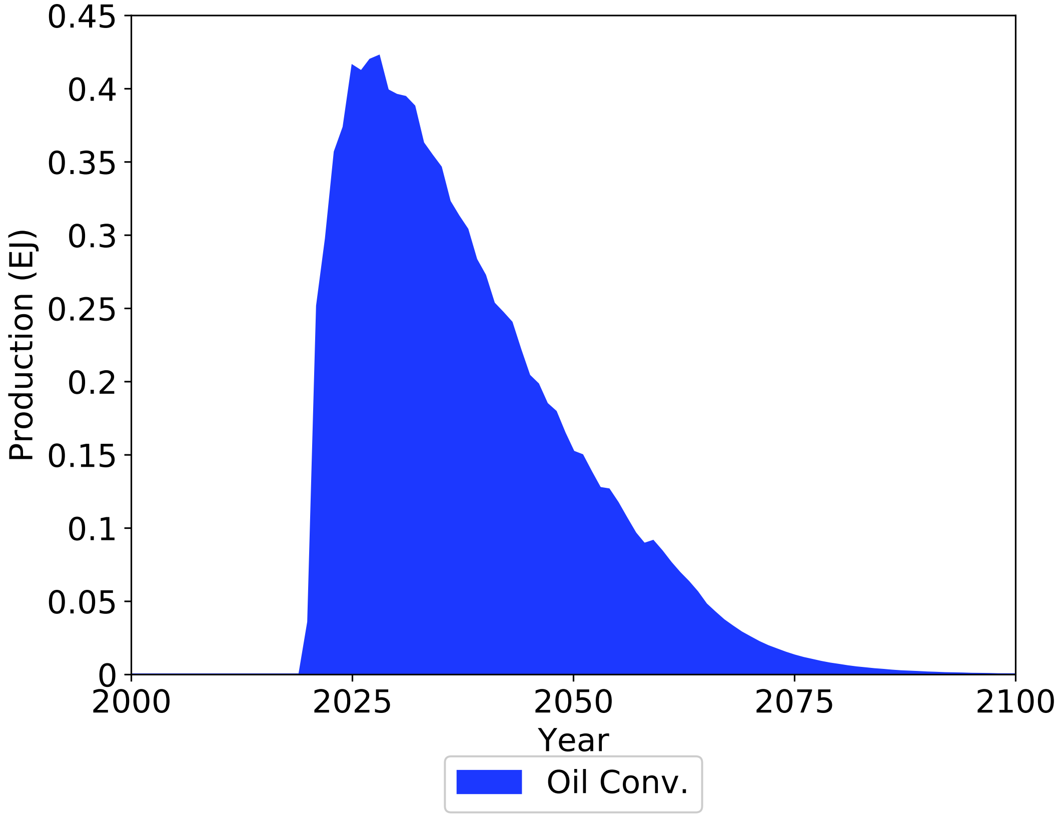

Figure 1.67: Sierra Leone projections capped at 16

Table 1.67: Peak years - All

Name	URR	Peak Year	Peak Rate
Oil Conv.	10.95	2028	0.42
**Total**	**10.95**	**2028**	**0.42**

1.34.2 By Mineral

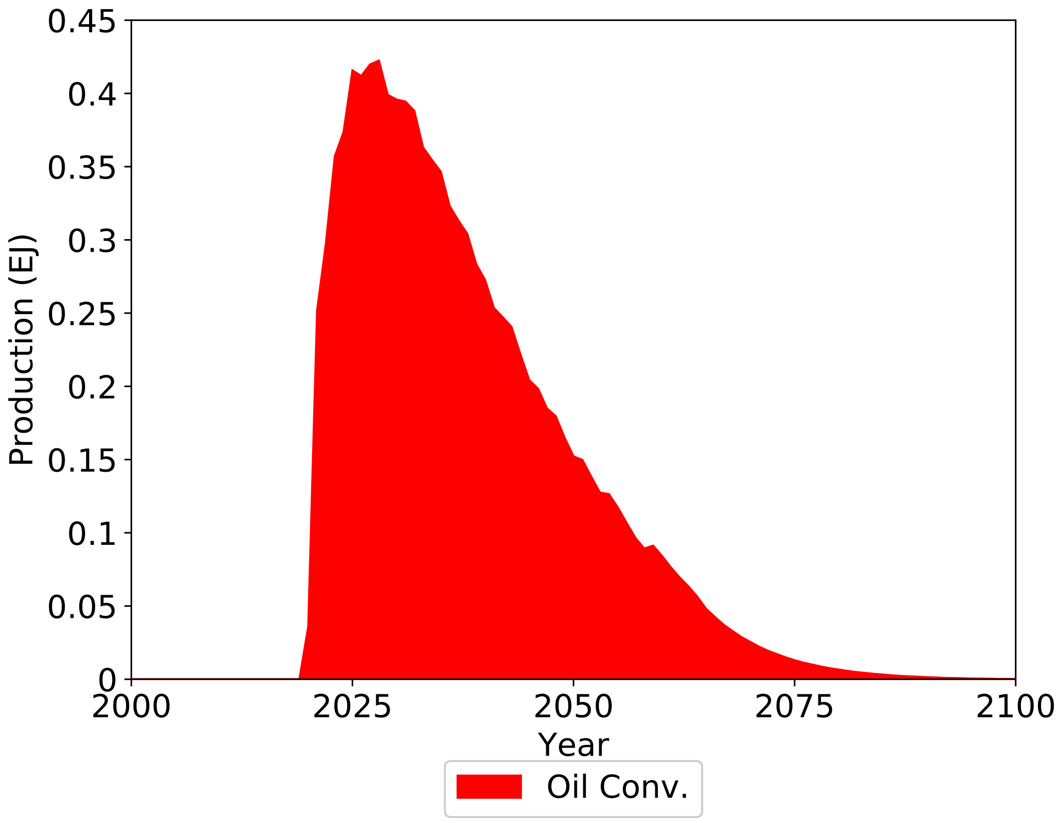

Figure 1.68: Sierra Leone projection by mineral type

Table 1.68: Peak years - Minerals

Name	URR	Peak Year	Peak Rate
Oil Conv.	10.95	2028	0.42
**Total**	**10.95**	**2028**	**0.42**

1.35 Somalia

1.35.1 All Projections

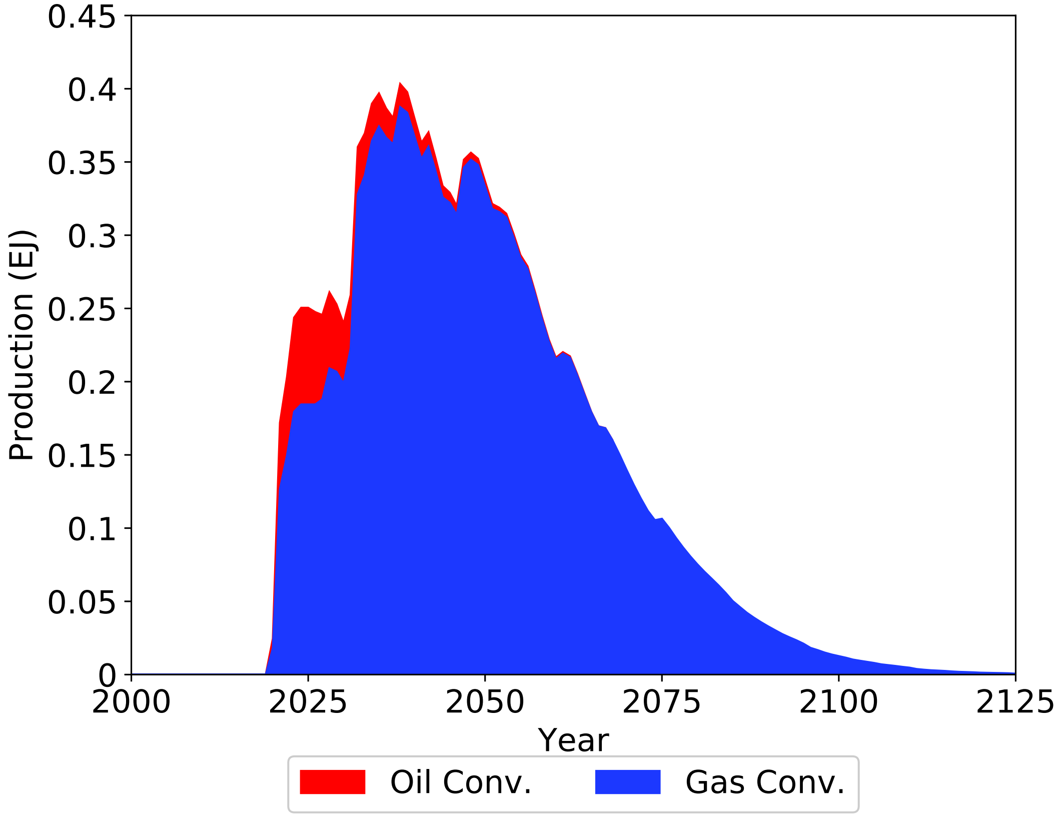

Figure 1.69: Somalia projections capped at 16

Table 1.69: Peak years - All

Name	URR	Peak Year	Peak Rate
Gas Conv.	15.1	2038	0.39
Oil Conv.	0.88	2024	0.07
**Total**	**15.98**	**2038**	**0.4**

1.35.2 By Mineral

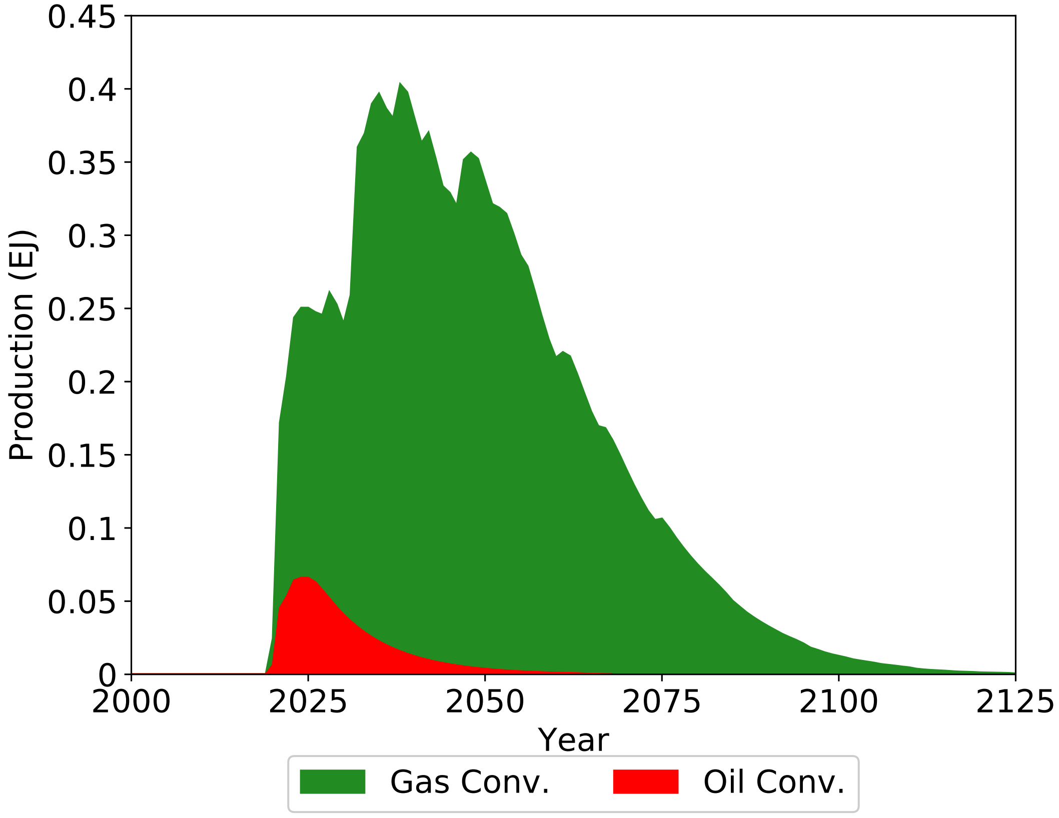

Figure 1.70: Somalia projection by mineral type

Table 1.70: Peak years - Minerals

Name	URR	Peak Year	Peak Rate
Oil Conv.	0.88	2024	0.07
Gas Conv.	15.1	2038	0.39
**Total**	**15.98**	**2038**	**0.4**

1.36 South Africa

1.36.1 All Projections

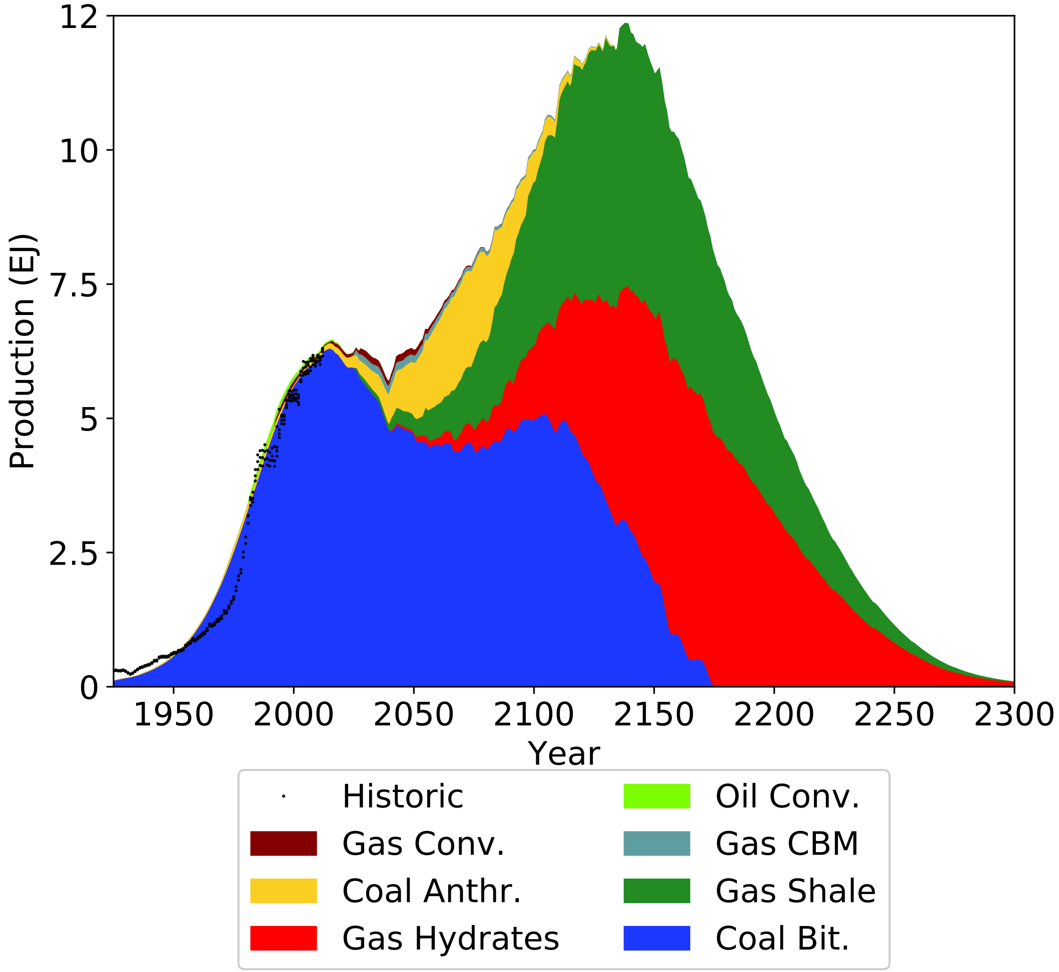

Figure 1.71: South Africa projections capped at 16

Table 1.71: Peak years - All

**Name**	**URR**	**Peak Year**	**Peak Rate**
Coal Bit.	859.57	2015	6.28
Gas Hydrates	521.5	2158	5.14
Gas Shale	509.34	2137	4.93
Coal Anthr.	95.02	2071	1.8
Gas CBM	8.4	2039	0.16
Gas Conv.	6.3	2045	0.14
Oil Conv.	3.34	1988	0.15
**Total**	**2003.47**	**2138**	**12.35**

1.36.2 By Mineral

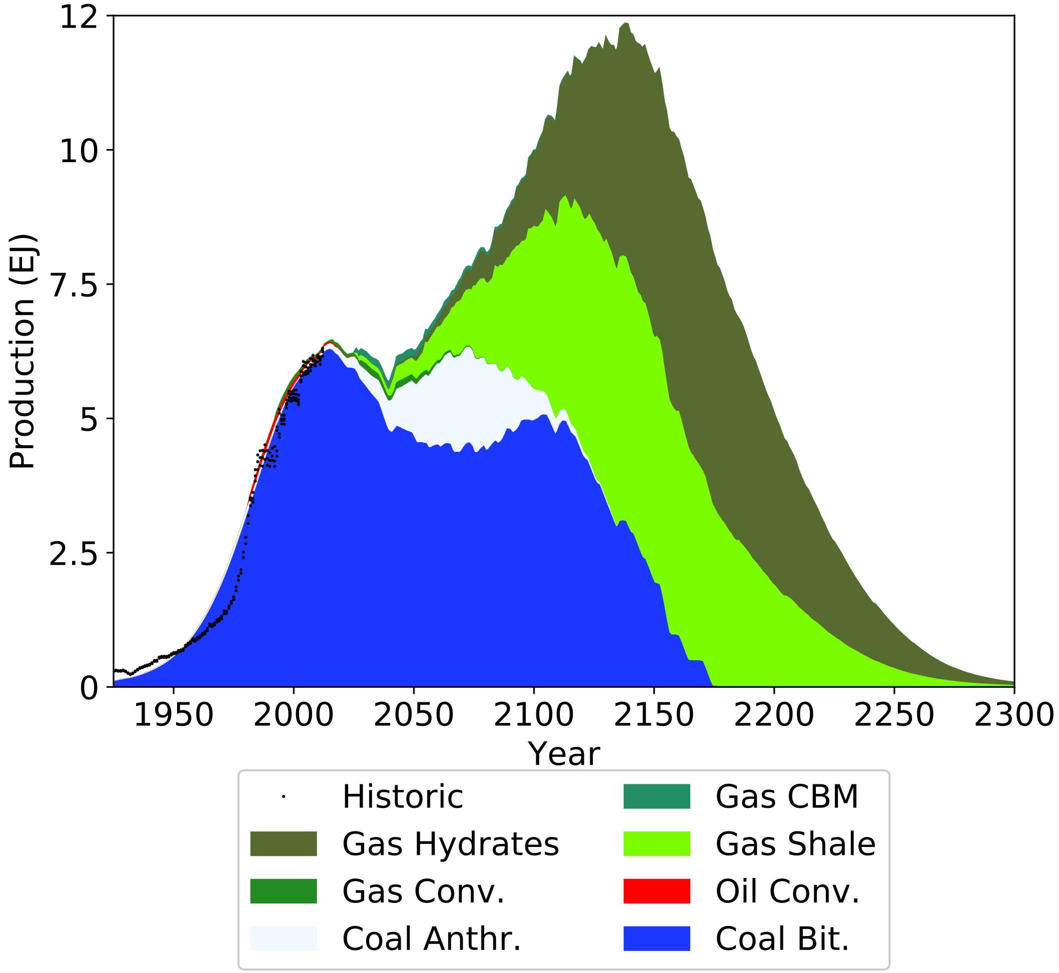

Figure 1.72: South Africa projection by mineral type

Table 1.72: Peak years - Minerals

**Name**	**URR**	**Peak Year**	**Peak Rate**
Coal Bit.	859.57	2015	6.28
Coal Anthr.	95.02	2071	1.8
Oil Conv.	3.34	1988	0.15
Gas Conv.	6.3	2045	0.14
Gas Shale	509.34	2137	4.93
Gas Hydrates	521.5	2158	5.14
Gas CBM	8.4	2039	0.16
**Total**	**2003.47**	**2138**	**12.35**

1.37 Sudan

1.37.1 All Projections

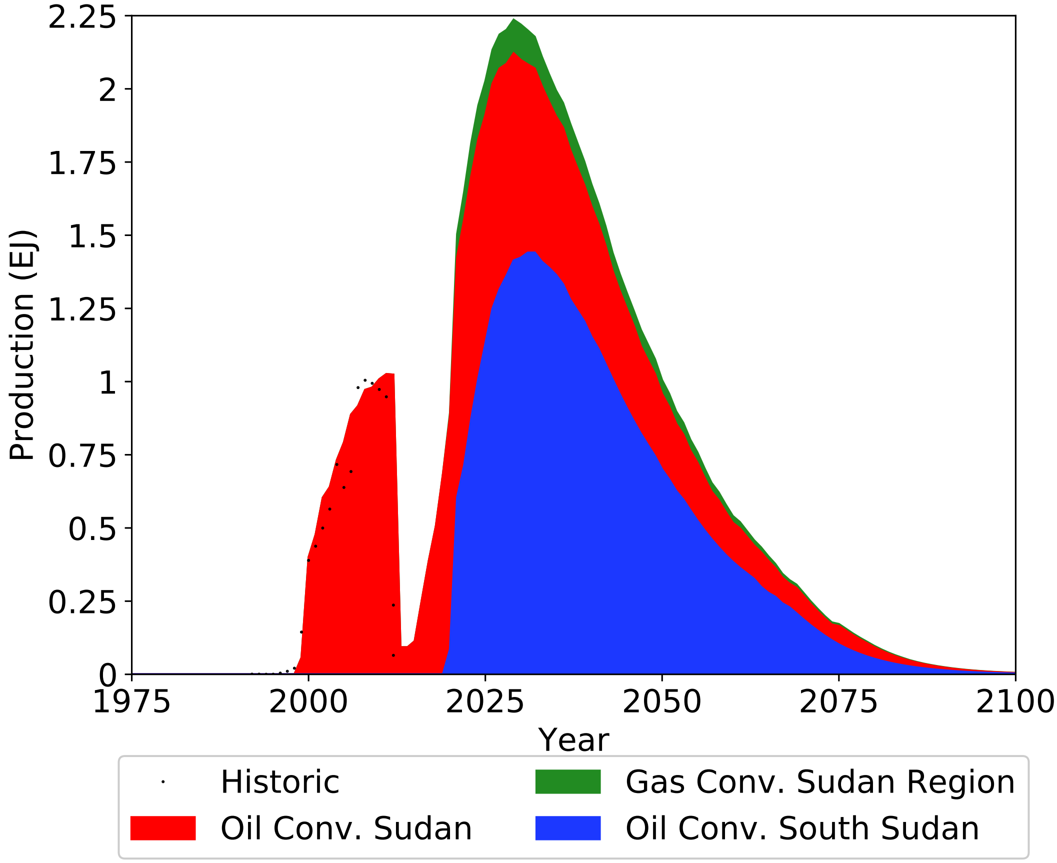

Figure 1.73: Sudan projections capped at 16

Table 1.73: Peak years - All

**Name**	**URR**	**Peak Year**	**Peak Rate**
Oil Conv. South Sudan	42.69	2032	1.44
Oil Conv. Sudan	33.66	2011	1.03
Gas Conv. Sudan Region	3.2	2030	0.12
**Total**	**79.55**	**2029**	**2.24**

1.37.2 By Mineral

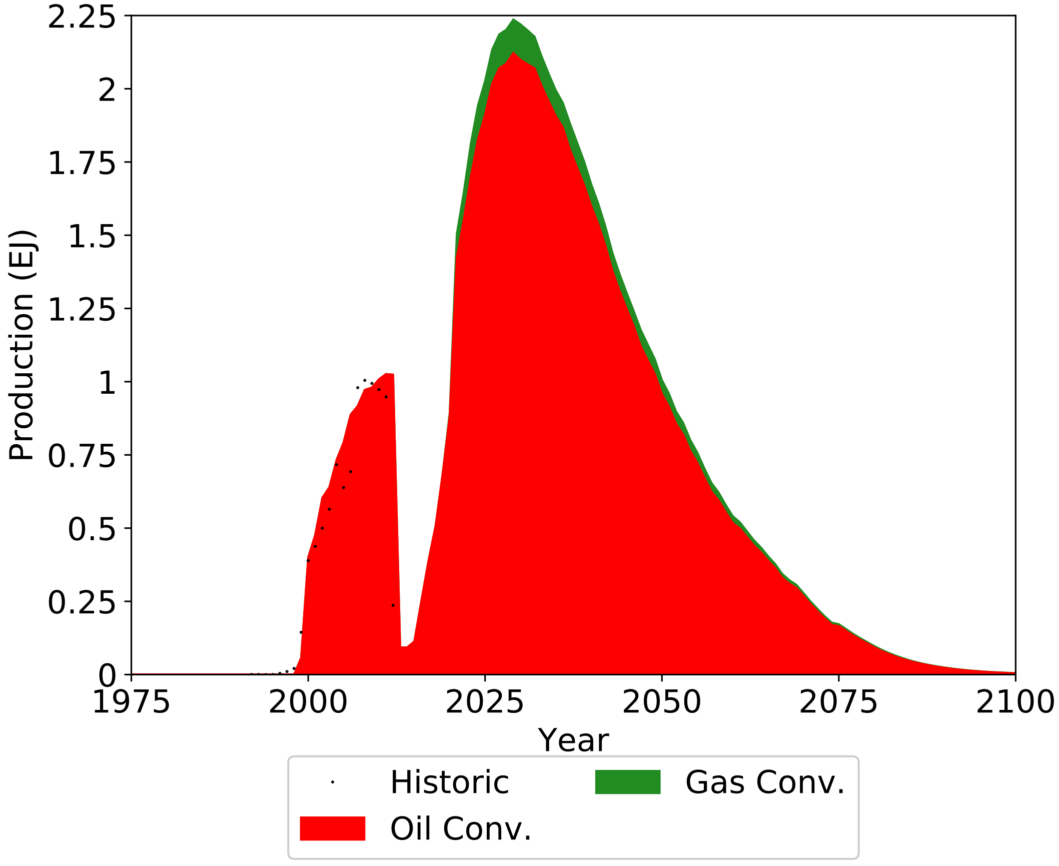

Figure 1.74: Sudan projection by mineral type

Table 1.74: Peak years - Minerals

Name	URR	Peak Year	Peak Rate
Oil Conv.	76.35	2029	2.12
Gas Conv.	3.2	2030	0.12
**Total**	**79.55**	**2029**	**2.24**

1.37.3 Regional Projections

South Sudan

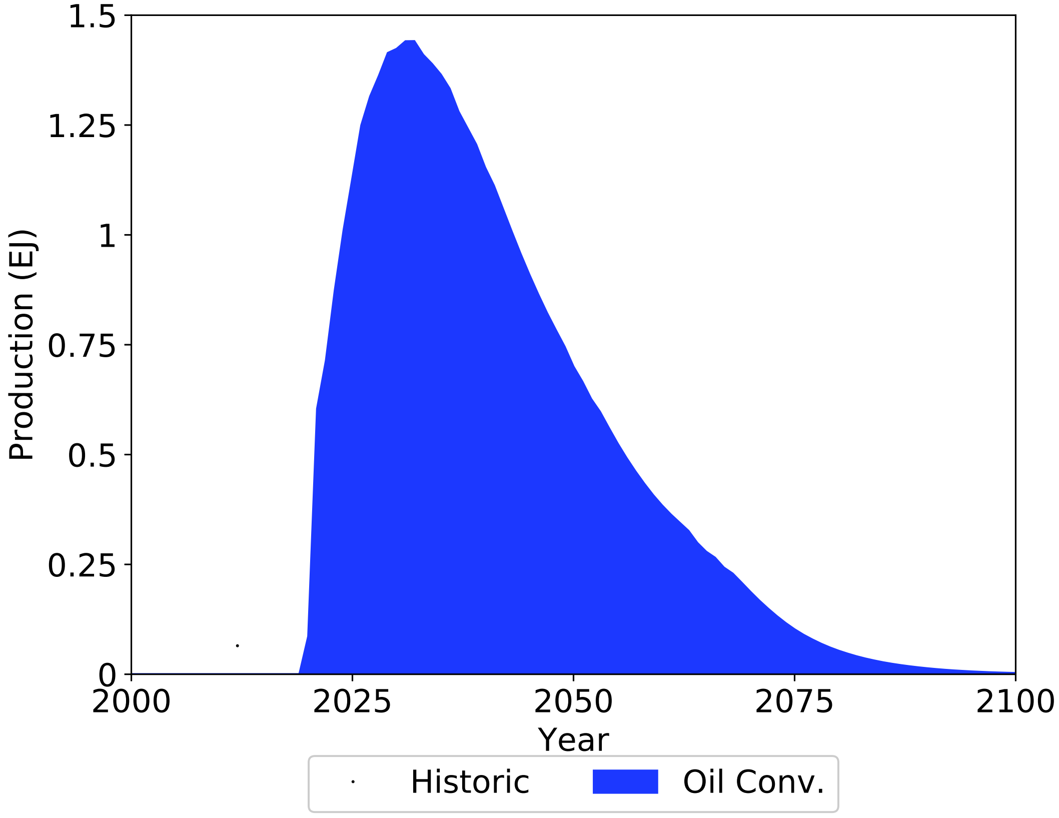

Figure 1.75: Sudan - South Sudan projections capped at 16

Table 1.75: Peak years - All

Name	URR	Peak Year	Peak Rate
Oil Conv. South Sudan	42.69	2032	1.44
**Total**	**42.69**	**2032**	**1.44**

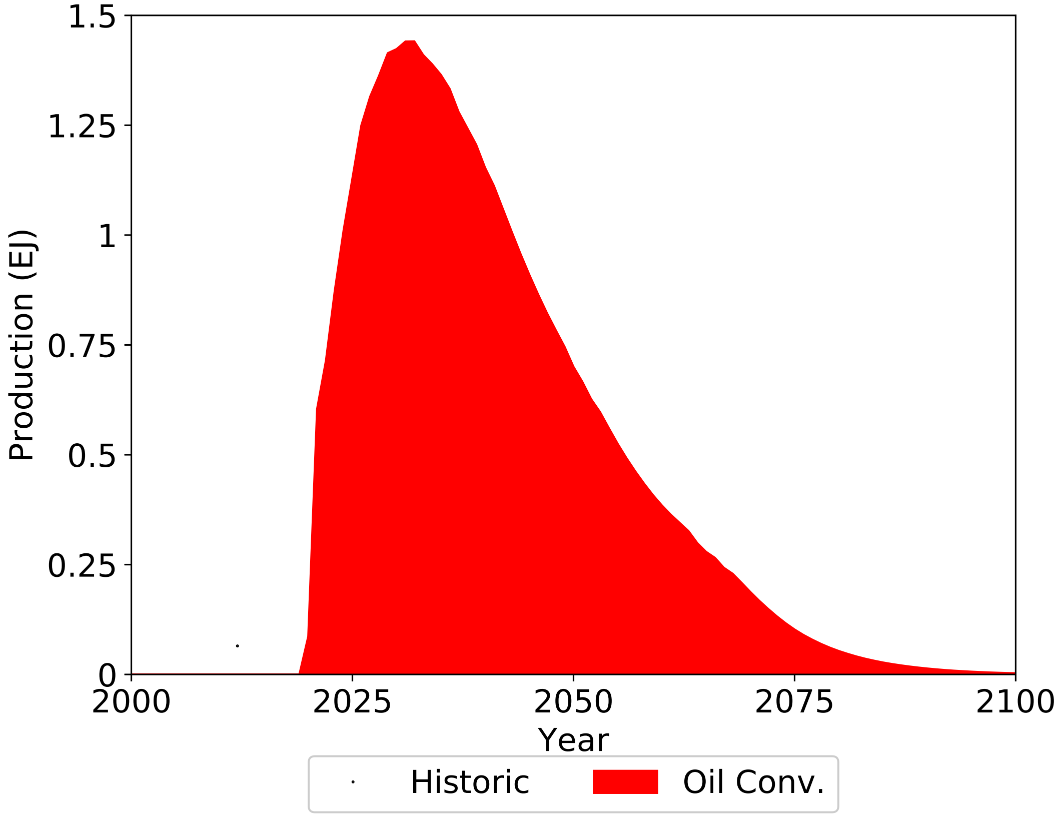

Figure 1.76: Sudan - South Sudan projection by mineral type

Table 1.76: Peak years - Minerals

Name	URR	Peak Year	Peak Rate
Oil Conv.	42.69	2032	1.44
**Total**	**42.69**	**2032**	**1.44**

Sudan

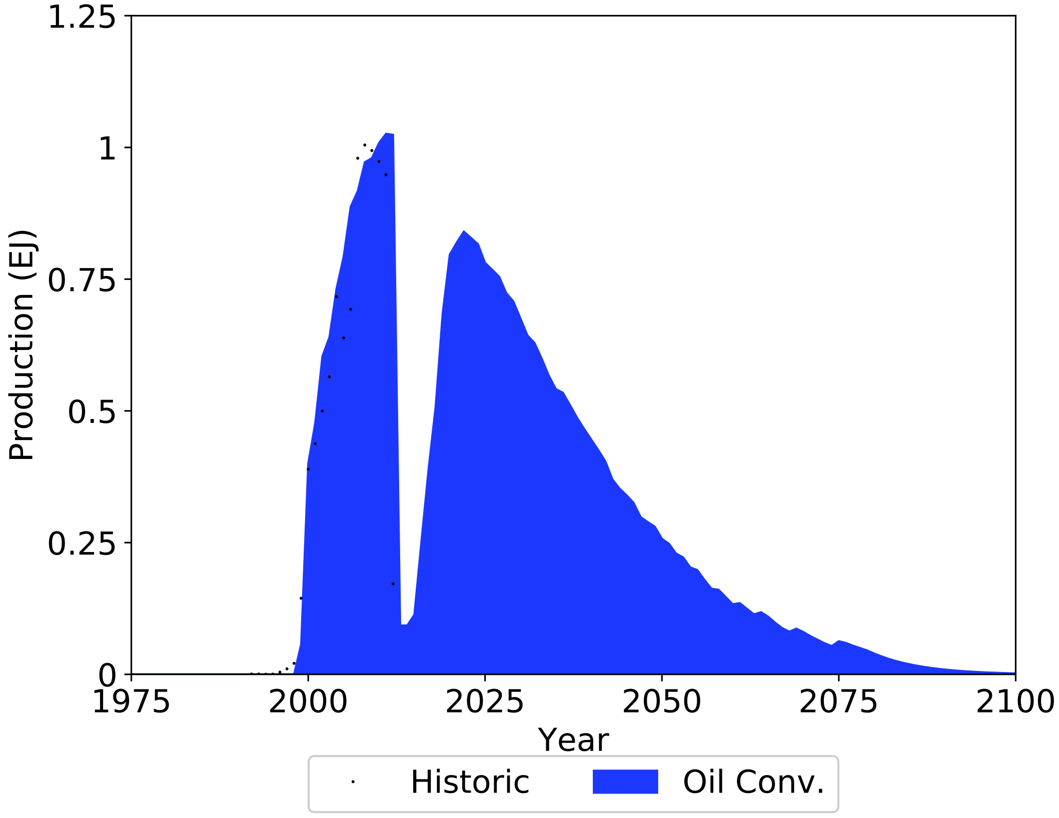

Figure 1.77: Sudan - Sudan projections capped at 16

Table 1.77: Peak years - All

Name	URR	Peak Year	Peak Rate
Oil Conv. Sudan	33.66	2011	1.03
**Total**	**33.66**	**2011**	**1.03**

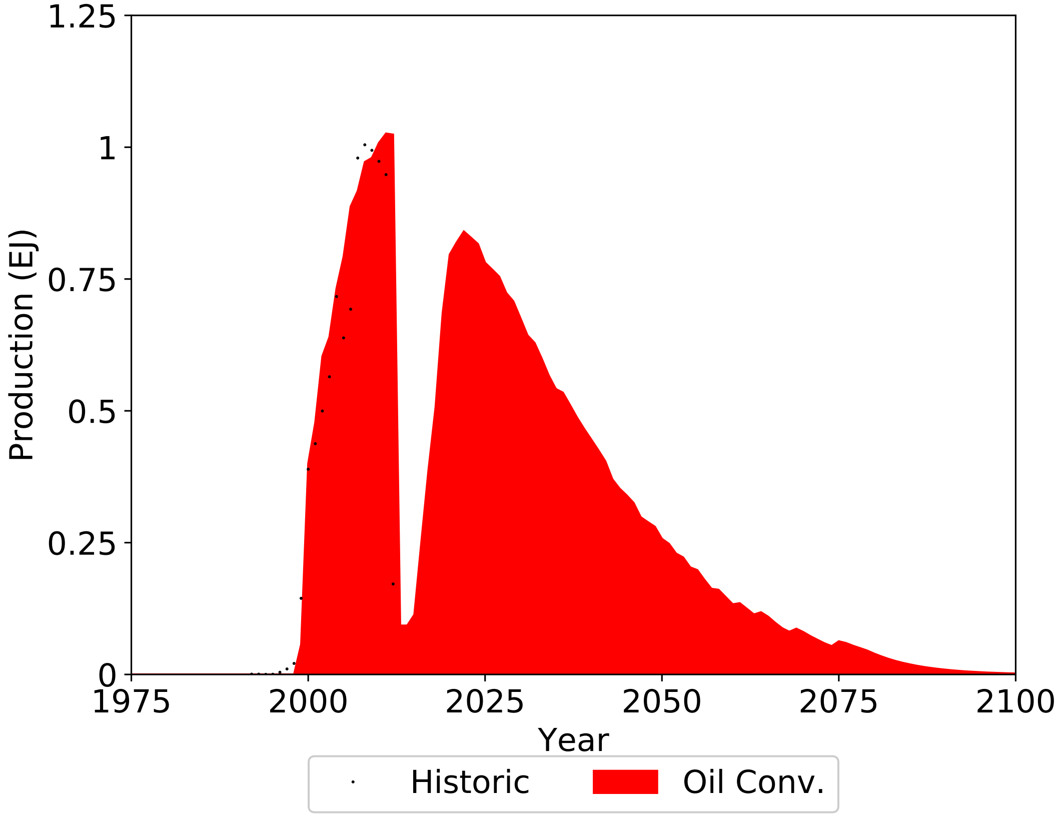

Figure 1.78: Sudan - Sudan projection by mineral type

Table 1.78: Peak years - Minerals

Name	URR	Peak Year	Peak Rate
Oil Conv.	33.66	2011	1.03
**Total**	**33.66**	**2011**	**1.03**

Sudan Region

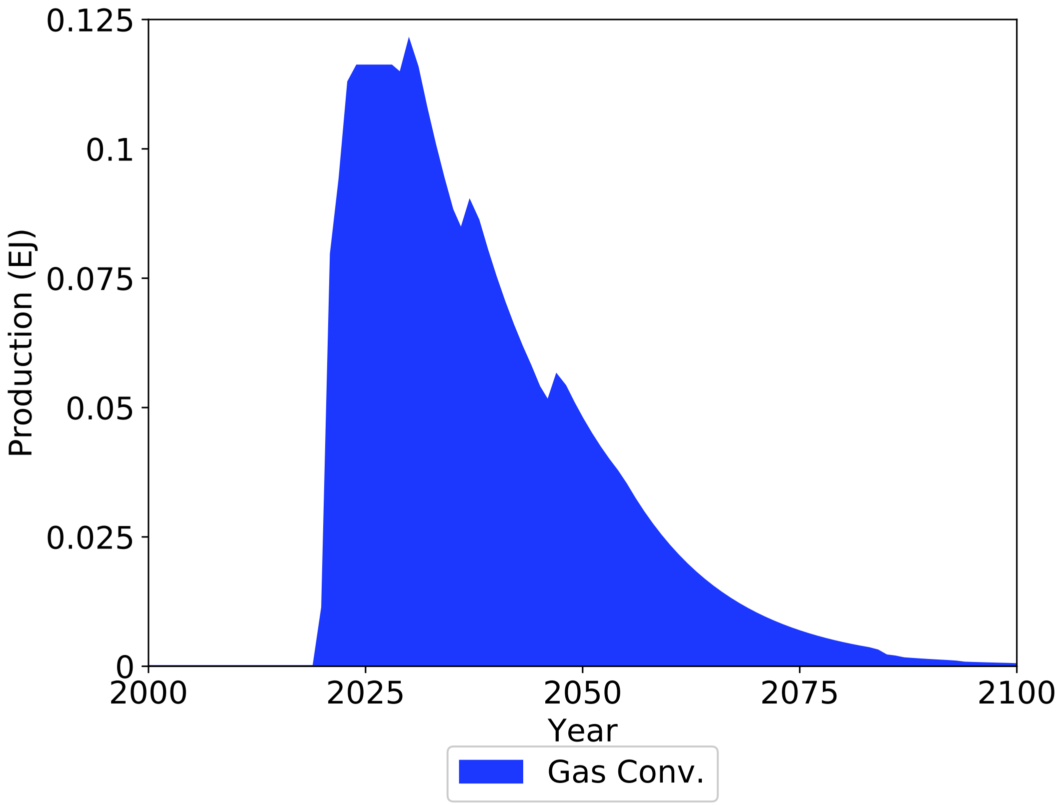

Figure 1.79: Sudan - Sudan Region projections capped at 16

Table 1.79: Peak years - All

Name	URR	Peak Year	Peak Rate
Gas Conv. Sudan Region	3.2	2030	0.12
**Total**	**3.2**	**2030**	**0.12**

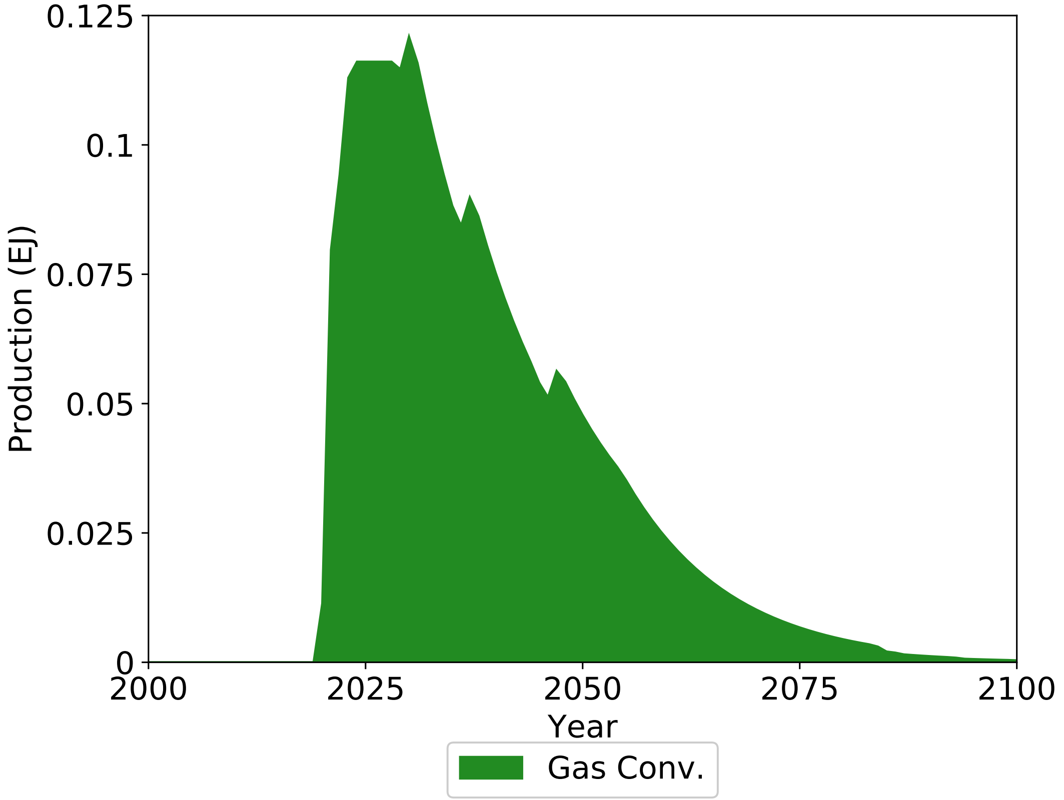

Figure 1.80: Sudan - Sudan Region projection by mineral type

Table 1.80: Peak years - Minerals

Name	URR	Peak Year	Peak Rate
Gas Conv.	3.2	2030	0.12
**Total**	**3.2**	**2030**	**0.12**

1.37.4 Projection by region

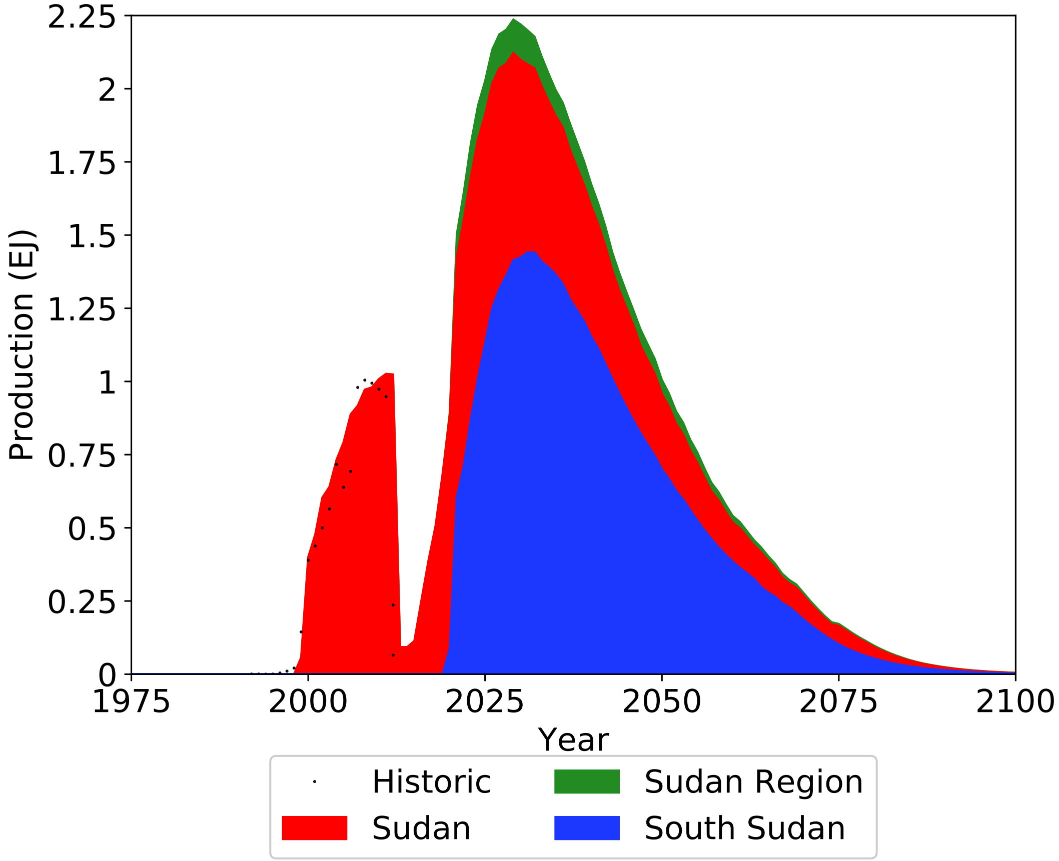

Figure 1.81: Sudan by region projections capped at 16

Table 1.81: Peak years - All

**Name**	**URR**	**Peak Year**	**Peak Rate**
South Sudan	42.69	2032	1.44
Sudan	33.66	2011	1.03
Sudan Region	3.2	2030	0.12
**Total**	**79.55**	**2029**	**2.24**

1.38 Swaziland

1.38.1 All Projections

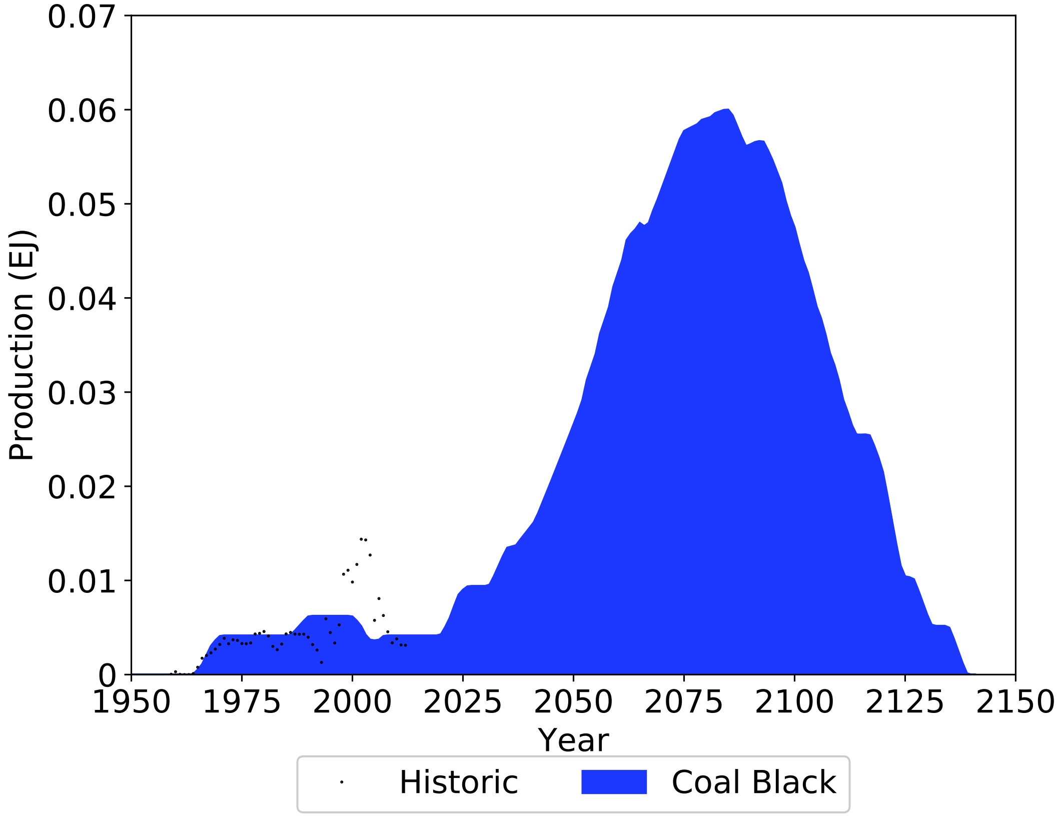

Figure 1.82: Swaziland projections capped at 16

Table 1.82: Peak years - All

Name	URR	Peak Year	Peak Rate
Coal Black	3.98	2085	0.06
**Total**	**3.98**	**2085**	**0.06**

1.38.2 By Mineral

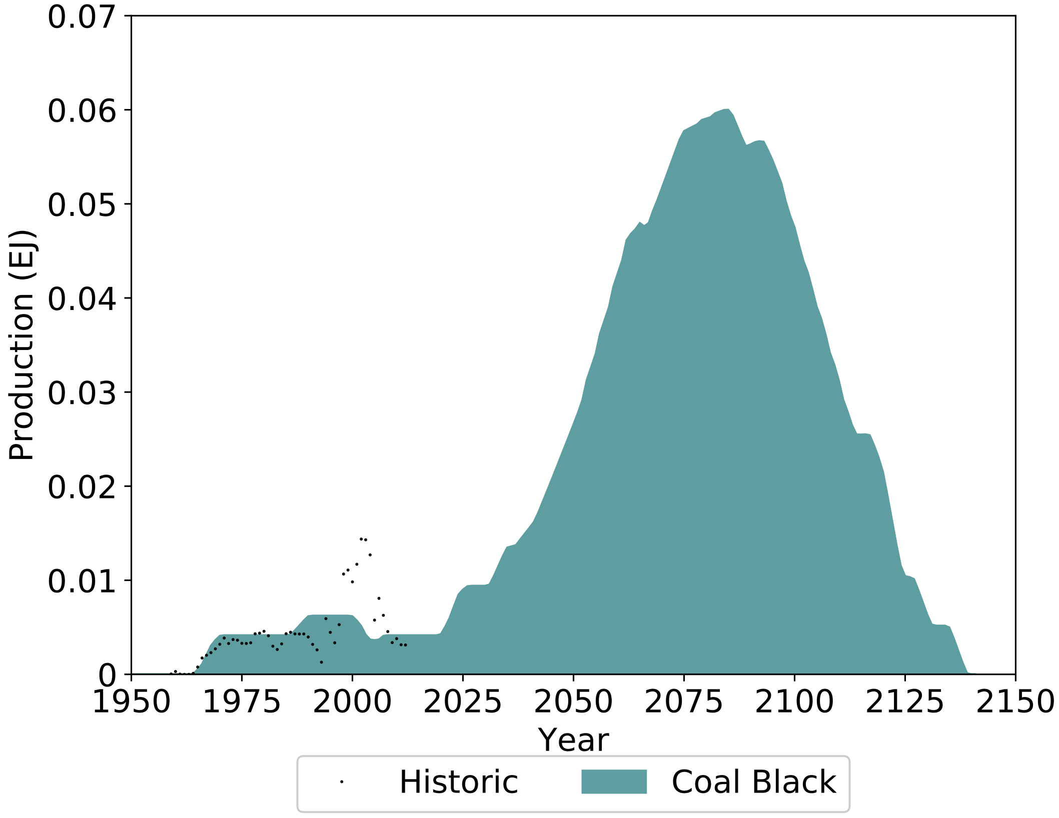

Figure 1.83: Swaziland projection by mineral type

Table 1.83: Peak years - Minerals

Name	URR	Peak Year	Peak Rate
Coal Black	3.98	2085	0.06
**Total**	**3.98**	**2085**	**0.06**

1.39 Tanzania

1.39.1 All Projections

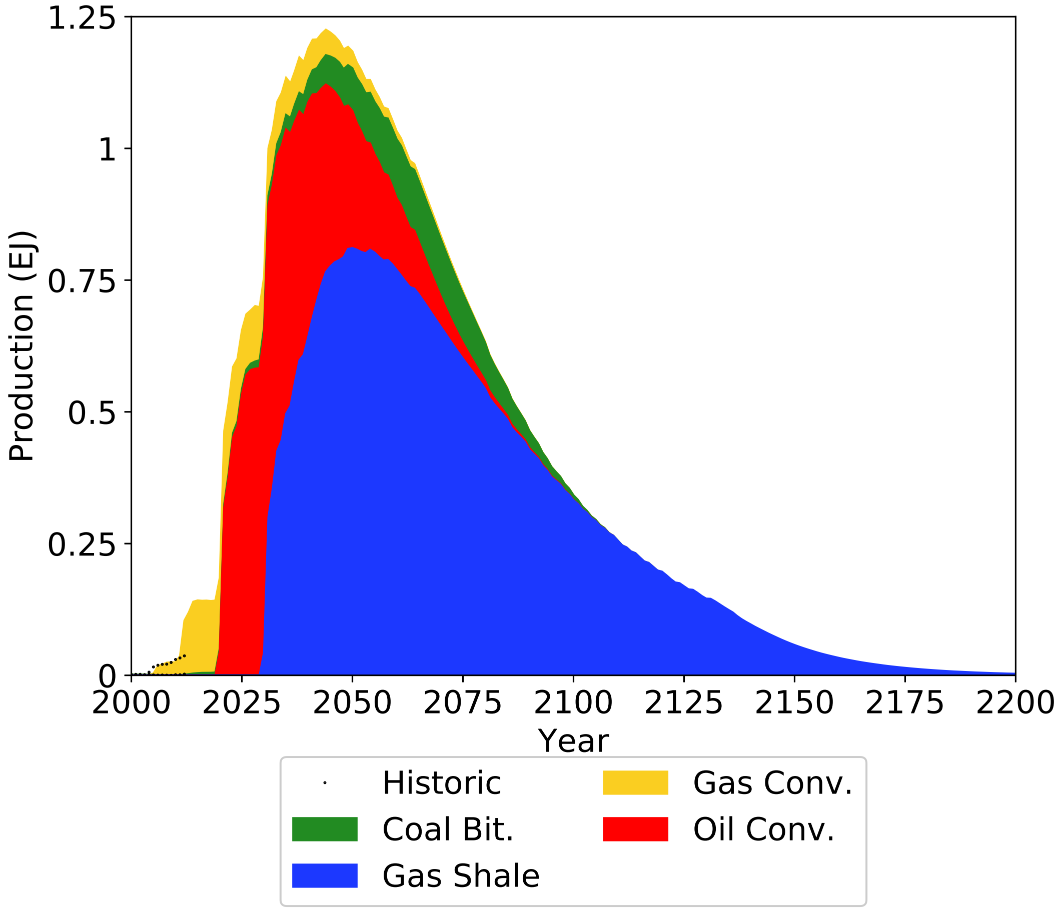

Figure 1.84: Tanzania projections capped at 16

Table 1.84: Peak years - All

**Name**	**URR**	**Peak Year**	**Peak Rate**
Gas Shale	51.91	2050	0.81
Oil Conv.	16.85	2030	0.6
Coal Bit.	4.83	2064	0.12
Gas Conv.	4.0	2021	0.14
**Total**	**77.59**	**2044**	**1.23**

1.39.2 By Mineral

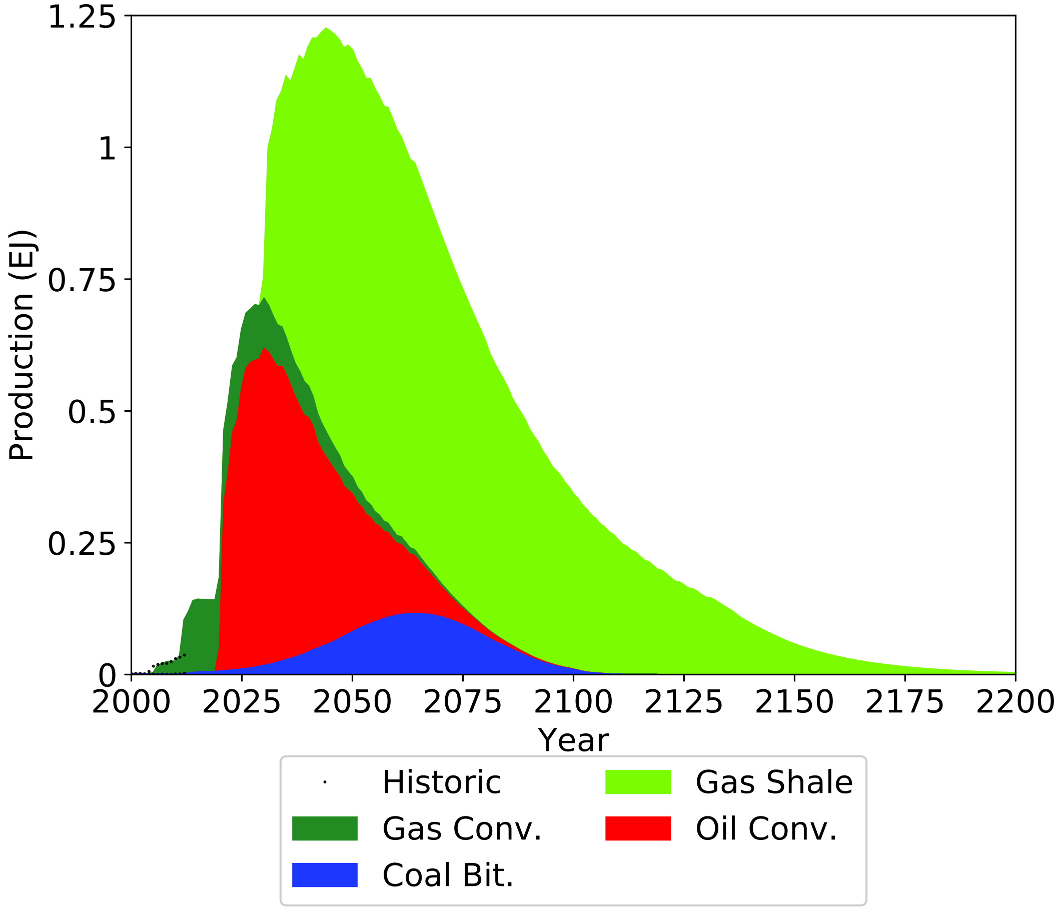

Figure 1.85: Tanzania projection by mineral type

Table 1.85: Peak years - Minerals

Name	URR	Peak Year	Peak Rate
Coal Bit.	4.83	2064	0.12
Oil Conv.	16.85	2030	0.6
Gas Conv.	4.0	2021	0.14
Gas Shale	51.91	2050	0.81
**Total**	**77.59**	**2044**	**1.23**

1.40 Togo

1.40.1 All Projections

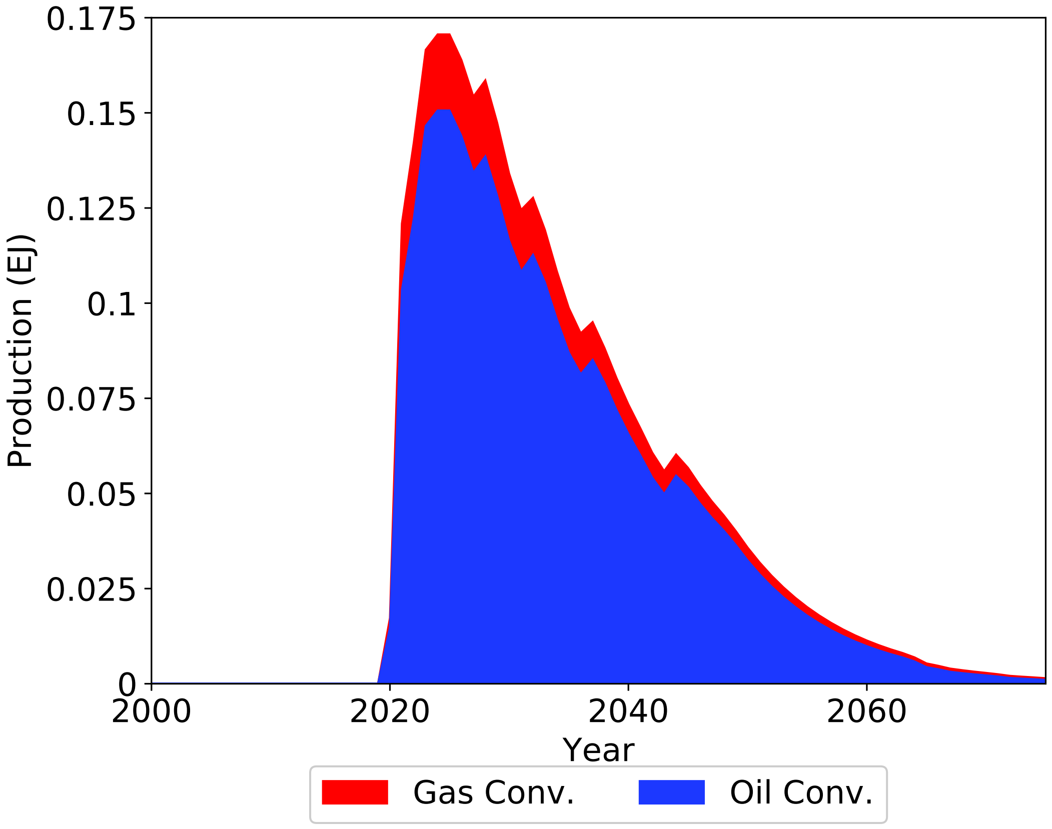

Figure 1.86: Togo projections capped at 16

Table 1.86: Peak years - All

Name	URR	Peak Year	Peak Rate
Oil Conv.	2.95	2024	0.15
Gas Conv.	0.4	2022	0.02
**Total**	**3.35**	**2024**	**0.17**

1.40.2 By Mineral

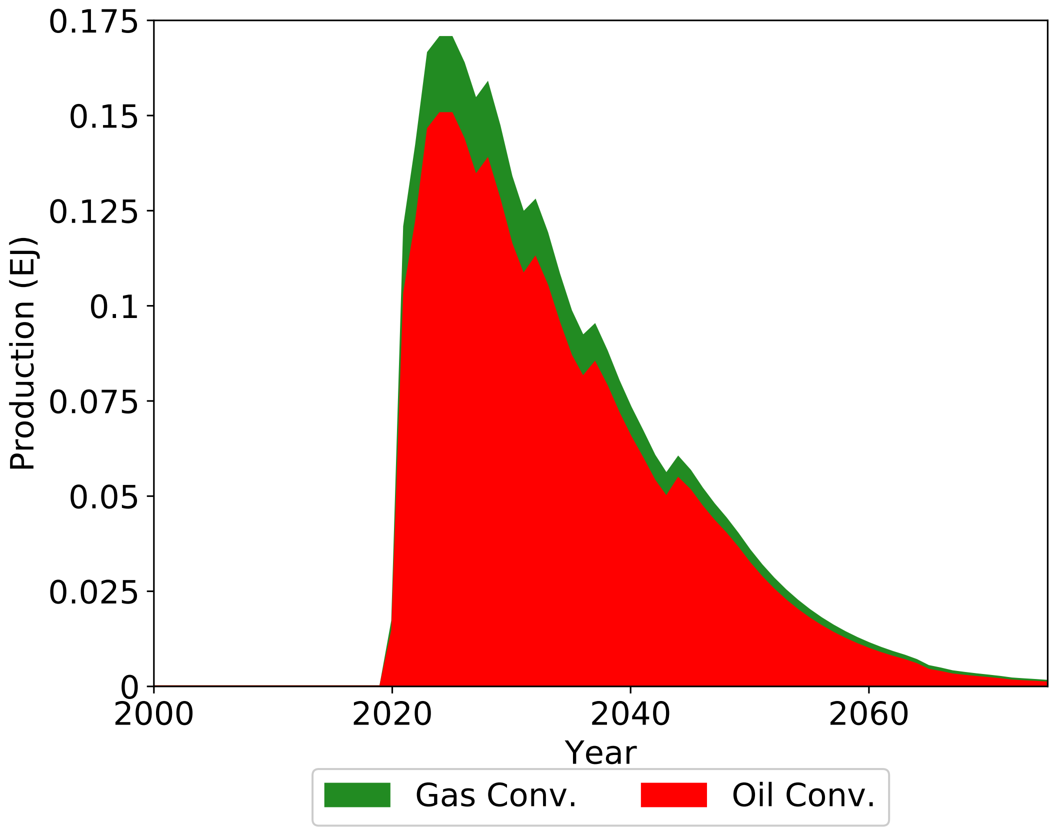

Figure 1.87: Togo projection by mineral type

Table 1.87: Peak years - Minerals

Name	URR	Peak Year	Peak Rate
Oil Conv.	2.95	2024	0.15
Gas Conv.	0.4	2022	0.02
**Total**	**3.35**	**2024**	**0.17**

1.41 Tunisia

1.41.1 All Projections

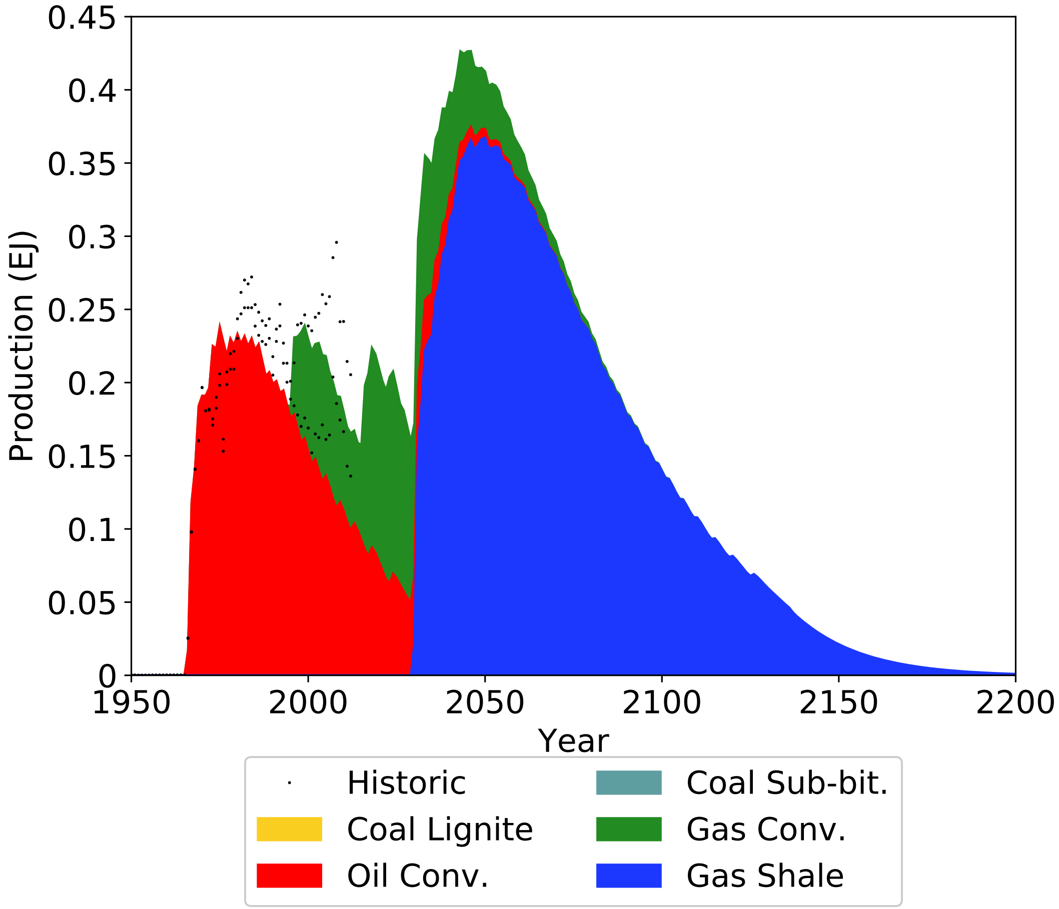

Figure 1.88: Tunisia projections capped at 16

Table 1.88: Peak years - All

**Name**	**URR**	**Peak Year**	**Peak Rate**
Gas Shale	22.62	2050	0.37
Oil Conv.	10.07	1975	0.24
Gas Conv.	5.3	2023	0.14
Coal Lignite	0.01	1943	–
Coal Sub-bit.	–	1919	–
**Total**	**38.0**	**2046**	**0.43**

1.41.2 By Mineral

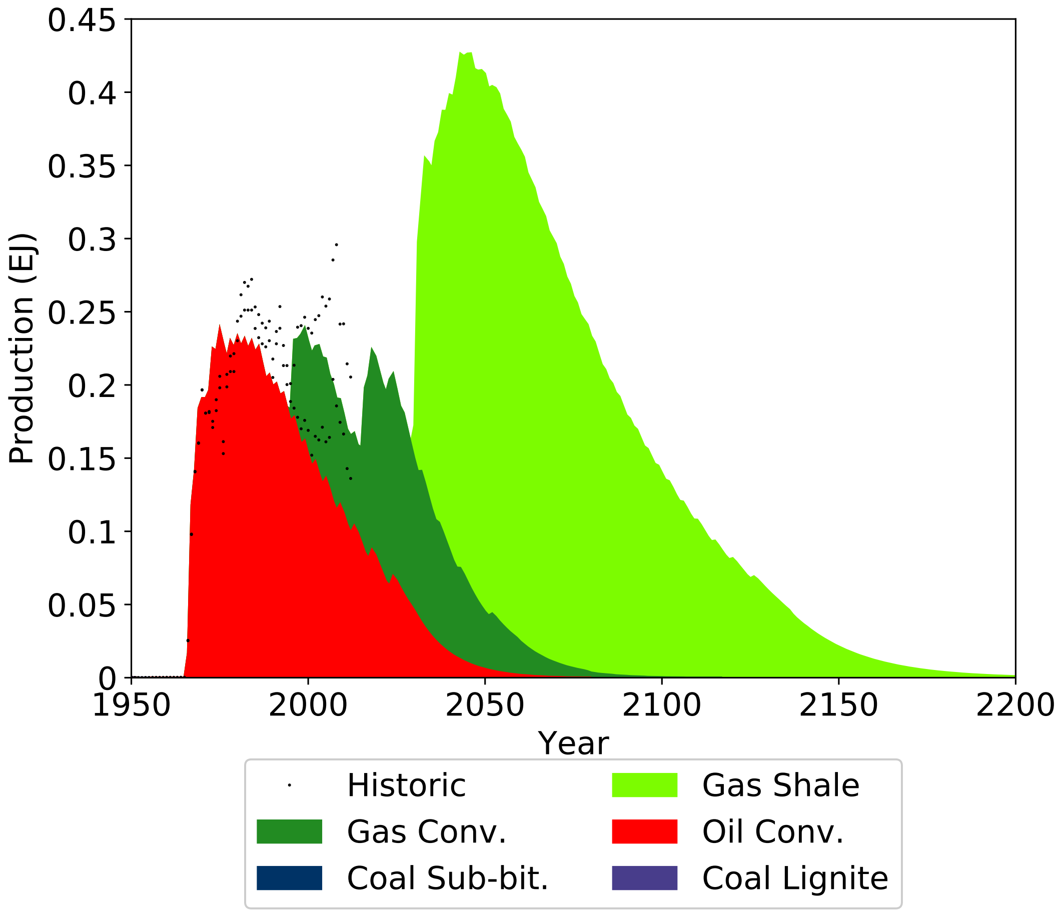

Figure 1.89: Tunisia projection by mineral type

Table 1.89: Peak years - Minerals

**Name**	**URR**	**Peak Year**	**Peak Rate**
Coal Lignite	0.01	1943	–
Coal Sub-bit.	–	1919	–
Oil Conv.	10.07	1975	0.24
Gas Conv.	5.3	2023	0.14
Gas Shale	22.62	2050	0.37
**Total**	**38.0**	**2046**	**0.43**

1.42 Uganda

1.42.1 All Projections

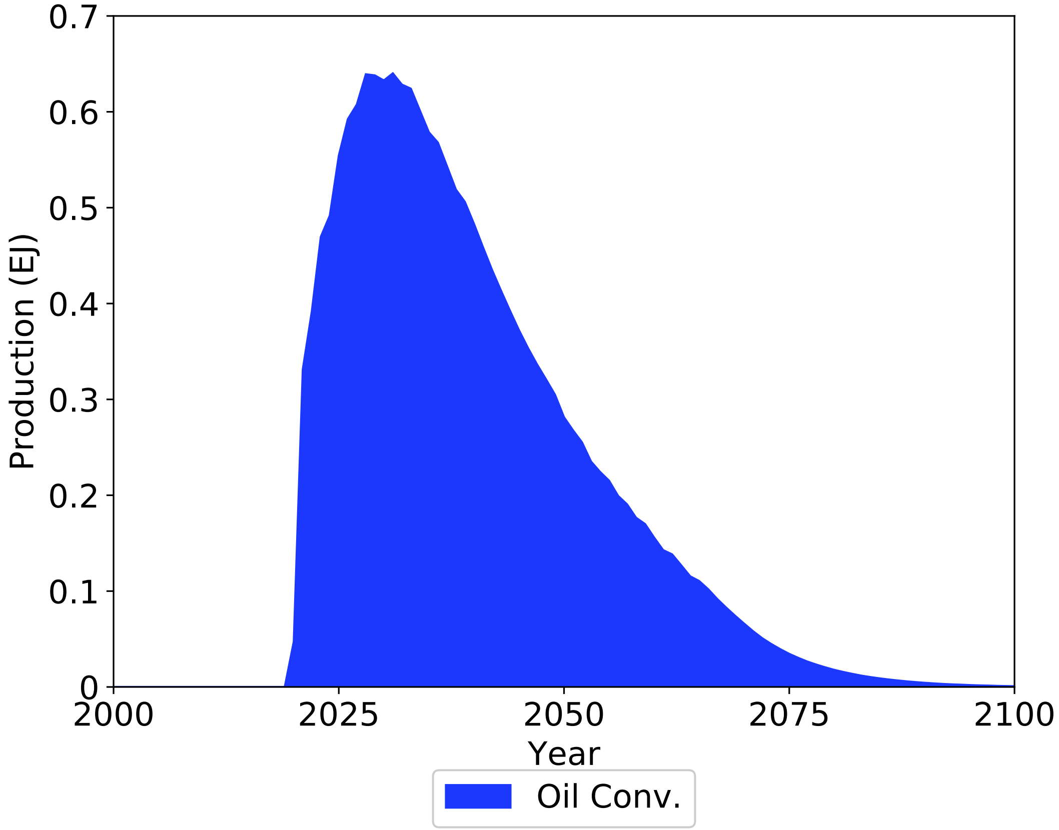

Figure 1.90: Uganda projections capped at 16

Table 1.90: Peak years - All

Name	URR	Peak Year	Peak Rate
Oil Conv.	18.36	2031	0.64
**Total**	**18.36**	**2031**	**0.64**

1.42.2 By Mineral

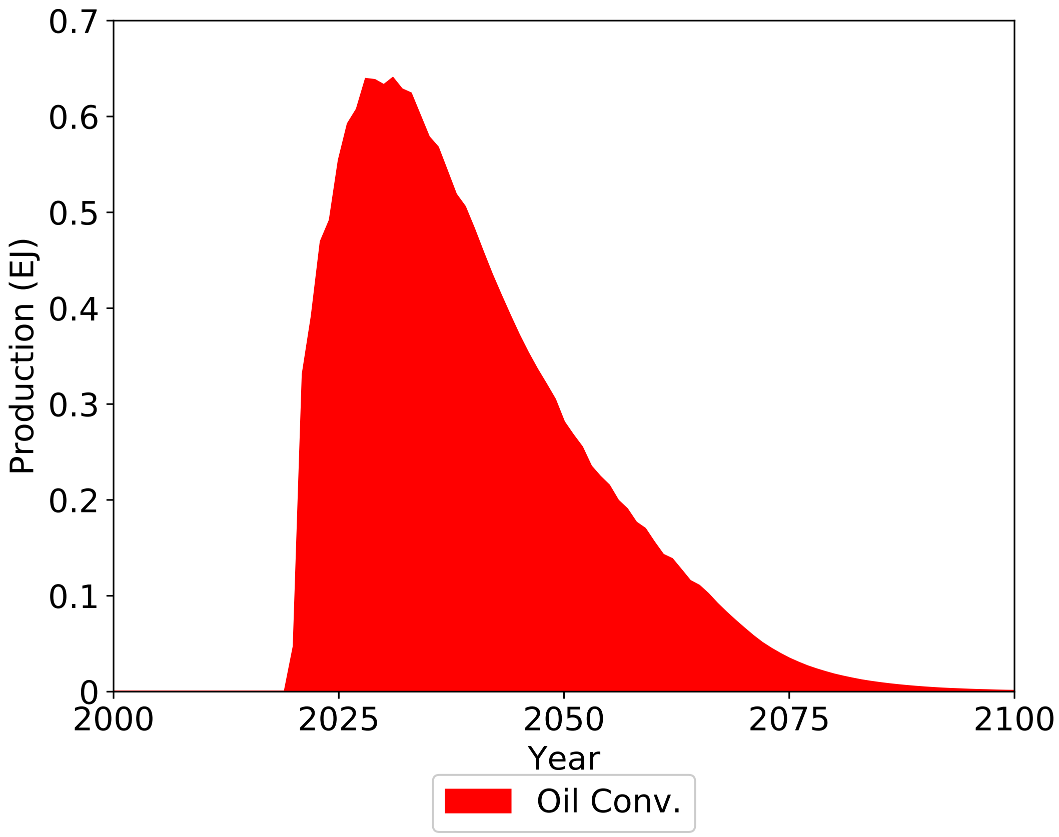

Figure 1.91: Uganda projection by mineral type

Table 1.91: Peak years - Minerals

Name	URR	Peak Year	Peak Rate
Oil Conv.	18.36	2031	0.64
**Total**	**18.36**	**2031**	**0.64**

1.43 Western Sahara

1.43.1 All Projections

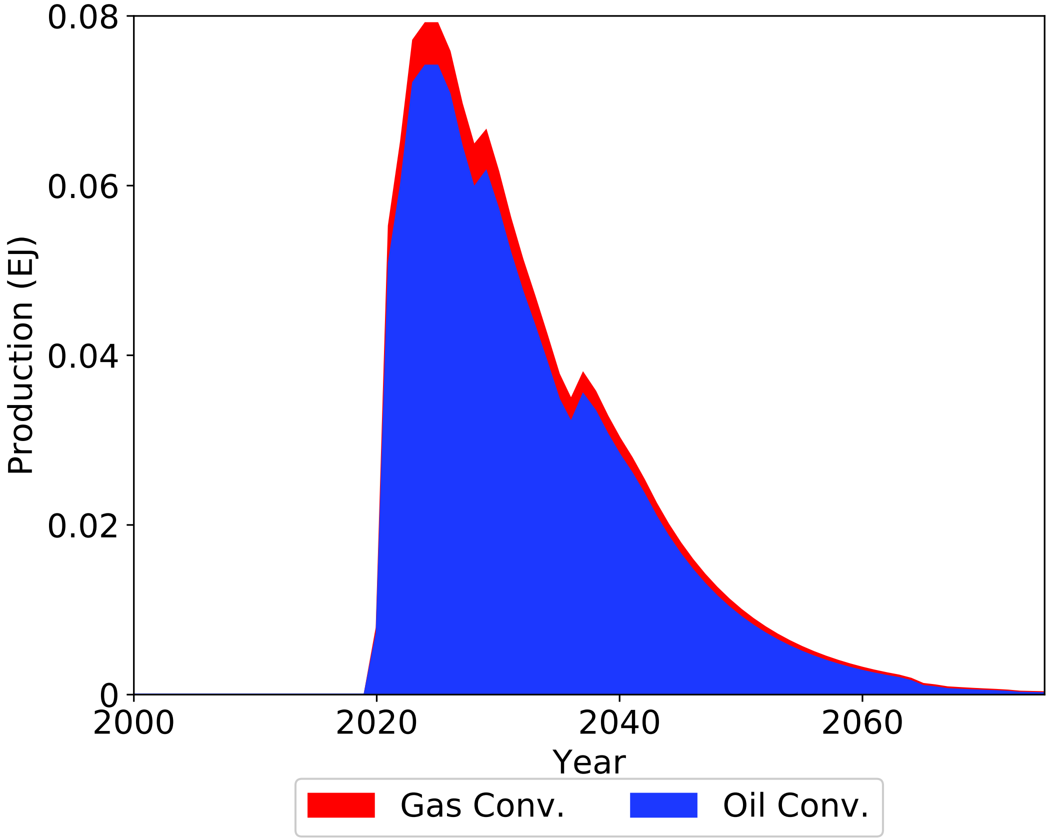

Figure 1.92: Western Sahara projections capped at 16

Table 1.92: Peak years - All

Name	URR	Peak Year	Peak Rate
Oil Conv.	1.26	2024	0.07
Gas Conv.	0.1	2022	0.01
**Total**	**1.36**	**2024**	**0.08**

1.43.2 By Mineral

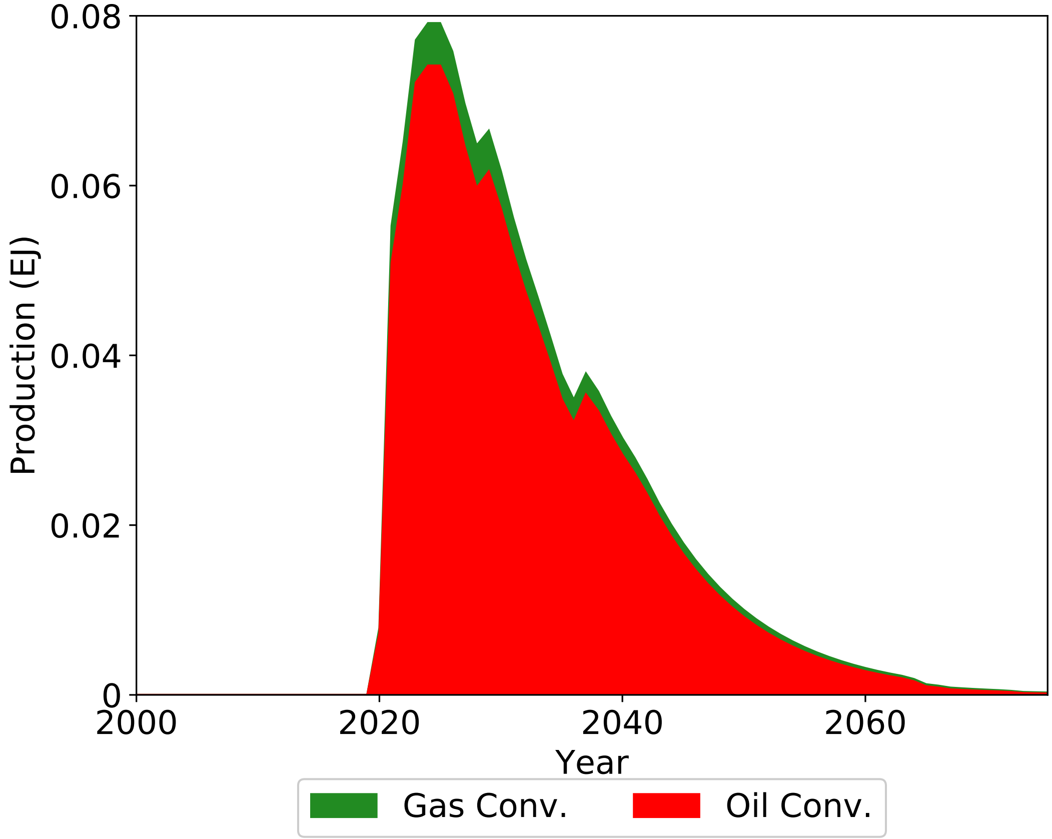

Figure 1.93: Western Sahara projection by mineral type

Table 1.93: Peak years - Minerals

Name	URR	Peak Year	Peak Rate
Oil Conv.	1.26	2024	0.07
Gas Conv.	0.1	2022	0.01
**Total**	**1.36**	**2024**	**0.08**

1.44 Zaire

1.44.1 All Projections

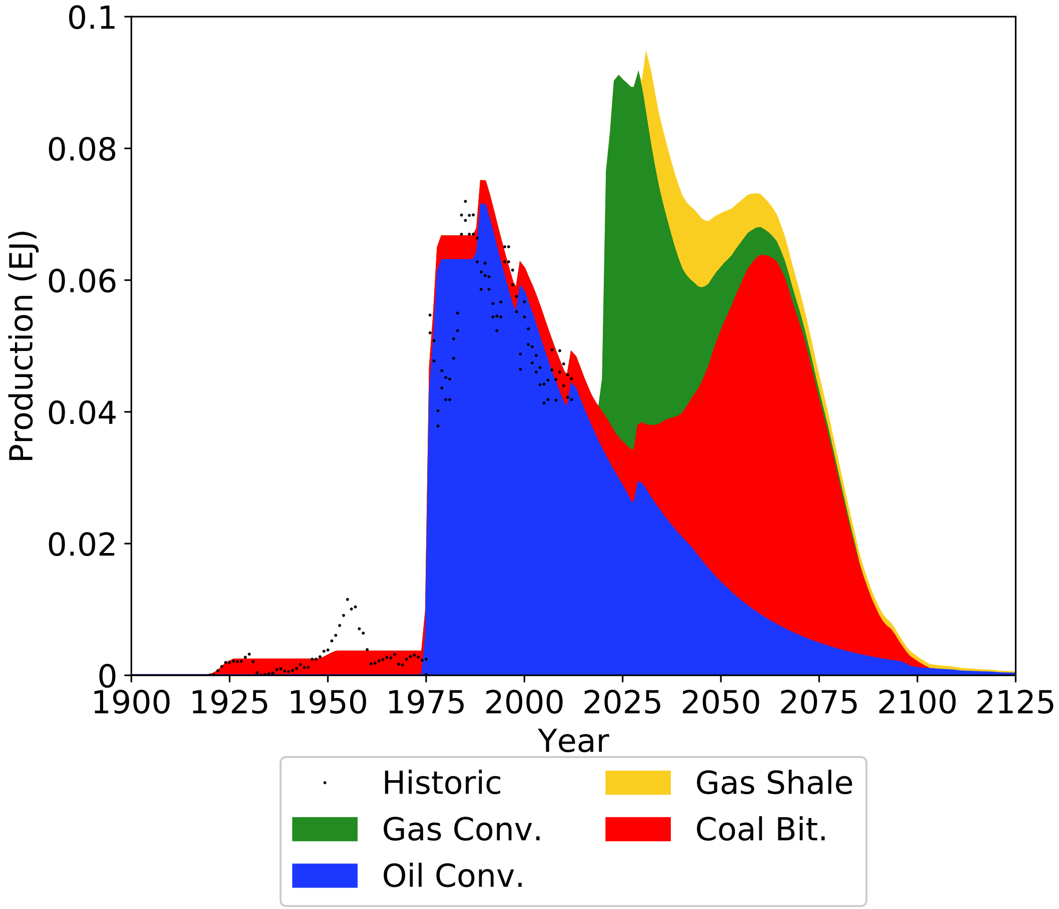

Figure 1.94: Zaire projections capped at 16

Table 1.94: Peak years - All

**Name**	**URR**	**Peak Year**	**Peak Rate**
Oil Conv.	3.43	1989	0.07
Coal Bit.	2.37	2063	0.06
Gas Conv.	1.1	2024	0.06
Gas Shale	0.37	2032	0.01
**Total**	**7.27**	**2031**	**0.09**

1.44.2 By Mineral

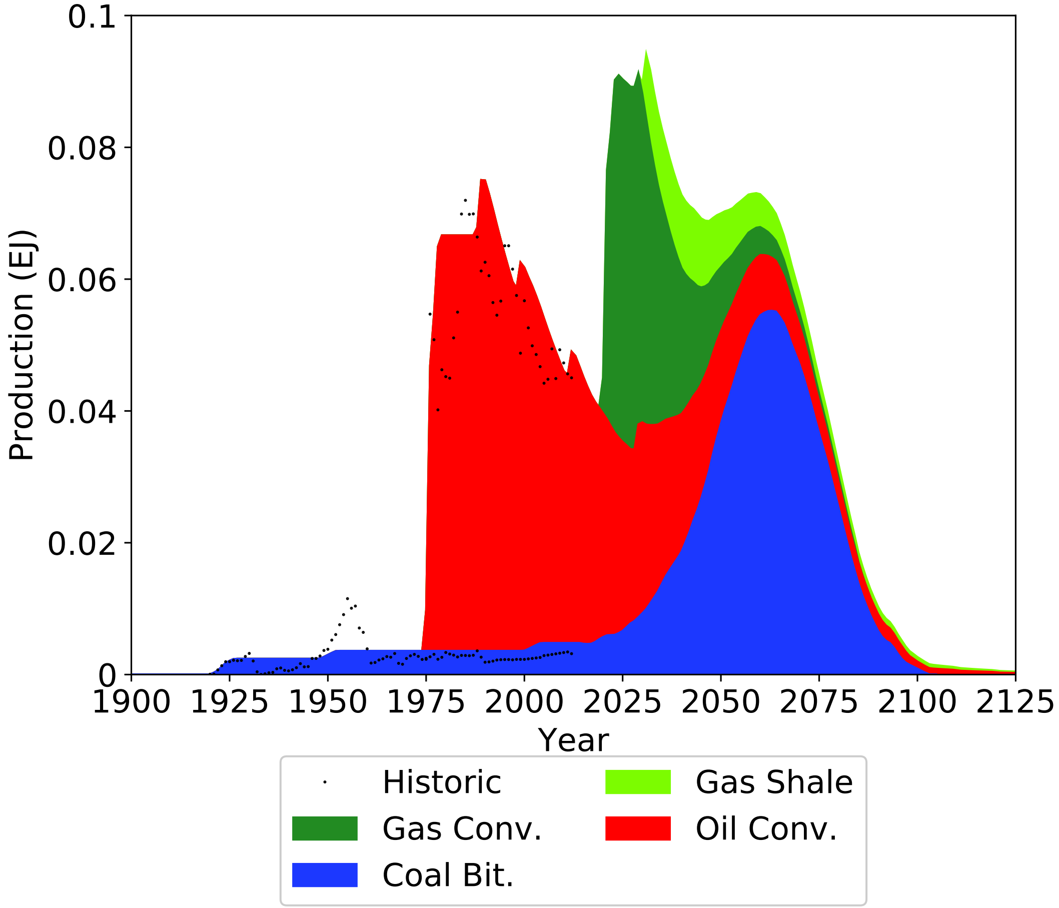

Figure 1.95: Zaire projection by mineral type

Table 1.95: Peak years - Minerals

**Name**	**URR**	**Peak Year**	**Peak Rate**
Coal Bit.	2.37	2063	0.06
Oil Conv.	3.43	1989	0.07
Gas Conv.	1.1	2024	0.06
Gas Shale	0.37	2032	0.01
**Total**	**7.27**	**2031**	**0.09**

1.45 Zambia

1.45.1 All Projections

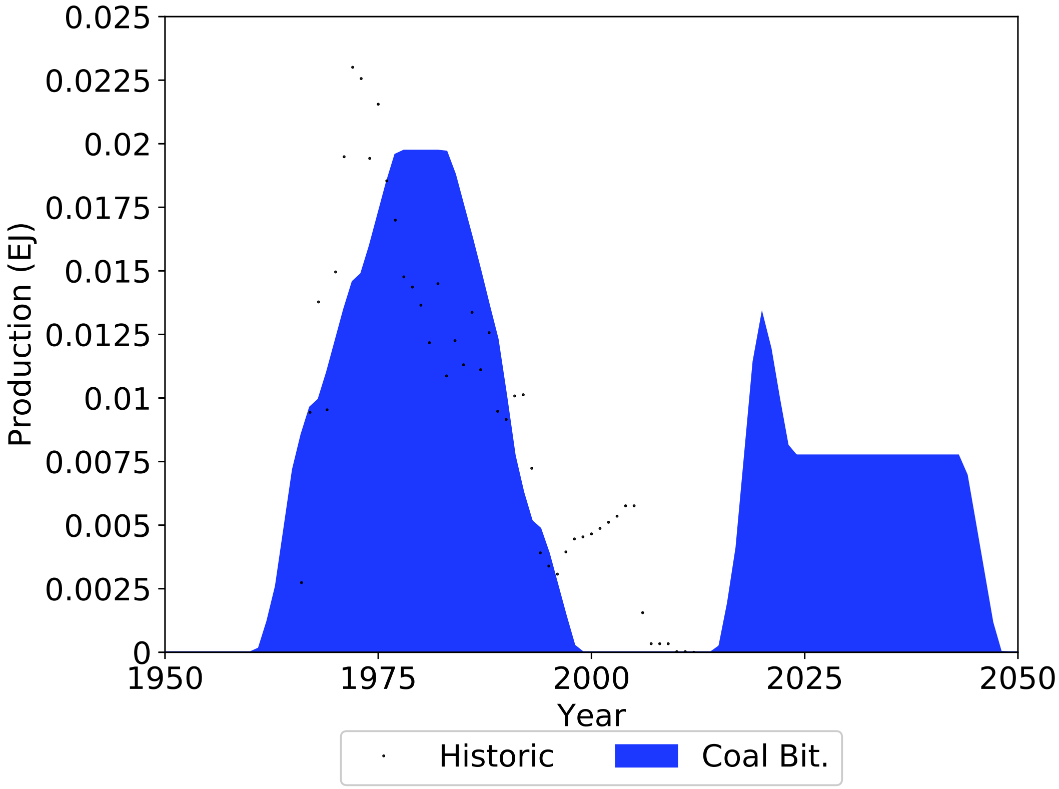

Figure 1.96: Zambia projections capped at 16

Table 1.96: Peak years - All

Name	URR	Peak Year	Peak Rate
Coal Bit.	0.68	1978	0.02
**Total**	**0.68**	**1978**	**0.02**

1.45.2 By Mineral

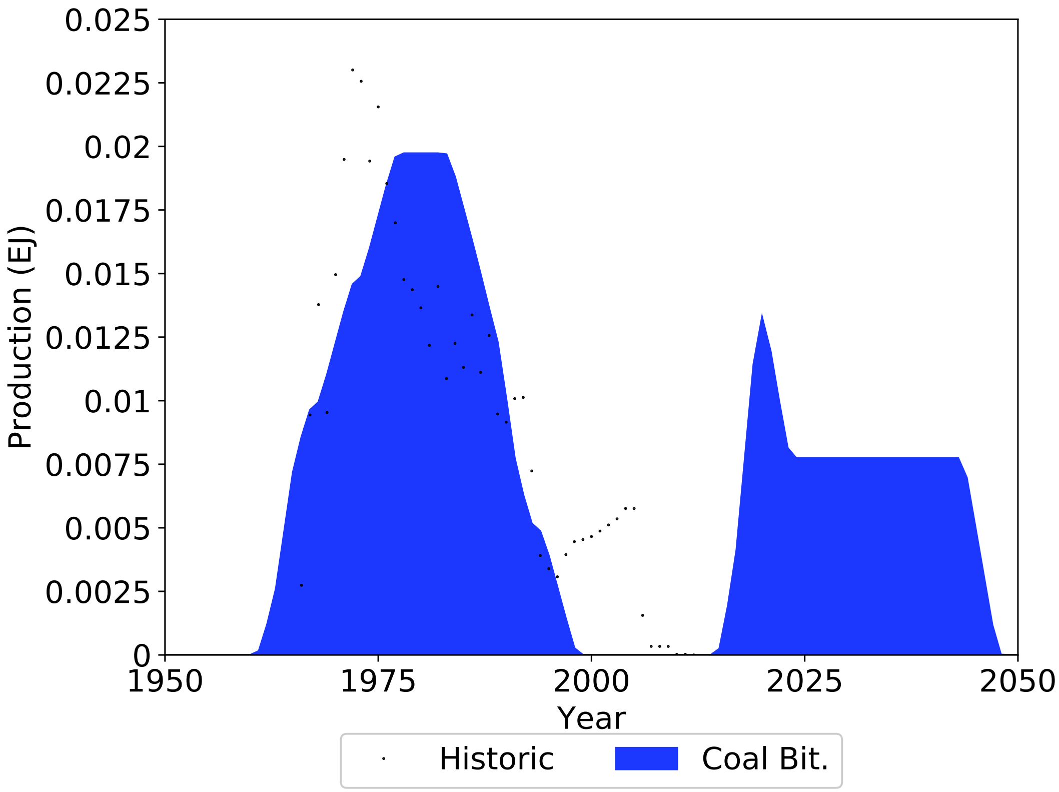

Figure 1.97: Zambia projection by mineral type

Table 1.97: Peak years - Minerals

Name	URR	Peak Year	Peak Rate
Coal Bit.	0.68	1978	0.02
**Total**	**0.68**	**1978**	**0.02**

1.46 Zimbabwe

1.46.1 All Projections

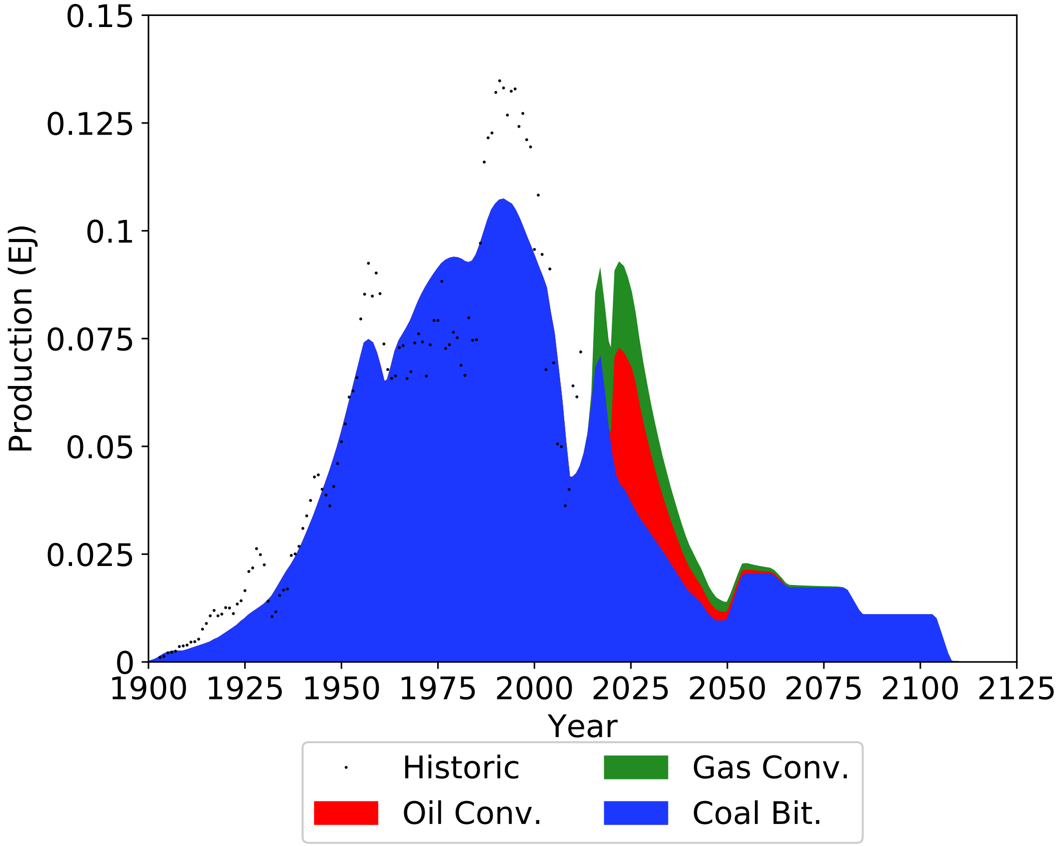

Figure 1.98: Zimbabwe projections capped at 16

Table 1.98: Peak years - All

Name	URR	Peak Year	Peak Rate
Coal Bit.	7.88	1992	0.11
Oil Conv.	0.42	2022	0.03
Gas Conv.	0.4	2017	0.02
**Total**	**8.7**	**1992**	**0.11**

1.46.2 By Mineral

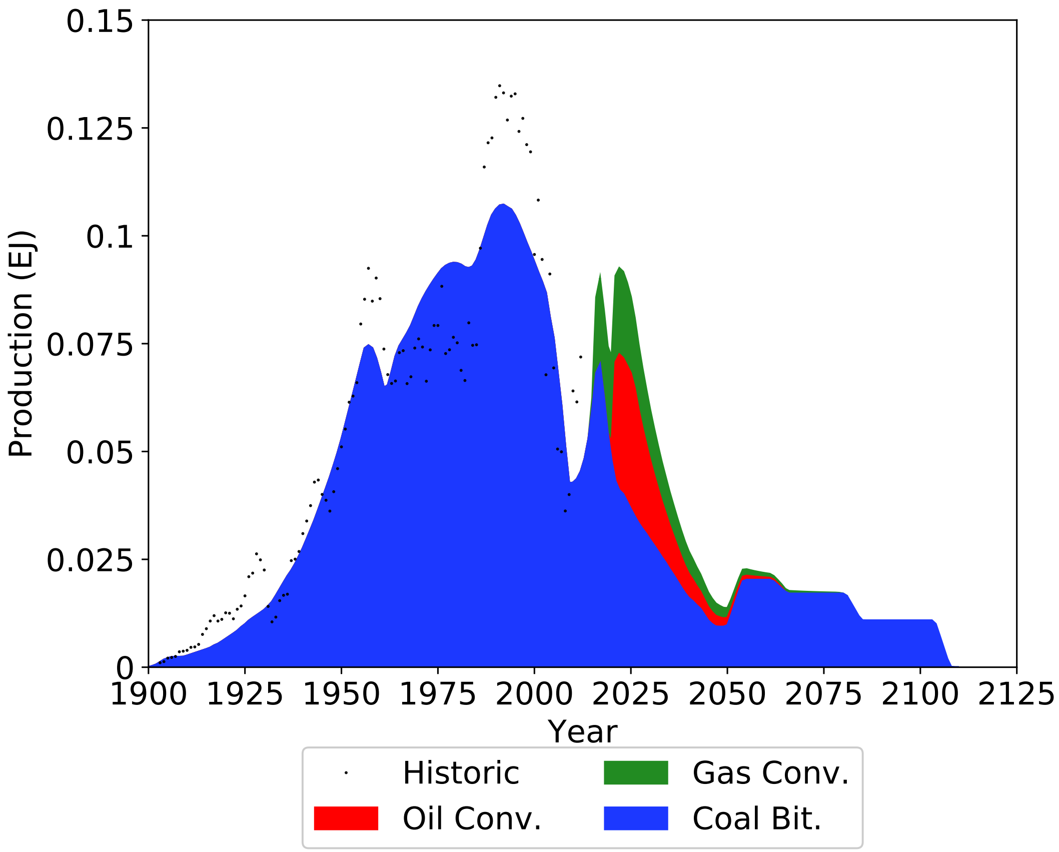

Figure 1.99: Zimbabwe projection by mineral type

Table 1.99: Peak years - Minerals

Name	URR	Peak Year	Peak Rate
Coal Bit.	7.88	1992	0.11
Oil Conv.	0.42	2022	0.03
Gas Conv.	0.4	2017	0.02
**Total**	**8.7**	**1992**	**0.11**

1.47 Total

1.47.1 By country

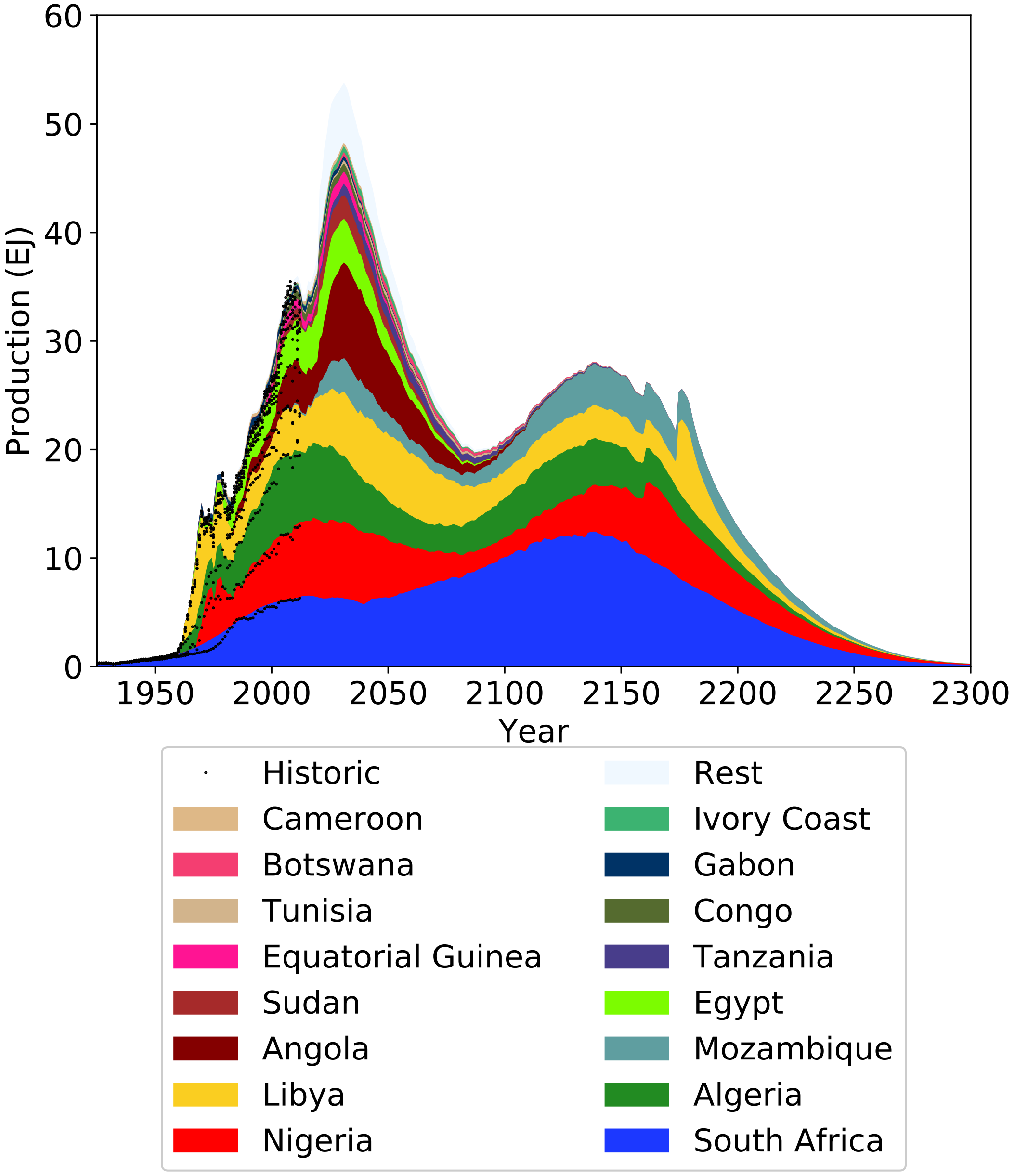

Figure 1.100: Africa projections by country

Table 1.100: Peak years - All

Name	URR	Peak Year	Peak Rate
South Africa	2003.47	2138	12.35
Nigeria	1125.36	2018	7.24
Algeria	937.76	2007	7.16
Libya	927.75	1970	7.51
Mozambique	490.74	2144	3.81
Angola	403.79	2032	8.87
Egypt	252.84	2024	4.42
Sudan	79.55	2029	2.24
Tanzania	77.59	2044	1.23
Equatorial Guinea	45.31	2025	1.32
Congo	42.64	2023	0.91
Tunisia	38.0	2046	0.43
Gabon	37.65	1994	0.77
Botswana	35.23	2050	0.59
Ivory Coast	30.93	2037	0.63
Cameroon	21.86	2021	0.4
Seychelles	20.5	2028	0.72
Namibia	19.7	2028	0.63
Uganda	18.36	2031	0.64
Ghana	17.83	2020	0.56
Somalia	15.98	2038	0.4
Sierra Leone	10.95	2028	0.42
Kenya	10.53	2028	0.41
Zimbabwe	8.7	1992	0.11
Sao Tome and Principe	7.58	2026	0.31
Zaire	7.27	2031	0.09
Liberia	6.74	2028	0.26
Guinea	6.32	2026	0.28
Eritrea	6.02	2028	0.21
Senegal	6.0	2026	0.26
Chad	4.17	2005	0.35
Swaziland	3.98	2085	0.06
Togo	3.35	2024	0.17
Niger	3.13	2016	0.09
Rwanda	2.9	2030	0.11
Ethiopia	2.55	2024	0.14
Mauritania	2.34	2024	0.08
Madagascar	2.3	2024	0.12
Morocco	2.02	1972	0.03
Guinea-Bissau	1.88	2026	0.08
Western Sahara	1.36	2024	0.08
Benin	0.97	2024	0.04
Gambia	0.84	2024	0.05
Zambia	0.68	1978	0.02
Malawi	0.08	2020	–
Central African Republic	0.03	2026	–
**Total**	**6745.5**	**2031**	**53.63**

1.47.2 By mineral

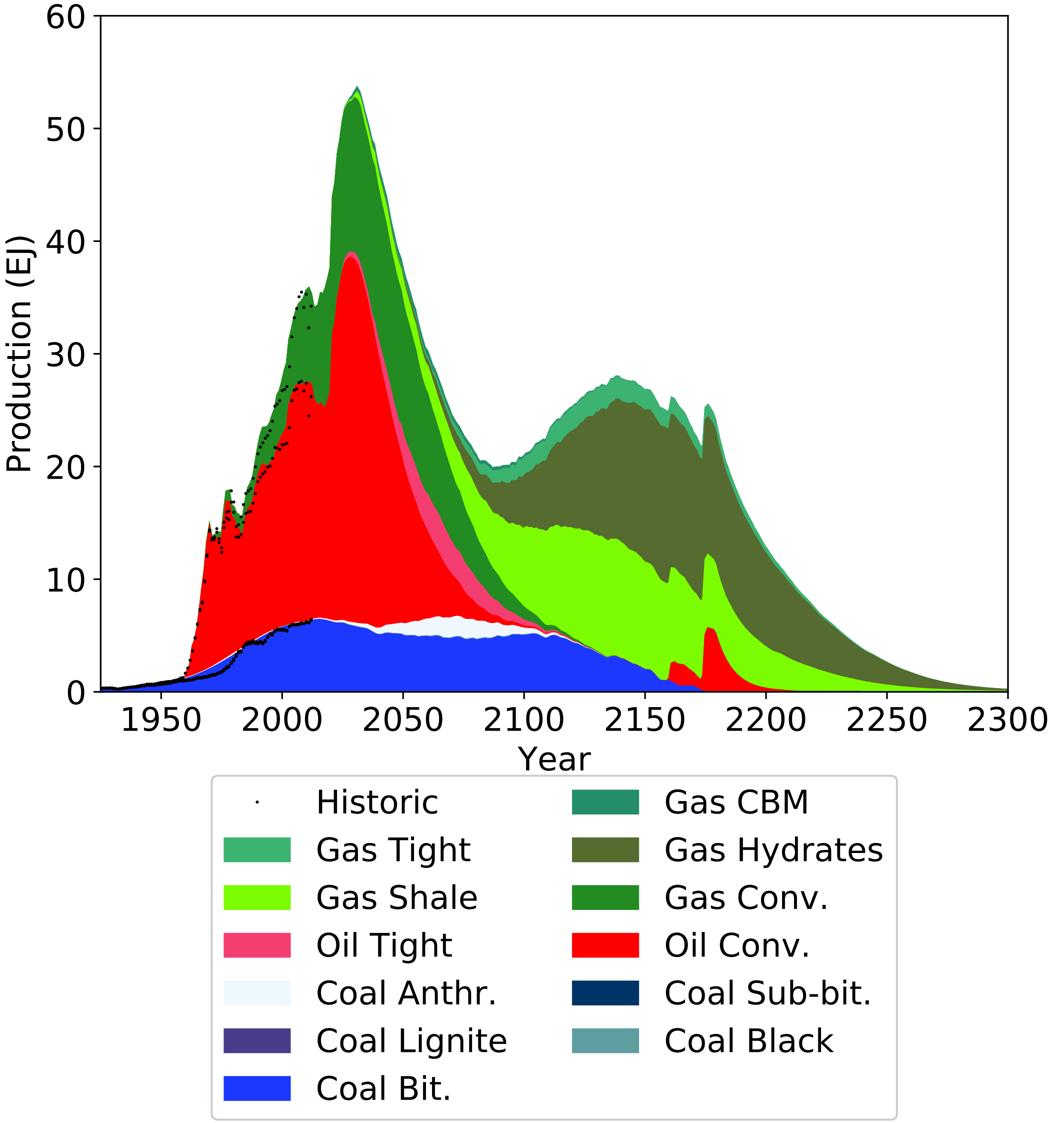

Figure 1.101: Africa projection by mineral type

Table 1.101: Peak years - Minerals

**Name**	**URR**	**Peak Year**	**Peak Rate**
Coal Bit.	887.34	2015	6.38
Coal Black	3.98	2085	0.06
Coal Lignite	0.42	2055	0.01
Coal Sub-bit.	0.54	1961	0.01
Coal Anthr.	95.9	2071	1.8
Oil Conv.	1894.53	2028	32.4
Oil Tight	149.55	2064	3.37
Gas Conv.	901.44	2032	14.04
Gas Shale	1130.32	2132	10.47
Gas Hydrates	1438.0	2158	13.8
Gas Tight	203.94	2128	2.16
Gas CBM	39.55	2044	0.65
**Total**	**6745.5**	**2031**	**53.63**

Chapter 2

Asia

2.1 Afghanistan

2.1.1 All Projections

Table 2.1: Peak years - All

Name	URR	Peak Year	Peak Rate
Gas Conv.	24.3	2032	0.57
Oil Conv.	12.21	2028	0.45
Coal Bit.	1.79	2029	0.06
**Total**	**38.3**	**2030**	**1.06**

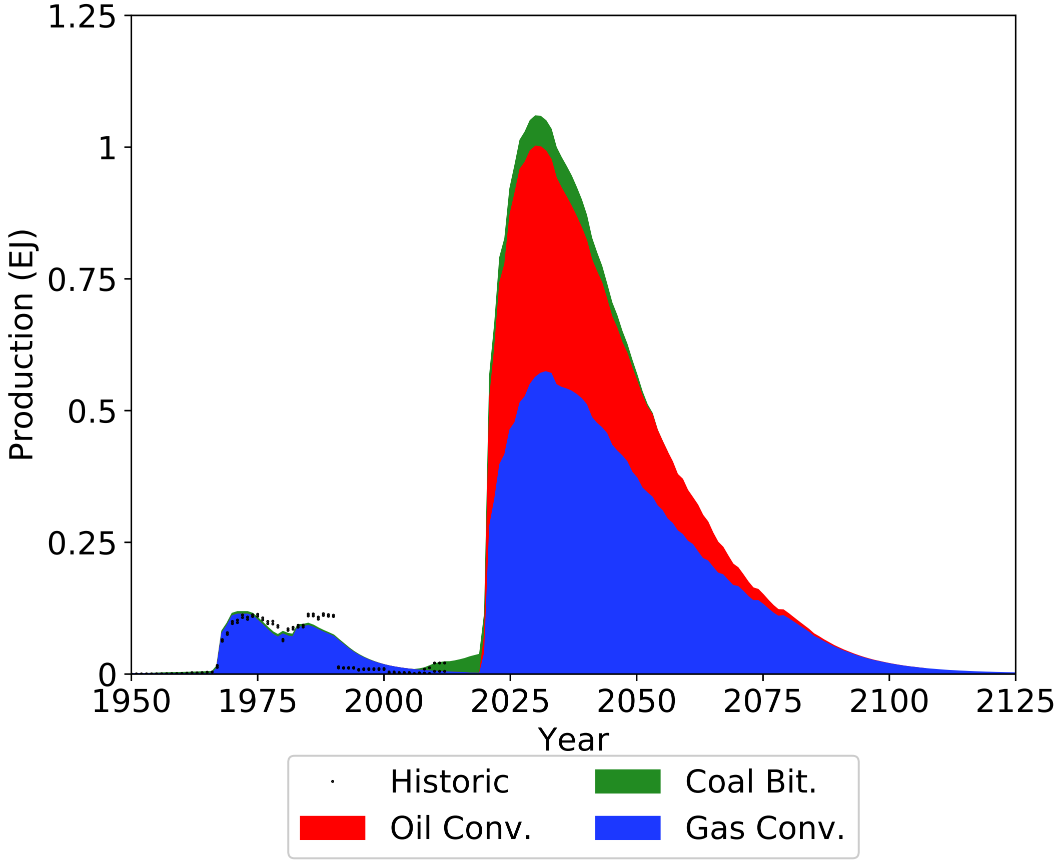

Figure 2.1: Afghanistan projections capped at 16

2.1.2 By Mineral

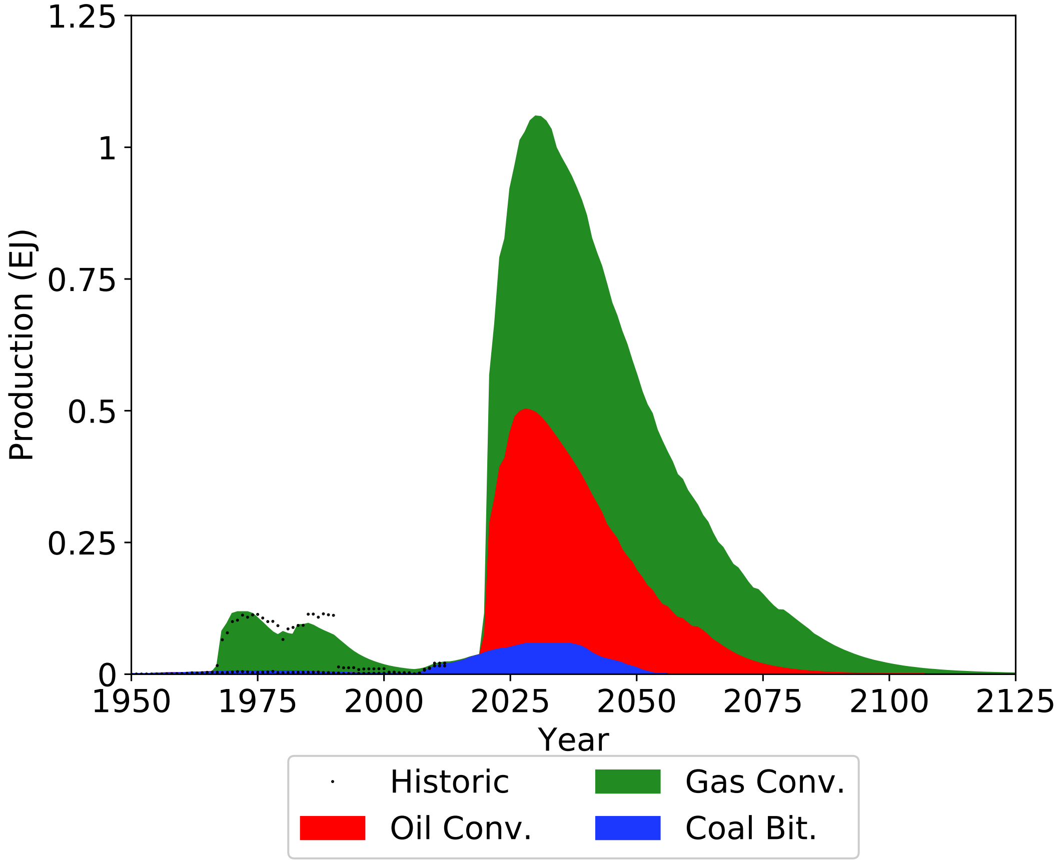

Figure 2.2: Afghanistan projection by mineral type

Table 2.2: Peak years - Minerals

Name	URR	Peak Year	Peak Rate
Coal Bit.	1.79	2029	0.06
Oil Conv.	12.21	2028	0.45
Gas Conv.	24.3	2032	0.57
**Total**	**38.3**	**2030**	**1.06**

2.2 Australia

2.2.1 All Projections

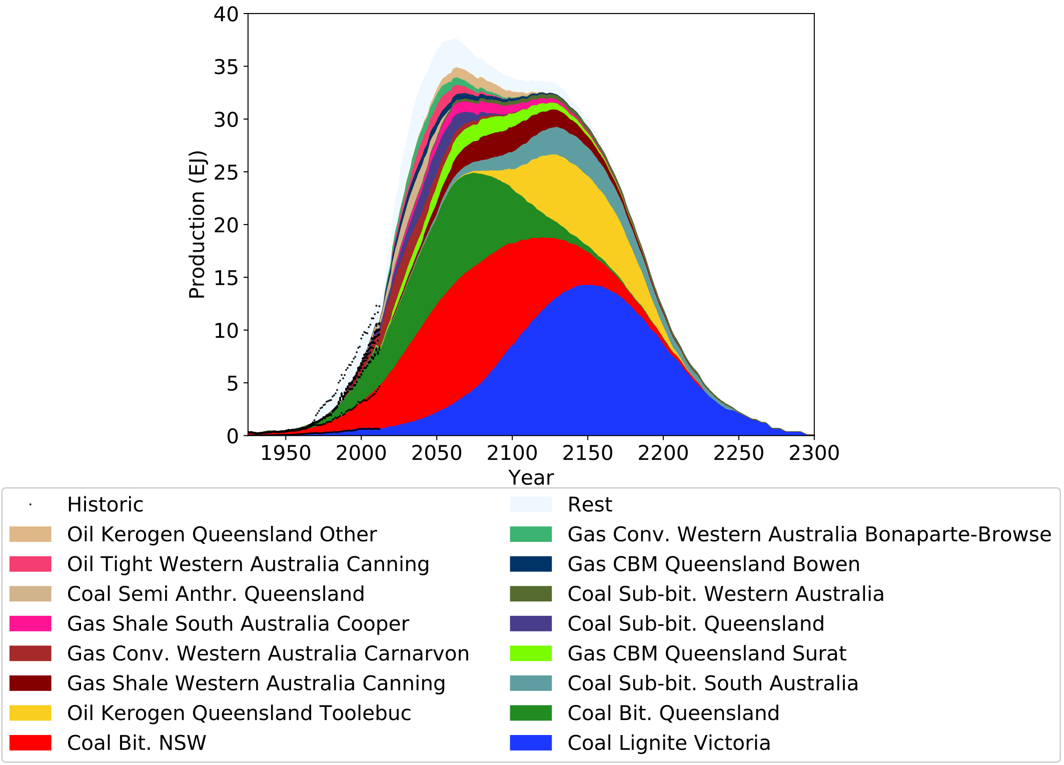

Figure 2.3: Australia projections capped at 16

Table 2.3: Peak years - All

**Name**	**URR**	**Peak Year**	**Peak Rate**
Coal Lignite Victoria	1797.05	2152	14.22
Coal Bit. NSW	1320.1	2072	11.64
Coal Bit. Queensland	839.09	2062	9.52
Oil Kerogen Queensland Toolebuc	515.7	2140	6.73
Coal Sub-bit. South Australia	302.33	2145	2.77
Gas Shale Western Australia Canning	223.63	2100	2.37
Gas CBM Queensland Surat	155.71	2061	1.79
Gas Conv. Western Australia Carnarvon	113.35	2023	2.43
Coal Sub-bit. Queensland	104.07	2049	2.29
Gas Shale South Australia Cooper	88.5	2060	1.06
Coal Sub-bit. Western Australia	75.74	2143	0.42
Coal Semi Anthr. Queensland	60.7	2033	1.9
Gas CBM Queensland Bowen	56.68	2034	0.87
Oil Tight Western Australia Canning	55.58	2041	1.44
Gas Conv. Western Australia Bonaparte-Browse	54.9	2038	1.15
Oil Kerogen Queensland Other	53.37	2072	1.03
Gas Shale Northern Territory Beetaloo	41.87	2045	0.66
Coal Lignite South Australia	41.8	2130	0.43
Gas Shale Western Australia Perth	31.4	2046	0.5
Oil Conv. Victoria Gippsland	30.0	1980	0.96
Oil Conv. Western Australia Carnarvon	28.44	2003	0.82
Oil Tight Northern Territory Beetaloo	26.93	2049	0.74
Gas Tight Australia	21.0	2041	0.35
Gas Shale Queensland Maryborough	18.08	2042	0.31
Coal Lignite Western Australia	17.58	2126	0.24
Gas Conv. Victoria Gippsland	17.53	2001	0.26
Gas CBM NSW	15.96	2031	0.27
Oil Conv. Northern Territory Browse-Bonaparte	13.84	2028	0.49
Gas Shale Northern Territory Georgina	12.37	2042	0.22
Coal Bit. Tasmania	6.7	2099	0.06
Gas Conv. South Australia Cooper-Eromanga	6.69	1977	0.2
Gas Conv. JPDA Timor Gap	6.29	2015	0.18
Oil Tight South Australia Cooper	5.73	2024	0.25
Oil Tight Northern Territory Georgina	5.73	2026	0.25
Oil Tight Western Australia Perth	2.87	2025	0.14
Oil Tight Queensland Cooper	2.87	2022	0.14
Gas CBM Queensland Clarence	2.65	2040	0.06
Oil Conv. South Australia Cooper-Eromanga	2.54	1987	0.11
Gas Conv. Victoria Otway	2.3	2010	0.11
Gas Conv. Queensland Cooper-Eromanga	2.23	1997	0.16
Oil Conv. JPDA Timor Gap	1.45	2017	0.07
Oil Conv. Queensland Cooper-Eromanga	1.17	1985	0.04
Gas Conv. Queensland Surat-Bowen	1.12	1990	0.02
Oil Conv. Northern Territory Timor Sea	1.09	2001	0.16
Coal Lignite Tasmania	1.05	2055	0.05
Oil Kerogen South Australia	1.03	2036	0.03
Gas Conv. Western Australia Perth	0.94	1981	0.03
Gas Conv. Tasmania Bass	0.84	2019	0.04
Gas Conv. Northern Territory Amadeus	0.69	1997	0.02

Table 2.3: Peak years - All – Continued

**Name**	**URR**	**Peak Year**	**Peak Rate**
Oil Conv. Tasmania Bass	0.68	2024	0.04
Coal Bit. Victoria	0.55	1917	0.02
Gas Conv. Queensland Denison	0.41	1992	0.02
Oil Conv. Queensland Surat-Bowen	0.35	1964	0.02
Oil Conv. Western Australia Perth	0.21	2005	0.03
Oil Conv. Northern Territory Amadeus	0.16	2024	0.01
Oil Conv. Western Australia Timor Sea	0.12	2002	0.03
Oil Conv. Victoria Otway	0.12	2010	0.01
Oil Kerogen NSW	0.09	2030	0.01
Oil Kerogen Tasmania	0.08	2020	0.01
Gas Conv. South Australia Otway	0.08	2000	0.01
Gas Conv. Queensland Clarence	0.07	2022	–
Gas Conv. Queensland Adavale	0.03	1997	–
Oil Conv. Western Australia Canning	0.02	1987	–
Gas Conv. Western Australia Canning	0.01	2022	–
Gas Conv. NSW Gunnedah	0.01	2017	–
Oil Conv. South Australia Otway	–	1994	–
Coal Sub-bit. NSW	–	1944	–
Coal Semi Anthr. Tasmania	–	1953	–
Gas CBM Western Australia	–	2008	–
**Total**	**6192.26**	**2061**	**37.62**

2.2.2 By Mineral

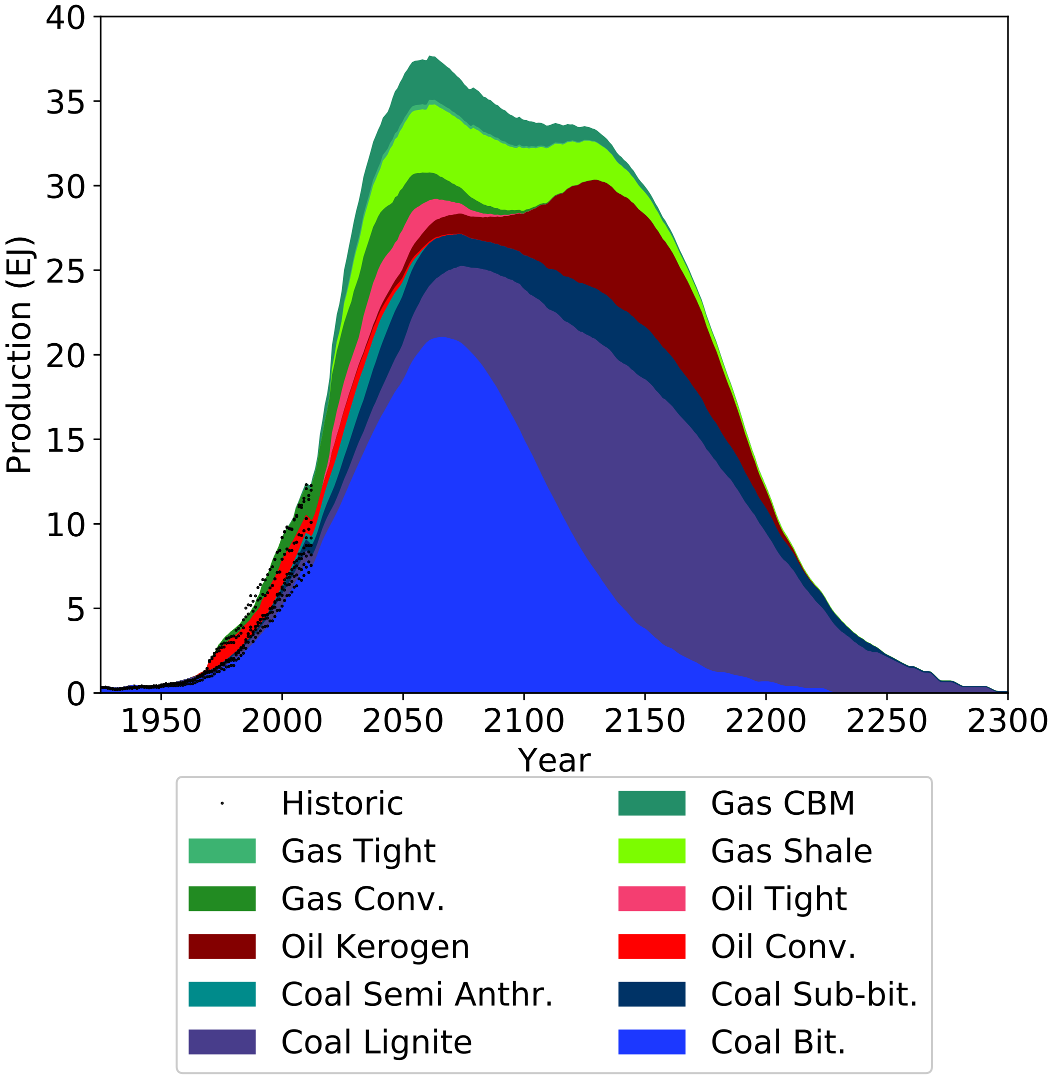

Figure 2.4: Australia projection by mineral type

2.2.3 Regional Projections

Table 2.4: Peak years - Minerals

**Name**	**URR**	**Peak Year**	**Peak Rate**
Coal Bit.	2166.44	2066	21.0
Coal Lignite	1857.48	2152	14.74
Coal Sub-bit.	482.14	2143	3.19
Coal Semi Anthr.	60.7	2033	1.9
Oil Conv.	80.17	2001	1.55
Oil Kerogen	570.28	2140	6.73
Oil Tight	99.71	2040	2.52
Gas Conv.	207.49	2032	3.59
Gas Shale	415.85	2081	4.2
Gas Tight	21.0	2041	0.35
Gas CBM	231.0	2058	2.65
**Total**	**6192.26**	**2061**	**37.62**

Australia

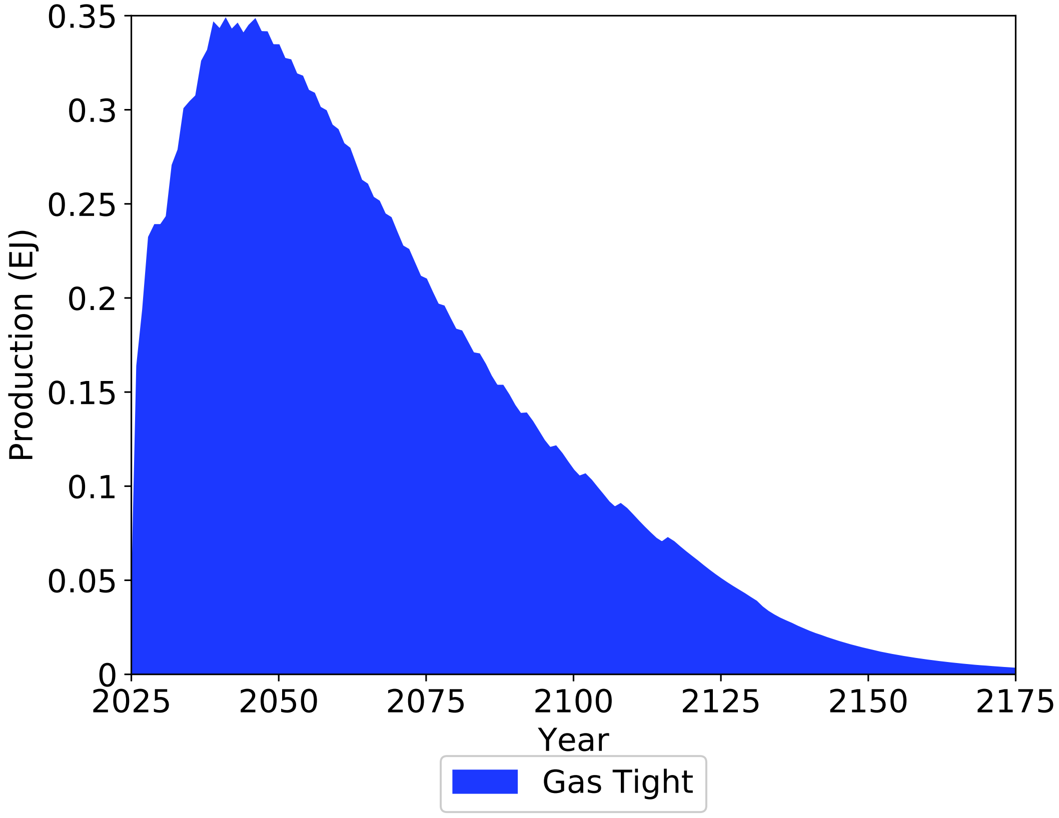

Figure 2.5: Australia - Australia projections capped at 16

Table 2.5: Peak years - All

Name	URR	Peak Year	Peak Rate
Gas Tight Australia	21.0	2041	0.35
**Total**	**21.0**	**2041**	**0.35**

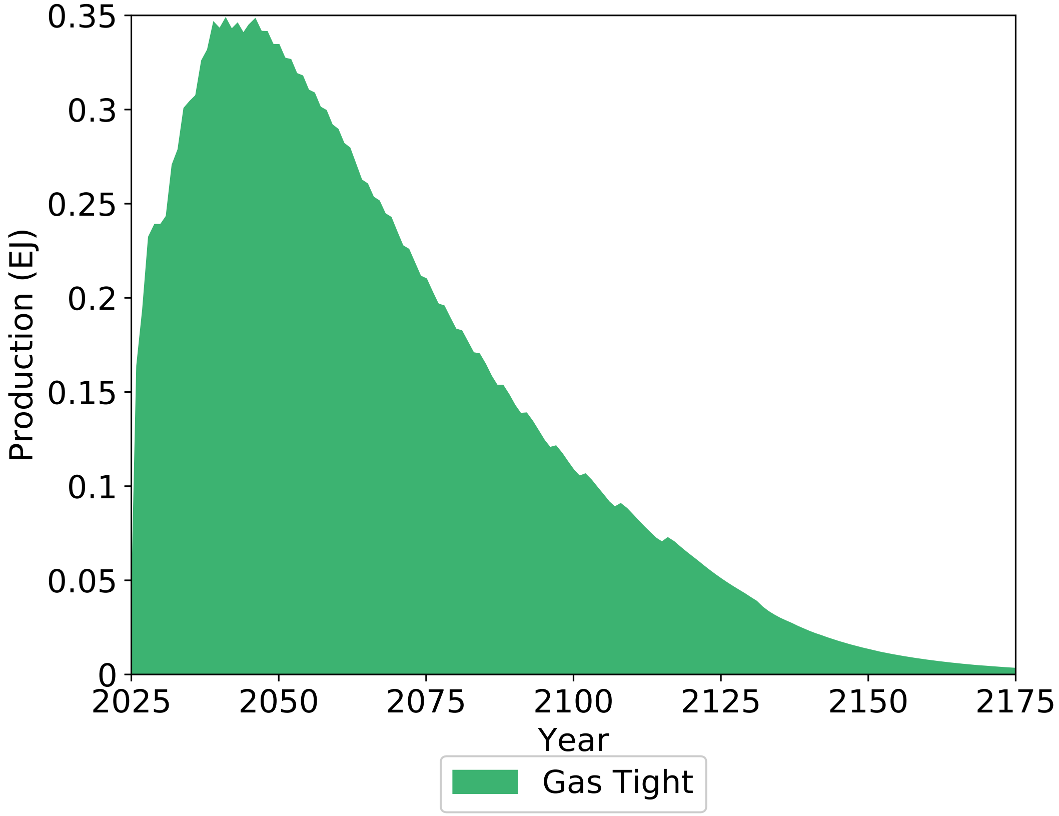

Figure 2.6: Australia - Australia projection by mineral type

Table 2.6: Peak years - Minerals

**Name**	**URR**	**Peak Year**	**Peak Rate**
Gas Tight	21.0	2041	0.35
**Total**	**21.0**	**2041**	**0.35**

JPDA

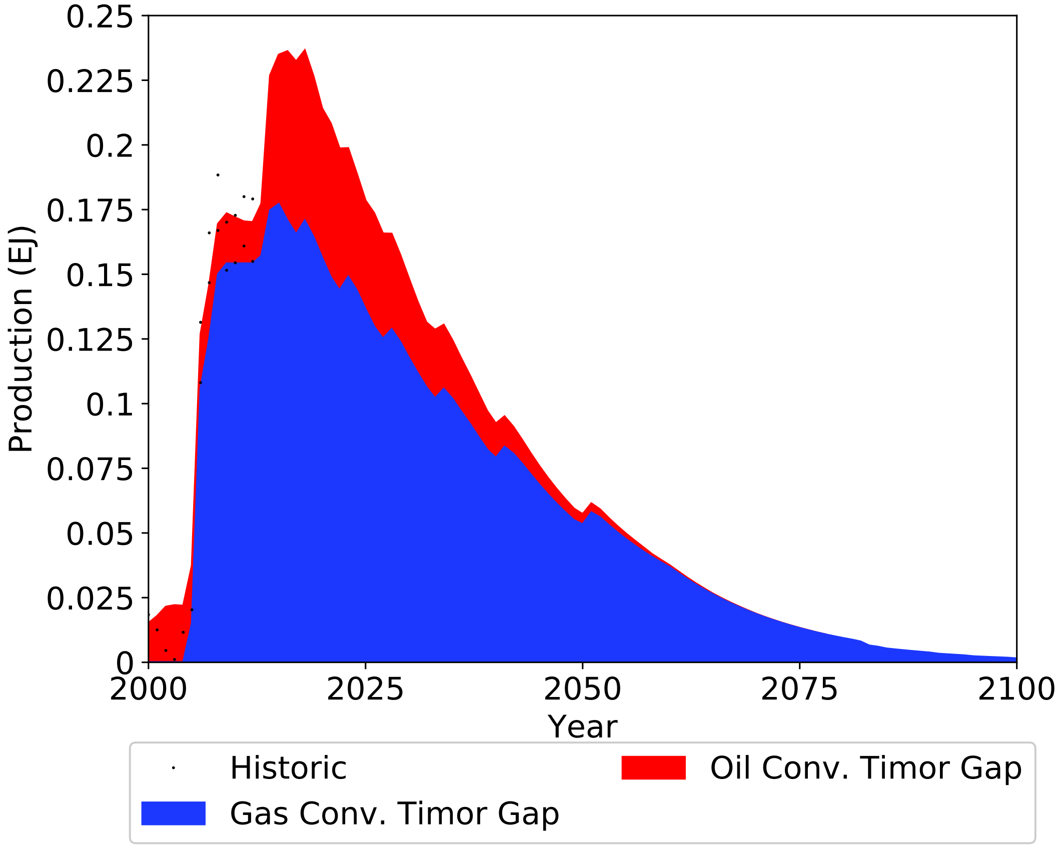

Figure 2.7: Australia - JPDA projections capped at 16

Table 2.7: Peak years - All

Name	URR	Peak Year	Peak Rate
Gas Conv. JPDA Timor Gap	6.29	2015	0.18
Oil Conv. JPDA Timor Gap	1.45	2017	0.07
**Total**	**7.74**	**2018**	**0.24**

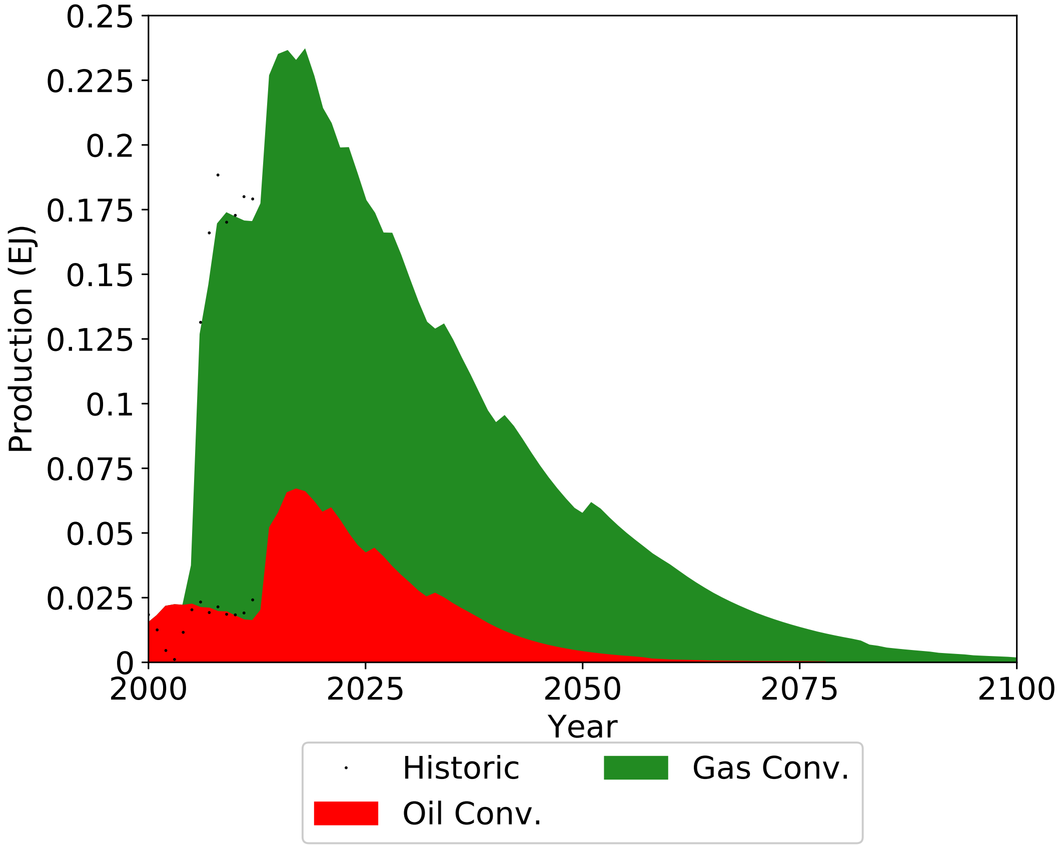

Figure 2.8: Australia - JPDA projection by mineral type

Table 2.8: Peak years - Minerals

**Name**	**URR**	**Peak Year**	**Peak Rate**
Oil Conv.	1.45	2017	0.07
Gas Conv.	6.29	2015	0.18
**Total**	**7.74**	**2018**	**0.24**

NSW

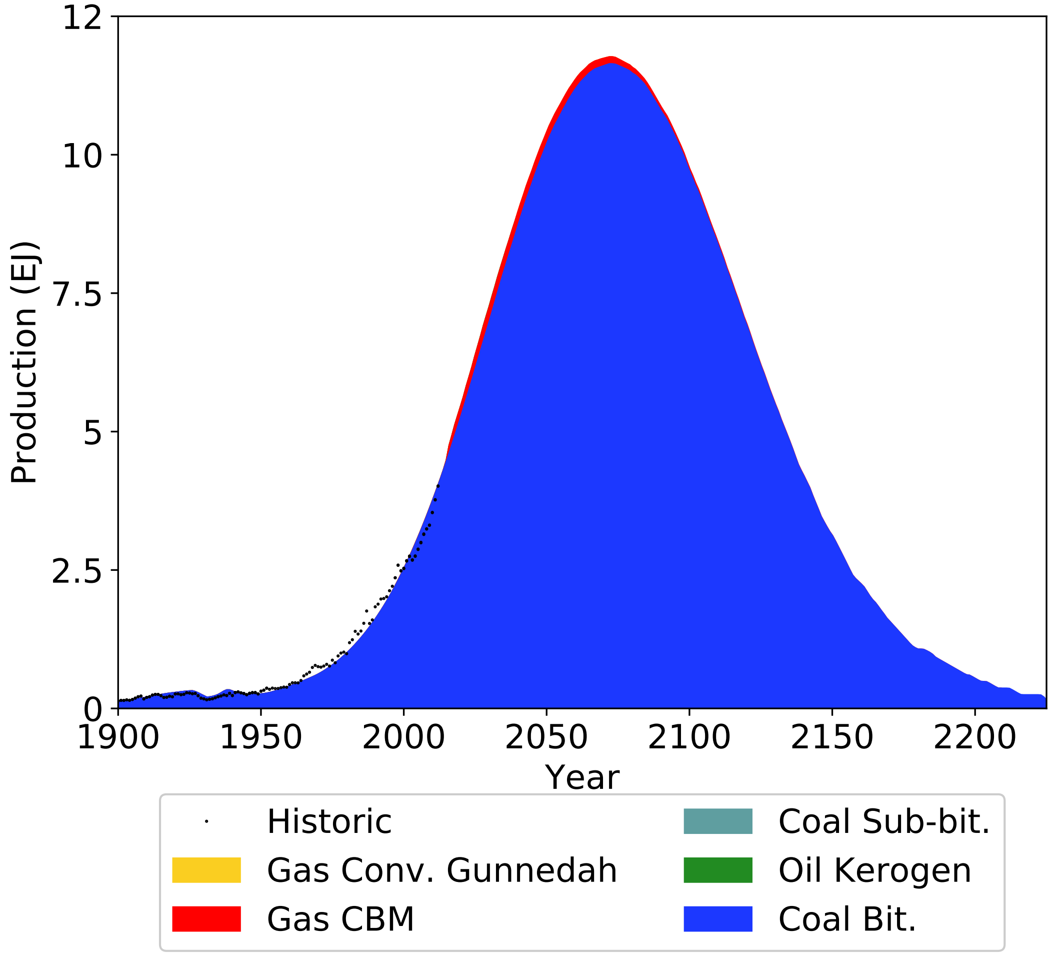

Figure 2.9: Australia - NSW projections capped at 16

Table 2.9: Peak years - All

Name	URR	Peak Year	Peak Rate
Coal Bit. NSW	1320.1	2072	11.64
Gas CBM NSW	15.96	2031	0.27
Oil Kerogen NSW	0.09	2030	0.01
Gas Conv. NSW Gunnedah	0.01	2017	–
Coal Sub-bit. NSW	–	1944	–
**Total**	**1336.16**	**2072**	**11.76**

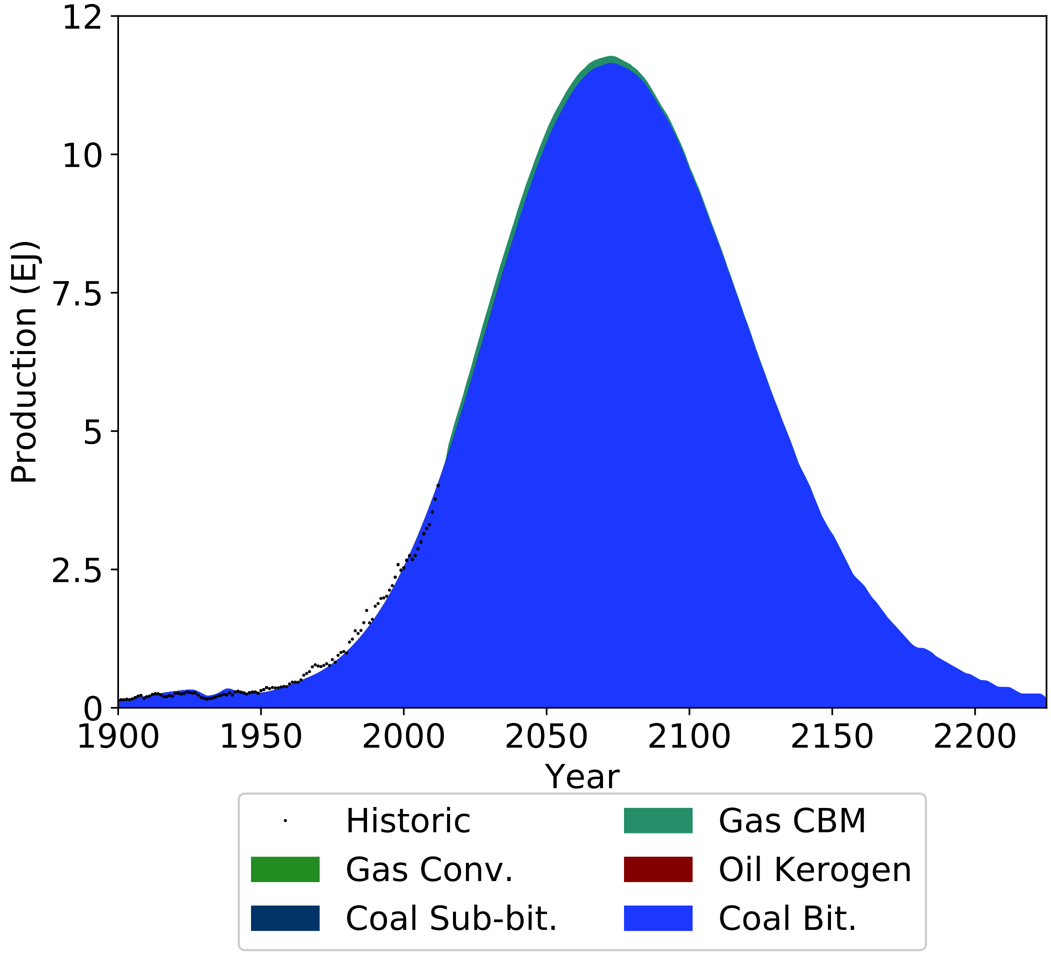

Figure 2.10: Australia - NSW projection by mineral type

Table 2.10: Peak years - Minerals

**Name**	**URR**	**Peak Year**	**Peak Rate**
Coal Bit.	1320.1	2072	11.64
Coal Sub-bit.	–	1944	–
Oil Kerogen	0.09	2030	0.01
Gas Conv.	0.01	2017	–
Gas CBM	15.96	2031	0.27
**Total**	**1336.16**	**2072**	**11.76**

Northern Territory

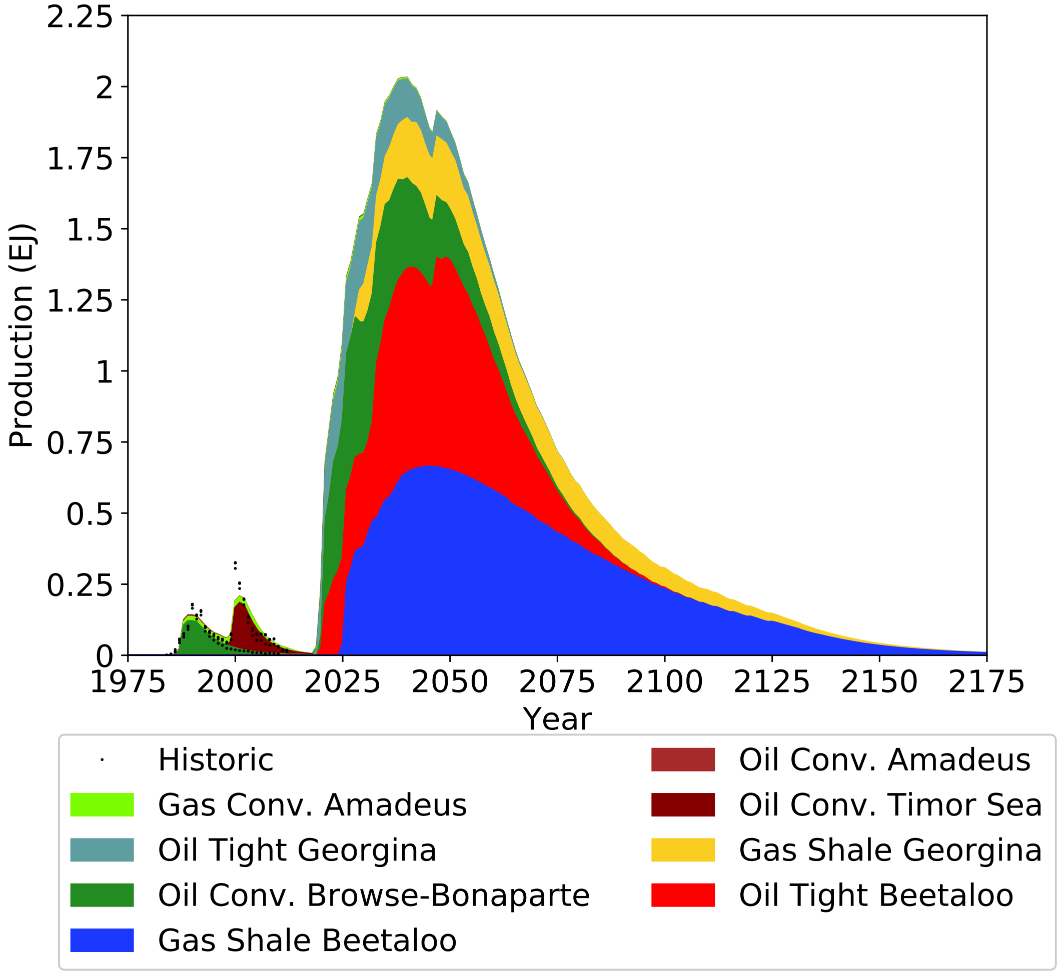

Figure 2.11: Australia - Northern Territory projections capped at 16

Table 2.11: Peak years - All

**Name**	**URR**	**Peak Year**	**Peak Rate**
Gas Shale Northern Territory Beetaloo	41.87	2045	0.66
Oil Tight Northern Territory Beetaloo	26.93	2049	0.74
Oil Conv. Northern Territory Browse-Bonaparte	13.84	2028	0.49
Gas Shale Northern Territory Georgina	12.37	2042	0.22
Oil Tight Northern Territory Georgina	5.73	2026	0.25
Oil Conv. Northern Territory Timor Sea	1.09	2001	0.16
Gas Conv. Northern Territory Amadeus	0.69	1997	0.02
Oil Conv. Northern Territory Amadeus	0.16	2024	0.01
**Total**	**102.68**	**2040**	**2.03**

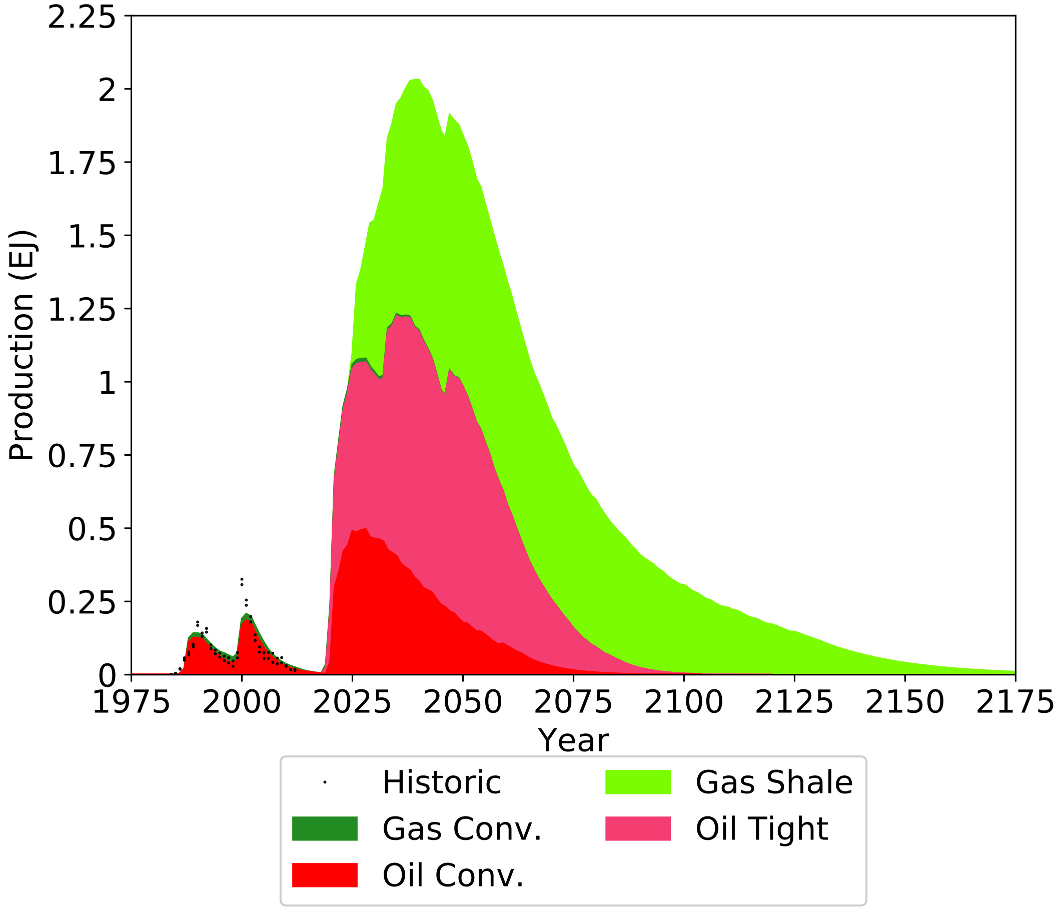

Figure 2.12: Australia - Northern Territory projection by mineral type

Table 2.12: Peak years - Minerals

**Name**	**URR**	**Peak Year**	**Peak Rate**
Oil Conv.	15.09	2028	0.5
Oil Tight	32.66	2038	0.86
Gas Conv.	0.69	1997	0.02
Gas Shale	54.24	2045	0.88
**Total**	**102.68**	**2040**	**2.03**

Queensland

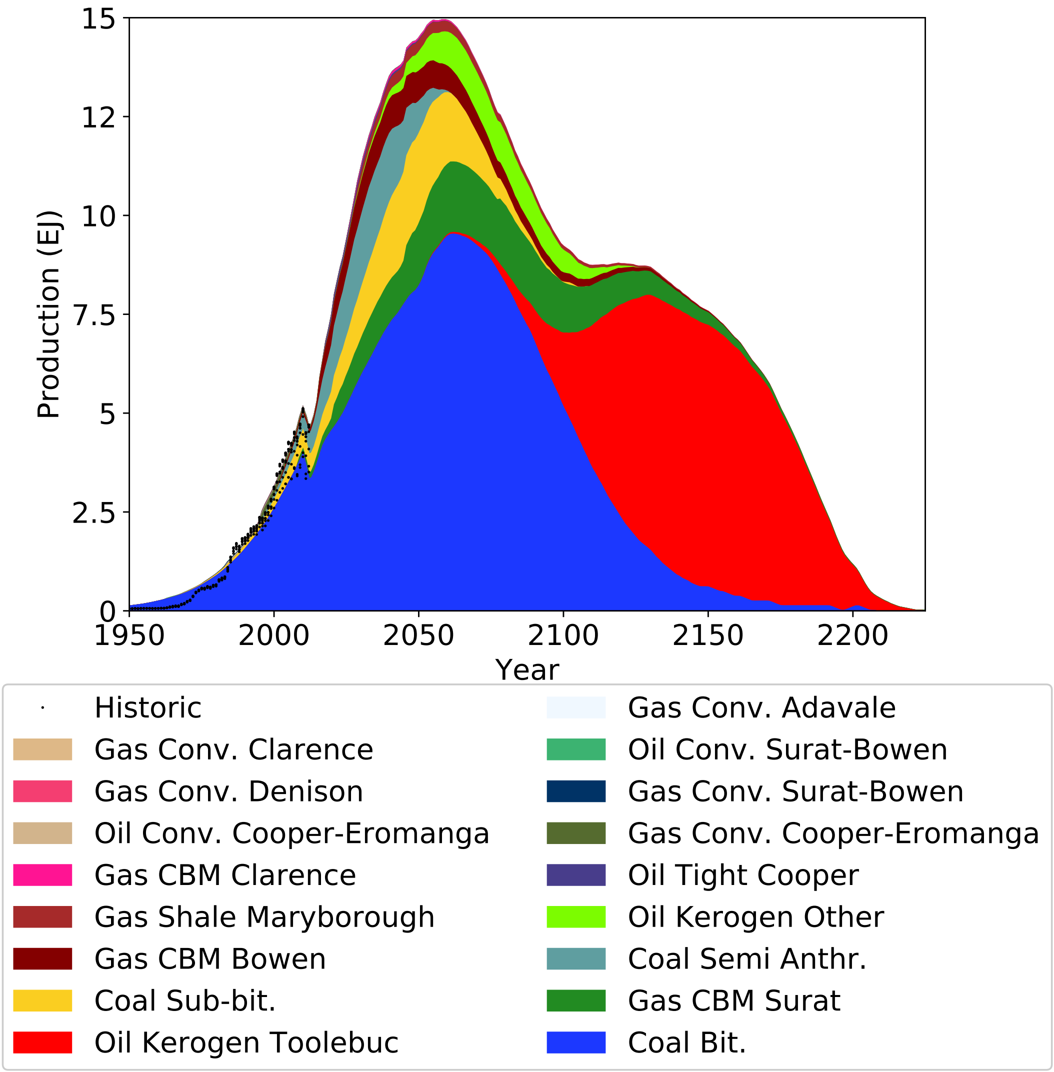

Figure 2.13: Australia - Queensland projections capped at 16

Table 2.13: Peak years - All

Name	URR	Peak Year	Peak Rate
Coal Bit. Queensland	839.09	2062	9.52
Oil Kerogen Queensland Toolebuc	515.7	2140	6.73
Gas CBM Queensland Surat	155.71	2061	1.79
Coal Sub-bit. Queensland	104.07	2049	2.29
Coal Semi Anthr. Queensland	60.7	2033	1.9
Gas CBM Queensland Bowen	56.68	2034	0.87
Oil Kerogen Queensland Other	53.37	2072	1.03
Gas Shale Queensland Maryborough	18.08	2042	0.31
Oil Tight Queensland Cooper	2.87	2022	0.14
Gas CBM Queensland Clarence	2.65	2040	0.06
Gas Conv. Queensland Cooper-Eromanga	2.23	1997	0.16
Oil Conv. Queensland Cooper-Eromanga	1.17	1985	0.04
Gas Conv. Queensland Surat-Bowen	1.12	1990	0.02
Gas Conv. Queensland Denison	0.41	1992	0.02
Oil Conv. Queensland Surat-Bowen	0.35	1964	0.02
Gas Conv. Queensland Clarence	0.07	2022	–
Gas Conv. Queensland Adavale	0.03	1997	–
**Total**	**1814.3**	**2059**	**14.94**

Table 2.14: Peak years - Minerals

Name	URR	Peak Year	Peak Rate
Coal Bit.	839.09	2062	9.52
Coal Sub-bit.	104.07	2049	2.29
Coal Semi Anthr.	60.7	2033	1.9
Oil Conv.	1.52	1985	0.05
Oil Kerogen	569.07	2140	6.73
Oil Tight	2.87	2022	0.14
Gas Conv.	3.86	1997	0.2
Gas Shale	18.08	2042	0.31
Gas CBM	215.04	2058	2.47
**Total**	**1814.3**	**2059**	**14.94**

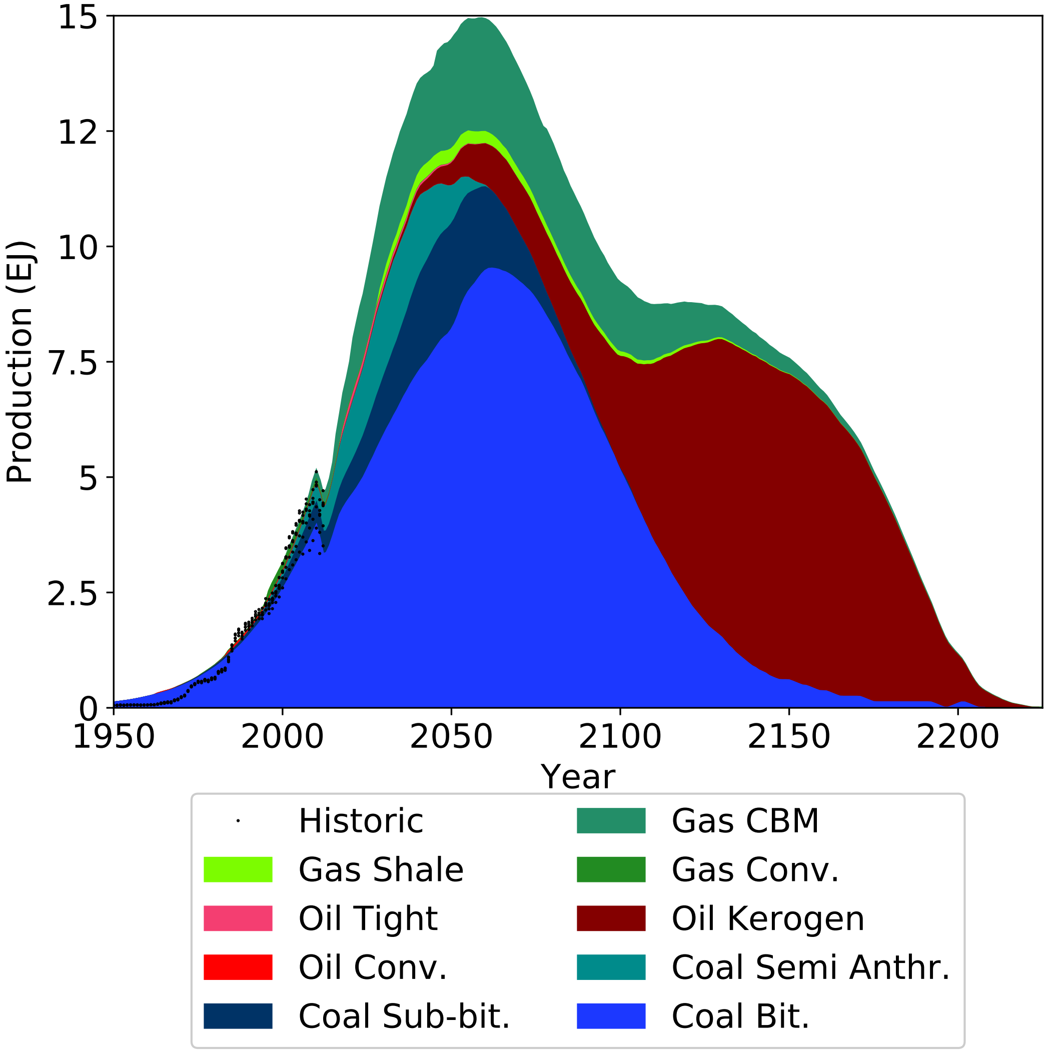

Figure 2.14: Australia - Queensland projection by mineral type

South Australia

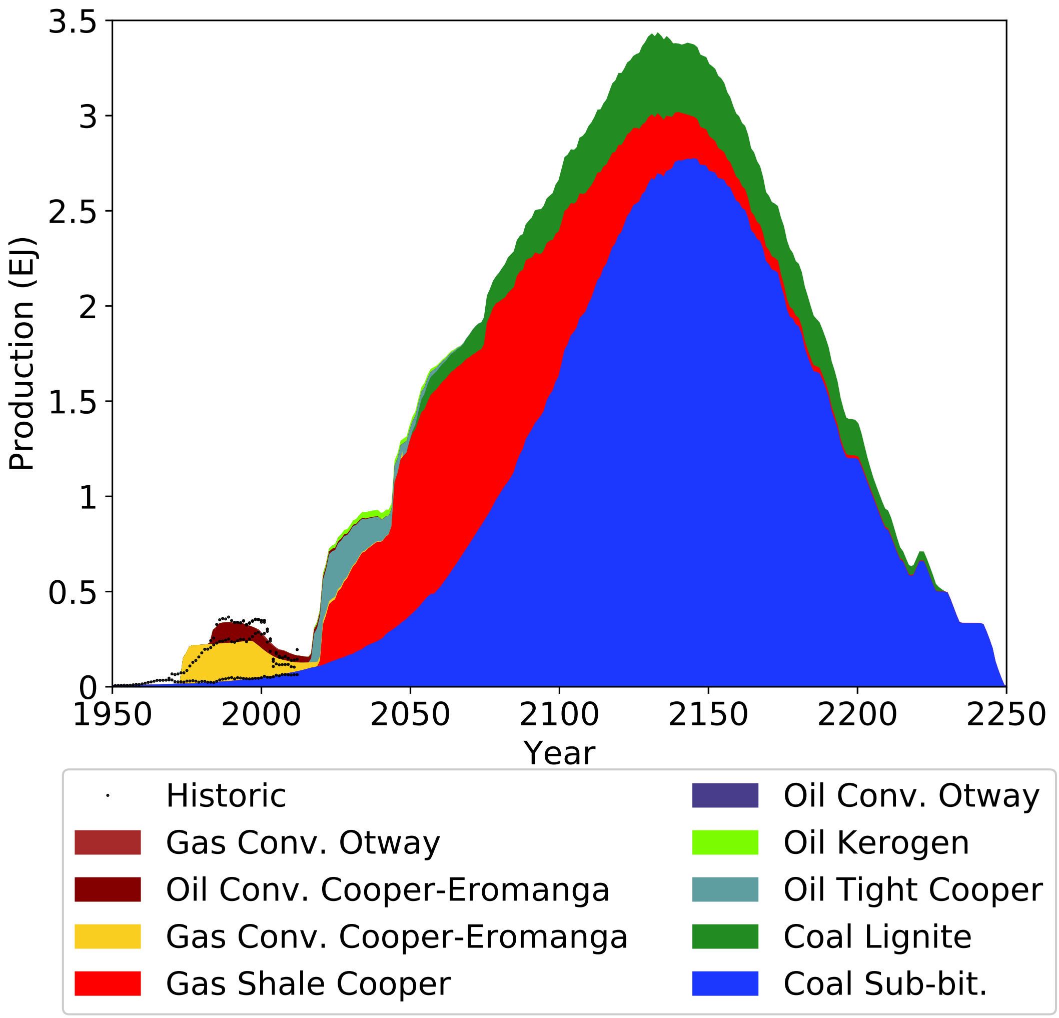

Figure 2.15: Australia - South Australia projections capped at 16

Table 2.15: Peak years - All

Name	URR	Peak Year	Peak Rate
Coal Sub-bit. South Australia	302.33	2145	2.77
Gas Shale South Australia Cooper	88.5	2060	1.06
Coal Lignite South Australia	41.8	2130	0.43
Gas Conv. South Australia Cooper-Eromanga	6.69	1977	0.2
Oil Tight South Australia Cooper	5.73	2024	0.25
Oil Conv. South Australia Cooper-Eromanga	2.54	1987	0.11
Oil Kerogen South Australia	1.03	2036	0.03
Gas Conv. South Australia Otway	0.08	2000	0.01
Oil Conv. South Australia Otway	–	1994	–
**Total**	**448.7**	**2133**	**3.43**

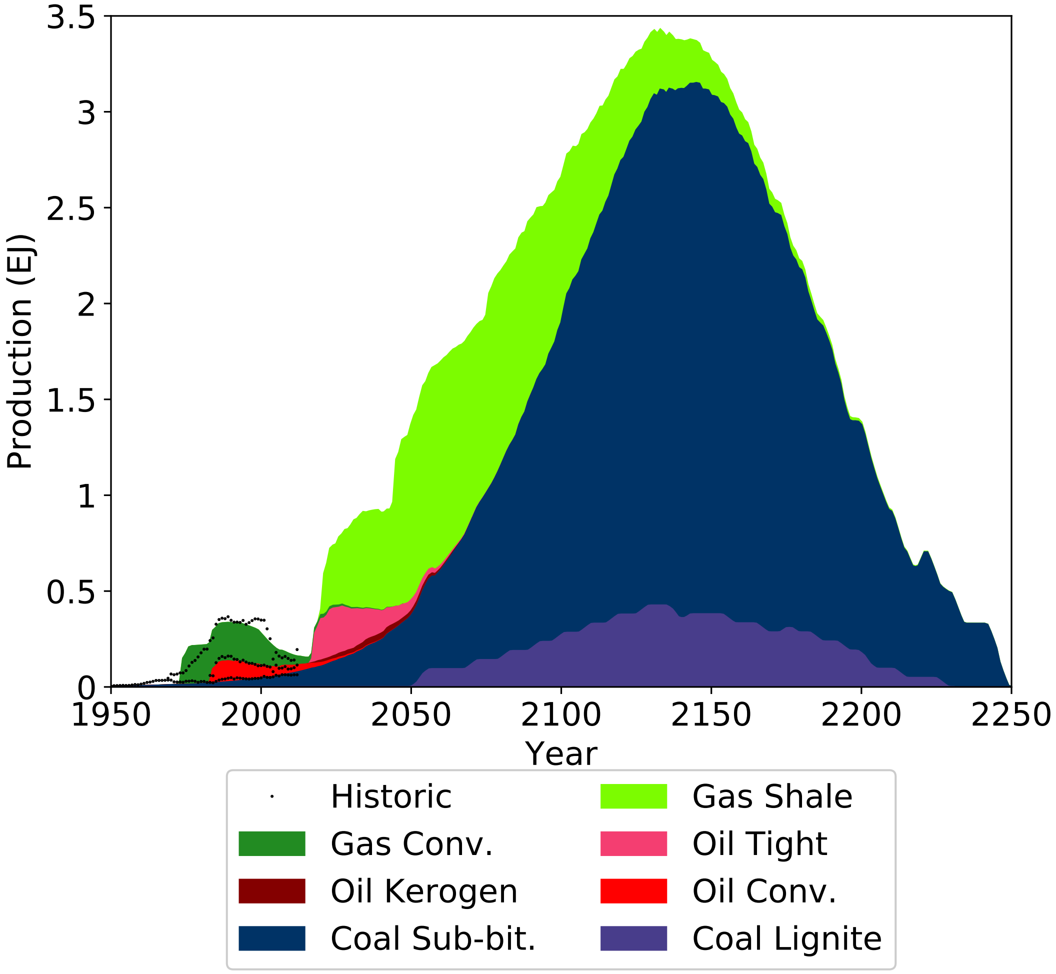

Figure 2.16: Australia - South Australia projection by mineral type

Table 2.16: Peak years - Minerals

**Name**	**URR**	**Peak Year**	**Peak Rate**
Coal Lignite	41.8	2130	0.43
Coal Sub-bit.	302.33	2145	2.77
Oil Conv.	2.54	1987	0.11
Oil Kerogen	1.03	2036	0.03
Oil Tight	5.73	2024	0.25
Gas Conv.	6.77	1977	0.2
Gas Shale	88.5	2060	1.06
**Total**	**448.7**	**2133**	**3.43**

Tasmania

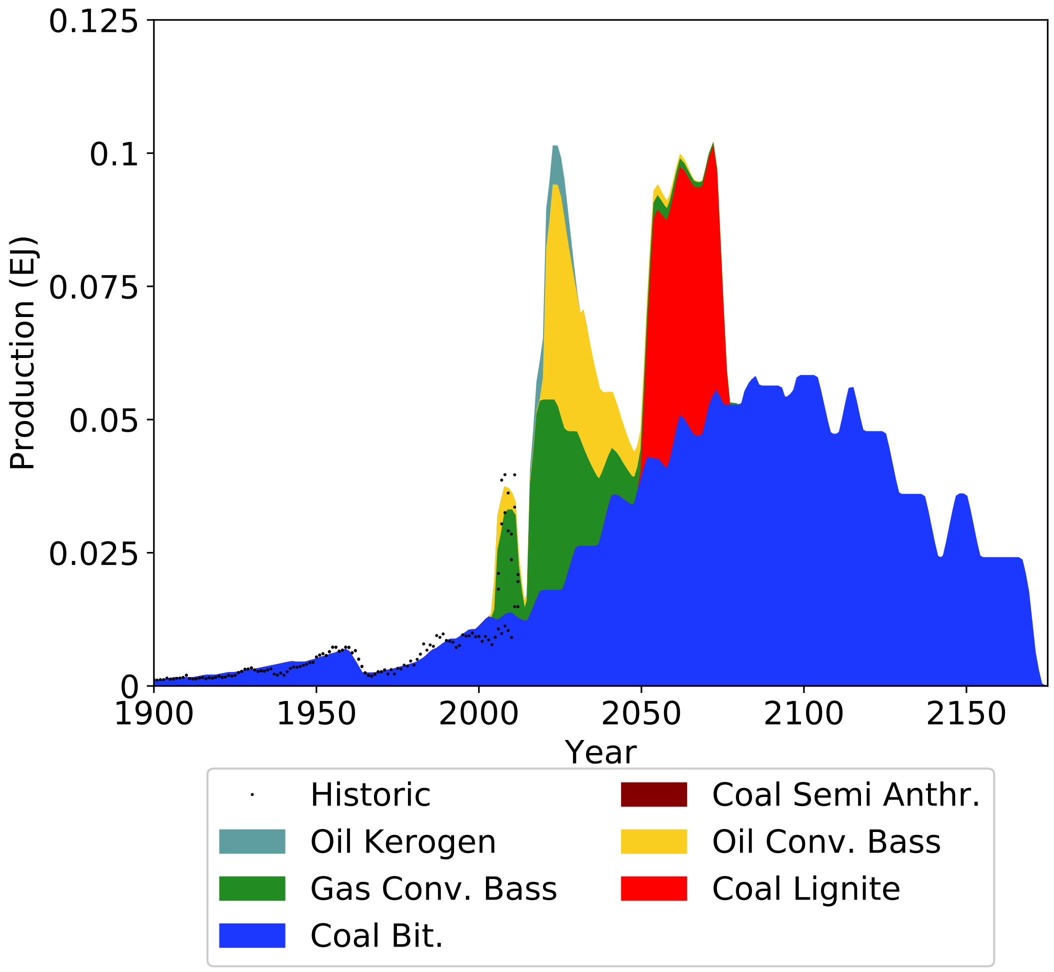

Figure 2.17: Australia - Tasmania projections capped at 16

Table 2.17: Peak years - All

Name	URR	Peak Year	Peak Rate
Coal Bit. Tasmania	6.7	2099	0.06
Coal Lignite Tasmania	1.05	2055	0.05
Gas Conv. Tasmania Bass	0.84	2019	0.04
Oil Conv. Tasmania Bass	0.68	2024	0.04
Oil Kerogen Tasmania	0.08	2020	0.01
Coal Semi Anthr. Tasmania	–	1953	–
**Total**	**9.35**	**2072**	**0.1**

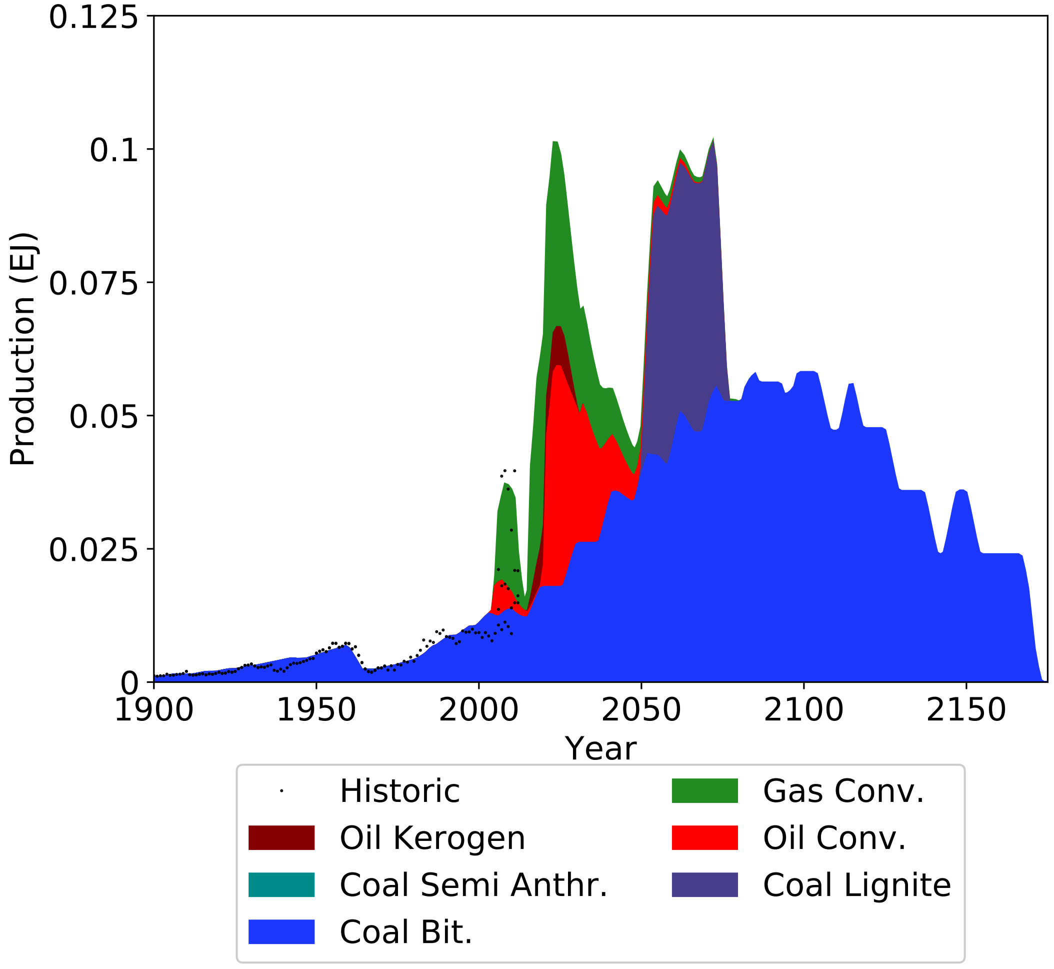

Figure 2.18: Australia - Tasmania projection by mineral type

Table 2.18: Peak years - Minerals

**Name**	**URR**	**Peak Year**	**Peak Rate**
Coal Bit.	6.7	2099	0.06
Coal Lignite	1.05	2055	0.05
Coal Semi Anthr.	–	1953	–
Oil Conv.	0.68	2024	0.04
Oil Kerogen	0.08	2020	0.01
Gas Conv.	0.84	2019	0.04
**Total**	**9.35**	**2072**	**0.1**

Victoria

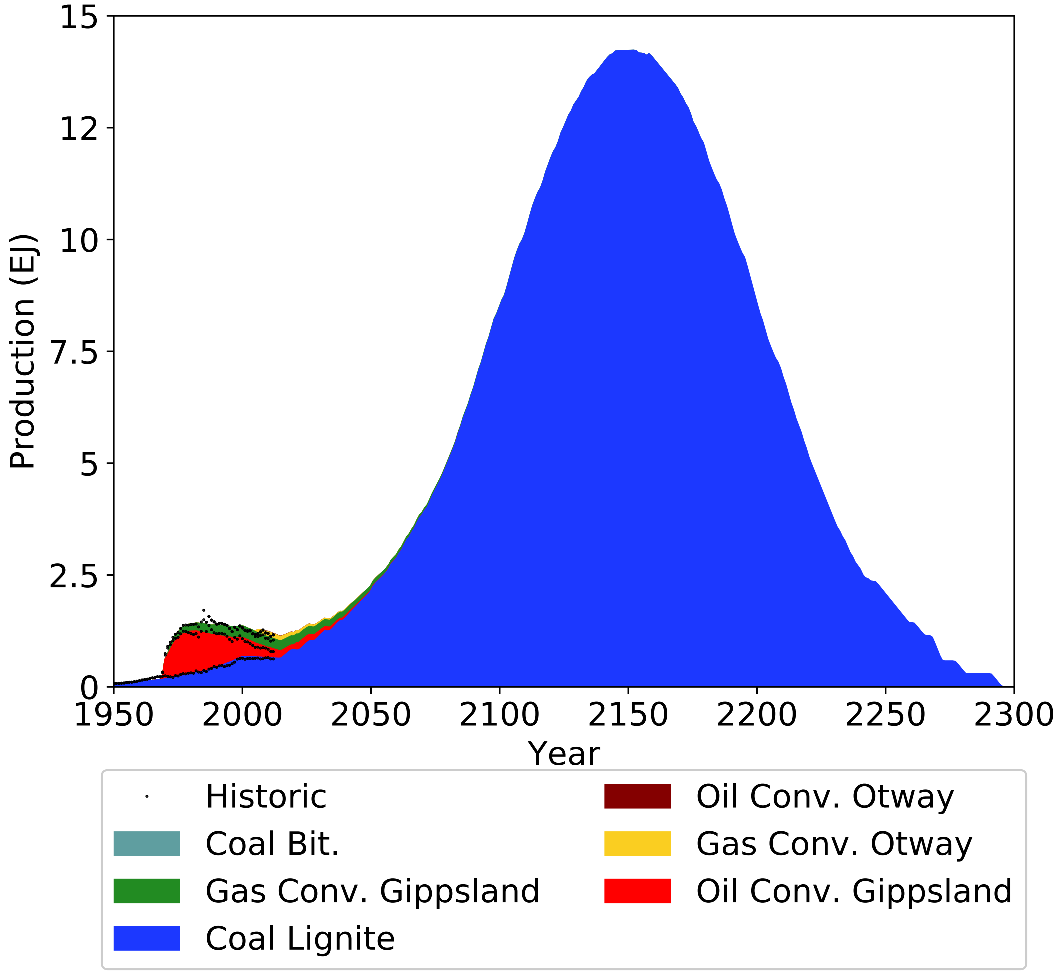

Figure 2.19: Australia - Victoria projections capped at 16

Table 2.19: Peak years - All

Name	URR	Peak Year	Peak Rate
Coal Lignite Victoria	1797.05	2152	14.22
Oil Conv. Victoria Gippsland	30.0	1980	0.96
Gas Conv. Victoria Gippsland	17.53	2001	0.26
Gas Conv. Victoria Otway	2.3	2010	0.11
Coal Bit. Victoria	0.55	1917	0.02
Oil Conv. Victoria Otway	0.12	2010	0.01
**Total**	**1847.55**	**2152**	**14.22**

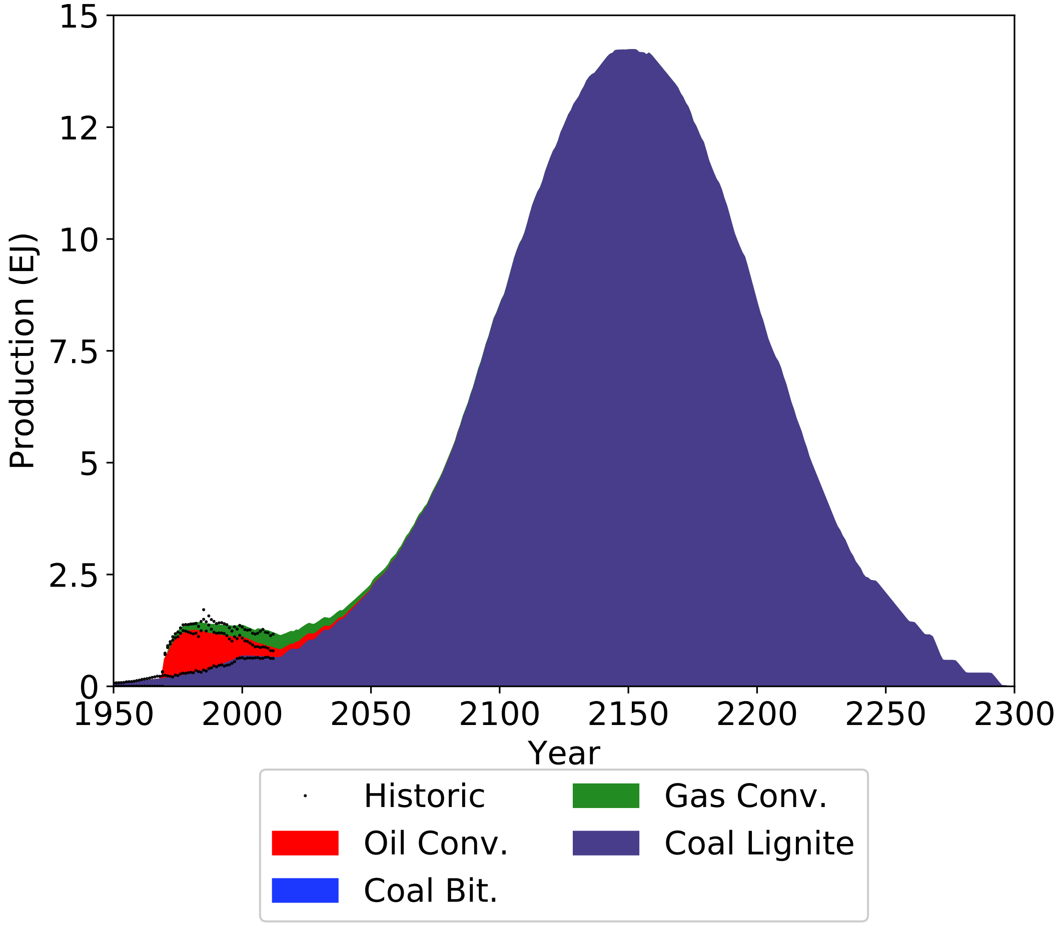

Figure 2.20: Australia - Victoria projection by mineral type

Table 2.20: Peak years - Minerals

Name	URR	Peak Year	Peak Rate
Coal Bit.	0.55	1917	0.02
Coal Lignite	1797.05	2152	14.22
Oil Conv.	30.12	1980	0.96
Gas Conv.	19.83	2010	0.35
**Total**	**1847.55**	**2152**	**14.22**

Western Australia

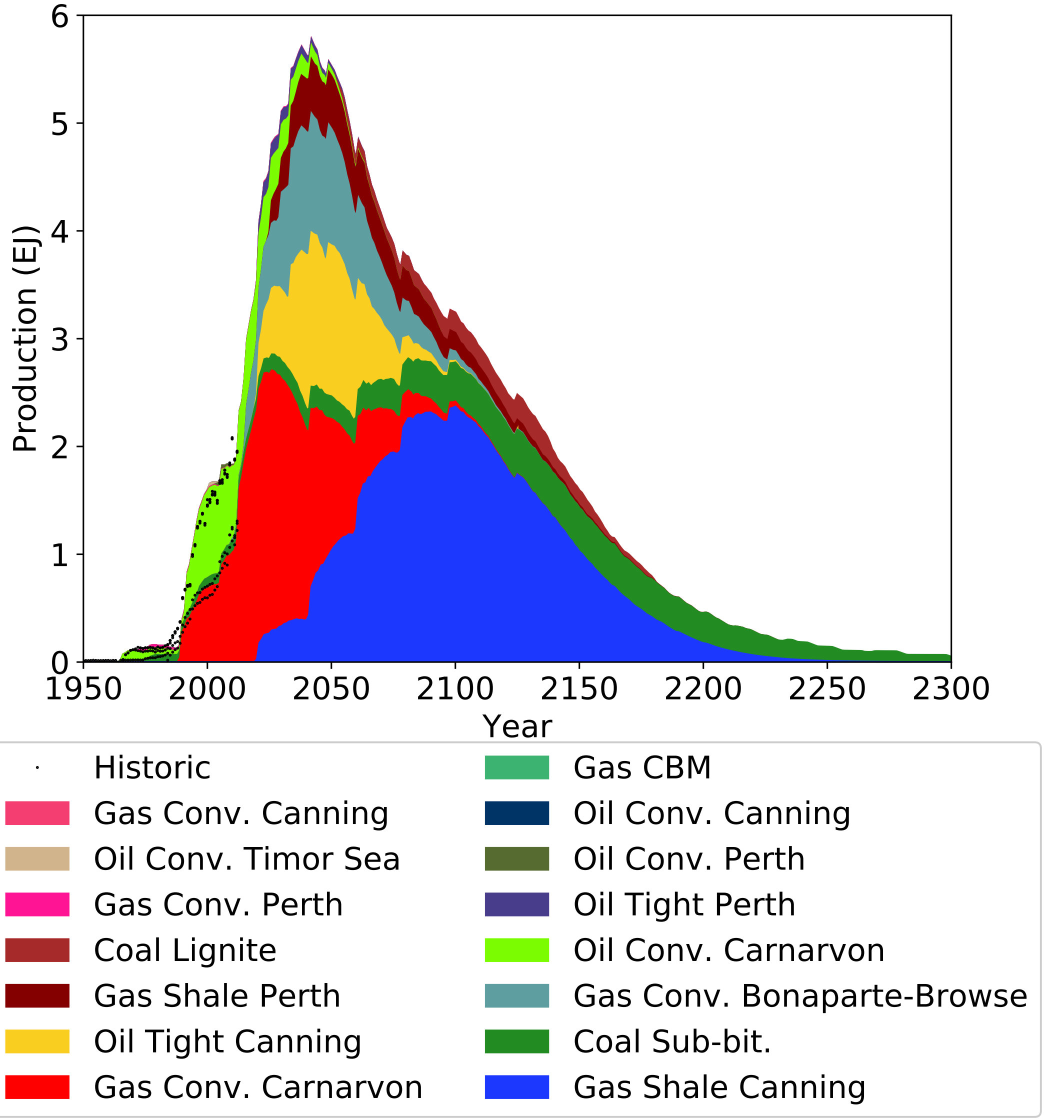

Figure 2.21: Australia - Western Australia projections capped at 16

Table 2.21: Peak years - All

Name	URR	Peak Year	Peak Rate
Gas Shale Western Australia Canning	223.63	2100	2.37
Gas Conv. Western Australia Carnarvon	113.35	2023	2.43
Coal Sub-bit. Western Australia	75.74	2143	0.42
Oil Tight Western Australia Canning	55.58	2041	1.44
Gas Conv. Western Australia Bonaparte-Browse	54.9	2038	1.15
Gas Shale Western Australia Perth	31.4	2046	0.5
Oil Conv. Western Australia Carnarvon	28.44	2003	0.82
Coal Lignite Western Australia	17.58	2126	0.24
Oil Tight Western Australia Perth	2.87	2025	0.14
Gas Conv. Western Australia Perth	0.94	1981	0.03
Oil Conv. Western Australia Perth	0.21	2005	0.03
Oil Conv. Western Australia Timor Sea	0.12	2002	0.03
Oil Conv. Western Australia Canning	0.02	1987	–
Gas Conv. Western Australia Canning	0.01	2022	–
Gas CBM Western Australia	–	2008	–
**Total**	**604.78**	**2042**	**5.77**

Table 2.22: Peak years - Minerals

Name	URR	Peak Year	Peak Rate
Coal Lignite	17.58	2126	0.24
Coal Sub-bit.	75.74	2143	0.42
Oil Conv.	28.78	2001	0.85
Oil Tight	58.45	2041	1.5
Gas Conv.	169.2	2032	3.24
Gas Shale	255.03	2085	2.55
Gas CBM	–	2008	–
**Total**	**604.78**	**2042**	**5.77**

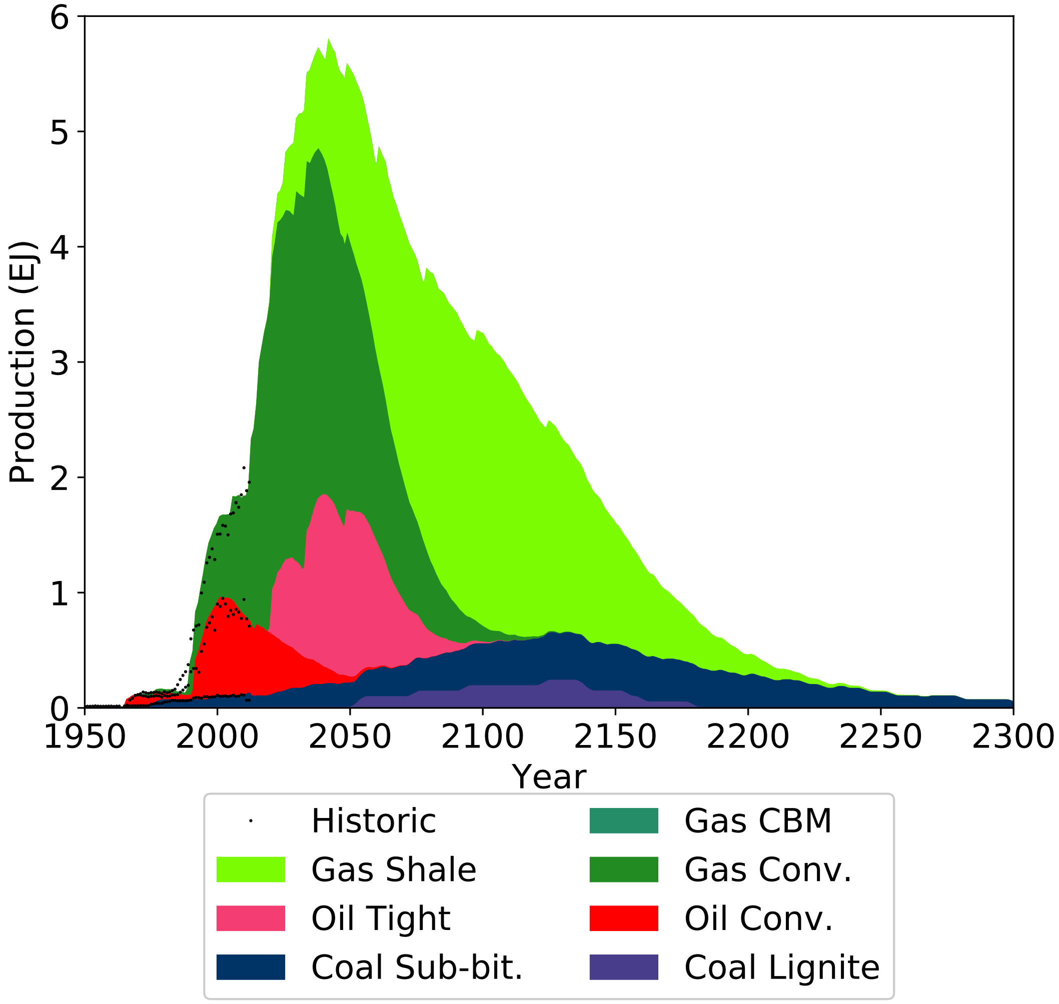

Figure 2.22: Australia - Western Australia projection by mineral type

2.2.4 Projection by region

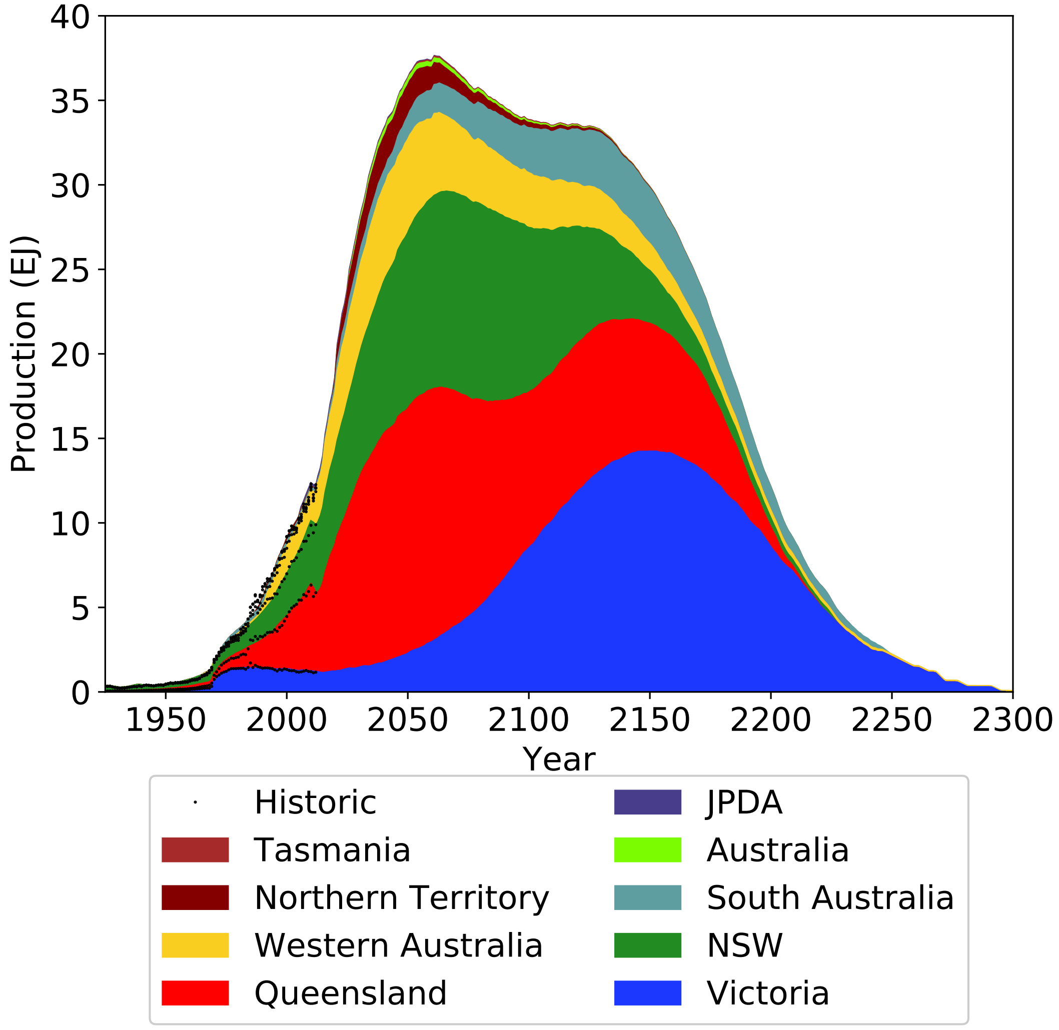

Figure 2.23: Australia by region projections capped at 16

Table 2.23: Peak years - All

**Name**	**URR**	**Peak Year**	**Peak Rate**
Victoria	1847.55	2152	14.22
Queensland	1814.3	2059	14.94
NSW	1336.16	2072	11.76
Western Australia	604.78	2042	5.77
South Australia	448.7	2133	3.43
Northern Territory	102.68	2040	2.03
Australia	21.0	2041	0.35
Tasmania	9.35	2072	0.1
JPDA	7.74	2018	0.24
**Total**	**6192.26**	**2061**	**37.62**

2.3 Bangladesh

2.3.1 All Projections

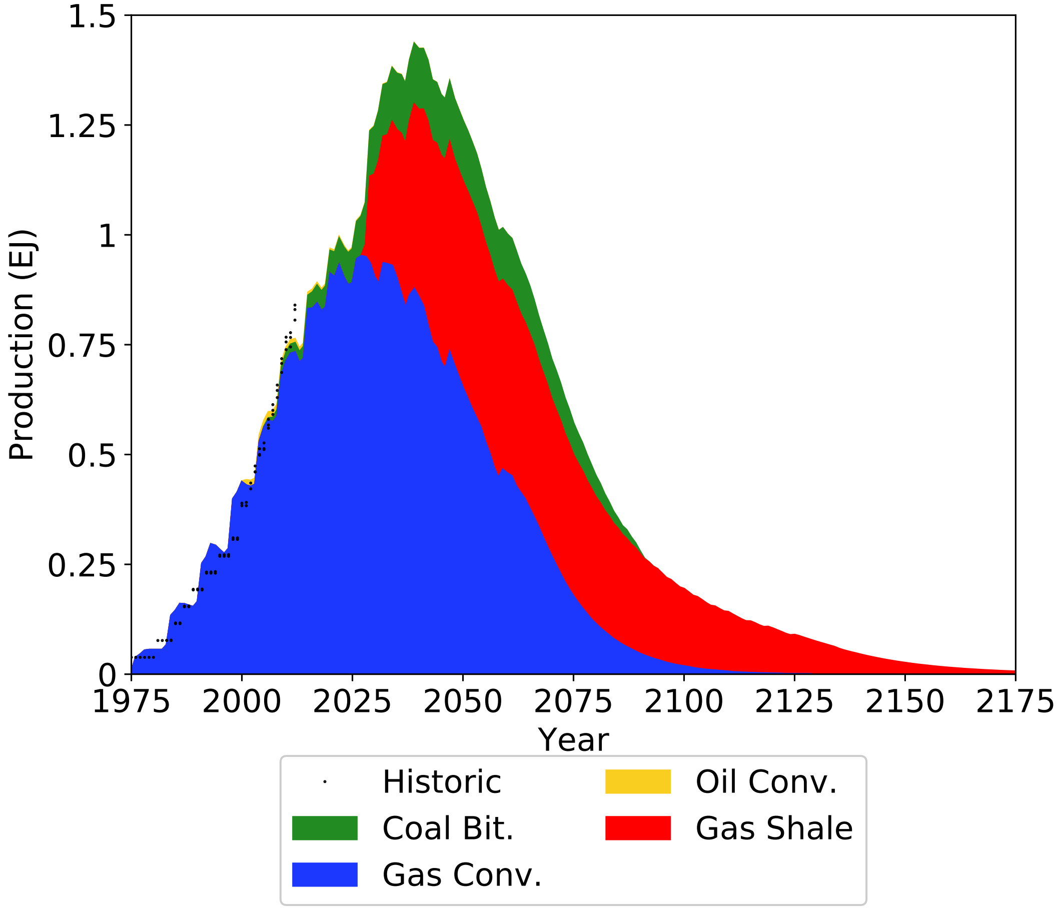

Figure 2.24: Bangladesh projections capped at 16

Table 2.24: Peak years - All

**Name**	**URR**	**Peak Year**	**Peak Rate**
Gas Conv.	55.4	2027	0.95
Gas Shale	29.66	2047	0.48
Coal Bit.	7.15	2038	0.14
Oil Conv.	0.25	2002	0.01
**Total**	**92.46**	**2039**	**1.44**

2.3.2 By Mineral

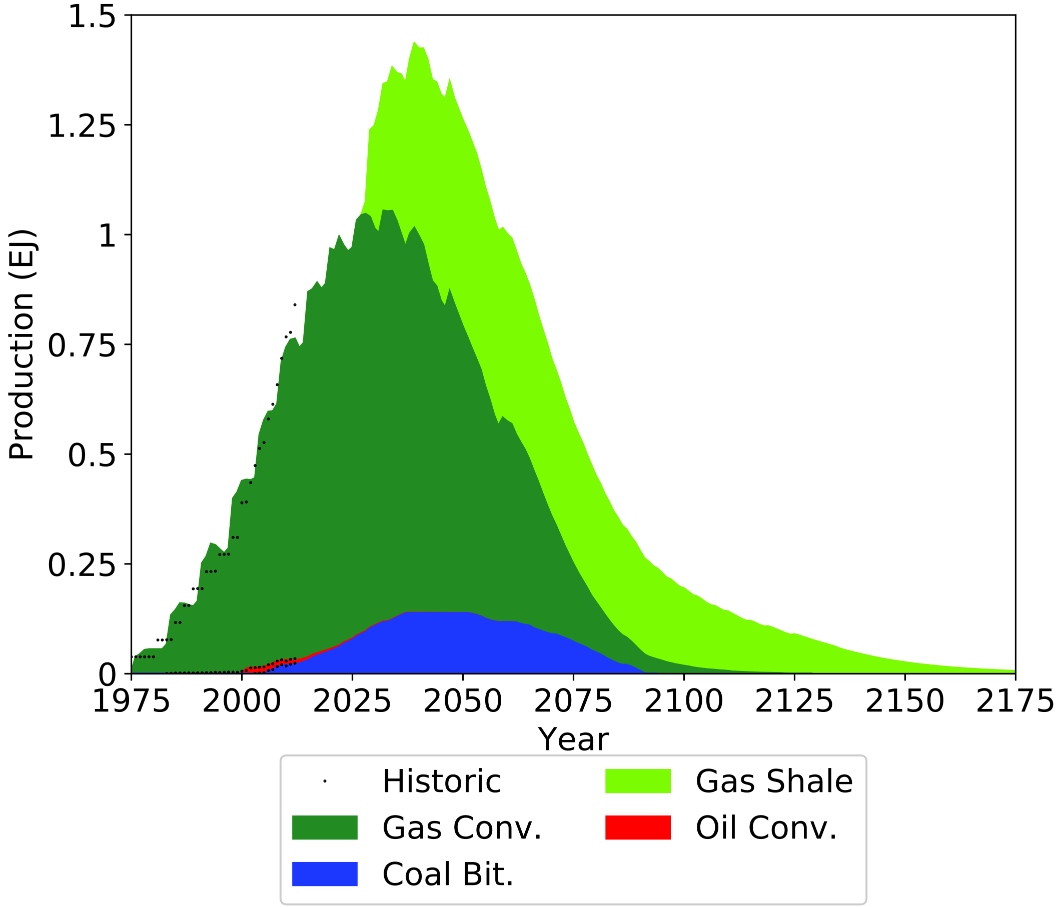

Figure 2.25: Bangladesh projection by mineral type

Table 2.25: Peak years - Minerals

**Name**	**URR**	**Peak Year**	**Peak Rate**
Coal Bit.	7.15	2038	0.14
Oil Conv.	0.25	2002	0.01
Gas Conv.	55.4	2027	0.95
Gas Shale	29.66	2047	0.48
**Total**	**92.46**	**2039**	**1.44**

2.4 Bhutan

2.4.1 All Projections

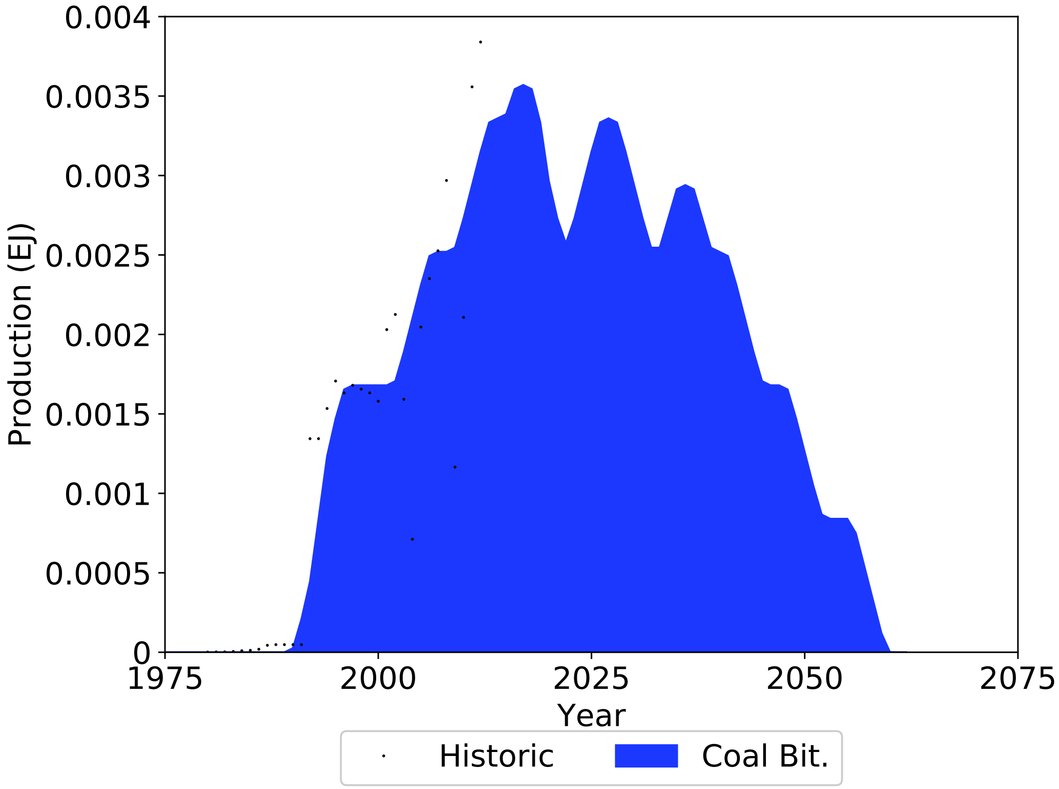

Figure 2.26: Bhutan projections capped at 16

Table 2.26: Peak years - All

**Name**	**URR**	**Peak Year**	**Peak Rate**
Coal Bit.	0.15	2017	–
**Total**	**0.15**	**2017**	–

2.4.2 By Mineral

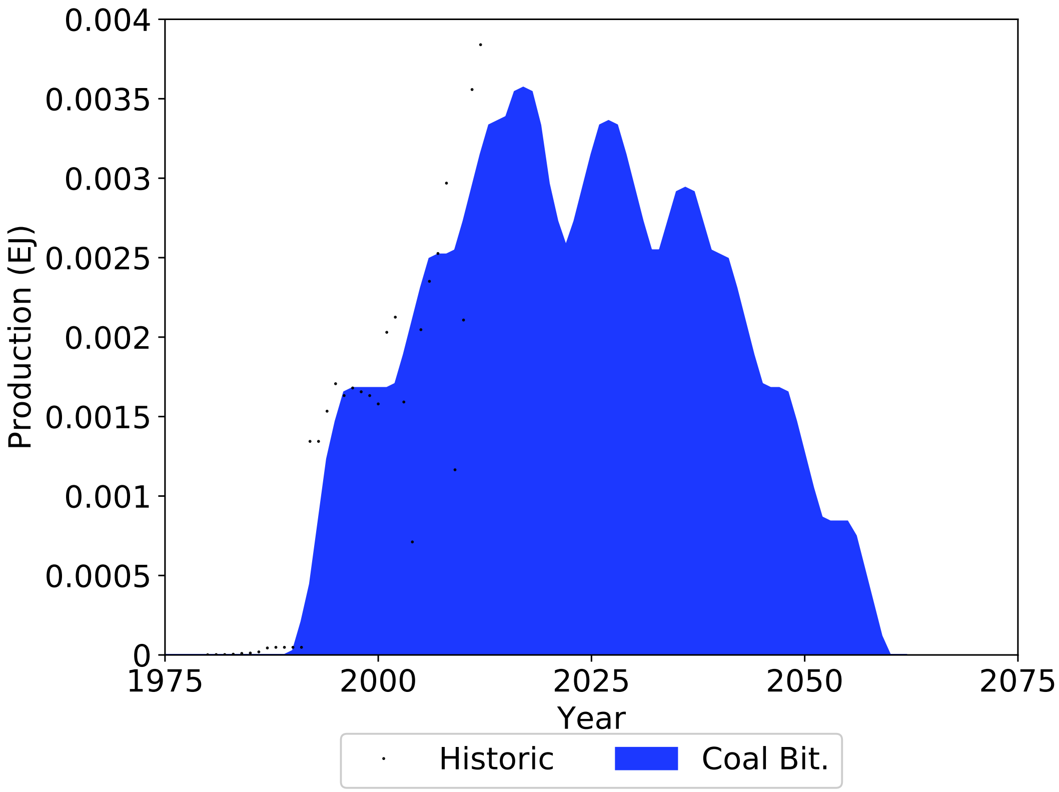

Figure 2.27: Bhutan projection by mineral type

Table 2.27: Peak years - Minerals

Name	URR	Peak Year	Peak Rate
Coal Bit.	0.15	2017	–
**Total**	**0.15**	**2017**	–

2.5 Brunei

2.5.1 All Projections

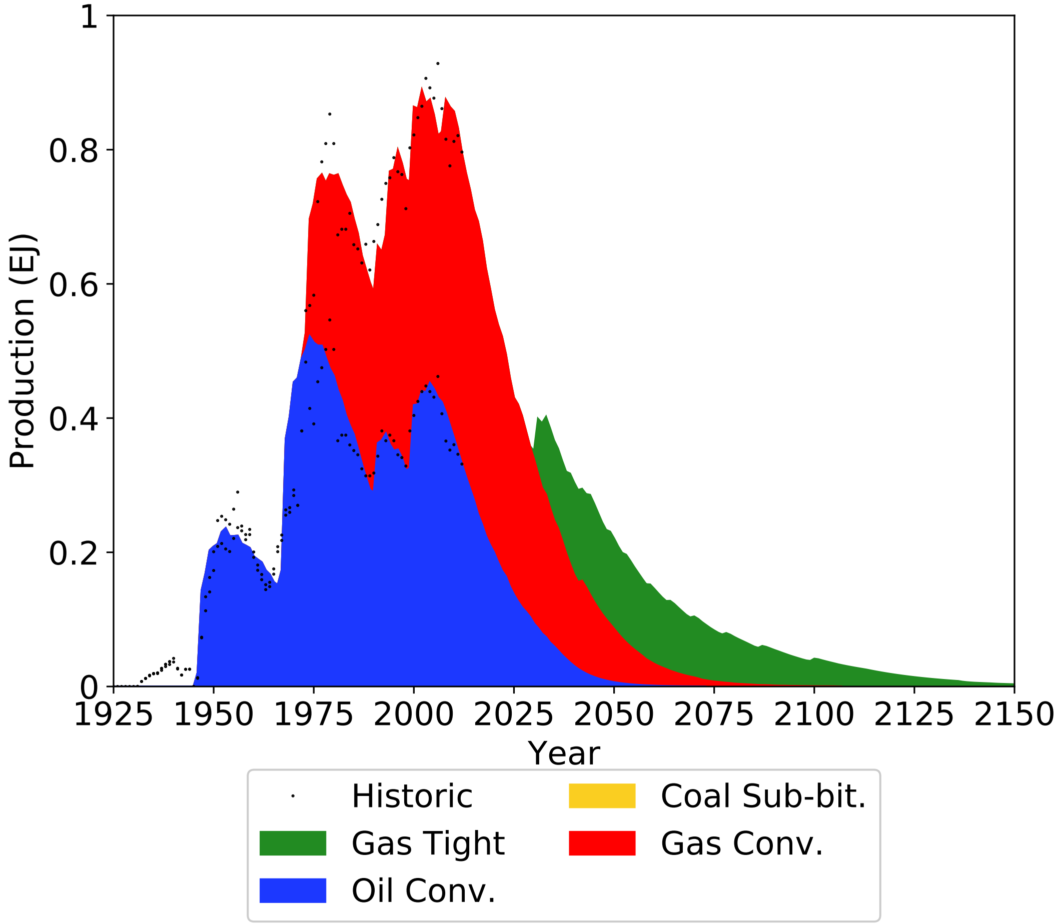

Figure 2.28: Brunei projections capped at 16

Table 2.28: Peak years - All

**Name**	**URR**	**Peak Year**	**Peak Rate**
Oil Conv.	26.5	1974	0.52
Gas Conv.	24.2	2010	0.49
Gas Tight	7.42	2044	0.15
Coal Sub-bit.	0.01	1893	–
**Total**	**58.13**	**2002**	**0.89**

2.5.2 By Mineral

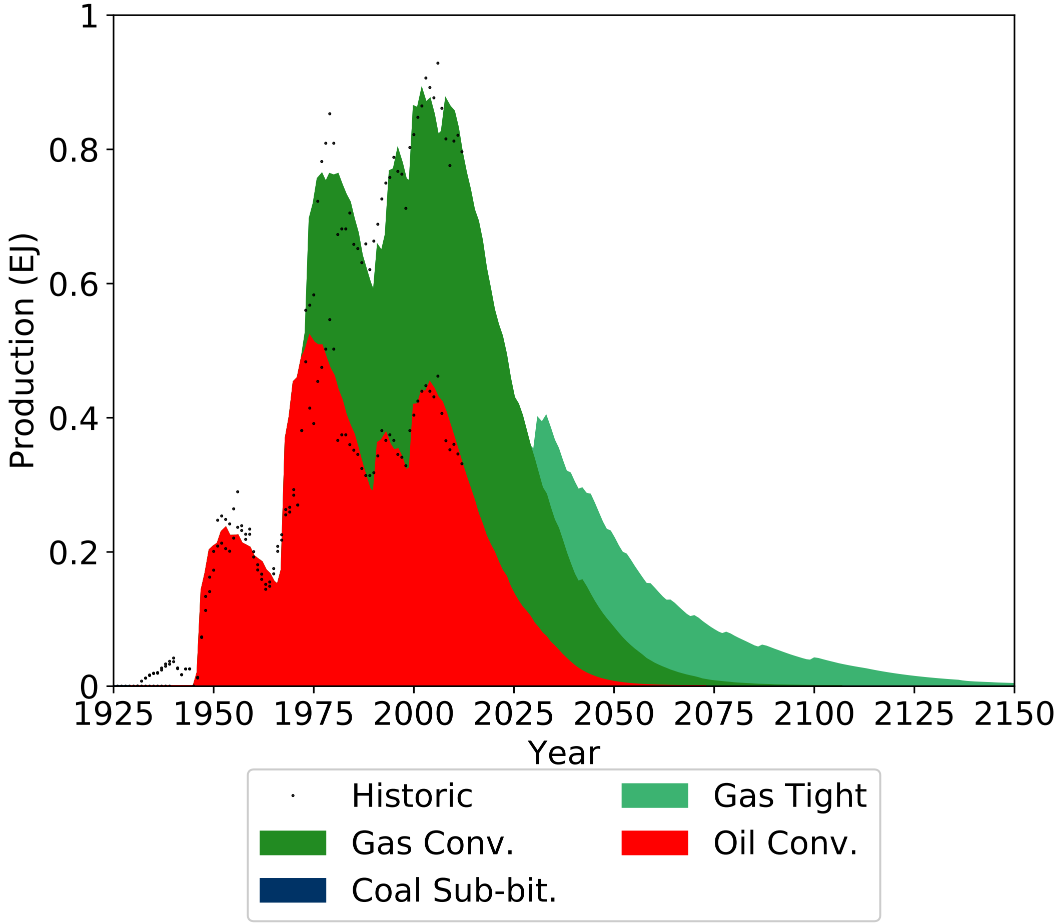

Figure 2.29: Brunei projection by mineral type

Table 2.29: Peak years - Minerals

**Name**	**URR**	**Peak Year**	**Peak Rate**
Coal Sub-bit.	0.01	1893	–
Oil Conv.	26.5	1974	0.52
Gas Conv.	24.2	2010	0.49
Gas Tight	7.42	2044	0.15
**Total**	**58.13**	**2002**	**0.89**

2.6 Burma

2.6.1 All Projections

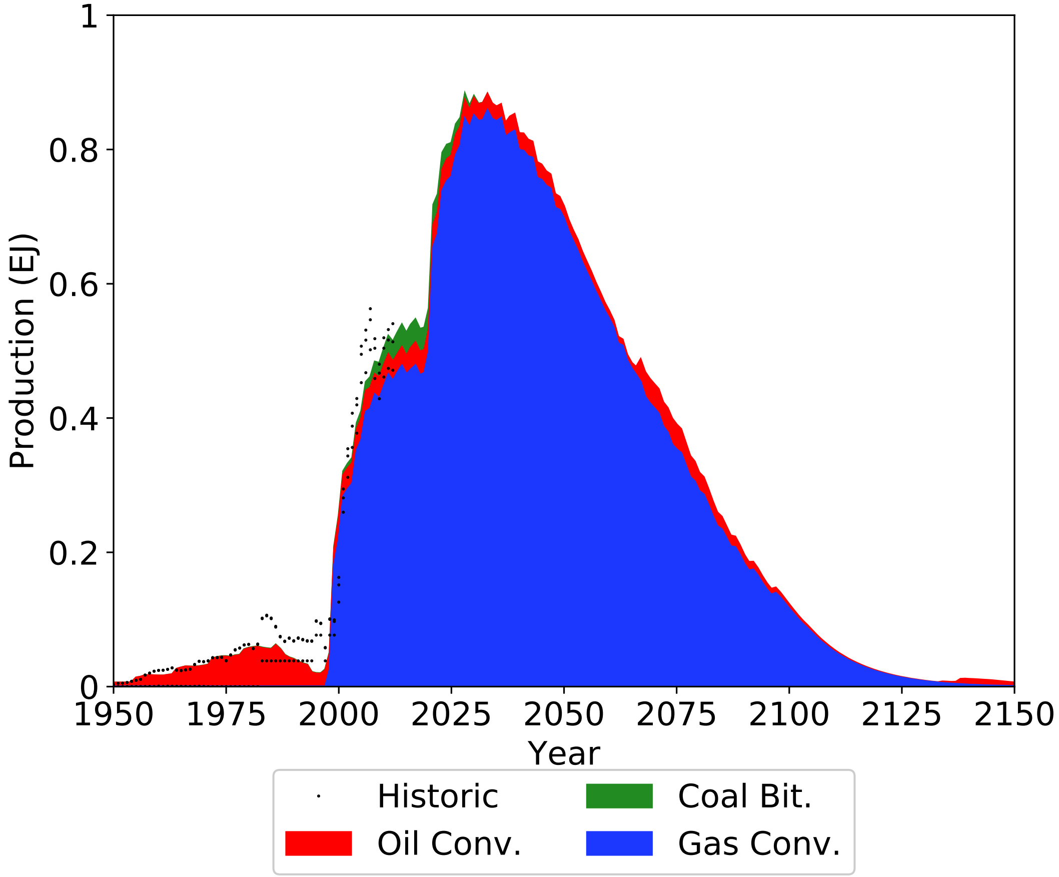

Figure 2.30: Burma projections capped at 16

Table 2.30: Peak years - All

Name	URR	Peak Year	Peak Rate
Gas Conv.	51.8	2033	0.86
Oil Conv.	4.25	1986	0.06
Coal Bit.	0.66	2016	0.03
**Total**	**56.71**	**2033**	**0.88**

2.6.2 By Mineral

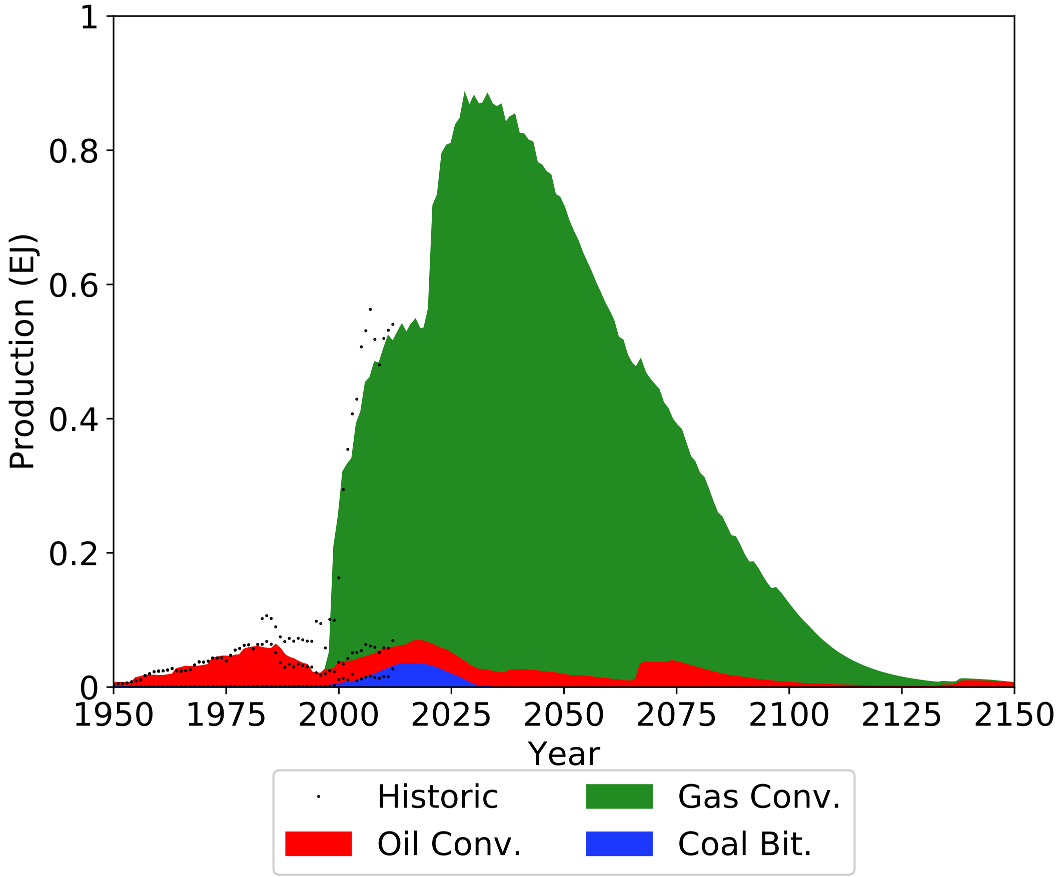

Figure 2.31: Burma projection by mineral type

Table 2.31: Peak years - Minerals

Name	URR	Peak Year	Peak Rate
Coal Bit.	0.66	2016	0.03
Oil Conv.	4.25	1986	0.06
Gas Conv.	51.8	2033	0.86
**Total**	**56.71**	**2033**	**0.88**

2.7 Cambodia

2.7.1 All Projections

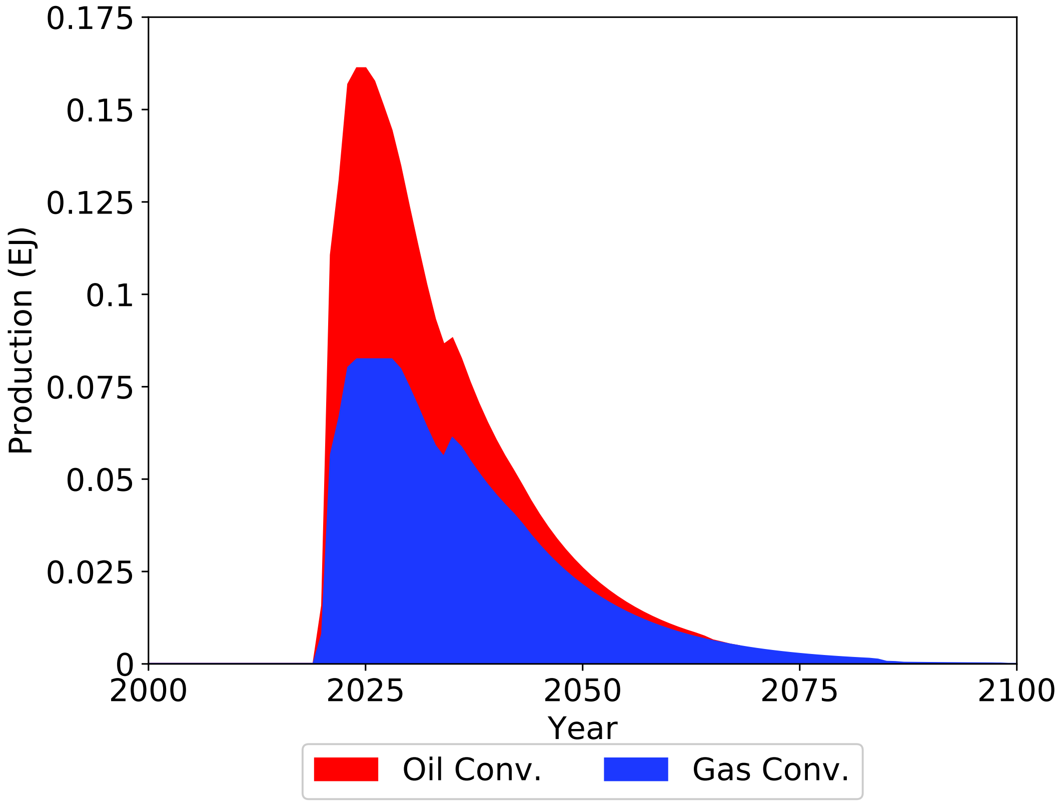

Figure 2.32: Cambodia projections capped at 16

Table 2.32: Peak years - All

**Name**	**URR**	**Peak Year**	**Peak Rate**
Gas Conv.	1.9	2024	0.08
Oil Conv.	1.05	2024	0.08
**Total**	**2.95**	**2024**	**0.16**

2.7.2 By Mineral

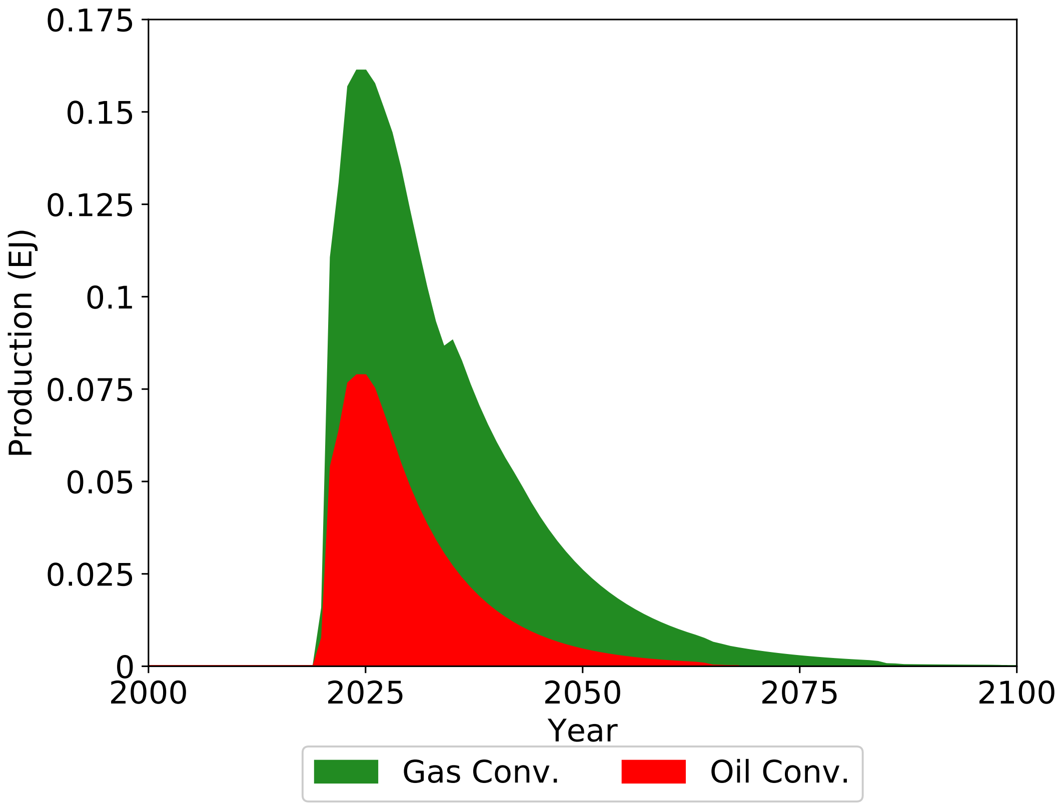

Figure 2.33: Cambodia projection by mineral type

Table 2.33: Peak years - Minerals

**Name**	**URR**	**Peak Year**	**Peak Rate**
Oil Conv.	1.05	2024	0.08
Gas Conv.	1.9	2024	0.08
**Total**	**2.95**	**2024**	**0.16**

2.8 China

2.8.1 All Projections

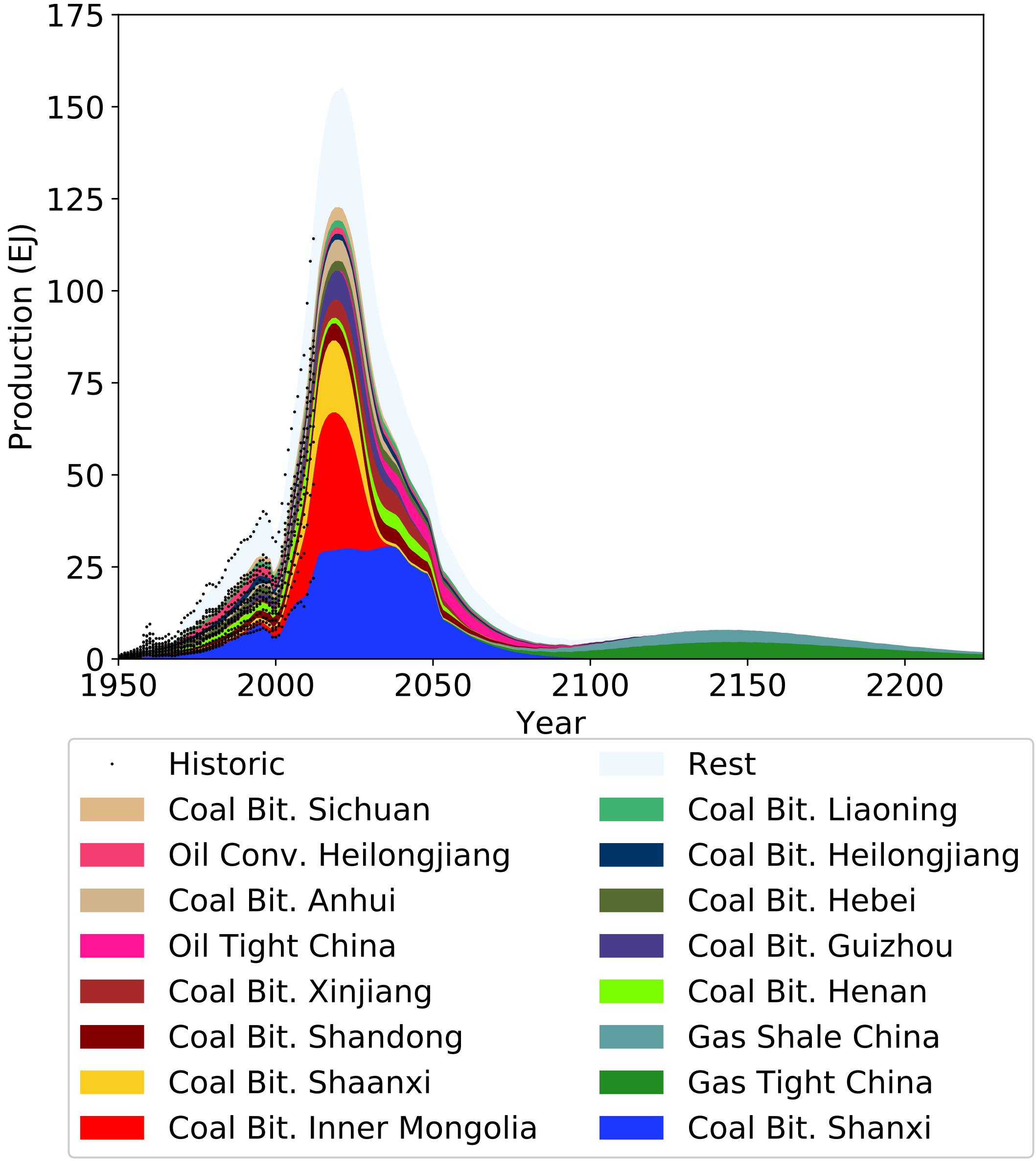

Figure 2.34: China projections capped at 16

Table 2.34: Peak years - All

**Name**	**URR**	**Peak Year**	**Peak Rate**
Coal Bit. Shanxi	1543.23	2036	30.33
Coal Bit. Inner Mongolia	696.15	2018	37.44
Gas Tight China	444.97	2143	4.35
Coal Bit. Shaanxi	346.35	2018	19.53
Gas Shale China	318.89	2140	3.33
Coal Bit. Shandong	278.09	2025	4.84
Coal Bit. Henan	241.25	2009	5.53
Coal Bit. Xinjiang	235.83	2031	6.85
Coal Bit. Guizhou	221.34	2024	8.5
Oil Tight China	184.51	2044	4.62
Coal Bit. Hebei	170.41	2030	2.91
Coal Bit. Anhui	162.36	2020	5.77
Coal Bit. Heilongjiang	157.4	2008	2.28
Oil Conv. Heilongjiang	156.83	1989	2.67
Coal Bit. Liaoning	141.13	2022	1.95
Coal Bit. Sichuan	126.66	2019	3.59
Coal Bit. Yunnan	113.89	2022	3.28
Gas CBM China	105.0	2067	1.31
Coal Bit. Ningxia	99.05	2021	5.0
Oil Conv. Shandong	85.59	1988	1.37
Gas Conv. Sichuan	83.72	2024	1.84
Gas Conv. Xinjiang	75.82	2026	1.39
Oil Conv. Shaanxi	69.46	2022	2.04
Coal Bit. Gansu	66.67	2022	1.26
Oil Conv. Xinjiang	65.09	2015	1.13
Gas Conv. Inner Mongolia	62.94	2023	1.51
Coal Bit. Hunan	56.4	2013	2.15
Coal Bit. Chongqing	53.62	2017	1.16
Gas Conv. Shaanxi	53.01	2019	1.42
Coal Bit. Jilin	52.66	2018	1.76
Oil Conv. Tianjin	44.06	2012	1.41
Oil Conv. Hebei	40.88	2028	0.81
Coal Bit. Jiangsu	37.45	2000	0.66
Gas Conv. Qinghai	37.32	2032	0.91
Oil Conv. Liaoning	35.68	1995	0.68
Oil Conv. Guangdong	28.79	2024	0.69
Coal Bit. Jiangxi	26.85	2008	0.79
Coal Bit. Qinghai	26.45	2022	1.25
Coal Bit. Historic	25.96	1937	0.94
Gas Conv. Offshore	21.38	2034	0.54
Oil Conv. Jilin	20.15	2016	0.4
Coal Bit. Beijing	19.54	2048	0.27
Gas Conv. Guangdong	17.26	2020	0.36
Gas Conv. Chongqing	17.06	2025	0.4
Oil Conv. Gansu	16.32	2028	0.49
Coal Bit. Fujian	15.95	2009	0.67
Gas Conv. Heilongjiang	15.95	2030	0.34
Oil Conv. Henan	15.83	1989	0.36
Coal Bit. Hubei	13.44	2009	0.28

Table 2.34: Peak years - All – Continued

**Name**	**URR**	**Peak Year**	**Peak Rate**
Coal Bit. Guangxi	11.04	1993	0.25
Coal Bit. Guangdong	7.83	1972	0.24
Coal Bit. Tianjin	7.13	2042	0.27
Oil Conv. Inner Mongolia	6.9	2019	0.26
Oil Conv. Qinghai	6.55	2023	0.18
Gas Conv. Jilin	6.09	2020	0.19
Oil Kerogen China	5.73	2033	0.14
Gas Conv. Shandong	5.23	2028	0.13
Oil Extra Heavy China	5.01	2030	0.15
Gas Conv. Liaoning	4.99	2027	0.1
Oil Conv. Jiangsu	4.2	2016	0.11
Gas Conv. Tianjin	3.96	2015	0.07
Gas Conv. Hebei	3.13	2023	0.1
Oil Conv. Hubei	3.09	2014	0.05
Gas Conv. Henan	2.91	2001	0.07
Oil Conv. Ningxia	2.51	1999	0.11
Coal Bit. Zhejiang	2.2	1975	0.04
Gas Conv. Ningxia	2.0	2030	0.07
Gas Conv. Gansu	1.71	2030	0.06
Coal Bit. Hainan	1.6	2052	0.05
Oil Nat. Bitumen China	1.05	2054	0.03
Oil Conv. Sichuan	0.68	2024	0.03
Gas Conv. Hubei	0.52	2022	0.02
Gas Conv. Shanghai	0.36	2004	0.02
Gas Conv. Hainan	0.34	2015	0.02
Oil Conv. Anhui	0.3	2024	0.02
Oil Conv. Hainan	0.26	2009	0.01
Coal Bit. Shanghai	0.25	1982	0.08
Gas Conv. Jiangsu	0.24	2024	0.01
Oil Conv. Shanghai	0.22	2001	0.02
Coal Bit. Tibet	0.19	2048	–
Oil Conv. Chongqing	0.19	2024	0.01
Oil Conv. Guangxi	0.15	2020	0.01
Gas Conv. Guizhou	0.13	2024	–
Gas Conv. Yunnan	0.04	2019	–
Gas Conv. Guangxi	0.03	2022	–
Gas Conv. Jiangxi	0.01	2007	–
Oil Conv. Yunnan	0.01	2022	–
Gas Conv. Anhui	0.01	2022	–
**Total**	**7043.41**	**2021**	**155.03**

2.8.2 By Mineral

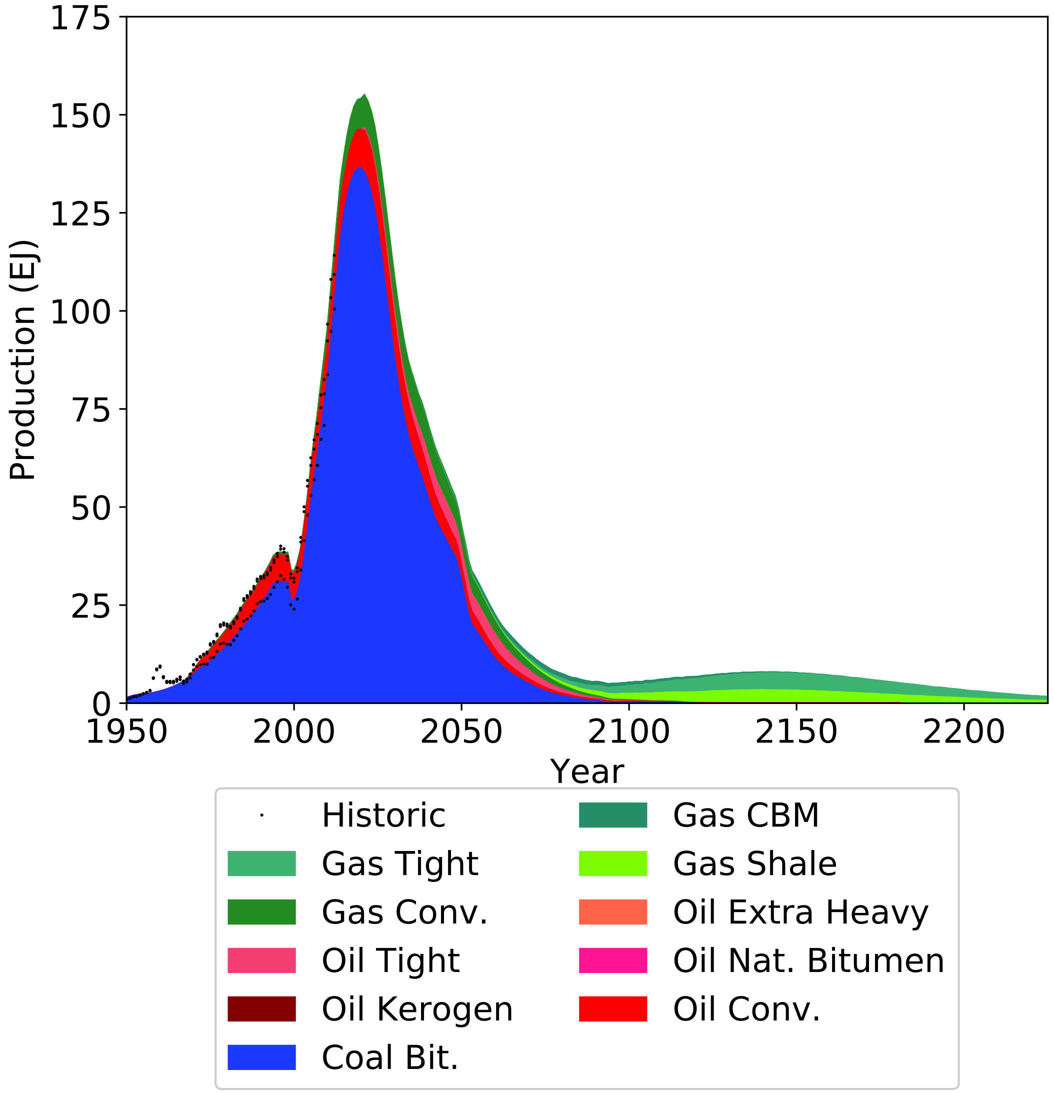

Figure 2.35: China projection by mineral type

2.8.3 Regional Projections

Table 2.35: Peak years - Minerals

Name	URR	Peak Year	Peak Rate
Coal Bit.	4958.36	2019	136.27
Oil Conv.	603.74	2023	10.74
Oil Kerogen	5.73	2033	0.14
Oil Nat. Bitumen	1.05	2054	0.03
Oil Tight	184.51	2044	4.62
Oil Extra Heavy	5.01	2030	0.15
Gas Conv.	416.15	2027	8.97
Gas Shale	318.89	2140	3.33
Gas Tight	444.97	2143	4.35
Gas CBM	105.0	2067	1.31
**Total**	**7043.41**	**2021**	**155.03**

Anhui

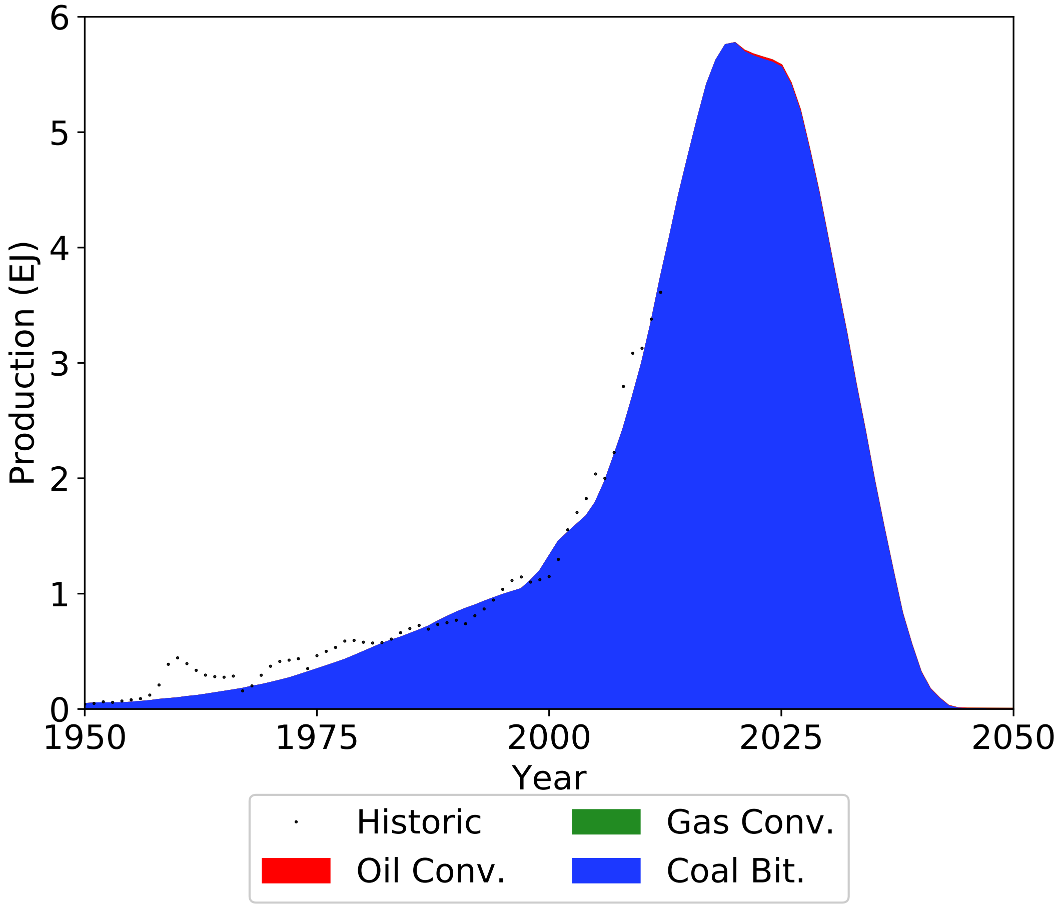

Figure 2.36: China - Anhui projections capped at 16

Table 2.36: Peak years - All

**Name**	**URR**	**Peak Year**	**Peak Rate**
Coal Bit. Anhui	162.36	2020	5.77
Oil Conv. Anhui	0.3	2024	0.02
Gas Conv. Anhui	0.01	2022	–
**Total**	**162.67**	**2020**	**5.77**

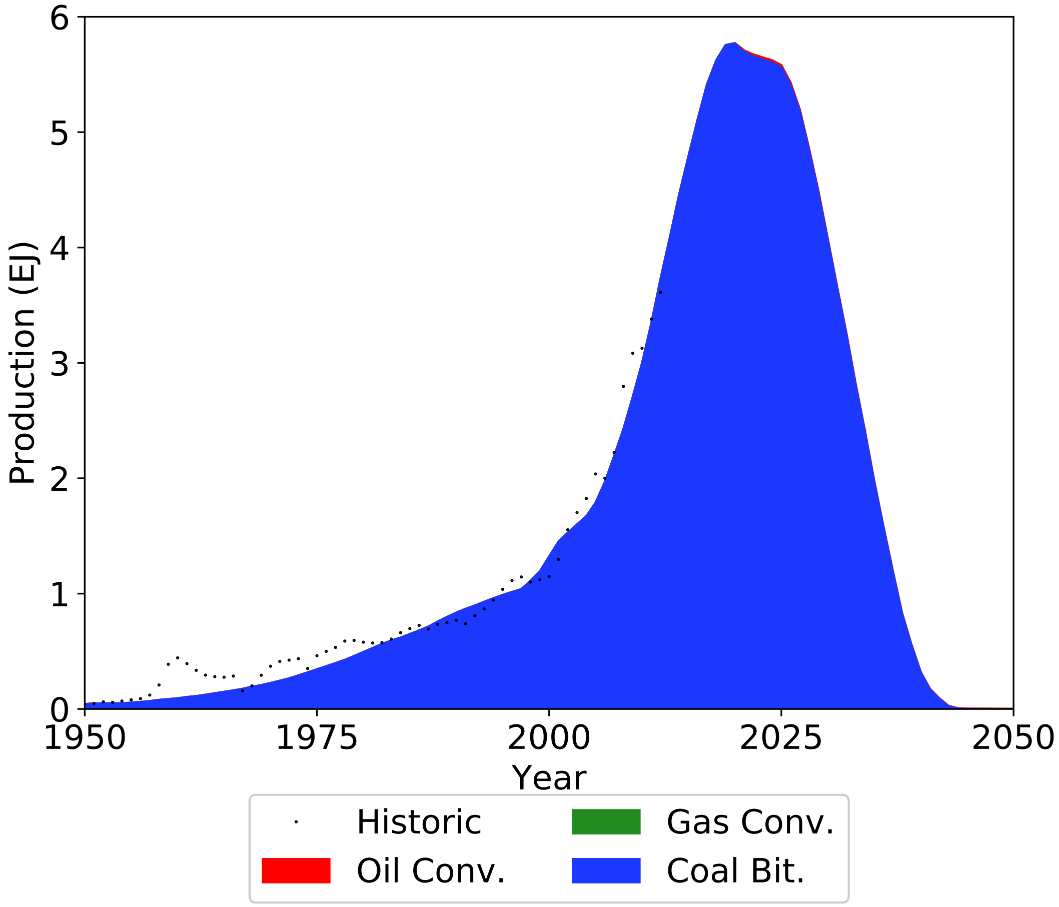

Figure 2.37: China - Anhui projection by mineral type

Table 2.37: Peak years - Minerals

Name	URR	Peak Year	Peak Rate
Coal Bit.	162.36	2020	5.77
Oil Conv.	0.3	2024	0.02
Gas Conv.	0.01	2022	–
**Total**	**162.67**	**2020**	**5.77**

Beijing

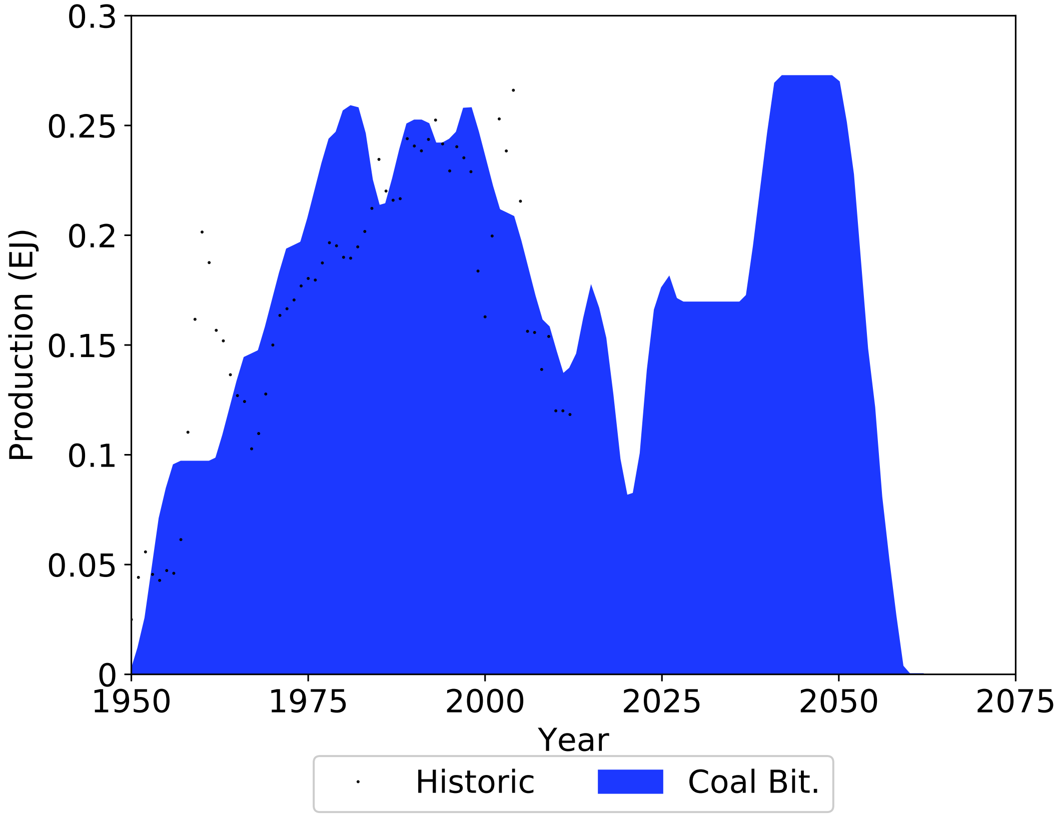

Figure 2.38: China - Beijing projections capped at 16

Table 2.38: Peak years - All

**Name**	**URR**	**Peak Year**	**Peak Rate**
Coal Bit. Beijing	19.54	2048	0.27
**Total**	**19.54**	**2048**	**0.27**

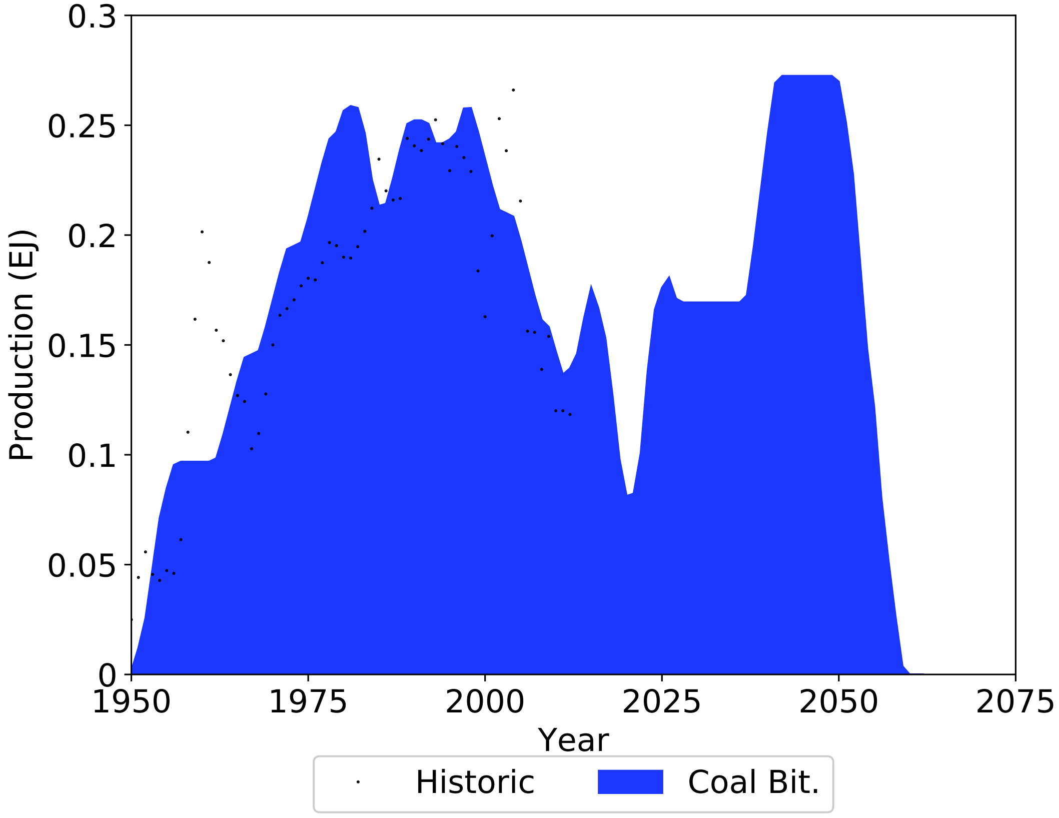

Figure 2.39: China - Beijing projection by mineral type

Table 2.39: Peak years - Minerals

Name	URR	Peak Year	Peak Rate
Coal Bit.	19.54	2048	0.27
**Total**	**19.54**	**2048**	**0.27**

China

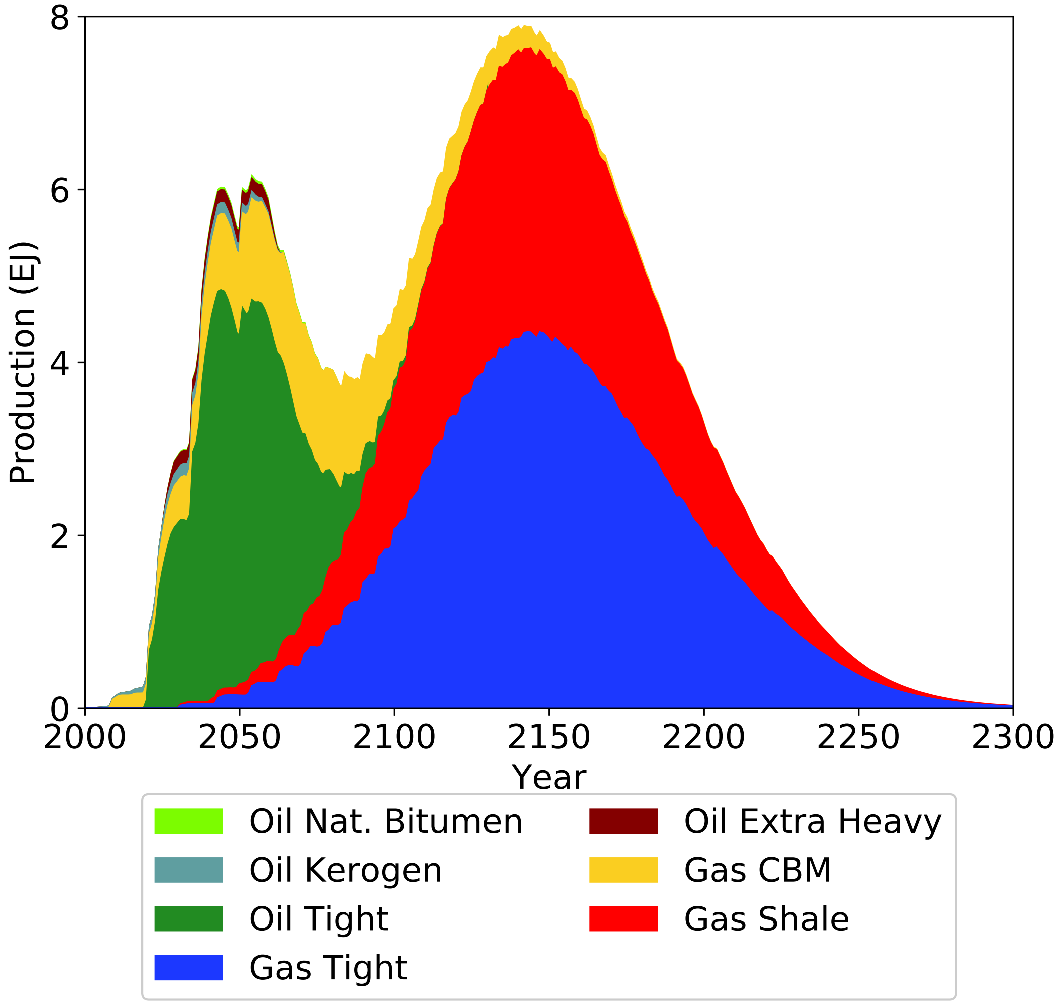

Figure 2.40: China - China projections capped at 16

Table 2.40: Peak years - All

Name	URR	Peak Year	Peak Rate
Gas Tight China	444.97	2143	4.35
Gas Shale China	318.89	2140	3.33
Oil Tight China	184.51	2044	4.62
Gas CBM China	105.0	2067	1.31
Oil Kerogen China	5.73	2033	0.14
Oil Extra Heavy China	5.01	2030	0.15
Oil Nat. Bitumen China	1.05	2054	0.03
**Total**	**1065.16**	**2142**	**7.89**

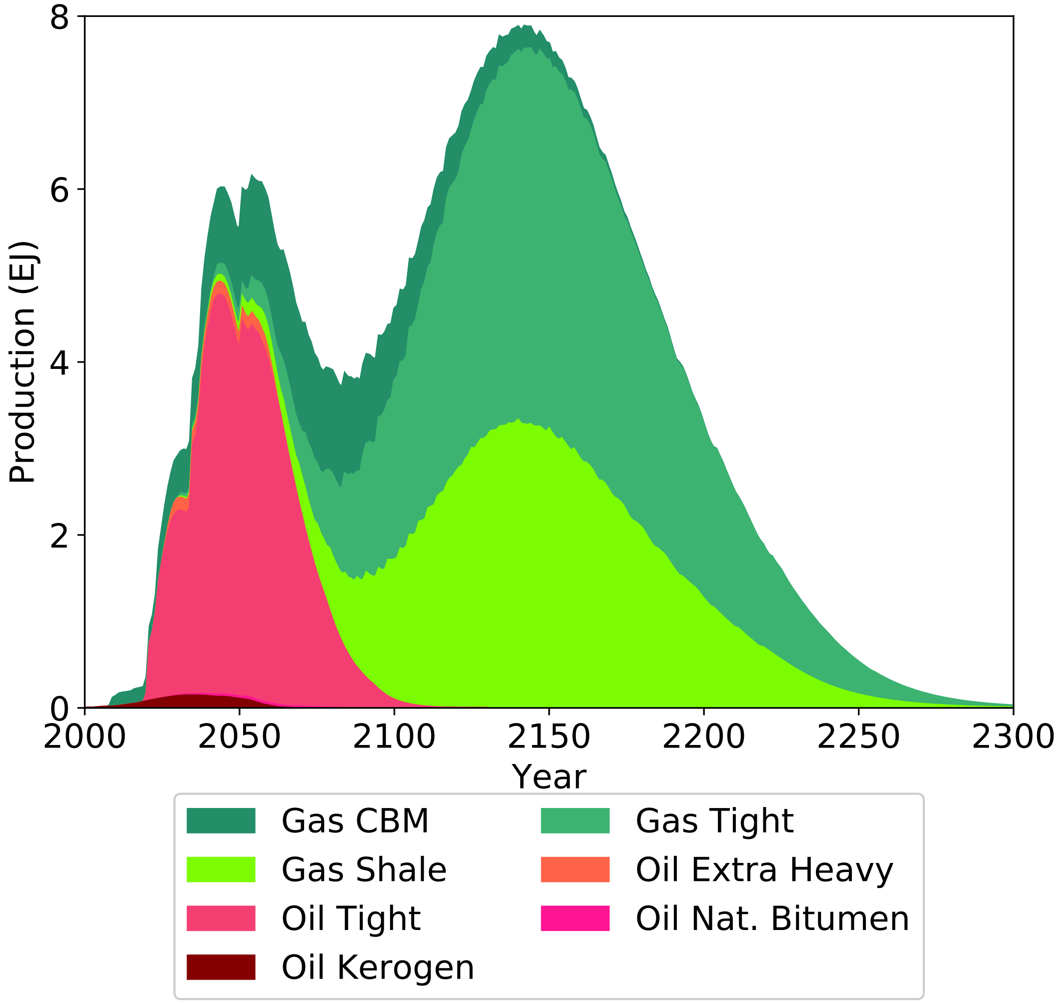

Figure 2.41: China - China projection by mineral type

Table 2.41: Peak years - Minerals

**Name**	**URR**	**Peak Year**	**Peak Rate**
Oil Kerogen	5.73	2033	0.14
Oil Nat. Bitumen	1.05	2054	0.03
Oil Tight	184.51	2044	4.62
Oil Extra Heavy	5.01	2030	0.15
Gas Shale	318.89	2140	3.33
Gas Tight	444.97	2143	4.35
Gas CBM	105.0	2067	1.31
**Total**	**1065.16**	**2142**	**7.89**

Chongqing

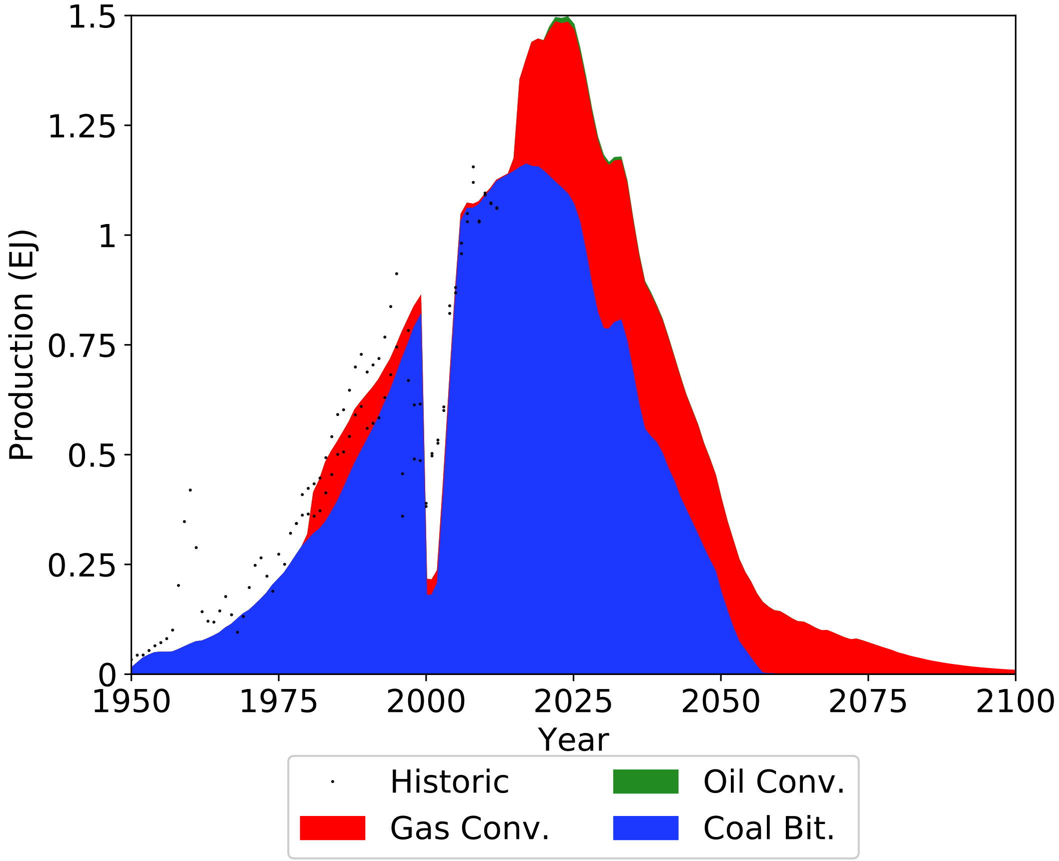

Figure 2.42: China - Chongqing projections capped at 16

Table 2.42: Peak years - All

**Name**	**URR**	**Peak Year**	**Peak Rate**
Coal Bit. Chongqing	53.62	2017	1.16
Gas Conv. Chongqing	17.06	2025	0.4
Oil Conv. Chongqing	0.19	2024	0.01
**Total**	**70.87**	**2024**	**1.5**

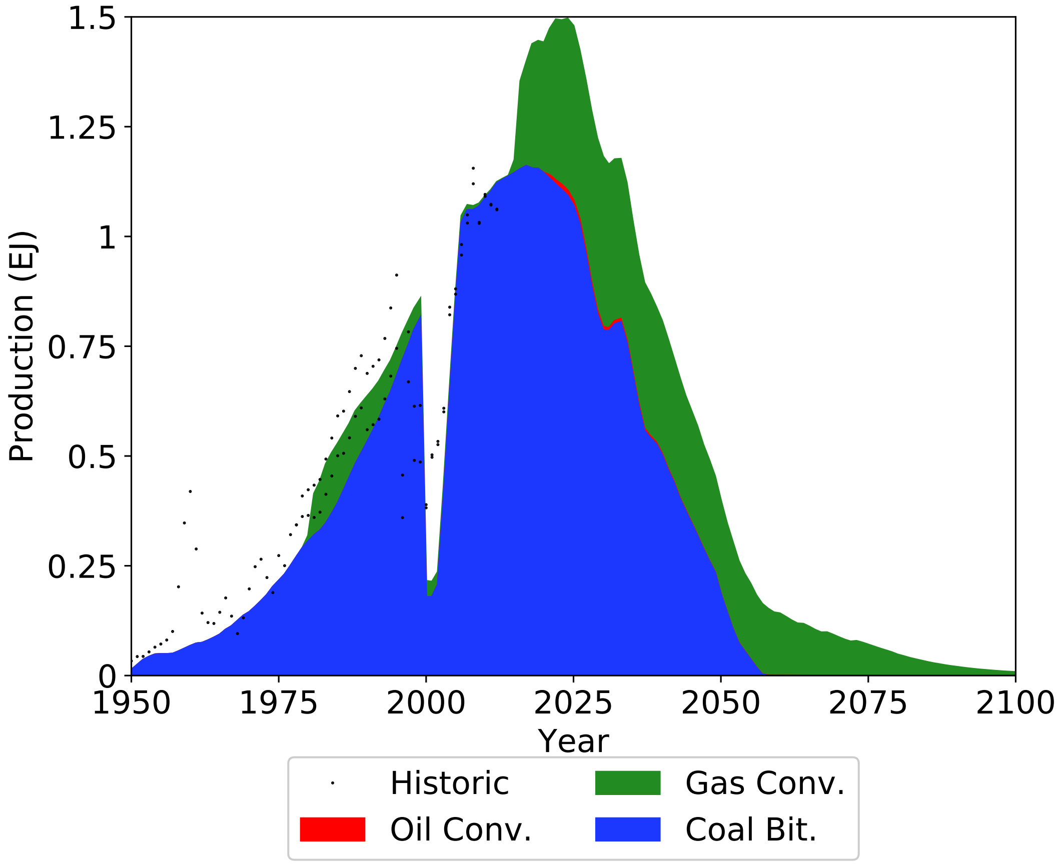

Figure 2.43: China - Chongqing projection by mineral type

Table 2.43: Peak years - Minerals

Name	URR	Peak Year	Peak Rate
Coal Bit.	53.62	2017	1.16
Oil Conv.	0.19	2024	0.01
Gas Conv.	17.06	2025	0.4
**Total**	**70.87**	**2024**	**1.5**

Fujian

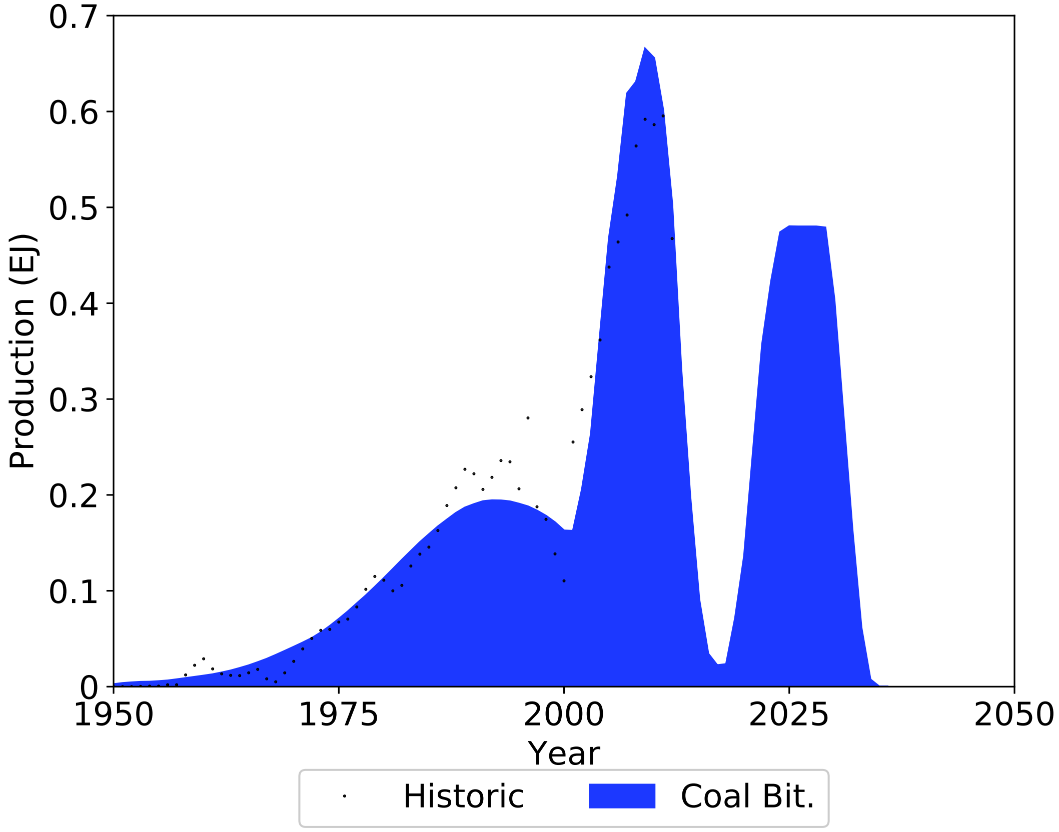

Figure 2.44: China - Fujian projections capped at 16

Table 2.44: Peak years - All

Name	URR	Peak Year	Peak Rate
Coal Bit. Fujian	15.95	2009	0.67
**Total**	**15.95**	**2009**	**0.67**

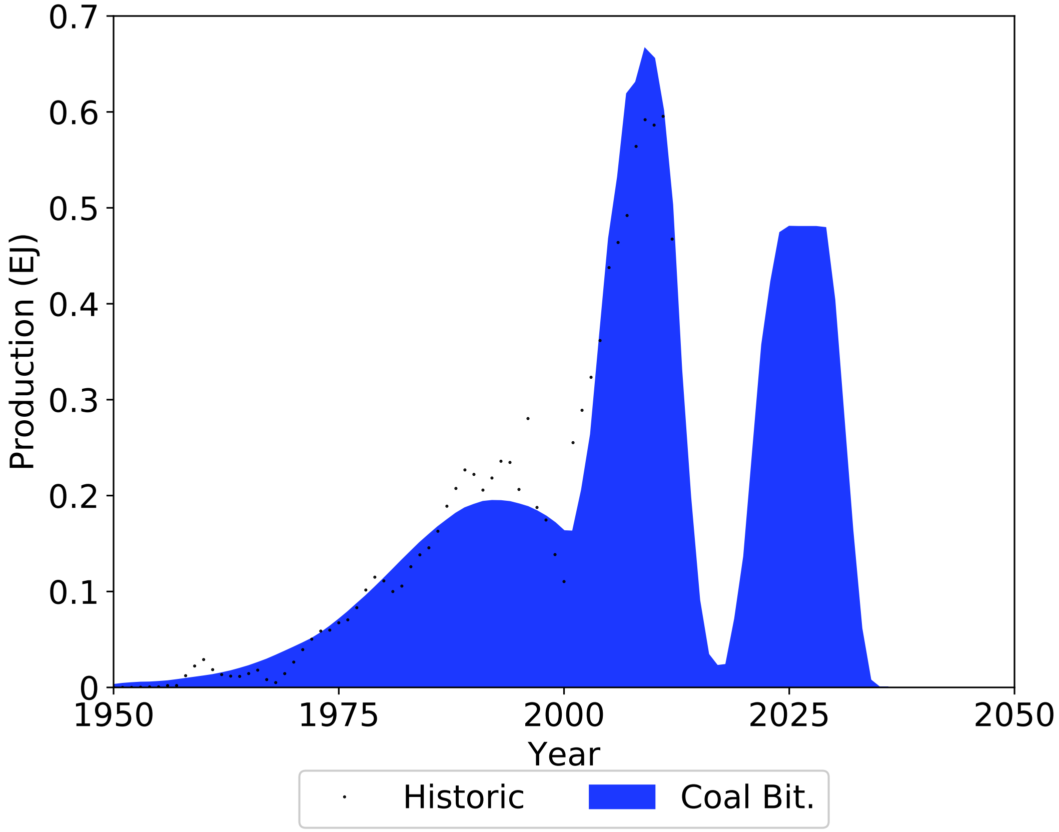

Figure 2.45: China - Fujian projection by mineral type

Table 2.45: Peak years - Minerals

Name	URR	Peak Year	Peak Rate
Coal Bit.	15.95	2009	0.67
**Total**	**15.95**	**2009**	**0.67**

Gansu

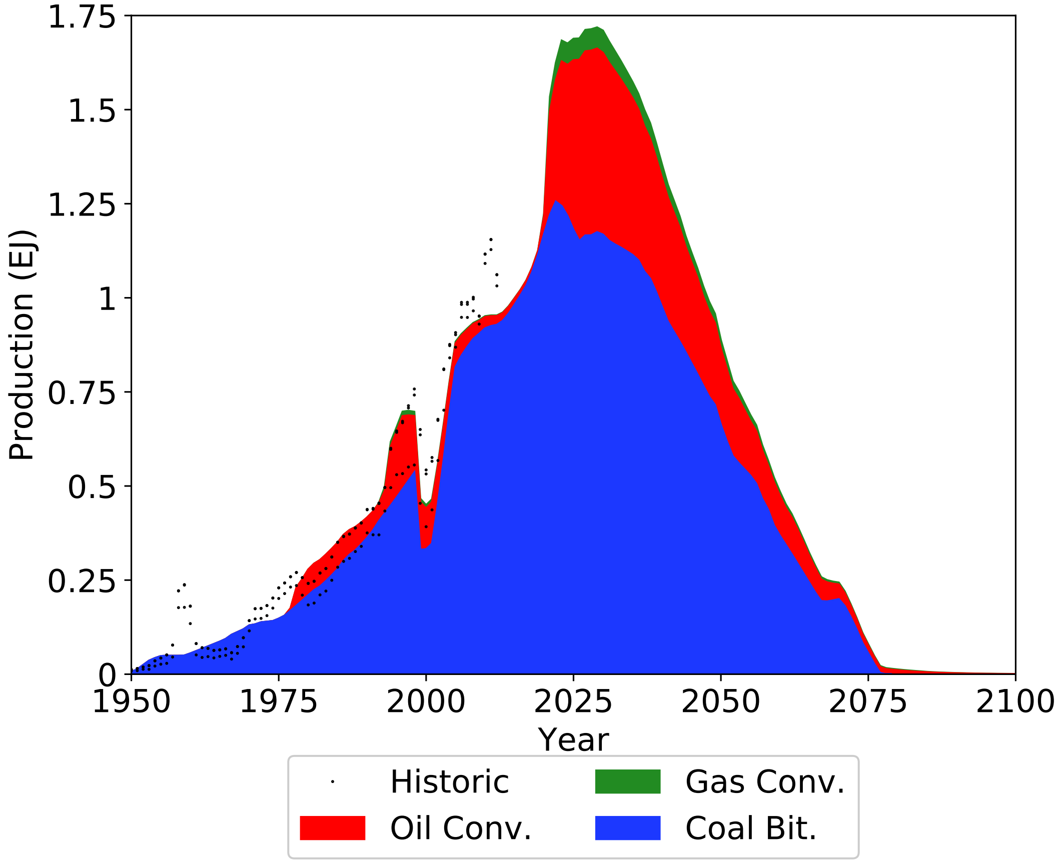

Figure 2.46: China - Gansu projections capped at 16

Table 2.46: Peak years - All

Name	URR	Peak Year	Peak Rate
Coal Bit. Gansu	66.67	2022	1.26
Oil Conv. Gansu	16.32	2028	0.49
Gas Conv. Gansu	1.71	2030	0.06
**Total**	**84.7**	**2029**	**1.72**

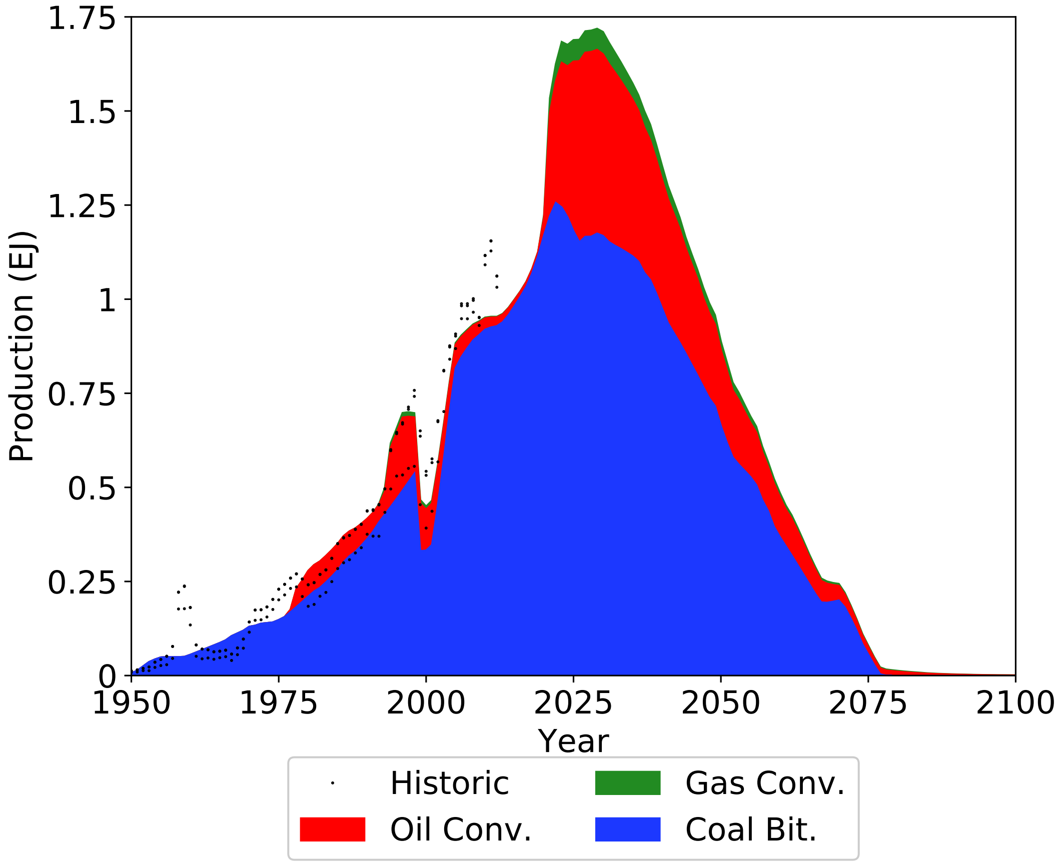

Figure 2.47: China - Gansu projection by mineral type

Table 2.47: Peak years - Minerals

Name	URR	Peak Year	Peak Rate
Coal Bit.	66.67	2022	1.26
Oil Conv.	16.32	2028	0.49
Gas Conv.	1.71	2030	0.06
**Total**	**84.7**	**2029**	**1.72**

Guangdong

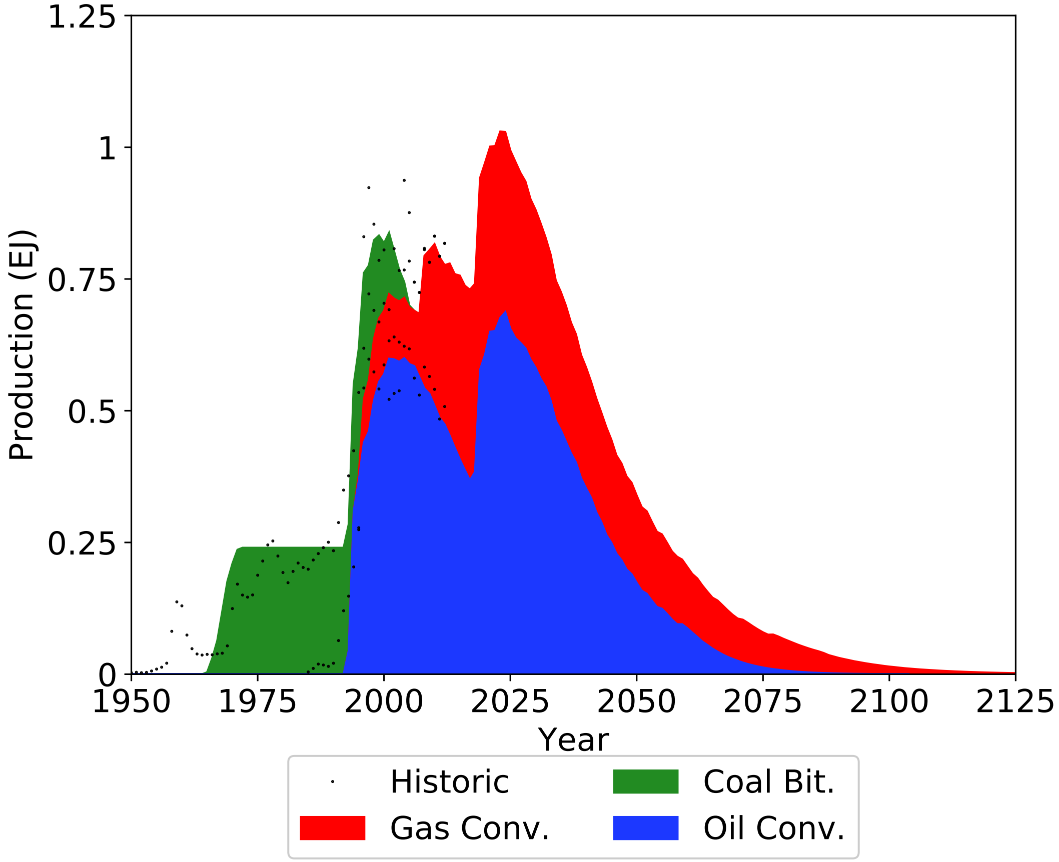

Figure 2.48: China - Guangdong projections capped at 16

Table 2.48: Peak years - All

Name	URR	Peak Year	Peak Rate
Oil Conv. Guangdong	28.79	2024	0.69
Gas Conv. Guangdong	17.26	2020	0.36
Coal Bit. Guangdong	7.83	1972	0.24
**Total**	**53.88**	**2023**	**1.03**

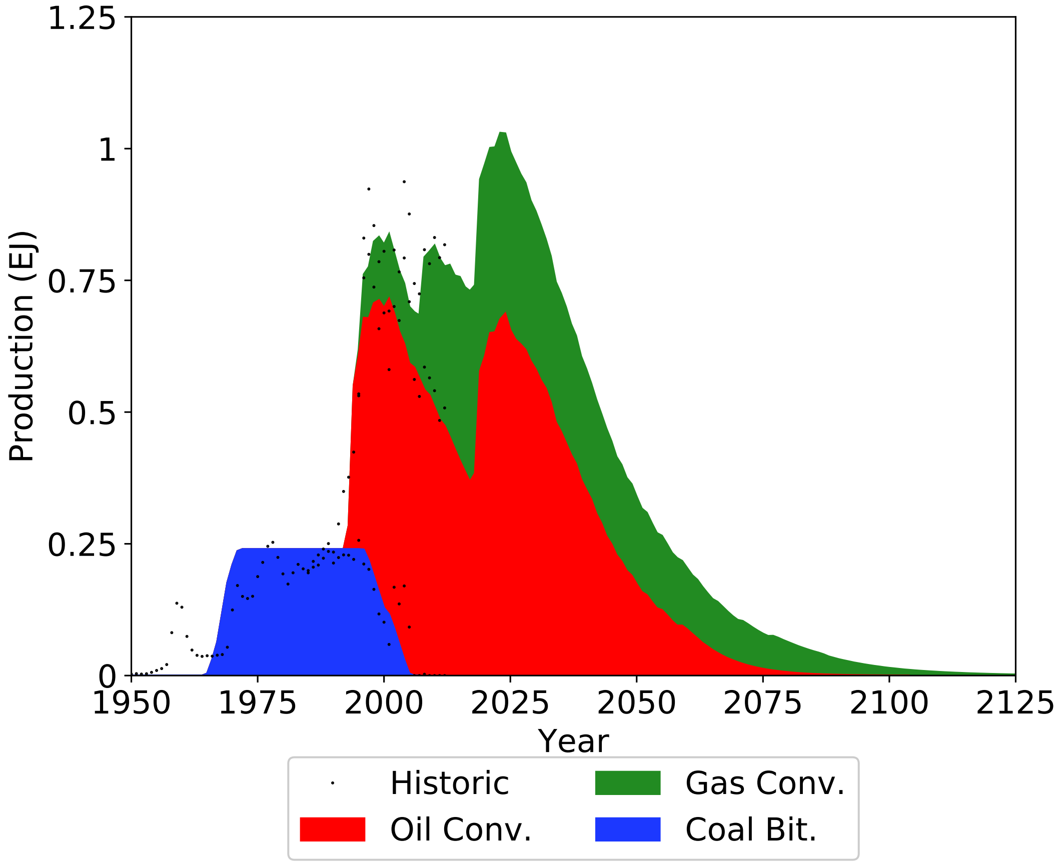

Figure 2.49: China - Guangdong projection by mineral type

Table 2.49: Peak years - Minerals

Name	URR	Peak Year	Peak Rate
Coal Bit.	7.83	1972	0.24
Oil Conv.	28.79	2024	0.69
Gas Conv.	17.26	2020	0.36
**Total**	**53.88**	**2023**	**1.03**

Guangxi

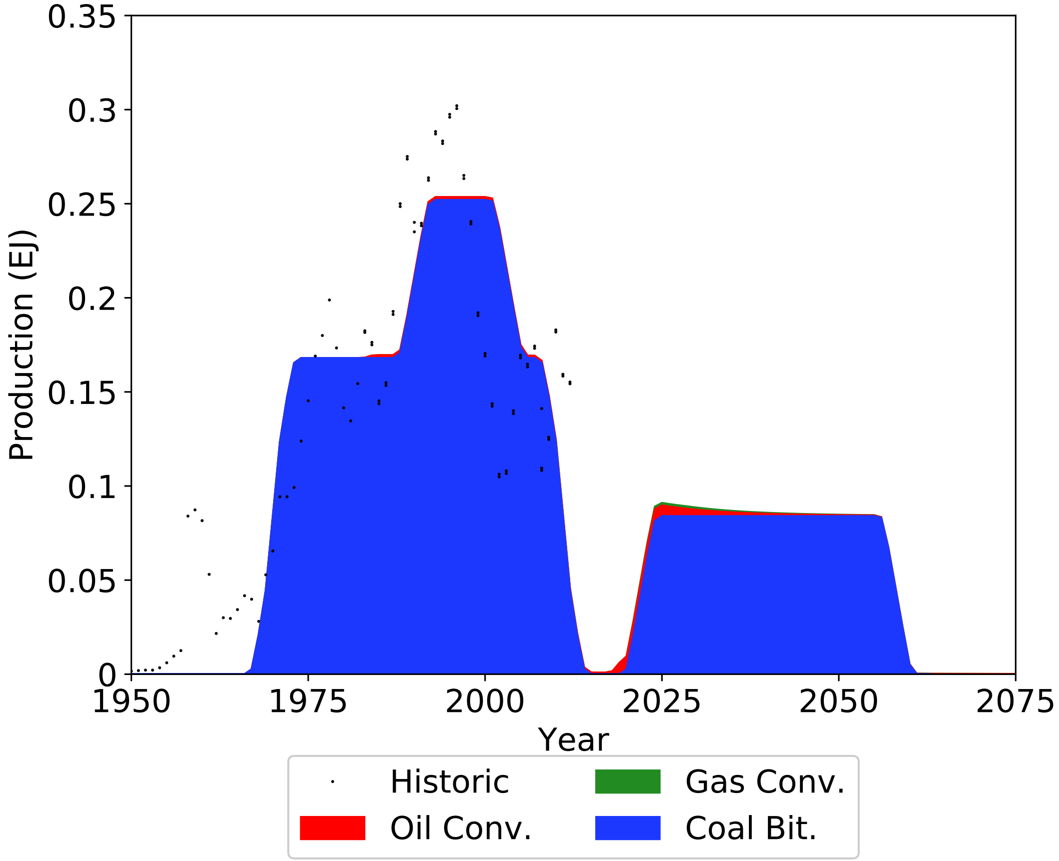

Figure 2.50: China - Guangxi projections capped at 16

Table 2.50: Peak years - All

Name	URR	Peak Year	Peak Rate
Coal Bit. Guangxi	11.04	1993	0.25
Oil Conv. Guangxi	0.15	2020	0.01
Gas Conv. Guangxi	0.03	2022	–
**Total**	**11.22**	**1993**	**0.25**

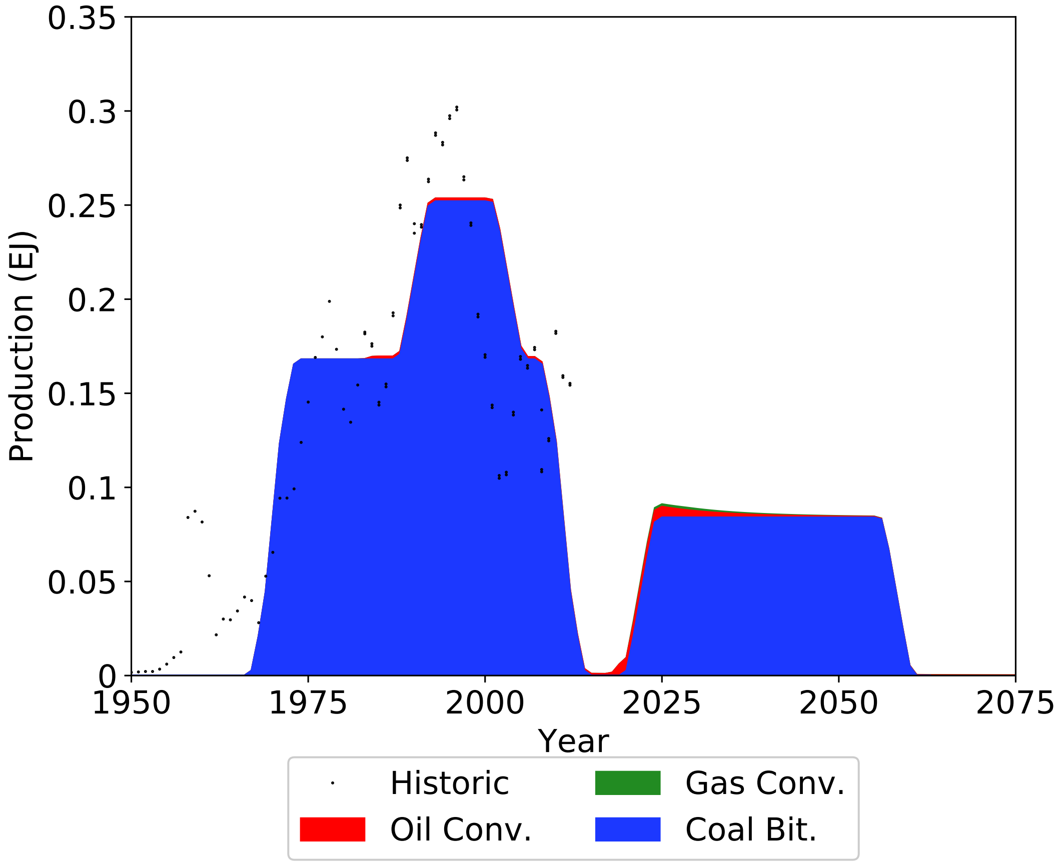

Figure 2.51: China - Guangxi projection by mineral type

Table 2.51: Peak years - Minerals

Name	URR	Peak Year	Peak Rate
Coal Bit.	11.04	1993	0.25
Oil Conv.	0.15	2020	0.01
Gas Conv.	0.03	2022	–
**Total**	**11.22**	**1993**	**0.25**

Guizhou

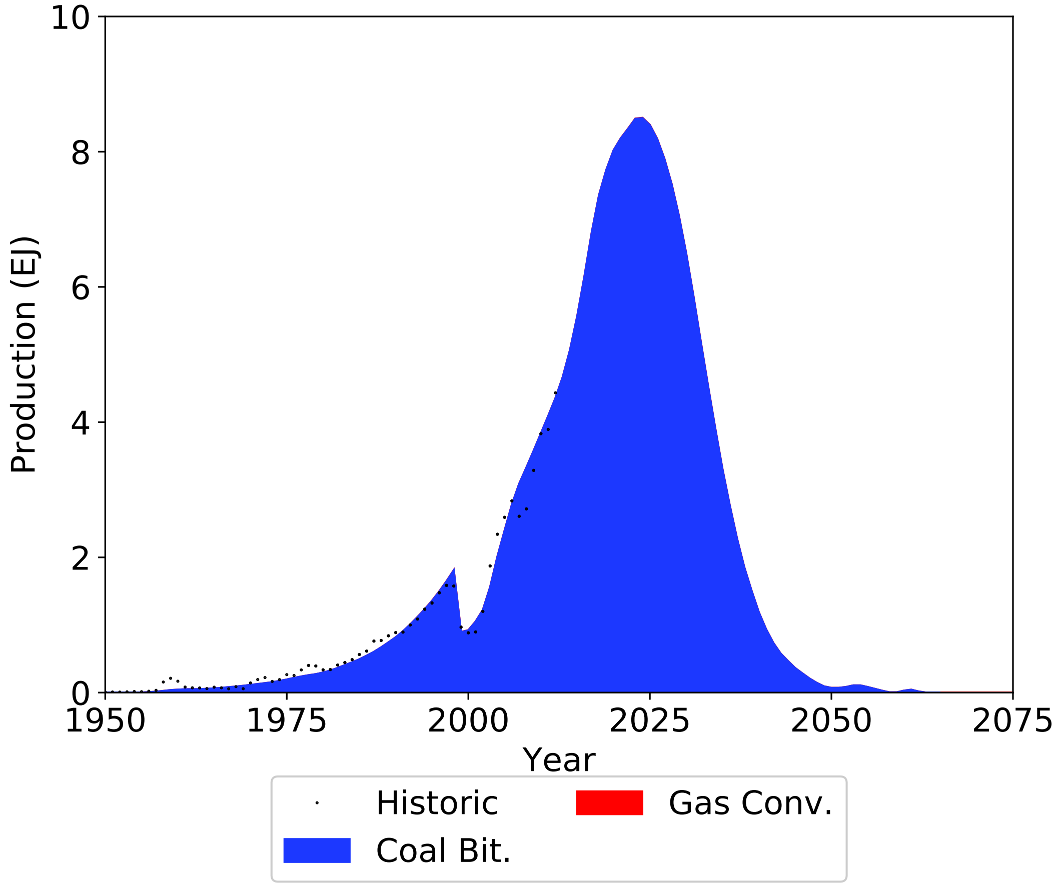

Figure 2.52: China - Guizhou projections capped at 16

Table 2.52: Peak years - All

**Name**	**URR**	**Peak Year**	**Peak Rate**
Coal Bit. Guizhou	221.34	2024	8.5
Gas Conv. Guizhou	0.13	2024	–
**Total**	**221.47**	**2024**	**8.5**

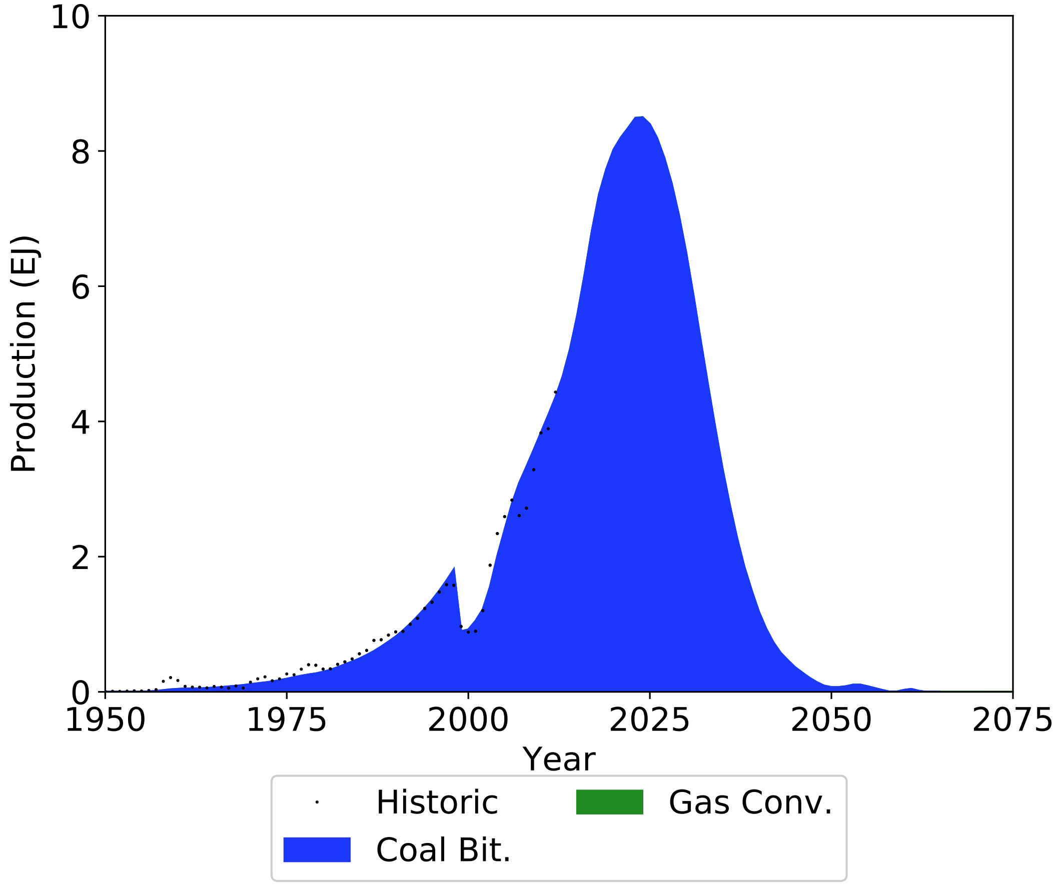

Figure 2.53: China - Guizhou projection by mineral type

Table 2.53: Peak years - Minerals

Name	URR	Peak Year	Peak Rate
Coal Bit.	221.34	2024	8.5
Gas Conv.	0.13	2024	–
**Total**	**221.47**	**2024**	**8.5**

Hainan

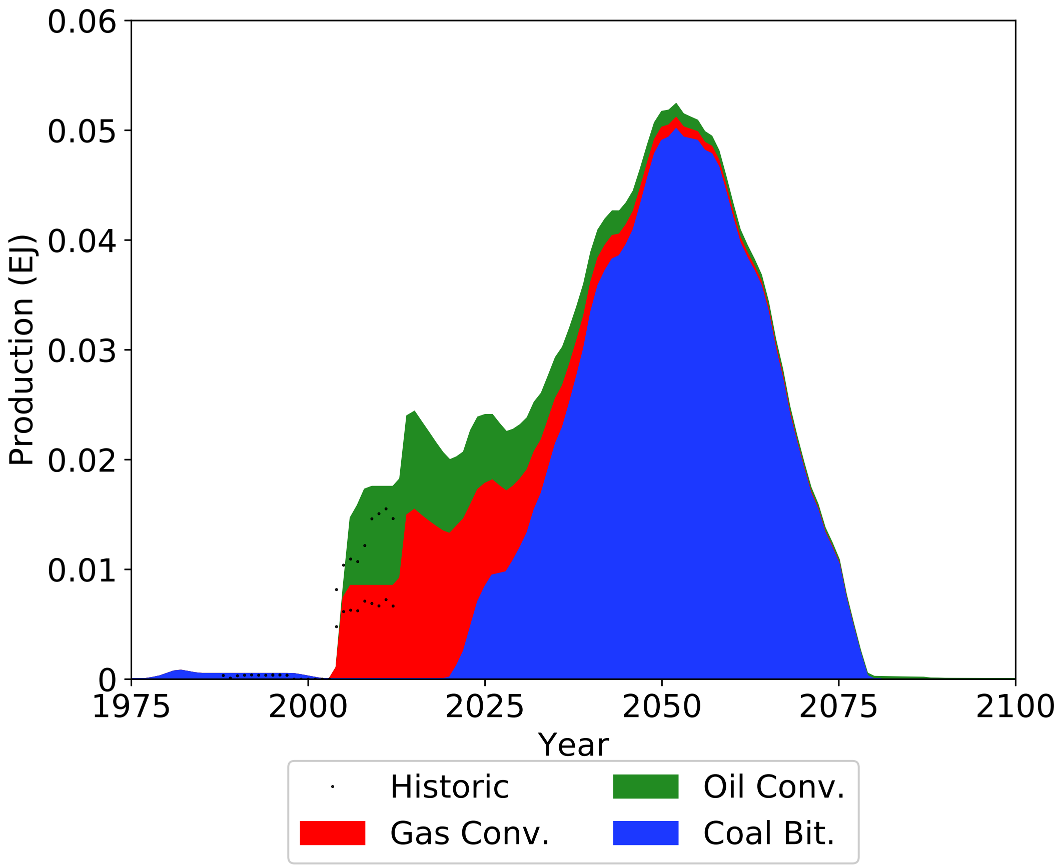

Figure 2.54: China - Hainan projections capped at 16

Table 2.54: Peak years - All

**Name**	**URR**	**Peak Year**	**Peak Rate**
Coal Bit. Hainan	1.6	2052	0.05
Gas Conv. Hainan	0.34	2015	0.02
Oil Conv. Hainan	0.26	2009	0.01
**Total**	**2.2**	**2052**	**0.05**

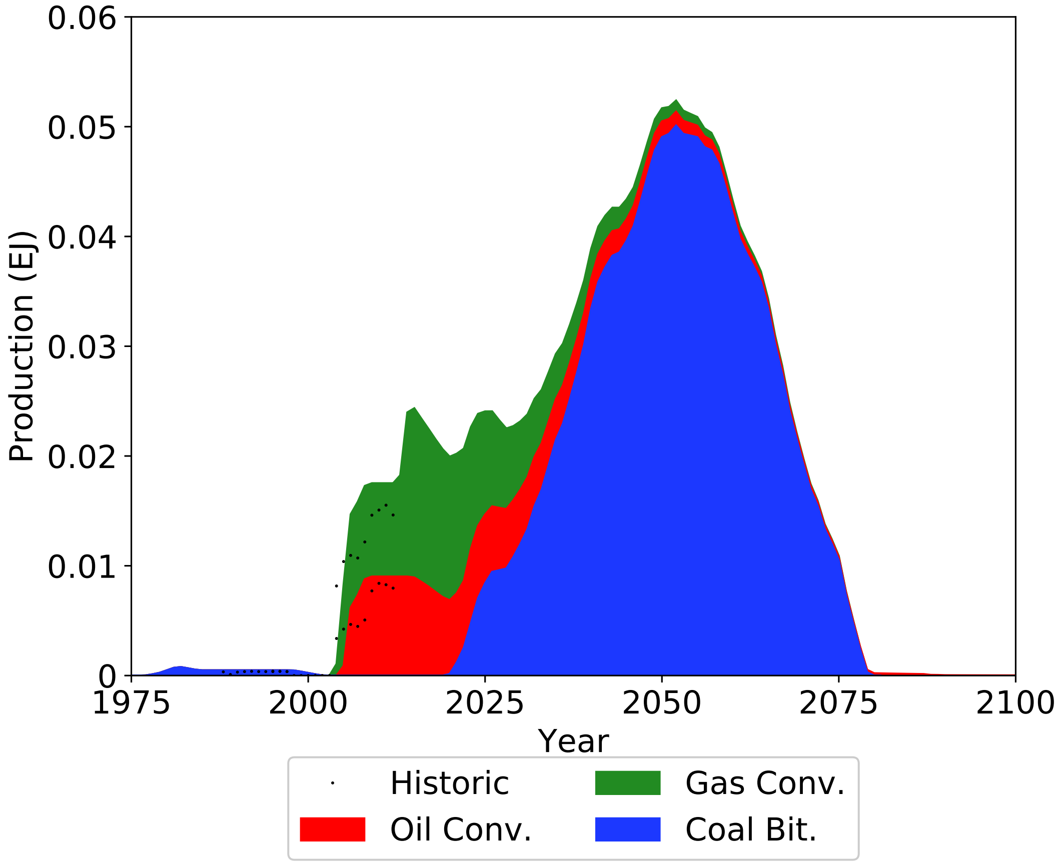

Figure 2.55: China - Hainan projection by mineral type

Table 2.55: Peak years - Minerals

Name	URR	Peak Year	Peak Rate
Coal Bit.	1.6	2052	0.05
Oil Conv.	0.26	2009	0.01
Gas Conv.	0.34	2015	0.02
**Total**	**2.19**	**2052**	**0.05**

Hebei

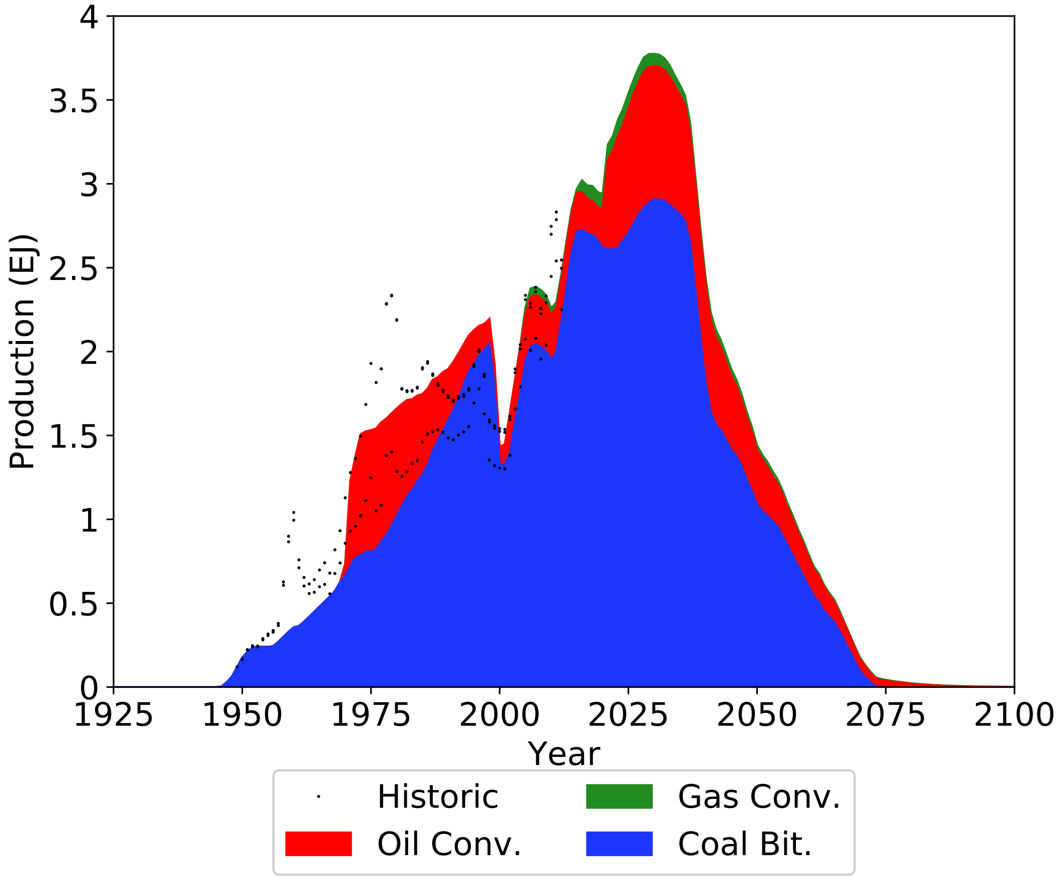

Figure 2.56: China - Hebei projections capped at 16

Table 2.56: Peak years - All

Name	URR	Peak Year	Peak Rate
Coal Bit. Hebei	170.41	2030	2.91
Oil Conv. Hebei	40.88	2028	0.81
Gas Conv. Hebei	3.13	2023	0.1
**Total**	**214.42**	**2030**	**3.77**

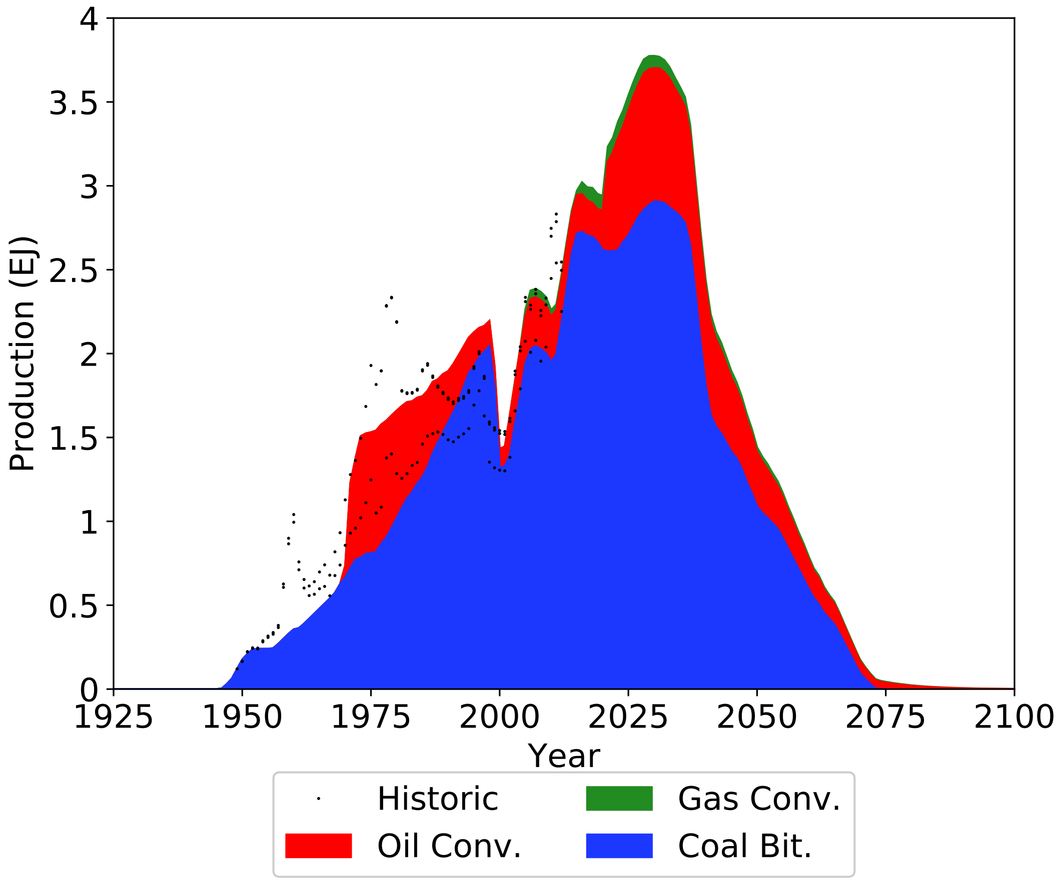

Figure 2.57: China - Hebei projection by mineral type

Table 2.57: Peak years - Minerals

Name	URR	Peak Year	Peak Rate
Coal Bit.	170.41	2030	2.91
Oil Conv.	40.88	2028	0.81
Gas Conv.	3.13	2023	0.1
**Total**	**214.42**	**2030**	**3.77**

Heilongjiang

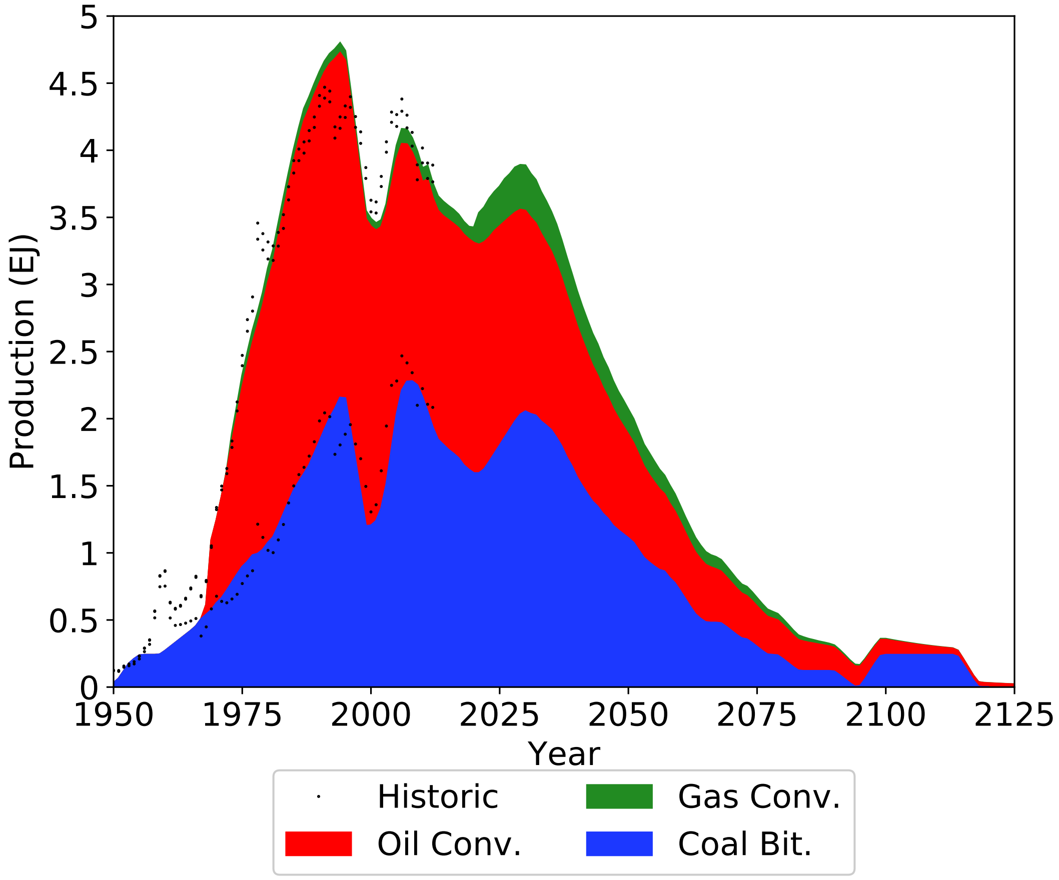

Figure 2.58: China - Heilongjiang projections capped at 16

Table 2.58: Peak years - All

**Name**	**URR**	**Peak Year**	**Peak Rate**
Coal Bit. Heilongjiang	157.4	2008	2.28
Oil Conv. Heilongjiang	156.83	1989	2.67
Gas Conv. Heilongjiang	15.95	2030	0.34
**Total**	**330.18**	**1994**	**4.8**

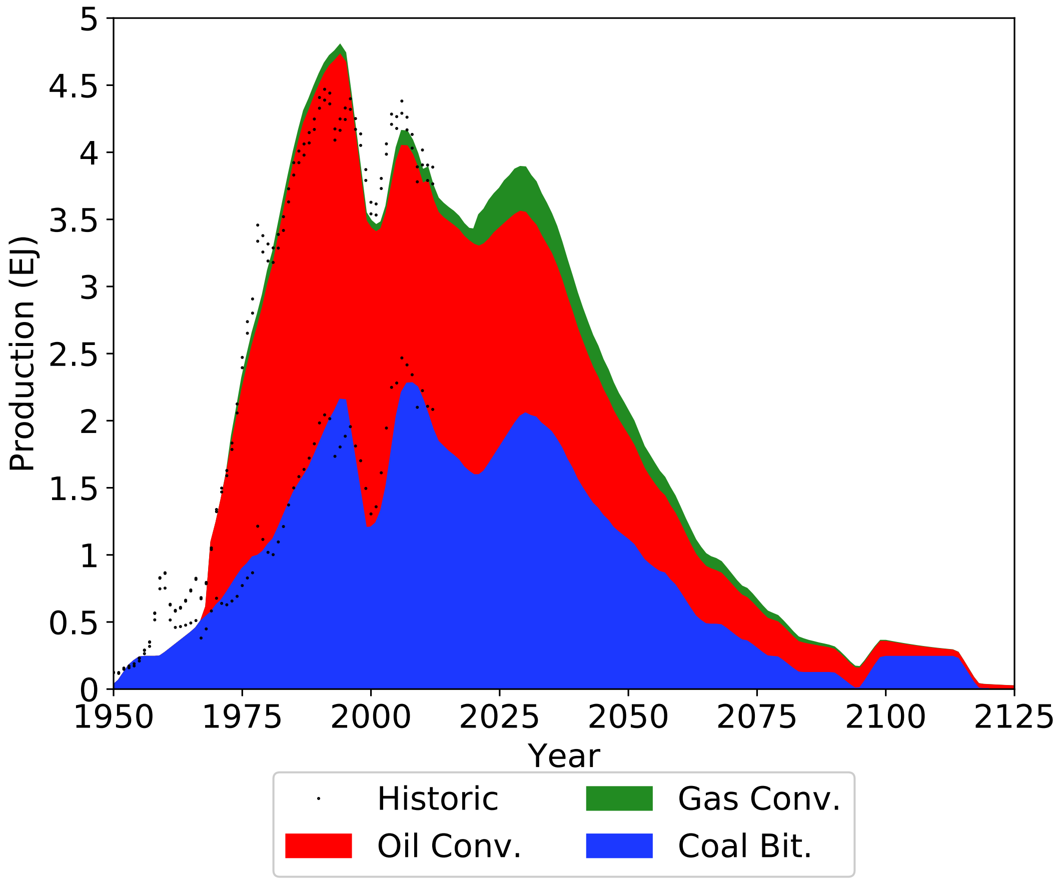

Figure 2.59: China - Heilongjiang projection by mineral type

Table 2.59: Peak years - Minerals

Name	URR	Peak Year	Peak Rate
Coal Bit.	157.4	2008	2.28
Oil Conv.	156.83	1989	2.67
Gas Conv.	15.95	2030	0.34
**Total**	**330.18**	**1994**	**4.8**

Henan

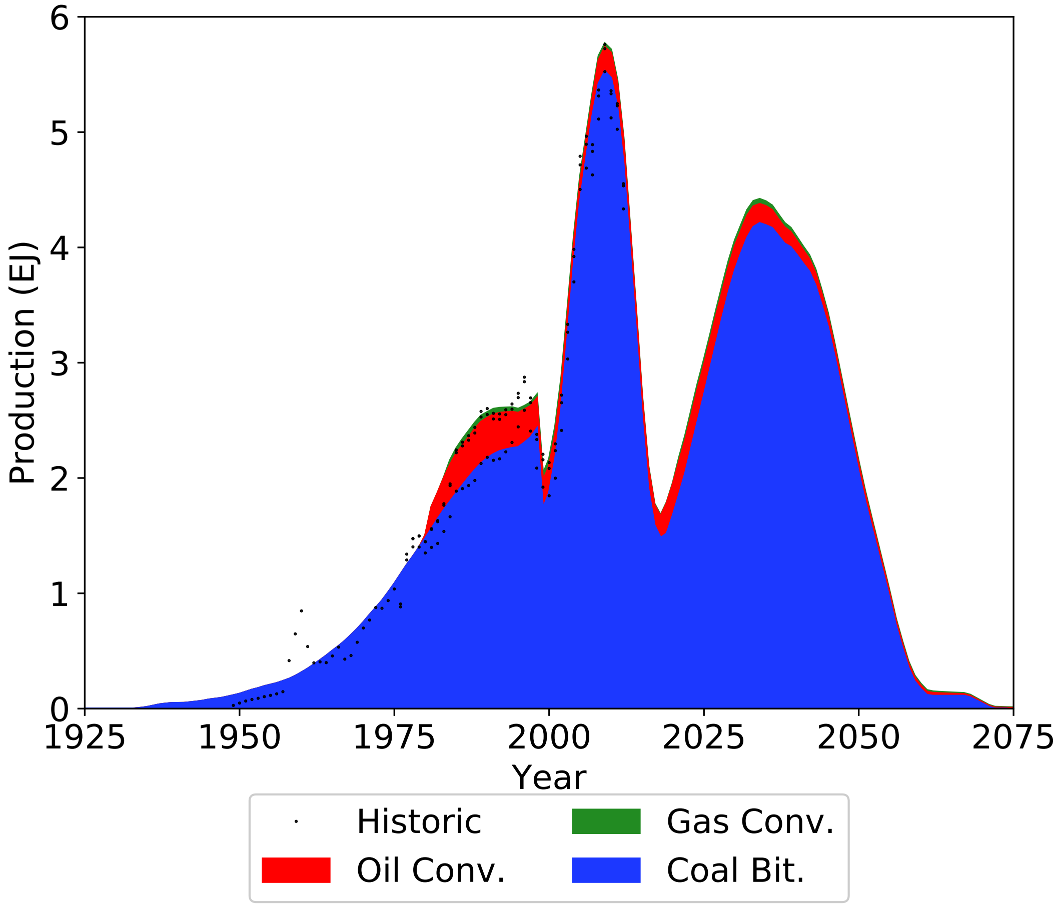

Figure 2.60: China - Henan projections capped at 16

Table 2.60: Peak years - All

**Name**	**URR**	**Peak Year**	**Peak Rate**
Coal Bit. Henan	241.25	2009	5.53
Oil Conv. Henan	15.83	1989	0.36
Gas Conv. Henan	2.91	2001	0.07
**Total**	**259.99**	**2009**	**5.77**

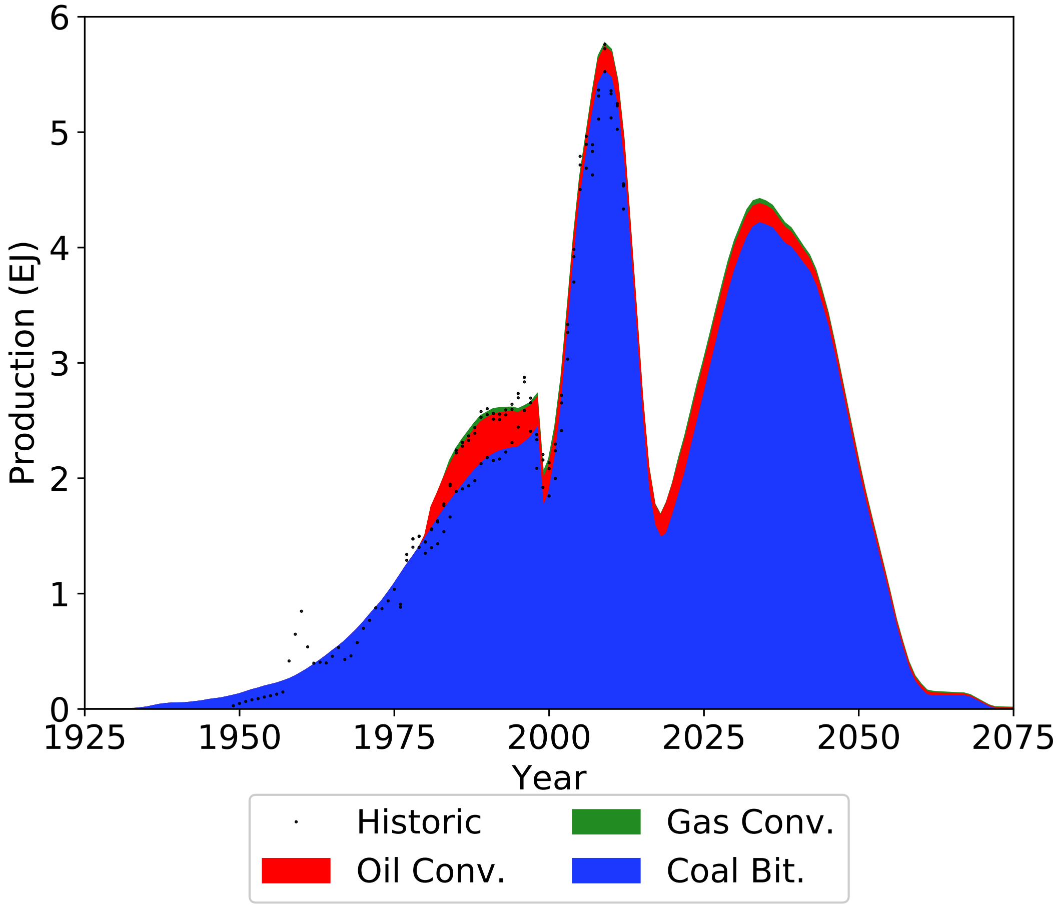

Figure 2.61: China - Henan projection by mineral type

Table 2.61: Peak years - Minerals

Name	URR	Peak Year	Peak Rate
Coal Bit.	241.25	2009	5.53
Oil Conv.	15.83	1989	0.36
Gas Conv.	2.91	2001	0.07
**Total**	**259.99**	**2009**	**5.77**

Historic

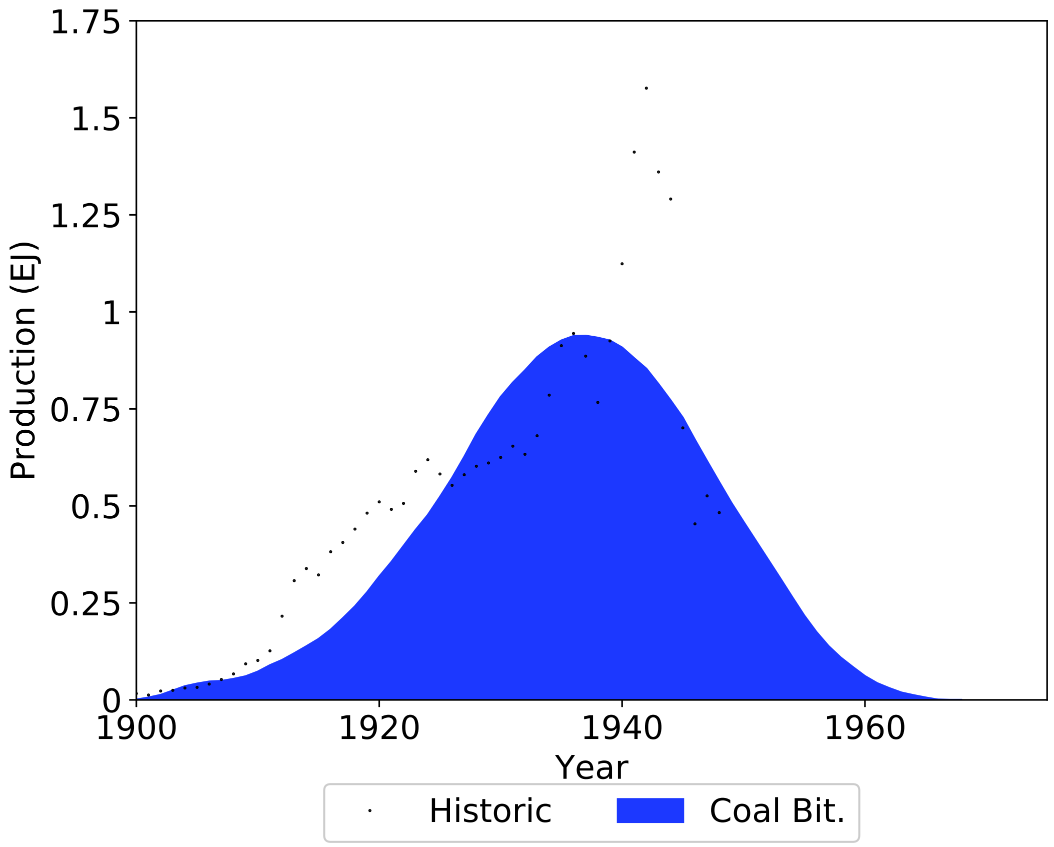

Figure 2.62: China - Historic projections capped at 16

Table 2.62: Peak years - All

Name	URR	Peak Year	Peak Rate
Coal Bit. Historic	25.96	1937	0.94
**Total**	**25.96**	**1937**	**0.94**

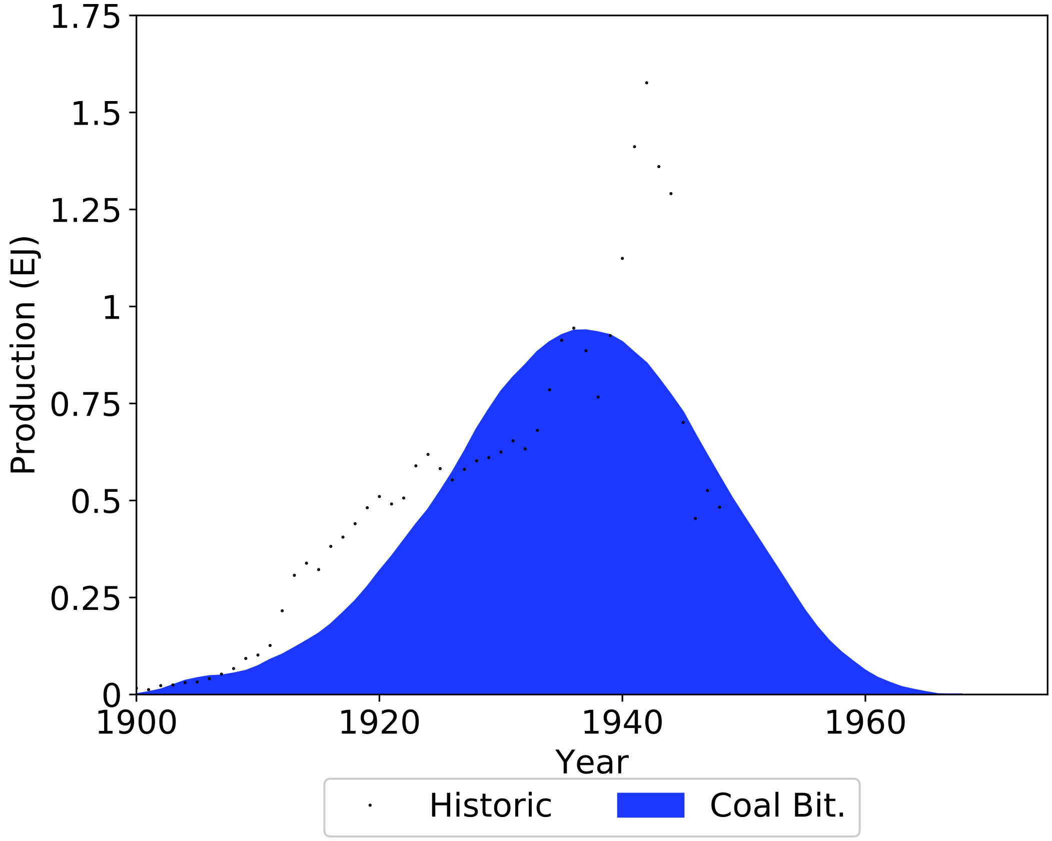

Figure 2.63: China - Historic projection by mineral type

Table 2.63: Peak years - Minerals

Name	URR	Peak Year	Peak Rate
Coal Bit.	25.96	1937	0.94
**Total**	**25.96**	**1937**	**0.94**

Hubei

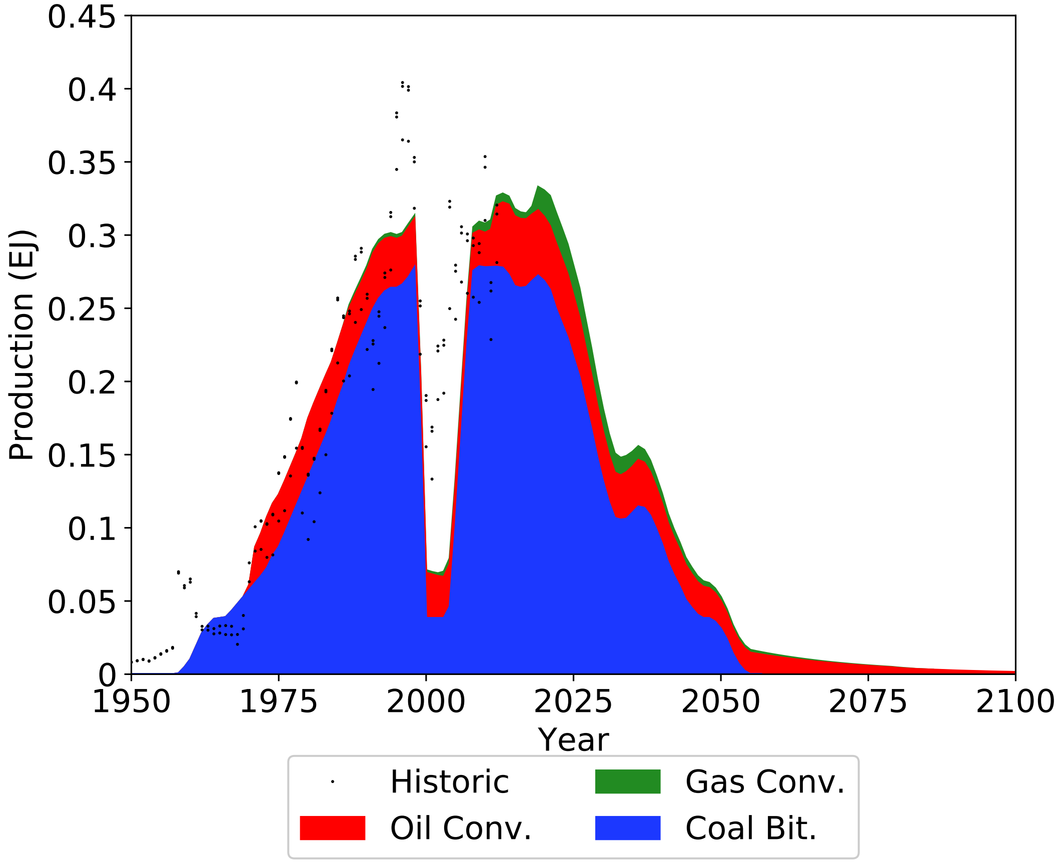

Figure 2.64: China - Hubei projections capped at 16

Table 2.64: Peak years - All

Name	URR	Peak Year	Peak Rate
Coal Bit. Hubei	13.44	2009	0.28
Oil Conv. Hubei	3.09	2014	0.05
Gas Conv. Hubei	0.52	2022	0.02
**Total**	**17.05**	**2019**	**0.33**

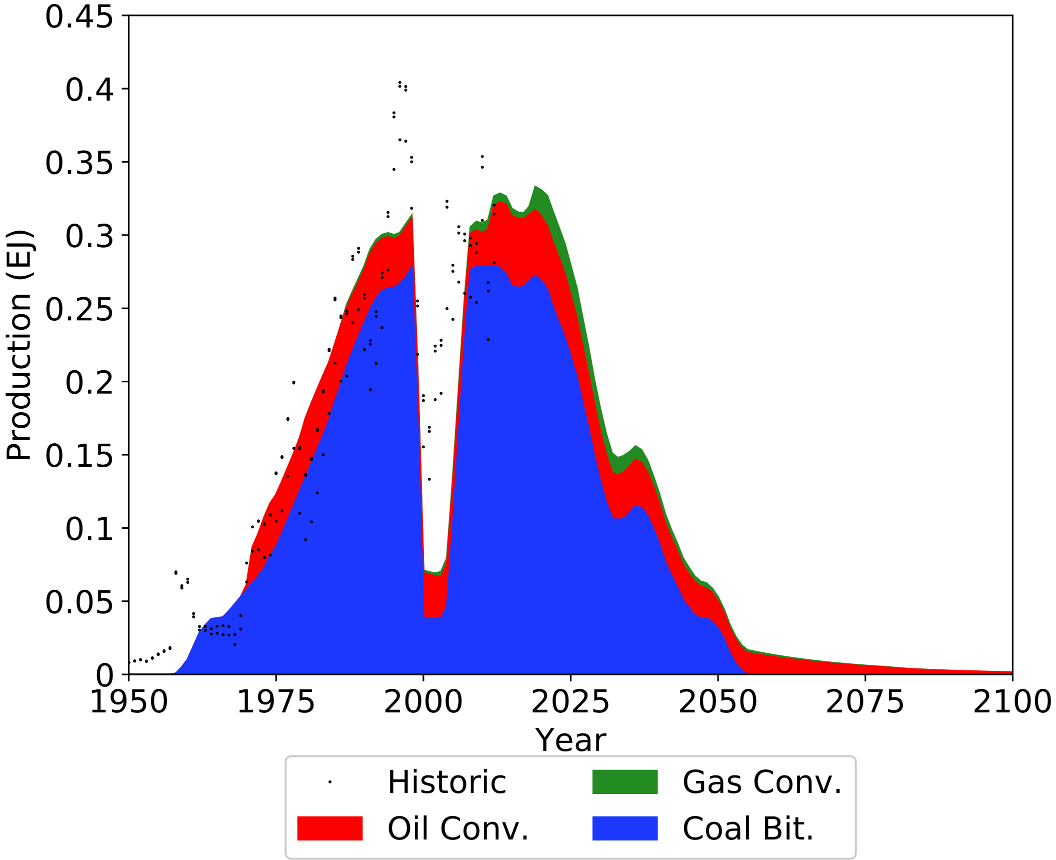

Figure 2.65: China - Hubei projection by mineral type

Table 2.65: Peak years - Minerals

Name	URR	Peak Year	Peak Rate
Coal Bit.	13.44	2009	0.28
Oil Conv.	3.09	2014	0.05
Gas Conv.	0.52	2022	0.02
**Total**	**17.05**	**2019**	**0.33**

Hunan

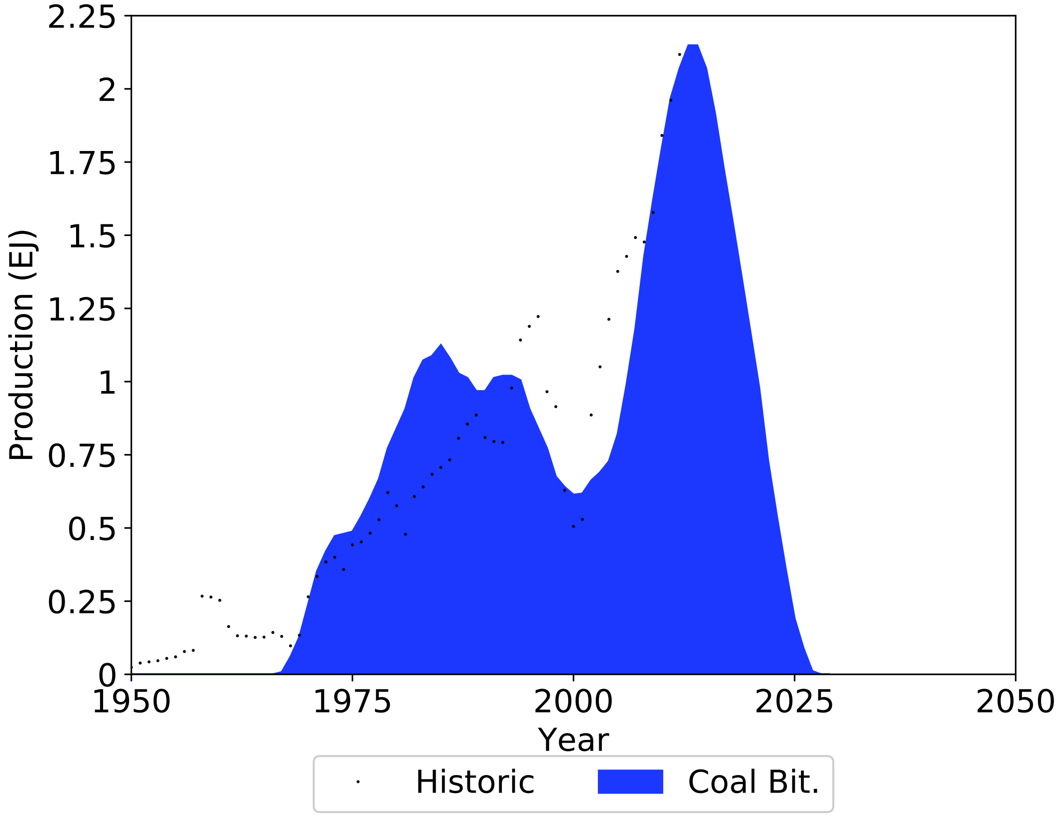

Figure 2.66: China - Hunan projections capped at 16

Table 2.66: Peak years - All

**Name**	**URR**	**Peak Year**	**Peak Rate**
Coal Bit. Hunan	56.4	2013	2.15
**Total**	**56.4**	**2013**	**2.15**

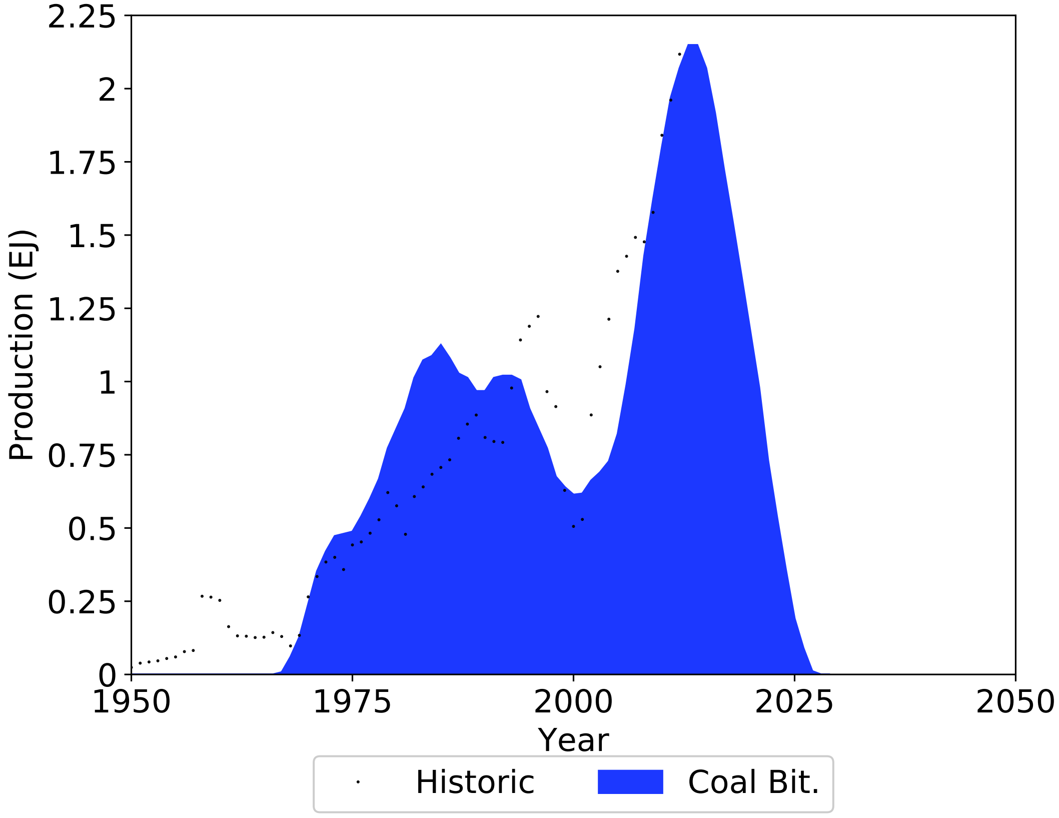

Figure 2.67: China - Hunan projection by mineral type

Table 2.67: Peak years - Minerals

Name	URR	Peak Year	Peak Rate
Coal Bit.	56.4	2013	2.15
**Total**	**56.4**	**2013**	**2.15**

Inner Mongolia

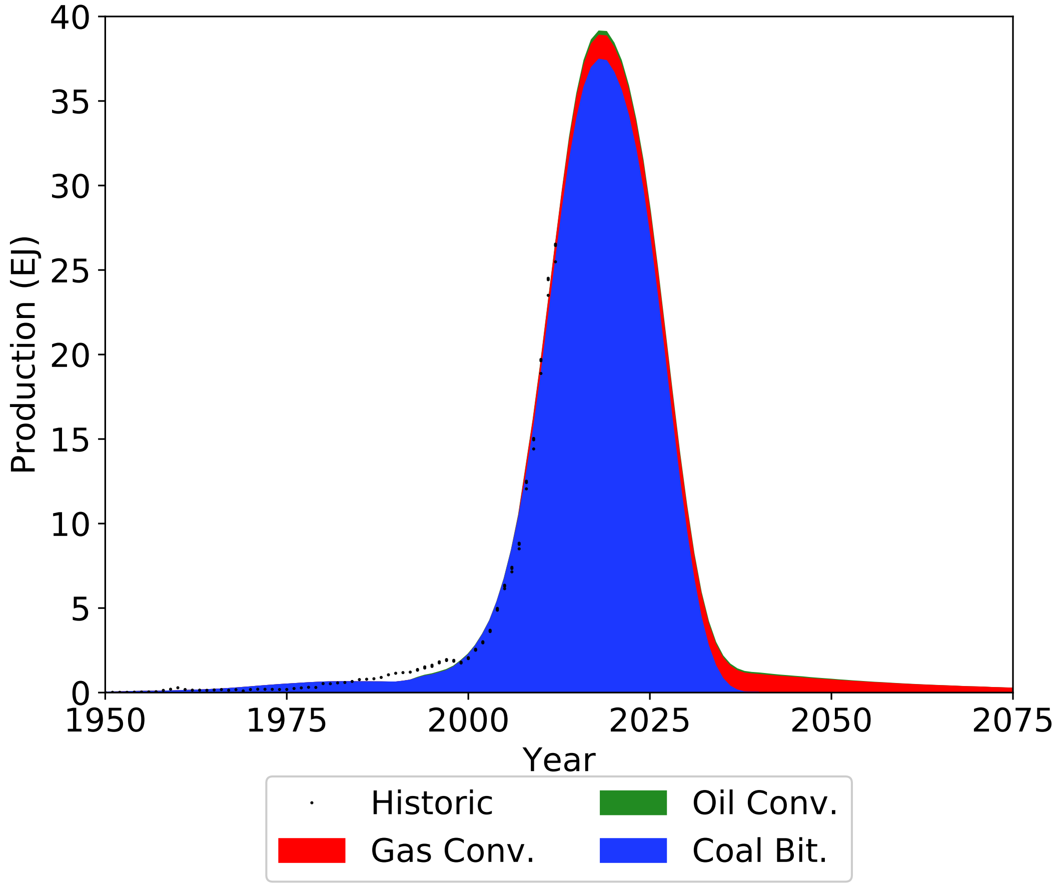

Figure 2.68: China - Inner Mongolia projections capped at 16

Table 2.68: Peak years - All

Name	URR	Peak Year	Peak Rate
Coal Bit. Inner Mongolia	696.15	2018	37.44
Gas Conv. Inner Mongolia	62.94	2023	1.51
Oil Conv. Inner Mongolia	6.9	2019	0.26
**Total**	**765.99**	**2018**	**39.11**

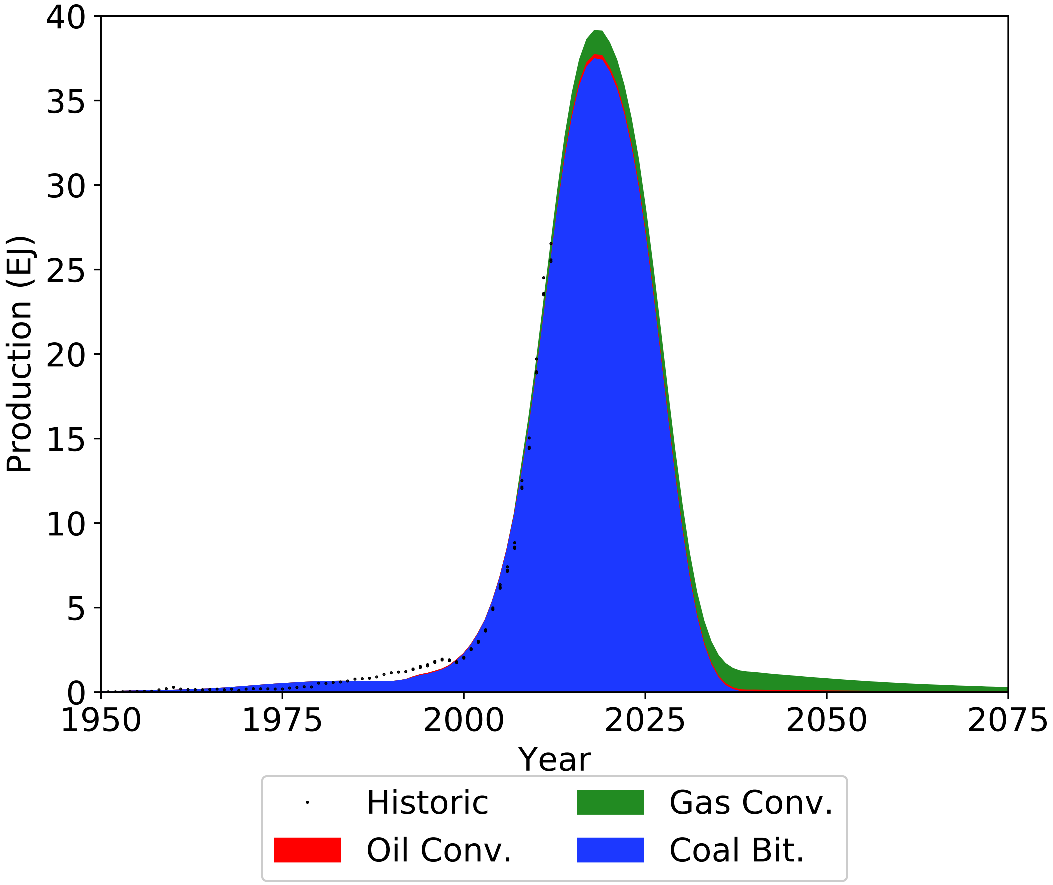

Figure 2.69: China - Inner Mongolia projection by mineral type

Table 2.69: Peak years - Minerals

Name	URR	Peak Year	Peak Rate
Coal Bit.	696.15	2018	37.44
Oil Conv.	6.9	2019	0.26
Gas Conv.	62.94	2023	1.51
**Total**	**765.99**	**2018**	**39.11**

Jiangsu

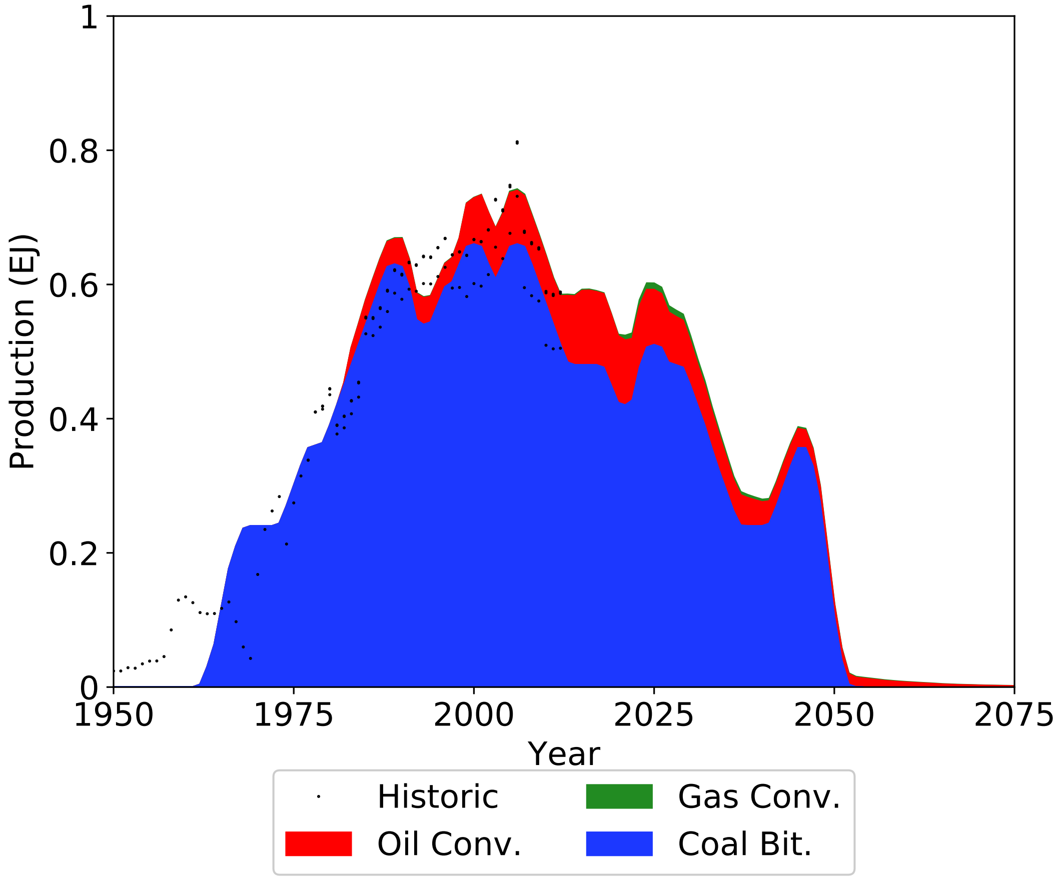

Figure 2.70: China - Jiangsu projections capped at 16

Table 2.70: Peak years - All

Name	URR	Peak Year	Peak Rate
Coal Bit. Jiangsu	37.45	2000	0.66
Oil Conv. Jiangsu	4.2	2016	0.11
Gas Conv. Jiangsu	0.24	2024	0.01
**Total**	**41.89**	**2006**	**0.74**

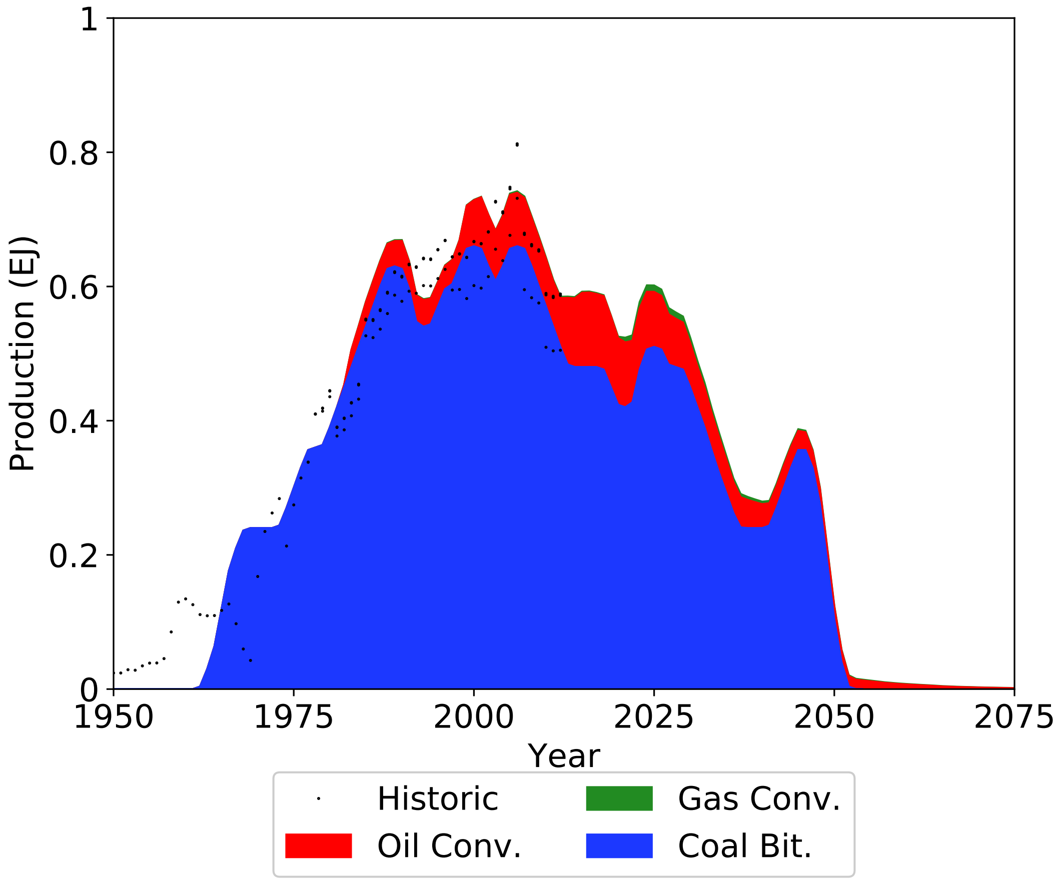

Figure 2.71: China - Jiangsu projection by mineral type

Table 2.71: Peak years - Minerals

Name	URR	Peak Year	Peak Rate
Coal Bit.	37.45	2000	0.66
Oil Conv.	4.2	2016	0.11
Gas Conv.	0.24	2024	0.01
**Total**	**41.89**	**2006**	**0.74**

Jiangxi

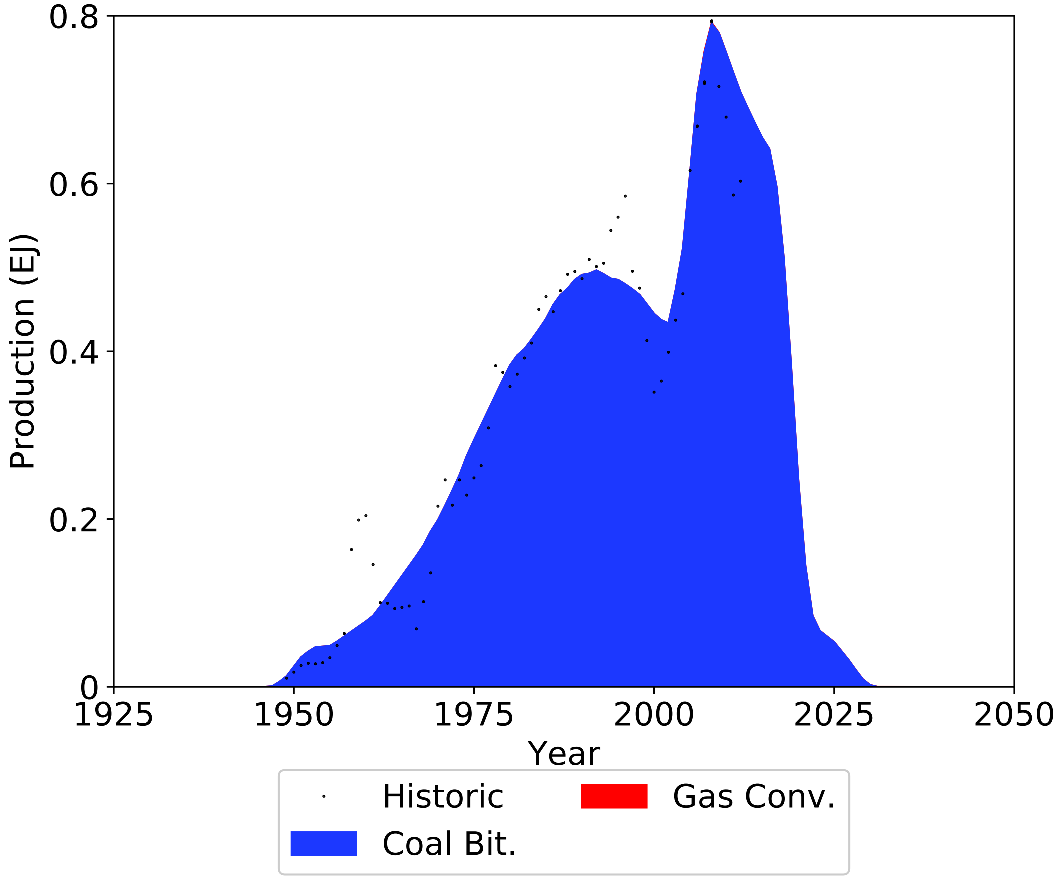

Figure 2.72: China - Jiangxi projections capped at 16

Table 2.72: Peak years - All

**Name**	**URR**	**Peak Year**	**Peak Rate**
Coal Bit. Jiangxi	26.85	2008	0.79
Gas Conv. Jiangxi	0.01	2007	–
**Total**	**26.86**	**2008**	**0.79**

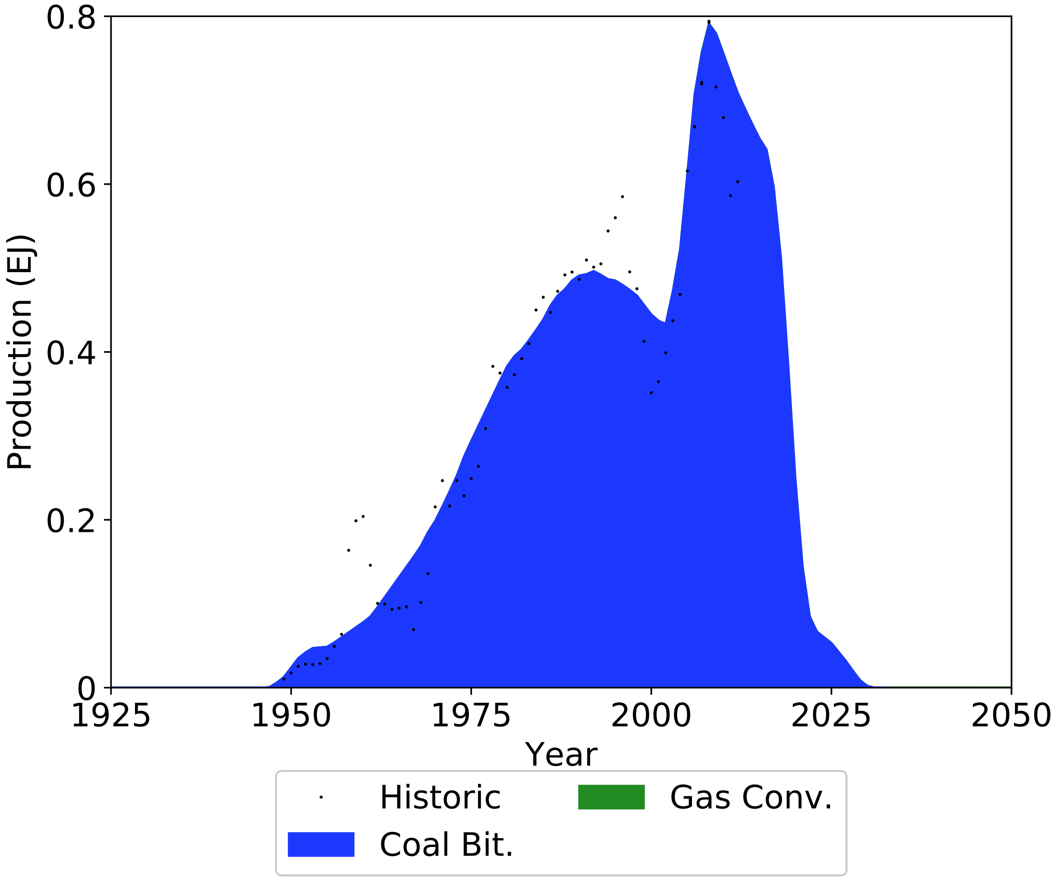

Figure 2.73: China - Jiangxi projection by mineral type

Table 2.73: Peak years - Minerals

Name	URR	Peak Year	Peak Rate
Coal Bit.	26.85	2008	0.79
Gas Conv.	0.01	2007	–
**Total**	**26.86**	**2008**	**0.79**

Jilin

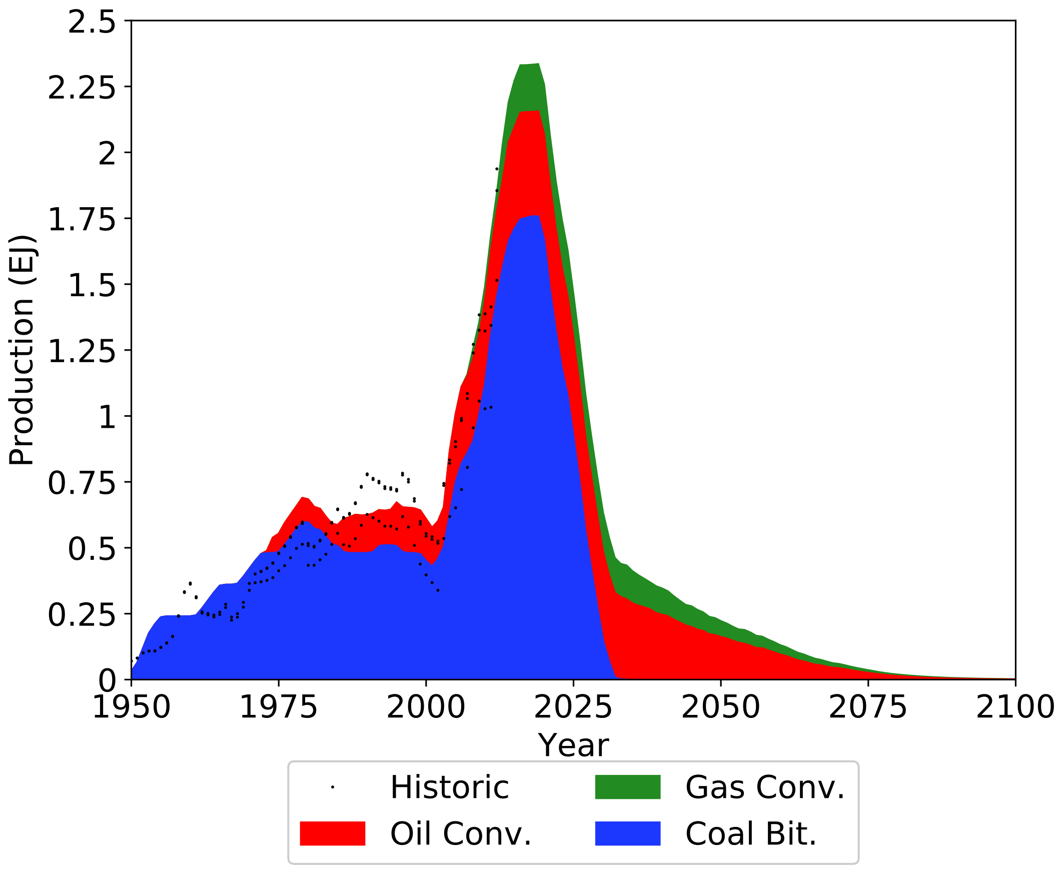

Figure 2.74: China - Jilin projections capped at 16

Table 2.74: Peak years - All

Name	URR	Peak Year	Peak Rate
Coal Bit. Jilin	52.66	2018	1.76
Oil Conv. Jilin	20.15	2016	0.4
Gas Conv. Jilin	6.09	2020	0.19
**Total**	**78.9**	**2019**	**2.33**

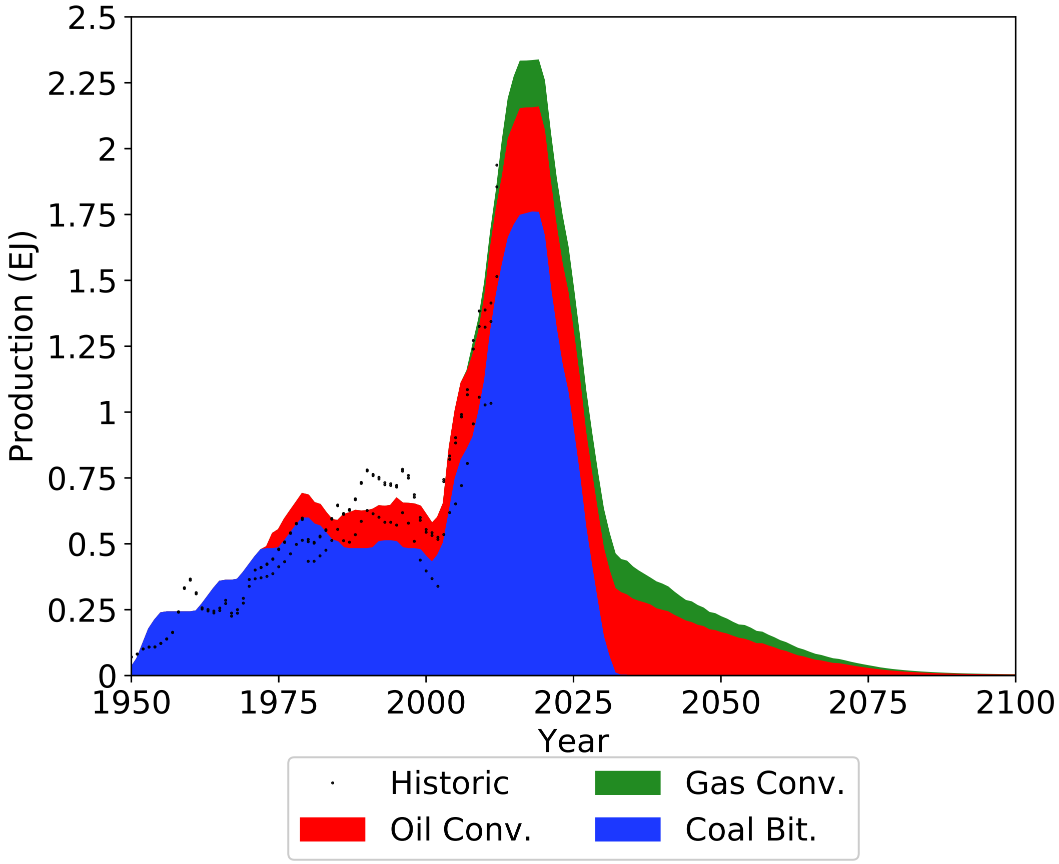

Figure 2.75: China - Jilin projection by mineral type

Table 2.75: Peak years - Minerals

Name	URR	Peak Year	Peak Rate
Coal Bit.	52.66	2018	1.76
Oil Conv.	20.15	2016	0.4
Gas Conv.	6.09	2020	0.19
**Total**	**78.9**	**2019**	**2.33**

Liaoning

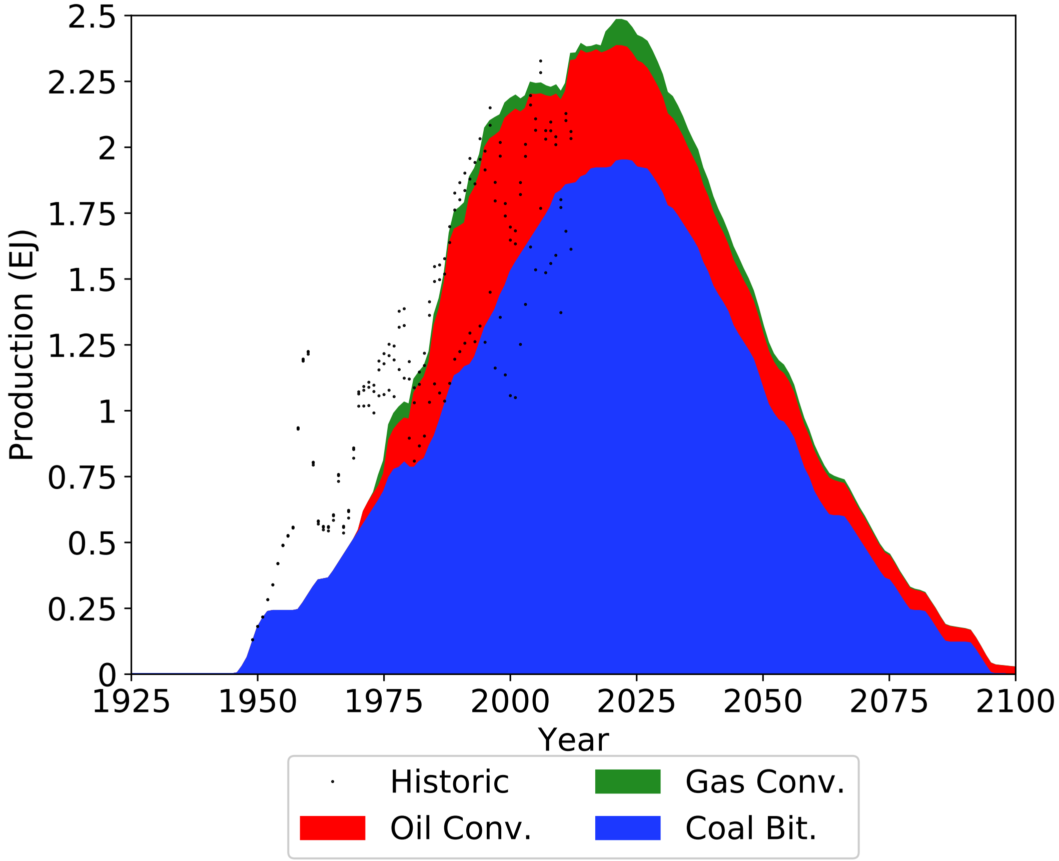

Figure 2.76: China - Liaoning projections capped at 16

Table 2.76: Peak years - All

Name	URR	Peak Year	Peak Rate
Coal Bit. Liaoning	141.13	2022	1.95
Oil Conv. Liaoning	35.68	1995	0.68
Gas Conv. Liaoning	4.99	2027	0.1
**Total**	**181.8**	**2022**	**2.48**

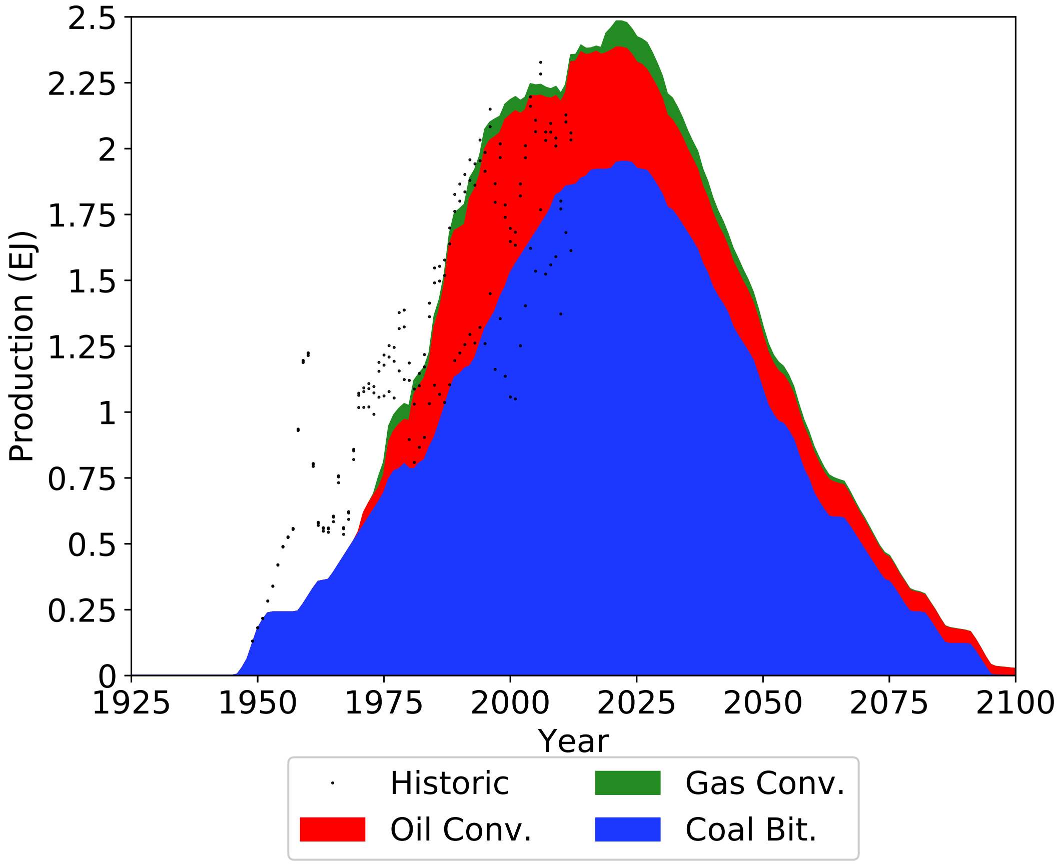

Figure 2.77: China - Liaoning projection by mineral type

Table 2.77: Peak years - Minerals

Name	URR	Peak Year	Peak Rate
Coal Bit.	141.13	2022	1.95
Oil Conv.	35.68	1995	0.68
Gas Conv.	4.99	2027	0.1
**Total**	**181.8**	**2022**	**2.48**

Ningxia

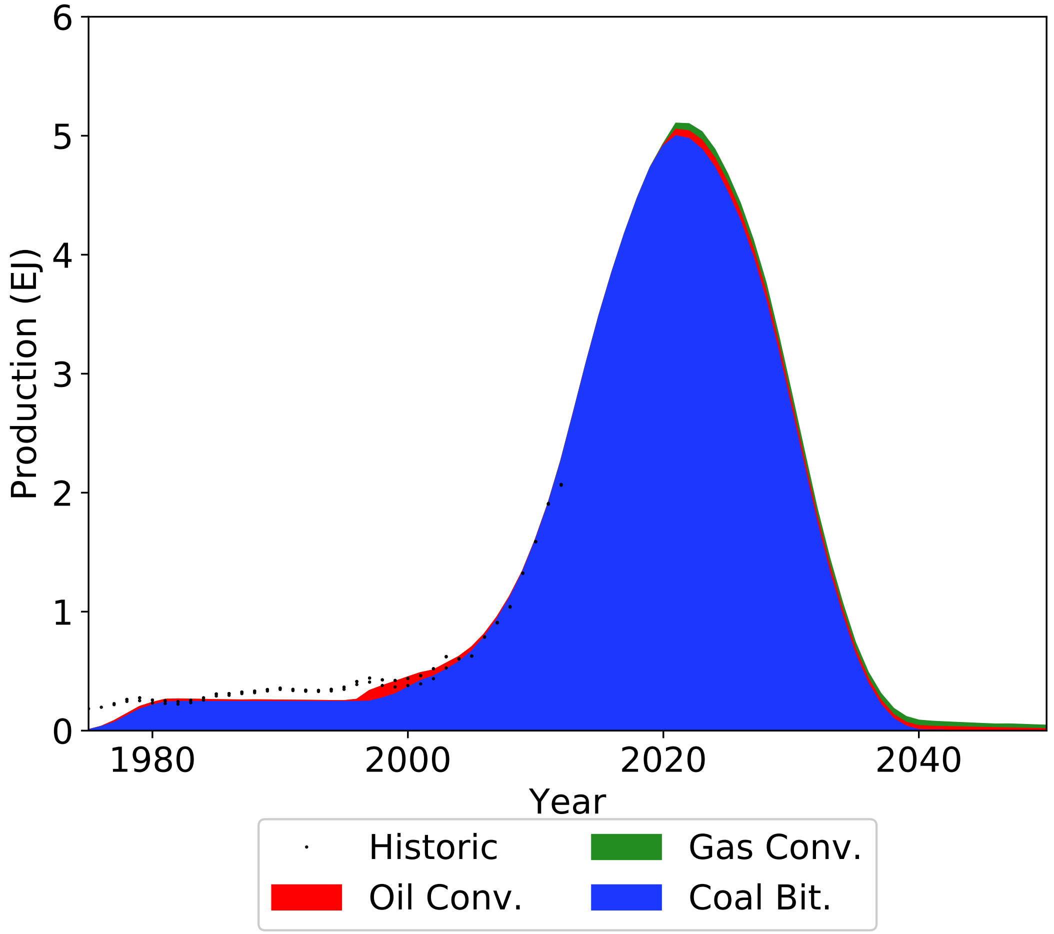

Figure 2.78: China - Ningxia projections capped at 16

Table 2.78: Peak years - All

**Name**	**URR**	**Peak Year**	**Peak Rate**
Coal Bit. Ningxia	99.05	2021	5.0
Oil Conv. Ningxia	2.51	1999	0.11
Gas Conv. Ningxia	2.0	2030	0.07
**Total**	**103.56**	**2021**	**5.1**

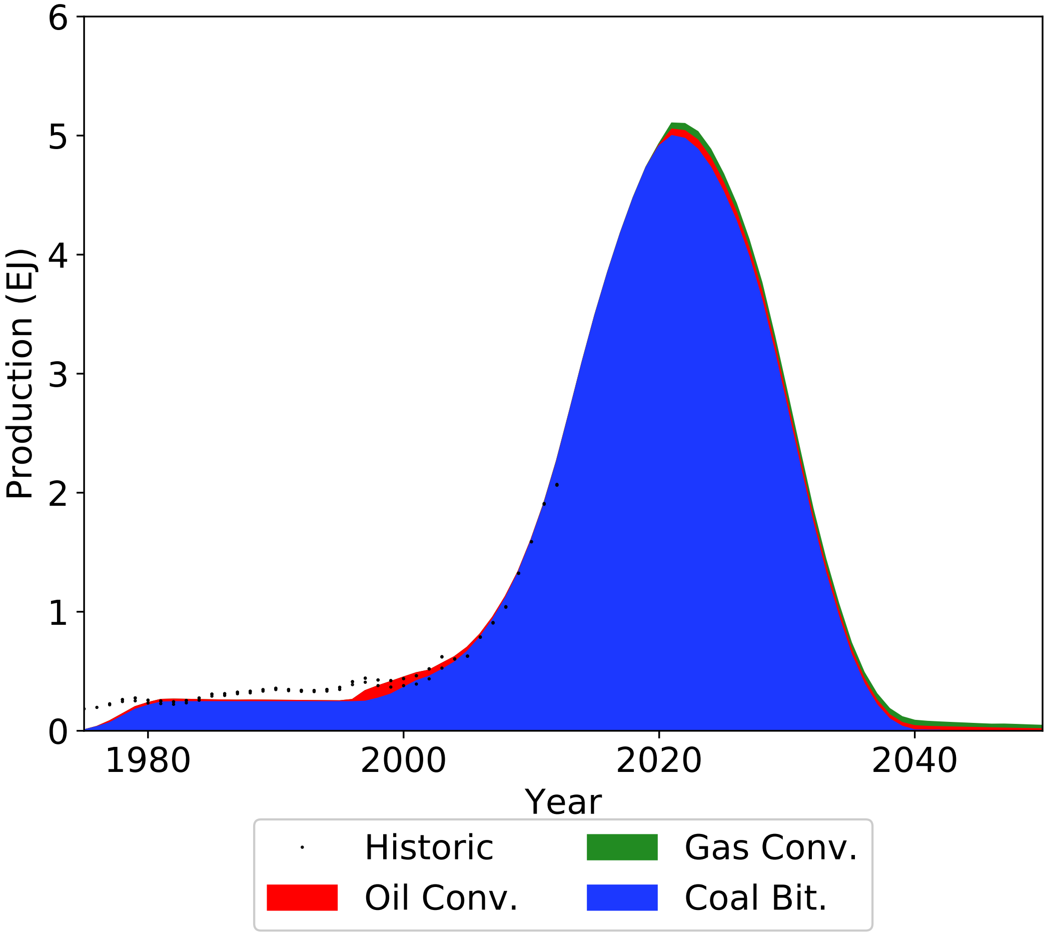

Figure 2.79: China - Ningxia projection by mineral type

Table 2.79: Peak years - Minerals

**Name**	**URR**	**Peak Year**	**Peak Rate**
Coal Bit.	99.05	2021	5.0
Oil Conv.	2.51	1999	0.11
Gas Conv.	2.0	2030	0.07
**Total**	**103.56**	**2021**	**5.1**

Offshore

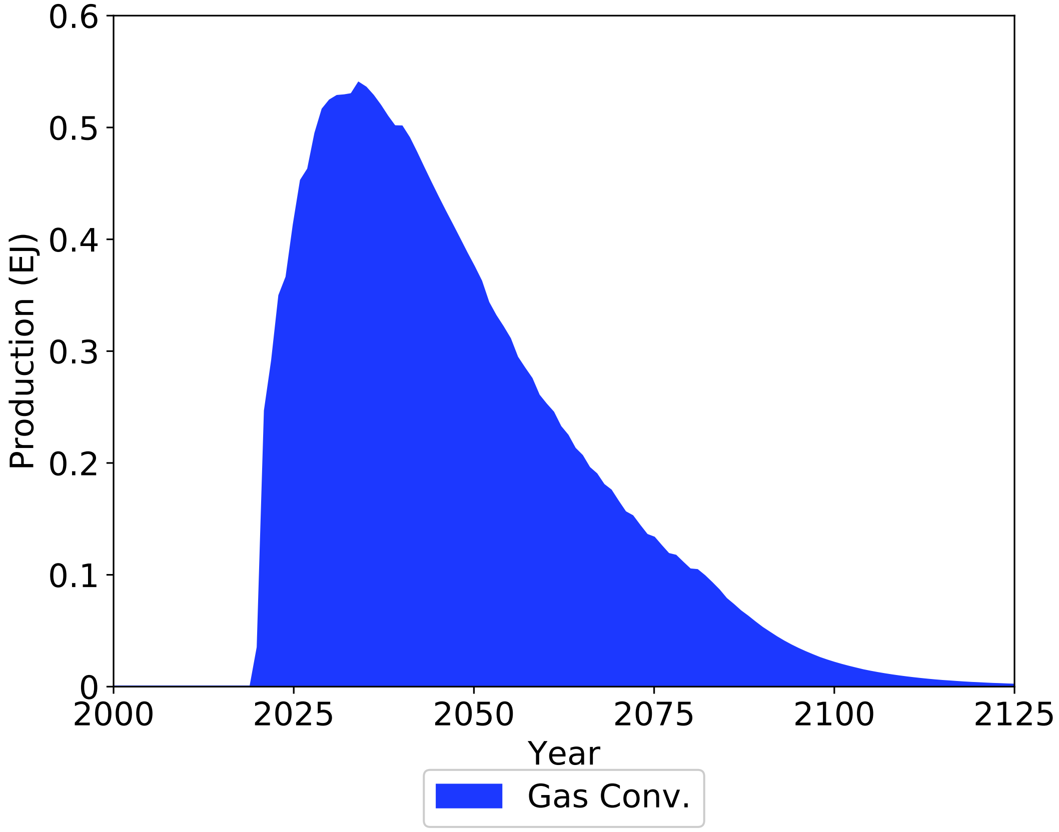

Figure 2.80: China - Offshore projections capped at 16

Table 2.80: Peak years - All

**Name**	**URR**	**Peak Year**	**Peak Rate**
Gas Conv. Offshore	21.38	2034	0.54
**Total**	**21.38**	**2034**	**0.54**

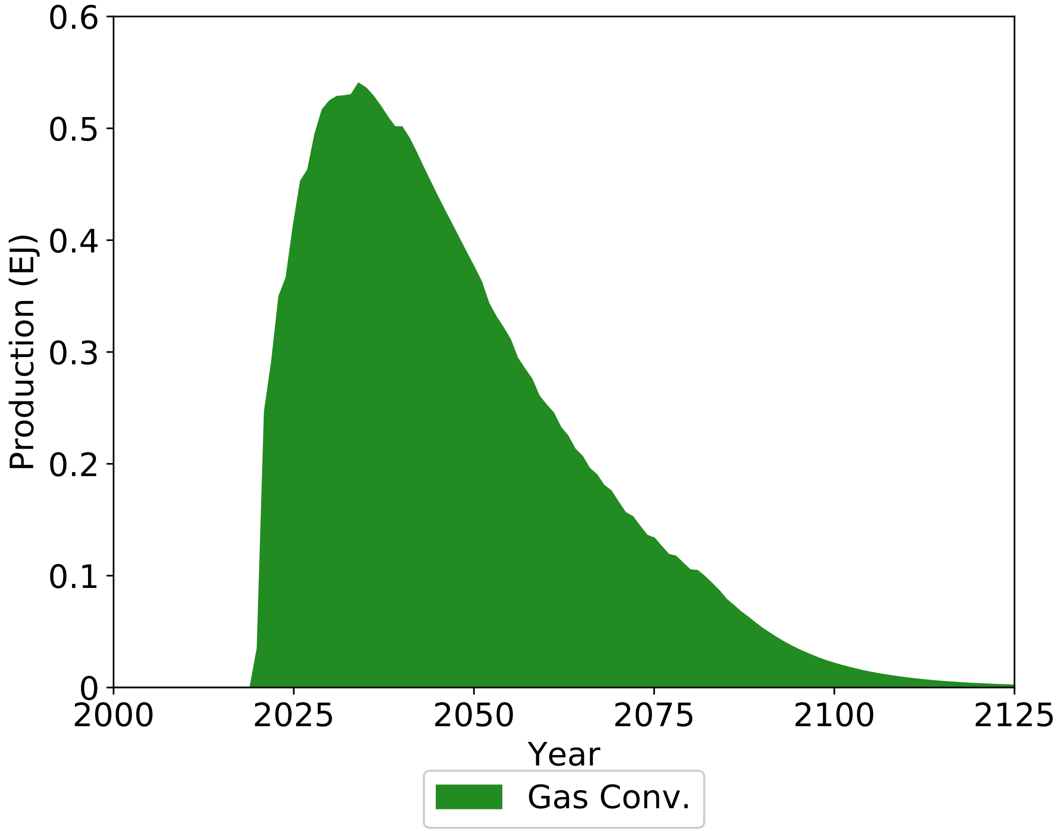

Figure 2.81: China - Offshore projection by mineral type

Table 2.81: Peak years - Minerals

**Name**	**URR**	**Peak Year**	**Peak Rate**
Gas Conv.	21.38	2034	0.54
**Total**	**21.38**	**2034**	**0.54**

Qinghai

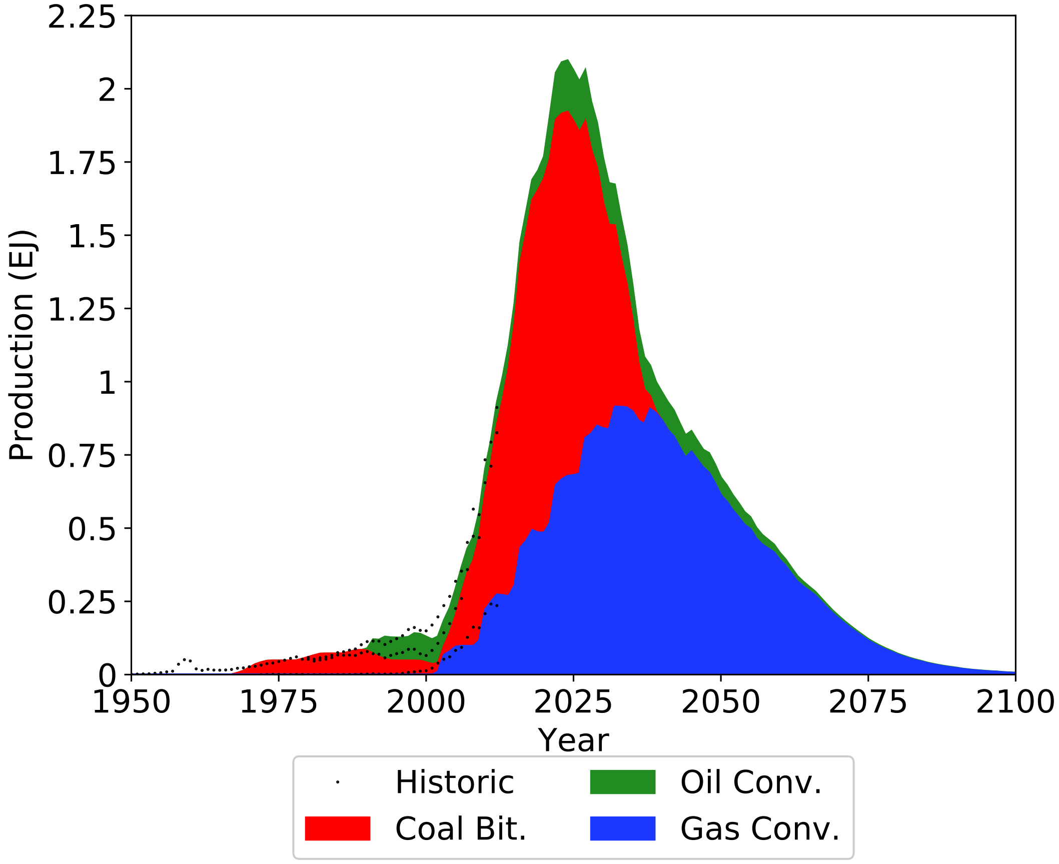

Figure 2.82: China - Qinghai projections capped at 16

Table 2.82: Peak years - All

**Name**	**URR**	**Peak Year**	**Peak Rate**
Gas Conv. Qinghai	37.32	2032	0.91
Coal Bit. Qinghai	26.45	2022	1.25
Oil Conv. Qinghai	6.55	2023	0.18
**Total**	**70.32**	**2024**	**2.1**

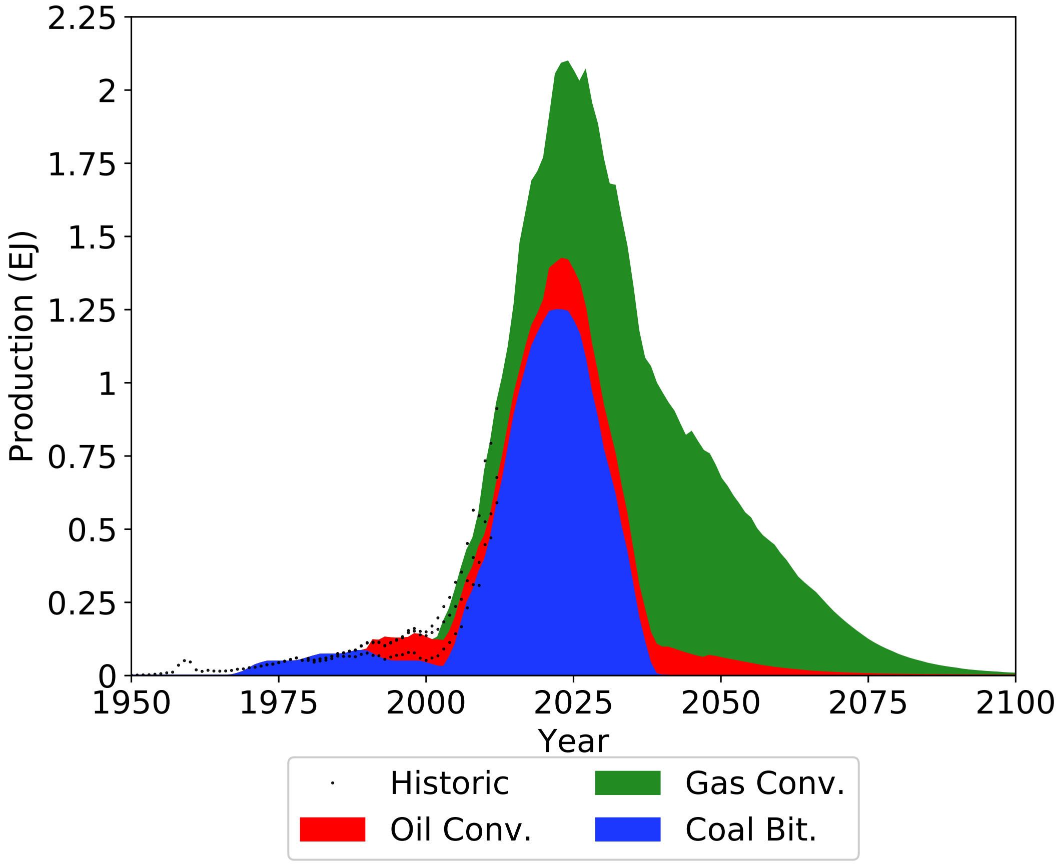

Figure 2.83: China - Qinghai projection by mineral type

Table 2.83: Peak years - Minerals

Name	URR	Peak Year	Peak Rate
Coal Bit.	26.45	2022	1.25
Oil Conv.	6.55	2023	0.18
Gas Conv.	37.32	2032	0.91
**Total**	**70.32**	**2024**	**2.1**

Shaanxi

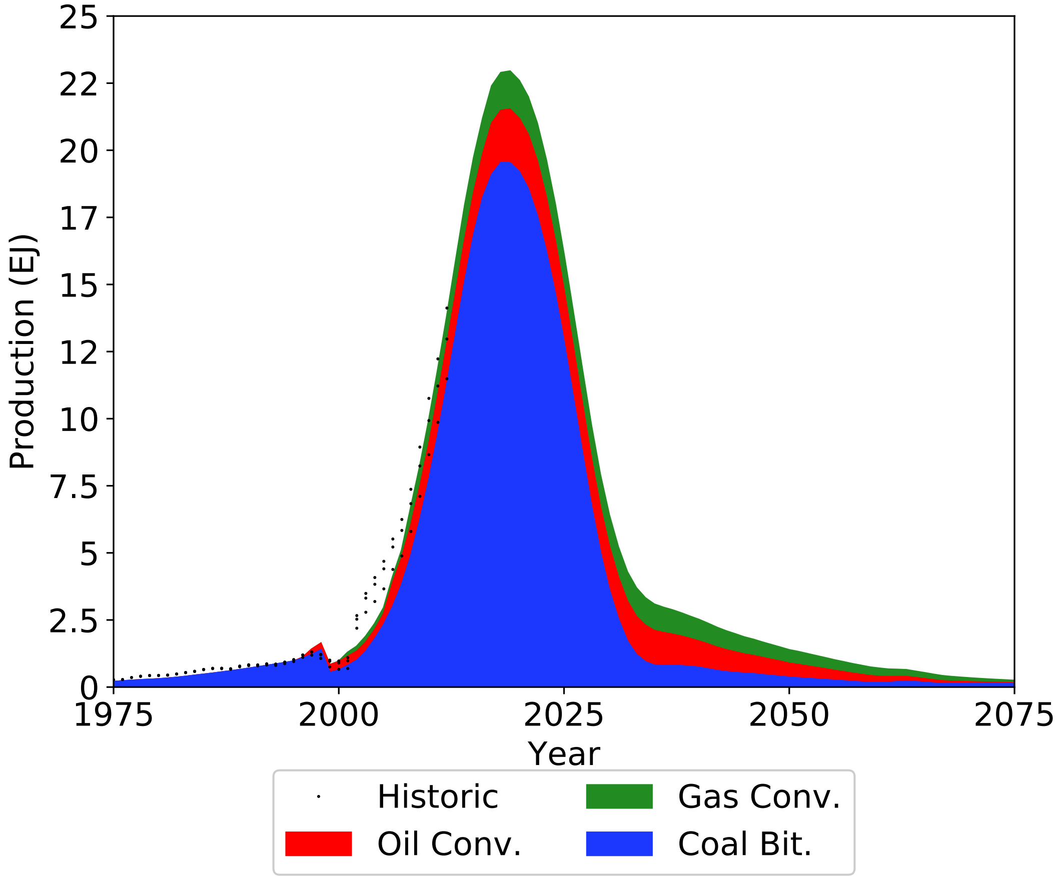

Figure 2.84: China - Shaanxi projections capped at 16

Table 2.84: Peak years - All

Name	URR	Peak Year	Peak Rate
Coal Bit. Shaanxi	346.35	2018	19.53
Oil Conv. Shaanxi	69.46	2022	2.04
Gas Conv. Shaanxi	53.01	2019	1.42
**Total**	**468.82**	**2019**	**22.94**

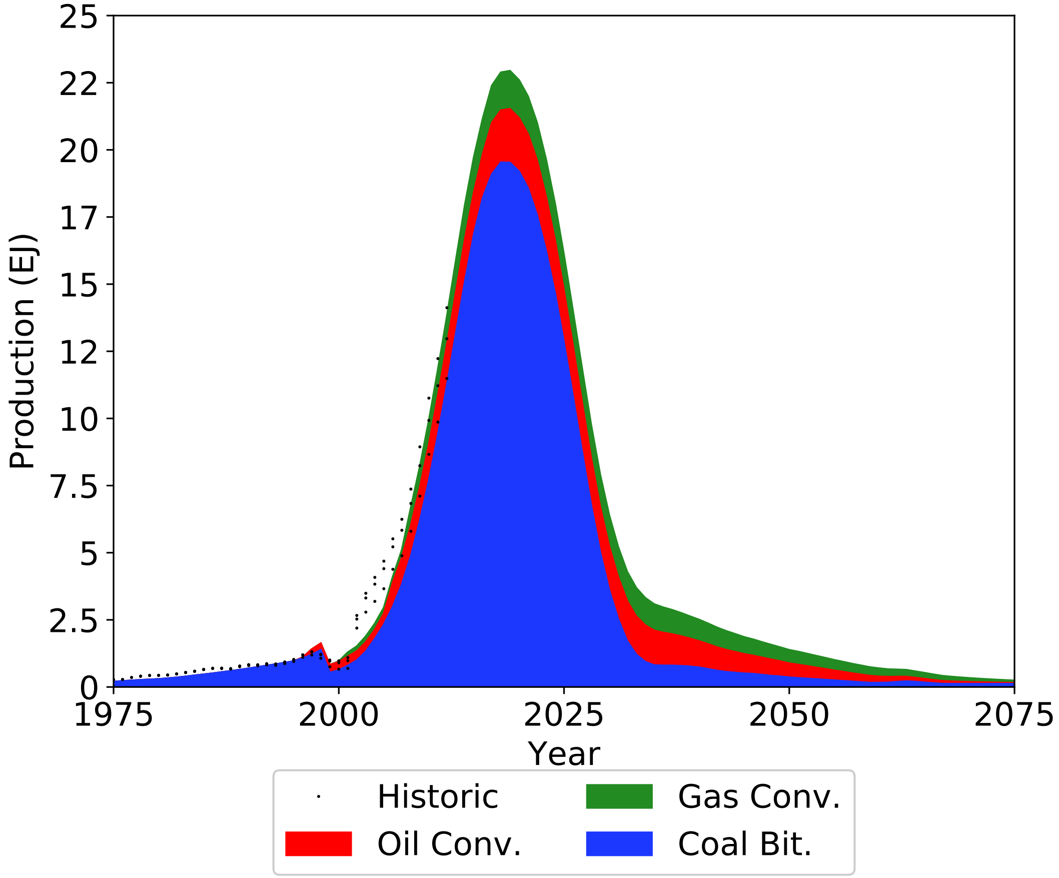

Figure 2.85: China - Shaanxi projection by mineral type

Table 2.85: Peak years - Minerals

Name	URR	Peak Year	Peak Rate
Coal Bit.	346.35	2018	19.53
Oil Conv.	69.46	2022	2.04
Gas Conv.	53.01	2019	1.42
**Total**	**468.82**	**2019**	**22.94**

Shandong

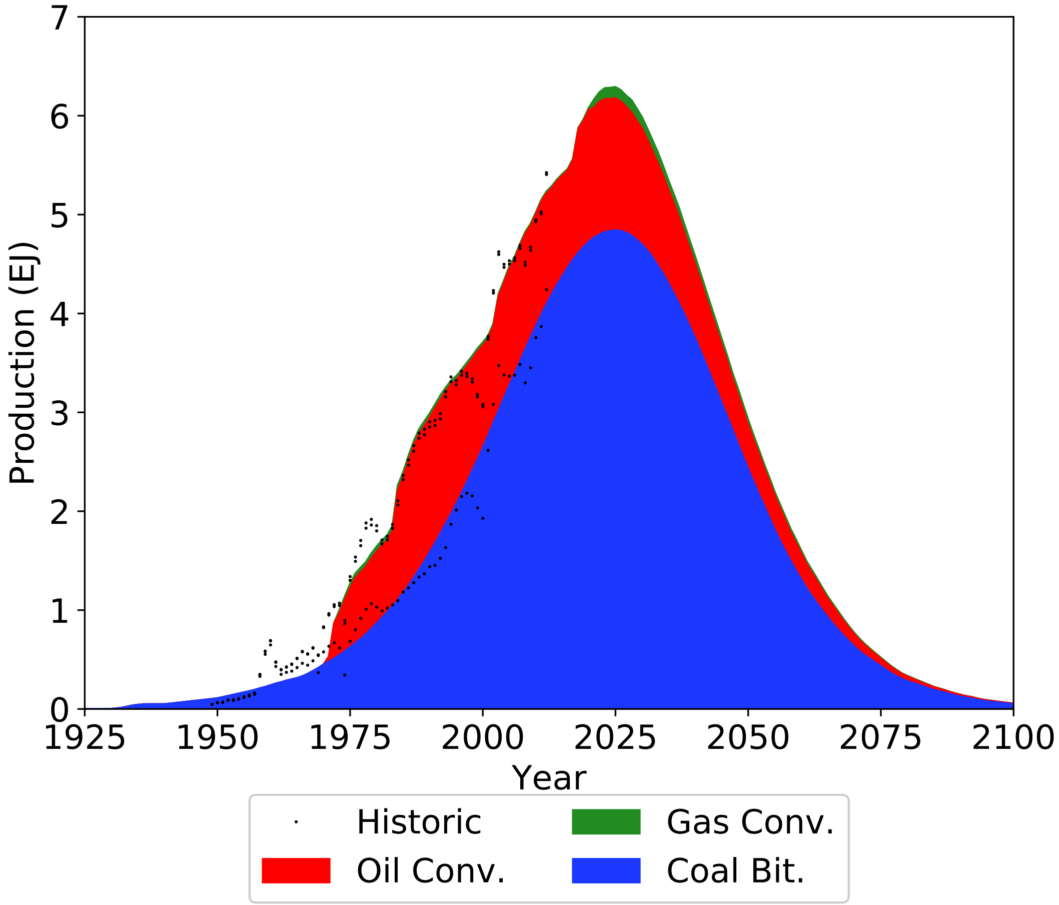

Figure 2.86: China - Shandong projections capped at 16

Table 2.86: Peak years - All

Name	URR	Peak Year	Peak Rate
Coal Bit. Shandong	278.09	2025	4.84
Oil Conv. Shandong	85.59	1988	1.37
Gas Conv. Shandong	5.23	2028	0.13
**Total**	**368.91**	**2025**	**6.29**

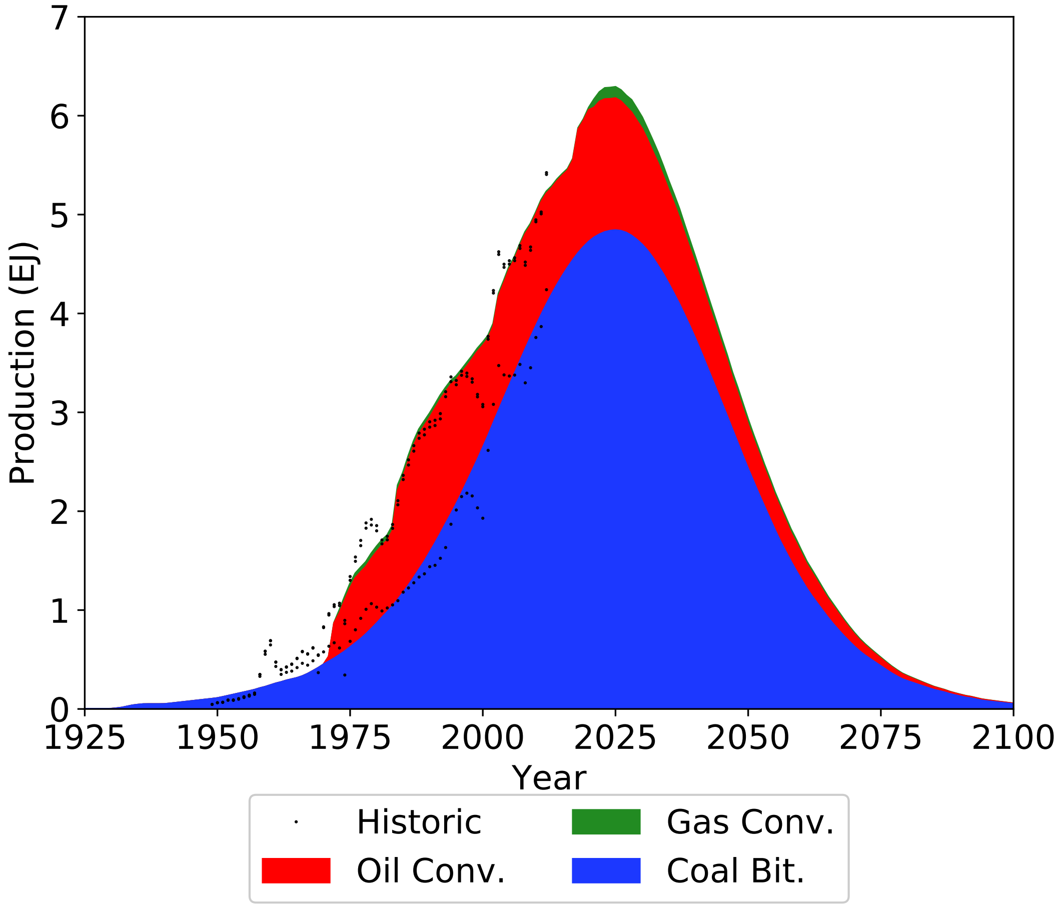

Figure 2.87: China - Shandong projection by mineral type

Table 2.87: Peak years - Minerals

Name	URR	Peak Year	Peak Rate
Coal Bit.	278.09	2025	4.84
Oil Conv.	85.59	1988	1.37
Gas Conv.	5.23	2028	0.13
**Total**	**368.91**	**2025**	**6.29**

Shanghai

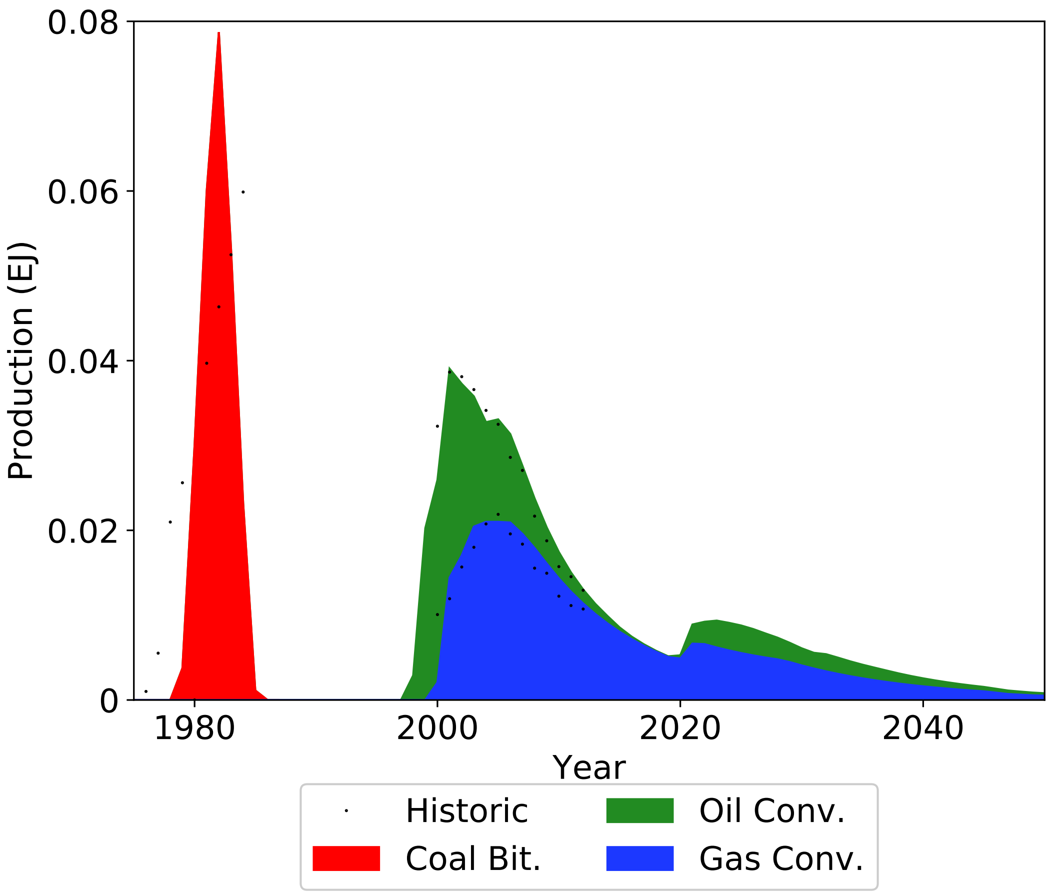

Figure 2.88: China - Shanghai projections capped at 16

Table 2.88: Peak years - All

Name	URR	Peak Year	Peak Rate
Gas Conv. Shanghai	0.36	2004	0.02
Coal Bit. Shanghai	0.25	1982	0.08
Oil Conv. Shanghai	0.22	2001	0.02
**Total**	**0.83**	**1982**	**0.08**

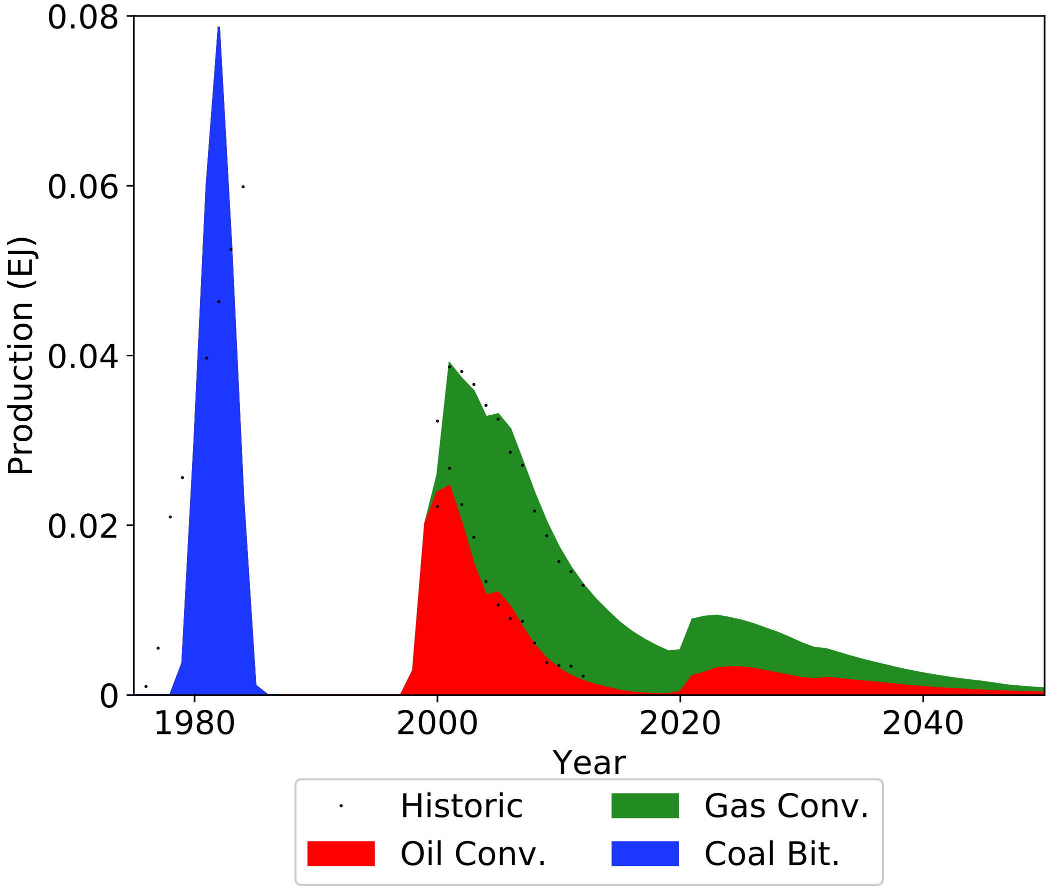

Figure 2.89: China - Shanghai projection by mineral type

Table 2.89: Peak years - Minerals

Name	URR	Peak Year	Peak Rate
Coal Bit.	0.25	1982	0.08
Oil Conv.	0.22	2001	0.02
Gas Conv.	0.36	2004	0.02
**Total**	**0.83**	**1982**	**0.08**

Shanxi

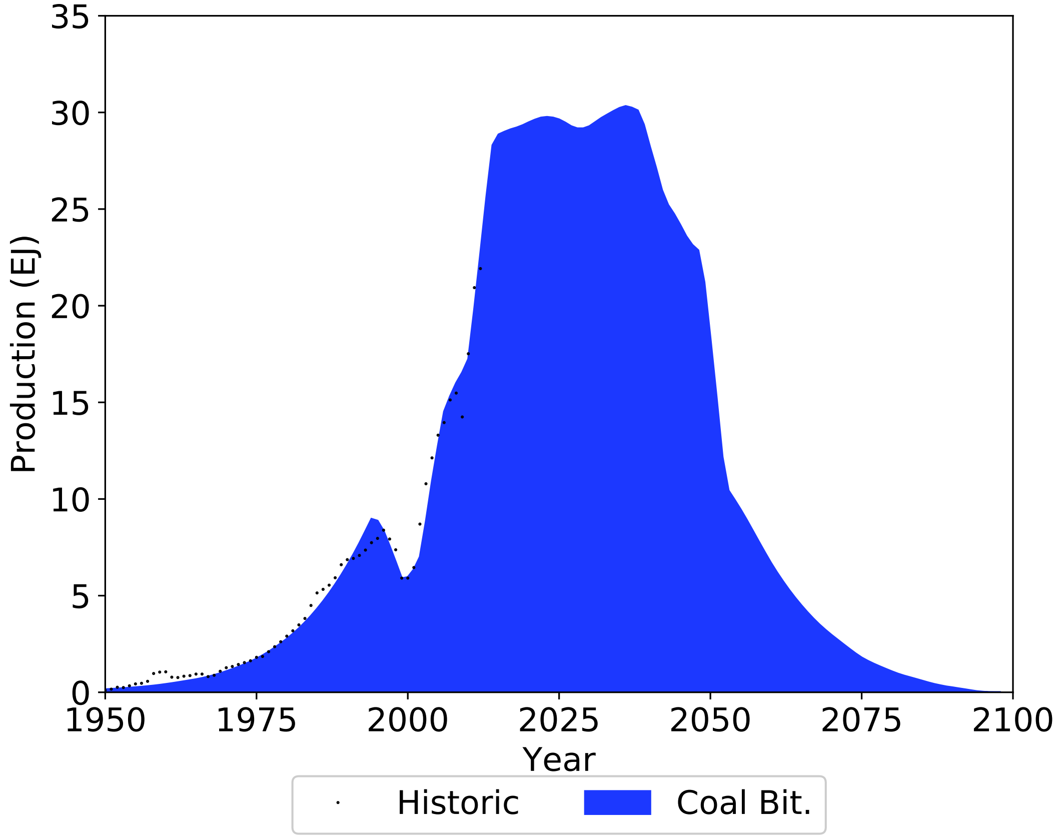

Figure 2.90: China - Shanxi projections capped at 16

Table 2.90: Peak years - All

Name	URR	Peak Year	Peak Rate
Coal Bit. Shanxi	1543.23	2036	30.33
**Total**	**1543.23**	**2036**	**30.33**

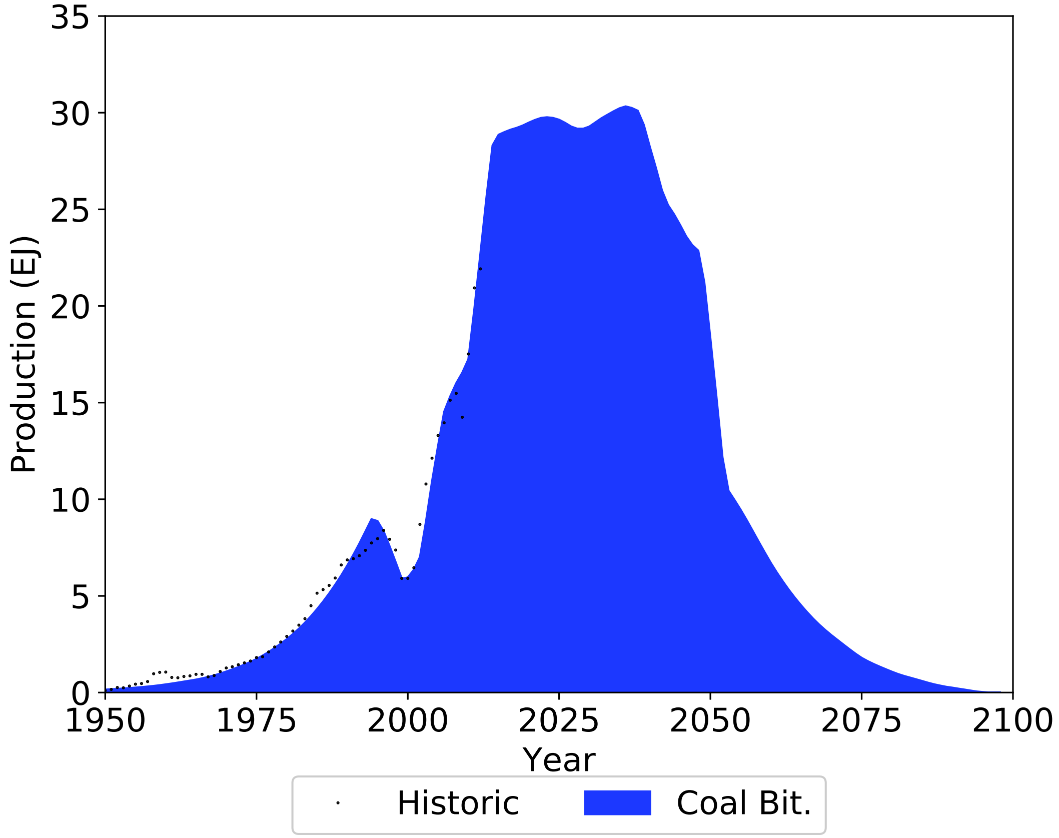

Figure 2.91: China - Shanxi projection by mineral type

Table 2.91: Peak years - Minerals

Name	URR	Peak Year	Peak Rate
Coal Bit.	1543.23	2036	30.33
**Total**	**1543.23**	**2036**	**30.33**

Sichuan

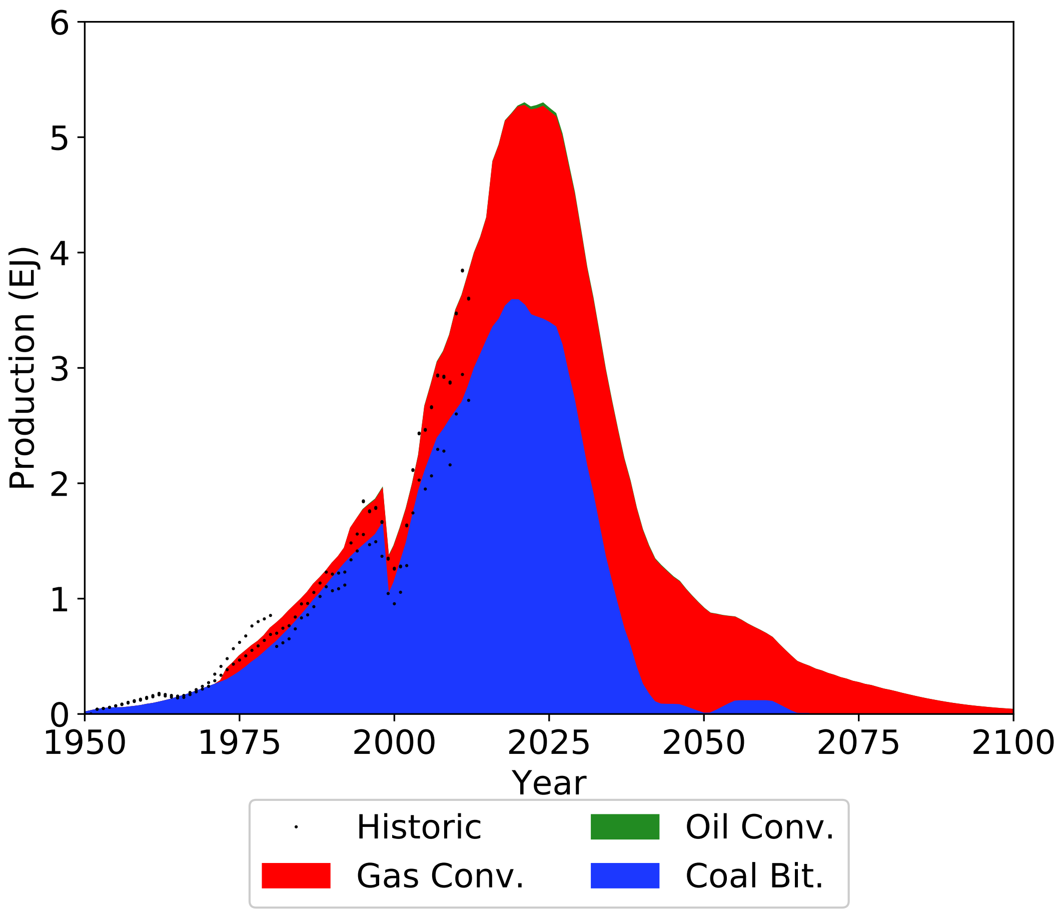

Figure 2.92: China - Sichuan projections capped at 16

Table 2.92: Peak years - All

Name	URR	Peak Year	Peak Rate
Coal Bit. Sichuan	126.66	2019	3.59
Gas Conv. Sichuan	83.72	2024	1.84
Oil Conv. Sichuan	0.68	2024	0.03
**Total**	**211.06**	**2021**	**5.29**

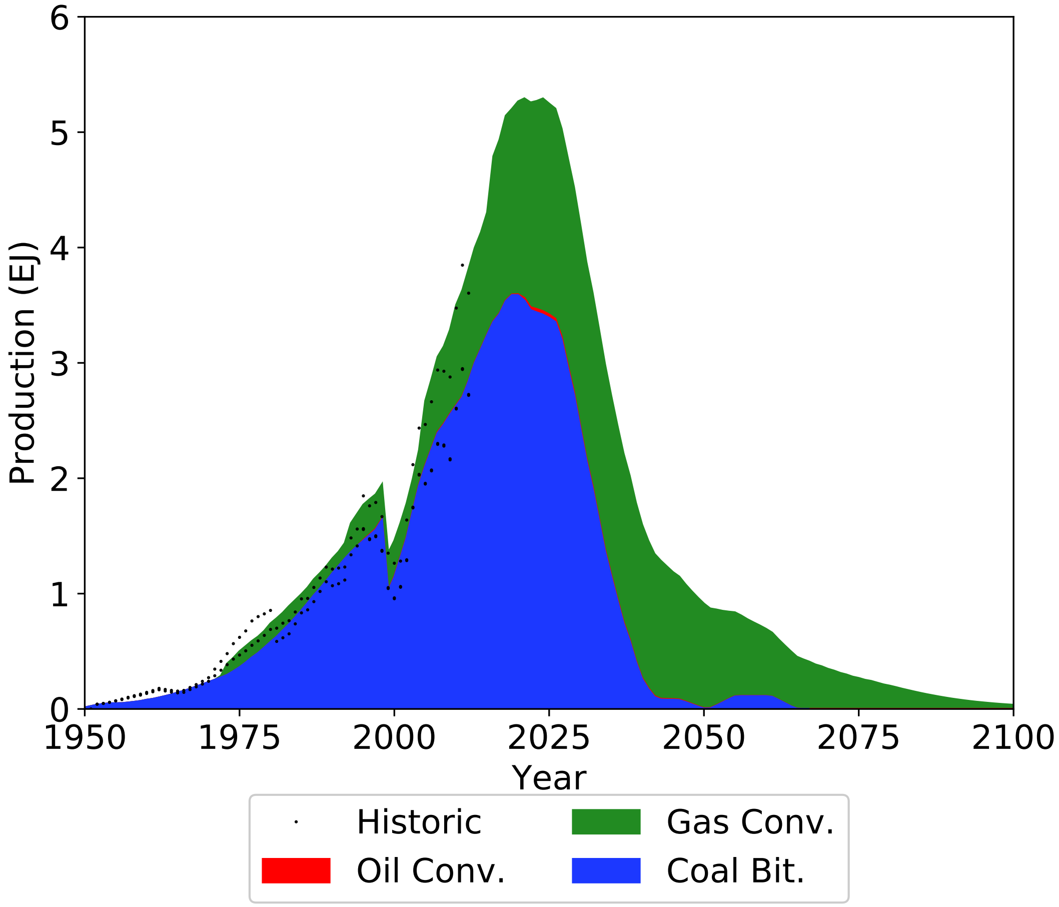

Figure 2.93: China - Sichuan projection by mineral type

Table 2.93: Peak years - Minerals

Name	URR	Peak Year	Peak Rate
Coal Bit.	126.66	2019	3.59
Oil Conv.	0.68	2024	0.03
Gas Conv.	83.72	2024	1.84
**Total**	**211.06**	**2021**	**5.29**

Tianjin

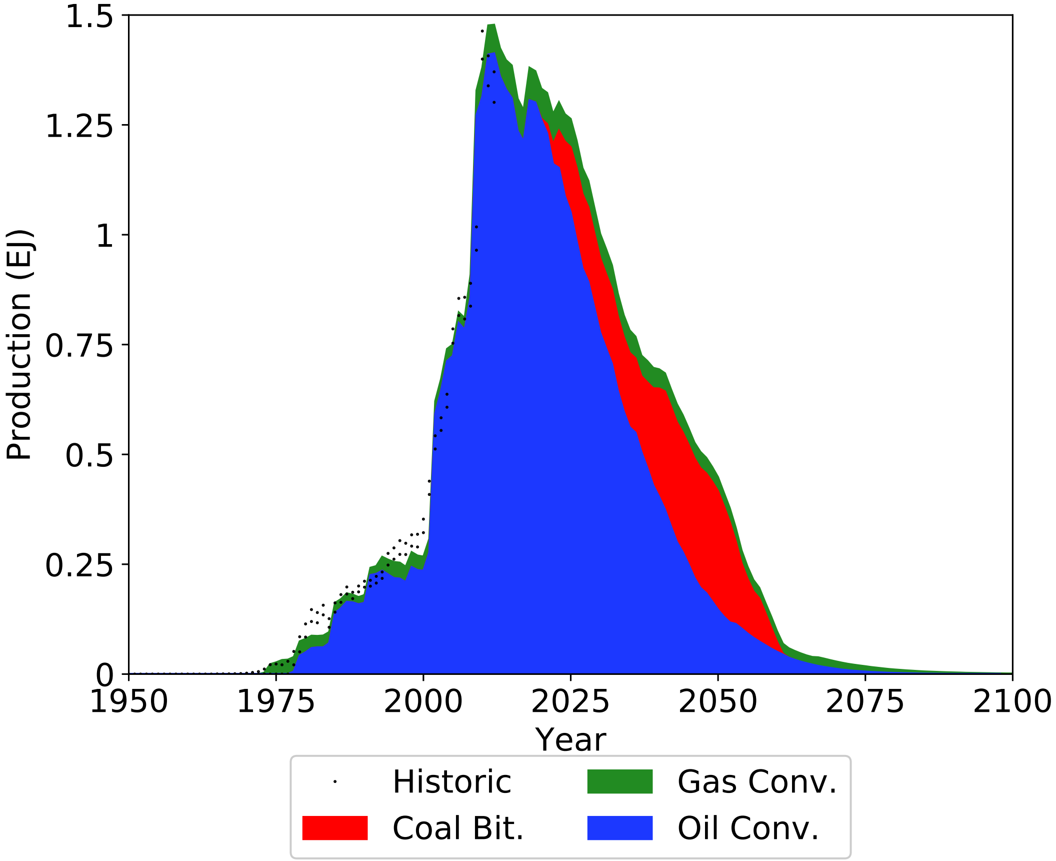

Figure 2.94: China - Tianjin projections capped at 16

Table 2.94: Peak years - All

Name	URR	Peak Year	Peak Rate
Oil Conv. Tianjin	44.06	2012	1.41
Coal Bit. Tianjin	7.13	2042	0.27
Gas Conv. Tianjin	3.96	2015	0.07
**Total**	**55.15**	**2012**	**1.48**

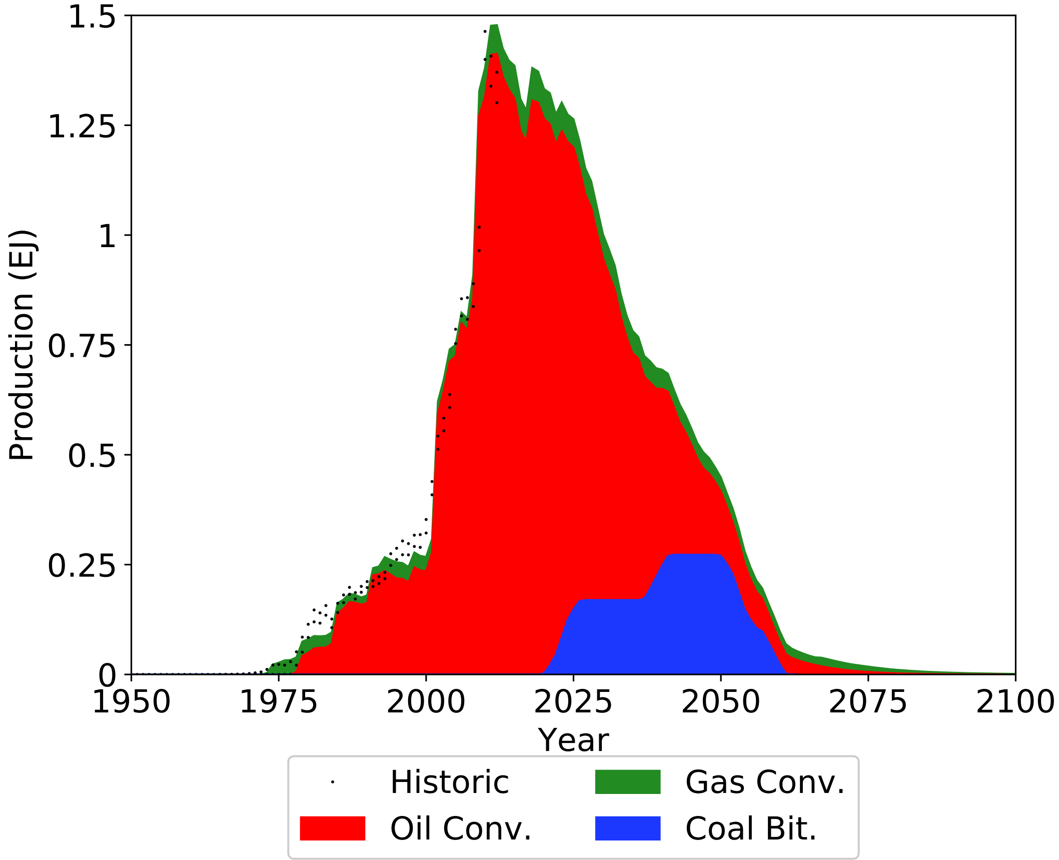

Figure 2.95: China - Tianjin projection by mineral type

Table 2.95: Peak years - Minerals

Name	URR	Peak Year	Peak Rate
Coal Bit.	7.13	2042	0.27
Oil Conv.	44.06	2012	1.41
Gas Conv.	3.96	2015	0.07
**Total**	**55.15**	**2012**	**1.48**

Tibet

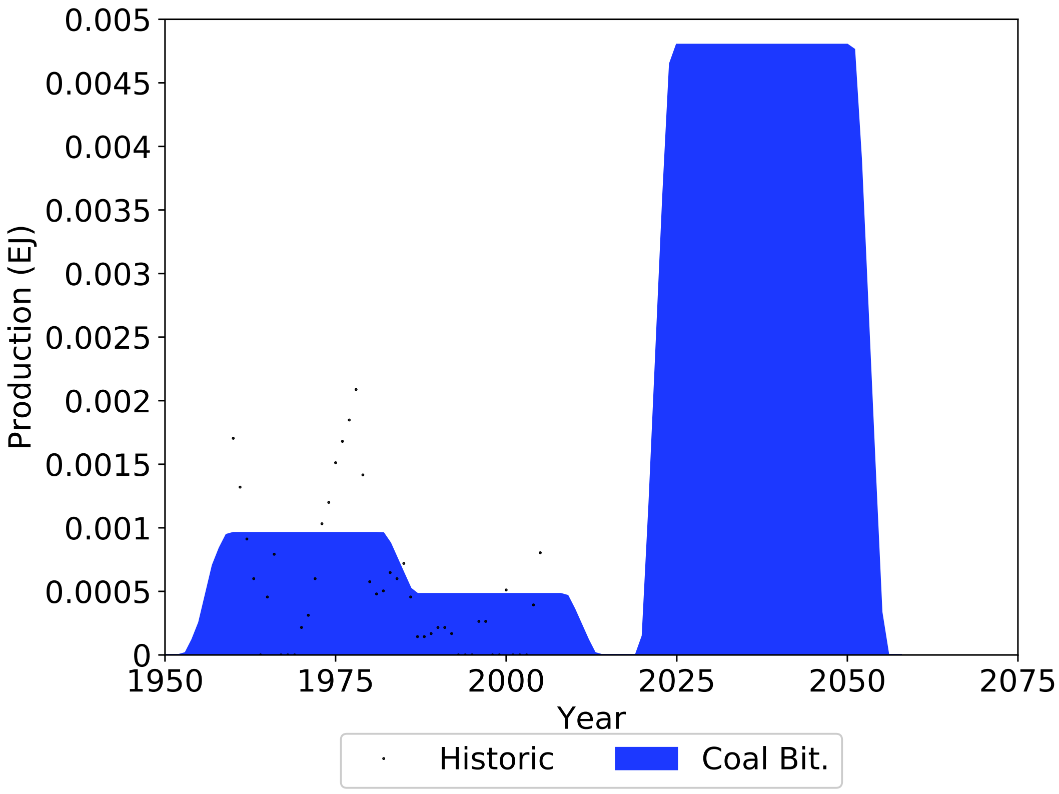

Figure 2.96: China - Tibet projections capped at 16

Table 2.96: Peak years - All

Name	URR	Peak Year	Peak Rate
Coal Bit. Tibet	0.19	2048	–
**Total**	**0.19**	**2048**	–

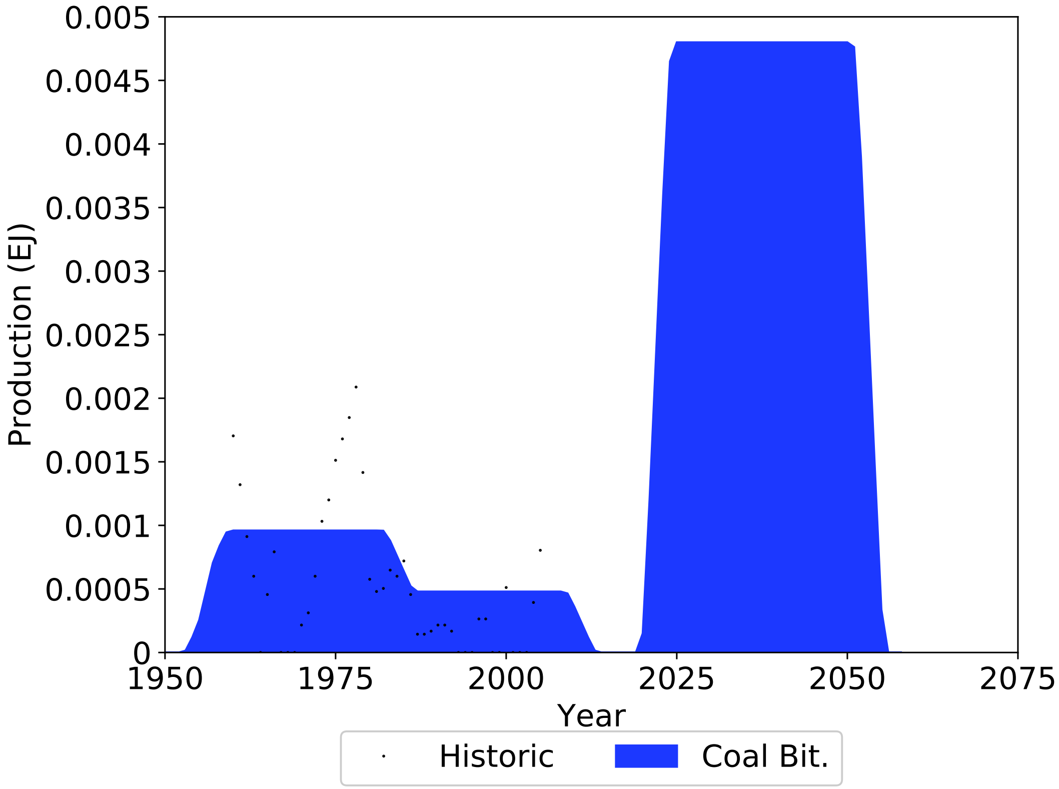

Figure 2.97: China - Tibet projection by mineral type

Table 2.97: Peak years - Minerals

Name	URR	Peak Year	Peak Rate
Coal Bit.	0.19	2048	–
**Total**	**0.19**	**2048**	–

Xinjiang

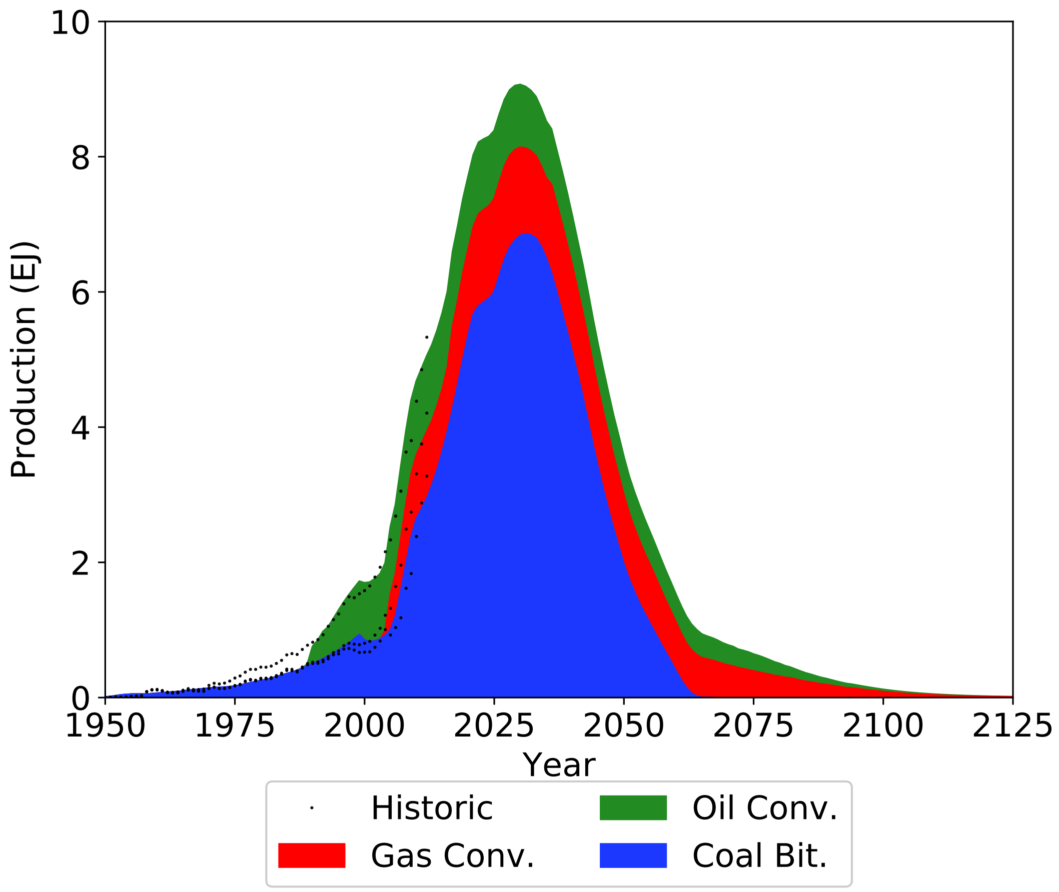

Figure 2.98: China - Xinjiang projections capped at 16

Table 2.98: Peak years - All

Name	URR	Peak Year	Peak Rate
Coal Bit. Xinjiang	235.83	2031	6.85
Gas Conv. Xinjiang	75.82	2026	1.39
Oil Conv. Xinjiang	65.09	2015	1.13
**Total**	**376.74**	**2030**	**9.07**

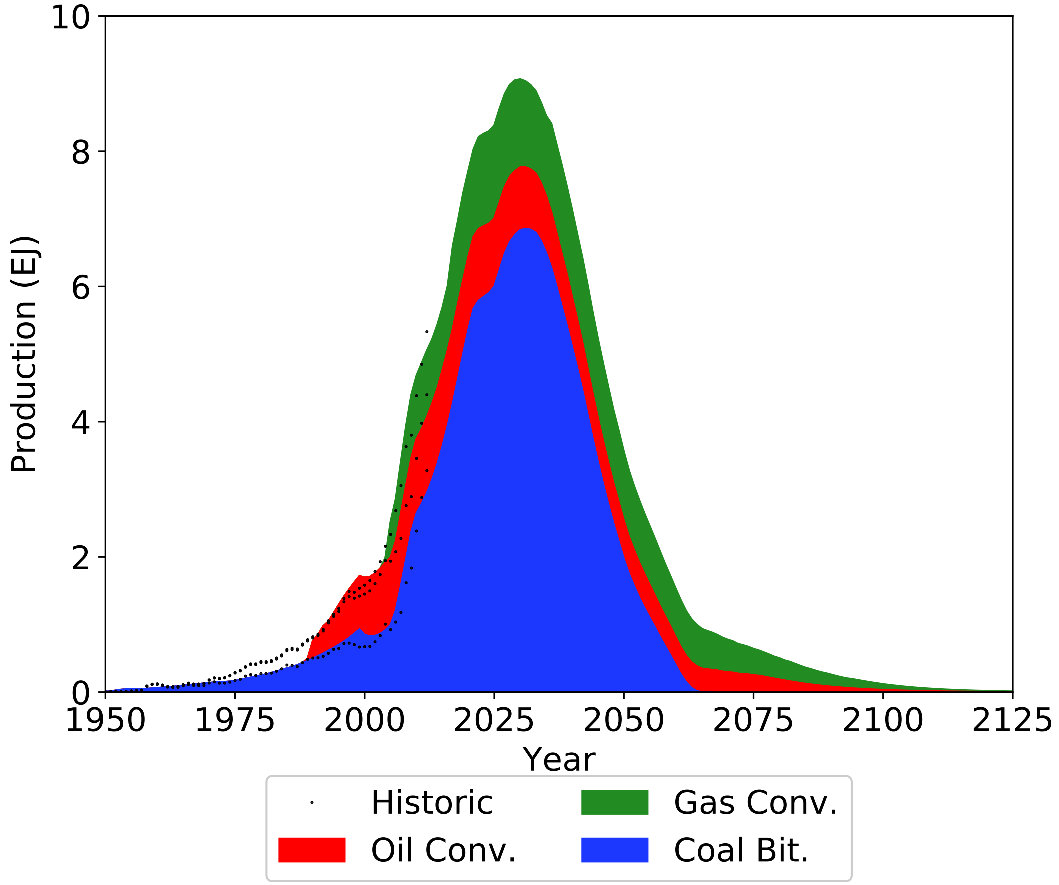

Figure 2.99: China - Xinjiang projection by mineral type

Table 2.99: Peak years - Minerals

Name	URR	Peak Year	Peak Rate
Coal Bit.	235.83	2031	6.85
Oil Conv.	65.09	2015	1.13
Gas Conv.	75.82	2026	1.39
**Total**	**376.74**	**2030**	**9.07**

Yunnan

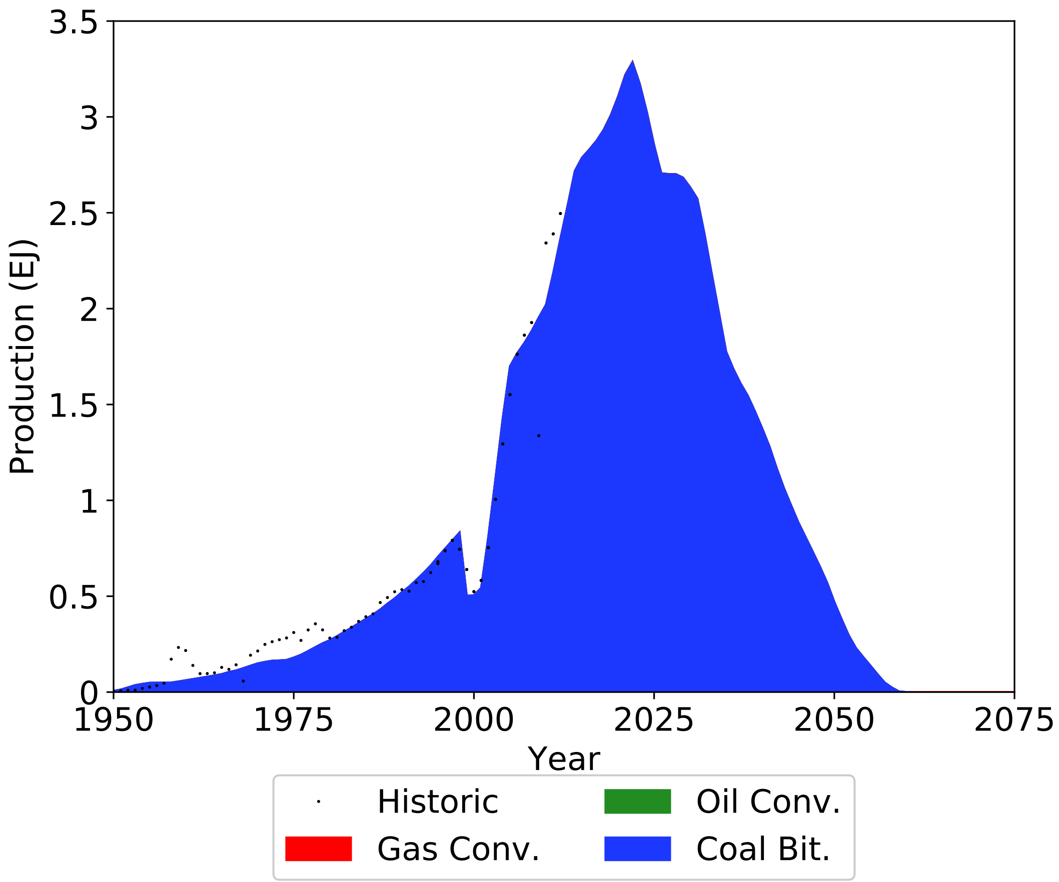

Figure 2.100: China - Yunnan projections capped at 16

Table 2.100: Peak years - All

**Name**	**URR**	**Peak Year**	**Peak Rate**
Coal Bit. Yunnan	113.89	2022	3.28
Gas Conv. Yunnan	0.04	2019	–
Oil Conv. Yunnan	0.01	2022	–
**Total**	**113.93**	**2022**	**3.29**

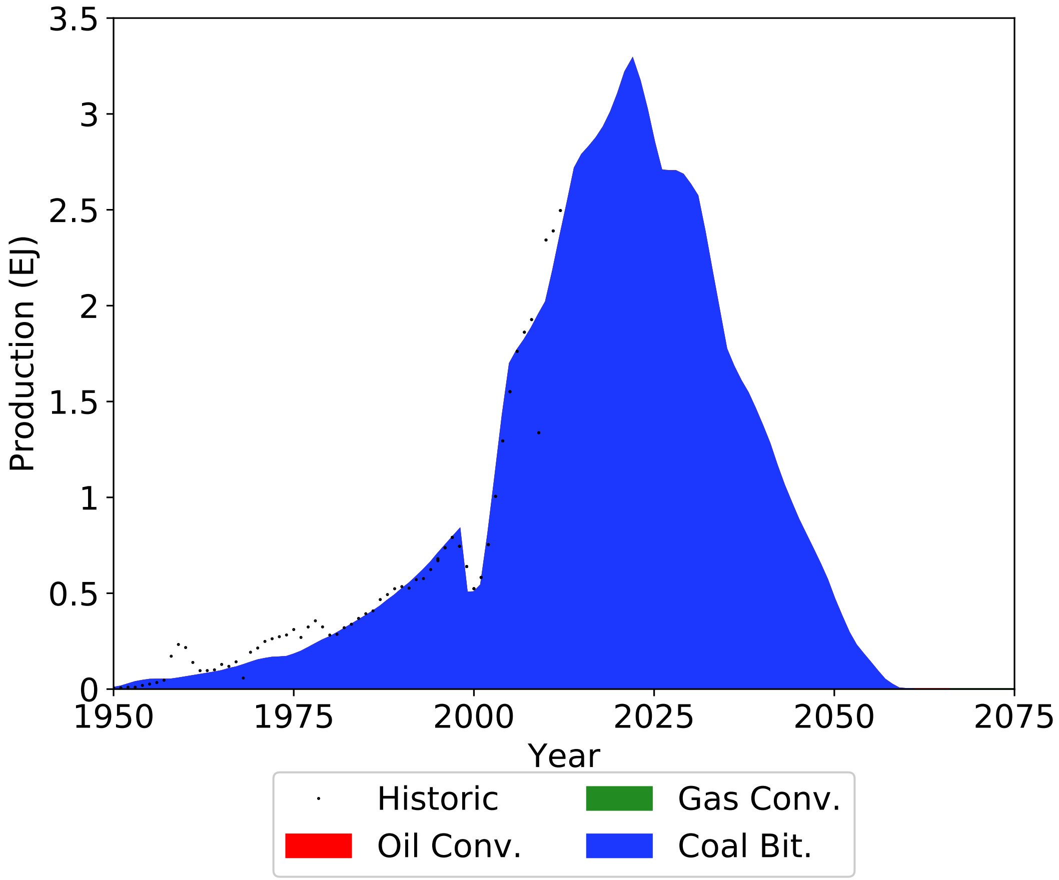

Figure 2.101: China - Yunnan projection by mineral type

Table 2.101: Peak years - Minerals

Name	URR	Peak Year	Peak Rate
Coal Bit.	113.89	2022	3.28
Oil Conv.	0.01	2022	–
Gas Conv.	0.04	2019	–
**Total**	**113.93**	**2022**	**3.29**

Zhejiang

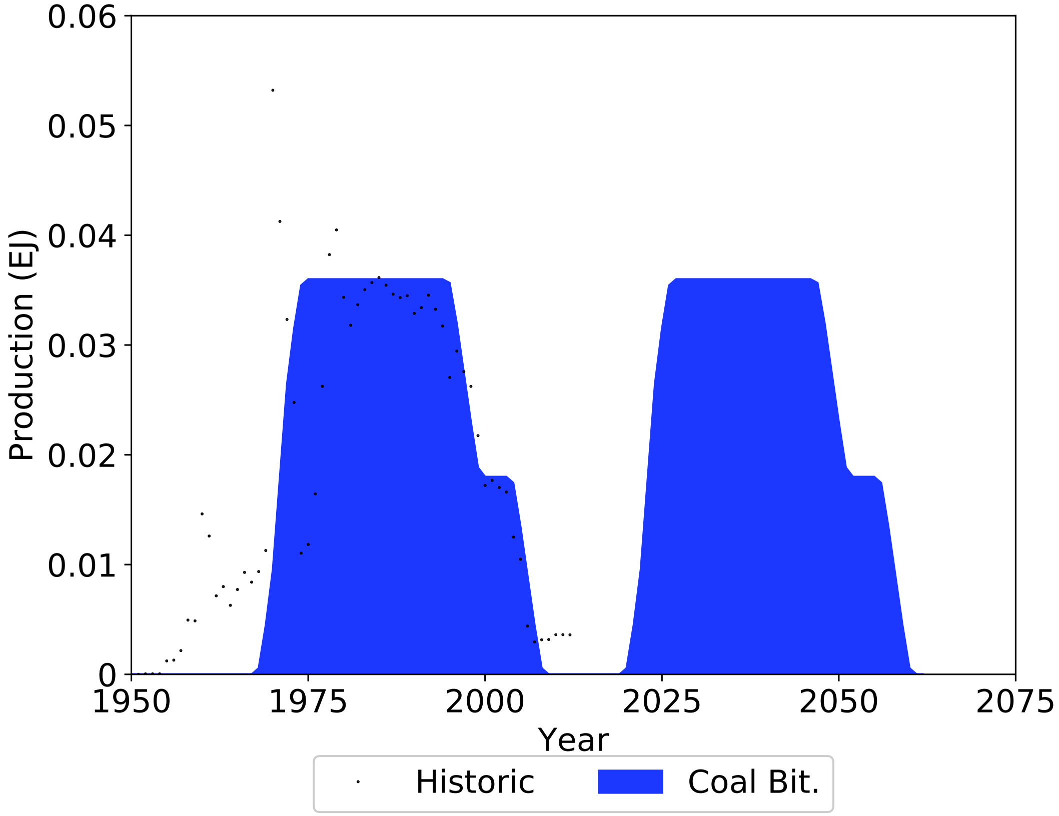

Figure 2.102: China - Zhejiang projections capped at 16

Table 2.102: Peak years - All

Name	URR	Peak Year	Peak Rate
Coal Bit. Zhejiang	2.2	1975	0.04
**Total**	**2.2**	**1975**	**0.04**

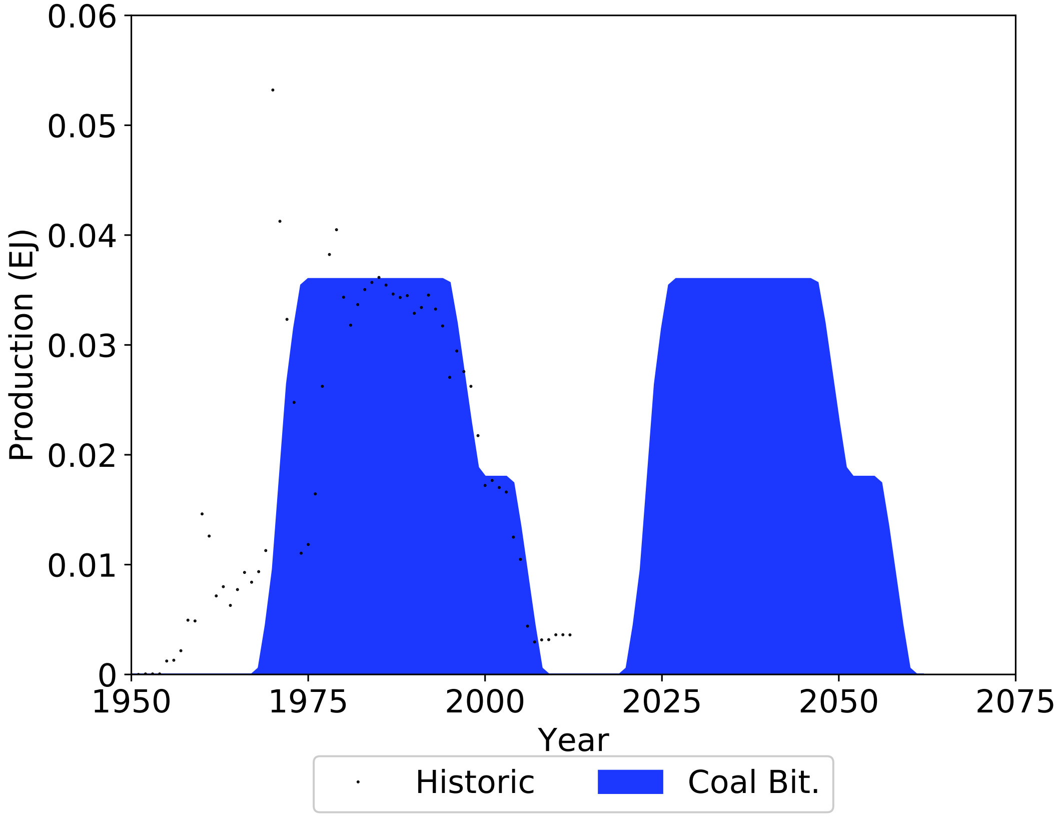

Figure 2.103: China - Zhejiang projection by mineral type

Table 2.103: Peak years - Minerals

Name	URR	Peak Year	Peak Rate
Coal Bit.	2.2	1975	0.04
**Total**	**2.2**	**1975**	**0.04**

2.8.4 Projection by region

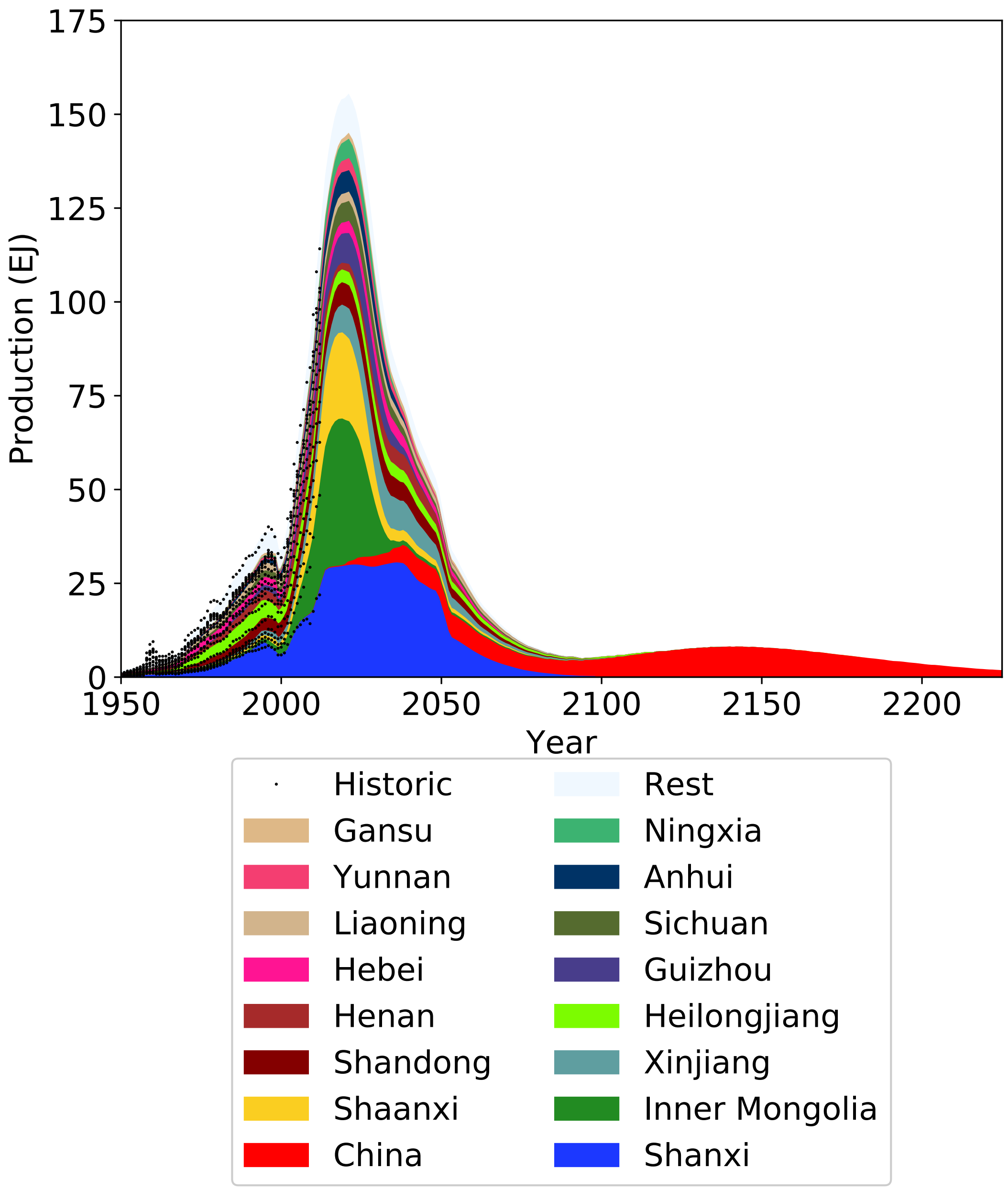

Figure 2.104: China by region projections capped at 16

Table 2.104: Peak years - All

**Name**	**URR**	**Peak Year**	**Peak Rate**
Shanxi	1543.23	2036	30.33
China	1065.16	2142	7.89
Inner Mongolia	765.99	2018	39.11
Shaanxi	468.82	2019	22.94
Xinjiang	376.74	2030	9.07
Shandong	368.91	2025	6.29
Heilongjiang	330.18	1994	4.8
Henan	259.99	2009	5.77
Guizhou	221.47	2024	8.5
Hebei	214.42	2030	3.77
Sichuan	211.06	2021	5.29
Liaoning	181.8	2022	2.48
Anhui	162.67	2020	5.77
Yunnan	113.93	2022	3.29
Ningxia	103.56	2021	5.1
Gansu	84.7	2029	1.72
Jilin	78.9	2019	2.33
Chongqing	70.87	2024	1.5
Qinghai	70.32	2024	2.1
Hunan	56.4	2013	2.15
Tianjin	55.15	2012	1.48
Guangdong	53.88	2023	1.03
Jiangsu	41.89	2006	0.74
Jiangxi	26.86	2008	0.79
Historic	25.96	1937	0.94
Offshore	21.38	2034	0.54
Beijing	19.54	2048	0.27
Hubei	17.05	2019	0.33
Fujian	15.95	2009	0.67
Guangxi	11.22	1993	0.25
Zhejiang	2.2	1975	0.04
Hainan	2.2	2052	0.05
Shanghai	0.83	1982	0.08
Tibet	0.19	2048	–
**Total**	**7043.41**	**2021**	**155.03**

2.9 East Timor

2.9.1 All Projections

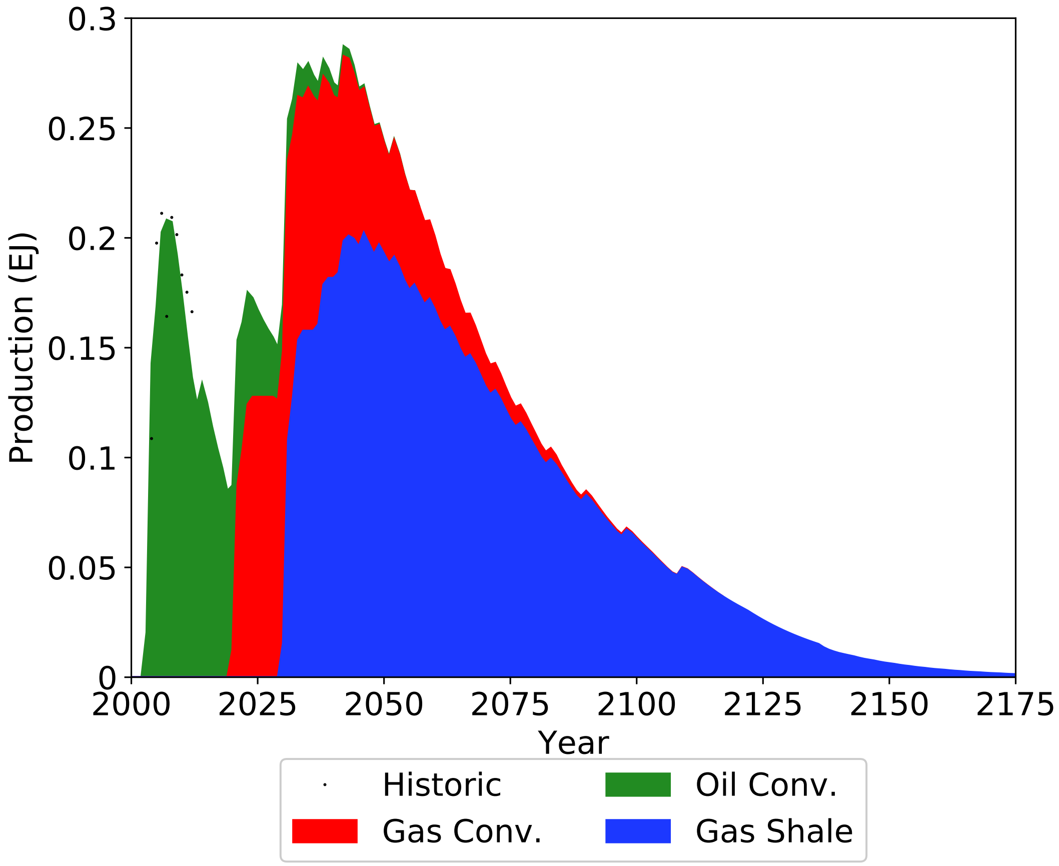

Figure 2.105: East Timor projections capped at 16

Table 2.105: Peak years - All

Name	URR	Peak Year	Peak Rate
Gas Shale	11.12	2046	0.2
Gas Conv.	3.75	2030	0.13
Oil Conv.	3.0	2007	0.21
**Total**	**17.87**	**2042**	**0.29**

2.9.2 By Mineral

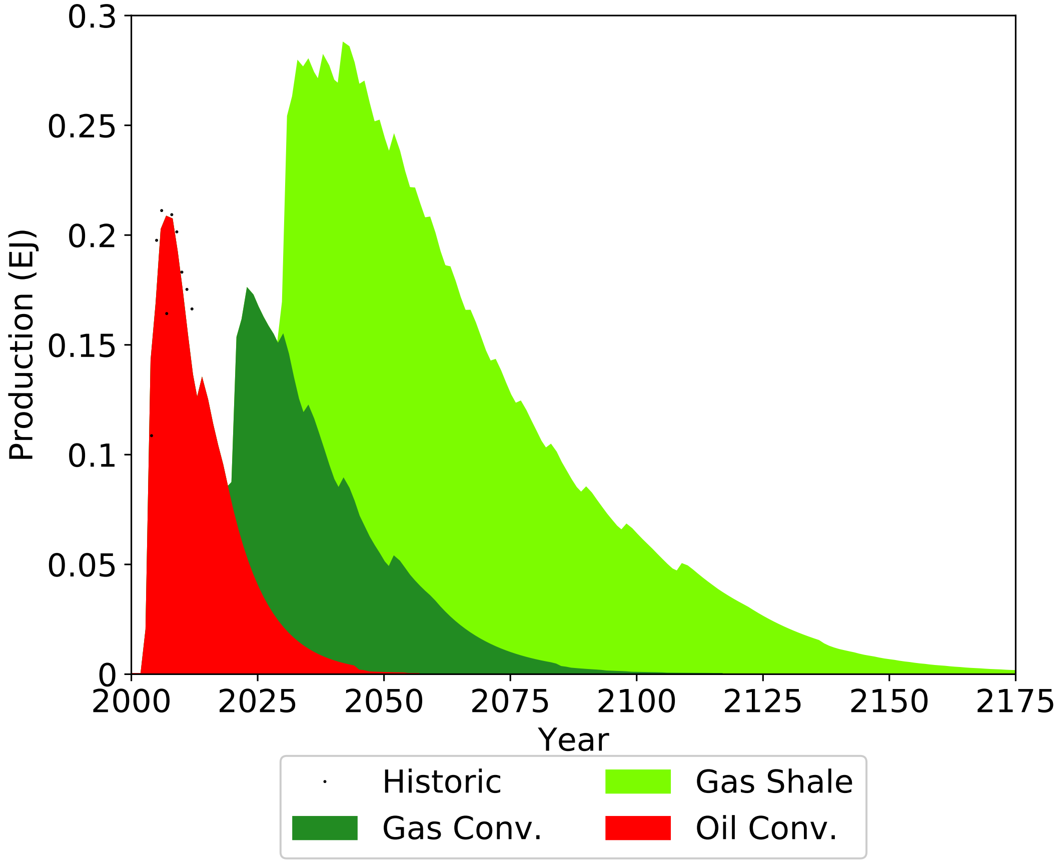

Figure 2.106: East Timor projection by mineral type

Table 2.106: Peak years - Minerals

Name	URR	Peak Year	Peak Rate
Oil Conv.	3.0	2007	0.21
Gas Conv.	3.75	2030	0.13
Gas Shale	11.12	2046	0.2
**Total**	**17.87**	**2042**	**0.29**

2.10 India

2.10.1 All Projections

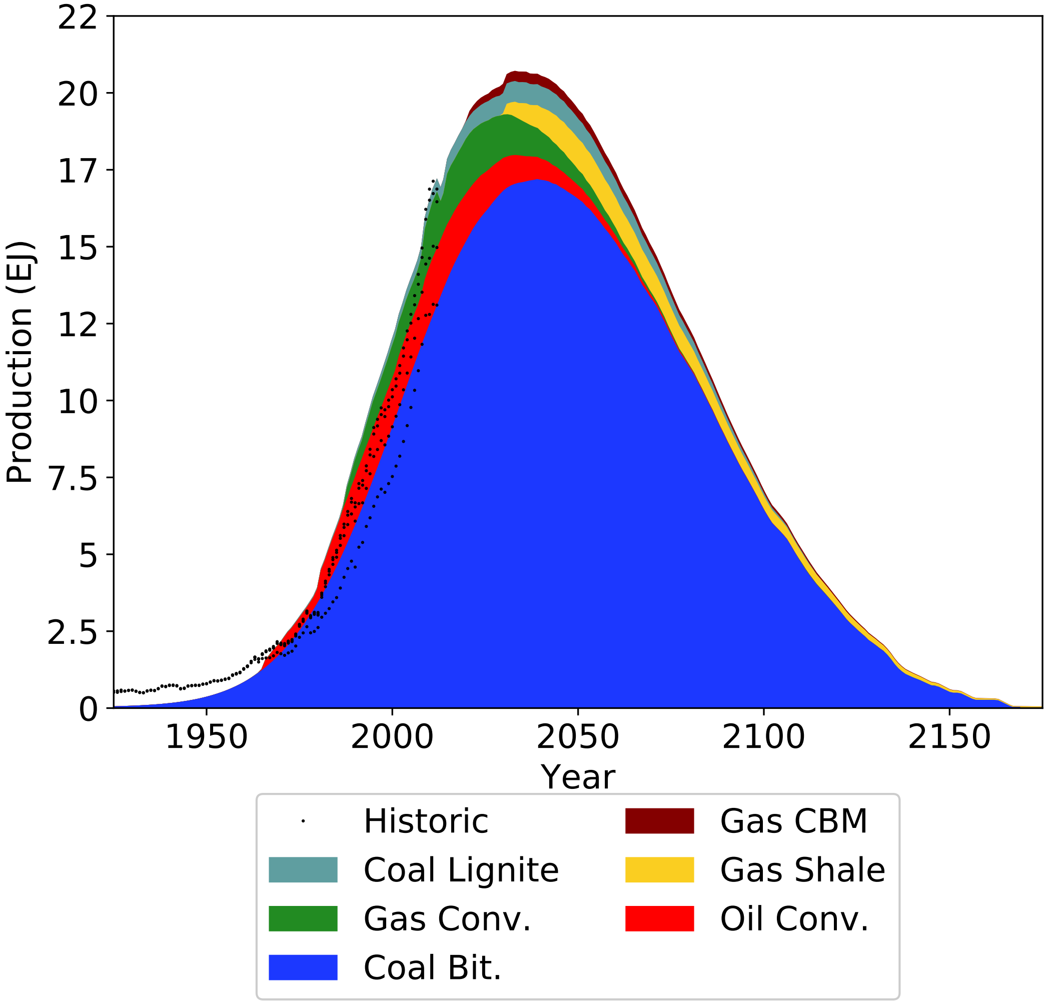

Figure 2.107: India projections capped at 16

Table 2.107: Peak years - All

**Name**	**URR**	**Peak Year**	**Peak Rate**
Coal Bit.	1652.7	2039	17.17
Oil Conv.	101.94	2011	1.9
Gas Conv.	78.8	2011	1.83
Gas Shale	66.15	2053	1.03
Coal Lignite	49.9	2042	0.69
Gas CBM	21.0	2036	0.35
**Total**	**1970.49**	**2033**	**20.68**

2.10.2 By Mineral

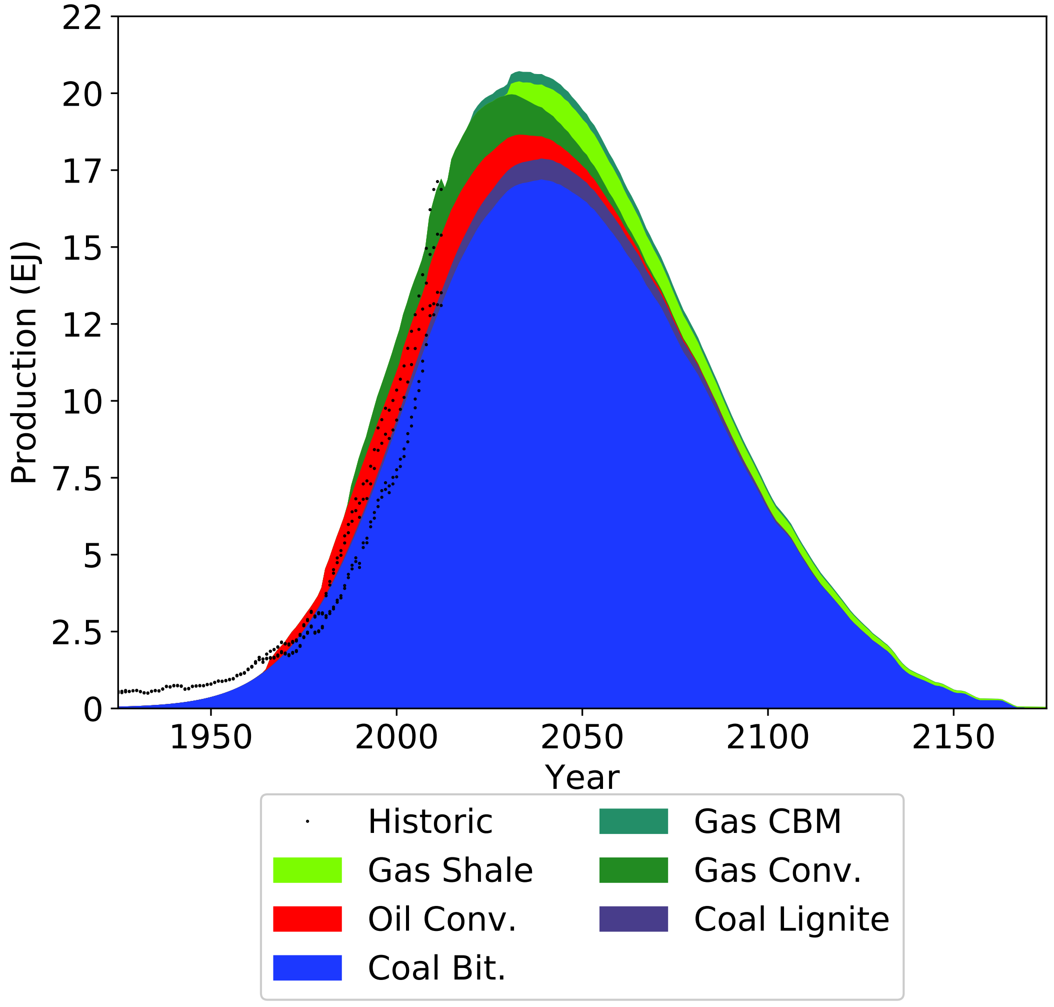

Figure 2.108: India projection by mineral type

Table 2.108: Peak years - Minerals

**Name**	**URR**	**Peak Year**	**Peak Rate**
Coal Bit.	1652.7	2039	17.17
Coal Lignite	49.9	2042	0.69
Oil Conv.	101.94	2011	1.9
Gas Conv.	78.8	2011	1.83
Gas Shale	66.15	2053	1.03
Gas CBM	21.0	2036	0.35
**Total**	**1970.49**	**2033**	**20.68**

2.11 Indonesia

2.11.1 All Projections

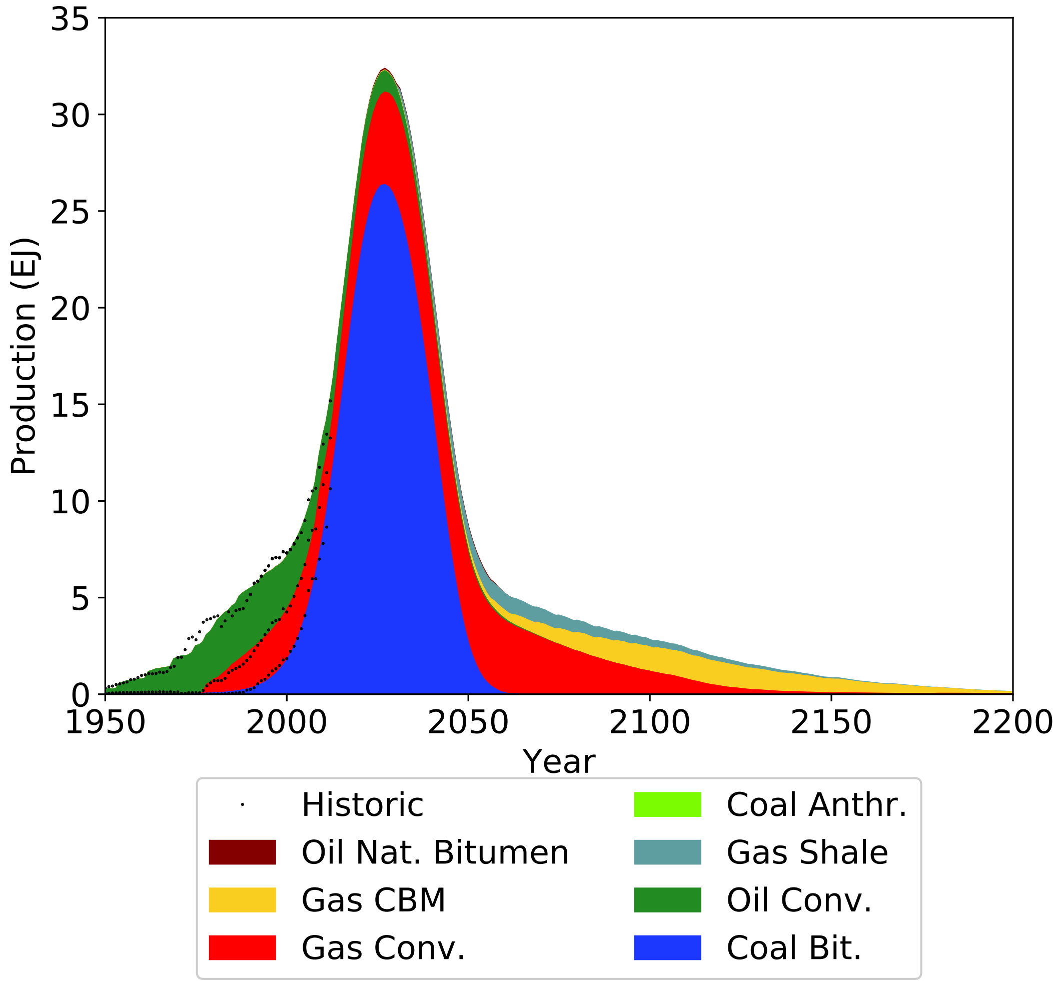

Figure 2.109: Indonesia projections capped at 16

Table 2.109: Peak years - All

**Name**	**URR**	**Peak Year**	**Peak Rate**
Coal Bit.	752.1	2027	26.35
Gas Conv.	403.4	2038	5.17
Oil Conv.	167.78	1988	3.28
Gas CBM	117.92	2109	1.33
Gas Shale	59.7	2053	0.93
Oil Nat. Bitumen	3.12	2048	0.08
Coal Anthr.	0.03	1992	–
**Total**	**1504.04**	**2027**	**32.36**

2.11.2 By Mineral

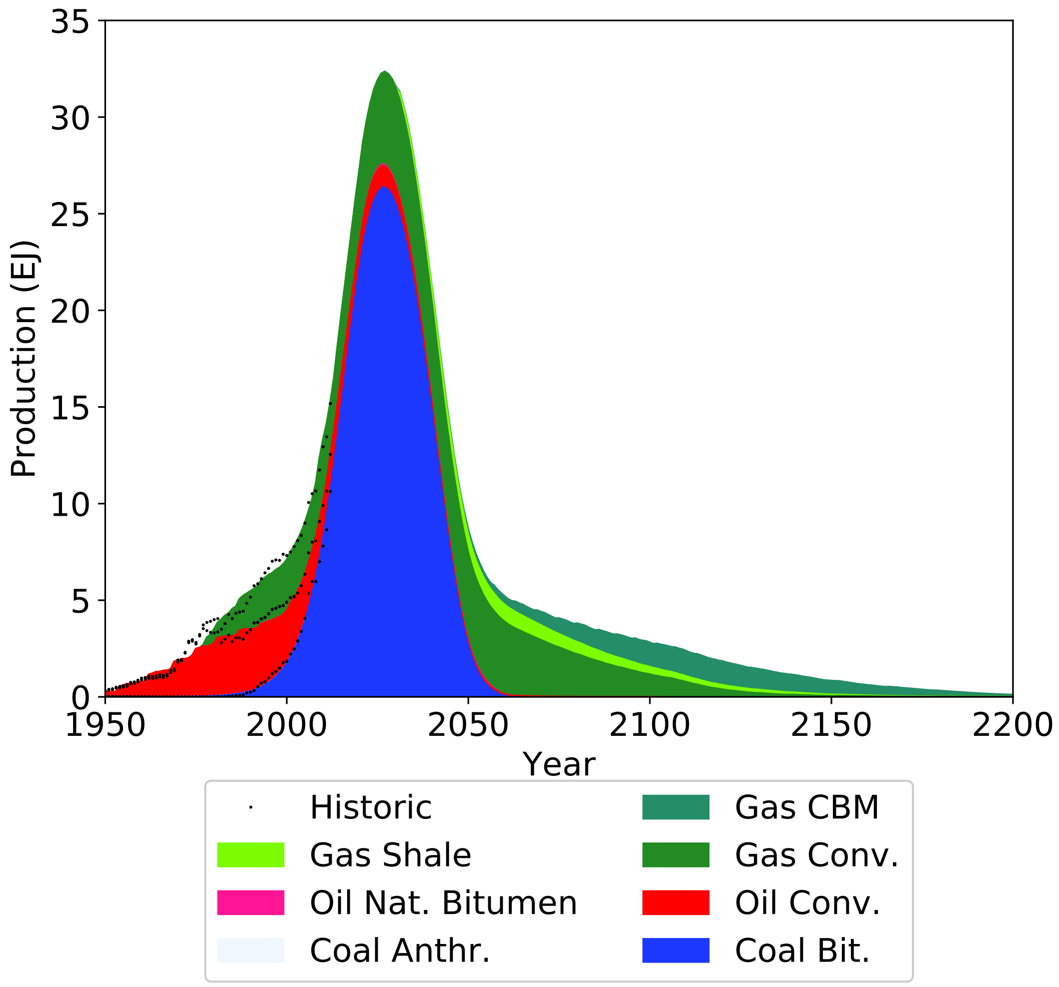

Figure 2.110: Indonesia projection by mineral type

Table 2.110: Peak years - Minerals

**Name**	**URR**	**Peak Year**	**Peak Rate**
Coal Bit.	752.1	2027	26.35
Coal Anthr.	0.03	1992	–
Oil Conv.	167.78	1988	3.28
Oil Nat. Bitumen	3.12	2048	0.08
Gas Conv.	403.4	2038	5.17
Gas Shale	59.7	2053	0.93
Gas CBM	117.92	2109	1.33
**Total**	**1504.04**	**2027**	**32.36**

2.12 Japan

2.12.1 All Projections

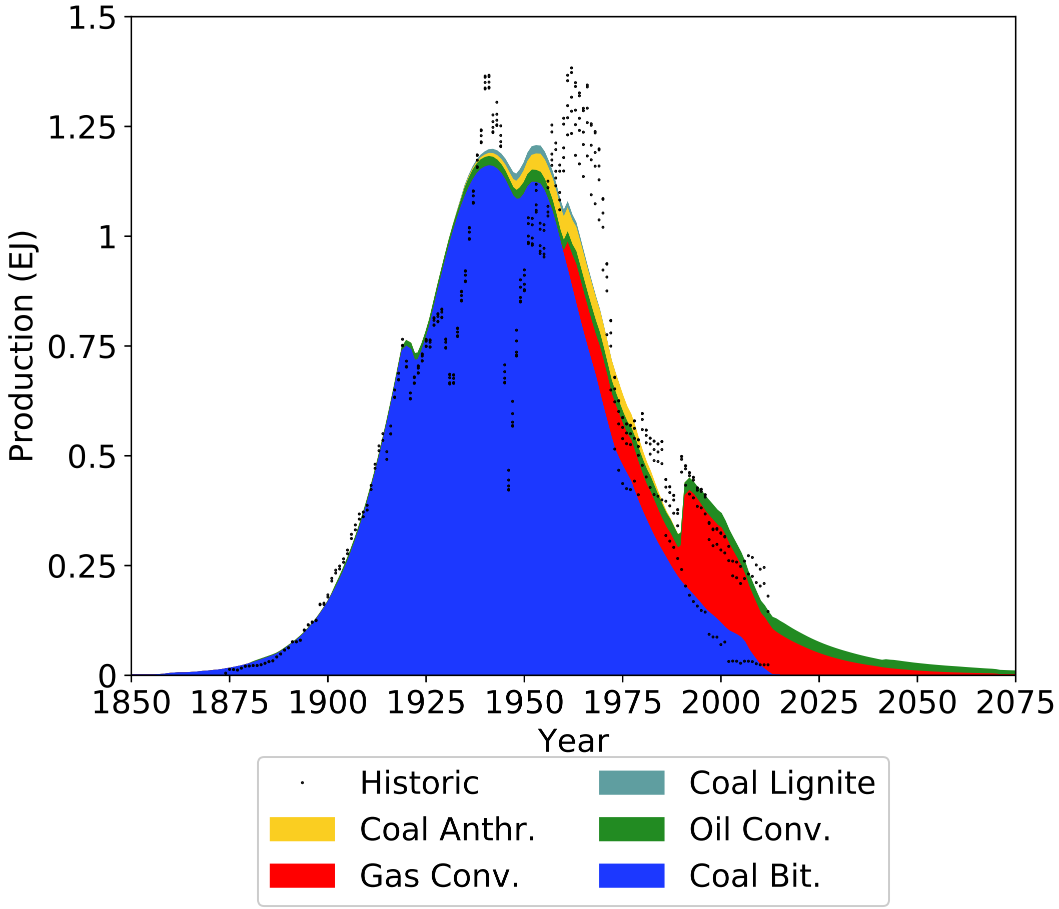

Figure 2.111: Japan projections capped at 16

Table 2.111: Peak years - All

**Name**	**URR**	**Peak Year**	**Peak Rate**
Coal Bit.	68.65	1941	1.16
Gas Conv.	8.26	1993	0.23
Oil Conv.	4.0	1997	0.03
Coal Anthr.	1.58	1964	0.06
Coal Lignite	0.42	1952	0.02
**Total**	**82.91**	**1953**	**1.21**

2.12.2 By Mineral

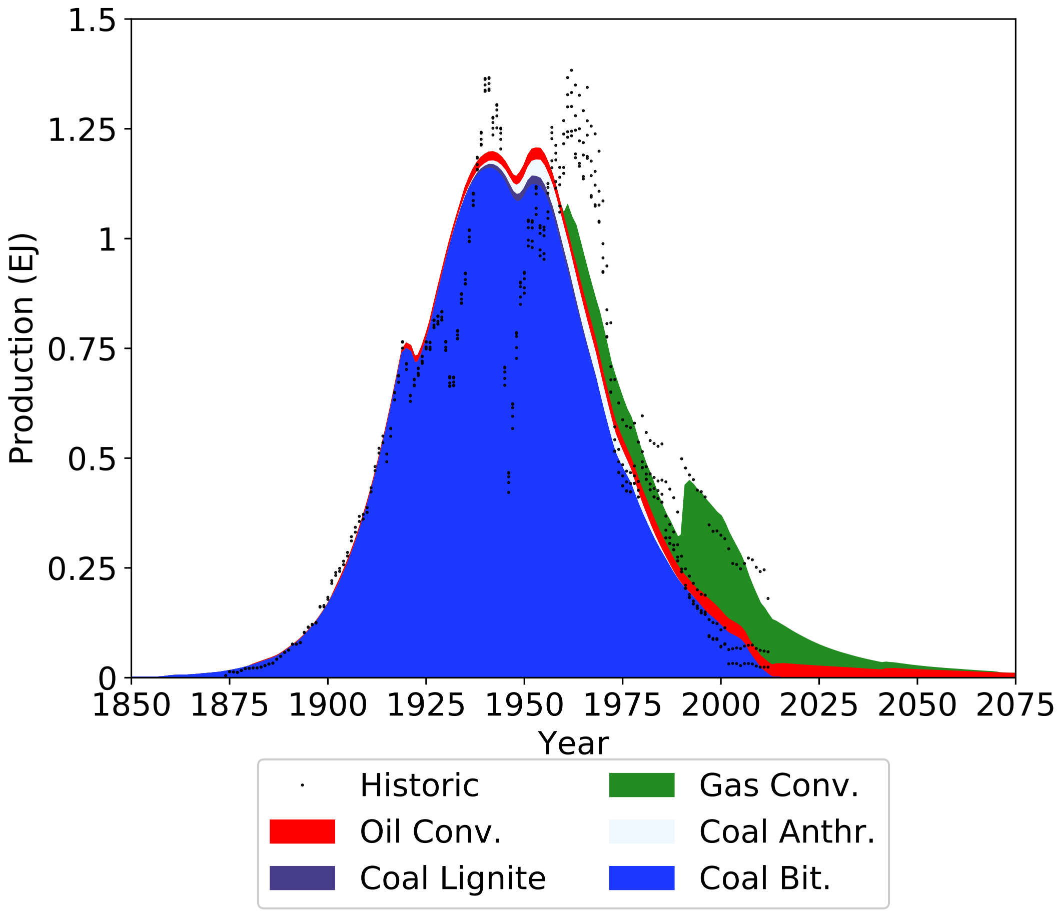

Figure 2.112: Japan projection by mineral type

Table 2.112: Peak years - Minerals

Name	URR	Peak Year	Peak Rate
Coal Bit.	68.65	1941	1.16
Coal Lignite	0.42	1952	0.02
Coal Anthr.	1.58	1964	0.06
Oil Conv.	4.0	1997	0.03
Gas Conv.	8.26	1993	0.23
**Total**	**82.91**	**1953**	**1.21**

2.13 Laos

2.13.1 All Projections

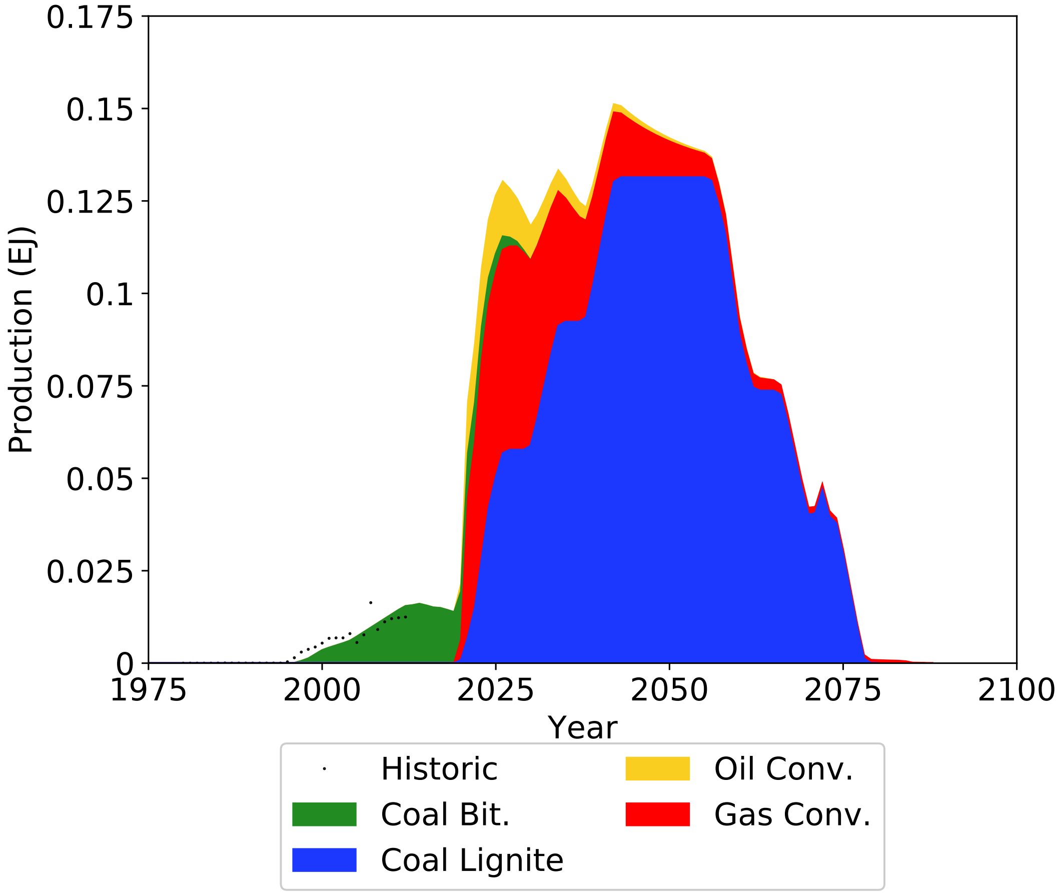

Figure 2.113: Laos projections capped at 16

Table 2.113: Peak years - All

Name	URR	Peak Year	Peak Rate
Coal Lignite	4.74	2043	0.13
Gas Conv.	1.1	2024	0.06
Coal Bit.	0.29	2014	0.02
Oil Conv.	0.21	2022	0.02
**Total**	**6.34**	**2042**	**0.15**

2.13.2 By Mineral

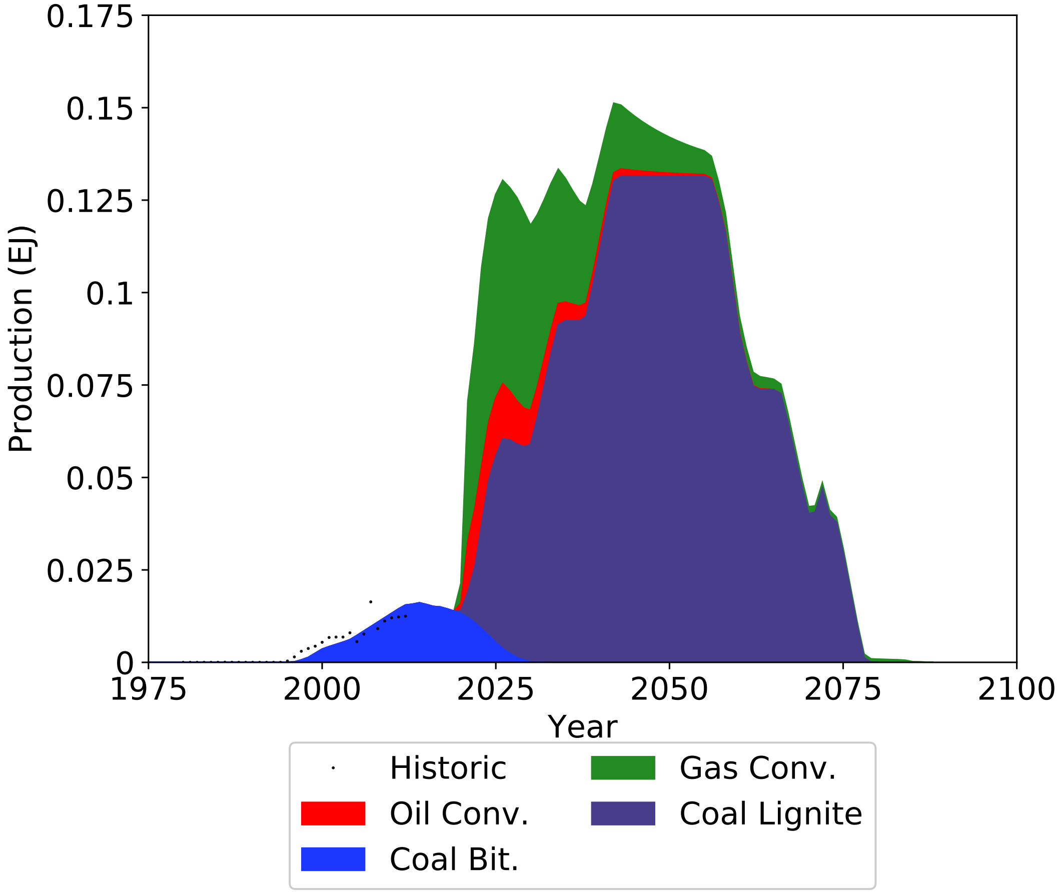

Figure 2.114: Laos projection by mineral type

Table 2.114: Peak years - Minerals

Name	URR	Peak Year	Peak Rate
Coal Bit.	0.29	2014	0.02
Coal Lignite	4.74	2043	0.13
Oil Conv.	0.21	2022	0.02
Gas Conv.	1.1	2024	0.06
**Total**	**6.34**	**2042**	**0.15**

2.14 Malaysia

2.14.1 All Projections

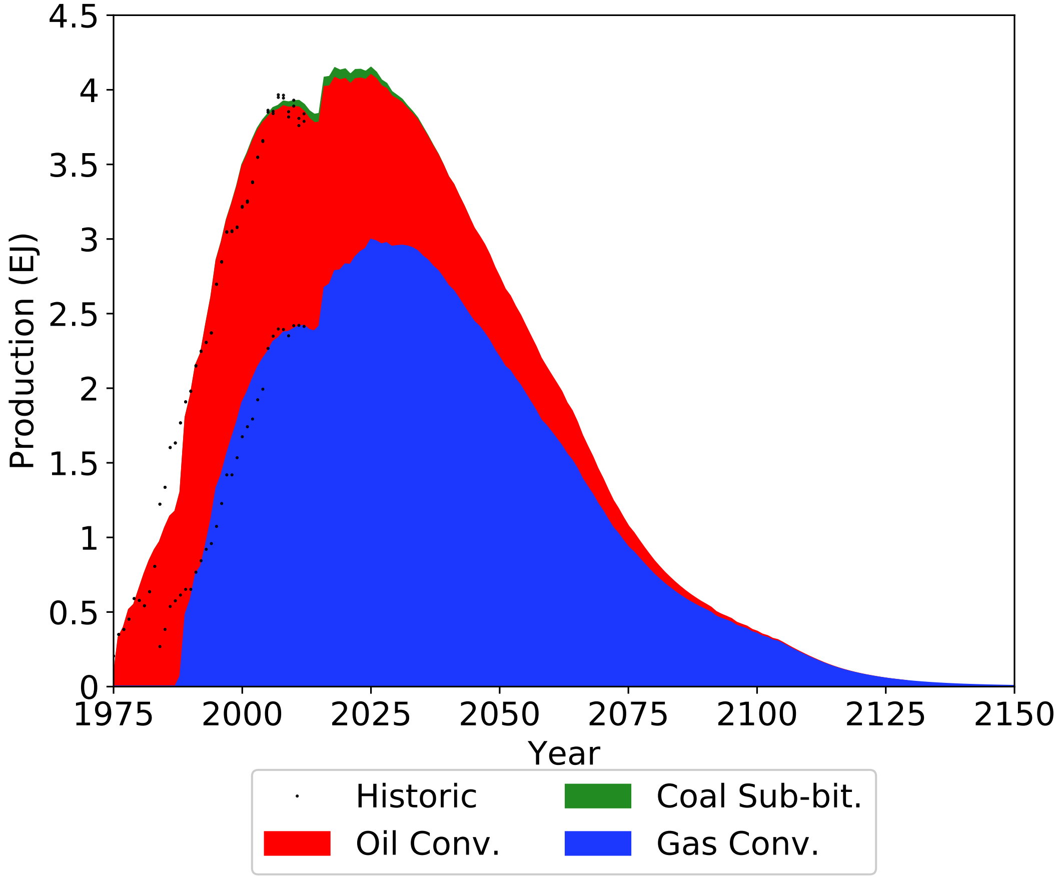

Figure 2.115: Malaysia projections capped at 16

Table 2.115: Peak years - All

Name	URR	Peak Year	Peak Rate
Gas Conv.	202.73	2025	3.0
Oil Conv.	92.9	2002	1.59
Coal Sub-bit.	1.69	2019	0.07
**Total**	**297.32**	**2025**	**4.15**

2.14.2 By Mineral

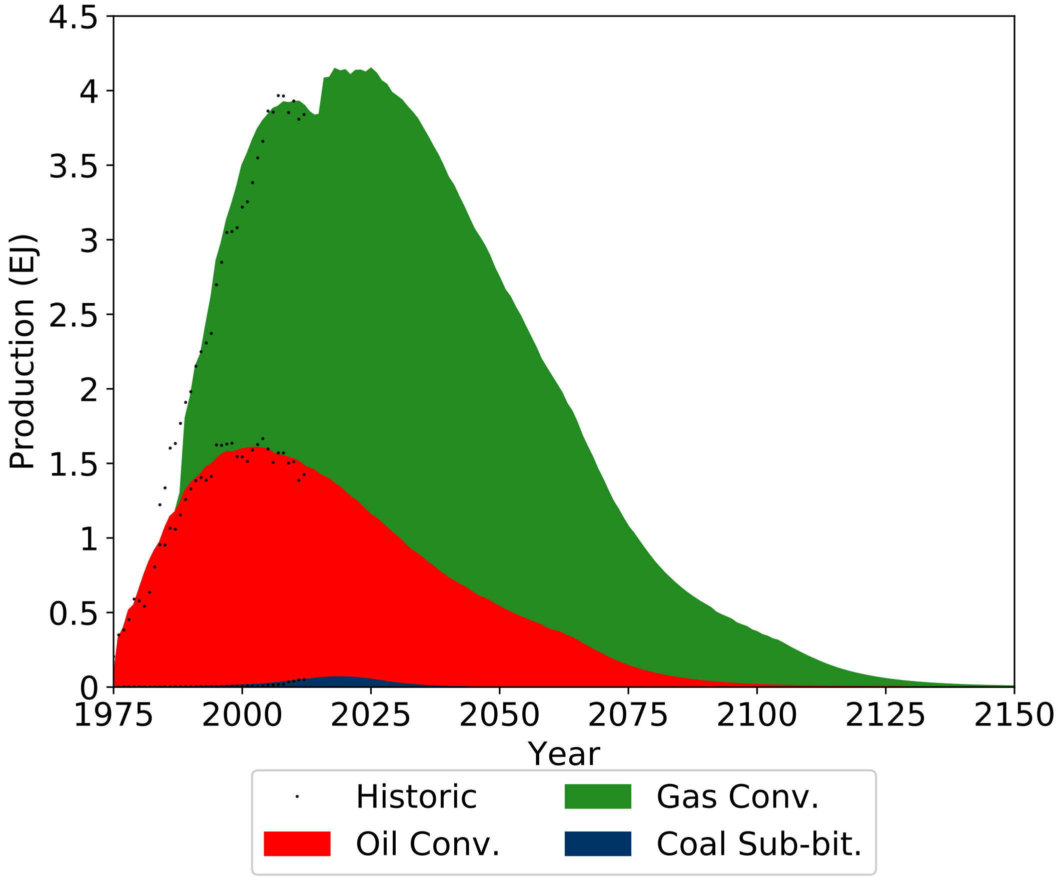

Figure 2.116: Malaysia projection by mineral type

Table 2.116: Peak years - Minerals

**Name**	**URR**	**Peak Year**	**Peak Rate**
Coal Sub-bit.	1.69	2019	0.07
Oil Conv.	92.9	2002	1.59
Gas Conv.	202.73	2025	3.0
**Total**	**297.32**	**2025**	**4.15**

2.15 Mongolia

2.15.1 All Projections

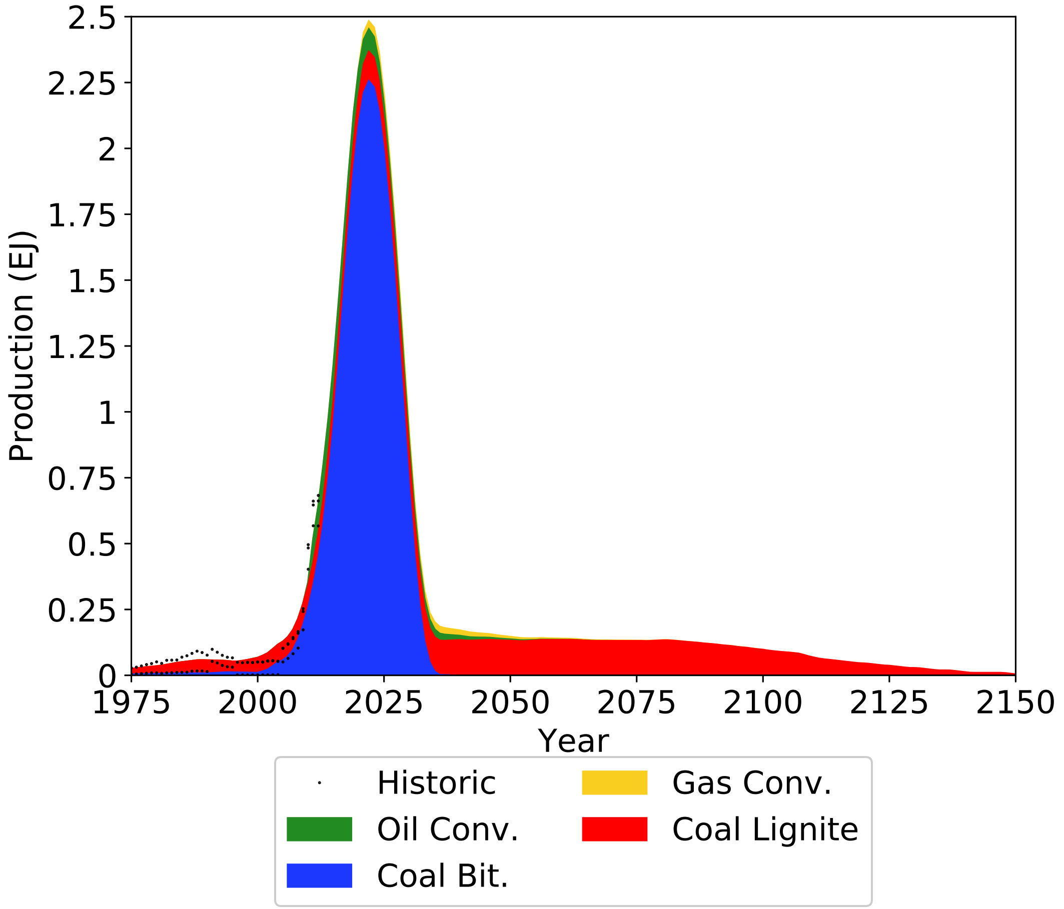

Figure 2.117: Mongolia projections capped at 16

Table 2.117: Peak years - All

**Name**	**URR**	**Peak Year**	**Peak Rate**
Coal Bit.	30.5	2022	2.26
Coal Lignite	15.0	2046	0.13
Oil Conv.	2.27	2014	0.13
Gas Conv.	0.85	2024	0.04
**Total**	**48.62**	**2022**	**2.48**

2.15.2 By Mineral

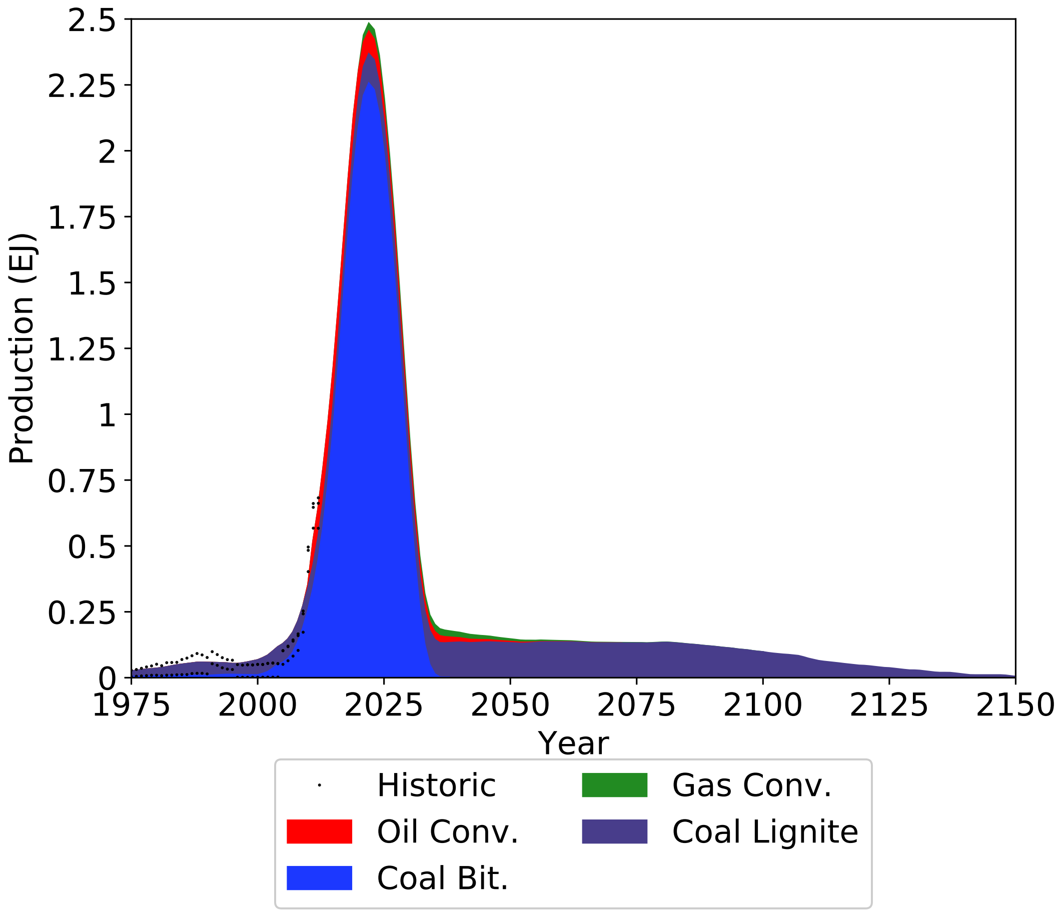

Figure 2.118: Mongolia projection by mineral type

Table 2.118: Peak years - Minerals

**Name**	**URR**	**Peak Year**	**Peak Rate**
Coal Bit.	30.5	2022	2.26
Coal Lignite	15.0	2046	0.13
Oil Conv.	2.27	2014	0.13
Gas Conv.	0.85	2024	0.04
**Total**	**48.62**	**2022**	**2.48**

2.16 Nepal

2.16.1 All Projections

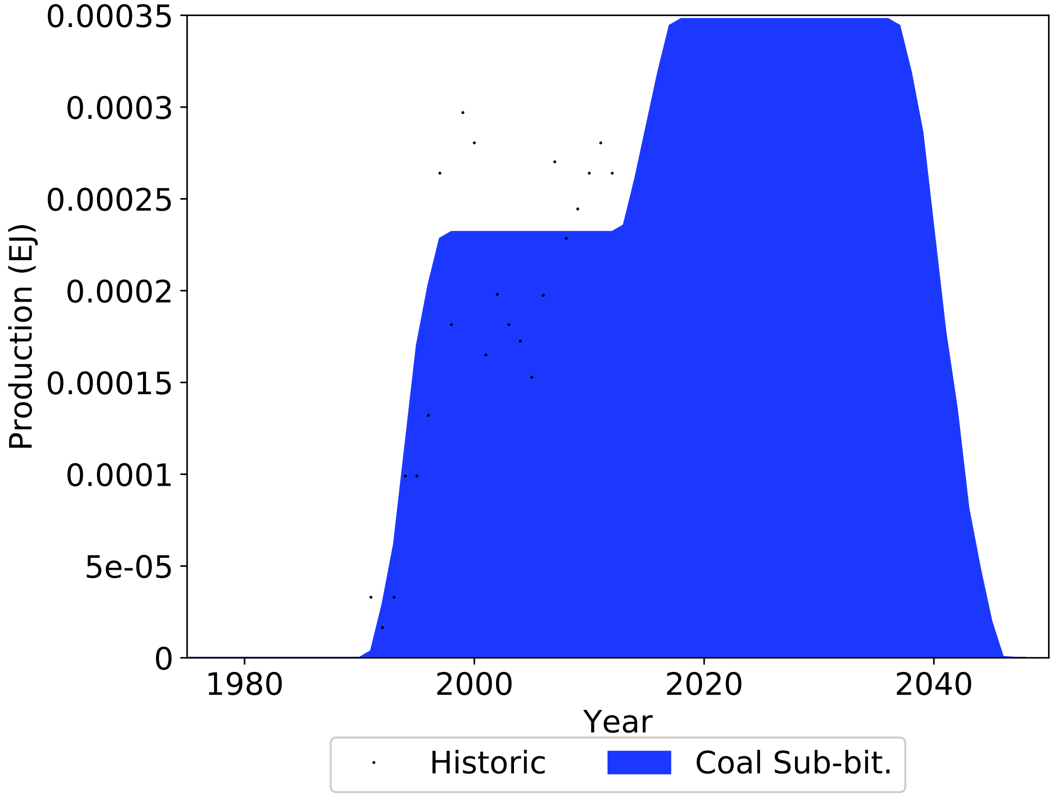

Figure 2.119: Nepal projections capped at 16

Table 2.119: Peak years - All

Name	URR	Peak Year	Peak Rate
Coal Sub-bit.	0.01	2018	–
**Total**	**0.01**	**2018**	–

2.16.2 By Mineral

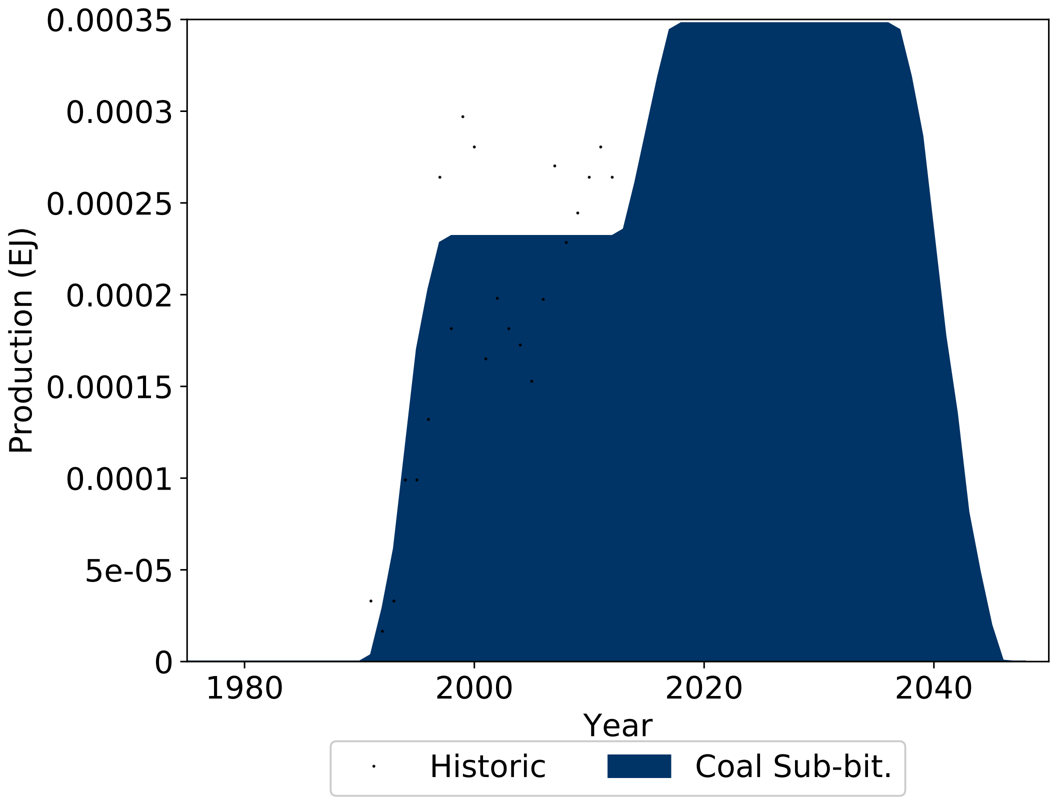

Figure 2.120: Nepal projection by mineral type

Table 2.120: Peak years - Minerals

Name	URR	Peak Year	Peak Rate
Coal Sub-bit.	0.01	2018	–
**Total**	**0.01**	**2018**	–

2.17 New Caledonia

2.17.1 All Projections

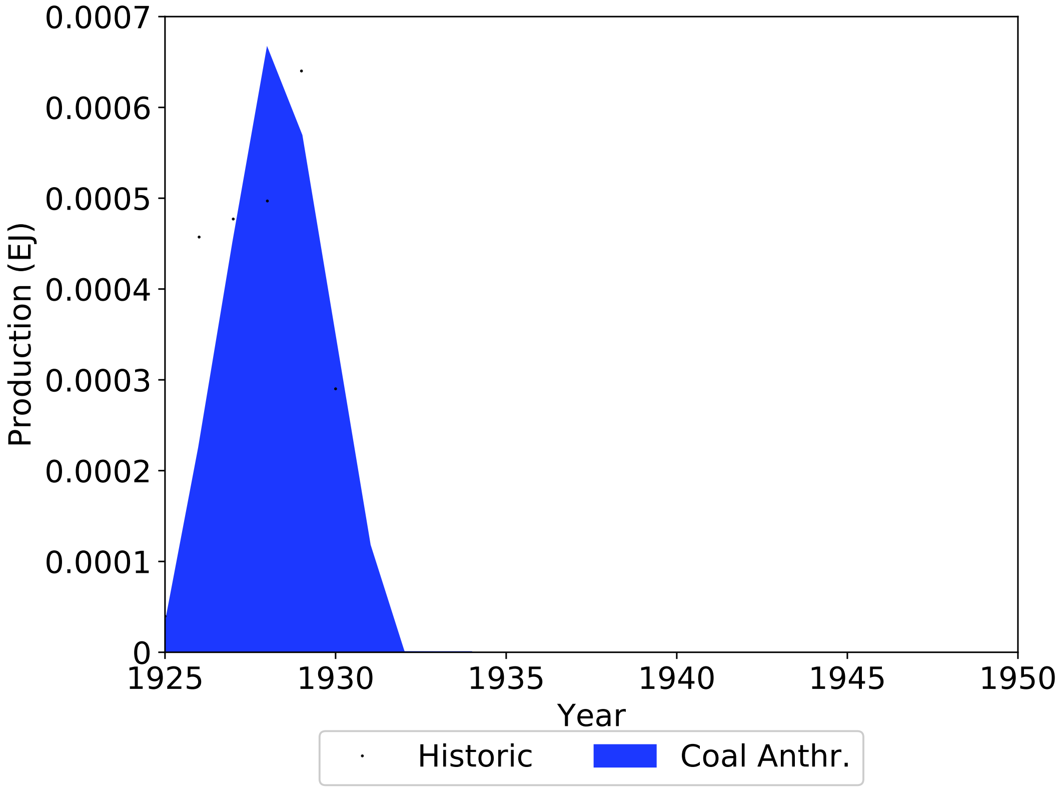

Figure 2.121: New Caledonia projections capped at 16

Table 2.121: Peak years - All

Name	URR	Peak Year	Peak Rate
Coal Anthr.	–	1928	–
**Total**	–	**1928**	–

2.17.2 By Mineral

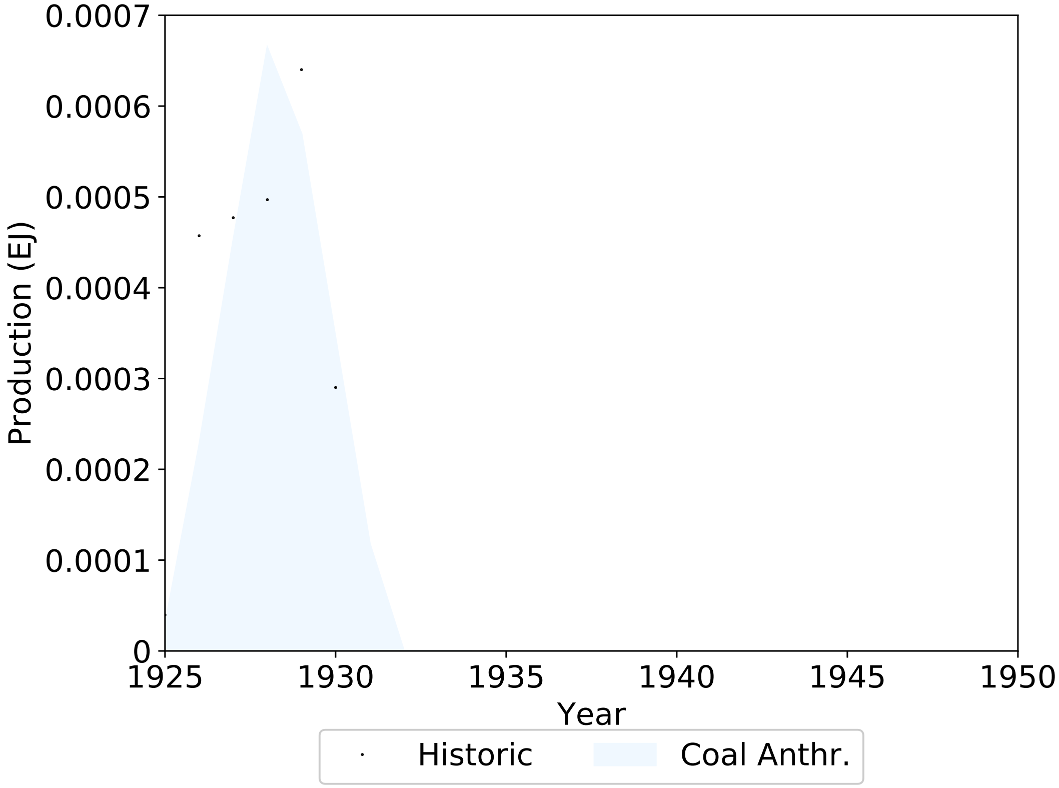

Figure 2.122: New Caledonia projection by mineral type

Table 2.122: Peak years - Minerals

Name	URR	Peak Year	Peak Rate
Coal Anthr.	–	1928	–
**Total**	–	**1928**	–

2.18 New Zealand

2.18.1 All Projections

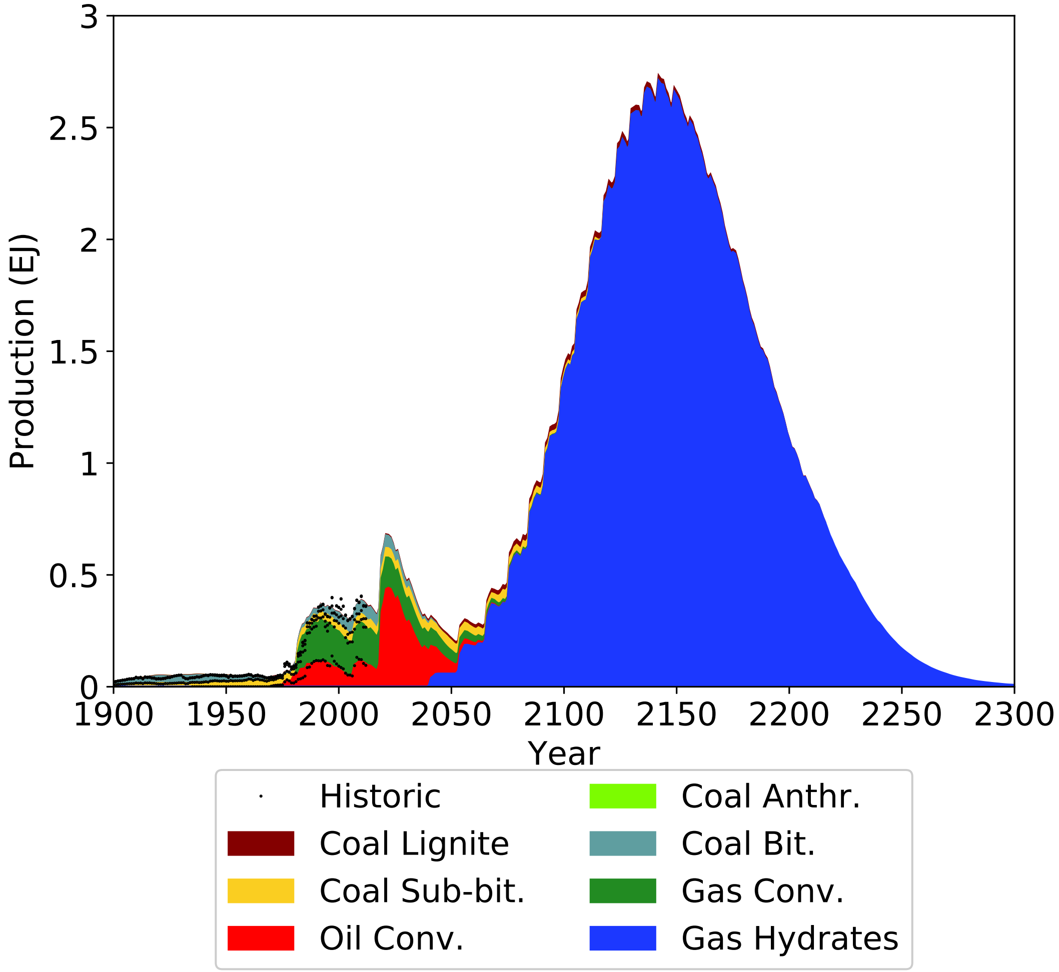

Figure 2.123: New Zealand projections capped at 16

Table 2.123: Peak years - All

**Name**	**URR**	**Peak Year**	**Peak Rate**
Gas Hydrates	266.5	2142	2.71
Oil Conv.	11.5	2022	0.44
Gas Conv.	9.9	1992	0.19
Coal Sub-bit.	6.2	2048	0.05
Coal Bit.	4.3	2011	0.06
Coal Lignite	3.38	2112	0.03
Coal Anthr.	–	1950	–
**Total**	**301.78**	**2142**	**2.73**

2.18.2 By Mineral

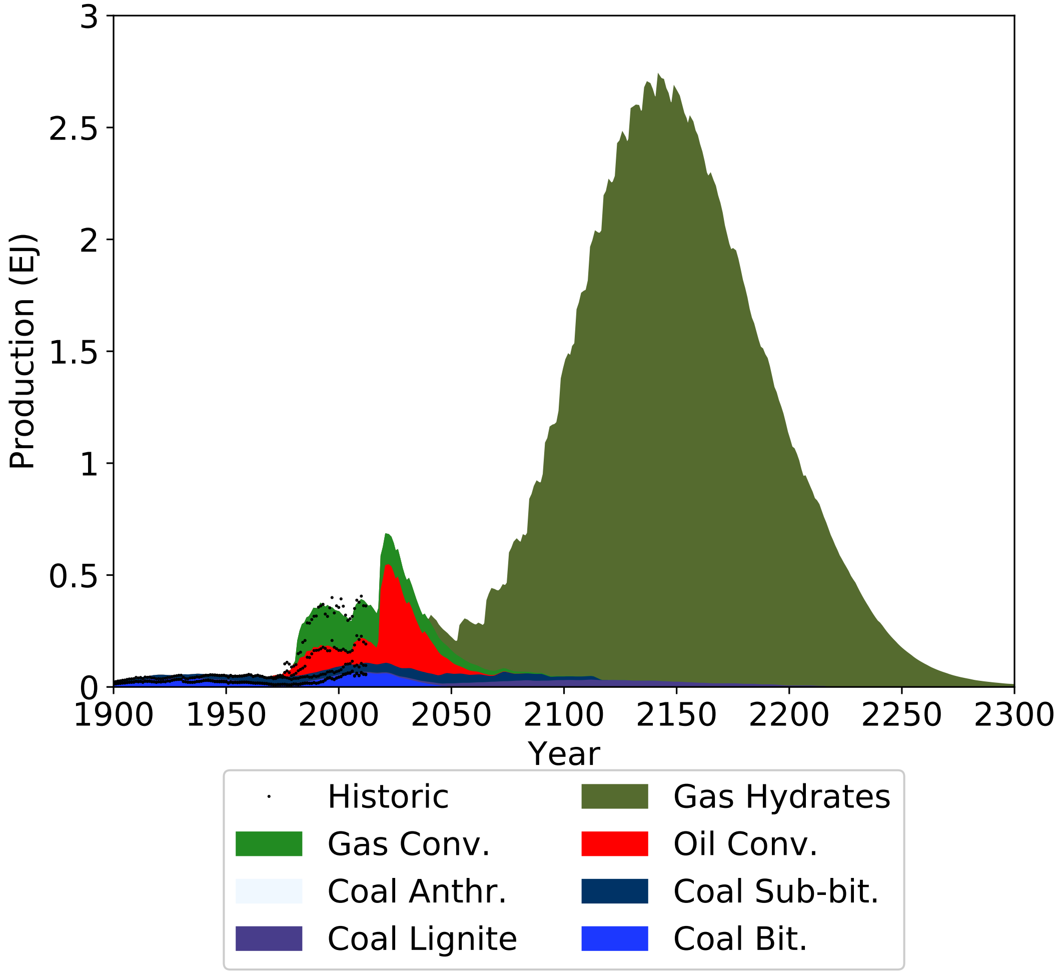

Figure 2.124: New Zealand projection by mineral type

Table 2.124: Peak years - Minerals

**Name**	**URR**	**Peak Year**	**Peak Rate**
Coal Bit.	4.3	2011	0.06
Coal Lignite	3.38	2112	0.03
Coal Sub-bit.	6.2	2048	0.05
Coal Anthr.	–	1950	–
Oil Conv.	11.5	2022	0.44
Gas Conv.	9.9	1992	0.19
Gas Hydrates	266.5	2142	2.71
**Total**	**301.78**	**2142**	**2.73**

2.19 North Korea

2.19.1 All Projections

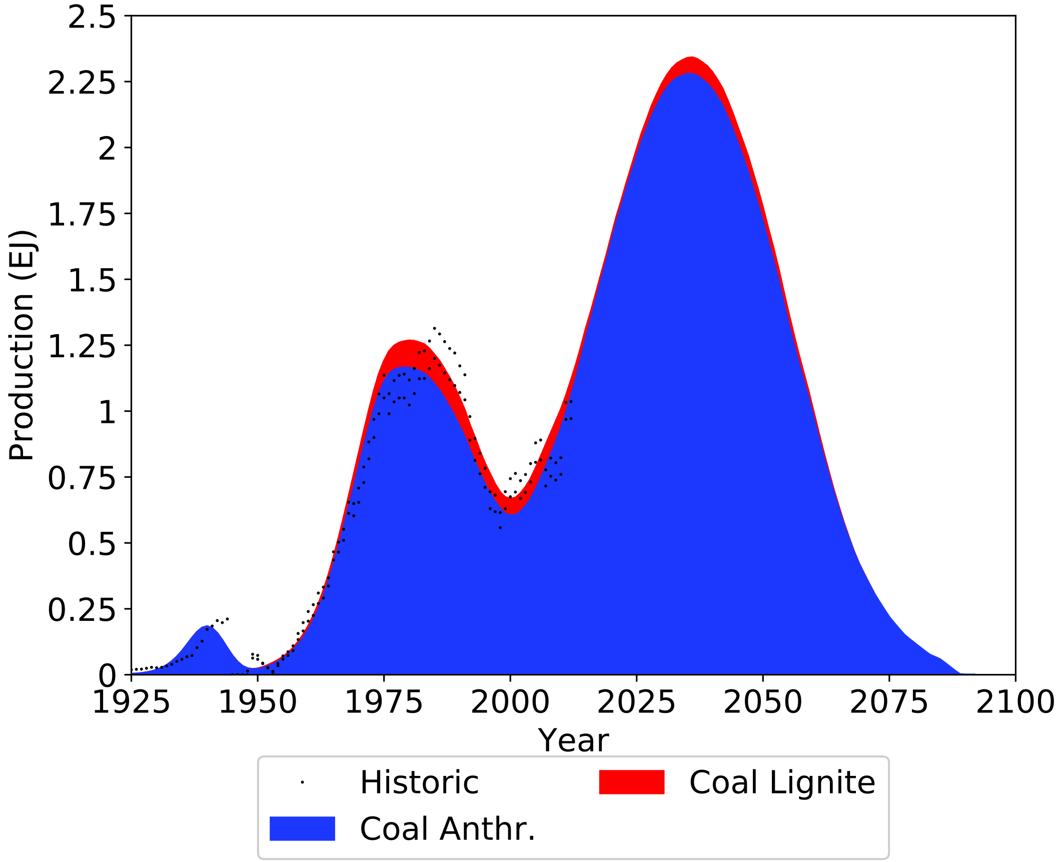

Figure 2.125: North Korea projections capped at 16

Table 2.125: Peak years - All

Name	URR	Peak Year	Peak Rate
Coal Anthr.	140.7	2035	2.28
Coal Lignite	6.27	1985	0.11
**Total**	**146.97**	**2036**	**2.34**

2.19.2 By Mineral

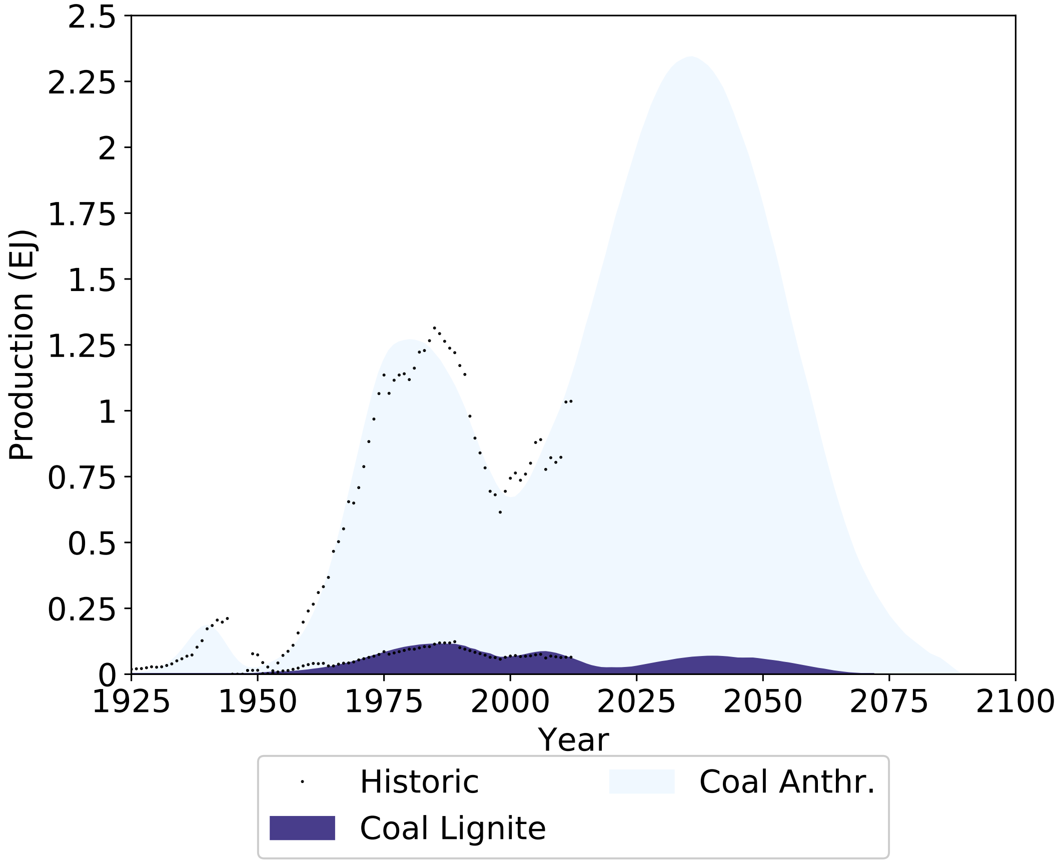

Figure 2.126: North Korea projection by mineral type

Table 2.126: Peak years - Minerals

Name	URR	Peak Year	Peak Rate
Coal Lignite	6.27	1985	0.11
Coal Anthr.	140.7	2035	2.28
**Total**	**146.97**	**2036**	**2.34**

2.20 PNG

2.20.1 All Projections

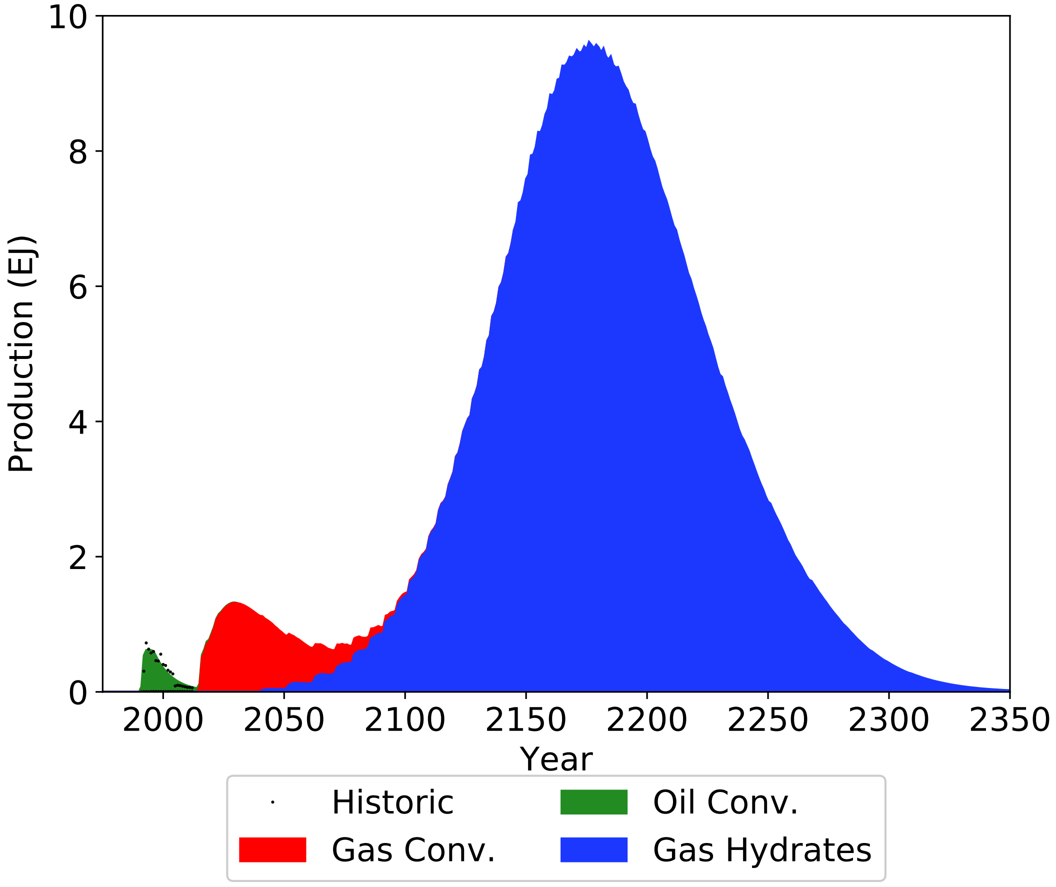

Figure 2.127: PNG projections capped at 16

Table 2.127: Peak years - All

Name	URR	Peak Year	Peak Rate
Gas Hydrates	1026.5	2176	9.62
Gas Conv.	53.55	2030	1.32
Oil Conv.	7.27	1993	0.62
**Total**	**1087.32**	**2176**	**9.62**

2.20.2 By Mineral

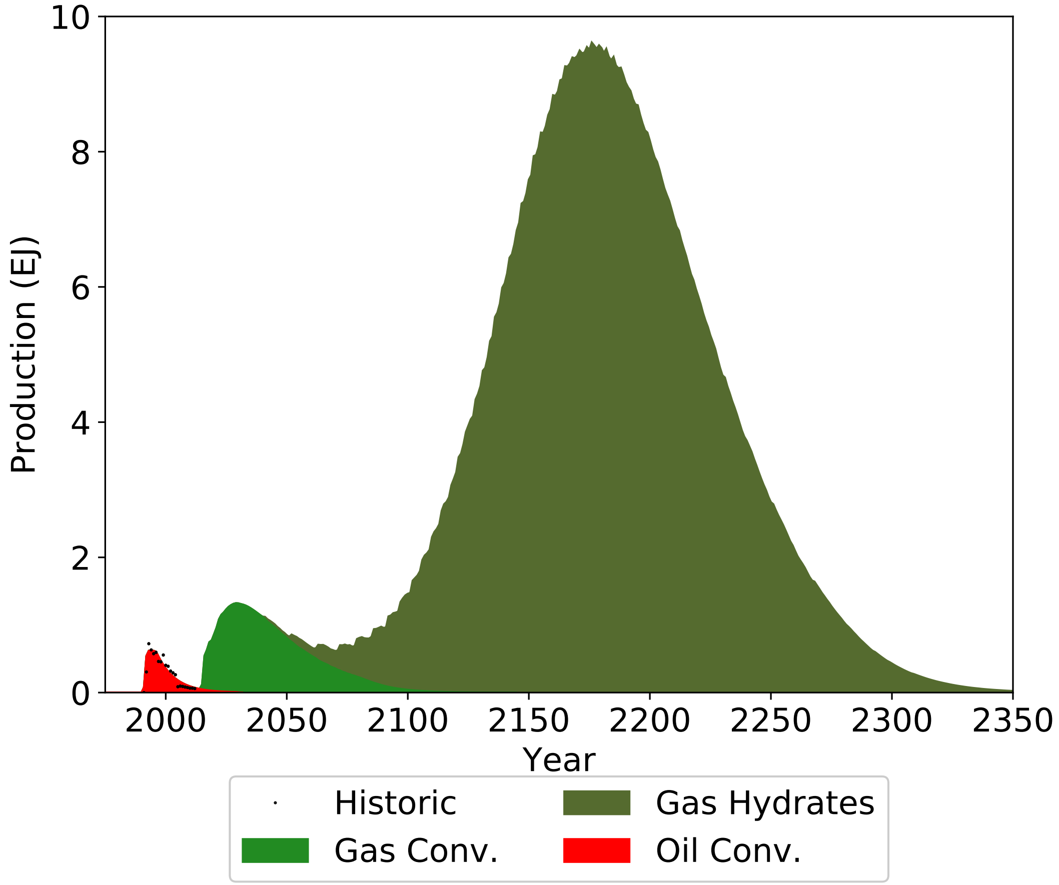

Figure 2.128: PNG projection by mineral type

Table 2.128: Peak years - Minerals

**Name**	**URR**	**Peak Year**	**Peak Rate**
Oil Conv.	7.27	1993	0.62
Gas Conv.	53.55	2030	1.32
Gas Hydrates	1026.5	2176	9.62
**Total**	**1087.32**	**2176**	**9.62**

2.21 Pakistan

2.21.1 All Projections

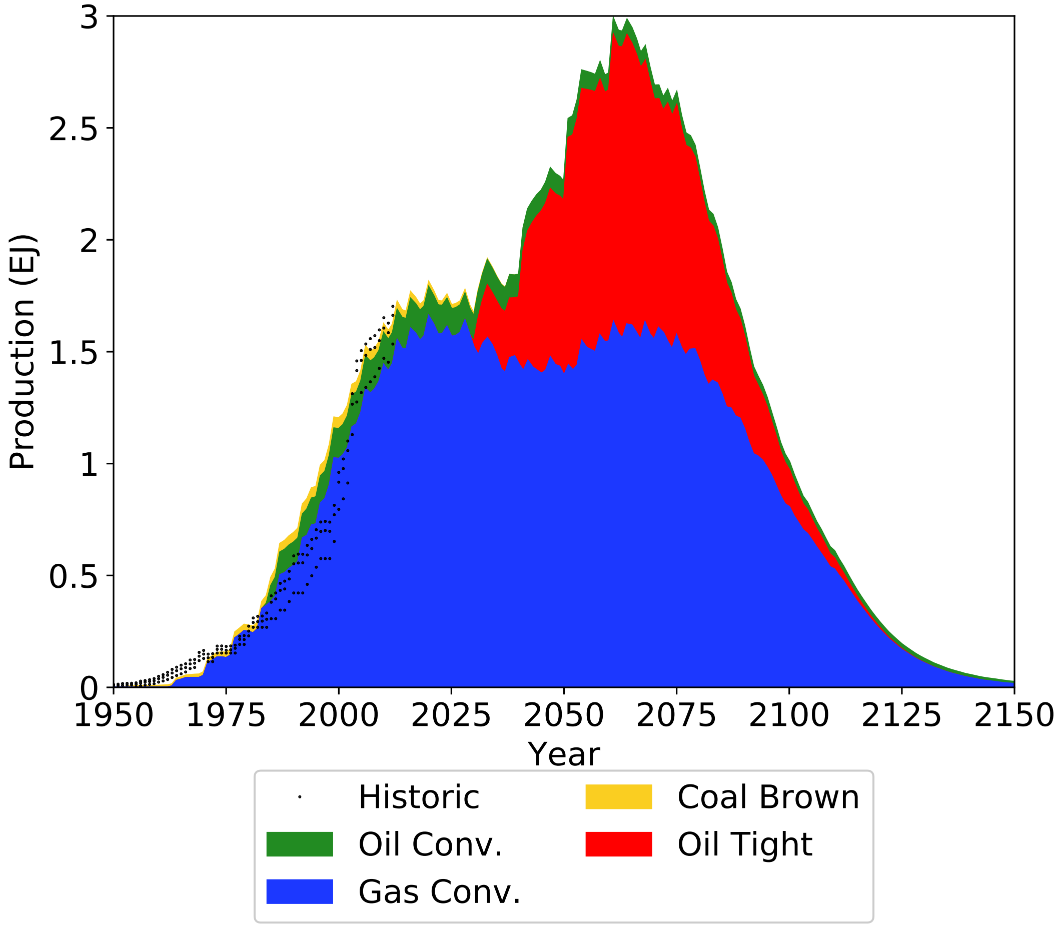

Figure 2.129: Pakistan projections capped at 16

Table 2.129: Peak years - All

**Name**	**URR**	**Peak Year**	**Peak Rate**
Gas Conv.	168.3	2020	1.66
Oil Tight	52.14	2063	1.3
Oil Conv.	12.08	2006	0.14
Coal Brown	2.2	1999	0.05
**Total**	**234.72**	**2061**	**2.98**

2.21.2 By Mineral

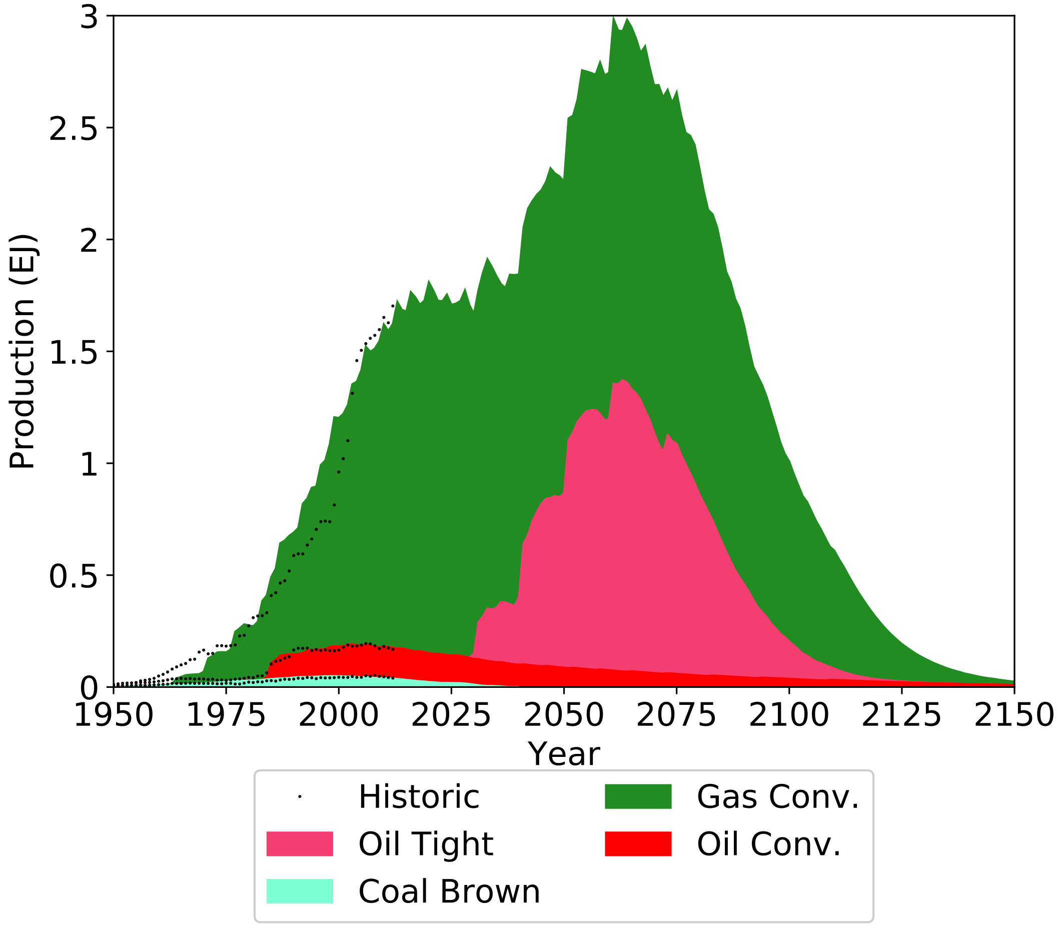

Figure 2.130: Pakistan projection by mineral type

Table 2.130: Peak years - Minerals

**Name**	**URR**	**Peak Year**	**Peak Rate**
Coal Brown	2.2	1999	0.05
Oil Conv.	12.08	2006	0.14
Oil Tight	52.14	2063	1.3
Gas Conv.	168.3	2020	1.66
**Total**	**234.72**	**2061**	**2.98**

2.22 Philippines

2.22.1 All Projections

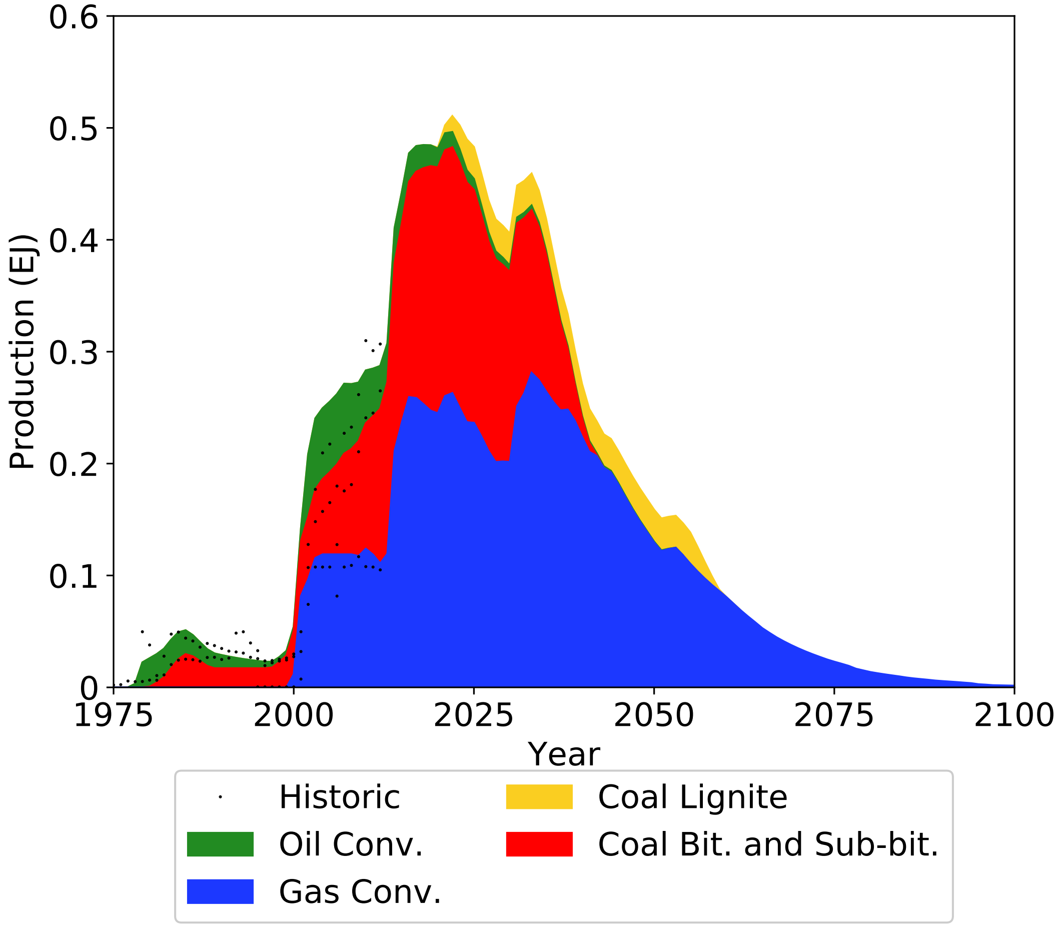

Figure 2.131: Philippines projections capped at 16

Table 2.131: Peak years - All

Name	URR	Peak Year	Peak Rate
Gas Conv.	11.7	2033	0.28
Coal Bit. and Sub-bit.	6.0	2020	0.22
Oil Conv.	1.28	2003	0.06
Coal Lignite	1.0	2025	0.03
**Total**	**19.98**	**2022**	**0.51**

2.22.2 By Mineral

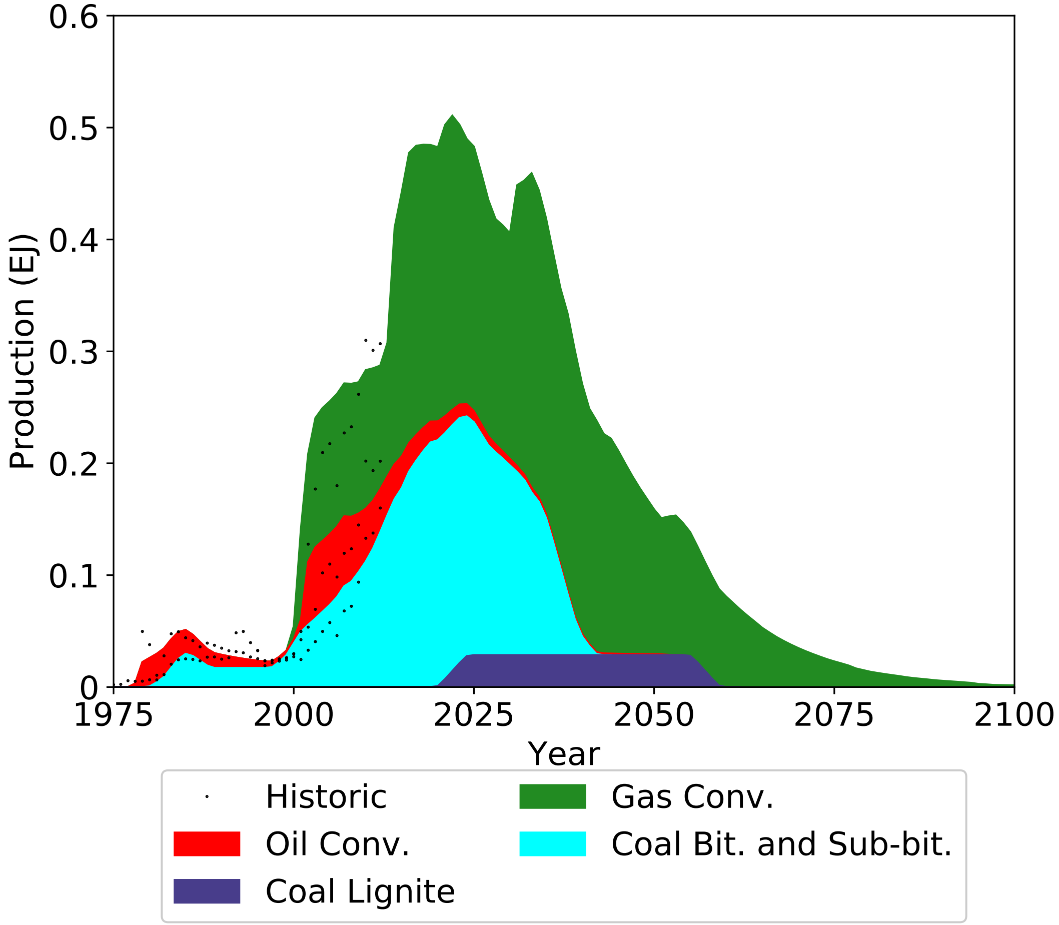

Figure 2.132: Philippines projection by mineral type

Table 2.132: Peak years - Minerals

**Name**	**URR**	**Peak Year**	**Peak Rate**
Coal Lignite	1.0	2025	0.03
Coal Bit. and Sub-bit.	6.0	2020	0.22
Oil Conv.	1.28	2003	0.06
Gas Conv.	11.7	2033	0.28
**Total**	**19.98**	**2022**	**0.51**

2.23 South Korea

2.23.1 All Projections

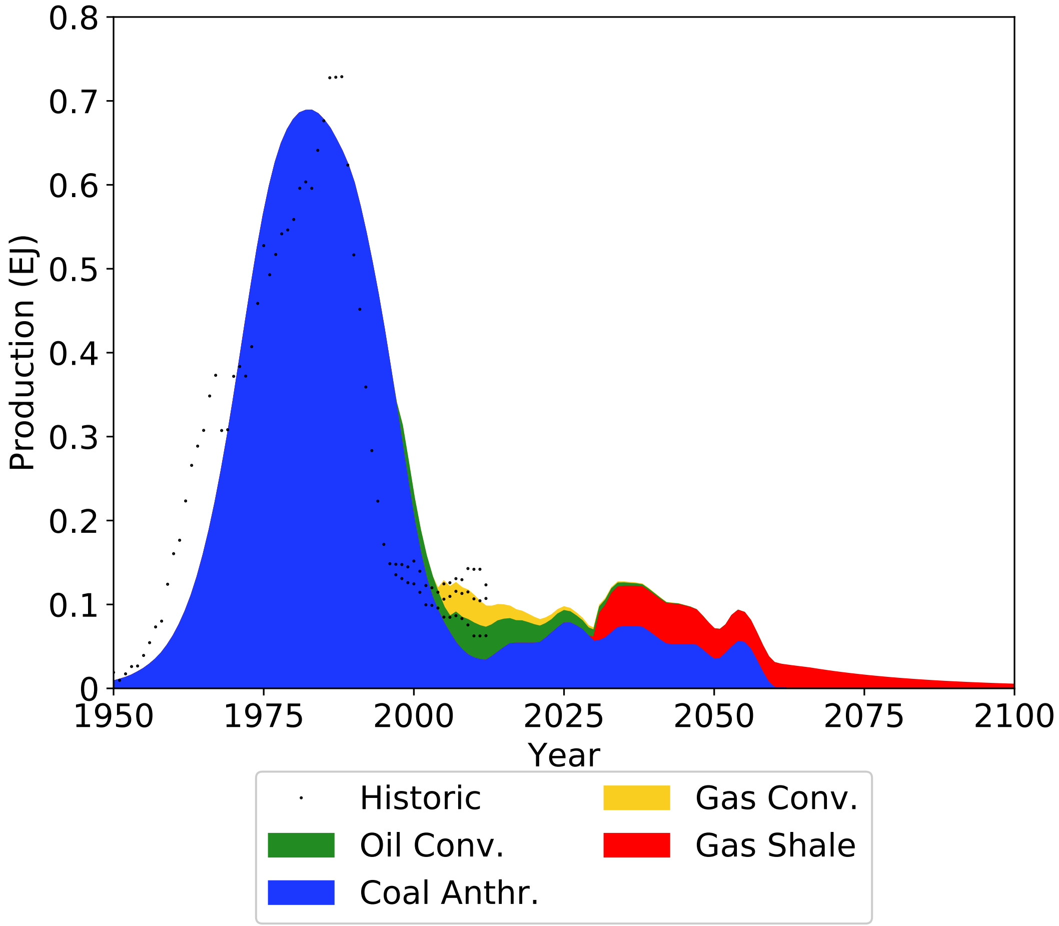

Figure 2.133: South Korea projections capped at 16

Table 2.133: Peak years - All

**Name**	**URR**	**Peak Year**	**Peak Rate**
Coal Anthr.	21.87	1983	0.69
Gas Shale	1.85	2034	0.05
Oil Conv.	0.88	2009	0.04
Gas Conv.	0.44	2006	0.04
**Total**	**25.04**	**1983**	**0.69**

2.23.2 By Mineral

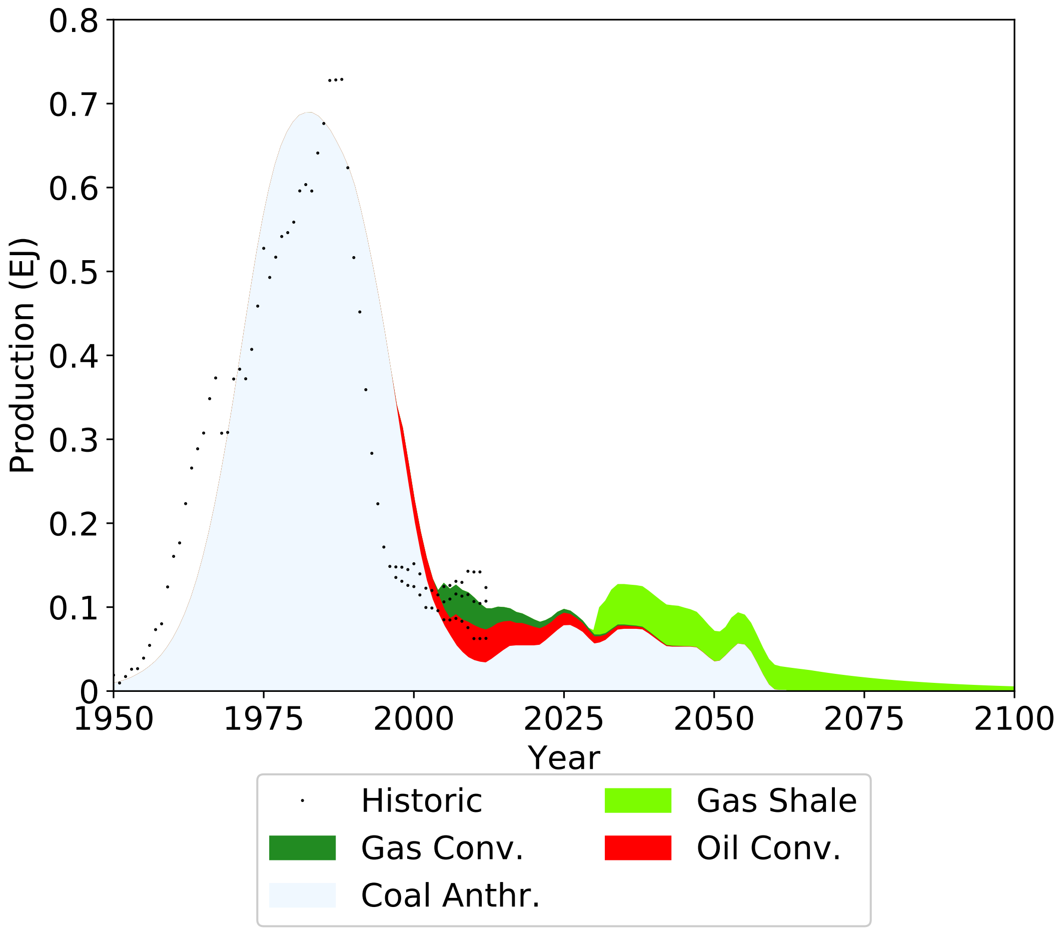

Figure 2.134: South Korea projection by mineral type

Table 2.134: Peak years - Minerals

**Name**	**URR**	**Peak Year**	**Peak Rate**
Coal Anthr.	21.87	1983	0.69
Oil Conv.	0.88	2009	0.04
Gas Conv.	0.44	2006	0.04
Gas Shale	1.85	2034	0.05
**Total**	**25.04**	**1983**	**0.69**

2.24 Sri Lanka

2.24.1 All Projections

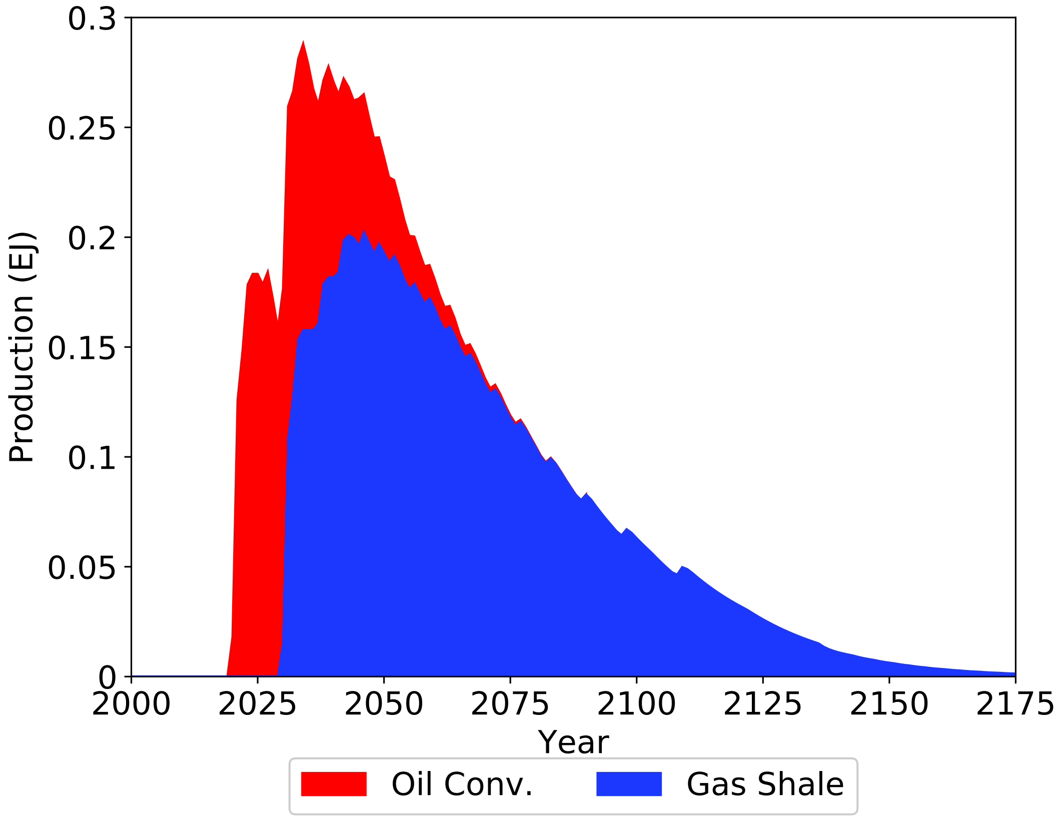

Figure 2.135: Sri Lanka projections capped at 16

Table 2.135: Peak years - All

Name	URR	Peak Year	Peak Rate
Gas Shale	11.12	2046	0.2
Oil Conv.	3.79	2027	0.18
**Total**	**14.91**	**2034**	**0.29**

2.24.2 By Mineral

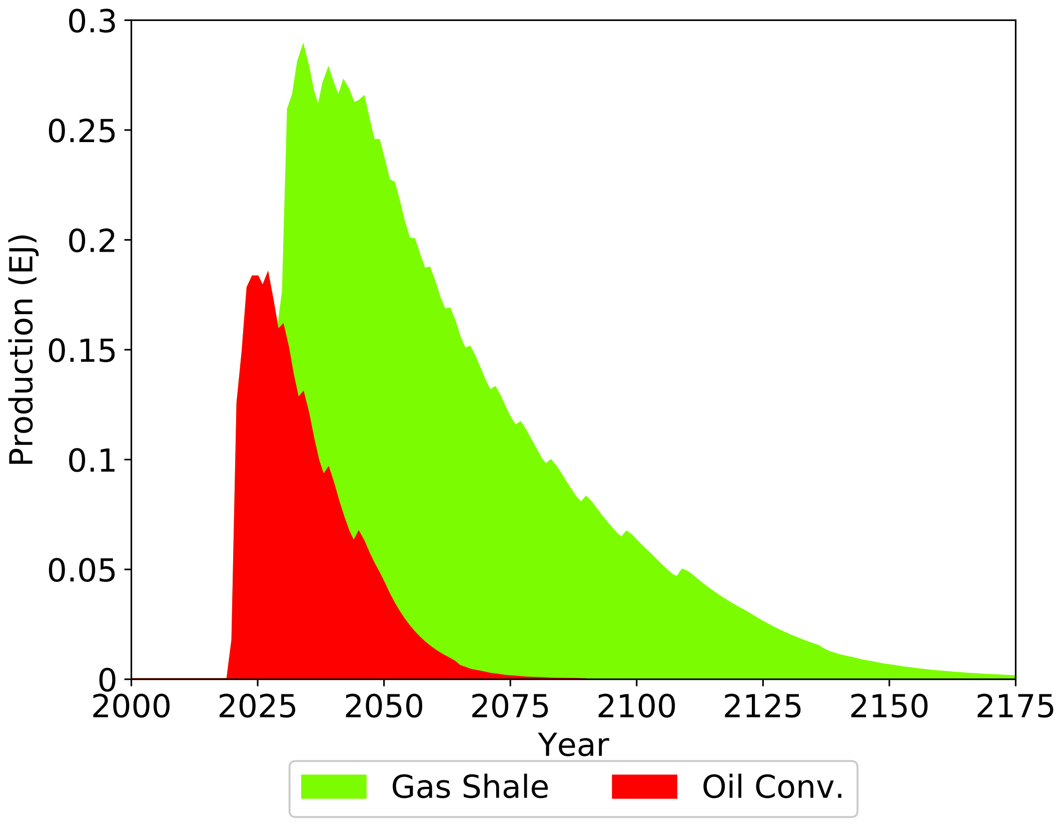

Figure 2.136: Sri Lanka projection by mineral type

Table 2.136: Peak years - Minerals

Name	URR	Peak Year	Peak Rate
Oil Conv.	3.79	2027	0.18
Gas Shale	11.12	2046	0.2
**Total**	**14.91**	**2034**	**0.29**

2.25 Taiwan

2.25.1 All Projections

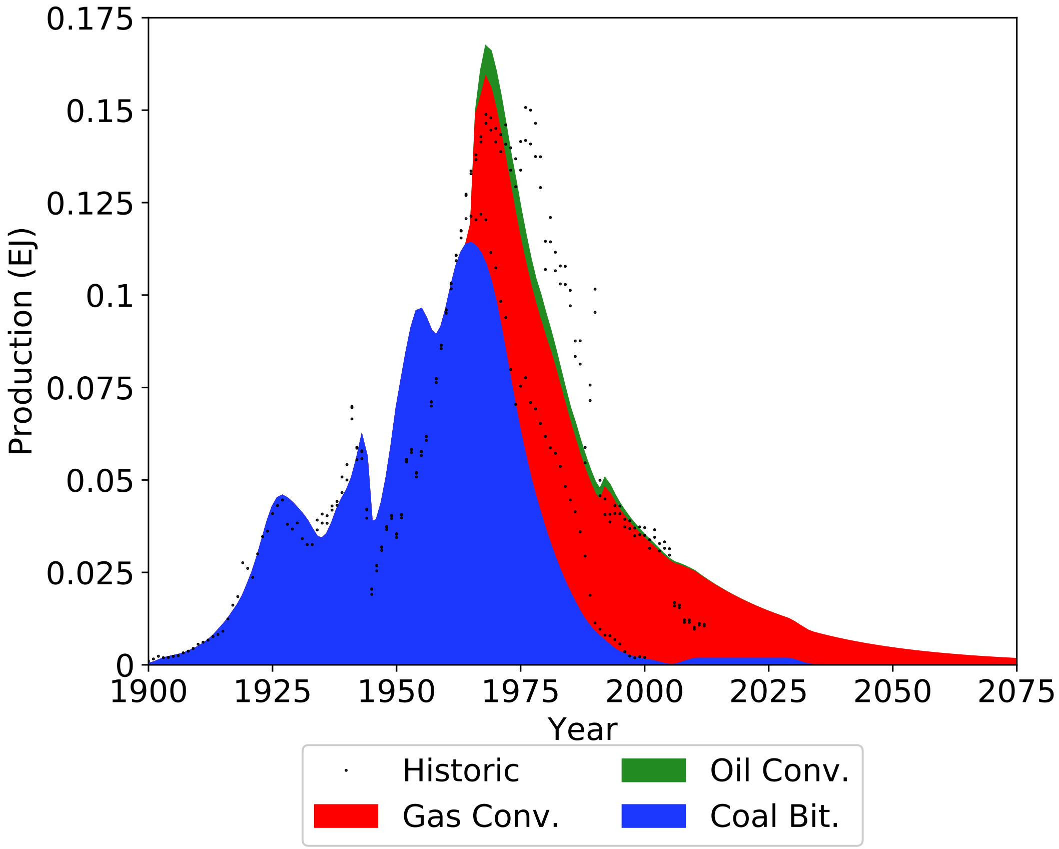

Figure 2.137: Taiwan projections capped at 16

Table 2.137: Peak years - All

Name	URR	Peak Year	Peak Rate
Coal Bit.	4.34	1965	0.11
Gas Conv.	2.4	1969	0.05
Oil Conv.	0.19	1970	0.01
**Total**	**6.93**	**1968**	**0.17**

2.25.2 By Mineral

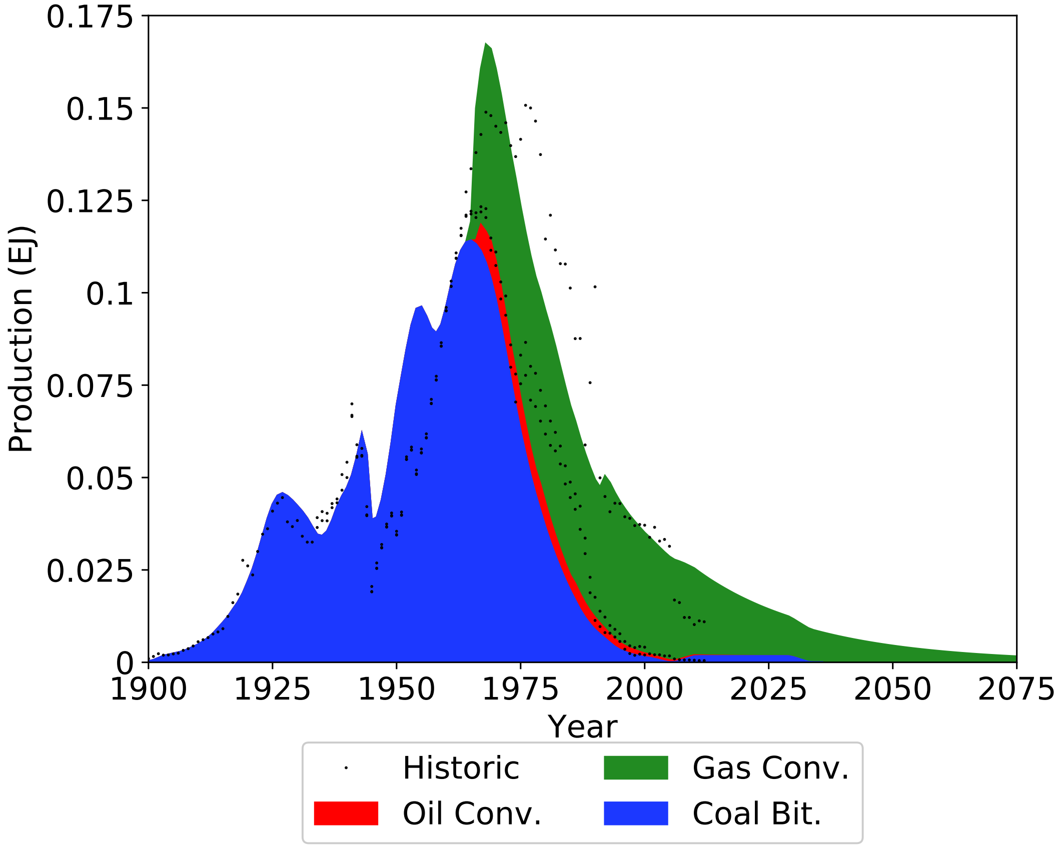

Figure 2.138: Taiwan projection by mineral type

Table 2.138: Peak years - Minerals

Name	URR	Peak Year	Peak Rate
Coal Bit.	4.34	1965	0.11
Oil Conv.	0.19	1970	0.01
Gas Conv.	2.4	1969	0.05
**Total**	**6.93**	**1968**	**0.17**

2.26 Thailand

2.26.1 All Projections

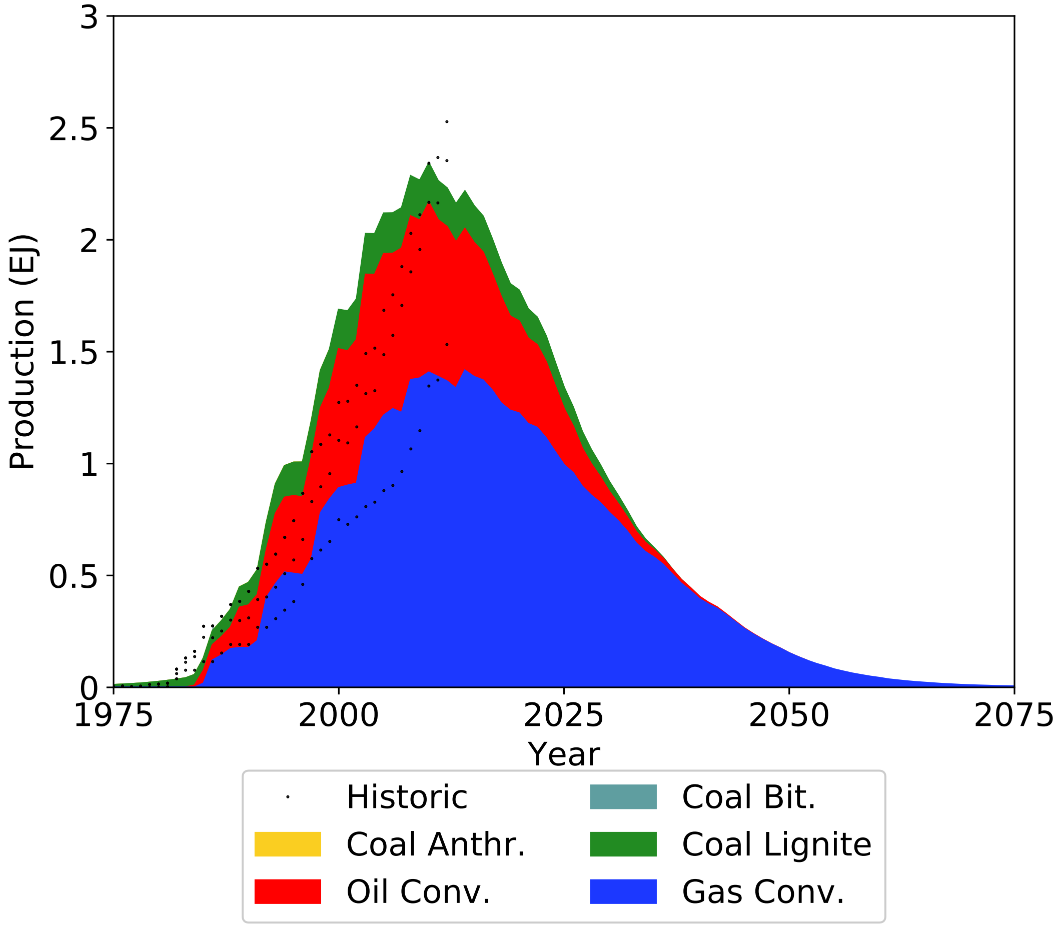

Figure 2.139: Thailand projections capped at 16

Table 2.139: Peak years - All

**Name**	**URR**	**Peak Year**	**Peak Rate**
Gas Conv.	50.8	2014	1.42
Oil Conv.	19.93	2010	0.76
Coal Lignite	6.66	2004	0.18
Coal Anthr.	–	1991	–
Coal Bit.	–	1982	–
**Total**	**77.39**	**2010**	**2.34**

2.26.2 By Mineral

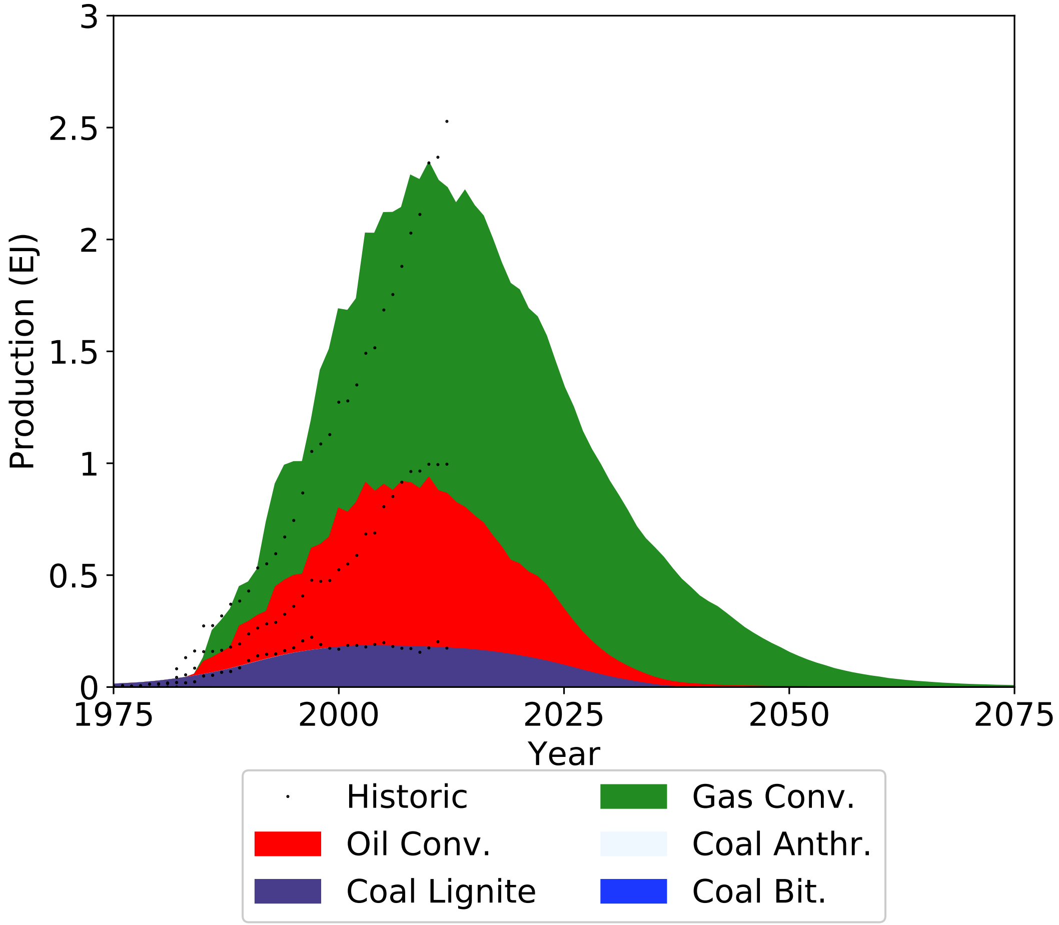

Figure 2.140: Thailand projection by mineral type

Table 2.140: Peak years - Minerals

**Name**	**URR**	**Peak Year**	**Peak Rate**
Coal Bit.	–	1982	–
Coal Lignite	6.66	2004	0.18
Coal Anthr.	–	1991	–
Oil Conv.	19.93	2010	0.76
Gas Conv.	50.8	2014	1.42
**Total**	**77.39**	**2010**	**2.34**

2.27 Vietnam

2.27.1 All Projections

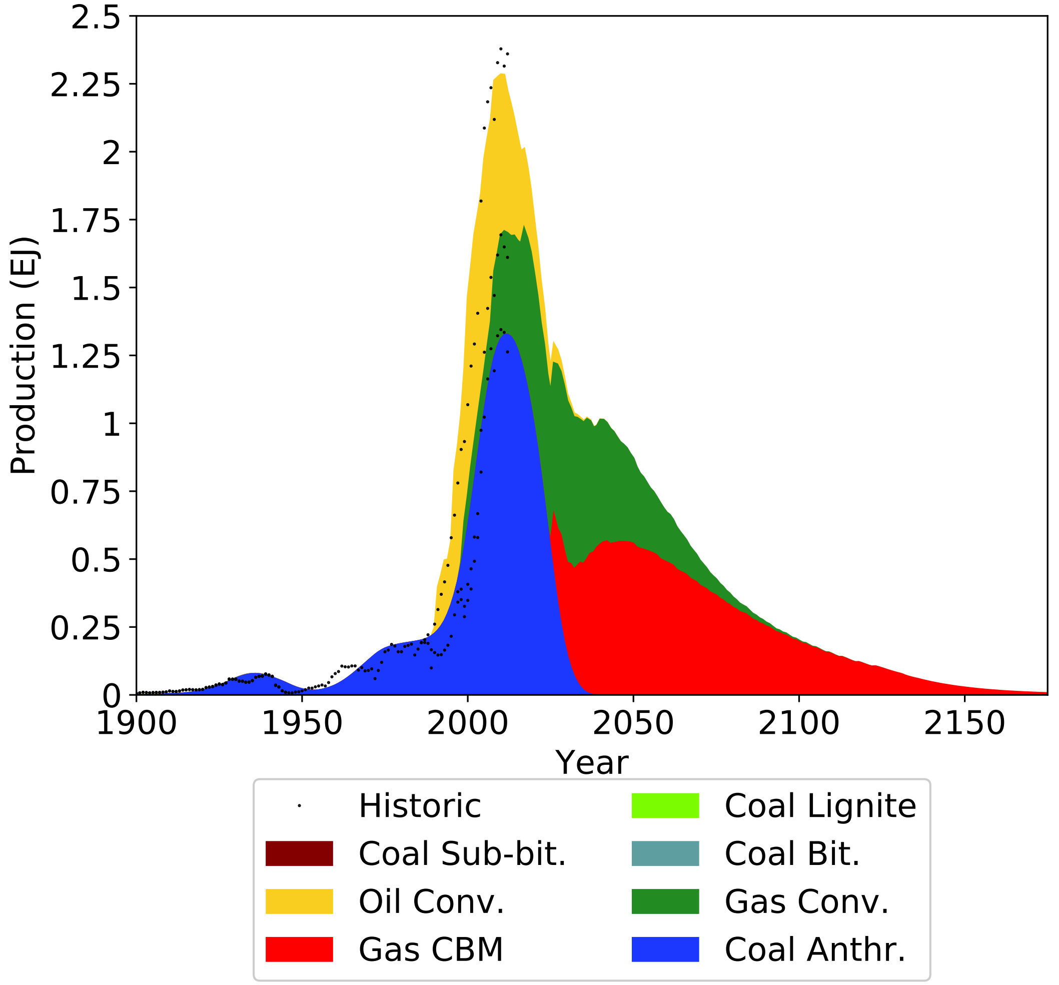

Figure 2.141: Vietnam projections capped at 16

Table 2.141: Peak years - All

**Name**	**URR**	**Peak Year**	**Peak Rate**
Coal Anthr.	38.0	2012	1.33
Gas CBM	35.5	2042	0.57
Gas Conv.	26.3	2029	0.61
Oil Conv.	15.76	2005	0.78
Coal Bit.	0.03	1914	–
Coal Sub-bit.	0.01	1931	–
Coal Lignite	–	1941	–
**Total**	**115.6**	**2010**	**2.28**

2.27.2 By Mineral

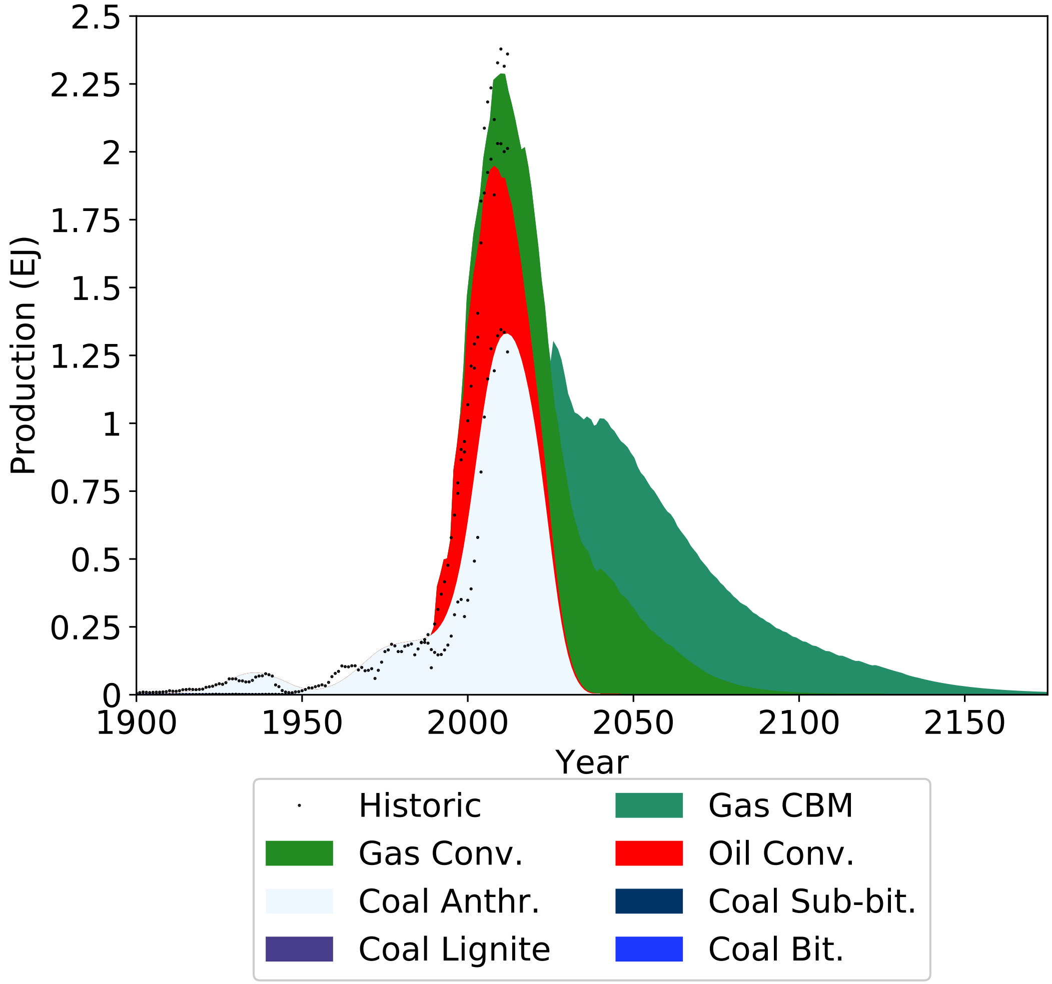

Figure 2.142: Vietnam projection by mineral type

Table 2.142: Peak years - Minerals

**Name**	**URR**	**Peak Year**	**Peak Rate**
Coal Bit.	0.03	1914	–
Coal Lignite	–	1941	–
Coal Sub-bit.	0.01	1931	–
Coal Anthr.	38.0	2012	1.33
Oil Conv.	15.76	2005	0.78
Gas Conv.	26.3	2029	0.61
Gas CBM	35.5	2042	0.57
**Total**	**115.6**	**2010**	**2.28**

2.28 Total

2.28.1 By country

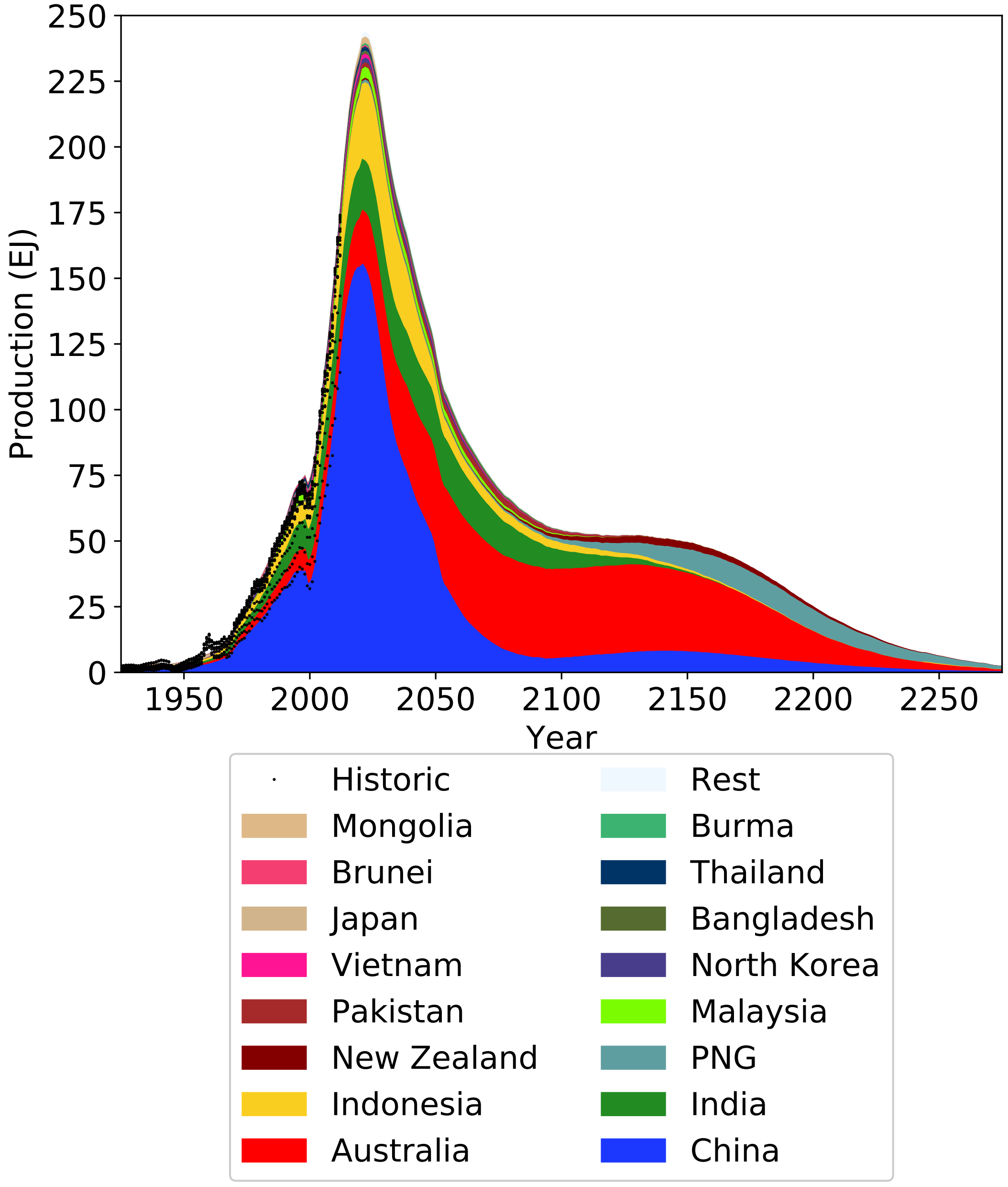

Figure 2.143: Asia projections by country

Table 2.143: Peak years - All

**Name**	**URR**	**Peak Year**	**Peak Rate**
China	7043.41	2021	155.03
Australia	6192.26	2061	37.62
India	1970.49	2033	20.68
Indonesia	1504.04	2027	32.36
PNG	1087.32	2176	9.62
New Zealand	301.78	2142	2.73
Malaysia	297.32	2025	4.15
Pakistan	234.72	2061	2.98
North Korea	146.97	2036	2.34
Vietnam	115.6	2010	2.28
Bangladesh	92.46	2039	1.44
Japan	82.91	1953	1.21
Thailand	77.39	2010	2.34
Brunei	58.13	2002	0.89
Burma	56.71	2033	0.88
Mongolia	48.62	2022	2.48
Afghanistan	38.3	2030	1.06
South Korea	25.04	1983	0.69
Philippines	19.98	2022	0.51
East Timor	17.87	2042	0.29
Sri Lanka	14.91	2034	0.29
Taiwan	6.93	1968	0.17
Laos	6.34	2042	0.15
Cambodia	2.95	2024	0.16
Bhutan	0.15	2017	–
Nepal	0.01	2018	–
New Caledonia	–	1928	–
**Total**	**19442.63**	**2022**	**243.41**

2.28.2 By mineral

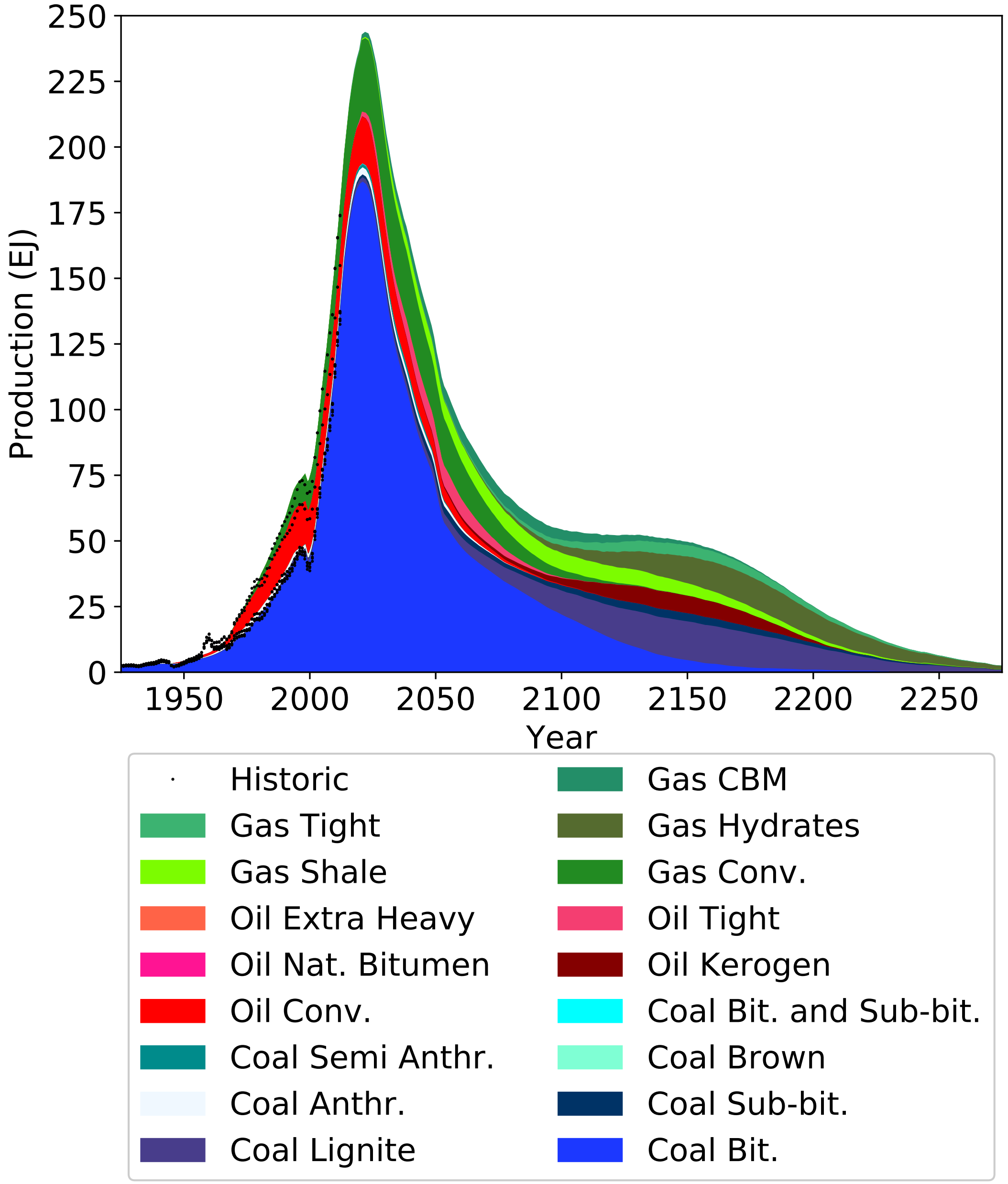

Figure 2.144: Asia projection by mineral type

Table 2.144: Peak years - Minerals

**Name**	**URR**	**Peak Year**	**Peak Rate**
Coal Bit.	9647.47	2021	186.37
Coal Lignite	1944.85	2147	14.76
Coal Sub-bit.	490.06	2143	3.19
Coal Anthr.	202.18	2020	2.68
Coal Brown	2.2	1999	0.05
Coal Semi Anthr.	60.7	2033	1.9
Coal Bit. and Sub-bit.	6.0	2020	0.22
Oil Conv.	1172.95	2023	17.83
Oil Kerogen	576.01	2140	6.73
Oil Nat. Bitumen	4.17	2053	0.12
Oil Tight	336.36	2043	7.67
Oil Extra Heavy	5.01	2030	0.15
Gas Conv.	1803.52	2028	29.36
Gas Shale	914.34	2064	6.89
Gas Hydrates	1293.0	2168	11.58
Gas Tight	473.39	2143	4.37
Gas CBM	510.42	2065	5.07
**Total**	**19442.63**	**2022**	**243.41**

Chapter 3

Europe

3.1 Albania

3.1.1 All Projections

Table 3.1: Peak years - All

Name	URR	Peak Year	Peak Rate
Oil Conv.	5.01	1978	0.09
Gas Conv.	1.4	2019	0.06
Coal Lignite	0.38	1983	0.02
Gas Shale	0.37	2032	0.01
**Total**	**7.16**	**2019**	**0.14**

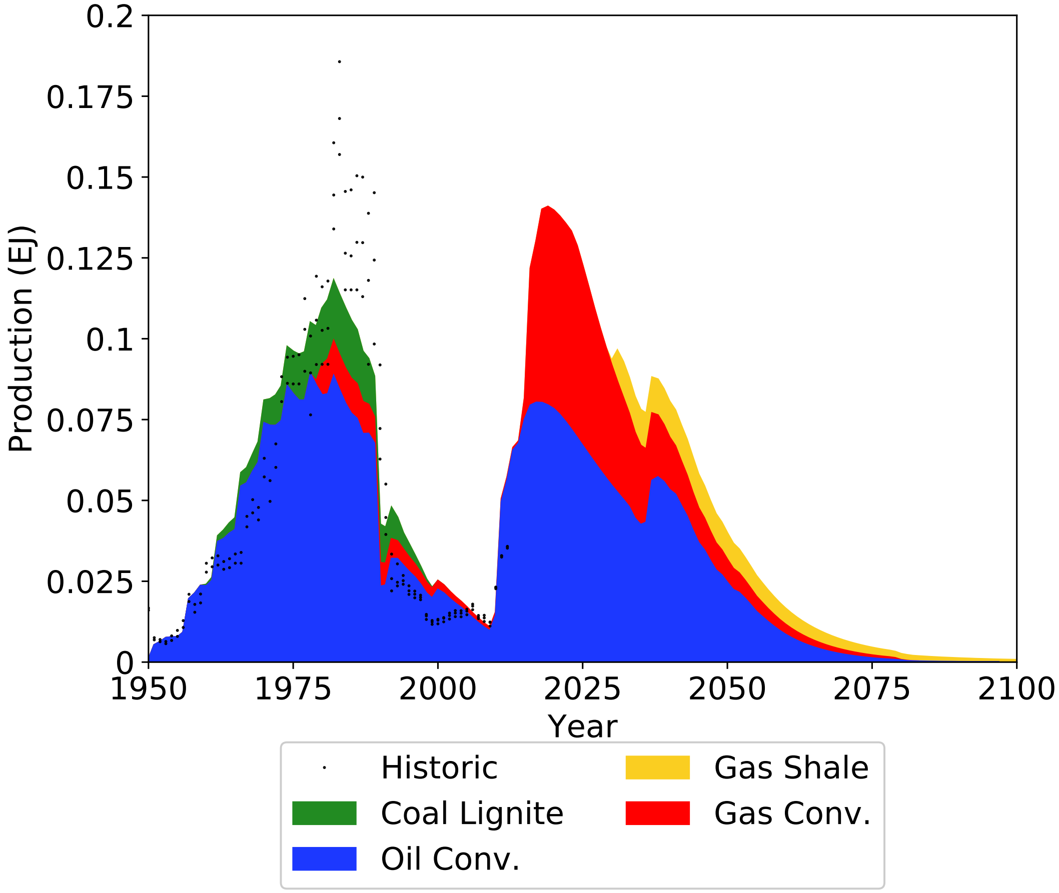

Figure 3.1: Albania projections capped at 16

3.1.2 By Mineral

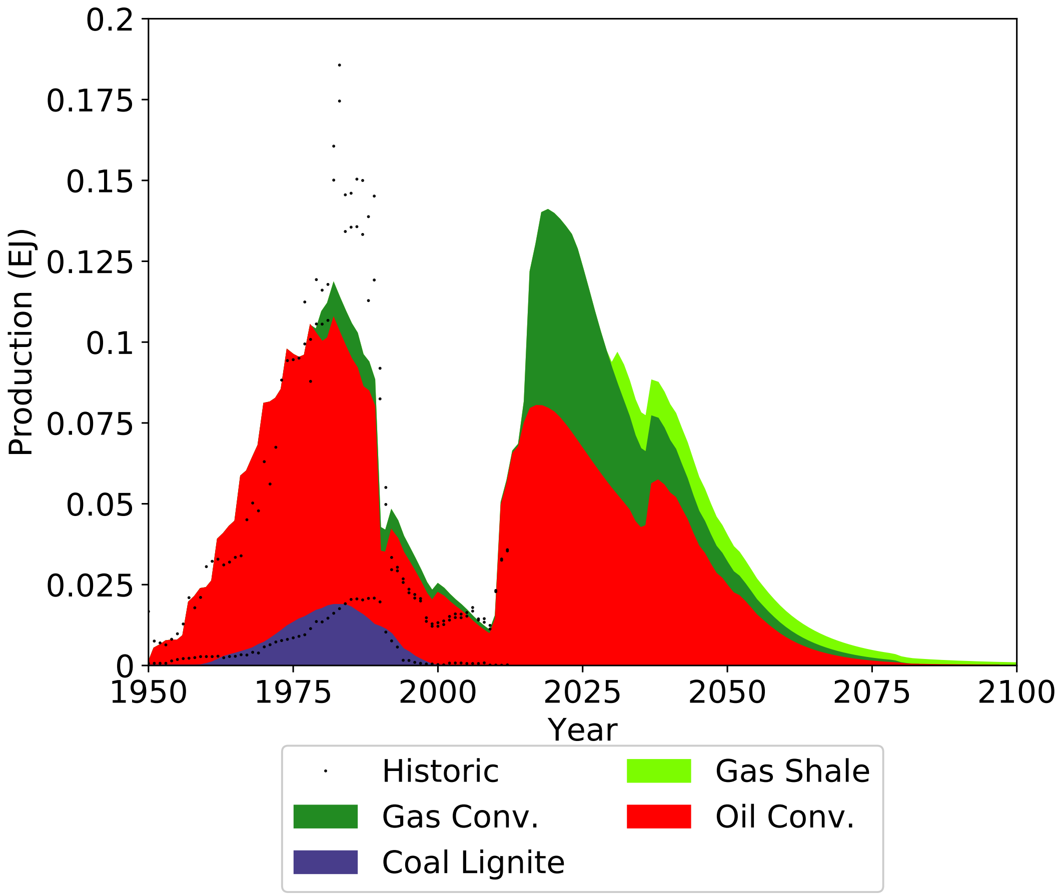

Figure 3.2: Albania projection by mineral type

Table 3.2: Peak years - Minerals

Name	URR	Peak Year	Peak Rate
Coal Lignite	0.38	1983	0.02
Oil Conv.	5.01	1978	0.09
Gas Conv.	1.4	2019	0.06
Gas Shale	0.37	2032	0.01
**Total**	**7.16**	**2019**	**0.14**

3.2 Austria

3.2.1 All Projections

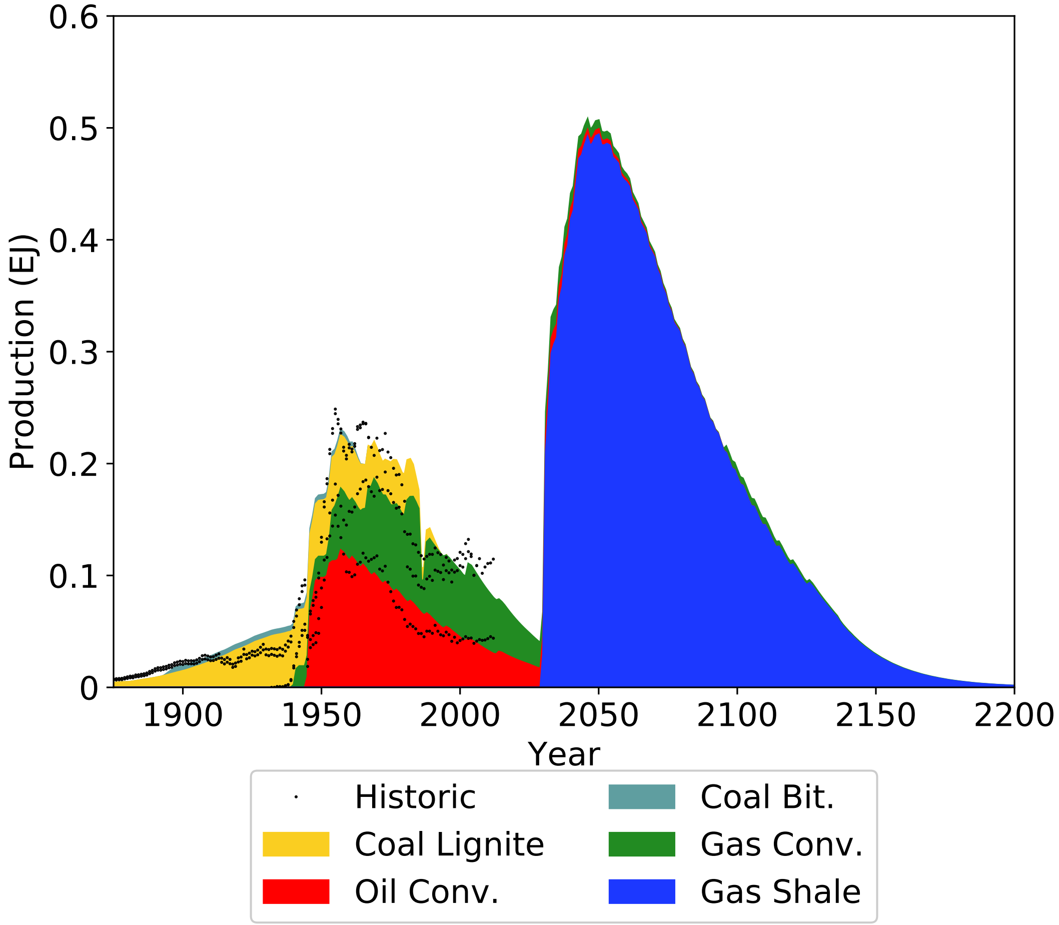

Figure 3.3: Austria projections capped at 16

Table 3.3: Peak years - All

**Name**	**URR**	**Peak Year**	**Peak Rate**
Gas Shale	30.41	2050	0.49
Oil Conv.	5.86	1957	0.12
Gas Conv.	5.3	1983	0.1
Coal Lignite	3.54	1944	0.05
Coal Bit.	0.32	1898	–
**Total**	**45.43**	**2046**	**0.51**

3.2.2 By Mineral

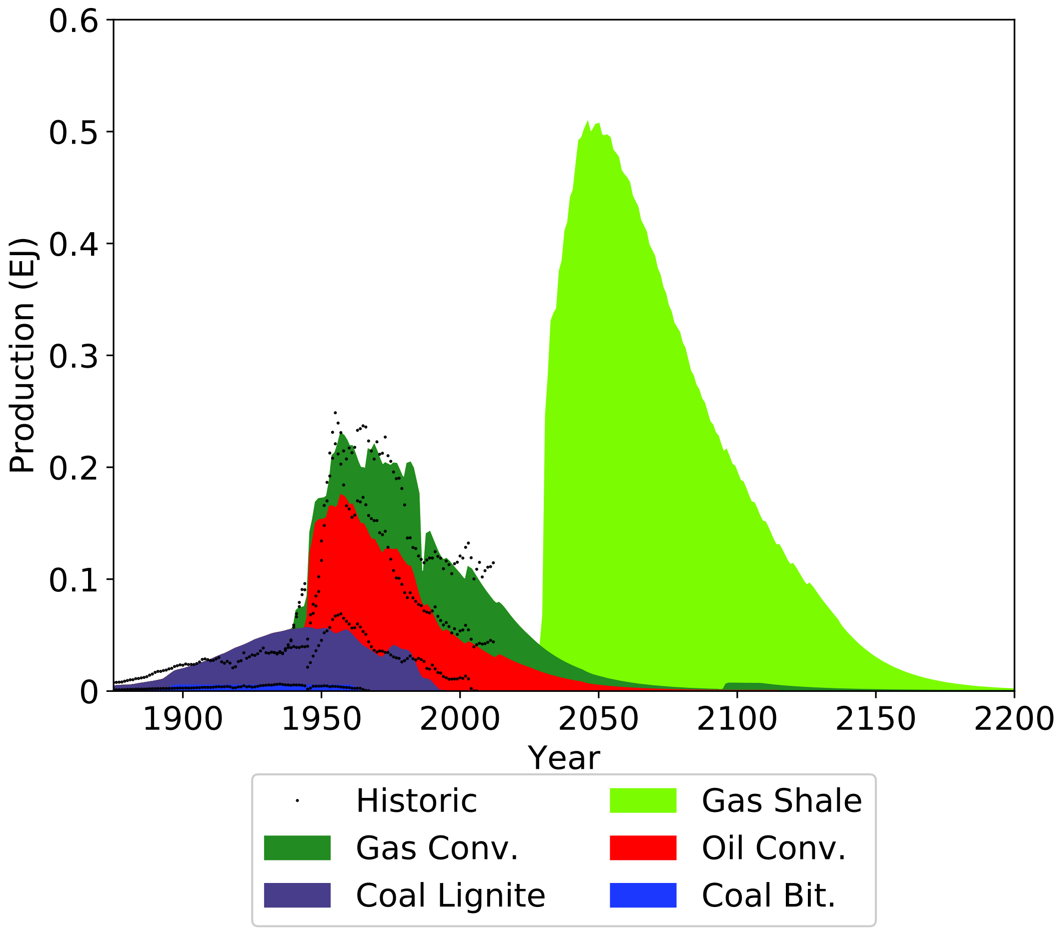

Figure 3.4: Austria projection by mineral type

Table 3.4: Peak years - Minerals

**Name**	**URR**	**Peak Year**	**Peak Rate**
Coal Bit.	0.32	1898	–
Coal Lignite	3.54	1944	0.05
Oil Conv.	5.86	1957	0.12
Gas Conv.	5.3	1983	0.1
Gas Shale	30.41	2050	0.49
**Total**	**45.43**	**2046**	**0.51**

3.3 Belgium

3.3.1 All Projections

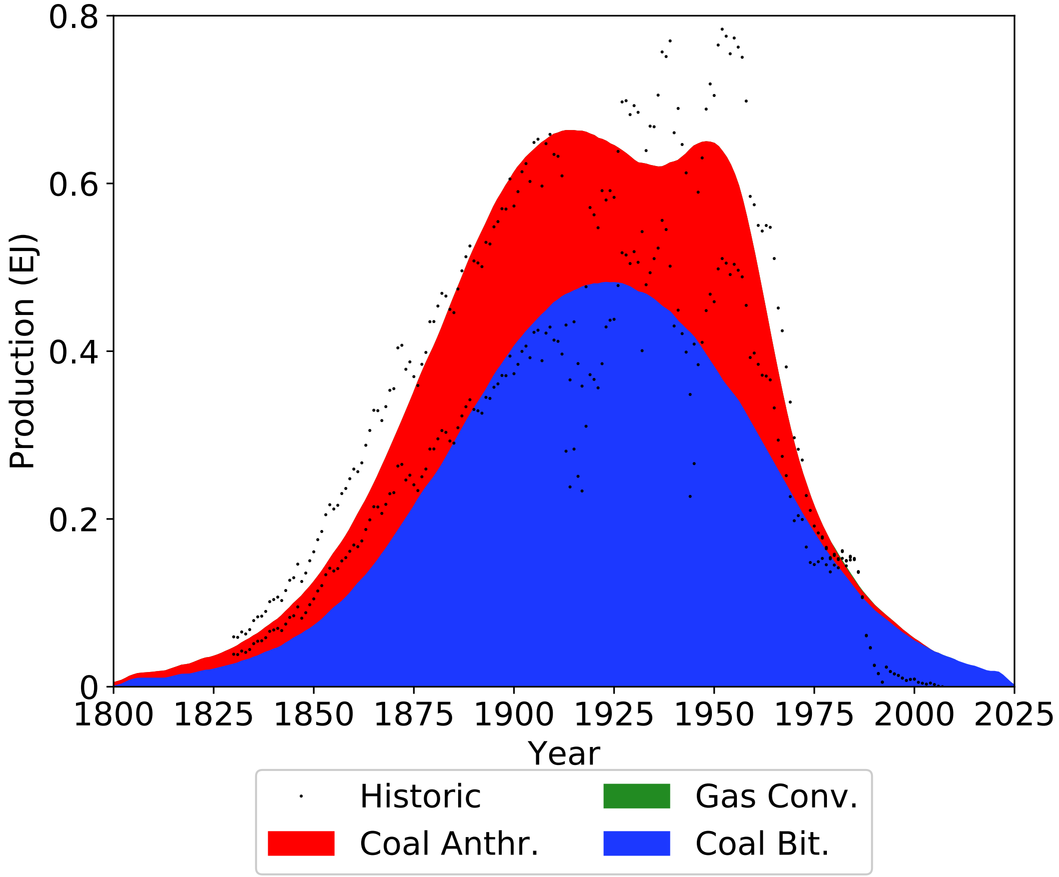

Figure 3.5: Belgium projections capped at 16

Table 3.5: Peak years - All

Name	URR	Peak Year	Peak Rate
Coal Bit.	45.5	1923	0.48
Coal Anthr.	21.6	1952	0.27
Gas Conv.	0.02	1982	–
**Total**	**67.12**	**1913**	**0.66**

3.3.2 By Mineral

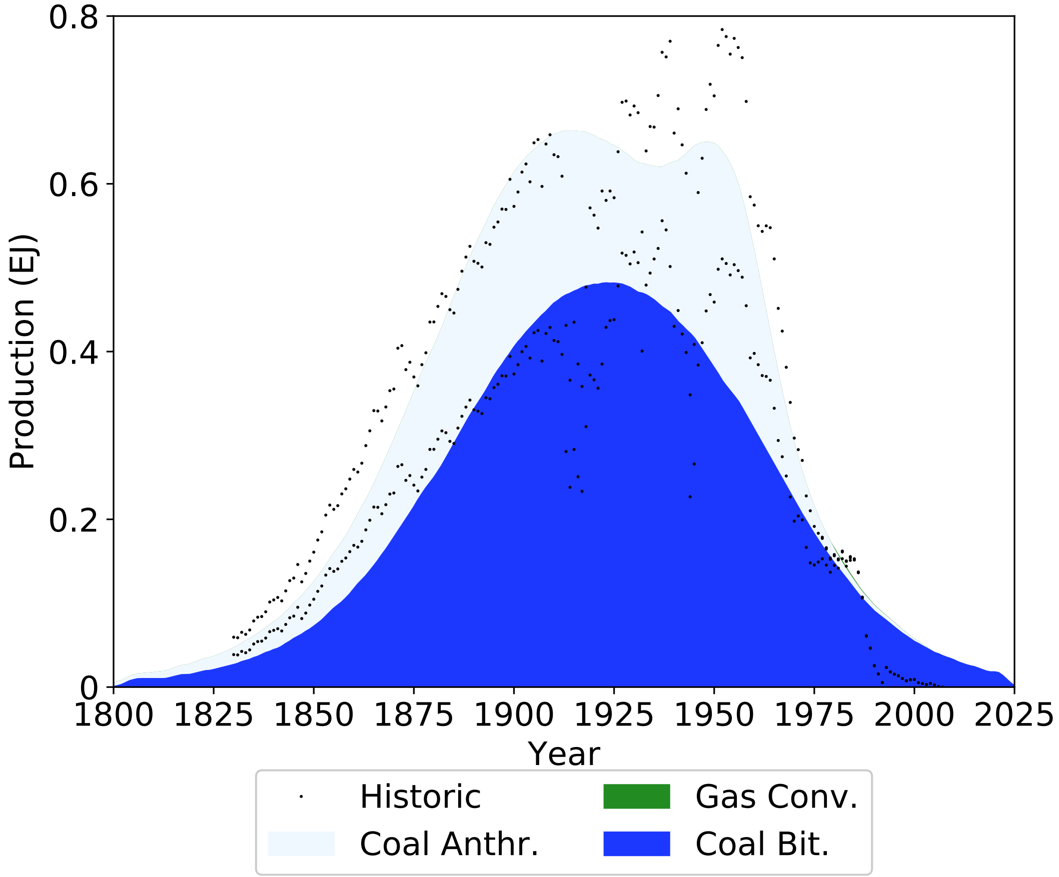

Figure 3.6: Belgium projection by mineral type

Table 3.6: Peak years - Minerals

Name	URR	Peak Year	Peak Rate
Coal Bit.	45.5	1923	0.48
Coal Anthr.	21.6	1952	0.27
Gas Conv.	0.02	1982	–
**Total**	**67.12**	**1913**	**0.66**

3.4 Bulgaria

3.4.1 All Projections

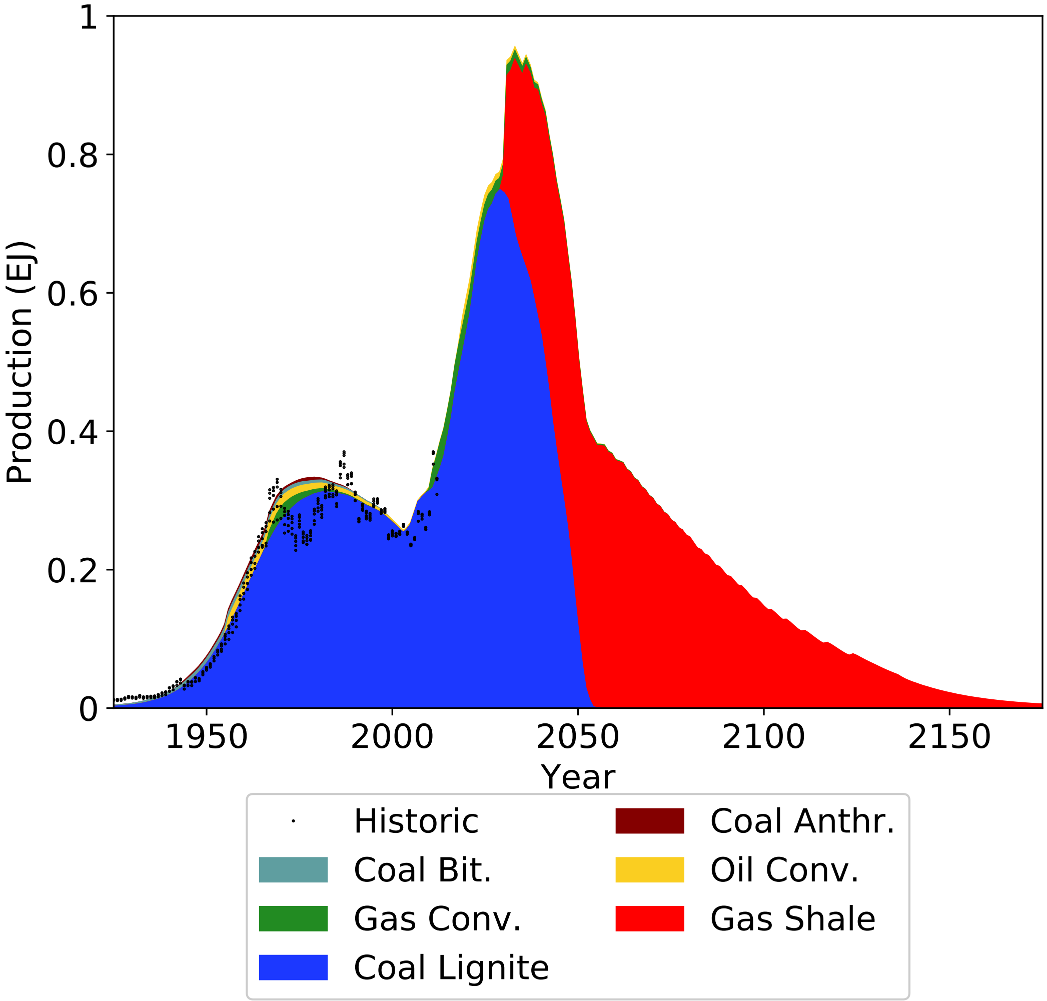

Figure 3.7: Bulgaria projections capped at 16

Table 3.7: Peak years - All

Name	URR	Peak Year	Peak Rate
Coal Lignite	36.3	2029	0.75
Gas Shale	24.29	2049	0.4
Gas Conv.	1.0	2014	0.04
Oil Conv.	0.7	2020	0.02
Coal Bit.	0.35	1953	0.01
Coal Anthr.	0.18	1964	0.01
**Total**	**62.82**	**2033**	**0.95**

3.4.2 By Mineral

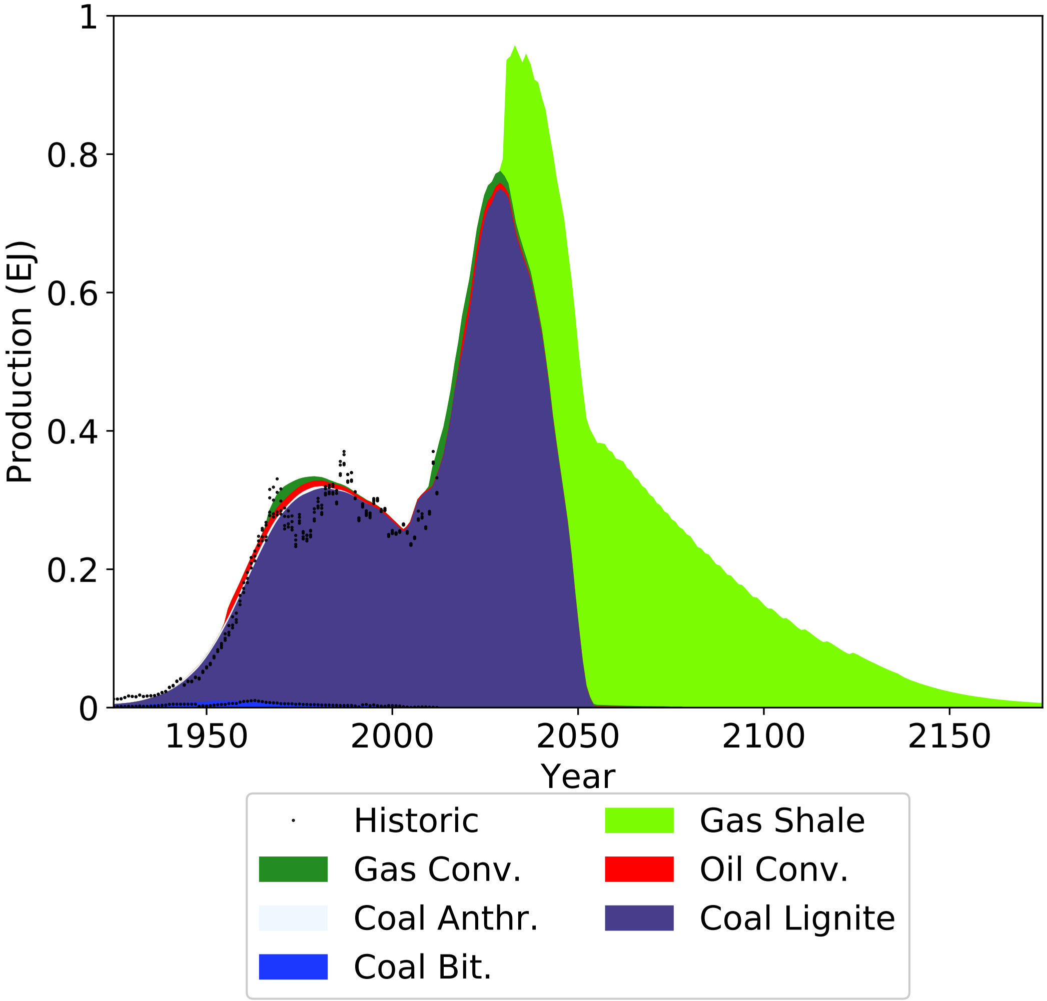

Figure 3.8: Bulgaria projection by mineral type

Table 3.8: Peak years - Minerals

**Name**	**URR**	**Peak Year**	**Peak Rate**
Coal Bit.	0.35	1953	0.01
Coal Lignite	36.3	2029	0.75
Coal Anthr.	0.18	1964	0.01
Oil Conv.	0.7	2020	0.02
Gas Conv.	1.0	2014	0.04
Gas Shale	24.29	2049	0.4
**Total**	**62.82**	**2033**	**0.95**

3.5 Cyprus

3.5.1 All Projections

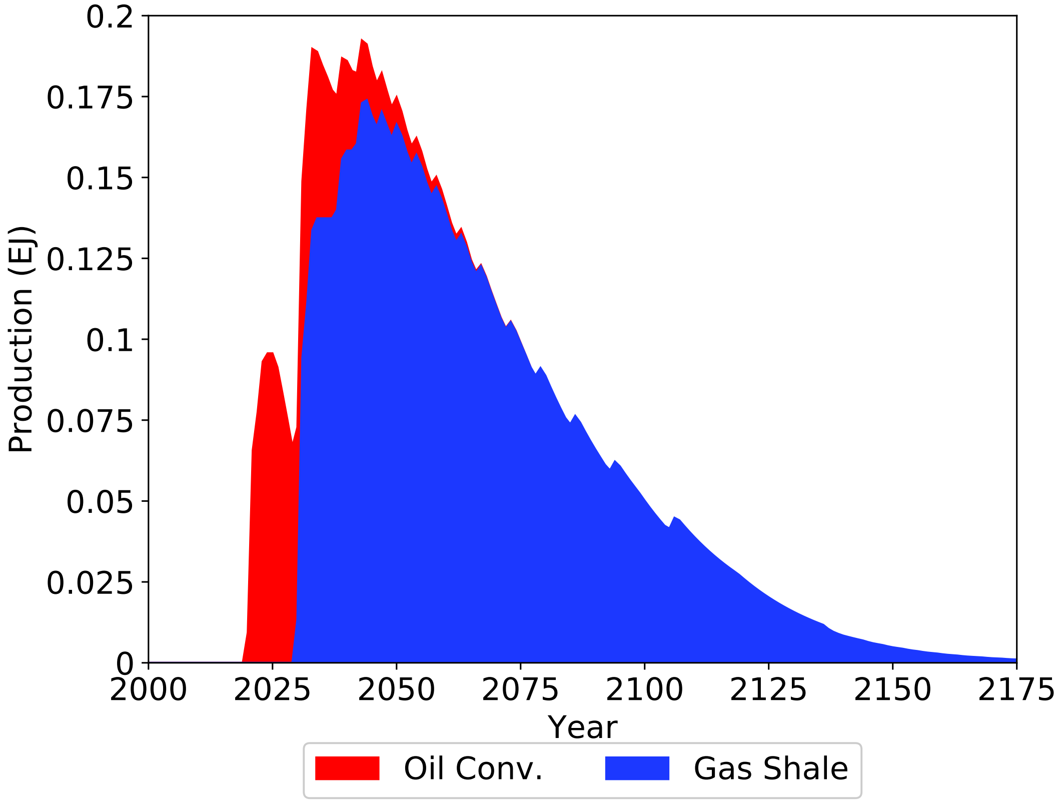

Figure 3.9: Cyprus projections capped at 16

Table 3.9: Peak years - All

**Name**	**URR**	**Peak Year**	**Peak Rate**
Gas Shale	9.27	2044	0.17
Oil Conv.	1.47	2024	0.1
**Total**	**10.74**	**2043**	**0.19**

3.5.2 By Mineral

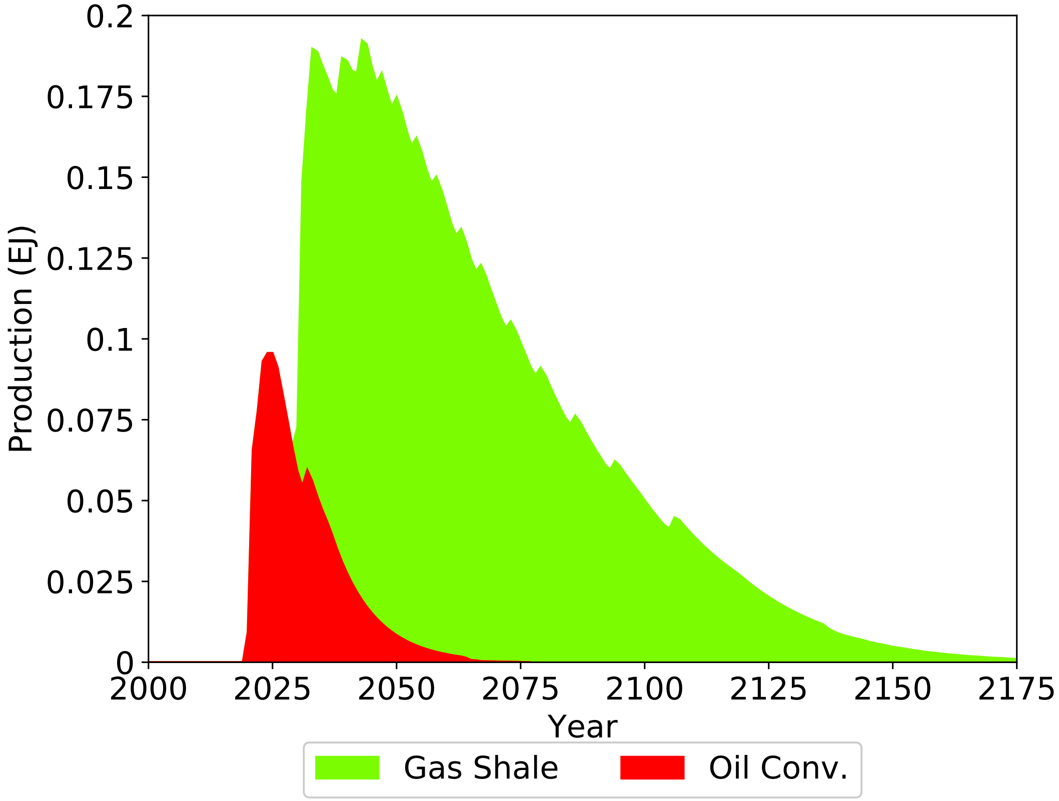

Figure 3.10: Cyprus projection by mineral type

Table 3.10: Peak years - Minerals

**Name**	**URR**	**Peak Year**	**Peak Rate**
Oil Conv.	1.47	2024	0.1
Gas Shale	9.27	2044	0.17
**Total**	**10.74**	**2043**	**0.19**

3.6 Czech Republic

3.6.1 All Projections

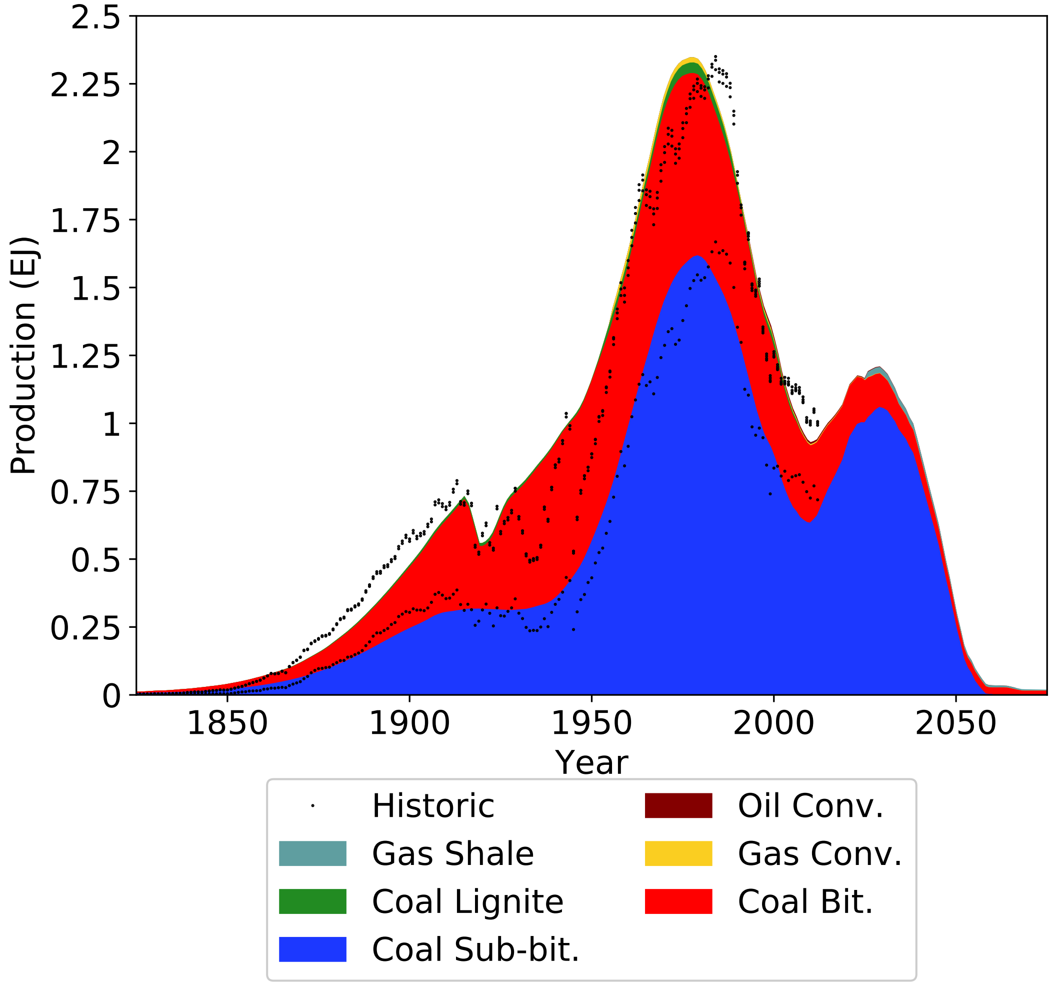

Figure 3.11: Czech Republic projections capped at 16

Table 3.11: Peak years - All

**Name**	**URR**	**Peak Year**	**Peak Rate**
Coal Sub-bit.	123.0	1979	1.61
Coal Bit.	64.86	1973	0.71
Coal Lignite	2.04	1973	0.04
Gas Conv.	0.91	1968	0.03
Gas Shale	0.74	2027	0.02
Oil Conv.	0.27	1999	0.01
**Total**	**191.82**	**1977**	**2.34**

3.6.2 By Mineral

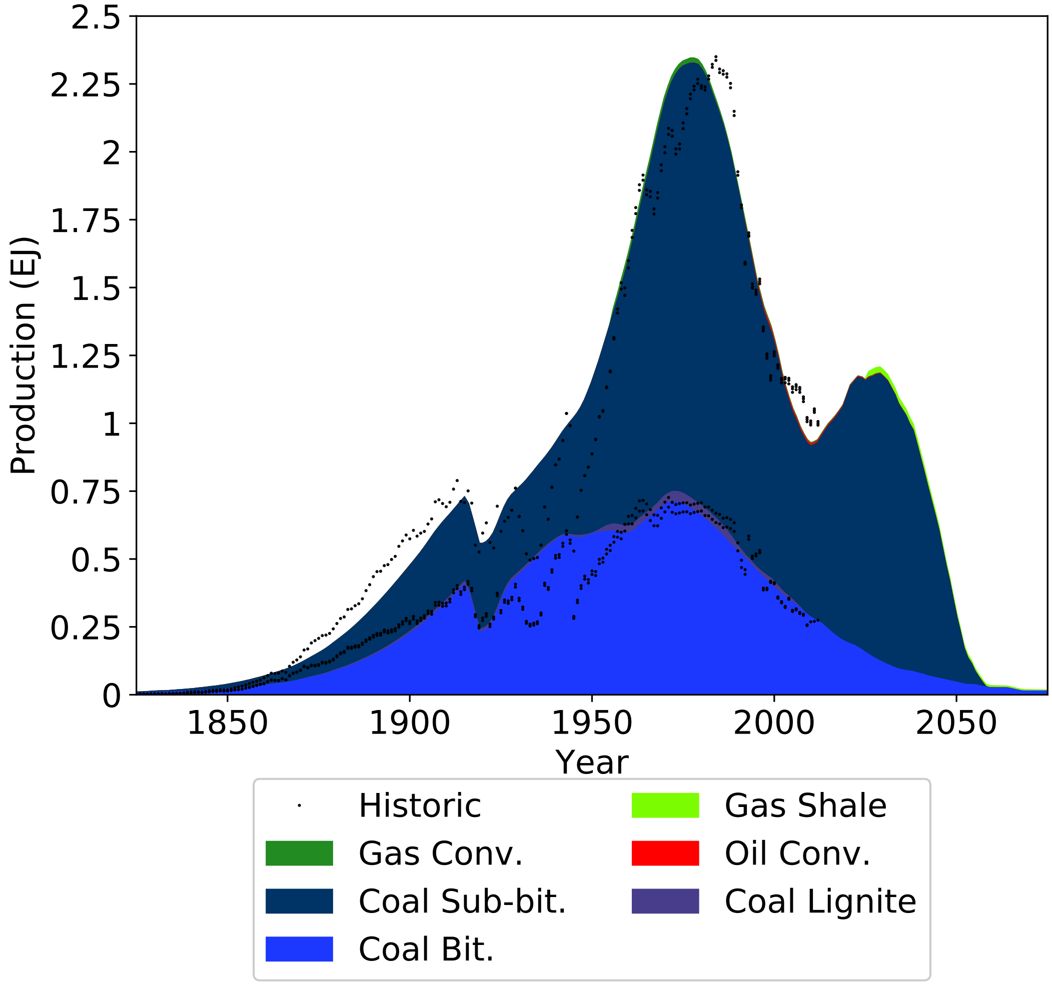

Figure 3.12: Czech Republic projection by mineral type

Table 3.12: Peak years - Minerals

**Name**	**URR**	**Peak Year**	**Peak Rate**
Coal Bit.	64.86	1973	0.71
Coal Lignite	2.04	1973	0.04
Coal Sub-bit.	123.0	1979	1.61
Oil Conv.	0.27	1999	0.01
Gas Conv.	0.91	1968	0.03
Gas Shale	0.74	2027	0.02
**Total**	**191.82**	**1977**	**2.34**

3.7 Denmark

3.7.1 All Projections

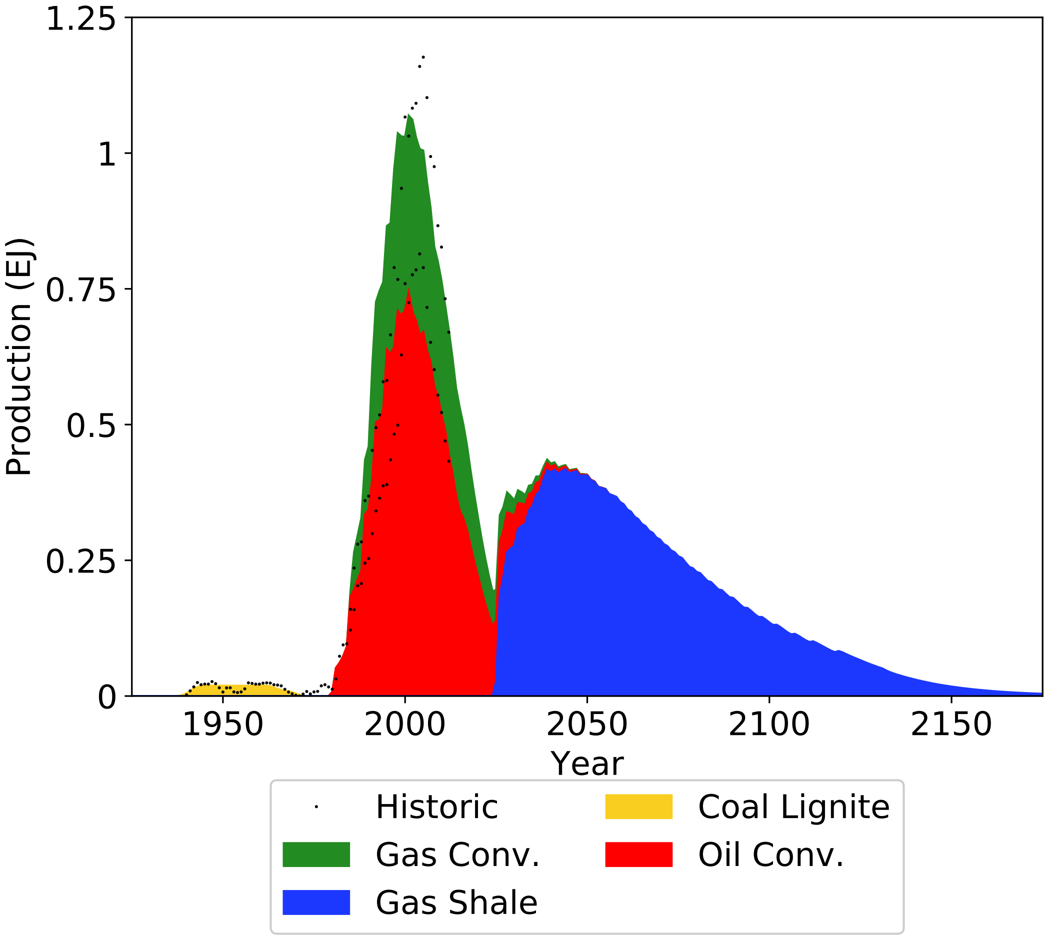

Figure 3.13: Denmark projections capped at 16

Table 3.13: Peak years - All

**Name**	**URR**	**Peak Year**	**Peak Rate**
Gas Shale	25.62	2044	0.42
Oil Conv.	19.0	2001	0.74
Gas Conv.	8.67	2002	0.35
Coal Lignite	0.5	1945	0.02
**Total**	**53.79**	**2001**	**1.07**

3.7.2 By Mineral

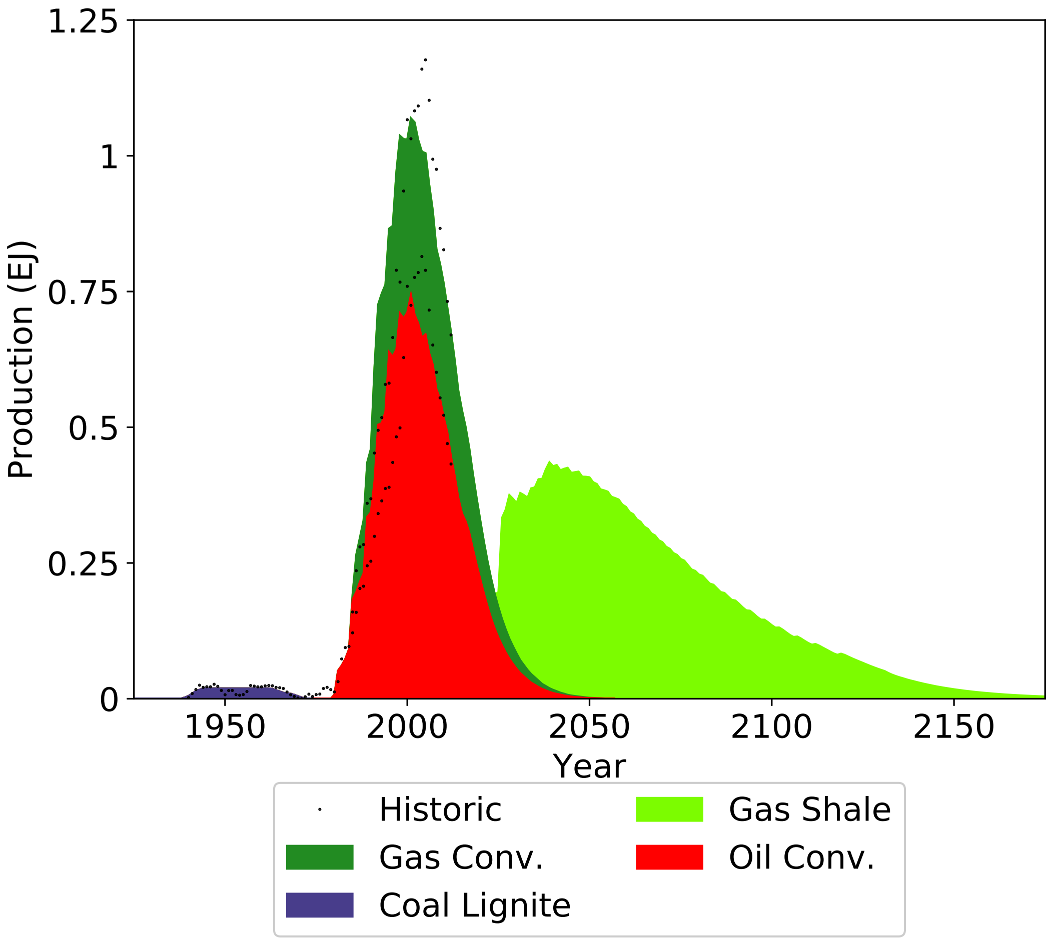

Figure 3.14: Denmark projection by mineral type

Table 3.14: Peak years - Minerals

**Name**	**URR**	**Peak Year**	**Peak Rate**
Coal Lignite	0.5	1945	0.02
Oil Conv.	19.0	2001	0.74
Gas Conv.	8.67	2002	0.35
Gas Shale	25.62	2044	0.42
**Total**	**53.79**	**2001**	**1.07**

3.8 France

3.8.1 All Projections

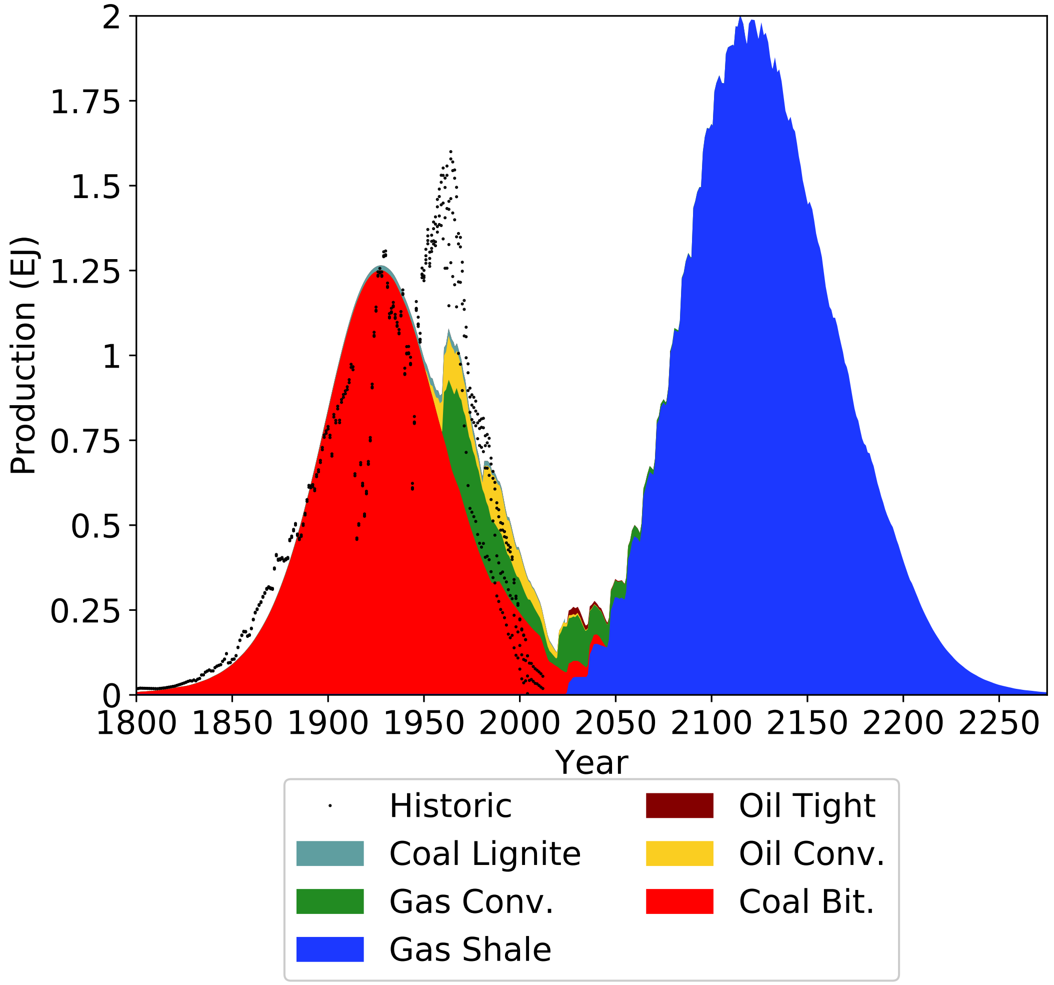

Figure 3.15: France projections capped at 16

Table 3.15: Peak years - All

**Name**	**URR**	**Peak Year**	**Peak Rate**
Gas Shale	189.04	2115	1.99
Coal Bit.	105.4	1927	1.25
Gas Conv.	12.6	1969	0.28
Oil Conv.	5.12	1985	0.14
Coal Lignite	1.8	1959	0.02
Oil Tight	0.3	2028	0.02
**Total**	**314.26**	**2115**	**1.99**

3.8.2 By Mineral

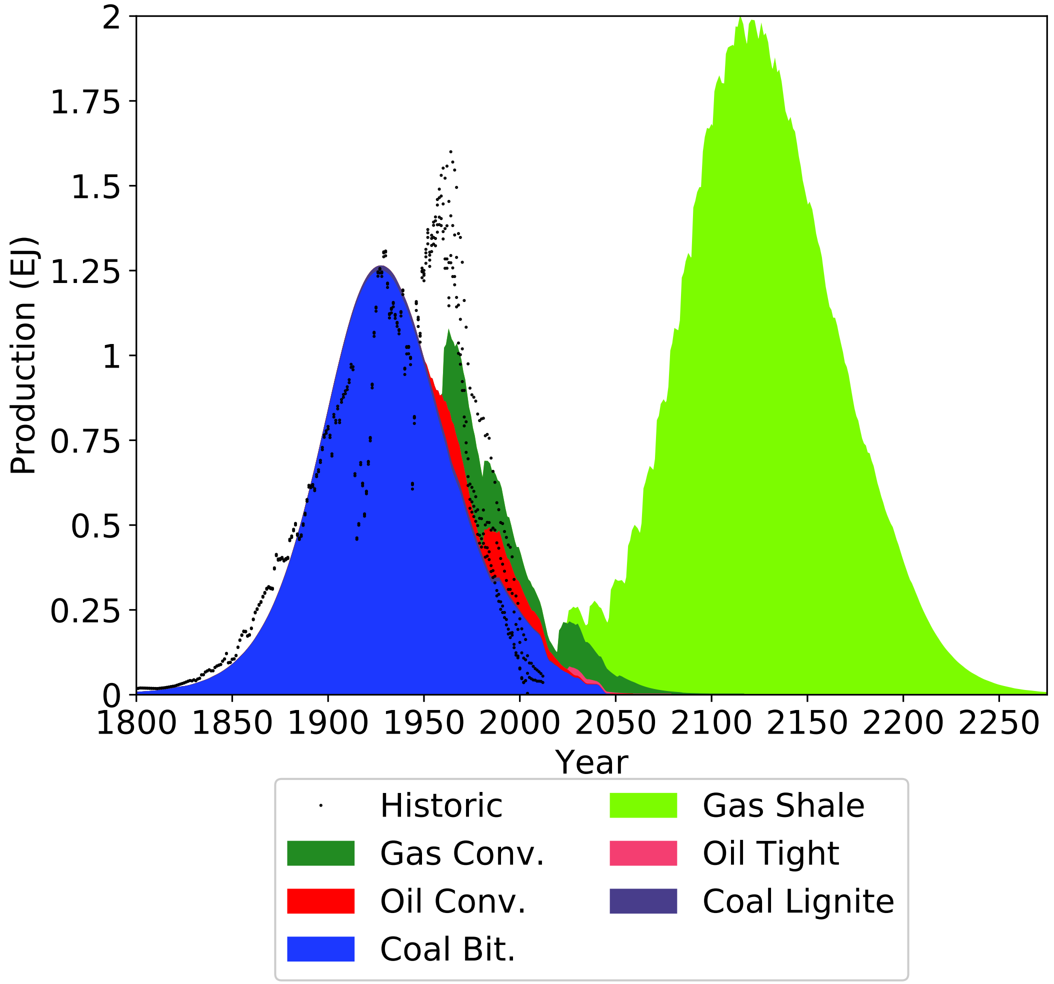

Figure 3.16: France projection by mineral type

Table 3.16: Peak years - Minerals

**Name**	**URR**	**Peak Year**	**Peak Rate**
Coal Bit.	105.4	1927	1.25
Coal Lignite	1.8	1959	0.02
Oil Conv.	5.12	1985	0.14
Oil Tight	0.3	2028	0.02
Gas Conv.	12.6	1969	0.28
Gas Shale	189.04	2115	1.99
**Total**	**314.26**	**2115**	**1.99**

3.9 Germany

3.9.1 All Projections

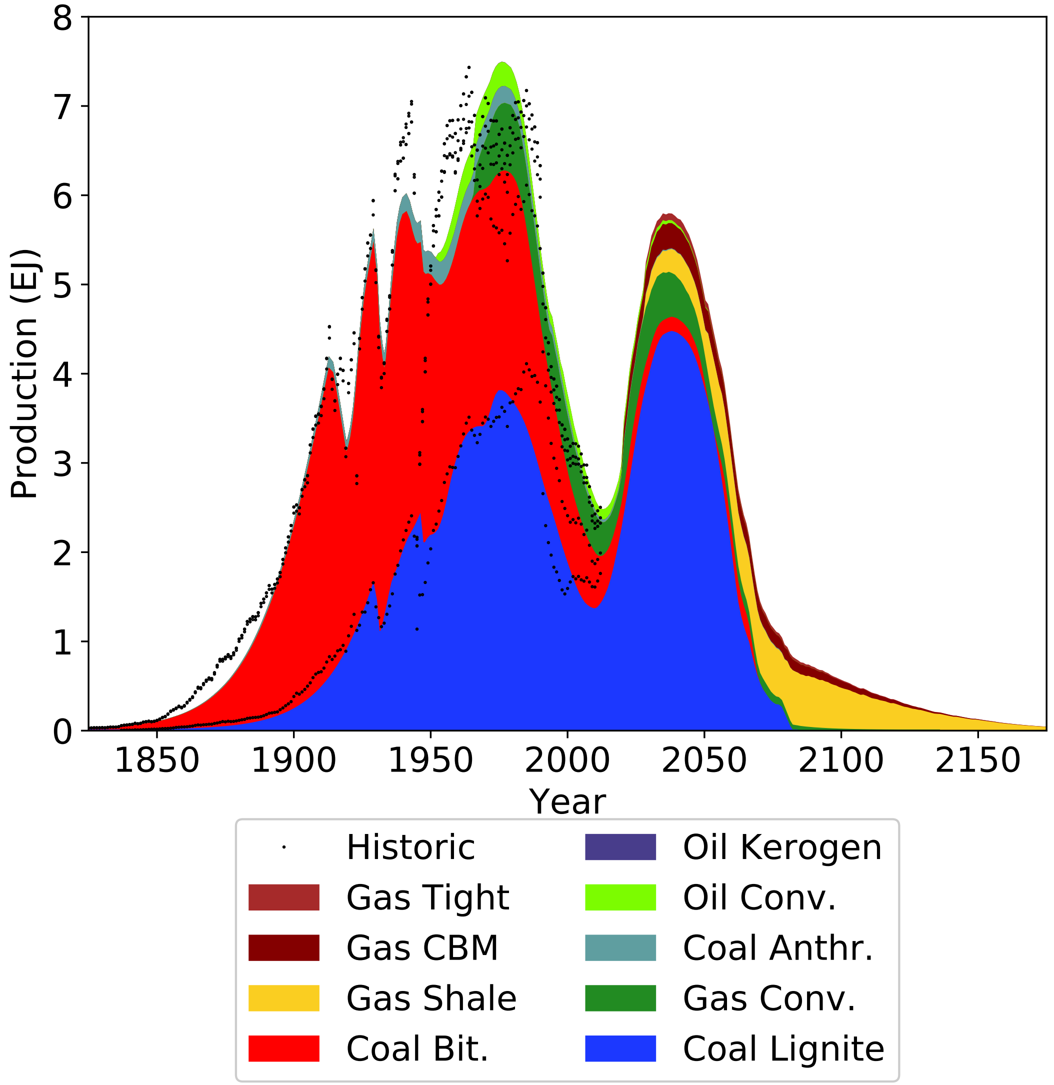

Figure 3.17: Germany projections capped at 16

Table 3.17: Peak years - All

**Name**	**URR**	**Peak Year**	**Peak Rate**
Coal Lignite	408.5	2038	4.47
Coal Bit.	318.3	1940	3.78
Gas Conv.	50.4	1979	0.76
Gas Shale	48.2	2063	0.62
Coal Anthr.	19.0	1956	0.26
Gas CBM	16.7	2037	0.29
Oil Conv.	14.48	1966	0.33
Gas Tight	3.71	2042	0.09
Oil Kerogen	0.07	1970	–
**Total**	**879.36**	**1976**	**7.49**

3.9.2 By Mineral

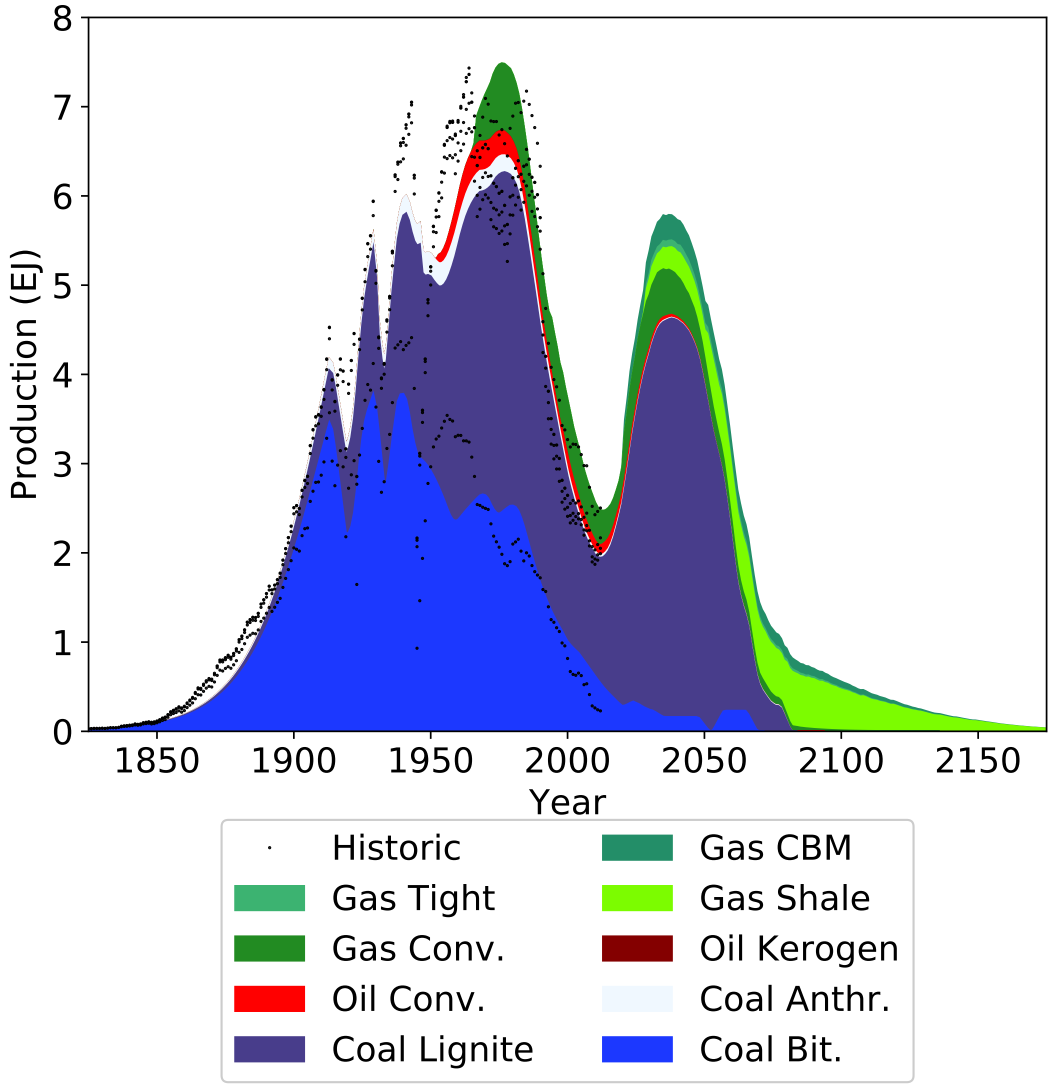

Figure 3.18: Germany projection by mineral type

Table 3.18: Peak years - Minerals

**Name**	**URR**	**Peak Year**	**Peak Rate**
Coal Bit.	318.3	1940	3.78
Coal Lignite	408.5	2038	4.47
Coal Anthr.	19.0	1956	0.26
Oil Conv.	14.48	1966	0.33
Oil Kerogen	0.07	1970	–
Gas Conv.	50.4	1979	0.76
Gas Shale	48.2	2063	0.62
Gas Tight	3.71	2042	0.09
Gas CBM	16.7	2037	0.29
**Total**	**879.36**	**1976**	**7.49**

3.10 Greece

3.10.1 All Projections

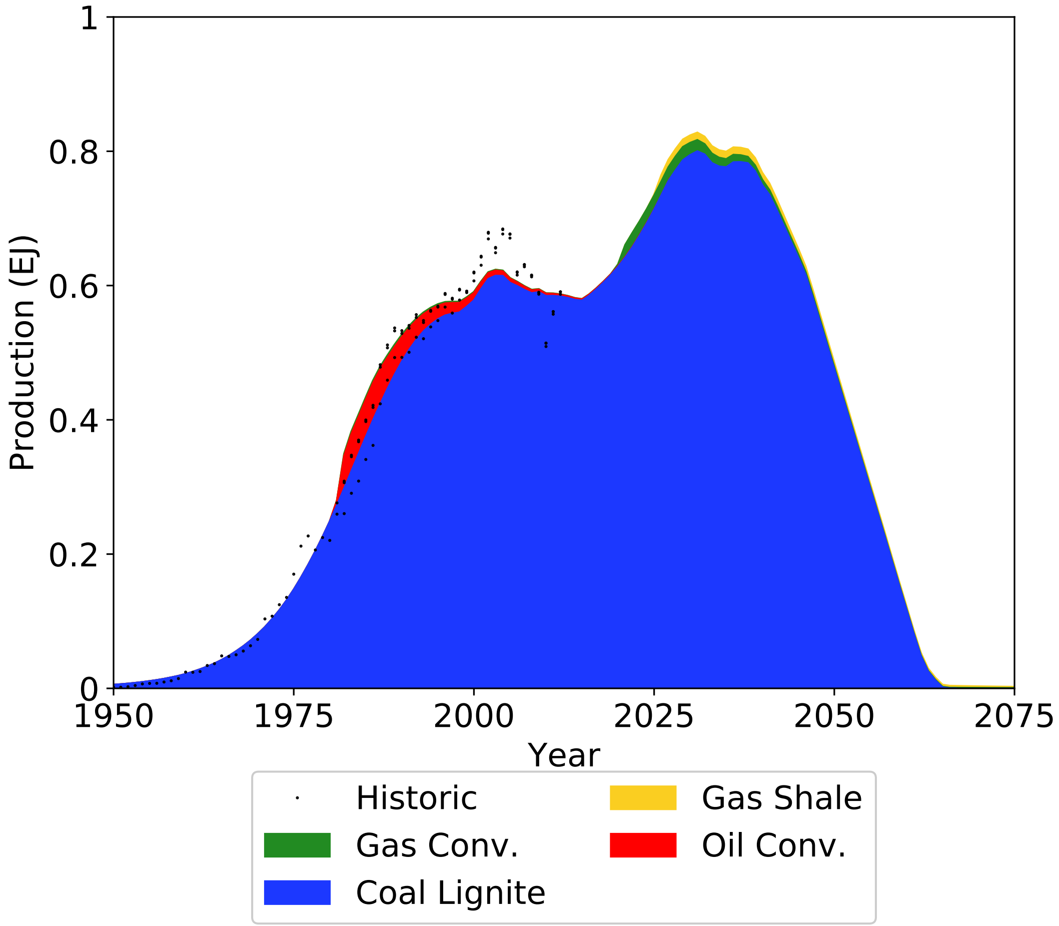

Figure 3.19: Greece projections capped at 16

Table 3.19: Peak years - All

**Name**	**URR**	**Peak Year**	**Peak Rate**
Coal Lignite	47.64	2031	0.8
Oil Conv.	0.74	1983	0.06
Gas Conv.	0.48	2022	0.02
Gas Shale	0.37	2027	0.01
**Total**	**49.23**	**2031**	**0.83**

3.10.2 By Mineral

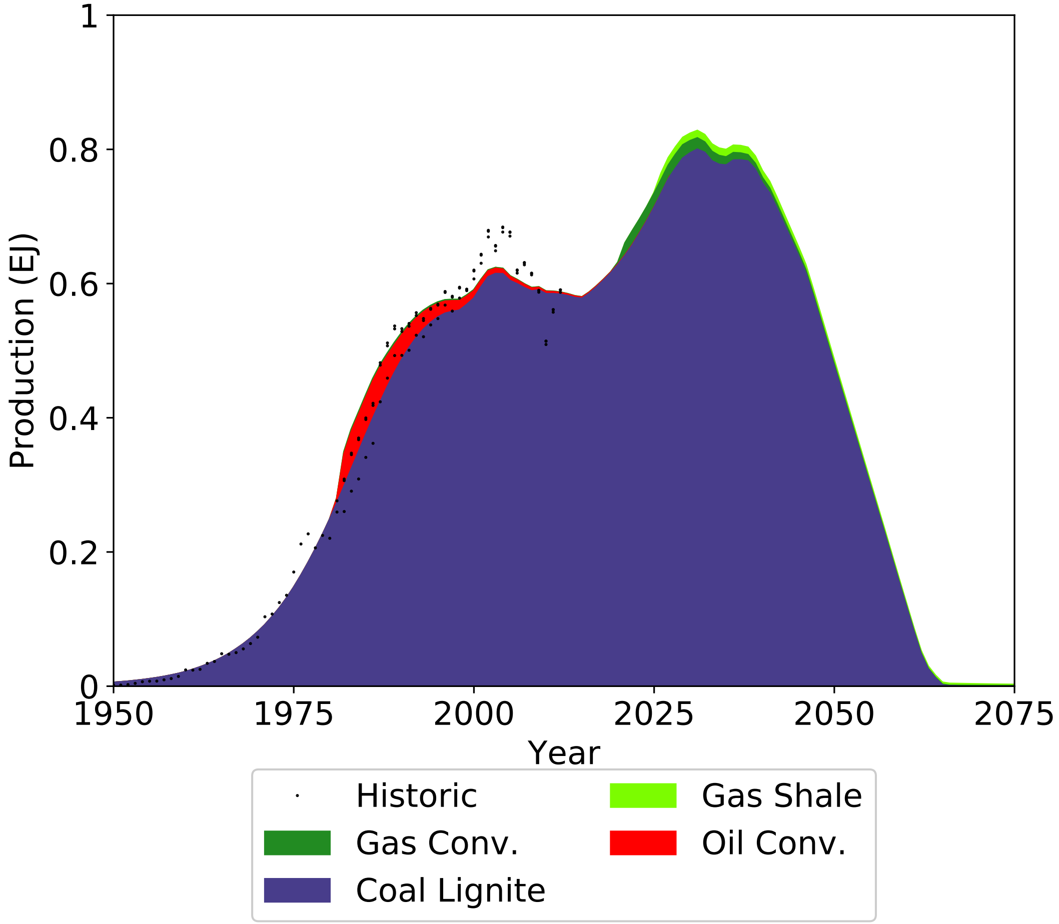

Figure 3.20: Greece projection by mineral type

Table 3.20: Peak years - Minerals

**Name**	**URR**	**Peak Year**	**Peak Rate**
Coal Lignite	47.64	2031	0.8
Oil Conv.	0.74	1983	0.06
Gas Conv.	0.48	2022	0.02
Gas Shale	0.37	2027	0.01
**Total**	**49.23**	**2031**	**0.83**

3.11 Greenland

3.11.1 All Projections

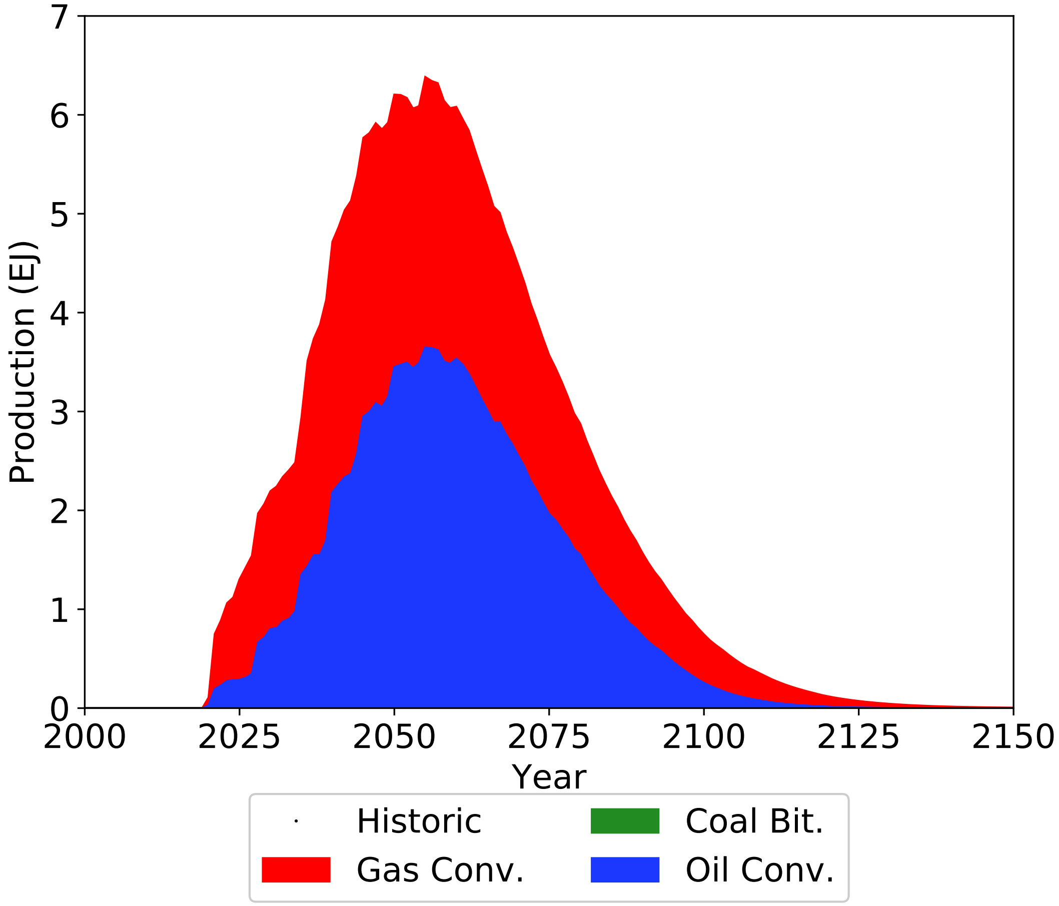

Figure 3.21: Greenland projections capped at 16

Table 3.21: Peak years - All

Name	URR	Peak Year	Peak Rate
Oil Conv.	147.4	2055	3.65
Gas Conv.	144.6	2047	2.83
Coal Bit.	0.02	1953	–
**Total**	**292.02**	**2055**	**6.38**

3.11.2 By Mineral

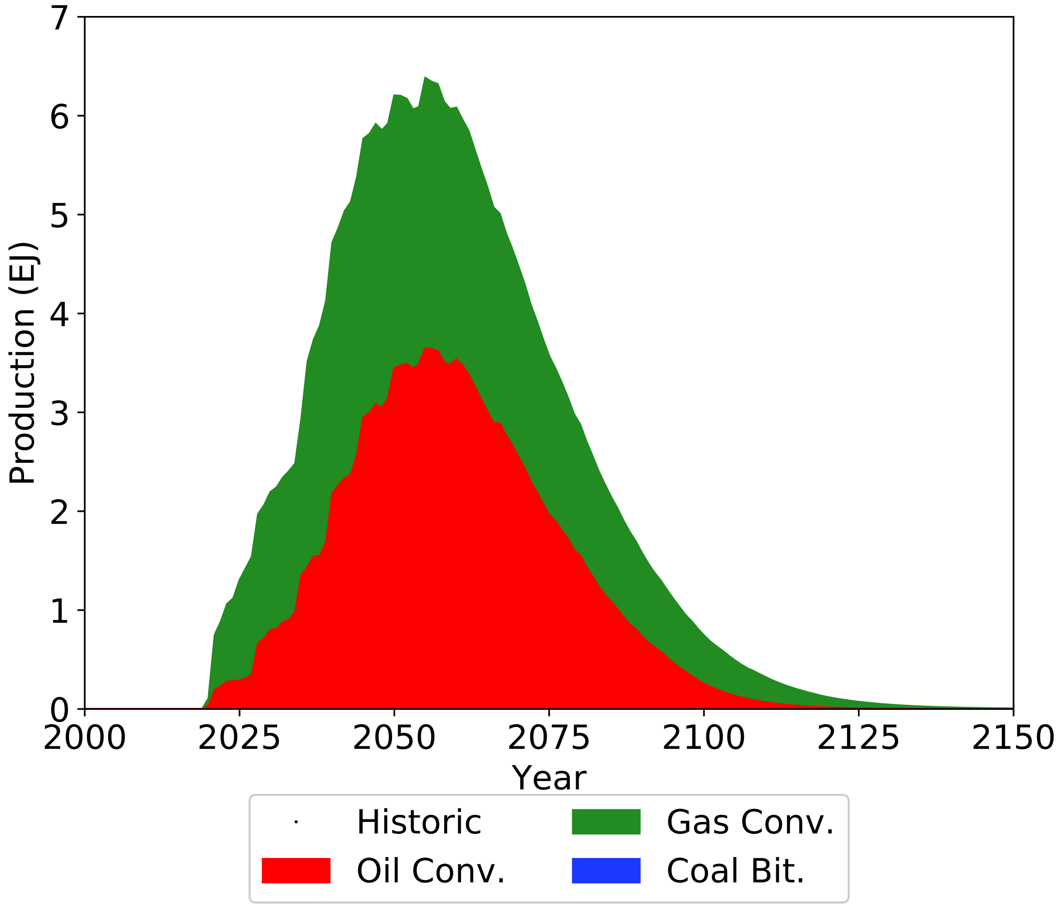

Figure 3.22: Greenland projection by mineral type

Table 3.22: Peak years - Minerals

Name	URR	Peak Year	Peak Rate
Coal Bit.	0.02	1953	–
Oil Conv.	147.4	2055	3.65
Gas Conv.	144.6	2047	2.83
**Total**	**292.02**	**2055**	**6.38**

3.12 Hungary

3.12.1 All Projections

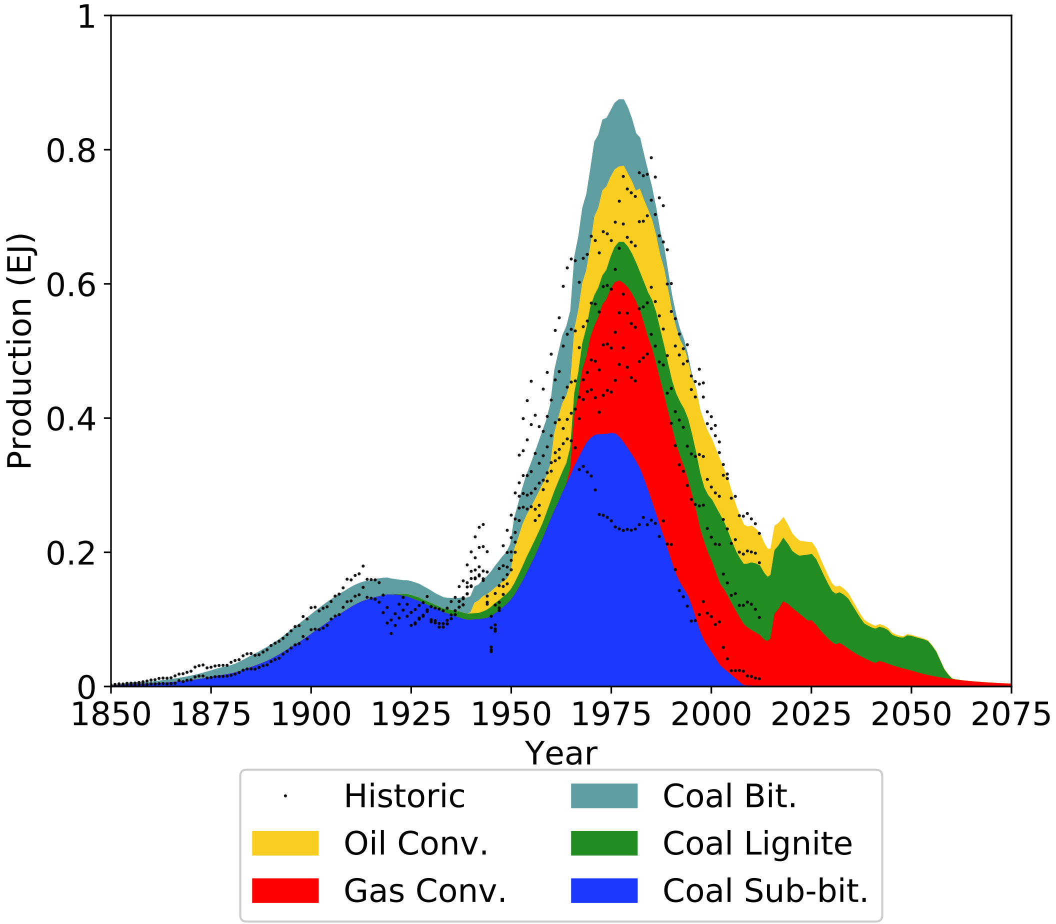

Figure 3.23: Hungary projections capped at 16

Table 3.23: Peak years - All

**Name**	**URR**	**Peak Year**	**Peak Rate**
Coal Sub-bit.	19.4	1975	0.38
Gas Conv.	10.5	1980	0.24
Coal Lignite	7.15	2012	0.1
Oil Conv.	6.36	1973	0.13
Coal Bit.	5.2	1970	0.11
**Total**	**48.61**	**1977**	**0.87**

3.12.2 By Mineral

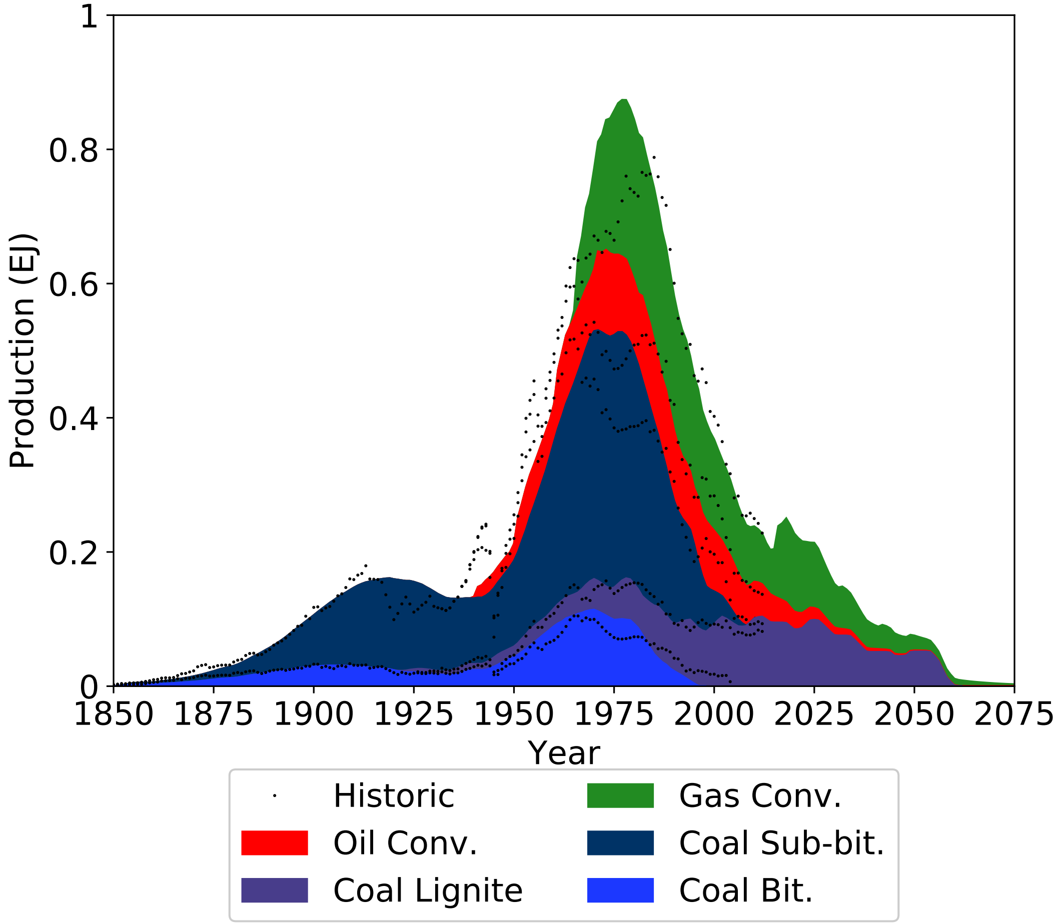

Figure 3.24: Hungary projection by mineral type

Table 3.24: Peak years - Minerals

Name	URR	Peak Year	Peak Rate
Coal Bit.	5.2	1970	0.11
Coal Lignite	7.15	2012	0.1
Coal Sub-bit.	19.4	1975	0.38
Oil Conv.	6.36	1973	0.13
Gas Conv.	10.5	1980	0.24
**Total**	**48.61**	**1977**	**0.87**

3.13 Ireland

3.13.1 All Projections

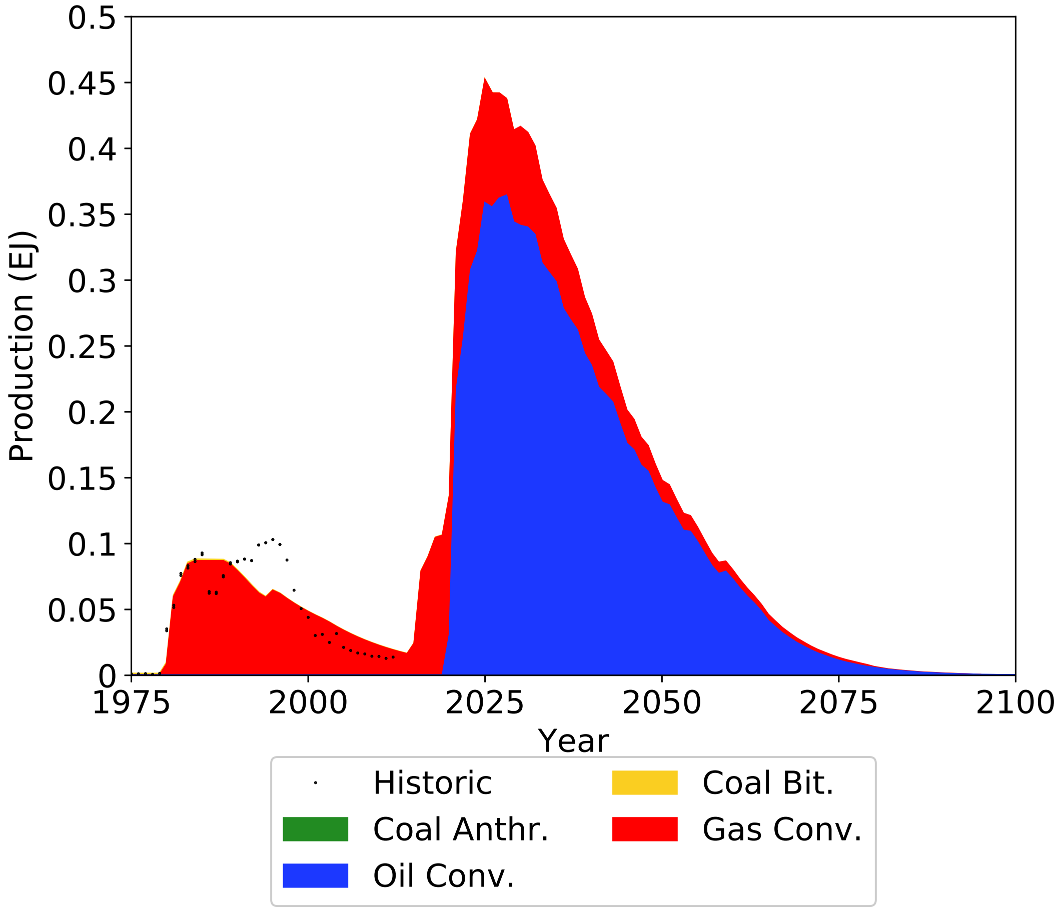

Figure 3.25: Ireland projections capped at 16

Table 3.25: Peak years - All

**Name**	**URR**	**Peak Year**	**Peak Rate**
Oil Conv.	9.44	2028	0.36
Gas Conv.	4.2	2019	0.11
Coal Anthr.	0.27	1882	–
Coal Bit.	0.12	1959	–
**Total**	**14.02**	**2025**	**0.45**

3.13.2 By Mineral

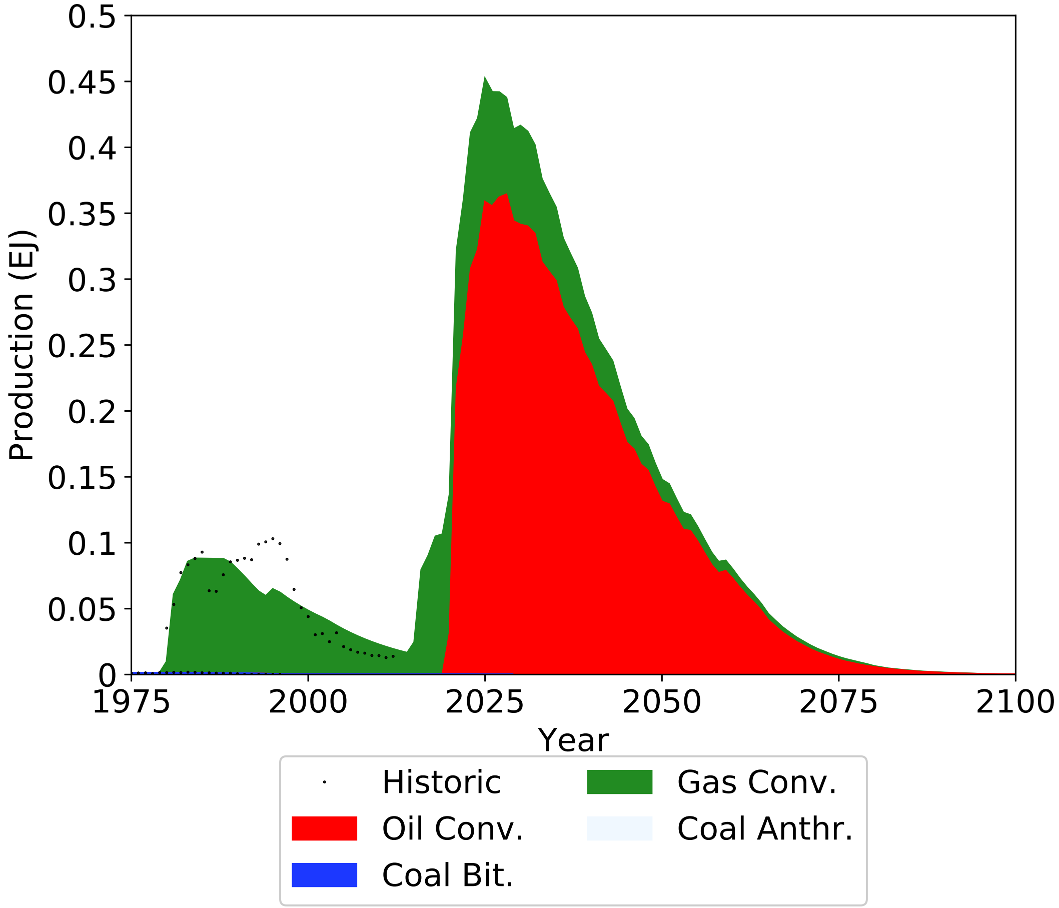

Figure 3.26: Ireland projection by mineral type

Table 3.26: Peak years - Minerals

**Name**	**URR**	**Peak Year**	**Peak Rate**
Coal Bit.	0.12	1959	–
Coal Anthr.	0.27	1882	–
Oil Conv.	9.44	2028	0.36
Gas Conv.	4.2	2019	0.11
**Total**	**14.02**	**2025**	**0.45**

3.14 Italy

3.14.1 All Projections

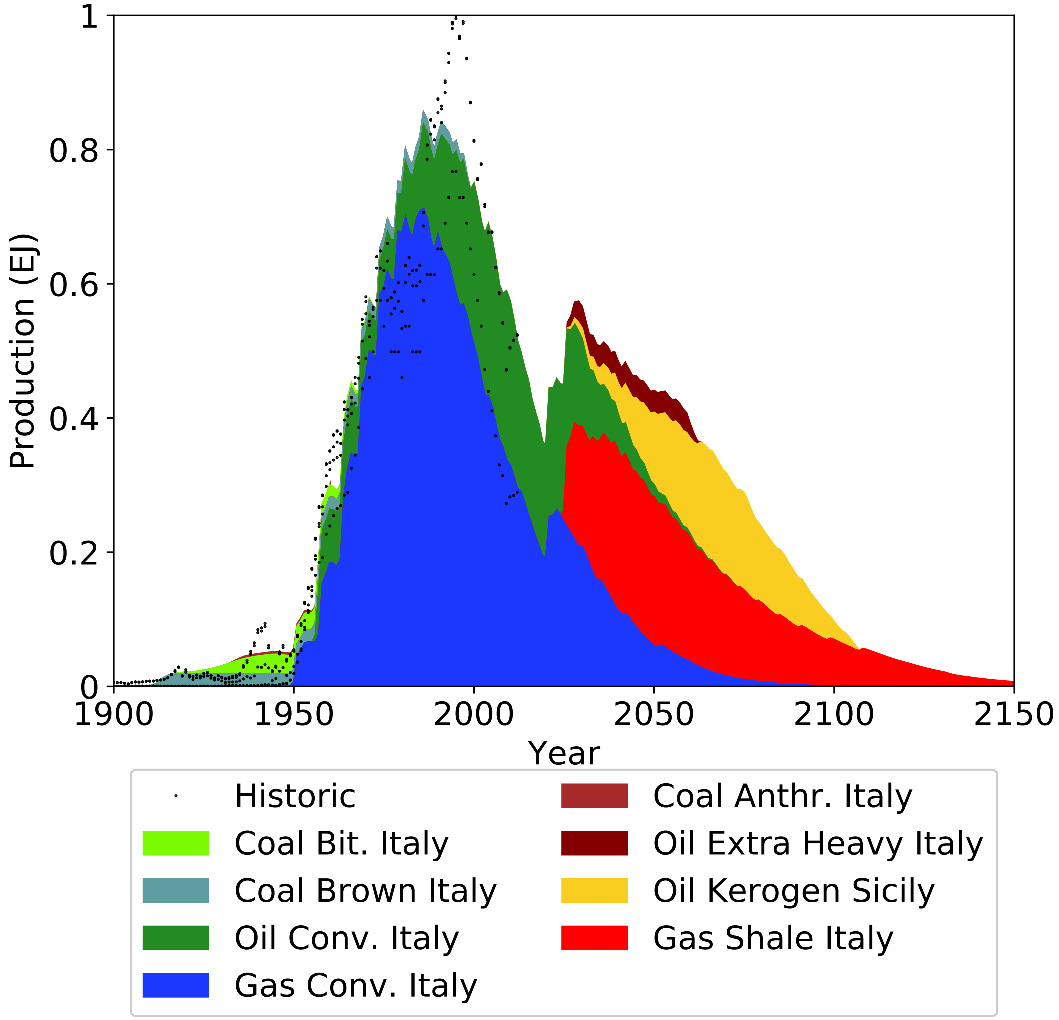

Figure 3.27: Italy projections capped at 16

Table 3.27: Peak years - All

**Name**	**URR**	**Peak Year**	**Peak Rate**
Gas Conv. Italy	34.7	1986	0.71
Gas Shale Italy	13.57	2039	0.24
Oil Conv. Italy	12.08	2004	0.26
Oil Kerogen Sicily	6.9	2065	0.16
Coal Brown Italy	1.53	1917	0.02
Oil Extra Heavy Italy	1.1	2030	0.03
Coal Bit. Italy	0.9	1945	0.03
Coal Anthr. Italy	0.08	1937	–
**Total**	**70.85**	**1986**	**0.86**

3.14.2 By Mineral

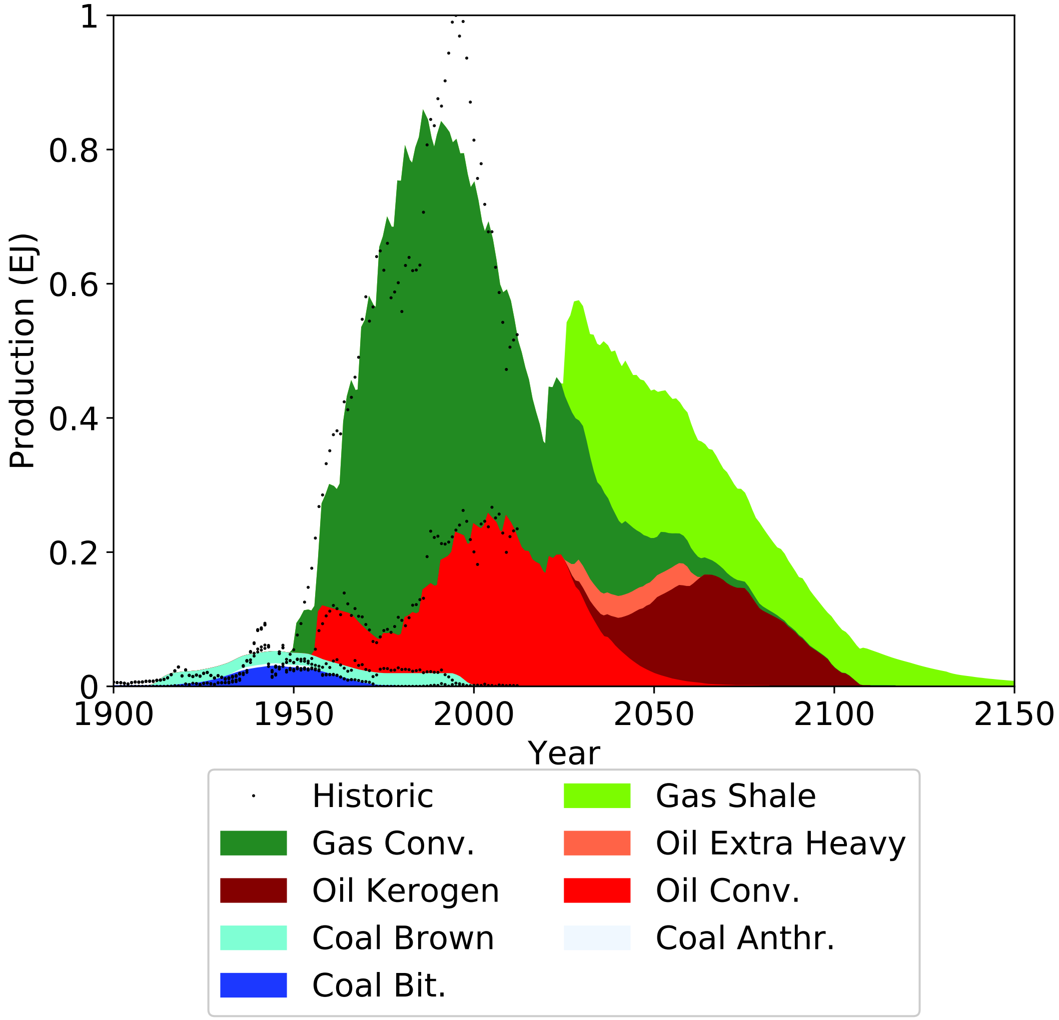

Figure 3.28: Italy projection by mineral type

3.14.3 Regional Projections

Table 3.28: Peak years - Minerals

**Name**	**URR**	**Peak Year**	**Peak Rate**
Coal Bit.	0.9	1945	0.03
Coal Anthr.	0.08	1937	–
Coal Brown	1.53	1917	0.02
Oil Conv.	12.08	2004	0.26
Oil Kerogen	6.9	2065	0.16
Oil Extra Heavy	1.1	2030	0.03
Gas Conv.	34.7	1986	0.71
Gas Shale	13.57	2039	0.24
**Total**	**70.85**	**1986**	**0.86**

Italy

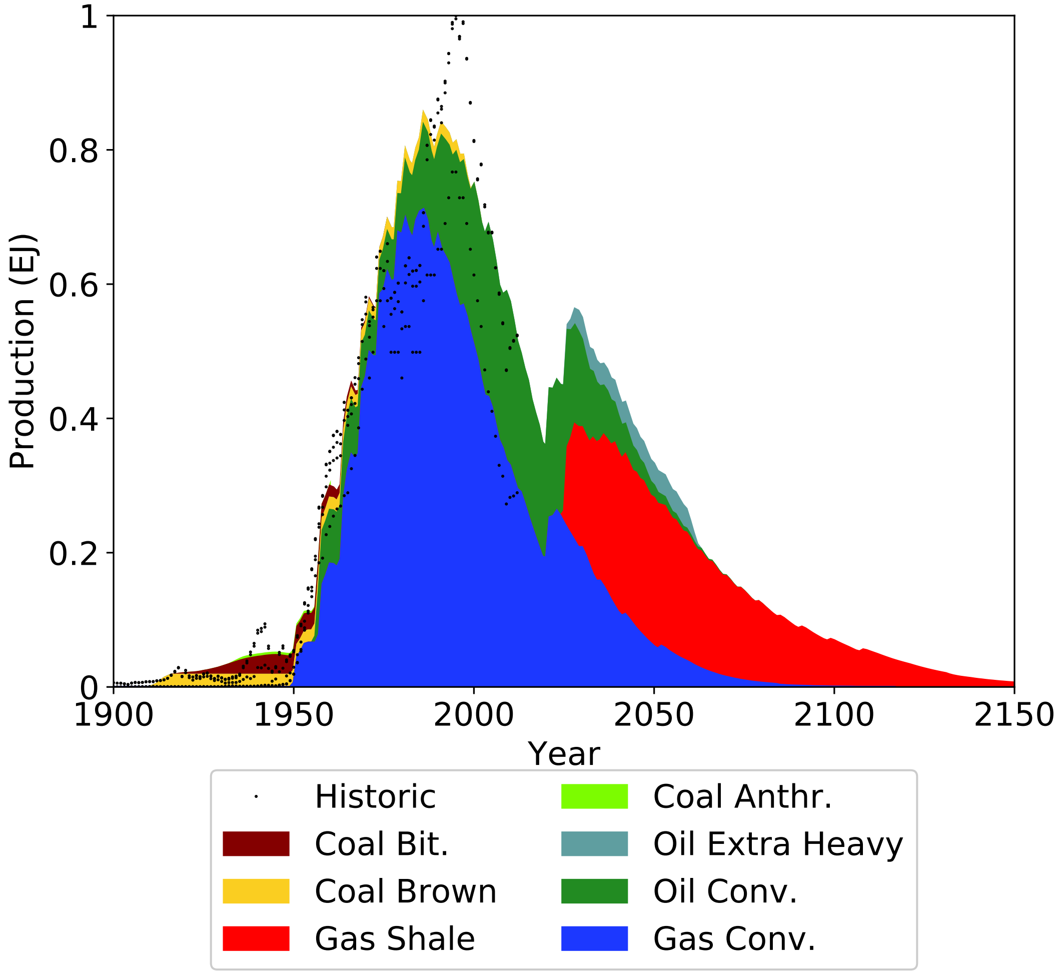

Figure 3.29: Italy - Italy projections capped at 16

Table 3.29: Peak years - All

Name	URR	Peak Year	Peak Rate
Gas Conv. Italy	34.7	1986	0.71
Gas Shale Italy	13.57	2039	0.24
Oil Conv. Italy	12.08	2004	0.26
Coal Brown Italy	1.53	1917	0.02
Oil Extra Heavy Italy	1.1	2030	0.03
Coal Bit. Italy	0.9	1945	0.03
Coal Anthr. Italy	0.08	1937	–
**Total**	**63.95**	**1986**	**0.86**

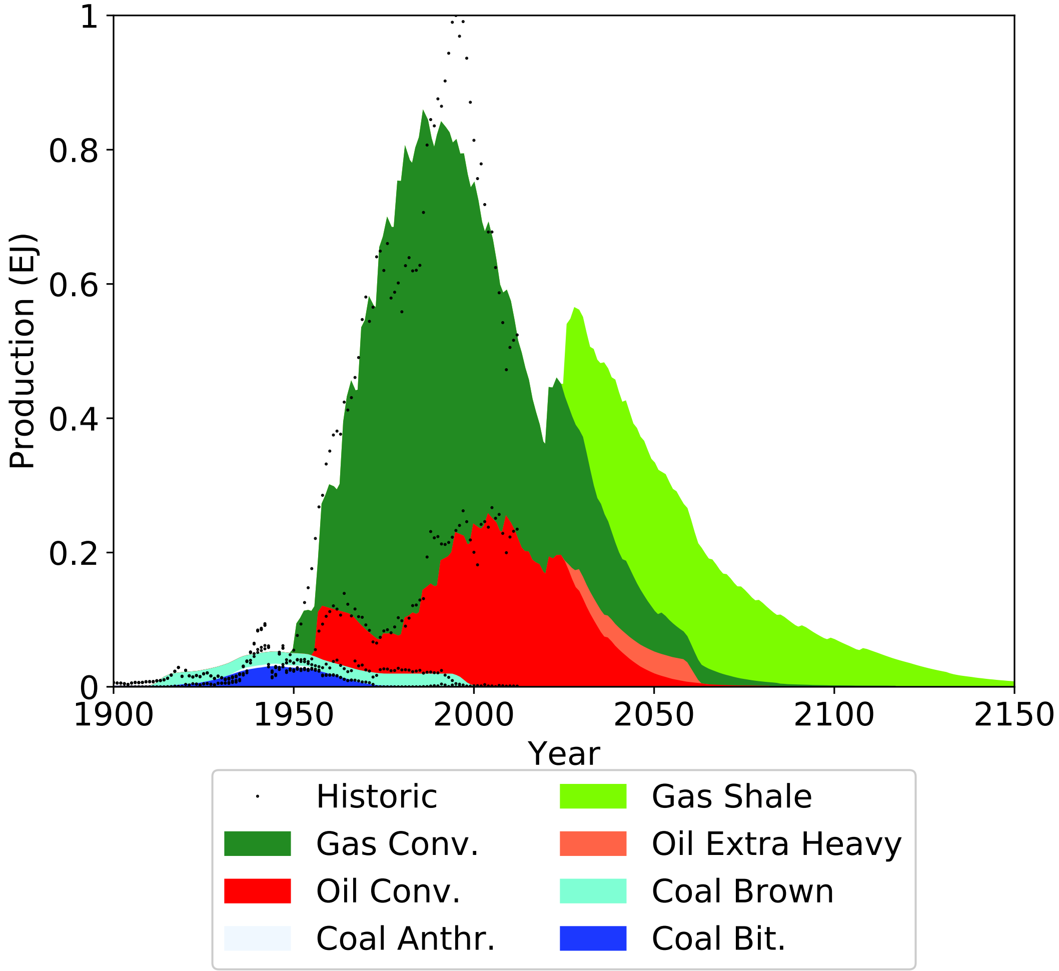

Figure 3.30: Italy - Italy projection by mineral type

Table 3.30: Peak years - Minerals

**Name**	**URR**	**Peak Year**	**Peak Rate**
Coal Bit.	0.9	1945	0.03
Coal Anthr.	0.08	1937	–
Coal Brown	1.53	1917	0.02
Oil Conv.	12.08	2004	0.26
Oil Extra Heavy	1.1	2030	0.03
Gas Conv.	34.7	1986	0.71
Gas Shale	13.57	2039	0.24
**Total**	**63.95**	**1986**	**0.86**

Sicily

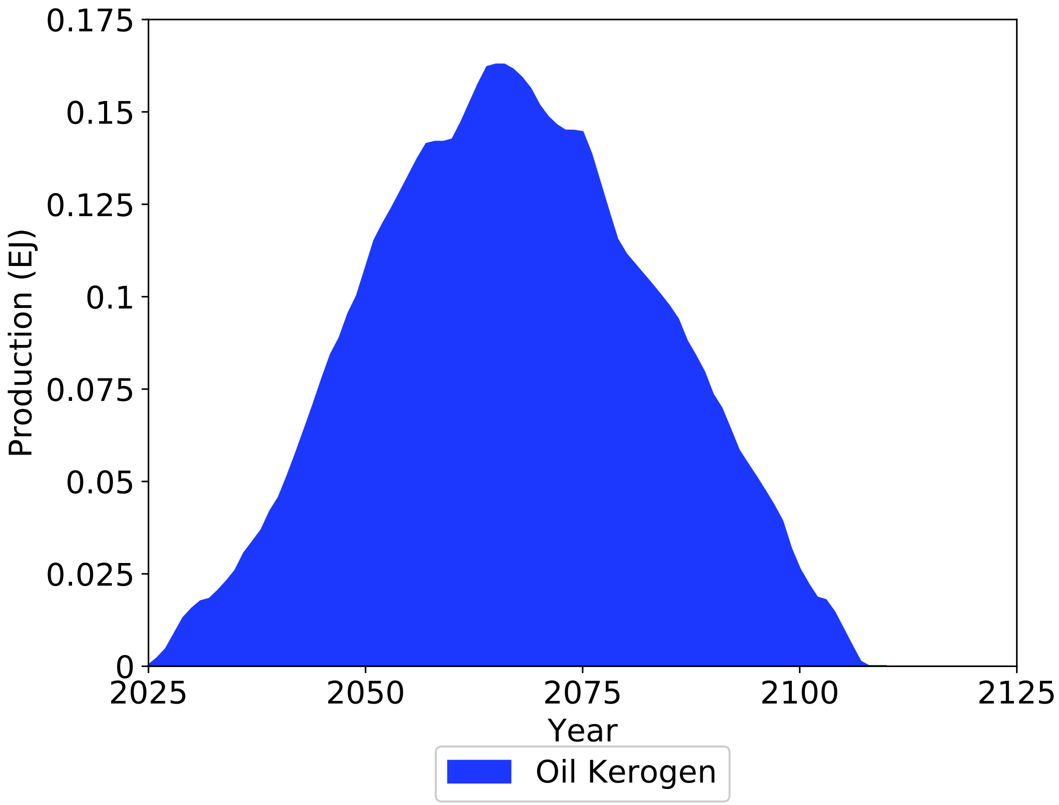

Figure 3.31: Italy - Sicily projections capped at 16

Table 3.31: Peak years - All

Name	URR	Peak Year	Peak Rate
Oil Kerogen Sicily	6.9	2065	0.16
**Total**	**6.9**	**2065**	**0.16**

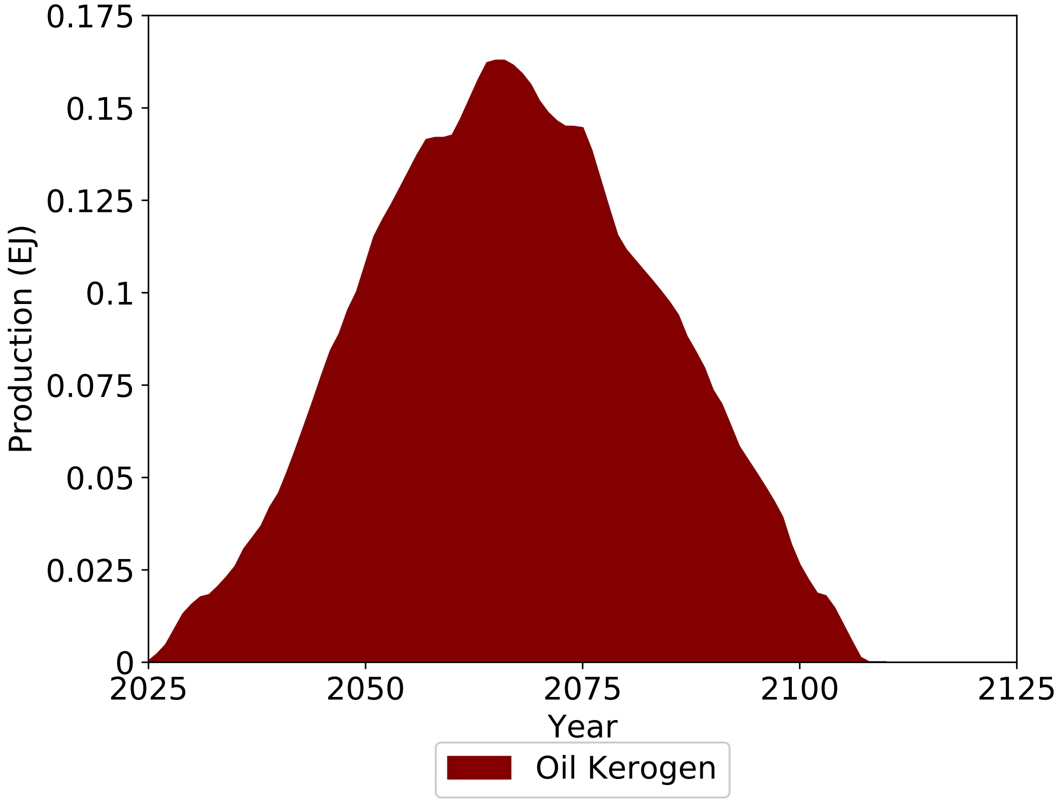

Figure 3.32: Italy - Sicily projection by mineral type

Table 3.32: Peak years - Minerals

Name	URR	Peak Year	Peak Rate
Oil Kerogen	6.9	2065	0.16
**Total**	**6.9**	**2065**	**0.16**

3.14.4 Projection by region

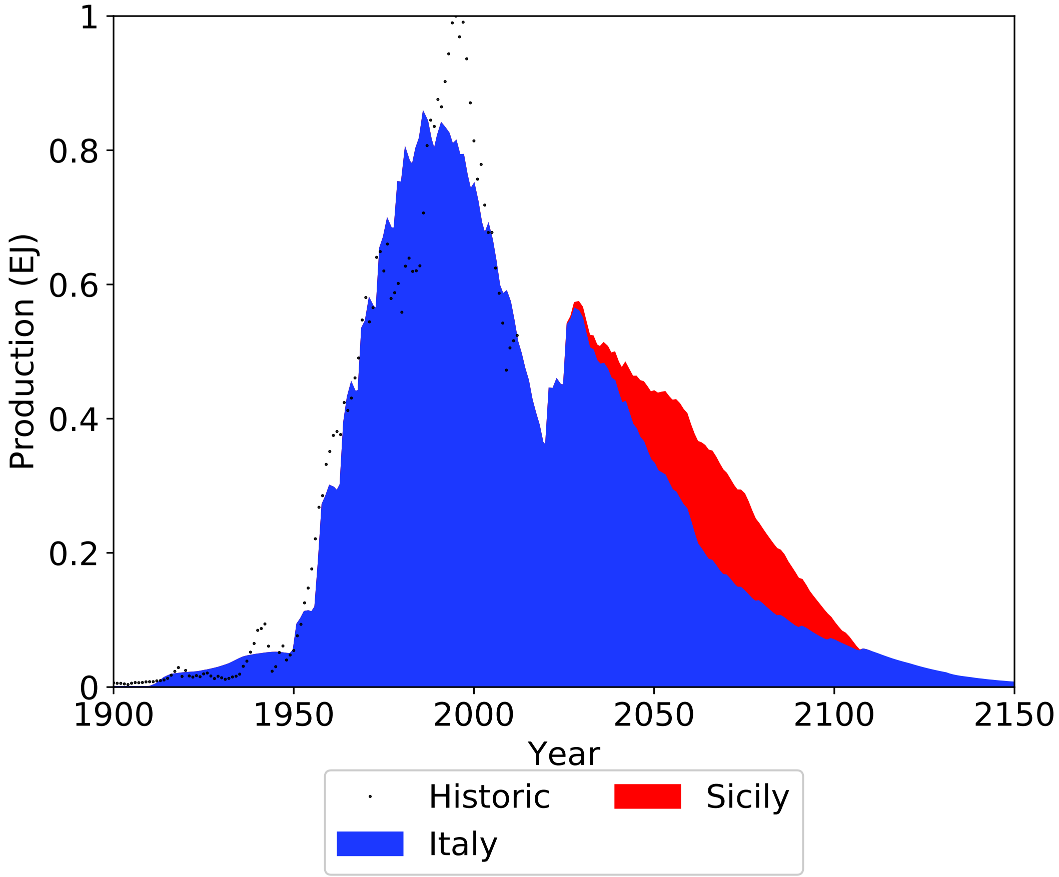

Figure 3.33: Italy by region projections capped at 16

Table 3.33: Peak years - All

Name	URR	Peak Year	Peak Rate
Italy	63.95	1986	0.86
Sicily	6.9	2065	0.16
**Total**	**70.85**	**1986**	**0.86**

3.15 Malta

3.15.1 All Projections

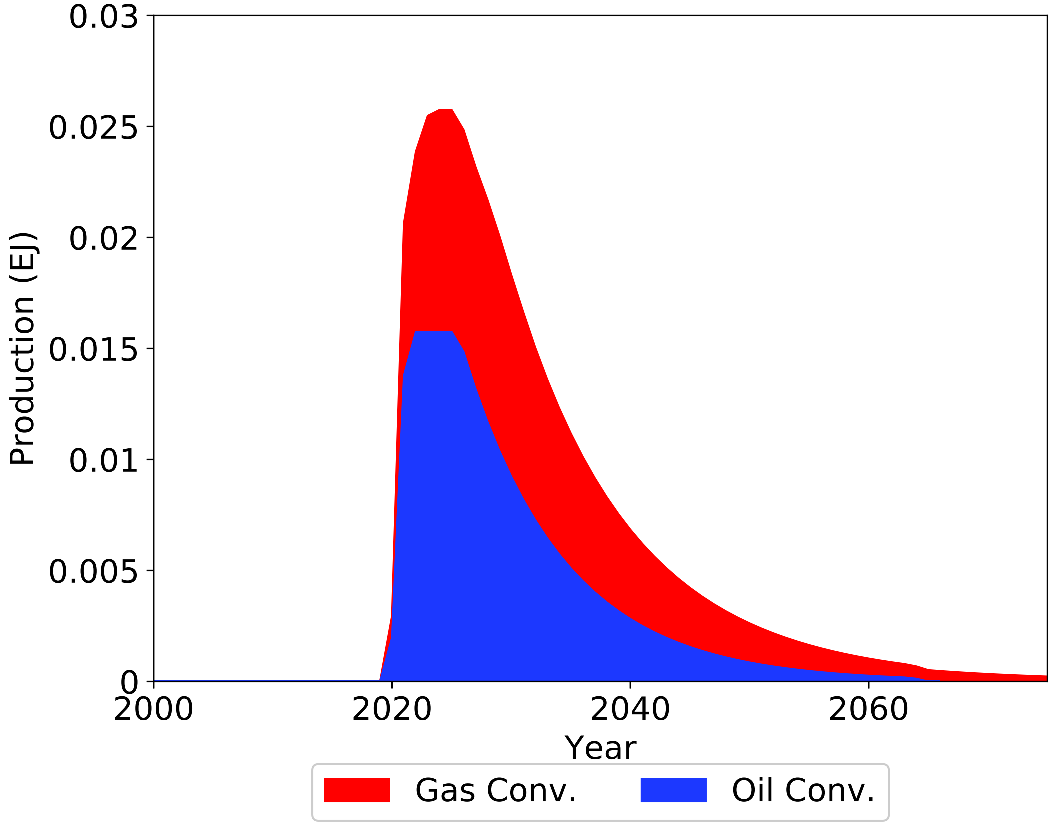

Figure 3.34: Malta projections capped at 16

Table 3.34: Peak years - All

Name	URR	Peak Year	Peak Rate
Oil Conv.	0.21	2022	0.02
Gas Conv.	0.2	2024	0.01
**Total**	**0.41**	**2024**	**0.03**

3.15.2 By Mineral

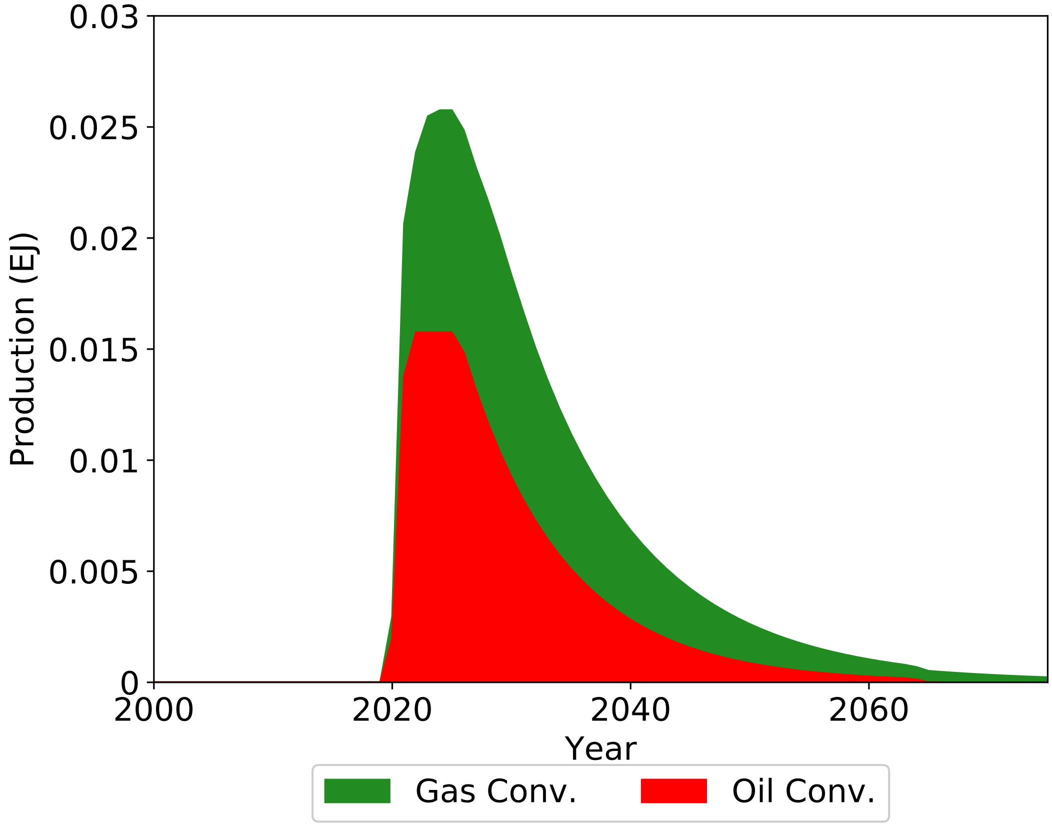

Figure 3.35: Malta projection by mineral type

Table 3.35: Peak years - Minerals

**Name**	**URR**	**Peak Year**	**Peak Rate**
Oil Conv.	0.21	2022	0.02
Gas Conv.	0.2	2024	0.01
**Total**	**0.41**	**2024**	**0.03**

3.16 Netherlands

3.16.1 All Projections

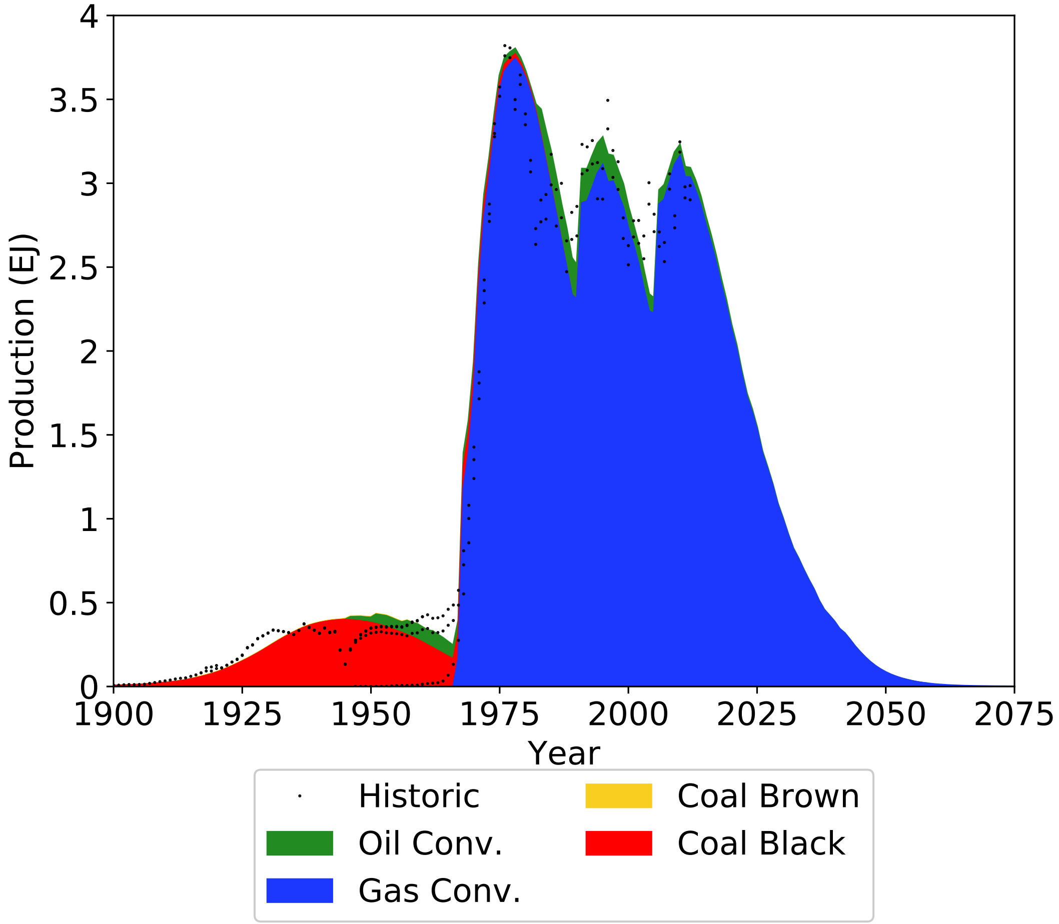

Figure 3.36: Netherlands projections capped at 16

Table 3.36: Peak years - All

**Name**	**URR**	**Peak Year**	**Peak Rate**
Gas Conv.	173.3	1978	3.74
Coal Black	14.9	1945	0.4
Oil Conv.	7.18	1988	0.23
Coal Brown	0.17	1918	–
**Total**	**195.55**	**1978**	**3.8**

3.16.2 By Mineral

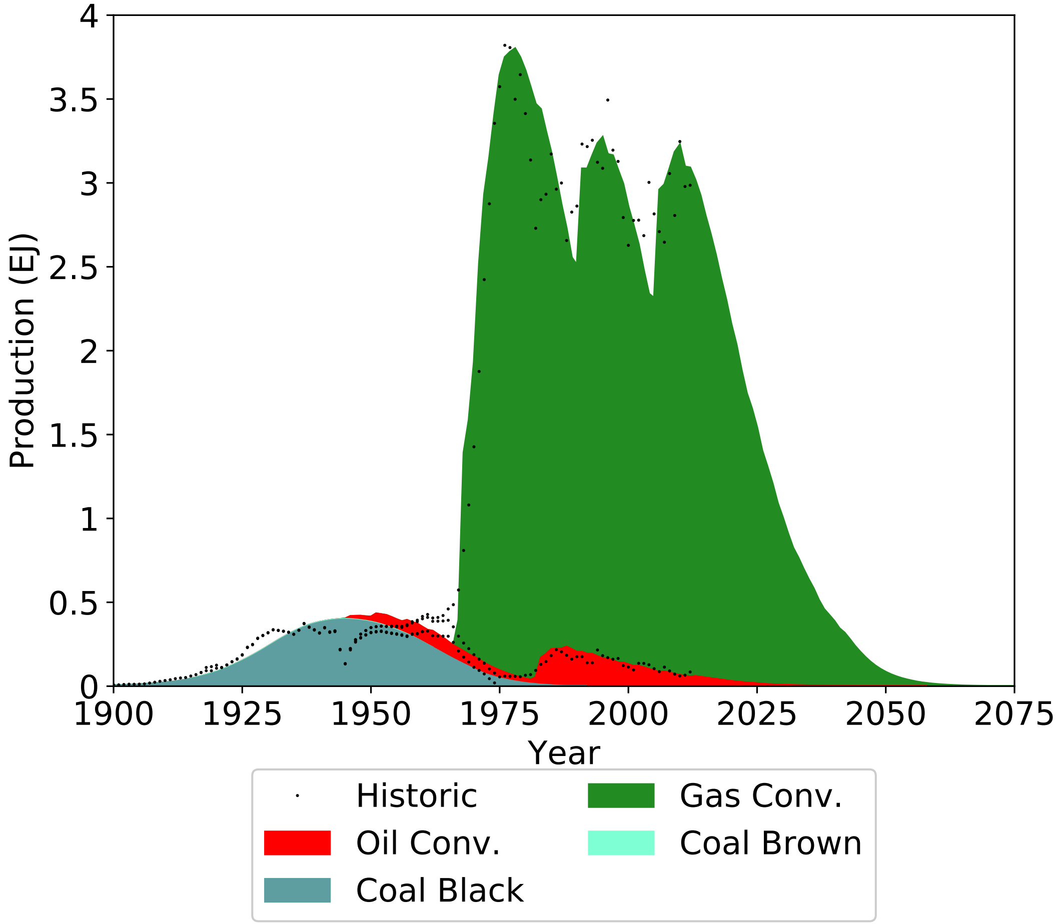

Figure 3.37: Netherlands projection by mineral type

Table 3.37: Peak years - Minerals

**Name**	**URR**	**Peak Year**	**Peak Rate**
Coal Black	14.9	1945	0.4
Coal Brown	0.17	1918	–
Oil Conv.	7.18	1988	0.23
Gas Conv.	173.3	1978	3.74
**Total**	**195.55**	**1978**	**3.8**

3.17 Norway

3.17.1 All Projections

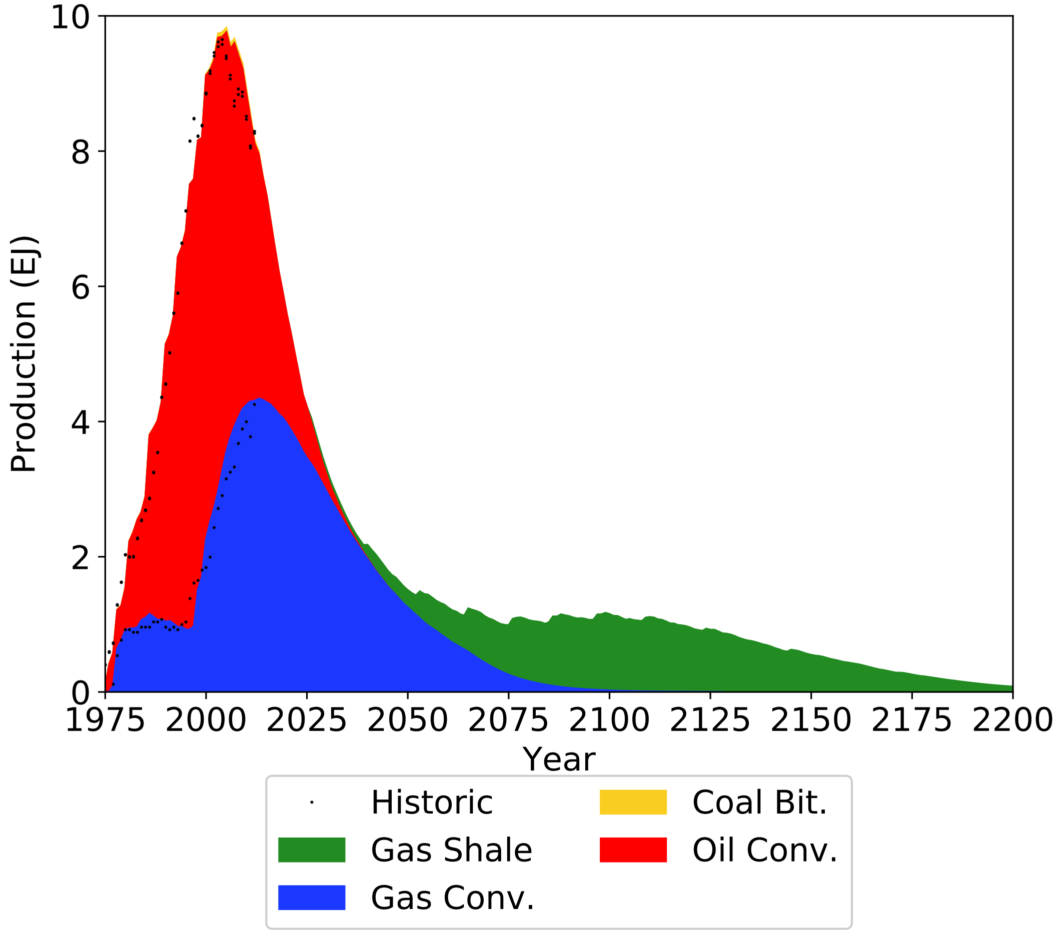

Figure 3.38: Norway projections capped at 16

Table 3.38: Peak years - All

**Name**	**URR**	**Peak Year**	**Peak Rate**
Gas Conv.	195.19	2013	4.34
Oil Conv.	180.8	2000	6.86
Gas Shale	100.7	2099	1.15
Coal Bit.	1.51	2004	0.07
**Total**	**478.2**	**2005**	**9.82**

3.17.2 By Mineral

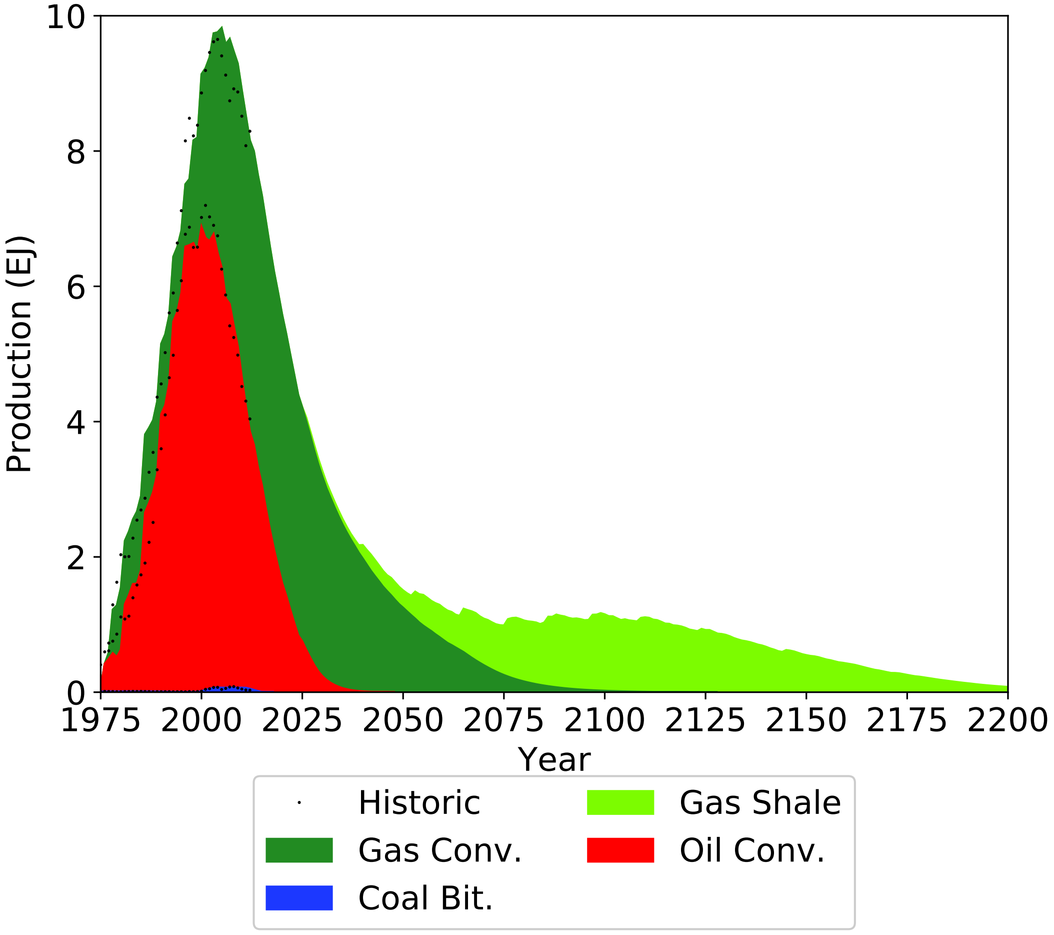

Figure 3.39: Norway projection by mineral type

Table 3.39: Peak years - Minerals

**Name**	**URR**	**Peak Year**	**Peak Rate**
Coal Bit.	1.51	2004	0.07
Oil Conv.	180.8	2000	6.86
Gas Conv.	195.19	2013	4.34
Gas Shale	100.7	2099	1.15
**Total**	**478.2**	**2005**	**9.82**

3.18 Poland

3.18.1 All Projections

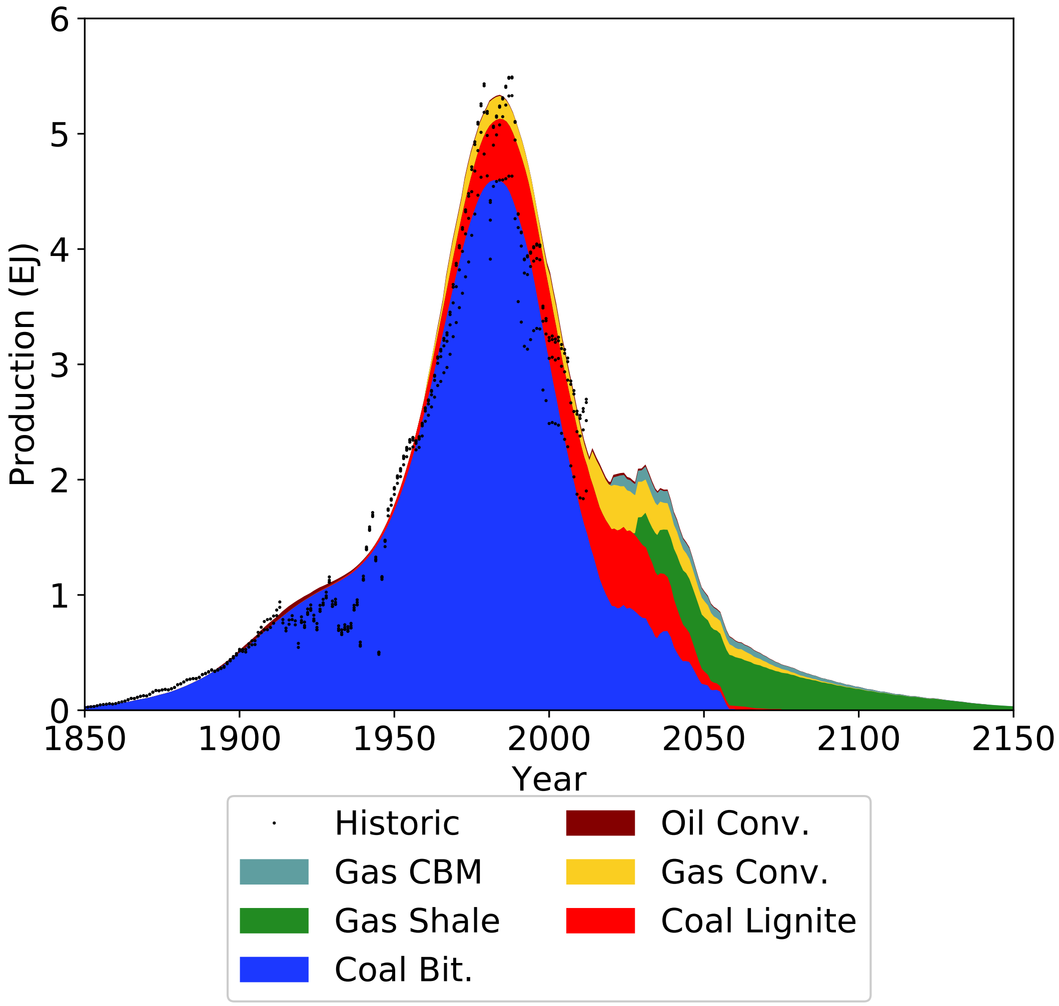

Figure 3.40: Poland projections capped at 16

Table 3.40: Peak years - All

**Name**	**URR**	**Peak Year**	**Peak Rate**
Coal Bit.	291.9	1983	4.59
Coal Lignite	46.0	2023	0.68
Gas Shale	29.55	2048	0.48
Gas Conv.	20.2	2022	0.39
Gas CBM	5.25	2032	0.11
Oil Conv.	3.08	1916	0.05
**Total**	**395.98**	**1984**	**5.33**

3.18.2 By Mineral

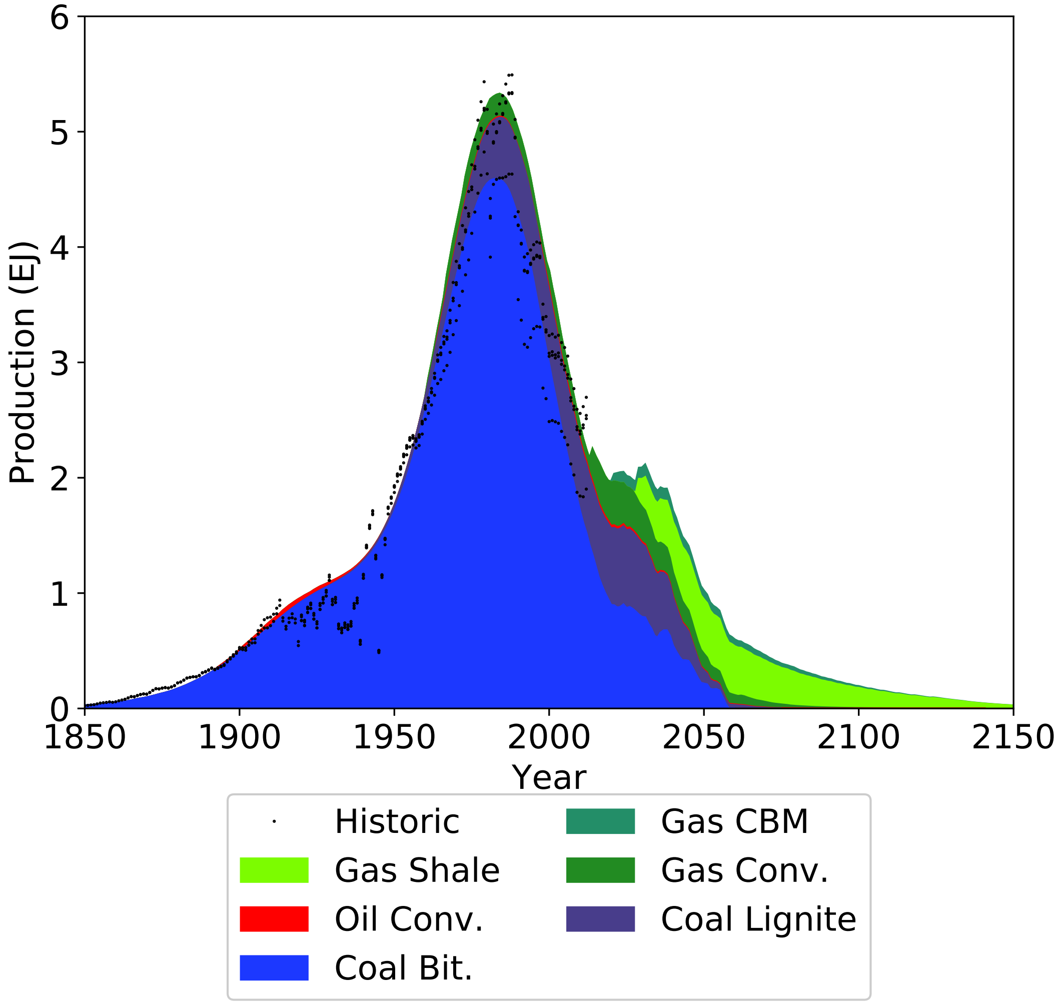

Figure 3.41: Poland projection by mineral type

Table 3.41: Peak years - Minerals

**Name**	**URR**	**Peak Year**	**Peak Rate**
Coal Bit.	291.9	1983	4.59
Coal Lignite	46.0	2023	0.68
Oil Conv.	3.08	1916	0.05
Gas Conv.	20.2	2022	0.39
Gas Shale	29.55	2048	0.48
Gas CBM	5.25	2032	0.11
**Total**	**395.98**	**1984**	**5.33**

3.19 Portugal

3.19.1 All Projections

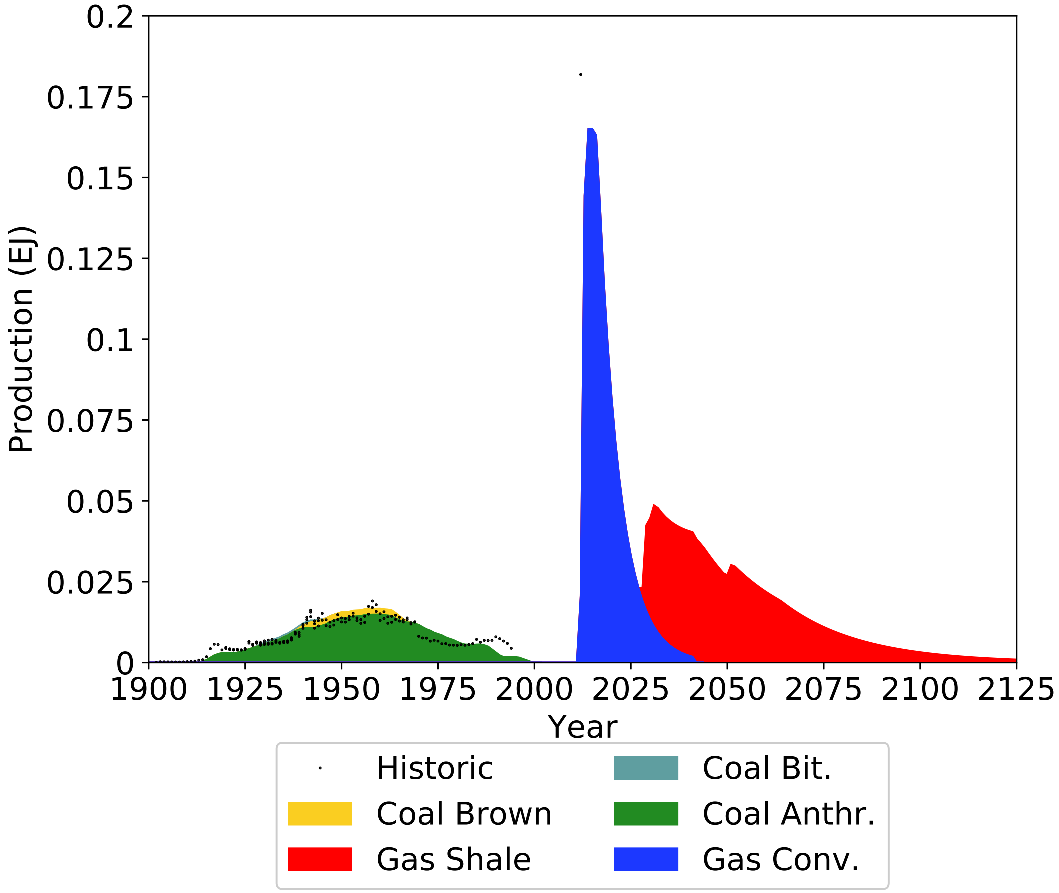

Figure 3.42: Portugal projections capped at 16

Table 3.42: Peak years - All

**Name**	**URR**	**Peak Year**	**Peak Rate**
Gas Conv.	1.5	2014	0.17
Gas Shale	1.48	2032	0.04
Coal Anthr.	0.68	1960	0.01
Coal Brown	0.05	1943	–
Coal Bit.	0.01	1932	–
**Total**	**3.72**	**2014**	**0.17**

3.19.2 By Mineral

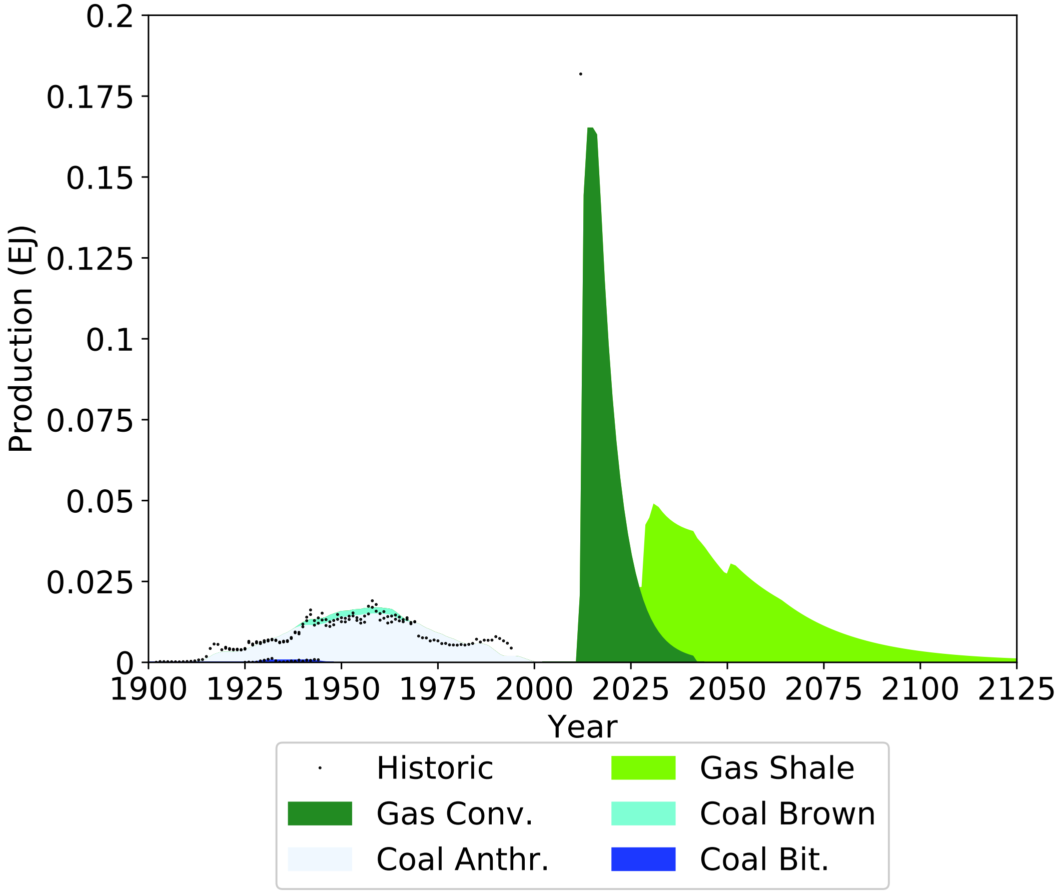

Figure 3.43: Portugal projection by mineral type

Table 3.43: Peak years - Minerals

Name	URR	Peak Year	Peak Rate
Coal Bit.	0.01	1932	–
Coal Anthr.	0.68	1960	0.01
Coal Brown	0.05	1943	–
Gas Conv.	1.5	2014	0.17
Gas Shale	1.48	2032	0.04
**Total**	**3.72**	**2014**	**0.17**

3.20 Romania

3.20.1 All Projections

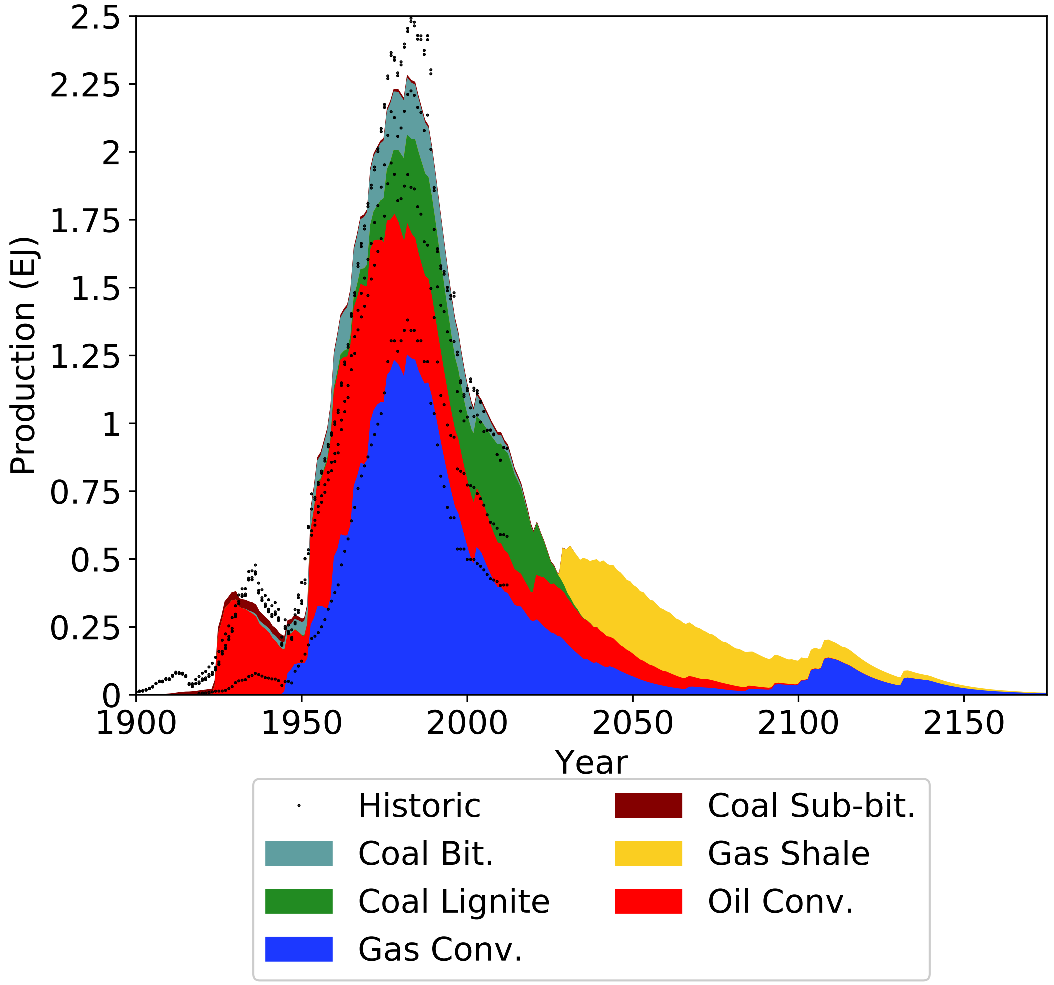

Figure 3.44: Romania projections capped at 16

Table 3.44: Peak years - All

**Name**	**URR**	**Peak Year**	**Peak Rate**
Gas Conv.	57.8	1982	1.25
Oil Conv.	38.65	1965	0.66
Coal Lignite	15.8	1987	0.38
Gas Shale	15.57	2044	0.27
Coal Bit.	9.0	1978	0.21
Coal Sub-bit.	1.6	1936	0.04
**Total**	**138.42**	**1982**	**2.28**

3.20.2 By Mineral

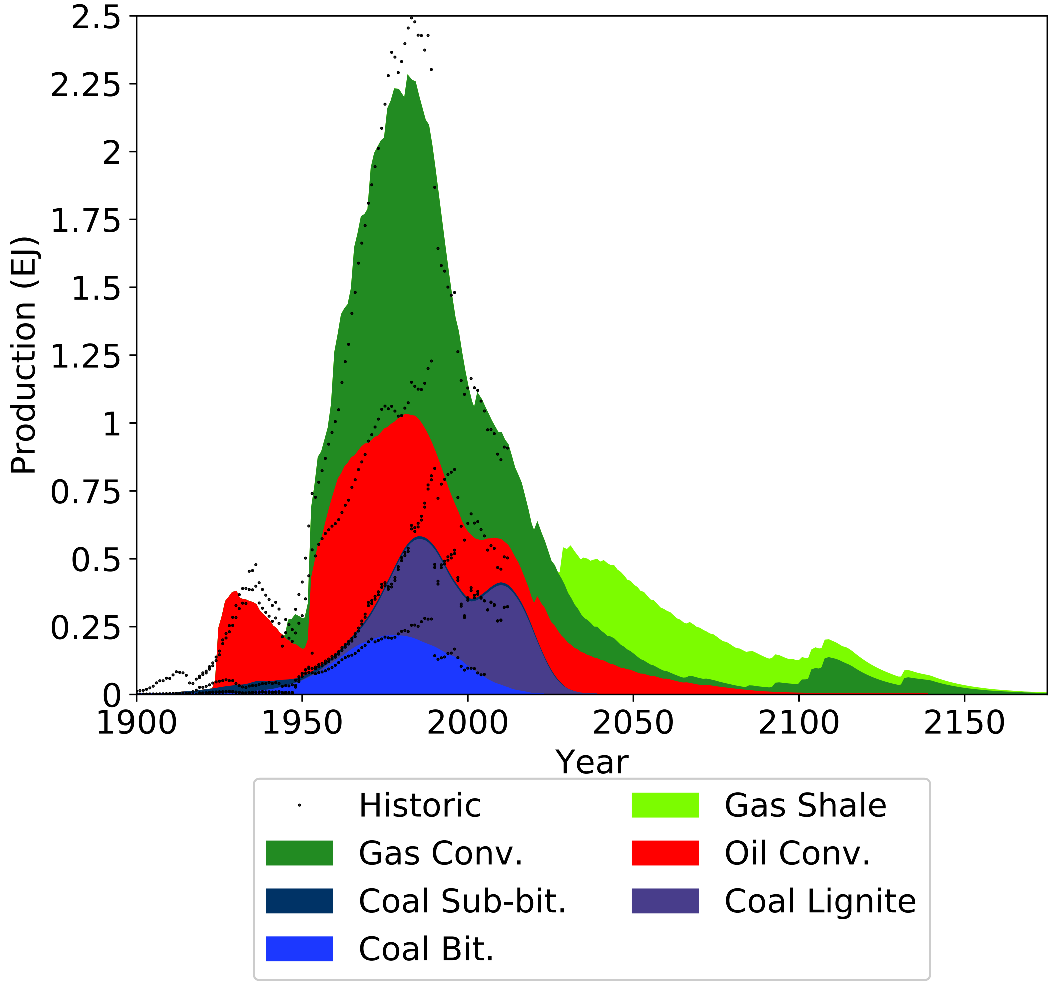

Figure 3.45: Romania projection by mineral type

Table 3.45: Peak years - Minerals

**Name**	**URR**	**Peak Year**	**Peak Rate**
Coal Bit.	9.0	1978	0.21
Coal Lignite	15.8	1987	0.38
Coal Sub-bit.	1.6	1936	0.04
Oil Conv.	38.65	1965	0.66
Gas Conv.	57.8	1982	1.25
Gas Shale	15.57	2044	0.27
**Total**	**138.42**	**1982**	**2.28**

3.21 Slovakia

3.21.1 All Projections

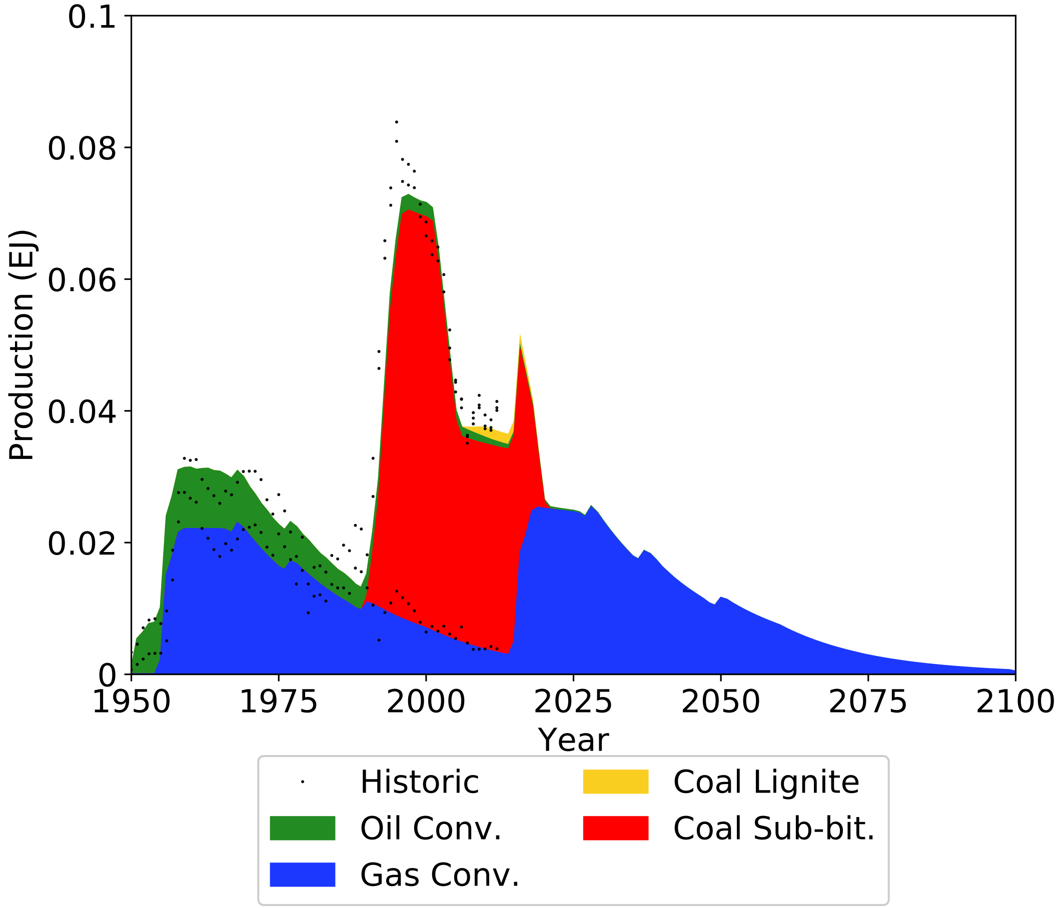

Figure 3.46: Slovakia projections capped at 16

Table 3.46: Peak years - All

**Name**	**URR**	**Peak Year**	**Peak Rate**
Gas Conv.	1.64	2028	0.03
Coal Sub-bit.	1.1	1997	0.06
Oil Conv.	0.32	1958	0.01
Coal Lignite	0.02	2011	–
**Total**	**3.08**	**1997**	**0.07**

3.21.2 By Mineral

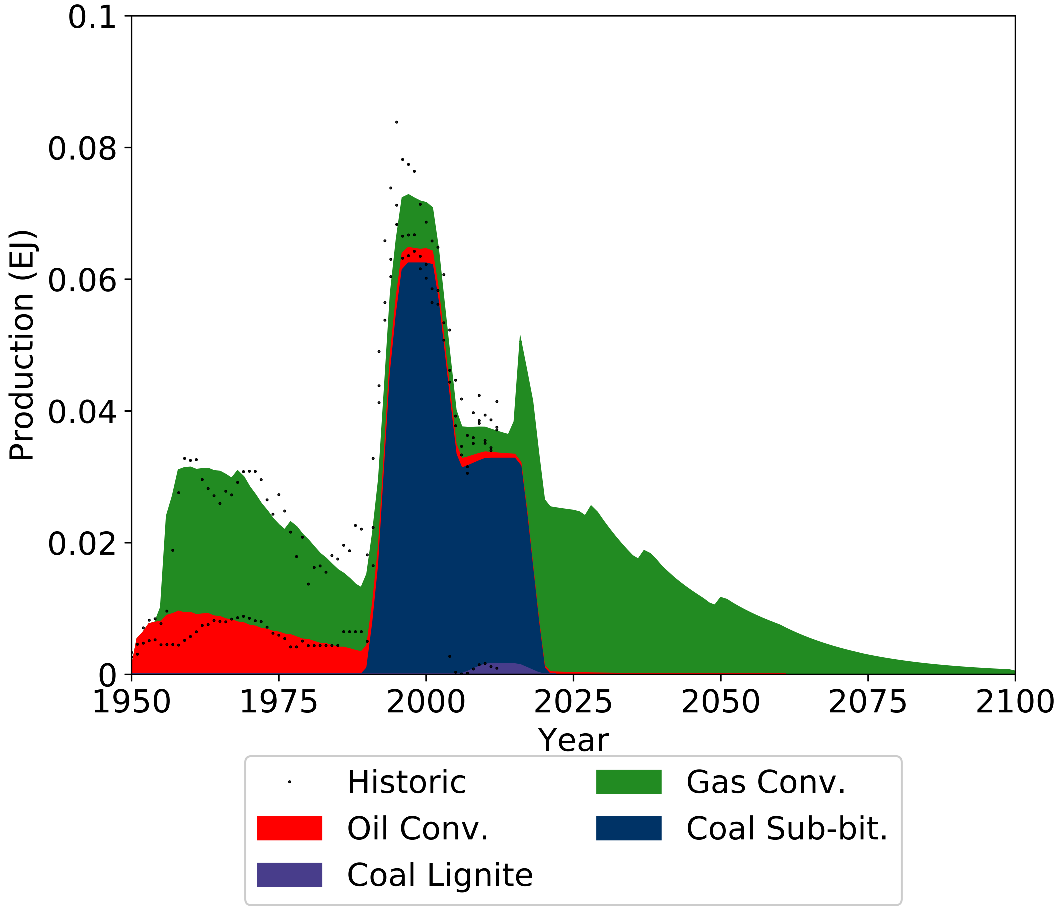

Figure 3.47: Slovakia projection by mineral type

Table 3.47: Peak years - Minerals

**Name**	**URR**	**Peak Year**	**Peak Rate**
Coal Lignite	0.02	2011	–
Coal Sub-bit.	1.1	1997	0.06
Oil Conv.	0.32	1958	0.01
Gas Conv.	1.64	2028	0.03
**Total**	**3.08**	**1997**	**0.07**

3.22 Spain

3.22.1 All Projections

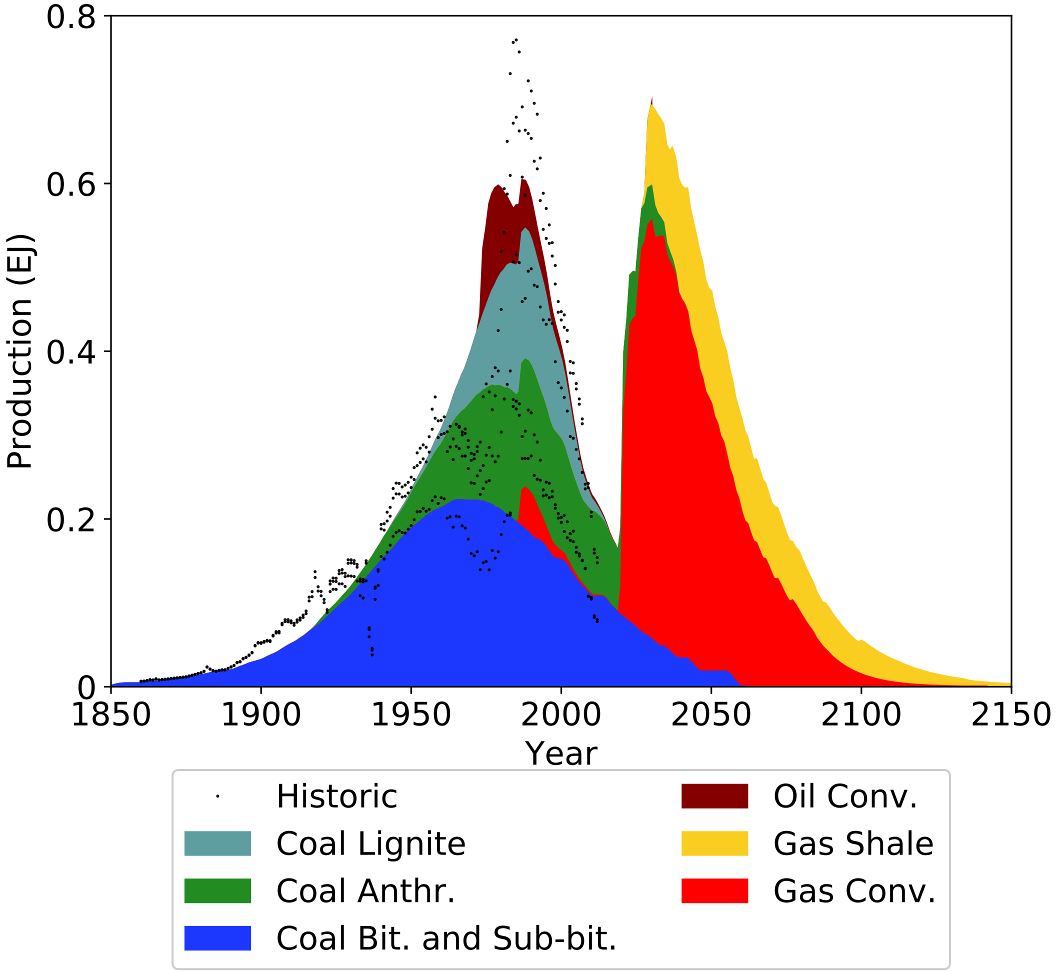

Figure 3.48: Spain projections capped at 16

Table 3.48: Peak years - All

**Name**	**URR**	**Peak Year**	**Peak Rate**
Coal Bit. and Sub-bit.	19.4	1966	0.22
Gas Conv.	19.1	2030	0.5
Coal Anthr.	9.03	1989	0.15
Gas Shale	7.56	2042	0.15
Coal Lignite	4.8	1987	0.16
Oil Conv.	1.78	1977	0.12
**Total**	**61.67**	**2030**	**0.69**

3.22.2 By Mineral

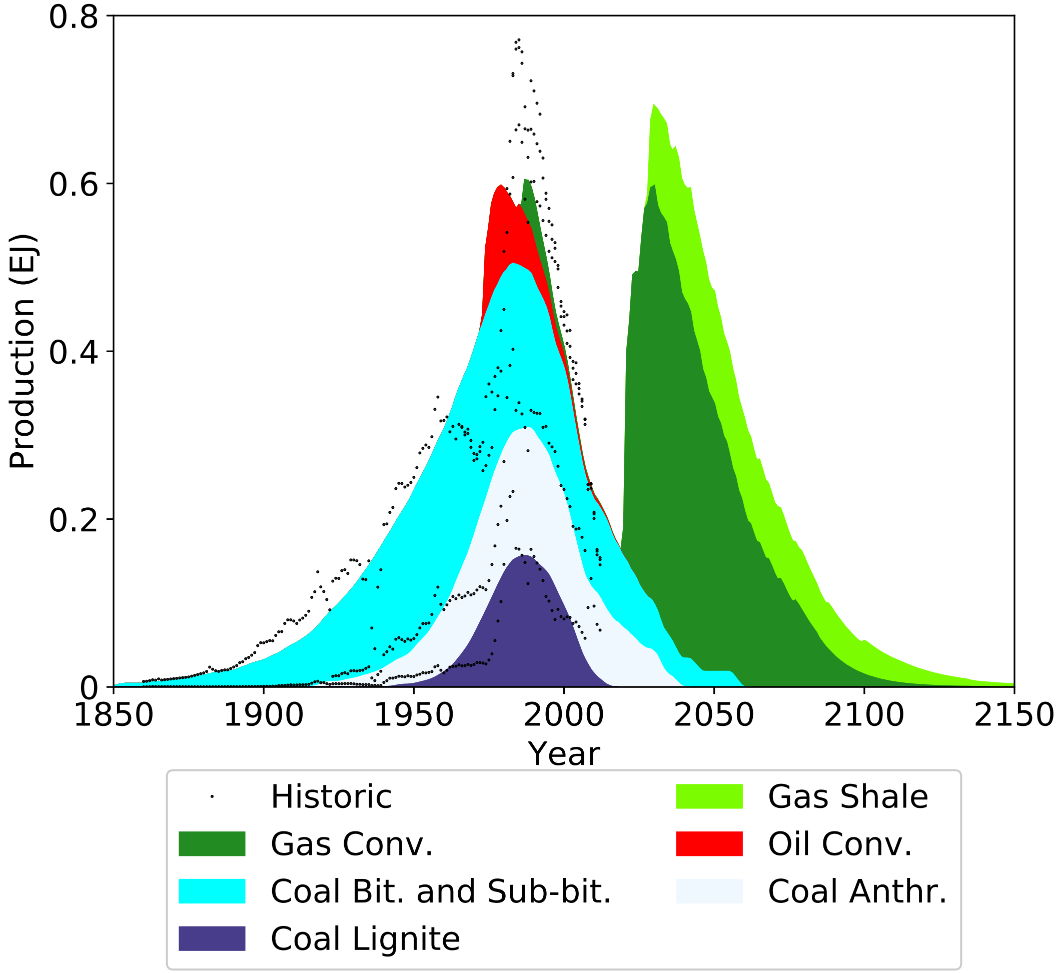

Figure 3.49: Spain projection by mineral type

Table 3.49: Peak years - Minerals

**Name**	**URR**	**Peak Year**	**Peak Rate**
Coal Lignite	4.8	1987	0.16
Coal Anthr.	9.03	1989	0.15
Coal Bit. and Sub-bit.	19.4	1966	0.22
Oil Conv.	1.78	1977	0.12
Gas Conv.	19.1	2030	0.5
Gas Shale	7.56	2042	0.15
**Total**	**61.67**	**2030**	**0.69**

3.23 Sweden

3.23.1 All Projections

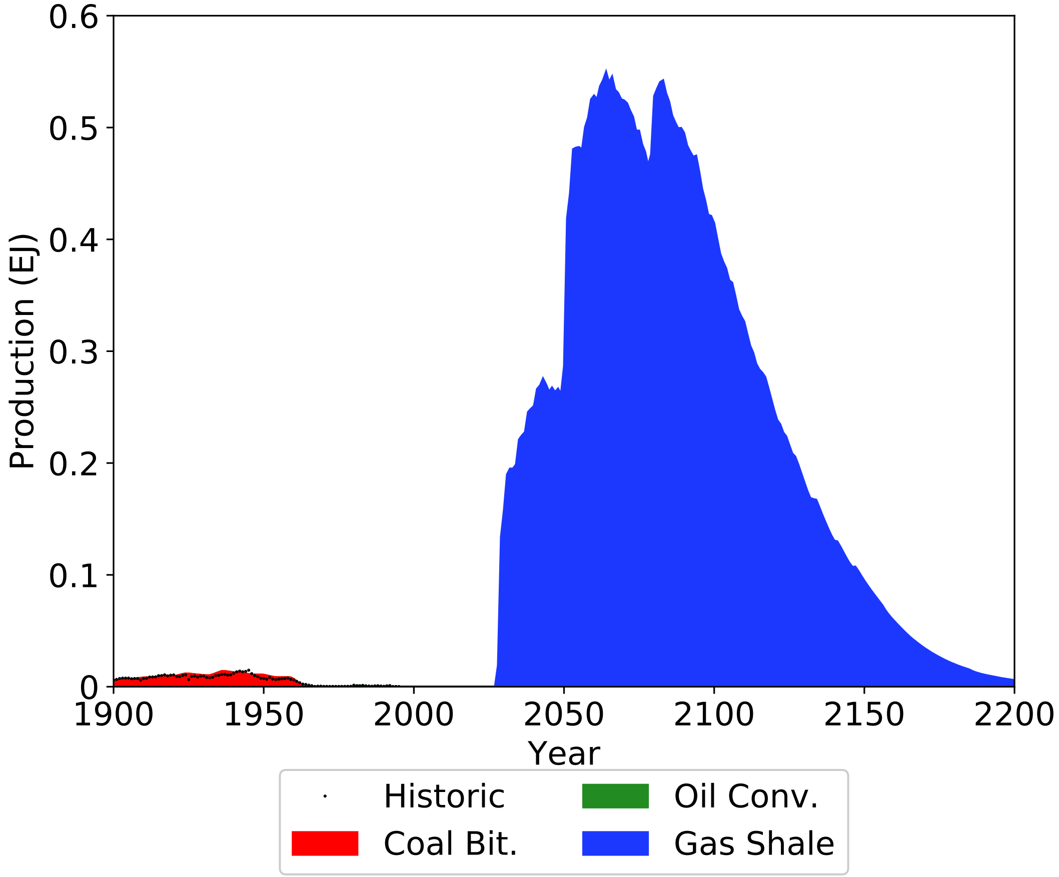

Figure 3.50: Sweden projections capped at 16

Table 3.50: Peak years - All

Name	URR	Peak Year	Peak Rate
Gas Shale	43.05	2064	0.55
Coal Bit.	0.69	1937	0.01
Oil Conv.	–	1982	–
**Total**	**43.74**	**2064**	**0.55**

3.23.2 By Mineral

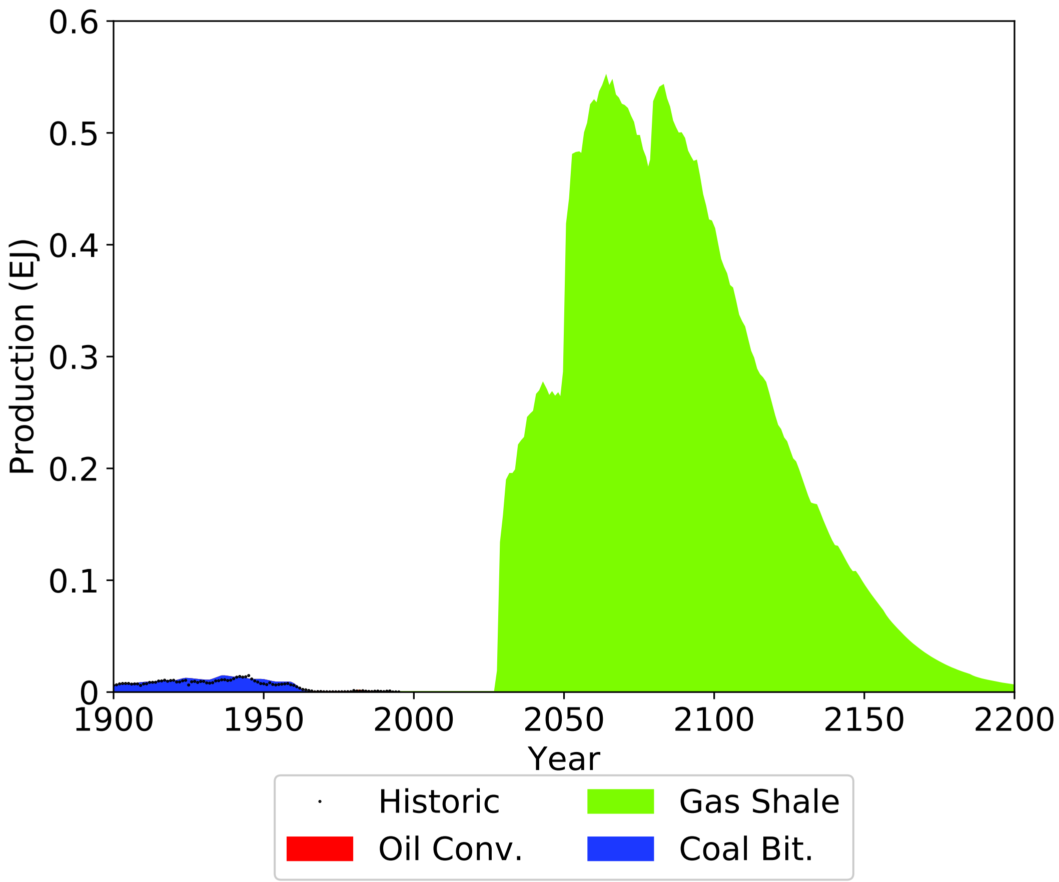

Figure 3.51: Sweden projection by mineral type

Table 3.51: Peak years - Minerals

Name	URR	Peak Year	Peak Rate
Coal Bit.	0.69	1937	0.01
Oil Conv.	–	1982	–
Gas Shale	43.05	2064	0.55
**Total**	**43.74**	**2064**	**0.55**

3.24 Switzerland

3.24.1 All Projections

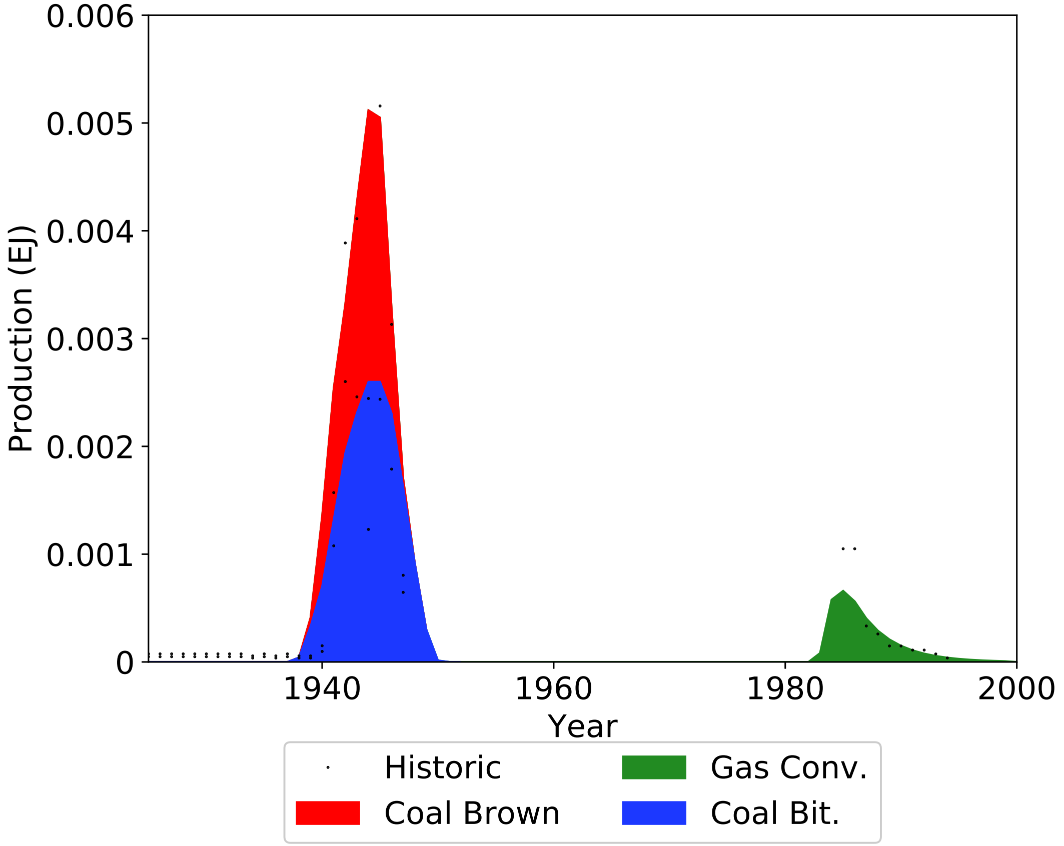

Figure 3.52: Switzerland projections capped at 16

Table 3.52: Peak years - All

Name	URR	Peak Year	Peak Rate
Coal Bit.	0.02	1944	–
Coal Brown	0.01	1944	–
Gas Conv.	–	1985	–
**Total**	**0.03**	**1944**	**0.01**

3.24.2 By Mineral

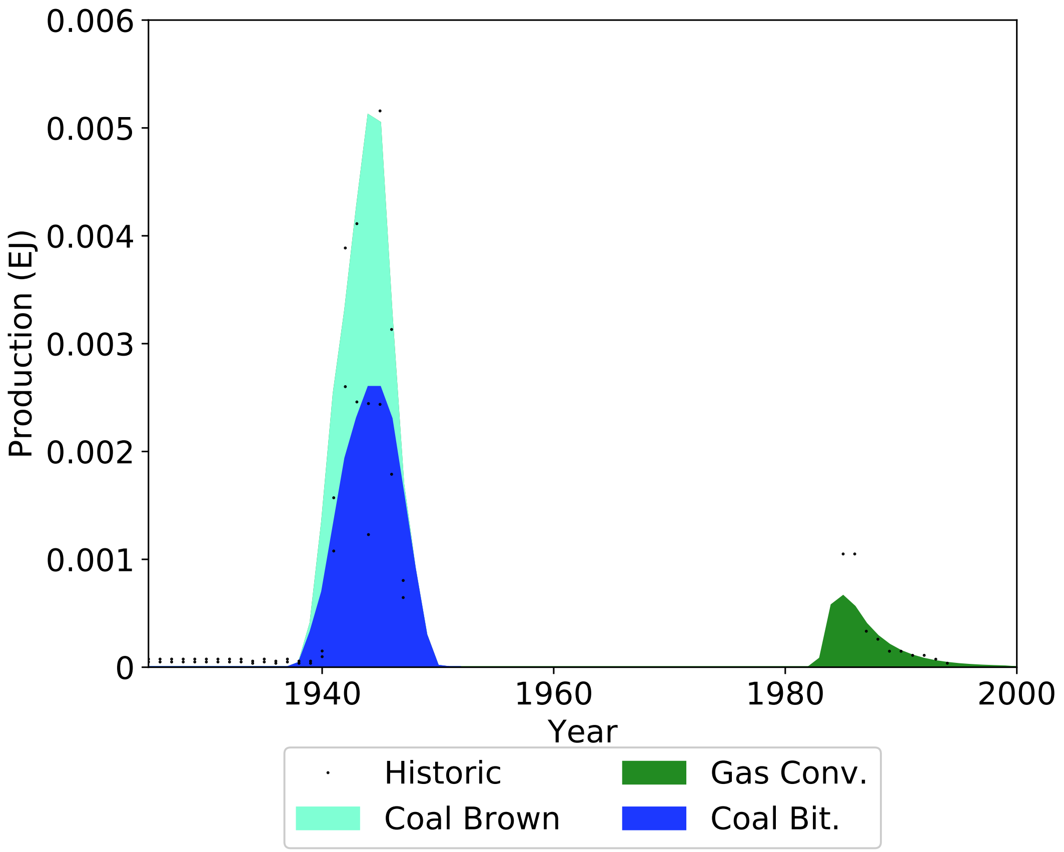

Figure 3.53: Switzerland projection by mineral type

Table 3.53: Peak years - Minerals

Name	URR	Peak Year	Peak Rate
Coal Bit.	0.02	1944	–
Coal Brown	0.01	1944	–
Gas Conv.	–	1985	–
**Total**	**0.03**	**1944**	**0.01**

3.25 Turkey

3.25.1 All Projections

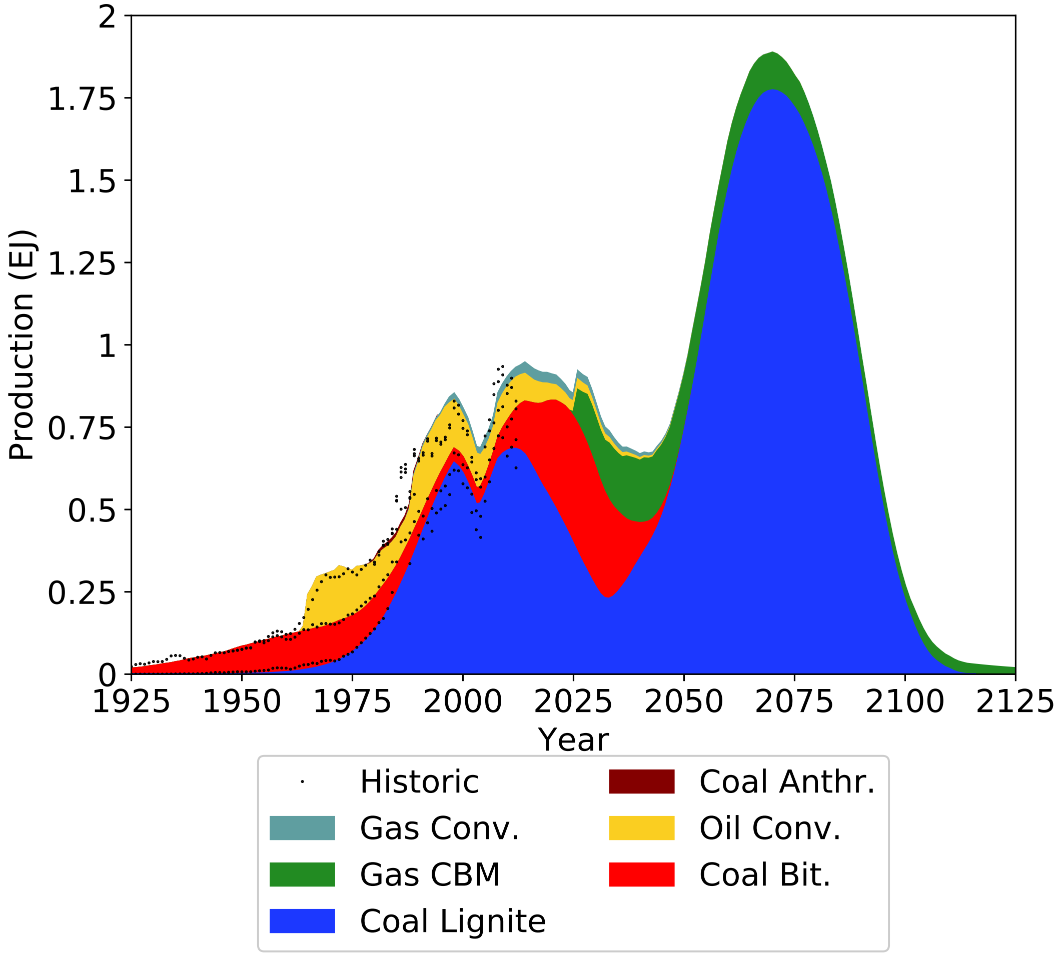

Figure 3.54: Turkey projections capped at 16

Table 3.54: Peak years - All

**Name**	**URR**	**Peak Year**	**Peak Rate**
Coal Lignite	96.4	2070	1.77
Coal Bit.	14.8	2027	0.39
Gas CBM	10.5	2041	0.2
Oil Conv.	7.32	1991	0.19
Gas Conv.	1.3	2014	0.03
Coal Anthr.	0.13	1983	0.01
**Total**	**130.45**	**2070**	**1.89**

3.25.2 By Mineral

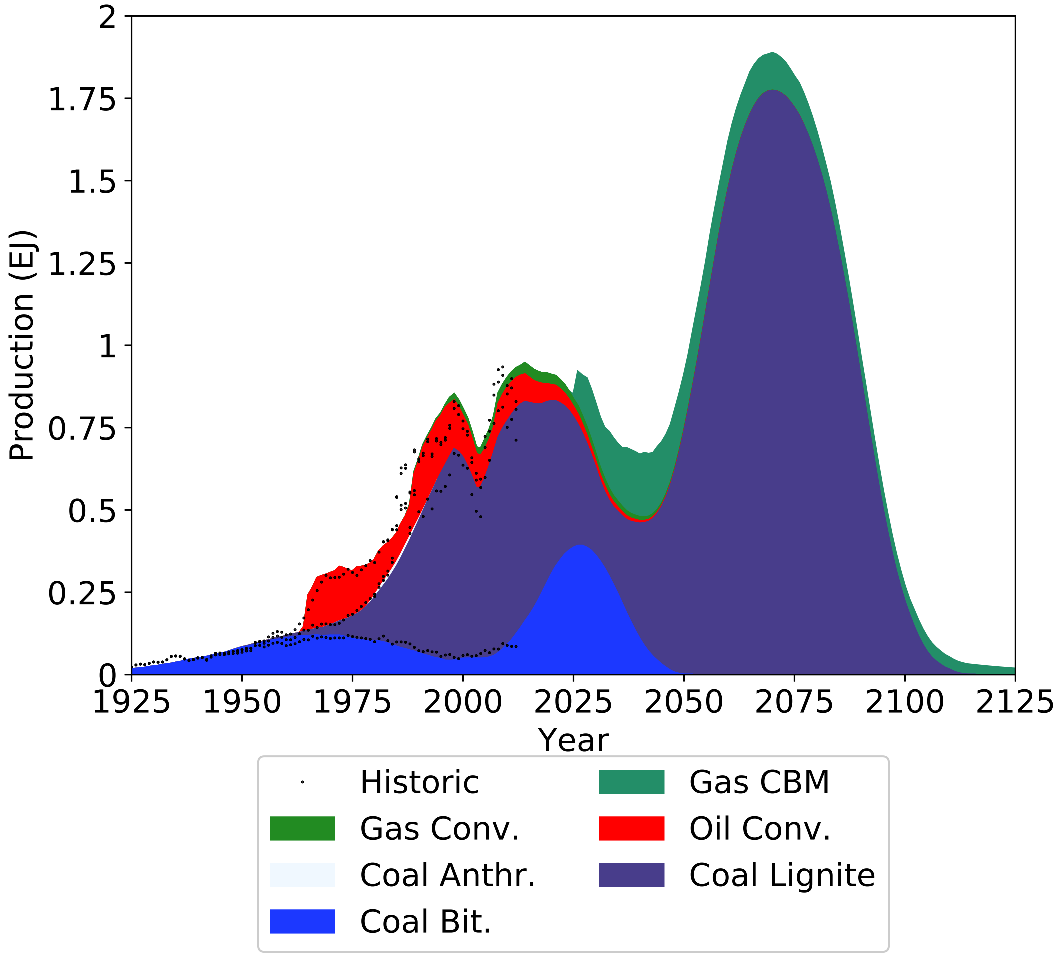

Figure 3.55: Turkey projection by mineral type

Table 3.55: Peak years - Minerals

Name	URR	Peak Year	Peak Rate
Coal Bit.	14.8	2027	0.39
Coal Lignite	96.4	2070	1.77
Coal Anthr.	0.13	1983	0.01
Oil Conv.	7.32	1991	0.19
Gas Conv.	1.3	2014	0.03
Gas CBM	10.5	2041	0.2
**Total**	**130.45**	**2070**	**1.89**

3.26 UK

3.26.1 All Projections

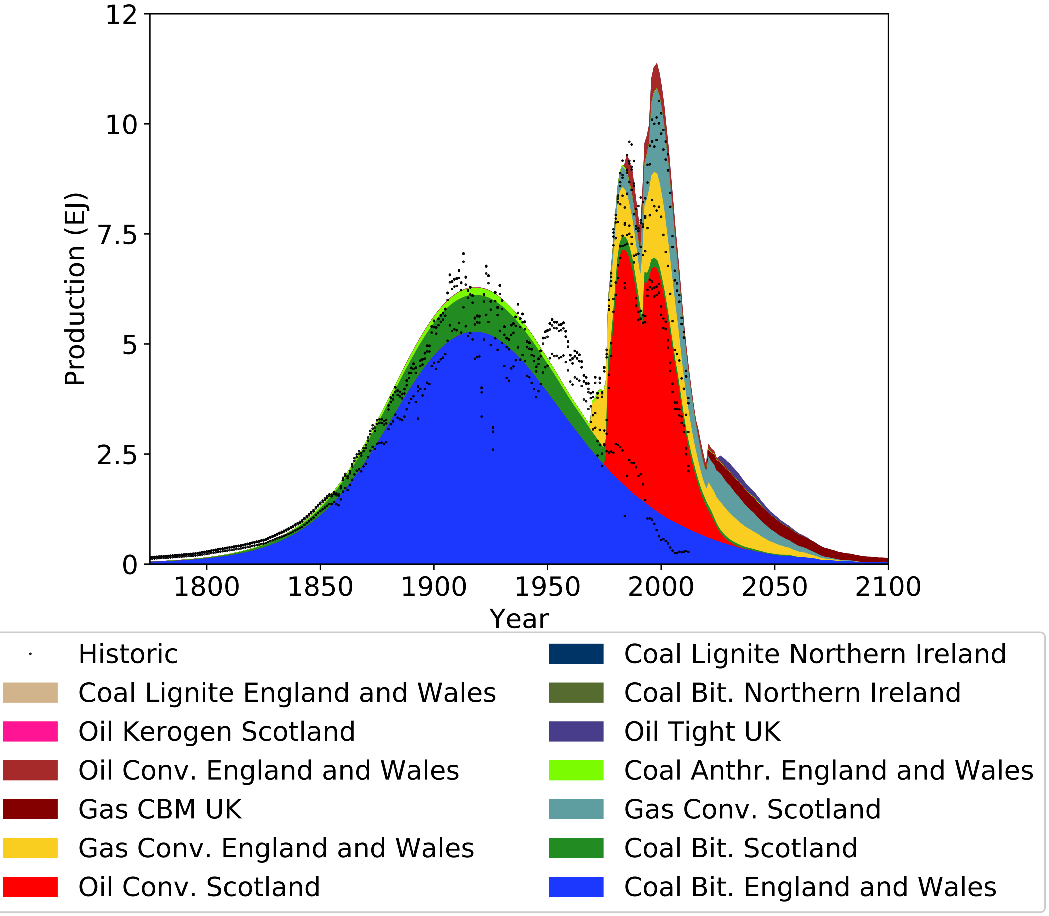

Figure 3.56: UK projections capped at 16

Table 3.56: Peak years - All

**Name**	**URR**	**Peak Year**	**Peak Rate**
Coal Bit. England and Wales	554.9	1918	5.26
Oil Conv. Scotland	164.26	1997	5.52
Coal Bit. Scotland	88.7	1918	0.84
Gas Conv. England and Wales	73.94	1998	1.96
Gas Conv. Scotland	57.36	1999	1.94
Gas CBM UK	21.0	2036	0.35
Coal Anthr. England and Wales	17.0	1921	0.17
Oil Conv. England and Wales	13.28	1987	0.58
Oil Tight UK	4.01	2032	0.18
Oil Kerogen Scotland	0.68	1887	0.01
Coal Bit. Northern Ireland	–	1933	–
Coal Lignite England and Wales	–	1947	–
Coal Lignite Northern Ireland	–	1944	–
**Total**	**995.13**	**1998**	**11.34**

3.26.2 By Mineral

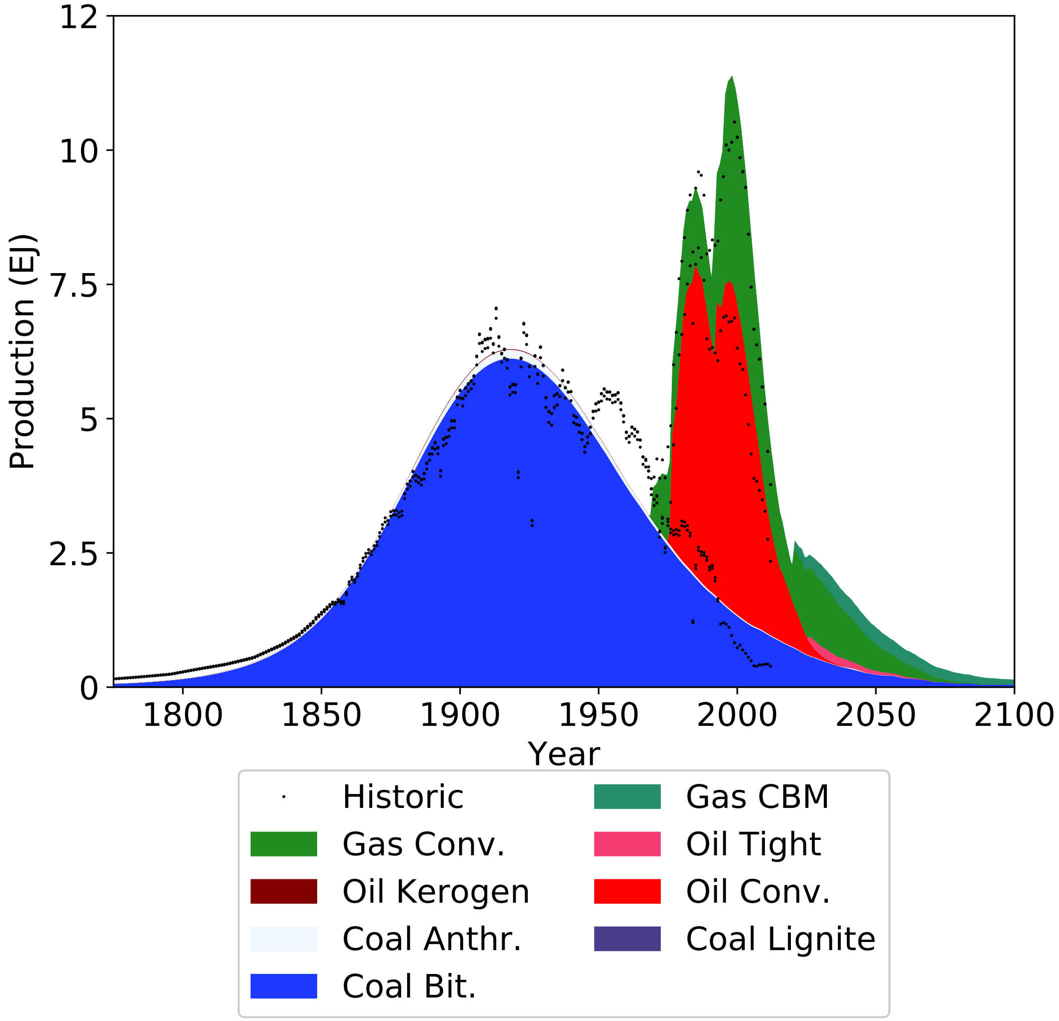

Figure 3.57: UK projection by mineral type

3.26.3 Regional Projections

Table 3.57: Peak years - Minerals

Name	URR	Peak Year	Peak Rate
Coal Bit.	643.6	1918	6.1
Coal Lignite	–	1947	–
Coal Anthr.	17.0	1921	0.17
Oil Conv.	177.54	1997	6.09
Oil Kerogen	0.68	1887	0.01
Oil Tight	4.01	2032	0.18
Gas Conv.	131.3	1998	3.86
Gas CBM	21.0	2036	0.35
**Total**	**995.13**	**1998**	**11.34**

England and Wales

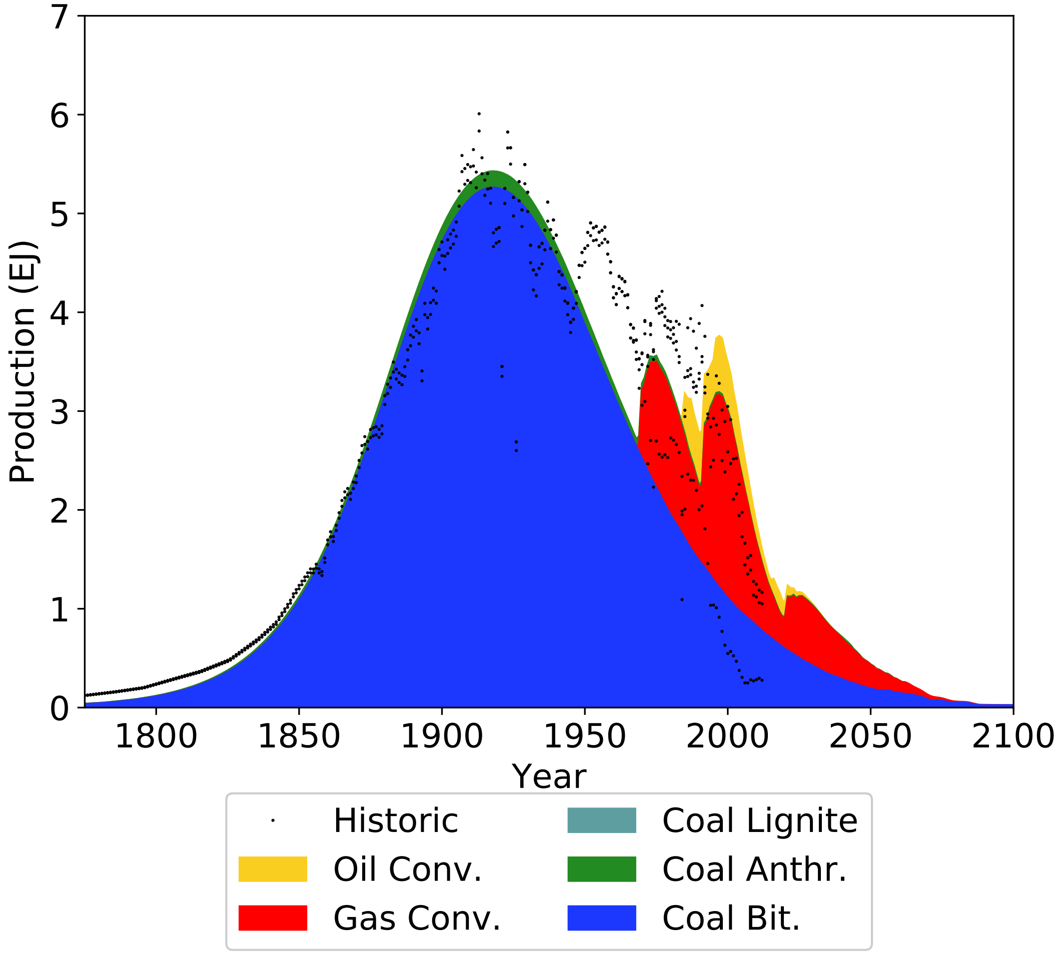

Figure 3.58: UK - England and Wales projections capped at 16

Table 3.58: Peak years - All

Name	URR	Peak Year	Peak Rate
Coal Bit. England and Wales	554.9	1918	5.26
Gas Conv. England and Wales	73.94	1998	1.96
Coal Anthr. England and Wales	17.0	1921	0.17
Oil Conv. England and Wales	13.28	1987	0.58
Coal Lignite England and Wales	–	1947	–
**Total**	**659.12**	**1918**	**5.43**

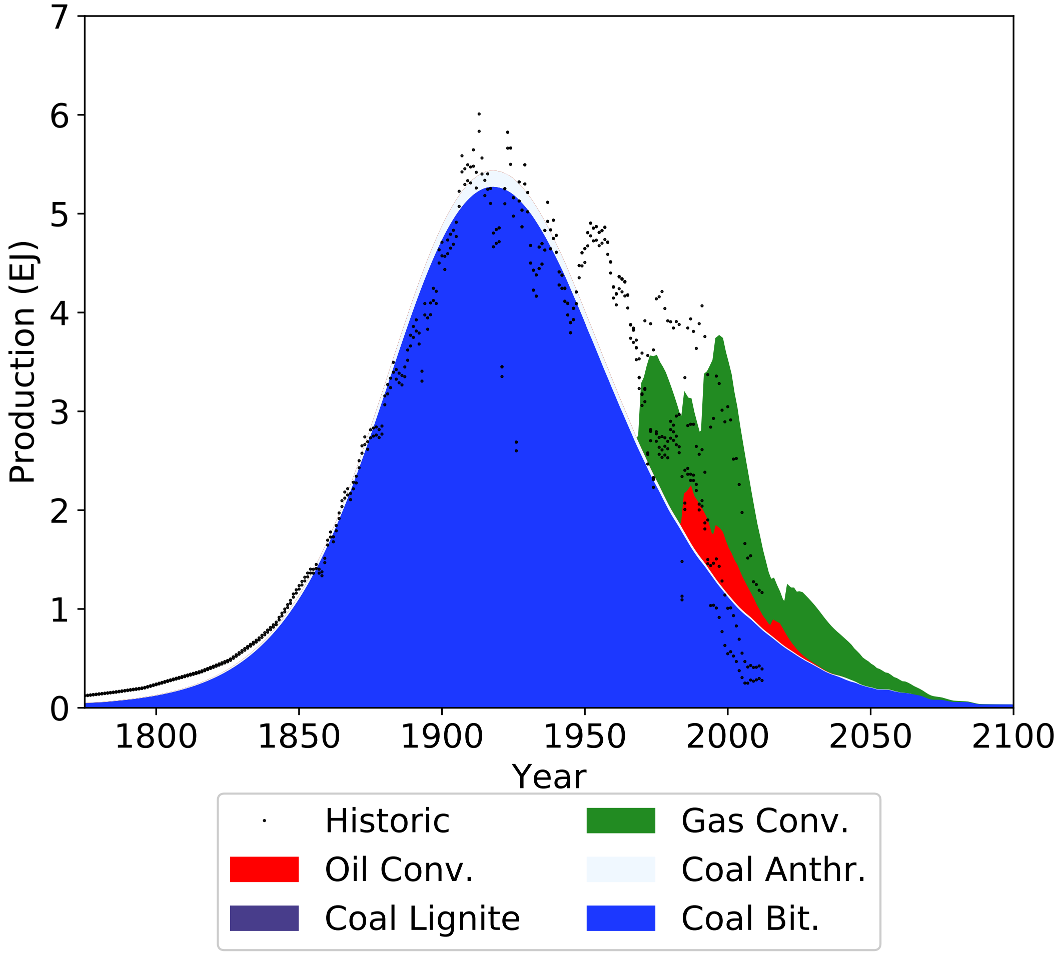

Figure 3.59: UK - England and Wales projection by mineral type

Table 3.59: Peak years - Minerals

**Name**	**URR**	**Peak Year**	**Peak Rate**
Coal Bit.	554.9	1918	5.26
Coal Lignite	–	1947	–
Coal Anthr.	17.0	1921	0.17
Oil Conv.	13.28	1987	0.58
Gas Conv.	73.94	1998	1.96
**Total**	**659.12**	**1918**	**5.43**

Northern Ireland

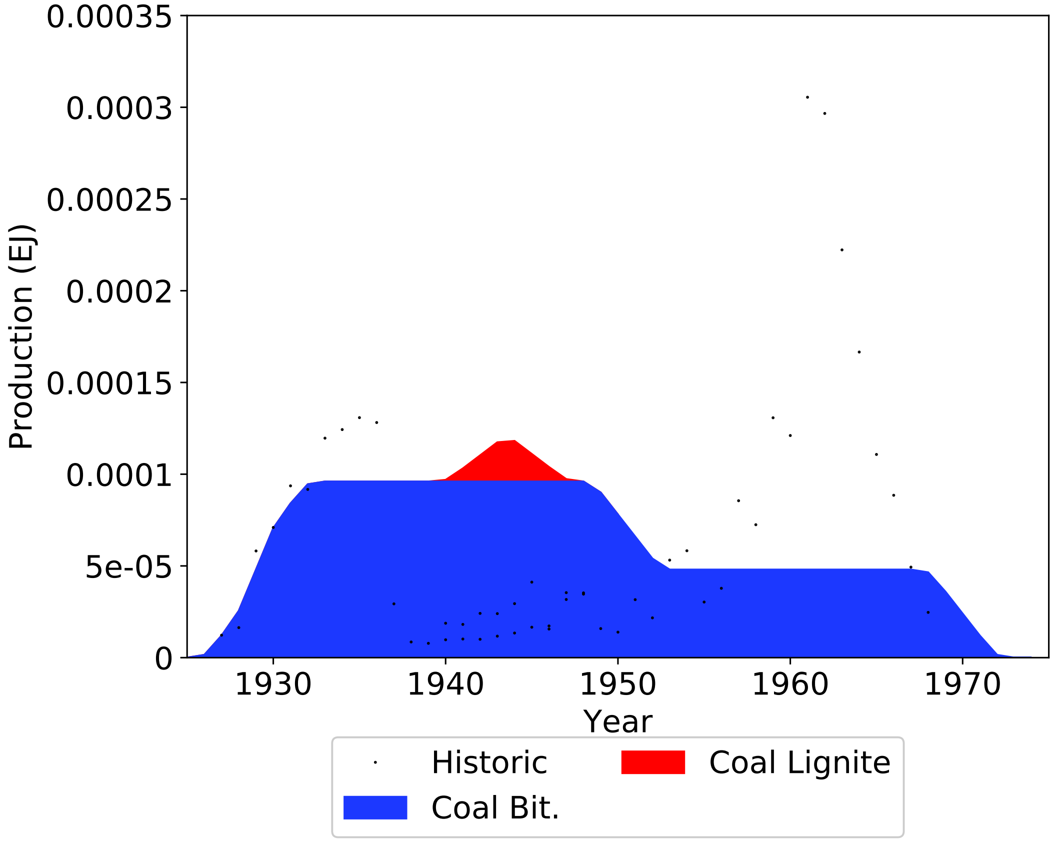

Figure 3.60: UK - Northern Ireland projections capped at 16

Table 3.60: Peak years - All

Name	URR	Peak Year	Peak Rate
Coal Bit. Northern Ireland	–	1933	–
Coal Lignite Northern Ireland	–	1944	–
**Total**	–	**1944**	–

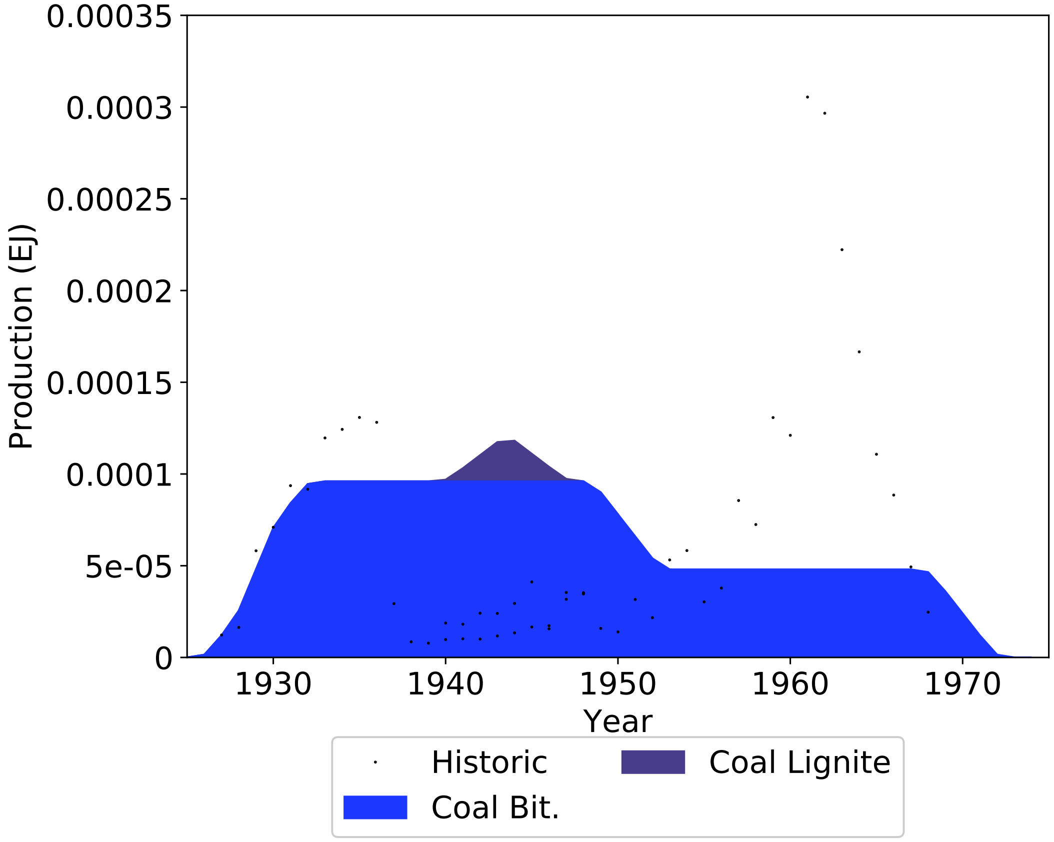

Figure 3.61: UK - Northern Ireland projection by mineral type

Table 3.61: Peak years - Minerals

Name	URR	Peak Year	Peak Rate
Coal Bit.	–	1933	–
Coal Lignite	–	1944	–
**Total**	–	**1944**	–

Scotland

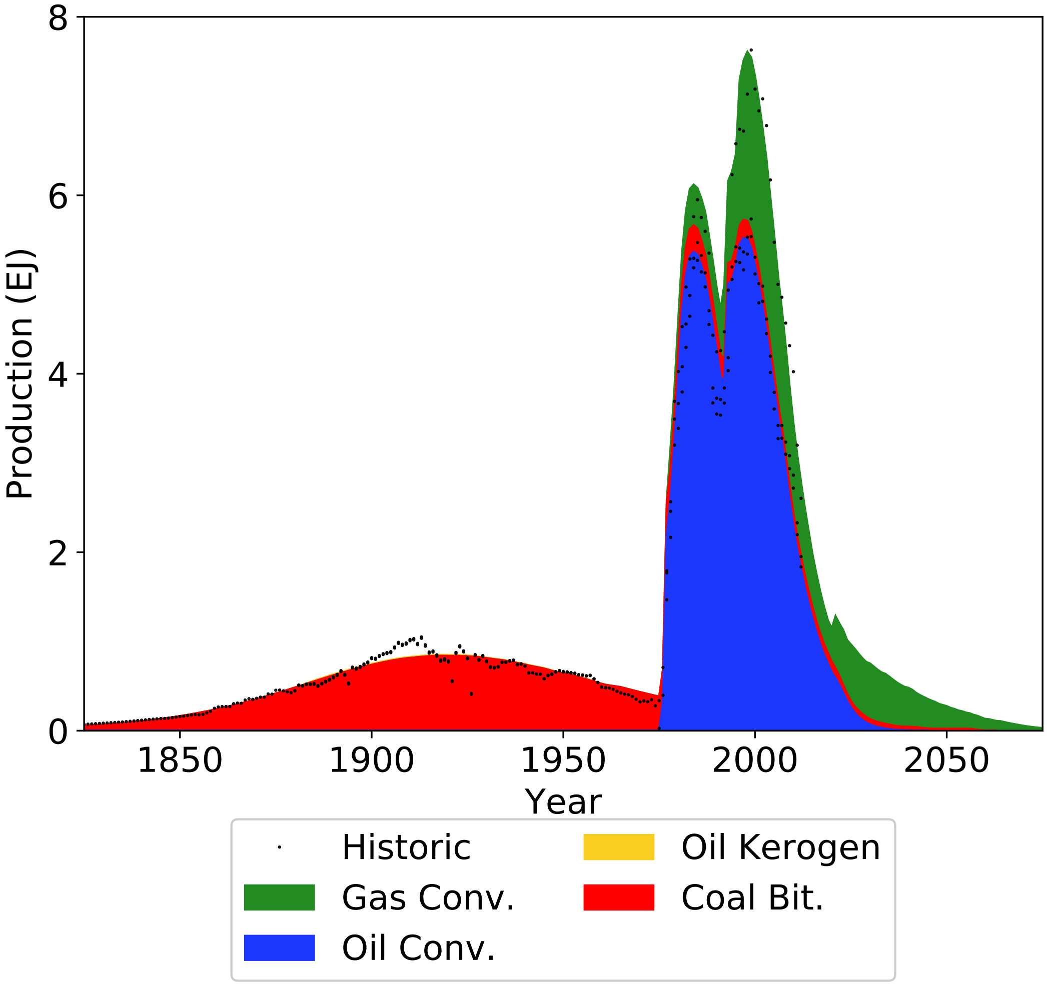

Figure 3.62: UK - Scotland projections capped at 16

Table 3.62: Peak years - All

Name	URR	Peak Year	Peak Rate
Oil Conv. Scotland	164.26	1997	5.52
Coal Bit. Scotland	88.7	1918	0.84
Gas Conv. Scotland	57.36	1999	1.94
Oil Kerogen Scotland	0.68	1887	0.01
**Total**	**311.0**	**1998**	**7.61**

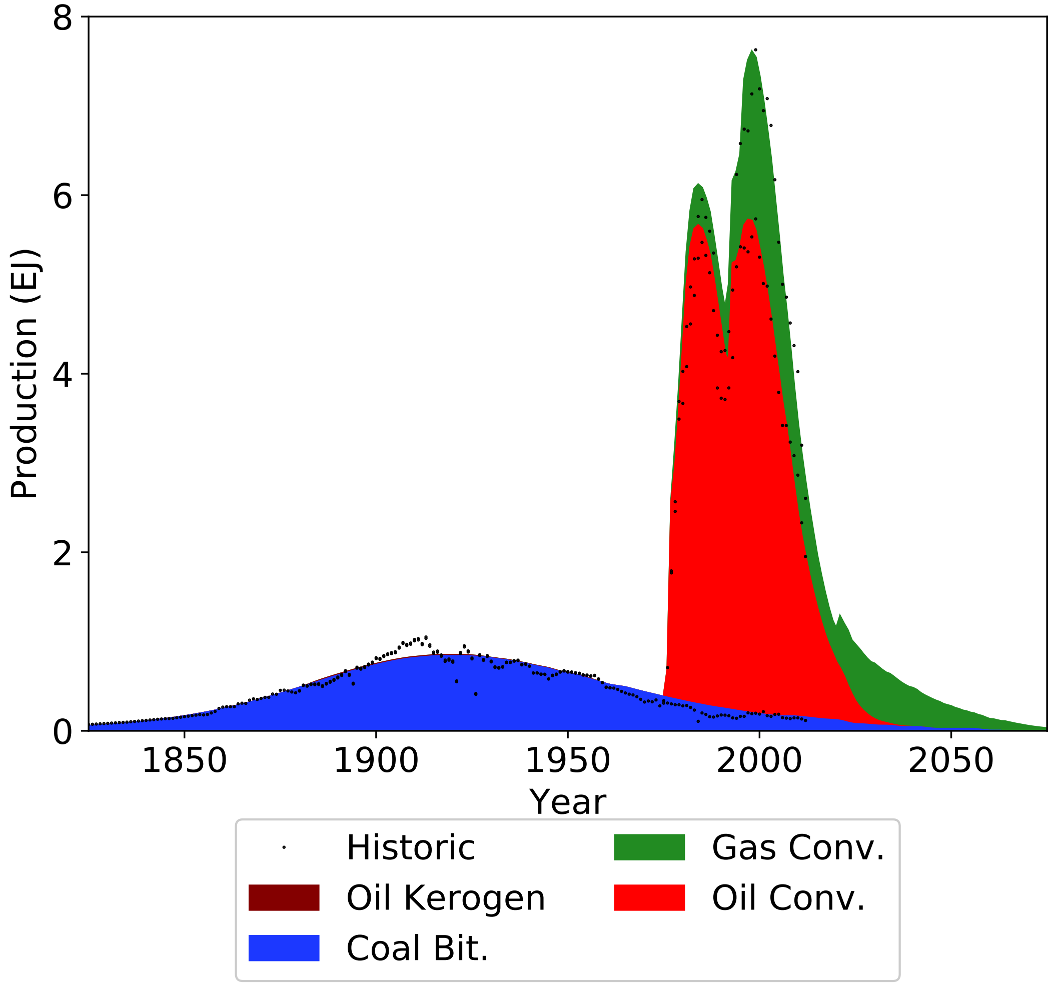

Figure 3.63: UK - Scotland projection by mineral type

Table 3.63: Peak years - Minerals

Name	URR	Peak Year	Peak Rate
Coal Bit.	88.7	1918	0.84
Oil Conv.	164.26	1997	5.52
Oil Kerogen	0.68	1887	0.01
Gas Conv.	57.36	1999	1.94
**Total**	**311.0**	**1998**	**7.61**

UK

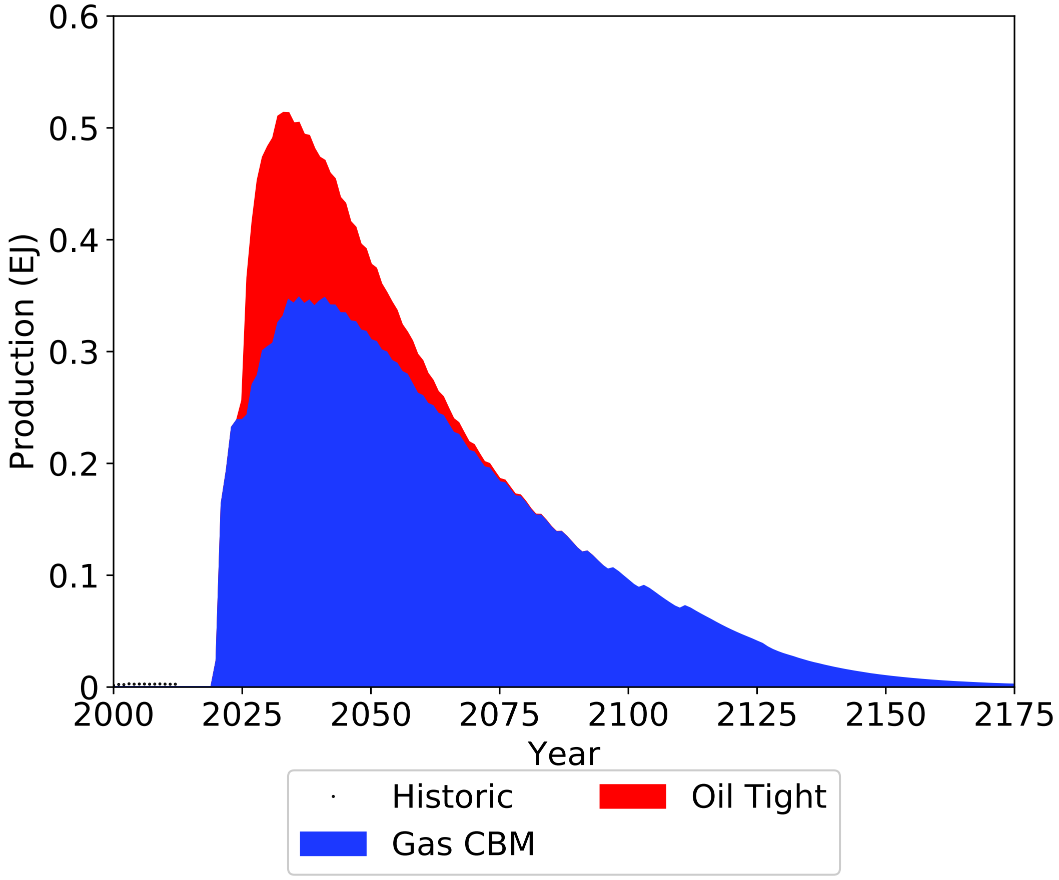

Figure 3.64: UK - UK projections capped at 16

Table 3.64: Peak years - All

Name	URR	Peak Year	Peak Rate
Gas CBM UK	21.0	2036	0.35
Oil Tight UK	4.01	2032	0.18
**Total**	**25.01**	**2033**	**0.51**

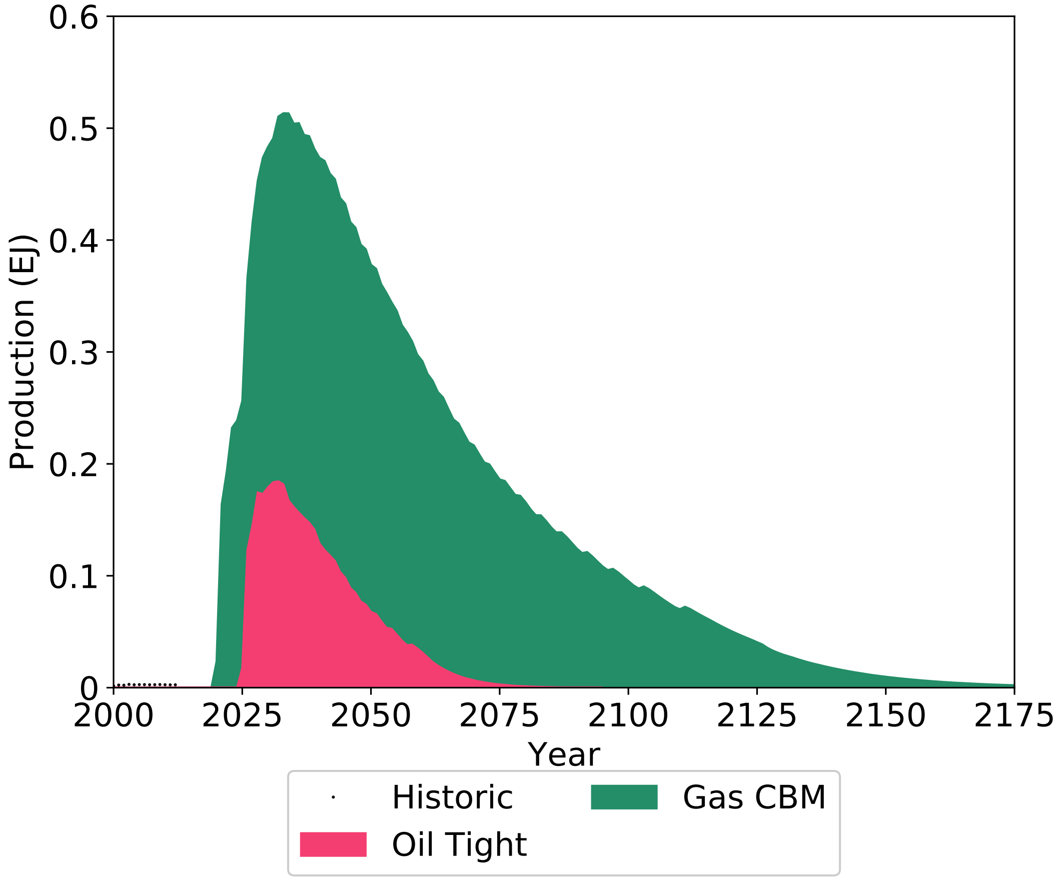

Figure 3.65: UK - UK projection by mineral type

Table 3.65: Peak years - Minerals

Name	URR	Peak Year	Peak Rate
Oil Tight	4.01	2032	0.18
Gas CBM	21.0	2036	0.35
**Total**	**25.01**	**2033**	**0.51**

3.26.4 Projection by region

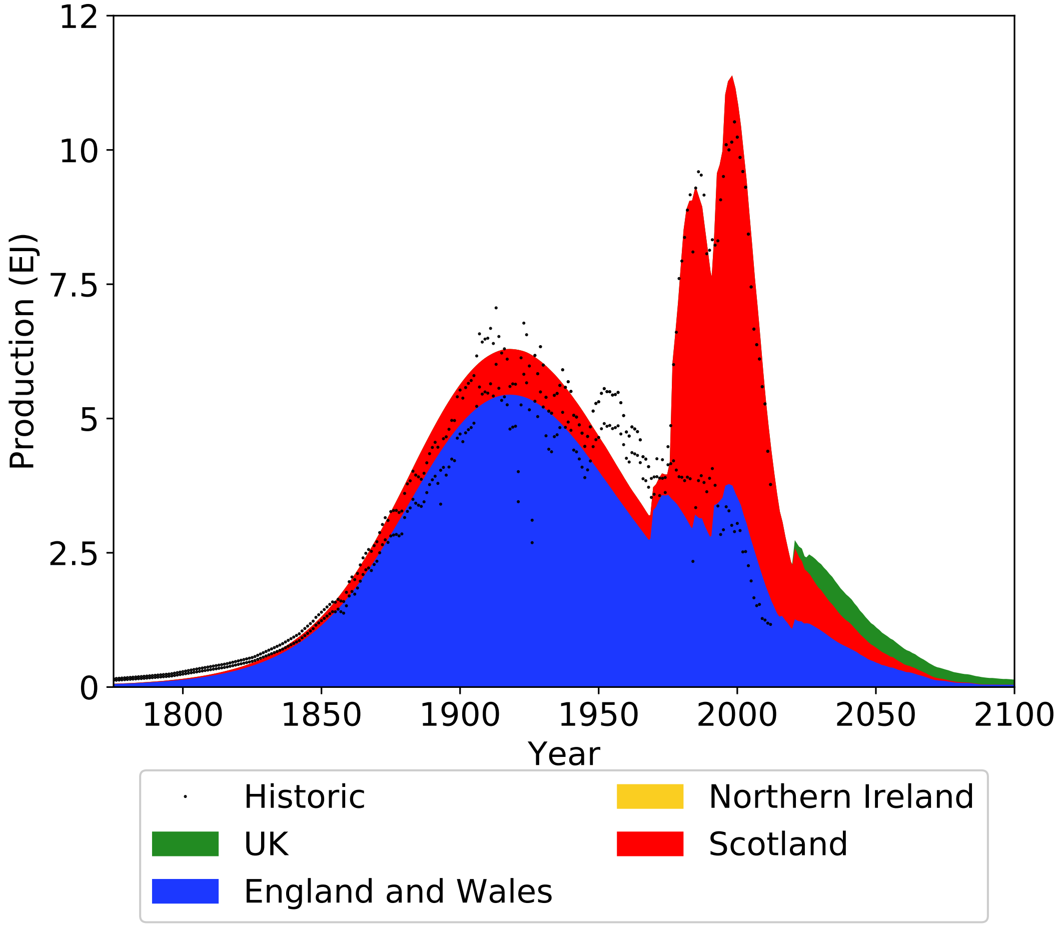

Figure 3.66: UK by region projections capped at 16

Table 3.66: Peak years - All

**Name**	**URR**	**Peak Year**	**Peak Rate**
England and Wales	659.12	1918	5.43
Scotland	311.0	1998	7.61
UK	25.01	2033	0.51
Northern Ireland	–	1944	–
**Total**	**995.13**	**1998**	**11.34**

3.27 Yugoslavia

3.27.1 All Projections

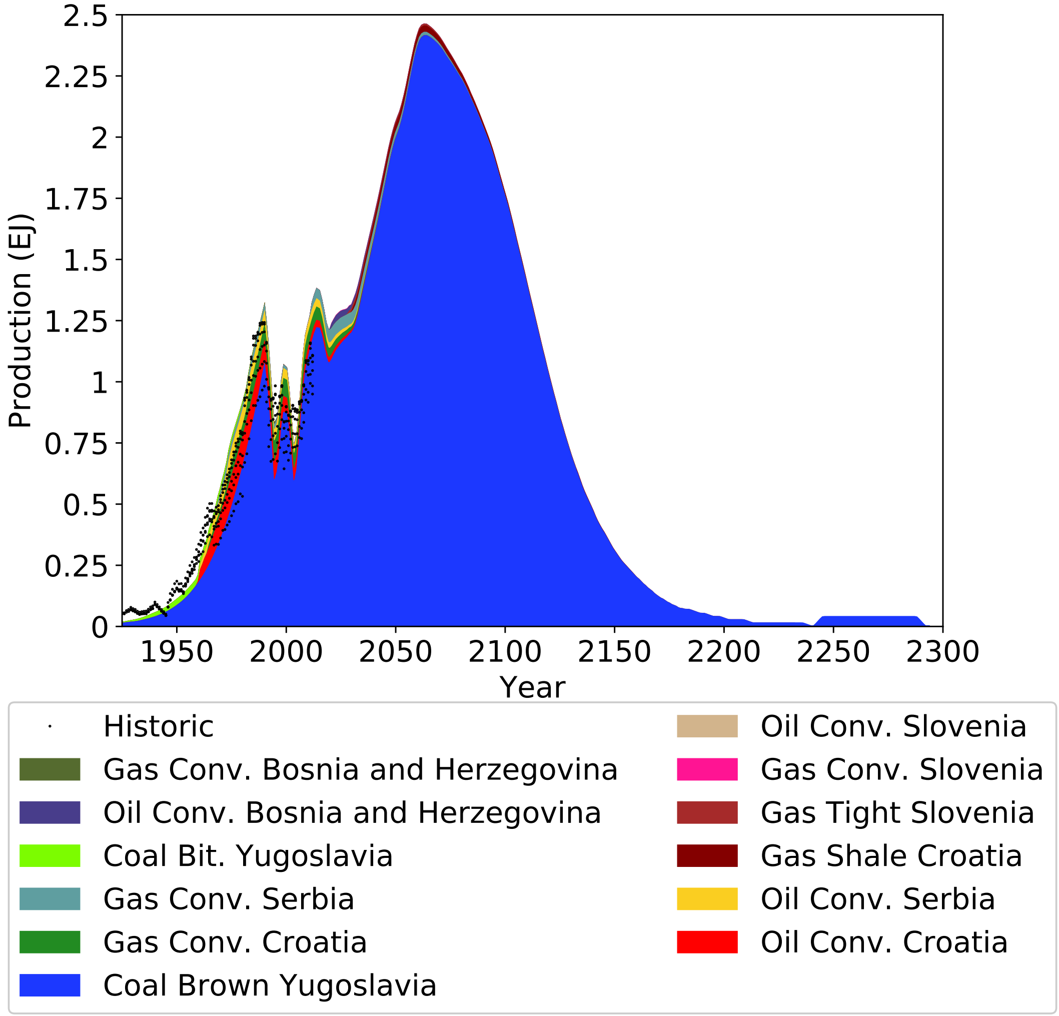

Figure 3.67: Yugoslavia projections capped at 16

Table 3.67: Peak years - All

**Name**	**URR**	**Peak Year**	**Peak Rate**
Coal Brown Yugoslavia	251.5	2064	2.41
Oil Conv. Croatia	4.73	1976	0.13
Gas Conv. Croatia	3.43	1993	0.08
Oil Conv. Serbia	2.9	1978	0.06
Gas Conv. Serbia	2.81	2022	0.05
Gas Shale Croatia	1.85	2049	0.03
Coal Bit. Yugoslavia	1.16	1956	0.03
Gas Tight Slovenia	0.56	2032	0.01
Oil Conv. Bosnia and Herzegovina	0.42	2024	0.03
Gas Conv. Slovenia	0.04	2022	–
Gas Conv. Bosnia and Herzegovina	0.03	1995	0.01
Oil Conv. Slovenia	–	1996	–
**Total**	**269.43**	**2063**	**2.46**

3.27.2 By Mineral

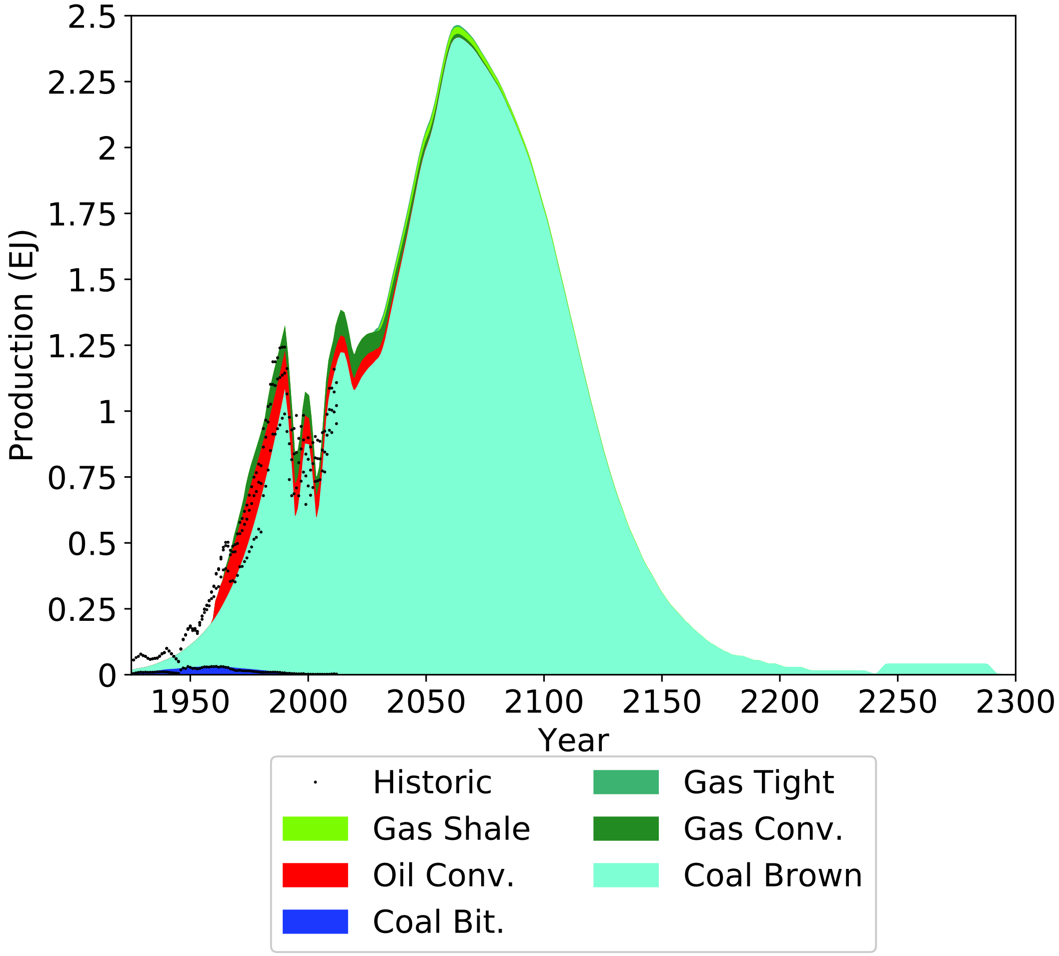

Figure 3.68: Yugoslavia projection by mineral type

3.27.3 Regional Projections

Table 3.68: Peak years - Minerals

**Name**	**URR**	**Peak Year**	**Peak Rate**
Coal Bit.	1.16	1956	0.03
Coal Brown	251.5	2064	2.41
Oil Conv.	8.05	1977	0.19
Gas Conv.	6.32	1994	0.11
Gas Shale	1.85	2049	0.03
Gas Tight	0.56	2032	0.01
**Total**	**269.43**	**2063**	**2.46**

Bosnia and Herzegovina

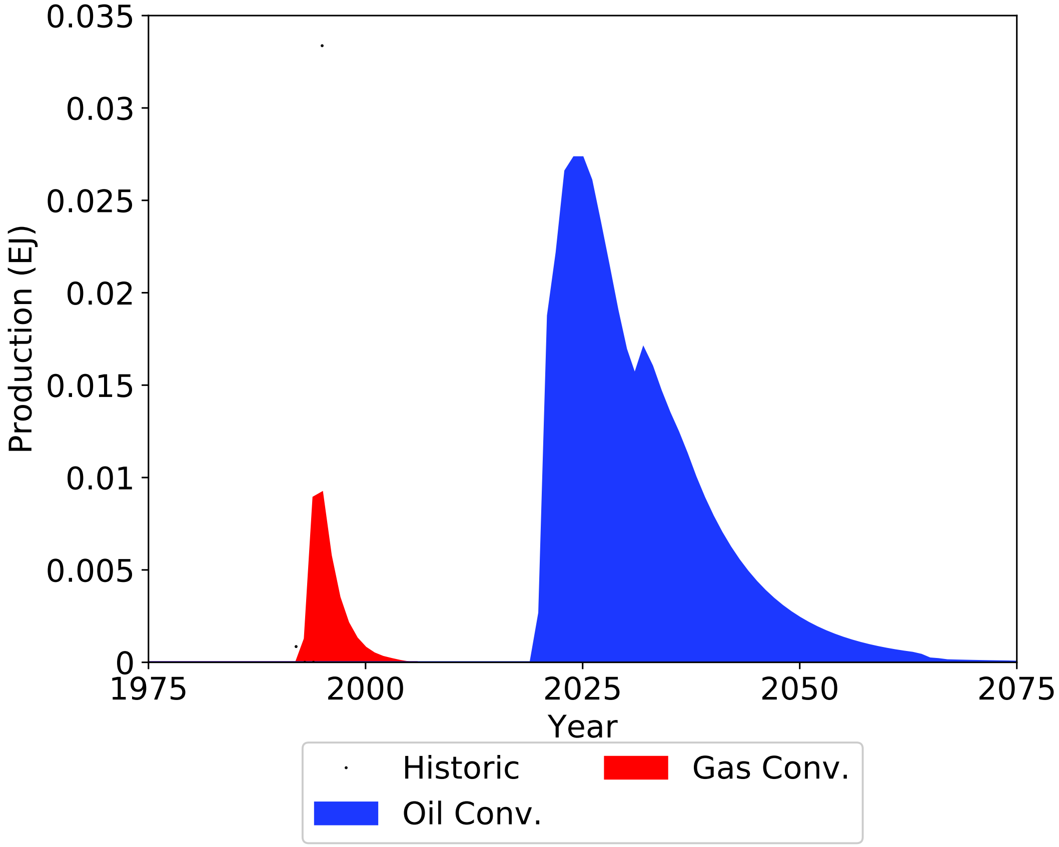

Figure 3.69: Yugoslavia - Bosnia and Herzegovina projections capped at 16

Table 3.69: Peak years - All

Name	URR	Peak Year	Peak Rate
Oil Conv. Bosnia and Herzegovina	0.42	2024	0.03
Gas Conv. Bosnia and Herzegovina	0.03	1995	0.01
**Total**	**0.45**	**2024**	**0.03**

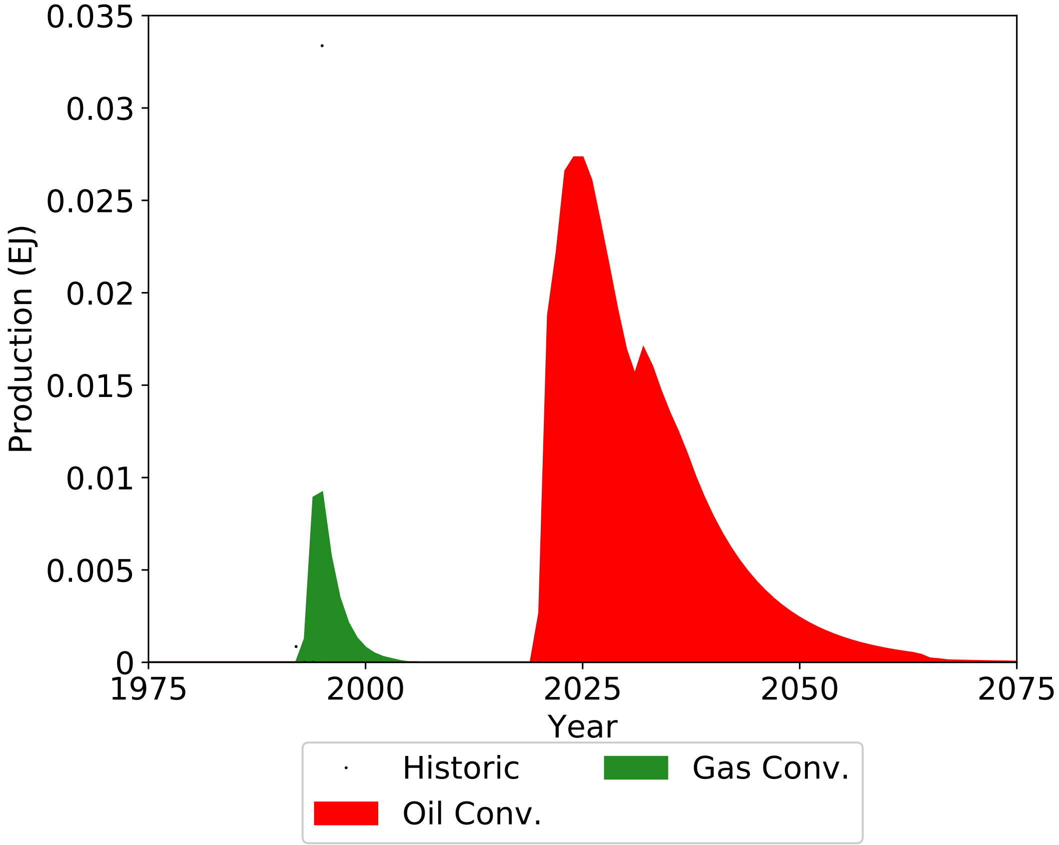

Figure 3.70: Yugoslavia - Bosnia and Herzegovina projection by mineral type

Table 3.70: Peak years - Minerals

Name	URR	Peak Year	Peak Rate
Oil Conv.	0.42	2024	0.03
Gas Conv.	0.03	1995	0.01
**Total**	**0.45**	**2024**	**0.03**

Croatia

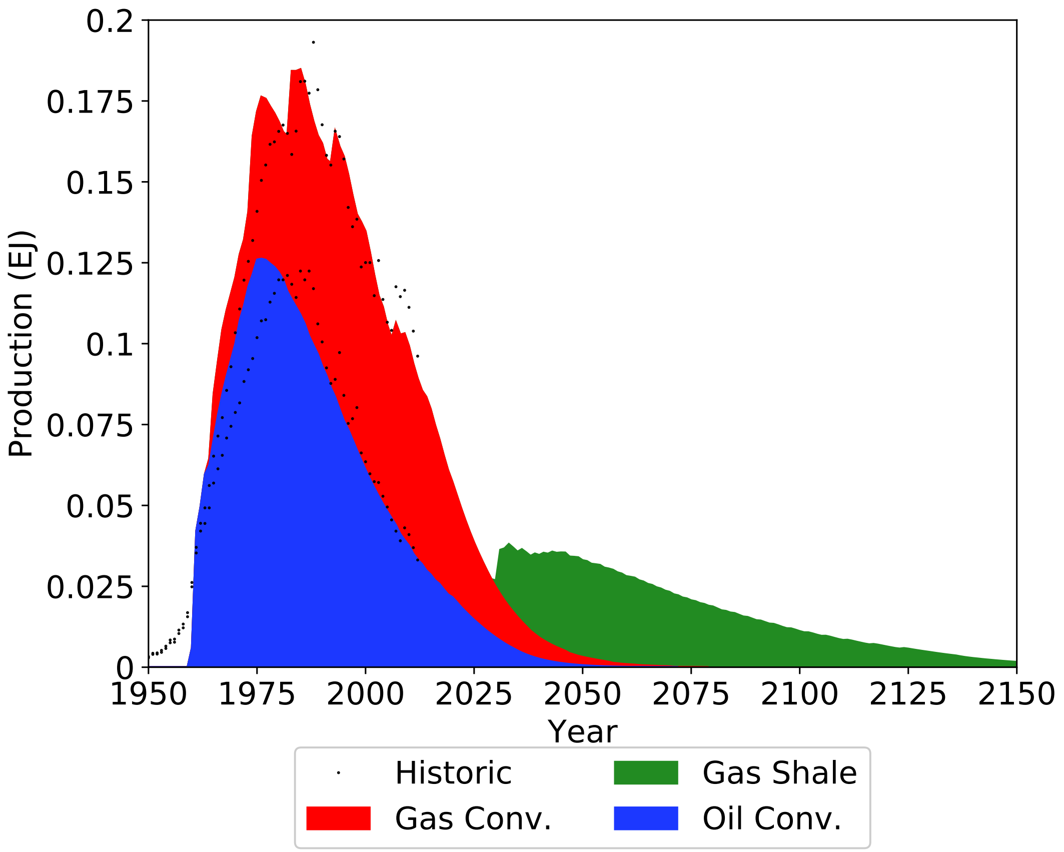

Figure 3.71: Yugoslavia - Croatia projections capped at 16

Table 3.71: Peak years - All

Name	URR	Peak Year	Peak Rate
Oil Conv. Croatia	4.73	1976	0.13
Gas Conv. Croatia	3.43	1993	0.08
Gas Shale Croatia	1.85	2049	0.03
**Total**	**10.01**	**1985**	**0.18**

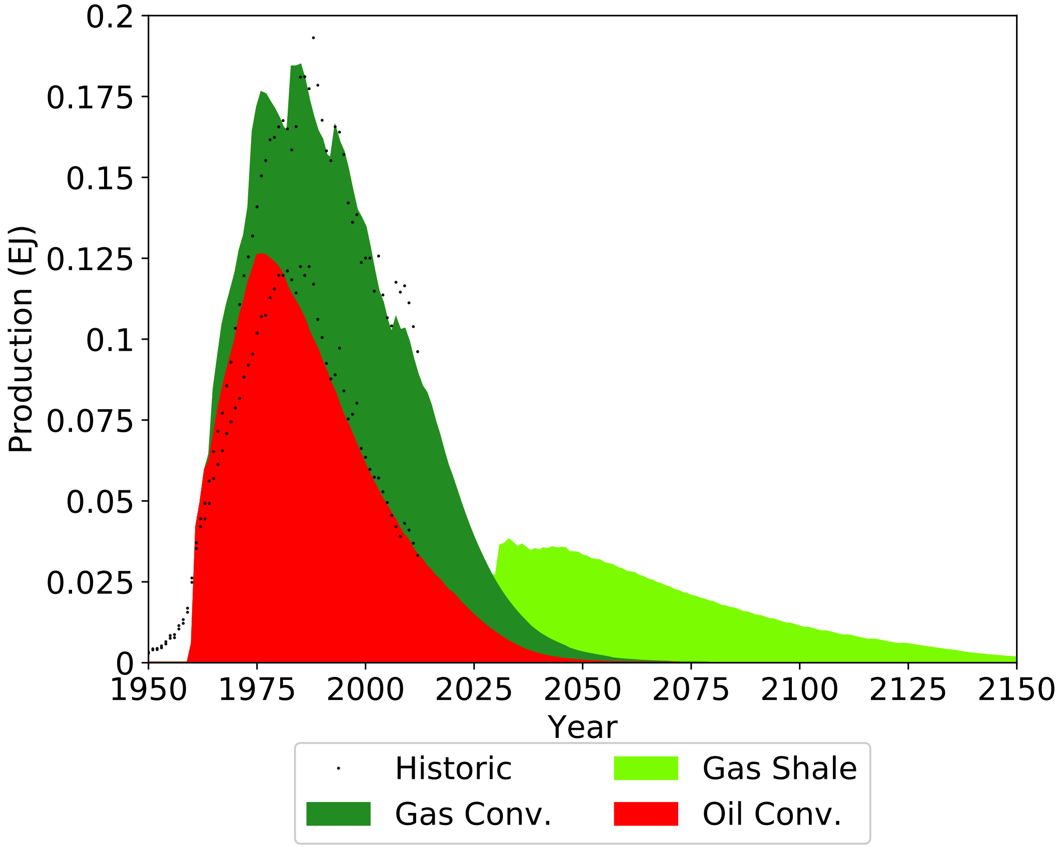

Figure 3.72: Yugoslavia - Croatia projection by mineral type

Table 3.72: Peak years - Minerals

Name	URR	Peak Year	Peak Rate
Oil Conv.	4.73	1976	0.13
Gas Conv.	3.43	1993	0.08
Gas Shale	1.85	2049	0.03
**Total**	**10.01**	**1985**	**0.18**

Serbia

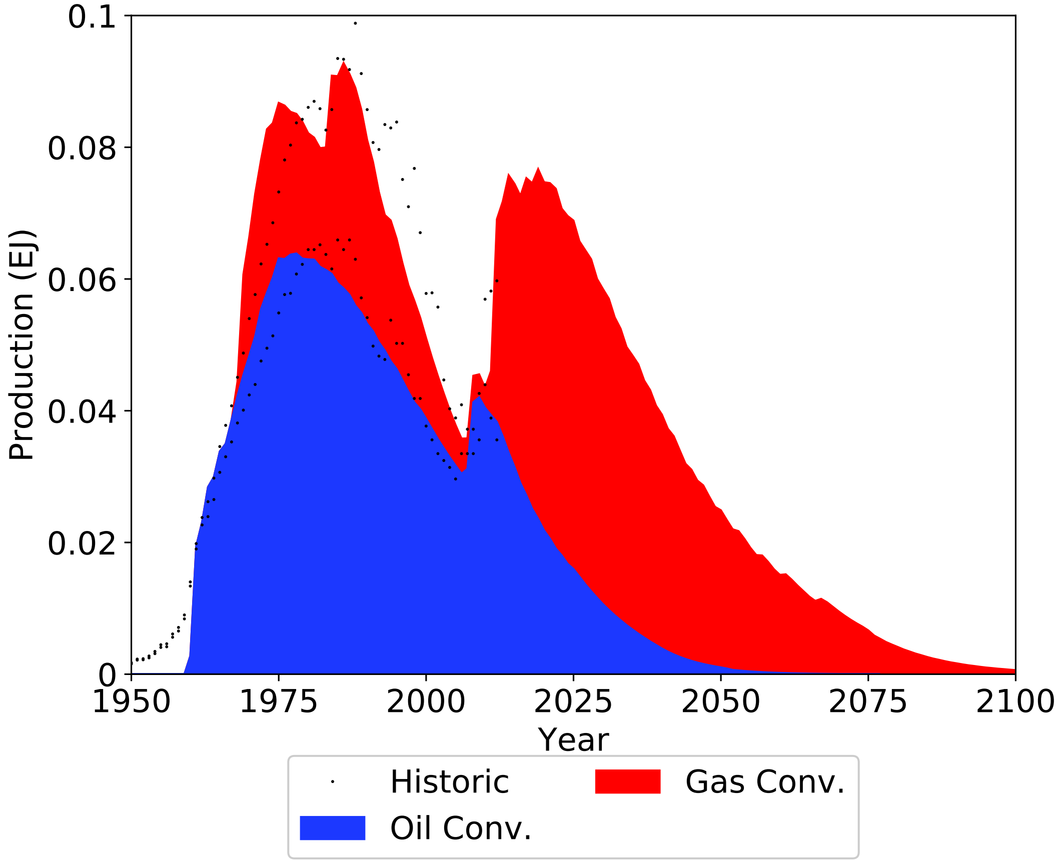

Figure 3.73: Yugoslavia - Serbia projections capped at 16

Table 3.73: Peak years - All

Name	URR	Peak Year	Peak Rate
Oil Conv. Serbia	2.9	1978	0.06
Gas Conv. Serbia	2.81	2022	0.05
**Total**	**5.71**	**1986**	**0.09**

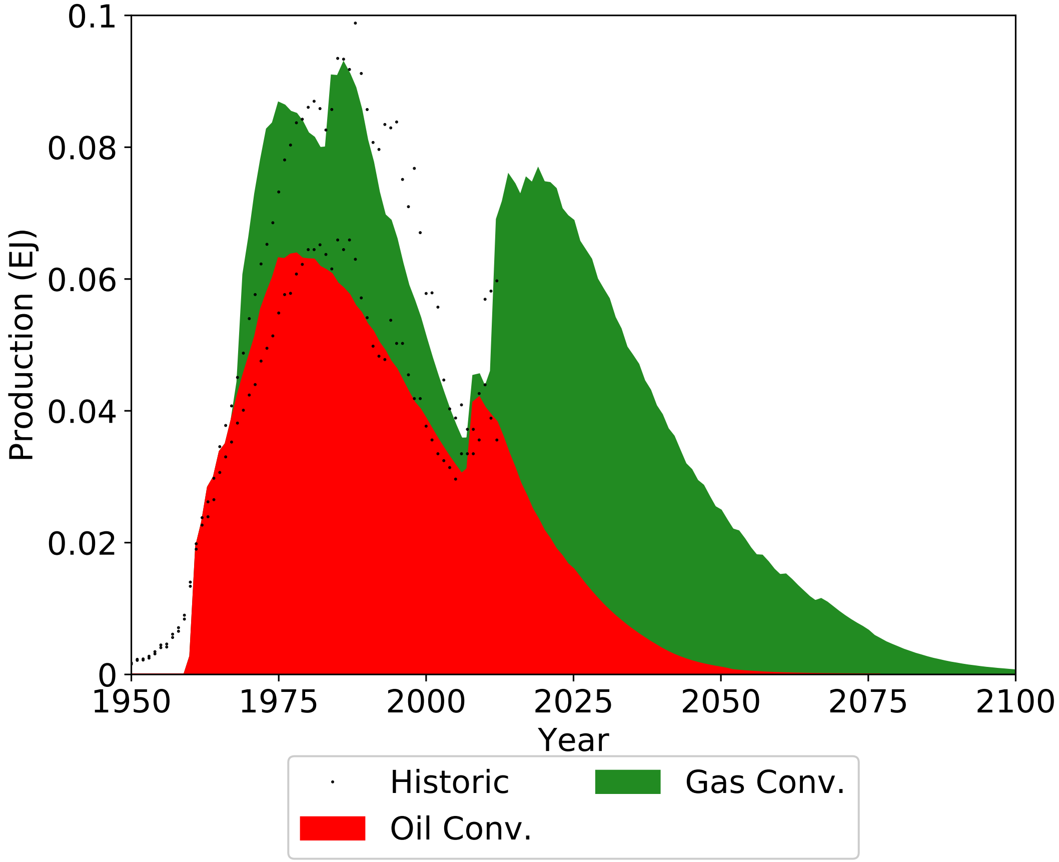

Figure 3.74: Yugoslavia - Serbia projection by mineral type

Table 3.74: Peak years - Minerals

Name	URR	Peak Year	Peak Rate
Oil Conv.	2.9	1978	0.06
Gas Conv.	2.81	2022	0.05
**Total**	**5.71**	**1986**	**0.09**

Slovenia

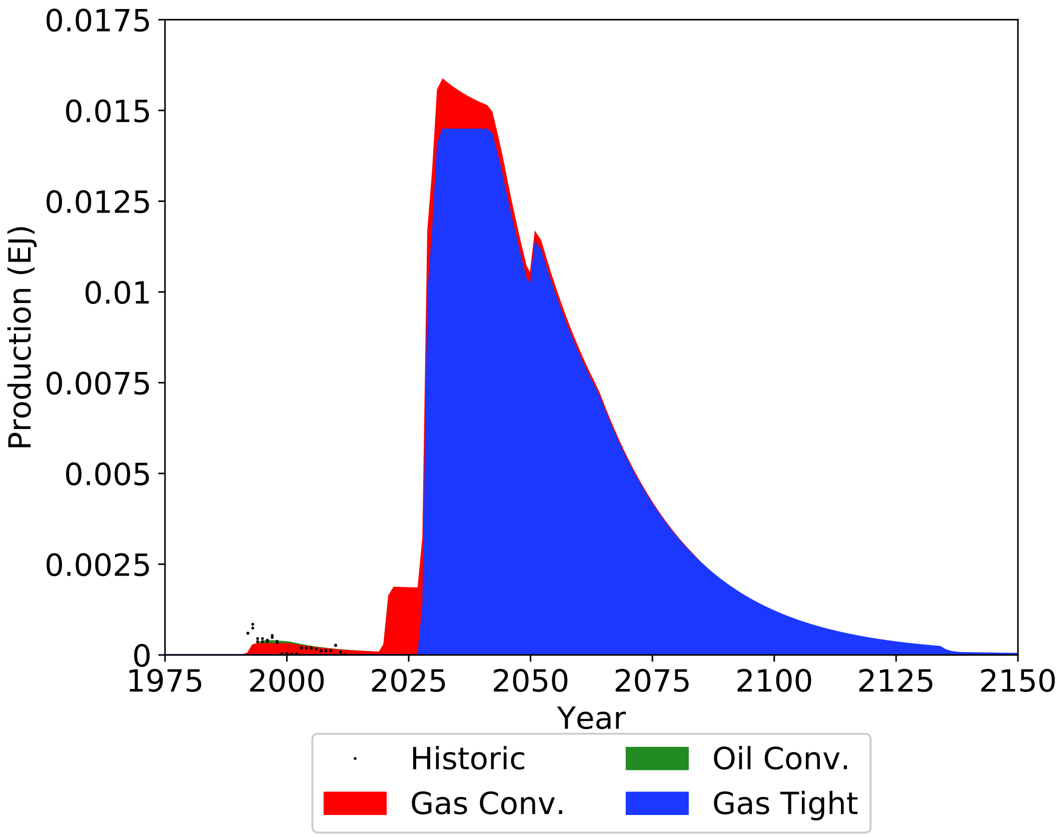

Figure 3.75: Yugoslavia - Slovenia projections capped at 16

Table 3.75: Peak years - All

Name	URR	Peak Year	Peak Rate
Gas Tight Slovenia	0.56	2032	0.01
Gas Conv. Slovenia	0.04	2022	–
Oil Conv. Slovenia	–	1996	–
**Total**	**0.6**	**2032**	**0.02**

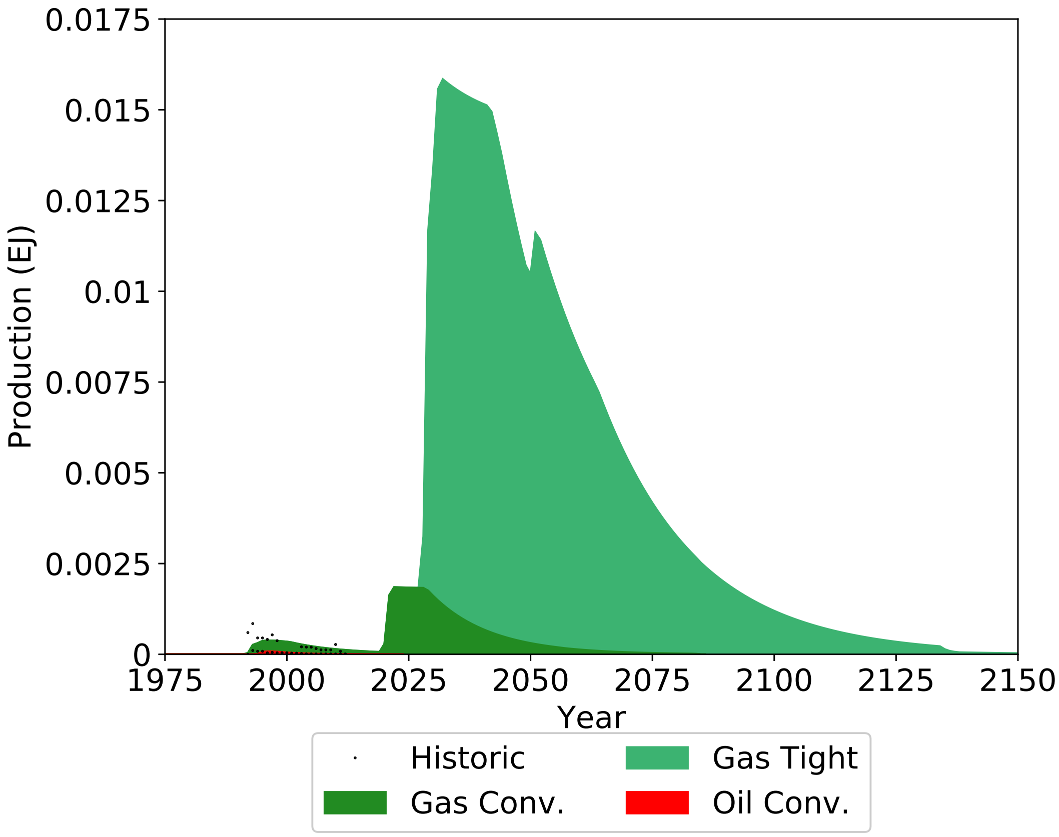

Figure 3.76: Yugoslavia - Slovenia projection by mineral type

Table 3.76: Peak years - Minerals

Name	URR	Peak Year	Peak Rate
Oil Conv.	–	1996	–
Gas Conv.	0.04	2022	–
Gas Tight	0.56	2032	0.01
**Total**	**0.6**	**2032**	**0.02**

Yugoslavia

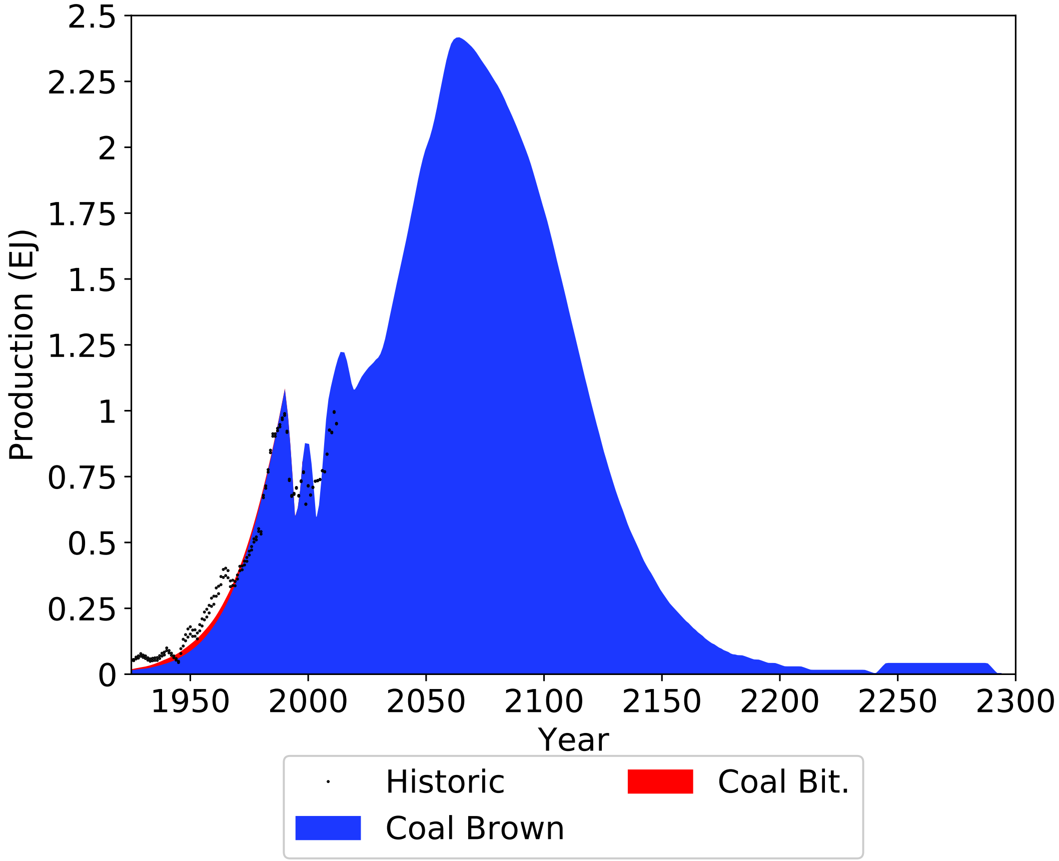

Figure 3.77: Yugoslavia - Yugoslavia projections capped at 16

Table 3.77: Peak years - All

Name	URR	Peak Year	Peak Rate
Coal Brown Yugoslavia	251.5	2064	2.41
Coal Bit. Yugoslavia	1.16	1956	0.03
**Total**	**252.66**	**2064**	**2.41**

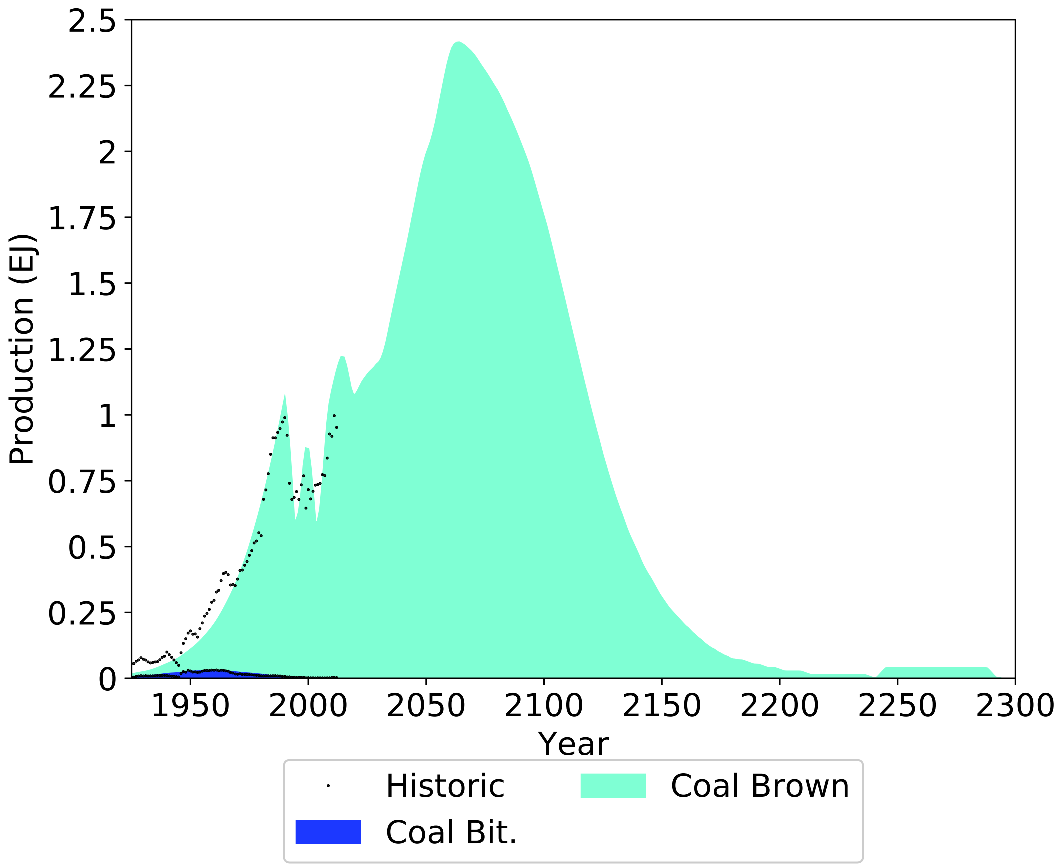

Figure 3.78: Yugoslavia - Yugoslavia projection by mineral type

Table 3.78: Peak years - Minerals

**Name**	**URR**	**Peak Year**	**Peak Rate**
Coal Bit.	1.16	1956	0.03
Coal Brown	251.5	2064	2.41
**Total**	**252.66**	**2064**	**2.41**

3.27.4 Projection by region

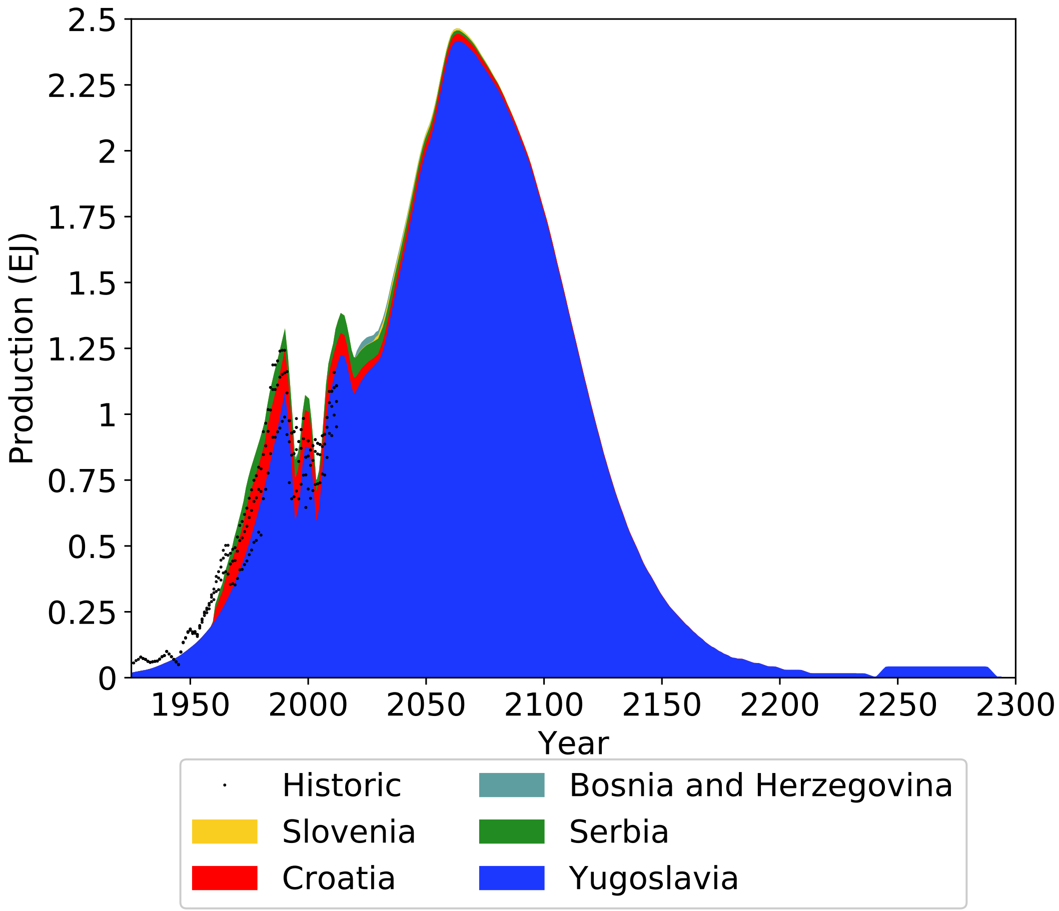

Figure 3.79: Yugoslavia by region projections capped at 16

Table 3.79: Peak years - All

**Name**	**URR**	**Peak Year**	**Peak Rate**
Yugoslavia	252.66	2064	2.41
Croatia	10.01	1985	0.18
Serbia	5.71	1986	0.09
Slovenia	0.6	2032	0.02
Bosnia and Herzegovina	0.45	2024	0.03
**Total**	**269.43**	**2063**	**2.46**

3.28 Total

3.28.1 By country

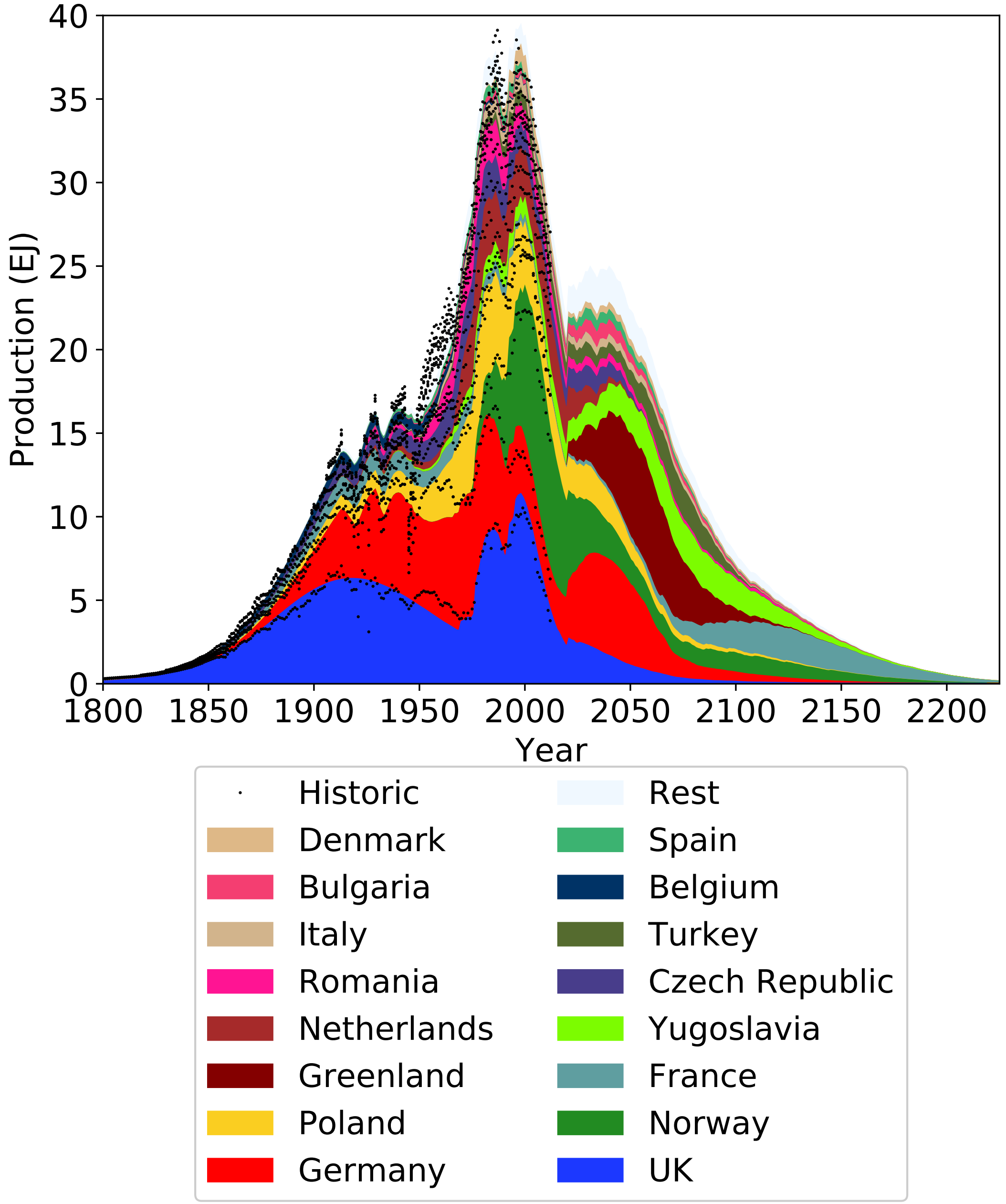

Figure 3.80: Europe projections by country

Table 3.80: Peak years - All

**Name**	**URR**	**Peak Year**	**Peak Rate**
UK	995.13	1998	11.34
Germany	879.36	1976	7.49
Norway	478.2	2005	9.82
Poland	395.98	1984	5.33
France	314.26	2115	1.99
Greenland	292.02	2055	6.38
Yugoslavia	269.43	2063	2.46
Netherlands	195.55	1978	3.8
Czech Republic	191.82	1977	2.34
Romania	138.42	1982	2.28
Turkey	130.45	2070	1.89
Italy	70.85	1986	0.86
Belgium	67.12	1913	0.66
Bulgaria	62.82	2033	0.95
Spain	61.67	2030	0.69
Denmark	53.79	2001	1.07
Greece	49.23	2031	0.83
Hungary	48.61	1977	0.87
Austria	45.43	2046	0.51
Sweden	43.74	2064	0.55
Ireland	14.02	2025	0.45
Cyprus	10.74	2043	0.19
Albania	7.16	2019	0.14
Portugal	3.72	2014	0.17
Slovakia	3.08	1997	0.07
Malta	0.41	2024	0.03
Switzerland	0.03	1944	0.01
**Total**	**4823.03**	**1998**	**39.37**

3.28.2 By mineral

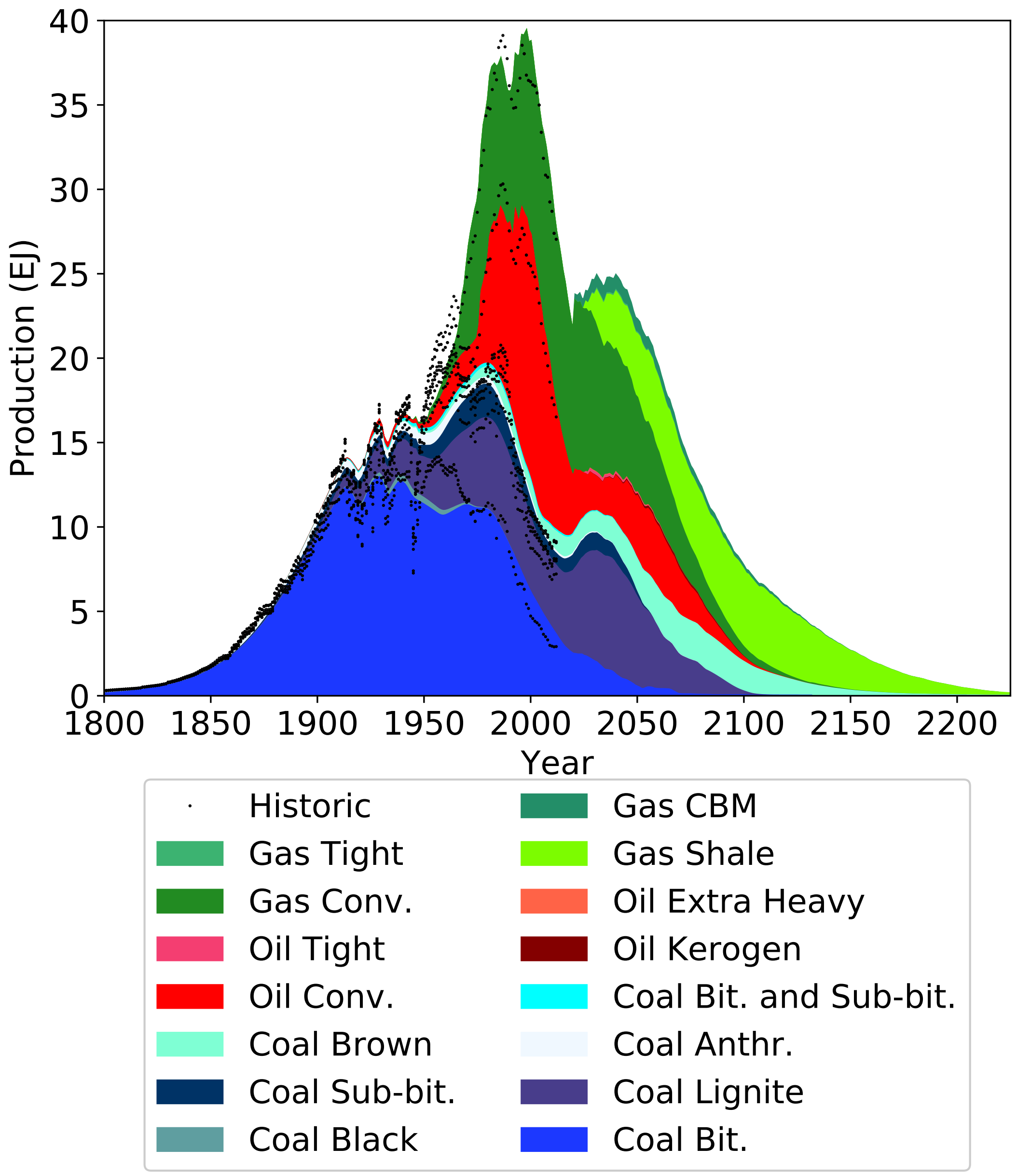

Figure 3.81: Europe projection by mineral type

Table 3.81: Peak years - Minerals

**Name**	**URR**	**Peak Year**	**Peak Rate**
Coal Bit.	1503.66	1929	12.95
Coal Black	14.9	1945	0.4
Coal Lignite	670.86	2035	6.71
Coal Sub-bit.	145.1	1978	1.98
Coal Anthr.	67.96	1953	0.72
Coal Brown	253.25	2064	2.41
Coal Bit. and Sub-bit.	19.4	1966	0.22
Oil Conv.	652.87	1998	14.76
Oil Kerogen	7.65	2065	0.16
Oil Tight	4.31	2032	0.2
Oil Extra Heavy	1.1	2030	0.03
Gas Conv.	882.63	2006	11.52
Gas Shale	541.64	2088	4.68
Gas Tight	4.27	2042	0.1
Gas CBM	53.45	2038	0.93
**Total**	**4823.03**	**1998**	**39.37**

Chapter 4

FSU

4.1 Azerbaijan

4.1.1 All Projections

Table 4.1: Peak years - All

Name	URR	Peak Year	Peak Rate
Oil Conv.	176.3	2035	2.47
Gas Conv.	70.41	2030	0.82
**Total**	**246.71**	**2034**	**3.23**

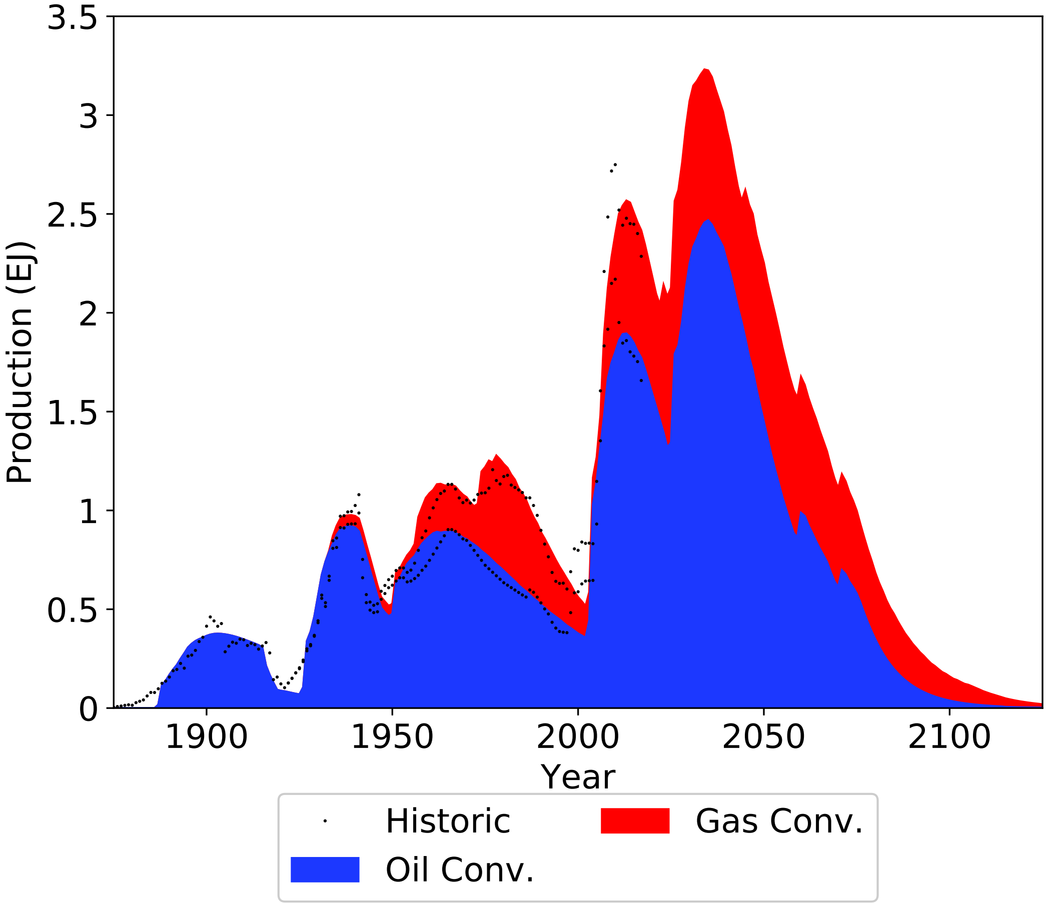

Figure 4.1: Azerbaijan projections capped at 16

4.1.2 By Mineral

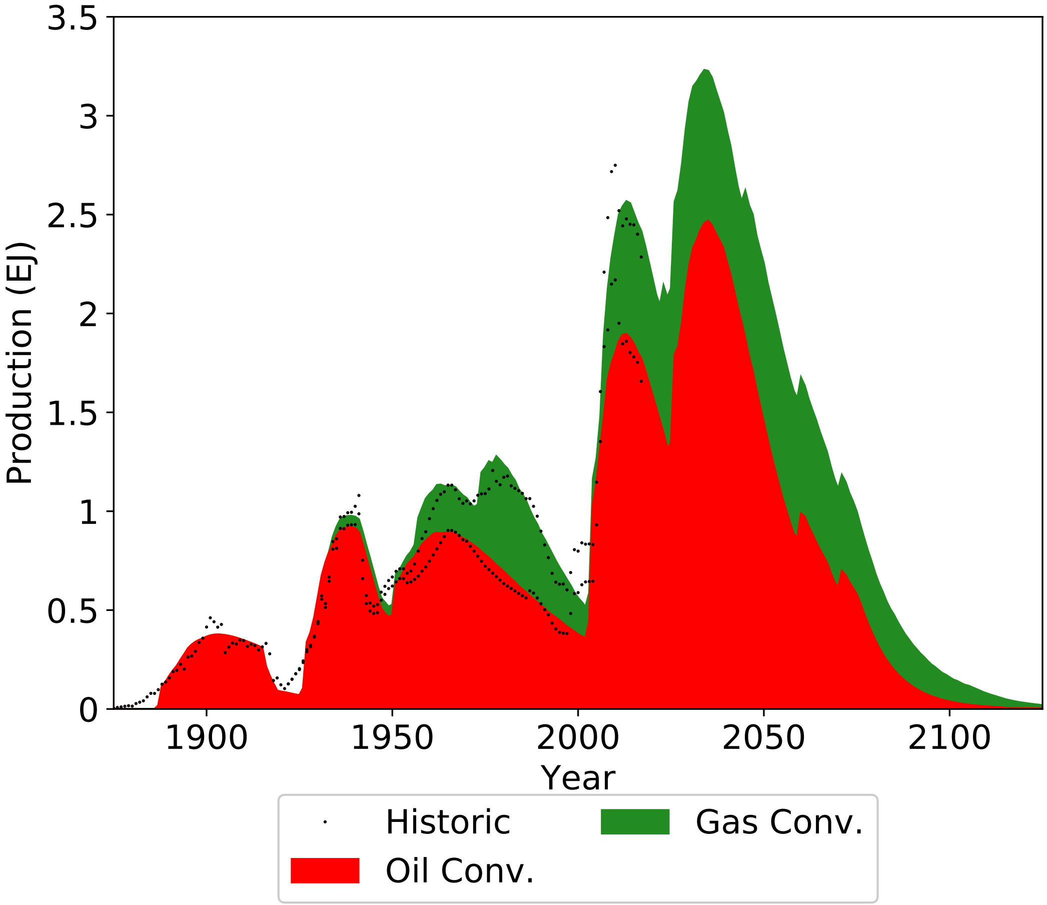

Figure 4.2: Azerbaijan projection by mineral type

Table 4.2: Peak years - Minerals

Name	URR	Peak Year	Peak Rate
Oil Conv.	176.3	2035	2.47
Gas Conv.	70.41	2030	0.82
**Total**	**246.71**	**2034**	**3.23**

4.2 Belarus

4.2.1 All Projections

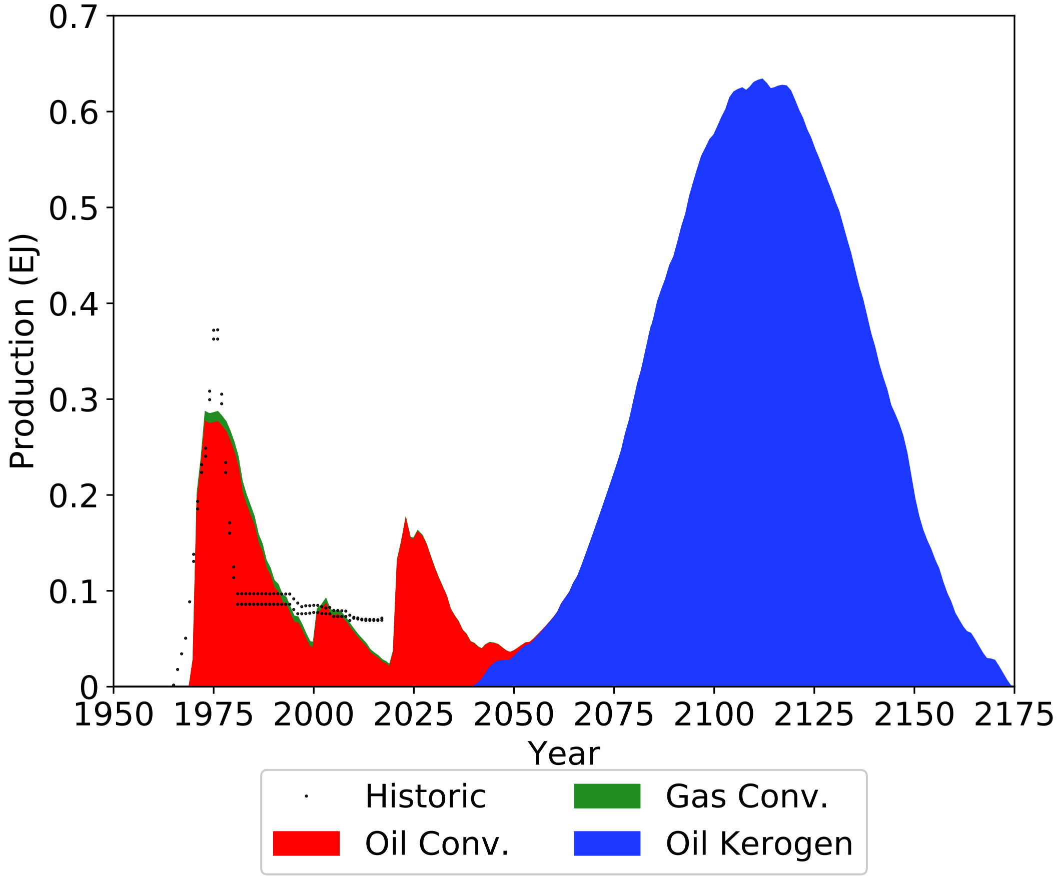

Figure 4.3: Belarus projections capped at 16

Table 4.3: Peak years - All

Name	URR	Peak Year	Peak Rate
Oil Kerogen	40.04	2112	0.63
Oil Conv.	8.36	1976	0.28
Gas Conv.	0.36	1982	0.01
**Total**	**48.76**	**2112**	**0.63**

4.2.2 By Mineral

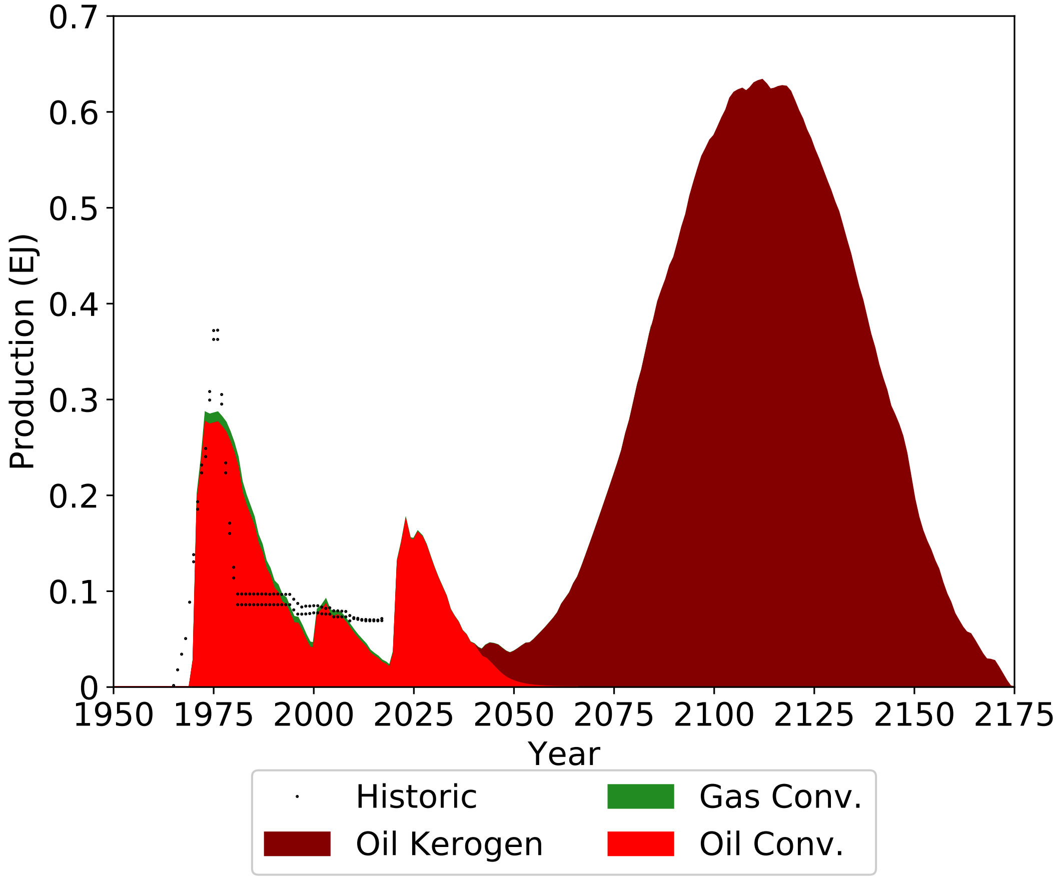

Figure 4.4: Belarus projection by mineral type

Table 4.4: Peak years - Minerals

Name	URR	Peak Year	Peak Rate
Oil Conv.	8.36	1976	0.28
Oil Kerogen	40.04	2112	0.63
Gas Conv.	0.36	1982	0.01
**Total**	**48.76**	**2112**	**0.63**

4.3 Crimea

4.3.1 All Projections

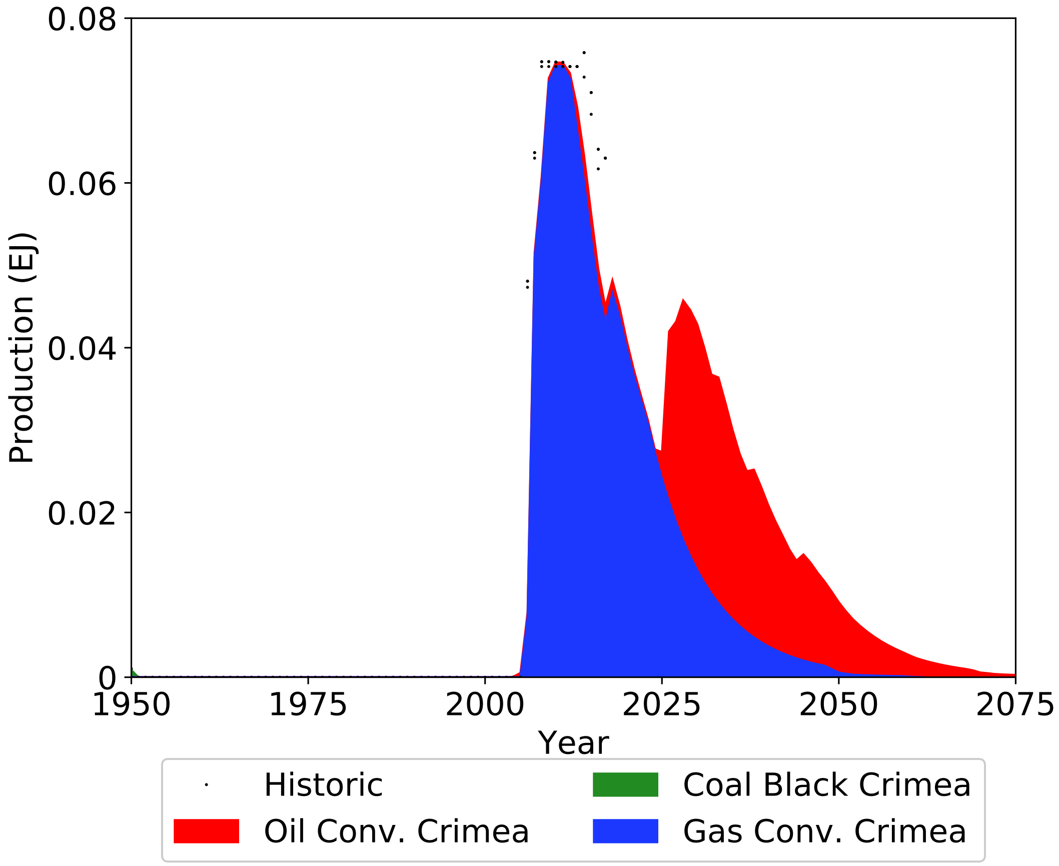

Figure 4.5: Crimea projections capped at 16

Table 4.5: Peak years - All

**Name**	**URR**	**Peak Year**	**Peak Rate**
Gas Conv. Crimea	1.14	2010	0.07
Oil Conv. Crimea	0.57	2029	0.03
Coal Black Crimea	–	1950	–
**Total**	**1.71**	**2010**	**0.07**

4.3.2 By Mineral

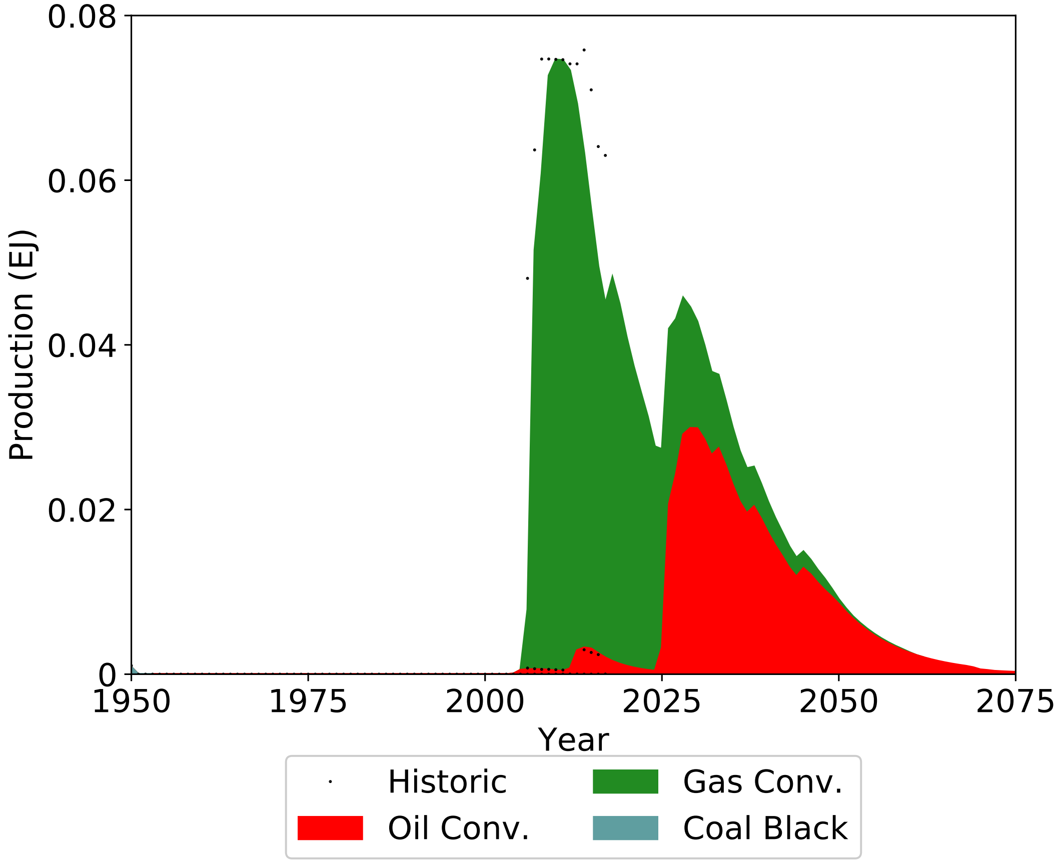

Figure 4.6: Crimea projection by mineral type

Table 4.6: Peak years - Minerals

**Name**	**URR**	**Peak Year**	**Peak Rate**
Coal Black	–	1950	–
Oil Conv.	0.57	2029	0.03
Gas Conv.	1.14	2010	0.07
**Total**	**1.71**	**2010**	**0.07**

4.3.3 Regional Projections

Crimea

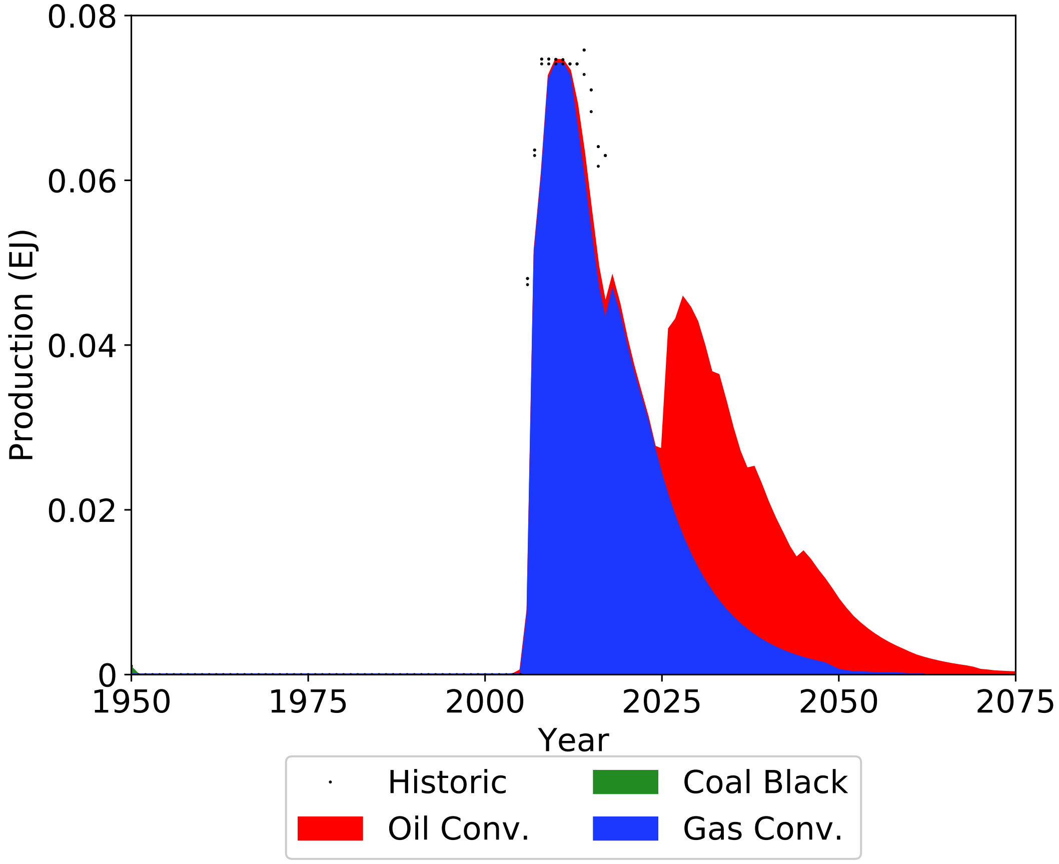

Figure 4.7: Crimea - Crimea projections capped at 16

Table 4.7: Peak years - All

Name	URR	Peak Year	Peak Rate
Gas Conv. Crimea	1.14	2010	0.07
Oil Conv. Crimea	0.57	2029	0.03
Coal Black Crimea	–	1950	–
**Total**	**1.71**	**2010**	**0.07**

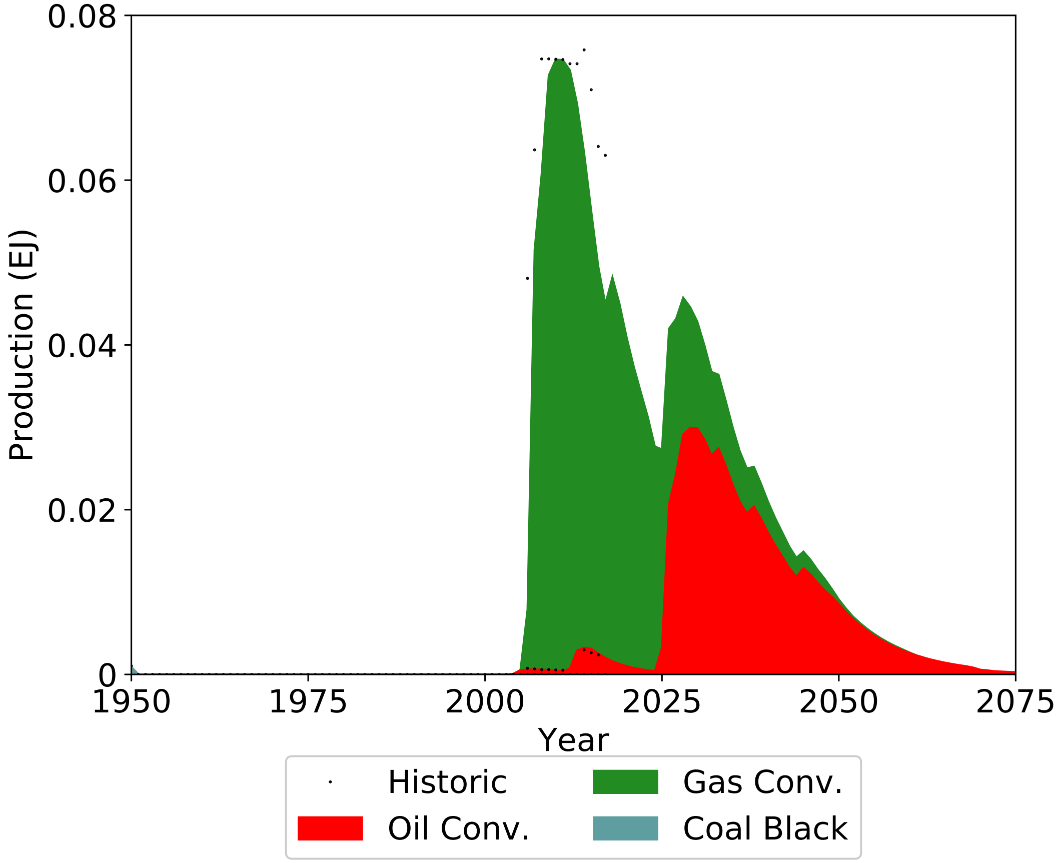

Figure 4.8: Crimea - Crimea projection by mineral type

Table 4.8: Peak years - Minerals

Name	URR	Peak Year	Peak Rate
Coal Black	–	1950	–
Oil Conv.	0.57	2029	0.03
Gas Conv.	1.14	2010	0.07
**Total**	**1.71**	**2010**	**0.07**

4.3.4 Projection by region

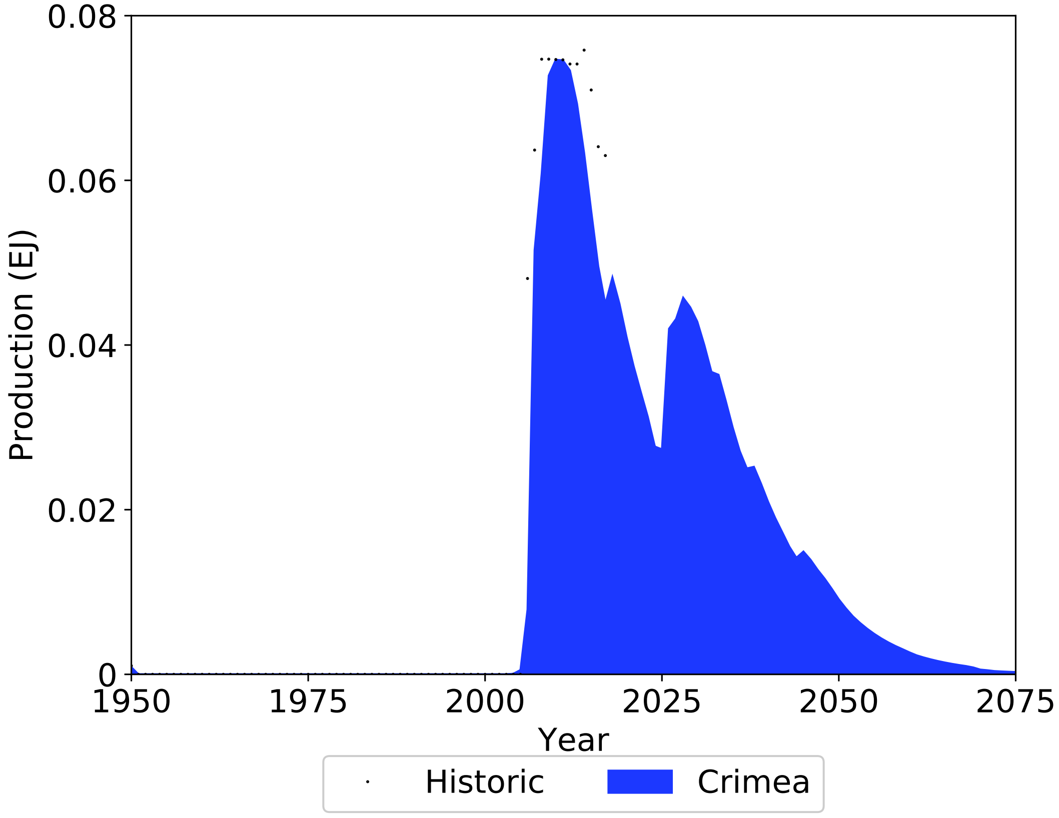

Figure 4.9: Crimea by region projections capped at 16

Table 4.9: Peak years - All

Name	URR	Peak Year	Peak Rate
Crimea	1.71	2010	0.07
**Total**	**1.71**	**2010**	**0.07**

4.4 Donetsk

4.4.1 All Projections

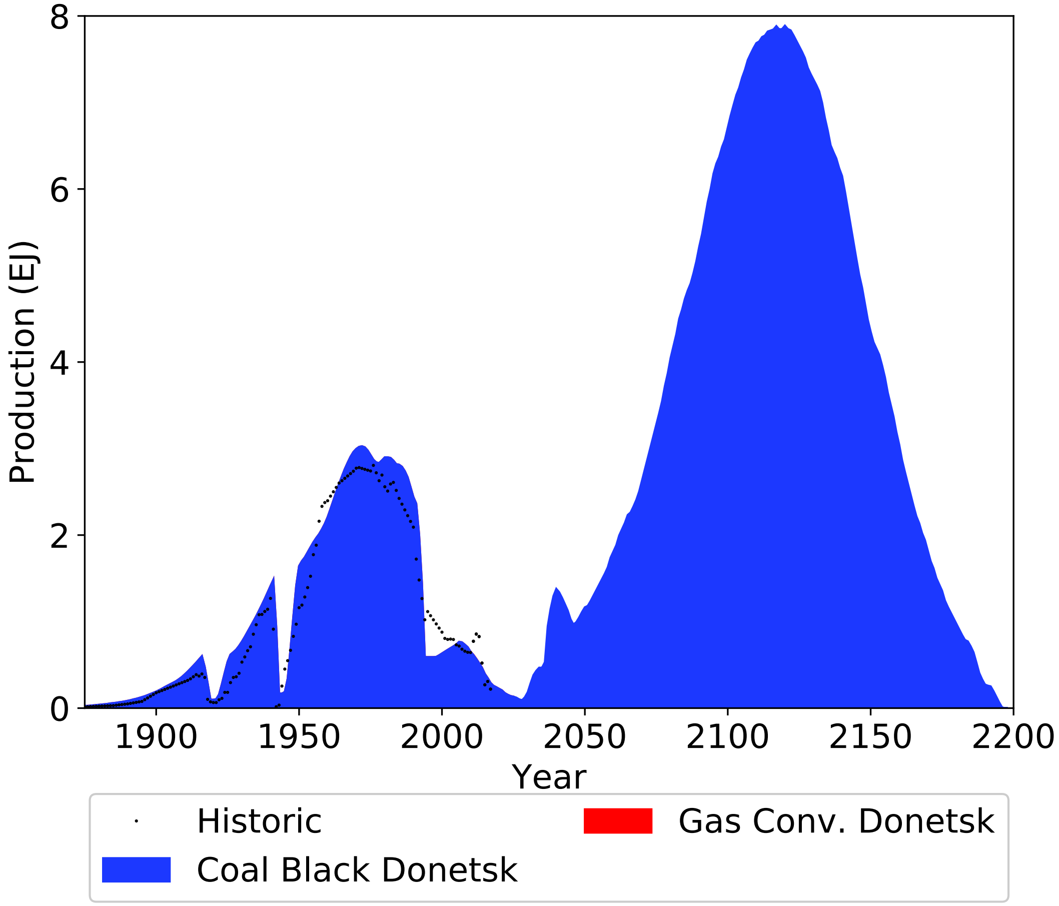

Figure 4.10: Donetsk projections capped at 16

Table 4.10: Peak years - All

**Name**	**URR**	**Peak Year**	**Peak Rate**
Coal Black Donetsk	783.02	2120	7.89
Gas Conv. Donetsk	0.01	2012	–
**Total**	**783.03**	**2120**	**7.89**

4.4.2 By Mineral

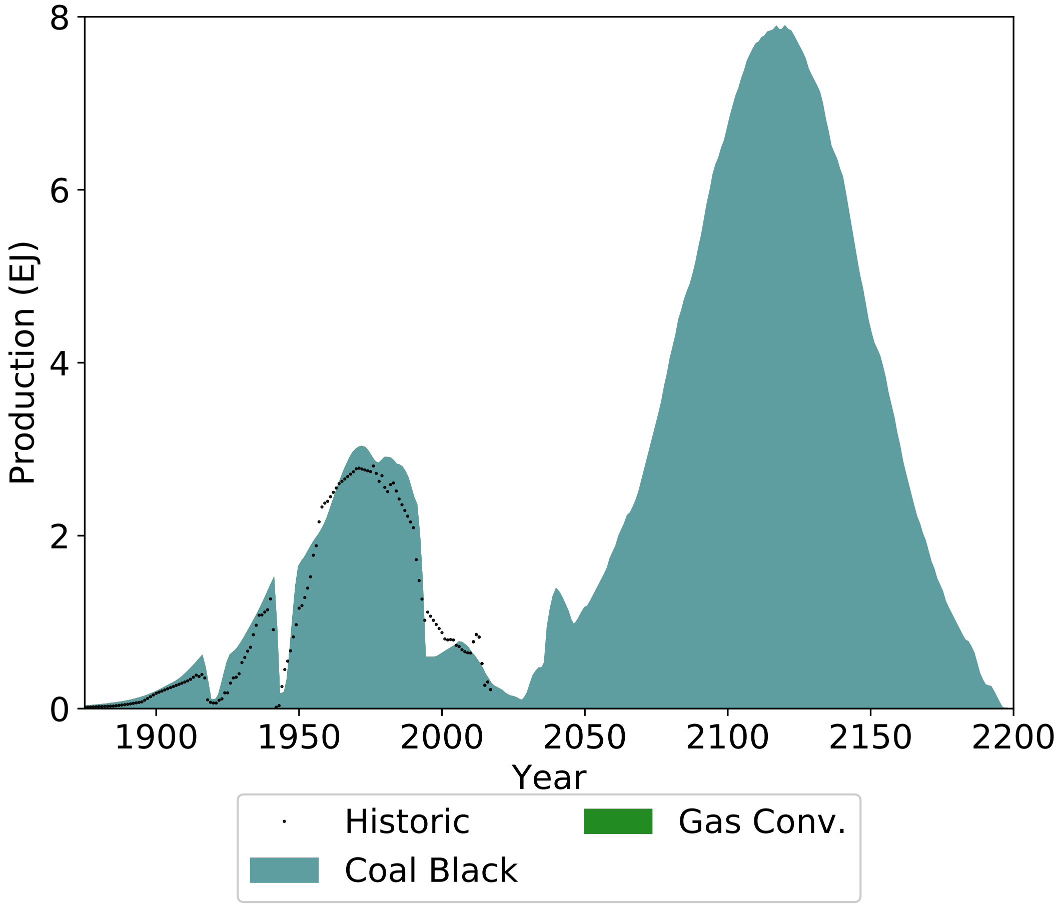

Figure 4.11: Donetsk projection by mineral type

Table 4.11: Peak years - Minerals

Name	URR	Peak Year	Peak Rate
Coal Black	783.02	2120	7.89
Gas Conv.	0.01	2012	–
**Total**	**783.03**	**2120**	**7.89**

4.4.3 Regional Projections

Donetsk

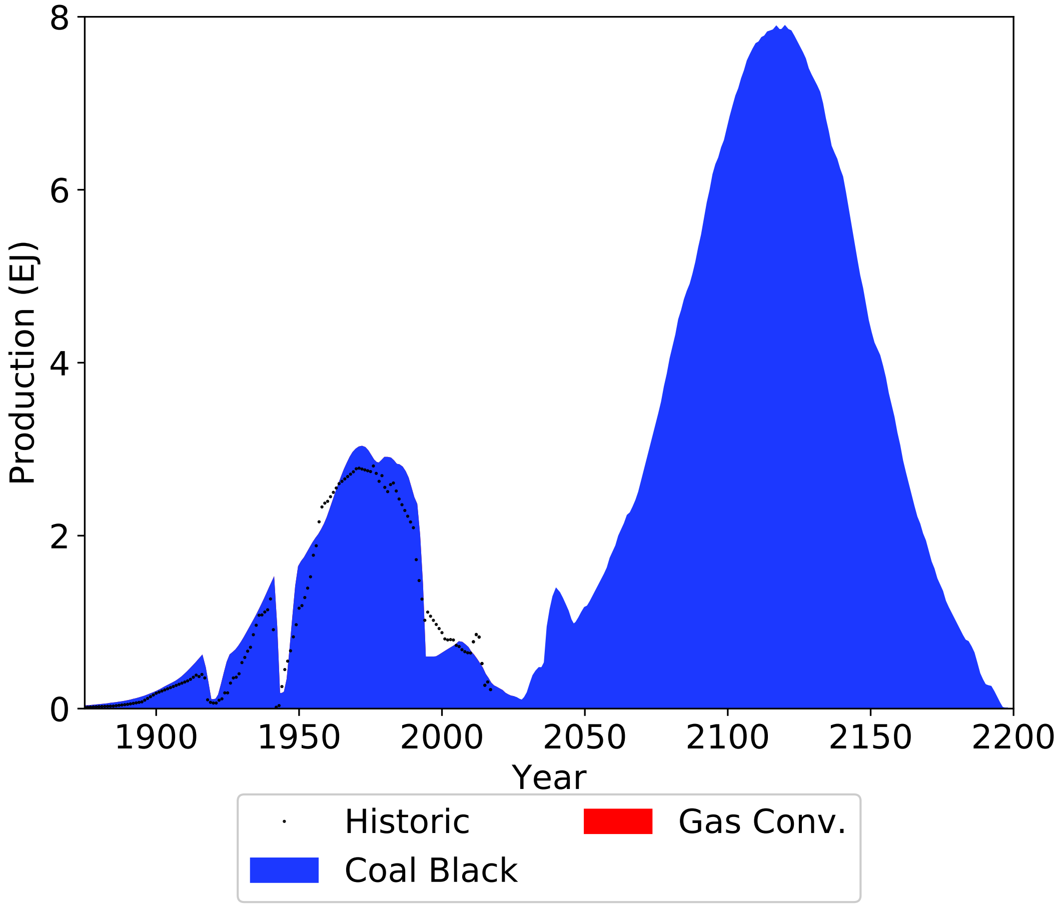

Figure 4.12: Donetsk - Donetsk projections capped at 16

Table 4.12: Peak years - All

Name	URR	Peak Year	Peak Rate
Coal Black Donetsk	783.02	2120	7.89
Gas Conv. Donetsk	0.01	2012	–
**Total**	**783.03**	**2120**	**7.89**

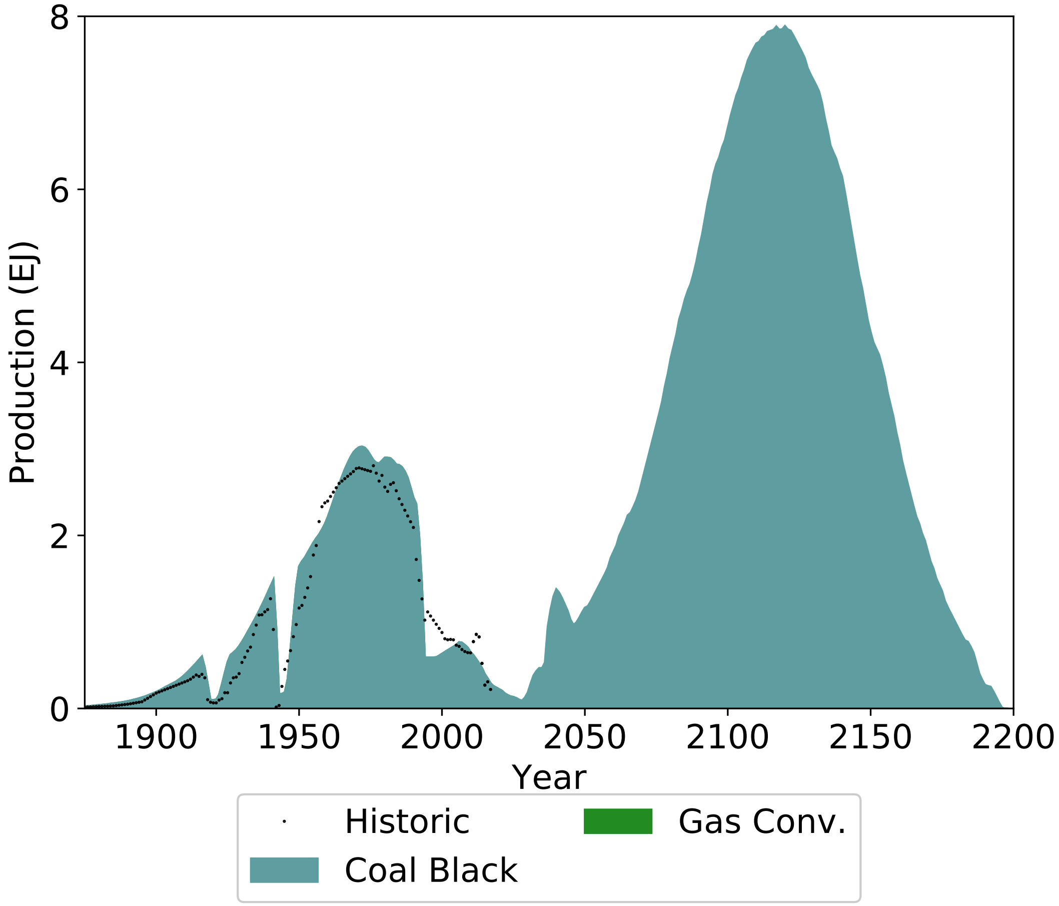

Figure 4.13: Donetsk - Donetsk projection by mineral type

Table 4.13: Peak years - Minerals

**Name**	**URR**	**Peak Year**	**Peak Rate**
Coal Black	783.02	2120	7.89
Gas Conv.	0.01	2012	–
**Total**	**783.03**	**2120**	**7.89**

4.4.4 Projection by region

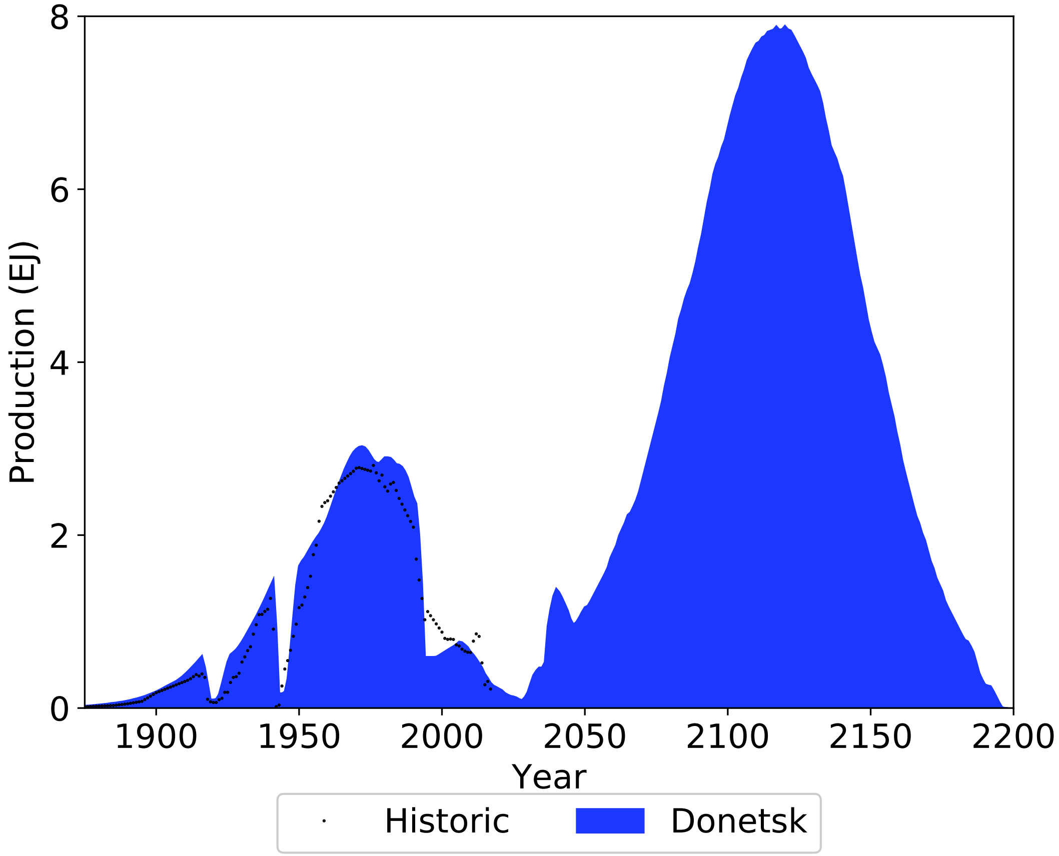

Figure 4.14: Donetsk by region projections capped at 16

Table 4.14: Peak years - All

**Name**	**URR**	**Peak Year**	**Peak Rate**
Donetsk	783.03	2120	7.89
**Total**	**783.03**	**2120**	**7.89**

4.5 Estonia

4.5.1 All Projections

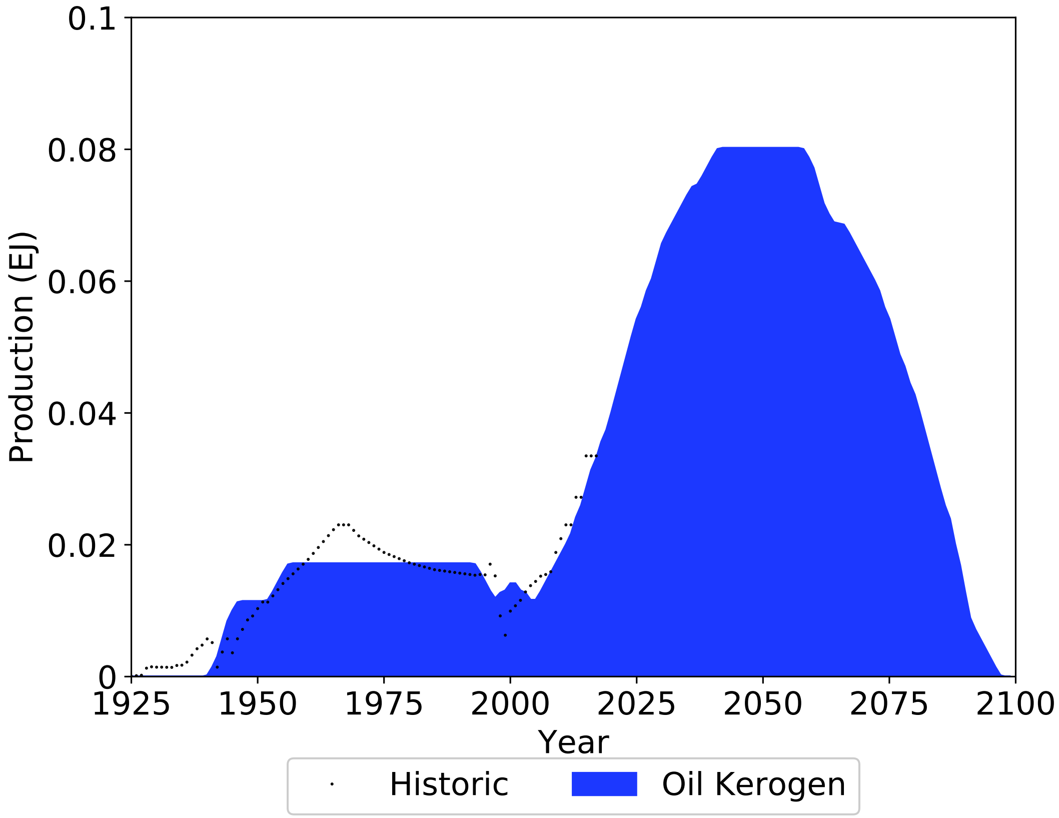

Figure 4.15: Estonia projections capped at 16

Table 4.15: Peak years - All

**Name**	**URR**	**Peak Year**	**Peak Rate**
Oil Kerogen	5.73	2048	0.08
**Total**	**5.73**	**2048**	**0.08**

4.5.2 By Mineral

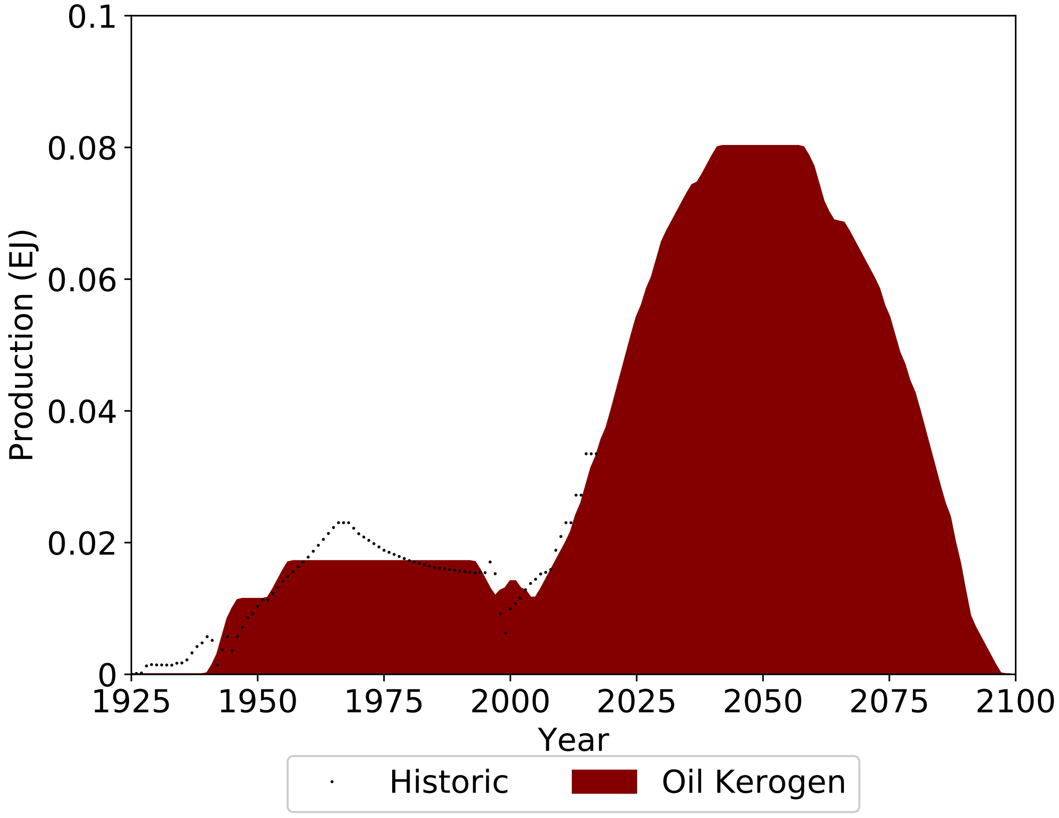

Figure 4.16: Estonia projection by mineral type

Table 4.16: Peak years - Minerals

Name	URR	Peak Year	Peak Rate
Oil Kerogen	5.73	2048	0.08
**Total**	**5.73**	**2048**	**0.08**

4.6 Georgia

4.6.1 All Projections

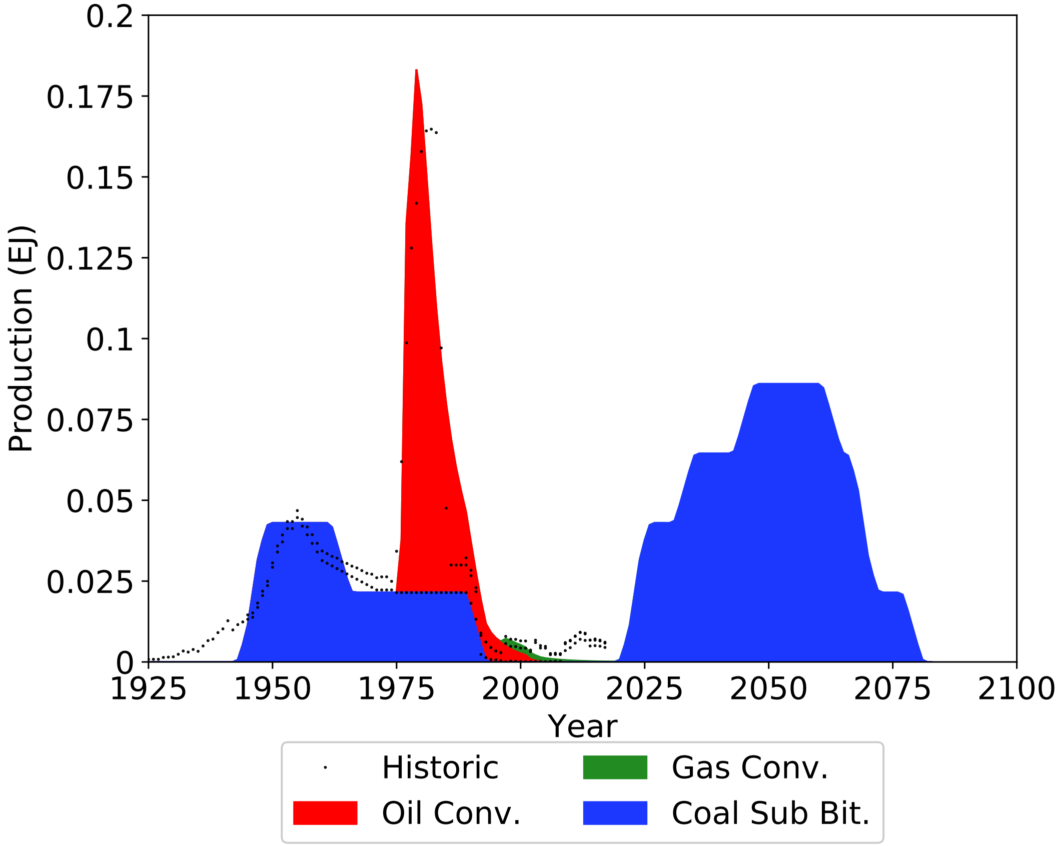

Figure 4.17: Georgia projections capped at 16

Table 4.17: Peak years - All

Name	URR	Peak Year	Peak Rate
Coal Sub Bit.	4.7	2048	0.09
Oil Conv.	1.28	1979	0.16
Gas Conv.	0.03	1998	–
**Total**	**6.01**	**1979**	**0.18**

4.6.2 By Mineral

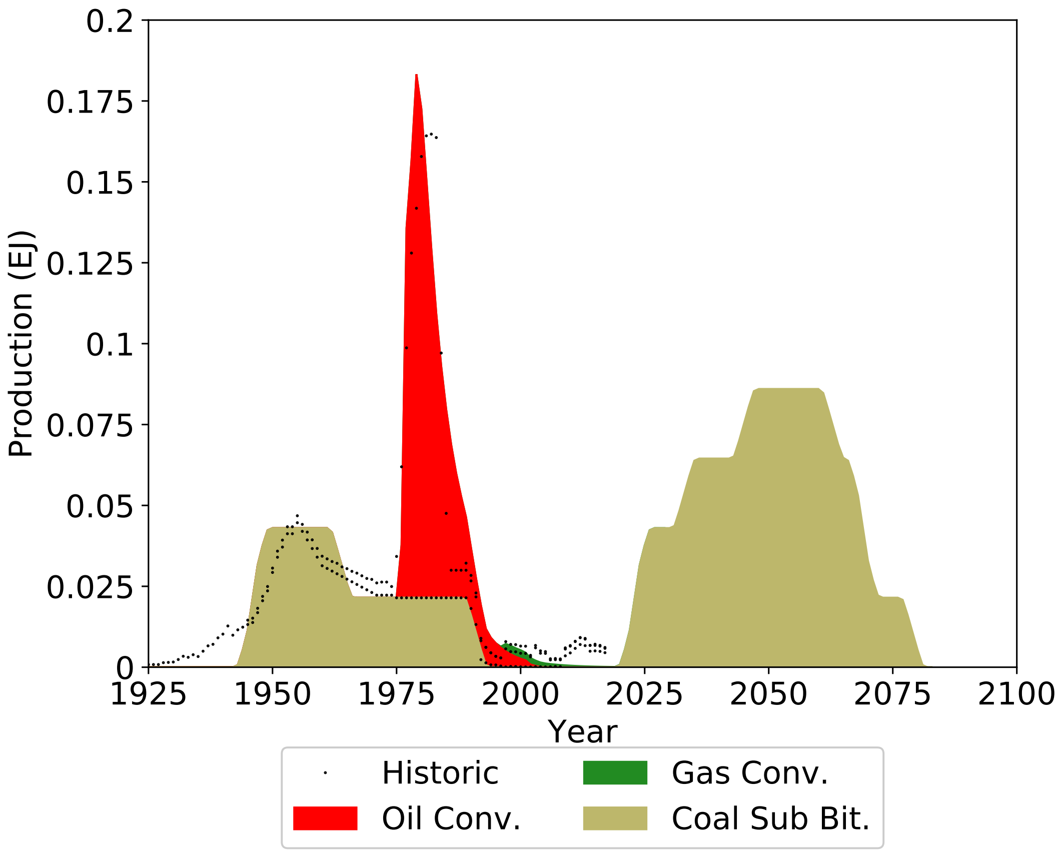

Figure 4.18: Georgia projection by mineral type

Table 4.18: Peak years - Minerals

Name	URR	Peak Year	Peak Rate
Coal Sub Bit.	4.7	2048	0.09
Oil Conv.	1.28	1979	0.16
Gas Conv.	0.03	1998	–
**Total**	**6.01**	**1979**	**0.18**

4.7 Kazakhstan

4.7.1 All Projections

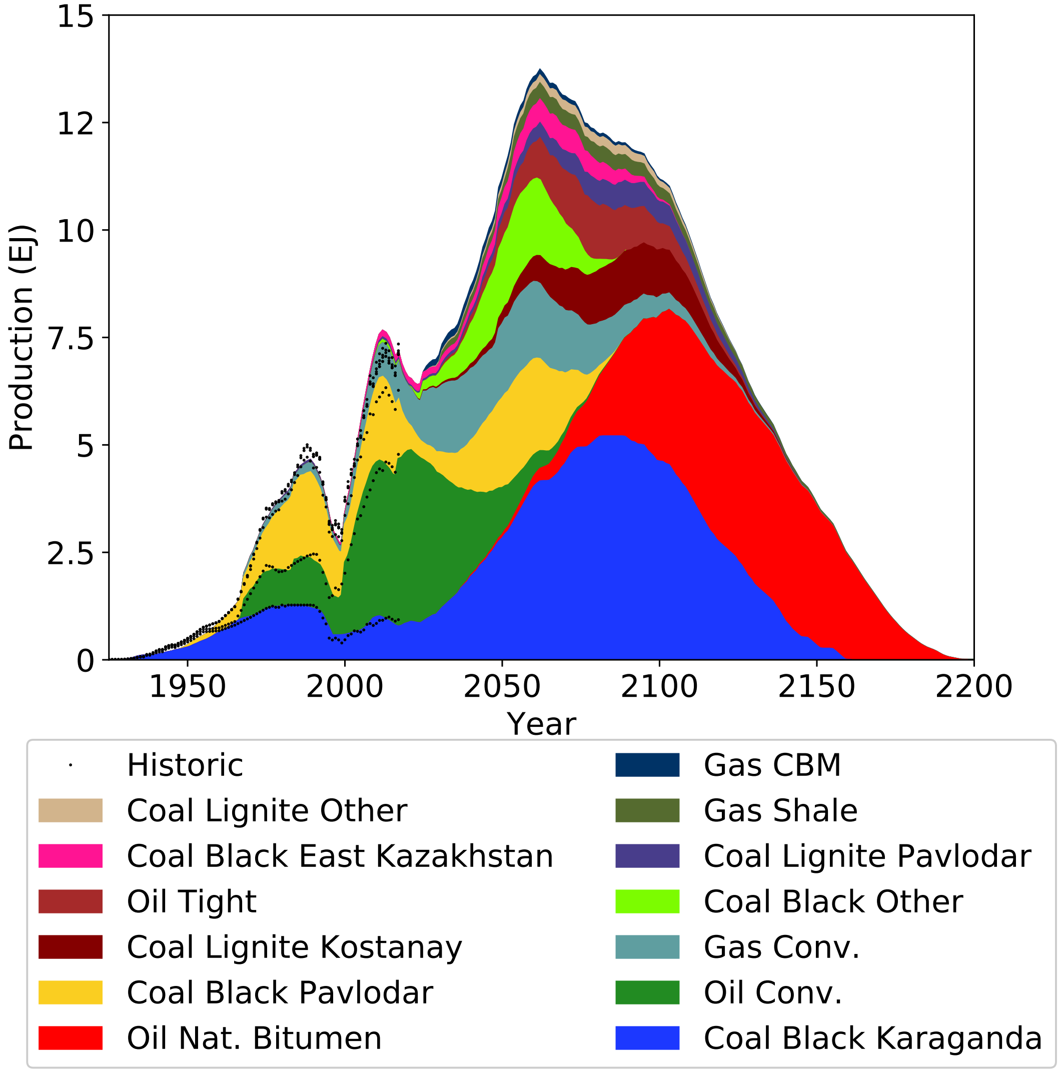

Figure 4.19: Kazakhstan projections capped at 16

Table 4.19: Peak years - All

**Name**	**URR**	**Peak Year**	**Peak Rate**
Coal Black Karaganda	456.95	2082	5.2
Oil Nat. Bitumen	312.53	2125	4.05
Oil Conv.	184.5	2020	4.0
Coal Black Pavlodar	154.66	2056	2.31
Gas Conv.	131.2	2057	1.84
Coal Lignite Kostanay	67.07	2086	1.26
Coal Black Other	62.09	2056	1.88
Oil Tight	60.74	2077	1.38
Coal Lignite Pavlodar	37.26	2088	0.59
Coal Black East Kazakhstan	28.97	2066	0.57
Gas Shale	28.91	2067	0.39
Coal Lignite Other	12.62	2070	0.23
Gas CBM	10.5	2041	0.2
**Total**	**1548.0**	**2062**	**13.73**

4.7.2 By Mineral

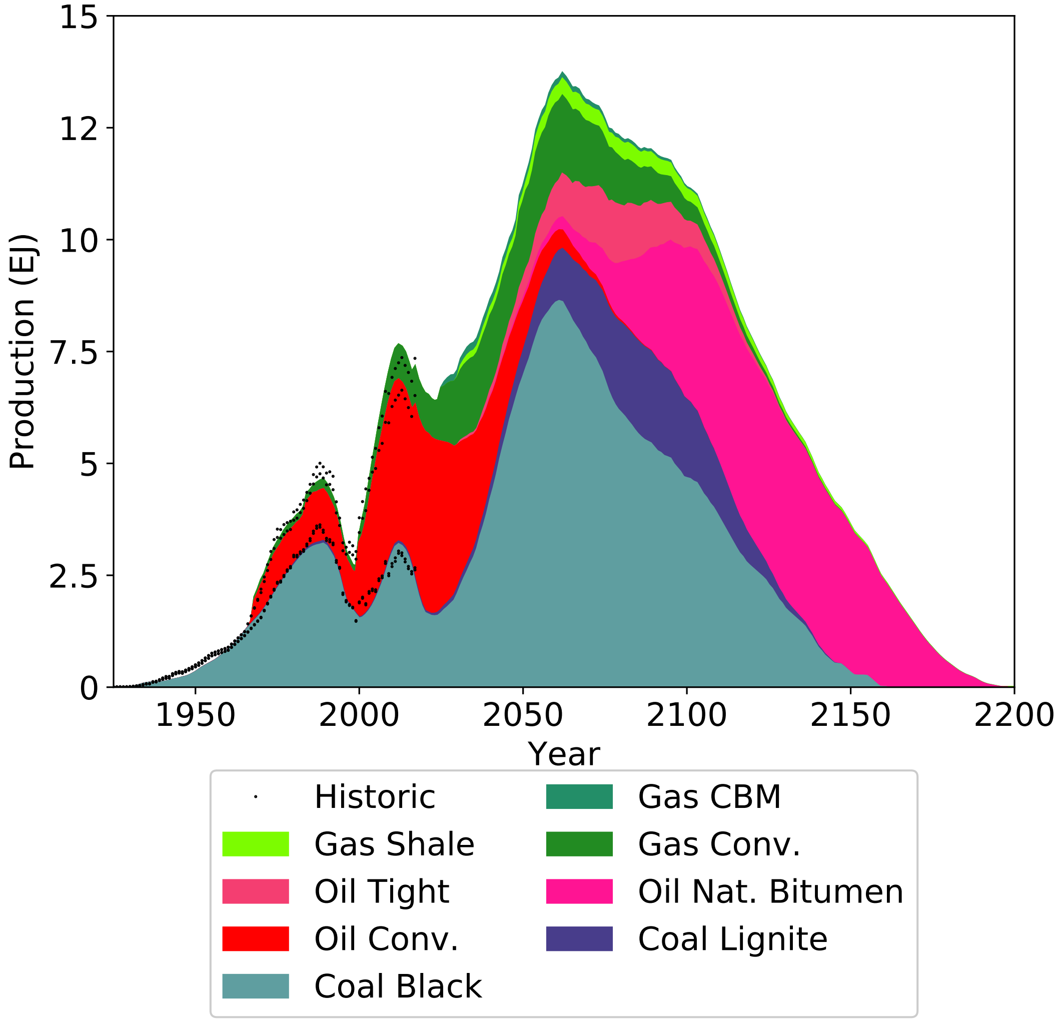

Figure 4.20: Kazakhstan projection by mineral type

4.7.3 Regional Projections

Table 4.20: Peak years - Minerals

**Name**	**URR**	**Peak Year**	**Peak Rate**
Coal Black	702.67	2061	8.63
Coal Lignite	116.95	2086	2.08
Oil Conv.	184.5	2020	4.0
Oil Nat. Bitumen	312.53	2125	4.05
Oil Tight	60.74	2077	1.38
Gas Conv.	131.2	2057	1.84
Gas Shale	28.91	2067	0.39
Gas CBM	10.5	2041	0.2
**Total**	**1548.0**	**2062**	**13.73**

All

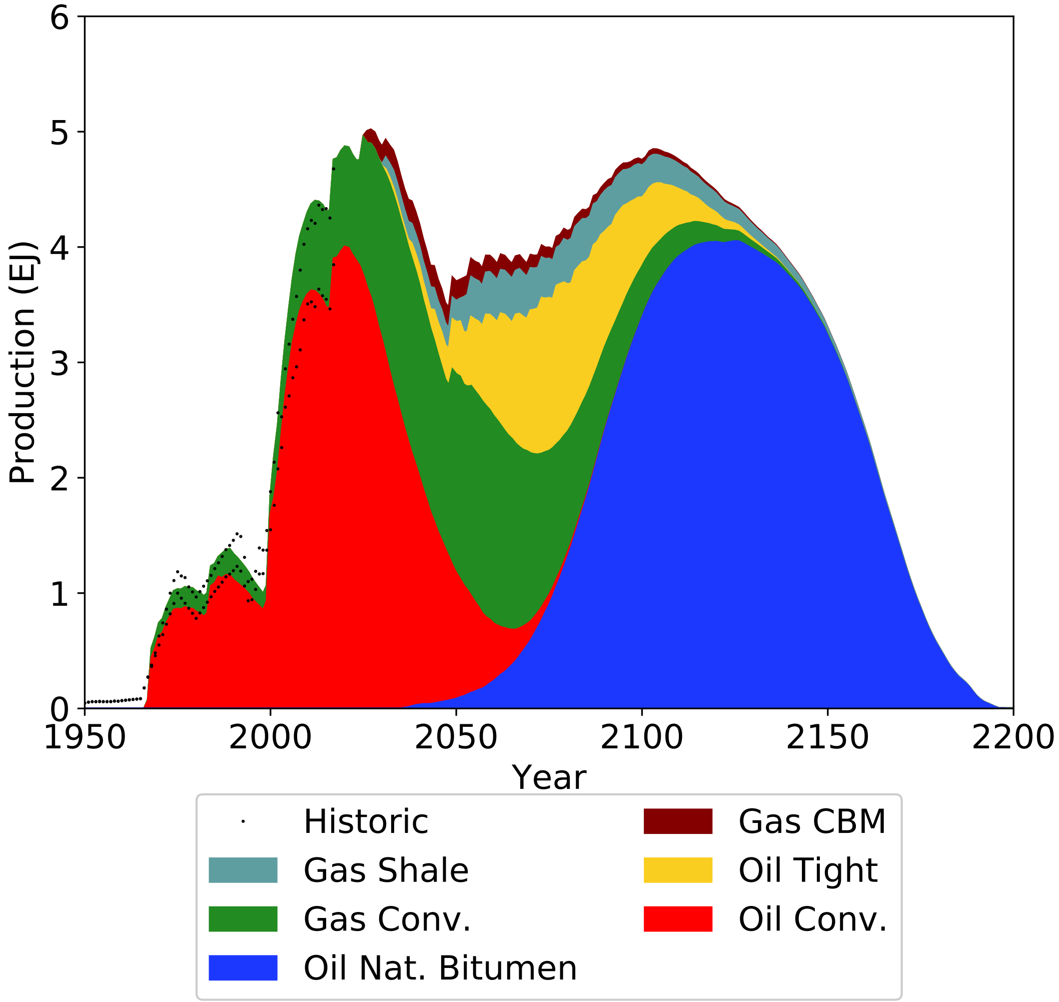

Figure 4.21: Kazakhstan - All projections capped at 16

Table 4.21: Peak years - All

Name	URR	Peak Year	Peak Rate
Oil Nat. Bitumen	312.53	2125	4.05
Oil Conv.	184.5	2020	4.0
Gas Conv.	131.2	2057	1.84
Oil Tight	60.74	2077	1.38
Gas Shale	28.91	2067	0.39
Gas CBM	10.5	2041	0.2
**Total**	**728.38**	**2027**	**5.02**

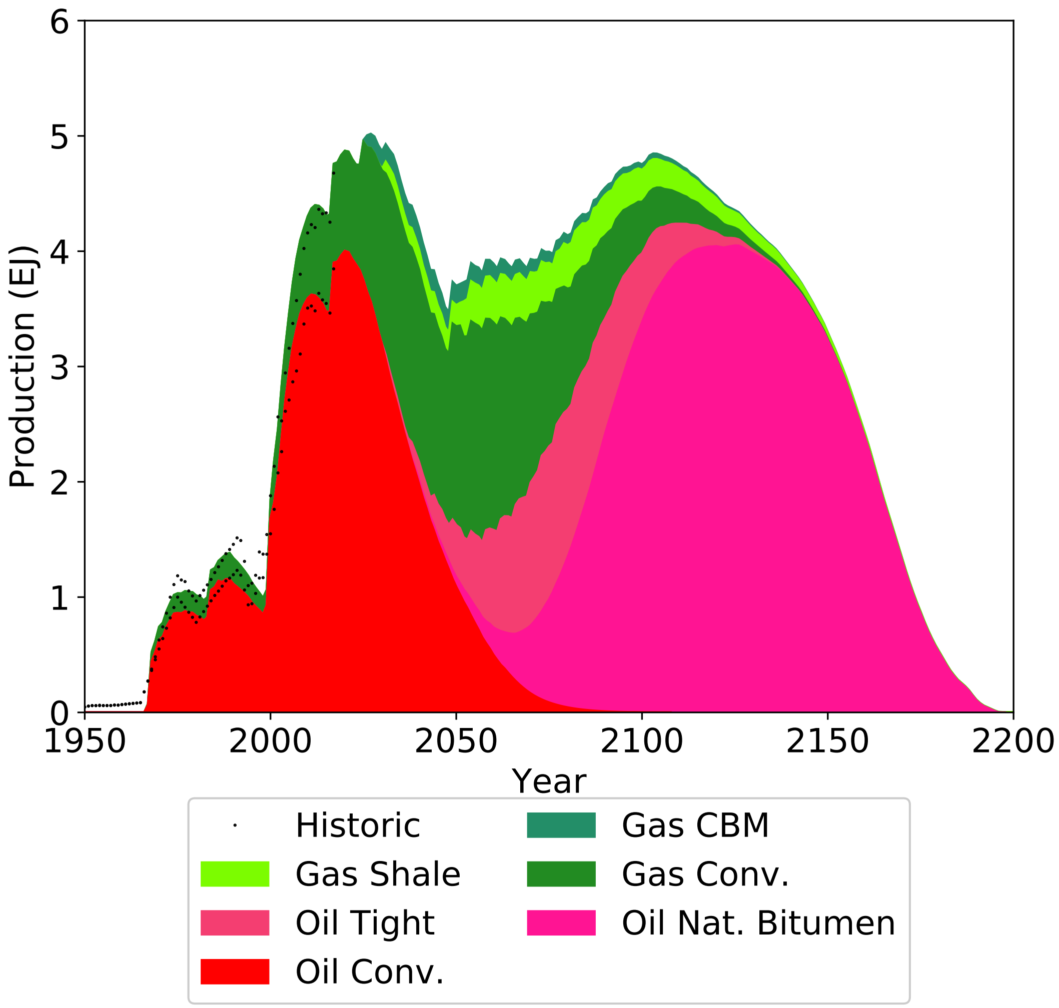

Figure 4.22: Kazakhstan - All projection by mineral type

Table 4.22: Peak years - Minerals

**Name**	**URR**	**Peak Year**	**Peak Rate**
Oil Conv.	184.5	2020	4.0
Oil Nat. Bitumen	312.53	2125	4.05
Oil Tight	60.74	2077	1.38
Gas Conv.	131.2	2057	1.84
Gas Shale	28.91	2067	0.39
Gas CBM	10.5	2041	0.2
**Total**	**728.38**	**2027**	**5.02**

East Kazakhstan

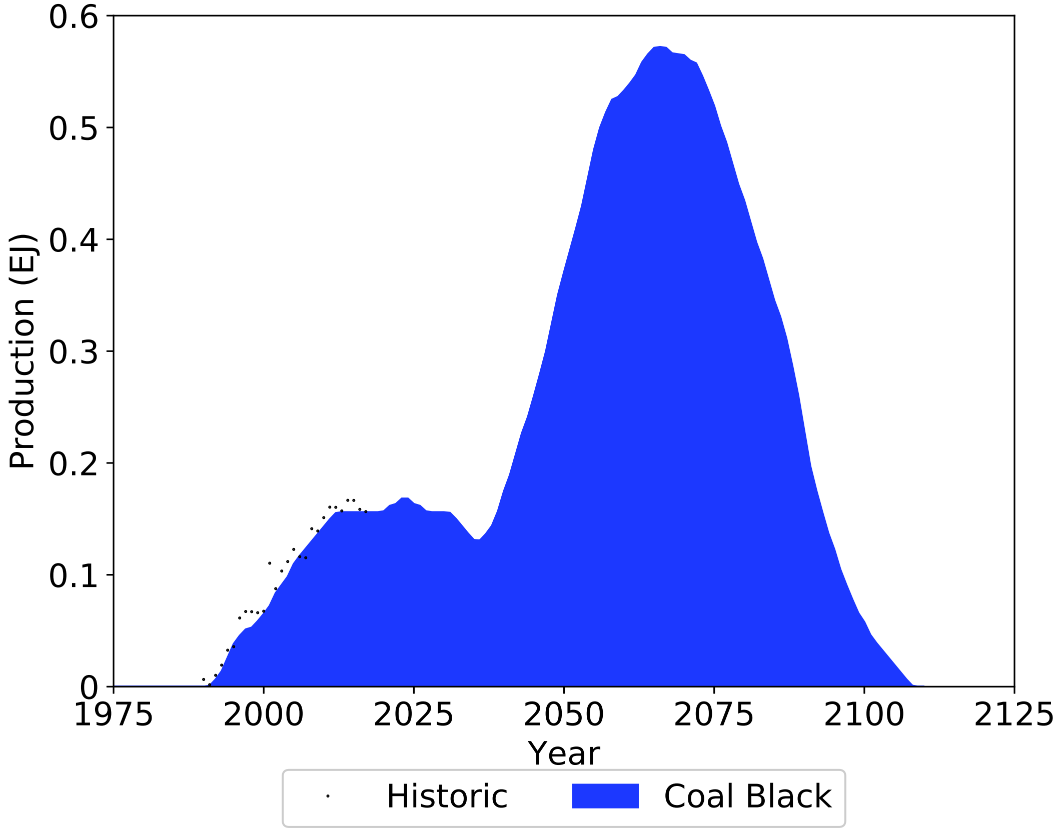

Figure 4.23: Kazakhstan - East Kazakhstan projections capped at 16

Table 4.23: Peak years - All

**Name**	**URR**	**Peak Year**	**Peak Rate**
Coal Black East Kazakhstan	28.97	2066	0.57
**Total**	**28.97**	**2066**	**0.57**

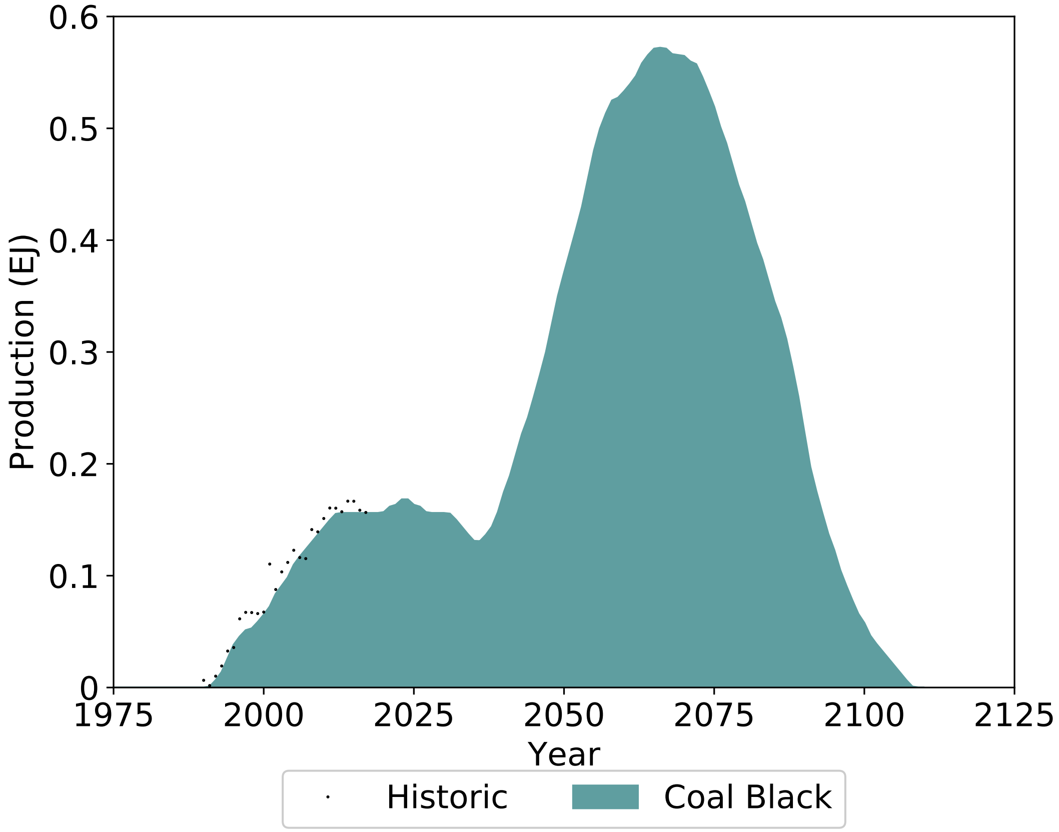

Figure 4.24: Kazakhstan - East Kazakhstan projection by mineral type

Table 4.24: Peak years - Minerals

Name	URR	Peak Year	Peak Rate
Coal Black	28.97	2066	0.57
**Total**	**28.97**	**2066**	**0.57**

Karaganda

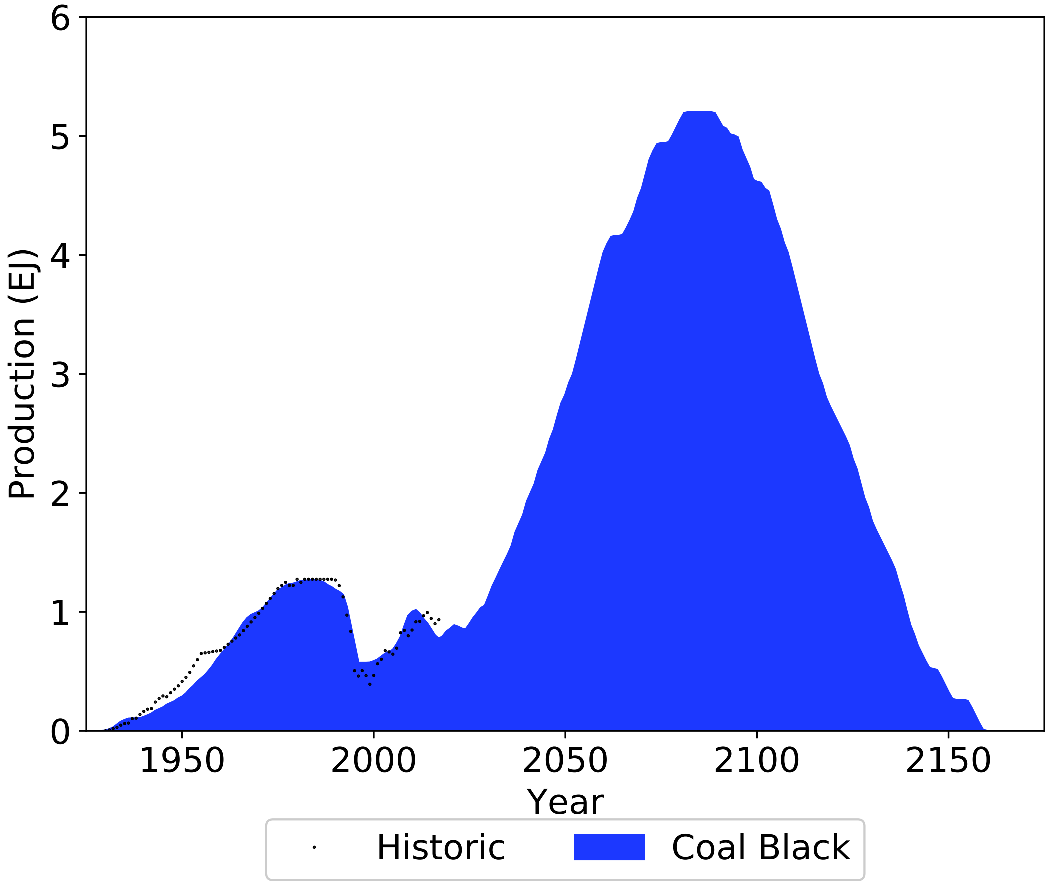

Figure 4.25: Kazakhstan - Karaganda projections capped at 16

Table 4.25: Peak years - All

Name	URR	Peak Year	Peak Rate
Coal Black Karaganda	456.95	2082	5.2
**Total**	**456.95**	**2082**	**5.2**

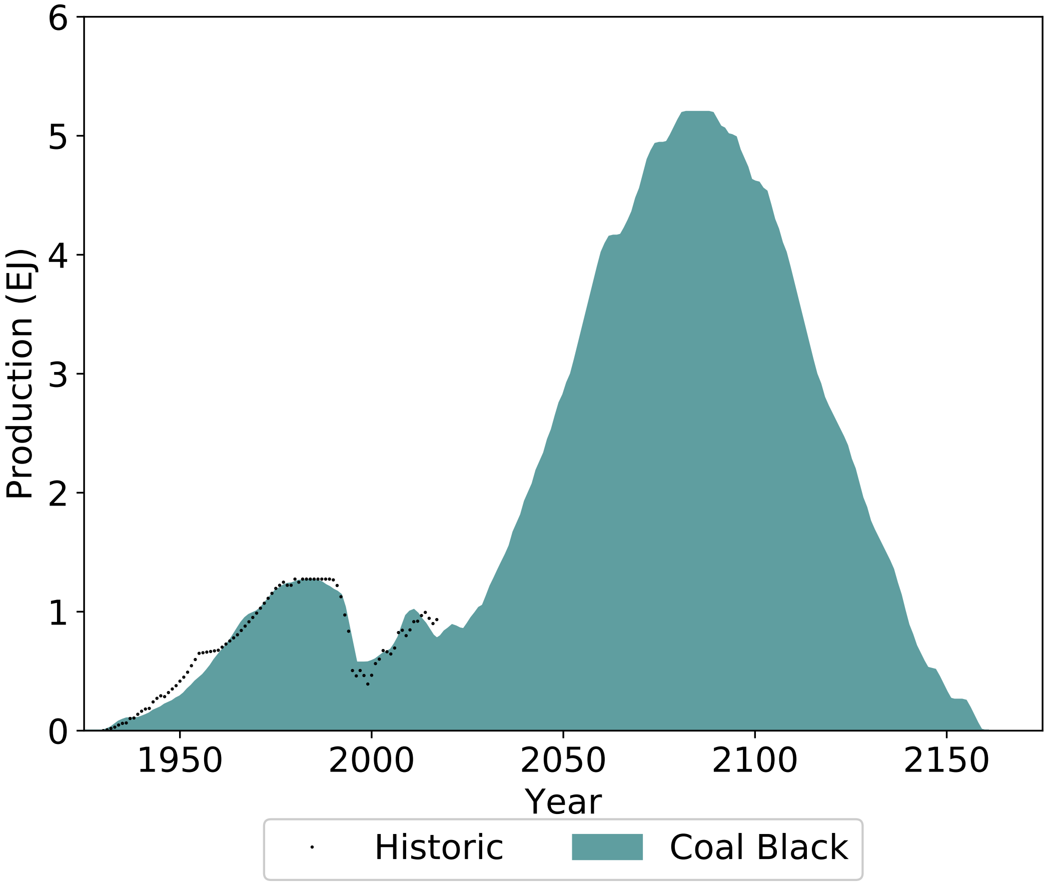

Figure 4.26: Kazakhstan - Karaganda projection by mineral type

Table 4.26: Peak years - Minerals

Name	URR	Peak Year	Peak Rate
Coal Black	456.95	2082	5.2
**Total**	**456.95**	**2082**	**5.2**

Kostanay

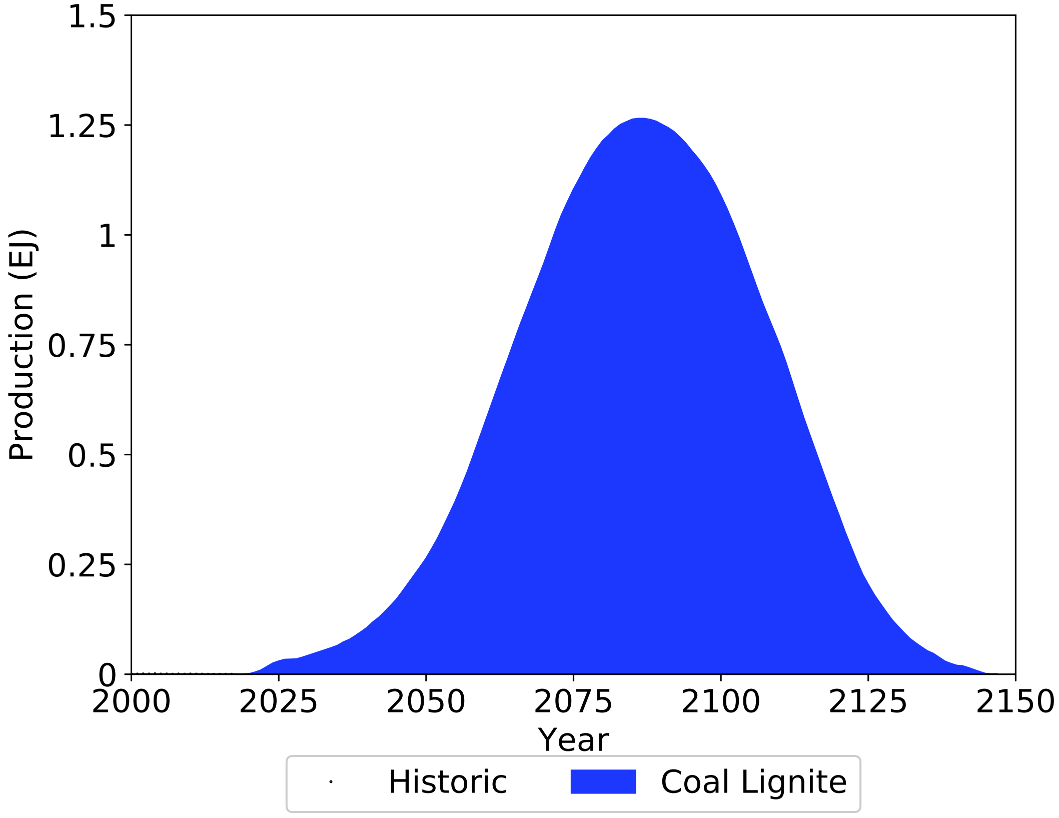

Figure 4.27: Kazakhstan - Kostanay projections capped at 16

Table 4.27: Peak years - All

Name	URR	Peak Year	Peak Rate
Coal Lignite Kostanay	67.07	2086	1.26
**Total**	**67.07**	**2086**	**1.26**

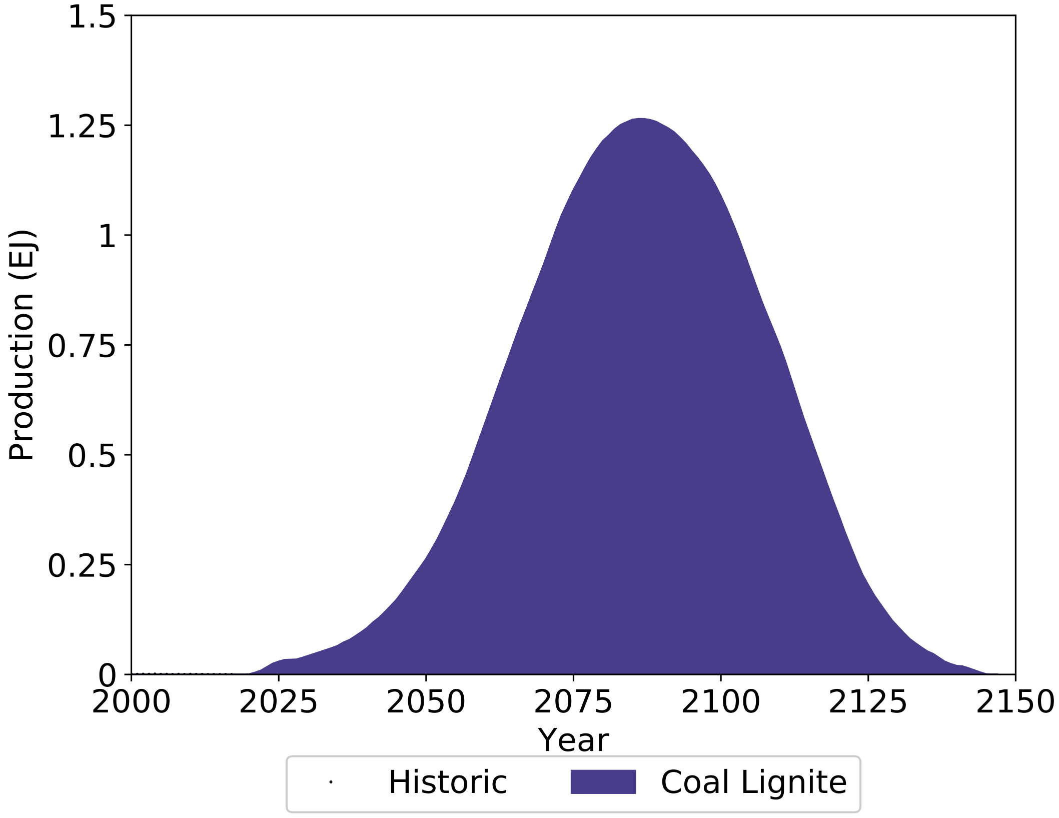

Figure 4.28: Kazakhstan - Kostanay projection by mineral type

Table 4.28: Peak years - Minerals

**Name**	**URR**	**Peak Year**	**Peak Rate**
Coal Lignite	67.07	2086	1.26
**Total**	**67.07**	**2086**	**1.26**

Other

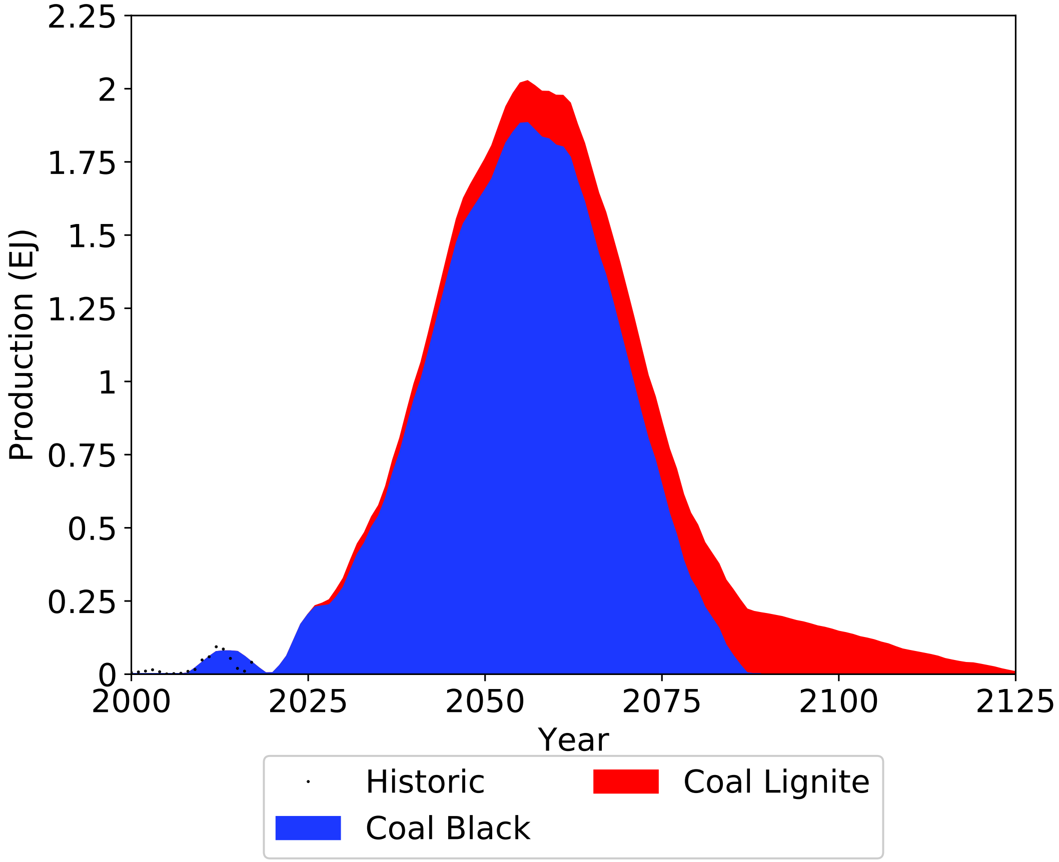

Figure 4.29: Kazakhstan - Other projections capped at 16

Table 4.29: Peak years - All

Name	URR	Peak Year	Peak Rate
Coal Black Other	62.09	2056	1.88
Coal Lignite Other	12.62	2070	0.23
**Total**	**74.71**	**2056**	**2.03**

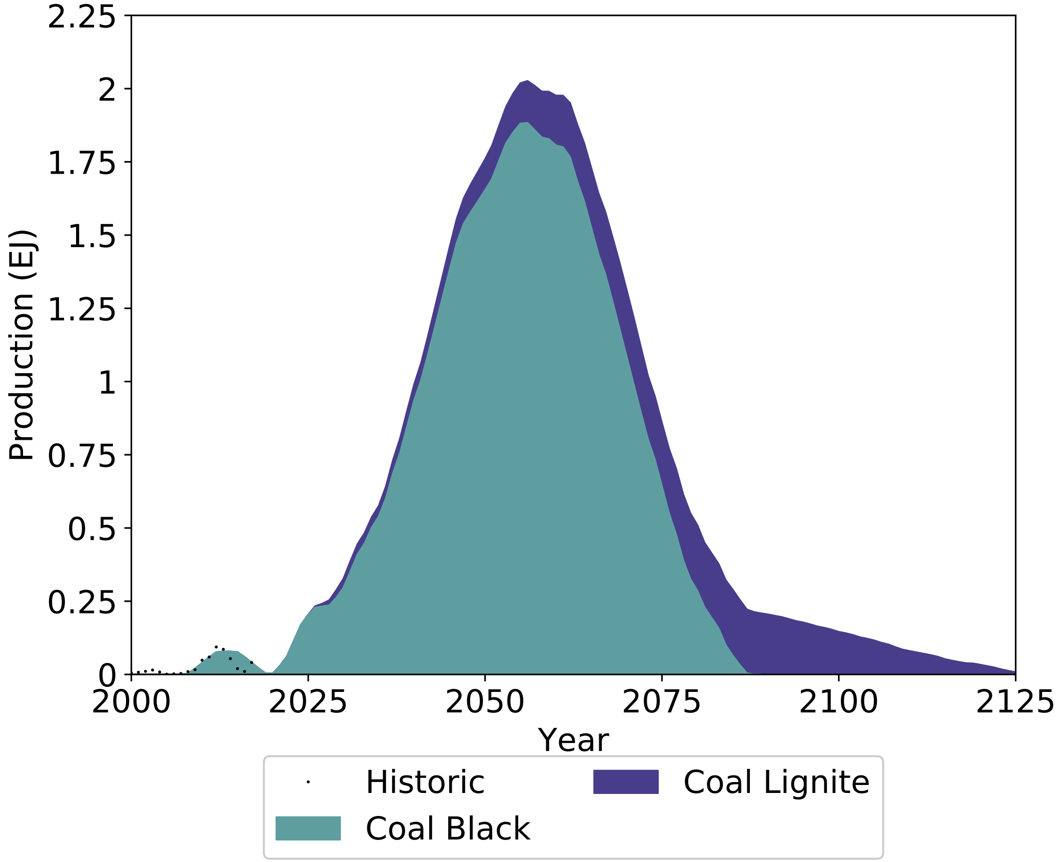

Figure 4.30: Kazakhstan - Other projection by mineral type

Table 4.30: Peak years - Minerals

Name	URR	Peak Year	Peak Rate
Coal Black	62.09	2056	1.88
Coal Lignite	12.62	2070	0.23
**Total**	**74.71**	**2056**	**2.03**

Pavlodar

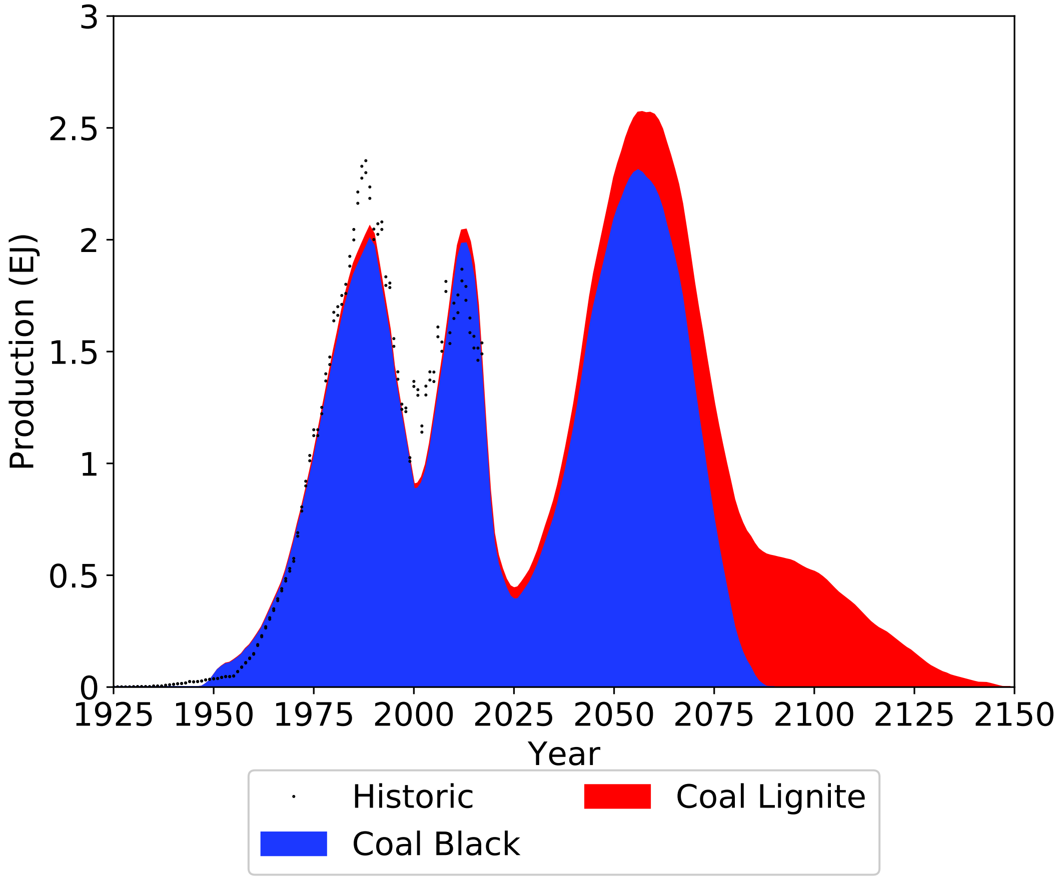

Figure 4.31: Kazakhstan - Pavlodar projections capped at 16

Table 4.31: Peak years - All

**Name**	**URR**	**Peak Year**	**Peak Rate**
Coal Black Pavlodar	154.66	2056	2.31
Coal Lignite Pavlodar	37.26	2088	0.59
**Total**	**191.92**	**2057**	**2.57**

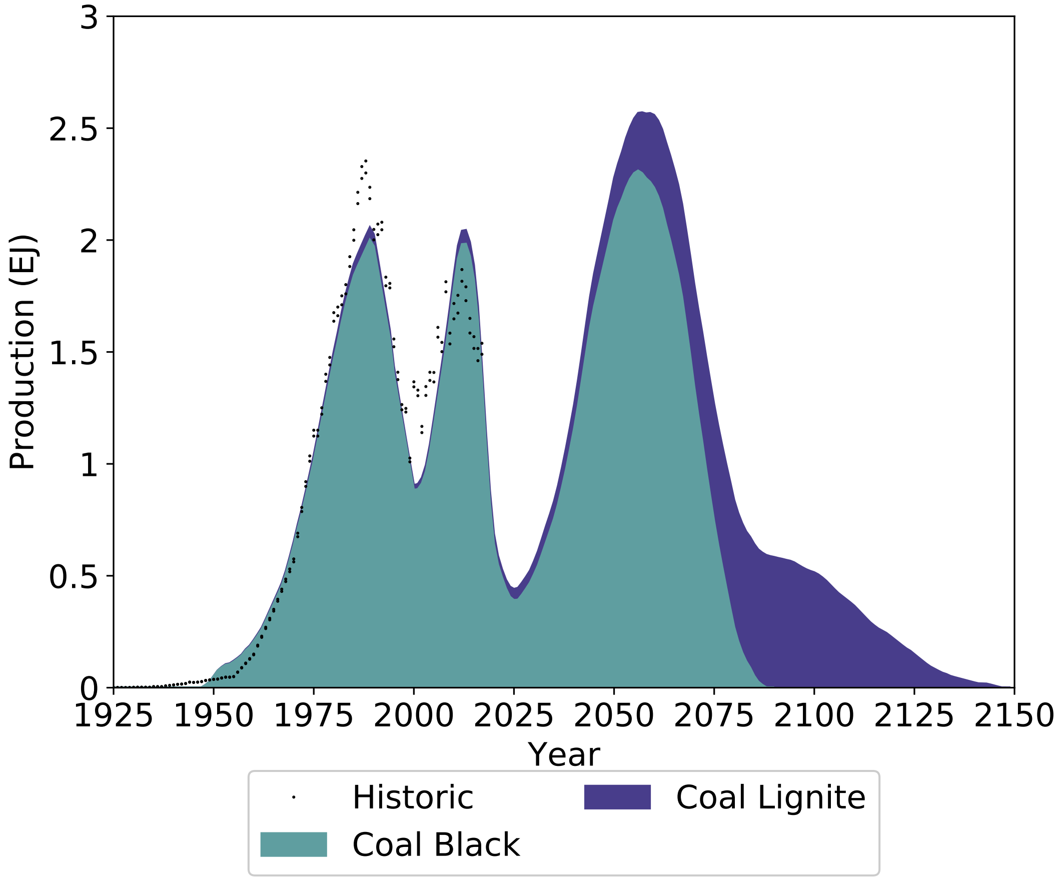

Figure 4.32: Kazakhstan - Pavlodar projection by mineral type

Table 4.32: Peak years - Minerals

Name	URR	Peak Year	Peak Rate
Coal Black	154.66	2056	2.31
Coal Lignite	37.26	2088	0.59
**Total**	**191.92**	**2057**	**2.57**

4.7.4 Projection by region

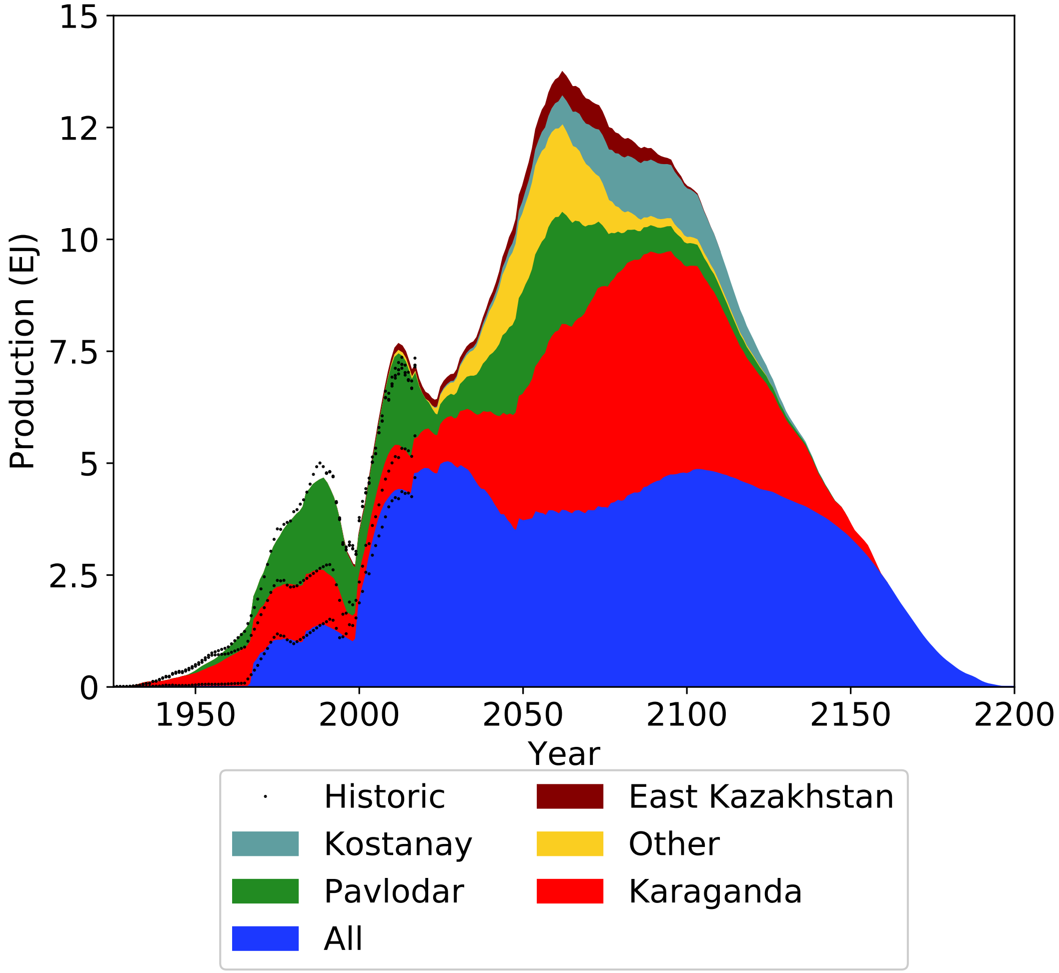

Figure 4.33: Kazakhstan by region projections capped at 16

Table 4.33: Peak years - All

Name	URR	Peak Year	Peak Rate
All	728.38	2027	5.02
Karaganda	456.95	2082	5.2
Pavlodar	191.92	2057	2.57
Other	74.71	2056	2.03
Kostanay	67.07	2086	1.26
East Kazakhstan	28.97	2066	0.57
**Total**	**1548.0**	**2062**	**13.73**

4.8 Kyrgyzstan

4.8.1 All Projections

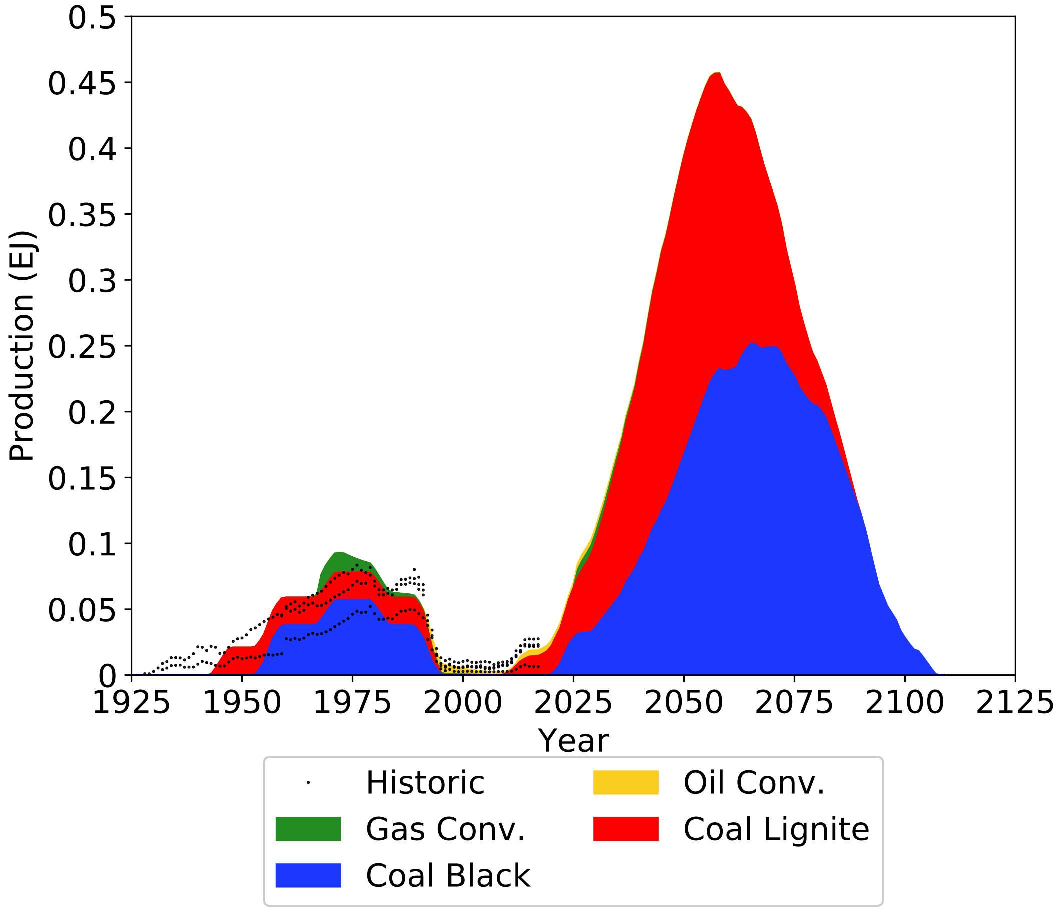

Figure 4.34: Kyrgyzstan projections capped at 16

Table 4.34: Peak years - All

Name	URR	Peak Year	Peak Rate
Coal Black	12.92	2066	0.25
Coal Lignite	9.32	2054	0.23
Gas Conv.	0.29	1969	0.02
Oil Conv.	0.21	1993	0.01
**Total**	**22.74**	**2058**	**0.46**

4.8.2 By Mineral

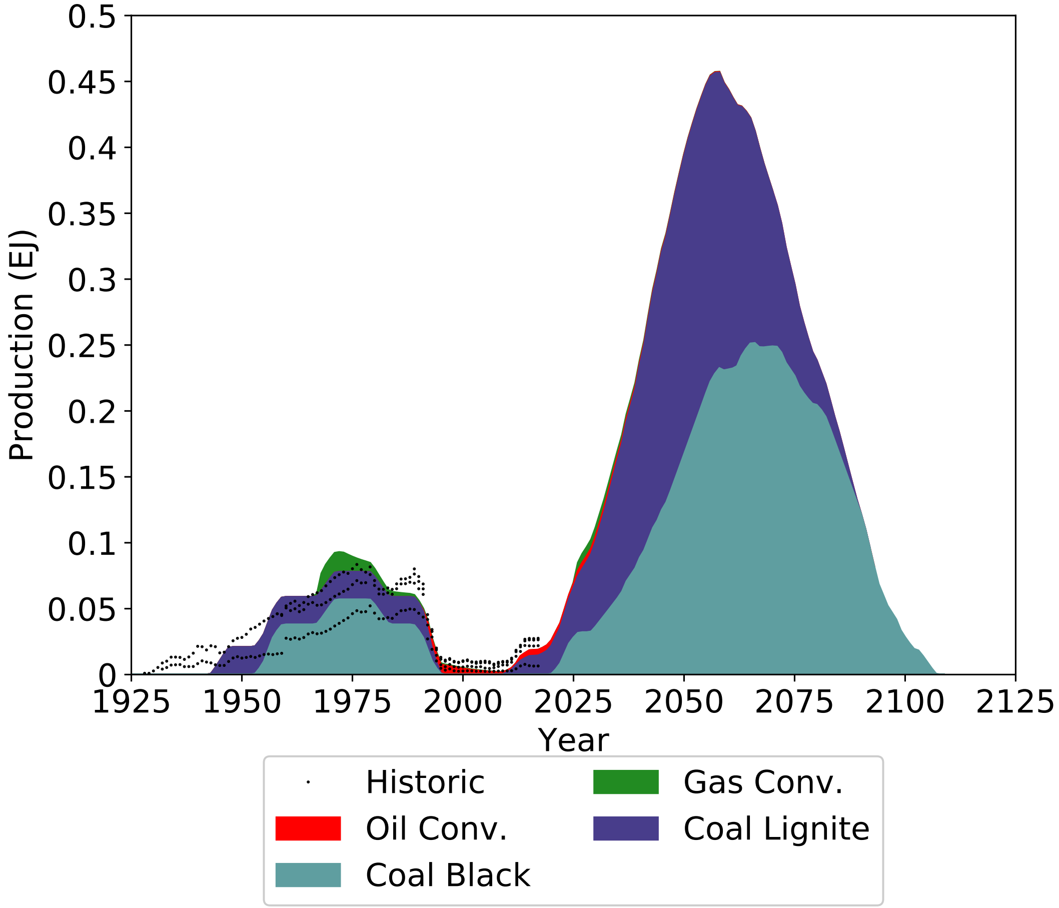

Figure 4.35: Kyrgyzstan projection by mineral type

Table 4.35: Peak years - Minerals

**Name**	**URR**	**Peak Year**	**Peak Rate**
Coal Black	12.92	2066	0.25
Coal Lignite	9.32	2054	0.23
Oil Conv.	0.21	1993	0.01
Gas Conv.	0.29	1969	0.02
**Total**	**22.74**	**2058**	**0.46**

4.9 Lithuania

4.9.1 All Projections

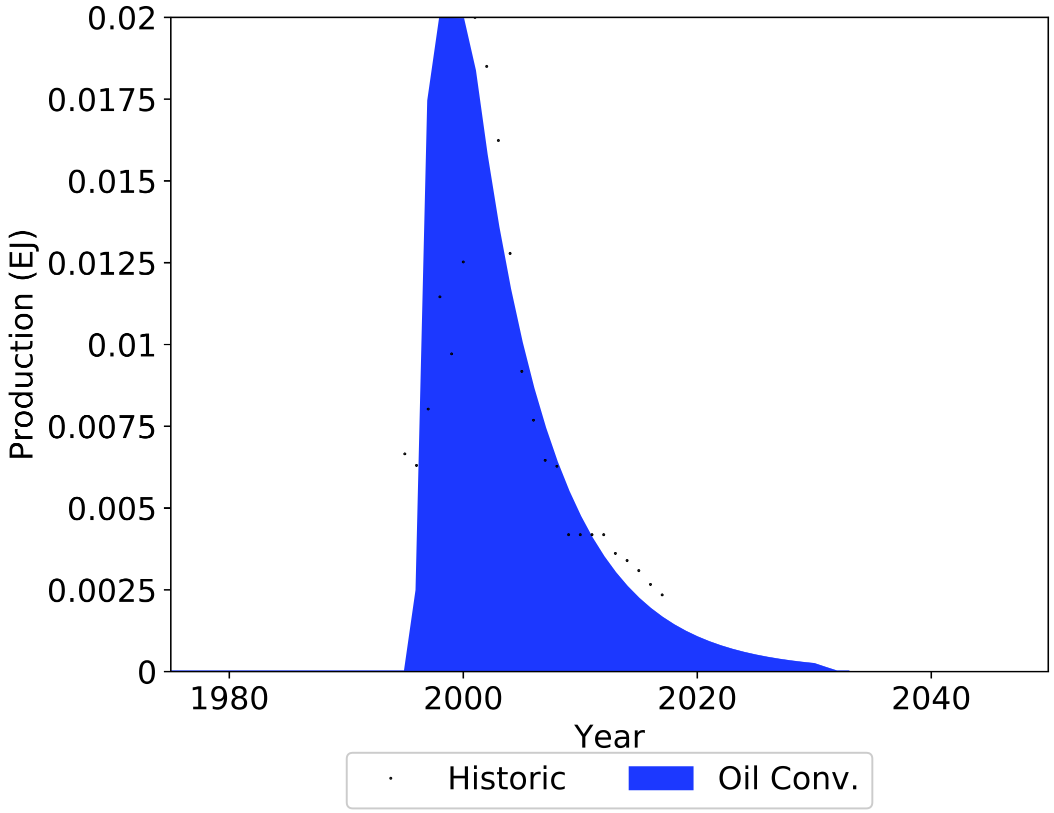

Figure 4.36: Lithuania projections capped at 16

Table 4.36: Peak years - All

Name	URR	Peak Year	Peak Rate
Oil Conv.	0.21	1998	0.02
**Total**	**0.21**	**1998**	**0.02**

4.9.2 By Mineral

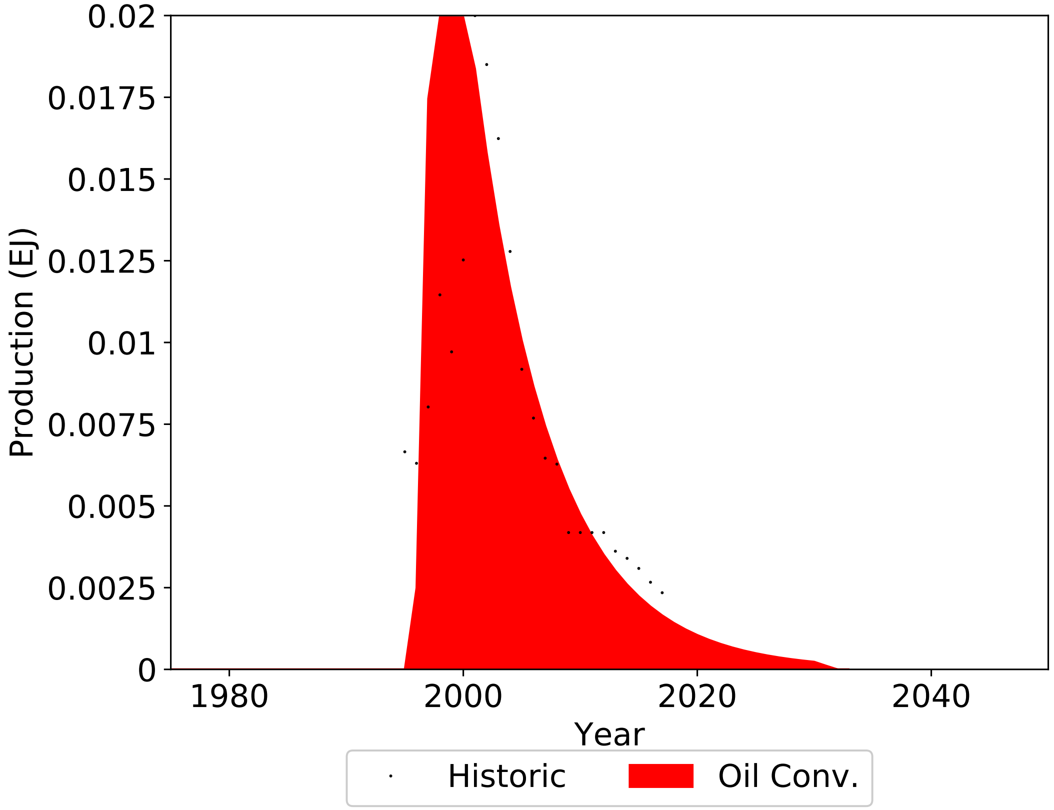

Figure 4.37: Lithuania projection by mineral type

Table 4.37: Peak years - Minerals

Name	URR	Peak Year	Peak Rate
Oil Conv.	0.21	1998	0.02
**Total**	**0.21**	**1998**	**0.02**

4.10 Luhansk

4.10.1 All Projections

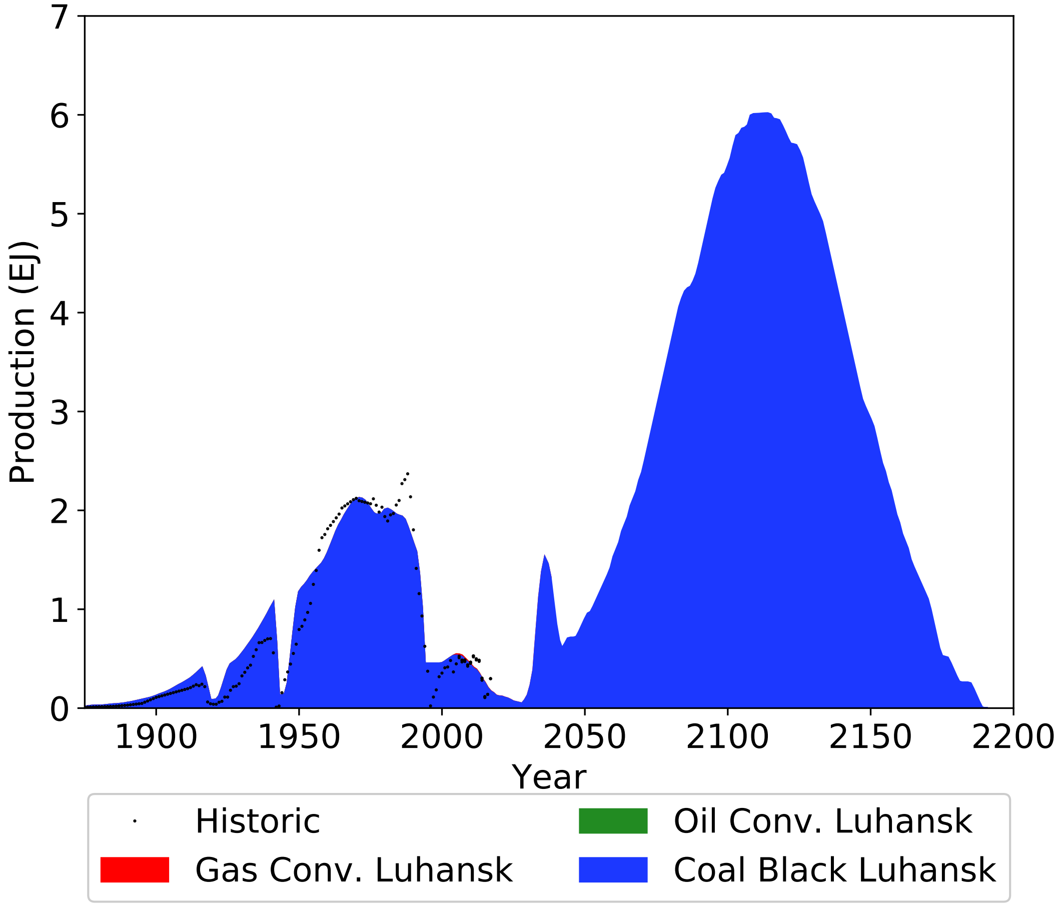

Figure 4.38: Luhansk projections capped at 16

Table 4.38: Peak years - All

**Name**	**URR**	**Peak Year**	**Peak Rate**
Coal Black Luhansk	582.48	2114	6.02
Gas Conv. Luhansk	0.14	2006	0.01
Oil Conv. Luhansk	–	2006	–
**Total**	**582.62**	**2114**	**6.02**

4.10.2 By Mineral

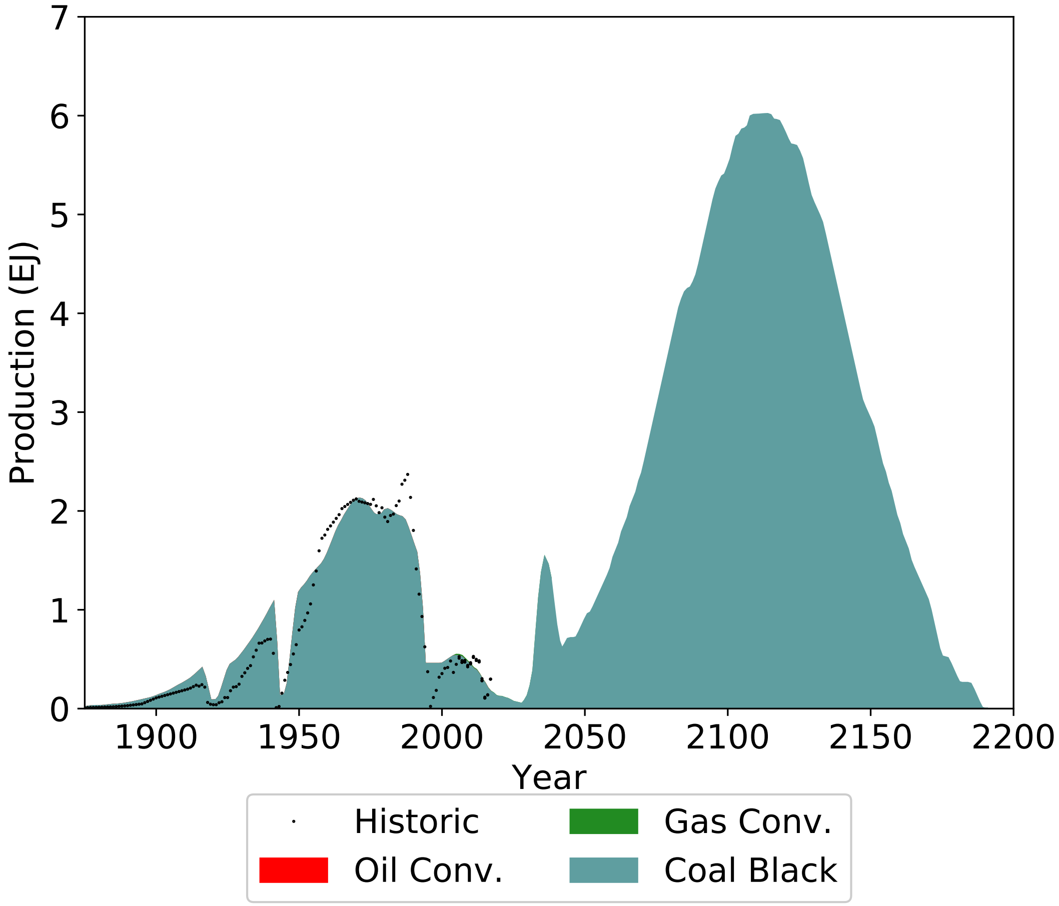

Figure 4.39: Luhansk projection by mineral type

Table 4.39: Peak years - Minerals

Name	URR	Peak Year	Peak Rate
Coal Black	582.48	2114	6.02
Oil Conv.	–	2006	–
Gas Conv.	0.14	2006	0.01
**Total**	**582.62**	**2114**	**6.02**

4.10.3 Regional Projections

Luhansk

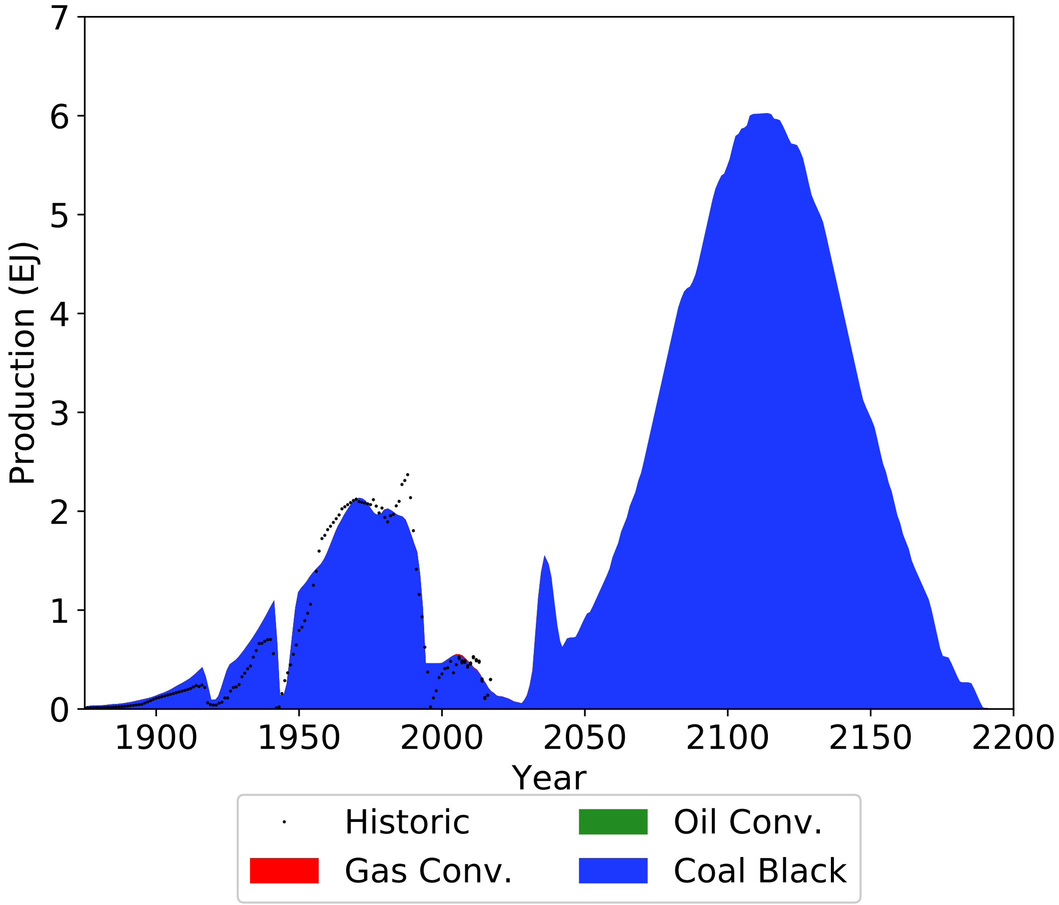

Figure 4.40: Luhansk - Luhansk projections capped at 16

Table 4.40: Peak years - All

Name	URR	Peak Year	Peak Rate
Coal Black Luhansk	582.48	2114	6.02
Gas Conv. Luhansk	0.14	2006	0.01
Oil Conv. Luhansk	–	2006	–
**Total**	**582.62**	**2114**	**6.02**

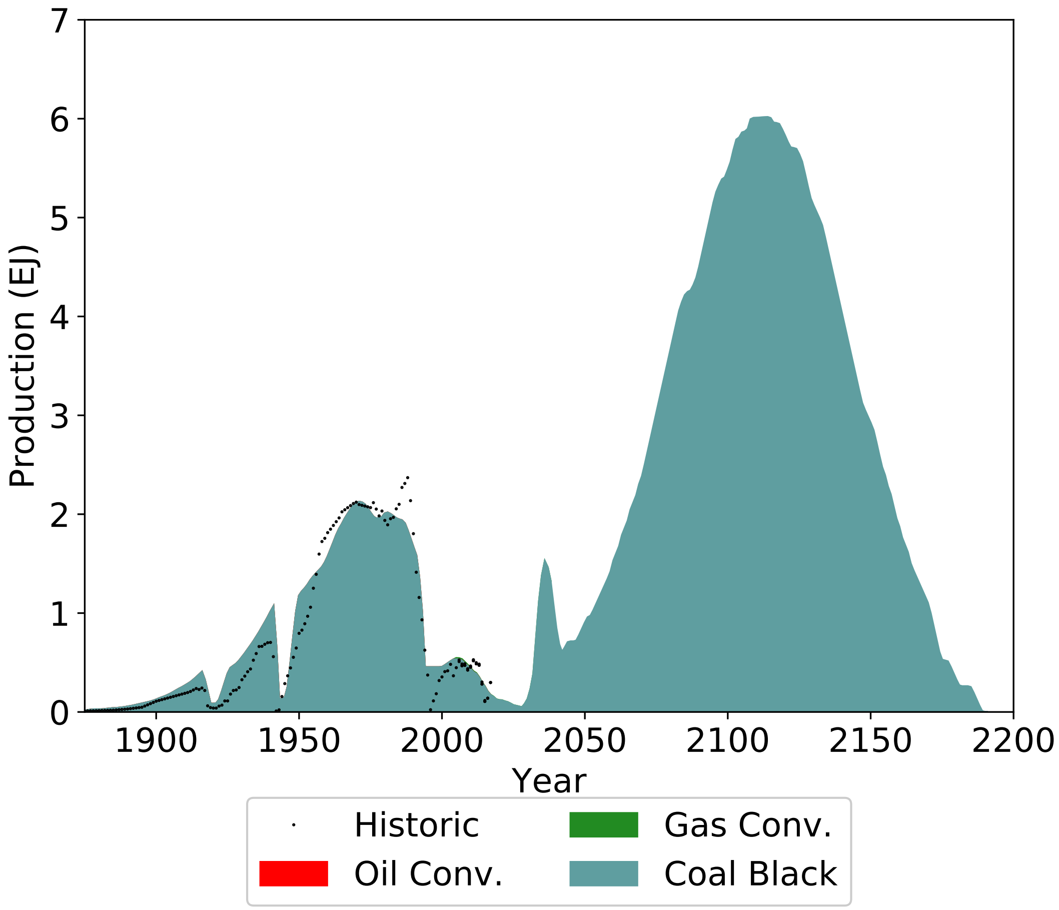

Figure 4.41: Luhansk - Luhansk projection by mineral type

Table 4.41: Peak years - Minerals

**Name**	**URR**	**Peak Year**	**Peak Rate**
Coal Black	582.48	2114	6.02
Oil Conv.	–	2006	–
Gas Conv.	0.14	2006	0.01
**Total**	**582.62**	**2114**	**6.02**

4.10.4 Projection by region

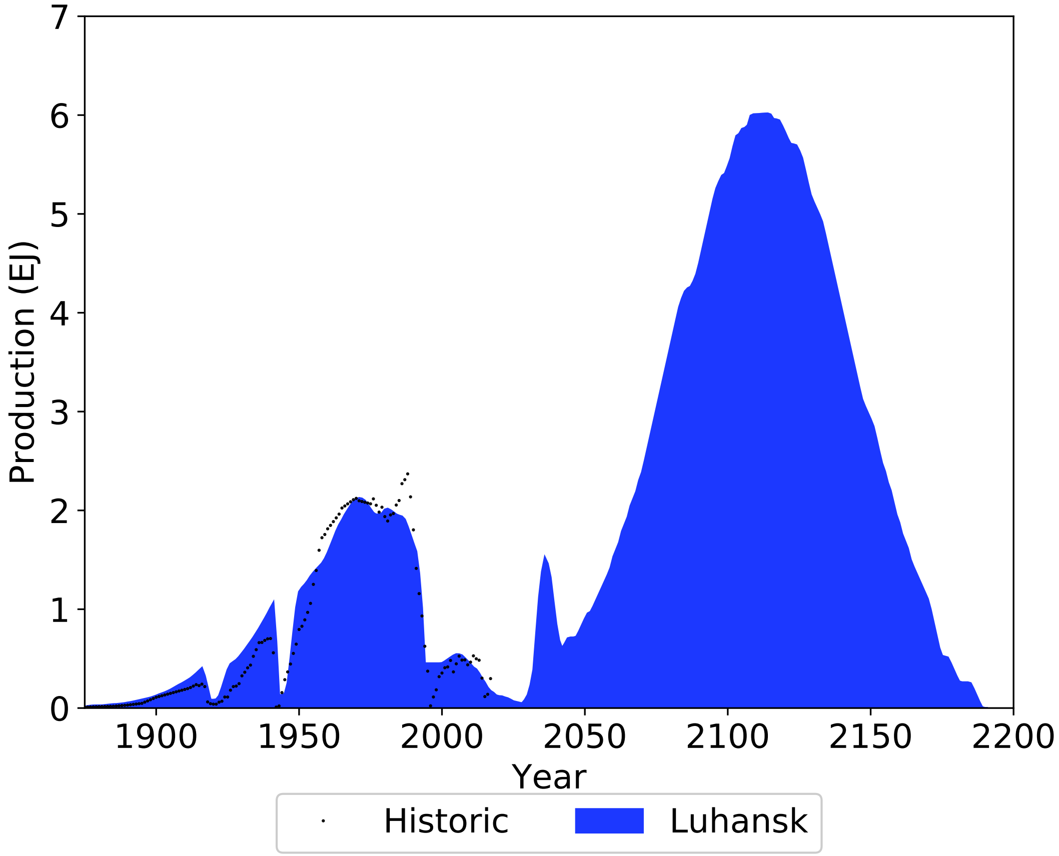

Figure 4.42: Luhansk by region projections capped at 16

Table 4.42: Peak years - All

**Name**	**URR**	**Peak Year**	**Peak Rate**
Luhansk	582.62	2114	6.02
**Total**	**582.62**	**2114**	**6.02**

4.11 Moldova

4.11.1 All Projections

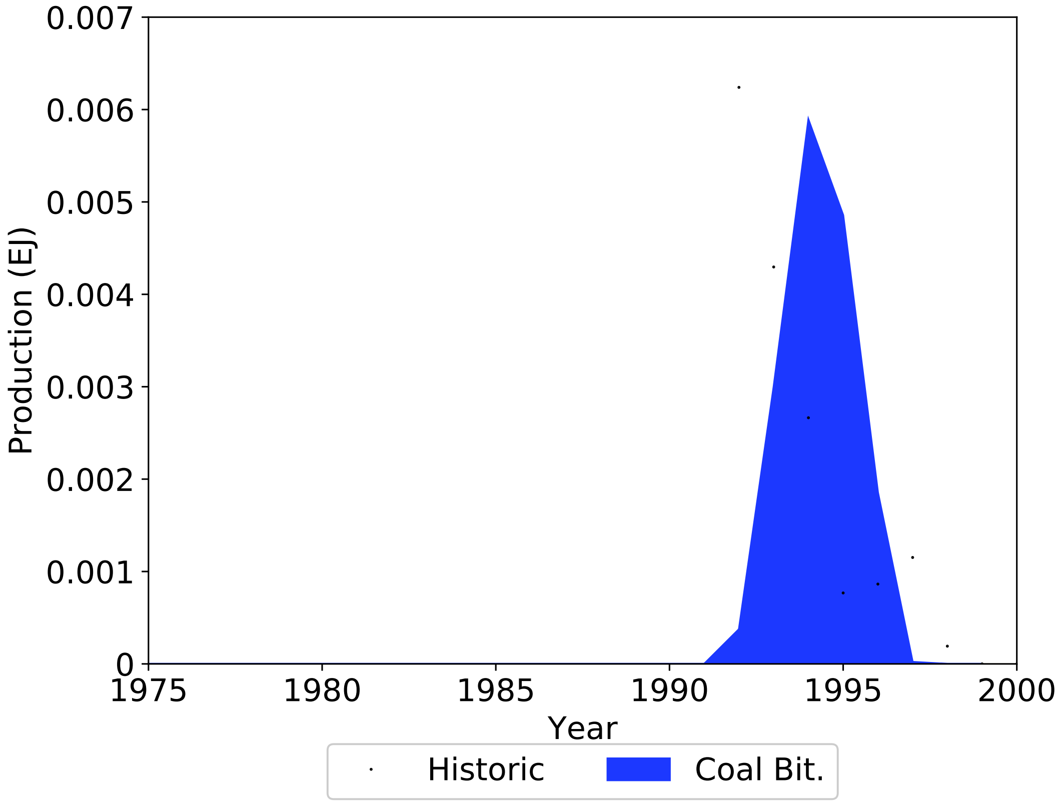

Figure 4.43: Moldova projections capped at 16

Table 4.43: Peak years - All

Name	URR	Peak Year	Peak Rate
Coal Bit.	0.02	1994	0.01
**Total**	**0.02**	**1994**	**0.01**

4.11.2 By Mineral

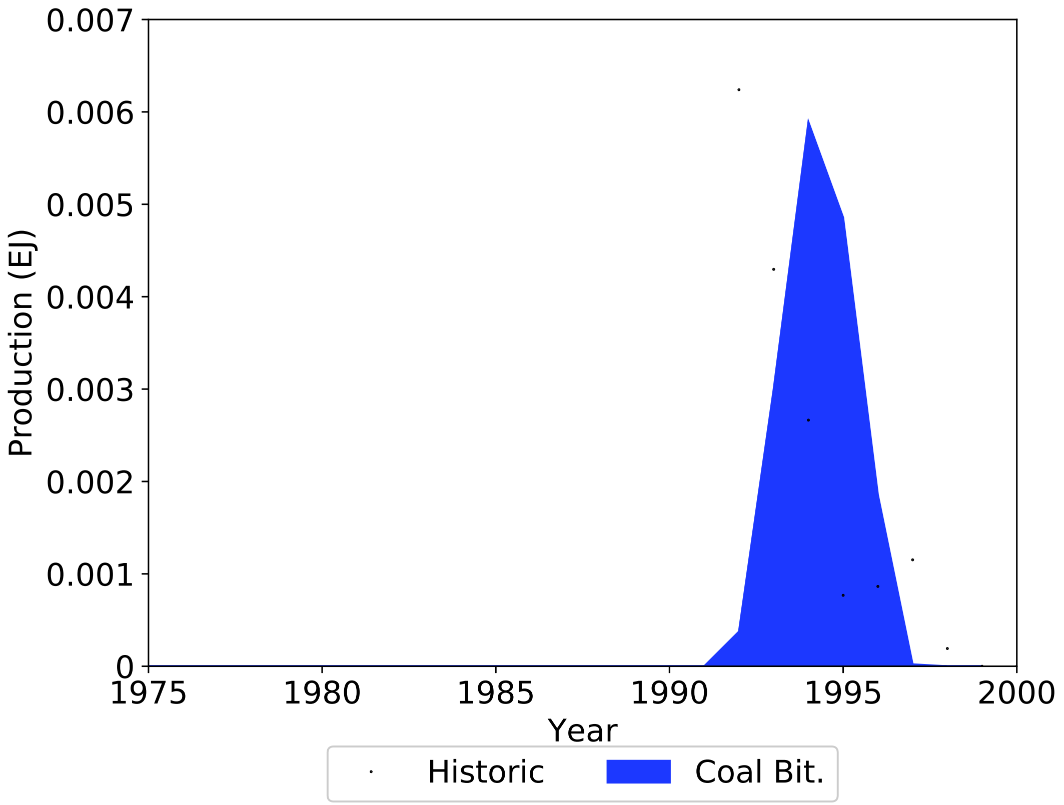

Figure 4.44: Moldova projection by mineral type

Table 4.44: Peak years - Minerals

Name	URR	Peak Year	Peak Rate
Coal Bit.	0.02	1994	0.01
**Total**	**0.02**	**1994**	**0.01**

4.12 Russia

4.12.1 All Projections

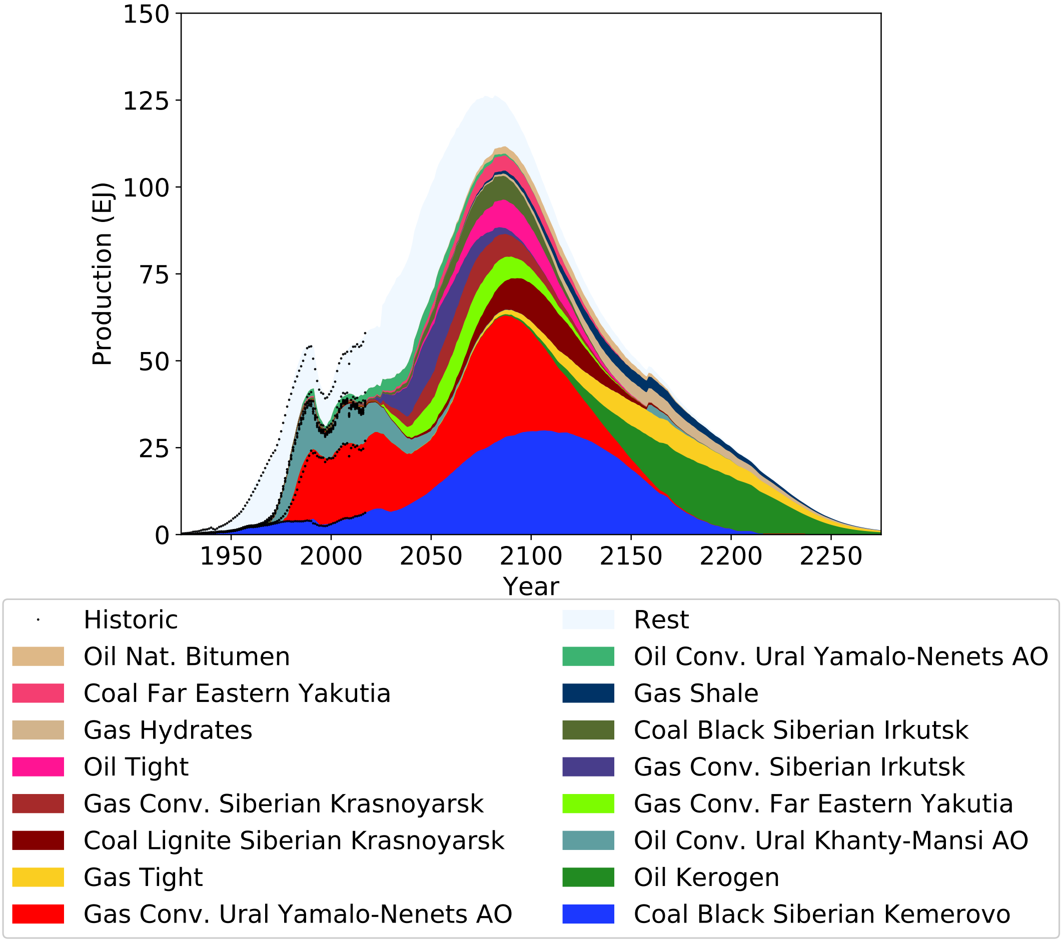

Figure 4.45: Russia projections capped at 16

Table 4.45: Peak years - All

Name	URR	Peak Year	Peak Rate
Coal Black Siberian Kemerovo	3378.92	2107	29.74
Gas Conv. Ural Yamalo-Nenets AO	3364.8	2085	35.37
Oil Kerogen	1421.06	2186	16.08
Gas Tight	741.3	2156	7.13
Oil Conv. Ural Khanty-Mansi AO	738.03	1985	15.34
Coal Lignite Siberian Krasnoyarsk	664.93	2105	11.18
Gas Conv. Far Eastern Yakutia	574.68	2065	10.52
Gas Conv. Siberian Krasnoyarsk	561.39	2065	10.09
Gas Conv. Siberian Irkutsk	496.29	2050	13.82
Oil Tight	432.61	2091	8.27
Coal Black Siberian Irkutsk	412.45	2081	7.11
Gas Hydrates	403.83	2155	4.07
Gas Shale	352.12	2143	3.55
Coal Far Eastern Yakutia	288.18	2087	4.34
Oil Conv. Ural Yamalo-Nenets AO	255.27	2045	4.41
Oil Nat. Bitumen	219.45	2114	2.9
Gas CBM	209.91	2108	2.21
Gas Conv. Far Eastern Sakhalin	195.44	2052	3.52
Oil Conv. Volga Tatarstan	184.67	1972	4.44
Gas Conv. Southern Astrakhan	176.28	2050	3.25
Coal Black Siberian Khakassia	153.37	2055	3.1
Gas Conv. Northwestern Nenets AO	122.36	2061	2.4
Oil Conv. Volga Samara	118.76	2042	1.65
Coal Black Siberian Tuva	99.86	2123	1.2
Gas Conv. Volga Orenburg	93.03	1983	1.76
Oil Conv. Volga Bashkortostan	90.64	1969	1.94
Coal Far Eastern Primorsky	87.56	2064	1.58
Oil Conv. Siberian Krasnoyarsk	86.64	2042	1.97
Gas Conv. Ural Khanty-Mansi AO	79.6	2016	1.26
Coal Black Far Eastern Sakhalin	77.12	2057	1.55
Oil Conv. Volga Perm	74.41	2052	0.86
Oil Conv. Volga Orenburg	72.11	2034	1.32
Coal Black Far Eastern Buryatia	71.78	2064	1.54
Oil Conv. Northwestern Komi	65.44	2037	0.91
Coal Black Far Eastern Khabarovsk	62.88	2070	1.06
Oil Conv. Northwestern Nenets AO	56.97	2041	1.2
Coal Brown Far Eastern Amur	55.72	2083	0.75
Coal Brown Far Eastern Zabaykalsky	55.6	2065	0.49
Coal Black Southern Rostov	48.65	1963	0.86
Oil Conv. Siberian Irkutsk	46.16	2031	1.22
Coal Black Northwestern Komi	42.48	1987	0.81
Coal Black Siberian Novosibirsk	39.44	2034	1.89
Oil Conv. Southern Astrakhan	37.77	2035	0.9
Oil Conv. Siberian Tomsk	37.17	2031	0.69
Gas Conv. Southern Other	36.55	1963	1.08
Oil Conv. Volga Udmurtia	34.67	1983	0.51
Oil Conv. Far Eastern Yakutia	32.37	2044	0.72
Gas Conv. North Caucasian	31.5	1963	1.58
Oil Conv. Far Eastern Sakhalin	29.53	2019	0.76

Table 4.45: Peak years - All – Continued

Name	URR	Peak Year	Peak Rate
Coal Ural Khanty-Mansi AO	26.33	2076	0.48
Oil Conv. Ural Tyumen	24.72	2033	0.67
Gas Conv. Far Eastern Kamchatka	24.15	2039	0.6
Gas Conv. Siberian Tomsk	20.05	2031	0.44
Oil Conv. North Caucasian Chechnya	18.92	1969	0.82
Coal Brown Ural Chelyabinsk	18.45	1966	0.28
Gas Conv. Northwestern Komi	17.86	1977	0.74
Coal Black Volga Perm	17.31	1958	0.34
Coal Lignite Central	15.38	1965	0.39
Oil Conv. Southern Volgograd	13.47	1965	0.3
Oil Conv. Southern Krasnodar	11.33	1962	0.25
Gas Conv. Volga Saratov	11.23	1963	0.27
Coal Brown Ural Sverdlovsk	10.02	1960	0.35
Oil Conv. Volga Saratov	8.46	2025	0.15
Gas Conv. Volga Other	6.26	2033	0.15
Oil Conv. North Caucasian Stavropol	5.89	1971	0.3
Oil Conv. Volga Ulyanovsk	4.77	2032	0.15
Coal Brown Far Eastern Kamchatka	3.93	2060	0.12
Coal Black Far Eastern Magadan	2.49	1987	0.06
Oil Conv. Northwestern Kaliningrad	2.19	1980	0.07
Oil Conv. North Caucasian Dagestan	1.78	1972	0.08
Coal Lignite Volga Bashkortostan	1.72	1976	0.08
Coal Black Far Eastern Chukotka AO	1.03	1989	0.03
Oil Conv. Siberian Novosibirsk	0.7	2004	0.09
Oil Conv. Southern Kalmykia	0.68	1977	0.03
Coal Brown Volga Orenburg	0.59	1985	0.04
Coal Black Northwestern Murmansk	0.47	1970	0.01
Oil Conv. Siberian Omsk	0.43	2005	0.04
Oil Conv. Southern Adygea	0.09	1971	0.01
Oil Conv. North Caucasian Ingushetia	0.07	1996	0.01
Coal Black North Caucasian Karachay-Cherkessia	0.06	1970	–
Oil Conv. Volga Penza	0.04	2002	0.01
Coal Brown Northwestern Novgorod	0.01	1951	0.01
Coal Brown Far Eastern Jewish AO	0.01	2004	–
Coal Brown Siberian Altai Krai	0.01	2009	–
Oil Conv. North Caucasian North Ossetia-Alania	–	2002	–
Oil Conv. North Caucasian Kabardino-Balkaria	–	1998	–
Oil Conv. Volga Kirov	–	2001	–
Coal Lignite Far Eastern Zabaykalsky	–	1997	–
Oil Conv. Central Yaroslavl	–	2005	–
**Total**	**17282.65**	**2082**	**126.08**

4.12.2 By Mineral

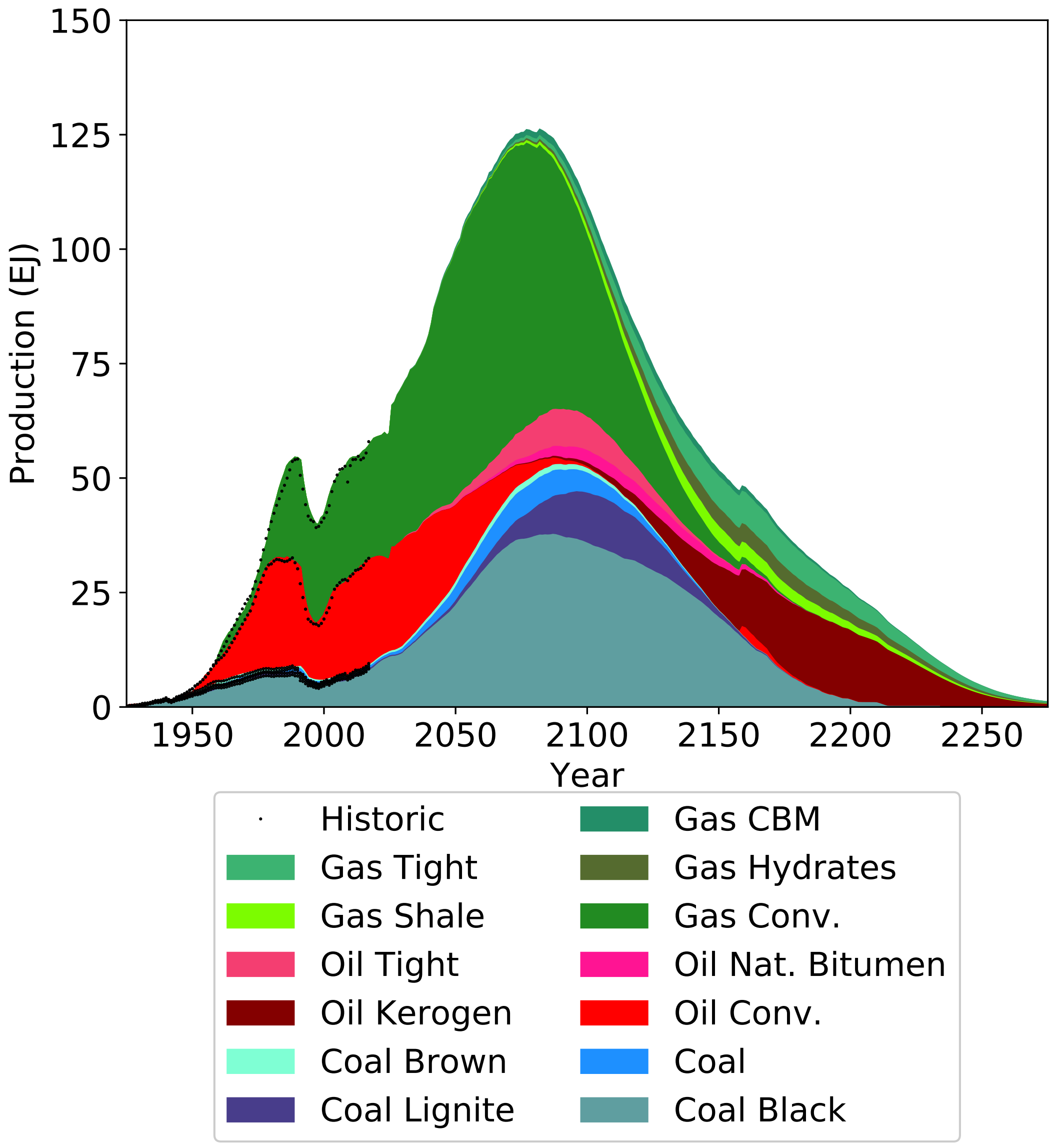

Figure 4.46: Russia projection by mineral type

4.12.3 Regional Projections

Table 4.46: Peak years - Minerals

**Name**	**URR**	**Peak Year**	**Peak Rate**
Coal Black	4408.31	2087	37.59
Coal Lignite	682.03	2105	11.18
Coal	402.07	2077	6.01
Coal Brown	144.33	2074	1.38
Oil Conv.	2054.16	1982	24.28
Oil Kerogen	1421.06	2186	16.08
Oil Nat. Bitumen	219.45	2114	2.9
Oil Tight	432.61	2091	8.27
Gas Conv.	5811.47	2070	63.65
Gas Shale	352.12	2143	3.55
Gas Hydrates	403.83	2155	4.07
Gas Tight	741.3	2156	7.13
Gas CBM	209.91	2108	2.21
**Total**	**17282.65**	**2082**	**126.08**

All

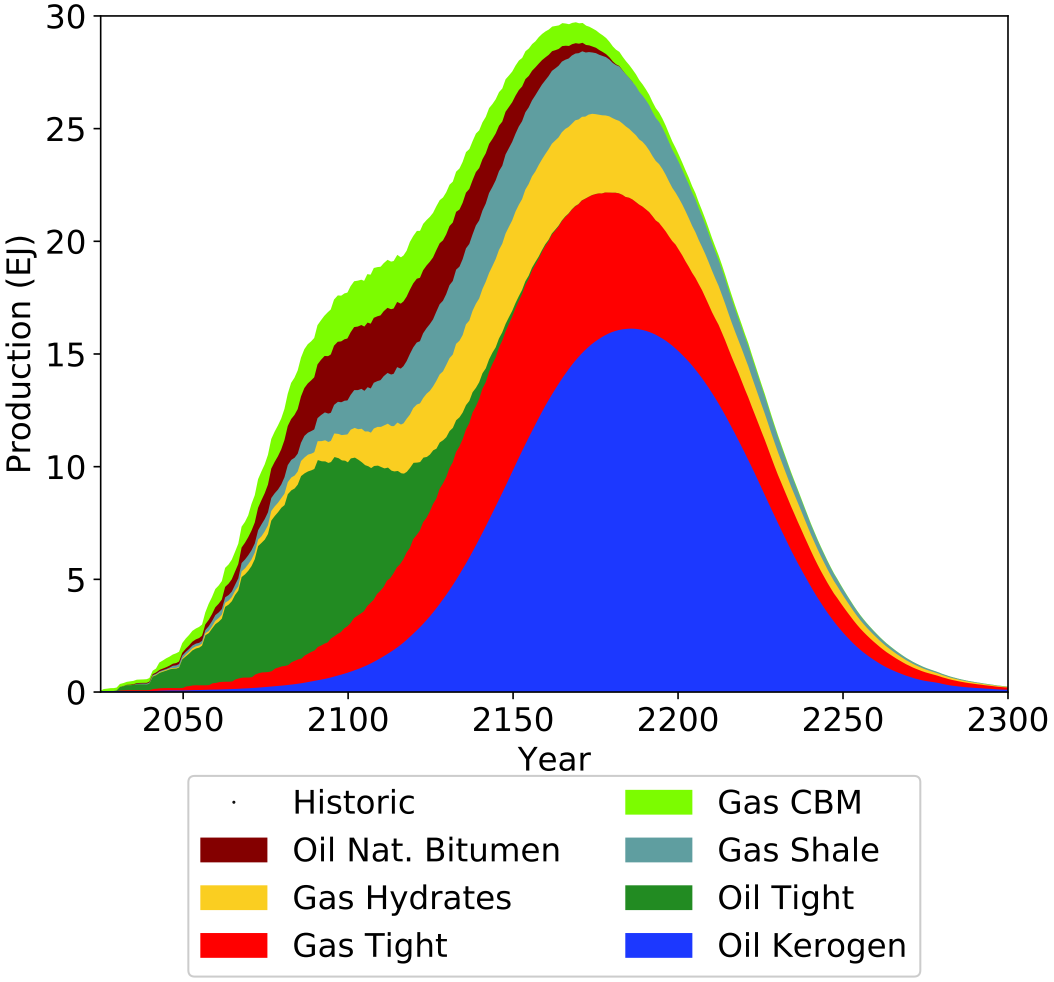

Figure 4.47: Russia - All projections capped at 16

Table 4.47: Peak years - All

Name	URR	Peak Year	Peak Rate
Oil Kerogen	1421.06	2186	16.08
Gas Tight	741.3	2156	7.13
Oil Tight	432.61	2091	8.27
Gas Hydrates	403.83	2155	4.07
Gas Shale	352.12	2143	3.55
Oil Nat. Bitumen	219.45	2114	2.9
Gas CBM	209.91	2108	2.21
**Total**	**3780.28**	**2169**	**29.67**

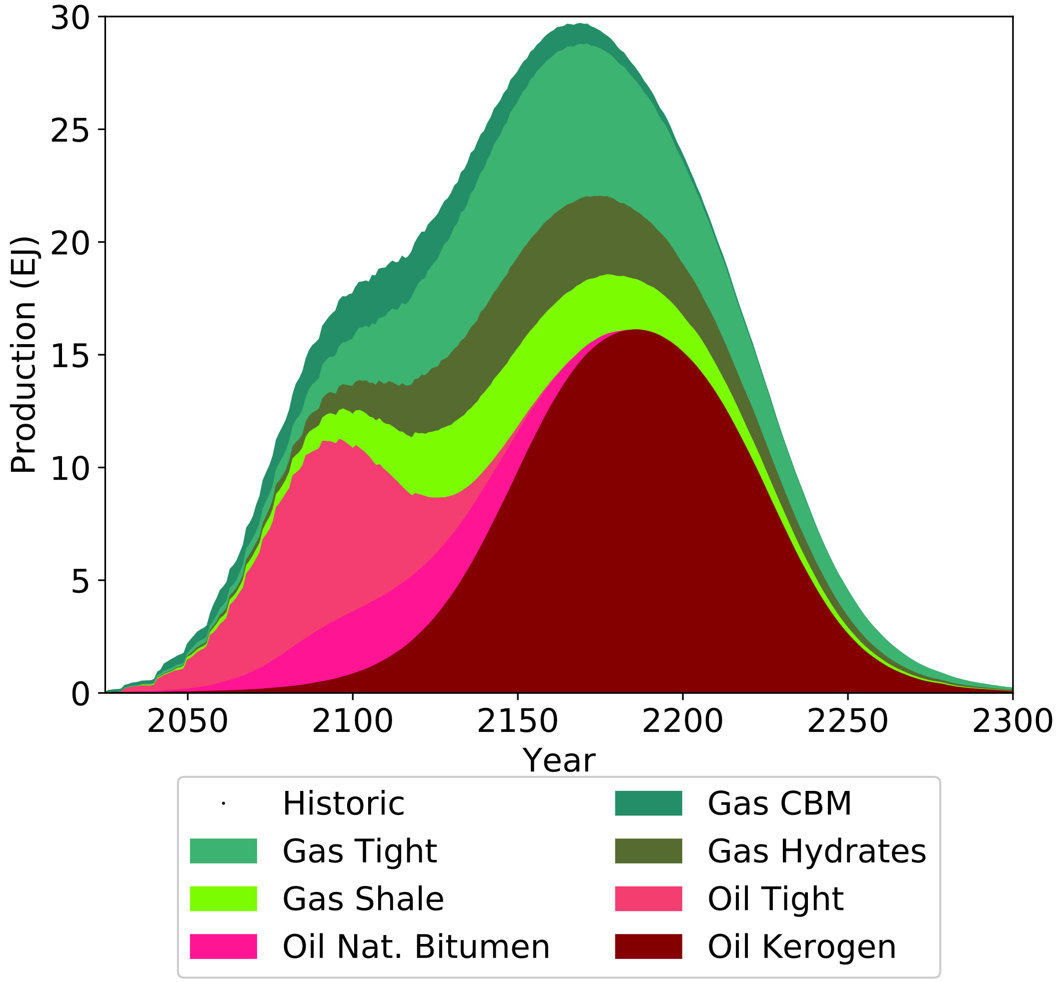

Figure 4.48: Russia - All projection by mineral type

Table 4.48: Peak years - Minerals

**Name**	**URR**	**Peak Year**	**Peak Rate**
Oil Kerogen	1421.06	2186	16.08
Oil Nat. Bitumen	219.45	2114	2.9
Oil Tight	432.61	2091	8.27
Gas Shale	352.12	2143	3.55
Gas Hydrates	403.83	2155	4.07
Gas Tight	741.3	2156	7.13
Gas CBM	209.91	2108	2.21
**Total**	**3780.28**	**2169**	**29.67**

Central

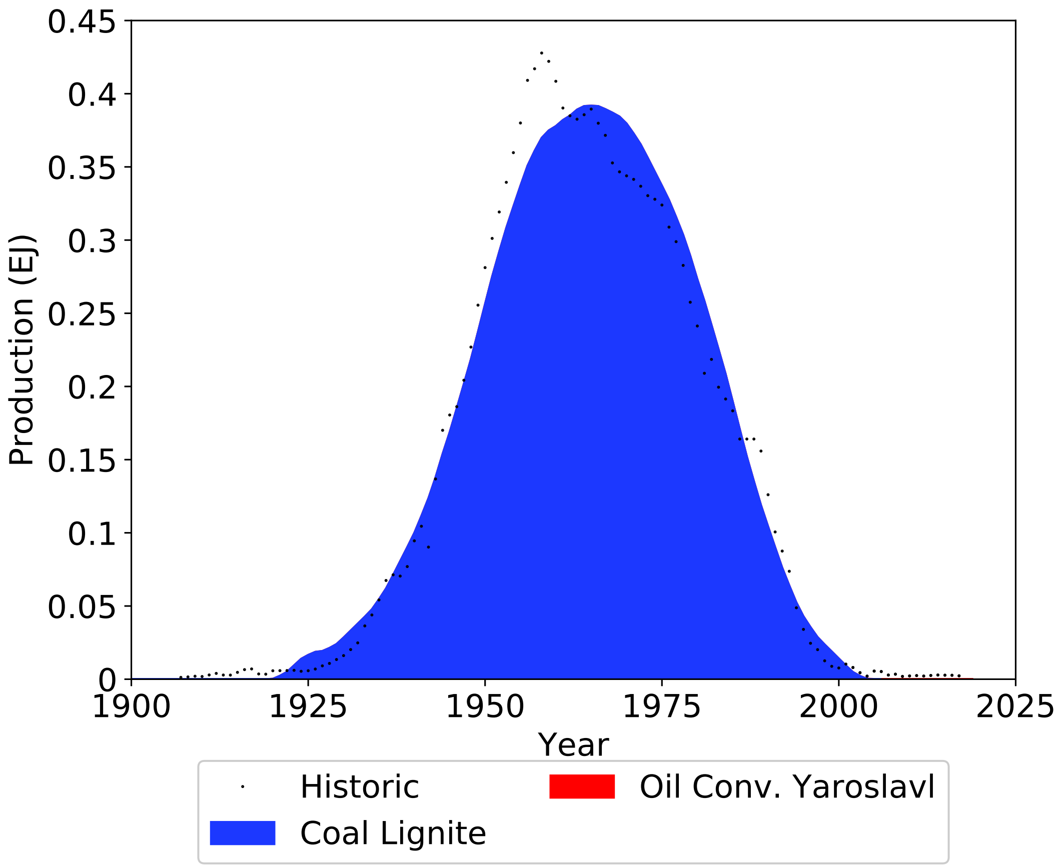

Figure 4.49: Russia - Central projections capped at 16

Table 4.49: Peak years - All

**Name**	**URR**	**Peak Year**	**Peak Rate**
Coal Lignite Central	15.38	1965	0.39
Oil Conv. Central Yaroslavl	–	2005	–
**Total**	**15.38**	**1965**	**0.39**

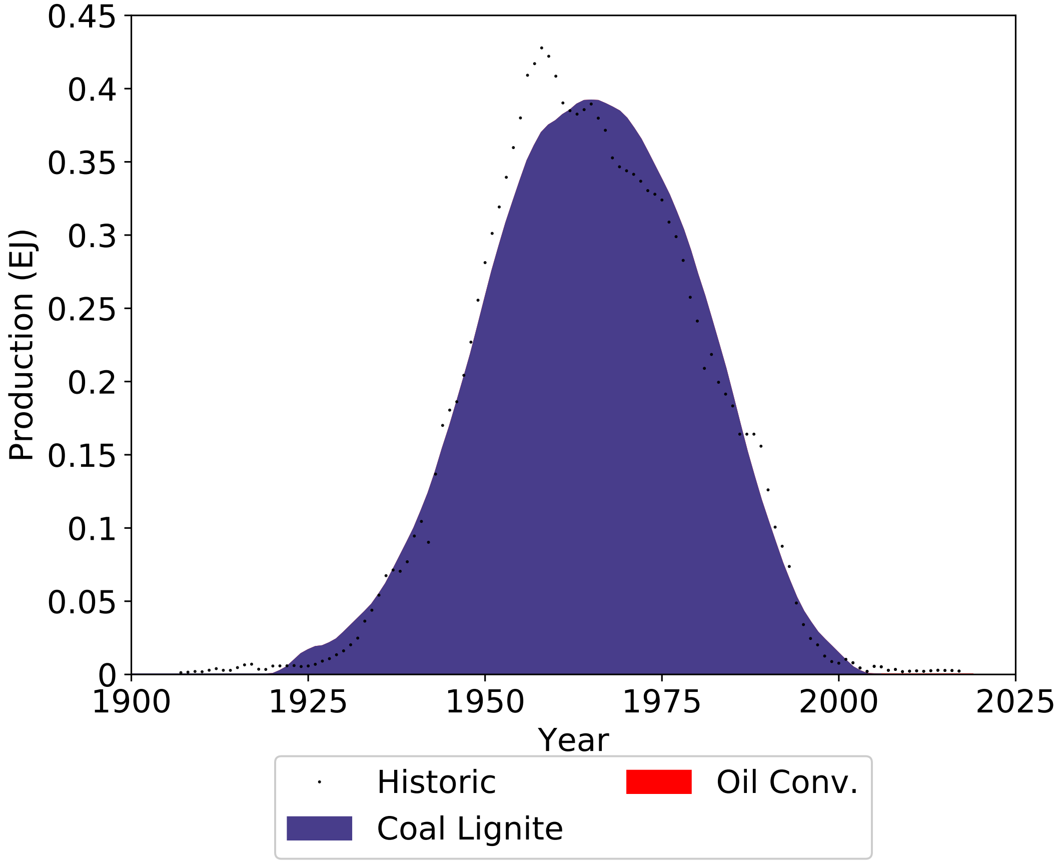

Figure 4.50: Russia - Central projection by mineral type

Table 4.50: Peak years - Minerals

**Name**	**URR**	**Peak Year**	**Peak Rate**
Coal Lignite	15.38	1965	0.39
Oil Conv.	–	2005	–
**Total**	**15.38**	**1965**	**0.39**

Far Eastern

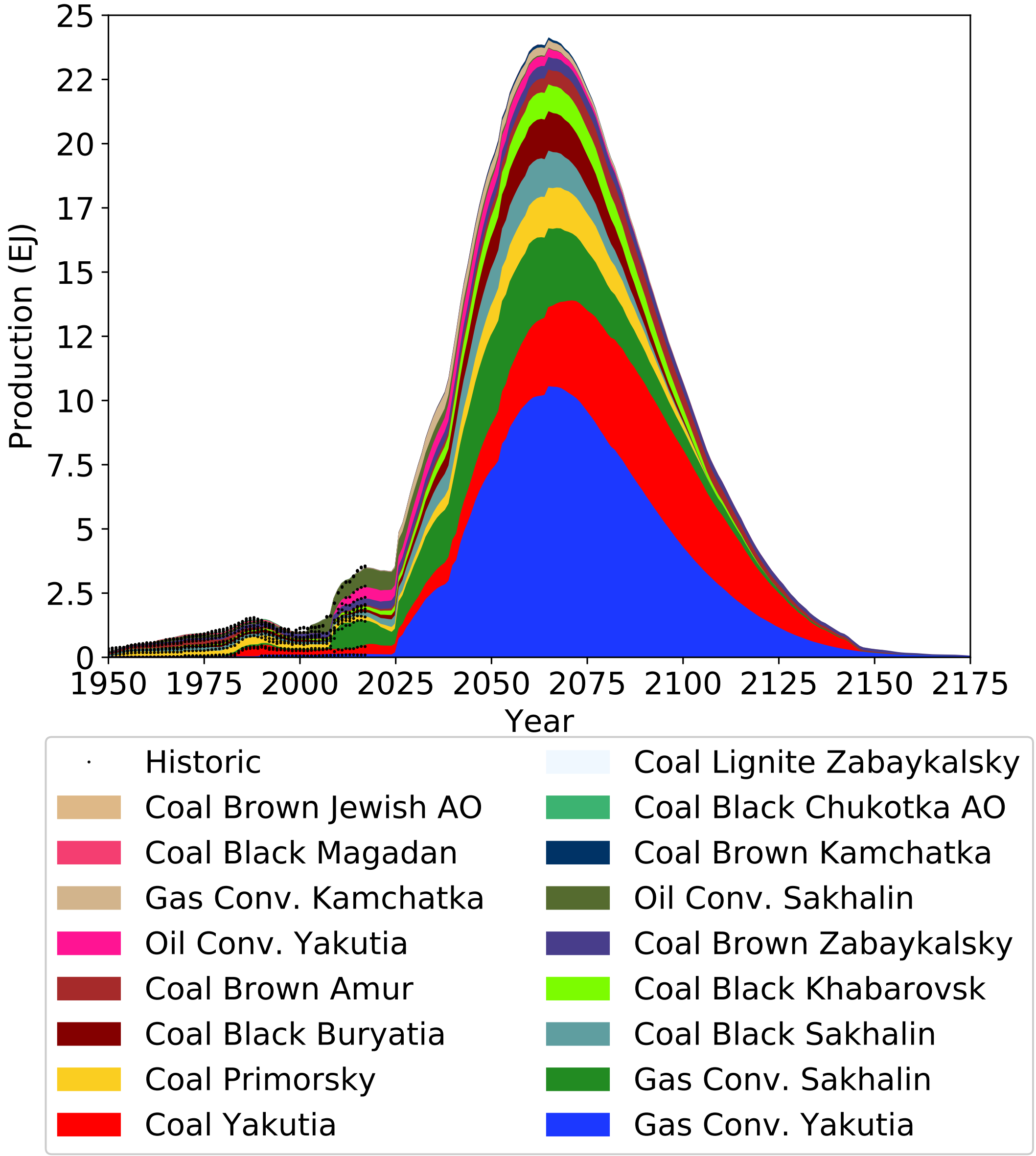

Figure 4.51: Russia - Far Eastern projections capped at 16

Table 4.51: Peak years - All

Name	URR	Peak Year	Peak Rate
Gas Conv. Far Eastern Yakutia	574.68	2065	10.52
Coal Far Eastern Yakutia	288.18	2087	4.34
Gas Conv. Far Eastern Sakhalin	195.44	2052	3.52
Coal Far Eastern Primorsky	87.56	2064	1.58
Coal Black Far Eastern Sakhalin	77.12	2057	1.55
Coal Black Far Eastern Buryatia	71.78	2064	1.54
Coal Black Far Eastern Khabarovsk	62.88	2070	1.06
Coal Brown Far Eastern Amur	55.72	2083	0.75
Coal Brown Far Eastern Zabaykalsky	55.6	2065	0.49
Oil Conv. Far Eastern Yakutia	32.37	2044	0.72
Oil Conv. Far Eastern Sakhalin	29.53	2019	0.76
Gas Conv. Far Eastern Kamchatka	24.15	2039	0.6
Coal Brown Far Eastern Kamchatka	3.93	2060	0.12
Coal Black Far Eastern Magadan	2.49	1987	0.06
Coal Black Far Eastern Chukotka AO	1.03	1989	0.03
Coal Brown Far Eastern Jewish AO	0.01	2004	–
Coal Lignite Far Eastern Zabaykalsky	–	1997	–
**Total**	**1562.47**	**2065**	**24.09**

Table 4.52: Peak years - Minerals

Name	URR	Peak Year	Peak Rate
Coal Black	215.3	2061	4.08
Coal Lignite	–	1997	–
Coal	375.74	2077	5.54
Coal Brown	115.26	2074	1.25
Oil Conv.	61.9	2019	1.2
Gas Conv.	794.27	2065	13.86
**Total**	**1562.47**	**2065**	**24.09**

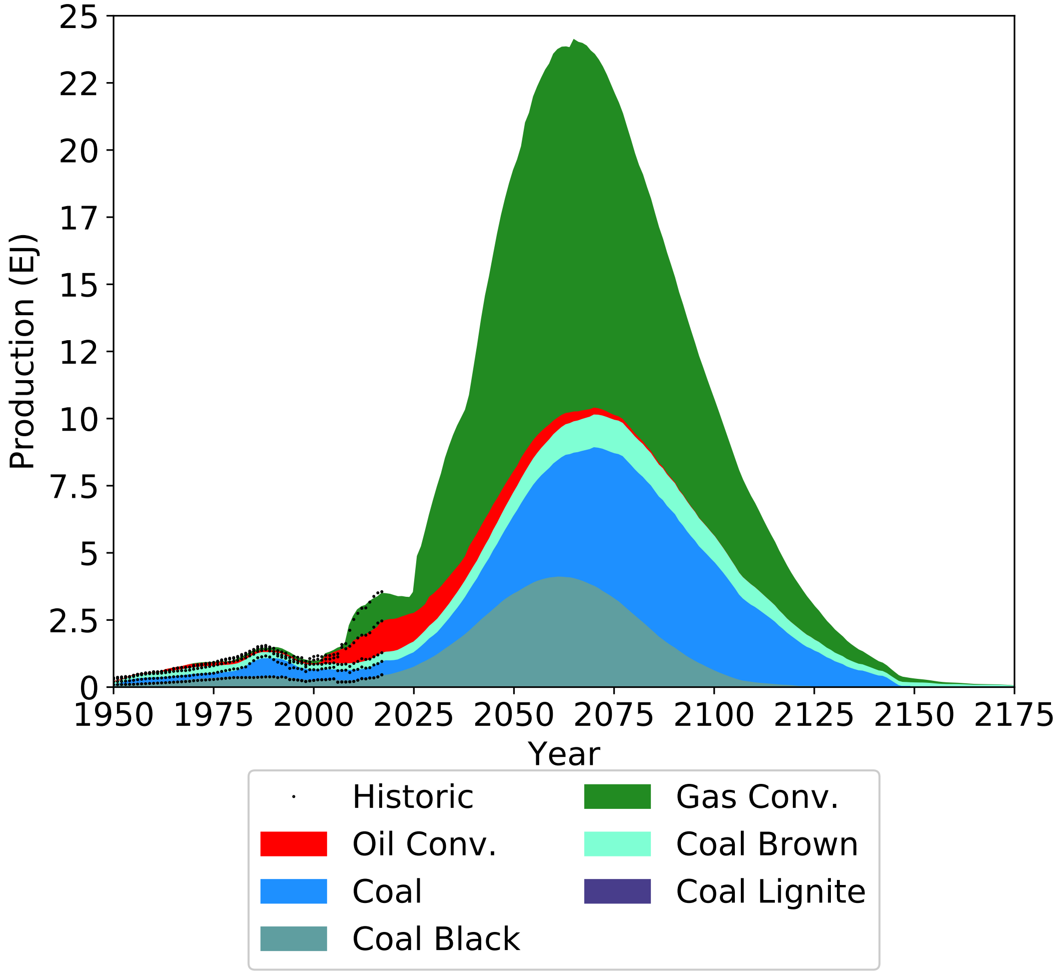

Figure 4.52: Russia - Far Eastern projection by mineral type

North Caucasian

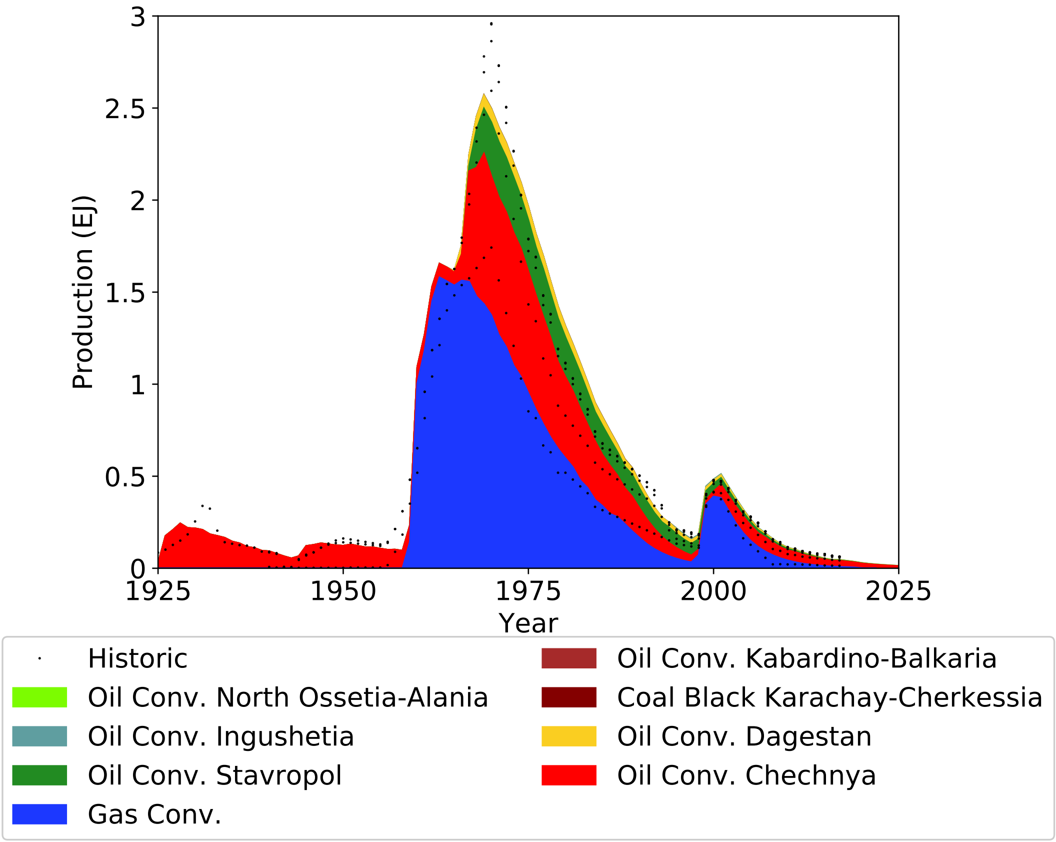

Figure 4.53: Russia - North Caucasian projections capped at 16

Table 4.53: Peak years - All

Name	URR	Peak Year	Peak Rate
Gas Conv. North Caucasian	31.5	1963	1.58
Oil Conv. North Caucasian Chechnya	18.92	1969	0.82
Oil Conv. North Caucasian Stavropol	5.89	1971	0.3
Oil Conv. North Caucasian Dagestan	1.78	1972	0.08
Oil Conv. North Caucasian Ingushetia	0.07	1996	0.01
Coal Black North Caucasian Karachay-Cherkessia	0.06	1970	–
Oil Conv. North Caucasian North Ossetia-Alania	–	2002	–
Oil Conv. North Caucasian Kabardino-Balkaria	–	1998	–
**Total**	**58.23**	**1969**	**2.57**

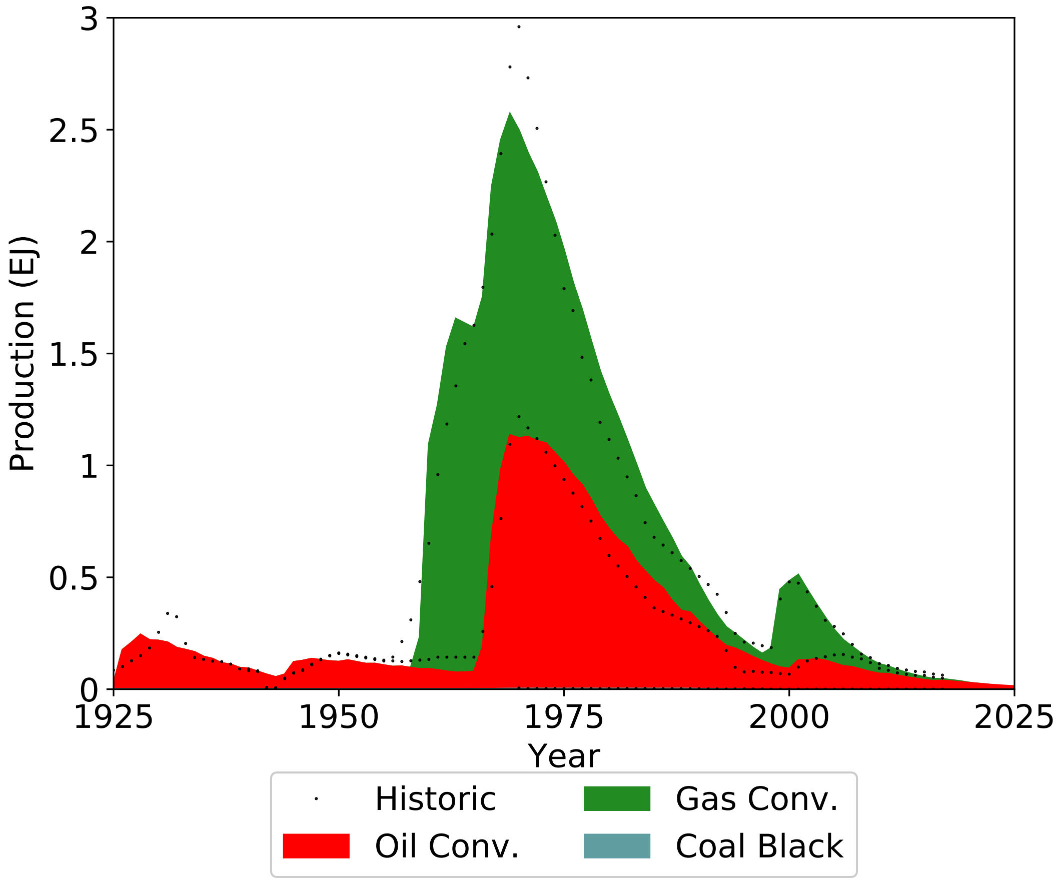

Figure 4.54: Russia - North Caucasian projection by mineral type

Table 4.54: Peak years - Minerals

Name	URR	Peak Year	Peak Rate
Coal Black	0.06	1970	–
Oil Conv.	26.67	1969	1.13
Gas Conv.	31.5	1963	1.58
**Total**	**58.23**	**1969**	**2.57**

Northwestern

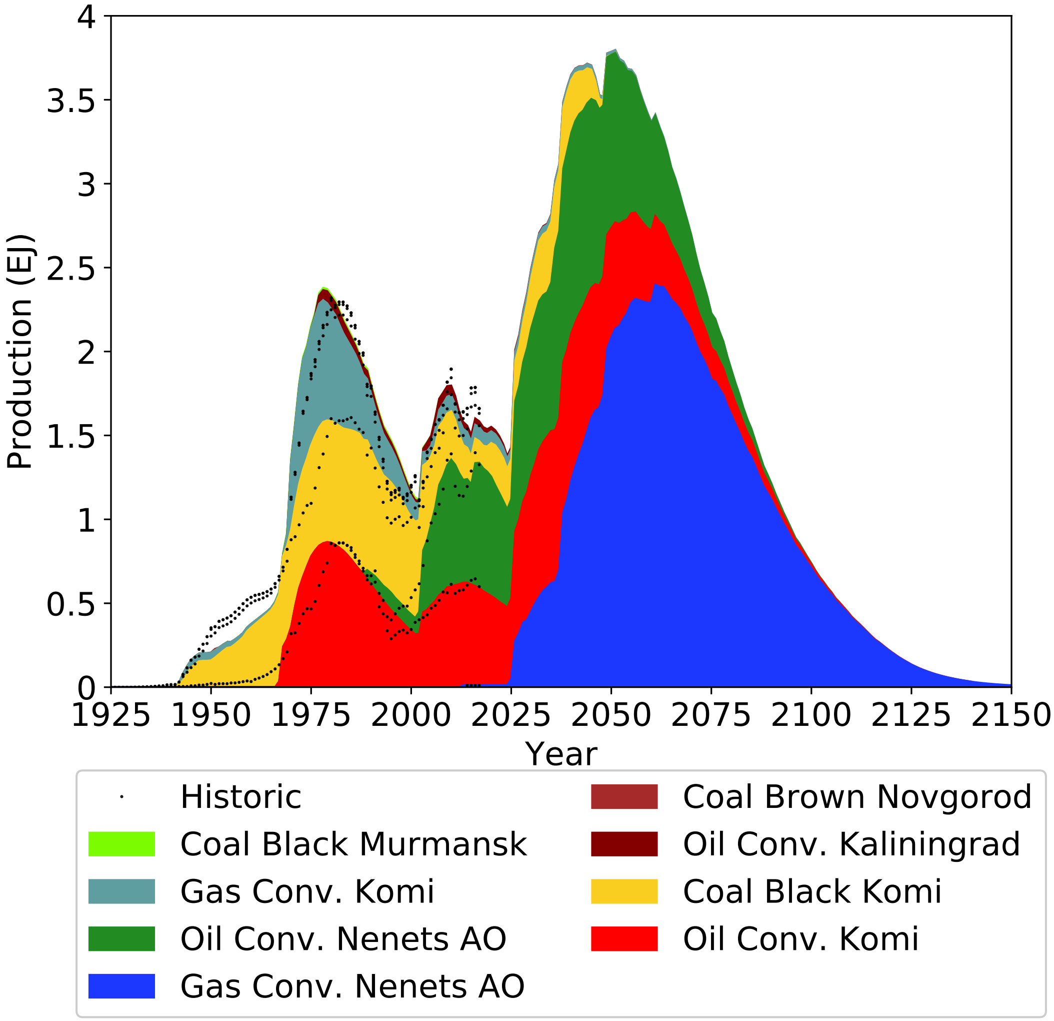

Figure 4.55: Russia - Northwestern projections capped at 16

Table 4.55: Peak years - All

Name	URR	Peak Year	Peak Rate
Gas Conv. Northwestern Nenets AO	122.36	2061	2.4
Oil Conv. Northwestern Komi	65.44	2037	0.91
Oil Conv. Northwestern Nenets AO	56.97	2041	1.2
Coal Black Northwestern Komi	42.48	1987	0.81
Gas Conv. Northwestern Komi	17.86	1977	0.74
Oil Conv. Northwestern Kaliningrad	2.19	1980	0.07
Coal Black Northwestern Murmansk	0.47	1970	0.01
Coal Brown Northwestern Novgorod	0.01	1951	0.01
**Total**	**307.78**	**2051**	**3.8**

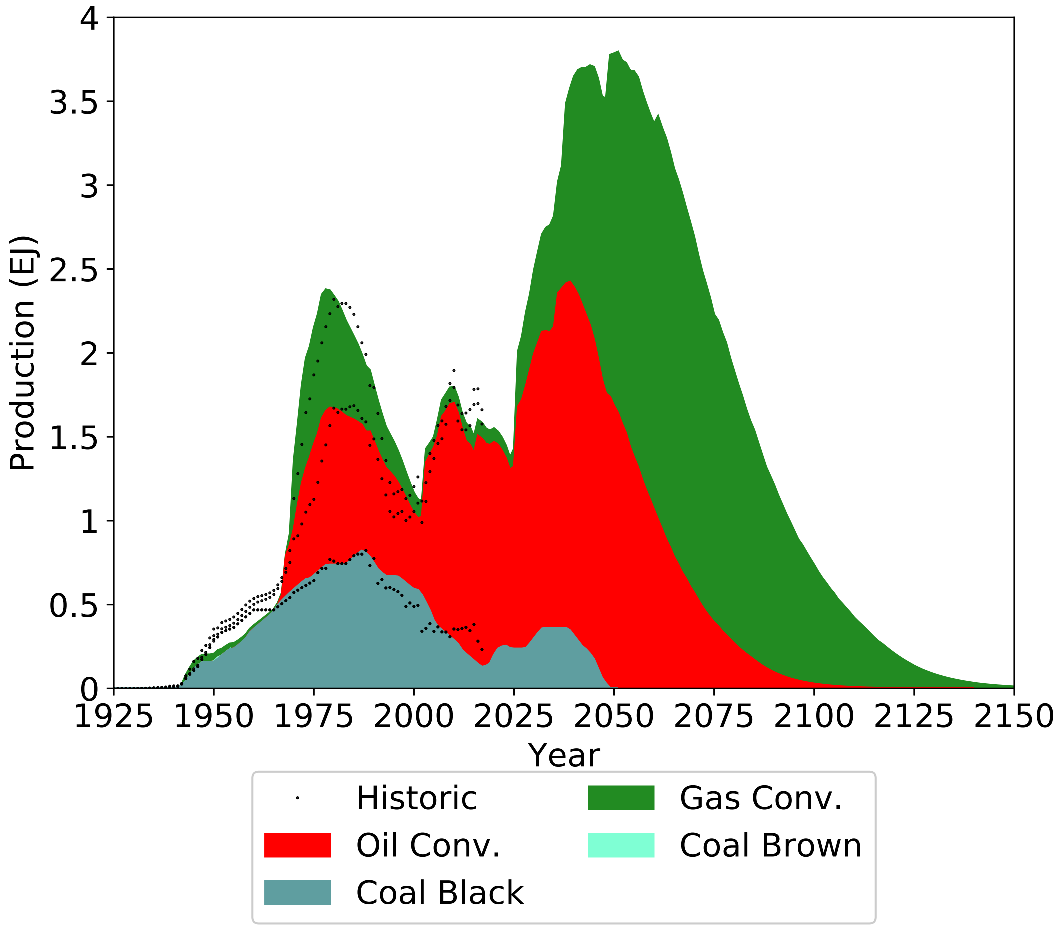

Figure 4.56: Russia - Northwestern projection by mineral type

Table 4.56: Peak years - Minerals

**Name**	**URR**	**Peak Year**	**Peak Rate**
Coal Black	42.95	1987	0.82
Coal Brown	0.01	1951	0.01
Oil Conv.	124.6	2039	2.08
Gas Conv.	140.22	2061	2.41
**Total**	**307.78**	**2051**	**3.8**

Siberian

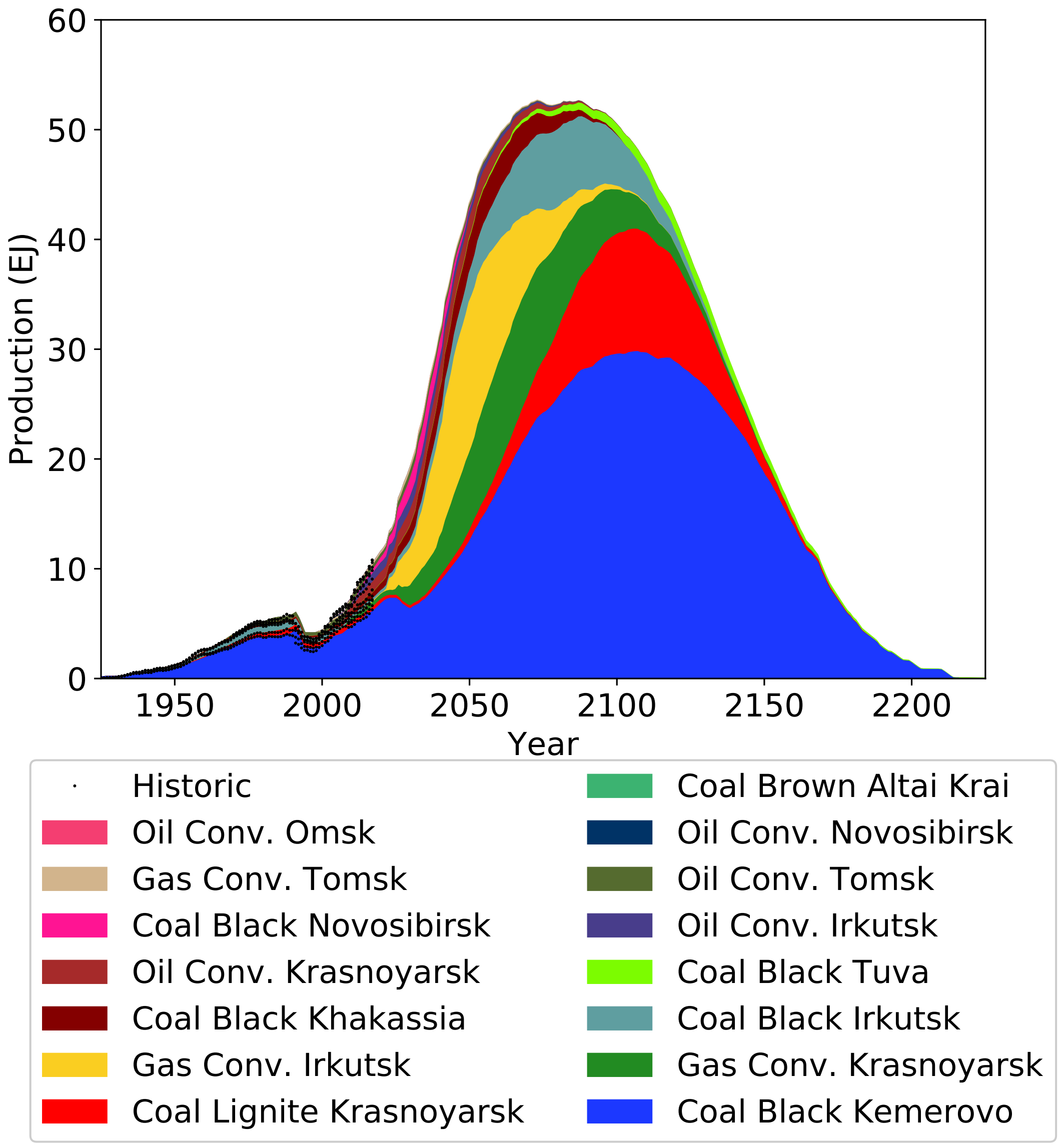

Figure 4.57: Russia - Siberian projections capped at 16

Table 4.57: Peak years - All

**Name**	**URR**	**Peak Year**	**Peak Rate**
Coal Black Siberian Kemerovo	3378.92	2107	29.74
Coal Lignite Siberian Krasnoyarsk	664.93	2105	11.18
Gas Conv. Siberian Krasnoyarsk	561.39	2065	10.09
Gas Conv. Siberian Irkutsk	496.29	2050	13.82
Coal Black Siberian Irkutsk	412.45	2081	7.11
Coal Black Siberian Khakassia	153.37	2055	3.1
Coal Black Siberian Tuva	99.86	2123	1.2
Oil Conv. Siberian Krasnoyarsk	86.64	2042	1.97
Oil Conv. Siberian Irkutsk	46.16	2031	1.22
Coal Black Siberian Novosibirsk	39.44	2034	1.89
Oil Conv. Siberian Tomsk	37.17	2031	0.69
Gas Conv. Siberian Tomsk	20.05	2031	0.44
Oil Conv. Siberian Novosibirsk	0.7	2004	0.09
Oil Conv. Siberian Omsk	0.43	2005	0.04
Coal Brown Siberian Altai Krai	0.01	2009	–
**Total**	**5997.81**	**2073**	**52.65**

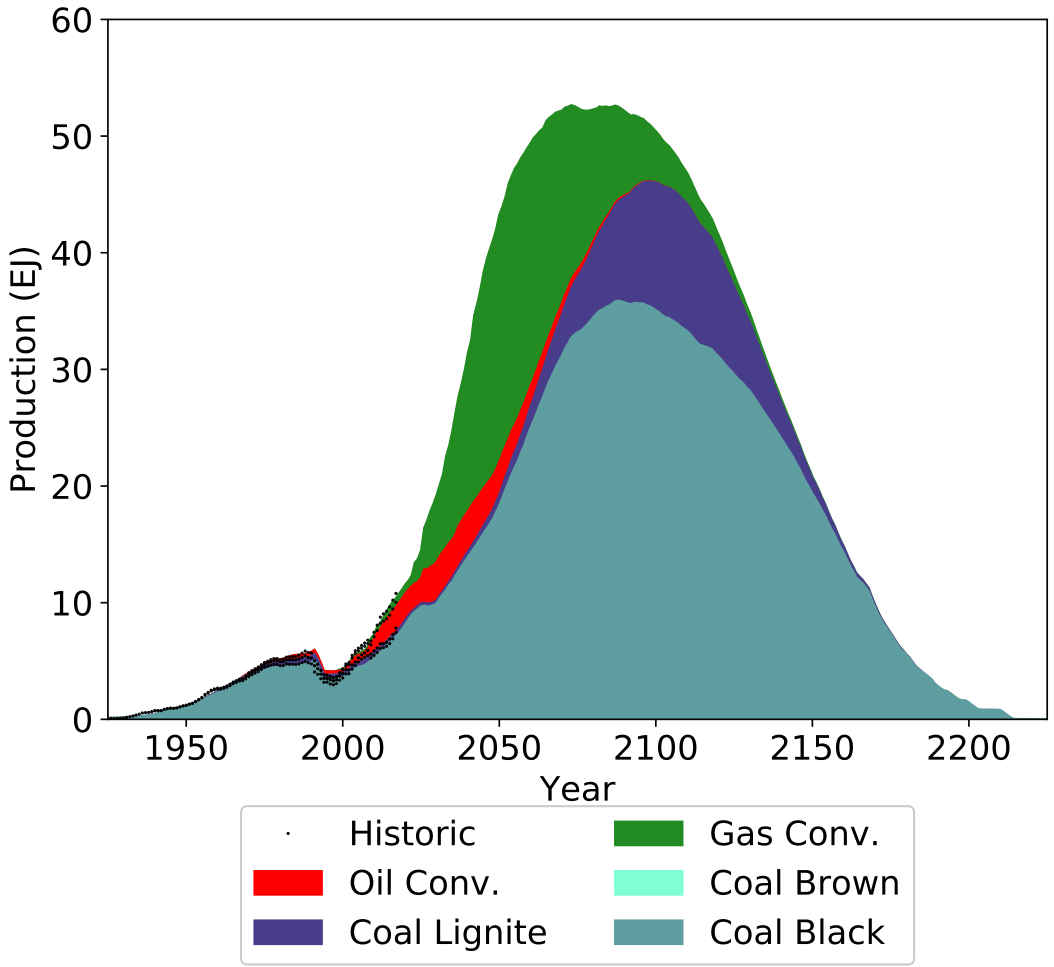

Figure 4.58: Russia - Siberian projection by mineral type

Table 4.58: Peak years - Minerals

**Name**	**URR**	**Peak Year**	**Peak Rate**
Coal Black	4084.04	2088	35.89
Coal Lignite	664.93	2105	11.18
Coal Brown	0.01	2009	–
Oil Conv.	171.1	2037	3.5
Gas Conv.	1077.73	2055	21.94
**Total**	**5997.81**	**2073**	**52.65**

Southern

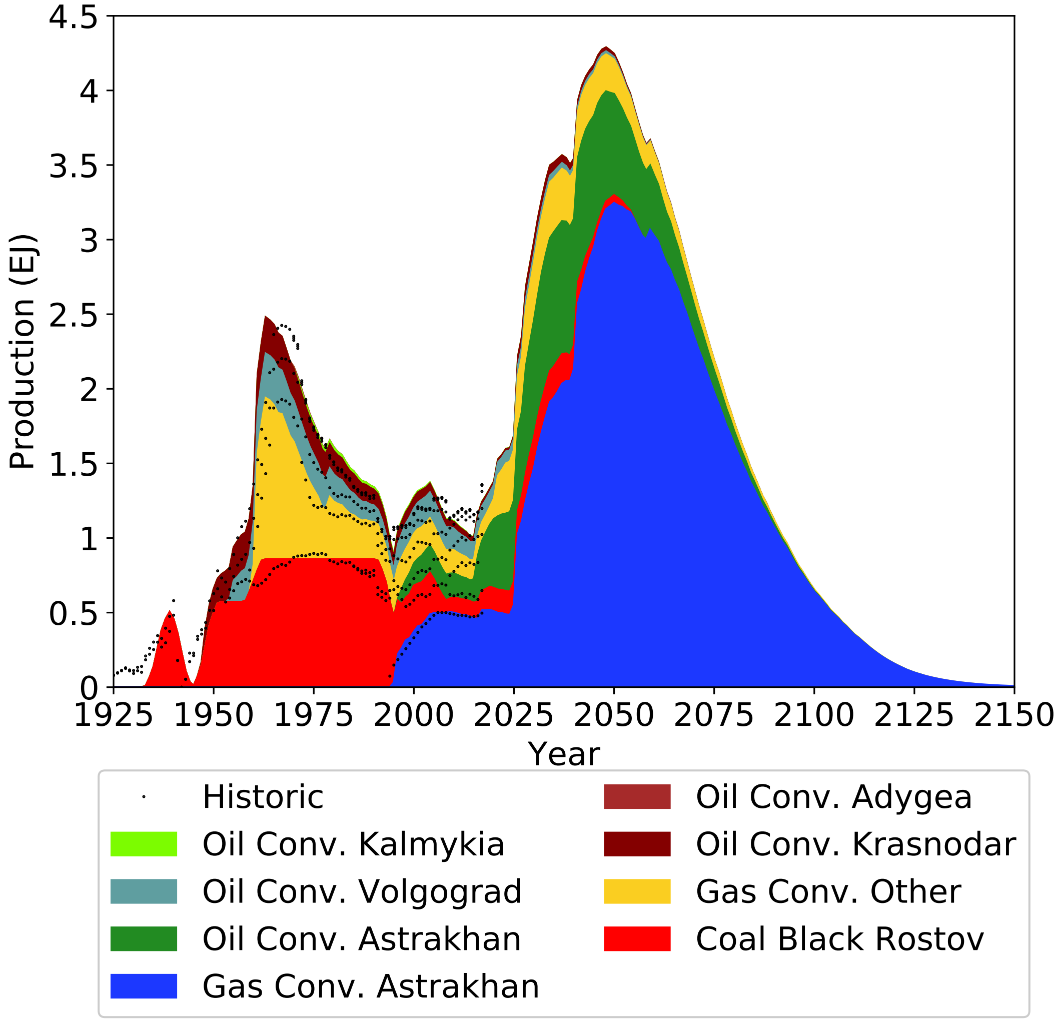

Figure 4.59: Russia - Southern projections capped at 16

Table 4.59: Peak years - All

**Name**	**URR**	**Peak Year**	**Peak Rate**
Gas Conv. Southern Astrakhan	176.28	2050	3.25
Coal Black Southern Rostov	48.65	1963	0.86
Oil Conv. Southern Astrakhan	37.77	2035	0.9
Gas Conv. Southern Other	36.55	1963	1.08
Oil Conv. Southern Volgograd	13.47	1965	0.3
Oil Conv. Southern Krasnodar	11.33	1962	0.25
Oil Conv. Southern Kalmykia	0.68	1977	0.03
Oil Conv. Southern Adygea	0.09	1971	0.01
**Total**	**324.82**	**2048**	**4.29**

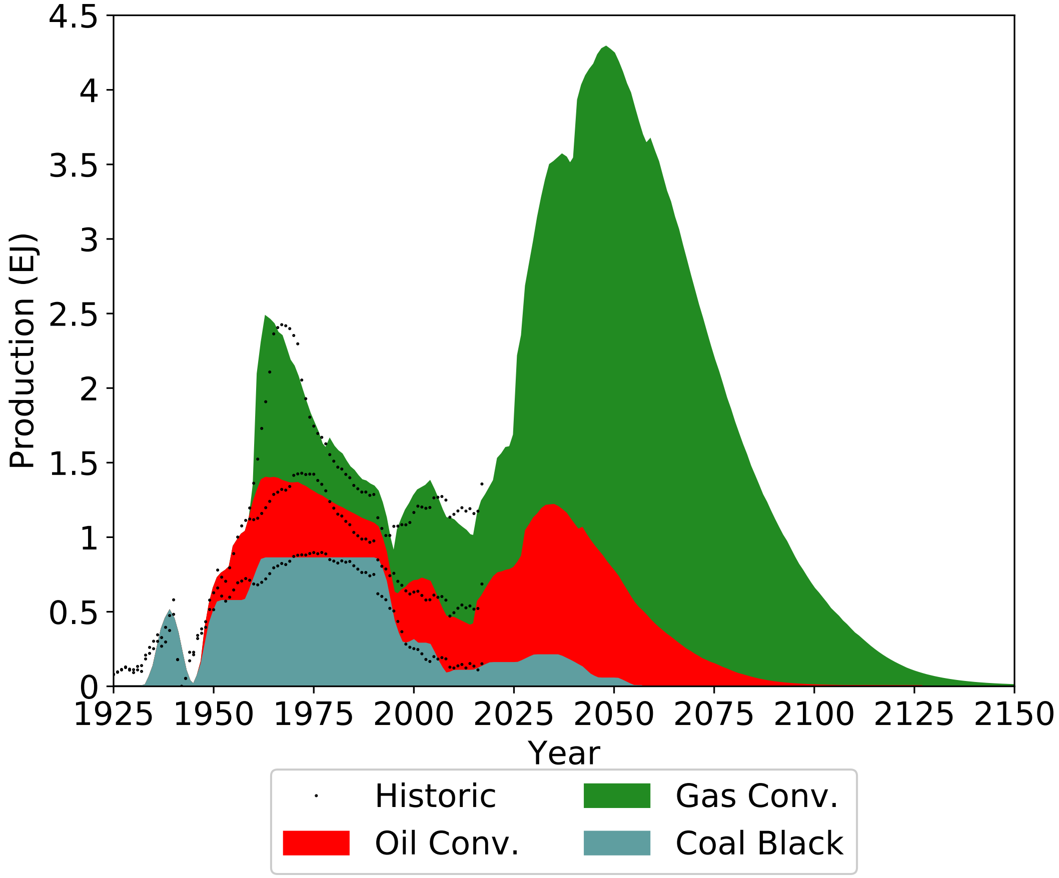

Figure 4.60: Russia - Southern projection by mineral type

Table 4.60: Peak years - Minerals

**Name**	**URR**	**Peak Year**	**Peak Rate**
Coal Black	48.65	1963	0.86
Oil Conv.	63.34	2035	1.01
Gas Conv.	212.83	2050	3.48
**Total**	**324.82**	**2048**	**4.29**

Ural

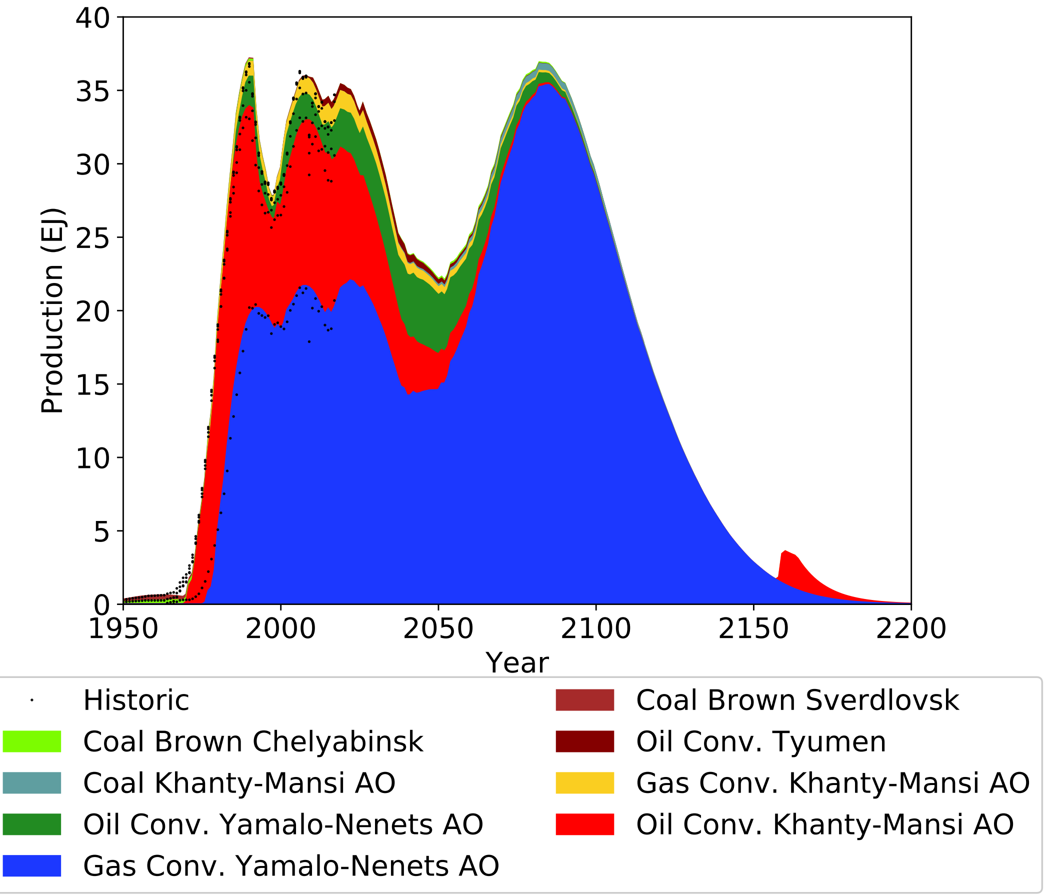

Figure 4.61: Russia - Ural projections capped at 16

Table 4.61: Peak years - All

Name	URR	Peak Year	Peak Rate
Gas Conv. Ural Yamalo-Nenets AO	3364.8	2085	35.37
Oil Conv. Ural Khanty-Mansi AO	738.03	1985	15.34
Oil Conv. Ural Yamalo-Nenets AO	255.27	2045	4.41
Gas Conv. Ural Khanty-Mansi AO	79.6	2016	1.26
Coal Ural Khanty-Mansi AO	26.33	2076	0.48
Oil Conv. Ural Tyumen	24.72	2033	0.67
Coal Brown Ural Chelyabinsk	18.45	1966	0.28
Coal Brown Ural Sverdlovsk	10.02	1960	0.35
**Total**	**4517.22**	**1990**	**37.19**

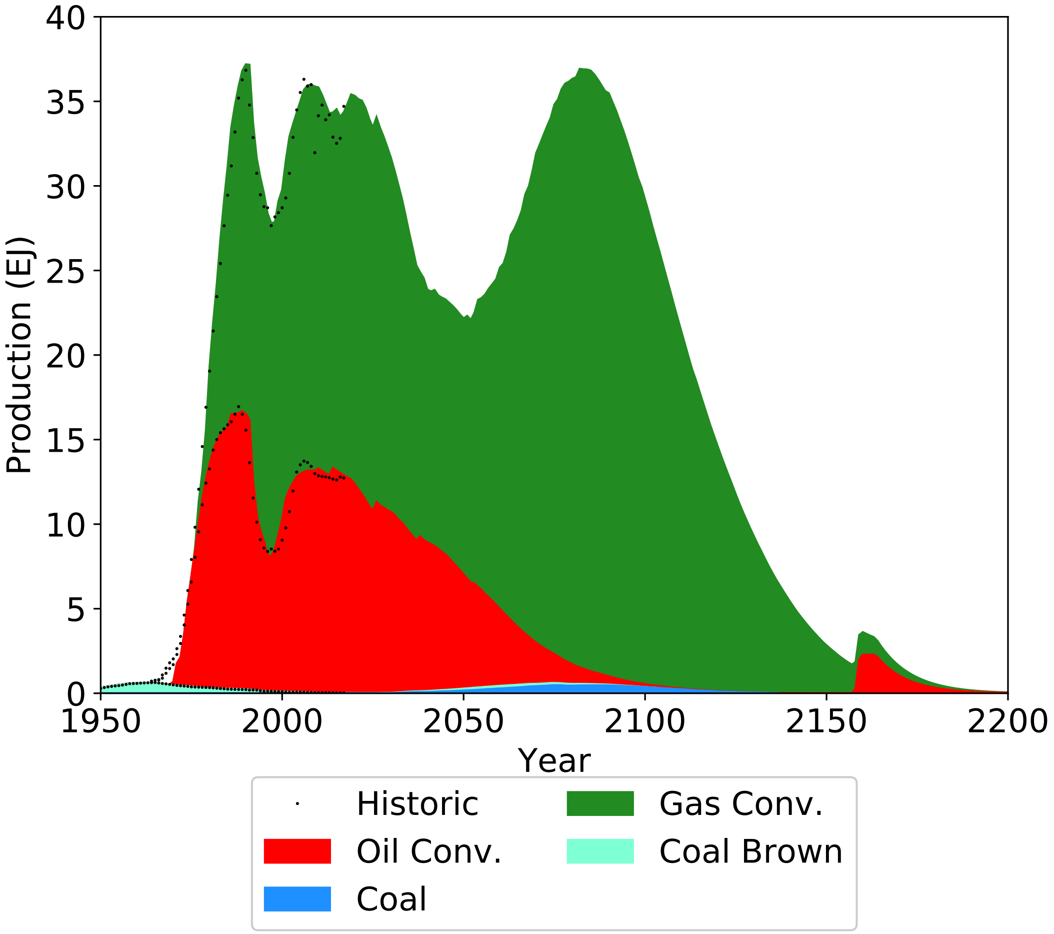

Figure 4.62: Russia - Ural projection by mineral type

Table 4.62: Peak years - Minerals

**Name**	**URR**	**Peak Year**	**Peak Rate**
Coal	26.33	2076	0.48
Coal Brown	28.47	1962	0.62
Oil Conv.	1018.02	1989	16.44
Gas Conv.	3444.4	2085	35.52
**Total**	**4517.22**	**1990**	**37.19**

Volga

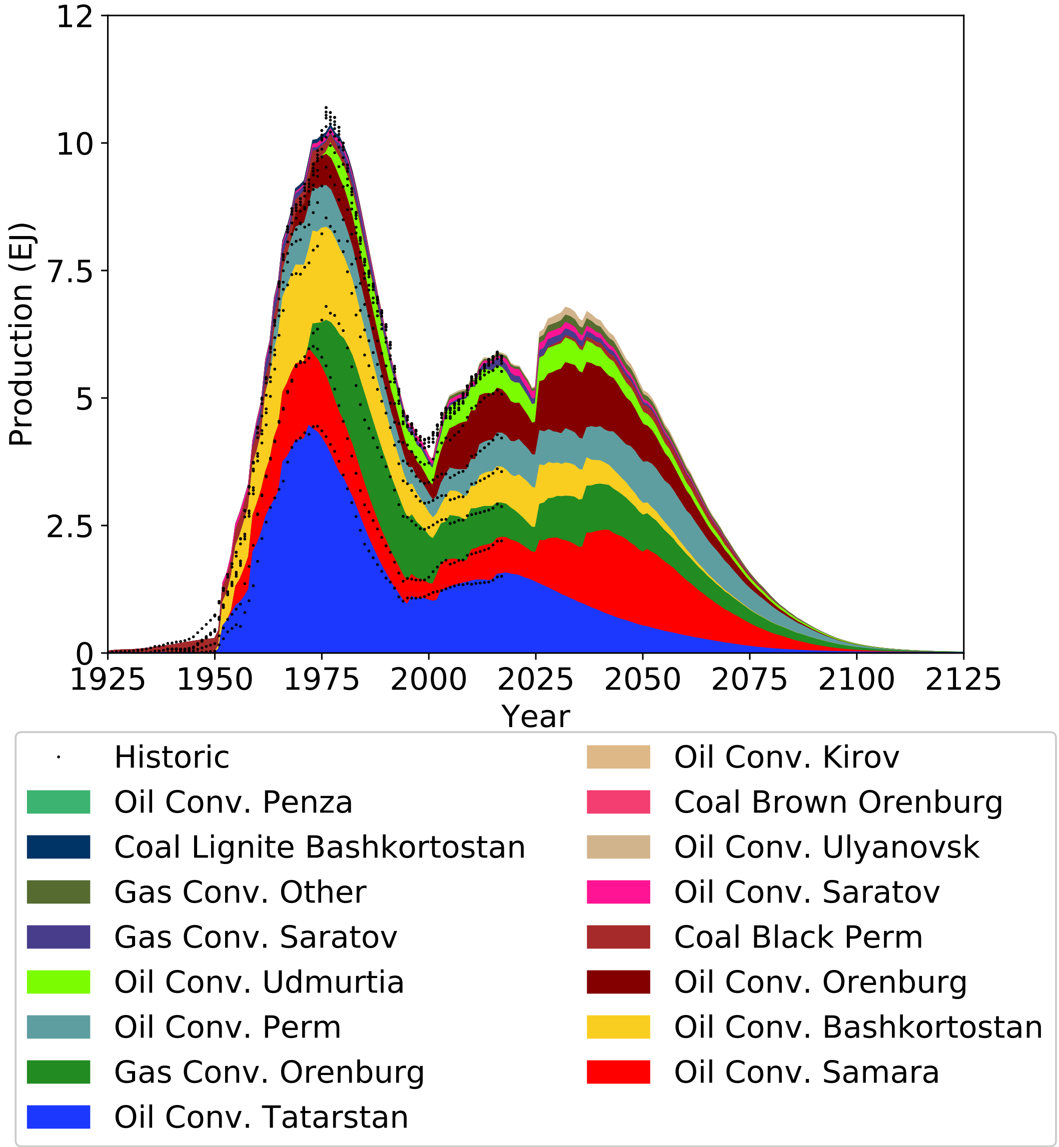

Figure 4.63: Russia - Volga projections capped at 16

Table 4.63: Peak years - All

Name	URR	Peak Year	Peak Rate
Oil Conv. Volga Tatarstan	184.67	1972	4.44
Oil Conv. Volga Samara	118.76	2042	1.65
Gas Conv. Volga Orenburg	93.03	1983	1.76
Oil Conv. Volga Bashkortostan	90.64	1969	1.94
Oil Conv. Volga Perm	74.41	2052	0.86
Oil Conv. Volga Orenburg	72.11	2034	1.32
Oil Conv. Volga Udmurtia	34.67	1983	0.51
Coal Black Volga Perm	17.31	1958	0.34
Gas Conv. Volga Saratov	11.23	1963	0.27
Oil Conv. Volga Saratov	8.46	2025	0.15
Gas Conv. Volga Other	6.26	2033	0.15
Oil Conv. Volga Ulyanovsk	4.77	2032	0.15
Coal Lignite Volga Bashkortostan	1.72	1976	0.08
Coal Brown Volga Orenburg	0.59	1985	0.04
Oil Conv. Volga Penza	0.04	2002	0.01
Oil Conv. Volga Kirov	–	2001	–
**Total**	**718.67**	**1977**	**10.33**

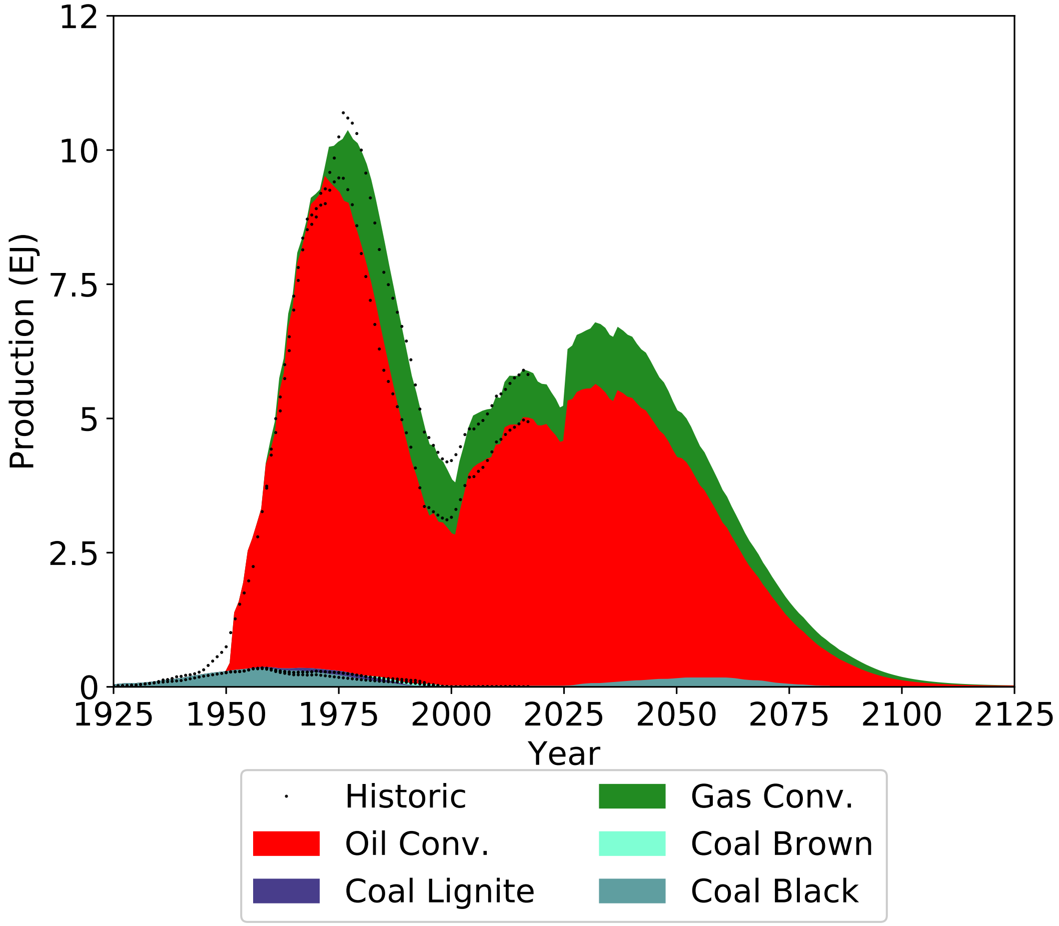

Figure 4.64: Russia - Volga projection by mineral type

Table 4.64: Peak years - Minerals

Name	URR	Peak Year	Peak Rate
Coal Black	17.31	1958	0.34
Coal Lignite	1.72	1976	0.08
Coal Brown	0.59	1985	0.04
Oil Conv.	588.53	1972	9.17
Gas Conv.	110.52	1984	1.95
**Total**	**718.67**	**1977**	**10.33**

4.12.4 Projection by region

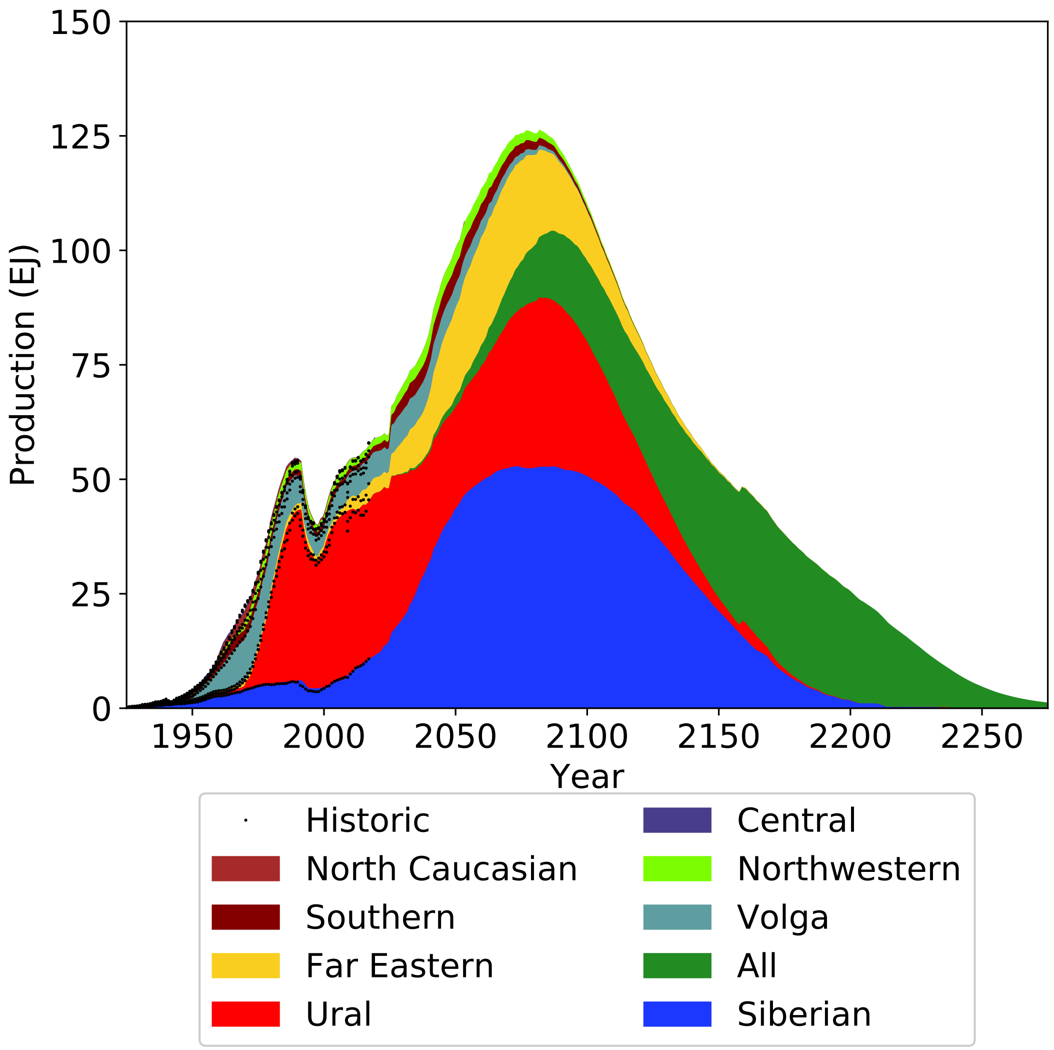

Figure 4.65: Russia by region projections capped at 16

Table 4.65: Peak years - All

**Name**	**URR**	**Peak Year**	**Peak Rate**
Siberian	5997.81	2073	52.65
Ural	4517.22	1990	37.19
All	3780.28	2169	29.67
Far Eastern	1562.47	2065	24.09
Volga	718.67	1977	10.33
Southern	324.82	2048	4.29
Northwestern	307.78	2051	3.8
North Caucasian	58.23	1969	2.57
Central	15.38	1965	0.39
**Total**	**17282.65**	**2082**	**126.08**

4.13 Tajikistan

4.13.1 All Projections

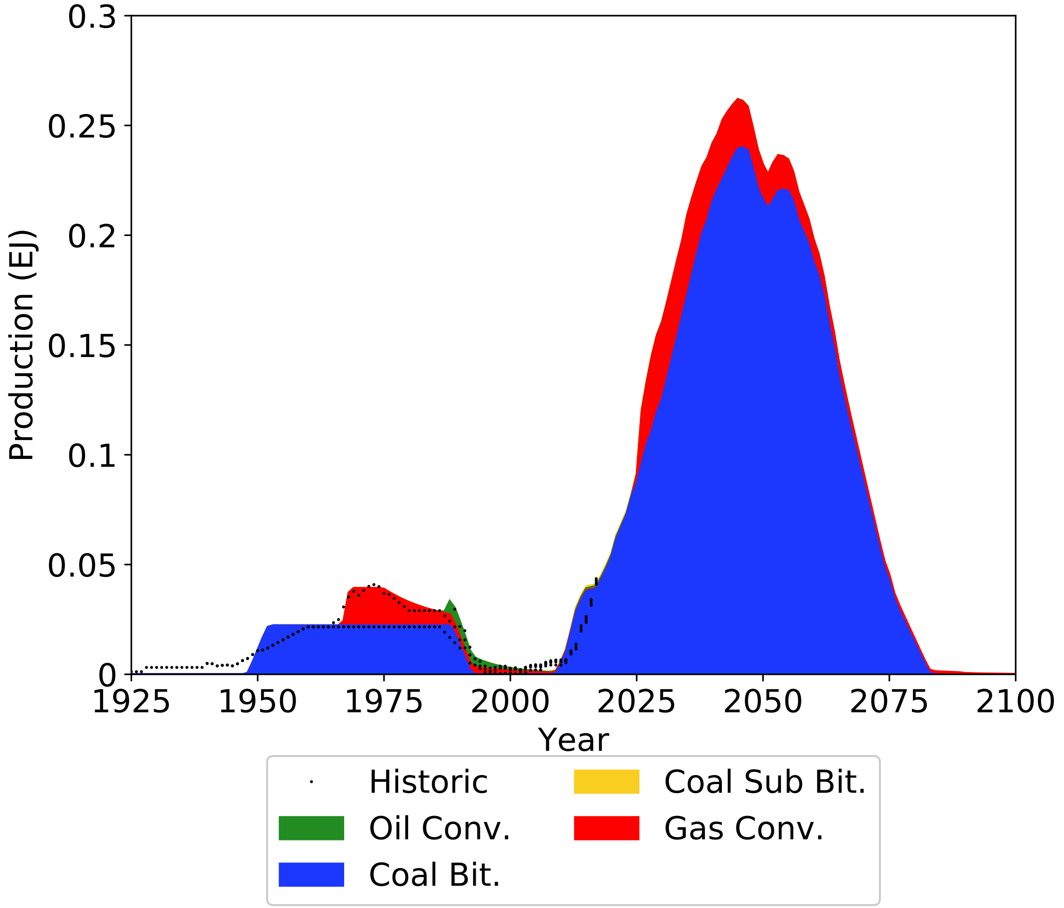

Figure 4.66: Tajikistan projections capped at 16

Table 4.66: Peak years - All

**Name**	**URR**	**Peak Year**	**Peak Rate**
Coal Bit.	10.1	2046	0.24
Gas Conv.	1.27	2035	0.04
Oil Conv.	0.08	1989	0.01
Coal Sub Bit.	0.02	2012	–
**Total**	**11.46**	**2045**	**0.26**

4.13.2 By Mineral

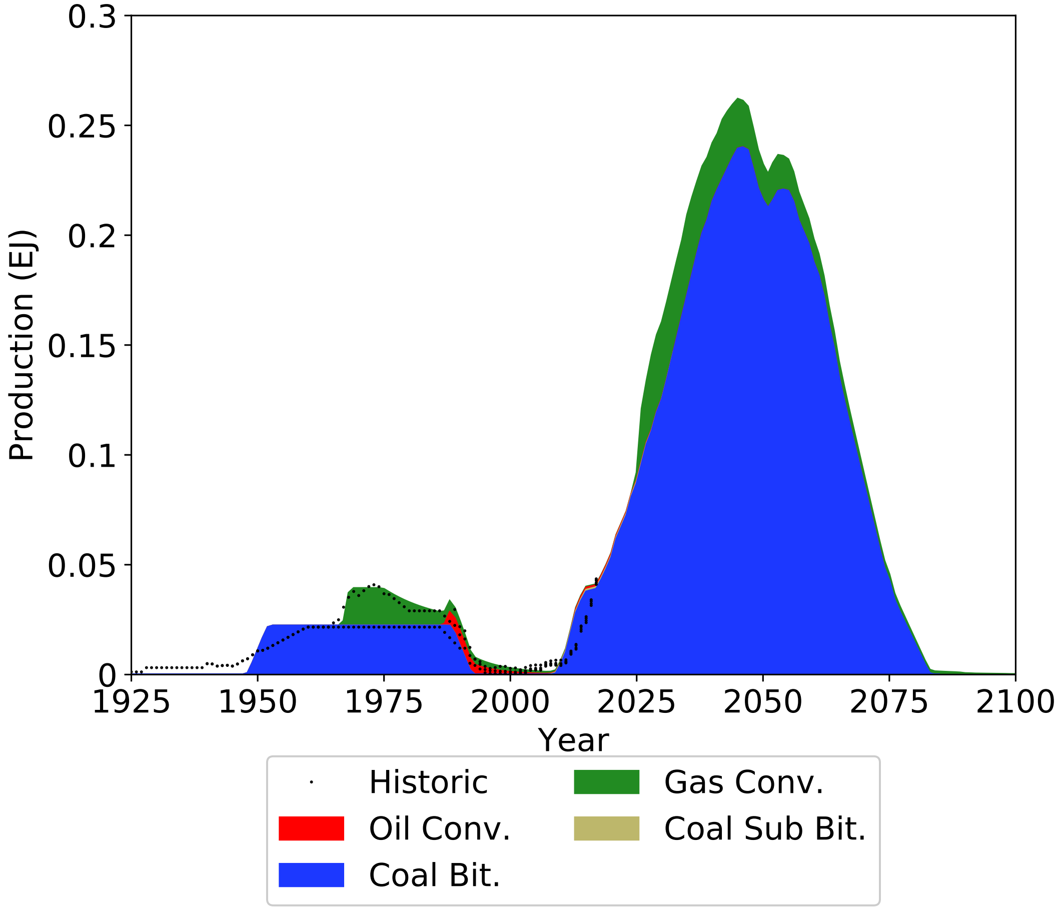

Figure 4.67: Tajikistan projection by mineral type

Table 4.67: Peak years - Minerals

**Name**	**URR**	**Peak Year**	**Peak Rate**
Coal Bit.	10.1	2046	0.24
Coal Sub Bit.	0.02	2012	–
Oil Conv.	0.08	1989	0.01
Gas Conv.	1.27	2035	0.04
**Total**	**11.46**	**2045**	**0.26**

4.14 Turkmenistan

4.14.1 All Projections

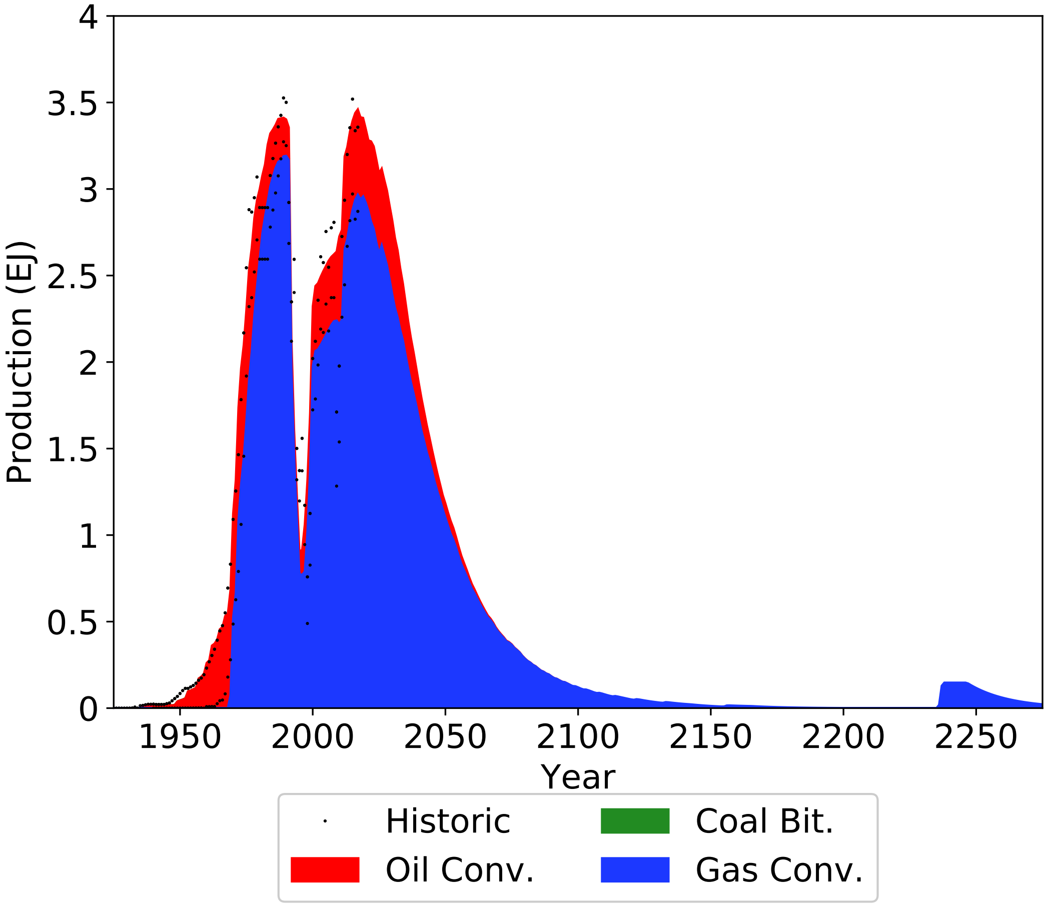

Figure 4.68: Turkmenistan projections capped at 16

Table 4.68: Peak years - All

Name	URR	Peak Year	Peak Rate
Gas Conv.	200.41	1990	3.19
Oil Conv.	35.51	1973	0.67
Coal Bit.	0.01	1947	–
**Total**	**235.93**	**2017**	**3.46**

4.14.2 By Mineral

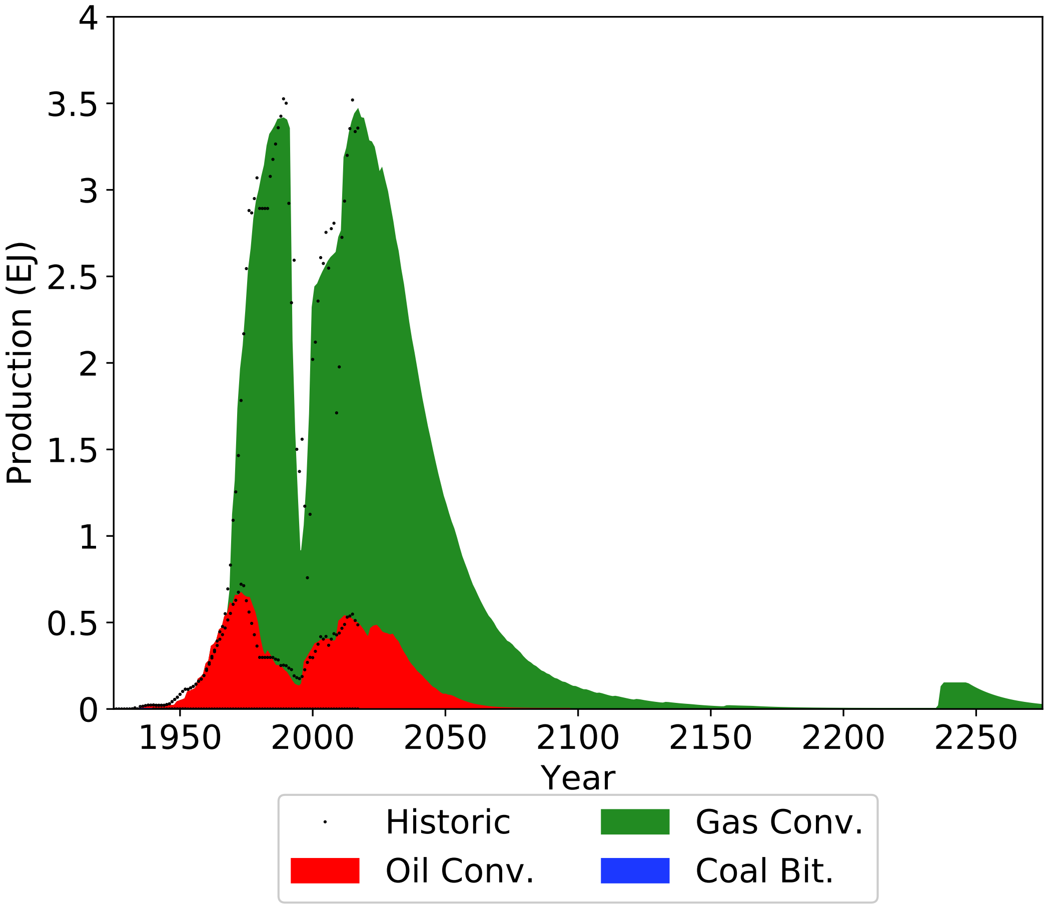

Figure 4.69: Turkmenistan projection by mineral type

Table 4.69: Peak years - Minerals

**Name**	**URR**	**Peak Year**	**Peak Rate**
Coal Bit.	0.01	1947	–
Oil Conv.	35.51	1973	0.67
Gas Conv.	200.41	1990	3.19
**Total**	**235.93**	**2017**	**3.46**

4.15 Ukraine

4.15.1 All Projections

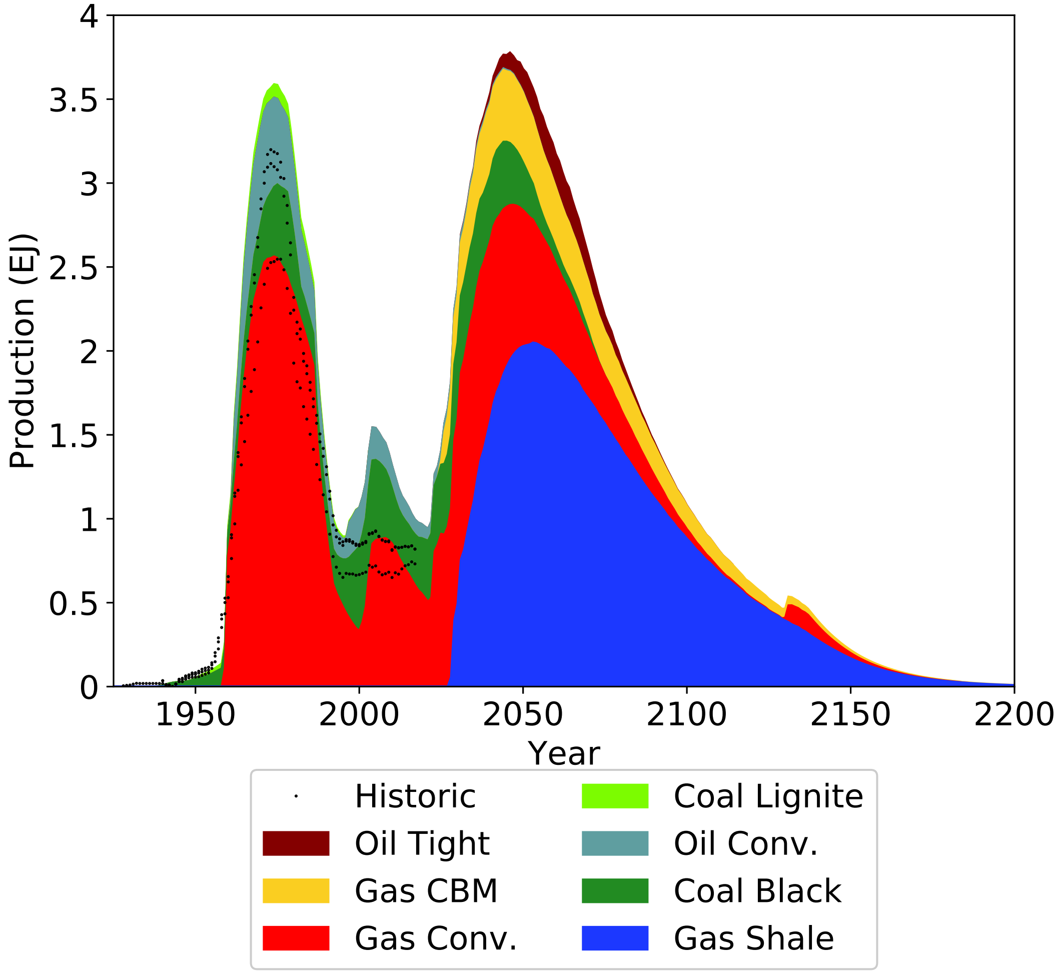

Figure 4.70: Ukraine projections capped at 16

Table 4.70: Peak years - All

**Name**	**URR**	**Peak Year**	**Peak Rate**
Gas Shale	134.55	2053	2.05
Gas Conv.	128.82	1974	2.56
Coal Black	34.81	2002	0.52
Gas CBM	26.24	2044	0.43
Oil Conv.	17.21	1970	0.57
Oil Tight	6.3	2061	0.21
Coal Lignite	2.3	1977	0.08
**Total**	**350.23**	**2046**	**3.78**

4.15.2 By Mineral

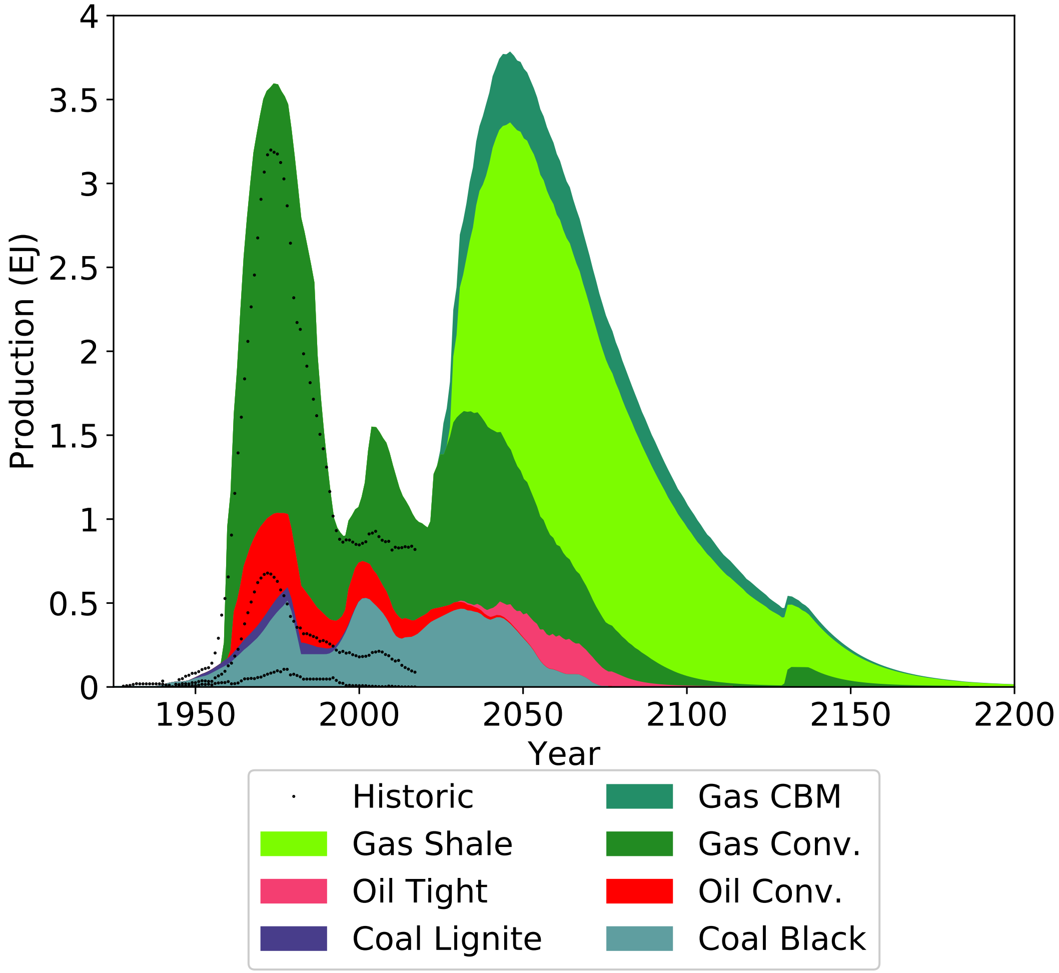

Figure 4.71: Ukraine projection by mineral type

Table 4.71: Peak years - Minerals

**Name**	**URR**	**Peak Year**	**Peak Rate**
Coal Black	34.81	2002	0.52
Coal Lignite	2.3	1977	0.08
Oil Conv.	17.21	1970	0.57
Oil Tight	6.3	2061	0.21
Gas Conv.	128.82	1974	2.56
Gas Shale	134.55	2053	2.05
Gas CBM	26.24	2044	0.43
**Total**	**350.23**	**2046**	**3.78**

4.16 Uzbekistan

4.16.1 All Projections

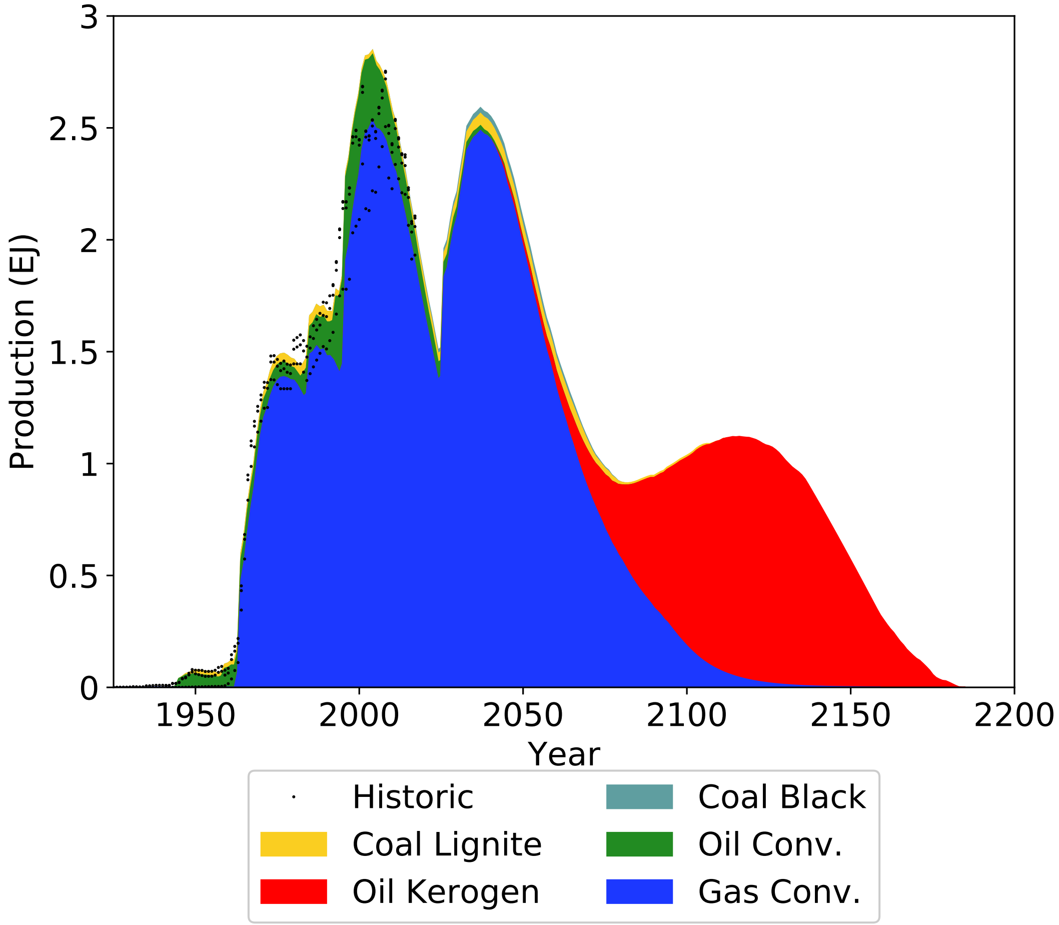

Figure 4.72: Uzbekistan projections capped at 16

Table 4.72: Peak years - All

**Name**	**URR**	**Peak Year**	**Peak Rate**
Gas Conv.	201.64	2004	2.53
Oil Kerogen	70.08	2119	1.08
Oil Conv.	12.07	1995	0.38
Coal Lignite	5.23	2039	0.06
Coal Black	1.33	2043	0.03
**Total**	**290.35**	**2004**	**2.84**

4.16.2 By Mineral

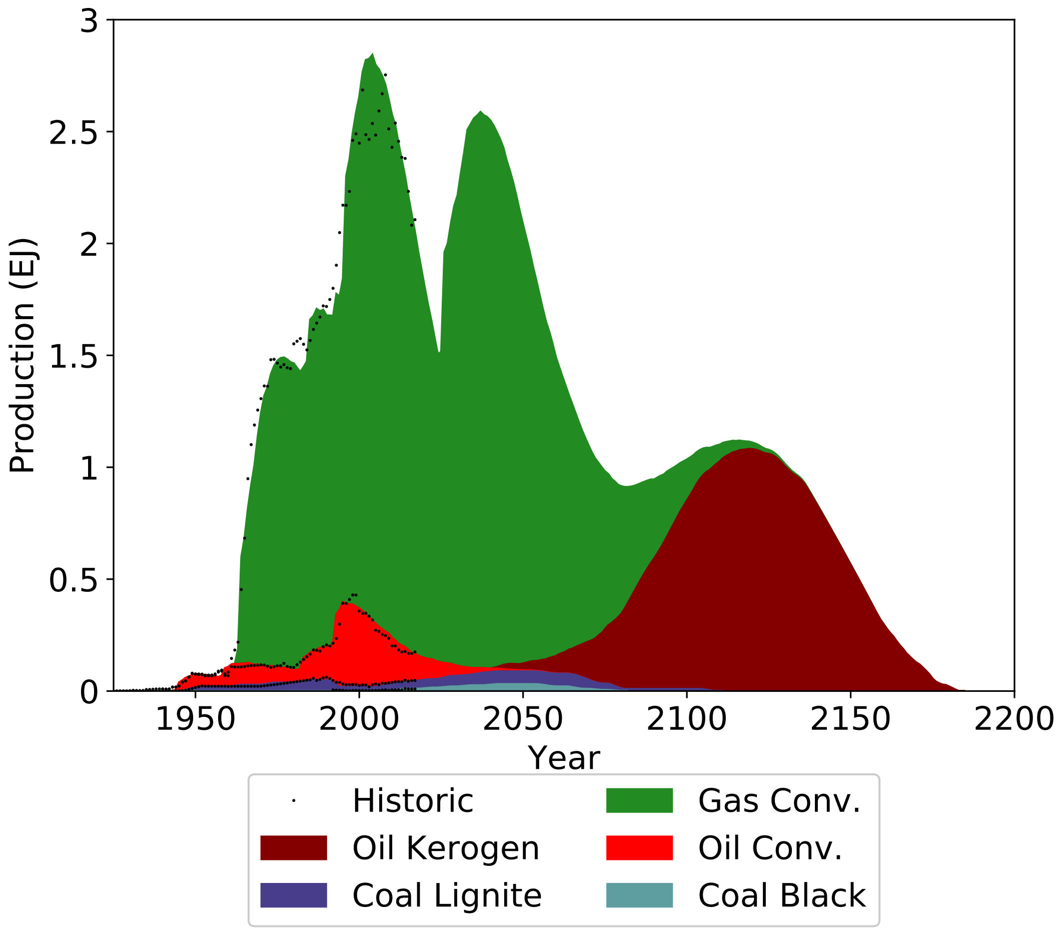

Figure 4.73: Uzbekistan projection by mineral type

Table 4.73: Peak years - Minerals

**Name**	**URR**	**Peak Year**	**Peak Rate**
Coal Black	1.33	2043	0.03
Coal Lignite	5.23	2039	0.06
Oil Conv.	12.07	1995	0.38
Oil Kerogen	70.08	2119	1.08
Gas Conv.	201.64	2004	2.53
**Total**	**290.35**	**2004**	**2.84**

4.17 Total

4.17.1 By country

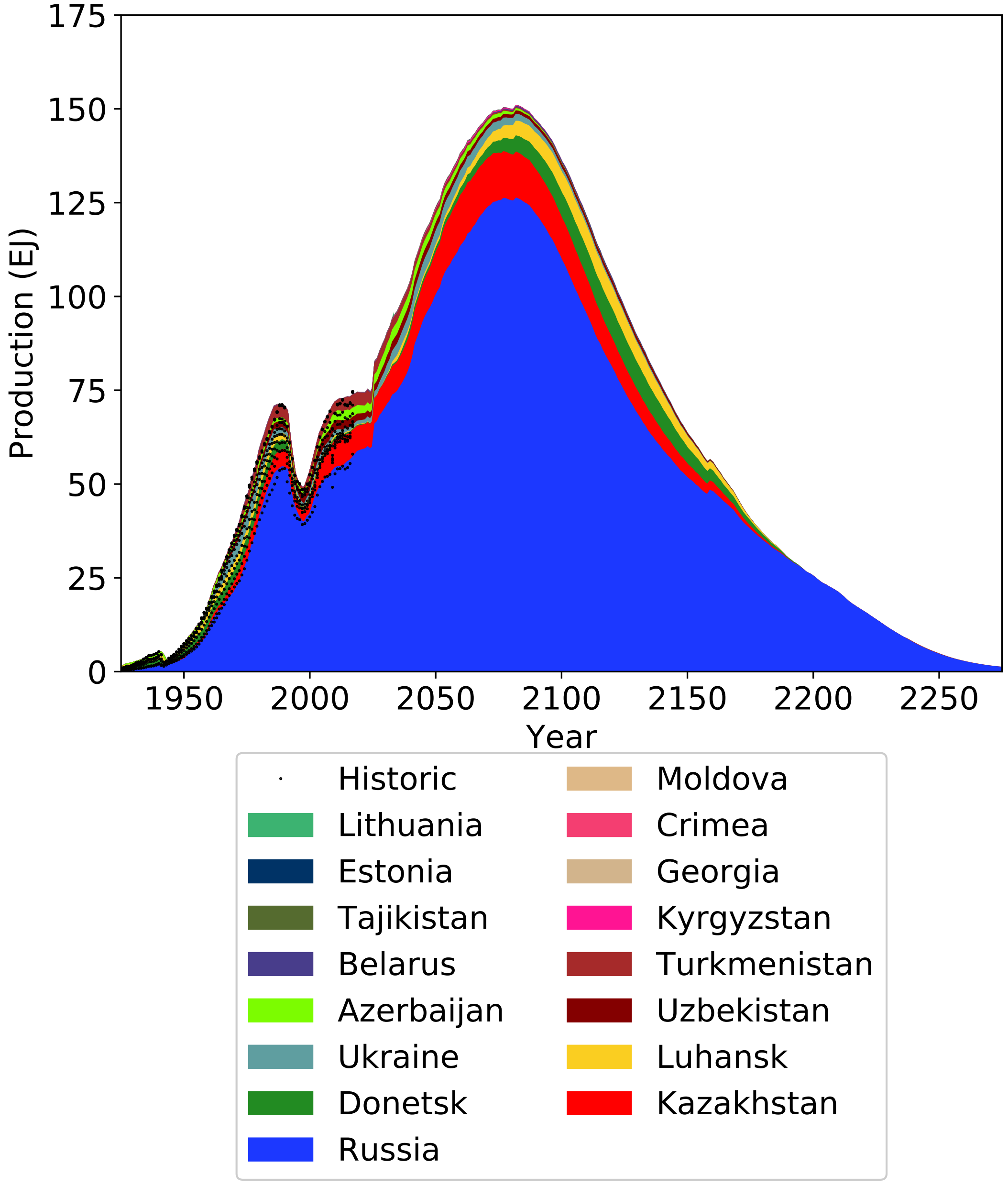

Figure 4.74: FSU projections by country

Table 4.74: Peak years - All

Name	URR	Peak Year	Peak Rate
Russia	17282.65	2082	126.08
Kazakhstan	1548.0	2062	13.73
Donetsk	783.03	2120	7.89
Luhansk	582.62	2114	6.02
Ukraine	350.23	2046	3.78
Uzbekistan	290.35	2004	2.84
Azerbaijan	246.71	2034	3.23
Turkmenistan	235.93	2017	3.46
Belarus	48.76	2112	0.63
Kyrgyzstan	22.74	2058	0.46
Tajikistan	11.46	2045	0.26
Georgia	6.01	1979	0.18
Estonia	5.73	2048	0.08
Crimea	1.71	2010	0.07
Lithuania	0.21	1998	0.02
Moldova	0.02	1994	0.01
**Total**	**21416.16**	**2082**	**150.77**

4.17.2 By mineral

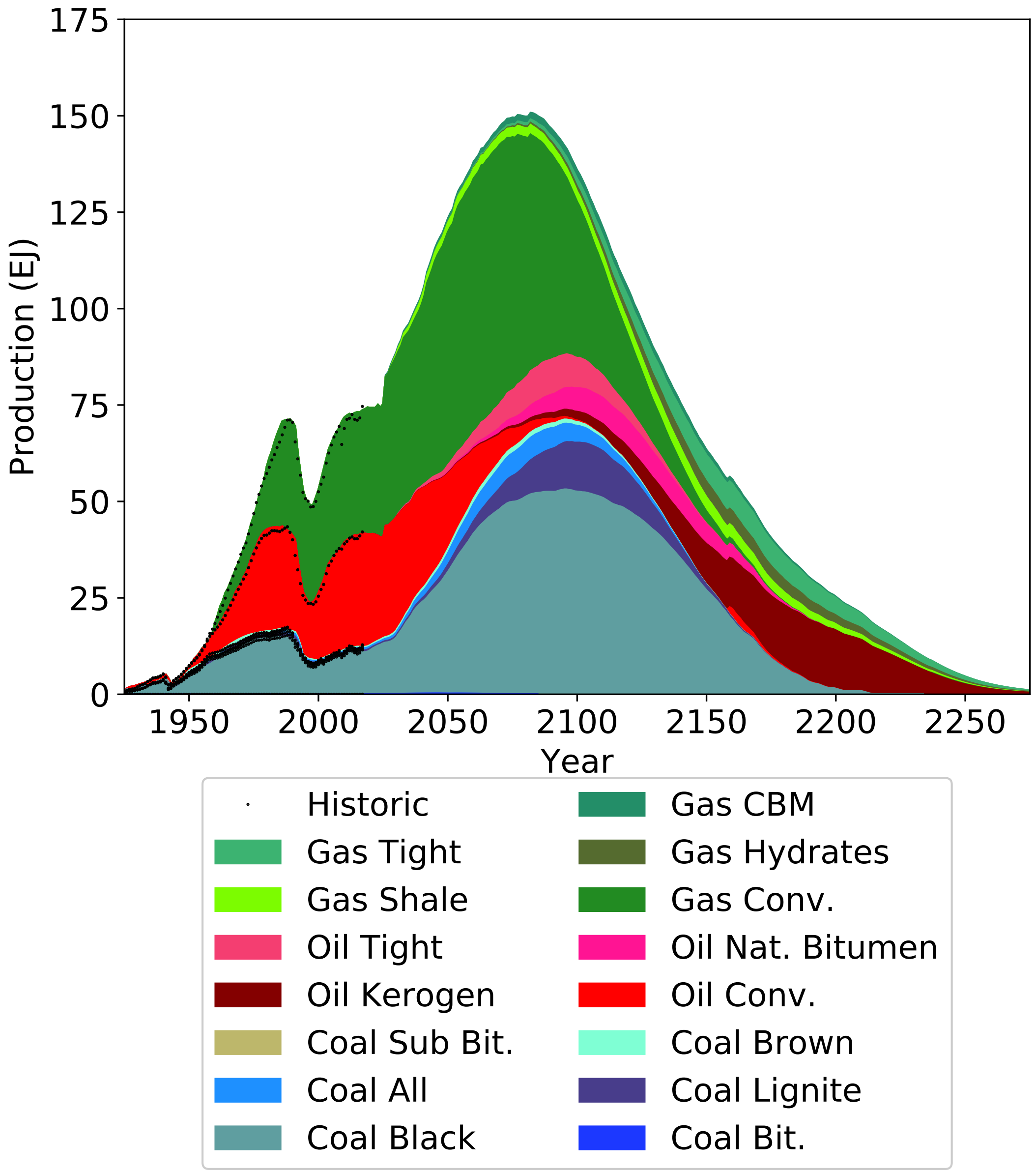

Figure 4.75: FSU projection by mineral type

Table 4.75: Peak years - Minerals

**Name**	**URR**	**Peak Year**	**Peak Rate**
Coal Bit.	10.12	2046	0.24
Coal Black	6525.54	2095	53.11
Coal Lignite	815.83	2102	12.77
Coal All	402.07	2077	6.01
Coal Brown	144.33	2074	1.38
Coal Sub Bit.	4.72	2048	0.09
Oil Conv.	2490.46	2017	29.52
Oil Kerogen	1536.91	2186	16.08
Oil Nat. Bitumen	531.98	2116	6.91
Oil Tight	499.65	2086	9.27
Gas Conv.	6547.19	2067	67.31
Gas Shale	515.58	2143	3.86
Gas Hydrates	403.83	2155	4.07
Gas Tight	741.3	2156	7.13
Gas CBM	246.65	2108	2.36
**Total**	**21416.16**	**2082**	**150.77**

Chapter 5

Middle East

5.1 Bahrain

5.1.1 All Projections

Table 5.1: Peak years - All

**Name**	**URR**	**Peak Year**	**Peak Rate**
Gas Conv.	25.8	2014	0.53
Oil Conv.	16.34	2001	0.1
**Total**	**42.14**	**2014**	**0.63**

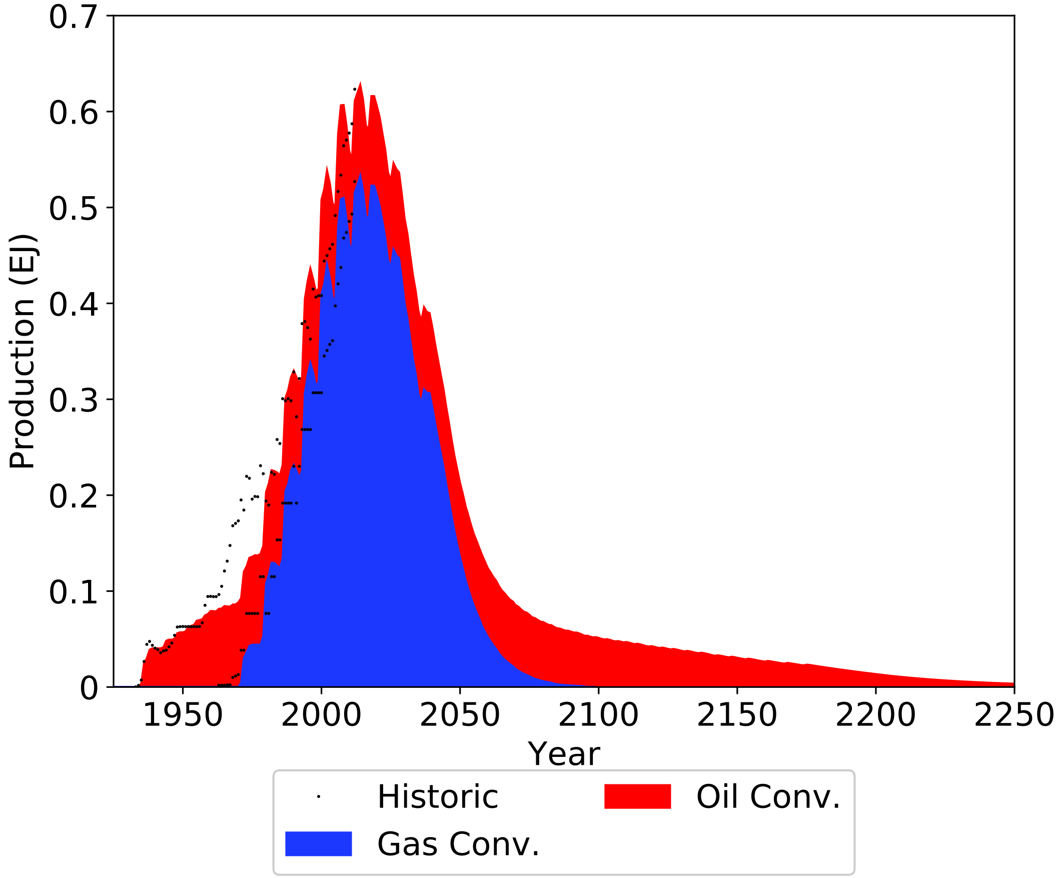

Figure 5.1: Bahrain projections capped at 16

5.1.2 By Mineral

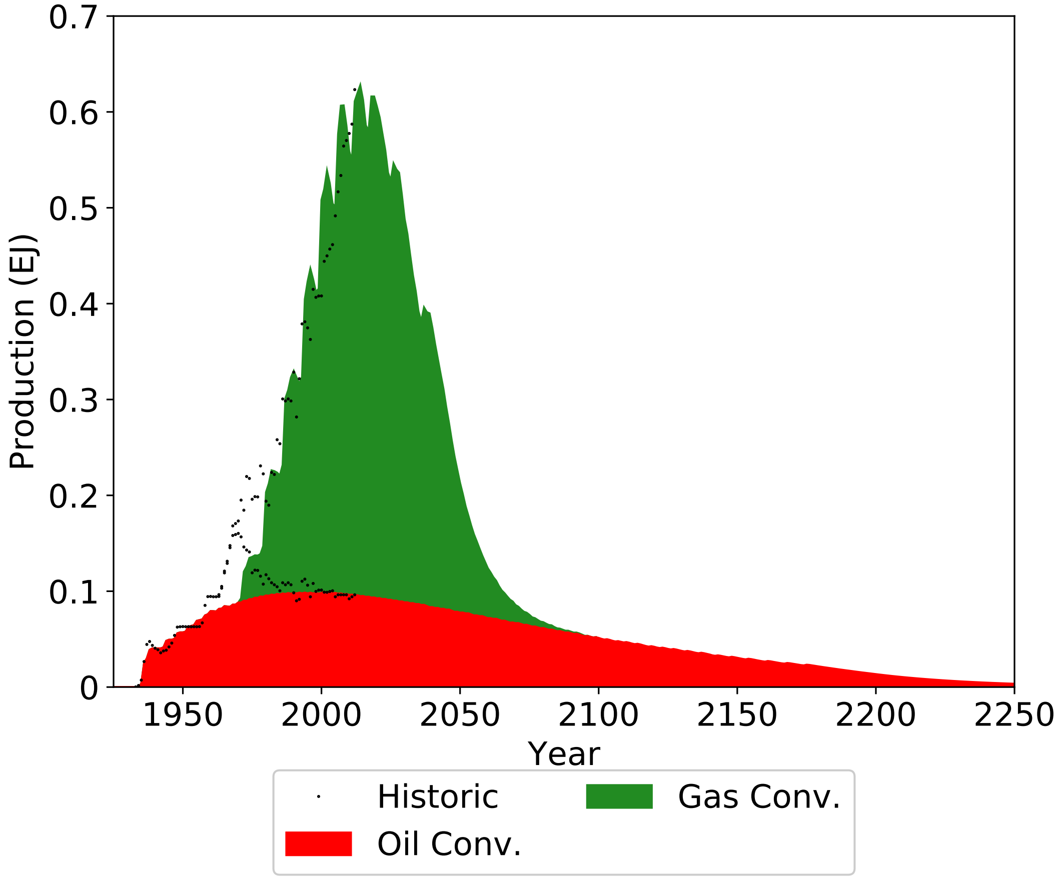

Figure 5.2: Bahrain projection by mineral type

Table 5.2: Peak years - Minerals

**Name**	**URR**	**Peak Year**	**Peak Rate**
Oil Conv.	16.34	2001	0.1
Gas Conv.	25.8	2014	0.53
**Total**	**42.14**	**2014**	**0.63**

5.2 Iran

5.2.1 All Projections

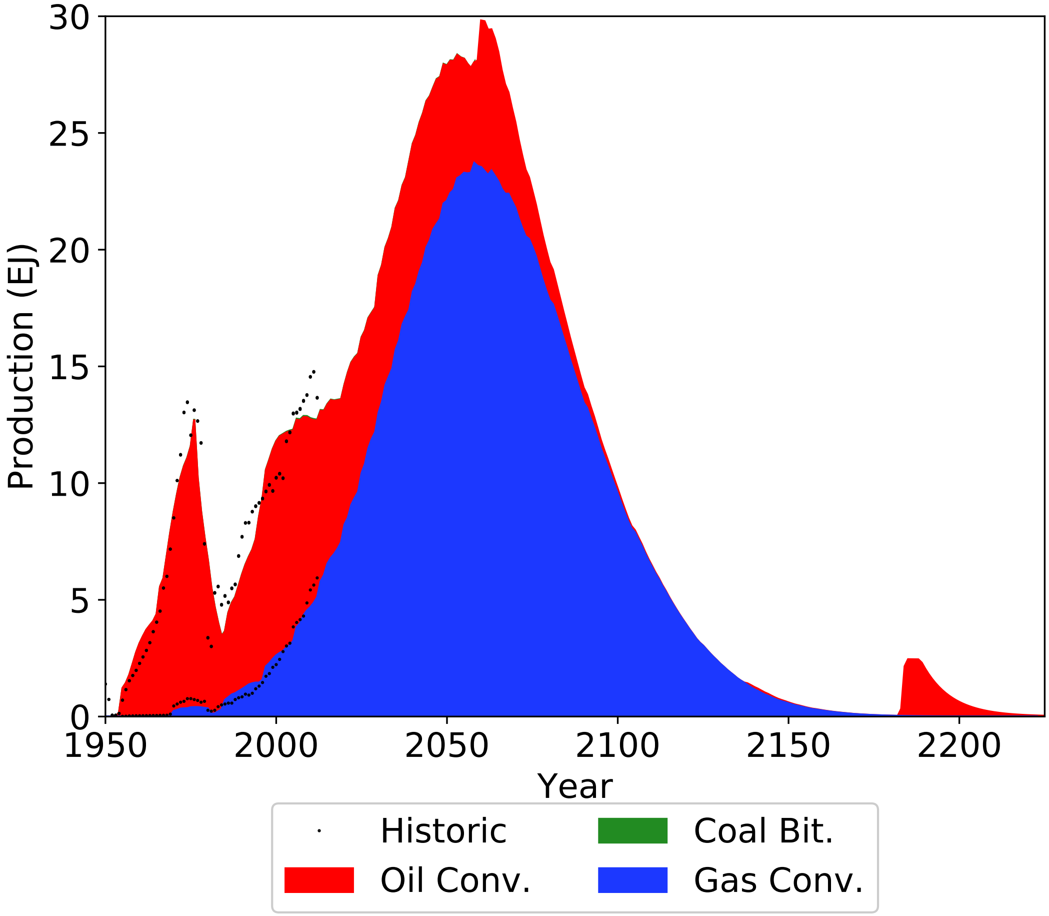

Figure 5.3: Iran projections capped at 16

Table 5.3: Peak years - All

Name	URR	Peak Year	Peak Rate
Gas Conv.	1712.1	2058	23.71
Oil Conv.	787.83	1976	12.32
Coal Bit.	2.3	2007	0.05
**Total**	**2502.23**	**2060**	**29.82**

5.2.2 By Mineral

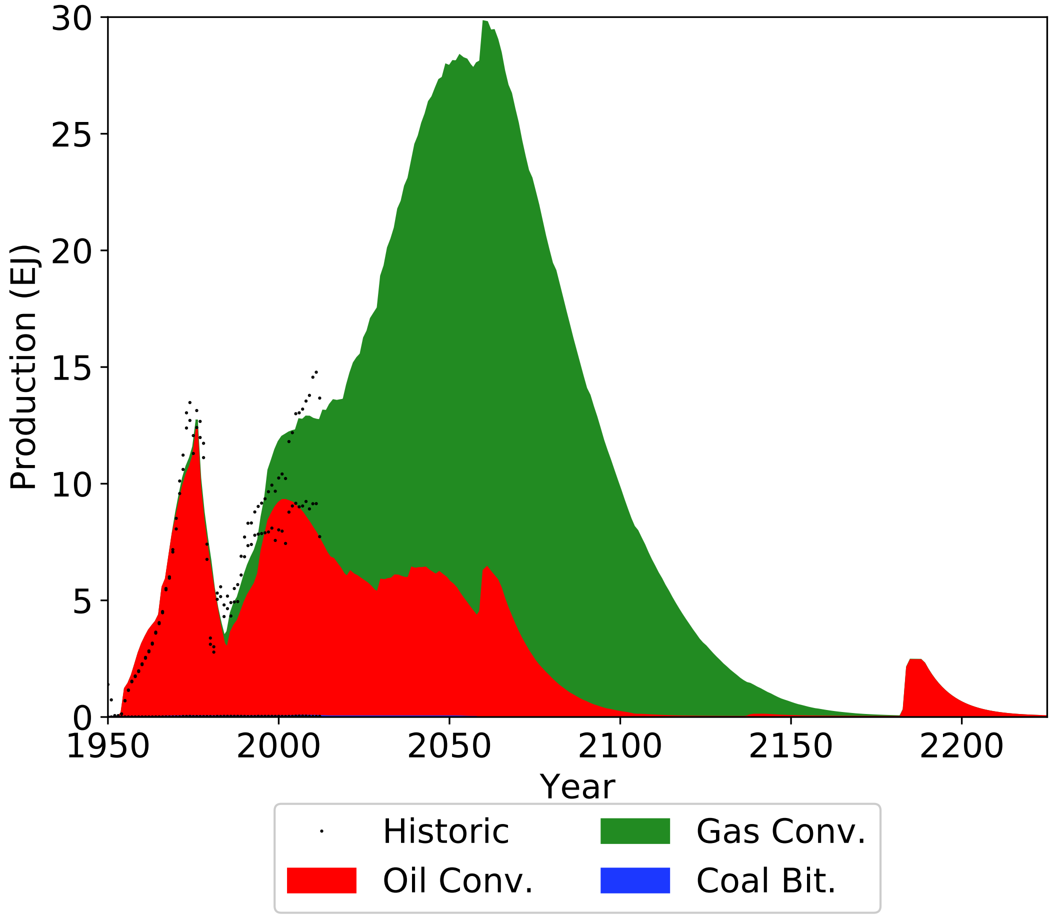

Figure 5.4: Iran projection by mineral type

Table 5.4: Peak years - Minerals

Name	URR	Peak Year	Peak Rate
Coal Bit.	2.3	2007	0.05
Oil Conv.	787.83	1976	12.32
Gas Conv.	1712.1	2058	23.71
**Total**	**2502.23**	**2060**	**29.82**

5.3 Iraq

5.3.1 All Projections

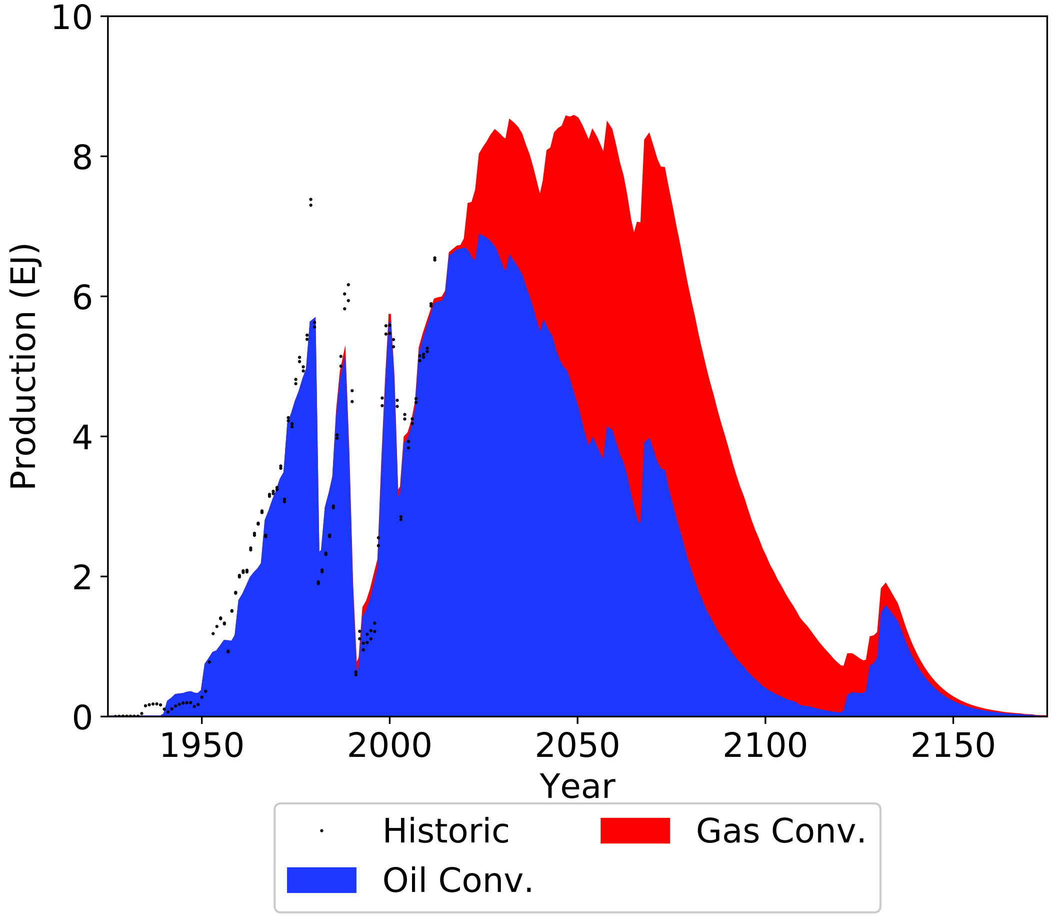

Figure 5.5: Iraq projections capped at 16

Table 5.5: Peak years - All

Name	URR	Peak Year	Peak Rate
Oil Conv.	573.0	2024	6.87
Gas Conv.	284.9	2055	4.42
**Total**	**857.9**	**2049**	**8.57**

5.3.2 By Mineral

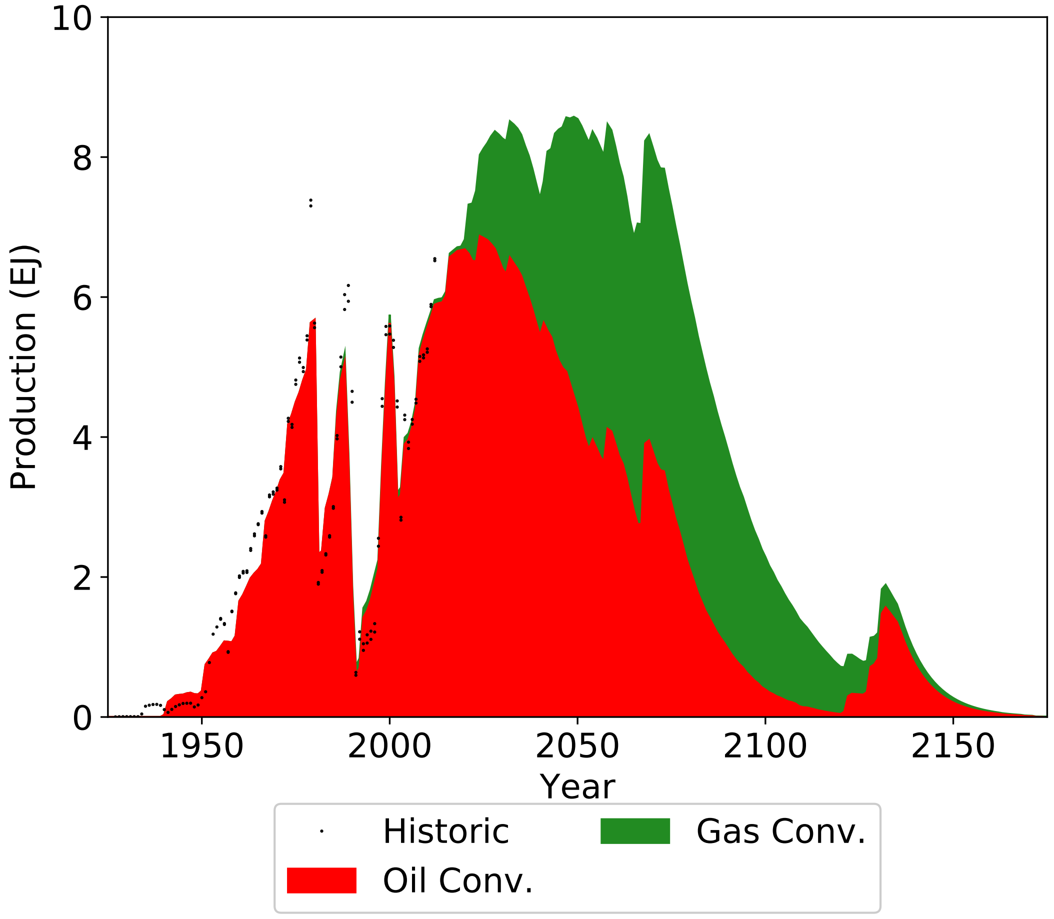

Figure 5.6: Iraq projection by mineral type

Table 5.6: Peak years - Minerals

**Name**	**URR**	**Peak Year**	**Peak Rate**
Oil Conv.	573.0	2024	6.87
Gas Conv.	284.9	2055	4.42
**Total**	**857.9**	**2049**	**8.57**

5.4 Israel

5.4.1 All Projections

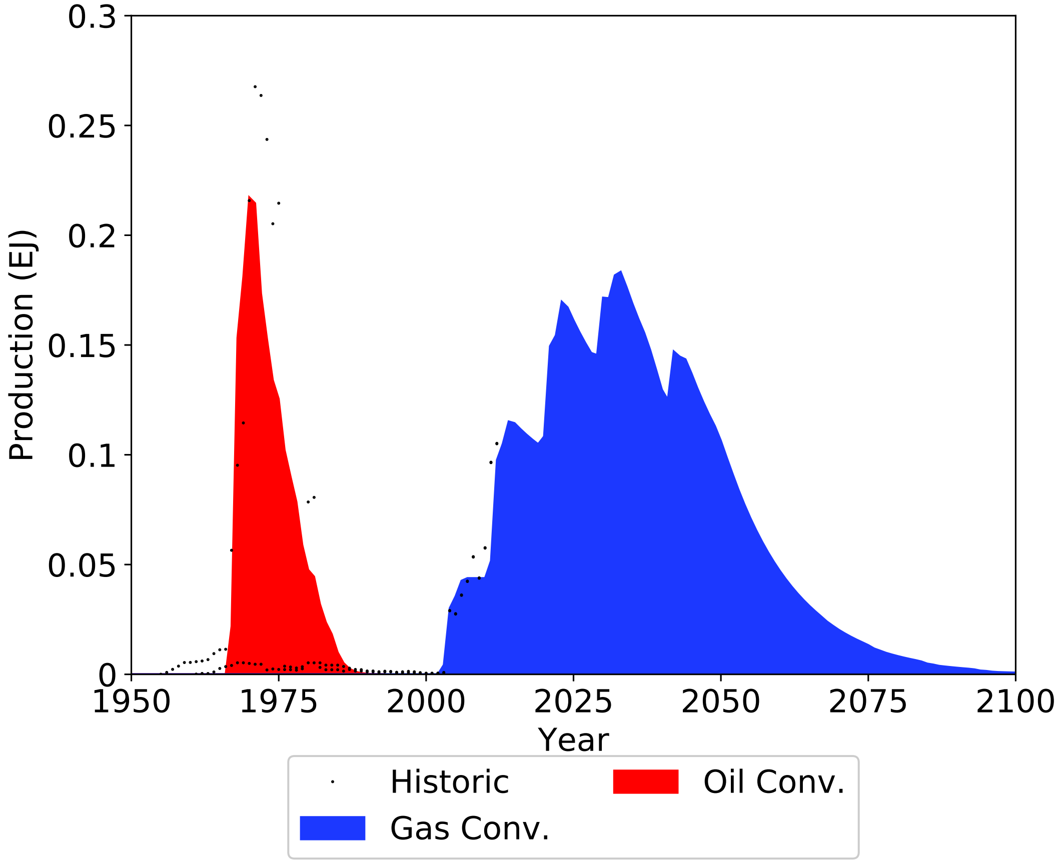

Figure 5.7: Israel projections capped at 16

Table 5.7: Peak years - All

Name	URR	Peak Year	Peak Rate
Gas Conv.	7.0	2033	0.18
Oil Conv.	1.89	1970	0.22
**Total**	**8.89**	**1970**	**0.22**

5.4.2 By Mineral

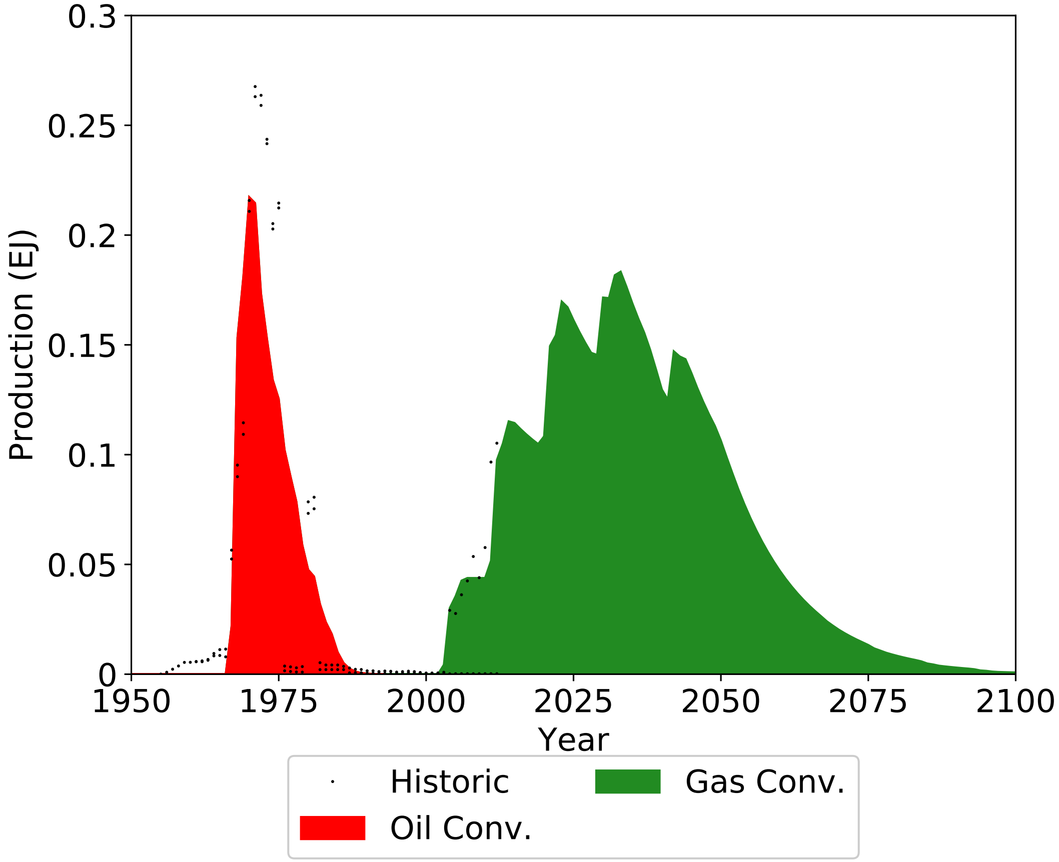

Figure 5.8: Israel projection by mineral type

Table 5.8: Peak years - Minerals

**Name**	**URR**	**Peak Year**	**Peak Rate**
Oil Conv.	1.89	1970	0.22
Gas Conv.	7.0	2033	0.18
**Total**	**8.89**	**1970**	**0.22**

5.5 Jordan

5.5.1 All Projections

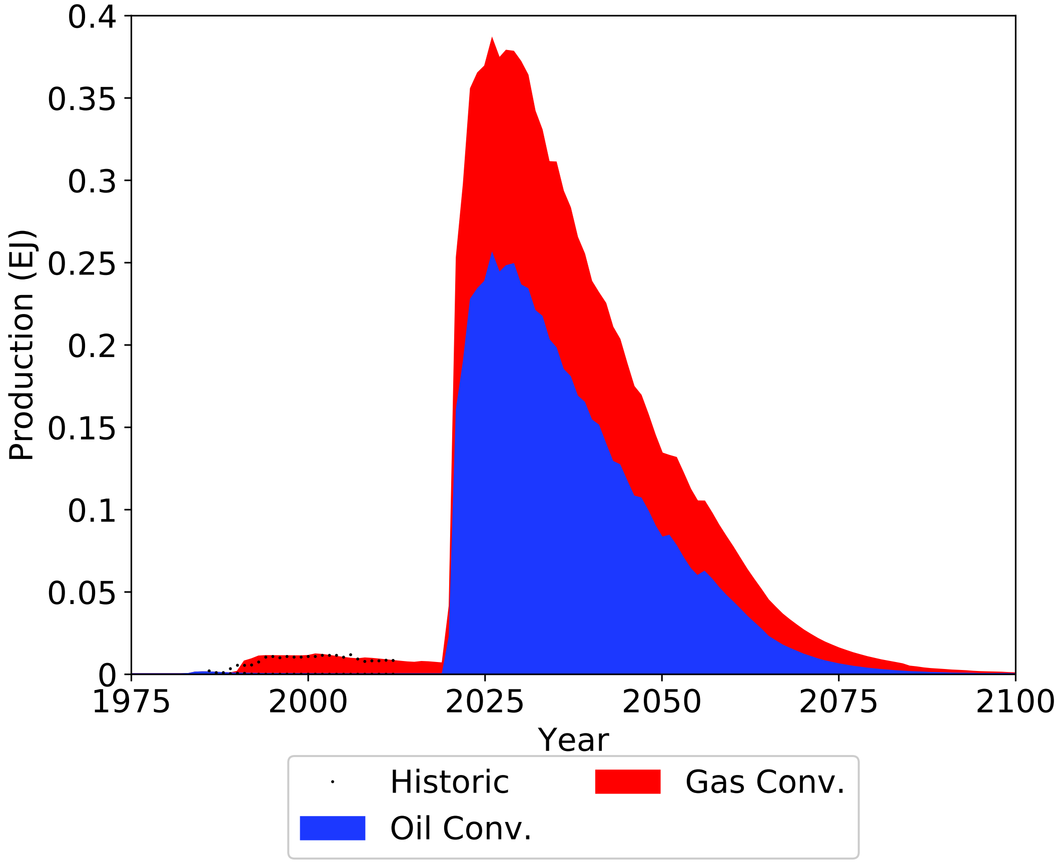

Figure 5.9: Jordan projections capped at 16

Table 5.9: Peak years - All

**Name**	**URR**	**Peak Year**	**Peak Rate**
Oil Conv.	6.33	2026	0.25
Gas Conv.	4.1	2030	0.14
**Total**	**10.43**	**2026**	**0.39**

5.5.2 By Mineral

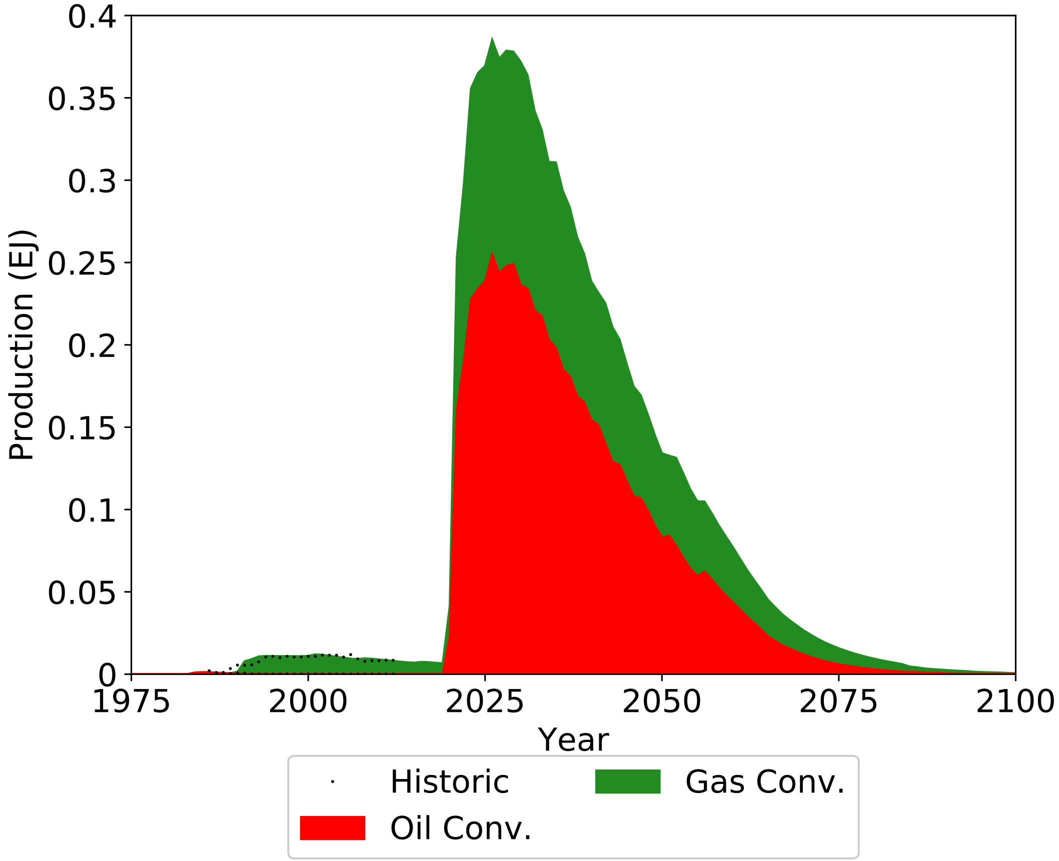

Figure 5.10: Jordan projection by mineral type

Table 5.10: Peak years - Minerals

Name	URR	Peak Year	Peak Rate
Oil Conv.	6.33	2026	0.25
Gas Conv.	4.1	2030	0.14
**Total**	**10.43**	**2026**	**0.39**

5.6 Kuwait

5.6.1 All Projections

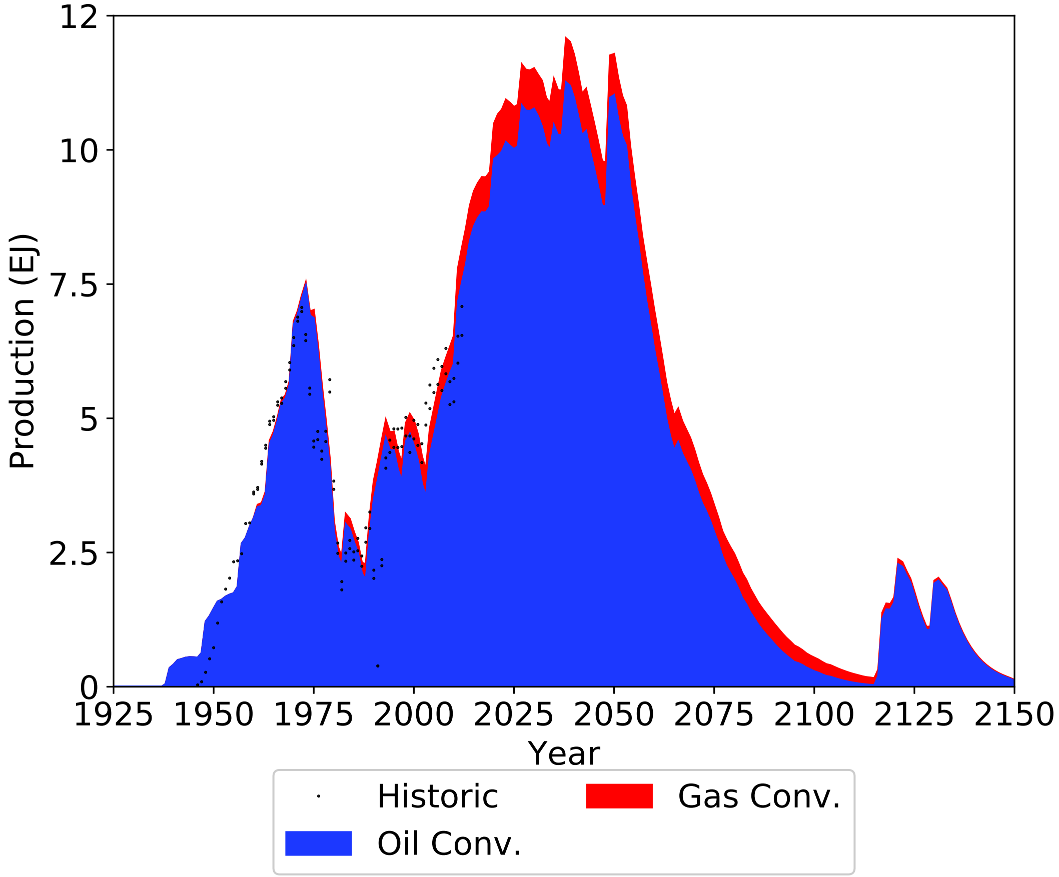

Figure 5.11: Kuwait projections capped at 16

Table 5.11: Peak years - All

**Name**	**URR**	**Peak Year**	**Peak Rate**
Oil Conv.	865.76	2038	11.26
Gas Conv.	74.0	2034	0.87
**Total**	**939.76**	**2038**	**12.08**

5.6.2 By Mineral

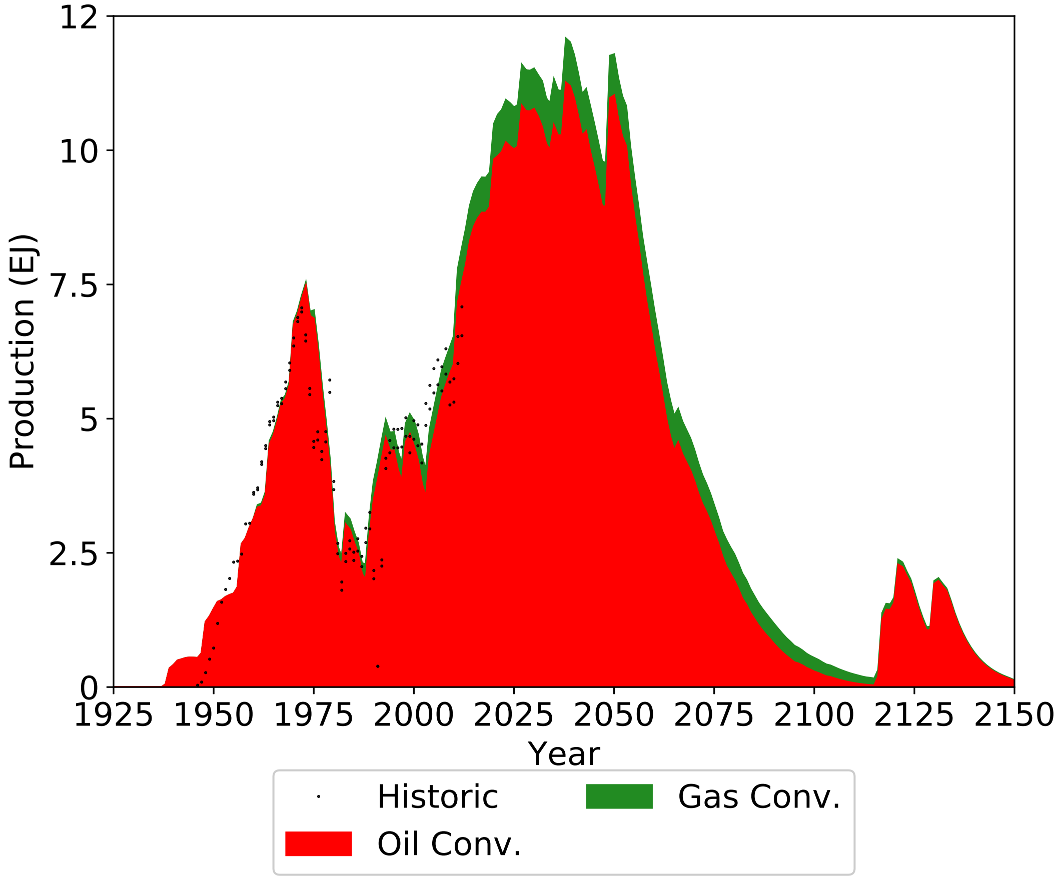

Figure 5.12: Kuwait projection by mineral type

Table 5.12: Peak years - Minerals

**Name**	**URR**	**Peak Year**	**Peak Rate**
Oil Conv.	865.76	2038	11.26
Gas Conv.	74.0	2034	0.87
**Total**	**939.76**	**2038**	**12.08**

5.7 Lebanon

5.7.1 All Projections

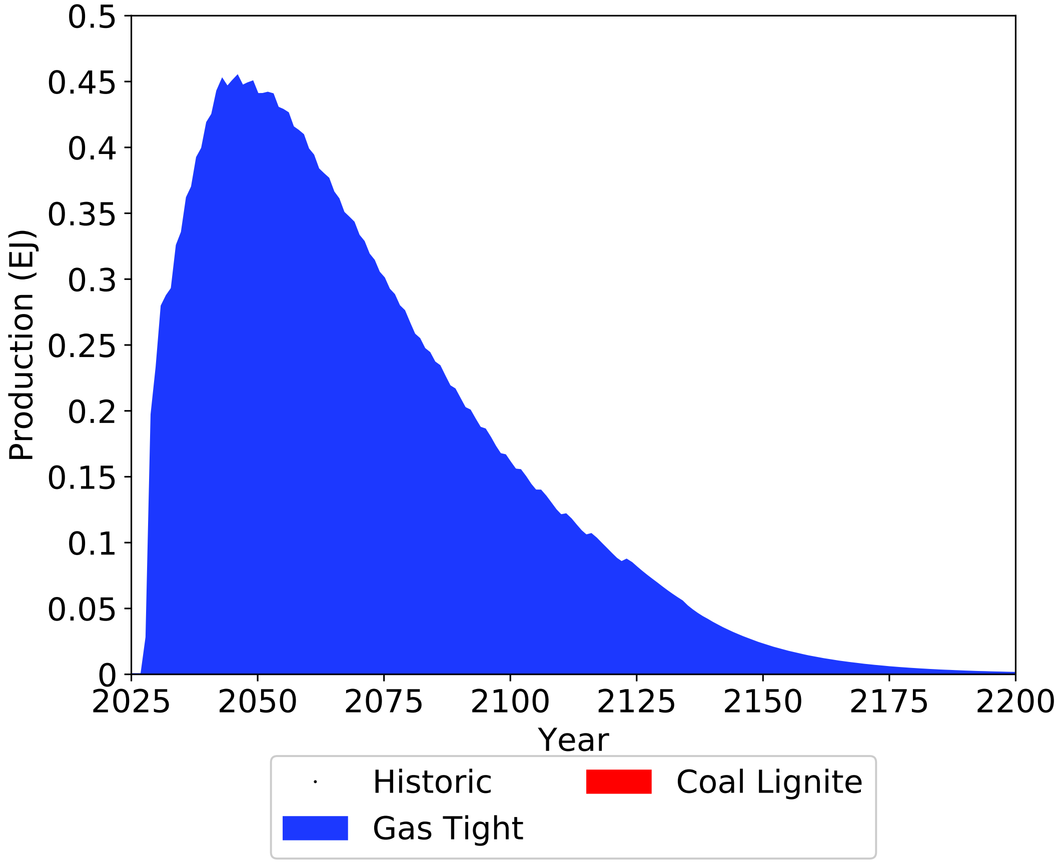

Figure 5.13: Lebanon projections capped at 16

Table 5.13: Peak years - All

Name	URR	Peak Year	Peak Rate
Gas Tight	27.81	2046	0.45
Coal Lignite	–	1942	–
**Total**	**27.81**	**2046**	**0.45**

5.7.2 By Mineral

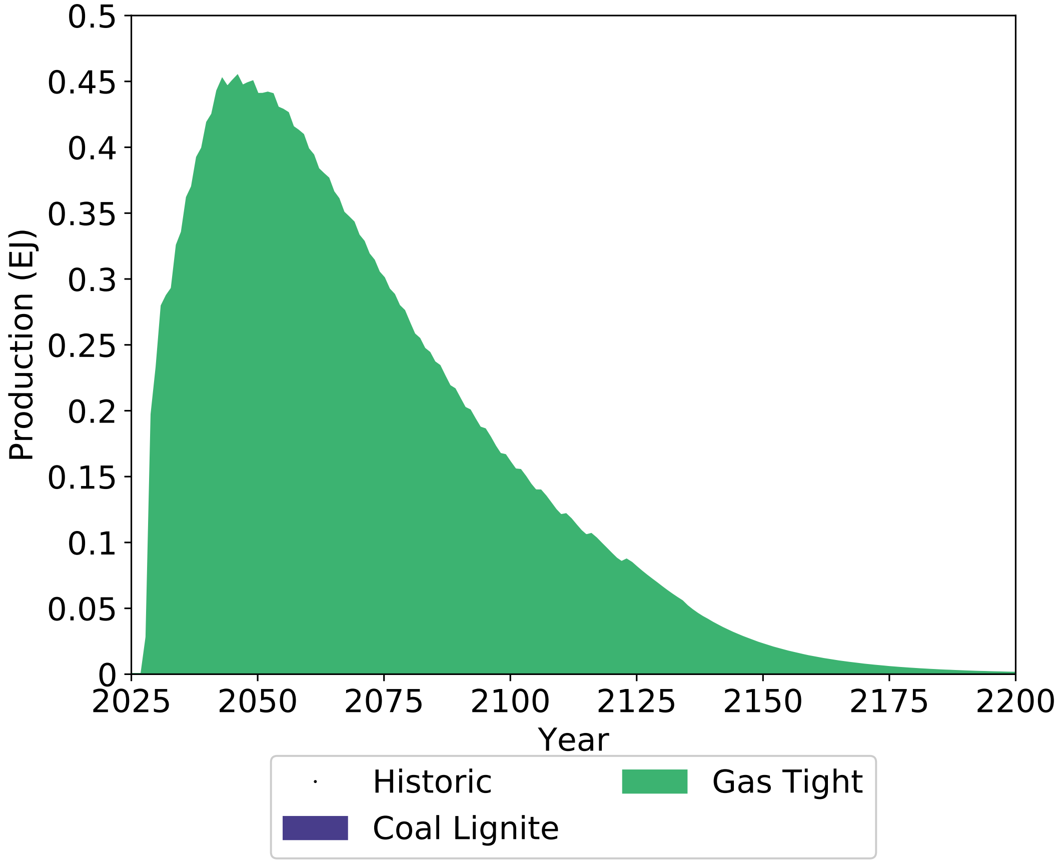

Figure 5.14: Lebanon projection by mineral type

Table 5.14: Peak years - Minerals

Name	URR	Peak Year	Peak Rate
Coal Lignite	–	1942	–
Gas Tight	27.81	2046	0.45
**Total**	**27.81**	**2046**	**0.45**

5.8 Oman

5.8.1 All Projections

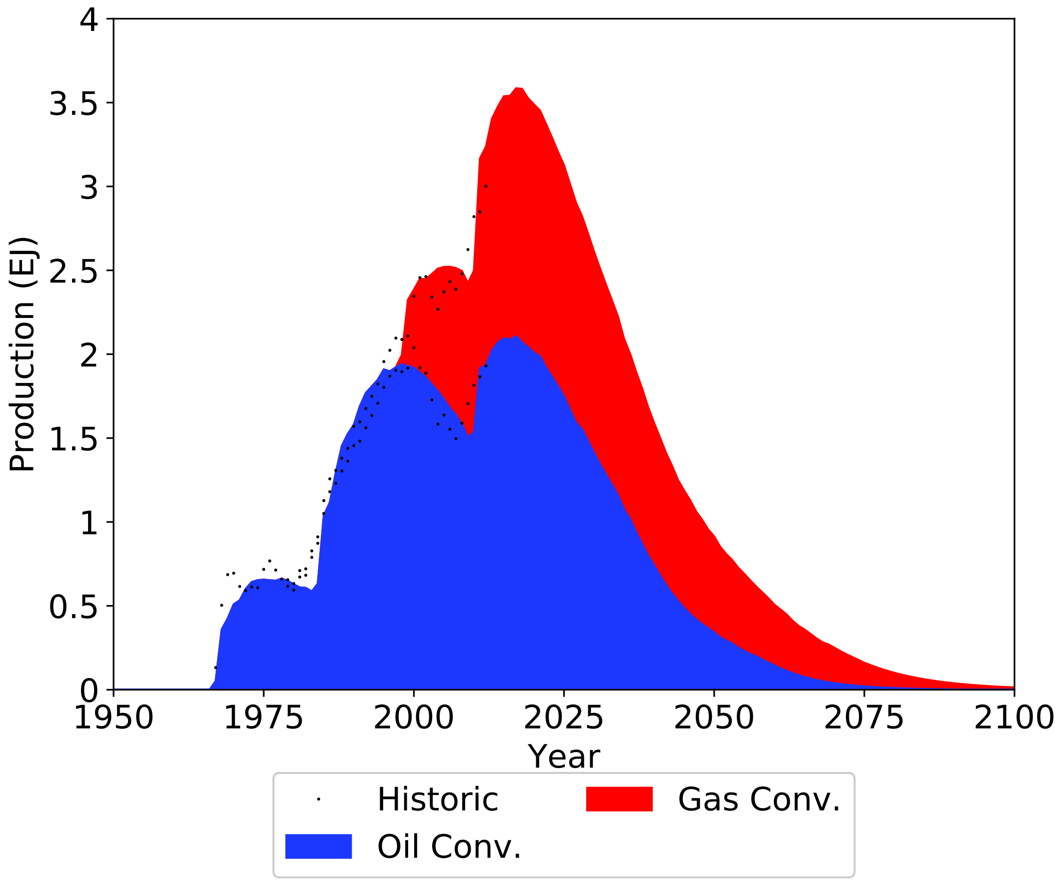

Figure 5.15: Oman projections capped at 16

Table 5.15: Peak years - All

**Name**	**URR**	**Peak Year**	**Peak Rate**
Oil Conv.	109.7	2017	2.11
Gas Conv.	63.0	2018	1.51
**Total**	**172.7**	**2017**	**3.58**

5.8.2 By Mineral

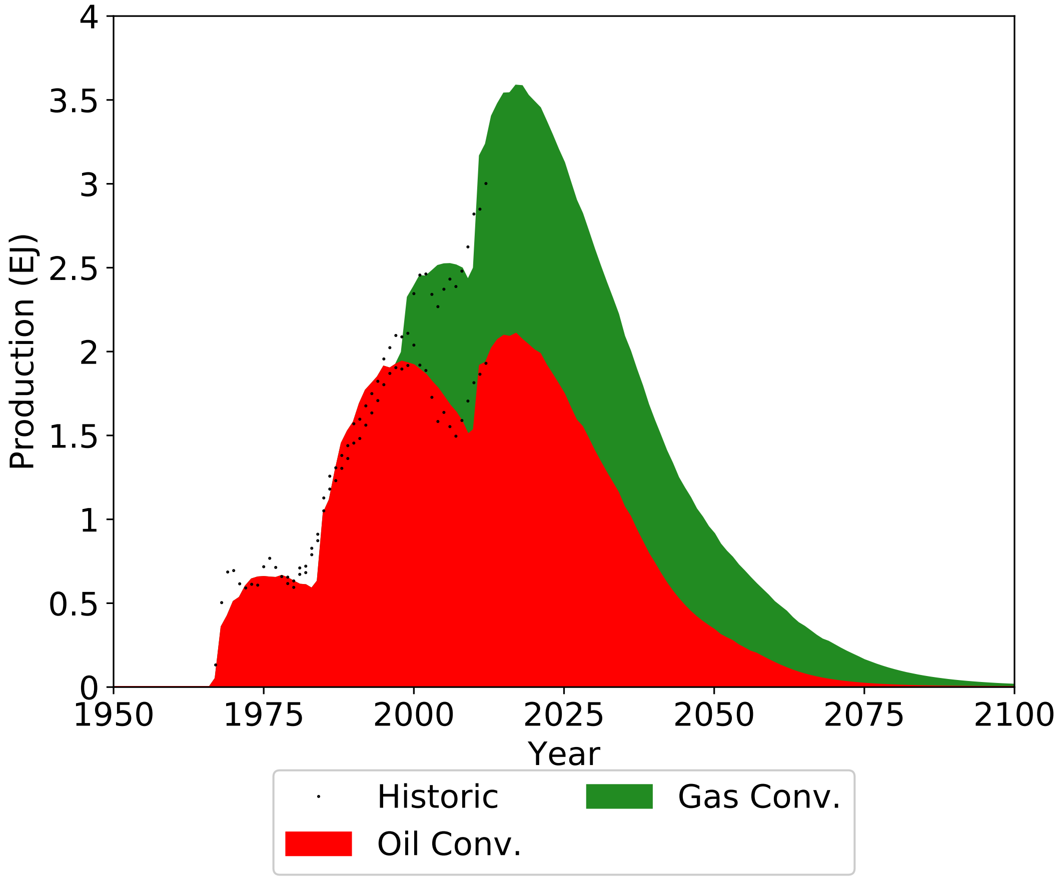

Figure 5.16: Oman projection by mineral type

Table 5.16: Peak years - Minerals

**Name**	**URR**	**Peak Year**	**Peak Rate**
Oil Conv.	109.7	2017	2.11
Gas Conv.	63.0	2018	1.51
**Total**	**172.7**	**2017**	**3.58**

5.9 Qatar

5.9.1 All Projections

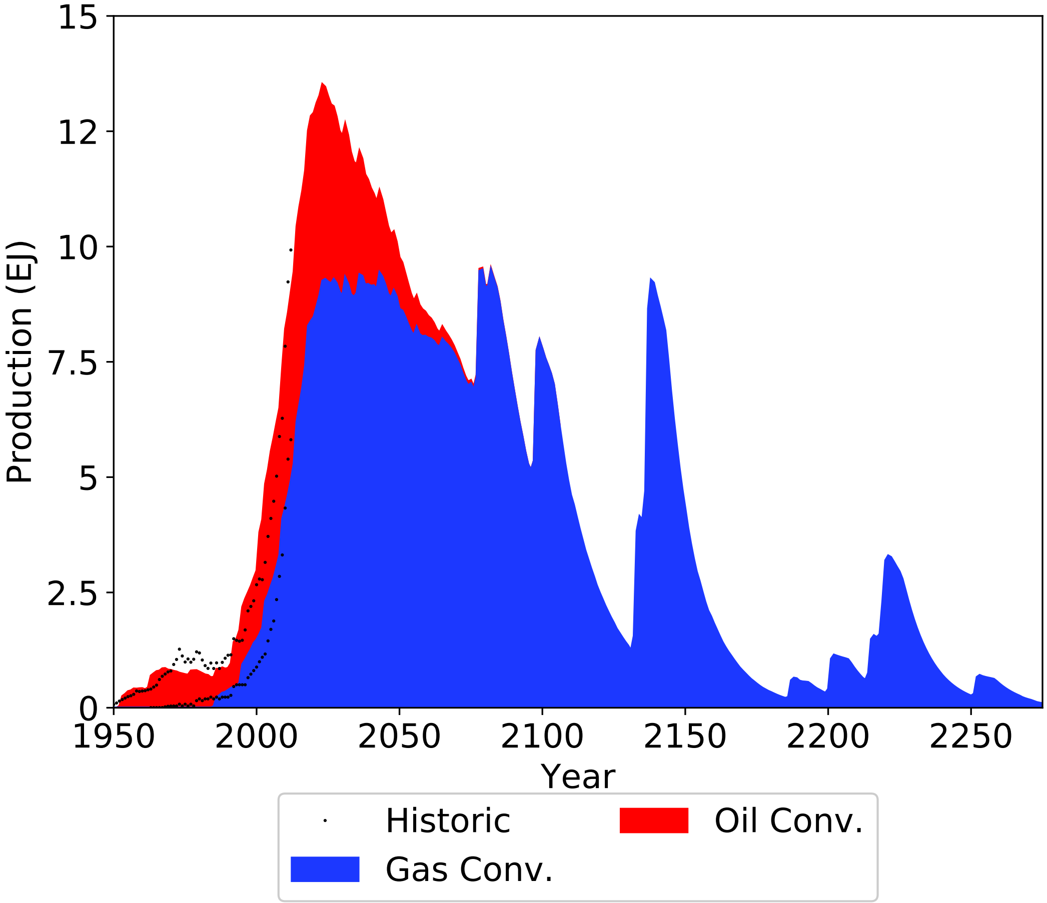

Figure 5.17: Qatar projections capped at 16

Table 5.17: Peak years - All

Name	URR	Peak Year	Peak Rate
Gas Conv.	1134.0	2082	9.5
Oil Conv.	200.55	2019	4.45
**Total**	**1334.55**	**2023**	**13.52**

5.9.2 By Mineral

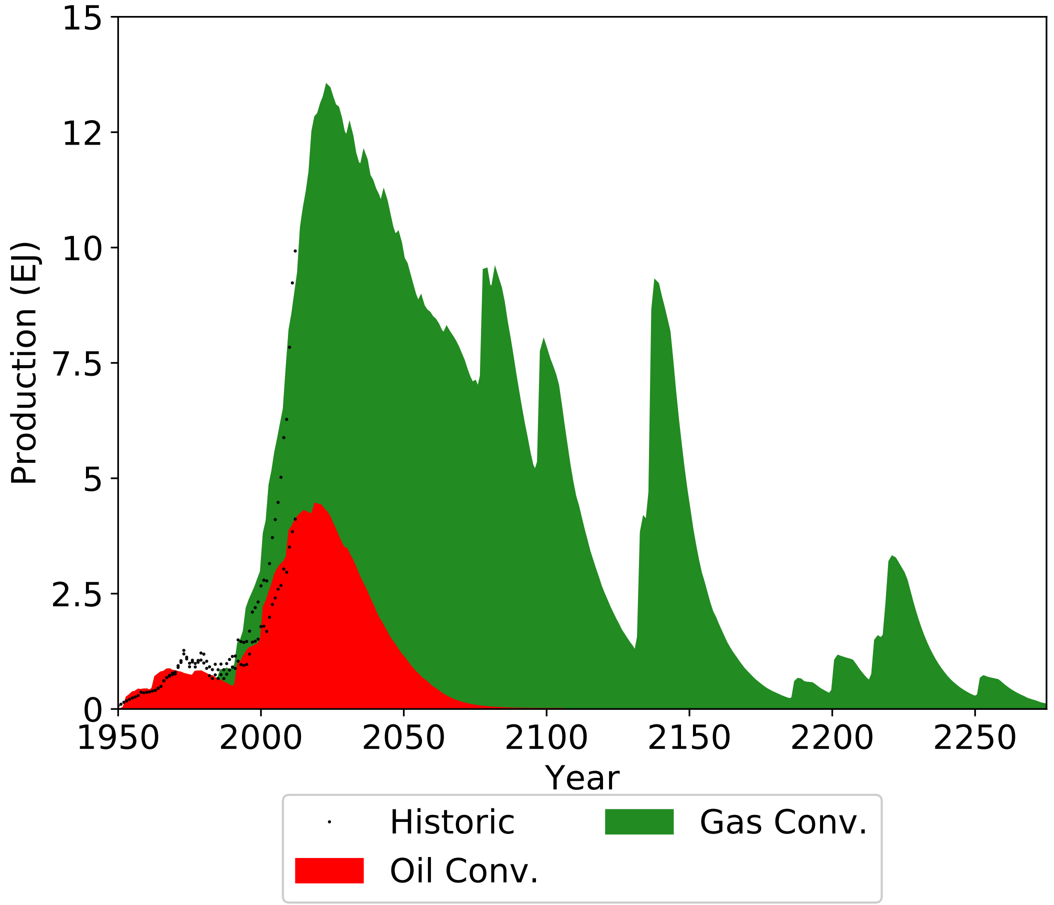

Figure 5.18: Qatar projection by mineral type

Table 5.18: Peak years - Minerals

Name	URR	Peak Year	Peak Rate
Oil Conv.	200.55	2019	4.45
Gas Conv.	1134.0	2082	9.5
**Total**	**1334.55**	**2023**	**13.52**

5.10 Saudi Arabia

5.10.1 All Projections

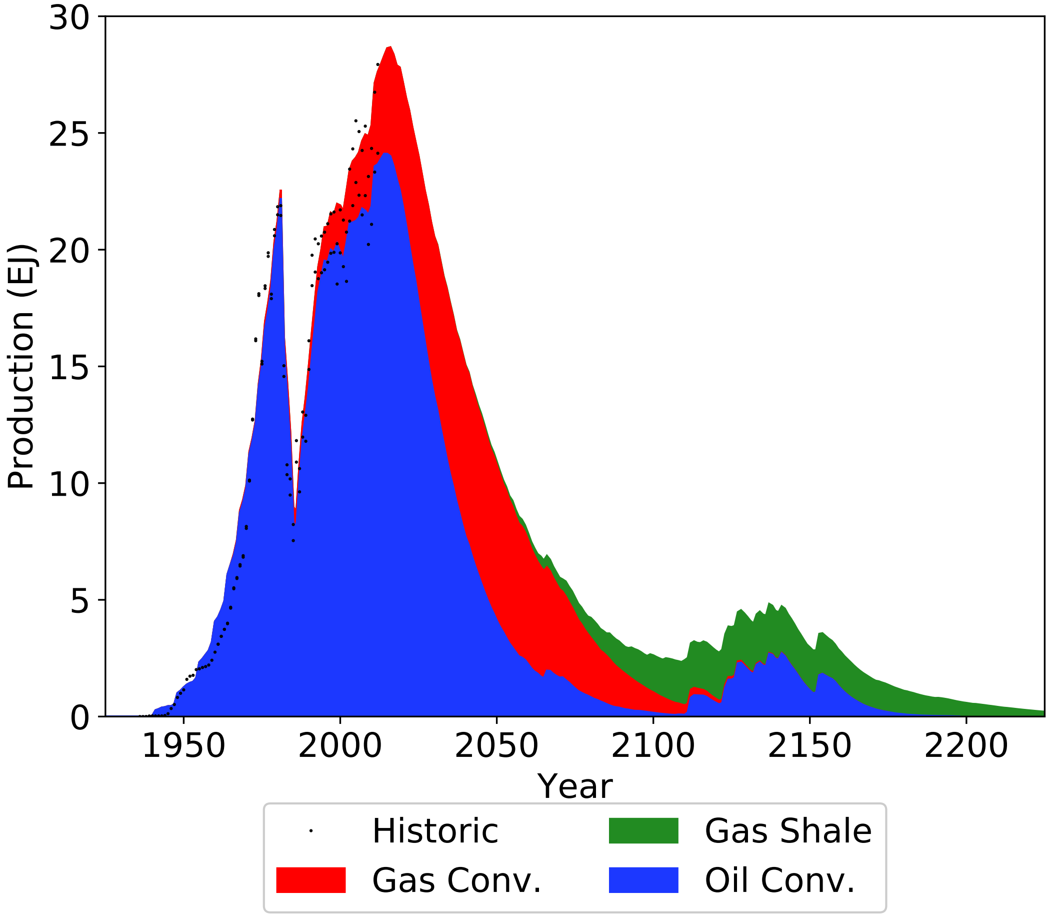

Figure 5.19: Saudi Arabia projections capped at 16

Table 5.19: Peak years - All

Name	URR	Peak Year	Peak Rate
Oil Conv.	1508.77	2014	24.11
Gas Conv.	478.3	2038	7.36
Gas Shale	210.0	2129	2.19
**Total**	**2197.07**	**2016**	**28.67**

5.10.2 By Mineral

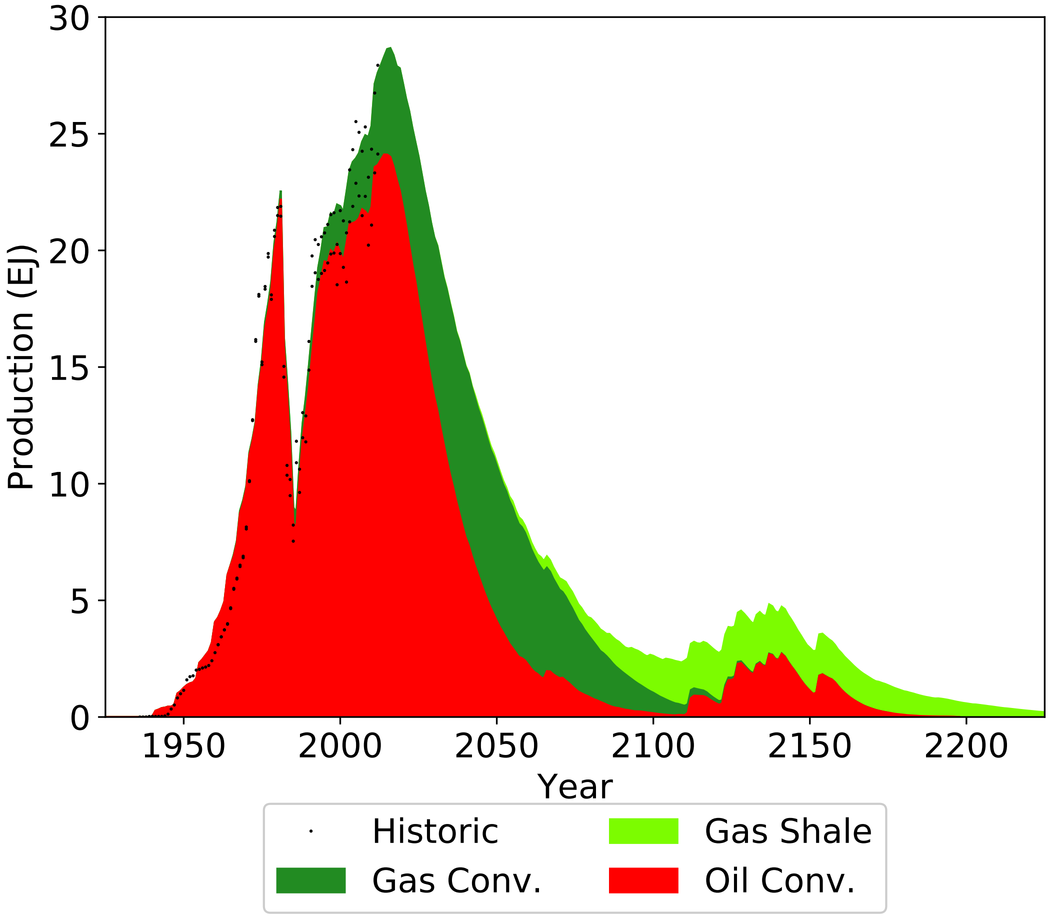

Figure 5.20: Saudi Arabia projection by mineral type

Table 5.20: Peak years - Minerals

Name	URR	Peak Year	Peak Rate
Oil Conv.	1508.77	2014	24.11
Gas Conv.	478.3	2038	7.36
Gas Shale	210.0	2129	2.19
**Total**	**2197.07**	**2016**	**28.67**

5.11 Syria

5.11.1 All Projections

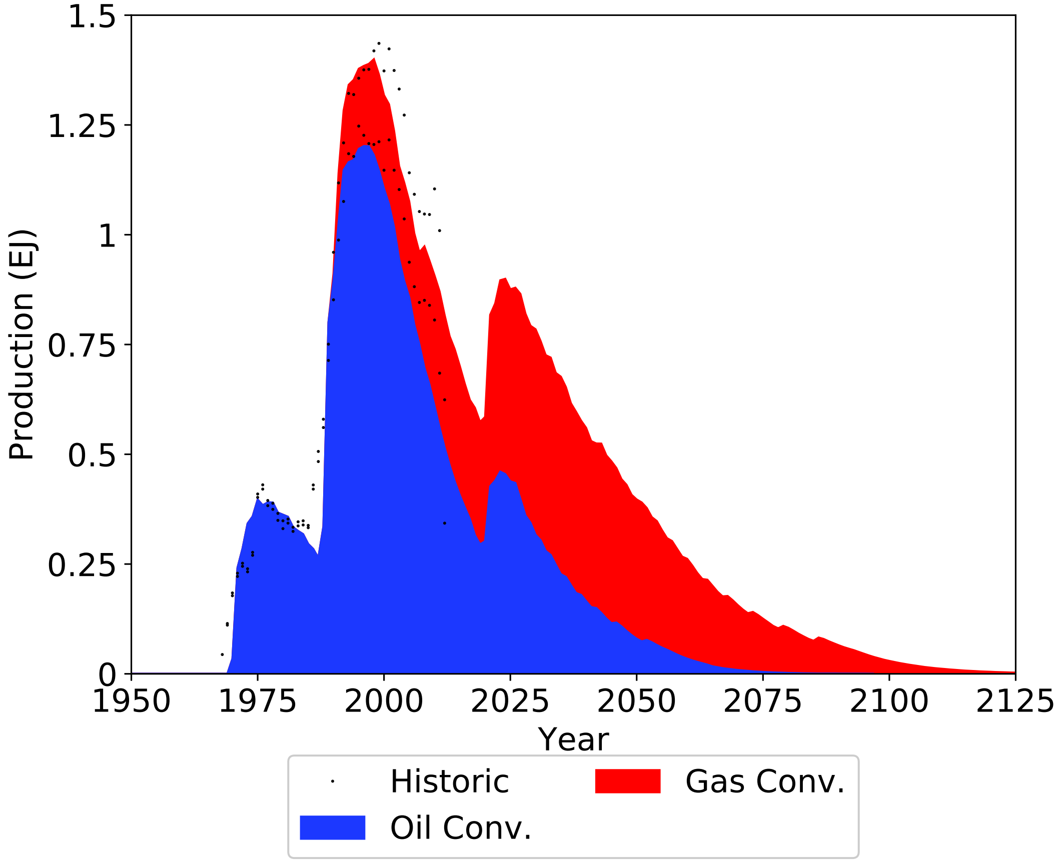

Figure 5.21: Syria projections capped at 16

Table 5.21: Peak years - All

Name	URR	Peak Year	Peak Rate
Oil Conv.	39.98	1996	1.2
Gas Conv.	26.5	2030	0.47
**Total**	**66.48**	**1998**	**1.4**

5.11.2 By Mineral

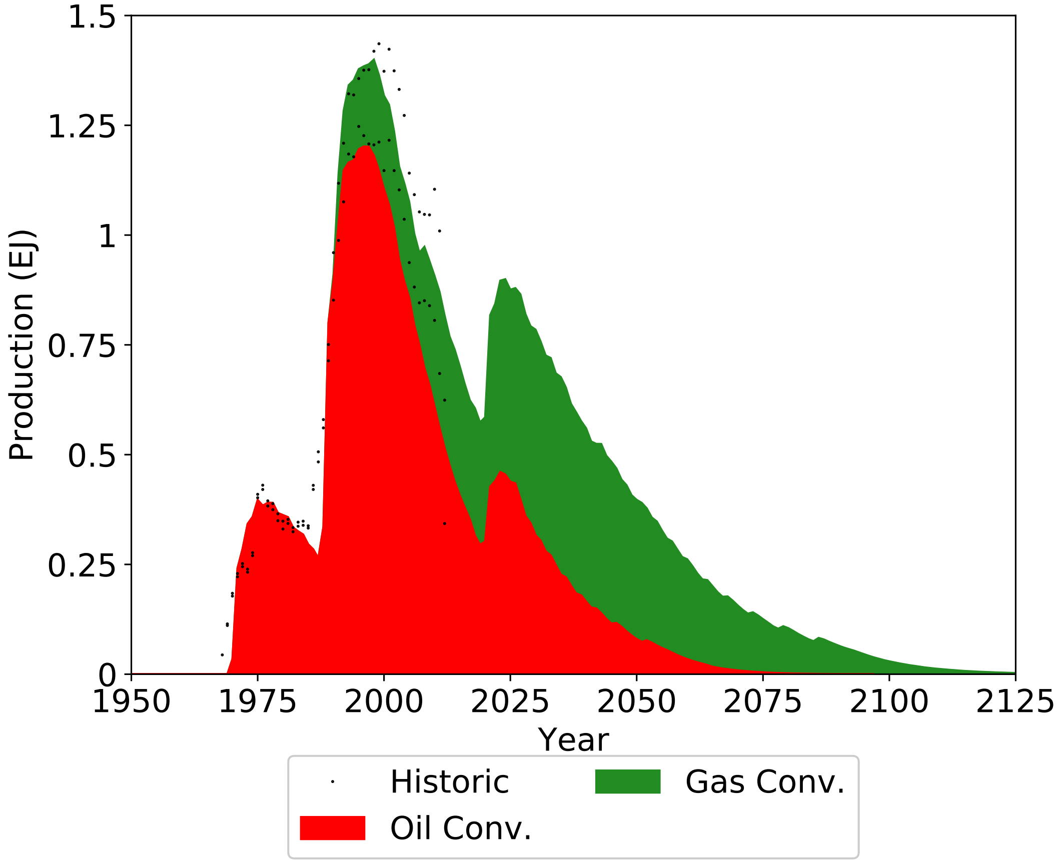

Figure 5.22: Syria projection by mineral type

Table 5.22: Peak years - Minerals

Name	URR	Peak Year	Peak Rate
Oil Conv.	39.98	1996	1.2
Gas Conv.	26.5	2030	0.47
**Total**	**66.48**	**1998**	**1.4**

5.12 UAE

5.12.1 All Projections

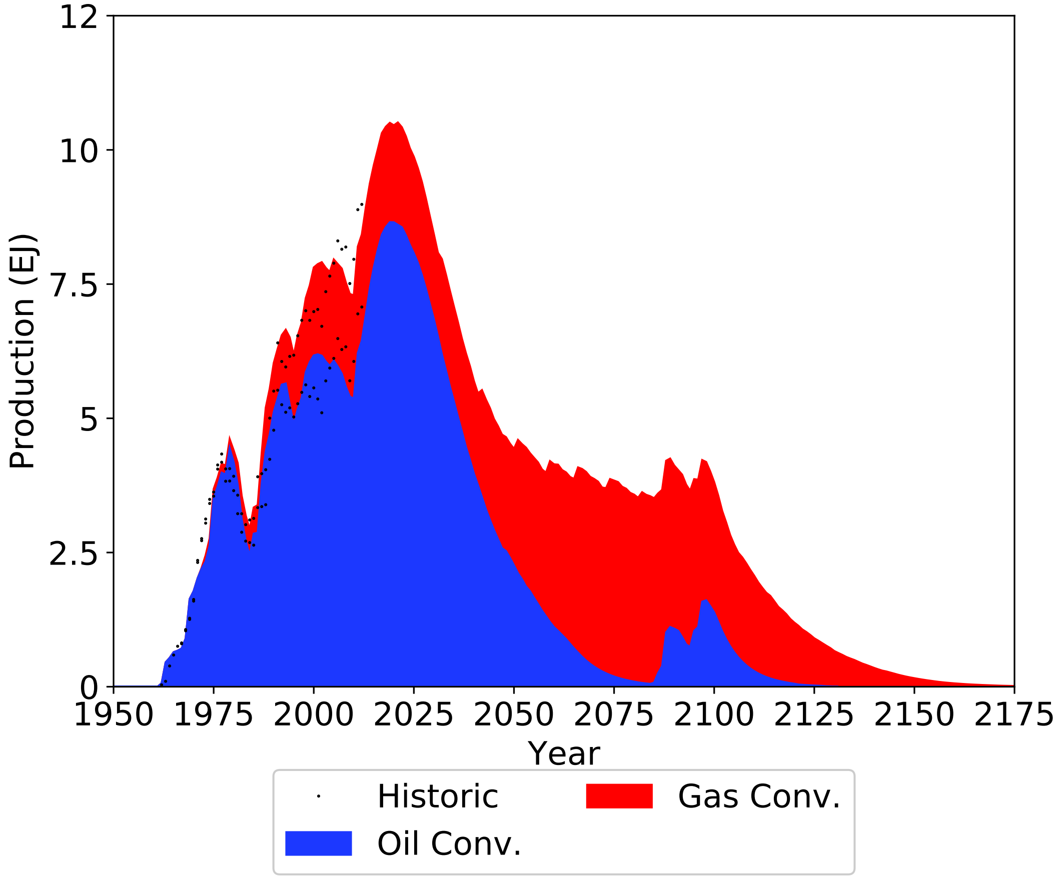

Figure 5.23: UAE projections capped at 16

Table 5.23: Peak years - All

Name	URR	Peak Year	Peak Rate
Oil Conv.	476.21	2019	8.65
Gas Conv.	321.1	2074	3.65
**Total**	**797.31**	**2021**	**10.51**

5.12.2 By Mineral

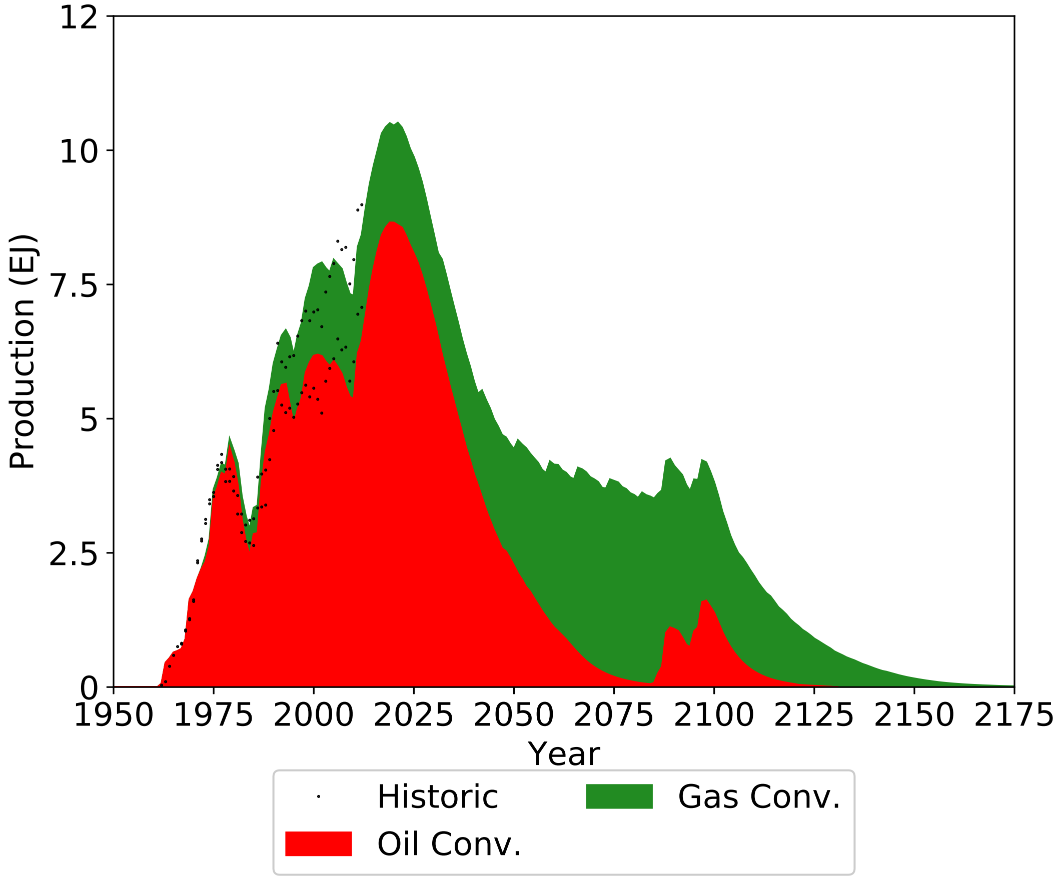

Figure 5.24: UAE projection by mineral type

Table 5.24: Peak years - Minerals

Name	URR	Peak Year	Peak Rate
Oil Conv.	476.21	2019	8.65
Gas Conv.	321.1	2074	3.65
**Total**	**797.31**	**2021**	**10.51**

5.13 Yemen

5.13.1 All Projections

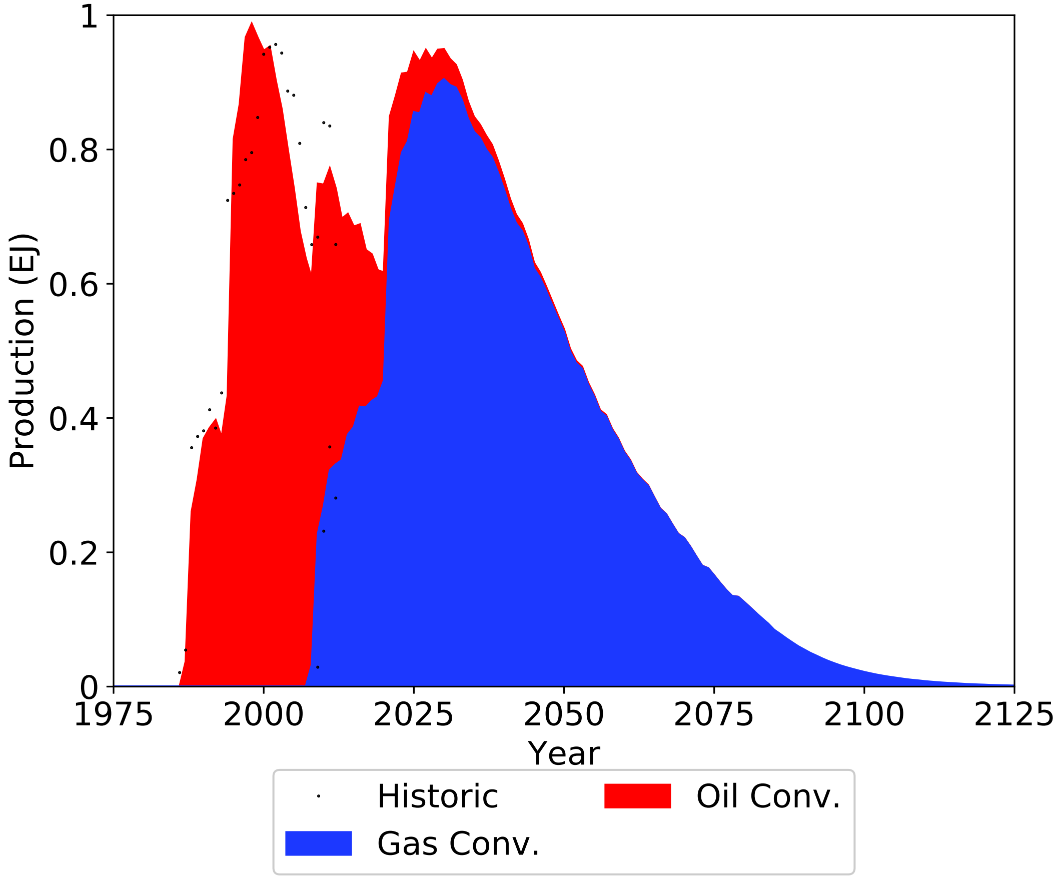

Figure 5.25: Yemen projections capped at 16

Table 5.25: Peak years - All

Name	URR	Peak Year	Peak Rate
Gas Conv.	37.2	2030	0.9
Oil Conv.	19.44	1998	0.99
**Total**	**56.64**	**1998**	**0.99**

5.13.2 By Mineral

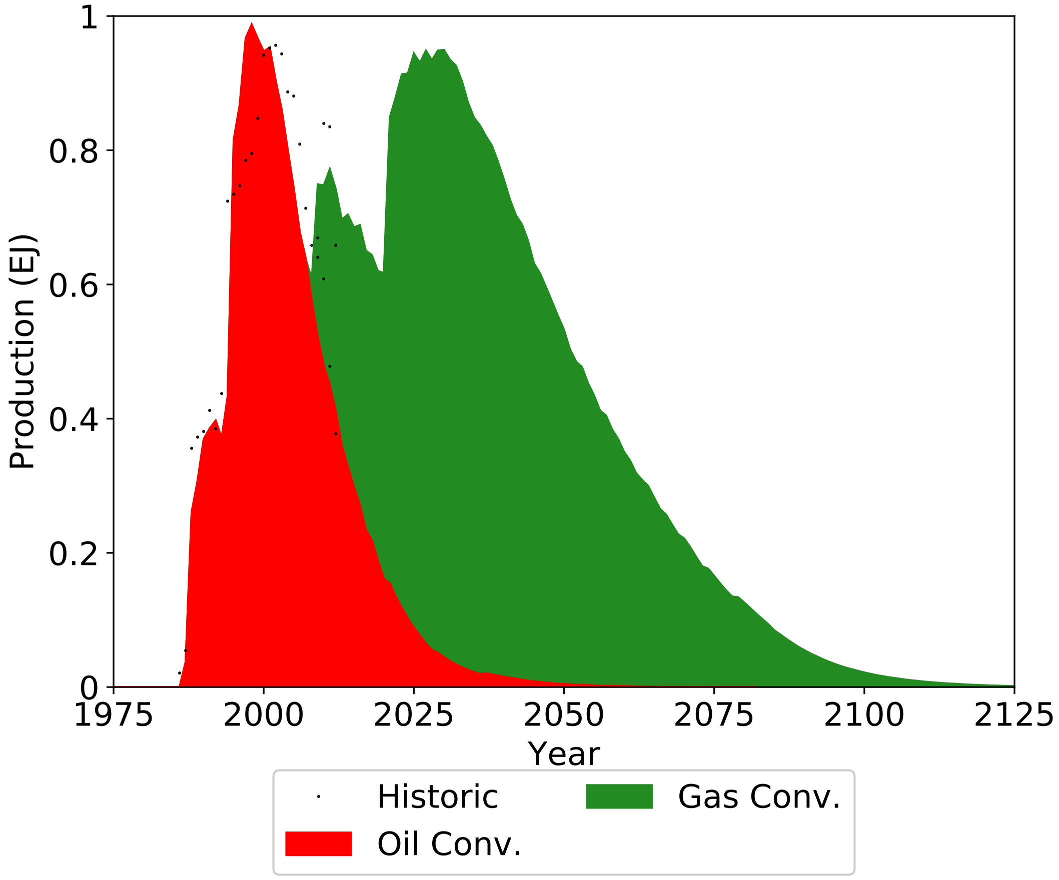

Figure 5.26: Yemen projection by mineral type

Table 5.26: Peak years - Minerals

Name	URR	Peak Year	Peak Rate
Oil Conv.	19.44	1998	0.99
Gas Conv.	37.2	2030	0.9
**Total**	**56.64**	**1998**	**0.99**

5.14 Total

5.14.1 By country

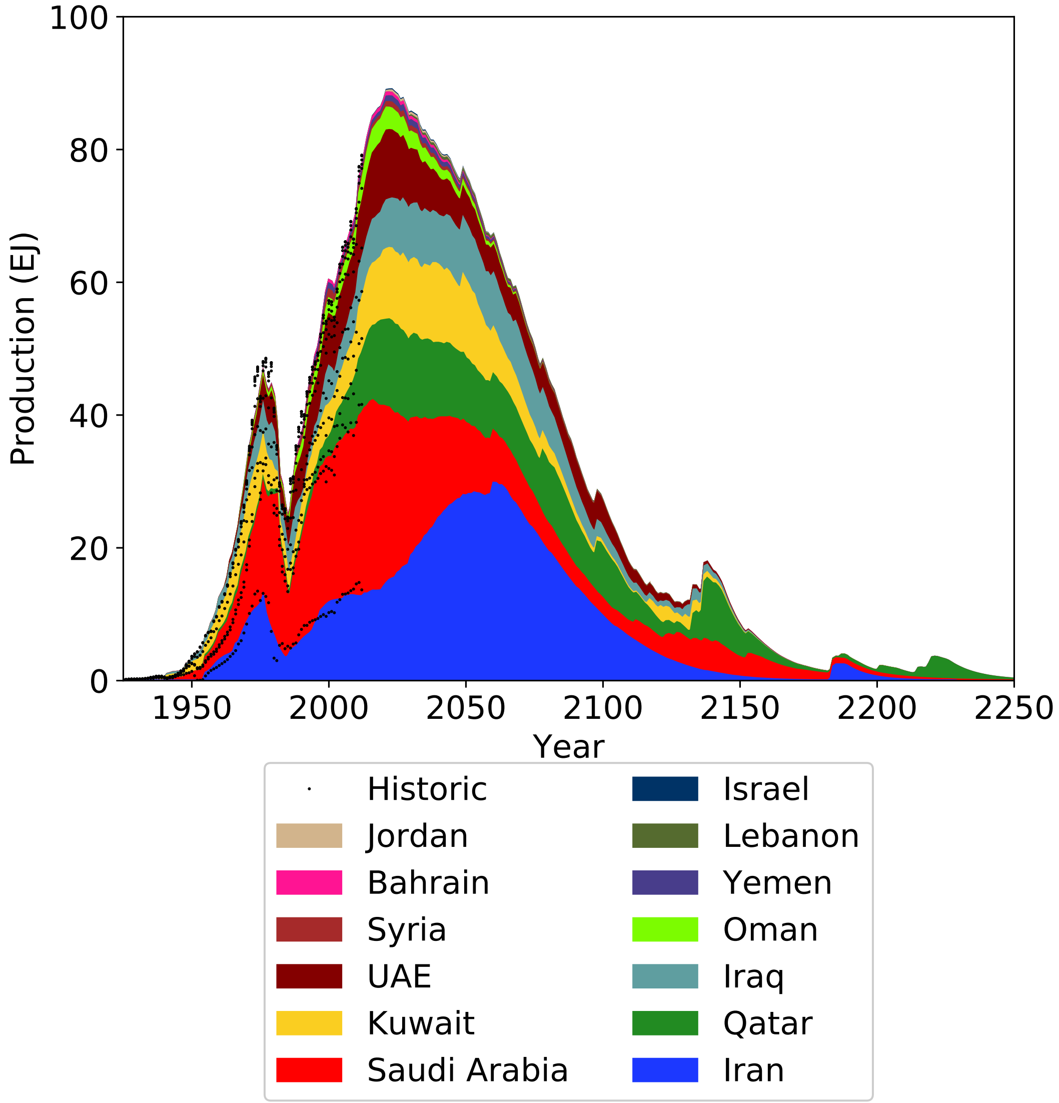

Figure 5.27: Middle East projections by country

5.14.2 By mineral

Table 5.27: Peak years - All

**Name**	**URR**	**Peak Year**	**Peak Rate**
Iran	2502.23	2060	29.82
Saudi Arabia	2197.07	2016	28.67
Qatar	1334.55	2023	13.52
Kuwait	939.76	2038	12.08
Iraq	857.9	2049	8.57
UAE	797.31	2021	10.51
Oman	172.7	2017	3.58
Syria	66.48	1998	1.4
Yemen	56.64	1998	0.99
Bahrain	42.14	2014	0.63
Lebanon	27.81	2046	0.45
Jordan	10.43	2026	0.39
Israel	8.89	1970	0.22
**Total**	**9013.91**	**2023**	**89.08**

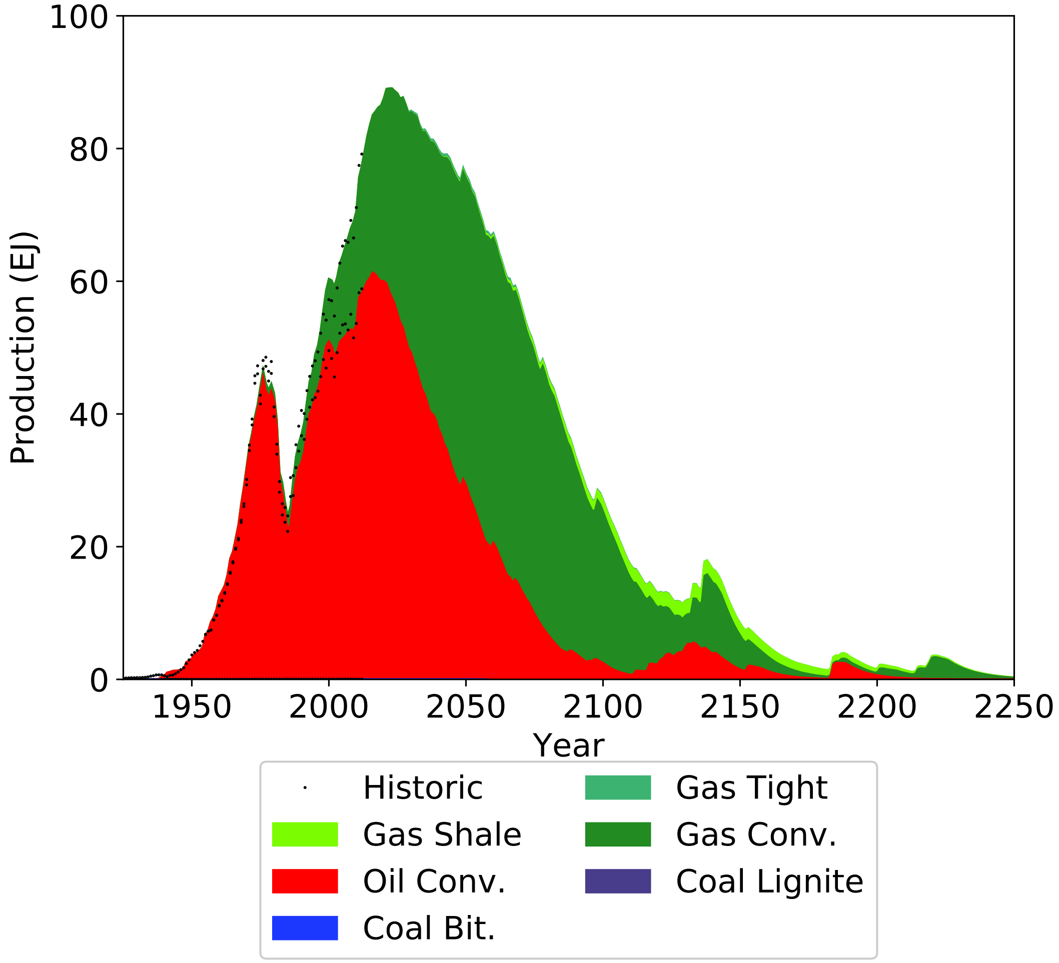

Figure 5.28: Middle East projection by mineral type

Table 5.28: Peak years - Minerals

**Name**	**URR**	**Peak Year**	**Peak Rate**
Coal Bit.	2.3	2007	0.05
Coal Lignite	–	1942	–
Oil Conv.	4605.8	2016	61.31
Gas Conv.	4168.0	2053	46.89
Gas Shale	210.0	2129	2.19
Gas Tight	27.81	2046	0.45
**Total**	**9013.91**	**2023**	**89.08**

Chapter 6

North America

6.1 Canada

6.1.1 All Projections

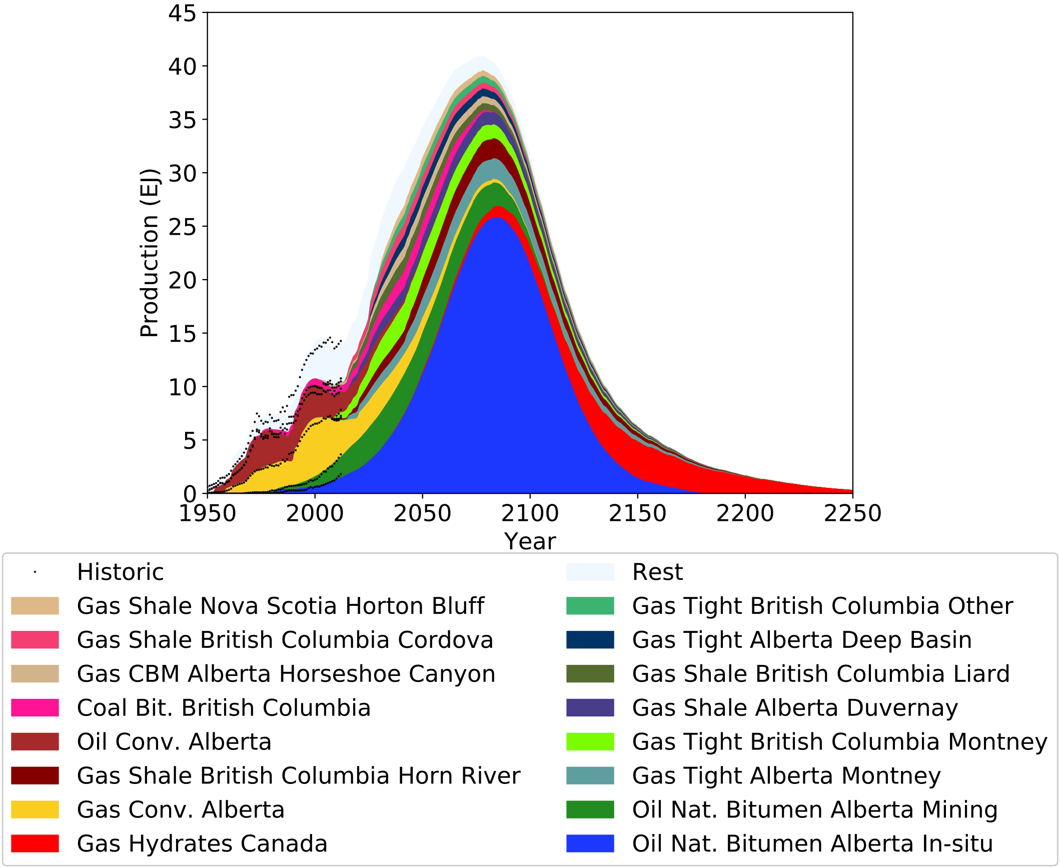

Figure 6.1: Canada projections capped at 16

Table 6.1: Peak years - All

Name	URR	Peak Year	Peak Rate
Oil Nat. Bitumen Alberta In-situ	1719.0	2084	25.77
Gas Hydrates Canada	356.0	2142	3.52
Oil Nat. Bitumen Alberta Mining	286.5	2043	3.72
Gas Conv. Alberta	270.34	2000	5.44
Gas Tight Alberta Montney	187.12	2060	2.12
Gas Shale British Columbia Horn River	178.85	2063	2.02
Gas Tight British Columbia Montney	175.74	2043	2.59
Oil Conv. Alberta	172.73	1976	3.3
Gas Shale Alberta Duvernay	118.65	2064	1.48
Coal Bit. British Columbia	94.22	2048	1.57
Gas Shale British Columbia Liard	82.95	2038	1.26
Gas CBM Alberta Horseshoe Canyon	72.93	2045	1.05
Gas Tight Alberta Deep Basin	72.45	2047	1.11
Gas Shale British Columbia Cordova	71.51	2039	1.1
Gas Tight British Columbia Other	61.95	2048	0.95
Gas Shale Nova Scotia Horton Bluff	51.45	2046	0.88
Oil Conv. Saskatchewan	46.99	2005	0.9
Gas Conv. British Columbia	44.77	2003	1.08
Gas Shale Quebec Utica	44.1	2043	0.69
Oil Tight Alberta	41.49	2039	1.13
Coal Sub-bit. Alberta	23.0	2008	0.52
Oil Kerogen Ontario	20.63	2075	0.4
Gas Shale Alberta Colorado	14.7	2046	0.25
Oil Conv. British Columbia	14.4	2018	0.28
Gas CBM Alberta Mannville	12.29	2040	0.21
Coal Bit. Nova Scotia	12.07	1929	0.15
Gas Tight British Columbia Jean Marie	11.55	2035	0.2
Gas Conv. Saskatchewan	11.19	2003	0.28
Oil Conv. East Coast Offshore	10.78	2003	0.66
Coal Bit. Alberta	9.23	1990	0.25
Oil Tight Saskatchewan	8.68	2019	0.33
Coal Lignite Saskatchewan	6.75	1997	0.13
Gas CBM British Columbia	5.71	2039	0.11
Gas Conv. East Coast Offshore	3.01	2002	0.17
Oil Kerogen Nova Scotia	2.93	2062	0.08
Gas CBM Nova Scotia	2.86	2037	0.06
Gas Conv. Ontario	2.03	2024	0.02
Oil Tight Manitoba	2.0	2015	0.1
Oil Conv. Northwest Territories	1.91	1988	0.07
Oil Conv. Manitoba	1.81	2007	0.05
Gas Conv. Northwest Territories	1.1	2000	0.04
Oil Kerogen Manitoba	1.07	2046	0.03
Oil Kerogen Saskatchewan	1.07	2046	0.03
Coal Bit. New Brunswick	0.94	1971	0.02
Gas CBM Saskatchewan	0.71	2029	0.02
Oil Conv. Ontario	0.53	1990	0.01
Oil Kerogen New Brunswick	0.49	2037	0.02
Oil Conv. Nova Scotia Offshore	0.24	2004	0.02
Gas Conv. New Brunswick	0.04	1918	–

Table 6.1: Peak years - All – Continued

**Name**	**URR**	**Peak Year**	**Peak Rate**
Oil Extra Heavy Saskatchewan	0.02	2029	0.01
Oil Extra Heavy Alberta	0.02	2029	0.01
Coal Bit. Yukon	0.01	1955	–
Oil Conv. New Brunswick	0.01	1934	–
Gas Conv. Quebec	–	1970	–
Coal Lignite Manitoba	–	1935	–
Gas Conv. Manitoba	–	1929	–
**Total**	**4333.53**	**2078**	**40.9**

6.1.2 By Mineral

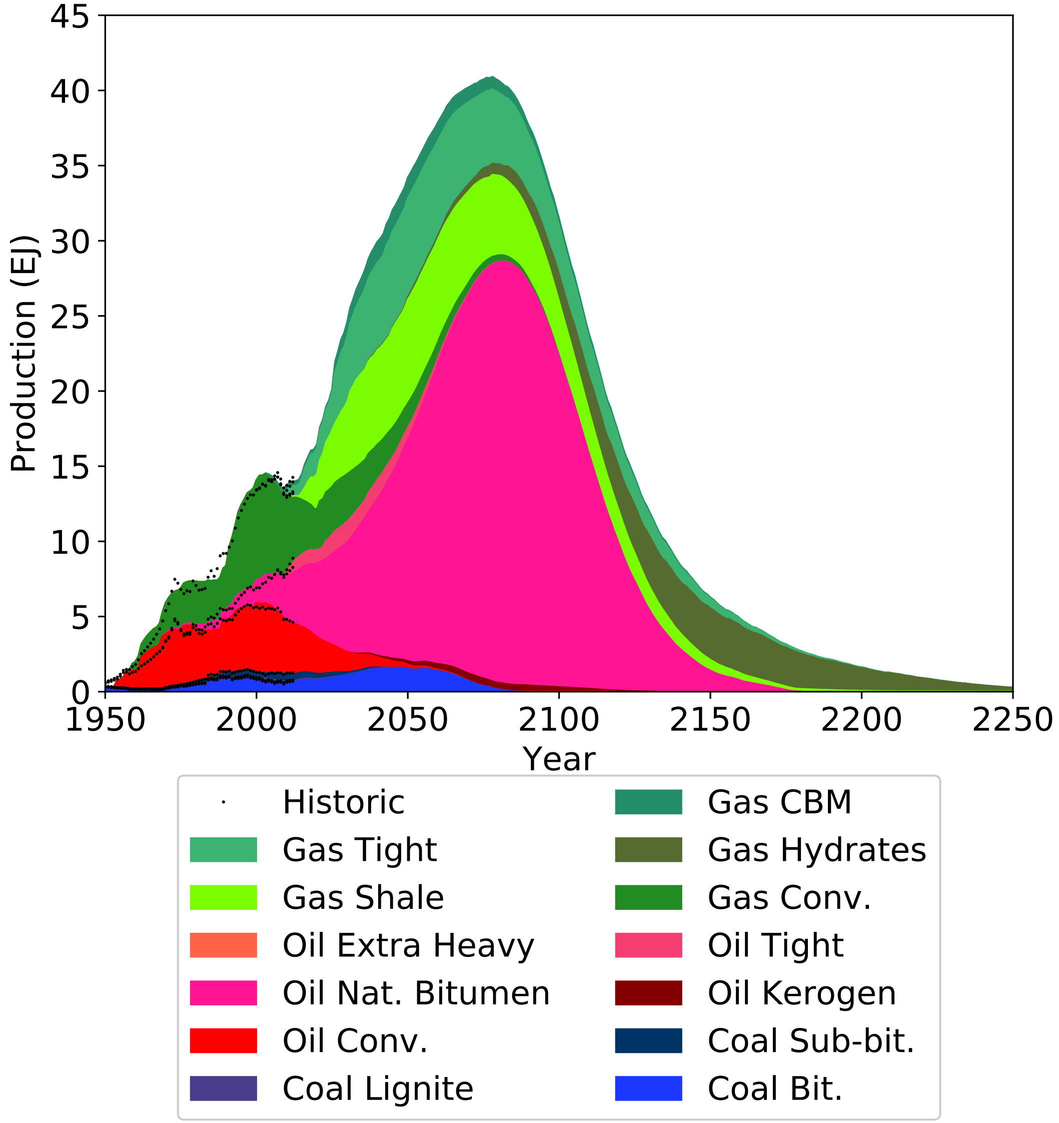

Figure 6.2: Canada projection by mineral type

6.1.3 Regional Projections

Table 6.2: Peak years - Minerals

**Name**	**URR**	**Peak Year**	**Peak Rate**
Coal Bit.	116.47	2048	1.57
Coal Lignite	6.75	1997	0.13
Coal Sub-bit.	23.0	2008	0.52
Oil Conv.	249.4	2003	4.62
Oil Kerogen	26.19	2073	0.5
Oil Nat. Bitumen	2005.5	2082	28.06
Oil Tight	52.17	2028	1.36
Oil Extra Heavy	0.04	2029	0.01
Gas Conv.	332.48	2001	6.88
Gas Shale	562.21	2052	6.97
Gas Hydrates	356.0	2142	3.52
Gas Tight	508.81	2051	6.57
Gas CBM	94.5	2045	1.42
**Total**	**4333.53**	**2078**	**40.9**

Alberta

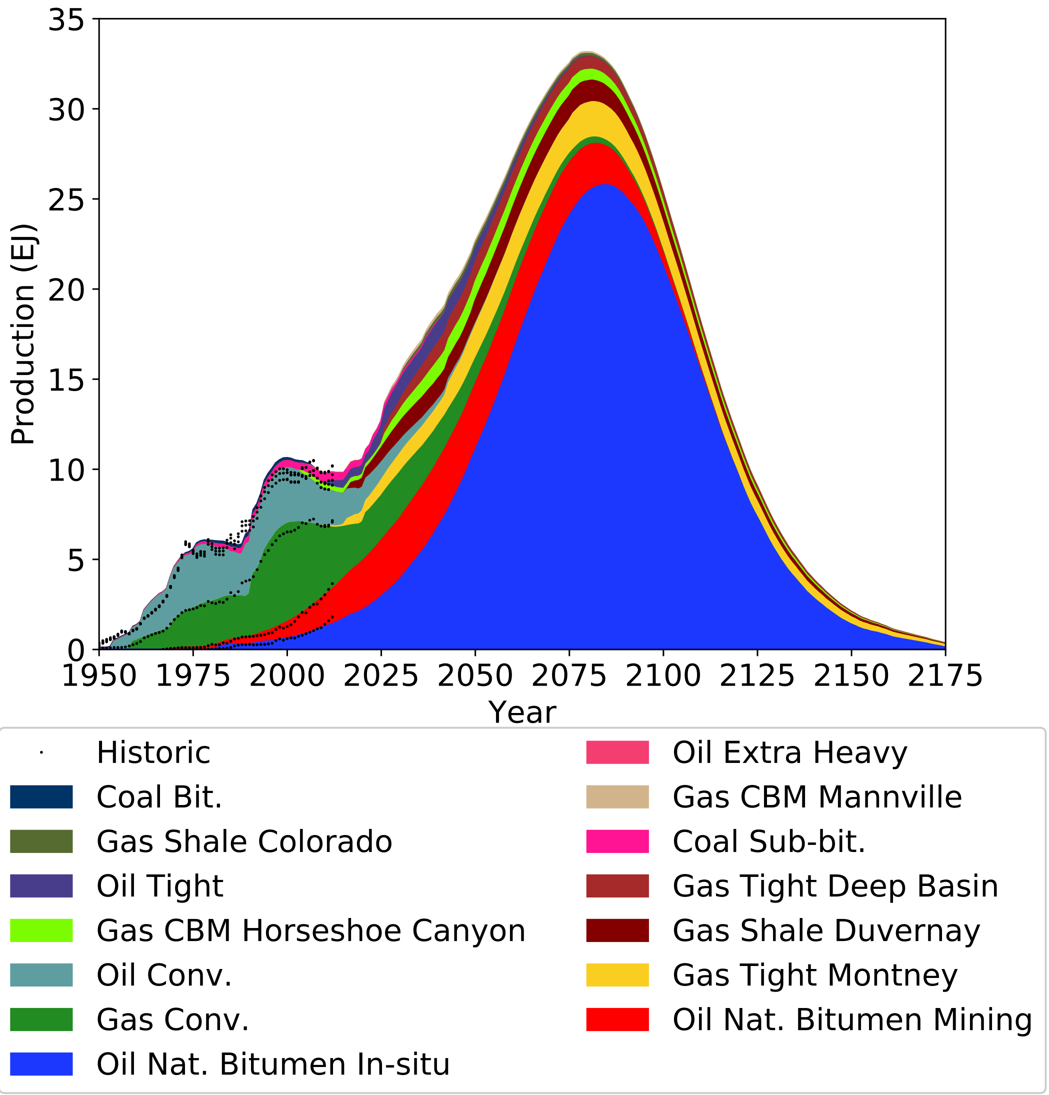

Figure 6.3: Canada - Alberta projections capped at 16

Table 6.3: Peak years - All

**Name**	**URR**	**Peak Year**	**Peak Rate**
Oil Nat. Bitumen Alberta In-situ	1719.0	2084	25.77
Oil Nat. Bitumen Alberta Mining	286.5	2043	3.72
Gas Conv. Alberta	270.34	2000	5.44
Gas Tight Alberta Montney	187.12	2060	2.12
Oil Conv. Alberta	172.73	1976	3.3
Gas Shale Alberta Duvernay	118.65	2064	1.48
Gas CBM Alberta Horseshoe Canyon	72.93	2045	1.05
Gas Tight Alberta Deep Basin	72.45	2047	1.11
Oil Tight Alberta	41.49	2039	1.13
Coal Sub-bit. Alberta	23.0	2008	0.52
Gas Shale Alberta Colorado	14.7	2046	0.25
Gas CBM Alberta Mannville	12.29	2040	0.21
Coal Bit. Alberta	9.23	1990	0.25
Oil Extra Heavy Alberta	0.02	2029	0.01
**Total**	**3000.45**	**2080**	**33.15**

Table 6.4: Peak years - Minerals

**Name**	**URR**	**Peak Year**	**Peak Rate**
Coal Bit.	9.23	1990	0.25
Coal Sub-bit.	23.0	2008	0.52
Oil Conv.	172.73	1976	3.3
Oil Nat. Bitumen	2005.5	2082	28.06
Oil Tight	41.49	2039	1.13
Oil Extra Heavy	0.02	2029	0.01
Gas Conv.	270.34	2000	5.44
Gas Shale	133.35	2062	1.69
Gas Tight	259.57	2056	3.14
Gas CBM	85.22	2045	1.25
**Total**	**3000.45**	**2080**	**33.15**

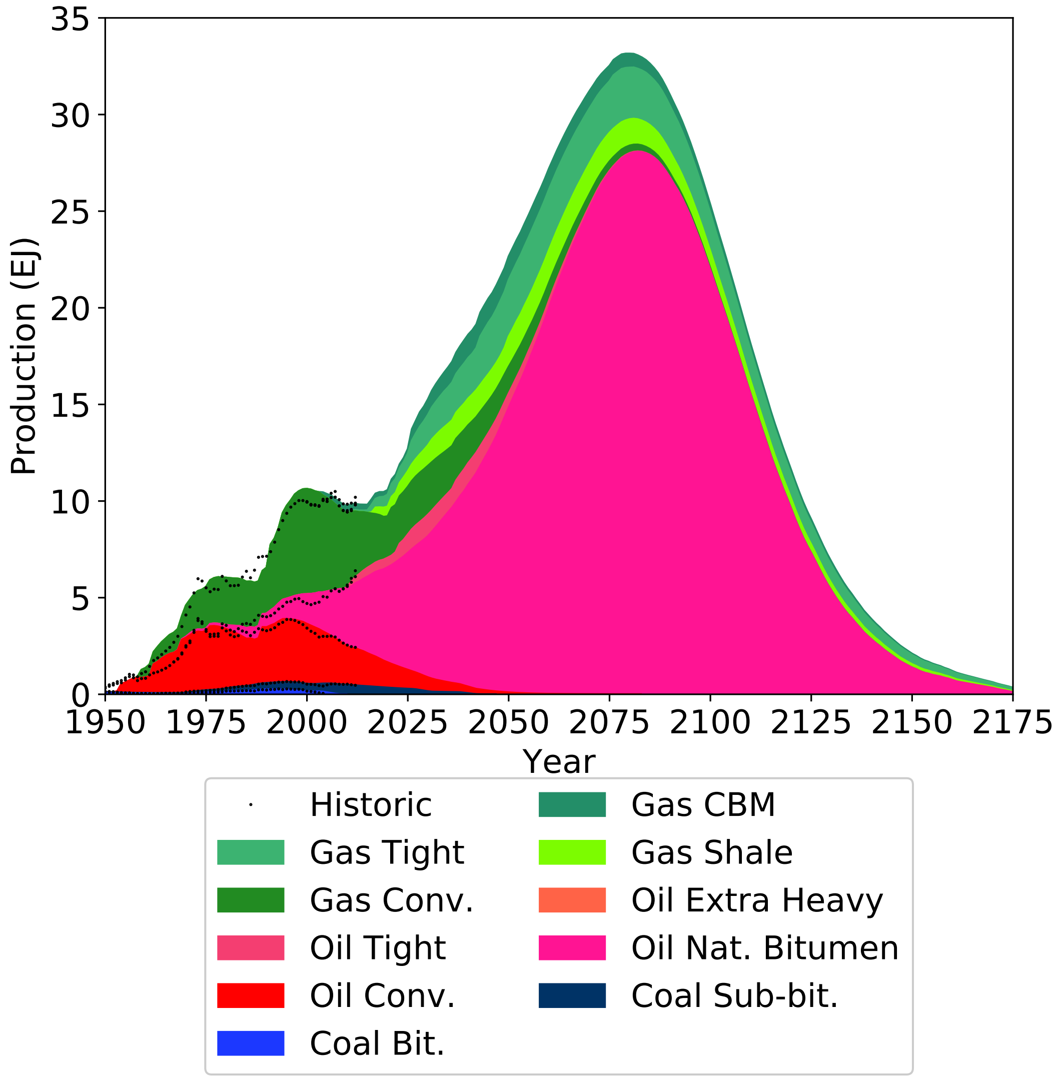

Figure 6.4: Canada - Alberta projection by mineral type

British Columbia

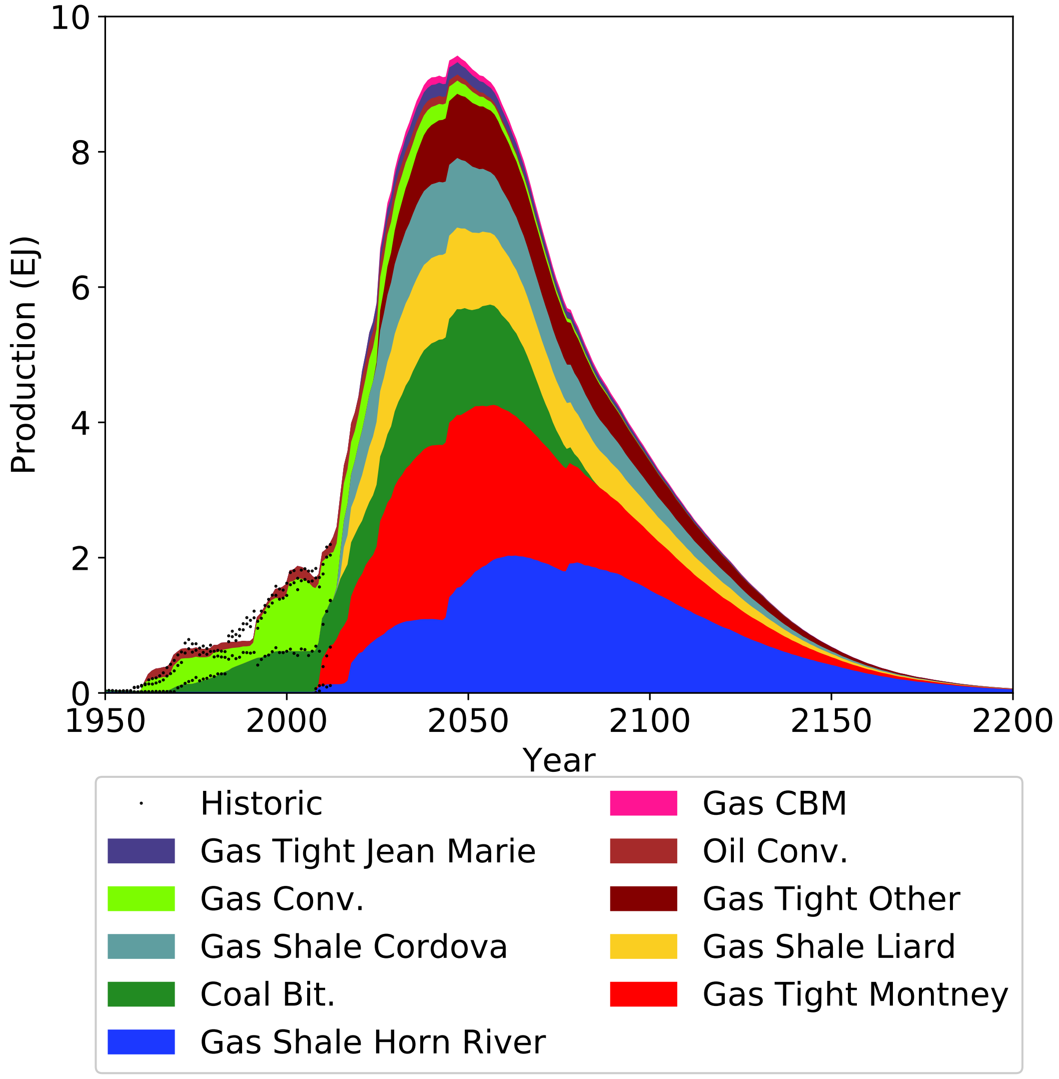

Figure 6.5: Canada - British Columbia projections capped at 16

Table 6.5: Peak years - All

Name	URR	Peak Year	Peak Rate
Gas Shale British Columbia Horn River	178.85	2063	2.02
Gas Tight British Columbia Montney	175.74	2043	2.59
Coal Bit. British Columbia	94.22	2048	1.57
Gas Shale British Columbia Liard	82.95	2038	1.26
Gas Shale British Columbia Cordova	71.51	2039	1.1
Gas Tight British Columbia Other	61.95	2048	0.95
Gas Conv. British Columbia	44.77	2003	1.08
Oil Conv. British Columbia	14.4	2018	0.28
Gas Tight British Columbia Jean Marie	11.55	2035	0.2
Gas CBM British Columbia	5.71	2039	0.11
**Total**	**741.65**	**2047**	**9.4**

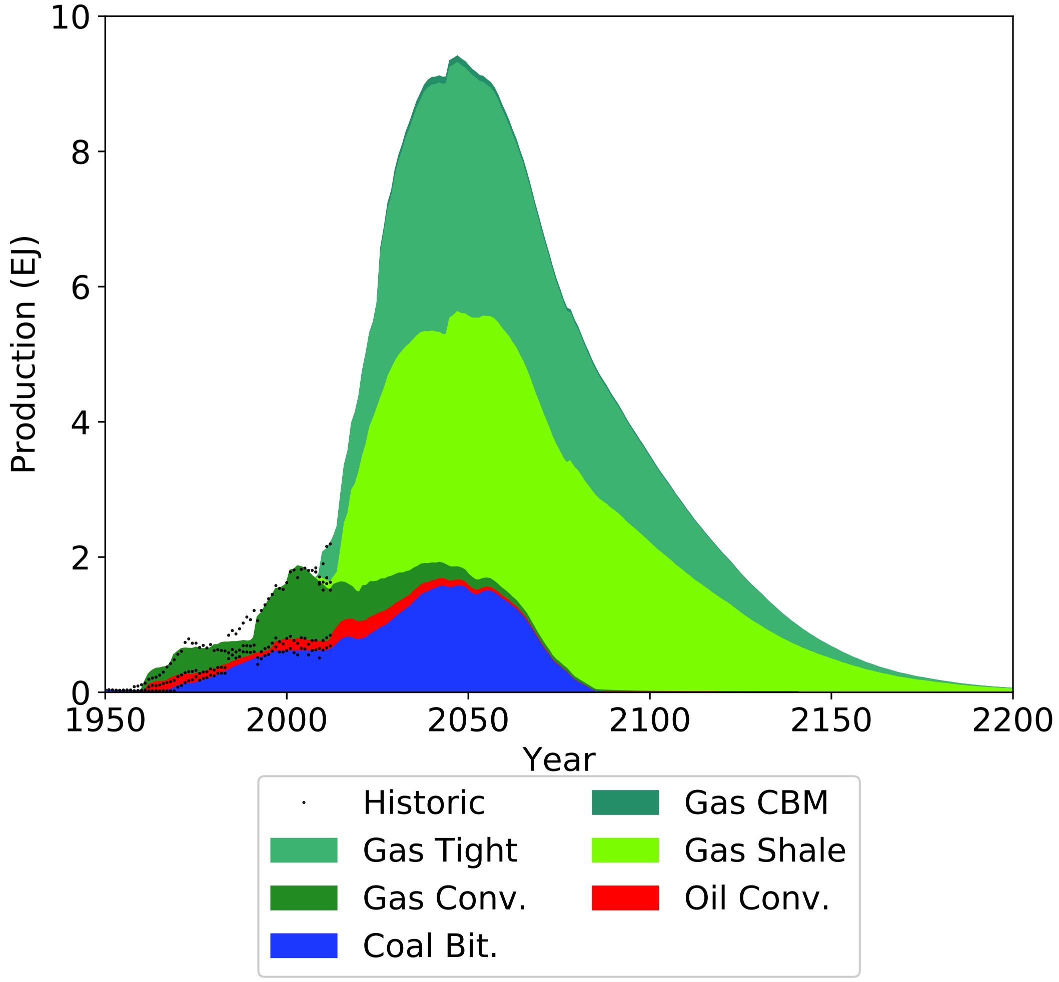

Figure 6.6: Canada - British Columbia projection by mineral type

Table 6.6: Peak years - Minerals

**Name**	**URR**	**Peak Year**	**Peak Rate**
Coal Bit.	94.22	2048	1.57
Oil Conv.	14.4	2018	0.28
Gas Conv.	44.77	2003	1.08
Gas Shale	333.31	2057	3.88
Gas Tight	249.24	2044	3.71
Gas CBM	5.71	2039	0.11
**Total**	**741.65**	**2047**	**9.4**

Canada

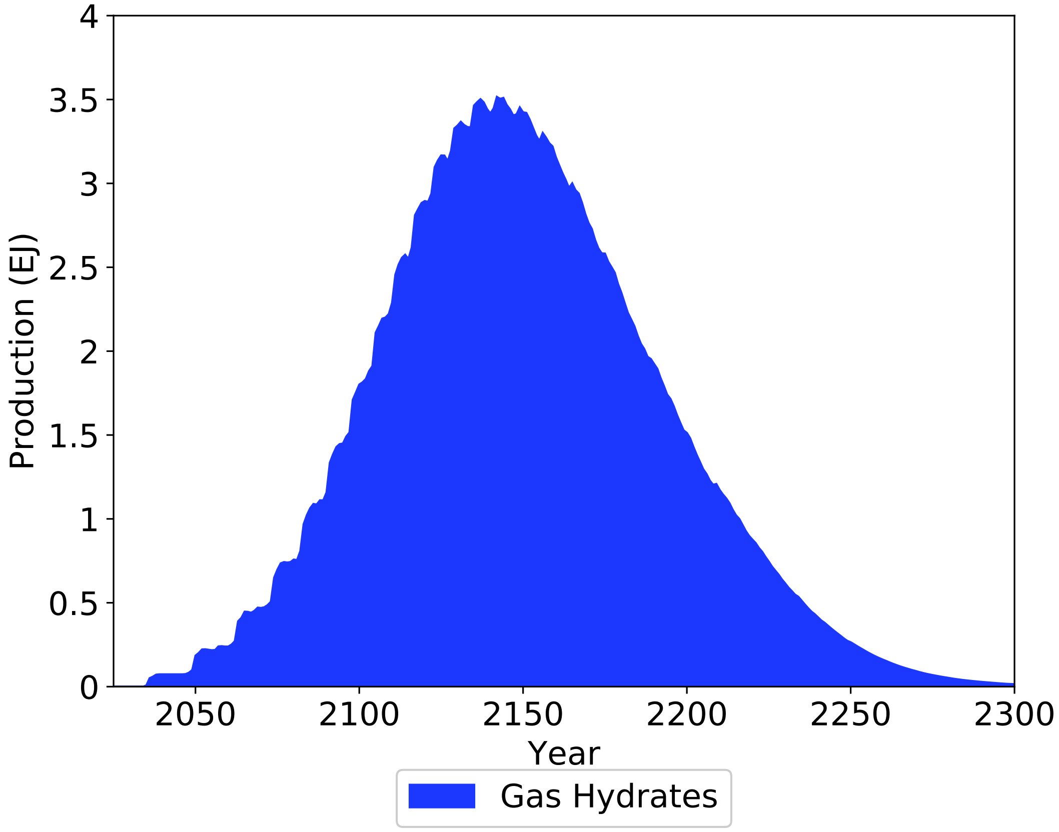

Figure 6.7: Canada - Canada projections capped at 16

Table 6.7: Peak years - All

Name	URR	Peak Year	Peak Rate
Gas Hydrates Canada	356.0	2142	3.52
**Total**	**356.0**	**2142**	**3.52**

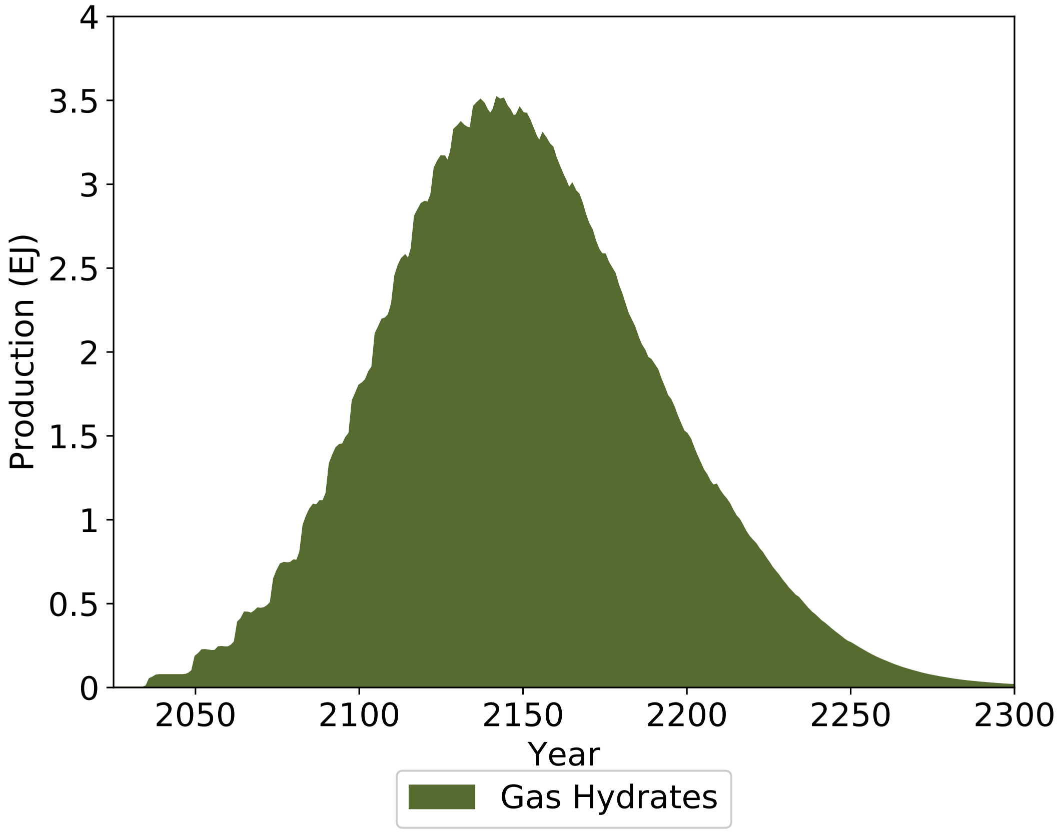

Figure 6.8: Canada - Canada projection by mineral type

Table 6.8: Peak years - Minerals

Name	URR	Peak Year	Peak Rate
Gas Hydrates	356.0	2142	3.52
**Total**	**356.0**	**2142**	**3.52**

East Coast Offshore

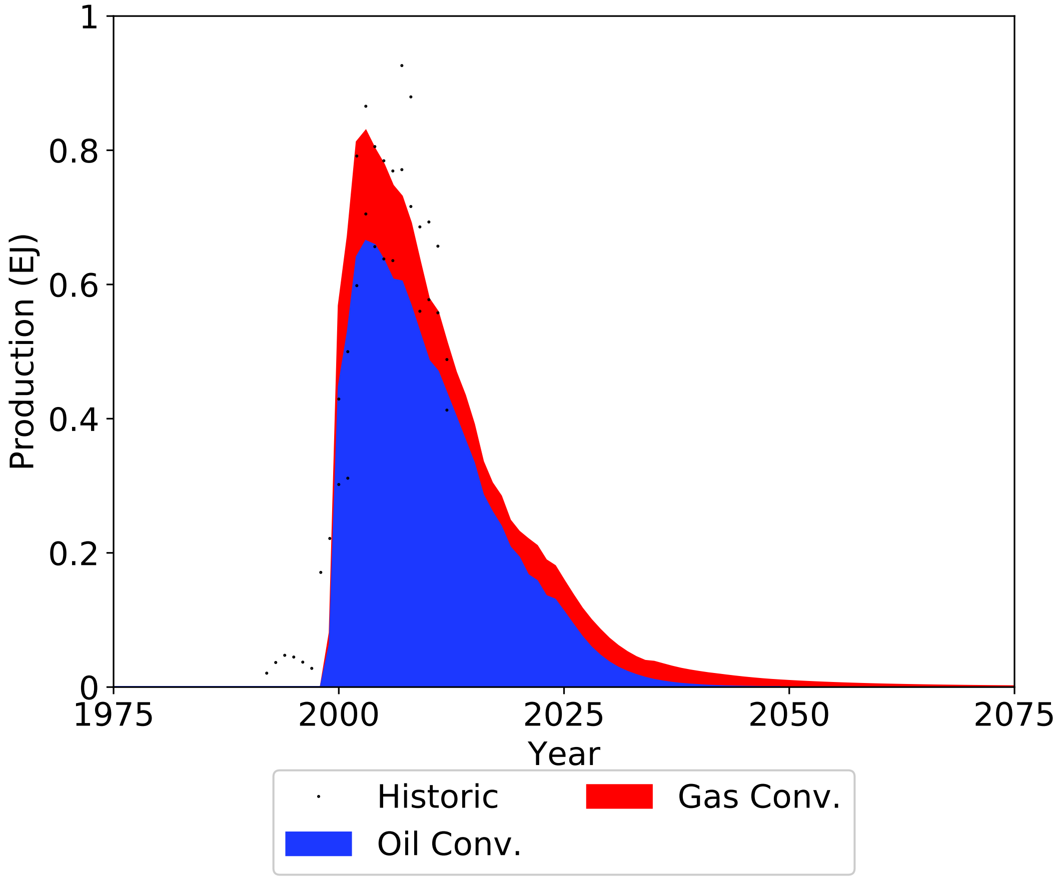

Figure 6.9: Canada - East Coast Offshore projections capped at 16

Table 6.9: Peak years - All

**Name**	**URR**	**Peak Year**	**Peak Rate**
Oil Conv. East Coast Offshore	10.78	2003	0.66
Gas Conv. East Coast Offshore	3.01	2002	0.17
**Total**	**13.79**	**2003**	**0.83**

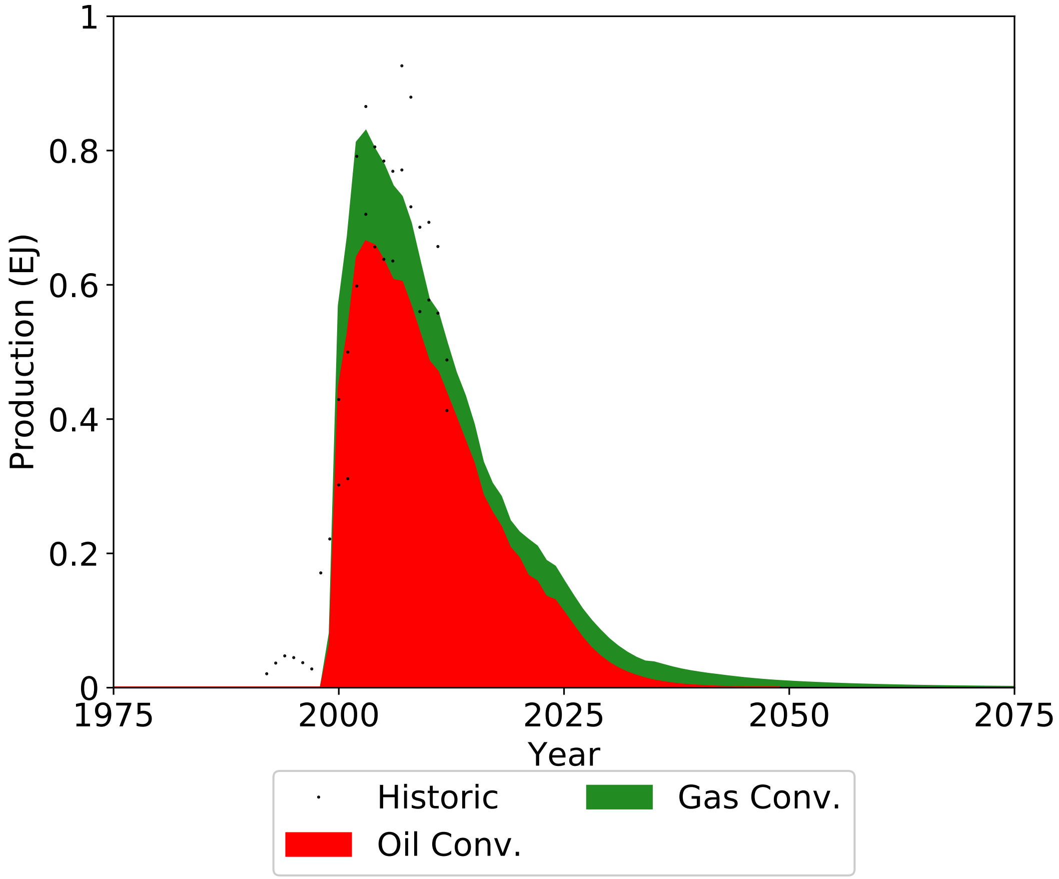

Figure 6.10: Canada - East Coast Offshore projection by mineral type

Table 6.10: Peak years - Minerals

Name	URR	Peak Year	Peak Rate
Oil Conv.	10.78	2003	0.66
Gas Conv.	3.01	2002	0.17
**Total**	**13.79**	**2003**	**0.83**

Manitoba

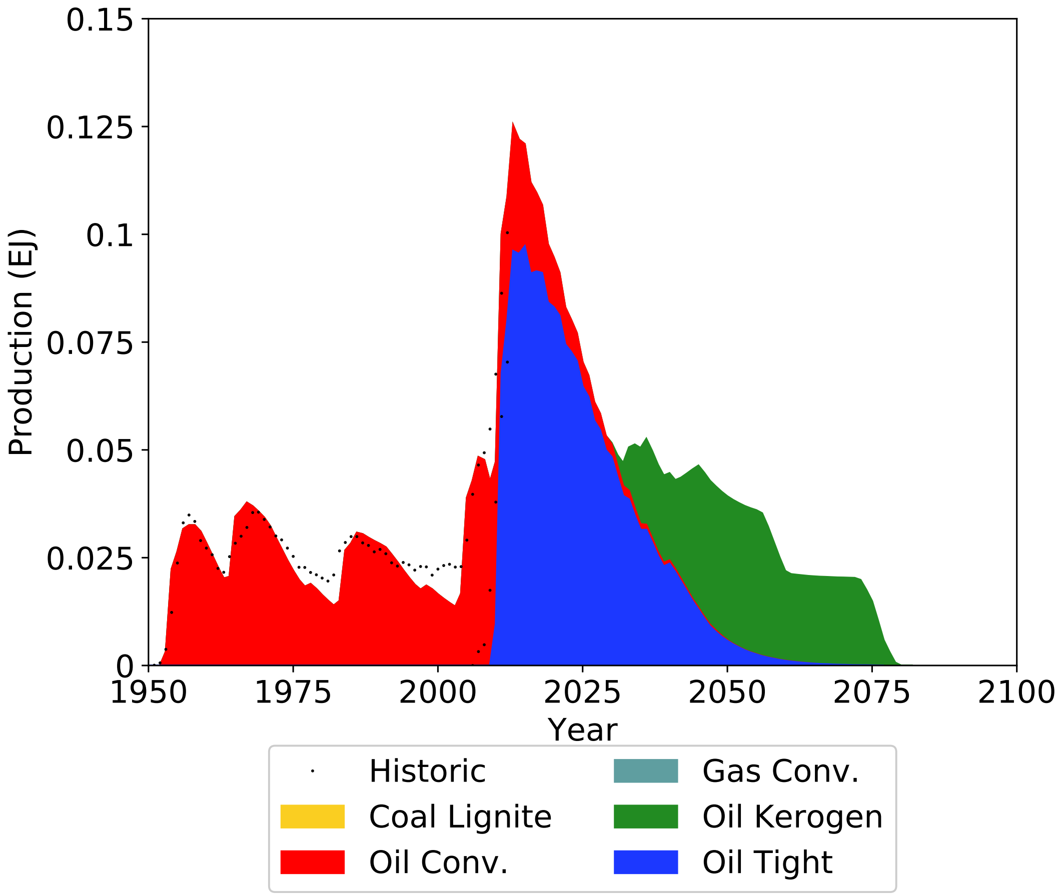

Figure 6.11: Canada - Manitoba projections capped at 16

Table 6.11: Peak years - All

Name	URR	Peak Year	Peak Rate
Oil Tight Manitoba	2.0	2015	0.1
Oil Conv. Manitoba	1.81	2007	0.05
Oil Kerogen Manitoba	1.07	2046	0.03
Coal Lignite Manitoba	–	1935	–
Gas Conv. Manitoba	–	1929	–
**Total**	**4.88**	**2013**	**0.13**

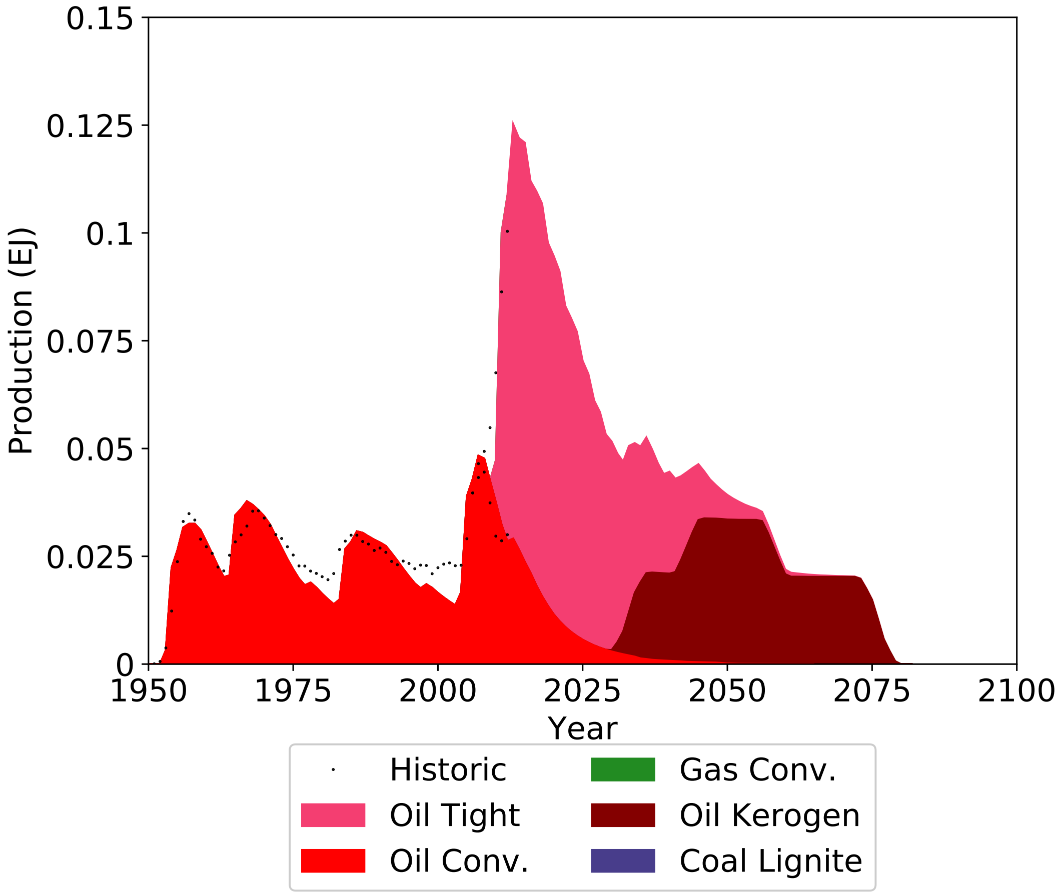

Figure 6.12: Canada - Manitoba projection by mineral type

Table 6.12: Peak years - Minerals

Name	URR	Peak Year	Peak Rate
Coal Lignite	–	1935	–
Oil Conv.	1.81	2007	0.05
Oil Kerogen	1.07	2046	0.03
Oil Tight	2.0	2015	0.1
Gas Conv.	–	1929	–
**Total**	**4.88**	**2013**	**0.13**

New Brunswick

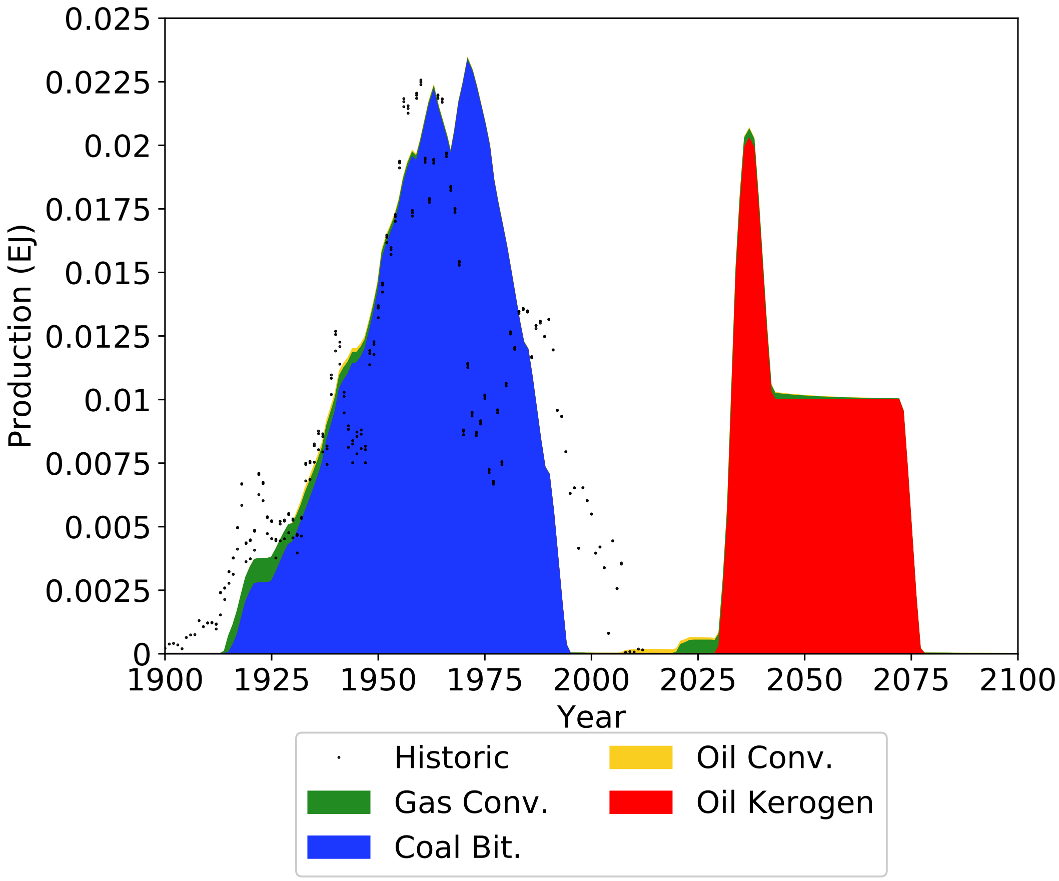

Figure 6.13: Canada - New Brunswick projections capped at 16

Table 6.13: Peak years - All

Name	URR	Peak Year	Peak Rate
Coal Bit. New Brunswick	0.94	1971	0.02
Oil Kerogen New Brunswick	0.49	2037	0.02
Gas Conv. New Brunswick	0.04	1918	–
Oil Conv. New Brunswick	0.01	1934	–
**Total**	**1.48**	**1971**	**0.02**

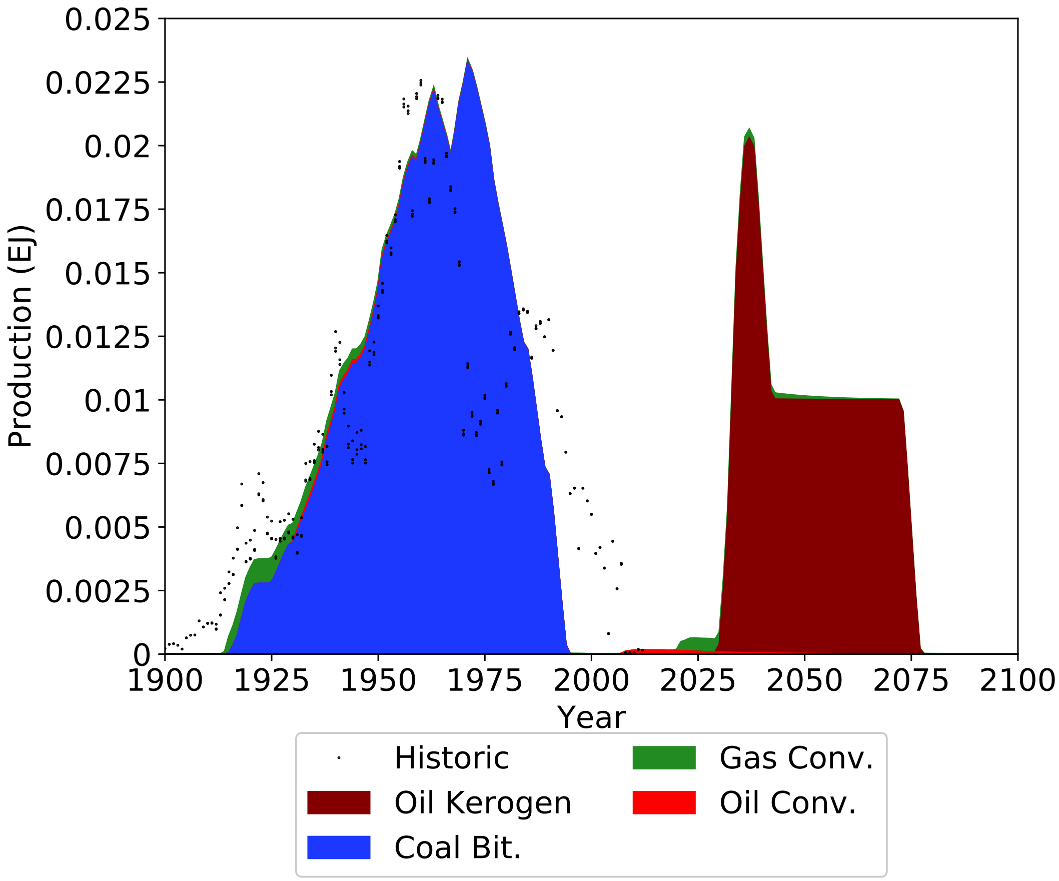

Figure 6.14: Canada - New Brunswick projection by mineral type

Table 6.14: Peak years - Minerals

Name	URR	Peak Year	Peak Rate
Coal Bit.	0.94	1971	0.02
Oil Conv.	0.01	1934	–
Oil Kerogen	0.49	2037	0.02
Gas Conv.	0.04	1918	–
**Total**	**1.48**	**1971**	**0.02**

Northwest Territories

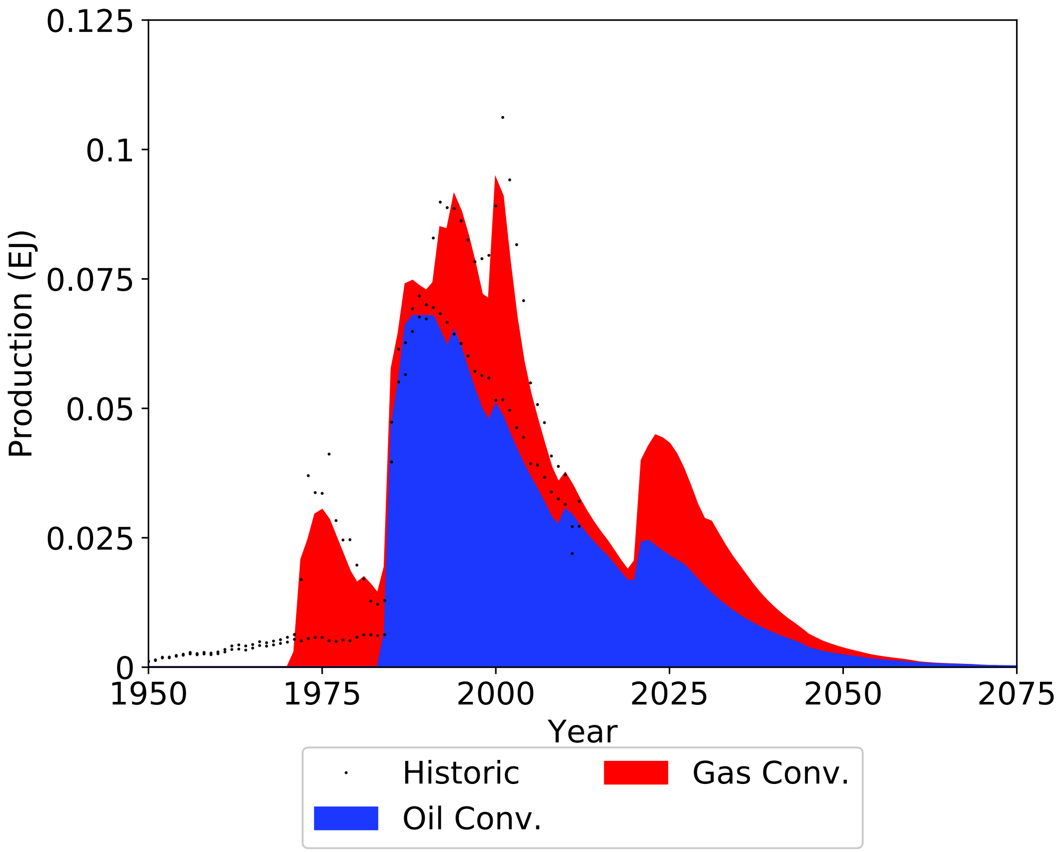

Figure 6.15: Canada - Northwest Territories projections capped at 16

Table 6.15: Peak years - All

Name	URR	Peak Year	Peak Rate
Oil Conv. Northwest Territories	1.91	1988	0.07
Gas Conv. Northwest Territories	1.1	2000	0.04
**Total**	**3.01**	**2000**	**0.09**

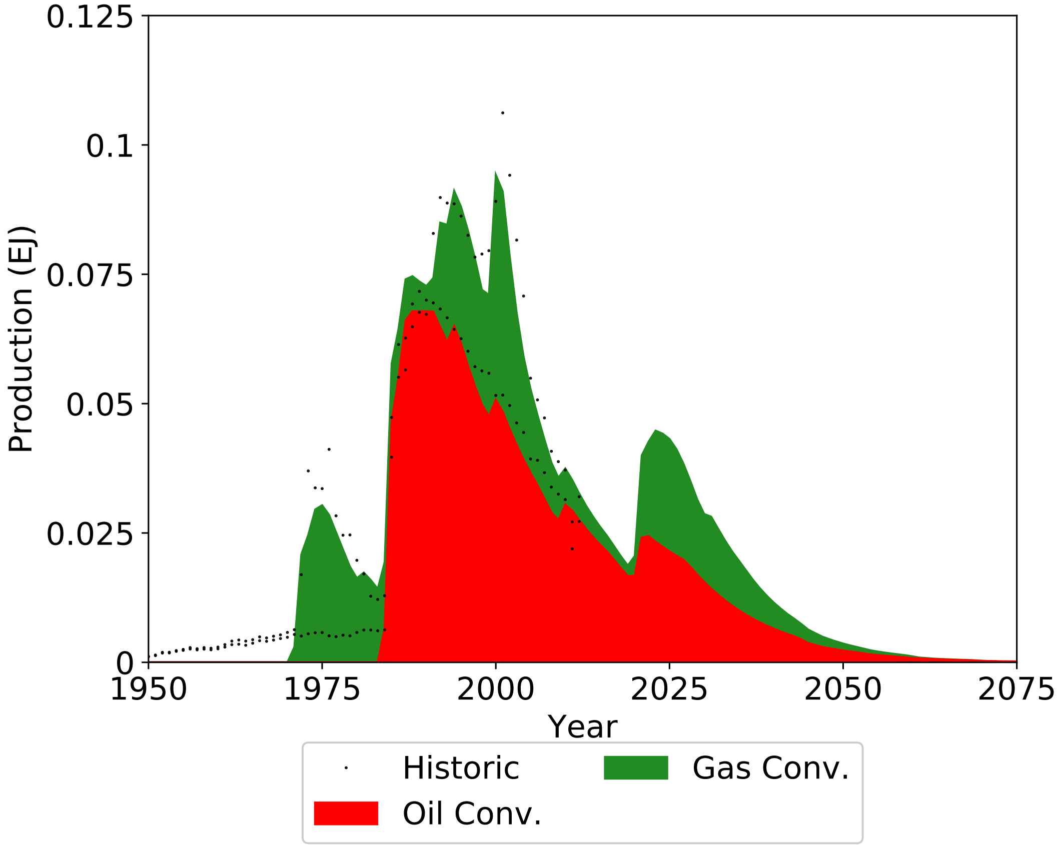

Figure 6.16: Canada - Northwest Territories projection by mineral type

Table 6.16: Peak years - Minerals

**Name**	**URR**	**Peak Year**	**Peak Rate**
Oil Conv.	1.91	1988	0.07
Gas Conv.	1.1	2000	0.04
**Total**	**3.01**	**2000**	**0.09**

Nova Scotia

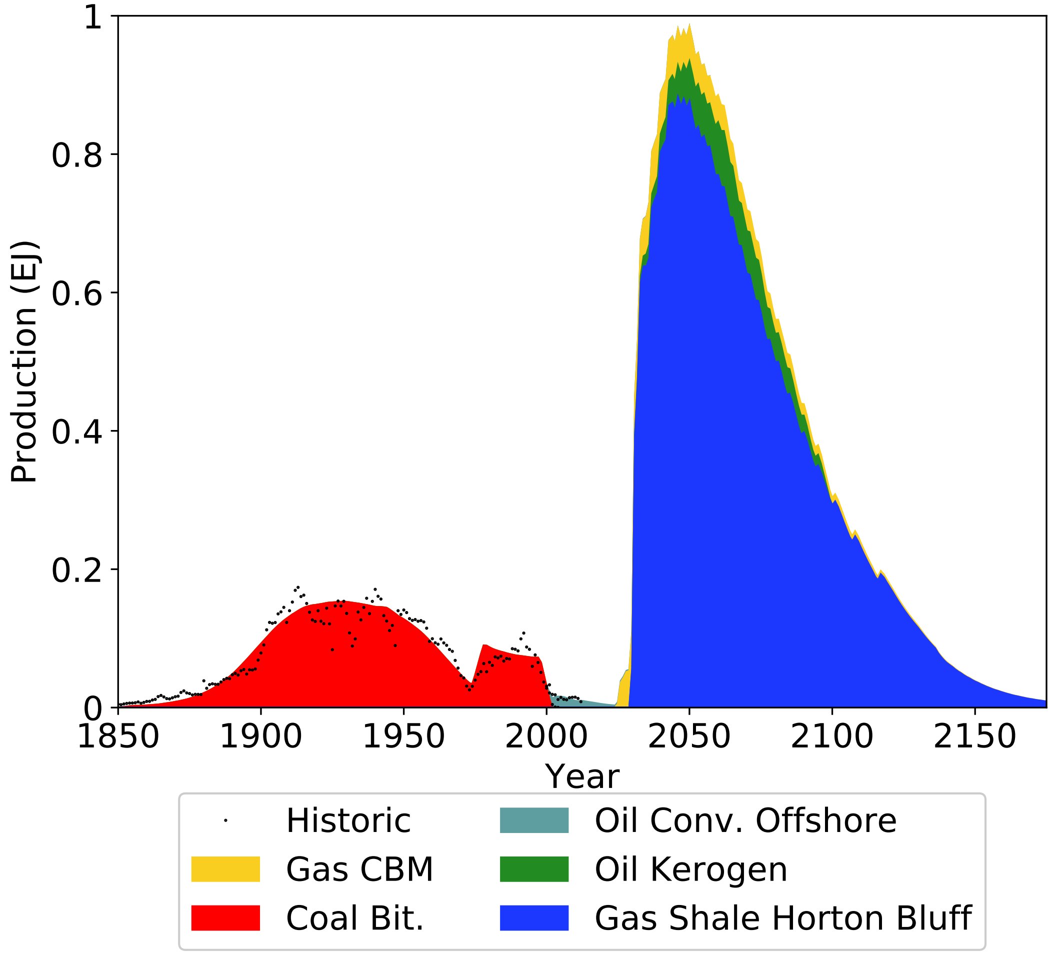

Figure 6.17: Canada - Nova Scotia projections capped at 16

Table 6.17: Peak years - All

Name	URR	Peak Year	Peak Rate
Gas Shale Nova Scotia Horton Bluff	51.45	2046	0.88
Coal Bit. Nova Scotia	12.07	1929	0.15
Oil Kerogen Nova Scotia	2.93	2062	0.08
Gas CBM Nova Scotia	2.86	2037	0.06
Oil Conv. Nova Scotia Offshore	0.24	2004	0.02
**Total**	**69.55**	**2050**	**0.98**

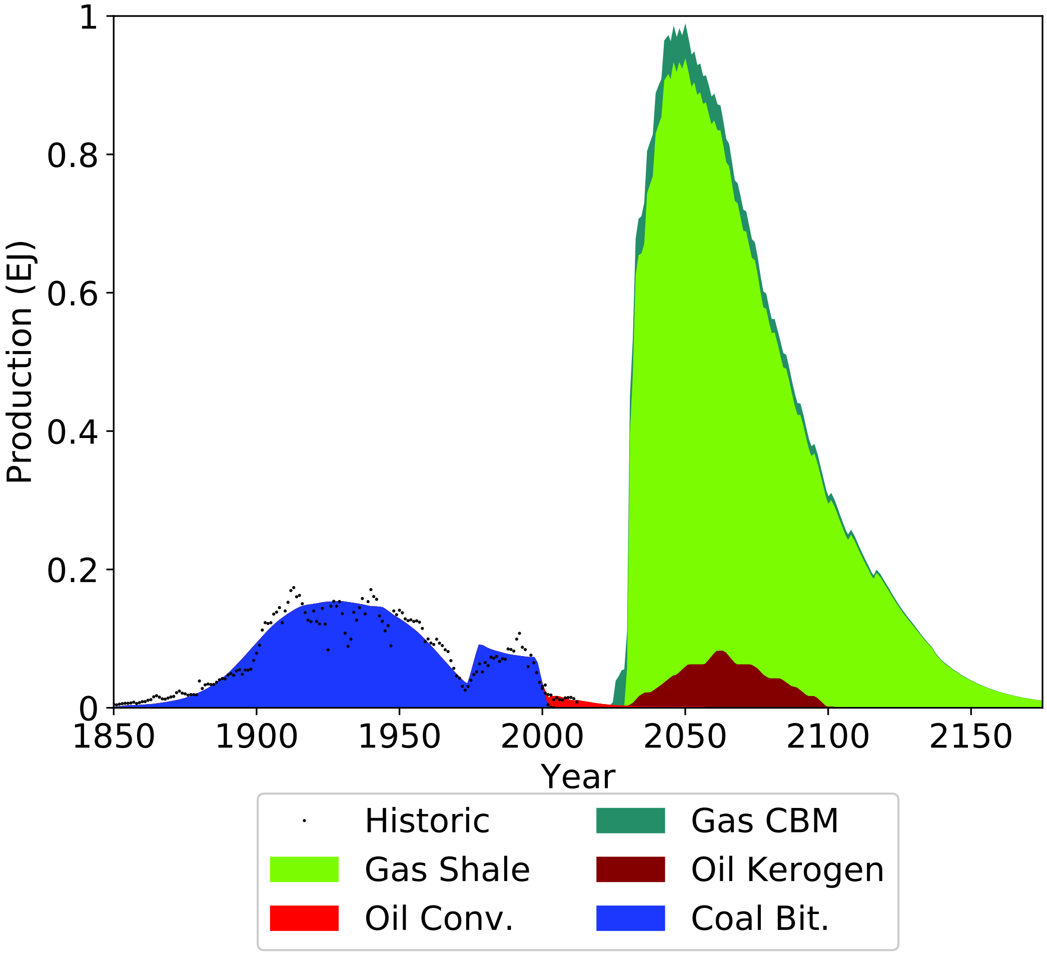

Figure 6.18: Canada - Nova Scotia projection by mineral type

Table 6.18: Peak years - Minerals

**Name**	**URR**	**Peak Year**	**Peak Rate**
Coal Bit.	12.07	1929	0.15
Oil Conv.	0.24	2004	0.02
Oil Kerogen	2.93	2062	0.08
Gas Shale	51.45	2046	0.88
Gas CBM	2.86	2037	0.06
**Total**	**69.55**	**2050**	**0.98**

Ontario

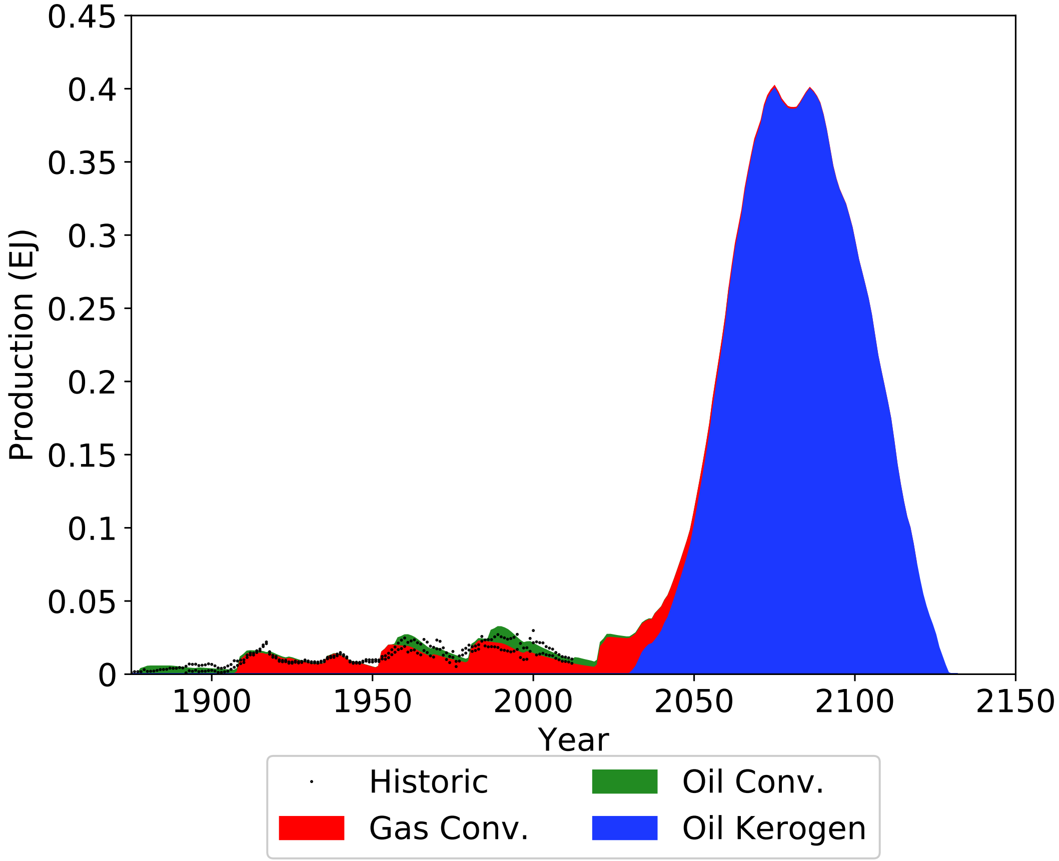

Figure 6.19: Canada - Ontario projections capped at 16

Table 6.19: Peak years - All

Name	URR	Peak Year	Peak Rate
Oil Kerogen Ontario	20.63	2075	0.4
Gas Conv. Ontario	2.03	2024	0.02
Oil Conv. Ontario	0.53	1990	0.01
**Total**	**23.19**	**2075**	**0.4**

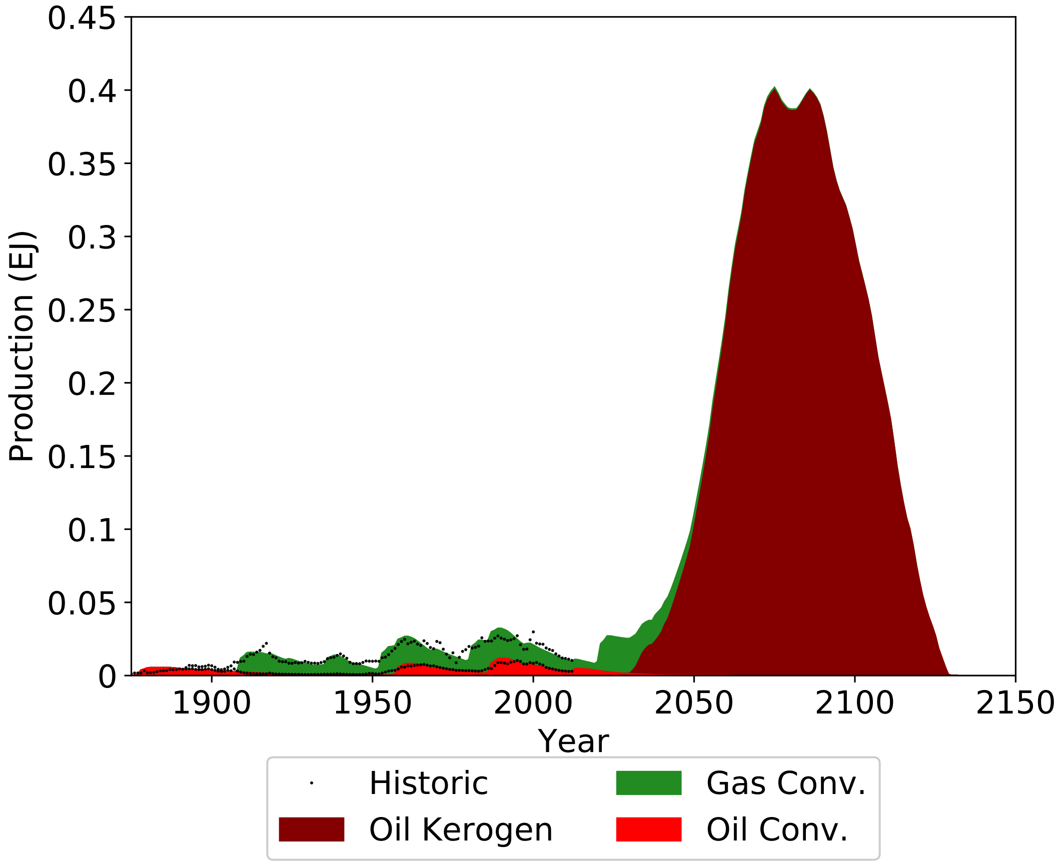

Figure 6.20: Canada - Ontario projection by mineral type

Table 6.20: Peak years - Minerals

Name	URR	Peak Year	Peak Rate
Oil Conv.	0.53	1990	0.01
Oil Kerogen	20.63	2075	0.4
Gas Conv.	2.03	2024	0.02
**Total**	**23.19**	**2075**	**0.4**

Quebec

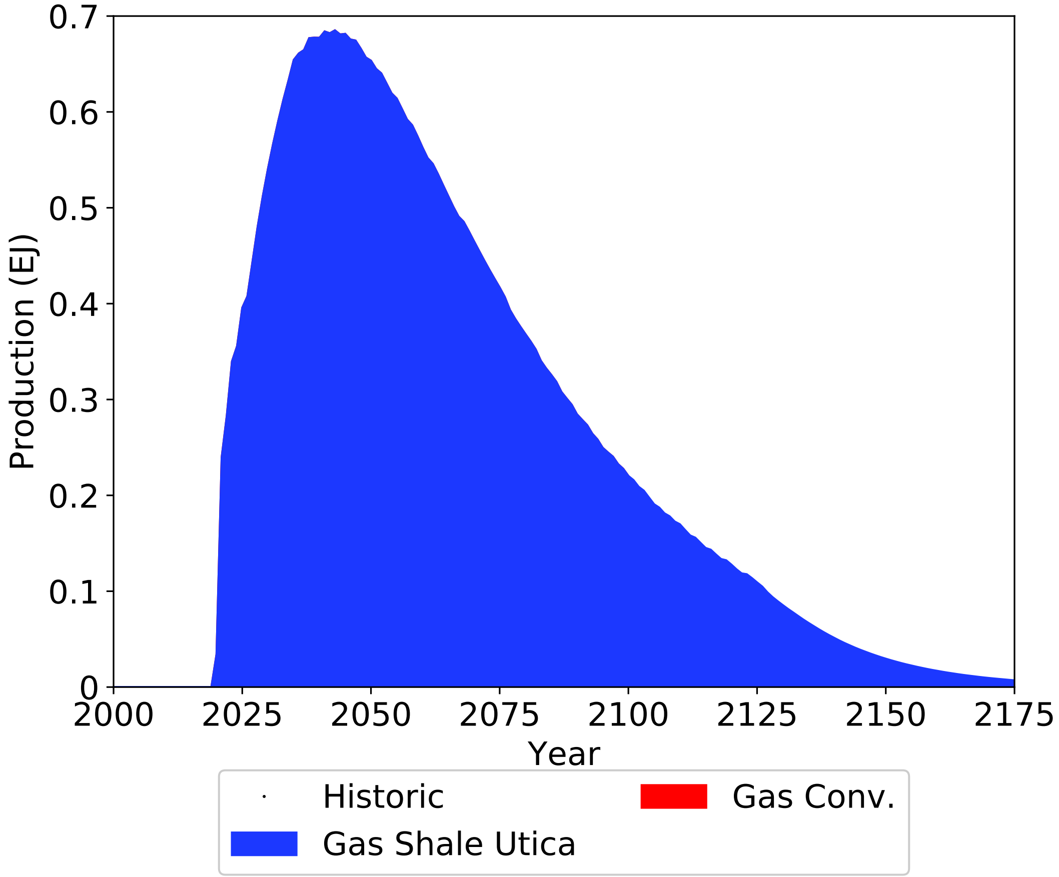

Figure 6.21: Canada - Quebec projections capped at 16

Table 6.21: Peak years - All

Name	URR	Peak Year	Peak Rate
Gas Shale Quebec Utica	44.1	2043	0.69
Gas Conv. Quebec	–	1970	–
**Total**	**44.1**	**2043**	**0.69**

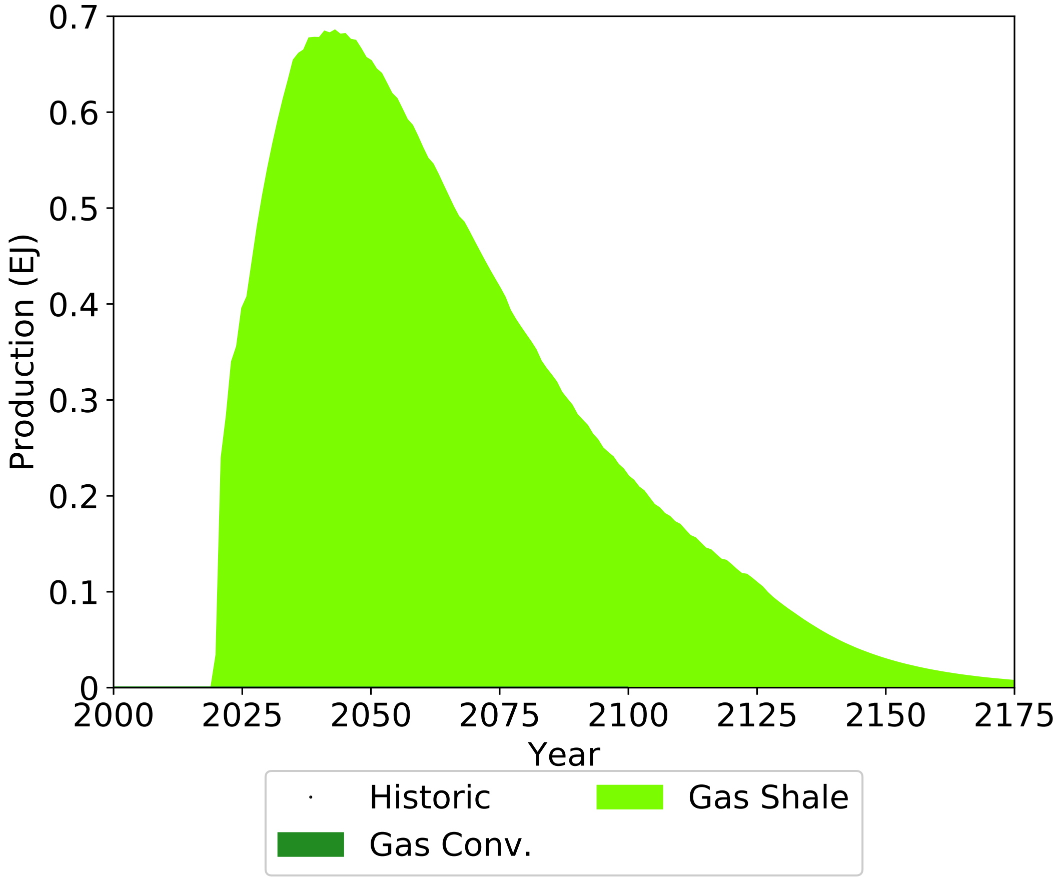

Figure 6.22: Canada - Quebec projection by mineral type

Table 6.22: Peak years - Minerals

Name	URR	Peak Year	Peak Rate
Gas Conv.	–	1970	–
Gas Shale	44.1	2043	0.69
**Total**	**44.1**	**2043**	**0.69**

Saskatchewan

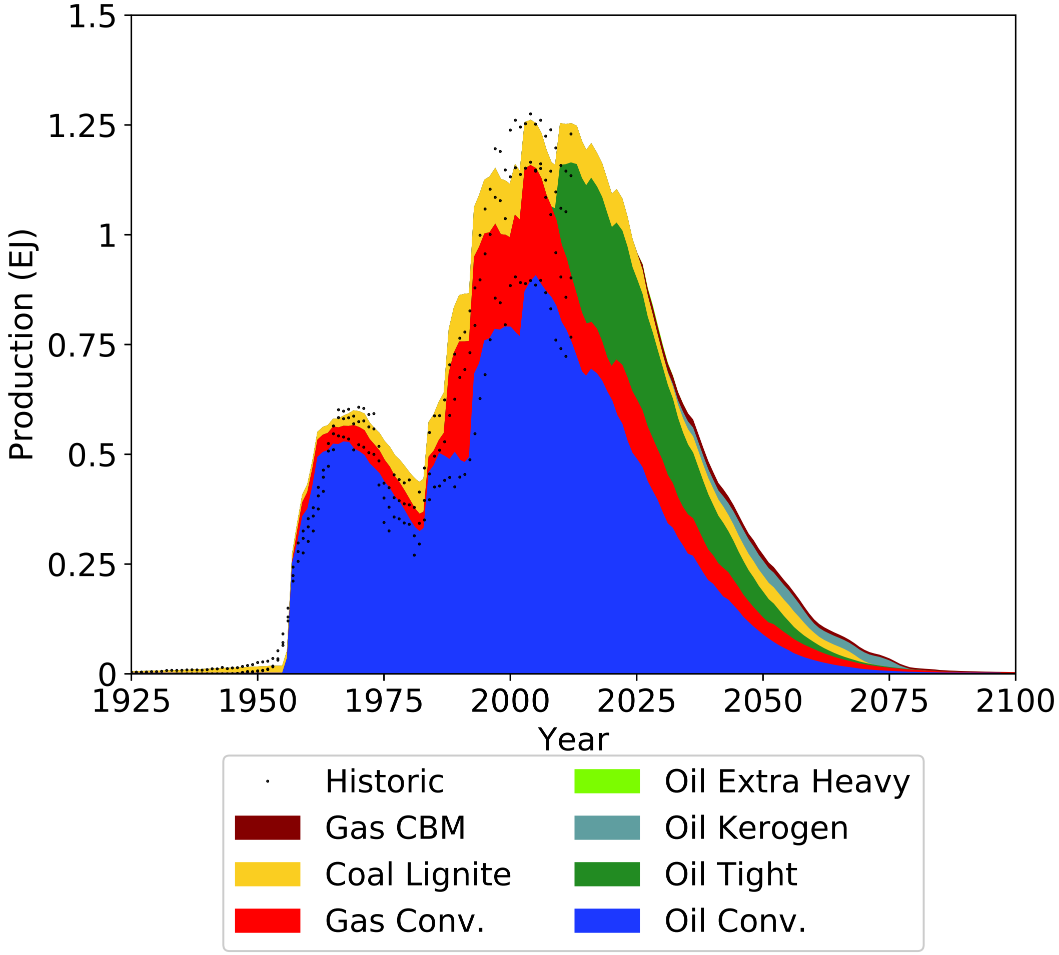

Figure 6.23: Canada - Saskatchewan projections capped at 16

Table 6.23: Peak years - All

Name	URR	Peak Year	Peak Rate
Oil Conv. Saskatchewan	46.99	2005	0.9
Gas Conv. Saskatchewan	11.19	2003	0.28
Oil Tight Saskatchewan	8.68	2019	0.33
Coal Lignite Saskatchewan	6.75	1997	0.13
Oil Kerogen Saskatchewan	1.07	2046	0.03
Gas CBM Saskatchewan	0.71	2029	0.02
Oil Extra Heavy Saskatchewan	0.02	2029	0.01
**Total**	**75.41**	**2004**	**1.26**

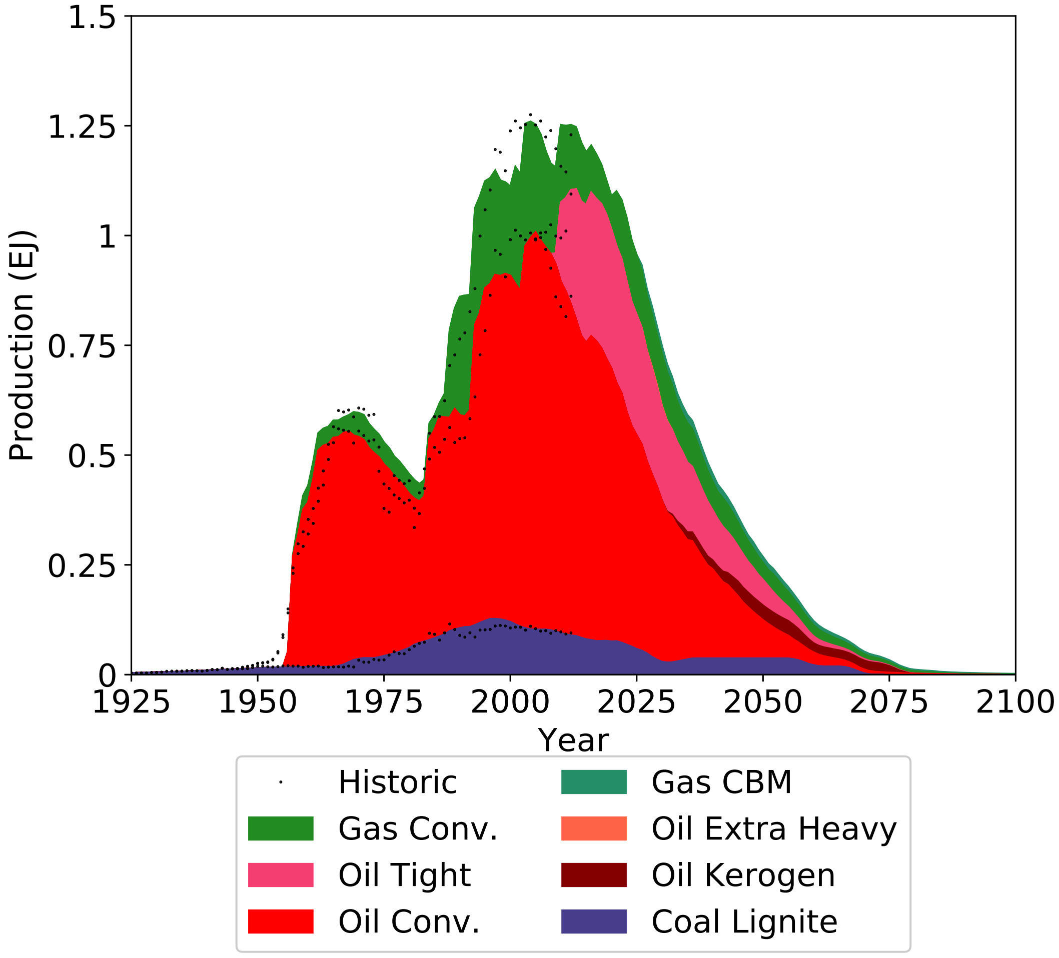

Figure 6.24: Canada - Saskatchewan projection by mineral type

Table 6.24: Peak years - Minerals

Name	URR	Peak Year	Peak Rate
Coal Lignite	6.75	1997	0.13
Oil Conv.	46.99	2005	0.9
Oil Kerogen	1.07	2046	0.03
Oil Tight	8.68	2019	0.33
Oil Extra Heavy	0.02	2029	0.01
Gas Conv.	11.19	2003	0.28
Gas CBM	0.71	2029	0.02
**Total**	**75.41**	**2004**	**1.26**

Yukon

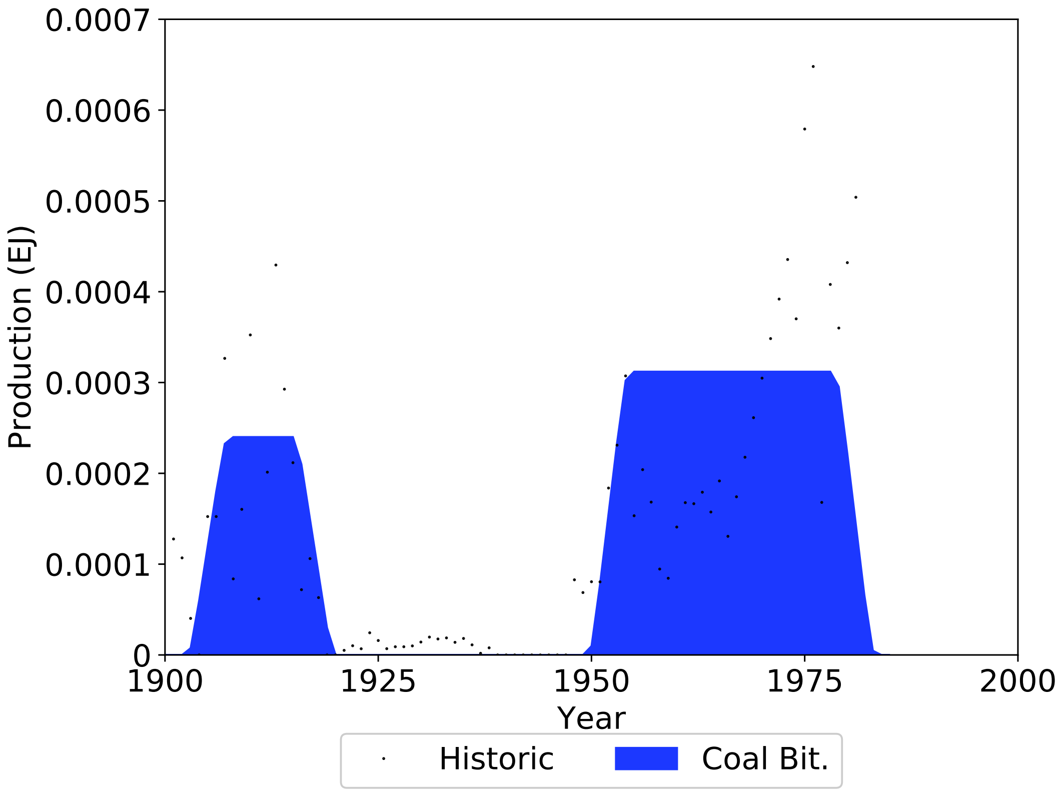

Figure 6.25: Canada - Yukon projections capped at 16

Table 6.25: Peak years - All

Name	URR	Peak Year	Peak Rate
Coal Bit. Yukon	0.01	1955	–
**Total**	**0.01**	**1955**	–

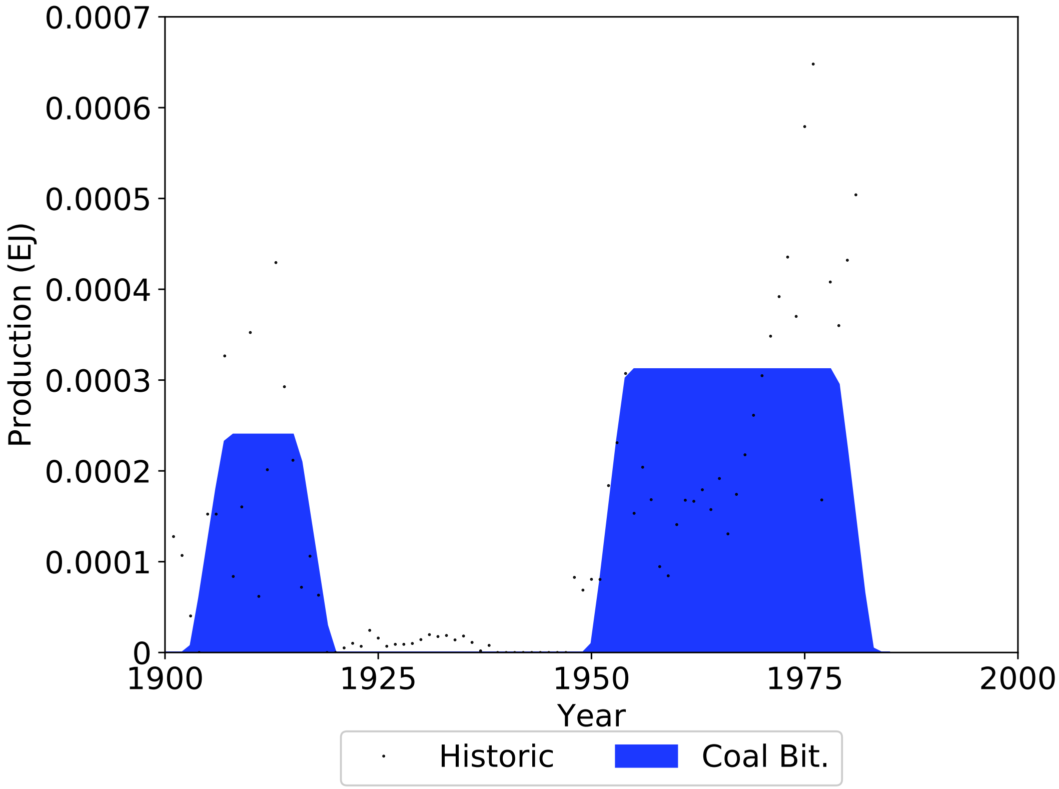

Figure 6.26: Canada - Yukon projection by mineral type

Table 6.26: Peak years - Minerals

Name	URR	Peak Year	Peak Rate
Coal Bit.	0.01	1955	–
**Total**	**0.01**	**1955**	–

6.1.4 Projection by region

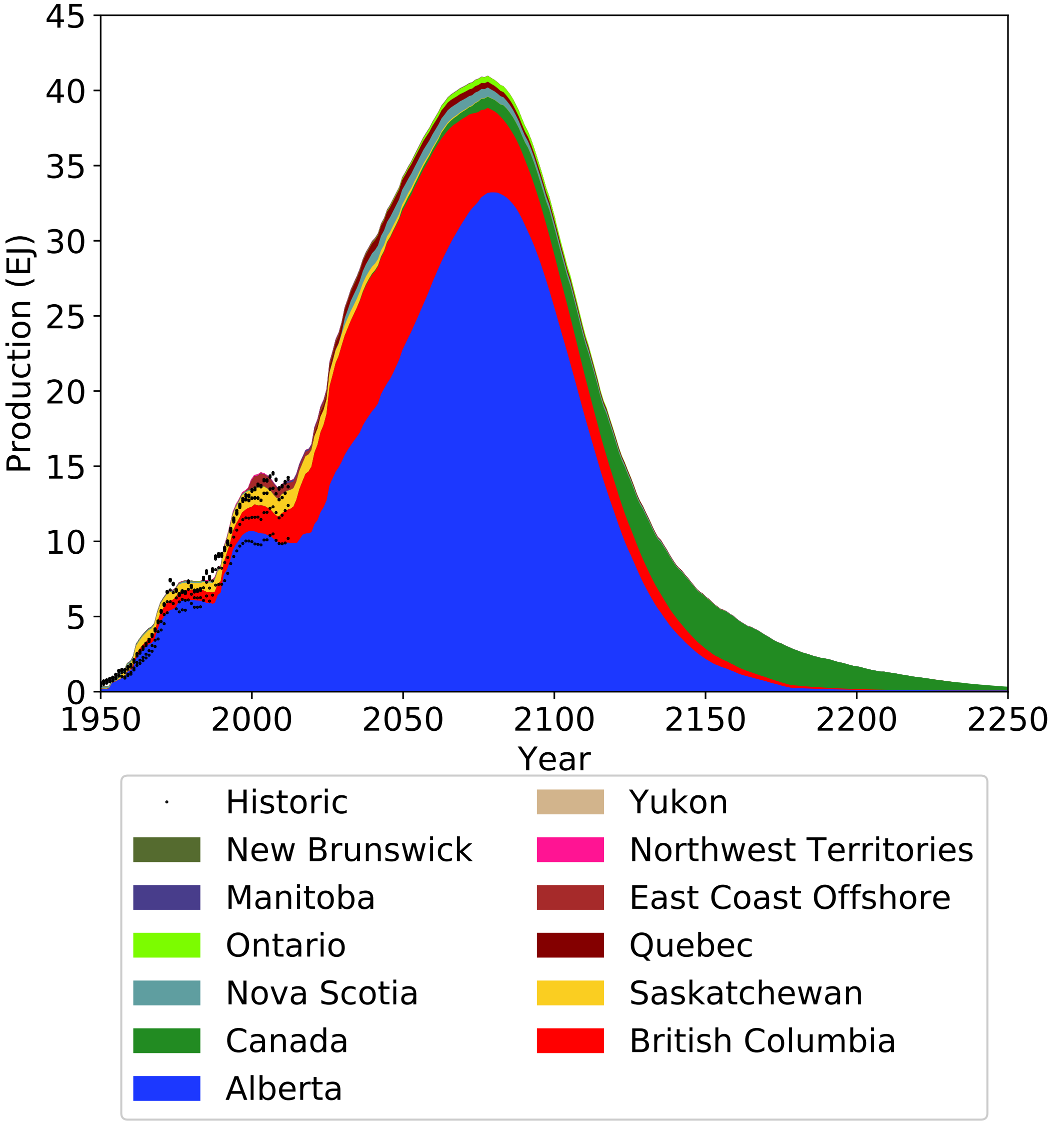

Figure 6.27: Canada by region projections capped at 16

Table 6.27: Peak years - All

**Name**	**URR**	**Peak Year**	**Peak Rate**
Alberta	3000.45	2080	33.15
British Columbia	741.65	2047	9.4
Canada	356.0	2142	3.52
Saskatchewan	75.41	2004	1.26
Nova Scotia	69.55	2050	0.98
Quebec	44.1	2043	0.69
Ontario	23.19	2075	0.4
East Coast Offshore	13.79	2003	0.83
Manitoba	4.88	2013	0.13
Northwest Territories	3.01	2000	0.09
New Brunswick	1.48	1971	0.02
Yukon	0.01	1955	–
**Total**	**4333.53**	**2078**	**40.9**

6.2 USA

6.2.1 All Projections

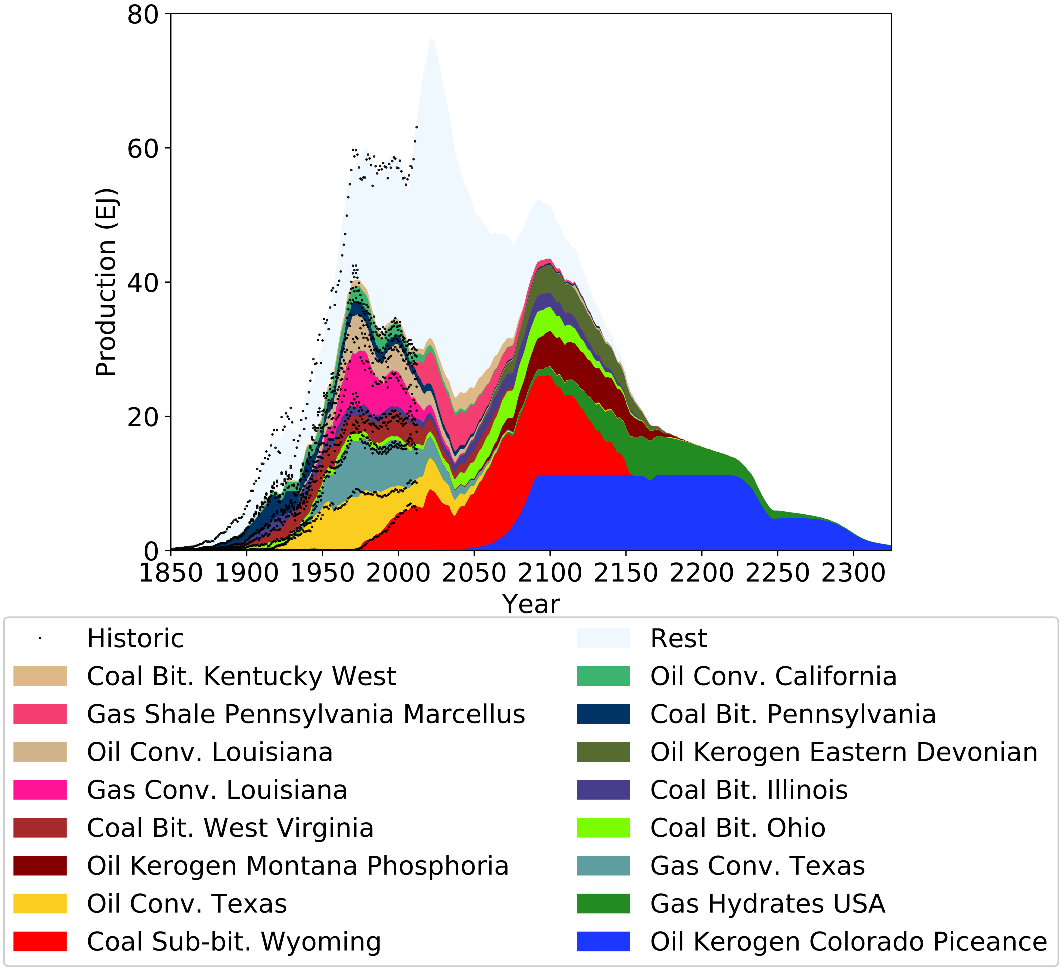

Figure 6.28: USA projections capped at 16

Table 6.28: Peak years - All

Name	URR	Peak Year	Peak Rate
Oil Kerogen Colorado Piceance	2092.7	2145	11.17
Coal Sub-bit. Wyoming	1489.93	2087	15.05
Gas Hydrates USA	614.5	2167	5.93
Oil Conv. Texas	603.57	1970	7.87
Gas Conv. Texas	526.44	1973	8.4
Oil Kerogen Montana Phosphoria	429.8	2112	5.59
Coal Bit. Ohio	414.76	2085	4.22
Coal Bit. West Virginia	389.11	1997	3.64
Coal Bit. Illinois	349.04	2093	2.39
Gas Conv. Louisiana	344.58	1974	8.36
Oil Kerogen Eastern Devonian	324.9	2111	4.28
Oil Conv. Louisiana	321.88	1972	5.74
Coal Bit. Pennsylvania	303.54	1917	3.91
Gas Shale Pennsylvania Marcellus	294.39	2030	5.73
Oil Conv. California	198.31	1969	2.35
Coal Bit. Kentucky West	180.45	2045	2.58
Coal Bit. Kentucky East	175.42	1991	2.71
Gas Shale Louisiana Haynesville	175.11	2024	4.84
Oil Conv. Oklahoma	156.34	1966	1.54
Coal Anthr. Pennsylvania	153.01	1919	2.73
Oil Conv. Alaska	144.15	1987	4.07
Gas Conv. Oklahoma	143.52	1985	2.33
Coal Bit. Indiana	132.5	2046	1.06
Gas Shale Texas Eagle Ford	126.25	2027	3.01
Coal Lignite North Dakota	118.29	2081	1.87
Oil Kerogen Utah Unita	112.1	2099	1.55
Gas Shale West Virginia Marcellus	97.52	2035	1.48
Gas Shale Texas Barnett	88.36	2020	2.29
Oil Tight North Dakota Bakken	87.48	2022	2.27
Gas Shale Oklahoma Woodford	82.9	2035	1.07
Oil Conv. New Mexico	82.6	2021	0.96
Gas Conv. New Mexico	81.47	1975	1.3
Oil Conv. Wyoming	76.56	1972	0.92
Oil Kerogen Wyoming Greater Green River	74.7	2092	1.06
Coal Sub-bit. New Mexico	68.57	2057	1.54
Coal Bit. Colorado	67.33	2045	1.31
Gas Conv. Colorado	63.0	2019	1.33
Coal Bit. Virginia	62.26	1975	0.84
Gas Conv. Wyoming	62.21	2012	1.67
Gas Shale Texas Haynesville	61.8	2037	0.8
Coal Bit. Alabama	61.43	1990	0.58
Oil Tight Texas Permian	61.13	2021	1.6
Gas Shale Colorado Niobrara	59.86	2038	0.92
Gas Shale Ohio Utica	58.28	2039	0.89
Gas Shale New York Utica	58.28	2044	0.89
Oil Conv. Kansas	57.63	1956	0.75
Oil Conv. Colorado	57.3	2023	0.9
Gas Shale Arkansas Fayetteville	54.17	2020	1.52
Gas Conv. Kansas	47.12	1970	0.89

Table 6.28: Peak years - All – Continued

Name	URR	Peak Year	Peak Rate
Gas Conv. California	44.43	1965	0.76
Oil Tight Texas Eagle Ford	39.84	2020	1.59
Coal Sub-bit. Montana	38.52	1993	0.59
Gas CBM New Mexico	38.36	1998	0.6
Gas CBM Colorado	38.03	2023	0.62
Coal Bit. New Mexico	35.07	2036	0.84
Gas Conv. Pennsylvania	31.5	2021	0.35
Gas Shale New York Other	30.45	2040	0.48
Gas Shale Texas Permian	28.68	2021	0.73
Oil Nat. Bitumen Utah	27.84	2077	0.45
Coal Bit. Utah	27.03	1998	0.55
Gas CBM Virginia	26.85	2034	0.42
Gas Conv. Alaska	25.4	1996	0.53
Gas Conv. West Virginia	23.49	1967	0.24
Gas CBM Wyoming	22.23	2013	0.56
Oil Conv. Illinois	21.98	1950	0.49
Coal Lignite Texas	21.9	1992	0.48
Gas Conv. Utah	21.0	2013	0.44
Oil Conv. North Dakota	20.8	1983	0.32
Oil Conv. Mississippi	20.09	1964	0.37
Oil Conv. Utah	17.19	2015	0.3
Coal Bit. Tennessee	16.08	1968	0.19
Gas Conv. Kentucky	15.75	2034	0.17
Gas Shale North Dakota Bakken	15.24	2031	0.22
Oil Conv. Montana	15.15	2015	0.22
Coal Bit. Arizona	14.62	2004	0.32
Oil Tight Ohio Utica	14.33	2022	0.62
Oil Conv. Arkansas	14.07	1923	0.39
Oil Nat. Bitumen Texas	13.0	2068	0.24
Oil Conv. Michigan	11.37	1977	0.29
Gas Conv. Ohio	10.99	1983	0.18
Oil Tight Oklahoma Woodford	10.95	2020	0.47
Gas Conv. Arkansas	10.85	1992	0.23
Oil Conv. Pennsylvania	10.19	1882	0.14
Gas Conv. Mississippi	10.15	1955	0.19
Oil Conv. Alabama	10.07	1984	0.17
Gas CBM Alabama	9.82	2029	0.19
Oil Conv. Kentucky	9.65	2020	0.13
Coal Bit. Maryland	9.63	1907	0.13
Gas Conv. Alabama	9.62	1996	0.48
Coal Bit. Missouri	9.53	1941	0.12
Gas Shale New Mexico Permian	8.96	2026	0.16
Coal Sub-bit. Alaska	8.51	2086	0.07
Coal Bit. Iowa	8.14	1914	0.15
Coal Bit. Oklahoma	7.62	1914	0.1
Oil Conv. Ohio	7.08	1899	0.12
Coal Sub-bit. Colorado	6.9	2006	0.13
Coal Bit. Kansas	6.64	1916	0.14
Oil Conv. West Virginia	6.6	1902	0.08

Table 6.28: Peak years - All – Continued

**Name**	**URR**	**Peak Year**	**Peak Rate**
Oil Tight Wyoming Niobrara	6.15	2022	0.19
Oil Tight Colorado Niobrara	6.15	2022	0.19
Oil Nat. Bitumen Alabama	6.05	2062	0.13
Oil Tight California Monterey	5.62	2016	0.23
Coal Bit. Arkansas	5.36	2069	0.06
Oil Nat. Bitumen Kentucky	5.34	2053	0.1
Gas Conv. Michigan	5.25	1998	0.15
Oil Conv. Florida	5.13	1976	0.35
Gas Shale Montana Bakken	5.06	2029	0.1
Gas Shale Michigan Antrim	4.67	1996	0.24
Coal Sub-bit. Washington	4.57	1975	0.07
Gas Conv. Montana	4.21	2004	0.09
Gas CBM Utah	3.99	2005	0.09
Oil Conv. Nebraska	3.96	1959	0.15
Oil Conv. Indiana	3.53	1956	0.07
Oil Extra Heavy California	3.33	2030	0.15
Oil Nat. Bitumen California	3.31	2050	0.07
Gas Conv. North Dakota	3.09	1990	0.07
Gas CBM Oklahoma	2.69	2006	0.07
Gas CBM Kansas	2.69	2023	0.07
Gas Conv. Virginia	2.56	1997	0.08
Oil Tight Texas Barnett	2.44	2018	0.07
Coal Lignite Louisiana	2.34	2028	0.05
Oil Tight West Virginia Marcellus	2.29	2022	0.1
Oil Tight Pennsylvania Marcellus	2.29	2022	0.1
Oil Tight Texas Austin Chalk	2.25	1999	0.12
Gas Conv. New York	1.8	2005	0.05
Oil Conv. New York	1.24	1941	0.04
Gas CBM West Virginia	1.21	2017	0.03
Gas Conv. Indiana	1.05	1893	0.03
Gas Conv. Florida	1.05	1976	0.04
Coal Bit. Michigan	1.04	1902	0.03
Oil Tight Texas Granite Wash	0.89	2011	0.07
Oil Tight Oklahoma Granite Wash	0.89	2011	0.07
Oil Conv. South Dakota	0.59	2007	0.01
Gas Conv. Illinois	0.56	1943	0.02
Gas CBM Pennsylvania	0.54	2024	0.01
Coal Lignite Mississippi	0.54	2005	0.03
Gas Shale New Mexico Lewis	0.53	2019	0.02
Gas Shale Colorado Lewis	0.53	2019	0.02
Coal Bit. Wyoming	0.48	1999	0.07
Oil Conv. Nevada	0.35	1980	0.02
Gas CBM Montana	0.3	2004	0.01
Gas CBM Arkansas	0.29	2027	0.01
Coal Bit. Georgia	0.26	1895	0.01
Gas Shale Kentucky New Albany	0.23	2025	0.01
Oil Conv. Tennessee	0.22	1973	–
Gas Conv. Oregon	0.21	2019	0.01
Gas Conv. Tennessee	0.14	2008	0.01

Table 6.28: Peak years - All – Continued

**Name**	**URR**	**Peak Year**	**Peak Rate**
Gas Conv. Nebraska	0.13	1969	0.01
Oil Conv. Arizona	0.13	1968	0.01
Coal Bit. Montana	0.11	2010	0.06
Gas Conv. South Dakota	0.11	1986	–
Coal Lignite Montana	0.11	1999	–
Oil Nat. Bitumen Wyoming	0.1	2040	0.01
Oil Conv. Missouri	0.07	2014	–
Coal Bit. Texas	0.05	1997	0.02
Gas Conv. Other	0.04	1887	–
Gas Conv. Arizona	0.03	1992	–
Coal Bit. Washington	0.02	1997	0.01
Coal Lignite South Dakota	0.01	1933	–
Gas Conv. Maryland	0.01	1969	–
Oil Conv. Virginia	0.01	1982	–
Gas CBM Louisiana	0.01	2008	–
Coal Anthr. Arkansas	–	1994	–
Gas Conv. Missouri	–	1903	–
Gas Conv. Nevada	–	1993	–
Oil Conv. Washington	–	1956	–
Gas Conv. Iowa	–	1919	–
**Total**	**12908.93**	**2022**	**76.17**

6.2.2 By Mineral

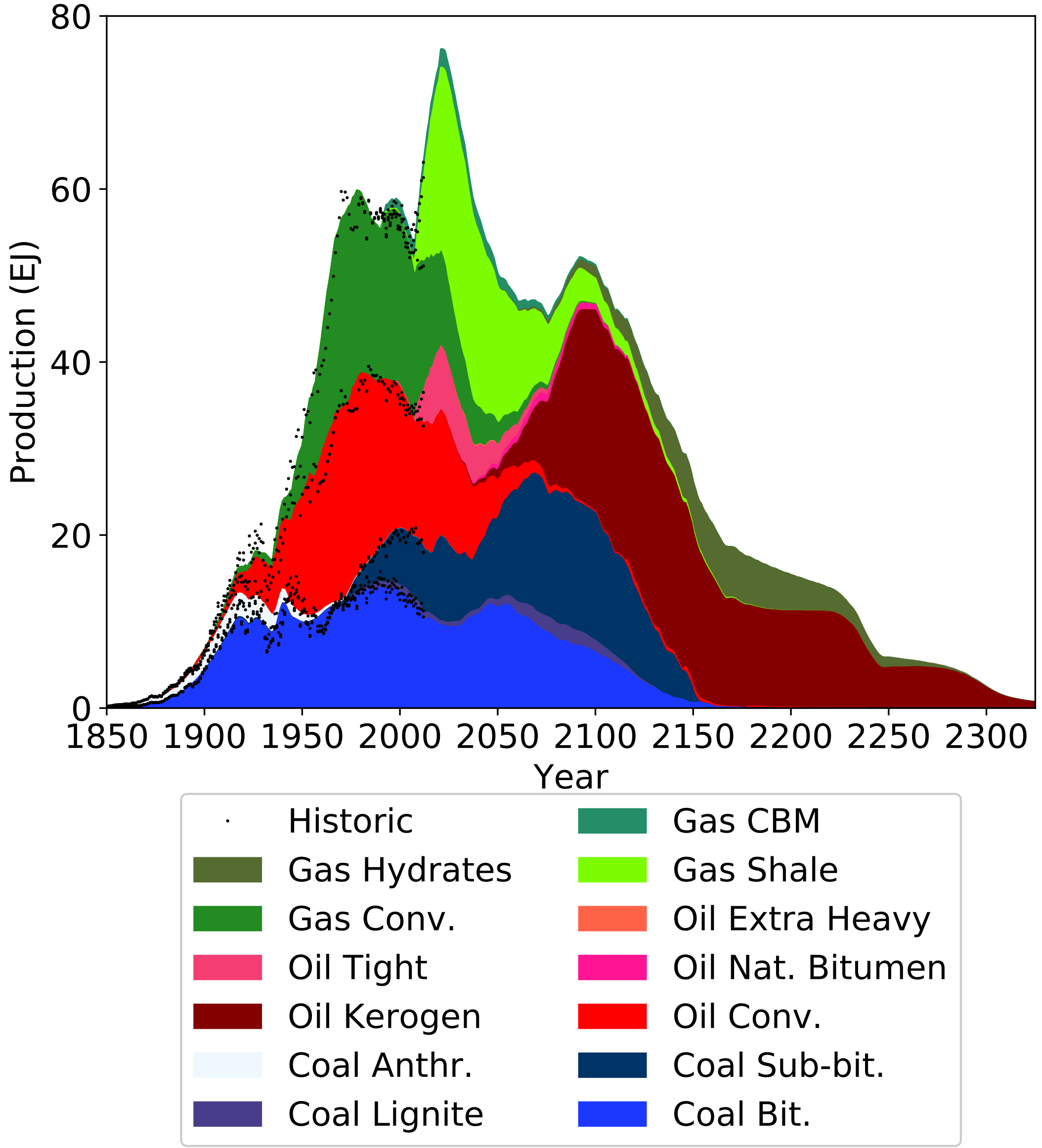

Figure 6.29: USA projection by mineral type

6.2.3 Regional Projections

Table 6.29: Peak years - Minerals

**Name**	**URR**	**Peak Year**	**Peak Rate**
Coal Bit.	2277.52	1989	13.88
Coal Lignite	143.19	2081	1.87
Coal Sub-bit.	1617.0	2070	15.83
Coal Anthr.	153.01	1919	2.73
Oil Conv.	1877.8	1980	22.88
Oil Kerogen	3034.2	2109	23.45
Oil Nat. Bitumen	55.64	2069	0.96
Oil Tight	242.7	2021	7.41
Oil Extra Heavy	3.33	2030	0.15
Gas Conv.	1491.76	1974	22.57
Gas Shale	1251.27	2028	23.63
Gas Hydrates	614.5	2167	5.93
Gas CBM	147.01	2023	2.27
**Total**	**12908.93**	**2022**	**76.17**

Alabama

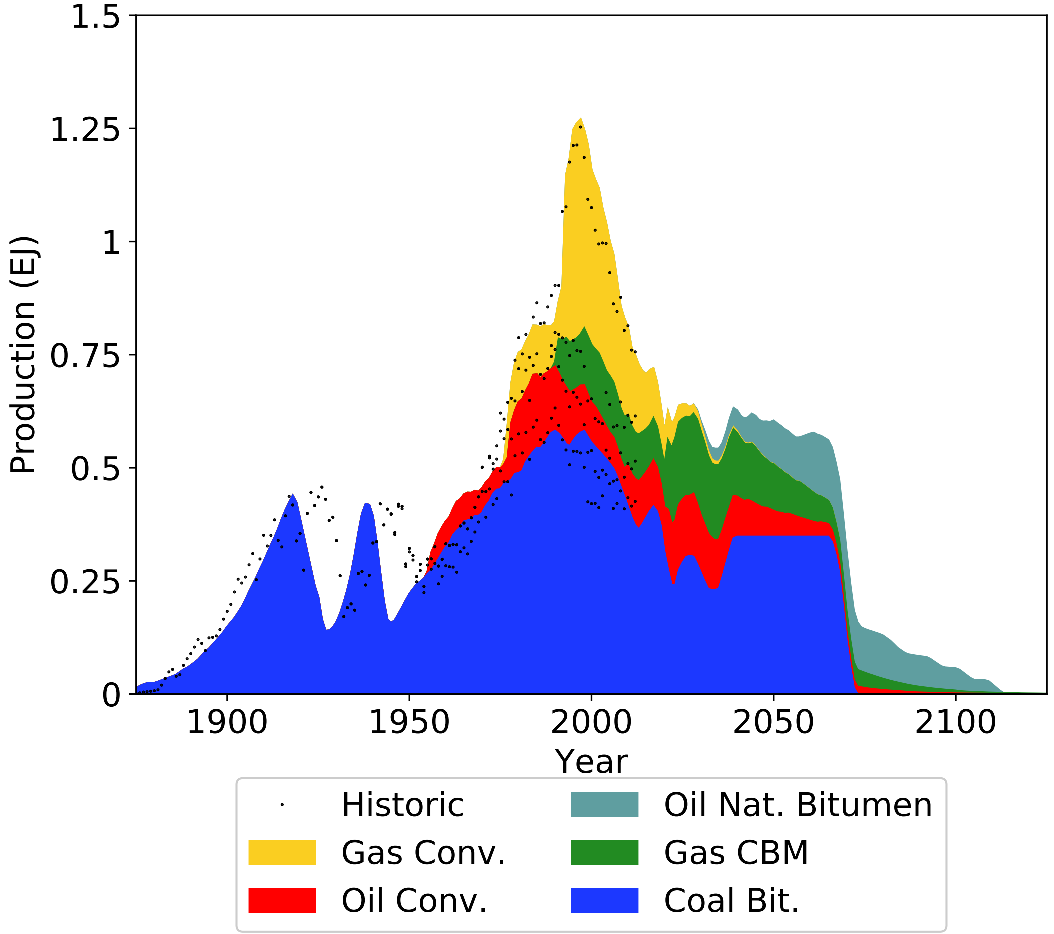

Figure 6.30: USA - Alabama projections capped at 16

Table 6.30: Peak years - All

Name	URR	Peak Year	Peak Rate
Coal Bit. Alabama	61.43	1990	0.58
Oil Conv. Alabama	10.07	1984	0.17
Gas CBM Alabama	9.82	2029	0.19
Gas Conv. Alabama	9.62	1996	0.48
Oil Nat. Bitumen Alabama	6.05	2062	0.13
**Total**	**96.99**	**1997**	**1.27**

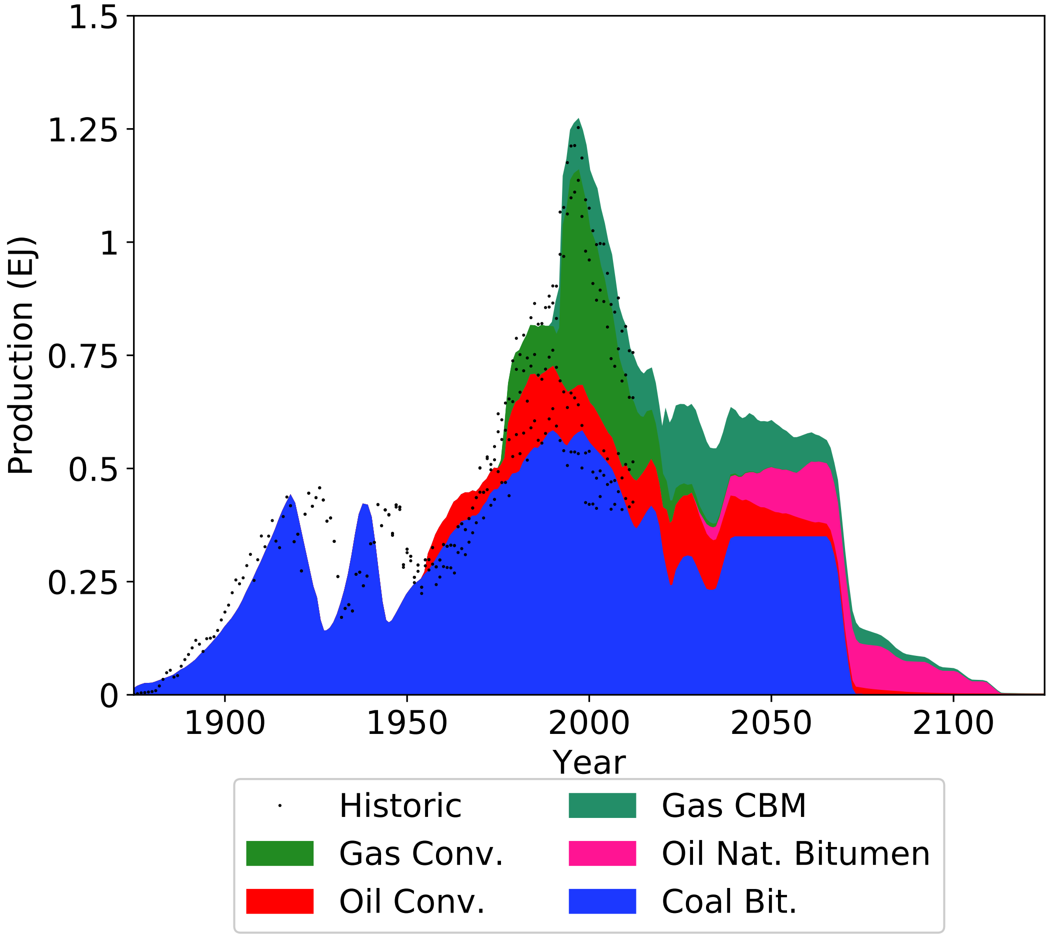

Figure 6.31: USA - Alabama projection by mineral type

Table 6.31: Peak years - Minerals

Name	URR	Peak Year	Peak Rate
Coal Bit.	61.43	1990	0.58
Oil Conv.	10.07	1984	0.17
Oil Nat. Bitumen	6.05	2062	0.13
Gas Conv.	9.62	1996	0.48
Gas CBM	9.82	2029	0.19
**Total**	**96.99**	**1997**	**1.27**

Alaska

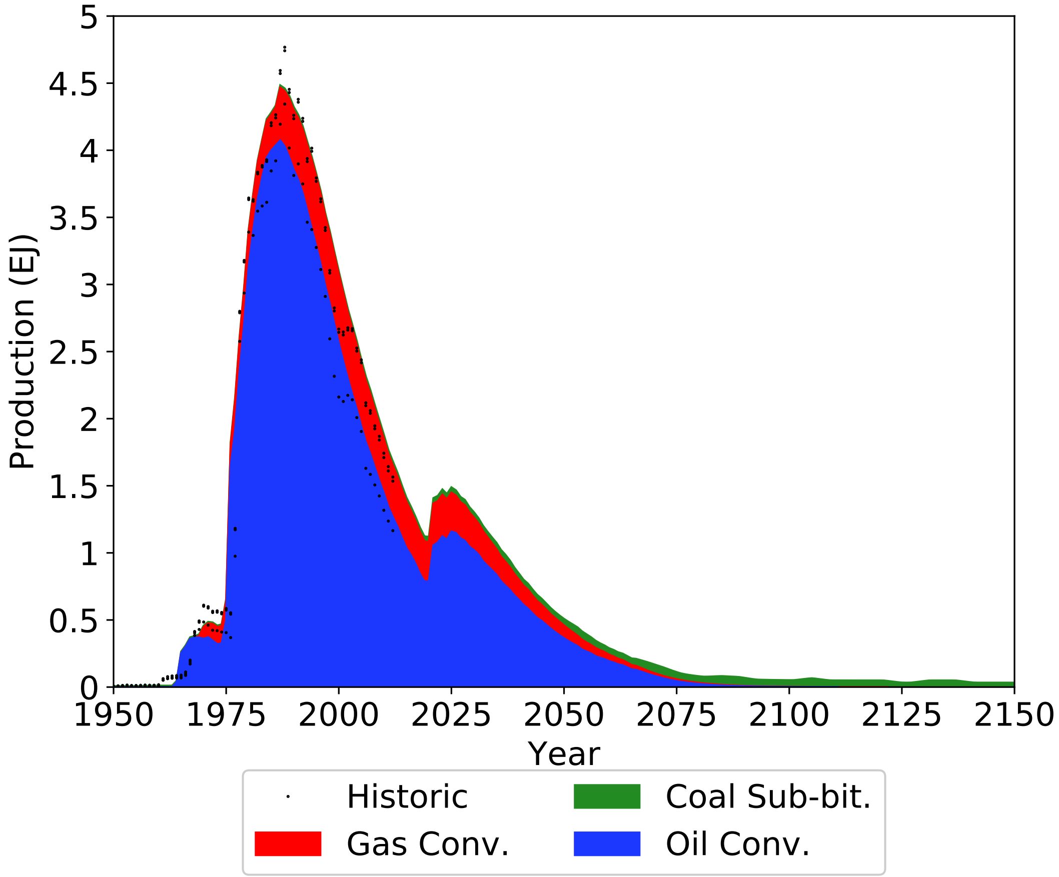

Figure 6.32: USA - Alaska projections capped at 16

Table 6.32: Peak years - All

Name	URR	Peak Year	Peak Rate
Oil Conv. Alaska	144.15	1987	4.07
Gas Conv. Alaska	25.4	1996	0.53
Coal Sub-bit. Alaska	8.51	2086	0.07
**Total**	**178.06**	**1987**	**4.48**

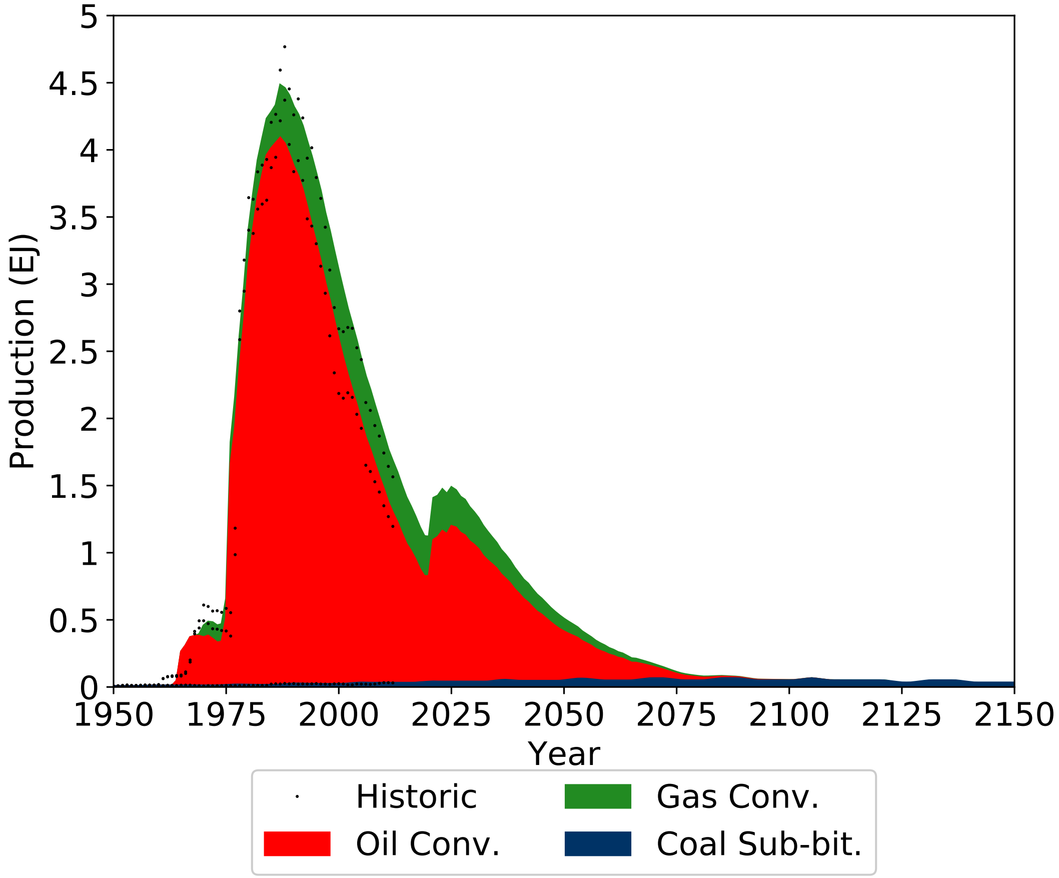

Figure 6.33: USA - Alaska projection by mineral type

Table 6.33: Peak years - Minerals

**Name**	**URR**	**Peak Year**	**Peak Rate**
Coal Sub-bit.	8.51	2086	0.07
Oil Conv.	144.15	1987	4.07
Gas Conv.	25.4	1996	0.53
**Total**	**178.06**	**1987**	**4.48**

Arizona

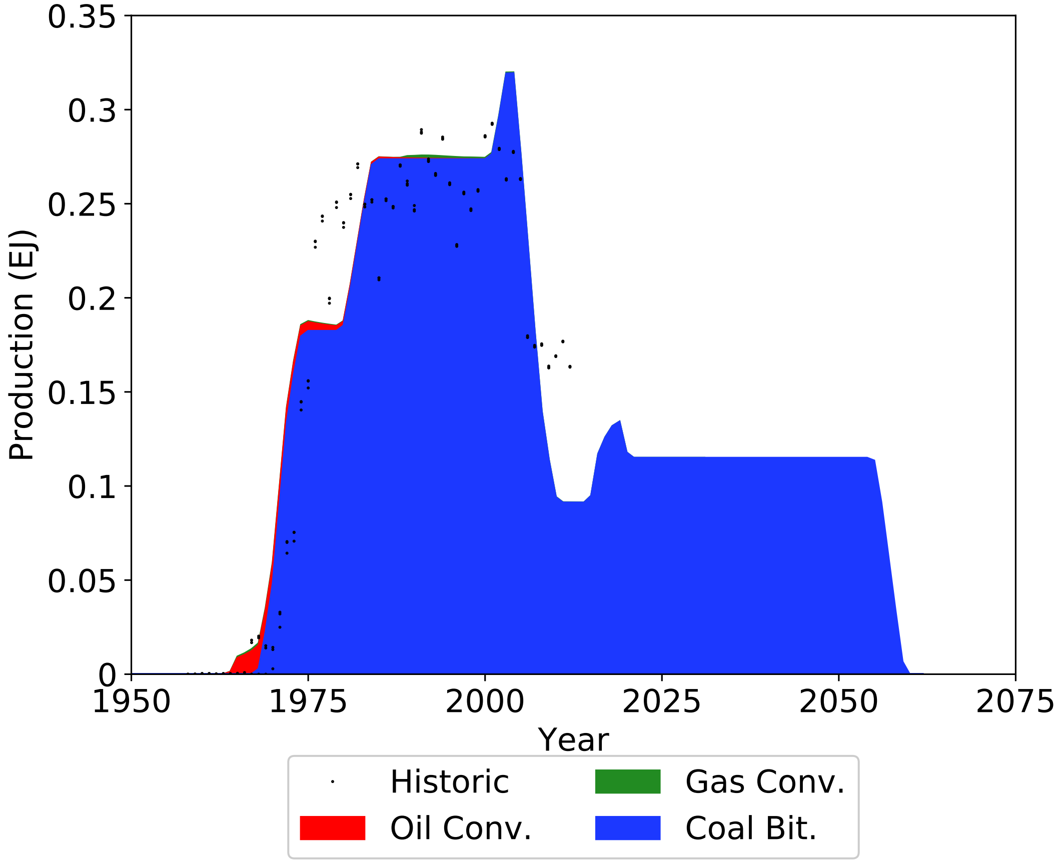

Figure 6.34: USA - Arizona projections capped at 16

Table 6.34: Peak years - All

Name	URR	Peak Year	Peak Rate
Coal Bit. Arizona	14.62	2004	0.32
Oil Conv. Arizona	0.13	1968	0.01
Gas Conv. Arizona	0.03	1992	–
**Total**	**14.78**	**2004**	**0.32**

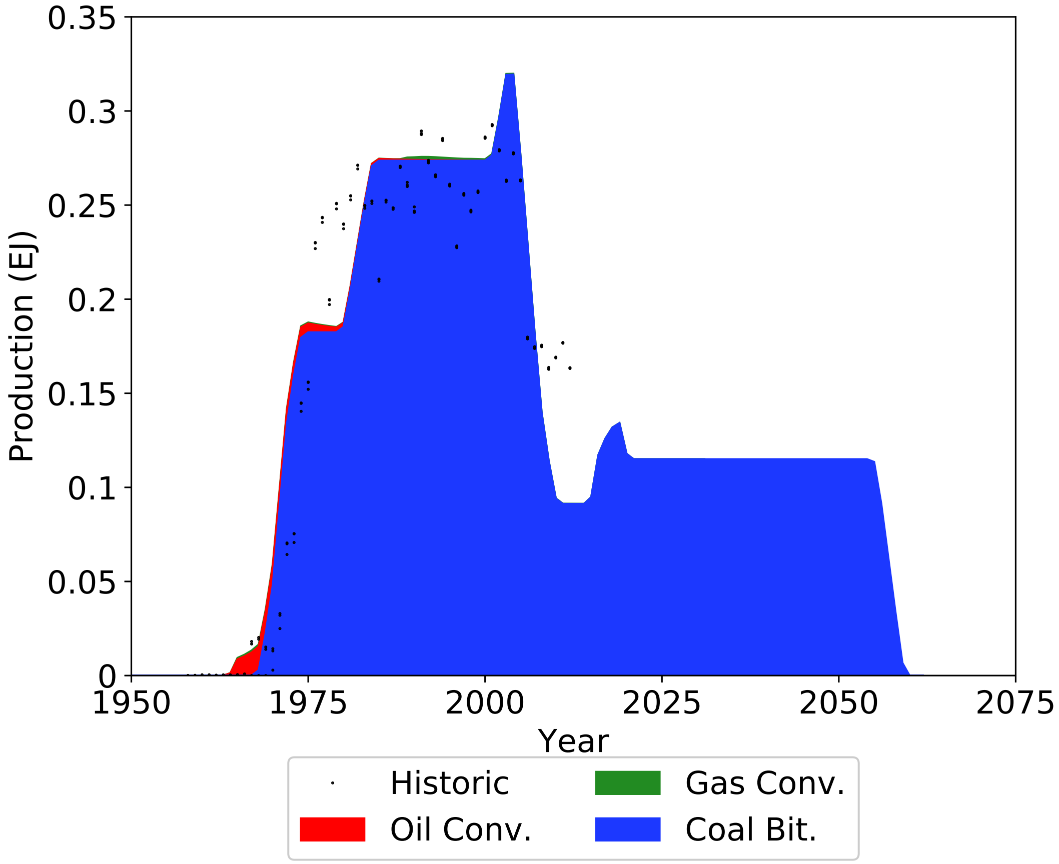

Figure 6.35: USA - Arizona projection by mineral type

Table 6.35: Peak years - Minerals

Name	URR	Peak Year	Peak Rate
Coal Bit.	14.62	2004	0.32
Oil Conv.	0.13	1968	0.01
Gas Conv.	0.03	1992	–
**Total**	**14.78**	**2004**	**0.32**

Arkansas

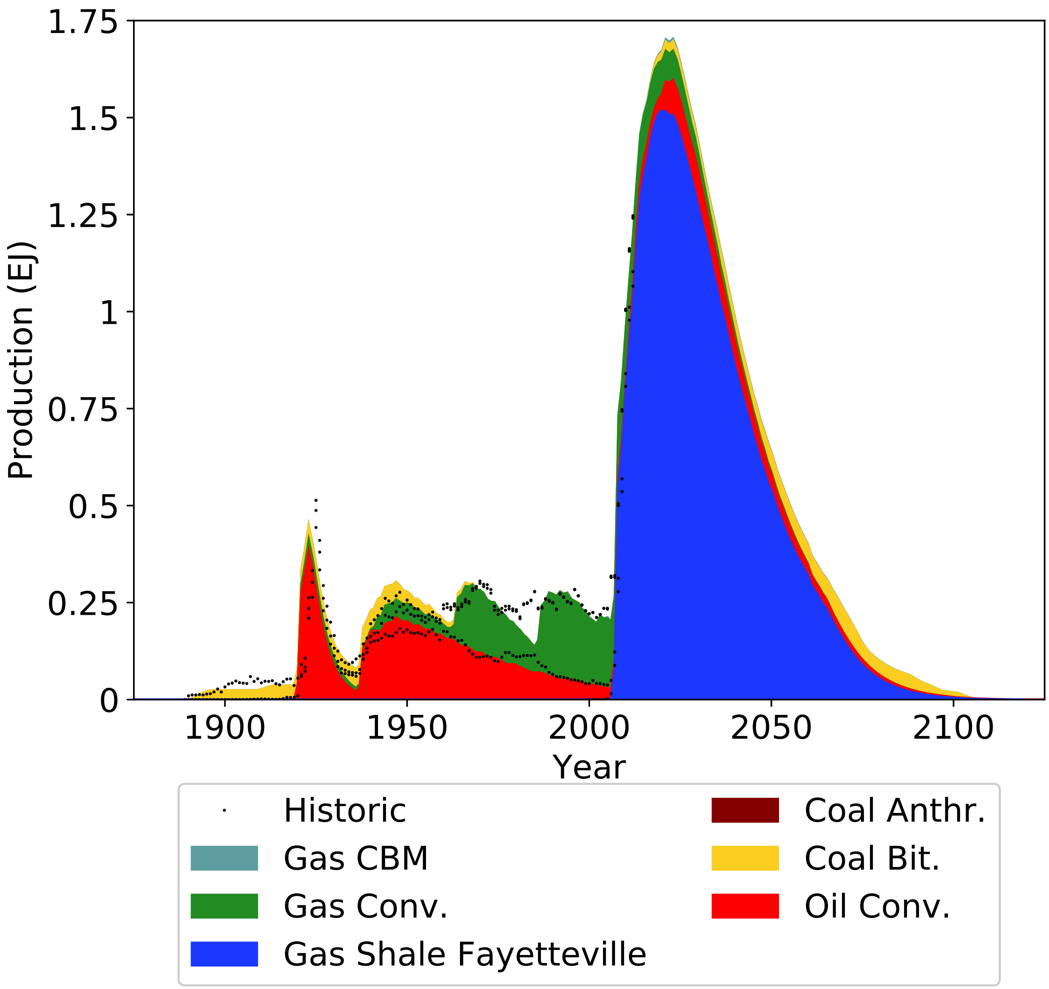

Figure 6.36: USA - Arkansas projections capped at 16

Table 6.36: Peak years - All

**Name**	**URR**	**Peak Year**	**Peak Rate**
Gas Shale Arkansas Fayetteville	54.17	2020	1.52
Oil Conv. Arkansas	14.07	1923	0.39
Gas Conv. Arkansas	10.85	1992	0.23
Coal Bit. Arkansas	5.36	2069	0.06
Gas CBM Arkansas	0.29	2027	0.01
Coal Anthr. Arkansas	–	1994	–
**Total**	**84.74**	**2023**	**1.7**

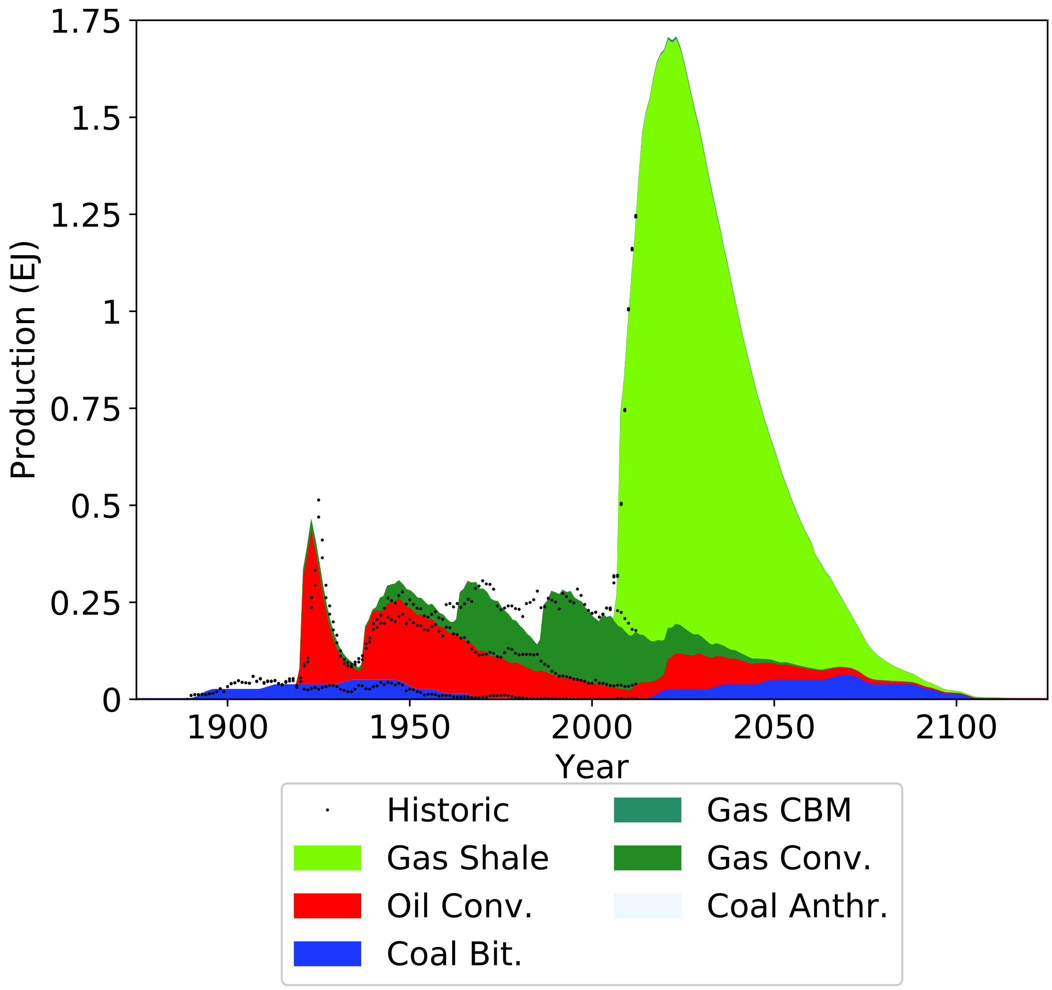

Figure 6.37: USA - Arkansas projection by mineral type

Table 6.37: Peak years - Minerals

**Name**	**URR**	**Peak Year**	**Peak Rate**
Coal Bit.	5.36	2069	0.06
Coal Anthr.	–	1994	–
Oil Conv.	14.07	1923	0.39
Gas Conv.	10.85	1992	0.23
Gas Shale	54.17	2020	1.52
Gas CBM	0.29	2027	0.01
**Total**	**84.74**	**2023**	**1.7**

California

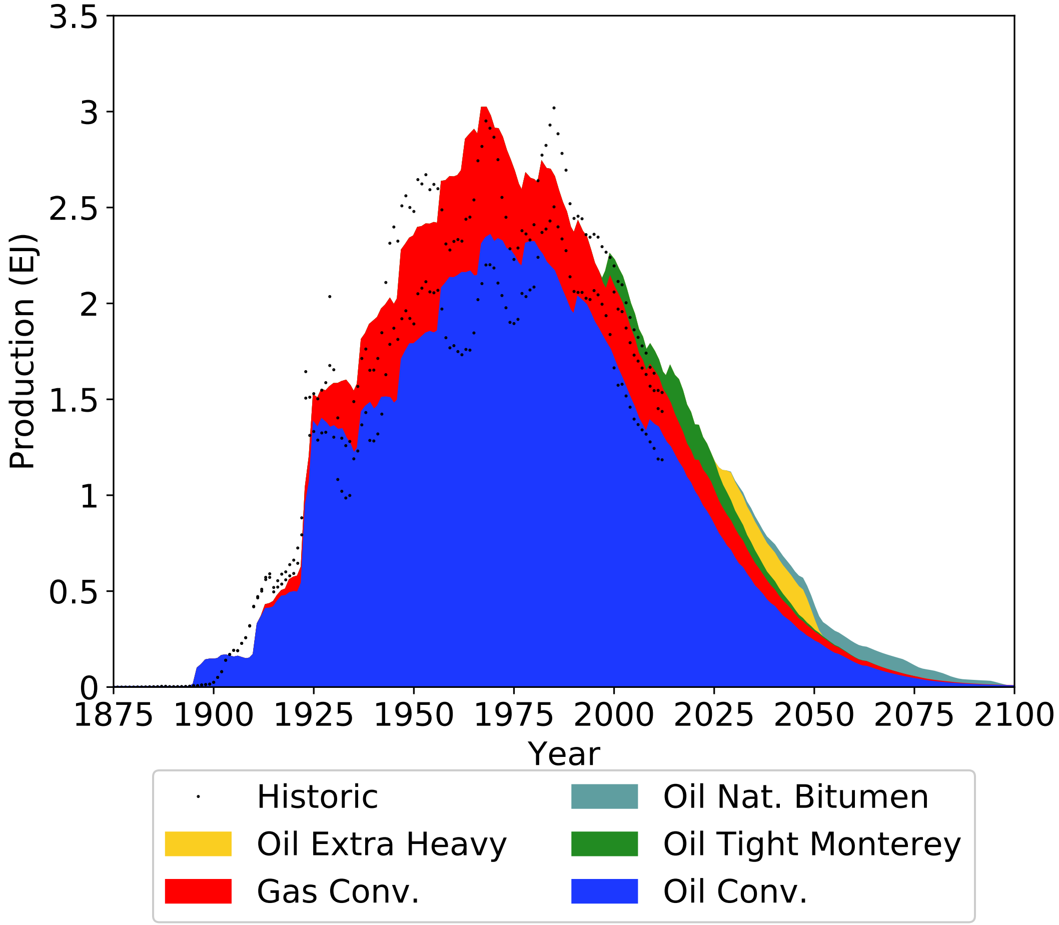

Figure 6.38: USA - California projections capped at 16

Table 6.38: Peak years - All

**Name**	**URR**	**Peak Year**	**Peak Rate**
Oil Conv. California	198.31	1969	2.35
Gas Conv. California	44.43	1965	0.76
Oil Tight California Monterey	5.62	2016	0.23
Oil Extra Heavy California	3.33	2030	0.15
Oil Nat. Bitumen California	3.31	2050	0.07
**Total**	**255.0**	**1968**	**3.02**

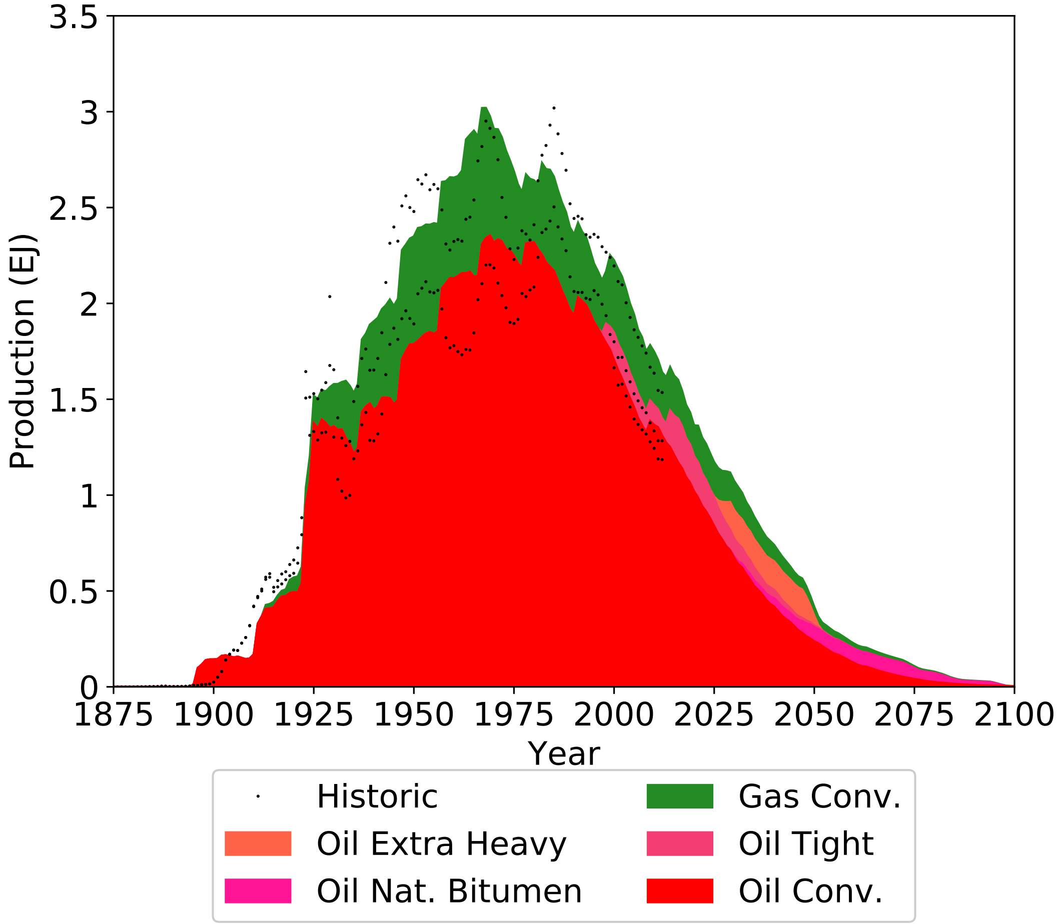

Figure 6.39: USA - California projection by mineral type

Table 6.39: Peak years - Minerals

**Name**	**URR**	**Peak Year**	**Peak Rate**
Oil Conv.	198.31	1969	2.35
Oil Nat. Bitumen	3.31	2050	0.07
Oil Tight	5.62	2016	0.23
Oil Extra Heavy	3.33	2030	0.15
Gas Conv.	44.43	1965	0.76
**Total**	**255.0**	**1968**	**3.02**

Colorado

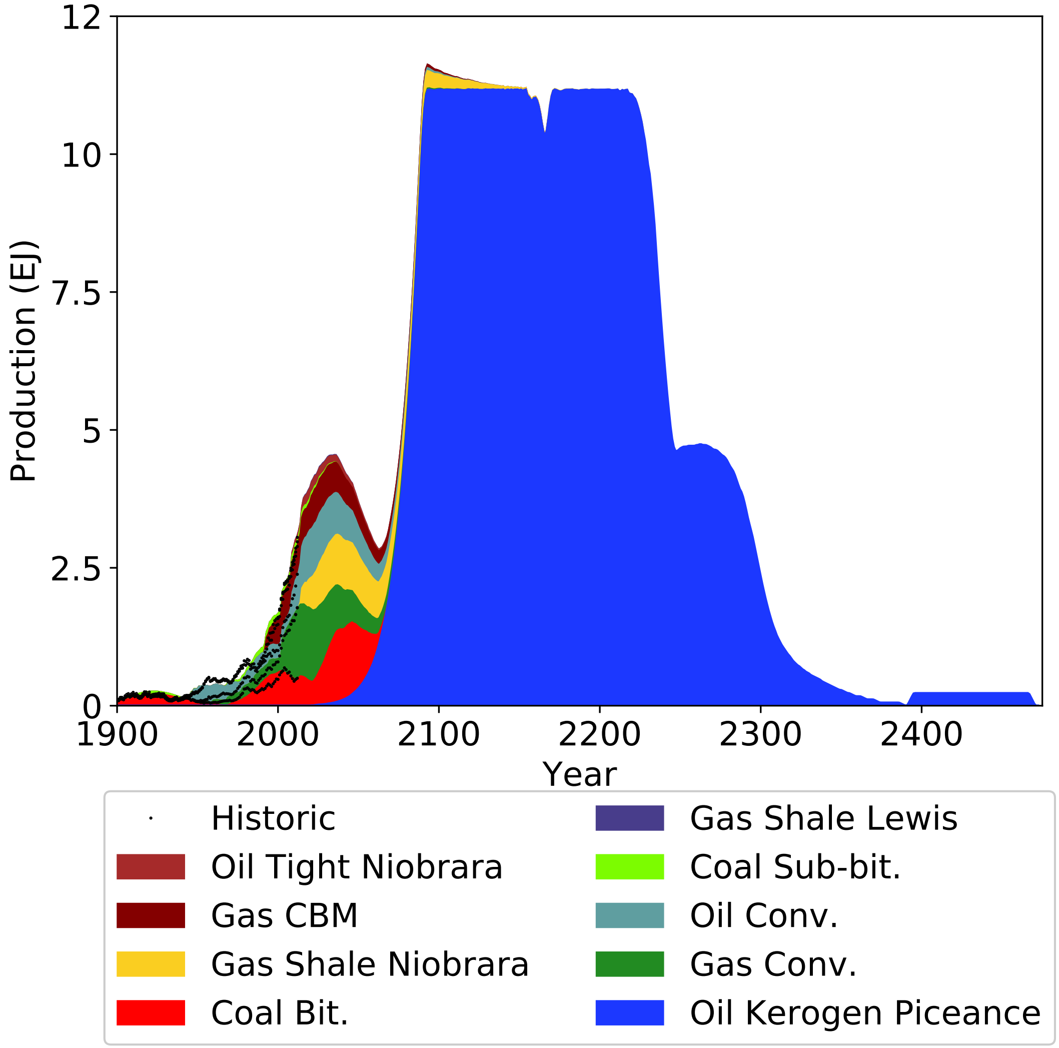

Figure 6.40: USA - Colorado projections capped at 16

Table 6.40: Peak years - All

Name	URR	Peak Year	Peak Rate
Oil Kerogen Colorado Piceance	2092.7	2145	11.17
Coal Bit. Colorado	67.33	2045	1.31
Gas Conv. Colorado	63.0	2019	1.33
Gas Shale Colorado Niobrara	59.86	2038	0.92
Oil Conv. Colorado	57.3	2023	0.9
Gas CBM Colorado	38.03	2023	0.62
Coal Sub-bit. Colorado	6.9	2006	0.13
Oil Tight Colorado Niobrara	6.15	2022	0.19
Gas Shale Colorado Lewis	0.53	2019	0.02
**Total**	**2391.8**	**2093**	**11.62**

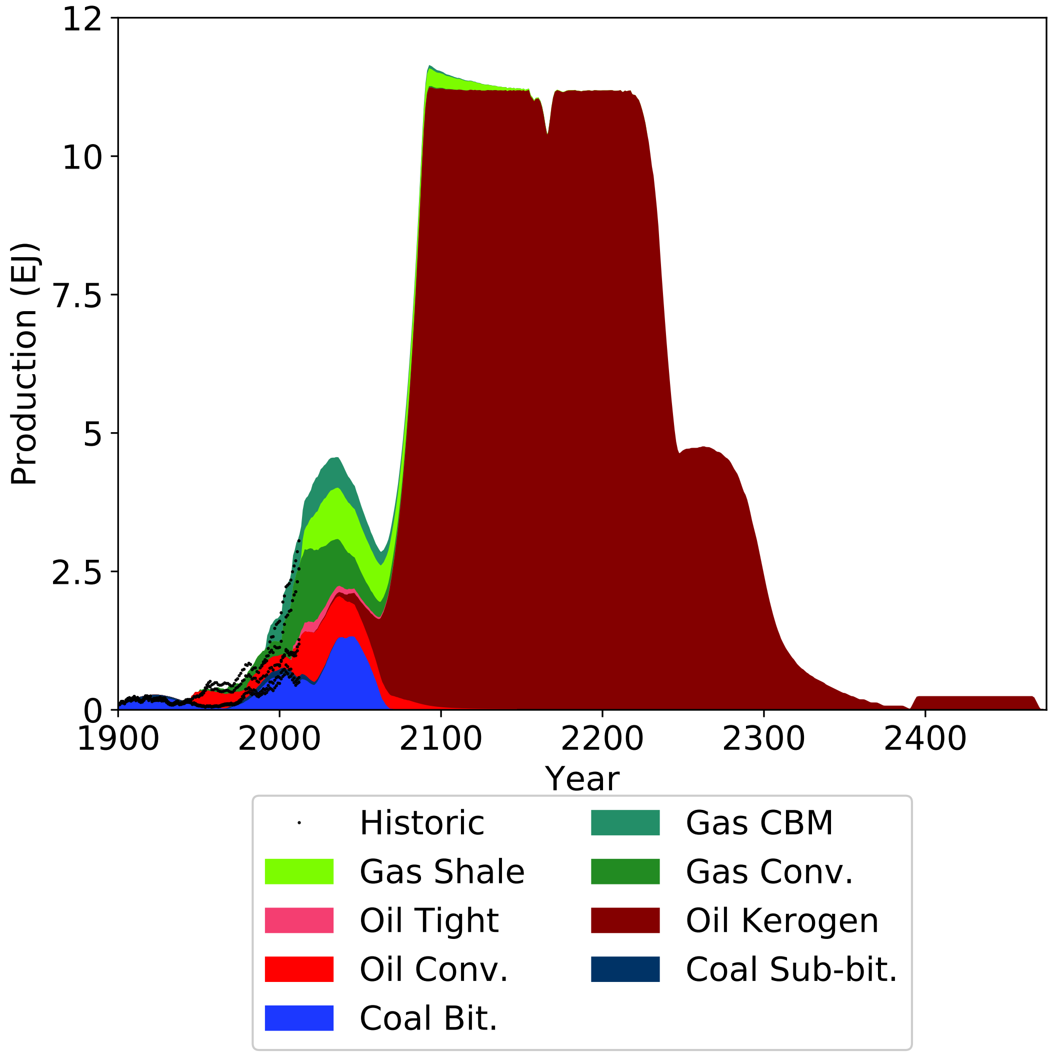

Figure 6.41: USA - Colorado projection by mineral type

Table 6.41: Peak years - Minerals

**Name**	**URR**	**Peak Year**	**Peak Rate**
Coal Bit.	67.33	2045	1.31
Coal Sub-bit.	6.9	2006	0.13
Oil Conv.	57.3	2023	0.9
Oil Kerogen	2092.7	2145	11.17
Oil Tight	6.15	2022	0.19
Gas Conv.	63.0	2019	1.33
Gas Shale	60.39	2037	0.93
Gas CBM	38.03	2023	0.62
**Total**	**2391.8**	**2093**	**11.62**

Eastern

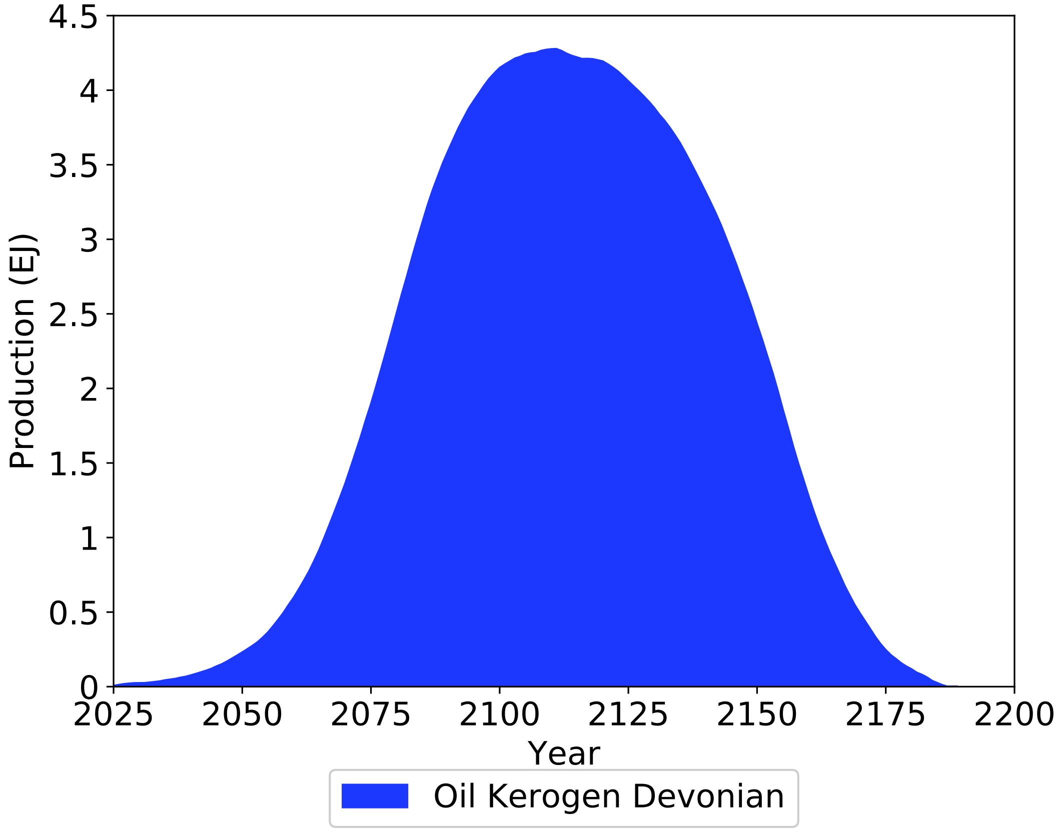

Figure 6.42: USA - Eastern projections capped at 16

Table 6.42: Peak years - All

Name	URR	Peak Year	Peak Rate
Oil Kerogen Eastern Devonian	324.9	2111	4.28
**Total**	**324.9**	**2111**	**4.28**

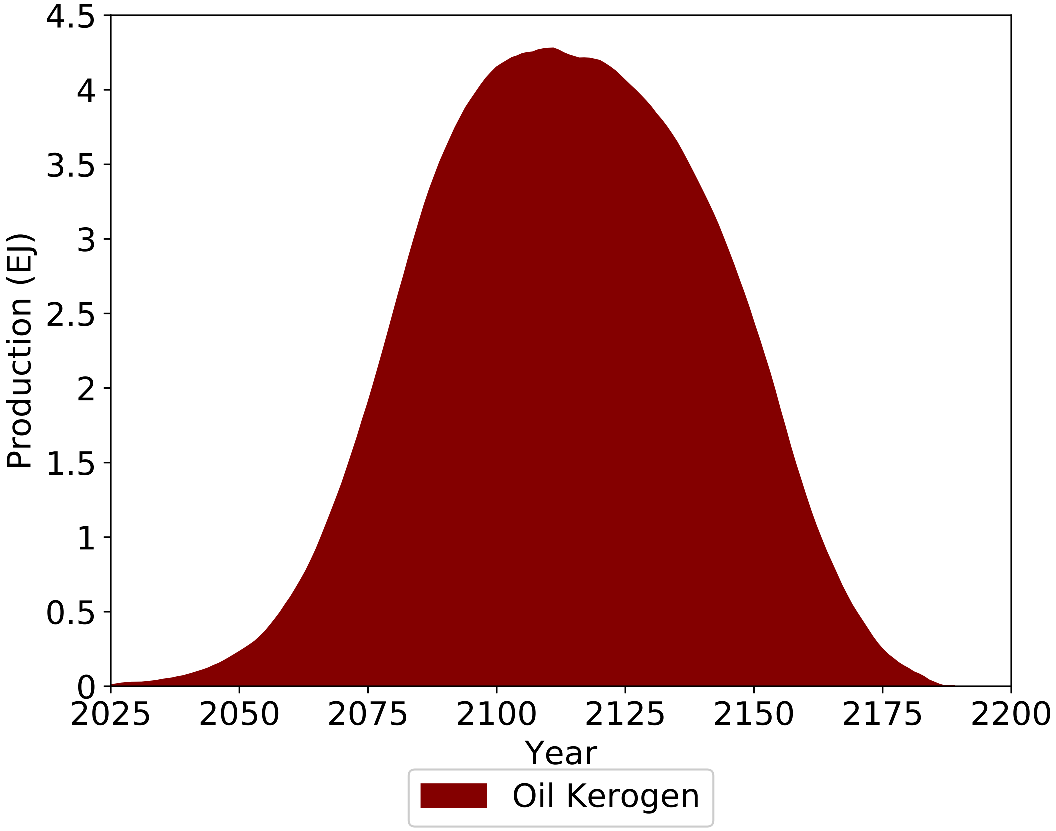

Figure 6.43: USA - Eastern projection by mineral type

Table 6.43: Peak years - Minerals

**Name**	**URR**	**Peak Year**	**Peak Rate**
Oil Kerogen	324.9	2111	4.28
**Total**	**324.9**	**2111**	**4.28**

Florida

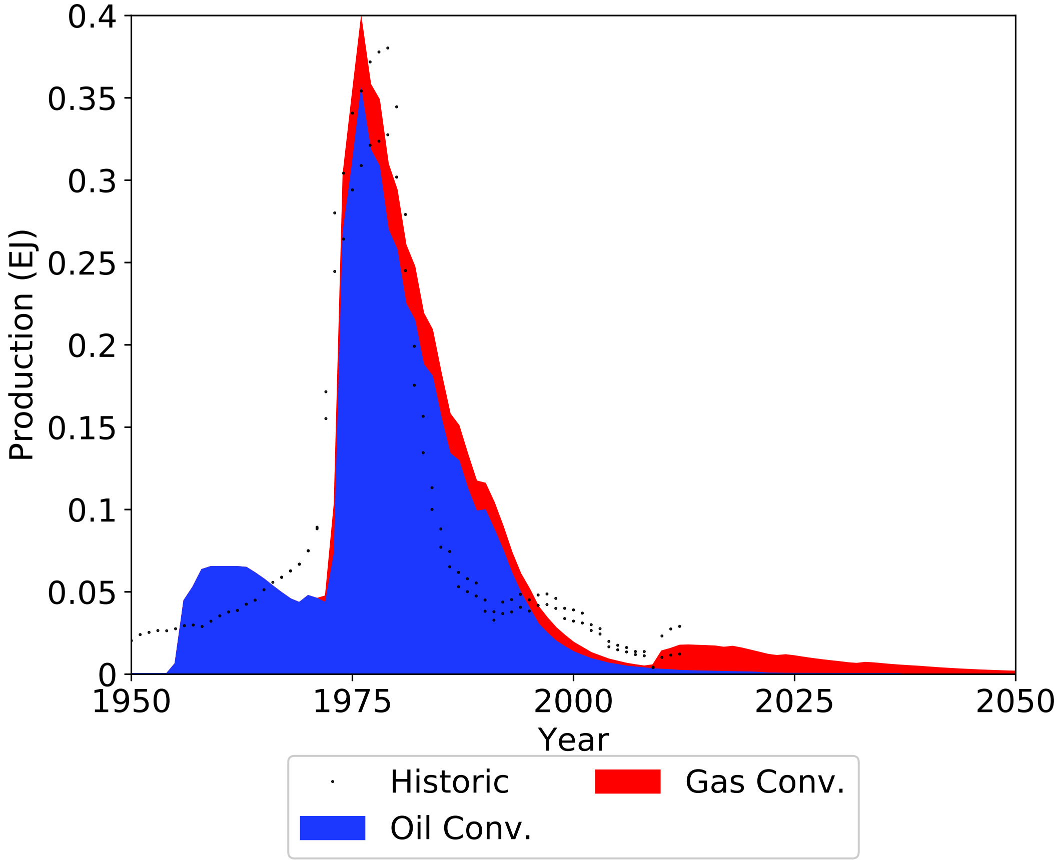

Figure 6.44: USA - Florida projections capped at 16

Table 6.44: Peak years - All

Name	URR	Peak Year	Peak Rate
Oil Conv. Florida	5.13	1976	0.35
Gas Conv. Florida	1.05	1976	0.04
**Total**	**6.18**	**1976**	**0.4**

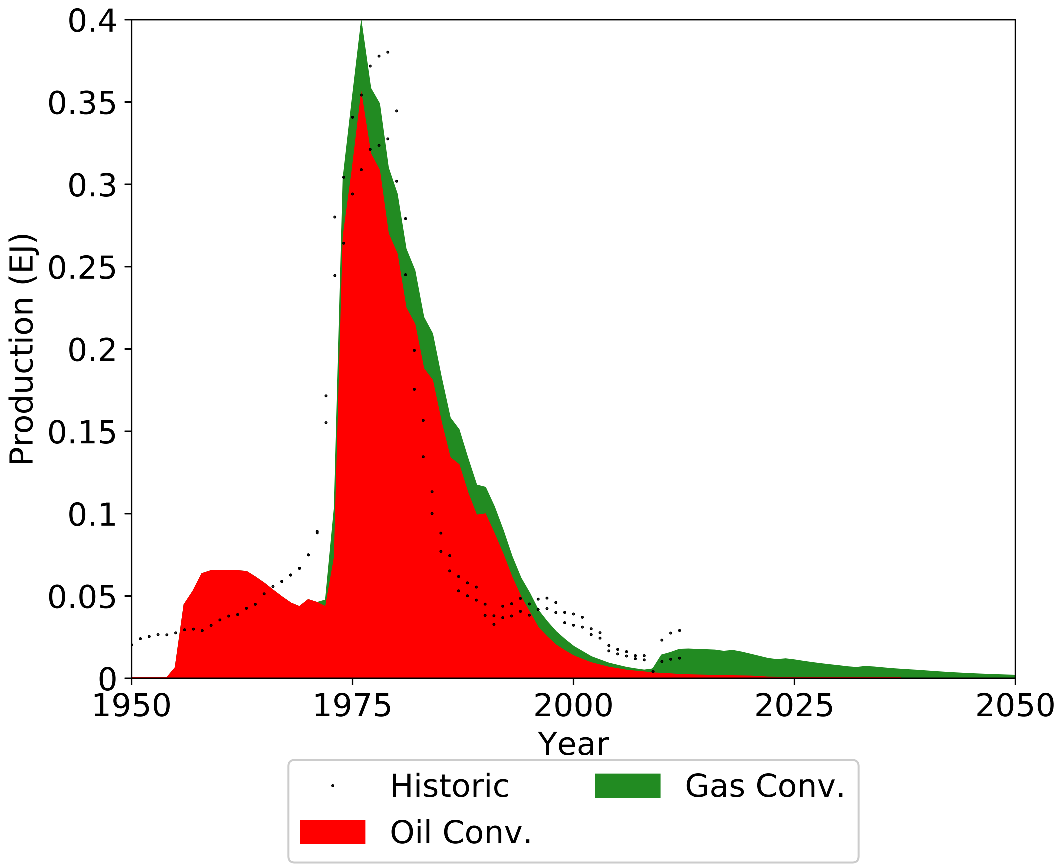

Figure 6.45: USA - Florida projection by mineral type

Table 6.45: Peak years - Minerals

Name	URR	Peak Year	Peak Rate
Oil Conv.	5.13	1976	0.35
Gas Conv.	1.05	1976	0.04
**Total**	**6.18**	**1976**	**0.4**

Georgia

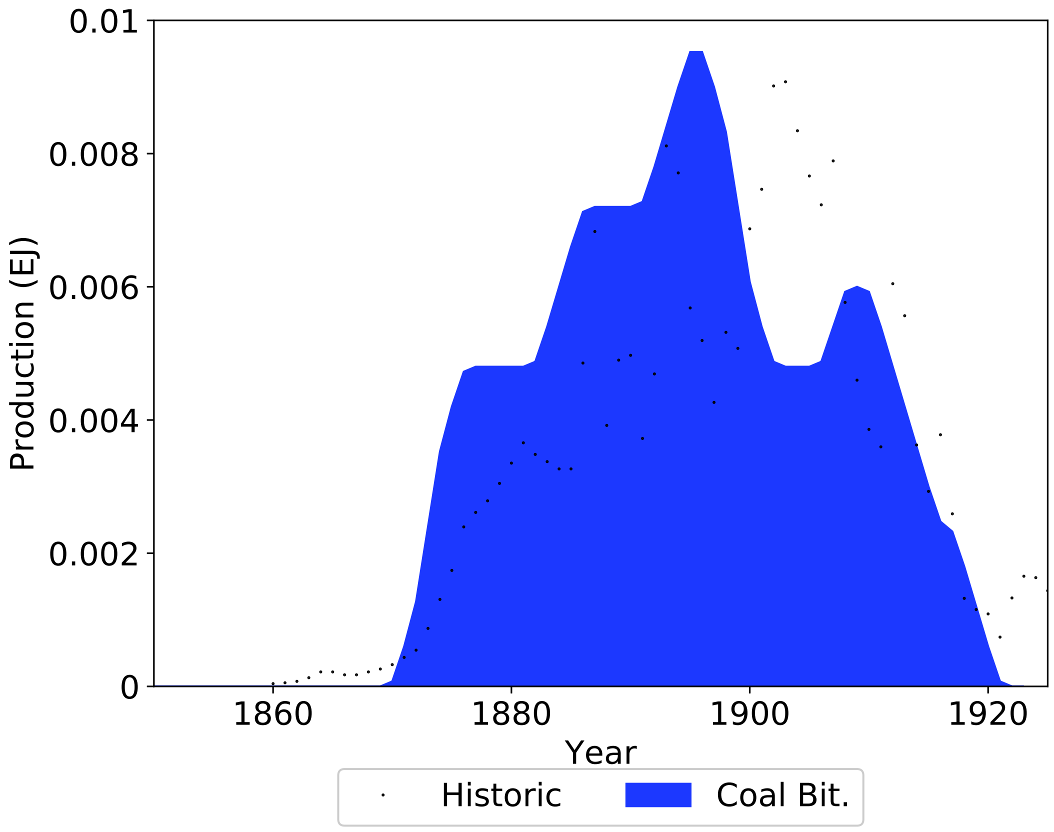

Figure 6.46: USA - Georgia projections capped at 16

Table 6.46: Peak years - All

Name	URR	Peak Year	Peak Rate
Coal Bit. Georgia	0.26	1895	0.01
**Total**	**0.26**	**1895**	**0.01**

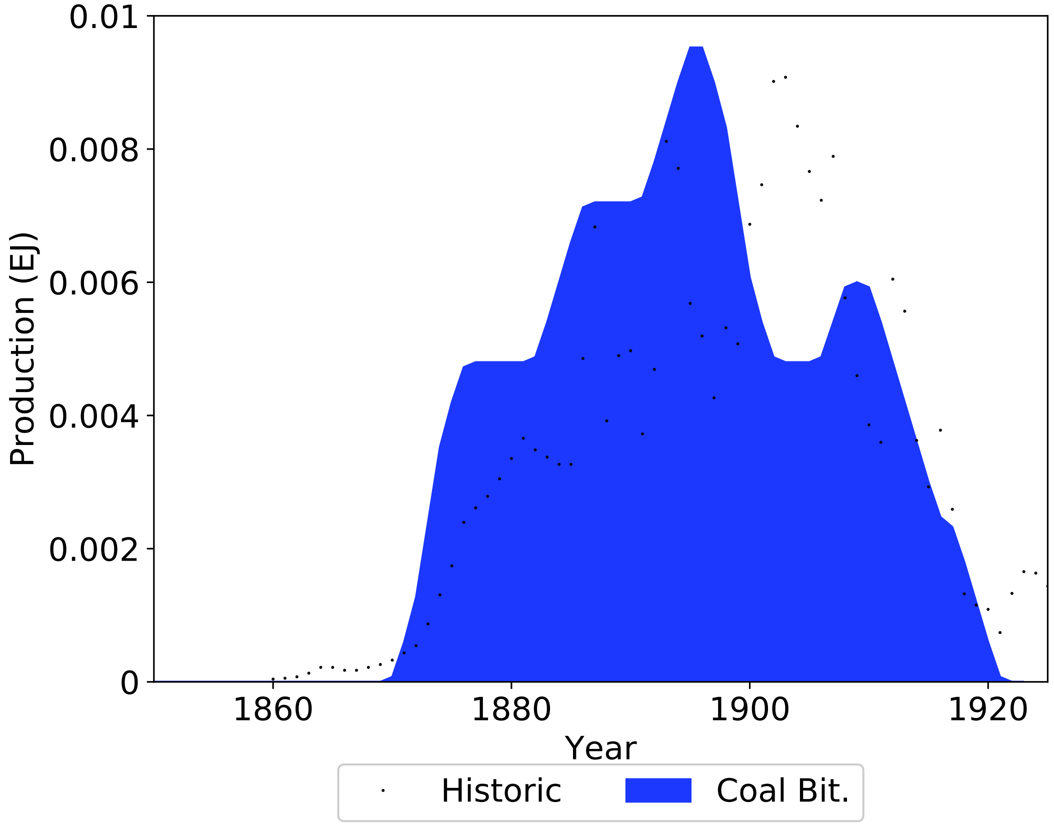

Figure 6.47: USA - Georgia projection by mineral type

Table 6.47: Peak years - Minerals

Name	URR	Peak Year	Peak Rate
Coal Bit.	0.26	1895	0.01
**Total**	**0.26**	**1895**	**0.01**

Illinois

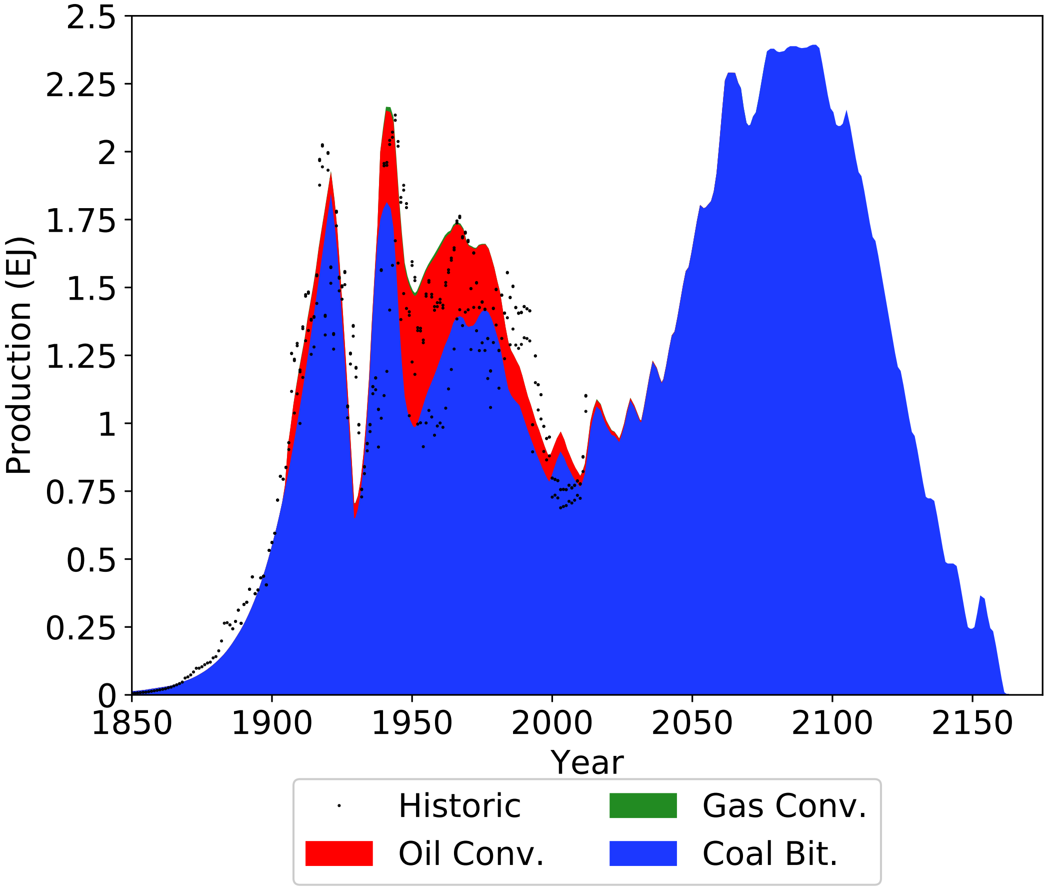

Figure 6.48: USA - Illinois projections capped at 16

Table 6.48: Peak years - All

Name	URR	Peak Year	Peak Rate
Coal Bit. Illinois	349.04	2093	2.39
Oil Conv. Illinois	21.98	1950	0.49
Gas Conv. Illinois	0.56	1943	0.02
**Total**	**371.58**	**2093**	**2.39**

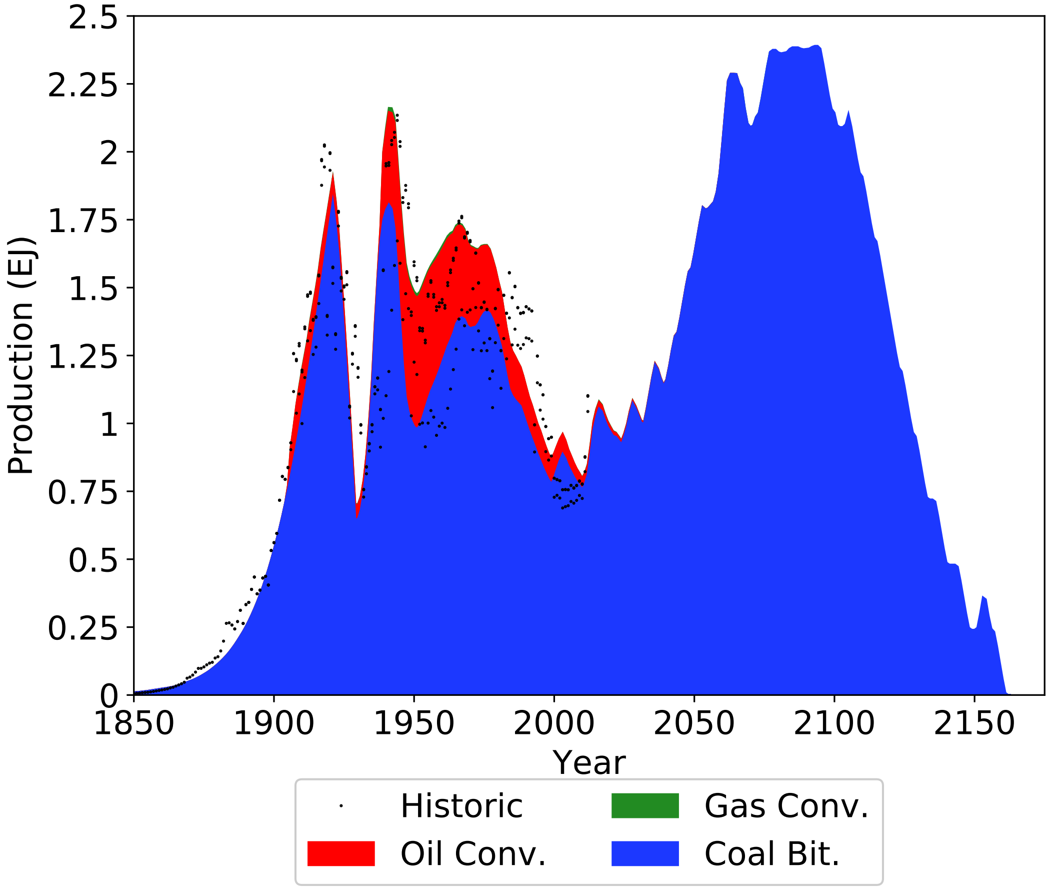

Figure 6.49: USA - Illinois projection by mineral type

Table 6.49: Peak years - Minerals

Name	URR	Peak Year	Peak Rate
Coal Bit.	349.04	2093	2.39
Oil Conv.	21.98	1950	0.49
Gas Conv.	0.56	1943	0.02
**Total**	**371.58**	**2093**	**2.39**

Indiana

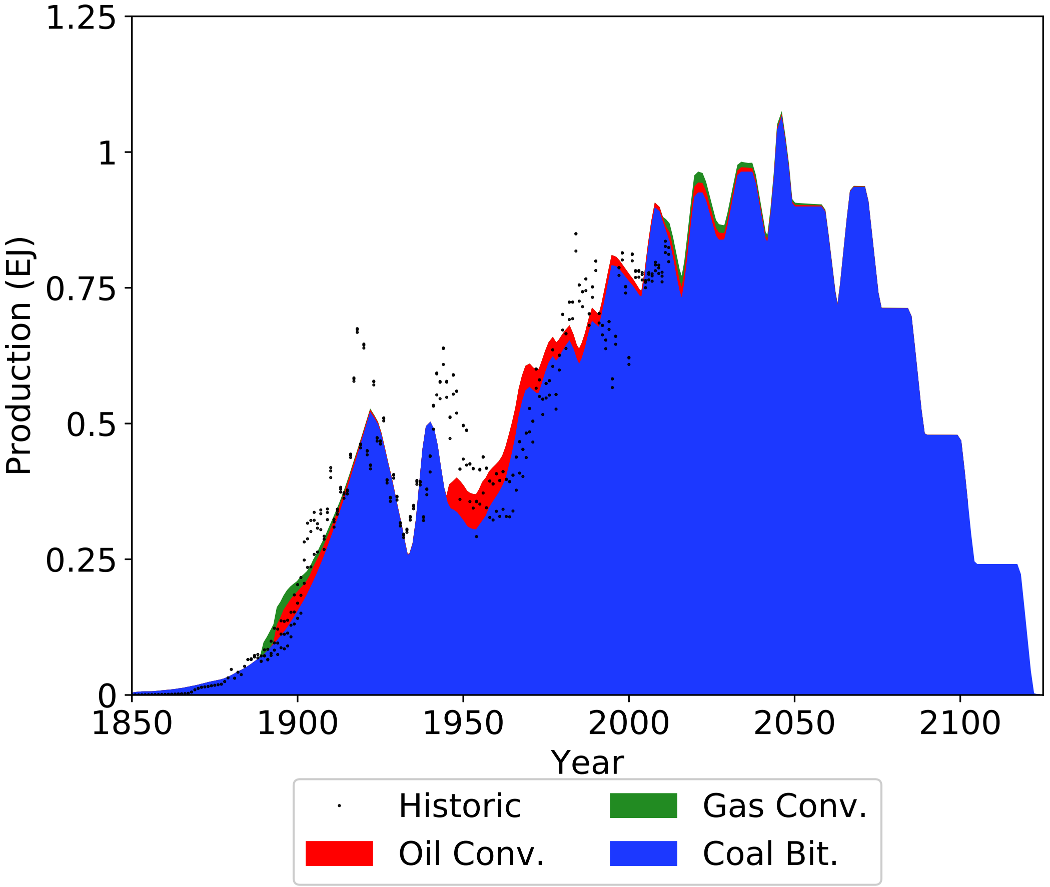

Figure 6.50: USA - Indiana projections capped at 16

Table 6.50: Peak years - All

Name	URR	Peak Year	Peak Rate
Coal Bit. Indiana	132.5	2046	1.06
Oil Conv. Indiana	3.53	1956	0.07
Gas Conv. Indiana	1.05	1893	0.03
**Total**	**137.08**	**2046**	**1.07**

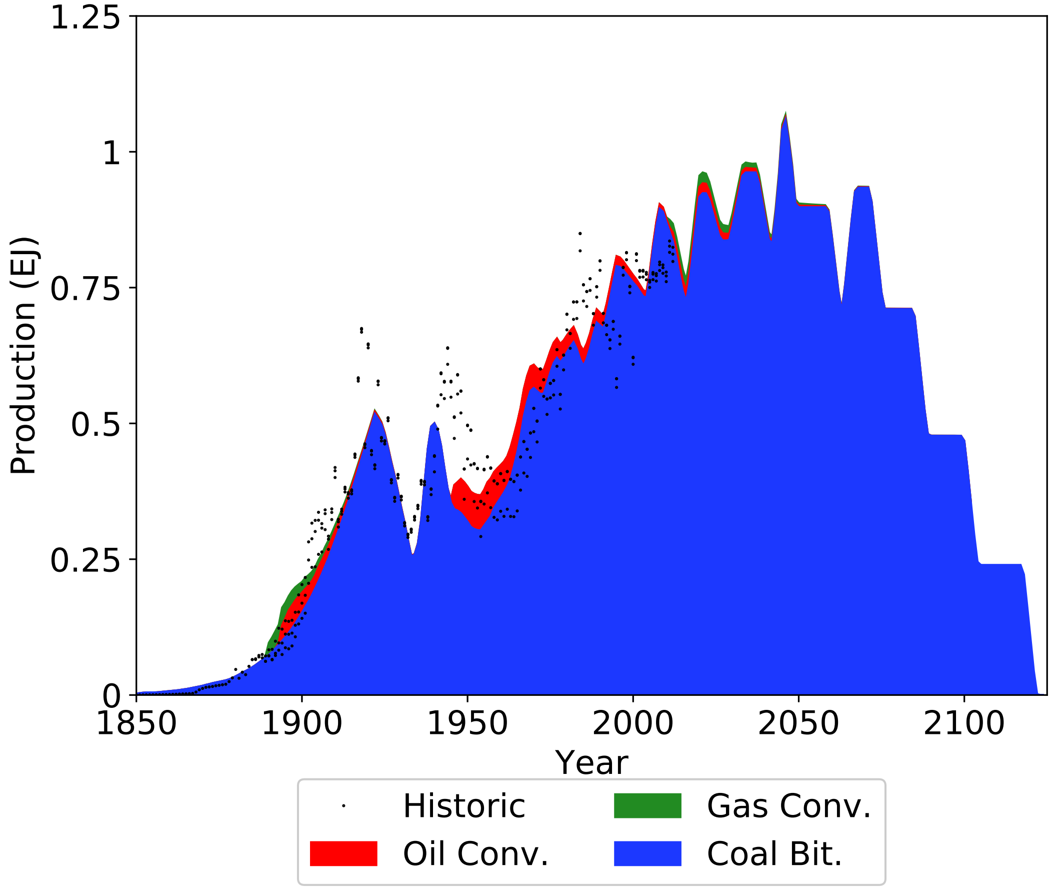

Figure 6.51: USA - Indiana projection by mineral type

Table 6.51: Peak years - Minerals

Name	URR	Peak Year	Peak Rate
Coal Bit.	132.5	2046	1.06
Oil Conv.	3.53	1956	0.07
Gas Conv.	1.05	1893	0.03
**Total**	**137.08**	**2046**	**1.07**

Iowa

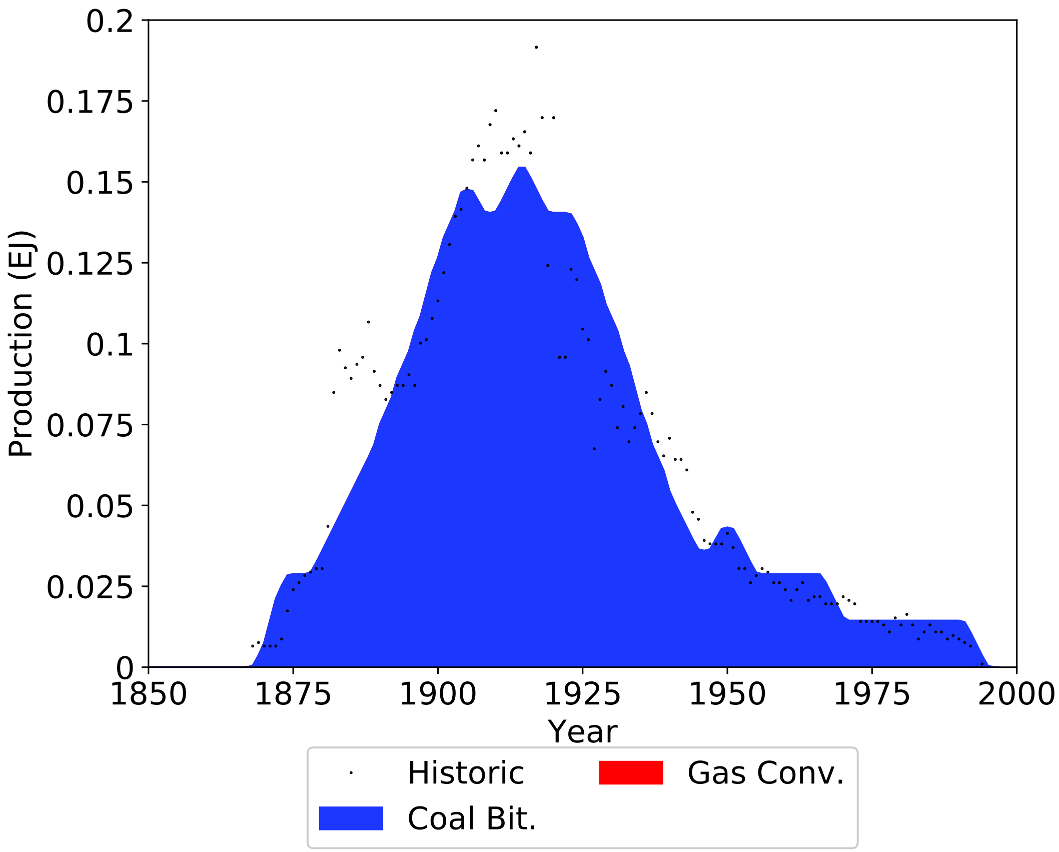

Figure 6.52: USA - Iowa projections capped at 16

Table 6.52: Peak years - All

Name	URR	Peak Year	Peak Rate
Coal Bit. Iowa	8.14	1914	0.15
Gas Conv. Iowa	–	1919	–
**Total**	**8.14**	**1914**	**0.15**

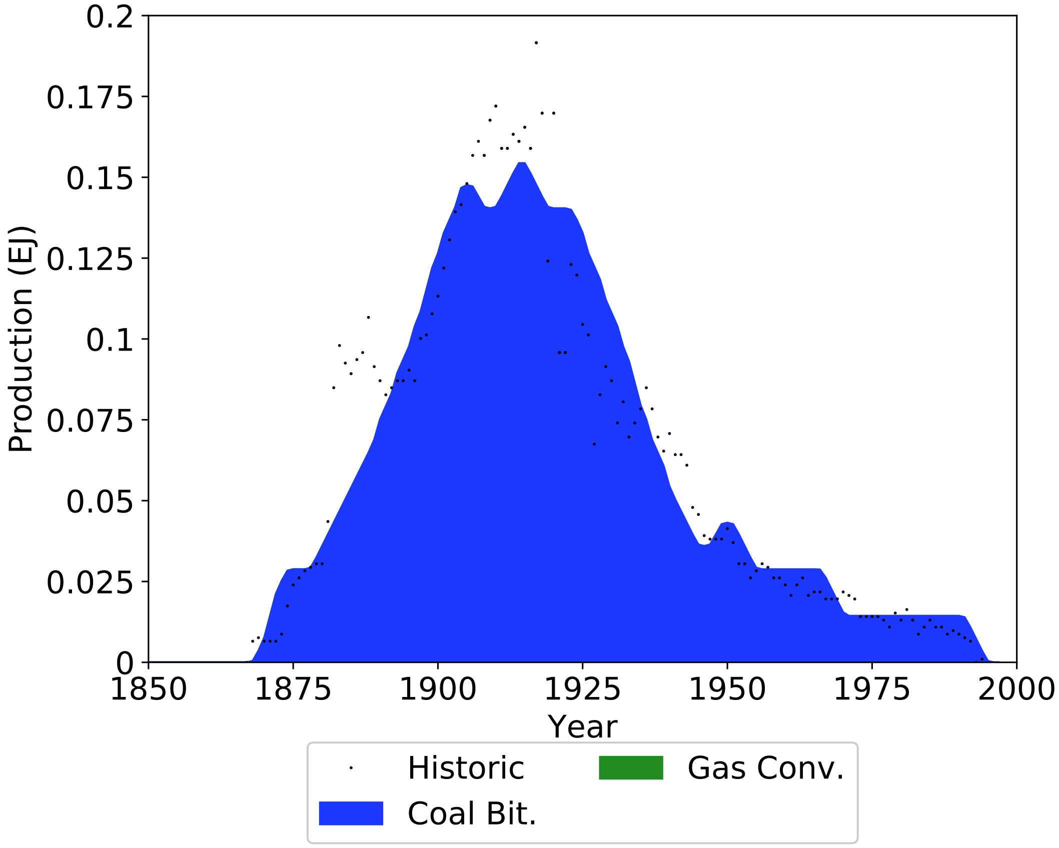

Figure 6.53: USA - Iowa projection by mineral type

Table 6.53: Peak years - Minerals

**Name**	**URR**	**Peak Year**	**Peak Rate**
Coal Bit.	8.14	1914	0.15
Gas Conv.	–	1919	–
**Total**	**8.14**	**1914**	**0.15**

Kansas

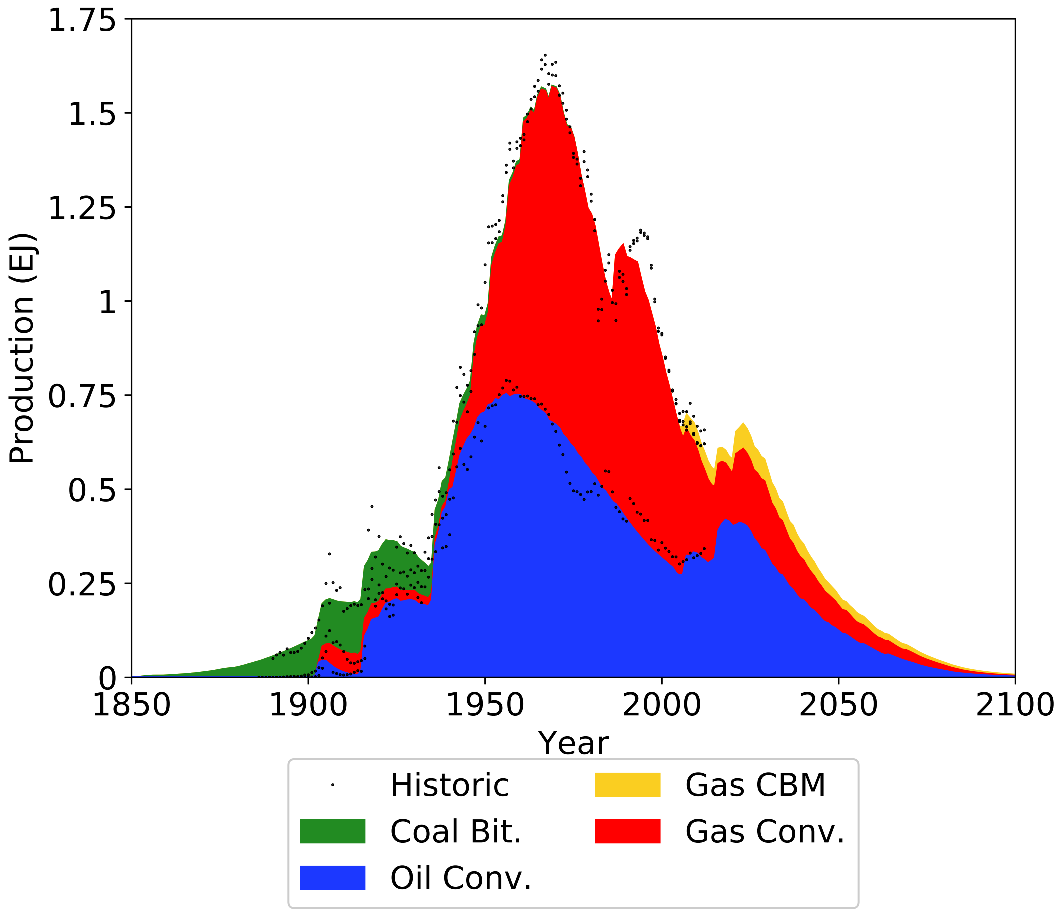

Figure 6.54: USA - Kansas projections capped at 16

Table 6.54: Peak years - All

Name	URR	Peak Year	Peak Rate
Oil Conv. Kansas	57.63	1956	0.75
Gas Conv. Kansas	47.12	1970	0.89
Coal Bit. Kansas	6.64	1916	0.14
Gas CBM Kansas	2.69	2023	0.07
**Total**	**114.08**	**1969**	**1.57**

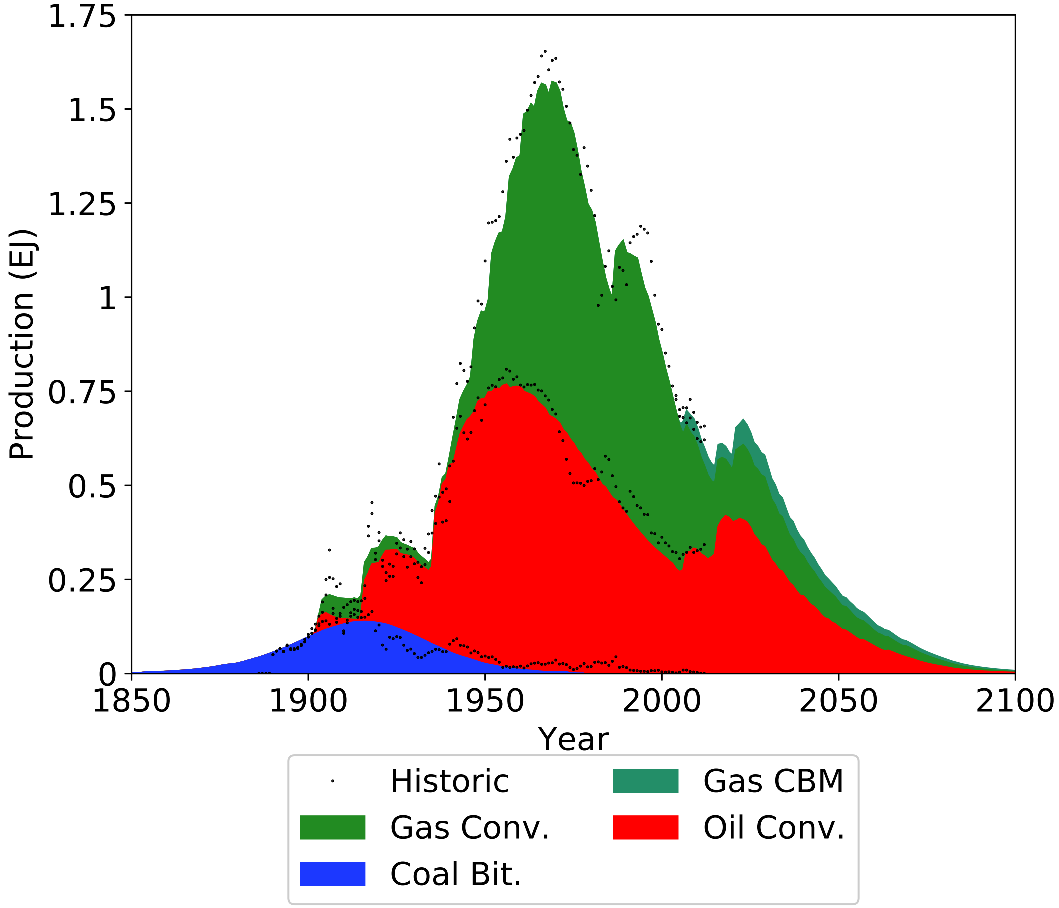

Figure 6.55: USA - Kansas projection by mineral type

Table 6.55: Peak years - Minerals

Name	URR	Peak Year	Peak Rate
Coal Bit.	6.64	1916	0.14
Oil Conv.	57.63	1956	0.75
Gas Conv.	47.12	1970	0.89
Gas CBM	2.69	2023	0.07
**Total**	**114.08**	**1969**	**1.57**

Kentucky

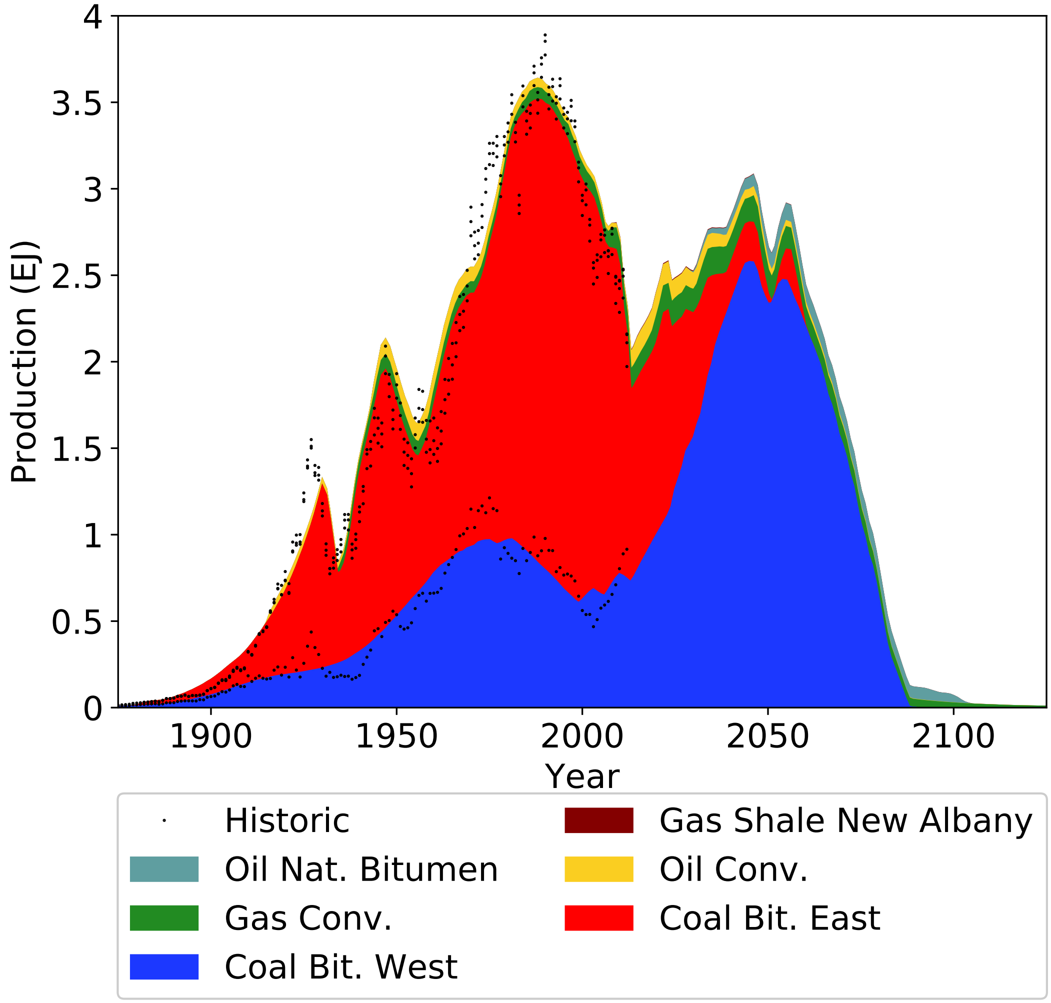

Figure 6.56: USA - Kentucky projections capped at 16

Table 6.56: Peak years - All

Name	URR	Peak Year	Peak Rate
Coal Bit. Kentucky West	180.45	2045	2.58
Coal Bit. Kentucky East	175.42	1991	2.71
Gas Conv. Kentucky	15.75	2034	0.17
Oil Conv. Kentucky	9.65	2020	0.13
Oil Nat. Bitumen Kentucky	5.34	2053	0.1
Gas Shale Kentucky New Albany	0.23	2025	0.01
**Total**	**386.84**	**1988**	**3.64**

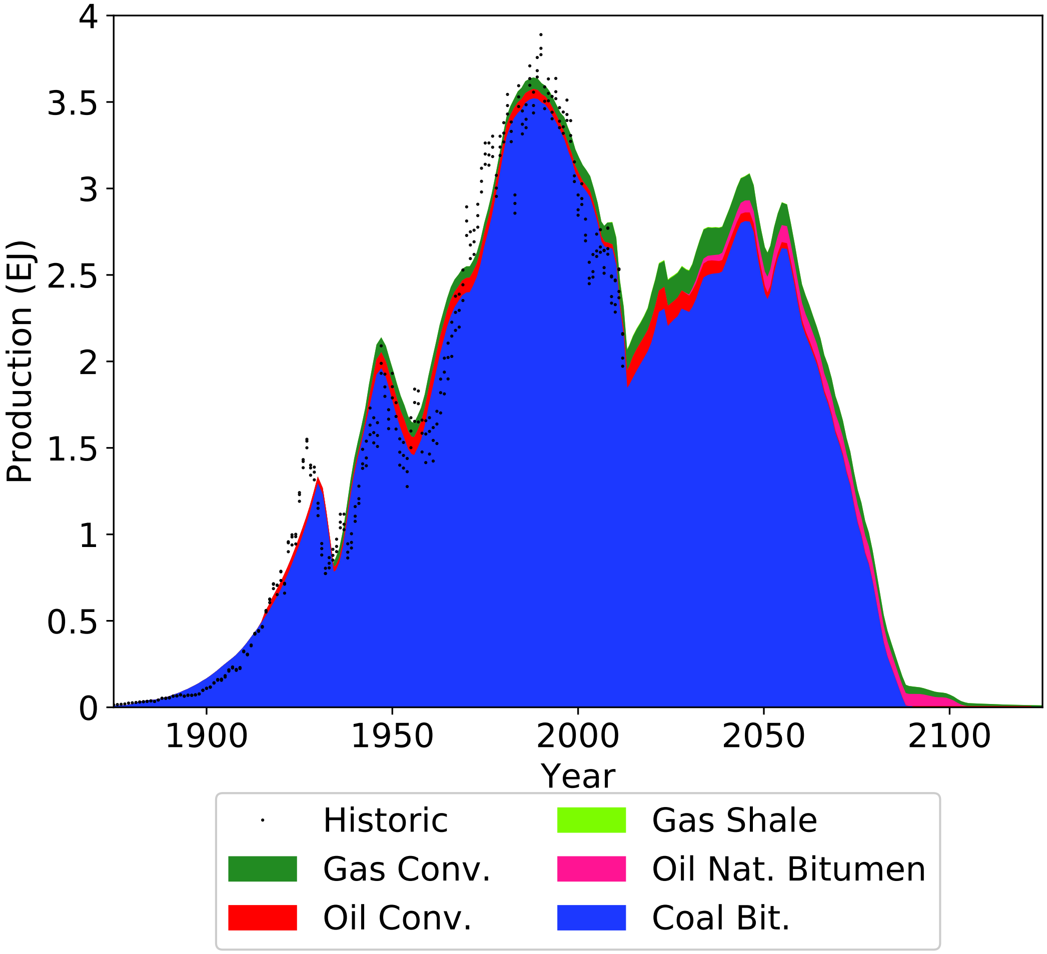

Figure 6.57: USA - Kentucky projection by mineral type

Table 6.57: Peak years - Minerals

**Name**	**URR**	**Peak Year**	**Peak Rate**
Coal Bit.	355.87	1988	3.52
Oil Conv.	9.65	2020	0.13
Oil Nat. Bitumen	5.34	2053	0.1
Gas Conv.	15.75	2034	0.17
Gas Shale	0.23	2025	0.01
**Total**	**386.84**	**1988**	**3.64**

Louisiana

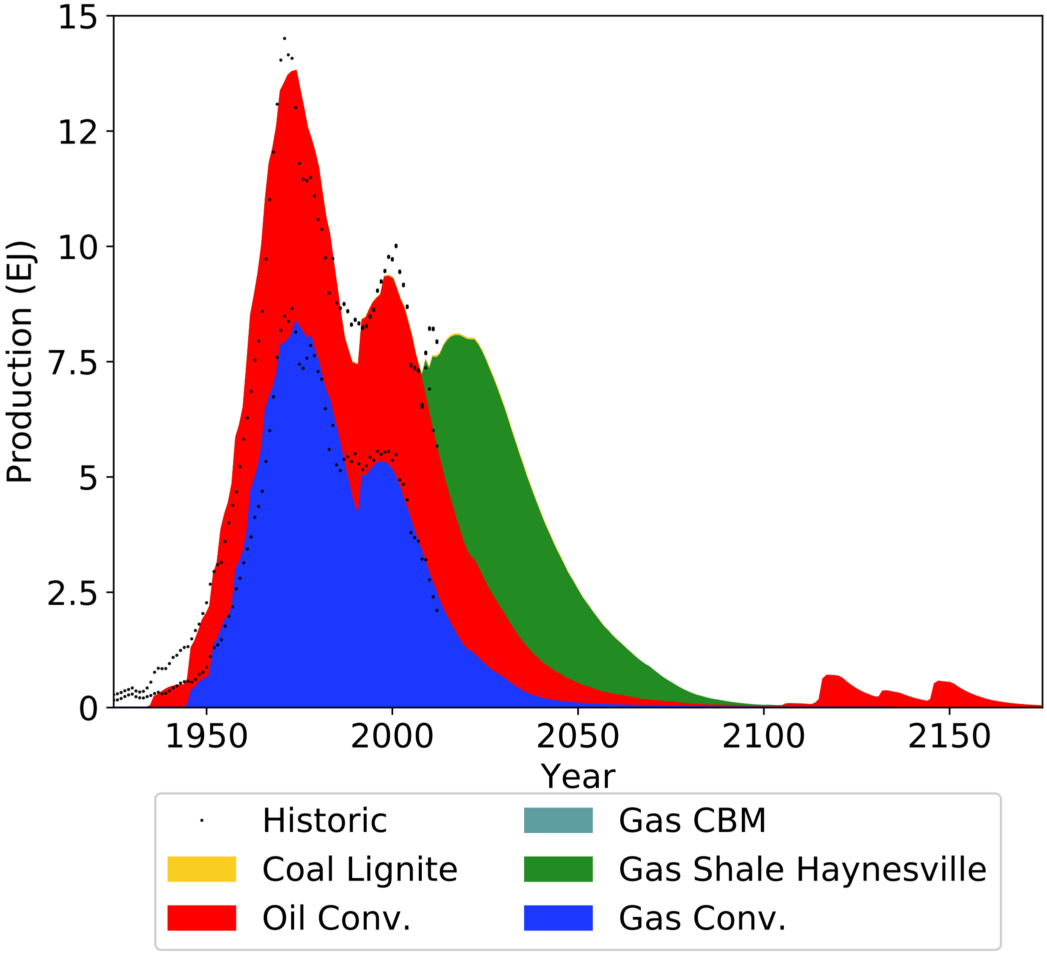

Figure 6.58: USA - Louisiana projections capped at 16

Table 6.58: Peak years - All

Name	URR	Peak Year	Peak Rate
Gas Conv. Louisiana	344.58	1974	8.36
Oil Conv. Louisiana	321.88	1972	5.74
Gas Shale Louisiana Haynesville	175.11	2024	4.84
Coal Lignite Louisiana	2.34	2028	0.05
Gas CBM Louisiana	0.01	2008	–
**Total**	**843.92**	**1974**	**13.8**

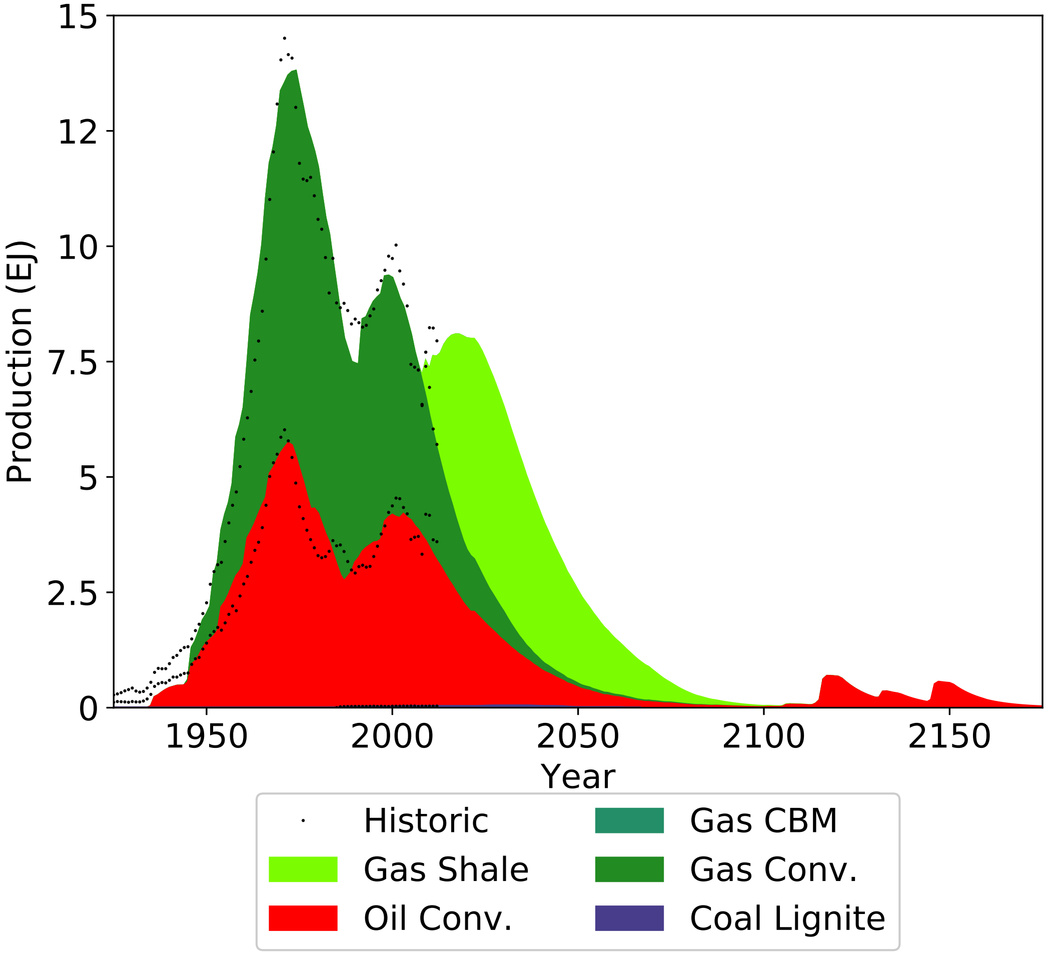

Figure 6.59: USA - Louisiana projection by mineral type

Table 6.59: Peak years - Minerals

Name	URR	Peak Year	Peak Rate
Coal Lignite	2.34	2028	0.05
Oil Conv.	321.88	1972	5.74
Gas Conv.	344.58	1974	8.36
Gas Shale	175.11	2024	4.84
Gas CBM	0.01	2008	–
**Total**	**843.92**	**1974**	**13.8**

Maryland

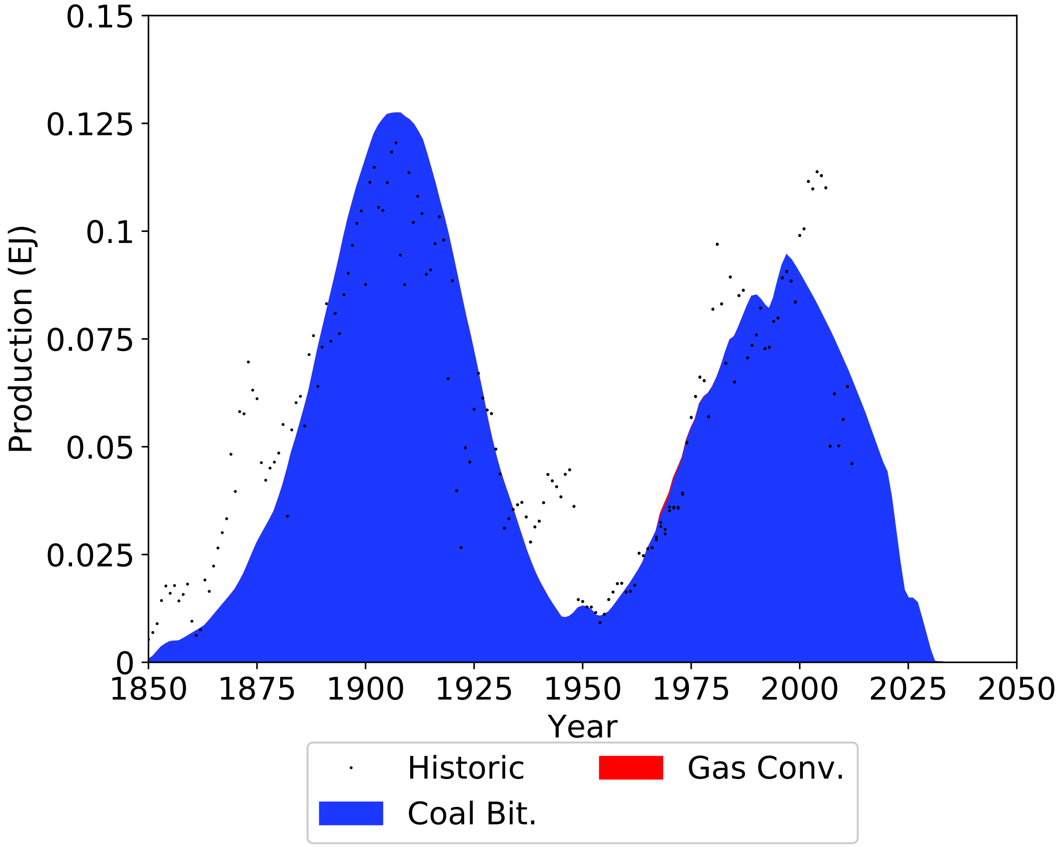

Figure 6.60: USA - Maryland projections capped at 16

Table 6.60: Peak years - All

**Name**	**URR**	**Peak Year**	**Peak Rate**
Coal Bit. Maryland	9.63	1907	0.13
Gas Conv. Maryland	0.01	1969	–
**Total**	**9.64**	**1907**	**0.13**

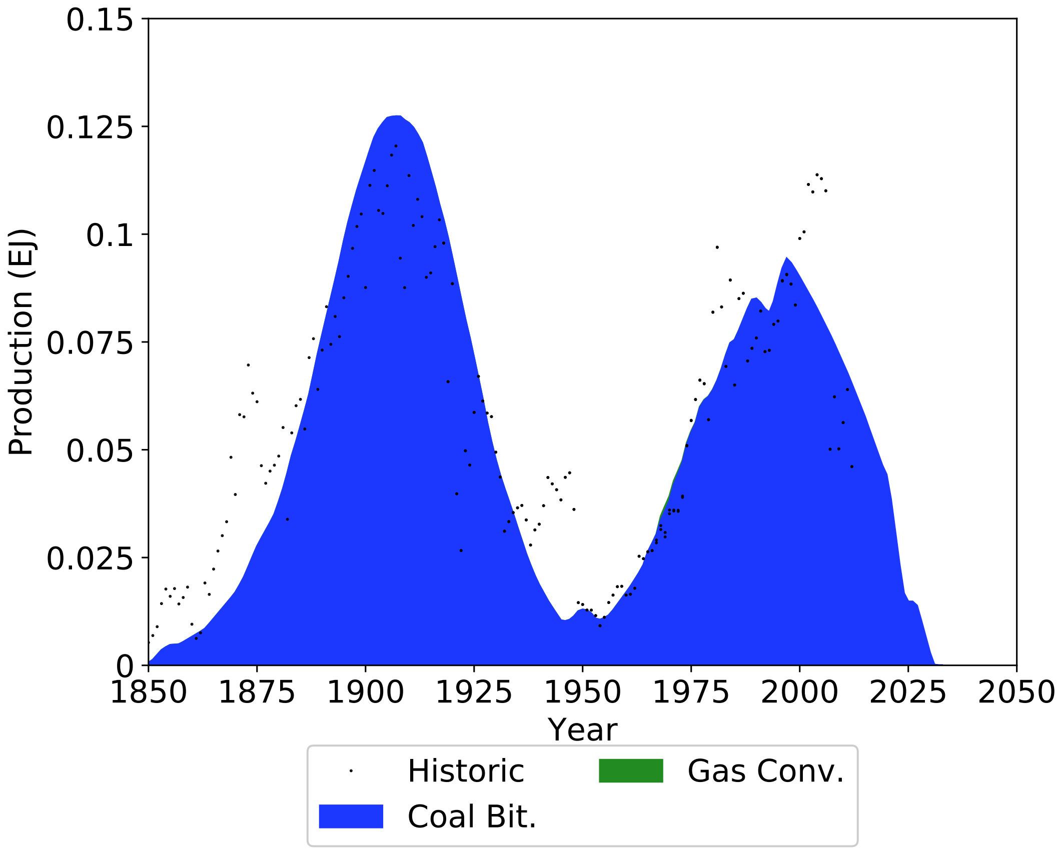

Figure 6.61: USA - Maryland projection by mineral type

Table 6.61: Peak years - Minerals

Name	URR	Peak Year	Peak Rate
Coal Bit.	9.63	1907	0.13
Gas Conv.	0.01	1969	–
**Total**	**9.64**	**1907**	**0.13**

Michigan

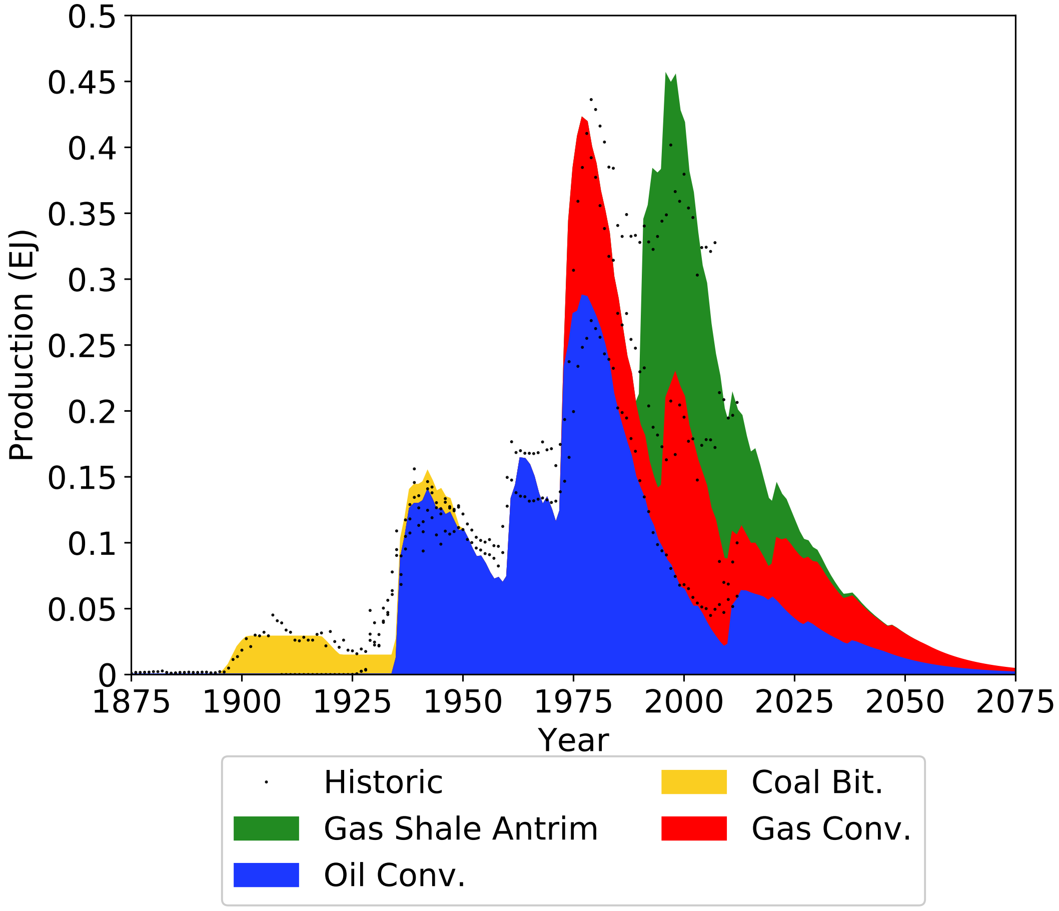

Figure 6.62: USA - Michigan projections capped at 16

Table 6.62: Peak years - All

Name	URR	Peak Year	Peak Rate
Oil Conv. Michigan	11.37	1977	0.29
Gas Conv. Michigan	5.25	1998	0.15
Gas Shale Michigan Antrim	4.67	1996	0.24
Coal Bit. Michigan	1.04	1902	0.03
**Total**	**22.33**	**1996**	**0.45**

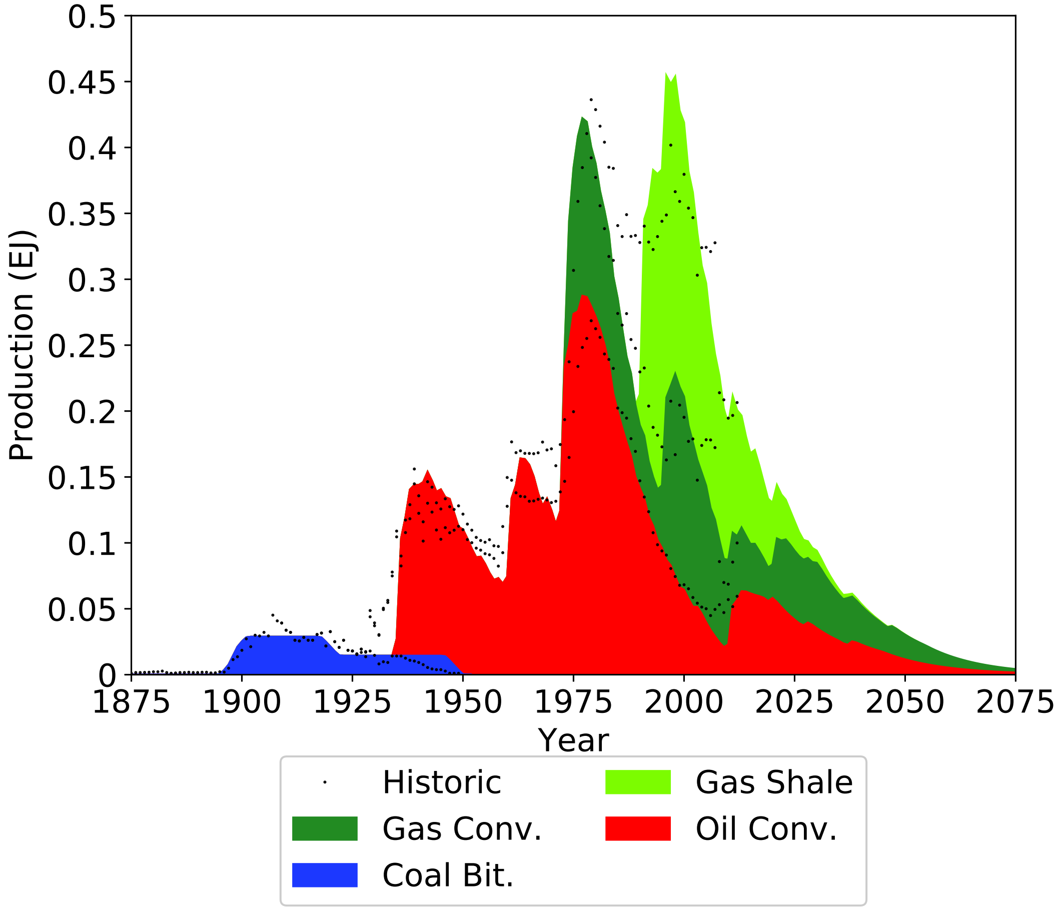

Figure 6.63: USA - Michigan projection by mineral type

Table 6.63: Peak years - Minerals

Name	URR	Peak Year	Peak Rate
Coal Bit.	1.04	1902	0.03
Oil Conv.	11.37	1977	0.29
Gas Conv.	5.25	1998	0.15
Gas Shale	4.67	1996	0.24
**Total**	**22.33**	**1996**	**0.45**

Mississippi

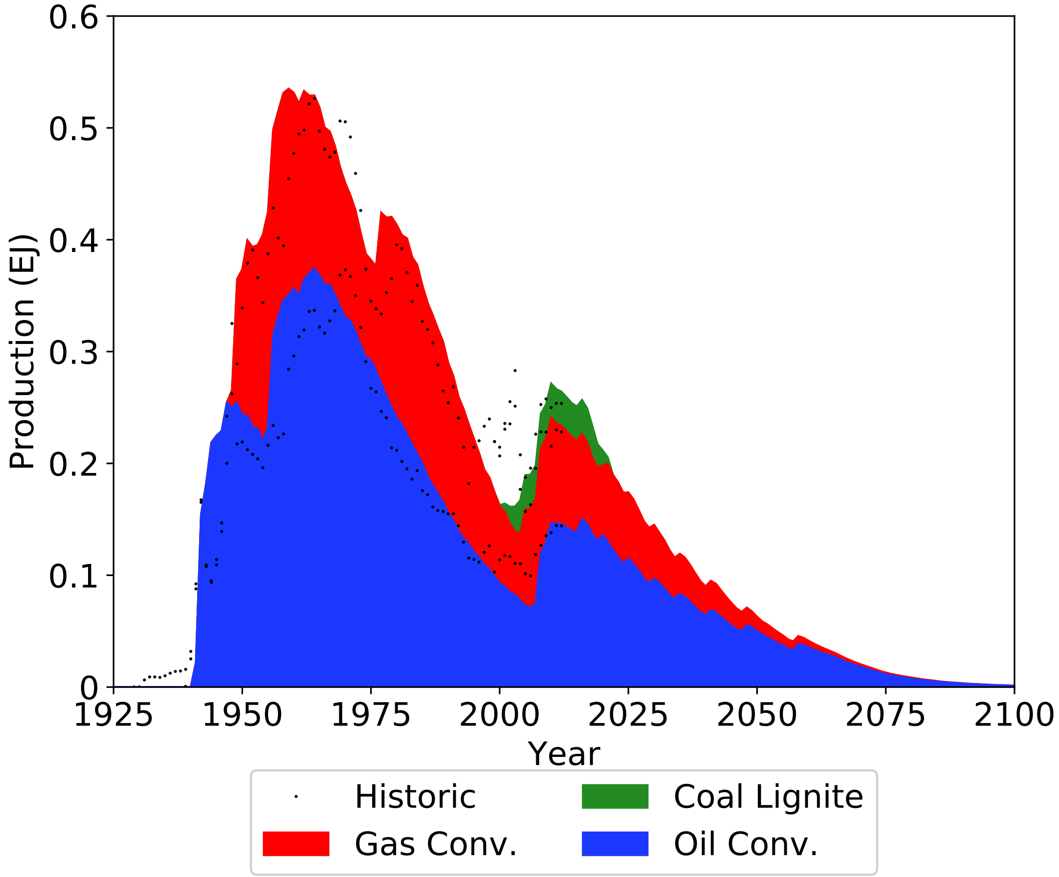

Figure 6.64: USA - Mississippi projections capped at 16

Table 6.64: Peak years - All

**Name**	**URR**	**Peak Year**	**Peak Rate**
Oil Conv. Mississippi	20.09	1964	0.37
Gas Conv. Mississippi	10.15	1955	0.19
Coal Lignite Mississippi	0.54	2005	0.03
**Total**	**30.78**	**1959**	**0.53**

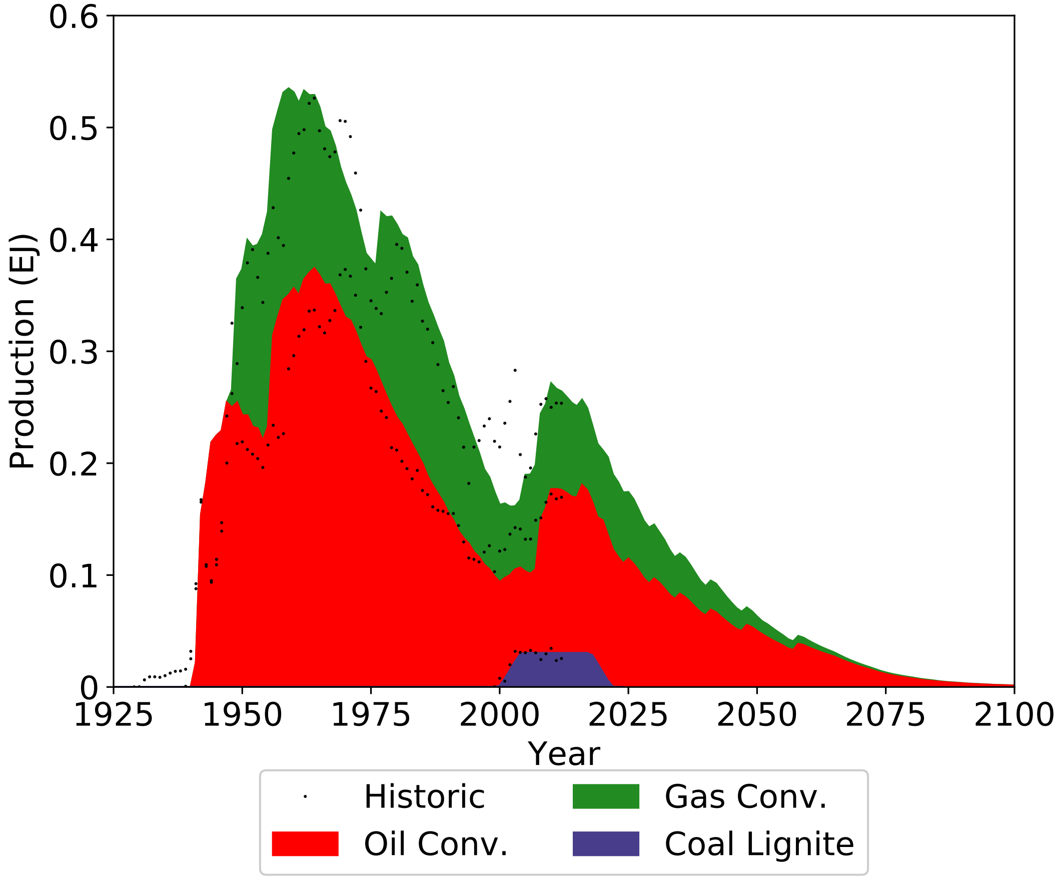

Figure 6.65: USA - Mississippi projection by mineral type

Table 6.65: Peak years - Minerals

**Name**	**URR**	**Peak Year**	**Peak Rate**
Coal Lignite	0.54	2005	0.03
Oil Conv.	20.09	1964	0.37
Gas Conv.	10.15	1955	0.19
**Total**	**30.78**	**1959**	**0.53**

Missouri

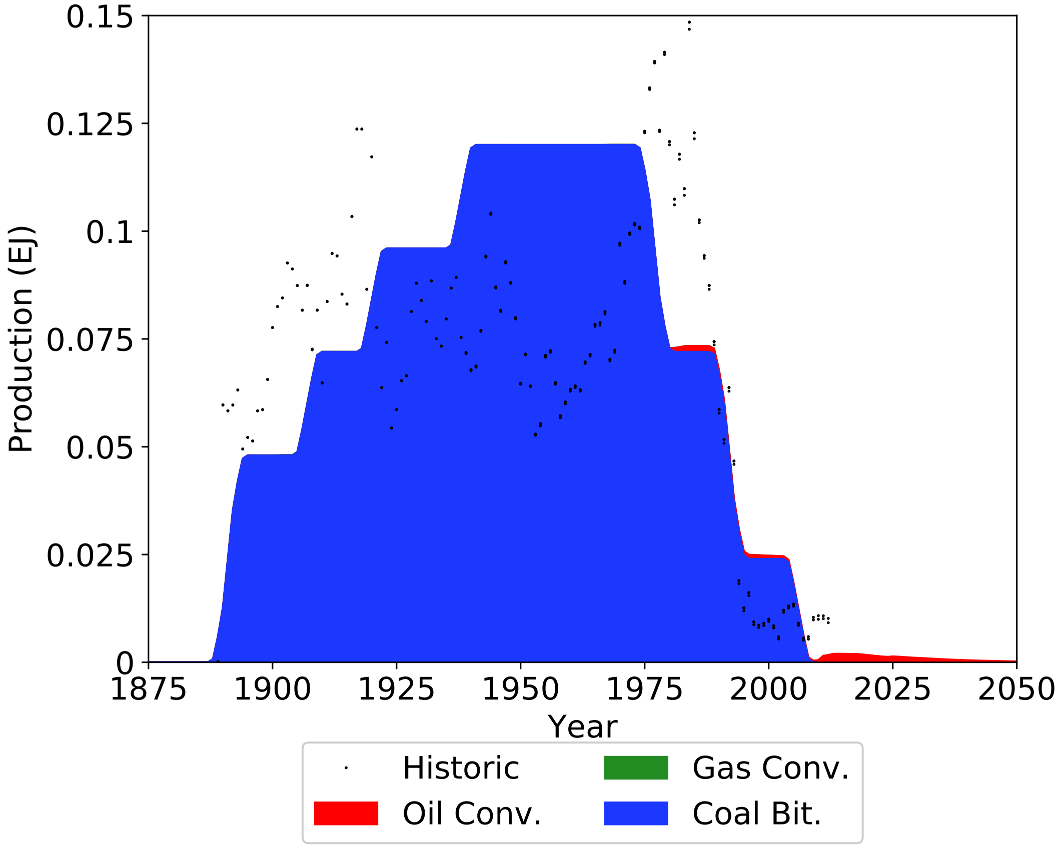

Figure 6.66: USA - Missouri projections capped at 16

Table 6.66: Peak years - All

Name	URR	Peak Year	Peak Rate
Coal Bit. Missouri	9.53	1941	0.12
Oil Conv. Missouri	0.07	2014	–
Gas Conv. Missouri	–	1903	–
**Total**	**9.6**	**1969**	**0.12**

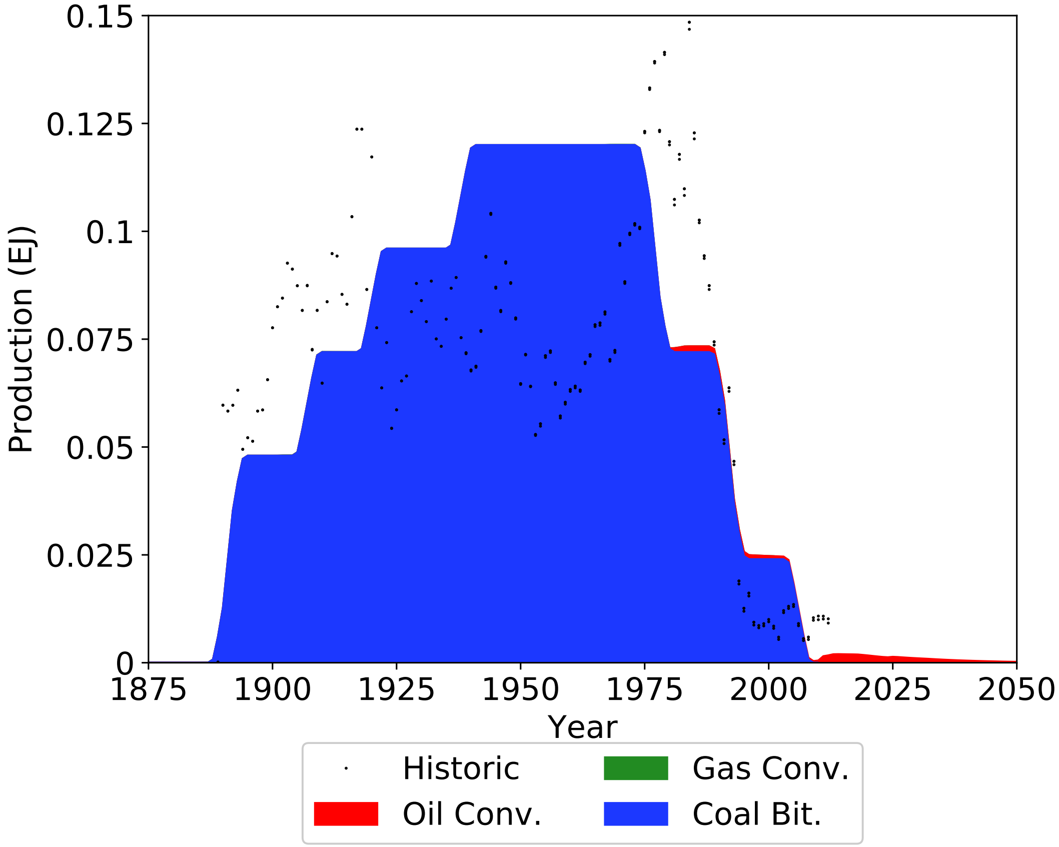

Figure 6.67: USA - Missouri projection by mineral type

Table 6.67: Peak years - Minerals

**Name**	**URR**	**Peak Year**	**Peak Rate**
Coal Bit.	9.53	1941	0.12
Oil Conv.	0.07	2014	–
Gas Conv.	–	1903	–
**Total**	**9.6**	**1969**	**0.12**

Montana

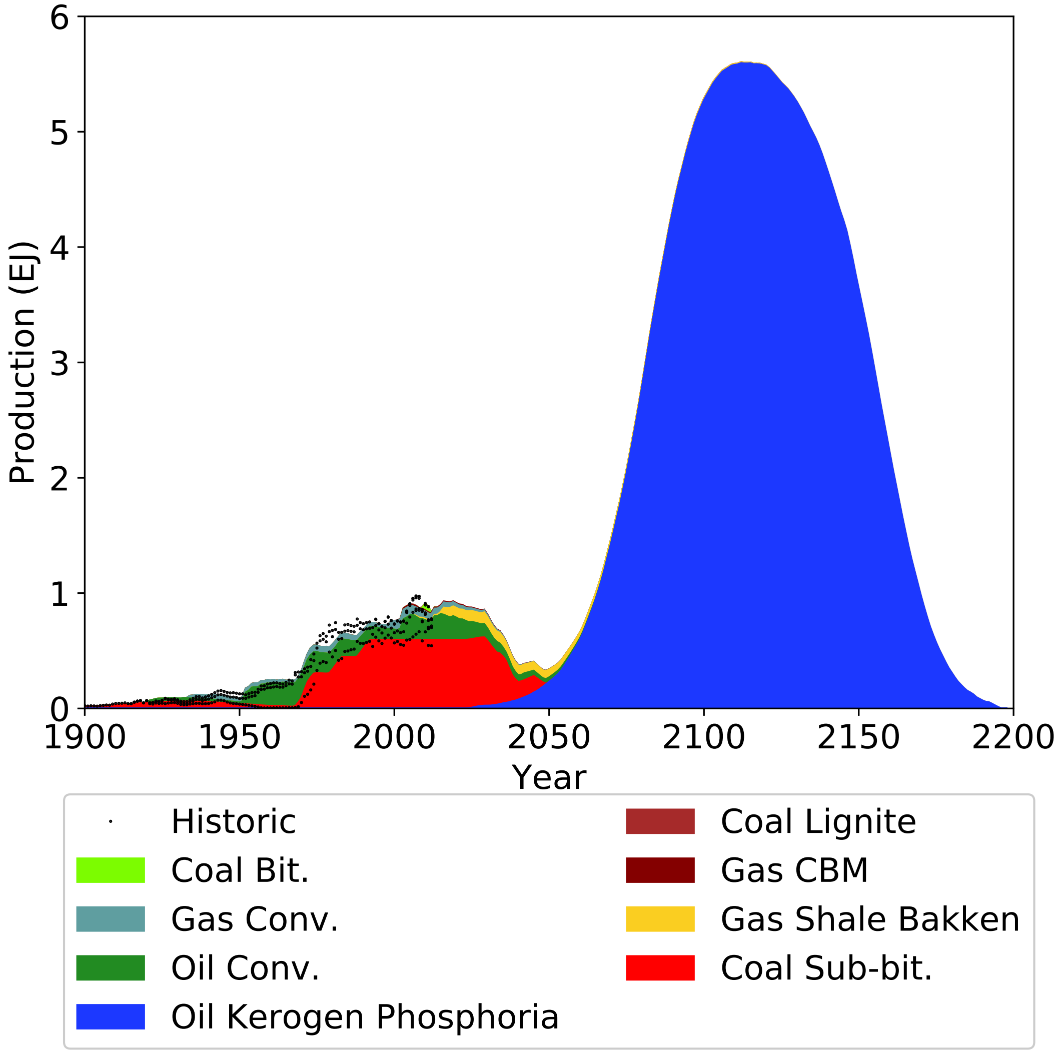

Figure 6.68: USA - Montana projections capped at 16

Table 6.68: Peak years - All

Name	URR	Peak Year	Peak Rate
Oil Kerogen Montana Phosphoria	429.8	2112	5.59
Coal Sub-bit. Montana	38.52	1993	0.59
Oil Conv. Montana	15.15	2015	0.22
Gas Shale Montana Bakken	5.06	2029	0.1
Gas Conv. Montana	4.21	2004	0.09
Gas CBM Montana	0.3	2004	0.01
Coal Bit. Montana	0.11	2010	0.06
Coal Lignite Montana	0.11	1999	–
**Total**	**493.26**	**2112**	**5.6**

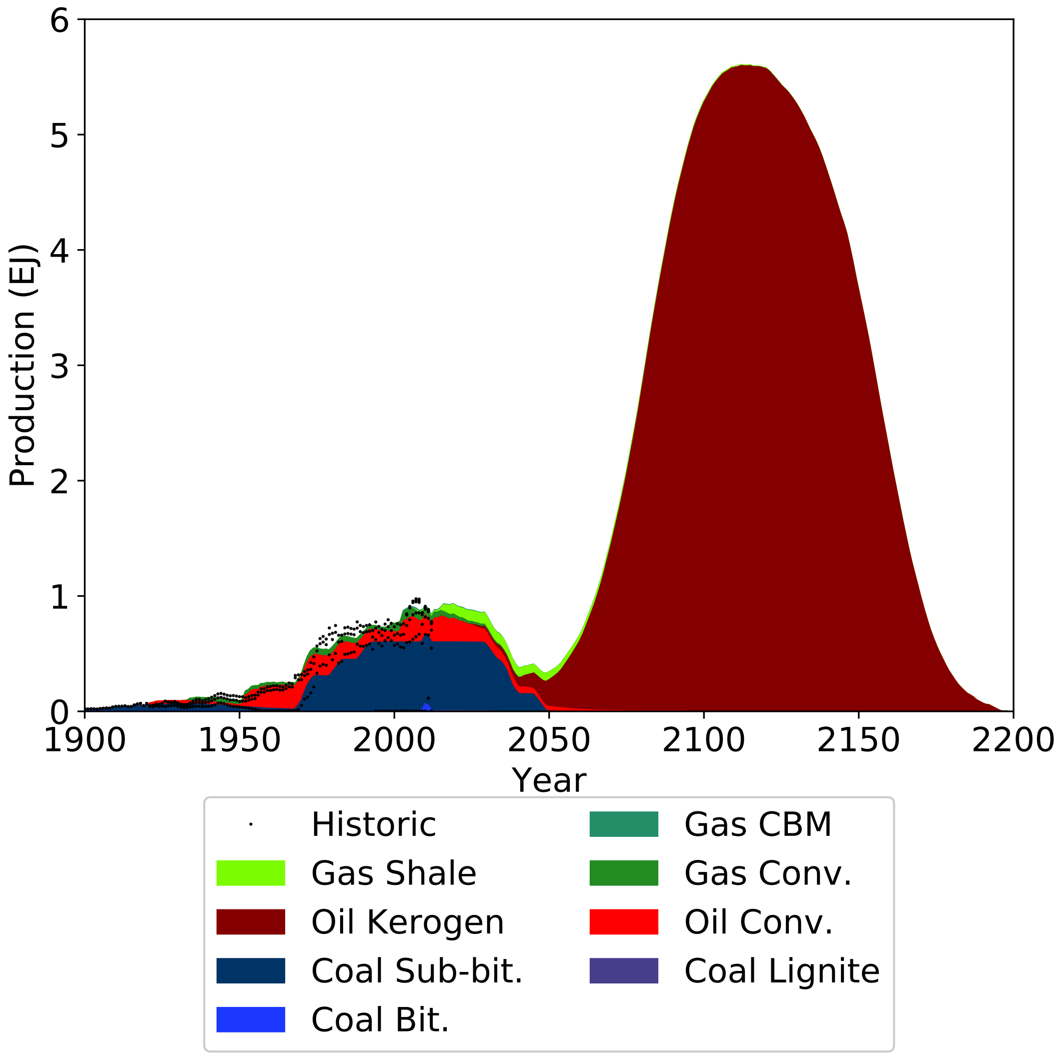

Figure 6.69: USA - Montana projection by mineral type

Table 6.69: Peak years - Minerals

**Name**	**URR**	**Peak Year**	**Peak Rate**
Coal Bit.	0.11	2010	0.06
Coal Lignite	0.11	1999	–
Coal Sub-bit.	38.52	1993	0.59
Oil Conv.	15.15	2015	0.22
Oil Kerogen	429.8	2112	5.59
Gas Conv.	4.21	2004	0.09
Gas Shale	5.06	2029	0.1
Gas CBM	0.3	2004	0.01
**Total**	**493.26**	**2112**	**5.6**

Nebraska

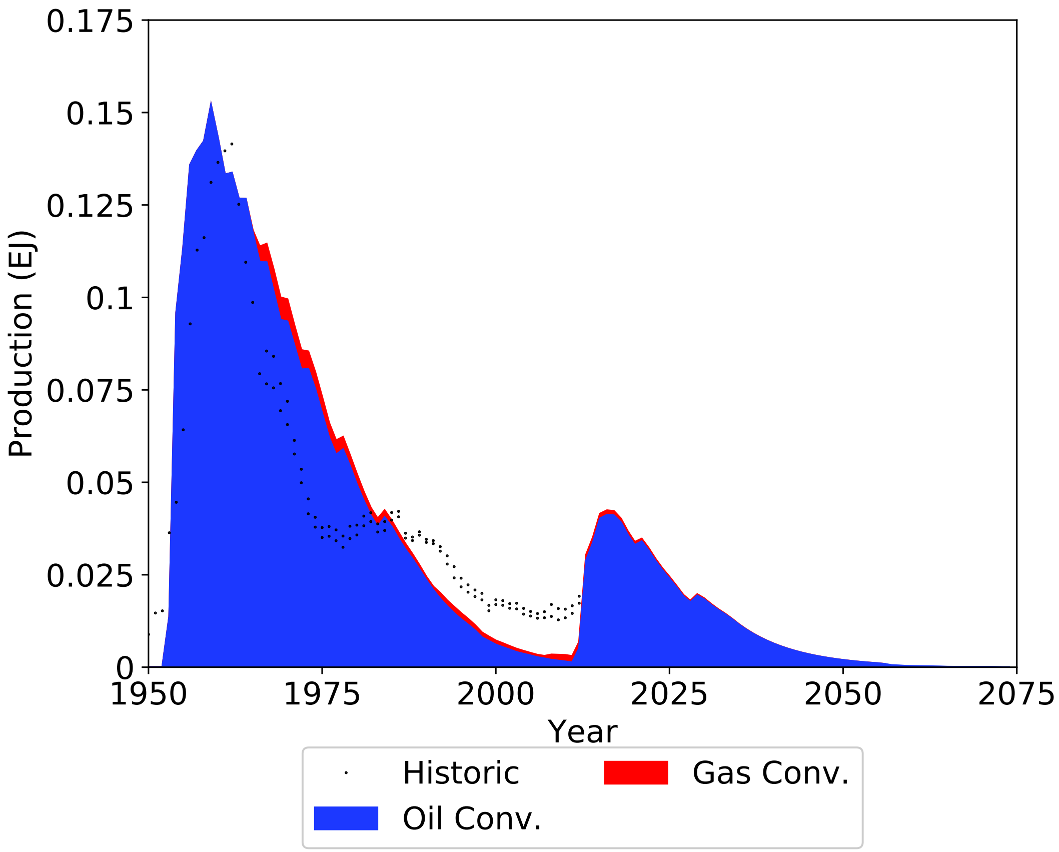

Figure 6.70: USA - Nebraska projections capped at 16

Table 6.70: Peak years - All

**Name**	**URR**	**Peak Year**	**Peak Rate**
Oil Conv. Nebraska	3.96	1959	0.15
Gas Conv. Nebraska	0.13	1969	0.01
**Total**	**4.09**	**1959**	**0.15**

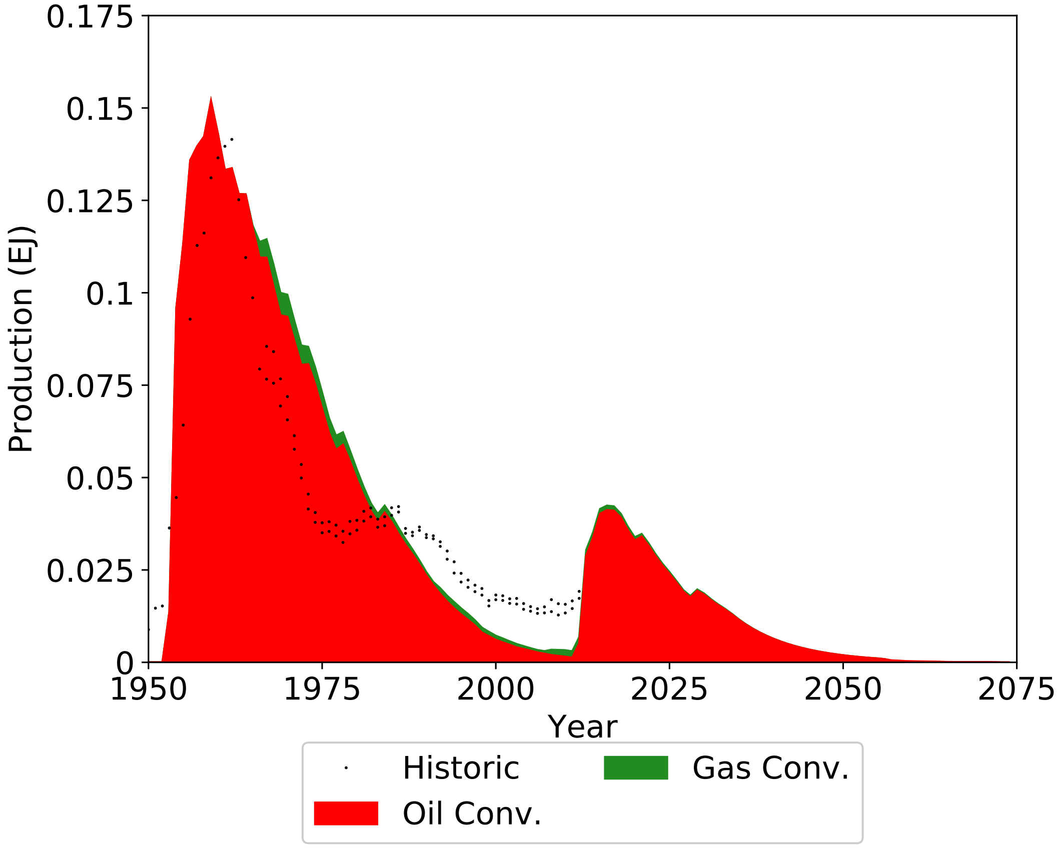

Figure 6.71: USA - Nebraska projection by mineral type

Table 6.71: Peak years - Minerals

**Name**	**URR**	**Peak Year**	**Peak Rate**
Oil Conv.	3.96	1959	0.15
Gas Conv.	0.13	1969	0.01
**Total**	**4.09**	**1959**	**0.15**

Nevada

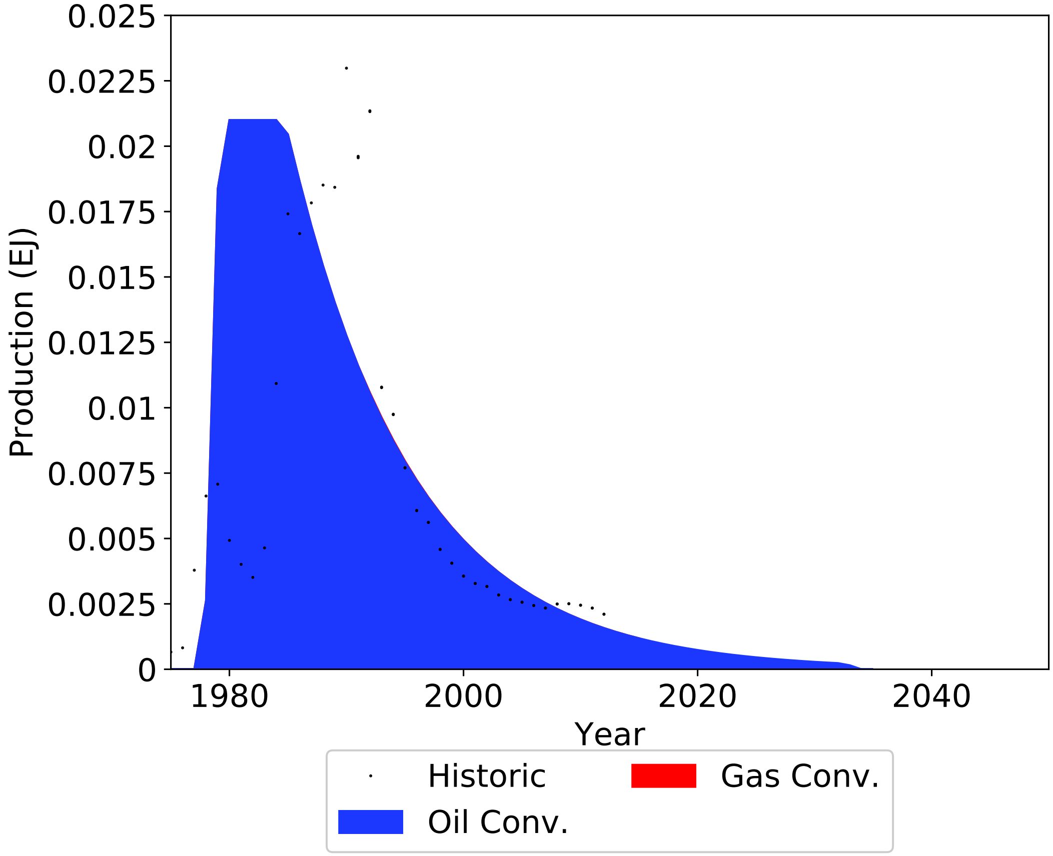

Figure 6.72: USA - Nevada projections capped at 16

Table 6.72: Peak years - All

Name	URR	Peak Year	Peak Rate
Oil Conv. Nevada	0.35	1980	0.02
Gas Conv. Nevada	–	1993	–
**Total**	**0.35**	**1980**	**0.02**

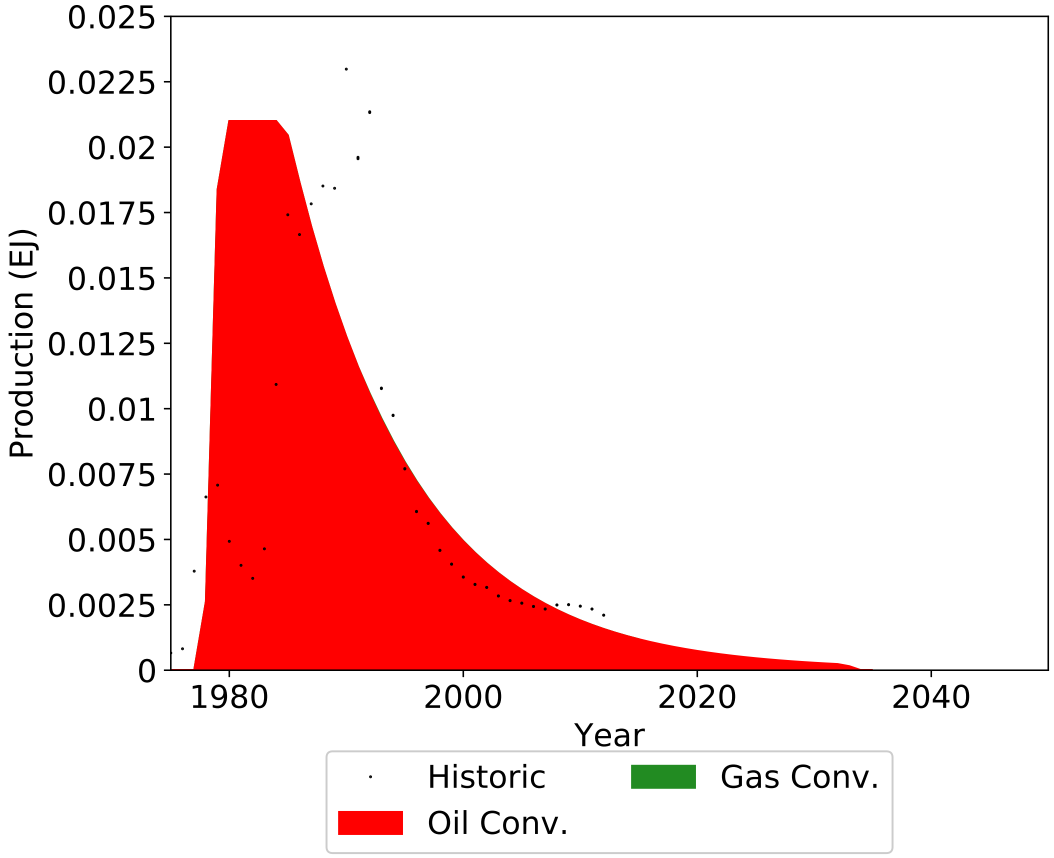

Figure 6.73: USA - Nevada projection by mineral type

Table 6.73: Peak years - Minerals

Name	URR	Peak Year	Peak Rate
Oil Conv.	0.35	1980	0.02
Gas Conv.	–	1993	–
**Total**	**0.35**	**1980**	**0.02**

New Mexico

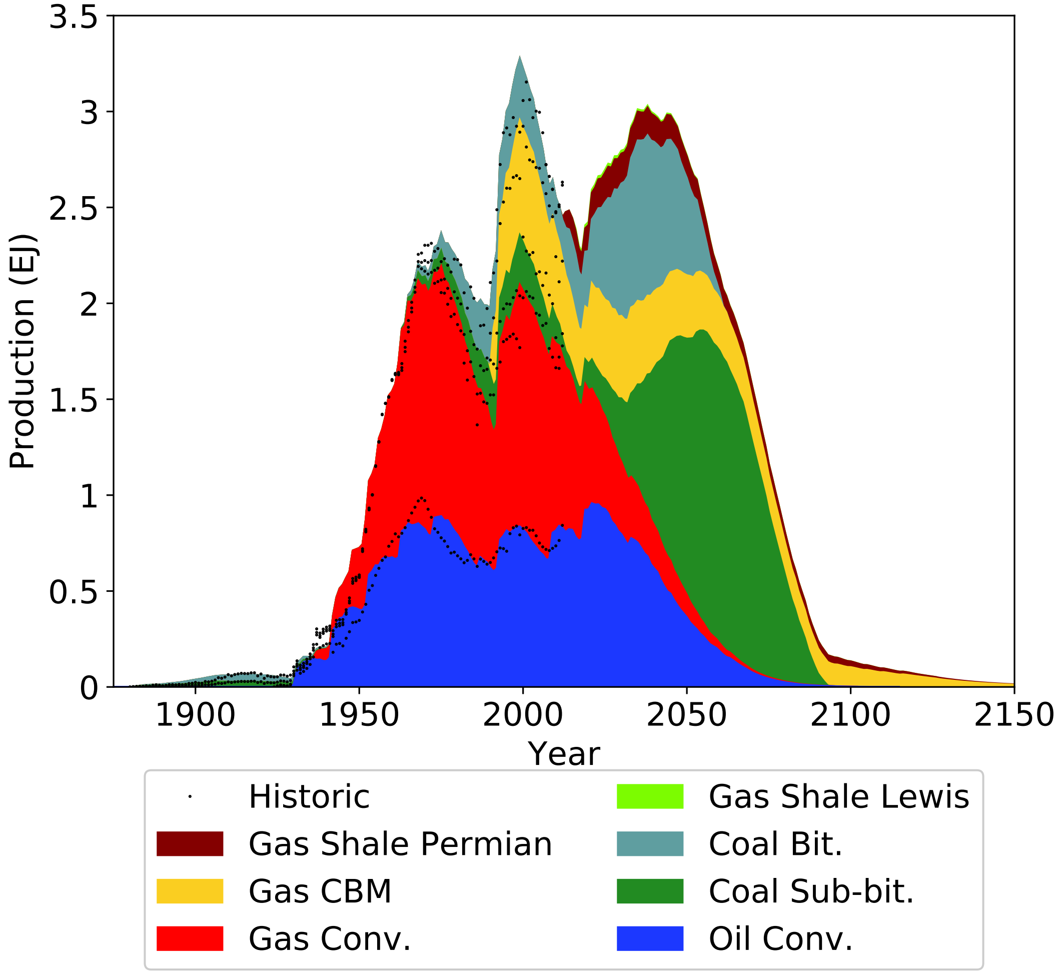

Figure 6.74: USA - New Mexico projections capped at 16

Table 6.74: Peak years - All

Name	URR	Peak Year	Peak Rate
Oil Conv. New Mexico	82.6	2021	0.96
Gas Conv. New Mexico	81.47	1975	1.3
Coal Sub-bit. New Mexico	68.57	2057	1.54
Gas CBM New Mexico	38.36	1998	0.6
Coal Bit. New Mexico	35.07	2036	0.84
Gas Shale New Mexico Permian	8.96	2026	0.16
Gas Shale New Mexico Lewis	0.53	2019	0.02
**Total**	**315.56**	**1999**	**3.27**

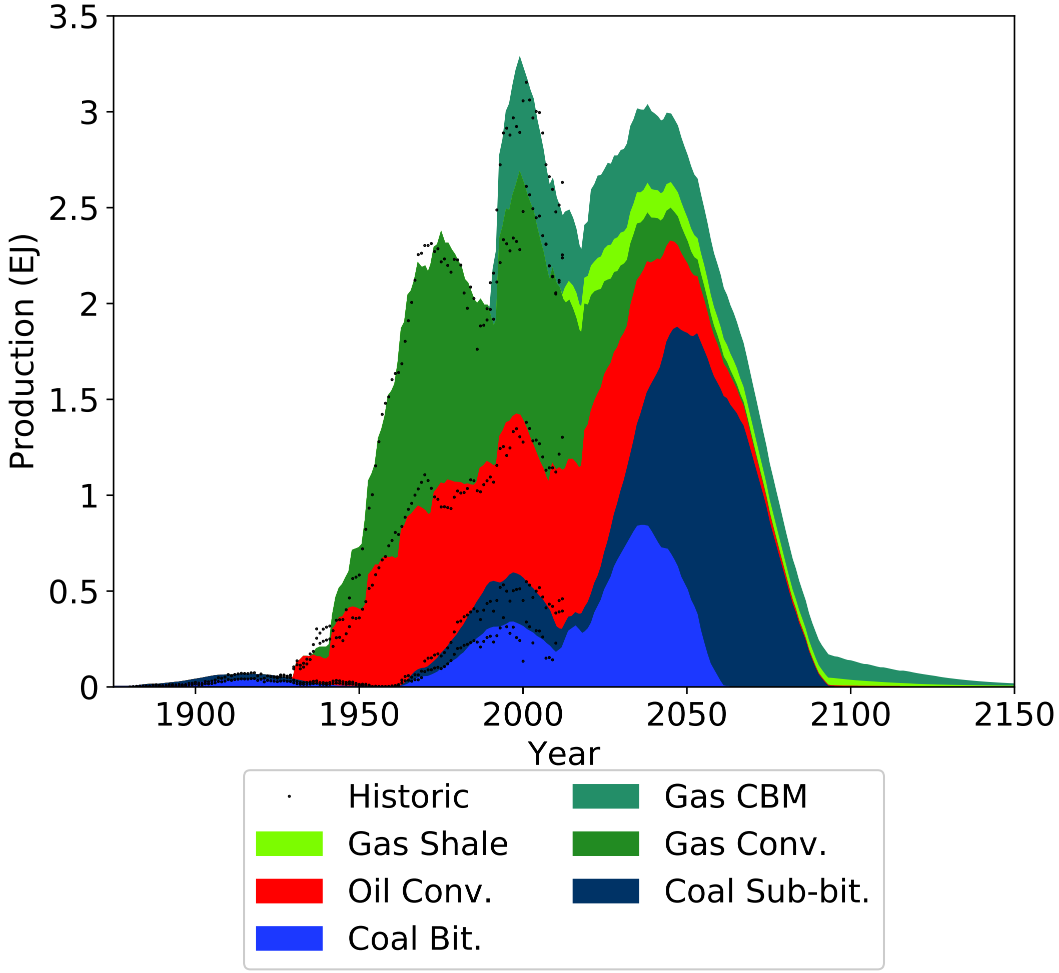

Figure 6.75: USA - New Mexico projection by mineral type

Table 6.75: Peak years - Minerals

**Name**	**URR**	**Peak Year**	**Peak Rate**
Coal Bit.	35.07	2036	0.84
Coal Sub-bit.	68.57	2057	1.54
Oil Conv.	82.6	2021	0.96
Gas Conv.	81.47	1975	1.3
Gas Shale	9.49	2026	0.18
Gas CBM	38.36	1998	0.6
**Total**	**315.56**	**1999**	**3.27**

New York

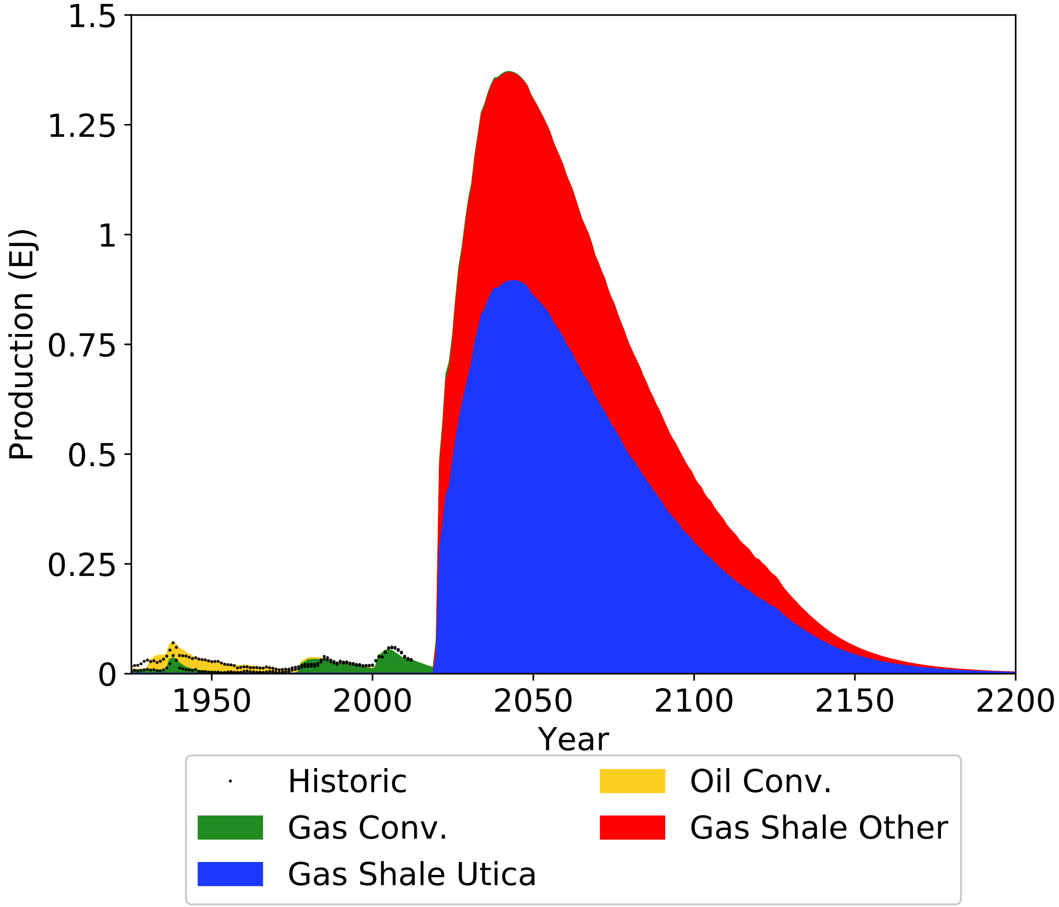

Figure 6.76: USA - New York projections capped at 16

Table 6.76: Peak years - All

Name	URR	Peak Year	Peak Rate
Gas Shale New York Utica	58.28	2044	0.89
Gas Shale New York Other	30.45	2040	0.48
Gas Conv. New York	1.8	2005	0.05
Oil Conv. New York	1.24	1941	0.04
**Total**	**91.77**	**2042**	**1.37**

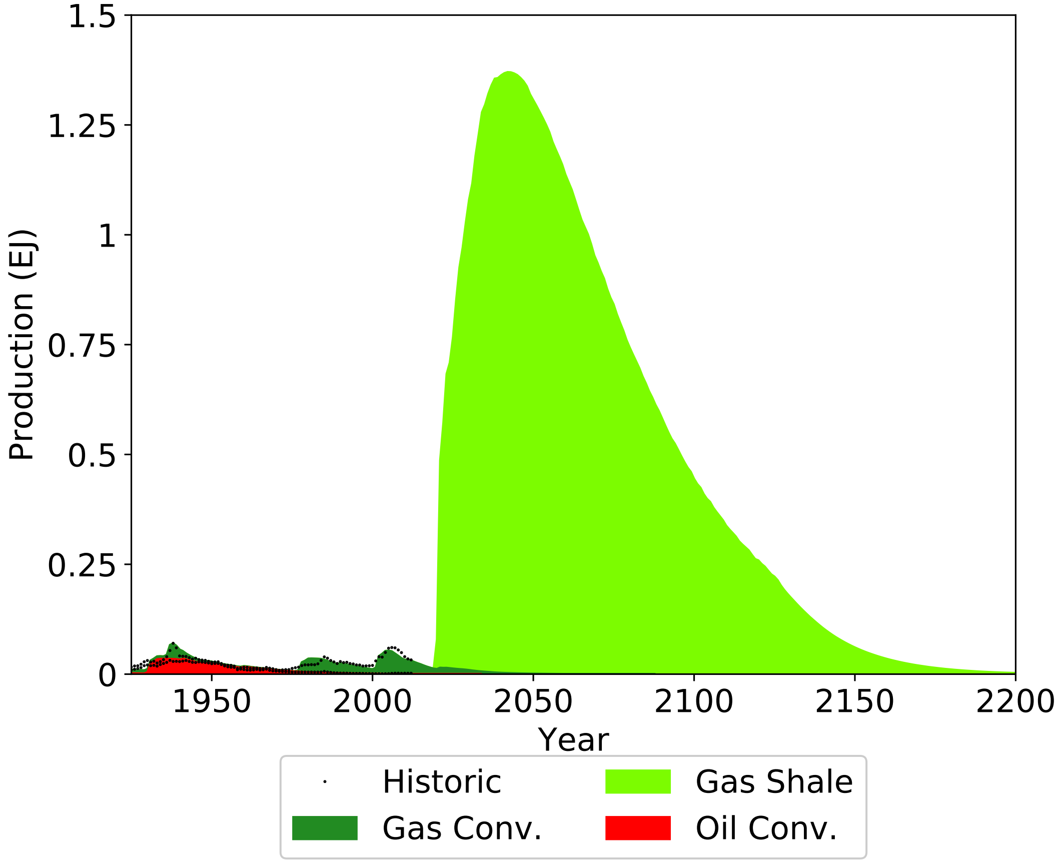

Figure 6.77: USA - New York projection by mineral type

Table 6.77: Peak years - Minerals

Name	URR	Peak Year	Peak Rate
Oil Conv.	1.24	1941	0.04
Gas Conv.	1.8	2005	0.05
Gas Shale	88.73	2042	1.37
**Total**	**91.77**	**2042**	**1.37**

North Dakota

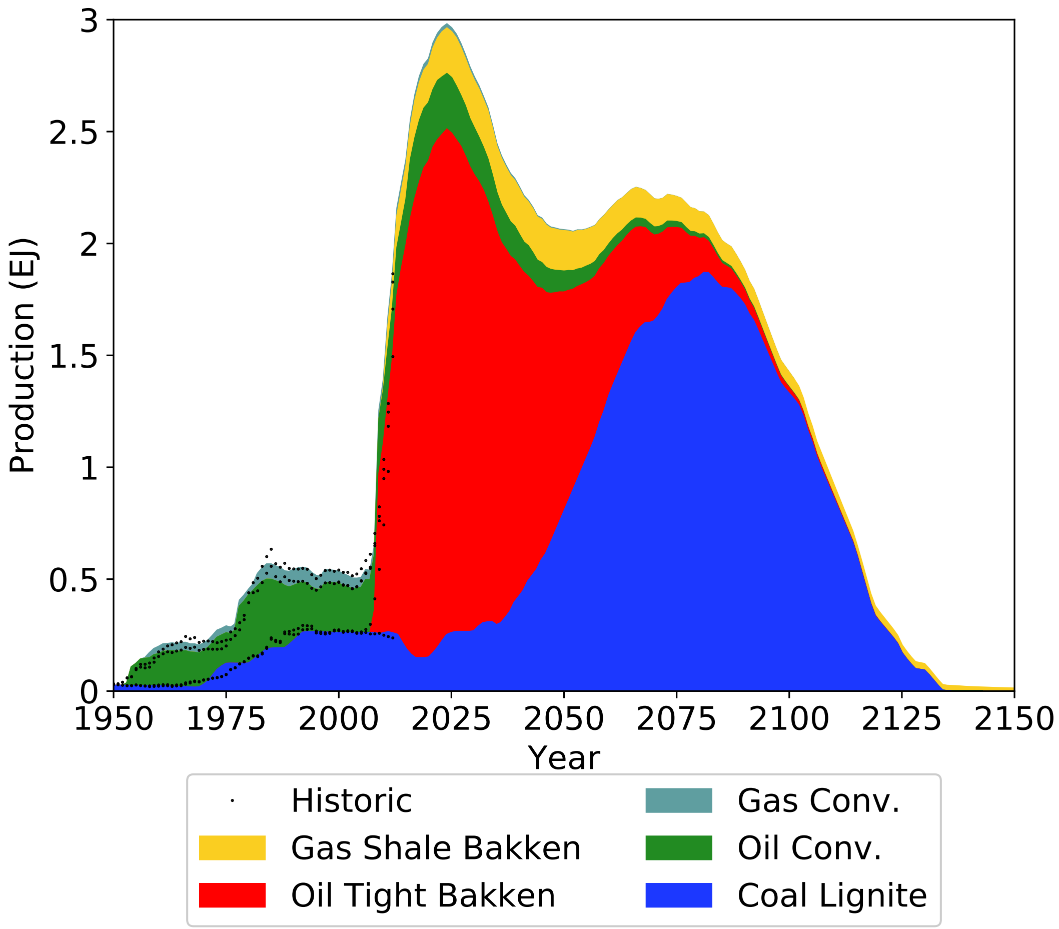

Figure 6.78: USA - North Dakota projections capped at 16

Table 6.78: Peak years - All

Name	URR	Peak Year	Peak Rate
Coal Lignite North Dakota	118.29	2081	1.87
Oil Tight North Dakota Bakken	87.48	2022	2.27
Oil Conv. North Dakota	20.8	1983	0.32
Gas Shale North Dakota Bakken	15.24	2031	0.22
Gas Conv. North Dakota	3.09	1990	0.07
**Total**	**244.9**	**2024**	**2.98**

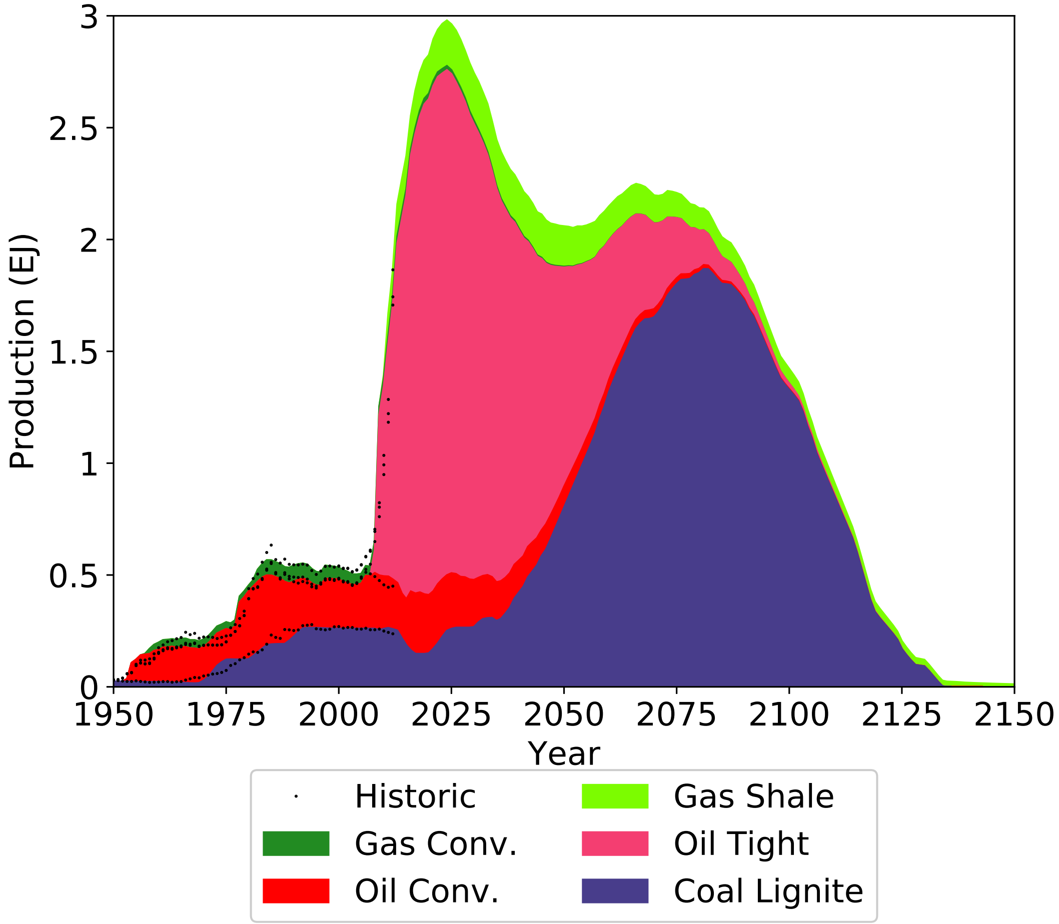

Figure 6.79: USA - North Dakota projection by mineral type

Table 6.79: Peak years - Minerals

Name	URR	Peak Year	Peak Rate
Coal Lignite	118.29	2081	1.87
Oil Conv.	20.8	1983	0.32
Oil Tight	87.48	2022	2.27
Gas Conv.	3.09	1990	0.07
Gas Shale	15.24	2031	0.22
**Total**	**244.9**	**2024**	**2.98**

Ohio

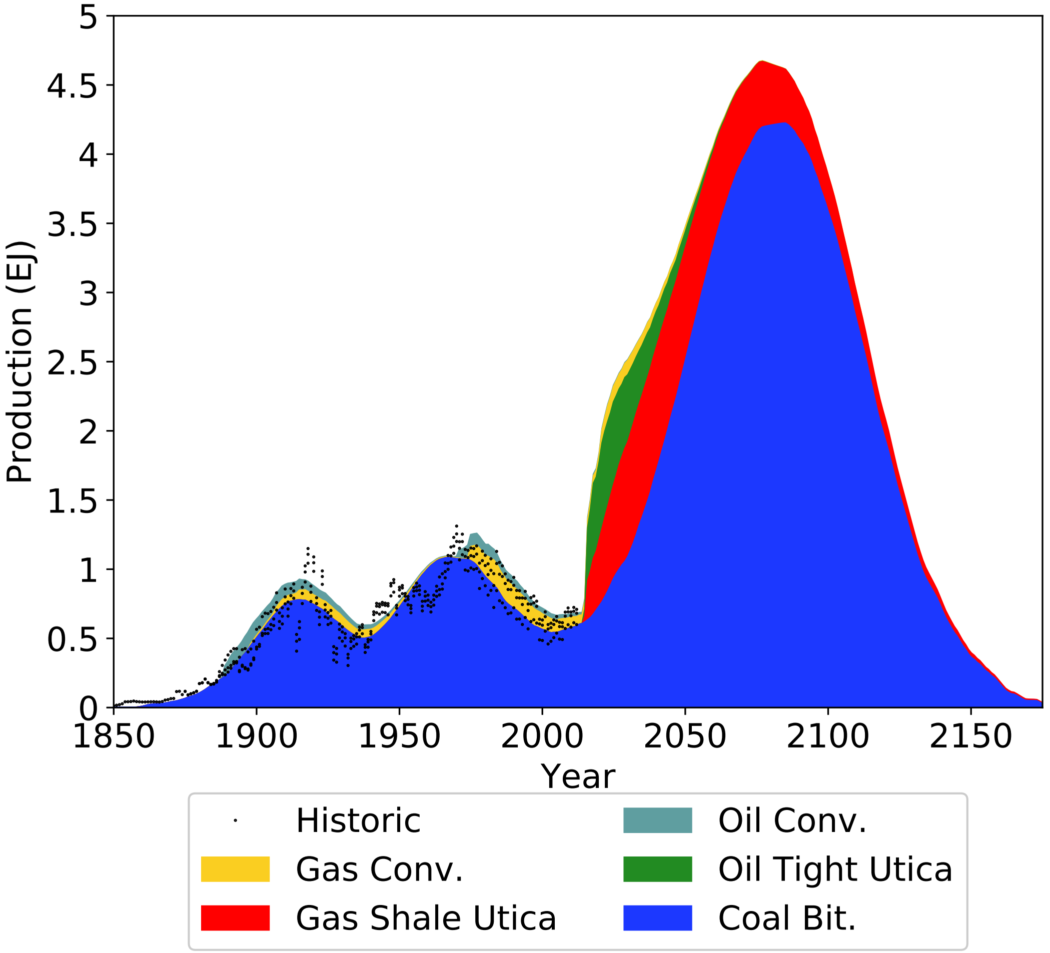

Figure 6.80: USA - Ohio projections capped at 16

Table 6.80: Peak years - All

Name	URR	Peak Year	Peak Rate
Coal Bit. Ohio	414.76	2085	4.22
Gas Shale Ohio Utica	58.28	2039	0.89
Oil Tight Ohio Utica	14.33	2022	0.62
Gas Conv. Ohio	10.99	1983	0.18
Oil Conv. Ohio	7.08	1899	0.12
**Total**	**505.44**	**2077**	**4.67**

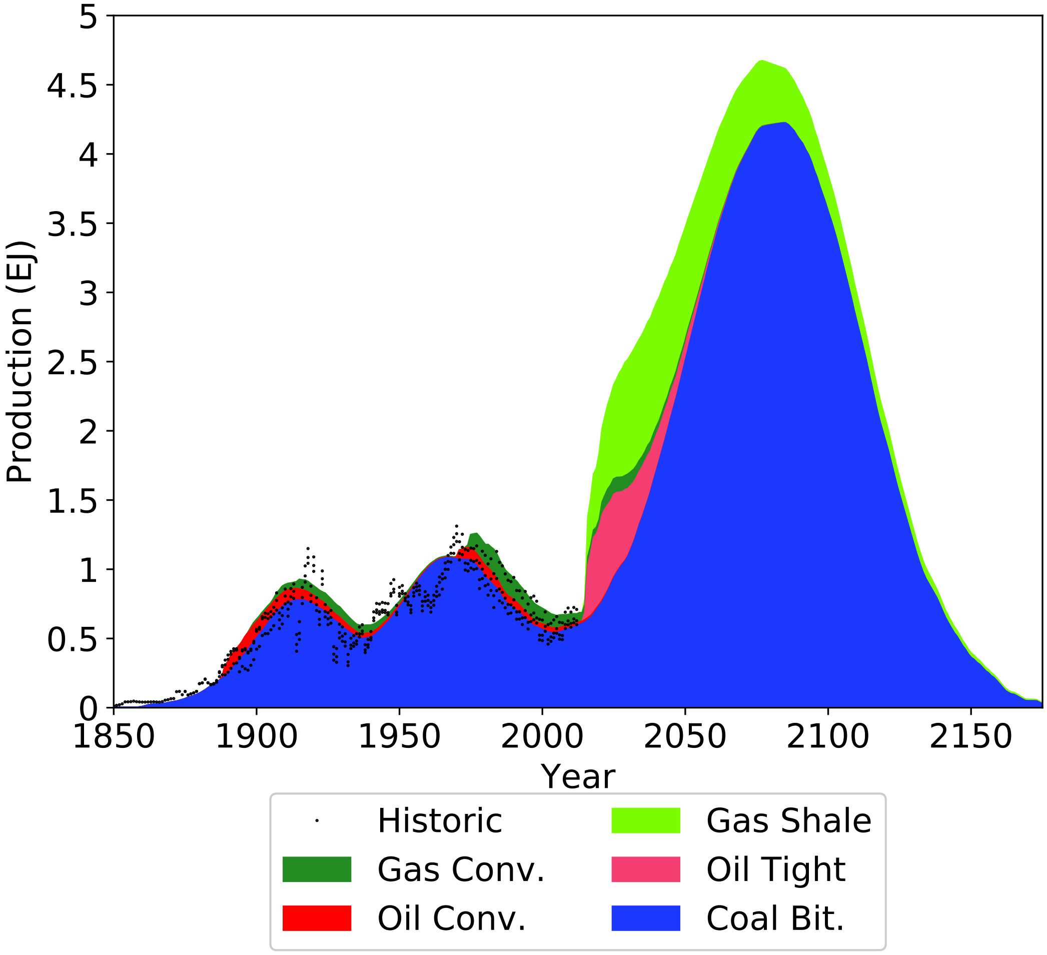

Figure 6.81: USA - Ohio projection by mineral type

Table 6.81: Peak years - Minerals

Name	URR	Peak Year	Peak Rate
Coal Bit.	414.76	2085	4.22
Oil Conv.	7.08	1899	0.12
Oil Tight	14.33	2022	0.62
Gas Conv.	10.99	1983	0.18
Gas Shale	58.28	2039	0.89
**Total**	**505.44**	**2077**	**4.67**

Oklahoma

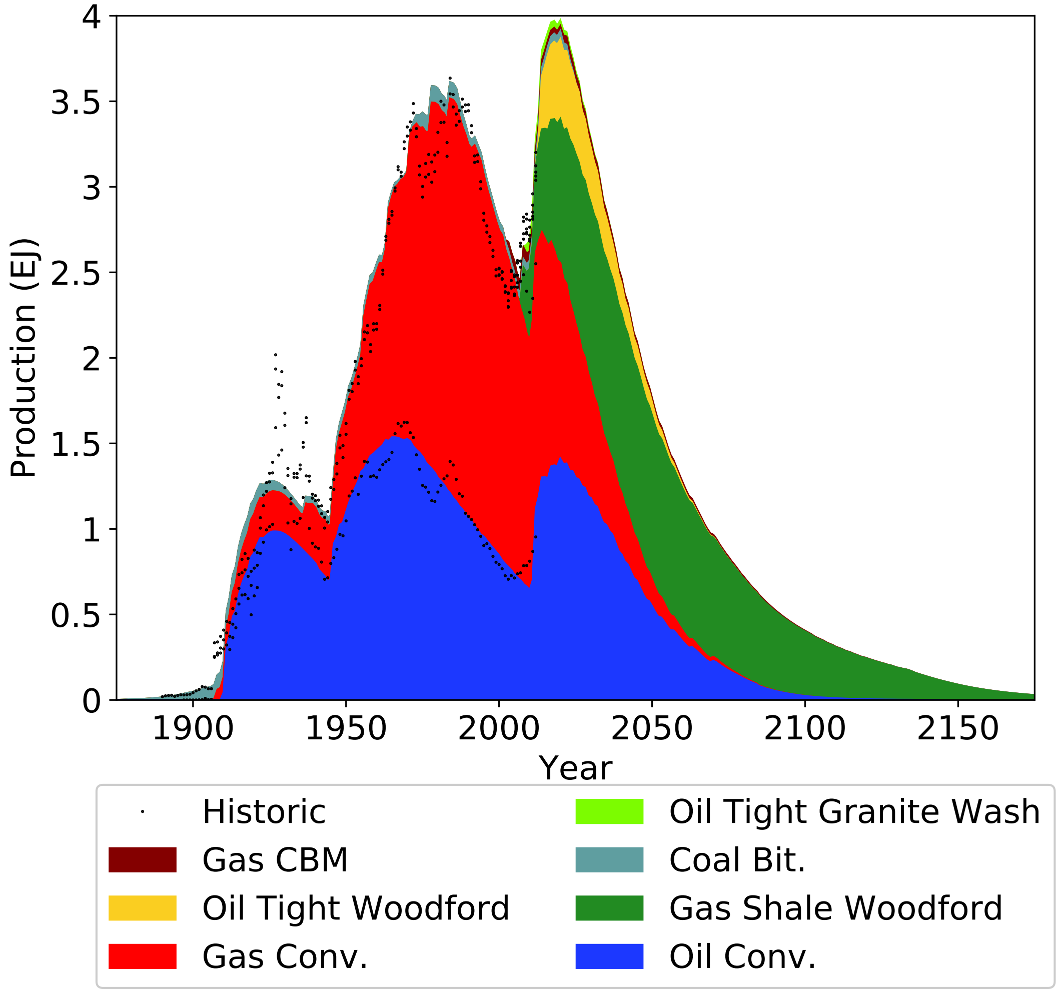

Figure 6.82: USA - Oklahoma projections capped at 16

Table 6.82: Peak years - All

Name	URR	Peak Year	Peak Rate
Oil Conv. Oklahoma	156.34	1966	1.54
Gas Conv. Oklahoma	143.52	1985	2.33
Gas Shale Oklahoma Woodford	82.9	2035	1.07
Oil Tight Oklahoma Woodford	10.95	2020	0.47
Coal Bit. Oklahoma	7.62	1914	0.1
Gas CBM Oklahoma	2.69	2006	0.07
Oil Tight Oklahoma Granite Wash	0.89	2011	0.07
**Total**	**404.91**	**2020**	**3.97**

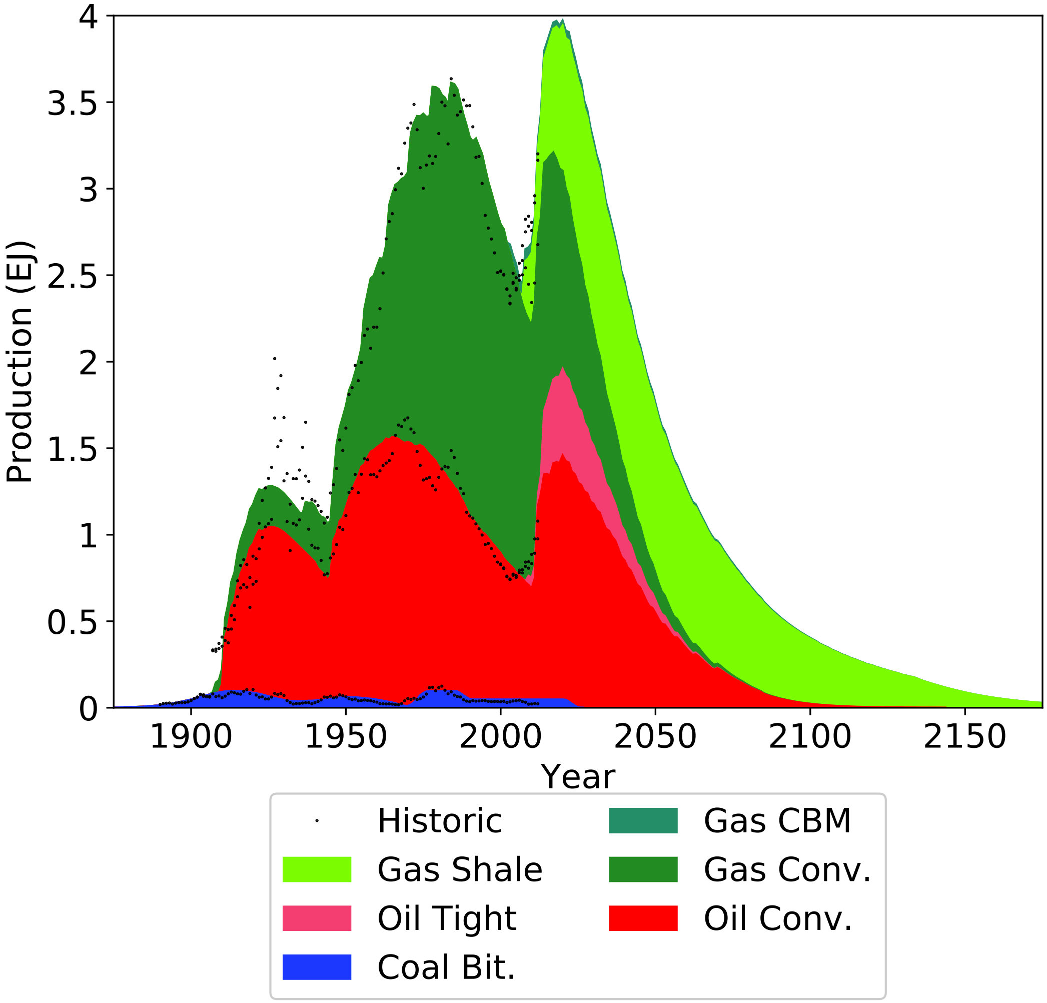

Figure 6.83: USA - Oklahoma projection by mineral type

Table 6.83: Peak years - Minerals

**Name**	**URR**	**Peak Year**	**Peak Rate**
Coal Bit.	7.62	1914	0.1
Oil Conv.	156.34	1966	1.54
Oil Tight	11.84	2020	0.5
Gas Conv.	143.52	1985	2.33
Gas Shale	82.9	2035	1.07
Gas CBM	2.69	2006	0.07
**Total**	**404.91**	**2020**	**3.97**

Oregon

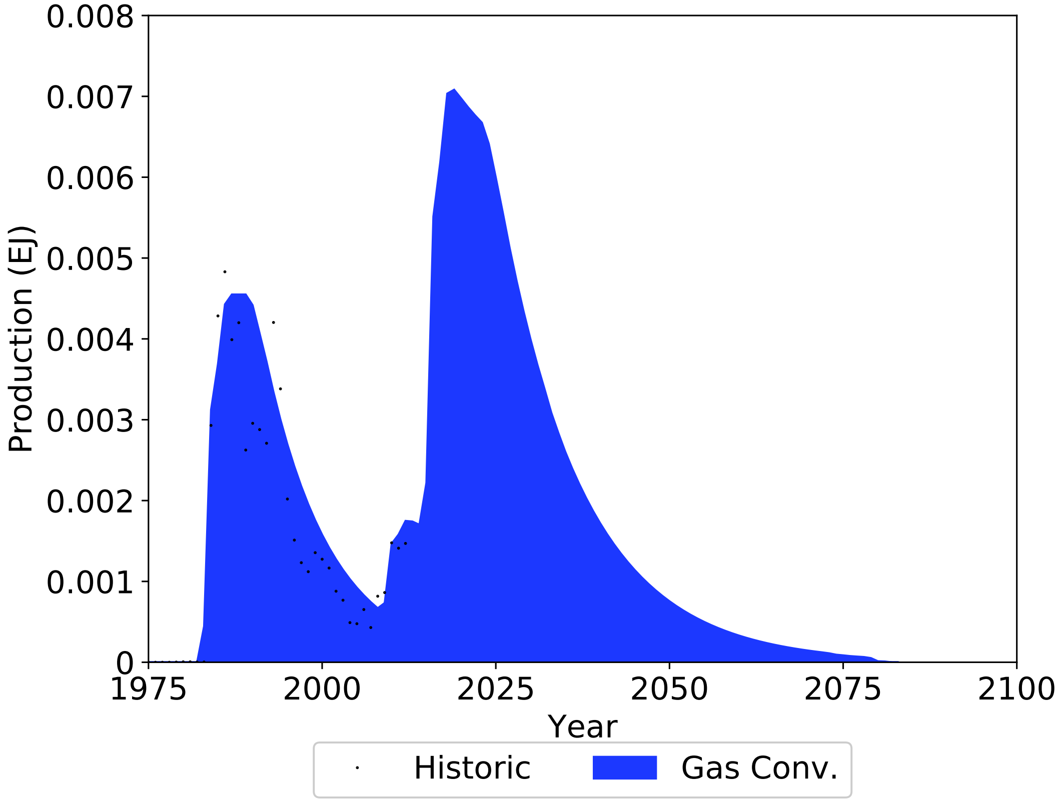

Figure 6.84: USA - Oregon projections capped at 16

Table 6.84: Peak years - All

Name	URR	Peak Year	Peak Rate
Gas Conv. Oregon	0.21	2019	0.01
**Total**	**0.21**	**2019**	**0.01**

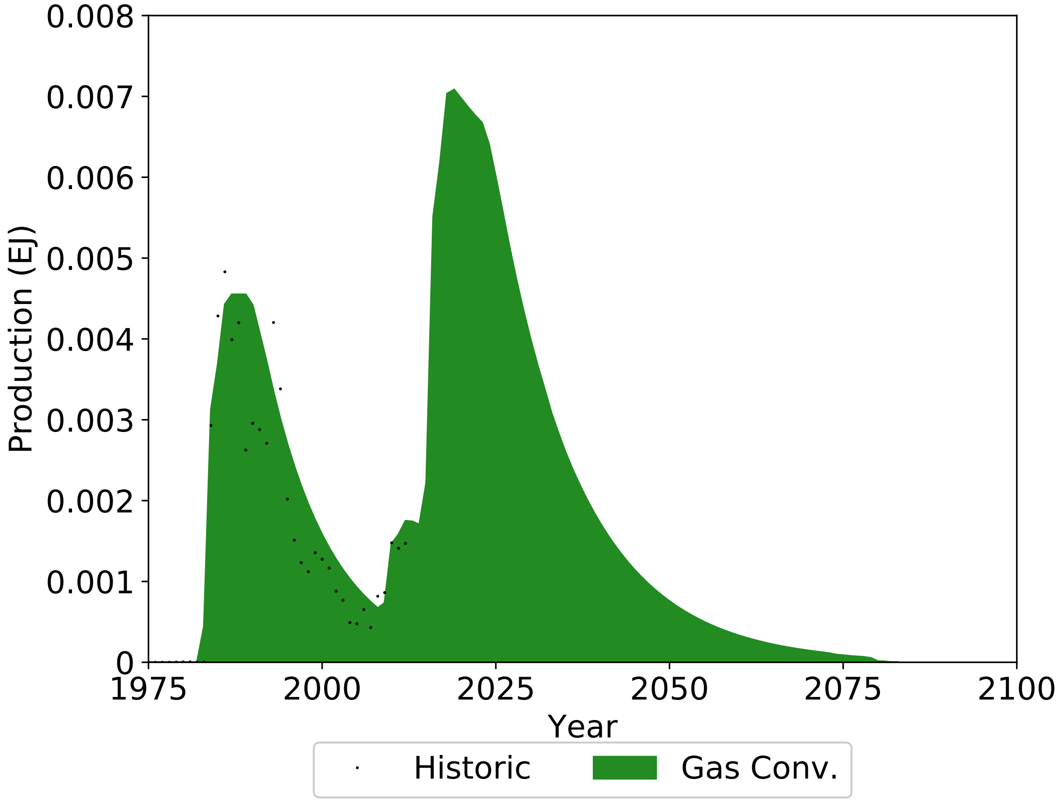

Figure 6.85: USA - Oregon projection by mineral type

Table 6.85: Peak years - Minerals

Name	URR	Peak Year	Peak Rate
Gas Conv.	0.21	2019	0.01
**Total**	**0.21**	**2019**	**0.01**

Other

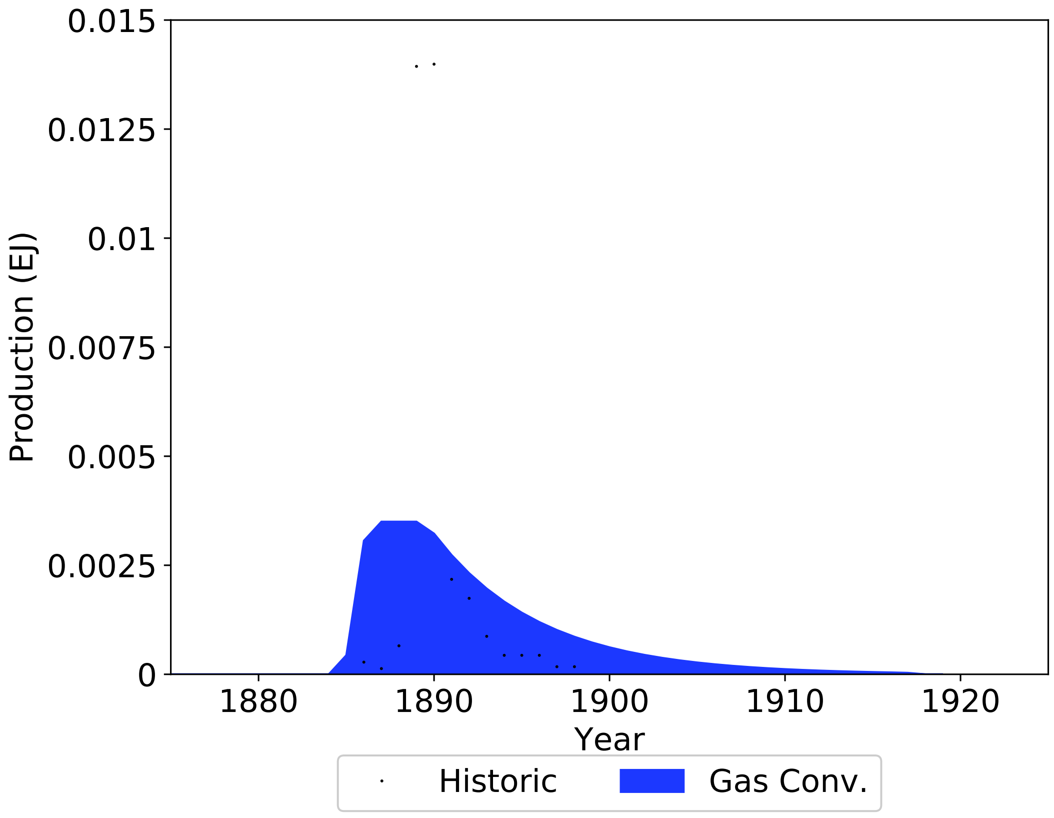

Figure 6.86: USA - Other projections capped at 16

Table 6.86: Peak years - All

Name	URR	Peak Year	Peak Rate
Gas Conv. Other	0.04	1887	–
**Total**	**0.04**	**1887**	–

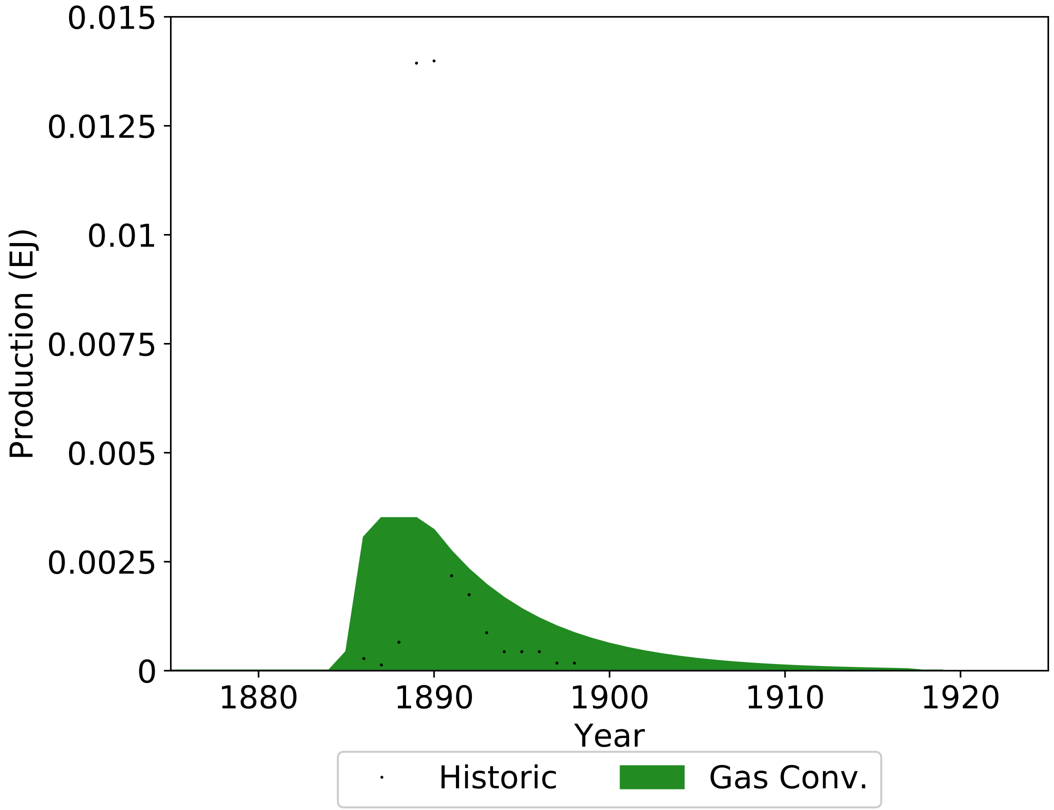

Figure 6.87: USA - Other projection by mineral type

Table 6.87: Peak years - Minerals

Name	URR	Peak Year	Peak Rate
Gas Conv.	0.04	1887	–
**Total**	**0.04**	**1887**	–

Pennsylvania

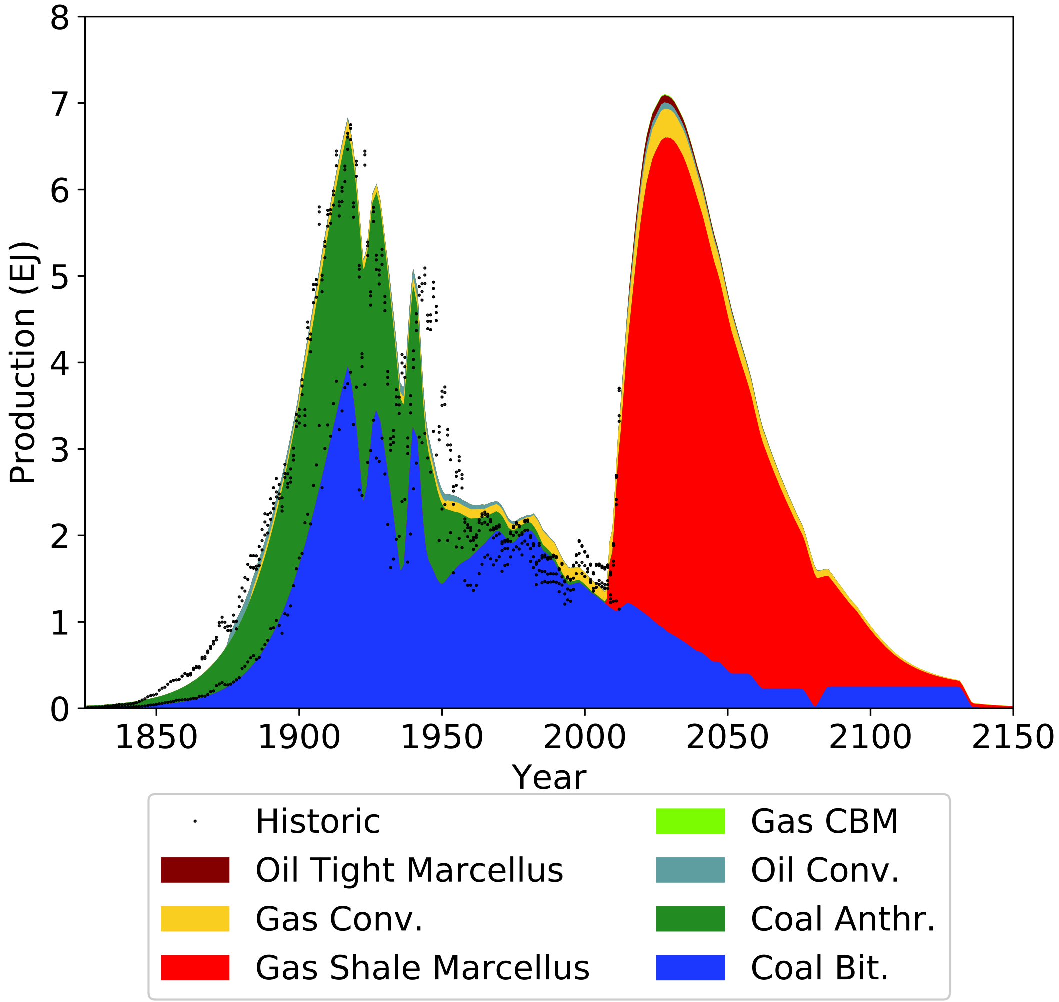

Figure 6.88: USA - Pennsylvania projections capped at 16

Table 6.88: Peak years - All

**Name**	**URR**	**Peak Year**	**Peak Rate**
Coal Bit. Pennsylvania	303.54	1917	3.91
Gas Shale Pennsylvania Marcellus	294.39	2030	5.73
Coal Anthr. Pennsylvania	153.01	1919	2.73
Gas Conv. Pennsylvania	31.5	2021	0.35
Oil Conv. Pennsylvania	10.19	1882	0.14
Oil Tight Pennsylvania Marcellus	2.29	2022	0.1
Gas CBM Pennsylvania	0.54	2024	0.01
**Total**	**795.46**	**2028**	**7.09**

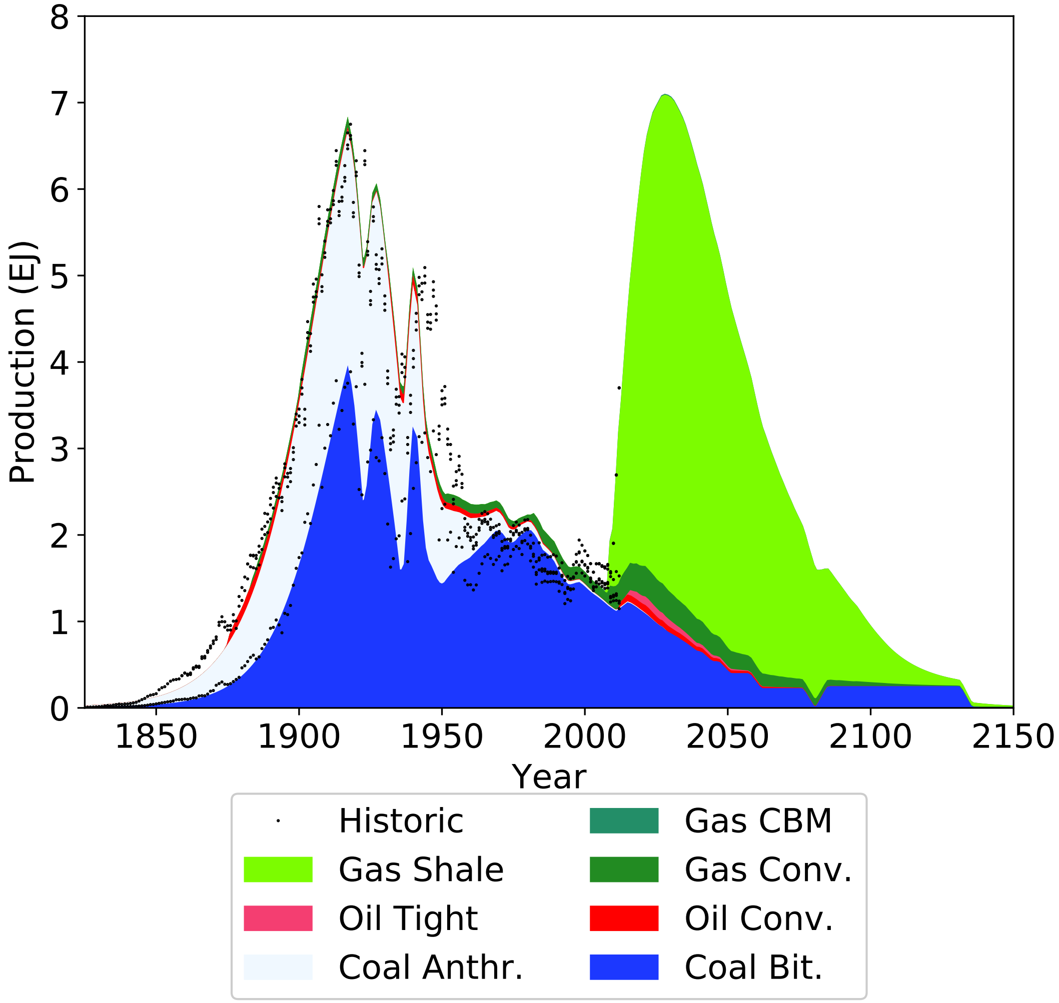

Figure 6.89: USA - Pennsylvania projection by mineral type

Table 6.89: Peak years - Minerals

**Name**	**URR**	**Peak Year**	**Peak Rate**
Coal Bit.	303.54	1917	3.91
Coal Anthr.	153.01	1919	2.73
Oil Conv.	10.19	1882	0.14
Oil Tight	2.29	2022	0.1
Gas Conv.	31.5	2021	0.35
Gas Shale	294.39	2030	5.73
Gas CBM	0.54	2024	0.01
**Total**	**795.46**	**2028**	**7.09**

South Dakota

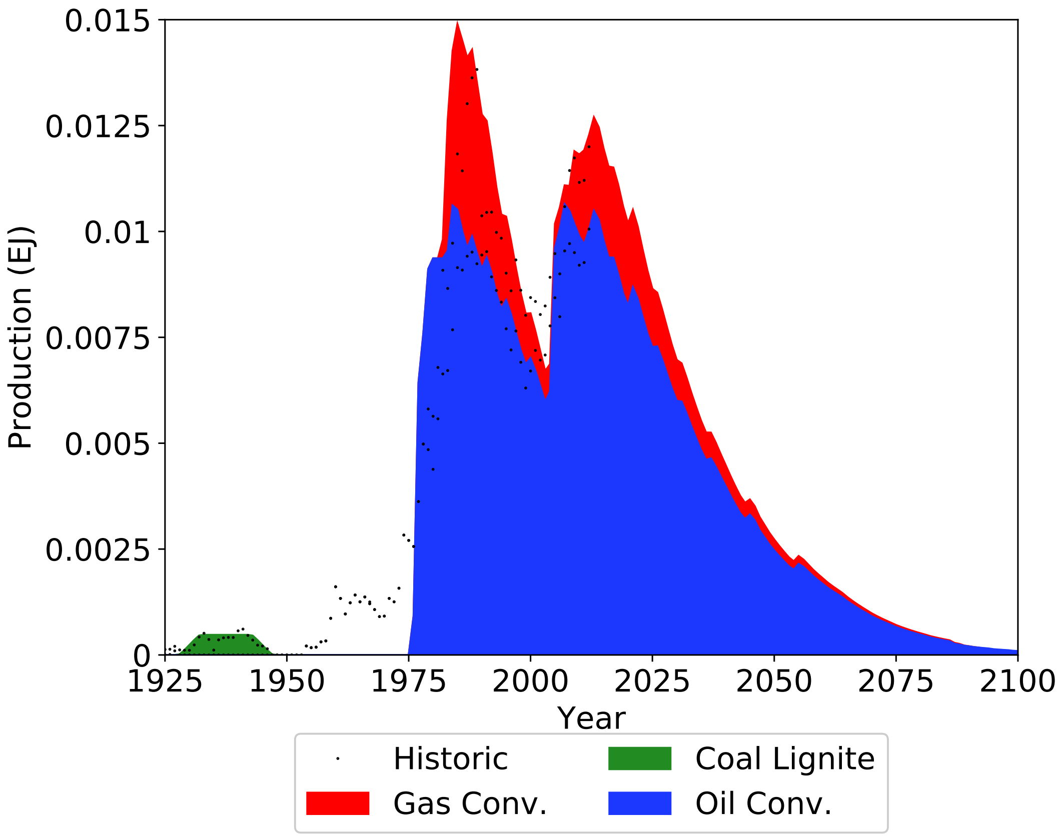

Figure 6.90: USA - South Dakota projections capped at 16

Table 6.90: Peak years - All

**Name**	**URR**	**Peak Year**	**Peak Rate**
Oil Conv. South Dakota	0.59	2007	0.01
Gas Conv. South Dakota	0.11	1986	–
Coal Lignite South Dakota	0.01	1933	–
**Total**	**0.71**	**1985**	**0.01**

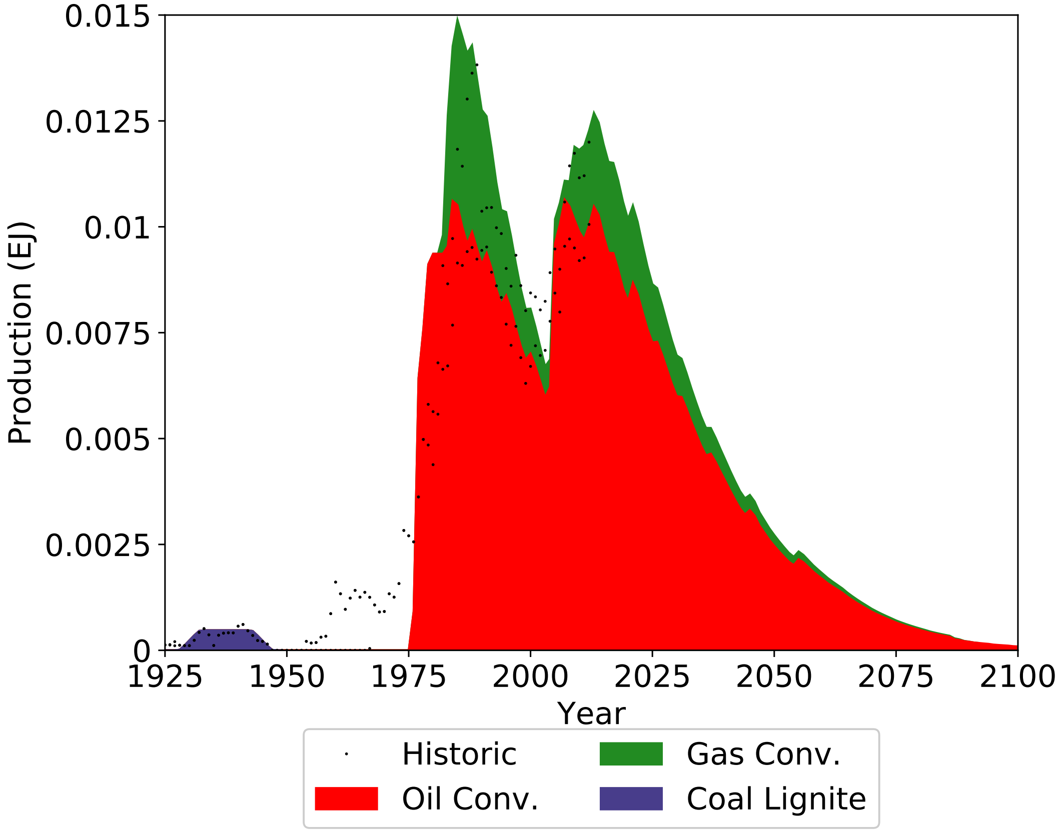

Figure 6.91: USA - South Dakota projection by mineral type

Table 6.91: Peak years - Minerals

Name	URR	Peak Year	Peak Rate
Coal Lignite	0.01	1933	–
Oil Conv.	0.59	2007	0.01
Gas Conv.	0.11	1986	–
**Total**	**0.71**	**1985**	**0.01**

Tennessee

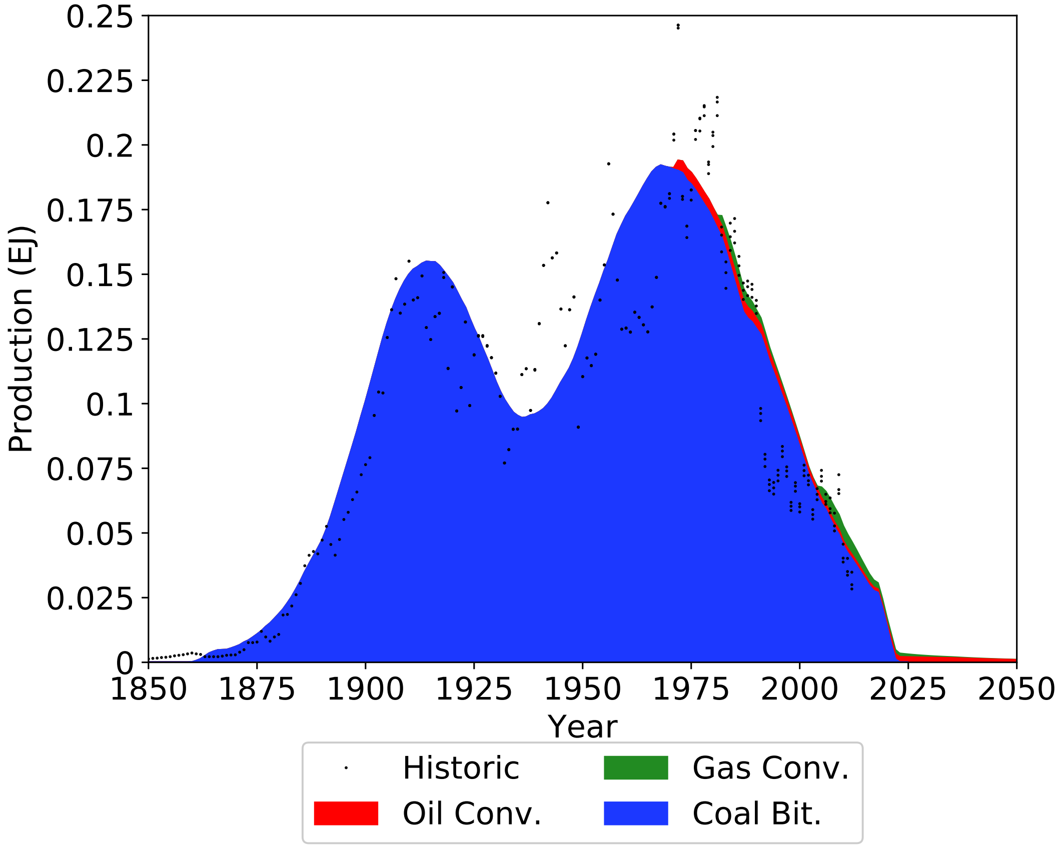

Figure 6.92: USA - Tennessee projections capped at 16

Table 6.92: Peak years - All

Name	URR	Peak Year	Peak Rate
Coal Bit. Tennessee	16.08	1968	0.19
Oil Conv. Tennessee	0.22	1973	–
Gas Conv. Tennessee	0.14	2008	0.01
**Total**	**16.44**	**1972**	**0.19**

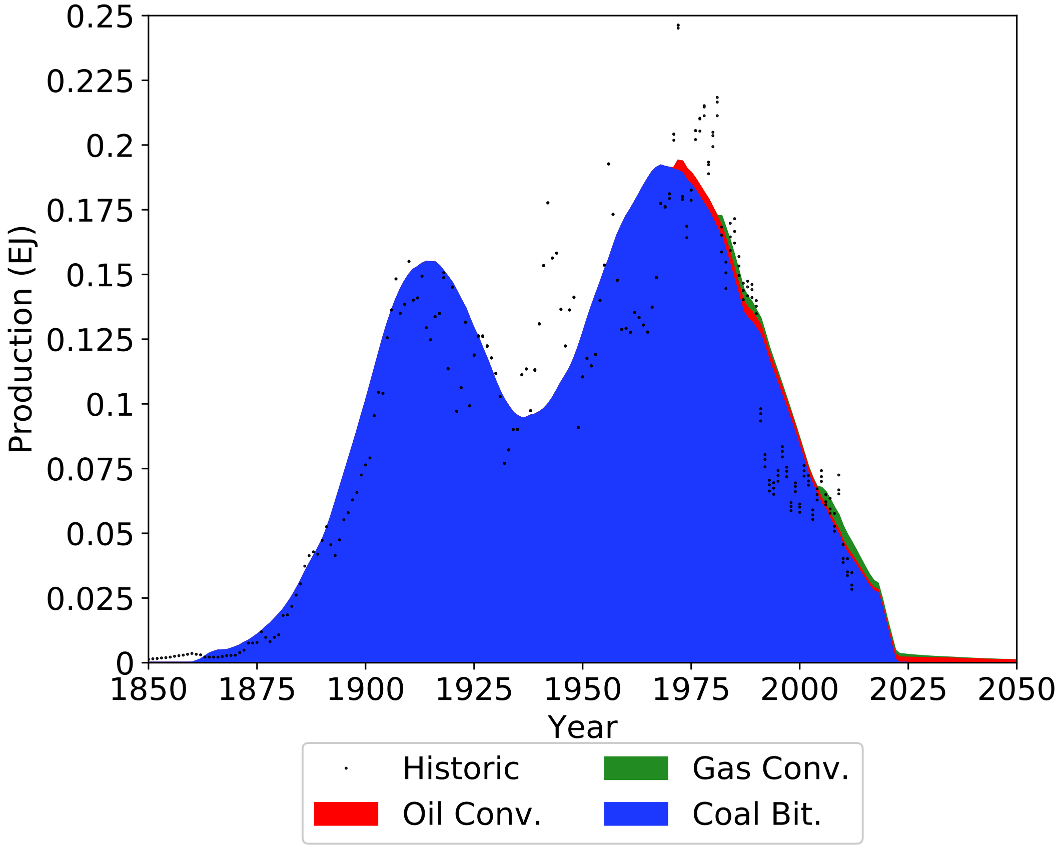

Figure 6.93: USA - Tennessee projection by mineral type

Table 6.93: Peak years - Minerals

**Name**	**URR**	**Peak Year**	**Peak Rate**
Coal Bit.	16.08	1968	0.19
Oil Conv.	0.22	1973	–
Gas Conv.	0.14	2008	0.01
**Total**	**16.44**	**1972**	**0.19**

Texas

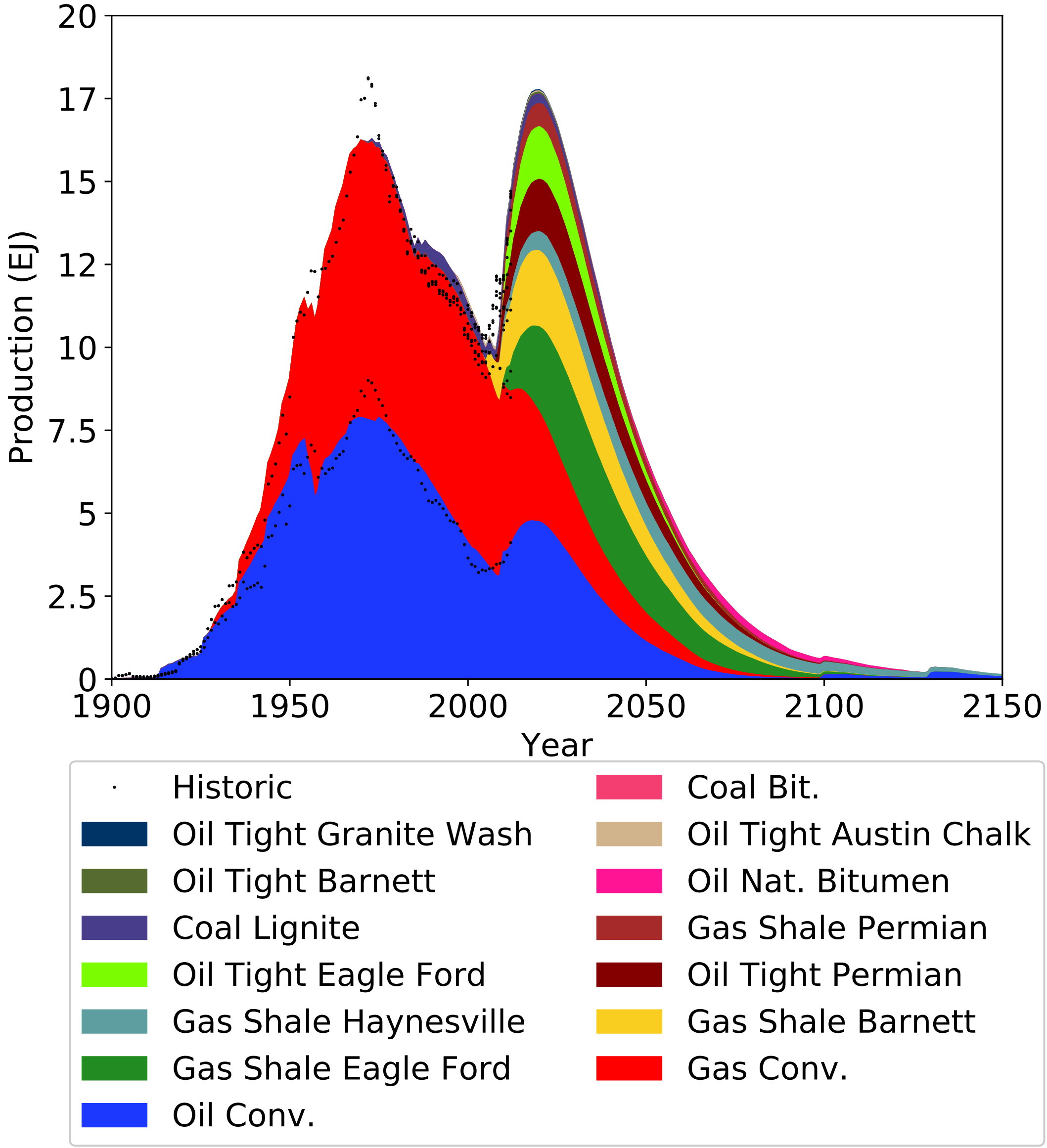

Figure 6.94: USA - Texas projections capped at 16

Table 6.94: Peak years - All

Name	URR	Peak Year	Peak Rate
Oil Conv. Texas	603.57	1970	7.87
Gas Conv. Texas	526.44	1973	8.4
Gas Shale Texas Eagle Ford	126.25	2027	3.01
Gas Shale Texas Barnett	88.36	2020	2.29
Gas Shale Texas Haynesville	61.8	2037	0.8
Oil Tight Texas Permian	61.13	2021	1.6
Oil Tight Texas Eagle Ford	39.84	2020	1.59
Gas Shale Texas Permian	28.68	2021	0.73
Coal Lignite Texas	21.9	1992	0.48
Oil Nat. Bitumen Texas	13.0	2068	0.24
Oil Tight Texas Barnett	2.44	2018	0.07
Oil Tight Texas Austin Chalk	2.25	1999	0.12
Oil Tight Texas Granite Wash	0.89	2011	0.07
Coal Bit. Texas	0.05	1997	0.02
**Total**	**1576.6**	**2020**	**17.76**

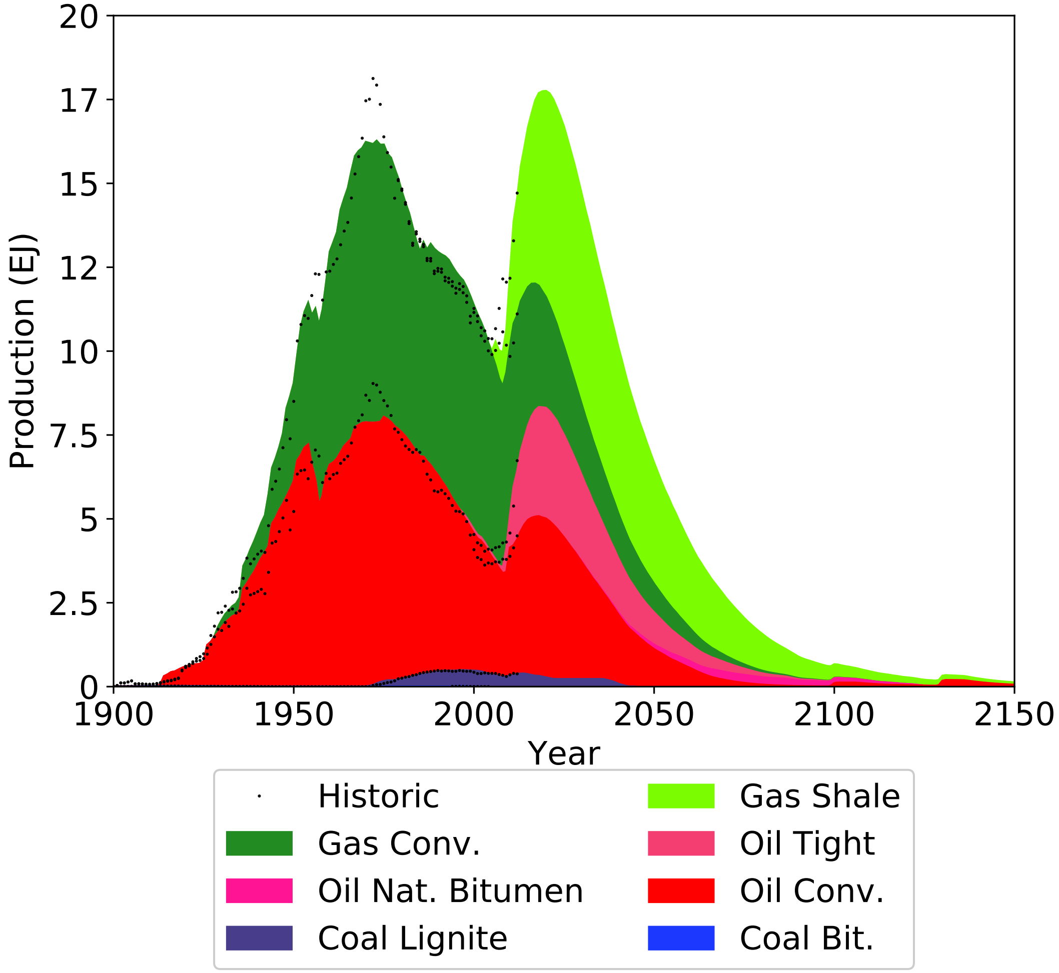

Figure 6.95: USA - Texas projection by mineral type

Table 6.95: Peak years - Minerals

Name	URR	Peak Year	Peak Rate
Coal Bit.	0.05	1997	0.02
Coal Lignite	21.9	1992	0.48
Oil Conv.	603.57	1970	7.87
Oil Nat. Bitumen	13.0	2068	0.24
Oil Tight	106.55	2020	3.3
Gas Conv.	526.44	1973	8.4
Gas Shale	305.09	2025	6.56
**Total**	**1576.6**	**2020**	**17.76**

USA

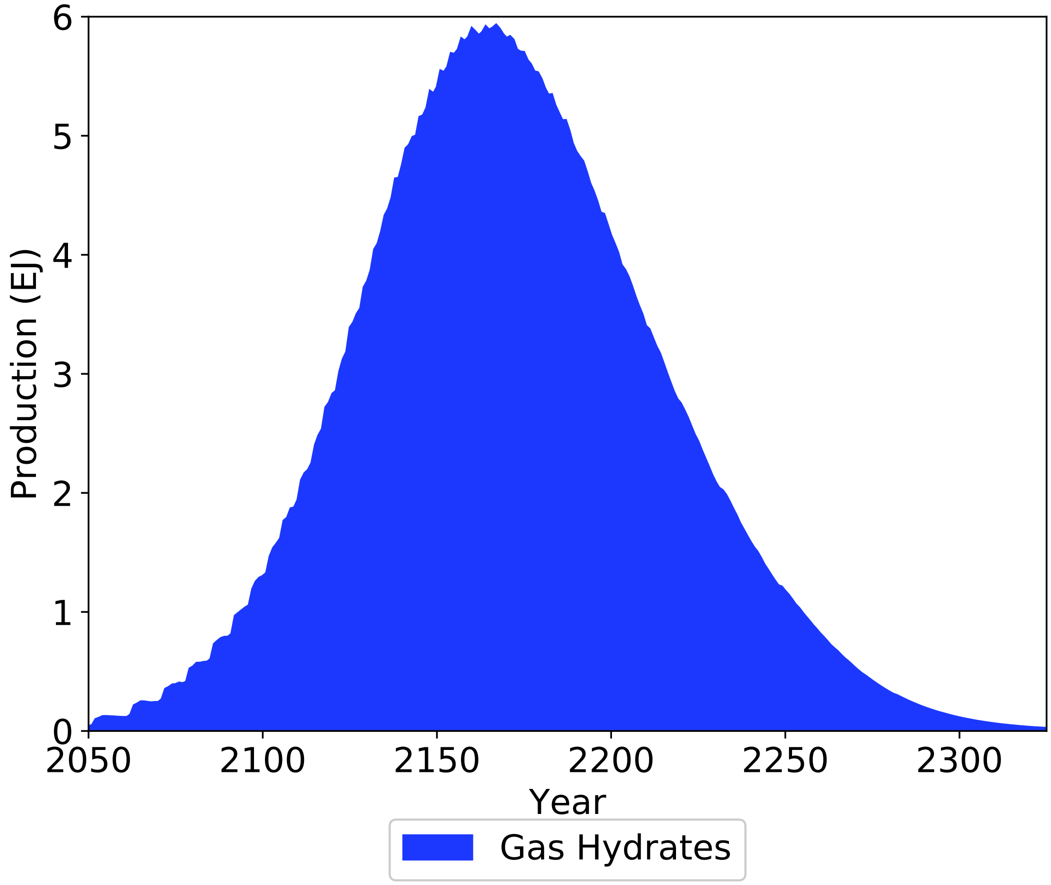

Figure 6.96: USA - USA projections capped at 16

Table 6.96: Peak years - All

Name	URR	Peak Year	Peak Rate
Gas Hydrates USA	614.5	2167	5.93
**Total**	**614.5**	**2167**	**5.93**

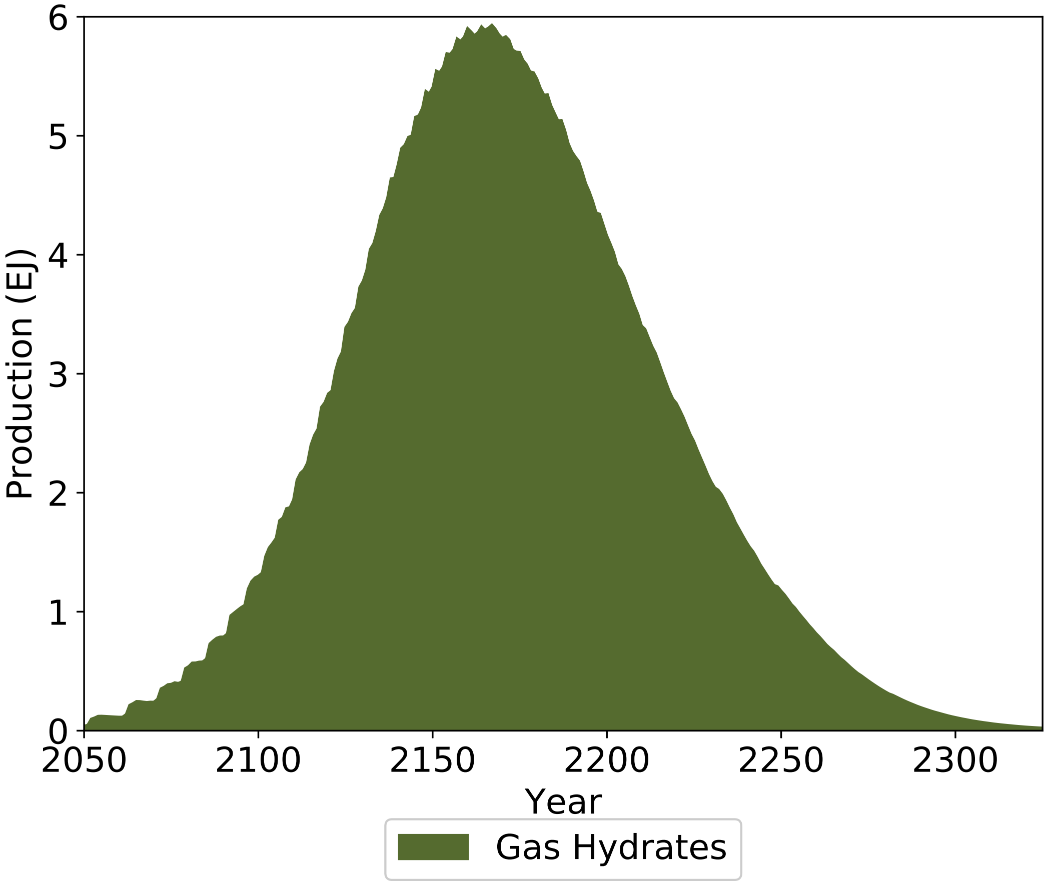

Figure 6.97: USA - USA projection by mineral type

Table 6.97: Peak years - Minerals

Name	URR	Peak Year	Peak Rate
Gas Hydrates	614.5	2167	5.93
**Total**	**614.5**	**2167**	**5.93**

Utah

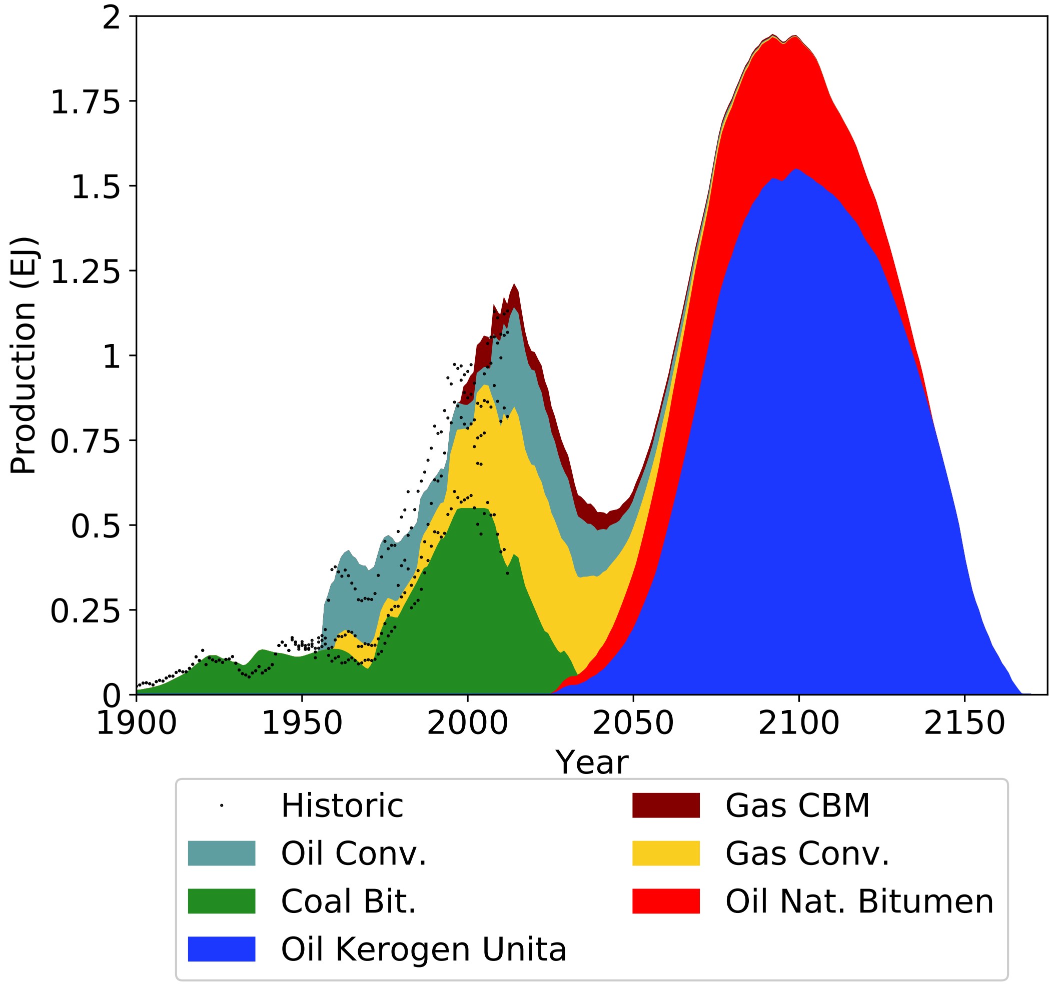

Figure 6.98: USA - Utah projections capped at 16

Table 6.98: Peak years - All

Name	URR	Peak Year	Peak Rate
Oil Kerogen Utah Unita	112.1	2099	1.55
Oil Nat. Bitumen Utah	27.84	2077	0.45
Coal Bit. Utah	27.03	1998	0.55
Gas Conv. Utah	21.0	2013	0.44
Oil Conv. Utah	17.19	2015	0.3
Gas CBM Utah	3.99	2005	0.09
**Total**	**209.15**	**2092**	**1.94**

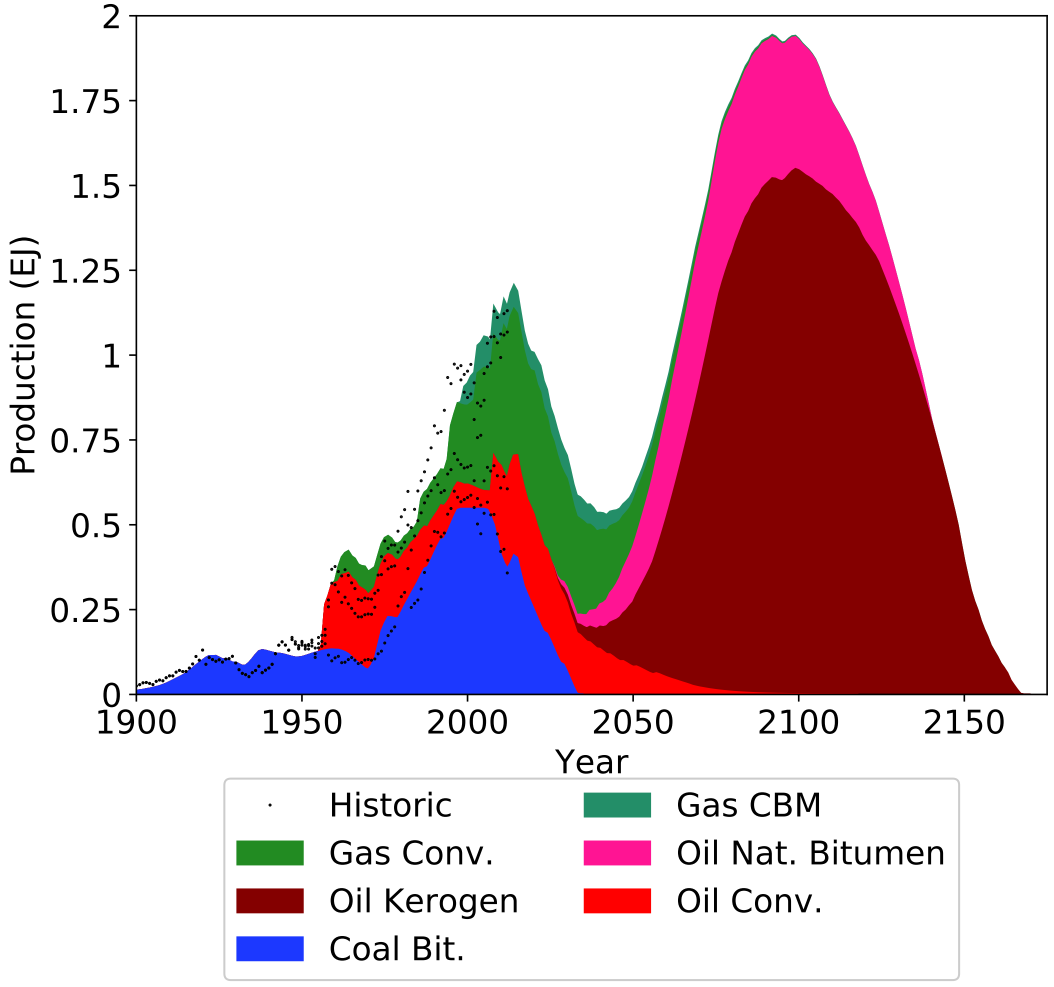

Figure 6.99: USA - Utah projection by mineral type

Table 6.99: Peak years - Minerals

**Name**	**URR**	**Peak Year**	**Peak Rate**
Coal Bit.	27.03	1998	0.55
Oil Conv.	17.19	2015	0.3
Oil Kerogen	112.1	2099	1.55
Oil Nat. Bitumen	27.84	2077	0.45
Gas Conv.	21.0	2013	0.44
Gas CBM	3.99	2005	0.09
**Total**	**209.15**	**2092**	**1.94**

Virginia

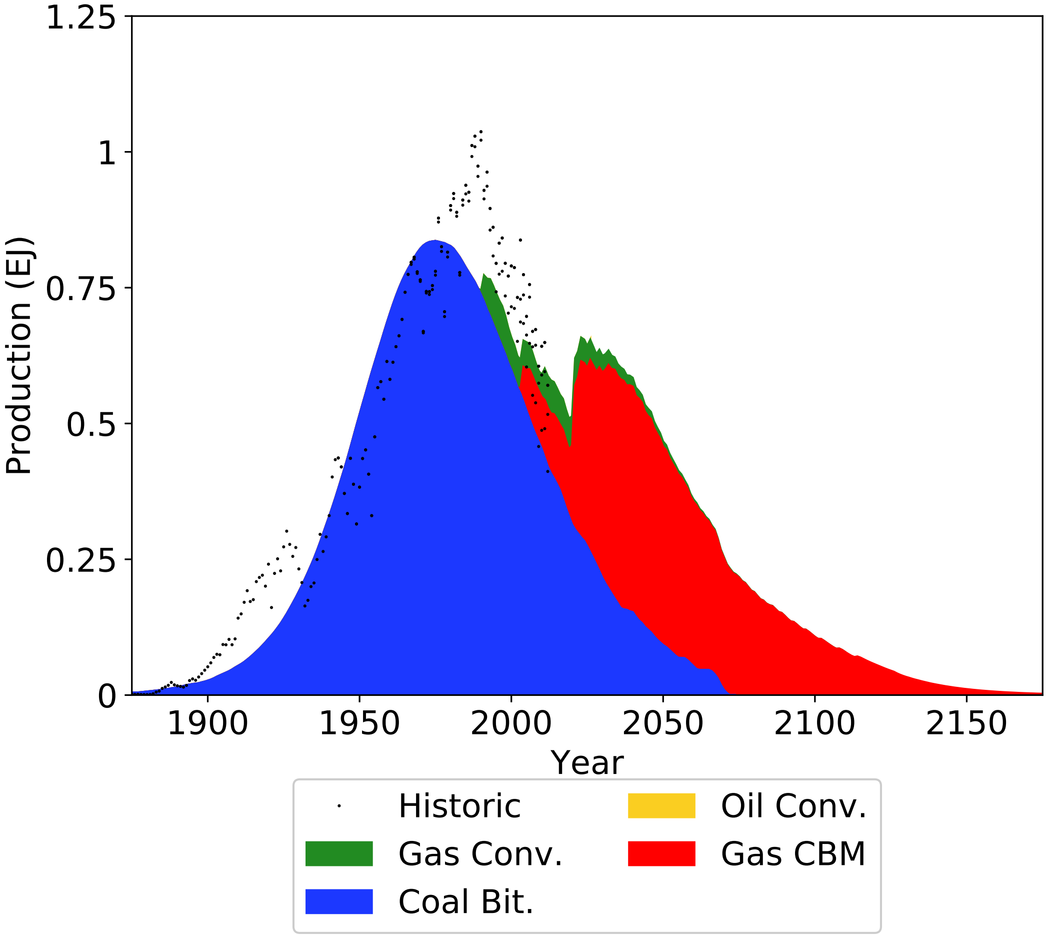

Figure 6.100: USA - Virginia projections capped at 16

Table 6.100: Peak years - All

Name	URR	Peak Year	Peak Rate
Coal Bit. Virginia	62.26	1975	0.84
Gas CBM Virginia	26.85	2034	0.42
Gas Conv. Virginia	2.56	1997	0.08
Oil Conv. Virginia	0.01	1982	–
**Total**	**91.68**	**1975**	**0.84**

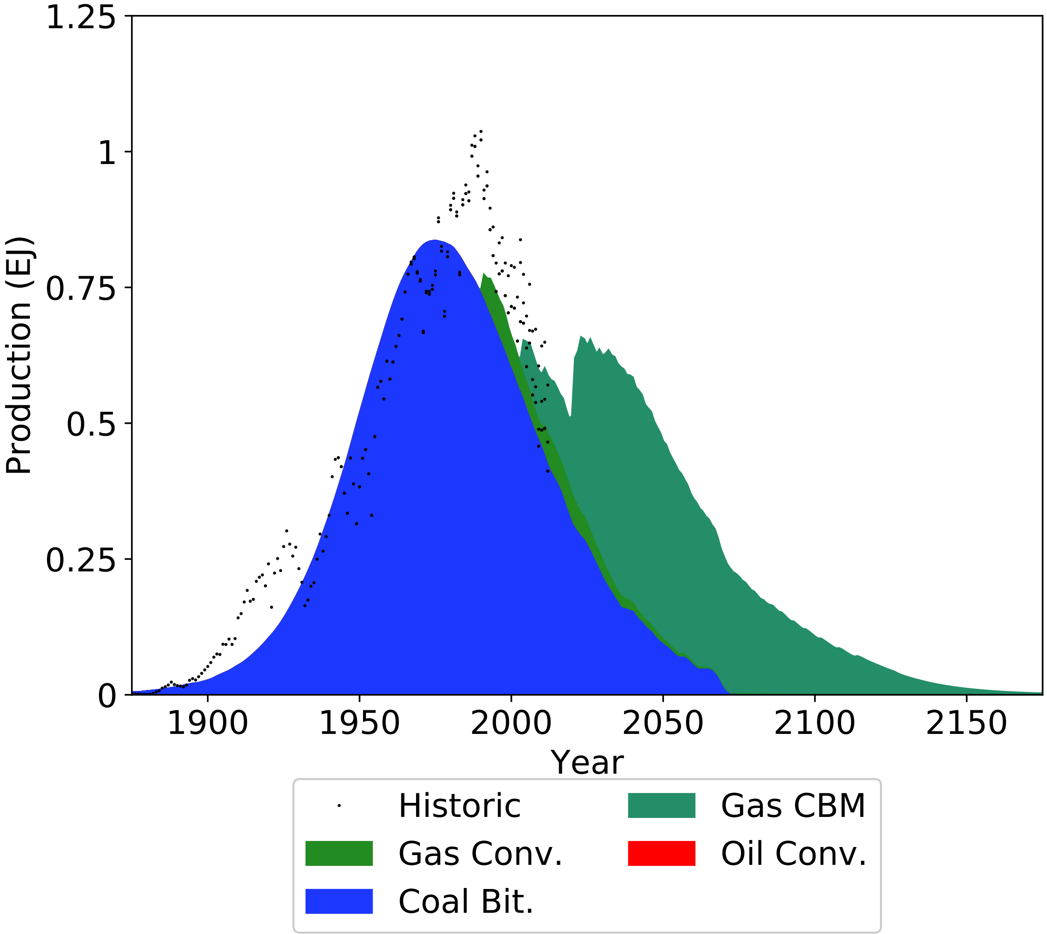

Figure 6.101: USA - Virginia projection by mineral type

Table 6.101: Peak years - Minerals

Name	URR	Peak Year	Peak Rate
Coal Bit.	62.26	1975	0.84
Oil Conv.	0.01	1982	–
Gas Conv.	2.56	1997	0.08
Gas CBM	26.85	2034	0.42
**Total**	**91.68**	**1975**	**0.84**

Washington

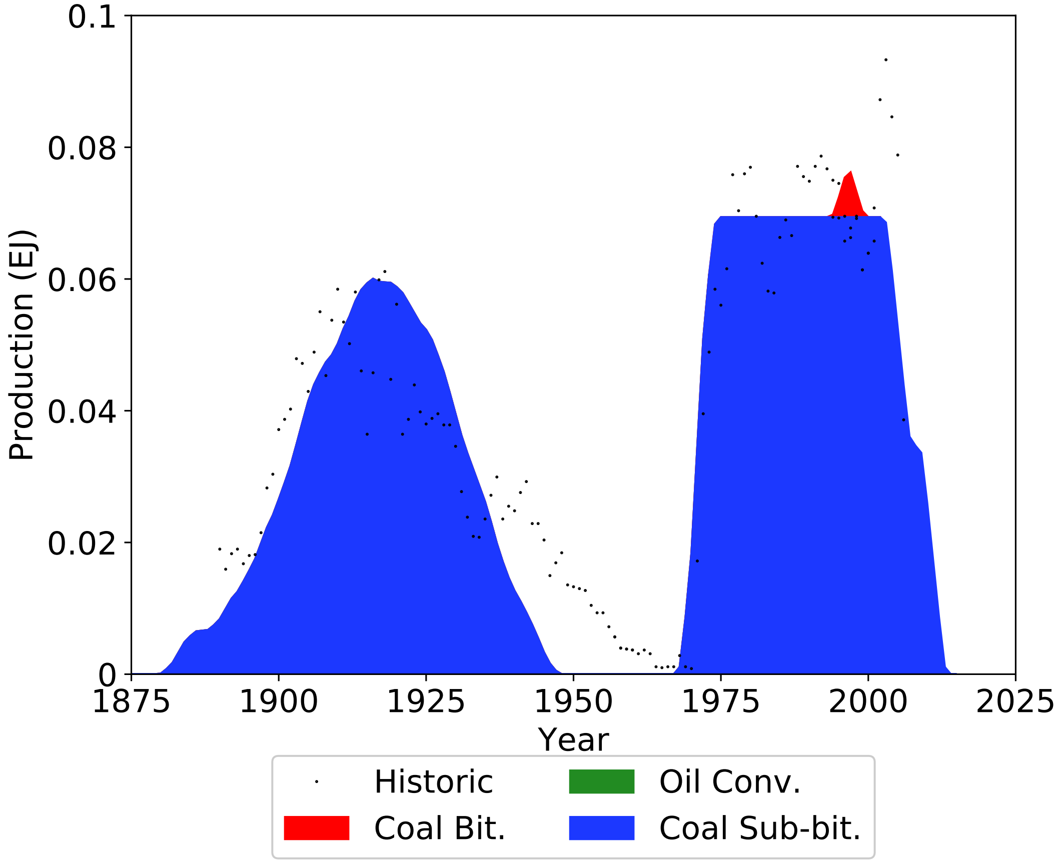

Figure 6.102: USA - Washington projections capped at 16

Table 6.102: Peak years - All

Name	URR	Peak Year	Peak Rate
Coal Sub-bit. Washington	4.57	1975	0.07
Coal Bit. Washington	0.02	1997	0.01
Oil Conv. Washington	–	1956	–
**Total**	**4.59**	**1997**	**0.08**

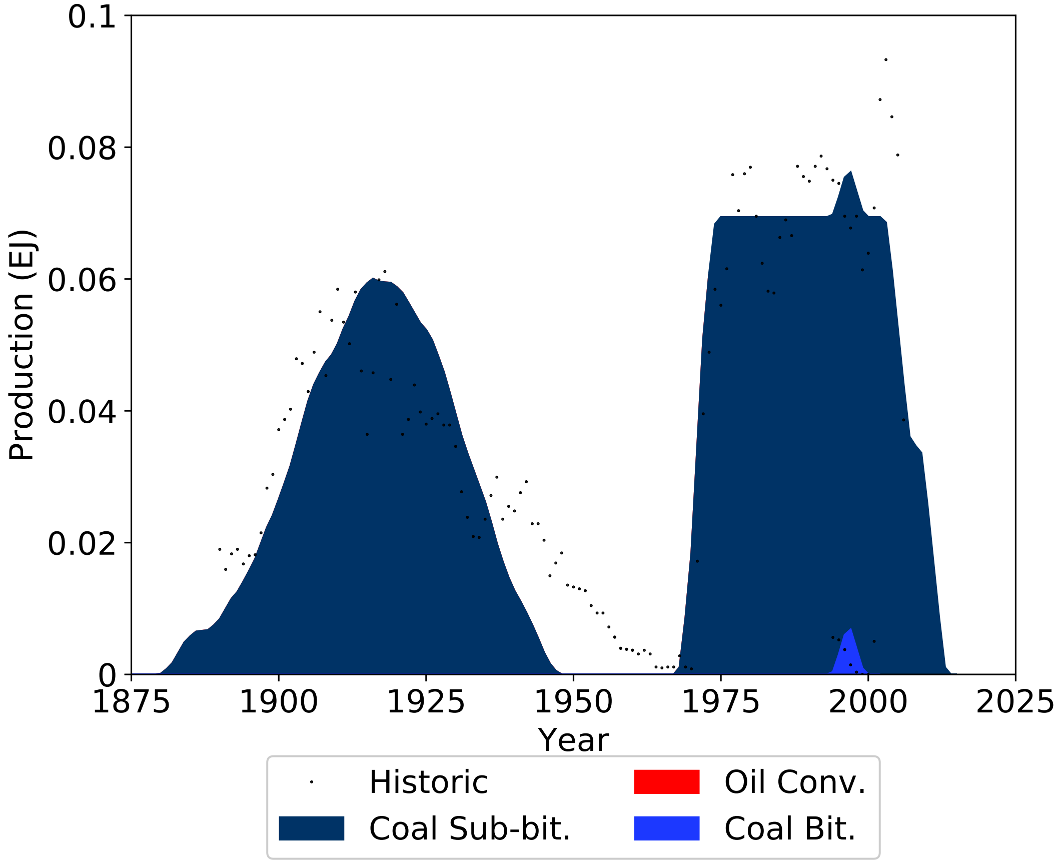

Figure 6.103: USA - Washington projection by mineral type

Table 6.103: Peak years - Minerals

Name	URR	Peak Year	Peak Rate
Coal Bit.	0.02	1997	0.01
Coal Sub-bit.	4.57	1975	0.07
Oil Conv.	–	1956	–
**Total**	**4.59**	**1997**	**0.08**

West Virginia

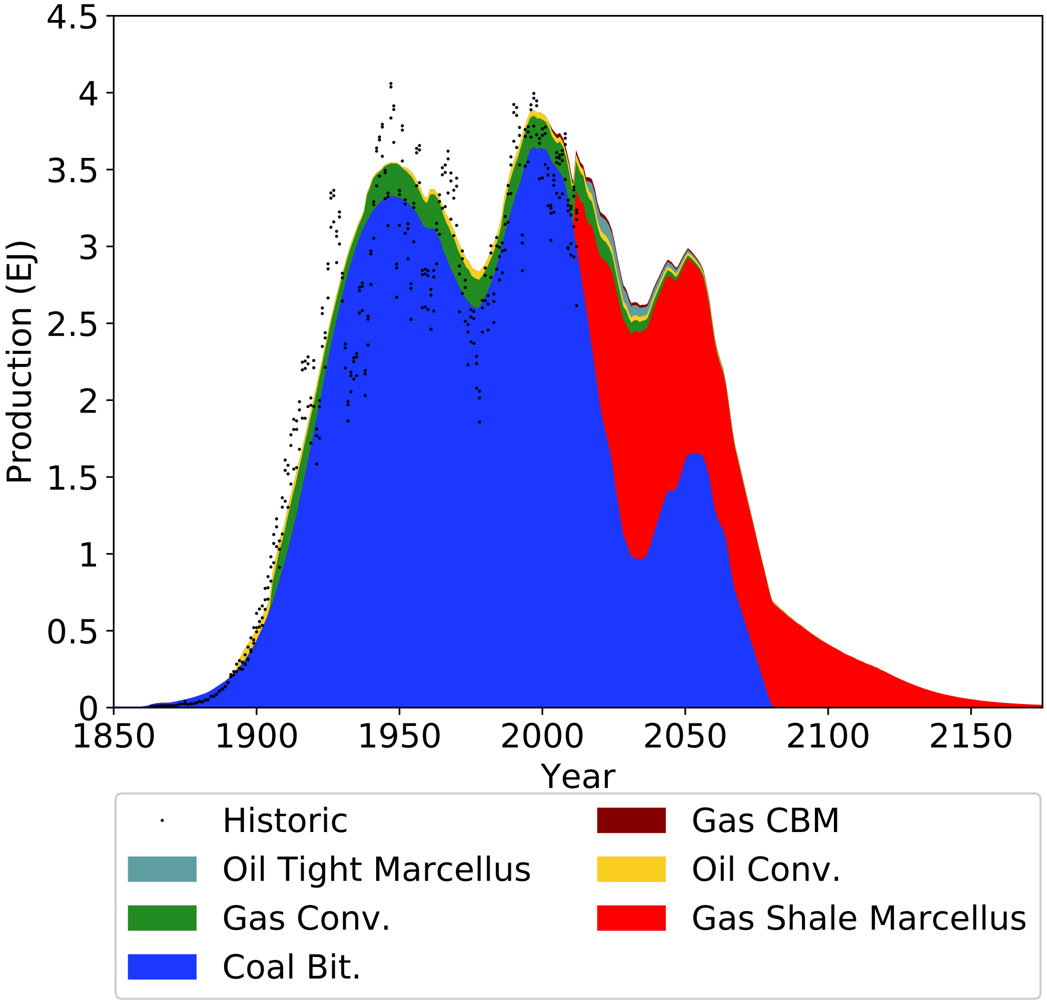

Figure 6.104: USA - West Virginia projections capped at 16

Table 6.104: Peak years - All

Name	URR	Peak Year	Peak Rate
Coal Bit. West Virginia	389.11	1997	3.64
Gas Shale West Virginia Marcellus	97.52	2035	1.48
Gas Conv. West Virginia	23.49	1967	0.24
Oil Conv. West Virginia	6.6	1902	0.08
Oil Tight West Virginia Marcellus	2.29	2022	0.1
Gas CBM West Virginia	1.21	2017	0.03
**Total**	**520.22**	**1997**	**3.88**

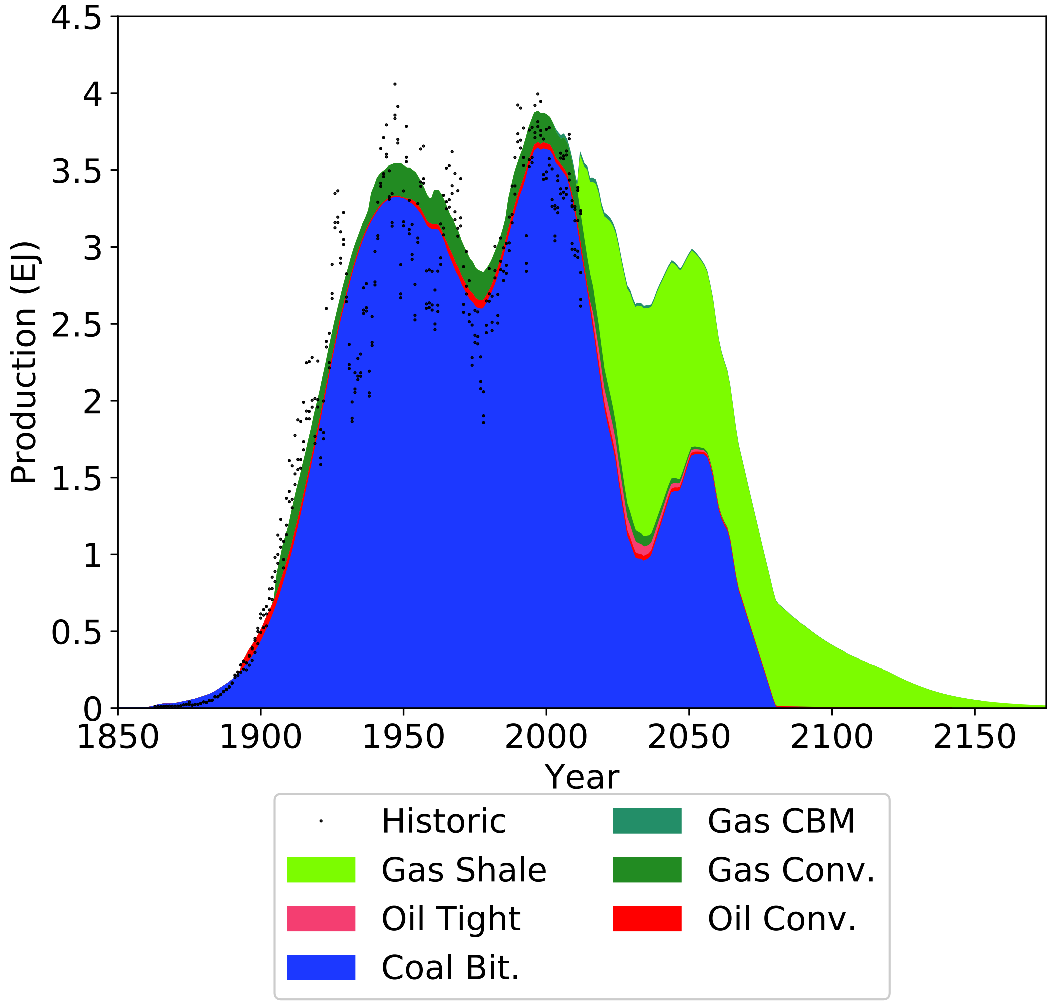

Figure 6.105: USA - West Virginia projection by mineral type

Table 6.105: Peak years - Minerals

**Name**	**URR**	**Peak Year**	**Peak Rate**
Coal Bit.	389.11	1997	3.64
Oil Conv.	6.6	1902	0.08
Oil Tight	2.29	2022	0.1
Gas Conv.	23.49	1967	0.24
Gas Shale	97.52	2035	1.48
Gas CBM	1.21	2017	0.03
**Total**	**520.22**	**1997**	**3.88**

Wyoming

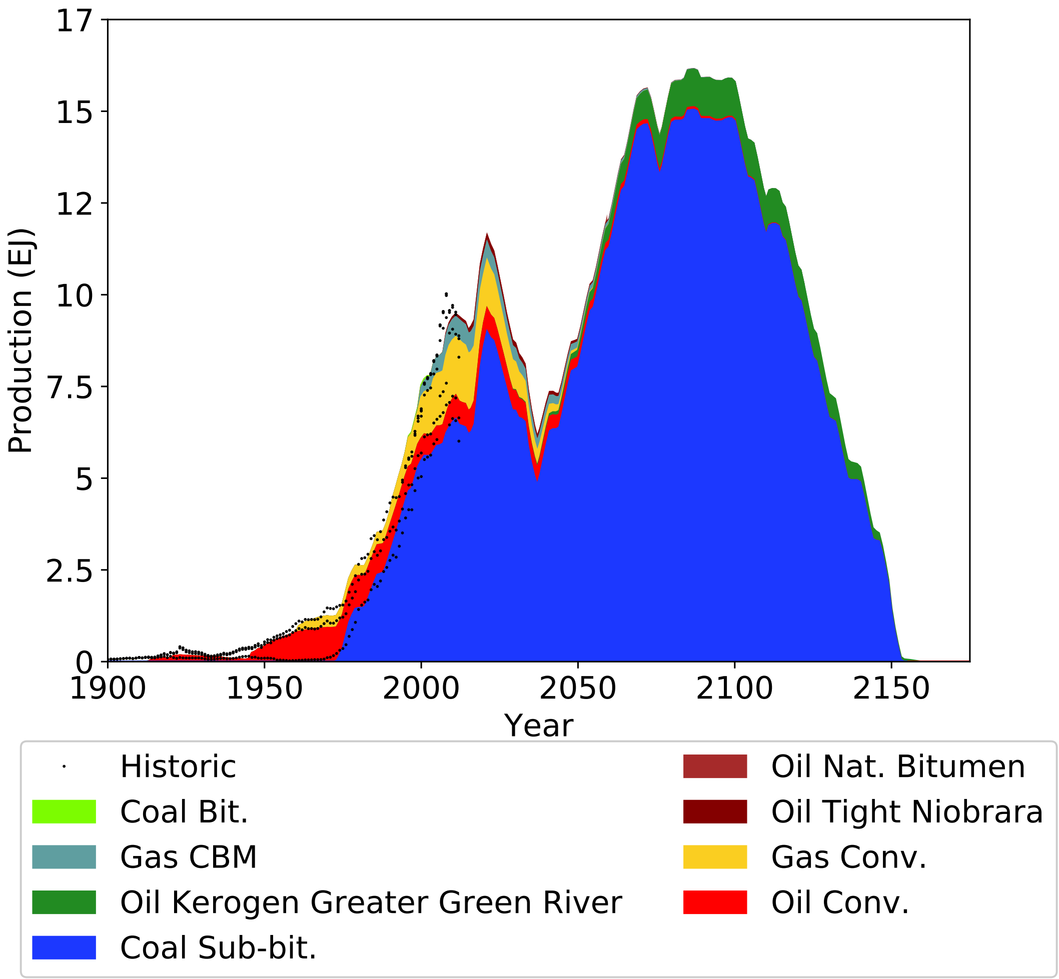

Figure 6.106: USA - Wyoming projections capped at 16

Table 6.106: Peak years - All

Name	URR	Peak Year	Peak Rate
Coal Sub-bit. Wyoming	1489.93	2087	15.05
Oil Conv. Wyoming	76.56	1972	0.92
Oil Kerogen Wyoming Greater Green River	74.7	2092	1.06
Gas Conv. Wyoming	62.21	2012	1.67
Gas CBM Wyoming	22.23	2013	0.56
Oil Tight Wyoming Niobrara	6.15	2022	0.19
Coal Bit. Wyoming	0.48	1999	0.07
Oil Nat. Bitumen Wyoming	0.1	2040	0.01
**Total**	**1732.36**	**2087**	**16.15**

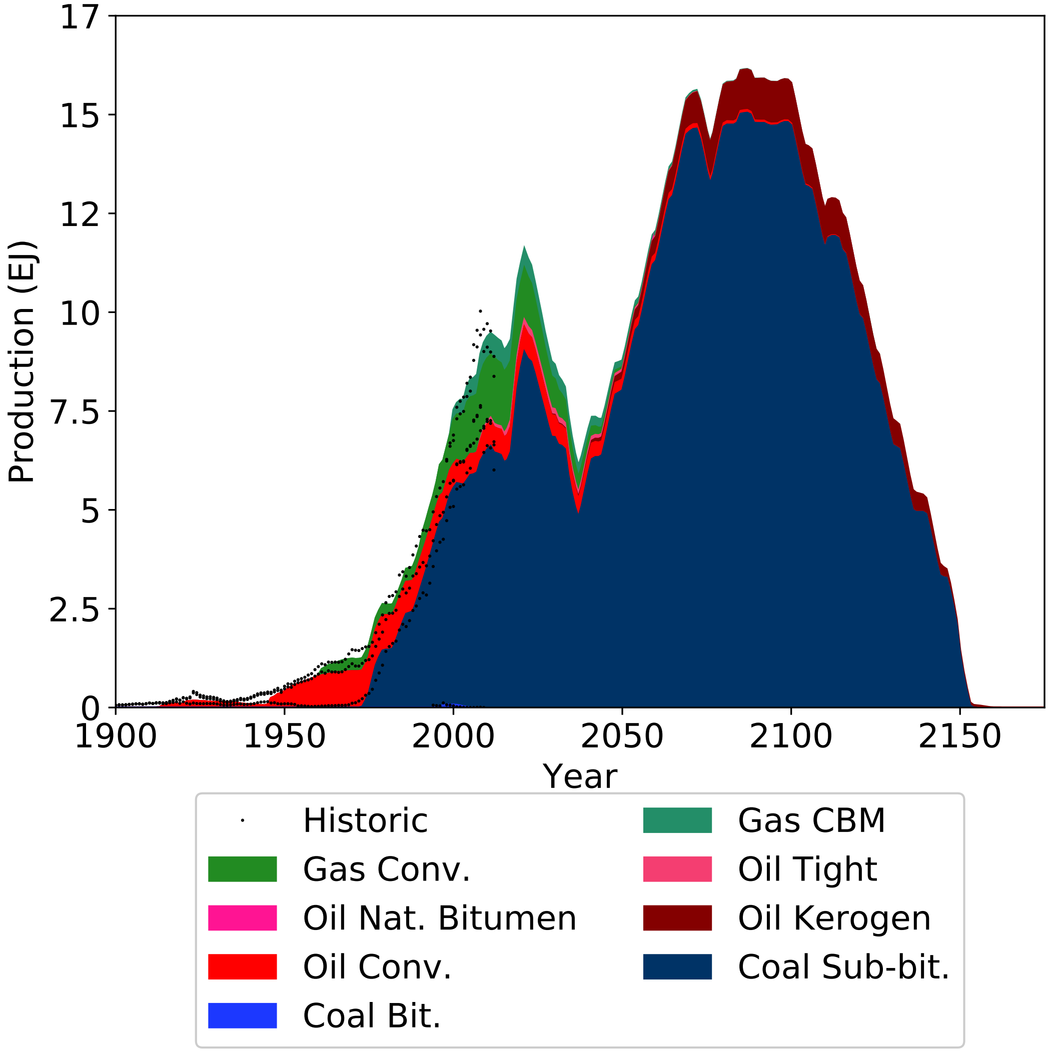

Figure 6.107: USA - Wyoming projection by mineral type

Table 6.107: Peak years - Minerals

**Name**	**URR**	**Peak Year**	**Peak Rate**
Coal Bit.	0.48	1999	0.07
Coal Sub-bit.	1489.93	2087	15.05
Oil Conv.	76.56	1972	0.92
Oil Kerogen	74.7	2092	1.06
Oil Nat. Bitumen	0.1	2040	0.01
Oil Tight	6.15	2022	0.19
Gas Conv.	62.21	2012	1.67
Gas CBM	22.23	2013	0.56
**Total**	**1732.36**	**2087**	**16.15**

6.2.4 Projection by region

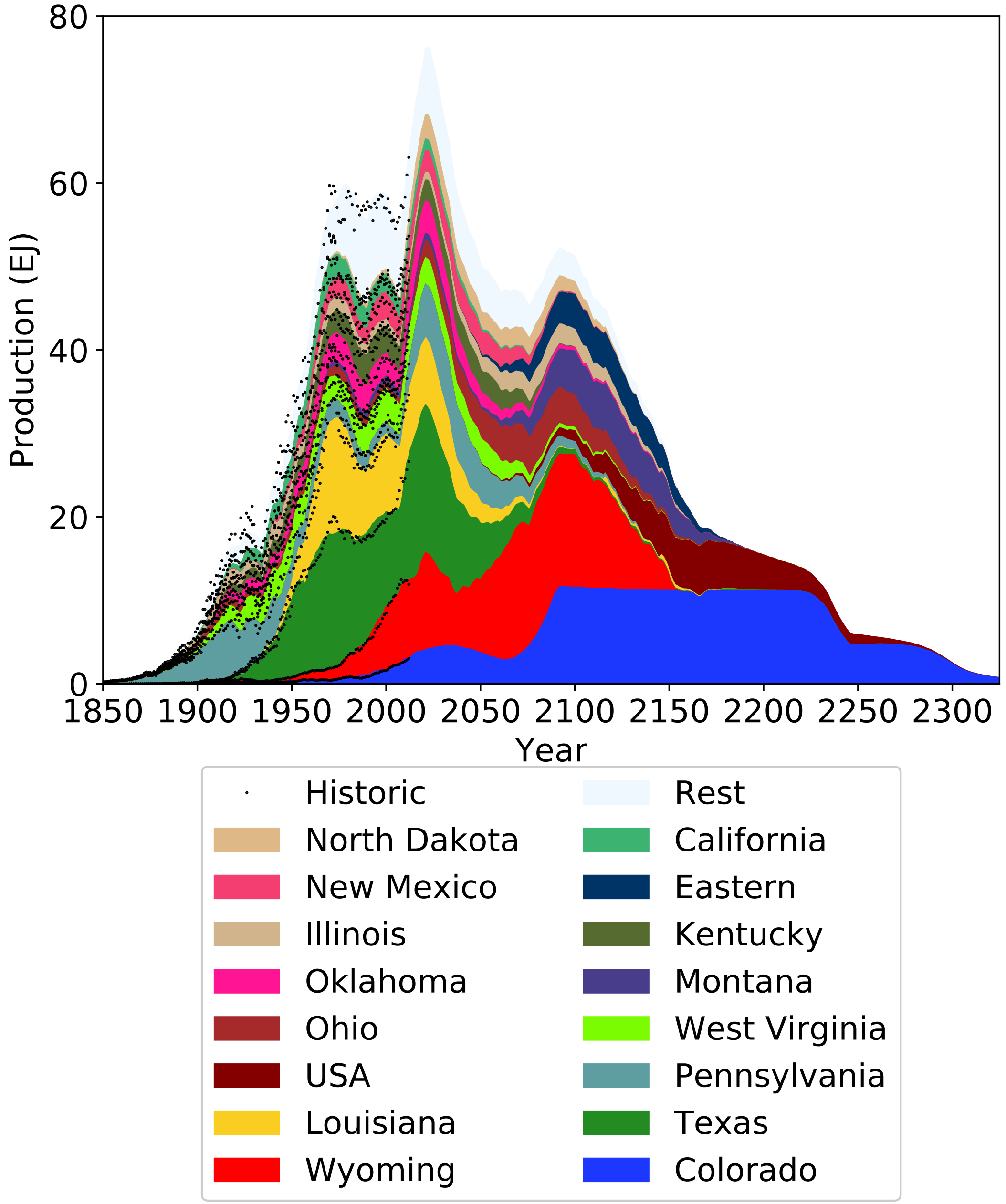

Figure 6.108: USA by region projections capped at 16

Table 6.108: Peak years - All

**Name**	**URR**	**Peak Year**	**Peak Rate**
Colorado	2391.8	2093	11.62
Wyoming	1732.36	2087	16.15
Texas	1576.6	2020	17.76
Louisiana	843.92	1974	13.8
Pennsylvania	795.46	2028	7.09
USA	614.5	2167	5.93
West Virginia	520.22	1997	3.88
Ohio	505.44	2077	4.67
Montana	493.26	2112	5.6
Oklahoma	404.91	2020	3.97
Kentucky	386.84	1988	3.64
Illinois	371.58	2093	2.39
Eastern	324.9	2111	4.28
New Mexico	315.56	1999	3.27
California	255.0	1968	3.02
North Dakota	244.9	2024	2.98
Utah	209.15	2092	1.94
Alaska	178.06	1987	4.48
Indiana	137.08	2046	1.07
Kansas	114.08	1969	1.57
Alabama	96.99	1997	1.27
New York	91.77	2042	1.37
Virginia	91.68	1975	0.84
Arkansas	84.74	2023	1.7
Mississippi	30.78	1959	0.53
Michigan	22.33	1996	0.45
Tennessee	16.44	1972	0.19
Arizona	14.78	2004	0.32
Maryland	9.64	1907	0.13
Missouri	9.6	1969	0.12
Iowa	8.14	1914	0.15
Florida	6.18	1976	0.4
Washington	4.59	1997	0.08
Nebraska	4.09	1959	0.15
South Dakota	0.71	1985	0.01
Nevada	0.35	1980	0.02
Georgia	0.26	1895	0.01
Oregon	0.21	2019	0.01
Other	0.04	1887	–
**Total**	**12908.93**	**2022**	**76.17**

6.3 Total

6.3.1 By country

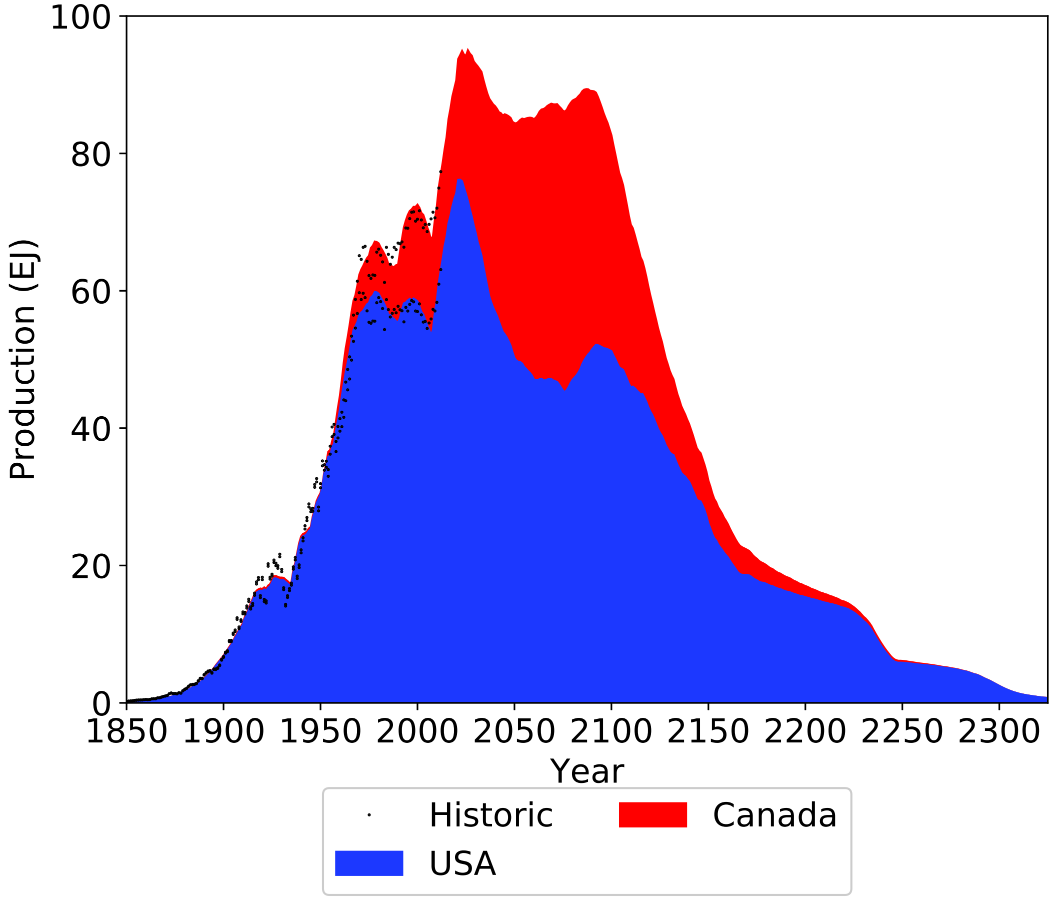

Figure 6.109: North America projections by country

Table 6.109: Peak years - All

Name	URR	Peak Year	Peak Rate
USA	12908.93	2022	76.17
Canada	4333.53	2078	40.9
**Total**	**17242.46**	**2026**	**95.01**

6.3.2 By mineral

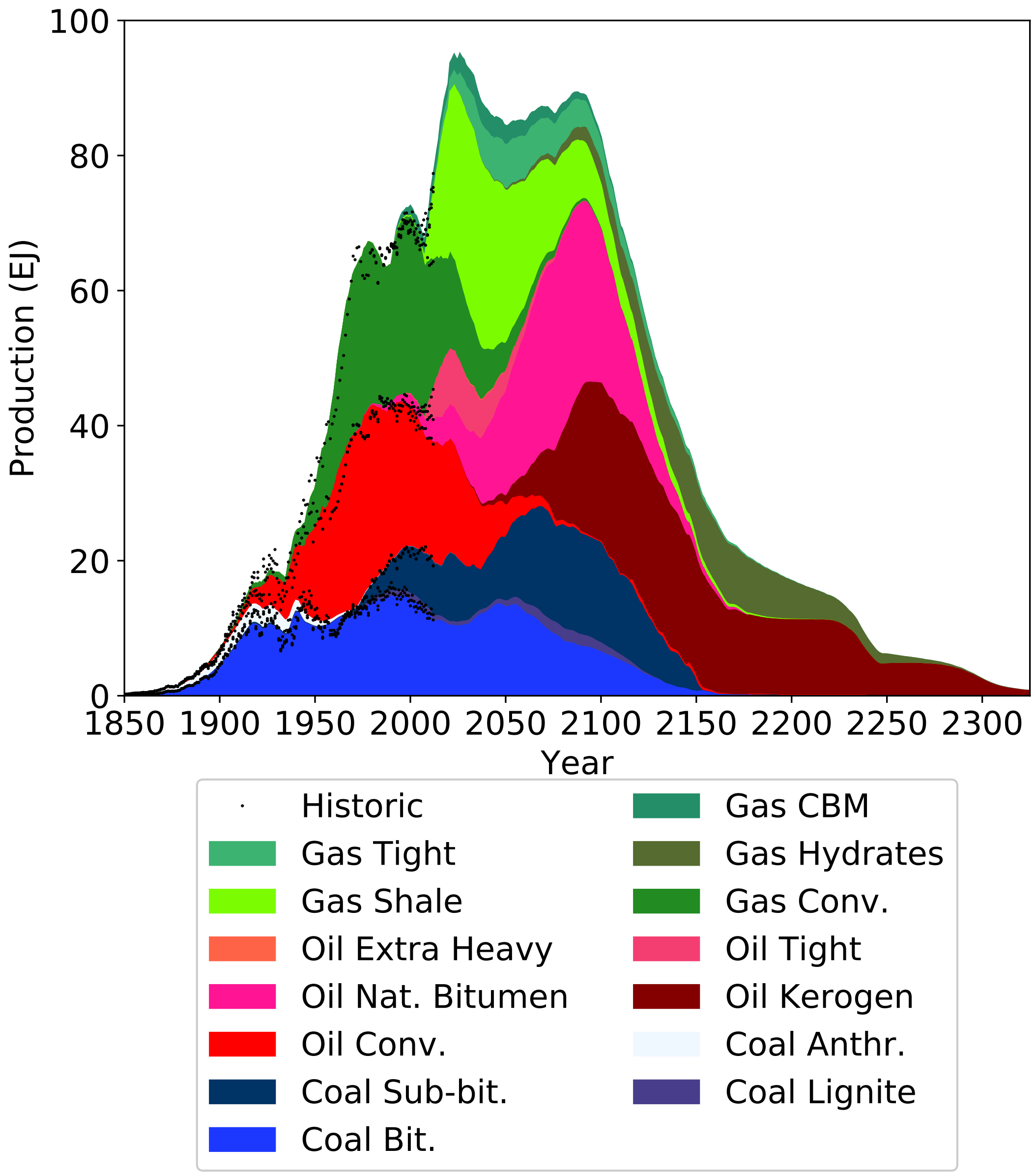

Figure 6.110: North America projection by mineral type

Table 6.110: Peak years - Minerals

**Name**	**URR**	**Peak Year**	**Peak Rate**
Coal Bit.	2394.0	1996	14.69
Coal Lignite	149.94	2081	1.87
Coal Sub-bit.	1640.0	2070	15.83
Coal Anthr.	153.01	1919	2.73
Oil Conv.	2127.2	1976	26.5
Oil Kerogen	3060.39	2106	23.66
Oil Nat. Bitumen	2061.14	2082	28.93
Oil Tight	294.87	2023	8.46
Oil Extra Heavy	3.37	2030	0.16
Gas Conv.	1824.24	1997	26.17
Gas Shale	1813.48	2032	28.71
Gas Hydrates	970.5	2157	9.09
Gas Tight	508.81	2051	6.57
Gas CBM	241.51	2039	3.3
**Total**	**17242.46**	**2026**	**95.01**

Chapter 7

South America

7.1 Argentina

7.1.1 All Projections

Table 7.1: Peak years - All

Name	URR	Peak Year	Peak Rate
Gas Shale	812.85	2160	7.76
Gas Hydrates	248.25	2141	2.54
Oil Tight	154.71	2075	3.23
Gas Conv.	88.4	2004	1.65
Oil Conv.	86.93	1999	2.03
Coal Bit.	13.7	2069	0.32
**Total**	**1404.84**	**2155**	**10.02**

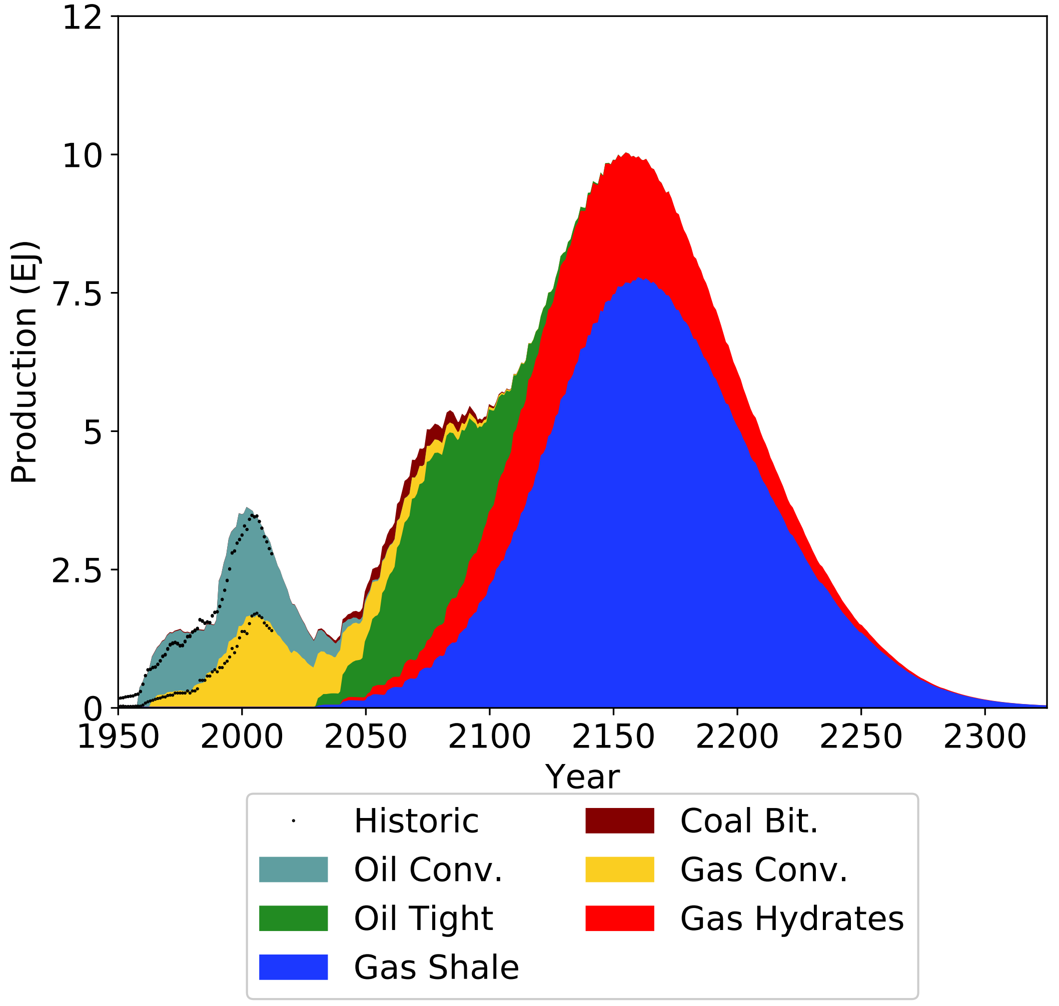

Figure 7.1: Argentina projections capped at 16

7.1.2 By Mineral

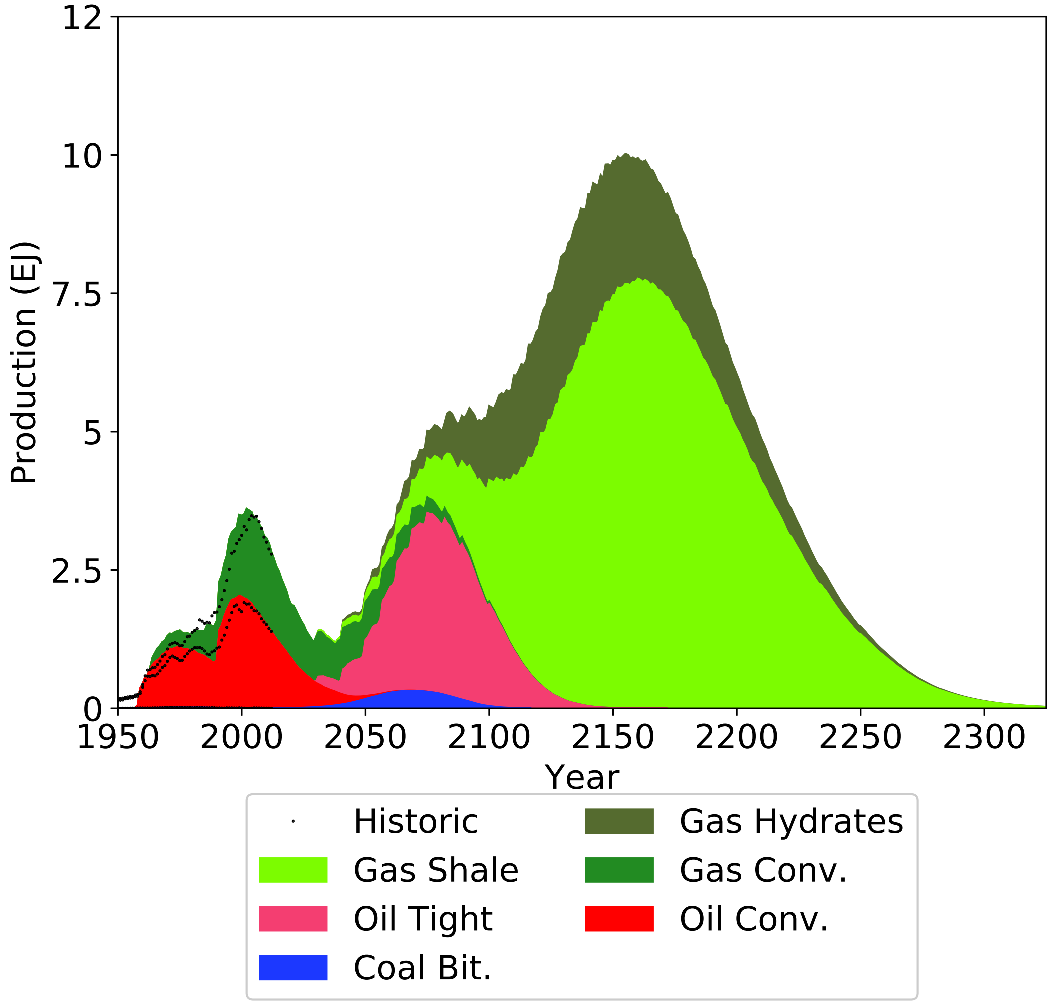

Figure 7.2: Argentina projection by mineral type

Table 7.2: Peak years - Minerals

**Name**	**URR**	**Peak Year**	**Peak Rate**
Coal Bit.	13.7	2069	0.32
Oil Conv.	86.93	1999	2.03
Oil Tight	154.71	2075	3.23
Gas Conv.	88.4	2004	1.65
Gas Shale	812.85	2160	7.76
Gas Hydrates	248.25	2141	2.54
**Total**	**1404.84**	**2155**	**10.02**

7.2 Barbados

7.2.1 All Projections

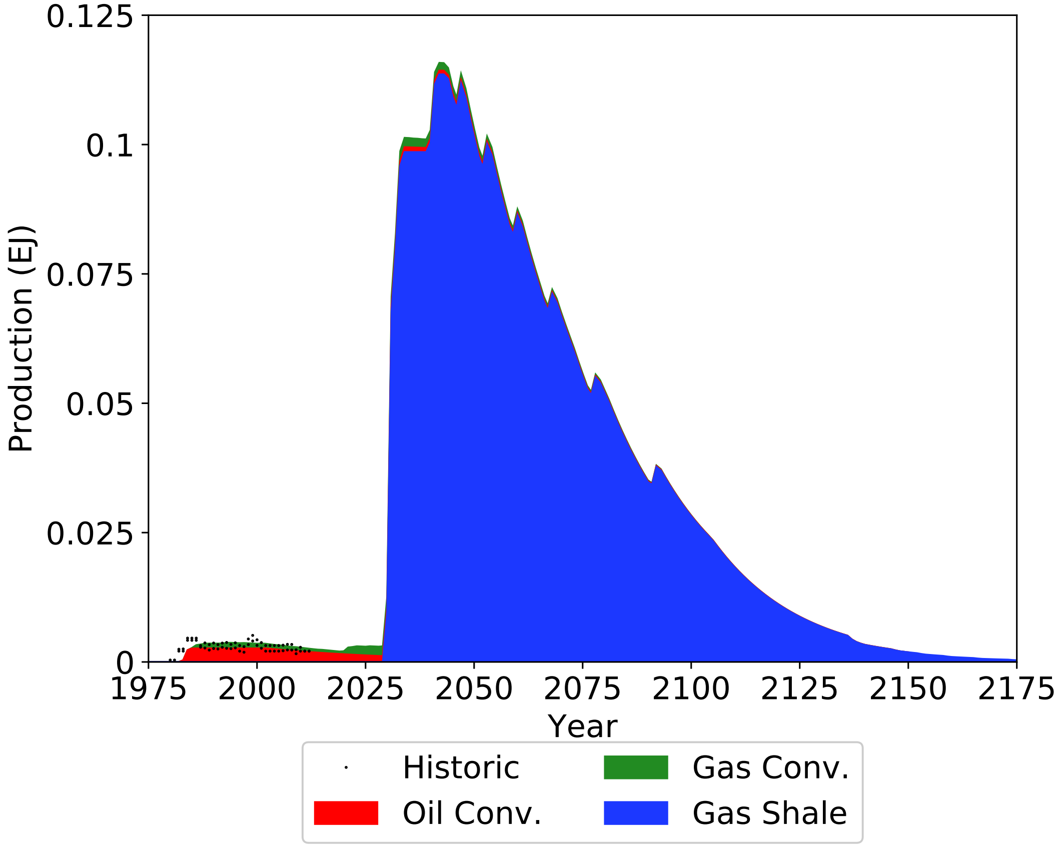

Figure 7.3: Barbados projections capped at 16

Table 7.3: Peak years - All

Name	URR	Peak Year	Peak Rate
Gas Shale	5.56	2042	0.11
Oil Conv.	0.13	1985	–
Gas Conv.	0.1	2031	–
**Total**	**5.79**	**2042**	**0.12**

7.2.2 By Mineral

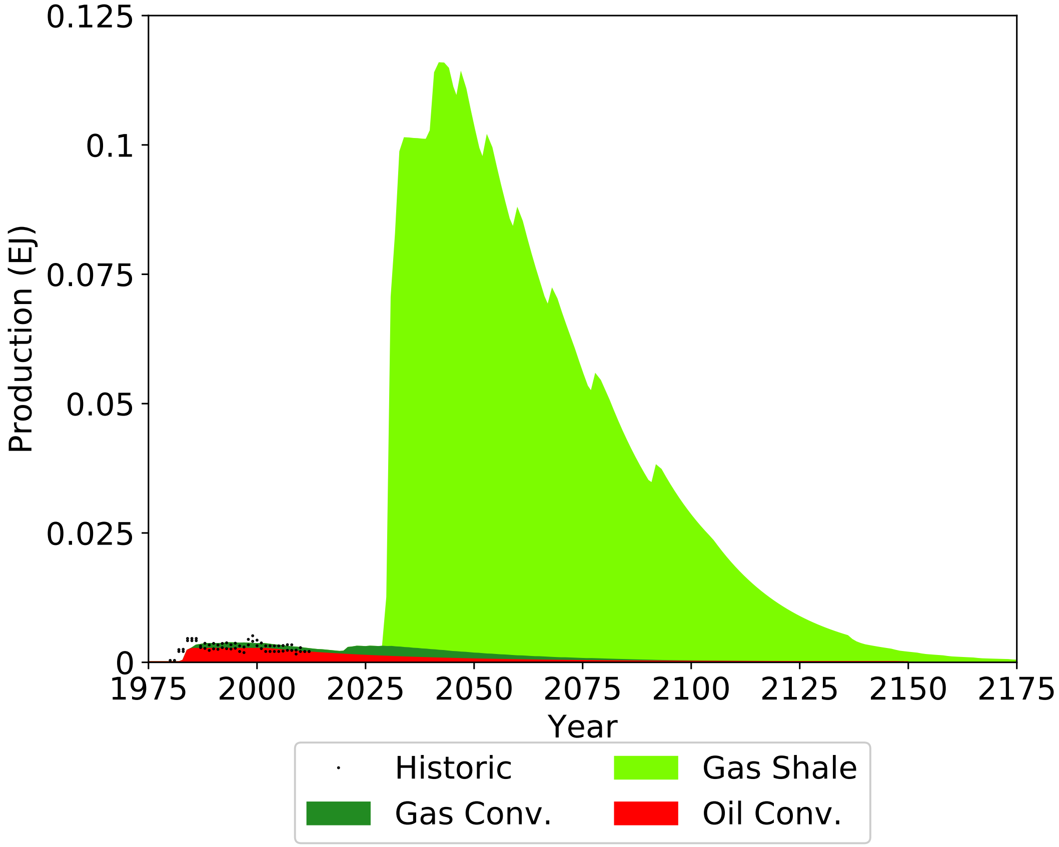

Figure 7.4: Barbados projection by mineral type

Table 7.4: Peak years - Minerals

Name	URR	Peak Year	Peak Rate
Oil Conv.	0.13	1985	–
Gas Conv.	0.1	2031	–
Gas Shale	5.56	2042	0.11
**Total**	**5.79**	**2042**	**0.12**

7.3 Belize

7.3.1 All Projections

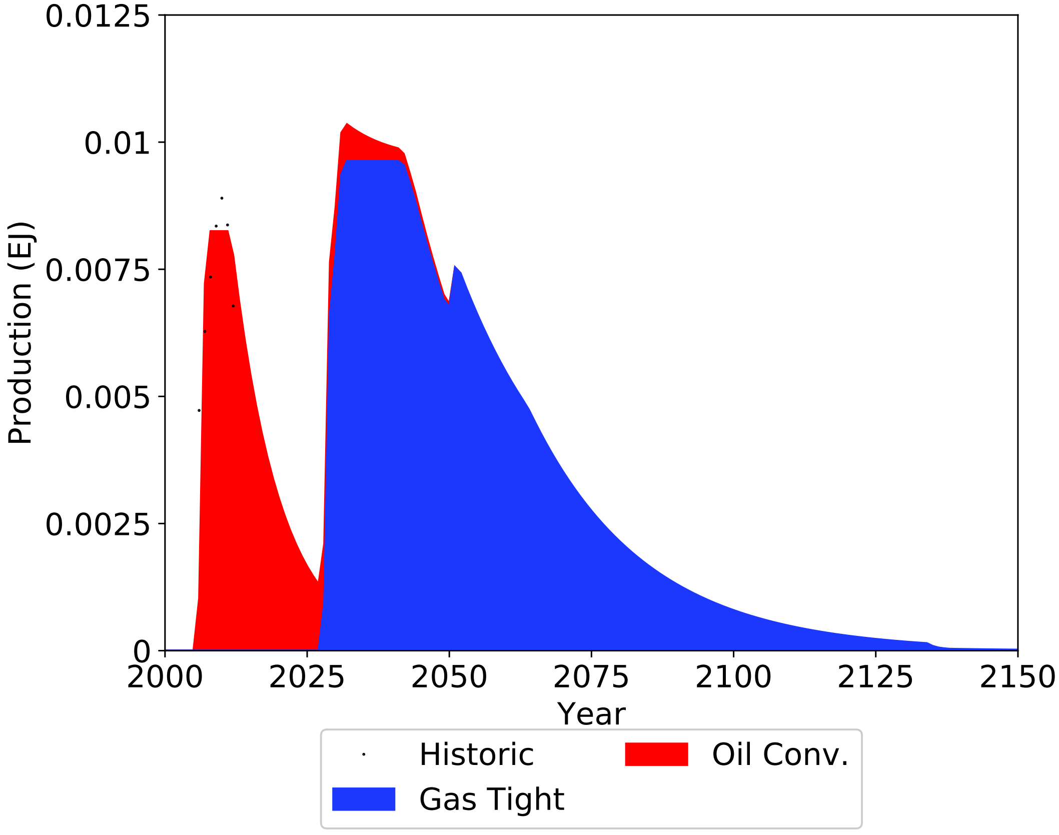

Figure 7.5: Belize projections capped at 16

Table 7.5: Peak years - All

Name	URR	Peak Year	Peak Rate
Gas Tight	0.37	2032	0.01
Oil Conv.	0.11	2008	0.01
**Total**	**0.48**	**2032**	**0.01**

7.3.2 By Mineral

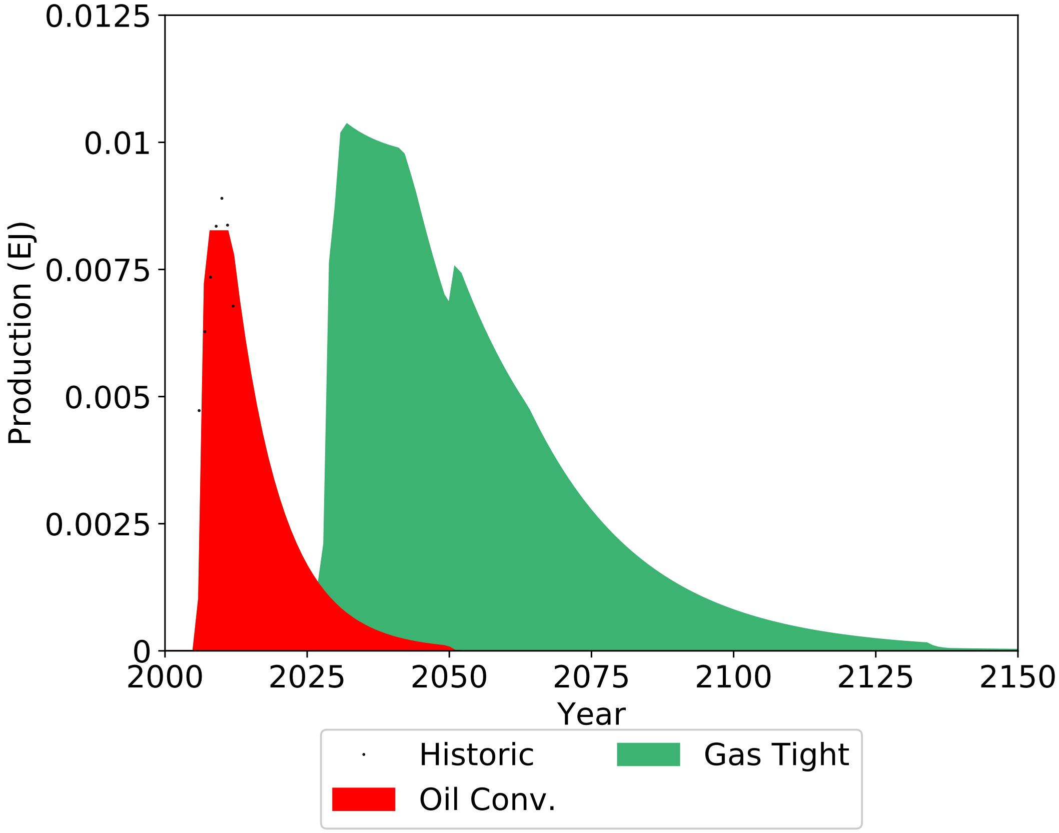

Figure 7.6: Belize projection by mineral type

Table 7.6: Peak years - Minerals

**Name**	**URR**	**Peak Year**	**Peak Rate**
Oil Conv.	0.11	2008	0.01
Gas Tight	0.37	2032	0.01
**Total**	**0.48**	**2032**	**0.01**

7.4 Bolivia

7.4.1 All Projections

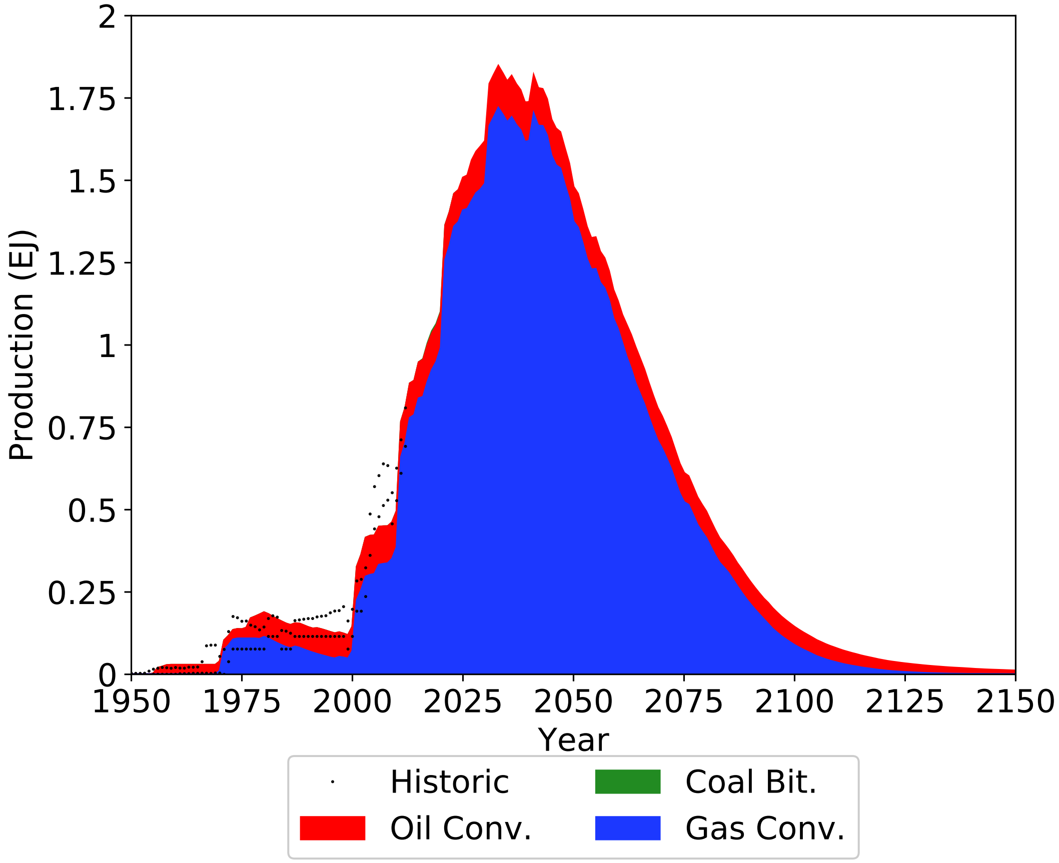

Figure 7.7: Bolivia projections capped at 16

Table 7.7: Peak years - All

**Name**	**URR**	**Peak Year**	**Peak Rate**
Gas Conv.	91.12	2033	1.72
Oil Conv.	13.9	2029	0.13
Coal Bit.	0.02	2018	0.01
**Total**	**105.04**	**2033**	**1.85**

7.4.2 By Mineral

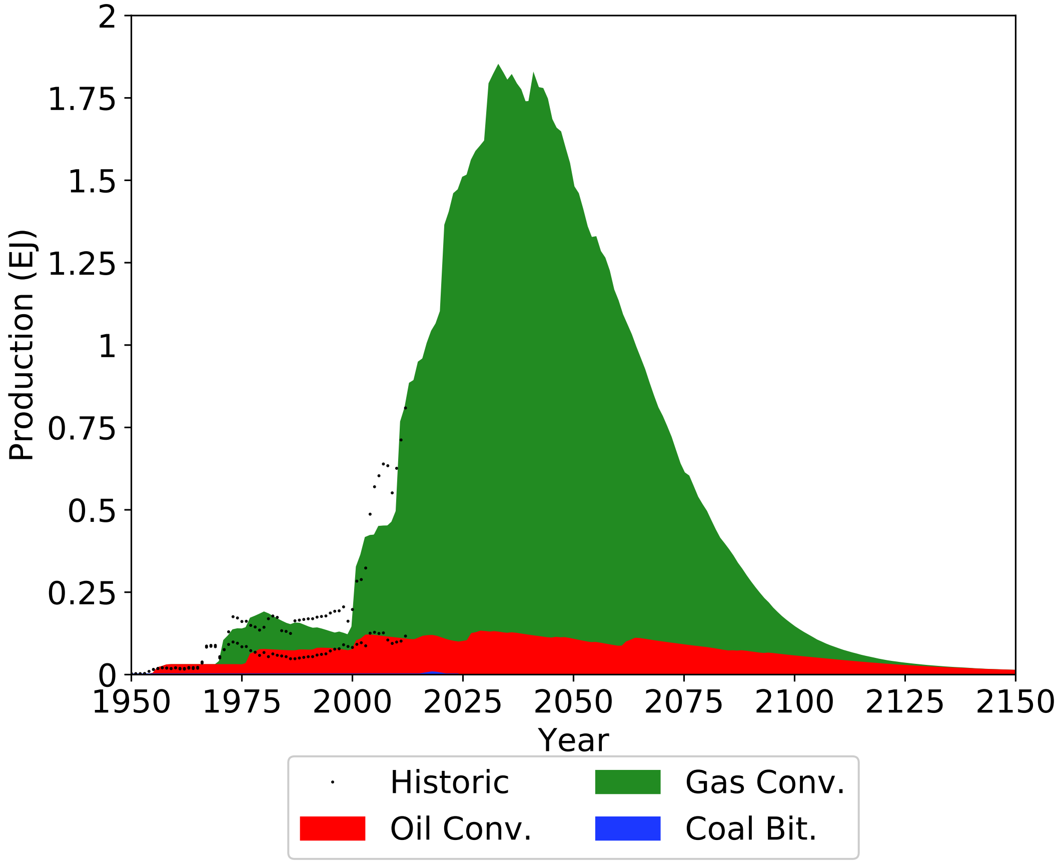

Figure 7.8: Bolivia projection by mineral type

Table 7.8: Peak years - Minerals

Name	URR	Peak Year	Peak Rate
Coal Bit.	0.02	2018	0.01
Oil Conv.	13.9	2029	0.13
Gas Conv.	91.12	2033	1.72
**Total**	**105.04**	**2033**	**1.85**

7.5 Brazil

7.5.1 All Projections

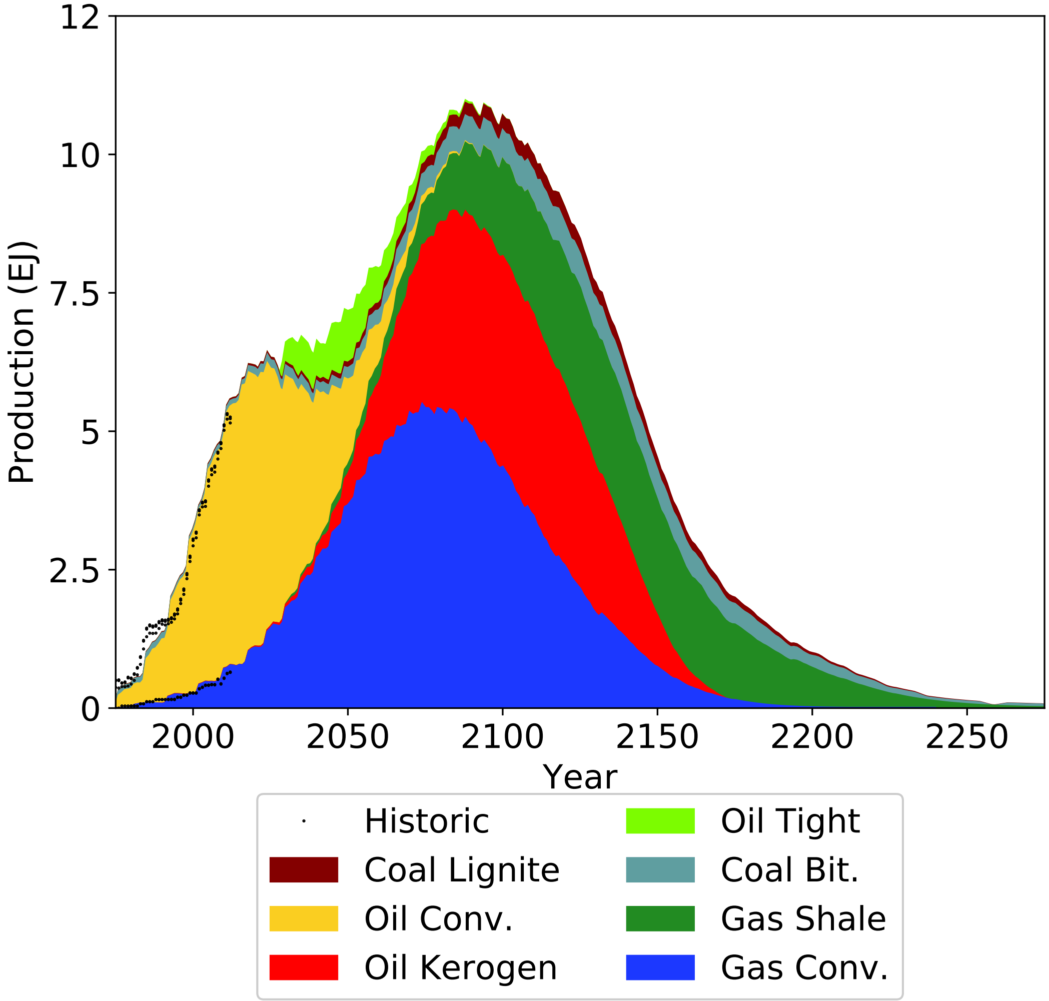

Figure 7.9: Brazil projections capped at 16

Table 7.9: Peak years - All

**Name**	**URR**	**Peak Year**	**Peak Rate**
Gas Conv.	452.15	2074	5.49
Oil Kerogen	282.07	2096	3.84
Gas Shale	237.35	2130	2.44
Oil Conv.	231.01	2016	5.05
Coal Bit.	85.2	2122	0.62
Coal Lignite	32.4	2119	0.28
Oil Tight	30.37	2049	0.95
**Total**	**1350.55**	**2088**	**10.98**

7.5.2 By Mineral

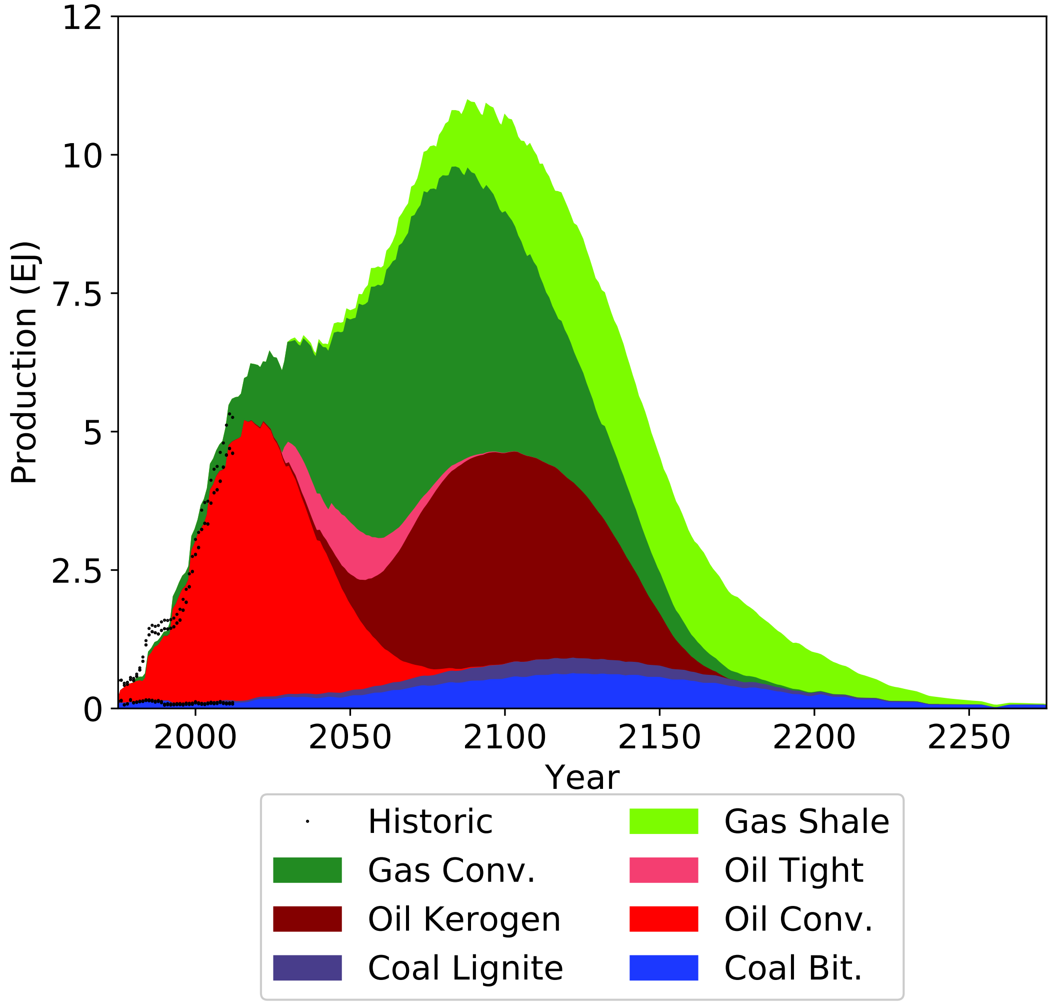

Figure 7.10: Brazil projection by mineral type

Table 7.10: Peak years - Minerals

**Name**	**URR**	**Peak Year**	**Peak Rate**
Coal Bit.	85.2	2122	0.62
Coal Lignite	32.4	2119	0.28
Oil Conv.	231.01	2016	5.05
Oil Kerogen	282.07	2096	3.84
Oil Tight	30.37	2049	0.95
Gas Conv.	452.15	2074	5.49
Gas Shale	237.35	2130	2.44
**Total**	**1350.55**	**2088**	**10.98**

7.6 Chile

7.6.1 All Projections

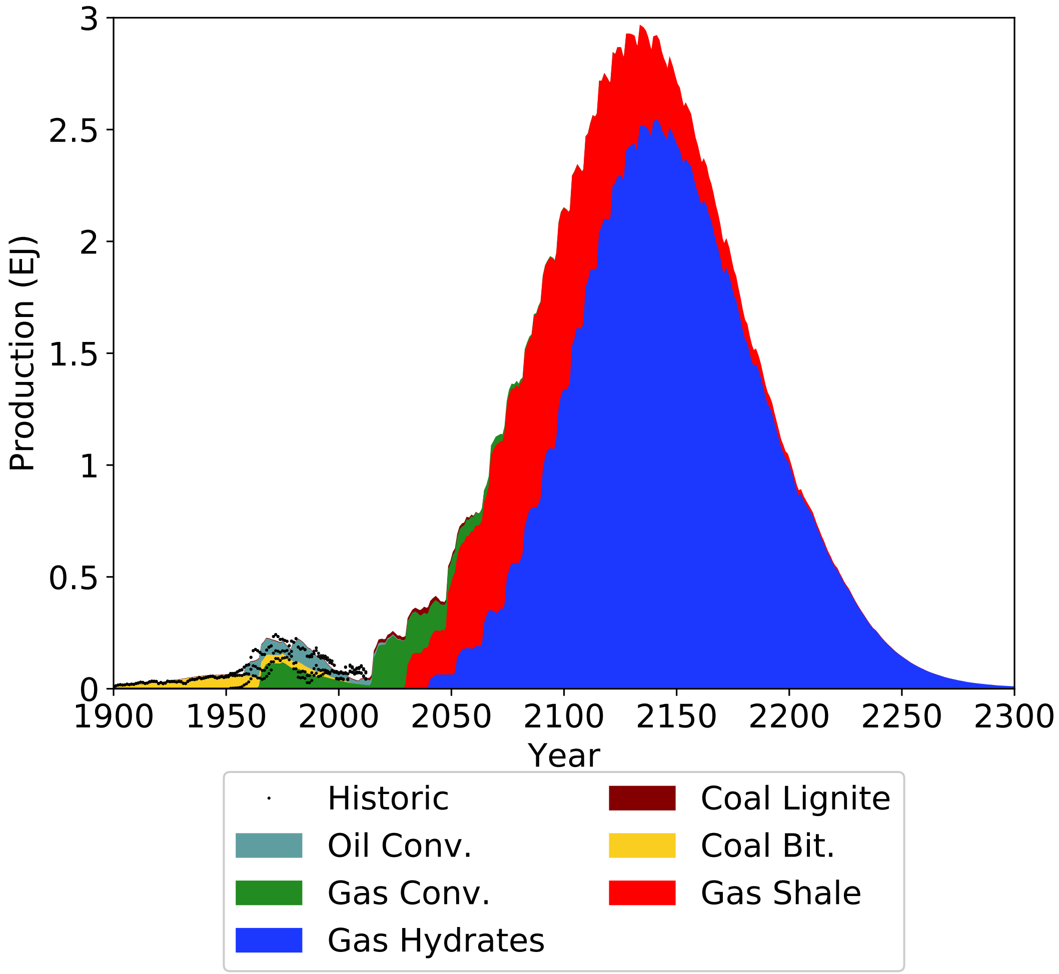

Figure 7.11: Chile projections capped at 16

Table 7.11: Peak years - All

**Name**	**URR**	**Peak Year**	**Peak Rate**
Gas Hydrates	248.25	2141	2.54
Gas Shale	72.79	2089	0.88
Gas Conv.	10.5	2024	0.23
Coal Bit.	3.4	1949	0.06
Oil Conv.	3.18	1982	0.1
Coal Lignite	1.02	2038	0.03
**Total**	**339.14**	**2134**	**2.96**

7.6.2 By Mineral

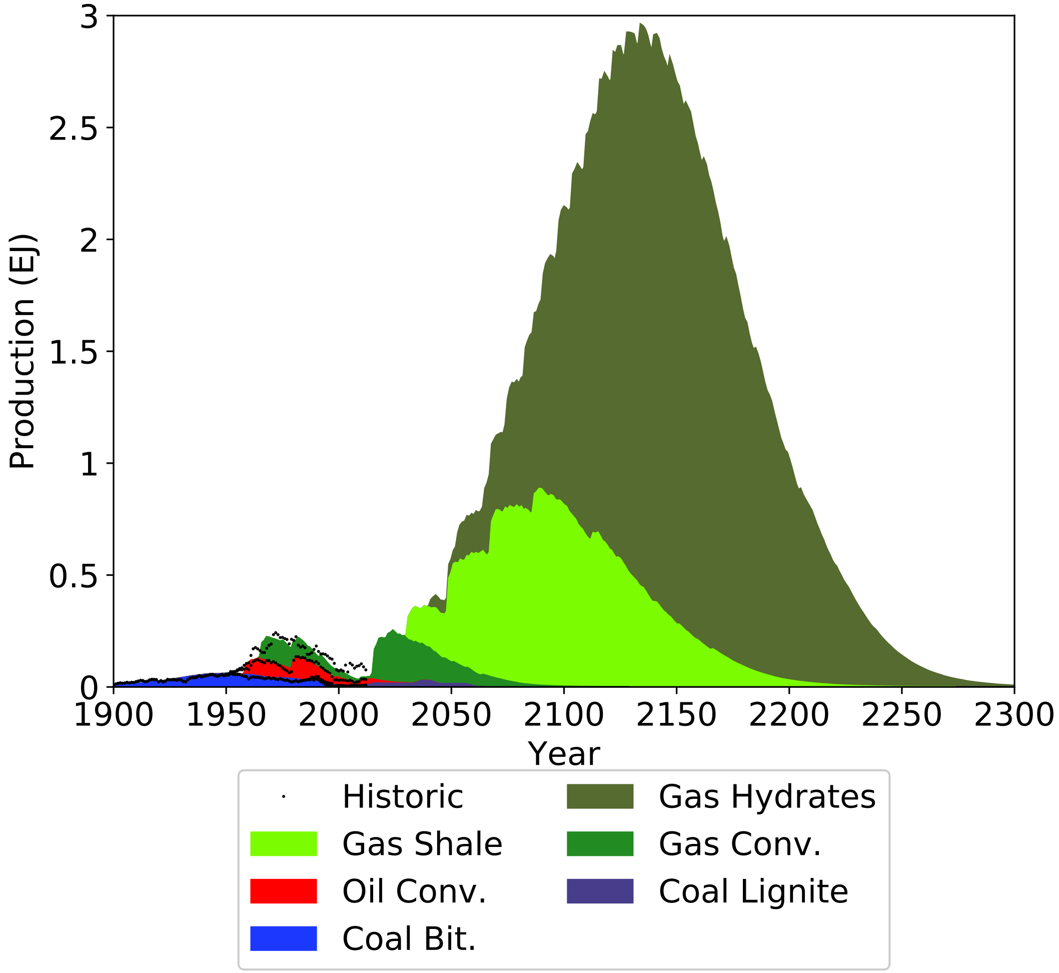

Figure 7.12: Chile projection by mineral type

Table 7.12: Peak years - Minerals

**Name**	**URR**	**Peak Year**	**Peak Rate**
Coal Bit.	3.4	1949	0.06
Coal Lignite	1.02	2038	0.03
Oil Conv.	3.18	1982	0.1
Gas Conv.	10.5	2024	0.23
Gas Shale	72.79	2089	0.88
Gas Hydrates	248.25	2141	2.54
**Total**	**339.14**	**2134**	**2.96**

7.7 Colombia

7.7.1 All Projections

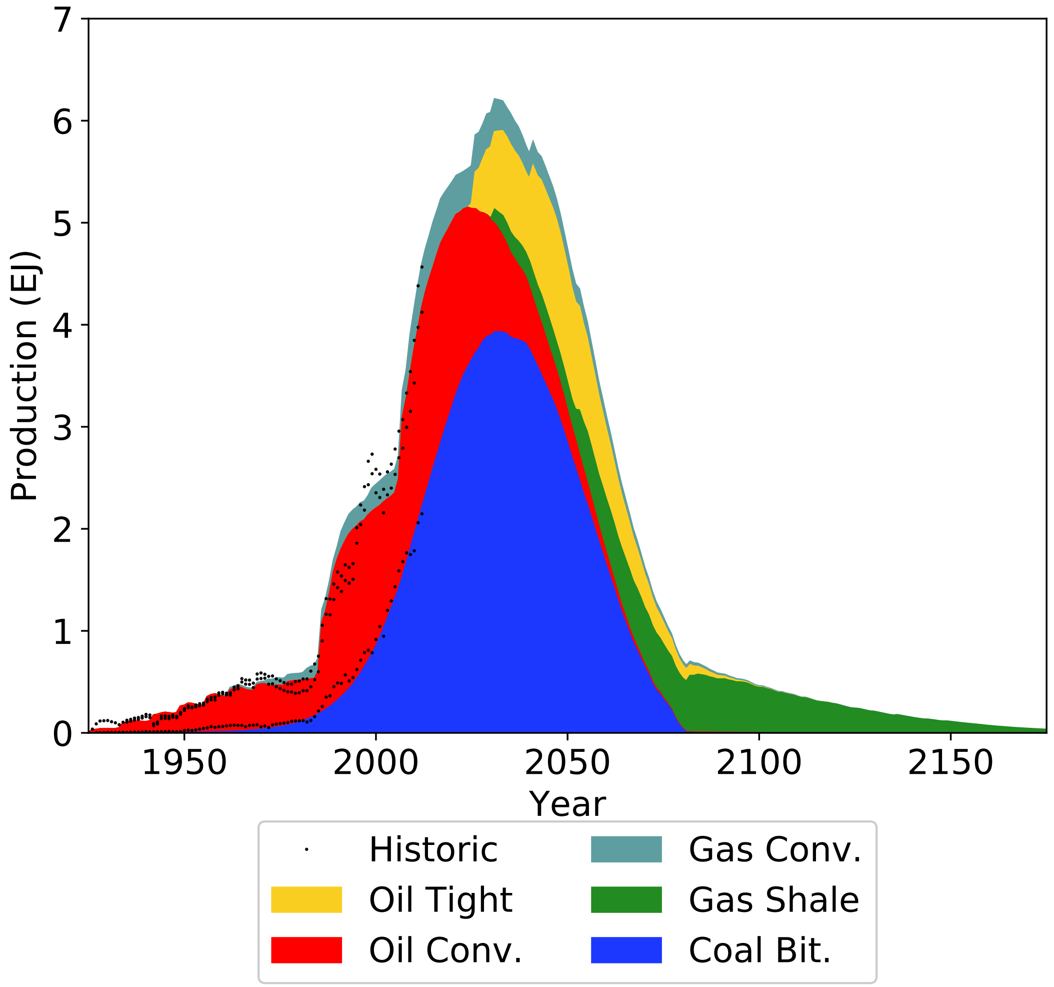

Figure 7.13: Colombia projections capped at 16

Table 7.13: Peak years - All

**Name**	**URR**	**Peak Year**	**Peak Rate**
Coal Bit.	192.3	2032	3.93
Oil Conv.	99.5	2014	1.99
Gas Shale	44.72	2066	0.57
Oil Tight	38.96	2047	1.2
Gas Conv.	23.1	2015	0.44
**Total**	**398.58**	**2031**	**6.21**

7.7.2 By Mineral

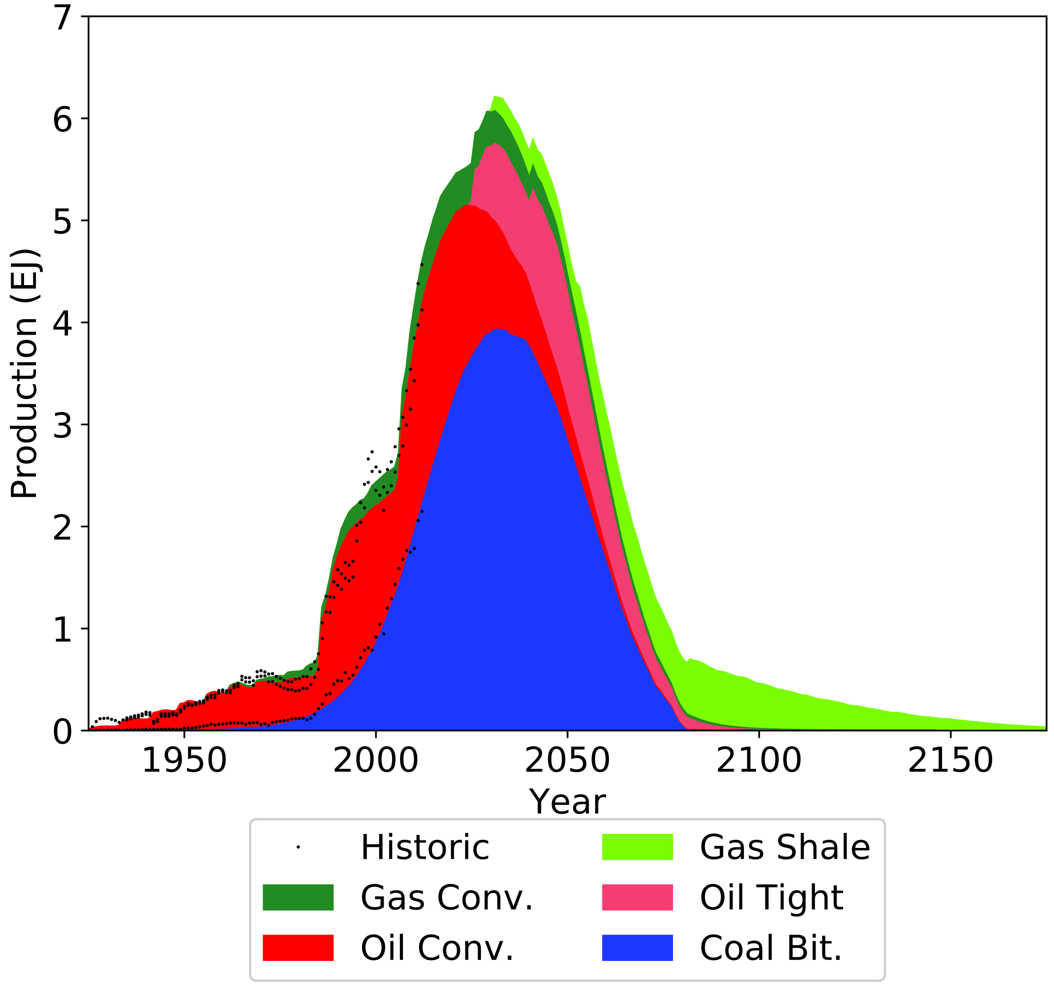

Figure 7.14: Colombia projection by mineral type

Table 7.14: Peak years - Minerals

**Name**	**URR**	**Peak Year**	**Peak Rate**
Coal Bit.	192.3	2032	3.93
Oil Conv.	99.5	2014	1.99
Oil Tight	38.96	2047	1.2
Gas Conv.	23.1	2015	0.44
Gas Shale	44.72	2066	0.57
**Total**	**398.58**	**2031**	**6.21**

7.8 Cuba

7.8.1 All Projections

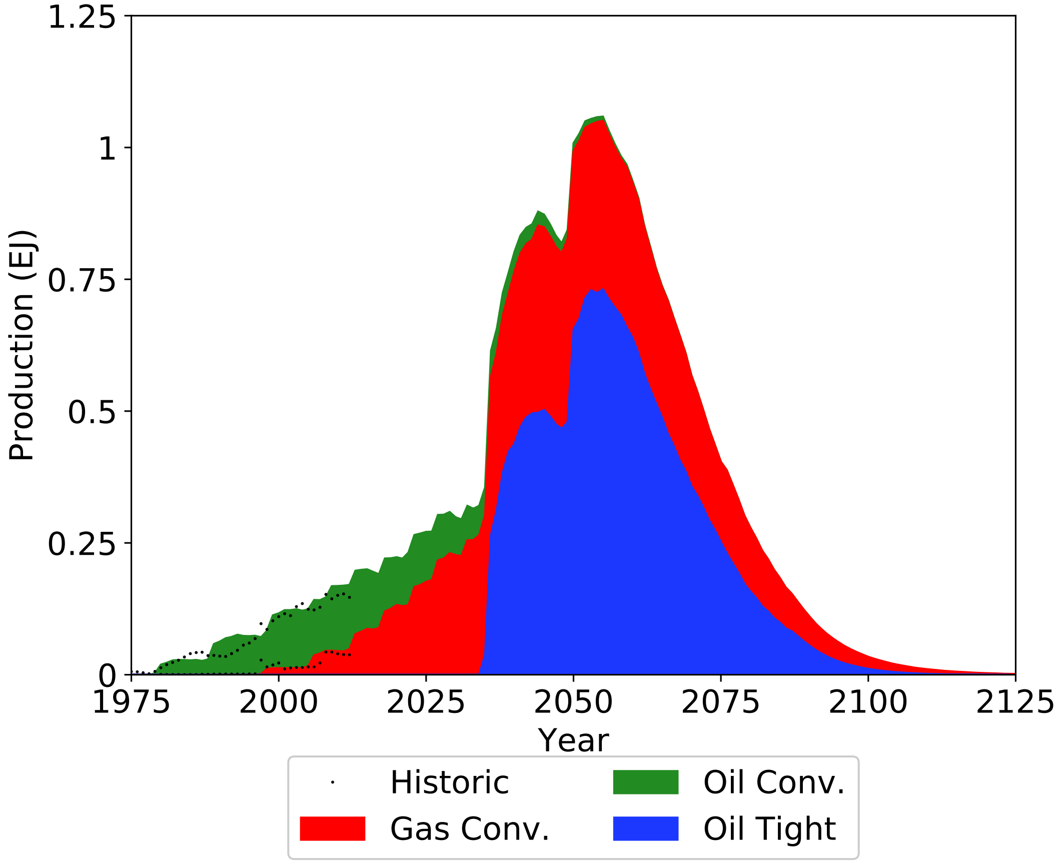

Figure 7.15: Cuba projections capped at 16

Table 7.15: Peak years - All

Name	URR	Peak Year	Peak Rate
Oil Tight	22.32	2055	0.73
Gas Conv.	17.83	2044	0.35
Oil Conv.	5.1	2011	0.12
**Total**	**45.25**	**2055**	**1.06**

7.8.2 By Mineral

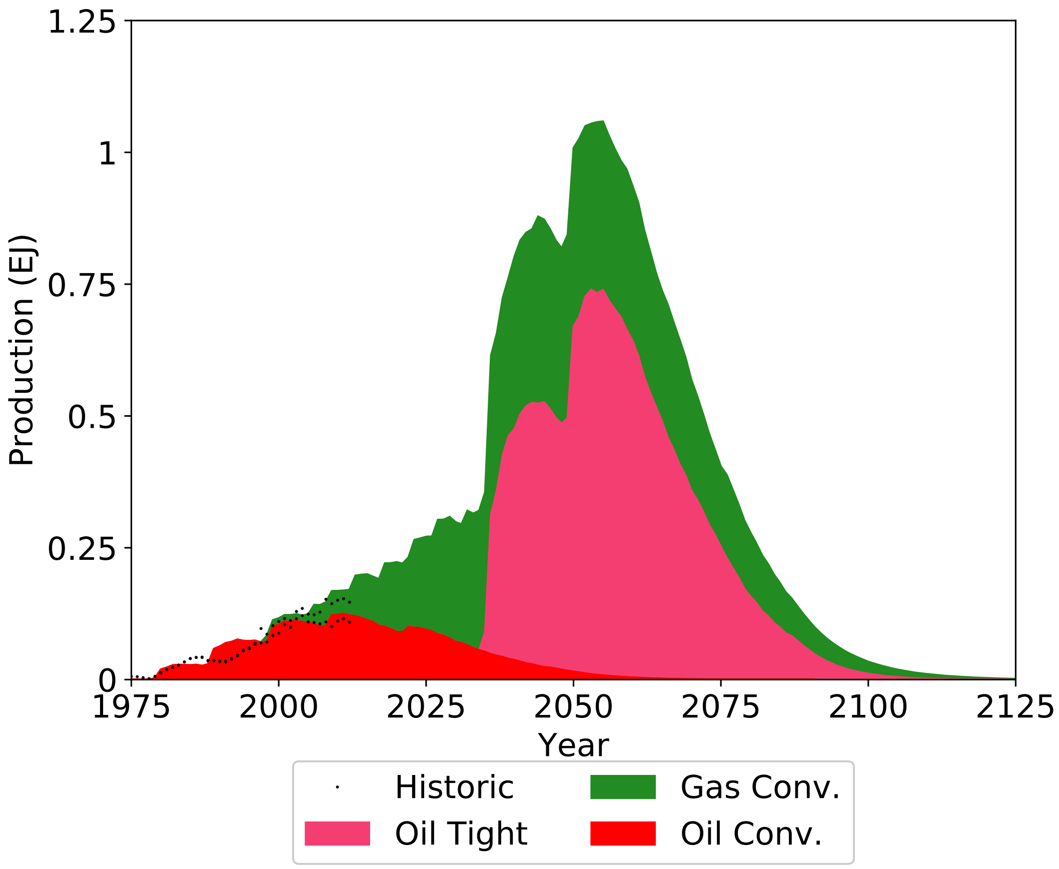

Figure 7.16: Cuba projection by mineral type

Table 7.16: Peak years - Minerals

**Name**	**URR**	**Peak Year**	**Peak Rate**
Oil Conv.	5.1	2011	0.12
Oil Tight	22.32	2055	0.73
Gas Conv.	17.83	2044	0.35
**Total**	**45.25**	**2055**	**1.06**

7.9 Dominican Republic

7.9.1 All Projections

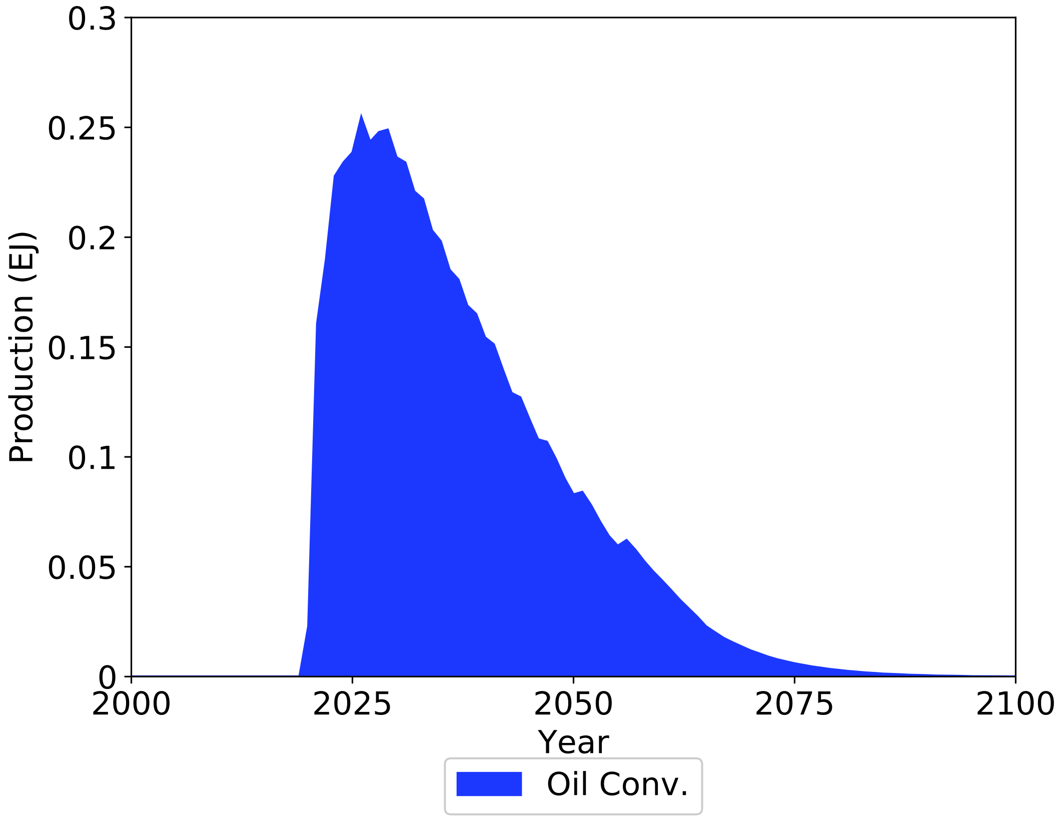

Figure 7.17: Dominican Republic projections capped at 16

Table 7.17: Peak years - All

Name	URR	Peak Year	Peak Rate
Oil Conv.	6.32	2026	0.25
**Total**	**6.32**	**2026**	**0.25**

7.9.2 By Mineral

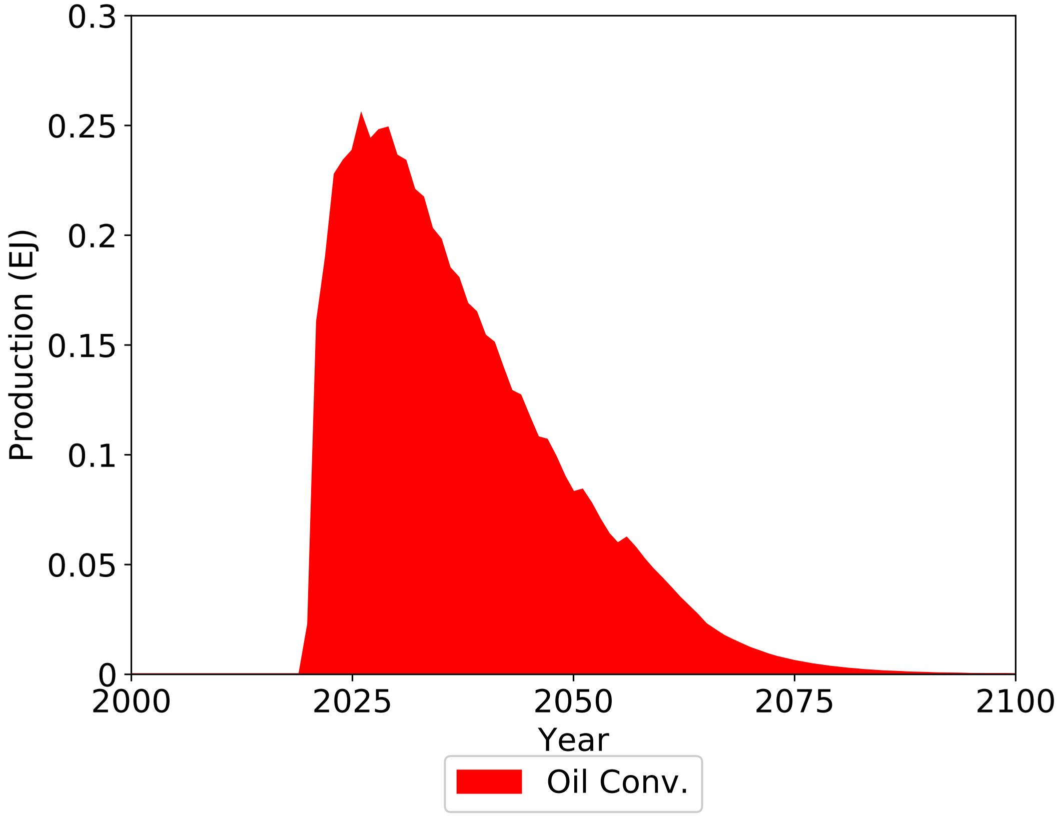

Figure 7.18: Dominican Republic projection by mineral type

Table 7.18: Peak years - Minerals

Name	URR	Peak Year	Peak Rate
Oil Conv.	6.32	2026	0.25
**Total**	**6.32**	**2026**	**0.25**

7.10 Ecuador

7.10.1 All Projections

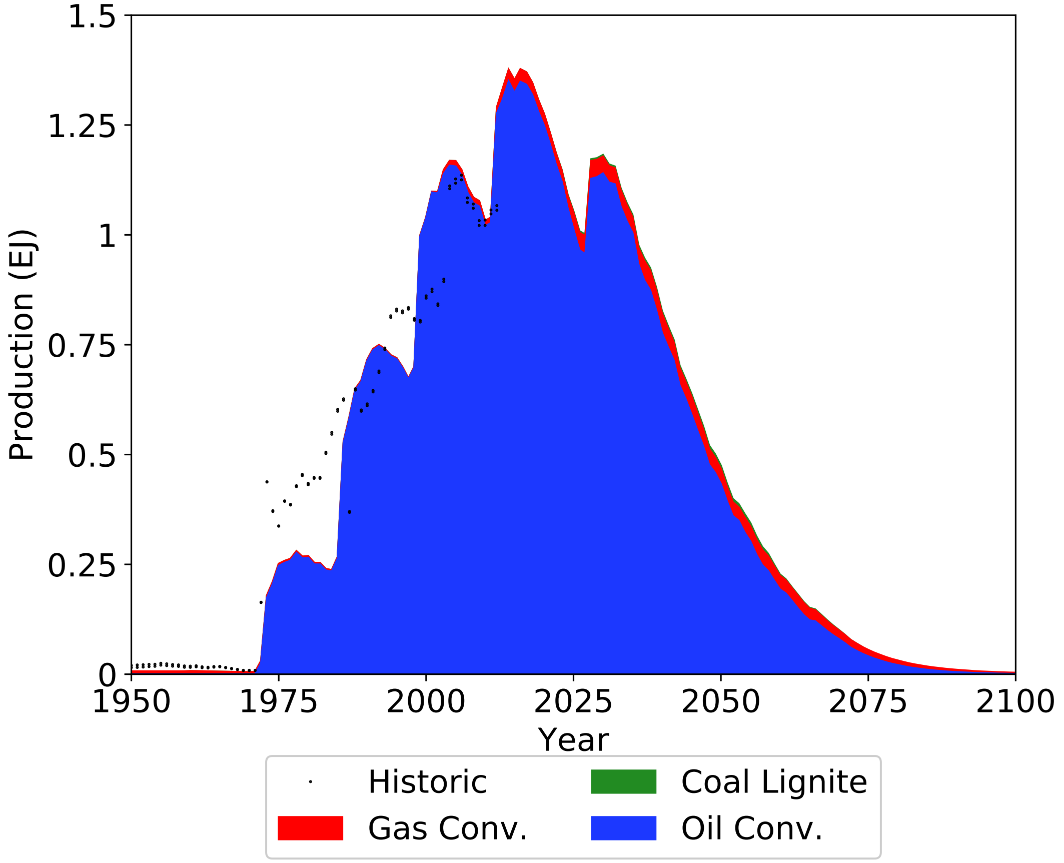

Figure 7.19: Ecuador projections capped at 16

Table 7.19: Peak years - All

Name	URR	Peak Year	Peak Rate
Oil Conv.	68.94	2014	1.35
Gas Conv.	2.6	2039	0.05
Coal Lignite	0.23	2049	0.01
**Total**	**71.77**	**2016**	**1.38**

7.10.2 By Mineral

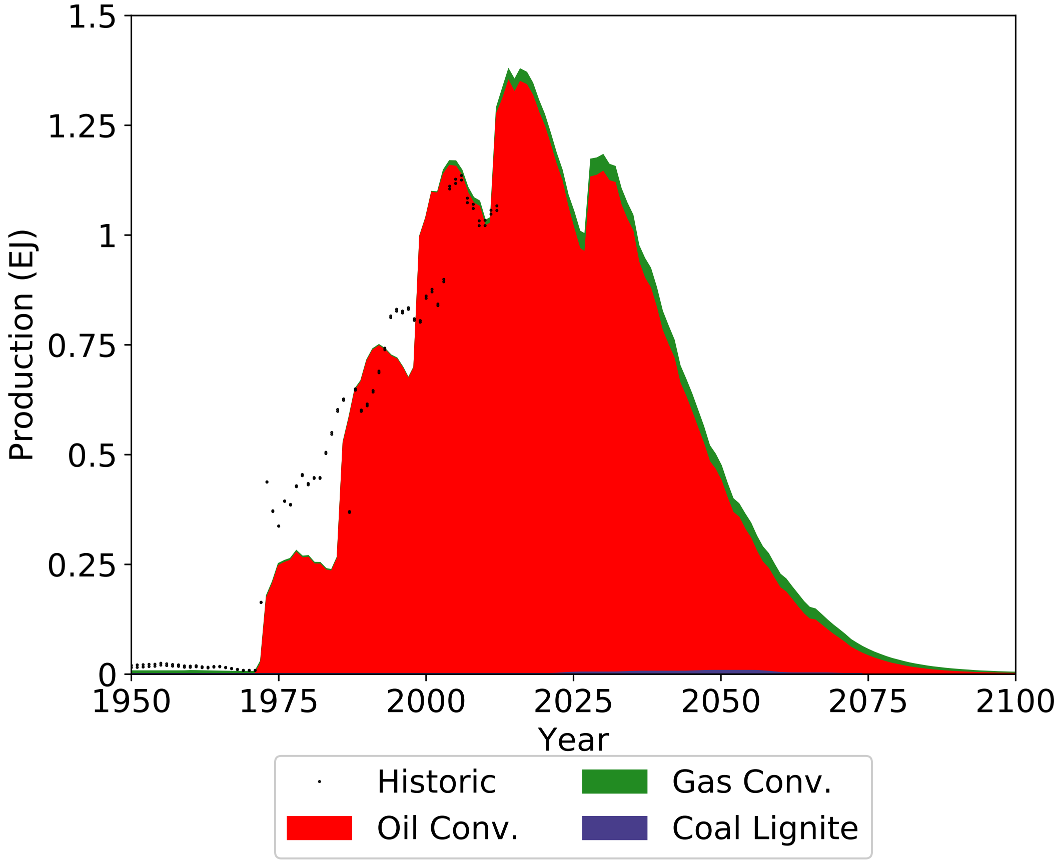

Figure 7.20: Ecuador projection by mineral type

Table 7.20: Peak years - Minerals

Name	URR	Peak Year	Peak Rate
Coal Lignite	0.23	2049	0.01
Oil Conv.	68.94	2014	1.35
Gas Conv.	2.6	2039	0.05
**Total**	**71.77**	**2016**	**1.38**

7.11 Falkland Islands

7.11.1 All Projections

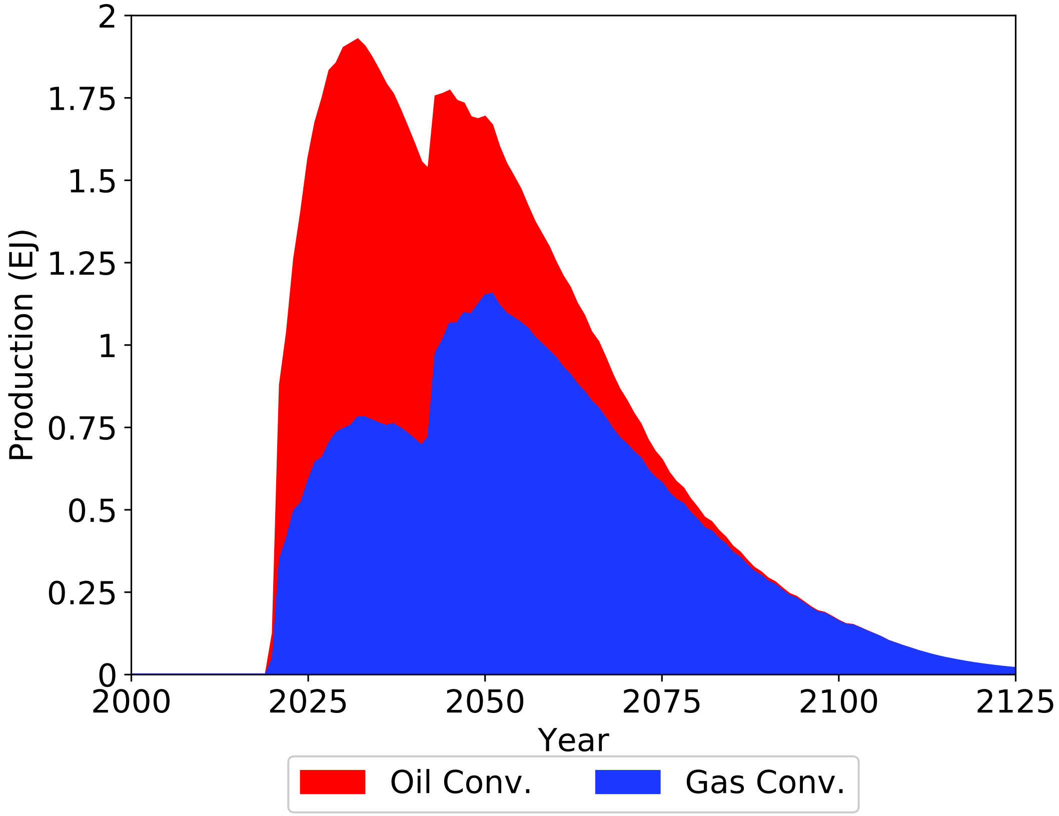

Figure 7.21: Falkland Islands projections capped at 16

Table 7.21: Peak years - All

Name	URR	Peak Year	Peak Rate
Gas Conv.	55.62	2051	1.16
Oil Conv.	33.69	2031	1.16
**Total**	**89.31**	**2032**	**1.93**

7.11.2 By Mineral

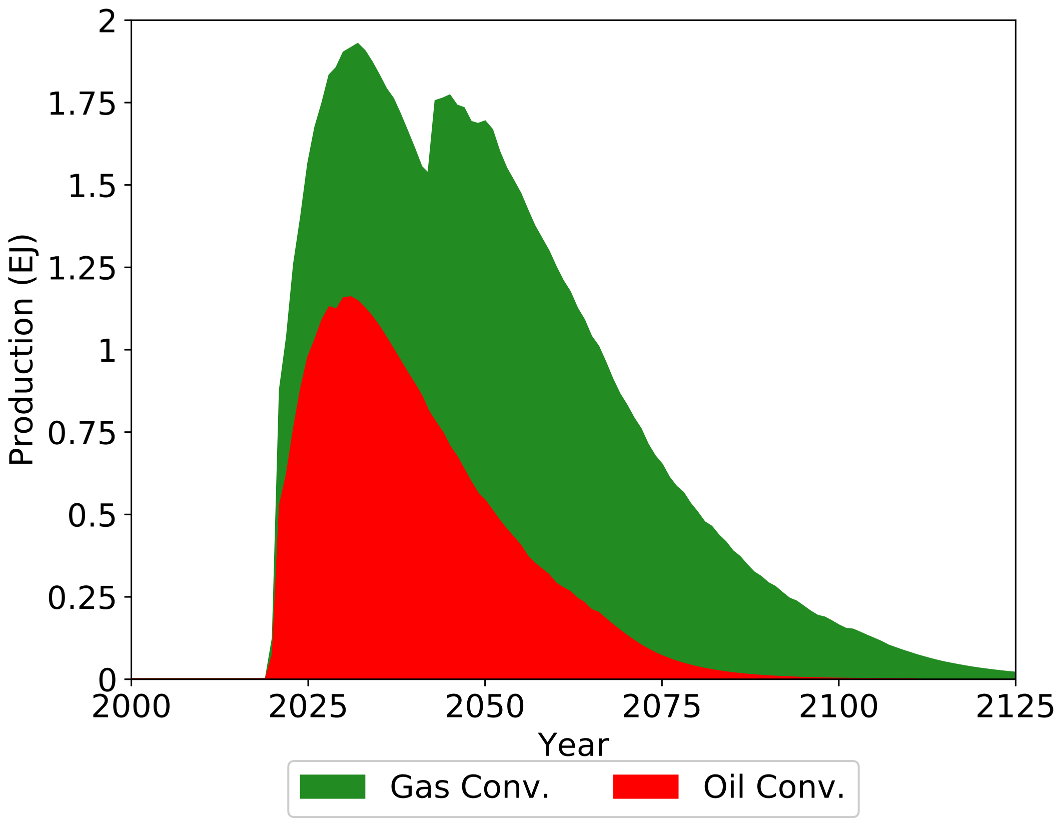

Figure 7.22: Falkland Islands projection by mineral type

Table 7.22: Peak years - Minerals

Name	URR	Peak Year	Peak Rate
Oil Conv.	33.69	2031	1.16
Gas Conv.	55.62	2051	1.16
**Total**	**89.31**	**2032**	**1.93**

7.12 French Guiana

7.12.1 All Projections

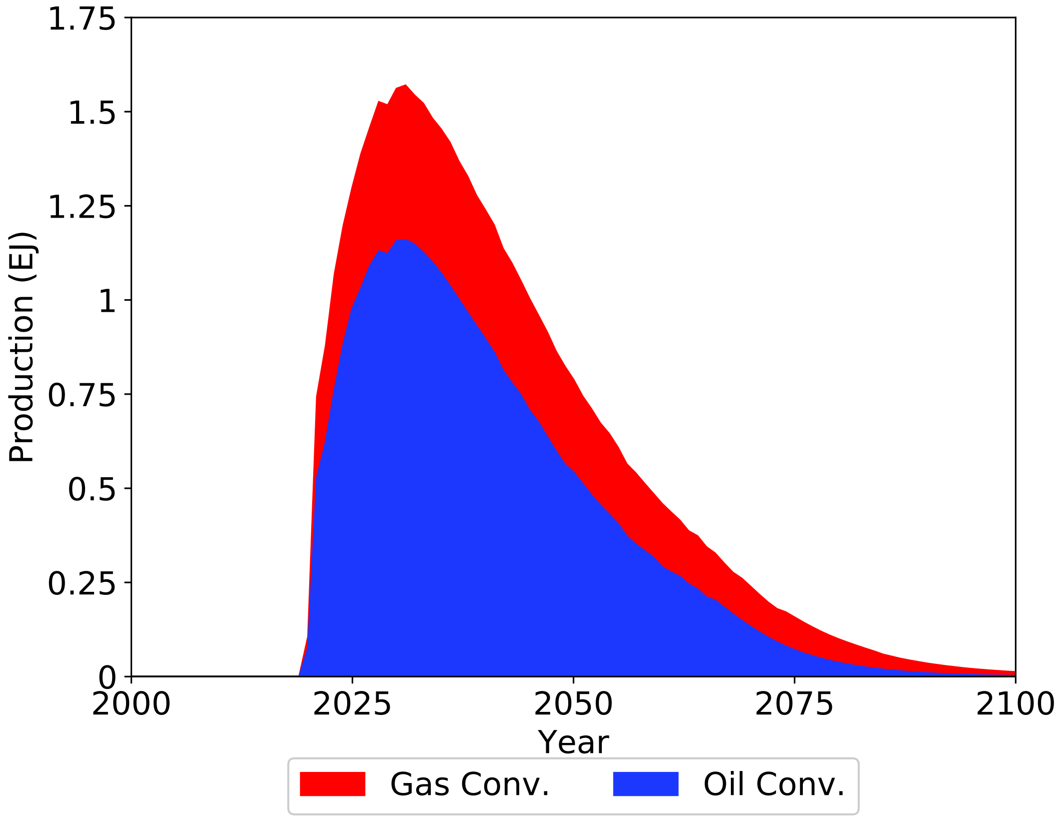

Figure 7.23: French Guiana projections capped at 16

Table 7.23: Peak years - All

Name	URR	Peak Year	Peak Rate
Oil Conv.	33.69	2031	1.16
Gas Conv.	14.8	2031	0.41
**Total**	**48.49**	**2031**	**1.57**

7.12.2 By Mineral

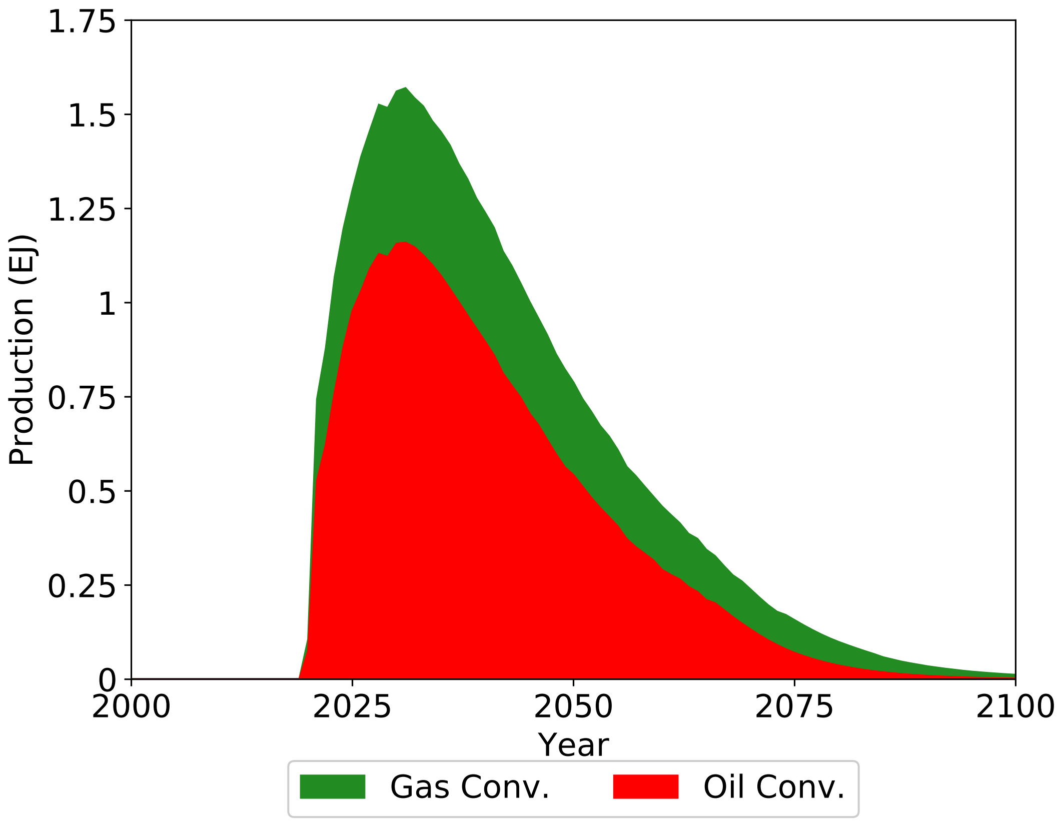

Figure 7.24: French Guiana projection by mineral type

Table 7.24: Peak years - Minerals

Name	URR	Peak Year	Peak Rate
Oil Conv.	33.69	2031	1.16
Gas Conv.	14.8	2031	0.41
**Total**	**48.49**	**2031**	**1.57**

7.13 Grenada

7.13.1 All Projections

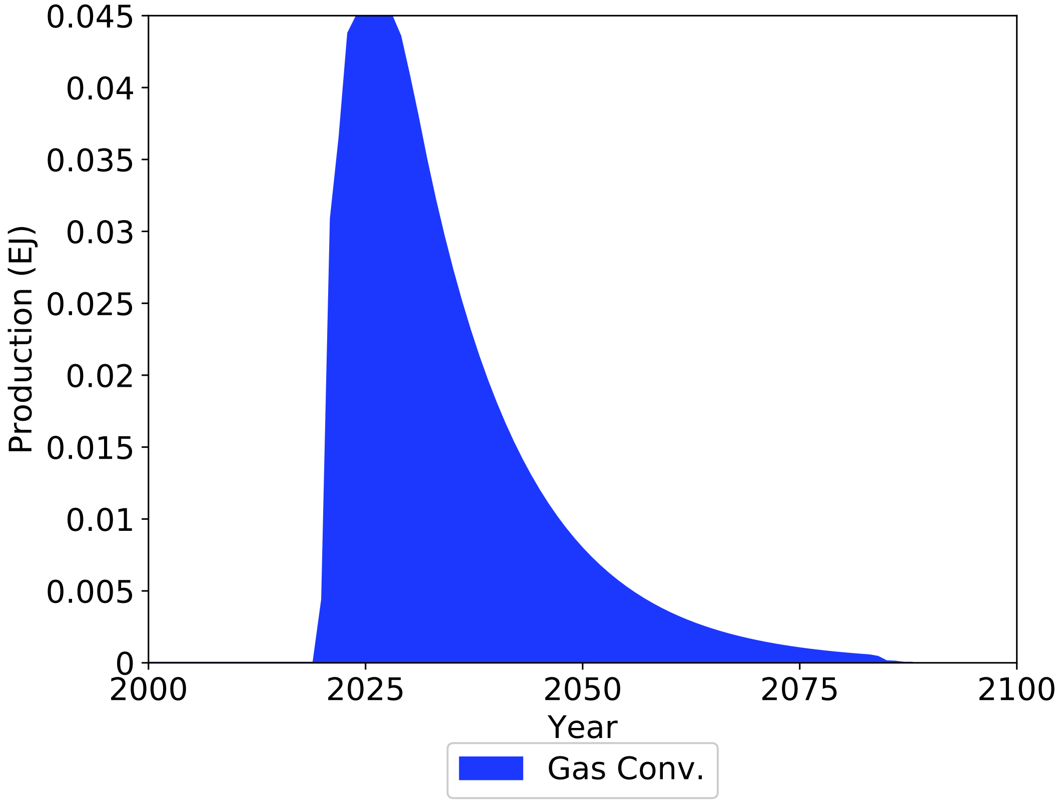

Figure 7.25: Grenada projections capped at 16

Table 7.25: Peak years - All

Name	URR	Peak Year	Peak Rate
Gas Conv.	0.9	2024	0.05
**Total**	**0.9**	**2024**	**0.05**

7.13.2 By Mineral

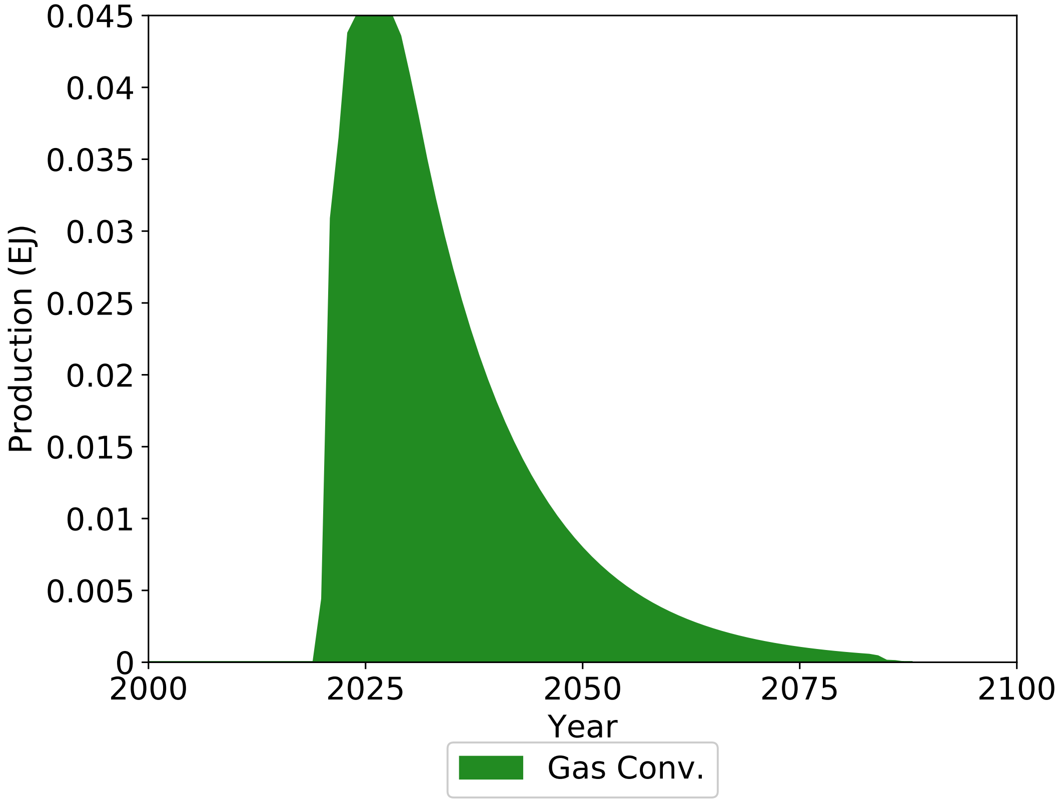

Figure 7.26: Grenada projection by mineral type

Table 7.26: Peak years - Minerals

**Name**	**URR**	**Peak Year**	**Peak Rate**
Gas Conv.	0.9	2024	0.05
**Total**	**0.9**	**2024**	**0.05**

7.14 Guatemala

7.14.1 All Projections

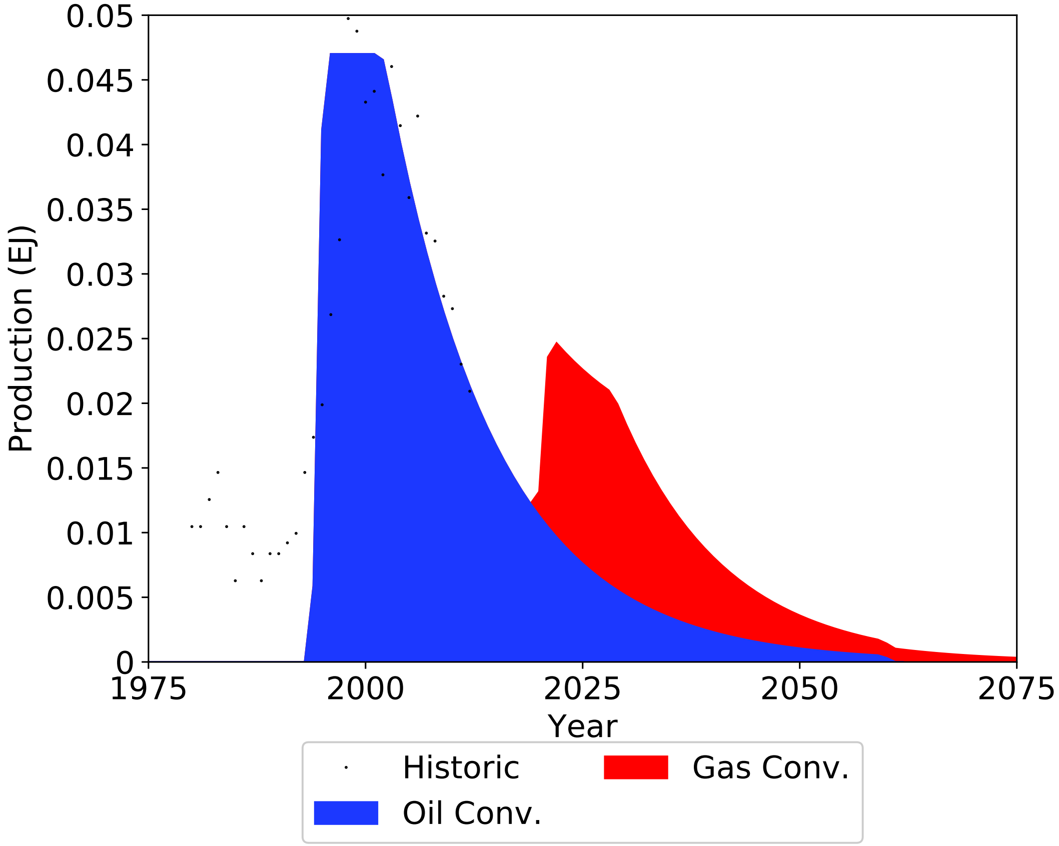

Figure 7.27: Guatemala projections capped at 16

Table 7.27: Peak years - All

Name	URR	Peak Year	Peak Rate
Oil Conv.	0.94	1996	0.05
Gas Conv.	0.3	2022	0.02
**Total**	**1.24**	**1996**	**0.05**

7.14.2 By Mineral

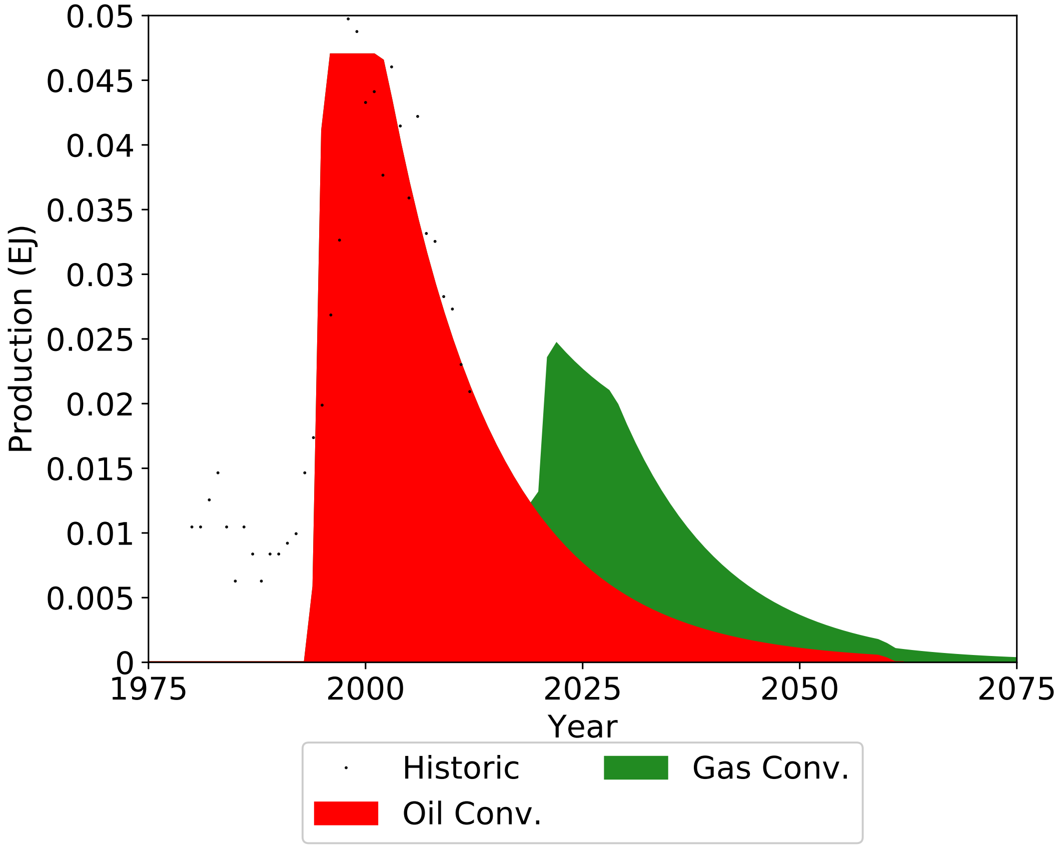

Figure 7.28: Guatemala projection by mineral type

Table 7.28: Peak years - Minerals

Name	URR	Peak Year	Peak Rate
Oil Conv.	0.94	1996	0.05
Gas Conv.	0.3	2022	0.02
**Total**	**1.24**	**1996**	**0.05**

7.15 Guyana

7.15.1 All Projections

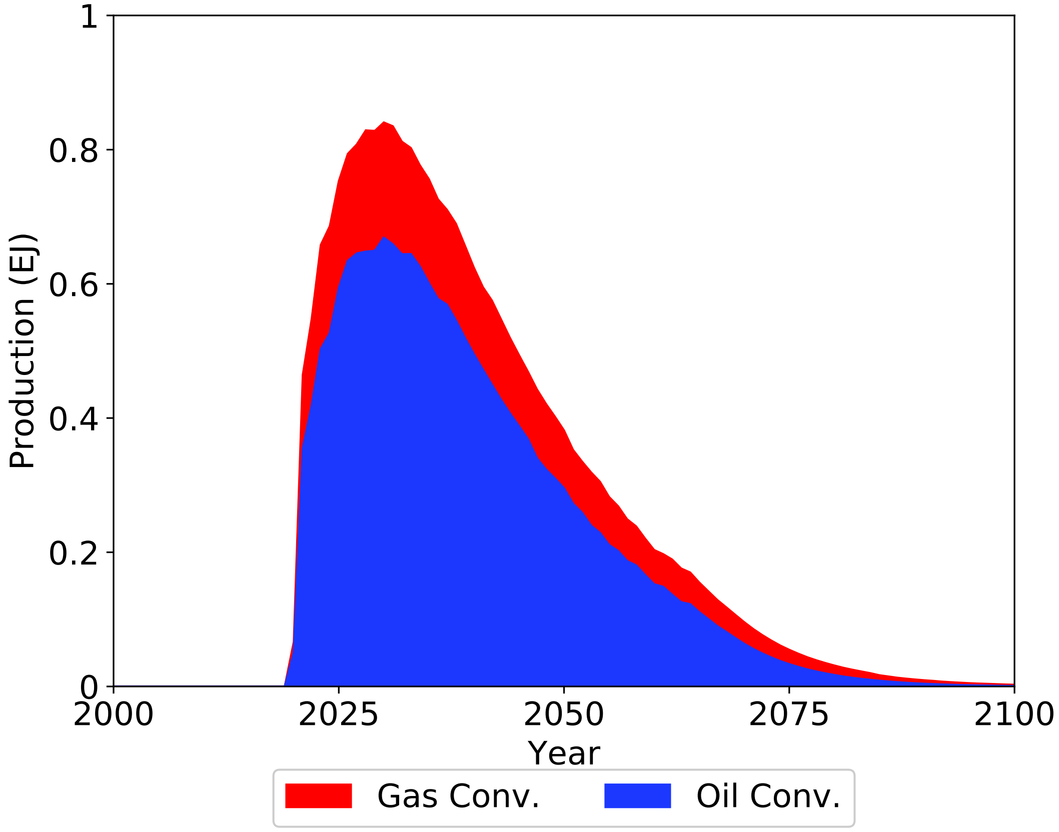

Figure 7.29: Guyana projections capped at 16

Table 7.29: Peak years - All

**Name**	**URR**	**Peak Year**	**Peak Rate**
Oil Conv.	18.95	2030	0.67
Gas Conv.	5.6	2028	0.18
**Total**	**24.55**	**2030**	**0.84**

7.15.2 By Mineral

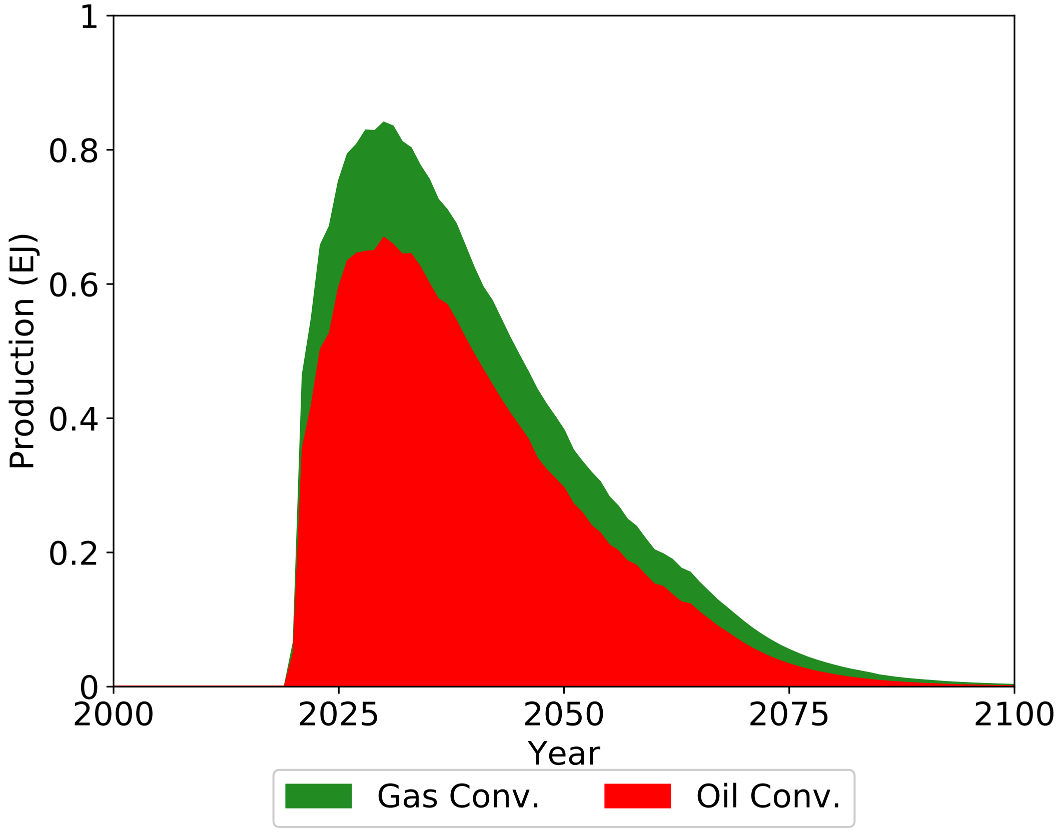

Figure 7.30: Guyana projection by mineral type

Table 7.30: Peak years - Minerals

**Name**	**URR**	**Peak Year**	**Peak Rate**
Oil Conv.	18.95	2030	0.67
Gas Conv.	5.6	2028	0.18
**Total**	**24.55**	**2030**	**0.84**

7.16 Haiti

7.16.1 All Projections

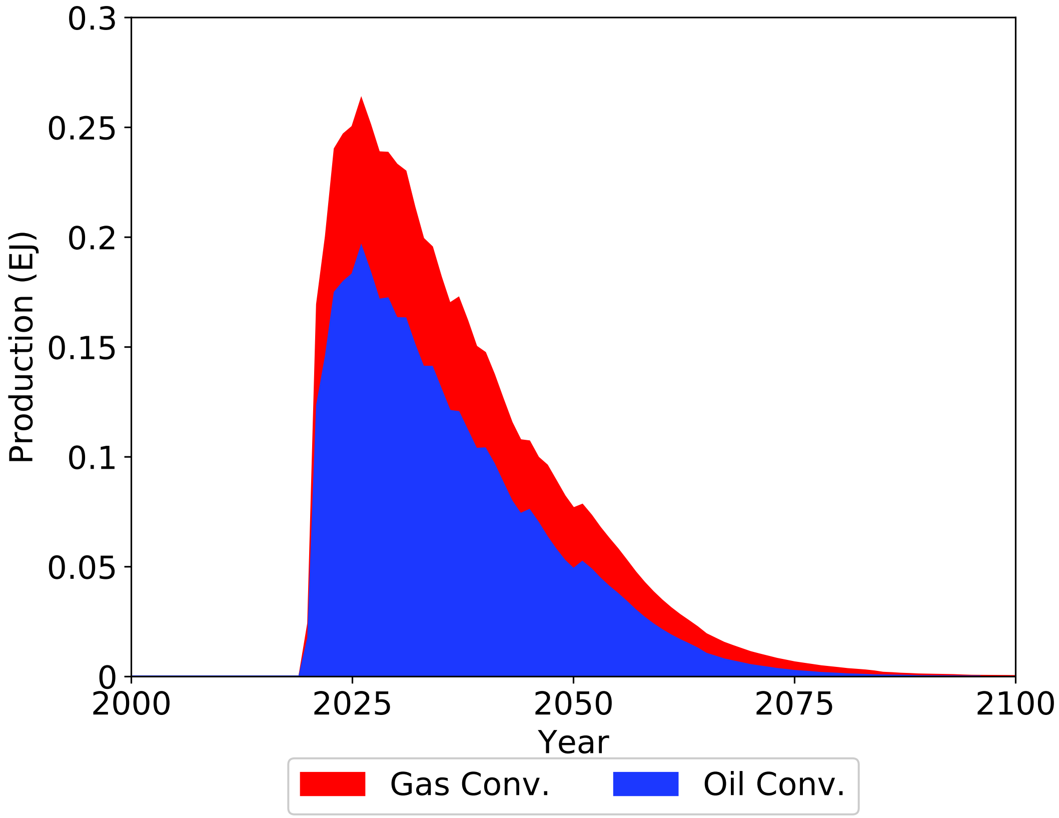

Figure 7.31: Haiti projections capped at 16

Table 7.31: Peak years - All

**Name**	**URR**	**Peak Year**	**Peak Rate**
Oil Conv.	4.21	2026	0.2
Gas Conv.	1.85	2030	0.07
**Total**	**6.06**	**2026**	**0.26**

7.16.2 By Mineral

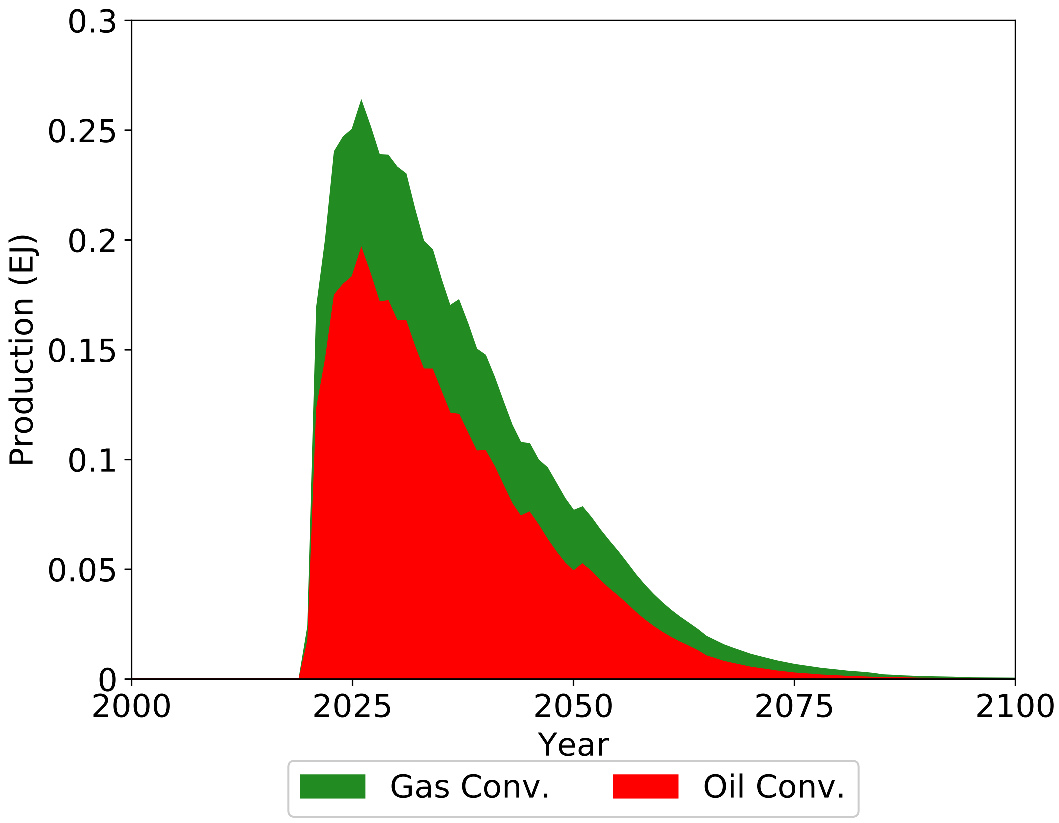

Figure 7.32: Haiti projection by mineral type

Table 7.32: Peak years - Minerals

**Name**	**URR**	**Peak Year**	**Peak Rate**
Oil Conv.	4.21	2026	0.2
Gas Conv.	1.85	2030	0.07
**Total**	**6.06**	**2026**	**0.26**

7.17 Mexico

7.17.1 All Projections

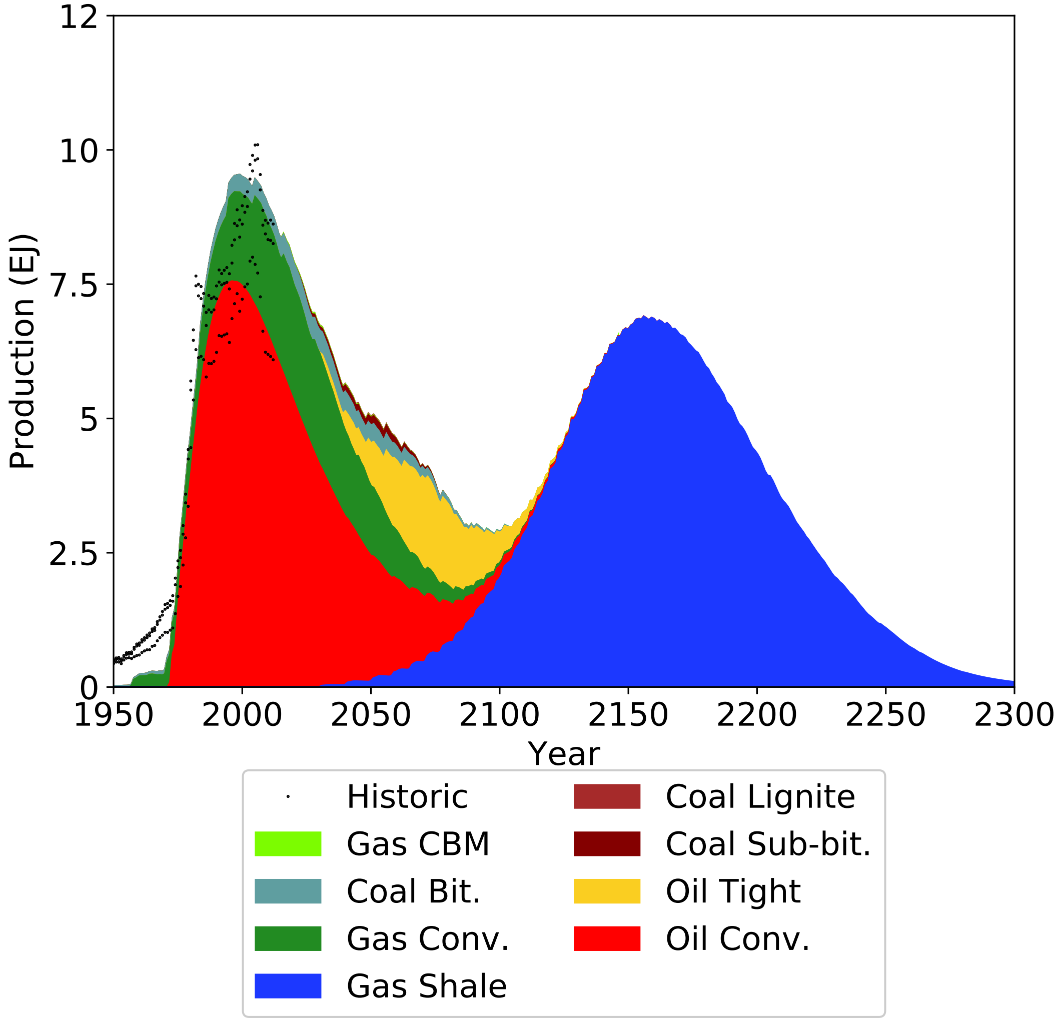

Figure 7.33: Mexico projections capped at 16

Table 7.33: Peak years - All

**Name**	**URR**	**Peak Year**	**Peak Rate**
Gas Shale	715.2	2156	6.9
Oil Conv.	445.73	1996	7.55
Gas Conv.	153.2	2018	2.24
Oil Tight	75.06	2072	1.7
Coal Bit.	32.7	2025	0.42
Coal Sub-bit.	4.95	2053	0.16
Gas CBM	1.11	2019	0.03
Coal Lignite	0.48	2055	0.01
**Total**	**1428.43**	**1999**	**9.54**

7.17.2 By Mineral

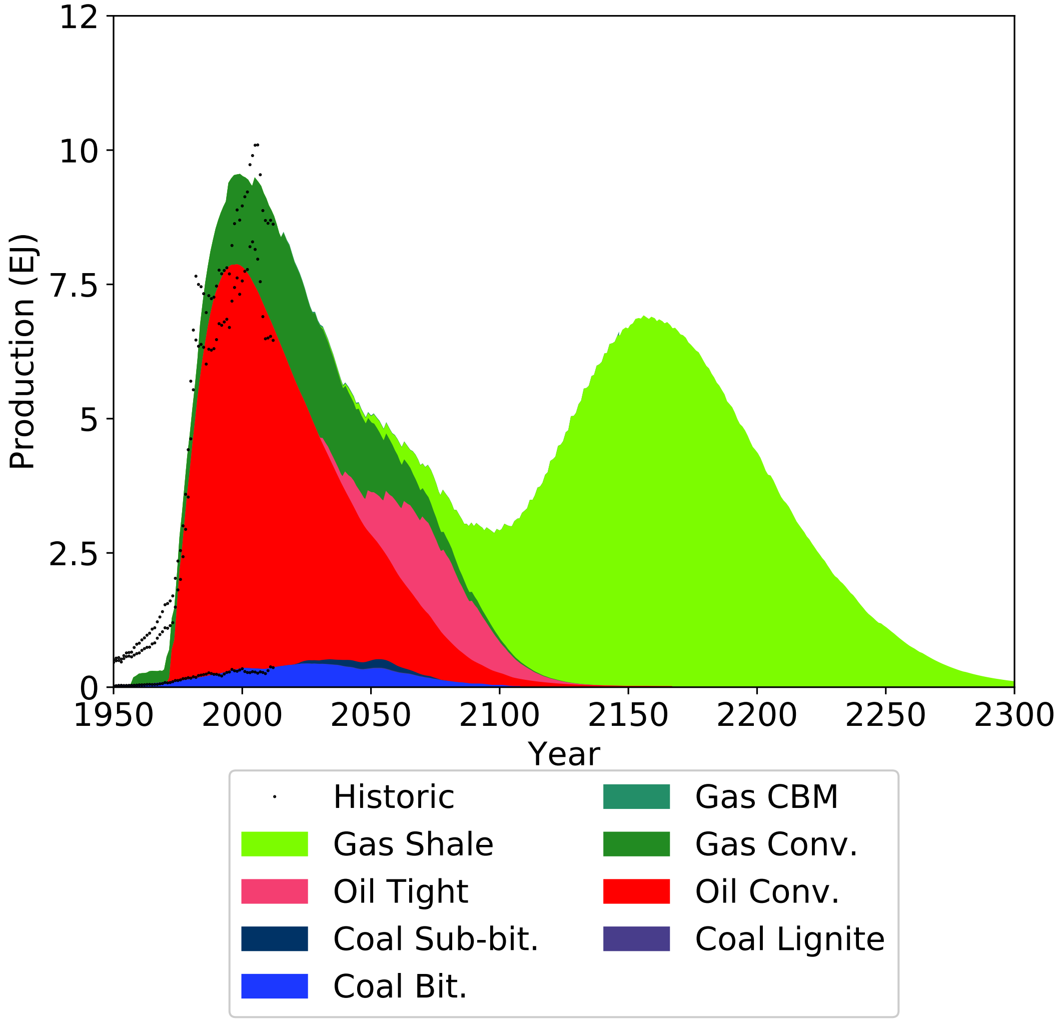

Figure 7.34: Mexico projection by mineral type

Table 7.34: Peak years - Minerals

**Name**	**URR**	**Peak Year**	**Peak Rate**
Coal Bit.	32.7	2025	0.42
Coal Lignite	0.48	2055	0.01
Coal Sub-bit.	4.95	2053	0.16
Oil Conv.	445.73	1996	7.55
Oil Tight	75.06	2072	1.7
Gas Conv.	153.2	2018	2.24
Gas Shale	715.2	2156	6.9
Gas CBM	1.11	2019	0.03
**Total**	**1428.43**	**1999**	**9.54**

7.18 Paraguay

7.18.1 All Projections

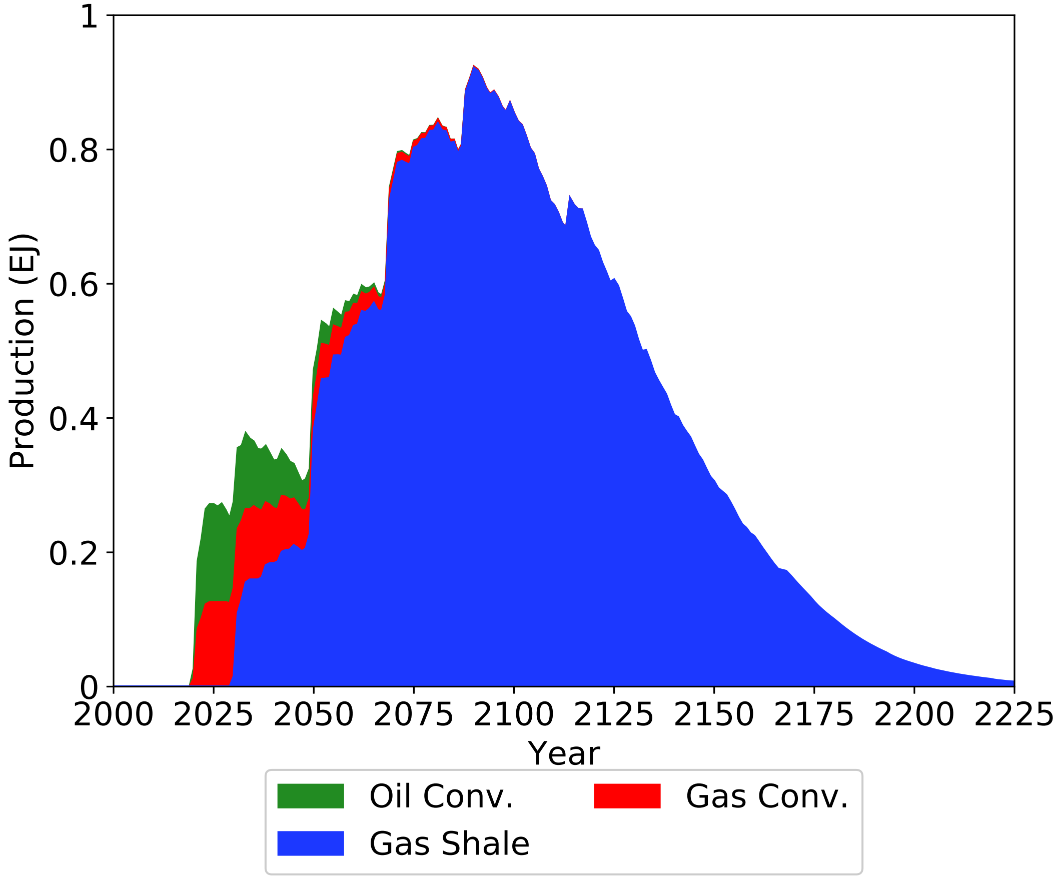

Figure 7.35: Paraguay projections capped at 16

Table 7.35: Peak years - All

Name	URR	Peak Year	Peak Rate
Gas Shale	76.2	2090	0.92
Gas Conv.	3.7	2030	0.13
Oil Conv.	3.16	2027	0.15
**Total**	**83.06**	**2090**	**0.92**

7.18.2 By Mineral

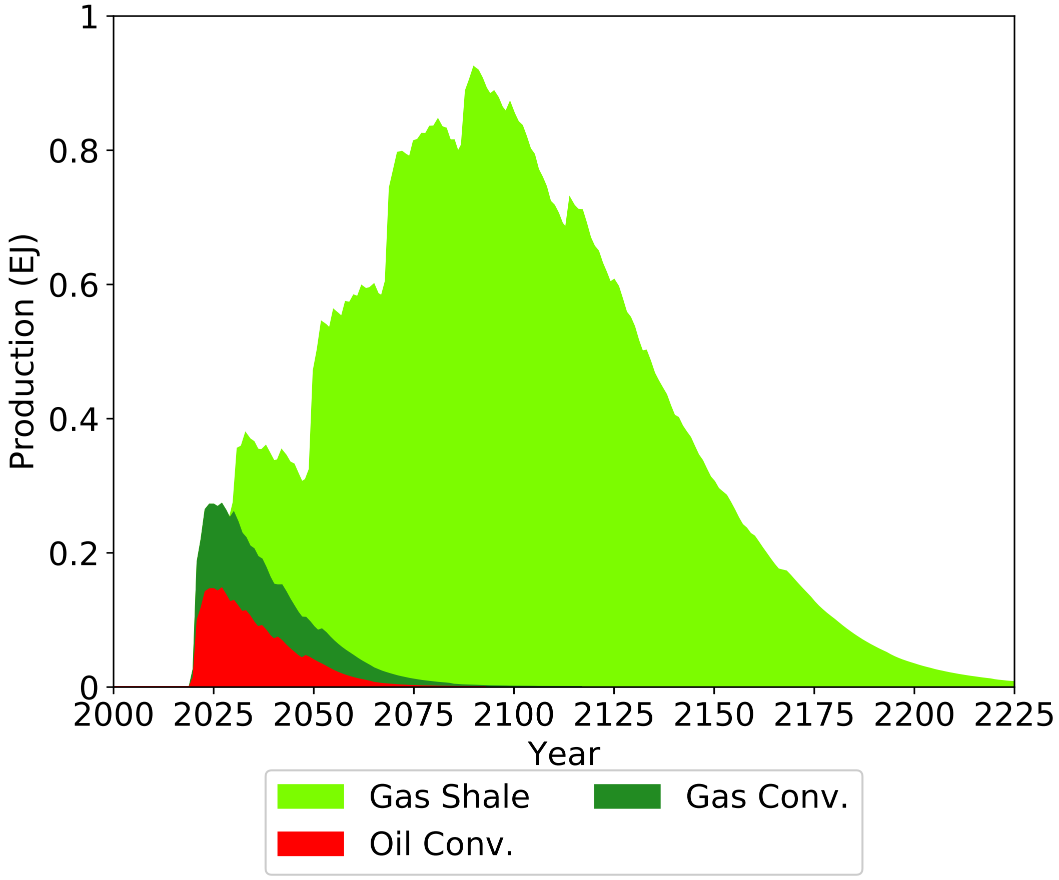

Figure 7.36: Paraguay projection by mineral type

Table 7.36: Peak years - Minerals

Name	URR	Peak Year	Peak Rate
Oil Conv.	3.16	2027	0.15
Gas Conv.	3.7	2030	0.13
Gas Shale	76.2	2090	0.92
**Total**	**83.06**	**2090**	**0.92**

7.19 Peru

7.19.1 All Projections

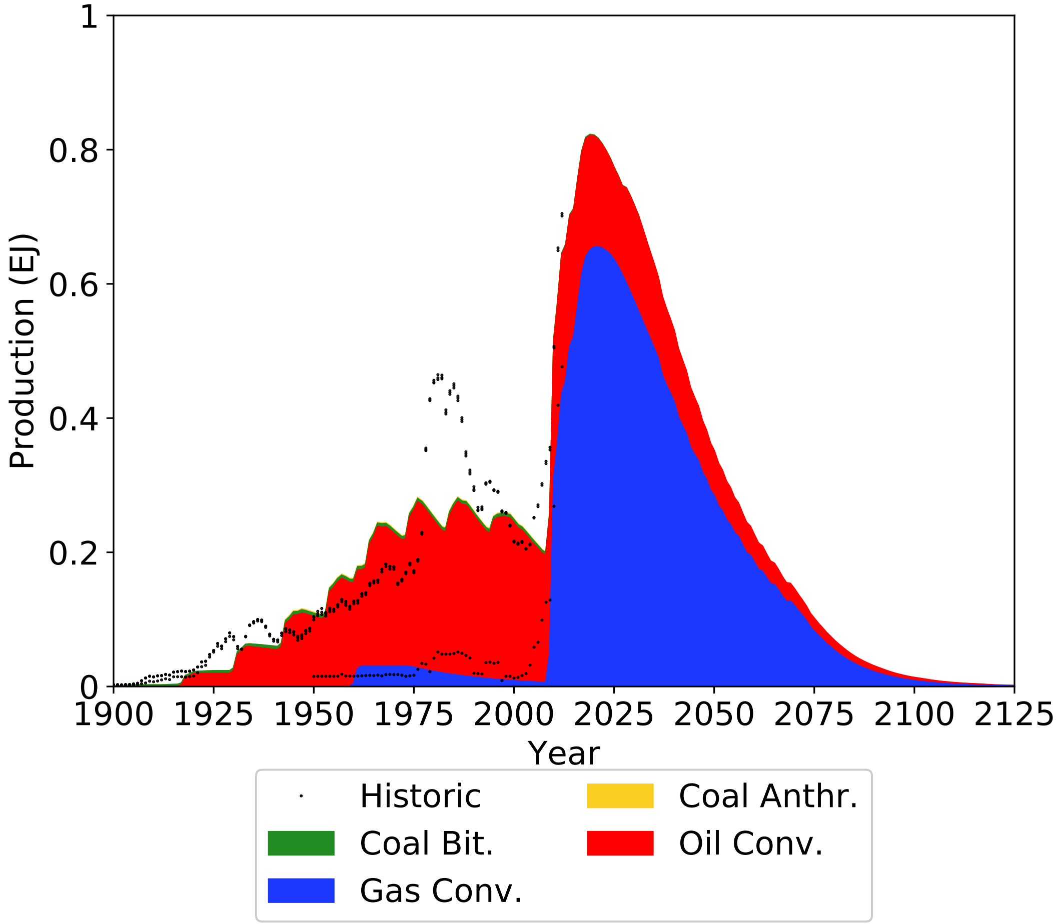

Figure 7.37: Peru projections capped at 16

Table 7.37: Peak years - All

**Name**	**URR**	**Peak Year**	**Peak Rate**
Gas Conv.	26.3	2021	0.65
Oil Conv.	20.64	1986	0.26
Coal Bit.	0.48	1938	–
Coal Anthr.	0.1	1944	–
**Total**	**47.52**	**2019**	**0.82**

7.19.2 By Mineral

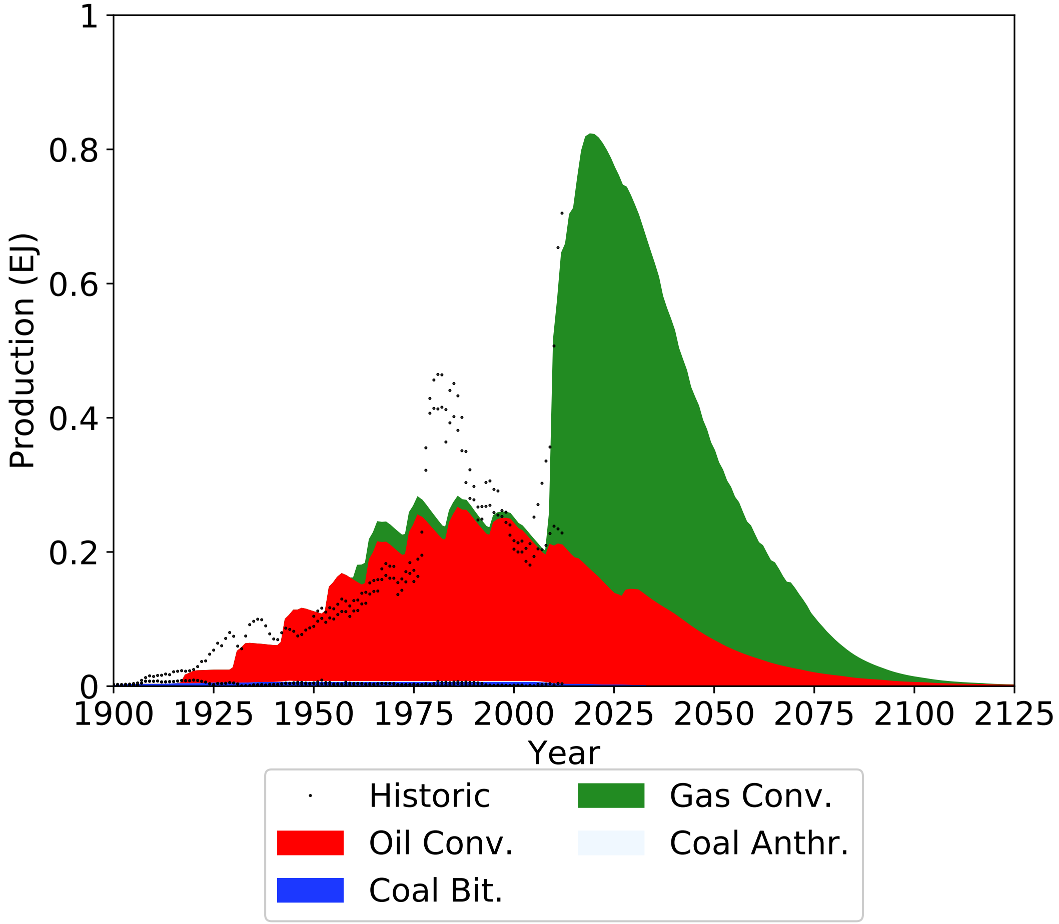

Figure 7.38: Peru projection by mineral type

Table 7.38: Peak years - Minerals

**Name**	**URR**	**Peak Year**	**Peak Rate**
Coal Bit.	0.48	1938	–
Coal Anthr.	0.1	1944	–
Oil Conv.	20.64	1986	0.26
Gas Conv.	26.3	2021	0.65
**Total**	**47.52**	**2019**	**0.82**

7.20 Puerto Rico

7.20.1 All Projections

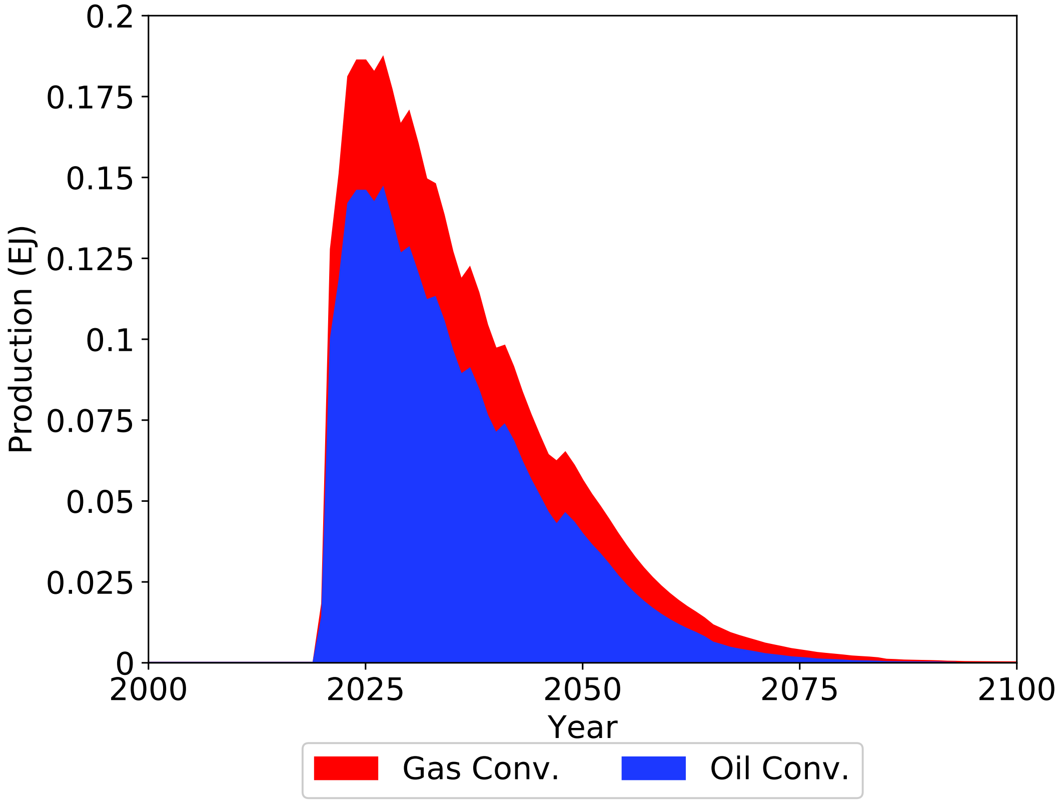

Figure 7.39: Puerto Rico projections capped at 16

Table 7.39: Peak years - All

Name	URR	Peak Year	Peak Rate
Oil Conv.	3.16	2027	0.15
Gas Conv.	1.11	2030	0.04
**Total**	**4.27**	**2027**	**0.19**

7.20.2 By Mineral

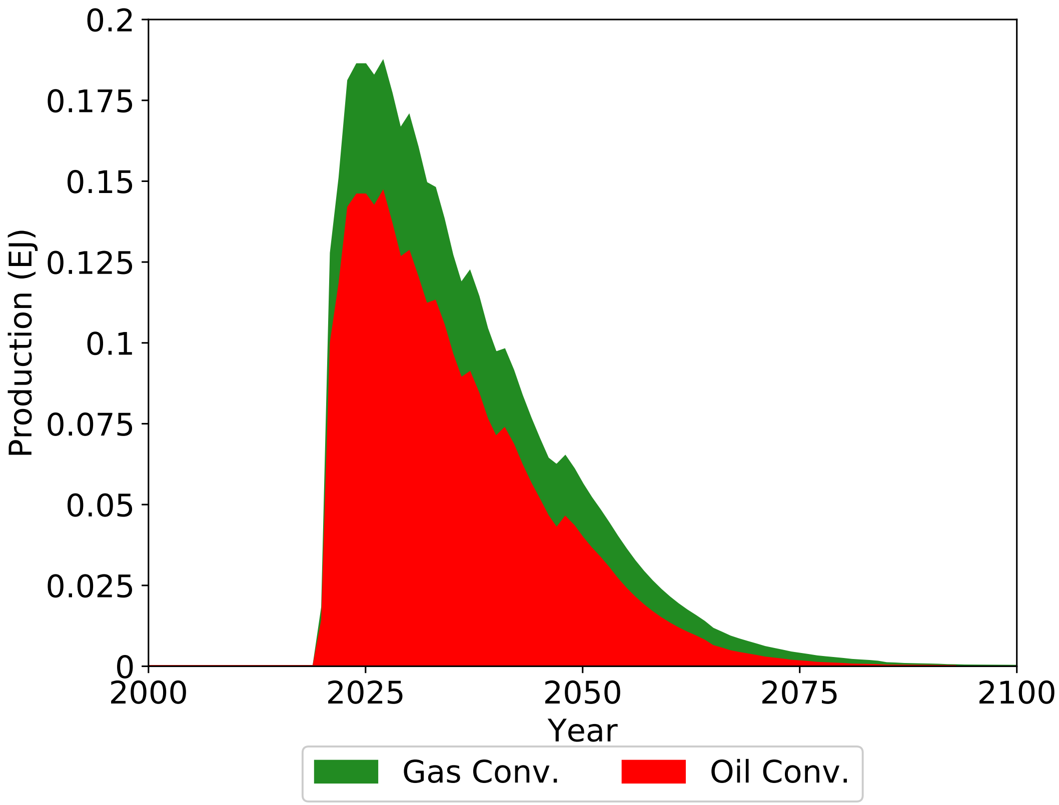

Figure 7.40: Puerto Rico projection by mineral type

Table 7.40: Peak years - Minerals

Name	URR	Peak Year	Peak Rate
Oil Conv.	3.16	2027	0.15
Gas Conv.	1.11	2030	0.04
**Total**	**4.27**	**2027**	**0.19**

7.21 Suriname

7.21.1 All Projections

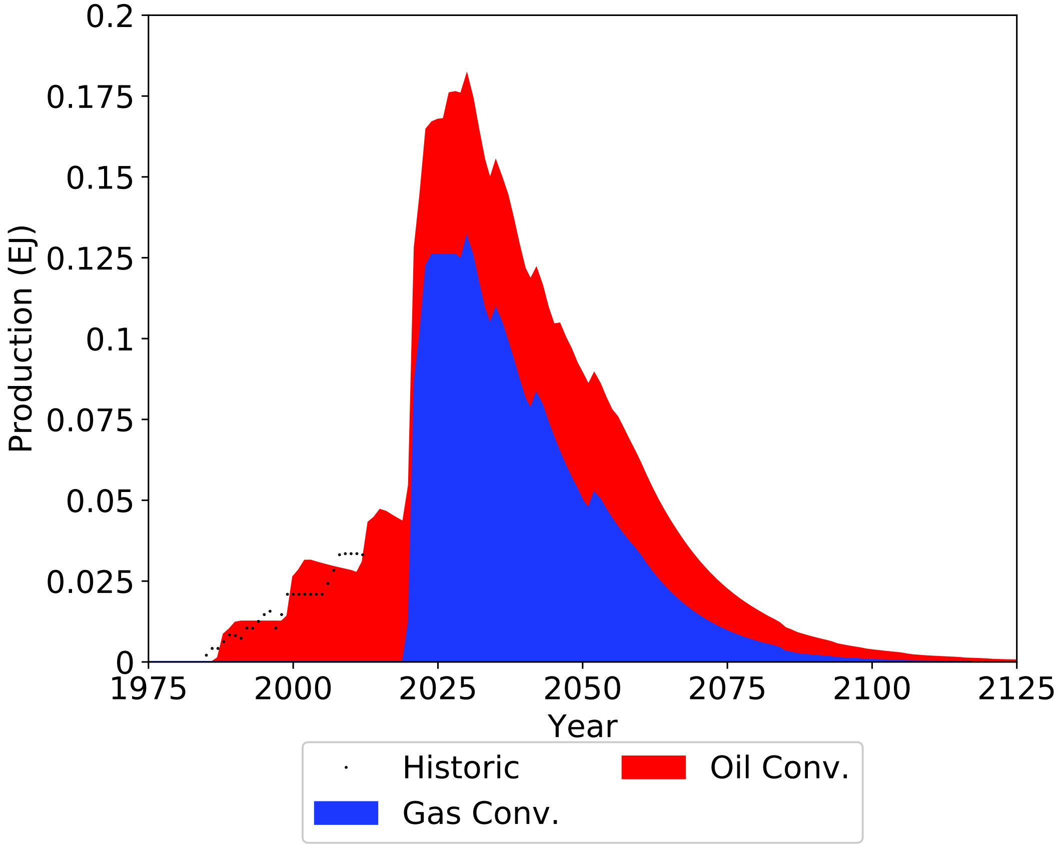

Figure 7.41: Suriname projections capped at 16

Table 7.41: Peak years - All

**Name**	**URR**	**Peak Year**	**Peak Rate**
Gas Conv.	3.7	2030	0.13
Oil Conv.	3.0	2029	0.05
**Total**	**6.7**	**2030**	**0.18**

7.21.2 By Mineral

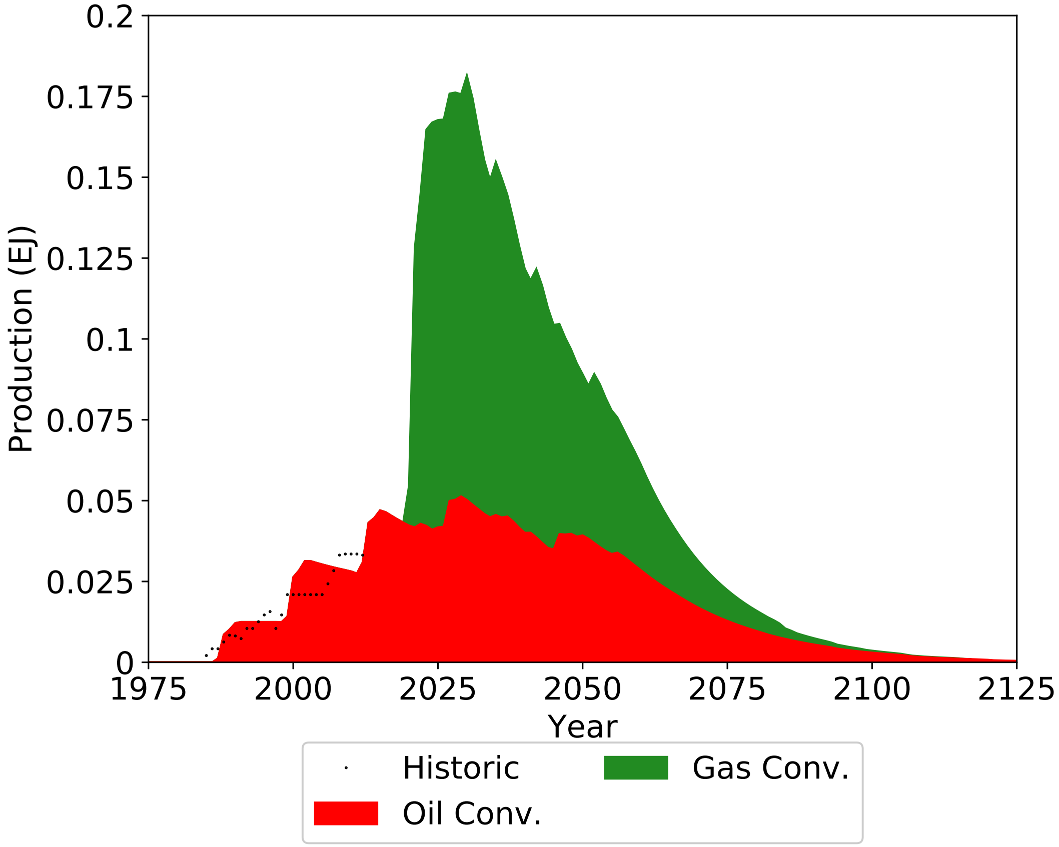

Figure 7.42: Suriname projection by mineral type

Table 7.42: Peak years - Minerals

Name	URR	Peak Year	Peak Rate
Oil Conv.	3.0	2029	0.05
Gas Conv.	3.7	2030	0.13
**Total**	**6.7**	**2030**	**0.18**

7.22 Trinidad and Tobago

7.22.1 All Projections

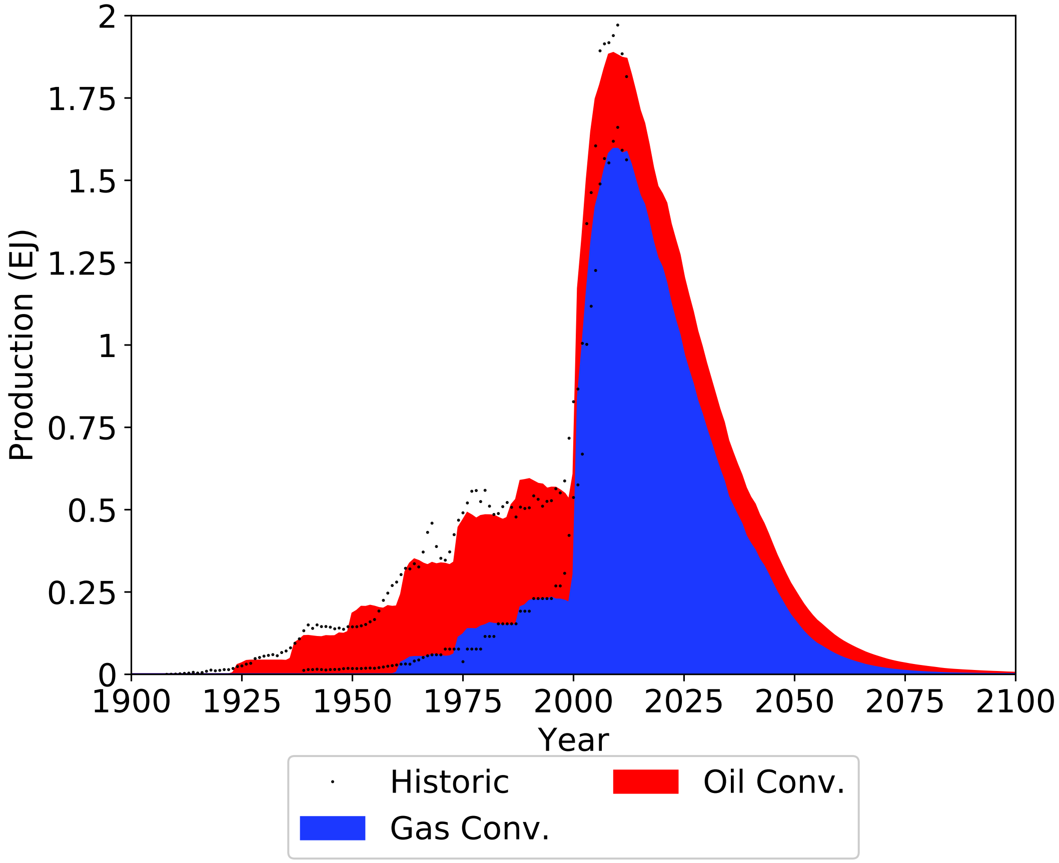

Figure 7.43: Trinidad and Tobago projections capped at 16

Table 7.43: Peak years - All

Name	URR	Peak Year	Peak Rate
Gas Conv.	52.5	2010	1.6
Oil Conv.	29.36	1988	0.38
**Total**	**81.86**	**2009**	**1.89**

7.22.2 By Mineral

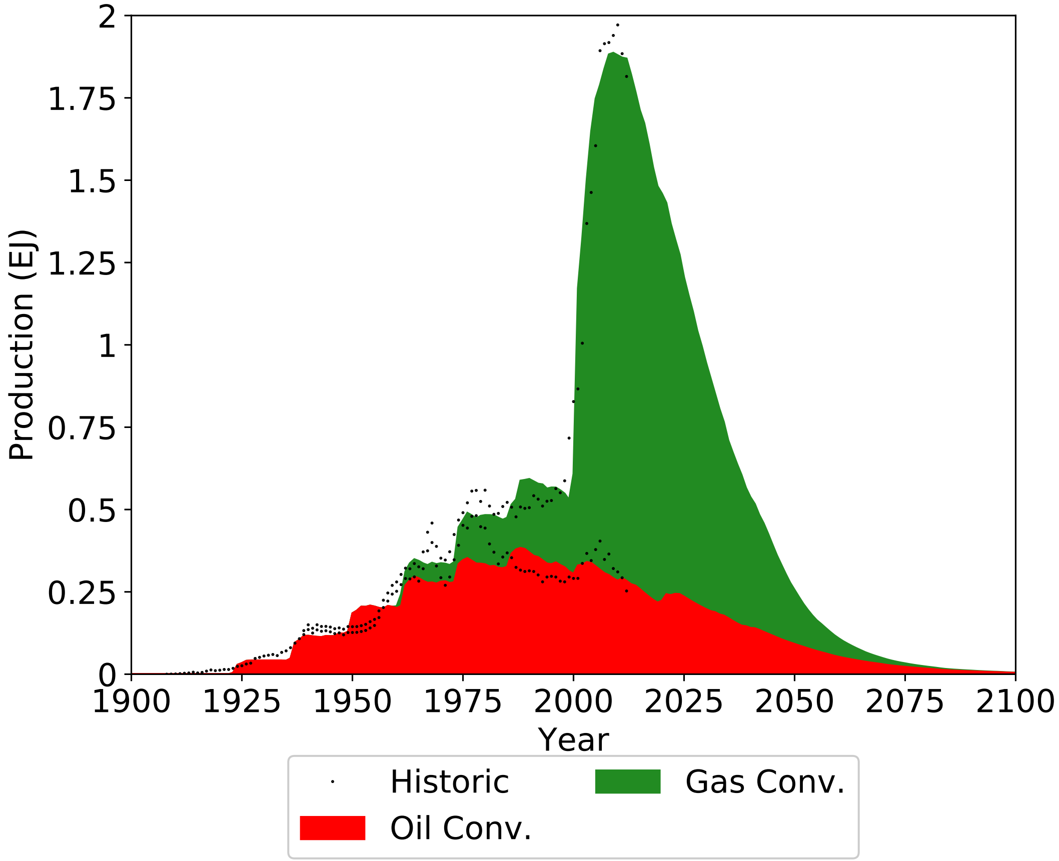

Figure 7.44: Trinidad and Tobago projection by mineral type

Table 7.44: Peak years - Minerals

Name	URR	Peak Year	Peak Rate
Oil Conv.	29.36	1988	0.38
Gas Conv.	52.5	2010	1.6
**Total**	**81.86**	**2009**	**1.89**

7.23 Uruguay

7.23.1 All Projections

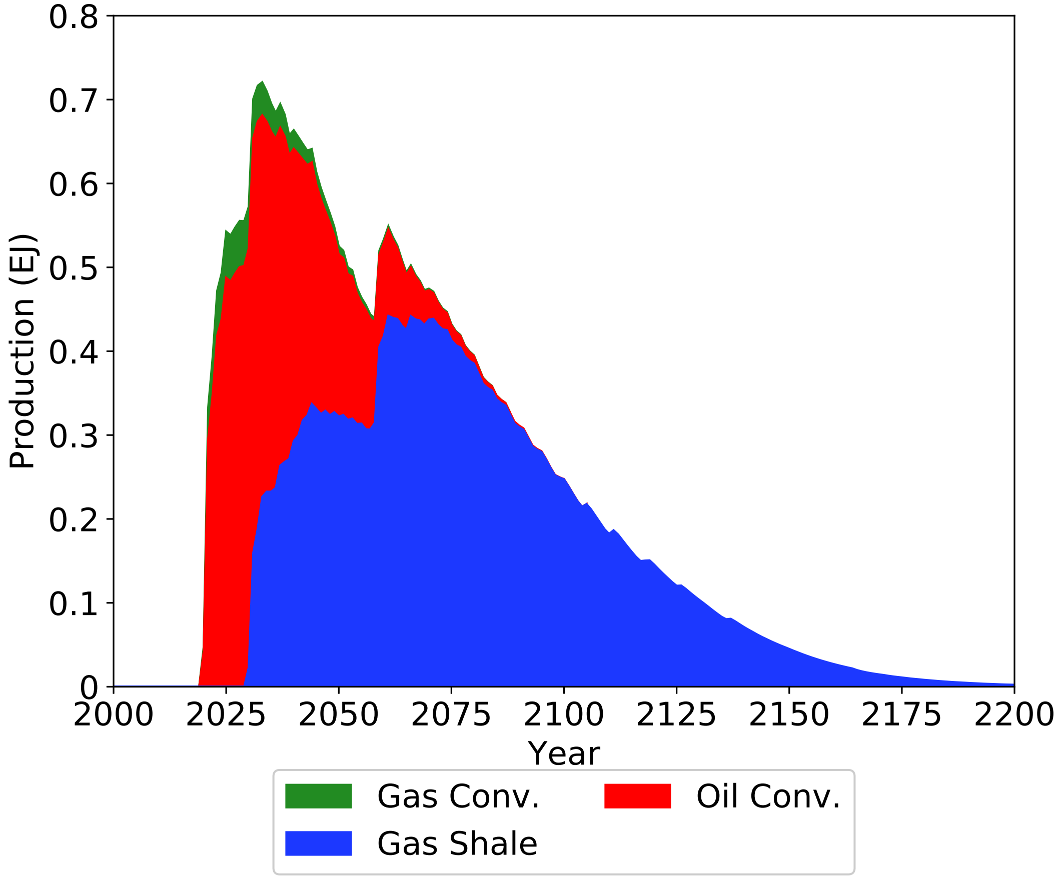

Figure 7.45: Uruguay projections capped at 16

Table 7.45: Peak years - All

Name	URR	Peak Year	Peak Rate
Gas Shale	30.7	2061	0.44
Oil Conv.	13.69	2029	0.5
Gas Conv.	1.1	2024	0.06
**Total**	**45.49**	**2033**	**0.72**

7.23.2 By Mineral

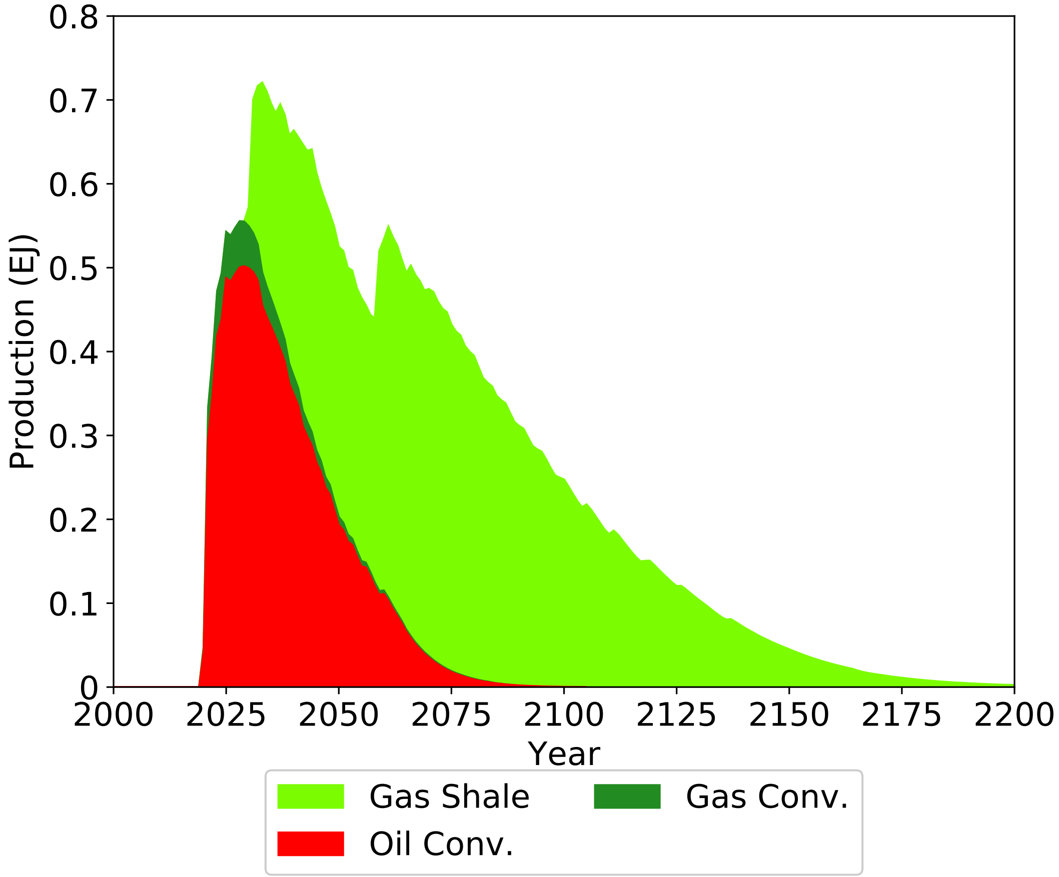

Figure 7.46: Uruguay projection by mineral type

Table 7.46: Peak years - Minerals

Name	URR	Peak Year	Peak Rate
Oil Conv.	13.69	2029	0.5
Gas Conv.	1.1	2024	0.06
Gas Shale	30.7	2061	0.44
**Total**	**45.49**	**2033**	**0.72**

7.24 Venezuela

7.24.1 All Projections

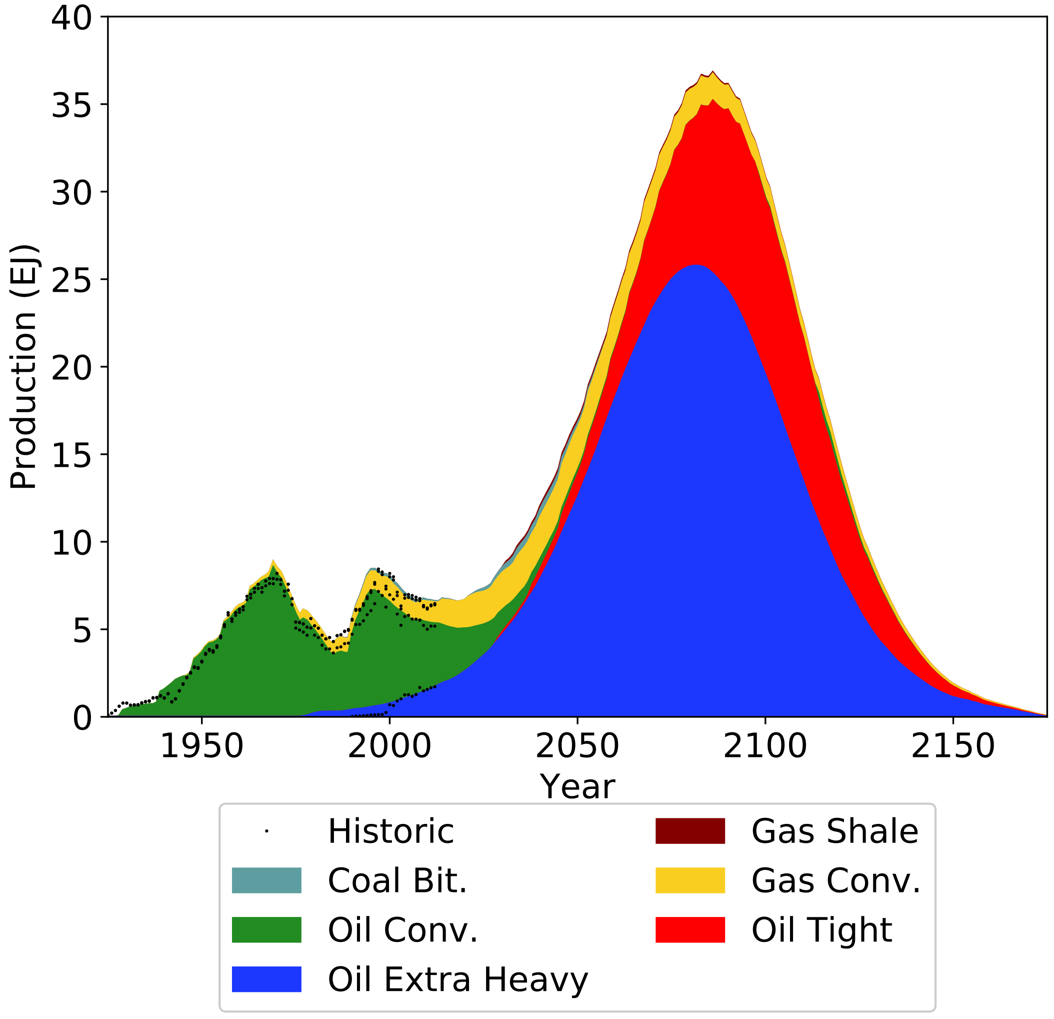

Figure 7.47: Venezuela projections capped at 16

Table 7.47: Peak years - All

**Name**	**URR**	**Peak Year**	**Peak Rate**
Oil Extra Heavy	1719.0	2081	25.77
Oil Tight	573.0	2093	10.68
Oil Conv.	435.0	1969	8.53
Gas Conv.	241.5	2048	2.49
Coal Bit.	14.5	2041	0.48
Gas Shale	11.57	2045	0.21
**Total**	**2994.57**	**2086**	**36.83**

7.24.2 By Mineral

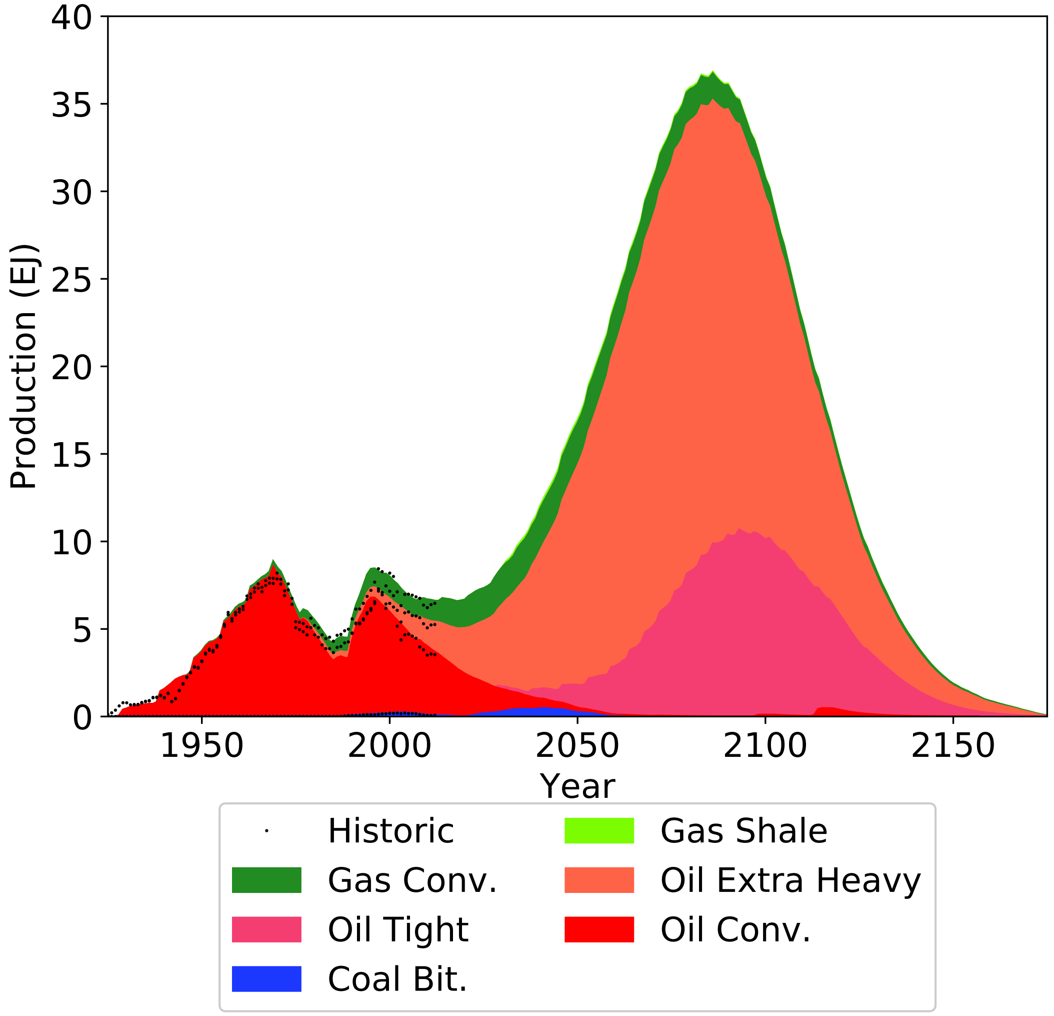

Figure 7.48: Venezuela projection by mineral type

Table 7.48: Peak years - Minerals

**Name**	**URR**	**Peak Year**	**Peak Rate**
Coal Bit.	14.5	2041	0.48
Oil Conv.	435.0	1969	8.53
Oil Tight	573.0	2093	10.68
Oil Extra Heavy	1719.0	2081	25.77
Gas Conv.	241.5	2048	2.49
Gas Shale	11.57	2045	0.21
**Total**	**2994.57**	**2086**	**36.83**

7.25 Total

7.25.1 By country

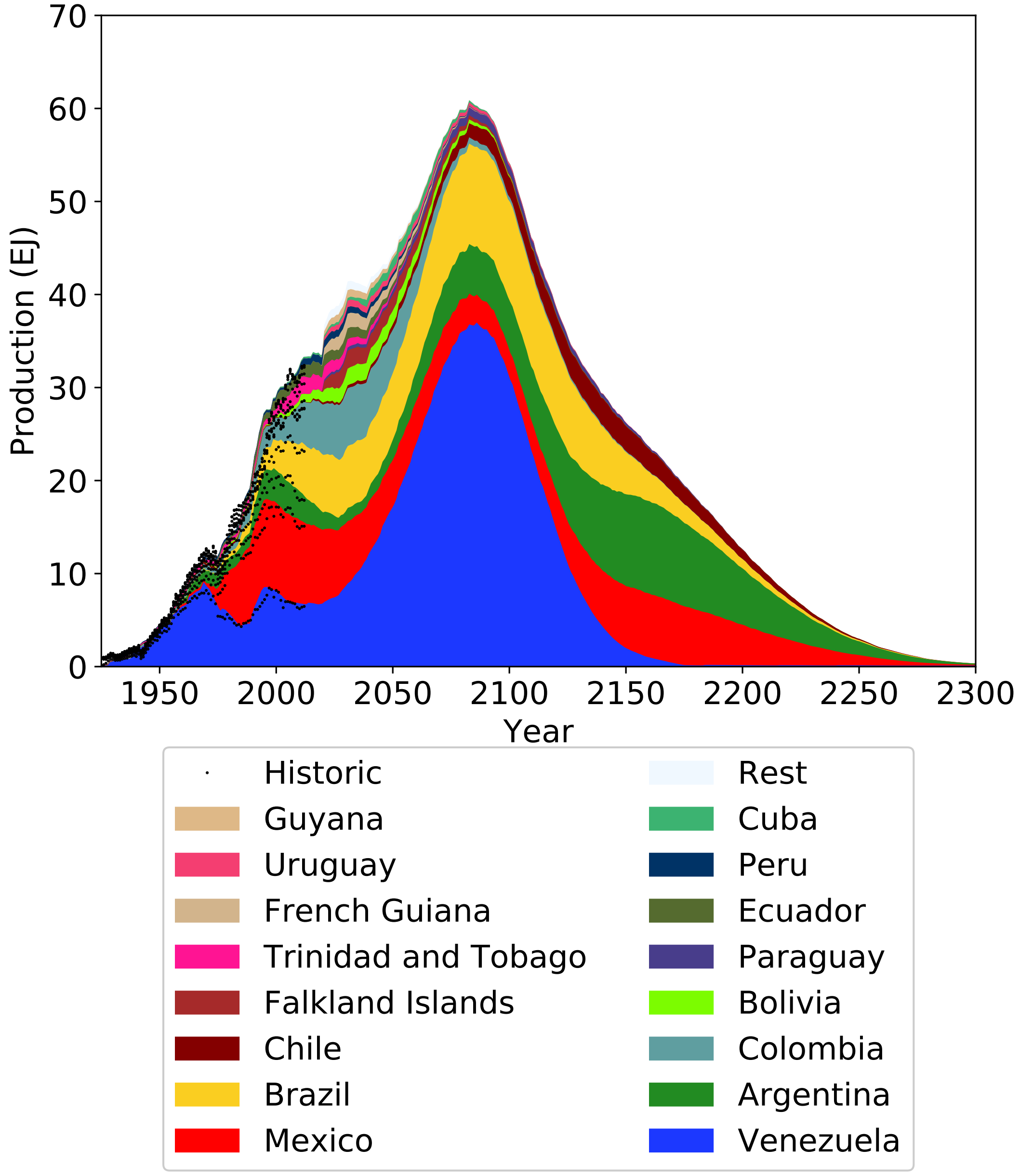

Figure 7.49: South America projections by country

Table 7.49: Peak years - All

**Name**	**URR**	**Peak Year**	**Peak Rate**
Venezuela	2994.57	2086	36.83
Mexico	1428.43	1999	9.54
Argentina	1404.84	2155	10.02
Brazil	1350.55	2088	10.98
Colombia	398.58	2031	6.21
Chile	339.14	2134	2.96
Bolivia	105.04	2033	1.85
Falkland Islands	89.31	2032	1.93
Paraguay	83.06	2090	0.92
Trinidad and Tobago	81.86	2009	1.89
Ecuador	71.77	2016	1.38
French Guiana	48.49	2031	1.57
Peru	47.52	2019	0.82
Uruguay	45.49	2033	0.72
Cuba	45.25	2055	1.06
Guyana	24.55	2030	0.84
Suriname	6.7	2030	0.18
Dominican Republic	6.32	2026	0.25
Haiti	6.06	2026	0.26
Barbados	5.79	2042	0.12
Puerto Rico	4.27	2027	0.19
Guatemala	1.24	1996	0.05
Grenada	0.9	2024	0.05
Belize	0.48	2032	0.01
**Total**	**8590.22**	**2083**	**60.78**

7.25.2 By mineral

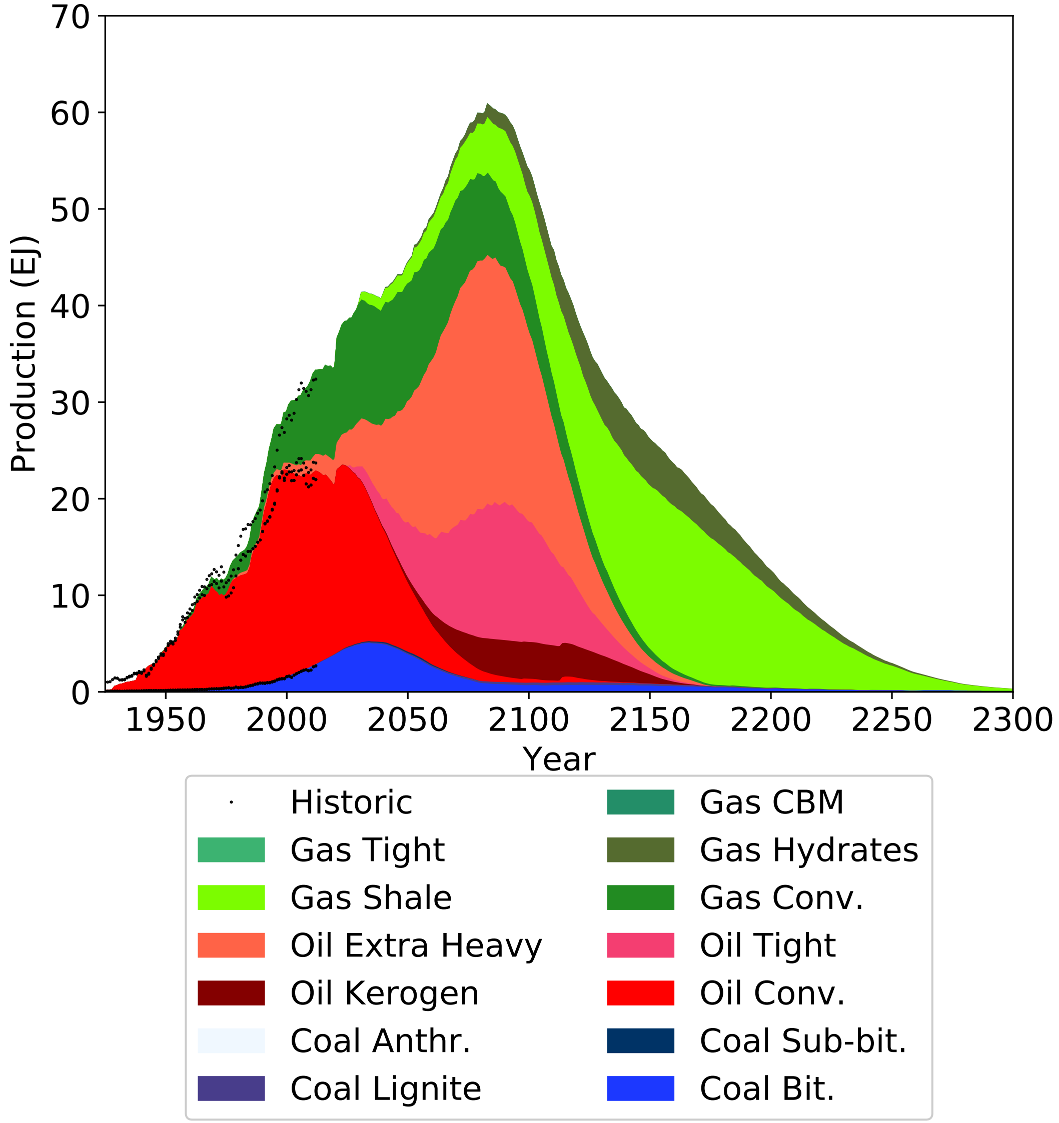

Figure 7.50: South America projection by mineral type

Table 7.50: Peak years - Minerals

**Name**	**URR**	**Peak Year**	**Peak Rate**
Coal Bit.	342.3	2034	4.96
Coal Lignite	34.13	2119	0.28
Coal Sub-bit.	4.95	2053	0.16
Coal Anthr.	0.1	1944	–
Oil Conv.	1560.34	1999	21.55
Oil Kerogen	282.07	2096	3.84
Oil Tight	894.43	2090	14.28
Oil Extra Heavy	1719.0	2081	25.77
Gas Conv.	1247.98	2049	12.38
Gas Shale	2006.94	2153	17.17
Gas Hydrates	496.5	2141	5.08
Gas Tight	0.37	2032	0.01
Gas CBM	1.11	2019	0.03
**Total**	**8590.22**	**2083**	**60.78**

Chapter 8

Total

8.1 By continent

Table 8.1: Peak years - All

Name	URR	Peak Year	Peak Rate
FSU	21416.16	2082	150.77
Asia	19442.63	2022	243.41
North America	17242.46	2026	95.01
Middle East	9013.91	2023	89.08
South America	8590.22	2083	60.78
Africa	6745.5	2031	53.63
Europe	4823.03	1998	39.37
**Total**	**87273.9**	**2023**	**611.61**

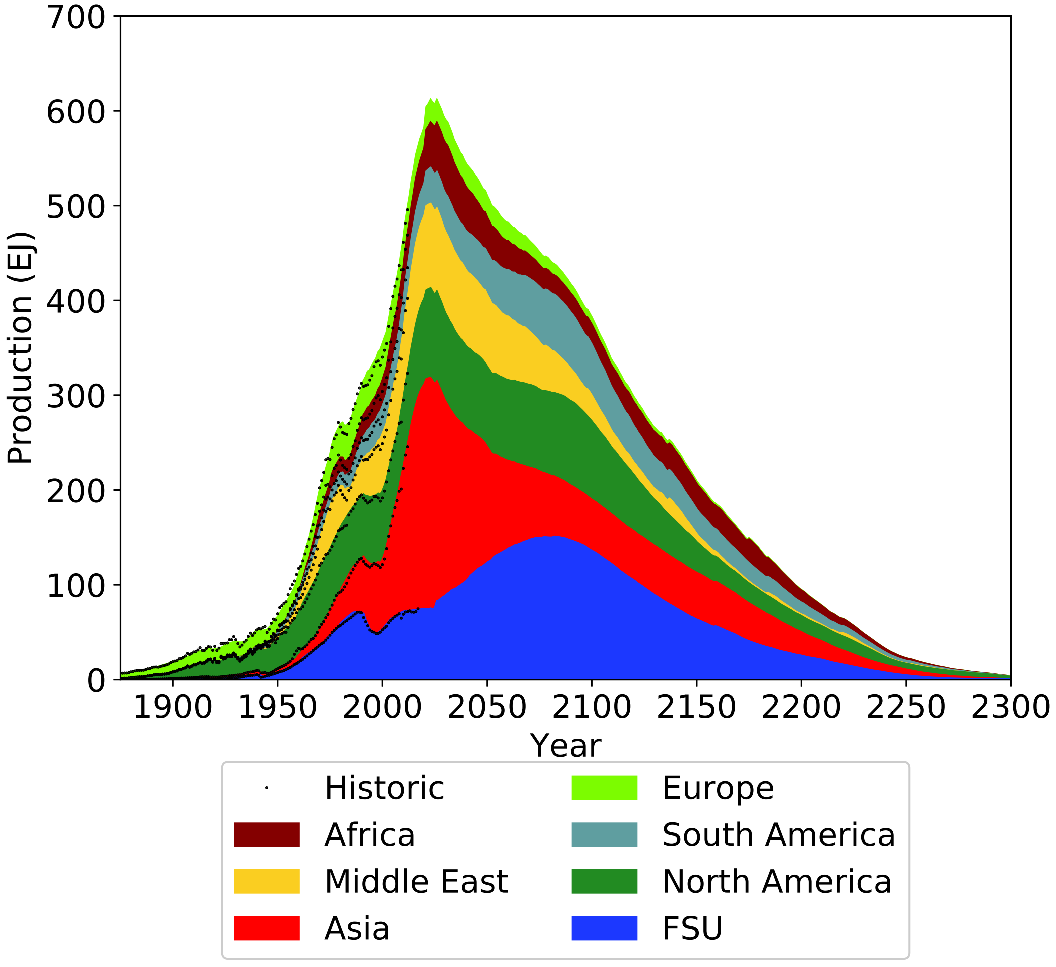

Figure 8.1: Total projections by continent

8.2 By mineral

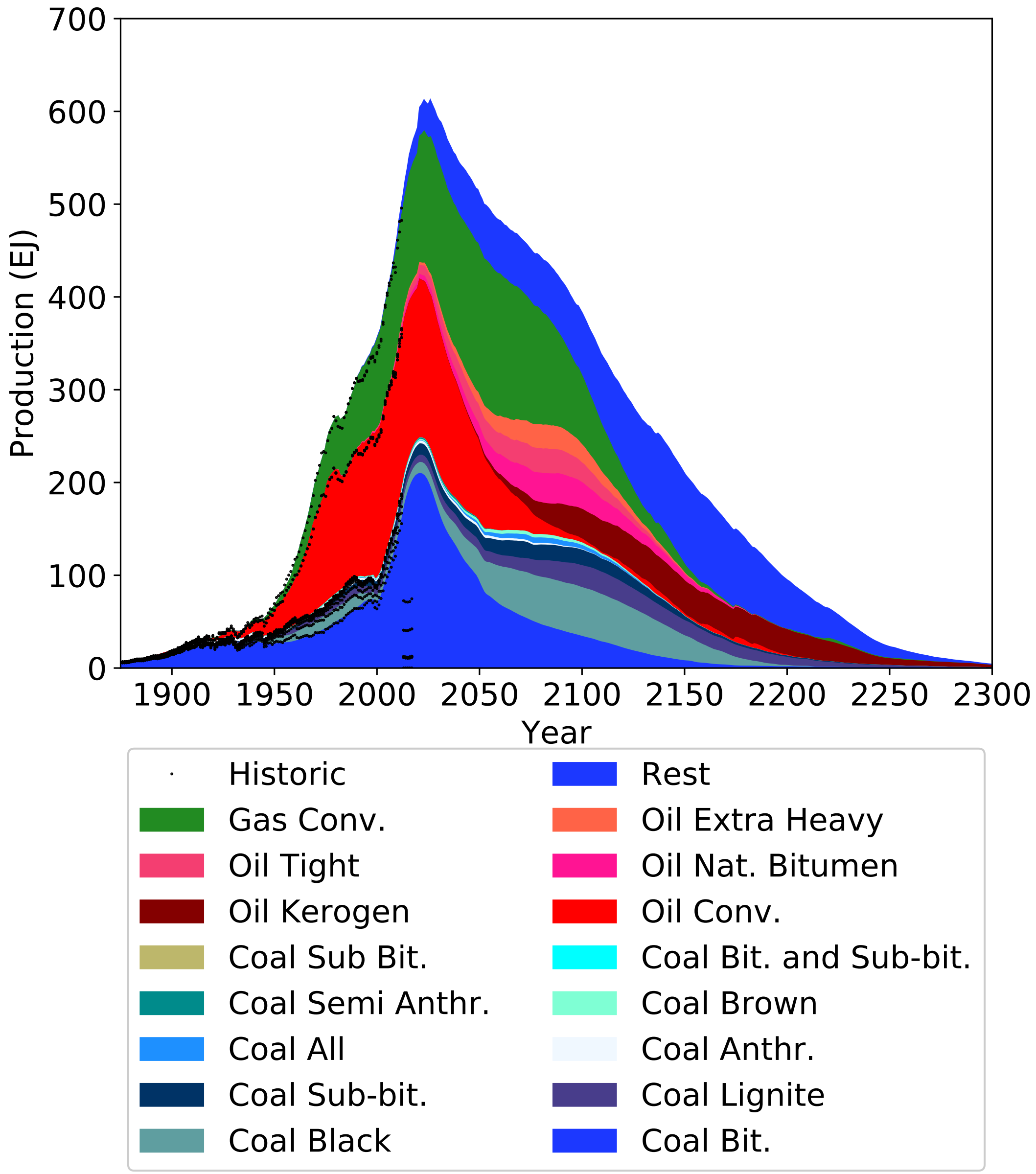

Figure 8.2: Total projection by mineral type

Table 8.2: Peak year by mineral

**Name**	**URR**	**Peak Year**	**Peak Rate**
Gas Conv.	17374.99	2049	160.65
Coal Bit.	14787.18	2021	209.32
Oil Conv.	14504.14	2021	170.63
Gas Shale	7132.3	2133	42.62
Coal Black	6544.43	2095	53.17
Oil Kerogen	5463.03	2133	37.05
Gas Hydrates	4601.83	2158	42.4
Coal Lignite	3616.03	2107	24.19
Oil Nat. Bitumen	2597.29	2086	32.55
Coal Sub-bit.	2280.65	2069	17.97
Oil Tight	2179.16	2083	26.71
Gas Tight	1959.89	2143	13.8
Oil Extra Heavy	1728.48	2081	25.77
Gas CBM	1092.69	2051	10.1
Coal Anthr.	519.15	1918	3.22
Coal All	402.07	2077	6.01
Coal Brown	399.79	2067	3.74
Coal Semi Anthr.	60.7	2033	1.9
Coal Bit. and Sub-bit.	25.4	2019	0.31
Coal Sub Bit.	4.72	2048	0.09
**Total**	**87273.9**	**2023**	**611.61**

8.3 By Country

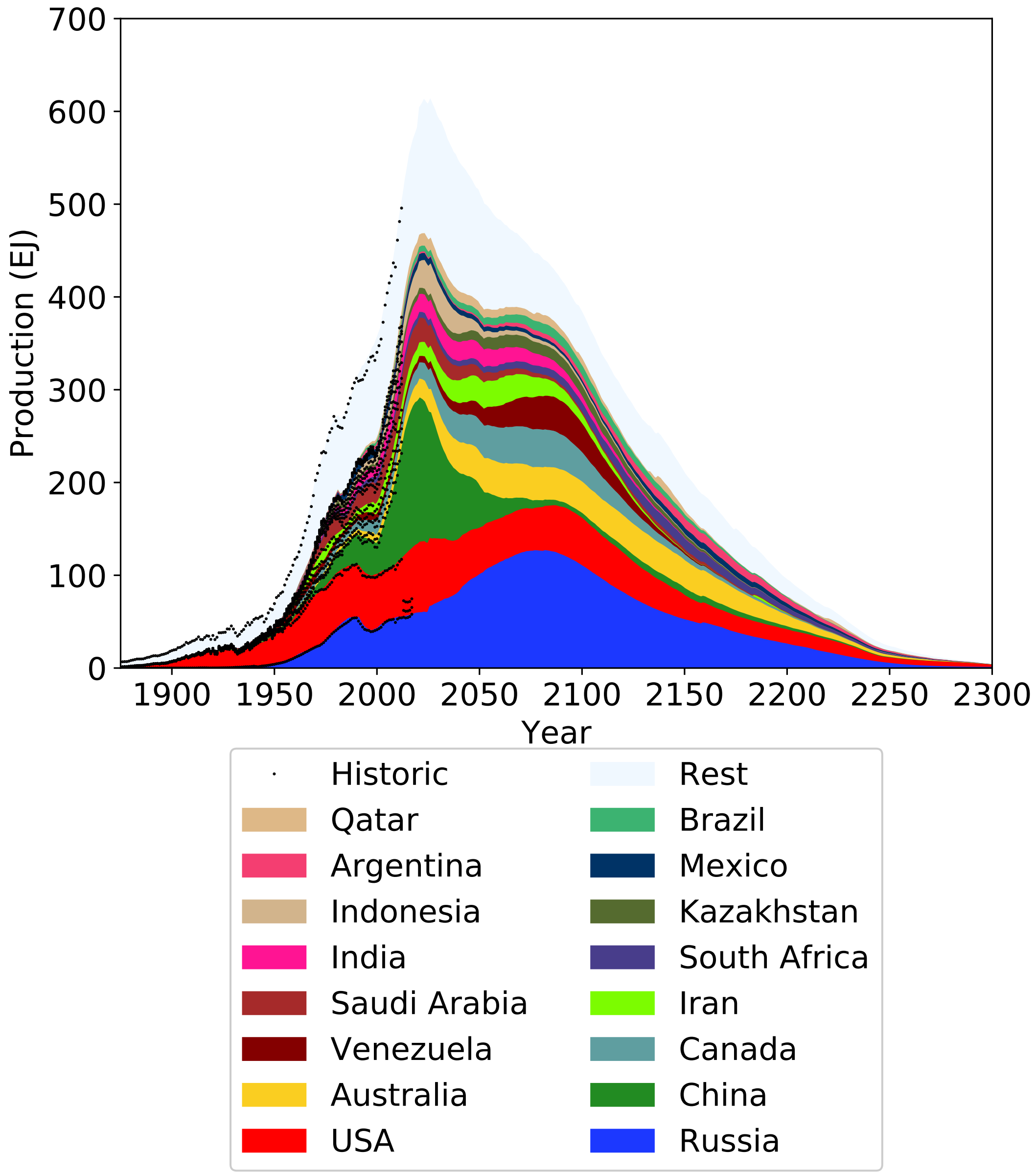

Figure 8.3: Total projection by country

Table 8.3: Peak year - Country

Name	URR	Peak Year	Peak Rate
Russia	17282.65	2082	126.08
USA	12908.93	2022	76.17
China	7043.41	2021	155.03
Australia	6192.26	2061	37.62
Canada	4333.53	2078	40.9
Venezuela	2994.57	2086	36.83
Iran	2502.23	2060	29.82
Saudi Arabia	2197.07	2016	28.67
South Africa	2003.47	2138	12.35
India	1970.49	2033	20.68
Kazakhstan	1548.0	2062	13.73
Indonesia	1504.04	2027	32.36
Mexico	1428.43	1999	9.54
Argentina	1404.84	2155	10.02
Brazil	1350.55	2088	10.98
Qatar	1334.55	2023	13.52
Nigeria	1125.36	2018	7.24
PNG	1087.32	2176	9.62
UK	995.13	1998	11.34
Kuwait	939.76	2038	12.08
Algeria	937.76	2007	7.16
Libya	927.75	1970	7.51
Germany	879.36	1976	7.49
Iraq	857.9	2049	8.57
UAE	797.31	2021	10.51
Donetsk	783.03	2120	7.89
Luhansk	582.62	2114	6.02
Mozambique	490.74	2144	3.81
Norway	478.2	2005	9.82
Angola	403.79	2032	8.87
Colombia	398.58	2031	6.21
Poland	395.98	1984	5.33
Ukraine	350.23	2046	3.78
Chile	339.14	2134	2.96
France	314.26	2115	1.99
New Zealand	301.78	2142	2.73
Malaysia	297.32	2025	4.15
Greenland	292.02	2055	6.38
Uzbekistan	290.35	2004	2.84
Yugoslavia	269.43	2063	2.46
Egypt	252.84	2024	4.42
Azerbaijan	246.71	2034	3.23
Turkmenistan	235.93	2017	3.46
Pakistan	234.72	2061	2.98
Netherlands	195.55	1978	3.8
Czech Republic	191.82	1977	2.34
Oman	172.7	2017	3.58
North Korea	146.97	2036	2.34
Romania	138.42	1982	2.28

Table 8.3: Peak year - Country – Continued

Name	URR	Peak Year	Peak Rate
Turkey	130.45	2070	1.89
Vietnam	115.6	2010	2.28
Bolivia	105.04	2033	1.85
Bangladesh	92.46	2039	1.44
Falkland Islands	89.31	2032	1.93
Paraguay	83.06	2090	0.92
Japan	82.91	1953	1.21
Trinidad and Tobago	81.86	2009	1.89
Sudan	79.55	2029	2.24
Tanzania	77.59	2044	1.23
Thailand	77.39	2010	2.34
Ecuador	71.77	2016	1.38
Italy	70.85	1986	0.86
Belgium	67.12	1913	0.66
Syria	66.48	1998	1.4
Bulgaria	62.82	2033	0.95
Spain	61.67	2030	0.69
Brunei	58.13	2002	0.89
Burma	56.71	2033	0.88
Yemen	56.64	1998	0.99
Denmark	53.79	2001	1.07
Greece	49.23	2031	0.83
Belarus	48.76	2112	0.63
Mongolia	48.62	2022	2.48
Hungary	48.61	1977	0.87
French Guiana	48.49	2031	1.57
Peru	47.52	2019	0.82
Uruguay	45.49	2033	0.72
Austria	45.43	2046	0.51
Equatorial Guinea	45.31	2025	1.32
Cuba	45.25	2055	1.06
Sweden	43.74	2064	0.55
Congo	42.64	2023	0.91
Bahrain	42.14	2014	0.63
Afghanistan	38.3	2030	1.06
Tunisia	38.0	2046	0.43
Gabon	37.65	1994	0.77
Botswana	35.23	2050	0.59
Ivory Coast	30.93	2037	0.63
Lebanon	27.81	2046	0.45
South Korea	25.04	1983	0.69
Guyana	24.55	2030	0.84
Kyrgyzstan	22.74	2058	0.46
Cameroon	21.86	2021	0.4
Seychelles	20.5	2028	0.72
Philippines	19.98	2022	0.51
Namibia	19.7	2028	0.63
Uganda	18.36	2031	0.64
East Timor	17.87	2042	0.29

Table 8.3: Peak year - Country – Continued

Name	URR	Peak Year	Peak Rate
Ghana	17.83	2020	0.56
Somalia	15.98	2038	0.4
Sri Lanka	14.91	2034	0.29
Ireland	14.02	2025	0.45
Tajikistan	11.46	2045	0.26
Sierra Leone	10.95	2028	0.42
Cyprus	10.74	2043	0.19
Kenya	10.53	2028	0.41
Jordan	10.43	2026	0.39
Israel	8.89	1970	0.22
Zimbabwe	8.7	1992	0.11
Sao Tome and Principe	7.58	2026	0.31
Zaire	7.27	2031	0.09
Albania	7.16	2019	0.14
Taiwan	6.93	1968	0.17
Liberia	6.74	2028	0.26
Suriname	6.7	2030	0.18
Laos	6.34	2042	0.15
Dominican Republic	6.32	2026	0.25
Guinea	6.32	2026	0.28
Haiti	6.06	2026	0.26
Eritrea	6.02	2028	0.21
Georgia	6.01	1979	0.18
Senegal	6.0	2026	0.26
Barbados	5.79	2042	0.12
Estonia	5.73	2048	0.08
Puerto Rico	4.27	2027	0.19
Chad	4.17	2005	0.35
Swaziland	3.98	2085	0.06
Portugal	3.72	2014	0.17
Togo	3.35	2024	0.17
Niger	3.13	2016	0.09
Slovakia	3.08	1997	0.07
Cambodia	2.95	2024	0.16
Rwanda	2.9	2030	0.11
Ethiopia	2.55	2024	0.14
Mauritania	2.34	2024	0.08
Madagascar	2.3	2024	0.12
Morocco	2.02	1972	0.03
Guinea-Bissau	1.88	2026	0.08
Crimea	1.71	2010	0.07
Western Sahara	1.36	2024	0.08
Guatemala	1.24	1996	0.05
Benin	0.97	2024	0.04
Grenada	0.9	2024	0.05
Gambia	0.84	2024	0.05
Zambia	0.68	1978	0.02
Belize	0.48	2032	0.01
Malta	0.41	2024	0.03

Table 8.3: Peak year - Country – Continued

**Name**	**URR**	**Peak Year**	**Peak Rate**
Lithuania	0.21	1998	0.02
Bhutan	0.15	2017	–
Malawi	0.08	2020	–
Switzerland	0.03	1944	0.01
Central African Republic	0.03	2026	–
Moldova	0.02	1994	0.01
Nepal	0.01	2018	–
New Caledonia	–	1928	–
**Total**	**87273.9**	**2023**	**611.61**
