# Supplementary material for: Projecting the global impact of fossil fuel production from the Former Soviet Union
Source: Int J Coal Sci Technol. 2021 Aug 9;8(6):1208–26. doi: 10.1007/s40789-021-00449-x (PMC8351787; doi:10.1007/s40789-021-00449-x)
Supplement: Supplementary file 7 — Supplementary file7 (PDF 22103 kb) [file 40789_2021_449_MOESM7_ESM.pdf]

GeRS-DeMo Post Processor

Compilation ran on scenario: Static Low

Date: 7/6/2020

Contents

1 Africa **20**

1.1 Algeria . . . 20
1.1.1 All Projections . . . 20
1.1.2 By Mineral . . . 22
1.2 Angola . . . 24
1.2.1 All Projections . . . 24
1.2.2 By Mineral . . . 25
1.3 Benin . . . 26
1.3.1 All Projections . . . 26
1.3.2 By Mineral . . . 27
1.4 Botswana . . . 28
1.4.1 All Projections . . . 28
1.4.2 By Mineral . . . 29
1.5 Cameroon . . . 30
1.5.1 All Projections . . . 30
1.5.2 By Mineral . . . 31
1.6 Chad . . . 32
1.6.1 All Projections . . . 32
1.6.2 By Mineral . . . 33
1.7 Congo . . . 34
1.7.1 All Projections . . . 34
1.7.2 By Mineral . . . 35
1.8 Egypt . . . 36
1.8.1 All Projections . . . 36
1.8.2 By Mineral . . . 38
1.9 Equatorial Guinea . . . 40
1.9.1 All Projections . . . 40
1.9.2 By Mineral . . . 41

1.10 Eritrea 42
1.10.1 All Projections 42
1.10.2 By Mineral 43
1.11 Ethiopia 44
1.11.1 All Projections 44
1.11.2 By Mineral 45
1.12 Gabon 46
1.12.1 All Projections 46
1.12.2 By Mineral 47
1.13 Ghana 48
1.13.1 All Projections 48
1.13.2 By Mineral 49
1.14 Guinea-Bissau 50
1.14.1 All Projections 50
1.14.2 By Mineral 51
1.15 Ivory Coast 52
1.15.1 All Projections 52
1.15.2 By Mineral 53
1.16 Libya 54
1.16.1 All Projections 54
1.16.2 By Mineral 56
1.17 Madagascar 58
1.17.1 All Projections 58
1.17.2 By Mineral 59
1.18 Malawi 60
1.18.1 All Projections 60
1.18.2 By Mineral 61
1.19 Mauritania 62
1.19.1 All Projections 62
1.19.2 By Mineral 63
1.20 Morocco 64
1.20.1 All Projections 64
1.20.2 By Mineral 66
1.21 Mozambique 68
1.21.1 All Projections 68
1.21.2 By Mineral 69

1.22 Namibia 70
1.22.1 All Projections 70
1.22.2 By Mineral 71
1.23 Niger 72
1.23.1 All Projections 72
1.23.2 By Mineral 73
1.24 Nigeria 74
1.24.1 All Projections 74
1.24.2 By Mineral 75
1.25 Rwanda 76
1.25.1 All Projections 76
1.25.2 By Mineral 77
1.26 Senegal 78
1.26.1 All Projections 78
1.26.2 By Mineral 79
1.27 Seychelles 80
1.27.1 All Projections 80
1.27.2 By Mineral 81
1.28 Somalia 82
1.28.1 All Projections 82
1.28.2 By Mineral 83
1.29 South Africa 84
1.29.1 All Projections 84
1.29.2 By Mineral 86
1.30 Sudan 88
1.30.1 All Projections 88
1.30.2 By Mineral 89
1.30.3 Regional Projections 89
South Sudan 90
Sudan 92
Sudan Region 94
1.30.4 Projection by region 96
1.31 Swaziland 97
1.31.1 All Projections 97
1.31.2 By Mineral 98
1.32 Tanzania 99

1.32.1 All Projections 99
1.32.2 By Mineral 100
1.33 Togo 101
1.33.1 All Projections 101
1.33.2 By Mineral 102
1.34 Tunisia 103
1.34.1 All Projections 103
1.34.2 By Mineral 105
1.35 Western Sahara 107
1.35.1 All Projections 107
1.35.2 By Mineral 108
1.36 Zaire 109
1.36.1 All Projections 109
1.36.2 By Mineral 111
1.37 Zambia 113
1.37.1 All Projections 113
1.37.2 By Mineral 114
1.38 Zimbabwe 115
1.38.1 All Projections 115
1.38.2 By Mineral 116
1.39 Total 117
1.39.1 By country 117
1.39.2 By mineral 119

2 Asia **121**
2.1 Afghanistan 121
2.1.1 All Projections 121
2.1.2 By Mineral 123
2.2 Australia 124
2.2.1 All Projections 124
2.2.2 By Mineral 127
2.2.3 Regional Projections 127
Australia 129
JPDA 131
NSW 133
Northern Territory 136

Queensland 139
South Australia 142
Tasmania 145
Victoria 147
Western Australia 149
2.2.4 Projection by region 152
2.3 Bangladesh 154
2.3.1 All Projections 154
2.3.2 By Mineral 156
2.4 Bhutan 158
2.4.1 All Projections 158
2.4.2 By Mineral 159
2.5 Brunei 160
2.5.1 All Projections 160
2.5.2 By Mineral 162
2.6 Burma 164
2.6.1 All Projections 164
2.6.2 By Mineral 166
2.7 Cambodia 168
2.7.1 All Projections 168
2.7.2 By Mineral 169
2.8 China 170
2.8.1 All Projections 170
2.8.2 By Mineral 173
2.8.3 Regional Projections 173
Anhui 175
Beijing 177
China 179
Chongqing 182
Fujian 184
Gansu 186
Guangdong 188
Guangxi 190
Guizhou 192
Hainan 194
Hebei 196

Heilongjiang . . . 198
Henan . . . 200
Historic . . . 202
Hubei . . . 204
Hunan . . . 206
Inner Mongolia . . . 208
Jiangsu . . . 210
Jiangxi . . . 212
Jilin . . . 214
Liaoning . . . 216
Ningxia . . . 218
Offshore . . . 220
Qinghai . . . 222
Shaanxi . . . 224
Shandong . . . 226
Shanghai . . . 228
Shanxi . . . 230
Sichuan . . . 232
Tianjin . . . 234
Tibet . . . 236
Xinjiang . . . 238
Yunnan . . . 240
Zhejiang . . . 242
2.8.4 Projection by region . . . 244
2.9 East Timor . . . 246
2.9.1 All Projections . . . 246
2.9.2 By Mineral . . . 247
2.10 India . . . 248
2.10.1 All Projections . . . 248
2.10.2 By Mineral . . . 250
2.11 Indonesia . . . 252
2.11.1 All Projections . . . 252
2.11.2 By Mineral . . . 254
2.12 Japan . . . 256
2.12.1 All Projections . . . 256
2.12.2 By Mineral . . . 258

2.13 Laos 260
2.13.1 All Projections 260
2.13.2 By Mineral 261
2.14 Malaysia 262
2.14.1 All Projections 262
2.14.2 By Mineral 263
2.15 Mongolia 264
2.15.1 All Projections 264
2.15.2 By Mineral 266
2.16 Nepal 268
2.16.1 All Projections 268
2.16.2 By Mineral 269
2.17 New Caledonia 270
2.17.1 All Projections 270
2.17.2 By Mineral 271
2.18 New Zealand 272
2.18.1 All Projections 272
2.18.2 By Mineral 274
2.19 North Korea 276
2.19.1 All Projections 276
2.19.2 By Mineral 277
2.20 PNG 278
2.20.1 All Projections 278
2.20.2 By Mineral 279
2.21 Pakistan 280
2.21.1 All Projections 280
2.21.2 By Mineral 282
2.22 Philippines 284
2.22.1 All Projections 284
2.22.2 By Mineral 285
2.23 South Korea 286
2.23.1 All Projections 286
2.23.2 By Mineral 288
2.24 Sri Lanka 290
2.24.1 All Projections 290
2.24.2 By Mineral 291

2.25 Taiwan . . . 292
2.25.1 All Projections . . . 292
2.25.2 By Mineral . . . 293
2.26 Thailand . . . 294
2.26.1 All Projections . . . 294
2.26.2 By Mineral . . . 296
2.27 Vietnam . . . 298
2.27.1 All Projections . . . 298
2.27.2 By Mineral . . . 300
2.28 Total . . . 302
2.28.1 By country . . . 302
2.28.2 By mineral . . . 304

3 Europe 306
3.1 Albania . . . 306
3.1.1 All Projections . . . 306
3.1.2 By Mineral . . . 308
3.2 Austria . . . 309
3.2.1 All Projections . . . 309
3.2.2 By Mineral . . . 311
3.3 Belgium . . . 313
3.3.1 All Projections . . . 313
3.3.2 By Mineral . . . 314
3.4 Bulgaria . . . 315
3.4.1 All Projections . . . 315
3.4.2 By Mineral . . . 317
3.5 Cyprus . . . 319
3.5.1 All Projections . . . 319
3.5.2 By Mineral . . . 320
3.6 Czech Republic . . . 321
3.6.1 All Projections . . . 321
3.6.2 By Mineral . . . 323
3.7 Denmark . . . 325
3.7.1 All Projections . . . 325
3.7.2 By Mineral . . . 327
3.8 France . . . 329

3.8.1 All Projections 329
3.8.2 By Mineral 331
3.9 Germany 333
3.9.1 All Projections 333
3.9.2 By Mineral 335
3.10 Greece 337
3.10.1 All Projections 337
3.10.2 By Mineral 339
3.11 Greenland 341
3.11.1 All Projections 341
3.11.2 By Mineral 342
3.12 Hungary 343
3.12.1 All Projections 343
3.12.2 By Mineral 345
3.13 Ireland 347
3.13.1 All Projections 347
3.13.2 By Mineral 348
3.14 Italy 349
3.14.1 All Projections 349
3.14.2 By Mineral 351
3.14.3 Regional Projections 351
Italy 353
3.14.4 Projection by region 356
3.15 Malta 357
3.15.1 All Projections 357
3.15.2 By Mineral 358
3.16 Netherlands 359
3.16.1 All Projections 359
3.16.2 By Mineral 361
3.17 Norway 363
3.17.1 All Projections 363
3.17.2 By Mineral 365
3.18 Poland 367
3.18.1 All Projections 367
3.18.2 By Mineral 369
3.19 Portugal 371

3.19.1 All Projections . . . 371
3.19.2 By Mineral . . . 373
3.20 Romania . . . 375
3.20.1 All Projections . . . 375
3.20.2 By Mineral . . . 377
3.21 Slovakia . . . 379
3.21.1 All Projections . . . 379
3.21.2 By Mineral . . . 380
3.22 Spain . . . 381
3.22.1 All Projections . . . 381
3.22.2 By Mineral . . . 383
3.23 Sweden . . . 385
3.23.1 All Projections . . . 385
3.23.2 By Mineral . . . 386
3.24 Switzerland . . . 387
3.24.1 All Projections . . . 387
3.24.2 By Mineral . . . 388
3.25 Turkey . . . 389
3.25.1 All Projections . . . 389
3.25.2 By Mineral . . . 391
3.26 UK . . . 393
3.26.1 All Projections . . . 393
3.26.2 By Mineral . . . 395
3.26.3 Regional Projections . . . 395
England and Wales . . . 397
Northern Ireland . . . 400
Scotland . . . 402
UK . . . 404
3.26.4 Projection by region . . . 406
3.27 Yugoslavia . . . 408
3.27.1 All Projections . . . 408
3.27.2 By Mineral . . . 410
3.27.3 Regional Projections . . . 410
Bosnia and Herzegovina . . . 412
Croatia . . . 414
Serbia . . . 416

Slovenia 418
Yugoslavia 420
3.27.4 Projection by region 422
3.28 Total 424
3.28.1 By country 424
3.28.2 By mineral 426

4 FSU **428**
4.1 Azerbaijan 428
4.1.1 All Projections 428
4.1.2 By Mineral 430
4.2 Belarus 431
4.2.1 All Projections 431
4.2.2 By Mineral 432
4.3 Crimea 433
4.3.1 All Projections 433
4.3.2 By Mineral 434
4.3.3 Regional Projections 434
Crimea 435
4.3.4 Projection by region 437
4.4 Donetsk 438
4.4.1 All Projections 438
4.4.2 By Mineral 439
4.4.3 Regional Projections 439
Donetsk 440
4.4.4 Projection by region 442
4.5 Estonia 443
4.5.1 All Projections 443
4.5.2 By Mineral 444
4.6 Georgia 445
4.6.1 All Projections 445
4.6.2 By Mineral 446
4.7 Kazakhstan 447
4.7.1 All Projections 447
4.7.2 By Mineral 449
4.7.3 Regional Projections 449

All . . . 451
East Kazakhstan . . . 454
Karaganda . . . 456
Kostanay . . . 458
Other . . . 460
Pavlodar . . . 462
4.7.4 Projection by region . . . 464
4.8 Kyrgyzstan . . . 466
4.8.1 All Projections . . . 466
4.8.2 By Mineral . . . 468
4.9 Lithuania . . . 470
4.9.1 All Projections . . . 470
4.9.2 By Mineral . . . 471
4.10 Luhansk . . . 472
4.10.1 All Projections . . . 472
4.10.2 By Mineral . . . 473
4.10.3 Regional Projections . . . 473
Luhansk . . . 474
4.10.4 Projection by region . . . 476
4.11 Moldova . . . 477
4.11.1 All Projections . . . 477
4.11.2 By Mineral . . . 478
4.12 Russia . . . 479
4.12.1 All Projections . . . 479
4.12.2 By Mineral . . . 482
4.12.3 Regional Projections . . . 482
All . . . 484
Central . . . 486
Far Eastern . . . 488
North Caucasian . . . 491
Northwestern . . . 493
Other . . . 496
Siberian . . . 498
Southern . . . 501
Ural . . . 503
Volga . . . 505

4.12.4 Projection by region 508
4.13 Tajikistan 510
4.13.1 All Projections 510
4.13.2 By Mineral 511
4.14 Turkmenistan 512
4.14.1 All Projections 512
4.14.2 By Mineral 513
4.15 Ukraine 514
4.15.1 All Projections 514
4.15.2 By Mineral 516
4.16 Uzbekistan 518
4.16.1 All Projections 518
4.16.2 By Mineral 520
4.17 Total 522
4.17.1 By country 522
4.17.2 By mineral 523

5 Middle East **526**
5.1 Bahrain 526
5.1.1 All Projections 526
5.1.2 By Mineral 528
5.2 Iran 529
5.2.1 All Projections 529
5.2.2 By Mineral 530
5.3 Iraq 531
5.3.1 All Projections 531
5.3.2 By Mineral 532
5.4 Israel 533
5.4.1 All Projections 533
5.4.2 By Mineral 534
5.5 Jordan 535
5.5.1 All Projections 535
5.5.2 By Mineral 536
5.6 Kuwait 537
5.6.1 All Projections 537
5.6.2 By Mineral 538

5.7 Lebanon 539
5.7.1 All Projections 539
5.7.2 By Mineral 540
5.8 Oman 541
5.8.1 All Projections 541
5.8.2 By Mineral 542
5.9 Qatar 543
5.9.1 All Projections 543
5.9.2 By Mineral 544
5.10 Saudi Arabia 545
5.10.1 All Projections 545
5.10.2 By Mineral 546
5.11 Syria 547
5.11.1 All Projections 547
5.11.2 By Mineral 548
5.12 UAE 549
5.12.1 All Projections 549
5.12.2 By Mineral 550
5.13 Yemen 551
5.13.1 All Projections 551
5.13.2 By Mineral 552
5.14 Total 553
5.14.1 By country 553
5.14.2 By mineral 553

6 North America 556
6.1 Canada 556
6.1.1 All Projections 556
6.1.2 By Mineral 560
6.1.3 Regional Projections 560
Alberta 562
British Columbia 565
East Coast Offshore 568
Manitoba 570
New Brunswick 572
Northwest Territories 574

Nova Scotia 576
Ontario 578
Quebec 580
Saskatchewan 582
Yukon 585
6.1.4 Projection by region 587
6.2 USA 589
6.2.1 All Projections 589
6.2.2 By Mineral 594
6.2.3 Regional Projections 594
Alabama 596
Alaska 598
Arizona 600
Arkansas 602
California 605
Colorado 607
Florida 610
Georgia 612
Illinois 614
Indiana 616
Iowa 618
Kansas 620
Kentucky 622
Louisiana 625
Maryland 628
Michigan 630
Mississippi 632
Missouri 634
Montana 636
Nebraska 639
Nevada 641
New Mexico 643
New York 646
North Dakota 648
Ohio 650
Oklahoma 652

Oregon 655
Other 657
Pennsylvania 659
South Dakota 662
Tennessee 664
Texas 666
Utah 669
Virginia 671
Washington 673
West Virginia 675
Wyoming 678
6.2.4 Projection by region 681
6.3 Total 683
6.3.1 By country 683
6.3.2 By mineral 684

7 South America **686**
7.1 Argentina 686
7.1.1 All Projections 686
7.1.2 By Mineral 688
7.2 Barbados 690
7.2.1 All Projections 690
7.2.2 By Mineral 691
7.3 Belize 692
7.3.1 All Projections 692
7.3.2 By Mineral 693
7.4 Bolivia 694
7.4.1 All Projections 694
7.4.2 By Mineral 695
7.5 Brazil 696
7.5.1 All Projections 696
7.5.2 By Mineral 698
7.6 Chile 700
7.6.1 All Projections 700
7.6.2 By Mineral 702
7.7 Colombia 704

7.7.1 All Projections 704
7.7.2 By Mineral 706
7.8 Cuba 708
7.8.1 All Projections 708
7.8.2 By Mineral 709
7.9 Ecuador 710
7.9.1 All Projections 710
7.9.2 By Mineral 711
7.10 Grenada 712
7.10.1 All Projections 712
7.10.2 By Mineral 713
7.11 Guatemala 714
7.11.1 All Projections 714
7.11.2 By Mineral 715
7.12 Guyana 716
7.12.1 All Projections 716
7.12.2 By Mineral 717
7.13 Mexico 718
7.13.1 All Projections 718
7.13.2 By Mineral 720
7.14 Paraguay 722
7.14.1 All Projections 722
7.14.2 By Mineral 723
7.15 Peru 724
7.15.1 All Projections 724
7.15.2 By Mineral 726
7.16 Suriname 728
7.16.1 All Projections 728
7.16.2 By Mineral 729
7.17 Trinidad and Tobago 730
7.17.1 All Projections 730
7.17.2 By Mineral 731
7.18 Uruguay 732
7.18.1 All Projections 732
7.18.2 By Mineral 733
7.19 Venezuela 734

7.19.1 All Projections 734
7.19.2 By Mineral 736
7.20 Total 738
7.20.1 By country 738
7.20.2 By mineral 739

8 Total 742

8.1 By continent 742
8.2 By mineral 744
8.3 By Country 746

Chapter 1

Africa

1.1 Algeria

1.1.1 All Projections

Table 1.1: Peak years - All

Name	URR	Peak Year	Peak Rate
Oil Conv.	261.15	2023	4.27
Gas Conv.	231.0	2008	3.26
Oil Tight	32.66	2030	1.28
Gas Shale	24.15	2039	0.4
Gas Tight	20.39	2041	0.35
Coal Bit.	0.13	1952	0.01
Coal Lignite	–	1942	–
**Total**	**569.48**	**2026**	**8.25**

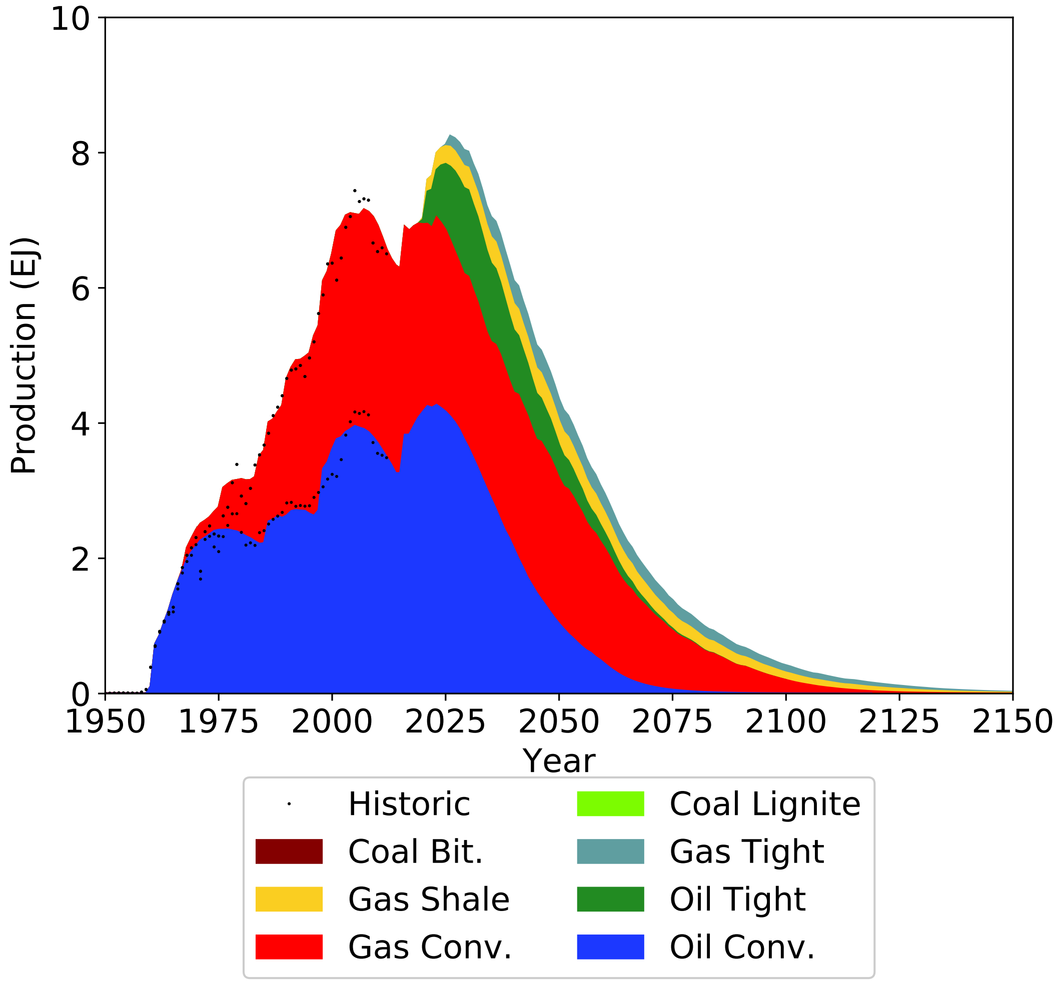

Figure 1.1: Algeria projections capped at 16

1.1.2 By Mineral

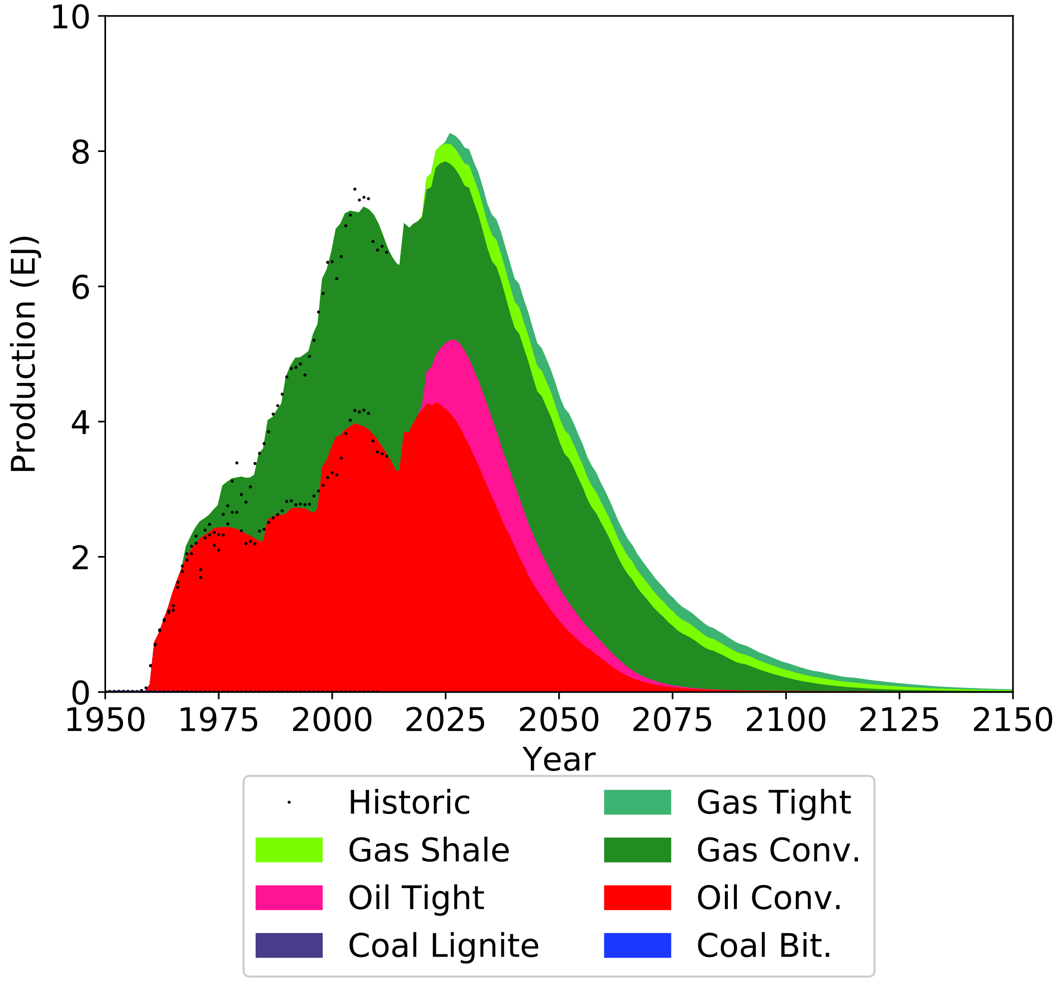

Figure 1.2: Algeria projection by mineral type

Table 1.2: Peak years - Minerals

**Name**	**URR**	**Peak Year**	**Peak Rate**
Coal Bit.	0.13	1952	0.01
Coal Lignite	–	1942	–
Oil Conv.	261.15	2023	4.27
Oil Tight	32.66	2030	1.28
Gas Conv.	231.0	2008	3.26
Gas Shale	24.15	2039	0.4
Gas Tight	20.39	2041	0.35
**Total**	**569.48**	**2026**	**8.25**

1.2 Angola

1.2.1 All Projections

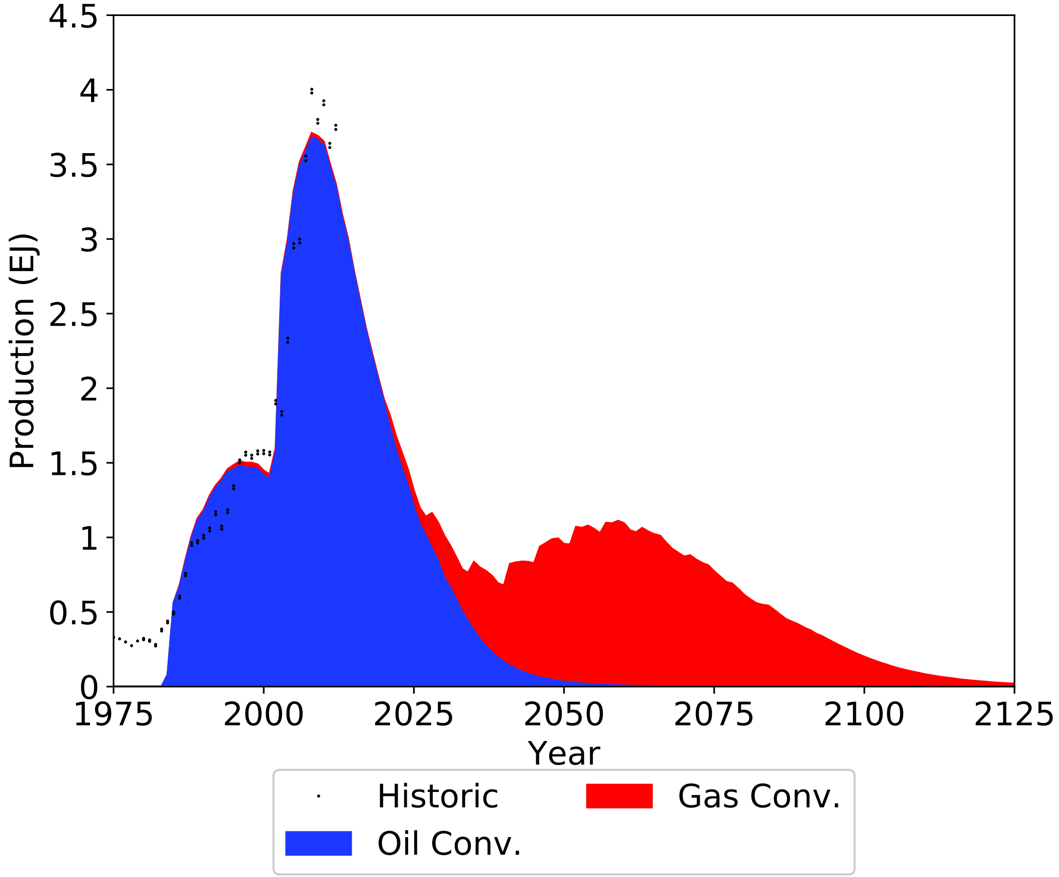

Figure 1.3: Angola projections capped at 16

Table 1.3: Peak years - All

**Name**	**URR**	**Peak Year**	**Peak Rate**
Oil Conv.	93.09	2008	3.68
Gas Conv.	52.5	2059	1.1
**Total**	**145.59**	**2008**	**3.71**

1.2.2 By Mineral

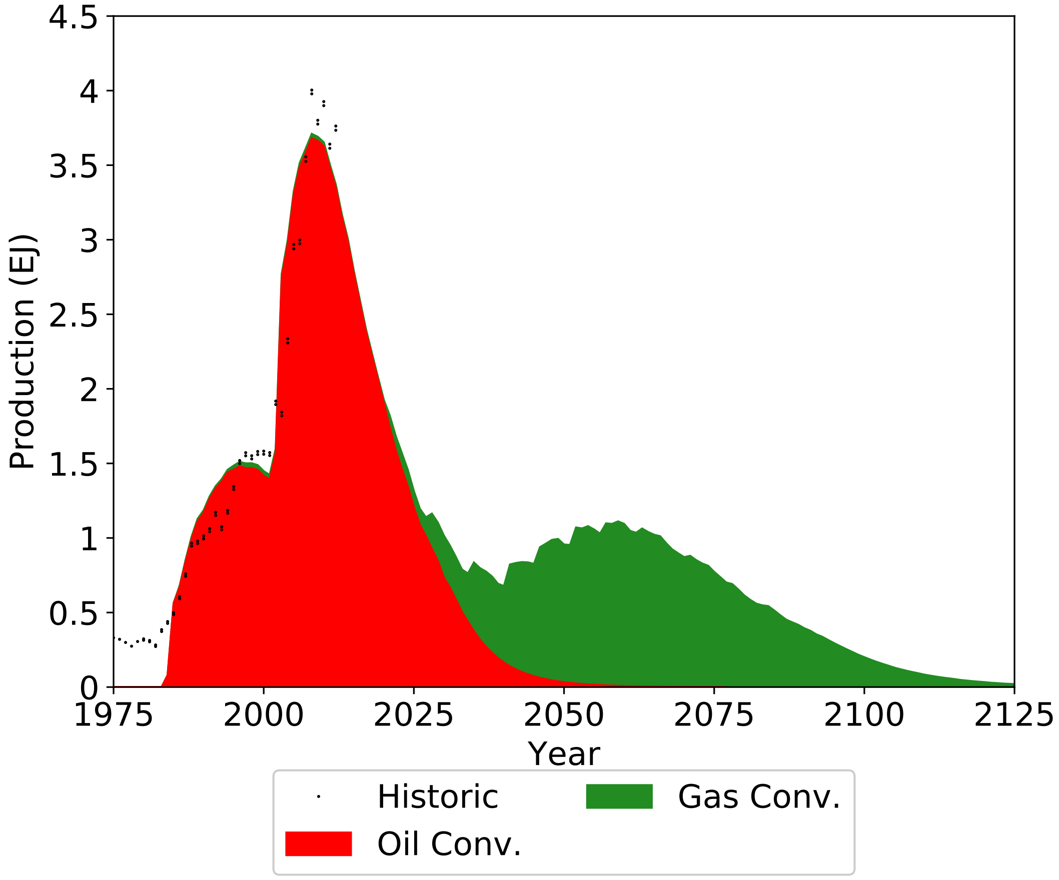

Figure 1.4: Angola projection by mineral type

Table 1.4: Peak years - Minerals

**Name**	**URR**	**Peak Year**	**Peak Rate**
Oil Conv.	93.09	2008	3.68
Gas Conv.	52.5	2059	1.1
**Total**	**145.59**	**2008**	**3.71**

1.3 Benin

1.3.1 All Projections

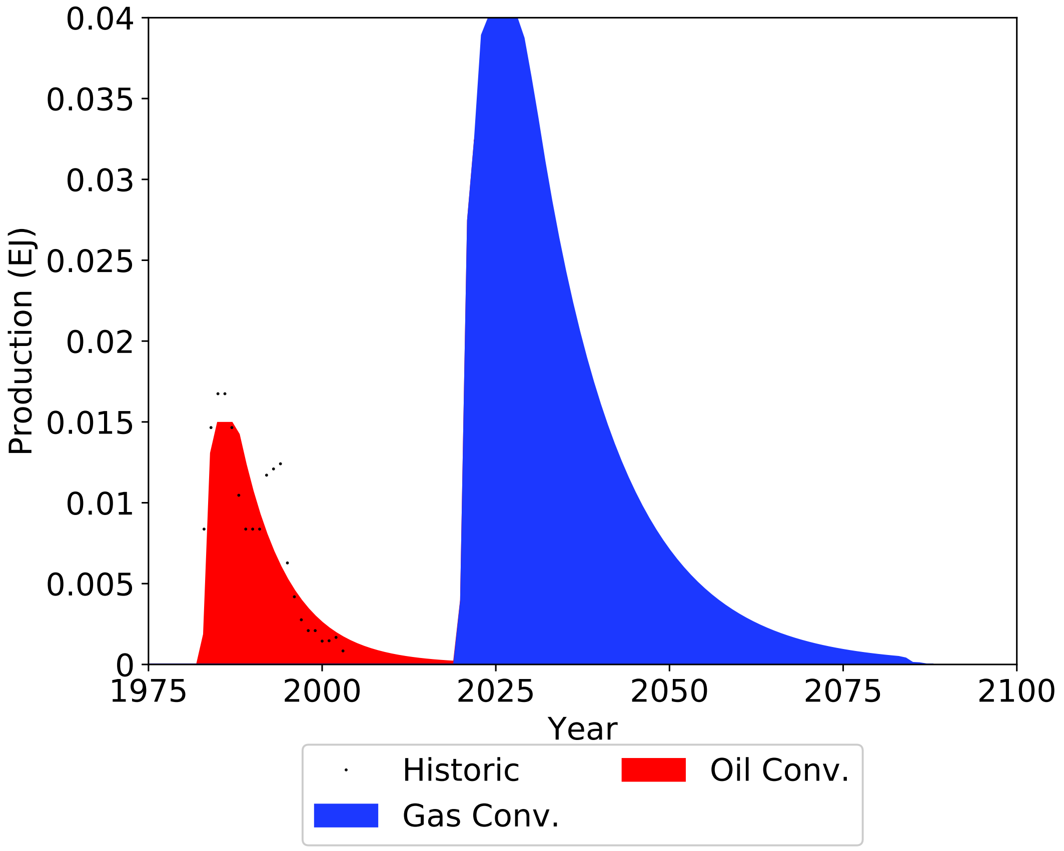

Figure 1.5: Benin projections capped at 16

Table 1.5: Peak years - All

Name	URR	Peak Year	Peak Rate
Gas Conv.	0.8	2024	0.04
Oil Conv.	0.17	1985	0.01
**Total**	**0.97**	**2024**	**0.04**

1.3.2 By Mineral

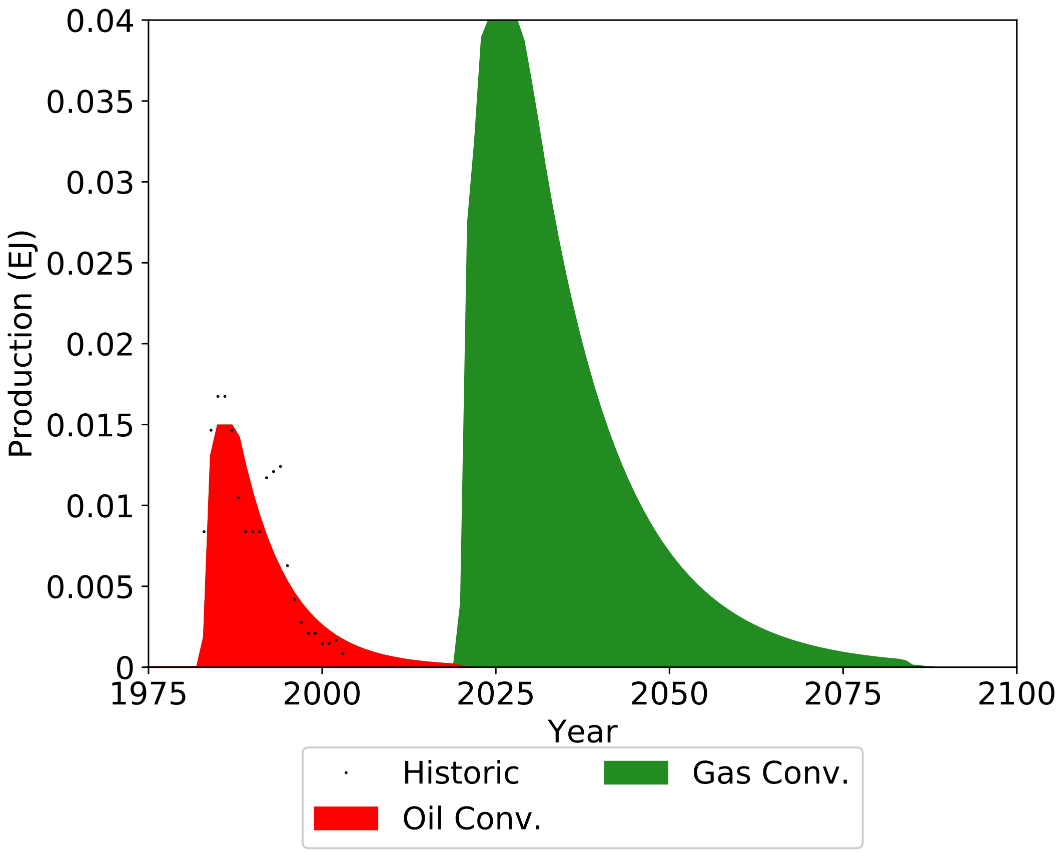

Figure 1.6: Benin projection by mineral type

Table 1.6: Peak years - Minerals

Name	URR	Peak Year	Peak Rate
Oil Conv.	0.17	1985	0.01
Gas Conv.	0.8	2024	0.04
**Total**	**0.97**	**2024**	**0.04**

1.4 Botswana

1.4.1 All Projections

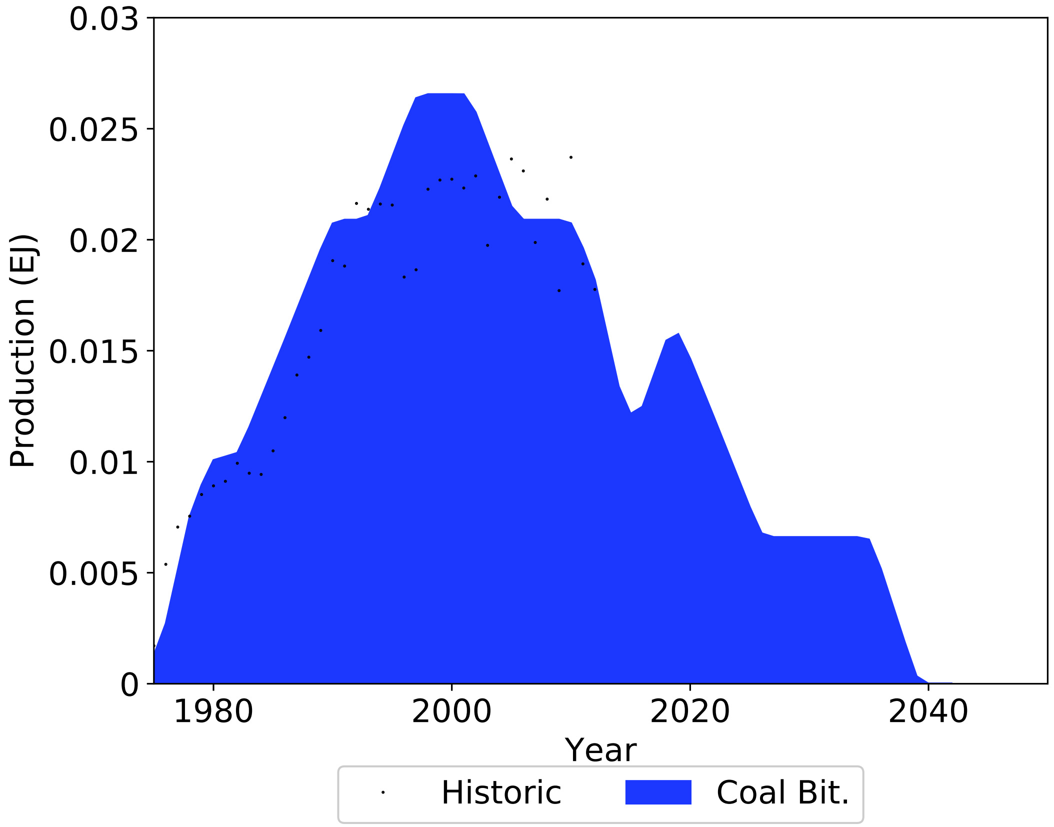

Figure 1.7: Botswana projections capped at 16

Table 1.7: Peak years - All

Name	URR	Peak Year	Peak Rate
Coal Bit.	0.93	1998	0.03
**Total**	**0.93**	**1998**	**0.03**

1.4.2 By Mineral

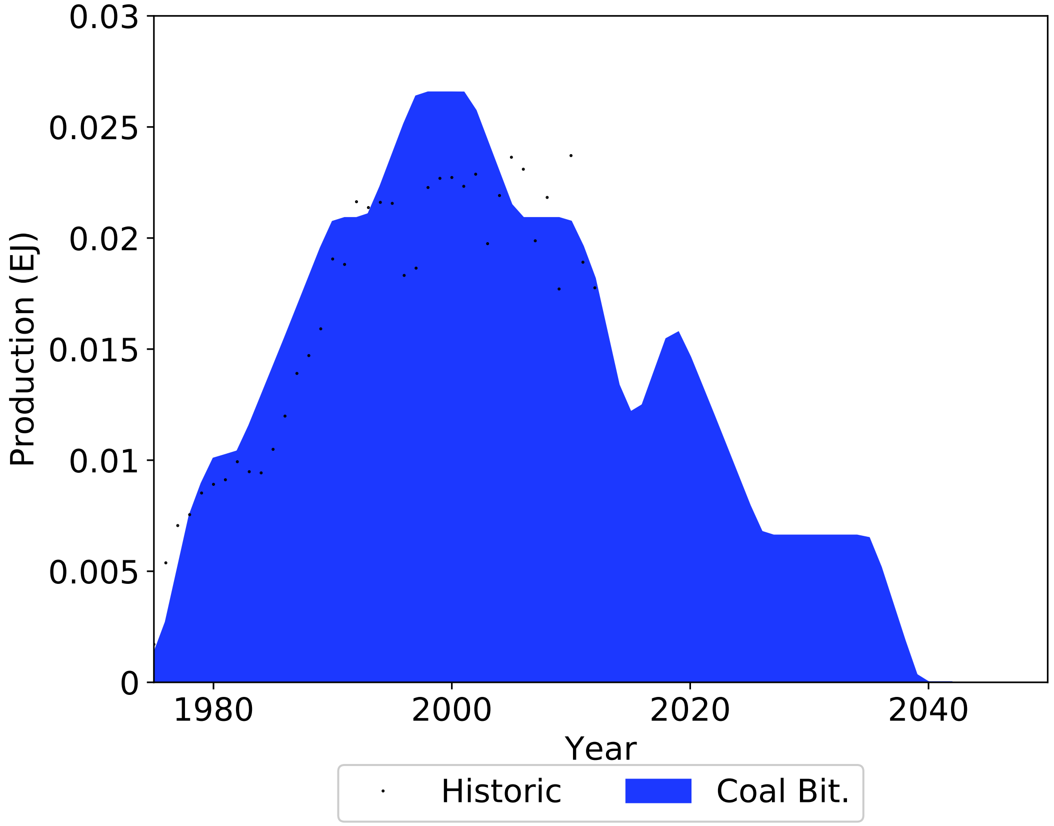

Figure 1.8: Botswana projection by mineral type

Table 1.8: Peak years - Minerals

Name	URR	Peak Year	Peak Rate
Coal Bit.	0.93	1998	0.03
**Total**	**0.93**	**1998**	**0.03**

1.5 Cameroon

1.5.1 All Projections

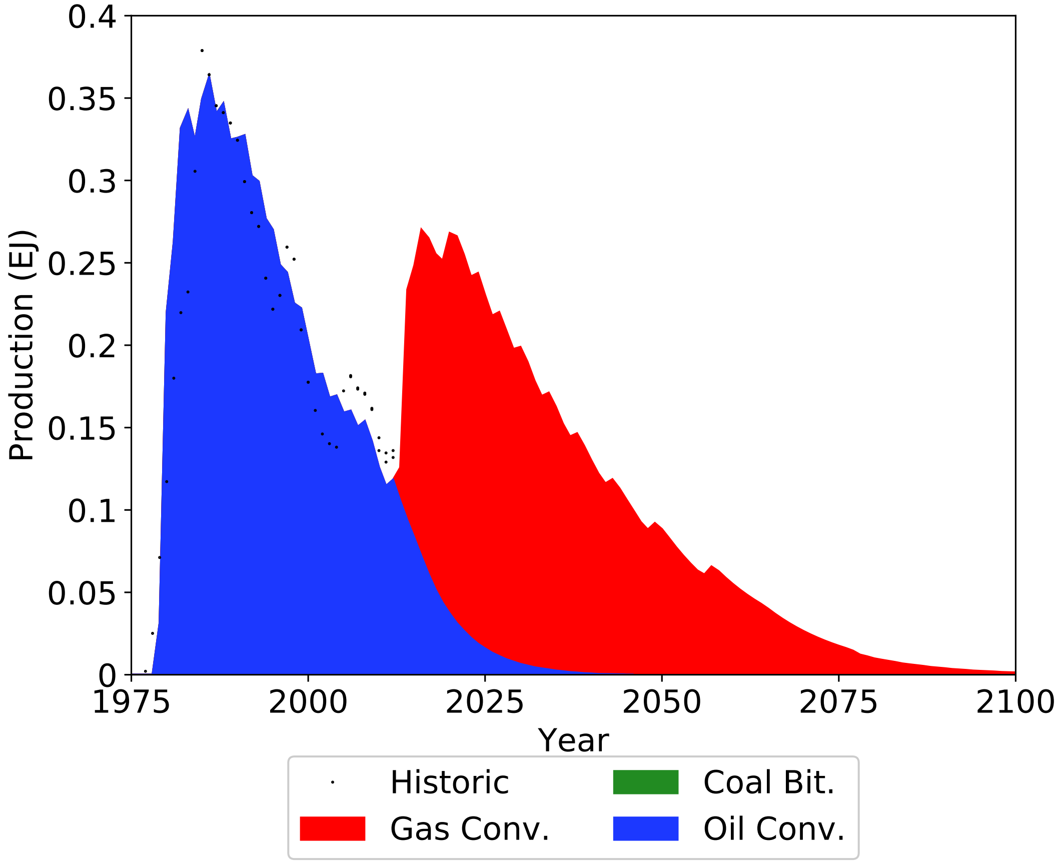

Figure 1.9: Cameroon projections capped at 16

Table 1.9: Peak years - All

Name	URR	Peak Year	Peak Rate
Oil Conv.	8.75	1986	0.36
Gas Conv.	7.4	2021	0.23
Coal Bit.	–	1988	–
**Total**	**16.15**	**1986**	**0.36**

1.5.2 By Mineral

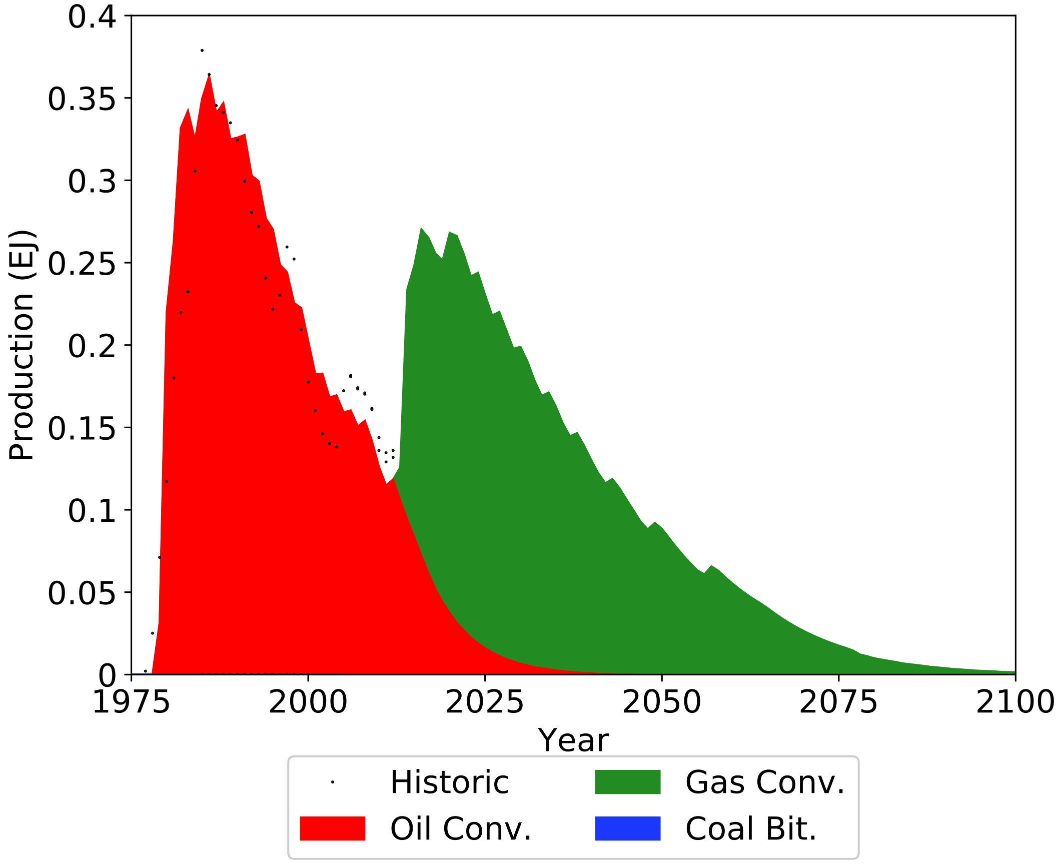

Figure 1.10: Cameroon projection by mineral type

Table 1.10: Peak years - Minerals

Name	URR	Peak Year	Peak Rate
Coal Bit.	–	1988	–
Oil Conv.	8.75	1986	0.36
Gas Conv.	7.4	2021	0.23
**Total**	**16.15**	**1986**	**0.36**

1.6 Chad

1.6.1 All Projections

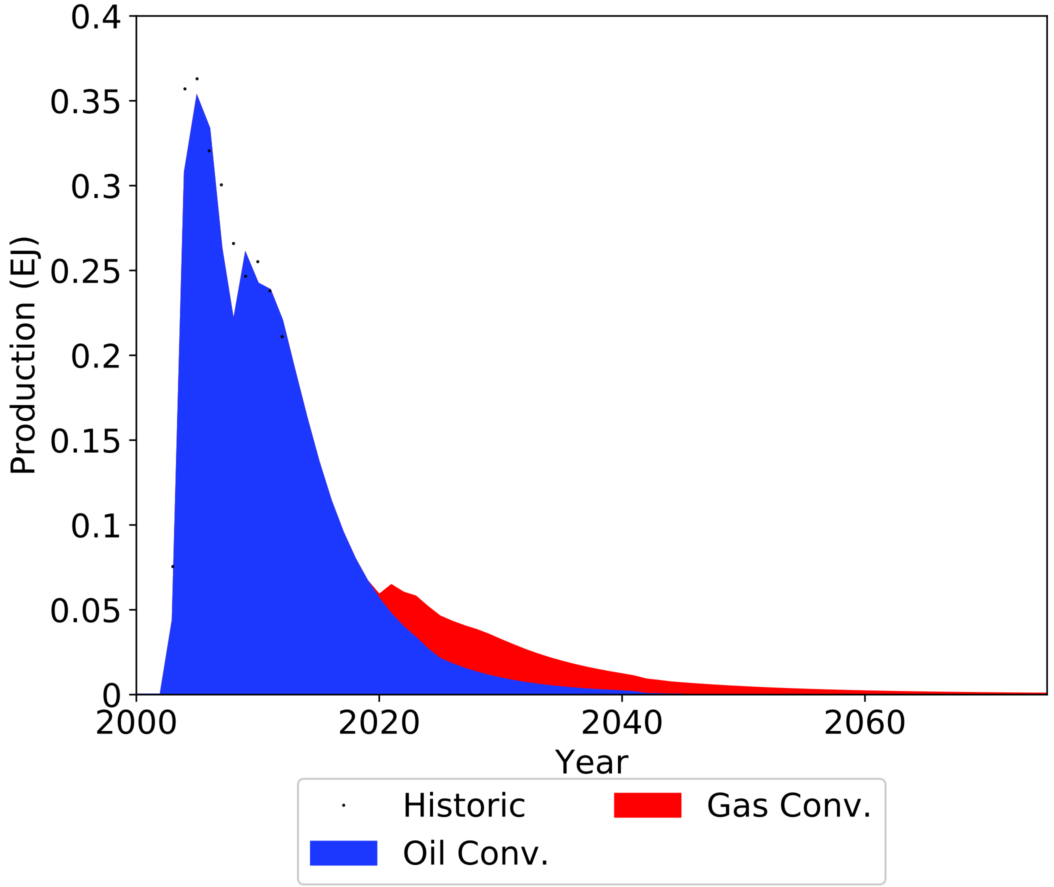

Figure 1.11: Chad projections capped at 16

Table 1.11: Peak years - All

Name	URR	Peak Year	Peak Rate
Oil Conv.	3.67	2005	0.35
Gas Conv.	0.5	2024	0.03
**Total**	**4.17**	**2005**	**0.35**

1.6.2 By Mineral

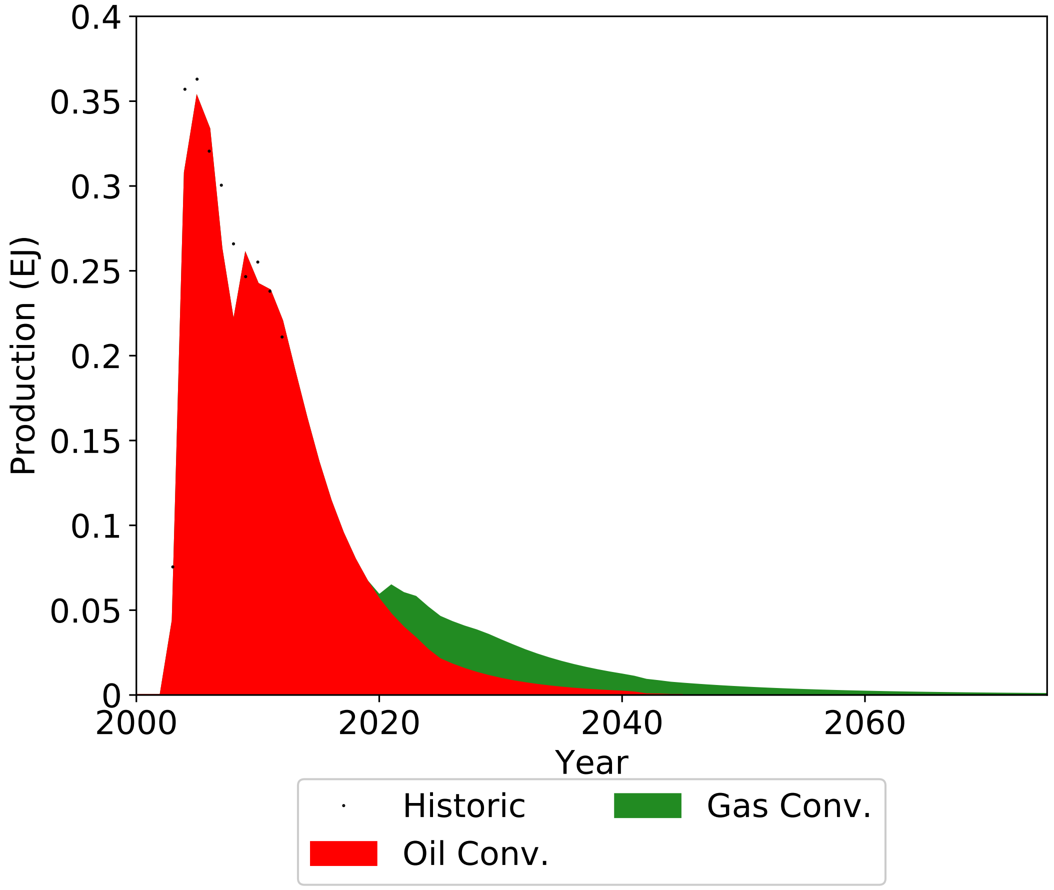

Figure 1.12: Chad projection by mineral type

Table 1.12: Peak years - Minerals

**Name**	**URR**	**Peak Year**	**Peak Rate**
Oil Conv.	3.67	2005	0.35
Gas Conv.	0.5	2024	0.03
**Total**	**4.17**	**2005**	**0.35**

1.7 Congo

1.7.1 All Projections

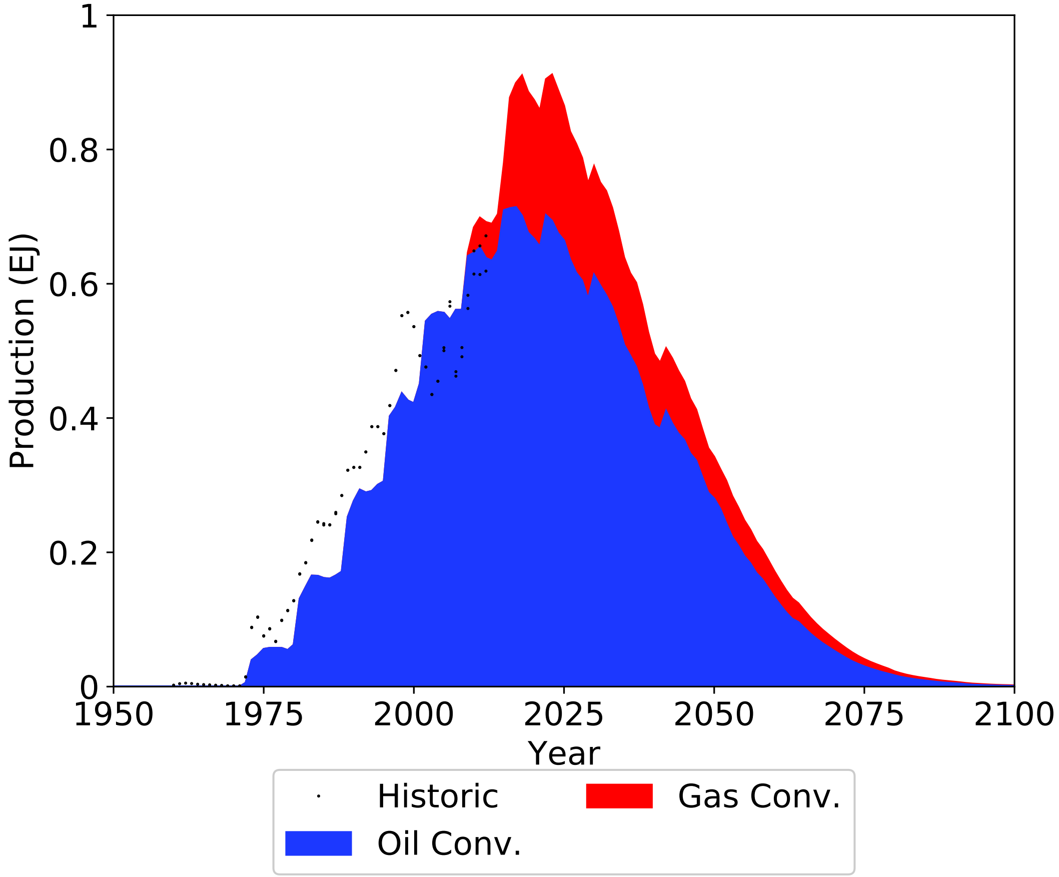

Figure 1.13: Congo projections capped at 16

Table 1.13: Peak years - All

Name	URR	Peak Year	Peak Rate
Oil Conv.	36.34	2017	0.71
Gas Conv.	6.3	2023	0.22
**Total**	**42.64**	**2023**	**0.91**

1.7.2 By Mineral

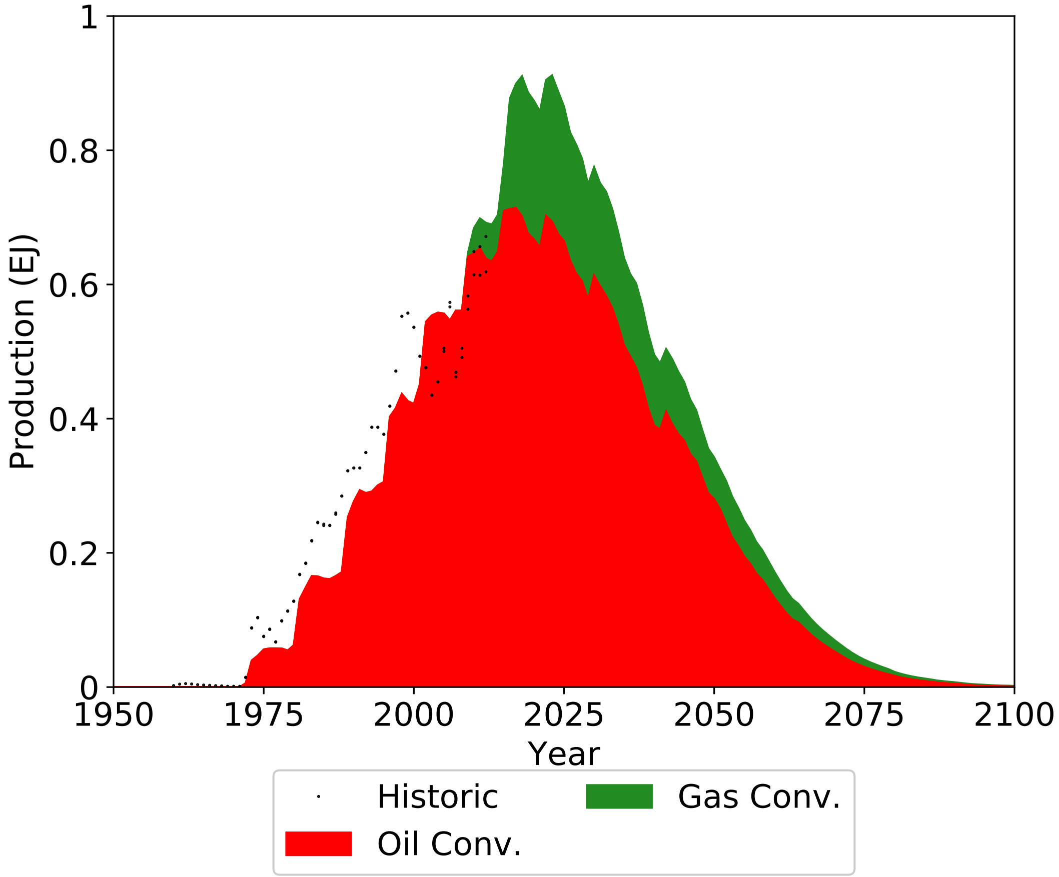

Figure 1.14: Congo projection by mineral type

Table 1.14: Peak years - Minerals

Name	URR	Peak Year	Peak Rate
Oil Conv.	36.34	2017	0.71
Gas Conv.	6.3	2023	0.22
**Total**	**42.64**	**2023**	**0.91**

1.8 Egypt

1.8.1 All Projections

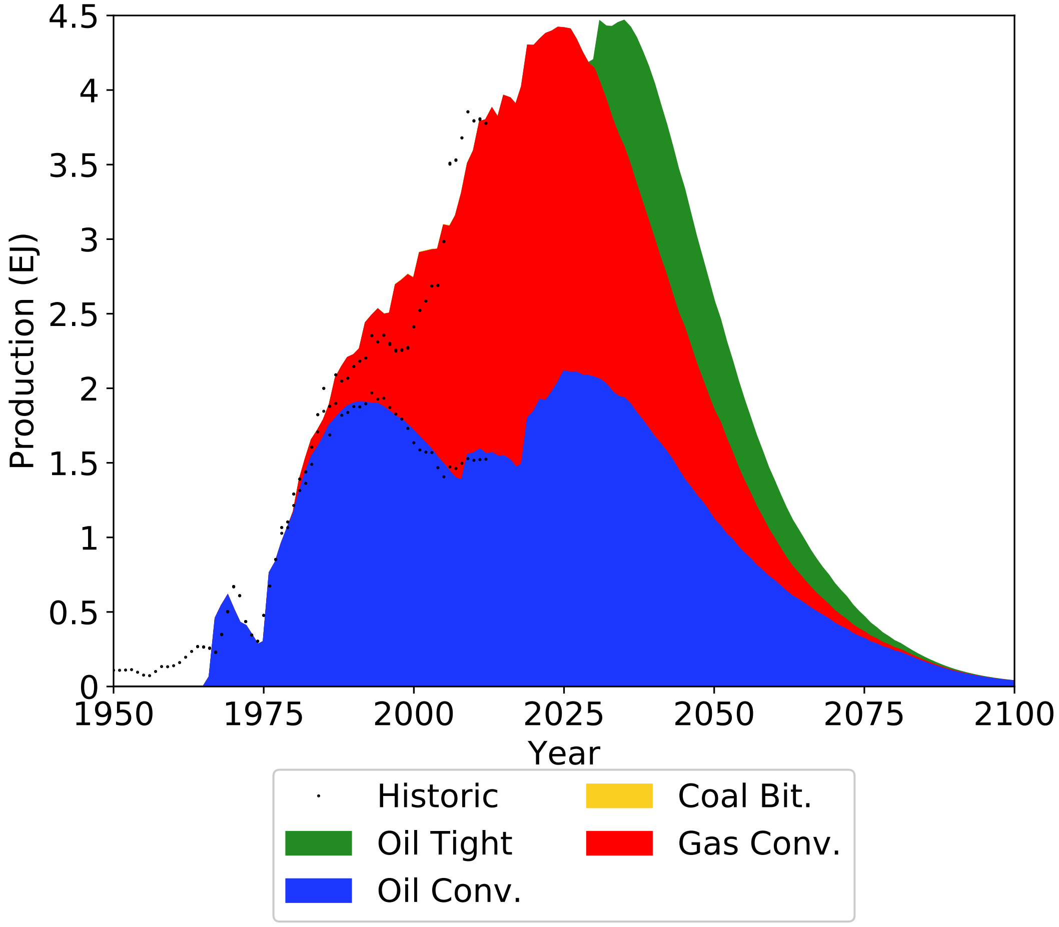

Figure 1.15: Egypt projections capped at 16

Table 1.15: Peak years - All

**Name**	**URR**	**Peak Year**	**Peak Rate**
Oil Conv.	147.78	2025	2.11
Gas Conv.	105.0	2018	2.53
Oil Tight	26.36	2040	1.04
Coal Bit.	0.06	1999	–
**Total**	**279.2**	**2035**	**4.46**

1.8.2 By Mineral

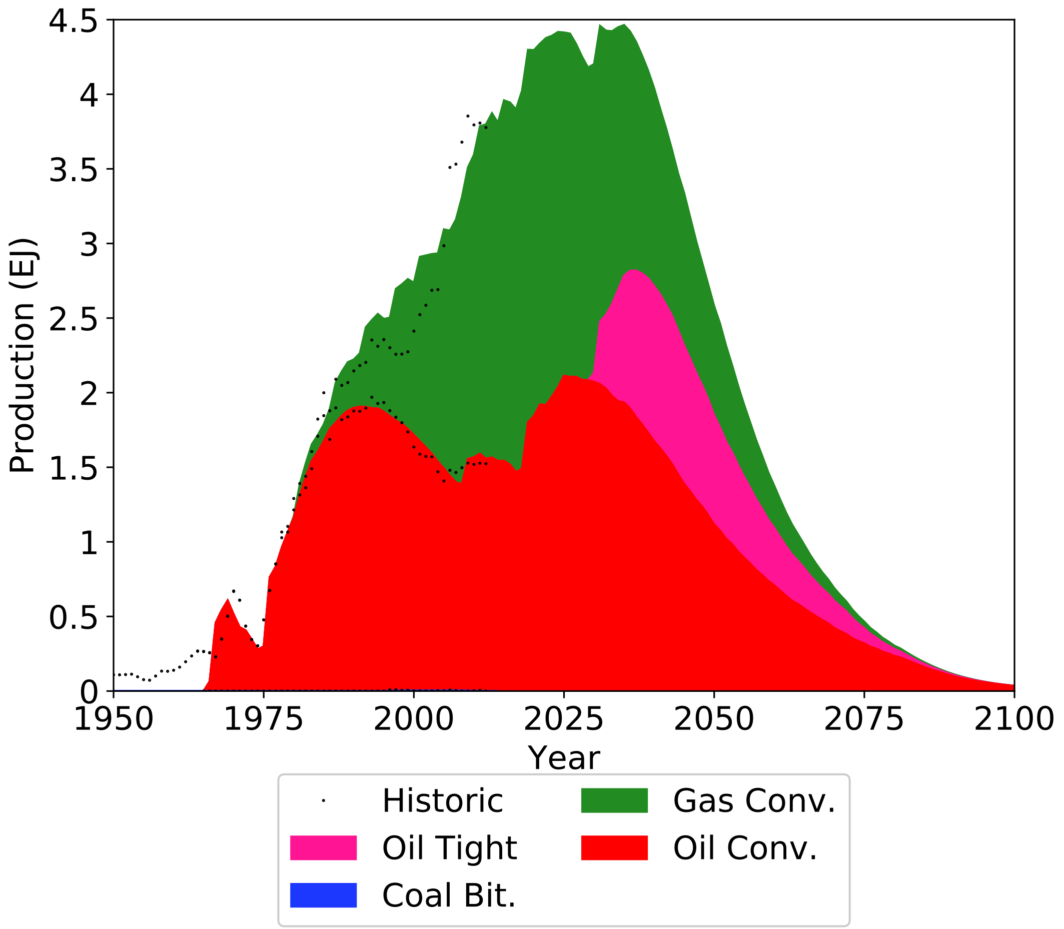

Figure 1.16: Egypt projection by mineral type

Table 1.16: Peak years - Minerals

**Name**	**URR**	**Peak Year**	**Peak Rate**
Coal Bit.	0.06	1999	–
Oil Conv.	147.78	2025	2.11
Oil Tight	26.36	2040	1.04
Gas Conv.	105.0	2018	2.53
**Total**	**279.2**	**2035**	**4.46**

1.9 Equatorial Guinea

1.9.1 All Projections

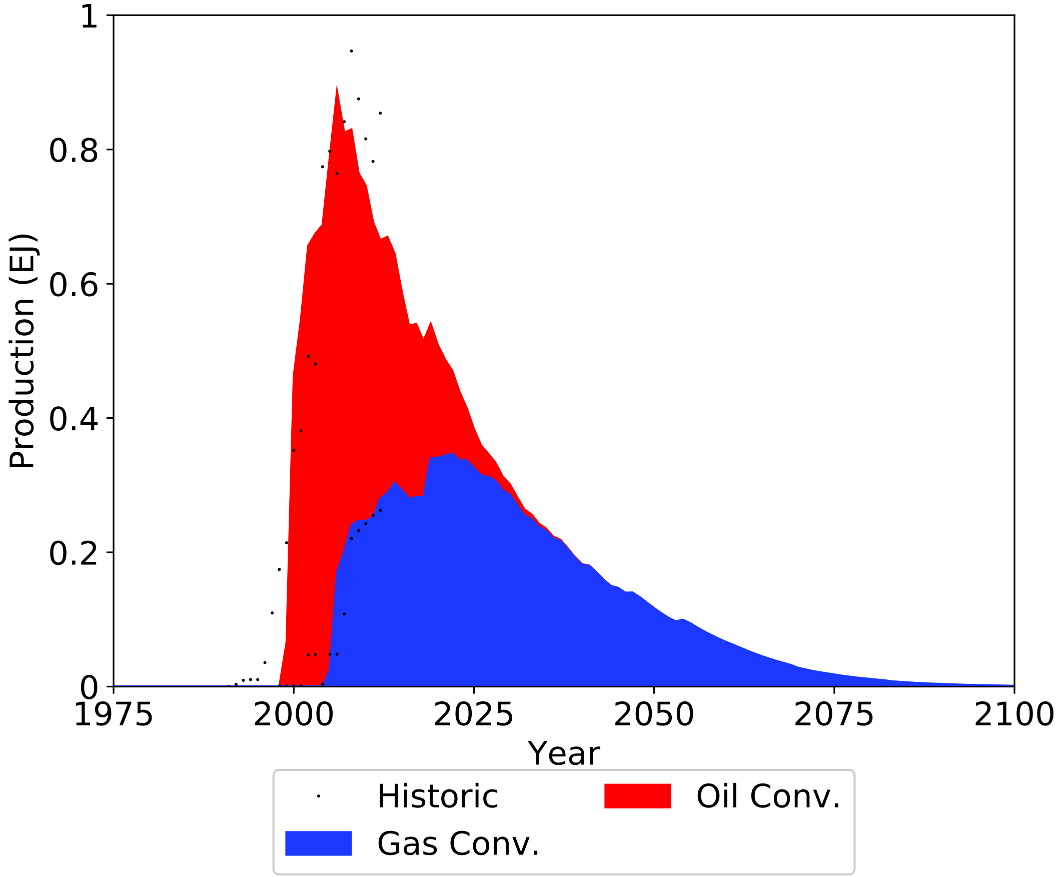

Figure 1.17: Equatorial Guinea projections capped at 16

Table 1.17: Peak years - All

**Name**	**URR**	**Peak Year**	**Peak Rate**
Gas Conv.	12.6	2022	0.35
Oil Conv.	10.46	2005	0.76
**Total**	**23.06**	**2006**	**0.89**

1.9.2 By Mineral

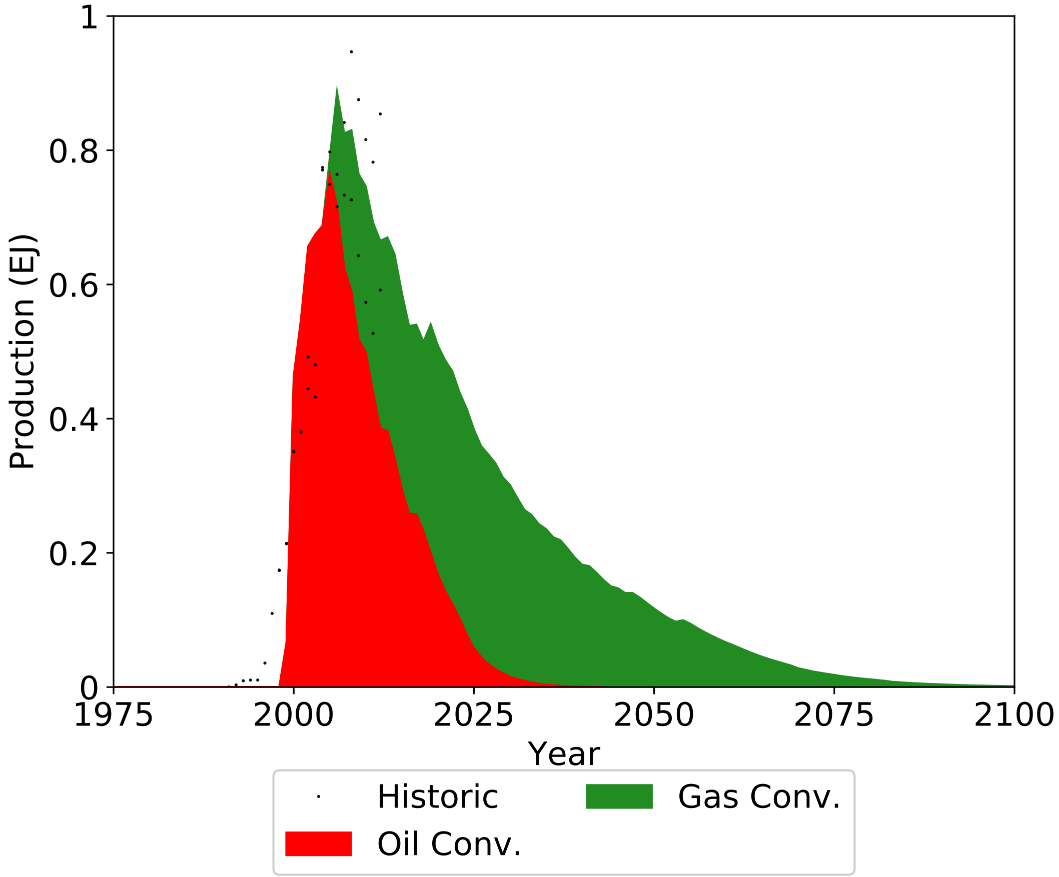

Figure 1.18: Equatorial Guinea projection by mineral type

Table 1.18: Peak years - Minerals

Name	URR	Peak Year	Peak Rate
Oil Conv.	10.46	2005	0.76
Gas Conv.	12.6	2022	0.35
**Total**	**23.06**	**2006**	**0.89**

1.10 Eritrea

1.10.1 All Projections

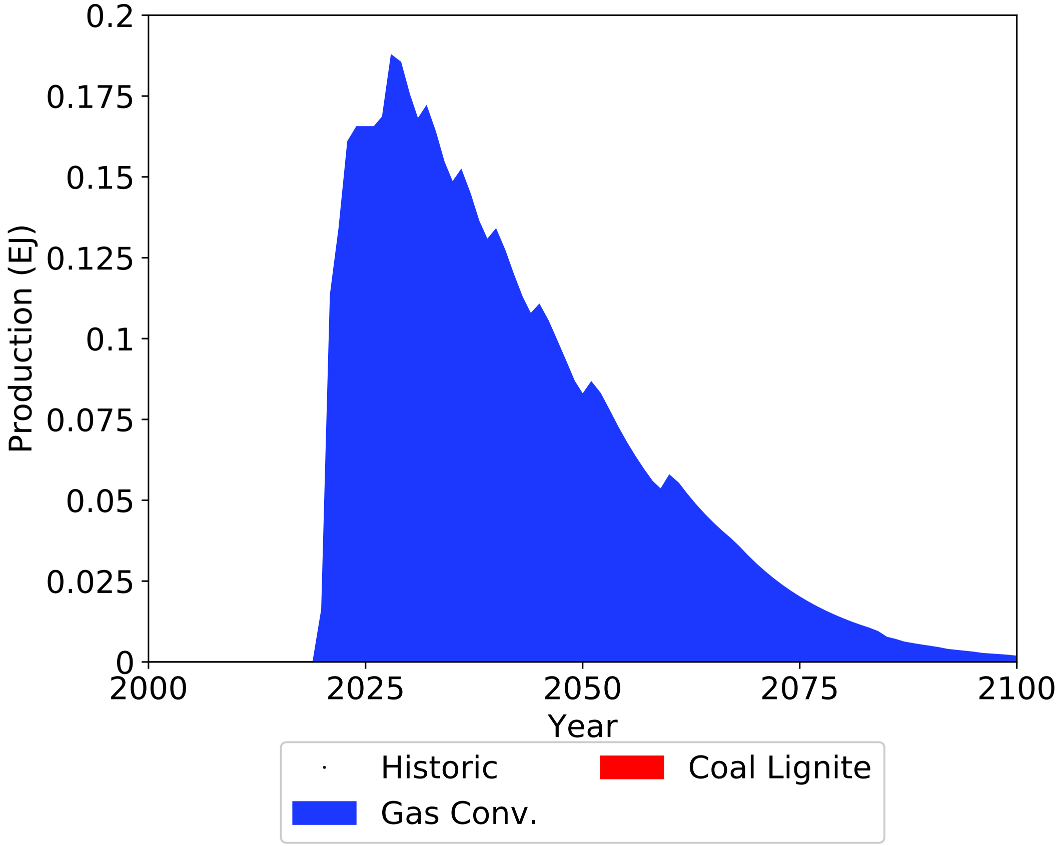

Figure 1.19: Eritrea projections capped at 16

Table 1.19: Peak years - All

Name	URR	Peak Year	Peak Rate
Gas Conv.	5.6	2028	0.19
Coal Lignite	–	1936	–
**Total**	**5.6**	**2028**	**0.19**

1.10.2 By Mineral

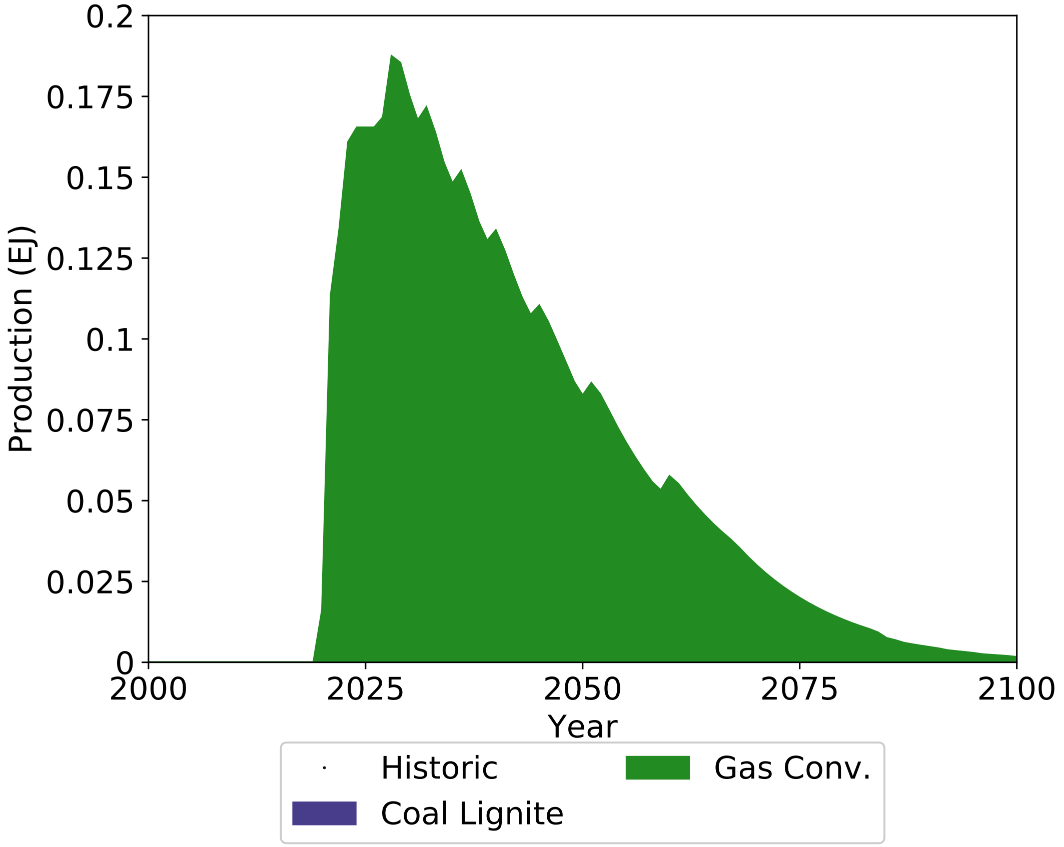

Figure 1.20: Eritrea projection by mineral type

Table 1.20: Peak years - Minerals

Name	URR	Peak Year	Peak Rate
Coal Lignite	–	1936	–
Gas Conv.	5.6	2028	0.19
**Total**	**5.6**	**2028**	**0.19**

1.11 Ethiopia

1.11.1 All Projections

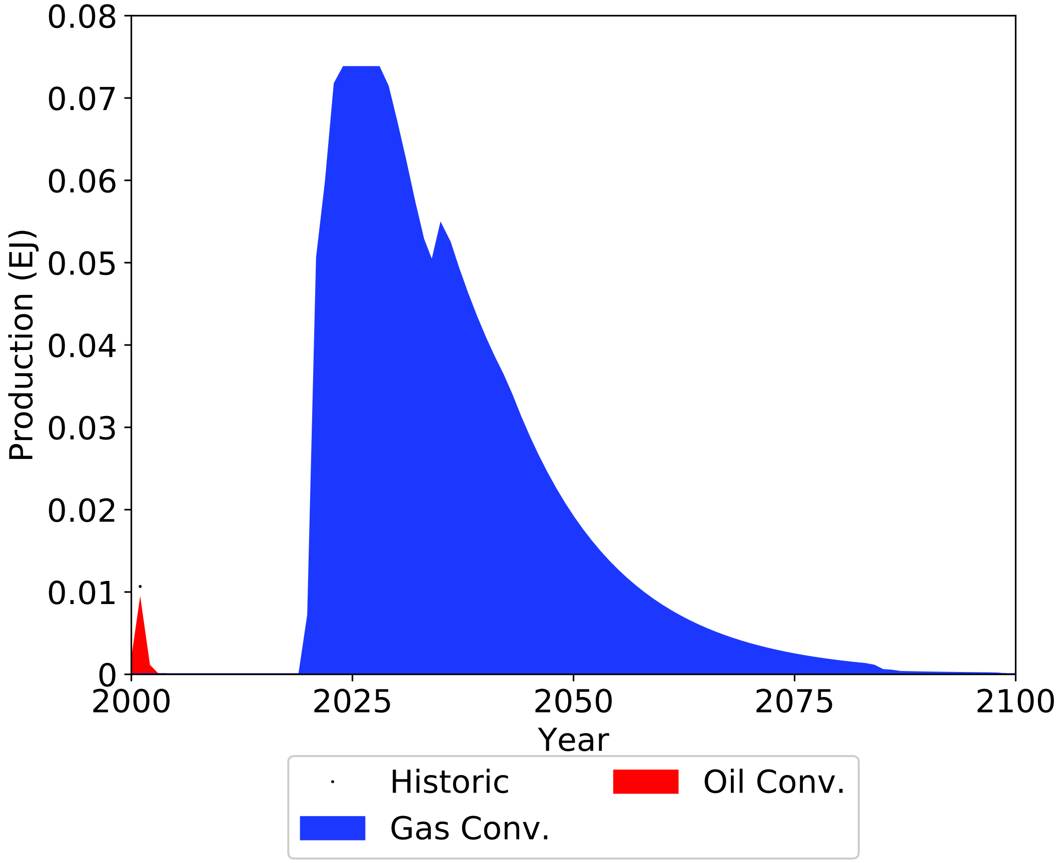

Figure 1.21: Ethiopia projections capped at 16

Table 1.21: Peak years - All

Name	URR	Peak Year	Peak Rate
Gas Conv.	1.7	2024	0.07
Oil Conv.	0.01	2001	0.01
**Total**	**1.71**	**2024**	**0.07**

1.11.2 By Mineral

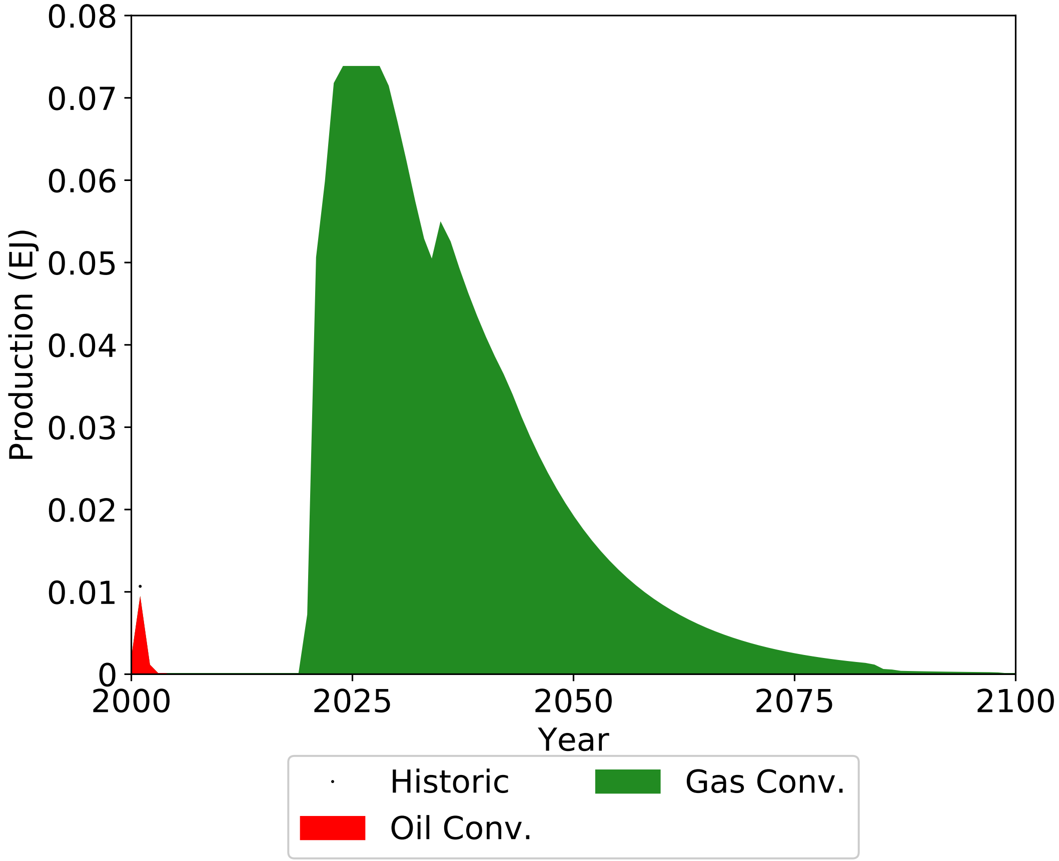

Figure 1.22: Ethiopia projection by mineral type

Table 1.22: Peak years - Minerals

Name	URR	Peak Year	Peak Rate
Oil Conv.	0.01	2001	0.01
Gas Conv.	1.7	2024	0.07
**Total**	**1.71**	**2024**	**0.07**

1.12 Gabon

1.12.1 All Projections

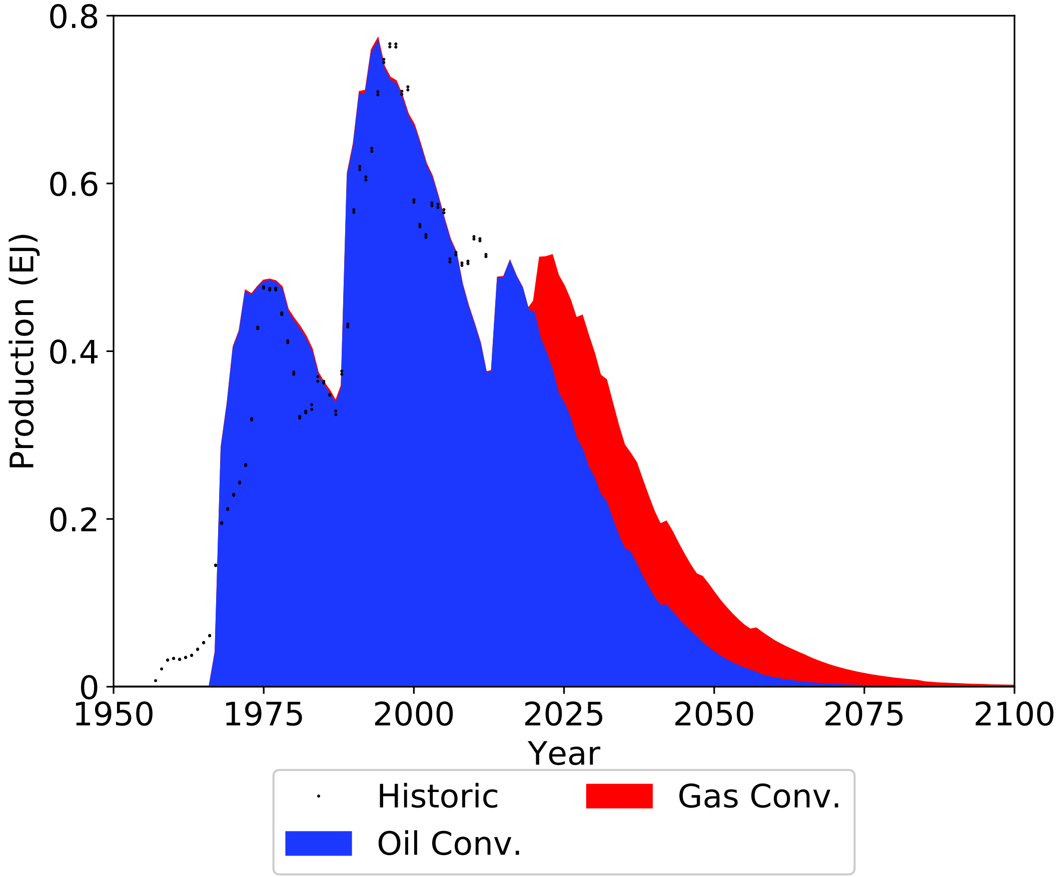

Figure 1.23: Gabon projections capped at 16

Table 1.23: Peak years - All

Name	URR	Peak Year	Peak Rate
Oil Conv.	32.95	1994	0.77
Gas Conv.	4.7	2028	0.16
**Total**	**37.65**	**1994**	**0.77**

1.12.2 By Mineral

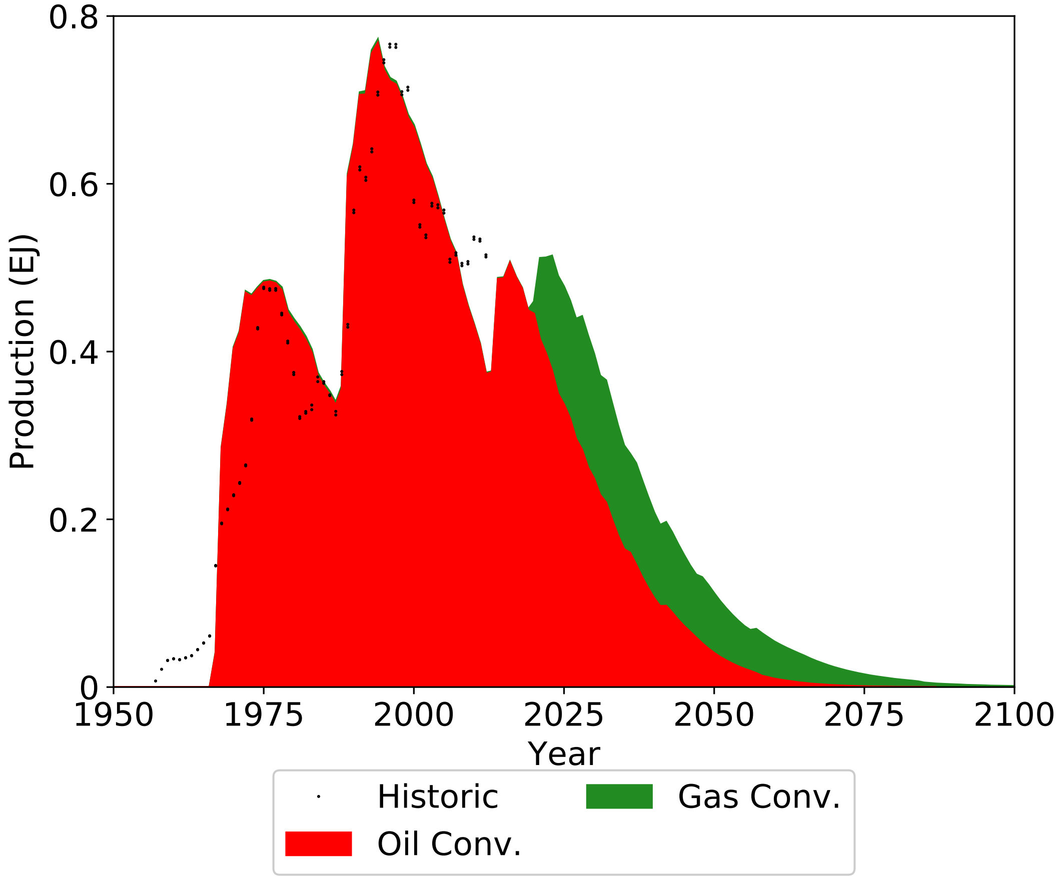

Figure 1.24: Gabon projection by mineral type

Table 1.24: Peak years - Minerals

Name	URR	Peak Year	Peak Rate
Oil Conv.	32.95	1994	0.77
Gas Conv.	4.7	2028	0.16
**Total**	**37.65**	**1994**	**0.77**

1.13 Ghana

1.13.1 All Projections

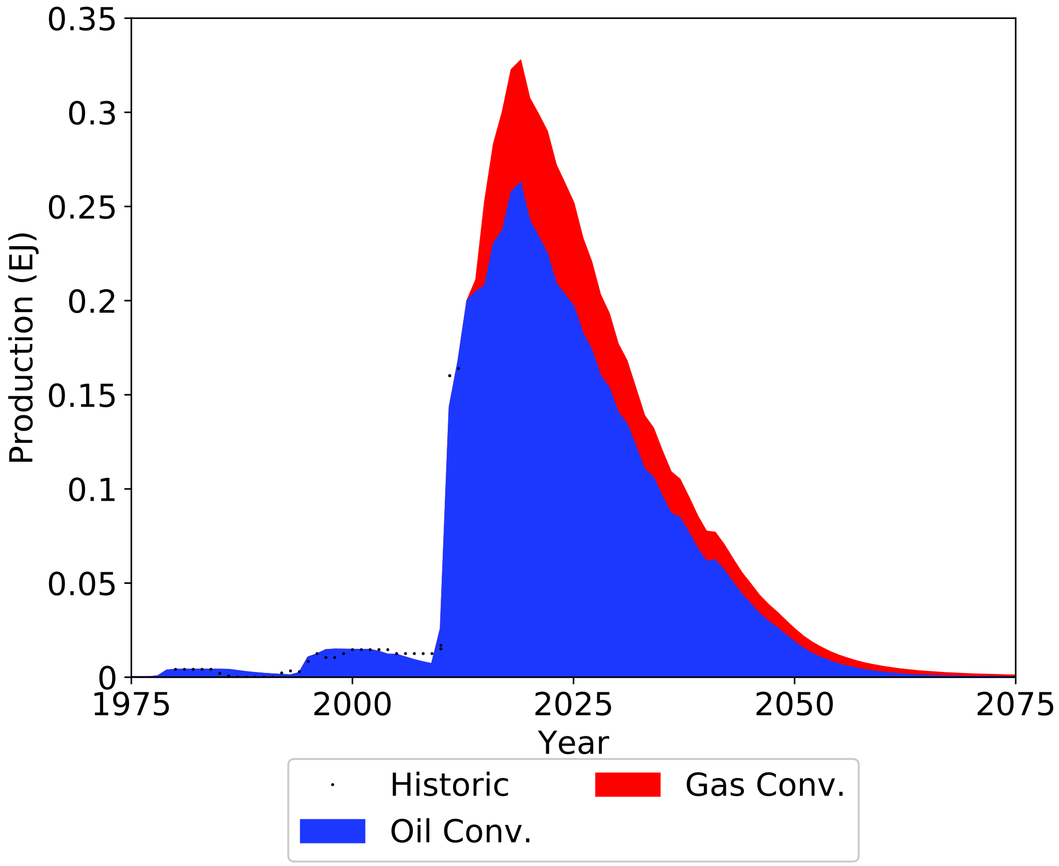

Figure 1.25: Ghana projections capped at 16

Table 1.25: Peak years - All

Name	URR	Peak Year	Peak Rate
Oil Conv.	5.7	2019	0.26
Gas Conv.	1.3	2018	0.07
**Total**	**7.0**	**2019**	**0.33**

1.13.2 By Mineral

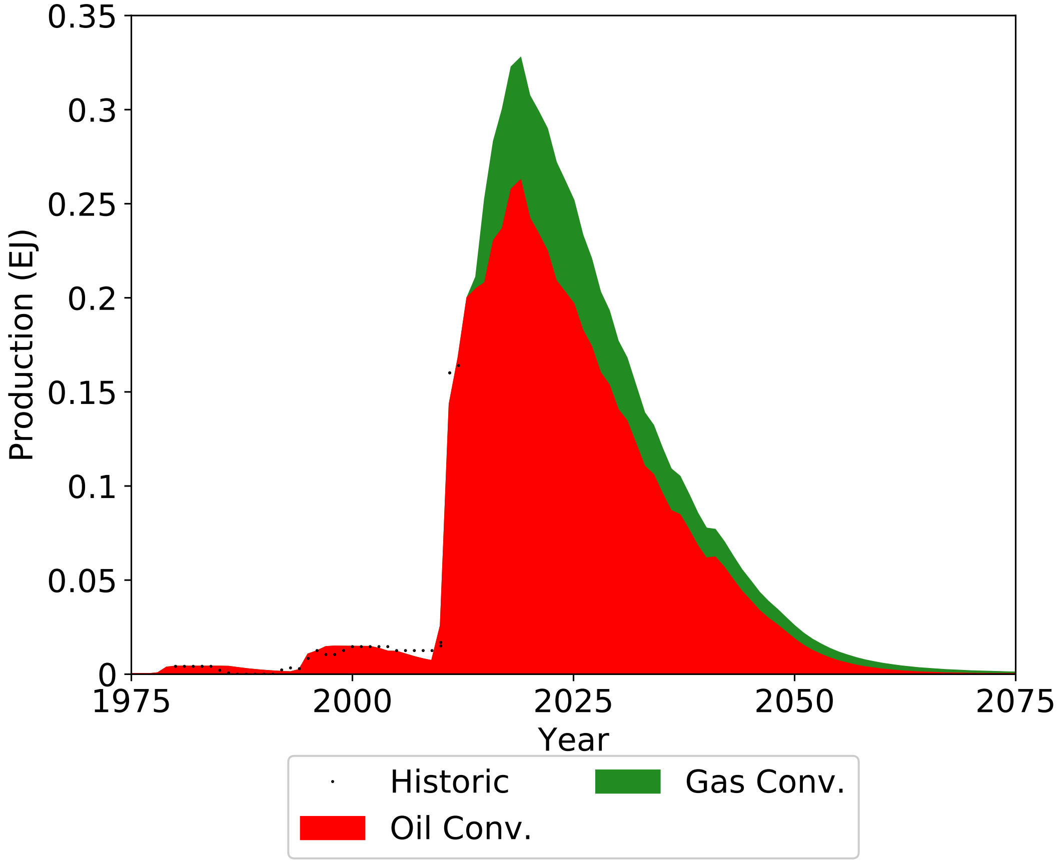

Figure 1.26: Ghana projection by mineral type

Table 1.26: Peak years - Minerals

Name	URR	Peak Year	Peak Rate
Oil Conv.	5.7	2019	0.26
Gas Conv.	1.3	2018	0.07
**Total**	**7.0**	**2019**	**0.33**

1.14 Guinea-Bissau

1.14.1 All Projections

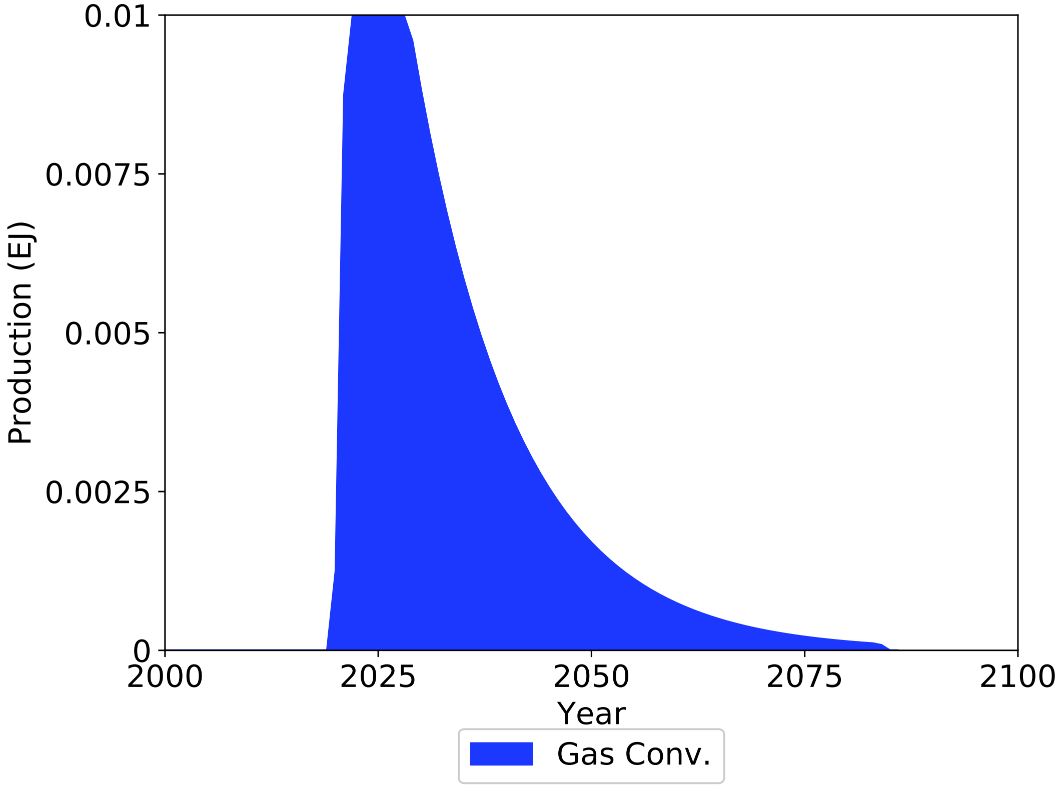

Figure 1.27: Guinea-Bissau projections capped at 16

Table 1.27: Peak years - All

Name	URR	Peak Year	Peak Rate
Gas Conv.	0.2	2022	0.01
**Total**	**0.2**	**2022**	**0.01**

1.14.2 By Mineral

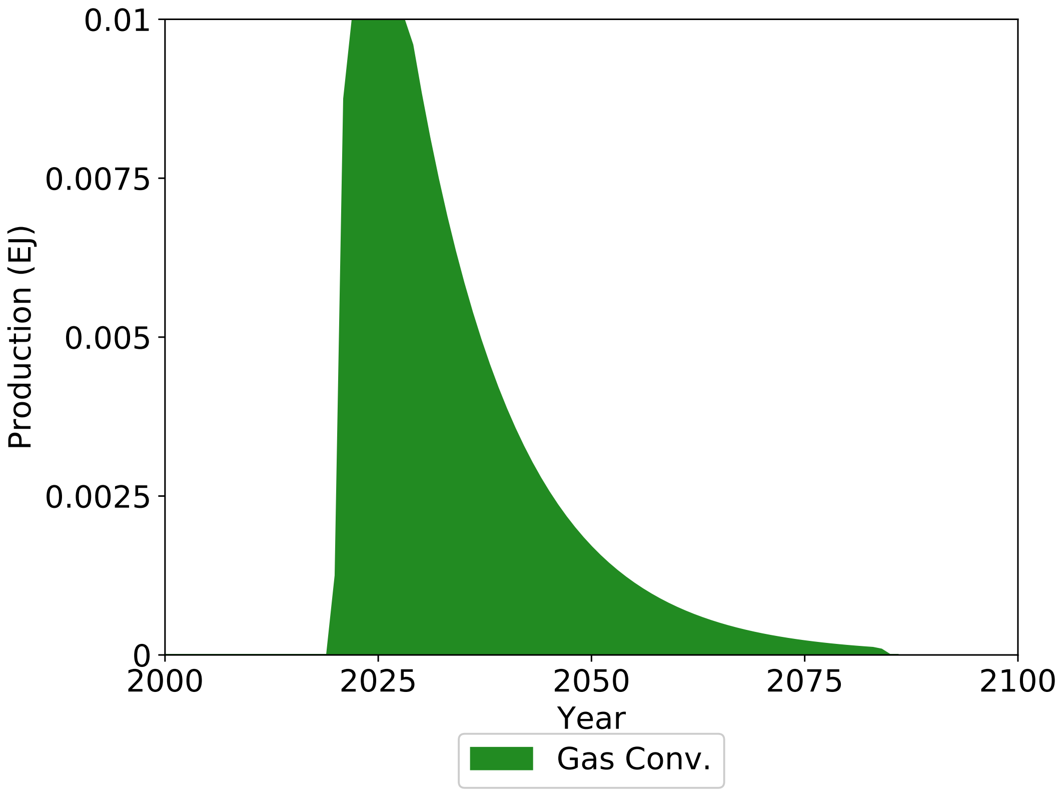

Figure 1.28: Guinea-Bissau projection by mineral type

Table 1.28: Peak years - Minerals

**Name**	**URR**	**Peak Year**	**Peak Rate**
Gas Conv.	0.2	2022	0.01
**Total**	**0.2**	**2022**	**0.01**

1.15 Ivory Coast

1.15.1 All Projections

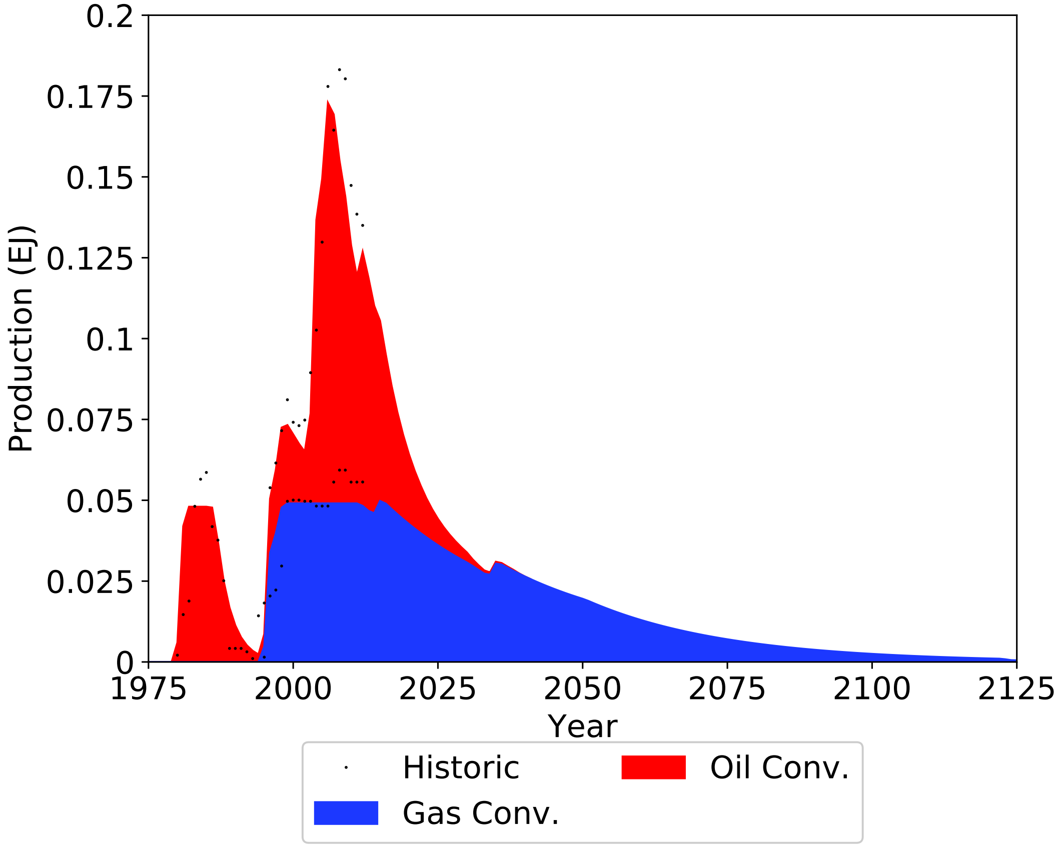

Figure 1.29: Ivory Coast projections capped at 16

Table 1.29: Peak years - All

Name	URR	Peak Year	Peak Rate
Gas Conv.	2.5	2015	0.05
Oil Conv.	1.88	2006	0.12
**Total**	**4.38**	**2006**	**0.17**

1.15.2 By Mineral

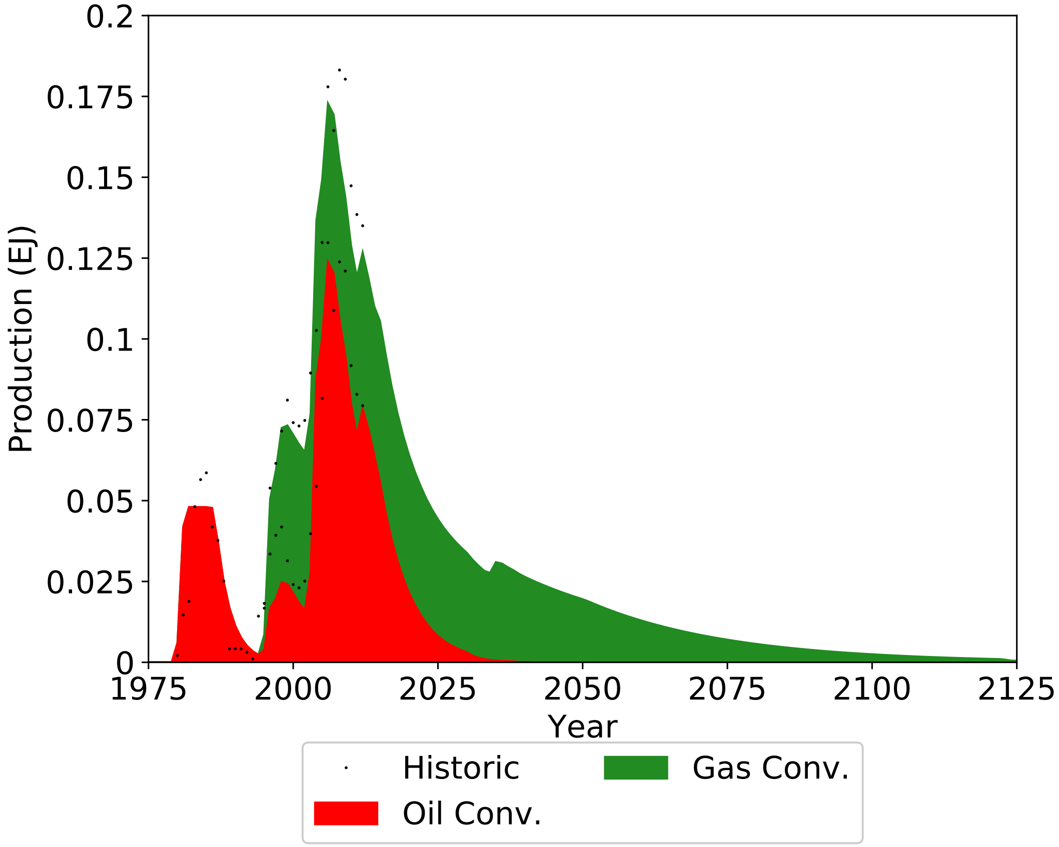

Figure 1.30: Ivory Coast projection by mineral type

Table 1.30: Peak years - Minerals

Name	URR	Peak Year	Peak Rate
Oil Conv.	1.88	2006	0.12
Gas Conv.	2.5	2015	0.05
**Total**	**4.38**	**2006**	**0.17**

1.16 Libya

1.16.1 All Projections

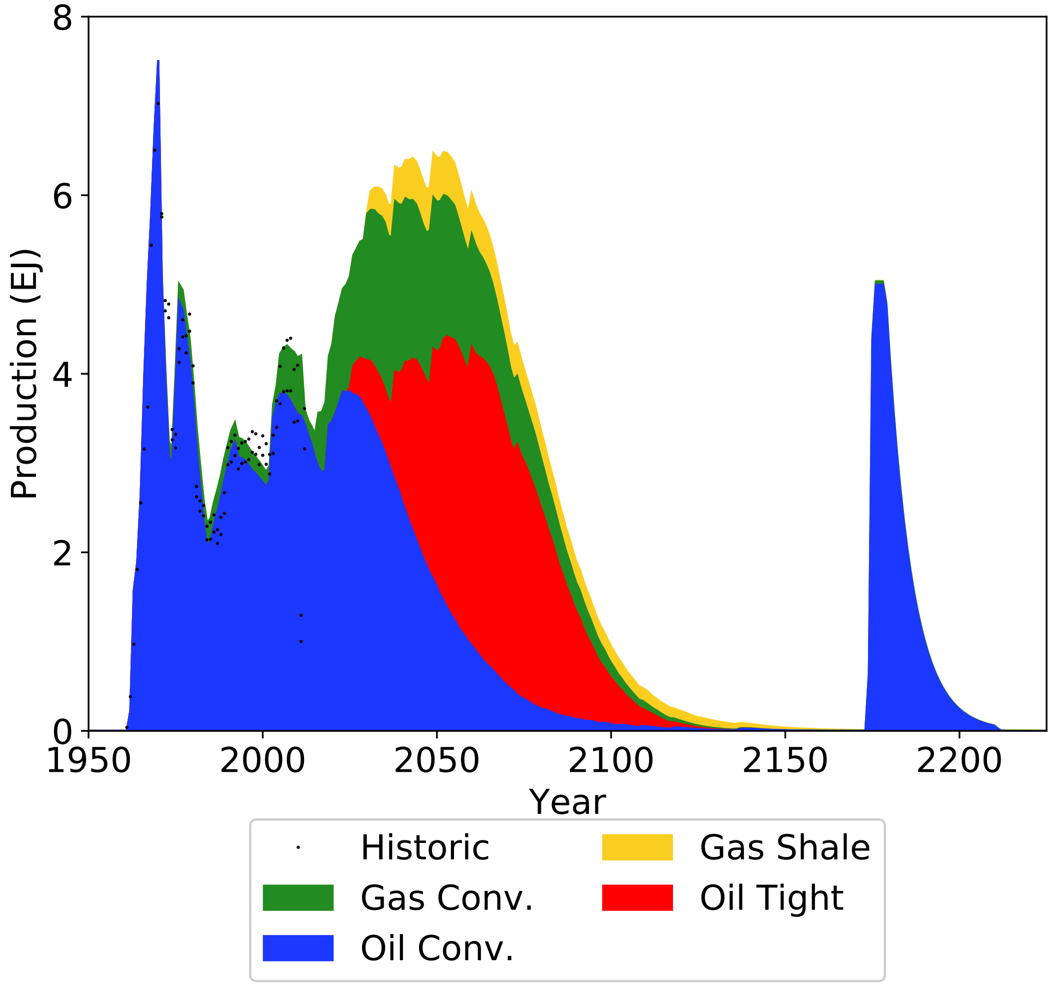

Figure 1.31: Libya projections capped at 16

Table 1.31: Peak years - All

**Name**	**URR**	**Peak Year**	**Peak Rate**
Oil Conv.	368.66	1970	7.51
Oil Tight	149.55	2064	3.37
Gas Conv.	105.0	2038	1.91
Gas Shale	30.45	2050	0.49
**Total**	**653.66**	**1970**	**7.51**

1.16.2 By Mineral

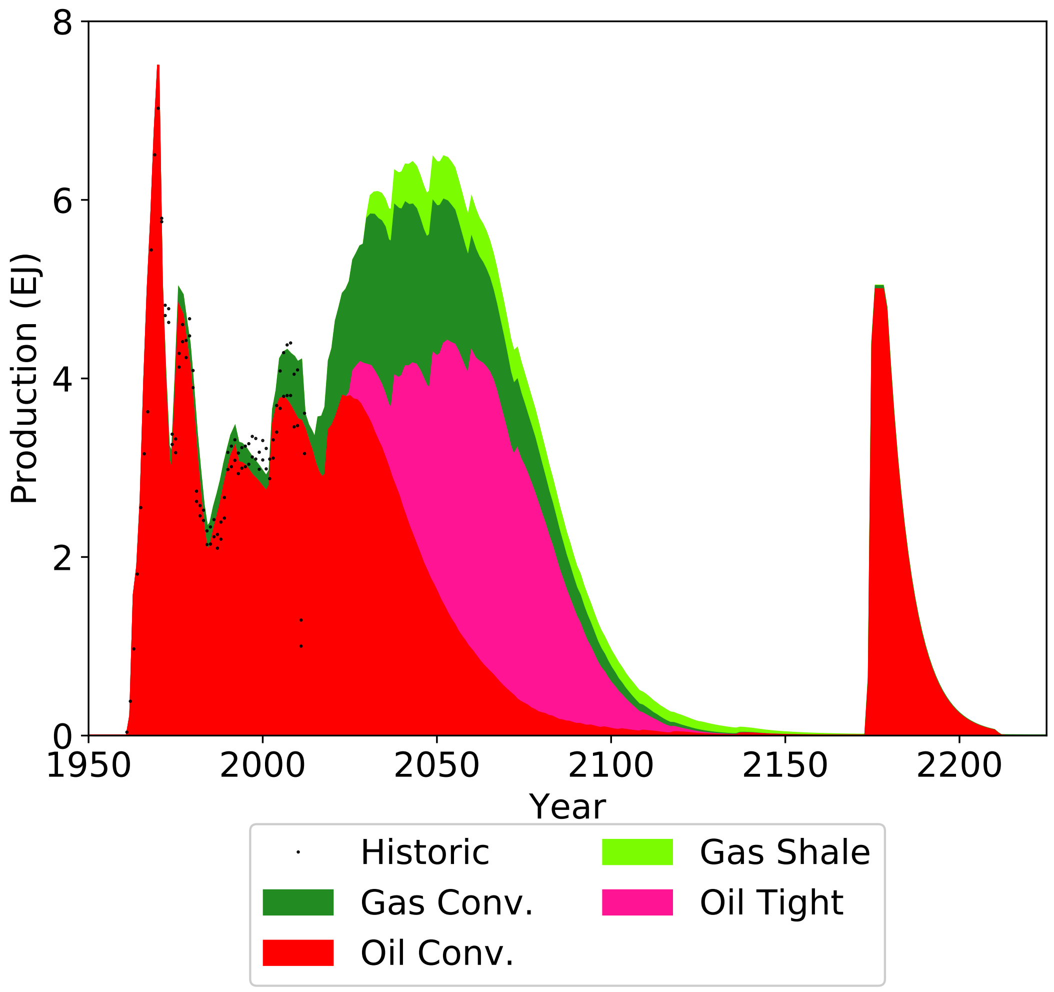

Figure 1.32: Libya projection by mineral type

Table 1.32: Peak years - Minerals

**Name**	**URR**	**Peak Year**	**Peak Rate**
Oil Conv.	368.66	1970	7.51
Oil Tight	149.55	2064	3.37
Gas Conv.	105.0	2038	1.91
Gas Shale	30.45	2050	0.49
**Total**	**653.66**	**1970**	**7.51**

1.17 Madagascar

1.17.1 All Projections

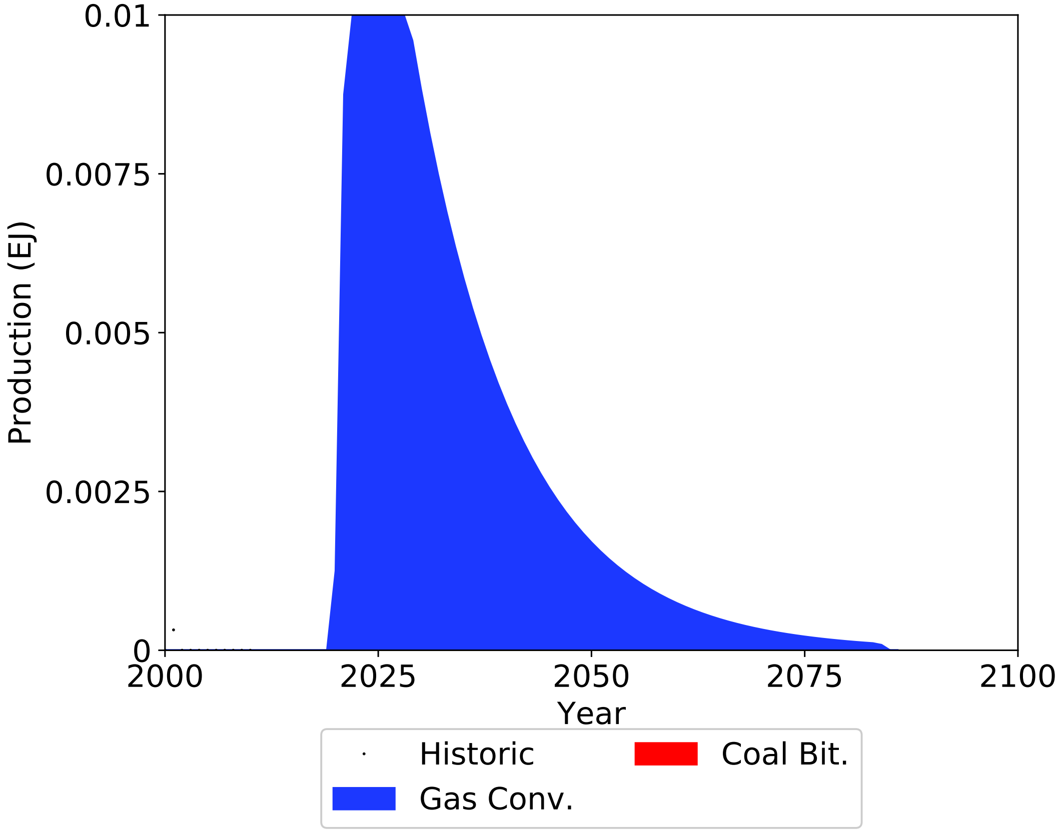

Figure 1.33: Madagascar projections capped at 16

Table 1.33: Peak years - All

Name	URR	Peak Year	Peak Rate
Gas Conv.	0.2	2022	0.01
Coal Bit.	–	1945	–
**Total**	**0.2**	**2022**	**0.01**

1.17.2 By Mineral

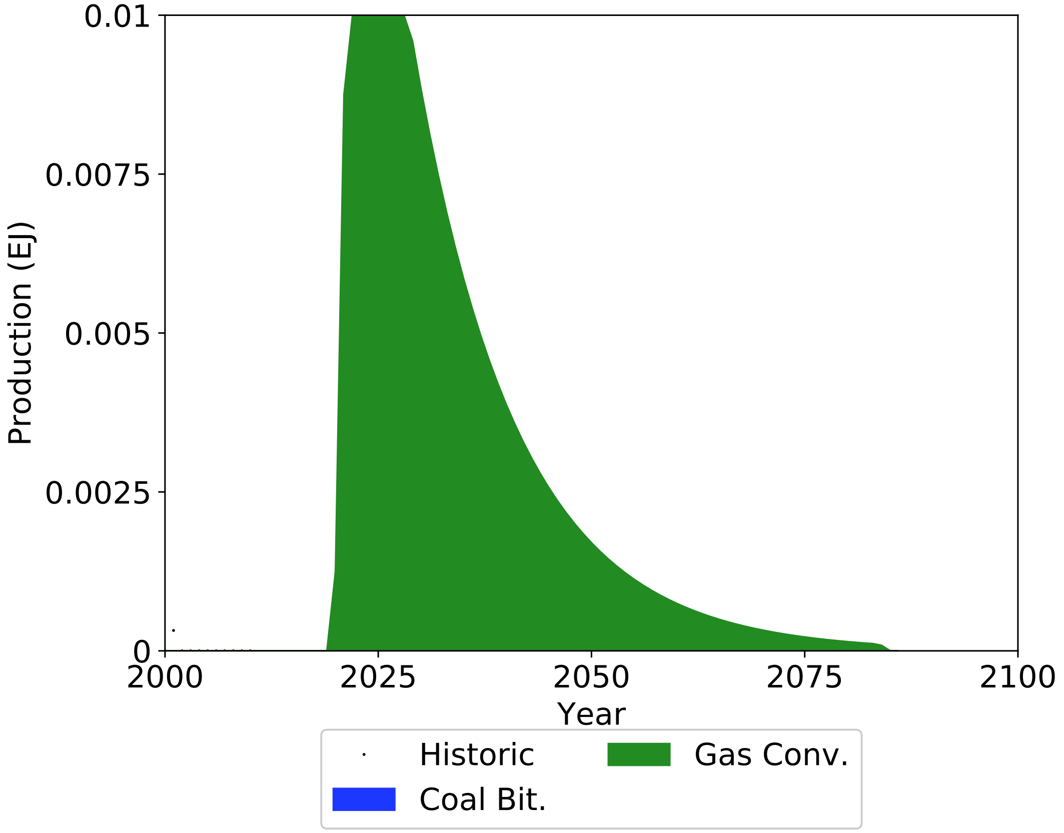

Figure 1.34: Madagascar projection by mineral type

Table 1.34: Peak years - Minerals

Name	URR	Peak Year	Peak Rate
Coal Bit.	–	1945	–
Gas Conv.	0.2	2022	0.01
**Total**	**0.2**	**2022**	**0.01**

1.18 Malawi

1.18.1 All Projections

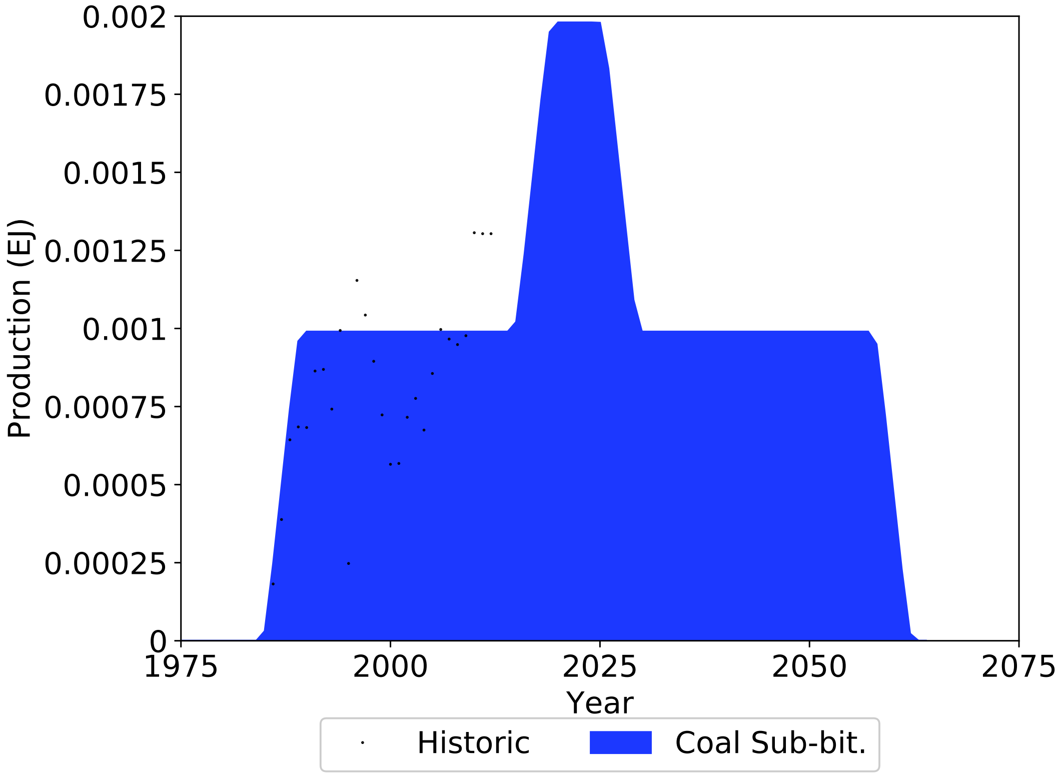

Figure 1.35: Malawi projections capped at 16

Table 1.35: Peak years - All

Name	URR	Peak Year	Peak Rate
Coal Sub-bit.	0.08	2020	–
**Total**	**0.08**	**2020**	–

1.18.2 By Mineral

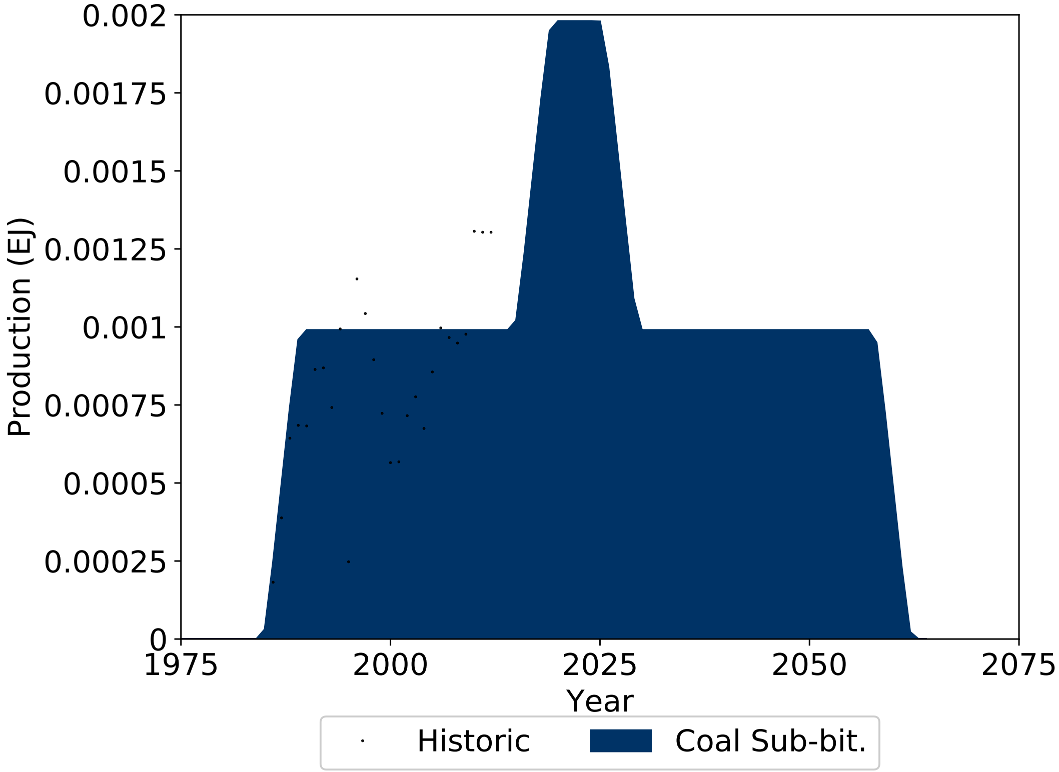

Figure 1.36: Malawi projection by mineral type

Table 1.36: Peak years - Minerals

Name	URR	Peak Year	Peak Rate
Coal Sub-bit.	0.08	2020	–
**Total**	**0.08**	**2020**	–

1.19 Mauritania

1.19.1 All Projections

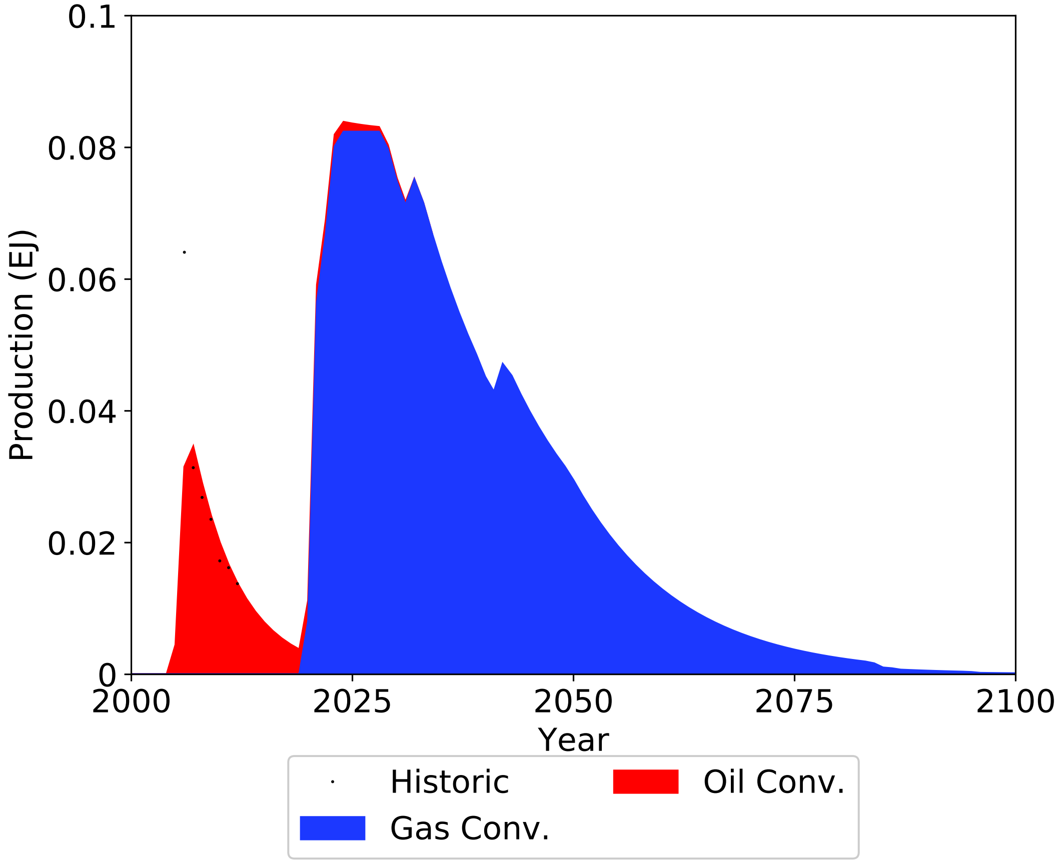

Figure 1.37: Mauritania projections capped at 16

Table 1.37: Peak years - All

Name	URR	Peak Year	Peak Rate
Gas Conv.	2.1	2024	0.08
Oil Conv.	0.24	2007	0.03
**Total**	**2.34**	**2024**	**0.08**

1.19.2 By Mineral

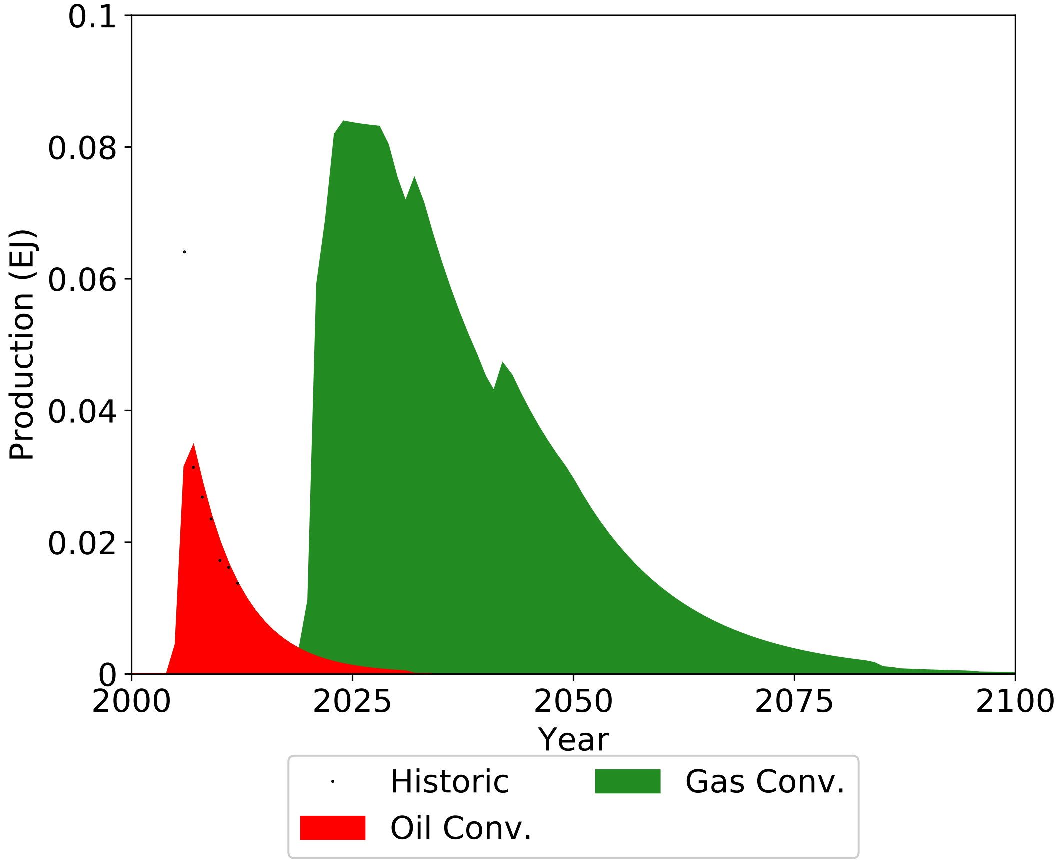

Figure 1.38: Mauritania projection by mineral type

Table 1.38: Peak years - Minerals

Name	URR	Peak Year	Peak Rate
Oil Conv.	0.24	2007	0.03
Gas Conv.	2.1	2024	0.08
**Total**	**2.34**	**2024**	**0.08**

1.20 Morocco

1.20.1 All Projections

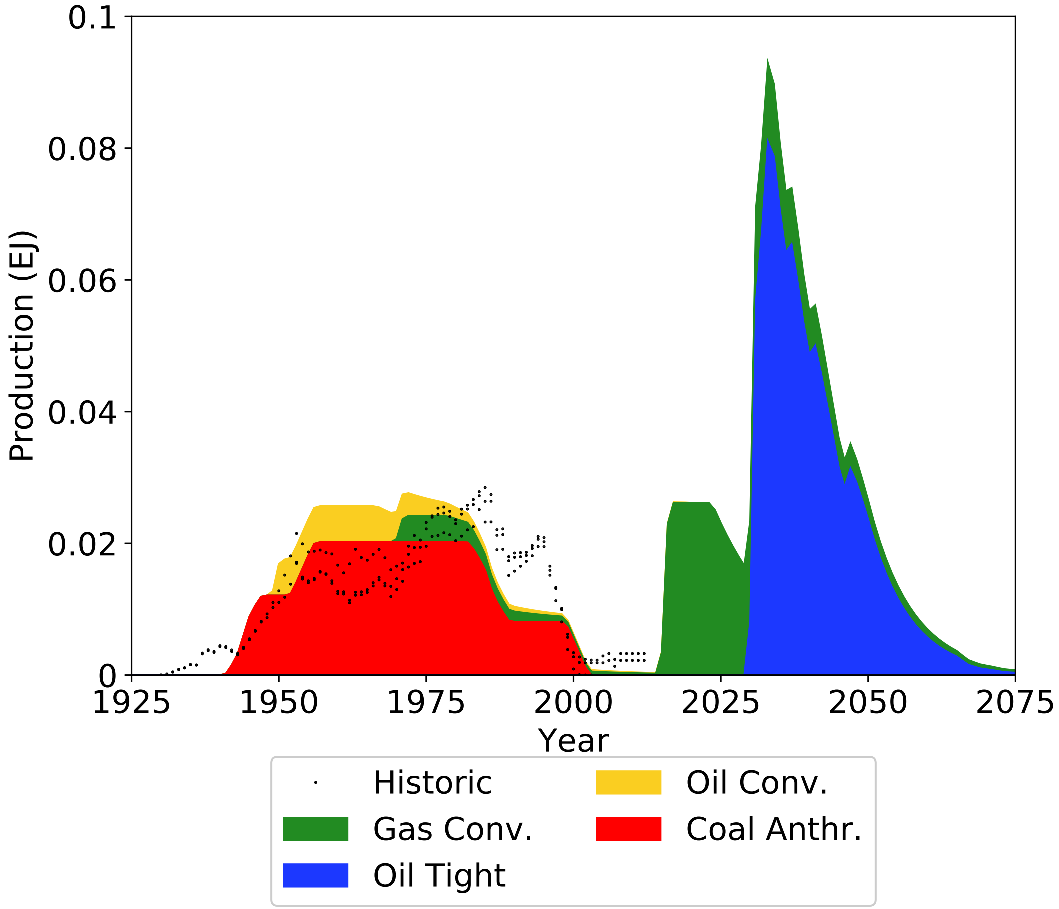

Figure 1.39: Morocco projections capped at 16

Table 1.39: Peak years - All

**Name**	**URR**	**Peak Year**	**Peak Rate**
Oil Tight	1.15	2033	0.08
Coal Anthr.	0.88	1957	0.02
Gas Conv.	0.6	2017	0.03
Oil Conv.	0.16	1951	0.01
**Total**	**2.78**	**2033**	**0.09**

1.20.2 By Mineral

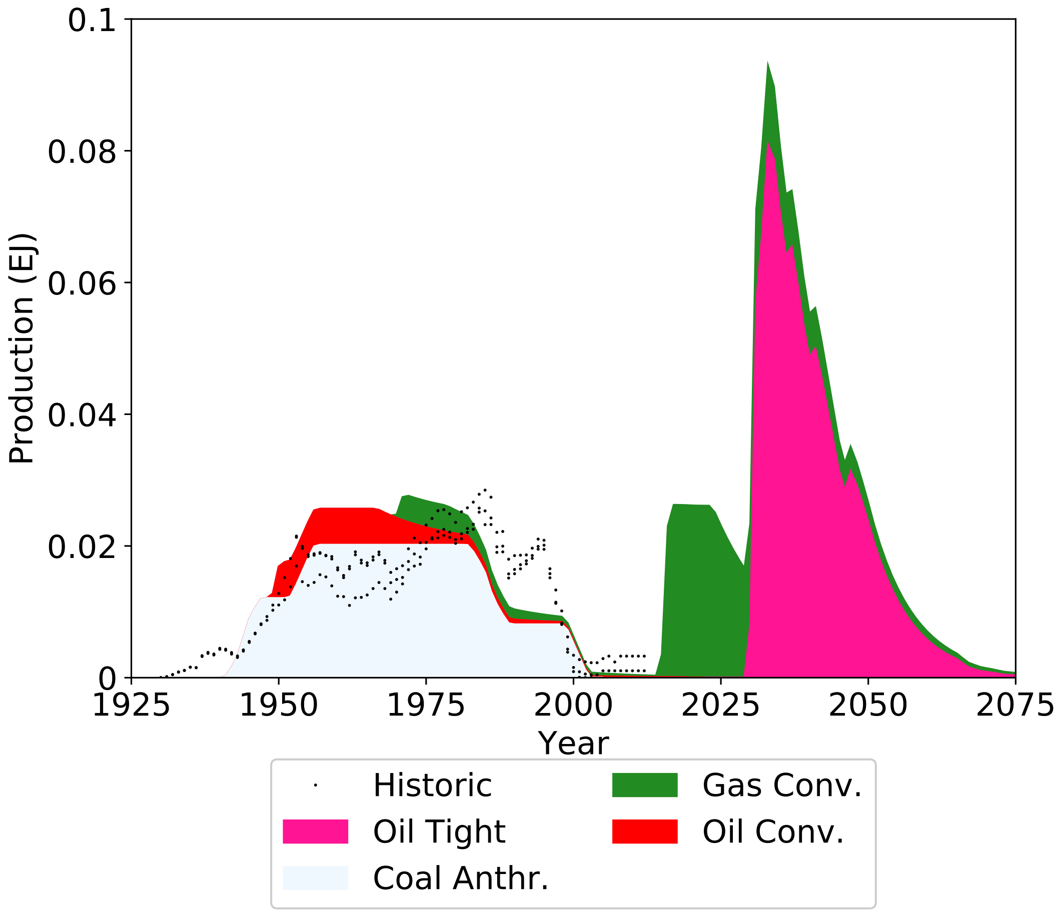

Figure 1.40: Morocco projection by mineral type

Table 1.40: Peak years - Minerals

Name	URR	Peak Year	Peak Rate
Coal Anthr.	0.88	1957	0.02
Oil Conv.	0.16	1951	0.01
Oil Tight	1.15	2033	0.08
Gas Conv.	0.6	2017	0.03
**Total**	**2.78**	**2033**	**0.09**

1.21 Mozambique

1.21.1 All Projections

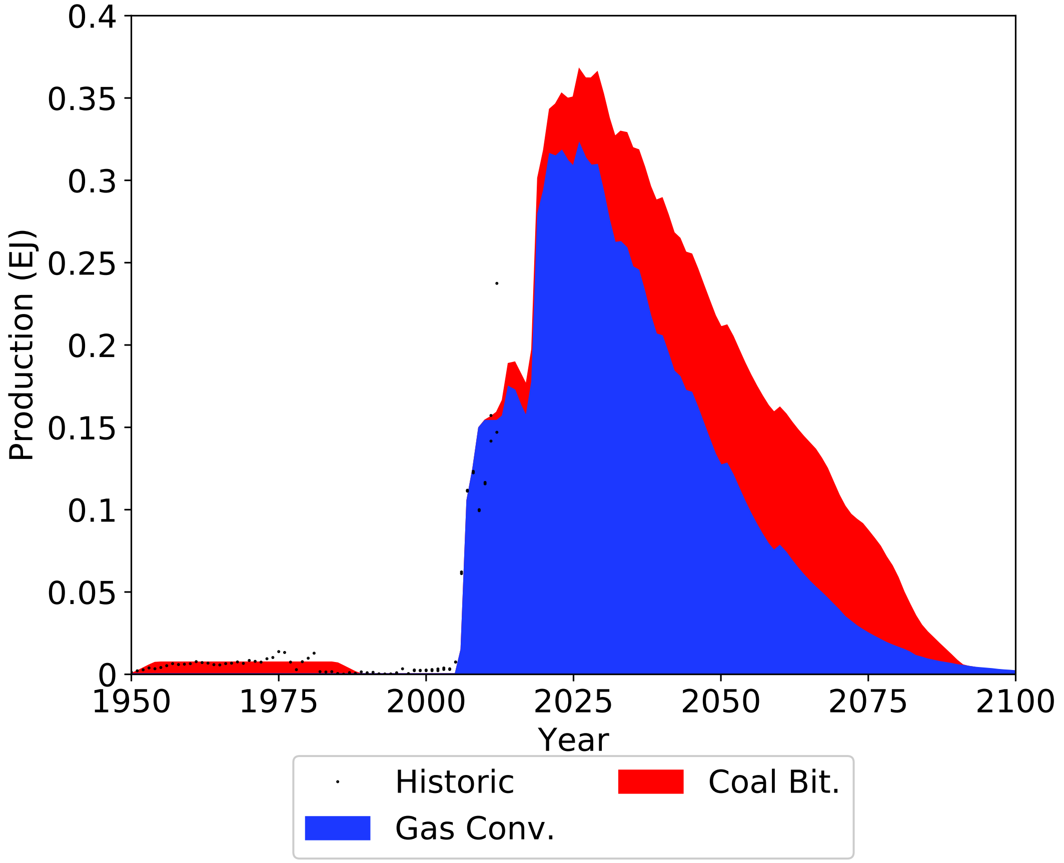

Figure 1.41: Mozambique projections capped at 16

Table 1.41: Peak years - All

Name	URR	Peak Year	Peak Rate
Gas Conv.	11.5	2026	0.32
Coal Bit.	4.8	2048	0.08
**Total**	**16.3**	**2026**	**0.37**

1.21.2 By Mineral

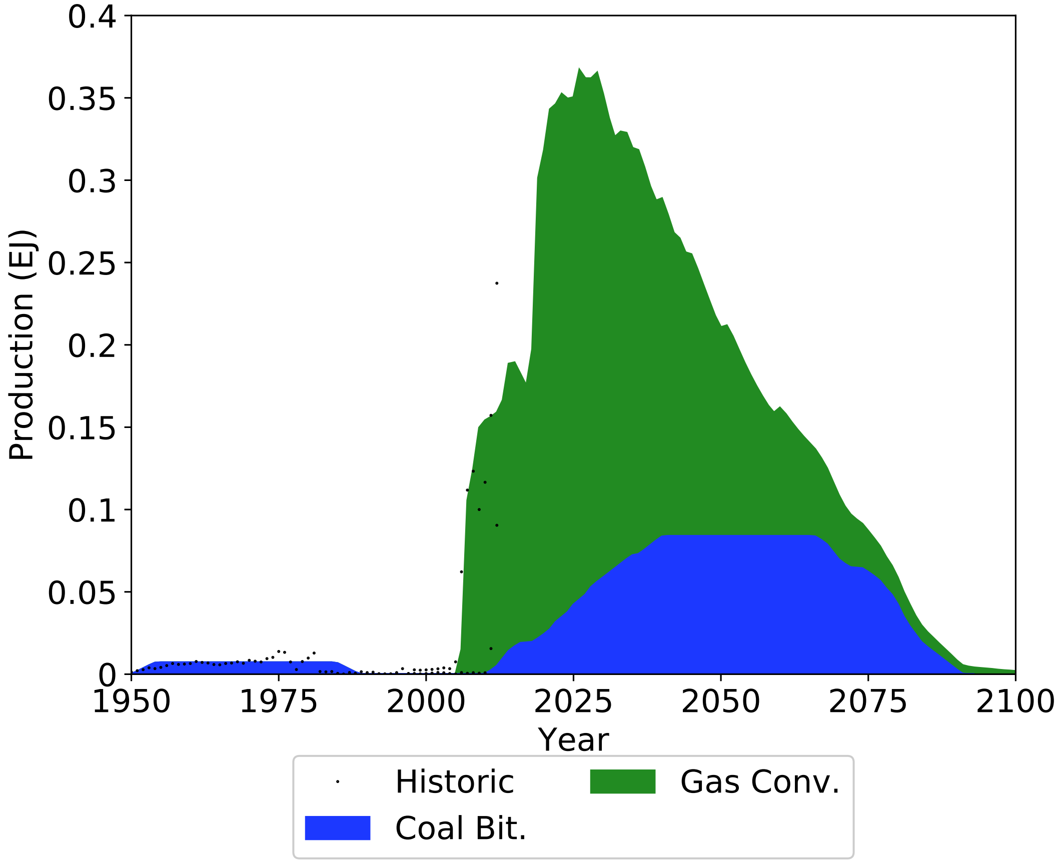

Figure 1.42: Mozambique projection by mineral type

Table 1.42: Peak years - Minerals

Name	URR	Peak Year	Peak Rate
Coal Bit.	4.8	2048	0.08
Gas Conv.	11.5	2026	0.32
**Total**	**16.3**	**2026**	**0.37**

1.22 Namibia

1.22.1 All Projections

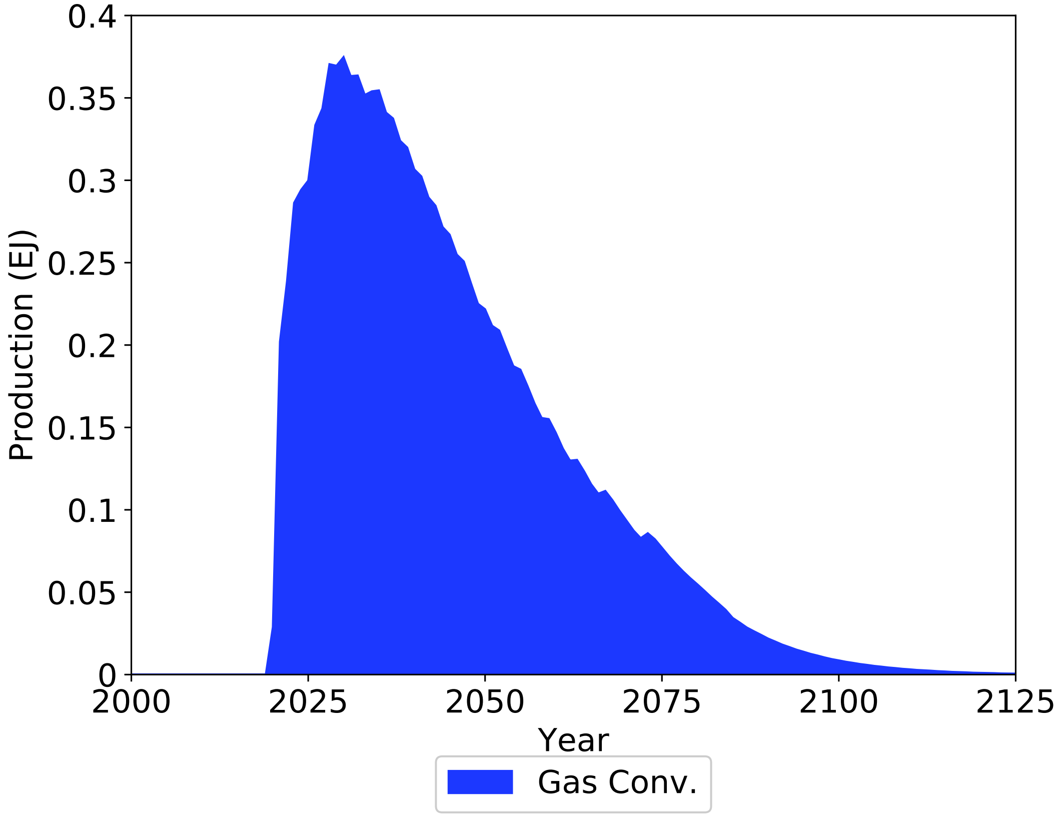

Figure 1.43: Namibia projections capped at 16

Table 1.43: Peak years - All

**Name**	**URR**	**Peak Year**	**Peak Rate**
Gas Conv.	13.4	2030	0.37
**Total**	**13.4**	**2030**	**0.37**

1.22.2 By Mineral

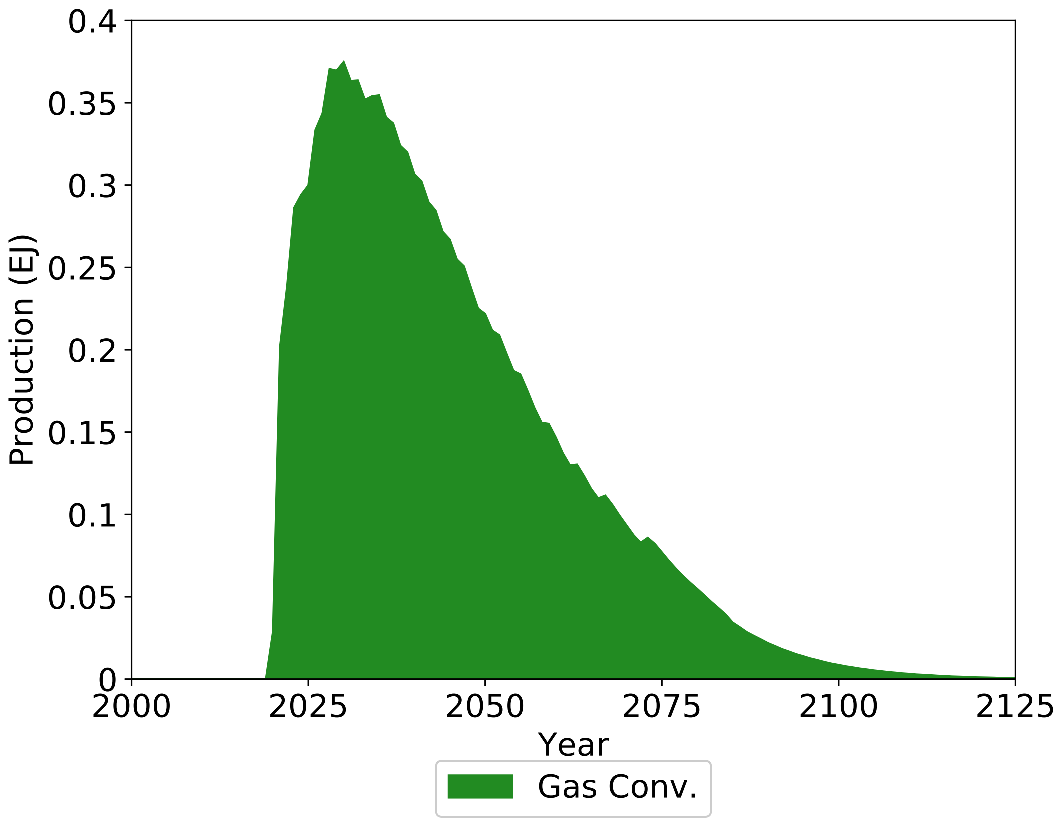

Figure 1.44: Namibia projection by mineral type

Table 1.44: Peak years - Minerals

Name	URR	Peak Year	Peak Rate
Gas Conv.	13.4	2030	0.37
**Total**	**13.4**	**2030**	**0.37**

1.23 Niger

1.23.1 All Projections

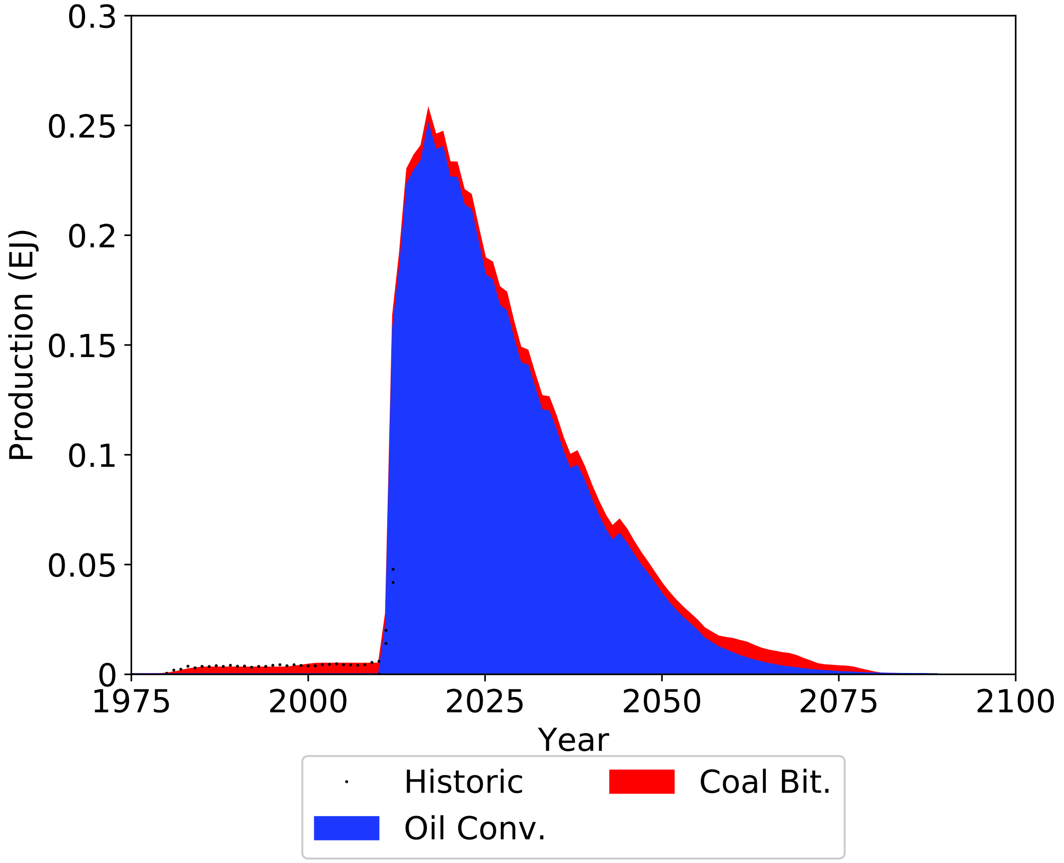

Figure 1.45: Niger projections capped at 16

Table 1.45: Peak years - All

Name	URR	Peak Year	Peak Rate
Oil Conv.	5.73	2017	0.25
Coal Bit.	0.51	2028	0.01
**Total**	**6.24**	**2017**	**0.26**

1.23.2 By Mineral

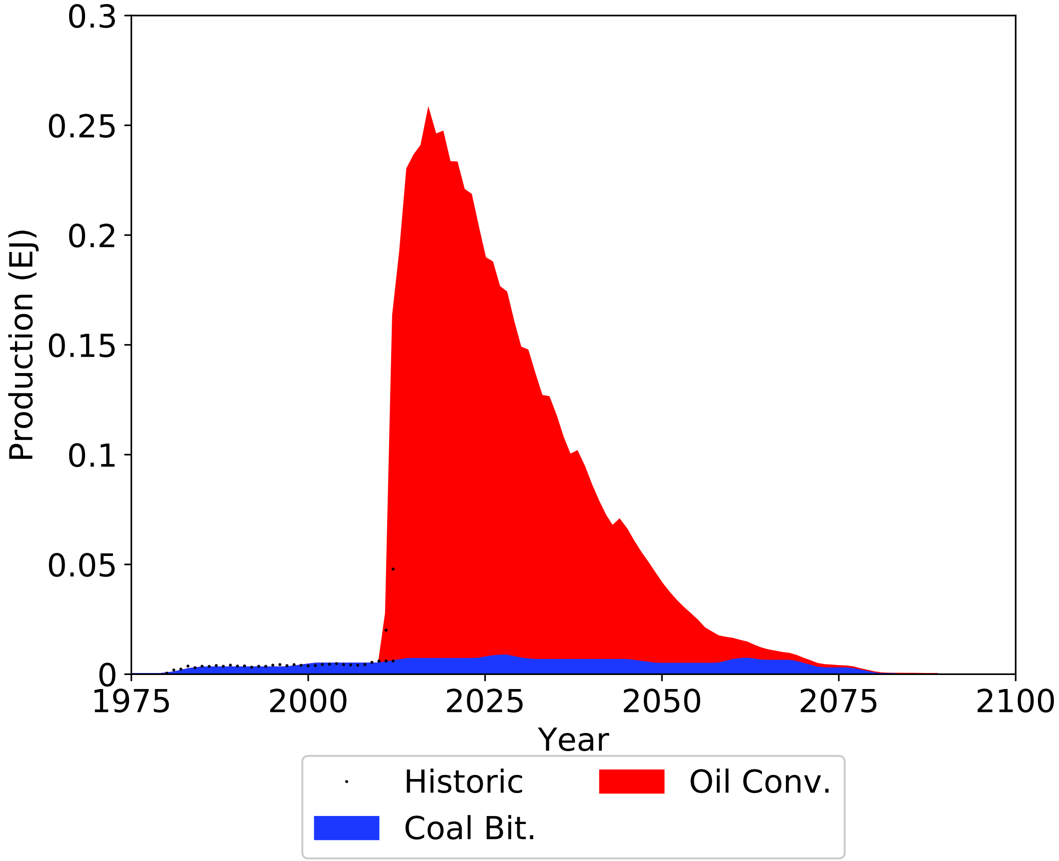

Figure 1.46: Niger projection by mineral type

Table 1.46: Peak years - Minerals

Name	URR	Peak Year	Peak Rate
Coal Bit.	0.51	2028	0.01
Oil Conv.	5.73	2017	0.25
**Total**	**6.24**	**2017**	**0.26**

1.24 Nigeria

1.24.1 All Projections

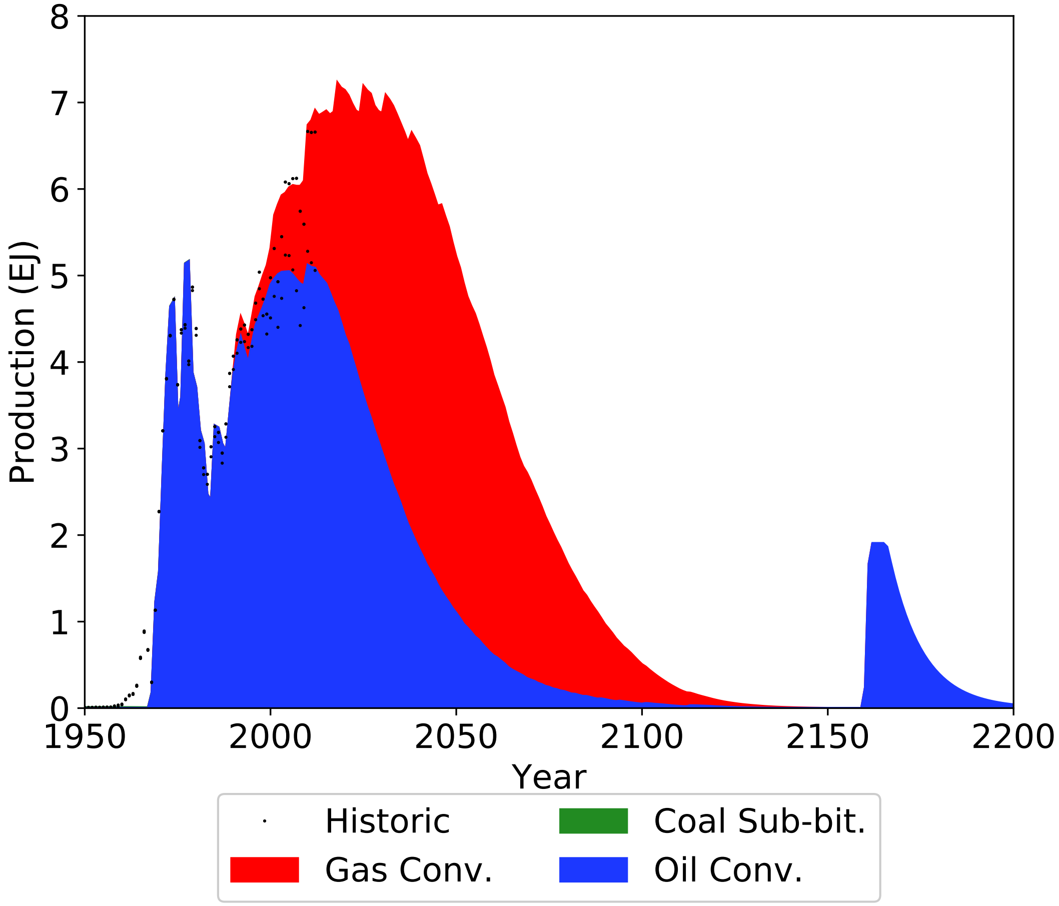

Figure 1.47: Nigeria projections capped at 16

Table 1.47: Peak years - All

Name	URR	Peak Year	Peak Rate
Oil Conv.	334.9	1978	5.17
Gas Conv.	262.5	2040	4.66
Coal Sub-bit.	0.45	1961	0.01
**Total**	**597.85**	**2018**	**7.23**

1.24.2 By Mineral

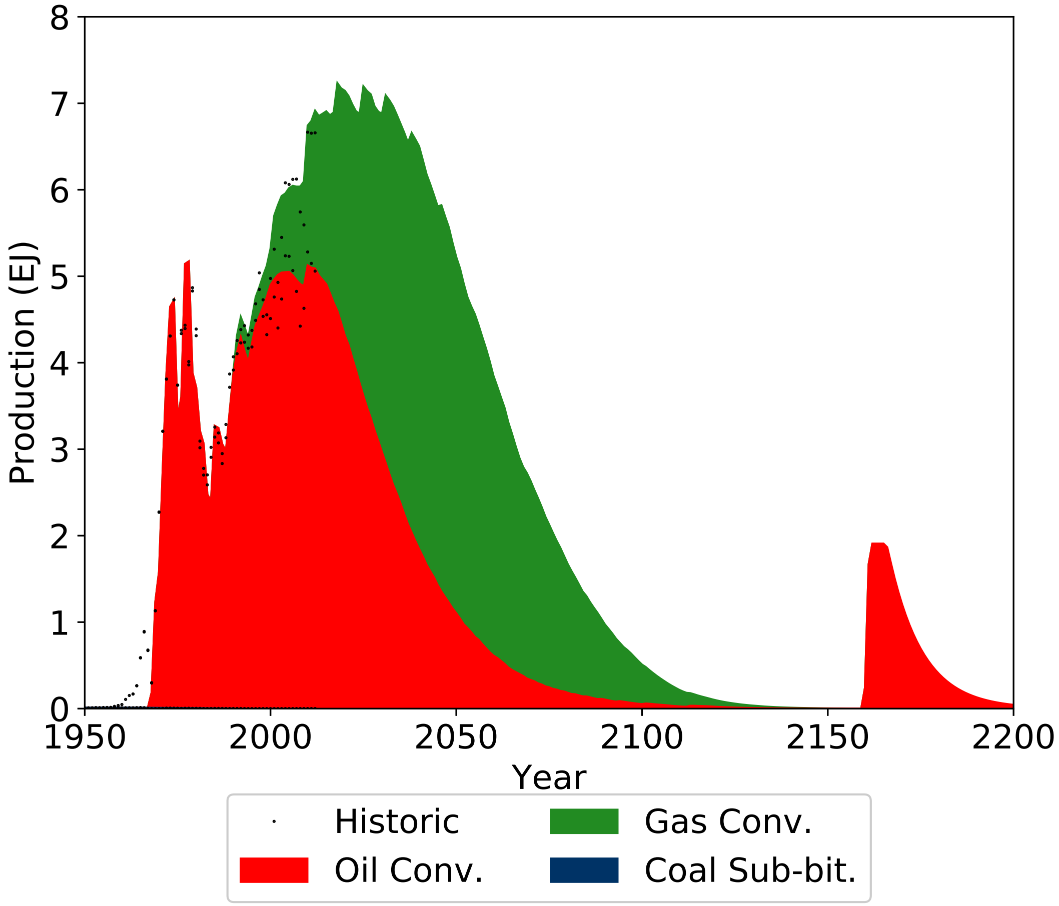

Figure 1.48: Nigeria projection by mineral type

Table 1.48: Peak years - Minerals

Name	URR	Peak Year	Peak Rate
Coal Sub-bit.	0.45	1961	0.01
Oil Conv.	334.9	1978	5.17
Gas Conv.	262.5	2040	4.66
**Total**	**597.85**	**2018**	**7.23**

1.25 Rwanda

1.25.1 All Projections

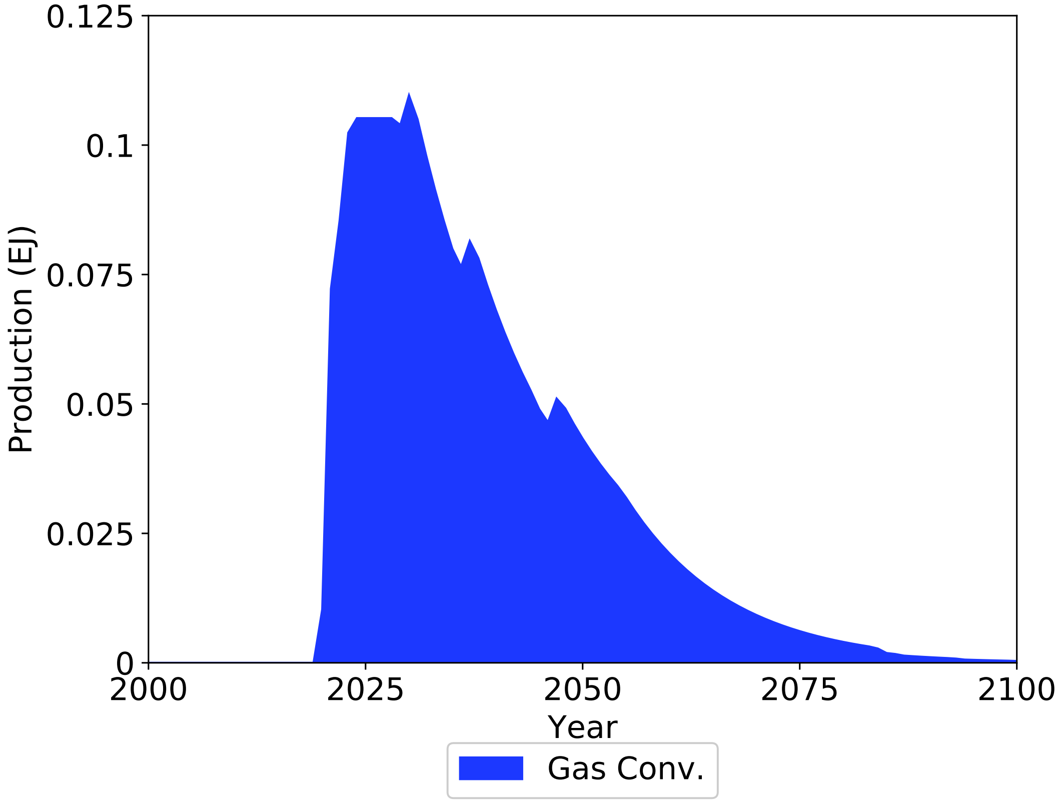

Figure 1.49: Rwanda projections capped at 16

Table 1.49: Peak years - All

Name	URR	Peak Year	Peak Rate
Gas Conv.	2.9	2030	0.11
**Total**	**2.9**	**2030**	**0.11**

1.25.2 By Mineral

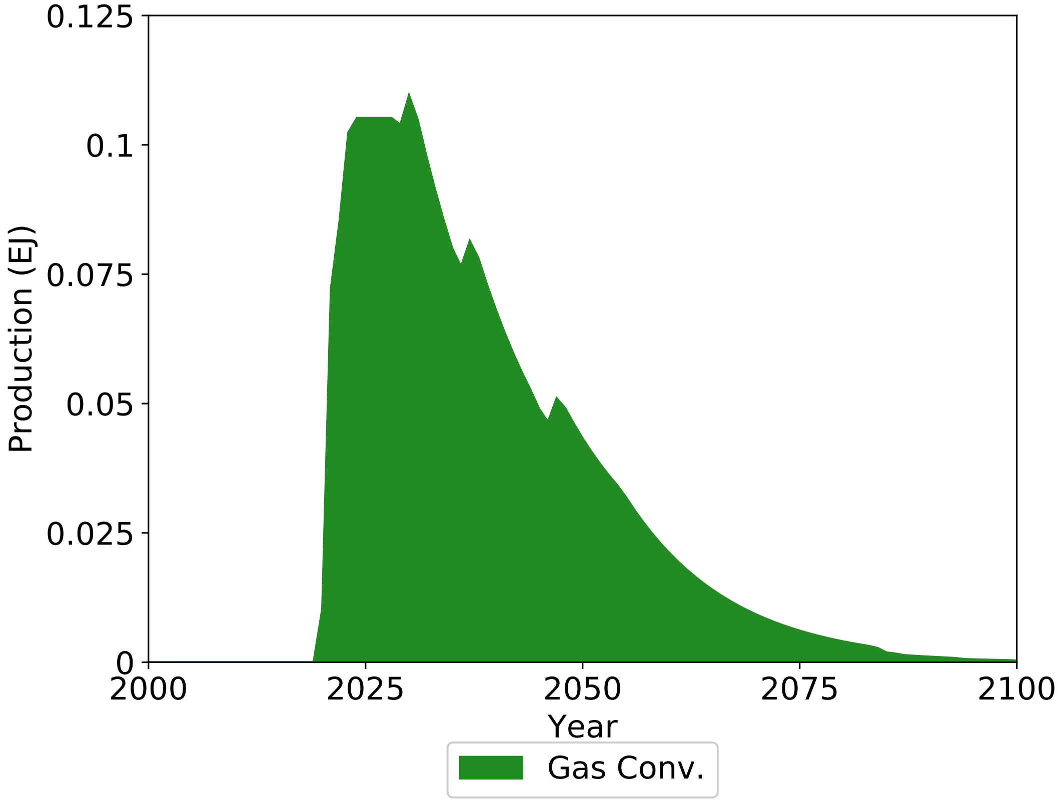

Figure 1.50: Rwanda projection by mineral type

Table 1.50: Peak years - Minerals

**Name**	**URR**	**Peak Year**	**Peak Rate**
Gas Conv.	2.9	2030	0.11
**Total**	**2.9**	**2030**	**0.11**

1.26 Senegal

1.26.1 All Projections

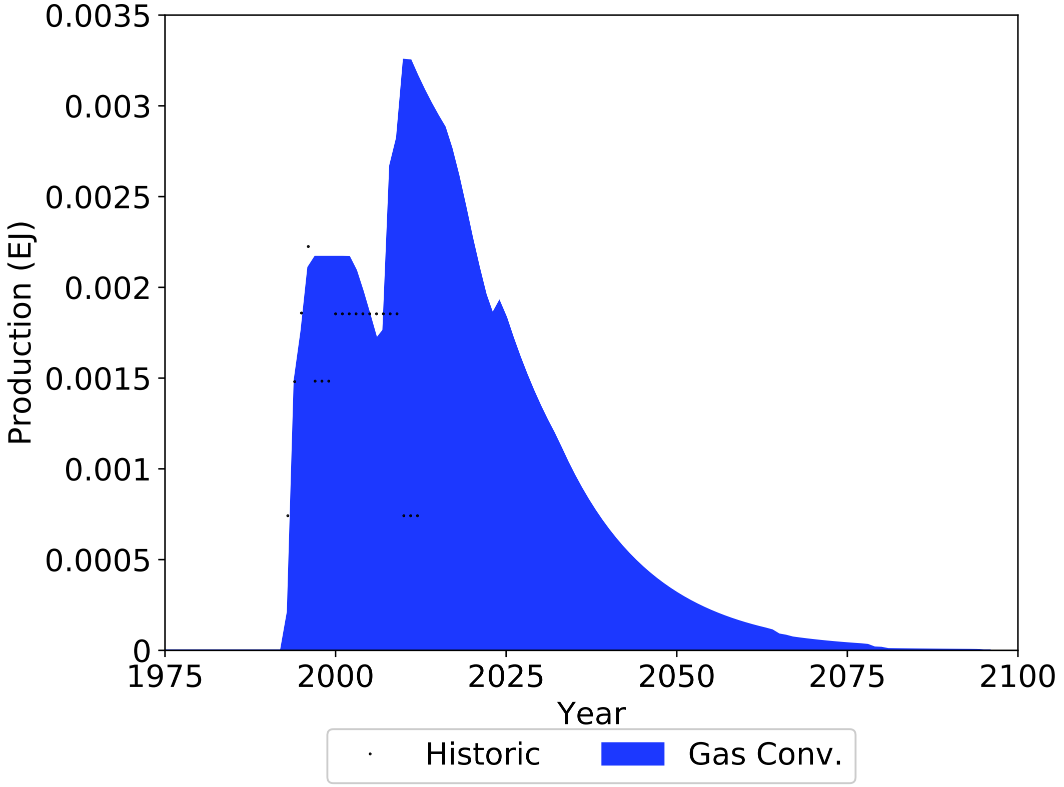

Figure 1.51: Senegal projections capped at 16

Table 1.51: Peak years - All

Name	URR	Peak Year	Peak Rate
Gas Conv.	0.1	2010	–
**Total**	**0.1**	**2010**	–

1.26.2 By Mineral

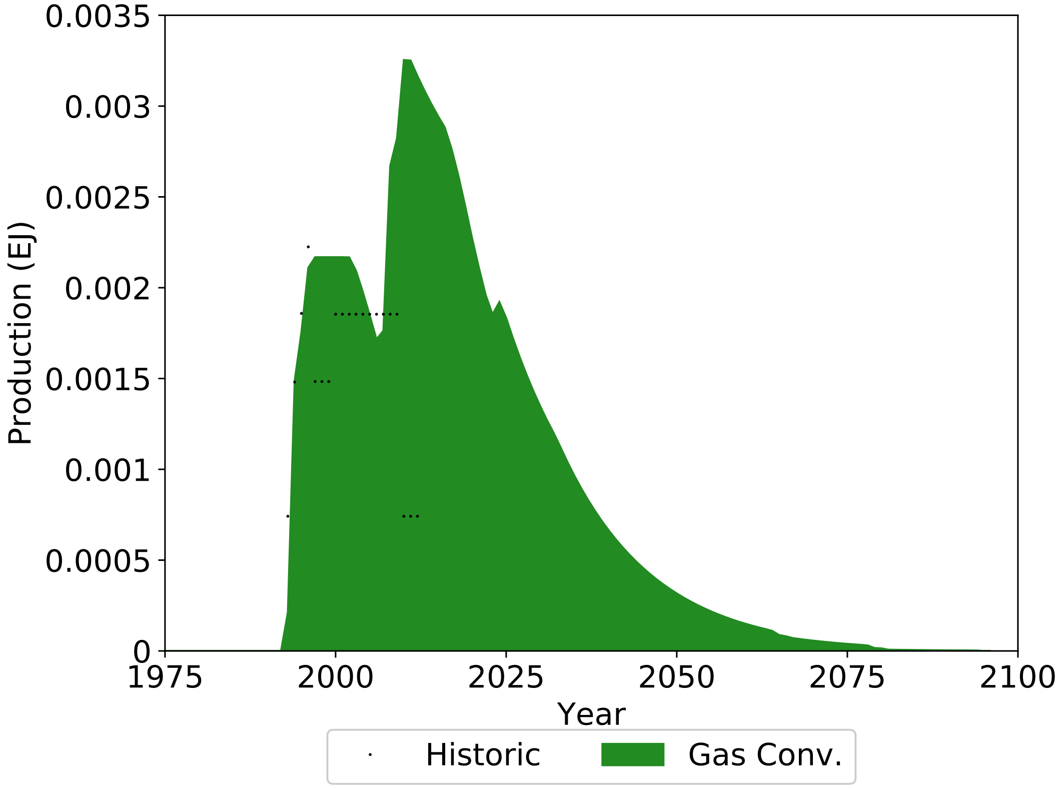

Figure 1.52: Senegal projection by mineral type

Table 1.52: Peak years - Minerals

Name	URR	Peak Year	Peak Rate
Gas Conv.	0.1	2010	–
**Total**	**0.1**	**2010**	–

1.27 Seychelles

1.27.1 All Projections

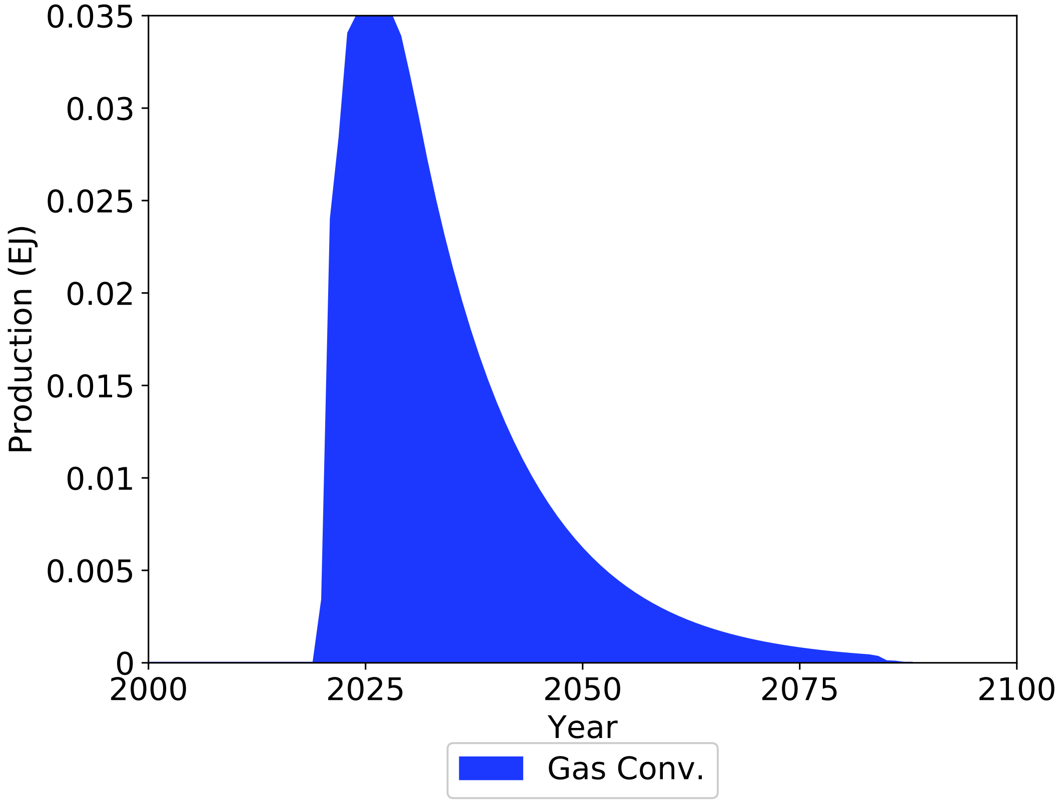

Figure 1.53: Seychelles projections capped at 16

Table 1.53: Peak years - All

Name	URR	Peak Year	Peak Rate
Gas Conv.	0.7	2024	0.04
**Total**	**0.7**	**2024**	**0.04**

1.27.2 By Mineral

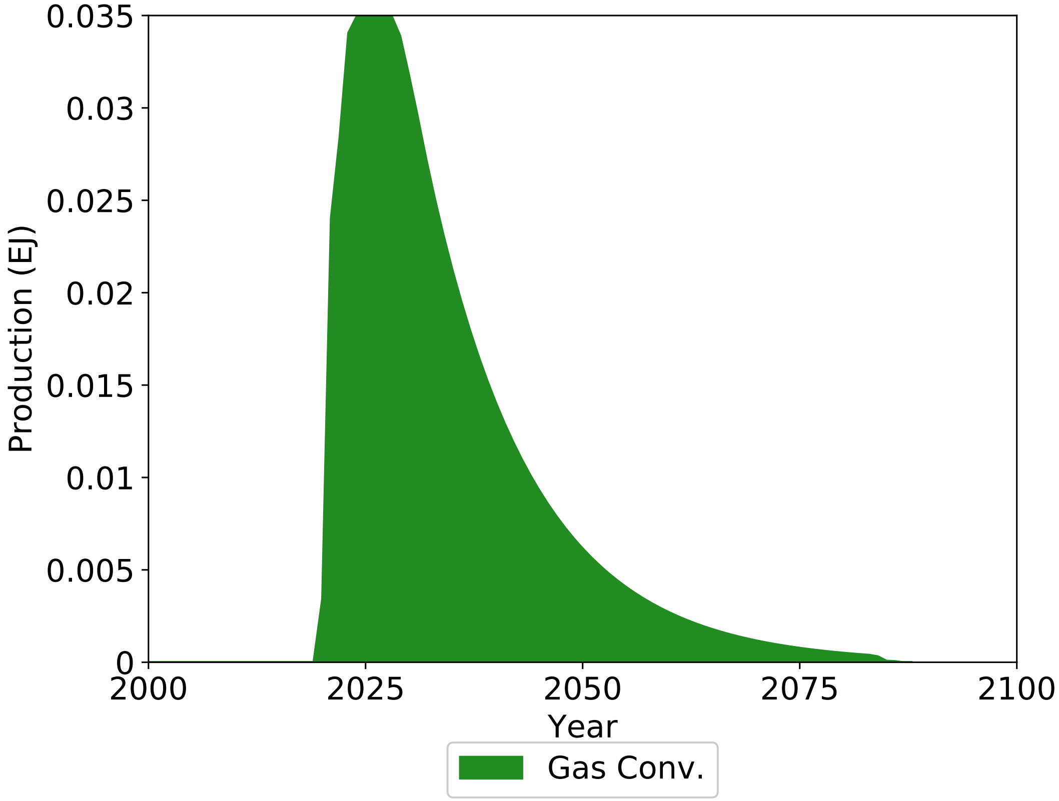

Figure 1.54: Seychelles projection by mineral type

Table 1.54: Peak years - Minerals

Name	URR	Peak Year	Peak Rate
Gas Conv.	0.7	2024	0.04
**Total**	**0.7**	**2024**	**0.04**

1.28 Somalia

1.28.1 All Projections

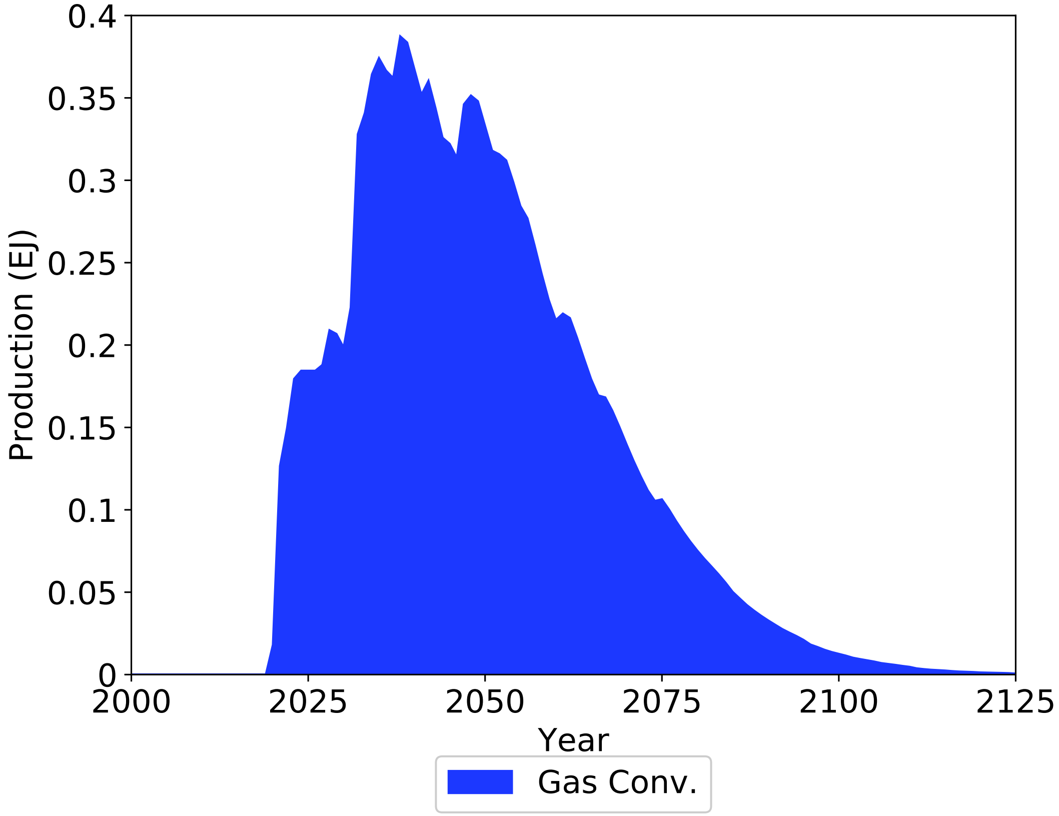

Figure 1.55: Somalia projections capped at 16

Table 1.55: Peak years - All

Name	URR	Peak Year	Peak Rate
Gas Conv.	15.1	2038	0.39
**Total**	**15.1**	**2038**	**0.39**

1.28.2 By Mineral

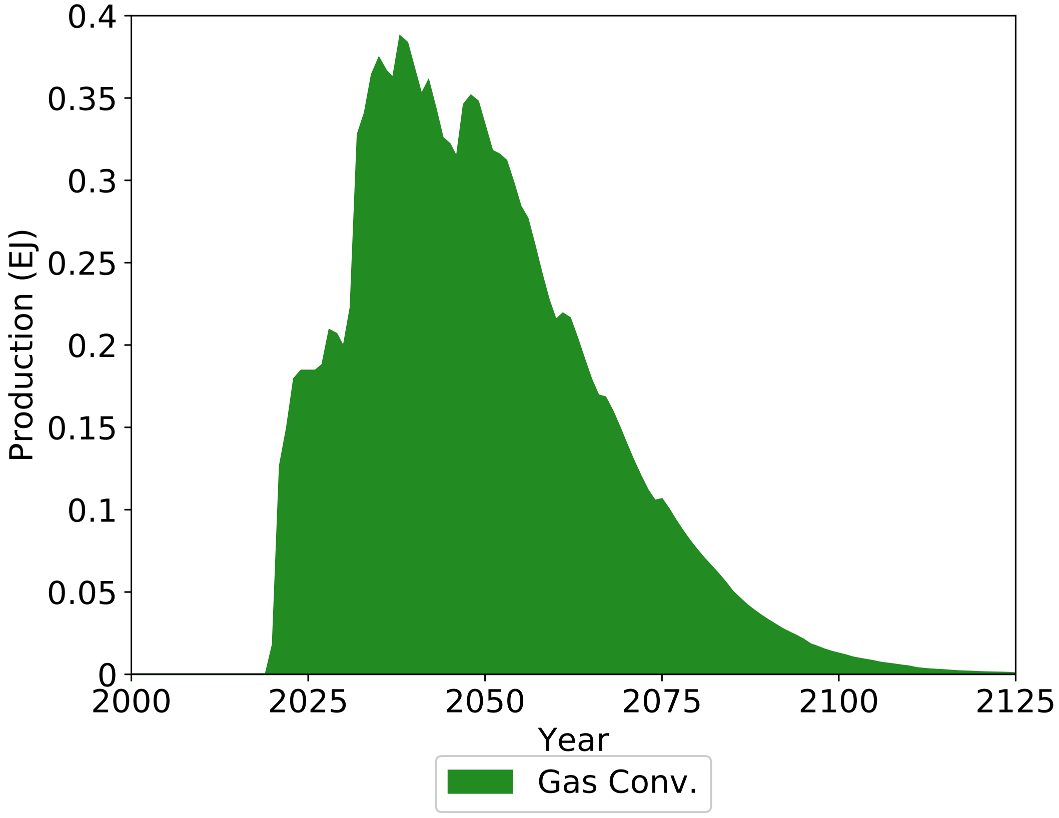

Figure 1.56: Somalia projection by mineral type

Table 1.56: Peak years - Minerals

Name	URR	Peak Year	Peak Rate
Gas Conv.	15.1	2038	0.39
**Total**	**15.1**	**2038**	**0.39**

1.29 South Africa

1.29.1 All Projections

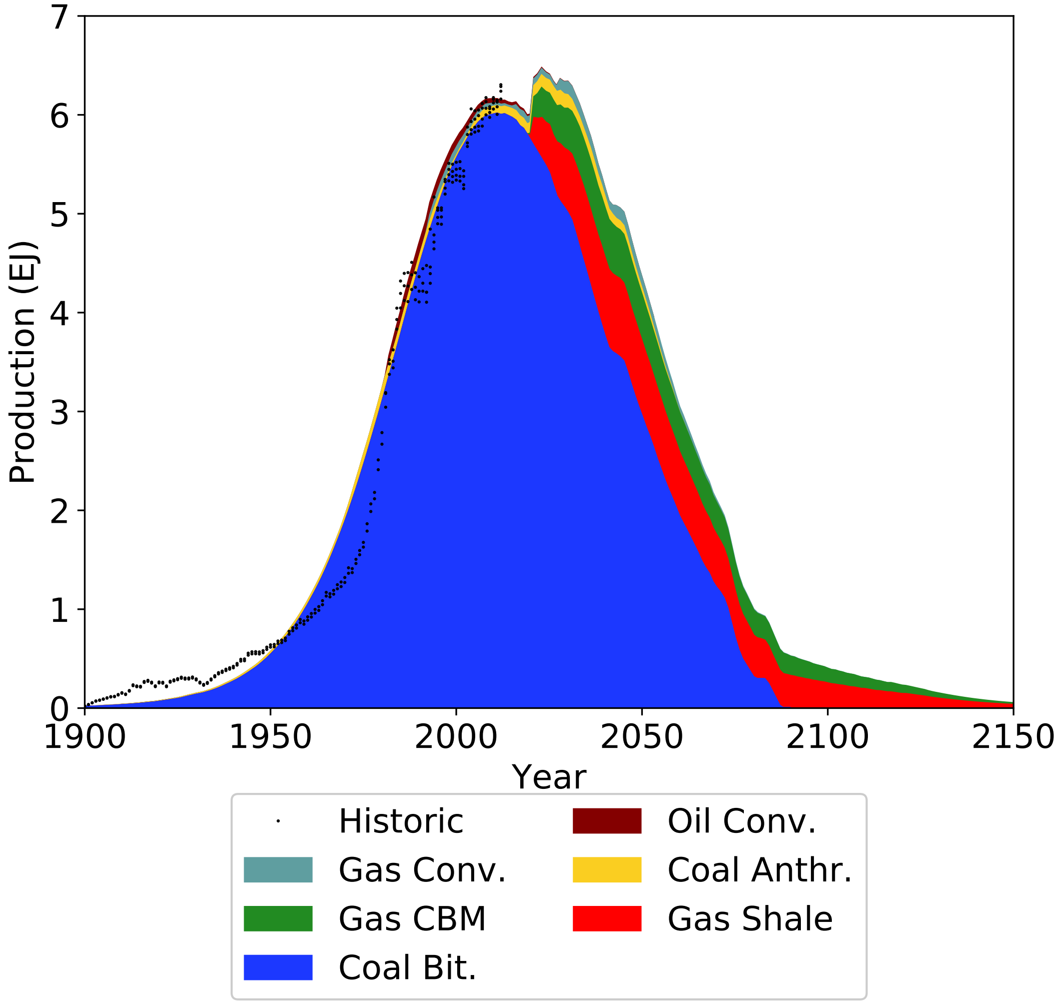

Figure 1.57: South Africa projections capped at 16

Table 1.57: Peak years - All

Name	URR	Peak Year	Peak Rate
Coal Bit.	440.96	2011	6.01
Gas Shale	50.93	2040	0.8
Gas CBM	31.5	2041	0.51
Coal Anthr.	9.11	2028	0.15
Gas Conv.	6.3	2045	0.14
Oil Conv.	3.34	1988	0.15
**Total**	**542.14**	**2023**	**6.47**

1.29.2 By Mineral

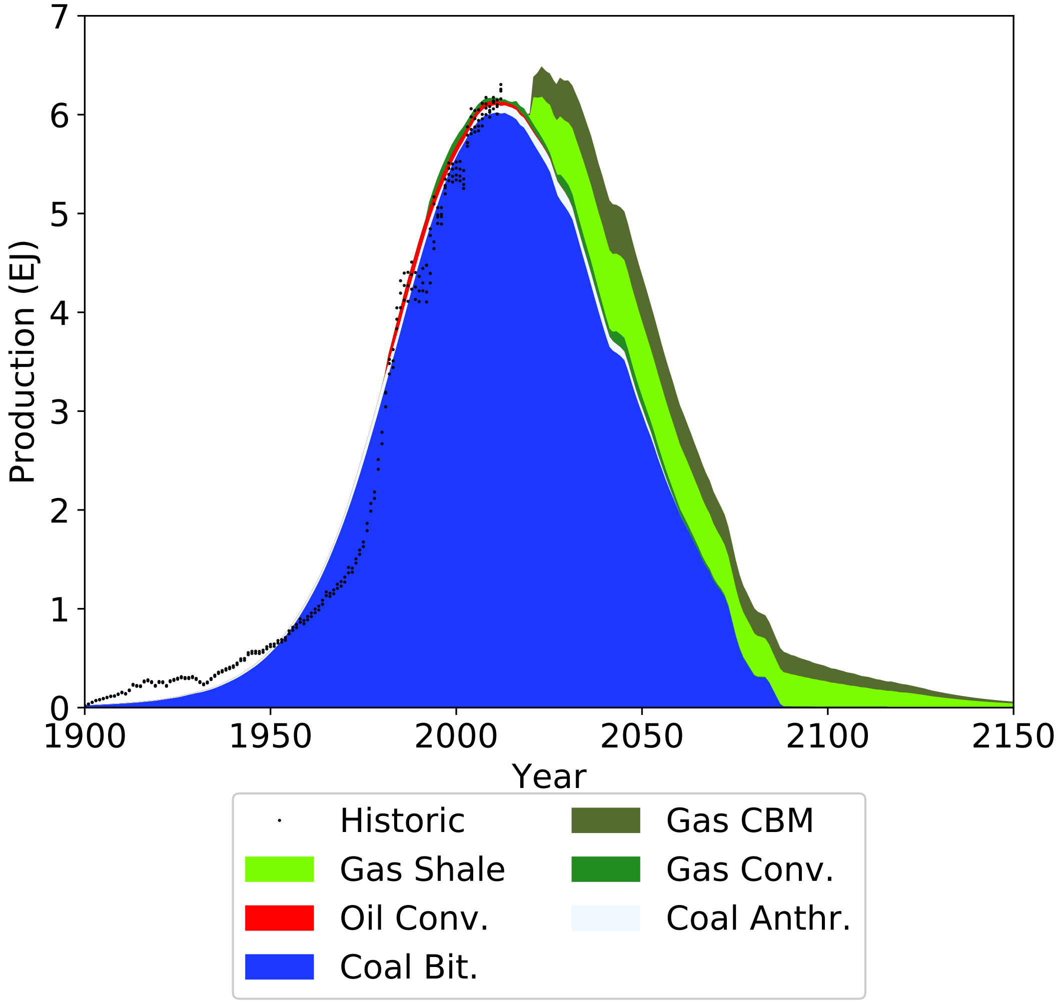

Figure 1.58: South Africa projection by mineral type

Table 1.58: Peak years - Minerals

**Name**	**URR**	**Peak Year**	**Peak Rate**
Coal Bit.	440.96	2011	6.01
Coal Anthr.	9.11	2028	0.15
Oil Conv.	3.34	1988	0.15
Gas Conv.	6.3	2045	0.14
Gas Shale	50.93	2040	0.8
Gas CBM	31.5	2041	0.51
**Total**	**542.14**	**2023**	**6.47**

1.30 Sudan

1.30.1 All Projections

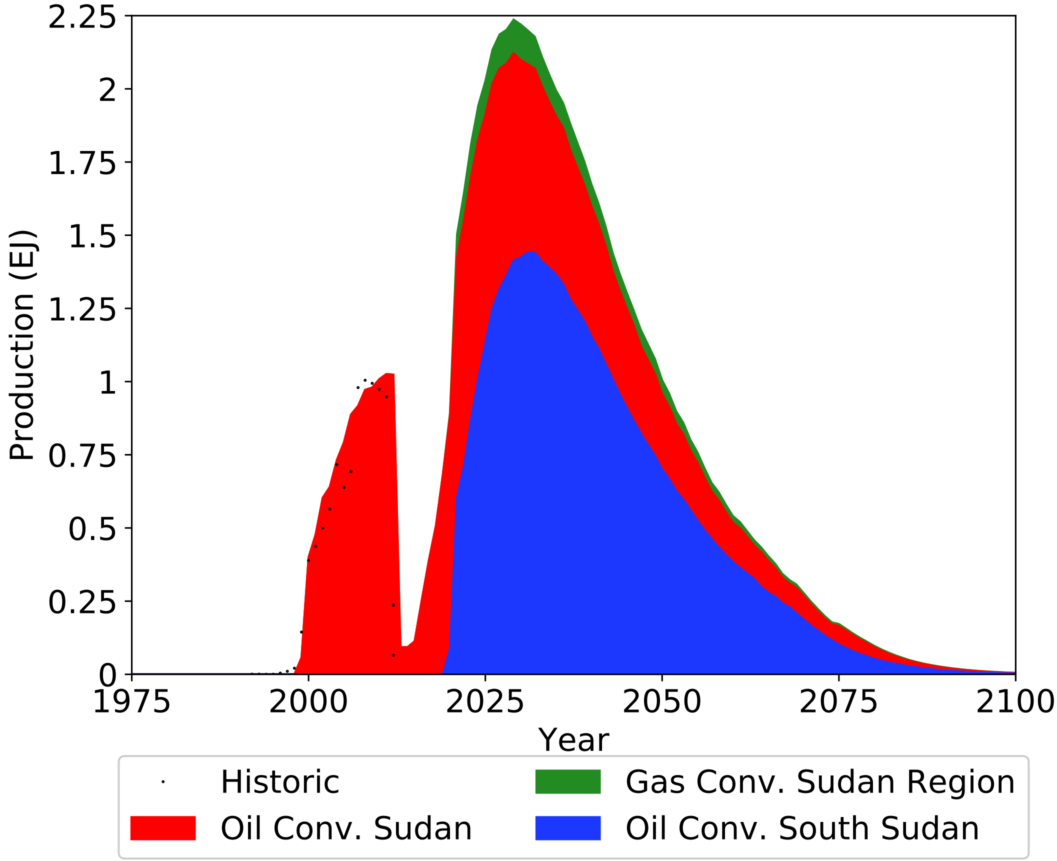

Figure 1.59: Sudan projections capped at 16

Table 1.59: Peak years - All

**Name**	**URR**	**Peak Year**	**Peak Rate**
Oil Conv. South Sudan	42.69	2032	1.44
Oil Conv. Sudan	33.66	2011	1.03
Gas Conv. Sudan Region	3.2	2030	0.12
**Total**	**79.55**	**2029**	**2.24**

1.30.2 By Mineral

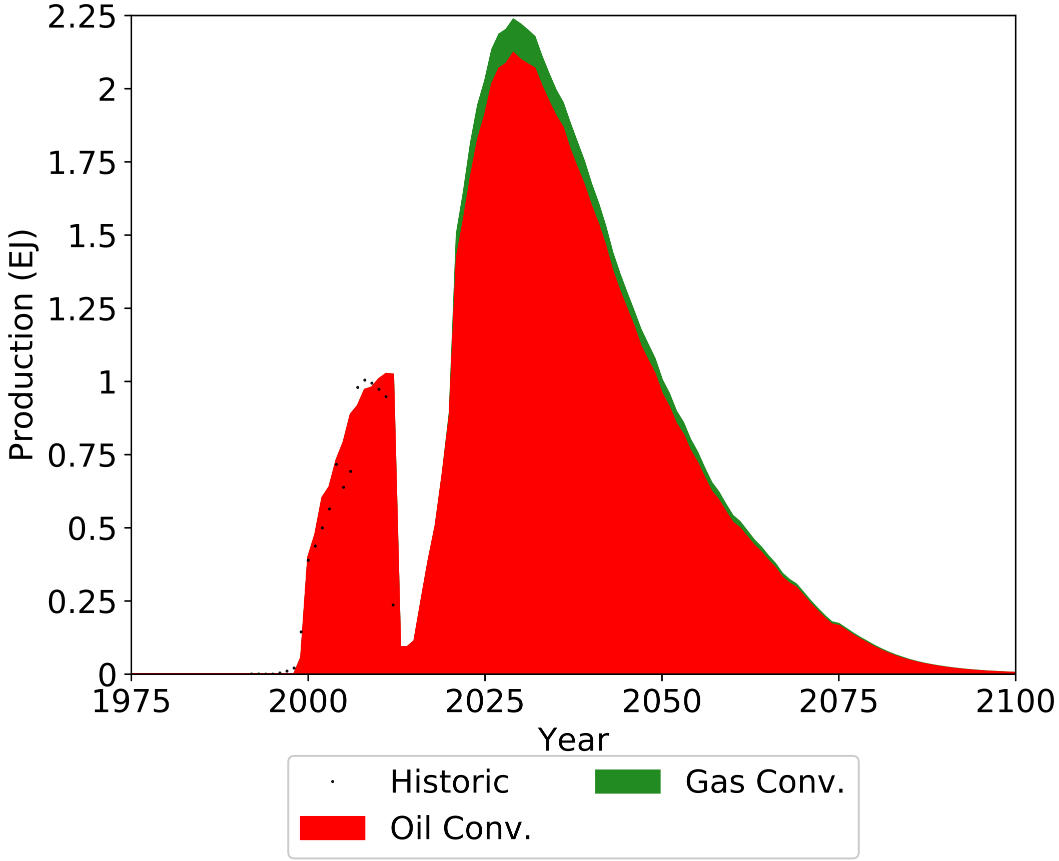

Figure 1.60: Sudan projection by mineral type

Table 1.60: Peak years - Minerals

Name	URR	Peak Year	Peak Rate
Oil Conv.	76.35	2029	2.12
Gas Conv.	3.2	2030	0.12
**Total**	**79.55**	**2029**	**2.24**

1.30.3 Regional Projections

South Sudan

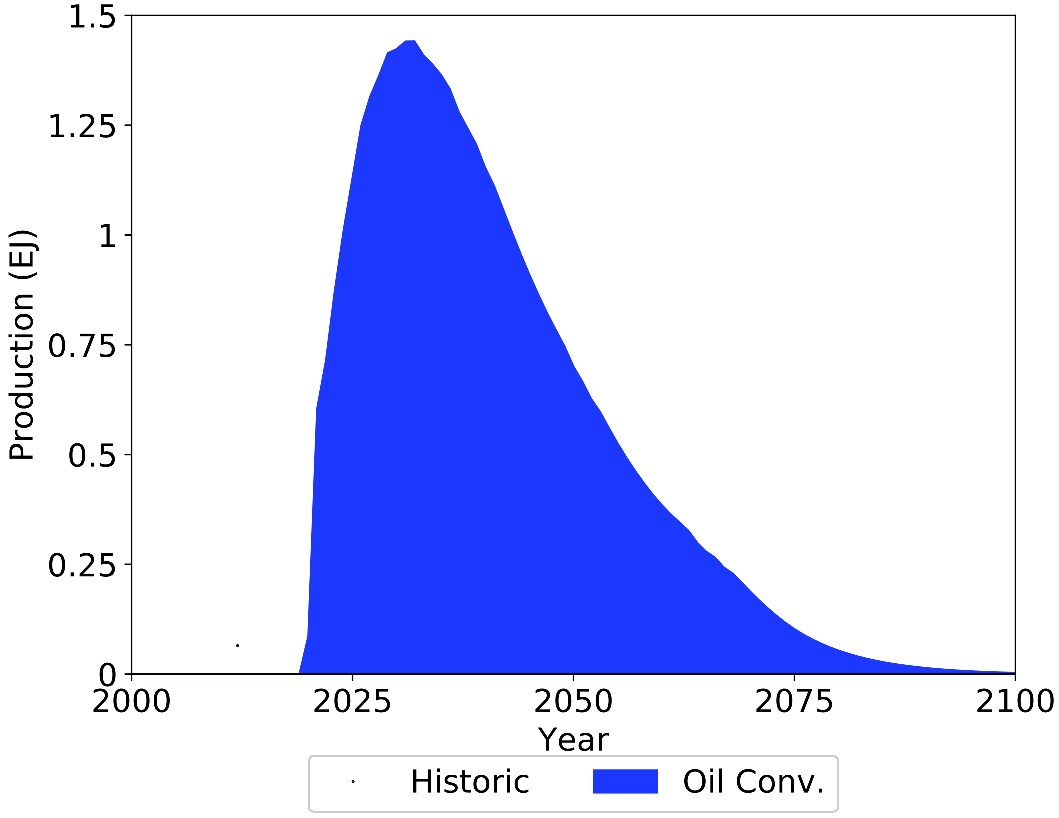

Figure 1.61: Sudan - South Sudan projections capped at 16

Table 1.61: Peak years - All

Name	URR	Peak Year	Peak Rate
Oil Conv. South Sudan	42.69	2032	1.44
**Total**	**42.69**	**2032**	**1.44**

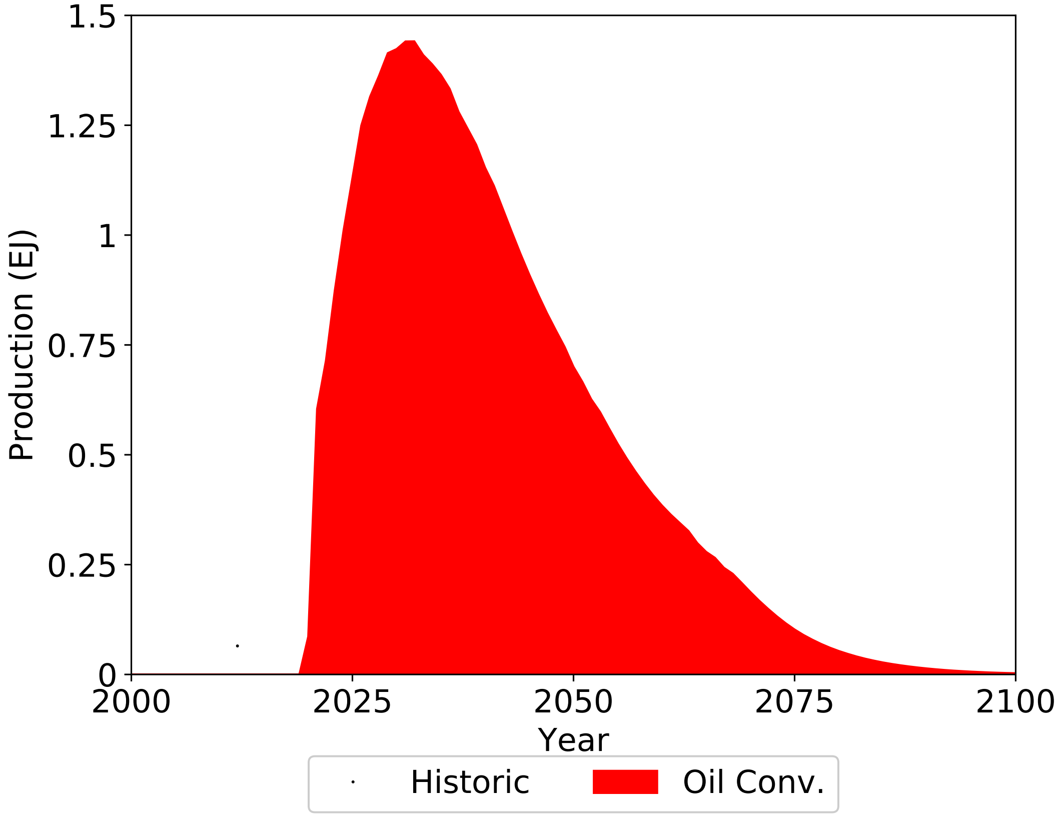

Figure 1.62: Sudan - South Sudan projection by mineral type

Table 1.62: Peak years - Minerals

Name	URR	Peak Year	Peak Rate
Oil Conv.	42.69	2032	1.44
**Total**	**42.69**	**2032**	**1.44**

Sudan

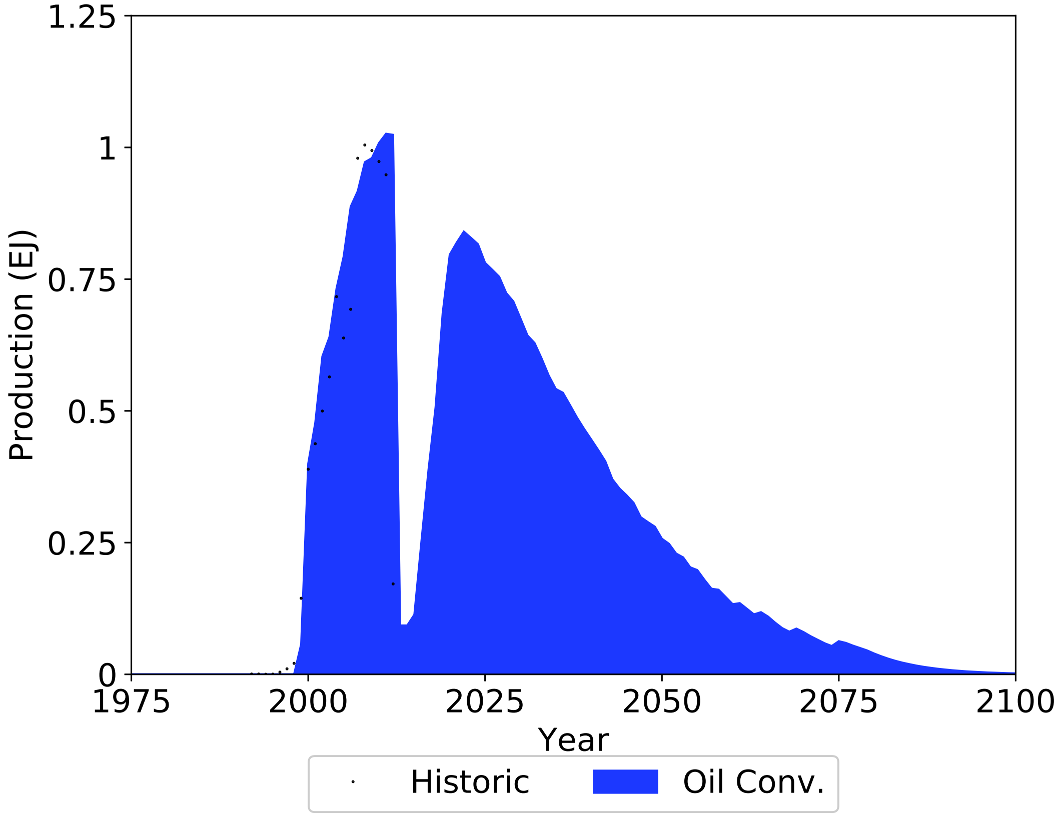

Figure 1.63: Sudan - Sudan projections capped at 16

Table 1.63: Peak years - All

Name	URR	Peak Year	Peak Rate
Oil Conv. Sudan	33.66	2011	1.03
**Total**	**33.66**	**2011**	**1.03**

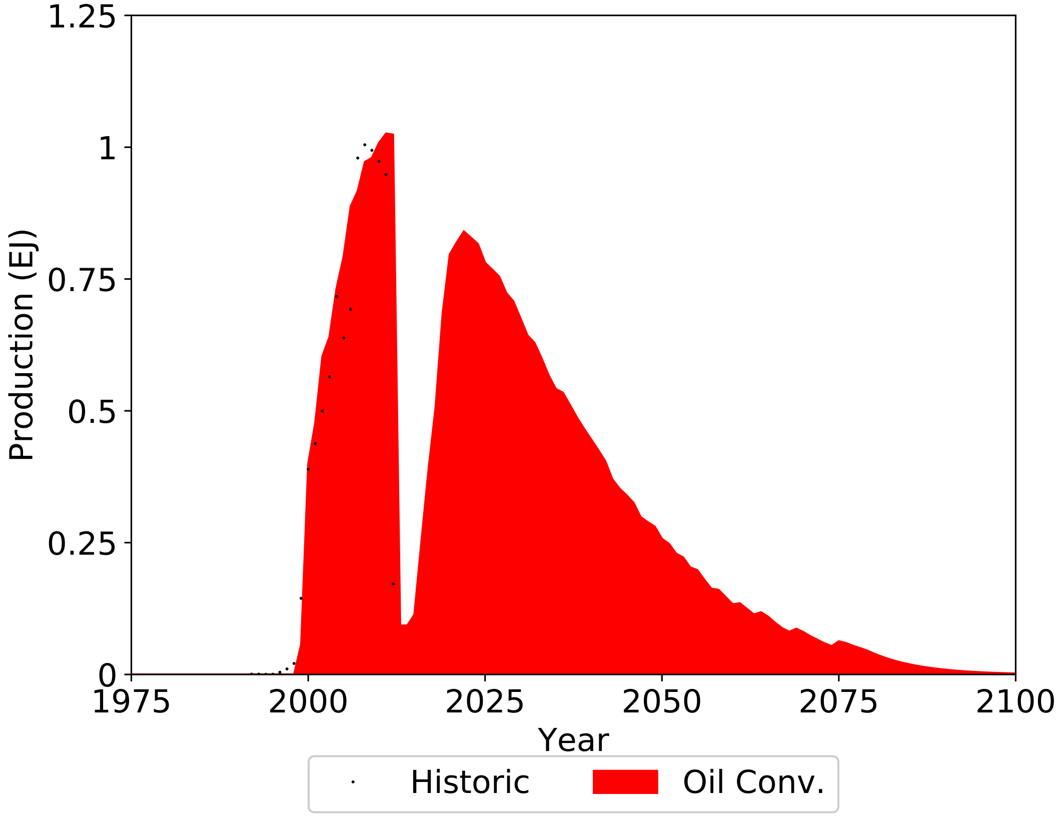

Figure 1.64: Sudan - Sudan projection by mineral type

Table 1.64: Peak years - Minerals

Name	URR	Peak Year	Peak Rate
Oil Conv.	33.66	2011	1.03
**Total**	**33.66**	**2011**	**1.03**

Sudan Region

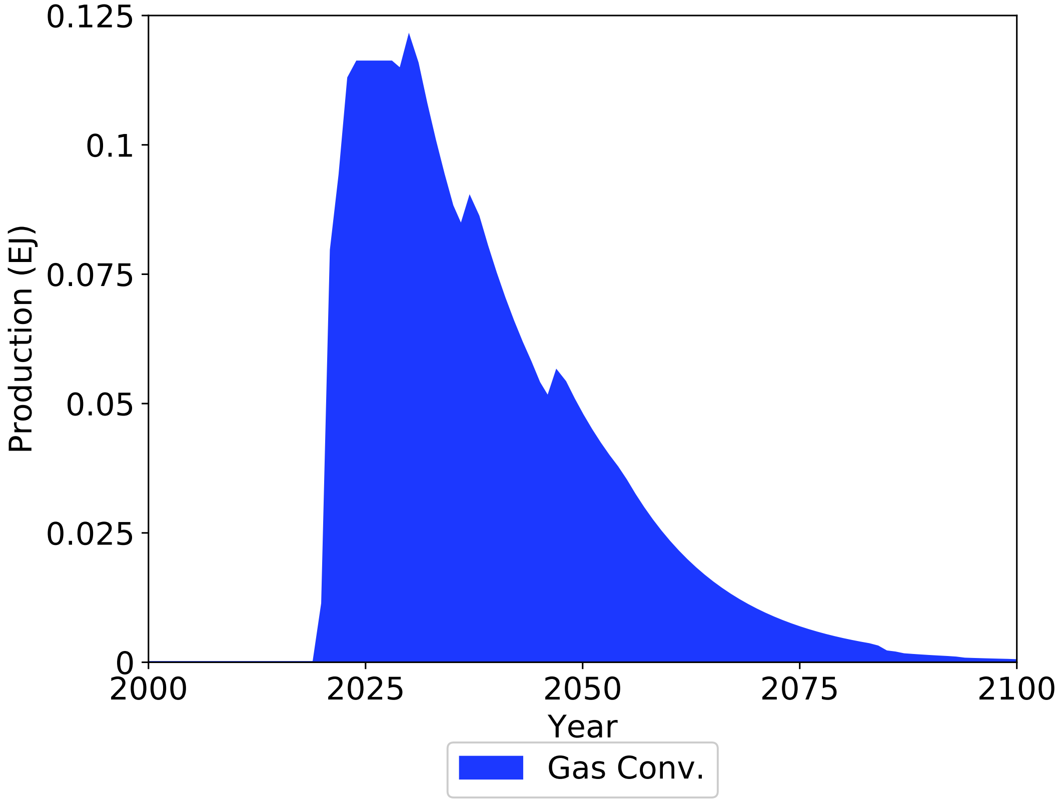

Figure 1.65: Sudan - Sudan Region projections capped at 16

Table 1.65: Peak years - All

**Name**	**URR**	**Peak Year**	**Peak Rate**
Gas Conv. Sudan Region	3.2	2030	0.12
**Total**	**3.2**	**2030**	**0.12**

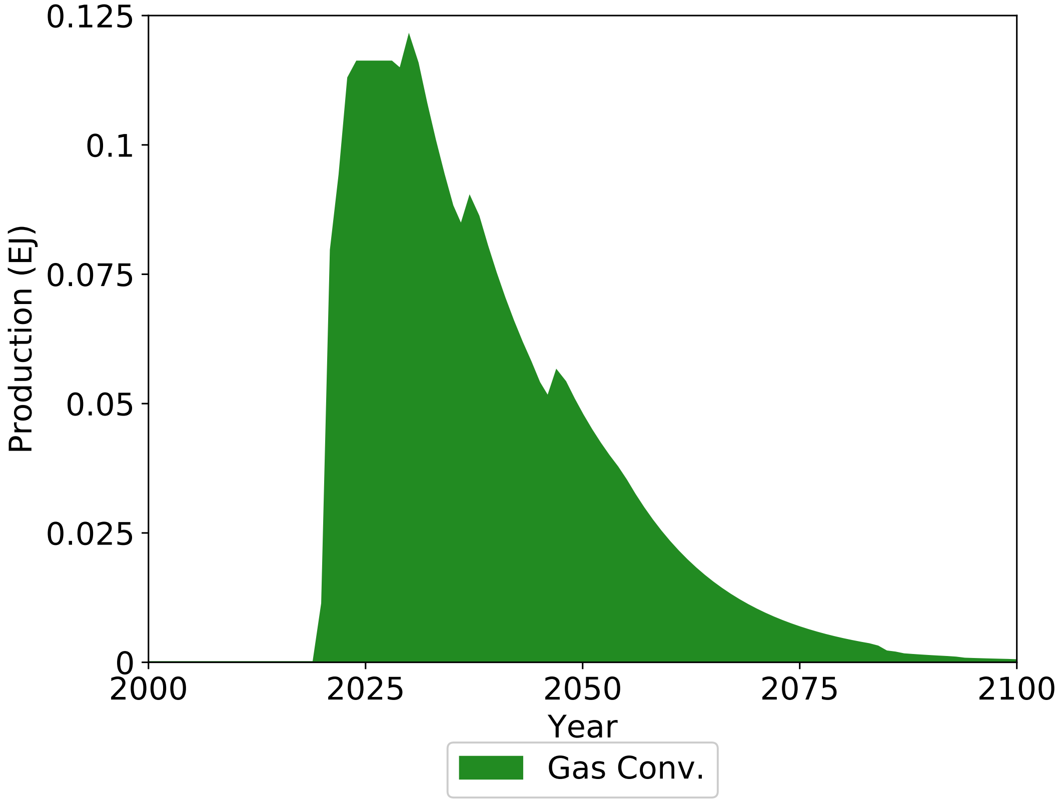

Figure 1.66: Sudan - Sudan Region projection by mineral type

Table 1.66: Peak years - Minerals

Name	URR	Peak Year	Peak Rate
Gas Conv.	3.2	2030	0.12
**Total**	**3.2**	**2030**	**0.12**

1.30.4 Projection by region

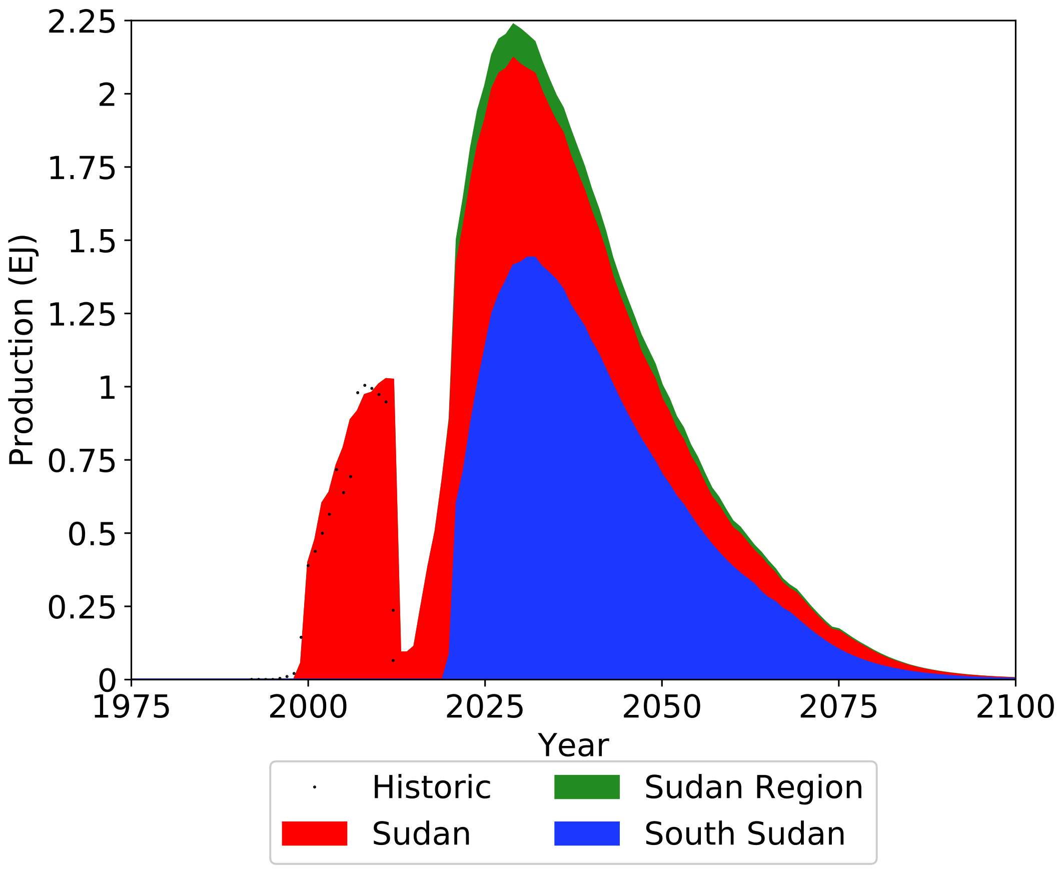

Figure 1.67: Sudan by region projections capped at 16

Table 1.67: Peak years - All

**Name**	**URR**	**Peak Year**	**Peak Rate**
South Sudan	42.69	2032	1.44
Sudan	33.66	2011	1.03
Sudan Region	3.2	2030	0.12
**Total**	**79.55**	**2029**	**2.24**

1.31 Swaziland

1.31.1 All Projections

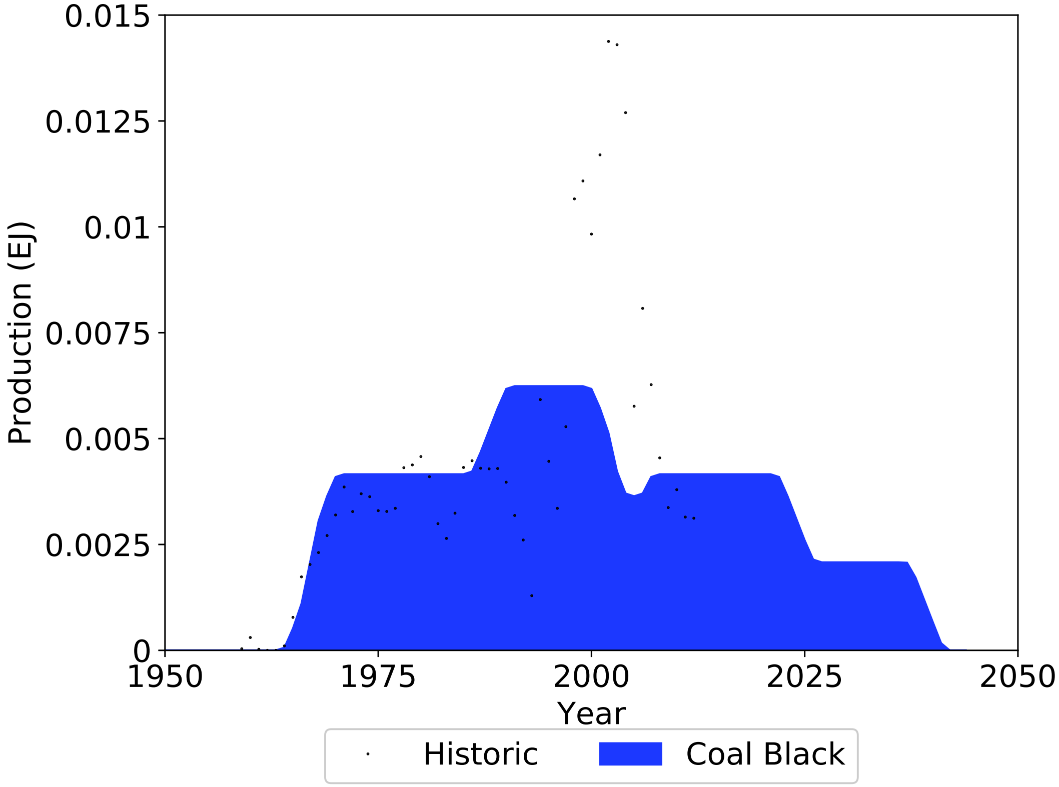

Figure 1.68: Swaziland projections capped at 16

Table 1.68: Peak years - All

**Name**	**URR**	**Peak Year**	**Peak Rate**
Coal Black	0.3	1991	0.01
**Total**	**0.3**	**1991**	**0.01**

1.31.2 By Mineral

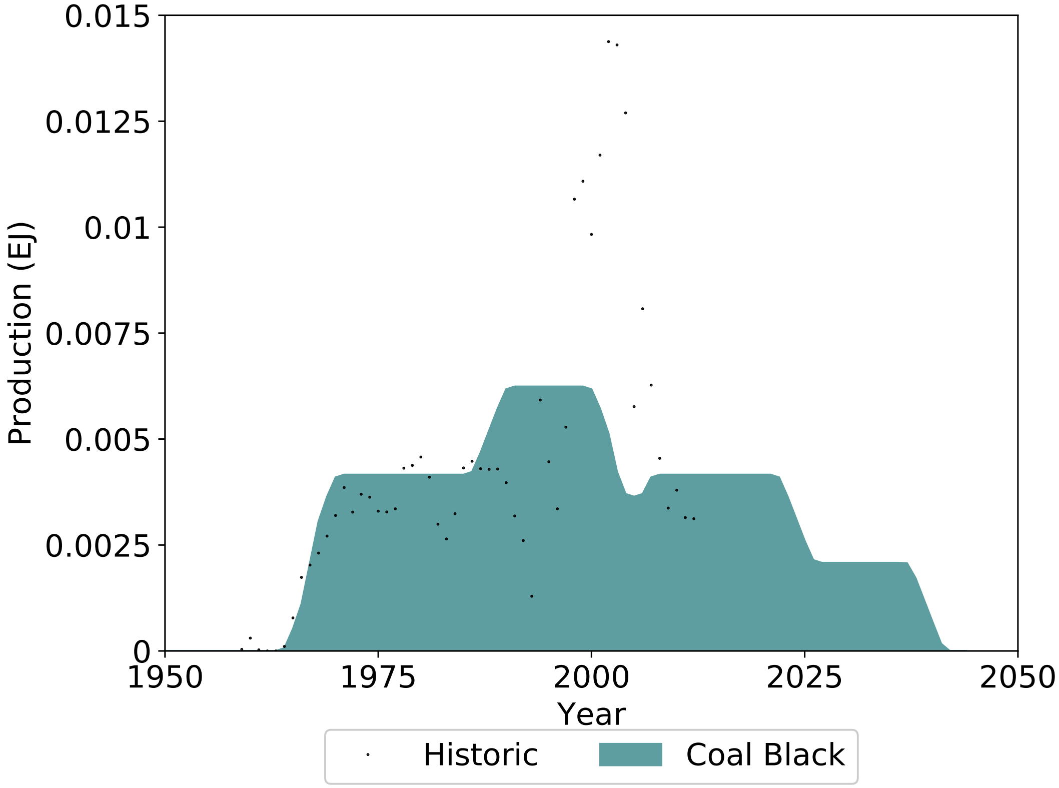

Figure 1.69: Swaziland projection by mineral type

Table 1.69: Peak years - Minerals

Name	URR	Peak Year	Peak Rate
Coal Black	0.3	1991	0.01
**Total**	**0.3**	**1991**	**0.01**

1.32 Tanzania

1.32.1 All Projections

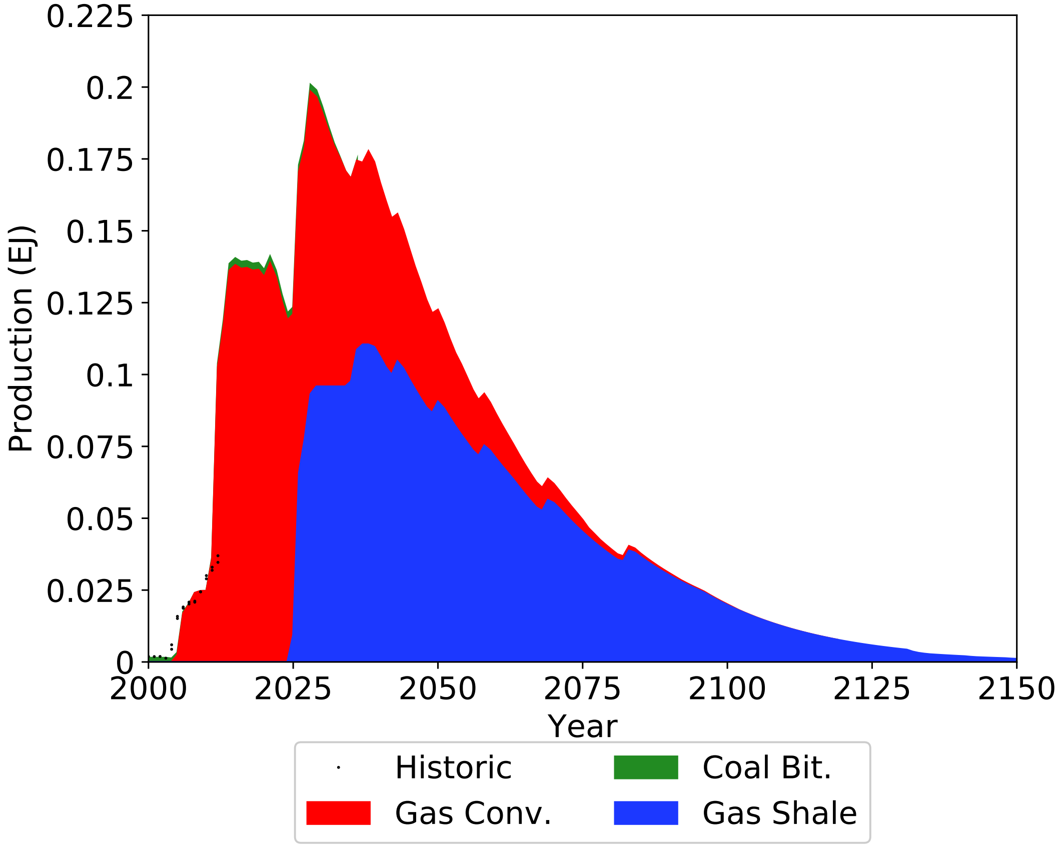

Figure 1.70: Tanzania projections capped at 16

Table 1.70: Peak years - All

**Name**	**URR**	**Peak Year**	**Peak Rate**
Gas Shale	5.19	2037	0.11
Gas Conv.	4.0	2021	0.14
Coal Bit.	0.07	2015	–
**Total**	**9.26**	**2028**	**0.2**

1.32.2 By Mineral

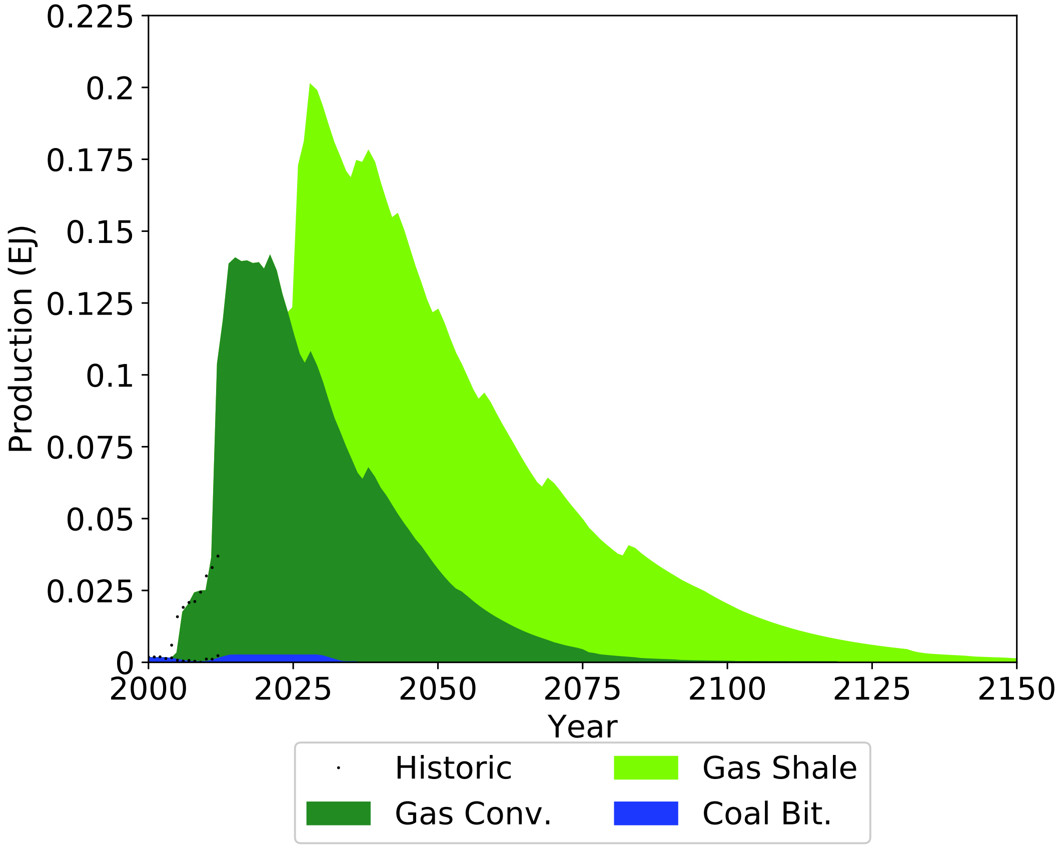

Figure 1.71: Tanzania projection by mineral type

Table 1.71: Peak years - Minerals

Name	URR	Peak Year	Peak Rate
Coal Bit.	0.07	2015	–
Gas Conv.	4.0	2021	0.14
Gas Shale	5.19	2037	0.11
**Total**	**9.26**	**2028**	**0.2**

1.33 Togo

1.33.1 All Projections

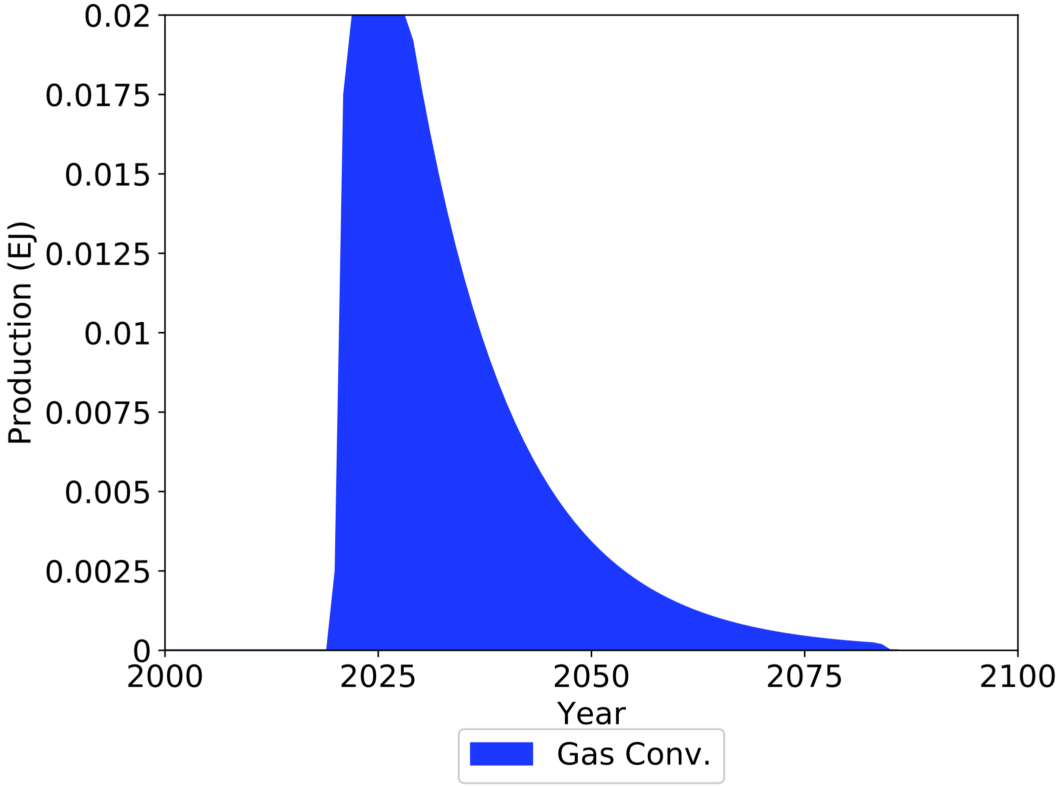

Figure 1.72: Togo projections capped at 16

Table 1.72: Peak years - All

Name	URR	Peak Year	Peak Rate
Gas Conv.	0.4	2022	0.02
**Total**	**0.4**	**2022**	**0.02**

1.33.2 By Mineral

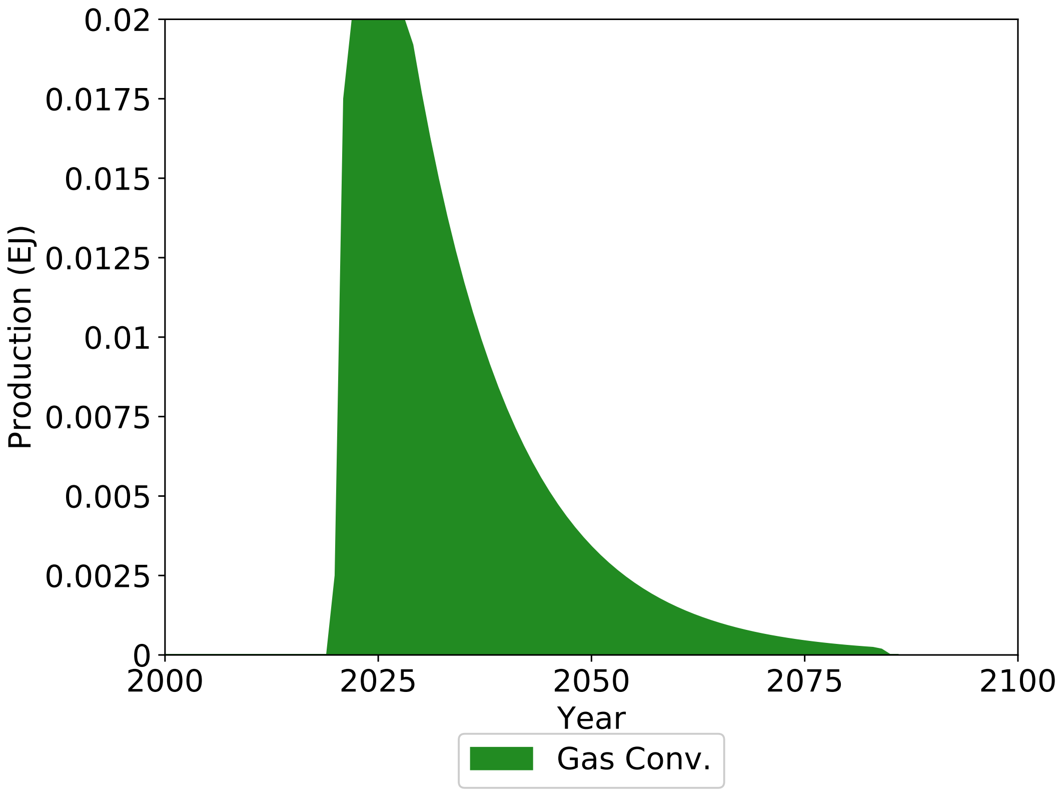

Figure 1.73: Togo projection by mineral type

Table 1.73: Peak years - Minerals

Name	URR	Peak Year	Peak Rate
Gas Conv.	0.4	2022	0.02
**Total**	**0.4**	**2022**	**0.02**

1.34 Tunisia

1.34.1 All Projections

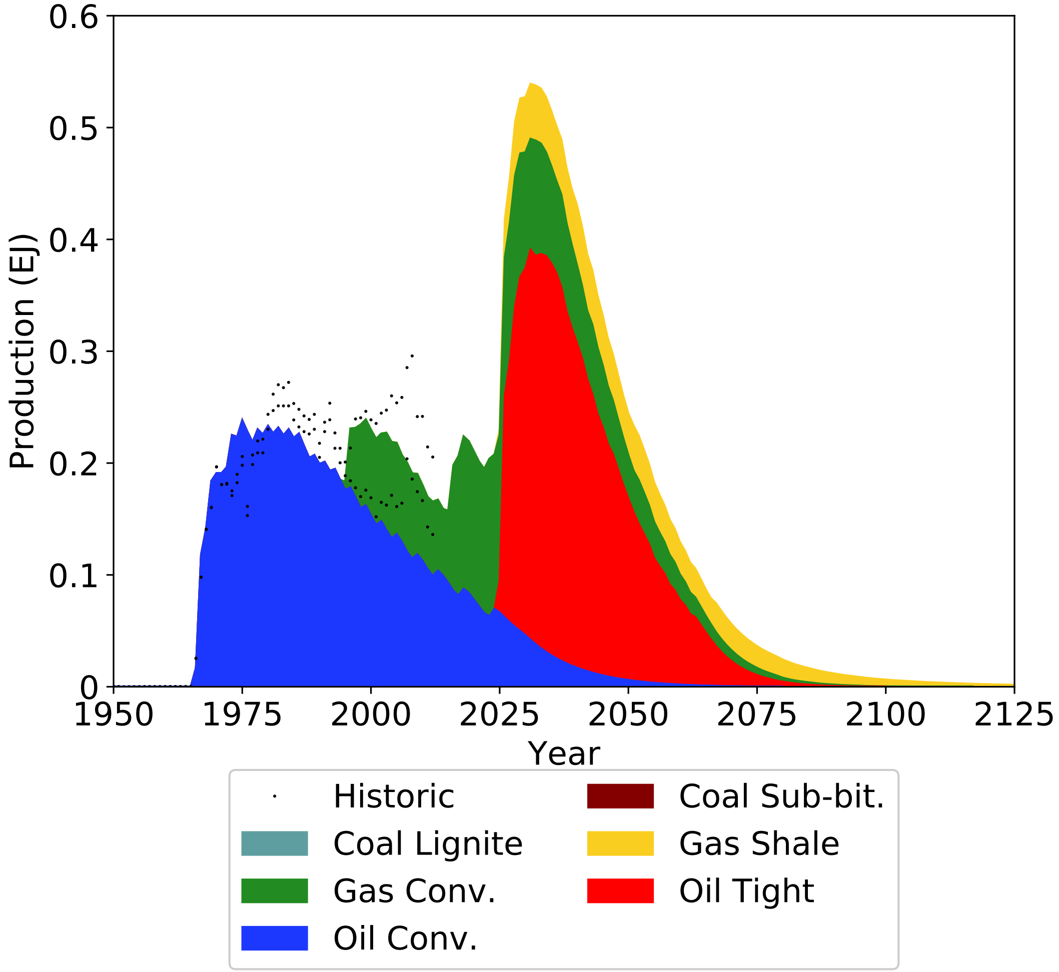

Figure 1.74: Tunisia projections capped at 16

Table 1.74: Peak years - All

**Name**	**URR**	**Peak Year**	**Peak Rate**
Oil Conv.	10.07	1975	0.24
Oil Tight	8.6	2034	0.35
Gas Conv.	5.3	2023	0.14
Gas Shale	2.26	2040	0.05
Coal Lignite	0.01	1943	–
Coal Sub-bit.	–	1919	–
**Total**	**26.24**	**2031**	**0.54**

1.34.2 By Mineral

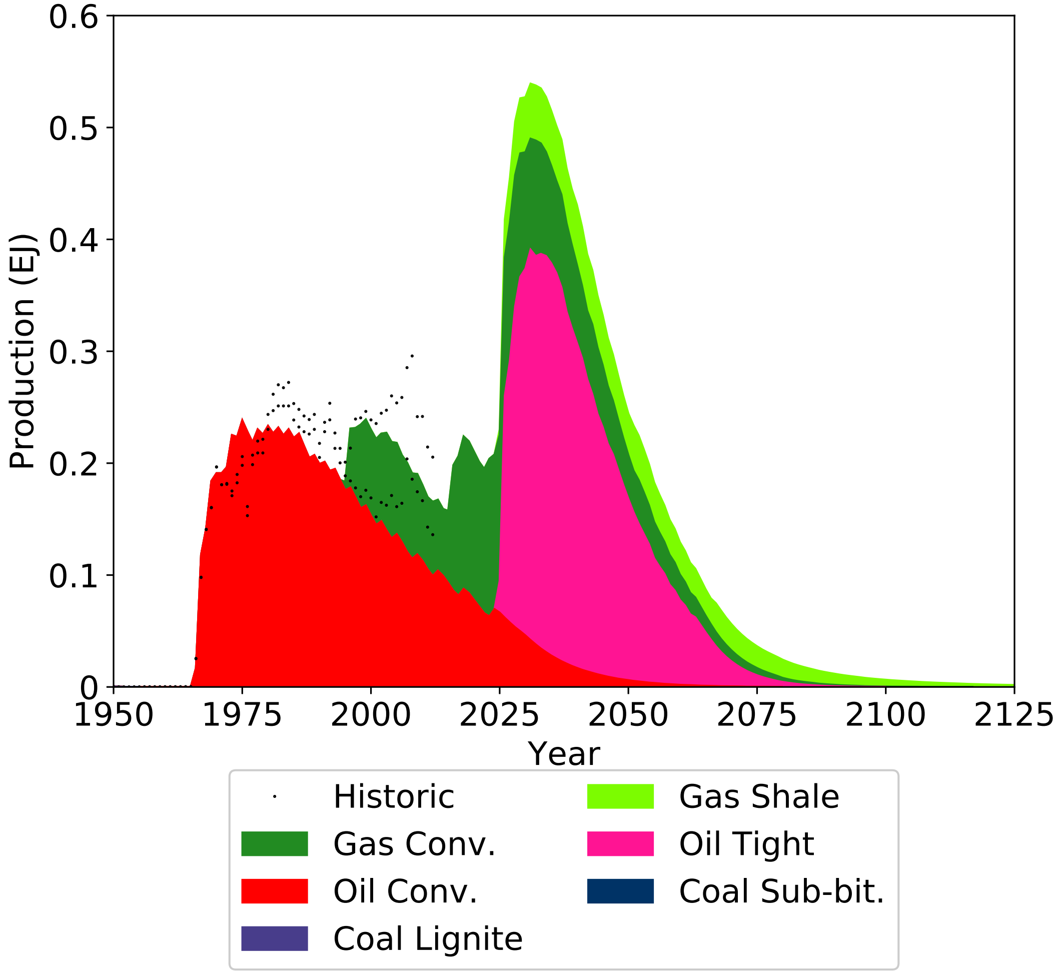

Figure 1.75: Tunisia projection by mineral type

Table 1.75: Peak years - Minerals

**Name**	**URR**	**Peak Year**	**Peak Rate**
Coal Lignite	0.01	1943	–
Coal Sub-bit.	–	1919	–
Oil Conv.	10.07	1975	0.24
Oil Tight	8.6	2034	0.35
Gas Conv.	5.3	2023	0.14
Gas Shale	2.26	2040	0.05
**Total**	**26.24**	**2031**	**0.54**

1.35 Western Sahara

1.35.1 All Projections

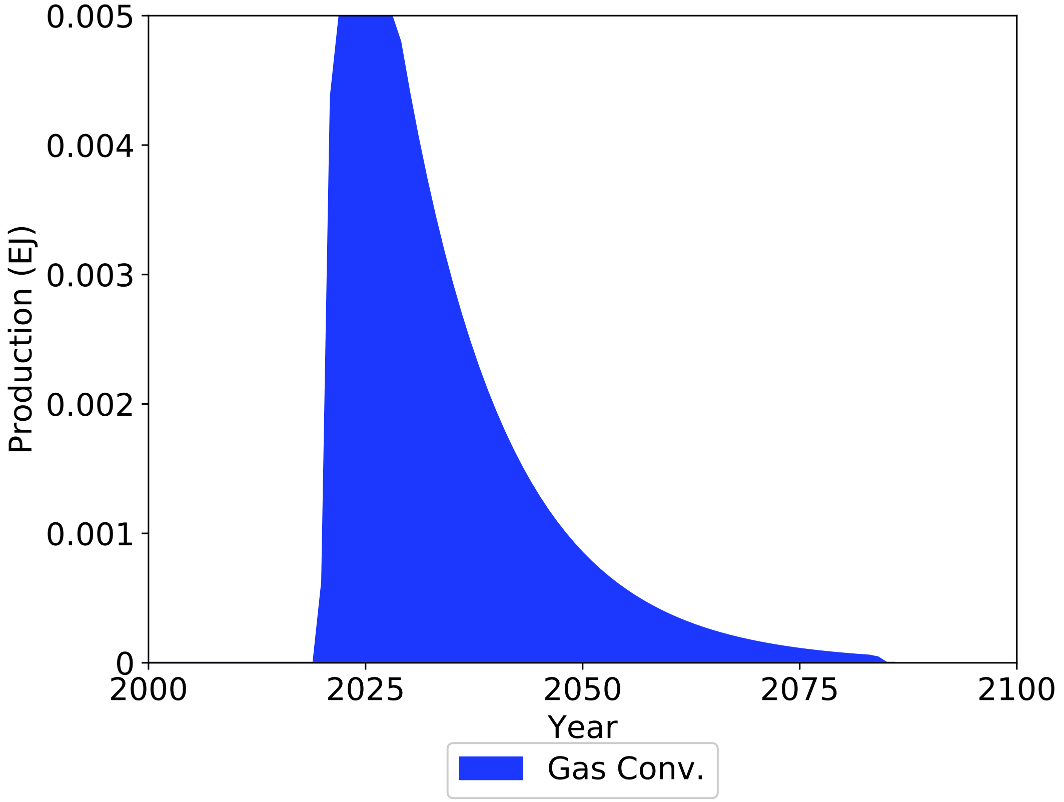

Figure 1.76: Western Sahara projections capped at 16

Table 1.76: Peak years - All

Name	URR	Peak Year	Peak Rate
Gas Conv.	0.1	2022	0.01
**Total**	**0.1**	**2022**	**0.01**

1.35.2 By Mineral

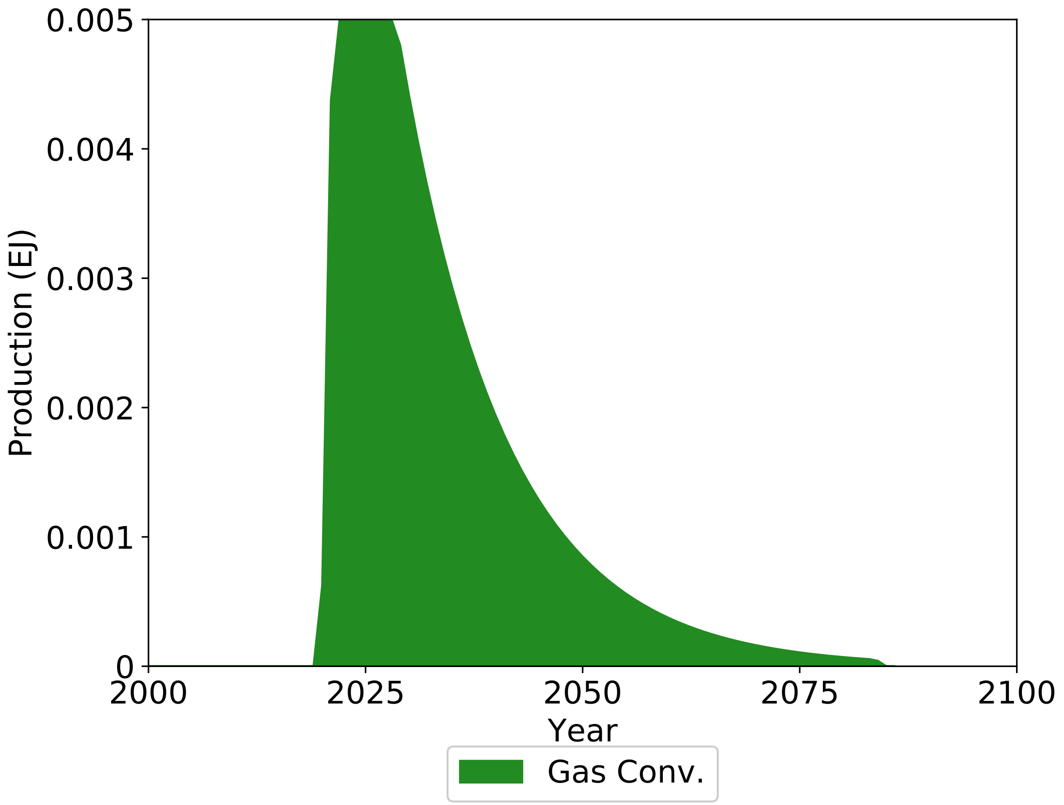

Figure 1.77: Western Sahara projection by mineral type

Table 1.77: Peak years - Minerals

**Name**	**URR**	**Peak Year**	**Peak Rate**
Gas Conv.	0.1	2022	0.01
**Total**	**0.1**	**2022**	**0.01**

1.36 Zaire

1.36.1 All Projections

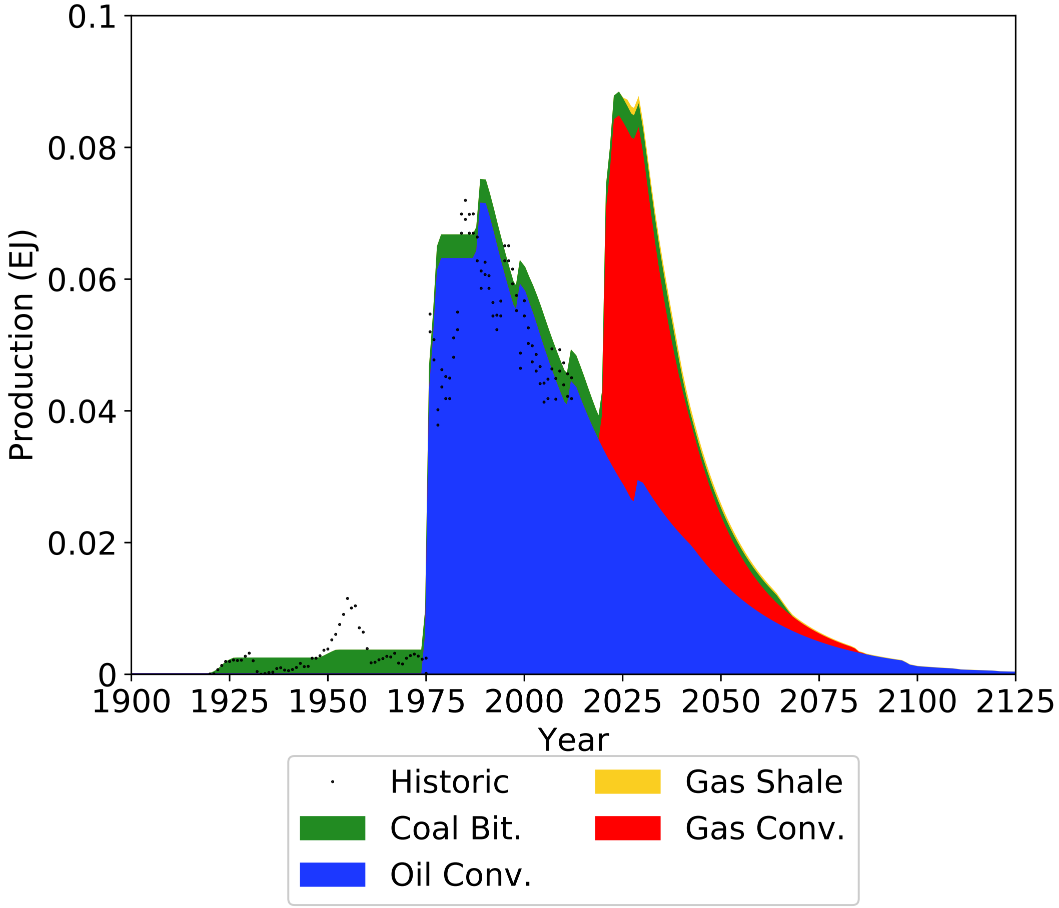

Figure 1.78: Zaire projections capped at 16

Table 1.78: Peak years - All

**Name**	**URR**	**Peak Year**	**Peak Rate**
Oil Conv.	3.43	1989	0.07
Gas Conv.	1.1	2024	0.06
Coal Bit.	0.44	2005	–
Gas Shale	0.04	2027	–
**Total**	**5.0**	**2024**	**0.09**

1.36.2 By Mineral

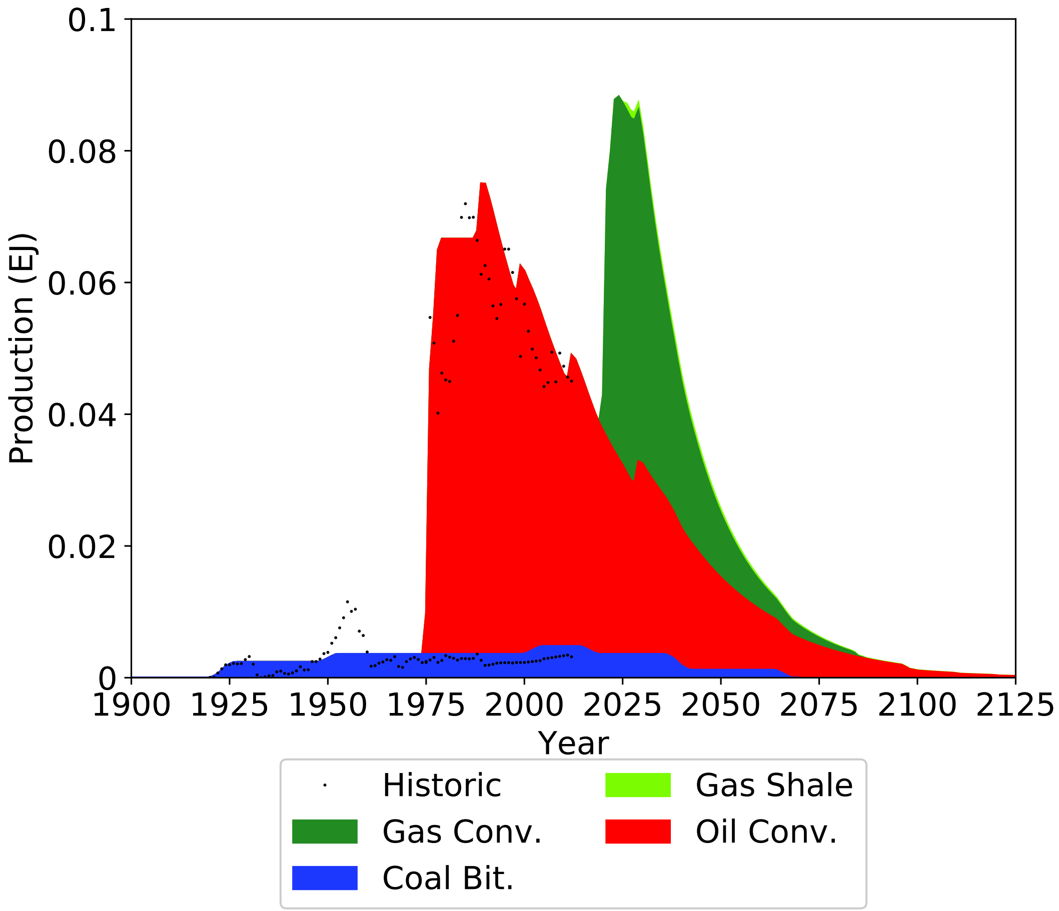

Figure 1.79: Zaire projection by mineral type

Table 1.79: Peak years - Minerals

Name	URR	Peak Year	Peak Rate
Coal Bit.	0.44	2005	–
Oil Conv.	3.43	1989	0.07
Gas Conv.	1.1	2024	0.06
Gas Shale	0.04	2027	–
**Total**	**5.0**	**2024**	**0.09**

1.37 Zambia

1.37.1 All Projections

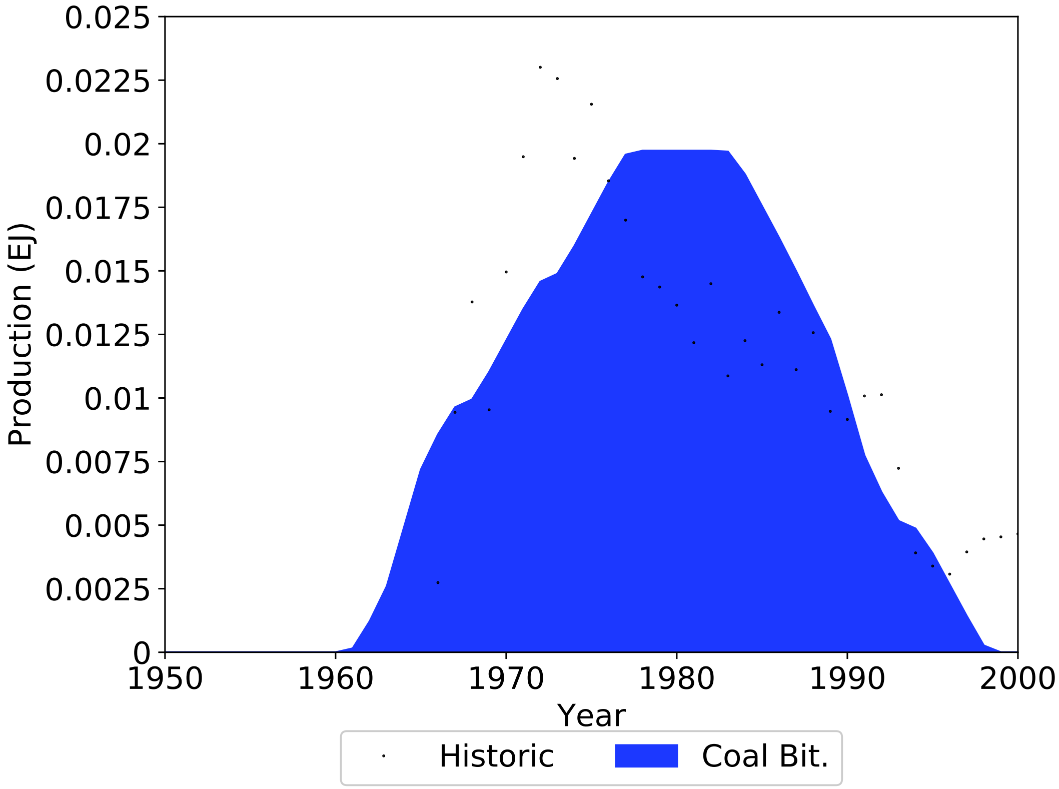

Figure 1.80: Zambia projections capped at 16

Table 1.80: Peak years - All

**Name**	**URR**	**Peak Year**	**Peak Rate**
Coal Bit.	0.44	1978	0.02
**Total**	**0.44**	**1978**	**0.02**

1.37.2 By Mineral

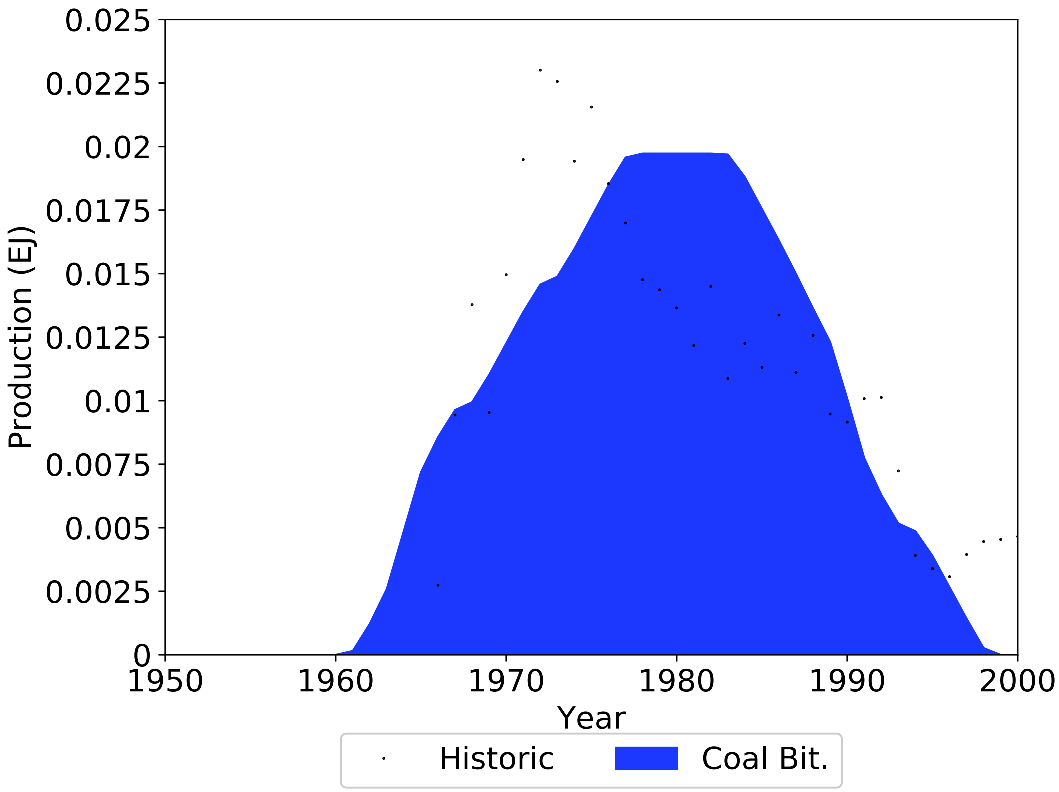

Figure 1.81: Zambia projection by mineral type

Table 1.81: Peak years - Minerals

Name	URR	Peak Year	Peak Rate
Coal Bit.	0.44	1978	0.02
**Total**	**0.44**	**1978**	**0.02**

1.38 Zimbabwe

1.38.1 All Projections

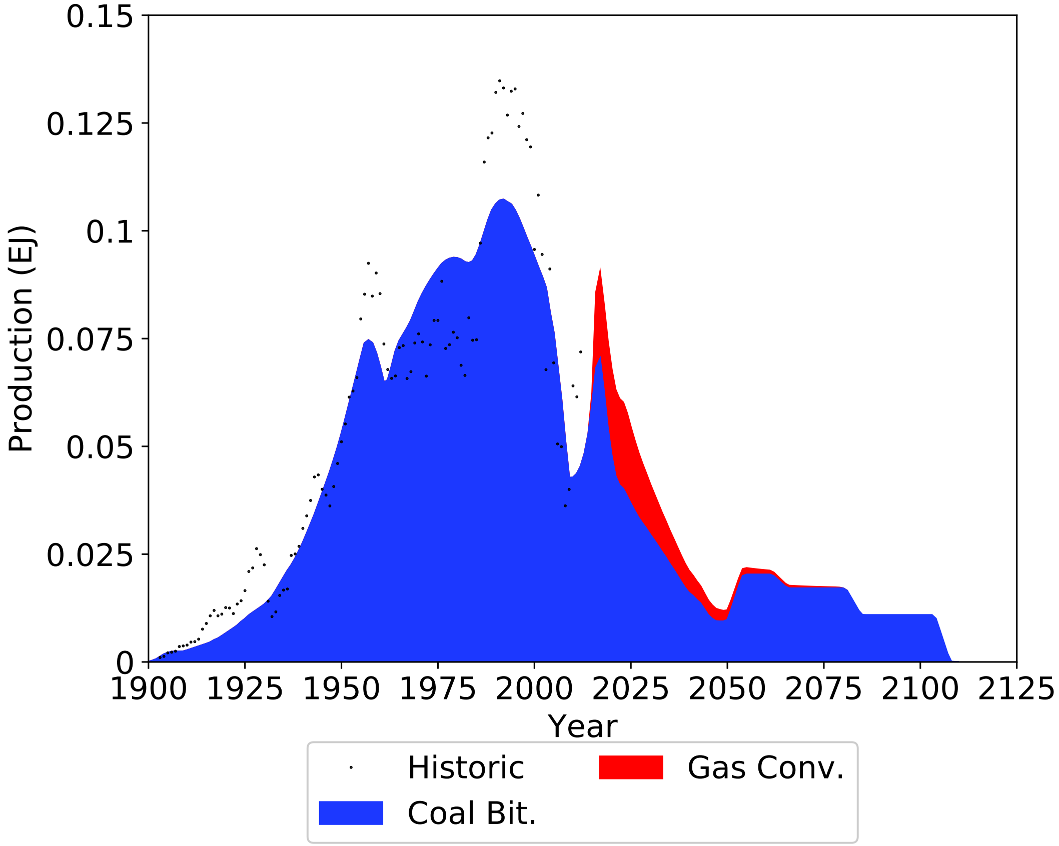

Figure 1.82: Zimbabwe projections capped at 16

Table 1.82: Peak years - All

Name	URR	Peak Year	Peak Rate
Coal Bit.	7.88	1992	0.11
Gas Conv.	0.4	2017	0.02
**Total**	**8.28**	**1992**	**0.11**

1.38.2 By Mineral

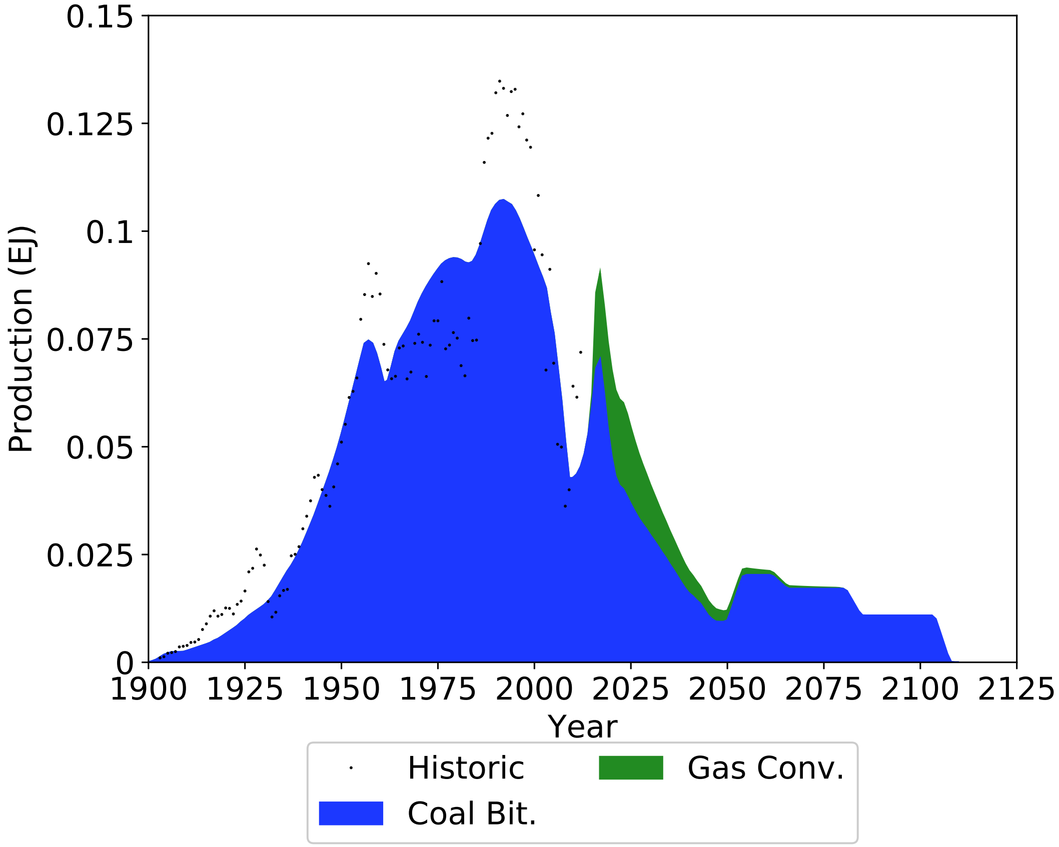

Figure 1.83: Zimbabwe projection by mineral type

Table 1.83: Peak years - Minerals

Name	URR	Peak Year	Peak Rate
Coal Bit.	7.88	1992	0.11
Gas Conv.	0.4	2017	0.02
**Total**	**8.28**	**1992**	**0.11**

1.39 Total

1.39.1 By country

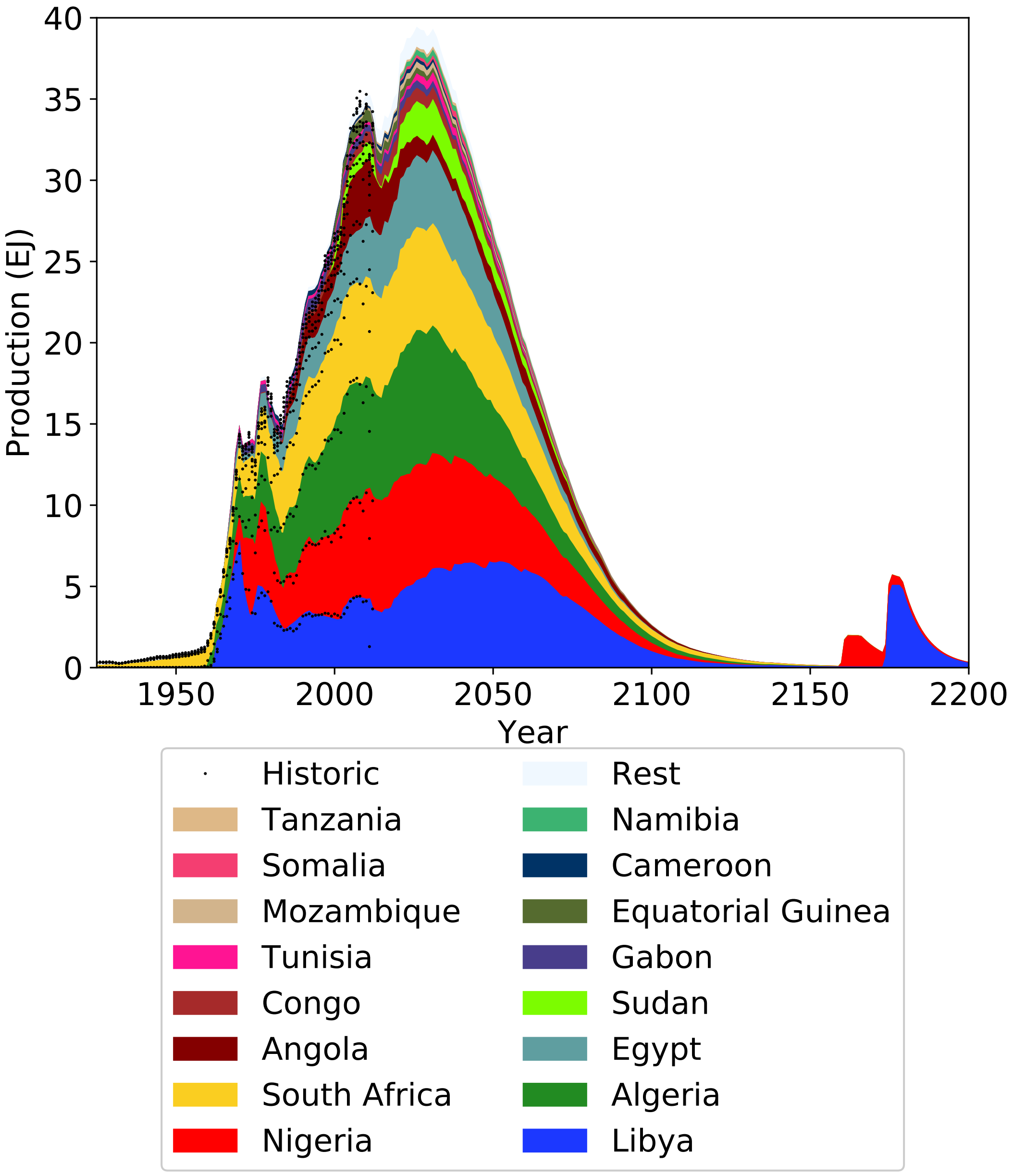

Figure 1.84: Africa projections by country

Table 1.84: Peak years - All

**Name**	**URR**	**Peak Year**	**Peak Rate**
Libya	653.66	1970	7.51
Nigeria	597.85	2018	7.23
Algeria	569.48	2026	8.25
South Africa	542.14	2023	6.47
Egypt	279.2	2035	4.46
Angola	145.59	2008	3.71
Sudan	79.55	2029	2.24
Congo	42.64	2023	0.91
Gabon	37.65	1994	0.77
Tunisia	26.24	2031	0.54
Equatorial Guinea	23.06	2006	0.89
Mozambique	16.3	2026	0.37
Cameroon	16.15	1986	0.36
Somalia	15.1	2038	0.39
Namibia	13.4	2030	0.37
Tanzania	9.26	2028	0.2
Zimbabwe	8.28	1992	0.11
Ghana	7.0	2019	0.33
Niger	6.24	2017	0.26
Eritrea	5.6	2028	0.19
Zaire	5.0	2024	0.09
Ivory Coast	4.38	2006	0.17
Chad	4.17	2005	0.35
Rwanda	2.9	2030	0.11
Morocco	2.78	2033	0.09
Mauritania	2.34	2024	0.08
Ethiopia	1.71	2024	0.07
Benin	0.97	2024	0.04
Botswana	0.93	1998	0.03
Seychelles	0.7	2024	0.04
Zambia	0.44	1978	0.02
Togo	0.4	2022	0.02
Swaziland	0.3	1991	0.01
Madagascar	0.2	2022	0.01
Guinea-Bissau	0.2	2022	0.01
Western Sahara	0.1	2022	0.01
Senegal	0.1	2010	–
Malawi	0.08	2020	–
**Total**	**3122.08**	**2026**	**39.36**

1.39.2 By mineral

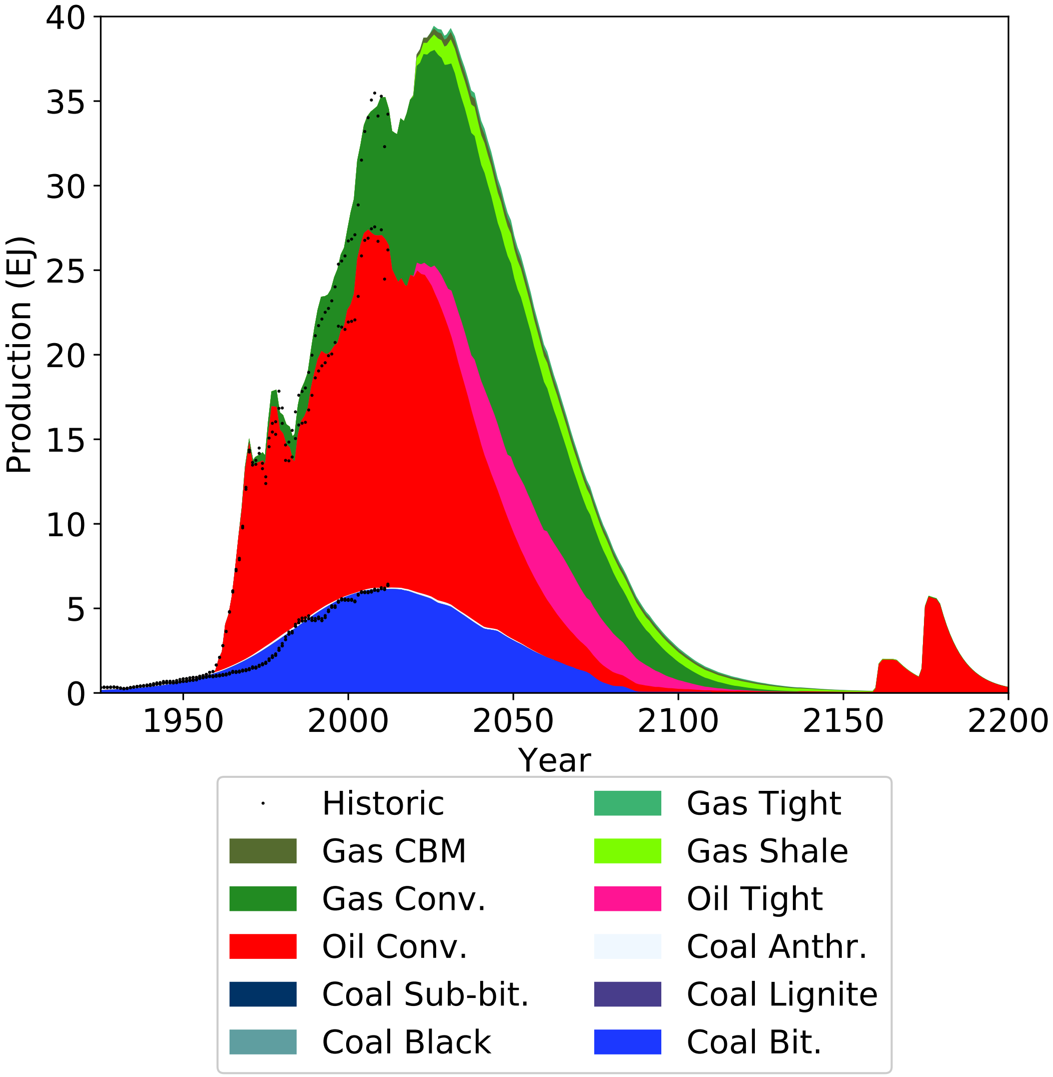

Figure 1.85: Africa projection by mineral type

Table 1.85: Peak years - Minerals

**Name**	**URR**	**Peak Year**	**Peak Rate**
Coal Bit.	456.2	2013	6.09
Coal Black	0.3	1991	0.01
Coal Lignite	0.01	1942	–
Coal Sub-bit.	0.54	1961	0.01
Coal Anthr.	9.99	1980	0.15
Oil Conv.	1404.82	2006	21.23
Oil Tight	218.31	2053	4.21
Gas Conv.	867.0	2032	13.46
Gas Shale	113.02	2044	1.8
Gas CBM	31.5	2041	0.51
Gas Tight	20.39	2041	0.35
**Total**	**3122.08**	**2026**	**39.36**

Chapter 2

Asia

2.1 Afghanistan

2.1.1 All Projections

Table 2.1: Peak years - All

**Name**	**URR**	**Peak Year**	**Peak Rate**
Gas Conv.	24.3	2032	0.57
Coal Bit.	0.38	2014	0.03
**Total**	**24.68**	**2032**	**0.57**

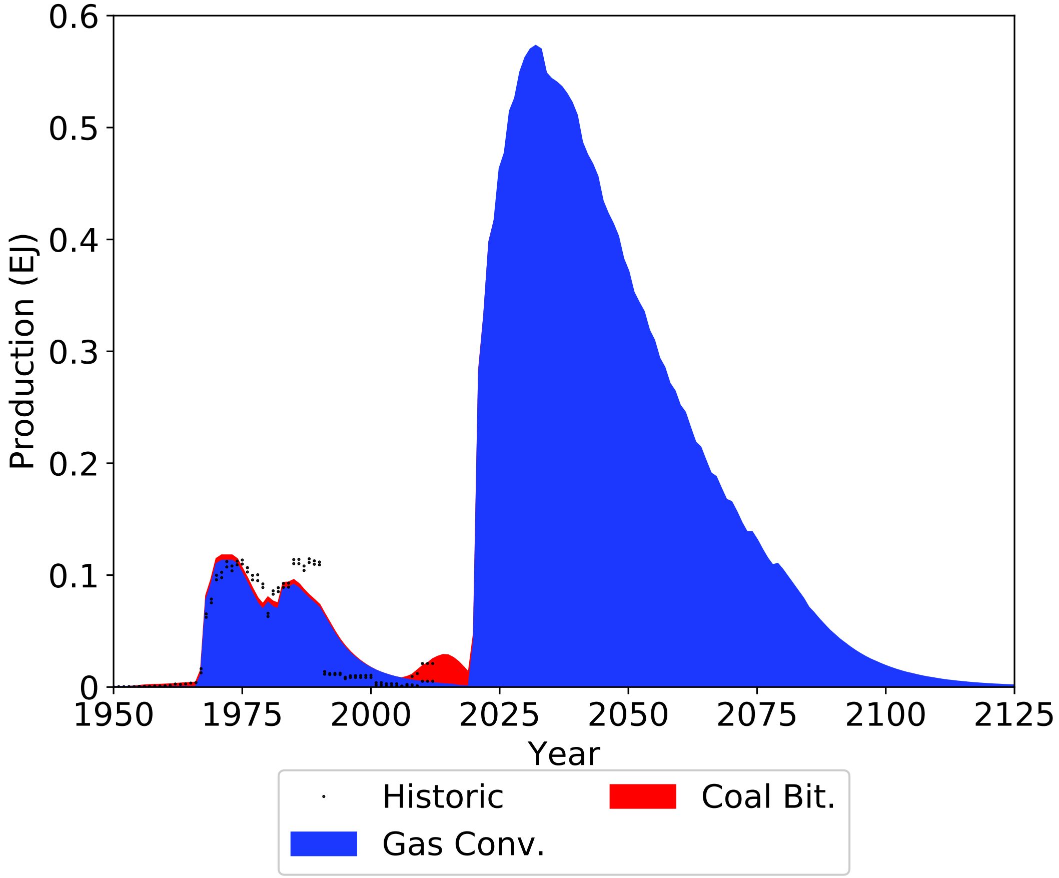

Figure 2.1: Afghanistan projections capped at 16

2.1.2 By Mineral

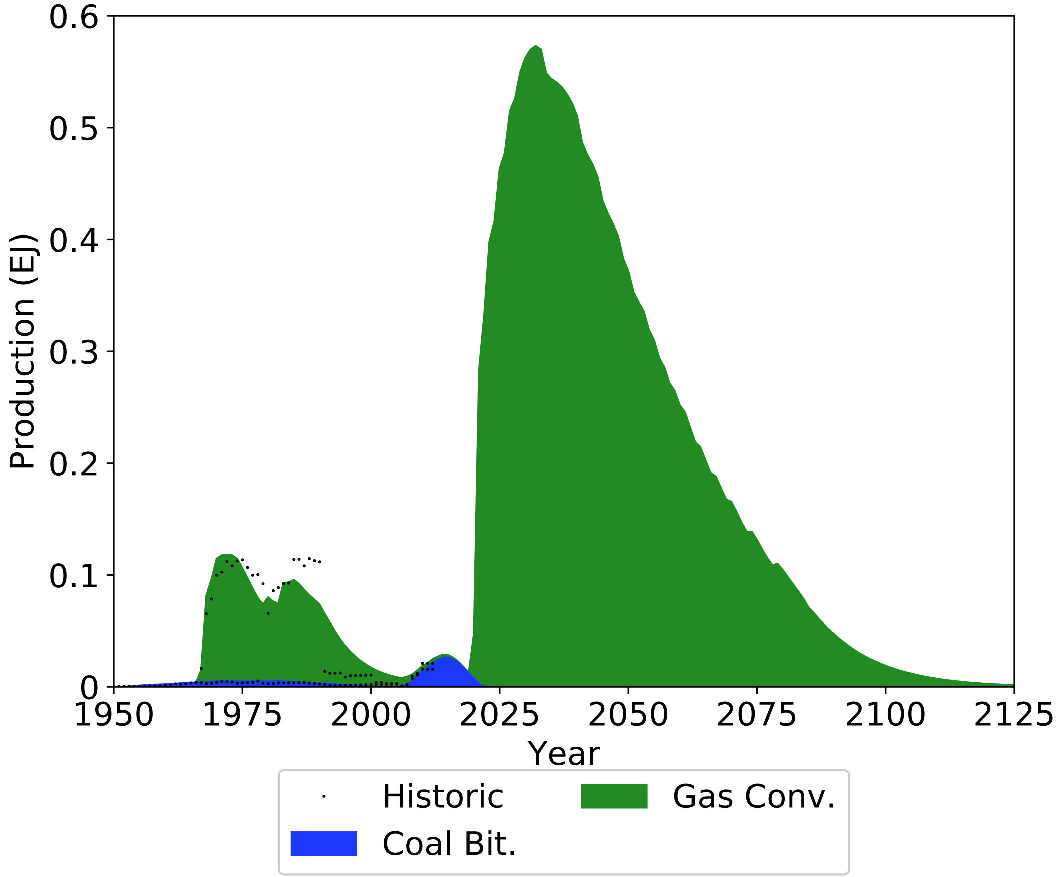

Figure 2.2: Afghanistan projection by mineral type

Table 2.2: Peak years - Minerals

Name	URR	Peak Year	Peak Rate
Coal Bit.	0.38	2014	0.03
Gas Conv.	24.3	2032	0.57
**Total**	**24.68**	**2032**	**0.57**

2.2 Australia

2.2.1 All Projections

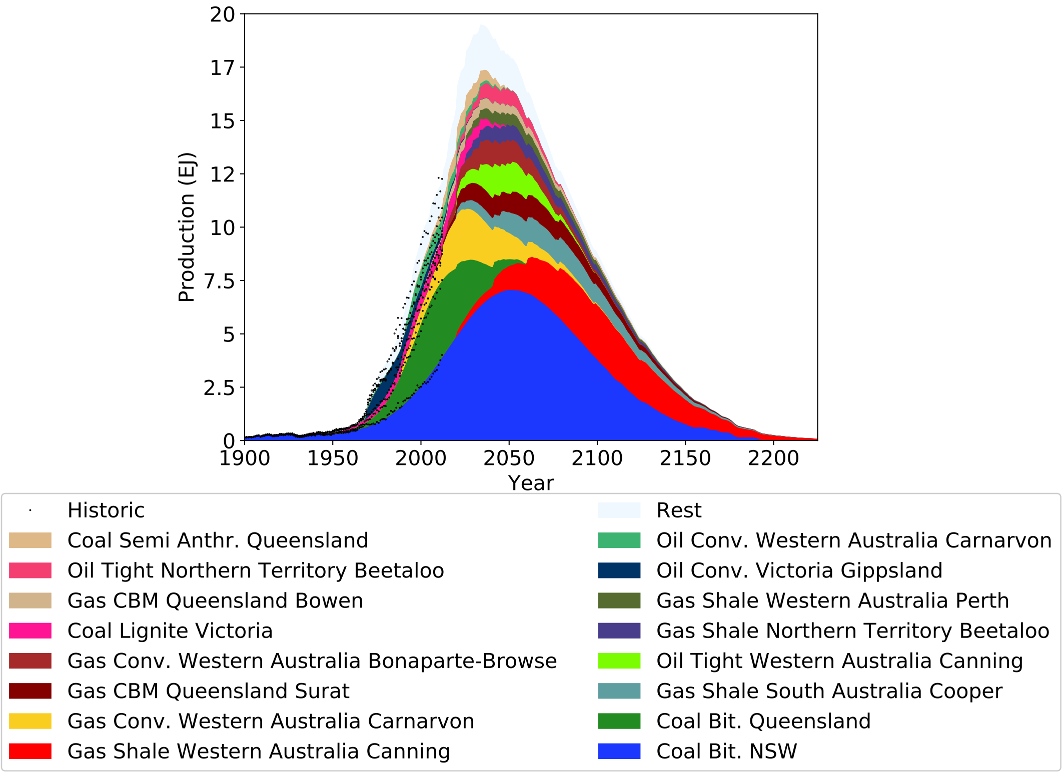

Figure 2.3: Australia projections capped at 16

Table 2.3: Peak years - All

**Name**	**URR**	**Peak Year**	**Peak Rate**
Coal Bit. NSW	723.66	2051	7.03
Gas Shale Western Australia Canning	246.75	2101	2.58
Coal Bit. Queensland	158.7	2011	3.61
Gas Conv. Western Australia Carnarvon	113.35	2023	2.43
Gas Shale South Australia Cooper	97.65	2059	1.16
Gas CBM Queensland Surat	84.93	2054	1.07
Oil Tight Western Australia Canning	55.58	2041	1.44
Gas Conv. Western Australia Bonaparte-Browse	54.9	2038	1.15
Gas Shale Northern Territory Beetaloo	46.2	2045	0.72
Coal Lignite Victoria	40.33	2017	0.77
Gas Shale Western Australia Perth	34.65	2046	0.55
Gas CBM Queensland Bowen	30.92	2033	0.5
Oil Conv. Victoria Gippsland	28.04	1980	0.96
Oil Tight Northern Territory Beetaloo	26.93	2049	0.74
Oil Conv. Western Australia Carnarvon	25.26	2003	0.82
Coal Semi Anthr. Queensland	22.82	2026	0.77
Gas Tight Australia	21.0	2041	0.35
Gas Shale Queensland Maryborough	19.95	2044	0.34
Gas Conv. Victoria Gippsland	17.53	2001	0.26
Coal Sub-bit. Queensland	14.97	2014	0.43
Gas Shale Northern Territory Georgina	13.65	2042	0.24
Gas CBM NSW	8.71	2029	0.17
Coal Sub-bit. South Australia	7.06	2051	0.14
Gas Conv. South Australia Cooper-Eromanga	6.69	1977	0.2
Gas Conv. JPDA Timor Gap	6.29	2015	0.18
Coal Sub-bit. Western Australia	6.25	2018	0.13
Oil Tight South Australia Cooper	5.73	2024	0.25
Oil Tight Northern Territory Georgina	5.73	2026	0.25
Oil Conv. South Australia Cooper-Eromanga	3.17	1987	0.11
Oil Tight Western Australia Perth	2.87	2025	0.14
Oil Tight Queensland Cooper	2.87	2022	0.14
Gas Conv. Victoria Otway	2.3	2010	0.11
Gas Conv. Queensland Cooper-Eromanga	2.23	1997	0.16
Gas CBM Queensland Clarence	1.44	2029	0.04
Oil Conv. Queensland Cooper-Eromanga	1.39	1985	0.05
Oil Conv. Northern Territory Timor Sea	1.17	2001	0.17
Gas Conv. Queensland Surat-Bowen	1.12	1990	0.02
Coal Bit. Tasmania	1.1	2026	0.02
Oil Conv. Northern Territory Browse-Bonaparte	1.09	1989	0.12
Gas Conv. Western Australia Perth	0.94	1981	0.03
Gas Conv. Tasmania Bass	0.84	2019	0.04
Gas Conv. Northern Territory Amadeus	0.69	1997	0.02
Coal Bit. Victoria	0.55	1917	0.02
Oil Conv. JPDA Timor Gap	0.44	2005	0.02
Gas Conv. Queensland Denison	0.41	1992	0.02
Oil Conv. Queensland Surat-Bowen	0.31	1964	0.02
Oil Conv. Western Australia Perth	0.19	2005	0.03
Oil Conv. Victoria Otway	0.13	2010	0.01
Oil Conv. Western Australia Timor Sea	0.12	2002	0.03

Table 2.3: Peak years - All – Continued

**Name**	**URR**	**Peak Year**	**Peak Rate**
Oil Conv. Northern Territory Amadeus	0.11	1989	0.01
Gas Conv. South Australia Otway	0.08	2000	0.01
Gas Conv. Queensland Clarence	0.07	2022	–
Oil Conv. Tasmania Bass	0.04	2006	0.01
Gas Conv. Queensland Adavale	0.03	1997	–
Oil Kerogen NSW	0.02	1943	–
Oil Conv. Western Australia Canning	0.02	1987	–
Oil Kerogen Queensland Other	0.01	2003	–
Gas Conv. Western Australia Canning	0.01	2022	–
Gas Conv. NSW Gunnedah	0.01	2017	–
Oil Conv. South Australia Otway	–	1994	–
Coal Sub-bit. NSW	–	1944	–
Coal Semi Anthr. Tasmania	–	1953	–
Gas CBM Western Australia	–	2008	–
Oil Kerogen Tasmania	–	1927	–
**Total**	**1950.01**	**2034**	**19.47**

2.2.2 By Mineral

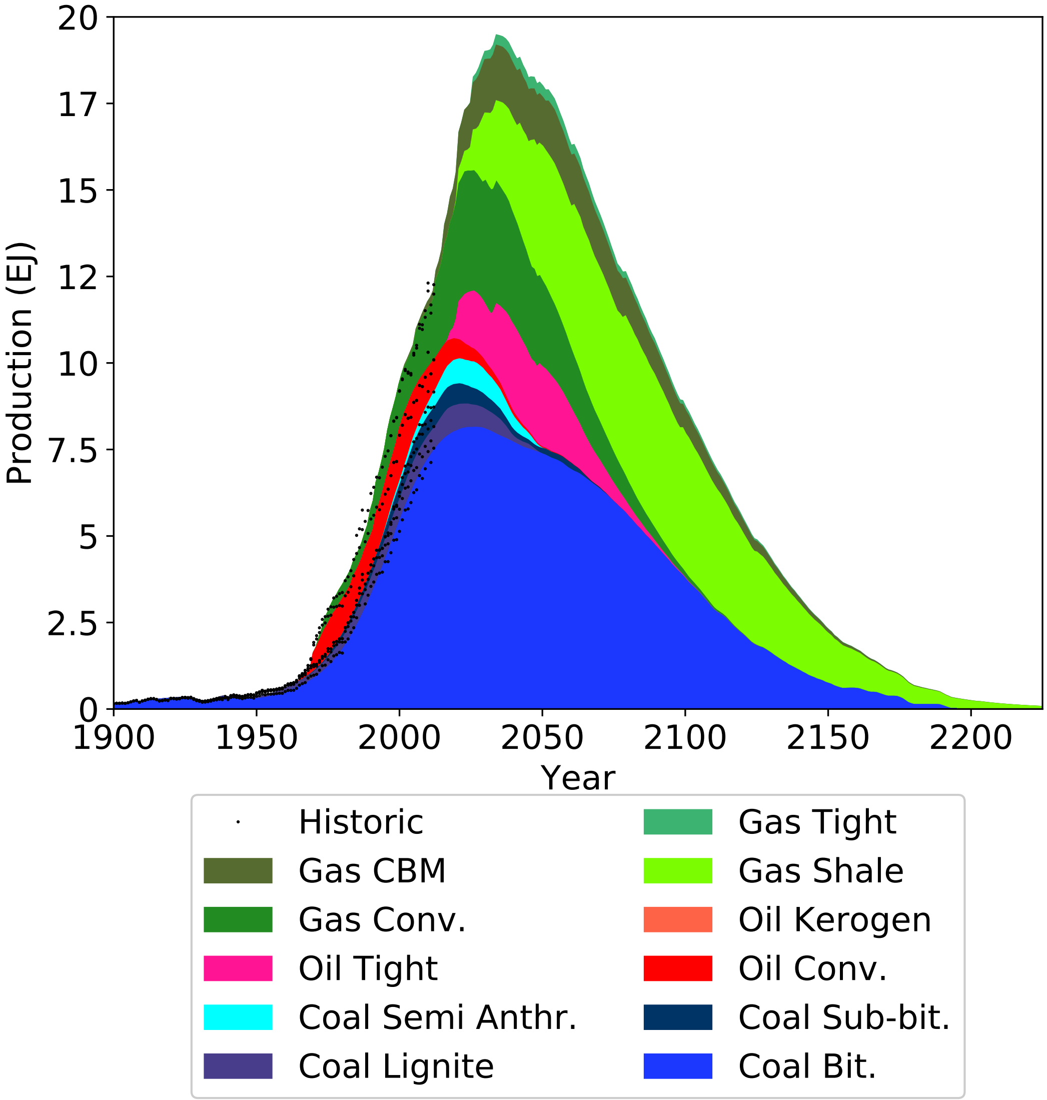

Figure 2.4: Australia projection by mineral type

2.2.3 Regional Projections

Table 2.4: Peak years - Minerals

**Name**	**URR**	**Peak Year**	**Peak Rate**
Coal Bit.	884.01	2027	8.13
Coal Lignite	40.33	2017	0.77
Coal Sub-bit.	28.28	2017	0.62
Coal Semi Anthr.	22.82	2026	0.77
Oil Conv.	61.48	2001	1.57
Oil Tight	99.71	2040	2.52
Oil Kerogen	0.04	2003	–
Gas Conv.	207.49	2032	3.59
Gas Shale	458.85	2080	4.63
Gas CBM	126.0	2036	1.61
Gas Tight	21.0	2041	0.35
**Total**	**1950.01**	**2034**	**19.47**

Australia

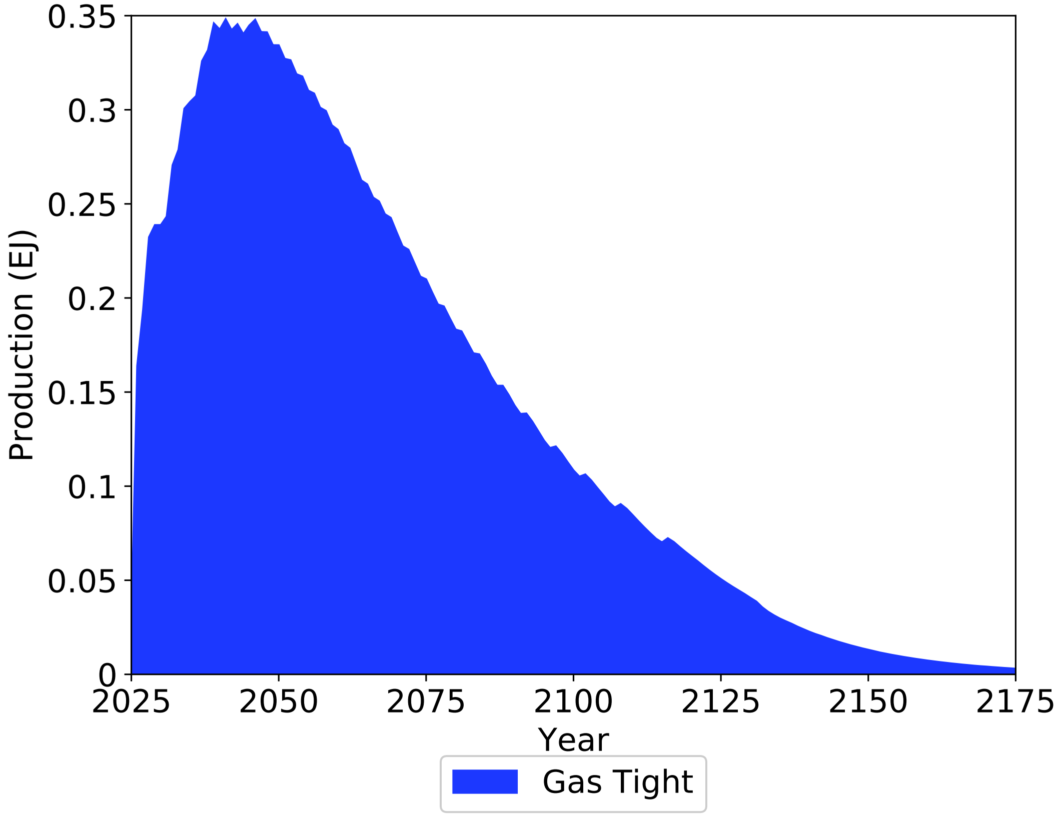

Figure 2.5: Australia - Australia projections capped at 16

Table 2.5: Peak years - All

Name	URR	Peak Year	Peak Rate
Gas Tight Australia	21.0	2041	0.35
**Total**	**21.0**	**2041**	**0.35**

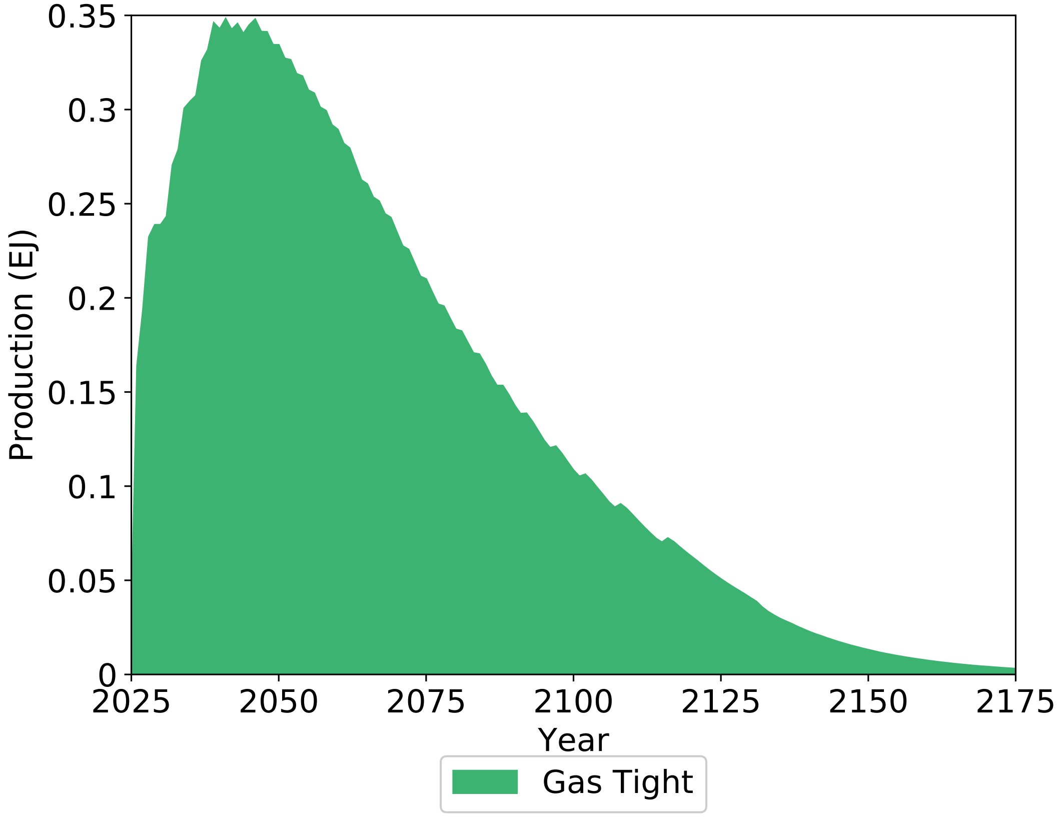

Figure 2.6: Australia - Australia projection by mineral type

Table 2.6: Peak years - Minerals

Name	URR	Peak Year	Peak Rate
Gas Tight	21.0	2041	0.35
**Total**	**21.0**	**2041**	**0.35**

JPDA

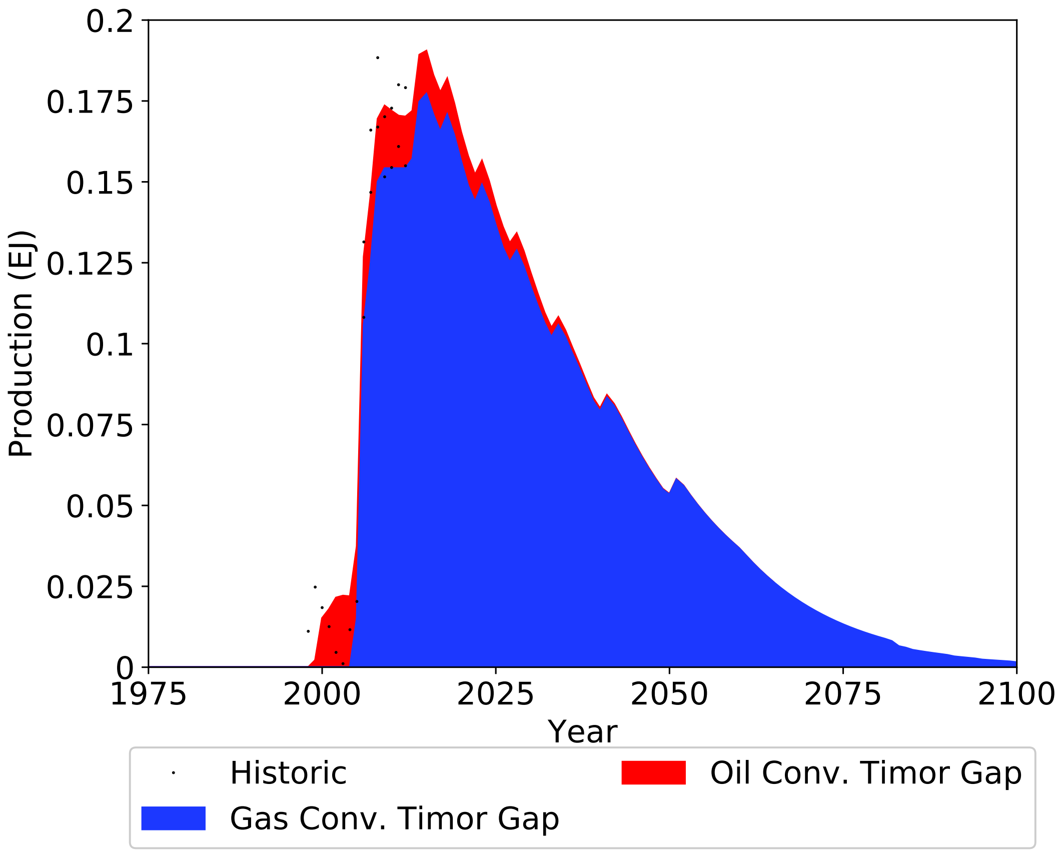

Figure 2.7: Australia - JPDA projections capped at 16

Table 2.7: Peak years - All

Name	URR	Peak Year	Peak Rate
Gas Conv. JPDA Timor Gap	6.29	2015	0.18
Oil Conv. JPDA Timor Gap	0.44	2005	0.02
**Total**	**6.73**	**2015**	**0.19**

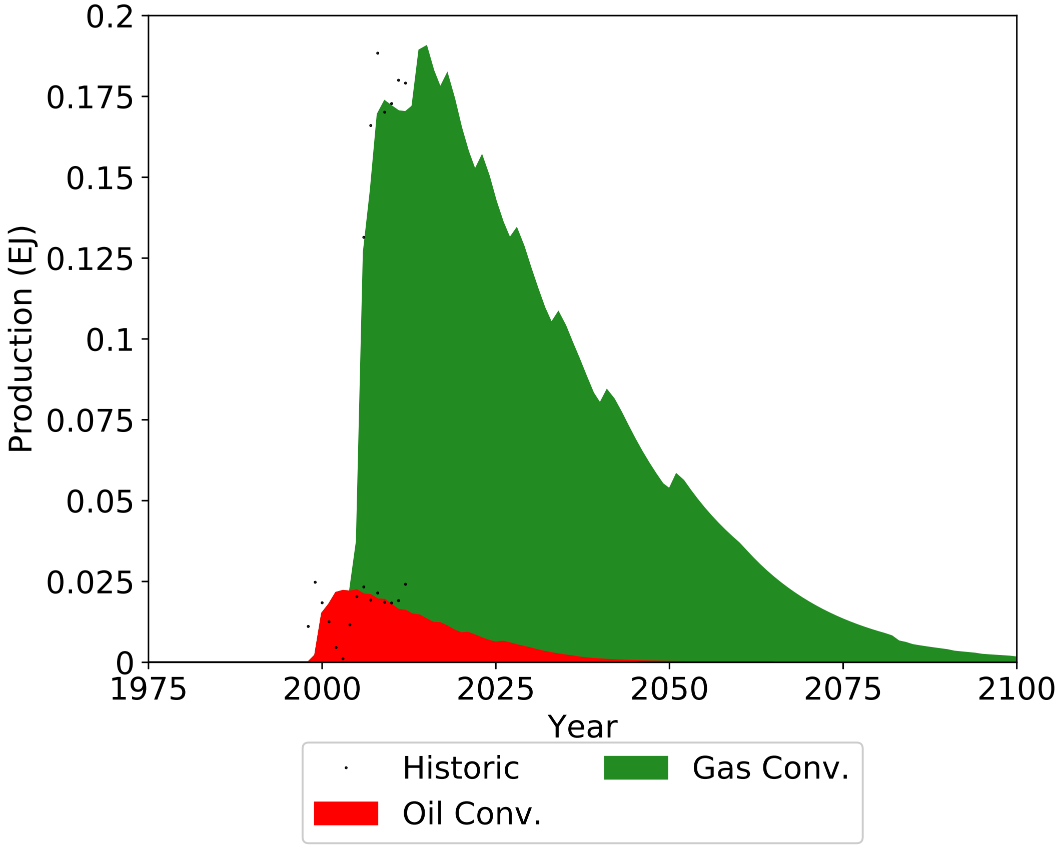

Figure 2.8: Australia - JPDA projection by mineral type

Table 2.8: Peak years - Minerals

Name	URR	Peak Year	Peak Rate
Oil Conv.	0.44	2005	0.02
Gas Conv.	6.29	2015	0.18
**Total**	**6.73**	**2015**	**0.19**

NSW

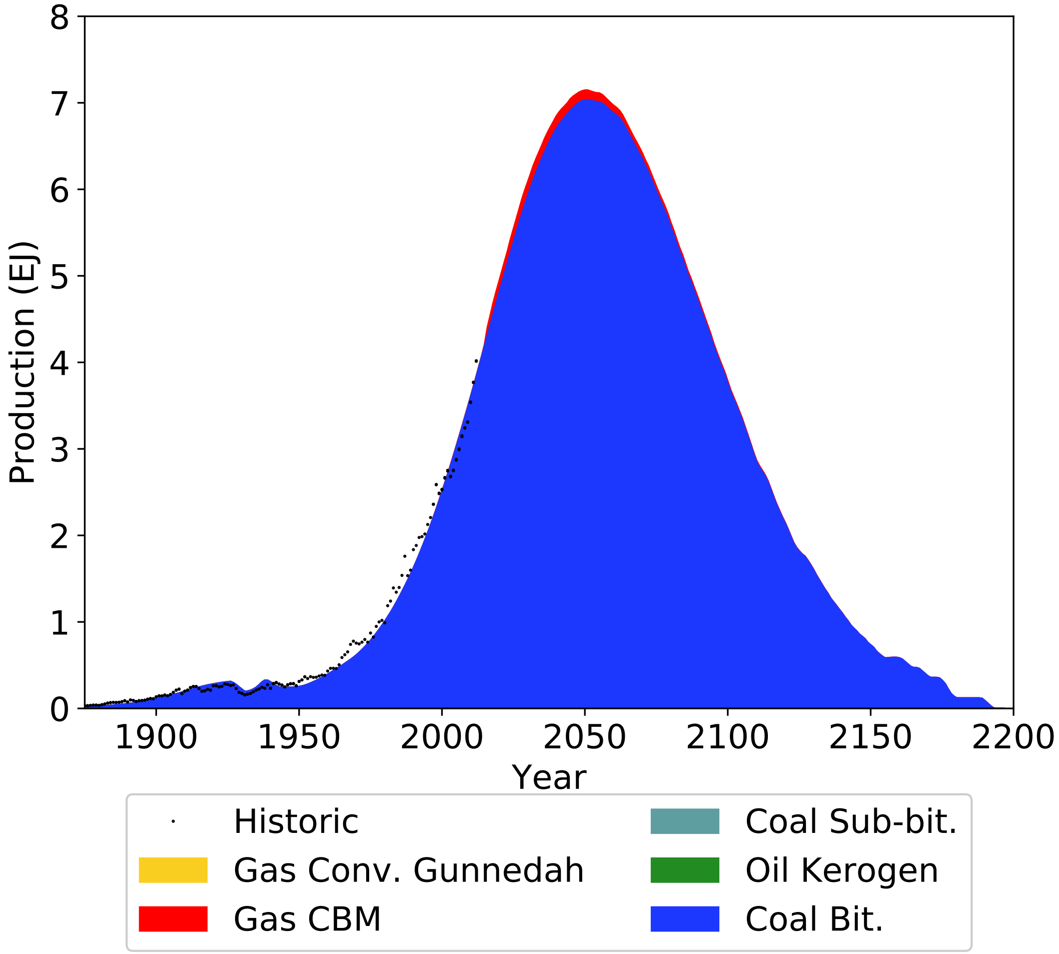

Figure 2.9: Australia - NSW projections capped at 16

Table 2.9: Peak years - All

**Name**	**URR**	**Peak Year**	**Peak Rate**
Coal Bit. NSW	723.66	2051	7.03
Gas CBM NSW	8.71	2029	0.17
Oil Kerogen NSW	0.02	1943	–
Gas Conv. NSW Gunnedah	0.01	2017	–
Coal Sub-bit. NSW	–	1944	–
**Total**	**732.41**	**2050**	**7.14**

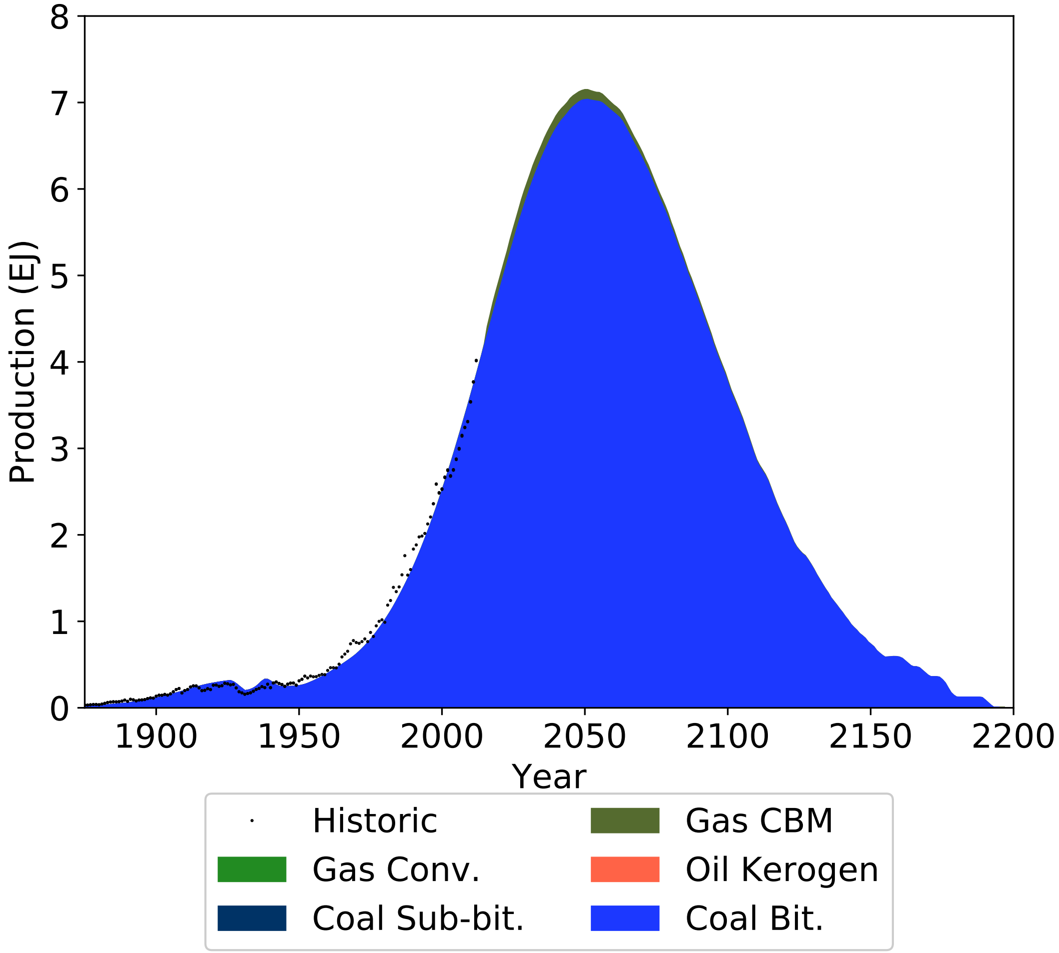

Figure 2.10: Australia - NSW projection by mineral type

Table 2.10: Peak years - Minerals

**Name**	**URR**	**Peak Year**	**Peak Rate**
Coal Bit.	723.66	2051	7.03
Coal Sub-bit.	–	1944	–
Oil Kerogen	0.02	1943	–
Gas Conv.	0.01	2017	–
Gas CBM	8.71	2029	0.17
**Total**	**732.41**	**2050**	**7.14**

Northern Territory

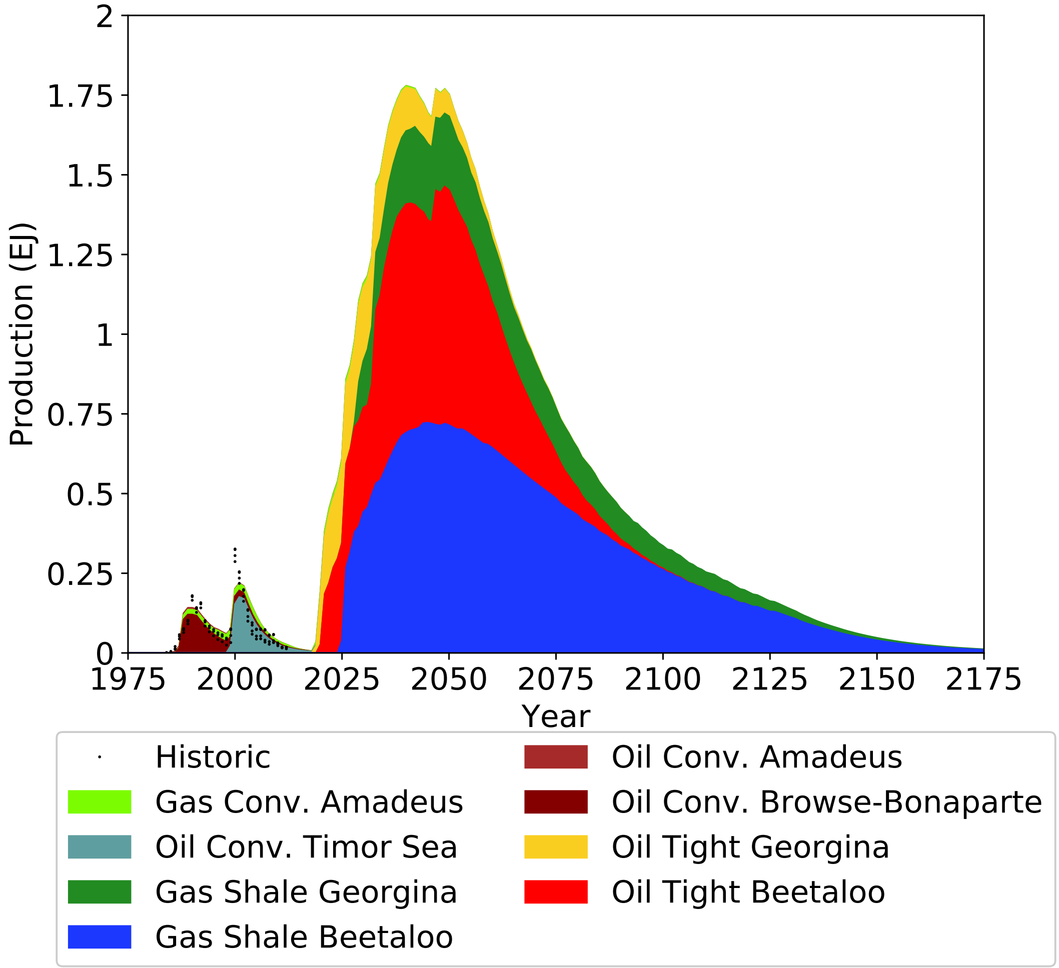

Figure 2.11: Australia - Northern Territory projections capped at 16

Table 2.11: Peak years - All

Name	URR	Peak Year	Peak Rate
Gas Shale Northern Territory Beetaloo	46.2	2045	0.72
Oil Tight Northern Territory Beetaloo	26.93	2049	0.74
Gas Shale Northern Territory Georgina	13.65	2042	0.24
Oil Tight Northern Territory Georgina	5.73	2026	0.25
Oil Conv. Northern Territory Timor Sea	1.17	2001	0.17
Oil Conv. Northern Territory Browse-Bonaparte	1.09	1989	0.12
Gas Conv. Northern Territory Amadeus	0.69	1997	0.02
Oil Conv. Northern Territory Amadeus	0.11	1989	0.01
**Total**	**95.56**	**2040**	**1.78**

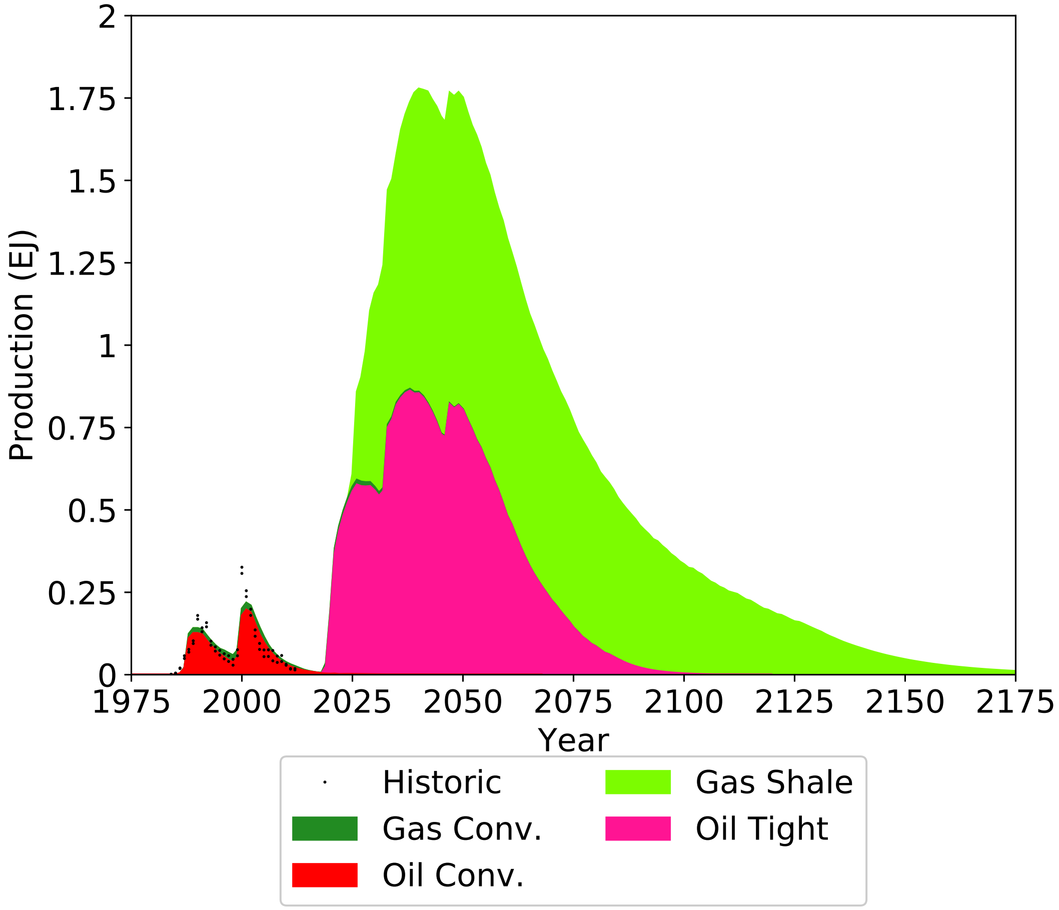

Figure 2.12: Australia - Northern Territory projection by mineral type

Table 2.12: Peak years - Minerals

Name	URR	Peak Year	Peak Rate
Oil Conv.	2.36	2001	0.2
Oil Tight	32.66	2038	0.86
Gas Conv.	0.69	1997	0.02
Gas Shale	59.85	2045	0.96
**Total**	**95.56**	**2040**	**1.78**

Queensland

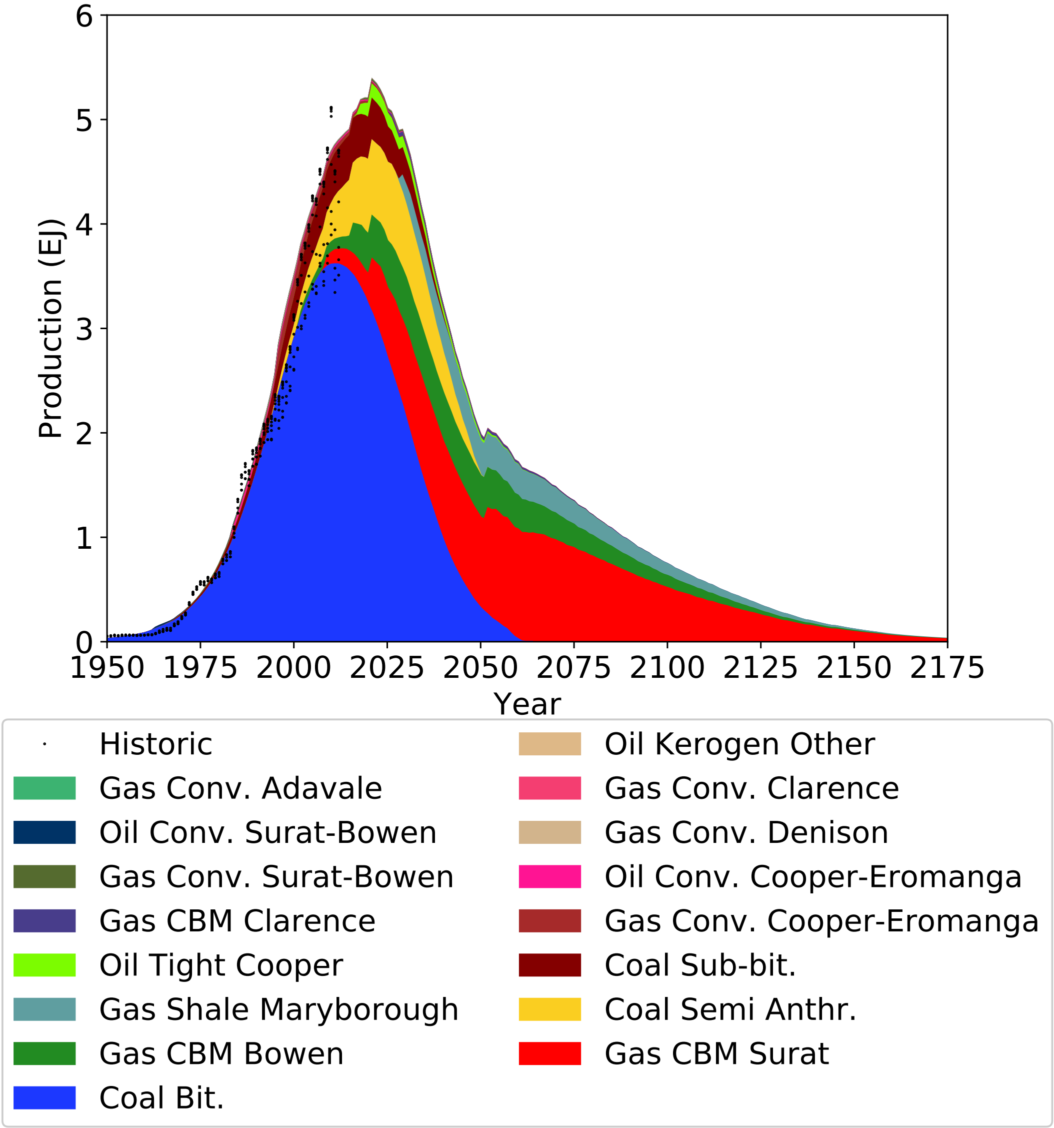

Figure 2.13: Australia - Queensland projections capped at 16

Table 2.13: Peak years - All

Name	URR	Peak Year	Peak Rate
Coal Bit. Queensland	158.7	2011	3.61
Gas CBM Queensland Surat	84.93	2054	1.07
Gas CBM Queensland Bowen	30.92	2033	0.5
Coal Semi Anthr. Queensland	22.82	2026	0.77
Gas Shale Queensland Maryborough	19.95	2044	0.34
Coal Sub-bit. Queensland	14.97	2014	0.43
Oil Tight Queensland Cooper	2.87	2022	0.14
Gas Conv. Queensland Cooper-Eromanga	2.23	1997	0.16
Gas CBM Queensland Clarence	1.44	2029	0.04
Oil Conv. Queensland Cooper-Eromanga	1.39	1985	0.05
Gas Conv. Queensland Surat-Bowen	1.12	1990	0.02
Gas Conv. Queensland Denison	0.41	1992	0.02
Oil Conv. Queensland Surat-Bowen	0.31	1964	0.02
Gas Conv. Queensland Clarence	0.07	2022	–
Gas Conv. Queensland Adavale	0.03	1997	–
Oil Kerogen Queensland Other	0.01	2003	–
**Total**	**342.17**	**2021**	**5.38**

Table 2.14: Peak years - Minerals

Name	URR	Peak Year	Peak Rate
Coal Bit.	158.7	2011	3.61
Coal Sub-bit.	14.97	2014	0.43
Coal Semi Anthr.	22.82	2026	0.77
Oil Conv.	1.7	1985	0.06
Oil Tight	2.87	2022	0.14
Oil Kerogen	0.01	2003	–
Gas Conv.	3.86	1997	0.2
Gas Shale	19.95	2044	0.34
Gas CBM	117.29	2035	1.46
**Total**	**342.17**	**2021**	**5.38**

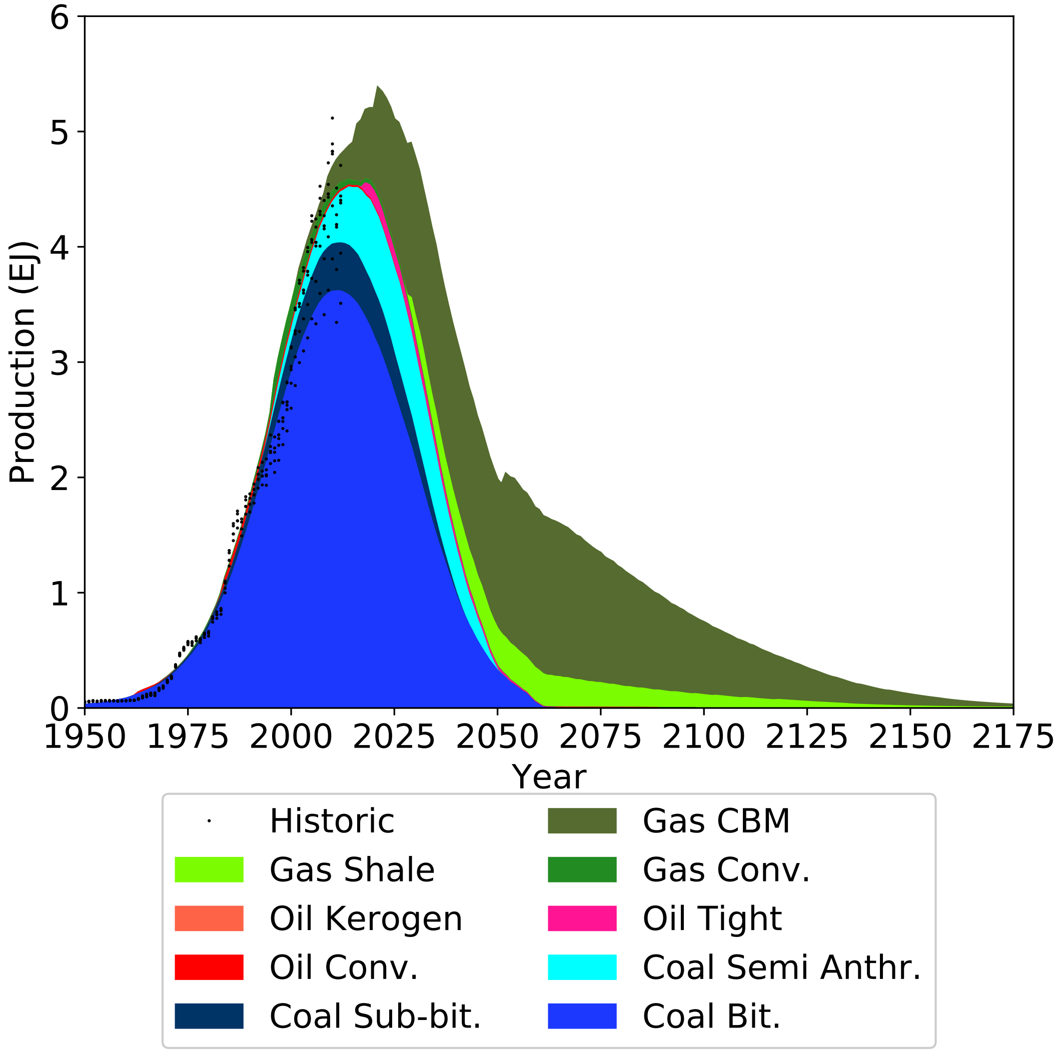

Figure 2.14: Australia - Queensland projection by mineral type

South Australia

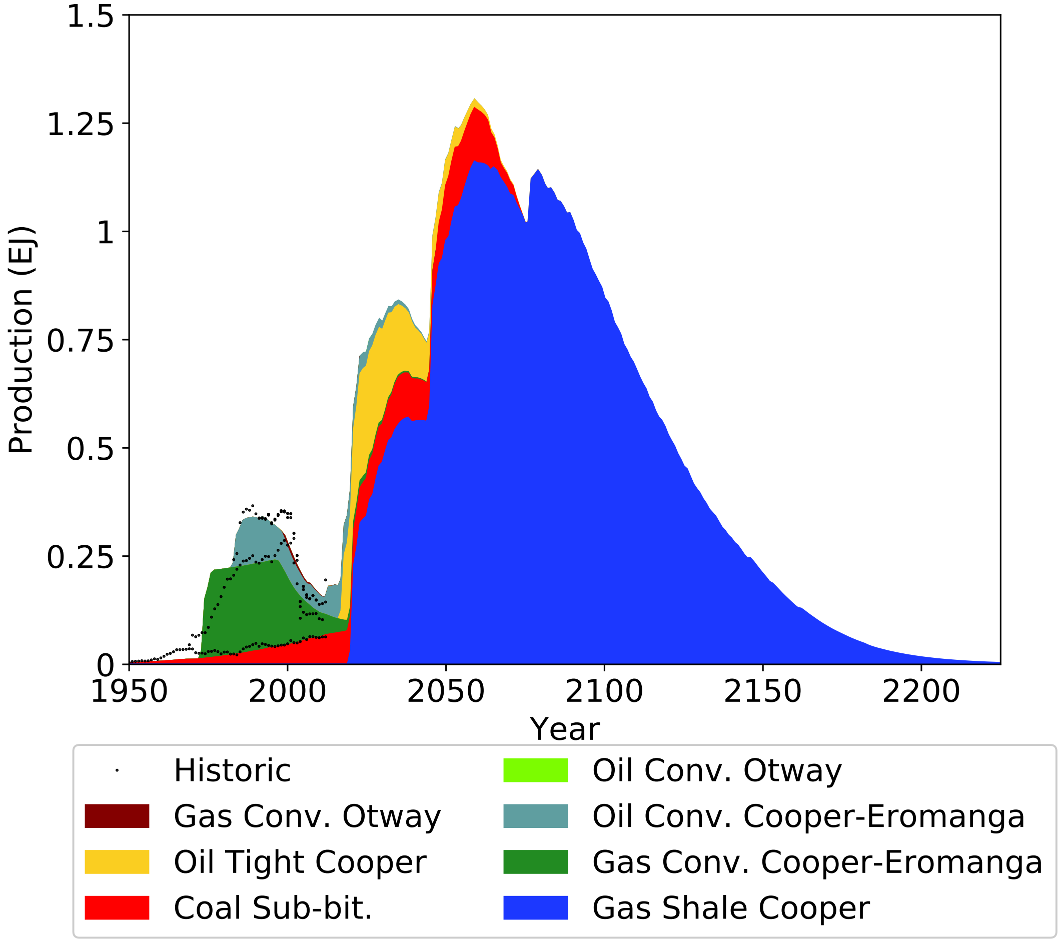

Figure 2.15: Australia - South Australia projections capped at 16

Table 2.15: Peak years - All

Name	URR	Peak Year	Peak Rate
Gas Shale South Australia Cooper	97.65	2059	1.16
Coal Sub-bit. South Australia	7.06	2051	0.14
Gas Conv. South Australia Cooper-Eromanga	6.69	1977	0.2
Oil Tight South Australia Cooper	5.73	2024	0.25
Oil Conv. South Australia Cooper-Eromanga	3.17	1987	0.11
Gas Conv. South Australia Otway	0.08	2000	0.01
Oil Conv. South Australia Otway	–	1994	–
**Total**	**120.38**	**2059**	**1.3**

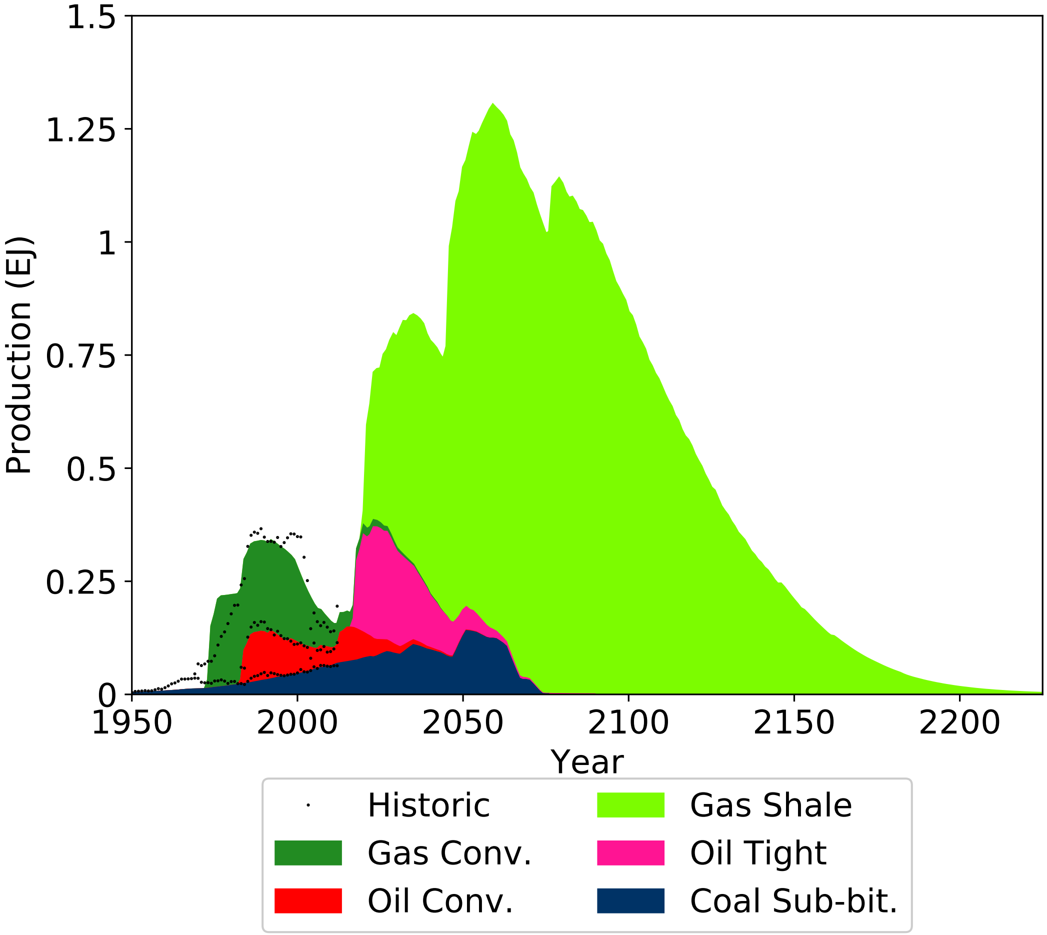

Figure 2.16: Australia - South Australia projection by mineral type

Table 2.16: Peak years - Minerals

**Name**	**URR**	**Peak Year**	**Peak Rate**
Coal Sub-bit.	7.06	2051	0.14
Oil Conv.	3.17	1987	0.11
Oil Tight	5.73	2024	0.25
Gas Conv.	6.77	1977	0.2
Gas Shale	97.65	2059	1.16
**Total**	**120.38**	**2059**	**1.3**

Tasmania

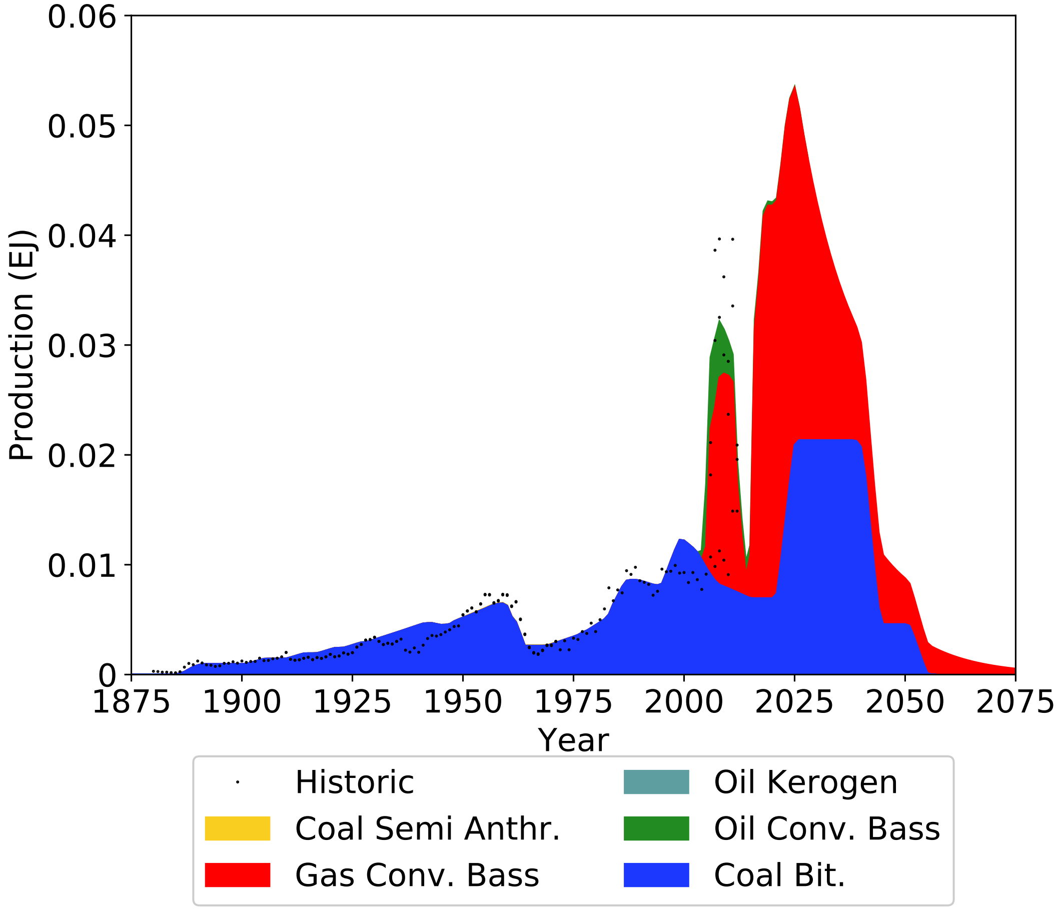

Figure 2.17: Australia - Tasmania projections capped at 16

Table 2.17: Peak years - All

Name	URR	Peak Year	Peak Rate
Coal Bit. Tasmania	1.1	2026	0.02
Gas Conv. Tasmania Bass	0.84	2019	0.04
Oil Conv. Tasmania Bass	0.04	2006	0.01
Coal Semi Anthr. Tasmania	–	1953	–
Oil Kerogen Tasmania	–	1927	–
**Total**	**1.98**	**2025**	**0.05**

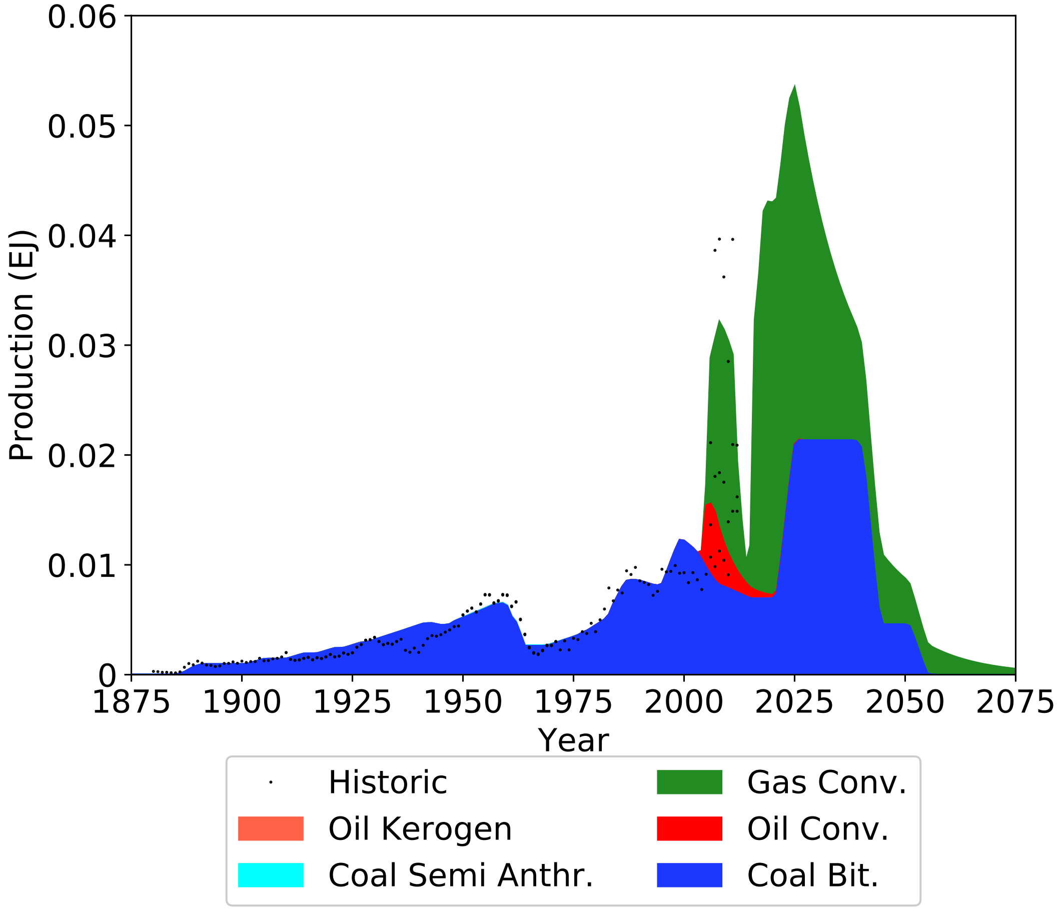

Figure 2.18: Australia - Tasmania projection by mineral type

Table 2.18: Peak years - Minerals

Name	URR	Peak Year	Peak Rate
Coal Bit.	1.1	2026	0.02
Coal Semi Anthr.	–	1953	–
Oil Conv.	0.04	2006	0.01
Oil Kerogen	–	1927	–
Gas Conv.	0.84	2019	0.04
**Total**	**1.98**	**2025**	**0.05**

Victoria

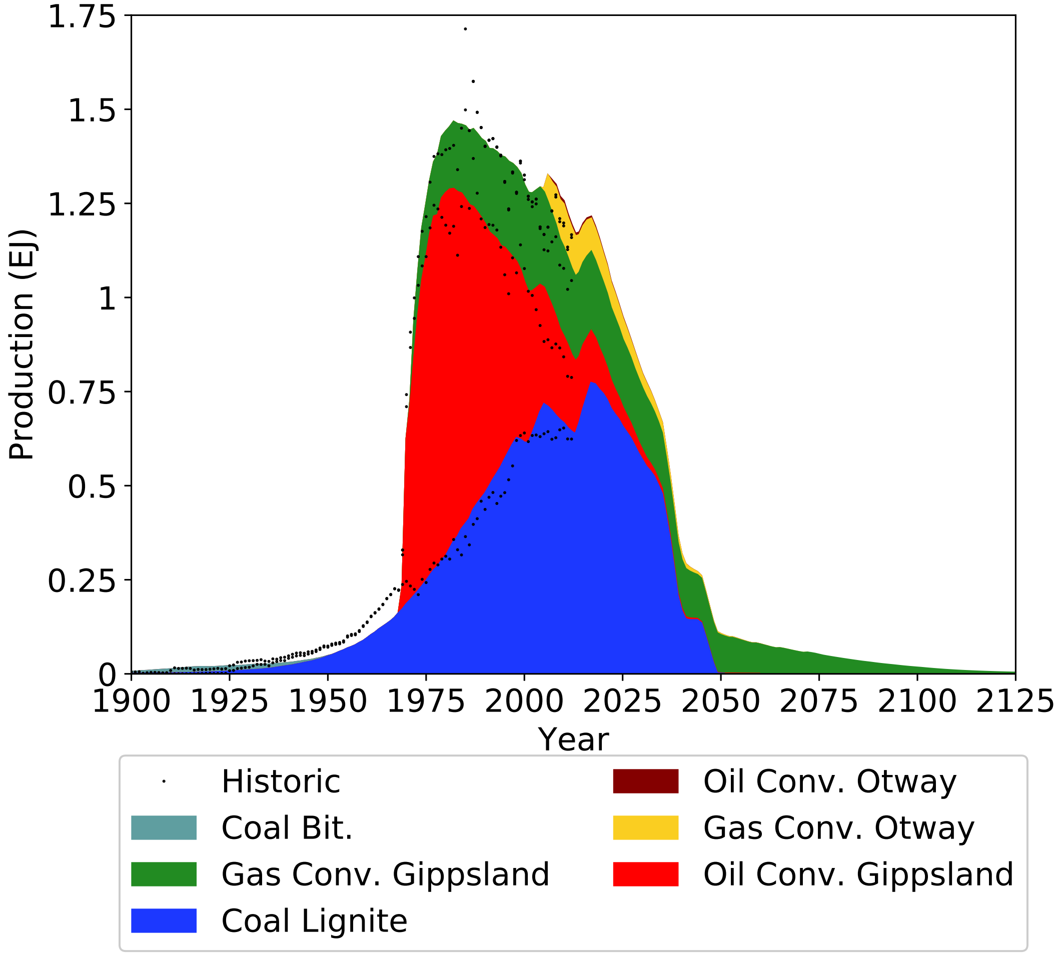

Figure 2.19: Australia - Victoria projections capped at 16

Table 2.19: Peak years - All

Name	URR	Peak Year	Peak Rate
Coal Lignite Victoria	40.33	2017	0.77
Oil Conv. Victoria Gippsland	28.04	1980	0.96
Gas Conv. Victoria Gippsland	17.53	2001	0.26
Gas Conv. Victoria Otway	2.3	2010	0.11
Coal Bit. Victoria	0.55	1917	0.02
Oil Conv. Victoria Otway	0.13	2010	0.01
**Total**	**88.88**	**1982**	**1.47**

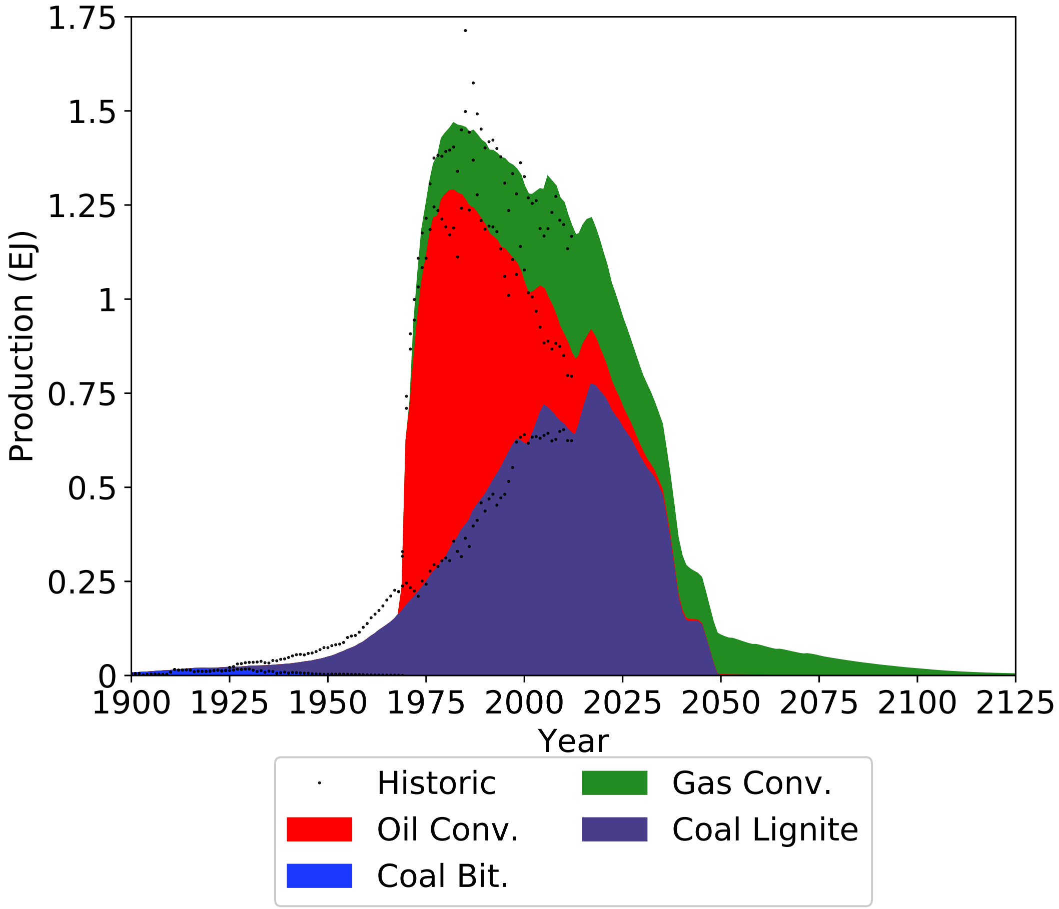

Figure 2.20: Australia - Victoria projection by mineral type

Table 2.20: Peak years - Minerals

Name	URR	Peak Year	Peak Rate
Coal Bit.	0.55	1917	0.02
Coal Lignite	40.33	2017	0.77
Oil Conv.	28.17	1980	0.96
Gas Conv.	19.83	2010	0.35
**Total**	**88.88**	**1982**	**1.47**

Western Australia

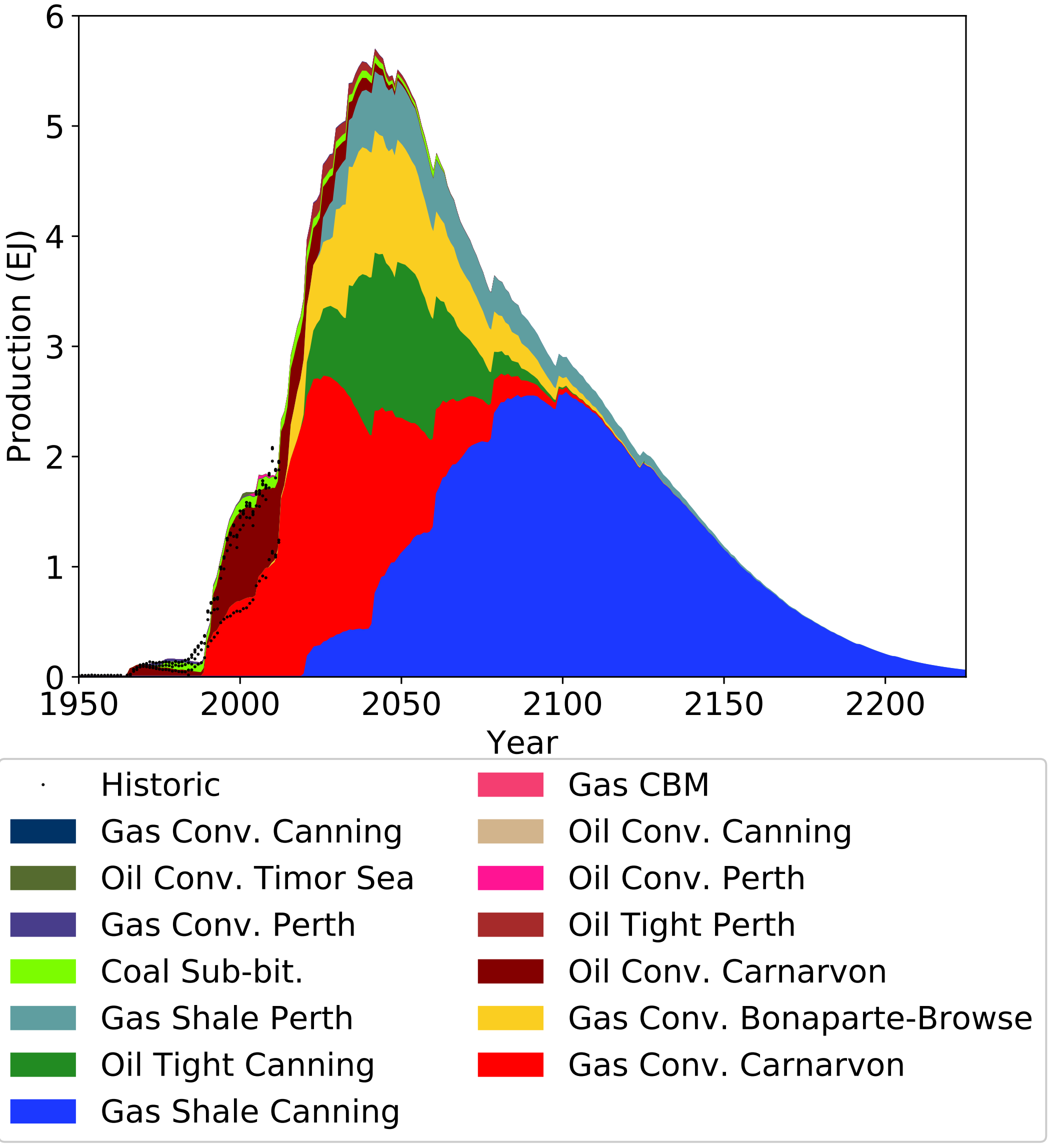

Figure 2.21: Australia - Western Australia projections capped at 16

Table 2.21: Peak years - All

**Name**	**URR**	**Peak Year**	**Peak Rate**
Gas Shale Western Australia Canning	246.75	2101	2.58
Gas Conv. Western Australia Carnarvon	113.35	2023	2.43
Oil Tight Western Australia Canning	55.58	2041	1.44
Gas Conv. Western Australia Bonaparte-Browse	54.9	2038	1.15
Gas Shale Western Australia Perth	34.65	2046	0.55
Oil Conv. Western Australia Carnarvon	25.26	2003	0.82
Coal Sub-bit. Western Australia	6.25	2018	0.13
Oil Tight Western Australia Perth	2.87	2025	0.14
Gas Conv. Western Australia Perth	0.94	1981	0.03
Oil Conv. Western Australia Perth	0.19	2005	0.03
Oil Conv. Western Australia Timor Sea	0.12	2002	0.03
Oil Conv. Western Australia Canning	0.02	1987	–
Gas Conv. Western Australia Canning	0.01	2022	–
Gas CBM Western Australia	–	2008	–
**Total**	**540.89**	**2042**	**5.68**

Table 2.22: Peak years - Minerals

**Name**	**URR**	**Peak Year**	**Peak Rate**
Coal Sub-bit.	6.25	2018	0.13
Oil Conv.	25.59	2001	0.85
Oil Tight	58.45	2041	1.5
Gas Conv.	169.2	2032	3.24
Gas Shale	281.4	2086	2.82
Gas CBM	–	2008	–
**Total**	**540.89**	**2042**	**5.68**

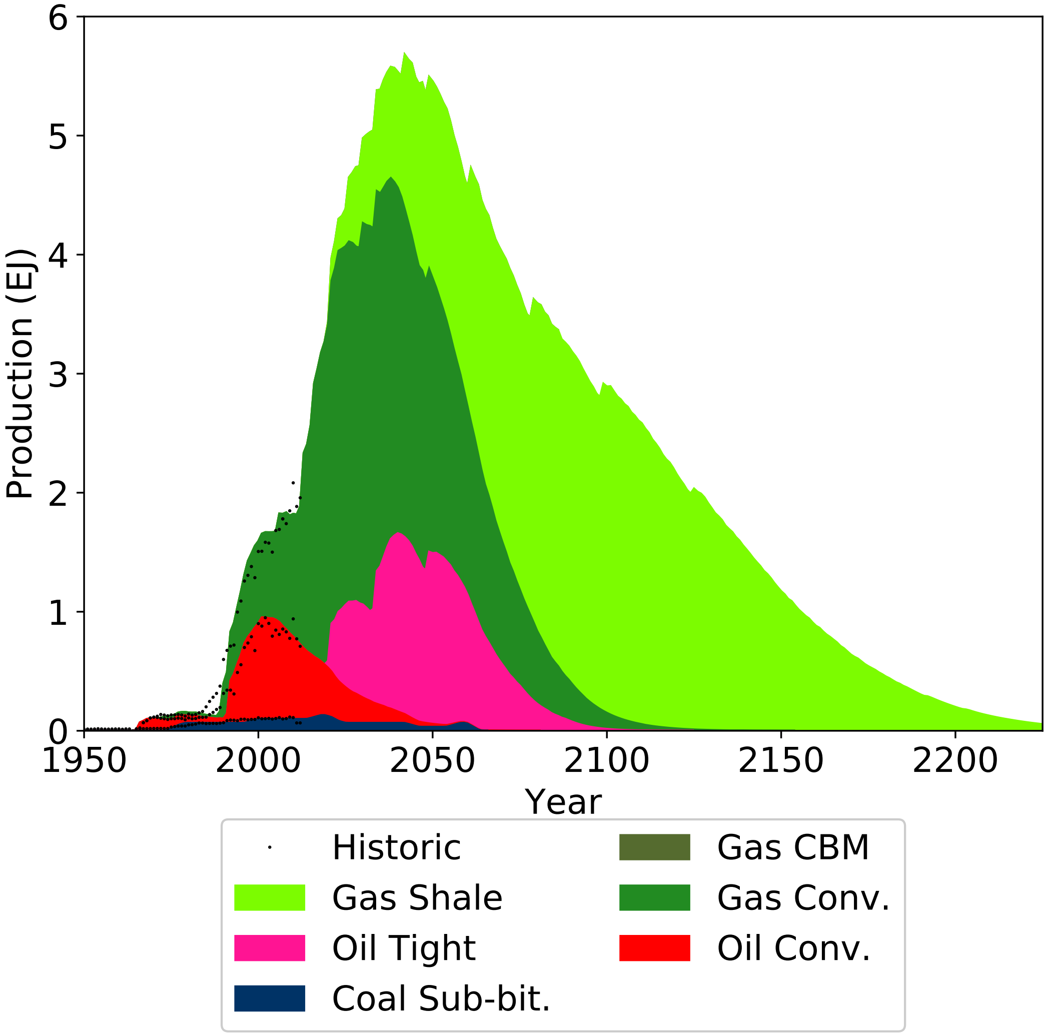

Figure 2.22: Australia - Western Australia projection by mineral type

2.2.4 Projection by region

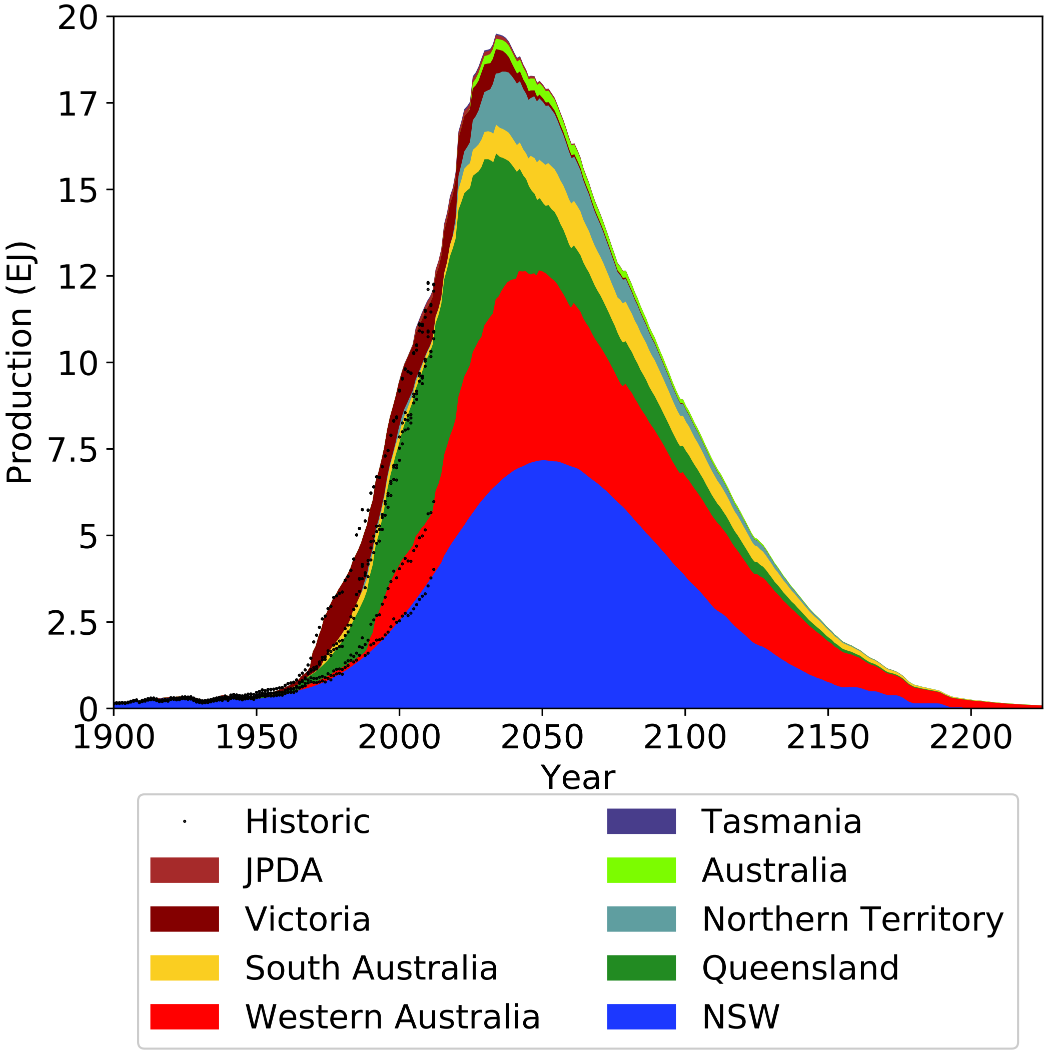

Figure 2.23: Australia by region projections capped at 16

Table 2.23: Peak years - All

**Name**	**URR**	**Peak Year**	**Peak Rate**
NSW	732.41	2050	7.14
Western Australia	540.89	2042	5.68
Queensland	342.17	2021	5.38
South Australia	120.38	2059	1.3
Northern Territory	95.56	2040	1.78
Victoria	88.88	1982	1.47
Australia	21.0	2041	0.35
JPDA	6.73	2015	0.19
Tasmania	1.98	2025	0.05
**Total**	**1950.01**	**2034**	**19.47**

2.3 Bangladesh

2.3.1 All Projections

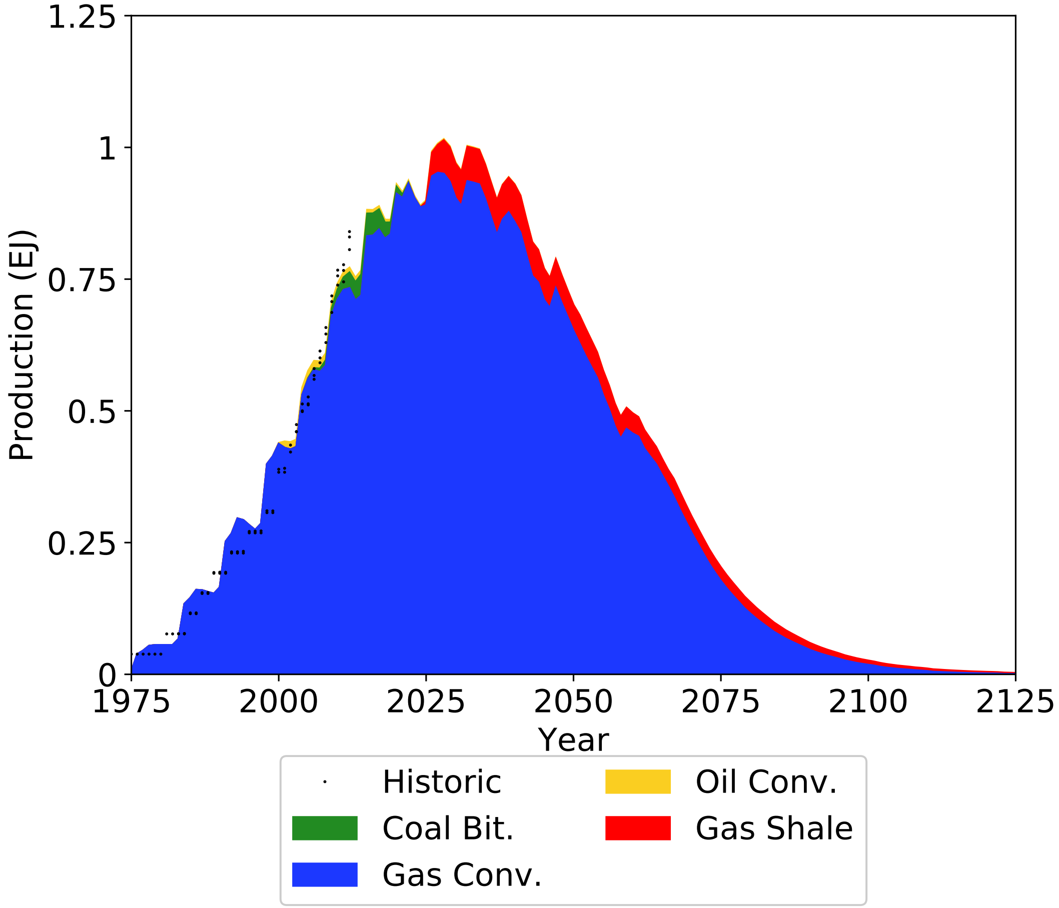

Figure 2.24: Bangladesh projections capped at 16

Table 2.24: Peak years - All

**Name**	**URR**	**Peak Year**	**Peak Rate**
Gas Conv.	55.4	2027	0.95
Gas Shale	3.0	2040	0.07
Coal Bit.	0.38	2015	0.04
Oil Conv.	0.25	2002	0.01
**Total**	**59.03**	**2028**	**1.02**

2.3.2 By Mineral

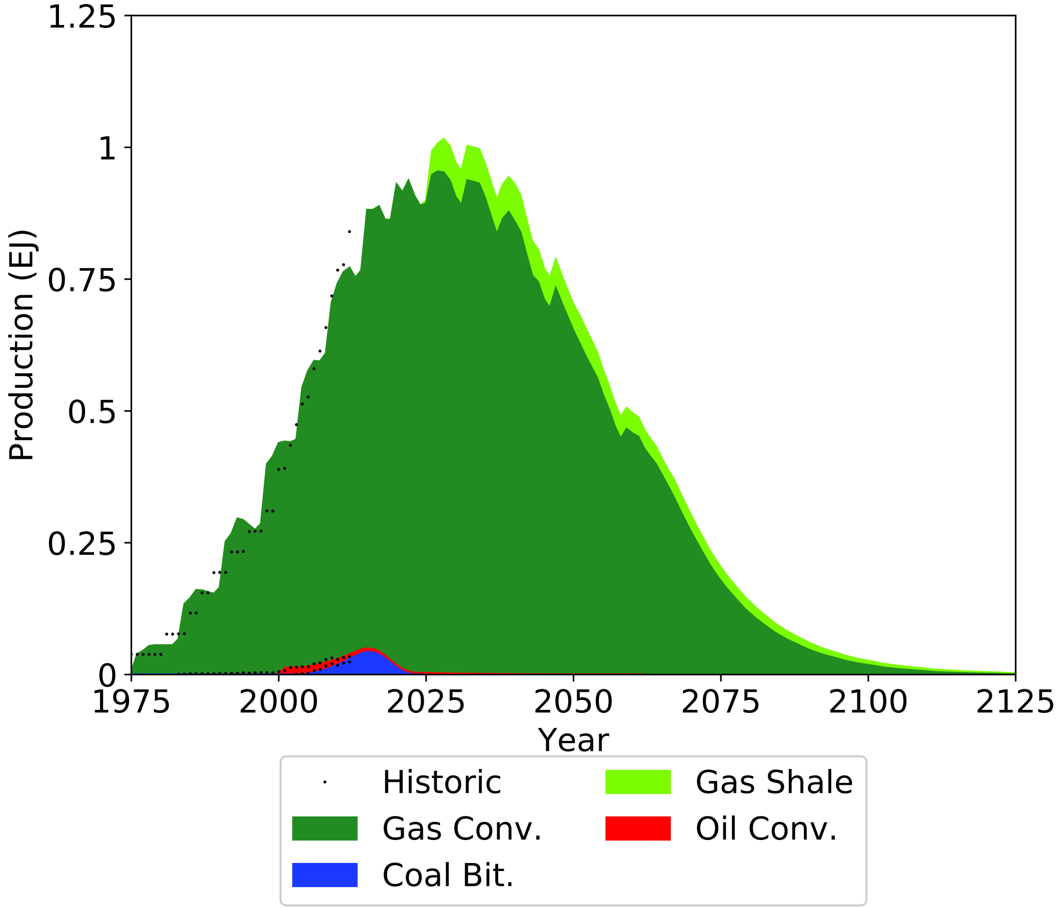

Figure 2.25: Bangladesh projection by mineral type

Table 2.25: Peak years - Minerals

**Name**	**URR**	**Peak Year**	**Peak Rate**
Coal Bit.	0.38	2015	0.04
Oil Conv.	0.25	2002	0.01
Gas Conv.	55.4	2027	0.95
Gas Shale	3.0	2040	0.07
**Total**	**59.03**	**2028**	**1.02**

2.4 Bhutan

2.4.1 All Projections

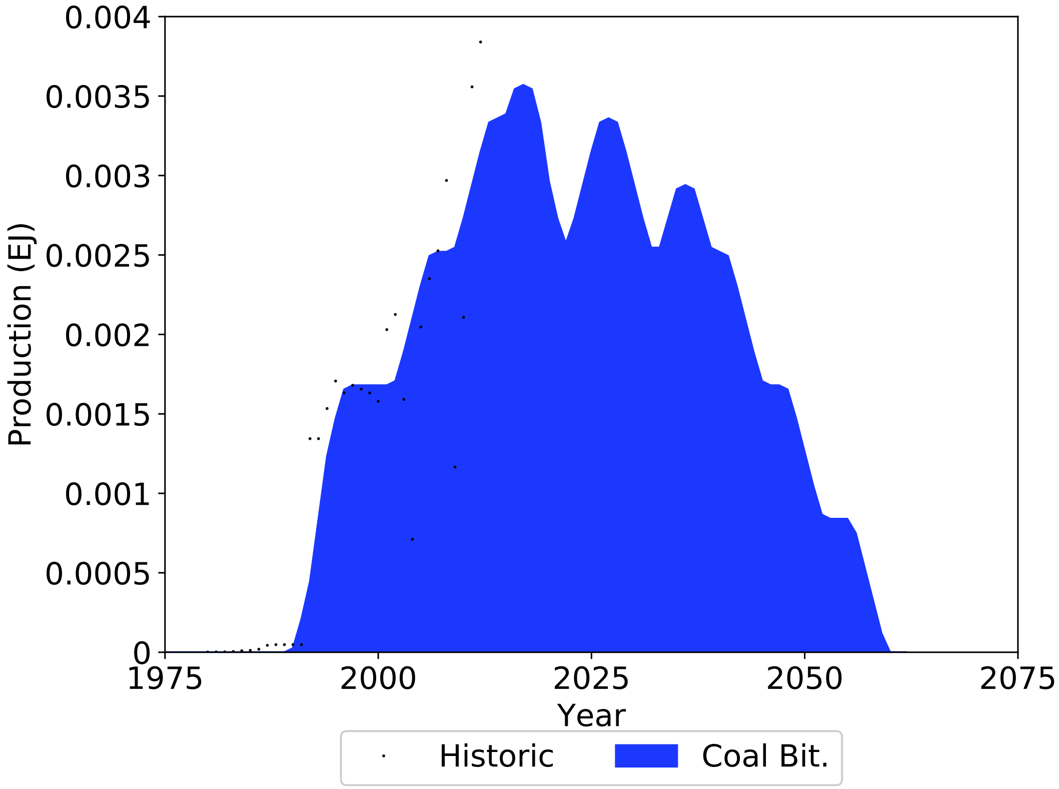

Figure 2.26: Bhutan projections capped at 16

Table 2.26: Peak years - All

Name	URR	Peak Year	Peak Rate
Coal Bit.	0.15	2017	–
**Total**	**0.15**	**2017**	–

2.4.2 By Mineral

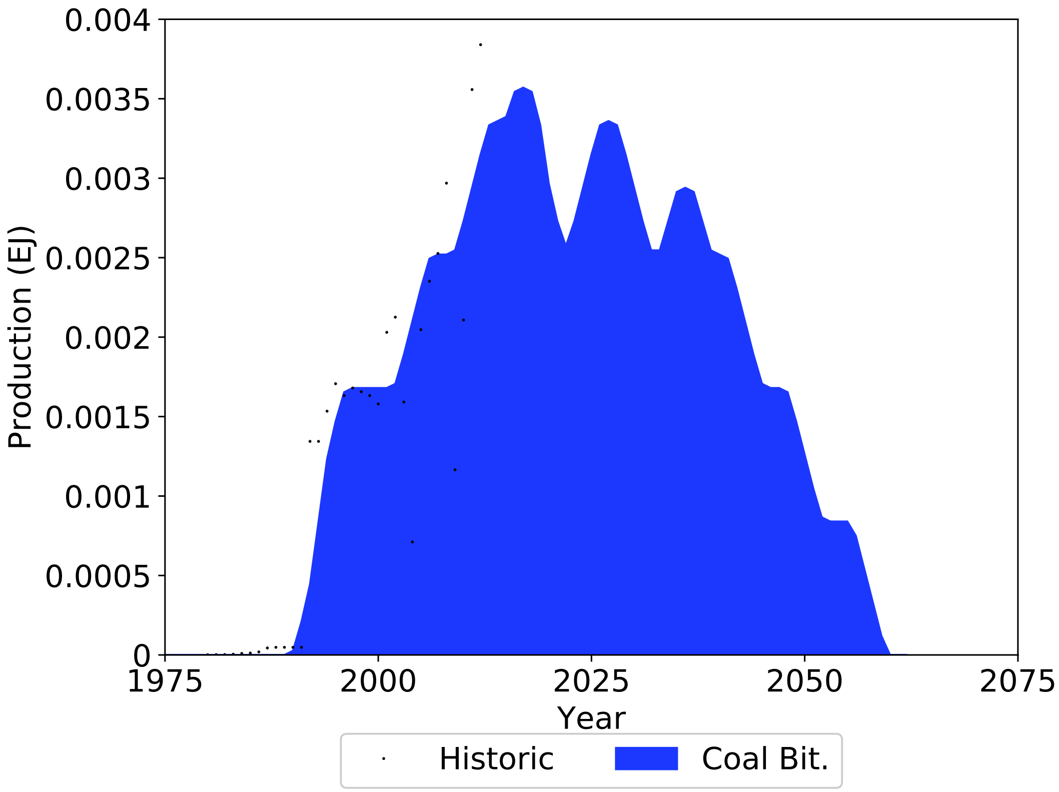

Figure 2.27: Bhutan projection by mineral type

Table 2.27: Peak years - Minerals

Name	URR	Peak Year	Peak Rate
Coal Bit.	0.15	2017	–
**Total**	**0.15**	**2017**	–

2.5 Brunei

2.5.1 All Projections

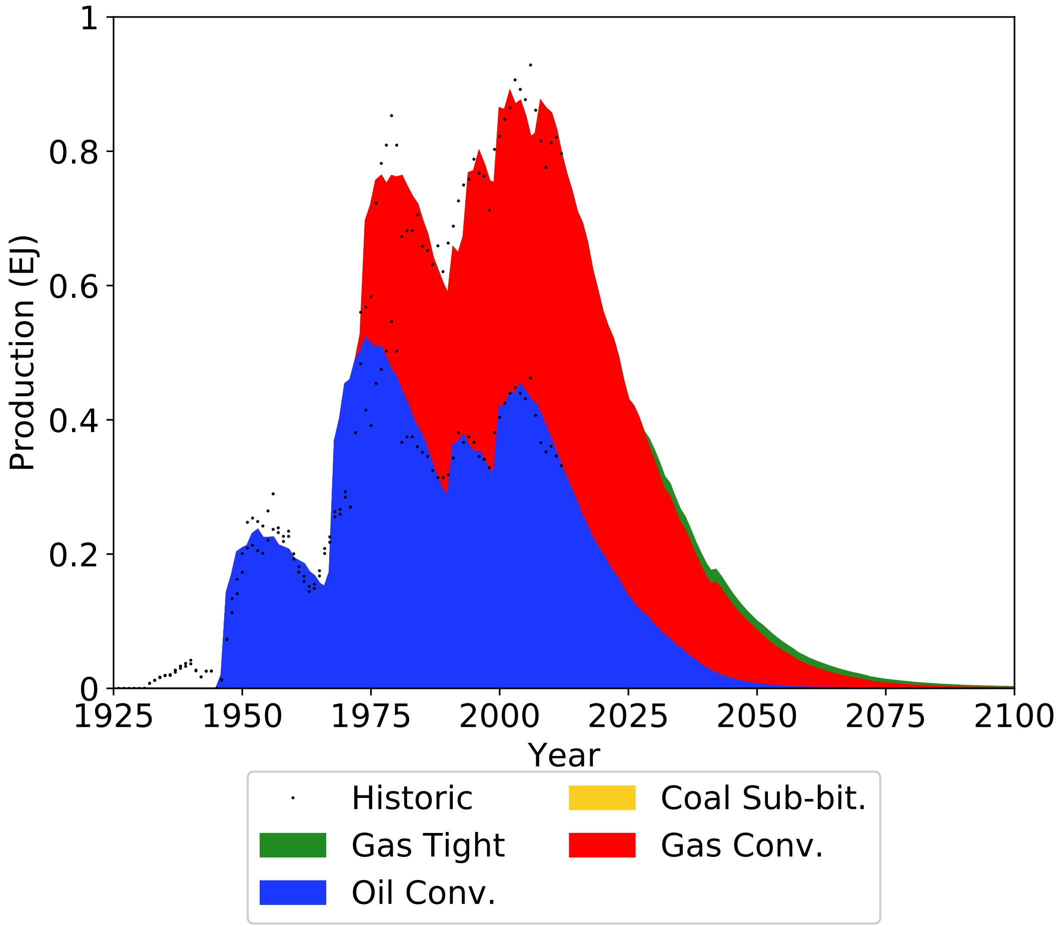

Figure 2.28: Brunei projections capped at 16

Table 2.28: Peak years - All

**Name**	**URR**	**Peak Year**	**Peak Rate**
Oil Conv.	26.5	1974	0.52
Gas Conv.	24.2	2010	0.49
Gas Tight	0.74	2032	0.02
Coal Sub-bit.	0.01	1893	–
**Total**	**51.45**	**2002**	**0.89**

2.5.2 By Mineral

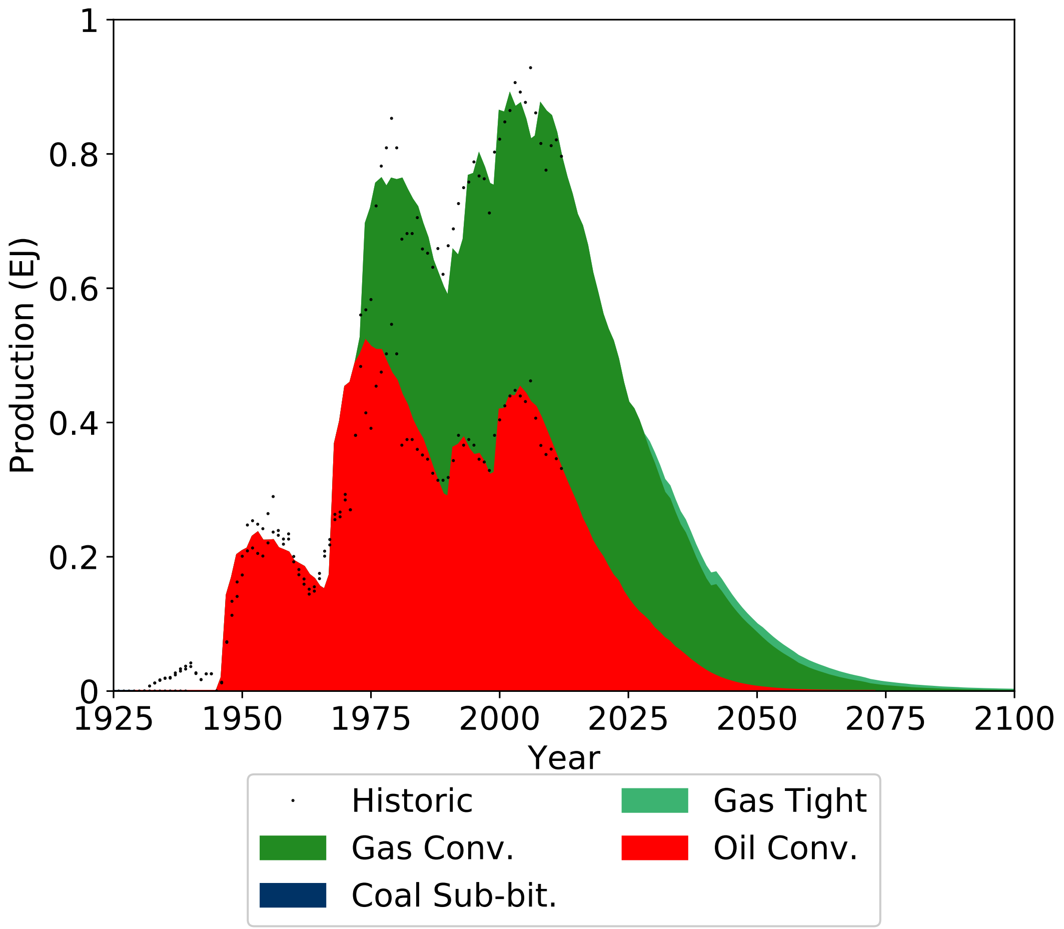

Figure 2.29: Brunei projection by mineral type

Table 2.29: Peak years - Minerals

**Name**	**URR**	**Peak Year**	**Peak Rate**
Coal Sub-bit.	0.01	1893	–
Oil Conv.	26.5	1974	0.52
Gas Conv.	24.2	2010	0.49
Gas Tight	0.74	2032	0.02
**Total**	**51.45**	**2002**	**0.89**

2.6 Burma

2.6.1 All Projections

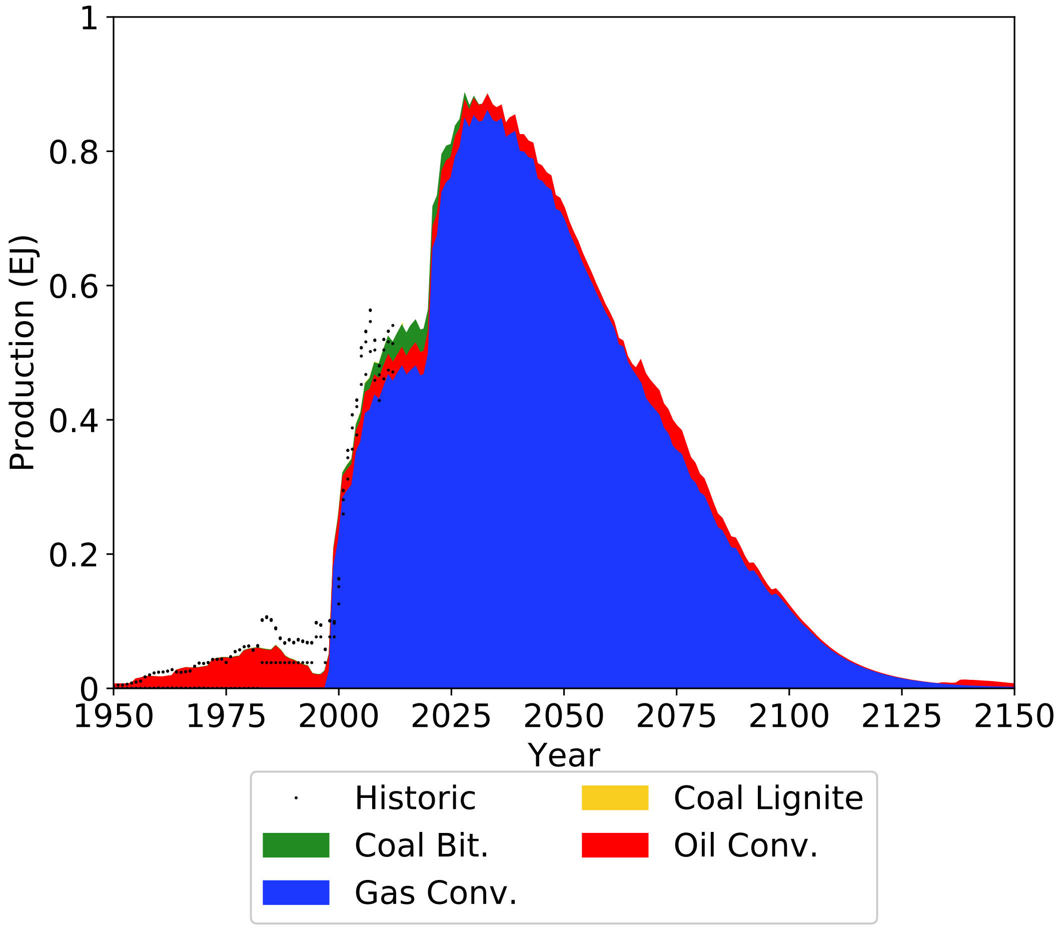

Figure 2.30: Burma projections capped at 16

Table 2.30: Peak years - All

Name	URR	Peak Year	Peak Rate
Gas Conv.	51.8	2033	0.86
Oil Conv.	4.25	1986	0.06
Coal Bit.	0.66	2016	0.03
Coal Lignite	0.01	2004	–
**Total**	**56.72**	**2033**	**0.88**

2.6.2 By Mineral

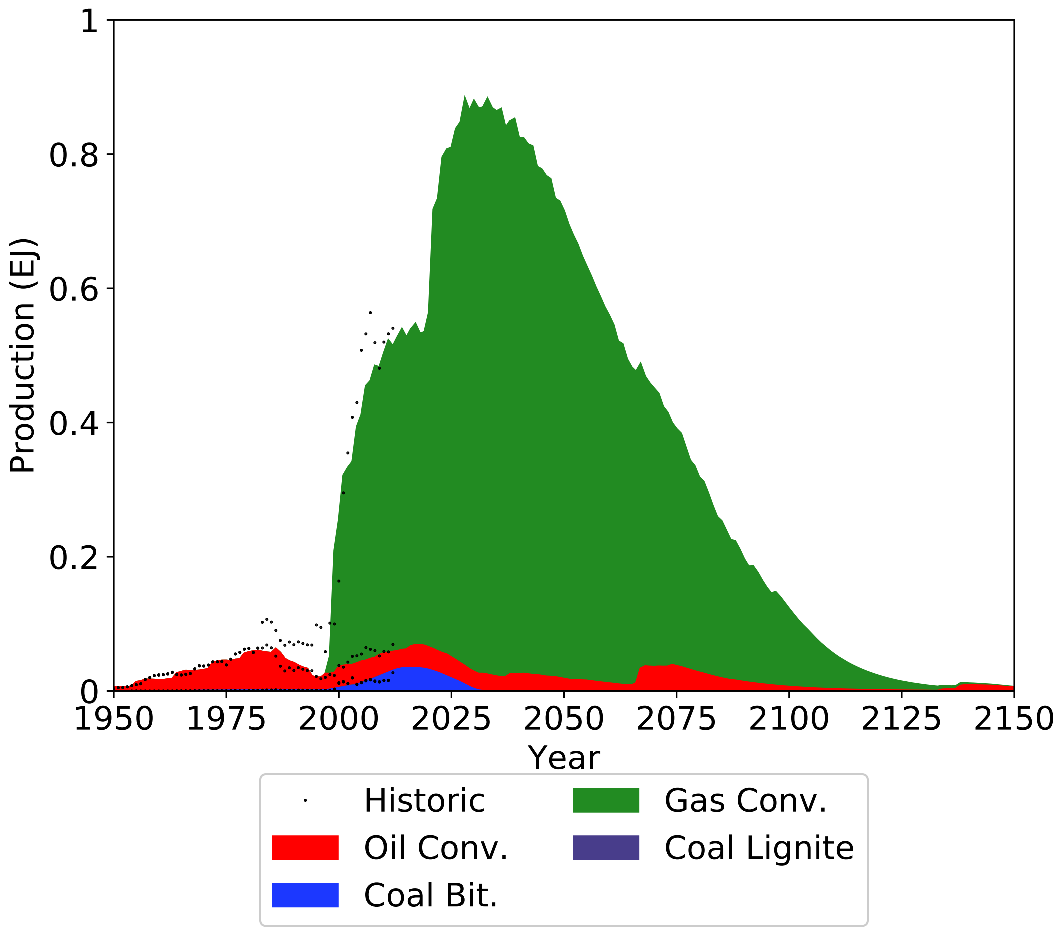

Figure 2.31: Burma projection by mineral type

Table 2.31: Peak years - Minerals

**Name**	**URR**	**Peak Year**	**Peak Rate**
Coal Bit.	0.66	2016	0.03
Coal Lignite	0.01	2004	–
Oil Conv.	4.25	1986	0.06
Gas Conv.	51.8	2033	0.86
**Total**	**56.72**	**2033**	**0.88**

2.7 Cambodia

2.7.1 All Projections

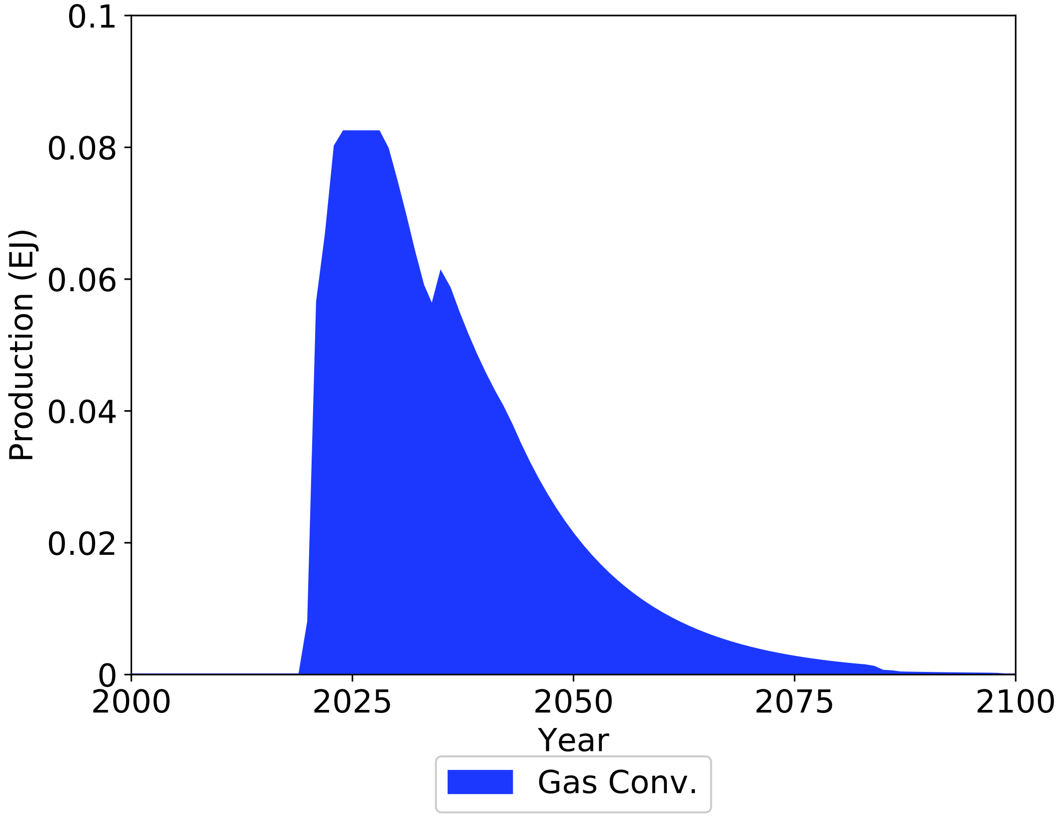

Figure 2.32: Cambodia projections capped at 16

Table 2.32: Peak years - All

Name	URR	Peak Year	Peak Rate
Gas Conv.	1.9	2024	0.08
**Total**	**1.9**	**2024**	**0.08**

2.7.2 By Mineral

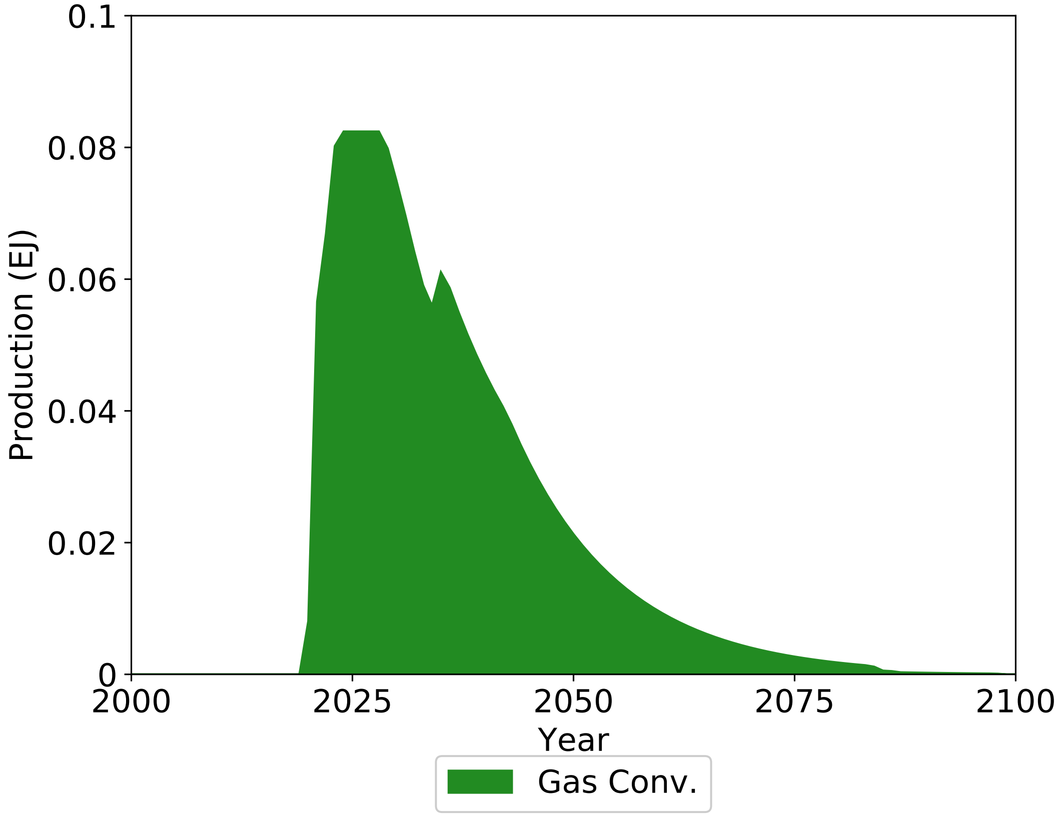

Figure 2.33: Cambodia projection by mineral type

Table 2.33: Peak years - Minerals

Name	URR	Peak Year	Peak Rate
Gas Conv.	1.9	2024	0.08
**Total**	**1.9**	**2024**	**0.08**

2.8 China

2.8.1 All Projections

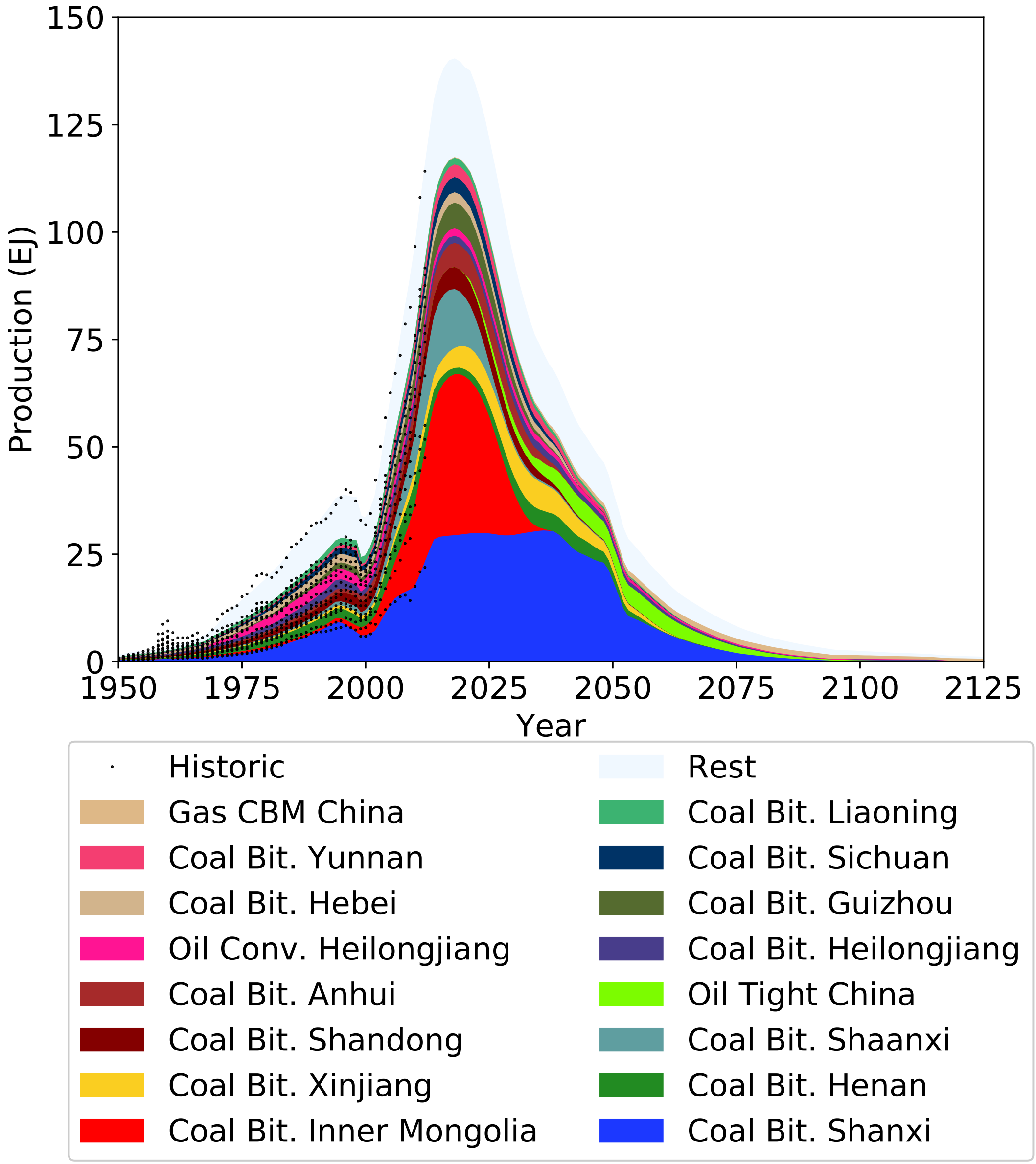

Figure 2.34: China projections capped at 16

Table 2.34: Peak years - All

**Name**	**URR**	**Peak Year**	**Peak Rate**
Coal Bit. Shanxi	1543.23	2036	30.33
Coal Bit. Inner Mongolia	696.15	2018	37.44
Coal Bit. Henan	241.25	2009	5.53
Coal Bit. Xinjiang	235.83	2031	6.85
Coal Bit. Shaanxi	230.57	2016	14.62
Coal Bit. Shandong	193.4	2020	5.19
Oil Tight China	184.51	2044	4.62
Coal Bit. Anhui	162.36	2020	5.77
Coal Bit. Heilongjiang	157.4	2008	2.28
Oil Conv. Heilongjiang	156.83	1989	2.67
Coal Bit. Guizhou	147.64	2019	6.01
Coal Bit. Hebei	128.44	2015	2.61
Coal Bit. Sichuan	126.66	2019	3.59
Coal Bit. Yunnan	113.89	2022	3.28
Coal Bit. Liaoning	107.22	2006	1.74
Gas CBM China	105.0	2067	1.31
Oil Conv. Shandong	85.59	1988	1.37
Gas Conv. Sichuan	83.72	2024	1.84
Gas Conv. Xinjiang	75.82	2026	1.39
Coal Bit. Gansu	66.67	2022	1.26
Oil Conv. Xinjiang	65.09	2015	1.13
Coal Bit. Ningxia	64.7	2019	3.21
Gas Conv. Inner Mongolia	62.94	2023	1.51
Coal Bit. Chongqing	53.62	2017	1.16
Gas Conv. Shaanxi	53.01	2019	1.42
Coal Bit. Hunan	49.38	2013	2.24
Gas Tight China	44.5	2085	0.58
Coal Bit. Jilin	42.23	2014	1.45
Oil Conv. Hebei	40.88	2028	0.81
Coal Bit. Jiangsu	37.45	2000	0.66
Oil Conv. Shaanxi	35.99	2012	1.37
Oil Conv. Liaoning	35.68	1995	0.68
Gas Shale China	31.89	2066	0.46
Coal Bit. Jiangxi	26.85	2008	0.79
Coal Bit. Qinghai	26.45	2022	1.25
Coal Bit. Historic	25.96	1937	0.94
Oil Conv. Tianjin	22.83	2011	1.35
Gas Conv. Offshore	21.38	2034	0.54
Oil Conv. Jilin	20.15	2016	0.4
Gas Conv. Chongqing	17.06	2025	0.4
Oil Conv. Gansu	16.32	2028	0.49
Coal Bit. Fujian	15.95	2009	0.67
Gas Conv. Heilongjiang	15.95	2030	0.34
Oil Conv. Henan	15.83	1989	0.36
Coal Bit. Beijing	15.6	1981	0.26
Oil Conv. Guangdong	14.92	2003	0.61
Coal Bit. Hubei	13.44	2009	0.28
Gas Conv. Qinghai	11.25	2017	0.3
Coal Bit. Guangxi	11.04	1993	0.25

Table 2.34: Peak years - All – Continued

**Name**	**URR**	**Peak Year**	**Peak Rate**
Coal Bit. Guangdong	7.83	1972	0.24
Oil Conv. Inner Mongolia	6.9	2019	0.26
Oil Conv. Qinghai	6.55	2023	0.18
Gas Conv. Jilin	6.09	2020	0.19
Oil Kerogen China	5.73	2033	0.14
Gas Conv. Shandong	5.23	2028	0.13
Gas Conv. Guangdong	5.2	2010	0.35
Gas Conv. Liaoning	4.99	2027	0.1
Oil Conv. Jiangsu	4.2	2016	0.11
Coal Bit. Tianjin	3.97	2027	0.17
Gas Conv. Tianjin	3.96	2015	0.07
Gas Conv. Hebei	3.13	2023	0.1
Oil Conv. Hubei	3.09	2014	0.05
Gas Conv. Henan	2.91	2001	0.07
Oil Conv. Ningxia	2.51	1999	0.11
Gas Conv. Ningxia	2.0	2030	0.07
Coal Bit. Zhejiang	1.74	1975	0.04
Gas Conv. Gansu	1.71	2030	0.06
Coal Bit. Hainan	1.6	2052	0.05
Oil Conv. Sichuan	0.68	2024	0.03
Gas Conv. Hubei	0.52	2022	0.02
Gas Conv. Shanghai	0.36	2004	0.02
Oil Conv. Hainan	0.26	2009	0.01
Coal Bit. Shanghai	0.25	1982	0.08
Gas Conv. Jiangsu	0.24	2024	0.01
Oil Conv. Shanghai	0.22	2001	0.02
Coal Bit. Tibet	0.19	2048	–
Oil Conv. Anhui	0.16	2024	0.01
Oil Conv. Guangxi	0.15	2020	0.01
Gas Conv. Guizhou	0.13	2024	–
Gas Conv. Hainan	0.1	2006	0.01
Oil Conv. Chongqing	0.1	2024	0.01
Gas Conv. Yunnan	0.04	2019	–
Gas Conv. Guangxi	0.01	2022	–
Gas Conv. Jiangxi	0.01	2007	–
Oil Conv. Yunnan	0.01	2022	–
Gas Conv. Anhui	–	2022	–
**Total**	**5833.28**	**2018**	**140.16**

2.8.2 By Mineral

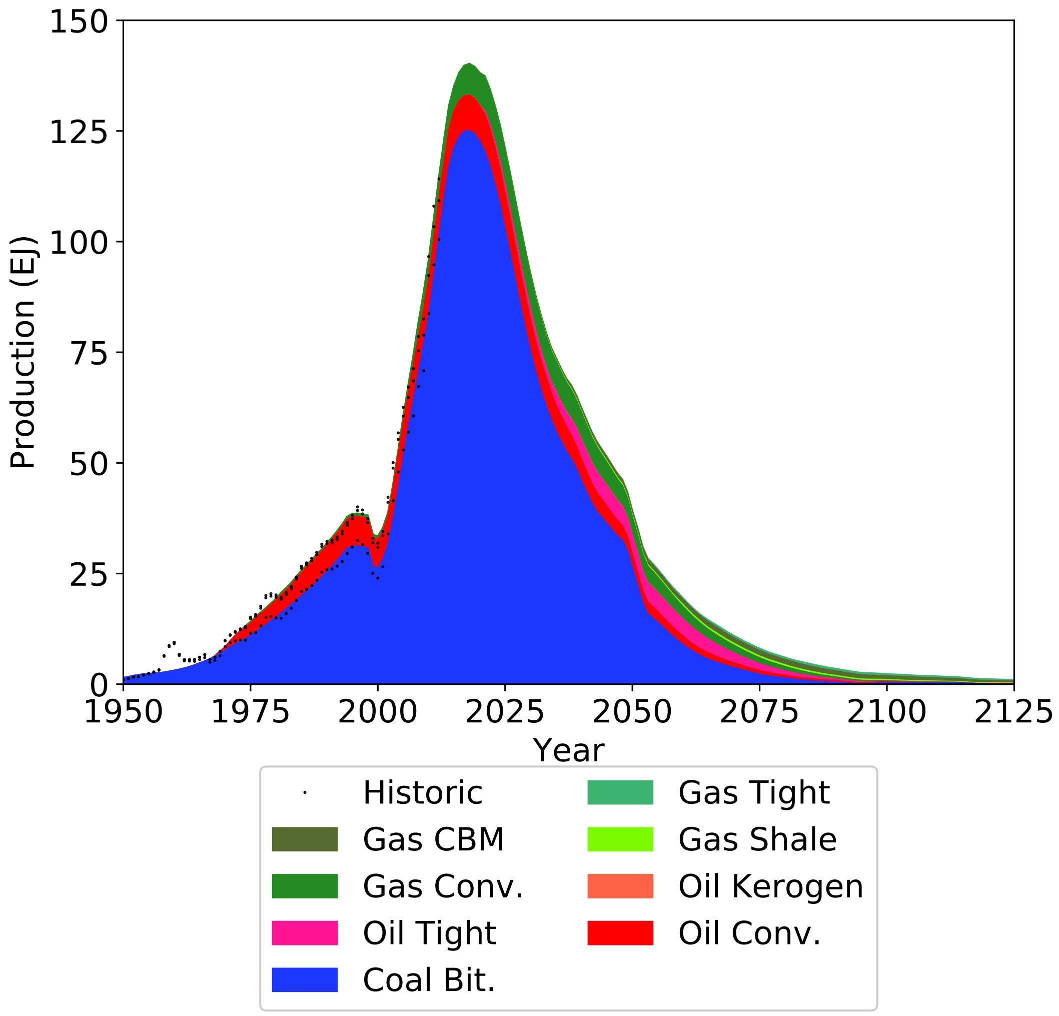

Figure 2.35: China projection by mineral type

2.8.3 Regional Projections

Table 2.35: Peak years - Minerals

**Name**	**URR**	**Peak Year**	**Peak Rate**
Coal Bit.	4548.96	2018	124.96
Oil Conv.	534.94	2012	8.62
Oil Tight	184.51	2044	4.62
Oil Kerogen	5.73	2033	0.14
Gas Conv.	377.75	2024	8.25
Gas Shale	31.89	2066	0.46
Gas CBM	105.0	2067	1.31
Gas Tight	44.5	2085	0.58
**Total**	**5833.28**	**2018**	**140.16**

Anhui

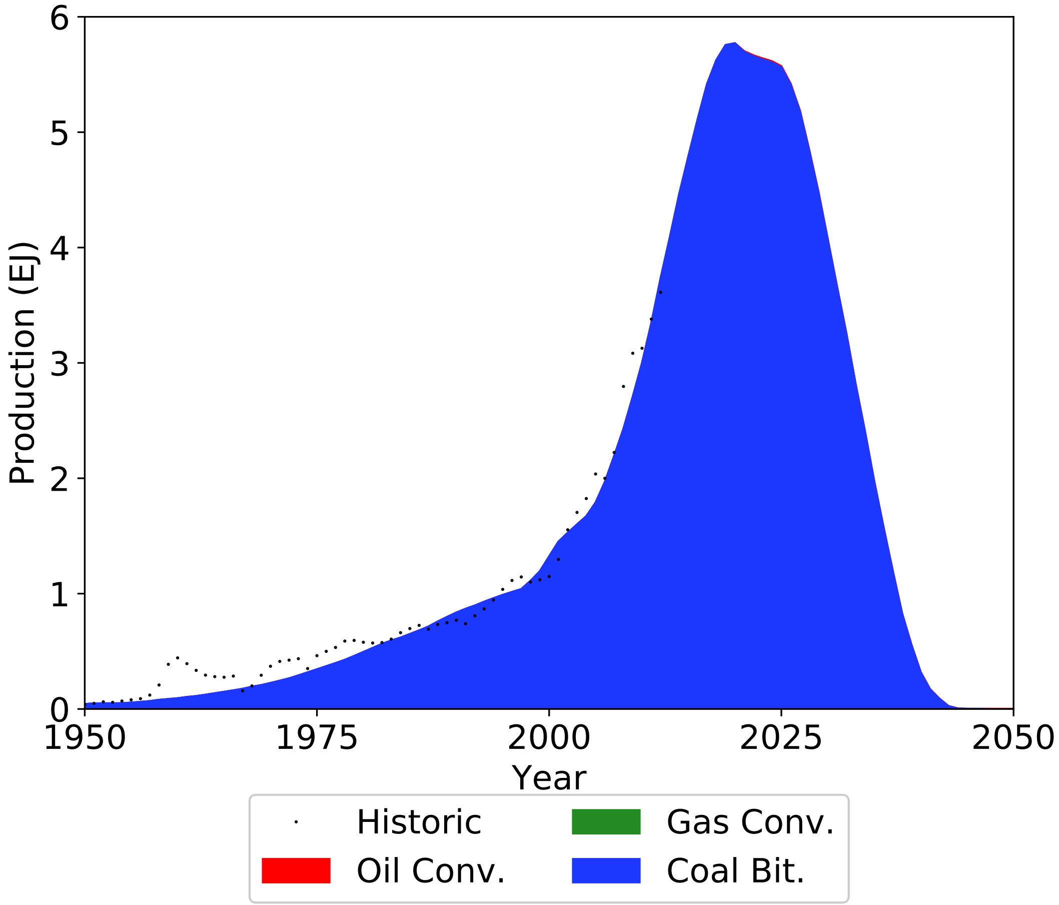

Figure 2.36: China - Anhui projections capped at 16

Table 2.36: Peak years - All

Name	URR	Peak Year	Peak Rate
Coal Bit. Anhui	162.36	2020	5.77
Oil Conv. Anhui	0.16	2024	0.01
Gas Conv. Anhui	–	2022	–
**Total**	**162.52**	**2020**	**5.77**

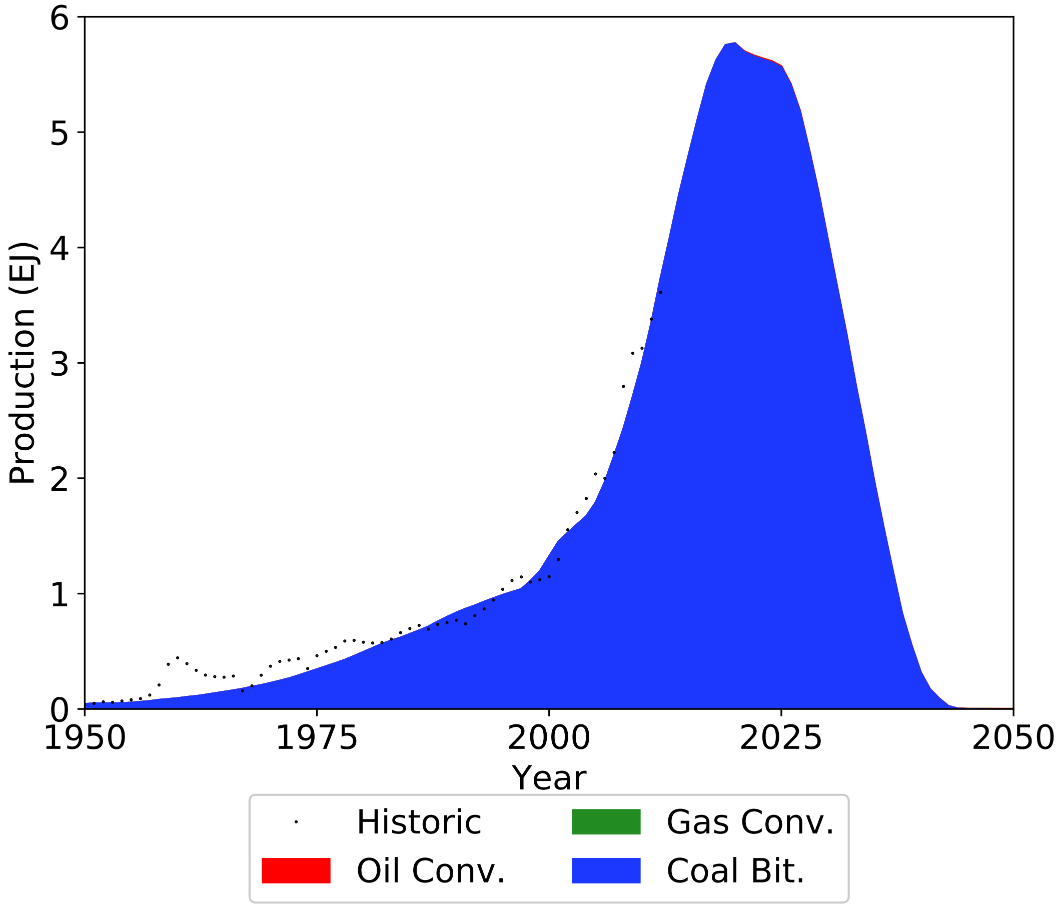

Figure 2.37: China - Anhui projection by mineral type

Table 2.37: Peak years - Minerals

Name	URR	Peak Year	Peak Rate
Coal Bit.	162.36	2020	5.77
Oil Conv.	0.16	2024	0.01
Gas Conv.	–	2022	–
**Total**	**162.52**	**2020**	**5.77**

Beijing

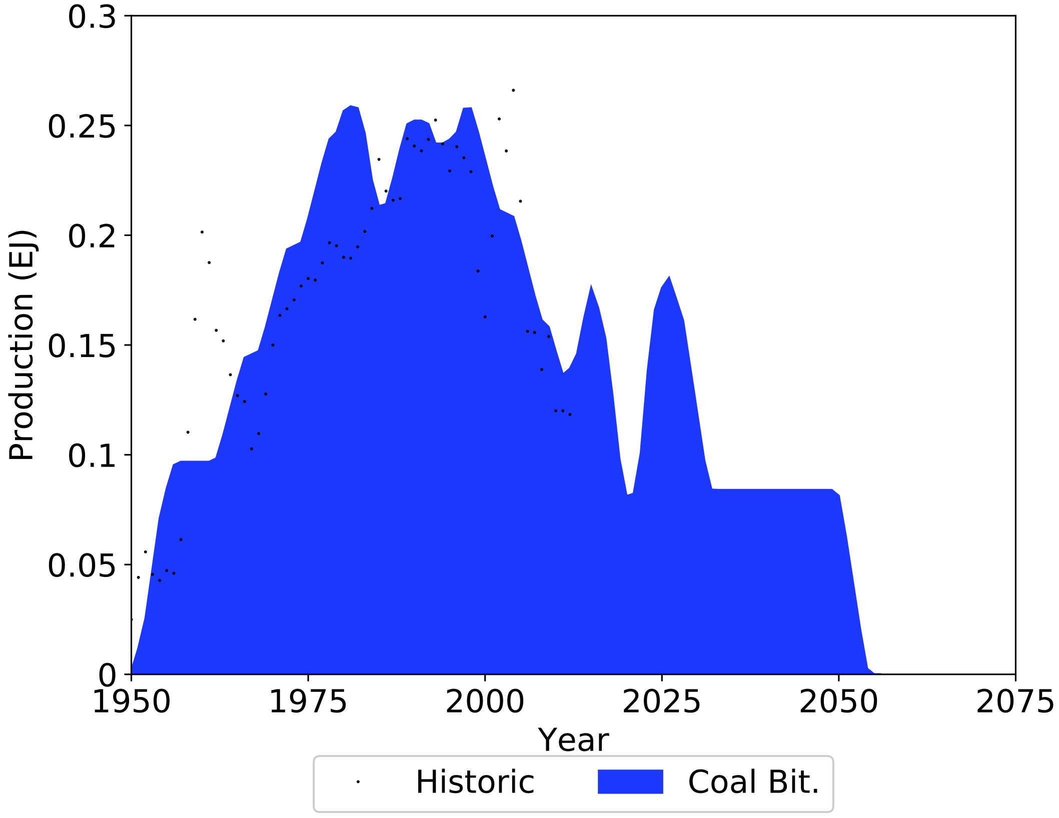

Figure 2.38: China - Beijing projections capped at 16

Table 2.38: Peak years - All

Name	URR	Peak Year	Peak Rate
Coal Bit. Beijing	15.6	1981	0.26
**Total**	**15.6**	**1981**	**0.26**

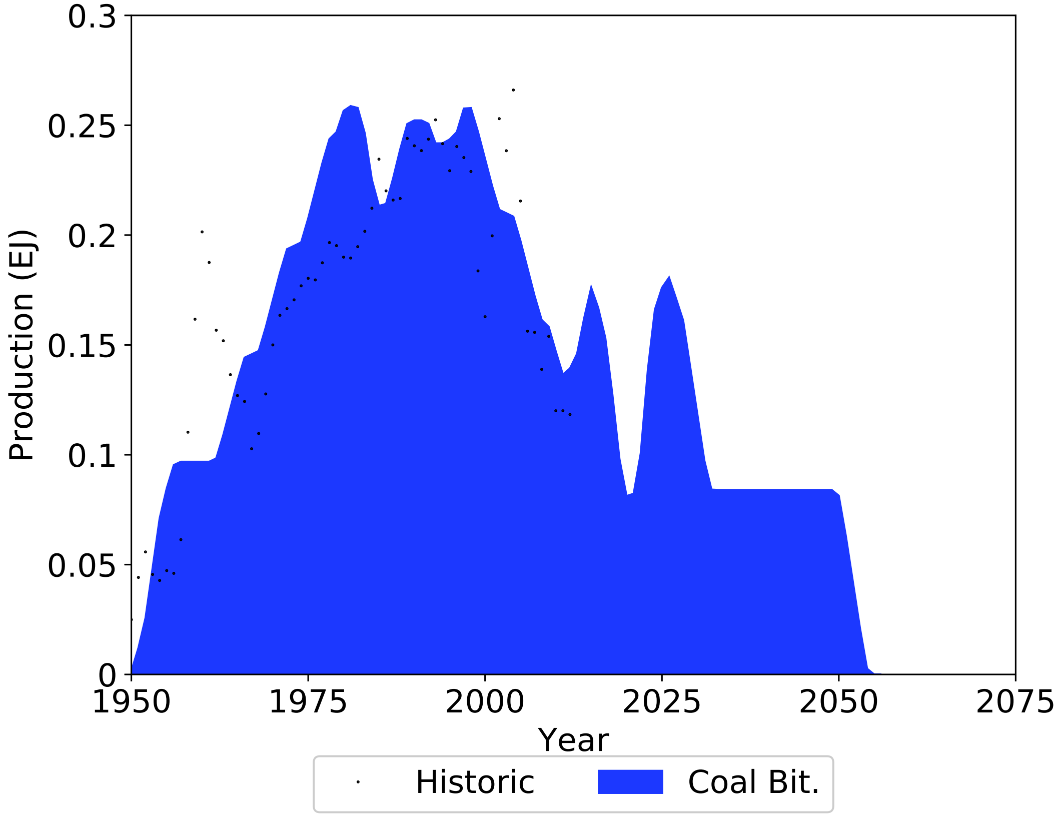

Figure 2.39: China - Beijing projection by mineral type

Table 2.39: Peak years - Minerals

Name	URR	Peak Year	Peak Rate
Coal Bit.	15.6	1981	0.26
**Total**	**15.6**	**1981**	**0.26**

China

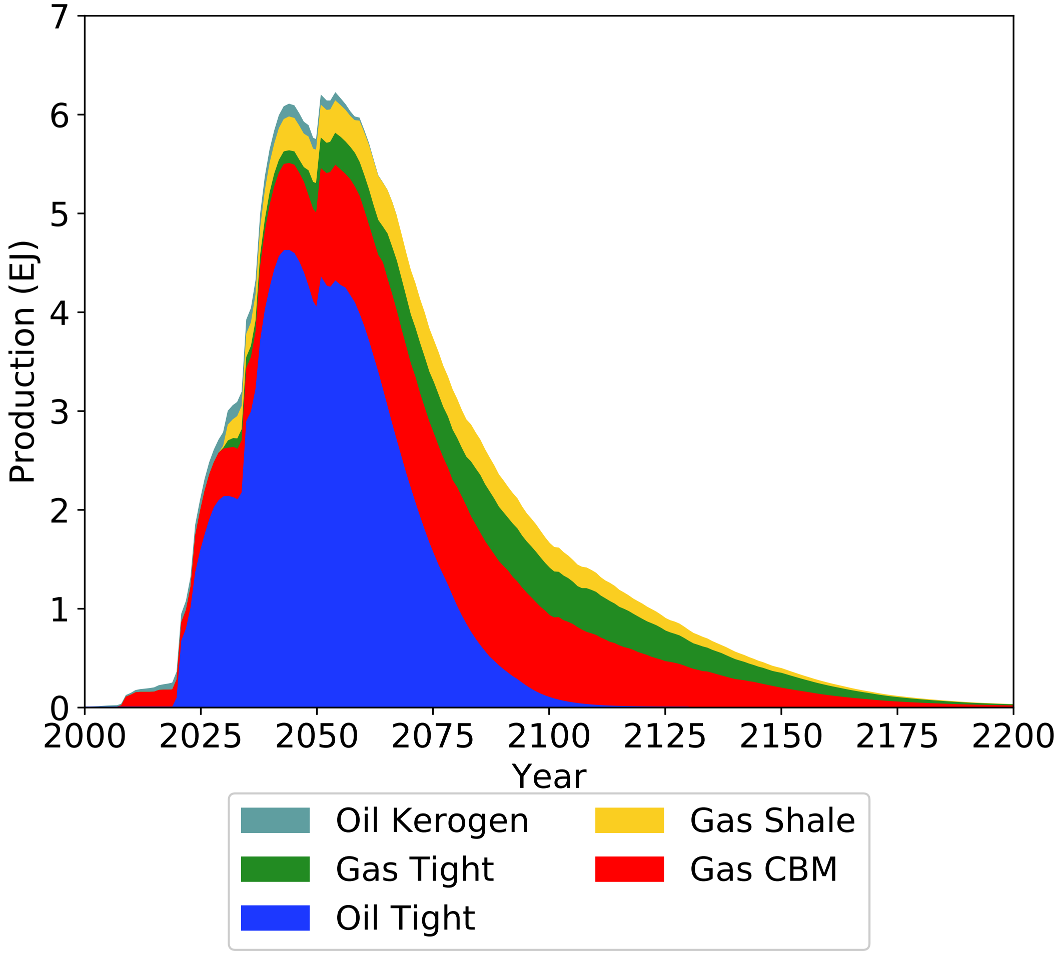

Figure 2.40: China - China projections capped at 16

Table 2.40: Peak years - All

**Name**	**URR**	**Peak Year**	**Peak Rate**
Oil Tight China	184.51	2044	4.62
Gas CBM China	105.0	2067	1.31
Gas Tight China	44.5	2085	0.58
Gas Shale China	31.89	2066	0.46
Oil Kerogen China	5.73	2033	0.14
**Total**	**371.63**	**2054**	**6.21**

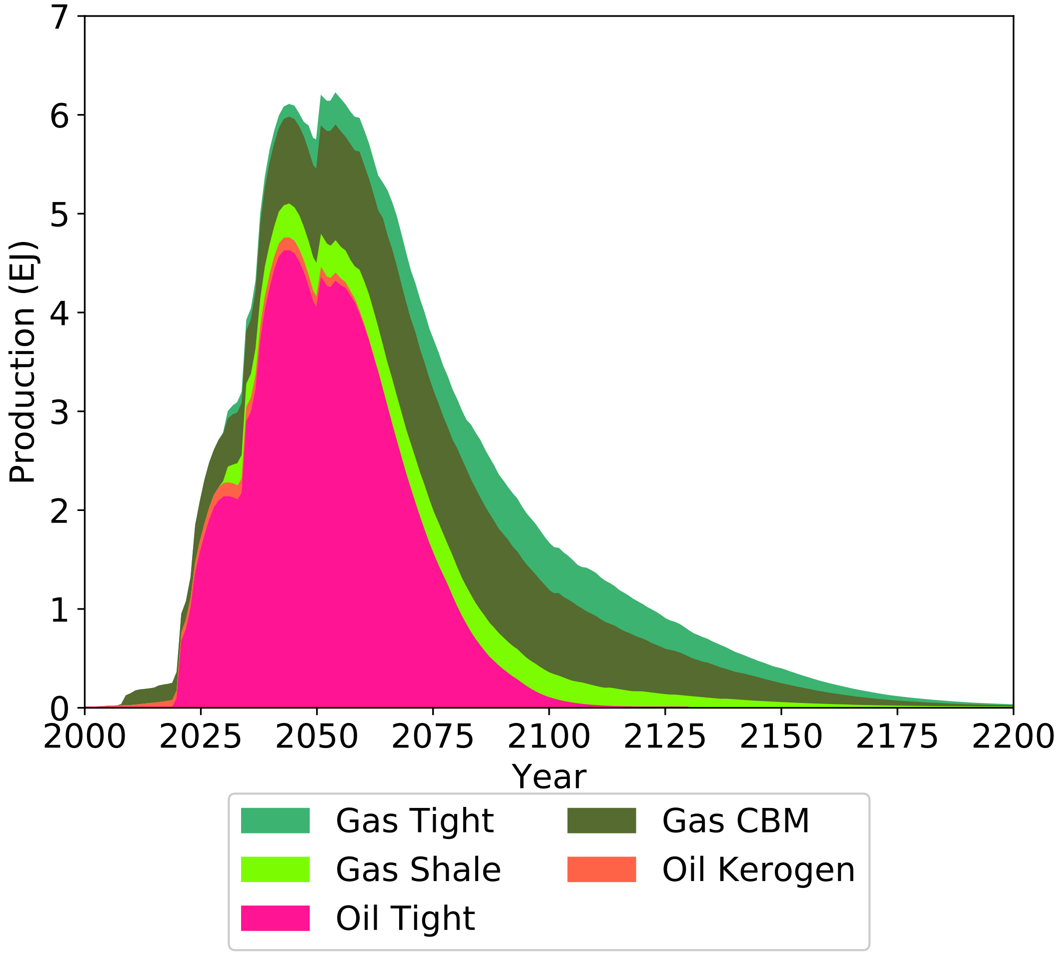

Figure 2.41: China - China projection by mineral type

Table 2.41: Peak years - Minerals

**Name**	**URR**	**Peak Year**	**Peak Rate**
Oil Tight	184.51	2044	4.62
Oil Kerogen	5.73	2033	0.14
Gas Shale	31.89	2066	0.46
Gas CBM	105.0	2067	1.31
Gas Tight	44.5	2085	0.58
**Total**	**371.63**	**2054**	**6.21**

Chongqing

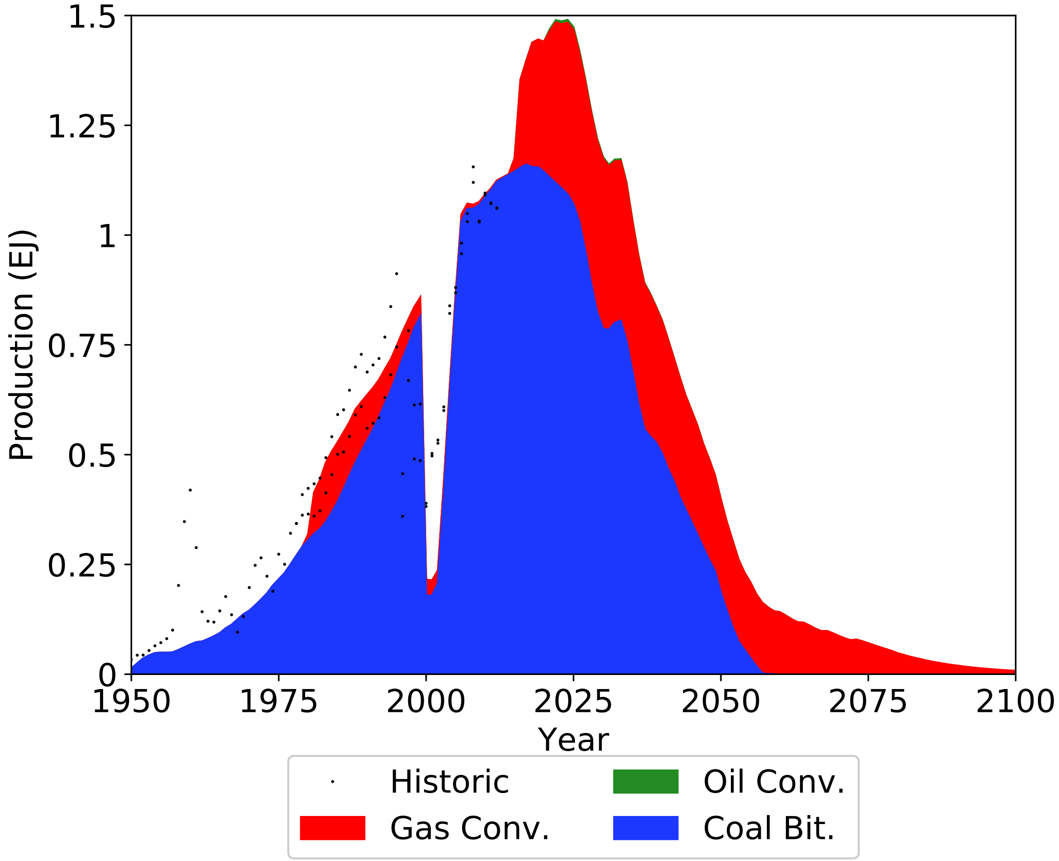

Figure 2.42: China - Chongqing projections capped at 16

Table 2.42: Peak years - All

Name	URR	Peak Year	Peak Rate
Coal Bit. Chongqing	53.62	2017	1.16
Gas Conv. Chongqing	17.06	2025	0.4
Oil Conv. Chongqing	0.1	2024	0.01
**Total**	**70.78**	**2024**	**1.49**

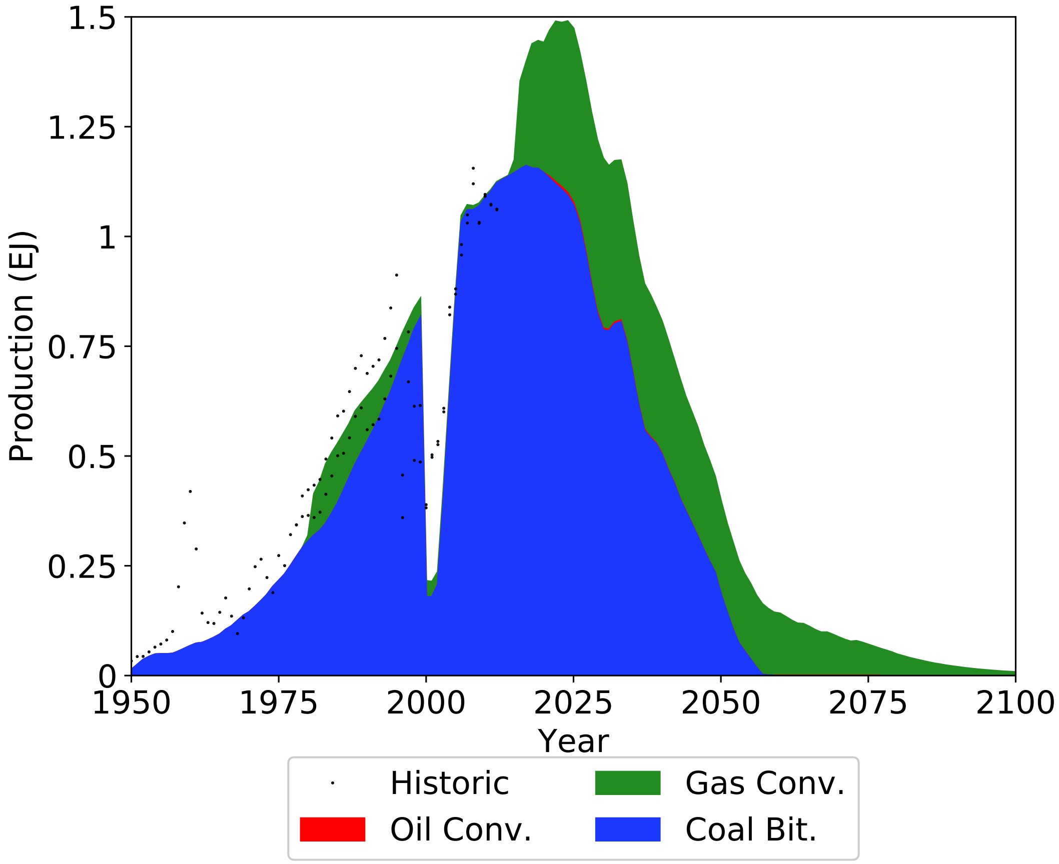

Figure 2.43: China - Chongqing projection by mineral type

Table 2.43: Peak years - Minerals

Name	URR	Peak Year	Peak Rate
Coal Bit.	53.62	2017	1.16
Oil Conv.	0.1	2024	0.01
Gas Conv.	17.06	2025	0.4
**Total**	**70.78**	**2024**	**1.49**

Fujian

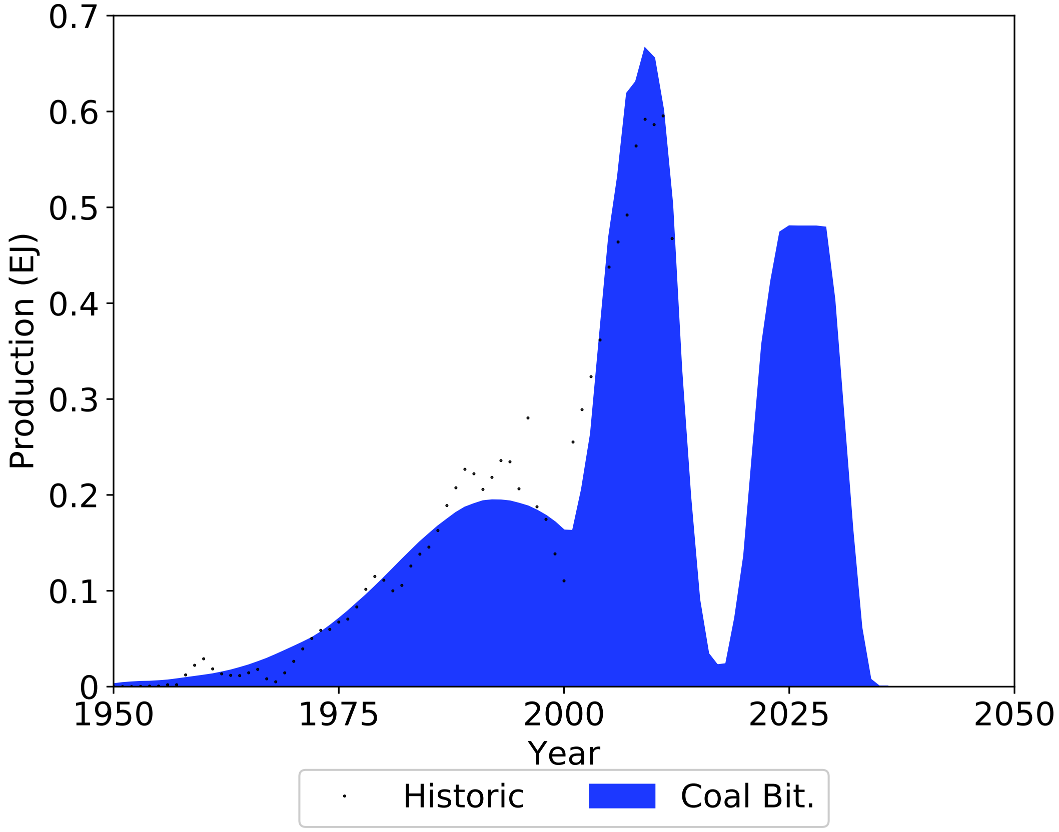

Figure 2.44: China - Fujian projections capped at 16

Table 2.44: Peak years - All

Name	URR	Peak Year	Peak Rate
Coal Bit. Fujian	15.95	2009	0.67
**Total**	**15.95**	**2009**	**0.67**

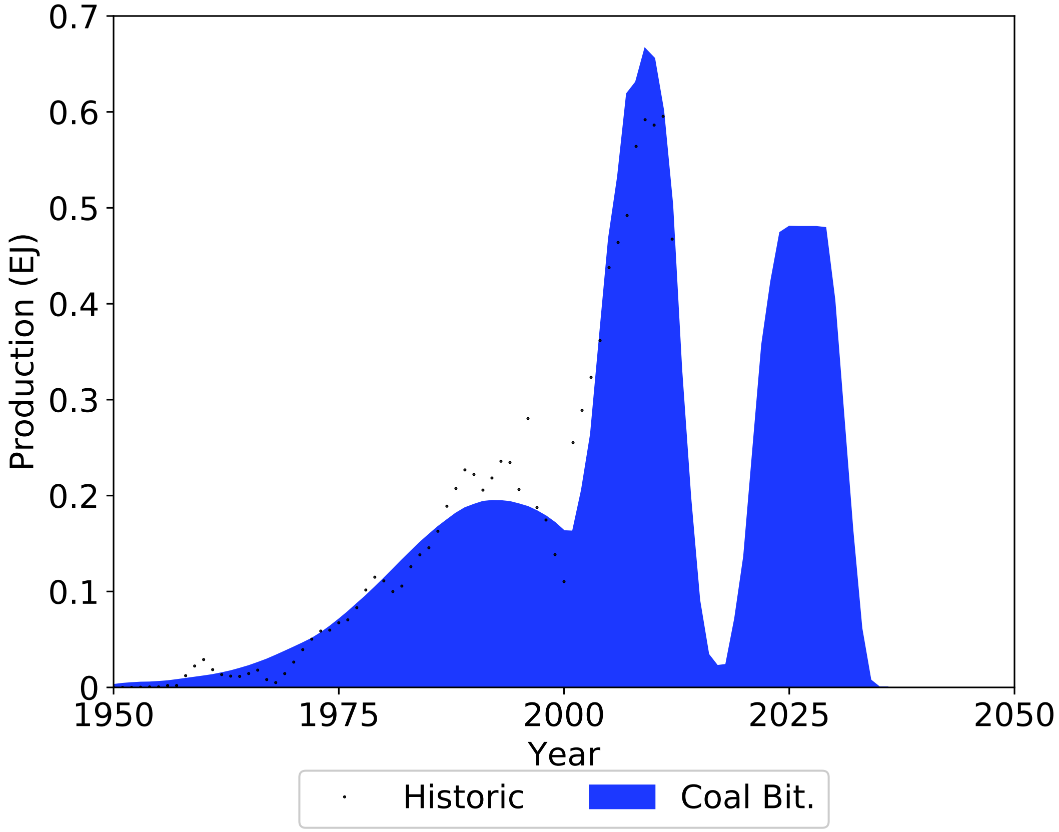

Figure 2.45: China - Fujian projection by mineral type

Table 2.45: Peak years - Minerals

Name	URR	Peak Year	Peak Rate
Coal Bit.	15.95	2009	0.67
**Total**	**15.95**	**2009**	**0.67**

Gansu

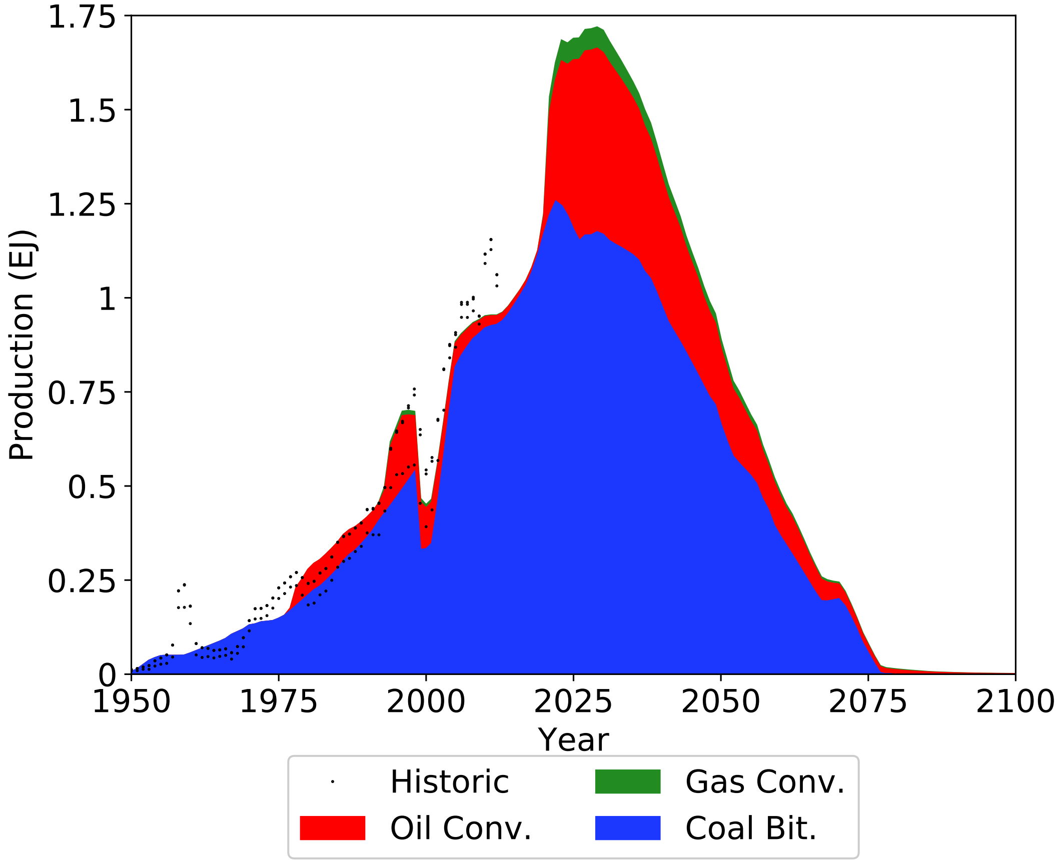

Figure 2.46: China - Gansu projections capped at 16

Table 2.46: Peak years - All

Name	URR	Peak Year	Peak Rate
Coal Bit. Gansu	66.67	2022	1.26
Oil Conv. Gansu	16.32	2028	0.49
Gas Conv. Gansu	1.71	2030	0.06
**Total**	**84.7**	**2029**	**1.72**

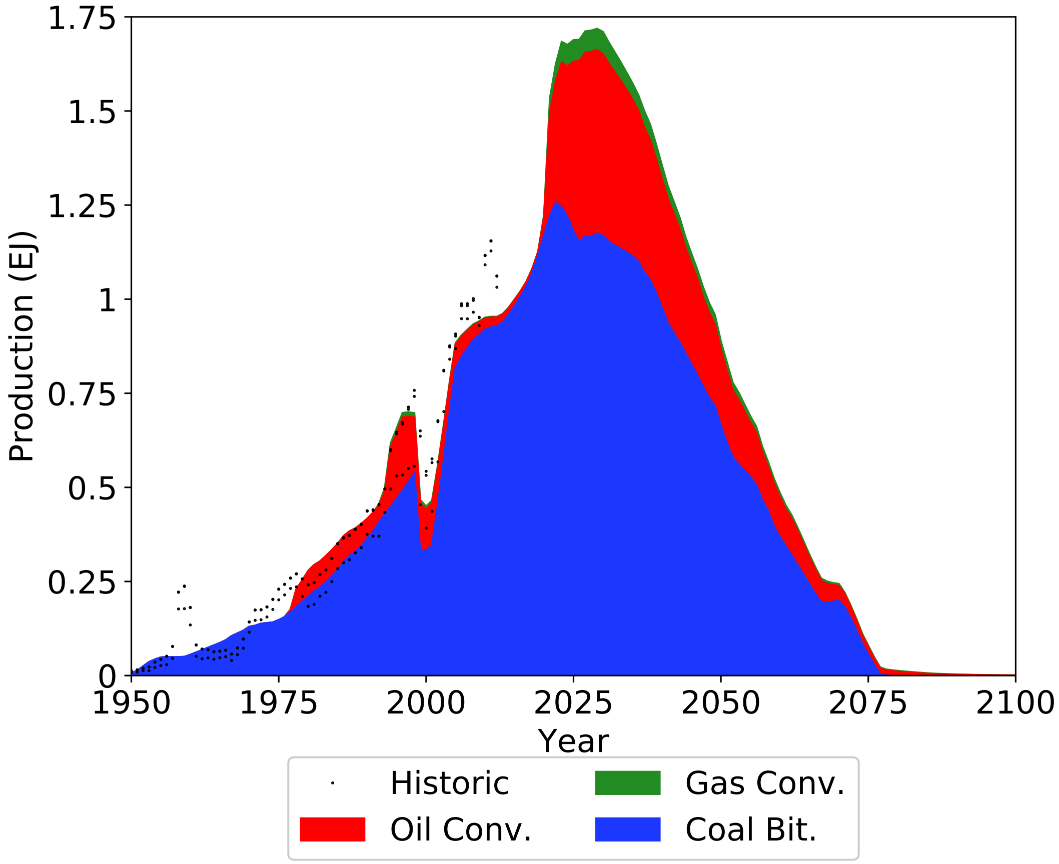

Figure 2.47: China - Gansu projection by mineral type

Table 2.47: Peak years - Minerals

**Name**	**URR**	**Peak Year**	**Peak Rate**
Coal Bit.	66.67	2022	1.26
Oil Conv.	16.32	2028	0.49
Gas Conv.	1.71	2030	0.06
**Total**	**84.7**	**2029**	**1.72**

Guangdong

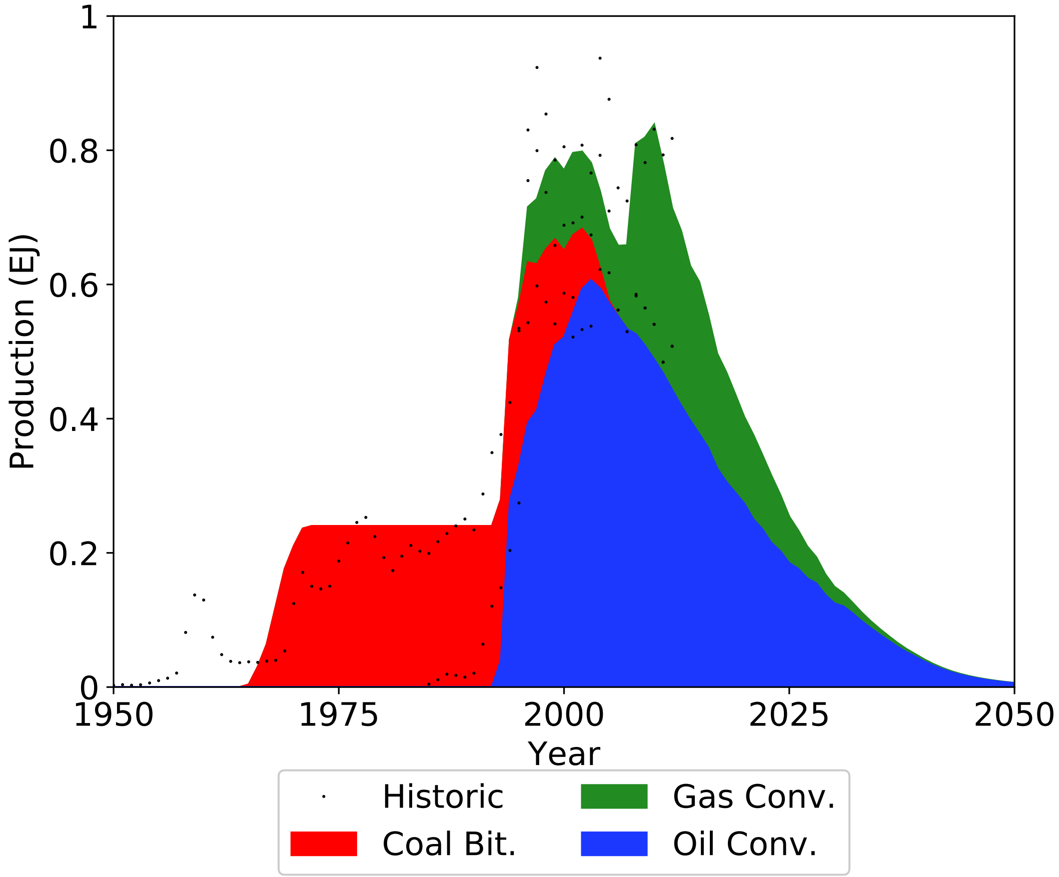

Figure 2.48: China - Guangdong projections capped at 16

Table 2.48: Peak years - All

Name	URR	Peak Year	Peak Rate
Oil Conv. Guangdong	14.92	2003	0.61
Coal Bit. Guangdong	7.83	1972	0.24
Gas Conv. Guangdong	5.2	2010	0.35
**Total**	**27.95**	**2010**	**0.84**

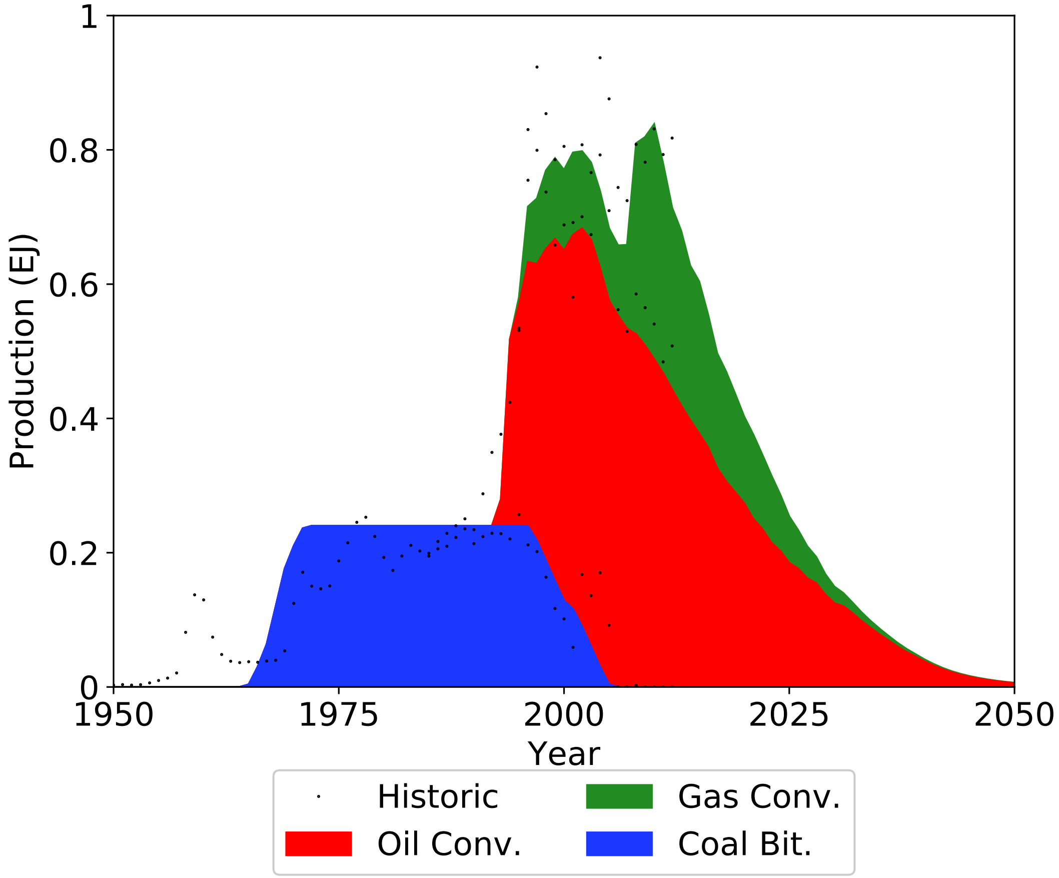

Figure 2.49: China - Guangdong projection by mineral type

Table 2.49: Peak years - Minerals

Name	URR	Peak Year	Peak Rate
Coal Bit.	7.83	1972	0.24
Oil Conv.	14.92	2003	0.61
Gas Conv.	5.2	2010	0.35
**Total**	**27.95**	**2010**	**0.84**

Guangxi

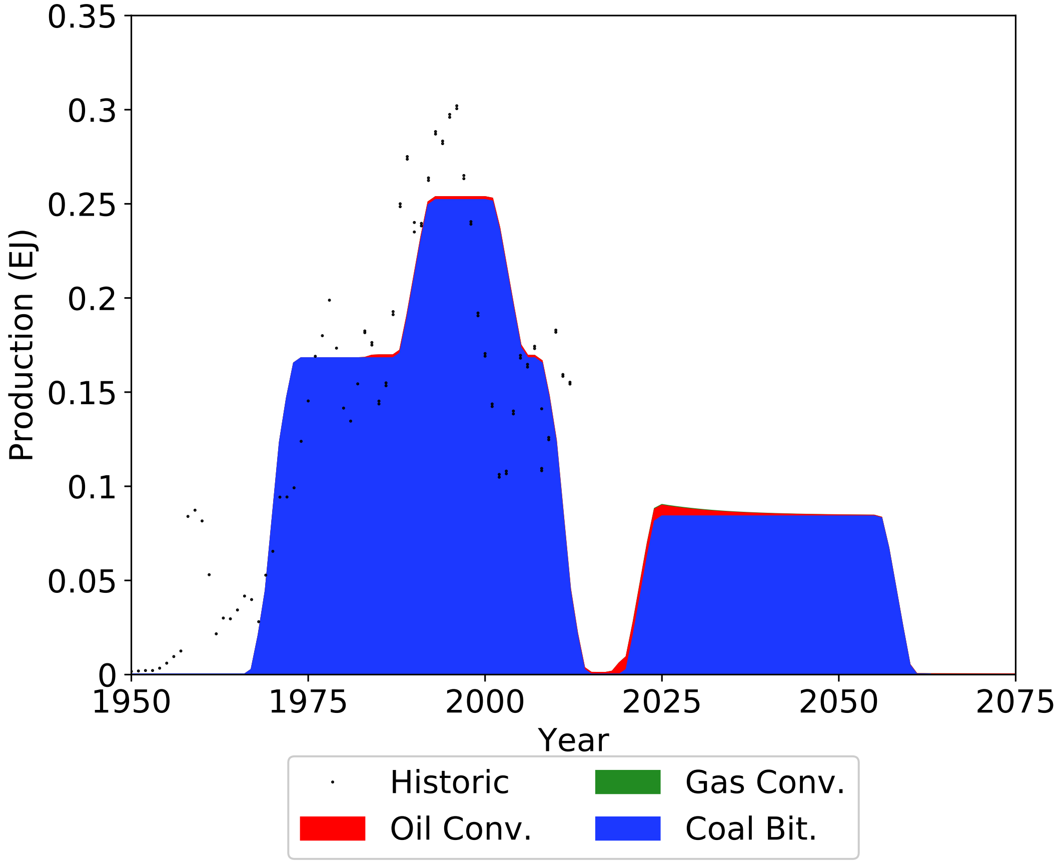

Figure 2.50: China - Guangxi projections capped at 16

Table 2.50: Peak years - All

Name	URR	Peak Year	Peak Rate
Coal Bit. Guangxi	11.04	1993	0.25
Oil Conv. Guangxi	0.15	2020	0.01
Gas Conv. Guangxi	0.01	2022	–
**Total**	**11.2**	**1993**	**0.25**

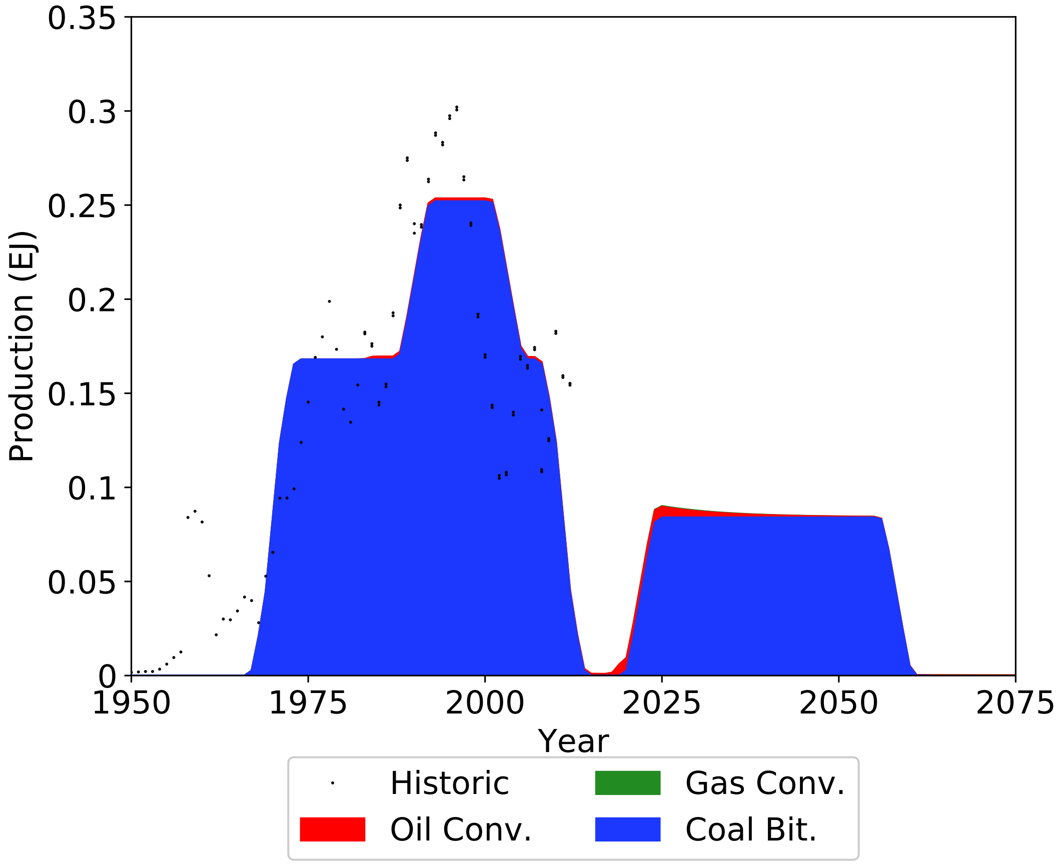

Figure 2.51: China - Guangxi projection by mineral type

Table 2.51: Peak years - Minerals

Name	URR	Peak Year	Peak Rate
Coal Bit.	11.04	1993	0.25
Oil Conv.	0.15	2020	0.01
Gas Conv.	0.01	2022	–
**Total**	**11.2**	**1993**	**0.25**

Guizhou

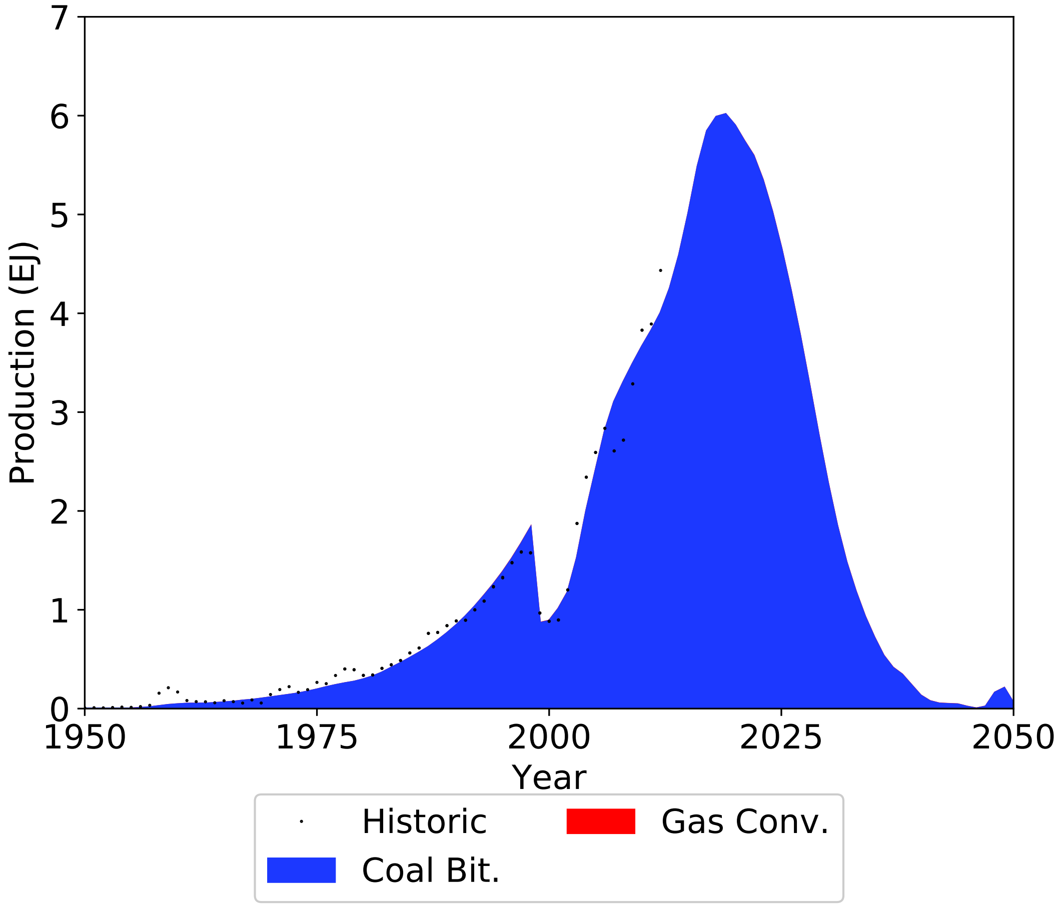

Figure 2.52: China - Guizhou projections capped at 16

Table 2.52: Peak years - All

Name	URR	Peak Year	Peak Rate
Coal Bit. Guizhou	147.64	2019	6.01
Gas Conv. Guizhou	0.13	2024	–
**Total**	**147.77**	**2019**	**6.01**

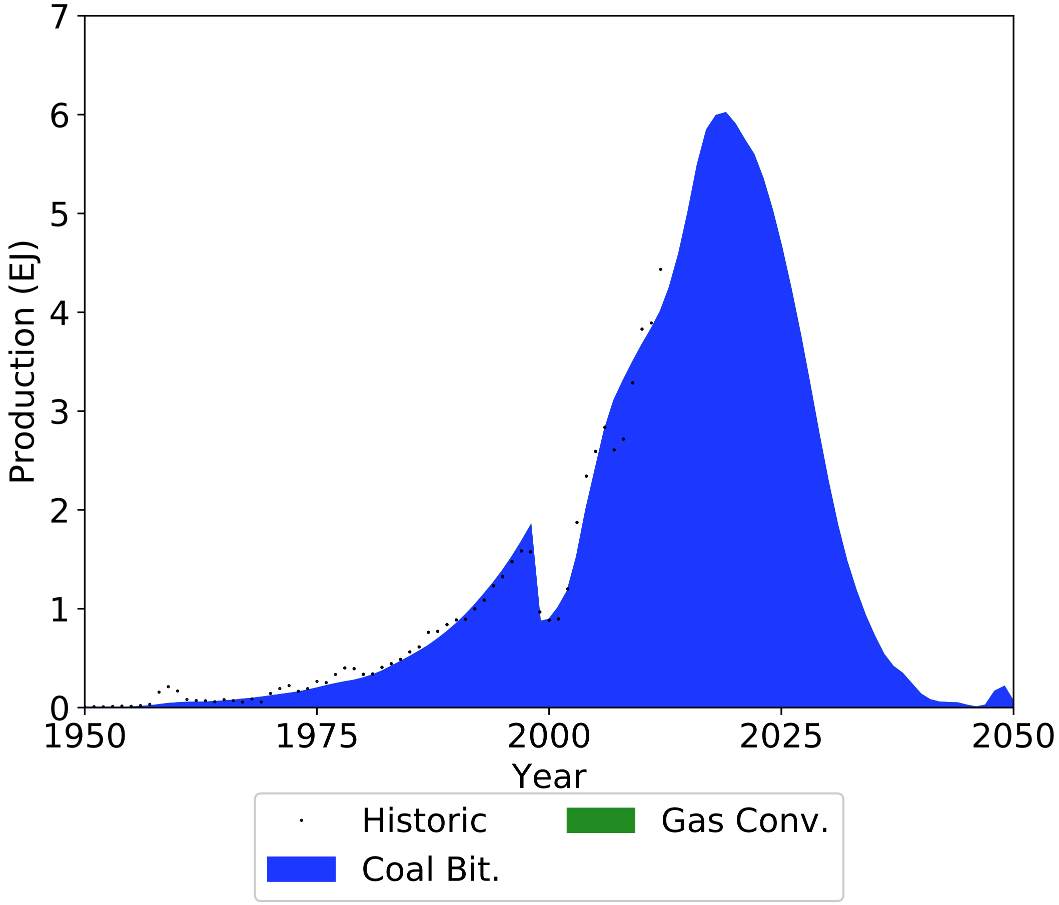

Figure 2.53: China - Guizhou projection by mineral type

Table 2.53: Peak years - Minerals

Name	URR	Peak Year	Peak Rate
Coal Bit.	147.64	2019	6.01
Gas Conv.	0.13	2024	–
**Total**	**147.77**	**2019**	**6.01**

Hainan

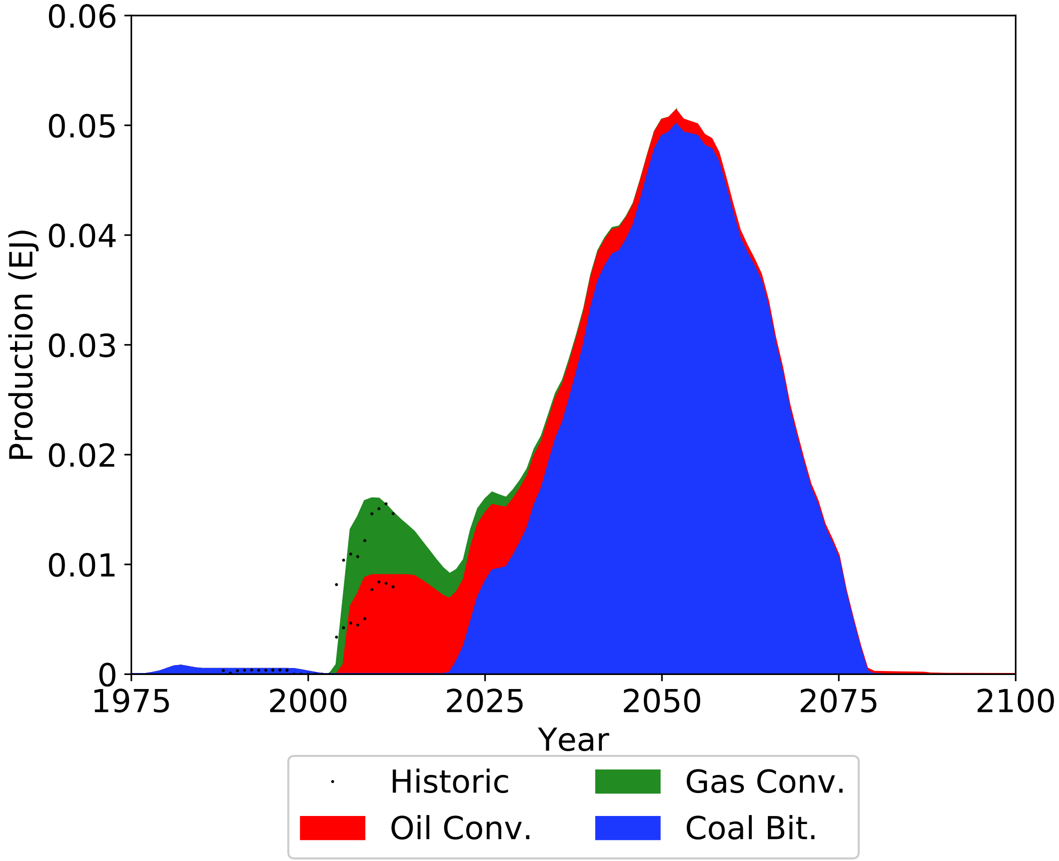

Figure 2.54: China - Hainan projections capped at 16

Table 2.54: Peak years - All

Name	URR	Peak Year	Peak Rate
Coal Bit. Hainan	1.6	2052	0.05
Oil Conv. Hainan	0.26	2009	0.01
Gas Conv. Hainan	0.1	2006	0.01
**Total**	**1.96**	**2052**	**0.05**

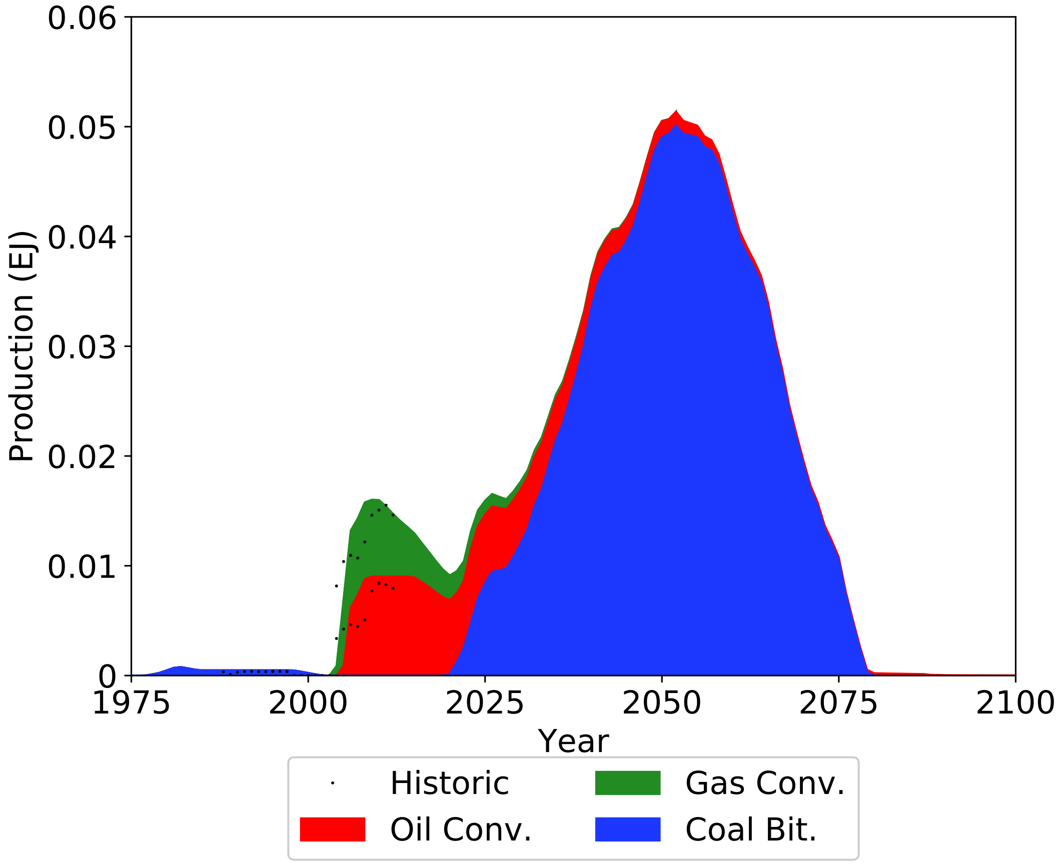

Figure 2.55: China - Hainan projection by mineral type

Table 2.55: Peak years - Minerals

Name	URR	Peak Year	Peak Rate
Coal Bit.	1.6	2052	0.05
Oil Conv.	0.26	2009	0.01
Gas Conv.	0.1	2006	0.01
**Total**	**1.96**	**2052**	**0.05**

Hebei

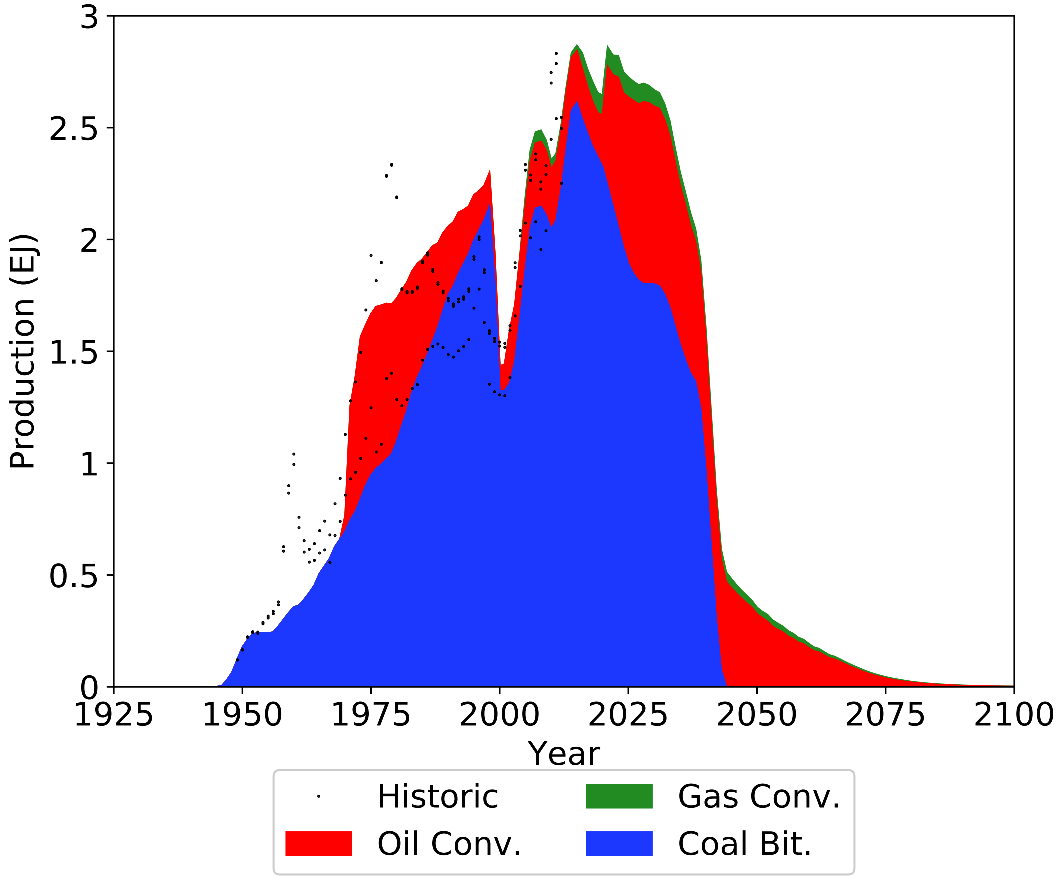

Figure 2.56: China - Hebei projections capped at 16

Table 2.56: Peak years - All

Name	URR	Peak Year	Peak Rate
Coal Bit. Hebei	128.44	2015	2.61
Oil Conv. Hebei	40.88	2028	0.81
Gas Conv. Hebei	3.13	2023	0.1
**Total**	**172.45**	**2015**	**2.87**

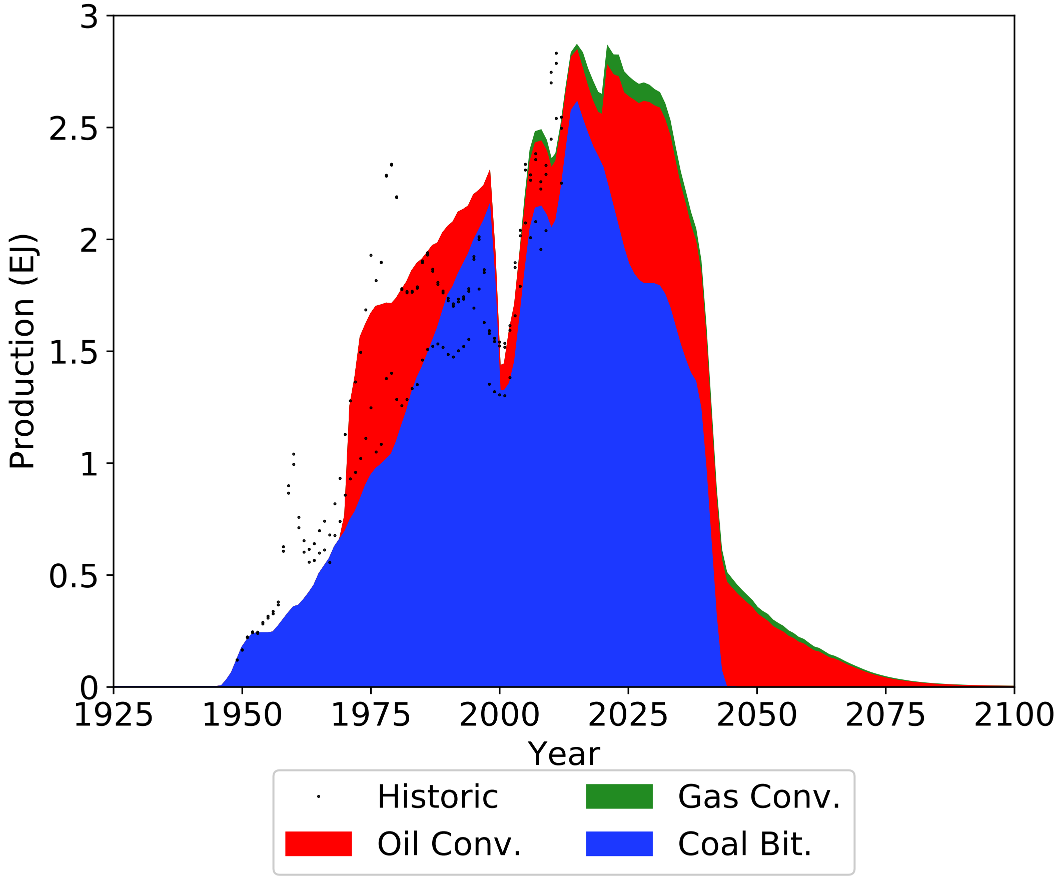

Figure 2.57: China - Hebei projection by mineral type

Table 2.57: Peak years - Minerals

**Name**	**URR**	**Peak Year**	**Peak Rate**
Coal Bit.	128.44	2015	2.61
Oil Conv.	40.88	2028	0.81
Gas Conv.	3.13	2023	0.1
**Total**	**172.45**	**2015**	**2.87**

Heilongjiang

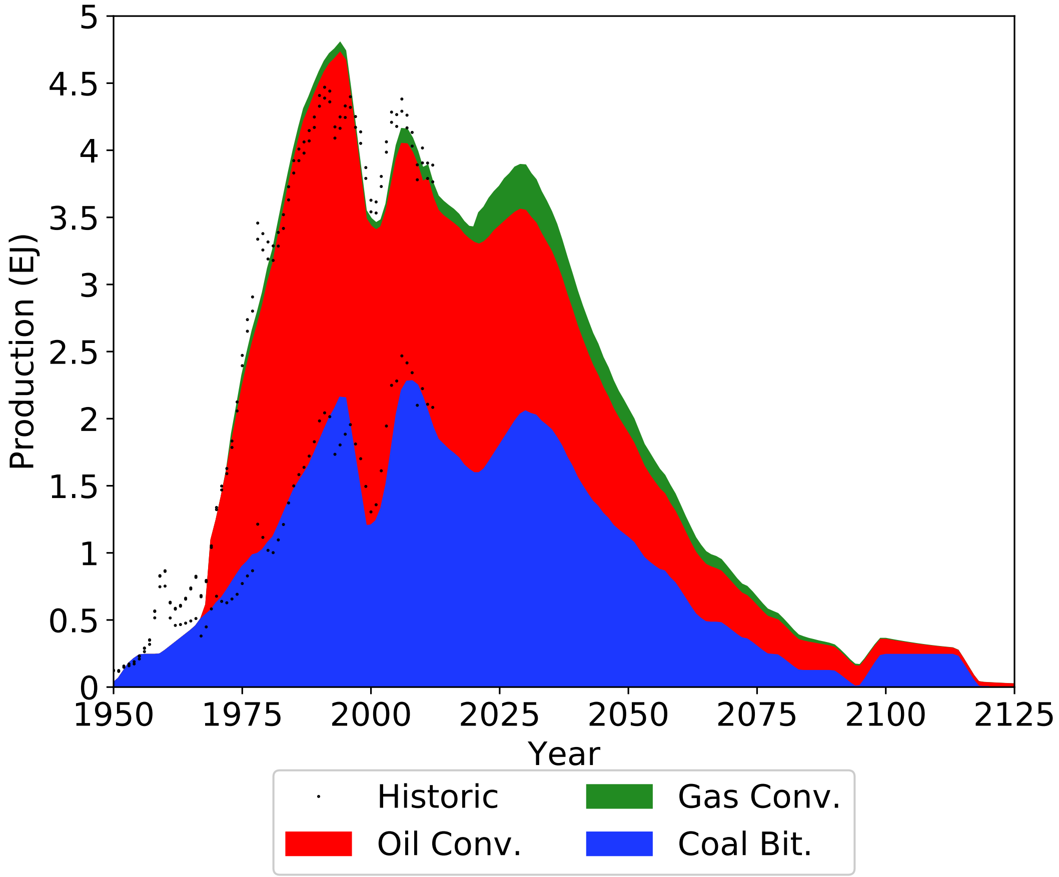

Figure 2.58: China - Heilongjiang projections capped at 16

Table 2.58: Peak years - All

Name	URR	Peak Year	Peak Rate
Coal Bit. Heilongjiang	157.4	2008	2.28
Oil Conv. Heilongjiang	156.83	1989	2.67
Gas Conv. Heilongjiang	15.95	2030	0.34
**Total**	**330.18**	**1994**	**4.8**

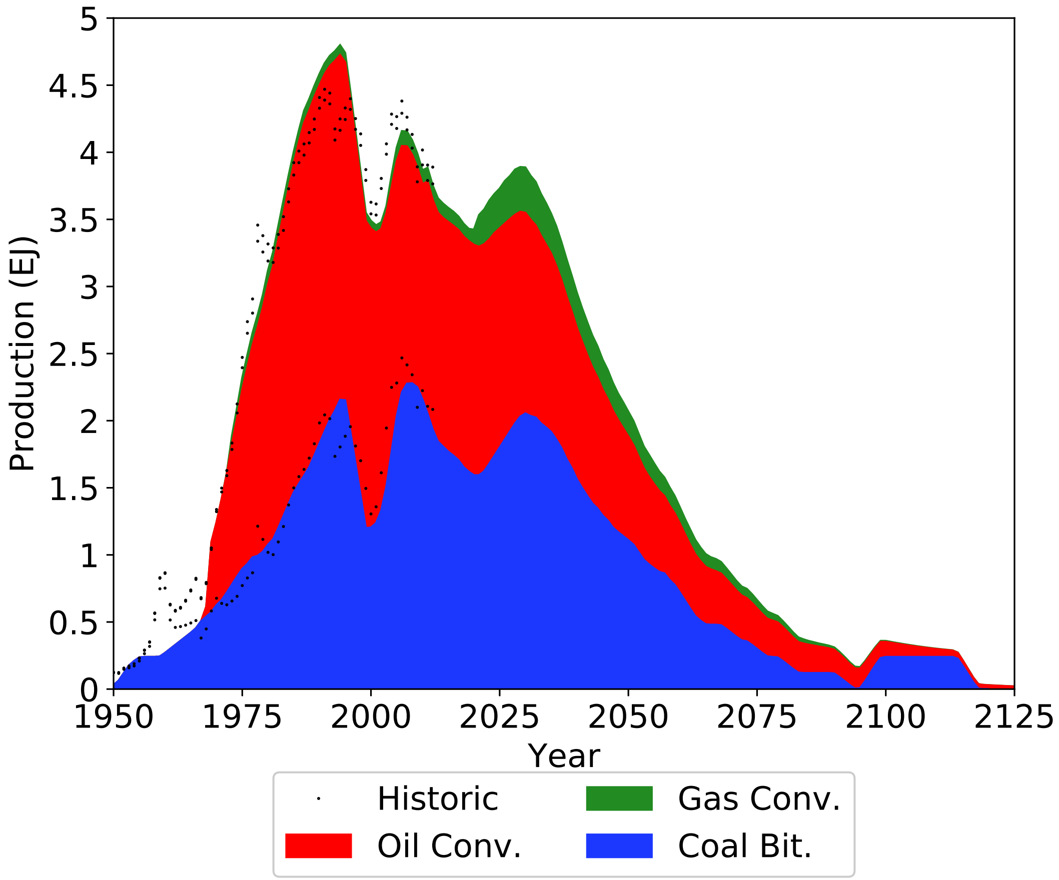

Figure 2.59: China - Heilongjiang projection by mineral type

Table 2.59: Peak years - Minerals

Name	URR	Peak Year	Peak Rate
Coal Bit.	157.4	2008	2.28
Oil Conv.	156.83	1989	2.67
Gas Conv.	15.95	2030	0.34
**Total**	**330.18**	**1994**	**4.8**

Henan

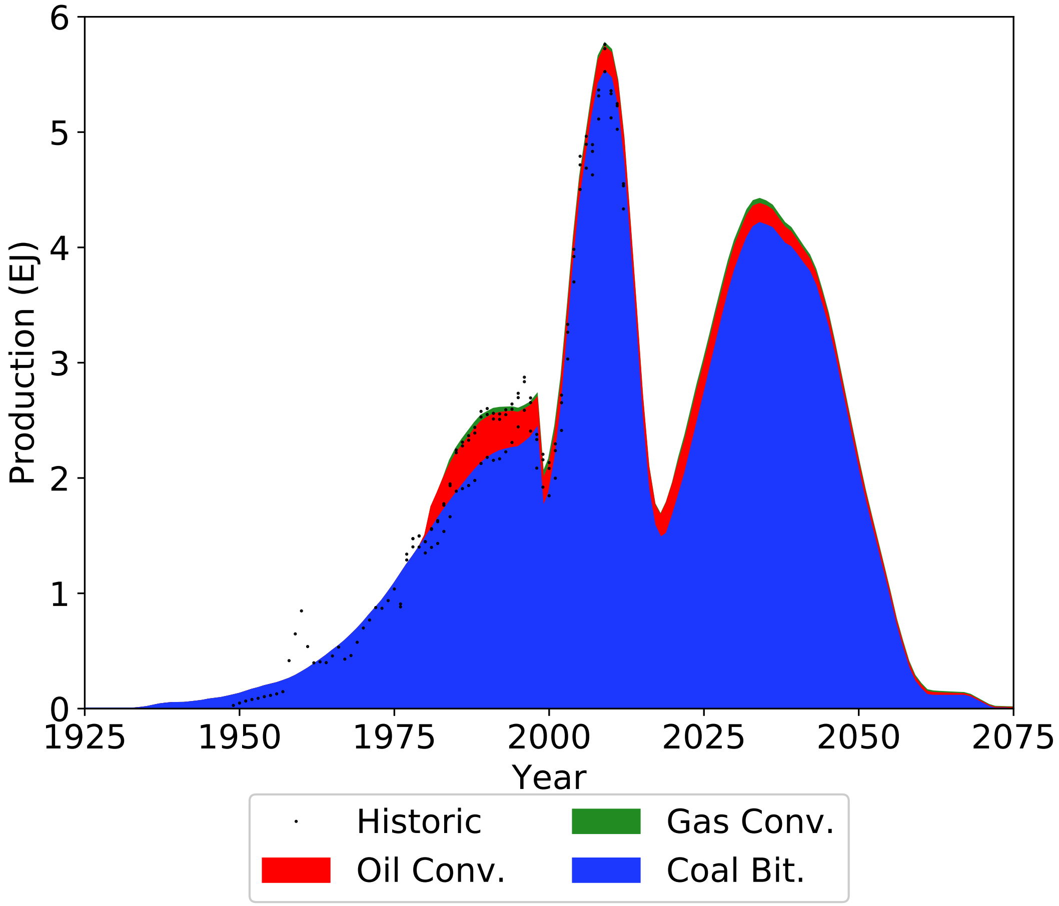

Figure 2.60: China - Henan projections capped at 16

Table 2.60: Peak years - All

Name	URR	Peak Year	Peak Rate
Coal Bit. Henan	241.25	2009	5.53
Oil Conv. Henan	15.83	1989	0.36
Gas Conv. Henan	2.91	2001	0.07
**Total**	**259.99**	**2009**	**5.77**

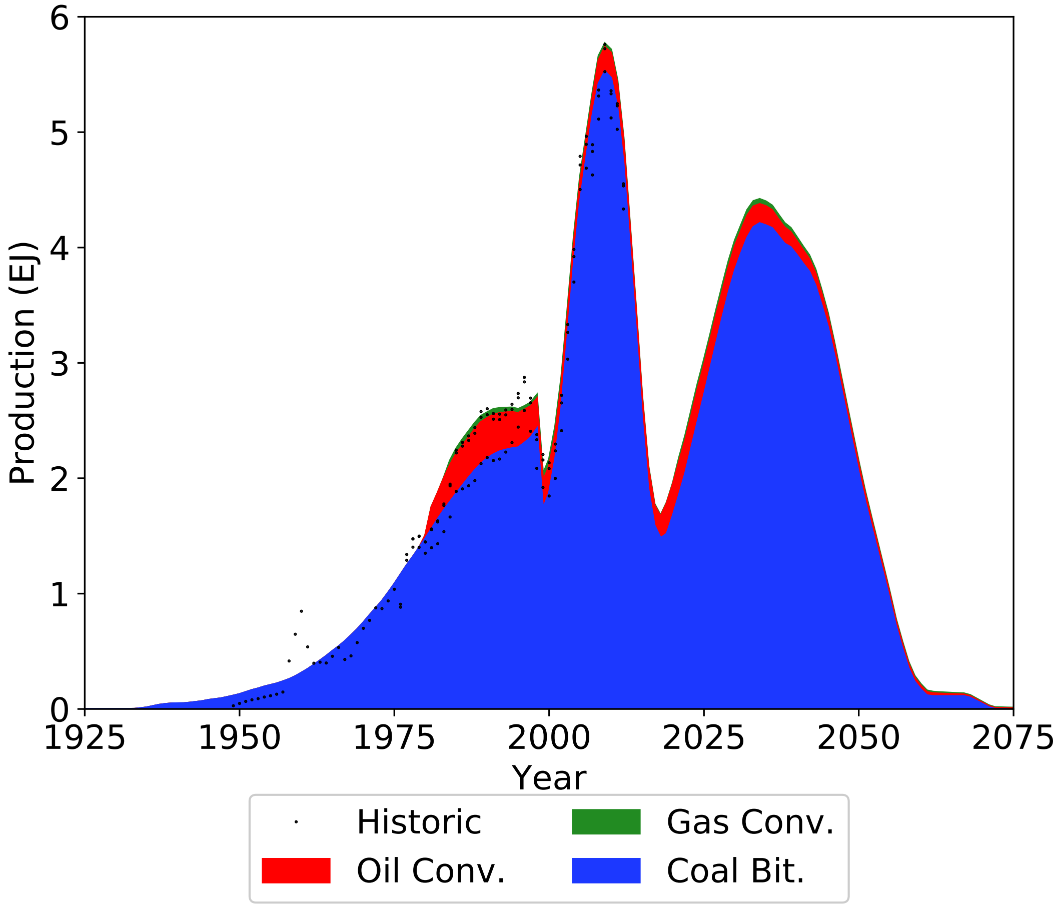

Figure 2.61: China - Henan projection by mineral type

Table 2.61: Peak years - Minerals

Name	URR	Peak Year	Peak Rate
Coal Bit.	241.25	2009	5.53
Oil Conv.	15.83	1989	0.36
Gas Conv.	2.91	2001	0.07
**Total**	**259.99**	**2009**	**5.77**

Historic

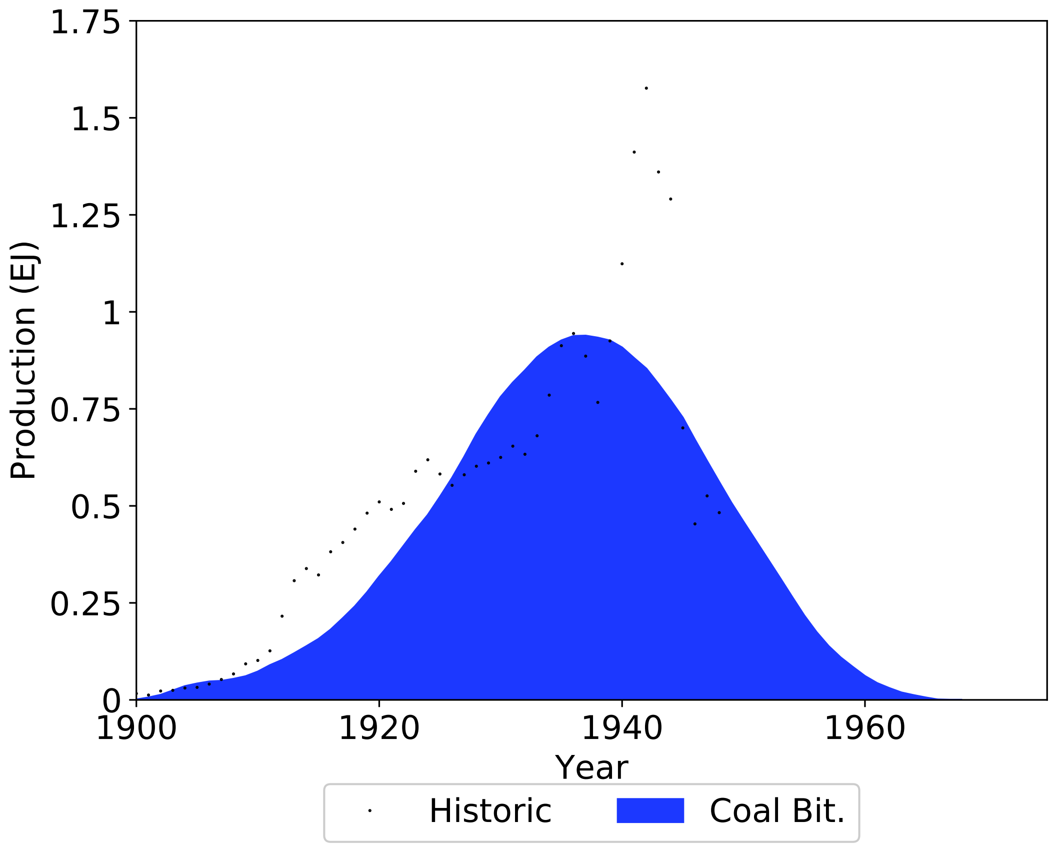

Figure 2.62: China - Historic projections capped at 16

Table 2.62: Peak years - All

Name	URR	Peak Year	Peak Rate
Coal Bit. Historic	25.96	1937	0.94
**Total**	**25.96**	**1937**	**0.94**

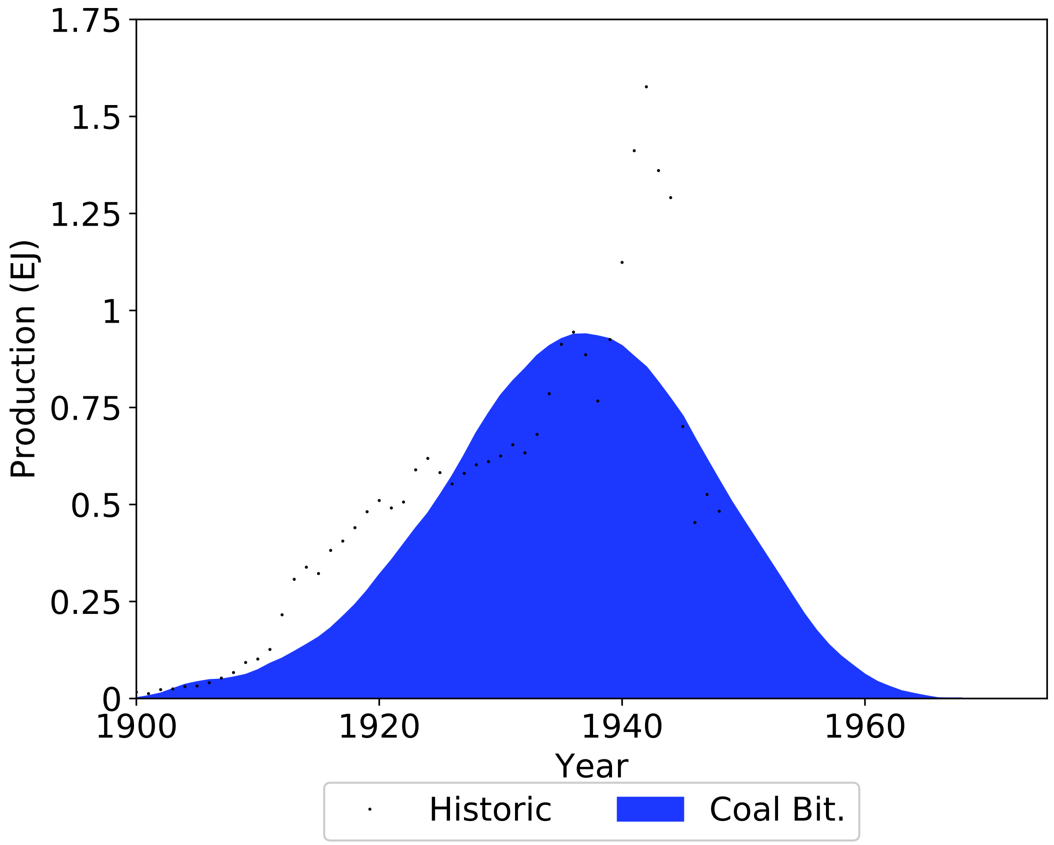

Figure 2.63: China - Historic projection by mineral type

Table 2.63: Peak years - Minerals

Name	URR	Peak Year	Peak Rate
Coal Bit.	25.96	1937	0.94
**Total**	**25.96**	**1937**	**0.94**

Hubei

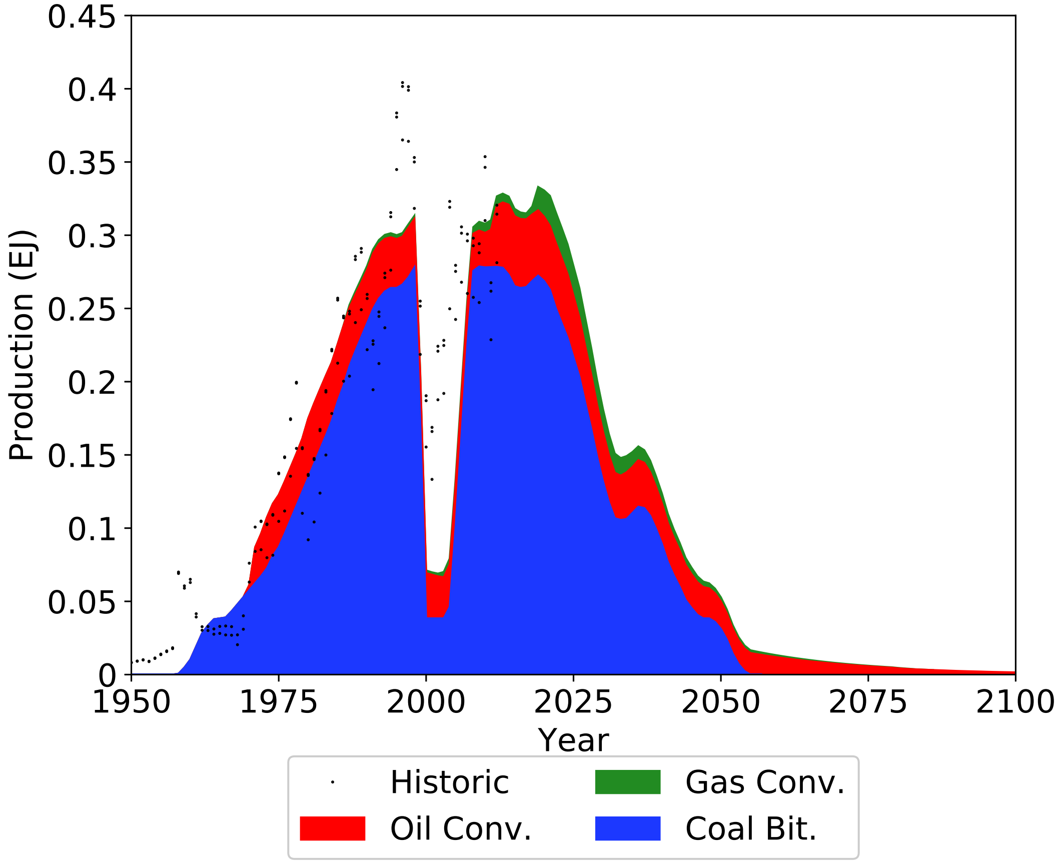

Figure 2.64: China - Hubei projections capped at 16

Table 2.64: Peak years - All

Name	URR	Peak Year	Peak Rate
Coal Bit. Hubei	13.44	2009	0.28
Oil Conv. Hubei	3.09	2014	0.05
Gas Conv. Hubei	0.52	2022	0.02
**Total**	**17.05**	**2019**	**0.33**

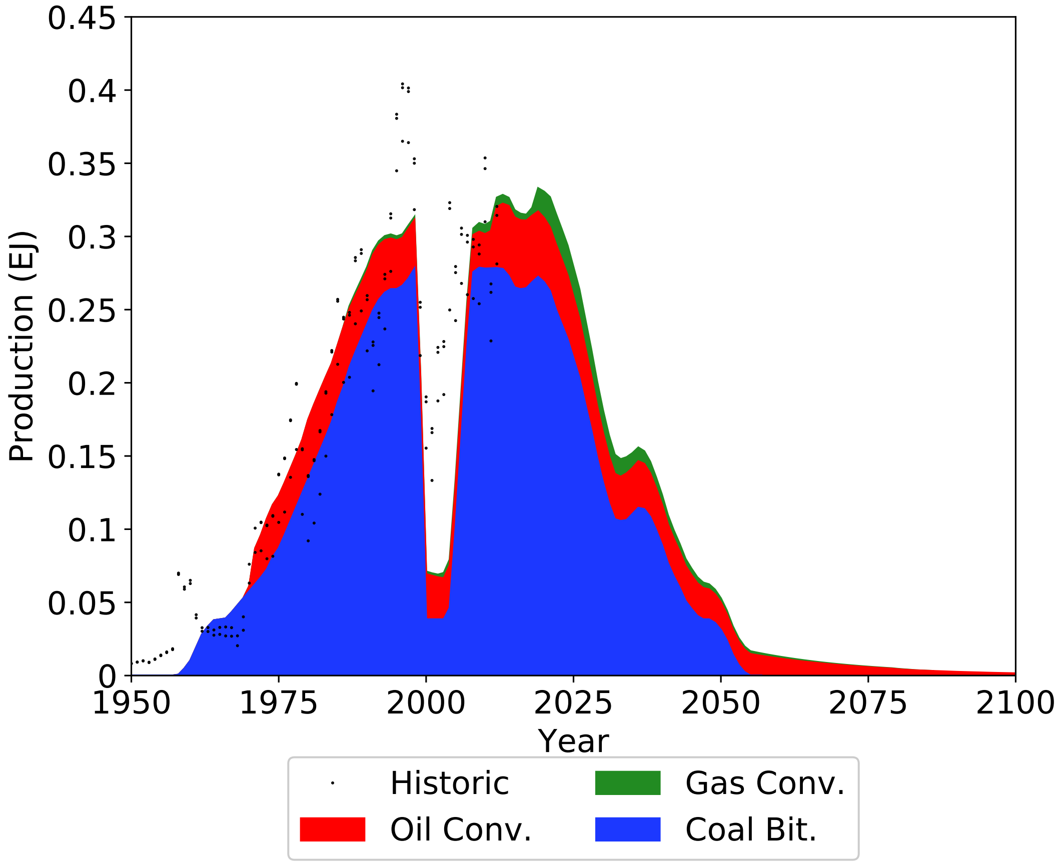

Figure 2.65: China - Hubei projection by mineral type

Table 2.65: Peak years - Minerals

Name	URR	Peak Year	Peak Rate
Coal Bit.	13.44	2009	0.28
Oil Conv.	3.09	2014	0.05
Gas Conv.	0.52	2022	0.02
**Total**	**17.05**	**2019**	**0.33**

Hunan

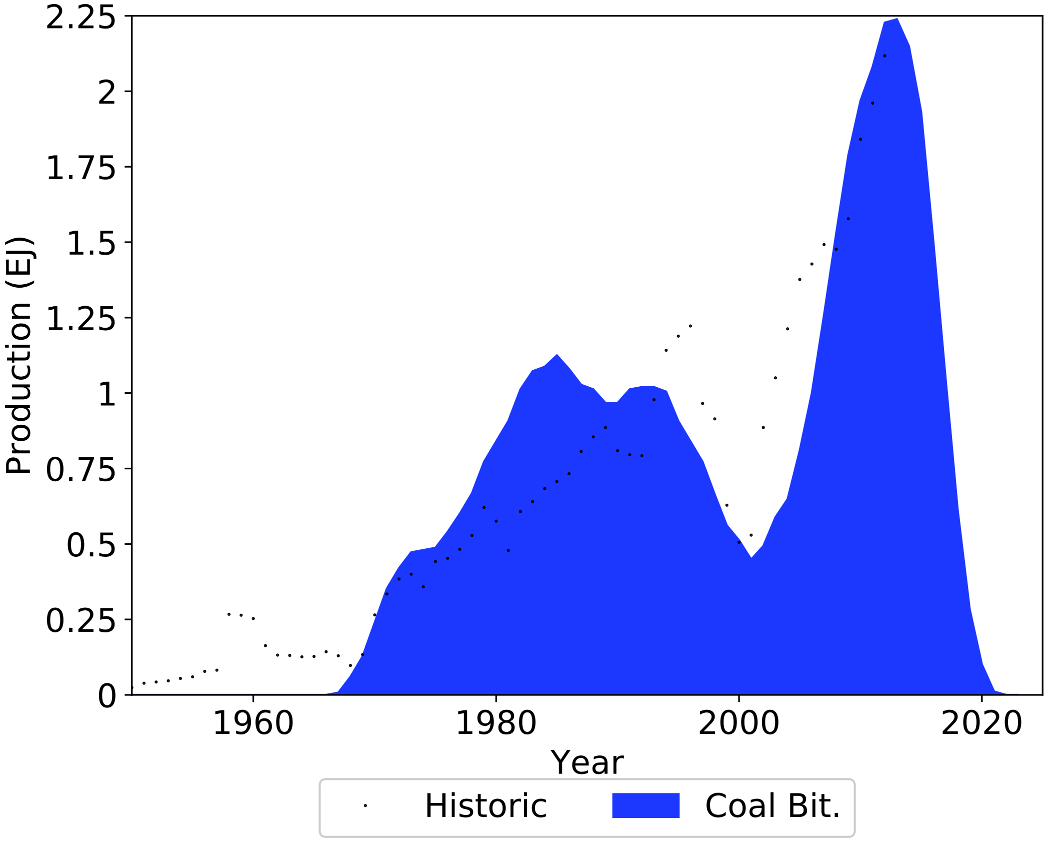

Figure 2.66: China - Hunan projections capped at 16

Table 2.66: Peak years - All

Name	URR	Peak Year	Peak Rate
Coal Bit. Hunan	49.38	2013	2.24
**Total**	**49.38**	**2013**	**2.24**

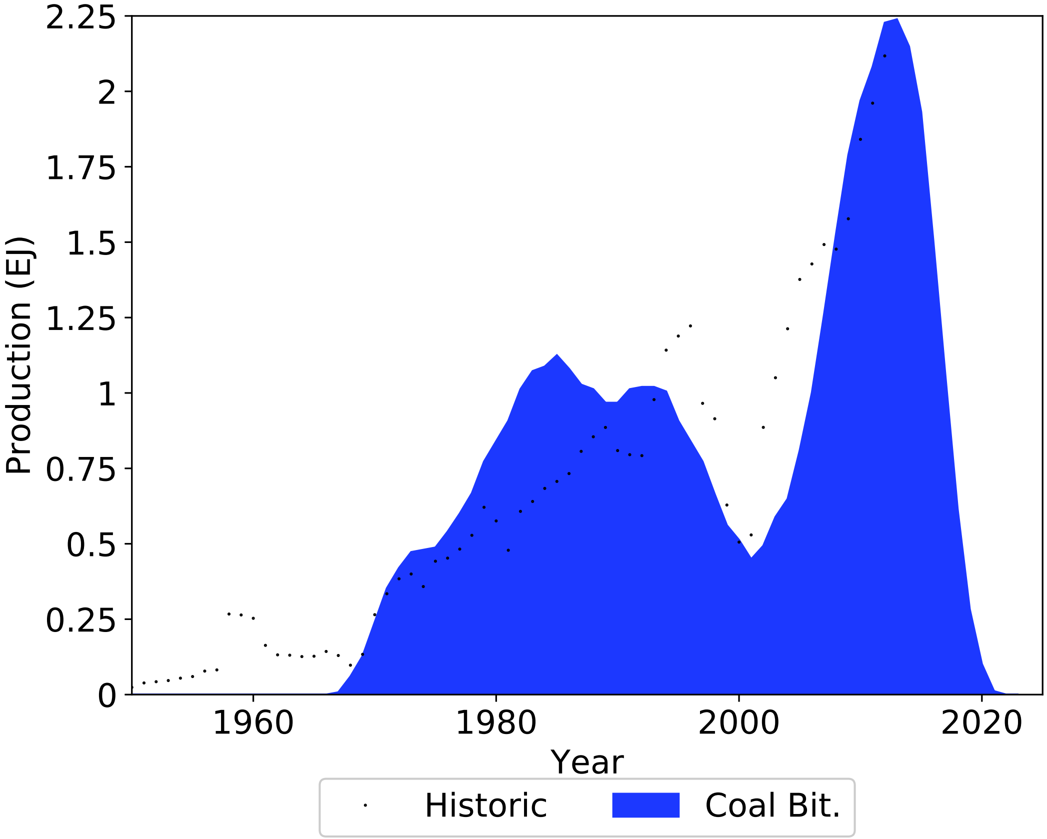

Figure 2.67: China - Hunan projection by mineral type

Table 2.67: Peak years - Minerals

Name	URR	Peak Year	Peak Rate
Coal Bit.	49.38	2013	2.24
**Total**	**49.38**	**2013**	**2.24**

Inner Mongolia

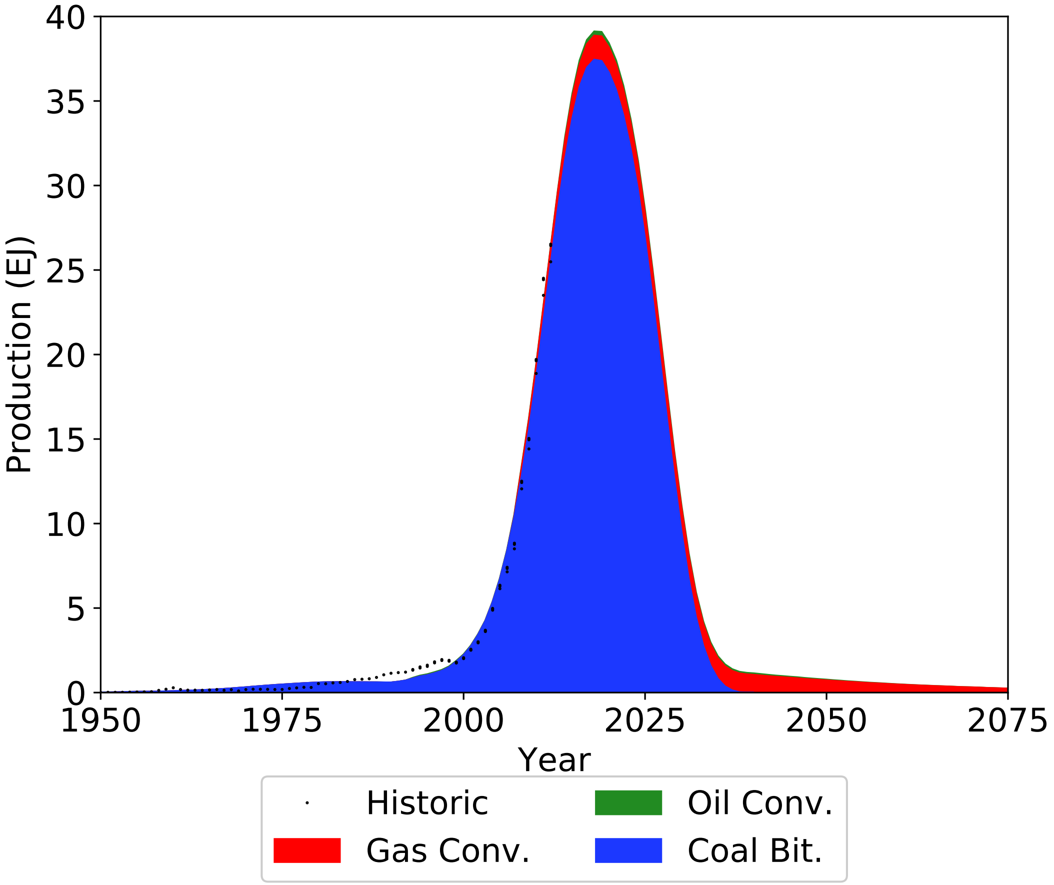

Figure 2.68: China - Inner Mongolia projections capped at 16

Table 2.68: Peak years - All

**Name**	**URR**	**Peak Year**	**Peak Rate**
Coal Bit. Inner Mongolia	696.15	2018	37.44
Gas Conv. Inner Mongolia	62.94	2023	1.51
Oil Conv. Inner Mongolia	6.9	2019	0.26
**Total**	**765.99**	**2018**	**39.11**

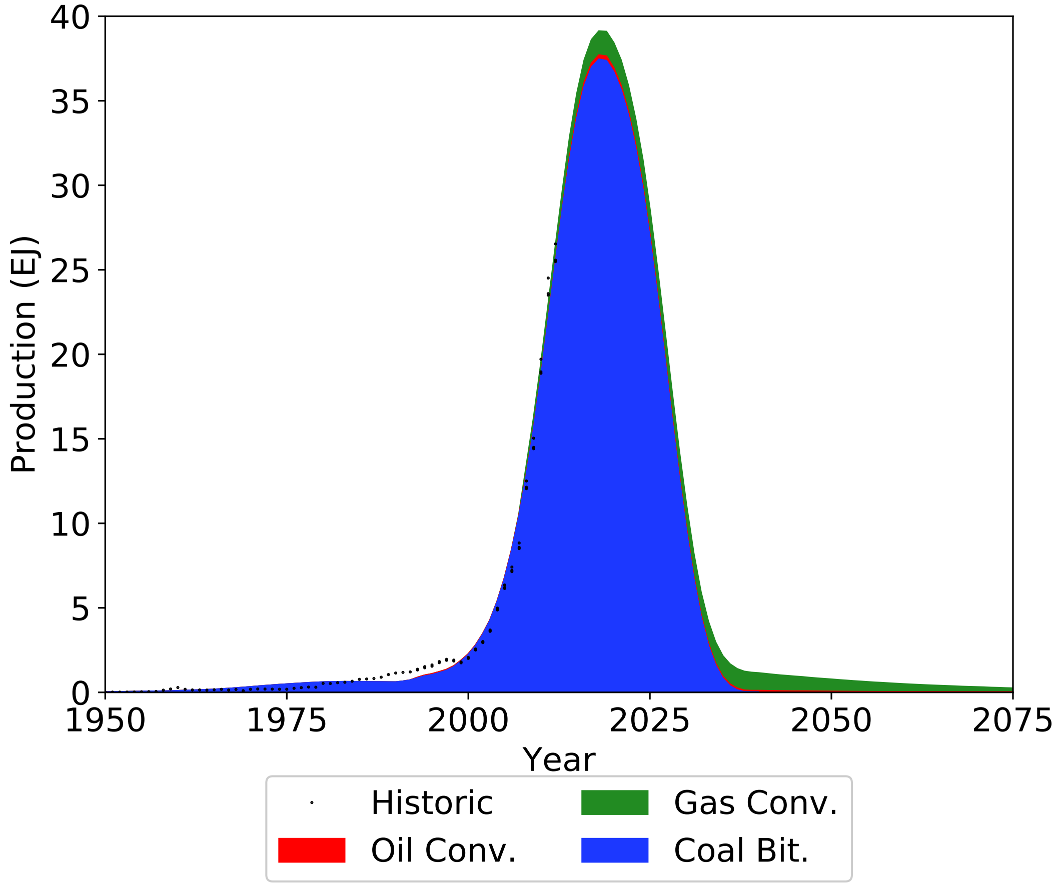

Figure 2.69: China - Inner Mongolia projection by mineral type

Table 2.69: Peak years - Minerals

**Name**	**URR**	**Peak Year**	**Peak Rate**
Coal Bit.	696.15	2018	37.44
Oil Conv.	6.9	2019	0.26
Gas Conv.	62.94	2023	1.51
**Total**	**765.99**	**2018**	**39.11**

Jiangsu

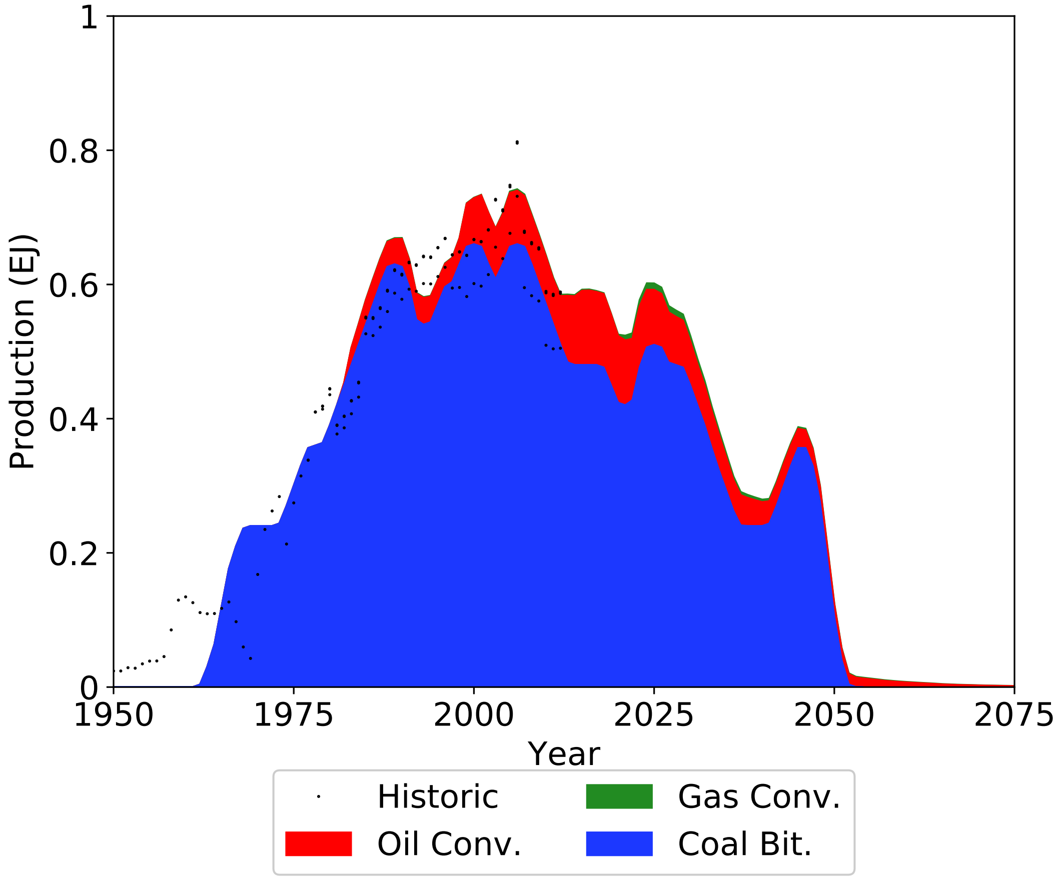

Figure 2.70: China - Jiangsu projections capped at 16

Table 2.70: Peak years - All

Name	URR	Peak Year	Peak Rate
Coal Bit. Jiangsu	37.45	2000	0.66
Oil Conv. Jiangsu	4.2	2016	0.11
Gas Conv. Jiangsu	0.24	2024	0.01
**Total**	**41.89**	**2006**	**0.74**

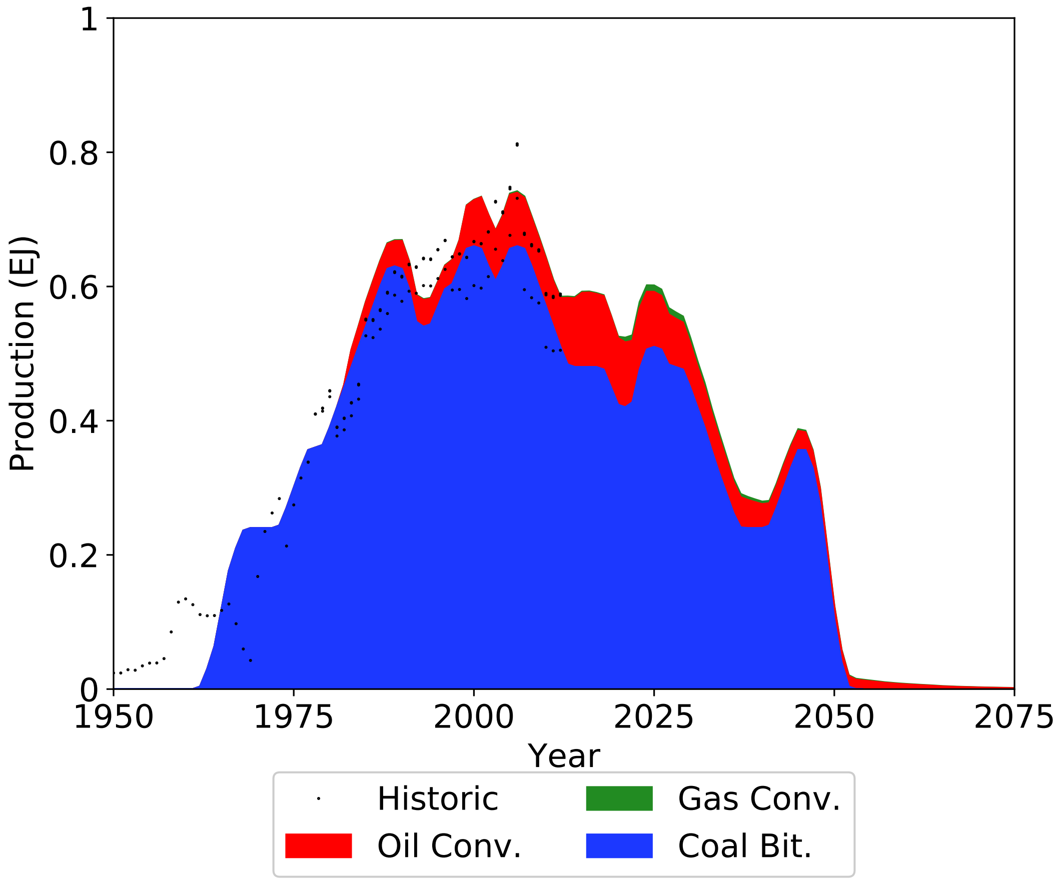

Figure 2.71: China - Jiangsu projection by mineral type

Table 2.71: Peak years - Minerals

Name	URR	Peak Year	Peak Rate
Coal Bit.	37.45	2000	0.66
Oil Conv.	4.2	2016	0.11
Gas Conv.	0.24	2024	0.01
**Total**	**41.89**	**2006**	**0.74**

Jiangxi

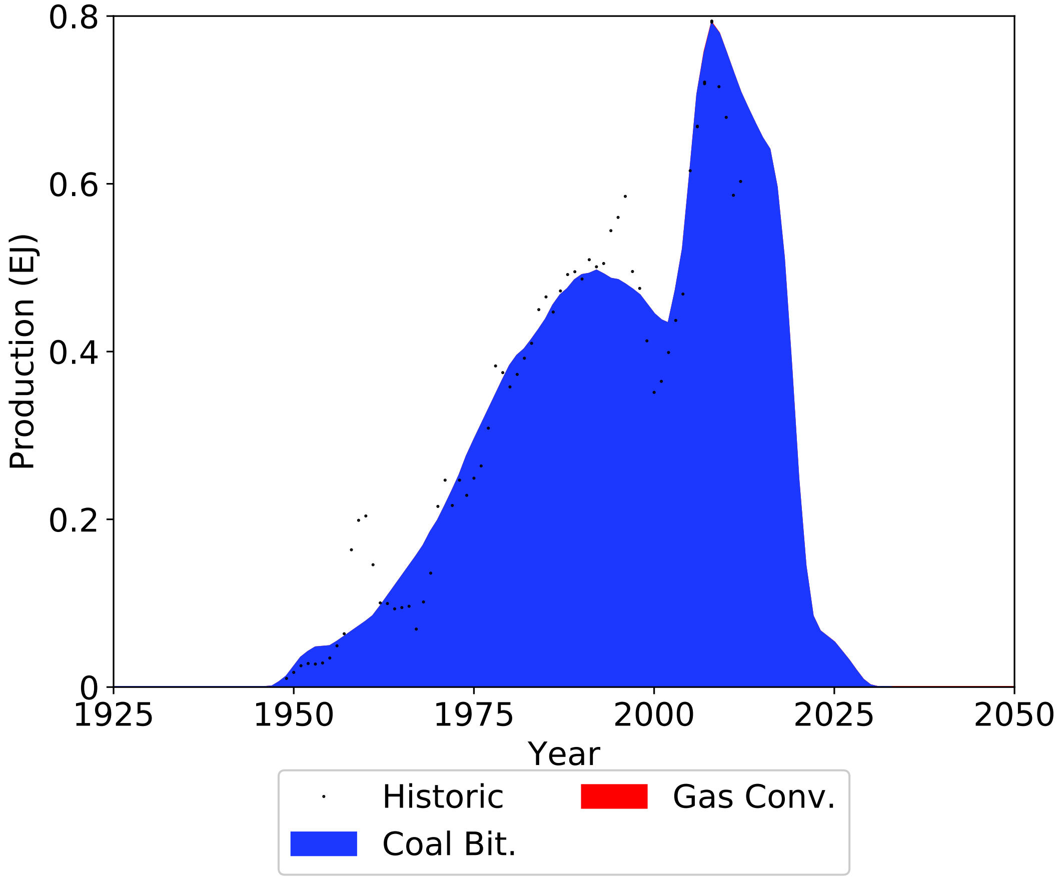

Figure 2.72: China - Jiangxi projections capped at 16

Table 2.72: Peak years - All

**Name**	**URR**	**Peak Year**	**Peak Rate**
Coal Bit. Jiangxi	26.85	2008	0.79
Gas Conv. Jiangxi	0.01	2007	–
**Total**	**26.86**	**2008**	**0.79**

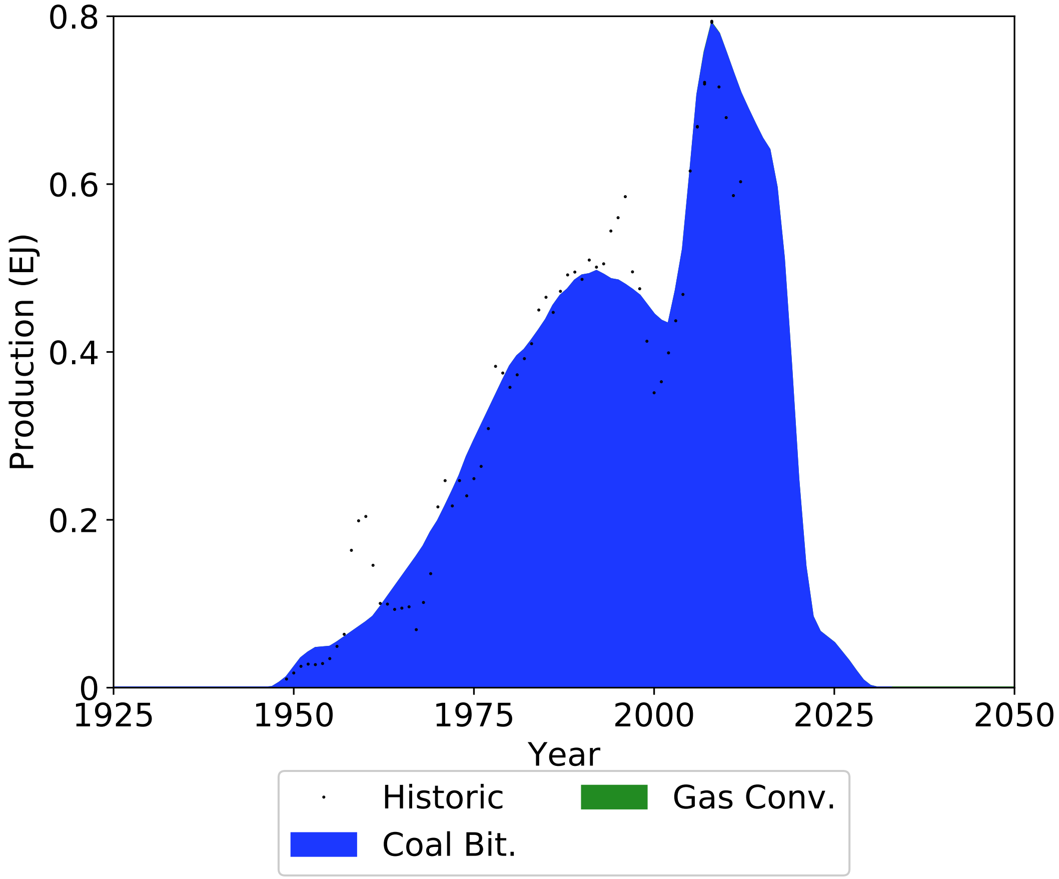

Figure 2.73: China - Jiangxi projection by mineral type

Table 2.73: Peak years - Minerals

Name	URR	Peak Year	Peak Rate
Coal Bit.	26.85	2008	0.79
Gas Conv.	0.01	2007	–
**Total**	**26.86**	**2008**	**0.79**

Jilin

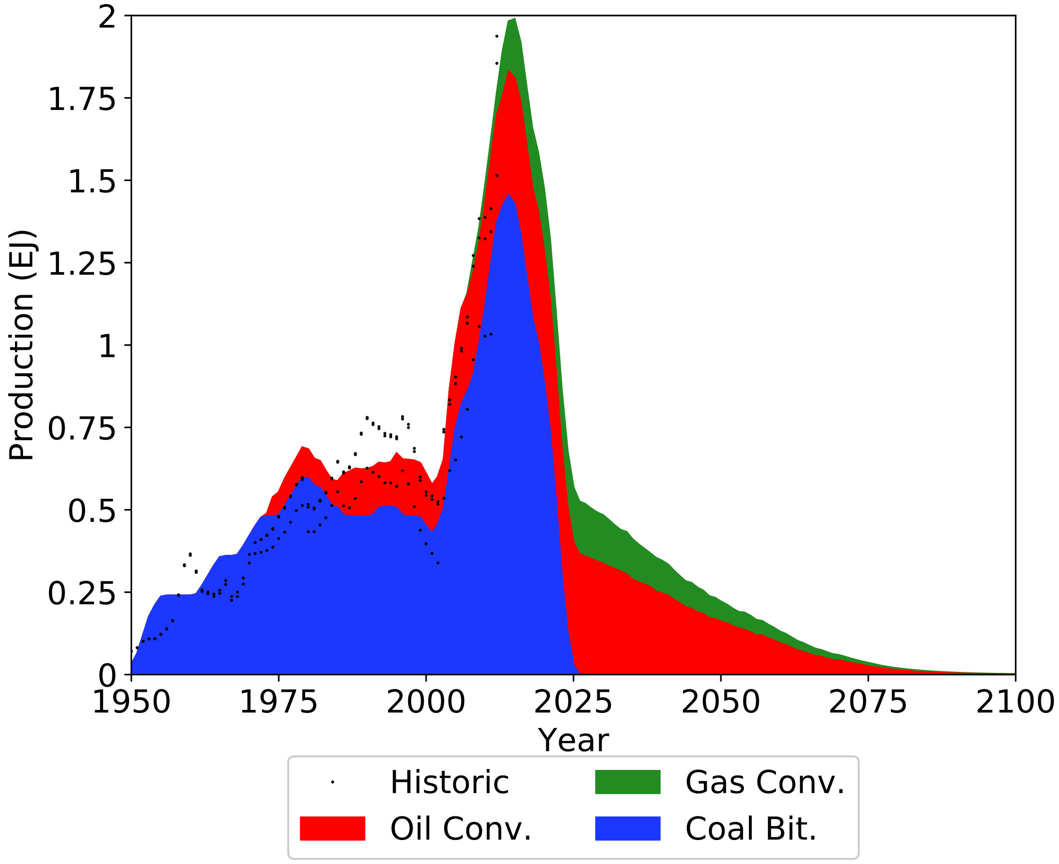

Figure 2.74: China - Jilin projections capped at 16

Table 2.74: Peak years - All

Name	URR	Peak Year	Peak Rate
Coal Bit. Jilin	42.23	2014	1.45
Oil Conv. Jilin	20.15	2016	0.4
Gas Conv. Jilin	6.09	2020	0.19
**Total**	**68.47**	**2015**	**1.99**

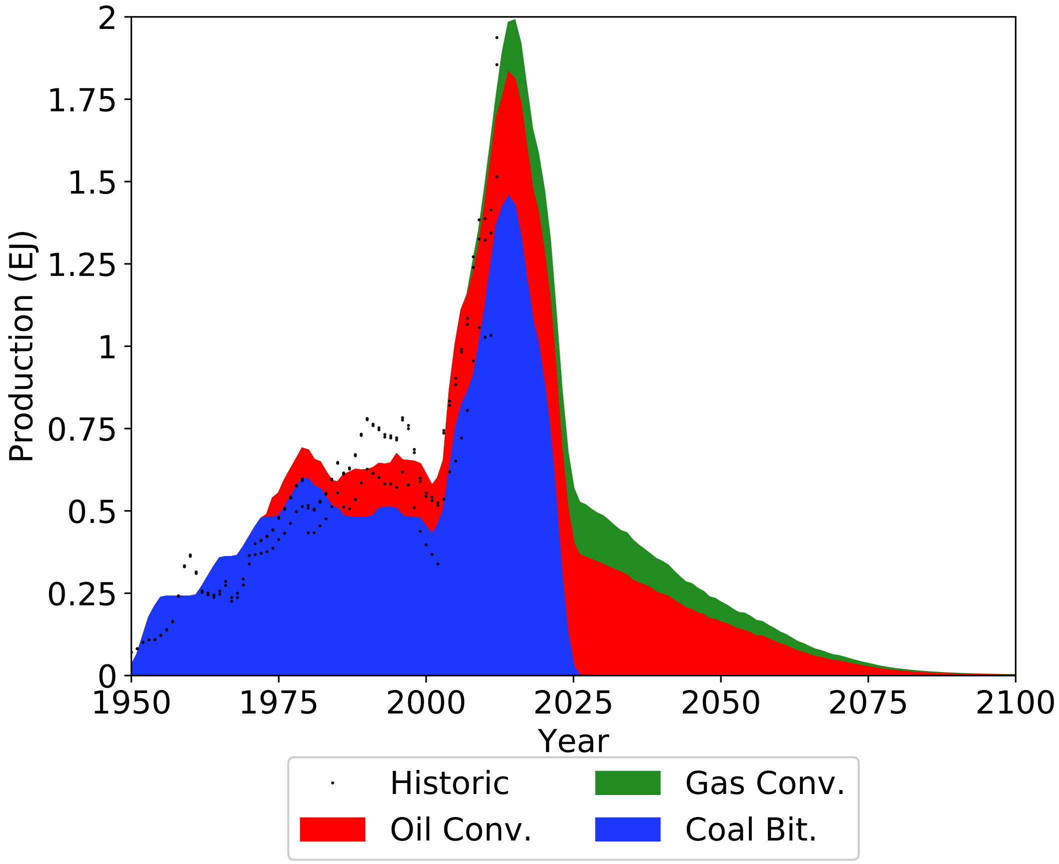

Figure 2.75: China - Jilin projection by mineral type

Table 2.75: Peak years - Minerals

Name	URR	Peak Year	Peak Rate
Coal Bit.	42.23	2014	1.45
Oil Conv.	20.15	2016	0.4
Gas Conv.	6.09	2020	0.19
**Total**	**68.47**	**2015**	**1.99**

Liaoning

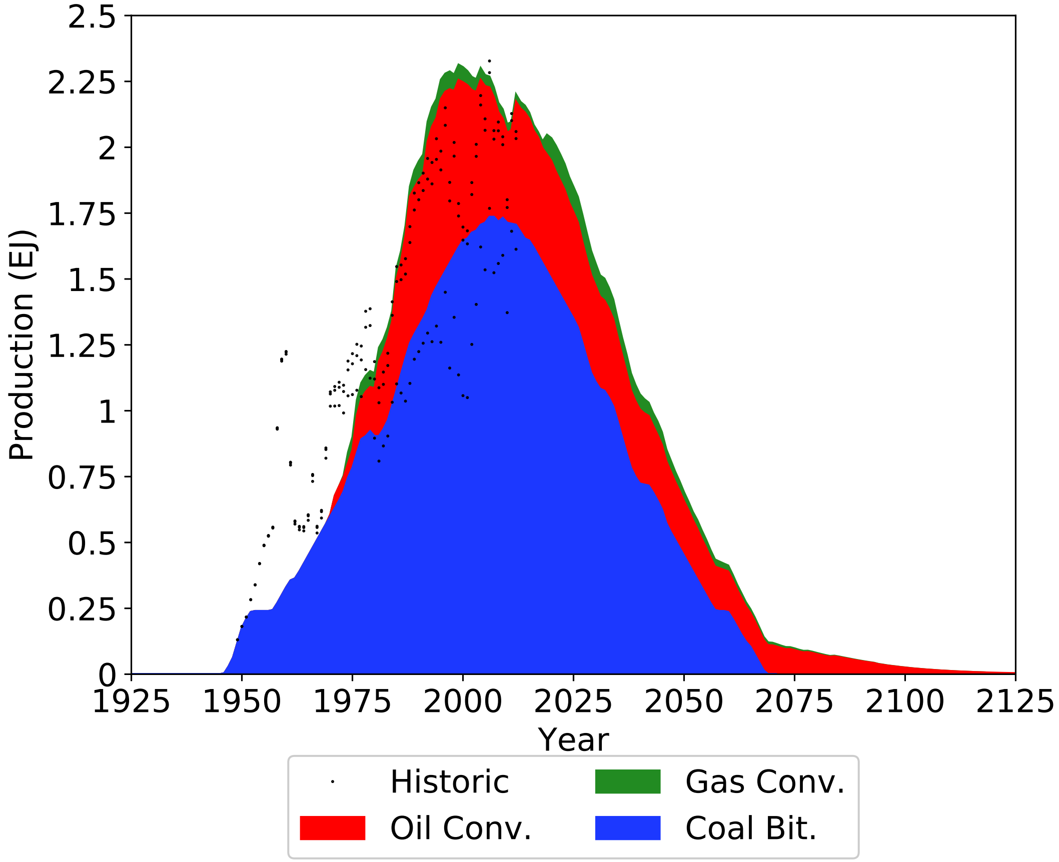

Figure 2.76: China - Liaoning projections capped at 16

Table 2.76: Peak years - All

Name	URR	Peak Year	Peak Rate
Coal Bit. Liaoning	107.22	2006	1.74
Oil Conv. Liaoning	35.68	1995	0.68
Gas Conv. Liaoning	4.99	2027	0.1
**Total**	**147.89**	**1999**	**2.31**

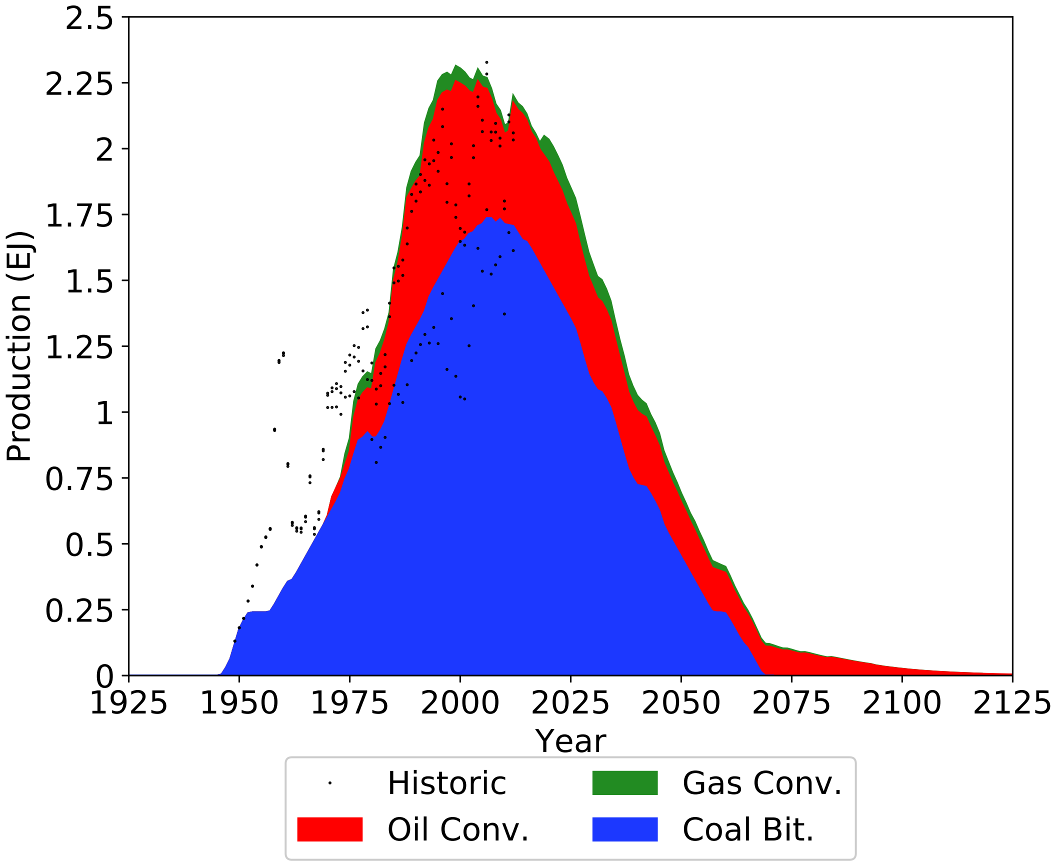

Figure 2.77: China - Liaoning projection by mineral type

Table 2.77: Peak years - Minerals

Name	URR	Peak Year	Peak Rate
Coal Bit.	107.22	2006	1.74
Oil Conv.	35.68	1995	0.68
Gas Conv.	4.99	2027	0.1
**Total**	**147.89**	**1999**	**2.31**

Ningxia

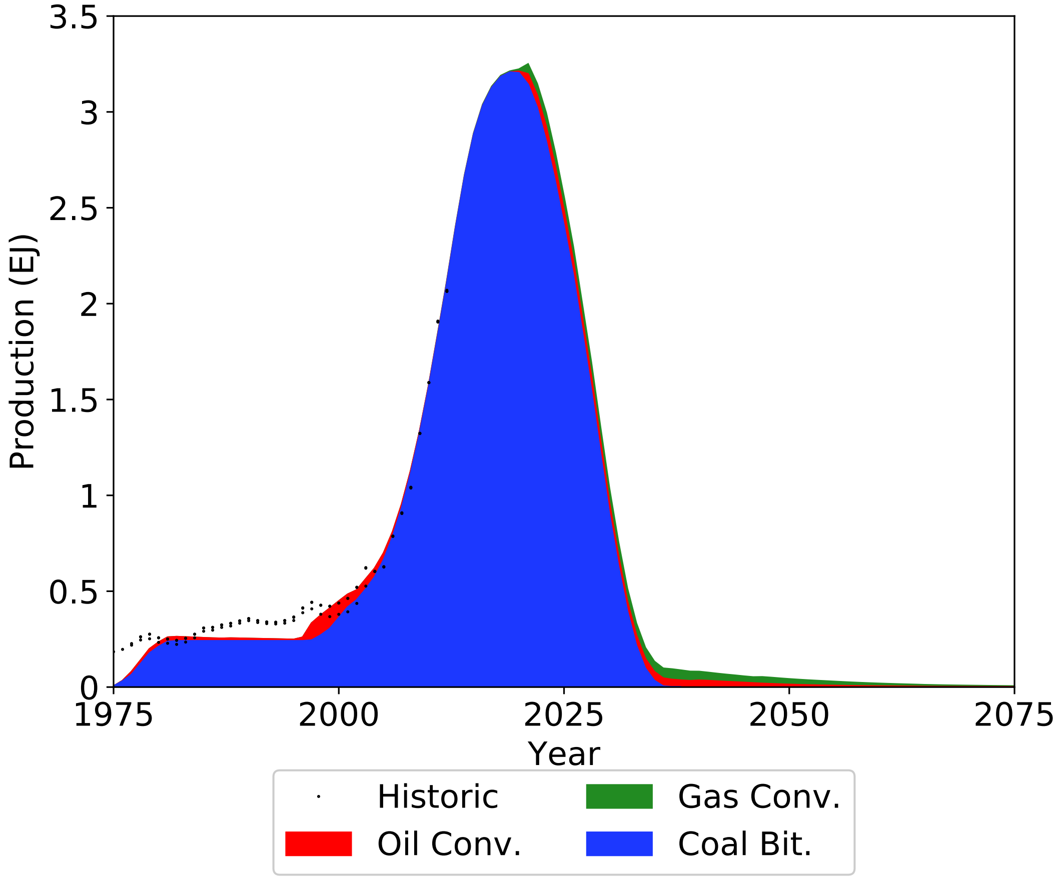

Figure 2.78: China - Ningxia projections capped at 16

Table 2.78: Peak years - All

Name	URR	Peak Year	Peak Rate
Coal Bit. Ningxia	64.7	2019	3.21
Oil Conv. Ningxia	2.51	1999	0.11
Gas Conv. Ningxia	2.0	2030	0.07
**Total**	**69.21**	**2021**	**3.25**

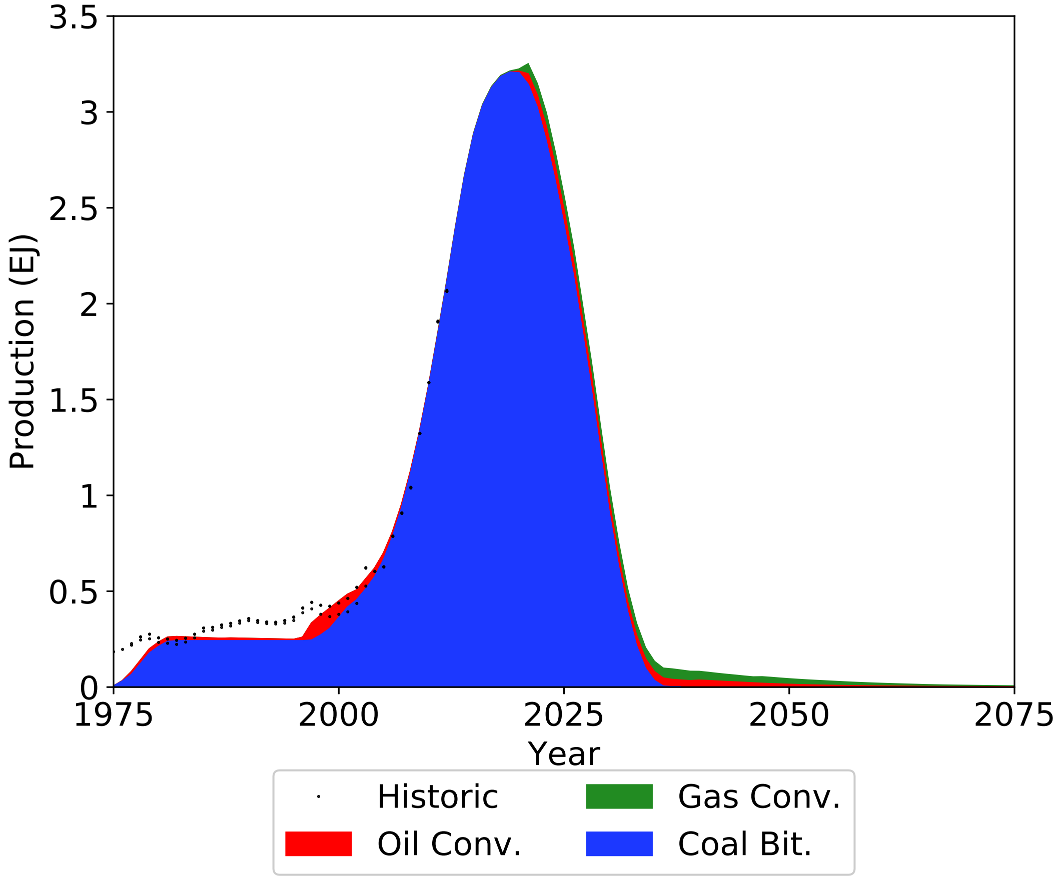

Figure 2.79: China - Ningxia projection by mineral type

Table 2.79: Peak years - Minerals

Name	URR	Peak Year	Peak Rate
Coal Bit.	64.7	2019	3.21
Oil Conv.	2.51	1999	0.11
Gas Conv.	2.0	2030	0.07
**Total**	**69.21**	**2021**	**3.25**

Offshore

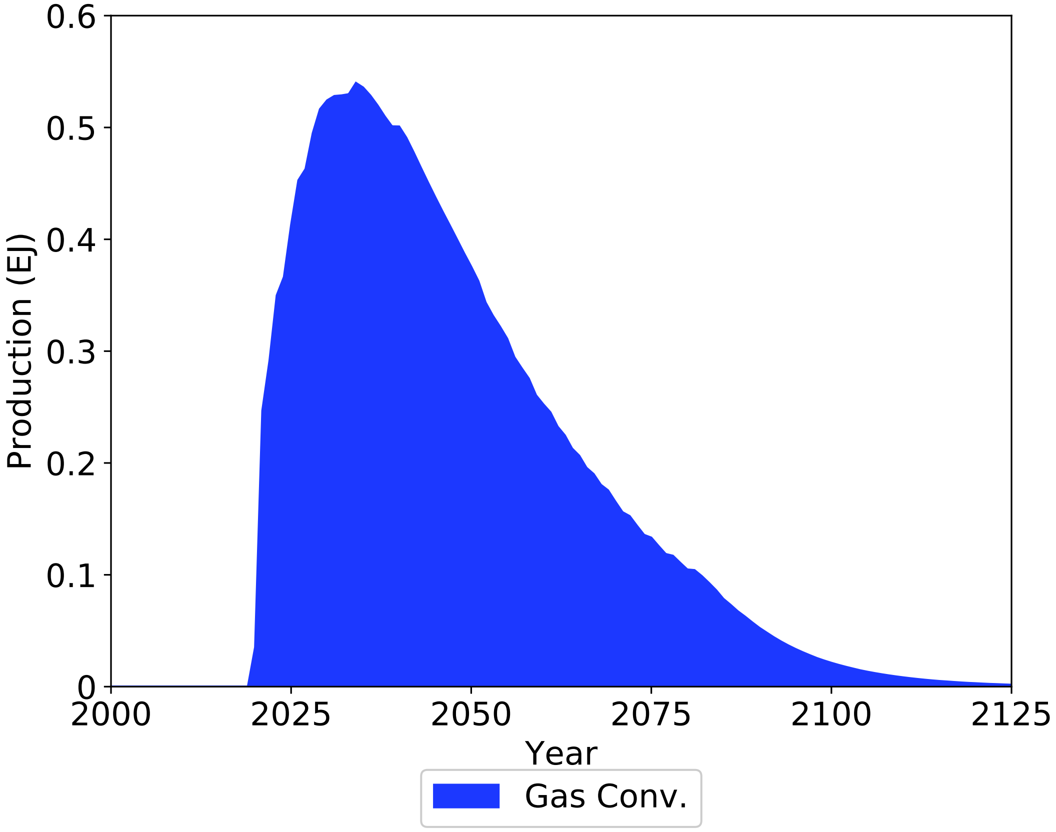

Figure 2.80: China - Offshore projections capped at 16

Table 2.80: Peak years - All

**Name**	**URR**	**Peak Year**	**Peak Rate**
Gas Conv. Offshore	21.38	2034	0.54
**Total**	**21.38**	**2034**	**0.54**

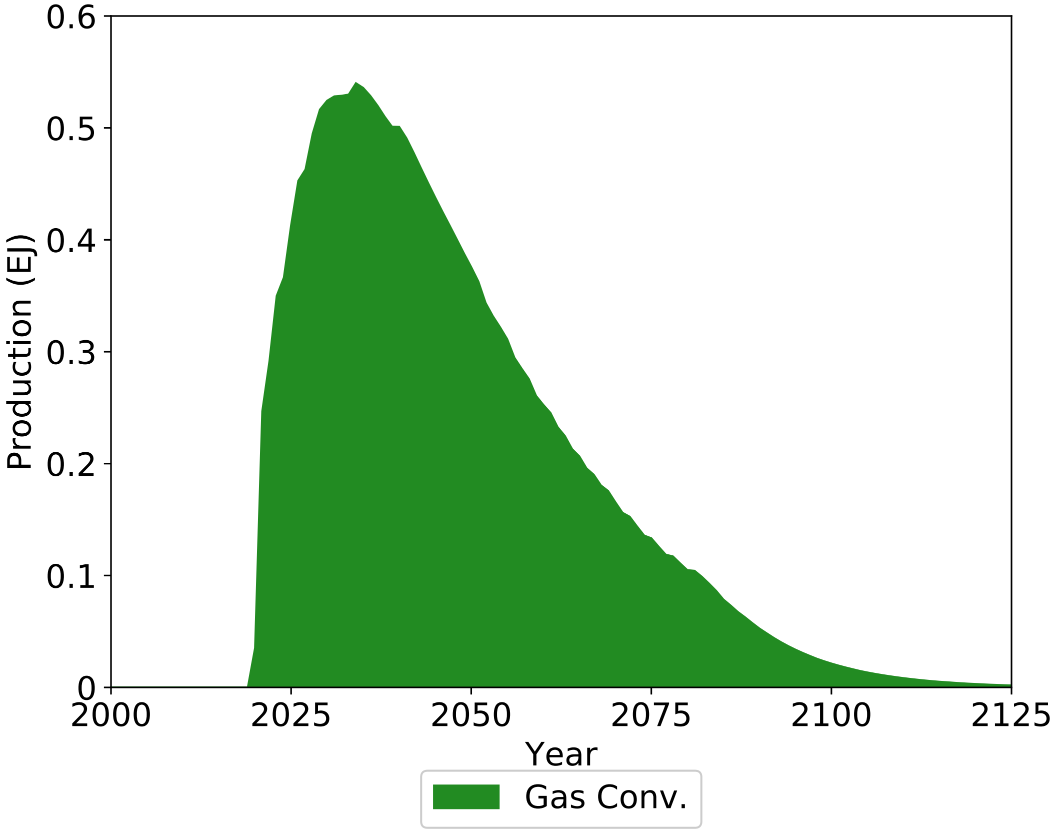

Figure 2.81: China - Offshore projection by mineral type

Table 2.81: Peak years - Minerals

Name	URR	Peak Year	Peak Rate
Gas Conv.	21.38	2034	0.54
**Total**	**21.38**	**2034**	**0.54**

Qinghai

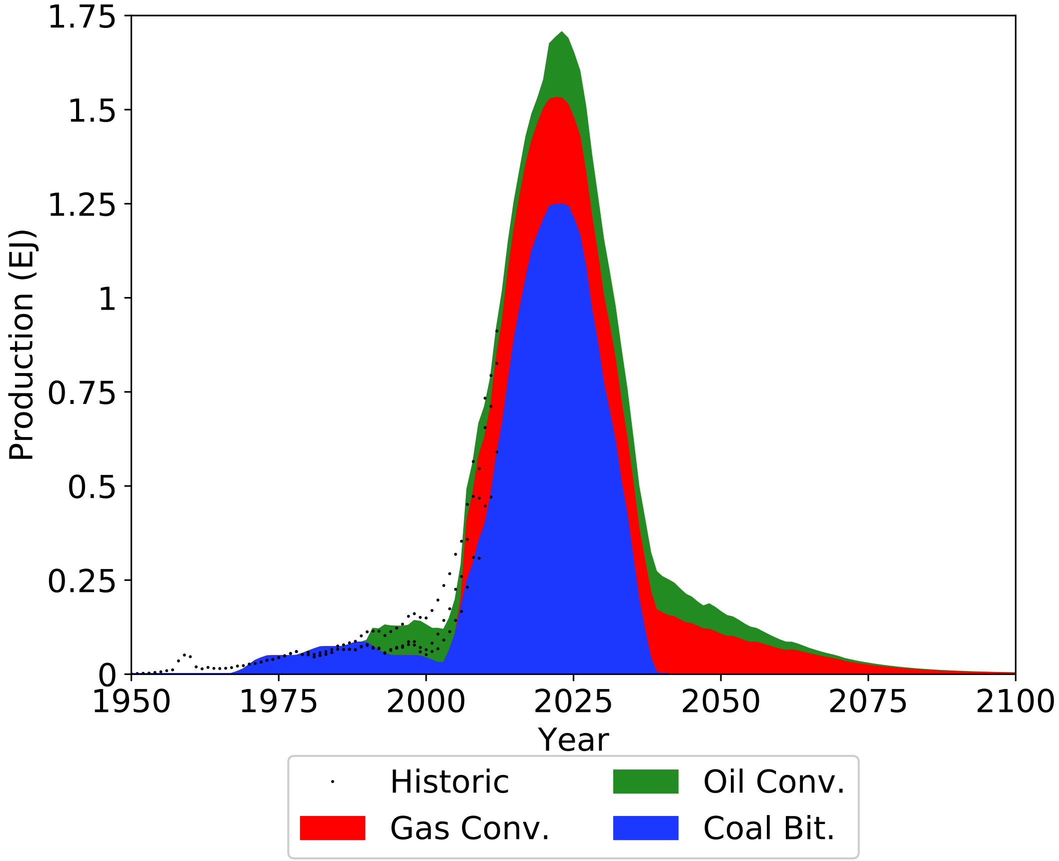

Figure 2.82: China - Qinghai projections capped at 16

Table 2.82: Peak years - All

Name	URR	Peak Year	Peak Rate
Coal Bit. Qinghai	26.45	2022	1.25
Gas Conv. Qinghai	11.25	2017	0.3
Oil Conv. Qinghai	6.55	2023	0.18
**Total**	**44.25**	**2023**	**1.71**

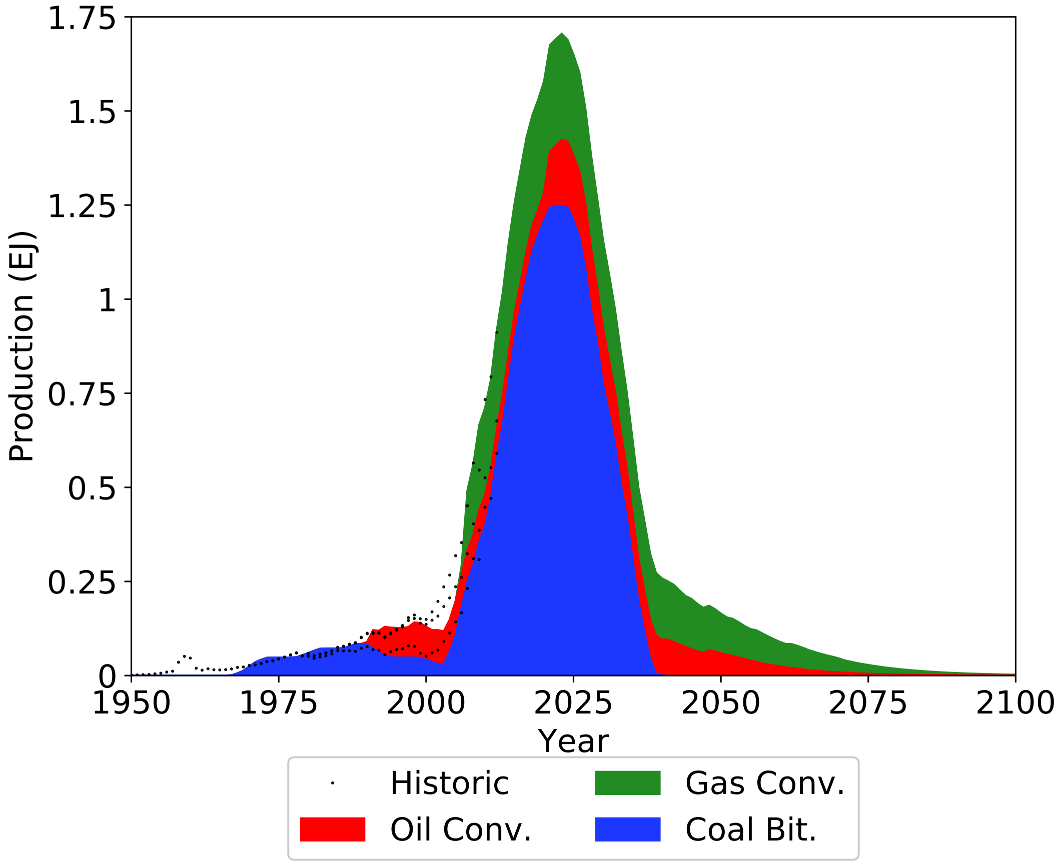

Figure 2.83: China - Qinghai projection by mineral type

Table 2.83: Peak years - Minerals

Name	URR	Peak Year	Peak Rate
Coal Bit.	26.45	2022	1.25
Oil Conv.	6.55	2023	0.18
Gas Conv.	11.25	2017	0.3
**Total**	**44.25**	**2023**	**1.71**

Shaanxi

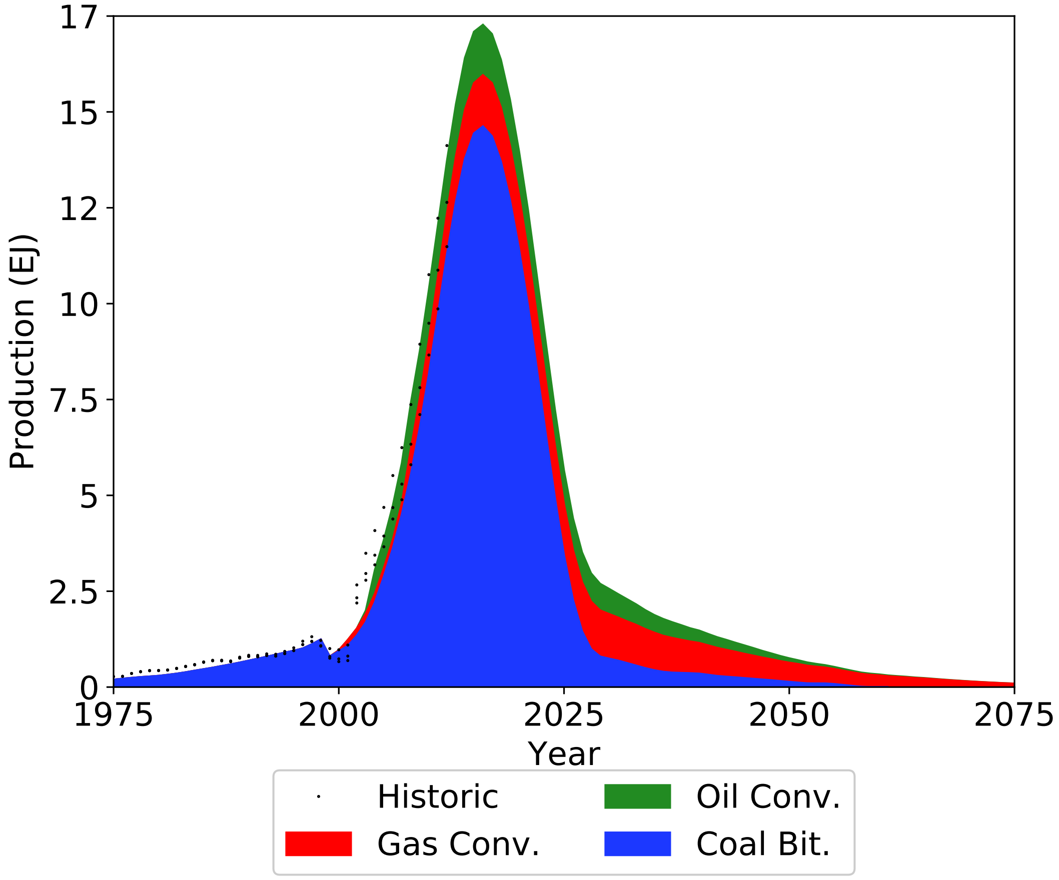

Figure 2.84: China - Shaanxi projections capped at 16

Table 2.84: Peak years - All

Name	URR	Peak Year	Peak Rate
Coal Bit. Shaanxi	230.57	2016	14.62
Gas Conv. Shaanxi	53.01	2019	1.42
Oil Conv. Shaanxi	35.99	2012	1.37
**Total**	**319.57**	**2016**	**17.27**

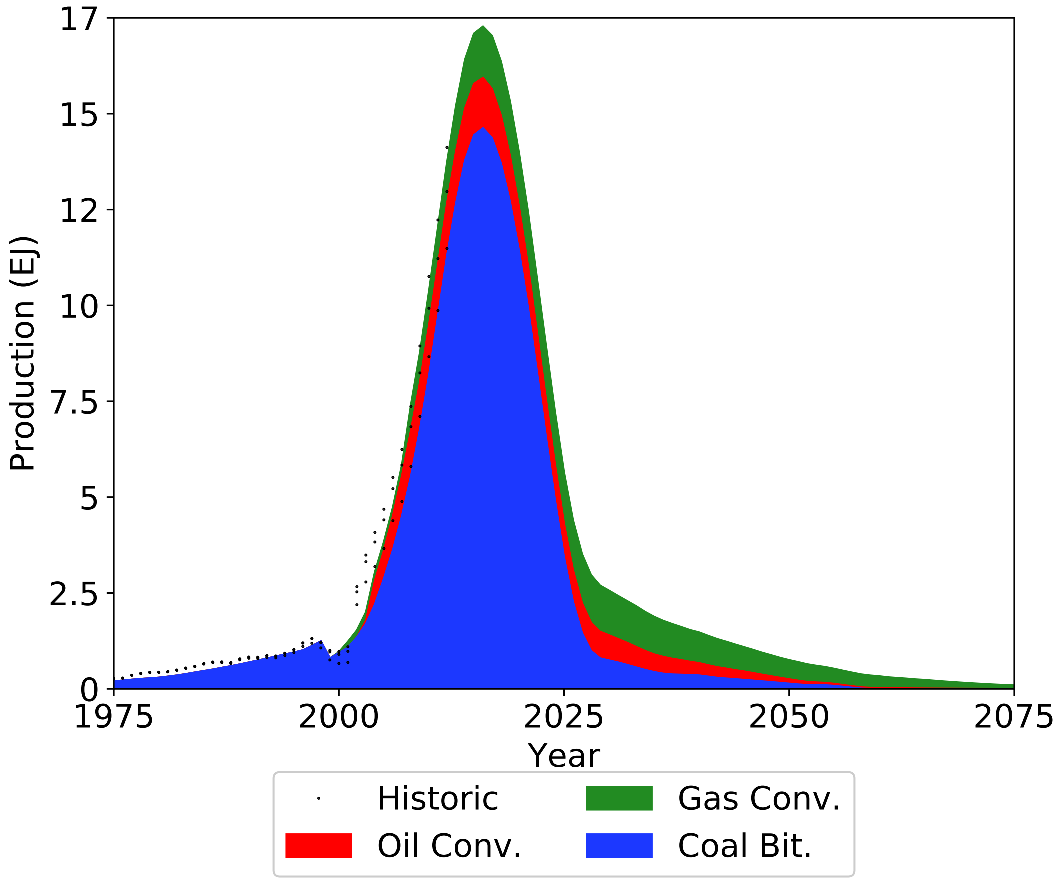

Figure 2.85: China - Shaanxi projection by mineral type

Table 2.85: Peak years - Minerals

Name	URR	Peak Year	Peak Rate
Coal Bit.	230.57	2016	14.62
Oil Conv.	35.99	2012	1.37
Gas Conv.	53.01	2019	1.42
**Total**	**319.57**	**2016**	**17.27**

Shandong

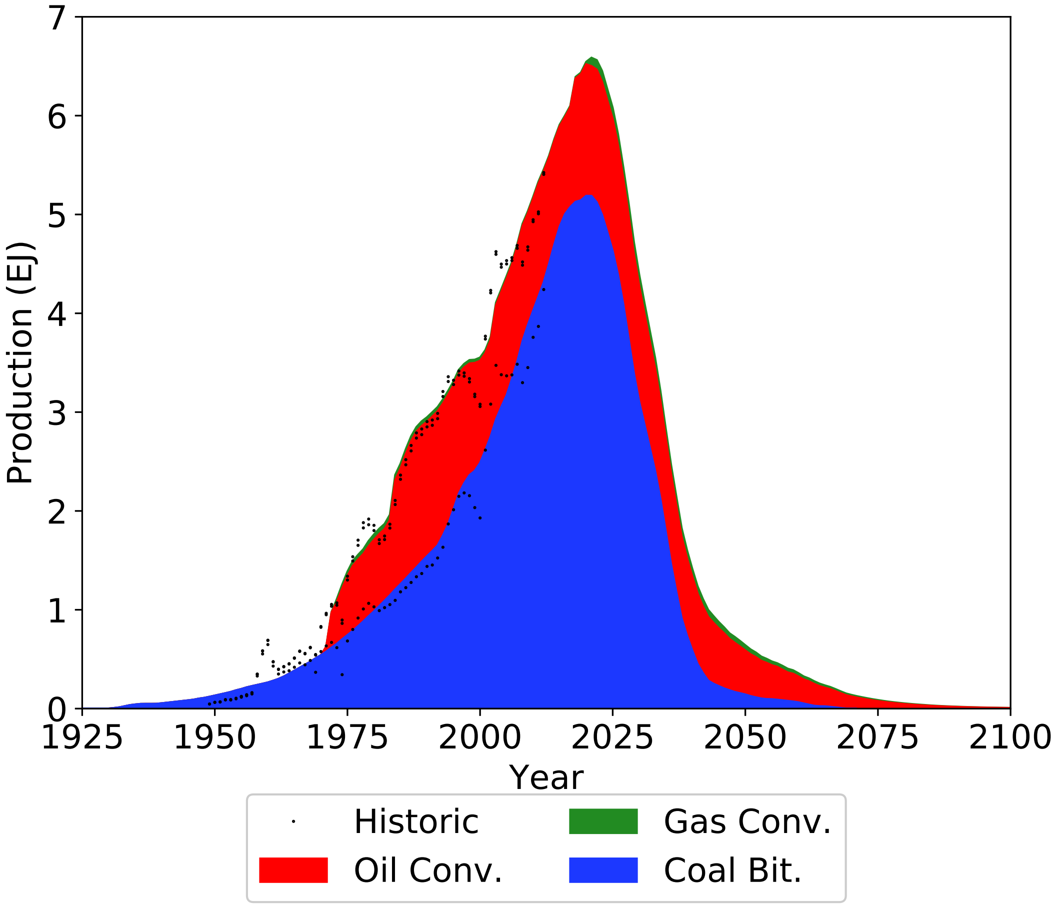

Figure 2.86: China - Shandong projections capped at 16

Table 2.86: Peak years - All

**Name**	**URR**	**Peak Year**	**Peak Rate**
Coal Bit. Shandong	193.4	2020	5.19
Oil Conv. Shandong	85.59	1988	1.37
Gas Conv. Shandong	5.23	2028	0.13
**Total**	**284.22**	**2021**	**6.59**

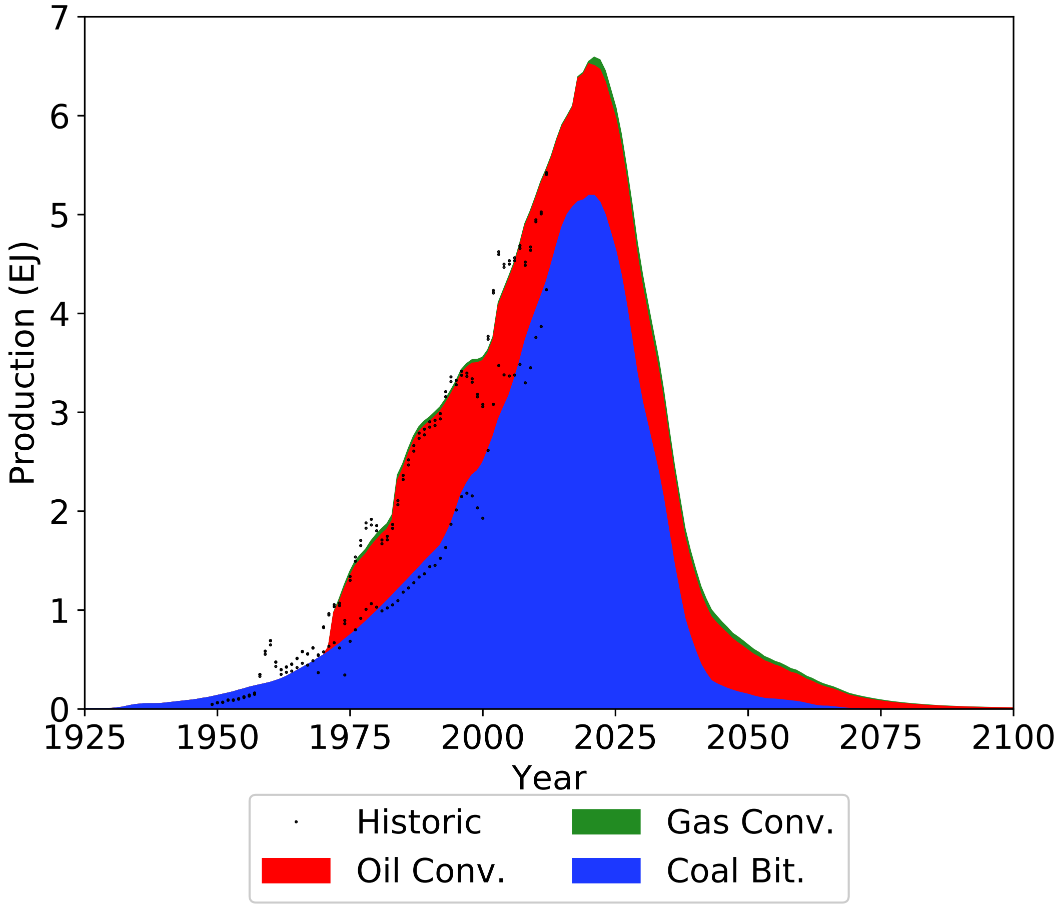

Figure 2.87: China - Shandong projection by mineral type

Table 2.87: Peak years - Minerals

Name	URR	Peak Year	Peak Rate
Coal Bit.	193.4	2020	5.19
Oil Conv.	85.59	1988	1.37
Gas Conv.	5.23	2028	0.13
**Total**	**284.22**	**2021**	**6.59**

Shanghai

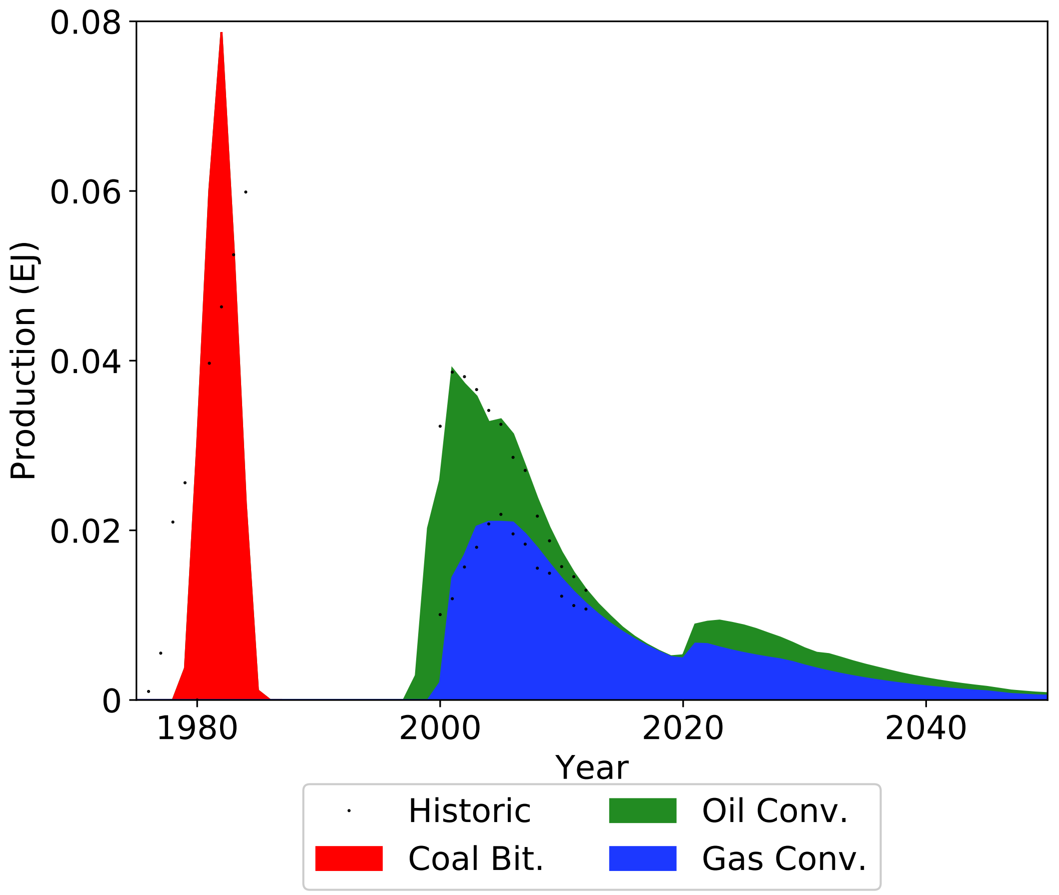

Figure 2.88: China - Shanghai projections capped at 16

Table 2.88: Peak years - All

Name	URR	Peak Year	Peak Rate
Gas Conv. Shanghai	0.36	2004	0.02
Coal Bit. Shanghai	0.25	1982	0.08
Oil Conv. Shanghai	0.22	2001	0.02
**Total**	**0.83**	**1982**	**0.08**

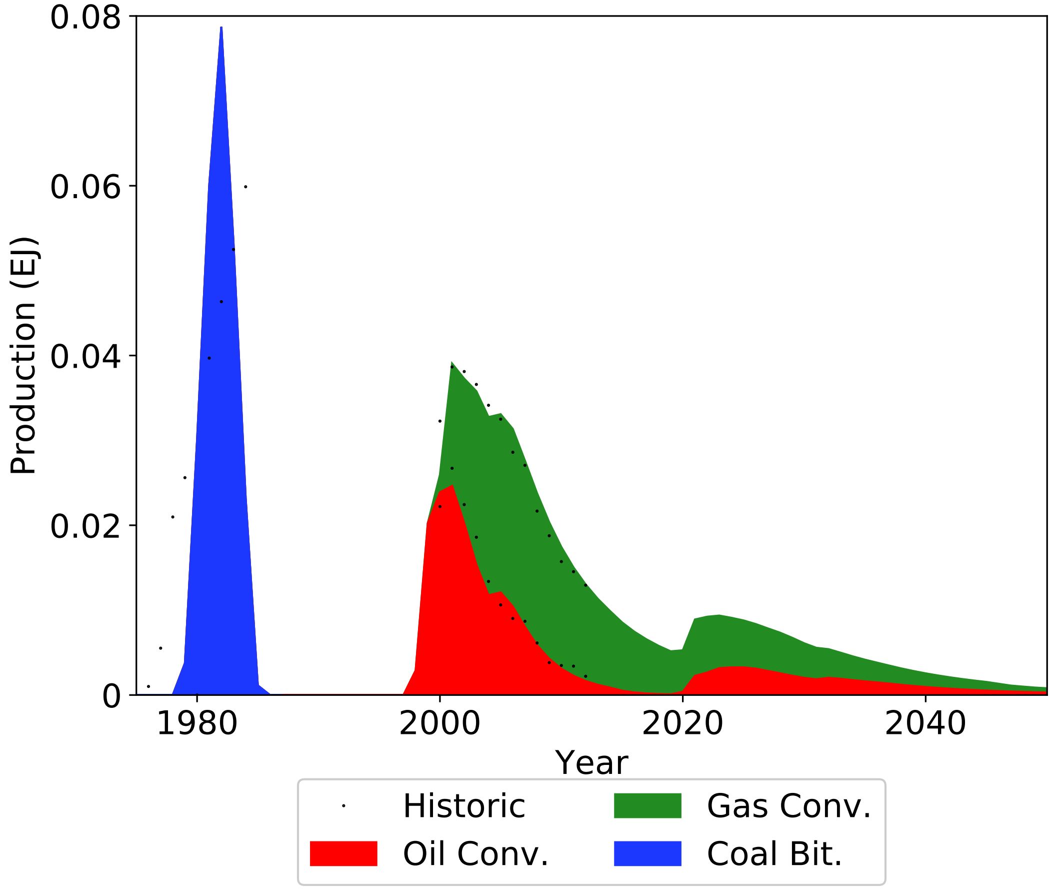

Figure 2.89: China - Shanghai projection by mineral type

Table 2.89: Peak years - Minerals

Name	URR	Peak Year	Peak Rate
Coal Bit.	0.25	1982	0.08
Oil Conv.	0.22	2001	0.02
Gas Conv.	0.36	2004	0.02
**Total**	**0.83**	**1982**	**0.08**

Shanxi

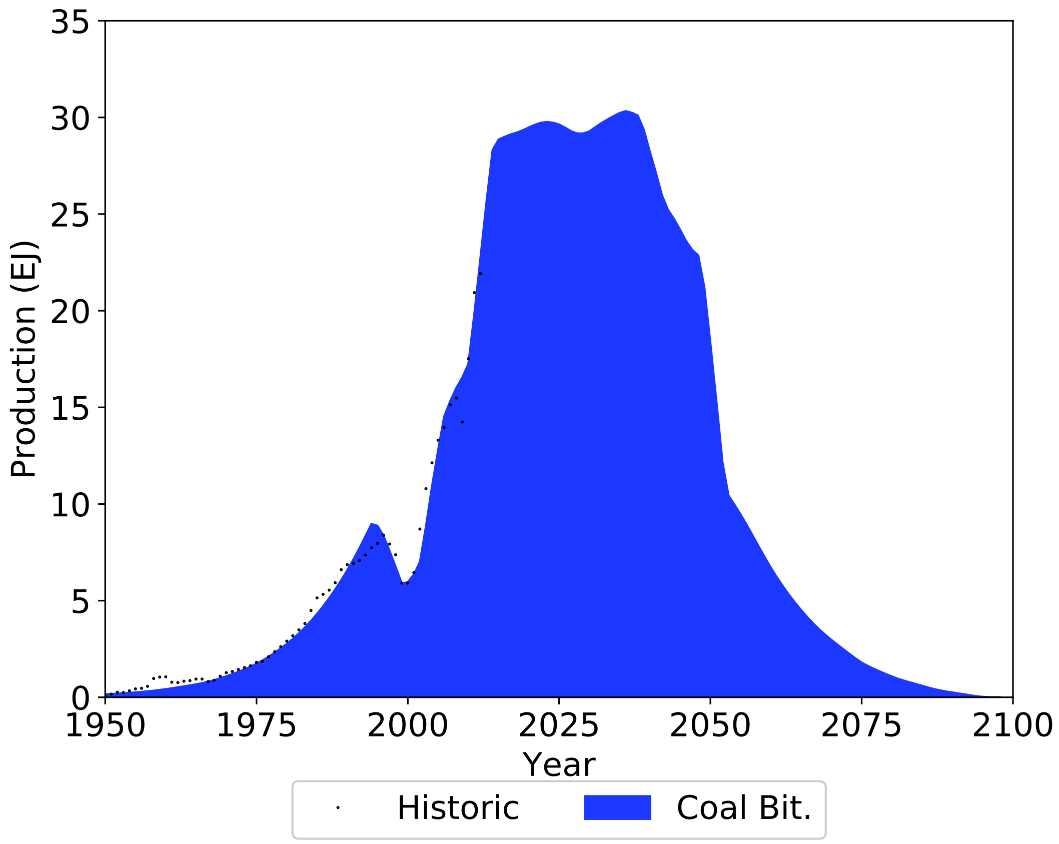

Figure 2.90: China - Shanxi projections capped at 16

Table 2.90: Peak years - All

**Name**	**URR**	**Peak Year**	**Peak Rate**
Coal Bit. Shanxi	1543.23	2036	30.33
**Total**	**1543.23**	**2036**	**30.33**

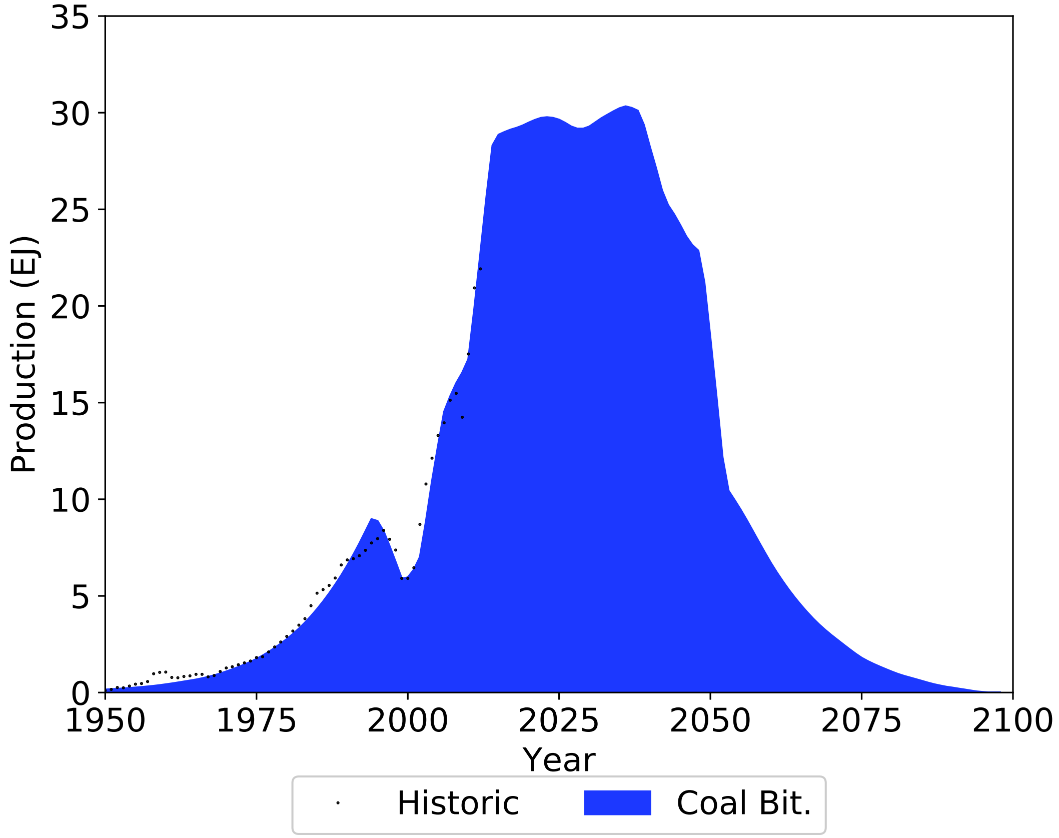

Figure 2.91: China - Shanxi projection by mineral type

Table 2.91: Peak years - Minerals

Name	URR	Peak Year	Peak Rate
Coal Bit.	1543.23	2036	30.33
**Total**	**1543.23**	**2036**	**30.33**

Sichuan

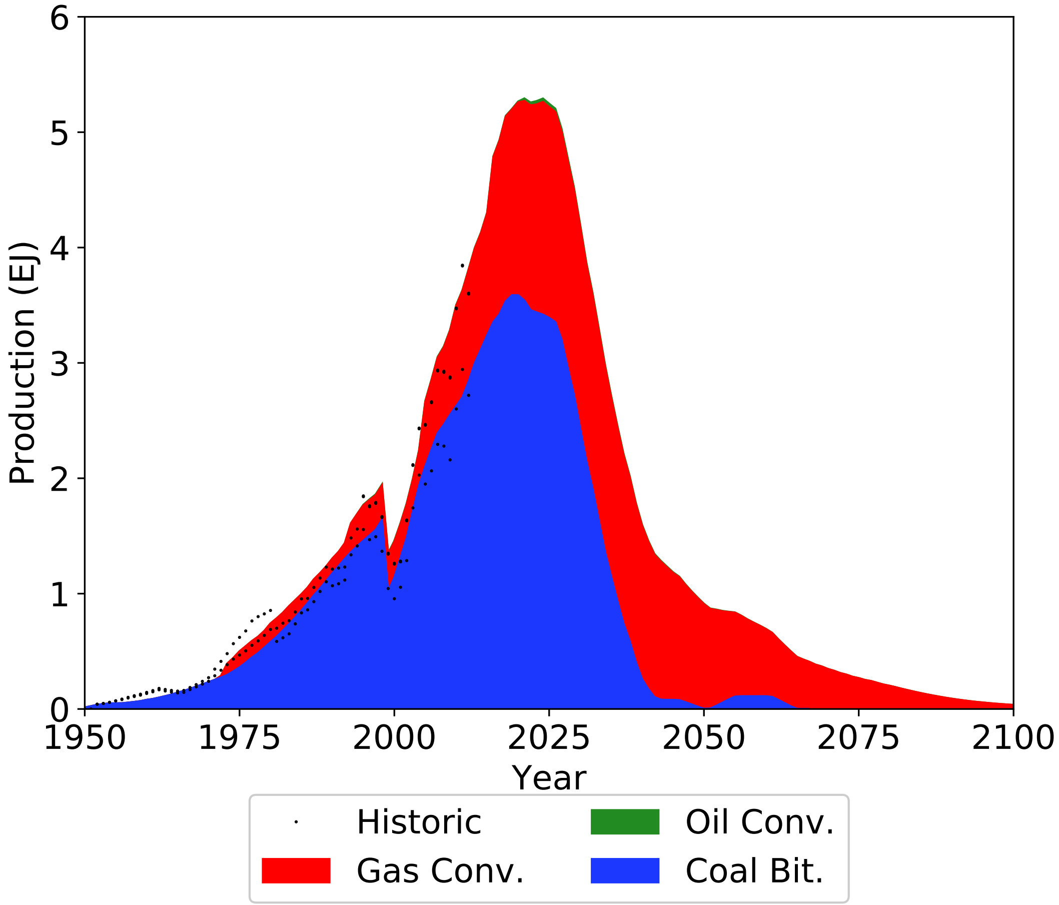

Figure 2.92: China - Sichuan projections capped at 16

Table 2.92: Peak years - All

Name	URR	Peak Year	Peak Rate
Coal Bit. Sichuan	126.66	2019	3.59
Gas Conv. Sichuan	83.72	2024	1.84
Oil Conv. Sichuan	0.68	2024	0.03
**Total**	**211.06**	**2021**	**5.29**

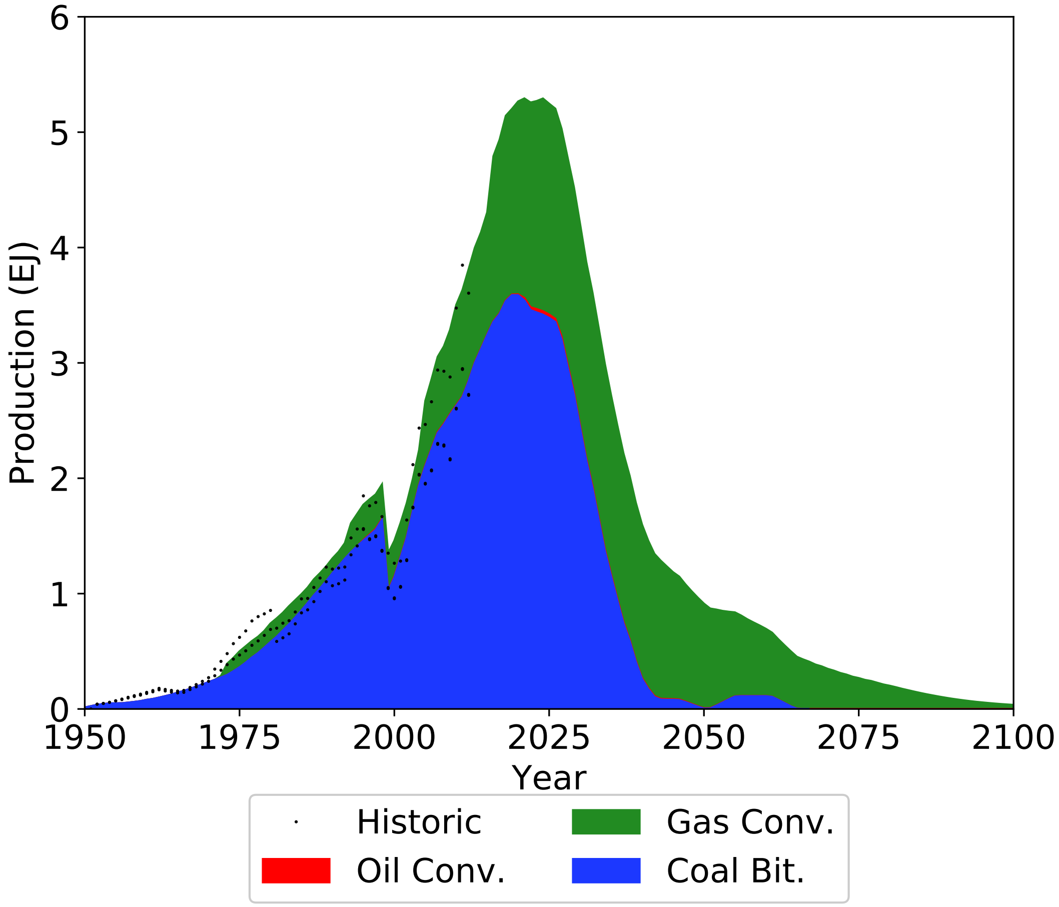

Figure 2.93: China - Sichuan projection by mineral type

Table 2.93: Peak years - Minerals

Name	URR	Peak Year	Peak Rate
Coal Bit.	126.66	2019	3.59
Oil Conv.	0.68	2024	0.03
Gas Conv.	83.72	2024	1.84
**Total**	**211.06**	**2021**	**5.29**

Tianjin

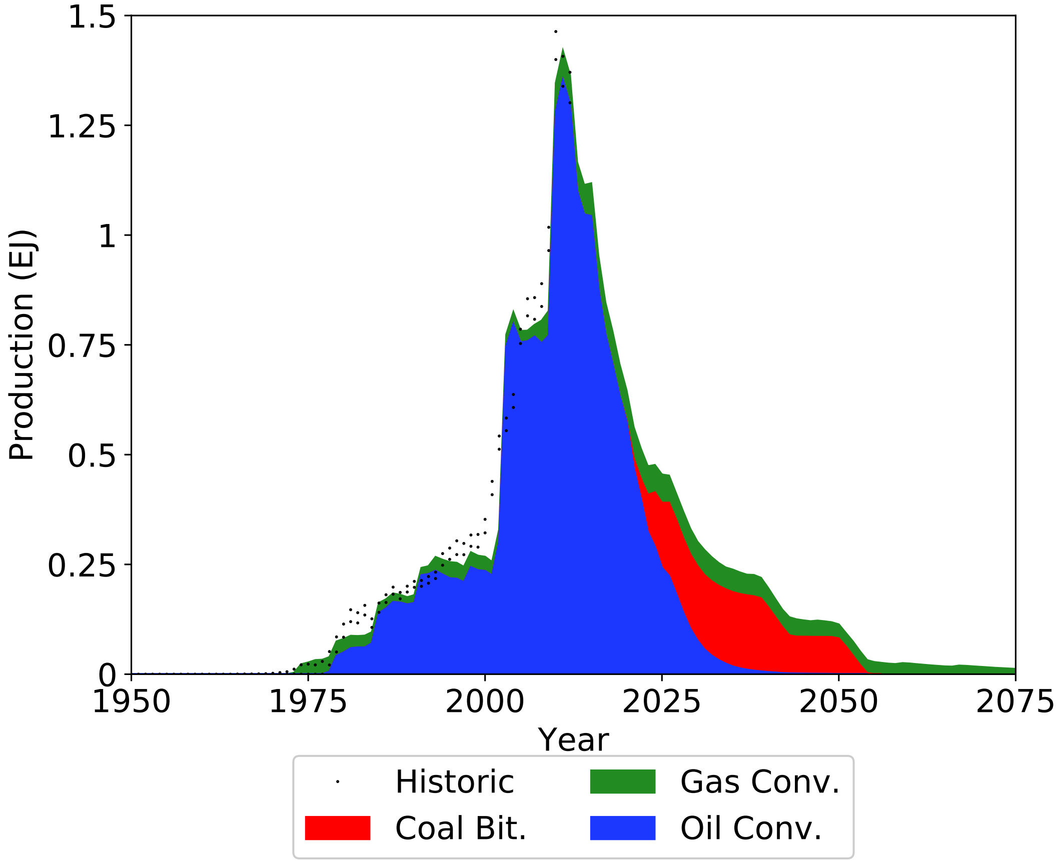

Figure 2.94: China - Tianjin projections capped at 16

Table 2.94: Peak years - All

**Name**	**URR**	**Peak Year**	**Peak Rate**
Oil Conv. Tianjin	22.83	2011	1.35
Coal Bit. Tianjin	3.97	2027	0.17
Gas Conv. Tianjin	3.96	2015	0.07
**Total**	**30.76**	**2011**	**1.42**

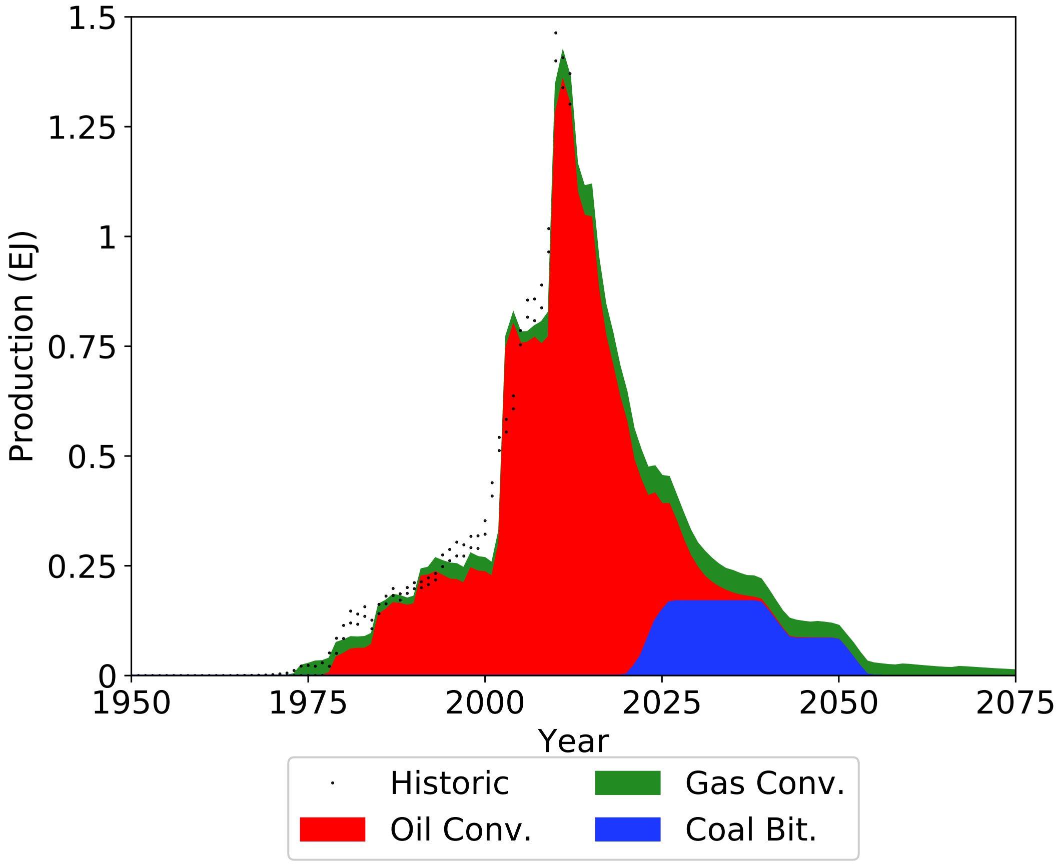

Figure 2.95: China - Tianjin projection by mineral type

Table 2.95: Peak years - Minerals

Name	URR	Peak Year	Peak Rate
Coal Bit.	3.97	2027	0.17
Oil Conv.	22.83	2011	1.35
Gas Conv.	3.96	2015	0.07
**Total**	**30.76**	**2011**	**1.42**

Tibet

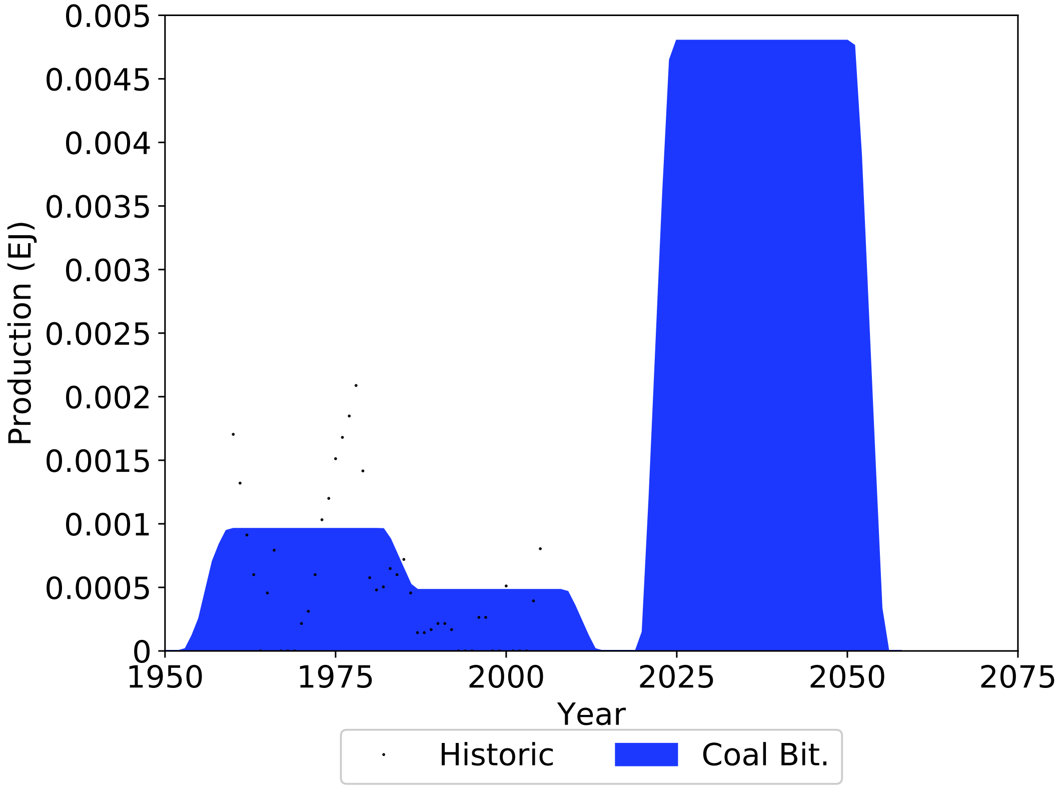

Figure 2.96: China - Tibet projections capped at 16

Table 2.96: Peak years - All

**Name**	**URR**	**Peak Year**	**Peak Rate**
Coal Bit. Tibet	0.19	2048	–
**Total**	**0.19**	**2048**	–

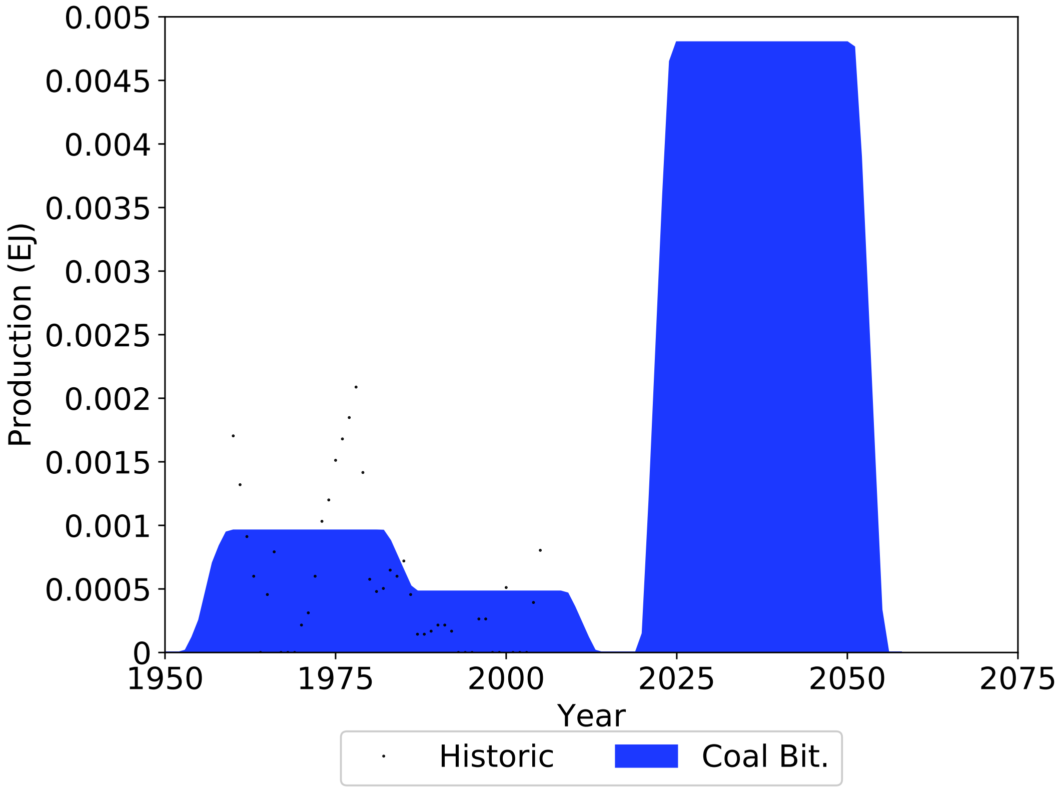

Figure 2.97: China - Tibet projection by mineral type

Table 2.97: Peak years - Minerals

Name	URR	Peak Year	Peak Rate
Coal Bit.	0.19	2048	–
**Total**	**0.19**	**2048**	–

Xinjiang

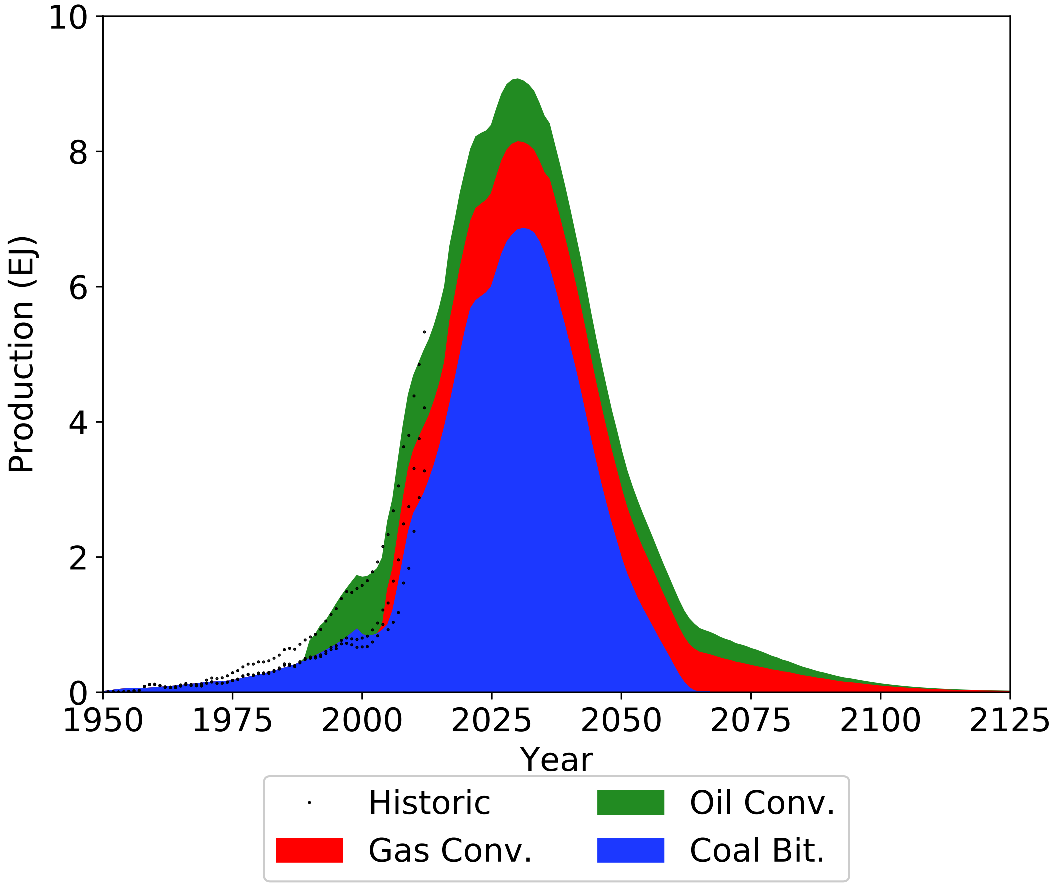

Figure 2.98: China - Xinjiang projections capped at 16

Table 2.98: Peak years - All

**Name**	**URR**	**Peak Year**	**Peak Rate**
Coal Bit. Xinjiang	235.83	2031	6.85
Gas Conv. Xinjiang	75.82	2026	1.39
Oil Conv. Xinjiang	65.09	2015	1.13
**Total**	**376.74**	**2030**	**9.07**

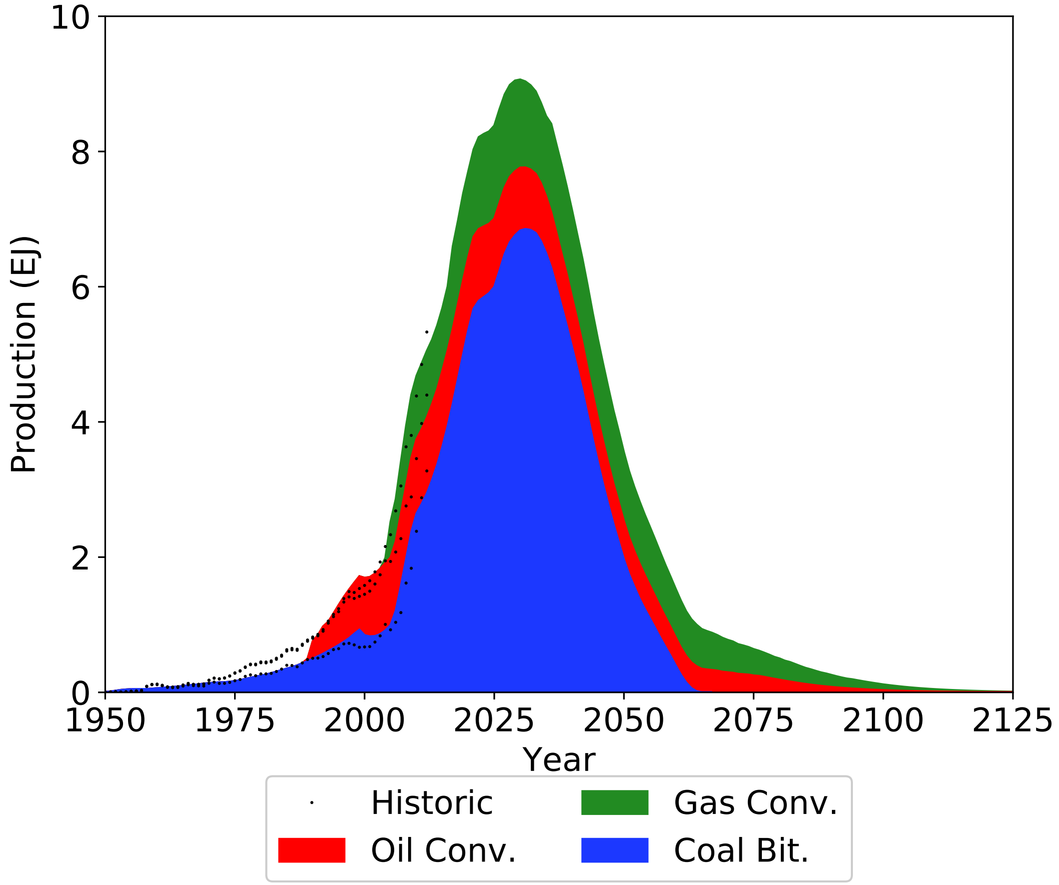

Figure 2.99: China - Xinjiang projection by mineral type

Table 2.99: Peak years - Minerals

Name	URR	Peak Year	Peak Rate
Coal Bit.	235.83	2031	6.85
Oil Conv.	65.09	2015	1.13
Gas Conv.	75.82	2026	1.39
**Total**	**376.74**	**2030**	**9.07**

Yunnan

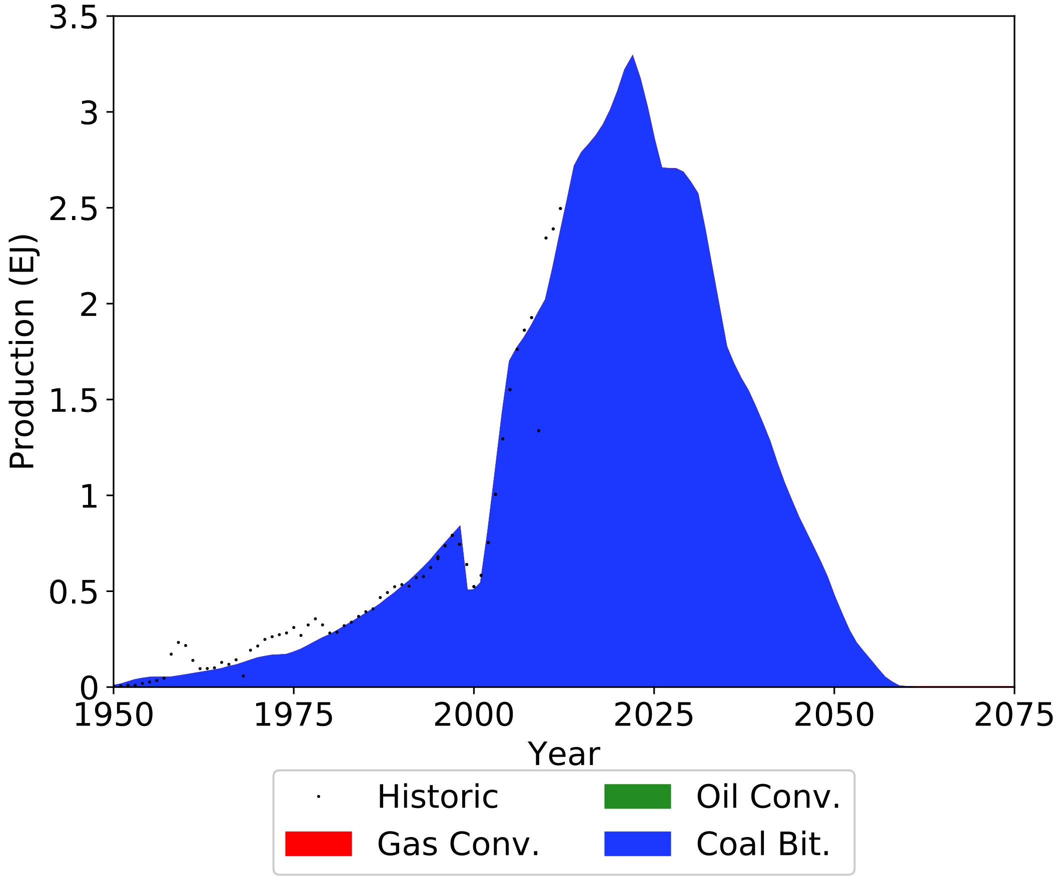

Figure 2.100: China - Yunnan projections capped at 16

Table 2.100: Peak years - All

Name	URR	Peak Year	Peak Rate
Coal Bit. Yunnan	113.89	2022	3.28
Gas Conv. Yunnan	0.04	2019	–
Oil Conv. Yunnan	0.01	2022	–
**Total**	**113.93**	**2022**	**3.29**

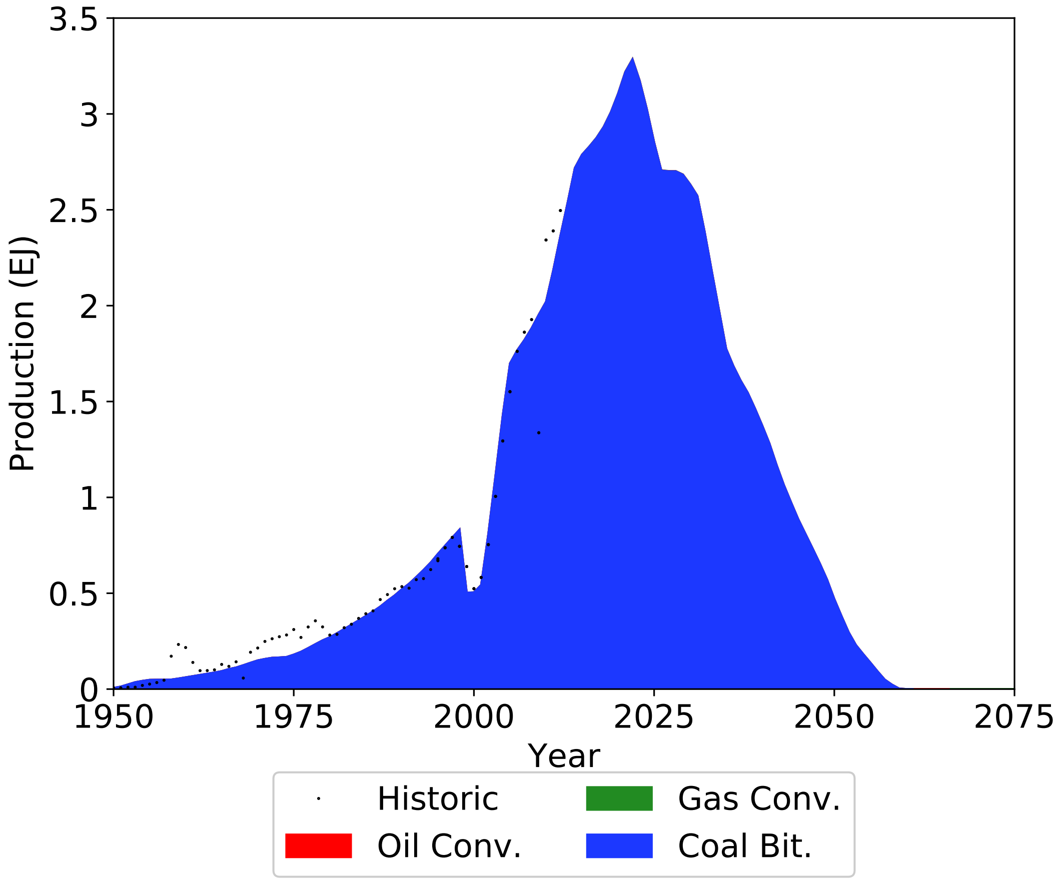

Figure 2.101: China - Yunnan projection by mineral type

Table 2.101: Peak years - Minerals

Name	URR	Peak Year	Peak Rate
Coal Bit.	113.89	2022	3.28
Oil Conv.	0.01	2022	–
Gas Conv.	0.04	2019	–
**Total**	**113.93**	**2022**	**3.29**

Zhejiang

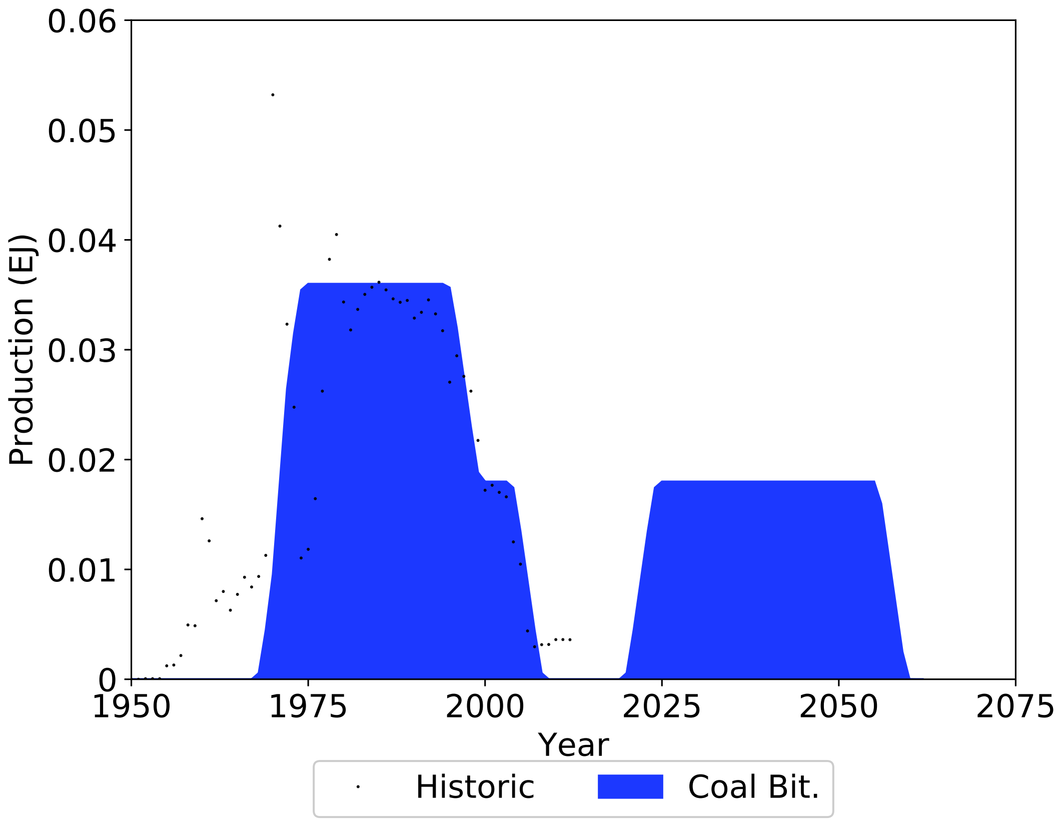

Figure 2.102: China - Zhejiang projections capped at 16

Table 2.102: Peak years - All

Name	URR	Peak Year	Peak Rate
Coal Bit. Zhejiang	1.74	1975	0.04
**Total**	**1.74**	**1975**	**0.04**

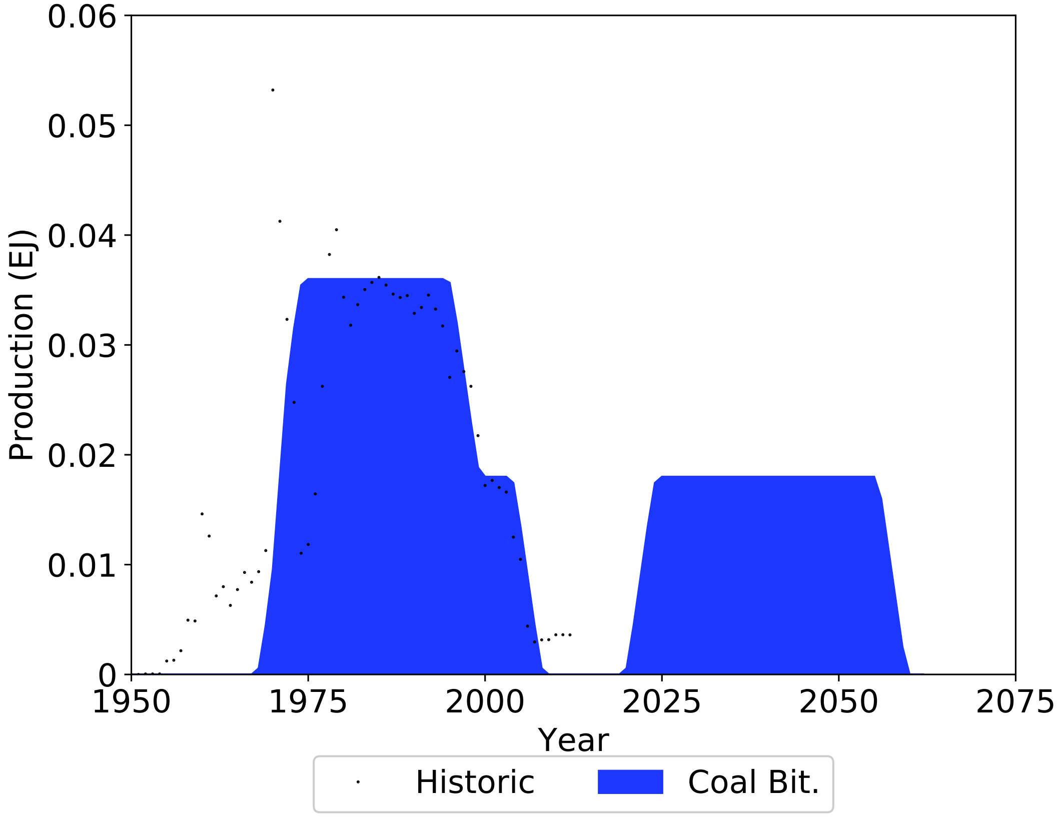

Figure 2.103: China - Zhejiang projection by mineral type

Table 2.103: Peak years - Minerals

Name	URR	Peak Year	Peak Rate
Coal Bit.	1.74	1975	0.04
**Total**	**1.74**	**1975**	**0.04**

2.8.4 Projection by region

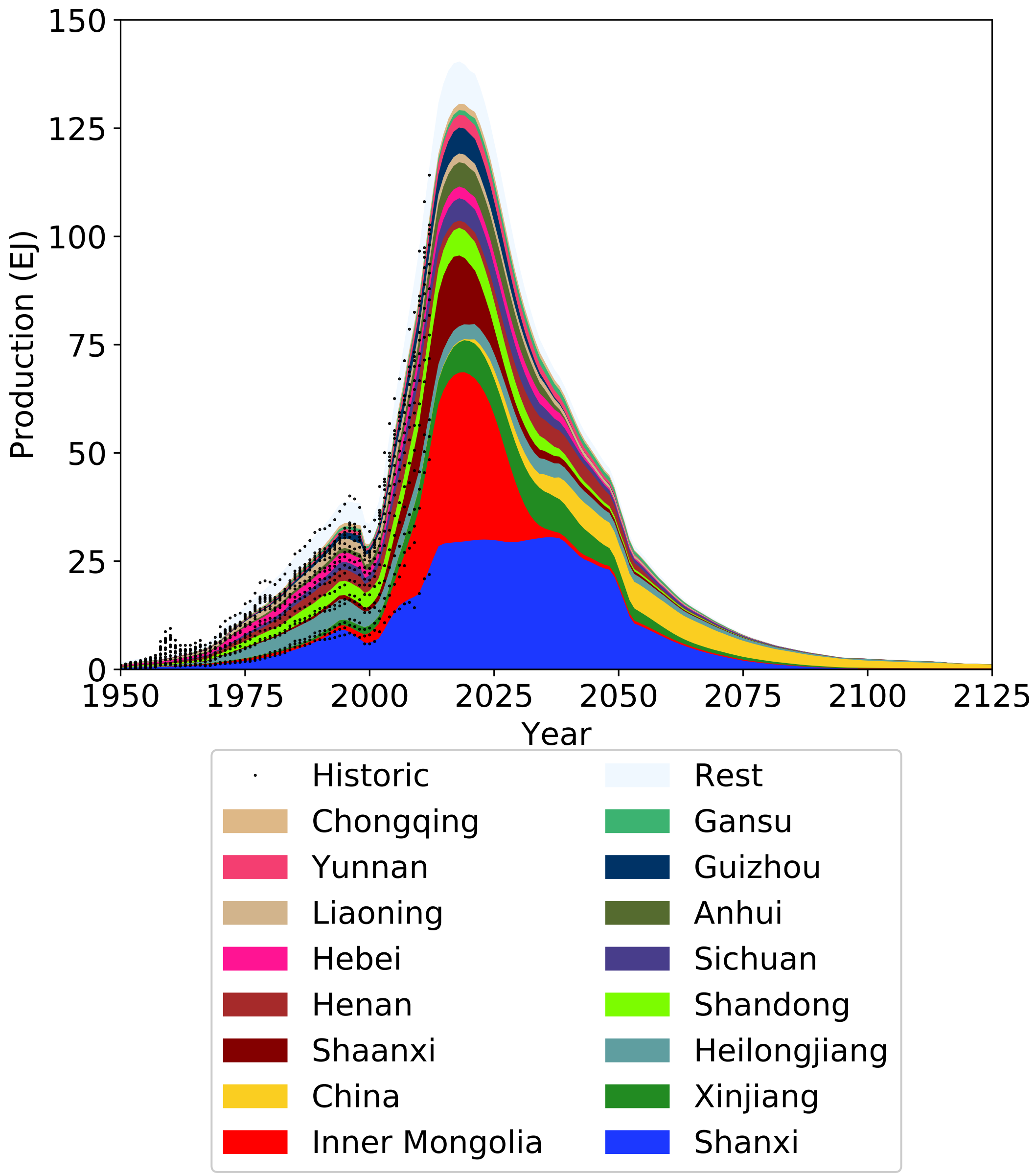

Figure 2.104: China by region projections capped at 16

Table 2.104: Peak years - All

**Name**	**URR**	**Peak Year**	**Peak Rate**
Shanxi	1543.23	2036	30.33
Inner Mongolia	765.99	2018	39.11
Xinjiang	376.74	2030	9.07
China	371.63	2054	6.21
Heilongjiang	330.18	1994	4.8
Shaanxi	319.57	2016	17.27
Shandong	284.22	2021	6.59
Henan	259.99	2009	5.77
Sichuan	211.06	2021	5.29
Hebei	172.45	2015	2.87
Anhui	162.52	2020	5.77
Liaoning	147.89	1999	2.31
Guizhou	147.77	2019	6.01
Yunnan	113.93	2022	3.29
Gansu	84.7	2029	1.72
Chongqing	70.78	2024	1.49
Ningxia	69.21	2021	3.25
Jilin	68.47	2015	1.99
Hunan	49.38	2013	2.24
Qinghai	44.25	2023	1.71
Jiangsu	41.89	2006	0.74
Tianjin	30.76	2011	1.42
Guangdong	27.95	2010	0.84
Jiangxi	26.86	2008	0.79
Historic	25.96	1937	0.94
Offshore	21.38	2034	0.54
Hubei	17.05	2019	0.33
Fujian	15.95	2009	0.67
Beijing	15.6	1981	0.26
Guangxi	11.2	1993	0.25
Hainan	1.96	2052	0.05
Zhejiang	1.74	1975	0.04
Shanghai	0.83	1982	0.08
Tibet	0.19	2048	–
**Total**	**5833.28**	**2018**	**140.16**

2.9 East Timor

2.9.1 All Projections

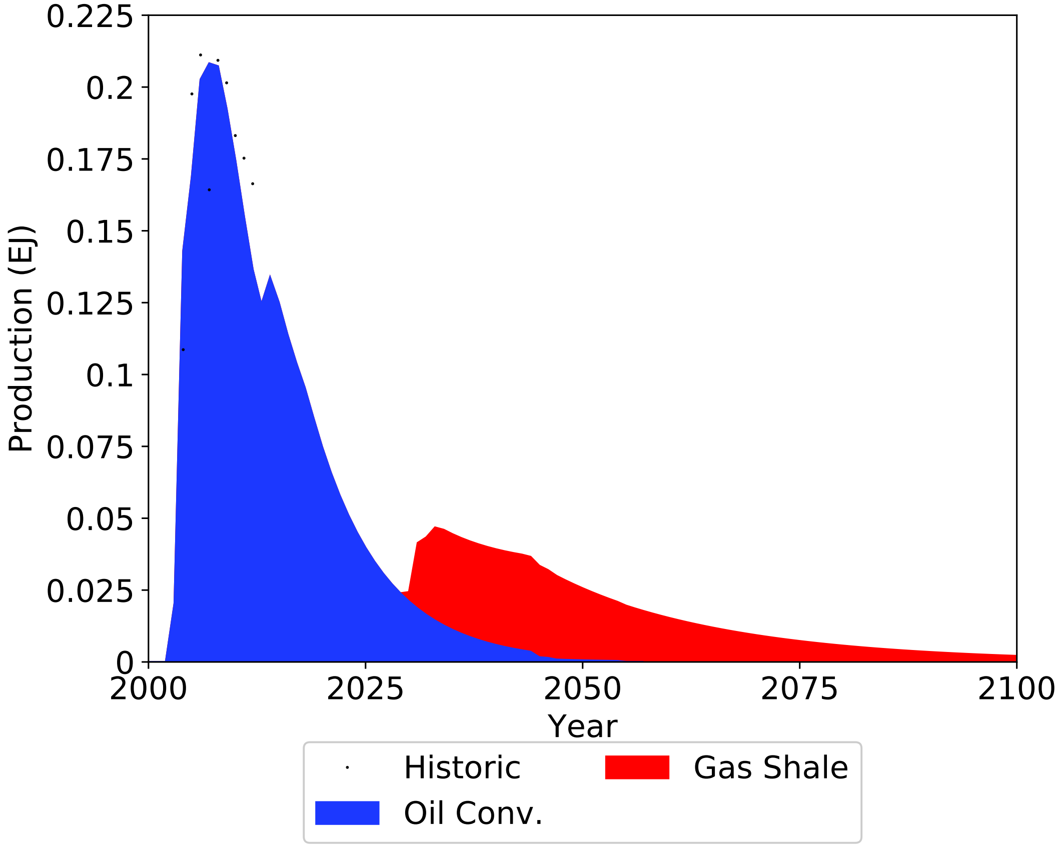

Figure 2.105: East Timor projections capped at 16

Table 2.105: Peak years - All

**Name**	**URR**	**Peak Year**	**Peak Rate**
Oil Conv.	3.0	2007	0.21
Gas Shale	1.11	2034	0.03
**Total**	**4.11**	**2007**	**0.21**

2.9.2 By Mineral

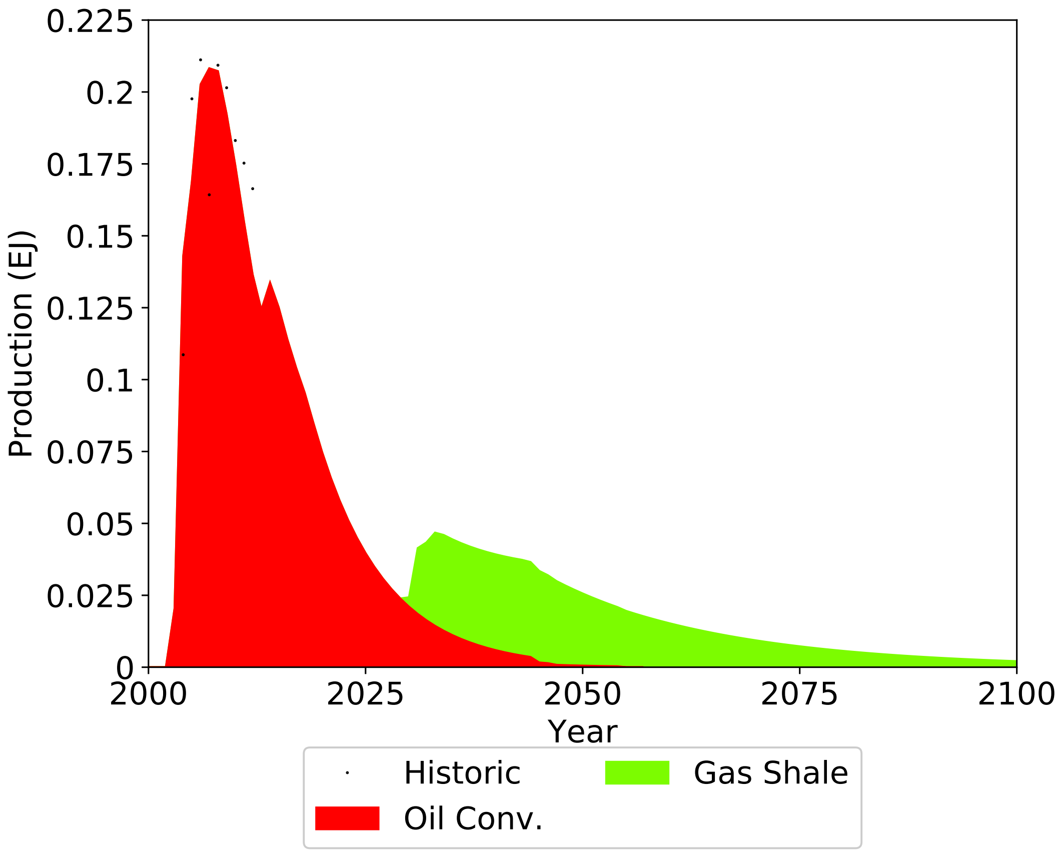

Figure 2.106: East Timor projection by mineral type

Table 2.106: Peak years - Minerals

Name	URR	Peak Year	Peak Rate
Oil Conv.	3.0	2007	0.21
Gas Shale	1.11	2034	0.03
**Total**	**4.11**	**2007**	**0.21**

2.10 India

2.10.1 All Projections

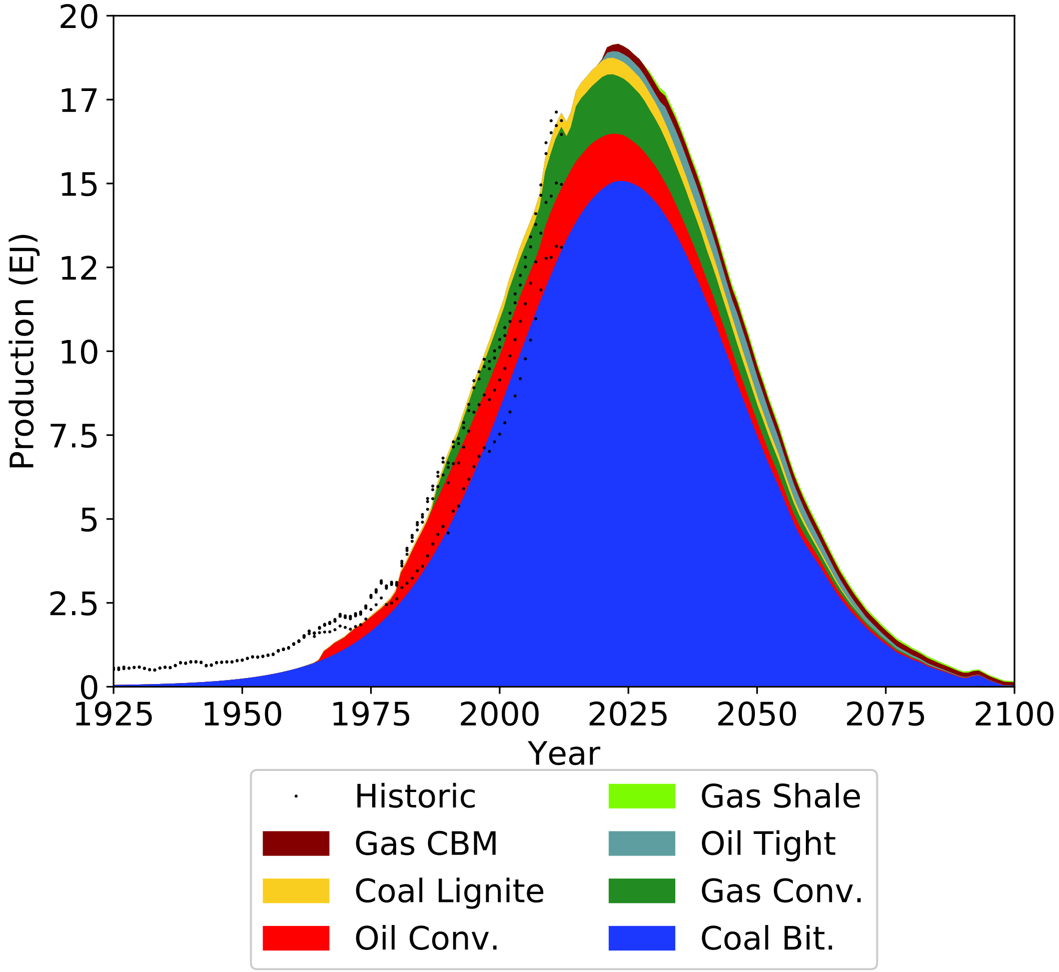

Figure 2.107: India projections capped at 16

Table 2.107: Peak years - All

**Name**	**URR**	**Peak Year**	**Peak Rate**
Coal Bit.	845.5	2024	15.05
Oil Conv.	101.94	2011	1.9
Gas Conv.	78.8	2011	1.83
Coal Lignite	26.05	2029	0.49
Oil Tight	21.77	2048	0.61
Gas CBM	21.0	2036	0.35
Gas Shale	6.6	2043	0.13
**Total**	**1101.66**	**2023**	**19.13**

2.10.2 By Mineral

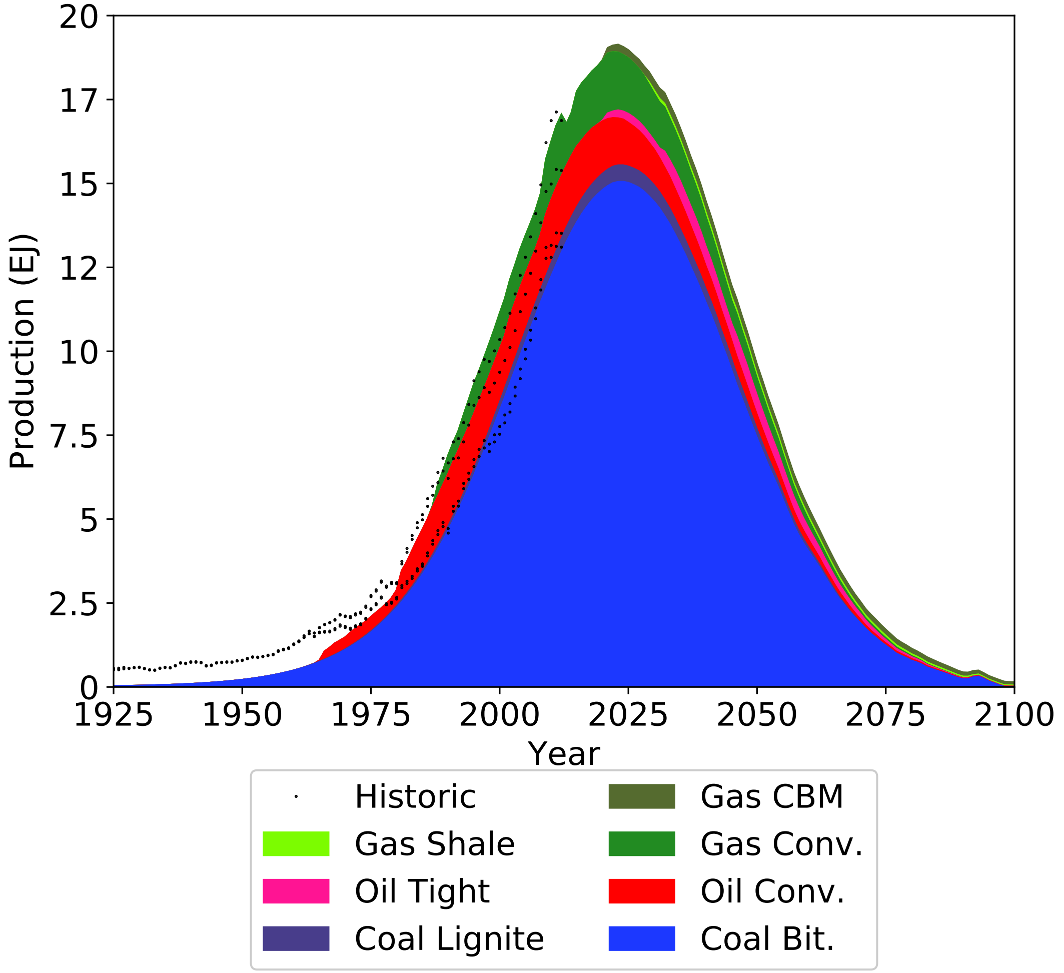

Figure 2.108: India projection by mineral type

Table 2.108: Peak years - Minerals

**Name**	**URR**	**Peak Year**	**Peak Rate**
Coal Bit.	845.5	2024	15.05
Coal Lignite	26.05	2029	0.49
Oil Conv.	101.94	2011	1.9
Oil Tight	21.77	2048	0.61
Gas Conv.	78.8	2011	1.83
Gas Shale	6.6	2043	0.13
Gas CBM	21.0	2036	0.35
**Total**	**1101.66**	**2023**	**19.13**

2.11 Indonesia

2.11.1 All Projections

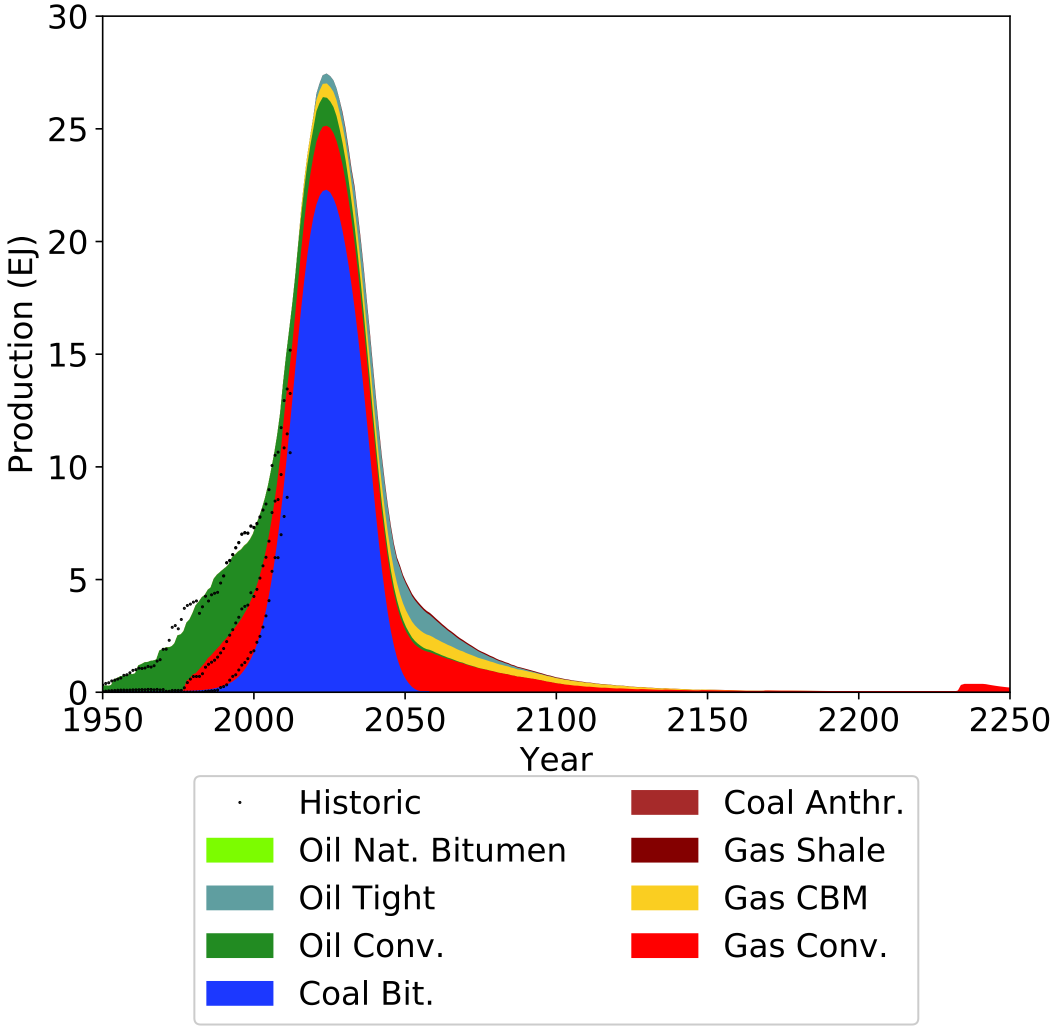

Figure 2.109: Indonesia projections capped at 16

Table 2.109: Peak years - All

**Name**	**URR**	**Peak Year**	**Peak Rate**
Coal Bit.	600.4	2024	22.25
Gas Conv.	241.5	2011	3.01
Oil Conv.	167.78	1988	3.28
Gas CBM	52.5	2038	0.82
Oil Tight	45.27	2040	1.2
Gas Shale	5.97	2040	0.12
Oil Nat. Bitumen	0.17	1995	0.02
Coal Anthr.	0.03	1992	–
**Total**	**1113.61**	**2024**	**27.4**

2.11.2 By Mineral

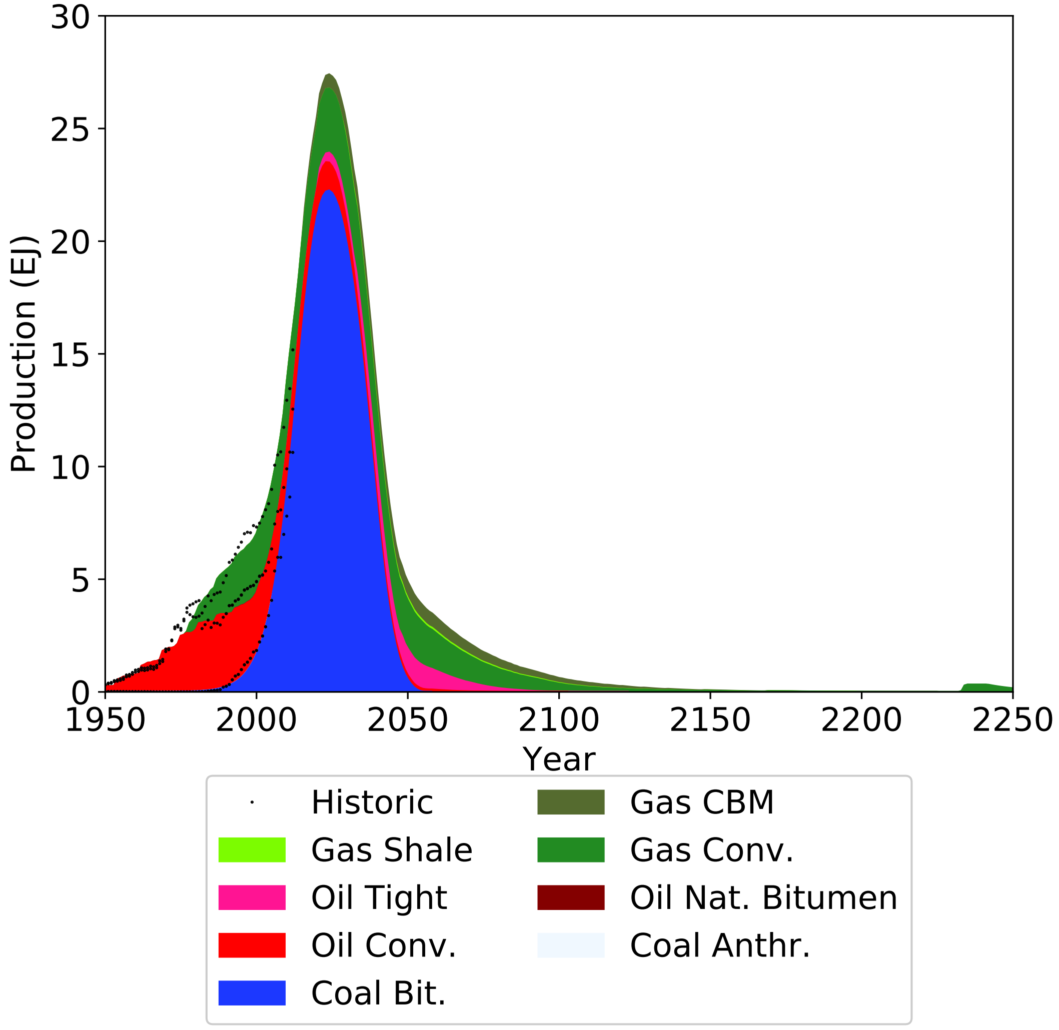

Figure 2.110: Indonesia projection by mineral type

Table 2.110: Peak years - Minerals

**Name**	**URR**	**Peak Year**	**Peak Rate**
Coal Bit.	600.4	2024	22.25
Coal Anthr.	0.03	1992	–
Oil Conv.	167.78	1988	3.28
Oil Nat. Bitumen	0.17	1995	0.02
Oil Tight	45.27	2040	1.2
Gas Conv.	241.5	2011	3.01
Gas Shale	5.97	2040	0.12
Gas CBM	52.5	2038	0.82
**Total**	**1113.61**	**2024**	**27.4**

2.12 Japan

2.12.1 All Projections

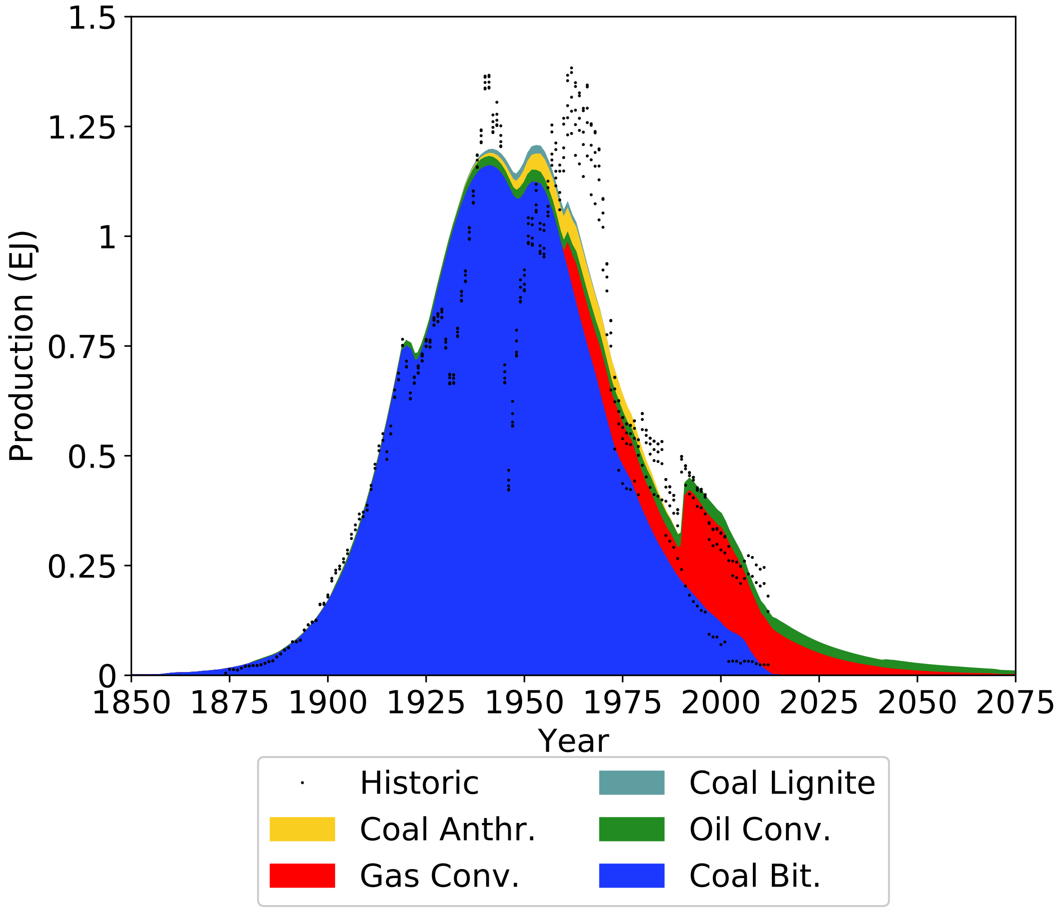

Figure 2.111: Japan projections capped at 16

Table 2.111: Peak years - All

**Name**	**URR**	**Peak Year**	**Peak Rate**
Coal Bit.	68.65	1941	1.16
Gas Conv.	8.26	1993	0.23
Oil Conv.	4.0	1997	0.03
Coal Anthr.	1.58	1964	0.06
Coal Lignite	0.42	1952	0.02
**Total**	**82.91**	**1953**	**1.21**

2.12.2 By Mineral

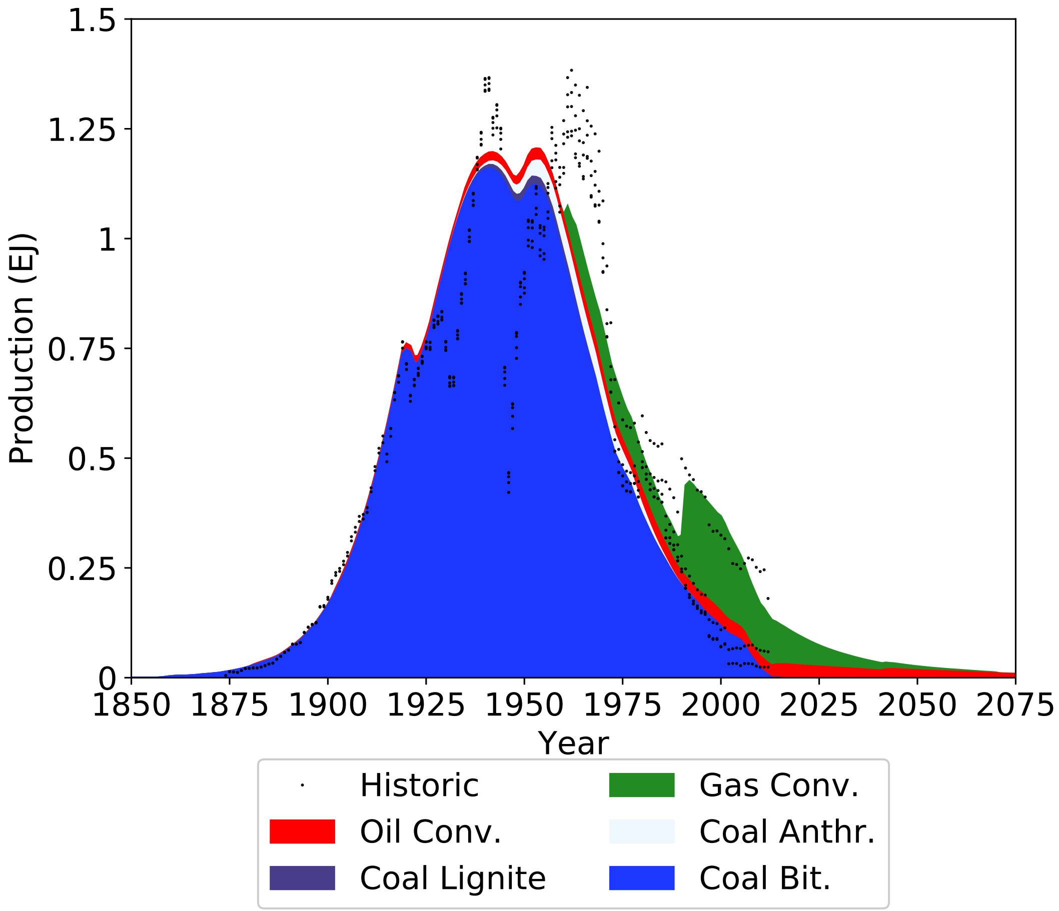

Figure 2.112: Japan projection by mineral type

Table 2.112: Peak years - Minerals

Name	URR	Peak Year	Peak Rate
Coal Bit.	68.65	1941	1.16
Coal Lignite	0.42	1952	0.02
Coal Anthr.	1.58	1964	0.06
Oil Conv.	4.0	1997	0.03
Gas Conv.	8.26	1993	0.23
**Total**	**82.91**	**1953**	**1.21**

2.13 Laos

2.13.1 All Projections

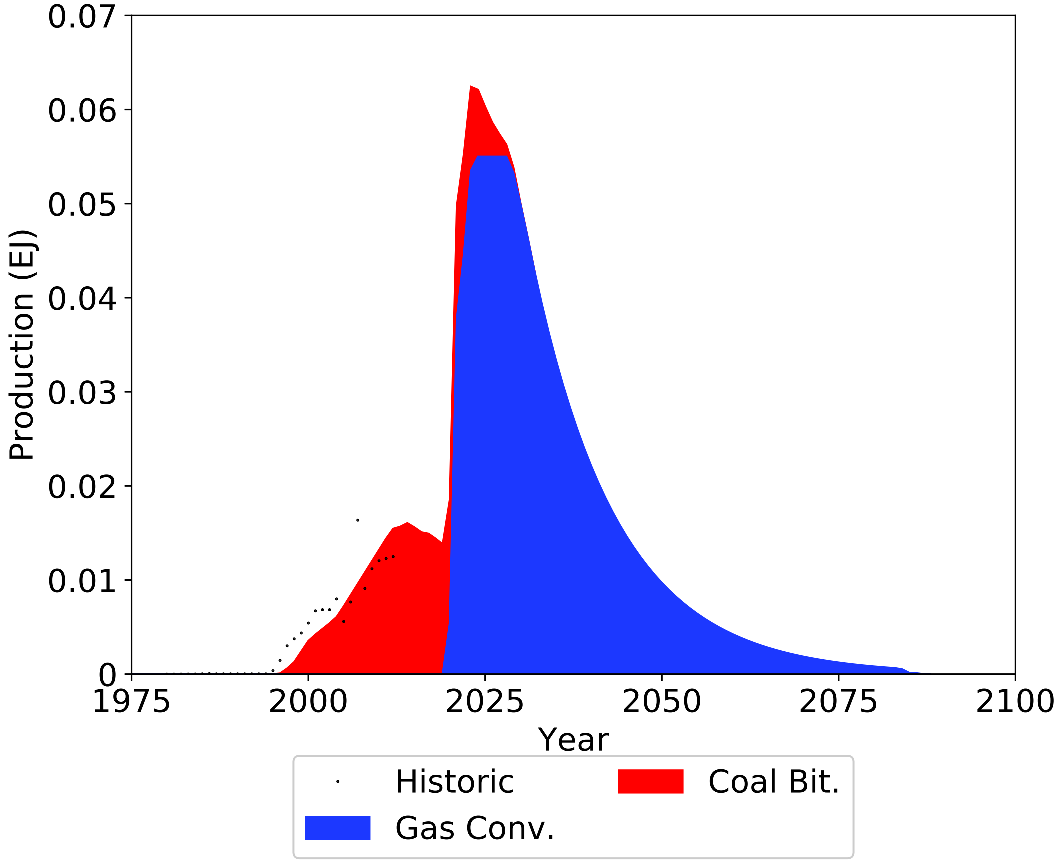

Figure 2.113: Laos projections capped at 16

Table 2.113: Peak years - All

Name	URR	Peak Year	Peak Rate
Gas Conv.	1.1	2024	0.06
Coal Bit.	0.29	2014	0.02
**Total**	**1.39**	**2023**	**0.06**

2.13.2 By Mineral

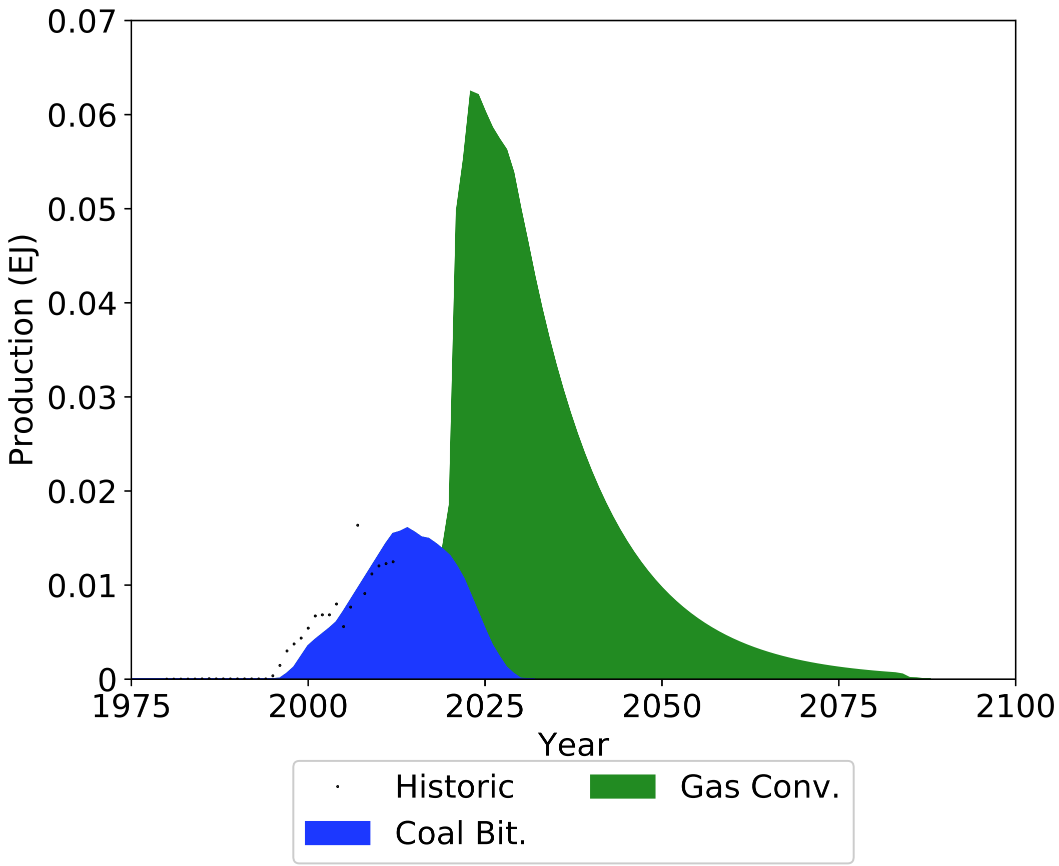

Figure 2.114: Laos projection by mineral type

Table 2.114: Peak years - Minerals

Name	URR	Peak Year	Peak Rate
Coal Bit.	0.29	2014	0.02
Gas Conv.	1.1	2024	0.06
**Total**	**1.39**	**2023**	**0.06**

2.14 Malaysia

2.14.1 All Projections

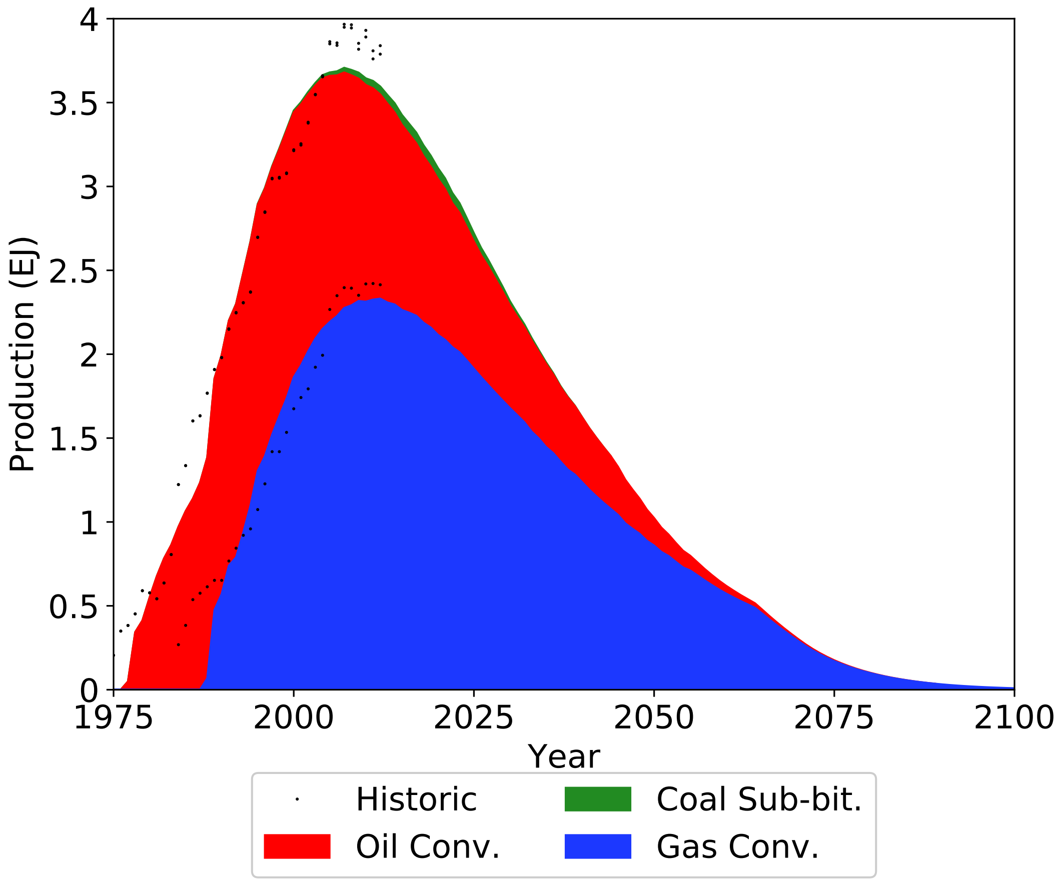

Figure 2.115: Malaysia projections capped at 16

Table 2.115: Peak years - All

Name	URR	Peak Year	Peak Rate
Gas Conv.	115.5	2012	2.33
Oil Conv.	69.33	1999	1.59
Coal Sub-bit.	1.69	2019	0.07
**Total**	**186.52**	**2007**	**3.71**

2.14.2 By Mineral

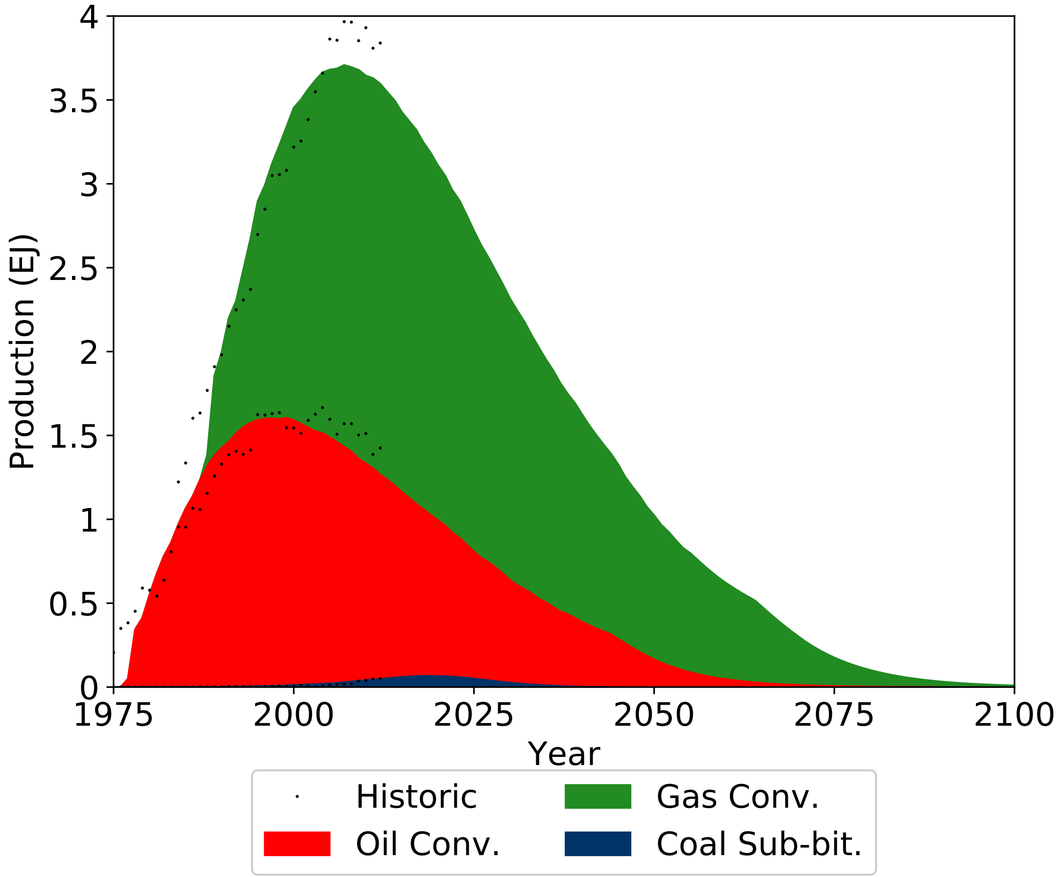

Figure 2.116: Malaysia projection by mineral type

Table 2.116: Peak years - Minerals

**Name**	**URR**	**Peak Year**	**Peak Rate**
Coal Sub-bit.	1.69	2019	0.07
Oil Conv.	69.33	1999	1.59
Gas Conv.	115.5	2012	2.33
**Total**	**186.52**	**2007**	**3.71**

2.15 Mongolia

2.15.1 All Projections

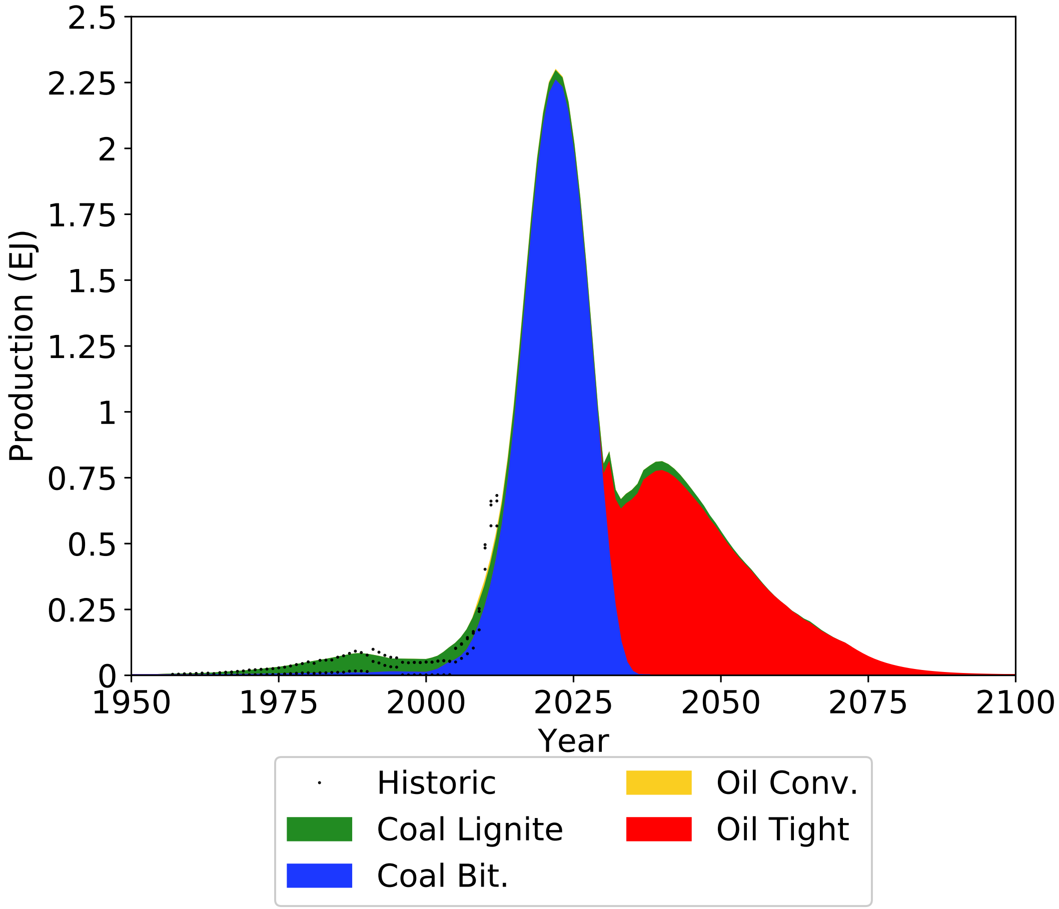

Figure 2.117: Mongolia projections capped at 16

Table 2.117: Peak years - All

Name	URR	Peak Year	Peak Rate
Coal Bit.	30.5	2022	2.26
Oil Tight	19.48	2040	0.78
Coal Lignite	3.8	2009	0.08
Oil Conv.	0.2	2010	0.02
**Total**	**53.98**	**2022**	**2.3**

2.15.2 By Mineral

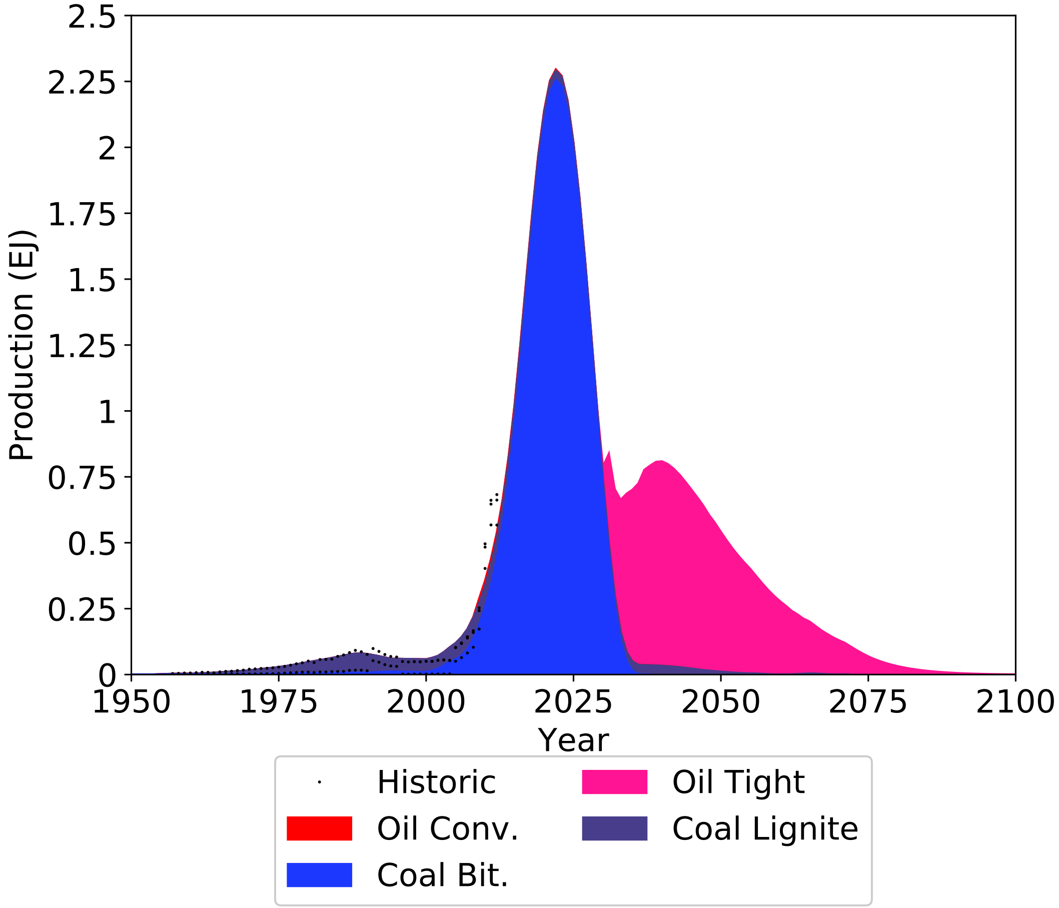

Figure 2.118: Mongolia projection by mineral type

Table 2.118: Peak years - Minerals

**Name**	**URR**	**Peak Year**	**Peak Rate**
Coal Bit.	30.5	2022	2.26
Coal Lignite	3.8	2009	0.08
Oil Conv.	0.2	2010	0.02
Oil Tight	19.48	2040	0.78
**Total**	**53.98**	**2022**	**2.3**

2.16 Nepal

2.16.1 All Projections

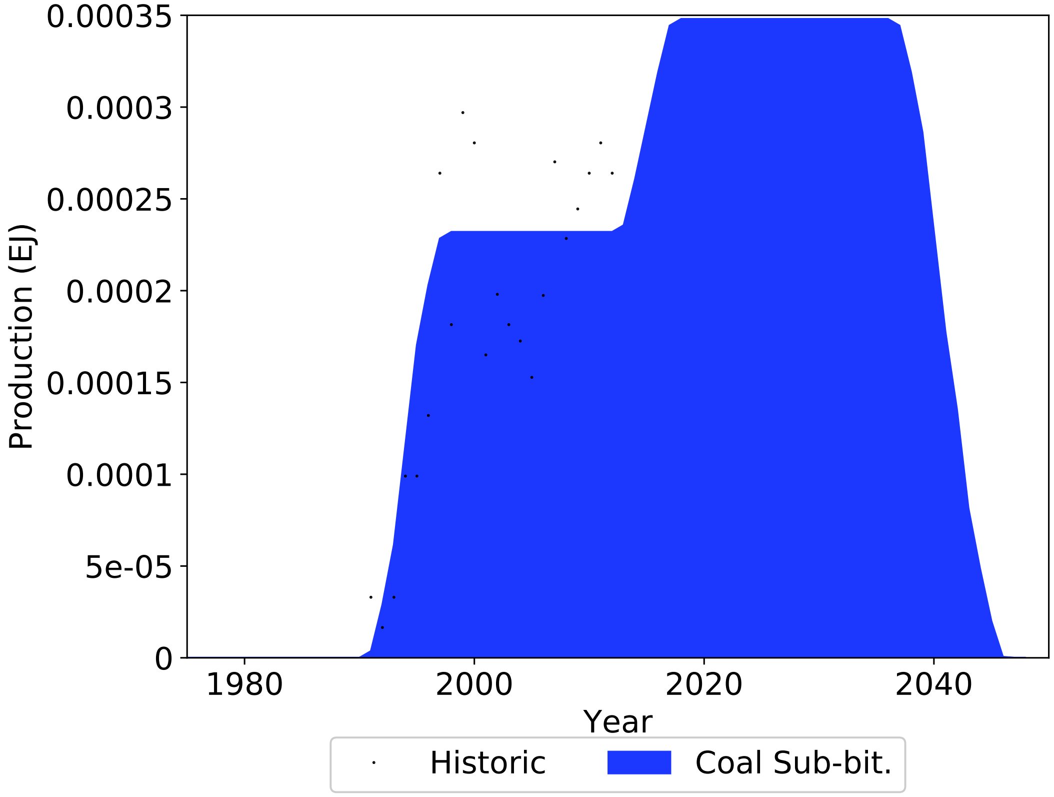

Figure 2.119: Nepal projections capped at 16

Table 2.119: Peak years - All

Name	URR	Peak Year	Peak Rate
Coal Sub-bit.	0.01	2018	–
**Total**	**0.01**	**2018**	–

2.16.2 By Mineral

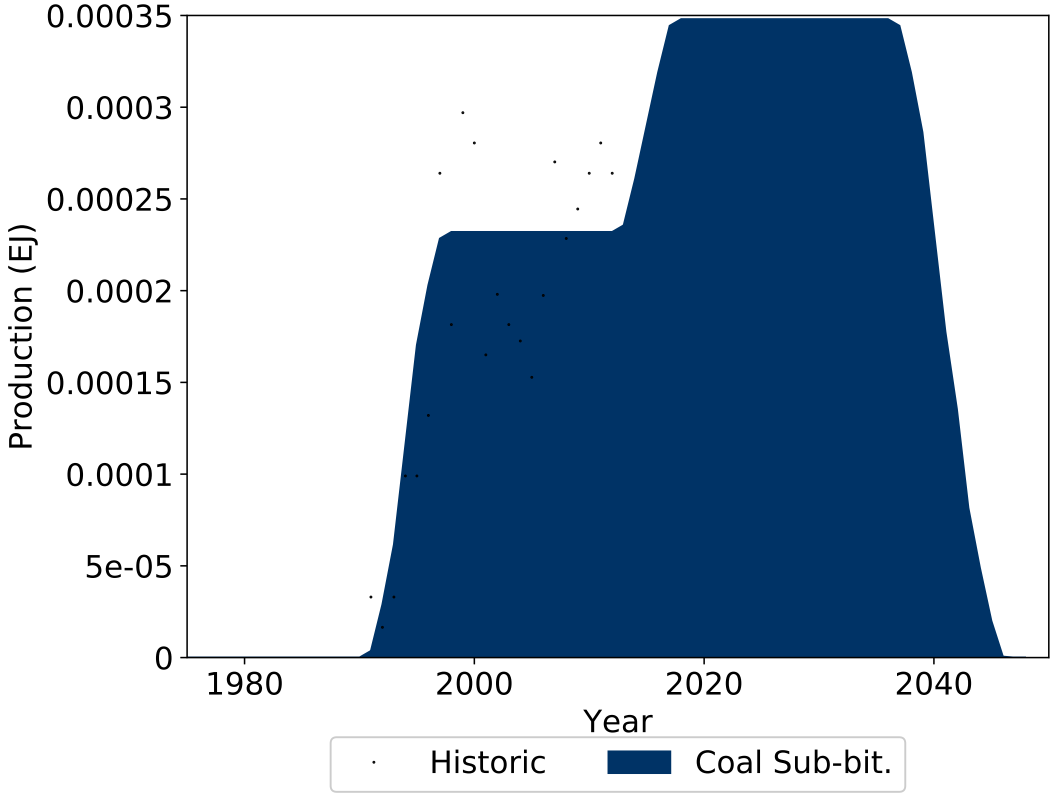

Figure 2.120: Nepal projection by mineral type

Table 2.120: Peak years - Minerals

**Name**	**URR**	**Peak Year**	**Peak Rate**
Coal Sub-bit.	0.01	2018	–
**Total**	**0.01**	**2018**	–

2.17 New Caledonia

2.17.1 All Projections

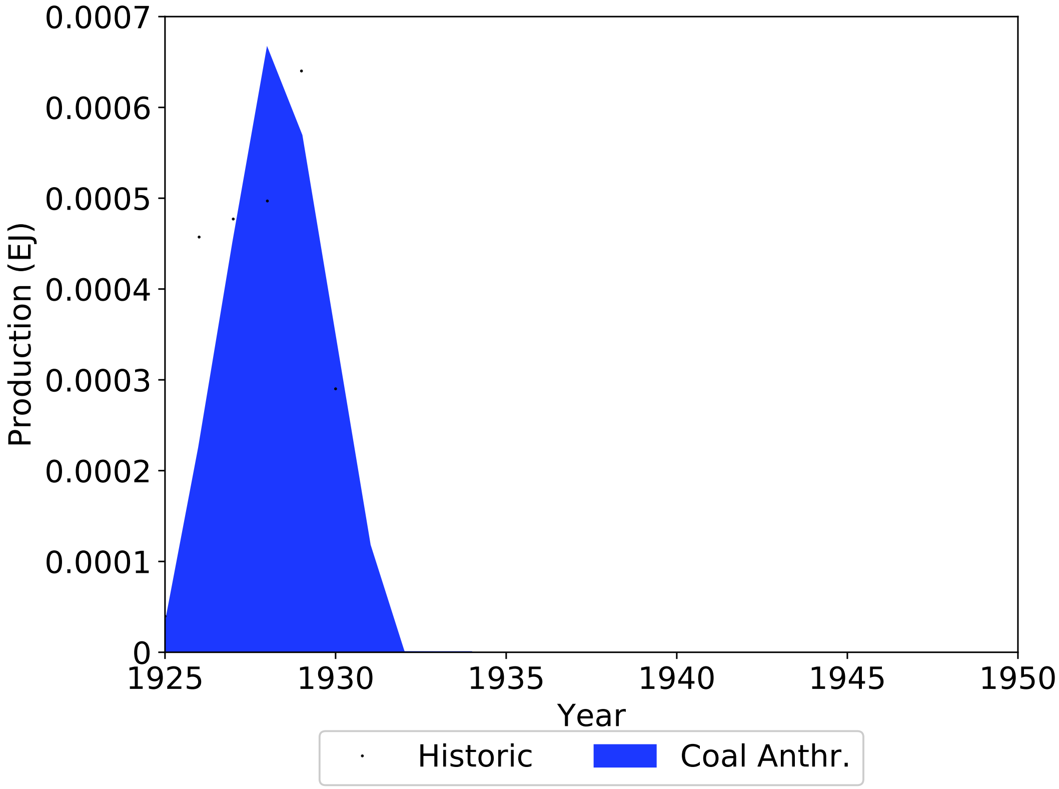

Figure 2.121: New Caledonia projections capped at 16

Table 2.121: Peak years - All

**Name**	**URR**	**Peak Year**	**Peak Rate**
Coal Anthr.	–	1928	–
**Total**	–	**1928**	–

2.17.2 By Mineral

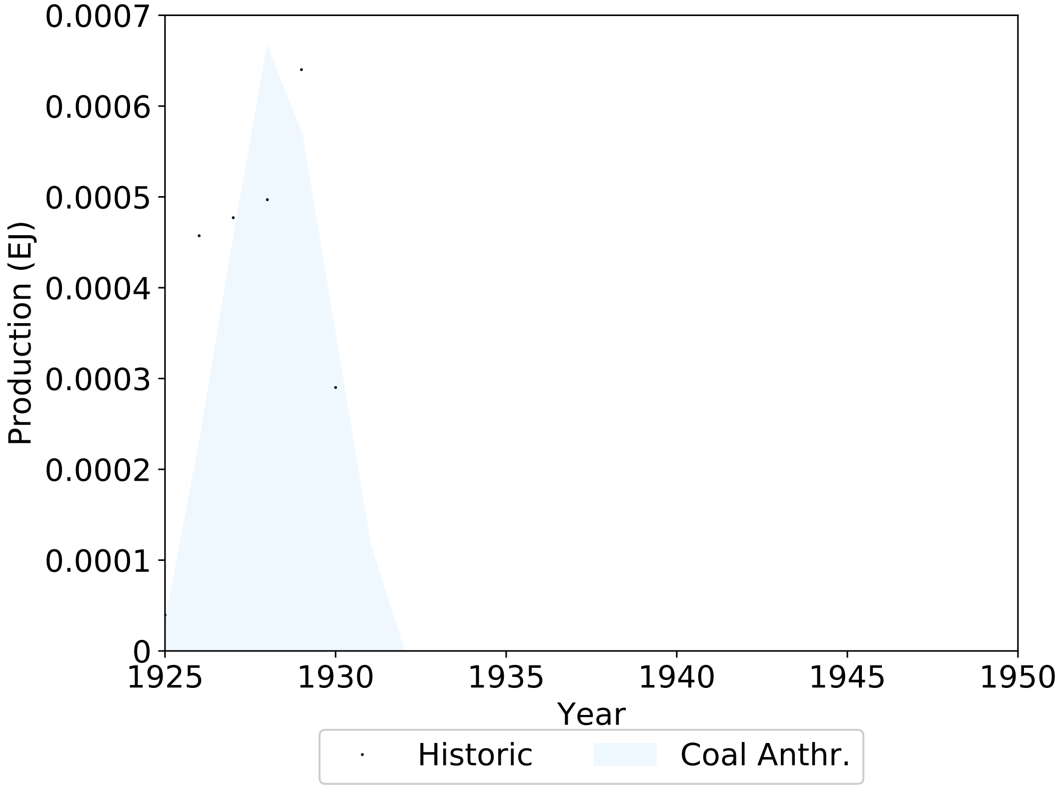

Figure 2.122: New Caledonia projection by mineral type

Table 2.122: Peak years - Minerals

Name	URR	Peak Year	Peak Rate
Coal Anthr.	–	1928	–
**Total**	–	**1928**	–

2.18 New Zealand

2.18.1 All Projections

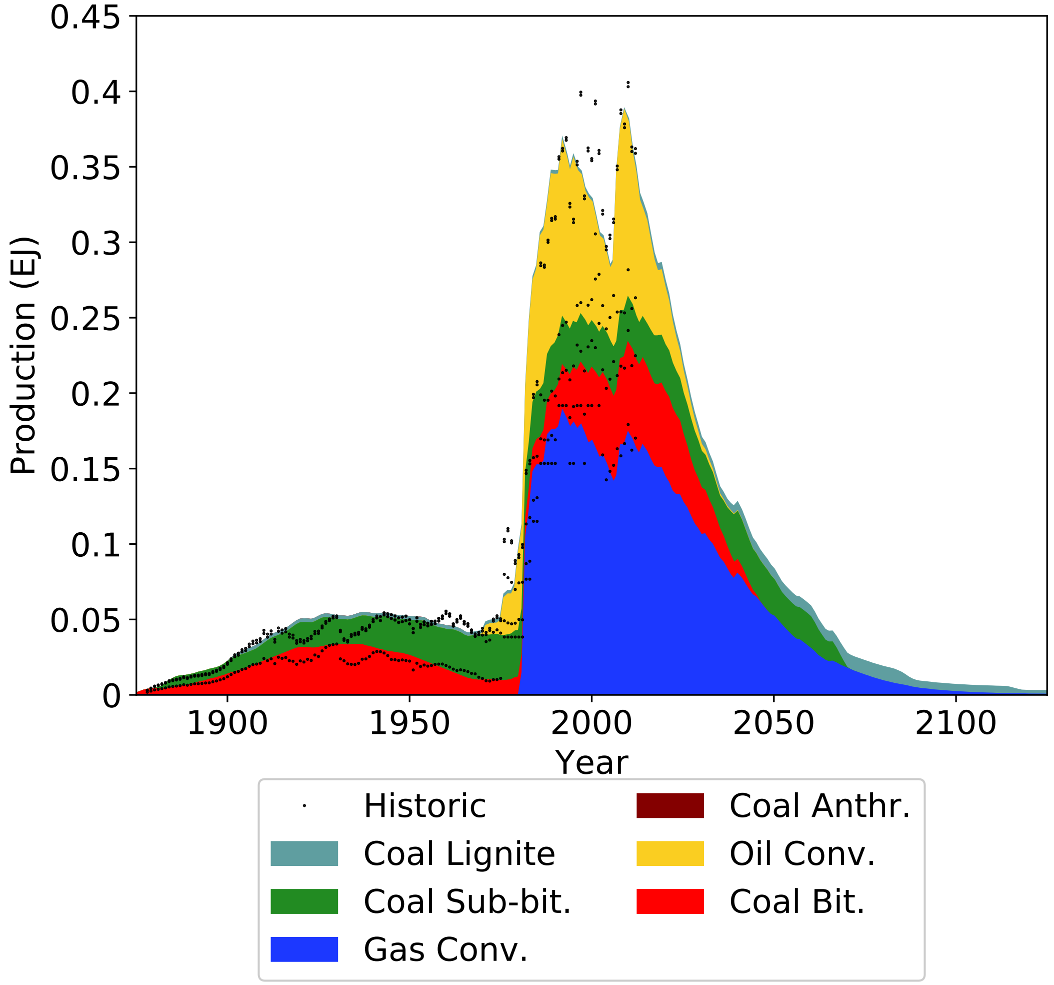

Figure 2.123: New Zealand projections capped at 16

Table 2.123: Peak years - All

**Name**	**URR**	**Peak Year**	**Peak Rate**
Gas Conv.	9.9	1992	0.19
Coal Bit.	4.3	2011	0.06
Coal Sub-bit.	4.15	2006	0.03
Oil Conv.	3.73	2009	0.13
Coal Lignite	0.96	2071	0.01
Coal Anthr.	–	1950	–
**Total**	**23.04**	**2009**	**0.39**

2.18.2 By Mineral

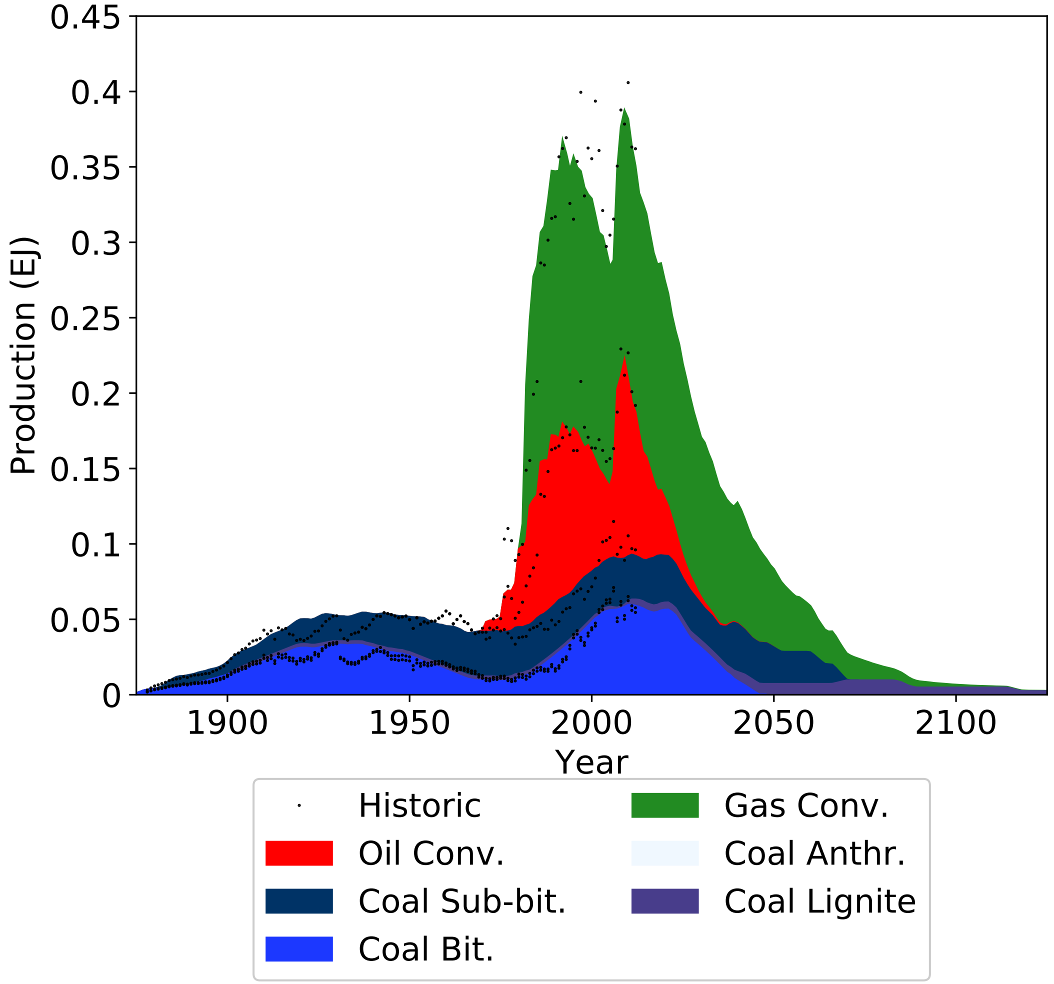

Figure 2.124: New Zealand projection by mineral type

Table 2.124: Peak years - Minerals

**Name**	**URR**	**Peak Year**	**Peak Rate**
Coal Bit.	4.3	2011	0.06
Coal Lignite	0.96	2071	0.01
Coal Sub-bit.	4.15	2006	0.03
Coal Anthr.	–	1950	–
Oil Conv.	3.73	2009	0.13
Gas Conv.	9.9	1992	0.19
**Total**	**23.04**	**2009**	**0.39**

2.19 North Korea

2.19.1 All Projections

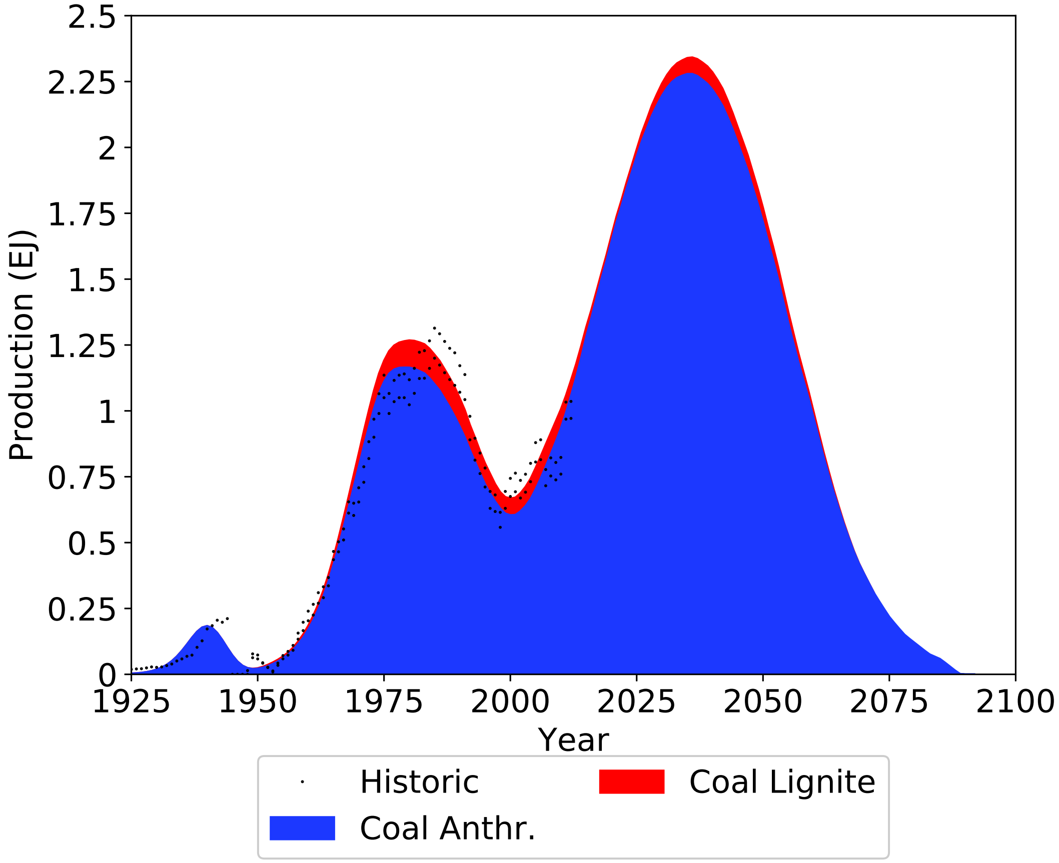

Figure 2.125: North Korea projections capped at 16

Table 2.125: Peak years - All

Name	URR	Peak Year	Peak Rate
Coal Anthr.	140.7	2035	2.28
Coal Lignite	6.27	1985	0.11
**Total**	**146.97**	**2036**	**2.34**

2.19.2 By Mineral

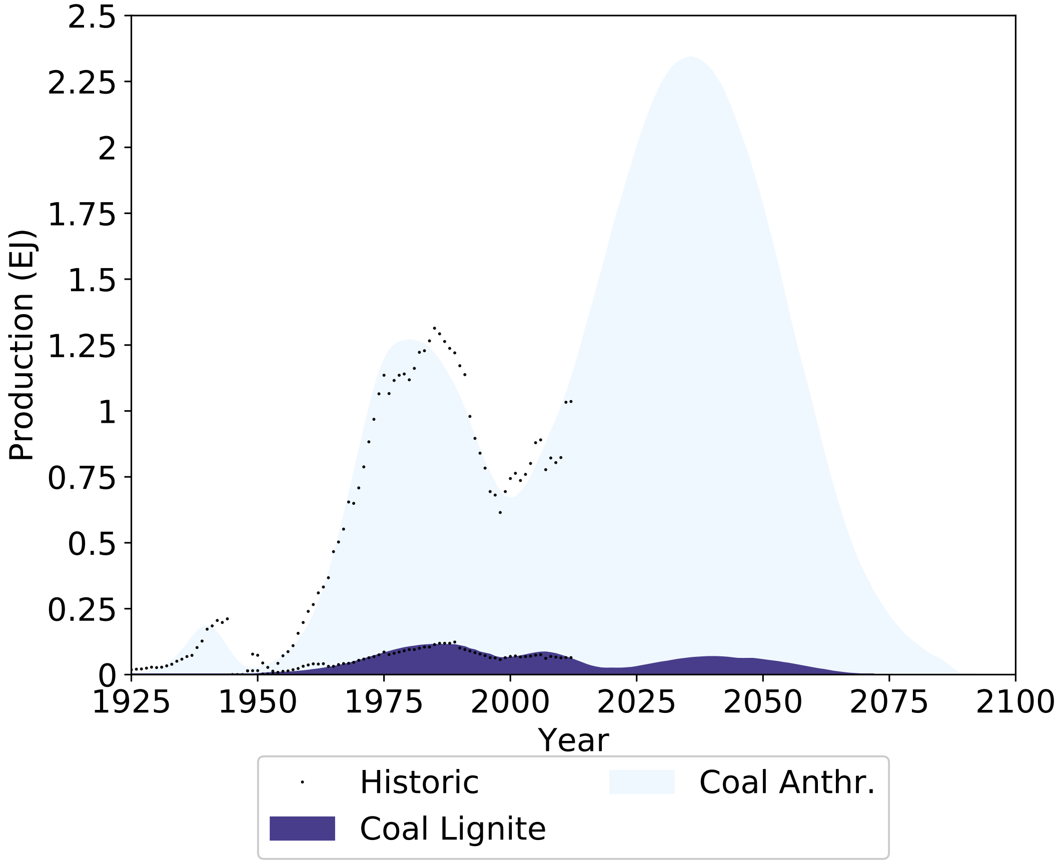

Figure 2.126: North Korea projection by mineral type

Table 2.126: Peak years - Minerals

Name	URR	Peak Year	Peak Rate
Coal Lignite	6.27	1985	0.11
Coal Anthr.	140.7	2035	2.28
**Total**	**146.97**	**2036**	**2.34**

2.20 PNG

2.20.1 All Projections

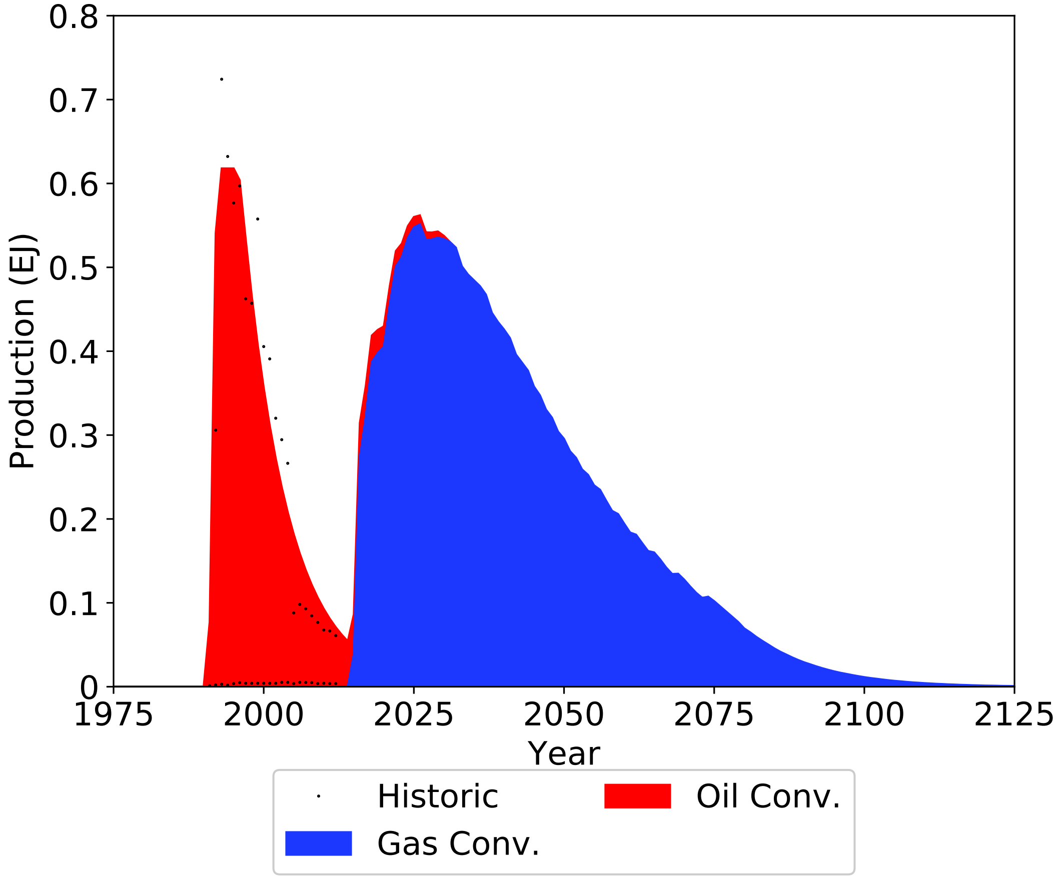

Figure 2.127: PNG projections capped at 16

Table 2.127: Peak years - All

Name	URR	Peak Year	Peak Rate
Gas Conv.	21.0	2026	0.55
Oil Conv.	7.27	1993	0.62
**Total**	**28.27**	**1993**	**0.62**

2.20.2 By Mineral

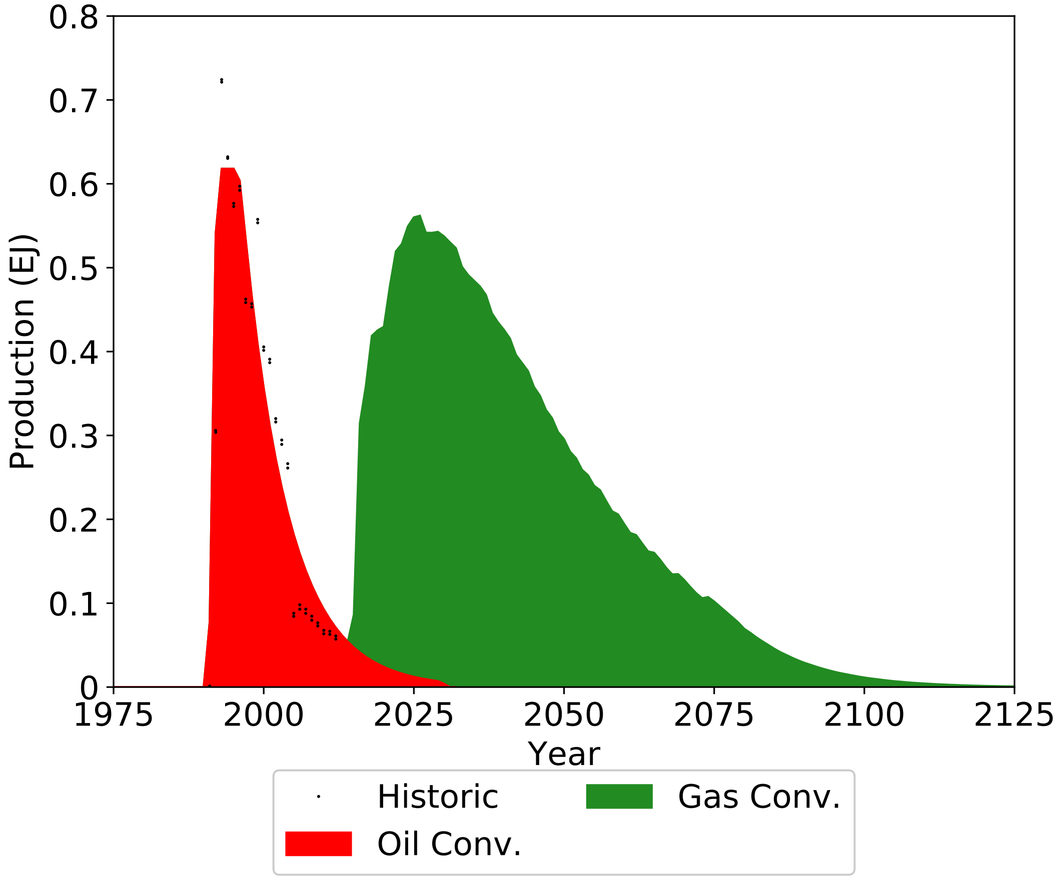

Figure 2.128: PNG projection by mineral type

Table 2.128: Peak years - Minerals

Name	URR	Peak Year	Peak Rate
Oil Conv.	7.27	1993	0.62
Gas Conv.	21.0	2026	0.55
**Total**	**28.27**	**1993**	**0.62**

2.21 Pakistan

2.21.1 All Projections

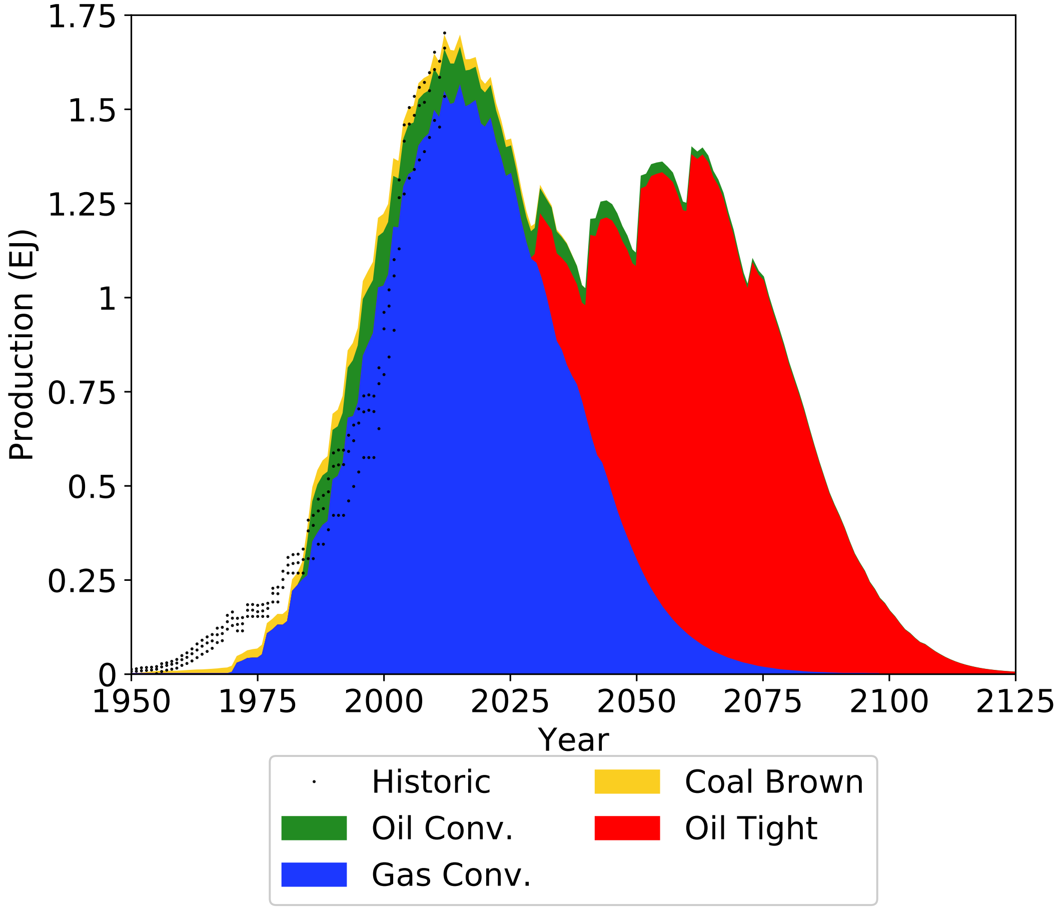

Figure 2.129: Pakistan projections capped at 16

Table 2.129: Peak years - All

**Name**	**URR**	**Peak Year**	**Peak Rate**
Gas Conv.	68.3	2015	1.56
Oil Tight	52.14	2063	1.3
Oil Conv.	6.74	1995	0.15
Coal Brown	2.2	1999	0.05
**Total**	**129.38**	**2012**	**1.69**

2.21.2 By Mineral

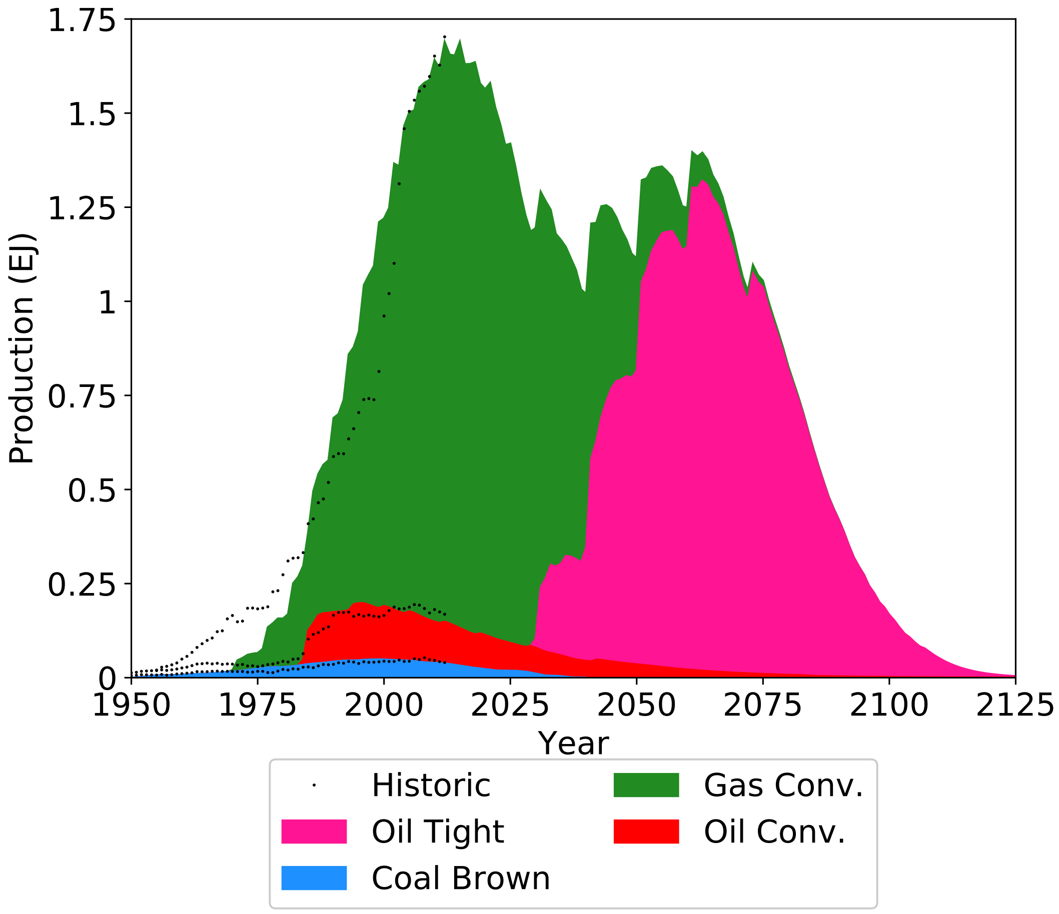

Figure 2.130: Pakistan projection by mineral type

Table 2.130: Peak years - Minerals

**Name**	**URR**	**Peak Year**	**Peak Rate**
Coal Brown	2.2	1999	0.05
Oil Conv.	6.74	1995	0.15
Oil Tight	52.14	2063	1.3
Gas Conv.	68.3	2015	1.56
**Total**	**129.38**	**2012**	**1.69**

2.22 Philippines

2.22.1 All Projections

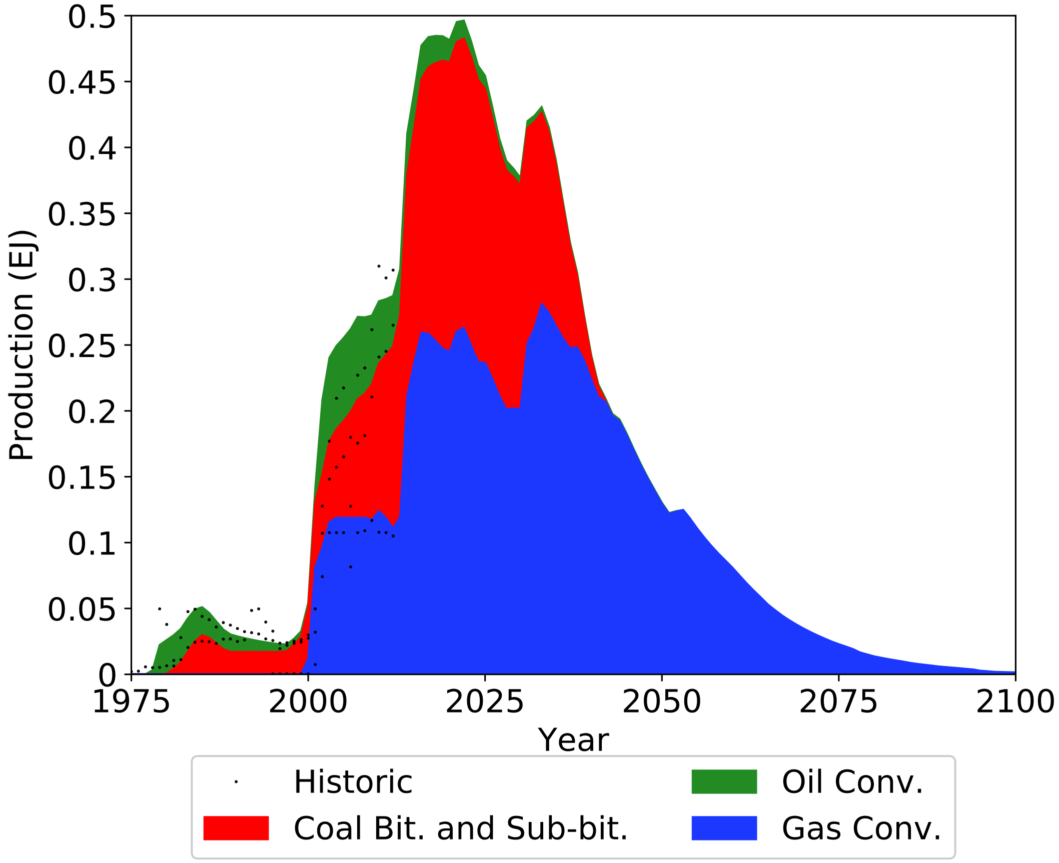

Figure 2.131: Philippines projections capped at 16

Table 2.131: Peak years - All

**Name**	**URR**	**Peak Year**	**Peak Rate**
Gas Conv.	11.7	2033	0.28
Coal Bit. and Sub-bit.	6.0	2020	0.22
Oil Conv.	1.28	2003	0.06
**Total**	**18.98**	**2022**	**0.5**

2.22.2 By Mineral

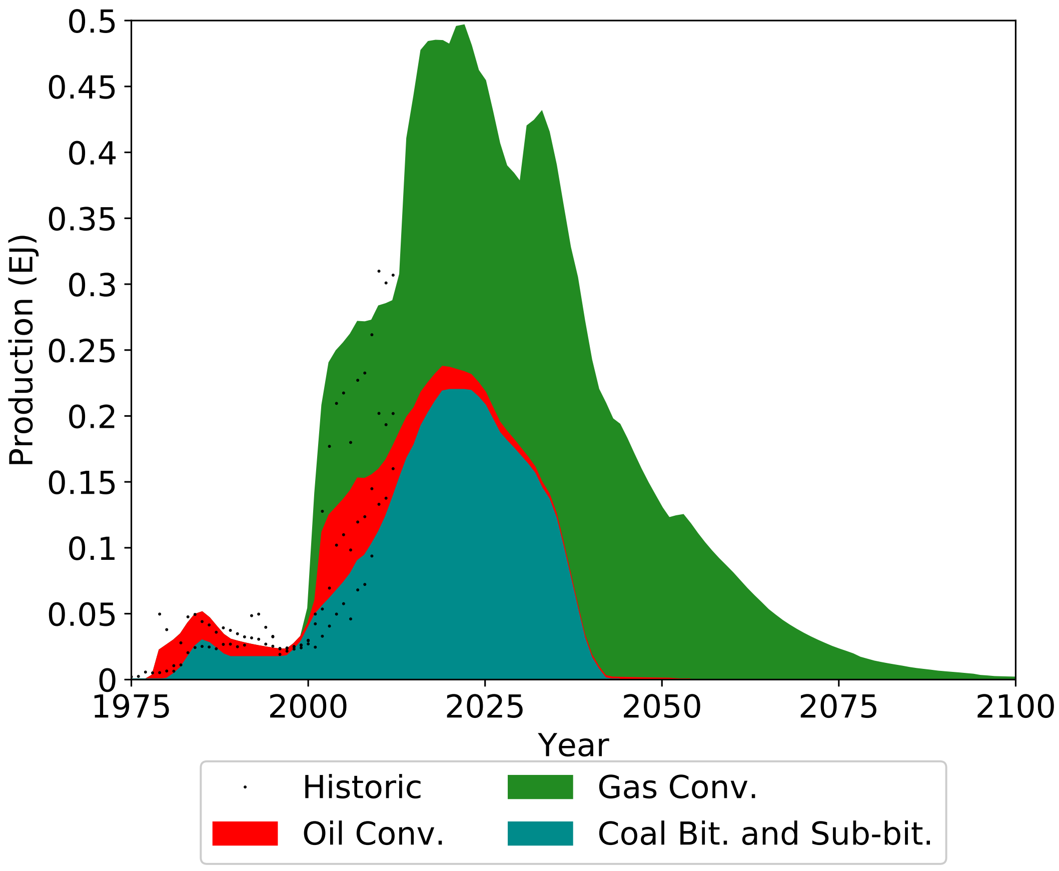

Figure 2.132: Philippines projection by mineral type

Table 2.132: Peak years - Minerals

Name	URR	Peak Year	Peak Rate
Coal Bit. and Sub-bit.	6.0	2020	0.22
Oil Conv.	1.28	2003	0.06
Gas Conv.	11.7	2033	0.28
**Total**	**18.98**	**2022**	**0.5**

2.23 South Korea

2.23.1 All Projections

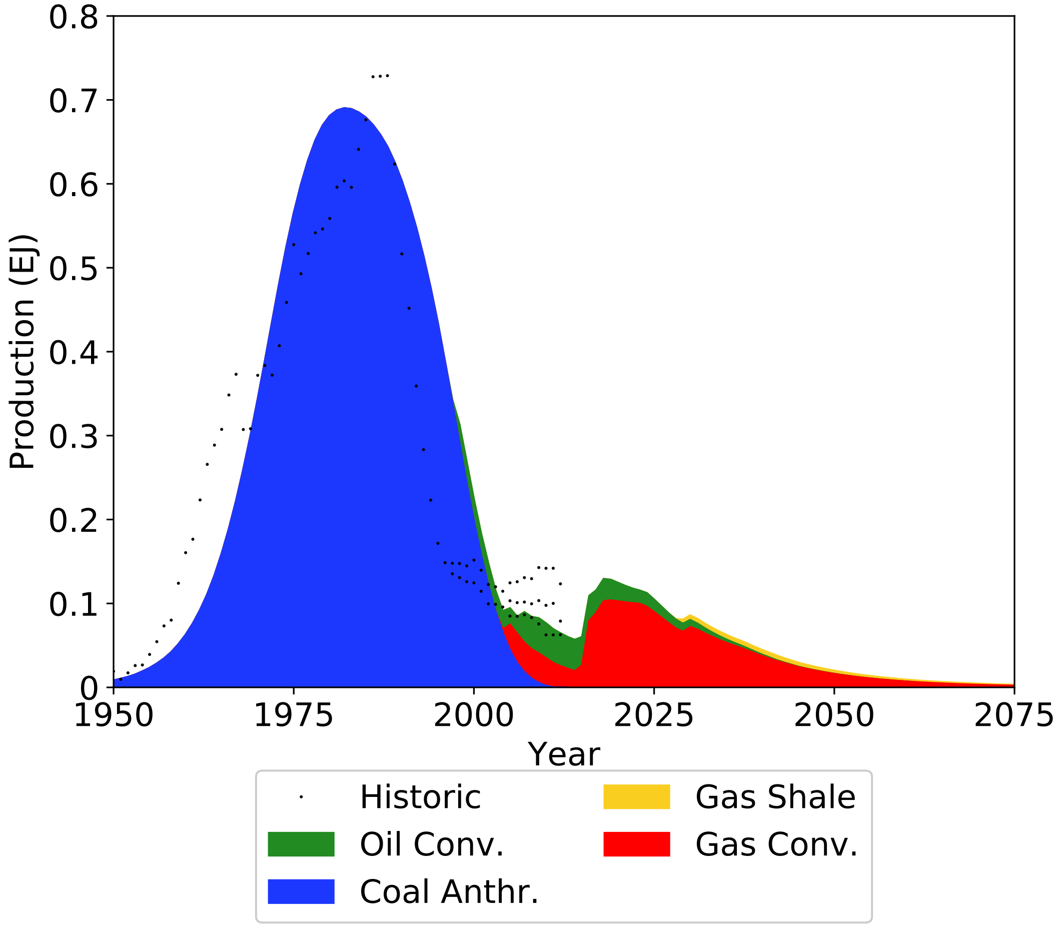

Figure 2.133: South Korea projections capped at 16

Table 2.133: Peak years - All

**Name**	**URR**	**Peak Year**	**Peak Rate**
Coal Anthr.	19.03	1982	0.69
Gas Conv.	2.6	2019	0.1
Oil Conv.	0.88	2009	0.04
Gas Shale	0.19	2030	0.01
**Total**	**22.7**	**1982**	**0.69**

2.23.2 By Mineral

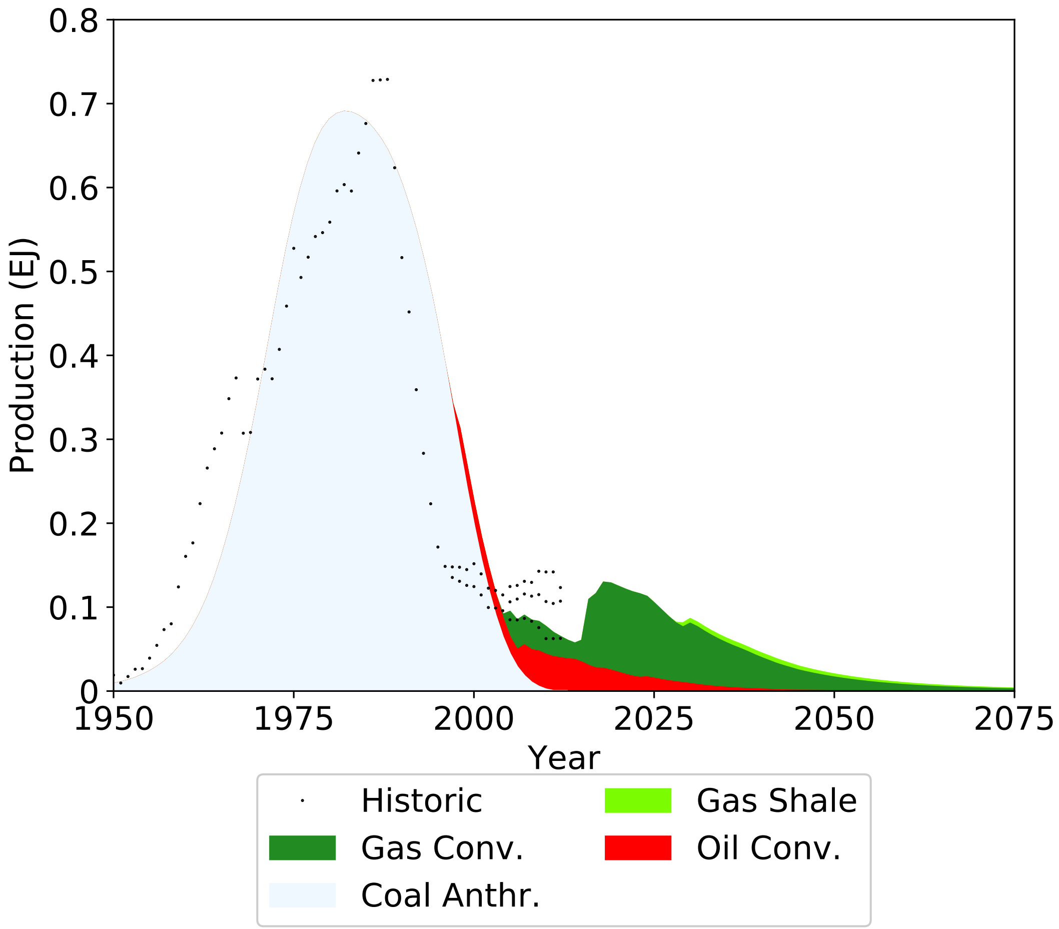

Figure 2.134: South Korea projection by mineral type

Table 2.134: Peak years - Minerals

**Name**	**URR**	**Peak Year**	**Peak Rate**
Coal Anthr.	19.03	1982	0.69
Oil Conv.	0.88	2009	0.04
Gas Conv.	2.6	2019	0.1
Gas Shale	0.19	2030	0.01
**Total**	**22.7**	**1982**	**0.69**

2.24 Sri Lanka

2.24.1 All Projections

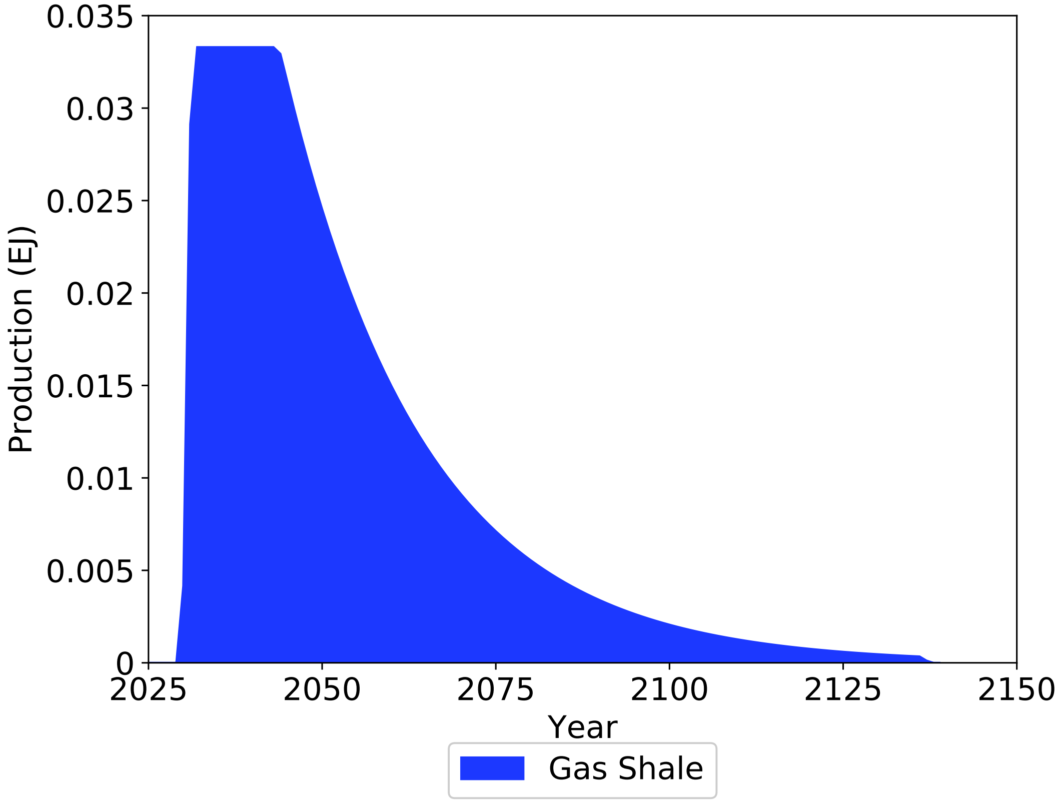

Figure 2.135: Sri Lanka projections capped at 16

Table 2.135: Peak years - All

Name	URR	Peak Year	Peak Rate
Gas Shale	1.11	2032	0.03
**Total**	**1.11**	**2032**	**0.03**

2.24.2 By Mineral

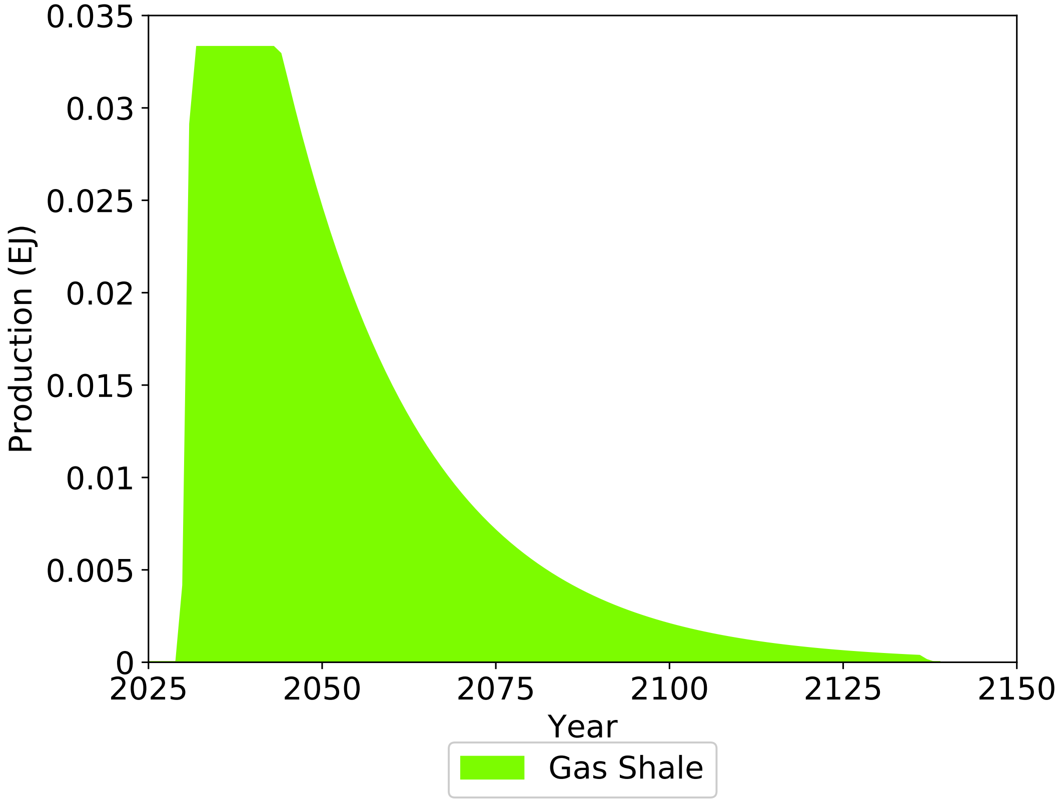

Figure 2.136: Sri Lanka projection by mineral type

Table 2.136: Peak years - Minerals

**Name**	**URR**	**Peak Year**	**Peak Rate**
Gas Shale	1.11	2032	0.03
**Total**	**1.11**	**2032**	**0.03**

2.25 Taiwan

2.25.1 All Projections

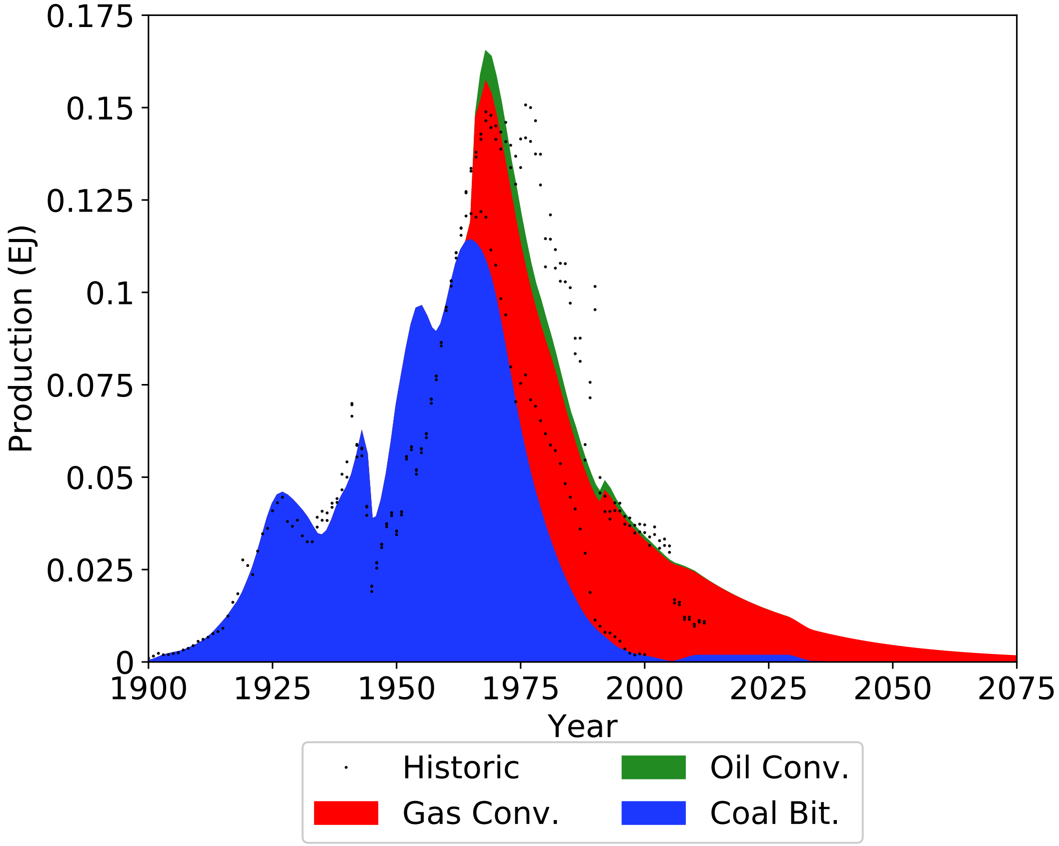

Figure 2.137: Taiwan projections capped at 16

Table 2.137: Peak years - All

Name	URR	Peak Year	Peak Rate
Coal Bit.	4.34	1965	0.11
Gas Conv.	2.3	1969	0.05
Oil Conv.	0.19	1970	0.01
**Total**	**6.83**	**1968**	**0.17**

2.25.2 By Mineral

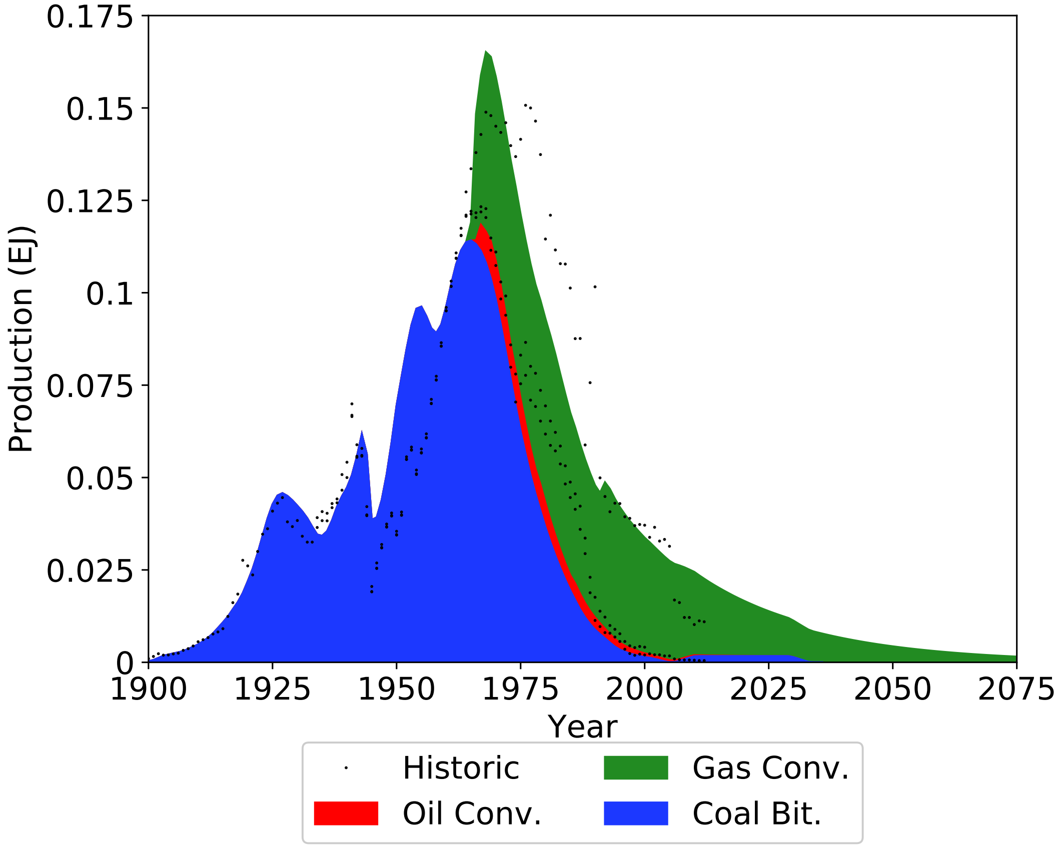

Figure 2.138: Taiwan projection by mineral type

Table 2.138: Peak years - Minerals

Name	URR	Peak Year	Peak Rate
Coal Bit.	4.34	1965	0.11
Oil Conv.	0.19	1970	0.01
Gas Conv.	2.3	1969	0.05
**Total**	**6.83**	**1968**	**0.17**

2.26 Thailand

2.26.1 All Projections

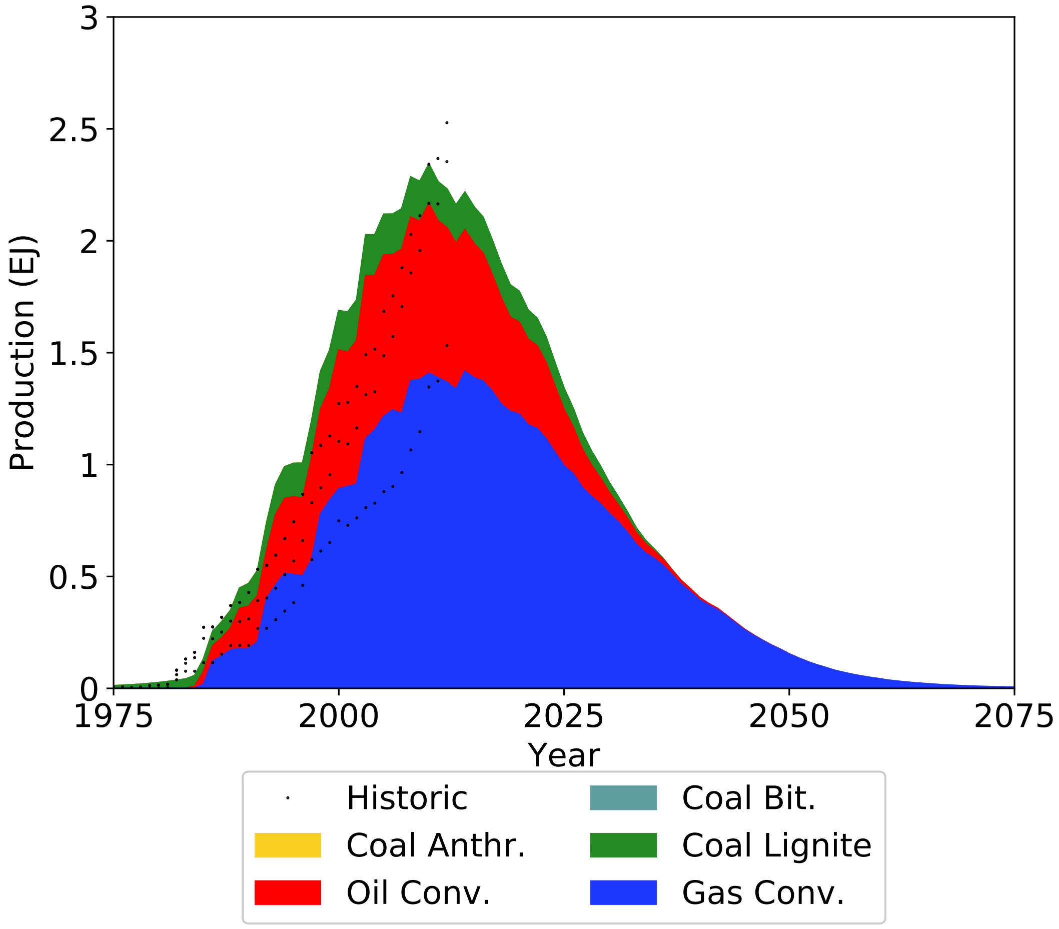

Figure 2.139: Thailand projections capped at 16

Table 2.139: Peak years - All

**Name**	**URR**	**Peak Year**	**Peak Rate**
Gas Conv.	50.8	2014	1.42
Oil Conv.	19.93	2010	0.76
Coal Lignite	6.66	2004	0.18
Coal Anthr.	–	1991	–
Coal Bit.	–	1982	–
**Total**	**77.39**	**2010**	**2.34**

2.26.2 By Mineral

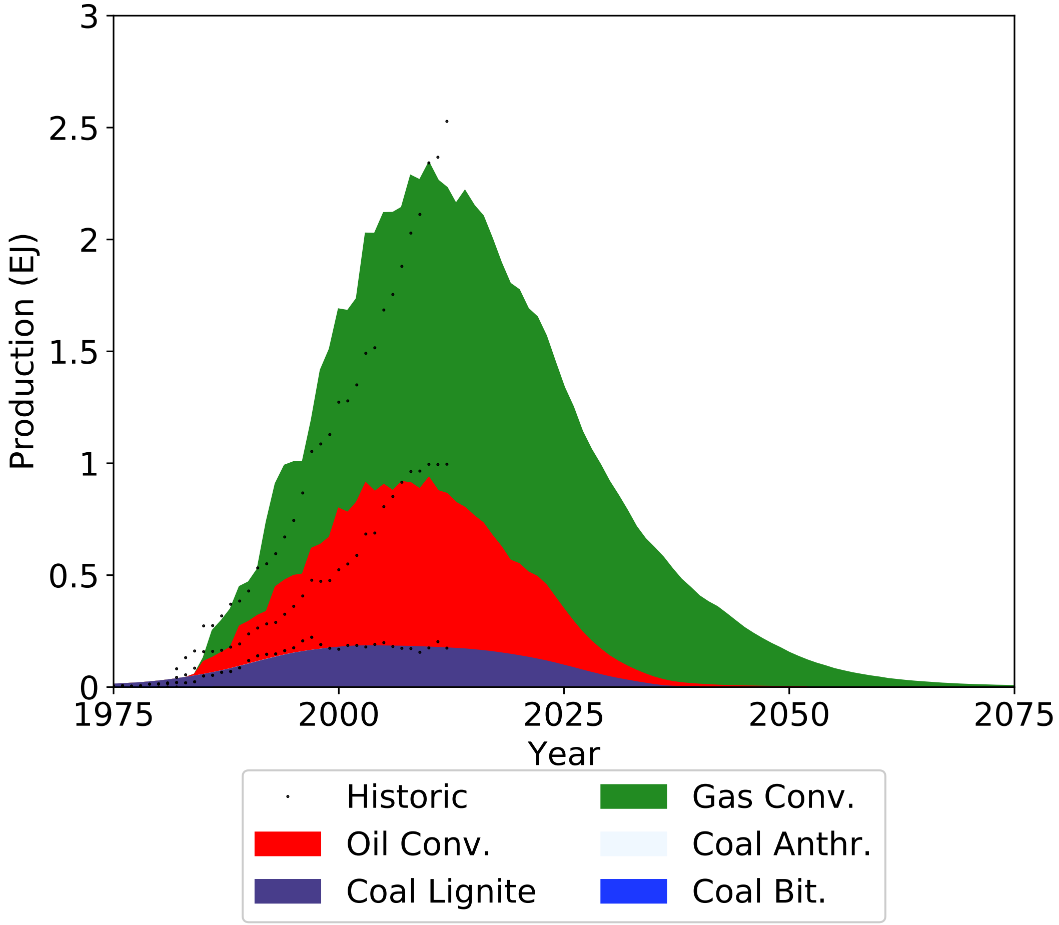

Figure 2.140: Thailand projection by mineral type

Table 2.140: Peak years - Minerals

**Name**	**URR**	**Peak Year**	**Peak Rate**
Coal Bit.	–	1982	–
Coal Lignite	6.66	2004	0.18
Coal Anthr.	–	1991	–
Oil Conv.	19.93	2010	0.76
Gas Conv.	50.8	2014	1.42
**Total**	**77.39**	**2010**	**2.34**

2.27 Vietnam

2.27.1 All Projections

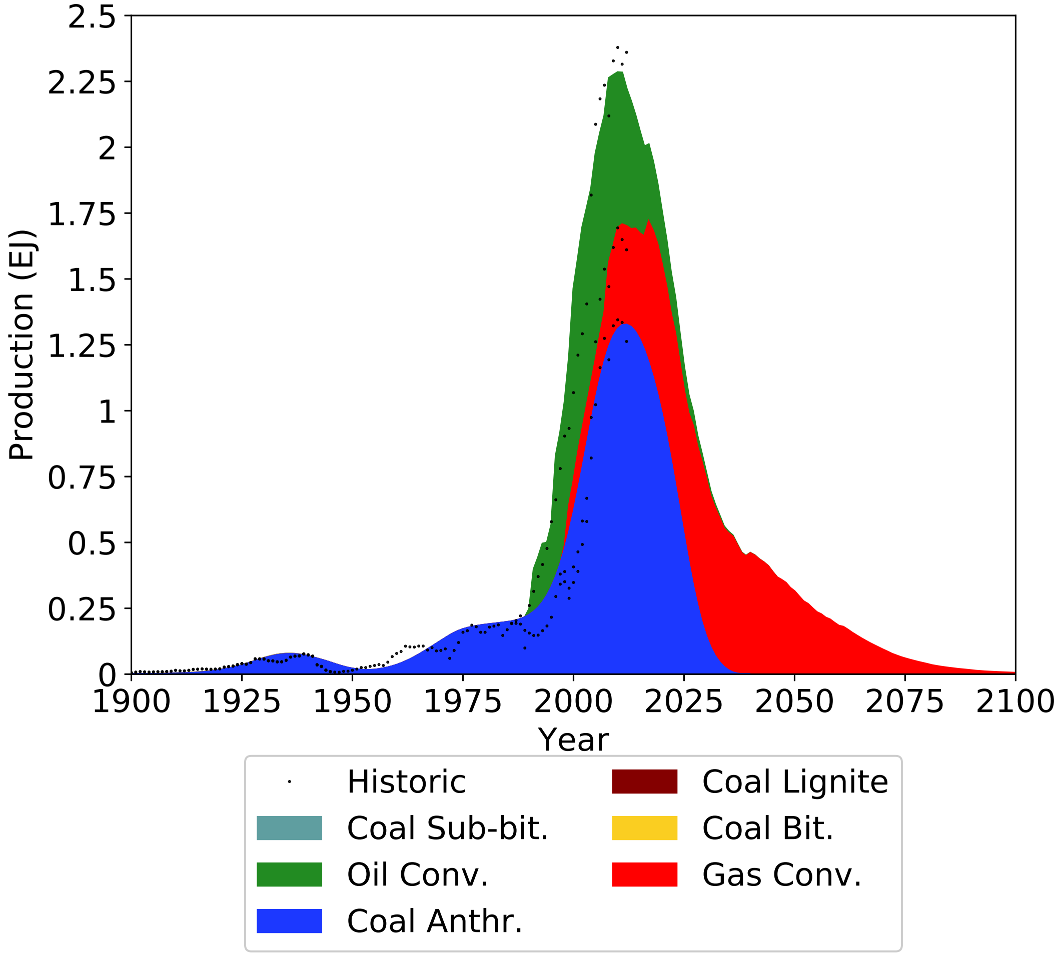

Figure 2.141: Vietnam projections capped at 16

Table 2.141: Peak years - All

**Name**	**URR**	**Peak Year**	**Peak Rate**
Coal Anthr.	38.0	2012	1.33
Gas Conv.	26.3	2029	0.61
Oil Conv.	15.76	2005	0.78
Coal Bit.	0.03	1914	–
Coal Sub-bit.	0.01	1931	–
Coal Lignite	–	1941	–
**Total**	**80.1**	**2010**	**2.28**

2.27.2 By Mineral

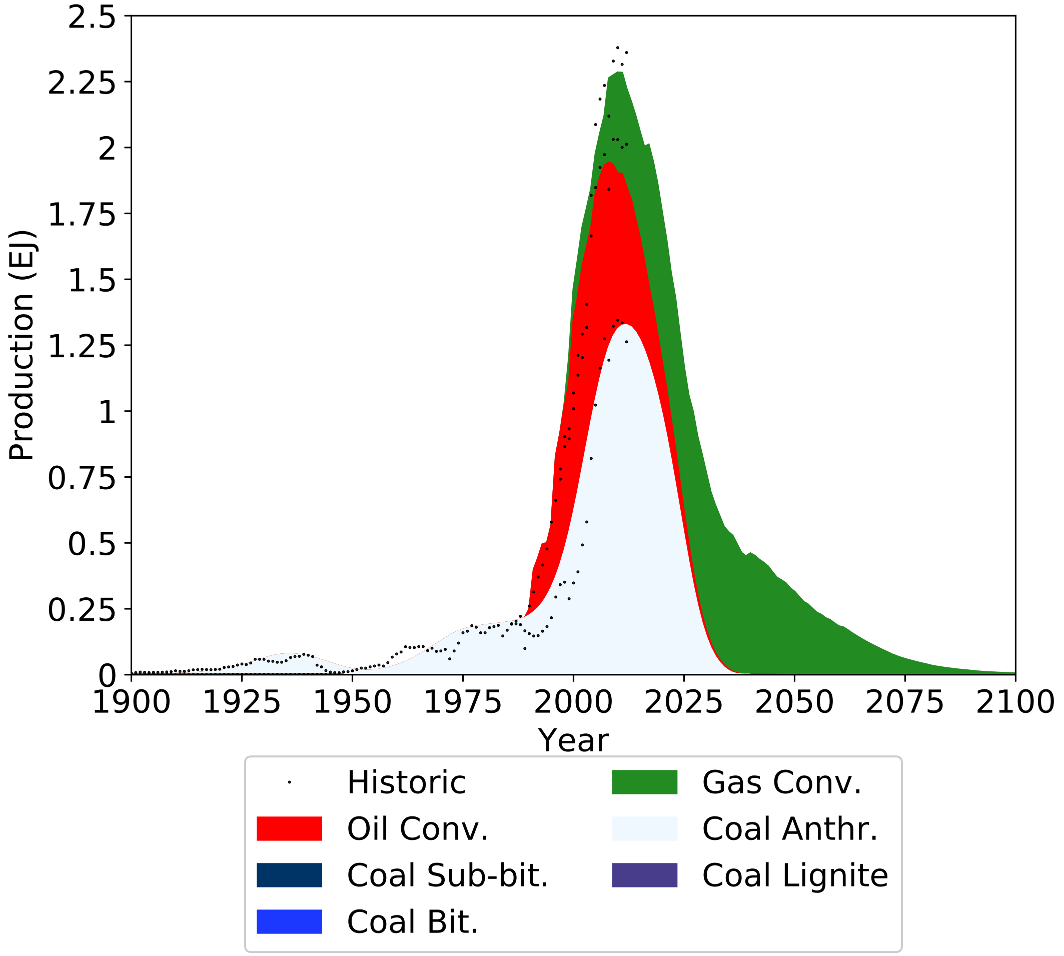

Figure 2.142: Vietnam projection by mineral type

Table 2.142: Peak years - Minerals

**Name**	**URR**	**Peak Year**	**Peak Rate**
Coal Bit.	0.03	1914	–
Coal Lignite	–	1941	–
Coal Sub-bit.	0.01	1931	–
Coal Anthr.	38.0	2012	1.33
Oil Conv.	15.76	2005	0.78
Gas Conv.	26.3	2029	0.61
**Total**	**80.1**	**2010**	**2.28**

2.28 Total

2.28.1 By country

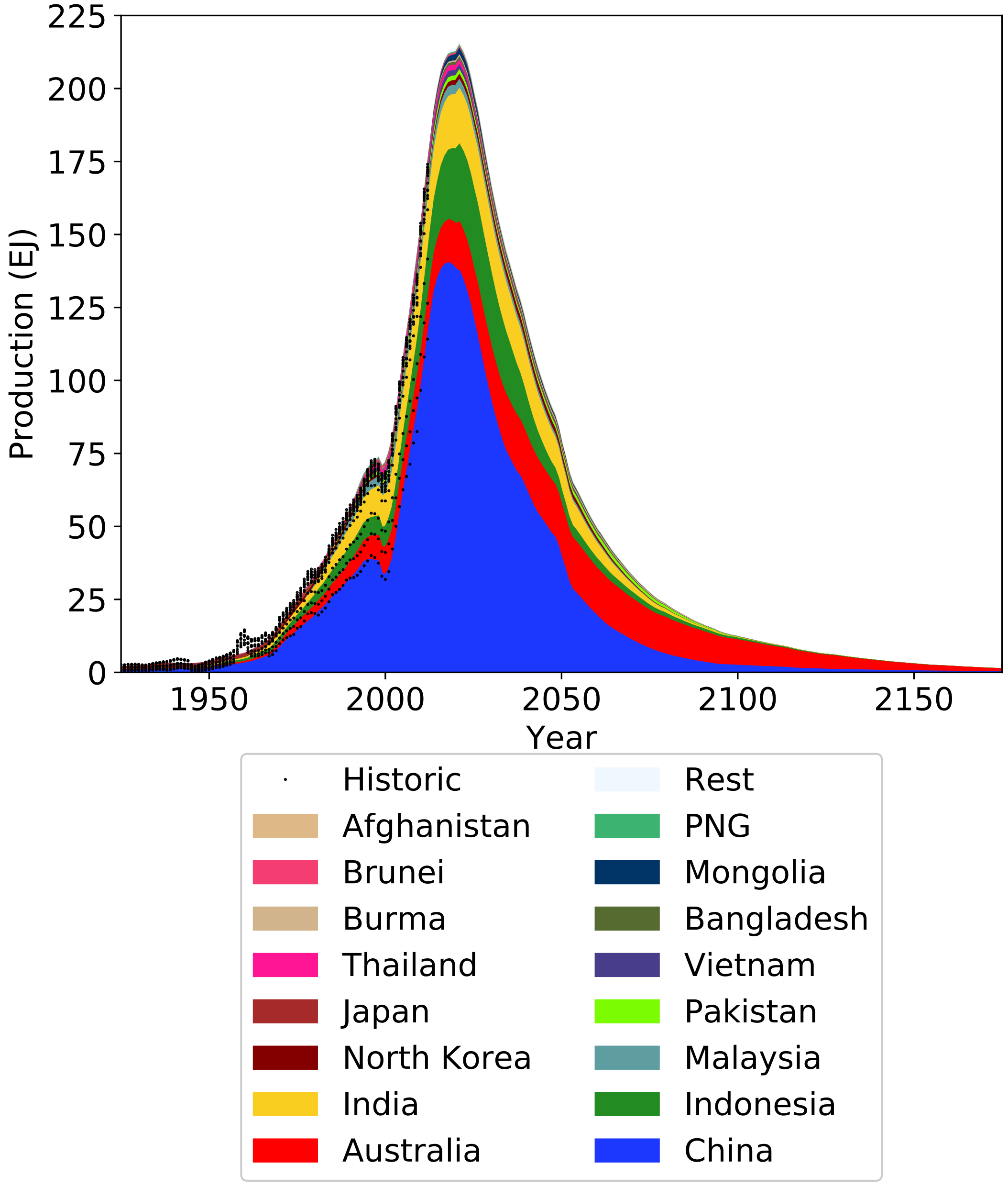

Figure 2.143: Asia projections by country

Table 2.143: Peak years - All

**Name**	**URR**	**Peak Year**	**Peak Rate**
China	5833.28	2018	140.16
Australia	1950.01	2034	19.47
Indonesia	1113.61	2024	27.4
India	1101.66	2023	19.13
Malaysia	186.52	2007	3.71
North Korea	146.97	2036	2.34
Pakistan	129.38	2012	1.69
Japan	82.91	1953	1.21
Vietnam	80.1	2010	2.28
Thailand	77.39	2010	2.34
Bangladesh	59.03	2028	1.02
Burma	56.72	2033	0.88
Mongolia	53.98	2022	2.3
Brunei	51.45	2002	0.89
PNG	28.27	1993	0.62
Afghanistan	24.68	2032	0.57
New Zealand	23.04	2009	0.39
South Korea	22.7	1982	0.69
Philippines	18.98	2022	0.5
Taiwan	6.83	1968	0.17
East Timor	4.11	2007	0.21
Cambodia	1.9	2024	0.08
Laos	1.39	2023	0.06
Sri Lanka	1.11	2032	0.03
Bhutan	0.15	2017	–
Nepal	0.01	2018	–
New Caledonia	–	1928	–
**Total**	**11056.18**	**2021**	**215.71**

2.28.2 By mineral

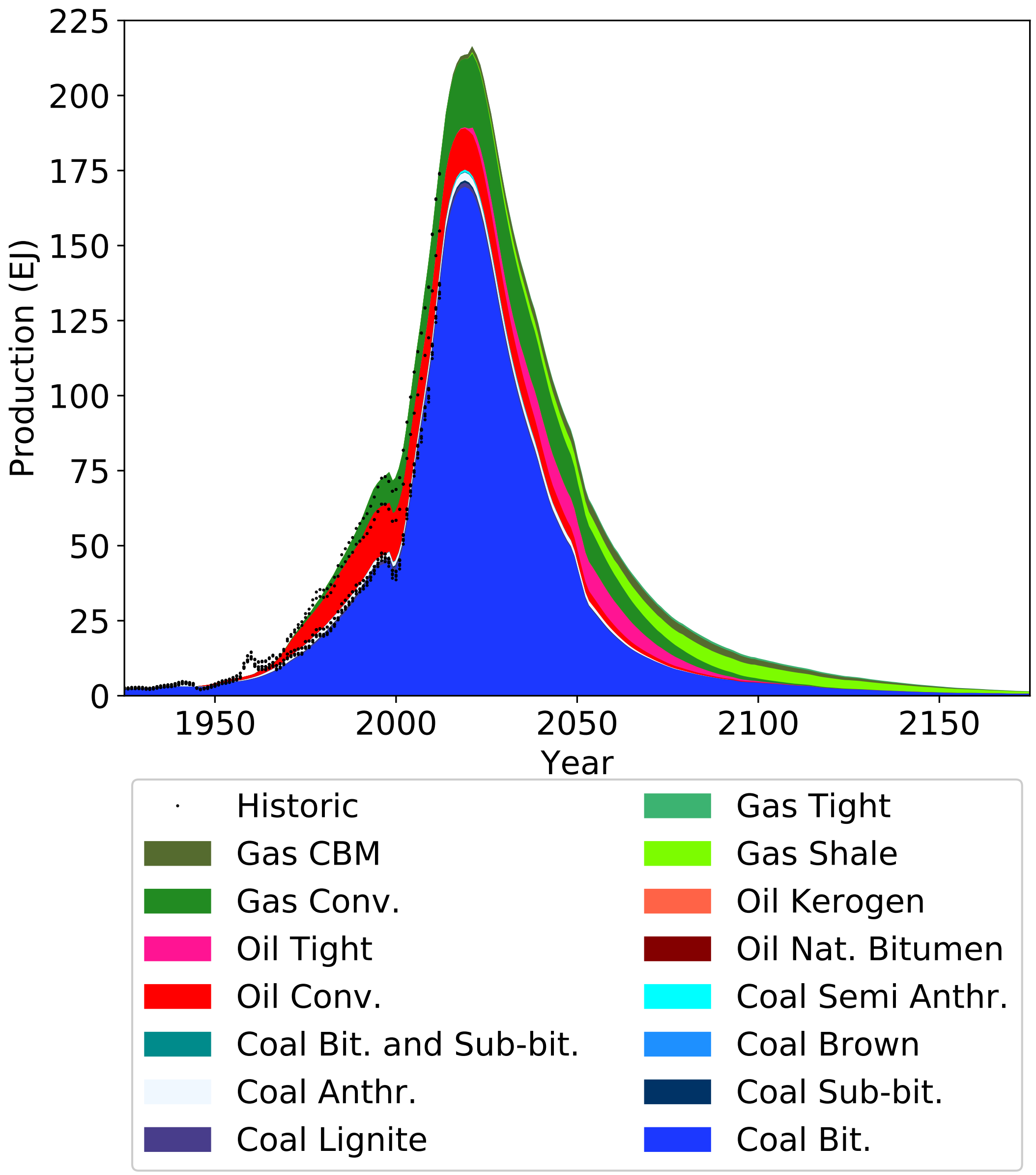

Figure 2.144: Asia projection by mineral type

Table 2.144: Peak years - Minerals

**Name**	**URR**	**Peak Year**	**Peak Rate**
Coal Bit.	6988.55	2019	169.19
Coal Lignite	84.51	2017	1.48
Coal Sub-bit.	34.15	2017	0.71
Coal Anthr.	199.34	2020	2.63
Coal Brown	2.2	1999	0.05
Coal Bit. and Sub-bit.	6.0	2020	0.22
Coal Semi Anthr.	22.82	2026	0.77
Oil Conv.	1029.45	2004	17.47
Oil Nat. Bitumen	0.17	1995	0.02
Oil Tight	422.88	2043	10.07
Oil Kerogen	5.77	2033	0.14
Gas Conv.	1380.9	2023	25.01
Gas Shale	508.72	2079	5.18
Gas CBM	304.5	2053	3.7
Gas Tight	66.24	2067	0.77
**Total**	**11056.18**	**2021**	**215.71**

Chapter 3

Europe

3.1 Albania

3.1.1 All Projections

Table 3.1: Peak years - All

Name	URR	Peak Year	Peak Rate
Oil Conv.	6.38	2018	0.13
Gas Conv.	1.4	2019	0.06
Coal Lignite	0.38	1983	0.02
Gas Shale	0.04	2032	–
**Total**	**8.2**	**2019**	**0.19**

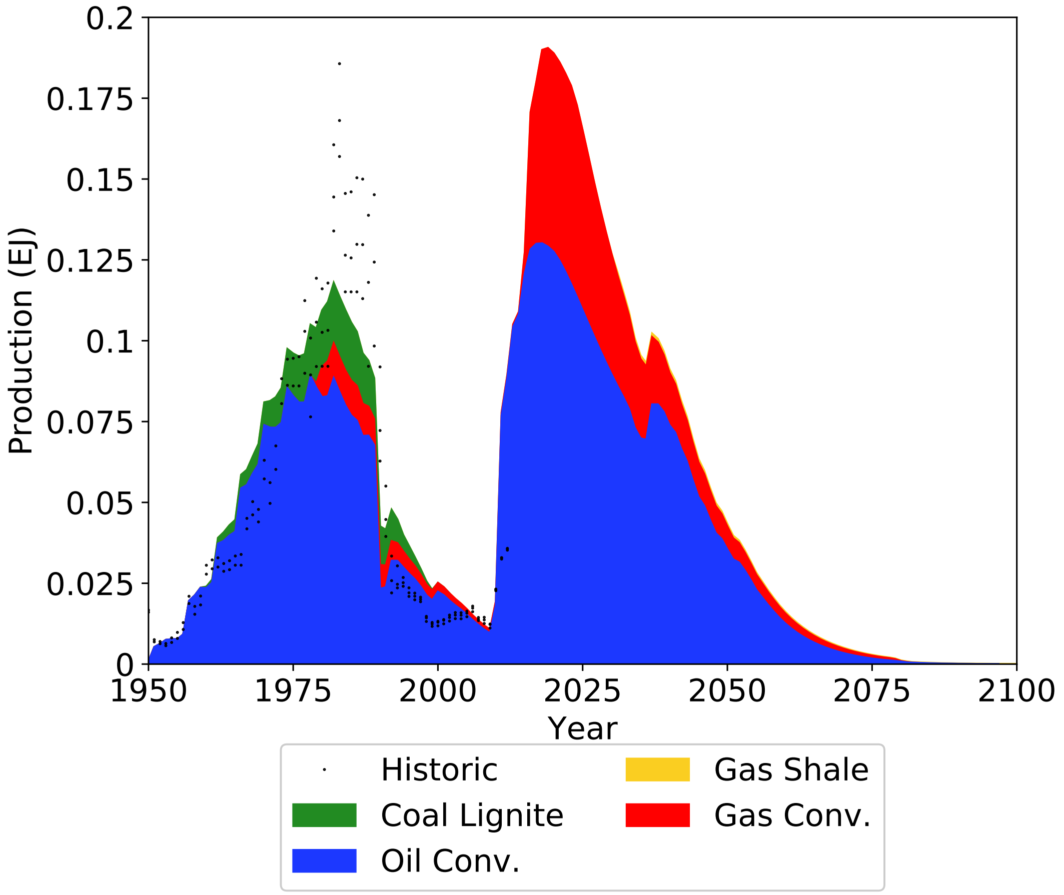

Figure 3.1: Albania projections capped at 16

3.1.2 By Mineral

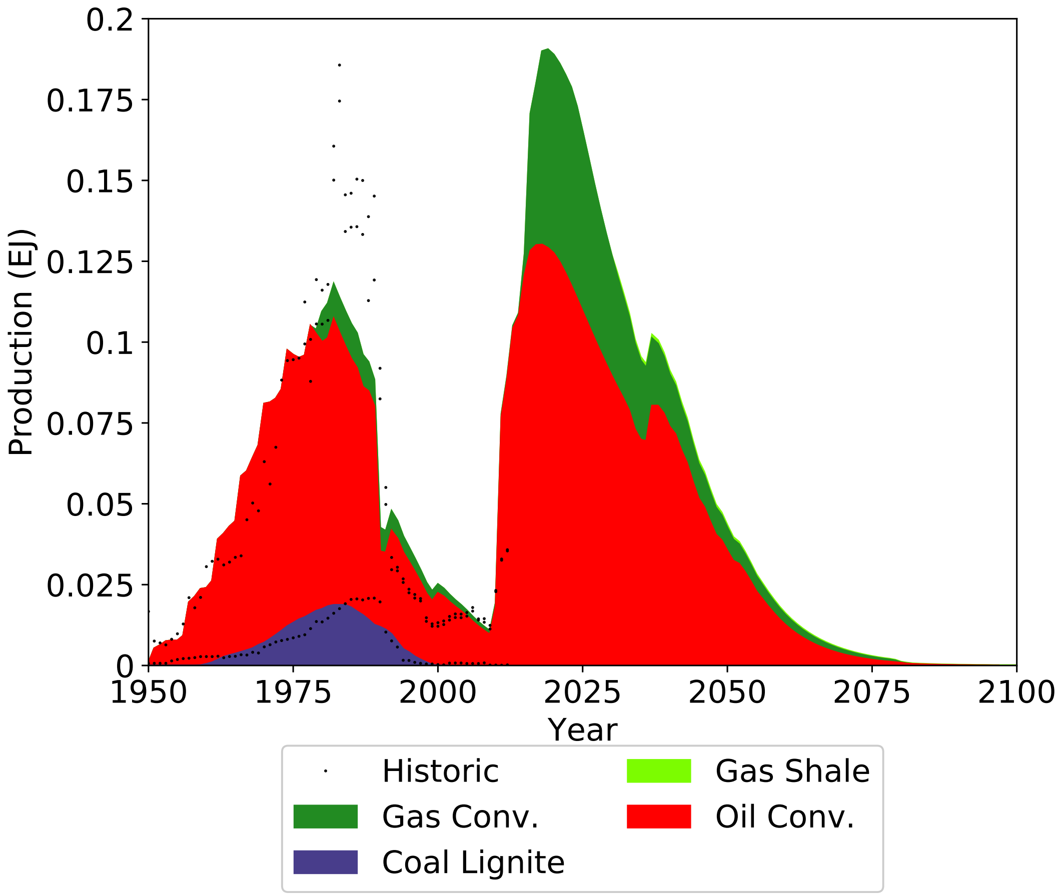

Figure 3.2: Albania projection by mineral type

Table 3.2: Peak years - Minerals

Name	URR	Peak Year	Peak Rate
Coal Lignite	0.38	1983	0.02
Oil Conv.	6.38	2018	0.13
Gas Conv.	1.4	2019	0.06
Gas Shale	0.04	2032	–
**Total**	**8.2**	**2019**	**0.19**

3.2 Austria

3.2.1 All Projections

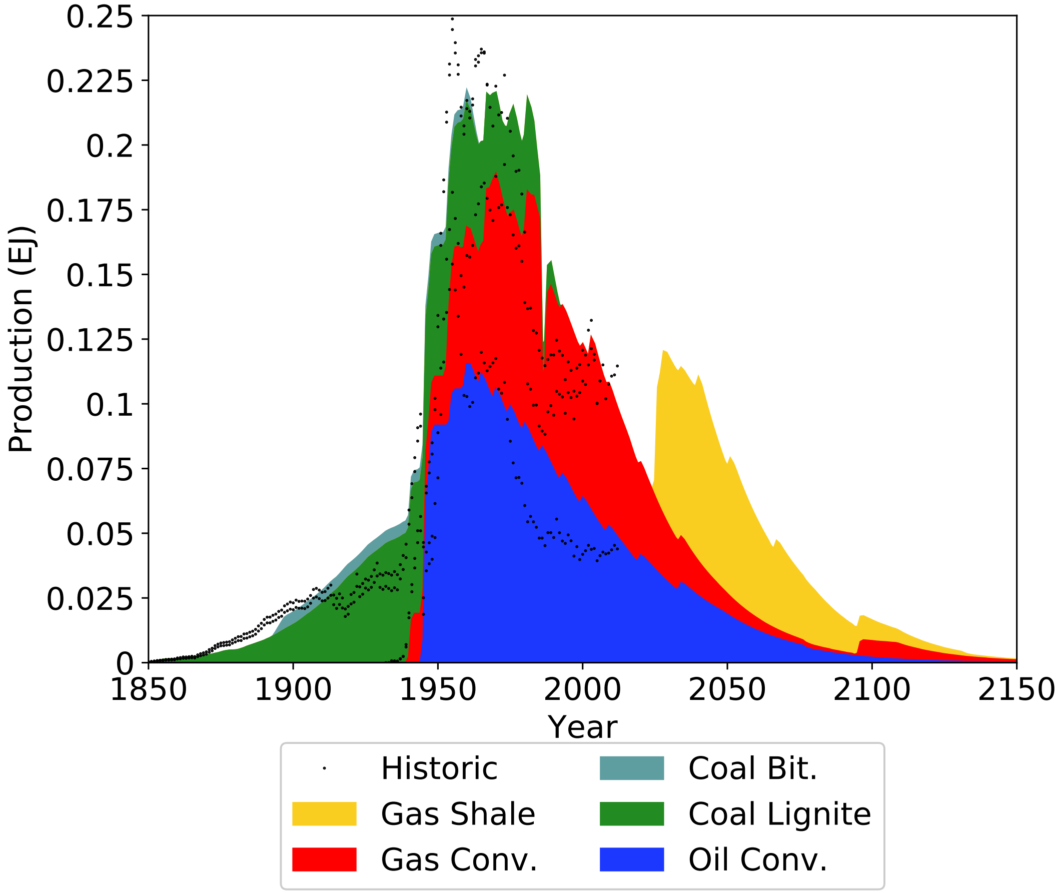

Figure 3.3: Austria projections capped at 16

Table 3.3: Peak years - All

**Name**	**URR**	**Peak Year**	**Peak Rate**
Oil Conv.	7.24	1960	0.12
Gas Conv.	5.3	1983	0.1
Coal Lignite	3.54	1944	0.05
Gas Shale	3.0	2040	0.07
Coal Bit.	0.32	1898	–
**Total**	**19.4**	**1960**	**0.22**

3.2.2 By Mineral

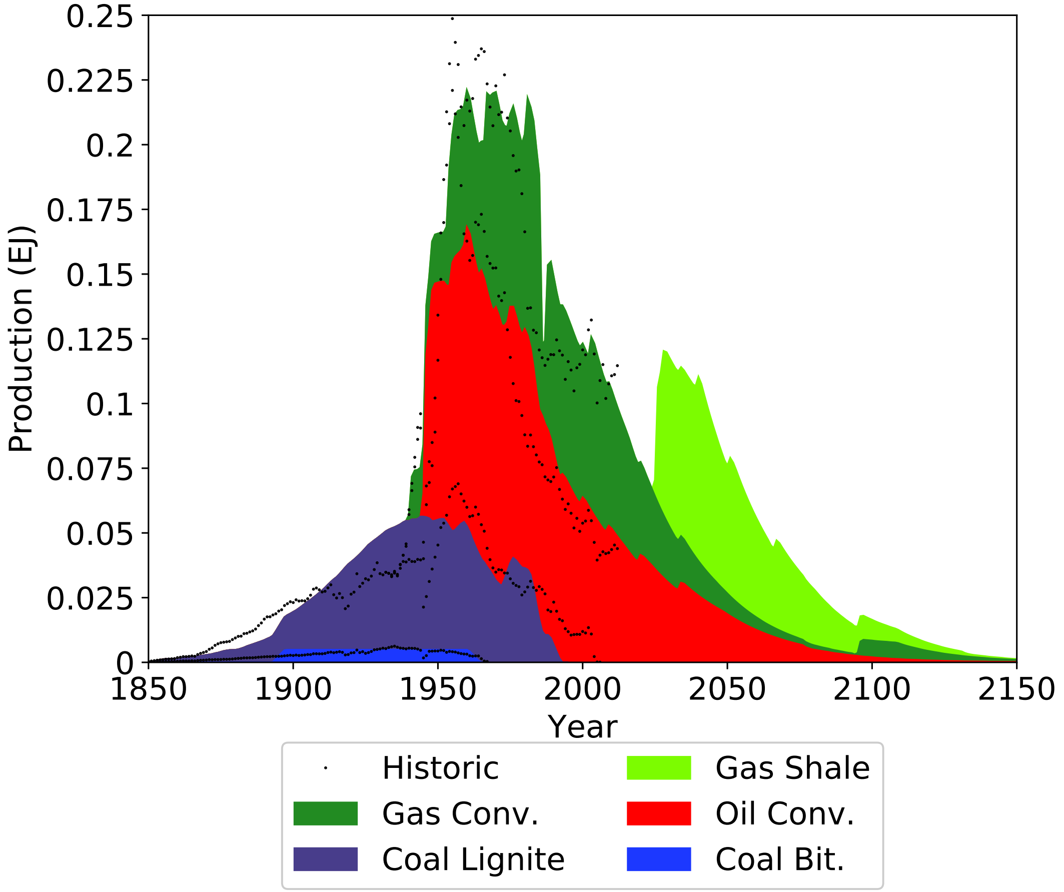

Figure 3.4: Austria projection by mineral type

Table 3.4: Peak years - Minerals

**Name**	**URR**	**Peak Year**	**Peak Rate**
Coal Bit.	0.32	1898	–
Coal Lignite	3.54	1944	0.05
Oil Conv.	7.24	1960	0.12
Gas Conv.	5.3	1983	0.1
Gas Shale	3.0	2040	0.07
**Total**	**19.4**	**1960**	**0.22**

3.3 Belgium

3.3.1 All Projections

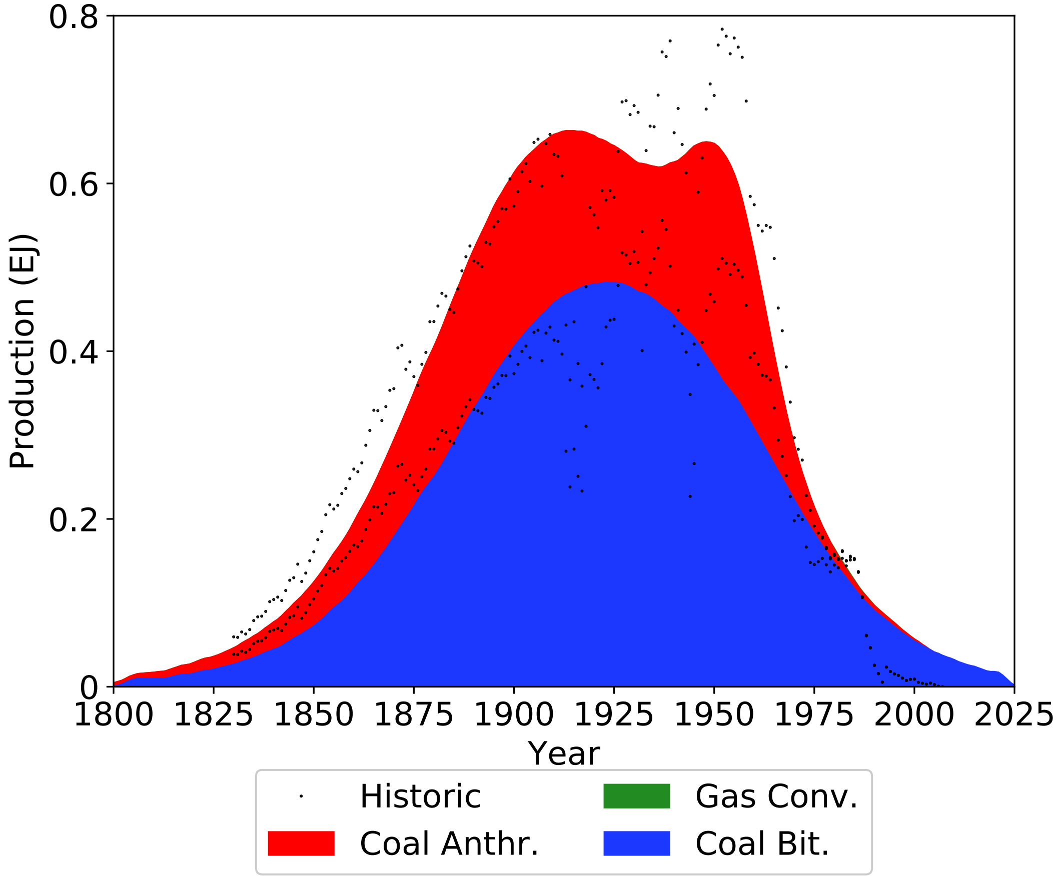

Figure 3.5: Belgium projections capped at 16

Table 3.5: Peak years - All

Name	URR	Peak Year	Peak Rate
Coal Bit.	45.5	1923	0.48
Coal Anthr.	21.6	1952	0.27
Gas Conv.	0.02	1982	–
**Total**	**67.12**	**1913**	**0.66**

3.3.2 By Mineral

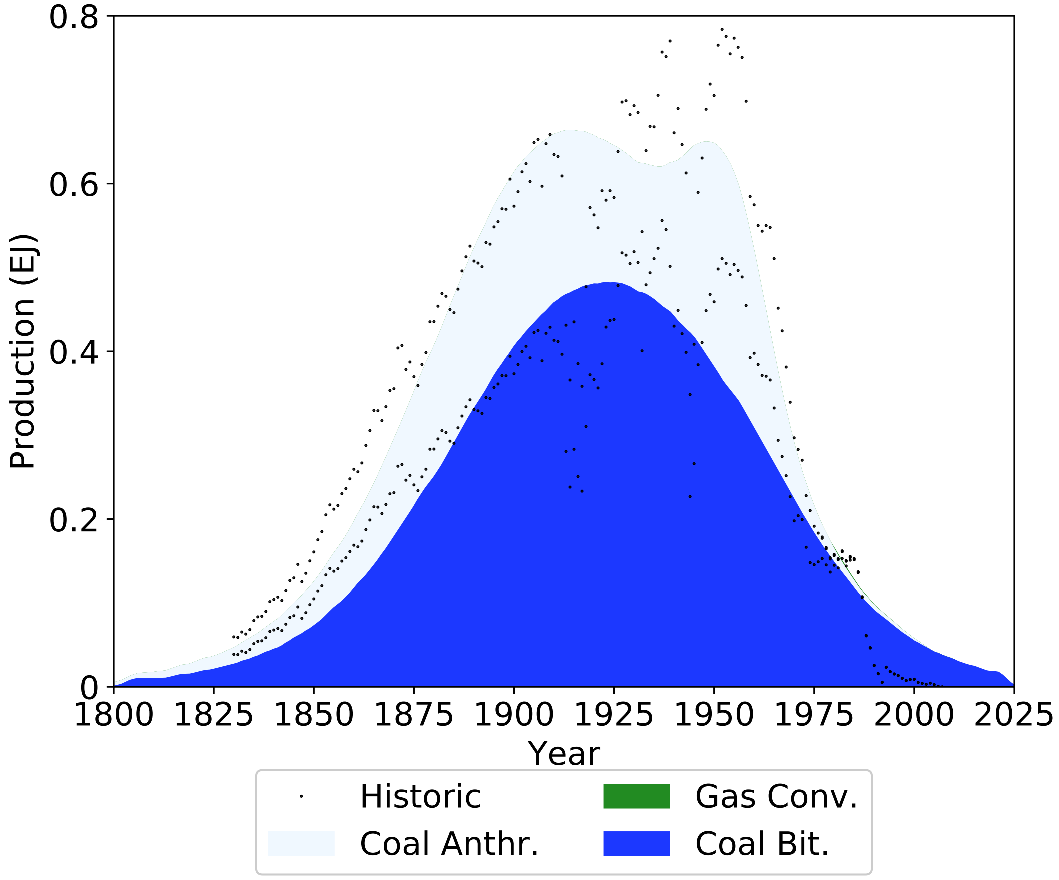

Figure 3.6: Belgium projection by mineral type

Table 3.6: Peak years - Minerals

**Name**	**URR**	**Peak Year**	**Peak Rate**
Coal Bit.	45.5	1923	0.48
Coal Anthr.	21.6	1952	0.27
Gas Conv.	0.02	1982	–
**Total**	**67.12**	**1913**	**0.66**

3.4 Bulgaria

3.4.1 All Projections

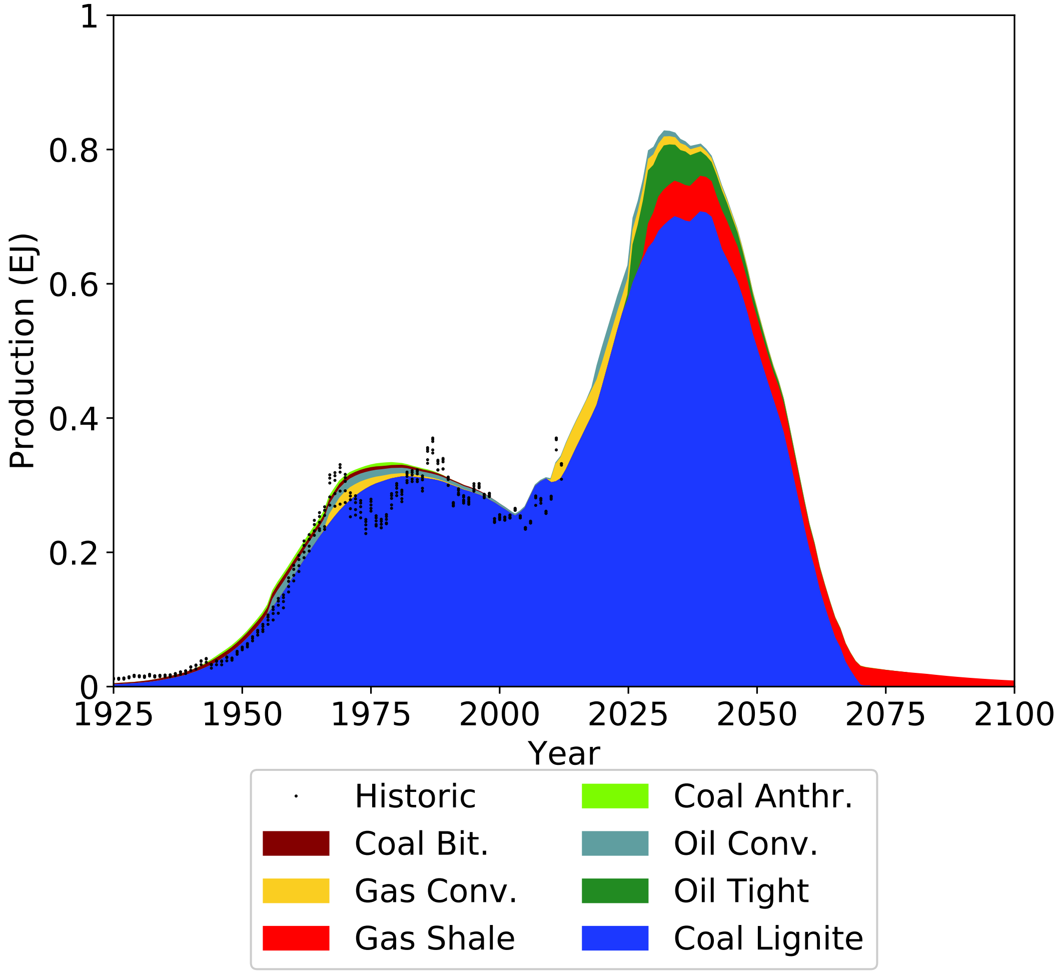

Figure 3.7: Bulgaria projections capped at 16

Table 3.7: Peak years - All

Name	URR	Peak Year	Peak Rate
Coal Lignite	42.3	2039	0.71
Gas Shale	2.43	2043	0.06
Oil Tight	1.15	2028	0.08
Gas Conv.	1.0	2014	0.04
Oil Conv.	0.8	2020	0.02
Coal Bit.	0.35	1953	0.01
Coal Anthr.	0.18	1964	0.01
**Total**	**48.2**	**2032**	**0.83**

3.4.2 By Mineral

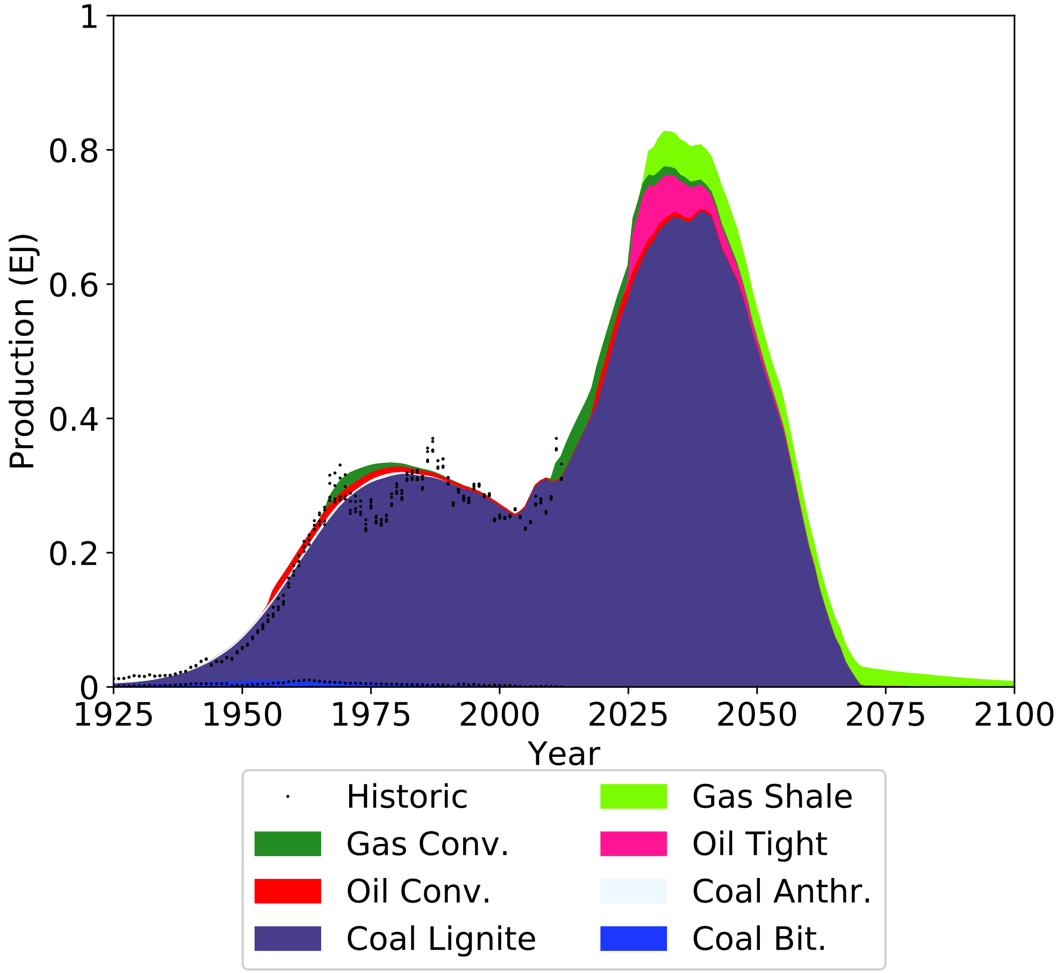

Figure 3.8: Bulgaria projection by mineral type

Table 3.8: Peak years - Minerals

**Name**	**URR**	**Peak Year**	**Peak Rate**
Coal Bit.	0.35	1953	0.01
Coal Lignite	42.3	2039	0.71
Coal Anthr.	0.18	1964	0.01
Oil Conv.	0.8	2020	0.02
Oil Tight	1.15	2028	0.08
Gas Conv.	1.0	2014	0.04
Gas Shale	2.43	2043	0.06
**Total**	**48.2**	**2032**	**0.83**

3.5 Cyprus

3.5.1 All Projections

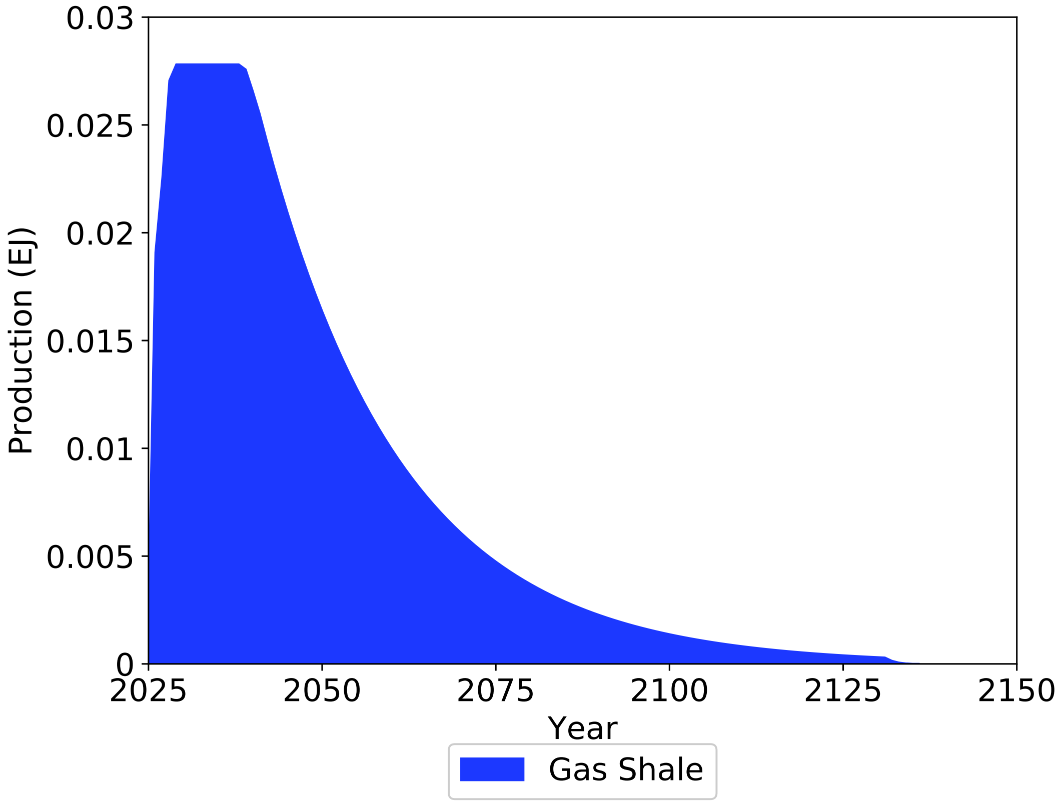

Figure 3.9: Cyprus projections capped at 16

Table 3.9: Peak years - All

Name	URR	Peak Year	Peak Rate
Gas Shale	0.93	2029	0.03
**Total**	**0.93**	**2029**	**0.03**

3.5.2 By Mineral

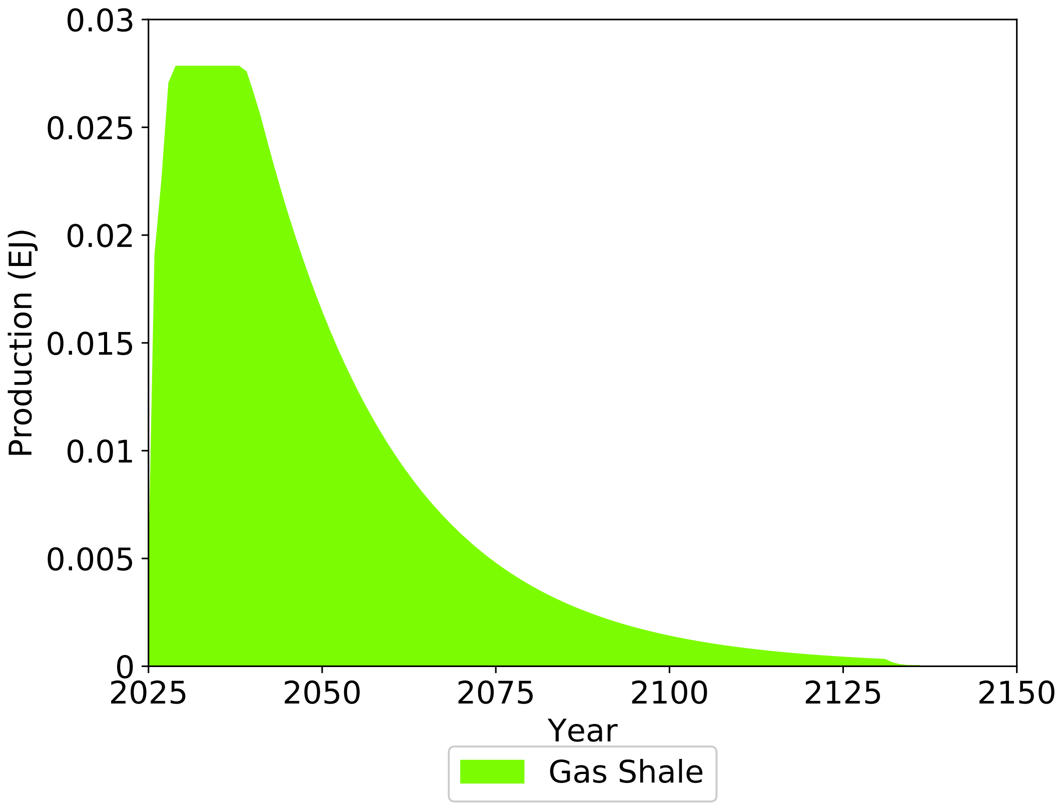

Figure 3.10: Cyprus projection by mineral type

Table 3.10: Peak years - Minerals

**Name**	**URR**	**Peak Year**	**Peak Rate**
Gas Shale	0.93	2029	0.03
**Total**	**0.93**	**2029**	**0.03**

3.6 Czech Republic

3.6.1 All Projections

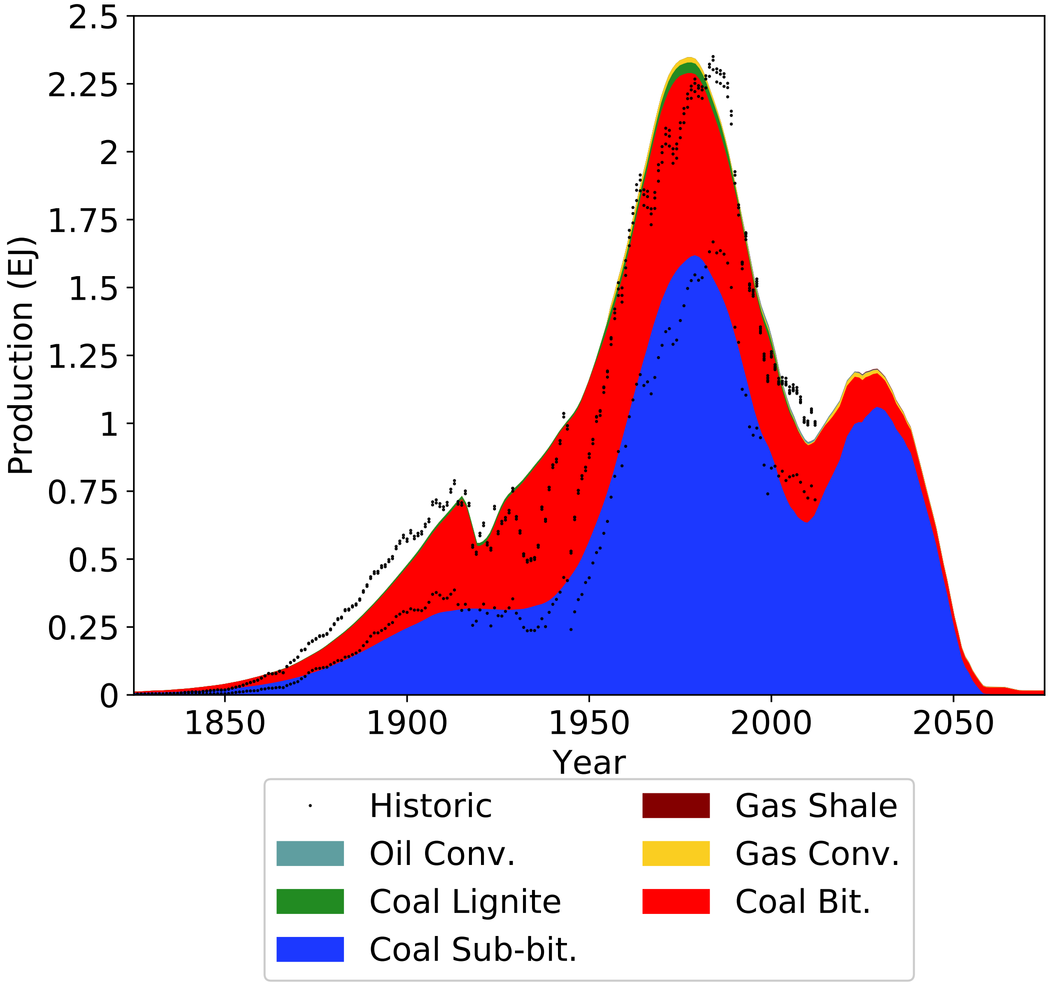

Figure 3.11: Czech Republic projections capped at 16

Table 3.11: Peak years - All

**Name**	**URR**	**Peak Year**	**Peak Rate**
Coal Sub-bit.	123.0	1979	1.61
Coal Bit.	64.86	1973	0.71
Coal Lignite	2.04	1973	0.04
Gas Conv.	1.3	1968	0.03
Oil Conv.	0.27	1999	0.01
Gas Shale	0.07	2025	–
**Total**	**191.54**	**1977**	**2.34**

3.6.2 By Mineral

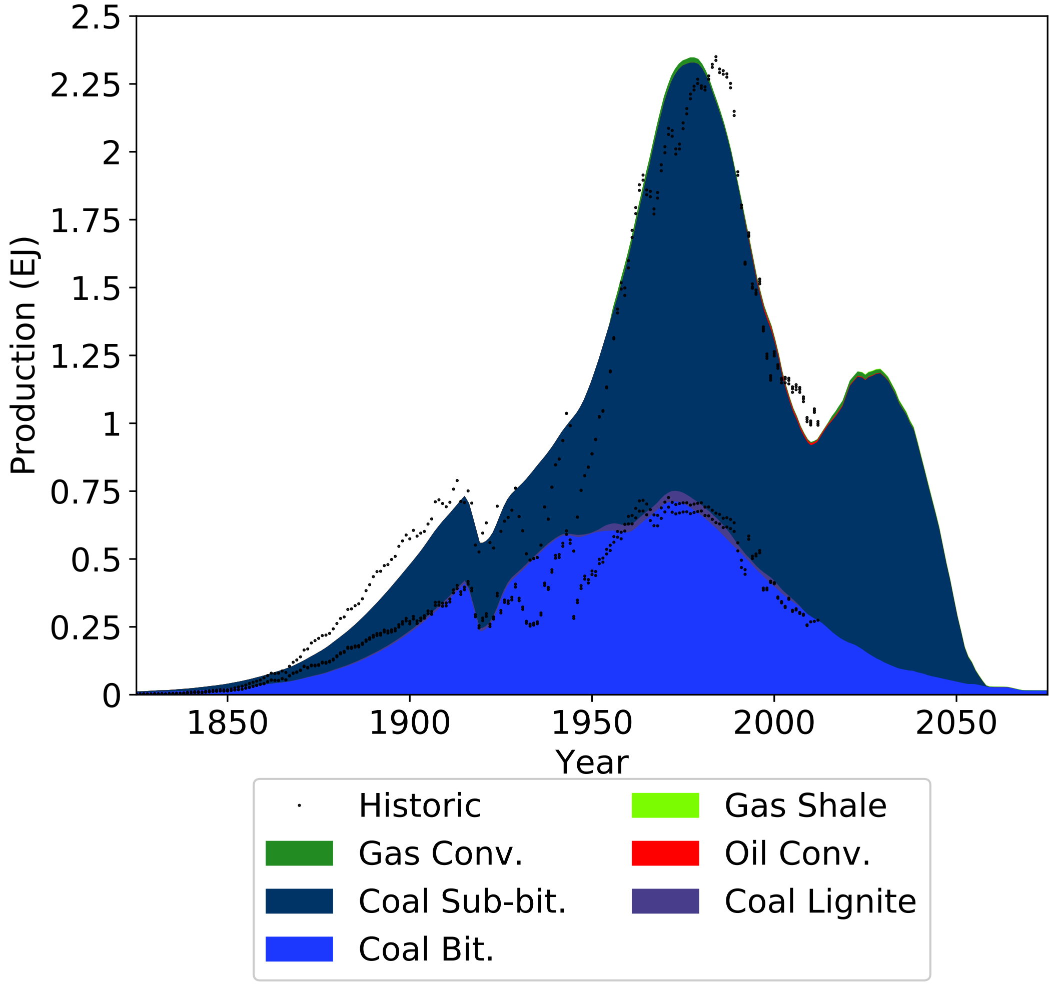

Figure 3.12: Czech Republic projection by mineral type

Table 3.12: Peak years - Minerals

**Name**	**URR**	**Peak Year**	**Peak Rate**
Coal Bit.	64.86	1973	0.71
Coal Lignite	2.04	1973	0.04
Coal Sub-bit.	123.0	1979	1.61
Oil Conv.	0.27	1999	0.01
Gas Conv.	1.3	1968	0.03
Gas Shale	0.07	2025	–
**Total**	**191.54**	**1977**	**2.34**

3.7 Denmark

3.7.1 All Projections

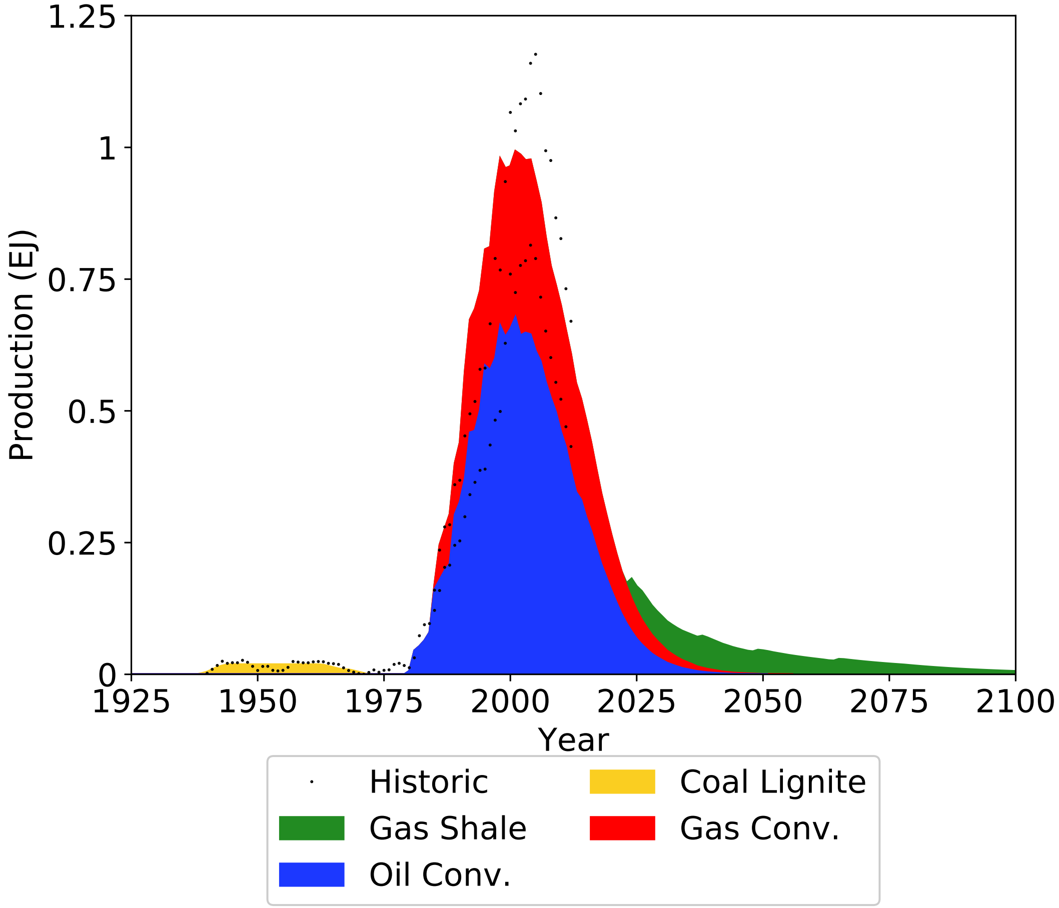

Figure 3.13: Denmark projections capped at 16

Table 3.13: Peak years - All

**Name**	**URR**	**Peak Year**	**Peak Rate**
Oil Conv.	16.68	2001	0.68
Gas Conv.	8.4	2002	0.34
Gas Shale	2.56	2038	0.06
Coal Lignite	0.5	1945	0.02
**Total**	**28.14**	**2001**	**0.99**

3.7.2 By Mineral

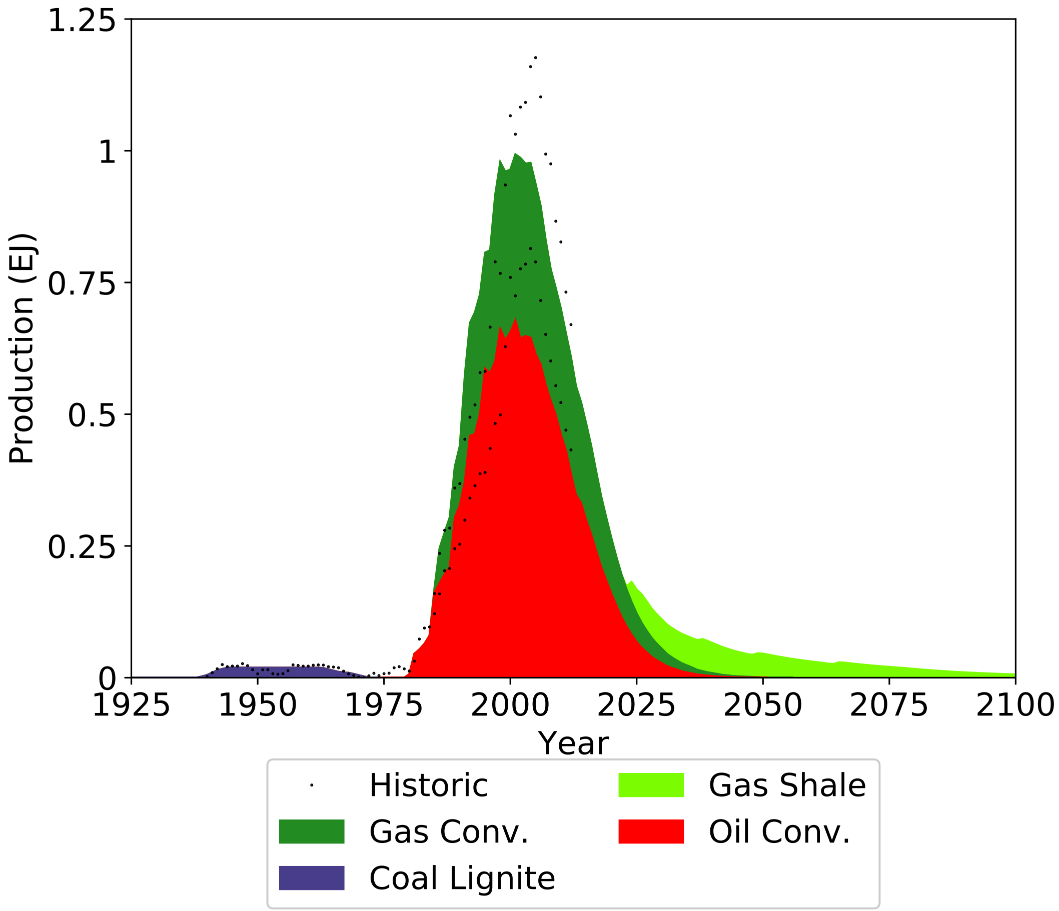

Figure 3.14: Denmark projection by mineral type

Table 3.14: Peak years - Minerals

**Name**	**URR**	**Peak Year**	**Peak Rate**
Coal Lignite	0.5	1945	0.02
Oil Conv.	16.68	2001	0.68
Gas Conv.	8.4	2002	0.34
Gas Shale	2.56	2038	0.06
**Total**	**28.14**	**2001**	**0.99**

3.8 France

3.8.1 All Projections

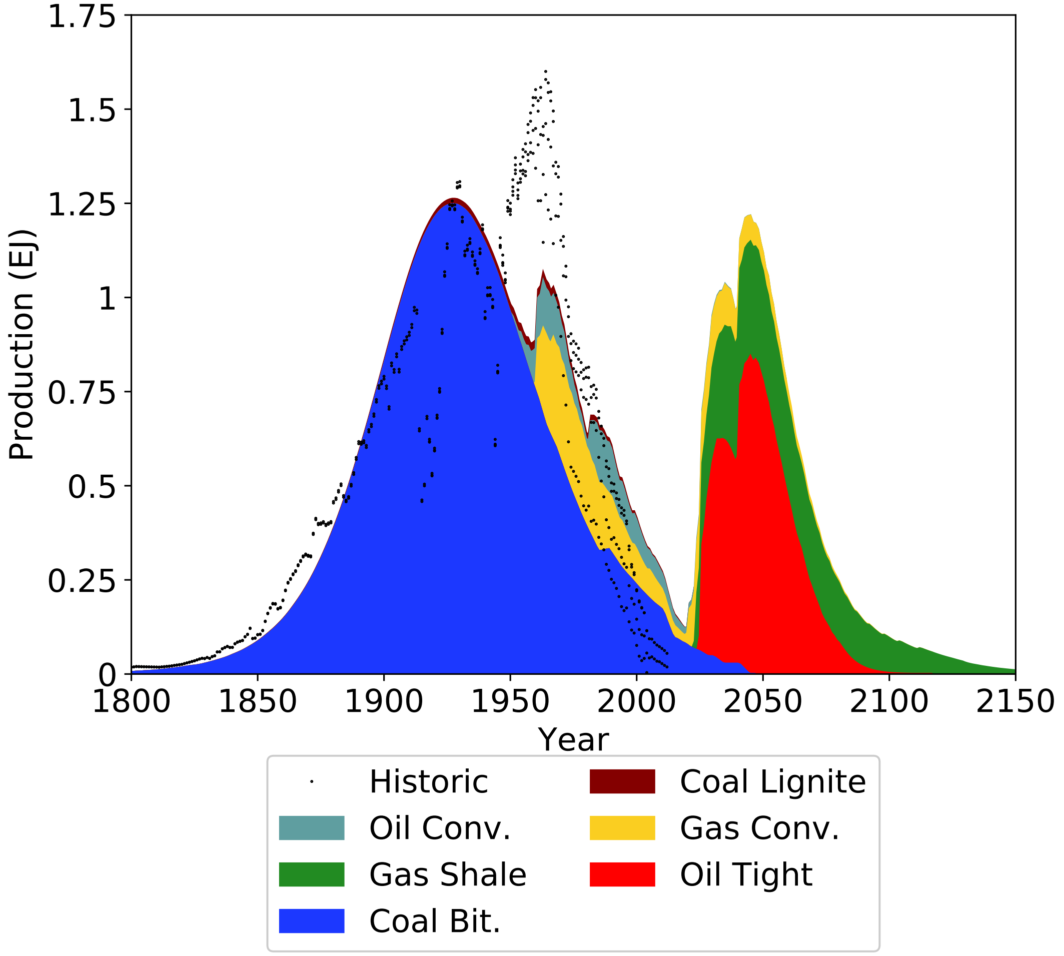

Figure 3.15: France projections capped at 16

Table 3.15: Peak years - All

**Name**	**URR**	**Peak Year**	**Peak Rate**
Coal Bit.	105.4	1927	1.25
Oil Tight	26.93	2045	0.84
Gas Shale	18.9	2037	0.32
Gas Conv.	12.6	1969	0.28
Oil Conv.	5.12	1985	0.14
Coal Lignite	1.8	1959	0.02
**Total**	**170.75**	**1927**	**1.26**

3.8.2 By Mineral

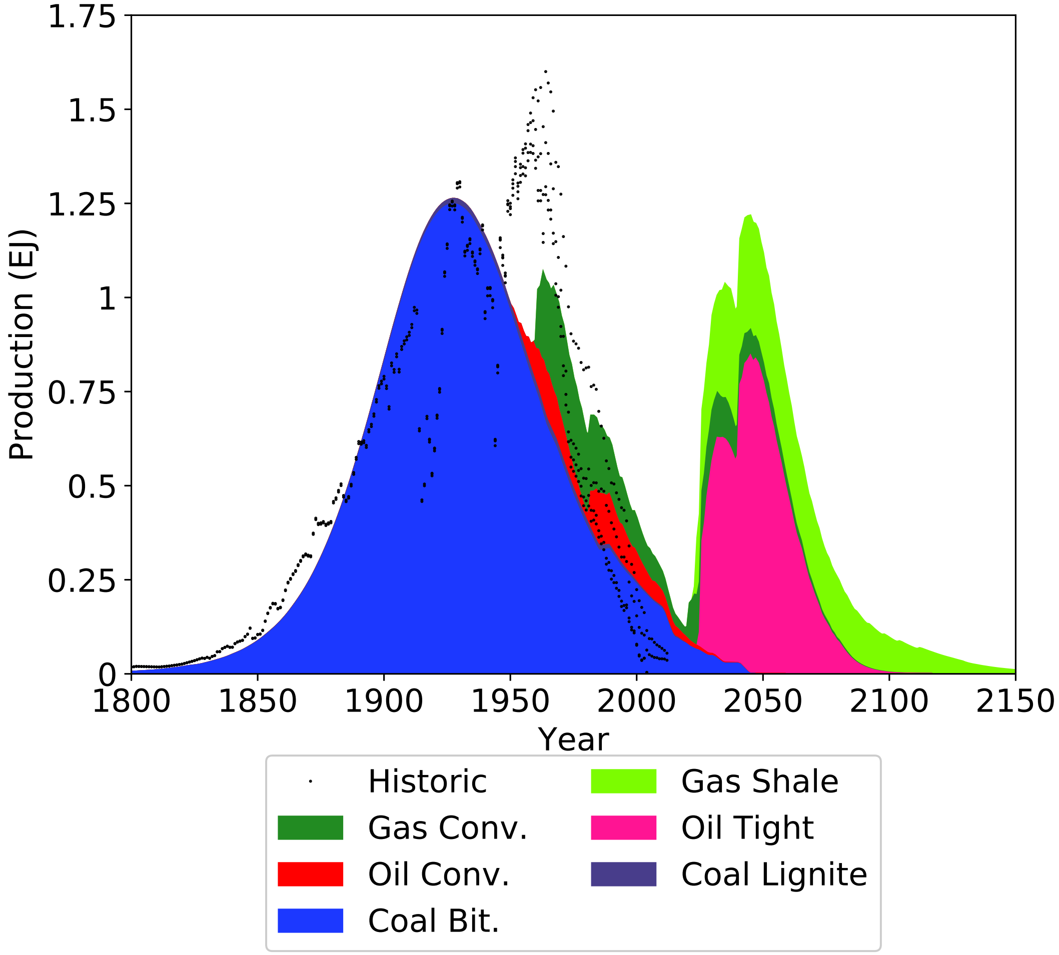

Figure 3.16: France projection by mineral type

Table 3.16: Peak years - Minerals

**Name**	**URR**	**Peak Year**	**Peak Rate**
Coal Bit.	105.4	1927	1.25
Coal Lignite	1.8	1959	0.02
Oil Conv.	5.12	1985	0.14
Oil Tight	26.93	2045	0.84
Gas Conv.	12.6	1969	0.28
Gas Shale	18.9	2037	0.32
**Total**	**170.75**	**1927**	**1.26**

3.9 Germany

3.9.1 All Projections

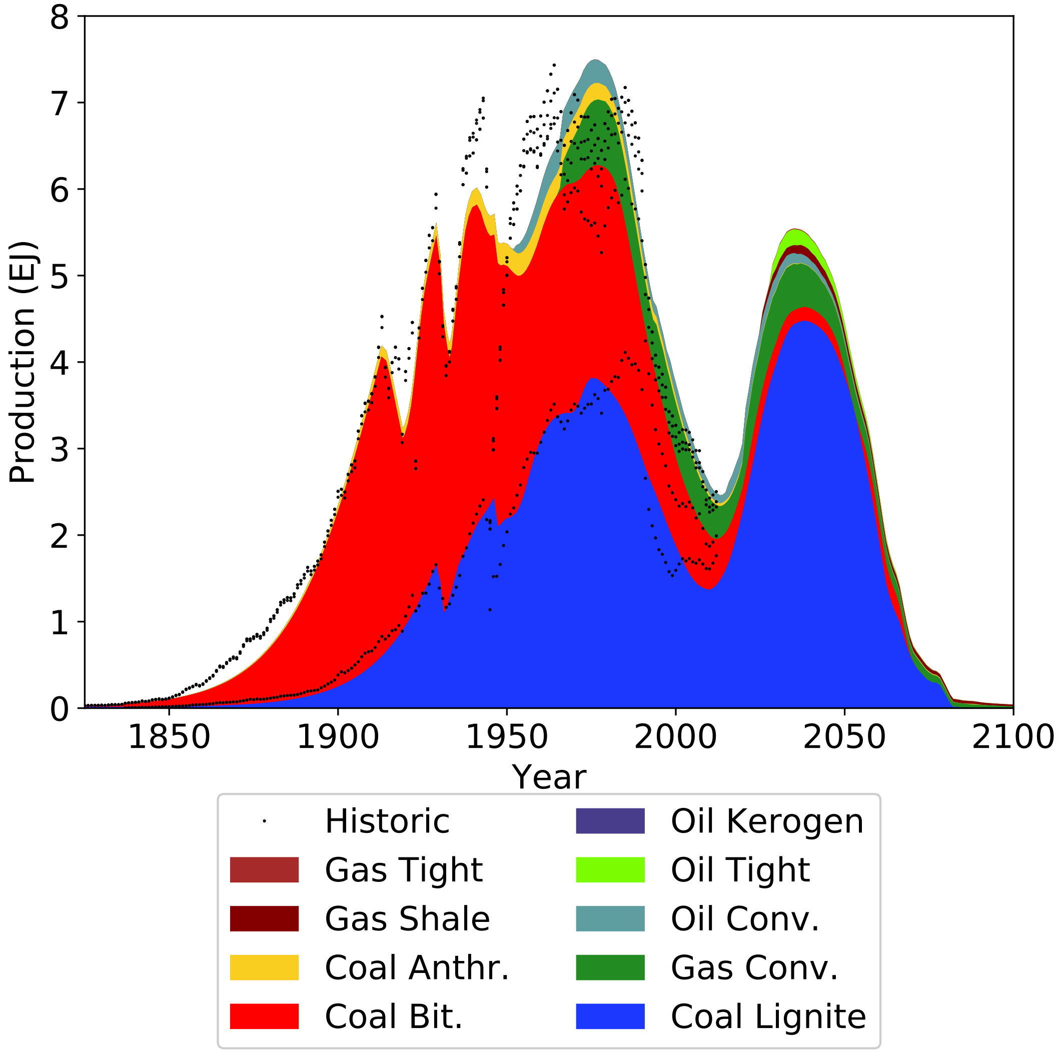

Figure 3.17: Germany projections capped at 16

Table 3.17: Peak years - All

Name	URR	Peak Year	Peak Rate
Coal Lignite	408.5	2038	4.47
Coal Bit.	318.3	1940	3.78
Gas Conv.	50.4	1979	0.76
Coal Anthr.	19.0	1956	0.26
Oil Conv.	17.53	1966	0.33
Gas Shale	4.82	2037	0.1
Oil Tight	4.01	2035	0.18
Gas Tight	0.37	2029	0.01
Oil Kerogen	0.07	1970	–
**Total**	**823.0**	**1976**	**7.49**

3.9.2 By Mineral

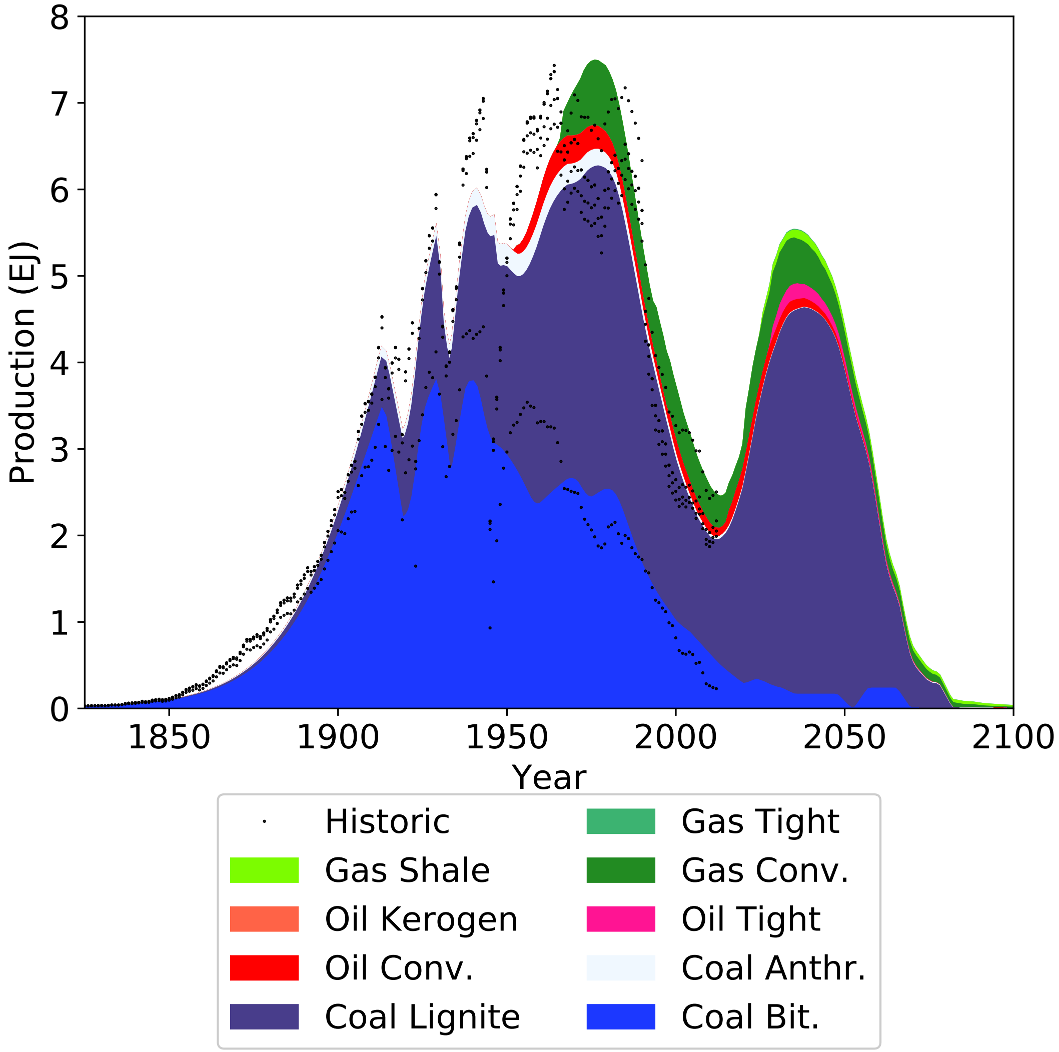

Figure 3.18: Germany projection by mineral type

Table 3.18: Peak years - Minerals

**Name**	**URR**	**Peak Year**	**Peak Rate**
Coal Bit.	318.3	1940	3.78
Coal Lignite	408.5	2038	4.47
Coal Anthr.	19.0	1956	0.26
Oil Conv.	17.53	1966	0.33
Oil Tight	4.01	2035	0.18
Oil Kerogen	0.07	1970	–
Gas Conv.	50.4	1979	0.76
Gas Shale	4.82	2037	0.1
Gas Tight	0.37	2029	0.01
**Total**	**823.0**	**1976**	**7.49**

3.10 Greece

3.10.1 All Projections

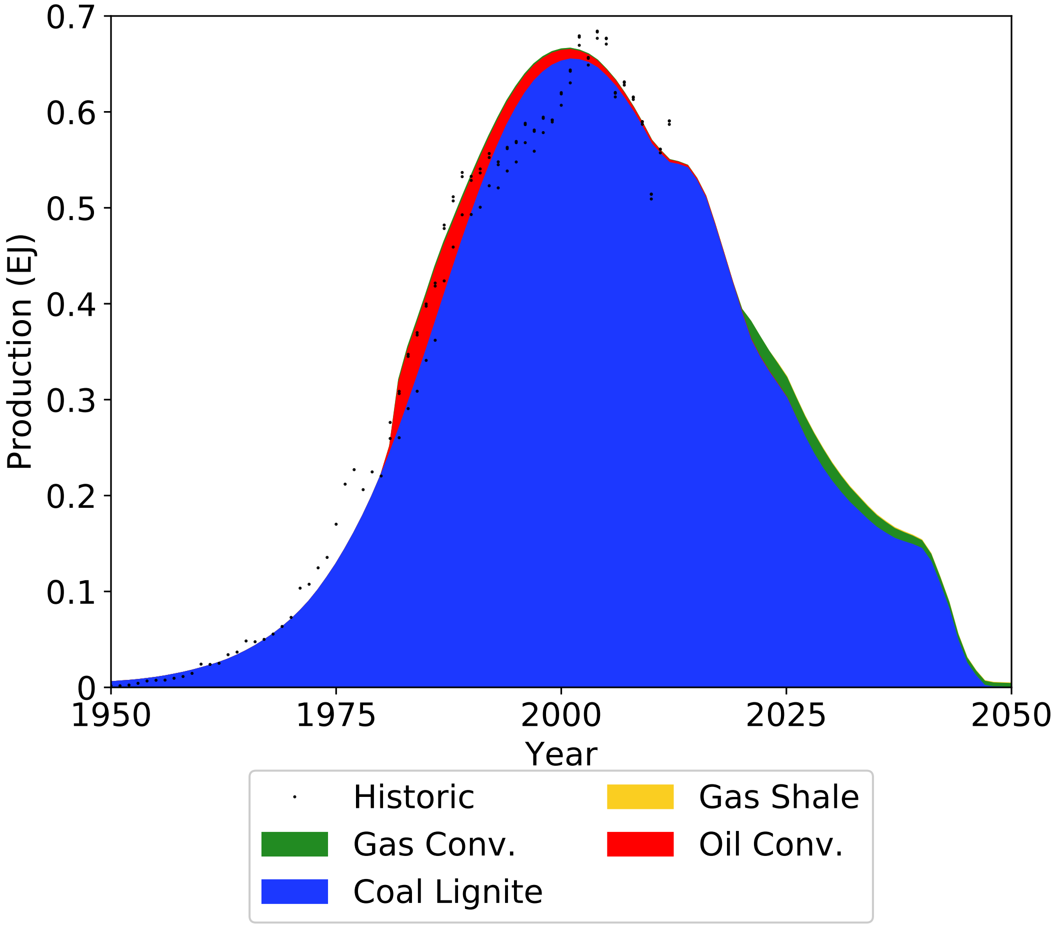

Figure 3.19: Greece projections capped at 16

Table 3.19: Peak years - All

**Name**	**URR**	**Peak Year**	**Peak Rate**
Coal Lignite	27.85	2001	0.65
Oil Conv.	0.74	1983	0.06
Gas Conv.	0.48	2022	0.02
Gas Shale	0.04	2025	–
**Total**	**29.11**	**2001**	**0.67**

3.10.2 By Mineral

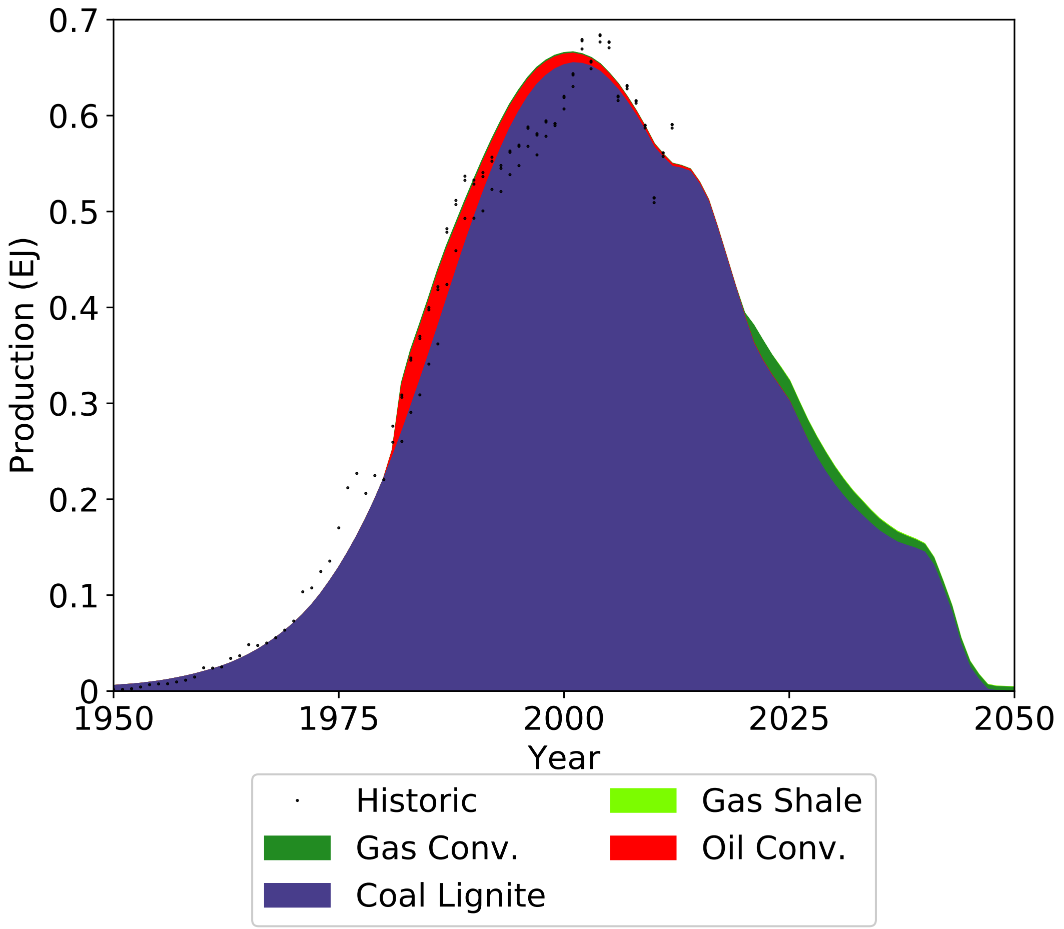

Figure 3.20: Greece projection by mineral type

Table 3.20: Peak years - Minerals

**Name**	**URR**	**Peak Year**	**Peak Rate**
Coal Lignite	27.85	2001	0.65
Oil Conv.	0.74	1983	0.06
Gas Conv.	0.48	2022	0.02
Gas Shale	0.04	2025	–
**Total**	**29.11**	**2001**	**0.67**

3.11 Greenland

3.11.1 All Projections

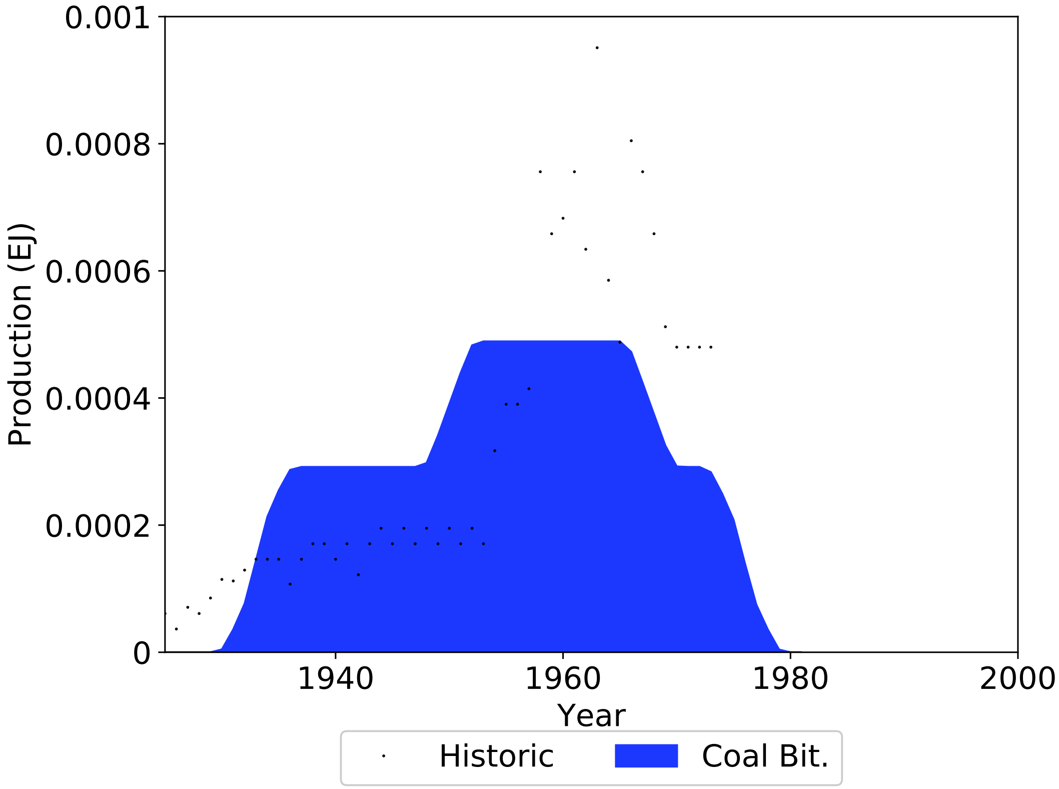

Figure 3.21: Greenland projections capped at 16

Table 3.21: Peak years - All

Name	URR	Peak Year	Peak Rate
Coal Bit.	0.02	1953	–
**Total**	**0.02**	**1953**	–

3.11.2 By Mineral

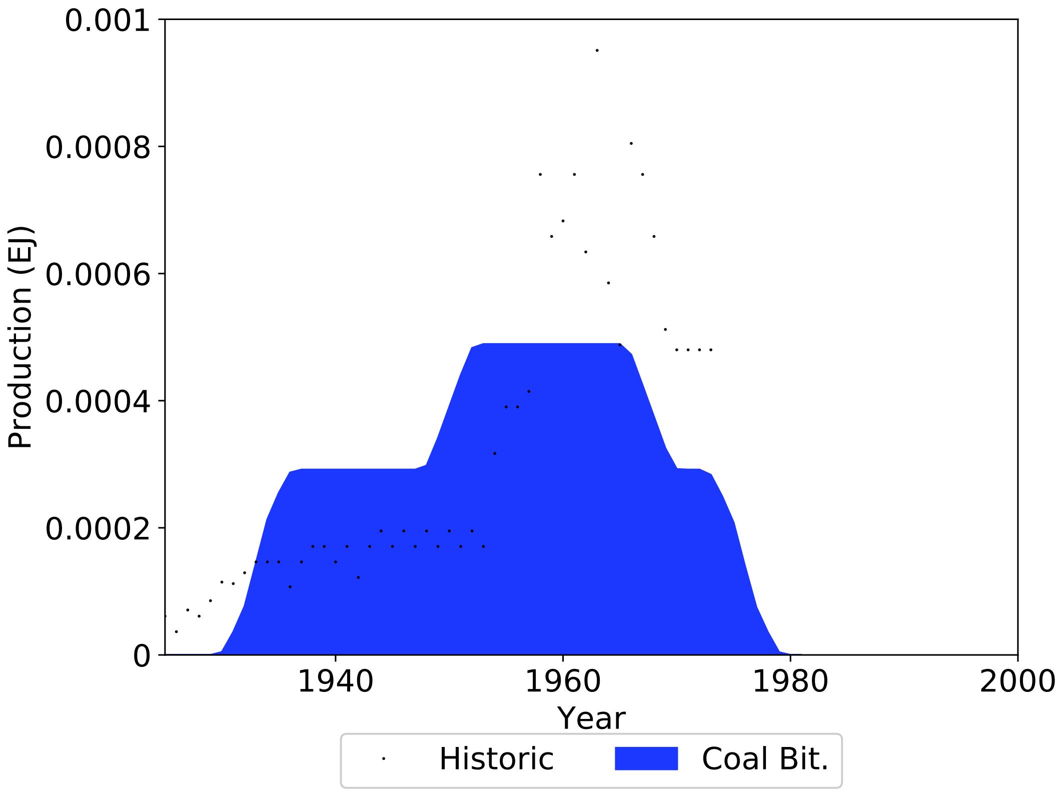

Figure 3.22: Greenland projection by mineral type

Table 3.22: Peak years - Minerals

Name	URR	Peak Year	Peak Rate
Coal Bit.	0.02	1953	–
**Total**	**0.02**	**1953**	–

3.12 Hungary

3.12.1 All Projections

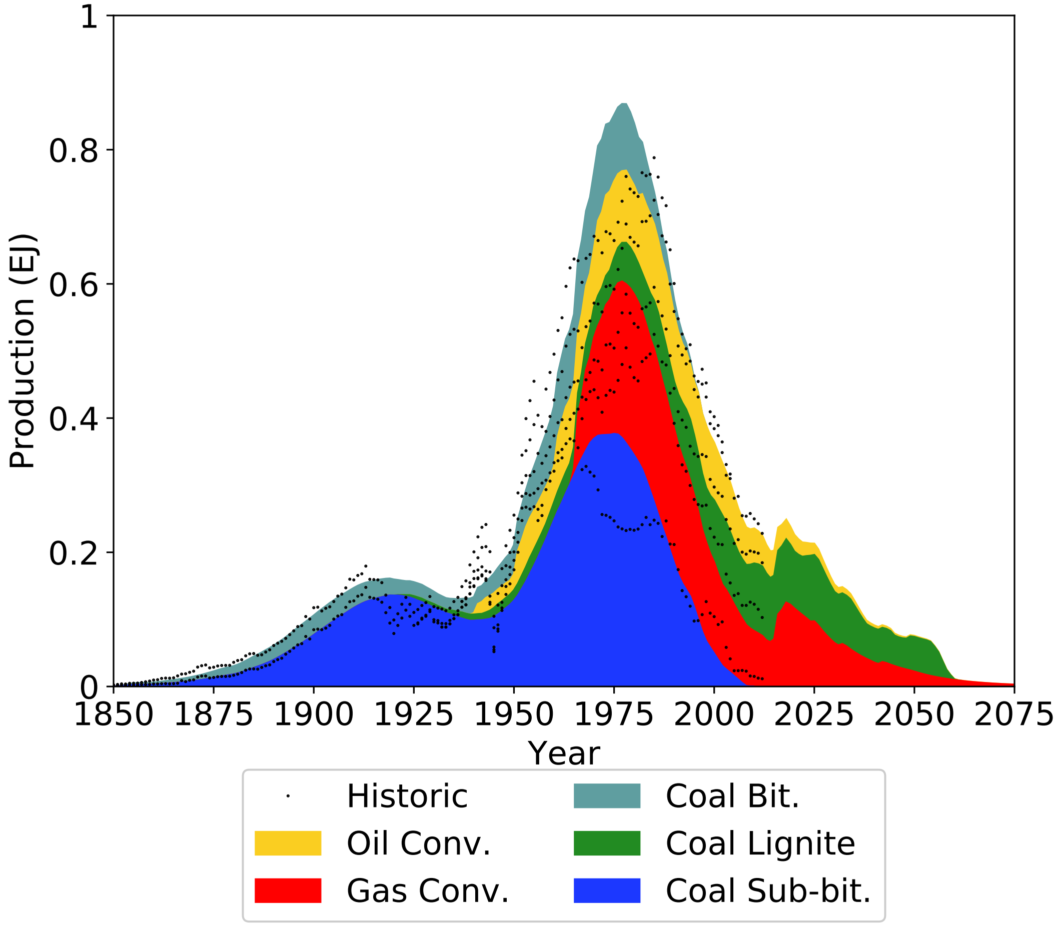

Figure 3.23: Hungary projections capped at 16

Table 3.23: Peak years - All

**Name**	**URR**	**Peak Year**	**Peak Rate**
Coal Sub-bit.	19.4	1975	0.38
Gas Conv.	10.5	1980	0.24
Coal Lignite	7.15	2012	0.1
Oil Conv.	6.04	1973	0.12
Coal Bit.	5.2	1970	0.11
**Total**	**48.29**	**1977**	**0.87**

3.12.2 By Mineral

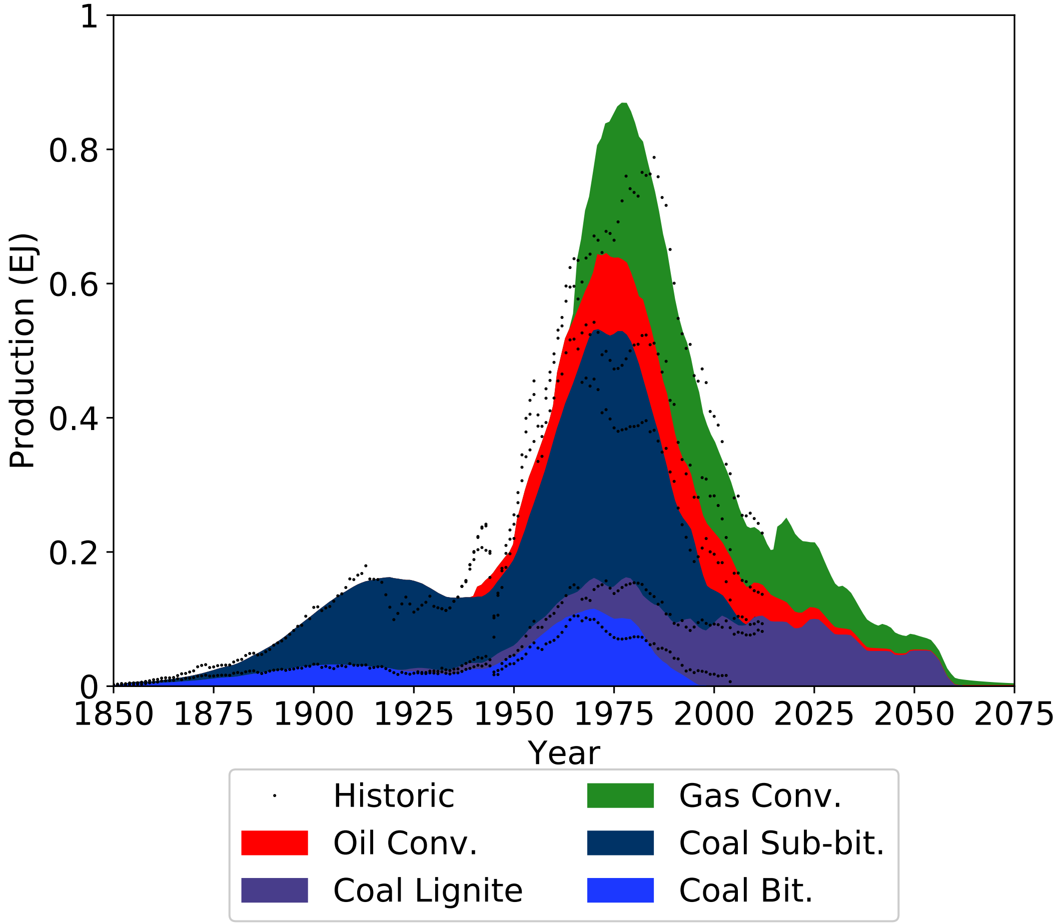

Figure 3.24: Hungary projection by mineral type

Table 3.24: Peak years - Minerals

**Name**	**URR**	**Peak Year**	**Peak Rate**
Coal Bit.	5.2	1970	0.11
Coal Lignite	7.15	2012	0.1
Coal Sub-bit.	19.4	1975	0.38
Oil Conv.	6.04	1973	0.12
Gas Conv.	10.5	1980	0.24
**Total**	**48.29**	**1977**	**0.87**

3.13 Ireland

3.13.1 All Projections

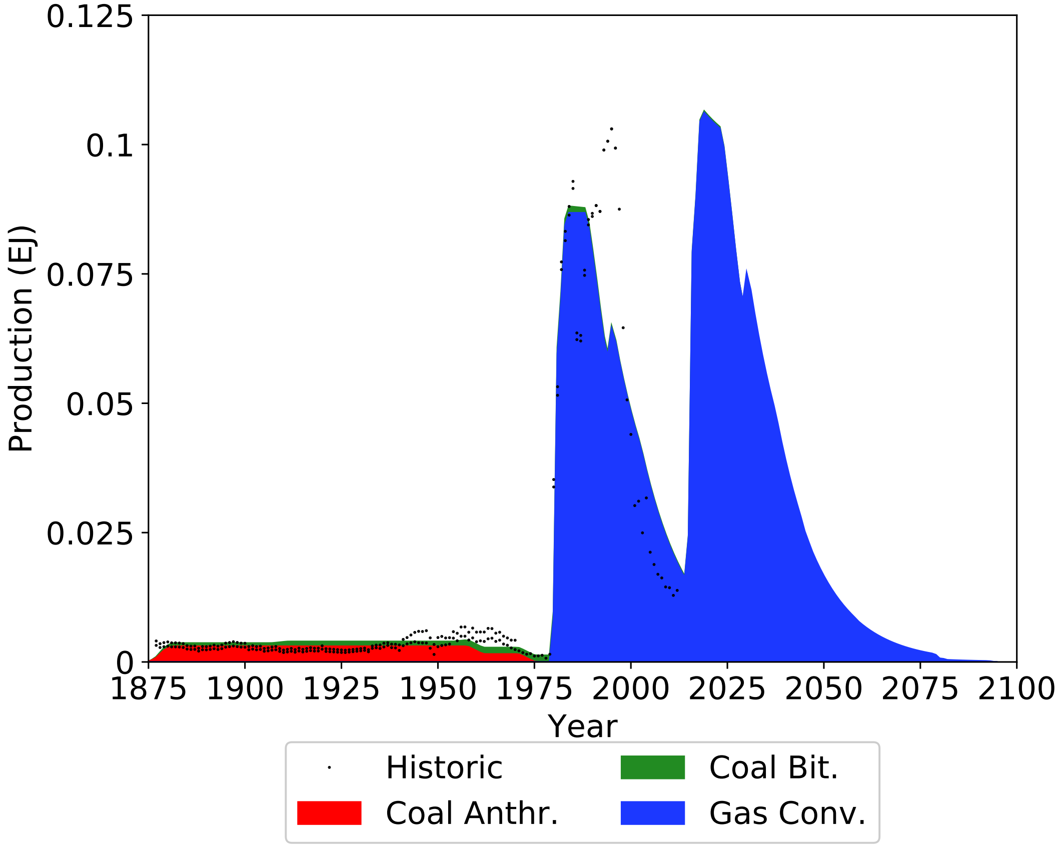

Figure 3.25: Ireland projections capped at 16

Table 3.25: Peak years - All

Name	URR	Peak Year	Peak Rate
Gas Conv.	4.2	2019	0.11
Coal Anthr.	0.27	1882	–
Coal Bit.	0.12	1959	–
**Total**	**4.58**	**2019**	**0.11**

3.13.2 By Mineral

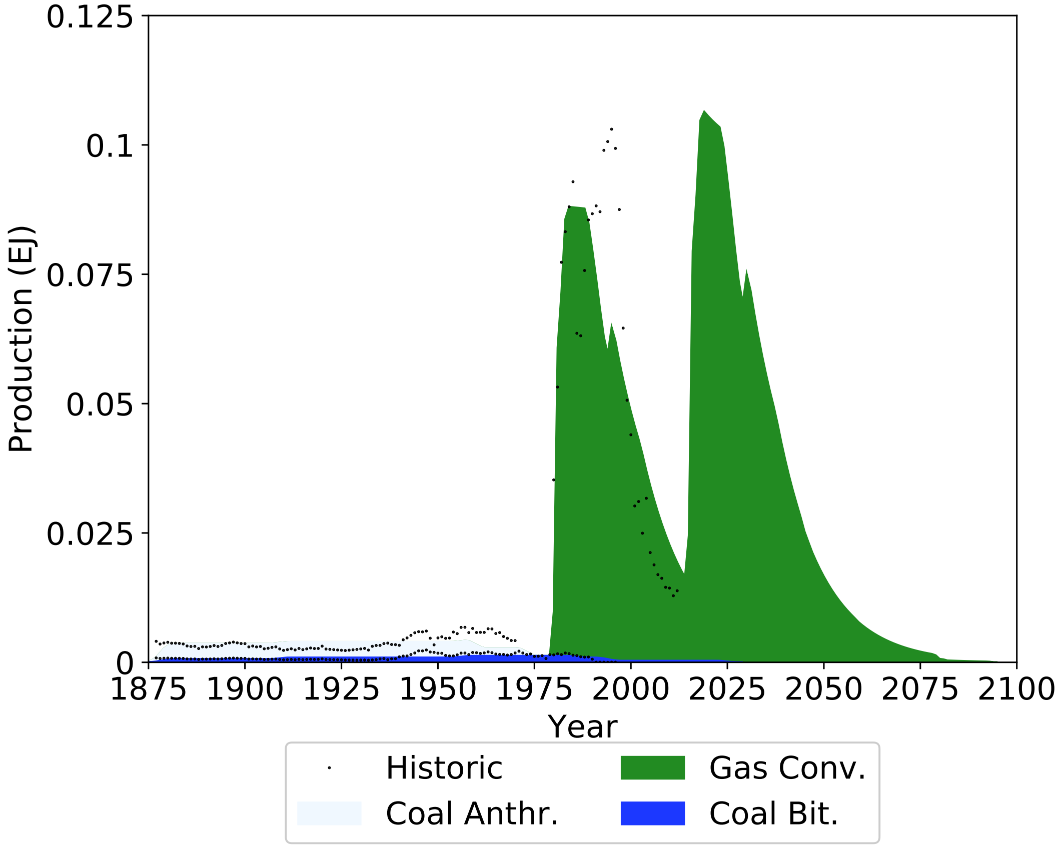

Figure 3.26: Ireland projection by mineral type

Table 3.26: Peak years - Minerals

Name	URR	Peak Year	Peak Rate
Coal Bit.	0.12	1959	–
Coal Anthr.	0.27	1882	–
Gas Conv.	4.2	2019	0.11
**Total**	**4.58**	**2019**	**0.11**

3.14 Italy

3.14.1 All Projections

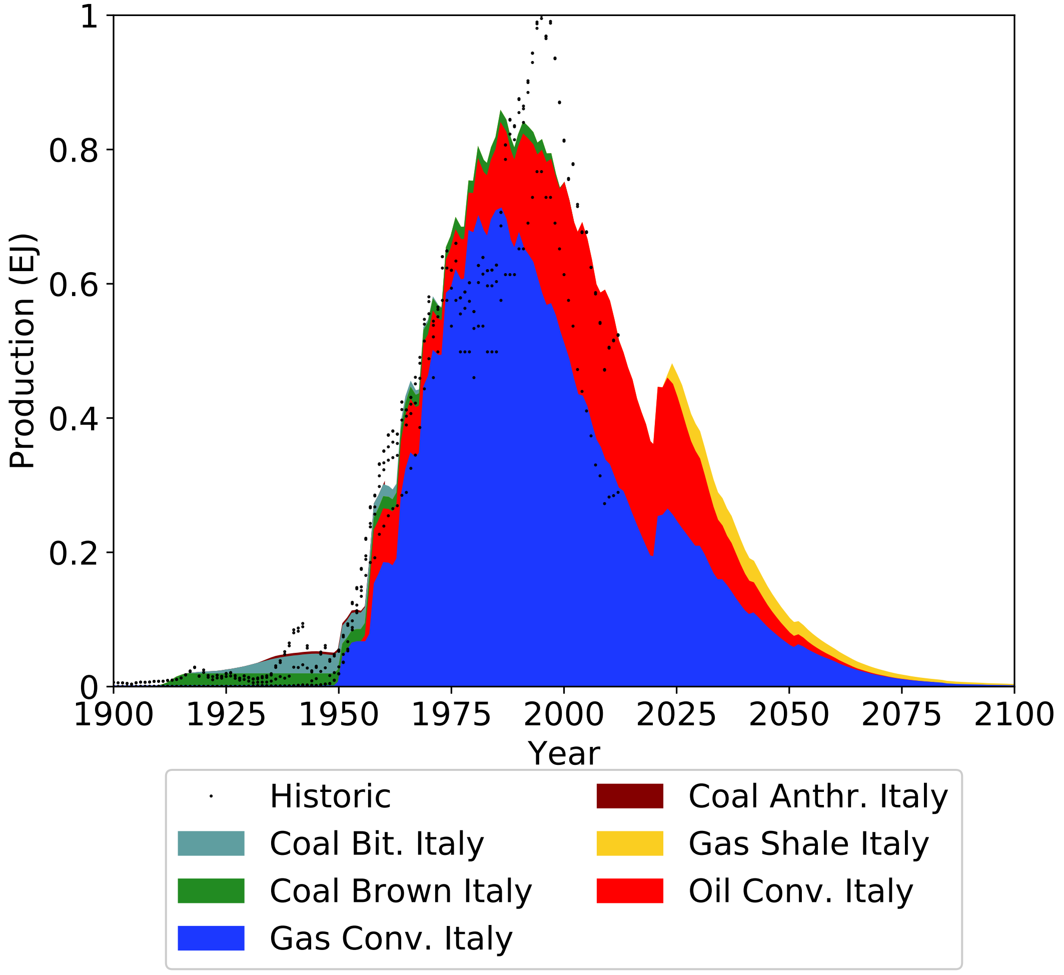

Figure 3.27: Italy projections capped at 16

Table 3.27: Peak years - All

**Name**	**URR**	**Peak Year**	**Peak Rate**
Gas Conv. Italy	34.7	1986	0.71
Oil Conv. Italy	12.08	2004	0.26
Coal Brown Italy	1.53	1917	0.02
Gas Shale Italy	1.36	2027	0.04
Coal Bit. Italy	0.9	1945	0.03
Coal Anthr. Italy	0.08	1937	–
**Total**	**50.65**	**1986**	**0.86**

3.14.2 By Mineral

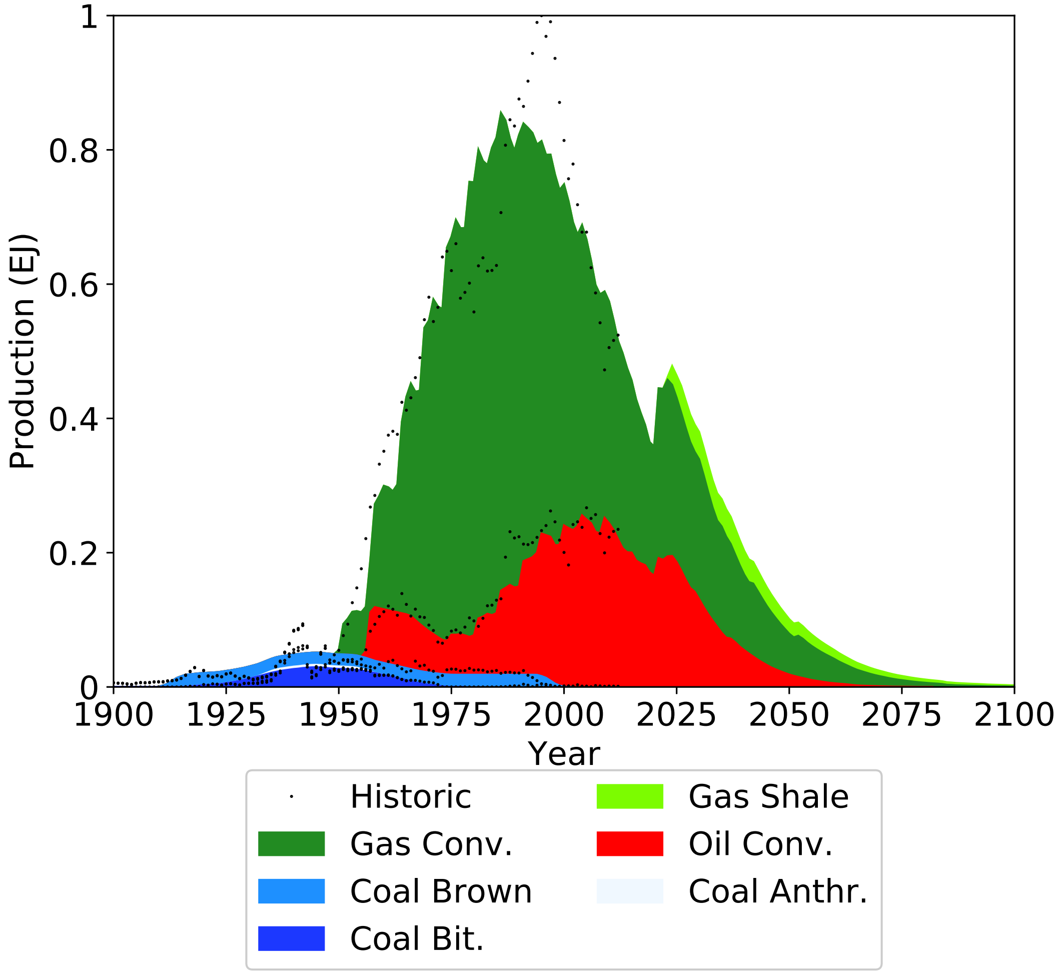

Figure 3.28: Italy projection by mineral type

3.14.3 Regional Projections

Table 3.28: Peak years - Minerals

Name	URR	Peak Year	Peak Rate
Coal Bit.	0.9	1945	0.03
Coal Anthr.	0.08	1937	–
Coal Brown	1.53	1917	0.02
Oil Conv.	12.08	2004	0.26
Gas Conv.	34.7	1986	0.71
Gas Shale	1.36	2027	0.04
**Total**	**50.65**	**1986**	**0.86**

Italy

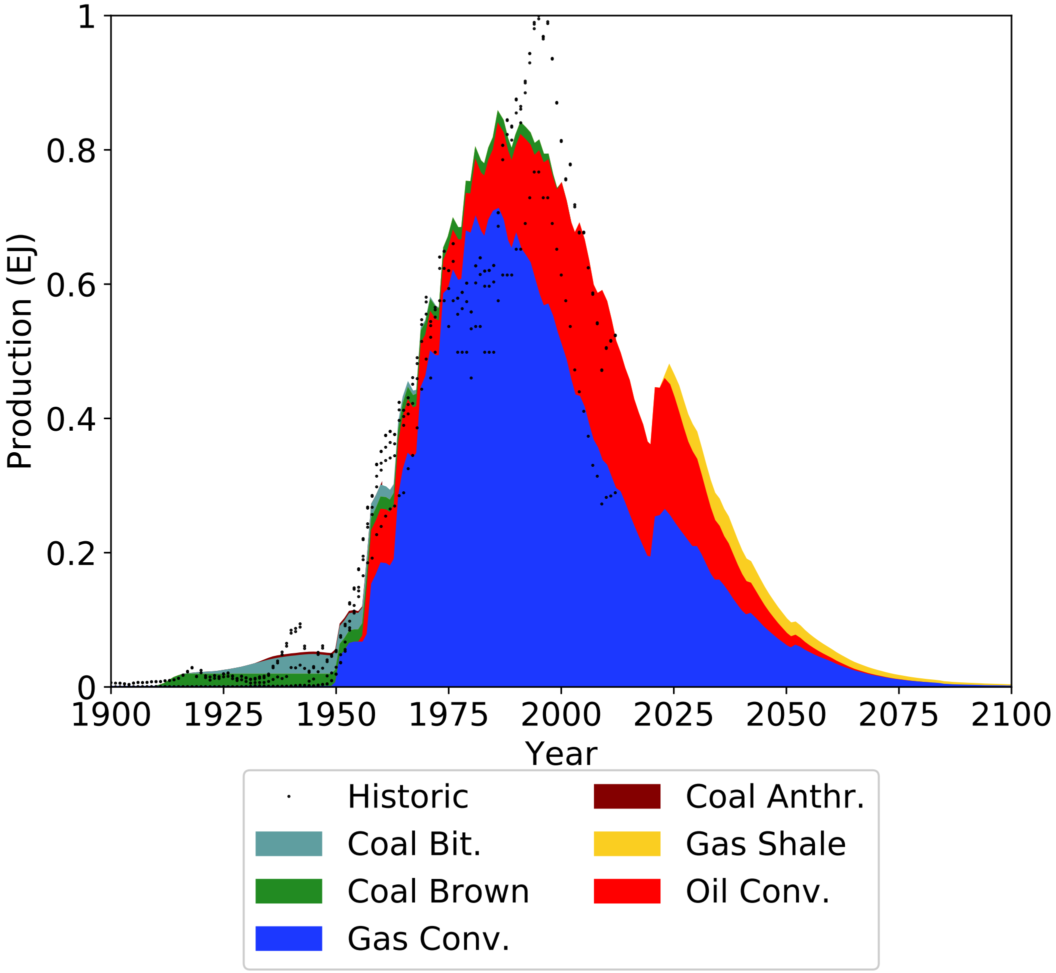

Figure 3.29: Italy - Italy projections capped at 16

Table 3.29: Peak years - All

**Name**	**URR**	**Peak Year**	**Peak Rate**
Gas Conv. Italy	34.7	1986	0.71
Oil Conv. Italy	12.08	2004	0.26
Coal Brown Italy	1.53	1917	0.02
Gas Shale Italy	1.36	2027	0.04
Coal Bit. Italy	0.9	1945	0.03
Coal Anthr. Italy	0.08	1937	–
**Total**	**50.65**	**1986**	**0.86**

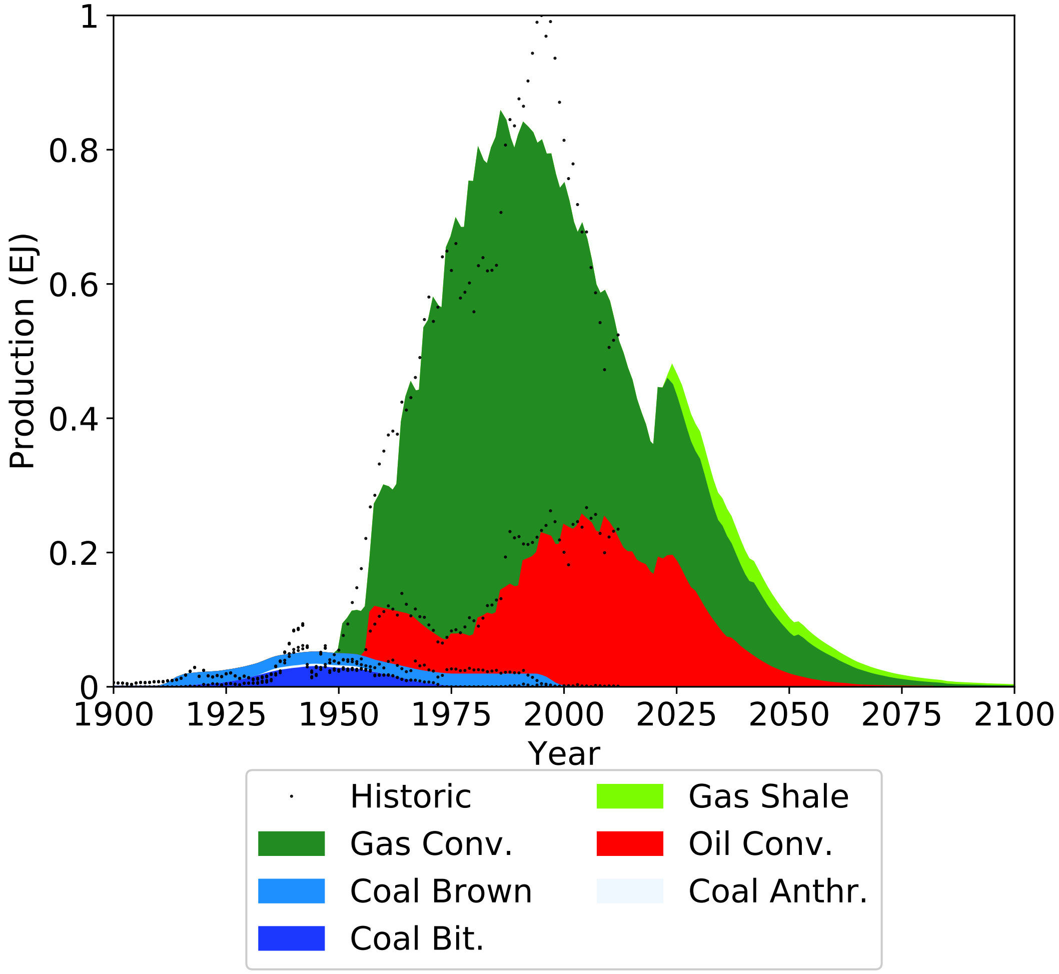

Figure 3.30: Italy - Italy projection by mineral type

Table 3.30: Peak years - Minerals

**Name**	**URR**	**Peak Year**	**Peak Rate**
Coal Bit.	0.9	1945	0.03
Coal Anthr.	0.08	1937	–
Coal Brown	1.53	1917	0.02
Oil Conv.	12.08	2004	0.26
Gas Conv.	34.7	1986	0.71
Gas Shale	1.36	2027	0.04
**Total**	**50.65**	**1986**	**0.86**

3.14.4 Projection by region

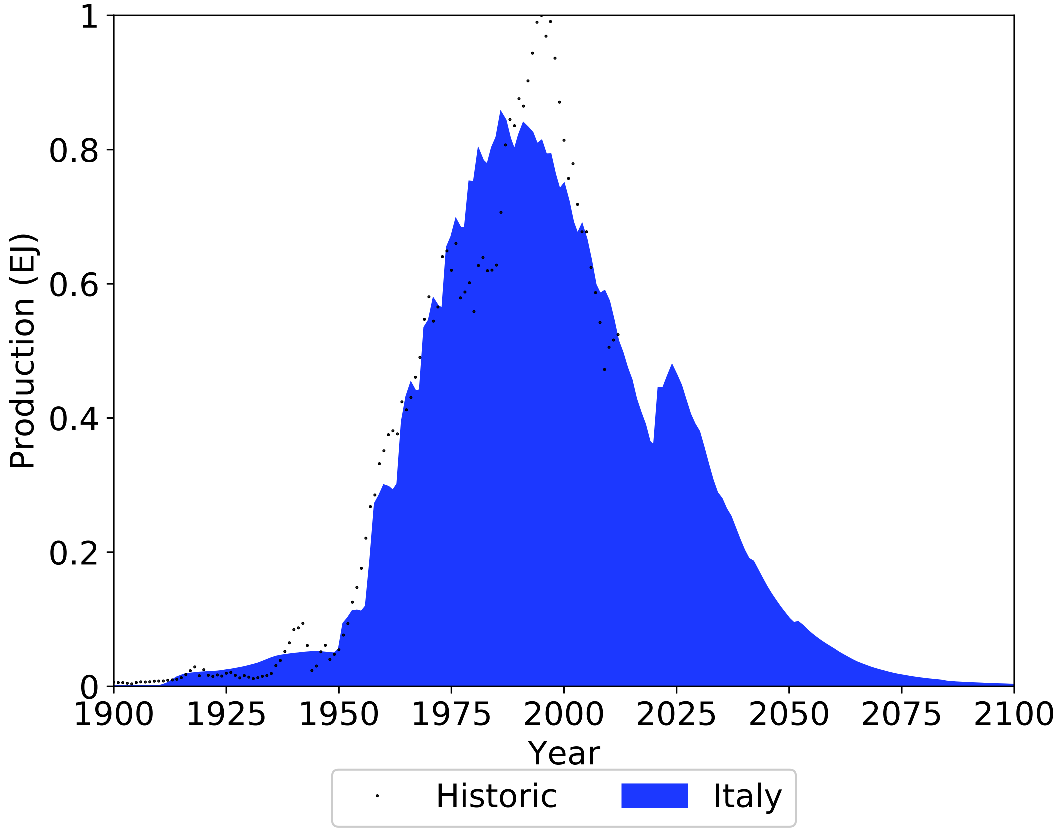

Figure 3.31: Italy by region projections capped at 16

Table 3.31: Peak years - All

Name	URR	Peak Year	Peak Rate
Italy	50.65	1986	0.86
**Total**	**50.65**	**1986**	**0.86**

3.15 Malta

3.15.1 All Projections

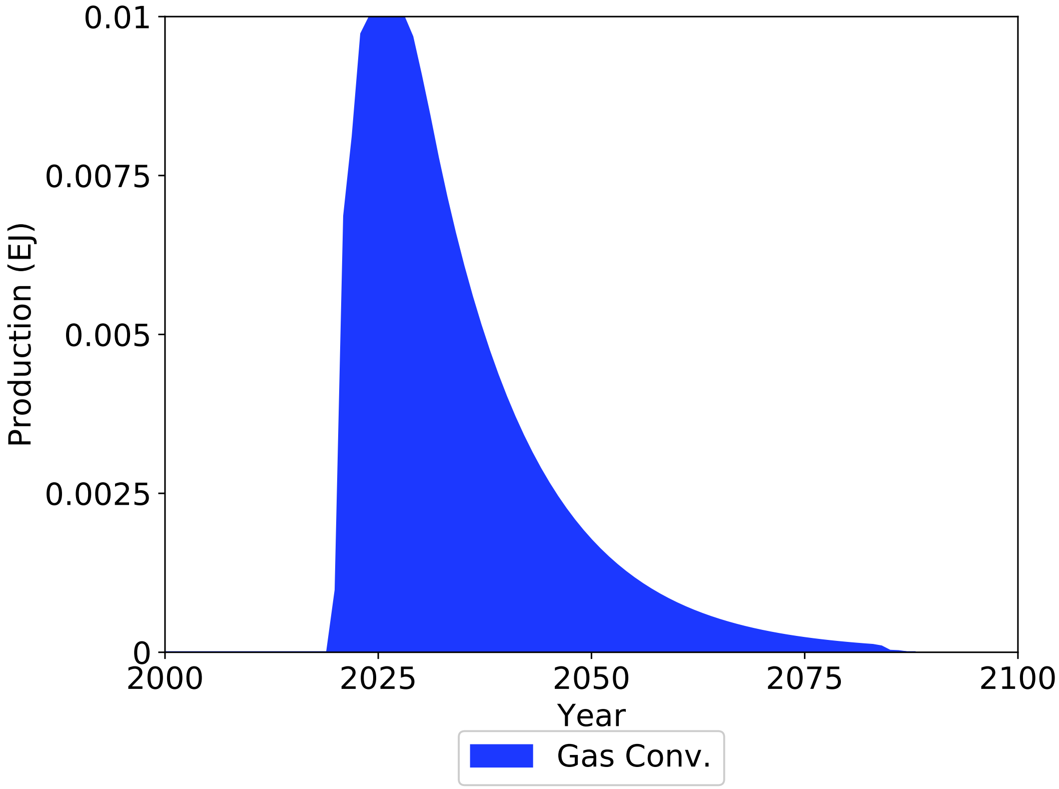

Figure 3.32: Malta projections capped at 16

Table 3.32: Peak years - All

Name	URR	Peak Year	Peak Rate
Gas Conv.	0.2	2024	0.01
**Total**	**0.2**	**2024**	**0.01**

3.15.2 By Mineral

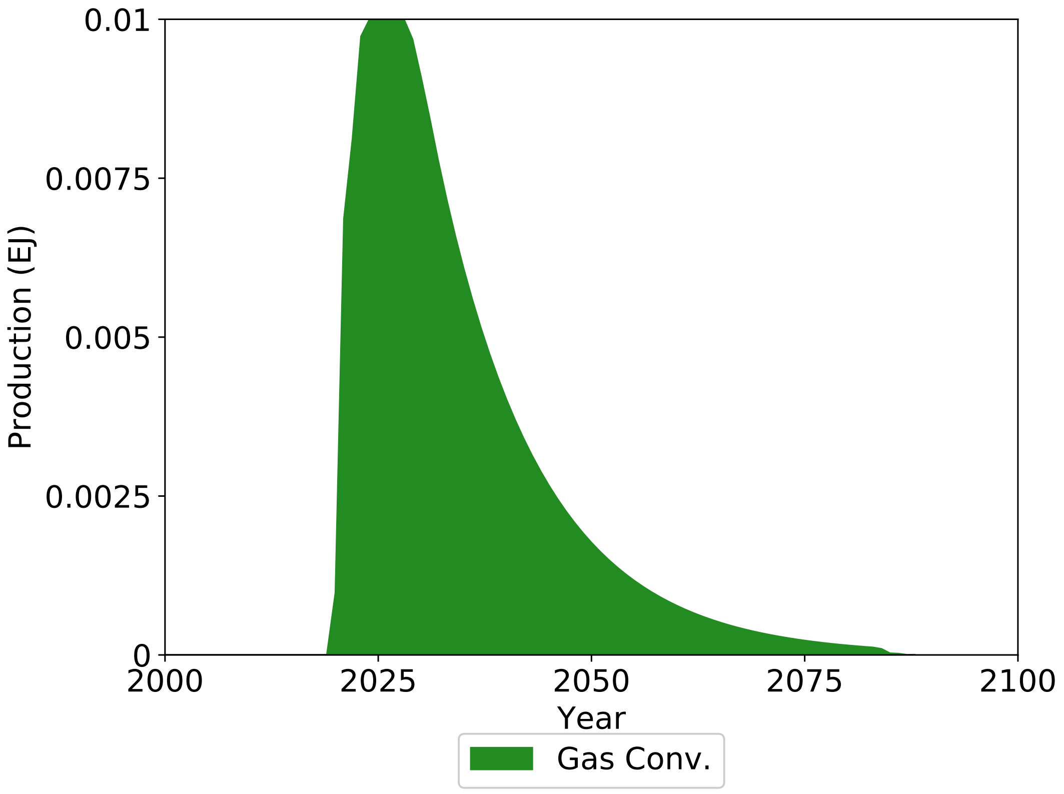

Figure 3.33: Malta projection by mineral type

Table 3.33: Peak years - Minerals

Name	URR	Peak Year	Peak Rate
Gas Conv.	0.2	2024	0.01
**Total**	**0.2**	**2024**	**0.01**

3.16 Netherlands

3.16.1 All Projections

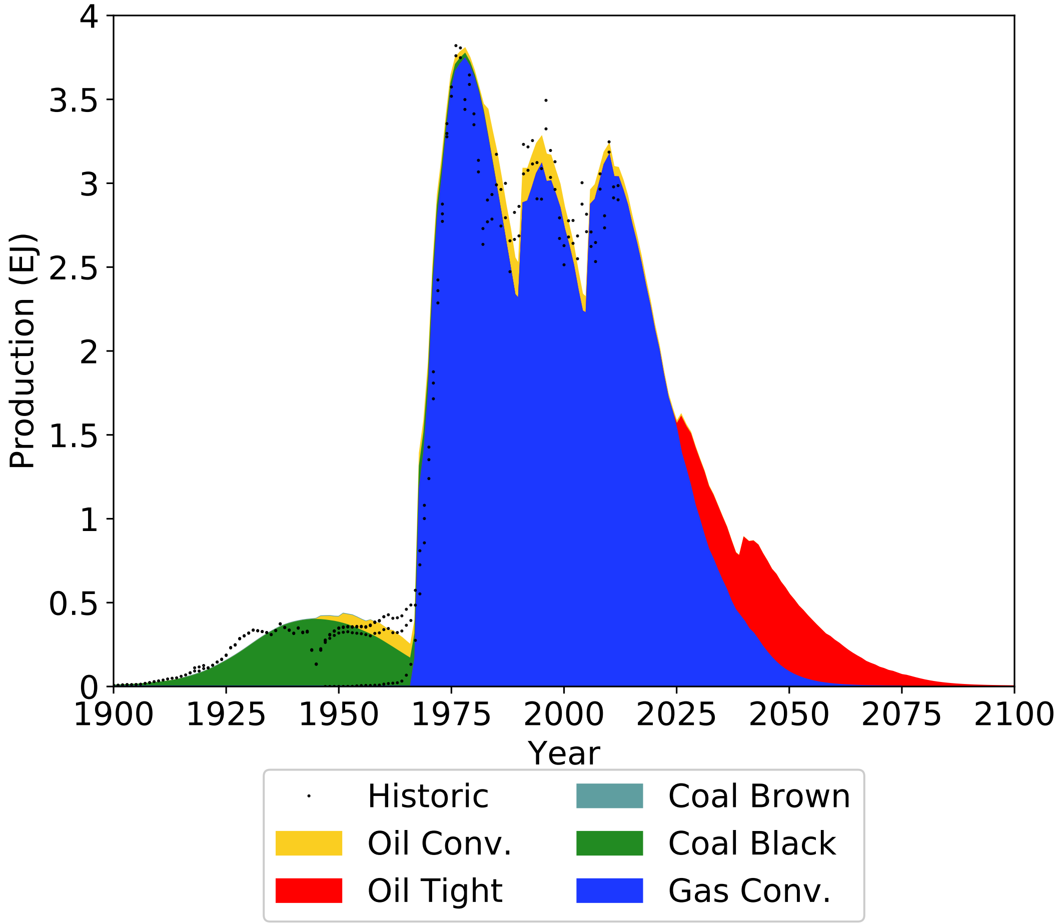

Figure 3.34: Netherlands projections capped at 16

Table 3.34: Peak years - All

**Name**	**URR**	**Peak Year**	**Peak Rate**
Gas Conv.	173.3	1978	3.74
Oil Tight	16.62	2043	0.56
Coal Black	14.9	1945	0.4
Oil Conv.	7.18	1988	0.23
Coal Brown	0.17	1918	–
**Total**	**212.16**	**1978**	**3.8**

3.16.2 By Mineral

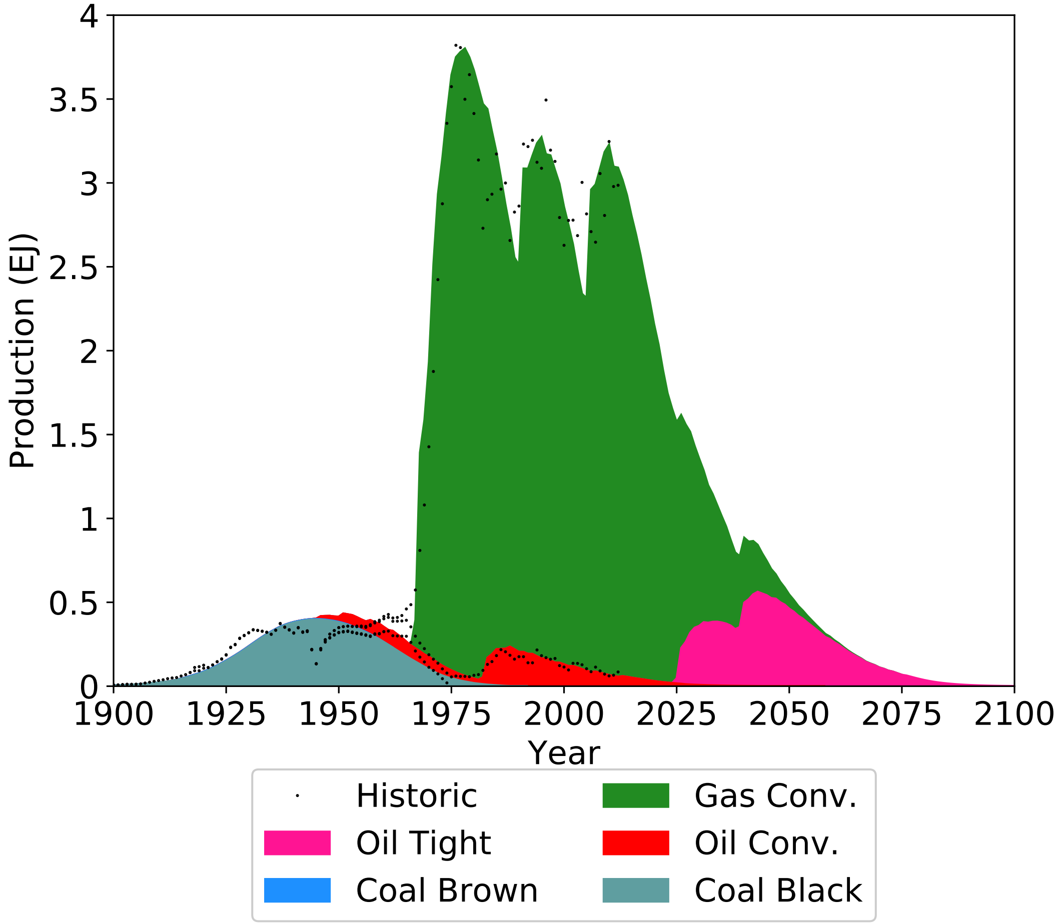

Figure 3.35: Netherlands projection by mineral type

Table 3.35: Peak years - Minerals

**Name**	**URR**	**Peak Year**	**Peak Rate**
Coal Black	14.9	1945	0.4
Coal Brown	0.17	1918	–
Oil Conv.	7.18	1988	0.23
Oil Tight	16.62	2043	0.56
Gas Conv.	173.3	1978	3.74
**Total**	**212.16**	**1978**	**3.8**

3.17 Norway

3.17.1 All Projections

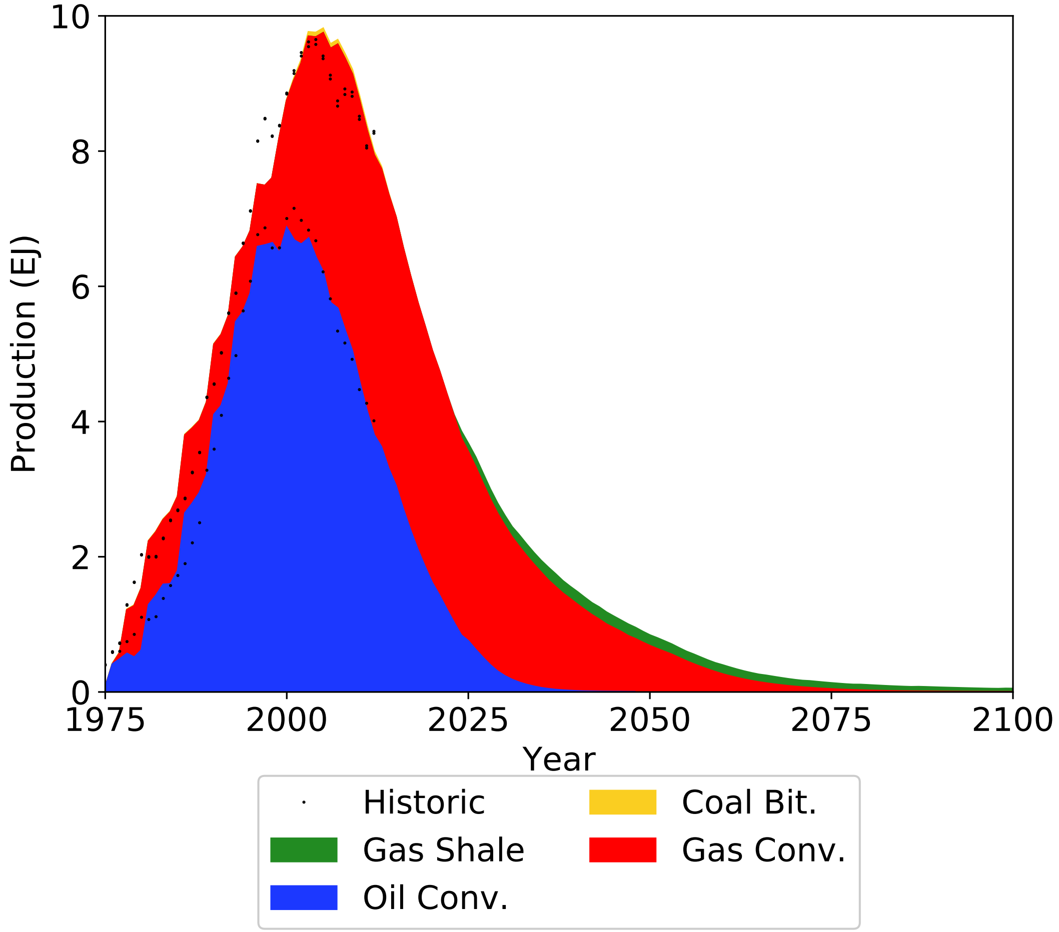

Figure 3.36: Norway projections capped at 16

Table 3.36: Peak years - All

**Name**	**URR**	**Peak Year**	**Peak Rate**
Oil Conv.	180.8	2000	6.86
Gas Conv.	157.5	2010	4.15
Gas Shale	10.07	2037	0.19
Coal Bit.	1.51	2004	0.07
**Total**	**349.88**	**2005**	**9.81**

3.17.2 By Mineral

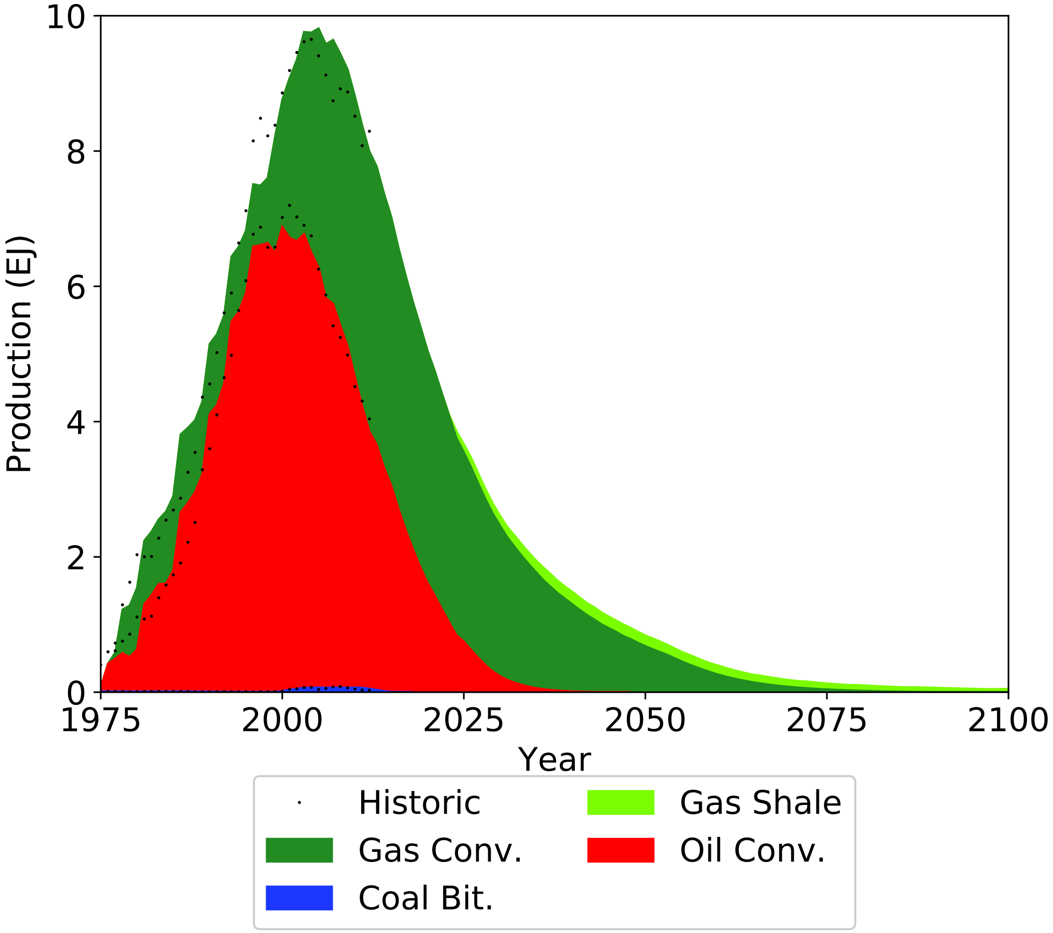

Figure 3.37: Norway projection by mineral type

Table 3.37: Peak years - Minerals

**Name**	**URR**	**Peak Year**	**Peak Rate**
Coal Bit.	1.51	2004	0.07
Oil Conv.	180.8	2000	6.86
Gas Conv.	157.5	2010	4.15
Gas Shale	10.07	2037	0.19
**Total**	**349.88**	**2005**	**9.81**

3.18 Poland

3.18.1 All Projections

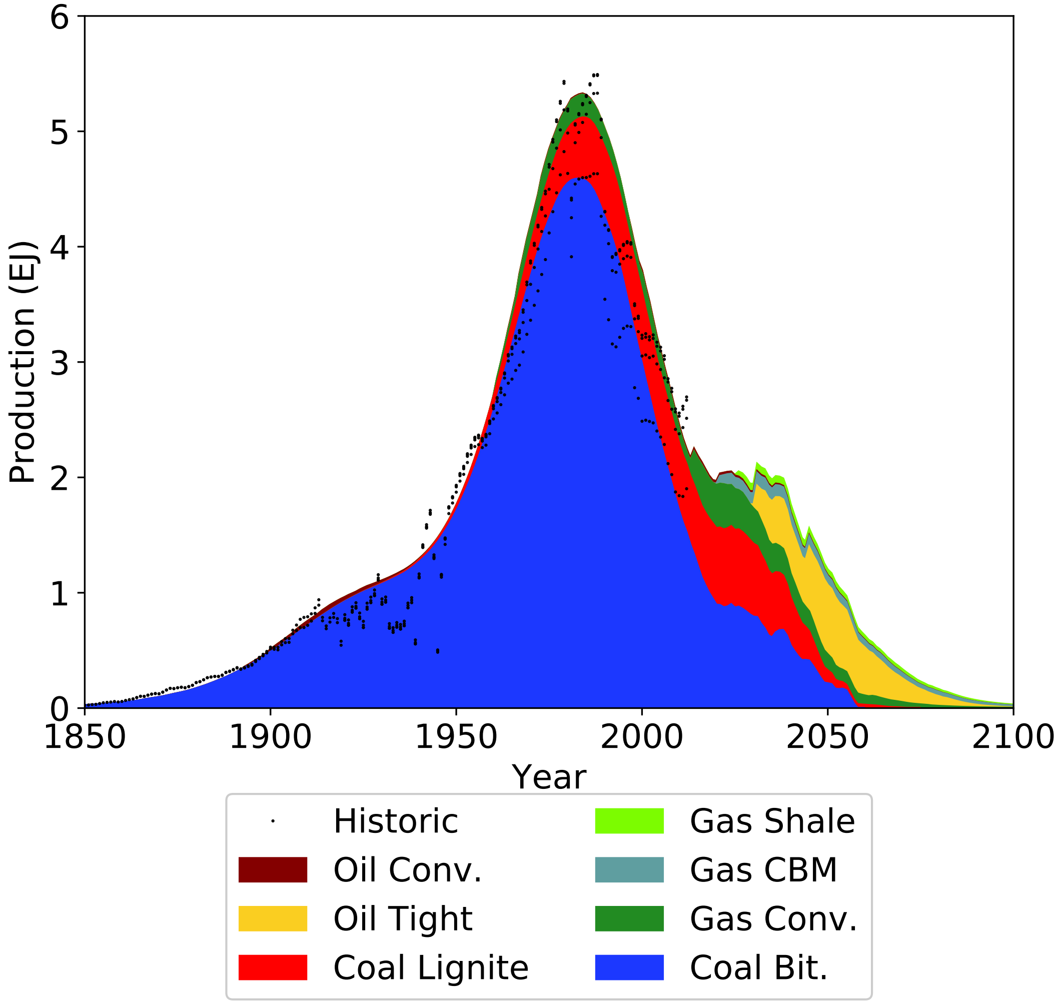

Figure 3.38: Poland projections capped at 16

Table 3.38: Peak years - All

Name	URR	Peak Year	Peak Rate
Coal Bit.	291.9	1983	4.59
Coal Lignite	46.0	2023	0.68
Gas Conv.	20.2	2022	0.39
Oil Tight	18.91	2048	0.62
Gas CBM	5.25	2032	0.11
Oil Conv.	3.08	1916	0.05
Gas Shale	2.96	2040	0.07
**Total**	**388.3**	**1984**	**5.33**

3.18.2 By Mineral

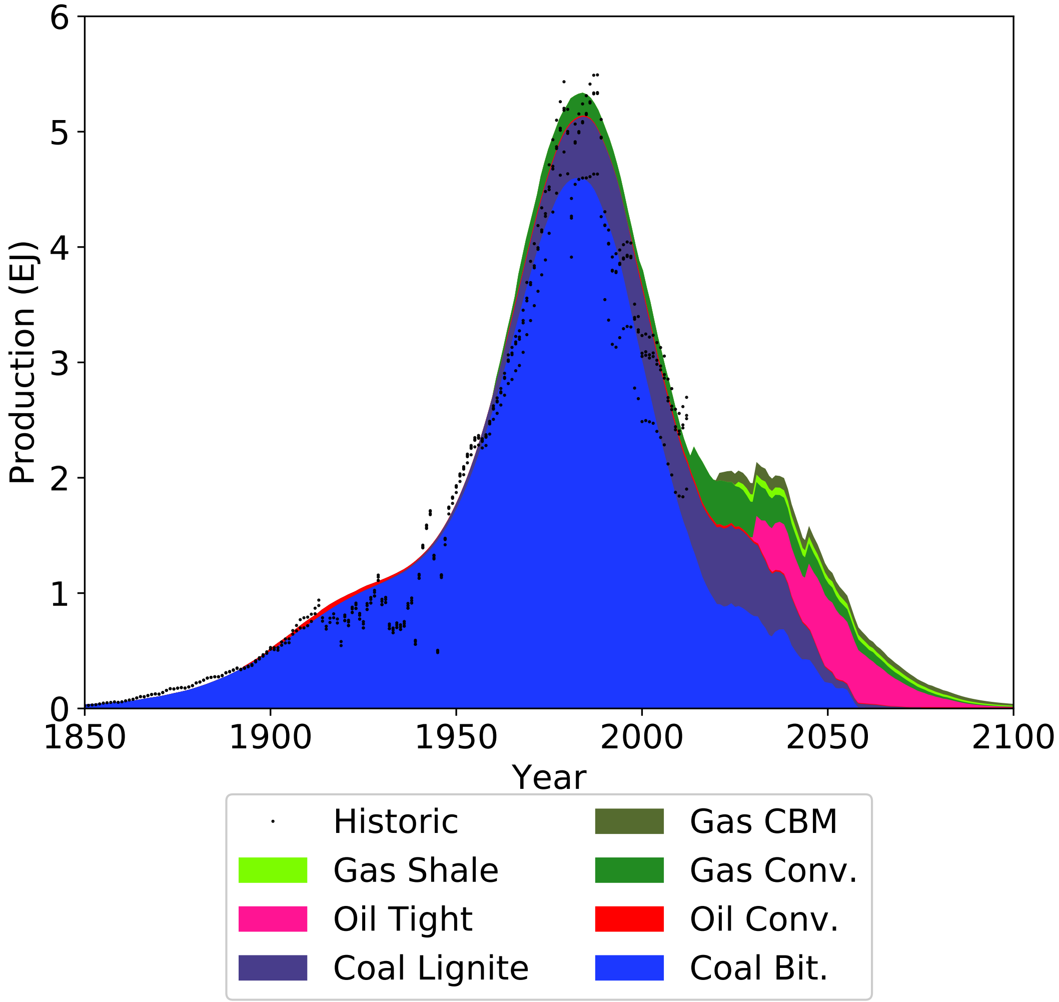

Figure 3.39: Poland projection by mineral type

Table 3.39: Peak years - Minerals

**Name**	**URR**	**Peak Year**	**Peak Rate**
Coal Bit.	291.9	1983	4.59
Coal Lignite	46.0	2023	0.68
Oil Conv.	3.08	1916	0.05
Oil Tight	18.91	2048	0.62
Gas Conv.	20.2	2022	0.39
Gas Shale	2.96	2040	0.07
Gas CBM	5.25	2032	0.11
**Total**	**388.3**	**1984**	**5.33**

3.19 Portugal

3.19.1 All Projections

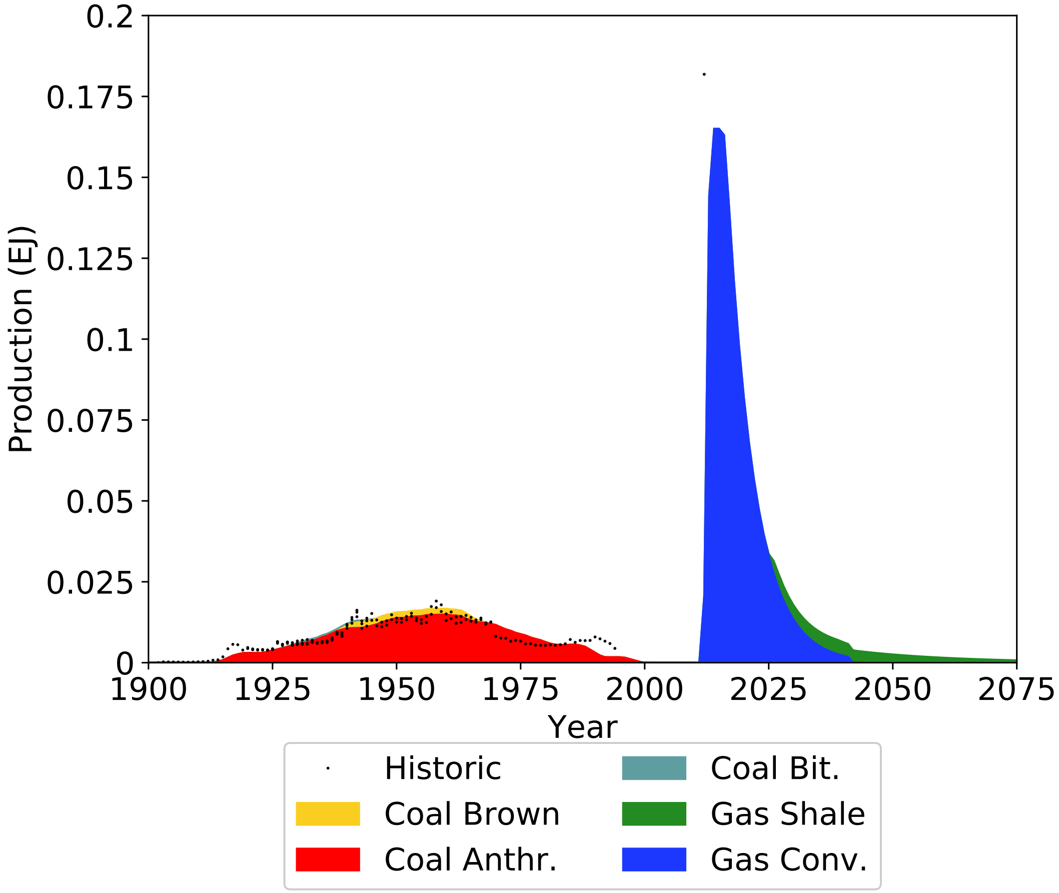

Figure 3.40: Portugal projections capped at 16

Table 3.40: Peak years - All

**Name**	**URR**	**Peak Year**	**Peak Rate**
Gas Conv.	1.5	2014	0.17
Coal Anthr.	0.68	1960	0.01
Gas Shale	0.15	2027	–
Coal Brown	0.05	1943	–
Coal Bit.	0.01	1932	–
**Total**	**2.39**	**2014**	**0.17**

3.19.2 By Mineral

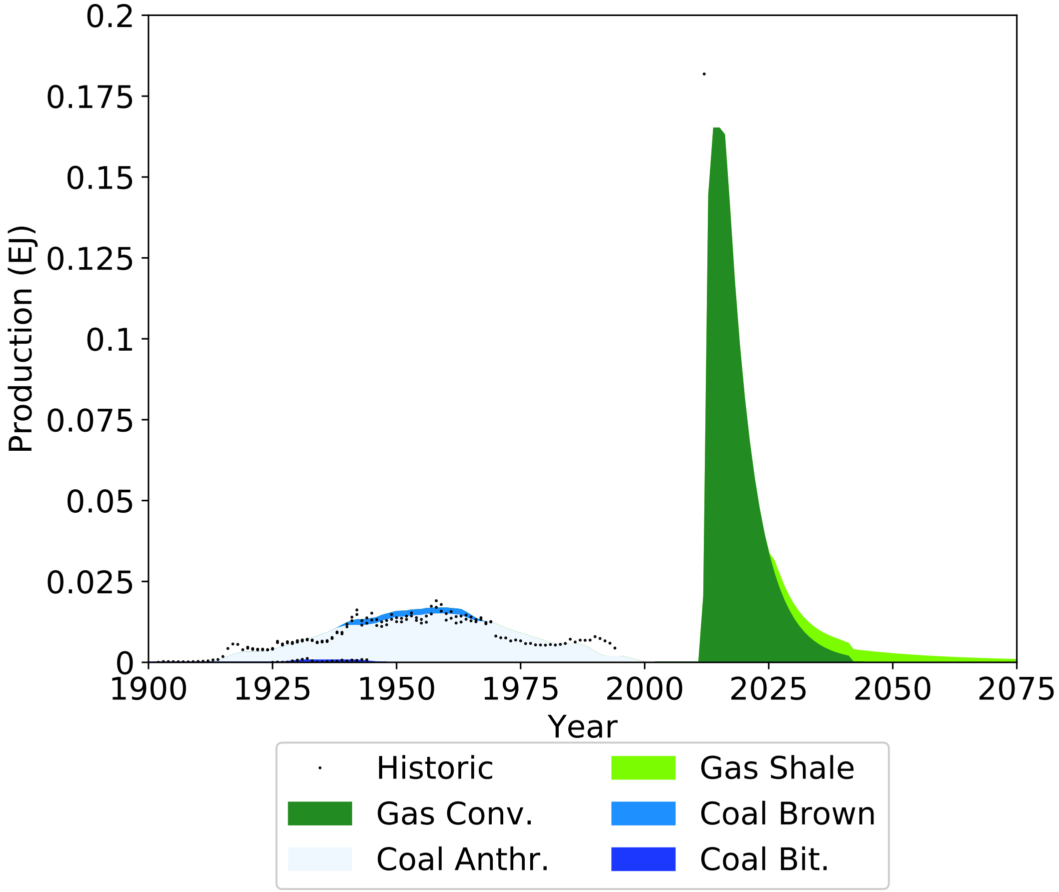

Figure 3.41: Portugal projection by mineral type

Table 3.41: Peak years - Minerals

**Name**	**URR**	**Peak Year**	**Peak Rate**
Coal Bit.	0.01	1932	–
Coal Anthr.	0.68	1960	0.01
Coal Brown	0.05	1943	–
Gas Conv.	1.5	2014	0.17
Gas Shale	0.15	2027	–
**Total**	**2.39**	**2014**	**0.17**

3.20 Romania

3.20.1 All Projections

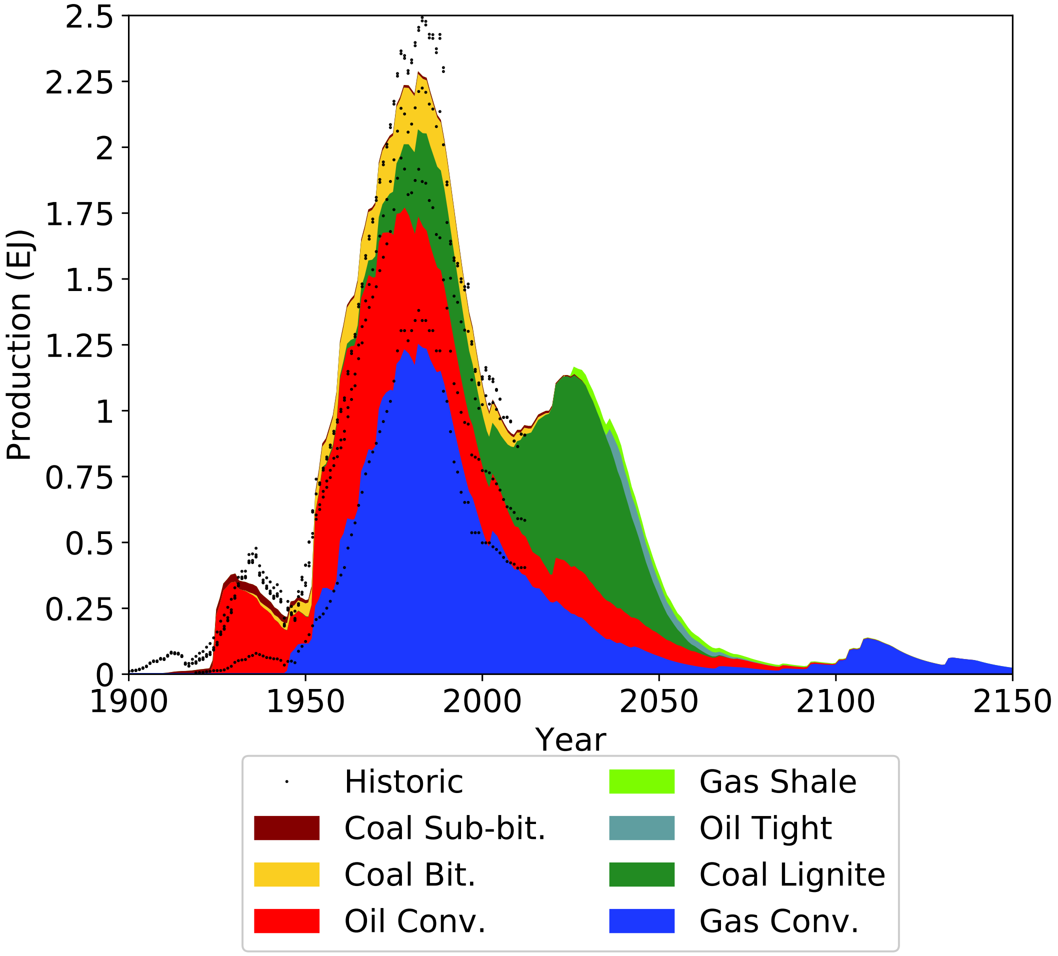

Figure 3.42: Romania projections capped at 16

Table 3.42: Peak years - All

**Name**	**URR**	**Peak Year**	**Peak Rate**
Gas Conv.	57.8	1982	1.25
Oil Conv.	38.65	1965	0.66
Coal Lignite	32.3	2027	0.73
Coal Bit.	9.0	1978	0.21
Oil Tight	1.72	2038	0.1
Coal Sub-bit.	1.6	1936	0.04
Gas Shale	1.56	2029	0.04
**Total**	**142.63**	**1982**	**2.28**

3.20.2 By Mineral

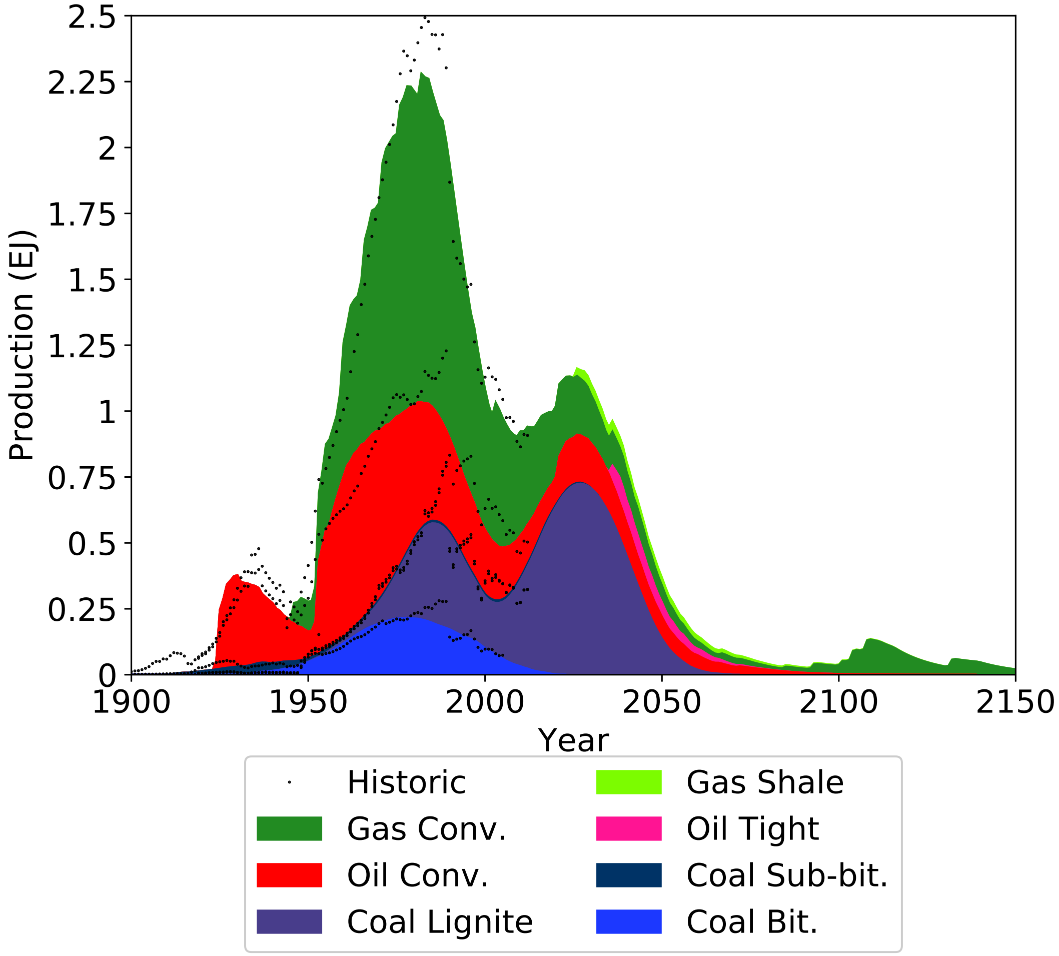

Figure 3.43: Romania projection by mineral type

Table 3.43: Peak years - Minerals

**Name**	**URR**	**Peak Year**	**Peak Rate**
Coal Bit.	9.0	1978	0.21
Coal Lignite	32.3	2027	0.73
Coal Sub-bit.	1.6	1936	0.04
Oil Conv.	38.65	1965	0.66
Oil Tight	1.72	2038	0.1
Gas Conv.	57.8	1982	1.25
Gas Shale	1.56	2029	0.04
**Total**	**142.63**	**1982**	**2.28**

3.21 Slovakia

3.21.1 All Projections

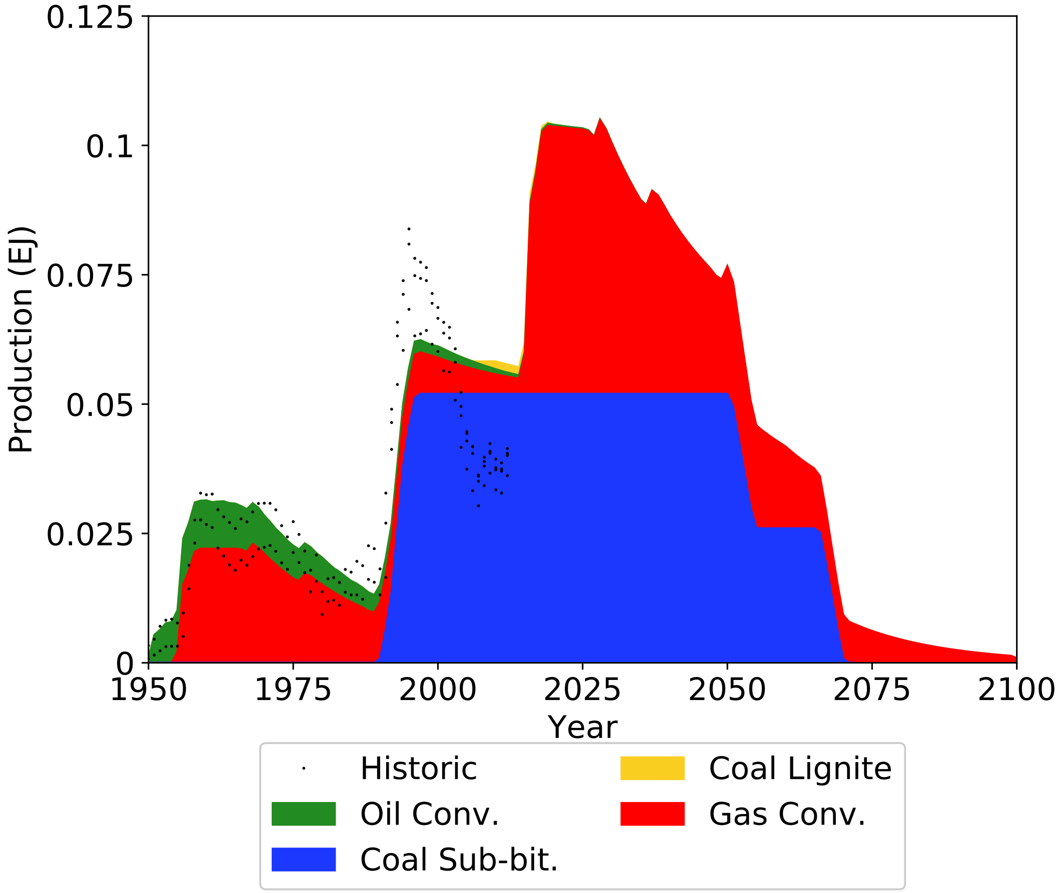

Figure 3.44: Slovakia projections capped at 16

Table 3.44: Peak years - All

Name	URR	Peak Year	Peak Rate
Coal Sub-bit.	3.5	1997	0.05
Gas Conv.	2.6	2028	0.05
Oil Conv.	0.32	1958	0.01
Coal Lignite	0.02	2011	–
**Total**	**6.44**	**2028**	**0.11**

3.21.2 By Mineral

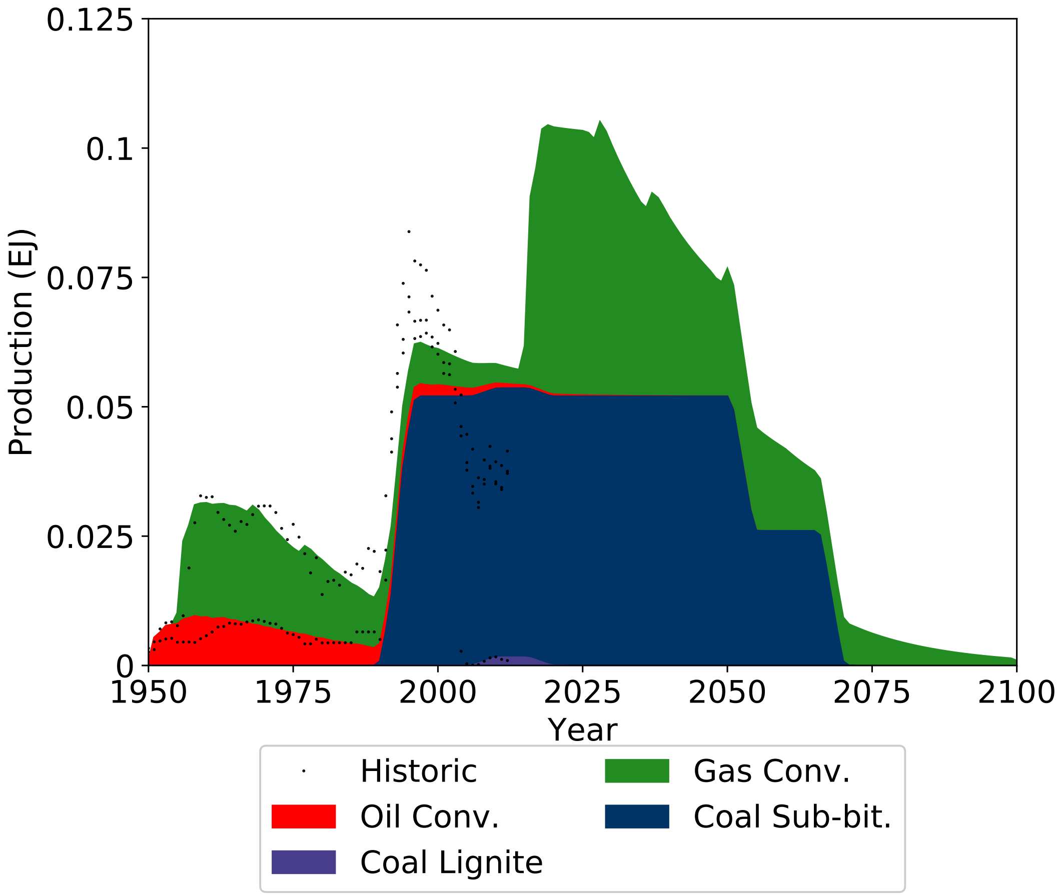

Figure 3.45: Slovakia projection by mineral type

Table 3.45: Peak years - Minerals

Name	URR	Peak Year	Peak Rate
Coal Lignite	0.02	2011	–
Coal Sub-bit.	3.5	1997	0.05
Oil Conv.	0.32	1958	0.01
Gas Conv.	2.6	2028	0.05
**Total**	**6.44**	**2028**	**0.11**

3.22 Spain

3.22.1 All Projections

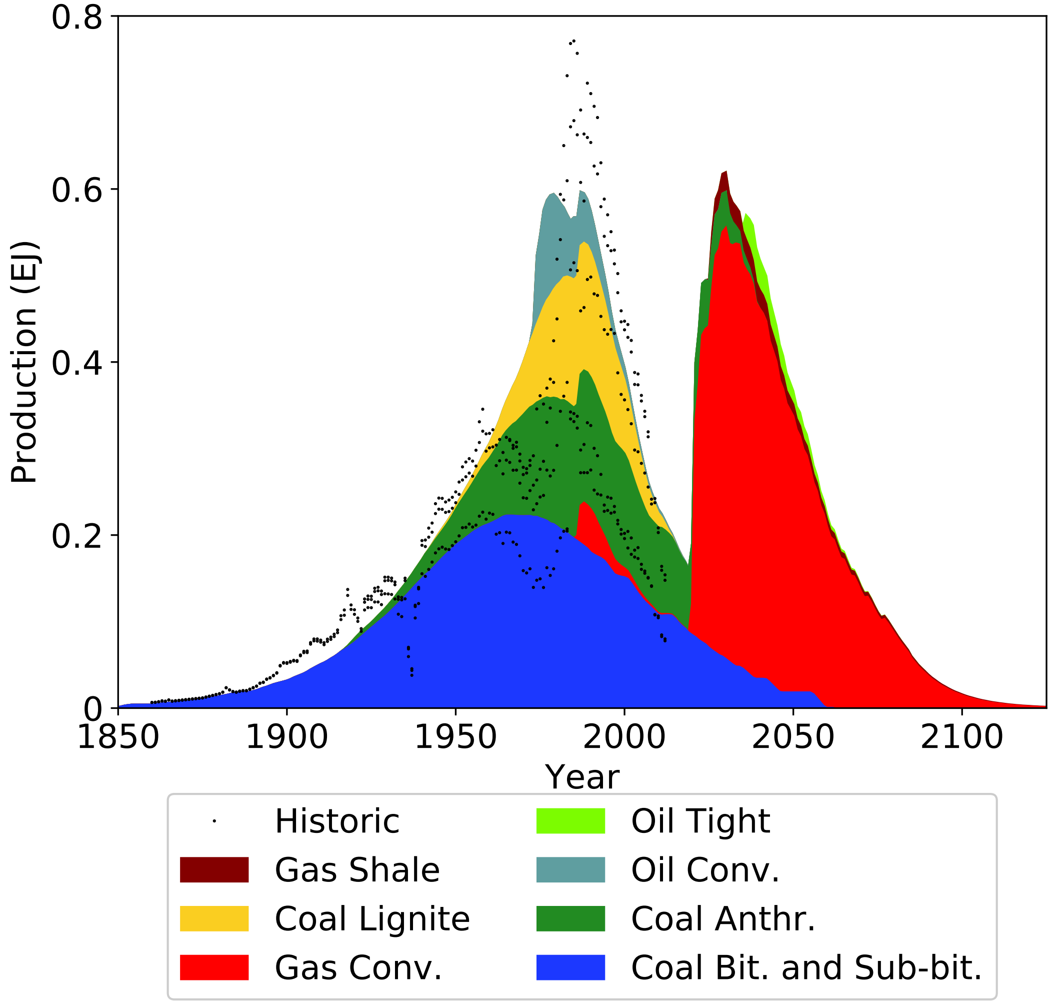

Figure 3.46: Spain projections capped at 16

Table 3.46: Peak years - All

**Name**	**URR**	**Peak Year**	**Peak Rate**
Coal Bit. and Sub-bit.	19.4	1966	0.22
Gas Conv.	19.1	2030	0.5
Coal Anthr.	9.02	1989	0.15
Coal Lignite	4.6	1987	0.15
Oil Conv.	1.78	1977	0.12
Gas Shale	0.76	2029	0.02
Oil Tight	0.57	2038	0.04
**Total**	**55.23**	**2030**	**0.62**

3.22.2 By Mineral

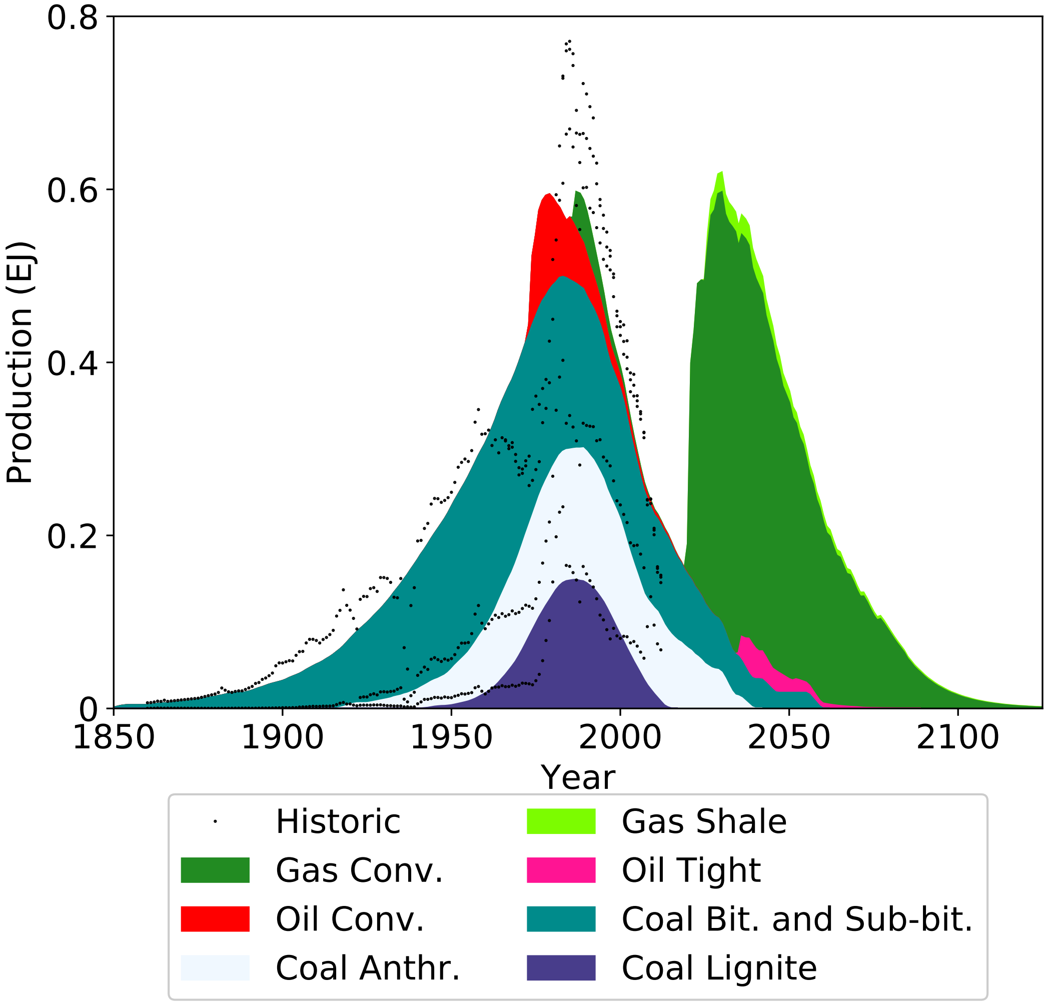

Figure 3.47: Spain projection by mineral type

Table 3.47: Peak years - Minerals

**Name**	**URR**	**Peak Year**	**Peak Rate**
Coal Lignite	4.6	1987	0.15
Coal Anthr.	9.02	1989	0.15
Coal Bit. and Sub-bit.	19.4	1966	0.22
Oil Conv.	1.78	1977	0.12
Oil Tight	0.57	2038	0.04
Gas Conv.	19.1	2030	0.5
Gas Shale	0.76	2029	0.02
**Total**	**55.23**	**2030**	**0.62**

3.23 Sweden

3.23.1 All Projections

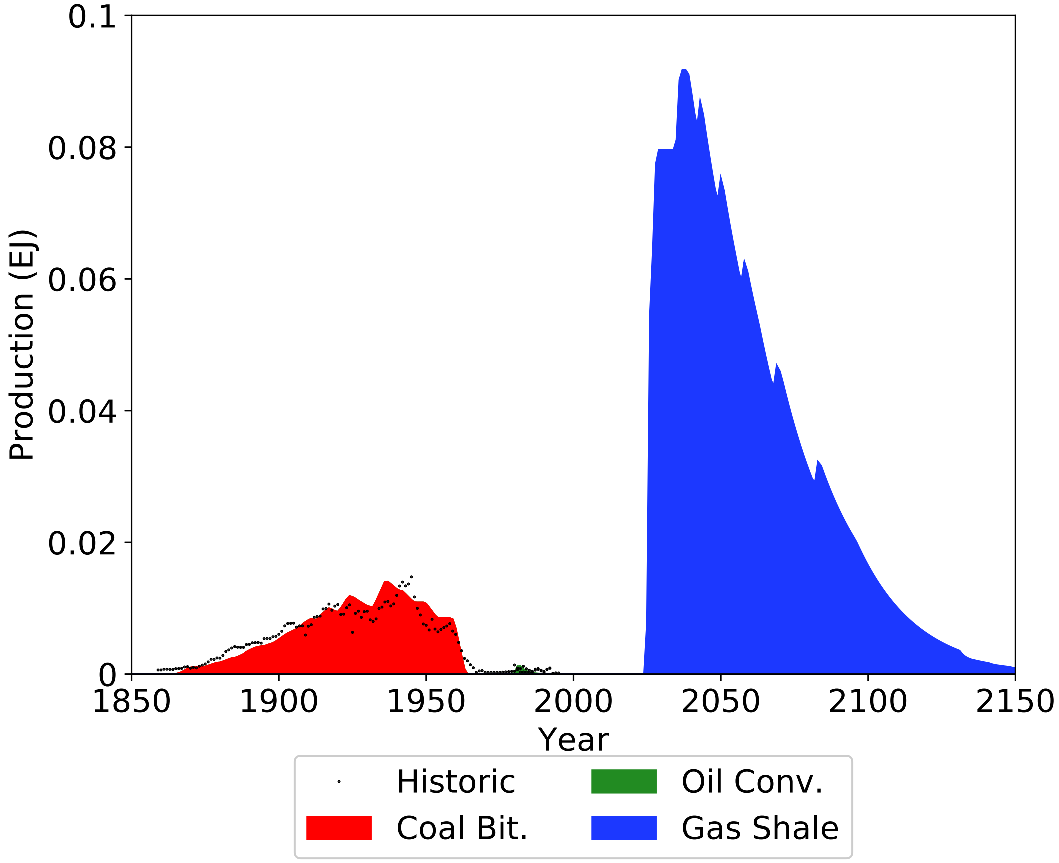

Figure 3.48: Sweden projections capped at 16

Table 3.48: Peak years - All

Name	URR	Peak Year	Peak Rate
Gas Shale	4.31	2037	0.09
Coal Bit.	0.69	1937	0.01
Oil Conv.	–	1982	–
**Total**	**5.0**	**2037**	**0.09**

3.23.2 By Mineral

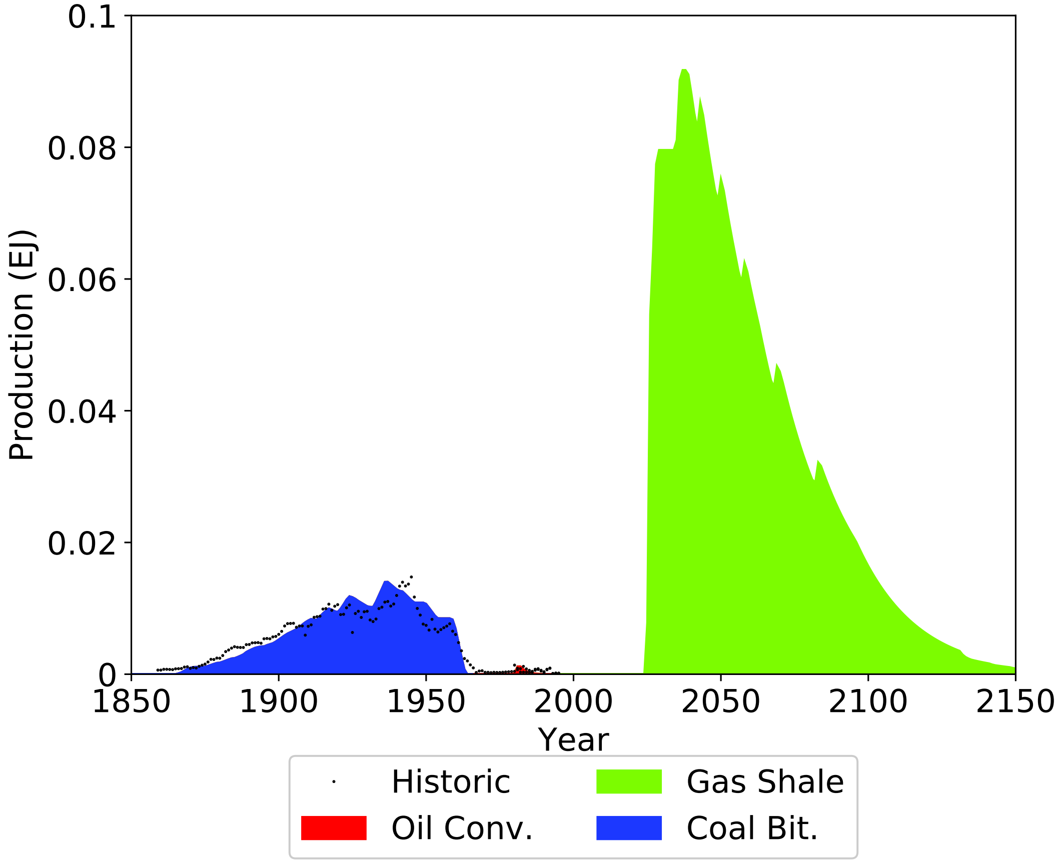

Figure 3.49: Sweden projection by mineral type

Table 3.49: Peak years - Minerals

Name	URR	Peak Year	Peak Rate
Coal Bit.	0.69	1937	0.01
Oil Conv.	–	1982	–
Gas Shale	4.31	2037	0.09
**Total**	**5.0**	**2037**	**0.09**

3.24 Switzerland

3.24.1 All Projections

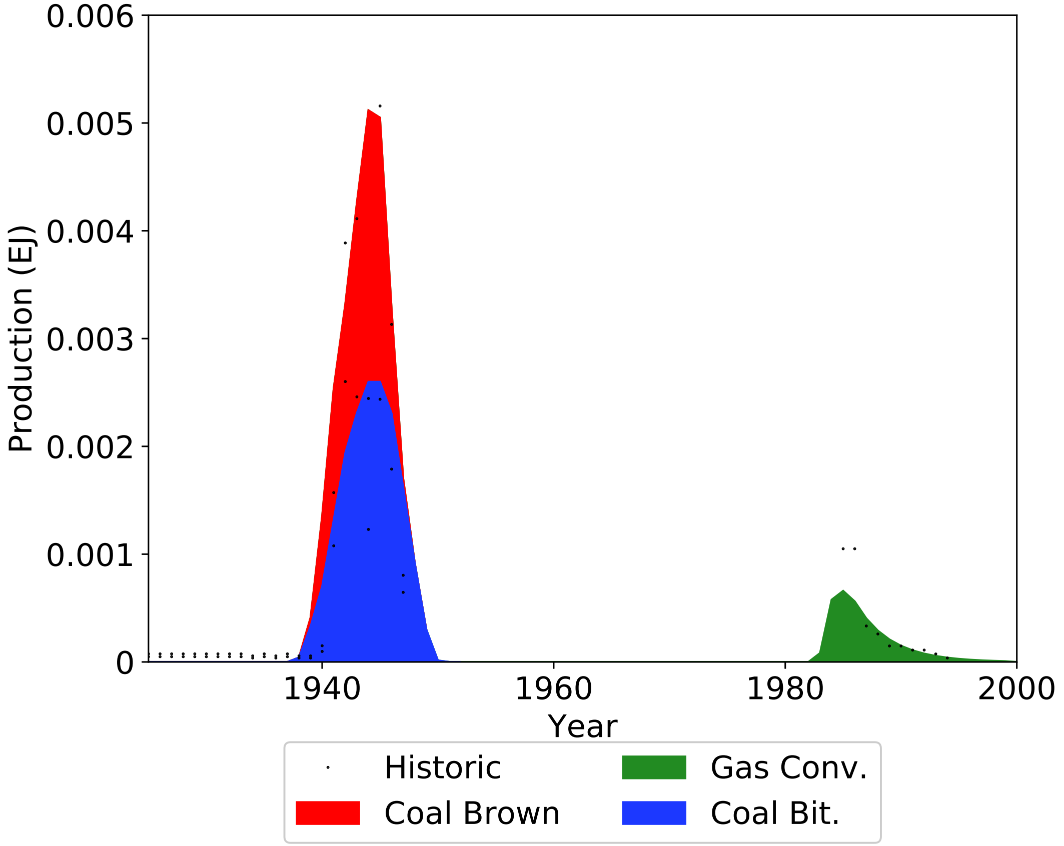

Figure 3.50: Switzerland projections capped at 16

Table 3.50: Peak years - All

Name	URR	Peak Year	Peak Rate
Coal Bit.	0.02	1944	–
Coal Brown	0.01	1944	–
Gas Conv.	–	1985	–
**Total**	**0.03**	**1944**	**0.01**

3.24.2 By Mineral

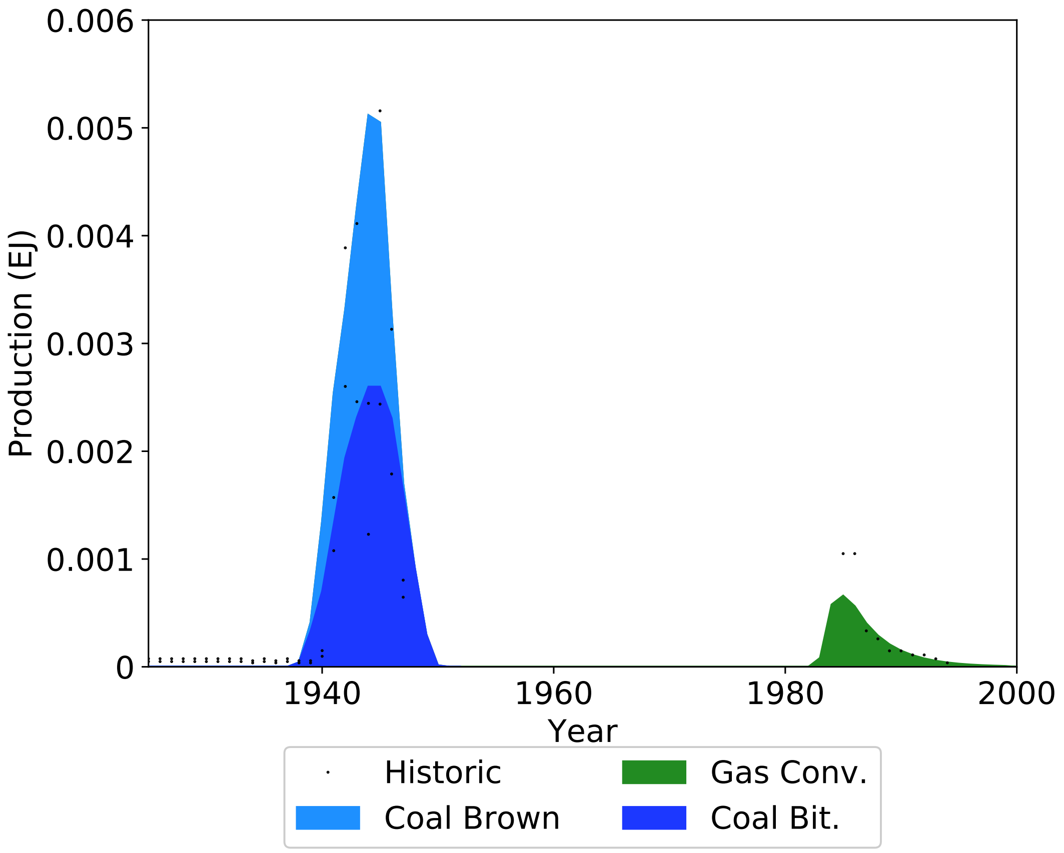

Figure 3.51: Switzerland projection by mineral type

Table 3.51: Peak years - Minerals

Name	URR	Peak Year	Peak Rate
Coal Bit.	0.02	1944	–
Coal Brown	0.01	1944	–
Gas Conv.	–	1985	–
**Total**	**0.03**	**1944**	**0.01**

3.25 Turkey

3.25.1 All Projections

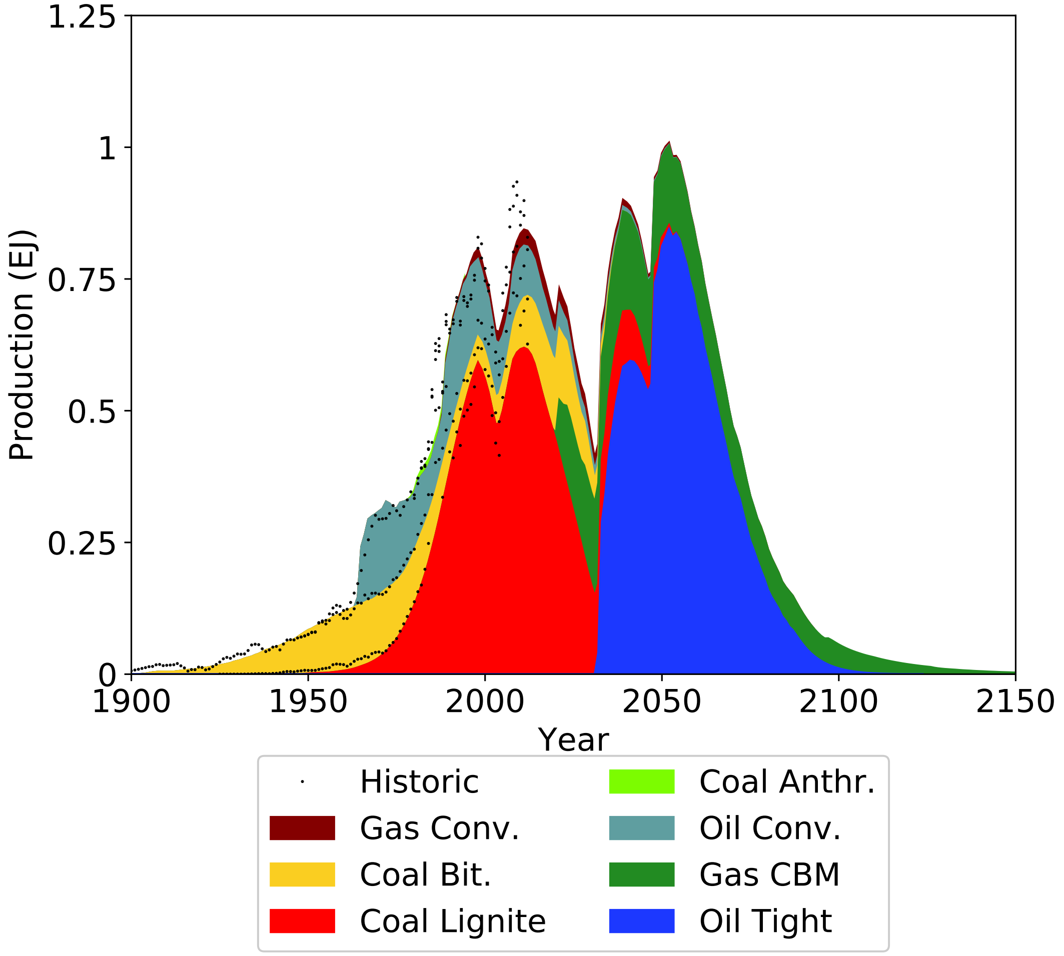

Figure 3.52: Turkey projections capped at 16

Table 3.52: Peak years - All

**Name**	**URR**	**Peak Year**	**Peak Rate**
Oil Tight	26.93	2052	0.84
Coal Lignite	24.3	2011	0.62
Gas CBM	10.5	2036	0.2
Coal Bit.	8.9	2020	0.14
Oil Conv.	7.32	1991	0.19
Gas Conv.	1.3	2014	0.03
Coal Anthr.	0.13	1983	0.01
**Total**	**79.38**	**2052**	**1.01**

3.25.2 By Mineral

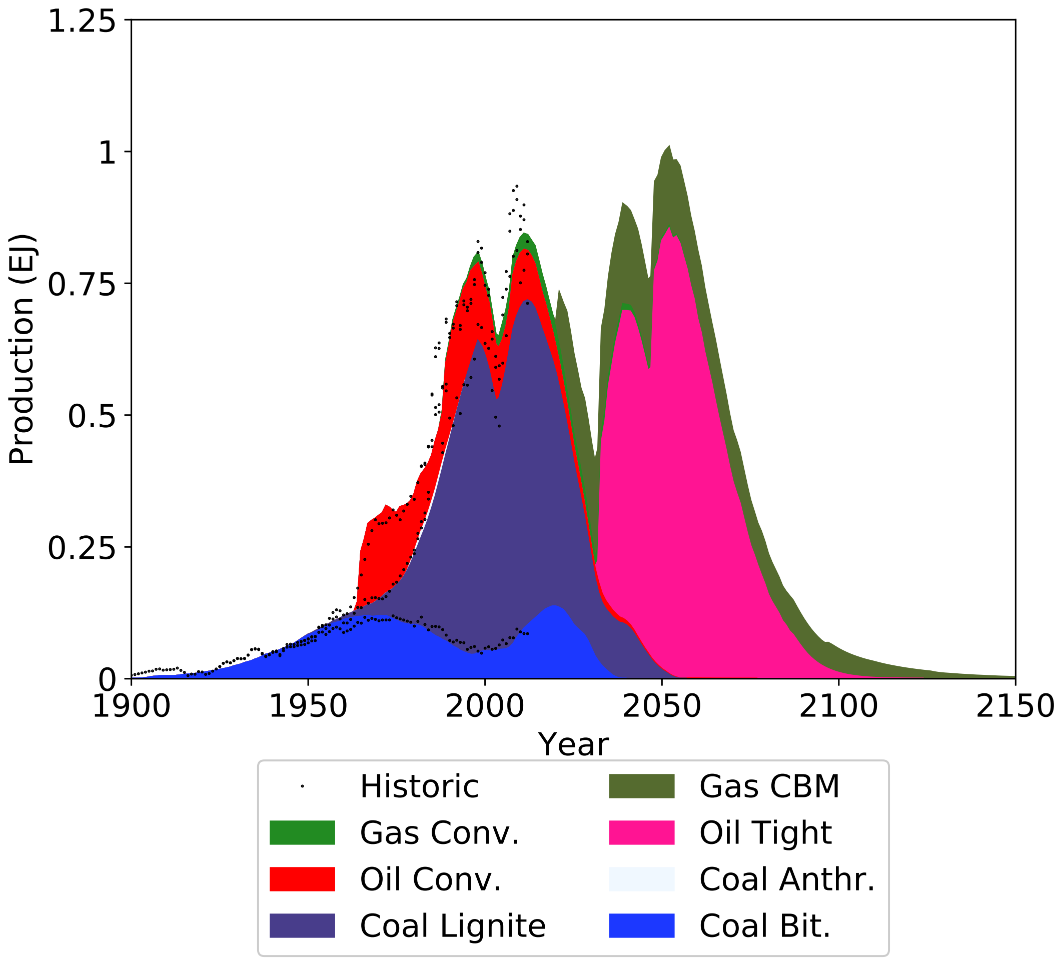

Figure 3.53: Turkey projection by mineral type

Table 3.53: Peak years - Minerals

**Name**	**URR**	**Peak Year**	**Peak Rate**
Coal Bit.	8.9	2020	0.14
Coal Lignite	24.3	2011	0.62
Coal Anthr.	0.13	1983	0.01
Oil Conv.	7.32	1991	0.19
Oil Tight	26.93	2052	0.84
Gas Conv.	1.3	2014	0.03
Gas CBM	10.5	2036	0.2
**Total**	**79.38**	**2052**	**1.01**

3.26 UK

3.26.1 All Projections

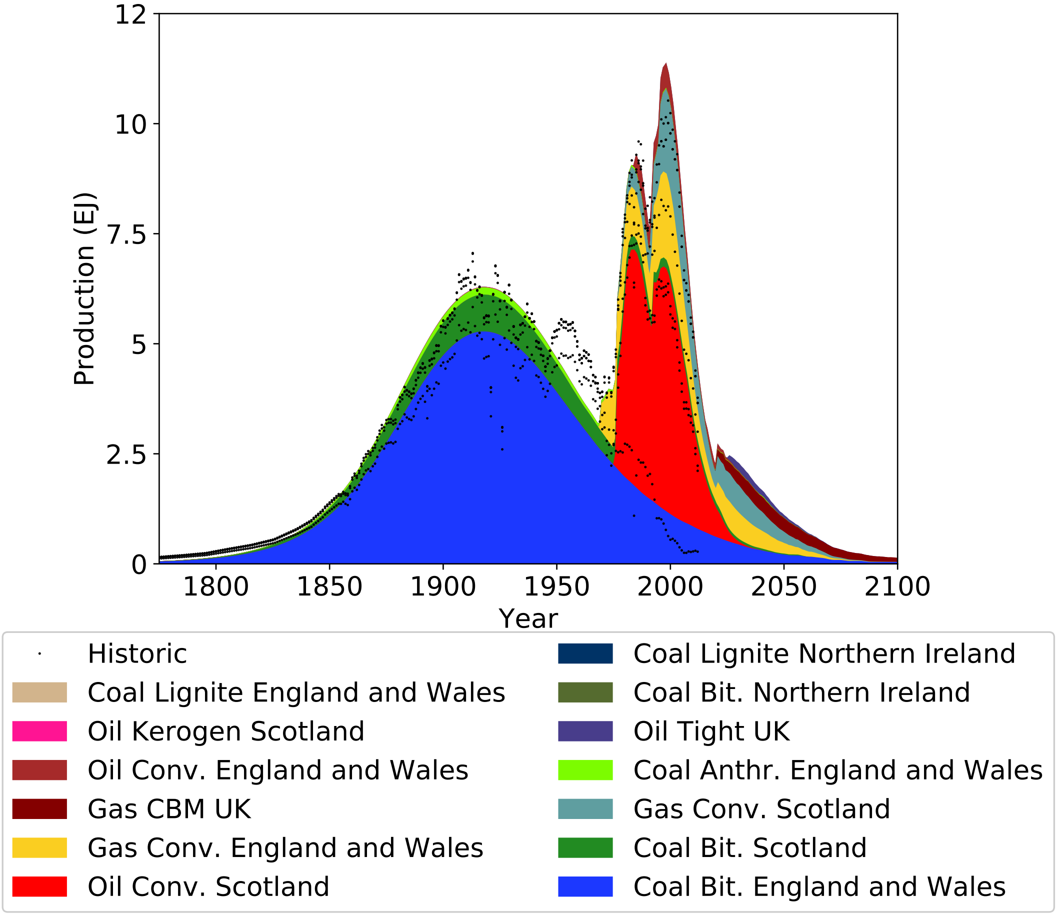

Figure 3.54: UK projections capped at 16

Table 3.54: Peak years - All

**Name**	**URR**	**Peak Year**	**Peak Rate**
Coal Bit. England and Wales	554.9	1918	5.26
Oil Conv. Scotland	164.26	1997	5.52
Coal Bit. Scotland	88.7	1918	0.84
Gas Conv. England and Wales	73.94	1998	1.96
Gas Conv. Scotland	57.36	1999	1.94
Gas CBM UK	21.0	2036	0.35
Coal Anthr. England and Wales	17.0	1921	0.17
Oil Conv. England and Wales	13.28	1987	0.58
Oil Tight UK	4.01	2032	0.18
Oil Kerogen Scotland	0.68	1887	0.01
Coal Bit. Northern Ireland	–	1933	–
Coal Lignite England and Wales	–	1947	–
Coal Lignite Northern Ireland	–	1944	–
**Total**	**995.13**	**1998**	**11.34**

3.26.2 By Mineral

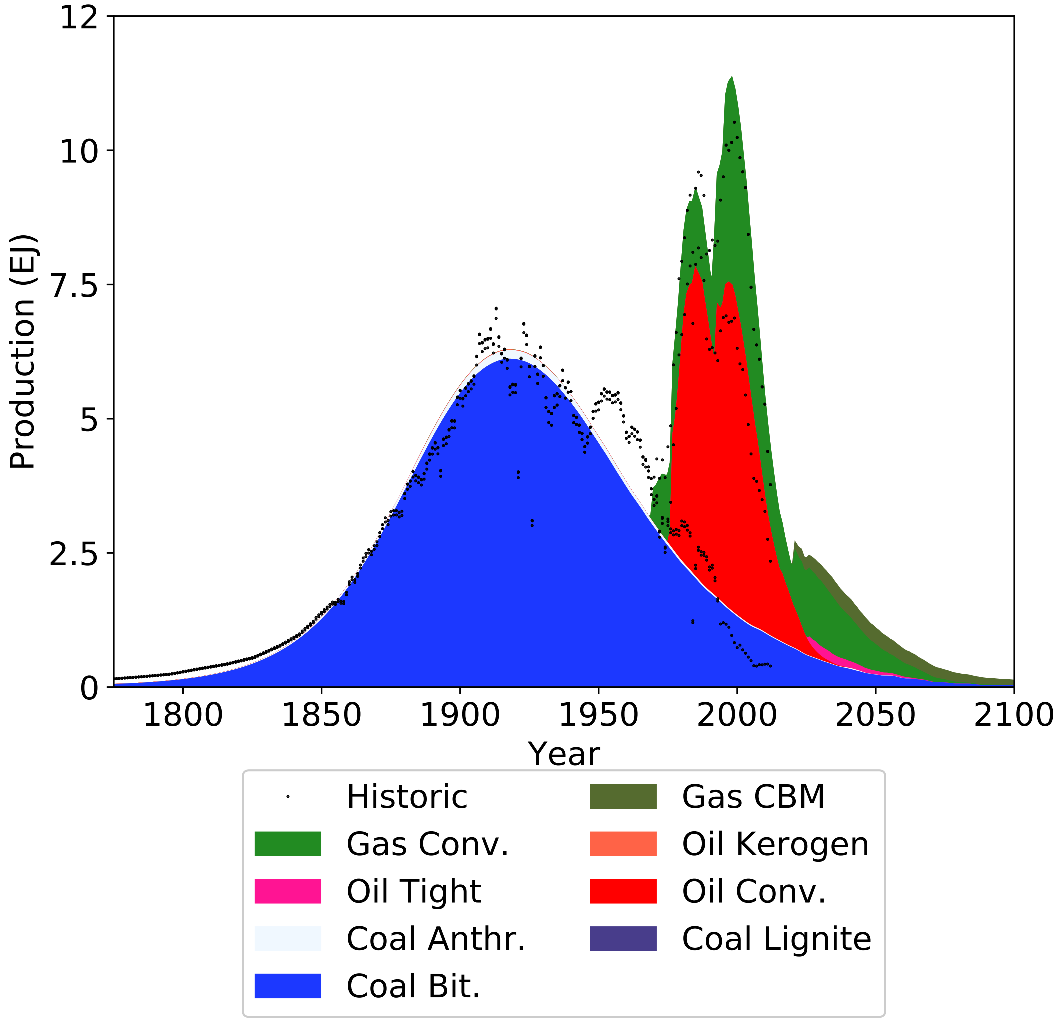

Figure 3.55: UK projection by mineral type

3.26.3 Regional Projections

Table 3.55: Peak years - Minerals

**Name**	**URR**	**Peak Year**	**Peak Rate**
Coal Bit.	643.6	1918	6.1
Coal Lignite	–	1947	–
Coal Anthr.	17.0	1921	0.17
Oil Conv.	177.54	1997	6.09
Oil Tight	4.01	2032	0.18
Oil Kerogen	0.68	1887	0.01
Gas Conv.	131.3	1998	3.86
Gas CBM	21.0	2036	0.35
**Total**	**995.13**	**1998**	**11.34**

England and Wales

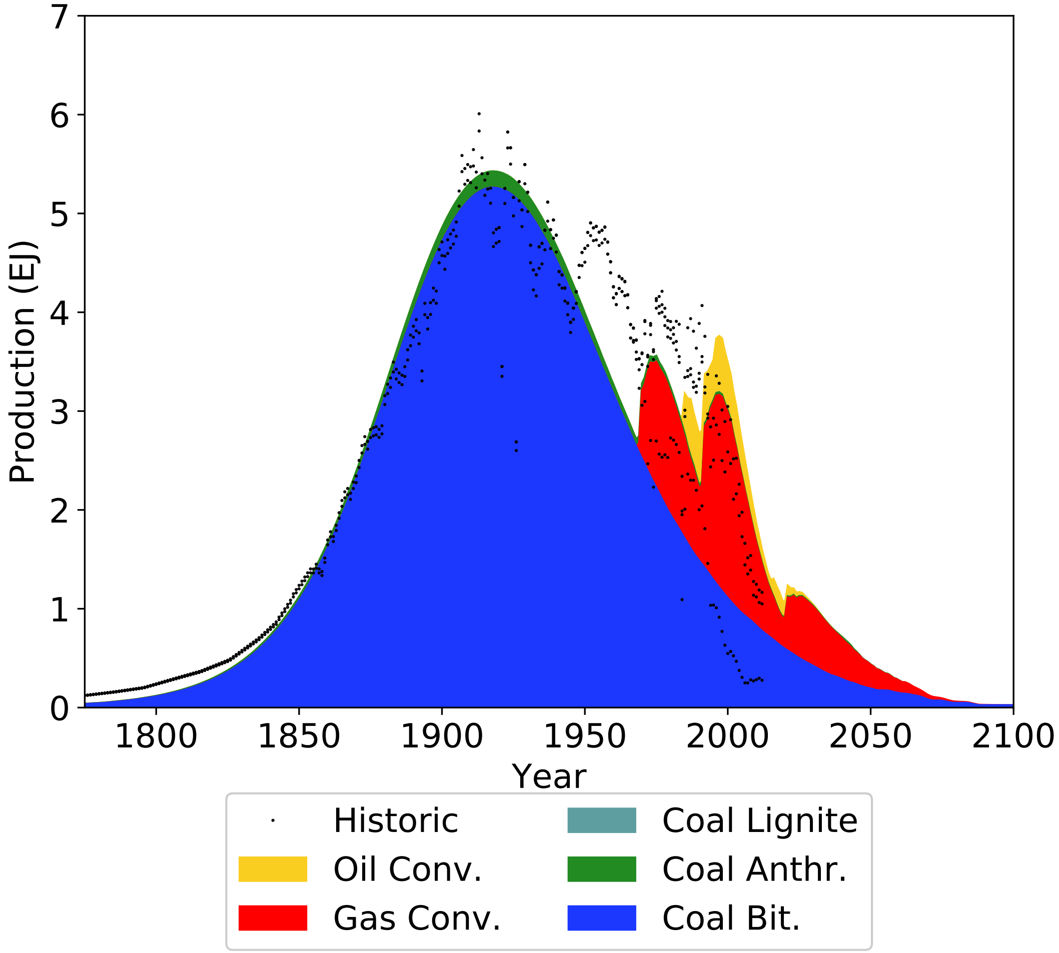

Figure 3.56: UK - England and Wales projections capped at 16

Table 3.56: Peak years - All

**Name**	**URR**	**Peak Year**	**Peak Rate**
Coal Bit. England and Wales	554.9	1918	5.26
Gas Conv. England and Wales	73.94	1998	1.96
Coal Anthr. England and Wales	17.0	1921	0.17
Oil Conv. England and Wales	13.28	1987	0.58
Coal Lignite England and Wales	–	1947	–
**Total**	**659.12**	**1918**	**5.43**

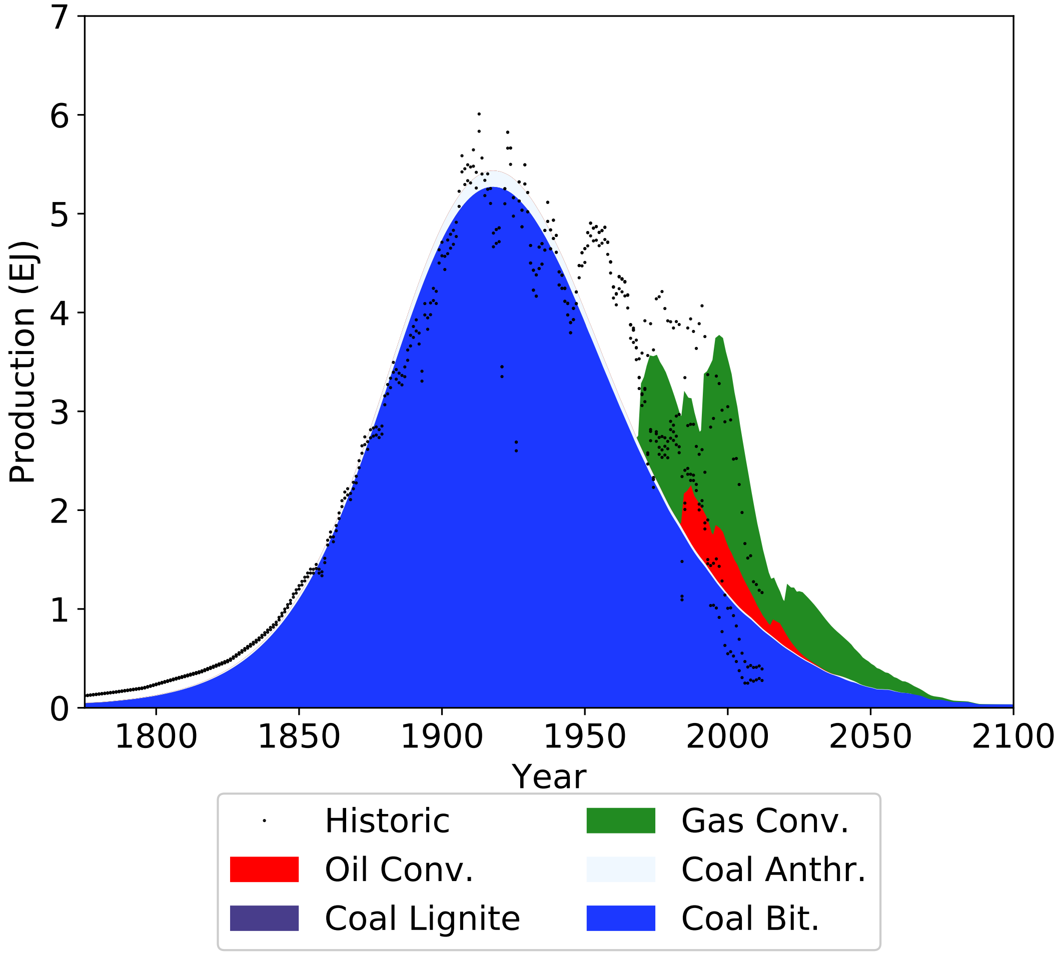

Figure 3.57: UK - England and Wales projection by mineral type

Table 3.57: Peak years - Minerals

**Name**	**URR**	**Peak Year**	**Peak Rate**
Coal Bit.	554.9	1918	5.26
Coal Lignite	–	1947	–
Coal Anthr.	17.0	1921	0.17
Oil Conv.	13.28	1987	0.58
Gas Conv.	73.94	1998	1.96
**Total**	**659.12**	**1918**	**5.43**

Northern Ireland

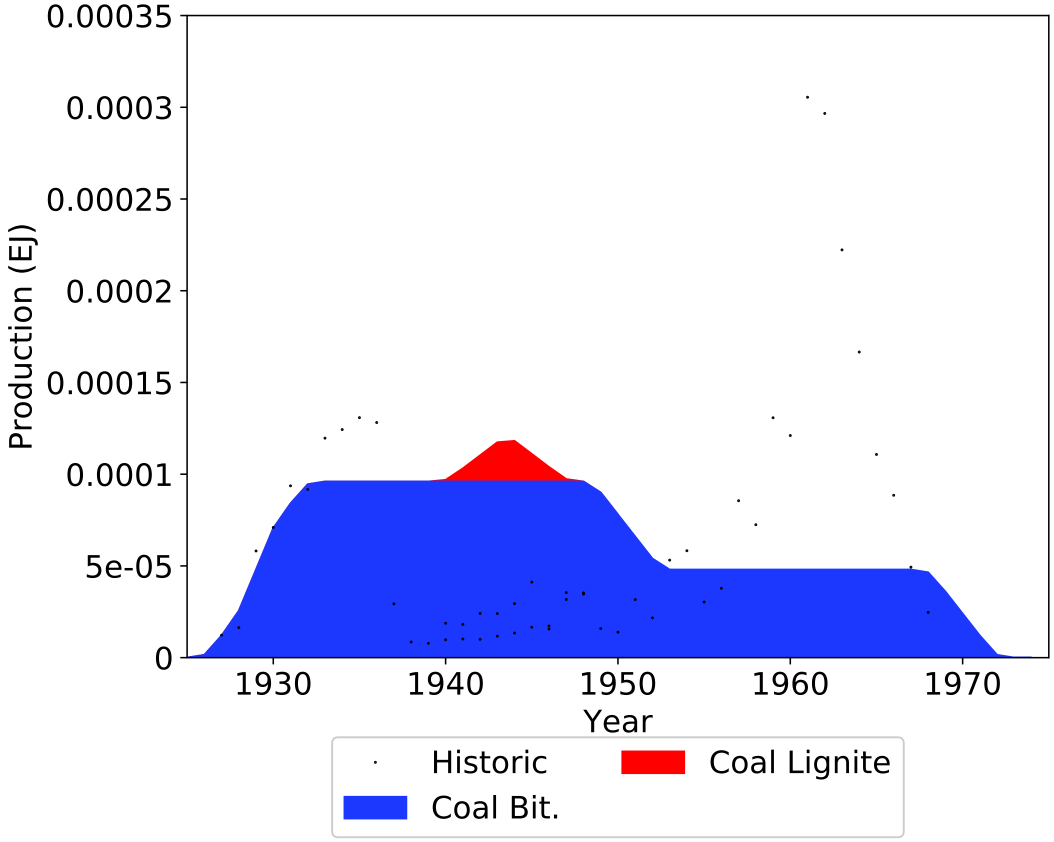

Figure 3.58: UK - Northern Ireland projections capped at 16

Table 3.58: Peak years - All

Name	URR	Peak Year	Peak Rate
Coal Bit. Northern Ireland	–	1933	–
Coal Lignite Northern Ireland	–	1944	–
**Total**	–	**1944**	–

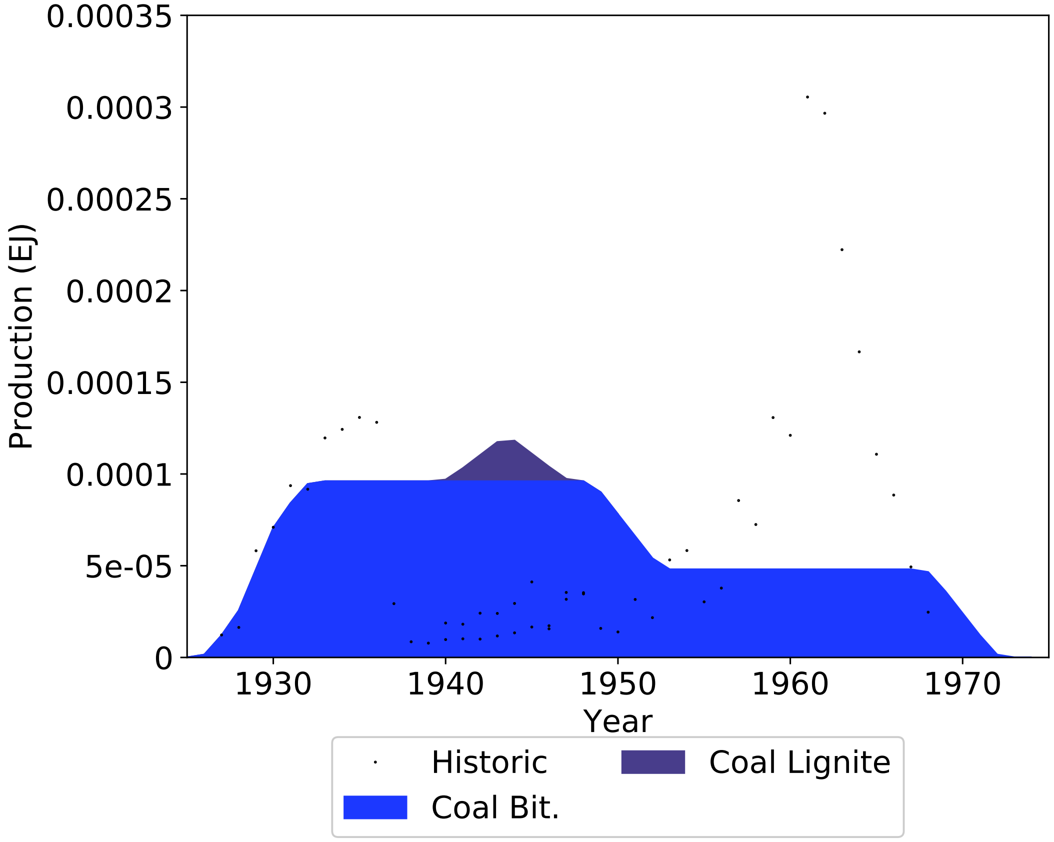

Figure 3.59: UK - Northern Ireland projection by mineral type

Table 3.59: Peak years - Minerals

Name	URR	Peak Year	Peak Rate
Coal Bit.	–	1933	–
Coal Lignite	–	1944	–
**Total**	–	**1944**	–

Scotland

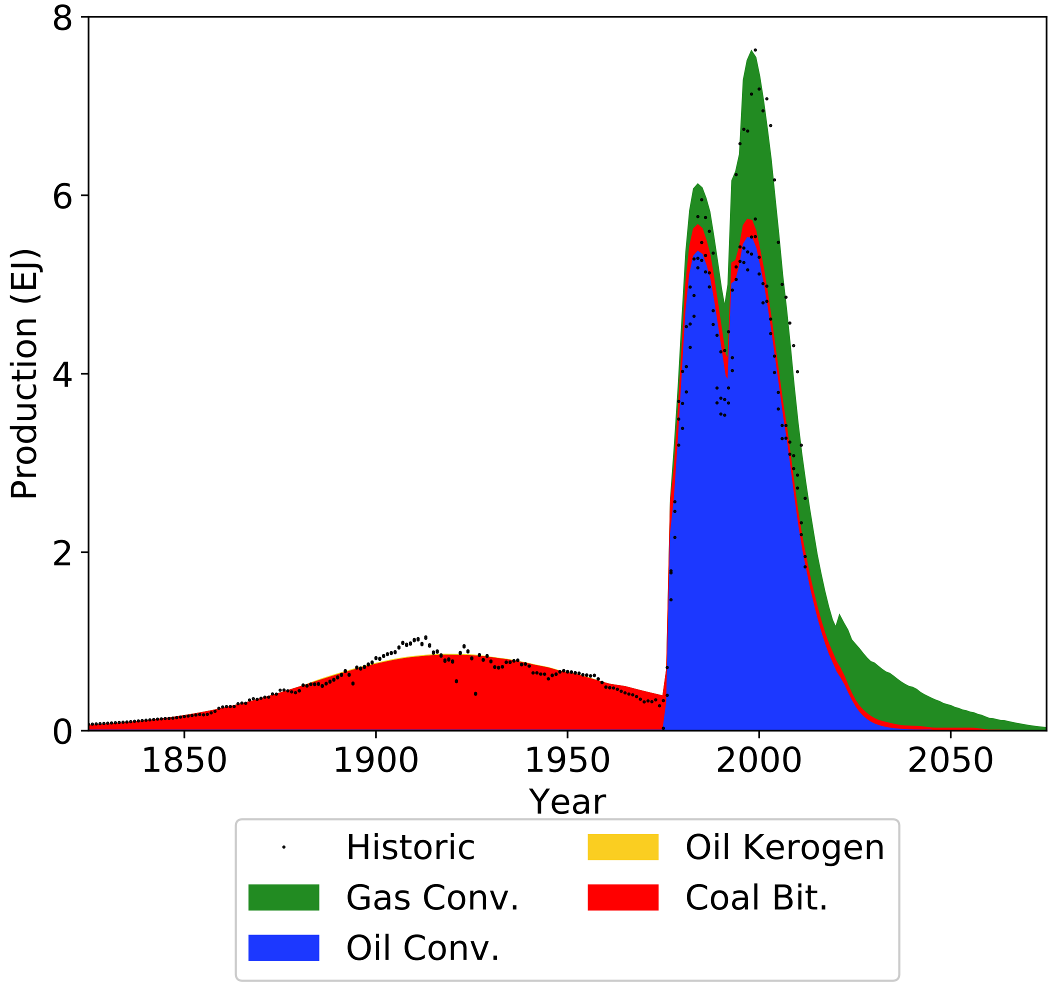

Figure 3.60: UK - Scotland projections capped at 16

Table 3.60: Peak years - All

Name	URR	Peak Year	Peak Rate
Oil Conv. Scotland	164.26	1997	5.52
Coal Bit. Scotland	88.7	1918	0.84
Gas Conv. Scotland	57.36	1999	1.94
Oil Kerogen Scotland	0.68	1887	0.01
**Total**	**311.0**	**1998**	**7.61**

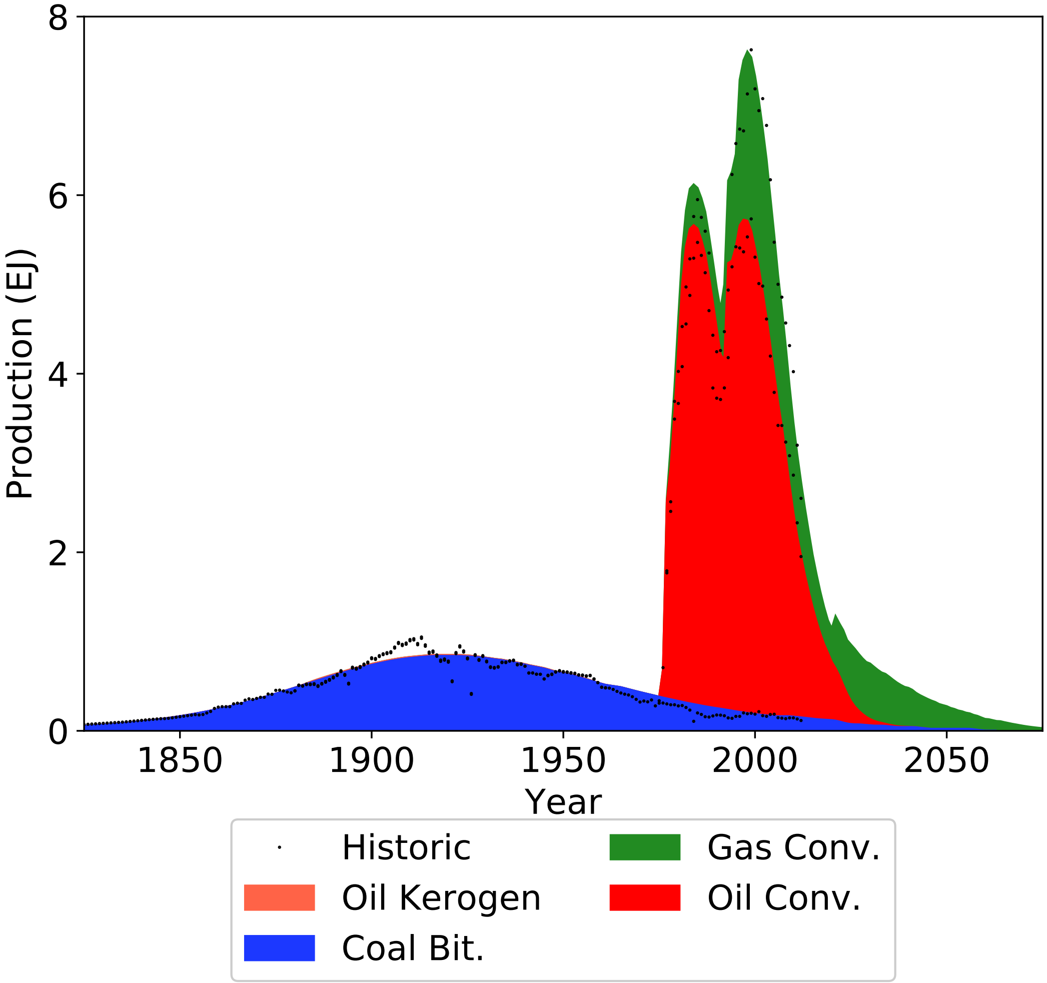

Figure 3.61: UK - Scotland projection by mineral type

Table 3.61: Peak years - Minerals

Name	URR	Peak Year	Peak Rate
Coal Bit.	88.7	1918	0.84
Oil Conv.	164.26	1997	5.52
Oil Kerogen	0.68	1887	0.01
Gas Conv.	57.36	1999	1.94
**Total**	**311.0**	**1998**	**7.61**

UK

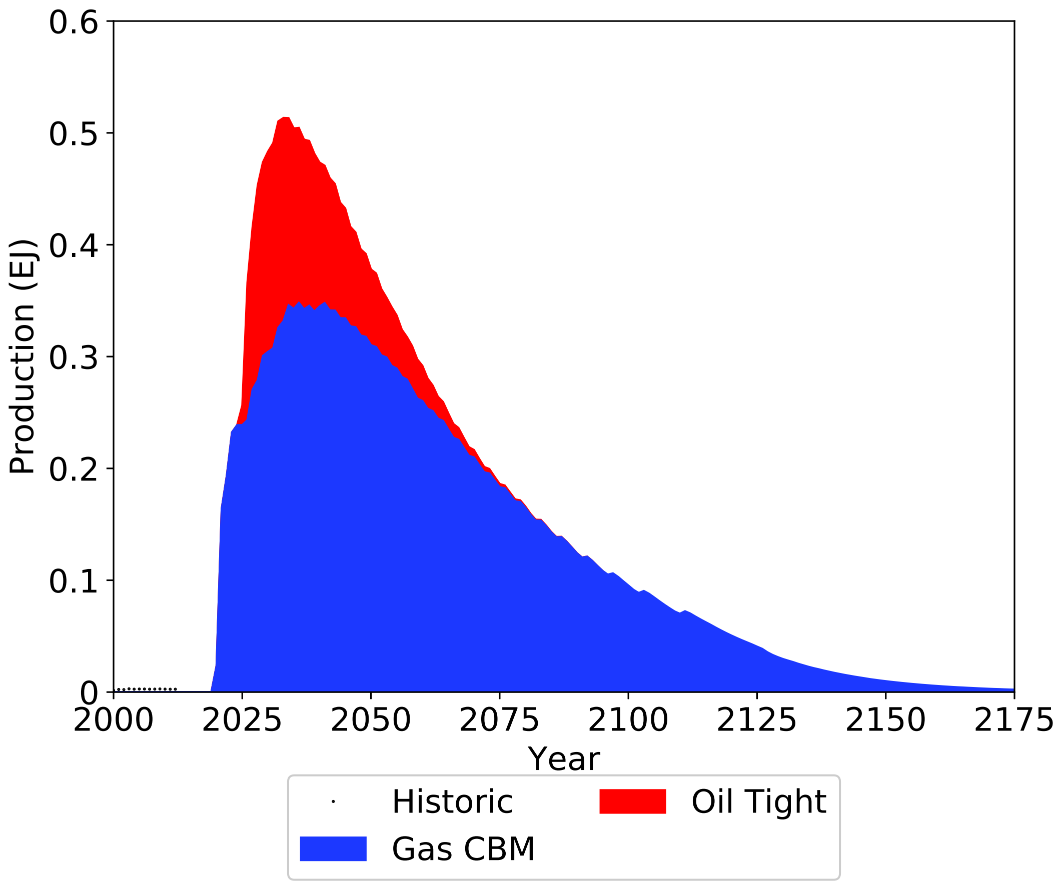

Figure 3.62: UK - UK projections capped at 16

Table 3.62: Peak years - All

Name	URR	Peak Year	Peak Rate
Gas CBM UK	21.0	2036	0.35
Oil Tight UK	4.01	2032	0.18
**Total**	**25.01**	**2033**	**0.51**

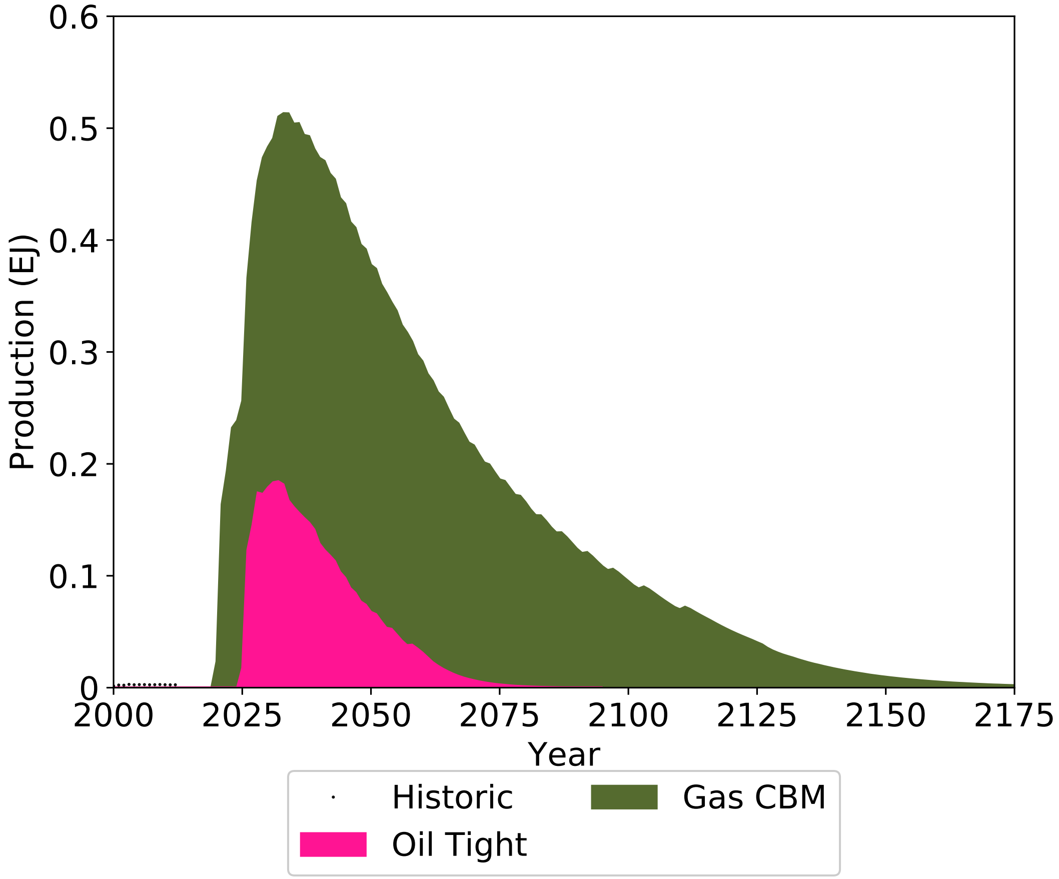

Figure 3.63: UK - UK projection by mineral type

Table 3.63: Peak years - Minerals

Name	URR	Peak Year	Peak Rate
Oil Tight	4.01	2032	0.18
Gas CBM	21.0	2036	0.35
**Total**	**25.01**	**2033**	**0.51**

3.26.4 Projection by region

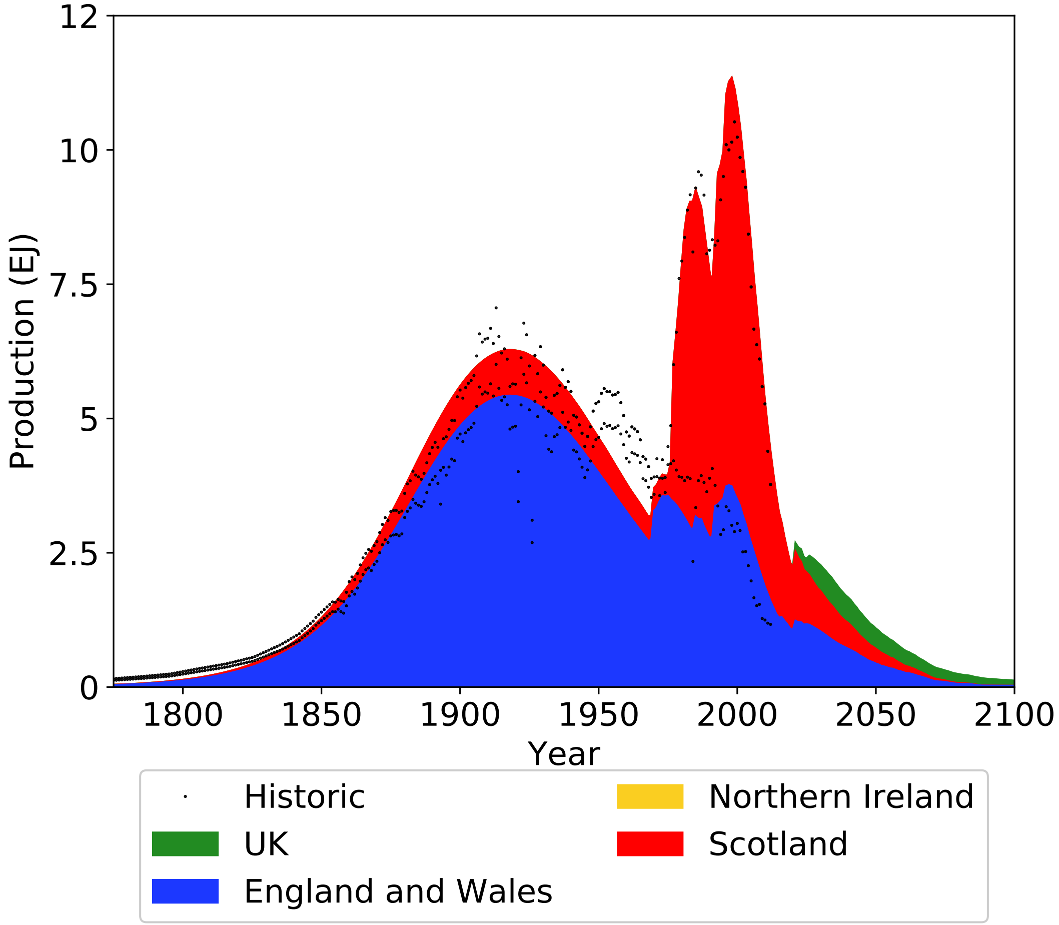

Figure 3.64: UK by region projections capped at 16

Table 3.64: Peak years - All

**Name**	**URR**	**Peak Year**	**Peak Rate**
England and Wales	659.12	1918	5.43
Scotland	311.0	1998	7.61
UK	25.01	2033	0.51
Northern Ireland	–	1944	–
**Total**	**995.13**	**1998**	**11.34**

3.27 Yugoslavia

3.27.1 All Projections

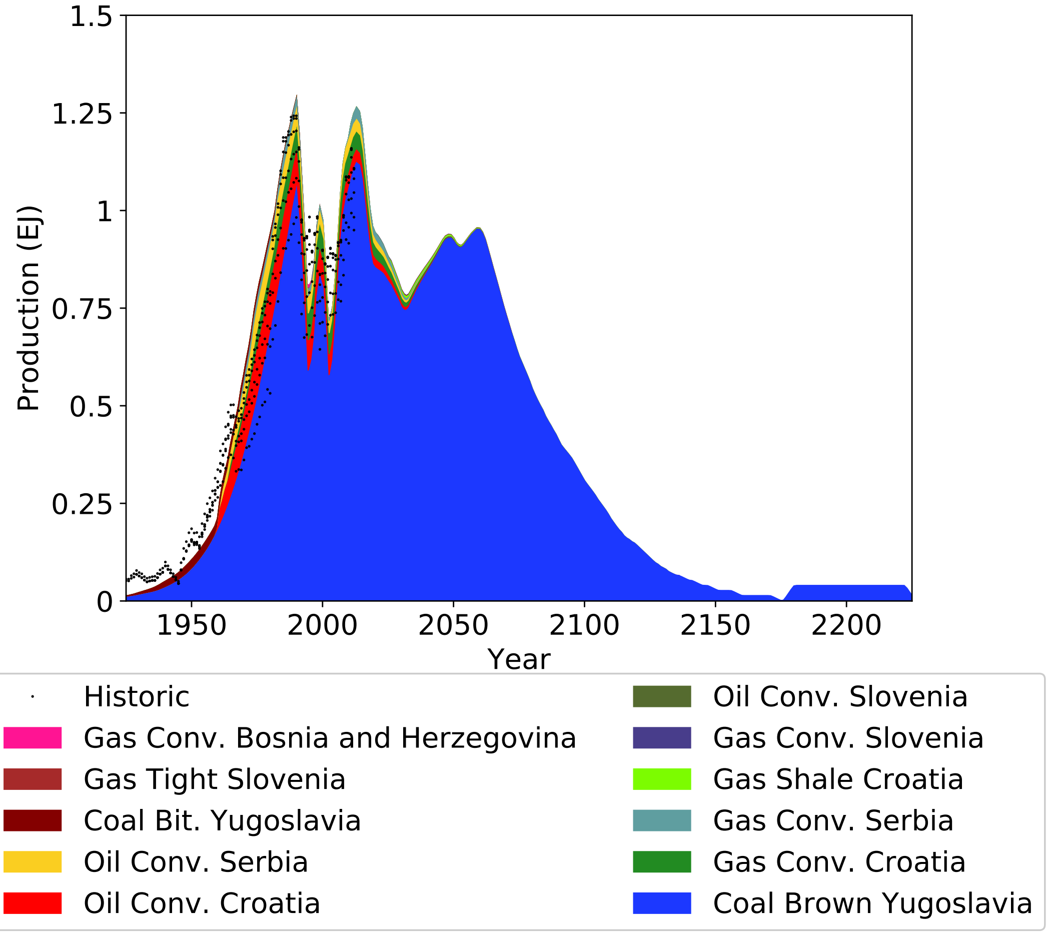

Figure 3.65: Yugoslavia projections capped at 16

Table 3.65: Peak years - All

**Name**	**URR**	**Peak Year**	**Peak Rate**
Coal Brown Yugoslavia	110.2	2013	1.12
Oil Conv. Croatia	4.73	1976	0.13
Gas Conv. Croatia	2.89	1993	0.07
Oil Conv. Serbia	2.7	1976	0.06
Gas Conv. Serbia	1.24	1986	0.03
Coal Bit. Yugoslavia	1.16	1956	0.03
Gas Shale Croatia	0.19	2027	0.01
Gas Tight Slovenia	0.06	2029	–
Gas Conv. Slovenia	0.04	2022	–
Gas Conv. Bosnia and Herzegovina	0.03	1995	0.01
Oil Conv. Slovenia	–	1996	–
**Total**	**123.24**	**1990**	**1.28**

3.27.2 By Mineral

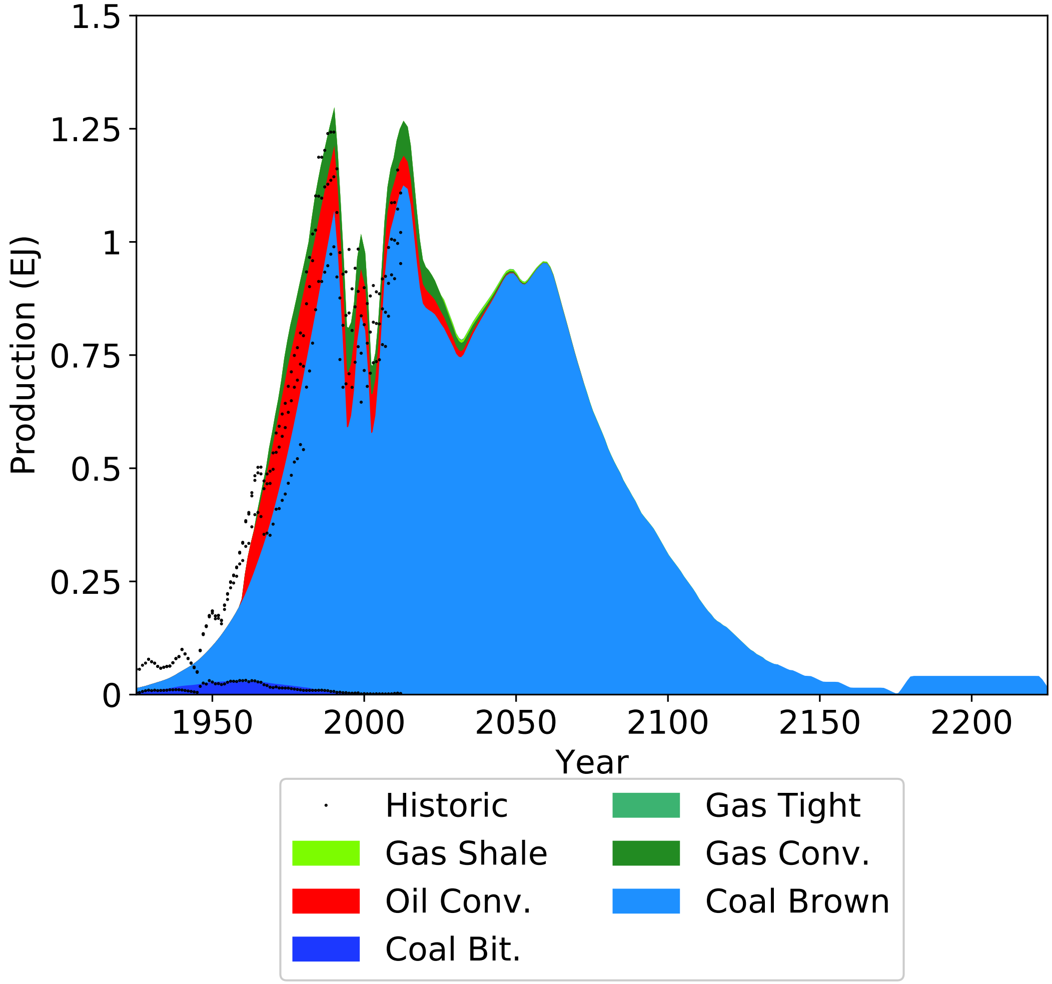

Figure 3.66: Yugoslavia projection by mineral type

3.27.3 Regional Projections

Table 3.66: Peak years - Minerals

**Name**	**URR**	**Peak Year**	**Peak Rate**
Coal Bit.	1.16	1956	0.03
Coal Brown	110.2	2013	1.12
Oil Conv.	7.43	1976	0.19
Gas Conv.	4.21	1994	0.1
Gas Shale	0.19	2027	0.01
Gas Tight	0.06	2029	–
**Total**	**123.24**	**1990**	**1.28**

Bosnia and Herzegovina

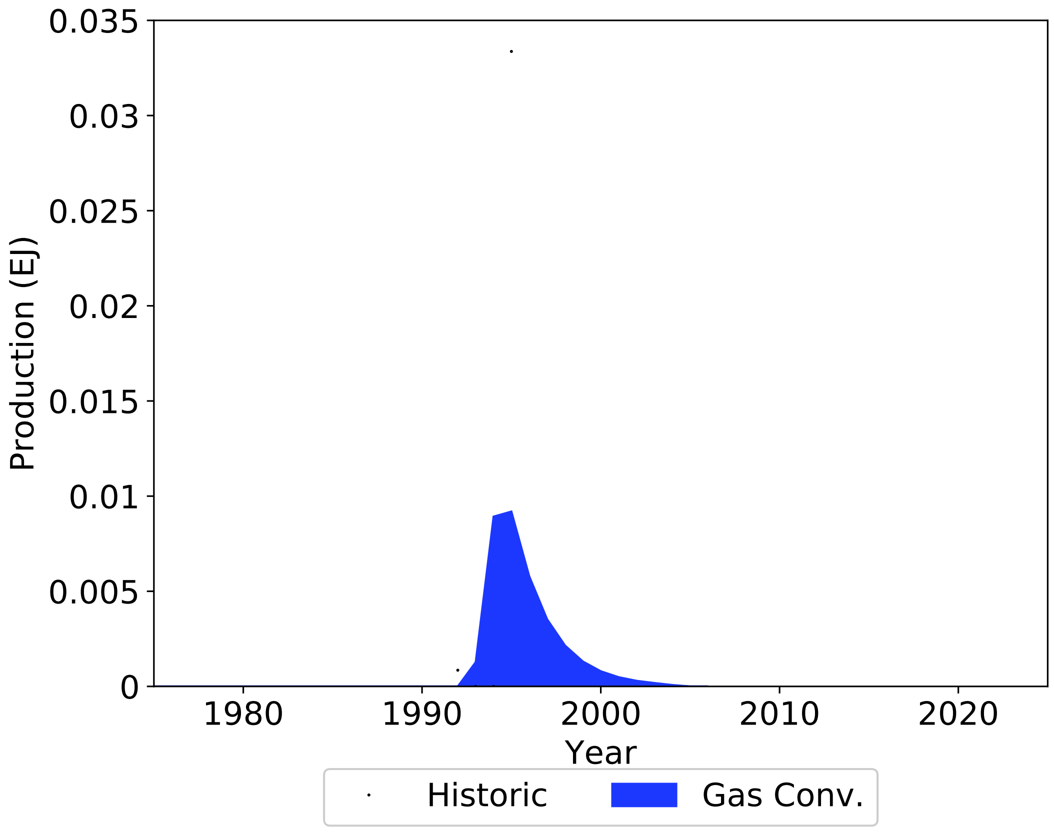

Figure 3.67: Yugoslavia - Bosnia and Herzegovina projections capped at 16

Table 3.67: Peak years - All

Name	URR	Peak Year	Peak Rate
Gas Conv. Bosnia and Herzegovina	0.03	1995	0.01
**Total**	**0.03**	**1995**	**0.01**

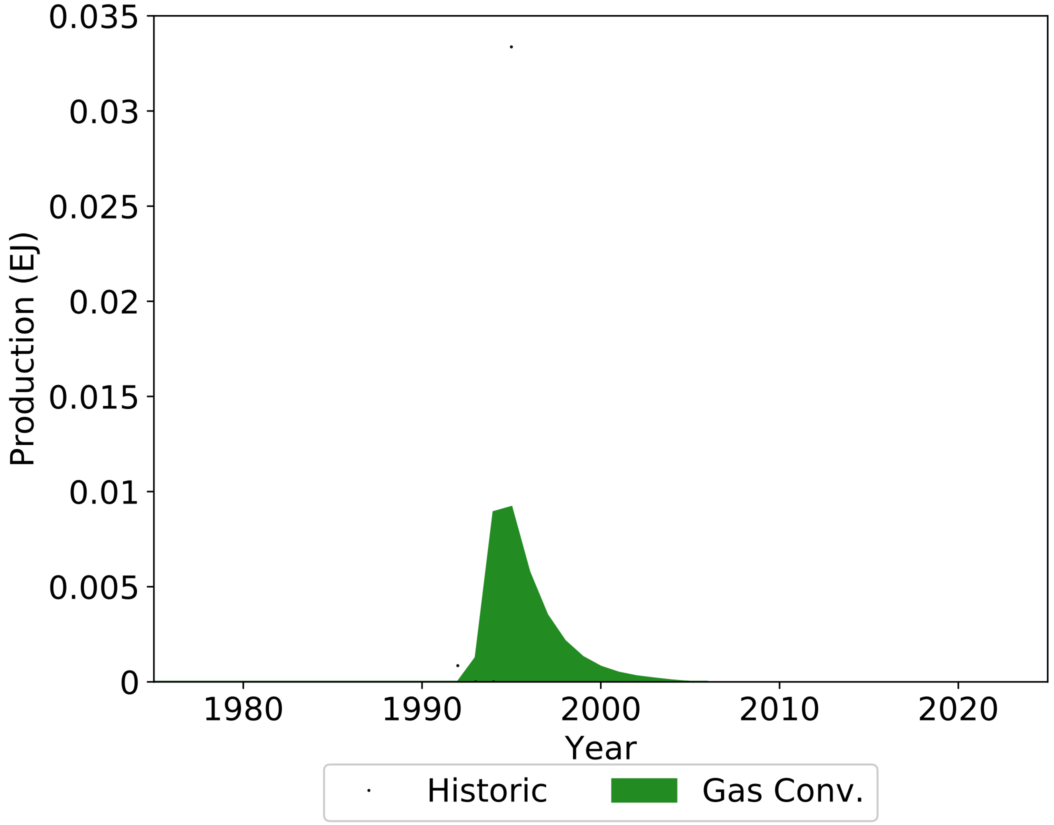

Figure 3.68: Yugoslavia - Bosnia and Herzegovina projection by mineral type

Table 3.68: Peak years - Minerals

**Name**	**URR**	**Peak Year**	**Peak Rate**
Gas Conv.	0.03	1995	0.01
**Total**	**0.03**	**1995**	**0.01**

Croatia

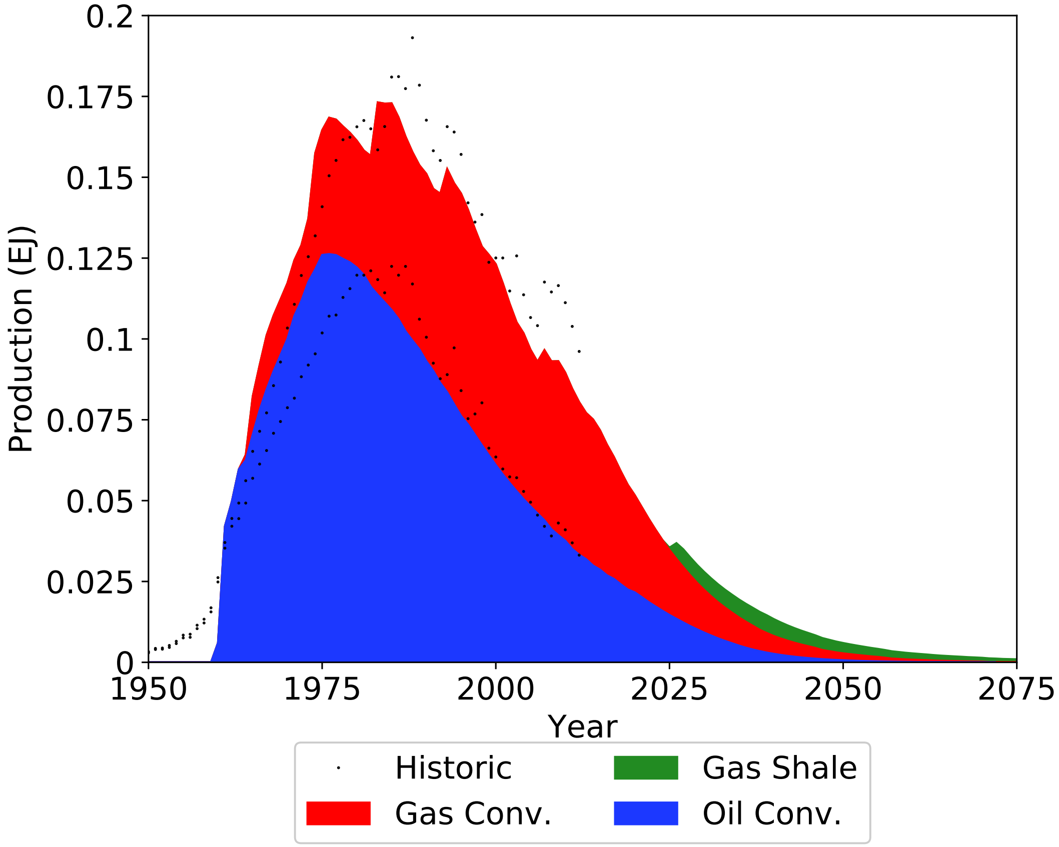

Figure 3.69: Yugoslavia - Croatia projections capped at 16

Table 3.69: Peak years - All

Name	URR	Peak Year	Peak Rate
Oil Conv. Croatia	4.73	1976	0.13
Gas Conv. Croatia	2.89	1993	0.07
Gas Shale Croatia	0.19	2027	0.01
**Total**	**7.81**	**1983**	**0.17**

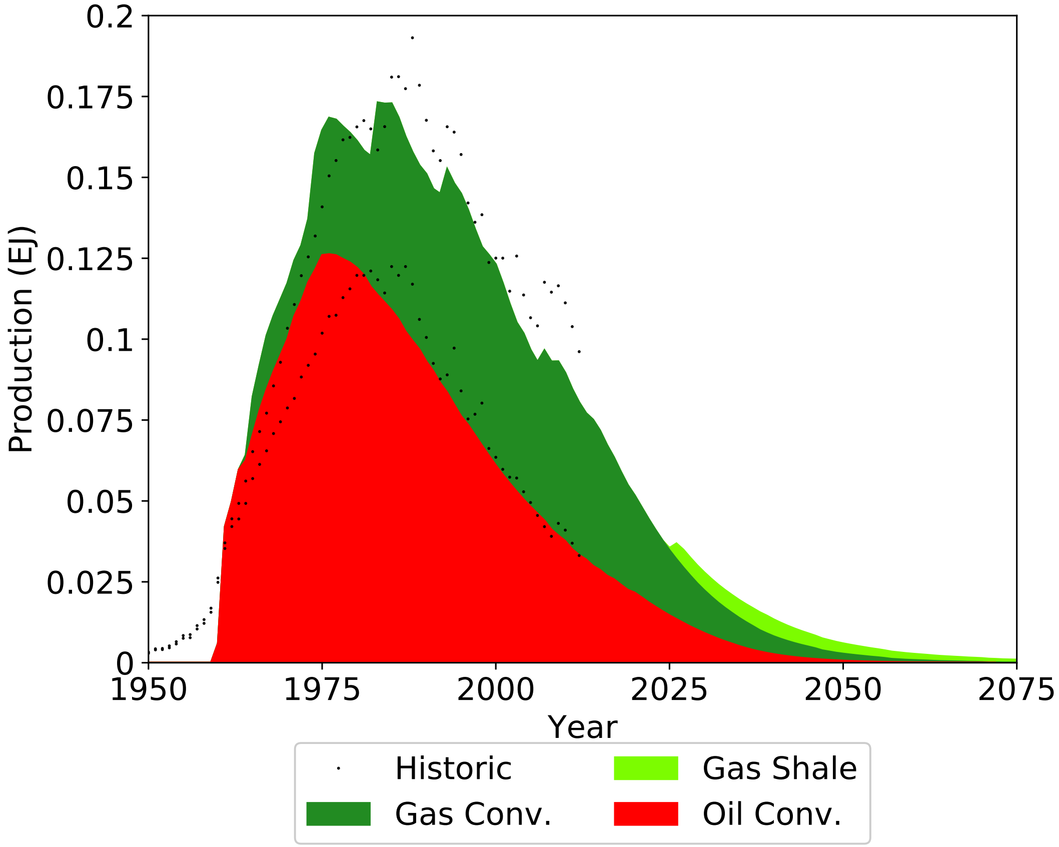

Figure 3.70: Yugoslavia - Croatia projection by mineral type

Table 3.70: Peak years - Minerals

Name	URR	Peak Year	Peak Rate
Oil Conv.	4.73	1976	0.13
Gas Conv.	2.89	1993	0.07
Gas Shale	0.19	2027	0.01
**Total**	**7.81**	**1983**	**0.17**

Serbia

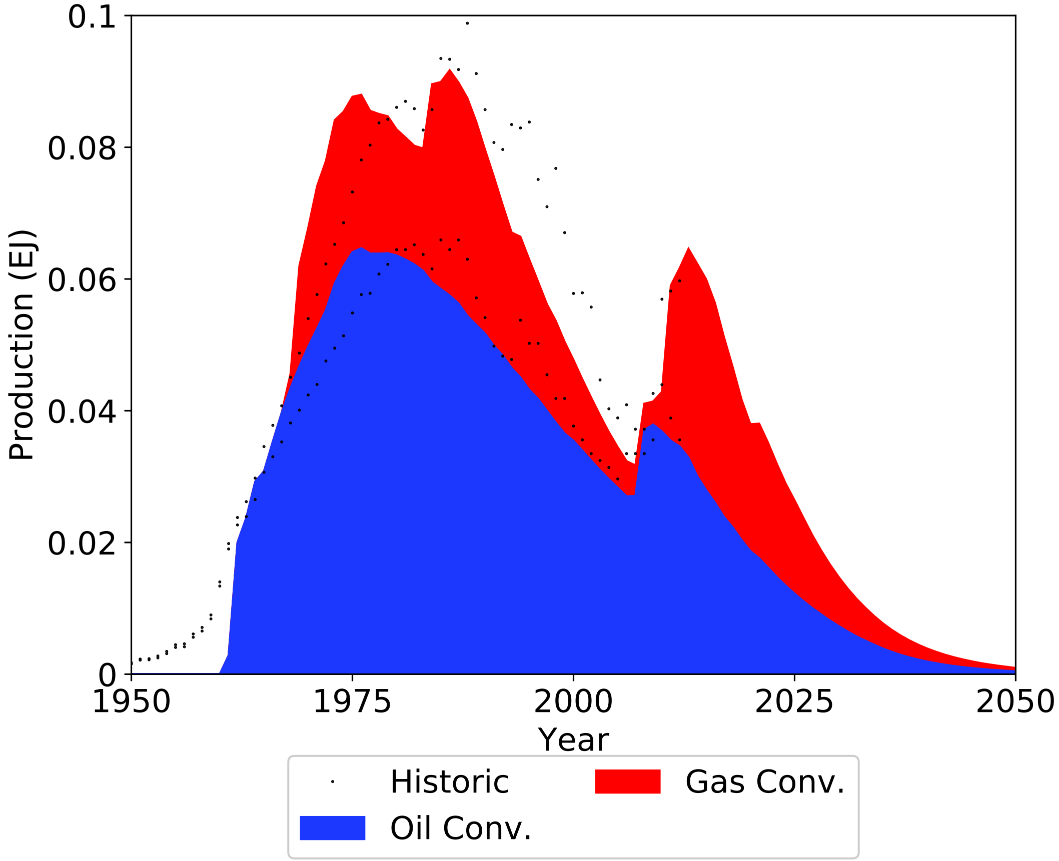

Figure 3.71: Yugoslavia - Serbia projections capped at 16

Table 3.71: Peak years - All

Name	URR	Peak Year	Peak Rate
Oil Conv. Serbia	2.7	1976	0.06
Gas Conv. Serbia	1.24	1986	0.03
**Total**	**3.94**	**1986**	**0.09**

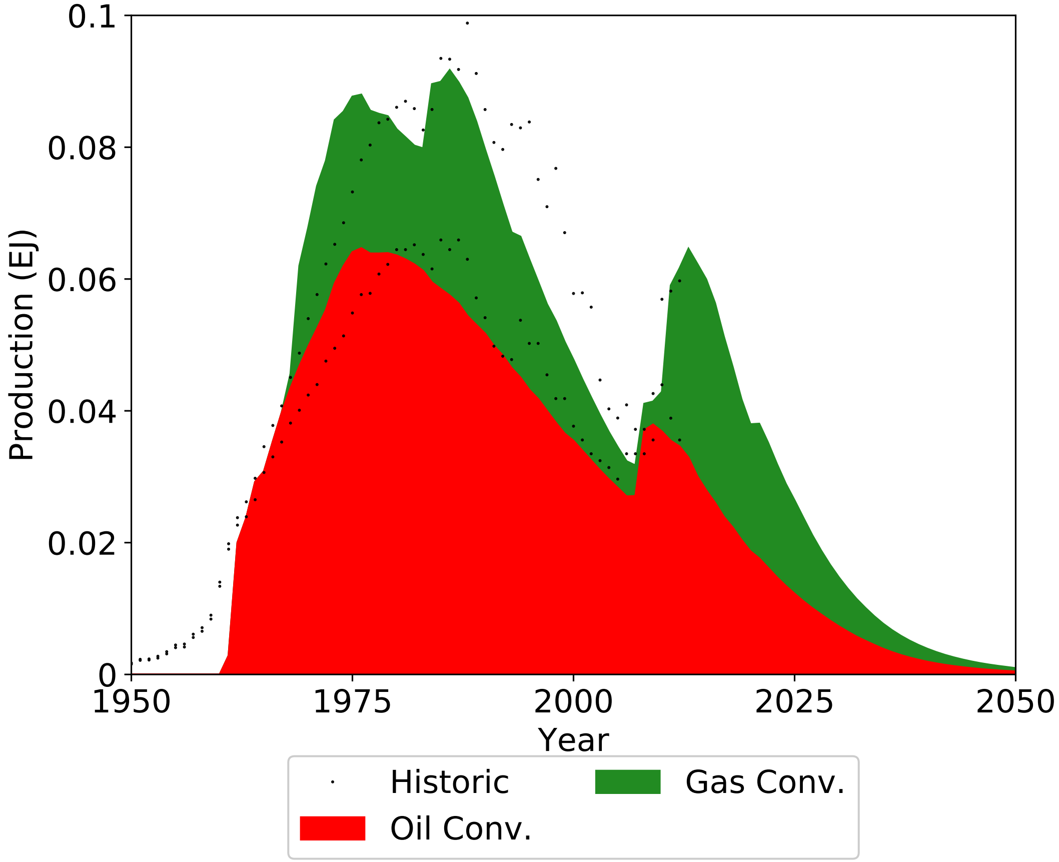

Figure 3.72: Yugoslavia - Serbia projection by mineral type

Table 3.72: Peak years - Minerals

Name	URR	Peak Year	Peak Rate
Oil Conv.	2.7	1976	0.06
Gas Conv.	1.24	1986	0.03
**Total**	**3.94**	**1986**	**0.09**

Slovenia

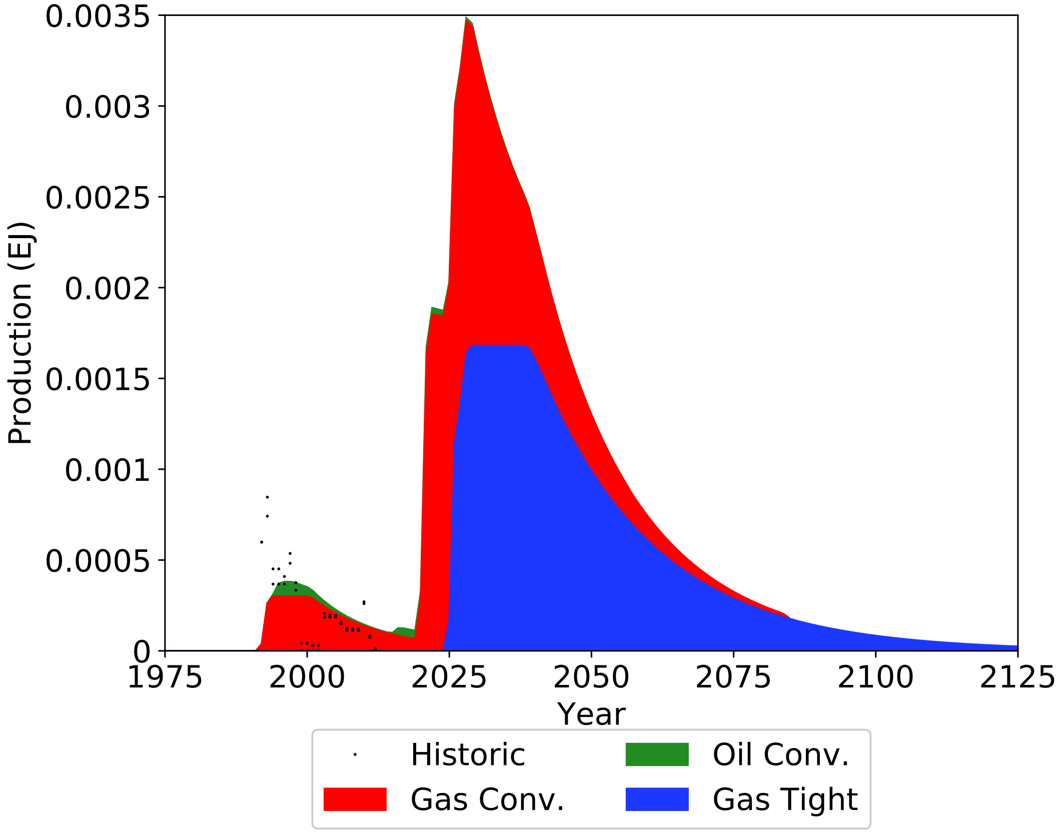

Figure 3.73: Yugoslavia - Slovenia projections capped at 16

Table 3.73: Peak years - All

**Name**	**URR**	**Peak Year**	**Peak Rate**
Gas Tight Slovenia	0.06	2029	–
Gas Conv. Slovenia	0.04	2022	–
Oil Conv. Slovenia	–	1996	–
**Total**	**0.1**	**2028**	–

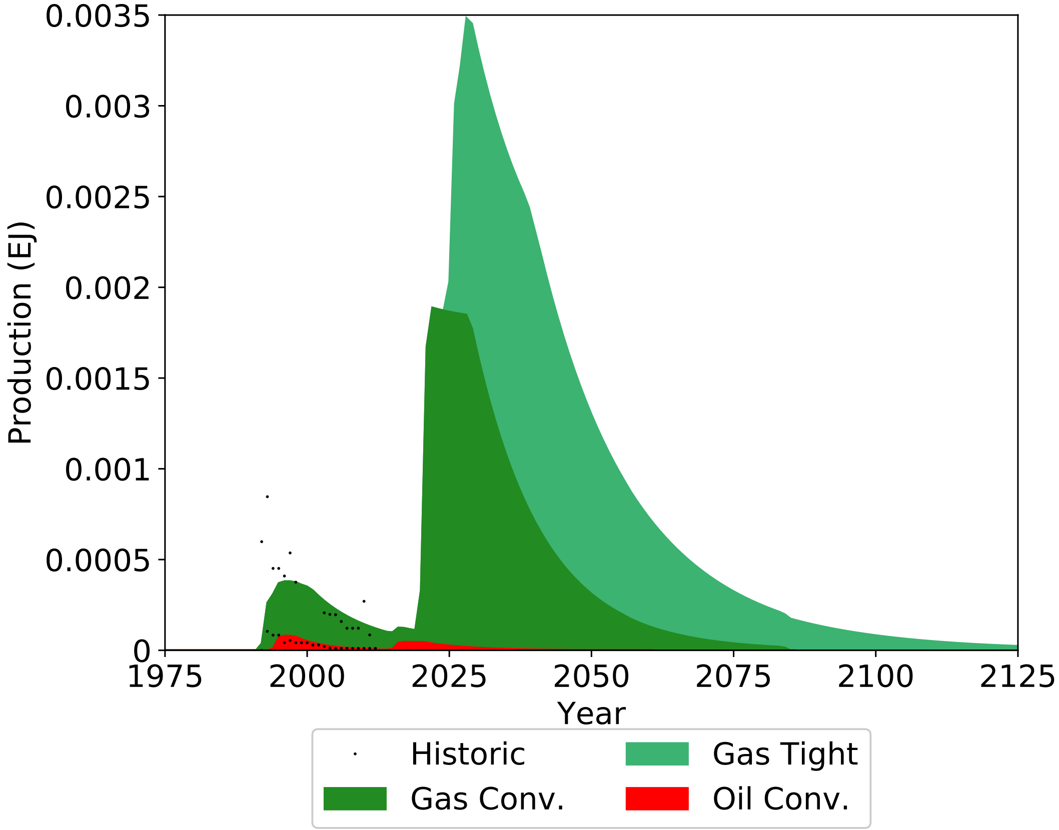

Figure 3.74: Yugoslavia - Slovenia projection by mineral type

Table 3.74: Peak years - Minerals

Name	URR	Peak Year	Peak Rate
Oil Conv.	–	1996	–
Gas Conv.	0.04	2022	–
Gas Tight	0.06	2029	–
**Total**	**0.1**	**2028**	–

Yugoslavia

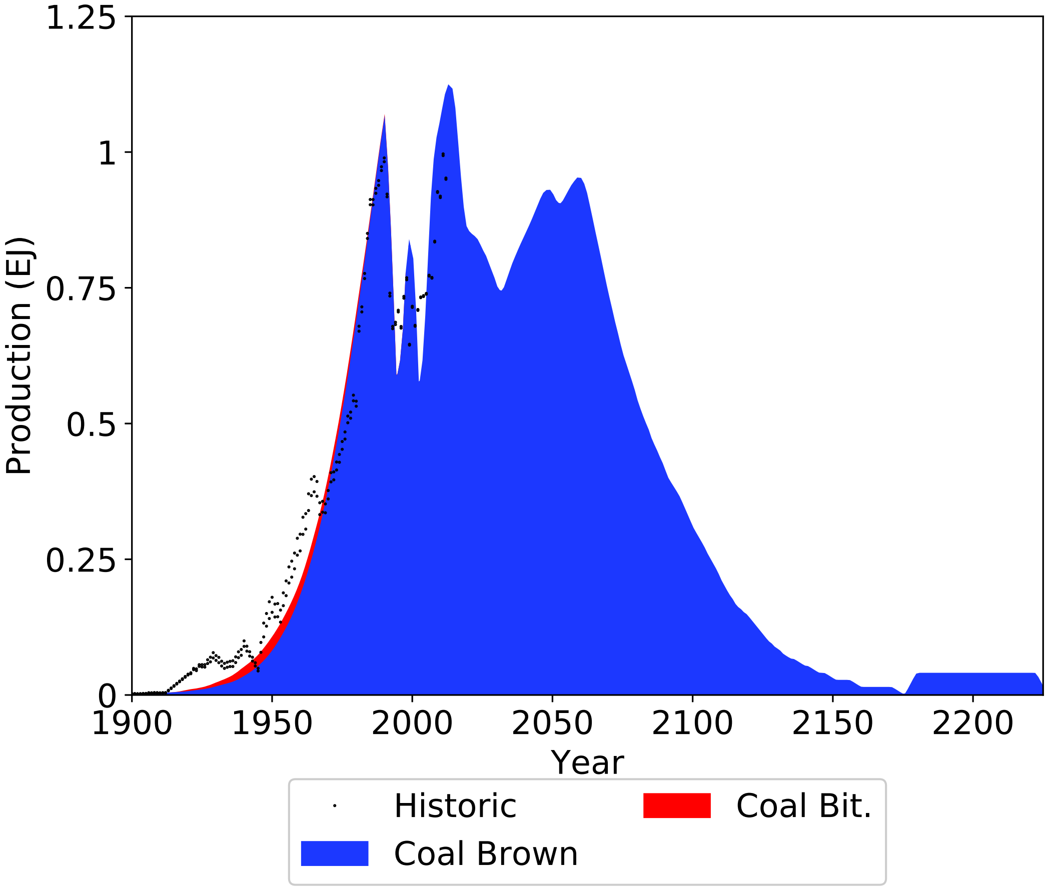

Figure 3.75: Yugoslavia - Yugoslavia projections capped at 16

Table 3.75: Peak years - All

Name	URR	Peak Year	Peak Rate
Coal Brown Yugoslavia	110.2	2013	1.12
Coal Bit. Yugoslavia	1.16	1956	0.03
**Total**	**111.36**	**2013**	**1.12**

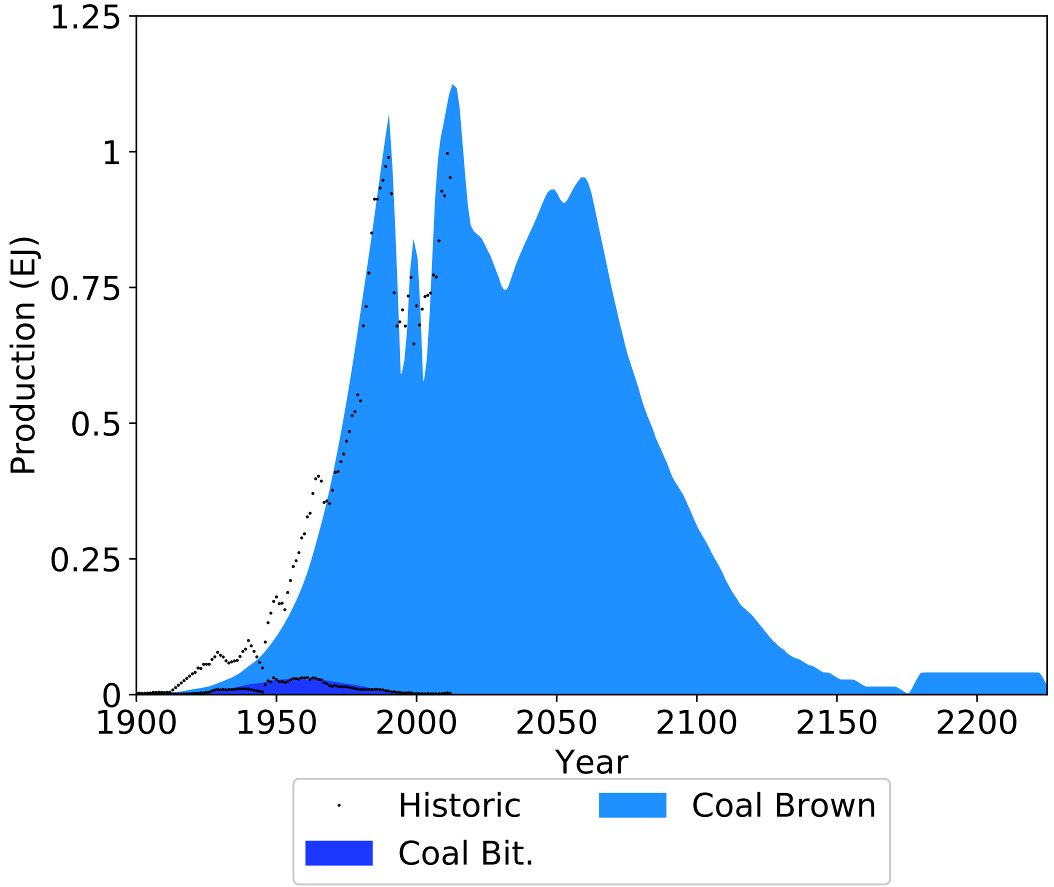

Figure 3.76: Yugoslavia - Yugoslavia projection by mineral type

Table 3.76: Peak years - Minerals

Name	URR	Peak Year	Peak Rate
Coal Bit.	1.16	1956	0.03
Coal Brown	110.2	2013	1.12
**Total**	**111.36**	**2013**	**1.12**

3.27.4 Projection by region

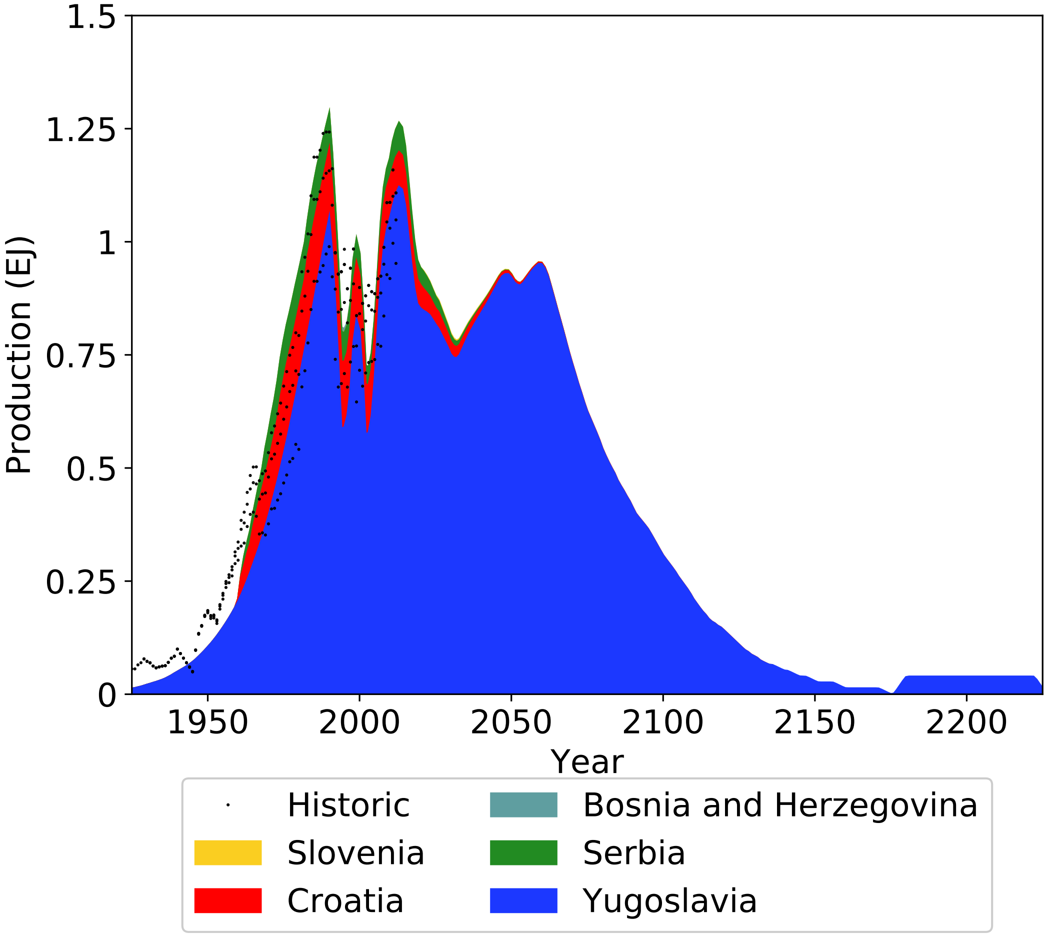

Figure 3.77: Yugoslavia by region projections capped at 16

Table 3.77: Peak years - All

**Name**	**URR**	**Peak Year**	**Peak Rate**
Yugoslavia	111.36	2013	1.12
Croatia	7.81	1983	0.17
Serbia	3.94	1986	0.09
Slovenia	0.1	2028	–
Bosnia and Herzegovina	0.03	1995	0.01
**Total**	**123.24**	**1990**	**1.28**

3.28 Total

3.28.1 By country

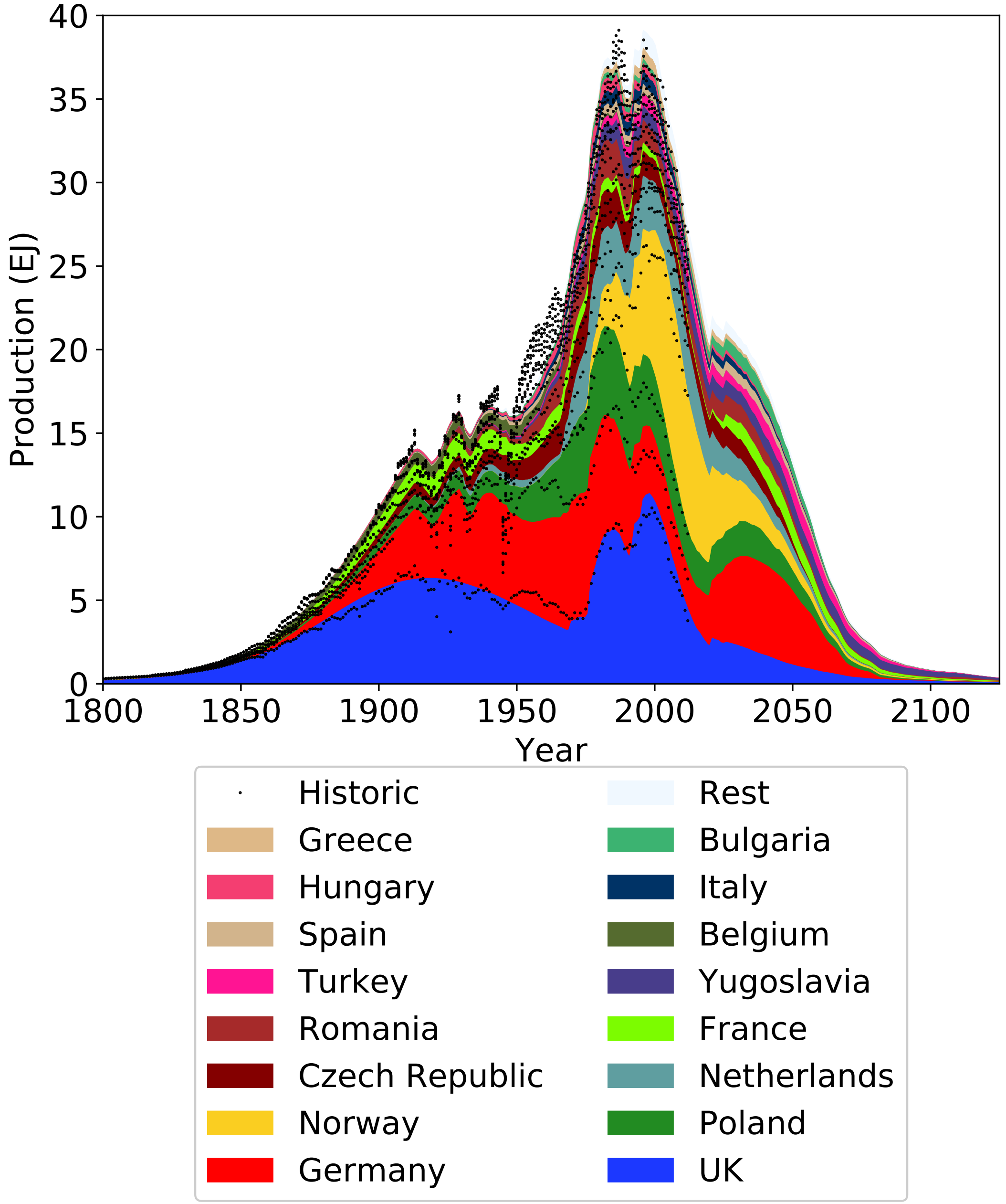

Figure 3.78: Europe projections by country

Table 3.78: Peak years - All

**Name**	**URR**	**Peak Year**	**Peak Rate**
UK	995.13	1998	11.34
Germany	823.0	1976	7.49
Poland	388.3	1984	5.33
Norway	349.88	2005	9.81
Netherlands	212.16	1978	3.8
Czech Republic	191.54	1977	2.34
France	170.75	1927	1.26
Romania	142.63	1982	2.28
Yugoslavia	123.24	1990	1.28
Turkey	79.38	2052	1.01
Belgium	67.12	1913	0.66
Spain	55.23	2030	0.62
Italy	50.65	1986	0.86
Hungary	48.29	1977	0.87
Bulgaria	48.2	2032	0.83
Greece	29.11	2001	0.67
Denmark	28.14	2001	0.99
Austria	19.4	1960	0.22
Albania	8.2	2019	0.19
Slovakia	6.44	2028	0.11
Sweden	5.0	2037	0.09
Ireland	4.58	2019	0.11
Portugal	2.39	2014	0.17
Cyprus	0.93	2029	0.03
Malta	0.2	2024	0.01
Switzerland	0.03	1944	0.01
Greenland	0.02	1953	–
**Total**	**3849.93**	**1996**	**39.05**

3.28.2 By mineral

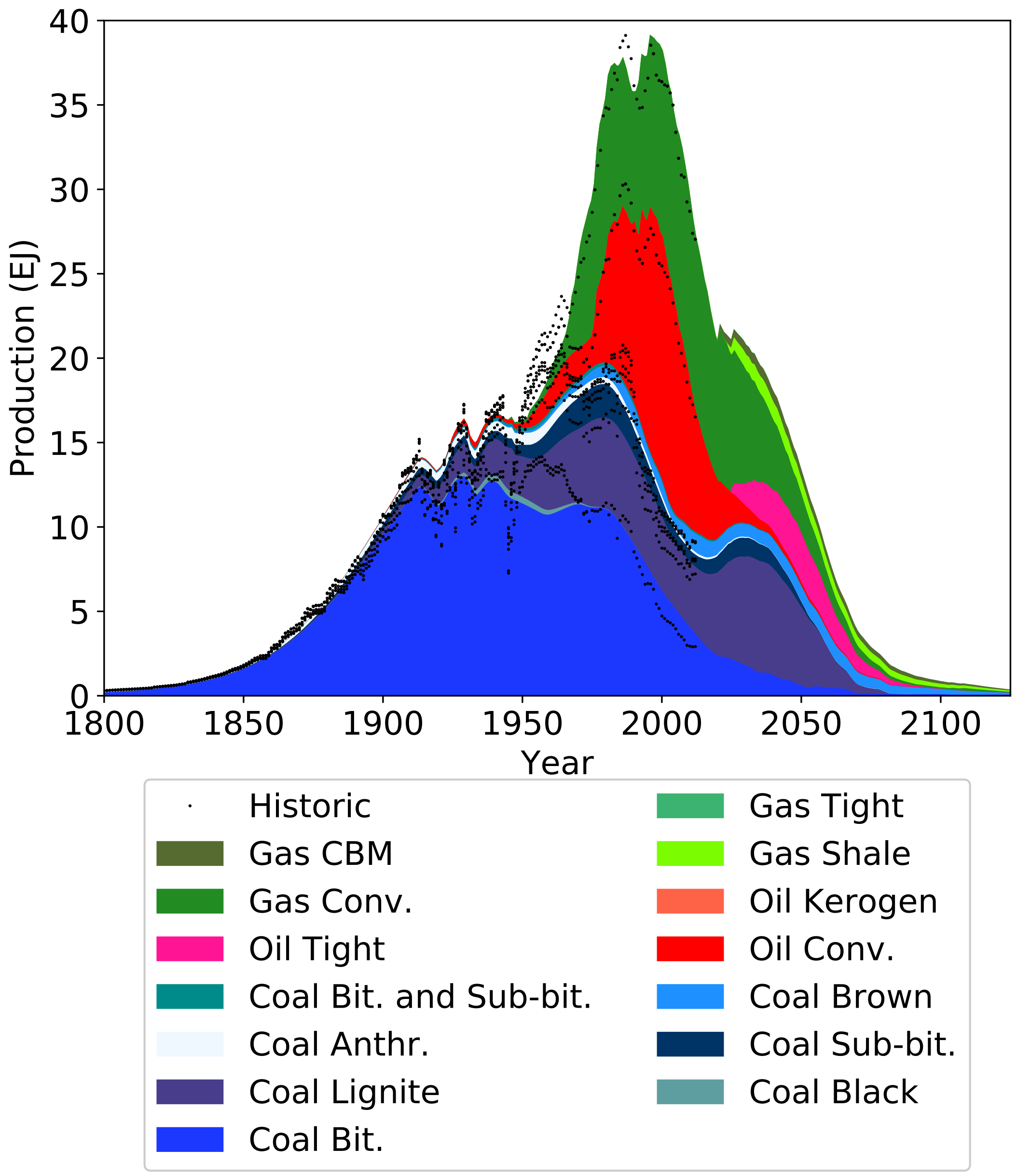

Figure 3.79: Europe projection by mineral type

Table 3.79: Peak years - Minerals

Name	URR	Peak Year	Peak Rate
Coal Bit.	1497.76	1929	12.95
Coal Black	14.9	1945	0.4
Coal Lignite	601.27	2034	6.64
Coal Sub-bit.	147.5	1978	1.98
Coal Anthr.	67.95	1953	0.72
Coal Brown	111.95	2013	1.12
Coal Bit. and Sub-bit.	19.4	1966	0.22
Oil Conv.	496.99	1998	14.72
Oil Tight	100.85	2048	2.97
Oil Kerogen	0.75	1887	0.01
Gas Conv.	699.31	2006	11.5
Gas Shale	54.14	2037	1.09
Gas CBM	36.75	2034	0.65
Gas Tight	0.43	2029	0.01
**Total**	**3849.93**	**1996**	**39.05**

Chapter 4

FSU

4.1 Azerbaijan

4.1.1 All Projections

Table 4.1: Peak years - All

Name	URR	Peak Year	Peak Rate
Oil Conv.	122.36	2013	1.93
Gas Conv.	70.41	2048	0.71
**Total**	**192.77**	**2013**	**2.61**

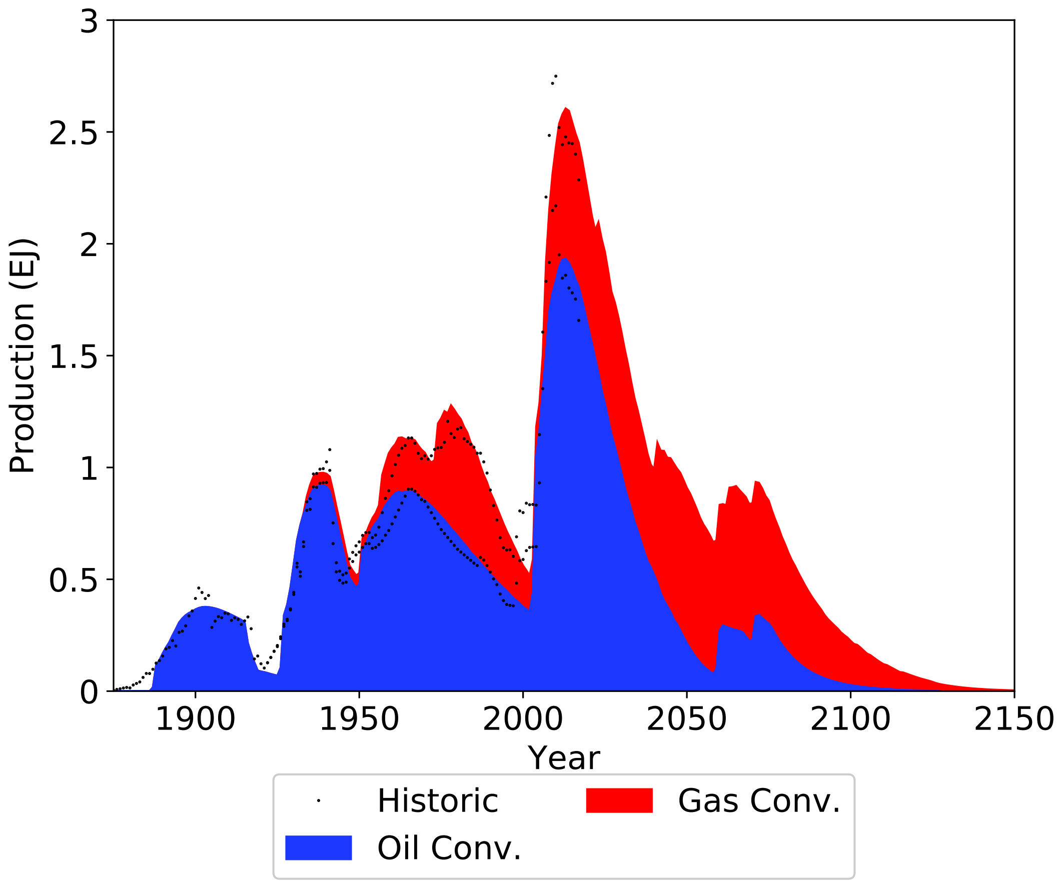

Figure 4.1: Azerbaijan projections capped at 16

4.1.2 By Mineral

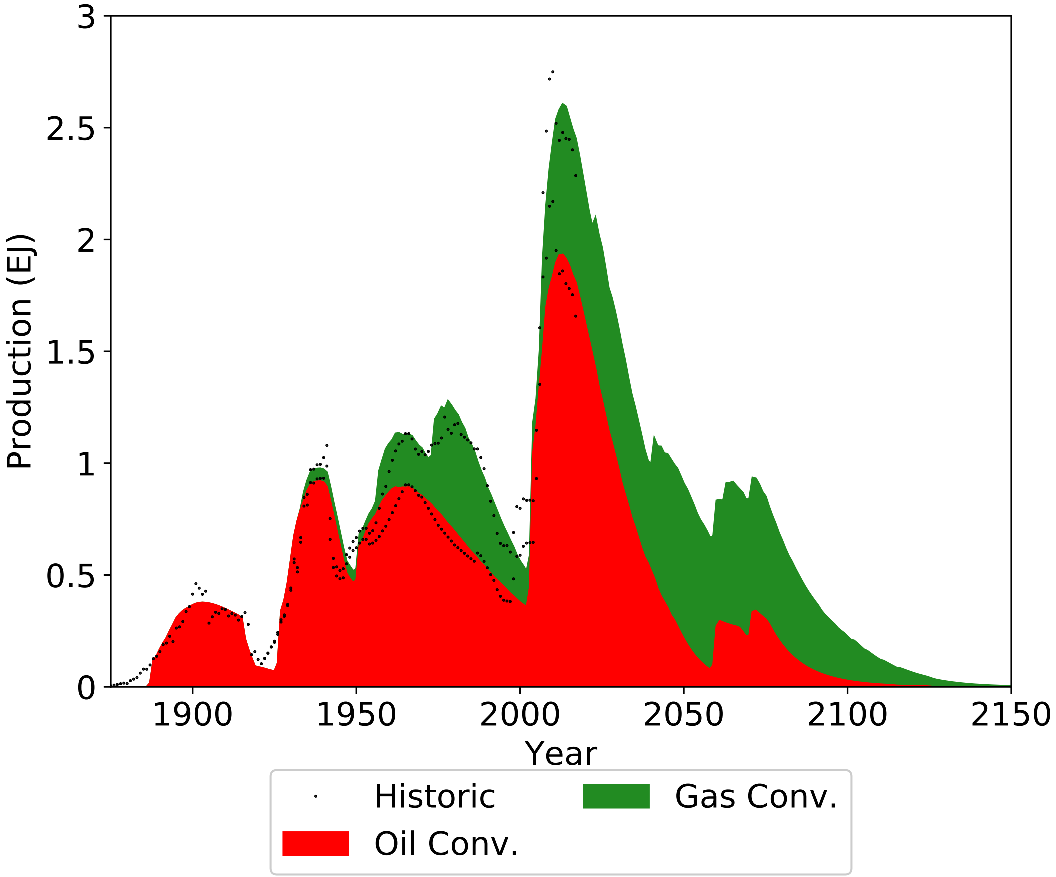

Figure 4.2: Azerbaijan projection by mineral type

Table 4.2: Peak years - Minerals

**Name**	**URR**	**Peak Year**	**Peak Rate**
Oil Conv.	122.36	2013	1.93
Gas Conv.	70.41	2048	0.71
**Total**	**192.77**	**2013**	**2.61**

4.2 Belarus

4.2.1 All Projections

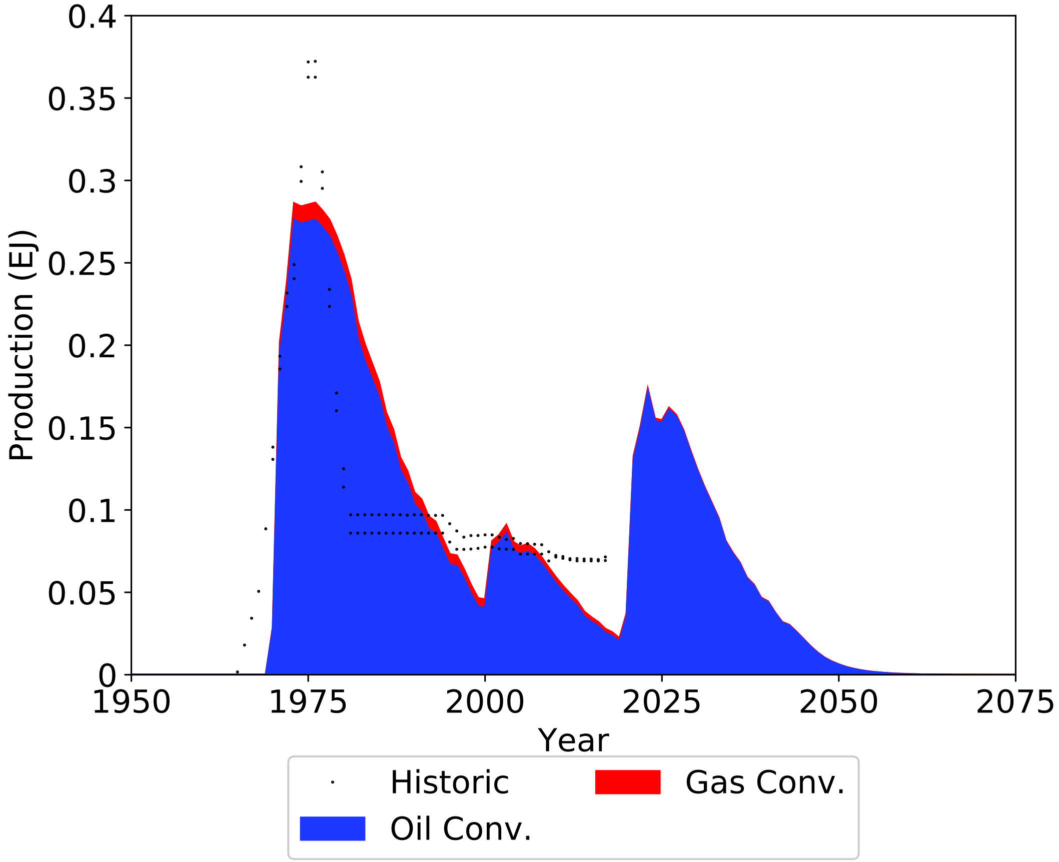

Figure 4.3: Belarus projections capped at 16

Table 4.3: Peak years - All

**Name**	**URR**	**Peak Year**	**Peak Rate**
Oil Conv.	8.36	1976	0.28
Gas Conv.	0.36	1982	0.01
**Total**	**8.72**	**1976**	**0.29**

4.2.2 By Mineral

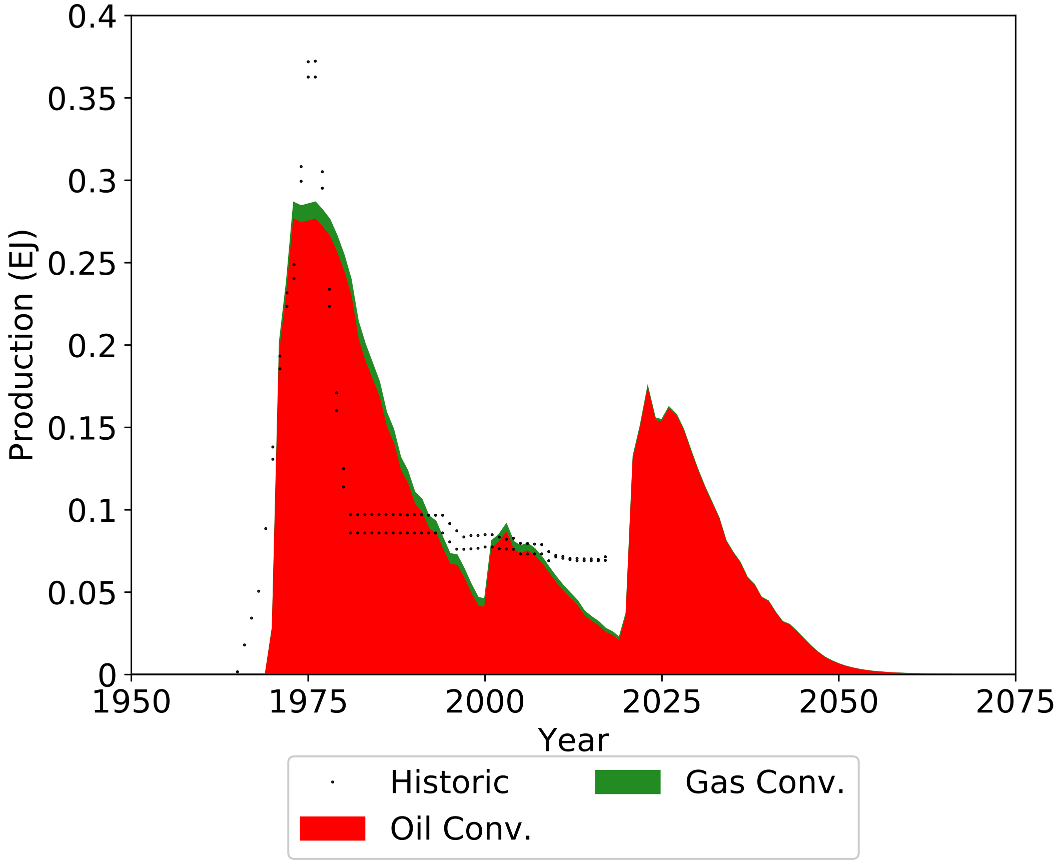

Figure 4.4: Belarus projection by mineral type

Table 4.4: Peak years - Minerals

Name	URR	Peak Year	Peak Rate
Oil Conv.	8.36	1976	0.28
Gas Conv.	0.36	1982	0.01
**Total**	**8.72**	**1976**	**0.29**

4.3 Crimea

4.3.1 All Projections

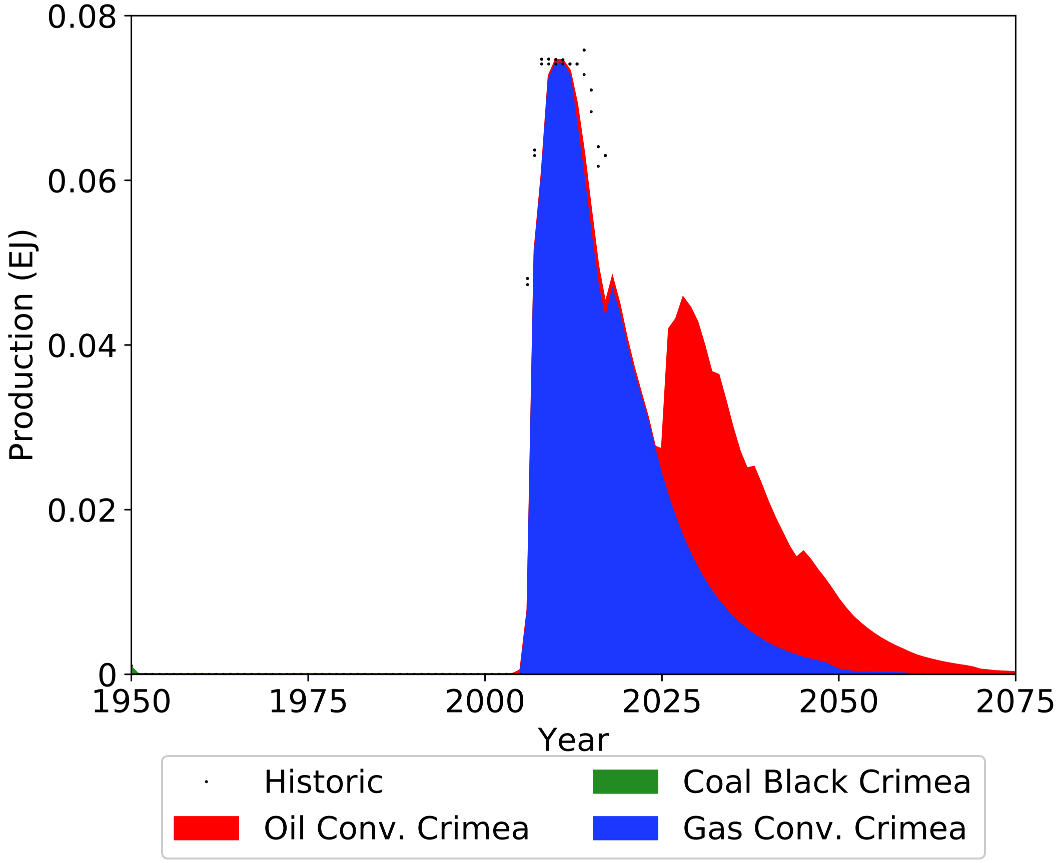

Figure 4.5: Crimea projections capped at 16

Table 4.5: Peak years - All

**Name**	**URR**	**Peak Year**	**Peak Rate**
Gas Conv. Crimea	1.14	2010	0.07
Oil Conv. Crimea	0.57	2029	0.03
Coal Black Crimea	–	1950	–
**Total**	**1.71**	**2010**	**0.07**

4.3.2 By Mineral

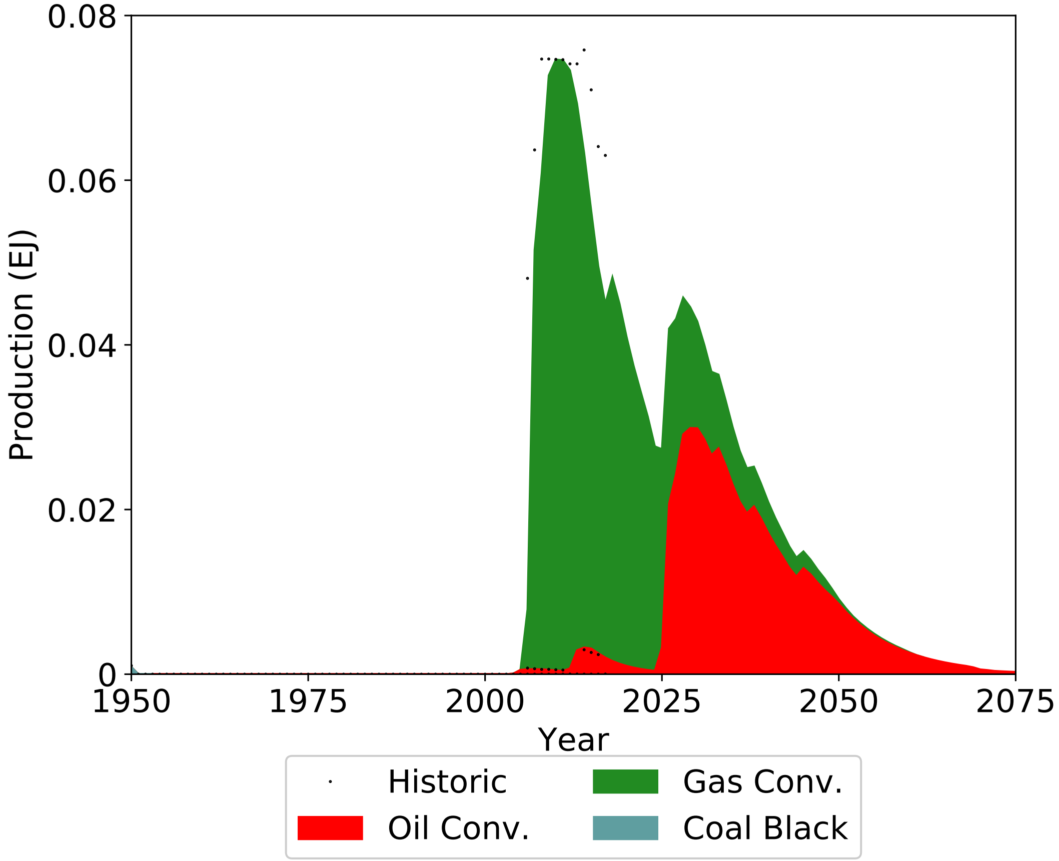

Figure 4.6: Crimea projection by mineral type

Table 4.6: Peak years - Minerals

Name	URR	Peak Year	Peak Rate
Coal Black	–	1950	–
Oil Conv.	0.57	2029	0.03
Gas Conv.	1.14	2010	0.07
**Total**	**1.71**	**2010**	**0.07**

4.3.3 Regional Projections

Crimea

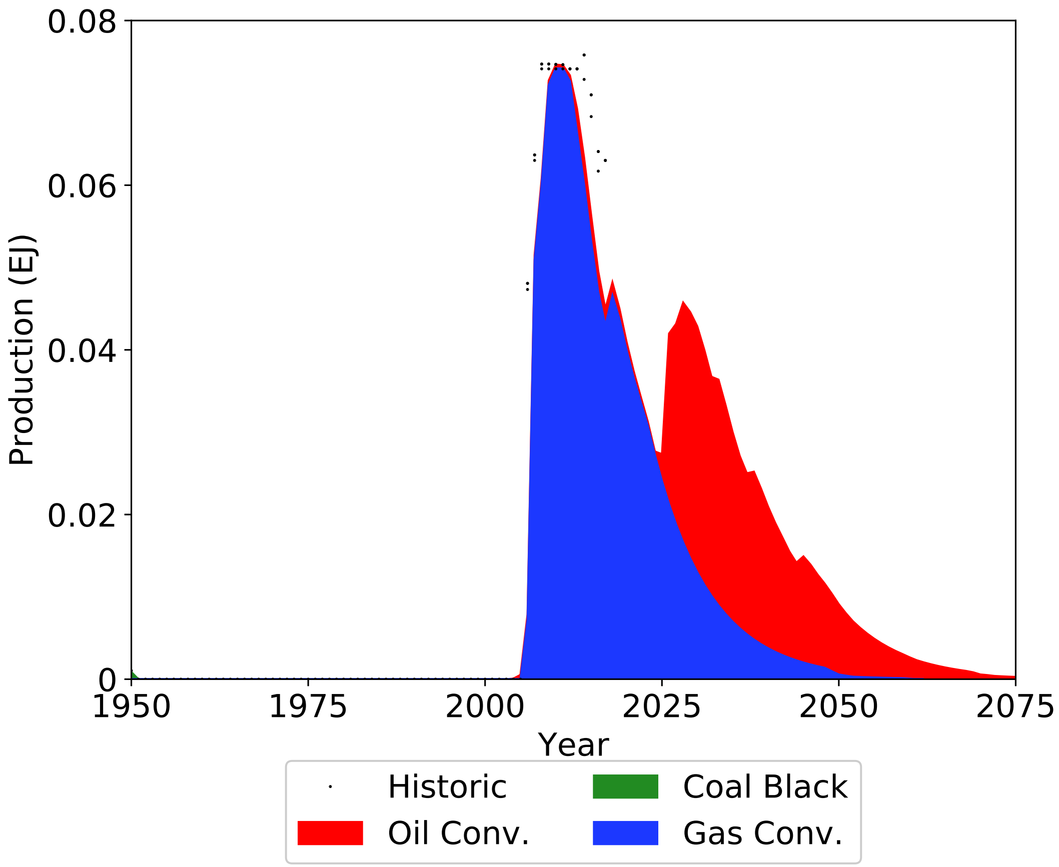

Figure 4.7: Crimea - Crimea projections capped at 16

Table 4.7: Peak years - All

**Name**	**URR**	**Peak Year**	**Peak Rate**
Gas Conv. Crimea	1.14	2010	0.07
Oil Conv. Crimea	0.57	2029	0.03
Coal Black Crimea	–	1950	–
**Total**	**1.71**	**2010**	**0.07**

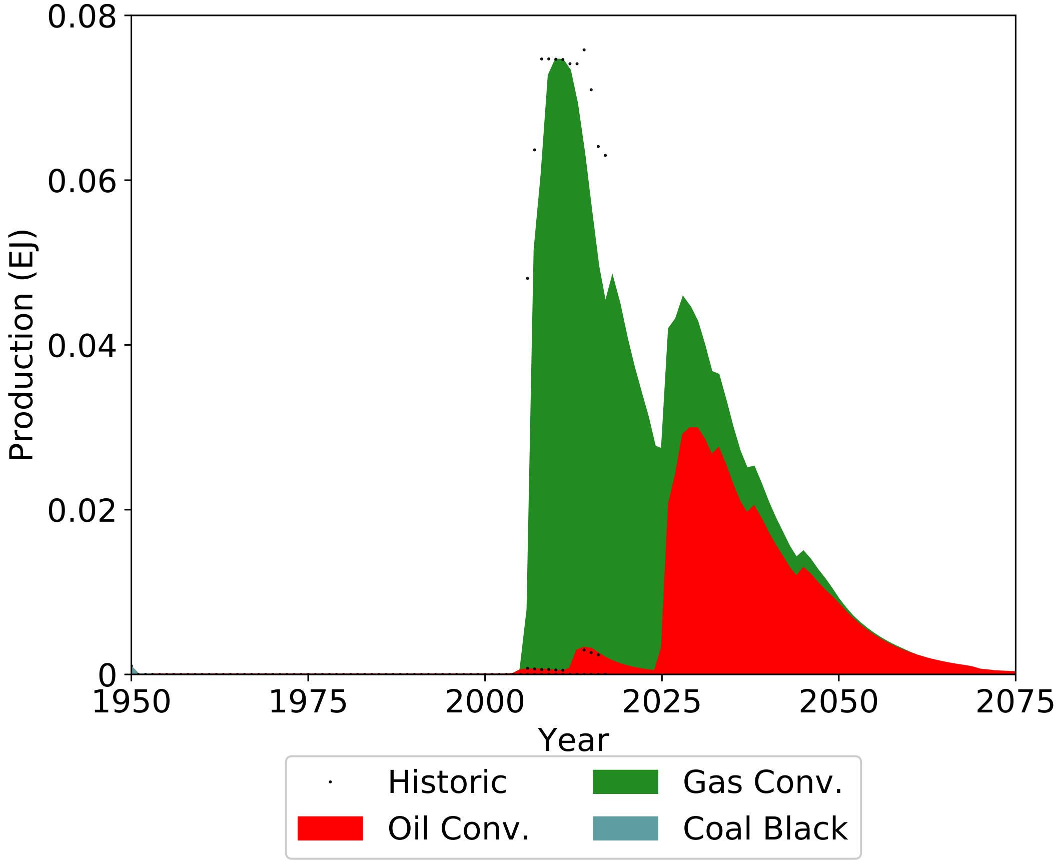

Figure 4.8: Crimea - Crimea projection by mineral type

Table 4.8: Peak years - Minerals

Name	URR	Peak Year	Peak Rate
Coal Black	–	1950	–
Oil Conv.	0.57	2029	0.03
Gas Conv.	1.14	2010	0.07
**Total**	**1.71**	**2010**	**0.07**

4.3.4 Projection by region

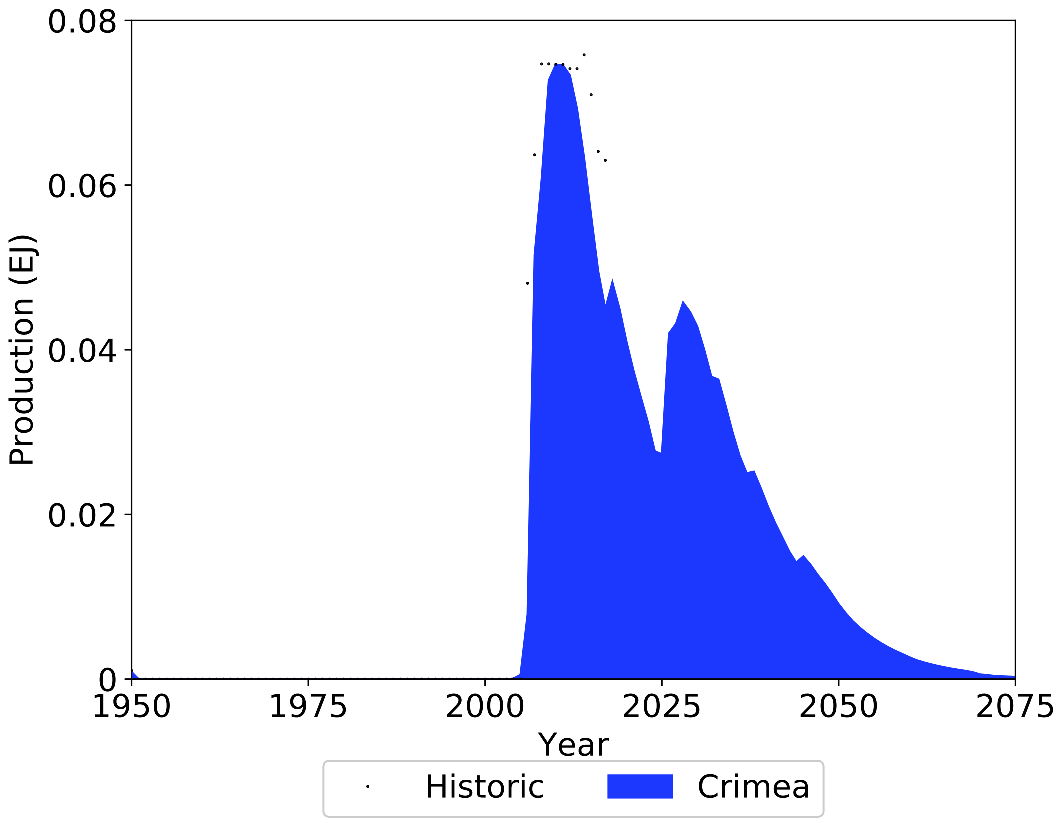

Figure 4.9: Crimea by region projections capped at 16

Table 4.9: Peak years - All

**Name**	**URR**	**Peak Year**	**Peak Rate**
Crimea	1.71	2010	0.07
**Total**	**1.71**	**2010**	**0.07**

4.4 Donetsk

4.4.1 All Projections

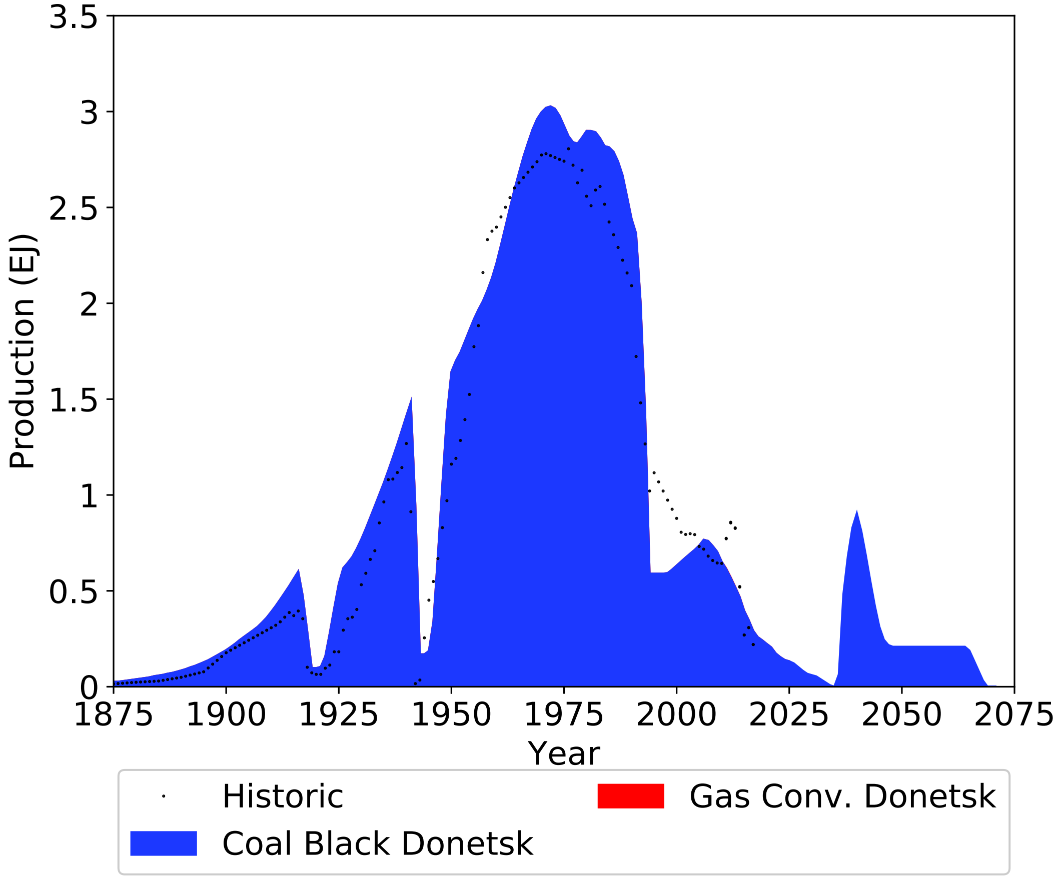

Figure 4.10: Donetsk projections capped at 16

Table 4.10: Peak years - All

**Name**	**URR**	**Peak Year**	**Peak Rate**
Coal Black Donetsk	169.0	1972	3.03
Gas Conv. Donetsk	0.01	2012	–
**Total**	**169.01**	**1972**	**3.03**

4.4.2 By Mineral

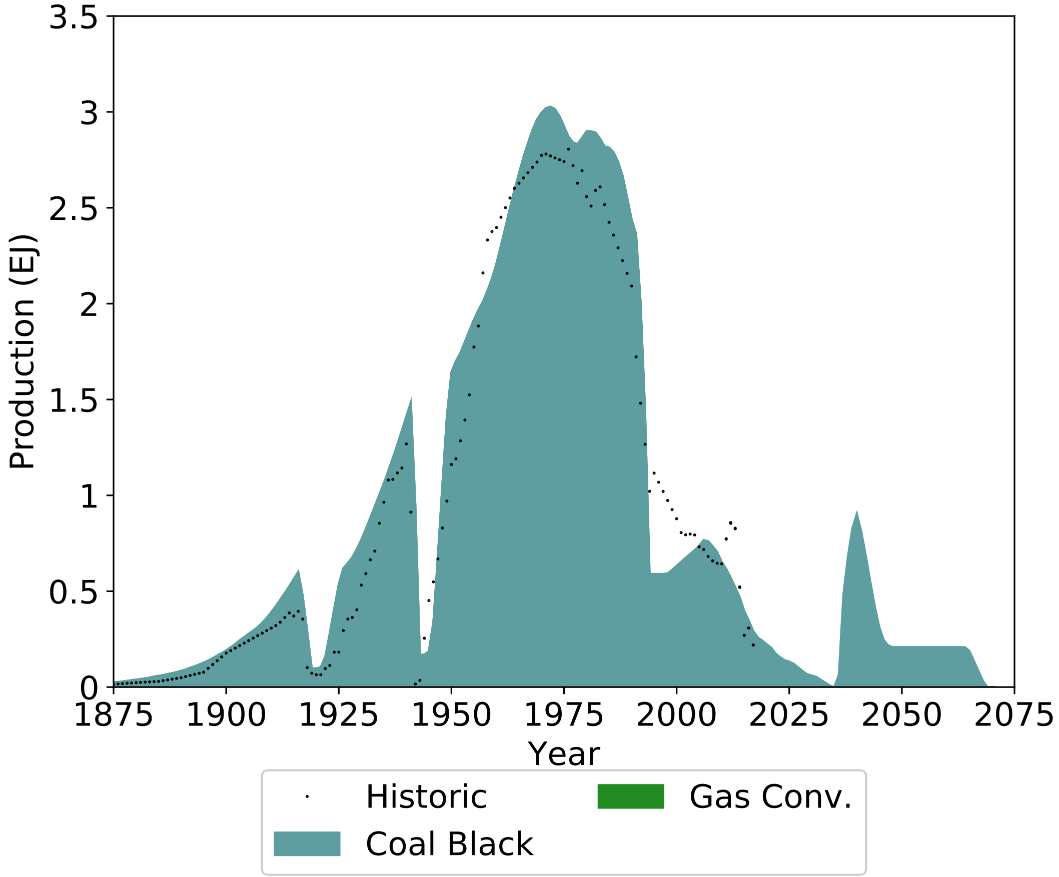

Figure 4.11: Donetsk projection by mineral type

Table 4.11: Peak years - Minerals

**Name**	**URR**	**Peak Year**	**Peak Rate**
Coal Black	169.0	1972	3.03
Gas Conv.	0.01	2012	–
**Total**	**169.01**	**1972**	**3.03**

4.4.3 Regional Projections

Donetsk

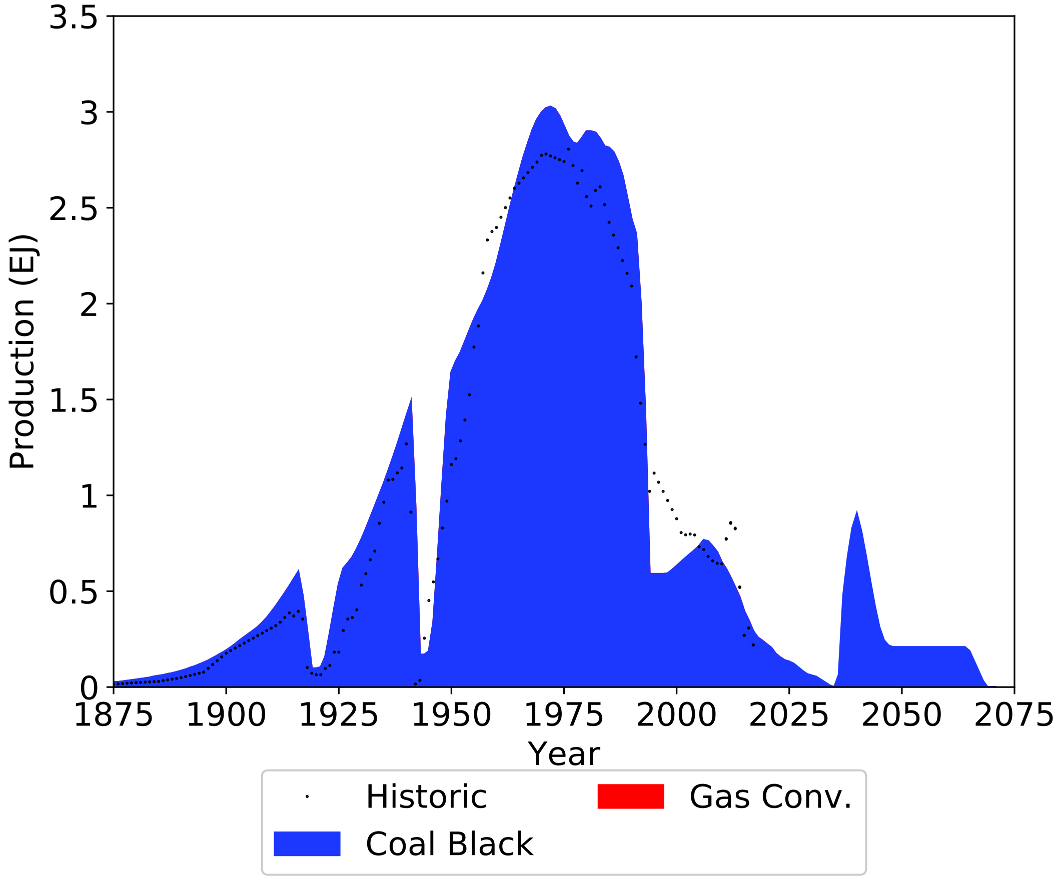

Figure 4.12: Donetsk - Donetsk projections capped at 16

Table 4.12: Peak years - All

**Name**	**URR**	**Peak Year**	**Peak Rate**
Coal Black Donetsk	169.0	1972	3.03
Gas Conv. Donetsk	0.01	2012	–
**Total**	**169.01**	**1972**	**3.03**

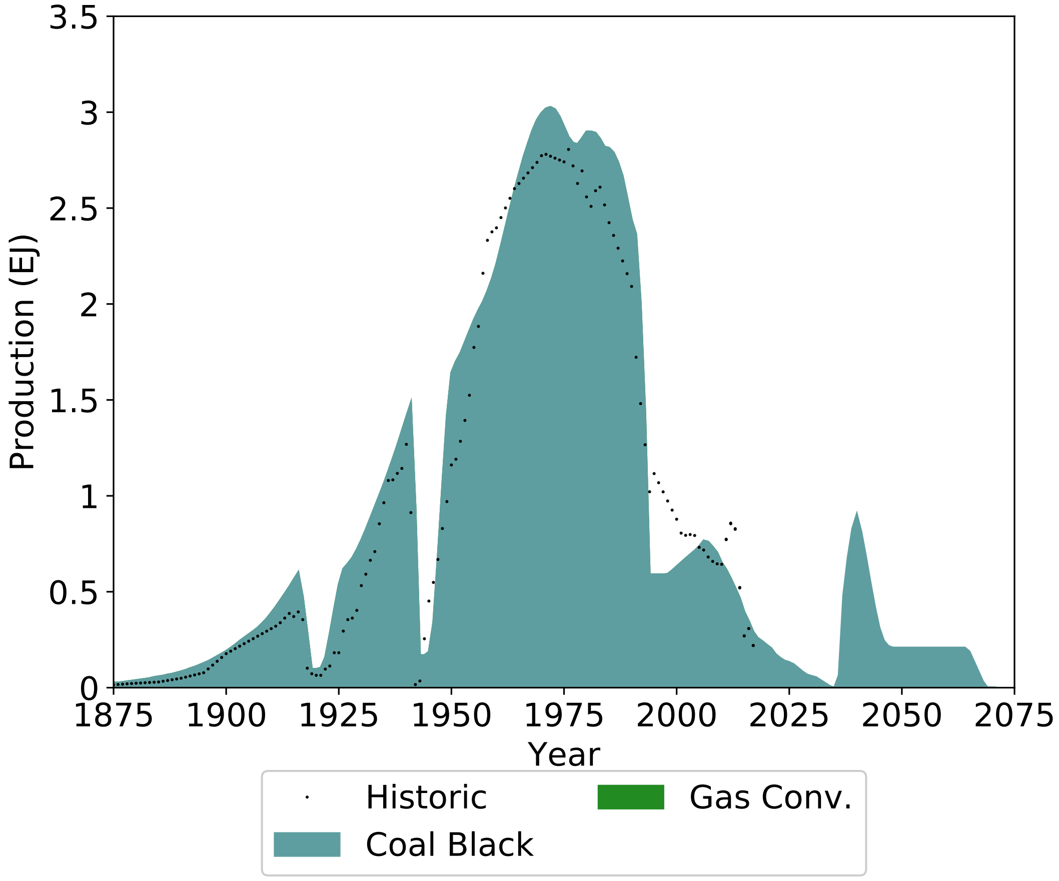

Figure 4.13: Donetsk - Donetsk projection by mineral type

Table 4.13: Peak years - Minerals

Name	URR	Peak Year	Peak Rate
Coal Black	169.0	1972	3.03
Gas Conv.	0.01	2012	–
**Total**	**169.01**	**1972**	**3.03**

4.4.4 Projection by region

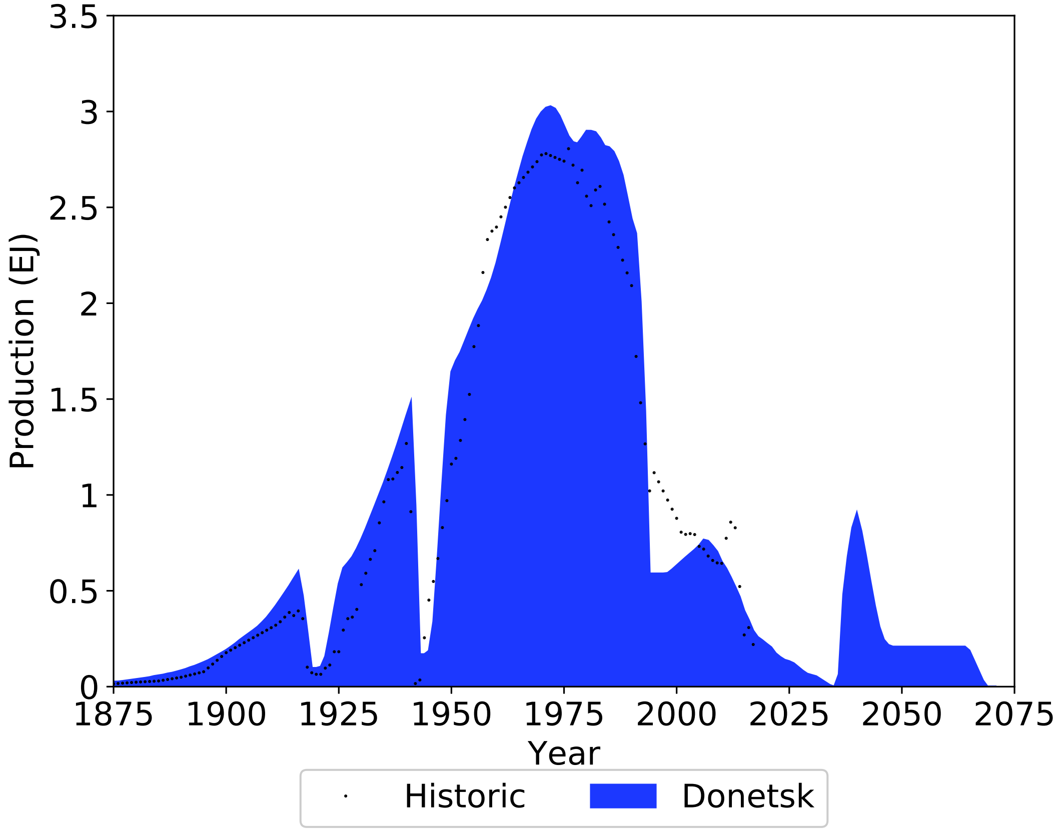

Figure 4.14: Donetsk by region projections capped at 16

Table 4.14: Peak years - All

Name	URR	Peak Year	Peak Rate
Donetsk	169.01	1972	3.03
**Total**	**169.01**	**1972**	**3.03**

4.5 Estonia

4.5.1 All Projections

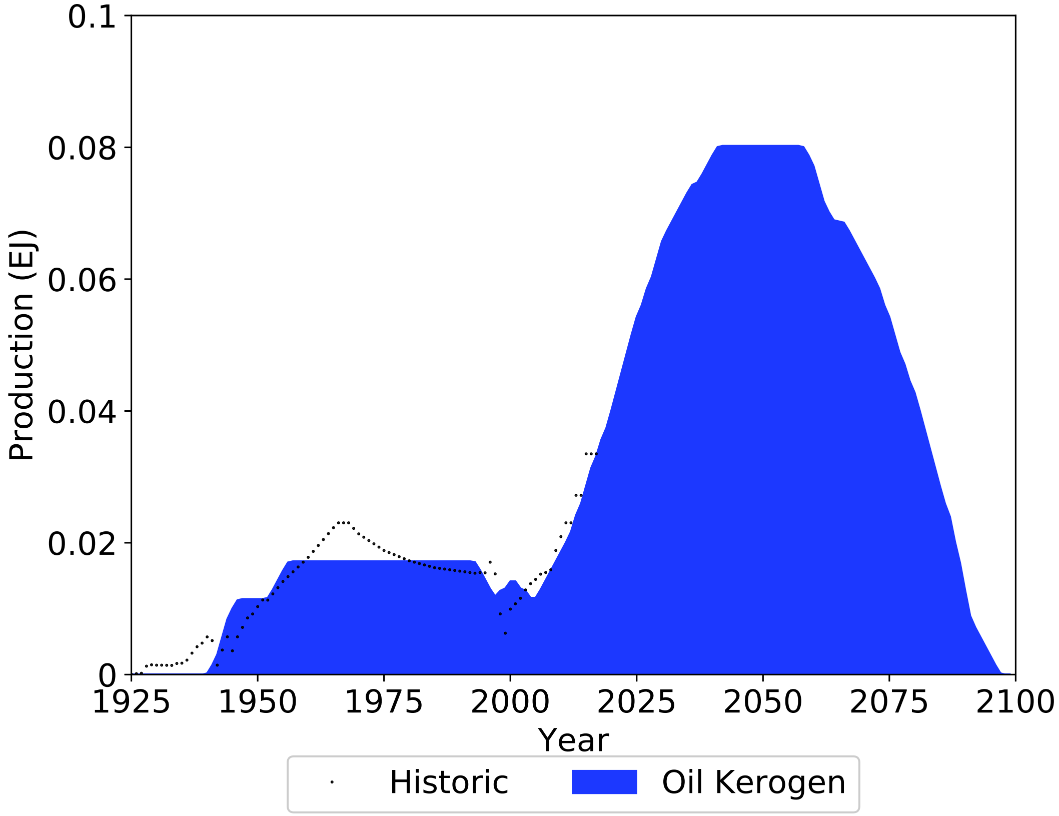

Figure 4.15: Estonia projections capped at 16

Table 4.15: Peak years - All

Name	URR	Peak Year	Peak Rate
Oil Kerogen	5.73	2048	0.08
**Total**	**5.73**	**2048**	**0.08**

4.5.2 By Mineral

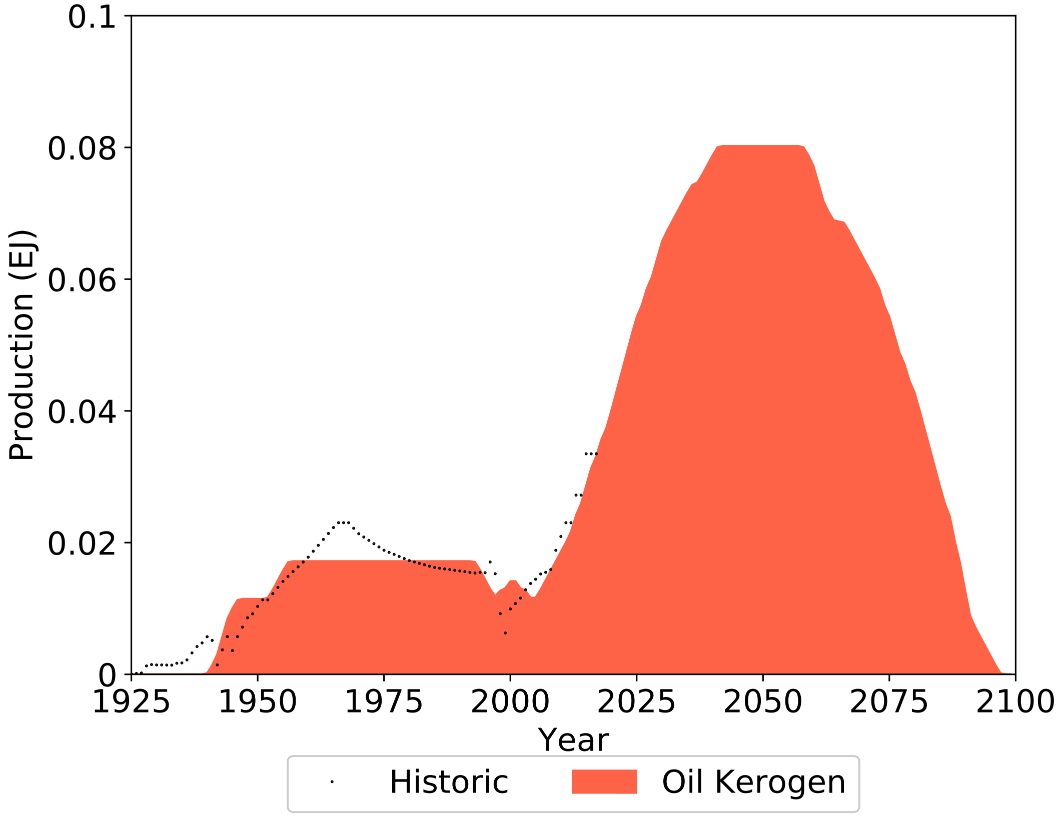

Figure 4.16: Estonia projection by mineral type

Table 4.16: Peak years - Minerals

Name	URR	Peak Year	Peak Rate
Oil Kerogen	5.73	2048	0.08
**Total**	**5.73**	**2048**	**0.08**

4.6 Georgia

4.6.1 All Projections

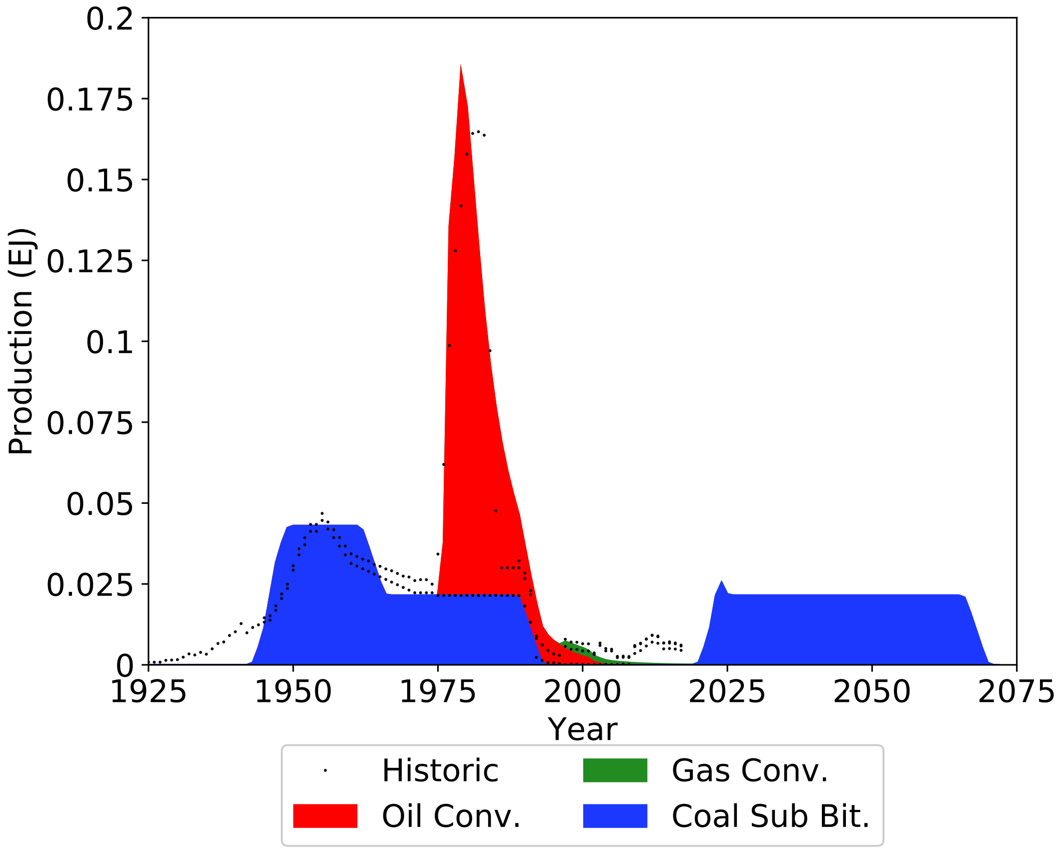

Figure 4.17: Georgia projections capped at 16

Table 4.17: Peak years - All

**Name**	**URR**	**Peak Year**	**Peak Rate**
Coal Sub Bit.	2.35	1950	0.04
Oil Conv.	1.28	1979	0.16
Gas Conv.	0.03	1998	–
**Total**	**3.66**	**1979**	**0.18**

4.6.2 By Mineral

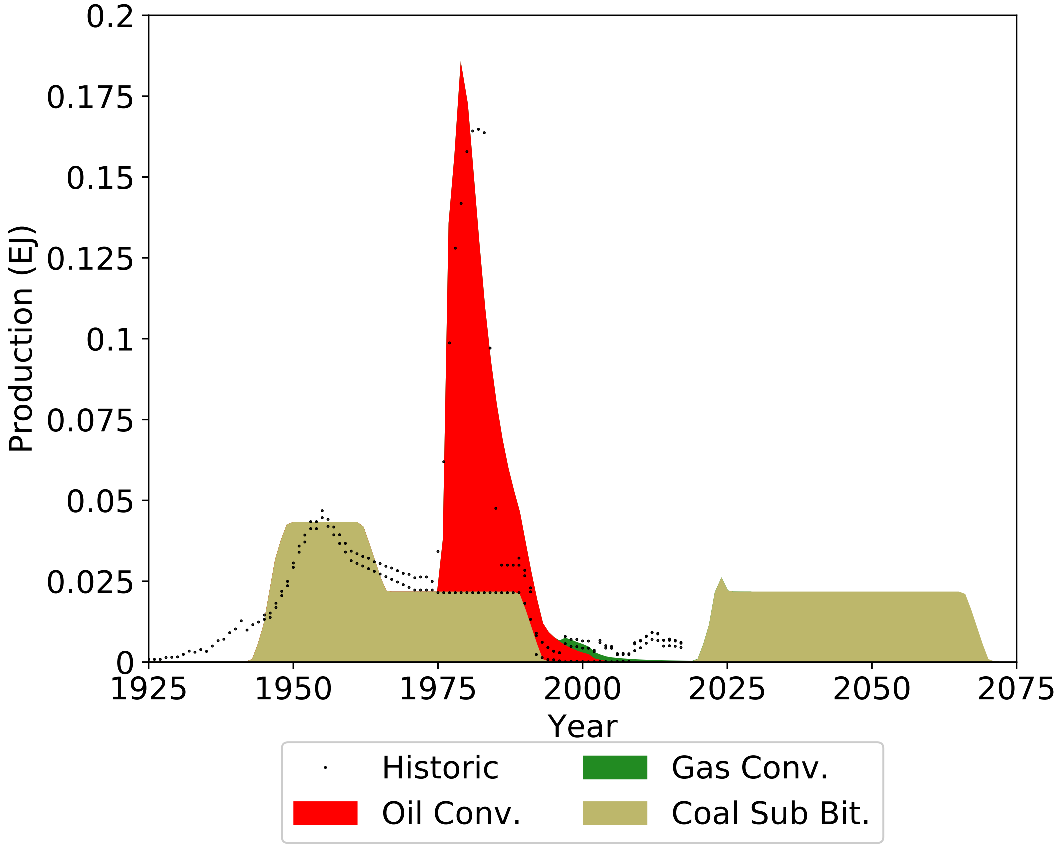

Figure 4.18: Georgia projection by mineral type

Table 4.18: Peak years - Minerals

Name	URR	Peak Year	Peak Rate
Coal Sub Bit.	2.35	1950	0.04
Oil Conv.	1.28	1979	0.16
Gas Conv.	0.03	1998	–
**Total**	**3.66**	**1979**	**0.18**

4.7 Kazakhstan

4.7.1 All Projections

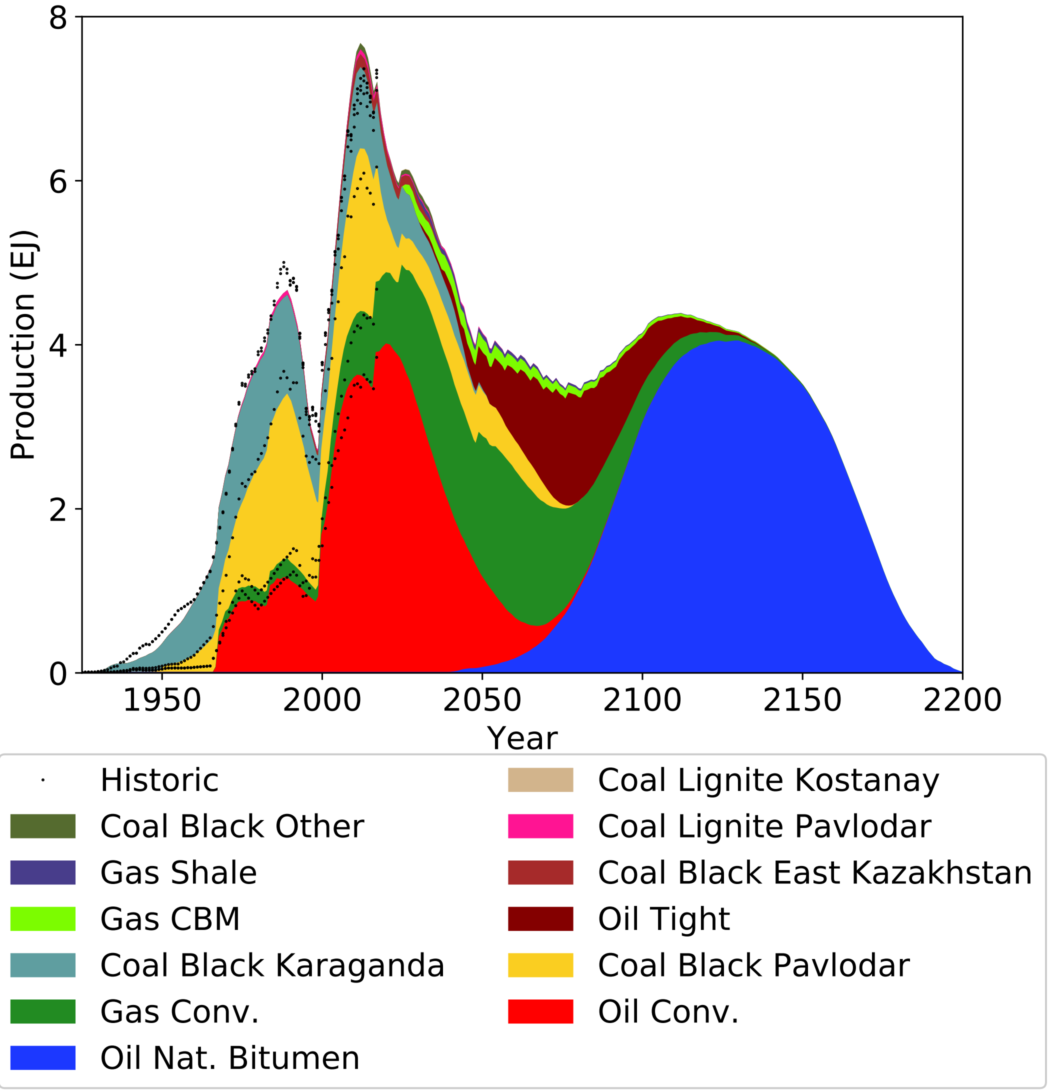

Figure 4.19: Kazakhstan projections capped at 16

Table 4.19: Peak years - All

**Name**	**URR**	**Peak Year**	**Peak Rate**
Oil Nat. Bitumen	312.53	2129	4.04
Oil Conv.	184.5	2020	4.0
Gas Conv.	131.2	2057	1.84
Coal Black Pavlodar	99.41	1989	2.0
Coal Black Karaganda	73.59	1984	1.27
Oil Tight	60.74	2077	1.38
Gas CBM	10.5	2041	0.2
Coal Black East Kazakhstan	4.5	2013	0.16
Gas Shale	2.89	2042	0.07
Coal Lignite Pavlodar	2.88	2015	0.06
Coal Black Other	1.26	2013	0.08
Coal Lignite Kostanay	0.1	2025	0.01
**Total**	**884.1**	**2012**	**7.66**

4.7.2 By Mineral

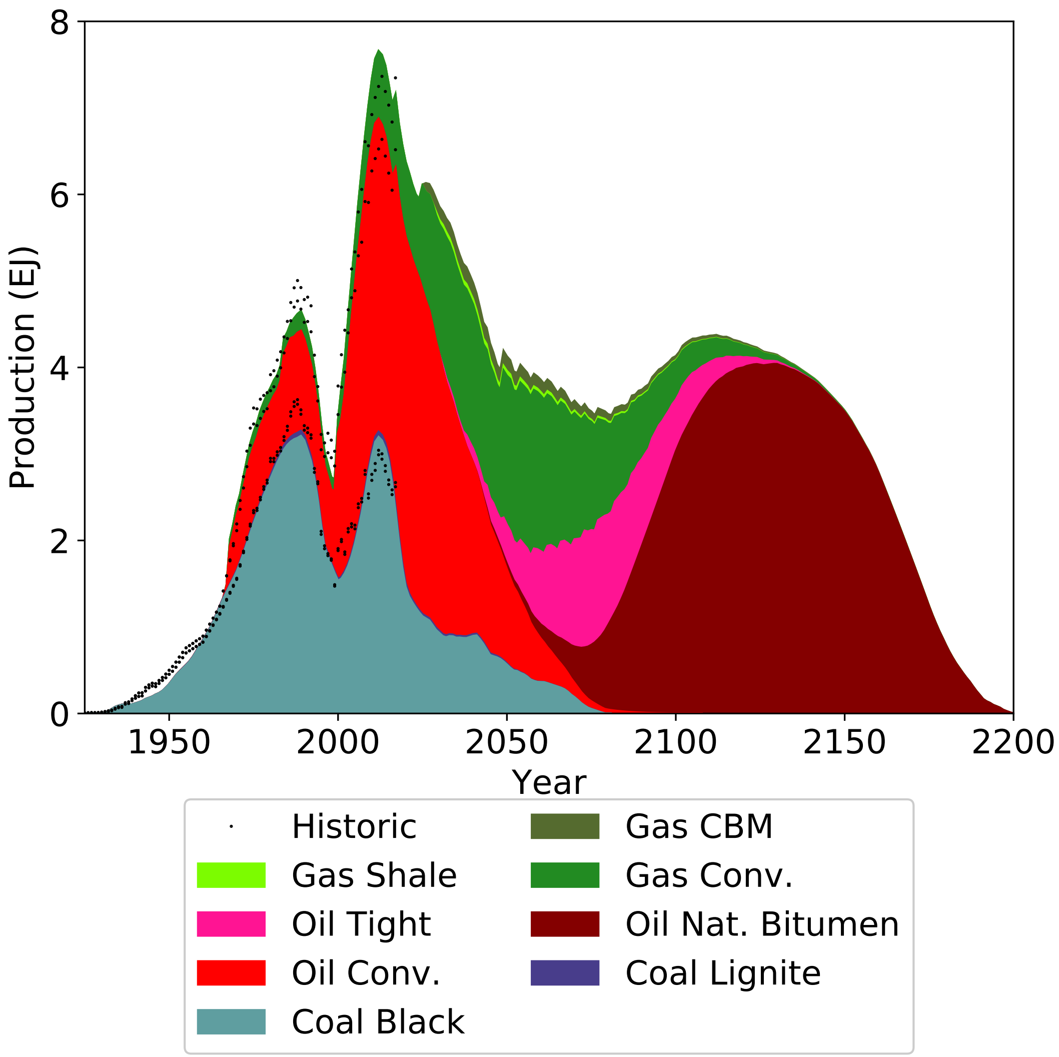

Figure 4.20: Kazakhstan projection by mineral type

4.7.3 Regional Projections

Table 4.20: Peak years - Minerals

**Name**	**URR**	**Peak Year**	**Peak Rate**
Coal Black	178.76	1989	3.21
Coal Lignite	2.98	2015	0.06
Oil Conv.	184.5	2020	4.0
Oil Nat. Bitumen	312.53	2129	4.04
Oil Tight	60.74	2077	1.38
Gas Conv.	131.2	2057	1.84
Gas Shale	2.89	2042	0.07
Gas CBM	10.5	2041	0.2
**Total**	**884.1**	**2012**	**7.66**

All

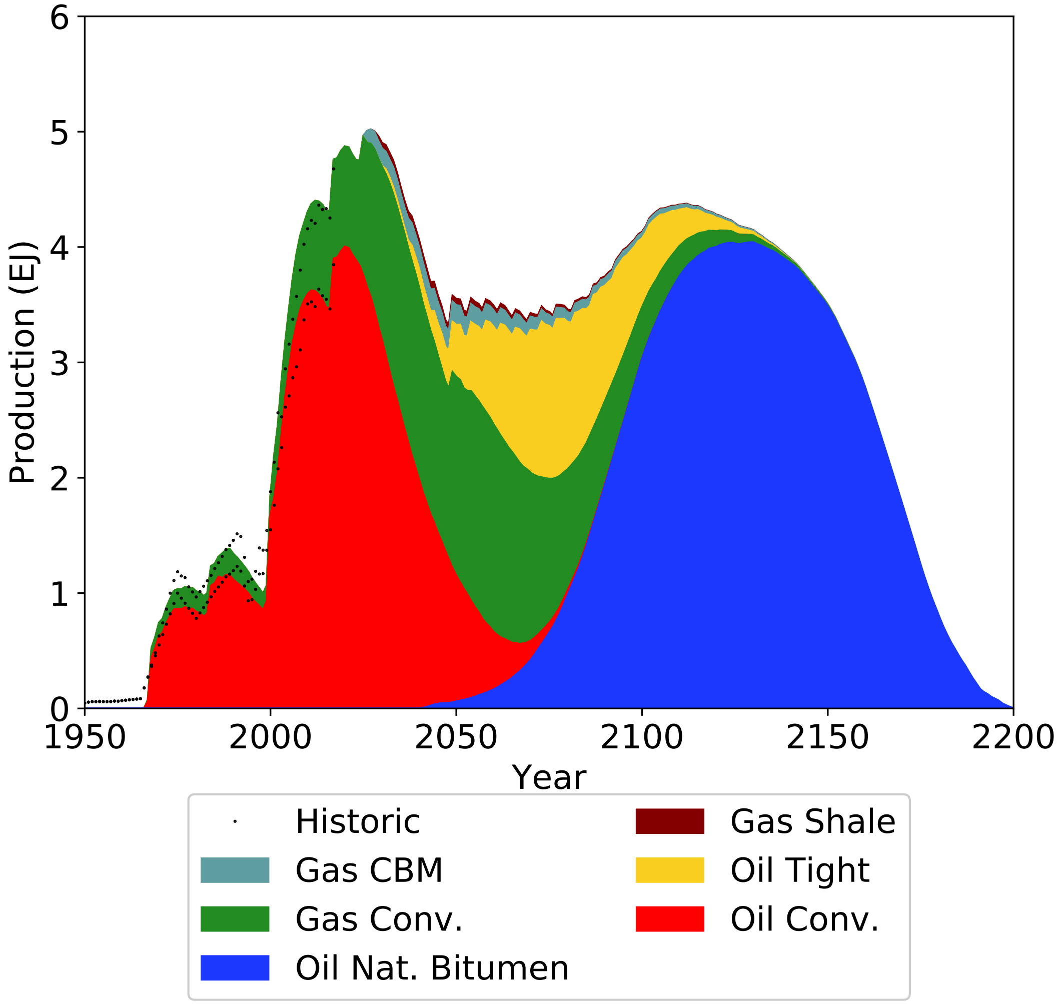

Figure 4.21: Kazakhstan - All projections capped at 16

Table 4.21: Peak years - All

Name	URR	Peak Year	Peak Rate
Oil Nat. Bitumen	312.53	2129	4.04
Oil Conv.	184.5	2020	4.0
Gas Conv.	131.2	2057	1.84
Oil Tight	60.74	2077	1.38
Gas CBM	10.5	2041	0.2
Gas Shale	2.89	2042	0.07
**Total**	**702.36**	**2027**	**5.02**

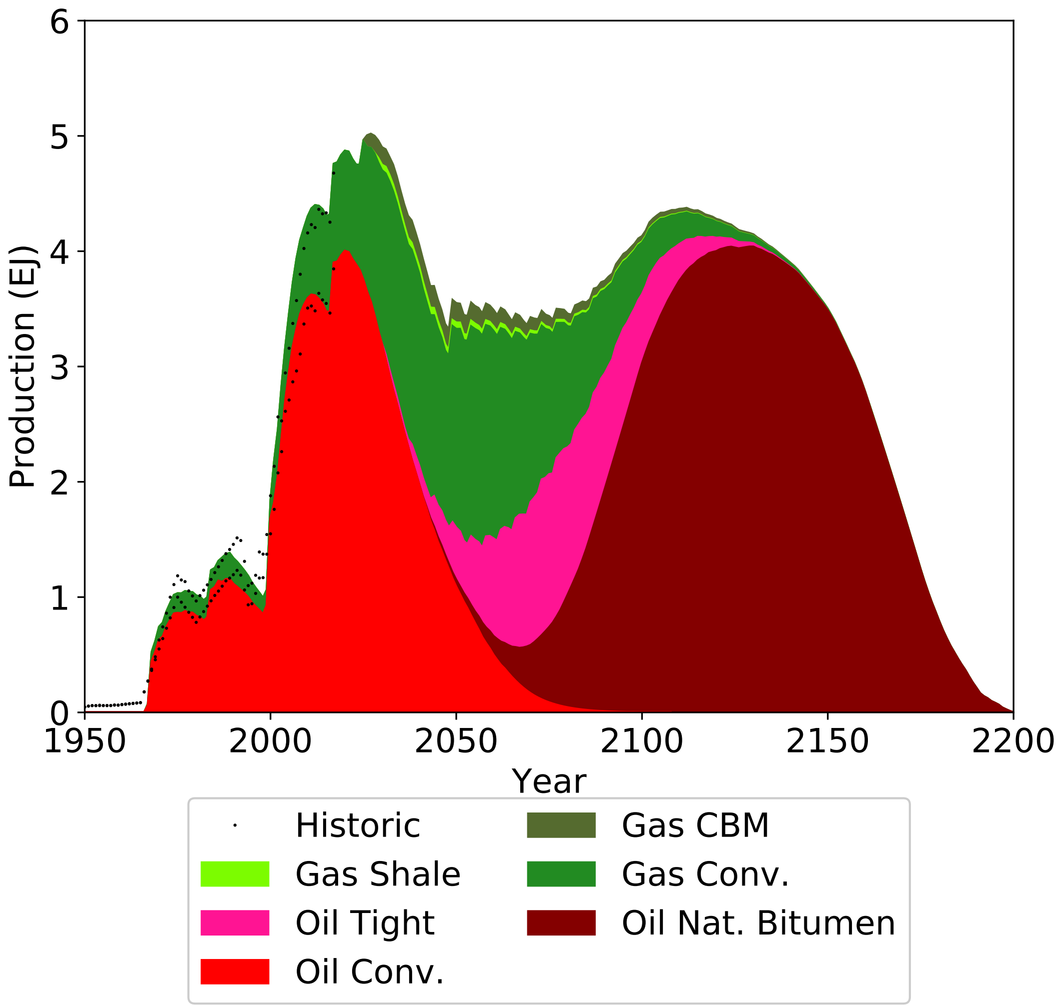

Figure 4.22: Kazakhstan - All projection by mineral type

Table 4.22: Peak years - Minerals

**Name**	**URR**	**Peak Year**	**Peak Rate**
Oil Conv.	184.5	2020	4.0
Oil Nat. Bitumen	312.53	2129	4.04
Oil Tight	60.74	2077	1.38
Gas Conv.	131.2	2057	1.84
Gas Shale	2.89	2042	0.07
Gas CBM	10.5	2041	0.2
**Total**	**702.36**	**2027**	**5.02**

East Kazakhstan

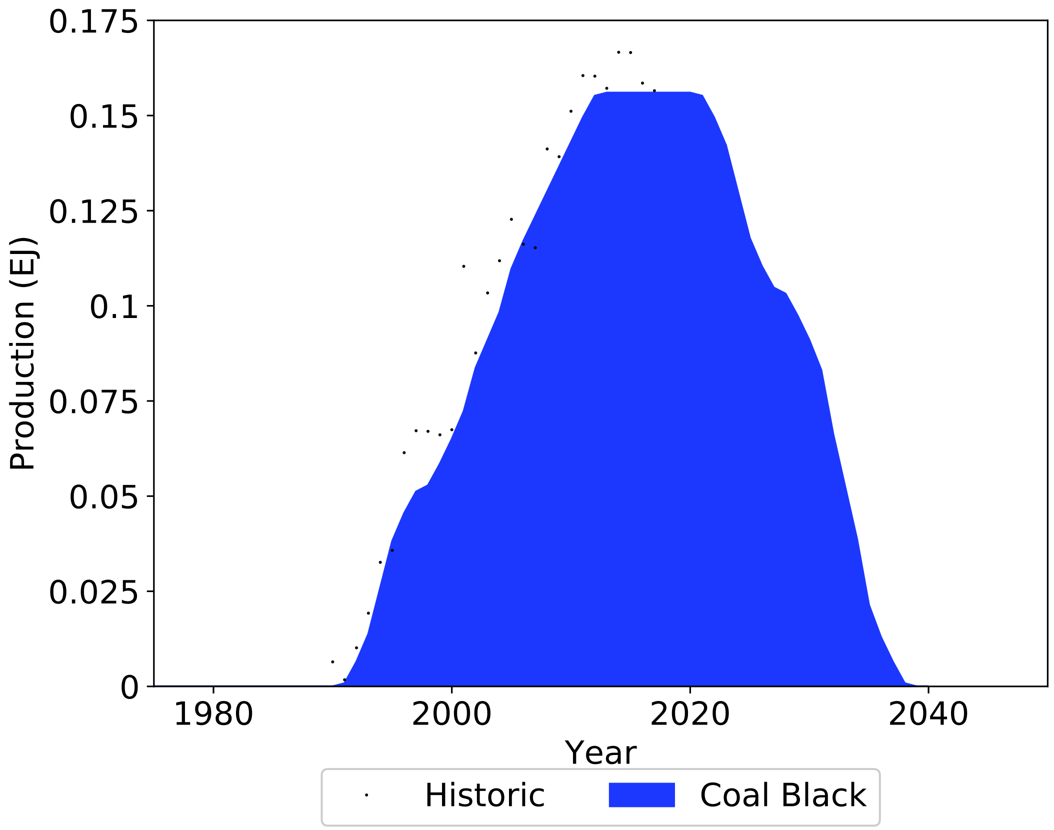

Figure 4.23: Kazakhstan - East Kazakhstan projections capped at 16

Table 4.23: Peak years - All

Name	URR	Peak Year	Peak Rate
Coal Black East Kazakhstan	4.5	2013	0.16
**Total**	**4.5**	**2013**	**0.16**

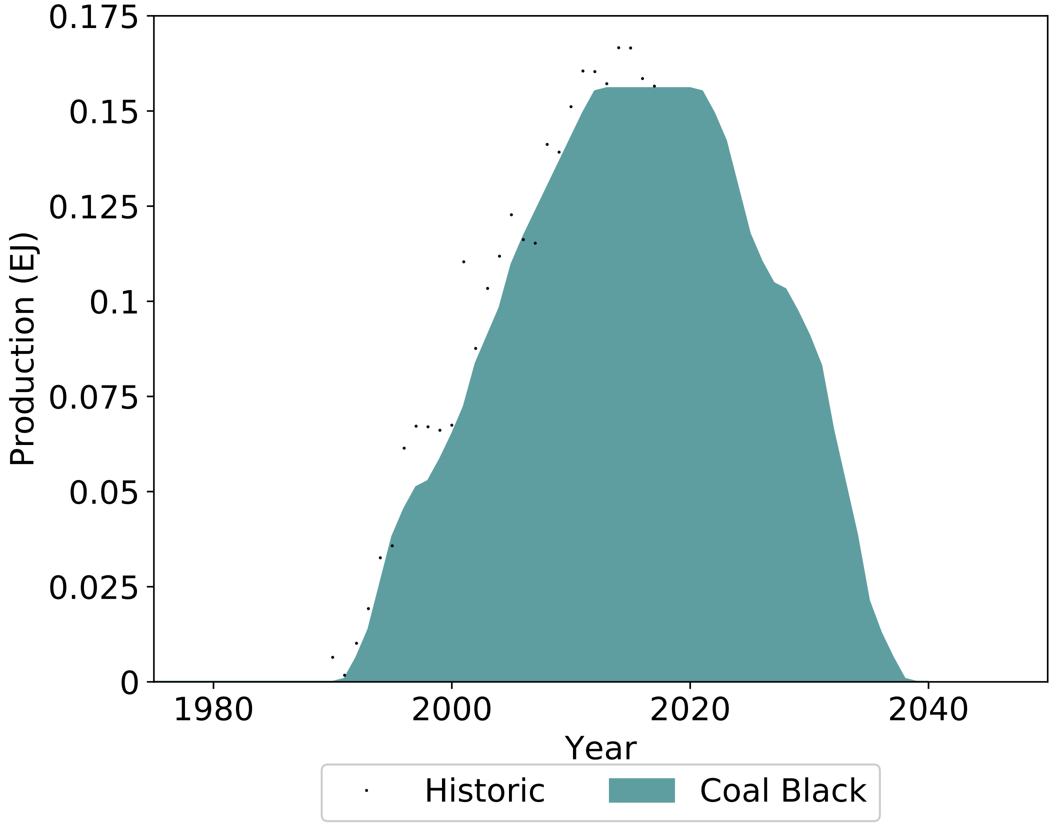

Figure 4.24: Kazakhstan - East Kazakhstan projection by mineral type

Table 4.24: Peak years - Minerals

Name	URR	Peak Year	Peak Rate
Coal Black	4.5	2013	0.16
**Total**	**4.5**	**2013**	**0.16**

Karaganda

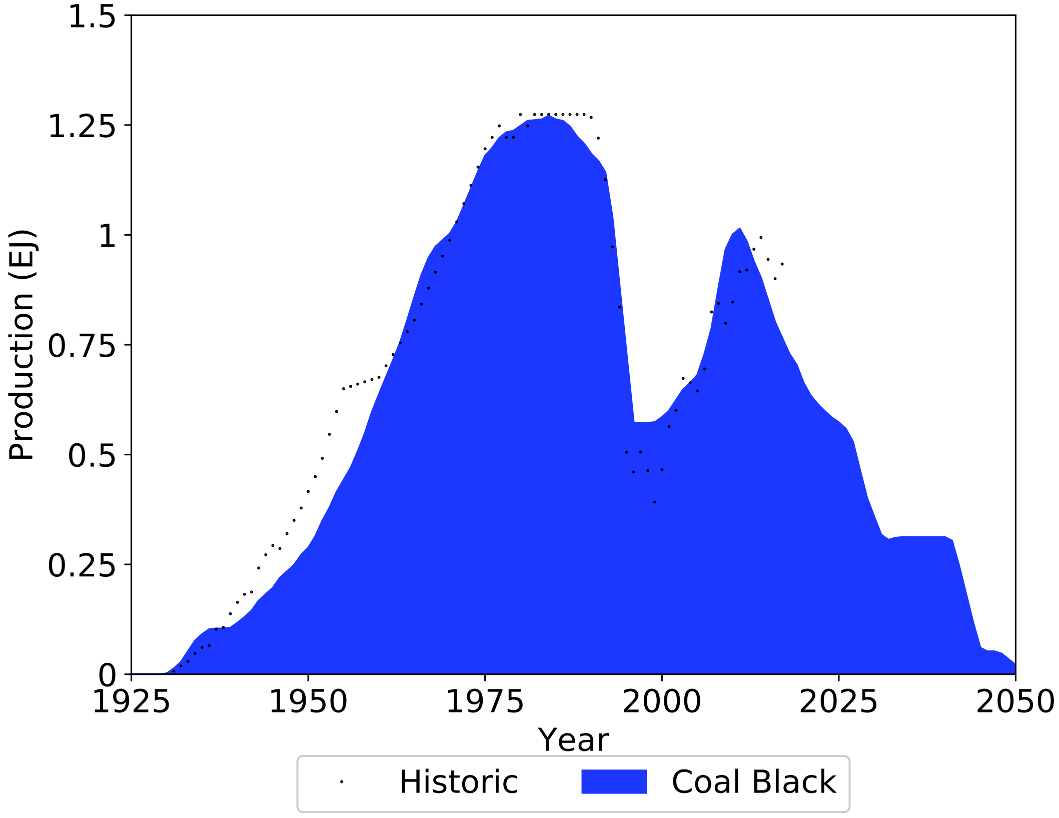

Figure 4.25: Kazakhstan - Karaganda projections capped at 16

Table 4.25: Peak years - All

Name	URR	Peak Year	Peak Rate
Coal Black Karaganda	73.59	1984	1.27
**Total**	**73.59**	**1984**	**1.27**

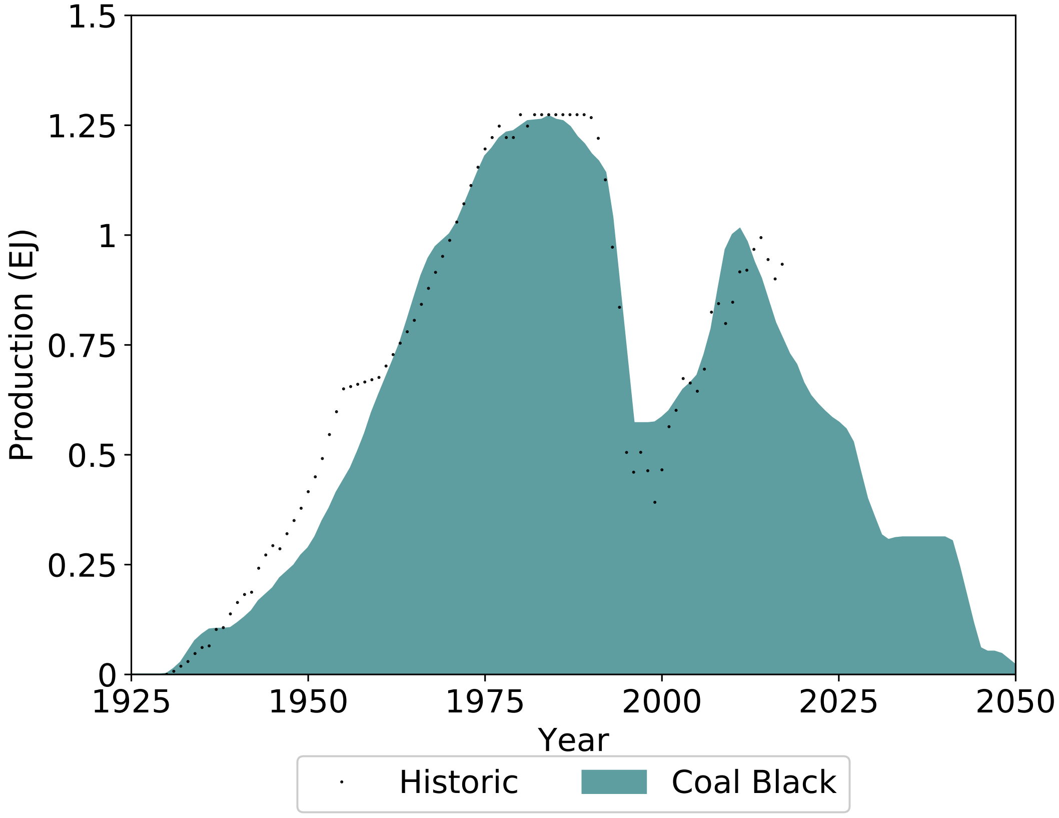

Figure 4.26: Kazakhstan - Karaganda projection by mineral type

Table 4.26: Peak years - Minerals

Name	URR	Peak Year	Peak Rate
Coal Black	73.59	1984	1.27
**Total**	**73.59**	**1984**	**1.27**

Kostanay

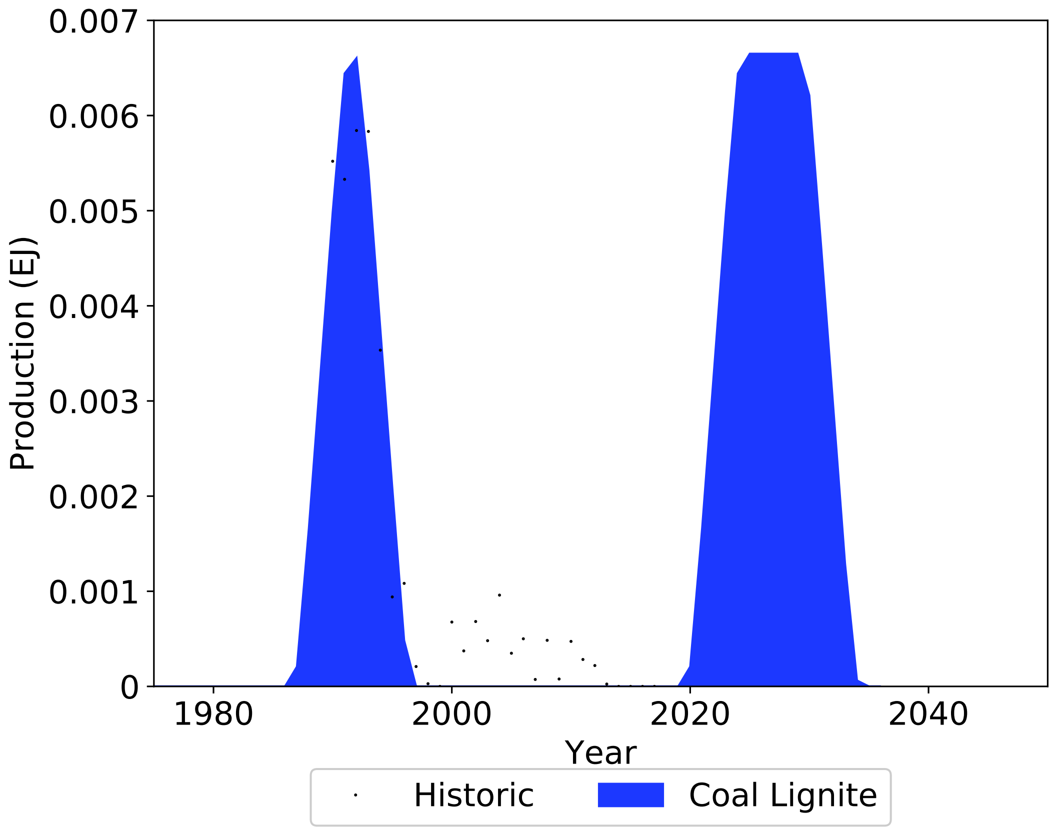

Figure 4.27: Kazakhstan - Kostanay projections capped at 16

Table 4.27: Peak years - All

Name	URR	Peak Year	Peak Rate
Coal Lignite Kostanay	0.1	2025	0.01
**Total**	**0.1**	**2025**	**0.01**

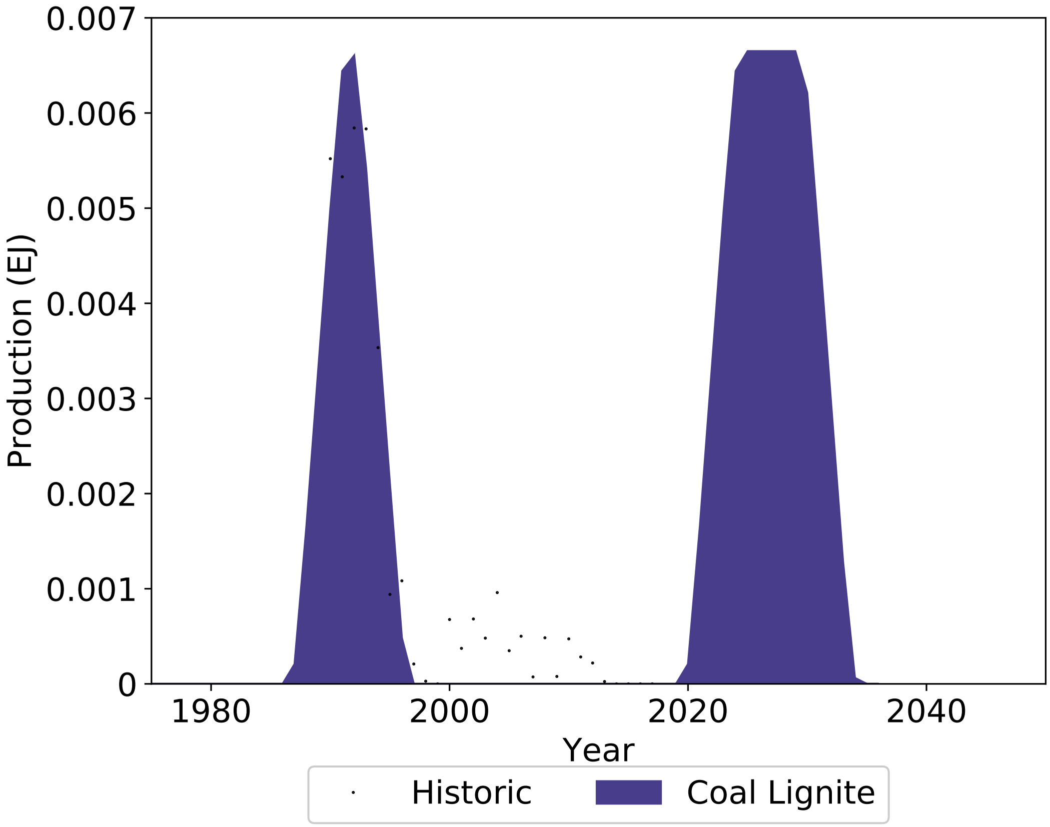

Figure 4.28: Kazakhstan - Kostanay projection by mineral type

Table 4.28: Peak years - Minerals

Name	URR	Peak Year	Peak Rate
Coal Lignite	0.1	2025	0.01
**Total**	**0.1**	**2025**	**0.01**

Other

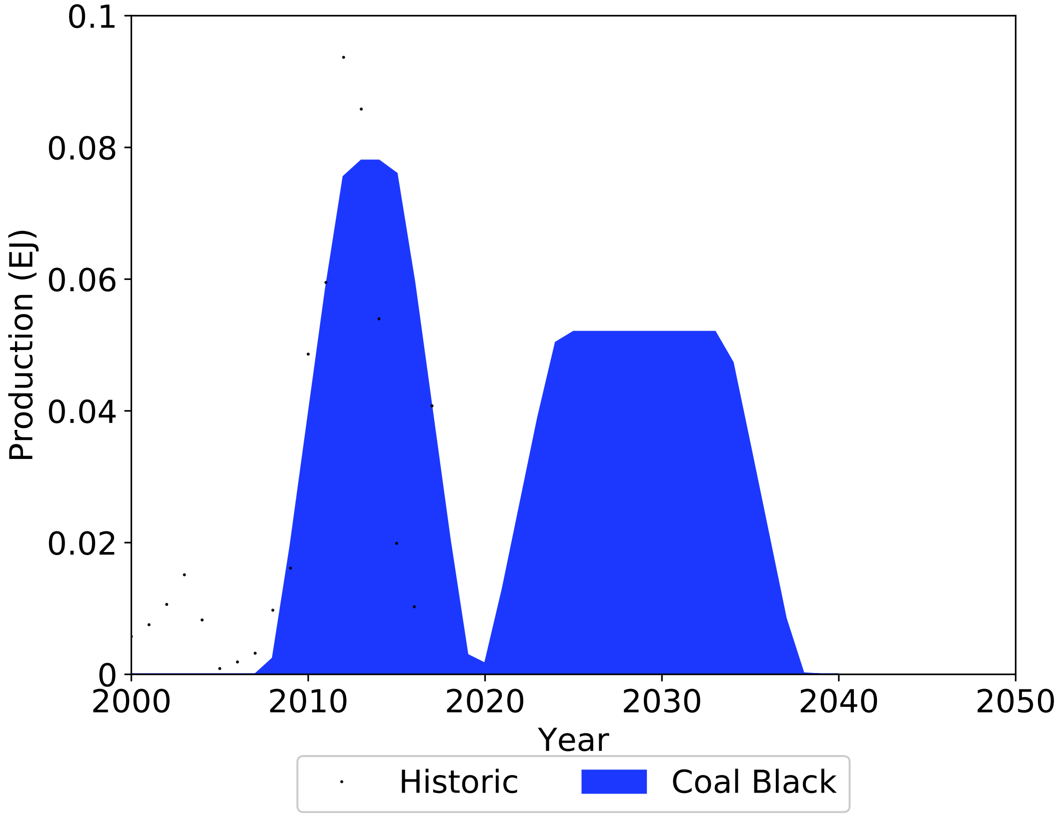

Figure 4.29: Kazakhstan - Other projections capped at 16

Table 4.29: Peak years - All

Name	URR	Peak Year	Peak Rate
Coal Black Other	1.26	2013	0.08
**Total**	**1.26**	**2013**	**0.08**

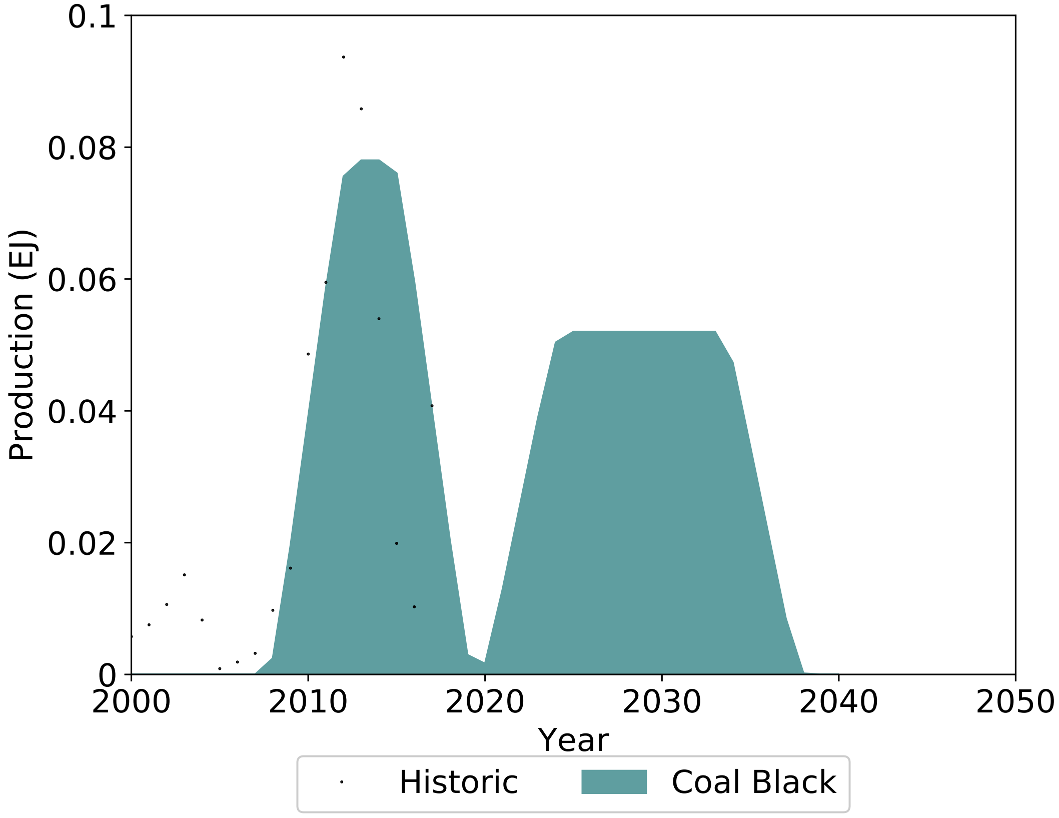

Figure 4.30: Kazakhstan - Other projection by mineral type

Table 4.30: Peak years - Minerals

Name	URR	Peak Year	Peak Rate
Coal Black	1.26	2013	0.08
**Total**	**1.26**	**2013**	**0.08**

Pavlodar

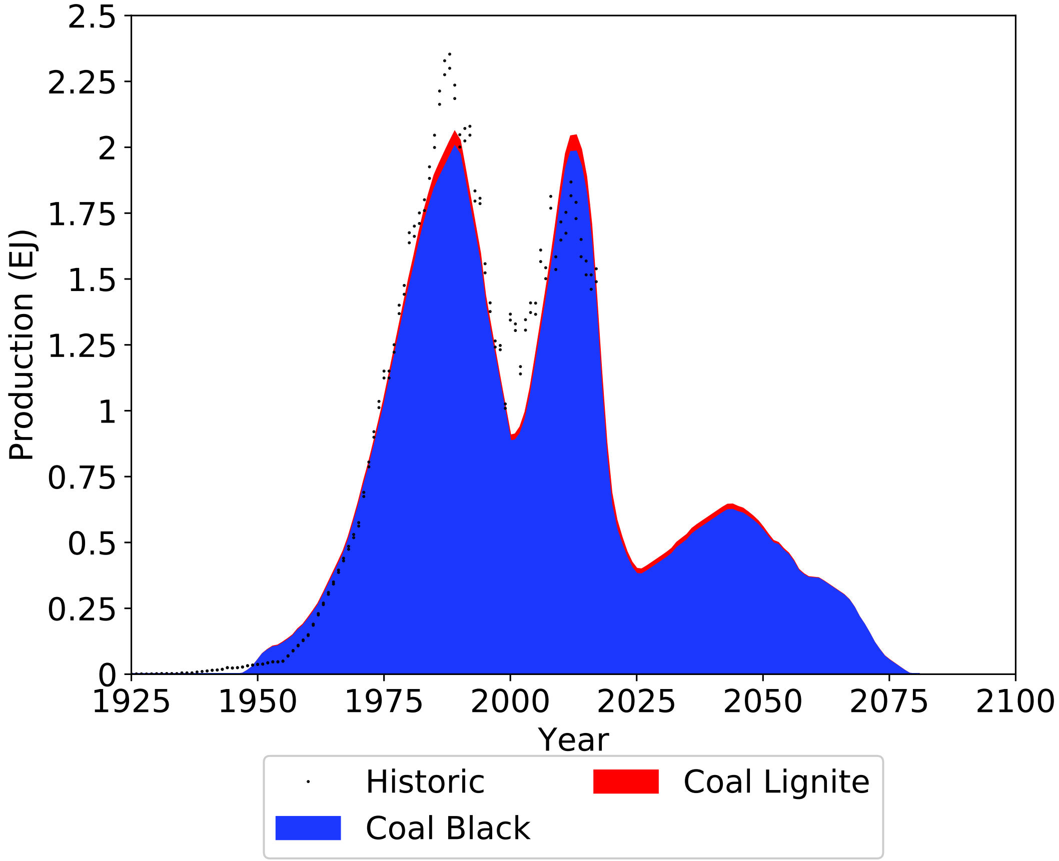

Figure 4.31: Kazakhstan - Pavlodar projections capped at 16

Table 4.31: Peak years - All

**Name**	**URR**	**Peak Year**	**Peak Rate**
Coal Black Pavlodar	99.41	1989	2.0
Coal Lignite Pavlodar	2.88	2015	0.06
**Total**	**102.29**	**1989**	**2.06**

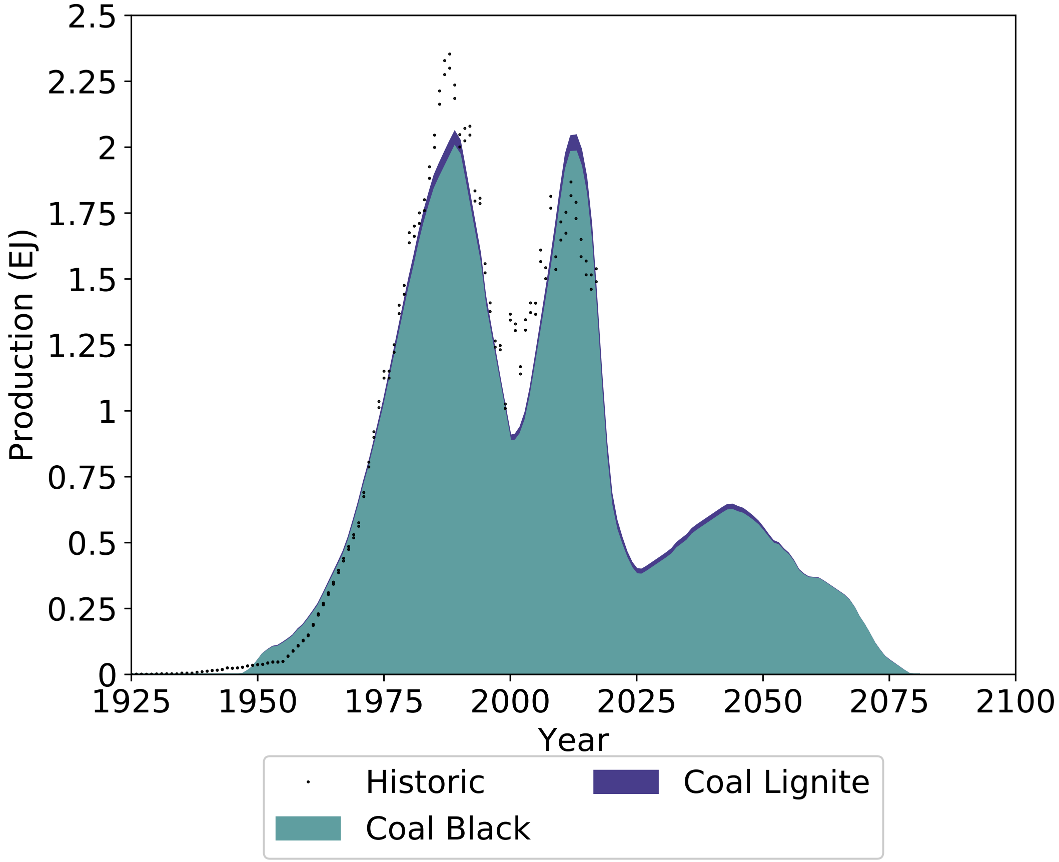

Figure 4.32: Kazakhstan - Pavlodar projection by mineral type

Table 4.32: Peak years - Minerals

Name	URR	Peak Year	Peak Rate
Coal Black	99.41	1989	2.0
Coal Lignite	2.88	2015	0.06
**Total**	**102.29**	**1989**	**2.06**

4.7.4 Projection by region

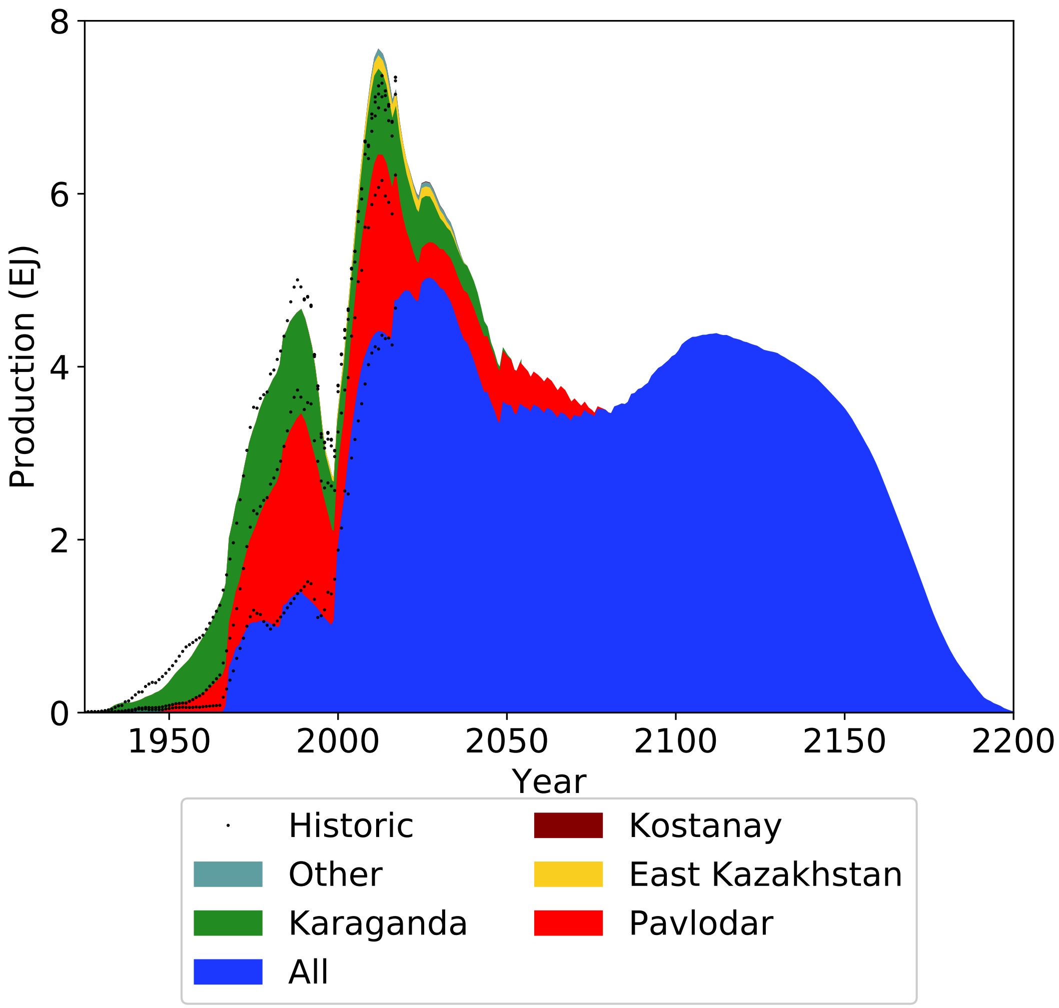

Figure 4.33: Kazakhstan by region projections capped at 16

Table 4.33: Peak years - All

**Name**	**URR**	**Peak Year**	**Peak Rate**
All	702.36	2027	5.02
Pavlodar	102.29	1989	2.06
Karaganda	73.59	1984	1.27
East Kazakhstan	4.5	2013	0.16
Other	1.26	2013	0.08
Kostanay	0.1	2025	0.01
**Total**	**884.1**	**2012**	**7.66**

4.8 Kyrgyzstan

4.8.1 All Projections

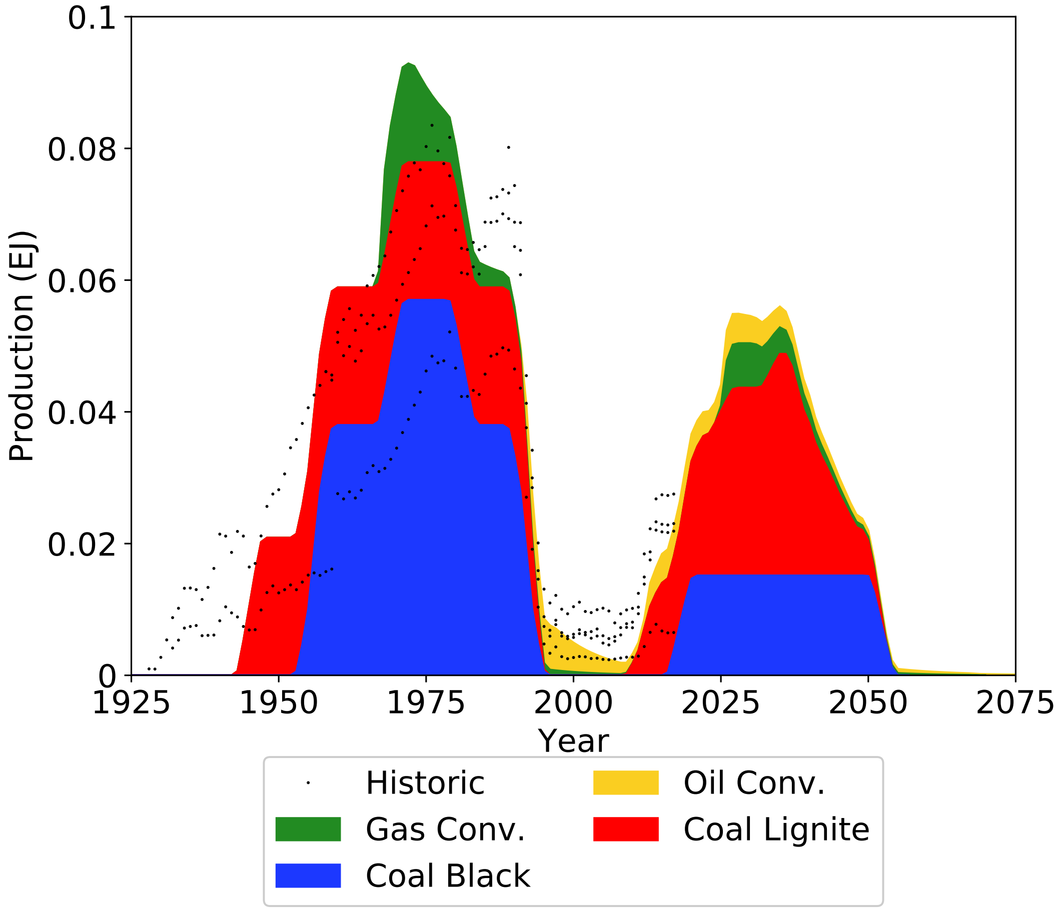

Figure 4.34: Kyrgyzstan projections capped at 16

Table 4.34: Peak years - All

**Name**	**URR**	**Peak Year**	**Peak Rate**
Coal Black	2.12	1972	0.06
Coal Lignite	1.8	2035	0.03
Gas Conv.	0.29	1969	0.02
Oil Conv.	0.21	1993	0.01
**Total**	**4.42**	**1972**	**0.09**

4.8.2 By Mineral

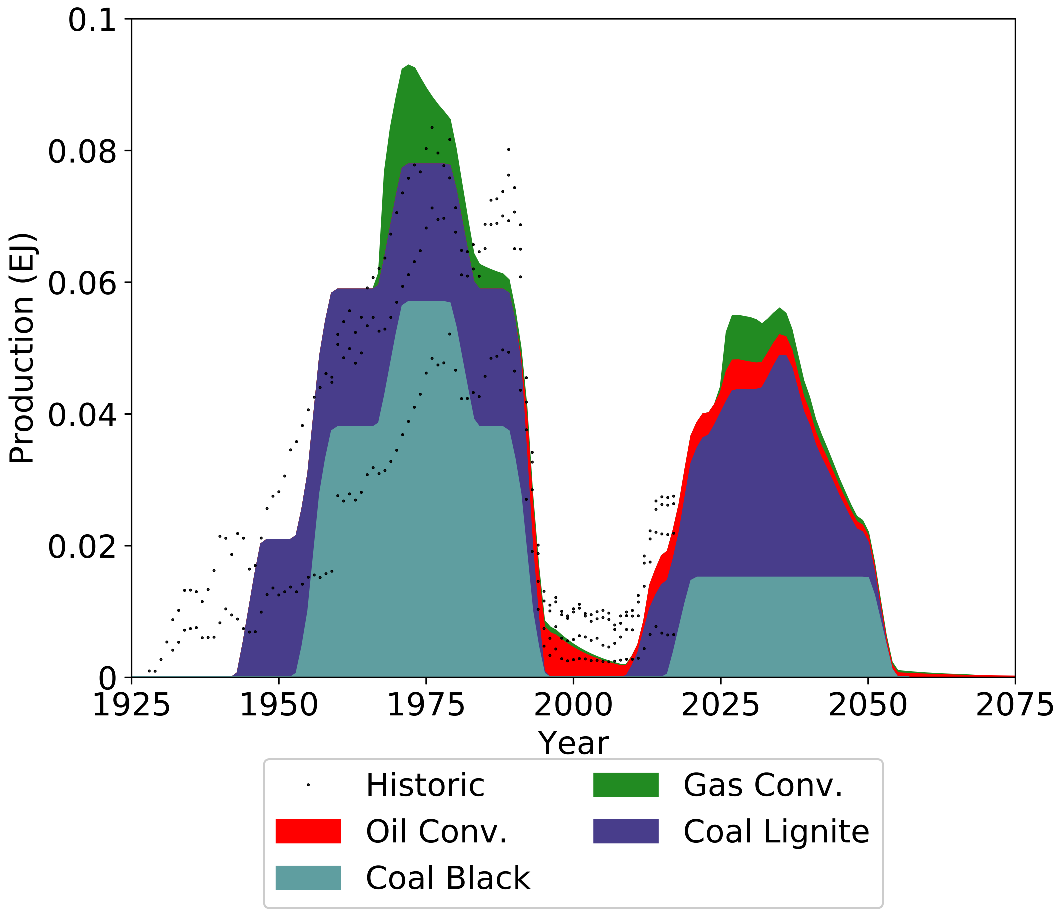

Figure 4.35: Kyrgyzstan projection by mineral type

Table 4.35: Peak years - Minerals

**Name**	**URR**	**Peak Year**	**Peak Rate**
Coal Black	2.12	1972	0.06
Coal Lignite	1.8	2035	0.03
Oil Conv.	0.21	1993	0.01
Gas Conv.	0.29	1969	0.02
**Total**	**4.42**	**1972**	**0.09**

4.9 Lithuania

4.9.1 All Projections

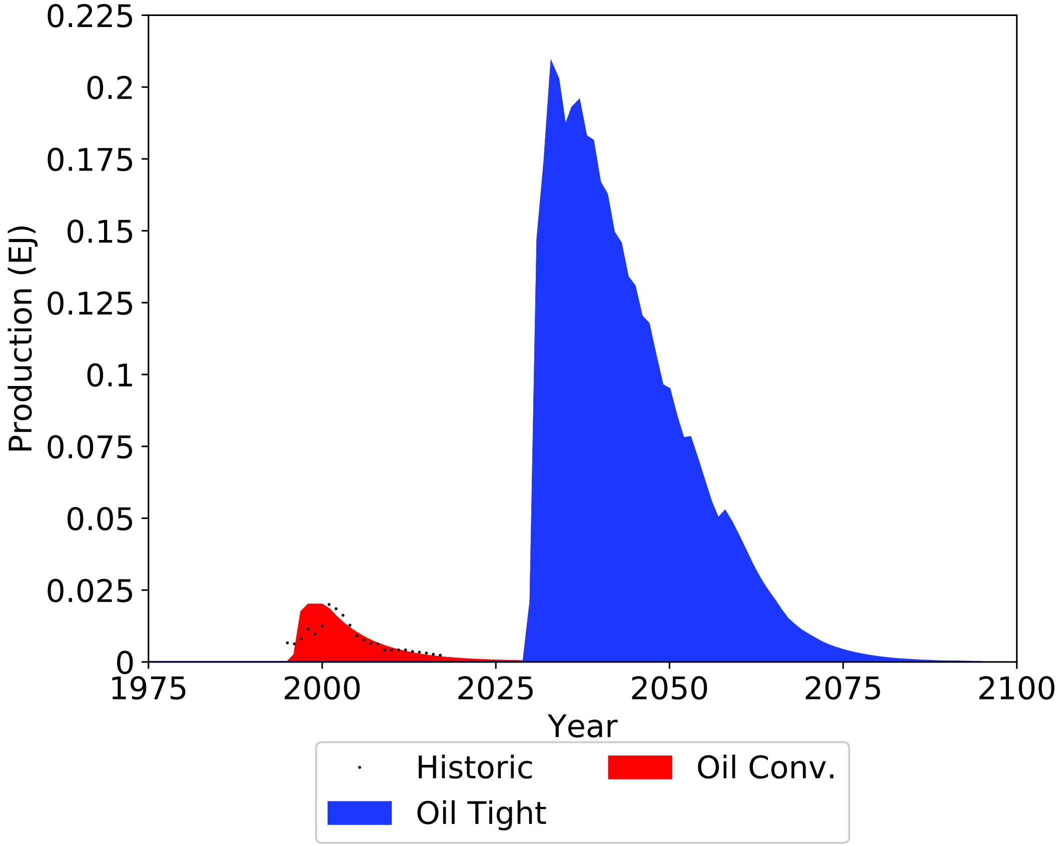

Figure 4.36: Lithuania projections capped at 16

Table 4.36: Peak years - All

Name	URR	Peak Year	Peak Rate
Oil Tight	4.01	2033	0.21
Oil Conv.	0.21	1998	0.02
**Total**	**4.22**	**2033**	**0.21**

4.9.2 By Mineral

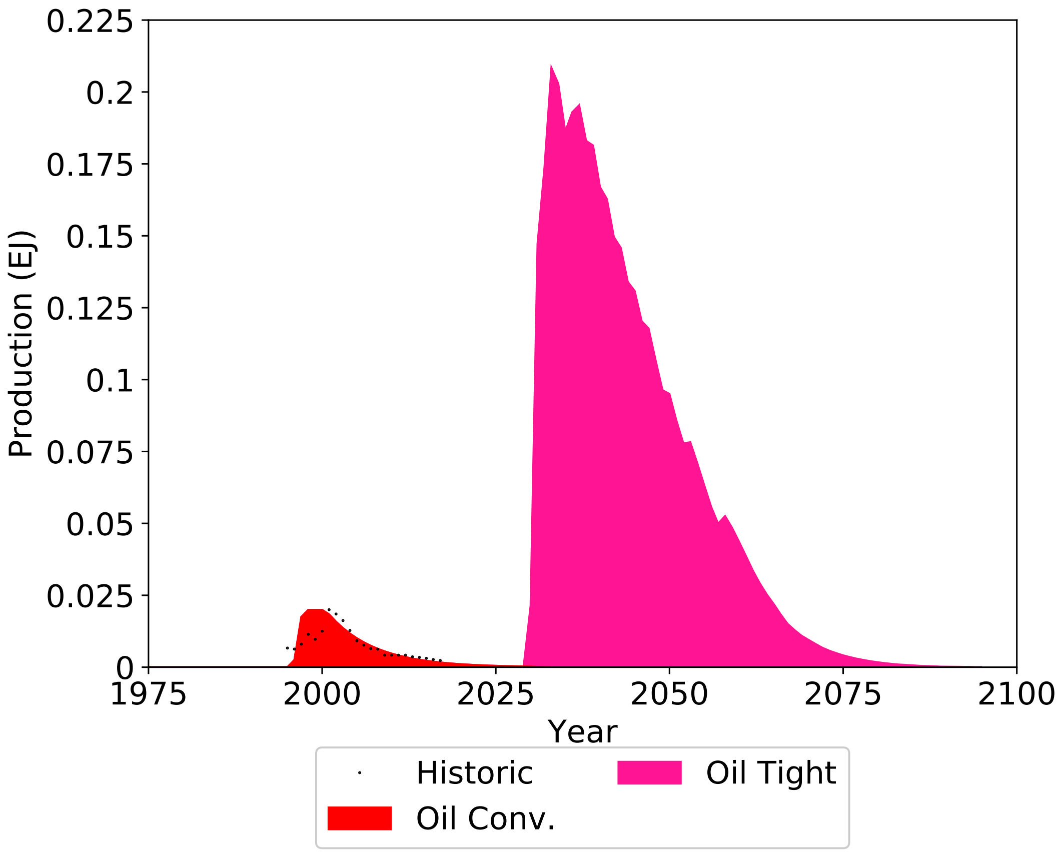

Figure 4.37: Lithuania projection by mineral type

Table 4.37: Peak years - Minerals

Name	URR	Peak Year	Peak Rate
Oil Conv.	0.21	1998	0.02
Oil Tight	4.01	2033	0.21
**Total**	**4.22**	**2033**	**0.21**

4.10 Luhansk

4.10.1 All Projections

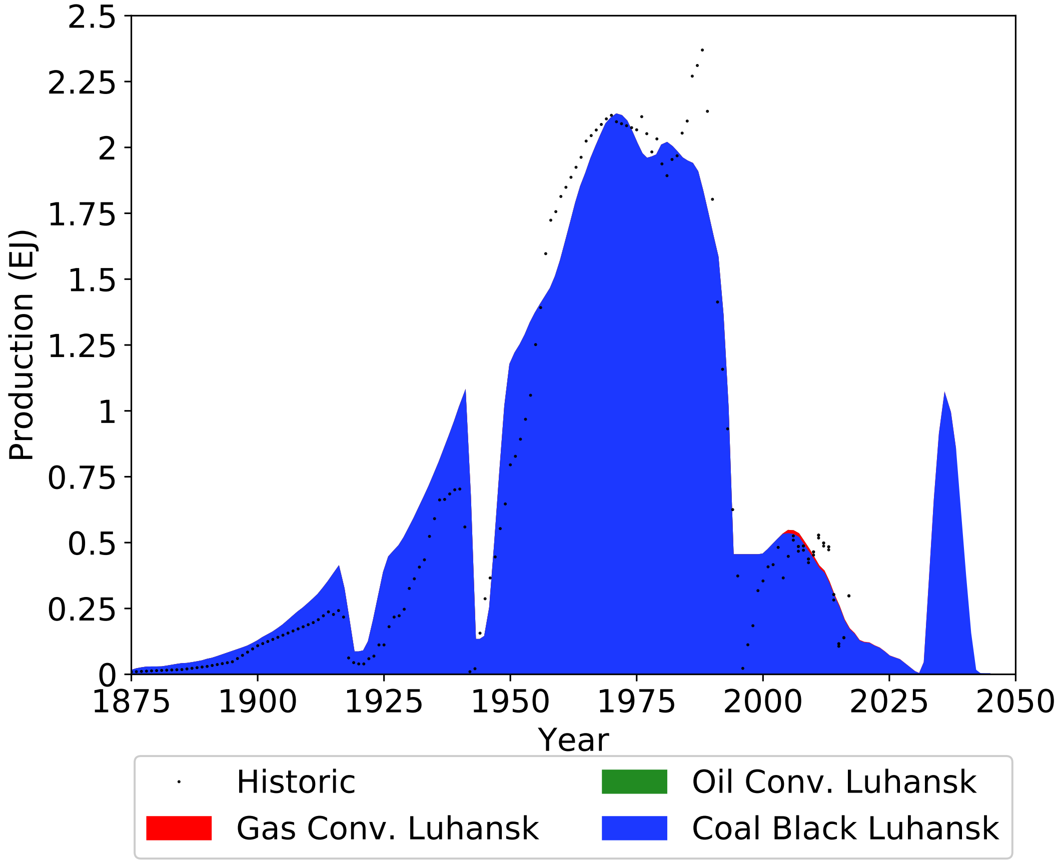

Figure 4.38: Luhansk projections capped at 16

Table 4.38: Peak years - All

**Name**	**URR**	**Peak Year**	**Peak Rate**
Coal Black Luhansk	117.0	1971	2.12
Gas Conv. Luhansk	0.14	2006	0.01
Oil Conv. Luhansk	–	2006	–
**Total**	**117.14**	**1971**	**2.12**

4.10.2 By Mineral

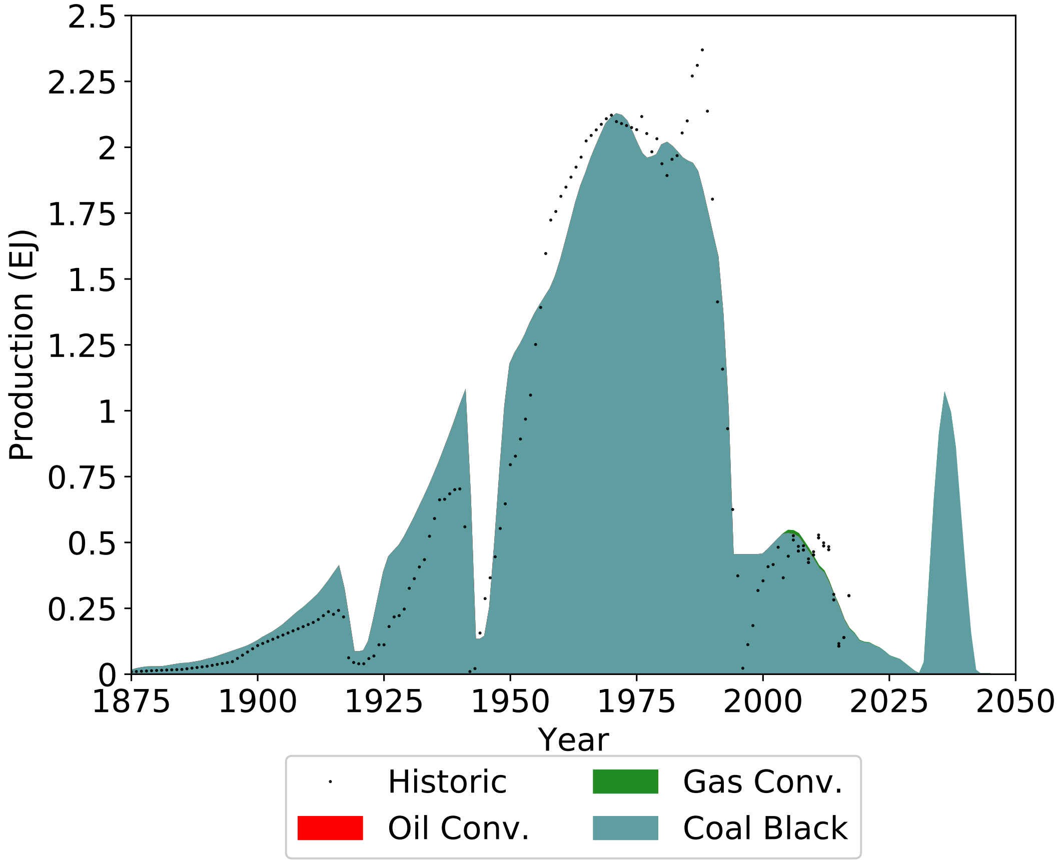

Figure 4.39: Luhansk projection by mineral type

Table 4.39: Peak years - Minerals

**Name**	**URR**	**Peak Year**	**Peak Rate**
Coal Black	117.0	1971	2.12
Oil Conv.	–	2006	–
Gas Conv.	0.14	2006	0.01
**Total**	**117.14**	**1971**	**2.12**

4.10.3 Regional Projections

Luhansk

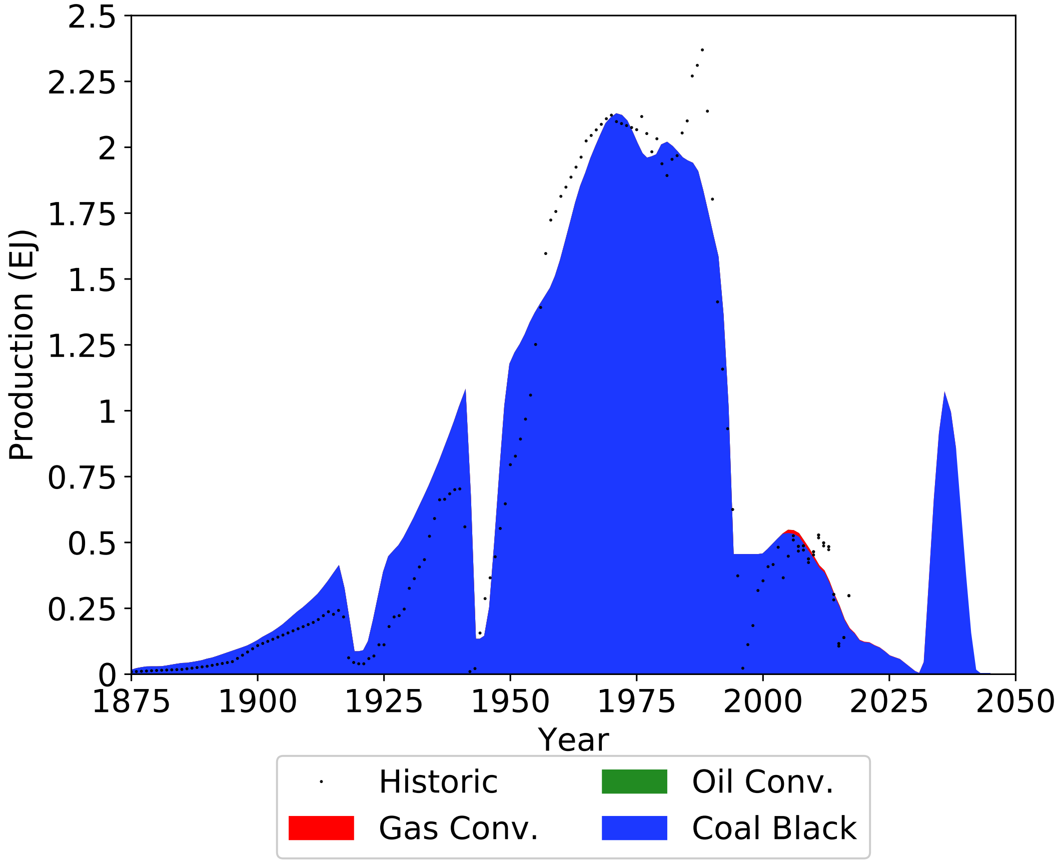

Figure 4.40: Luhansk - Luhansk projections capped at 16

Table 4.40: Peak years - All

**Name**	**URR**	**Peak Year**	**Peak Rate**
Coal Black Luhansk	117.0	1971	2.12
Gas Conv. Luhansk	0.14	2006	0.01
Oil Conv. Luhansk	–	2006	–
**Total**	**117.14**	**1971**	**2.12**

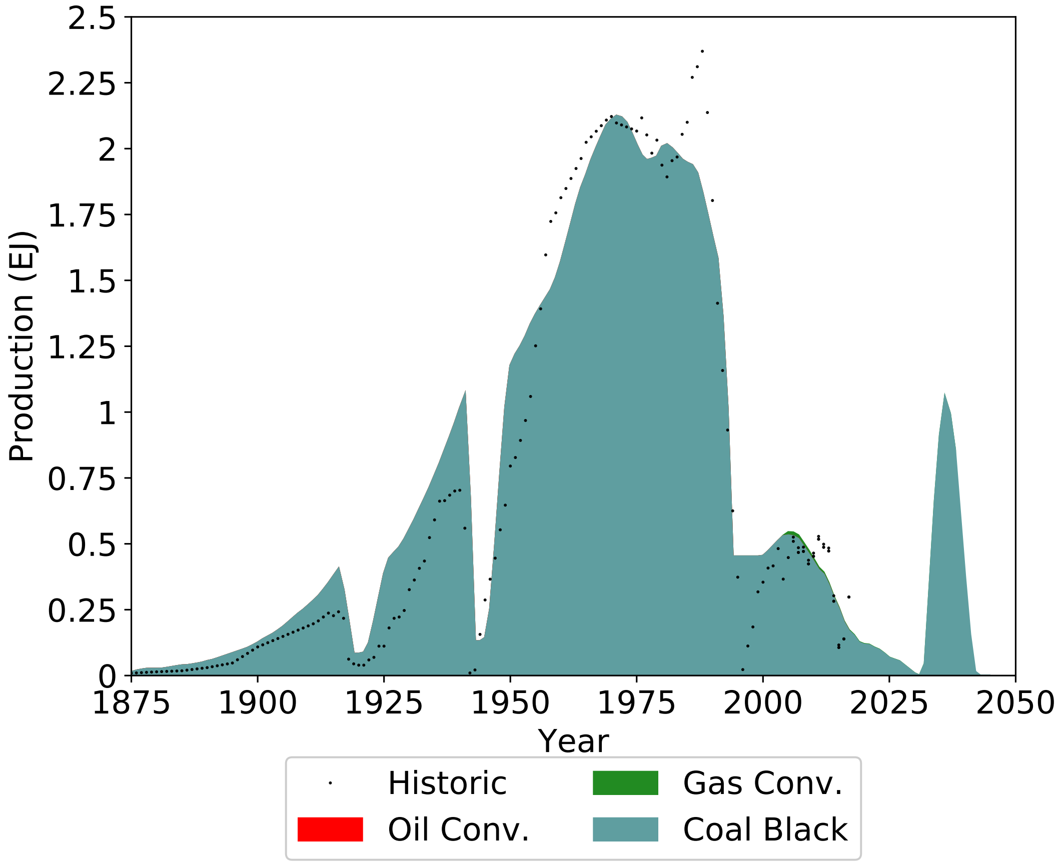

Figure 4.41: Luhansk - Luhansk projection by mineral type

Table 4.41: Peak years - Minerals

Name	URR	Peak Year	Peak Rate
Coal Black	117.0	1971	2.12
Oil Conv.	–	2006	–
Gas Conv.	0.14	2006	0.01
**Total**	**117.14**	**1971**	**2.12**

4.10.4 Projection by region

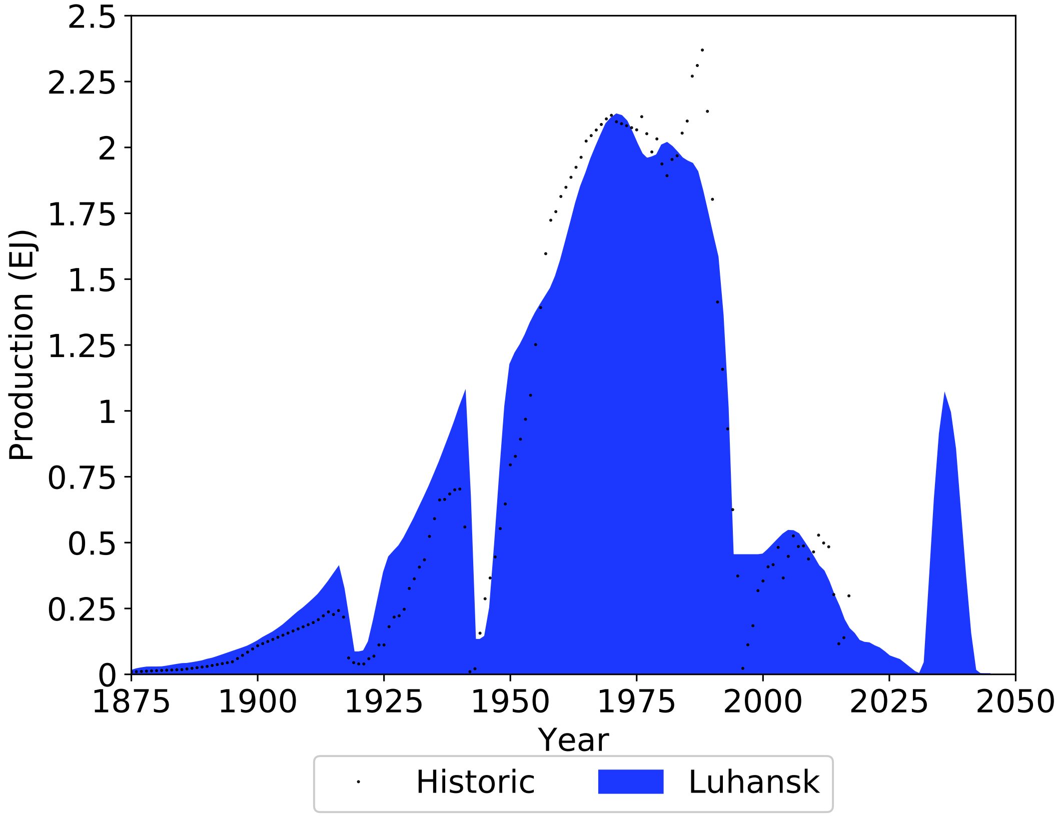

Figure 4.42: Luhansk by region projections capped at 16

Table 4.42: Peak years - All

**Name**	**URR**	**Peak Year**	**Peak Rate**
Luhansk	117.14	1971	2.12
**Total**	**117.14**	**1971**	**2.12**

4.11 Moldova

4.11.1 All Projections

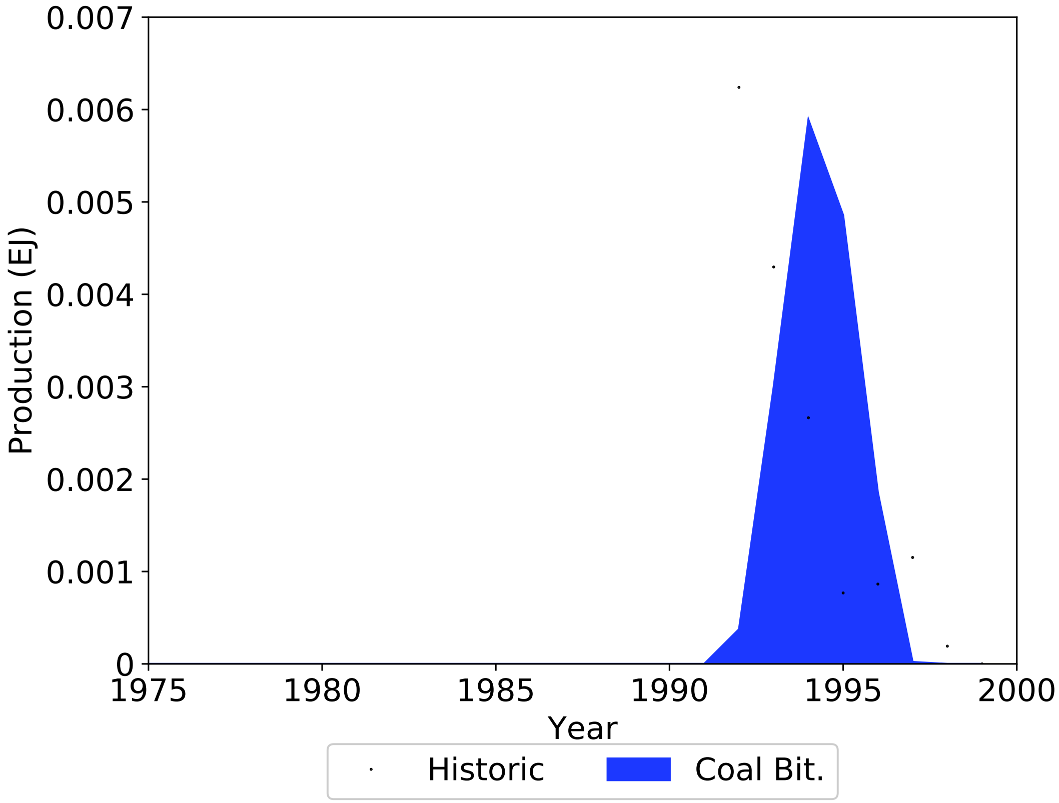

Figure 4.43: Moldova projections capped at 16

Table 4.43: Peak years - All

Name	URR	Peak Year	Peak Rate
Coal Bit.	0.02	1994	0.01
**Total**	**0.02**	**1994**	**0.01**

4.11.2 By Mineral

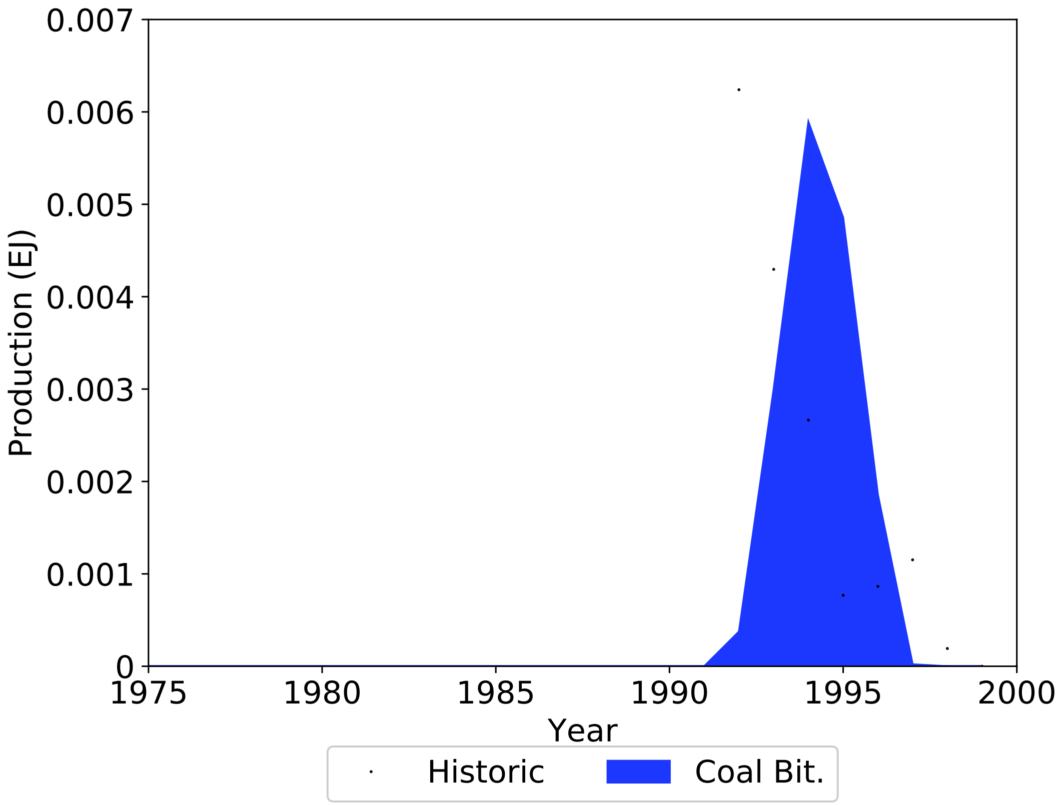

Figure 4.44: Moldova projection by mineral type

Table 4.44: Peak years - Minerals

**Name**	**URR**	**Peak Year**	**Peak Rate**
Coal Bit.	0.02	1994	0.01
**Total**	**0.02**	**1994**	**0.01**

4.12 Russia

4.12.1 All Projections

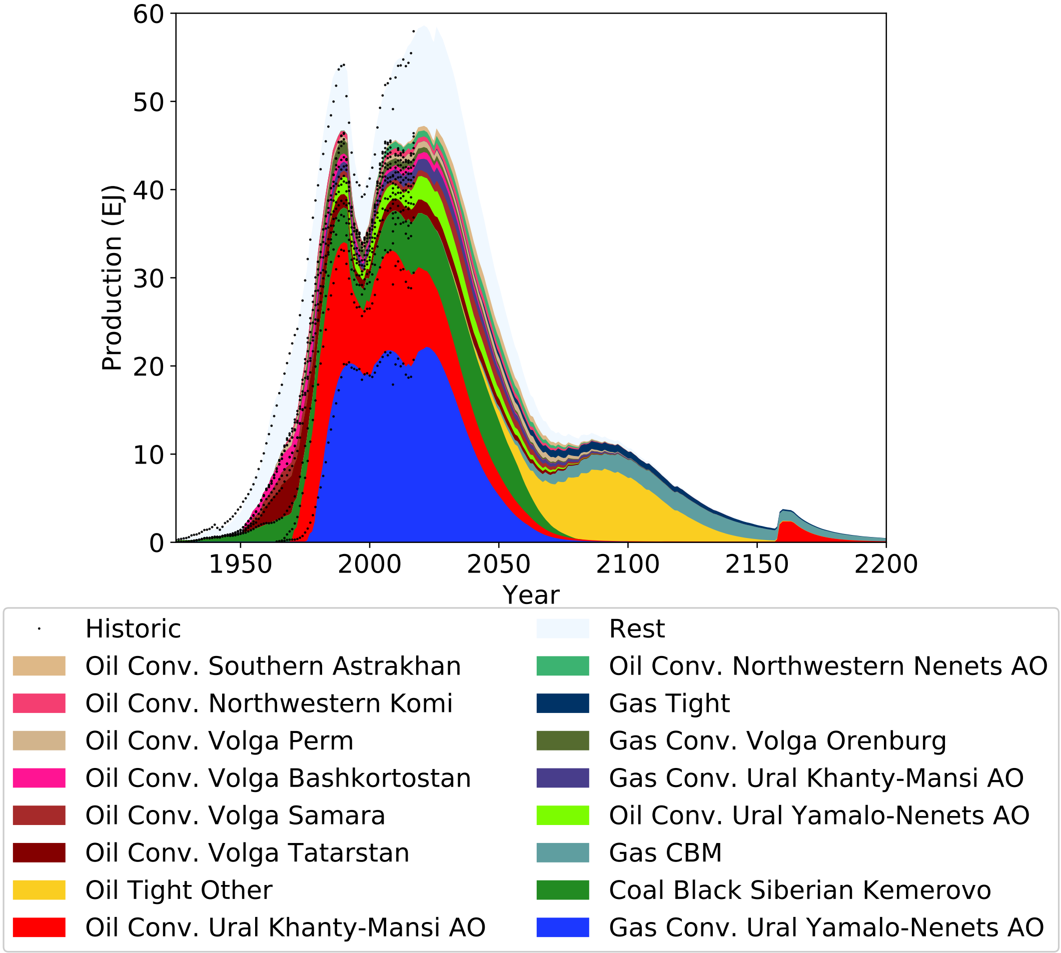

Figure 4.45: Russia projections capped at 16

Table 4.45: Peak years - All

Name	URR	Peak Year	Peak Rate
Gas Conv. Ural Yamalo-Nenets AO	1232.13	2022	22.07
Oil Conv. Ural Khanty-Mansi AO	738.03	1985	15.34
Coal Black Siberian Kemerovo	520.0	2037	7.51
Oil Tight Other	427.46	2091	8.18
Gas CBM	209.91	2108	2.21
Oil Conv. Volga Tatarstan	200.55	1972	4.44
Oil Conv. Ural Yamalo-Nenets AO	143.25	2026	2.93
Oil Conv. Volga Samara	118.76	2036	2.05
Gas Conv. Ural Khanty-Mansi AO	97.18	2023	1.52
Oil Conv. Volga Bashkortostan	90.39	1969	1.94
Gas Conv. Volga Orenburg	75.02	1983	1.76
Oil Conv. Volga Perm	74.41	2052	0.86
Gas Tight	74.13	2084	0.9
Oil Conv. Northwestern Komi	64.95	2037	0.89
Oil Conv. Northwestern Nenets AO	57.3	2041	1.21
Oil Conv. Southern Astrakhan	57.3	2040	1.4
Coal Black Southern Rostov	48.65	1963	0.86
Oil Conv. Volga Orenburg	48.36	2012	0.96
Gas Conv. Southern Other	47.48	1963	1.08
Coal Black Siberian Irkutsk	46.19	1975	0.66
Coal Black Northwestern Komi	42.48	1987	0.81
Coal Black Siberian Khakassia	39.0	2027	0.88
Gas Shale	35.21	2061	0.46
Oil Conv. Volga Udmurtia	33.9	1983	0.51
Coal Lignite Siberian Krasnoyarsk	33.83	1991	0.53
Gas Conv. North Caucasian	31.5	1963	1.58
Oil Conv. Siberian Tomsk	31.29	2007	0.54
Oil Conv. Siberian Krasnoyarsk	28.65	2020	1.0
Oil Conv. Siberian Irkutsk	28.65	2024	0.88
Oil Conv. Far Eastern Sakhalin	25.53	2017	0.77
Gas Conv. Southern Astrakhan	24.56	2019	0.52
Coal Far Eastern Yakutia	23.4	2018	0.35
Coal Far Eastern Primorsky	20.23	1984	0.29
Coal Brown Far Eastern Zabaykalsky	19.39	2016	0.24
Gas Conv. Far Eastern Sakhalin	19.33	2015	1.05
Oil Conv. North Caucasian Chechnya	18.92	1969	0.82
Gas Conv. Siberian Krasnoyarsk	18.0	2021	0.46
Gas Conv. Northwestern Komi	17.86	1977	0.74
Oil Conv. Far Eastern Yakutia	17.19	2020	0.49
Coal Lignite Central	15.38	1965	0.39
Oil Conv. Southern Volgograd	13.47	1965	0.3
Coal Black Siberian Novosibirsk	13.0	2026	0.78
Coal Black Far Eastern Khabarovsk	13.0	2035	0.27
Coal Black Far Eastern Sakhalin	13.0	2027	0.26
Oil Conv. Ural Tyumen	12.11	2015	0.48
Coal Black Volga Perm	12.11	1958	0.34
Coal Brown Ural Chelyabinsk	12.02	1966	0.28
Oil Conv. Southern Krasnodar	11.33	1962	0.25
Gas Conv. Siberian Tomsk	11.0	2021	0.25

Table 4.45: Peak years - All – Continued

Name	URR	Peak Year	Peak Rate
Coal Brown Ural Sverdlovsk	10.02	1960	0.35
Coal Brown Far Eastern Amur	9.54	1973	0.18
Gas Conv. Volga Saratov	9.27	1963	0.27
Oil Conv. North Caucasian Stavropol	5.89	1971	0.3
Coal Black Far Eastern Buryatia	5.2	1990	0.1
Oil Conv. Volga Saratov	5.16	1955	0.11
Gas Conv. Far Eastern Yakutia	4.25	2013	0.1
Oil Tight Northwestern Kalingrad	4.01	2033	0.21
Coal Black Far Eastern Magadan	2.49	1987	0.06
Oil Conv. Northwestern Kaliningrad	2.19	1980	0.07
Gas Conv. Volga Other	1.85	2003	0.06
Oil Conv. North Caucasian Dagestan	1.78	1972	0.08
Coal Lignite Volga Bashkortostan	1.72	1976	0.08
Coal Black Siberian Tuva	1.55	1982	0.03
Oil Conv. Volga Ulyanovsk	1.2	2010	0.03
Coal Black Far Eastern Chukotka AO	1.03	1989	0.03
Gas Conv. Siberian Irkutsk	0.93	2015	0.08
Oil Conv. Siberian Novosibirsk	0.7	2004	0.09
Oil Kerogen	0.69	1969	0.02
Oil Conv. Southern Kalmykia	0.68	1977	0.03
Coal Brown Volga Orenburg	0.59	1985	0.04
Coal Black Northwestern Murmansk	0.47	1970	0.01
Oil Conv. Siberian Omsk	0.43	2005	0.04
Gas Conv. Northwestern Nenets AO	0.37	2014	0.01
Gas Conv. Far Eastern Kamchatka	0.37	2016	0.02
Oil Conv. Southern Adygea	0.09	1971	0.01
Oil Conv. North Caucasian Ingushetia	0.07	1996	0.01
Coal Black North Caucasian Karachay-Cherkessia	0.06	1970	–
Oil Conv. Volga Penza	0.04	2002	0.01
Coal Brown Far Eastern Kamchatka	0.03	1999	–
Coal Brown Northwestern Novgorod	0.01	1951	0.01
Coal Brown Far Eastern Jewish AO	0.01	2004	–
Coal Brown Siberian Altai Krai	0.01	2009	–
Oil Conv. North Caucasian North Ossetia-Alania	–	2002	–
Oil Conv. North Caucasian Kabardino-Balkaria	–	1998	–
Oil Conv. Volga Kirov	–	2001	–
Coal Lignite Far Eastern Zabaykalsky	–	1997	–
Oil Conv. Central Yaroslavl	–	2005	–
**Total**	**5079.49**	**2021**	**58.51**

4.12.2 By Mineral

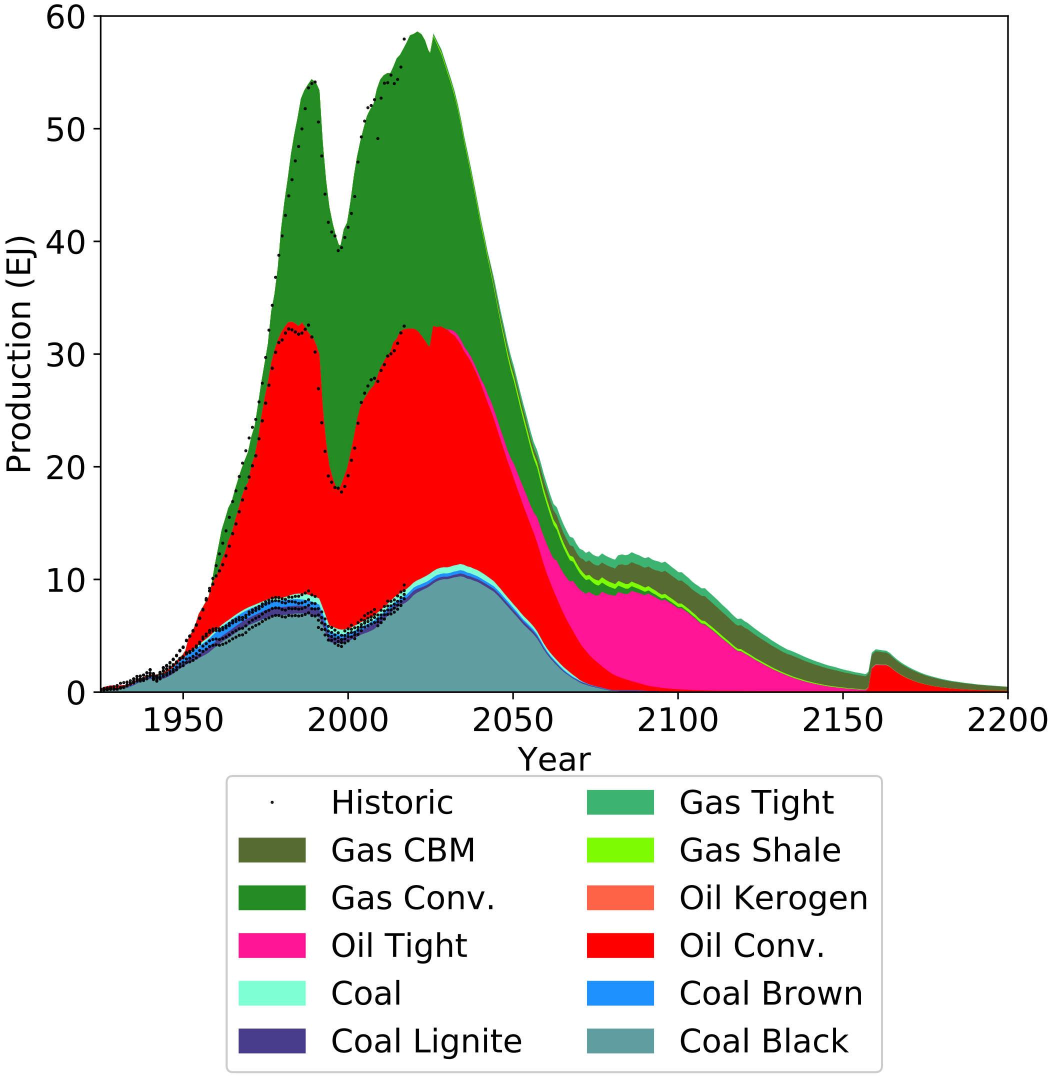

Figure 4.46: Russia projection by mineral type

4.12.3 Regional Projections

Table 4.46: Peak years - Minerals

**Name**	**URR**	**Peak Year**	**Peak Rate**
Coal Black	758.23	2034	10.19
Coal Lignite	50.93	1978	0.73
Coal Brown	51.61	1964	0.84
Coal	43.63	2025	0.55
Oil Conv.	1832.58	1982	24.28
Oil Tight	431.47	2091	8.18
Oil Kerogen	0.69	1969	0.02
Gas Conv.	1591.1	2022	26.76
Gas Shale	35.21	2061	0.46
Gas CBM	209.91	2108	2.21
Gas Tight	74.13	2084	0.9
**Total**	**5079.49**	**2021**	**58.51**

All

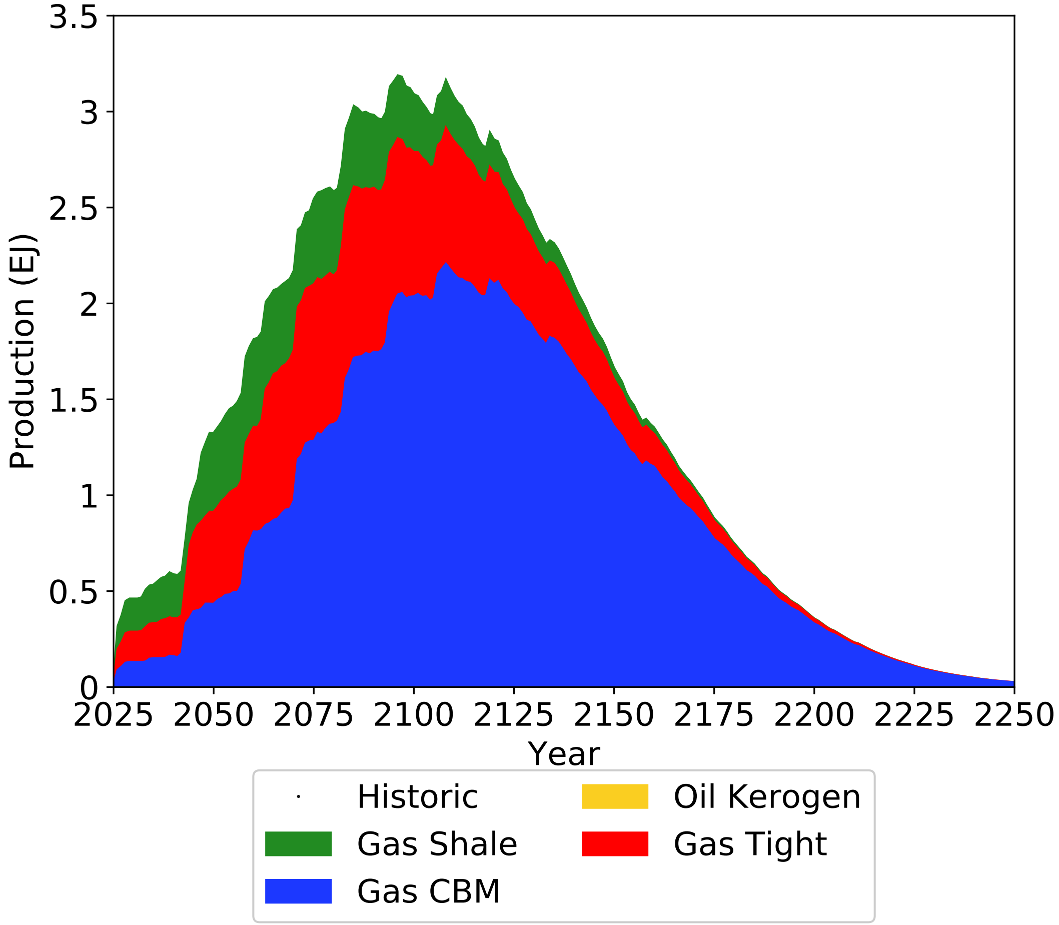

Figure 4.47: Russia - All projections capped at 16

Table 4.47: Peak years - All

Name	URR	Peak Year	Peak Rate
Gas CBM	209.91	2108	2.21
Gas Tight	74.13	2084	0.9
Gas Shale	35.21	2061	0.46
Oil Kerogen	0.69	1969	0.02
**Total**	**319.94**	**2096**	**3.19**

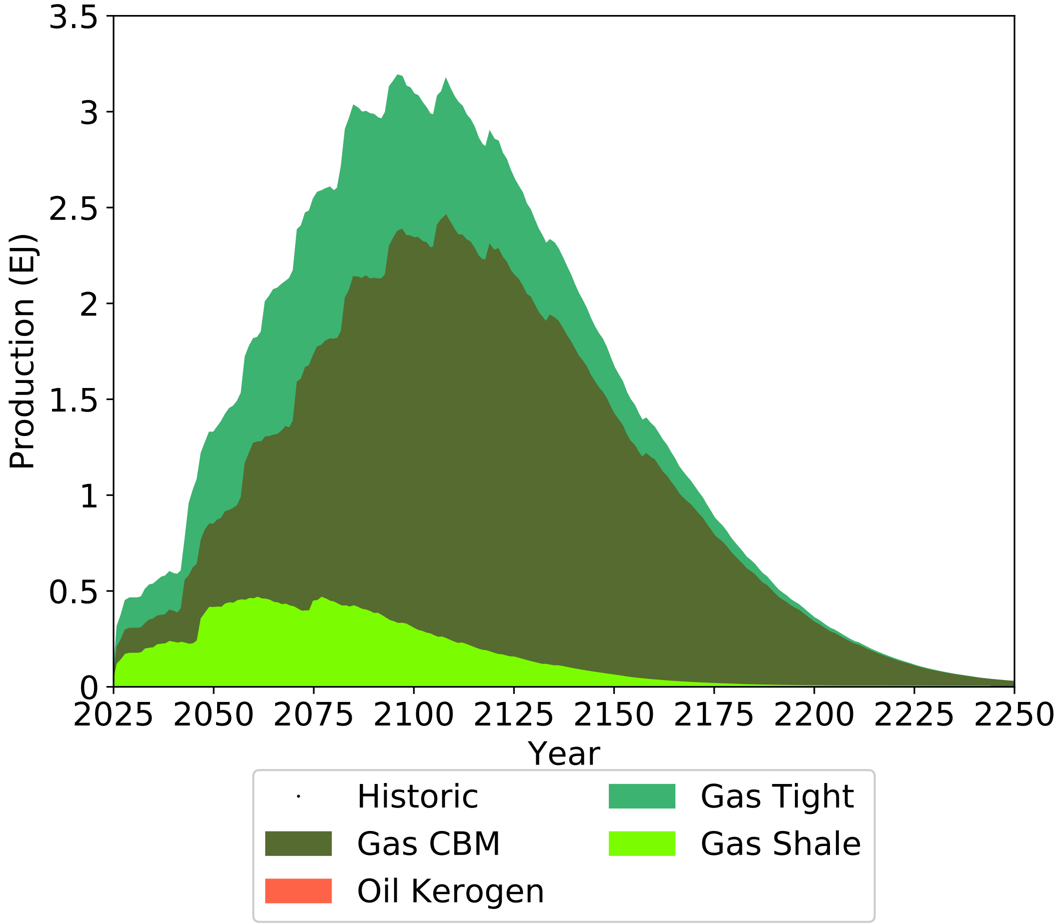

Figure 4.48: Russia - All projection by mineral type

Table 4.48: Peak years - Minerals

Name	URR	Peak Year	Peak Rate
Oil Kerogen	0.69	1969	0.02
Gas Shale	35.21	2061	0.46
Gas CBM	209.91	2108	2.21
Gas Tight	74.13	2084	0.9
**Total**	**319.94**	**2096**	**3.19**

Central

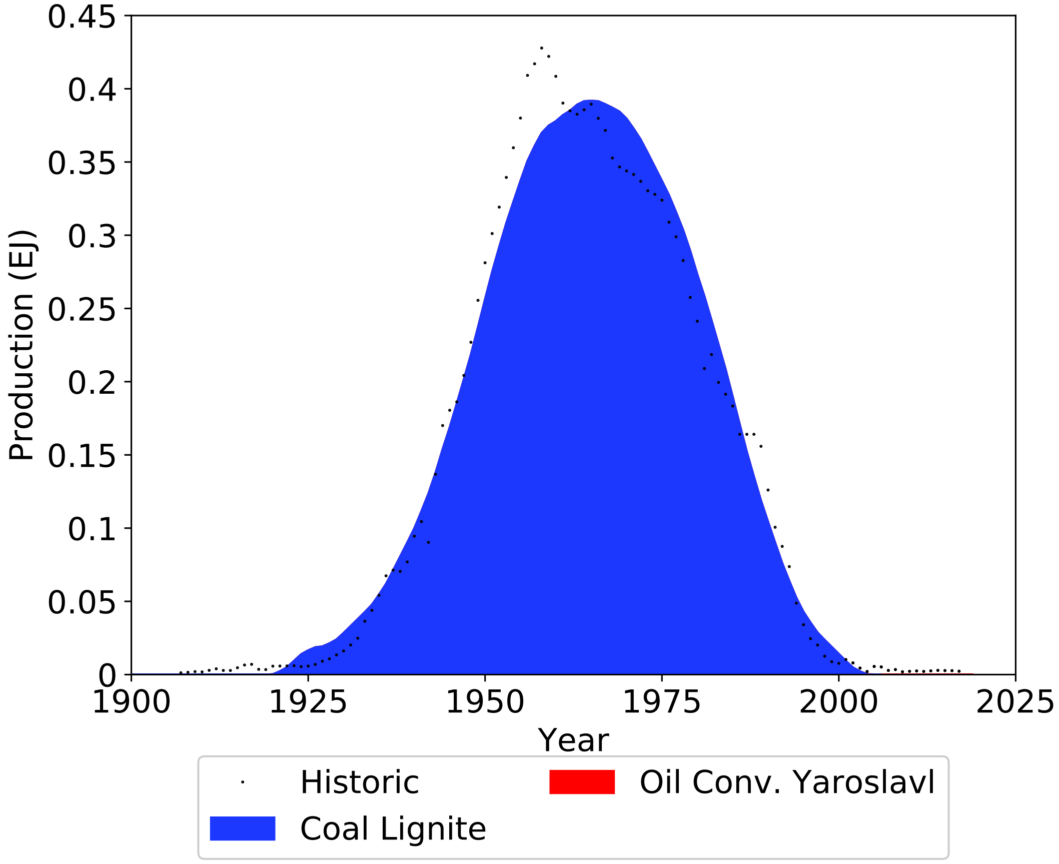

Figure 4.49: Russia - Central projections capped at 16

Table 4.49: Peak years - All

Name	URR	Peak Year	Peak Rate
Coal Lignite Central	15.38	1965	0.39
Oil Conv. Central Yaroslavl	–	2005	–
**Total**	**15.38**	**1965**	**0.39**

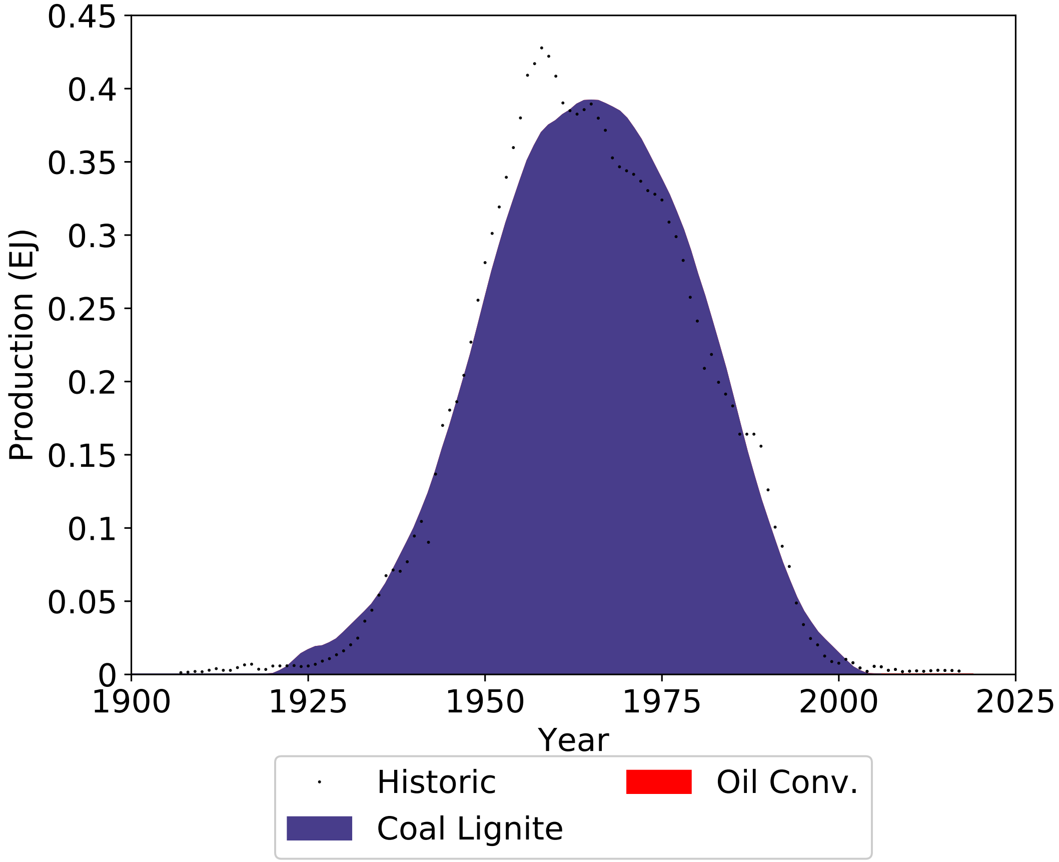

Figure 4.50: Russia - Central projection by mineral type

Table 4.50: Peak years - Minerals

Name	URR	Peak Year	Peak Rate
Coal Lignite	15.38	1965	0.39
Oil Conv.	–	2005	–
**Total**	**15.38**	**1965**	**0.39**

Far Eastern

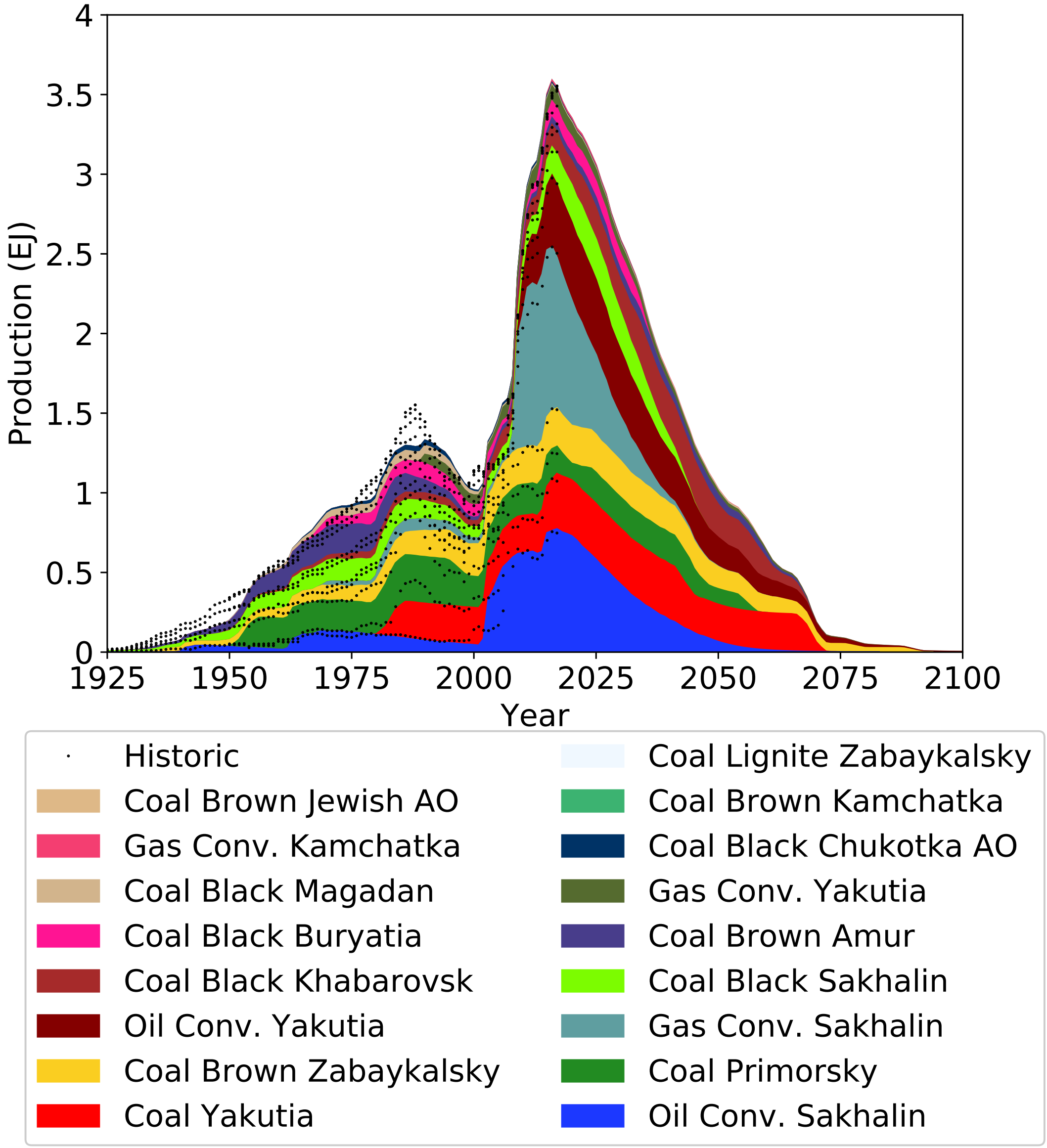

Figure 4.51: Russia - Far Eastern projections capped at 16

Table 4.51: Peak years - All

**Name**	**URR**	**Peak Year**	**Peak Rate**
Oil Conv. Far Eastern Sakhalin	25.53	2017	0.77
Coal Far Eastern Yakutia	23.4	2018	0.35
Coal Far Eastern Primorsky	20.23	1984	0.29
Coal Brown Far Eastern Zabaykalsky	19.39	2016	0.24
Gas Conv. Far Eastern Sakhalin	19.33	2015	1.05
Oil Conv. Far Eastern Yakutia	17.19	2020	0.49
Coal Black Far Eastern Sakhalin	13.0	2027	0.26
Coal Black Far Eastern Khabarovsk	13.0	2035	0.27
Coal Brown Far Eastern Amur	9.54	1973	0.18
Coal Black Far Eastern Buryatia	5.2	1990	0.1
Gas Conv. Far Eastern Yakutia	4.25	2013	0.1
Coal Black Far Eastern Magadan	2.49	1987	0.06
Coal Black Far Eastern Chukotka AO	1.03	1989	0.03
Gas Conv. Far Eastern Kamchatka	0.37	2016	0.02
Coal Brown Far Eastern Kamchatka	0.03	1999	–
Coal Brown Far Eastern Jewish AO	0.01	2004	–
Coal Lignite Far Eastern Zabaykalsky	–	1997	–
**Total**	**173.99**	**2016**	**3.59**

Table 4.52: Peak years - Minerals

**Name**	**URR**	**Peak Year**	**Peak Rate**
Coal Black	34.72	2027	0.57
Coal Lignite	–	1997	–
Coal Brown	28.97	2025	0.3
Coal	43.63	2025	0.55
Oil Conv.	42.72	2017	1.23
Gas Conv.	23.95	2015	1.16
**Total**	**173.99**	**2016**	**3.59**

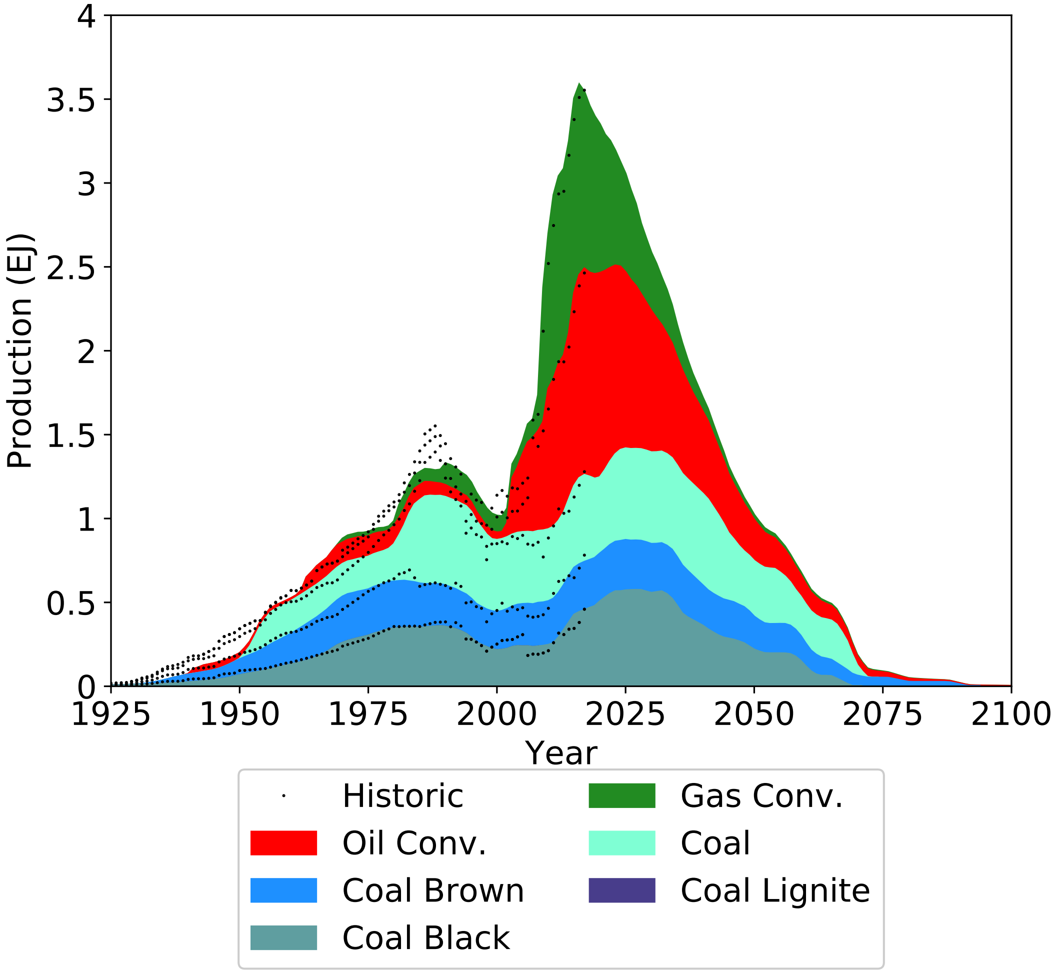

Figure 4.52: Russia - Far Eastern projection by mineral type

North Caucasian

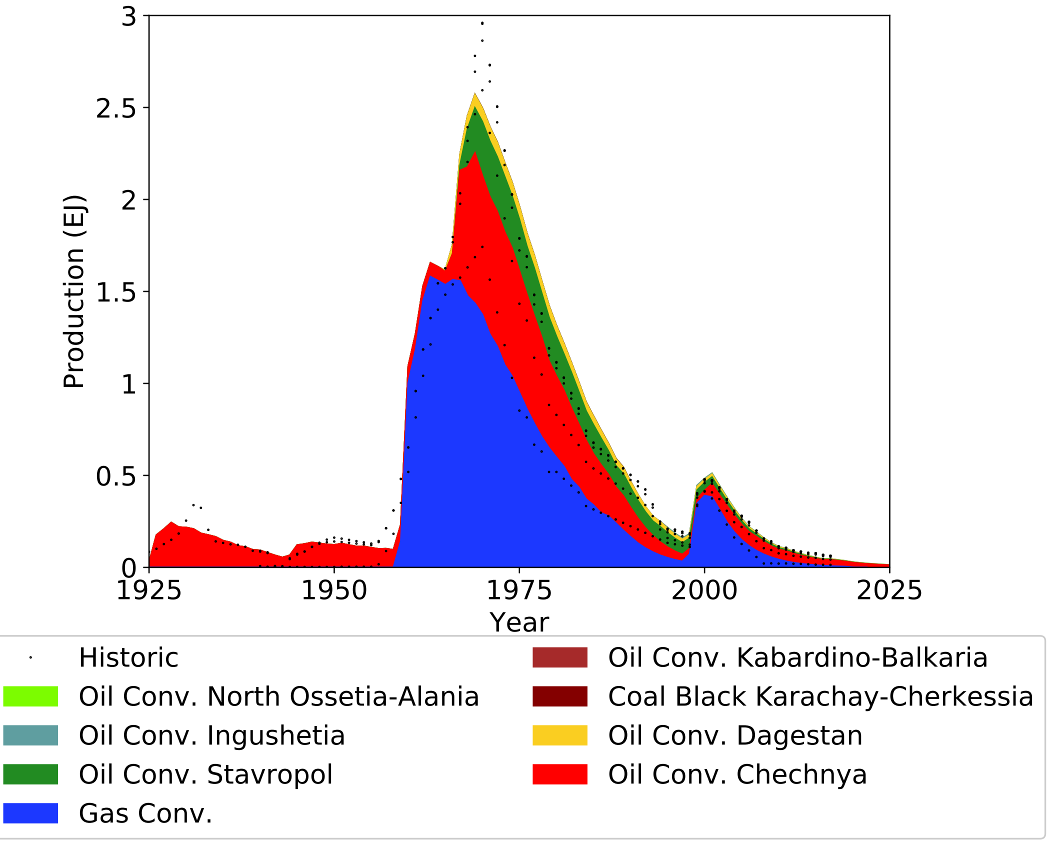

Figure 4.53: Russia - North Caucasian projections capped at 16

Table 4.53: Peak years - All

Name	URR	Peak Year	Peak Rate
Gas Conv. North Caucasian	31.5	1963	1.58
Oil Conv. North Caucasian Chechnya	18.92	1969	0.82
Oil Conv. North Caucasian Stavropol	5.89	1971	0.3
Oil Conv. North Caucasian Dagestan	1.78	1972	0.08
Oil Conv. North Caucasian Ingushetia	0.07	1996	0.01
Coal Black North Caucasian Karachay-Cherkessia	0.06	1970	–
Oil Conv. North Caucasian North Ossetia-Alania	–	2002	–
Oil Conv. North Caucasian Kabardino-Balkaria	–	1998	–
**Total**	**58.23**	**1969**	**2.57**

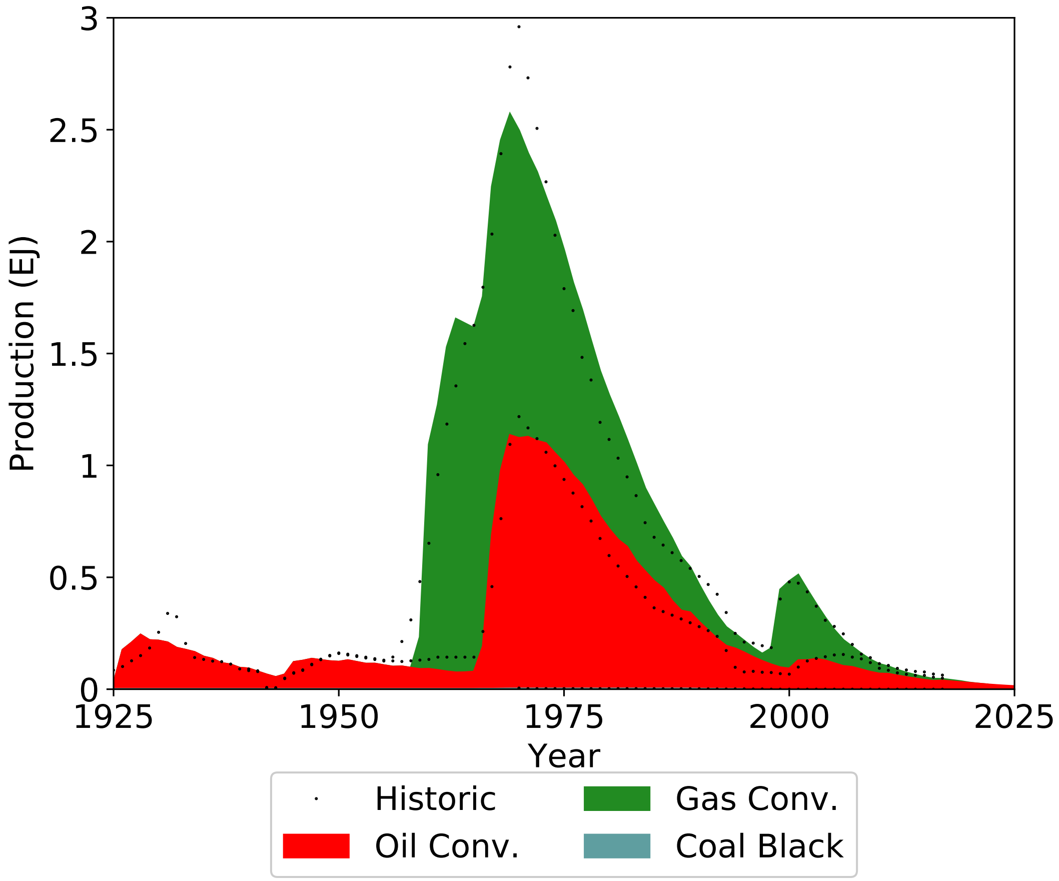

Figure 4.54: Russia - North Caucasian projection by mineral type

Table 4.54: Peak years - Minerals

Name	URR	Peak Year	Peak Rate
Coal Black	0.06	1970	–
Oil Conv.	26.67	1969	1.13
Gas Conv.	31.5	1963	1.58
**Total**	**58.23**	**1969**	**2.57**

Northwestern

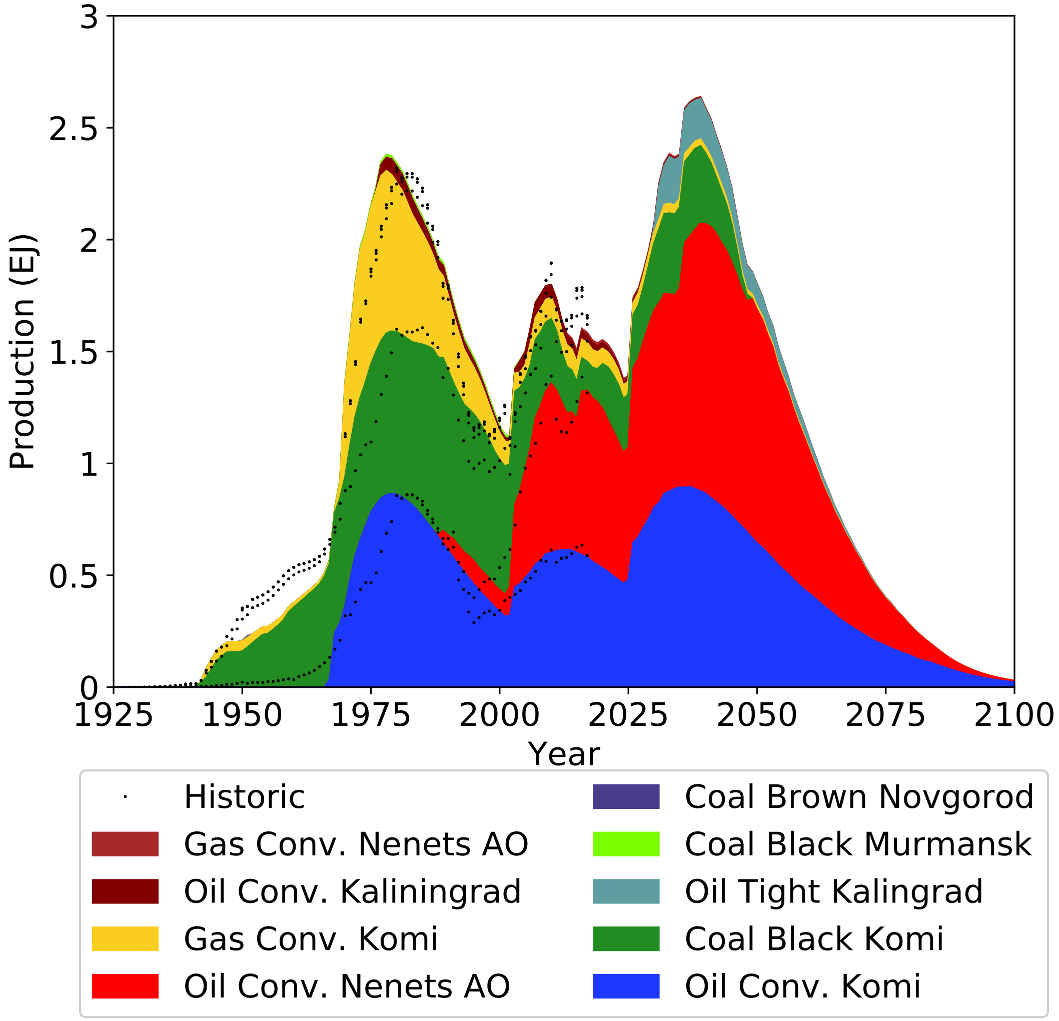

Figure 4.55: Russia - Northwestern projections capped at 16

Table 4.55: Peak years - All

Name	URR	Peak Year	Peak Rate
Oil Conv. Northwestern Komi	64.95	2037	0.89
Oil Conv. Northwestern Nenets AO	57.3	2041	1.21
Coal Black Northwestern Komi	42.48	1987	0.81
Gas Conv. Northwestern Komi	17.86	1977	0.74
Oil Tight Northwestern Kalingrad	4.01	2033	0.21
Oil Conv. Northwestern Kaliningrad	2.19	1980	0.07
Coal Black Northwestern Murmansk	0.47	1970	0.01
Gas Conv. Northwestern Nenets AO	0.37	2014	0.01
Coal Brown Northwestern Novgorod	0.01	1951	0.01
**Total**	**189.64**	**2039**	**2.64**

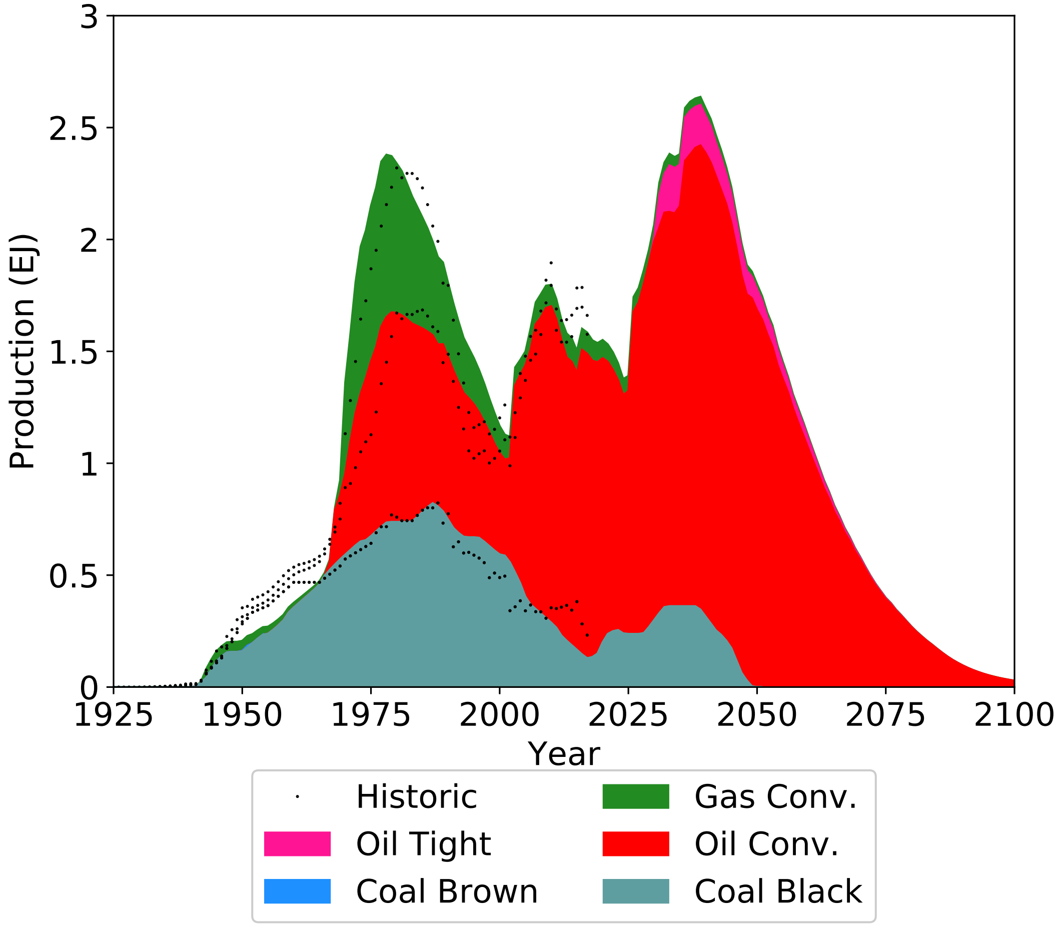

Figure 4.56: Russia - Northwestern projection by mineral type

Table 4.56: Peak years - Minerals

Name	URR	Peak Year	Peak Rate
Coal Black	42.95	1987	0.82
Coal Brown	0.01	1951	0.01
Oil Conv.	124.44	2039	2.07
Oil Tight	4.01	2033	0.21
Gas Conv.	18.23	1977	0.74
**Total**	**189.64**	**2039**	**2.64**

Other

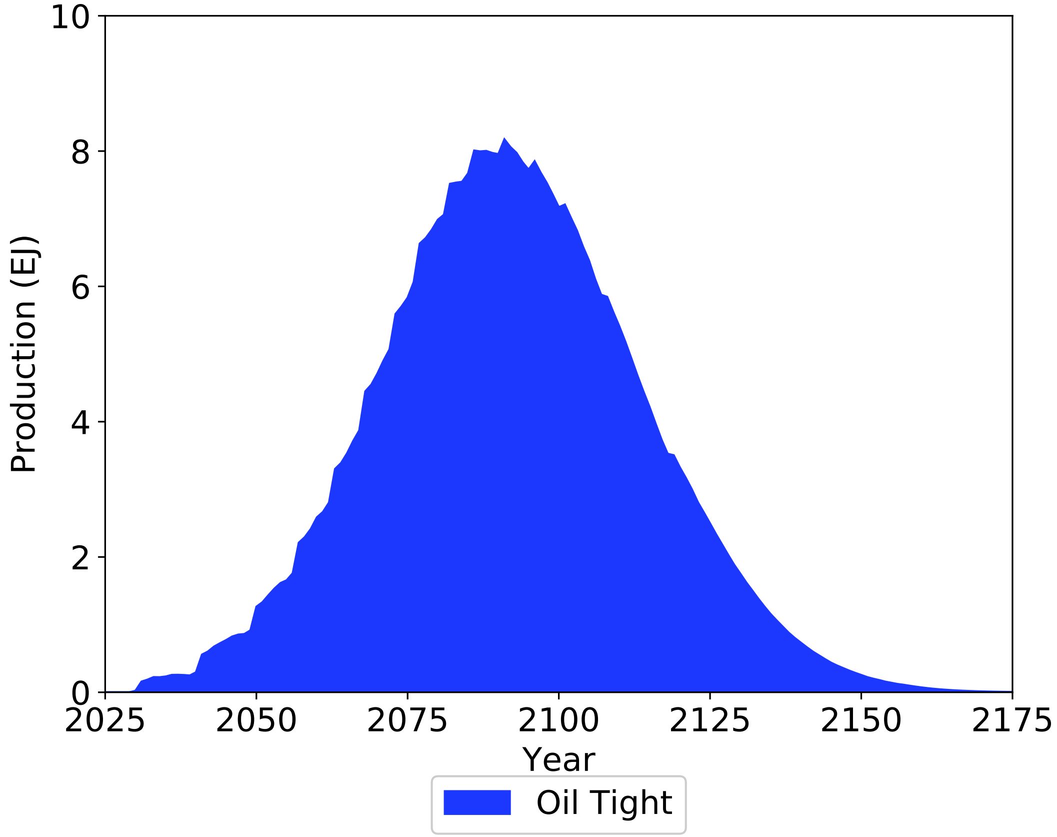

Figure 4.57: Russia - Other projections capped at 16

Table 4.57: Peak years - All

Name	URR	Peak Year	Peak Rate
Oil Tight Other	427.46	2091	8.18
**Total**	**427.46**	**2091**	**8.18**

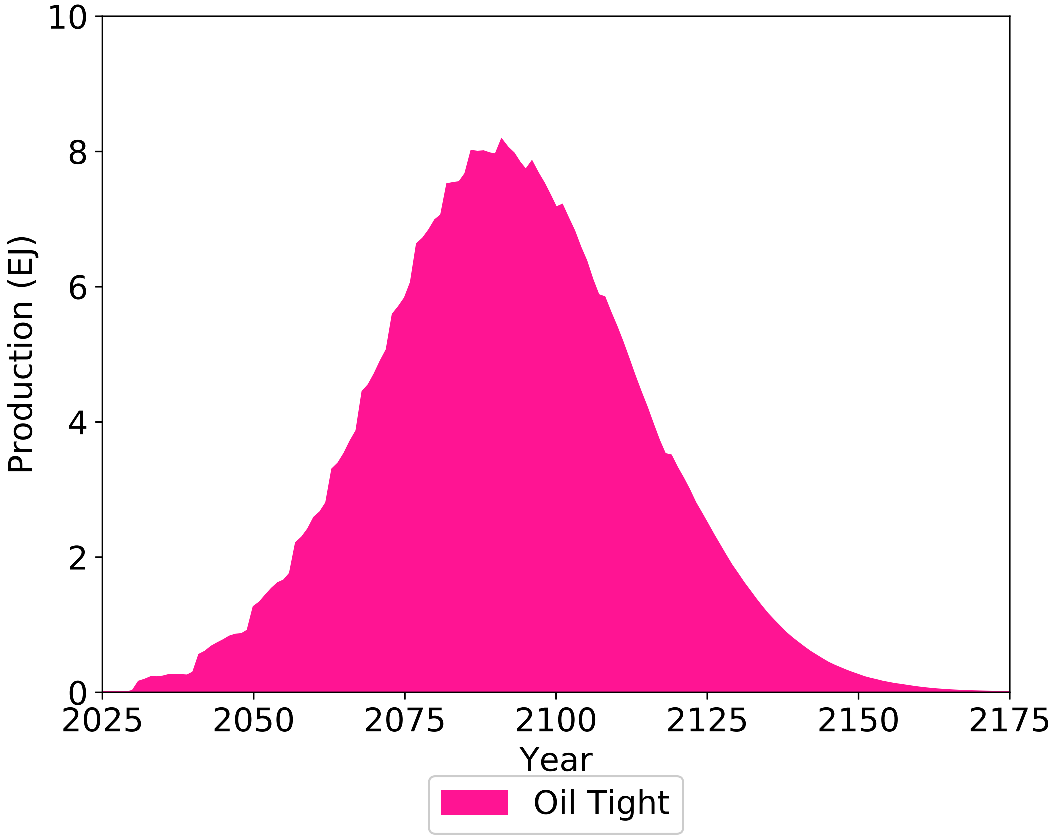

Figure 4.58: Russia - Other projection by mineral type

Table 4.58: Peak years - Minerals

Name	URR	Peak Year	Peak Rate
Oil Tight	427.46	2091	8.18
**Total**	**427.46**	**2091**	**8.18**

Siberian

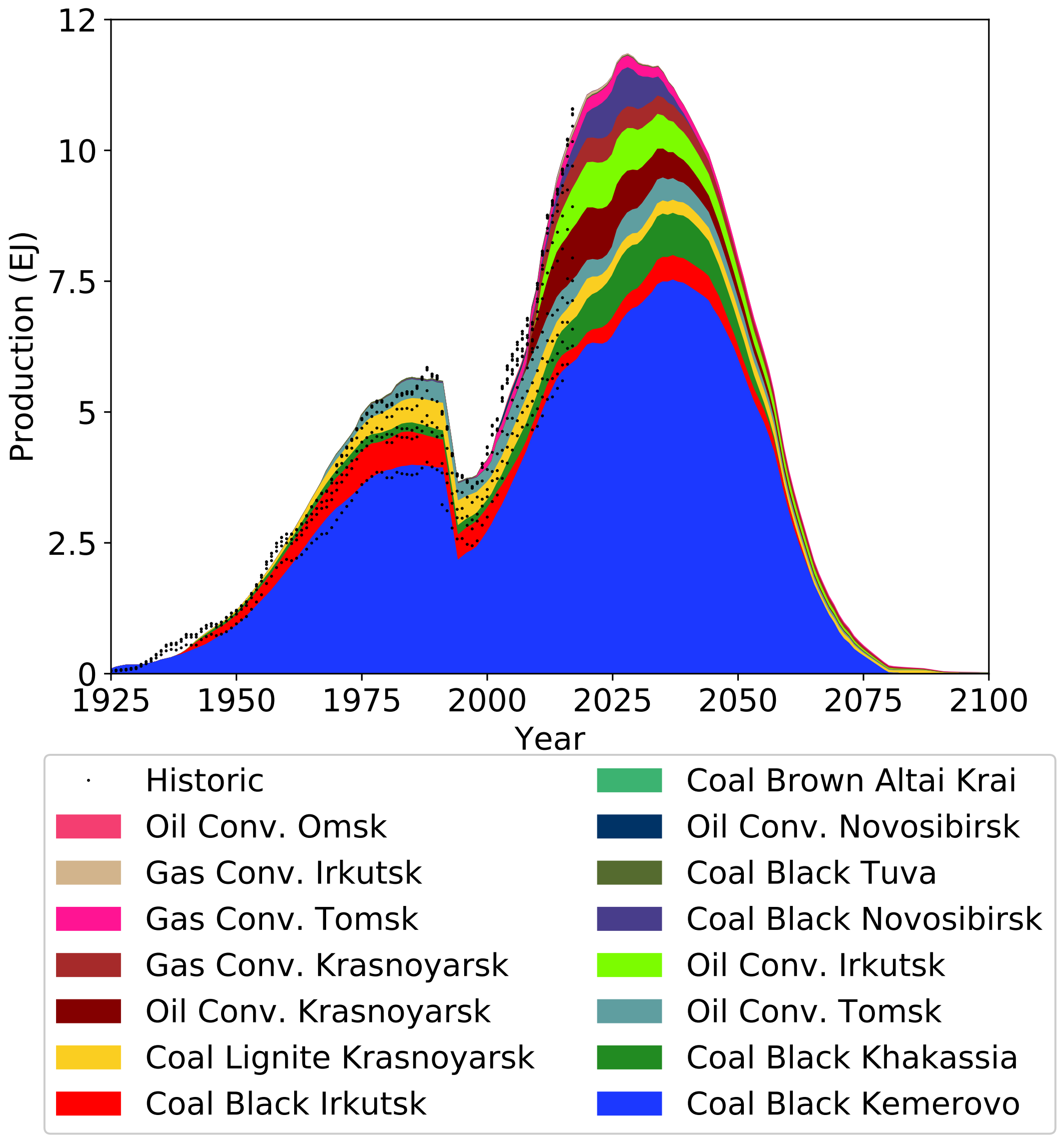

Figure 4.59: Russia - Siberian projections capped at 16

Table 4.59: Peak years - All

Name	URR	Peak Year	Peak Rate
Coal Black Siberian Kemerovo	520.0	2037	7.51
Coal Black Siberian Irkutsk	46.19	1975	0.66
Coal Black Siberian Khakassia	39.0	2027	0.88
Coal Lignite Siberian Krasnoyarsk	33.83	1991	0.53
Oil Conv. Siberian Tomsk	31.29	2007	0.54
Oil Conv. Siberian Krasnoyarsk	28.65	2020	1.0
Oil Conv. Siberian Irkutsk	28.65	2024	0.88
Gas Conv. Siberian Krasnoyarsk	18.0	2021	0.46
Coal Black Siberian Novosibirsk	13.0	2026	0.78
Gas Conv. Siberian Tomsk	11.0	2021	0.25
Coal Black Siberian Tuva	1.55	1982	0.03
Gas Conv. Siberian Irkutsk	0.93	2015	0.08
Oil Conv. Siberian Novosibirsk	0.7	2004	0.09
Oil Conv. Siberian Omsk	0.43	2005	0.04
Coal Brown Siberian Altai Krai	0.01	2009	–
**Total**	**773.23**	**2028**	**11.83**

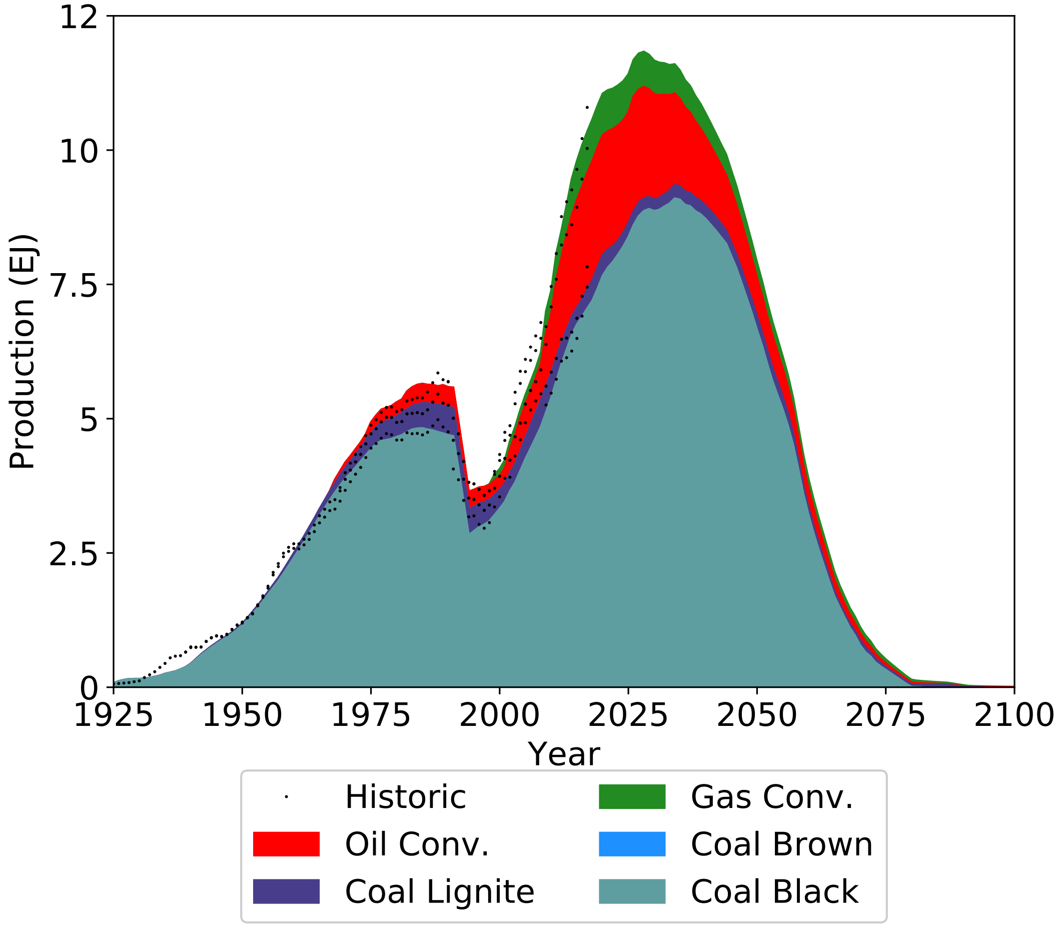

Figure 4.60: Russia - Siberian projection by mineral type

Table 4.60: Peak years - Minerals

**Name**	**URR**	**Peak Year**	**Peak Rate**
Coal Black	619.74	2034	9.1
Coal Lignite	33.83	1991	0.53
Coal Brown	0.01	2009	–
Oil Conv.	89.72	2020	2.24
Gas Conv.	29.93	2019	0.78
**Total**	**773.23**	**2028**	**11.83**

Southern

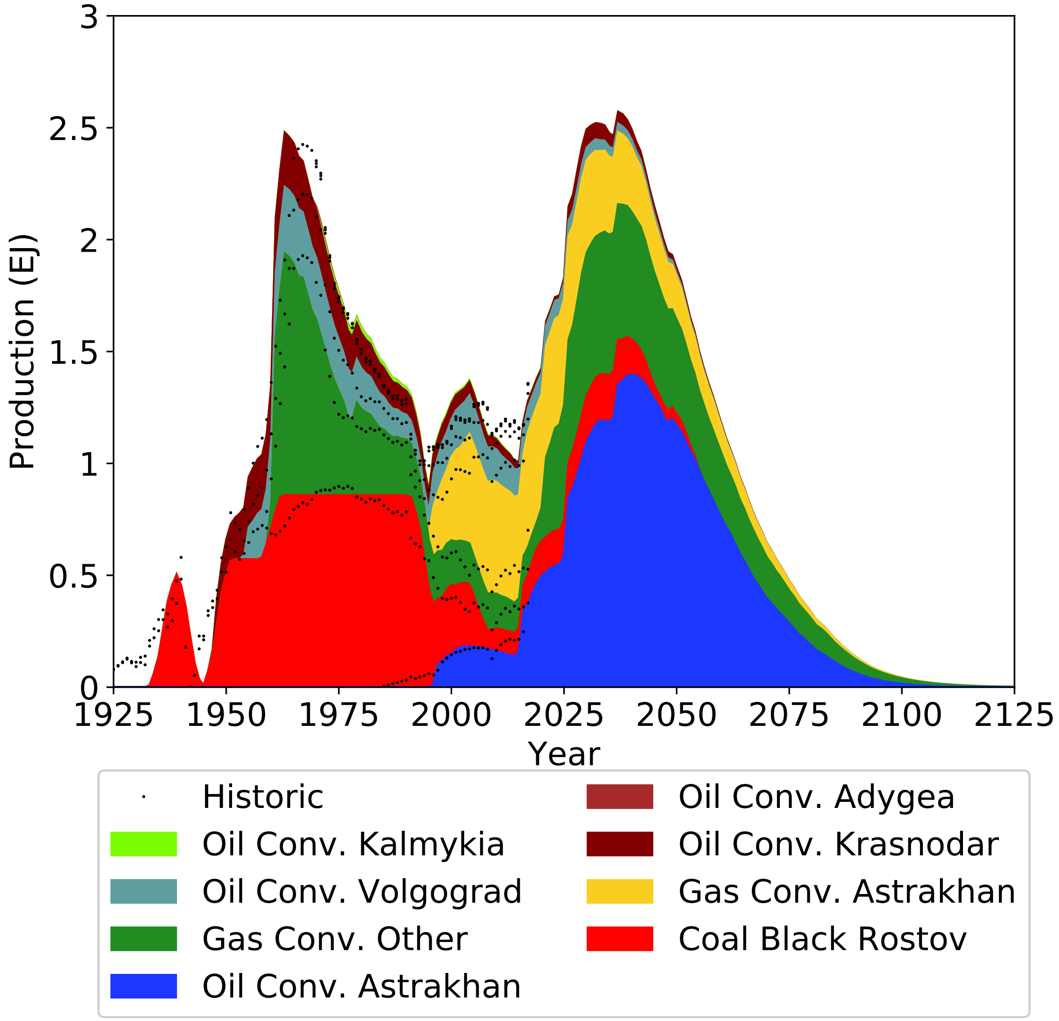

Figure 4.61: Russia - Southern projections capped at 16

Table 4.61: Peak years - All

Name	URR	Peak Year	Peak Rate
Oil Conv. Southern Astrakhan	57.3	2040	1.4
Coal Black Southern Rostov	48.65	1963	0.86
Gas Conv. Southern Other	47.48	1963	1.08
Gas Conv. Southern Astrakhan	24.56	2019	0.52
Oil Conv. Southern Volgograd	13.47	1965	0.3
Oil Conv. Southern Krasnodar	11.33	1962	0.25
Oil Conv. Southern Kalmykia	0.68	1977	0.03
Oil Conv. Southern Adygea	0.09	1971	0.01
**Total**	**203.56**	**2037**	**2.57**

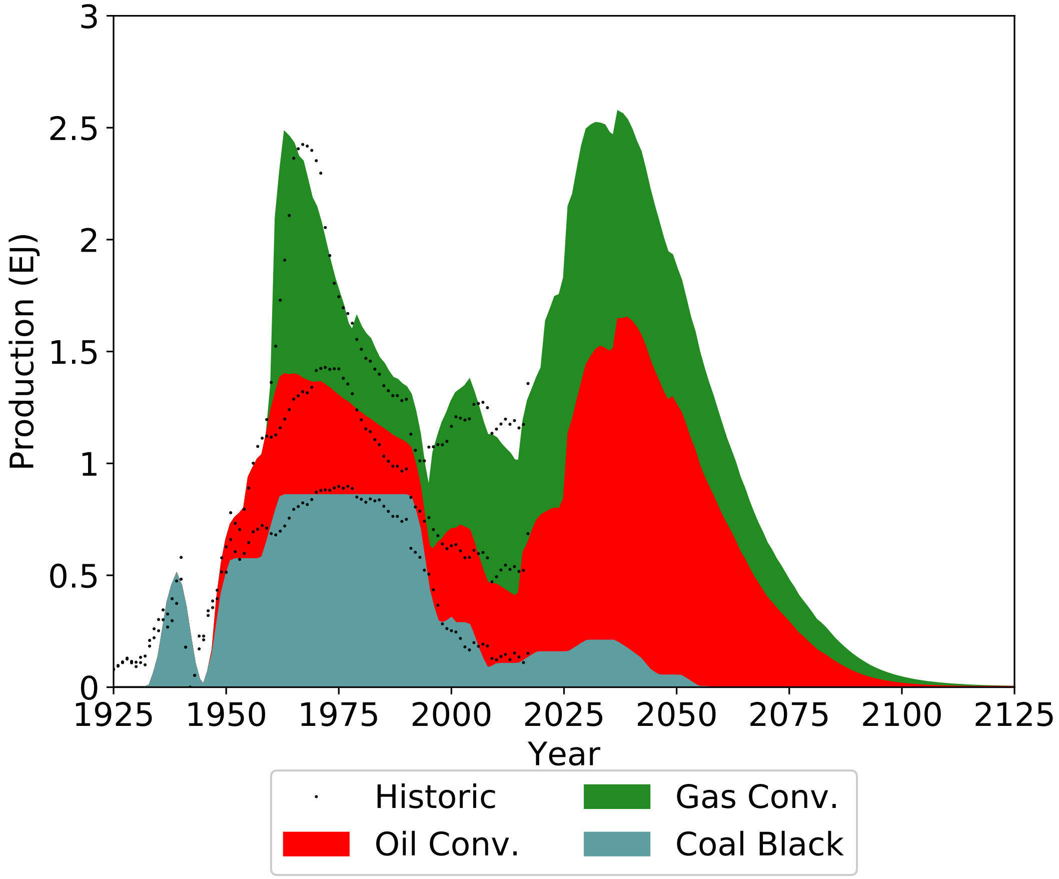

Figure 4.62: Russia - Southern projection by mineral type

Table 4.62: Peak years - Minerals

Name	URR	Peak Year	Peak Rate
Coal Black	48.65	1963	0.86
Oil Conv.	82.87	2039	1.48
Gas Conv.	72.04	1963	1.08
**Total**	**203.56**	**2037**	**2.57**

Ural

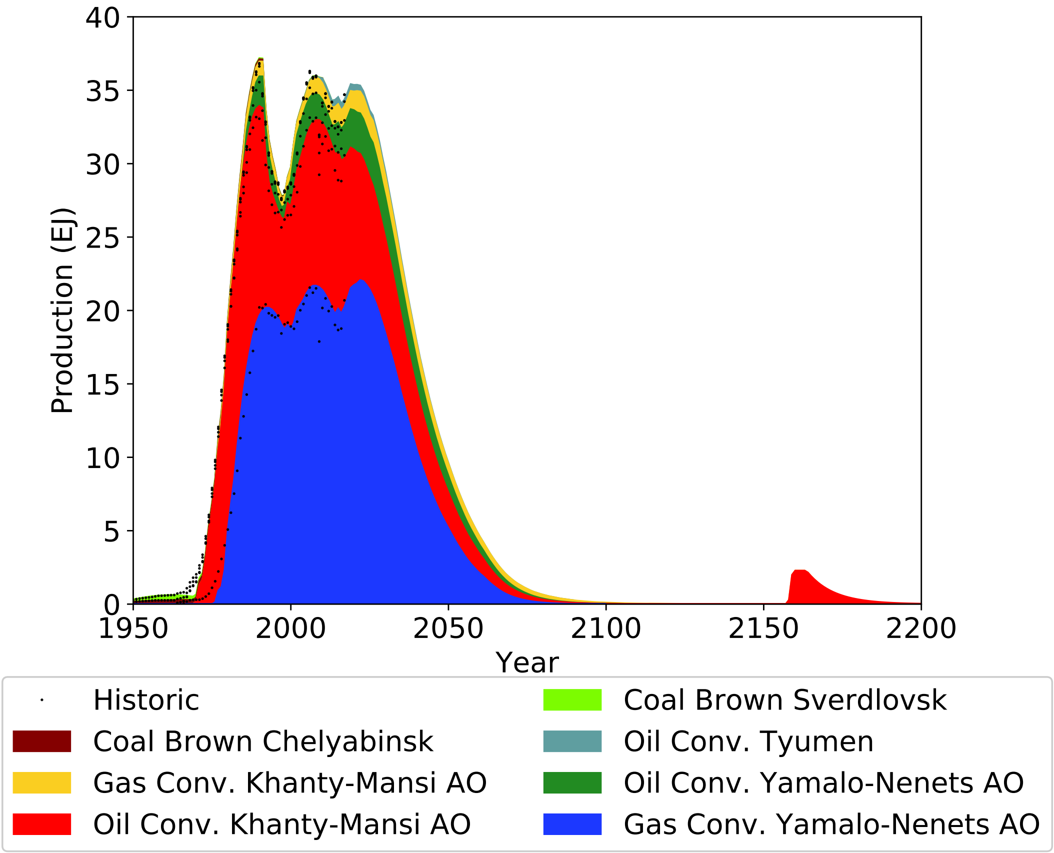

Figure 4.63: Russia - Ural projections capped at 16

Table 4.63: Peak years - All

Name	URR	Peak Year	Peak Rate
Gas Conv. Ural Yamalo-Nenets AO	1232.13	2022	22.07
Oil Conv. Ural Khanty-Mansi AO	738.03	1985	15.34
Oil Conv. Ural Yamalo-Nenets AO	143.25	2026	2.93
Gas Conv. Ural Khanty-Mansi AO	97.18	2023	1.52
Oil Conv. Ural Tyumen	12.11	2015	0.48
Coal Brown Ural Chelyabinsk	12.02	1966	0.28
Coal Brown Ural Sverdlovsk	10.02	1960	0.35
**Total**	**2244.74**	**1990**	**37.19**

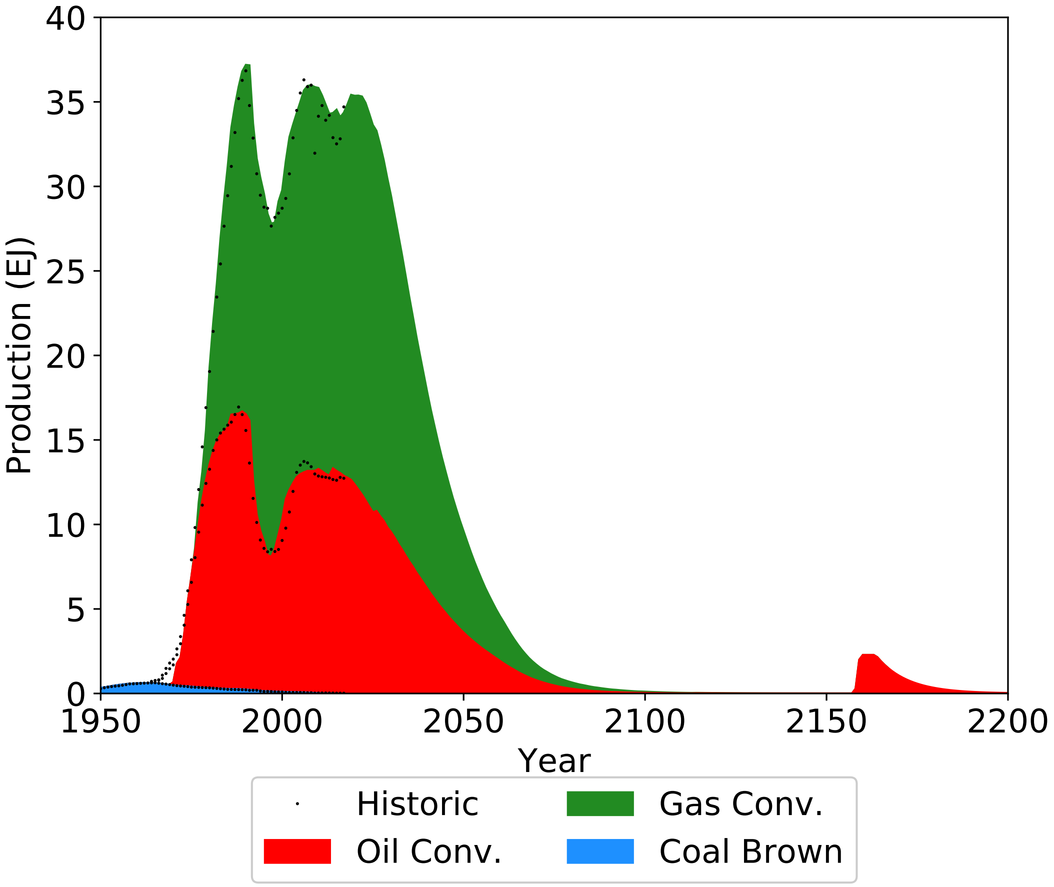

Figure 4.64: Russia - Ural projection by mineral type

Table 4.64: Peak years - Minerals

Name	URR	Peak Year	Peak Rate
Coal Brown	22.04	1962	0.62
Oil Conv.	893.39	1989	16.44
Gas Conv.	1329.31	2022	23.56
**Total**	**2244.74**	**1990**	**37.19**

Volga

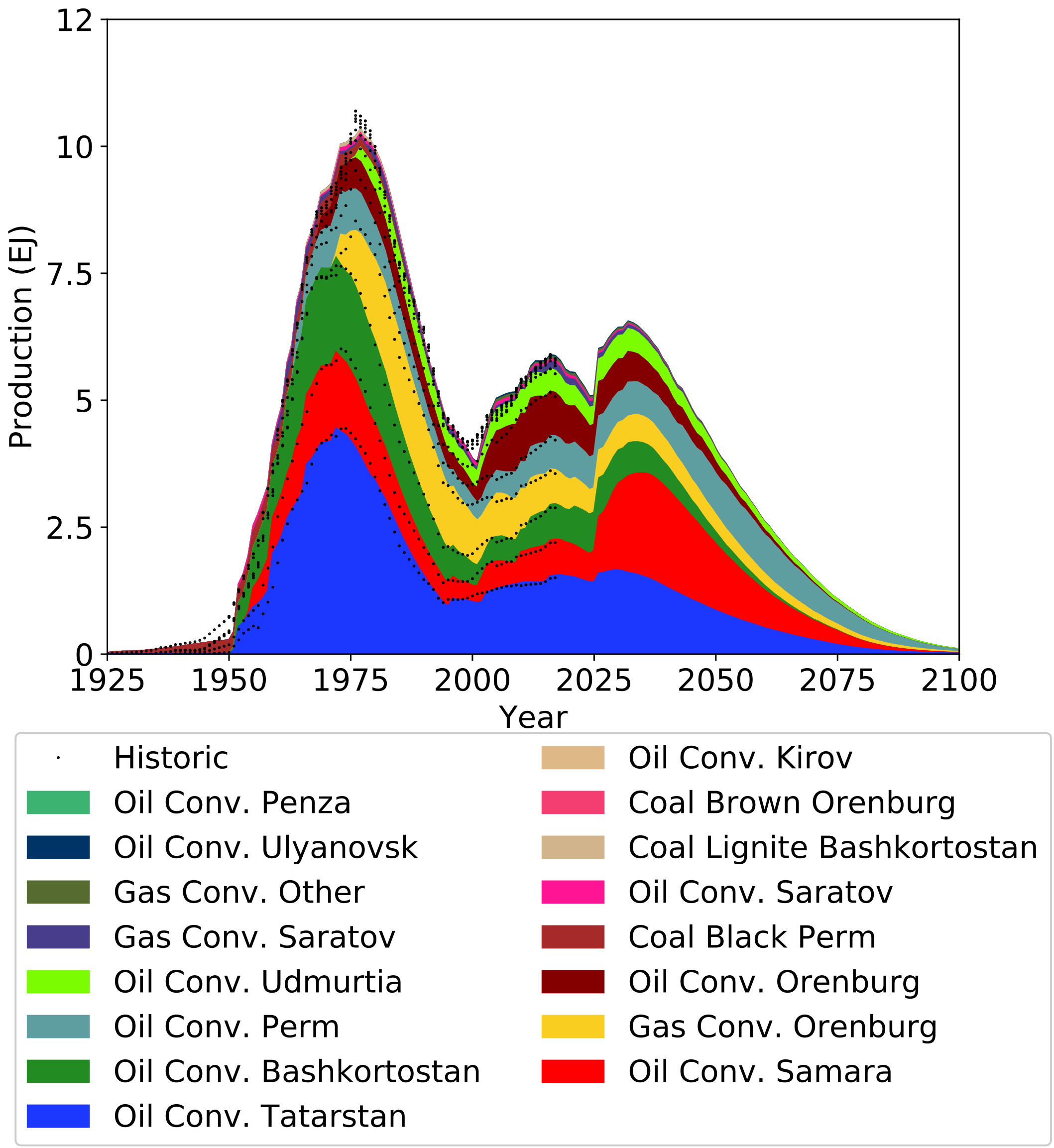

Figure 4.65: Russia - Volga projections capped at 16

Table 4.65: Peak years - All

Name	URR	Peak Year	Peak Rate
Oil Conv. Volga Tatarstan	200.55	1972	4.44
Oil Conv. Volga Samara	118.76	2036	2.05
Oil Conv. Volga Bashkortostan	90.39	1969	1.94
Gas Conv. Volga Orenburg	75.02	1983	1.76
Oil Conv. Volga Perm	74.41	2052	0.86
Oil Conv. Volga Orenburg	48.36	2012	0.96
Oil Conv. Volga Udmurtia	33.9	1983	0.51
Coal Black Volga Perm	12.11	1958	0.34
Gas Conv. Volga Saratov	9.27	1963	0.27
Oil Conv. Volga Saratov	5.16	1955	0.11
Gas Conv. Volga Other	1.85	2003	0.06
Coal Lignite Volga Bashkortostan	1.72	1976	0.08
Oil Conv. Volga Ulyanovsk	1.2	2010	0.03
Coal Brown Volga Orenburg	0.59	1985	0.04
Oil Conv. Volga Penza	0.04	2002	0.01
Oil Conv. Volga Kirov	–	2001	–
**Total**	**673.33**	**1977**	**10.33**

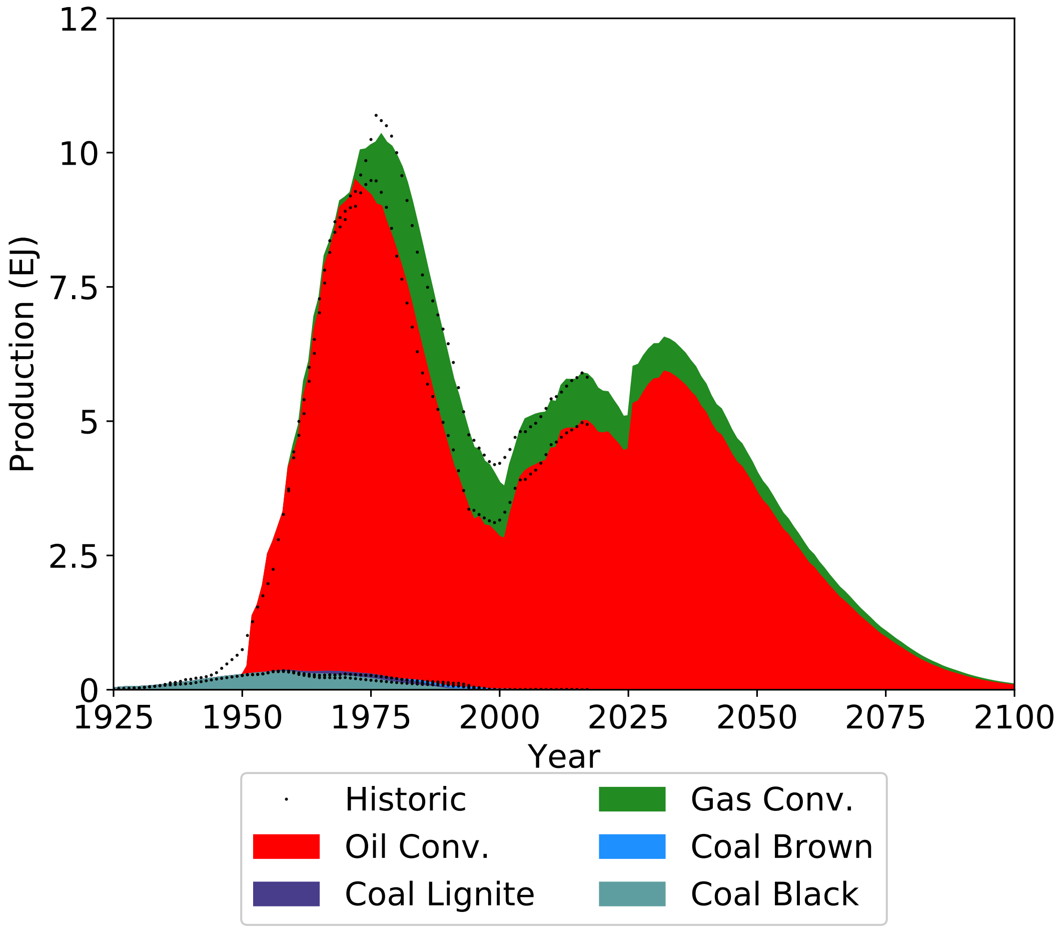

Figure 4.66: Russia - Volga projection by mineral type

Table 4.66: Peak years - Minerals

**Name**	**URR**	**Peak Year**	**Peak Rate**
Coal Black	12.11	1958	0.34
Coal Lignite	1.72	1976	0.08
Coal Brown	0.59	1985	0.04
Oil Conv.	572.77	1972	9.17
Gas Conv.	86.14	1984	1.95
**Total**	**673.33**	**1977**	**10.33**

4.12.4 Projection by region

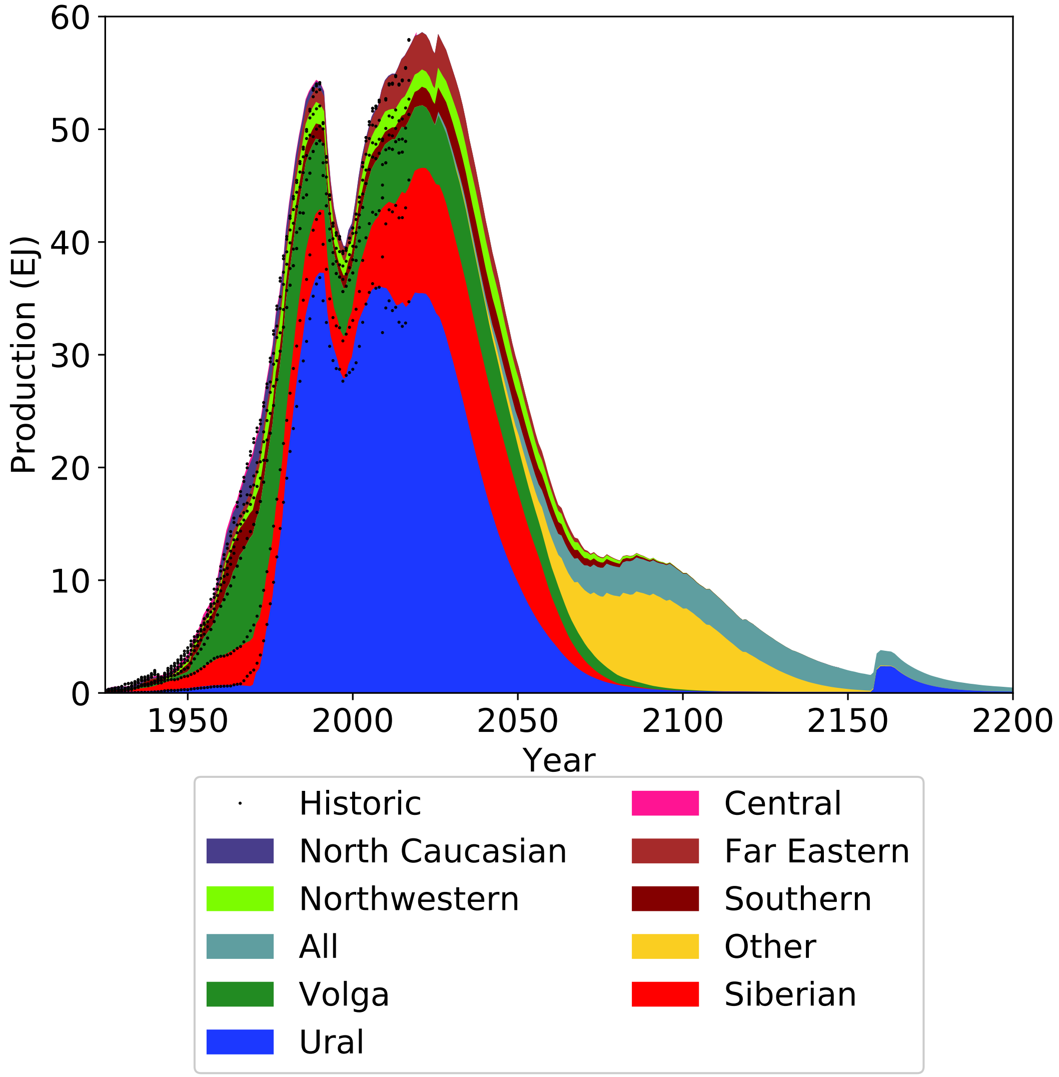

Figure 4.67: Russia by region projections capped at 16

Table 4.67: Peak years - All

**Name**	**URR**	**Peak Year**	**Peak Rate**
Ural	2244.74	1990	37.19
Siberian	773.23	2028	11.83
Volga	673.33	1977	10.33
Other	427.46	2091	8.18
All	319.94	2096	3.19
Southern	203.56	2037	2.57
Northwestern	189.64	2039	2.64
Far Eastern	173.99	2016	3.59
North Caucasian	58.23	1969	2.57
Central	15.38	1965	0.39
**Total**	**5079.49**	**2021**	**58.51**

4.13 Tajikistan

4.13.1 All Projections

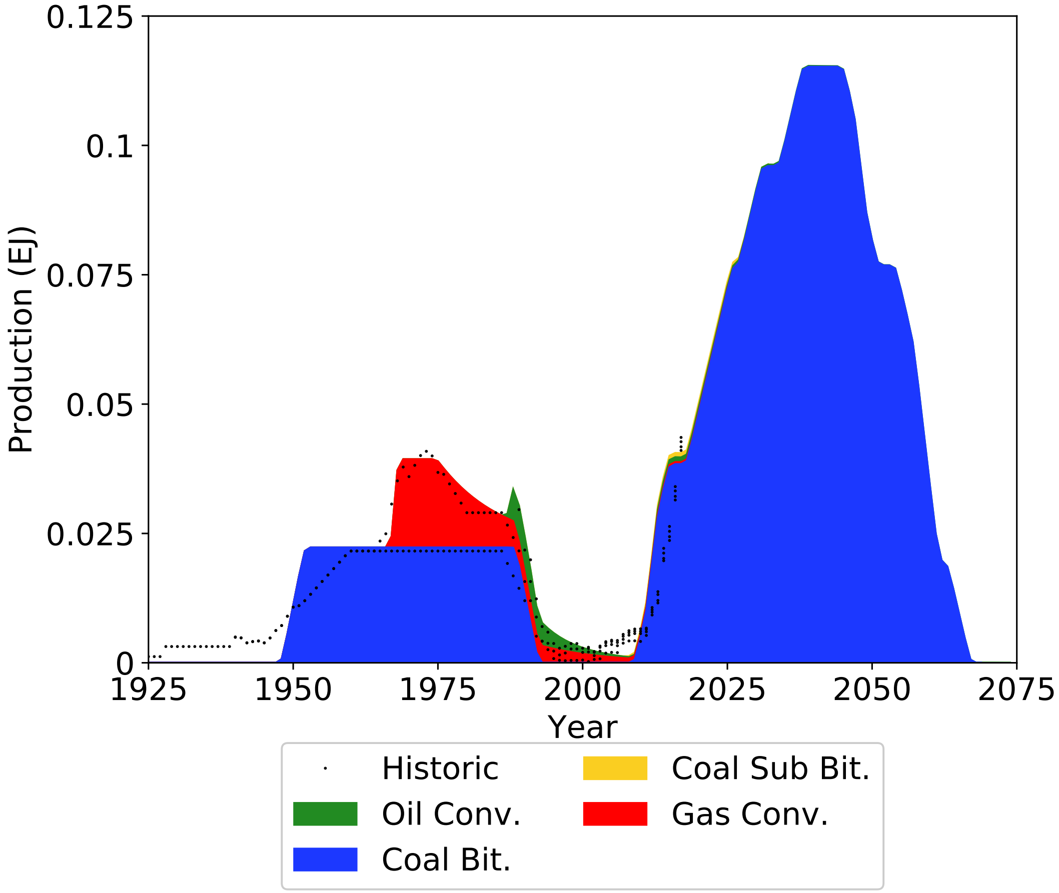

Figure 4.68: Tajikistan projections capped at 16

Table 4.68: Peak years - All

Name	URR	Peak Year	Peak Rate
Coal Bit.	4.8	2039	0.12
Gas Conv.	0.31	1969	0.02
Oil Conv.	0.08	1989	0.01
Coal Sub Bit.	0.02	2012	–
**Total**	**5.2**	**2039**	**0.12**

4.13.2 By Mineral

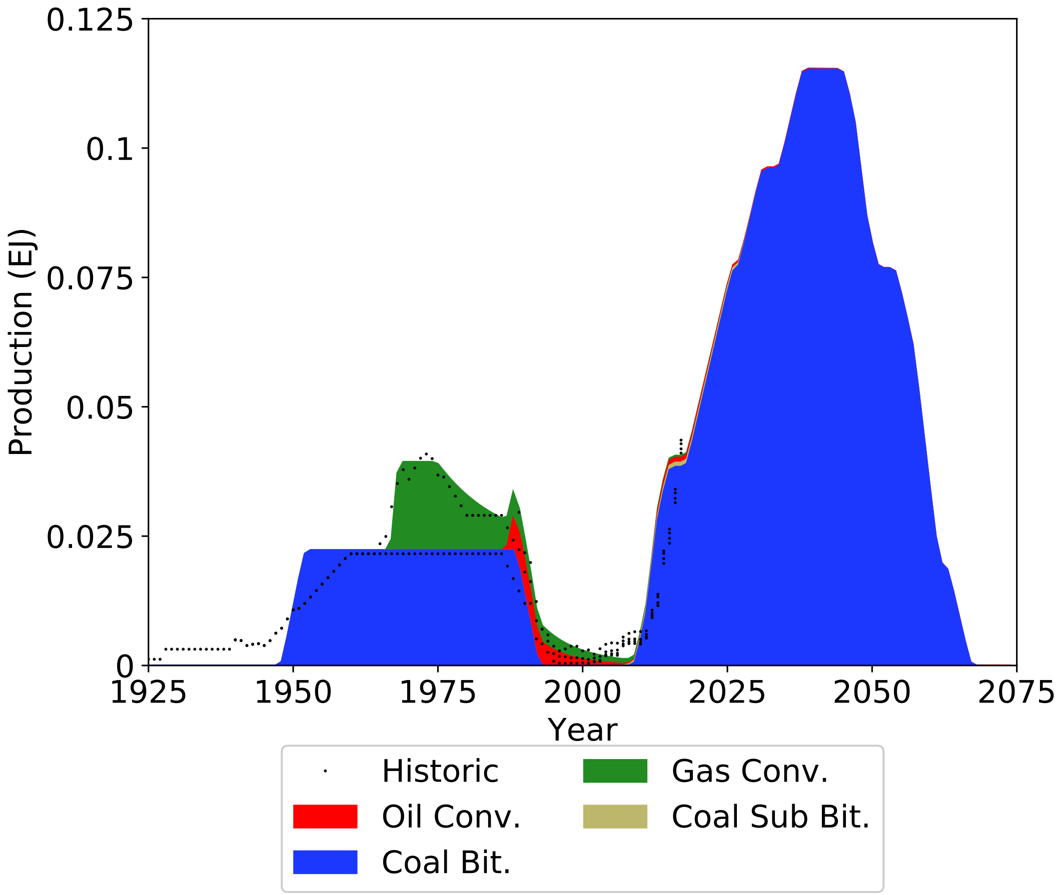

Figure 4.69: Tajikistan projection by mineral type

Table 4.69: Peak years - Minerals

Name	URR	Peak Year	Peak Rate
Coal Bit.	4.8	2039	0.12
Coal Sub Bit.	0.02	2012	–
Oil Conv.	0.08	1989	0.01
Gas Conv.	0.31	1969	0.02
**Total**	**5.2**	**2039**	**0.12**

4.14 Turkmenistan

4.14.1 All Projections

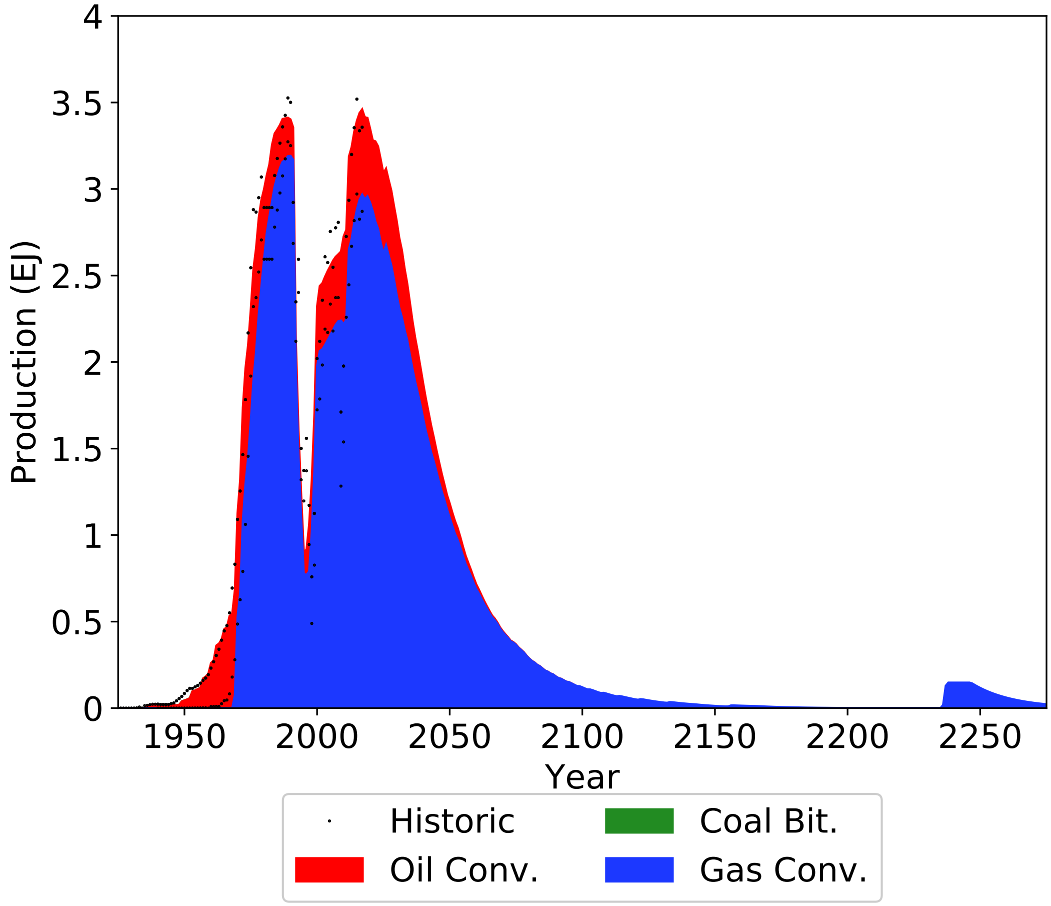

Figure 4.70: Turkmenistan projections capped at 16

Table 4.70: Peak years - All

Name	URR	Peak Year	Peak Rate
Gas Conv.	200.41	1990	3.19
Oil Conv.	35.51	1973	0.67
Coal Bit.	0.01	1947	–
**Total**	**235.93**	**2017**	**3.46**

4.14.2 By Mineral

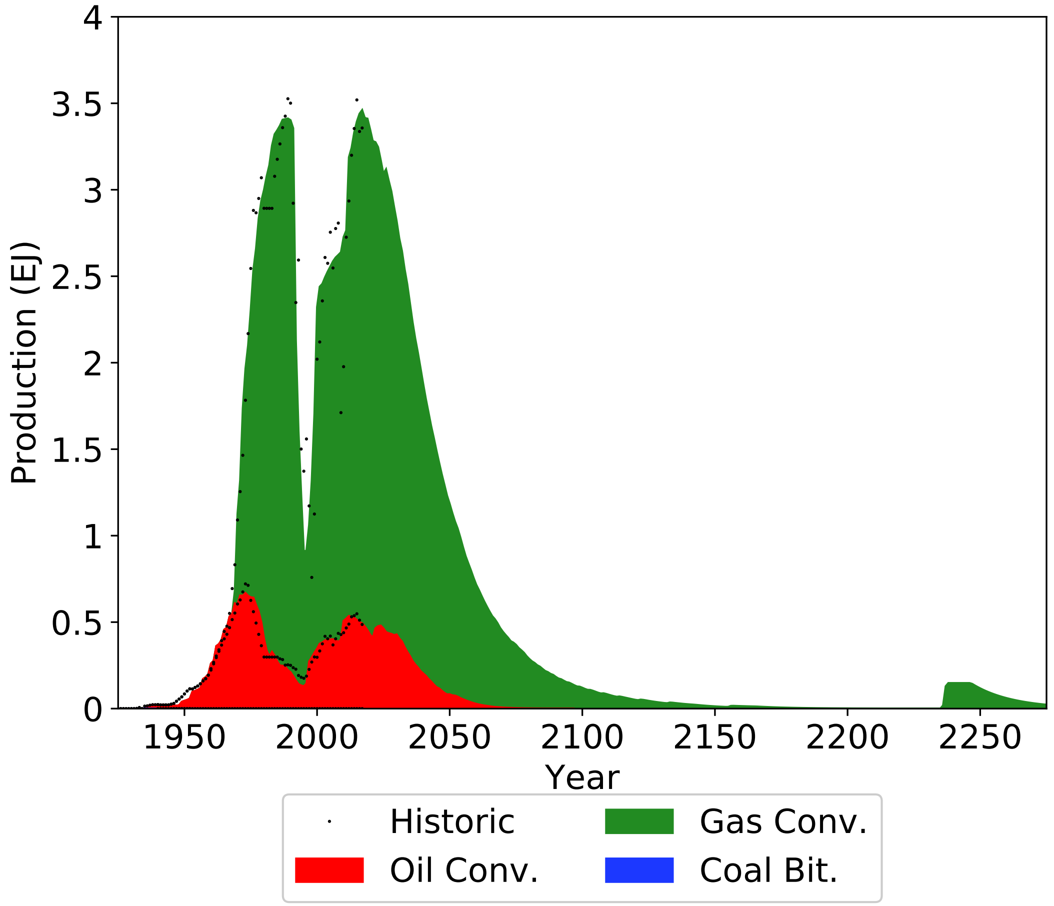

Figure 4.71: Turkmenistan projection by mineral type

Table 4.71: Peak years - Minerals

Name	URR	Peak Year	Peak Rate
Coal Bit.	0.01	1947	–
Oil Conv.	35.51	1973	0.67
Gas Conv.	200.41	1990	3.19
**Total**	**235.93**	**2017**	**3.46**

4.15 Ukraine

4.15.1 All Projections

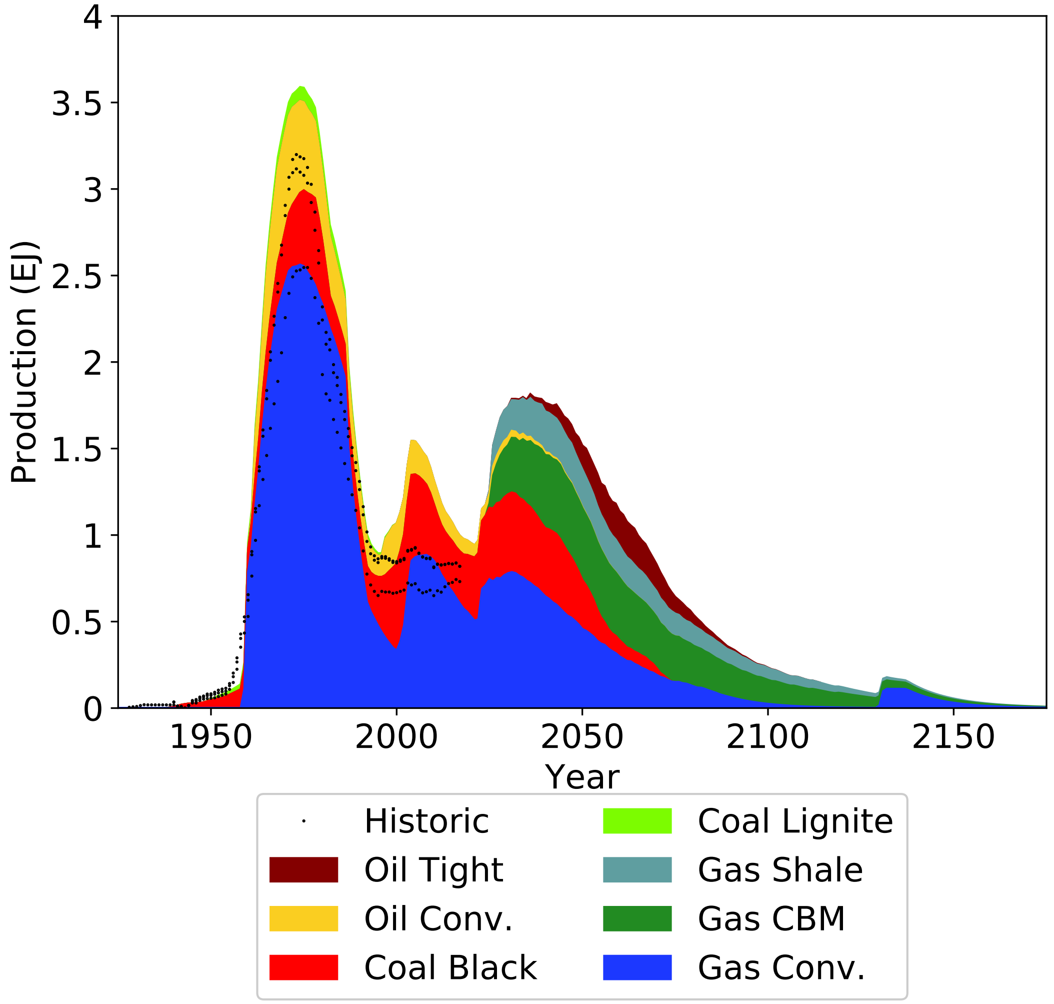

Figure 4.72: Ukraine projections capped at 16

Table 4.72: Peak years - All

**Name**	**URR**	**Peak Year**	**Peak Rate**
Gas Conv.	111.2	1974	2.56
Coal Black	34.81	2002	0.52
Gas CBM	26.24	2044	0.43
Oil Conv.	17.21	1970	0.57
Gas Shale	13.45	2039	0.24
Oil Tight	6.3	2061	0.21
Coal Lignite	2.3	1977	0.08
**Total**	**211.51**	**1974**	**3.59**

4.15.2 By Mineral

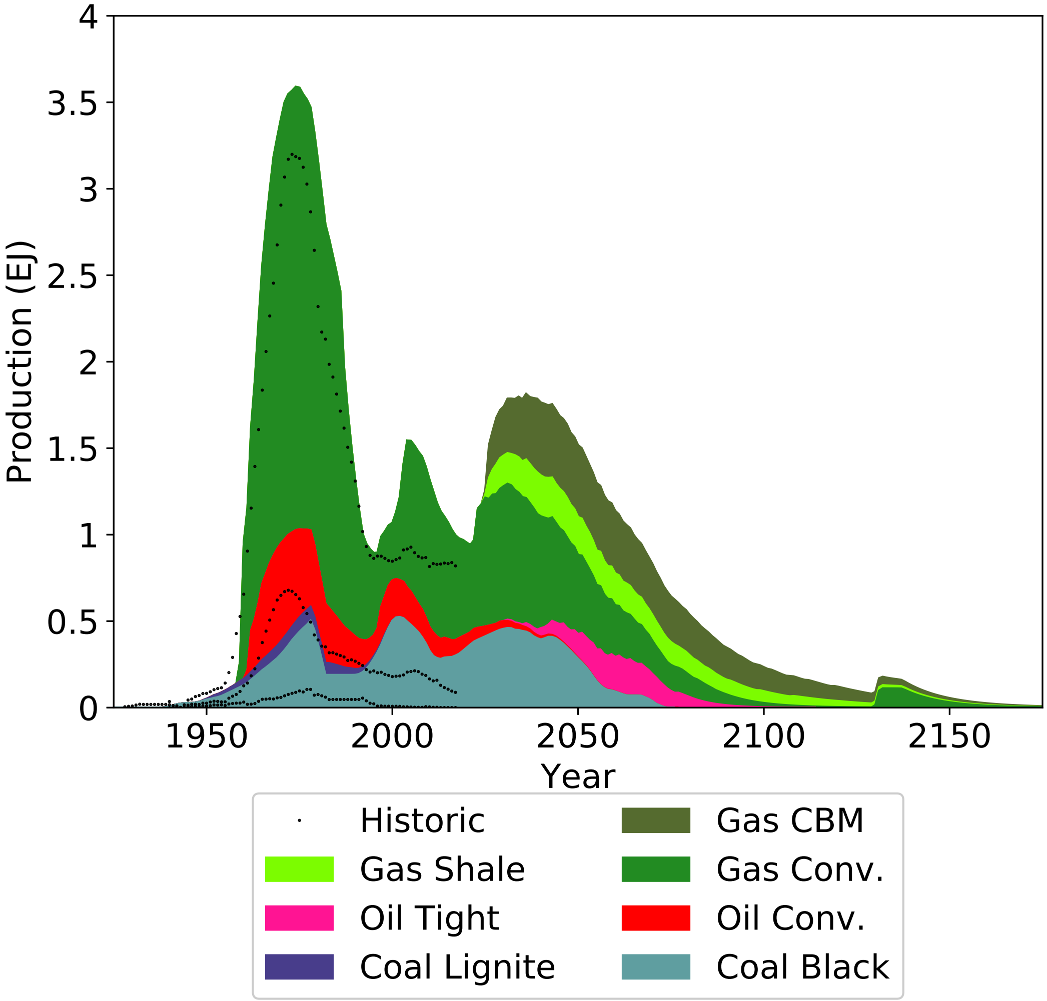

Figure 4.73: Ukraine projection by mineral type

Table 4.73: Peak years - Minerals

**Name**	**URR**	**Peak Year**	**Peak Rate**
Coal Black	34.81	2002	0.52
Coal Lignite	2.3	1977	0.08
Oil Conv.	17.21	1970	0.57
Oil Tight	6.3	2061	0.21
Gas Conv.	111.2	1974	2.56
Gas Shale	13.45	2039	0.24
Gas CBM	26.24	2044	0.43
**Total**	**211.51**	**1974**	**3.59**

4.16 Uzbekistan

4.16.1 All Projections

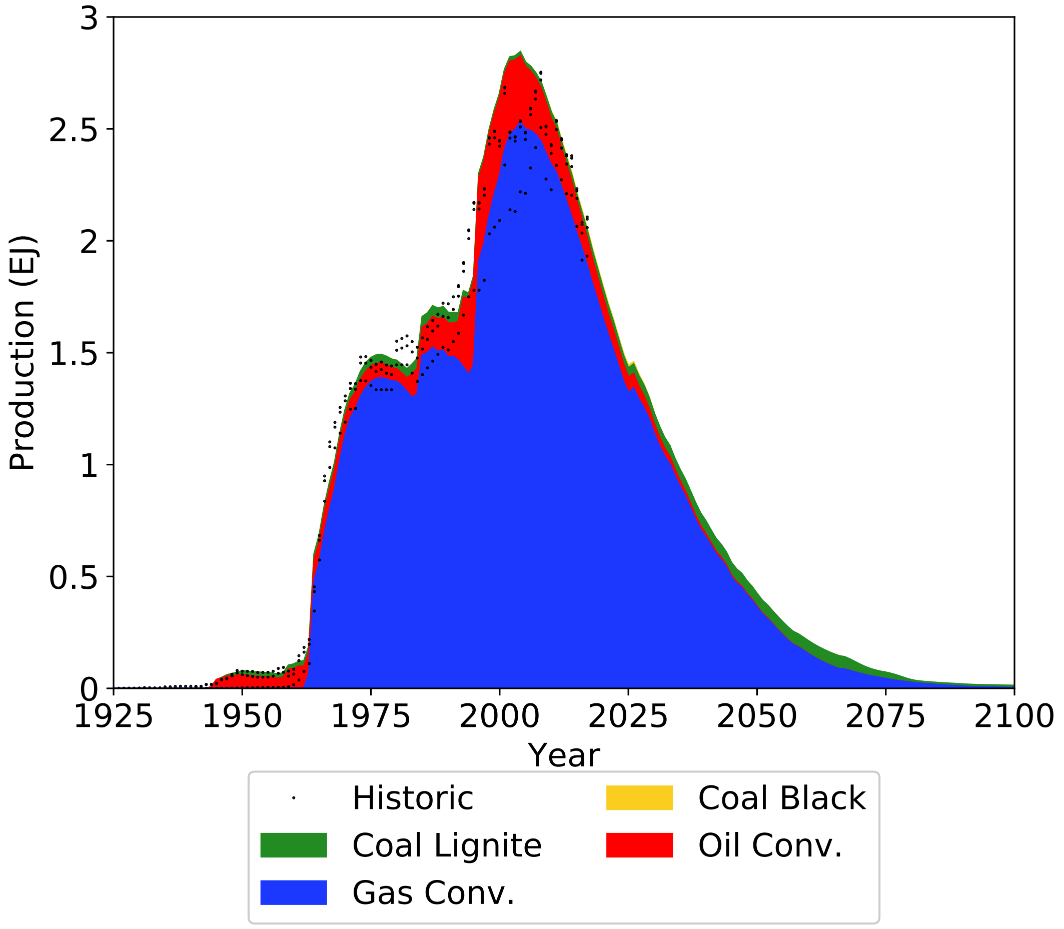

Figure 4.74: Uzbekistan projections capped at 16

Table 4.74: Peak years - All

**Name**	**URR**	**Peak Year**	**Peak Rate**
Gas Conv.	126.55	2004	2.53
Oil Conv.	12.07	1995	0.38
Coal Lignite	5.23	2039	0.06
Coal Black	0.19	2013	0.01
**Total**	**144.04**	**2004**	**2.84**

4.16.2 By Mineral

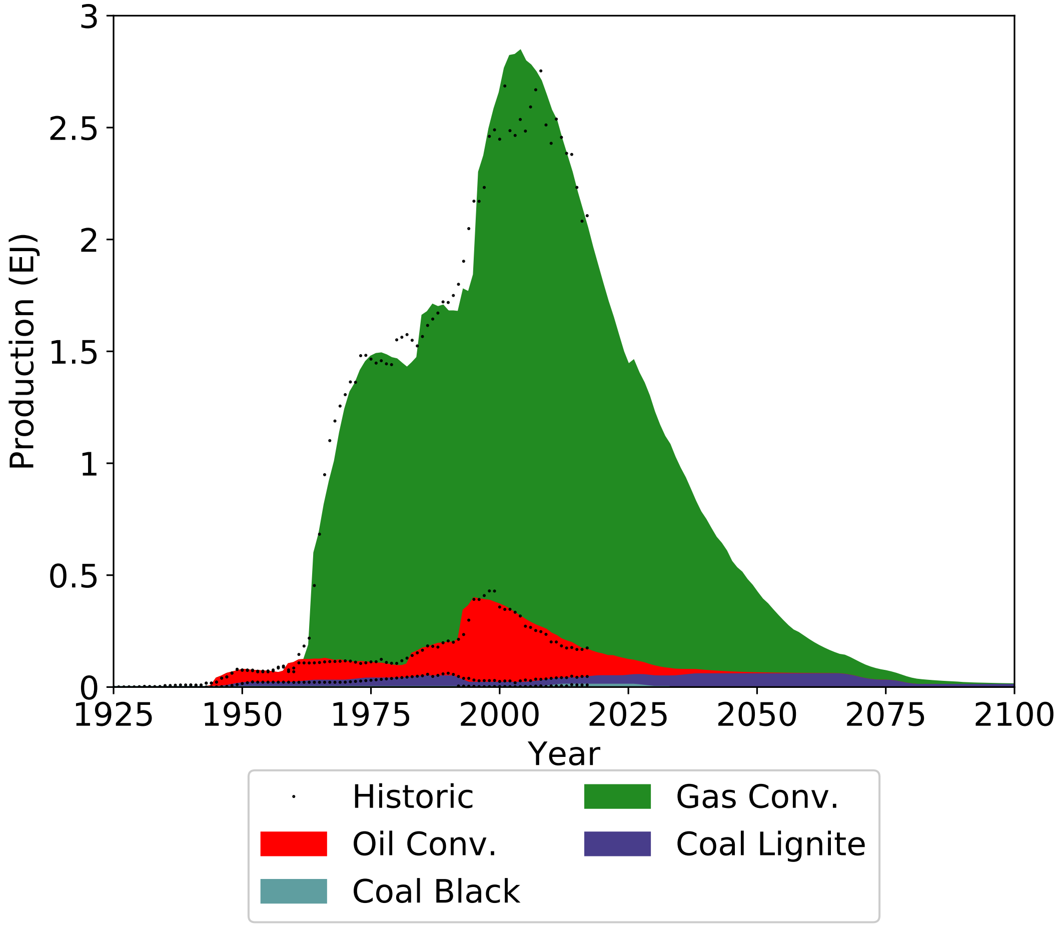

Figure 4.75: Uzbekistan projection by mineral type

Table 4.75: Peak years - Minerals

**Name**	**URR**	**Peak Year**	**Peak Rate**
Coal Black	0.19	2013	0.01
Coal Lignite	5.23	2039	0.06
Oil Conv.	12.07	1995	0.38
Gas Conv.	126.55	2004	2.53
**Total**	**144.04**	**2004**	**2.84**

4.17 Total

4.17.1 By country

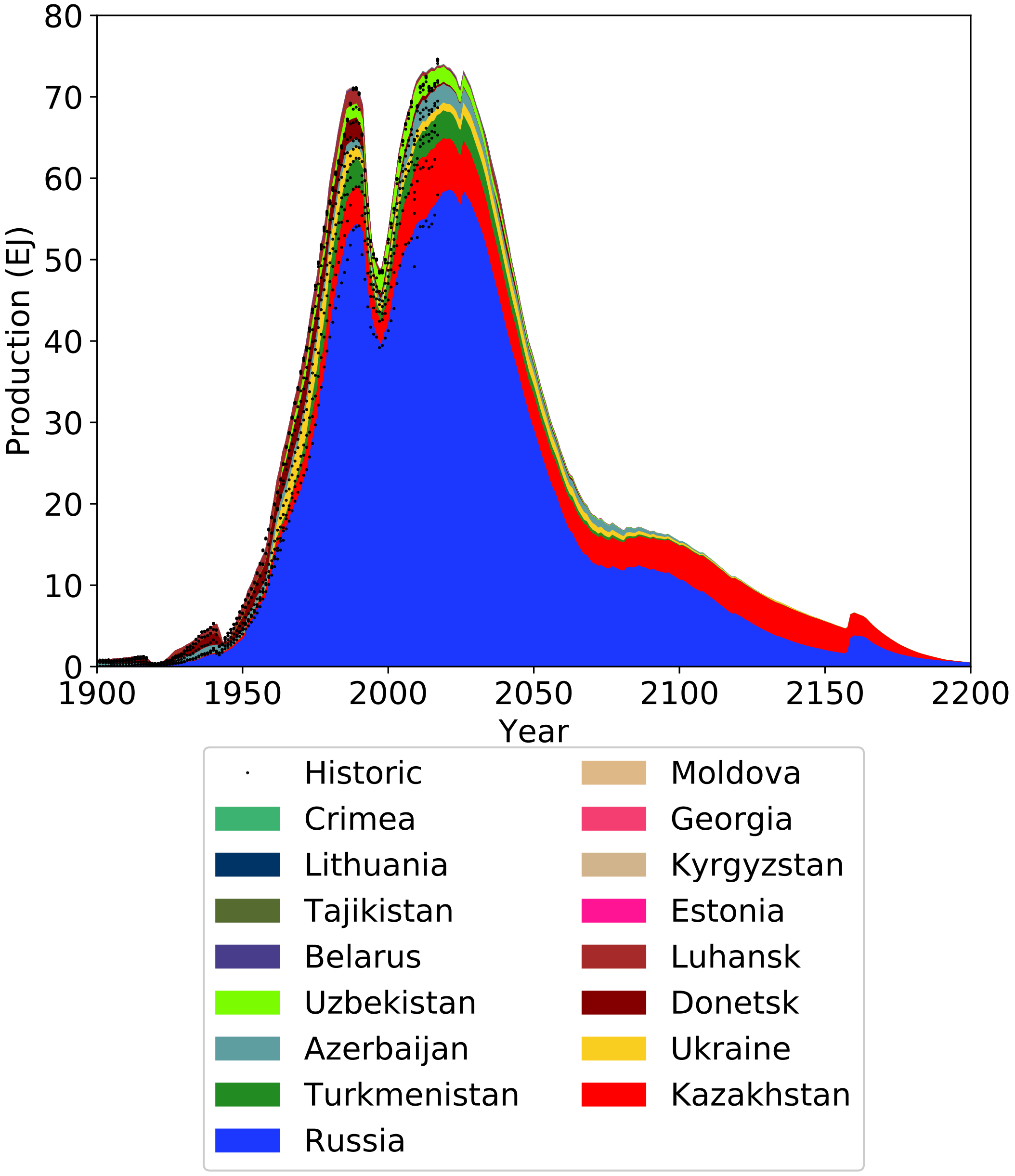

Figure 4.76: FSU projections by country

Table 4.76: Peak years - All

Name	URR	Peak Year	Peak Rate
Russia	5079.49	2021	58.51
Kazakhstan	884.1	2012	7.66
Turkmenistan	235.93	2017	3.46
Ukraine	211.51	1974	3.59
Azerbaijan	192.77	2013	2.61
Donetsk	169.01	1972	3.03
Uzbekistan	144.04	2004	2.84
Luhansk	117.14	1971	2.12
Belarus	8.72	1976	0.29
Estonia	5.73	2048	0.08
Tajikistan	5.2	2039	0.12
Kyrgyzstan	4.42	1972	0.09
Lithuania	4.22	2033	0.21
Georgia	3.66	1979	0.18
Crimea	1.71	2010	0.07
Moldova	0.02	1994	0.01
**Total**	**7067.67**	**2019**	**73.91**

4.17.2 By mineral

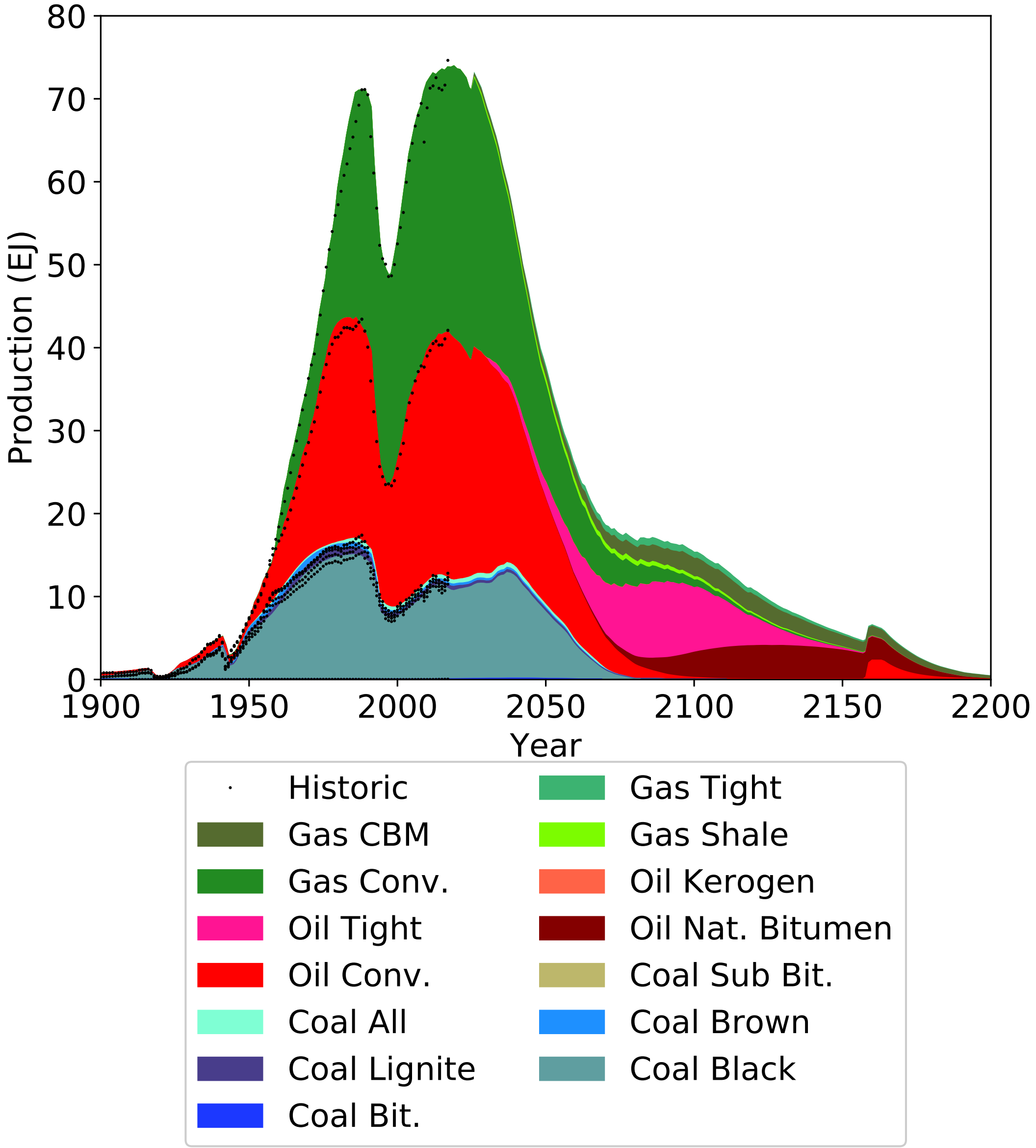

Figure 4.77: FSU projection by mineral type

Table 4.77: Peak years - Minerals

**Name**	**URR**	**Peak Year**	**Peak Rate**
Coal Bit.	4.82	2039	0.12
Coal Black	1260.11	1986	14.98
Coal Lignite	63.24	1978	0.9
Coal Brown	51.61	1964	0.84
Coal All	43.63	2025	0.55
Coal Sub Bit.	2.37	1950	0.04
Oil Conv.	2214.94	2017	29.64
Oil Nat. Bitumen	312.53	2129	4.04
Oil Tight	502.52	2087	9.16
Oil Kerogen	6.42	2048	0.08
Gas Conv.	2233.15	2023	33.13
Gas Shale	51.55	2056	0.69
Gas CBM	246.65	2108	2.36
Gas Tight	74.13	2084	0.9
**Total**	**7067.67**	**2019**	**73.91**

Chapter 5

Middle East

5.1 Bahrain

5.1.1 All Projections

Table 5.1: Peak years - All

Name	URR	Peak Year	Peak Rate
Gas Conv.	25.8	2014	0.53
Oil Conv.	11.4	1976	0.12
**Total**	**37.2**	**2014**	**0.61**

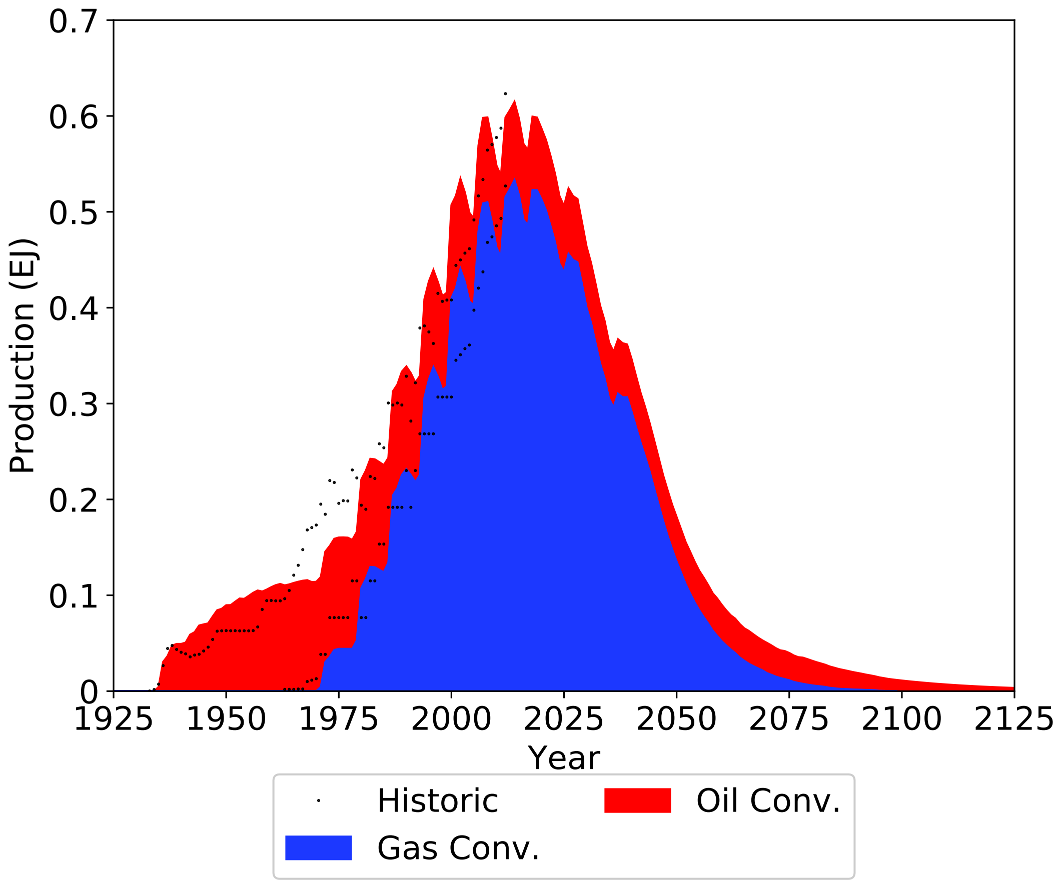

Figure 5.1: Bahrain projections capped at 16

5.1.2 By Mineral

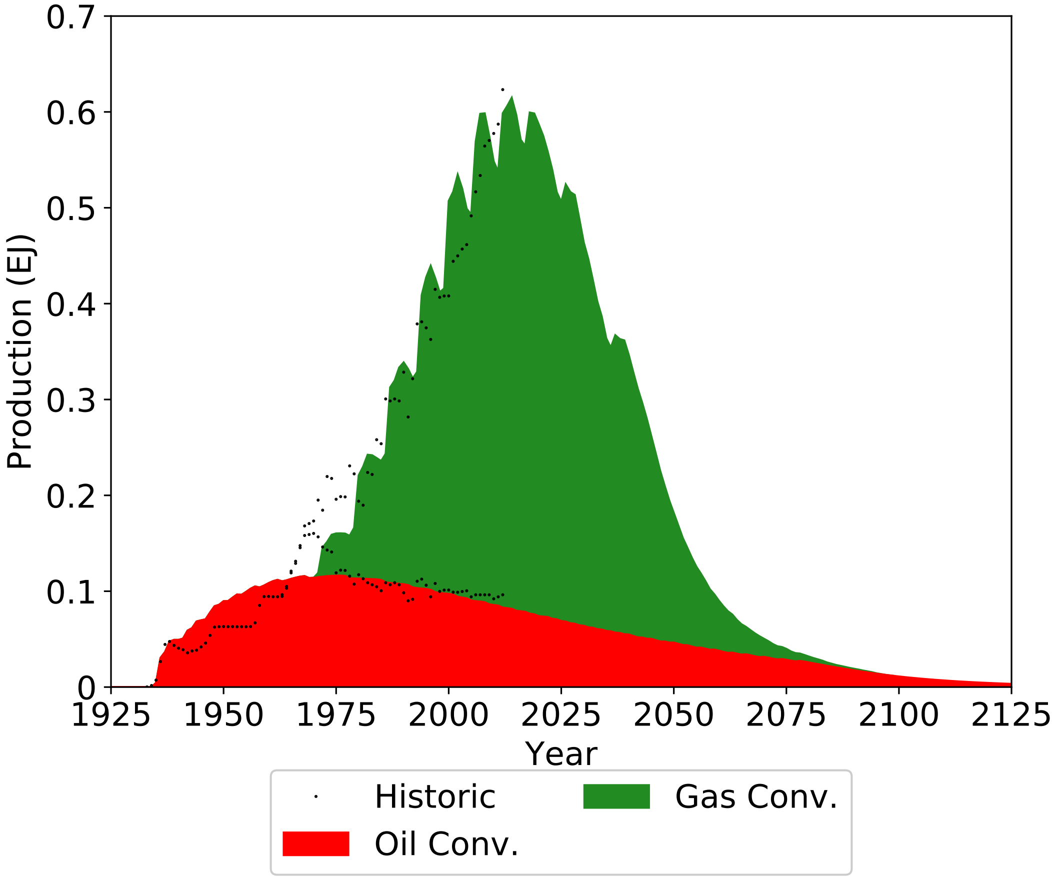

Figure 5.2: Bahrain projection by mineral type

Table 5.2: Peak years - Minerals

Name	URR	Peak Year	Peak Rate
Oil Conv.	11.4	1976	0.12
Gas Conv.	25.8	2014	0.53
**Total**	**37.2**	**2014**	**0.61**

5.2 Iran

5.2.1 All Projections

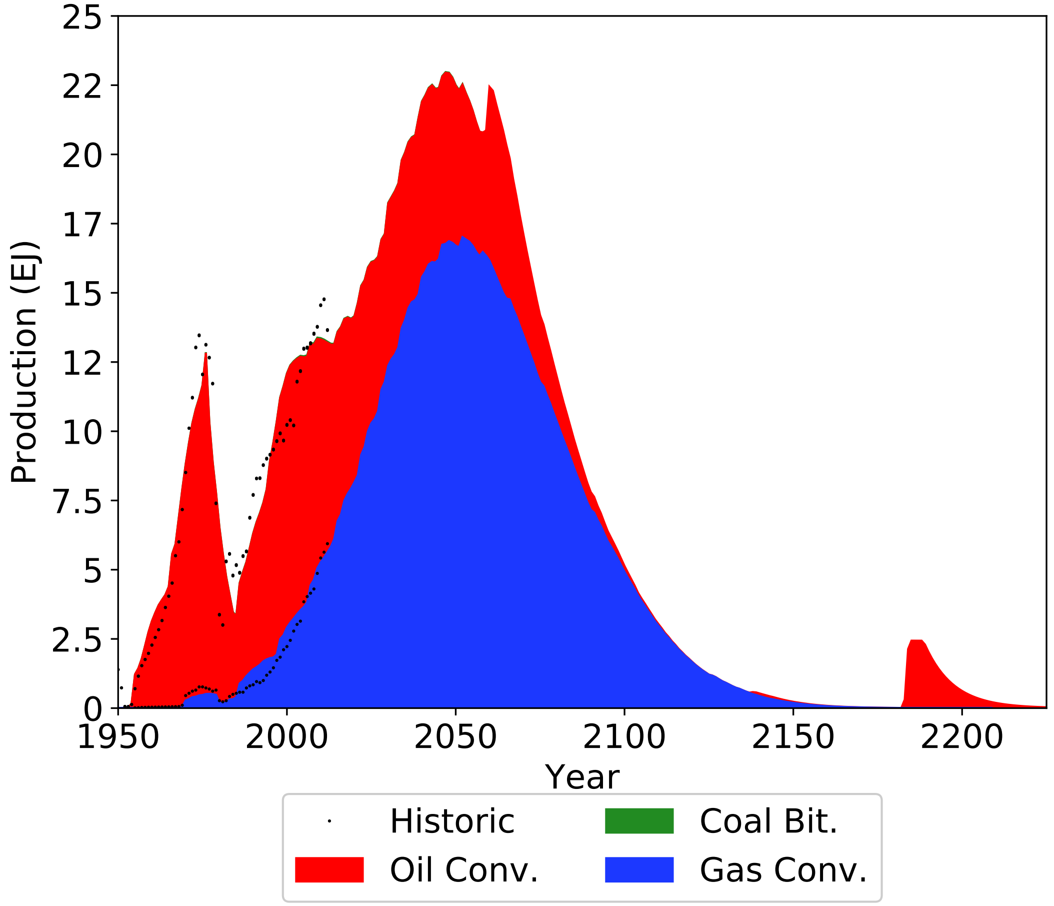

Figure 5.3: Iran projections capped at 16

Table 5.3: Peak years - All

Name	URR	Peak Year	Peak Rate
Gas Conv.	1207.5	2052	17.01
Oil Conv.	787.83	1976	12.32
Coal Bit.	2.3	2007	0.05
**Total**	**1997.63**	**2047**	**22.97**

5.2.2 By Mineral

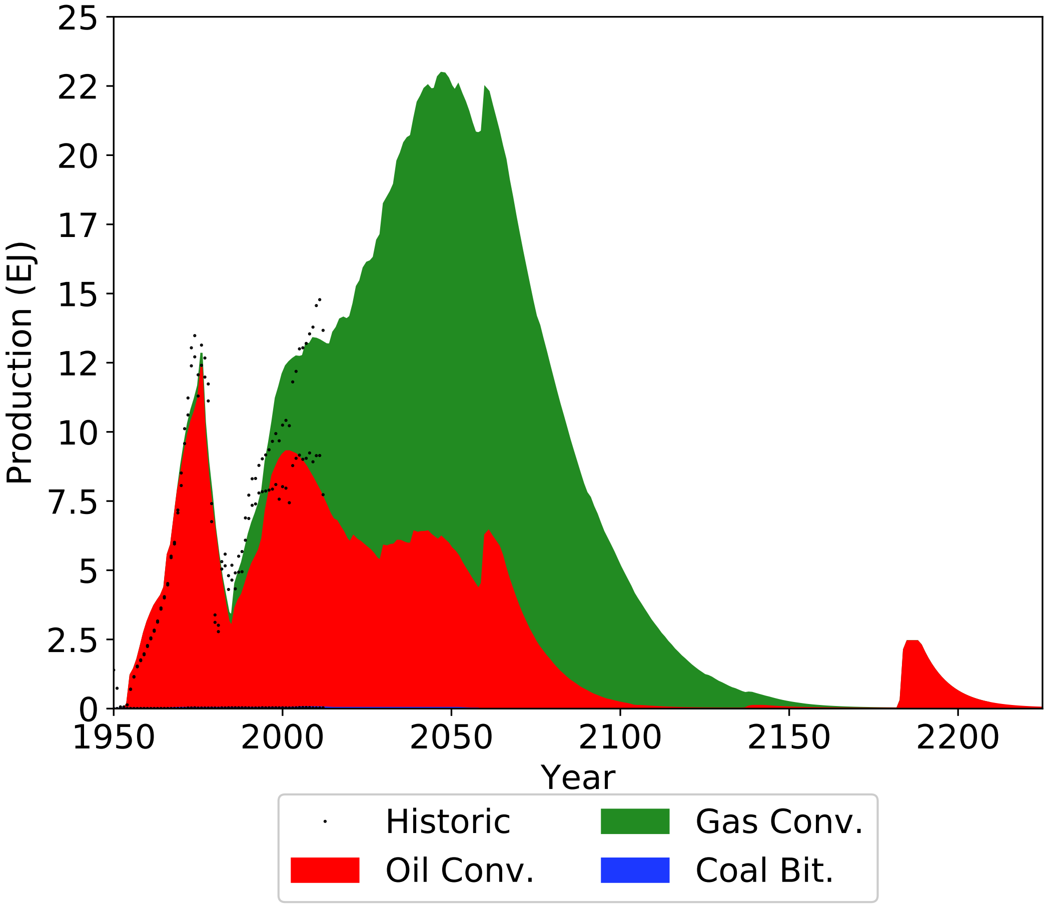

Figure 5.4: Iran projection by mineral type

Table 5.4: Peak years - Minerals

**Name**	**URR**	**Peak Year**	**Peak Rate**
Coal Bit.	2.3	2007	0.05
Oil Conv.	787.83	1976	12.32
Gas Conv.	1207.5	2052	17.01
**Total**	**1997.63**	**2047**	**22.97**

5.3 Iraq

5.3.1 All Projections

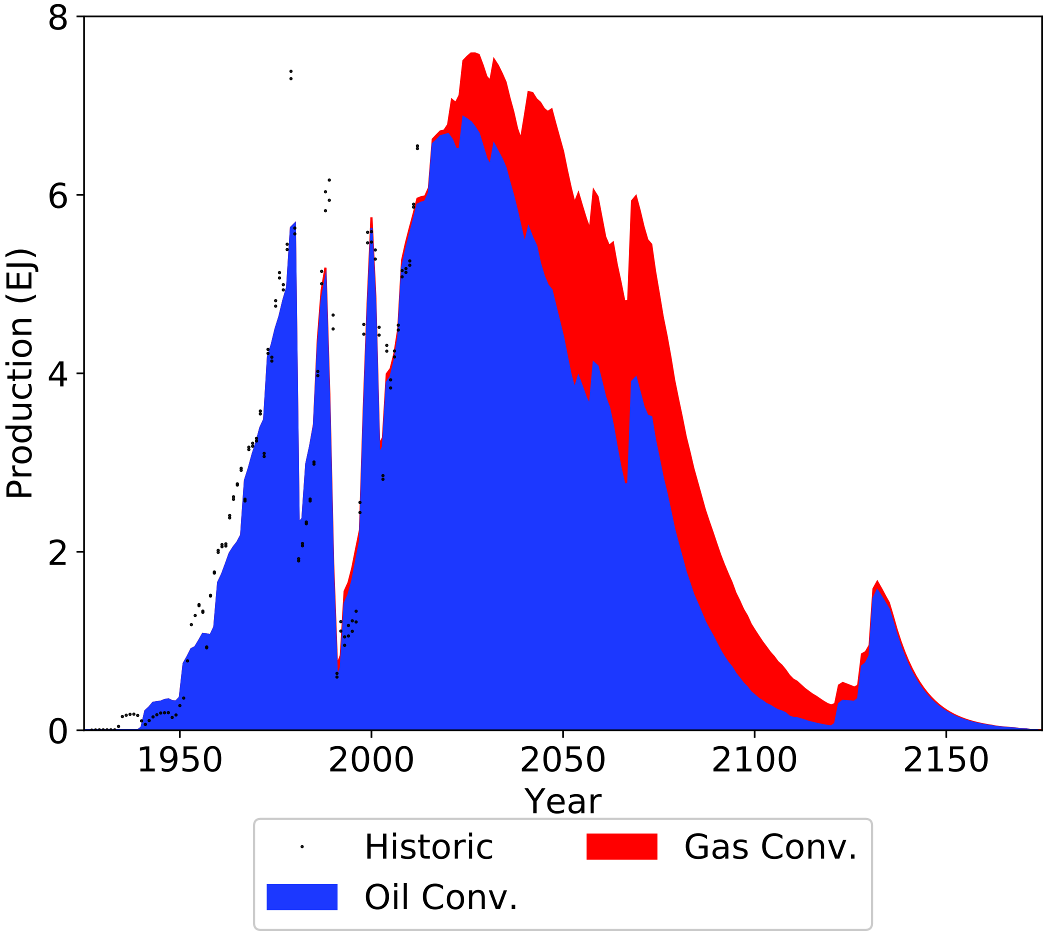

Figure 5.5: Iraq projections capped at 16

Table 5.5: Peak years - All

Name	URR	Peak Year	Peak Rate
Oil Conv.	573.0	2024	6.87
Gas Conv.	131.3	2052	2.1
**Total**	**704.3**	**2027**	**7.59**

5.3.2 By Mineral

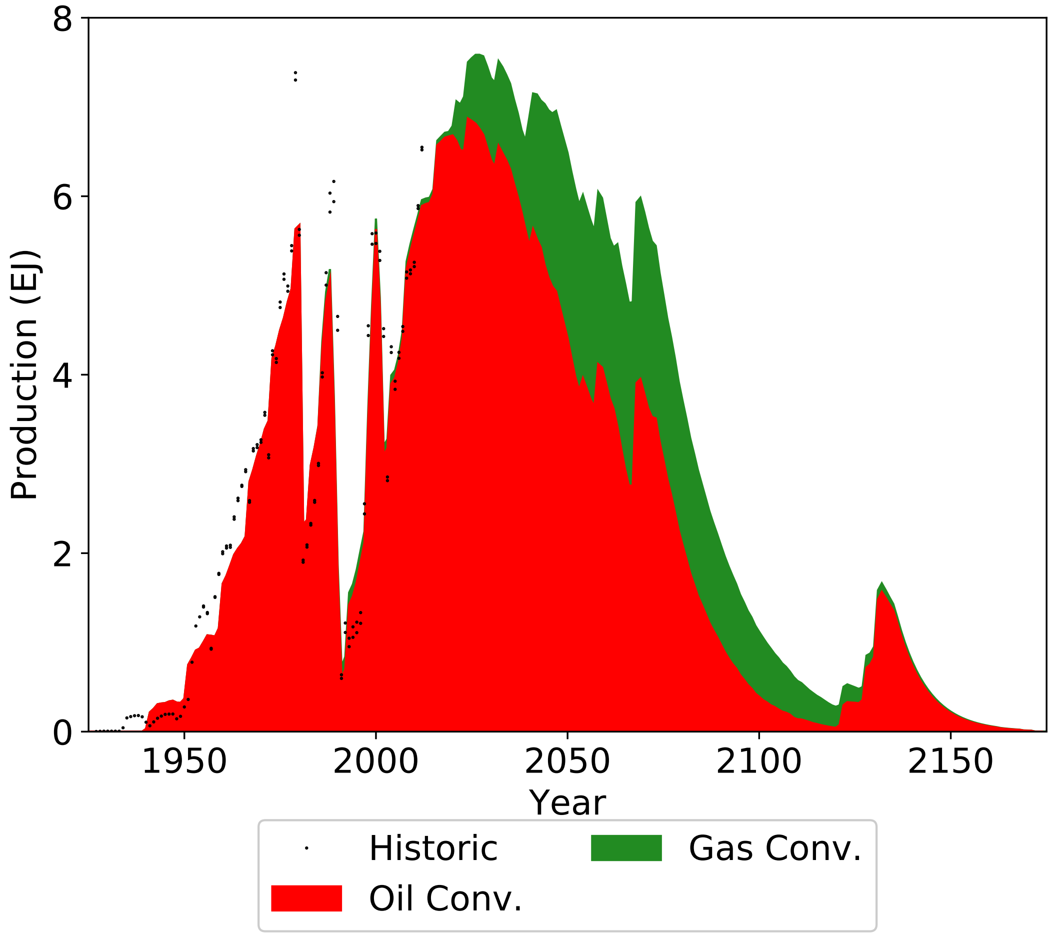

Figure 5.6: Iraq projection by mineral type

Table 5.6: Peak years - Minerals

Name	URR	Peak Year	Peak Rate
Oil Conv.	573.0	2024	6.87
Gas Conv.	131.3	2052	2.1
**Total**	**704.3**	**2027**	**7.59**

5.4 Israel

5.4.1 All Projections

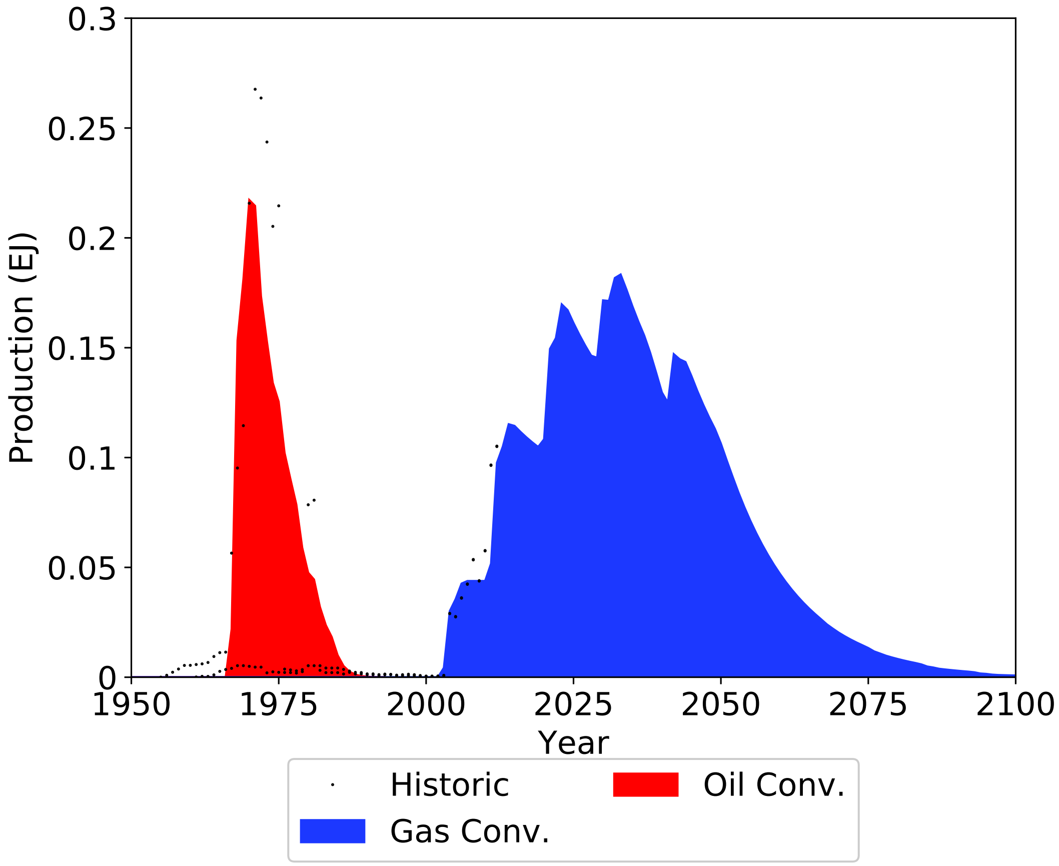

Figure 5.7: Israel projections capped at 16

Table 5.7: Peak years - All

Name	URR	Peak Year	Peak Rate
Gas Conv.	7.0	2033	0.18
Oil Conv.	1.89	1970	0.22
**Total**	**8.89**	**1970**	**0.22**

5.4.2 By Mineral

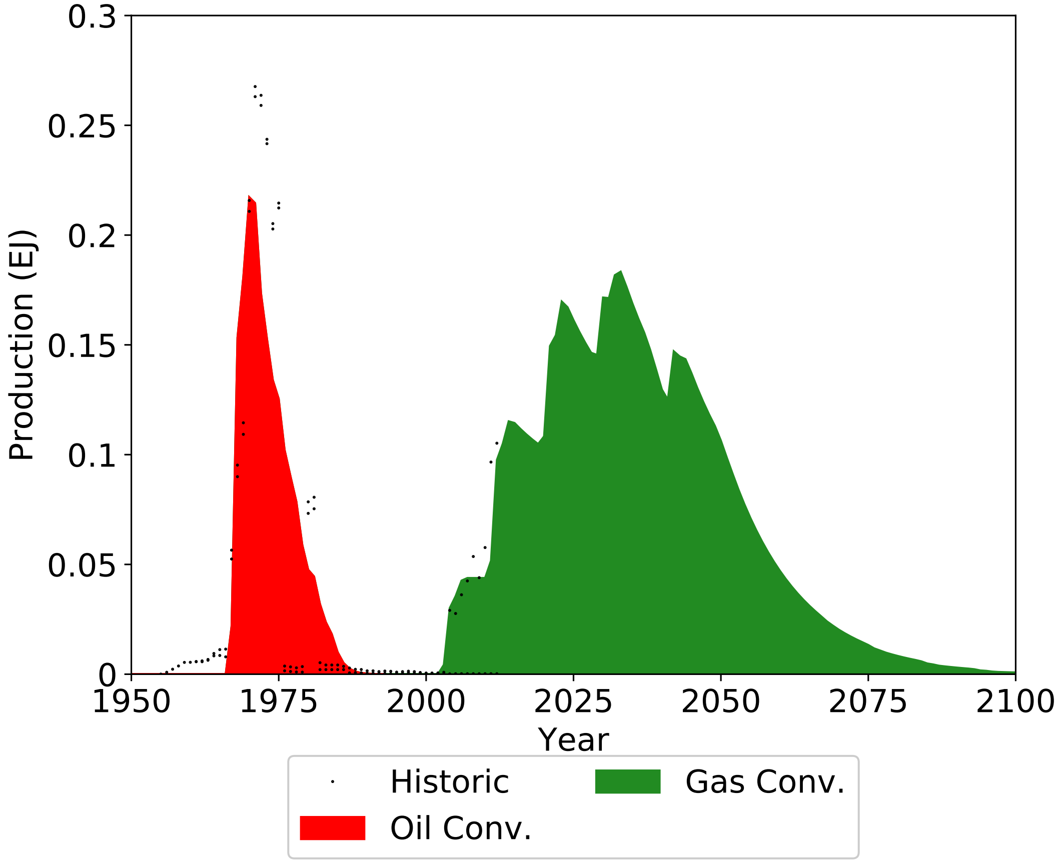

Figure 5.8: Israel projection by mineral type

Table 5.8: Peak years - Minerals

Name	URR	Peak Year	Peak Rate
Oil Conv.	1.89	1970	0.22
Gas Conv.	7.0	2033	0.18
**Total**	**8.89**	**1970**	**0.22**

5.5 Jordan

5.5.1 All Projections

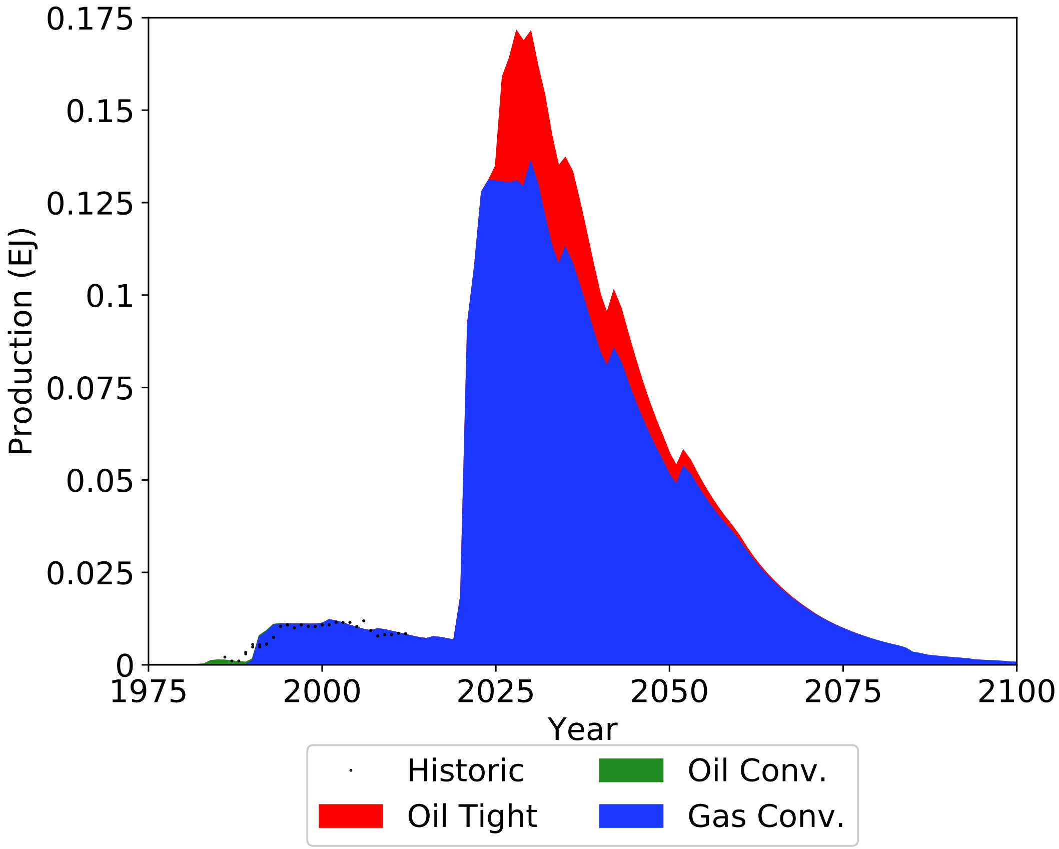

Figure 5.9: Jordan projections capped at 16

Table 5.9: Peak years - All

**Name**	**URR**	**Peak Year**	**Peak Rate**
Gas Conv.	4.1	2030	0.14
Oil Tight	0.57	2028	0.04
Oil Conv.	0.01	1985	–
**Total**	**4.68**	**2028**	**0.17**

5.5.2 By Mineral

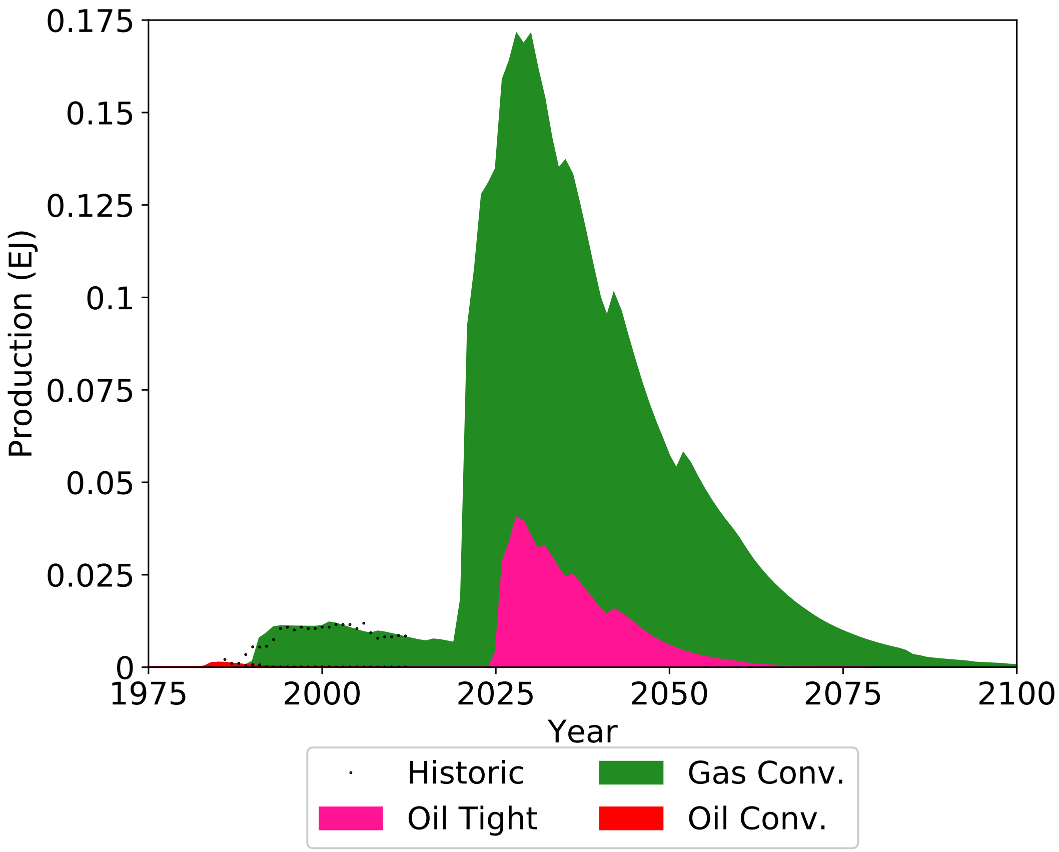

Figure 5.10: Jordan projection by mineral type

Table 5.10: Peak years - Minerals

Name	URR	Peak Year	Peak Rate
Oil Conv.	0.01	1985	–
Oil Tight	0.57	2028	0.04
Gas Conv.	4.1	2030	0.14
**Total**	**4.68**	**2028**	**0.17**

5.6 Kuwait

5.6.1 All Projections

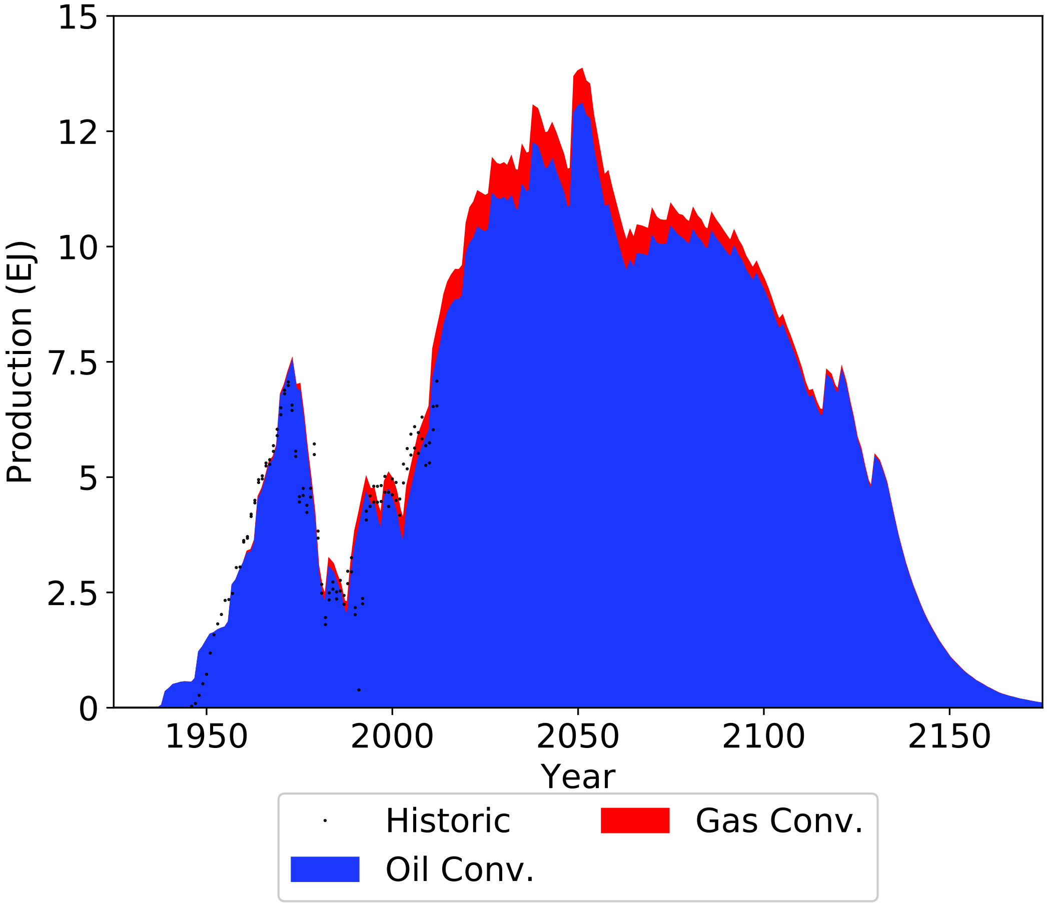

Figure 5.11: Kuwait projections capped at 16

Table 5.11: Peak years - All

Name	URR	Peak Year	Peak Rate
Oil Conv.	1460.81	2051	13.09
Gas Conv.	74.0	2034	0.87
**Total**	**1534.81**	**2051**	**13.85**

5.6.2 By Mineral

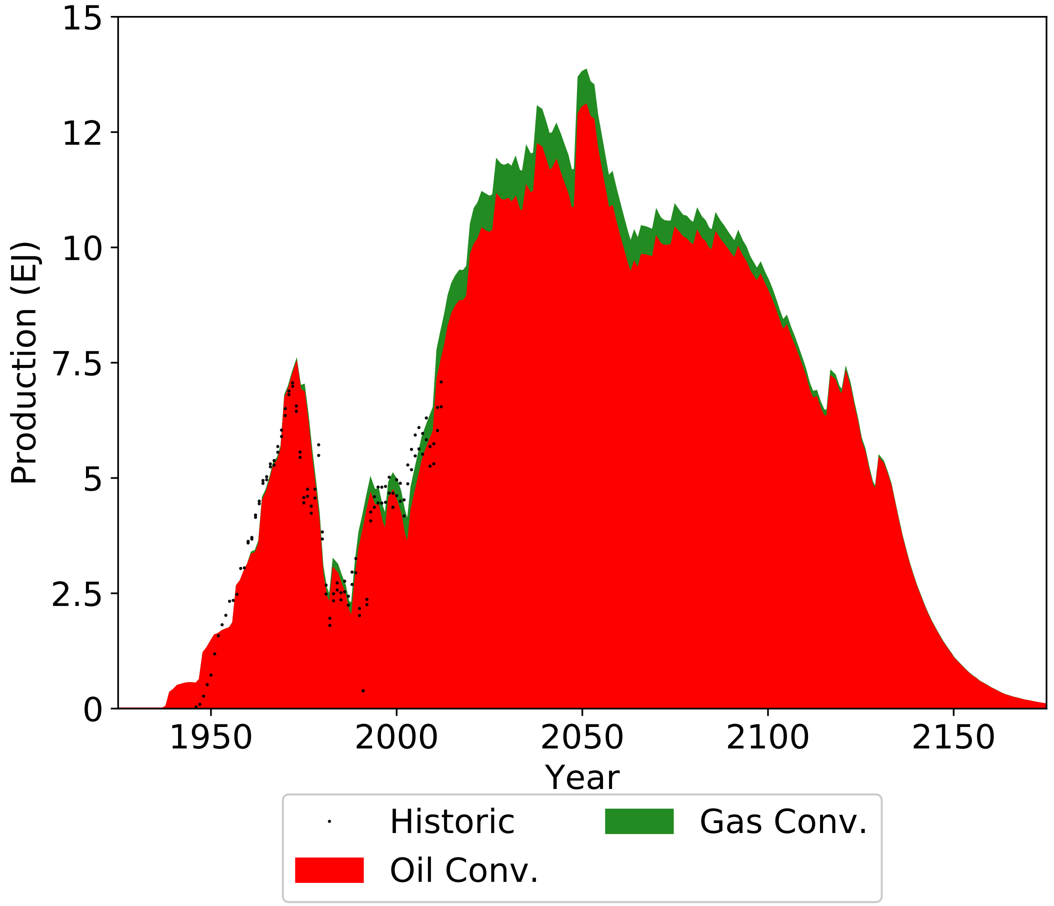

Figure 5.12: Kuwait projection by mineral type

Table 5.12: Peak years - Minerals

Name	URR	Peak Year	Peak Rate
Oil Conv.	1460.81	2051	13.09
Gas Conv.	74.0	2034	0.87
**Total**	**1534.81**	**2051**	**13.85**

5.7 Lebanon

5.7.1 All Projections

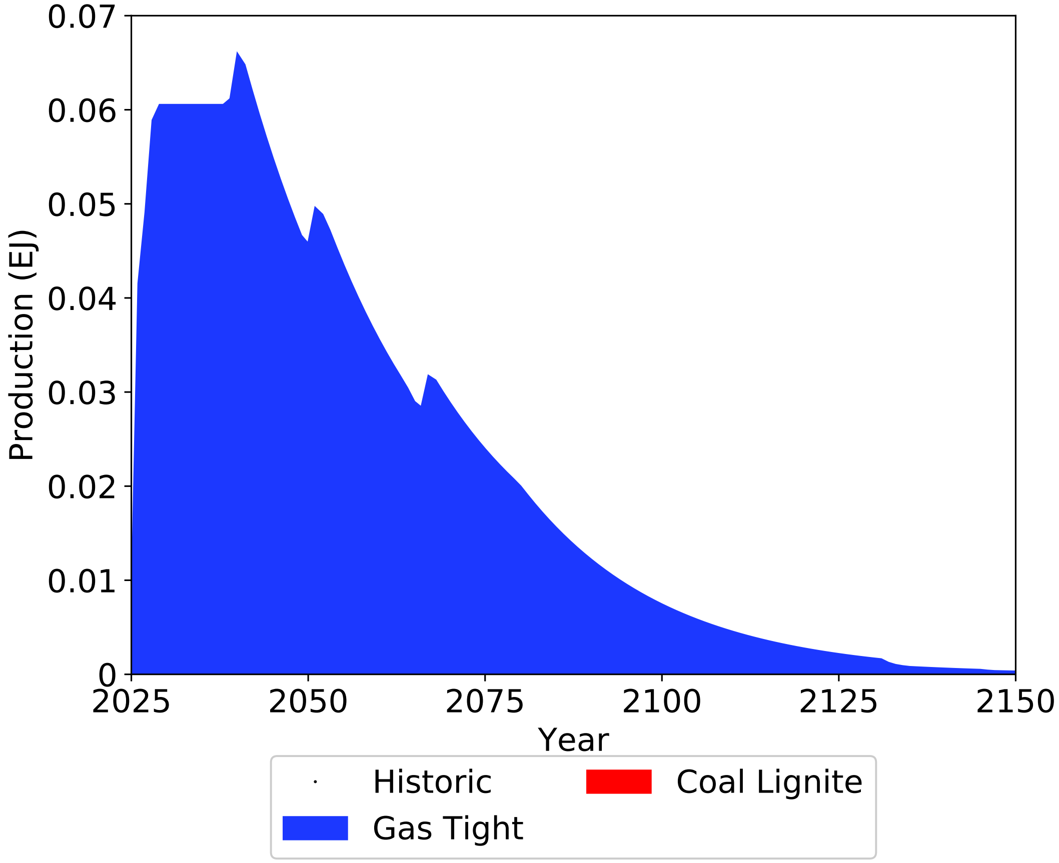

Figure 5.13: Lebanon projections capped at 16

Table 5.13: Peak years - All

**Name**	**URR**	**Peak Year**	**Peak Rate**
Gas Tight	2.78	2040	0.07
Coal Lignite	–	1942	–
**Total**	**2.78**	**2040**	**0.07**

5.7.2 By Mineral

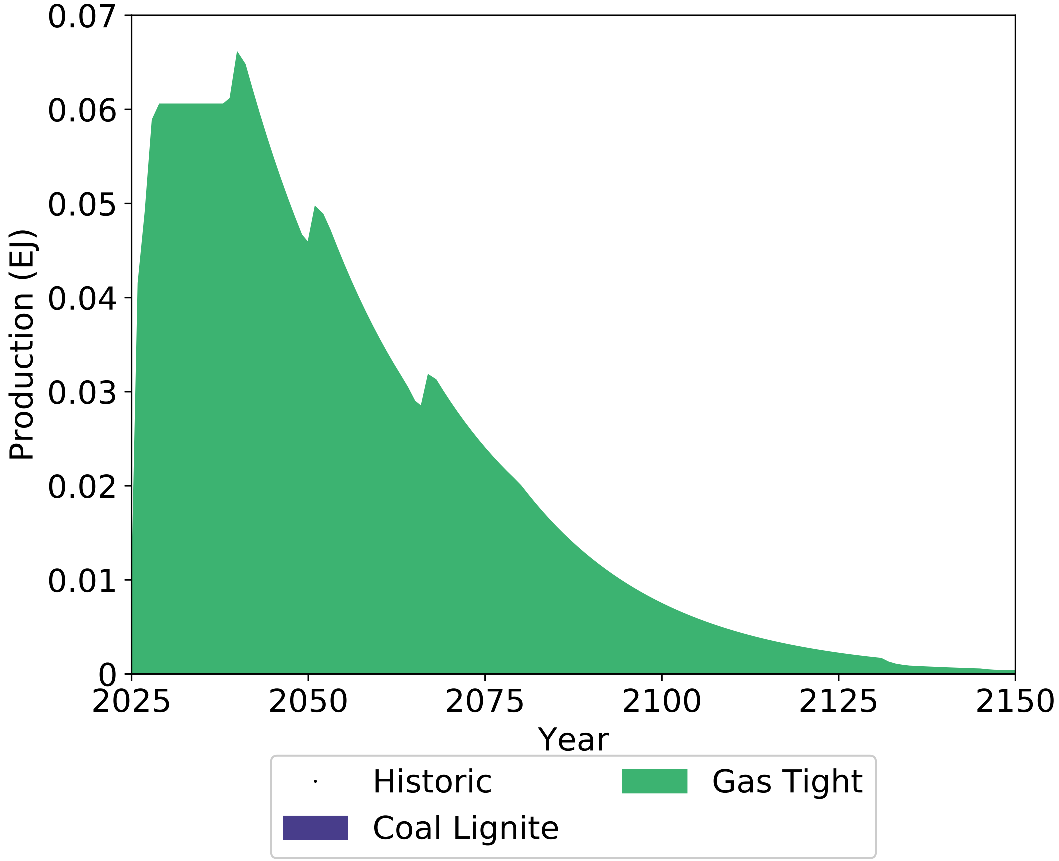

Figure 5.14: Lebanon projection by mineral type

Table 5.14: Peak years - Minerals

**Name**	**URR**	**Peak Year**	**Peak Rate**
Coal Lignite	–	1942	–
Gas Tight	2.78	2040	0.07
**Total**	**2.78**	**2040**	**0.07**

5.8 Oman

5.8.1 All Projections

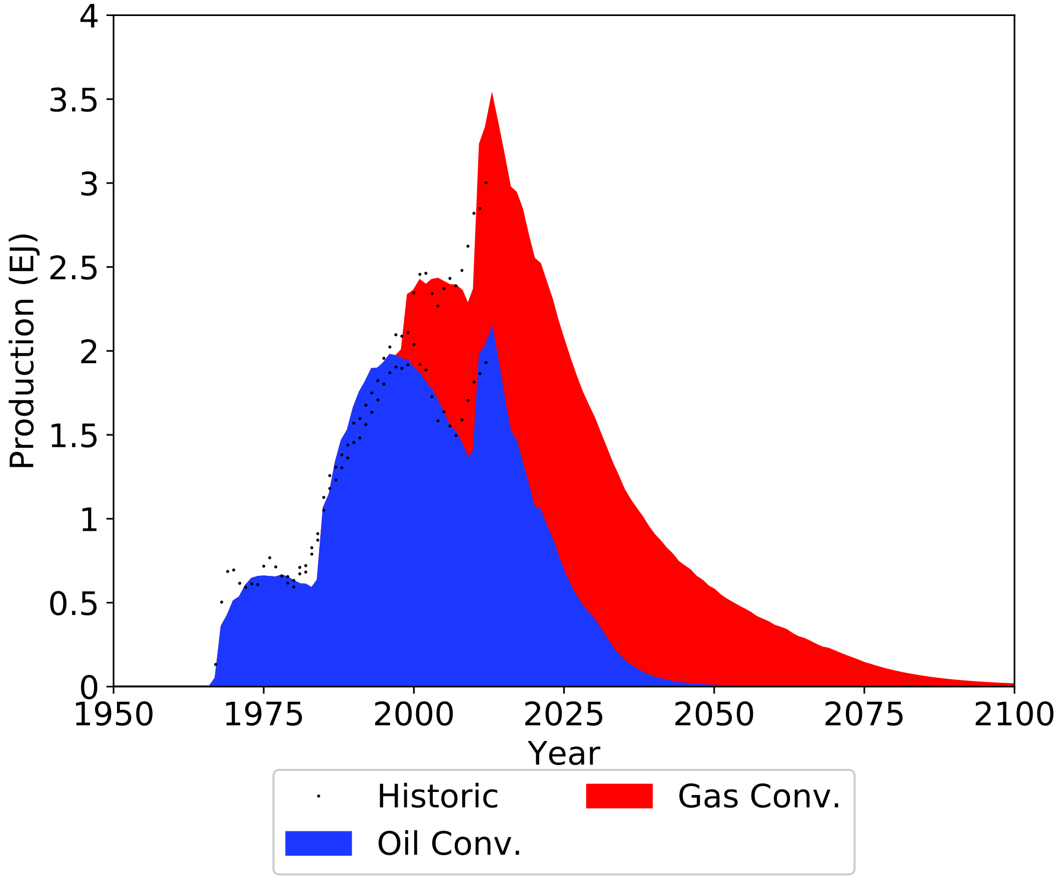

Figure 5.15: Oman projections capped at 16

Table 5.15: Peak years - All

Name	URR	Peak Year	Peak Rate
Oil Conv.	78.43	2013	2.13
Gas Conv.	63.0	2018	1.51
**Total**	**141.43**	**2013**	**3.52**

5.8.2 By Mineral

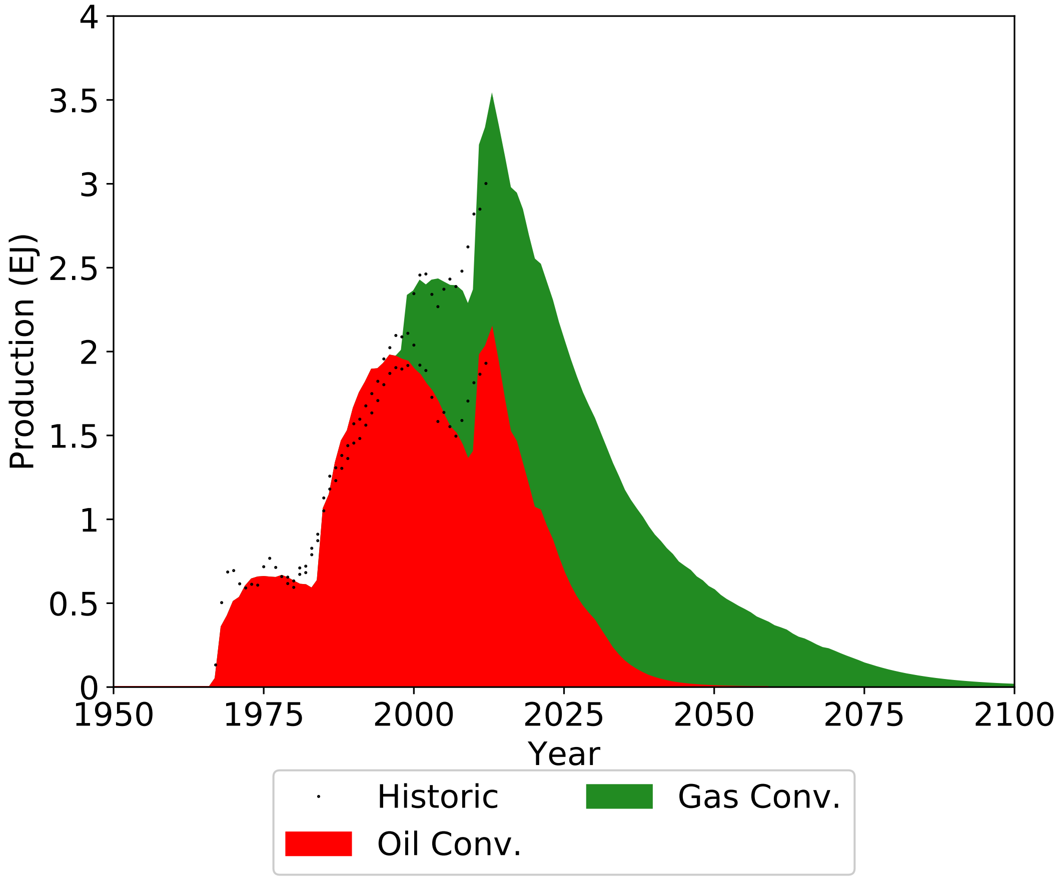

Figure 5.16: Oman projection by mineral type

Table 5.16: Peak years - Minerals

**Name**	**URR**	**Peak Year**	**Peak Rate**
Oil Conv.	78.43	2013	2.13
Gas Conv.	63.0	2018	1.51
**Total**	**141.43**	**2013**	**3.52**

5.9 Qatar

5.9.1 All Projections

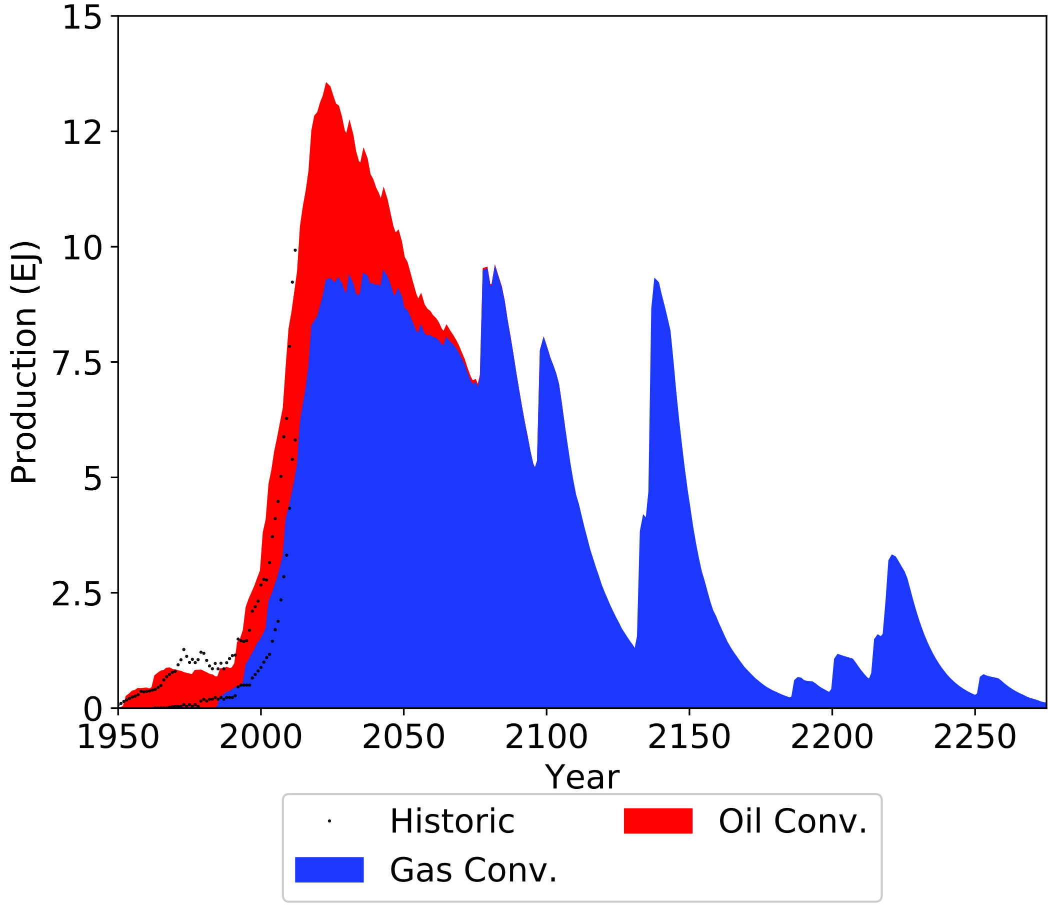

Figure 5.17: Qatar projections capped at 16

Table 5.17: Peak years - All

**Name**	**URR**	**Peak Year**	**Peak Rate**
Gas Conv.	1134.0	2082	9.5
Oil Conv.	200.55	2019	4.45
**Total**	**1334.55**	**2023**	**13.52**

5.9.2 By Mineral

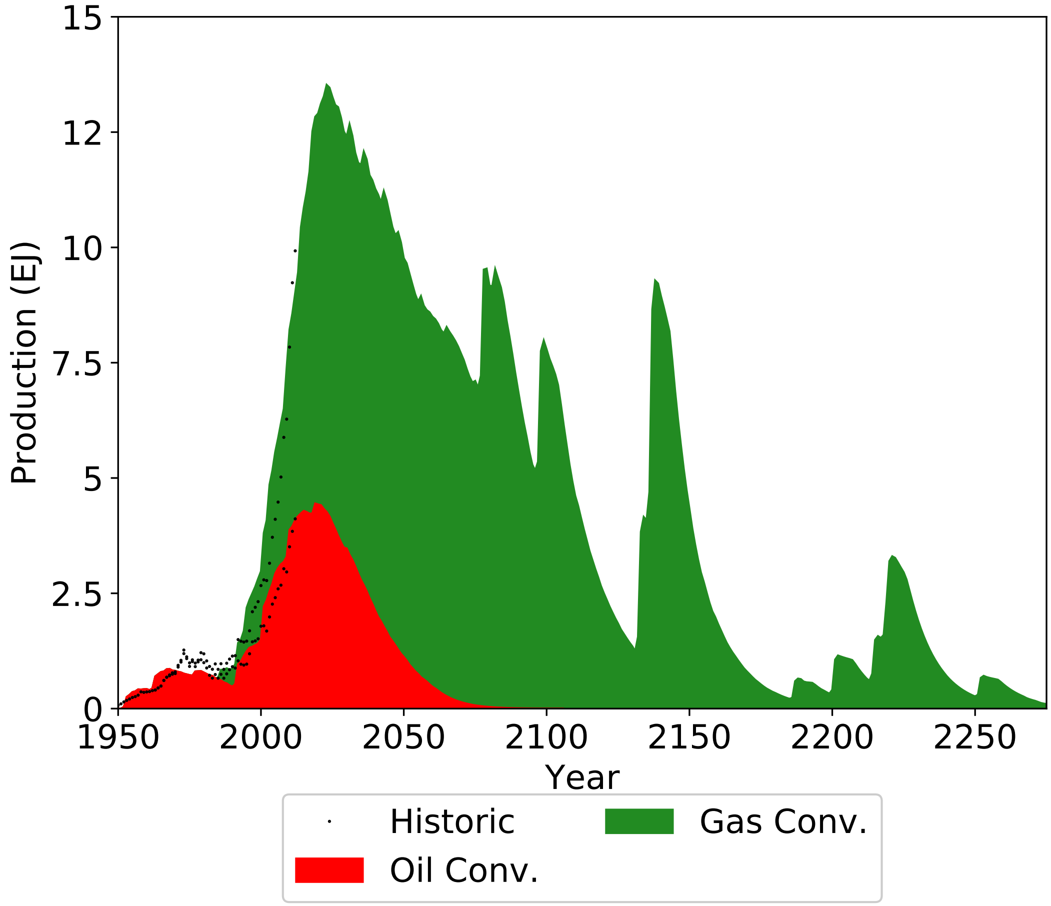

Figure 5.18: Qatar projection by mineral type

Table 5.18: Peak years - Minerals

Name	URR	Peak Year	Peak Rate
Oil Conv.	200.55	2019	4.45
Gas Conv.	1134.0	2082	9.5
**Total**	**1334.55**	**2023**	**13.52**

5.10 Saudi Arabia

5.10.1 All Projections

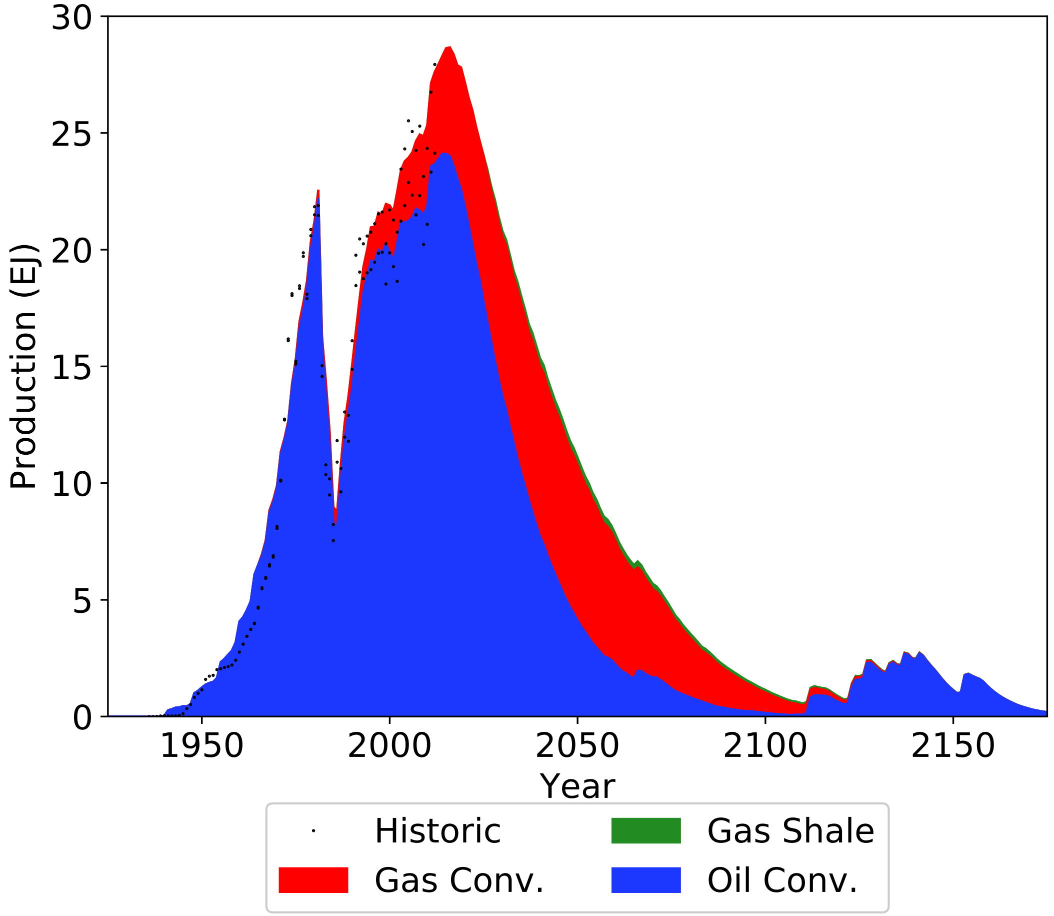

Figure 5.19: Saudi Arabia projections capped at 16

Table 5.19: Peak years - All

Name	URR	Peak Year	Peak Rate
Oil Conv.	1508.77	2014	24.11
Gas Conv.	478.3	2038	7.36
Gas Shale	21.0	2041	0.35
**Total**	**2008.07**	**2016**	**28.67**

5.10.2 By Mineral

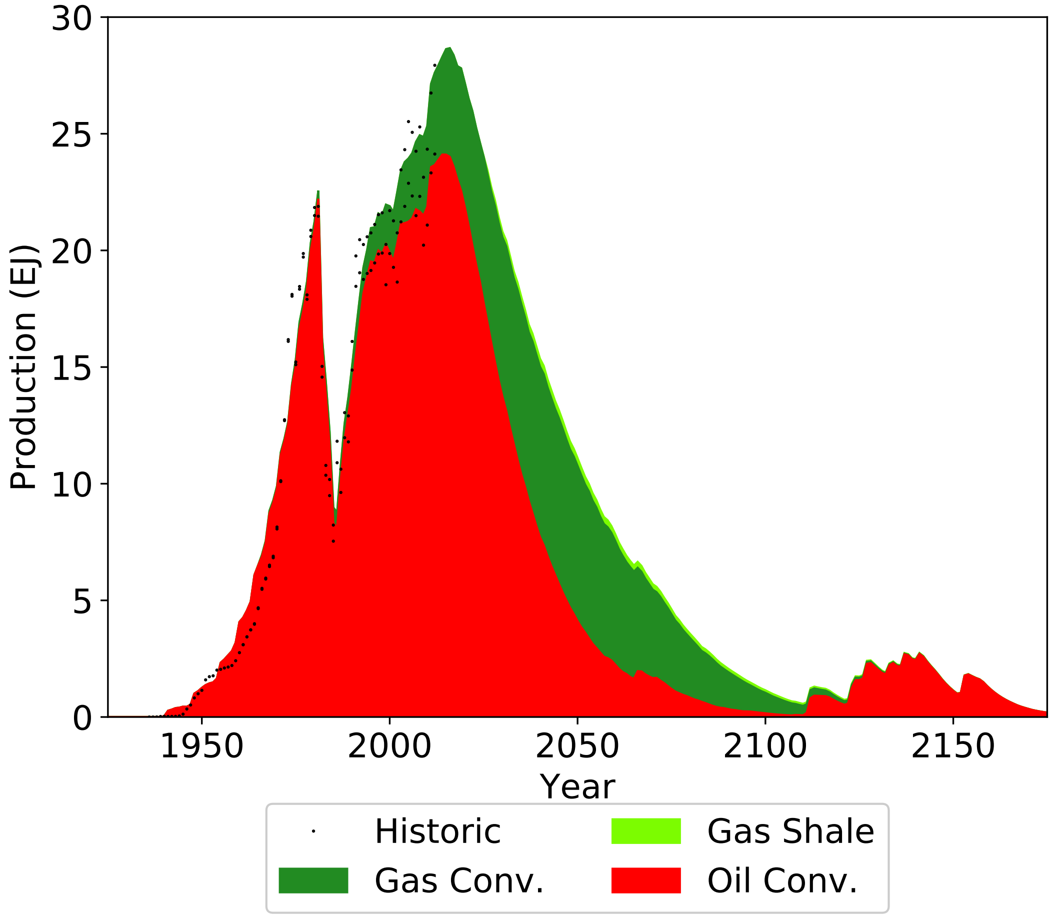

Figure 5.20: Saudi Arabia projection by mineral type

Table 5.20: Peak years - Minerals

**Name**	**URR**	**Peak Year**	**Peak Rate**
Oil Conv.	1508.77	2014	24.11
Gas Conv.	478.3	2038	7.36
Gas Shale	21.0	2041	0.35
**Total**	**2008.07**	**2016**	**28.67**

5.11 Syria

5.11.1 All Projections

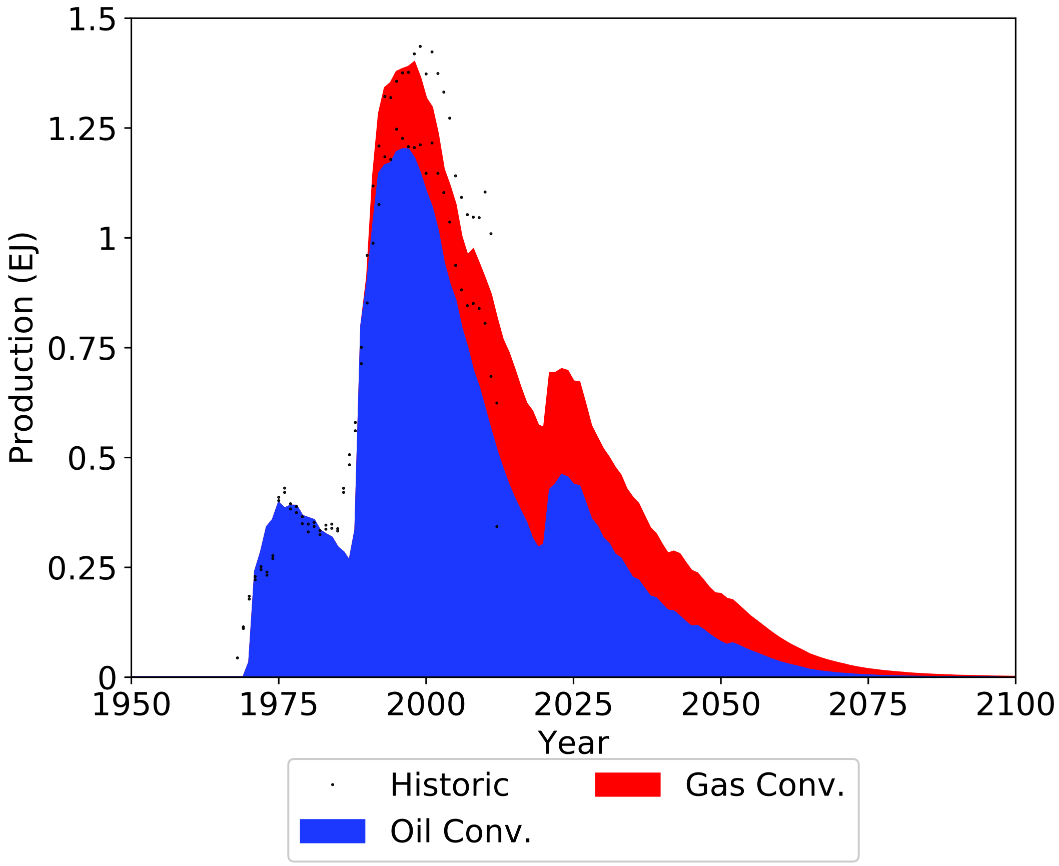

Figure 5.21: Syria projections capped at 16

Table 5.21: Peak years - All

Name	URR	Peak Year	Peak Rate
Oil Conv.	39.98	1996	1.2
Gas Conv.	13.7	2011	0.31
**Total**	**53.68**	**1998**	**1.4**

5.11.2 By Mineral

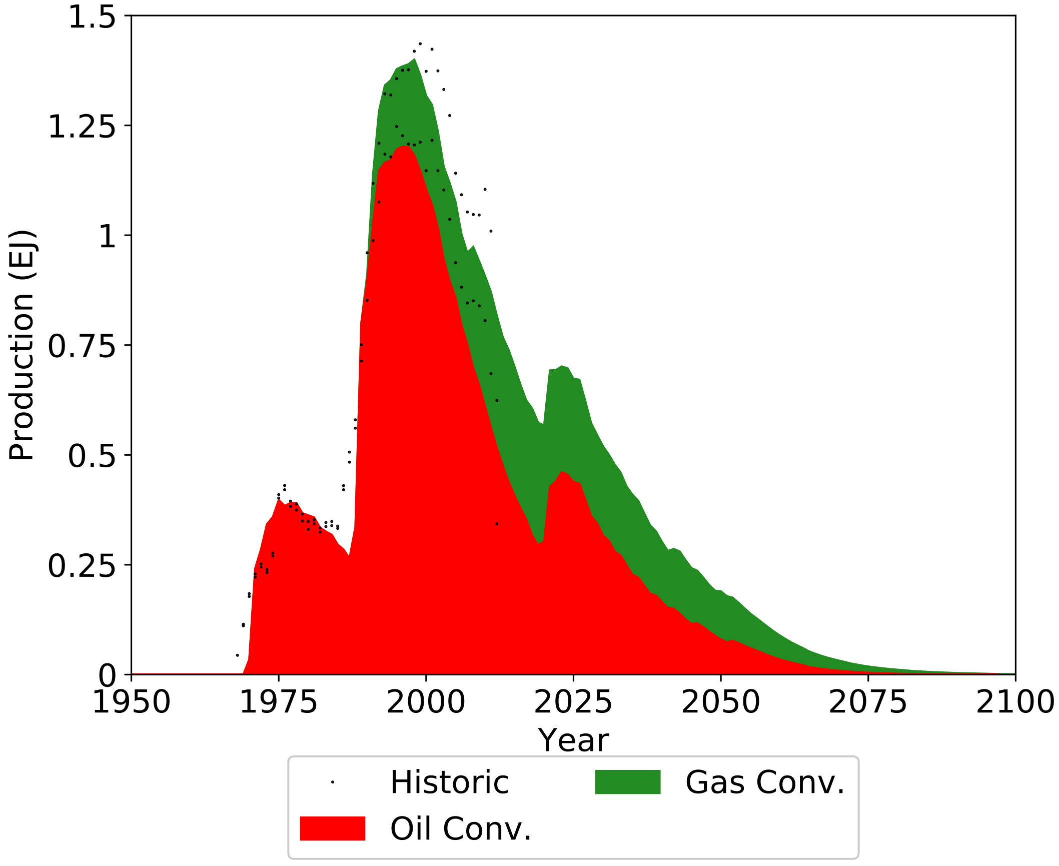

Figure 5.22: Syria projection by mineral type

Table 5.22: Peak years - Minerals

**Name**	**URR**	**Peak Year**	**Peak Rate**
Oil Conv.	39.98	1996	1.2
Gas Conv.	13.7	2011	0.31
**Total**	**53.68**	**1998**	**1.4**

5.12 UAE

5.12.1 All Projections

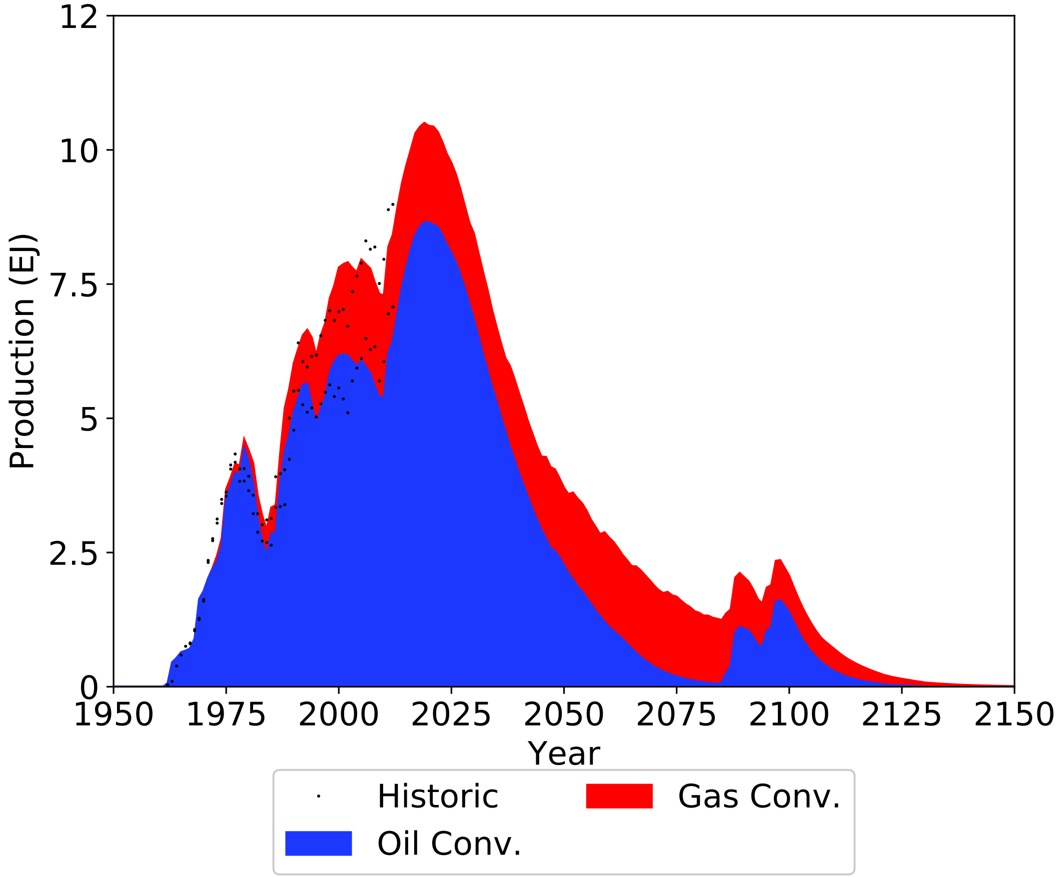

Figure 5.23: UAE projections capped at 16

Table 5.23: Peak years - All

Name	URR	Peak Year	Peak Rate
Oil Conv.	476.21	2019	8.65
Gas Conv.	178.5	2013	2.0
**Total**	**654.71**	**2019**	**10.5**

5.12.2 By Mineral

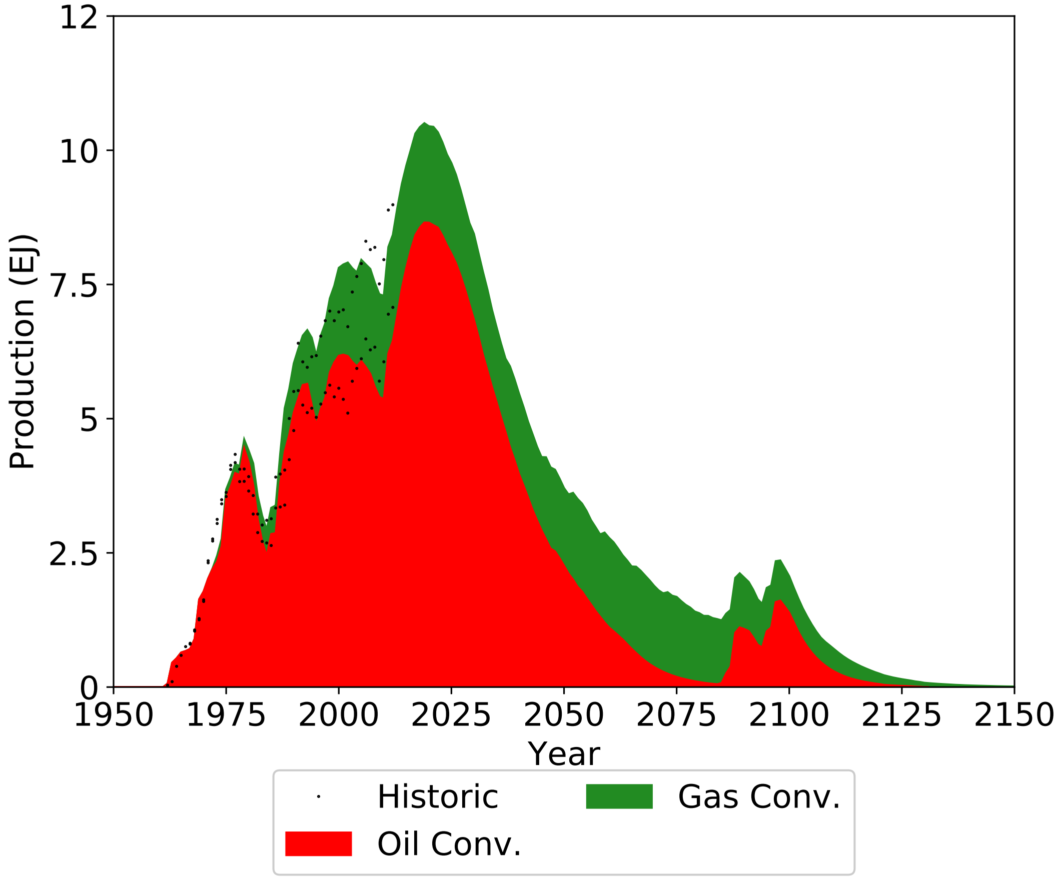

Figure 5.24: UAE projection by mineral type

Table 5.24: Peak years - Minerals

Name	URR	Peak Year	Peak Rate
Oil Conv.	476.21	2019	8.65
Gas Conv.	178.5	2013	2.0
**Total**	**654.71**	**2019**	**10.5**

5.13 Yemen

5.13.1 All Projections

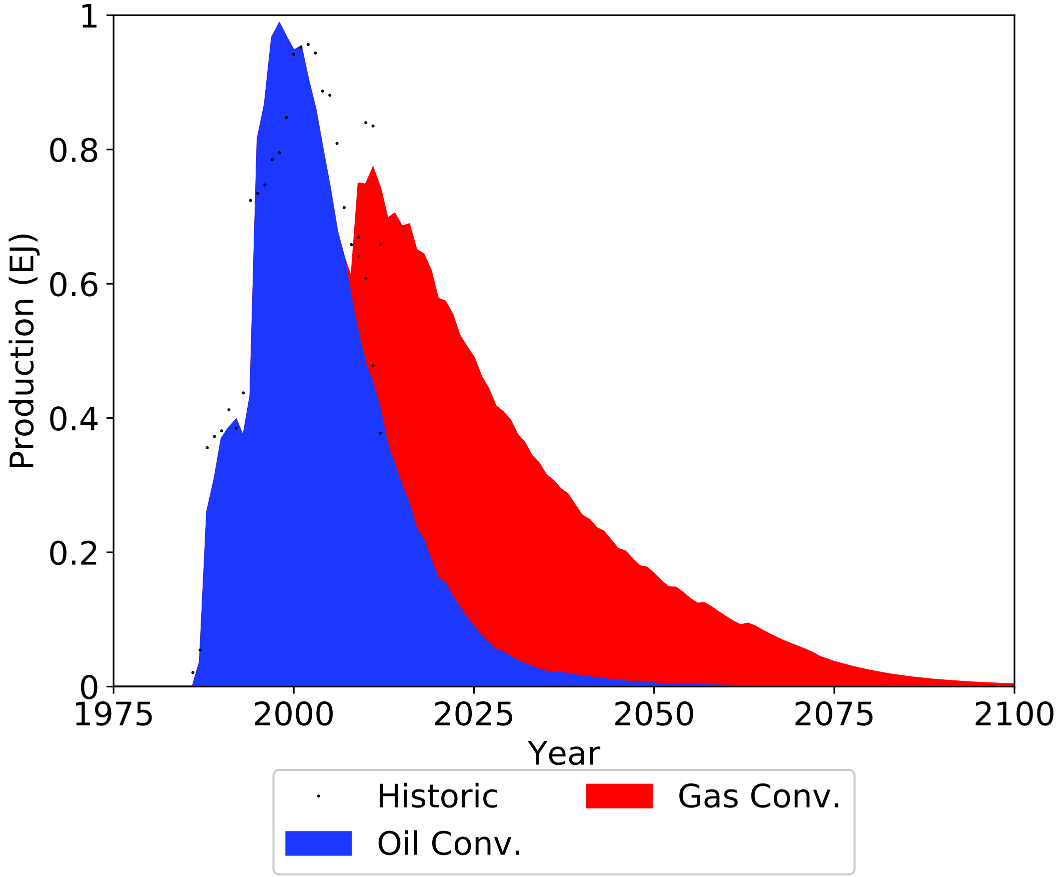

Figure 5.25: Yemen projections capped at 16

Table 5.25: Peak years - All

Name	URR	Peak Year	Peak Rate
Oil Conv.	19.44	1998	0.99
Gas Conv.	15.8	2019	0.43
**Total**	**35.24**	**1998**	**0.99**

5.13.2 By Mineral

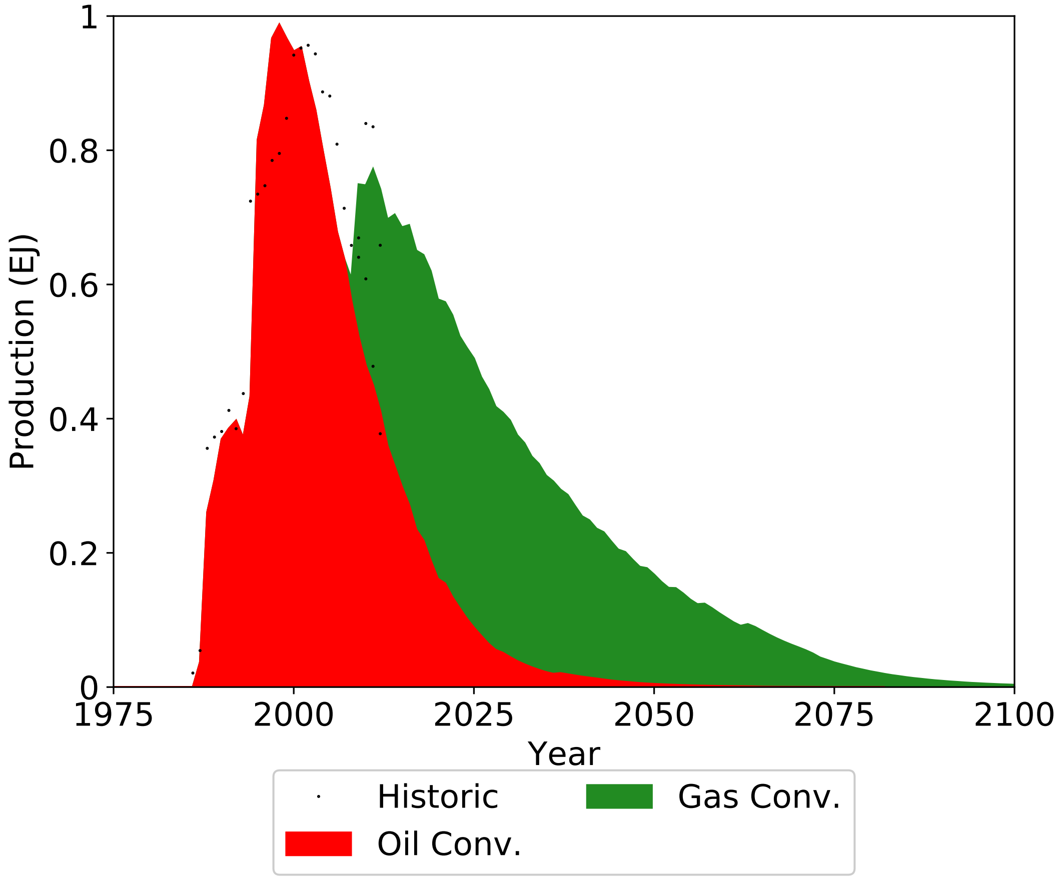

Figure 5.26: Yemen projection by mineral type

Table 5.26: Peak years - Minerals

Name	URR	Peak Year	Peak Rate
Oil Conv.	19.44	1998	0.99
Gas Conv.	15.8	2019	0.43
**Total**	**35.24**	**1998**	**0.99**

5.14 Total

5.14.1 By country

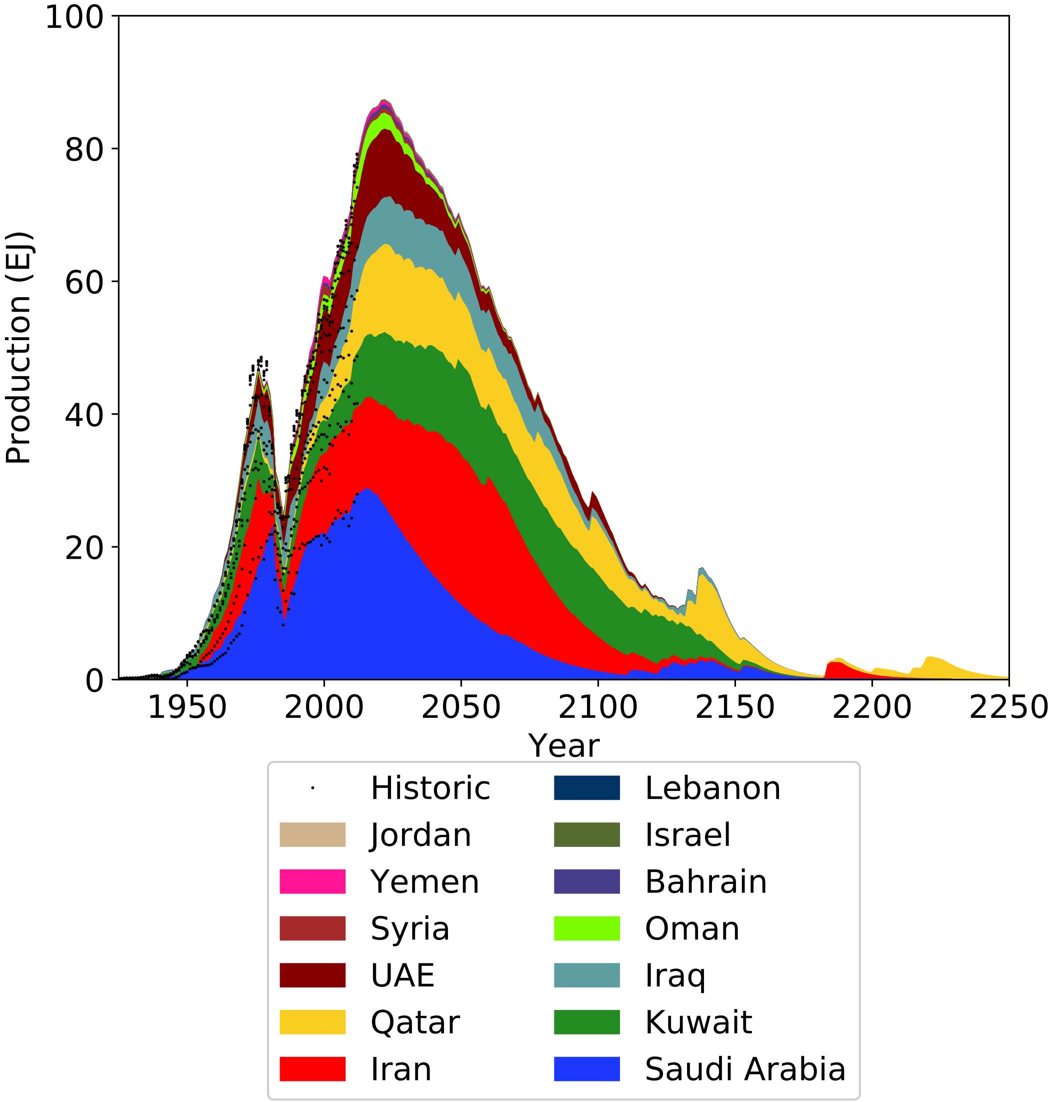

Figure 5.27: Middle East projections by country

5.14.2 By mineral

Table 5.27: Peak years - All

Name	URR	Peak Year	Peak Rate
Saudi Arabia	2008.07	2016	28.67
Iran	1997.63	2047	22.97
Kuwait	1534.81	2051	13.85
Qatar	1334.55	2023	13.52
Iraq	704.3	2027	7.59
UAE	654.71	2019	10.5
Oman	141.43	2013	3.52
Syria	53.68	1998	1.4
Bahrain	37.2	2014	0.61
Yemen	35.24	1998	0.99
Israel	8.89	1970	0.22
Jordan	4.68	2028	0.17
Lebanon	2.78	2040	0.07
**Total**	**8517.97**	**2022**	**87.32**

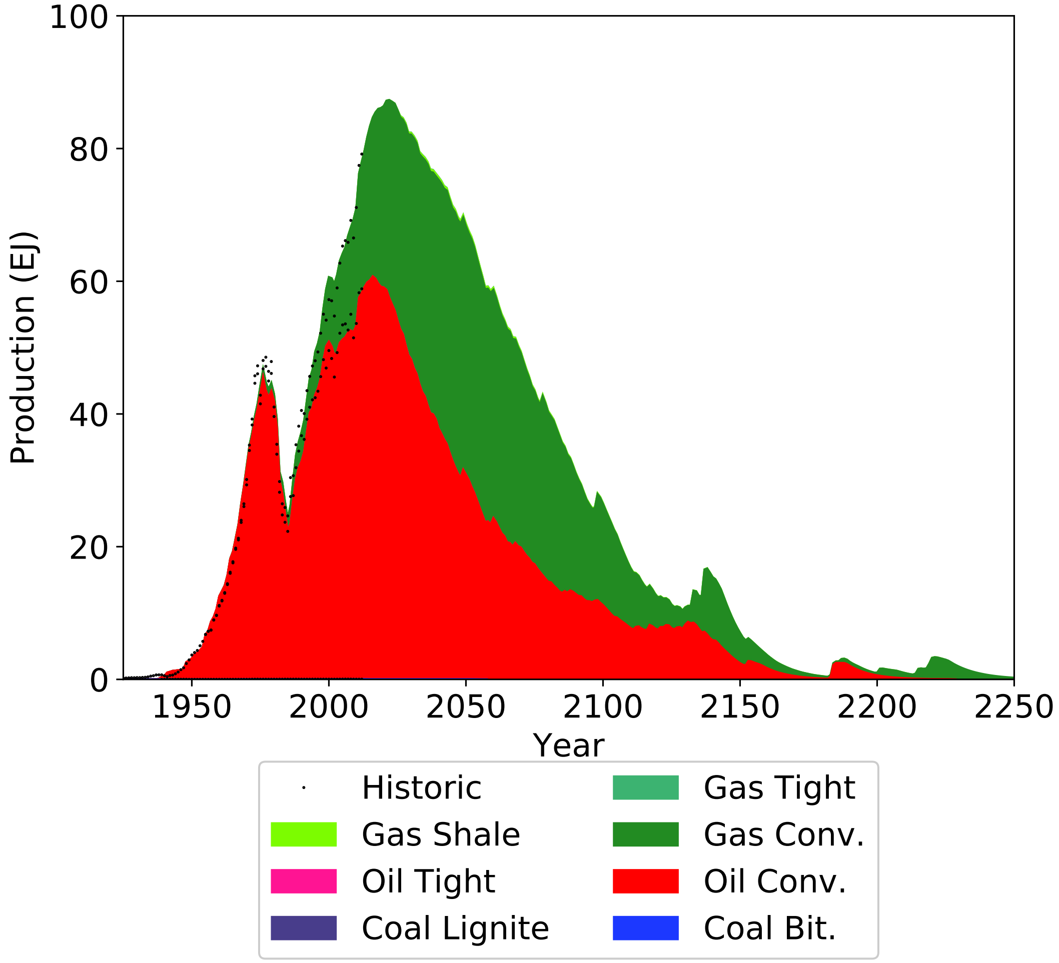

Figure 5.28: Middle East projection by mineral type

Table 5.28: Peak years - Minerals

**Name**	**URR**	**Peak Year**	**Peak Rate**
Coal Bit.	2.3	2007	0.05
Coal Lignite	–	1942	–
Oil Conv.	5158.32	2016	60.73
Oil Tight	0.57	2028	0.04
Gas Conv.	3333.0	2046	38.43
Gas Shale	21.0	2041	0.35
Gas Tight	2.78	2040	0.07
**Total**	**8517.97**	**2022**	**87.32**

Chapter 6

North America

6.1 Canada

6.1.1 All Projections

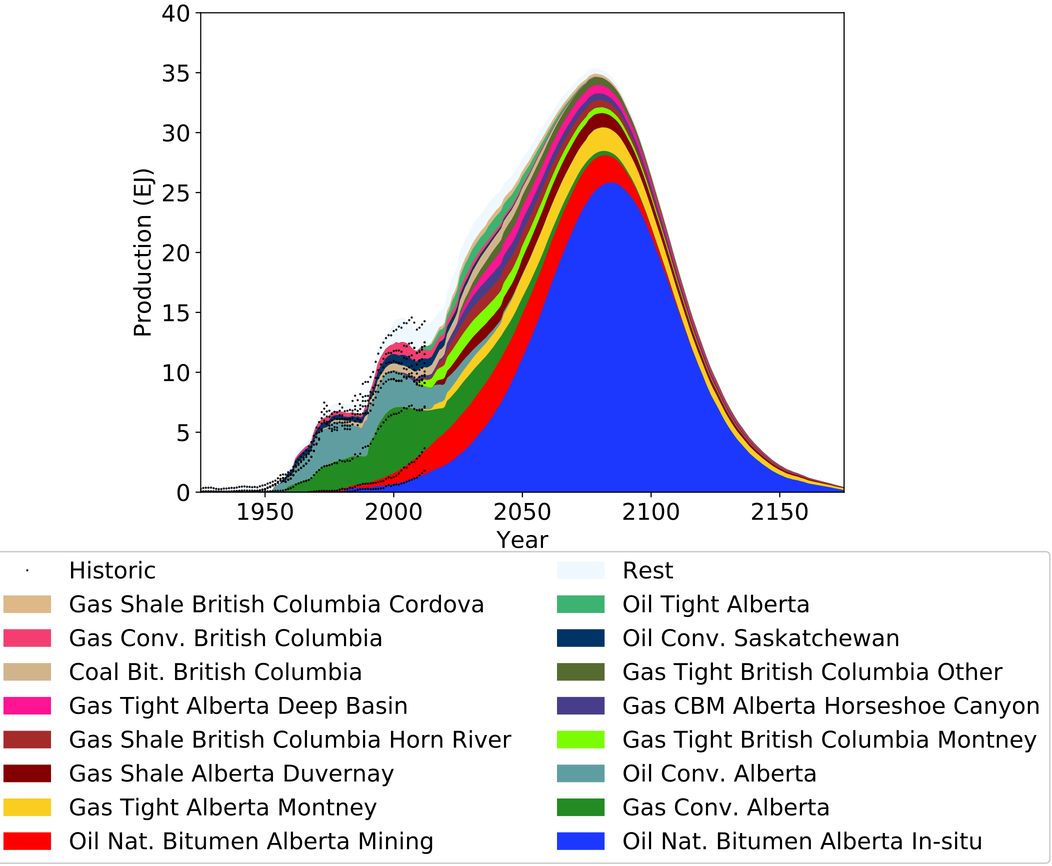

Figure 6.1: Canada projections capped at 16

Table 6.1: Peak years - All

Name	URR	Peak Year	Peak Rate
Oil Nat. Bitumen Alberta In-situ	1719.0	2084	25.77
Oil Nat. Bitumen Alberta Mining	286.5	2043	3.72
Gas Conv. Alberta	270.34	2000	5.44
Gas Tight Alberta Montney	187.12	2060	2.12
Oil Conv. Alberta	172.73	1976	3.3
Gas Shale Alberta Duvernay	118.65	2064	1.48
Gas Tight British Columbia Montney	82.29	2031	1.37
Gas Shale British Columbia Horn River	79.1	2038	1.2
Gas CBM Alberta Horseshoe Canyon	72.93	2040	1.07
Gas Tight Alberta Deep Basin	72.45	2047	1.11
Gas Tight British Columbia Other	61.95	2048	0.95
Coal Bit. British Columbia	60.93	2035	1.0
Oil Conv. Saskatchewan	46.99	2005	0.9
Gas Conv. British Columbia	44.77	2003	1.08
Oil Tight Alberta	41.49	2039	1.13
Gas Shale British Columbia Cordova	31.61	2034	0.5
Coal Sub-bit. Alberta	23.0	2008	0.52
Gas CBM Alberta Mannville	12.29	2035	0.21
Coal Bit. Nova Scotia	12.07	1929	0.15
Gas Shale Nova Scotia Horton Bluff	11.55	2045	0.2
Gas Tight British Columbia Jean Marie	11.55	2035	0.2
Gas Conv. Saskatchewan	11.19	2003	0.28
Oil Conv. East Coast Offshore	10.78	2003	0.66
Oil Conv. British Columbia	9.67	2003	0.18
Coal Bit. Alberta	9.23	1990	0.25
Oil Tight Saskatchewan	7.45	2016	0.31
Gas Shale Quebec Utica	7.35	2034	0.14
Coal Lignite Saskatchewan	6.75	1997	0.13
Gas CBM British Columbia	5.71	2039	0.11
Gas Shale Alberta Colorado	4.2	2041	0.08
Gas Conv. East Coast Offshore	3.01	2002	0.17
Gas CBM Nova Scotia	2.86	2037	0.06
Gas Conv. Ontario	2.03	2024	0.02
Oil Conv. Northwest Territories	1.91	1988	0.07
Oil Conv. Manitoba	1.81	2007	0.05
Oil Tight Manitoba	1.72	2013	0.1
Gas Conv. Northwest Territories	1.1	2000	0.04
Coal Bit. New Brunswick	0.94	1971	0.02
Gas CBM Saskatchewan	0.71	2029	0.02
Oil Conv. Ontario	0.53	1990	0.01
Oil Conv. Nova Scotia Offshore	0.24	2004	0.02
Gas Conv. New Brunswick	0.04	1918	–
Oil Extra Heavy Saskatchewan	0.02	2029	0.01
Oil Extra Heavy Alberta	0.02	2029	0.01
Coal Bit. Yukon	0.01	1955	–
Oil Conv. New Brunswick	0.01	1934	–
Gas Conv. Quebec	–	1970	–

Table 6.1: Peak years - All – Continued

**Name**	**URR**	**Peak Year**	**Peak Rate**
Coal Lignite Manitoba	–	1935	–
Gas Conv. Manitoba	–	1929	–
**Total**	**3508.61**	**2078**	**35.34**

6.1.2 By Mineral

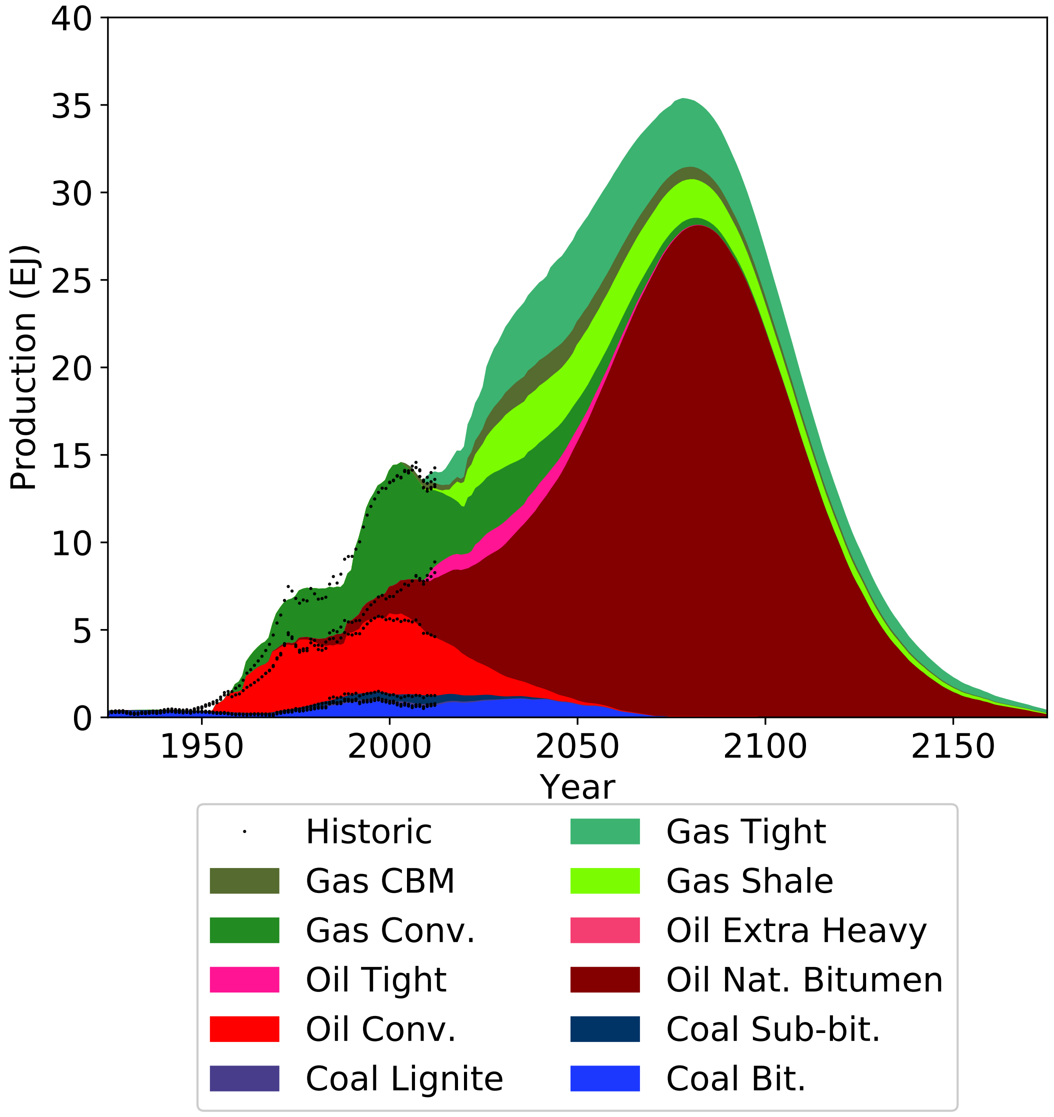

Figure 6.2: Canada projection by mineral type

6.1.3 Regional Projections

Table 6.2: Peak years - Minerals

Name	URR	Peak Year	Peak Rate
Coal Bit.	83.18	2035	1.0
Coal Lignite	6.75	1997	0.13
Coal Sub-bit.	23.0	2008	0.52
Oil Conv.	244.67	2003	4.62
Oil Nat. Bitumen	2005.5	2082	28.06
Oil Tight	50.66	2028	1.32
Oil Extra Heavy	0.04	2029	0.01
Gas Conv.	332.48	2001	6.88
Gas Shale	252.46	2040	3.25
Gas CBM	94.5	2039	1.46
Gas Tight	415.36	2056	5.16
**Total**	**3508.61**	**2078**	**35.34**

Alberta

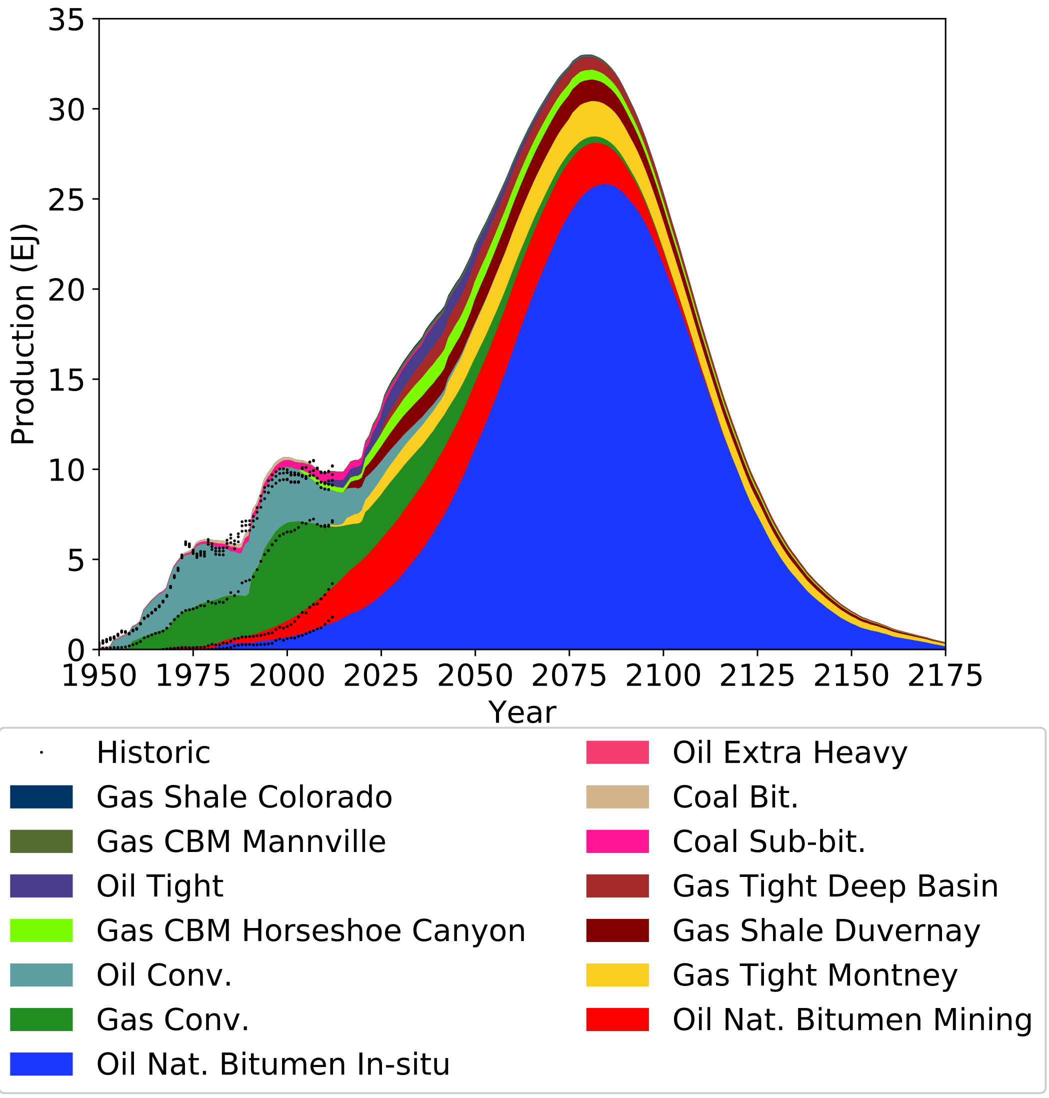

Figure 6.3: Canada - Alberta projections capped at 16

Table 6.3: Peak years - All

**Name**	**URR**	**Peak Year**	**Peak Rate**
Oil Nat. Bitumen Alberta In-situ	1719.0	2084	25.77
Oil Nat. Bitumen Alberta Mining	286.5	2043	3.72
Gas Conv. Alberta	270.34	2000	5.44
Gas Tight Alberta Montney	187.12	2060	2.12
Oil Conv. Alberta	172.73	1976	3.3
Gas Shale Alberta Duvernay	118.65	2064	1.48
Gas CBM Alberta Horseshoe Canyon	72.93	2040	1.07
Gas Tight Alberta Deep Basin	72.45	2047	1.11
Oil Tight Alberta	41.49	2039	1.13
Coal Sub-bit. Alberta	23.0	2008	0.52
Gas CBM Alberta Mannville	12.29	2035	0.21
Coal Bit. Alberta	9.23	1990	0.25
Gas Shale Alberta Colorado	4.2	2041	0.08
Oil Extra Heavy Alberta	0.02	2029	0.01
**Total**	**2989.95**	**2080**	**32.96**

Table 6.4: Peak years - Minerals

**Name**	**URR**	**Peak Year**	**Peak Rate**
Coal Bit.	9.23	1990	0.25
Coal Sub-bit.	23.0	2008	0.52
Oil Conv.	172.73	1976	3.3
Oil Nat. Bitumen	2005.5	2082	28.06
Oil Tight	41.49	2039	1.13
Oil Extra Heavy	0.02	2029	0.01
Gas Conv.	270.34	2000	5.44
Gas Shale	122.85	2064	1.53
Gas CBM	85.22	2040	1.27
Gas Tight	259.57	2056	3.14
**Total**	**2989.95**	**2080**	**32.96**

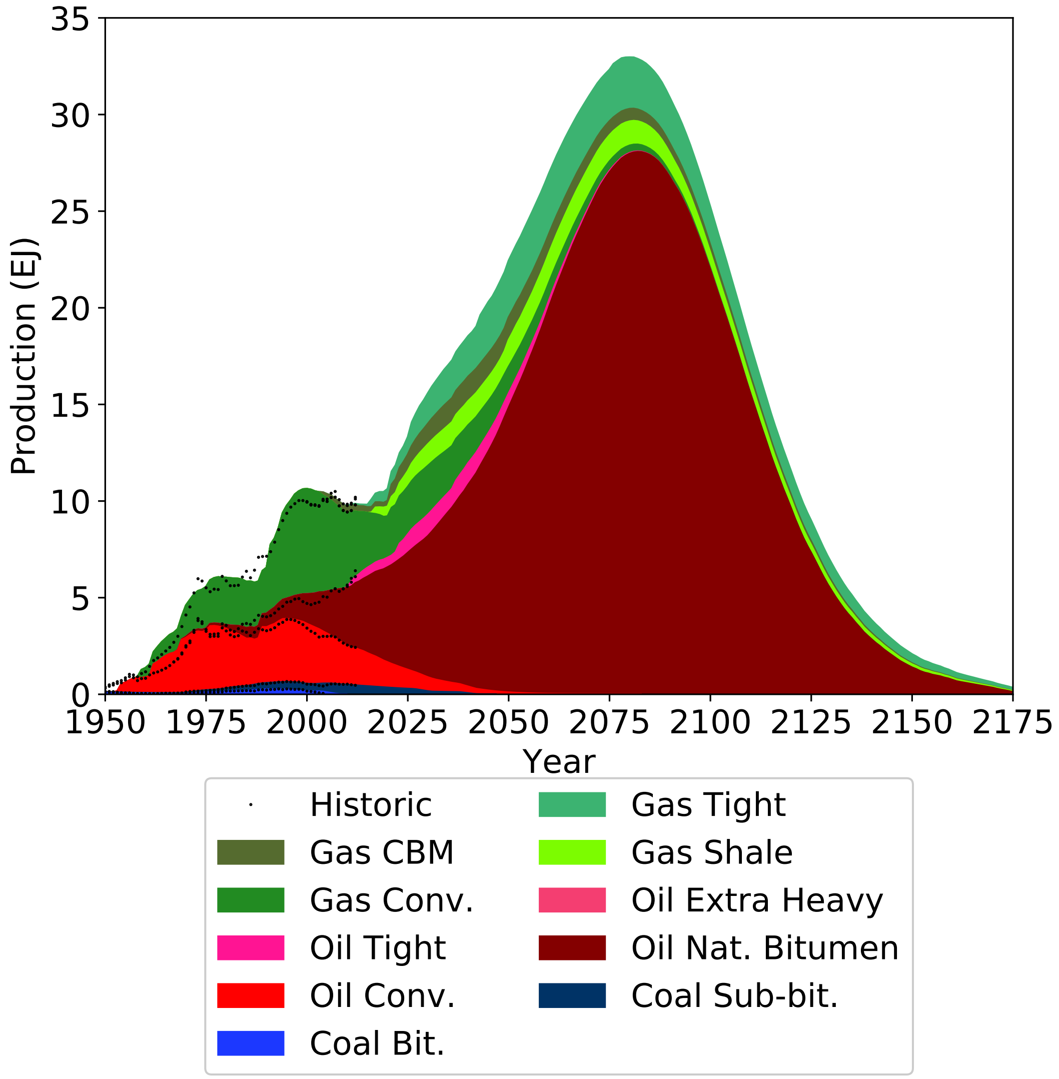

Figure 6.4: Canada - Alberta projection by mineral type

British Columbia

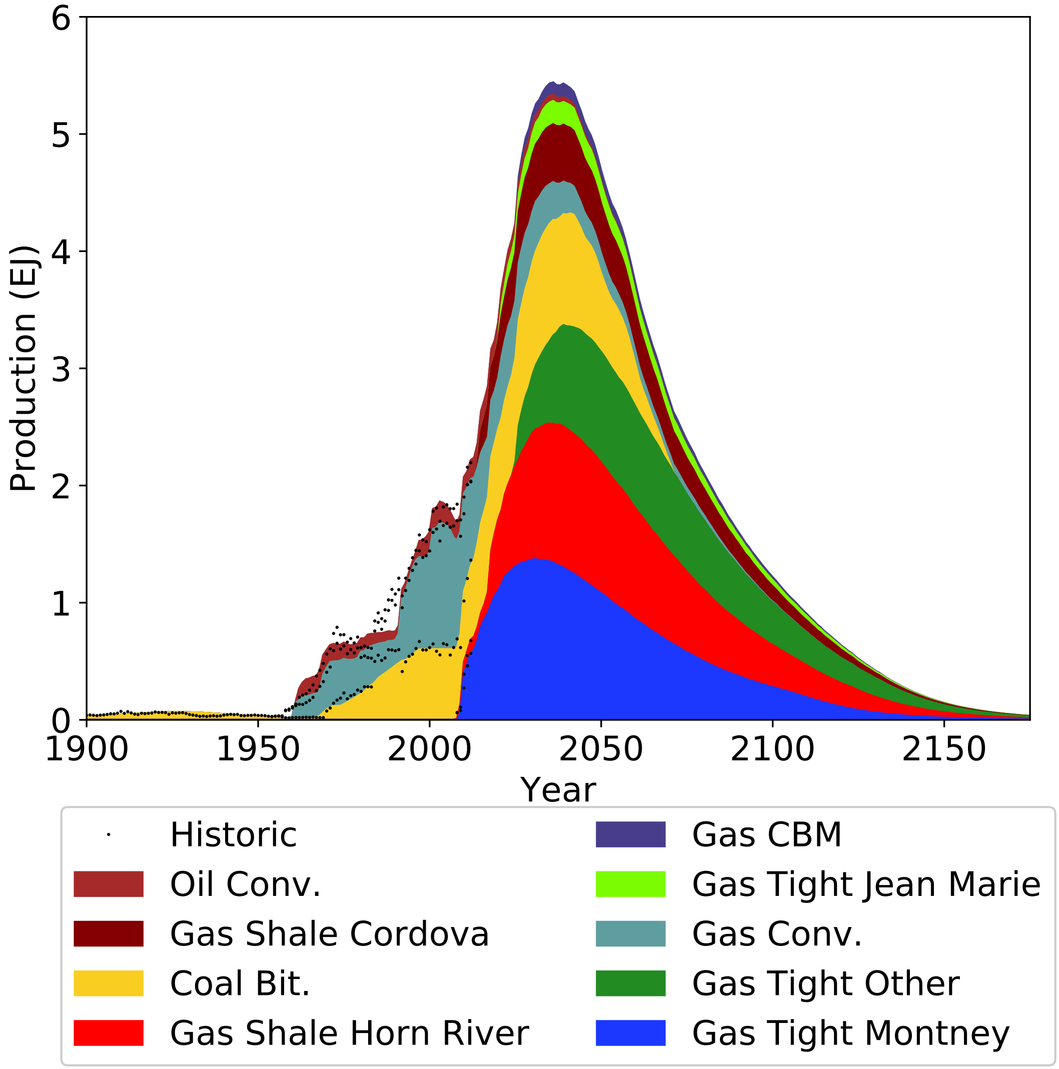

Figure 6.5: Canada - British Columbia projections capped at 16

Table 6.5: Peak years - All

Name	URR	Peak Year	Peak Rate
Gas Tight British Columbia Montney	82.29	2031	1.37
Gas Shale British Columbia Horn River	79.1	2038	1.2
Gas Tight British Columbia Other	61.95	2048	0.95
Coal Bit. British Columbia	60.93	2035	1.0
Gas Conv. British Columbia	44.77	2003	1.08
Gas Shale British Columbia Cordova	31.61	2034	0.5
Gas Tight British Columbia Jean Marie	11.55	2035	0.2
Oil Conv. British Columbia	9.67	2003	0.18
Gas CBM British Columbia	5.71	2039	0.11
**Total**	**387.58**	**2036**	**5.44**

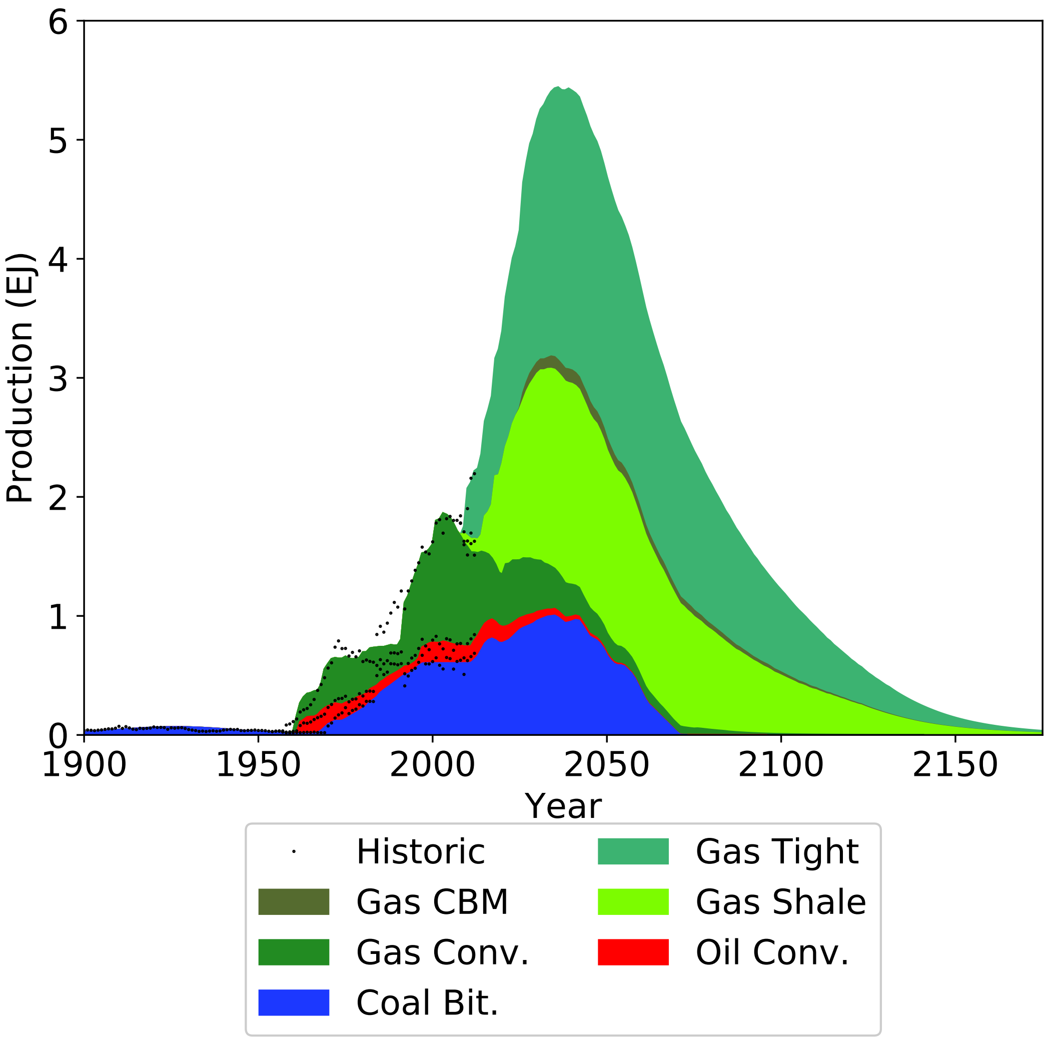

Figure 6.6: Canada - British Columbia projection by mineral type

Table 6.6: Peak years - Minerals

**Name**	**URR**	**Peak Year**	**Peak Rate**
Coal Bit.	60.93	2035	1.0
Oil Conv.	9.67	2003	0.18
Gas Conv.	44.77	2003	1.08
Gas Shale	110.71	2038	1.69
Gas CBM	5.71	2039	0.11
Gas Tight	155.79	2039	2.36
**Total**	**387.58**	**2036**	**5.44**

East Coast Offshore

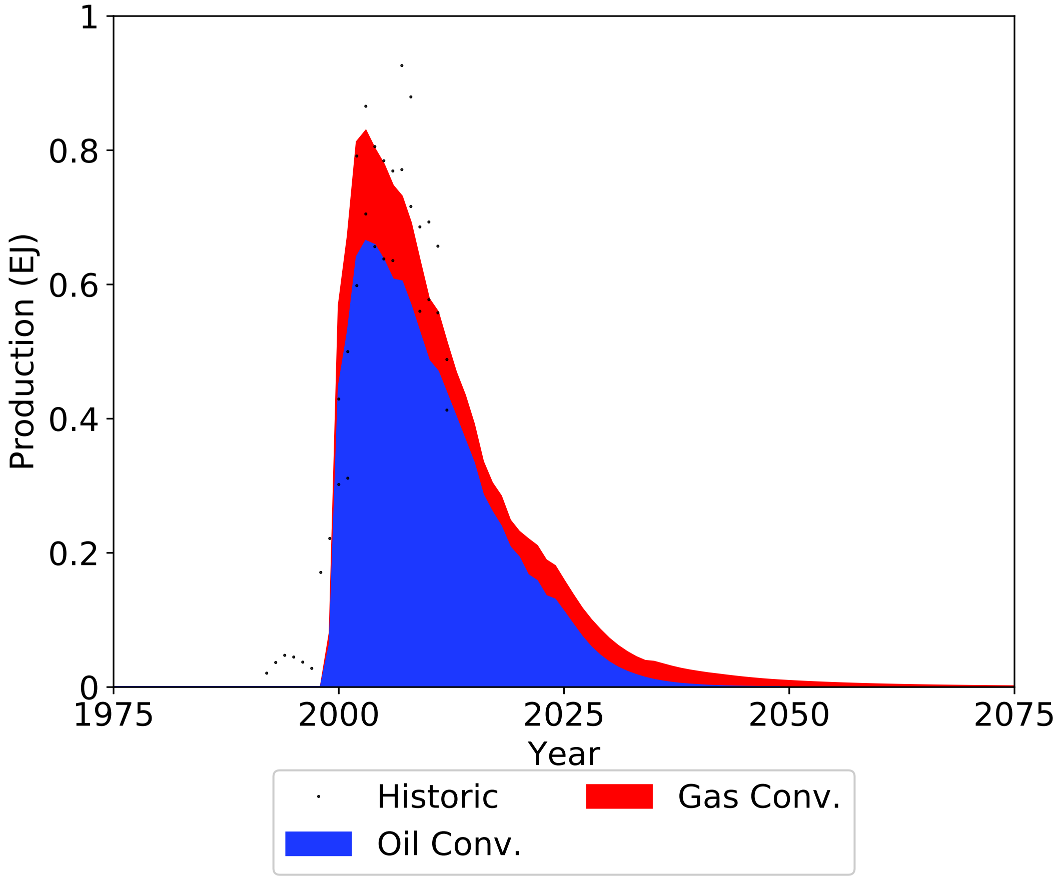

Figure 6.7: Canada - East Coast Offshore projections capped at 16

Table 6.7: Peak years - All

**Name**	**URR**	**Peak Year**	**Peak Rate**
Oil Conv. East Coast Offshore	10.78	2003	0.66
Gas Conv. East Coast Offshore	3.01	2002	0.17
**Total**	**13.79**	**2003**	**0.83**

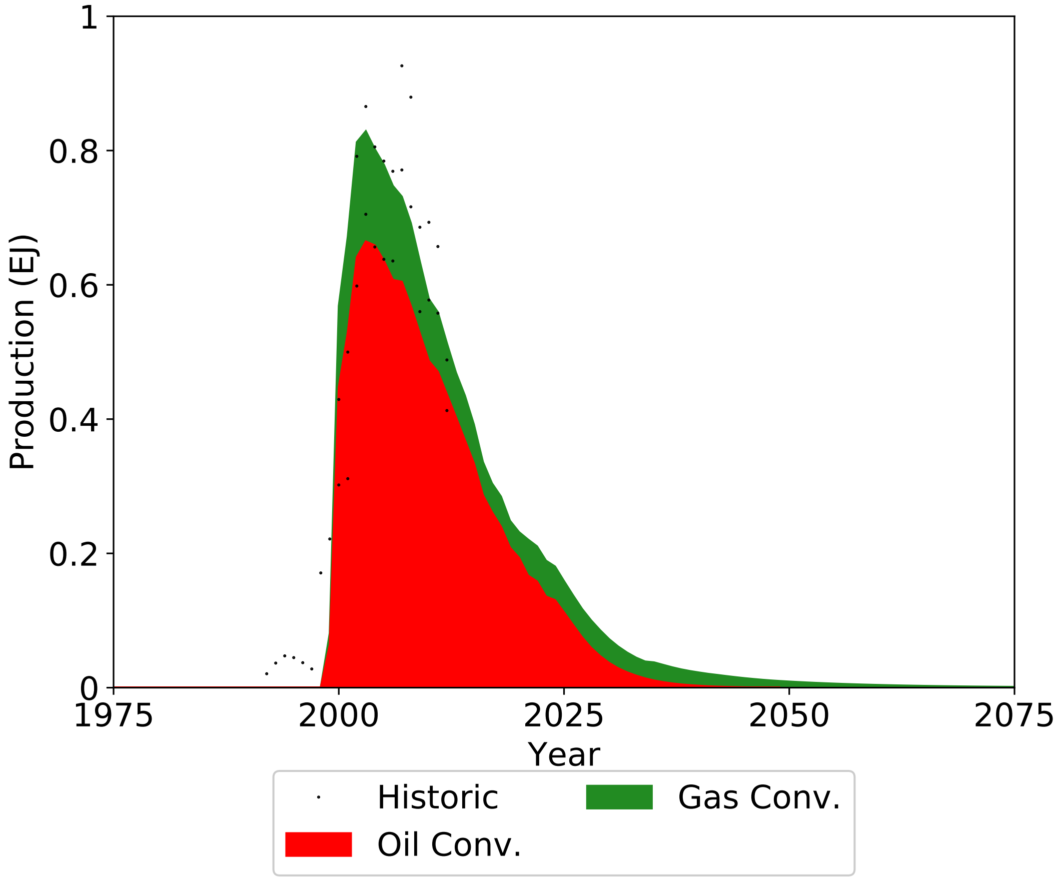

Figure 6.8: Canada - East Coast Offshore projection by mineral type

Table 6.8: Peak years - Minerals

Name	URR	Peak Year	Peak Rate
Oil Conv.	10.78	2003	0.66
Gas Conv.	3.01	2002	0.17
**Total**	**13.79**	**2003**	**0.83**

Manitoba

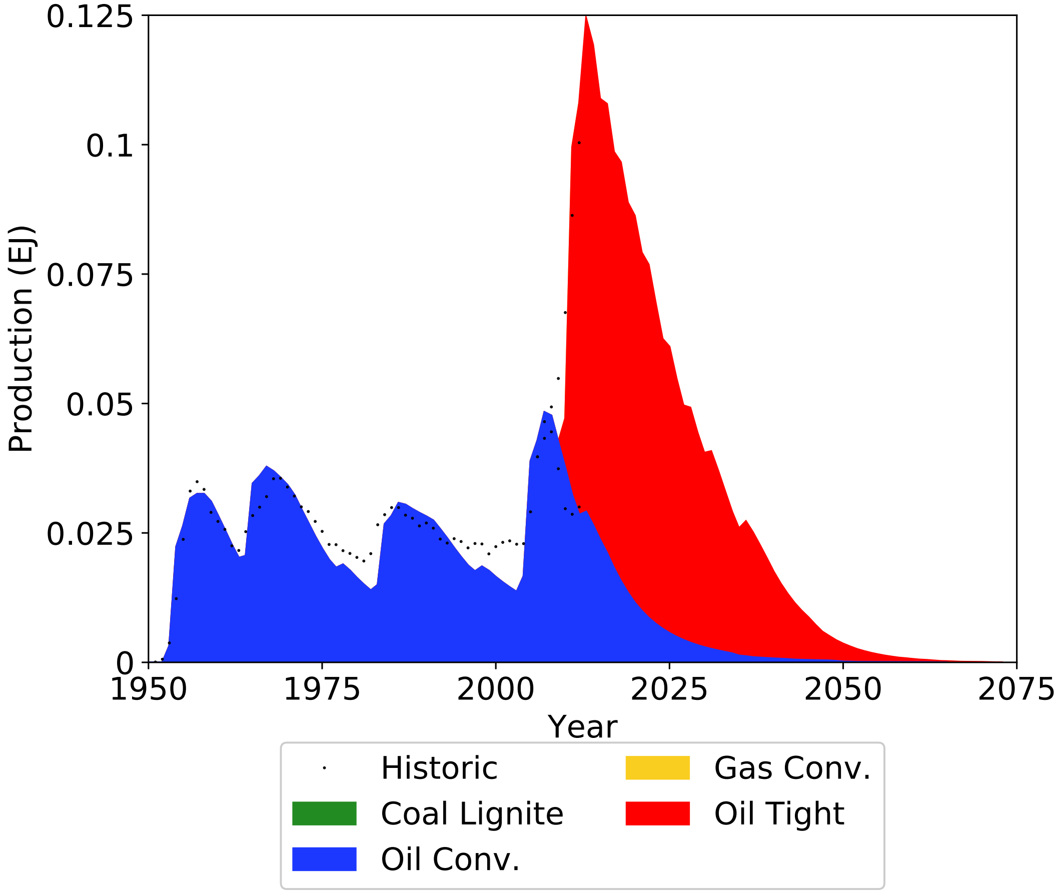

Figure 6.9: Canada - Manitoba projections capped at 16

Table 6.9: Peak years - All

Name	URR	Peak Year	Peak Rate
Oil Conv. Manitoba	1.81	2007	0.05
Oil Tight Manitoba	1.72	2013	0.1
Coal Lignite Manitoba	–	1935	–
Gas Conv. Manitoba	–	1929	–
**Total**	**3.53**	**2013**	**0.12**

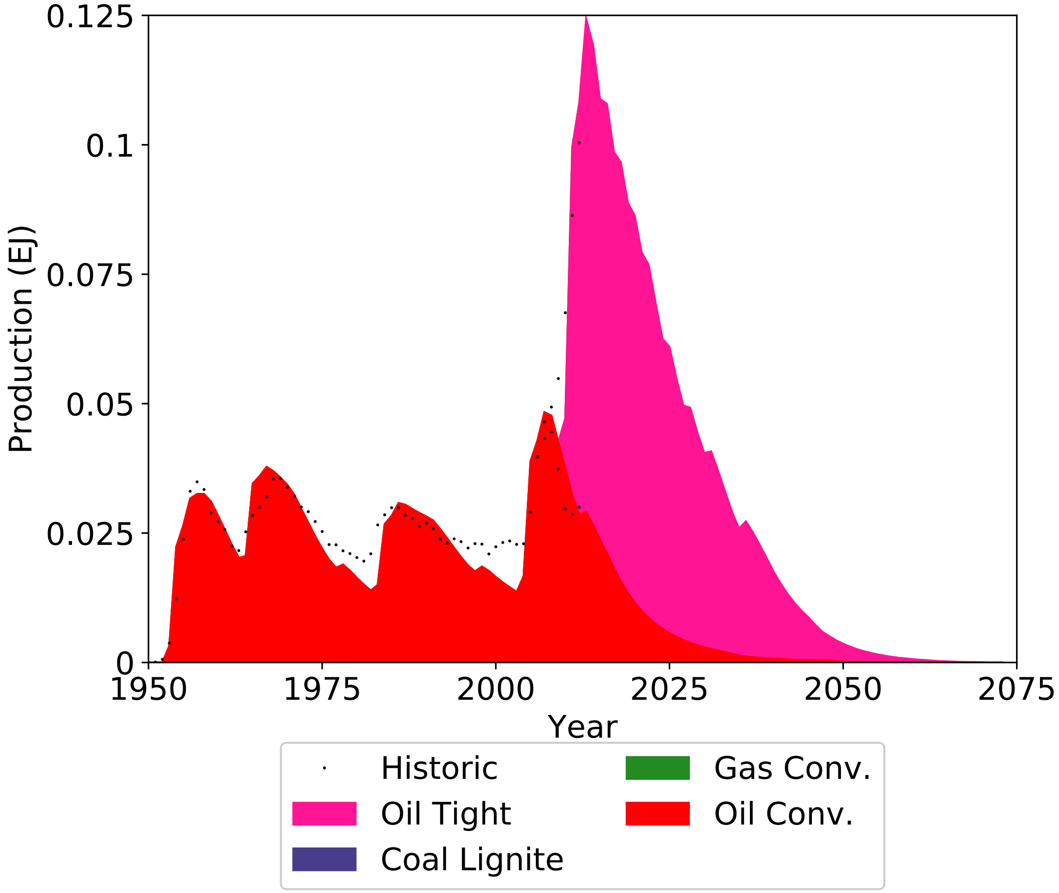

Figure 6.10: Canada - Manitoba projection by mineral type

Table 6.10: Peak years - Minerals

Name	URR	Peak Year	Peak Rate
Coal Lignite	–	1935	–
Oil Conv.	1.81	2007	0.05
Oil Tight	1.72	2013	0.1
Gas Conv.	–	1929	–
**Total**	**3.53**	**2013**	**0.12**

New Brunswick

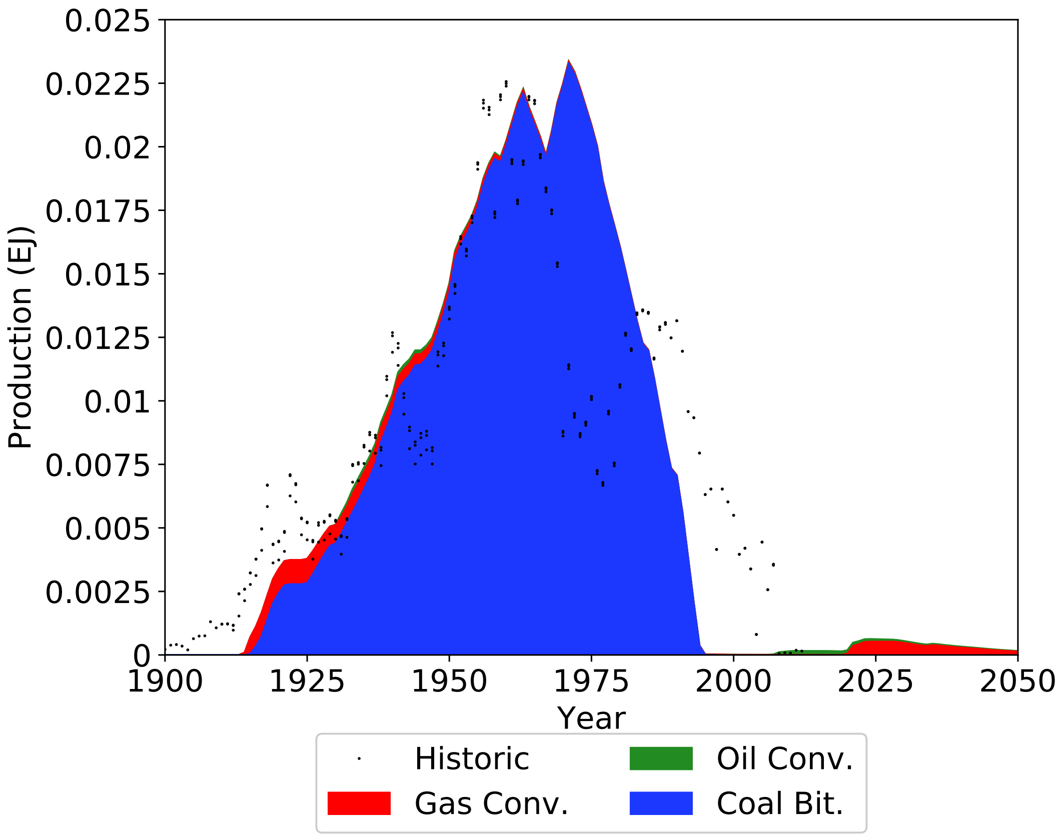

Figure 6.11: Canada - New Brunswick projections capped at 16

Table 6.11: Peak years - All

Name	URR	Peak Year	Peak Rate
Coal Bit. New Brunswick	0.94	1971	0.02
Gas Conv. New Brunswick	0.04	1918	–
Oil Conv. New Brunswick	0.01	1934	–
**Total**	**0.99**	**1971**	**0.02**

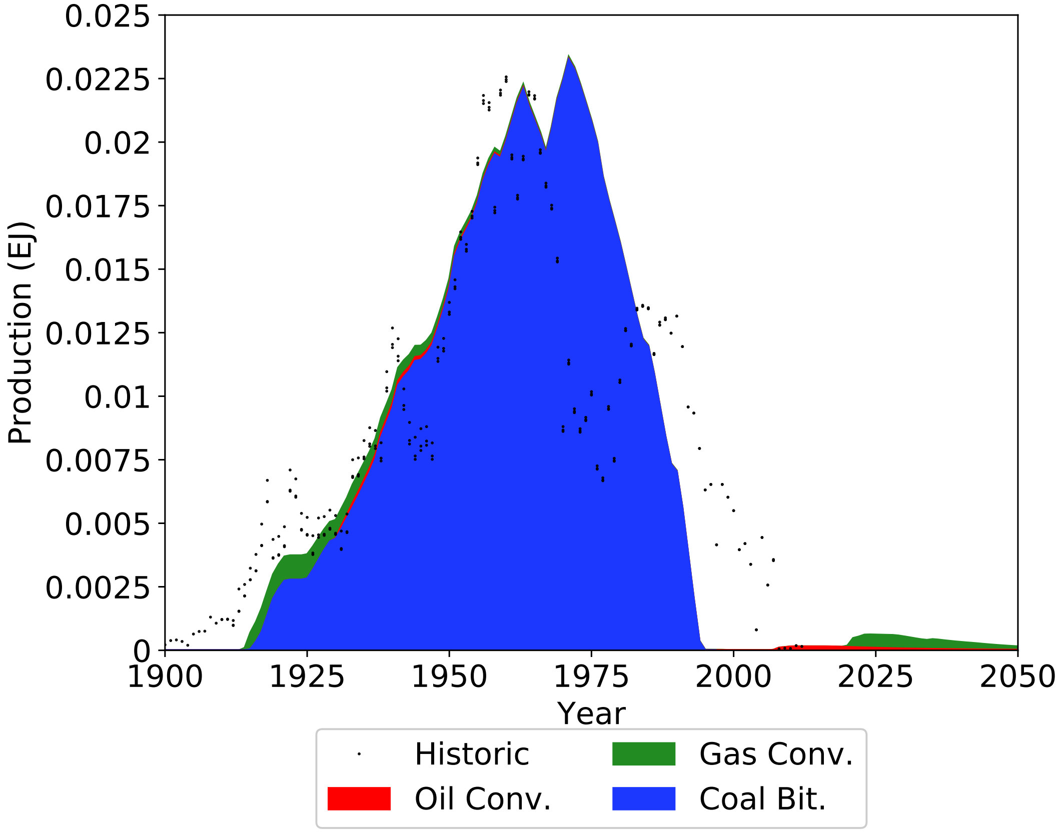

Figure 6.12: Canada - New Brunswick projection by mineral type

Table 6.12: Peak years - Minerals

Name	URR	Peak Year	Peak Rate
Coal Bit.	0.94	1971	0.02
Oil Conv.	0.01	1934	–
Gas Conv.	0.04	1918	–
**Total**	**0.99**	**1971**	**0.02**

Northwest Territories

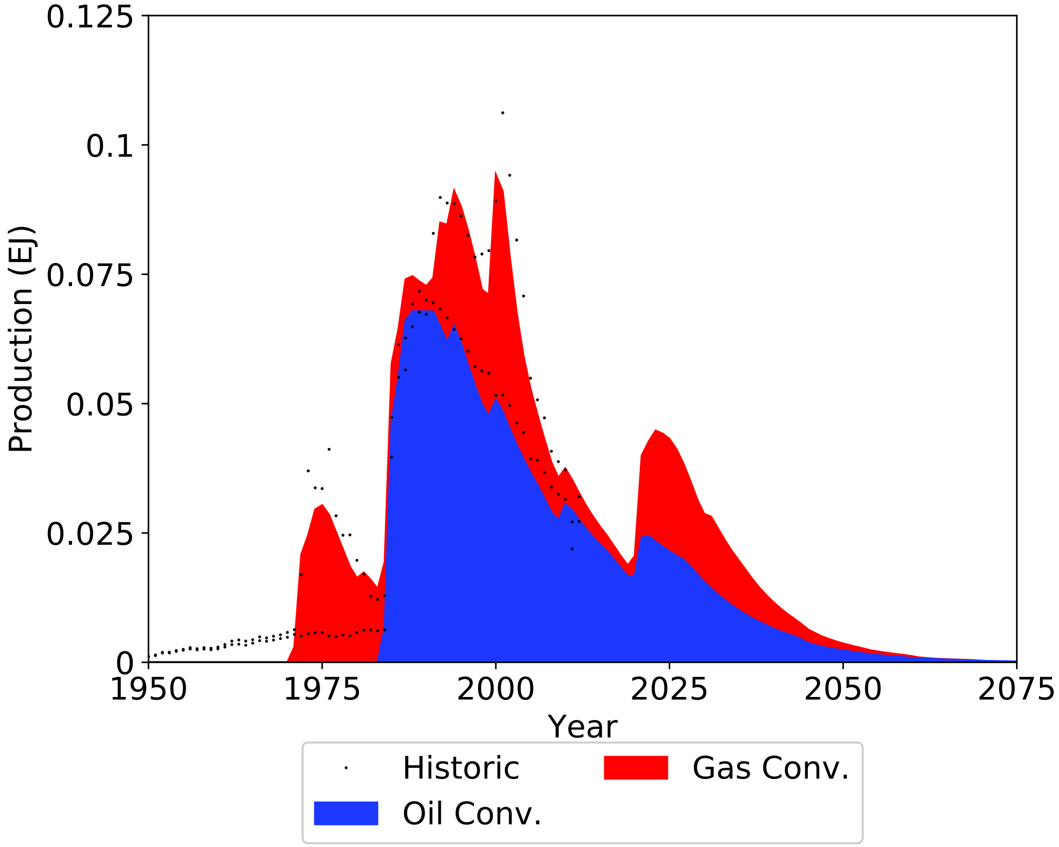

Figure 6.13: Canada - Northwest Territories projections capped at 16

Table 6.13: Peak years - All

Name	URR	Peak Year	Peak Rate
Oil Conv. Northwest Territories	1.91	1988	0.07
Gas Conv. Northwest Territories	1.1	2000	0.04
**Total**	**3.01**	**2000**	**0.09**

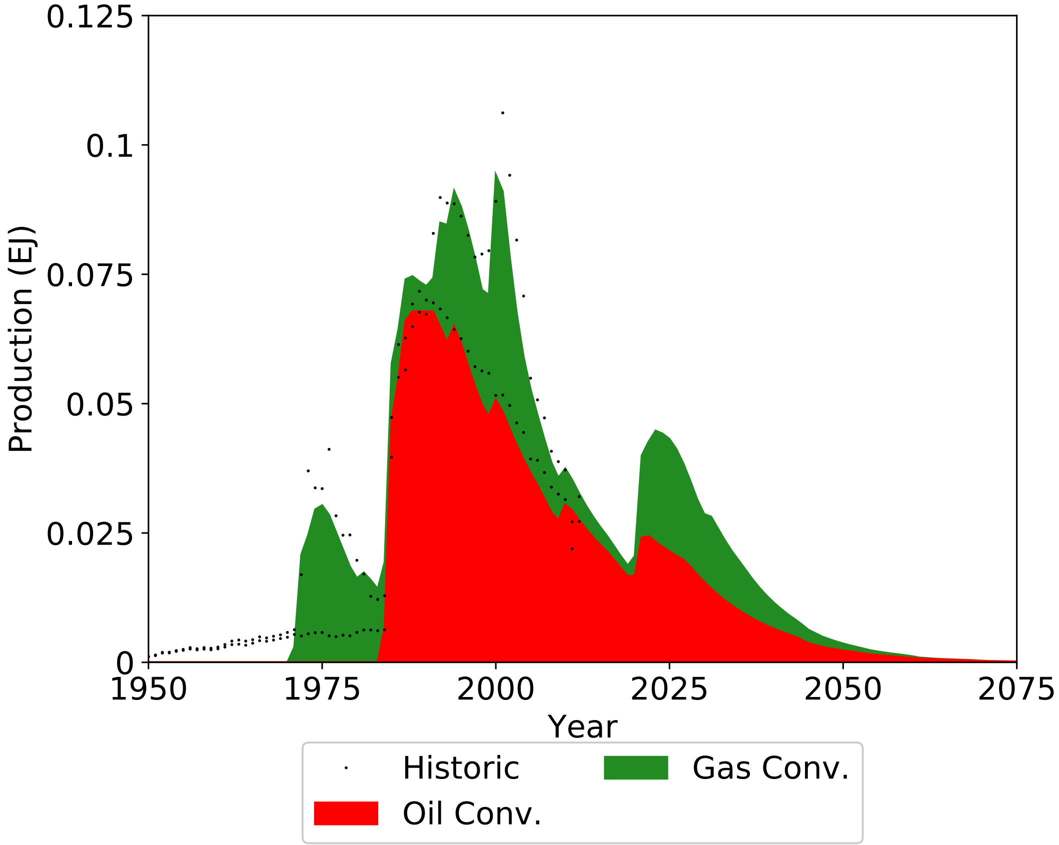

Figure 6.14: Canada - Northwest Territories projection by mineral type

Table 6.14: Peak years - Minerals

Name	URR	Peak Year	Peak Rate
Oil Conv.	1.91	1988	0.07
Gas Conv.	1.1	2000	0.04
**Total**	**3.01**	**2000**	**0.09**

Nova Scotia

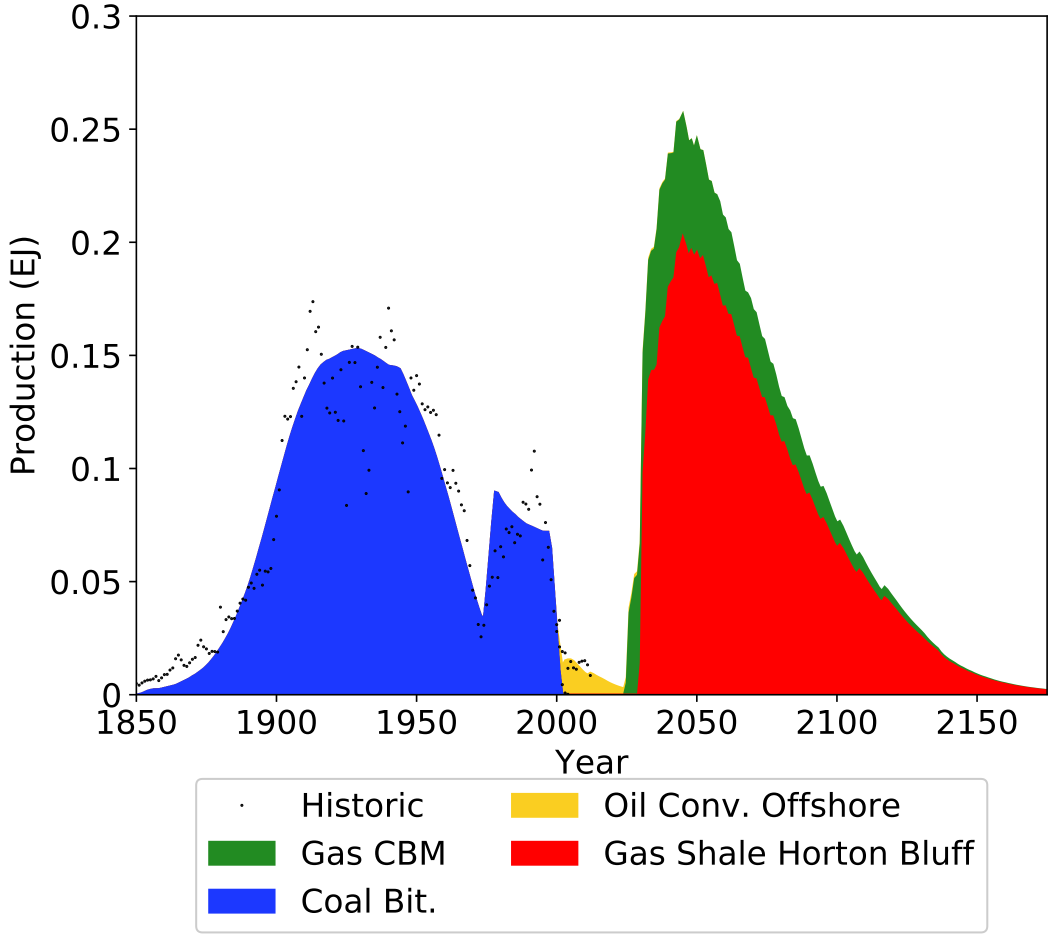

Figure 6.15: Canada - Nova Scotia projections capped at 16

Table 6.15: Peak years - All

Name	URR	Peak Year	Peak Rate
Coal Bit. Nova Scotia	12.07	1929	0.15
Gas Shale Nova Scotia Horton Bluff	11.55	2045	0.2
Gas CBM Nova Scotia	2.86	2037	0.06
Oil Conv. Nova Scotia Offshore	0.24	2004	0.02
**Total**	**26.72**	**2045**	**0.26**

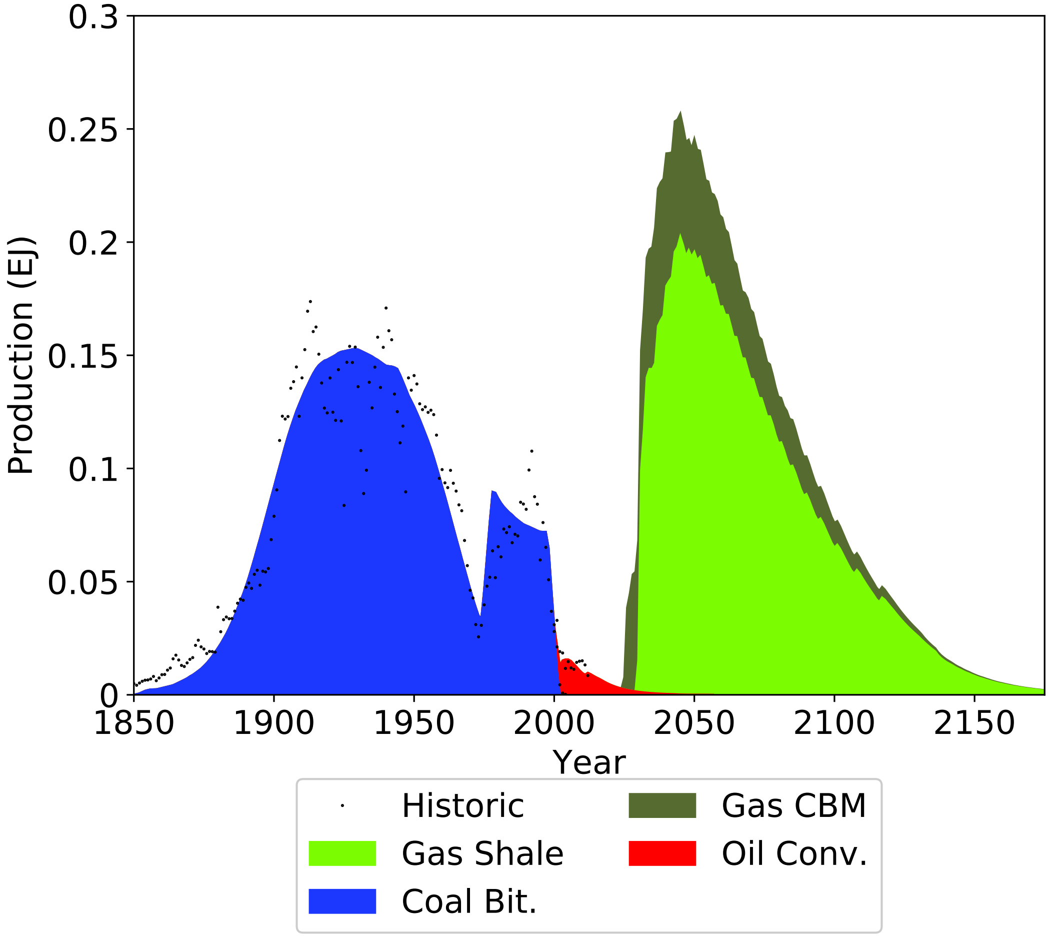

Figure 6.16: Canada - Nova Scotia projection by mineral type

Table 6.16: Peak years - Minerals

Name	URR	Peak Year	Peak Rate
Coal Bit.	12.07	1929	0.15
Oil Conv.	0.24	2004	0.02
Gas Shale	11.55	2045	0.2
Gas CBM	2.86	2037	0.06
**Total**	**26.72**	**2045**	**0.26**

Ontario

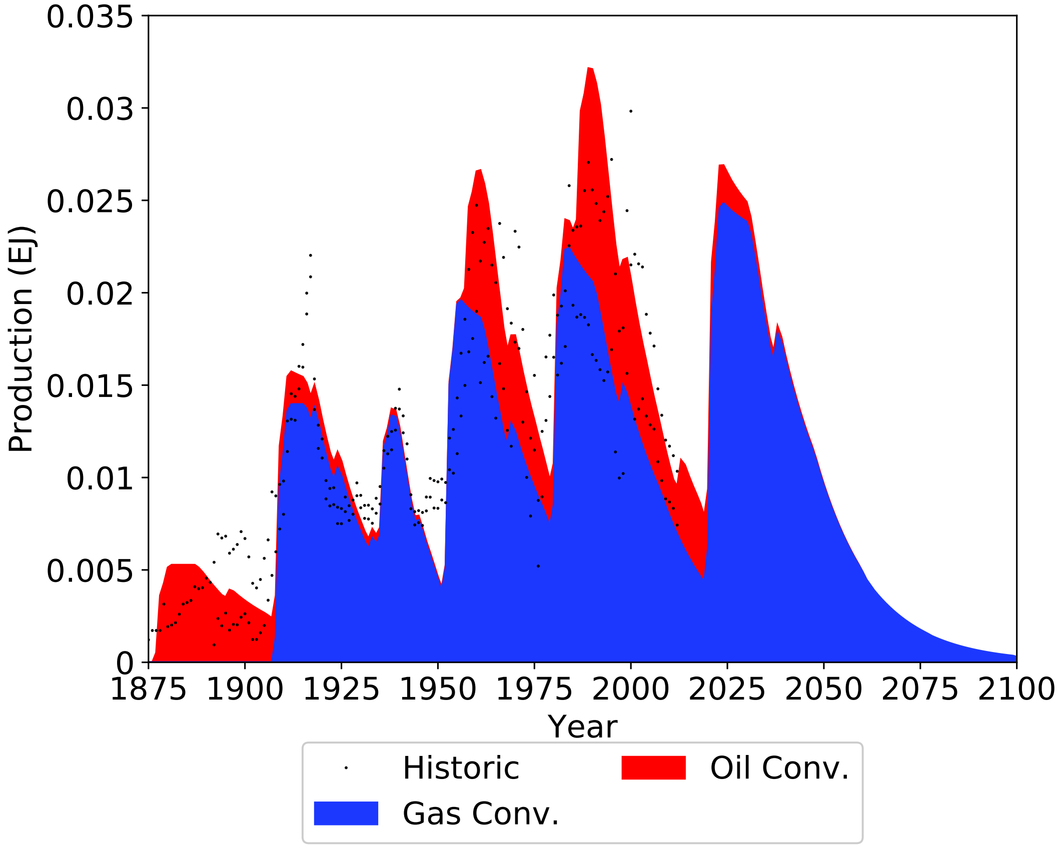

Figure 6.17: Canada - Ontario projections capped at 16

Table 6.17: Peak years - All

Name	URR	Peak Year	Peak Rate
Gas Conv. Ontario	2.03	2024	0.02
Oil Conv. Ontario	0.53	1990	0.01
**Total**	**2.56**	**1989**	**0.03**

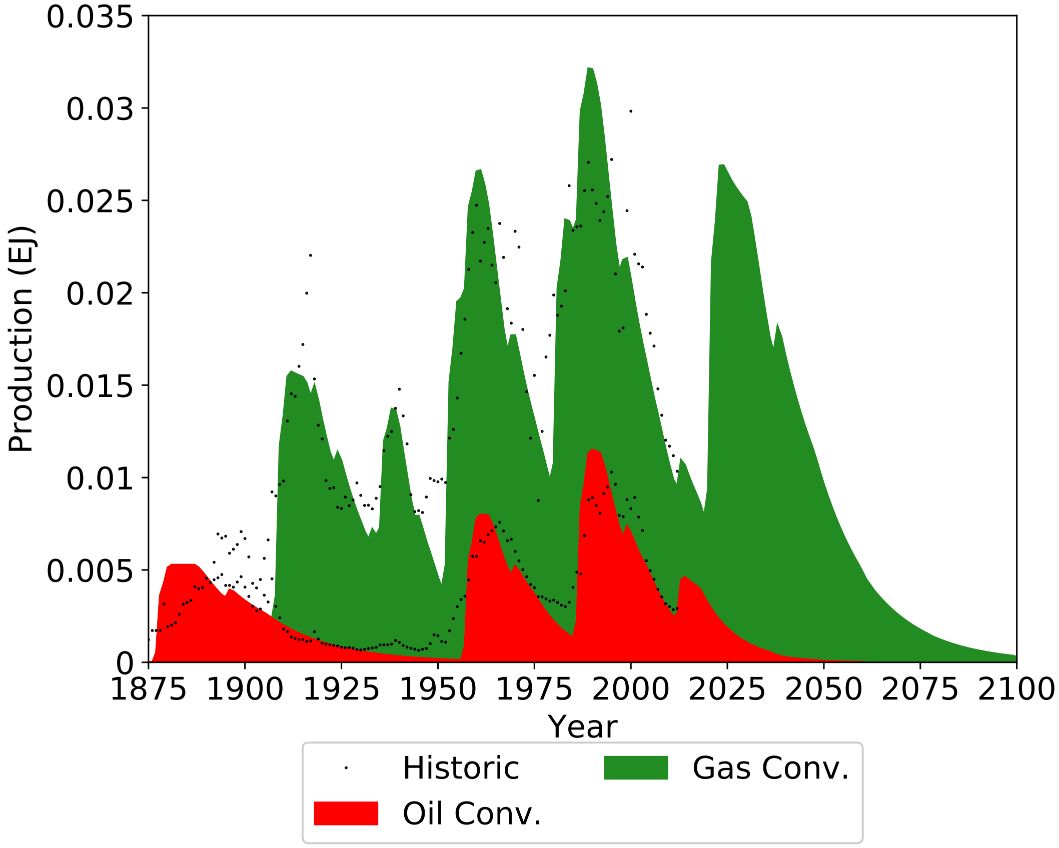

Figure 6.18: Canada - Ontario projection by mineral type

Table 6.18: Peak years - Minerals

Name	URR	Peak Year	Peak Rate
Oil Conv.	0.53	1990	0.01
Gas Conv.	2.03	2024	0.02
**Total**	**2.56**	**1989**	**0.03**

Quebec

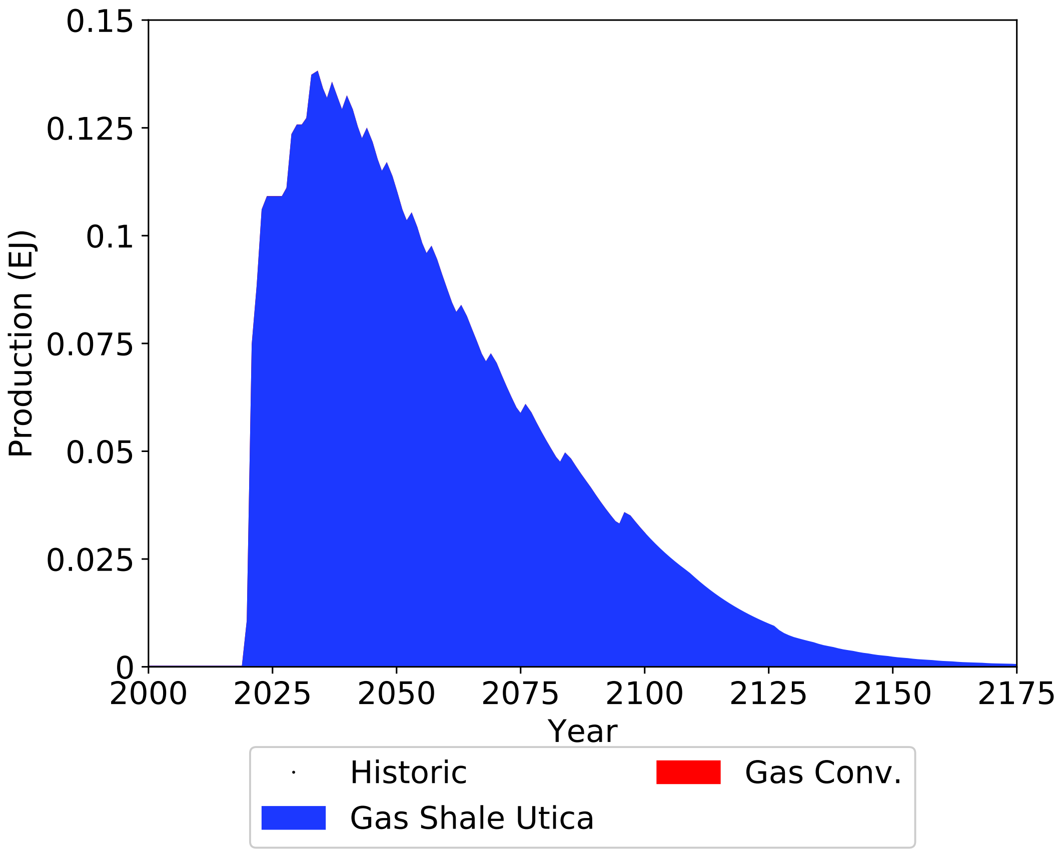

Figure 6.19: Canada - Quebec projections capped at 16

Table 6.19: Peak years - All

Name	URR	Peak Year	Peak Rate
Gas Shale Quebec Utica	7.35	2034	0.14
Gas Conv. Quebec	–	1970	–
**Total**	**7.35**	**2034**	**0.14**

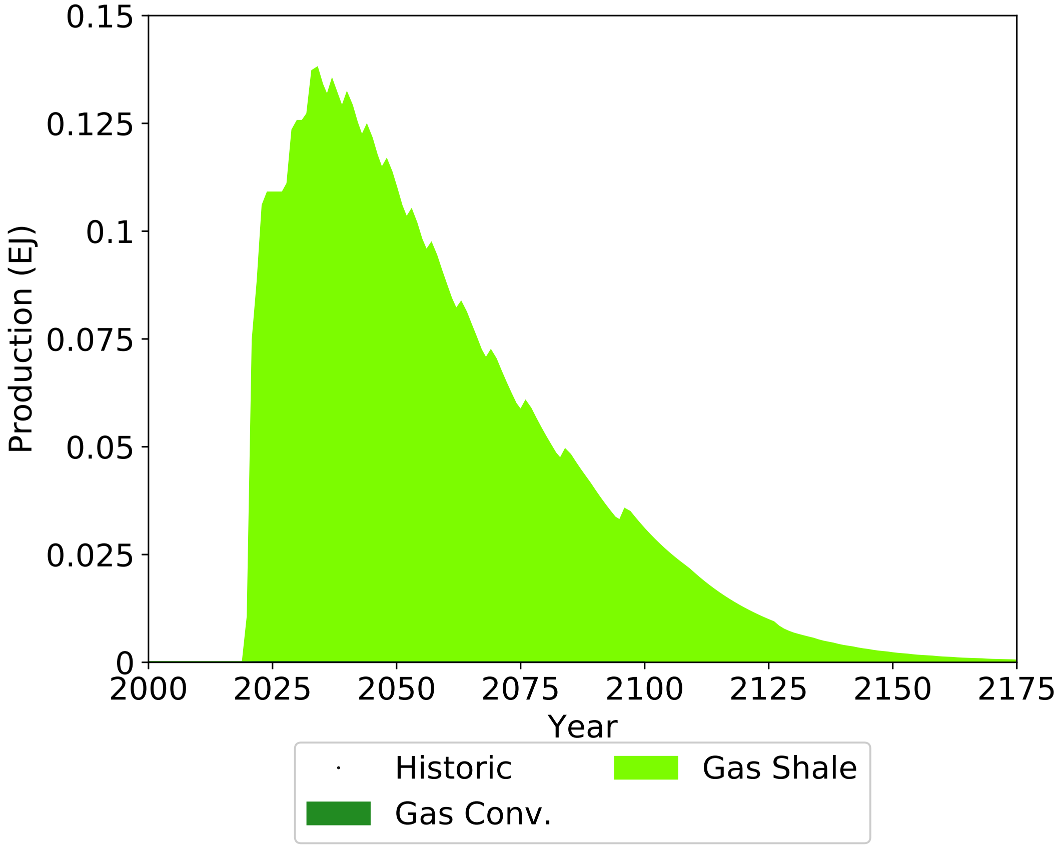

Figure 6.20: Canada - Quebec projection by mineral type

Table 6.20: Peak years - Minerals

Name	URR	Peak Year	Peak Rate
Gas Conv.	–	1970	–
Gas Shale	7.35	2034	0.14
**Total**	**7.35**	**2034**	**0.14**

Saskatchewan

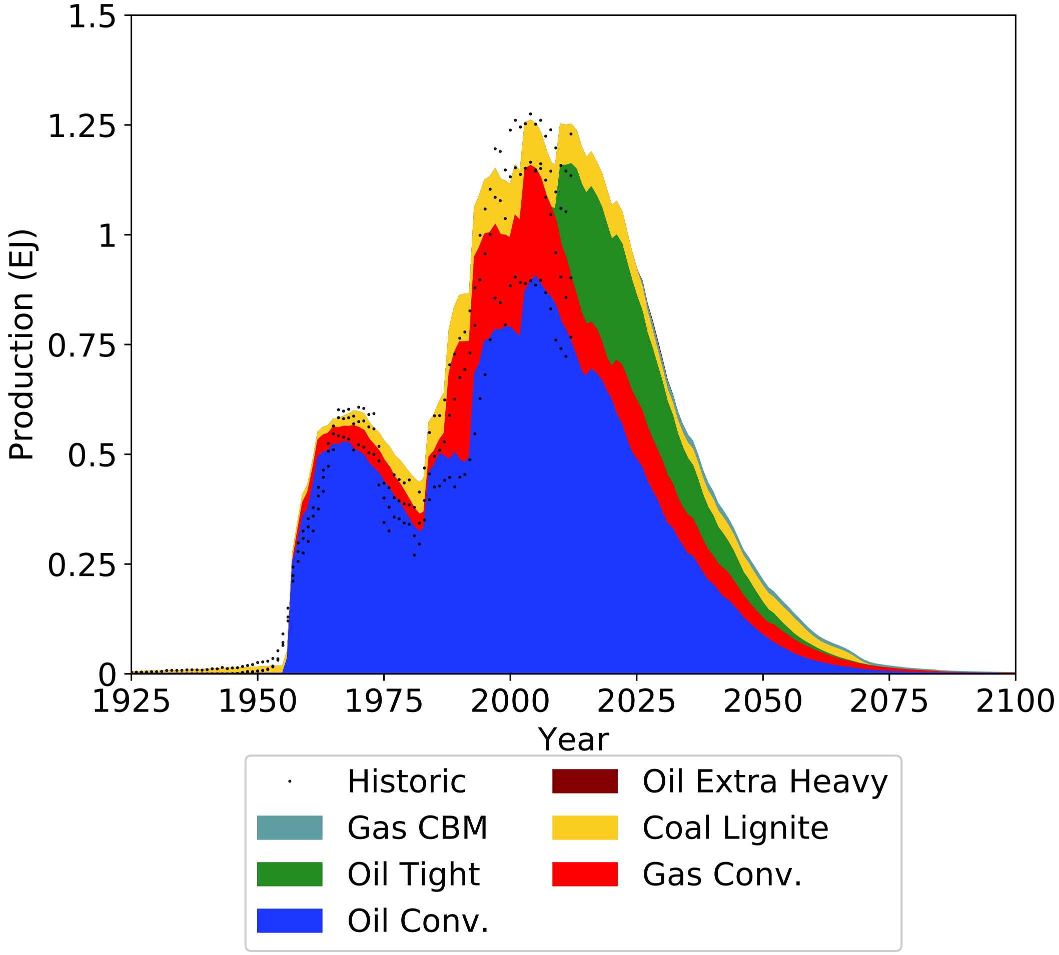

Figure 6.21: Canada - Saskatchewan projections capped at 16

Table 6.21: Peak years - All

Name	URR	Peak Year	Peak Rate
Oil Conv. Saskatchewan	46.99	2005	0.9
Gas Conv. Saskatchewan	11.19	2003	0.28
Oil Tight Saskatchewan	7.45	2016	0.31
Coal Lignite Saskatchewan	6.75	1997	0.13
Gas CBM Saskatchewan	0.71	2029	0.02
Oil Extra Heavy Saskatchewan	0.02	2029	0.01
**Total**	**73.11**	**2004**	**1.26**

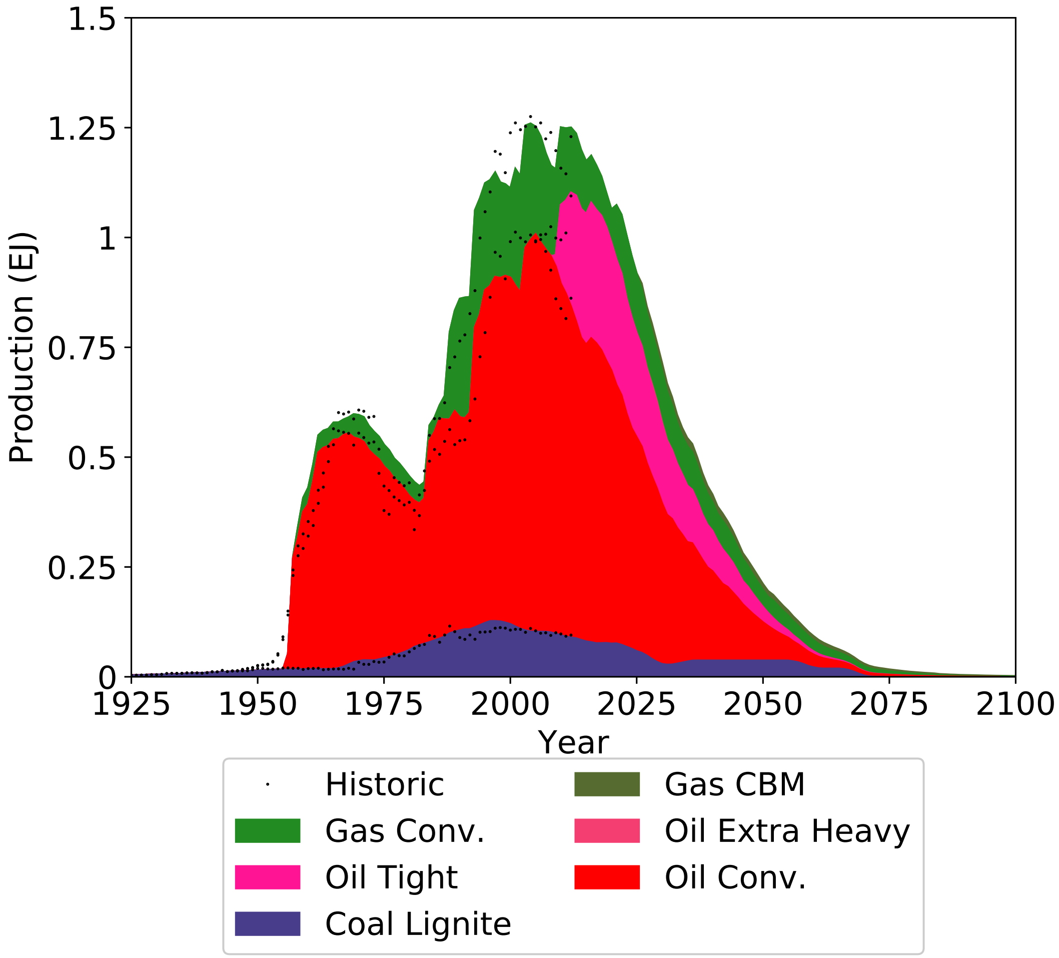

Figure 6.22: Canada - Saskatchewan projection by mineral type

Table 6.22: Peak years - Minerals

**Name**	**URR**	**Peak Year**	**Peak Rate**
Coal Lignite	6.75	1997	0.13
Oil Conv.	46.99	2005	0.9
Oil Tight	7.45	2016	0.31
Oil Extra Heavy	0.02	2029	0.01
Gas Conv.	11.19	2003	0.28
Gas CBM	0.71	2029	0.02
**Total**	**73.11**	**2004**	**1.26**

Yukon

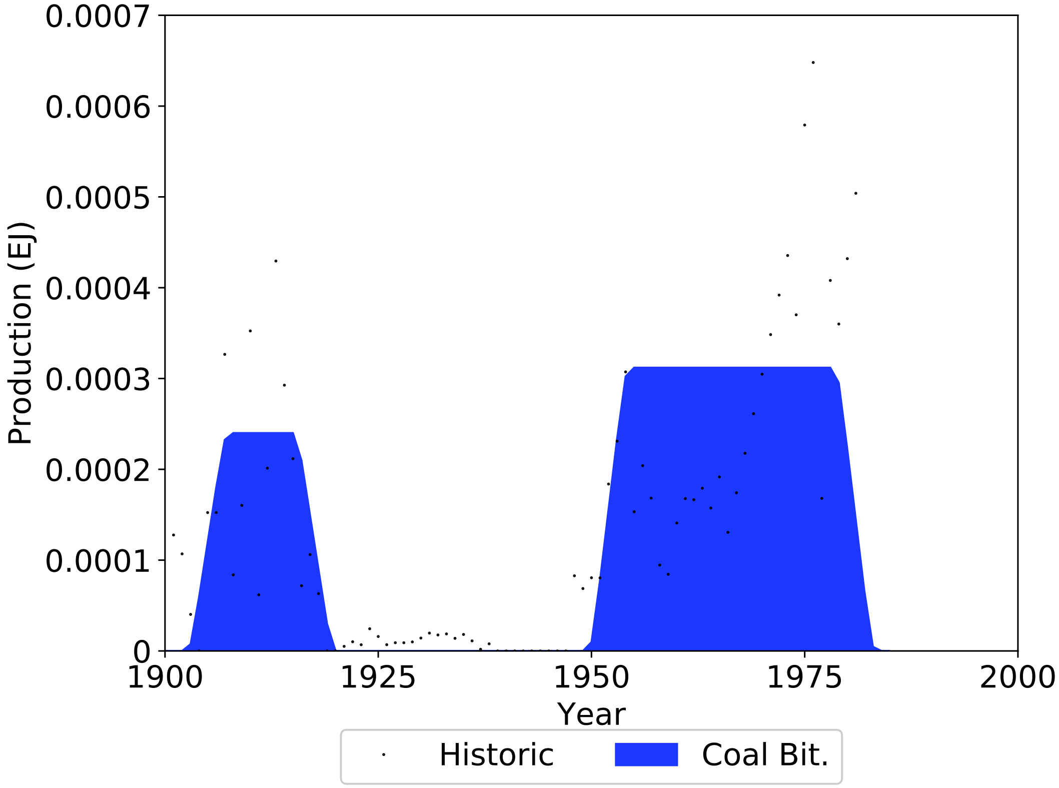

Figure 6.23: Canada - Yukon projections capped at 16

Table 6.23: Peak years - All

Name	URR	Peak Year	Peak Rate
Coal Bit. Yukon	0.01	1955	–
**Total**	**0.01**	**1955**	–

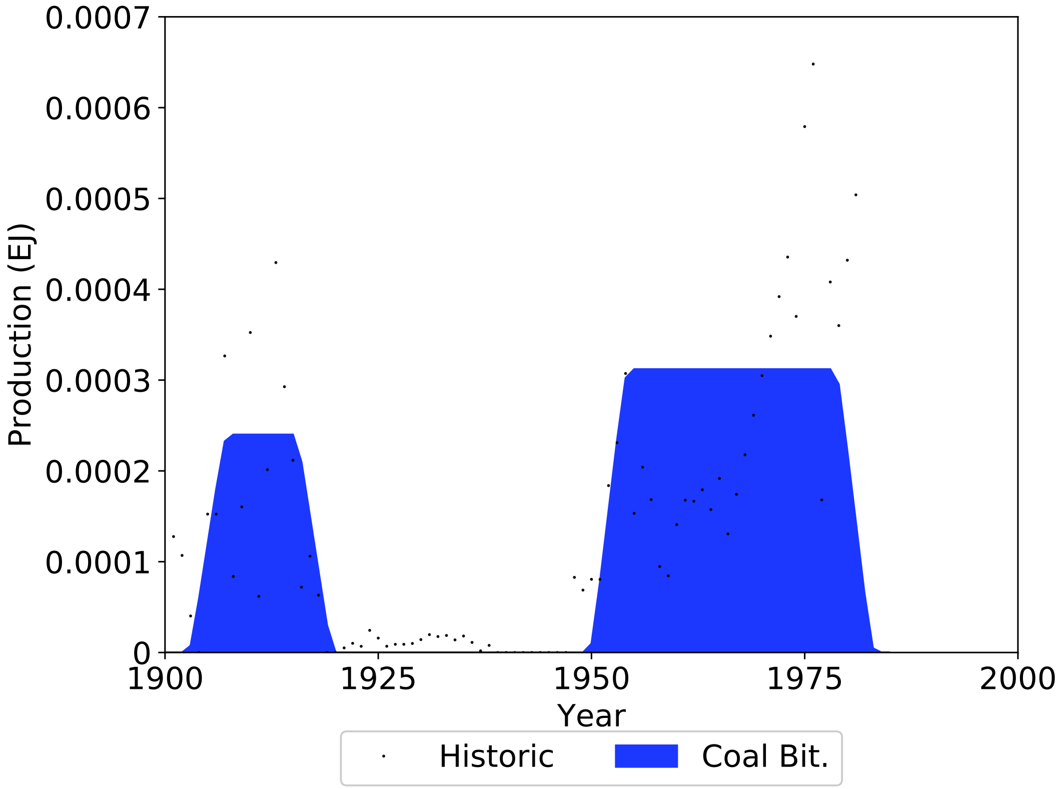

Figure 6.24: Canada - Yukon projection by mineral type

Table 6.24: Peak years - Minerals

Name	URR	Peak Year	Peak Rate
Coal Bit.	0.01	1955	–
**Total**	**0.01**	**1955**	–

6.1.4 Projection by region

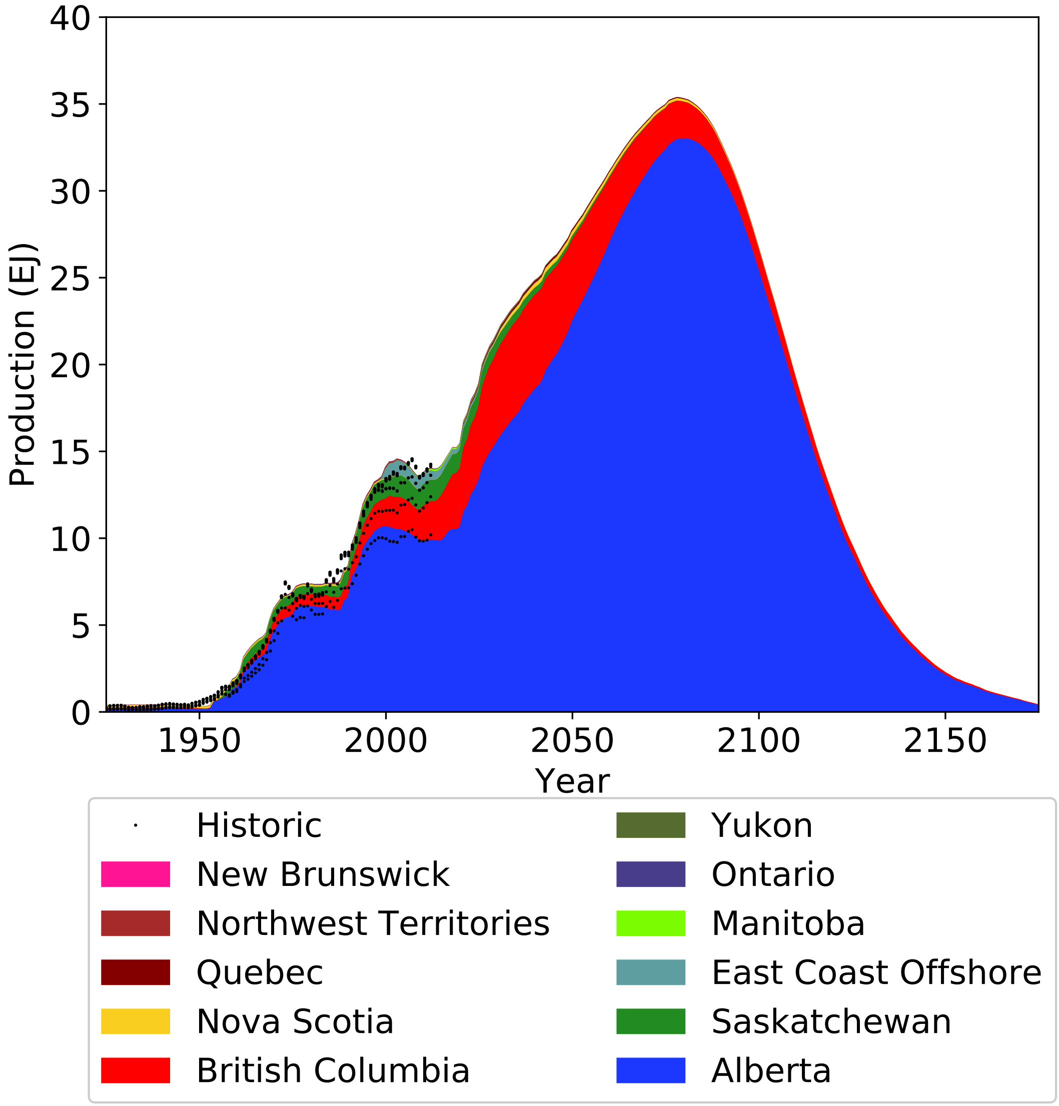

Figure 6.25: Canada by region projections capped at 16

Table 6.25: Peak years - All

Name	URR	Peak Year	Peak Rate
Alberta	2989.95	2080	32.96
British Columbia	387.58	2036	5.44
Saskatchewan	73.11	2004	1.26
Nova Scotia	26.72	2045	0.26
East Coast Offshore	13.79	2003	0.83
Quebec	7.35	2034	0.14
Manitoba	3.53	2013	0.12
Northwest Territories	3.01	2000	0.09
Ontario	2.56	1989	0.03
New Brunswick	0.99	1971	0.02
Yukon	0.01	1955	–
**Total**	**3508.61**	**2078**	**35.34**

6.2 USA

6.2.1 All Projections

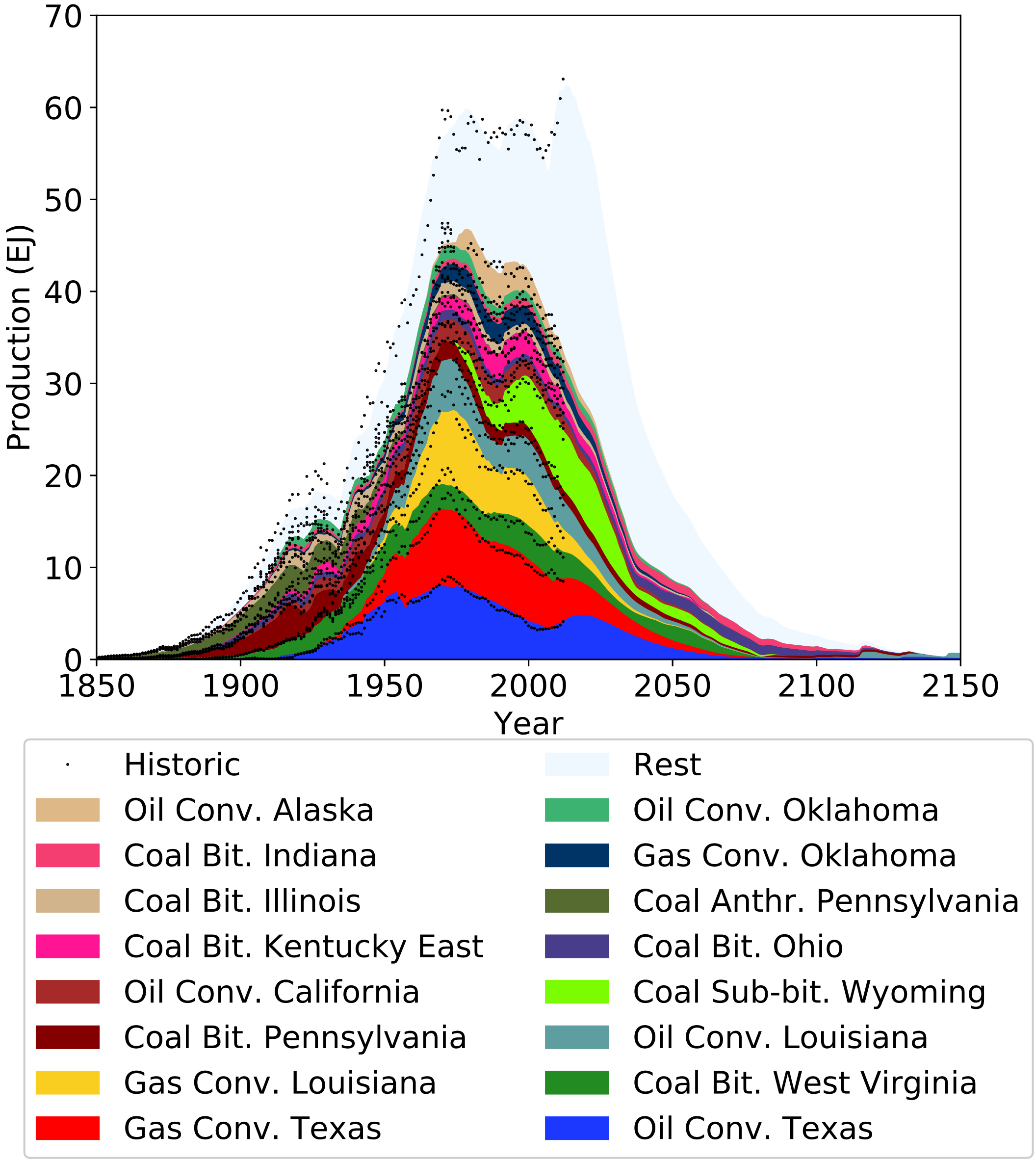

Figure 6.26: USA projections capped at 16

Table 6.26: Peak years - All

**Name**	**URR**	**Peak Year**	**Peak Rate**
Oil Conv. Texas	603.57	1970	7.87
Gas Conv. Texas	526.44	1973	8.4
Coal Bit. West Virginia	389.11	1997	3.64
Gas Conv. Louisiana	344.58	1974	8.36
Oil Conv. Louisiana	321.88	1972	5.74
Coal Bit. Pennsylvania	303.54	1917	3.91
Coal Sub-bit. Wyoming	297.1	2011	6.62
Oil Conv. California	198.31	1969	2.35
Coal Bit. Ohio	179.97	2059	1.27
Coal Bit. Kentucky East	175.42	1991	2.71
Coal Anthr. Pennsylvania	153.01	1919	2.73
Coal Bit. Illinois	151.45	1921	1.82
Gas Conv. Oklahoma	143.52	1985	2.33
Coal Bit. Indiana	132.5	2046	1.06
Oil Conv. Oklahoma	129.07	1966	1.54
Oil Conv. Alaska	119.01	1987	3.96
Oil Tight North Dakota Bakken	87.48	2022	2.27
Oil Conv. New Mexico	82.6	2021	0.96
Gas Conv. New Mexico	81.47	1975	1.3
Oil Tight Texas Eagle Ford	79.38	2024	2.21
Coal Bit. Kentucky West	78.3	1981	0.97
Oil Conv. Wyoming	76.56	1972	0.92
Gas Conv. Colorado	63.0	2019	1.33
Coal Bit. Virginia	62.26	1975	0.84
Gas Conv. Wyoming	62.21	2012	1.67
Coal Bit. Alabama	61.43	1990	0.58
Oil Tight Texas Permian	61.13	2021	1.6
Oil Conv. Kansas	57.63	1956	0.75
Oil Conv. Colorado	57.3	2023	0.9
Gas Conv. Kansas	47.12	1970	0.89
Gas Conv. California	44.43	1965	0.76
Coal Sub-bit. Montana	38.52	1993	0.59
Gas CBM New Mexico	38.36	1998	0.6
Gas CBM Colorado	38.03	2023	0.62
Gas Shale Pennsylvania Marcellus	32.87	2015	1.87
Gas Conv. Pennsylvania	31.5	2021	0.35
Coal Bit. Colorado	29.21	2002	0.63
Oil Nat. Bitumen Utah	27.84	2077	0.45
Coal Bit. Utah	27.03	1998	0.55
Gas CBM Virginia	26.85	2034	0.42
Gas Conv. Alaska	25.4	1996	0.53
Gas Conv. West Virginia	23.49	1967	0.24
Gas Shale Louisiana Haynesville	22.96	2013	2.08
Gas CBM Wyoming	22.23	2013	0.56
Oil Conv. Illinois	21.98	1950	0.49
Coal Lignite Texas	21.9	1992	0.48
Gas Conv. Utah	21.0	2013	0.44
Oil Conv. North Dakota	20.8	1983	0.32
Gas Shale Texas Barnett	20.32	2012	1.83

Table 6.26: Peak years - All – Continued

**Name**	**URR**	**Peak Year**	**Peak Rate**
Oil Conv. Mississippi	20.09	1964	0.37
Oil Conv. Utah	17.19	2015	0.3
Coal Bit. Tennessee	16.08	1968	0.19
Gas Conv. Kentucky	15.75	2034	0.17
Coal Bit. New Mexico	15.22	2022	0.36
Coal Lignite North Dakota	15.17	1993	0.26
Coal Bit. Arizona	14.62	2004	0.32
Oil Tight Ohio Utica	14.33	2022	0.62
Gas Shale Texas Eagle Ford	13.8	2017	0.83
Coal Sub-bit. New Mexico	13.67	1998	0.26
Oil Nat. Bitumen Texas	13.0	2068	0.24
Oil Conv. Montana	12.51	2006	0.22
Oil Tight Wyoming Niobrara	12.26	2024	0.36
Oil Tight Colorado Niobrara	12.26	2024	0.36
Oil Conv. Arkansas	11.62	1923	0.39
Oil Conv. Michigan	11.37	1977	0.29
Gas Conv. Ohio	10.99	1983	0.18
Oil Tight Oklahoma Woodford	10.95	2020	0.47
Gas Conv. Arkansas	10.85	1992	0.23
Gas Shale West Virginia Marcellus	10.35	2016	0.51
Gas Conv. Mississippi	10.15	1955	0.19
Gas Shale Oklahoma Woodford	10.13	2013	0.49
Oil Conv. Alabama	10.07	1984	0.17
Gas CBM Alabama	9.82	2029	0.19
Oil Conv. Kentucky	9.65	2020	0.13
Coal Bit. Maryland	9.63	1907	0.13
Gas Conv. Alabama	9.62	1996	0.48
Coal Bit. Missouri	9.53	1941	0.12
Gas Shale Arkansas Fayetteville	8.81	2010	0.95
Oil Conv. Pennsylvania	8.41	1882	0.14
Coal Bit. Iowa	8.14	1914	0.15
Gas Shale Texas Haynesville	7.94	2014	0.37
Coal Bit. Oklahoma	7.62	1914	0.1
Oil Conv. Ohio	7.08	1899	0.12
Coal Sub-bit. Colorado	6.9	2006	0.13
Coal Bit. Kansas	6.64	1916	0.14
Oil Conv. West Virginia	6.6	1902	0.08
Oil Nat. Bitumen Alabama	6.05	2054	0.1
Gas Shale Colorado Niobrara	6.0	2026	0.11
Gas Shale Ohio Utica	5.83	2029	0.12
Gas Shale New York Utica	5.83	2034	0.12
Oil Tight California Monterey	5.62	2016	0.23
Oil Nat. Bitumen Kentucky	5.34	2057	0.11
Gas Conv. Michigan	5.25	1998	0.15
Oil Conv. Florida	5.13	1976	0.35
Gas Shale Michigan Antrim	4.67	1996	0.24
Gas Shale Texas Permian	4.59	2010	0.59
Coal Sub-bit. Washington	4.57	1975	0.07
Gas Conv. Montana	4.21	2004	0.09

Table 6.26: Peak years - All – Continued

**Name**	**URR**	**Peak Year**	**Peak Rate**
Gas CBM Utah	3.99	2005	0.09
Oil Conv. Indiana	3.53	1956	0.07
Oil Nat. Bitumen California	3.31	2054	0.08
Oil Conv. Nebraska	3.27	1959	0.15
Gas Conv. North Dakota	3.09	1990	0.07
Gas Shale New York Other	3.05	2036	0.06
Gas CBM Oklahoma	2.69	2006	0.07
Gas CBM Kansas	2.69	2023	0.07
Gas Conv. Virginia	2.56	1997	0.08
Oil Tight Texas Barnett	2.44	2018	0.07
Coal Lignite Louisiana	2.34	2028	0.05
Coal Bit. Arkansas	2.33	1935	0.05
Oil Tight West Virginia Marcellus	2.29	2022	0.1
Oil Tight Pennsylvania Marcellus	2.29	2022	0.1
Gas Conv. New York	1.8	2005	0.05
Gas CBM West Virginia	1.78	2023	0.04
Gas Shale North Dakota Bakken	1.77	2014	0.13
Oil Tight Texas Austin Chalk	1.75	1999	0.12
Coal Sub-bit. Alaska	1.7	2004	0.03
Oil Conv. New York	1.24	1941	0.04
Gas Conv. Indiana	1.05	1893	0.03
Gas Conv. Florida	1.05	1976	0.04
Coal Bit. Michigan	1.04	1902	0.03
Gas Shale New Mexico Permian	0.93	2025	0.02
Oil Tight Texas Granite Wash	0.89	2011	0.07
Oil Tight Oklahoma Granite Wash	0.89	2011	0.07
Oil Conv. South Dakota	0.59	2007	0.01
Gas Shale Montana Bakken	0.57	2021	0.01
Gas Conv. Illinois	0.56	1943	0.02
Gas CBM Pennsylvania	0.54	2024	0.01
Coal Lignite Mississippi	0.54	2005	0.03
Coal Bit. Wyoming	0.48	1999	0.07
Oil Conv. Nevada	0.35	1980	0.02
Gas CBM Montana	0.3	2004	0.01
Gas CBM Arkansas	0.29	2027	0.01
Coal Bit. Georgia	0.26	1895	0.01
Gas Shale Kentucky New Albany	0.23	2025	0.01
Oil Conv. Tennessee	0.22	1973	–
Gas Conv. Oregon	0.21	2019	0.01
Gas Conv. Tennessee	0.14	2008	0.01
Gas Conv. Nebraska	0.13	1969	0.01
Oil Conv. Arizona	0.13	1968	0.01
Coal Bit. Montana	0.11	2010	0.06
Gas Conv. South Dakota	0.11	1986	–
Coal Lignite Montana	0.11	1999	–
Oil Nat. Bitumen Wyoming	0.1	2035	0.01
Oil Conv. Missouri	0.07	2014	–
Coal Bit. Texas	0.05	1997	0.02
Gas Shale New Mexico Lewis	0.05	2017	–

Table 6.26: Peak years - All – Continued

**Name**	**URR**	**Peak Year**	**Peak Rate**
Gas Shale Colorado Lewis	0.05	2017	–
Gas Conv. Other	0.04	1887	–
Gas Conv. Arizona	0.03	1992	–
Coal Bit. Washington	0.02	1997	0.01
Coal Lignite South Dakota	0.01	1933	–
Gas Conv. Maryland	0.01	1969	–
Oil Conv. Virginia	0.01	1982	–
Gas CBM Louisiana	0.01	2008	–
Coal Anthr. Arkansas	–	1994	–
Gas Conv. Missouri	–	1903	–
Gas Conv. Nevada	–	1993	–
Oil Conv. Washington	–	1956	–
Gas Conv. Iowa	–	1919	–
**Total**	**6205.04**	**2013**	**62.27**

6.2.2 By Mineral

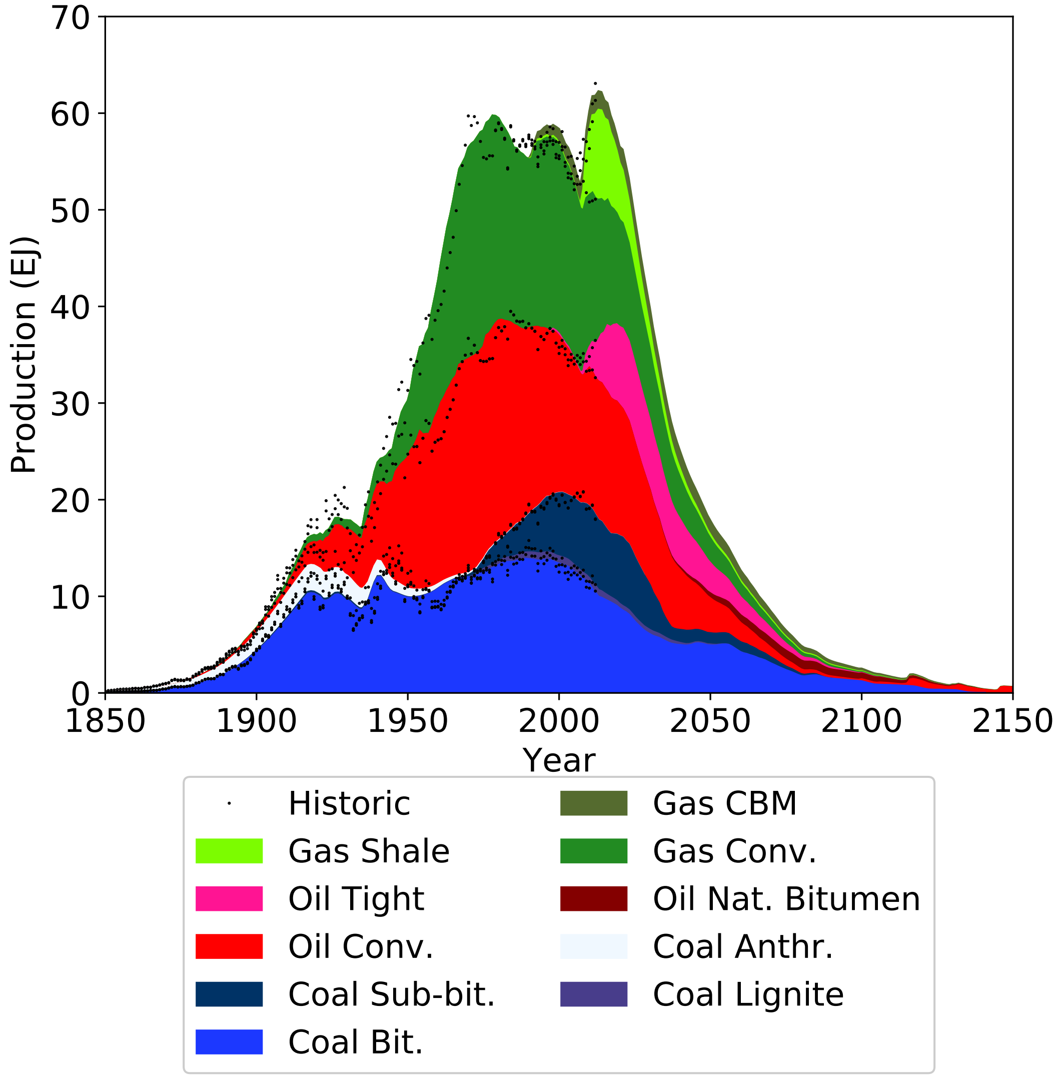

Figure 6.27: USA projection by mineral type

6.2.3 Regional Projections

Table 6.27: Peak years - Minerals

**Name**	**URR**	**Peak Year**	**Peak Rate**
Coal Bit.	1681.99	1989	13.88
Coal Lignite	40.07	2001	0.77
Coal Sub-bit.	362.46	2011	7.47
Coal Anthr.	153.01	1919	2.73
Oil Conv.	1817.83	1980	22.81
Oil Nat. Bitumen	55.64	2077	0.95
Oil Tight	293.96	2021	8.33
Gas Conv.	1491.76	1974	22.57
Gas Shale	160.75	2013	9.31
Gas CBM	147.58	2023	2.29
**Total**	**6205.04**	**2013**	**62.27**

Alabama

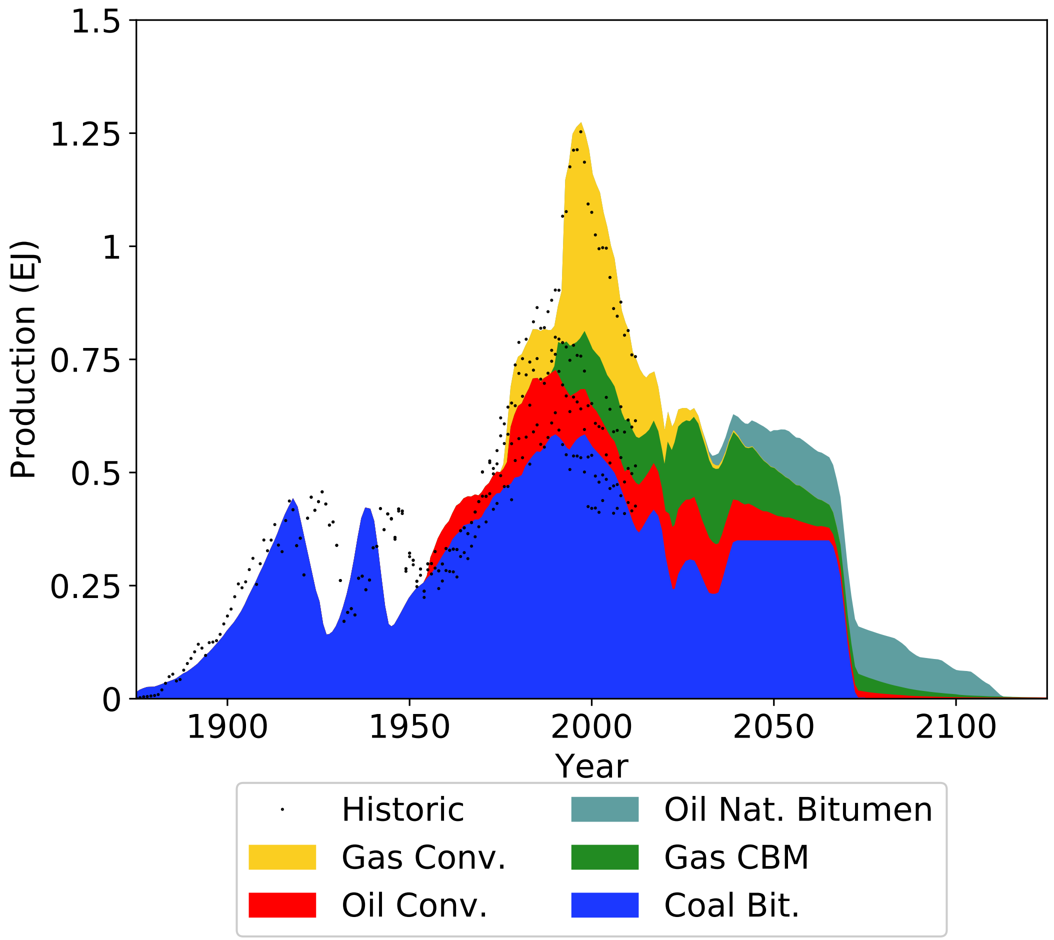

Figure 6.28: USA - Alabama projections capped at 16

Table 6.28: Peak years - All

Name	URR	Peak Year	Peak Rate
Coal Bit. Alabama	61.43	1990	0.58
Oil Conv. Alabama	10.07	1984	0.17
Gas CBM Alabama	9.82	2029	0.19
Gas Conv. Alabama	9.62	1996	0.48
Oil Nat. Bitumen Alabama	6.05	2054	0.1
**Total**	**96.99**	**1997**	**1.27**

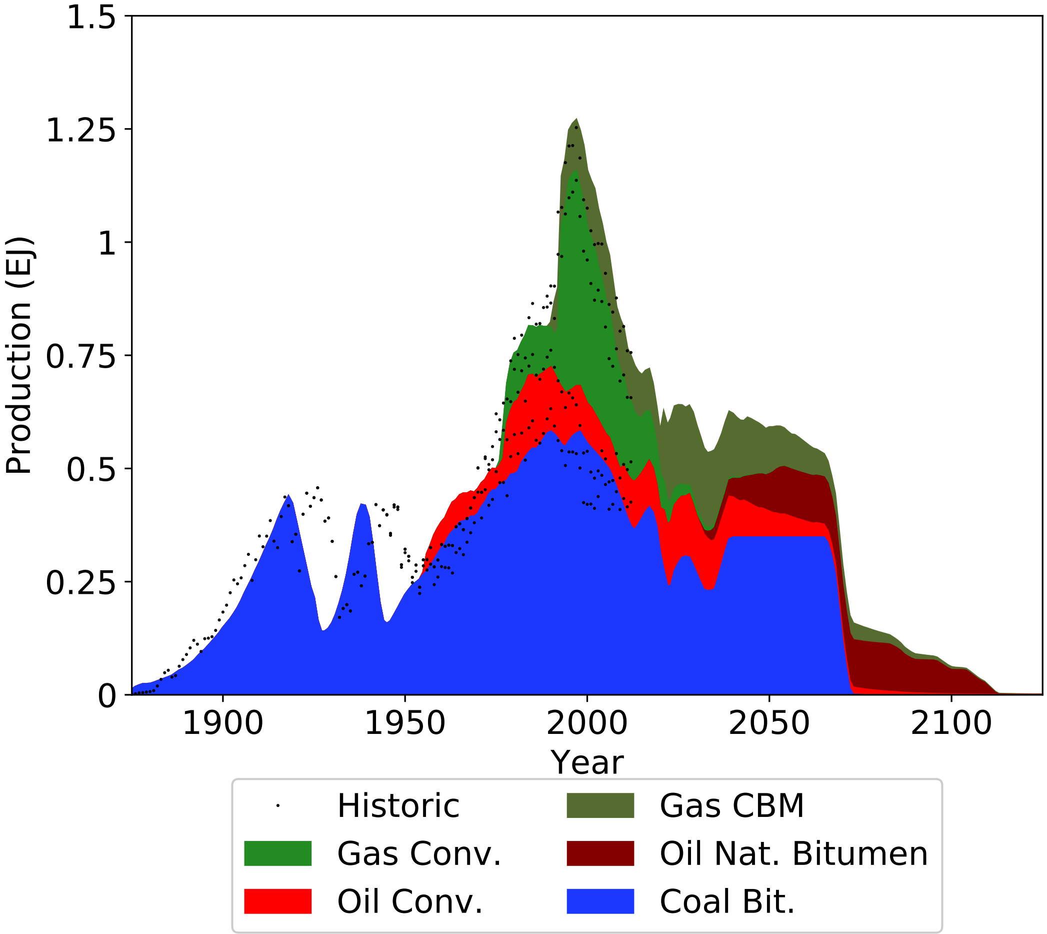

Figure 6.29: USA - Alabama projection by mineral type

Table 6.29: Peak years - Minerals

Name	URR	Peak Year	Peak Rate
Coal Bit.	61.43	1990	0.58
Oil Conv.	10.07	1984	0.17
Oil Nat. Bitumen	6.05	2054	0.1
Gas Conv.	9.62	1996	0.48
Gas CBM	9.82	2029	0.19
**Total**	**96.99**	**1997**	**1.27**

Alaska

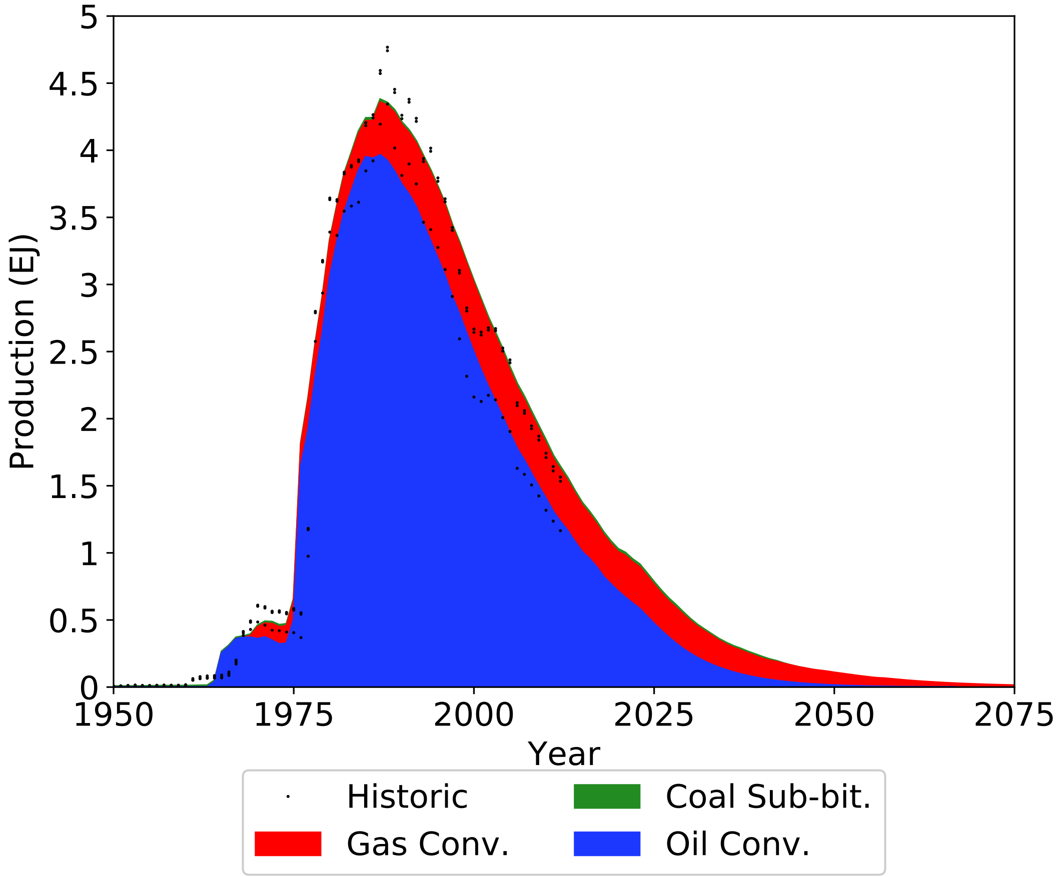

Figure 6.30: USA - Alaska projections capped at 16

Table 6.30: Peak years - All

**Name**	**URR**	**Peak Year**	**Peak Rate**
Oil Conv. Alaska	119.01	1987	3.96
Gas Conv. Alaska	25.4	1996	0.53
Coal Sub-bit. Alaska	1.7	2004	0.03
**Total**	**146.11**	**1987**	**4.38**

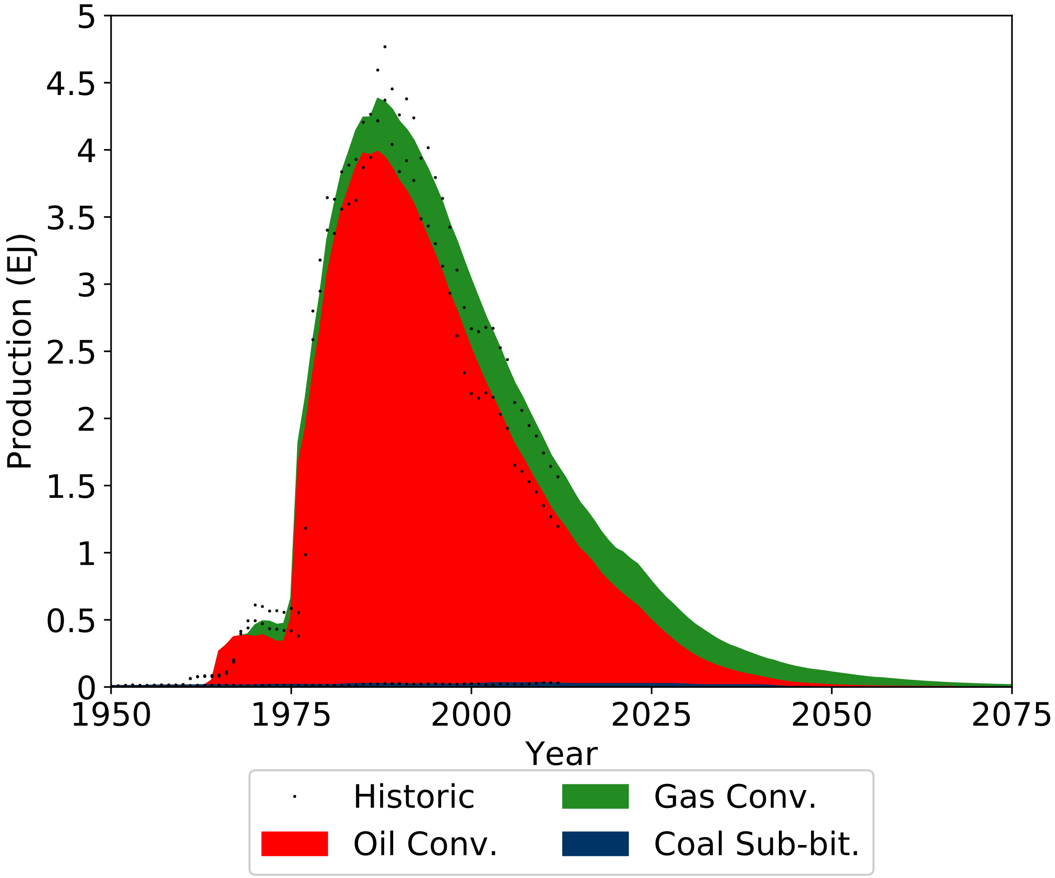

Figure 6.31: USA - Alaska projection by mineral type

Table 6.31: Peak years - Minerals

Name	URR	Peak Year	Peak Rate
Coal Sub-bit.	1.7	2004	0.03
Oil Conv.	119.01	1987	3.96
Gas Conv.	25.4	1996	0.53
**Total**	**146.11**	**1987**	**4.38**

Arizona

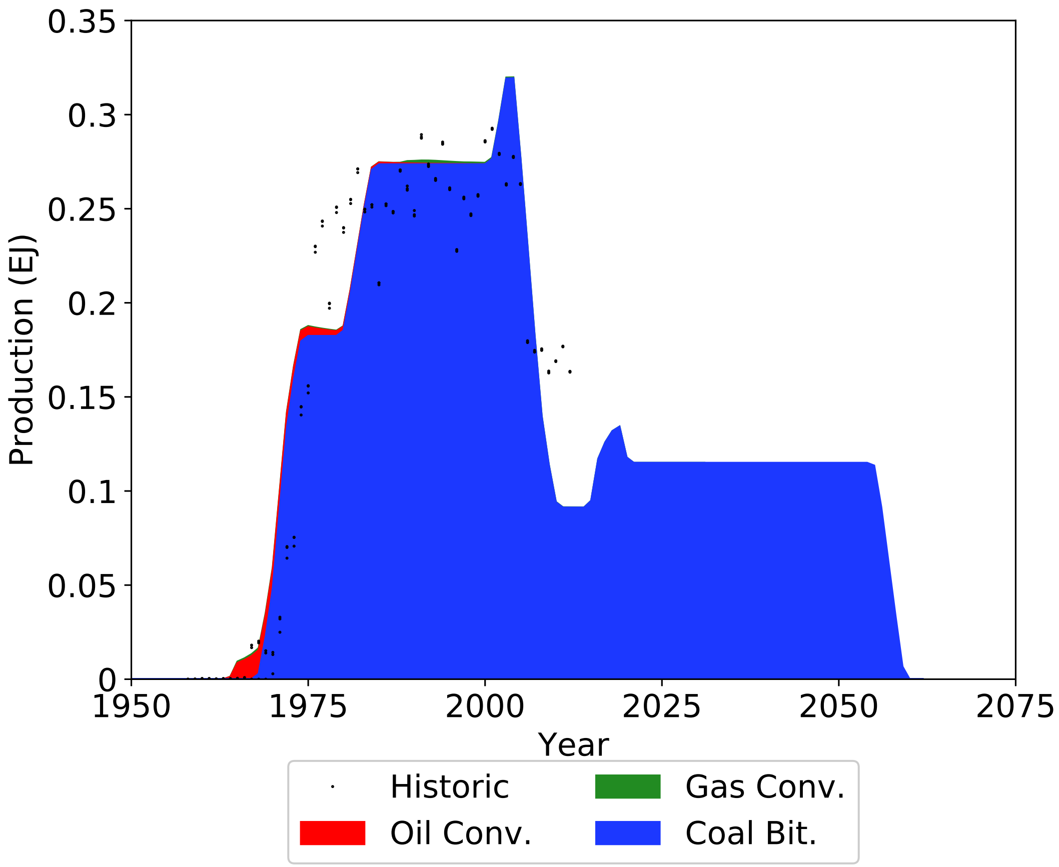

Figure 6.32: USA - Arizona projections capped at 16

Table 6.32: Peak years - All

Name	URR	Peak Year	Peak Rate
Coal Bit. Arizona	14.62	2004	0.32
Oil Conv. Arizona	0.13	1968	0.01
Gas Conv. Arizona	0.03	1992	–
**Total**	**14.78**	**2004**	**0.32**

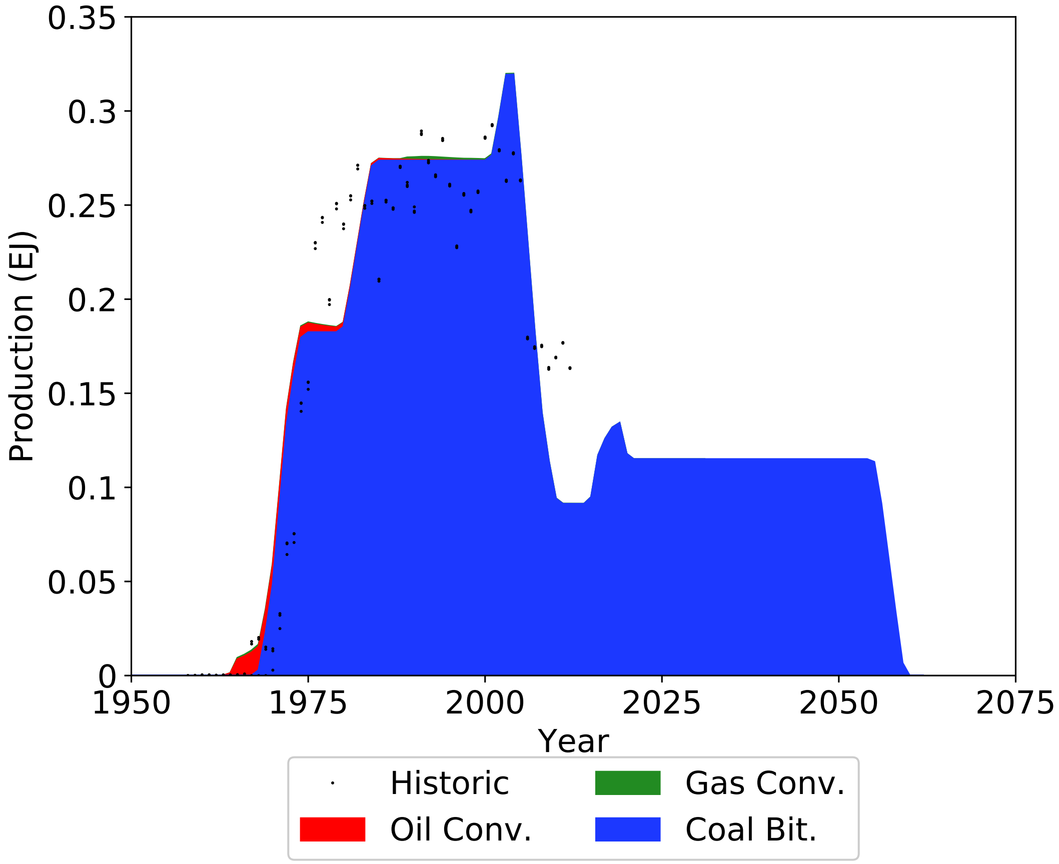

Figure 6.33: USA - Arizona projection by mineral type

Table 6.33: Peak years - Minerals

Name	URR	Peak Year	Peak Rate
Coal Bit.	14.62	2004	0.32
Oil Conv.	0.13	1968	0.01
Gas Conv.	0.03	1992	–
**Total**	**14.78**	**2004**	**0.32**

Arkansas

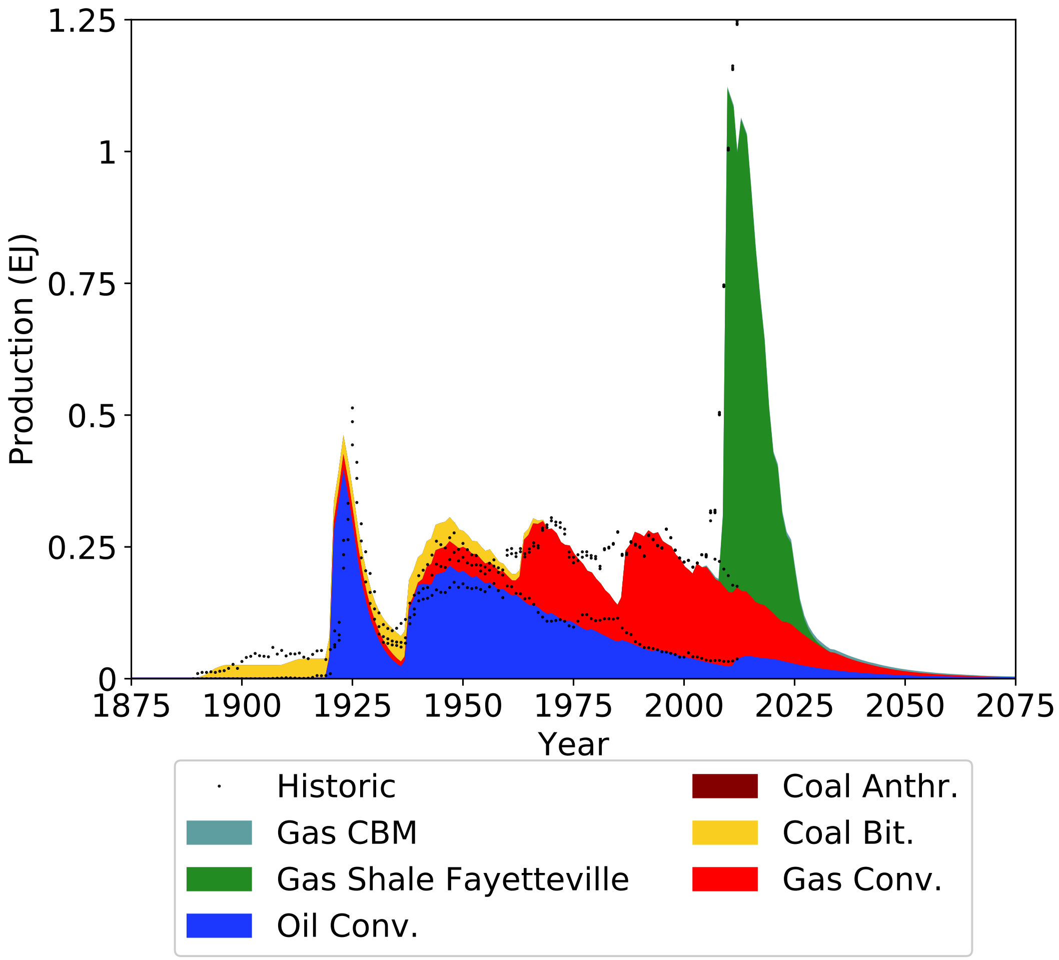

Figure 6.34: USA - Arkansas projections capped at 16

Table 6.34: Peak years - All

**Name**	**URR**	**Peak Year**	**Peak Rate**
Oil Conv. Arkansas	11.62	1923	0.39
Gas Conv. Arkansas	10.85	1992	0.23
Gas Shale Arkansas Fayetteville	8.81	2010	0.95
Coal Bit. Arkansas	2.33	1935	0.05
Gas CBM Arkansas	0.29	2027	0.01
Coal Anthr. Arkansas	–	1994	–
**Total**	**33.9**	**2010**	**1.11**

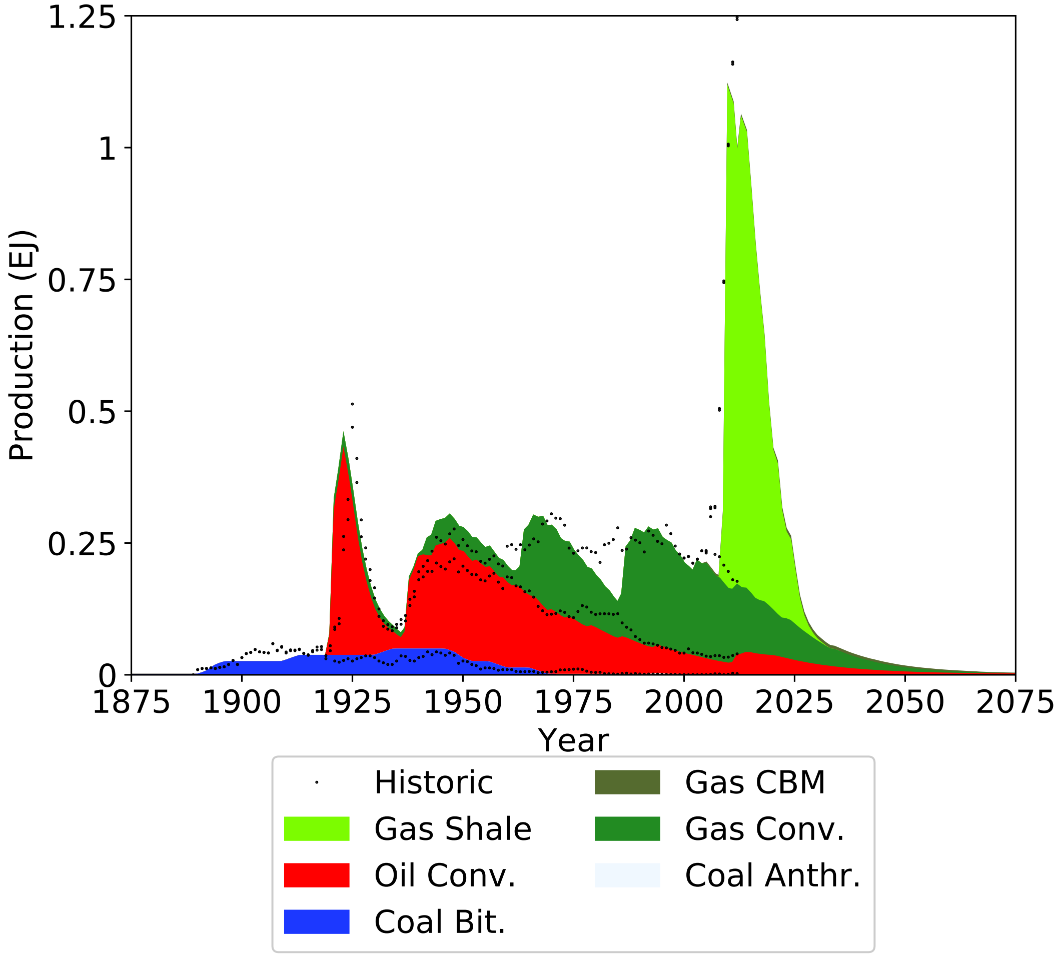

Figure 6.35: USA - Arkansas projection by mineral type

Table 6.35: Peak years - Minerals

**Name**	**URR**	**Peak Year**	**Peak Rate**
Coal Bit.	2.33	1935	0.05
Coal Anthr.	–	1994	–
Oil Conv.	11.62	1923	0.39
Gas Conv.	10.85	1992	0.23
Gas Shale	8.81	2010	0.95
Gas CBM	0.29	2027	0.01
**Total**	**33.9**	**2010**	**1.11**

California

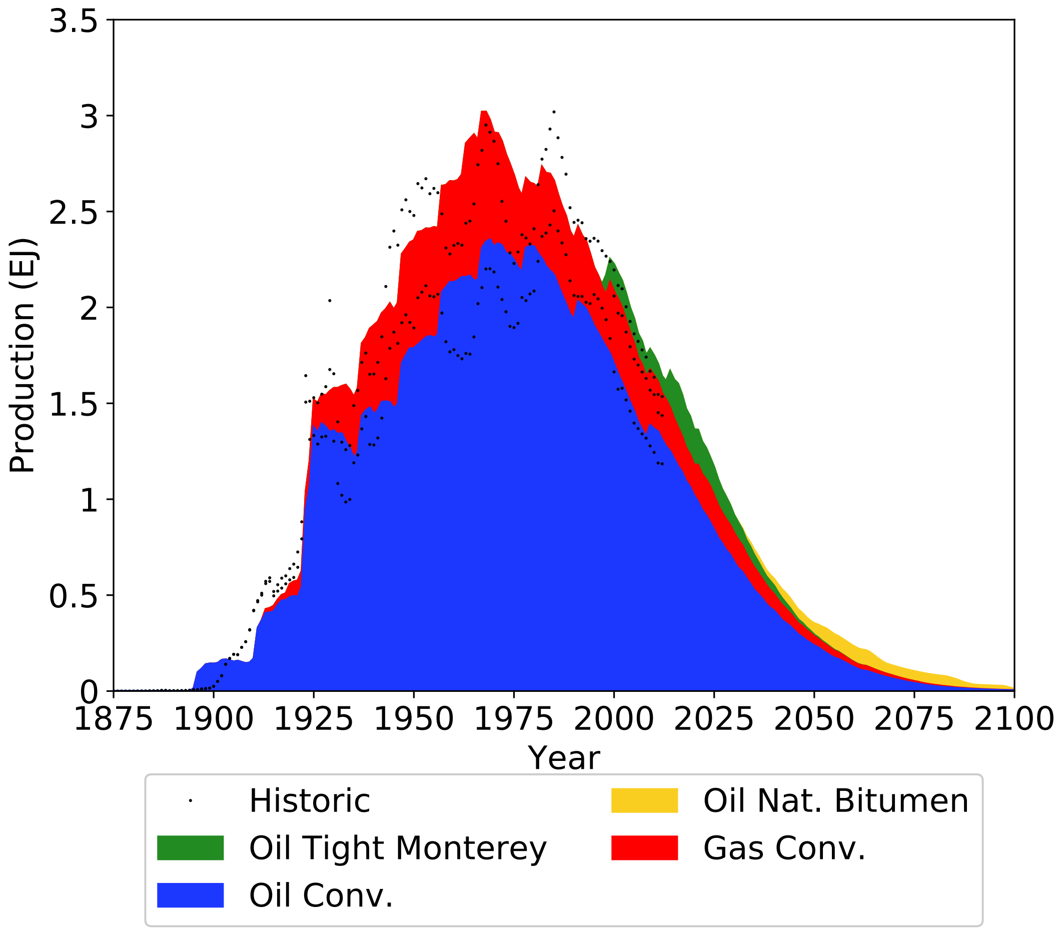

Figure 6.36: USA - California projections capped at 16

Table 6.36: Peak years - All

Name	URR	Peak Year	Peak Rate
Oil Conv. California	198.31	1969	2.35
Gas Conv. California	44.43	1965	0.76
Oil Tight California Monterey	5.62	2016	0.23
Oil Nat. Bitumen California	3.31	2054	0.08
**Total**	**251.67**	**1968**	**3.02**

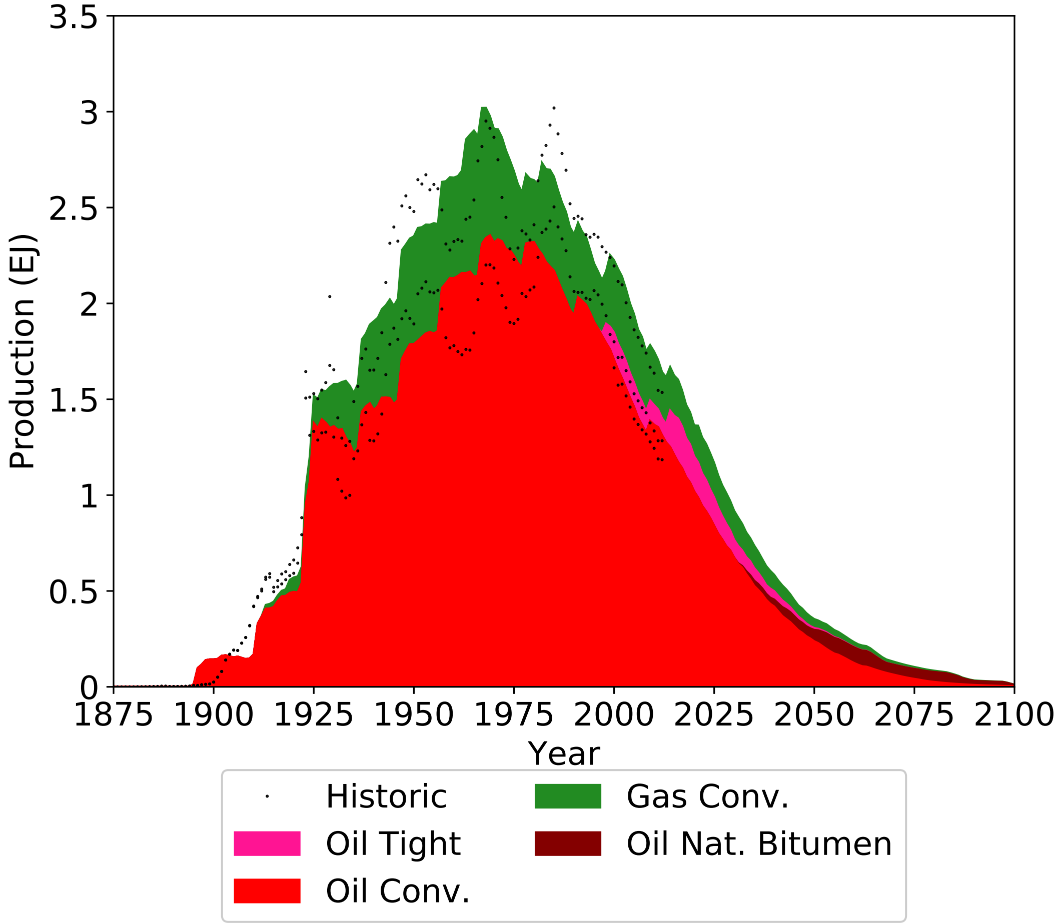

Figure 6.37: USA - California projection by mineral type

Table 6.37: Peak years - Minerals

Name	URR	Peak Year	Peak Rate
Oil Conv.	198.31	1969	2.35
Oil Nat. Bitumen	3.31	2054	0.08
Oil Tight	5.62	2016	0.23
Gas Conv.	44.43	1965	0.76
**Total**	**251.67**	**1968**	**3.02**

Colorado

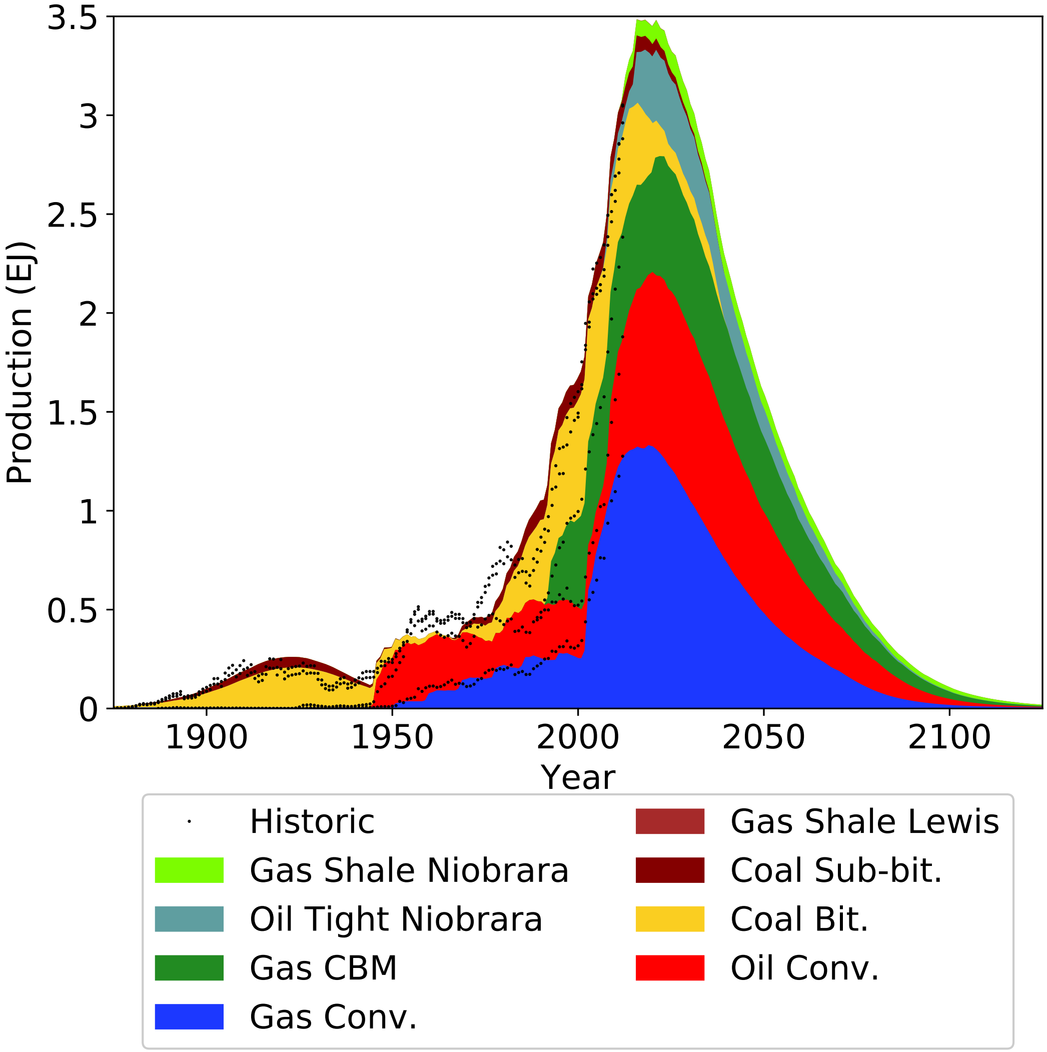

Figure 6.38: USA - Colorado projections capped at 16

Table 6.38: Peak years - All

Name	URR	Peak Year	Peak Rate
Gas Conv. Colorado	63.0	2019	1.33
Oil Conv. Colorado	57.3	2023	0.9
Gas CBM Colorado	38.03	2023	0.62
Coal Bit. Colorado	29.21	2002	0.63
Oil Tight Colorado Niobrara	12.26	2024	0.36
Coal Sub-bit. Colorado	6.9	2006	0.13
Gas Shale Colorado Niobrara	6.0	2026	0.11
Gas Shale Colorado Lewis	0.05	2017	–
**Total**	**212.75**	**2016**	**3.48**

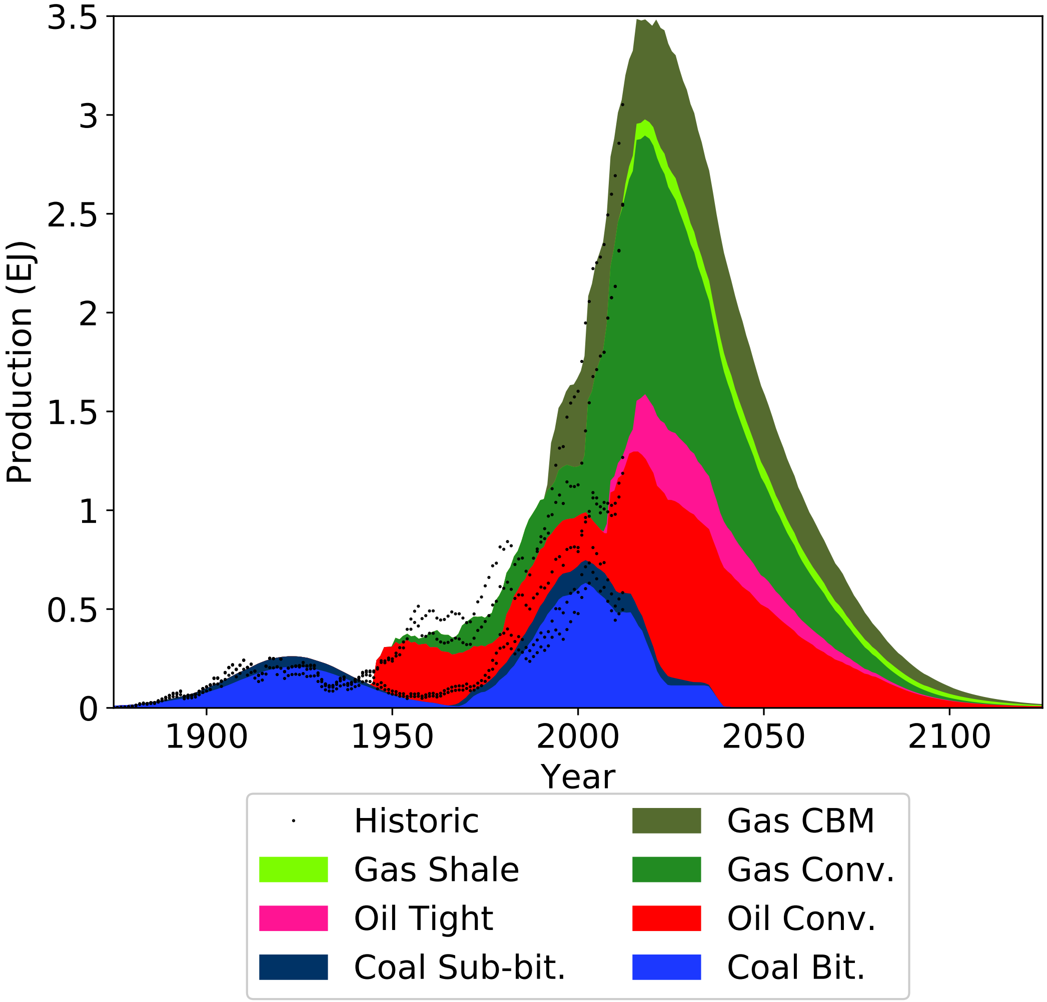

Figure 6.39: USA - Colorado projection by mineral type

Table 6.39: Peak years - Minerals

Name	URR	Peak Year	Peak Rate
Coal Bit.	29.21	2002	0.63
Coal Sub-bit.	6.9	2006	0.13
Oil Conv.	57.3	2023	0.9
Oil Tight	12.26	2024	0.36
Gas Conv.	63.0	2019	1.33
Gas Shale	6.05	2026	0.11
Gas CBM	38.03	2023	0.62
**Total**	**212.75**	**2016**	**3.48**

Florida

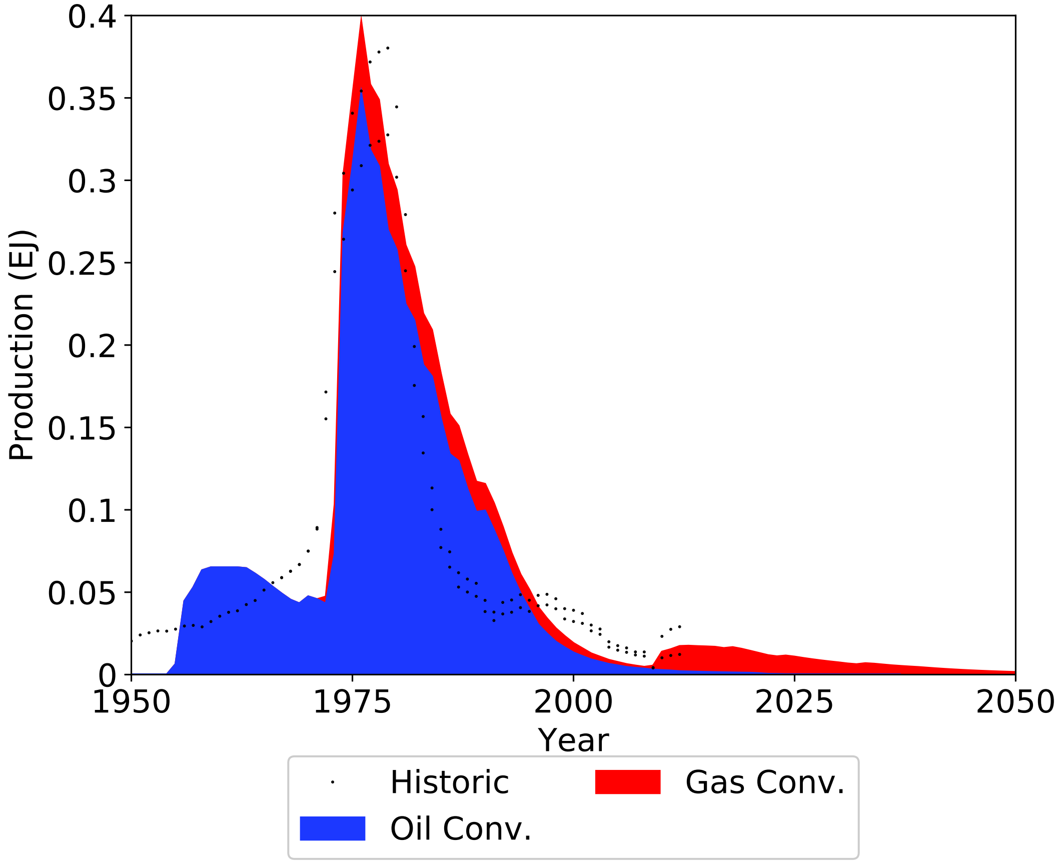

Figure 6.40: USA - Florida projections capped at 16

Table 6.40: Peak years - All

Name	URR	Peak Year	Peak Rate
Oil Conv. Florida	5.13	1976	0.35
Gas Conv. Florida	1.05	1976	0.04
**Total**	**6.18**	**1976**	**0.4**

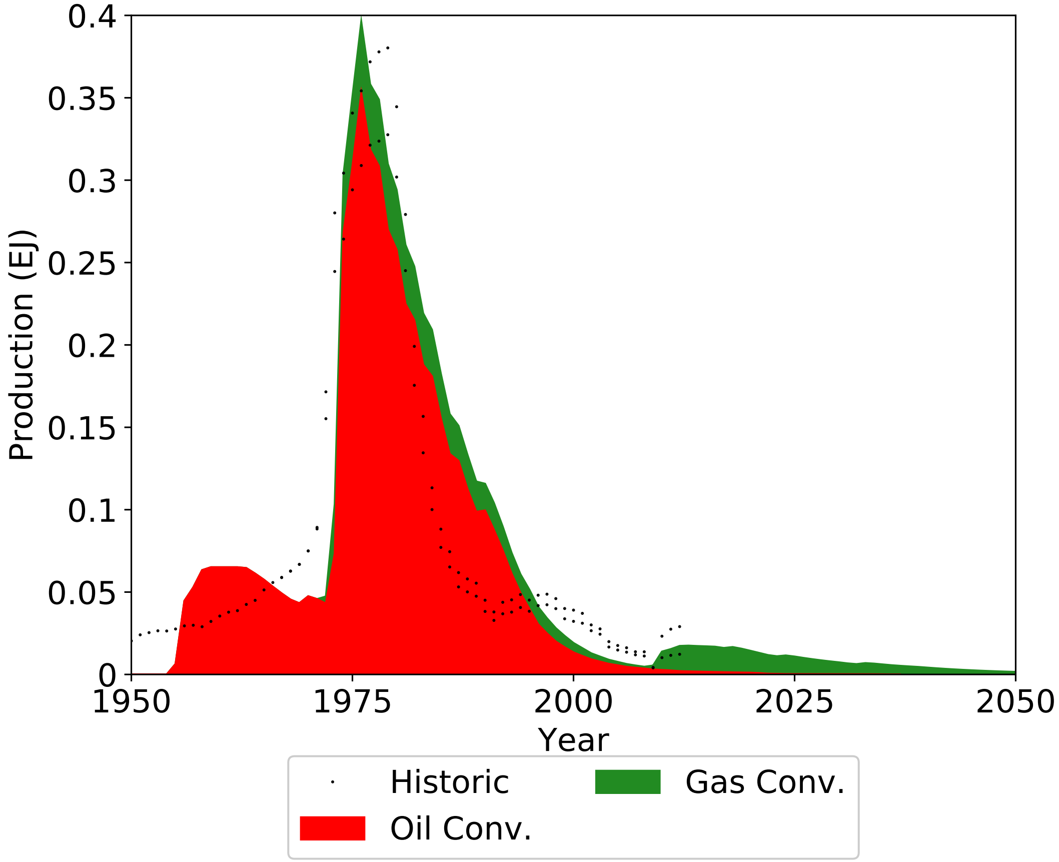

Figure 6.41: USA - Florida projection by mineral type

Table 6.41: Peak years - Minerals

Name	URR	Peak Year	Peak Rate
Oil Conv.	5.13	1976	0.35
Gas Conv.	1.05	1976	0.04
**Total**	**6.18**	**1976**	**0.4**

Georgia

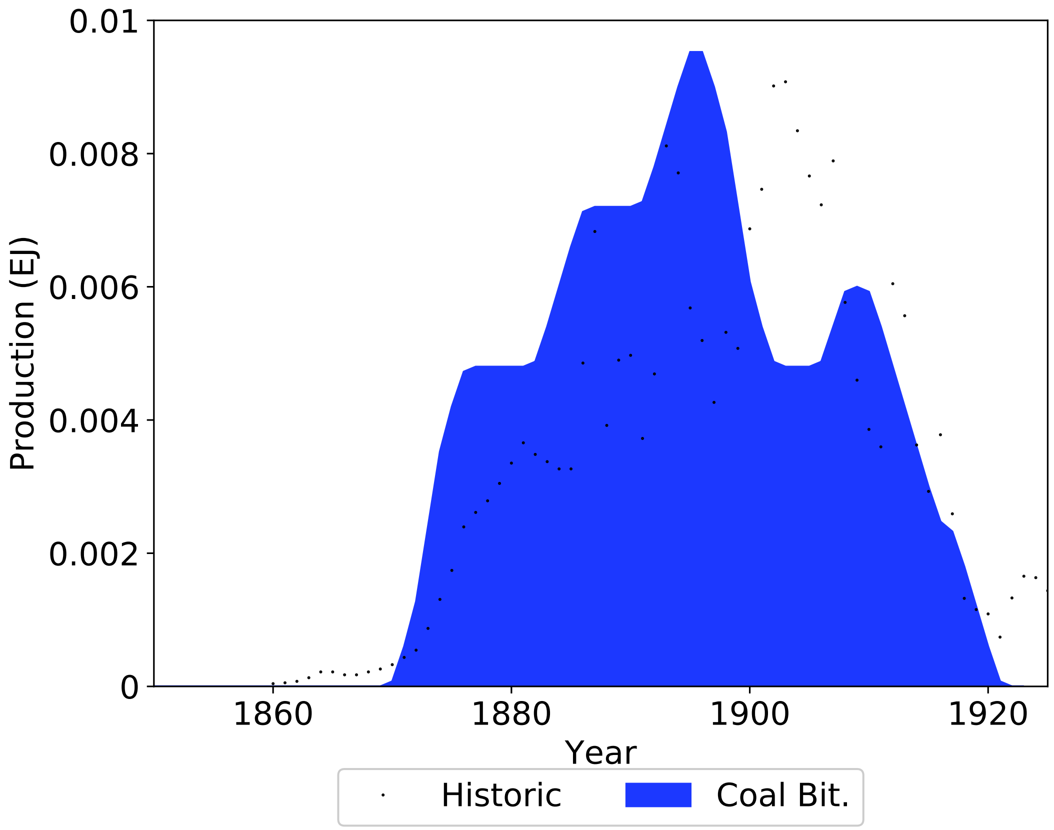

Figure 6.42: USA - Georgia projections capped at 16

Table 6.42: Peak years - All

Name	URR	Peak Year	Peak Rate
Coal Bit. Georgia	0.26	1895	0.01
**Total**	**0.26**	**1895**	**0.01**

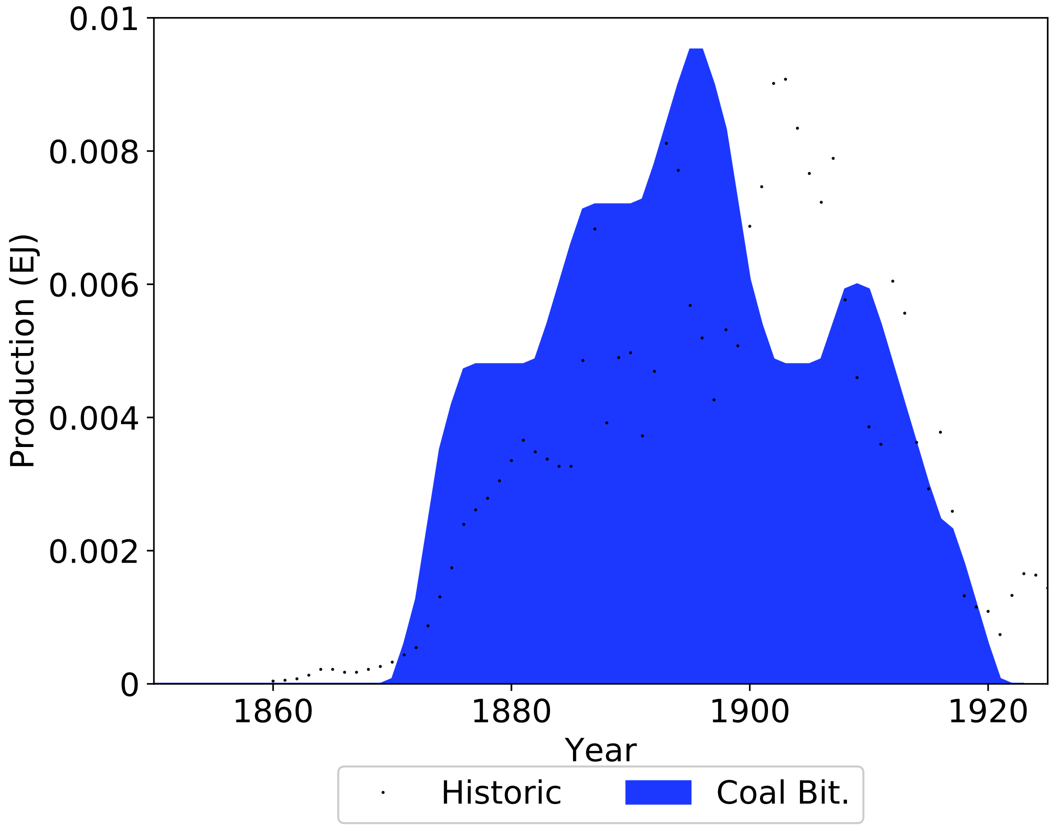

Figure 6.43: USA - Georgia projection by mineral type

Table 6.43: Peak years - Minerals

Name	URR	Peak Year	Peak Rate
Coal Bit.	0.26	1895	0.01
**Total**	**0.26**	**1895**	**0.01**

Illinois

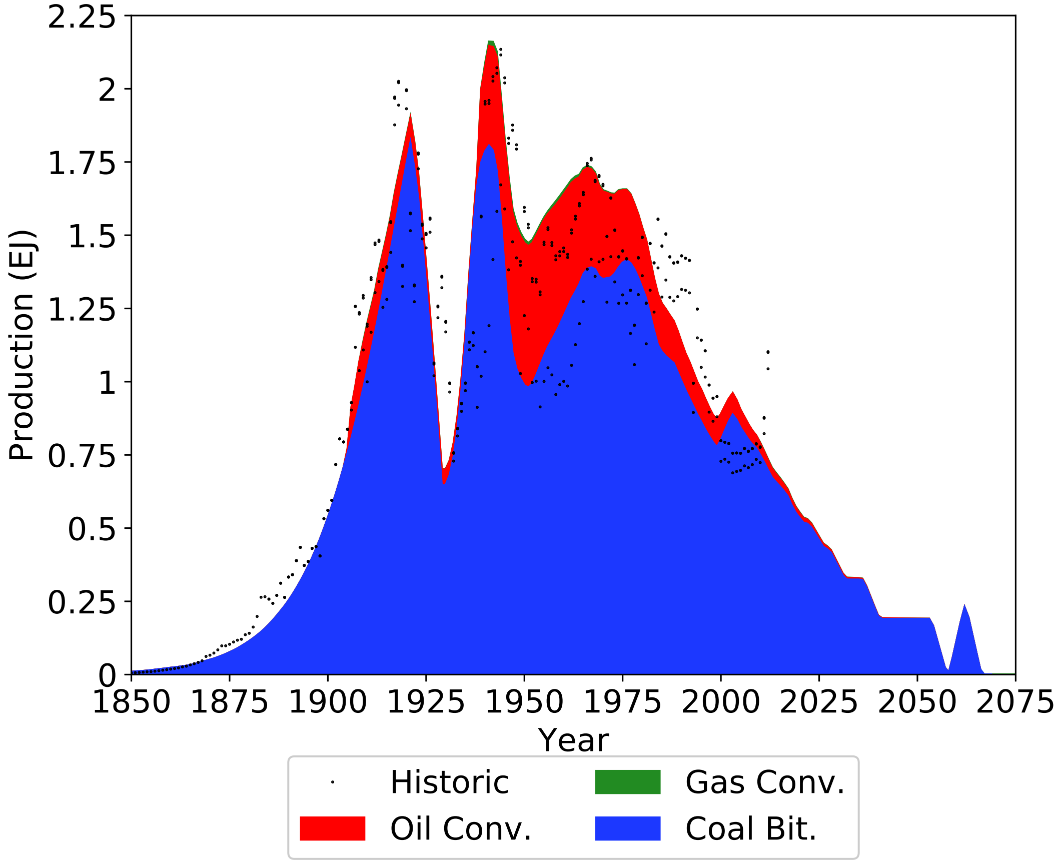

Figure 6.44: USA - Illinois projections capped at 16

Table 6.44: Peak years - All

Name	URR	Peak Year	Peak Rate
Coal Bit. Illinois	151.45	1921	1.82
Oil Conv. Illinois	21.98	1950	0.49
Gas Conv. Illinois	0.56	1943	0.02
**Total**	**173.99**	**1941**	**2.16**

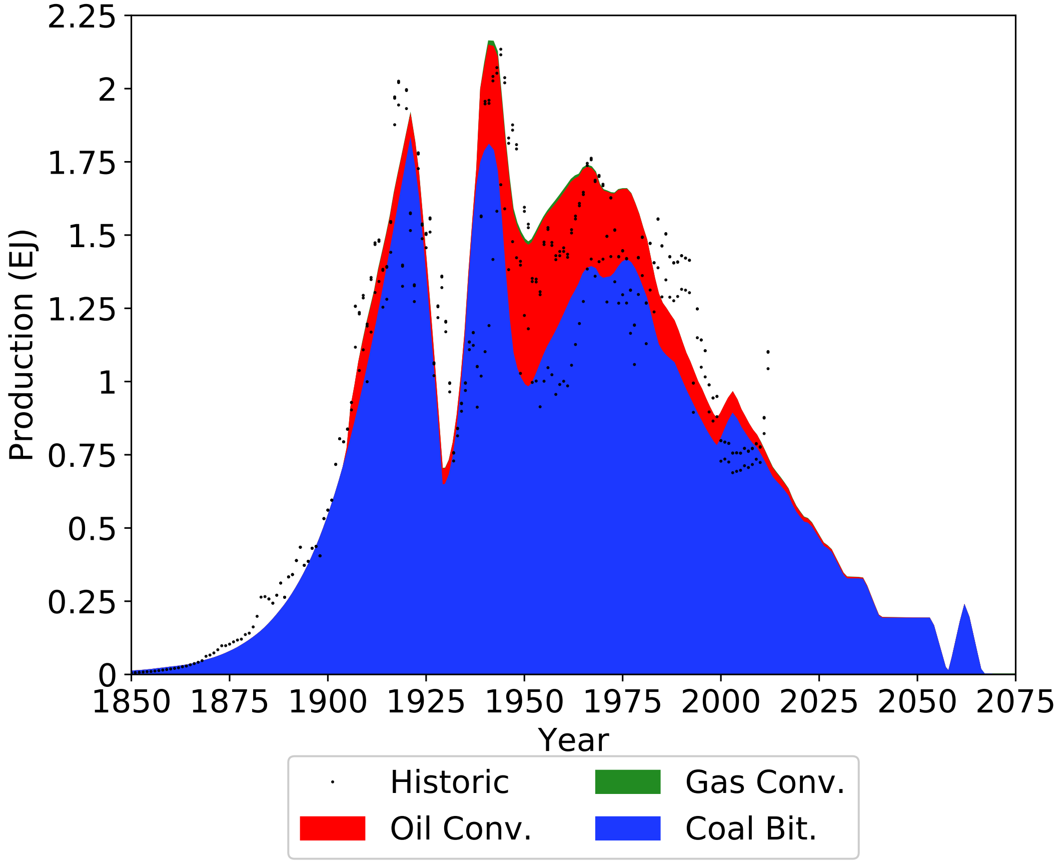

Figure 6.45: USA - Illinois projection by mineral type

Table 6.45: Peak years - Minerals

Name	URR	Peak Year	Peak Rate
Coal Bit.	151.45	1921	1.82
Oil Conv.	21.98	1950	0.49
Gas Conv.	0.56	1943	0.02
**Total**	**173.99**	**1941**	**2.16**

Indiana

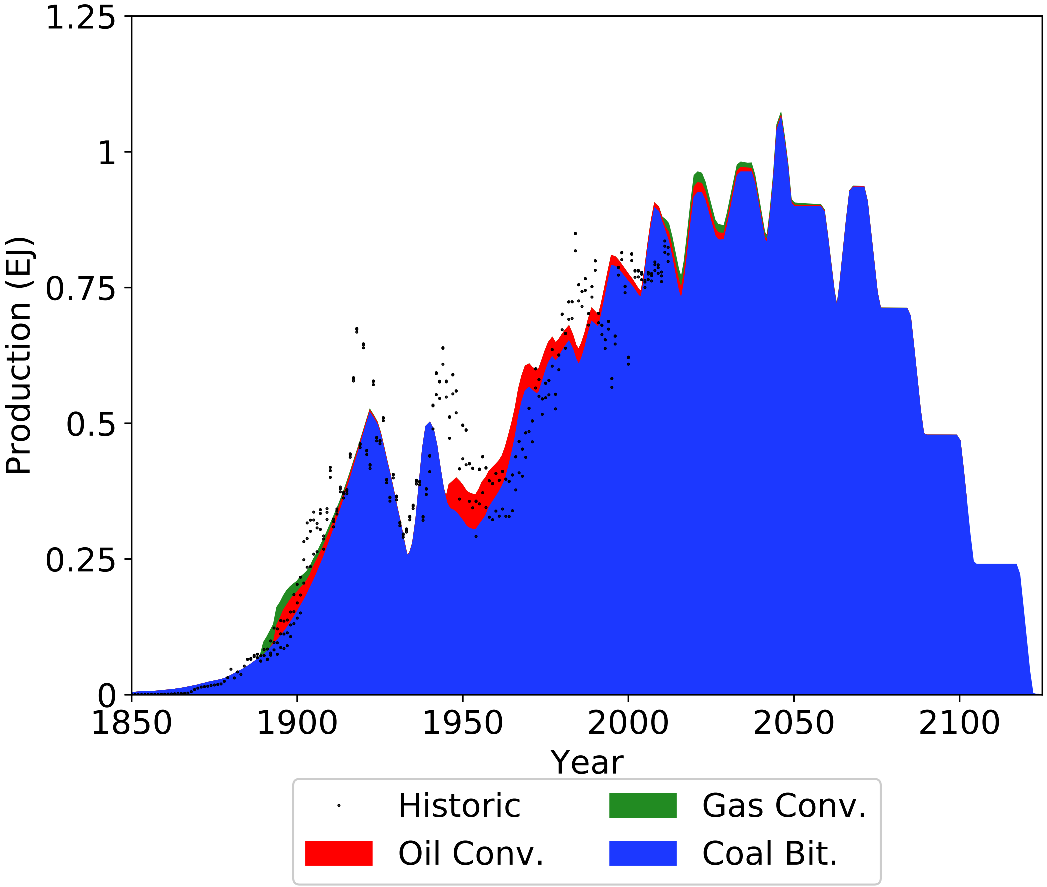

Figure 6.46: USA - Indiana projections capped at 16

Table 6.46: Peak years - All

Name	URR	Peak Year	Peak Rate
Coal Bit. Indiana	132.5	2046	1.06
Oil Conv. Indiana	3.53	1956	0.07
Gas Conv. Indiana	1.05	1893	0.03
**Total**	**137.08**	**2046**	**1.07**

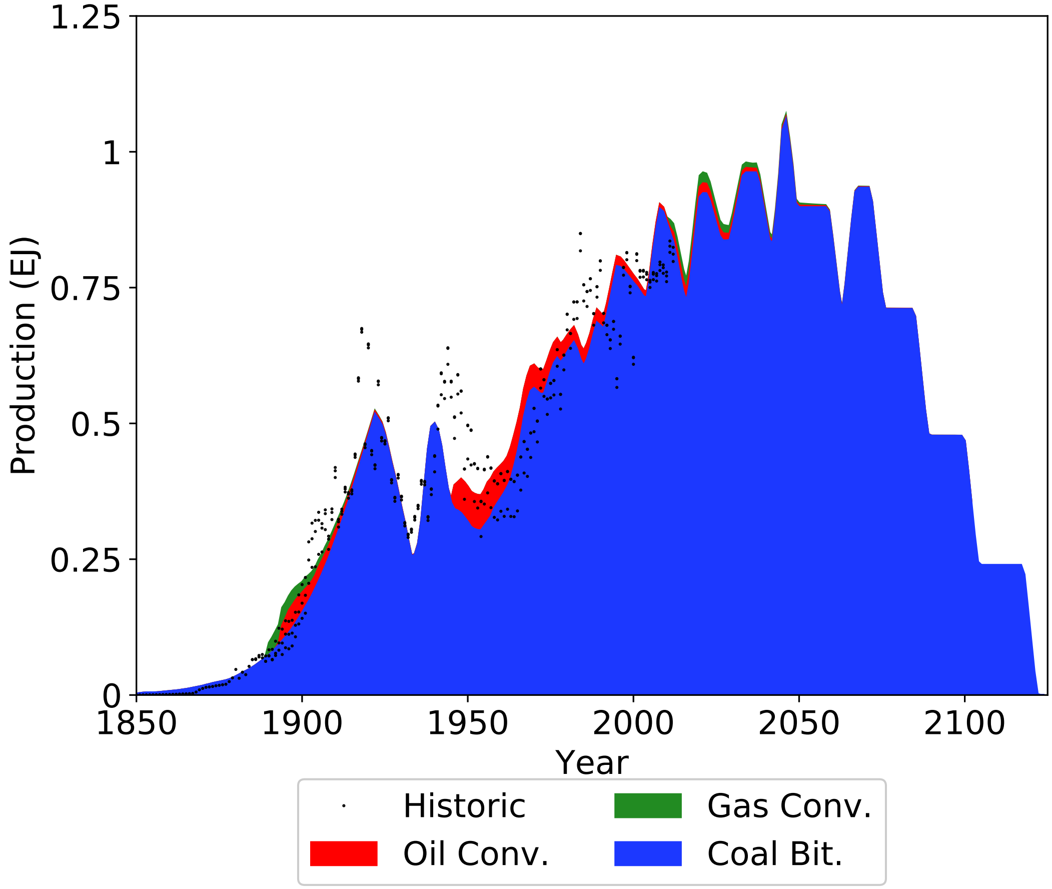

Figure 6.47: USA - Indiana projection by mineral type

Table 6.47: Peak years - Minerals

Name	URR	Peak Year	Peak Rate
Coal Bit.	132.5	2046	1.06
Oil Conv.	3.53	1956	0.07
Gas Conv.	1.05	1893	0.03
**Total**	**137.08**	**2046**	**1.07**

Iowa

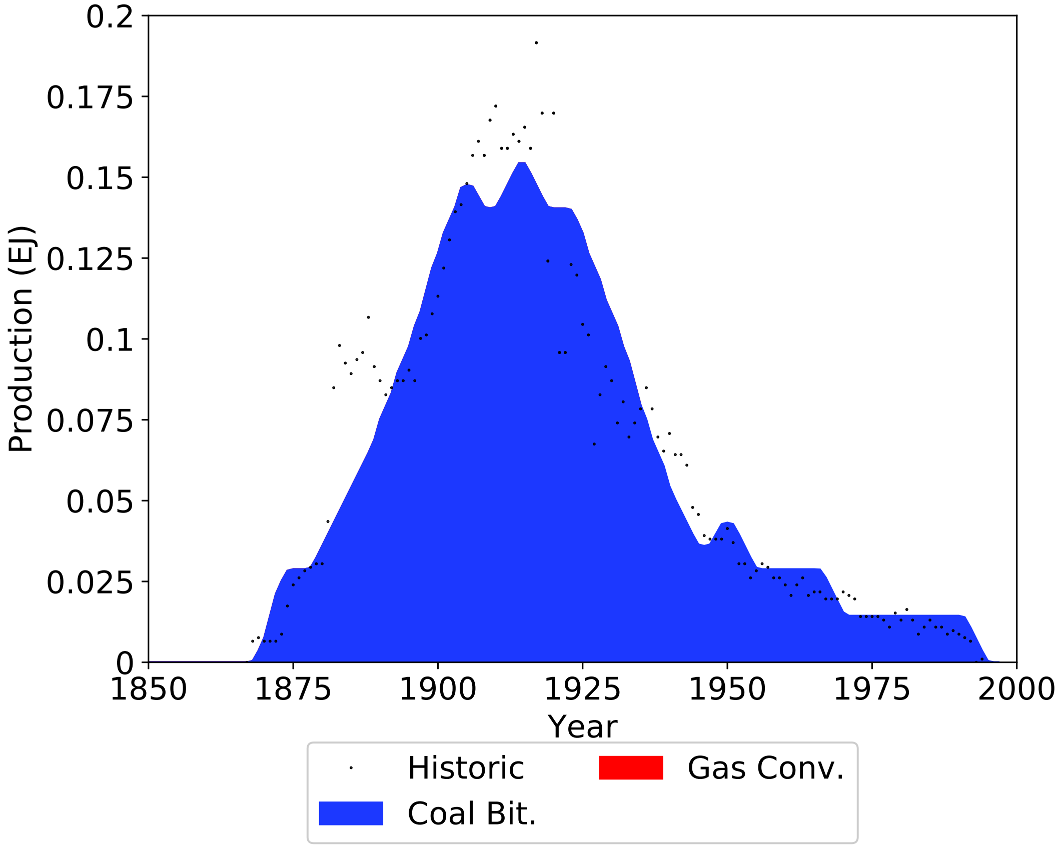

Figure 6.48: USA - Iowa projections capped at 16

Table 6.48: Peak years - All

Name	URR	Peak Year	Peak Rate
Coal Bit. Iowa	8.14	1914	0.15
Gas Conv. Iowa	–	1919	–
**Total**	**8.14**	**1914**	**0.15**

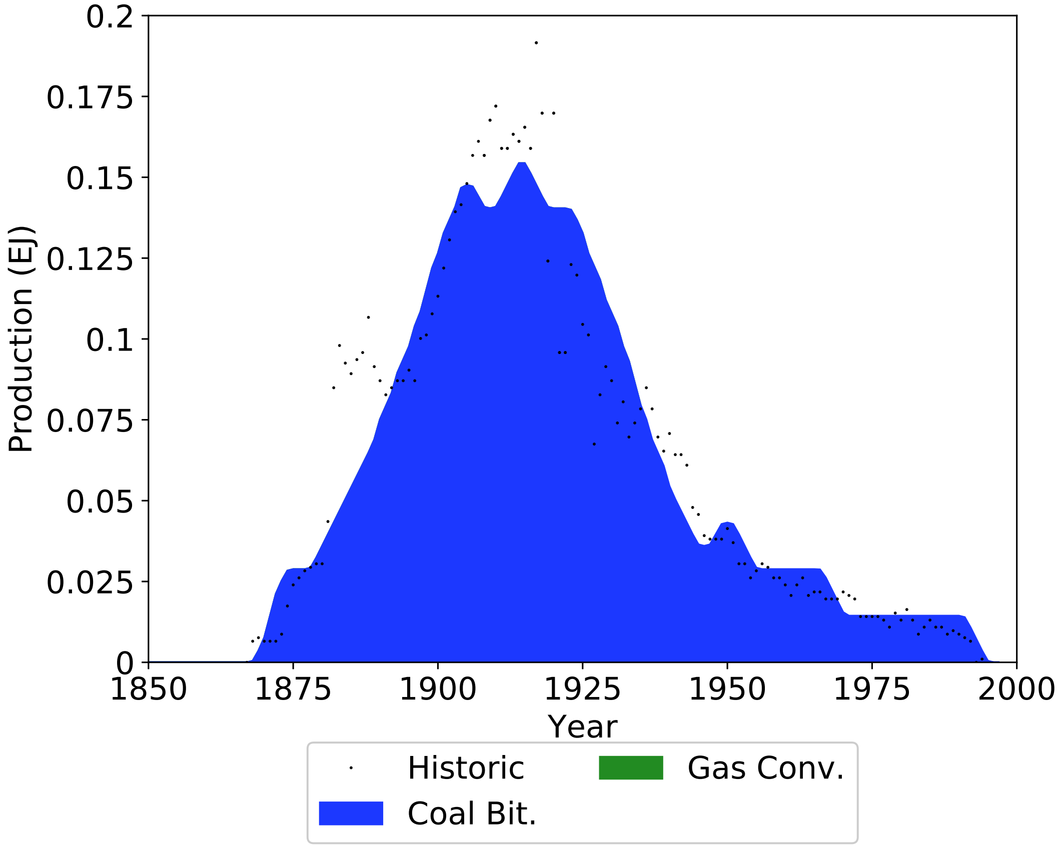

Figure 6.49: USA - Iowa projection by mineral type

Table 6.49: Peak years - Minerals

Name	URR	Peak Year	Peak Rate
Coal Bit.	8.14	1914	0.15
Gas Conv.	–	1919	–
**Total**	**8.14**	**1914**	**0.15**

Kansas

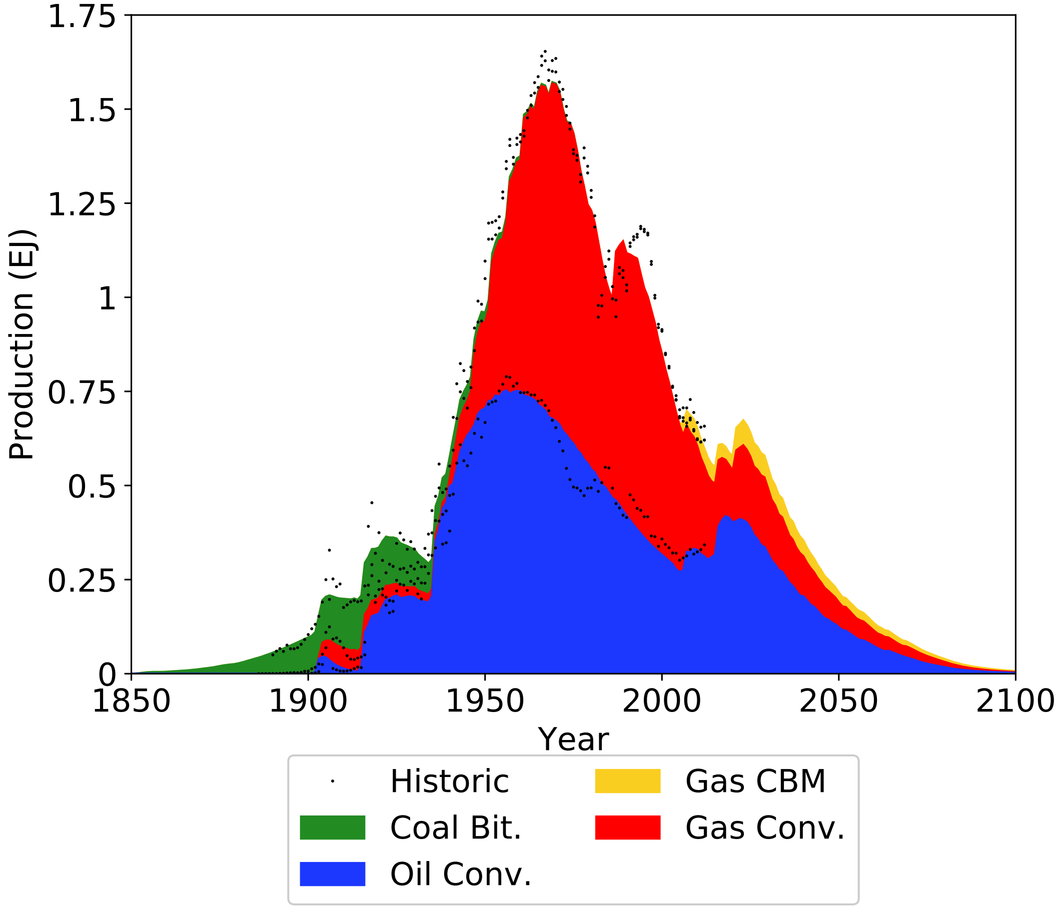

Figure 6.50: USA - Kansas projections capped at 16

Table 6.50: Peak years - All

Name	URR	Peak Year	Peak Rate
Oil Conv. Kansas	57.63	1956	0.75
Gas Conv. Kansas	47.12	1970	0.89
Coal Bit. Kansas	6.64	1916	0.14
Gas CBM Kansas	2.69	2023	0.07
**Total**	**114.08**	**1969**	**1.57**

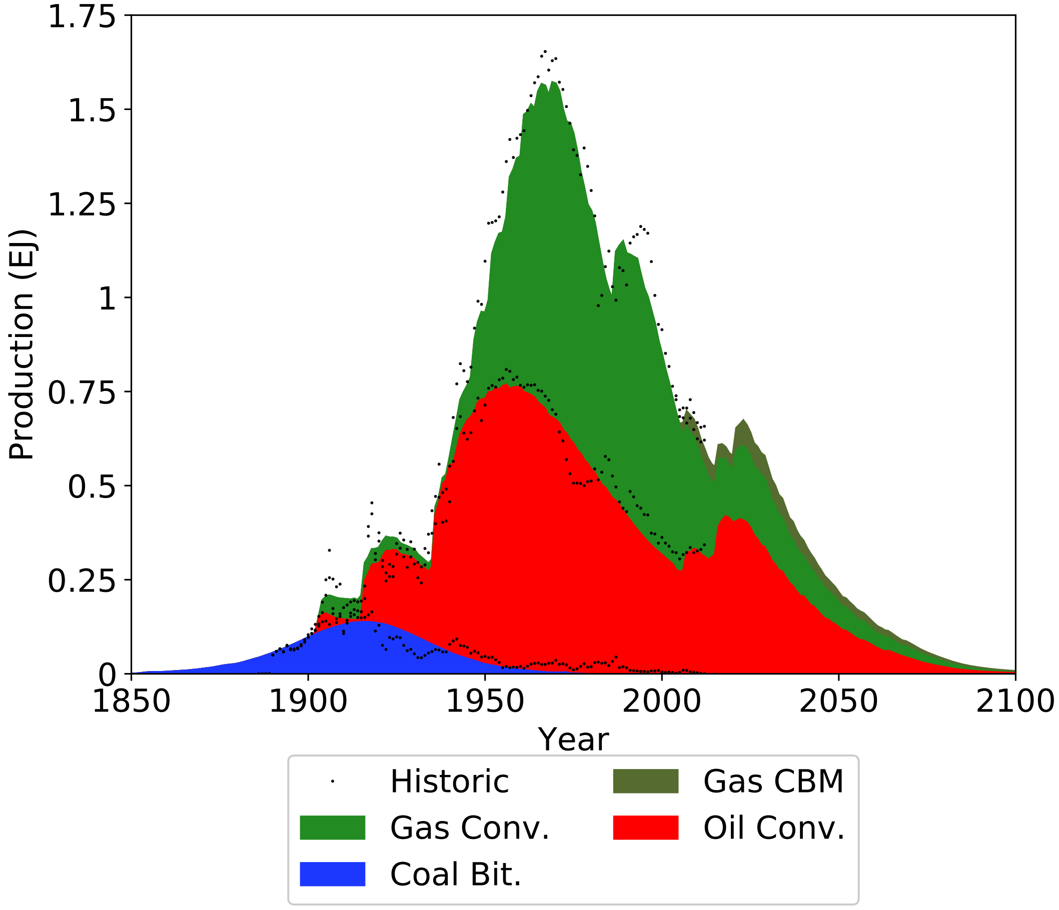

Figure 6.51: USA - Kansas projection by mineral type

Table 6.51: Peak years - Minerals

Name	URR	Peak Year	Peak Rate
Coal Bit.	6.64	1916	0.14
Oil Conv.	57.63	1956	0.75
Gas Conv.	47.12	1970	0.89
Gas CBM	2.69	2023	0.07
**Total**	**114.08**	**1969**	**1.57**

Kentucky

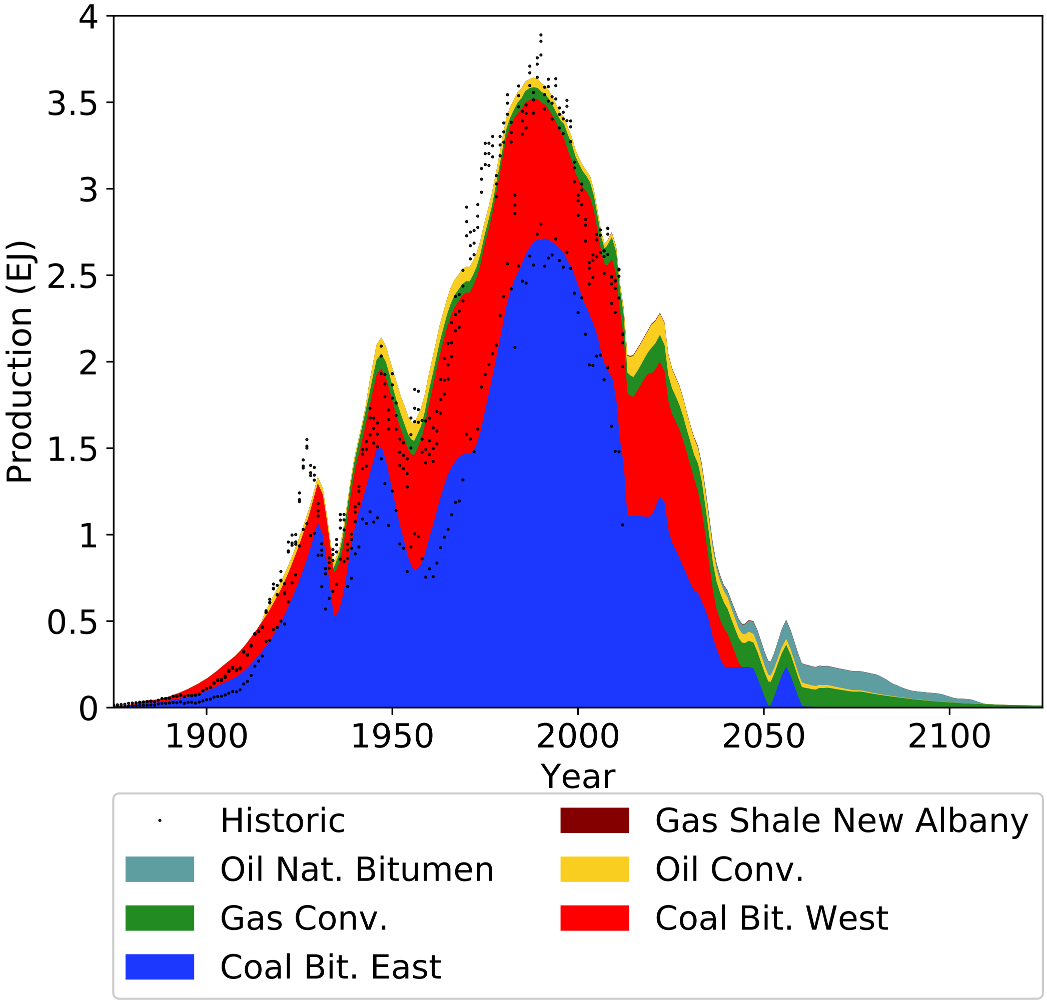

Figure 6.52: USA - Kentucky projections capped at 16

Table 6.52: Peak years - All

**Name**	**URR**	**Peak Year**	**Peak Rate**
Coal Bit. Kentucky East	175.42	1991	2.71
Coal Bit. Kentucky West	78.3	1981	0.97
Gas Conv. Kentucky	15.75	2034	0.17
Oil Conv. Kentucky	9.65	2020	0.13
Oil Nat. Bitumen Kentucky	5.34	2057	0.11
Gas Shale Kentucky New Albany	0.23	2025	0.01
**Total**	**284.69**	**1988**	**3.64**

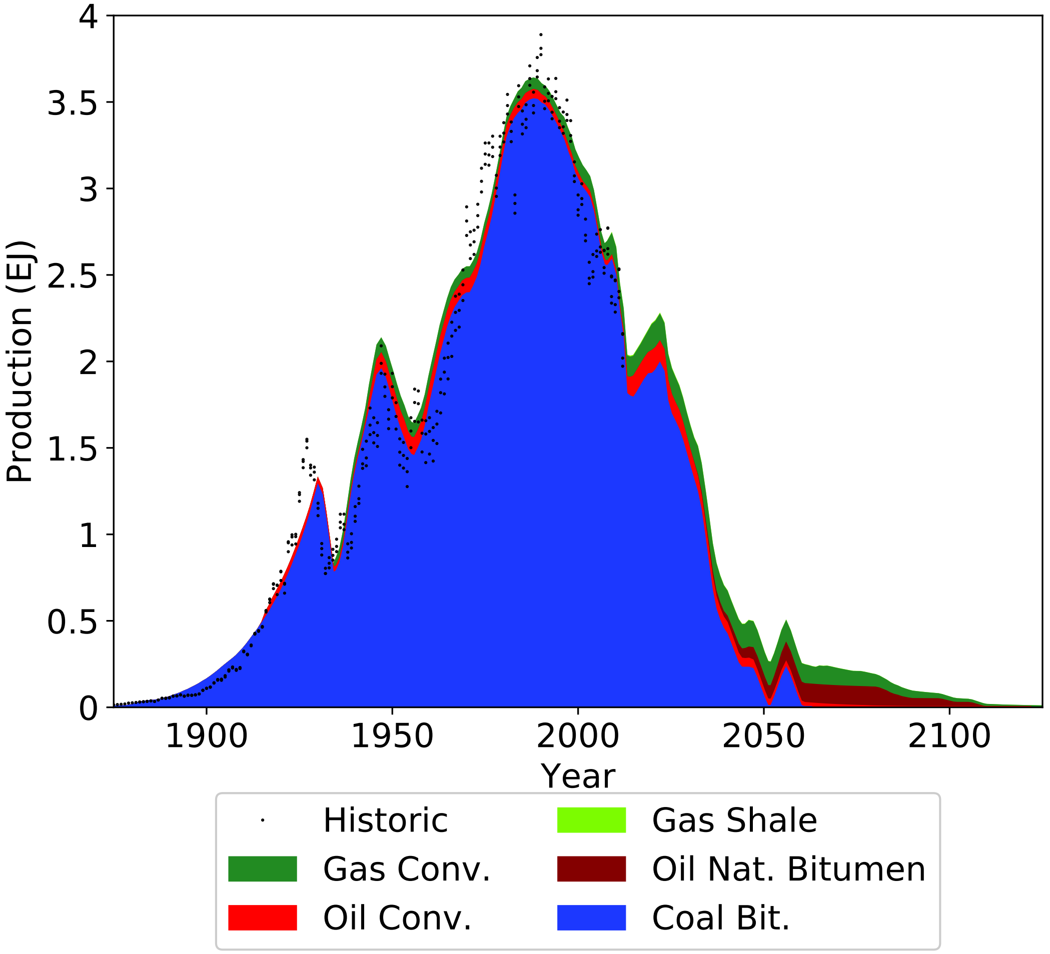

Figure 6.53: USA - Kentucky projection by mineral type

Table 6.53: Peak years - Minerals

**Name**	**URR**	**Peak Year**	**Peak Rate**
Coal Bit.	253.72	1988	3.52
Oil Conv.	9.65	2020	0.13
Oil Nat. Bitumen	5.34	2057	0.11
Gas Conv.	15.75	2034	0.17
Gas Shale	0.23	2025	0.01
**Total**	**284.69**	**1988**	**3.64**

Louisiana

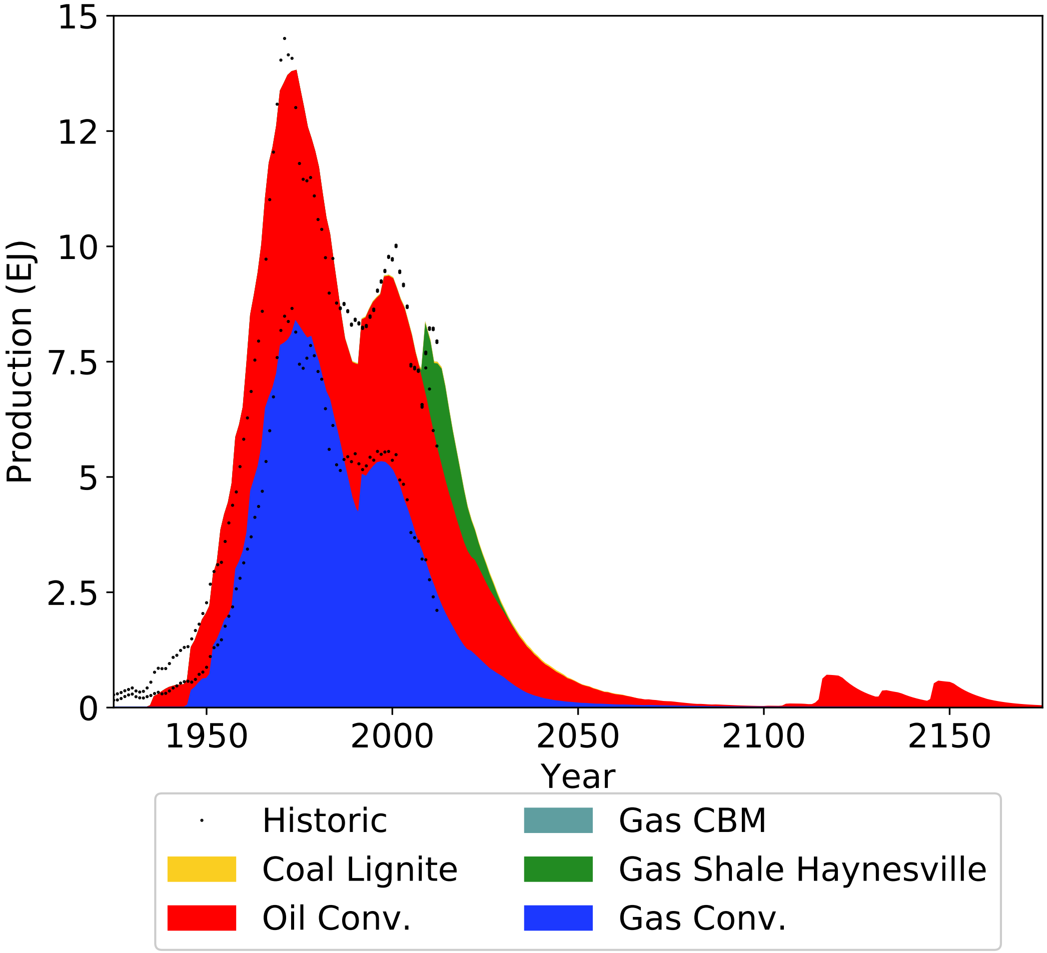

Figure 6.54: USA - Louisiana projections capped at 16

Table 6.54: Peak years - All

**Name**	**URR**	**Peak Year**	**Peak Rate**
Gas Conv. Louisiana	344.58	1974	8.36
Oil Conv. Louisiana	321.88	1972	5.74
Gas Shale Louisiana Haynesville	22.96	2013	2.08
Coal Lignite Louisiana	2.34	2028	0.05
Gas CBM Louisiana	0.01	2008	–
**Total**	**691.77**	**1974**	**13.8**

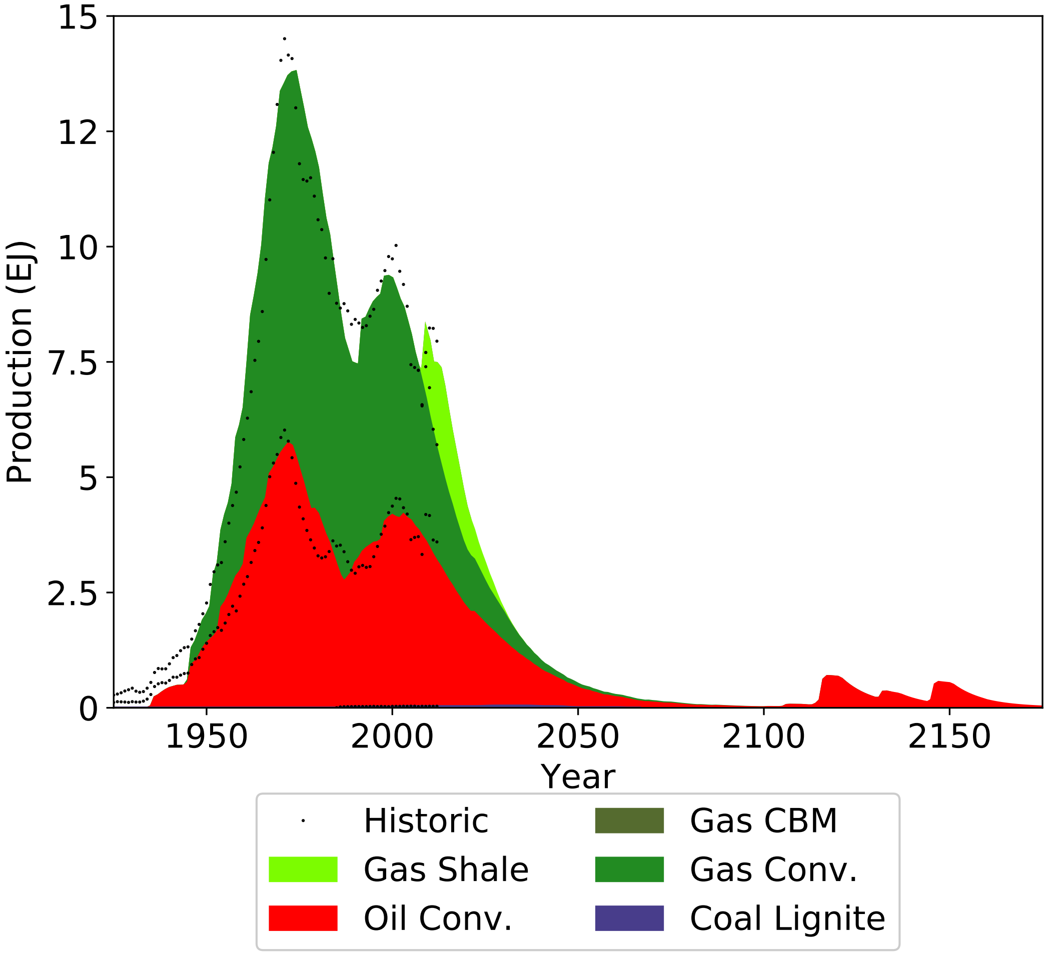

Figure 6.55: USA - Louisiana projection by mineral type

Table 6.55: Peak years - Minerals

**Name**	**URR**	**Peak Year**	**Peak Rate**
Coal Lignite	2.34	2028	0.05
Oil Conv.	321.88	1972	5.74
Gas Conv.	344.58	1974	8.36
Gas Shale	22.96	2013	2.08
Gas CBM	0.01	2008	–
**Total**	**691.77**	**1974**	**13.8**

Maryland

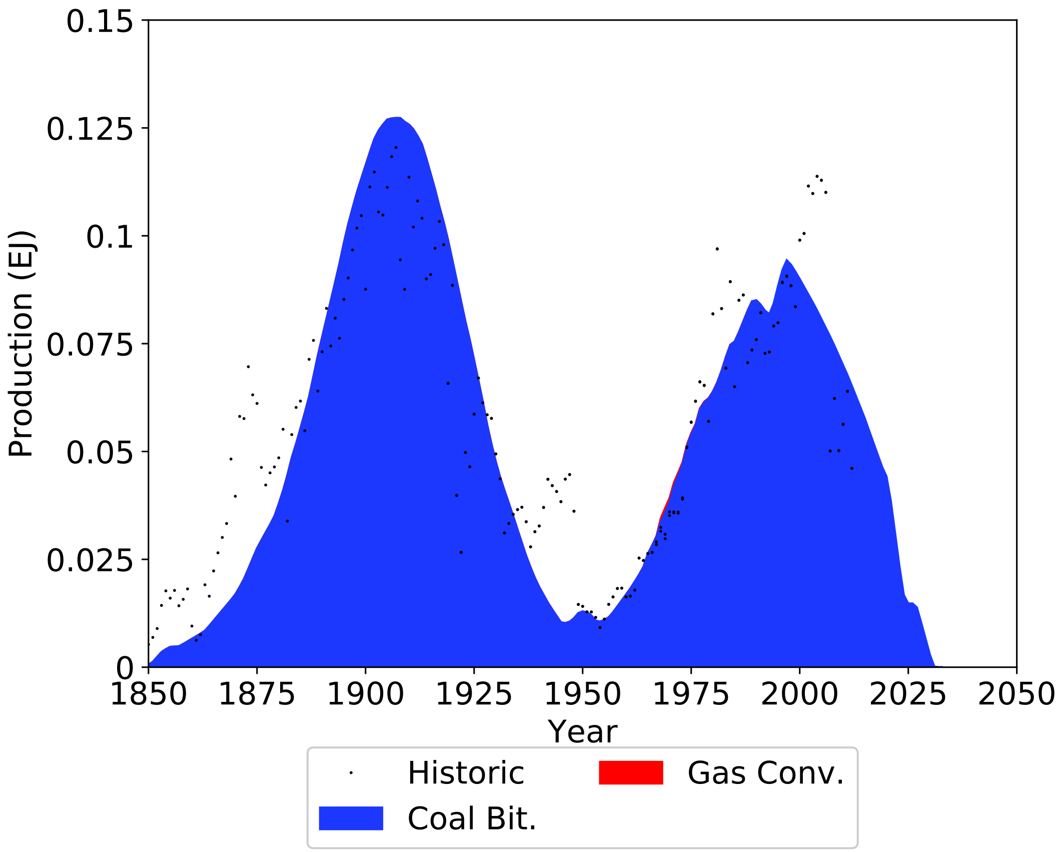

Figure 6.56: USA - Maryland projections capped at 16

Table 6.56: Peak years - All

Name	URR	Peak Year	Peak Rate
Coal Bit. Maryland	9.63	1907	0.13
Gas Conv. Maryland	0.01	1969	–
**Total**	**9.64**	**1907**	**0.13**

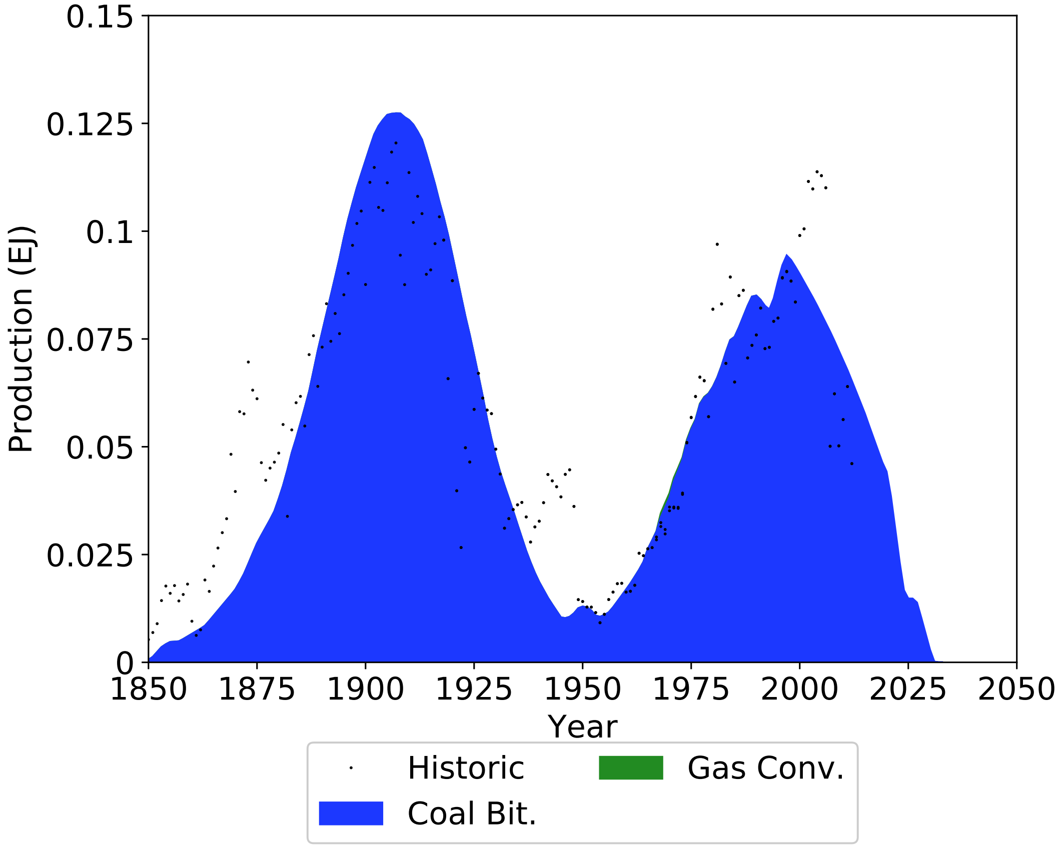

Figure 6.57: USA - Maryland projection by mineral type

Table 6.57: Peak years - Minerals

Name	URR	Peak Year	Peak Rate
Coal Bit.	9.63	1907	0.13
Gas Conv.	0.01	1969	–
**Total**	**9.64**	**1907**	**0.13**

Michigan

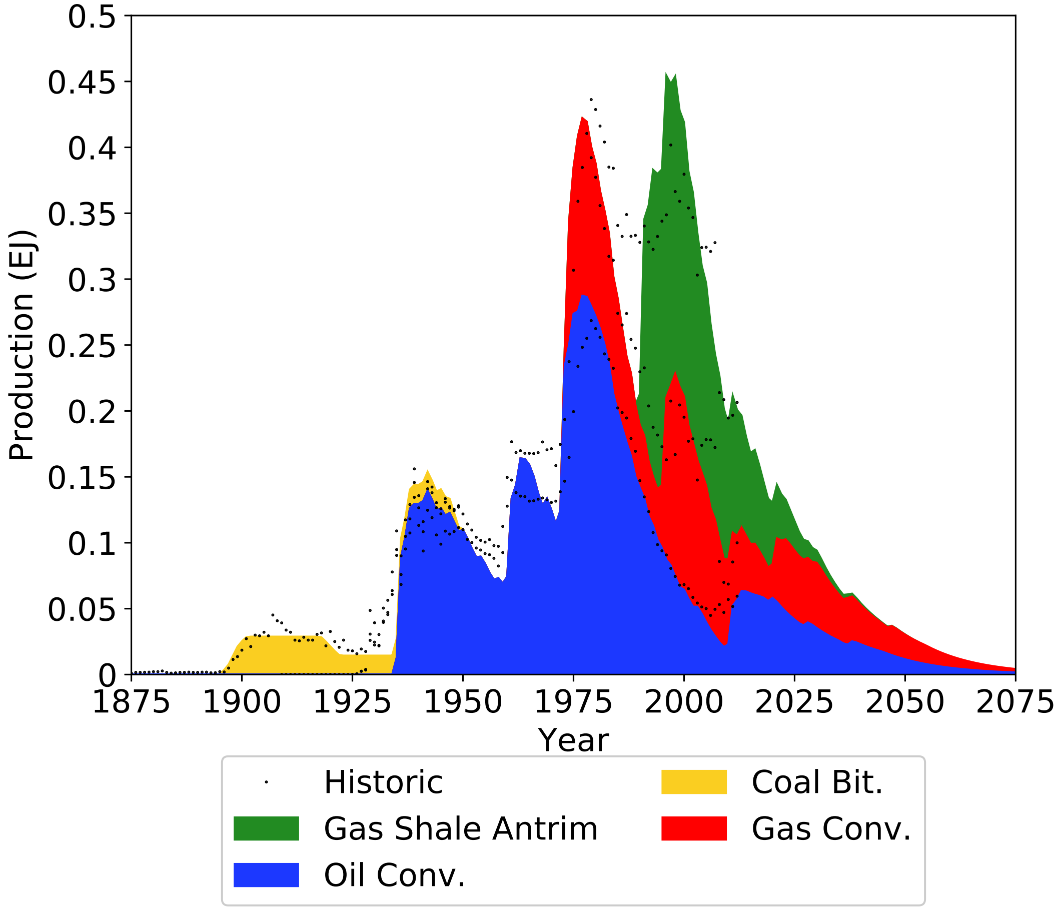

Figure 6.58: USA - Michigan projections capped at 16

Table 6.58: Peak years - All

Name	URR	Peak Year	Peak Rate
Oil Conv. Michigan	11.37	1977	0.29
Gas Conv. Michigan	5.25	1998	0.15
Gas Shale Michigan Antrim	4.67	1996	0.24
Coal Bit. Michigan	1.04	1902	0.03
**Total**	**22.33**	**1996**	**0.45**

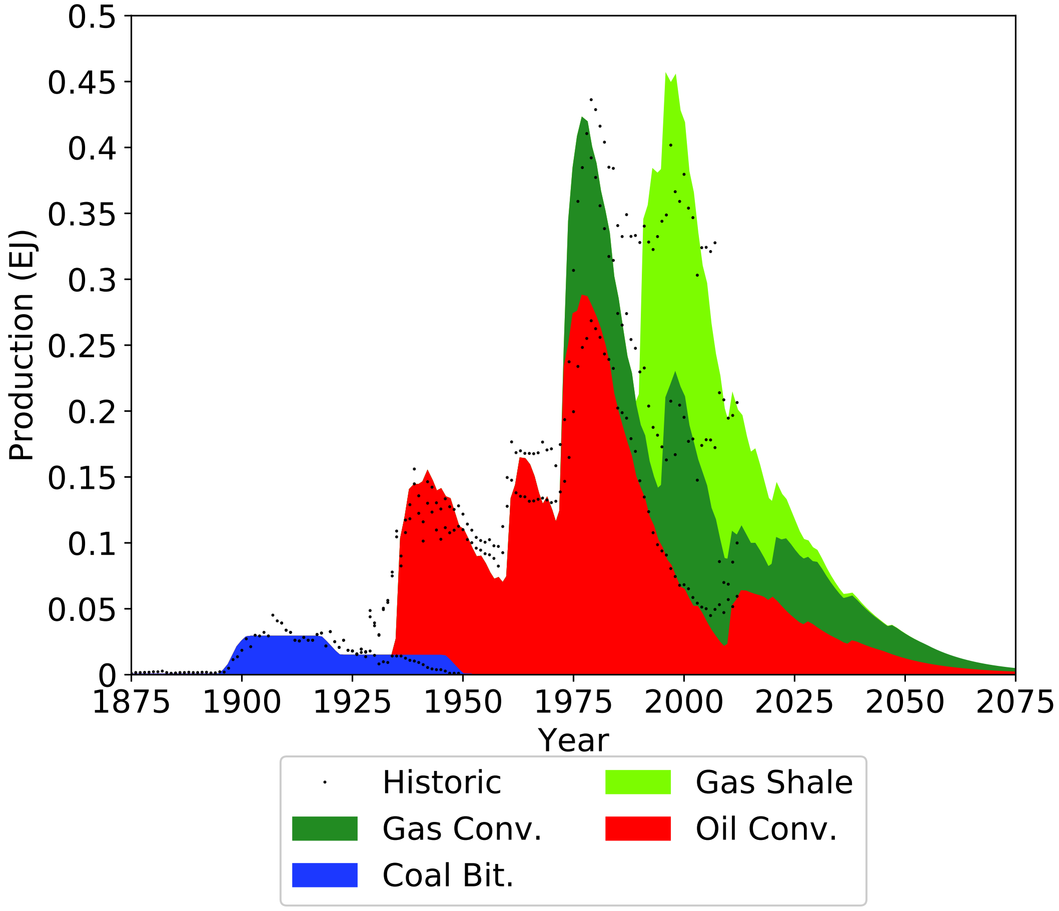

Figure 6.59: USA - Michigan projection by mineral type

Table 6.59: Peak years - Minerals

Name	URR	Peak Year	Peak Rate
Coal Bit.	1.04	1902	0.03
Oil Conv.	11.37	1977	0.29
Gas Conv.	5.25	1998	0.15
Gas Shale	4.67	1996	0.24
**Total**	**22.33**	**1996**	**0.45**

Mississippi

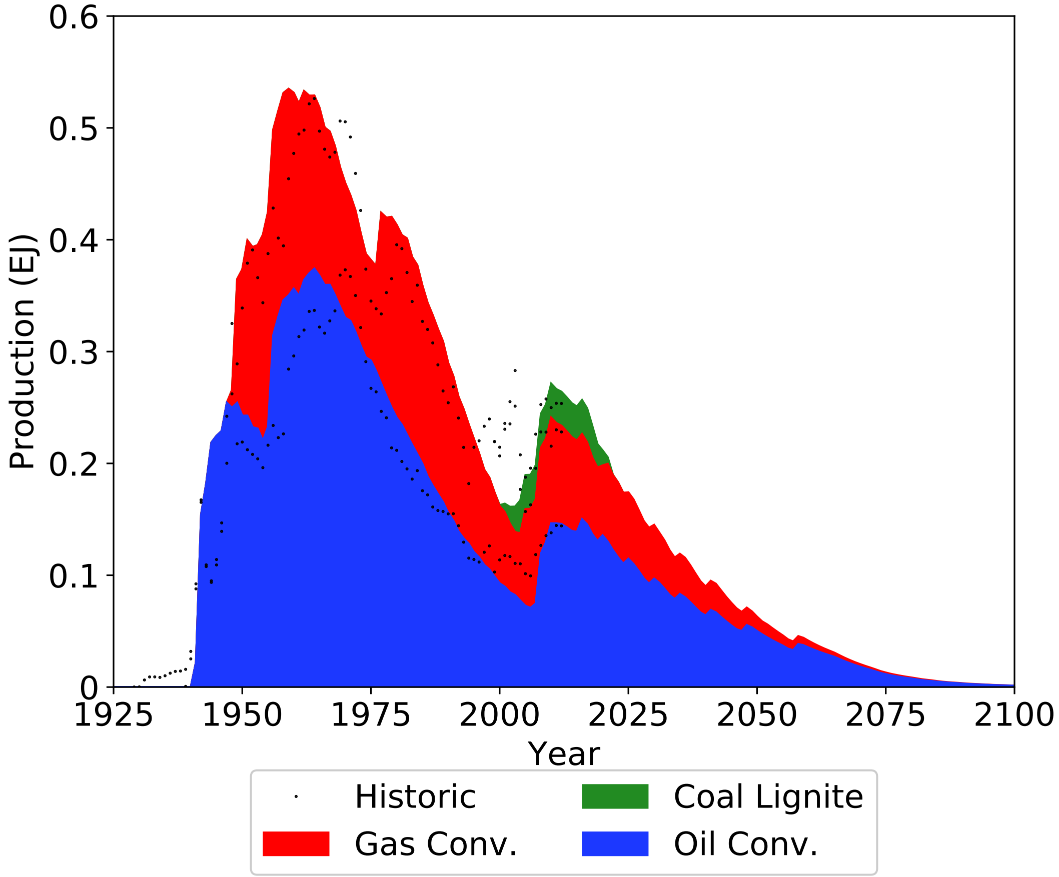

Figure 6.60: USA - Mississippi projections capped at 16

Table 6.60: Peak years - All

Name	URR	Peak Year	Peak Rate
Oil Conv. Mississippi	20.09	1964	0.37
Gas Conv. Mississippi	10.15	1955	0.19
Coal Lignite Mississippi	0.54	2005	0.03
**Total**	**30.78**	**1959**	**0.53**

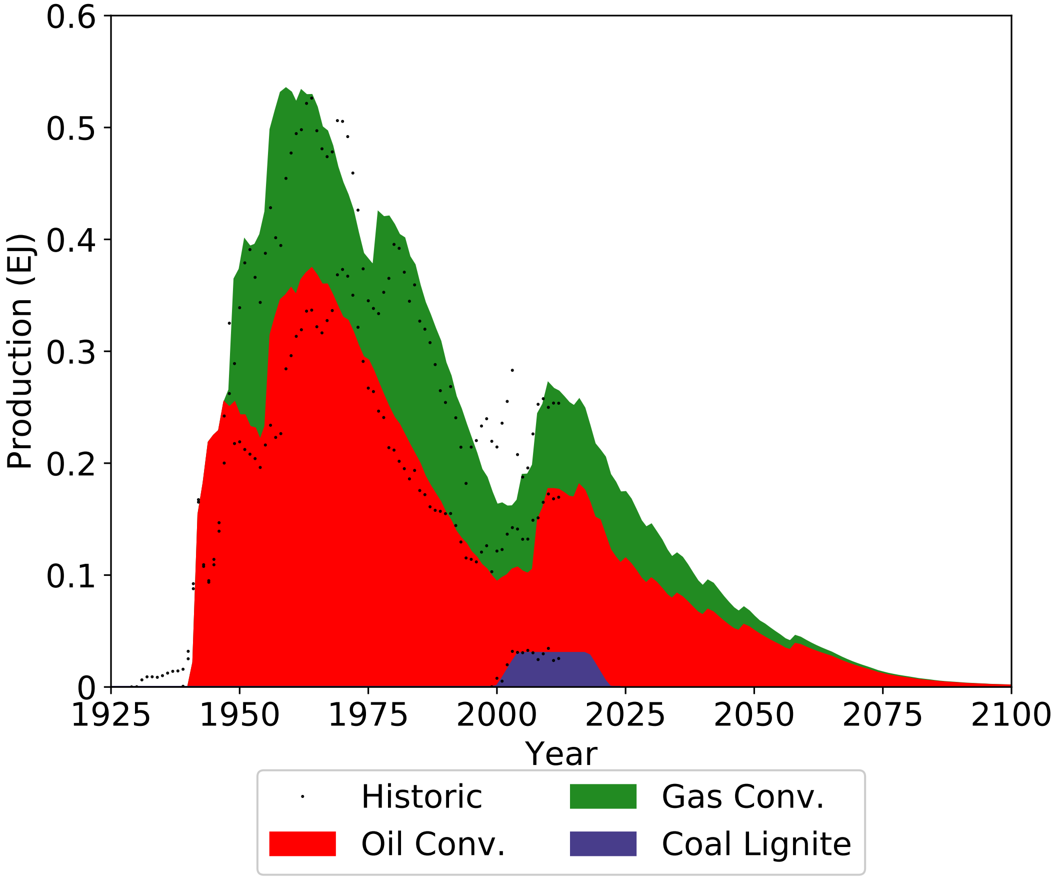

Figure 6.61: USA - Mississippi projection by mineral type

Table 6.61: Peak years - Minerals

Name	URR	Peak Year	Peak Rate
Coal Lignite	0.54	2005	0.03
Oil Conv.	20.09	1964	0.37
Gas Conv.	10.15	1955	0.19
**Total**	**30.78**	**1959**	**0.53**

Missouri

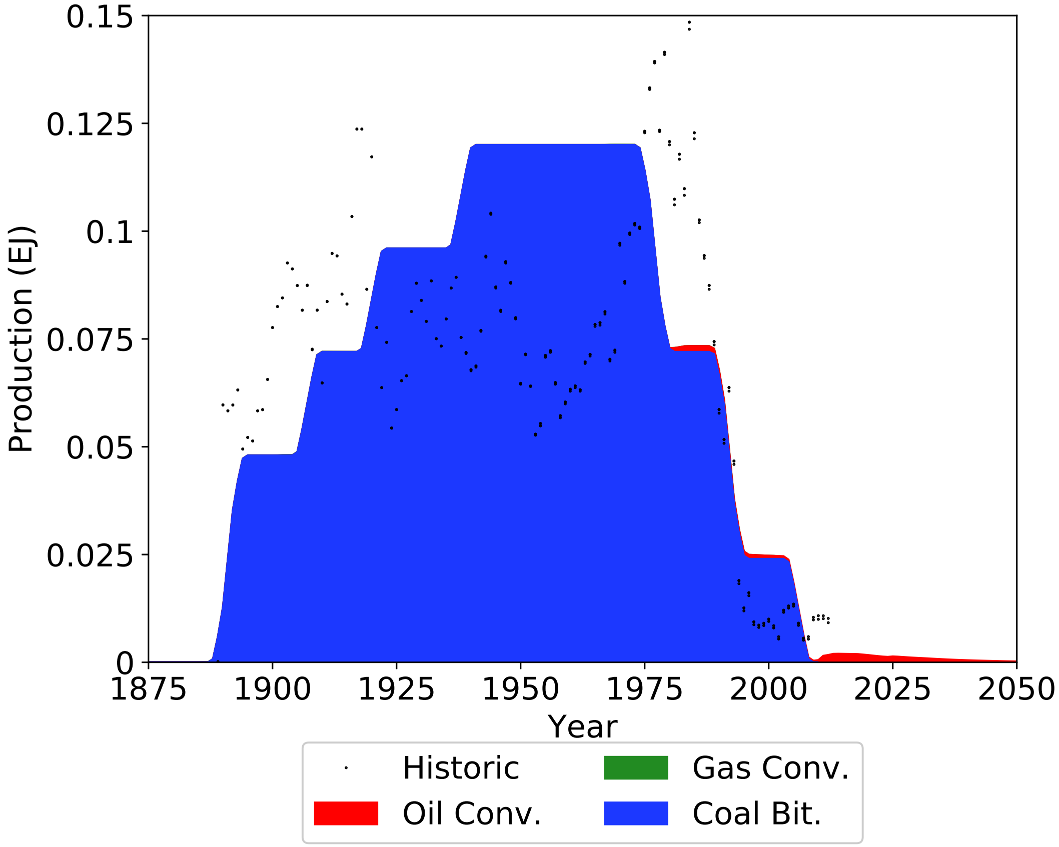

Figure 6.62: USA - Missouri projections capped at 16

Table 6.62: Peak years - All

Name	URR	Peak Year	Peak Rate
Coal Bit. Missouri	9.53	1941	0.12
Oil Conv. Missouri	0.07	2014	–
Gas Conv. Missouri	–	1903	–
**Total**	**9.6**	**1969**	**0.12**

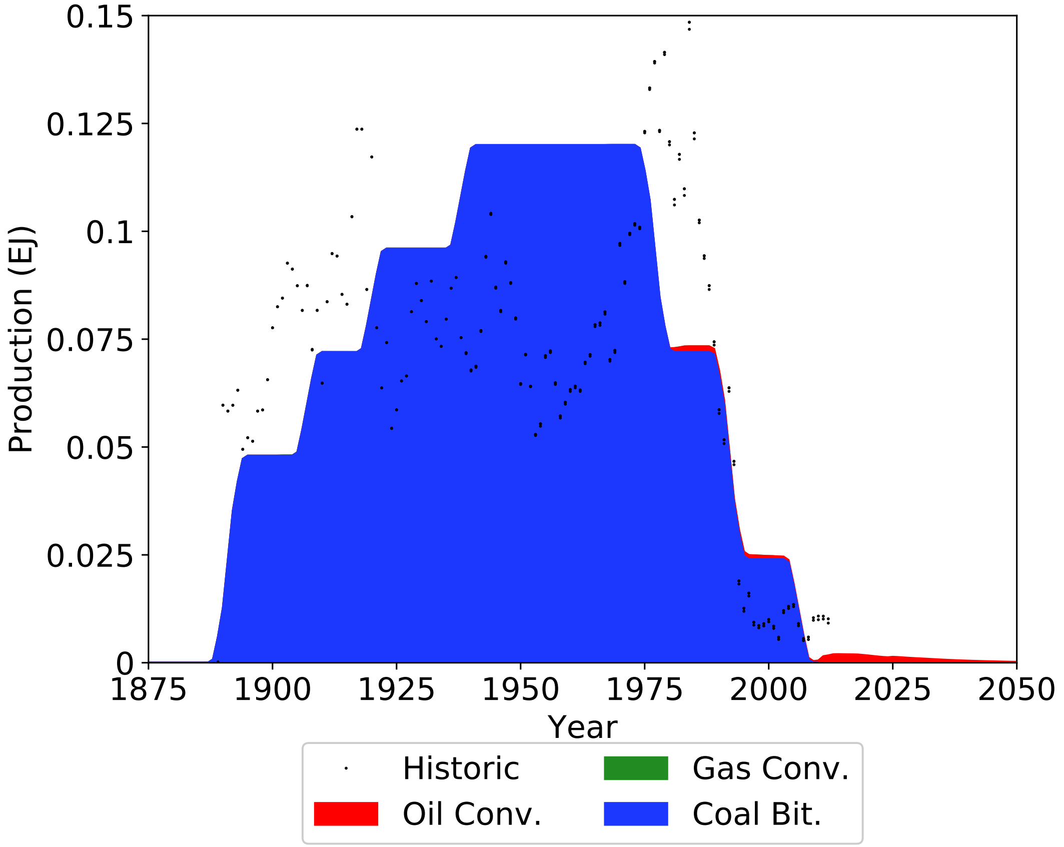

Figure 6.63: USA - Missouri projection by mineral type

Table 6.63: Peak years - Minerals

Name	URR	Peak Year	Peak Rate
Coal Bit.	9.53	1941	0.12
Oil Conv.	0.07	2014	–
Gas Conv.	–	1903	–
**Total**	**9.6**	**1969**	**0.12**

Montana

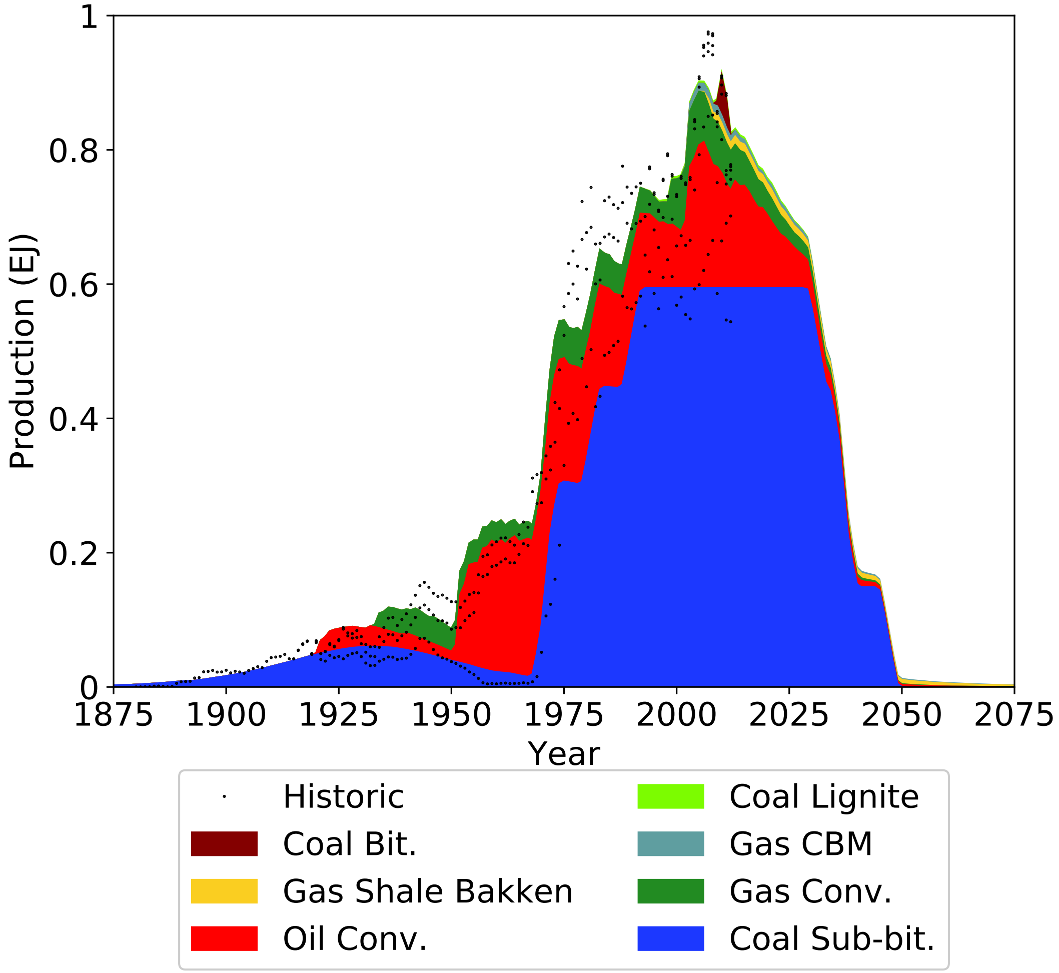

Figure 6.64: USA - Montana projections capped at 16

Table 6.64: Peak years - All

Name	URR	Peak Year	Peak Rate
Coal Sub-bit. Montana	38.52	1993	0.59
Oil Conv. Montana	12.51	2006	0.22
Gas Conv. Montana	4.21	2004	0.09
Gas Shale Montana Bakken	0.57	2021	0.01
Gas CBM Montana	0.3	2004	0.01
Coal Bit. Montana	0.11	2010	0.06
Coal Lignite Montana	0.11	1999	–
**Total**	**56.33**	**2010**	**0.91**

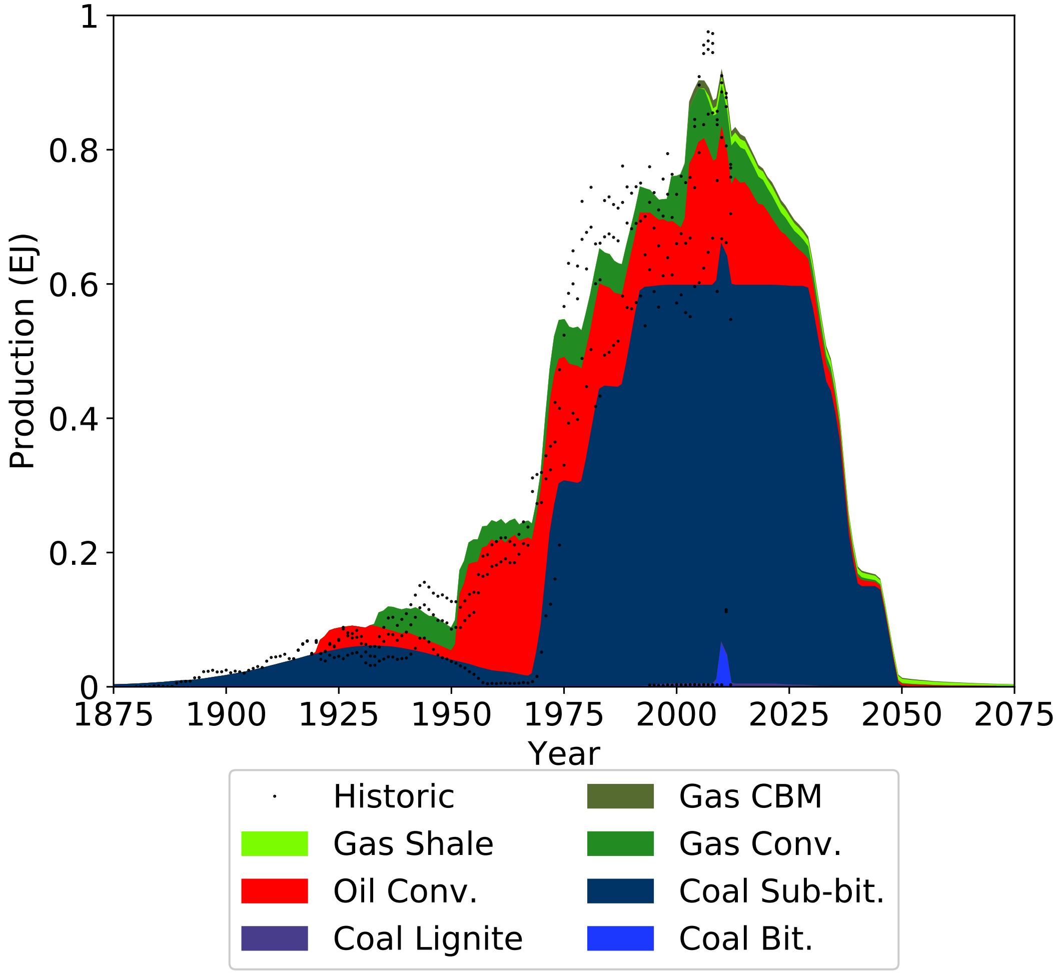

Figure 6.65: USA - Montana projection by mineral type

Table 6.65: Peak years - Minerals

**Name**	**URR**	**Peak Year**	**Peak Rate**
Coal Bit.	0.11	2010	0.06
Coal Lignite	0.11	1999	–
Coal Sub-bit.	38.52	1993	0.59
Oil Conv.	12.51	2006	0.22
Gas Conv.	4.21	2004	0.09
Gas Shale	0.57	2021	0.01
Gas CBM	0.3	2004	0.01
**Total**	**56.33**	**2010**	**0.91**

Nebraska

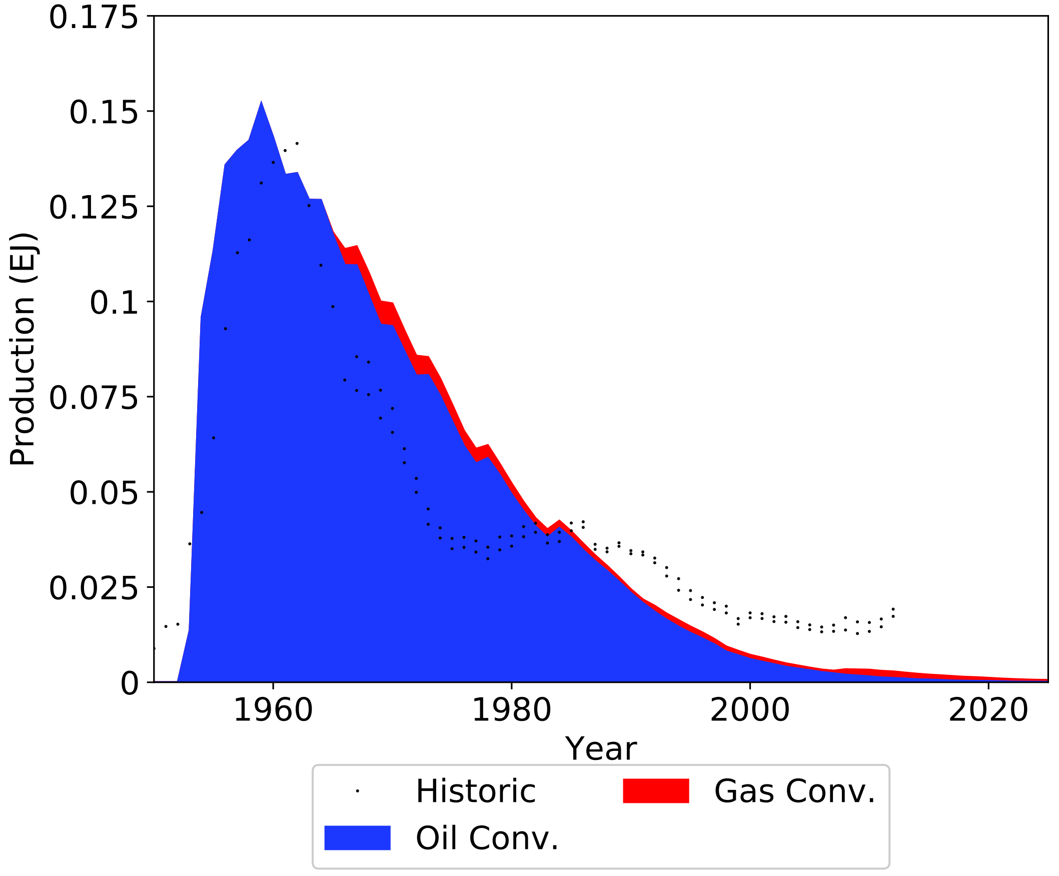

Figure 6.66: USA - Nebraska projections capped at 16

Table 6.66: Peak years - All

Name	URR	Peak Year	Peak Rate
Oil Conv. Nebraska	3.27	1959	0.15
Gas Conv. Nebraska	0.13	1969	0.01
**Total**	**3.4**	**1959**	**0.15**

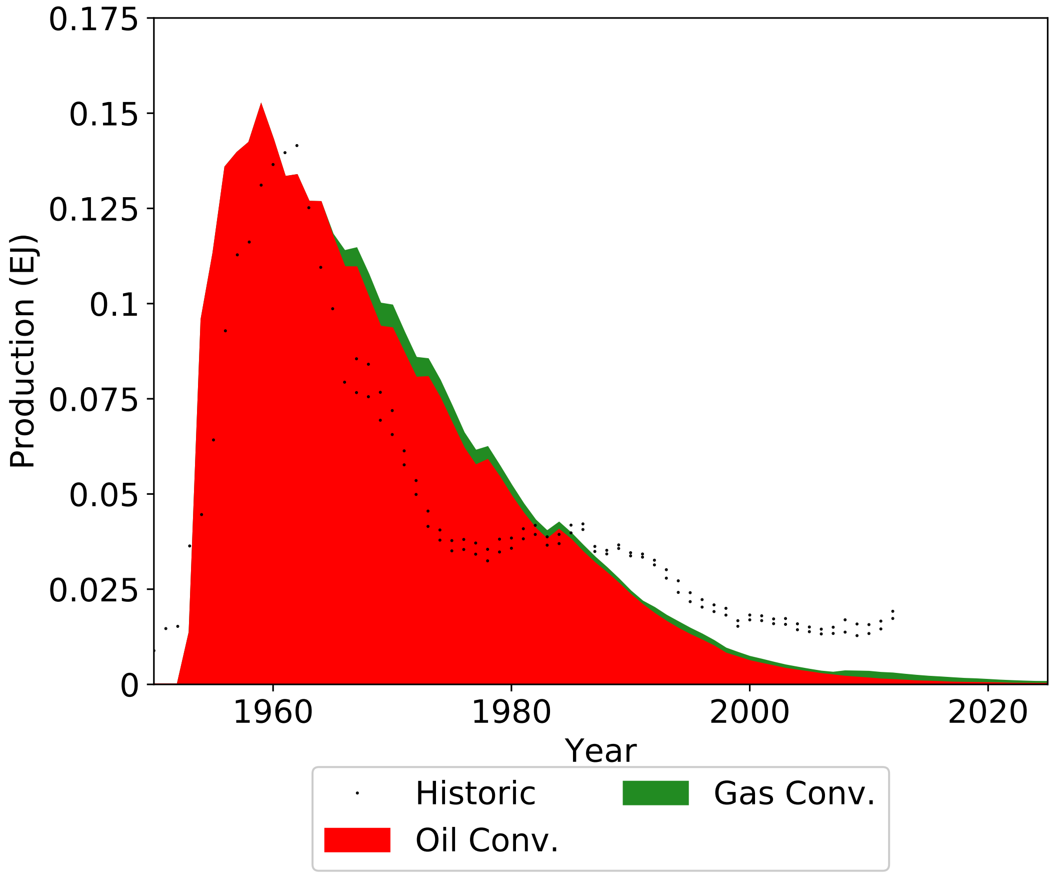

Figure 6.67: USA - Nebraska projection by mineral type

Table 6.67: Peak years - Minerals

Name	URR	Peak Year	Peak Rate
Oil Conv.	3.27	1959	0.15
Gas Conv.	0.13	1969	0.01
**Total**	**3.4**	**1959**	**0.15**

Nevada

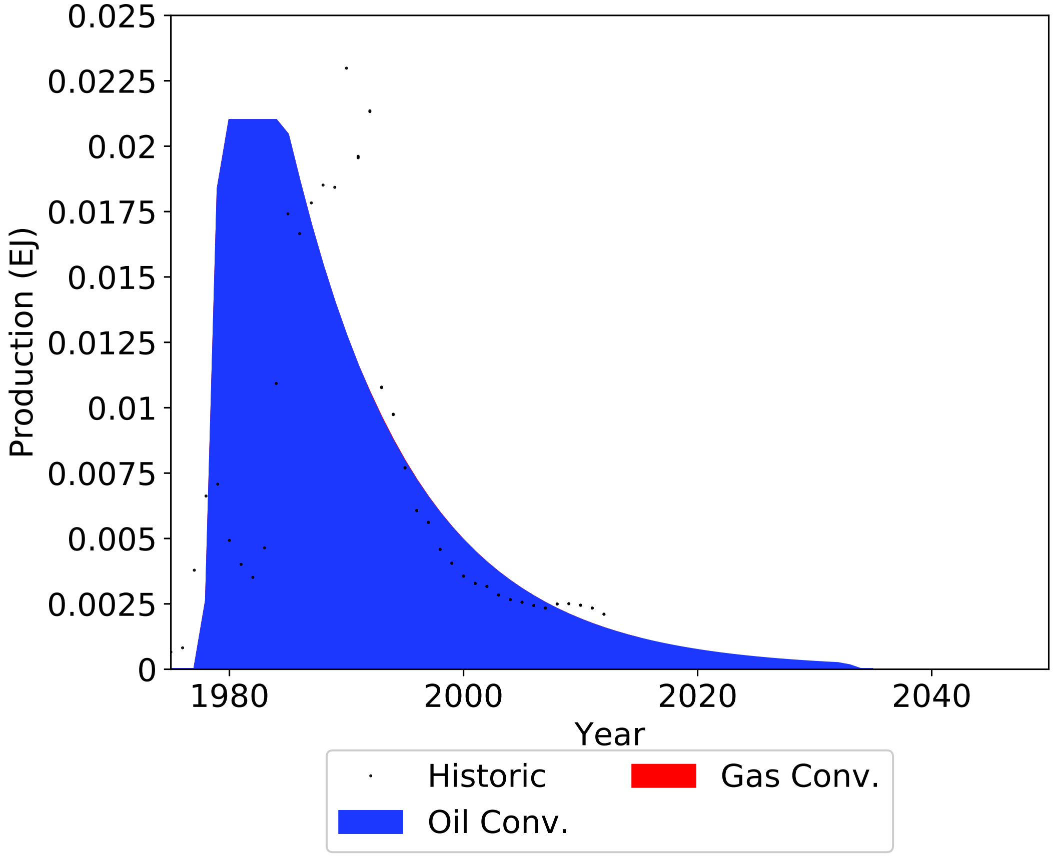

Figure 6.68: USA - Nevada projections capped at 16

Table 6.68: Peak years - All

Name	URR	Peak Year	Peak Rate
Oil Conv. Nevada	0.35	1980	0.02
Gas Conv. Nevada	–	1993	–
**Total**	**0.35**	**1980**	**0.02**

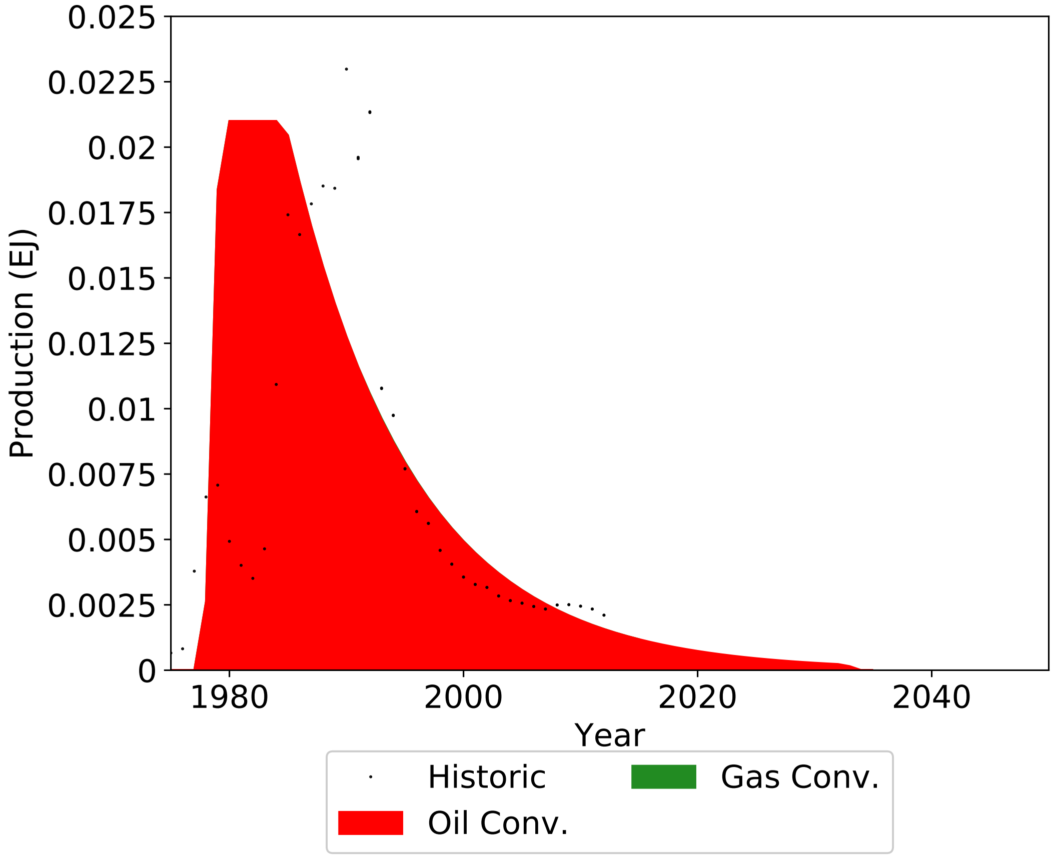

Figure 6.69: USA - Nevada projection by mineral type

Table 6.69: Peak years - Minerals

Name	URR	Peak Year	Peak Rate
Oil Conv.	0.35	1980	0.02
Gas Conv.	–	1993	–
**Total**	**0.35**	**1980**	**0.02**

New Mexico

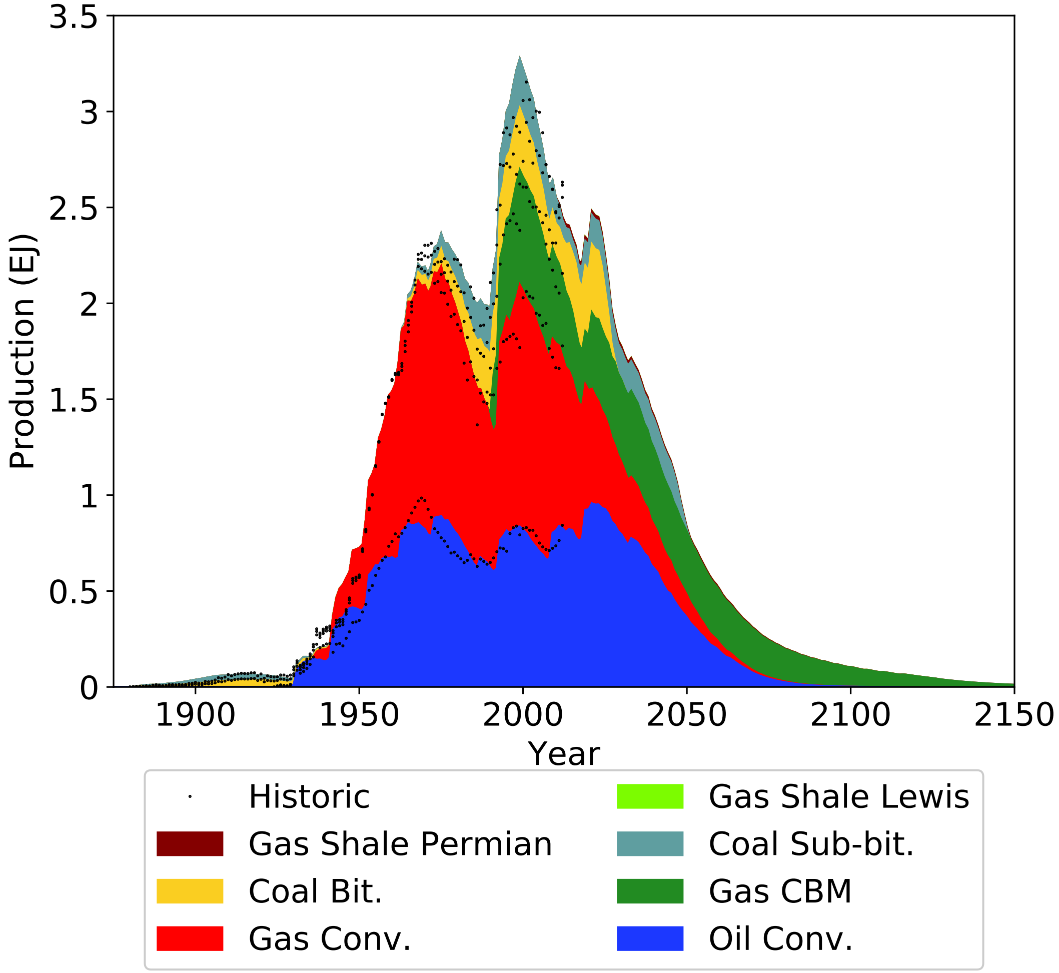

Figure 6.70: USA - New Mexico projections capped at 16

Table 6.70: Peak years - All

Name	URR	Peak Year	Peak Rate
Oil Conv. New Mexico	82.6	2021	0.96
Gas Conv. New Mexico	81.47	1975	1.3
Gas CBM New Mexico	38.36	1998	0.6
Coal Bit. New Mexico	15.22	2022	0.36
Coal Sub-bit. New Mexico	13.67	1998	0.26
Gas Shale New Mexico Permian	0.93	2025	0.02
Gas Shale New Mexico Lewis	0.05	2017	–
**Total**	**232.3**	**1999**	**3.27**

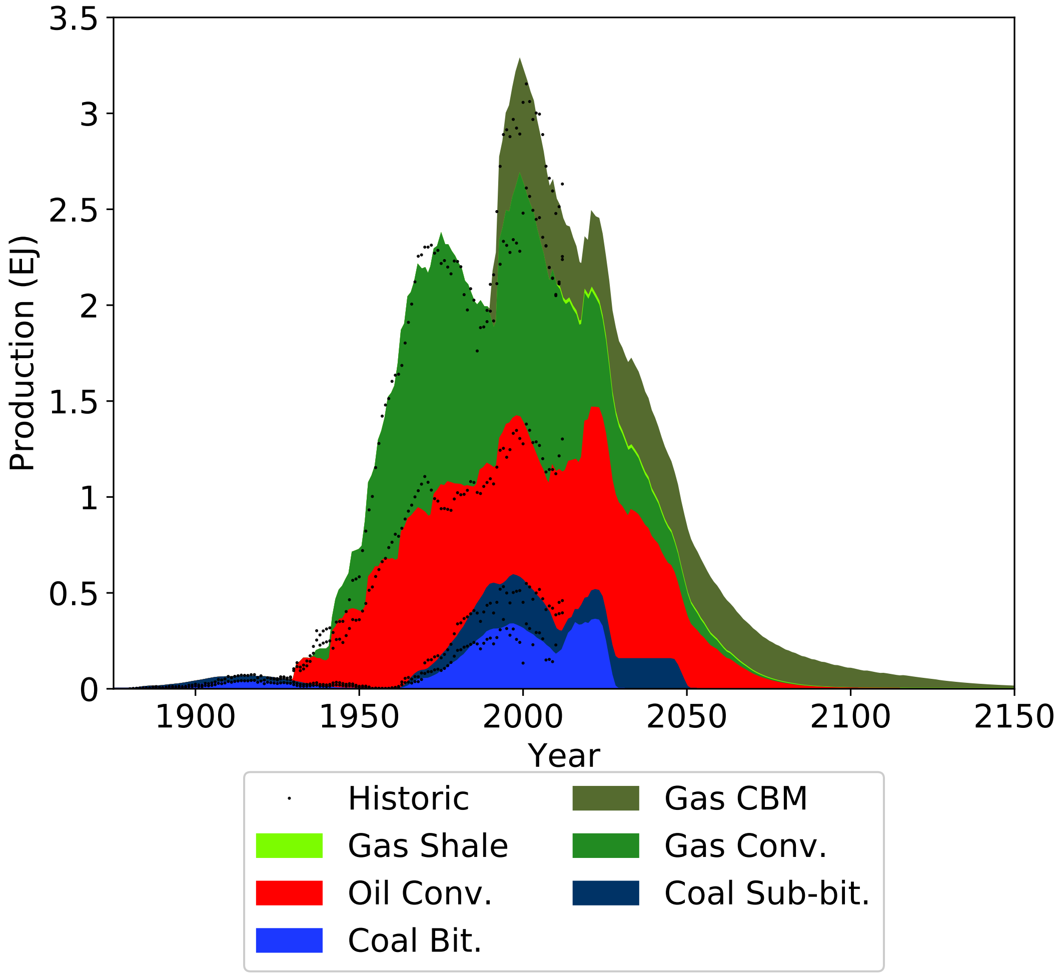

Figure 6.71: USA - New Mexico projection by mineral type

Table 6.71: Peak years - Minerals

**Name**	**URR**	**Peak Year**	**Peak Rate**
Coal Bit.	15.22	2022	0.36
Coal Sub-bit.	13.67	1998	0.26
Oil Conv.	82.6	2021	0.96
Gas Conv.	81.47	1975	1.3
Gas Shale	0.98	2025	0.02
Gas CBM	38.36	1998	0.6
**Total**	**232.3**	**1999**	**3.27**

New York

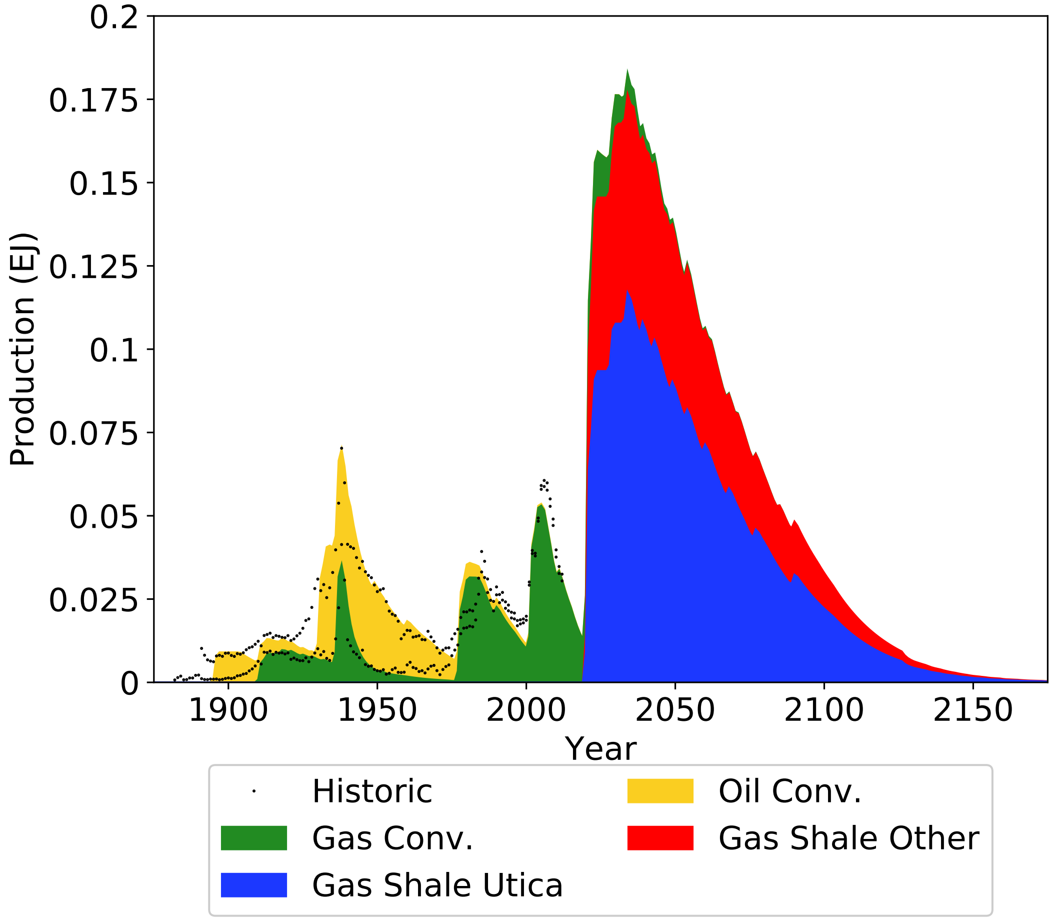

Figure 6.72: USA - New York projections capped at 16

Table 6.72: Peak years - All

Name	URR	Peak Year	Peak Rate
Gas Shale New York Utica	5.83	2034	0.12
Gas Shale New York Other	3.05	2036	0.06
Gas Conv. New York	1.8	2005	0.05
Oil Conv. New York	1.24	1941	0.04
**Total**	**11.92**	**2034**	**0.18**

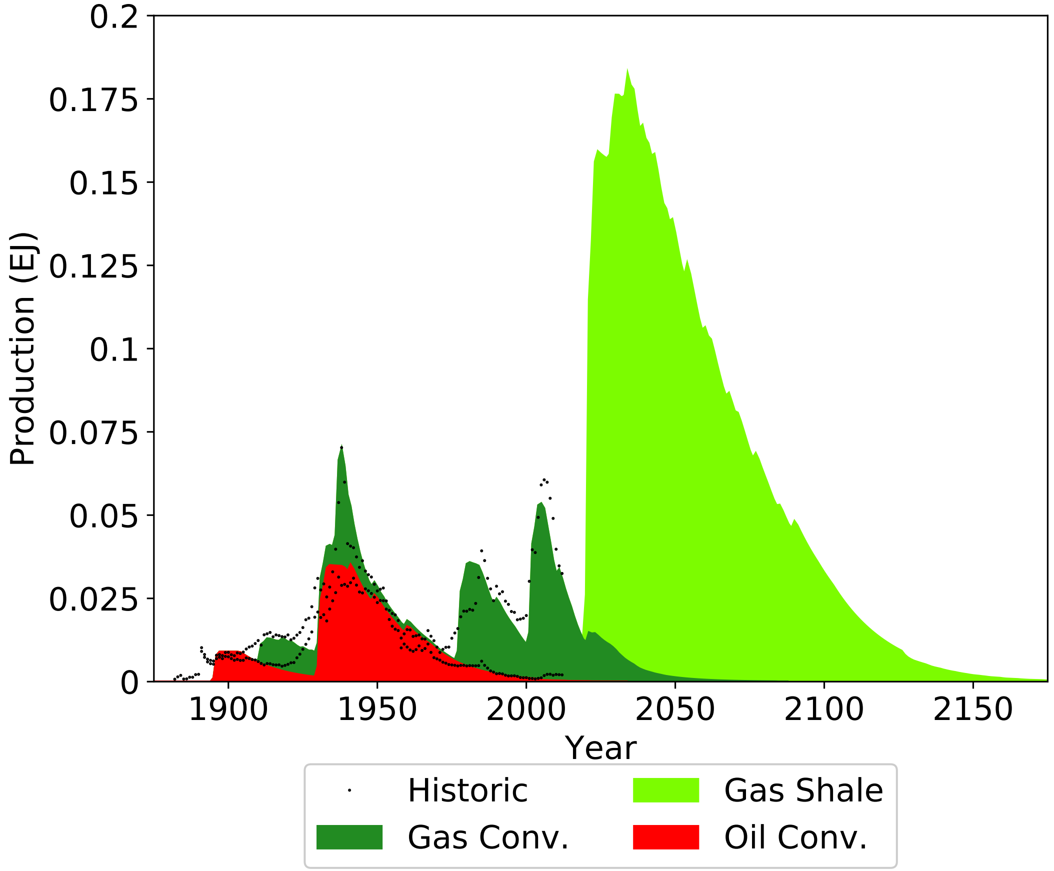

Figure 6.73: USA - New York projection by mineral type

Table 6.73: Peak years - Minerals

Name	URR	Peak Year	Peak Rate
Oil Conv.	1.24	1941	0.04
Gas Conv.	1.8	2005	0.05
Gas Shale	8.88	2034	0.18
**Total**	**11.92**	**2034**	**0.18**

North Dakota

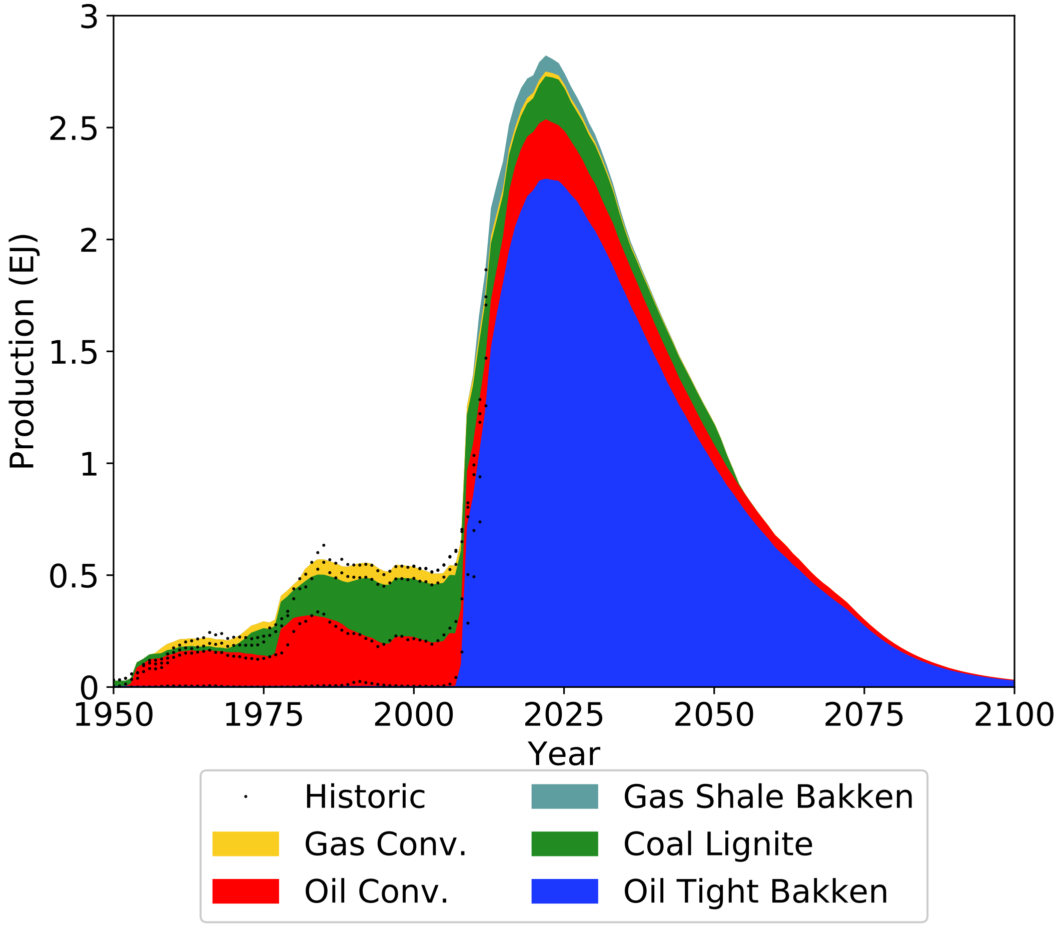

Figure 6.74: USA - North Dakota projections capped at 16

Table 6.74: Peak years - All

Name	URR	Peak Year	Peak Rate
Oil Tight North Dakota Bakken	87.48	2022	2.27
Oil Conv. North Dakota	20.8	1983	0.32
Coal Lignite North Dakota	15.17	1993	0.26
Gas Conv. North Dakota	3.09	1990	0.07
Gas Shale North Dakota Bakken	1.77	2014	0.13
**Total**	**128.31**	**2022**	**2.82**

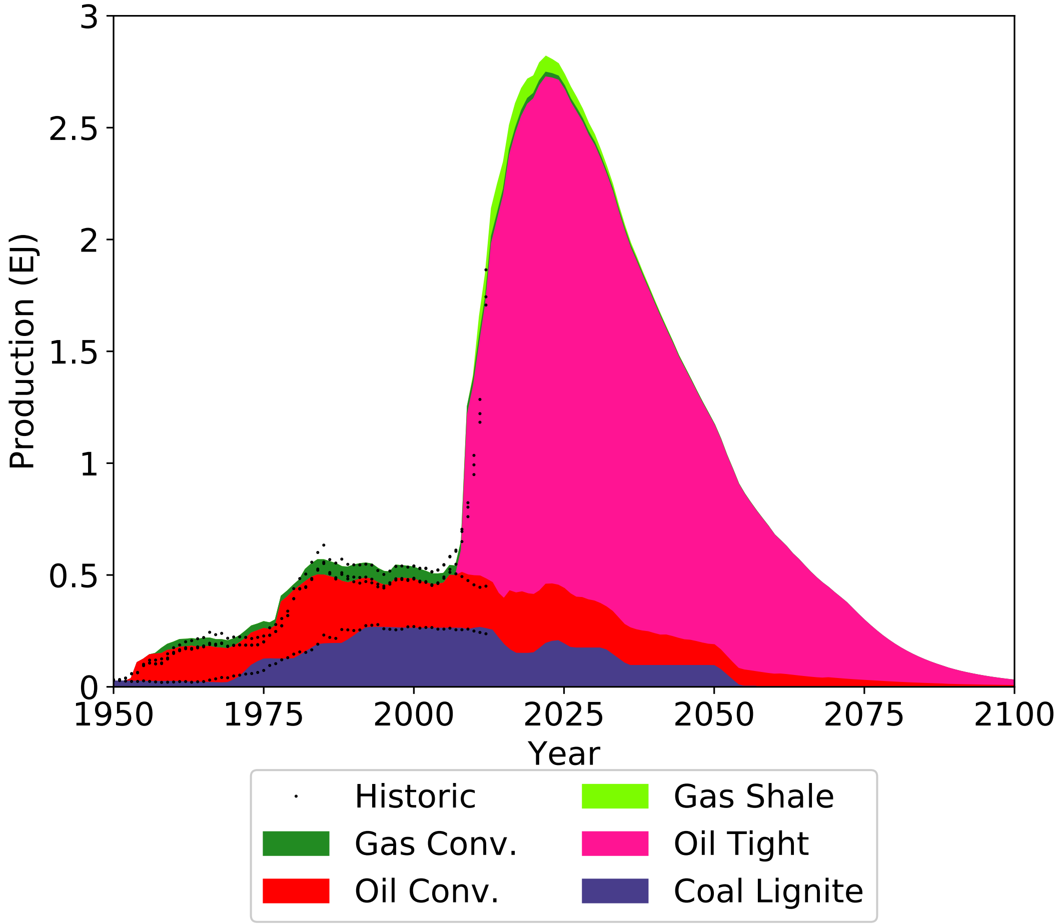

Figure 6.75: USA - North Dakota projection by mineral type

Table 6.75: Peak years - Minerals

Name	URR	Peak Year	Peak Rate
Coal Lignite	15.17	1993	0.26
Oil Conv.	20.8	1983	0.32
Oil Tight	87.48	2022	2.27
Gas Conv.	3.09	1990	0.07
Gas Shale	1.77	2014	0.13
**Total**	**128.31**	**2022**	**2.82**

Ohio

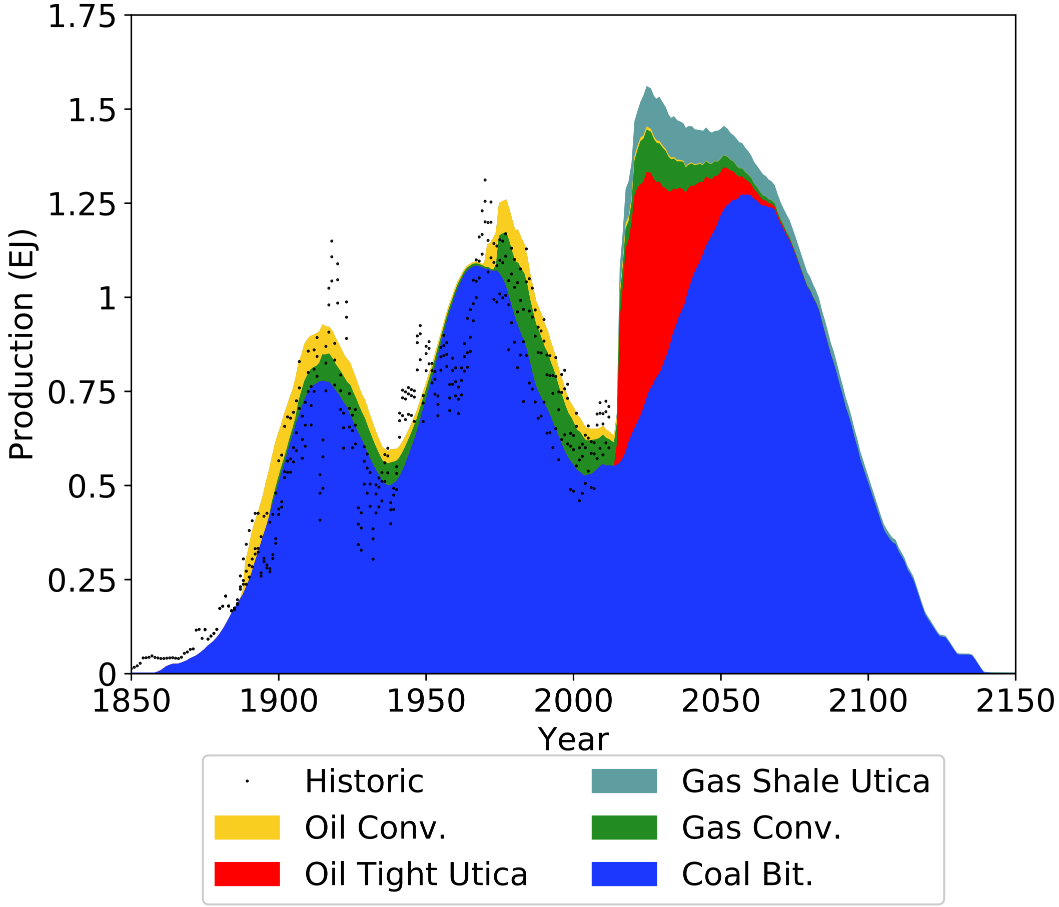

Figure 6.76: USA - Ohio projections capped at 16

Table 6.76: Peak years - All

Name	URR	Peak Year	Peak Rate
Coal Bit. Ohio	179.97	2059	1.27
Oil Tight Ohio Utica	14.33	2022	0.62
Gas Conv. Ohio	10.99	1983	0.18
Oil Conv. Ohio	7.08	1899	0.12
Gas Shale Ohio Utica	5.83	2029	0.12
**Total**	**218.2**	**2025**	**1.56**

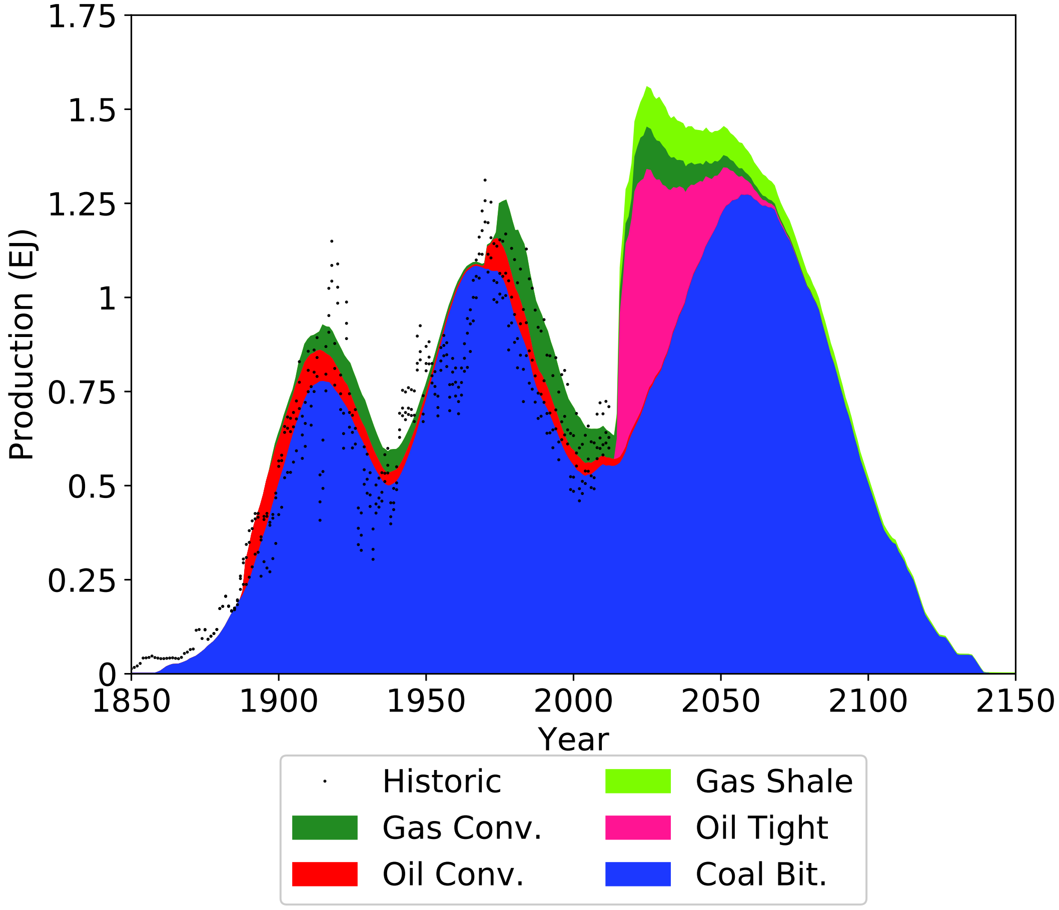

Figure 6.77: USA - Ohio projection by mineral type

Table 6.77: Peak years - Minerals

Name	URR	Peak Year	Peak Rate
Coal Bit.	179.97	2059	1.27
Oil Conv.	7.08	1899	0.12
Oil Tight	14.33	2022	0.62
Gas Conv.	10.99	1983	0.18
Gas Shale	5.83	2029	0.12
**Total**	**218.2**	**2025**	**1.56**

Oklahoma

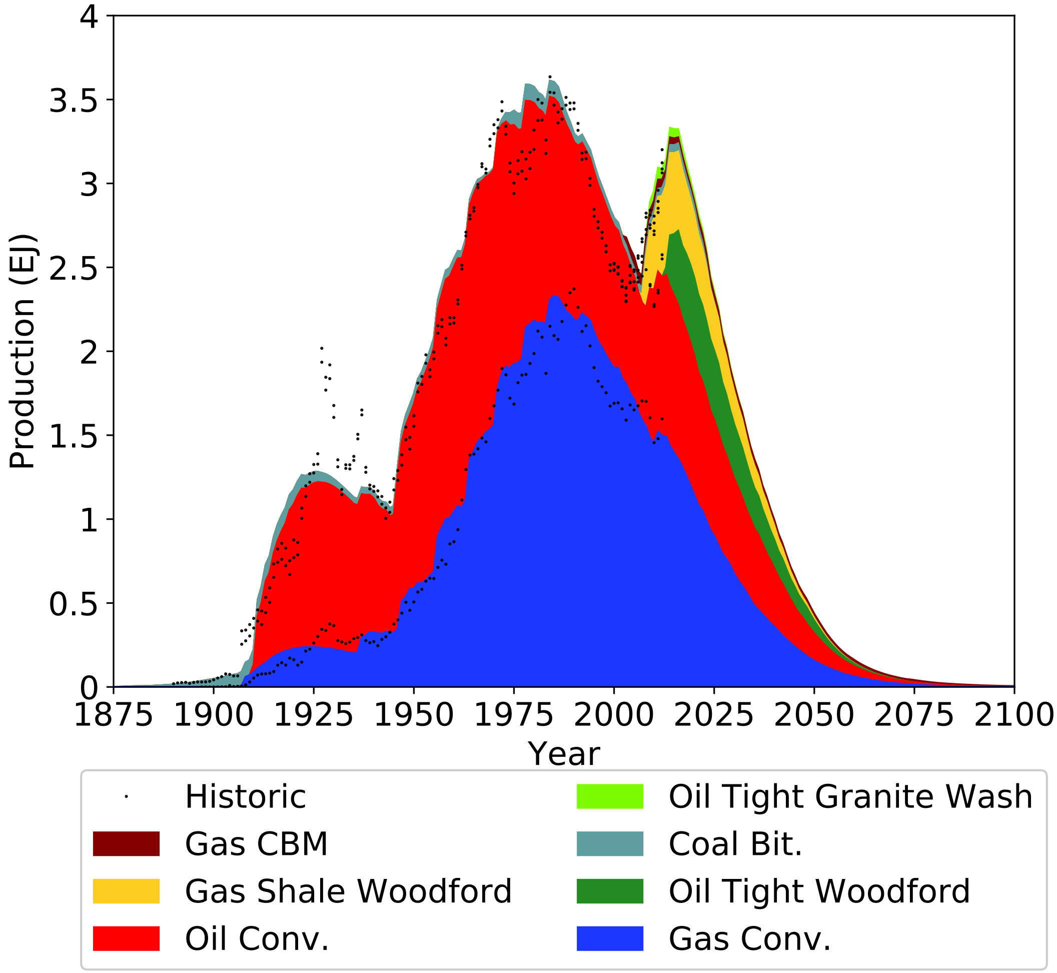

Figure 6.78: USA - Oklahoma projections capped at 16

Table 6.78: Peak years - All

Name	URR	Peak Year	Peak Rate
Gas Conv. Oklahoma	143.52	1985	2.33
Oil Conv. Oklahoma	129.07	1966	1.54
Oil Tight Oklahoma Woodford	10.95	2020	0.47
Gas Shale Oklahoma Woodford	10.13	2013	0.49
Coal Bit. Oklahoma	7.62	1914	0.1
Gas CBM Oklahoma	2.69	2006	0.07
Oil Tight Oklahoma Granite Wash	0.89	2011	0.07
**Total**	**304.87**	**1984**	**3.61**

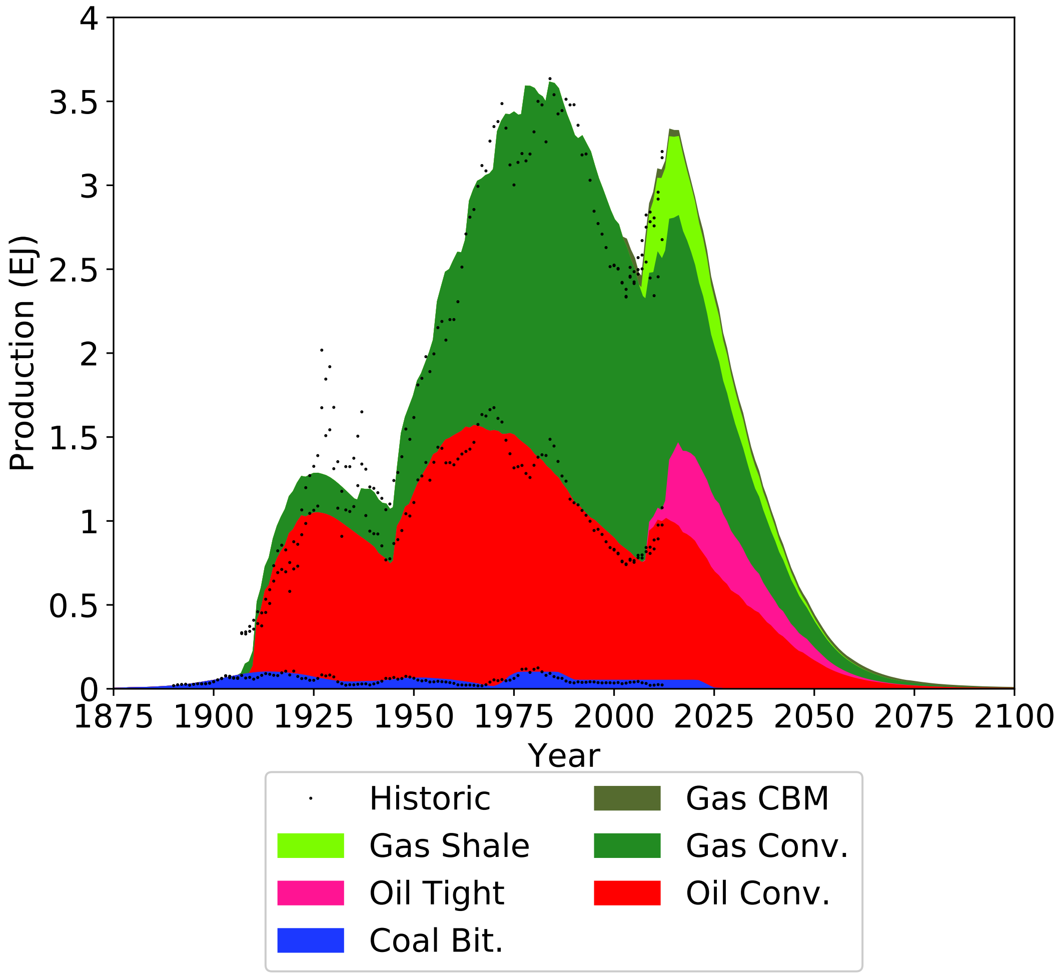

Figure 6.79: USA - Oklahoma projection by mineral type

Table 6.79: Peak years - Minerals

**Name**	**URR**	**Peak Year**	**Peak Rate**
Coal Bit.	7.62	1914	0.1
Oil Conv.	129.07	1966	1.54
Oil Tight	11.84	2020	0.5
Gas Conv.	143.52	1985	2.33
Gas Shale	10.13	2013	0.49
Gas CBM	2.69	2006	0.07
**Total**	**304.87**	**1984**	**3.61**

Oregon

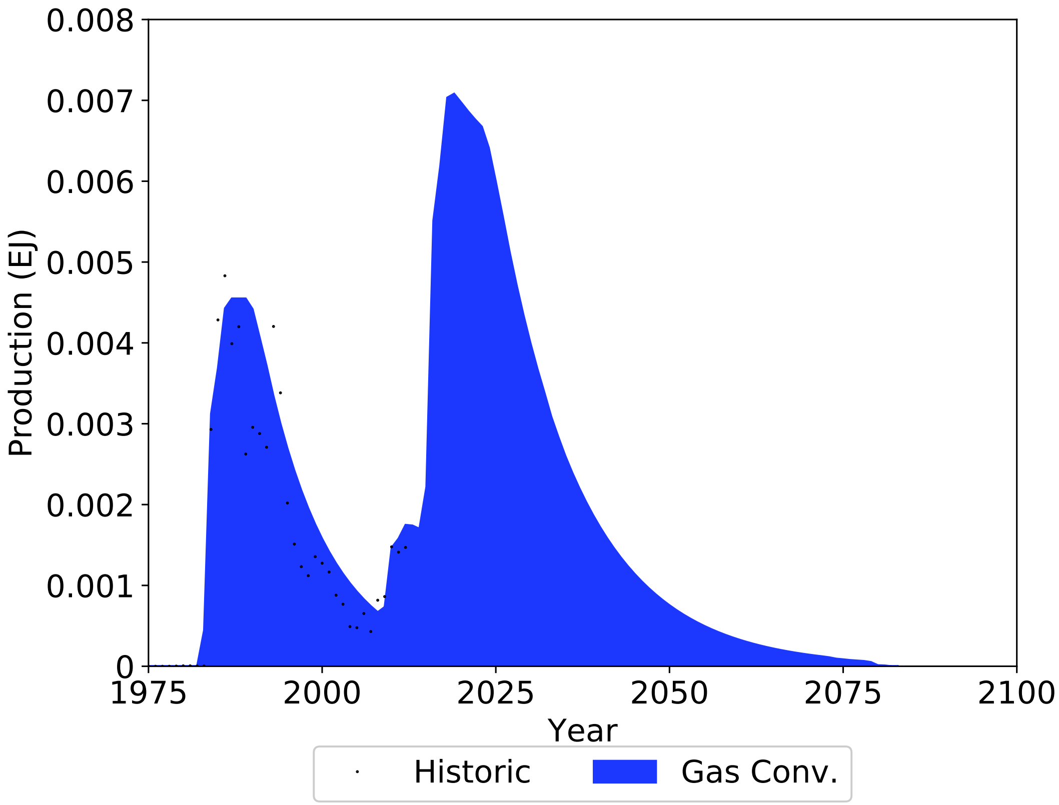

Figure 6.80: USA - Oregon projections capped at 16

Table 6.80: Peak years - All

Name	URR	Peak Year	Peak Rate
Gas Conv. Oregon	0.21	2019	0.01
**Total**	**0.21**	**2019**	**0.01**

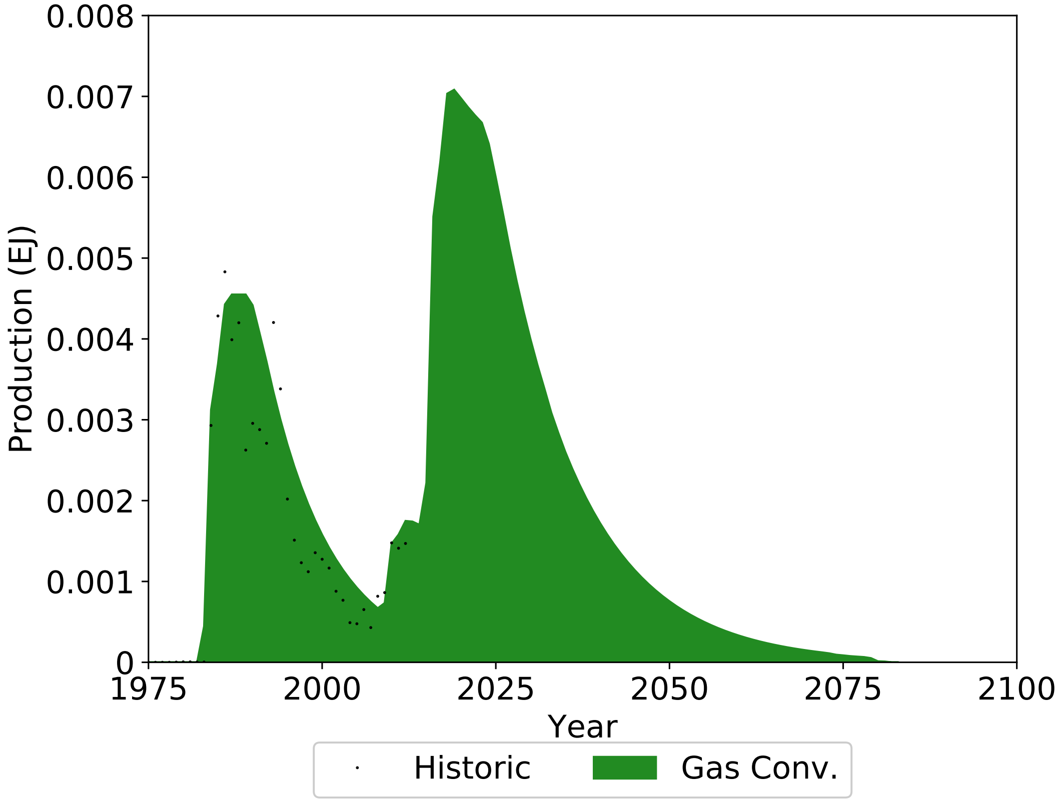

Figure 6.81: USA - Oregon projection by mineral type

Table 6.81: Peak years - Minerals

Name	URR	Peak Year	Peak Rate
Gas Conv.	0.21	2019	0.01
**Total**	**0.21**	**2019**	**0.01**

Other

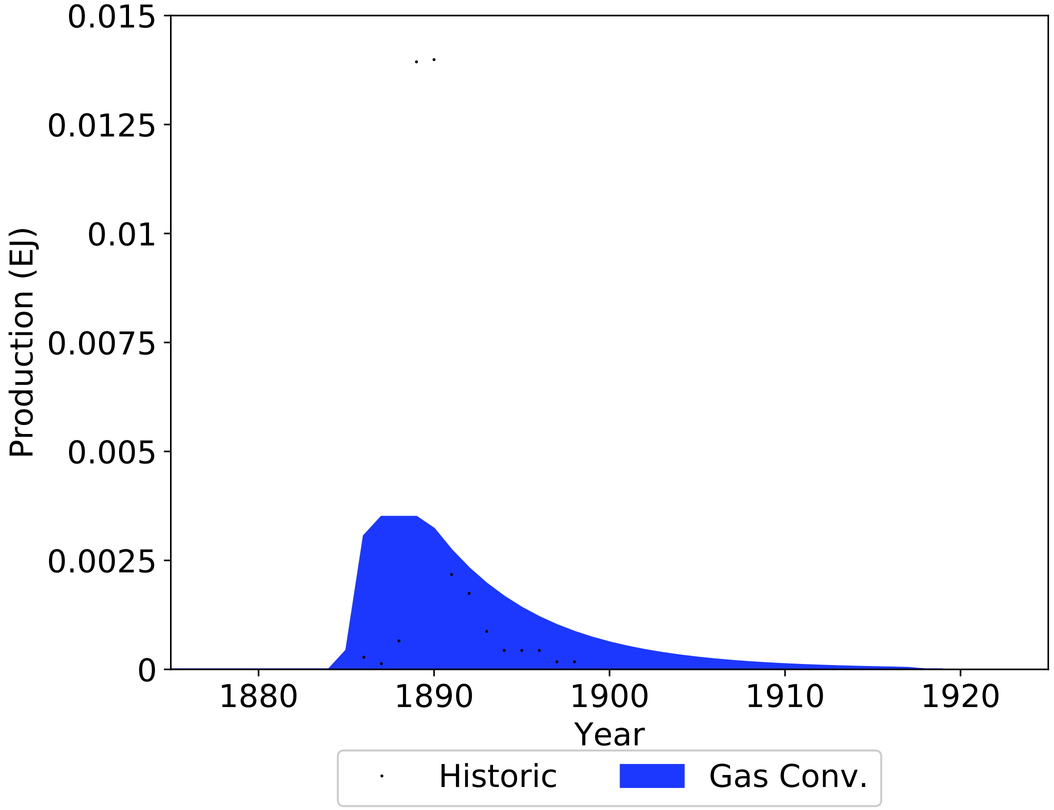

Figure 6.82: USA - Other projections capped at 16

Table 6.82: Peak years - All

**Name**	**URR**	**Peak Year**	**Peak Rate**
Gas Conv. Other	0.04	1887	–
**Total**	**0.04**	**1887**	–

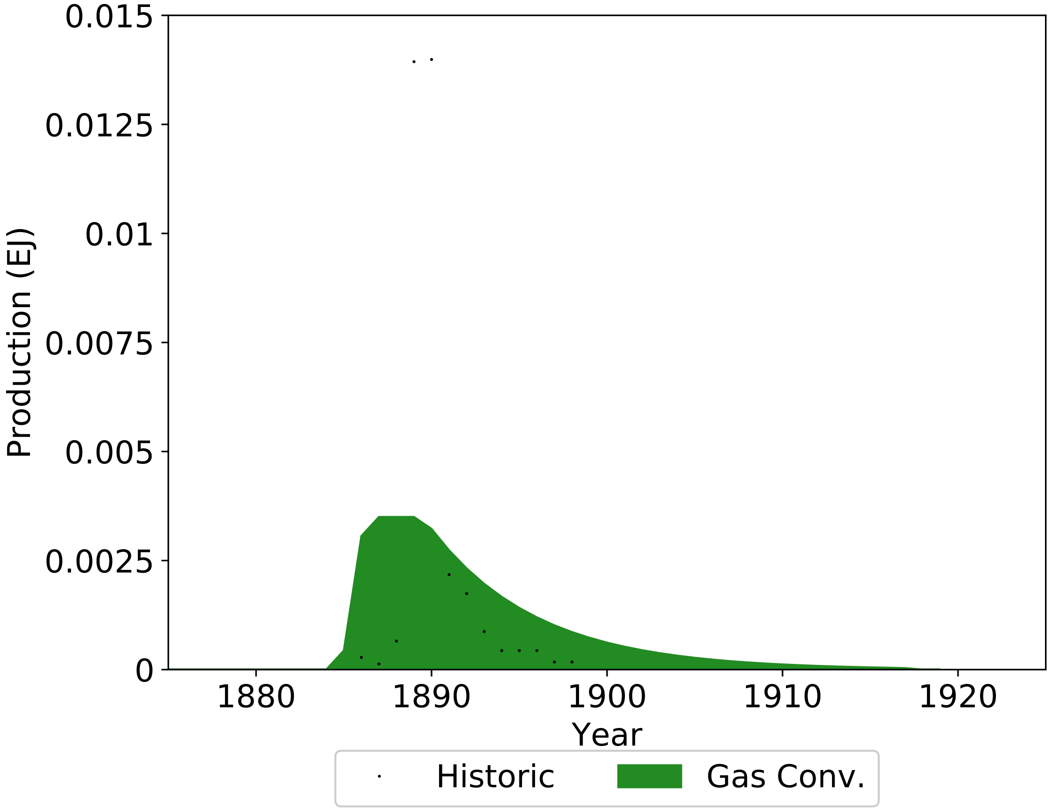

Figure 6.83: USA - Other projection by mineral type

Table 6.83: Peak years - Minerals

Name	URR	Peak Year	Peak Rate
Gas Conv.	0.04	1887	–
**Total**	**0.04**	**1887**	–

Pennsylvania

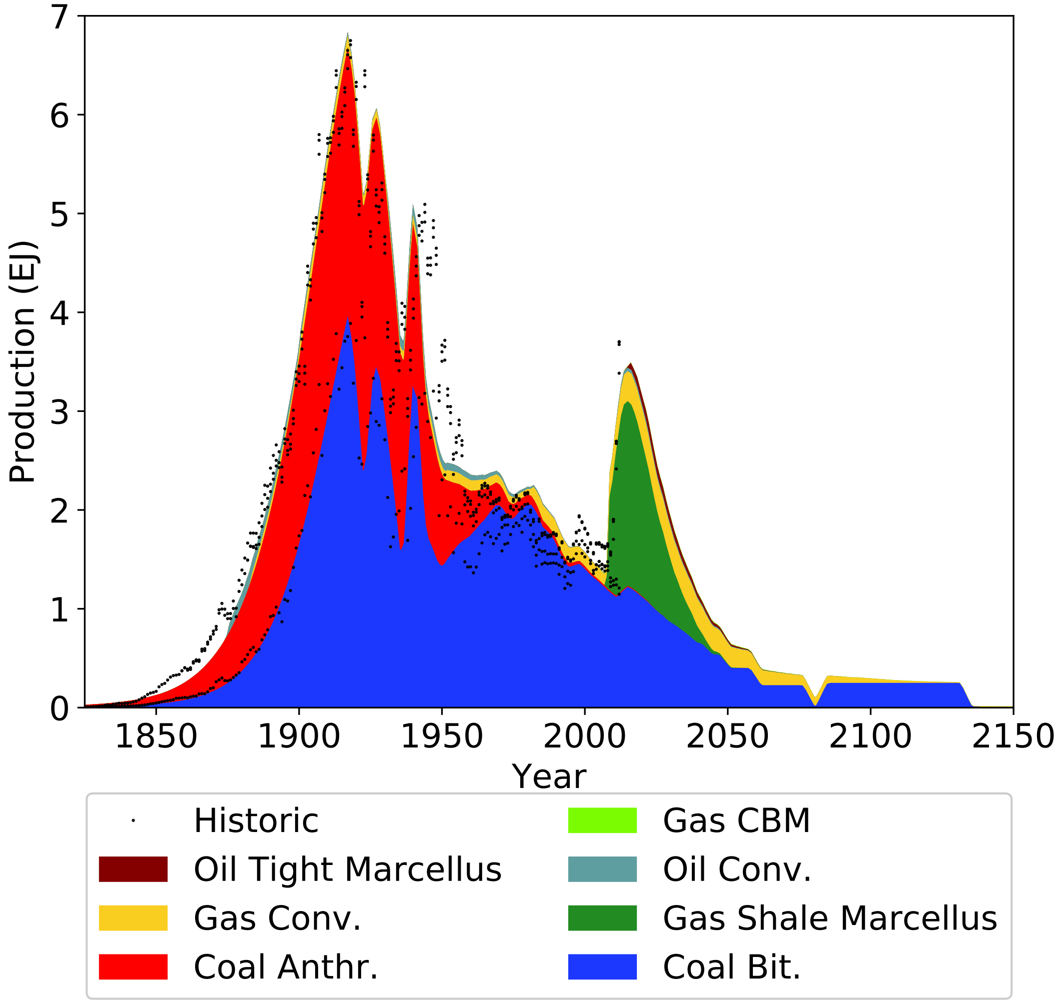

Figure 6.84: USA - Pennsylvania projections capped at 16

Table 6.84: Peak years - All

**Name**	**URR**	**Peak Year**	**Peak Rate**
Coal Bit. Pennsylvania	303.54	1917	3.91
Coal Anthr. Pennsylvania	153.01	1919	2.73
Gas Shale Pennsylvania Marcellus	32.87	2015	1.87
Gas Conv. Pennsylvania	31.5	2021	0.35
Oil Conv. Pennsylvania	8.41	1882	0.14
Oil Tight Pennsylvania Marcellus	2.29	2022	0.1
Gas CBM Pennsylvania	0.54	2024	0.01
**Total**	**532.16**	**1917**	**6.78**

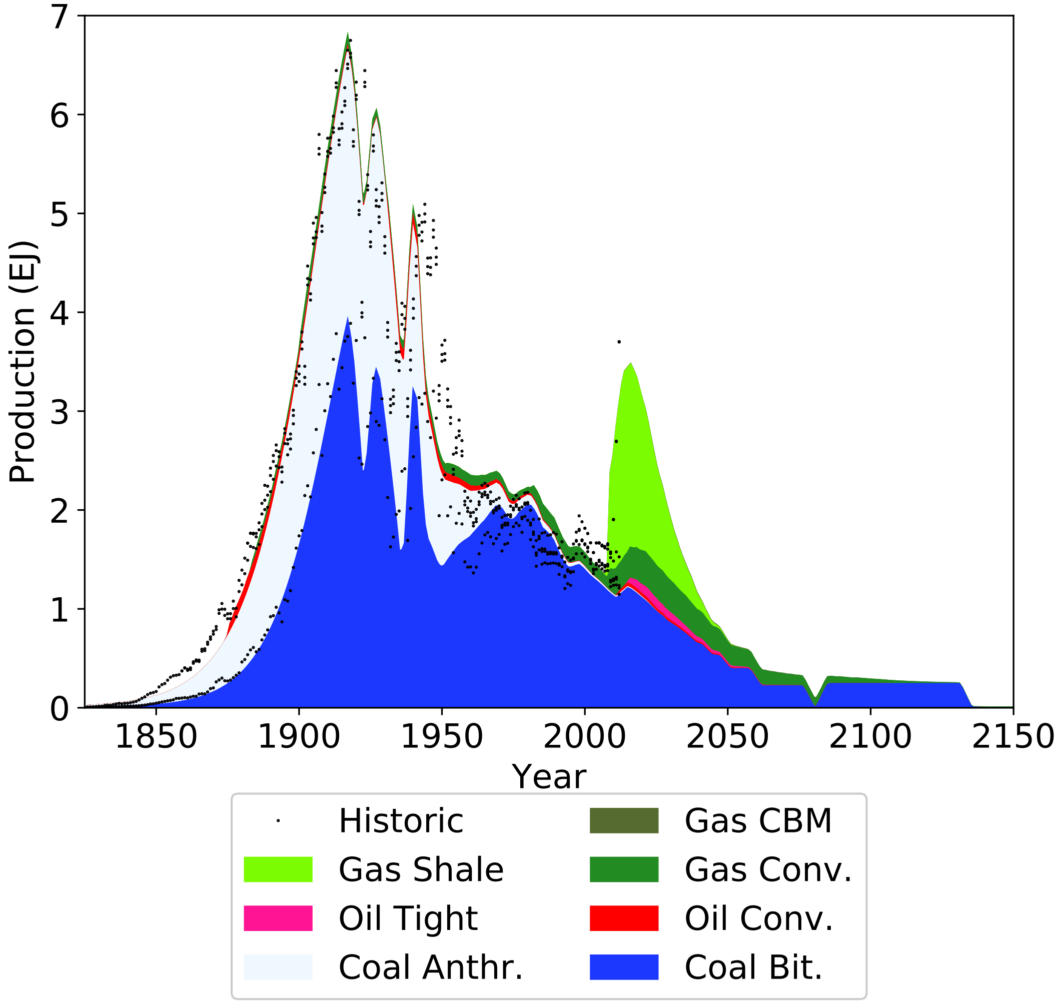

Figure 6.85: USA - Pennsylvania projection by mineral type

Table 6.85: Peak years - Minerals

**Name**	**URR**	**Peak Year**	**Peak Rate**
Coal Bit.	303.54	1917	3.91
Coal Anthr.	153.01	1919	2.73
Oil Conv.	8.41	1882	0.14
Oil Tight	2.29	2022	0.1
Gas Conv.	31.5	2021	0.35
Gas Shale	32.87	2015	1.87
Gas CBM	0.54	2024	0.01
**Total**	**532.16**	**1917**	**6.78**

South Dakota

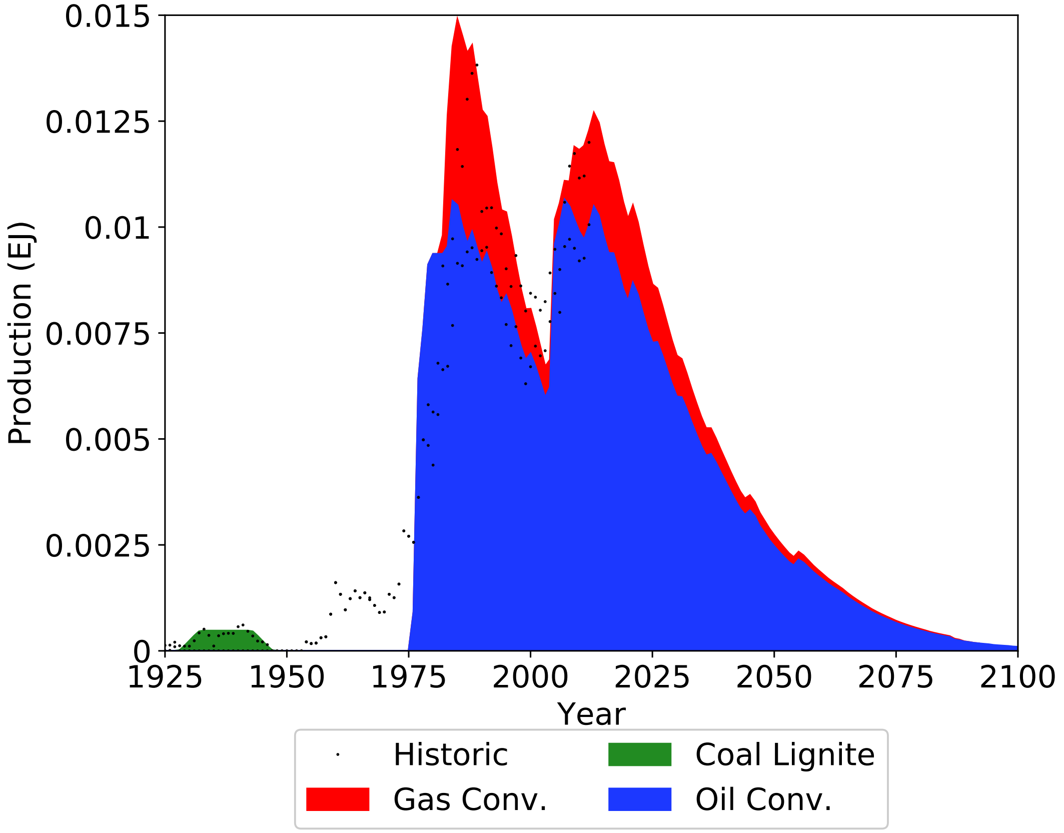

Figure 6.86: USA - South Dakota projections capped at 16

Table 6.86: Peak years - All

**Name**	**URR**	**Peak Year**	**Peak Rate**
Oil Conv. South Dakota	0.59	2007	0.01
Gas Conv. South Dakota	0.11	1986	–
Coal Lignite South Dakota	0.01	1933	–
**Total**	**0.71**	**1985**	**0.01**

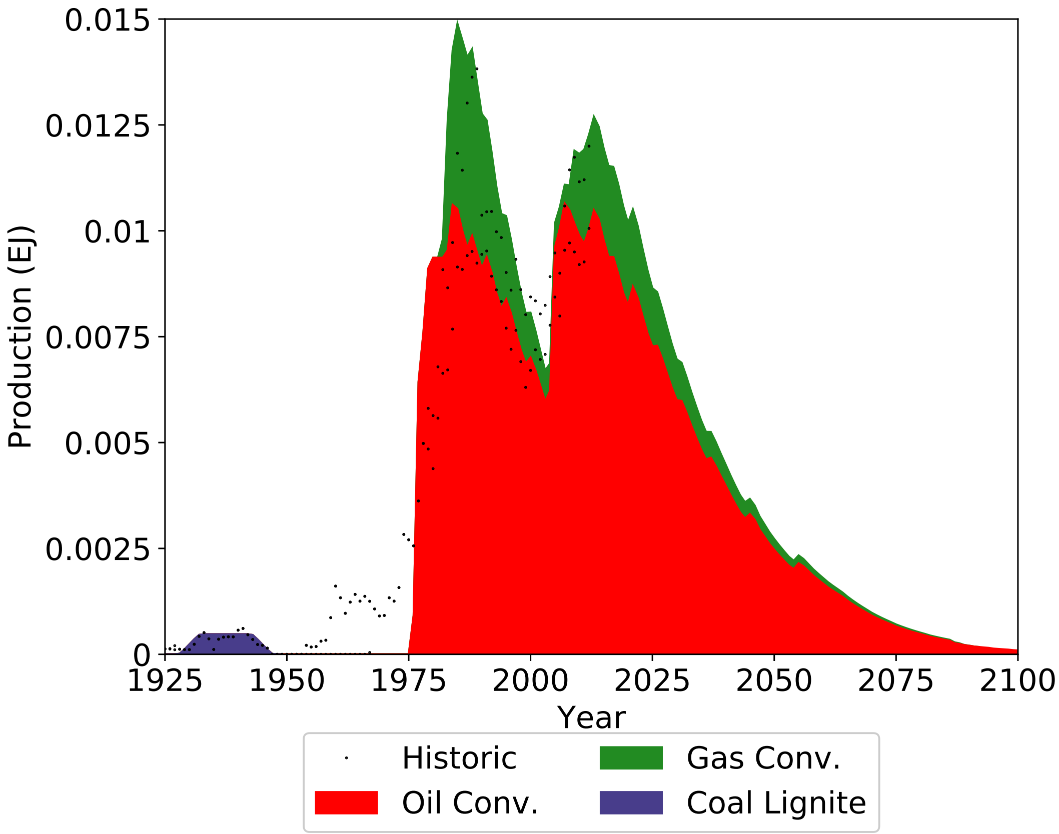

Figure 6.87: USA - South Dakota projection by mineral type

Table 6.87: Peak years - Minerals

Name	URR	Peak Year	Peak Rate
Coal Lignite	0.01	1933	–
Oil Conv.	0.59	2007	0.01
Gas Conv.	0.11	1986	–
**Total**	**0.71**	**1985**	**0.01**

Tennessee

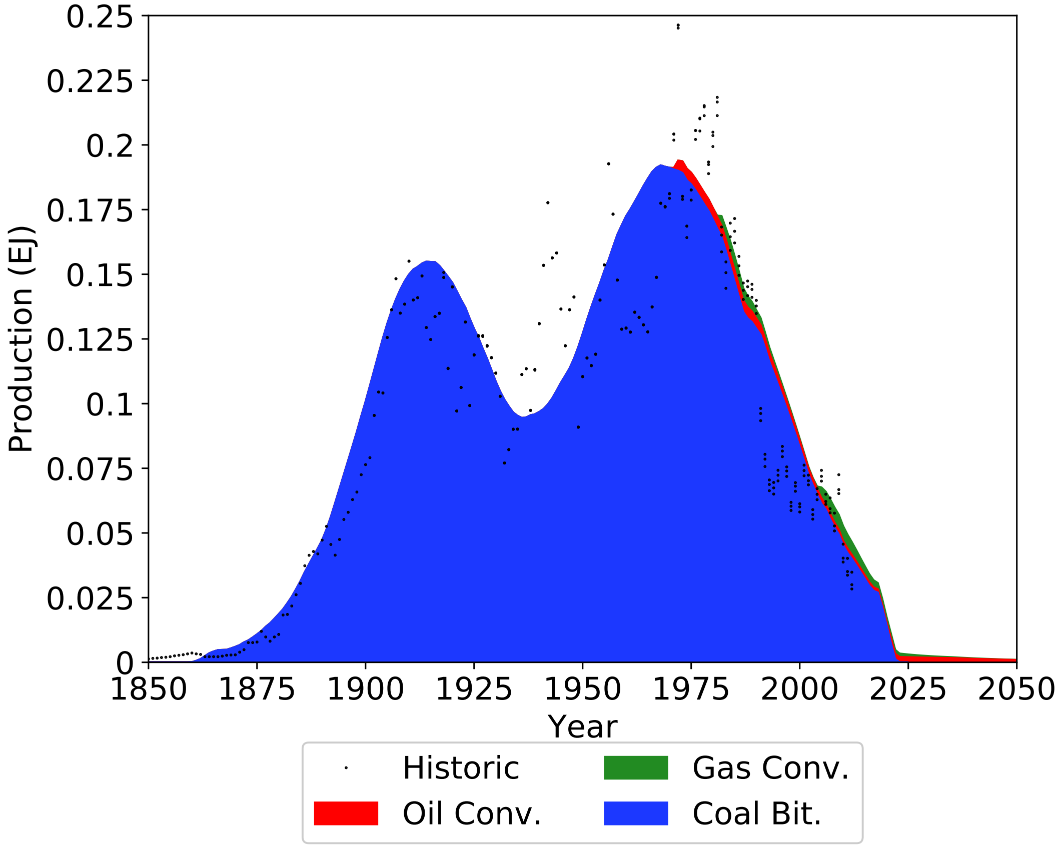

Figure 6.88: USA - Tennessee projections capped at 16

Table 6.88: Peak years - All

Name	URR	Peak Year	Peak Rate
Coal Bit. Tennessee	16.08	1968	0.19
Oil Conv. Tennessee	0.22	1973	–
Gas Conv. Tennessee	0.14	2008	0.01
**Total**	**16.44**	**1972**	**0.19**

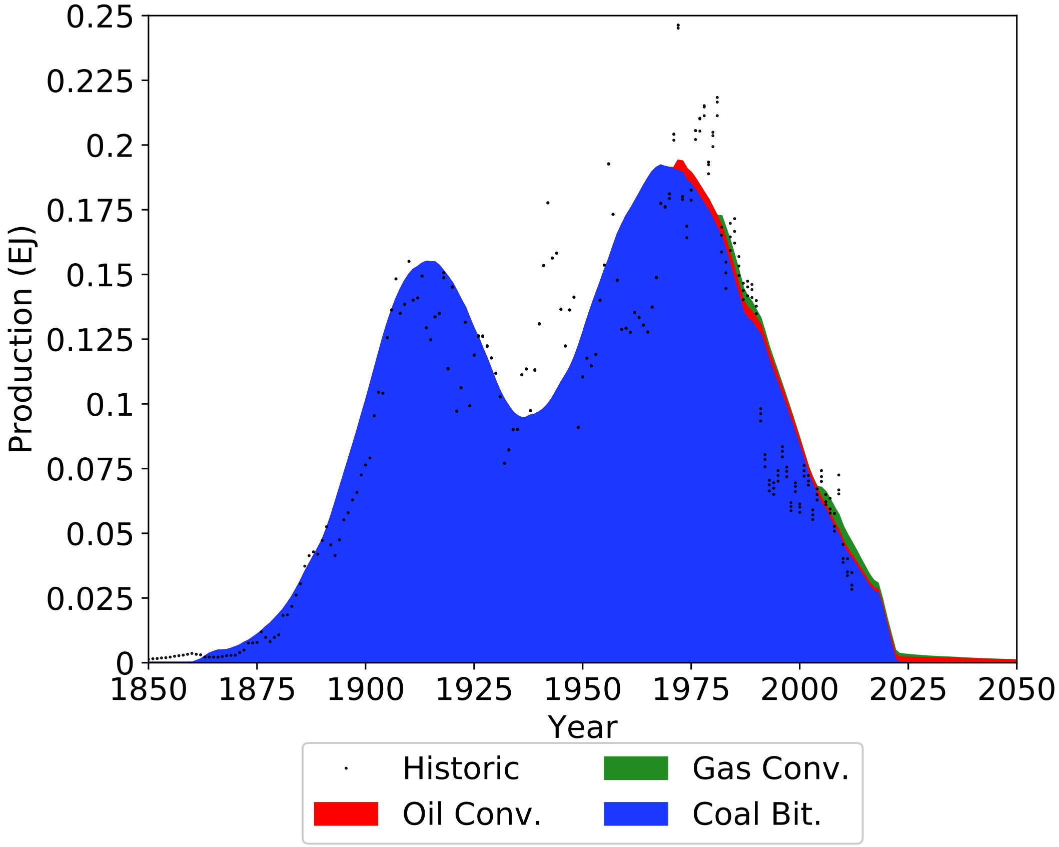

Figure 6.89: USA - Tennessee projection by mineral type

Table 6.89: Peak years - Minerals

Name	URR	Peak Year	Peak Rate
Coal Bit.	16.08	1968	0.19
Oil Conv.	0.22	1973	–
Gas Conv.	0.14	2008	0.01
**Total**	**16.44**	**1972**	**0.19**

Texas

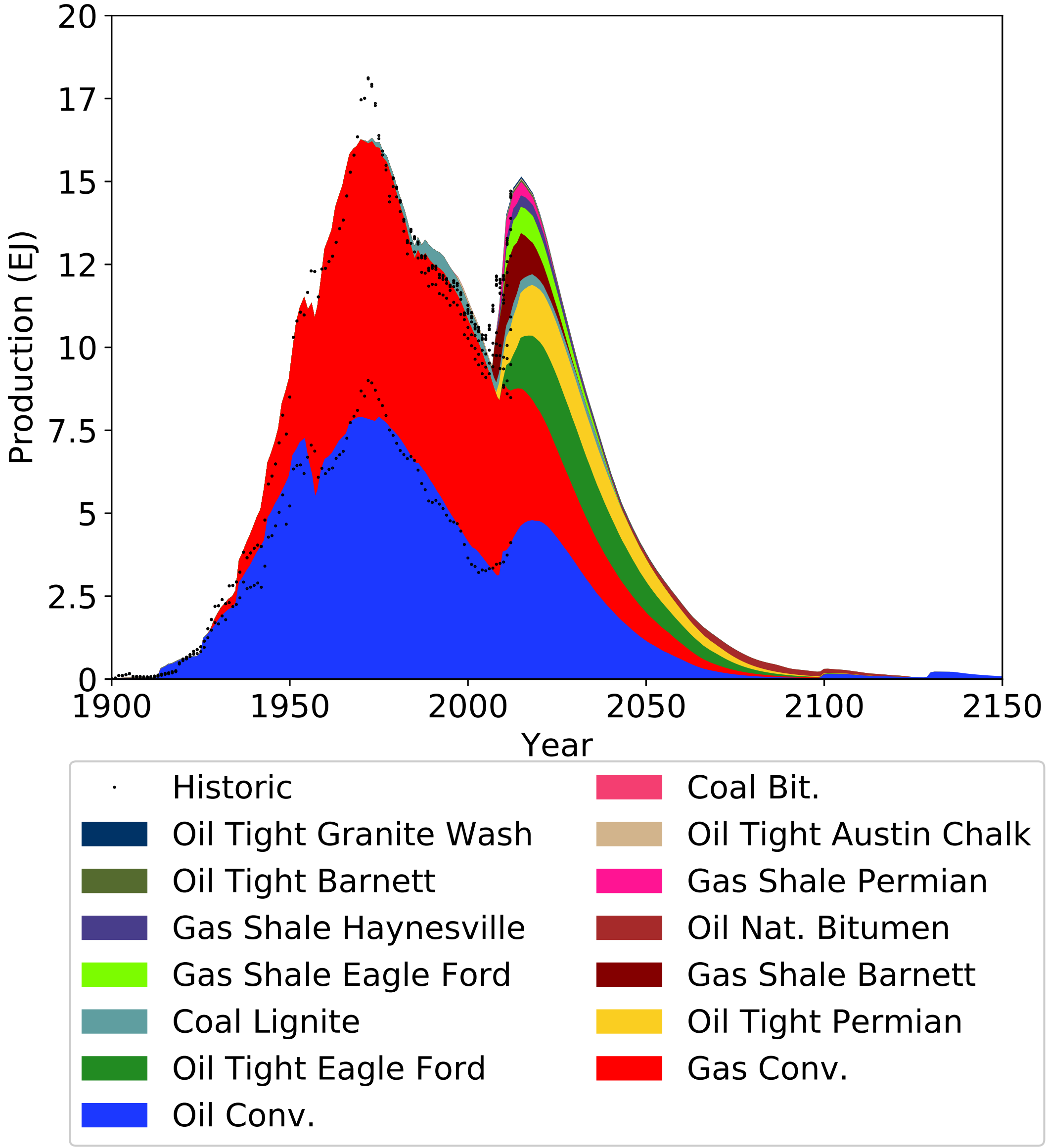

Figure 6.90: USA - Texas projections capped at 16

Table 6.90: Peak years - All

Name	URR	Peak Year	Peak Rate
Oil Conv. Texas	603.57	1970	7.87
Gas Conv. Texas	526.44	1973	8.4
Oil Tight Texas Eagle Ford	79.38	2024	2.21
Oil Tight Texas Permian	61.13	2021	1.6
Coal Lignite Texas	21.9	1992	0.48
Gas Shale Texas Barnett	20.32	2012	1.83
Gas Shale Texas Eagle Ford	13.8	2017	0.83
Oil Nat. Bitumen Texas	13.0	2068	0.24
Gas Shale Texas Haynesville	7.94	2014	0.37
Gas Shale Texas Permian	4.59	2010	0.59
Oil Tight Texas Barnett	2.44	2018	0.07
Oil Tight Texas Austin Chalk	1.75	1999	0.12
Oil Tight Texas Granite Wash	0.89	2011	0.07
Coal Bit. Texas	0.05	1997	0.02
**Total**	**1357.2**	**1973**	**16.27**

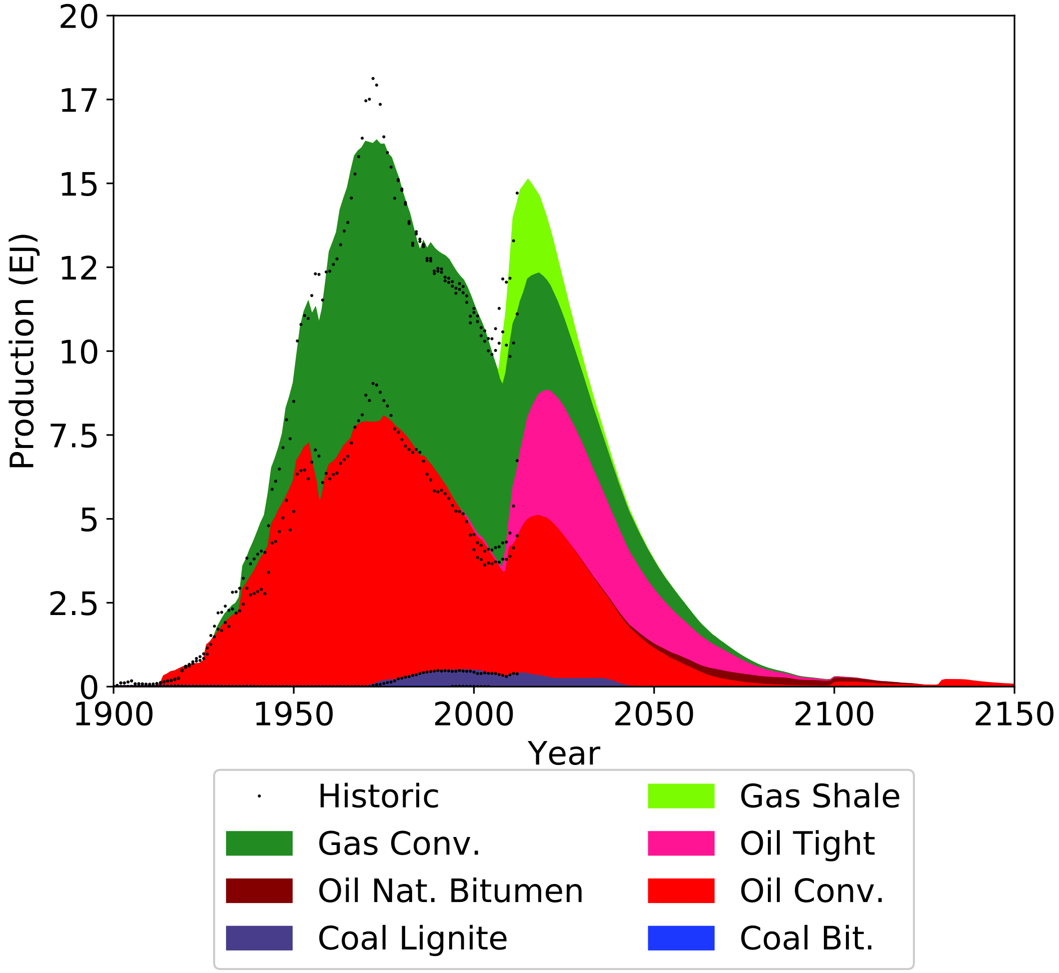

Figure 6.91: USA - Texas projection by mineral type

Table 6.91: Peak years - Minerals

**Name**	**URR**	**Peak Year**	**Peak Rate**
Coal Bit.	0.05	1997	0.02
Coal Lignite	21.9	1992	0.48
Oil Conv.	603.57	1970	7.87
Oil Nat. Bitumen	13.0	2068	0.24
Oil Tight	145.59	2023	3.89
Gas Conv.	526.44	1973	8.4
Gas Shale	46.65	2013	3.32
**Total**	**1357.2**	**1973**	**16.27**

Utah

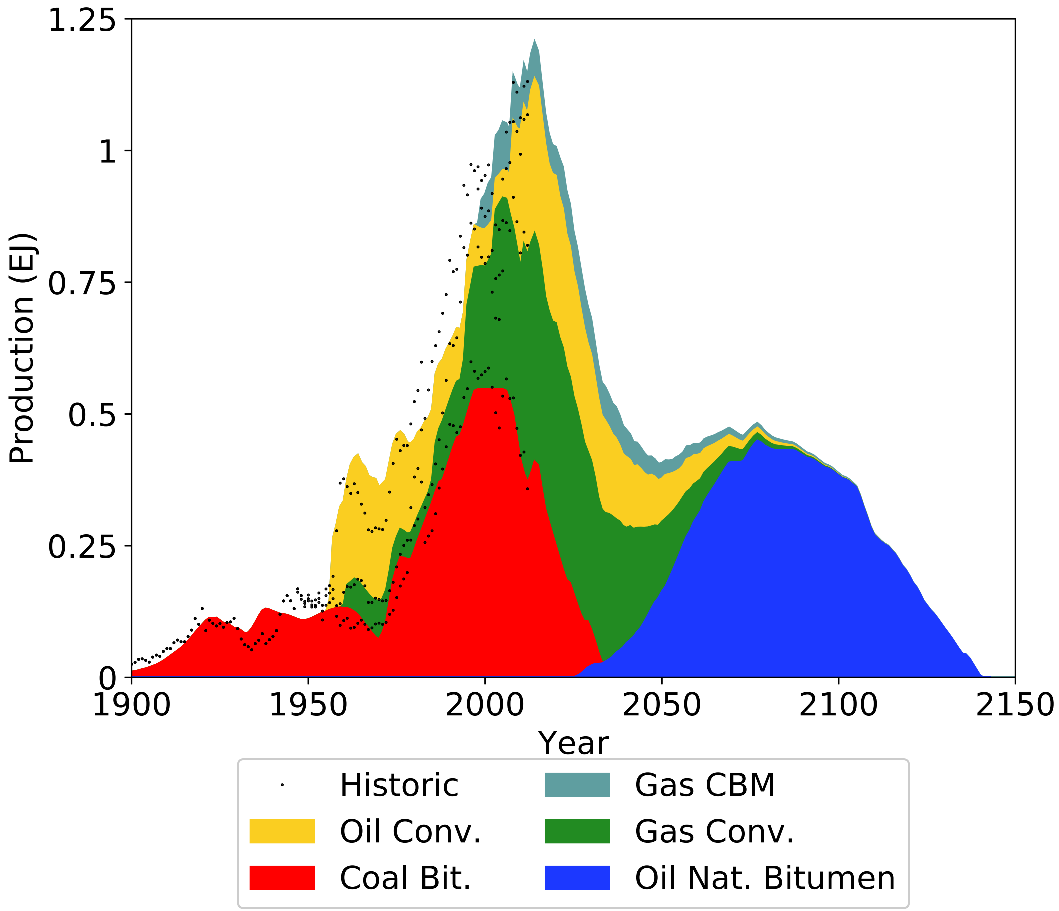

Figure 6.92: USA - Utah projections capped at 16

Name	URR	Peak Year	Peak Rate
Oil Nat. Bitumen Utah	27.84	2077	0.45
Coal Bit. Utah	27.03	1998	0.55
Gas Conv. Utah	21.0	2013	0.44
Oil Conv. Utah	17.19	2015	0.3
Gas CBM Utah	3.99	2005	0.09
**Total**	**97.05**	**2014**	**1.21**

Table 6.92: Peak years - All

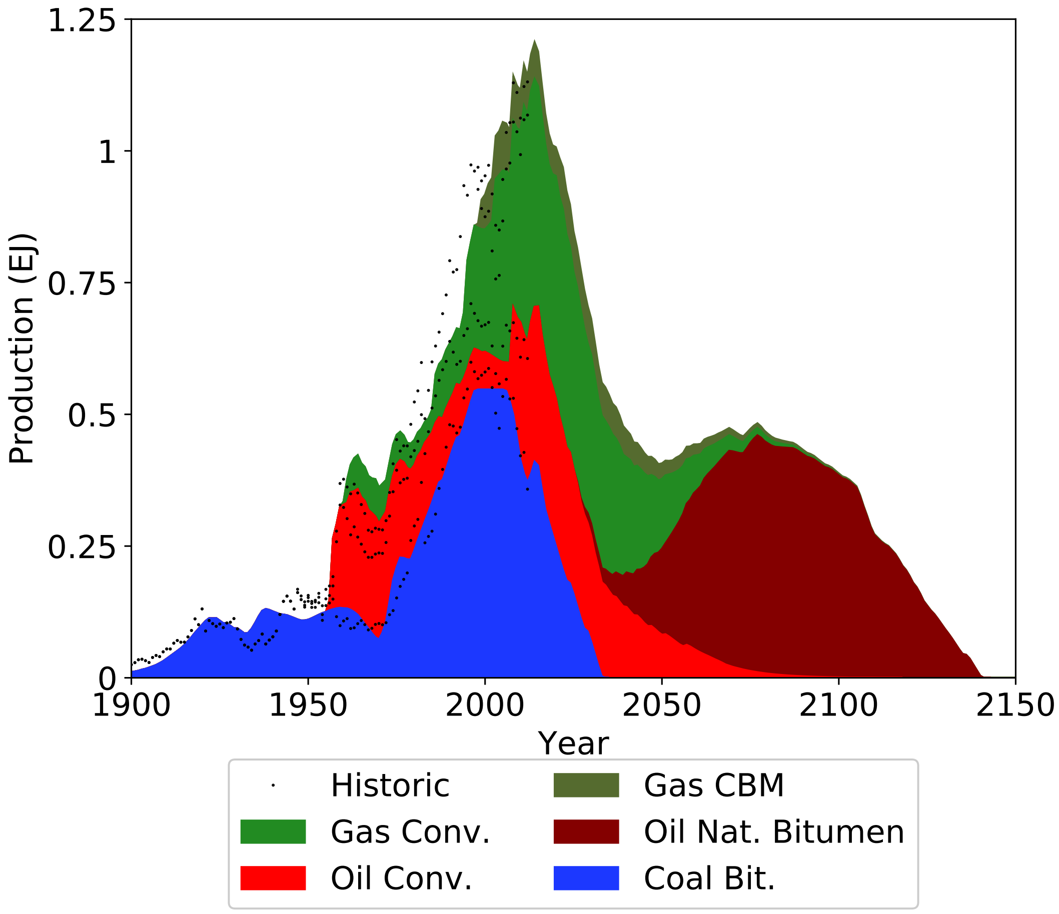

Figure 6.93: USA - Utah projection by mineral type

Table 6.93: Peak years - Minerals

Name	URR	Peak Year	Peak Rate
Coal Bit.	27.03	1998	0.55
Oil Conv.	17.19	2015	0.3
Oil Nat. Bitumen	27.84	2077	0.45
Gas Conv.	21.0	2013	0.44
Gas CBM	3.99	2005	0.09
**Total**	**97.05**	**2014**	**1.21**

Virginia

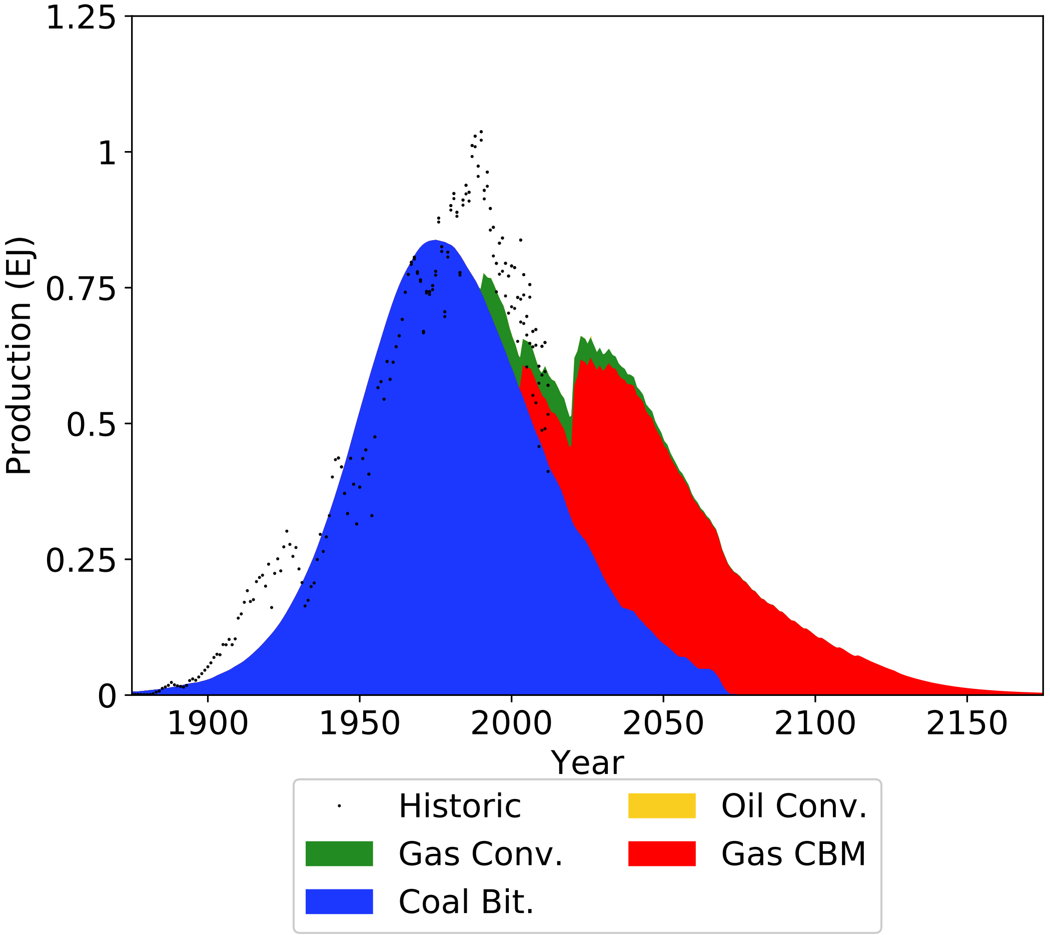

Figure 6.94: USA - Virginia projections capped at 16

Table 6.94: Peak years - All

Name	URR	Peak Year	Peak Rate
Coal Bit. Virginia	62.26	1975	0.84
Gas CBM Virginia	26.85	2034	0.42
Gas Conv. Virginia	2.56	1997	0.08
Oil Conv. Virginia	0.01	1982	–
**Total**	**91.68**	**1975**	**0.84**

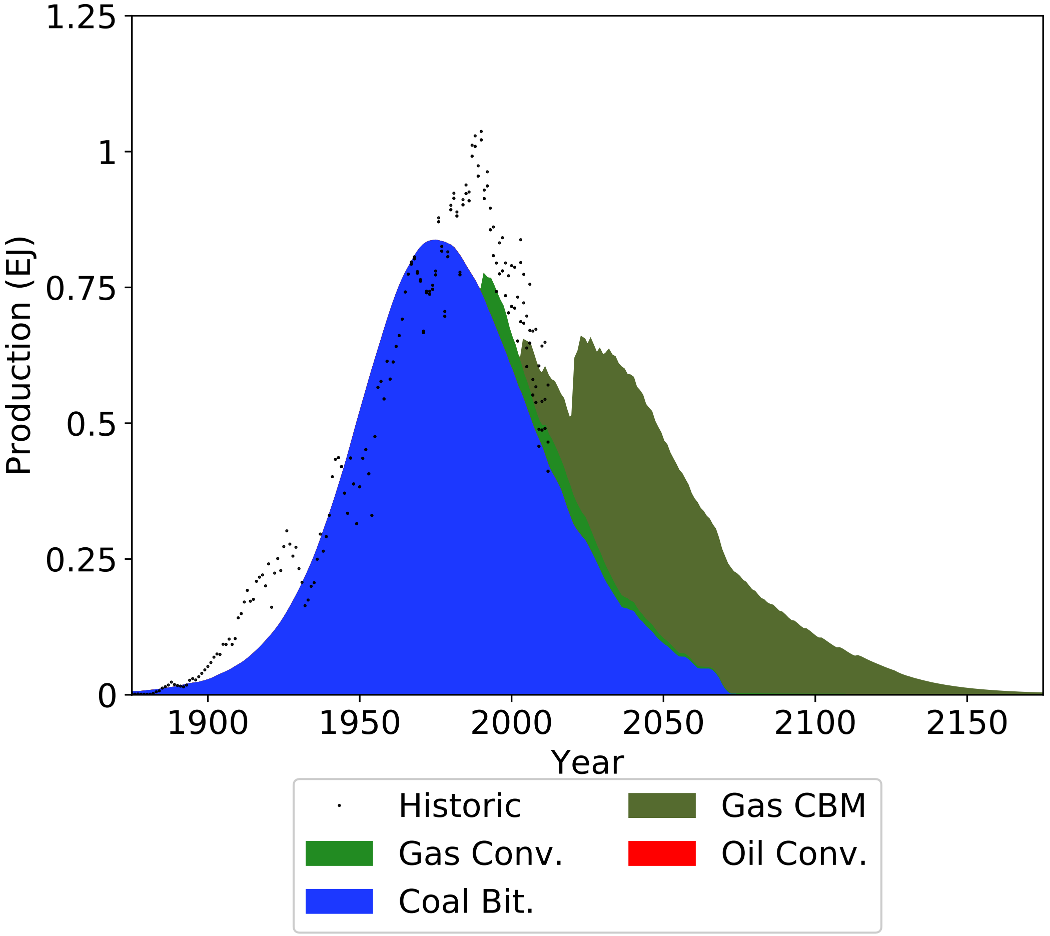

Figure 6.95: USA - Virginia projection by mineral type

Table 6.95: Peak years - Minerals

Name	URR	Peak Year	Peak Rate
Coal Bit.	62.26	1975	0.84
Oil Conv.	0.01	1982	–
Gas Conv.	2.56	1997	0.08
Gas CBM	26.85	2034	0.42
**Total**	**91.68**	**1975**	**0.84**

Washington

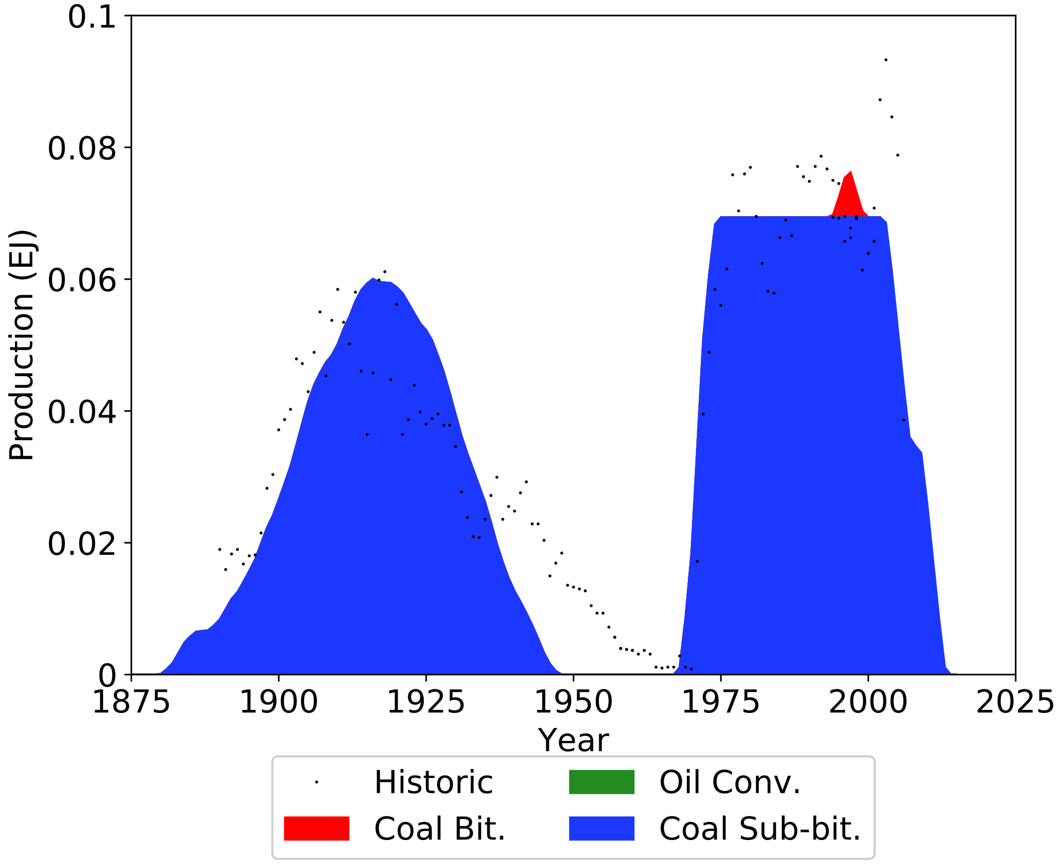

Figure 6.96: USA - Washington projections capped at 16

Table 6.96: Peak years - All

Name	URR	Peak Year	Peak Rate
Coal Sub-bit. Washington	4.57	1975	0.07
Coal Bit. Washington	0.02	1997	0.01
Oil Conv. Washington	–	1956	–
**Total**	**4.59**	**1997**	**0.08**

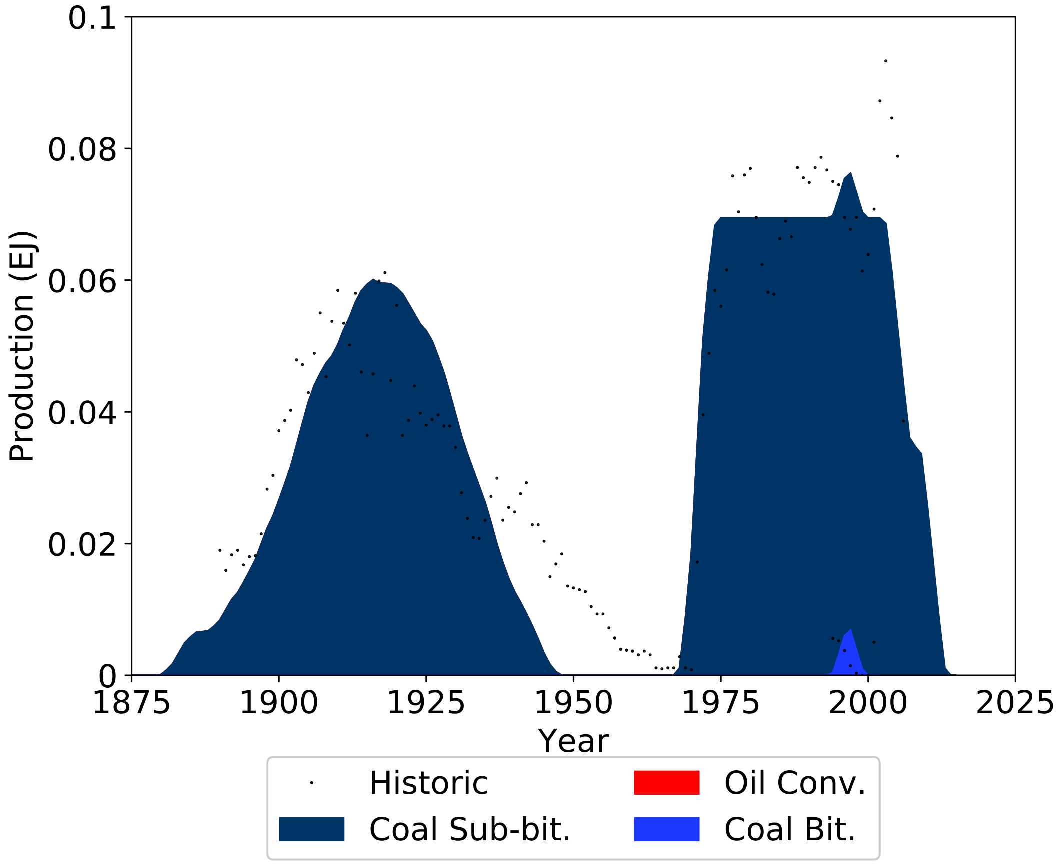

Figure 6.97: USA - Washington projection by mineral type

Table 6.97: Peak years - Minerals

Name	URR	Peak Year	Peak Rate
Coal Bit.	0.02	1997	0.01
Coal Sub-bit.	4.57	1975	0.07
Oil Conv.	–	1956	–
**Total**	**4.59**	**1997**	**0.08**

West Virginia

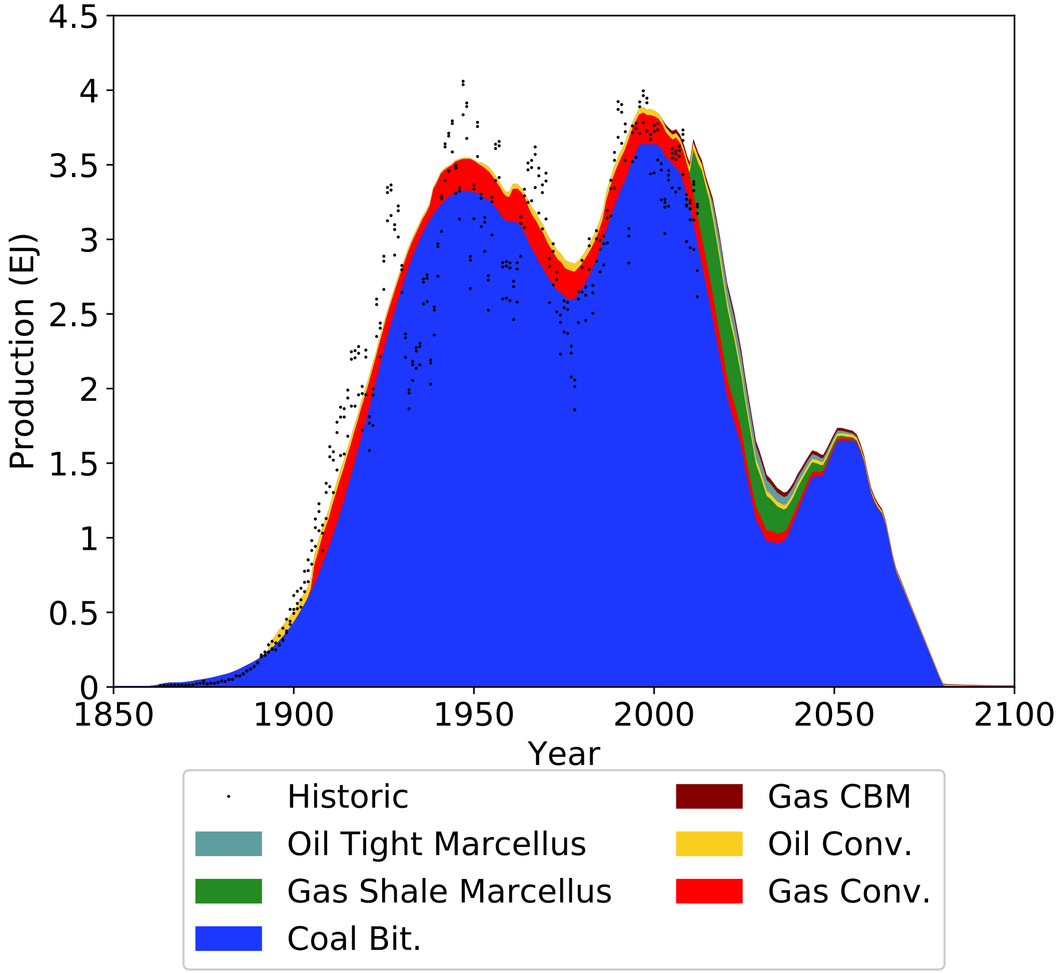

Figure 6.98: USA - West Virginia projections capped at 16

Table 6.98: Peak years - All

Name	URR	Peak Year	Peak Rate
Coal Bit. West Virginia	389.11	1997	3.64
Gas Conv. West Virginia	23.49	1967	0.24
Gas Shale West Virginia Marcellus	10.35	2016	0.51
Oil Conv. West Virginia	6.6	1902	0.08
Oil Tight West Virginia Marcellus	2.29	2022	0.1
Gas CBM West Virginia	1.78	2023	0.04
**Total**	**433.62**	**1997**	**3.88**

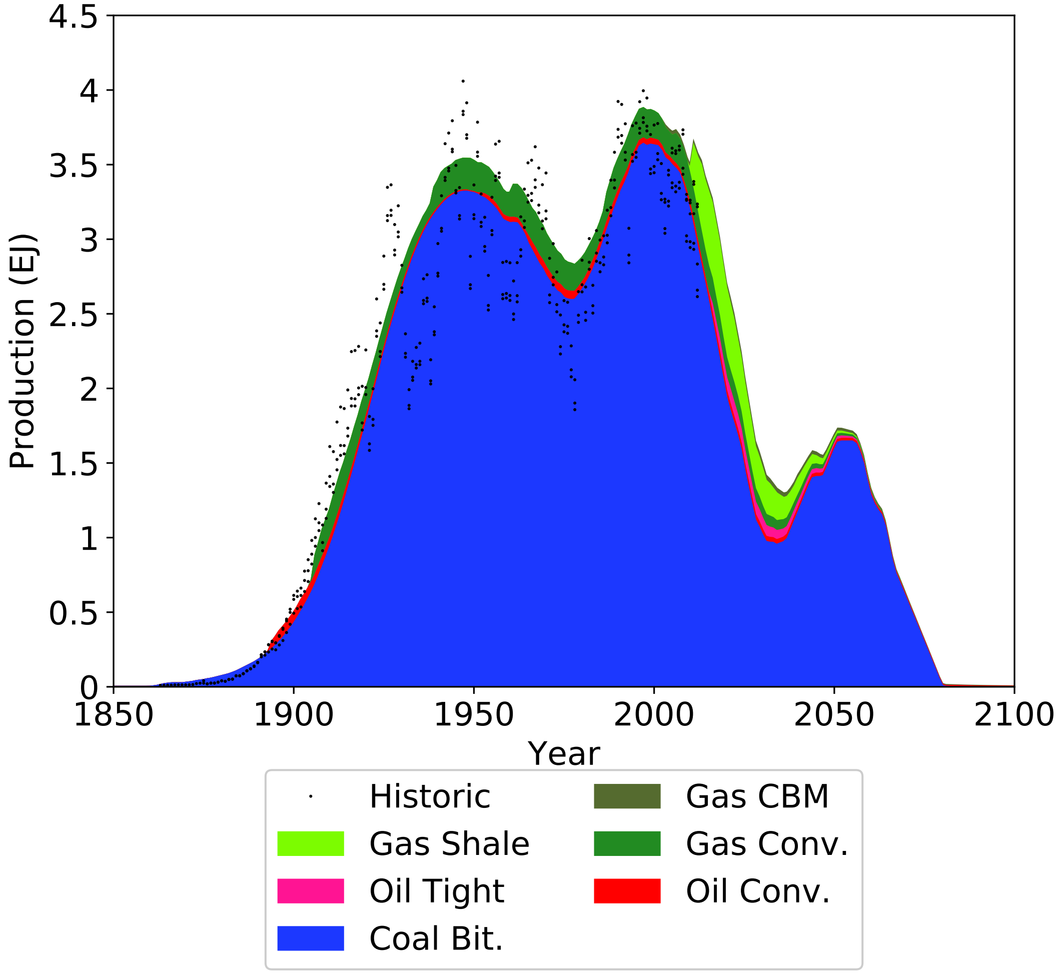

Figure 6.99: USA - West Virginia projection by mineral type

Table 6.99: Peak years - Minerals

**Name**	**URR**	**Peak Year**	**Peak Rate**
Coal Bit.	389.11	1997	3.64
Oil Conv.	6.6	1902	0.08
Oil Tight	2.29	2022	0.1
Gas Conv.	23.49	1967	0.24
Gas Shale	10.35	2016	0.51
Gas CBM	1.78	2023	0.04
**Total**	**433.62**	**1997**	**3.88**

Wyoming

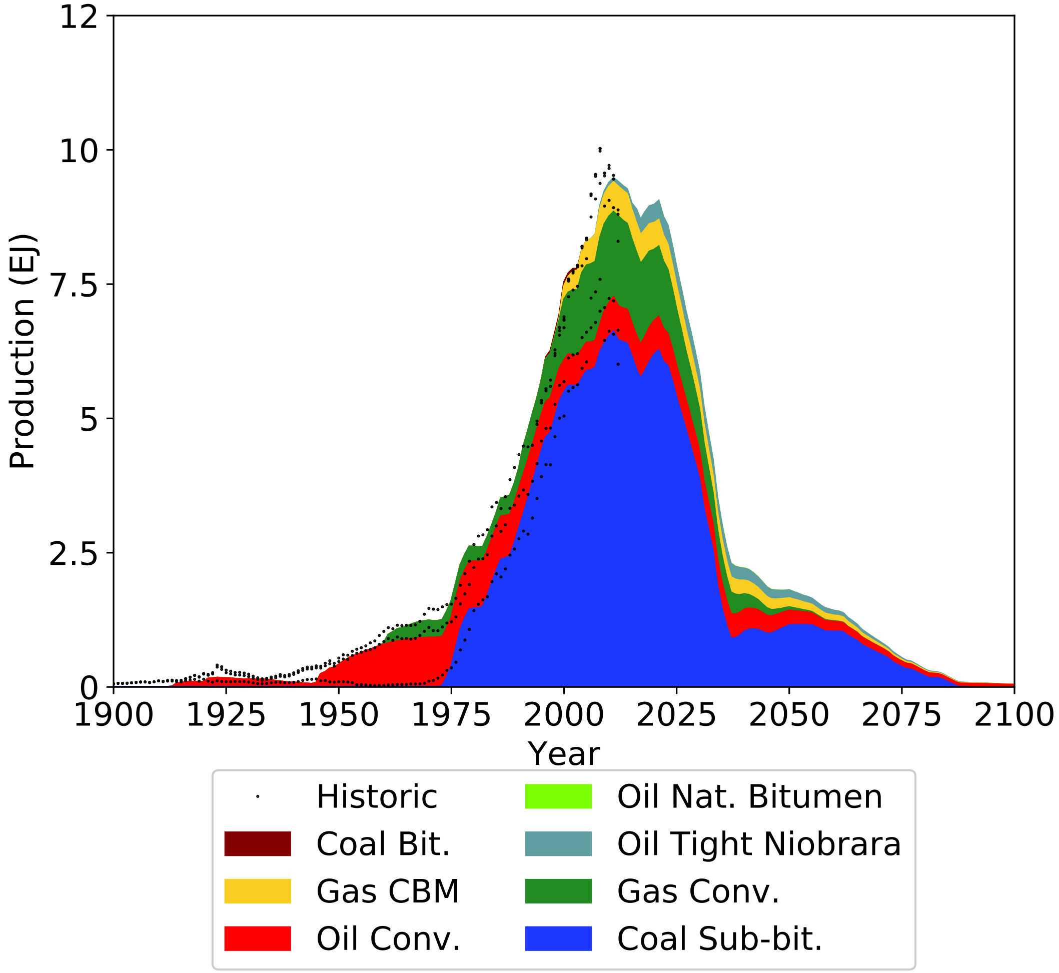

Figure 6.100: USA - Wyoming projections capped at 16

Table 6.100: Peak years - All

**Name**	**URR**	**Peak Year**	**Peak Rate**
Coal Sub-bit. Wyoming	297.1	2011	6.62
Oil Conv. Wyoming	76.56	1972	0.92
Gas Conv. Wyoming	62.21	2012	1.67
Gas CBM Wyoming	22.23	2013	0.56
Oil Tight Wyoming Niobrara	12.26	2024	0.36
Coal Bit. Wyoming	0.48	1999	0.07
Oil Nat. Bitumen Wyoming	0.1	2035	0.01
**Total**	**470.94**	**2011**	**9.47**

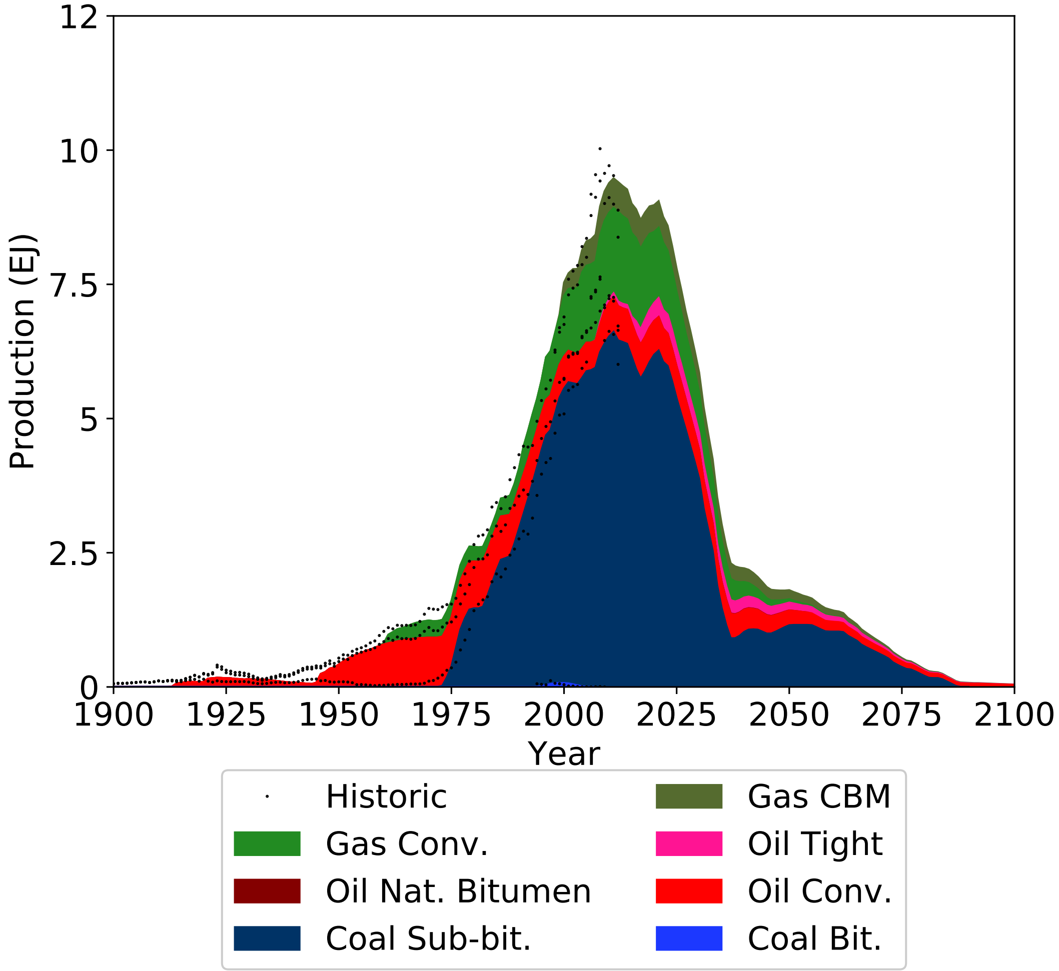

Figure 6.101: USA - Wyoming projection by mineral type

Table 6.101: Peak years - Minerals

**Name**	**URR**	**Peak Year**	**Peak Rate**
Coal Bit.	0.48	1999	0.07
Coal Sub-bit.	297.1	2011	6.62
Oil Conv.	76.56	1972	0.92
Oil Nat. Bitumen	0.1	2035	0.01
Oil Tight	12.26	2024	0.36
Gas Conv.	62.21	2012	1.67
Gas CBM	22.23	2013	0.56
**Total**	**470.94**	**2011**	**9.47**

6.2.4 Projection by region

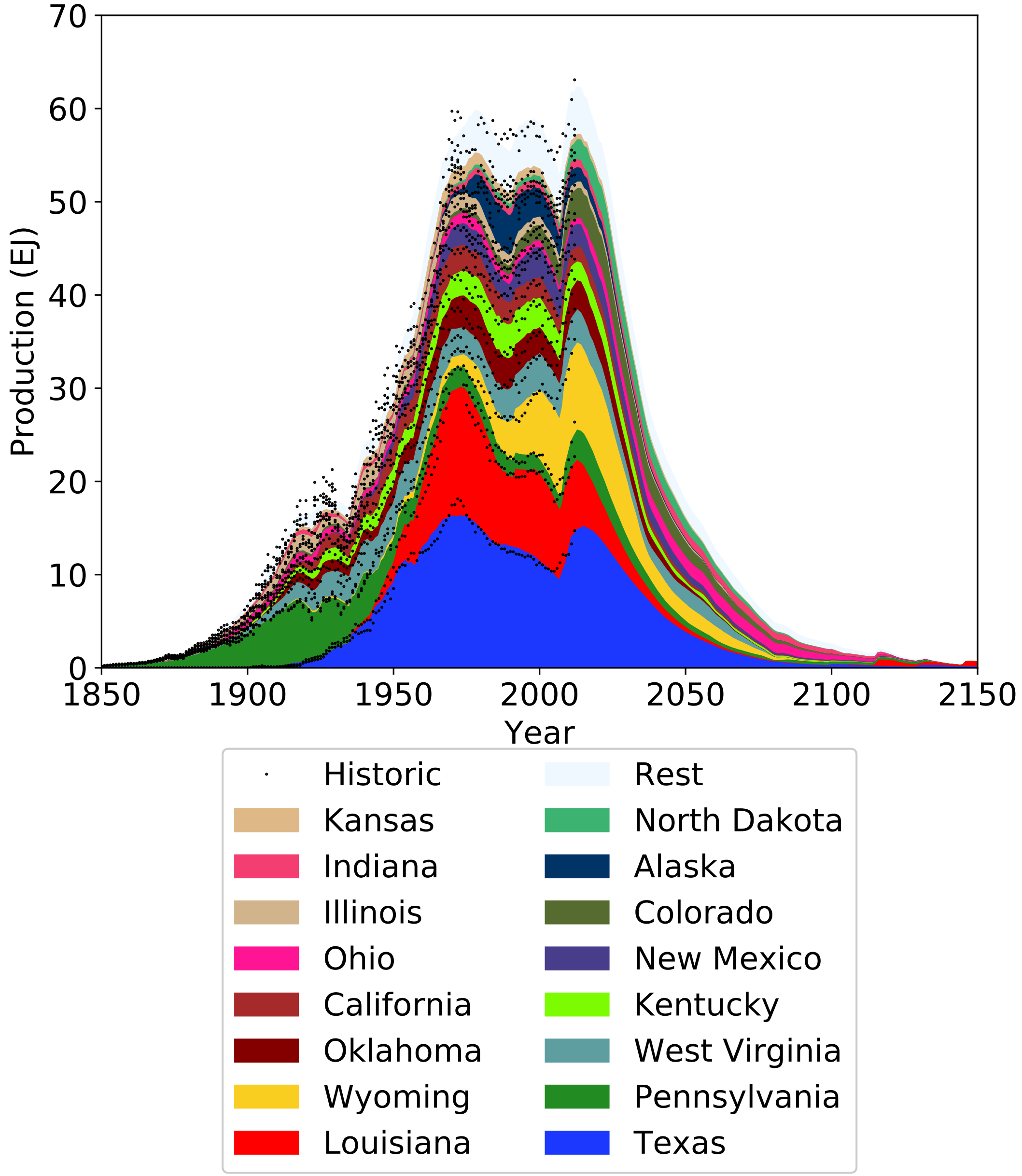

Figure 6.102: USA by region projections capped at 16

Table 6.102: Peak years - All

**Name**	**URR**	**Peak Year**	**Peak Rate**
Texas	1357.2	1973	16.27
Louisiana	691.77	1974	13.8
Pennsylvania	532.16	1917	6.78
Wyoming	470.94	2011	9.47
West Virginia	433.62	1997	3.88
Oklahoma	304.87	1984	3.61
Kentucky	284.69	1988	3.64
California	251.67	1968	3.02
New Mexico	232.3	1999	3.27
Ohio	218.2	2025	1.56
Colorado	212.75	2016	3.48
Illinois	173.99	1941	2.16
Alaska	146.11	1987	4.38
Indiana	137.08	2046	1.07
North Dakota	128.31	2022	2.82
Kansas	114.08	1969	1.57
Utah	97.05	2014	1.21
Alabama	96.99	1997	1.27
Virginia	91.68	1975	0.84
Montana	56.33	2010	0.91
Arkansas	33.9	2010	1.11
Mississippi	30.78	1959	0.53
Michigan	22.33	1996	0.45
Tennessee	16.44	1972	0.19
Arizona	14.78	2004	0.32
New York	11.92	2034	0.18
Maryland	9.64	1907	0.13
Missouri	9.6	1969	0.12
Iowa	8.14	1914	0.15
Florida	6.18	1976	0.4
Washington	4.59	1997	0.08
Nebraska	3.4	1959	0.15
South Dakota	0.71	1985	0.01
Nevada	0.35	1980	0.02
Georgia	0.26	1895	0.01
Oregon	0.21	2019	0.01
Other	0.04	1887	–
**Total**	**6205.04**	**2013**	**62.27**

6.3 Total

6.3.1 By country

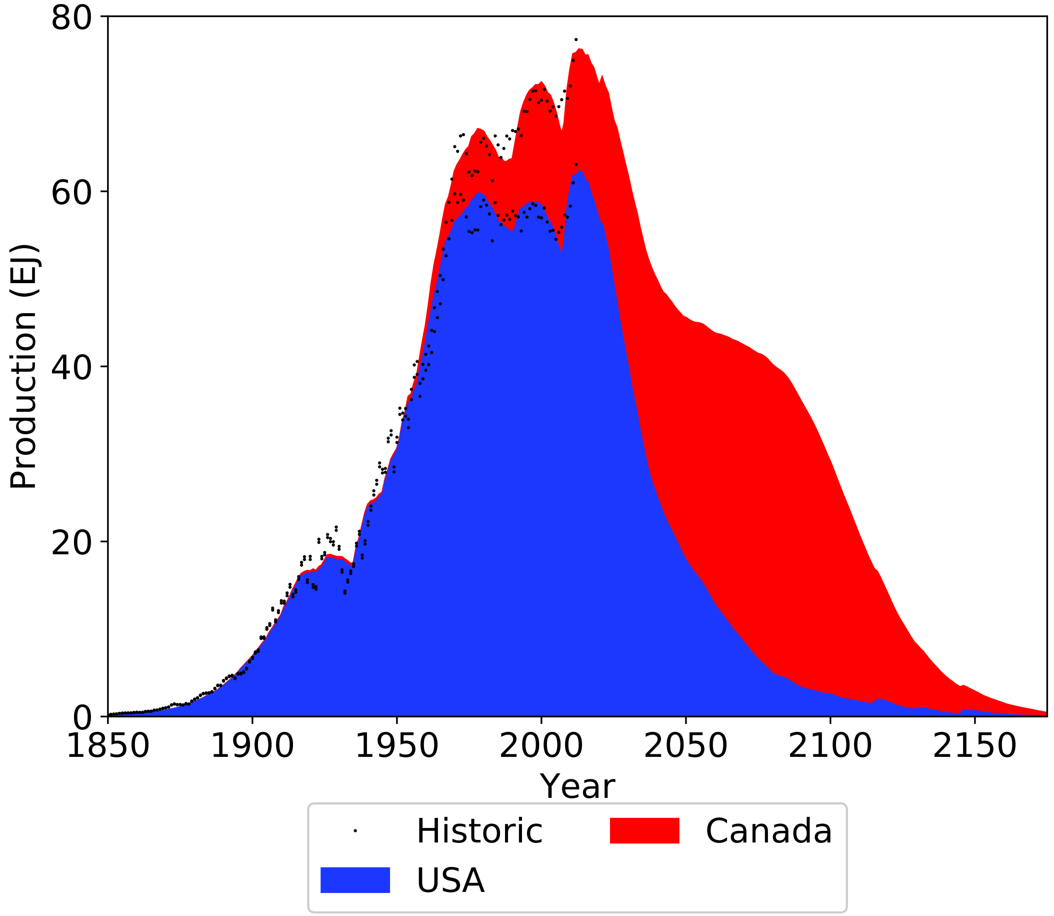

Figure 6.103: North America projections by country

Table 6.103: Peak years - All

Name	URR	Peak Year	Peak Rate
USA	6205.04	2013	62.27
Canada	3508.61	2078	35.34
**Total**	**9713.65**	**2013**	**76.21**

6.3.2 By mineral

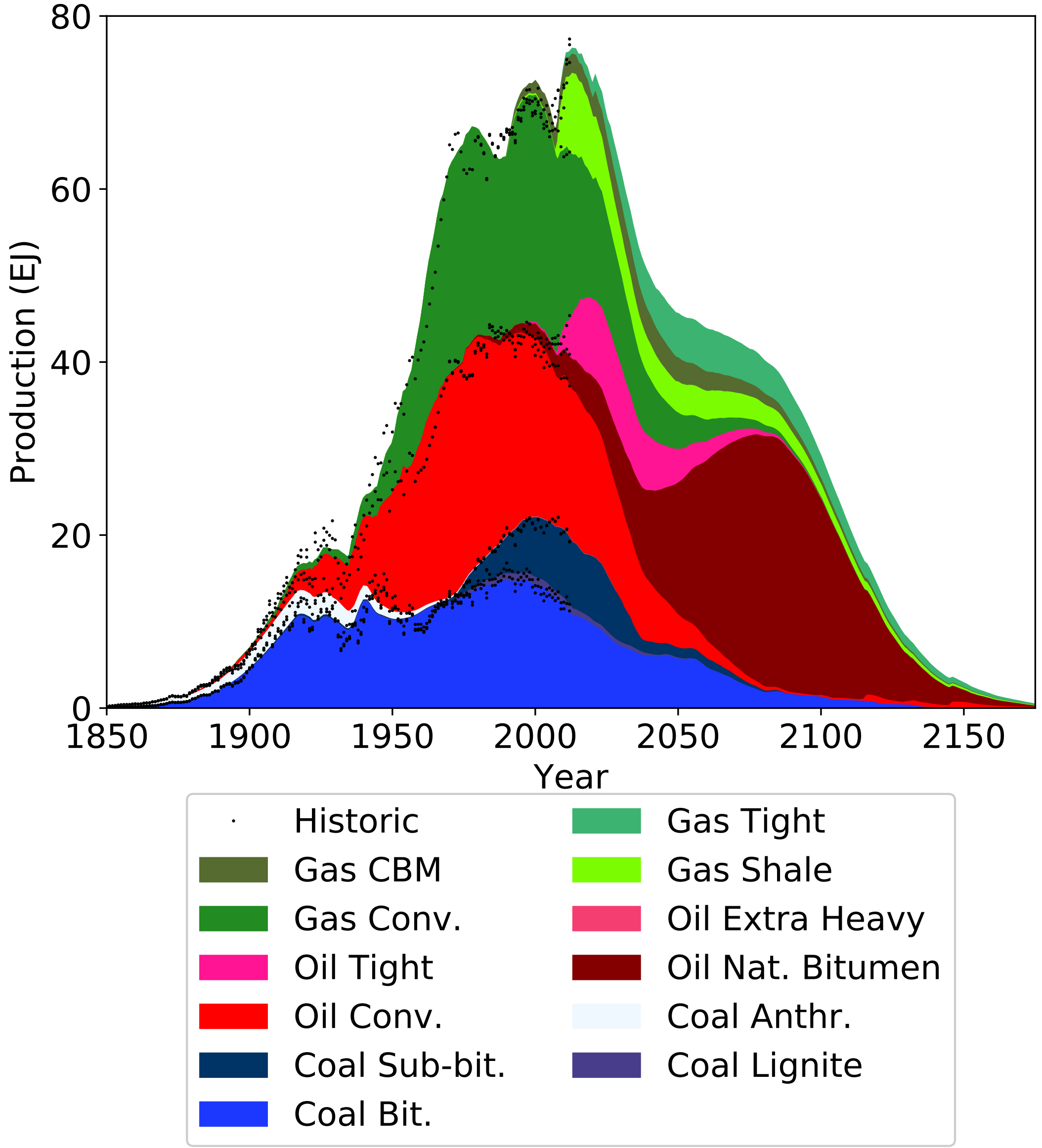

Figure 6.104: North America projection by mineral type

Table 6.104: Peak years - Minerals

Name	URR	Peak Year	Peak Rate
Coal Bit.	1765.17	1996	14.69
Coal Lignite	46.82	1996	0.89
Coal Sub-bit.	385.46	2011	7.95
Coal Anthr.	153.01	1919	2.73
Oil Conv.	2062.5	1976	26.5
Oil Nat. Bitumen	2061.14	2082	28.97
Oil Tight	344.62	2023	9.44
Oil Extra Heavy	0.04	2029	0.01
Gas Conv.	1824.24	1997	26.17
Gas Shale	413.21	2013	9.42
Gas CBM	242.08	2034	3.52
Gas Tight	415.36	2056	5.16
**Total**	**9713.65**	**2013**	**76.21**

Chapter 7

South America

7.1 Argentina

7.1.1 All Projections

Table 7.1: Peak years - All

Name	URR	Peak Year	Peak Rate
Oil Tight	154.71	2075	3.23
Oil Conv.	86.93	1999	2.03
Gas Shale	81.28	2086	0.97
Gas Conv.	78.8	2004	1.65
Coal Bit.	0.5	1974	0.02
**Total**	**402.22**	**2076**	**4.26**

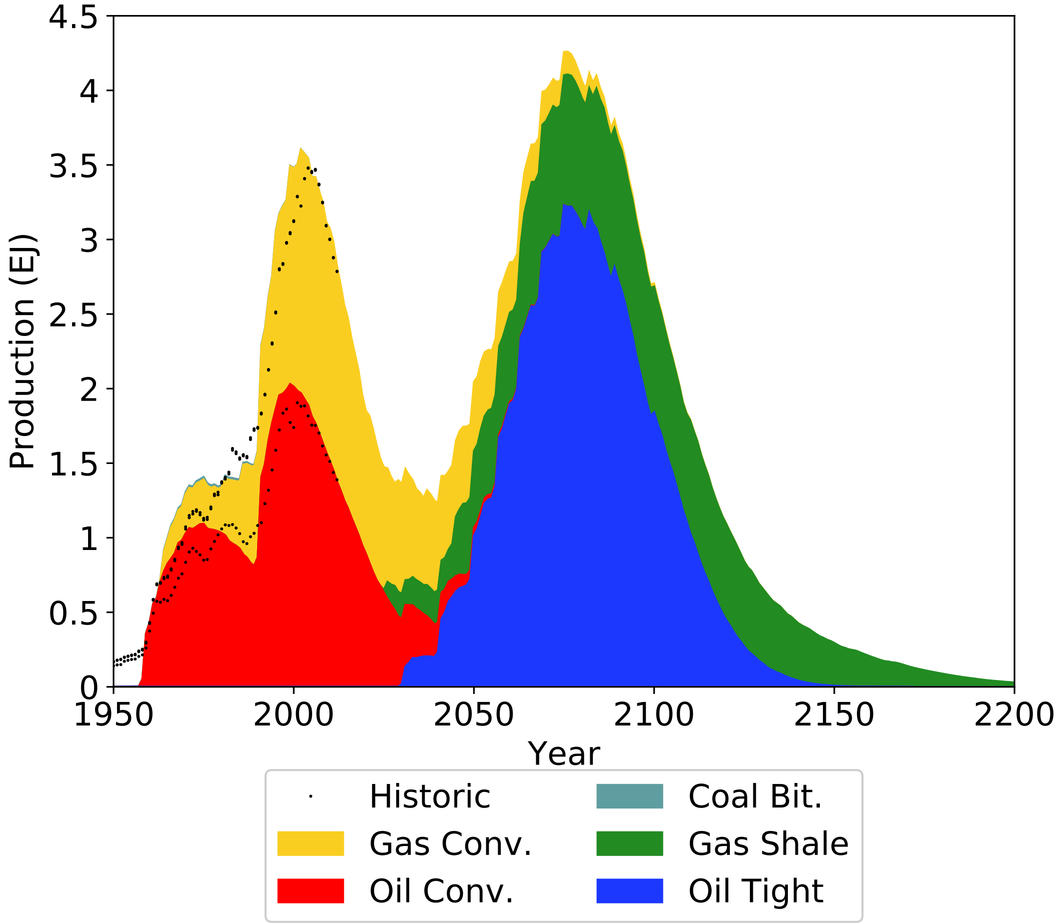

Figure 7.1: Argentina projections capped at 16

7.1.2 By Mineral

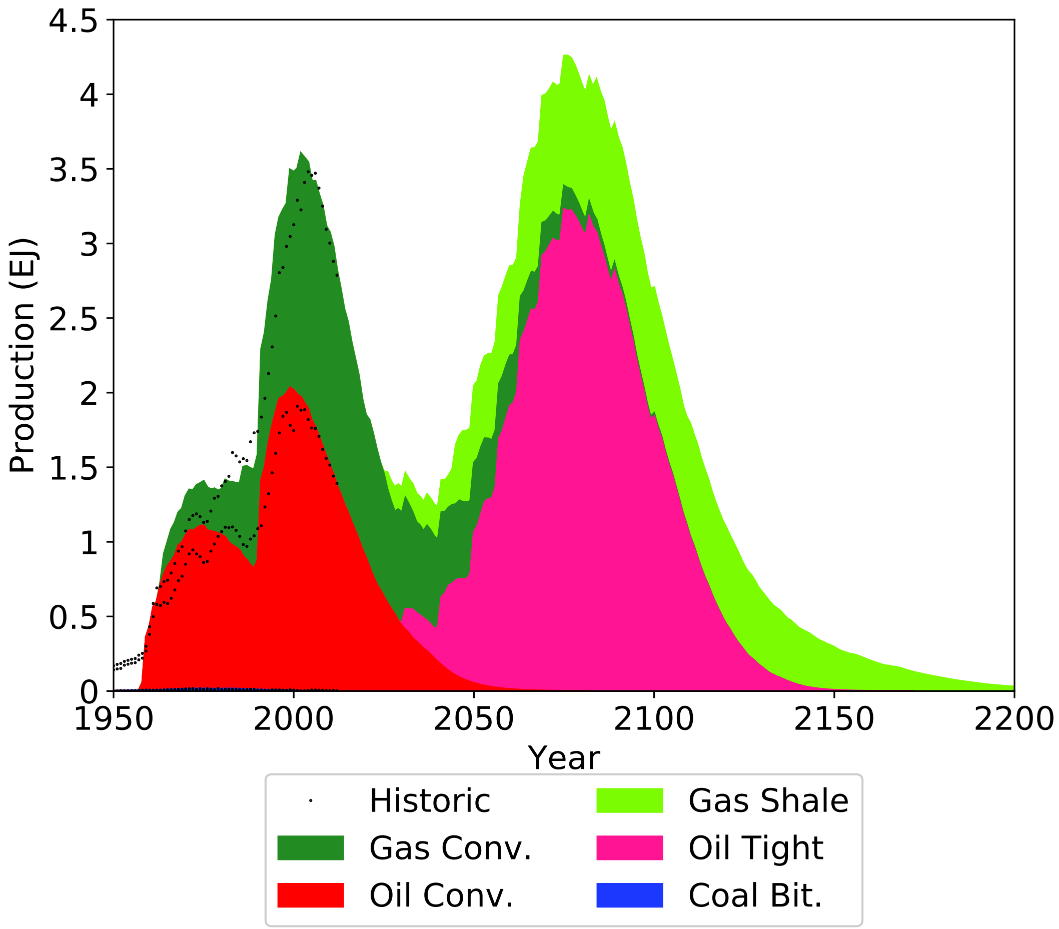

Figure 7.2: Argentina projection by mineral type

Table 7.2: Peak years - Minerals

**Name**	**URR**	**Peak Year**	**Peak Rate**
Coal Bit.	0.5	1974	0.02
Oil Conv.	86.93	1999	2.03
Oil Tight	154.71	2075	3.23
Gas Conv.	78.8	2004	1.65
Gas Shale	81.28	2086	0.97
**Total**	**402.22**	**2076**	**4.26**

7.2 Barbados

7.2.1 All Projections

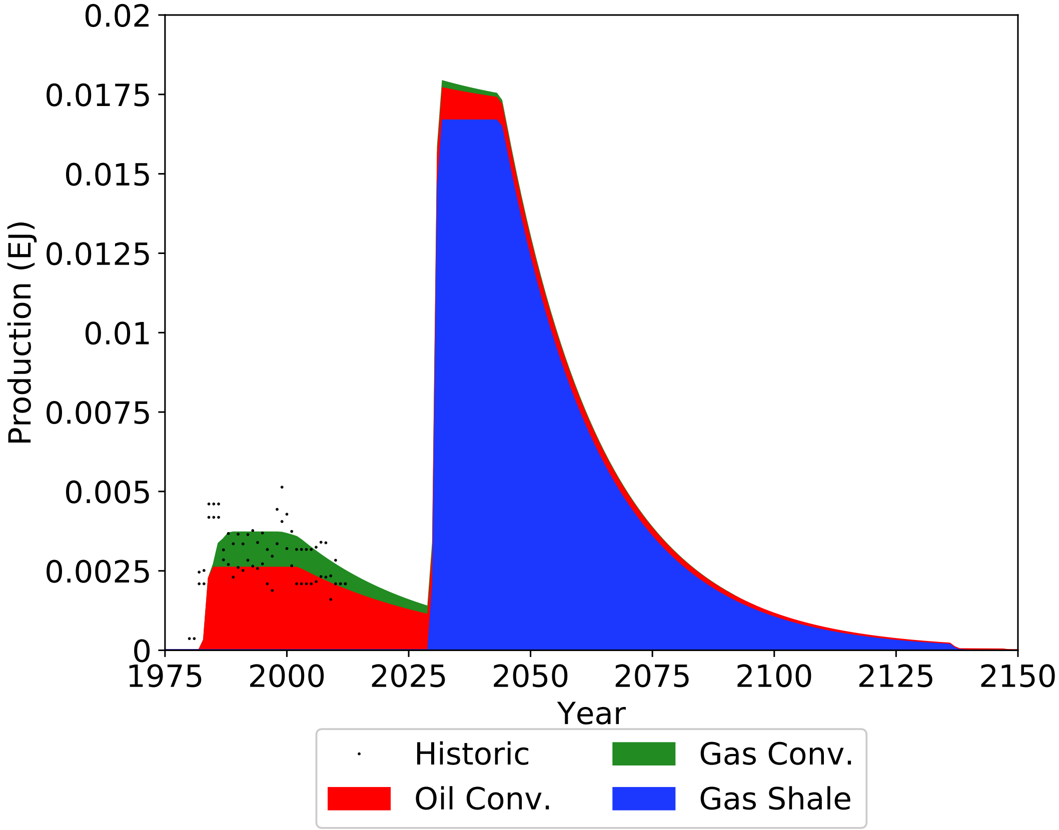

Figure 7.3: Barbados projections capped at 16

Table 7.3: Peak years - All

**Name**	**URR**	**Peak Year**	**Peak Rate**
Gas Shale	0.56	2032	0.02
Oil Conv.	0.13	1985	–
Gas Conv.	0.04	1989	–
**Total**	**0.72**	**2032**	**0.02**

7.2.2 By Mineral

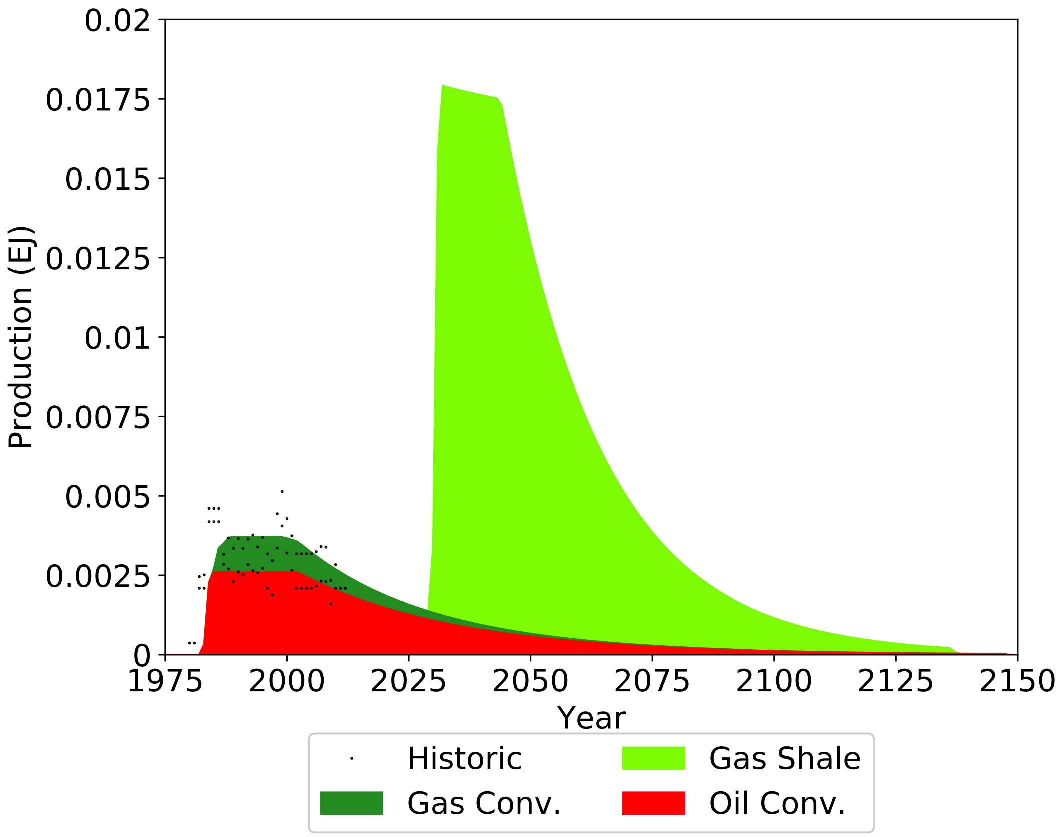

Figure 7.4: Barbados projection by mineral type

Table 7.4: Peak years - Minerals

**Name**	**URR**	**Peak Year**	**Peak Rate**
Oil Conv.	0.13	1985	–
Gas Conv.	0.04	1989	–
Gas Shale	0.56	2032	0.02
**Total**	**0.72**	**2032**	**0.02**

7.3 Belize

7.3.1 All Projections

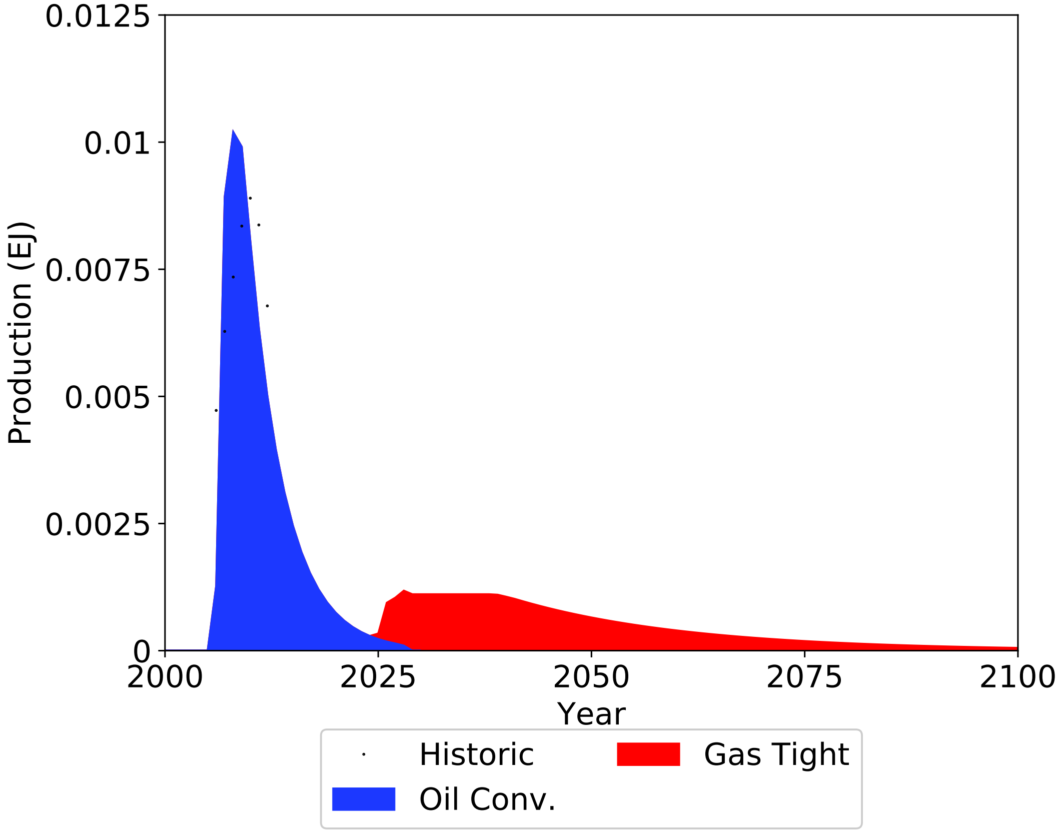

Figure 7.5: Belize projections capped at 16

Table 7.5: Peak years - All

Name	URR	Peak Year	Peak Rate
Oil Conv.	0.07	2008	0.01
Gas Tight	0.04	2029	–
**Total**	**0.11**	**2008**	**0.01**

7.3.2 By Mineral

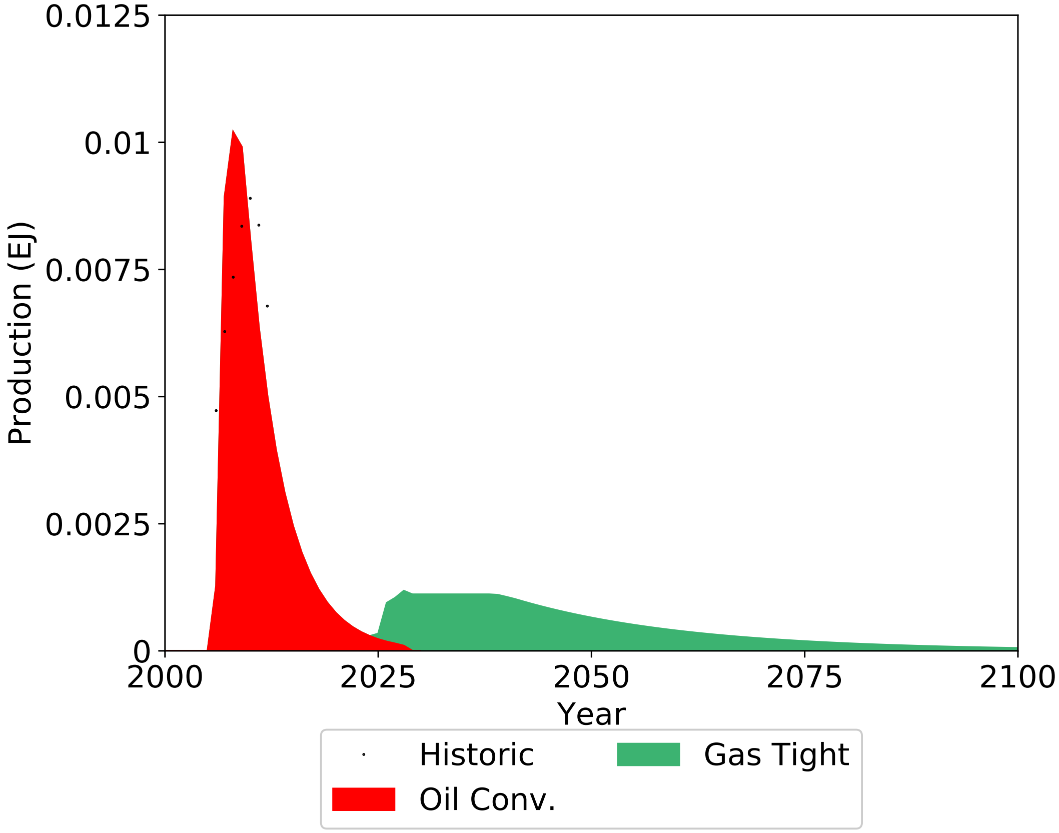

Figure 7.6: Belize projection by mineral type

Table 7.6: Peak years - Minerals

Name	URR	Peak Year	Peak Rate
Oil Conv.	0.07	2008	0.01
Gas Tight	0.04	2029	–
**Total**	**0.11**	**2008**	**0.01**

7.4 Bolivia

7.4.1 All Projections

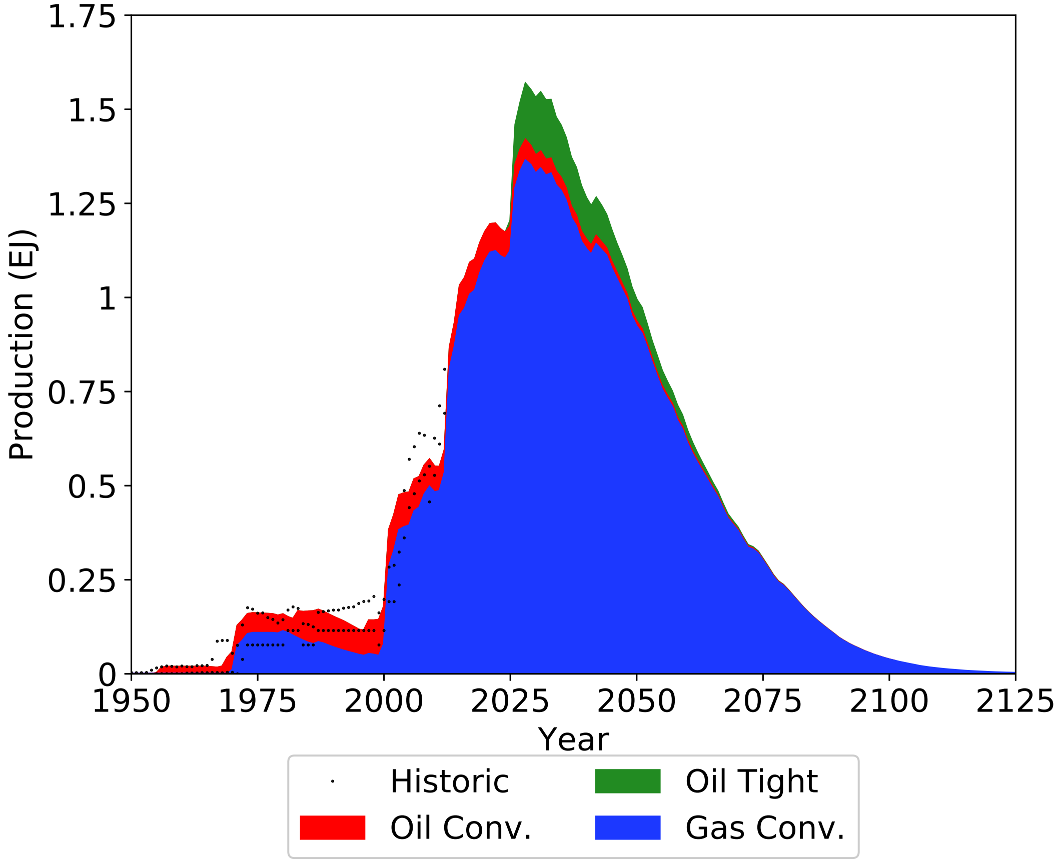

Figure 7.7: Bolivia projections capped at 16

Table 7.7: Peak years - All

Name	URR	Peak Year	Peak Rate
Gas Conv.	68.3	2028	1.36
Oil Conv.	5.3	2000	0.1
Oil Tight	3.44	2032	0.16
**Total**	**77.04**	**2028**	**1.57**

7.4.2 By Mineral

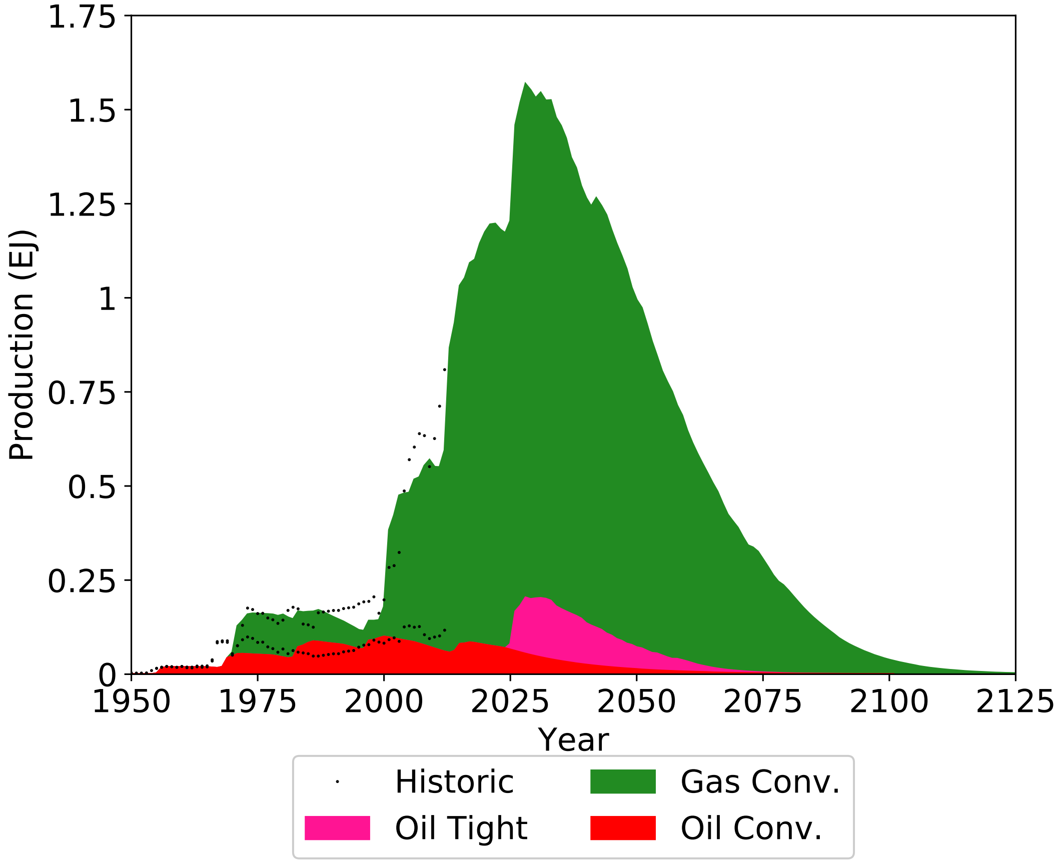

Figure 7.8: Bolivia projection by mineral type

Table 7.8: Peak years - Minerals

**Name**	**URR**	**Peak Year**	**Peak Rate**
Oil Conv.	5.3	2000	0.1
Oil Tight	3.44	2032	0.16
Gas Conv.	68.3	2028	1.36
**Total**	**77.04**	**2028**	**1.57**

7.5 Brazil

7.5.1 All Projections

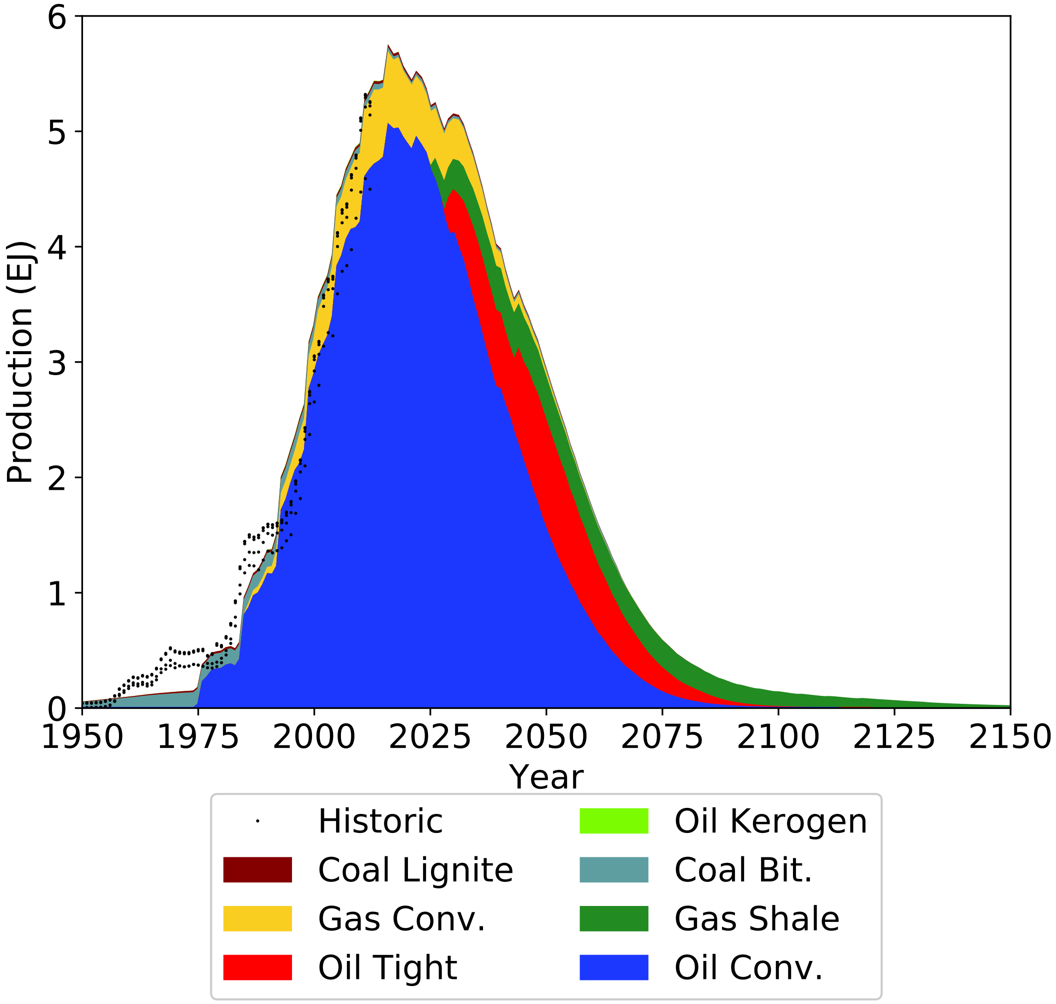

Figure 7.9: Brazil projections capped at 16

Table 7.9: Peak years - All

**Name**	**URR**	**Peak Year**	**Peak Rate**
Oil Conv.	231.01	2016	5.05
Oil Tight	30.37	2049	0.95
Gas Shale	23.74	2043	0.39
Gas Conv.	21.0	2013	0.64
Coal Bit.	8.0	1981	0.13
Coal Lignite	1.9	2000	0.02
Oil Kerogen	0.17	1993	0.01
**Total**	**316.19**	**2016**	**5.73**

7.5.2 By Mineral

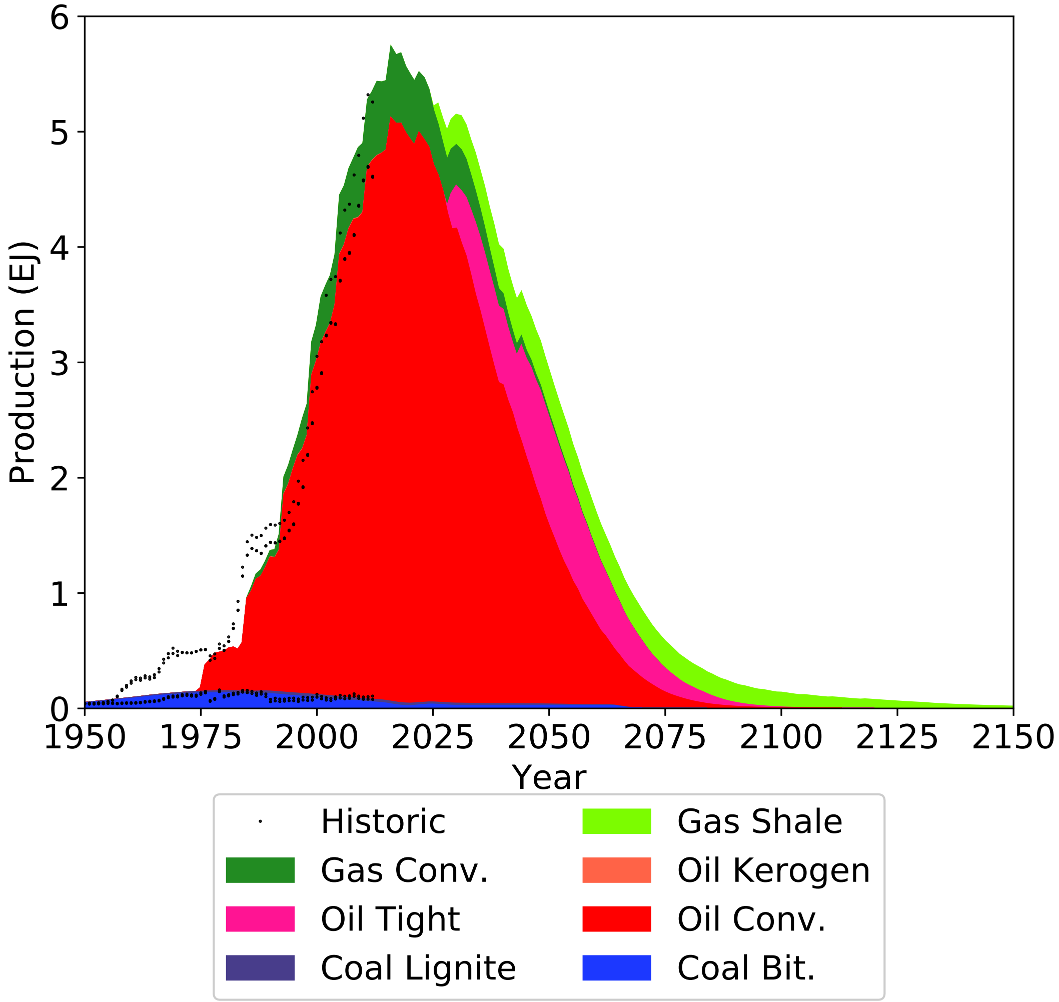

Figure 7.10: Brazil projection by mineral type

Table 7.10: Peak years - Minerals

**Name**	**URR**	**Peak Year**	**Peak Rate**
Coal Bit.	8.0	1981	0.13
Coal Lignite	1.9	2000	0.02
Oil Conv.	231.01	2016	5.05
Oil Tight	30.37	2049	0.95
Oil Kerogen	0.17	1993	0.01
Gas Conv.	21.0	2013	0.64
Gas Shale	23.74	2043	0.39
**Total**	**316.19**	**2016**	**5.73**

7.6 Chile

7.6.1 All Projections

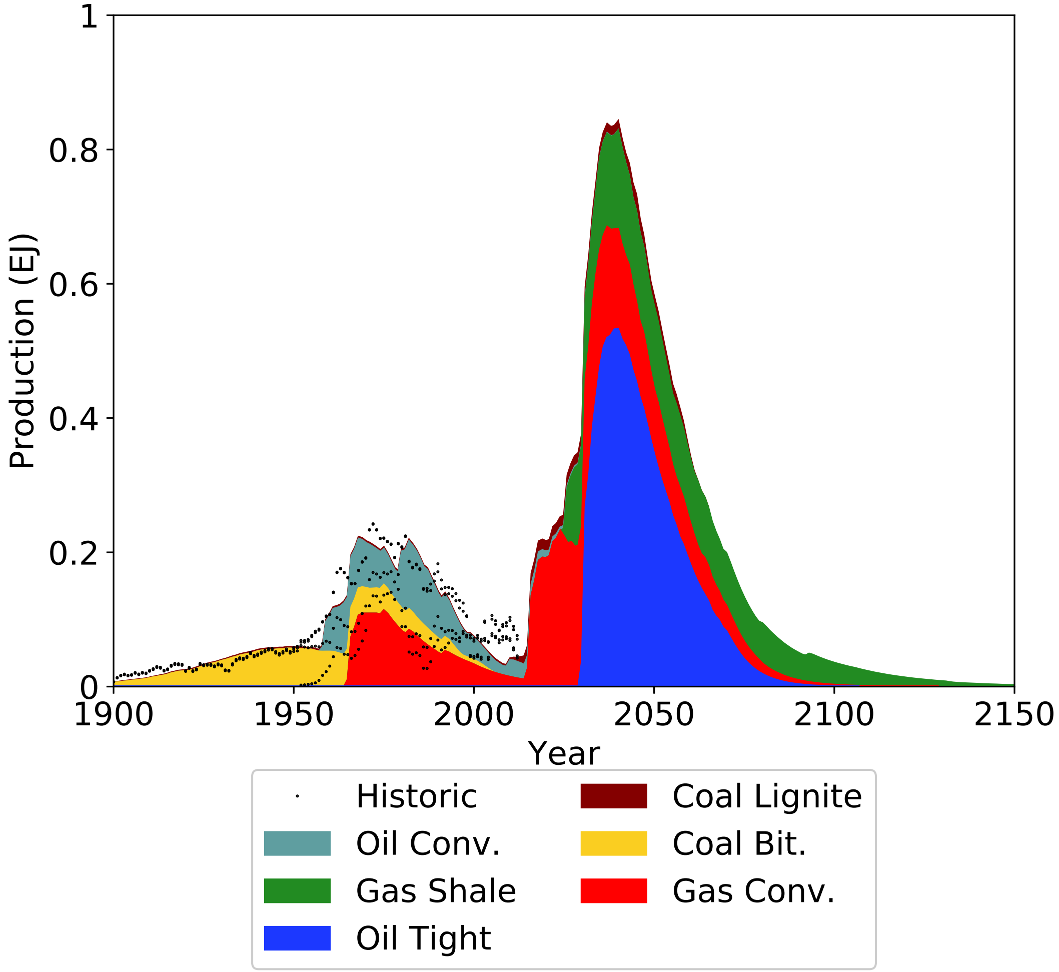

Figure 7.11: Chile projections capped at 16

Table 7.11: Peak years - All

**Name**	**URR**	**Peak Year**	**Peak Rate**
Oil Tight	13.18	2040	0.53
Gas Conv.	10.5	2024	0.23
Gas Shale	7.28	2040	0.15
Coal Bit.	3.4	1949	0.06
Oil Conv.	3.18	1982	0.1
Coal Lignite	0.95	2045	0.02
**Total**	**38.49**	**2040**	**0.84**

7.6.2 By Mineral

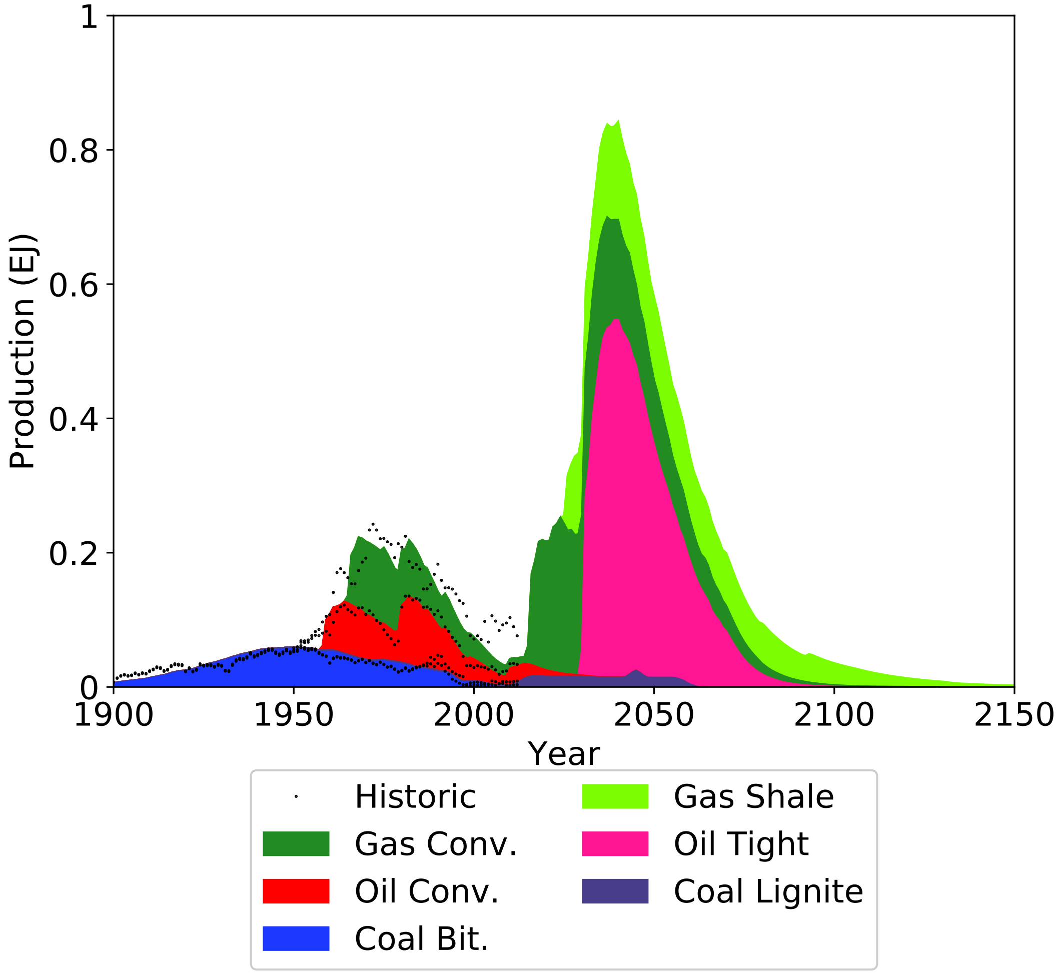

Figure 7.12: Chile projection by mineral type

Table 7.12: Peak years - Minerals

**Name**	**URR**	**Peak Year**	**Peak Rate**
Coal Bit.	3.4	1949	0.06
Coal Lignite	0.95	2045	0.02
Oil Conv.	3.18	1982	0.1
Oil Tight	13.18	2040	0.53
Gas Conv.	10.5	2024	0.23
Gas Shale	7.28	2040	0.15
**Total**	**38.49**	**2040**	**0.84**

7.7 Colombia

7.7.1 All Projections

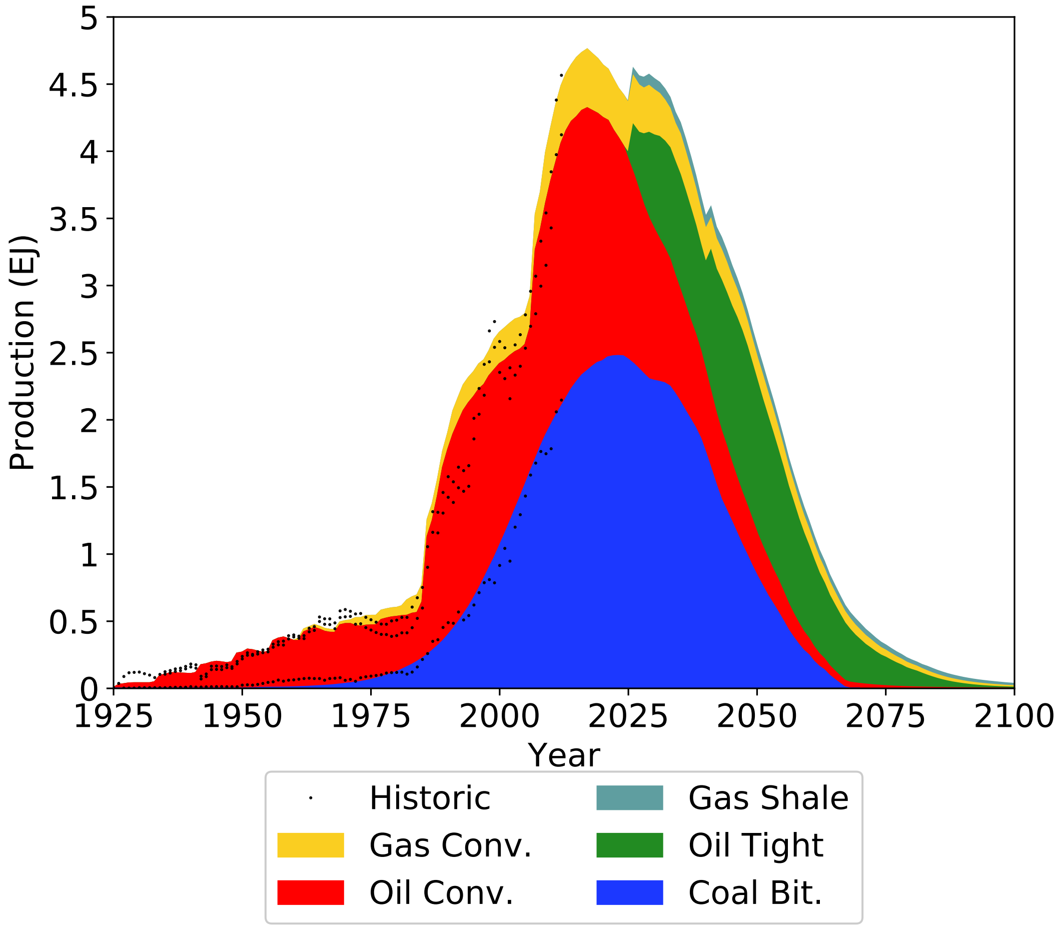

Figure 7.13: Colombia projections capped at 16

Table 7.13: Peak years - All

**Name**	**URR**	**Peak Year**	**Peak Rate**
Coal Bit.	111.6	2023	2.48
Oil Conv.	99.5	2014	1.99
Oil Tight	38.96	2047	1.2
Gas Conv.	23.1	2015	0.44
Gas Shale	4.47	2037	0.1
**Total**	**277.63**	**2017**	**4.76**

7.7.2 By Mineral

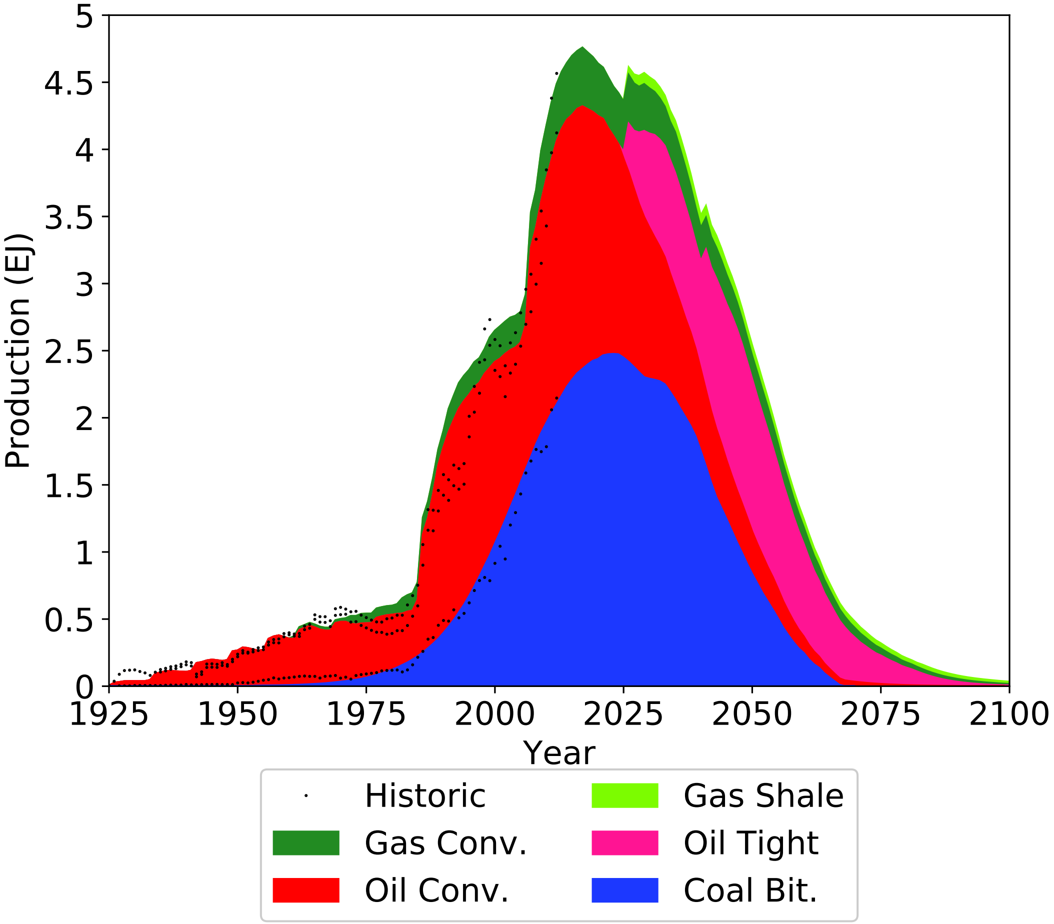

Figure 7.14: Colombia projection by mineral type

Table 7.14: Peak years - Minerals

**Name**	**URR**	**Peak Year**	**Peak Rate**
Coal Bit.	111.6	2023	2.48
Oil Conv.	99.5	2014	1.99
Oil Tight	38.96	2047	1.2
Gas Conv.	23.1	2015	0.44
Gas Shale	4.47	2037	0.1
**Total**	**277.63**	**2017**	**4.76**

7.8 Cuba

7.8.1 All Projections

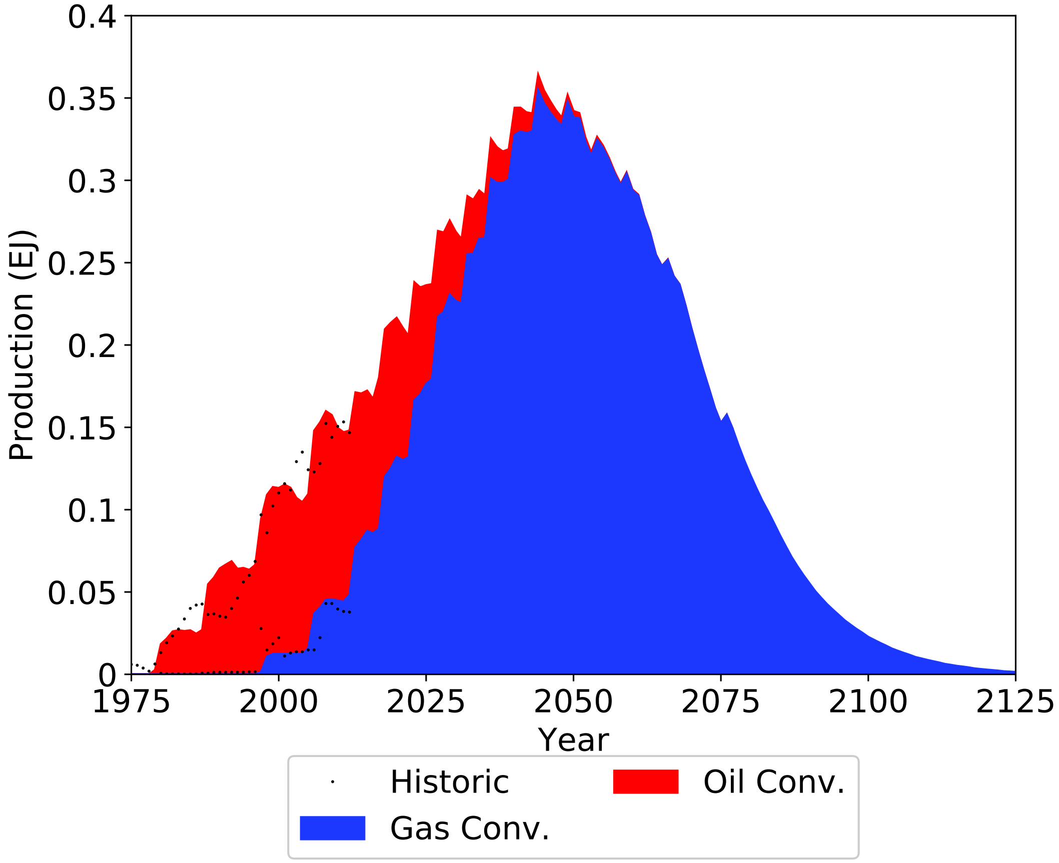

Figure 7.15: Cuba projections capped at 16

Table 7.15: Peak years - All

**Name**	**URR**	**Peak Year**	**Peak Rate**
Gas Conv.	17.83	2044	0.35
Oil Conv.	4.07	2008	0.11
**Total**	**21.9**	**2044**	**0.36**

7.8.2 By Mineral

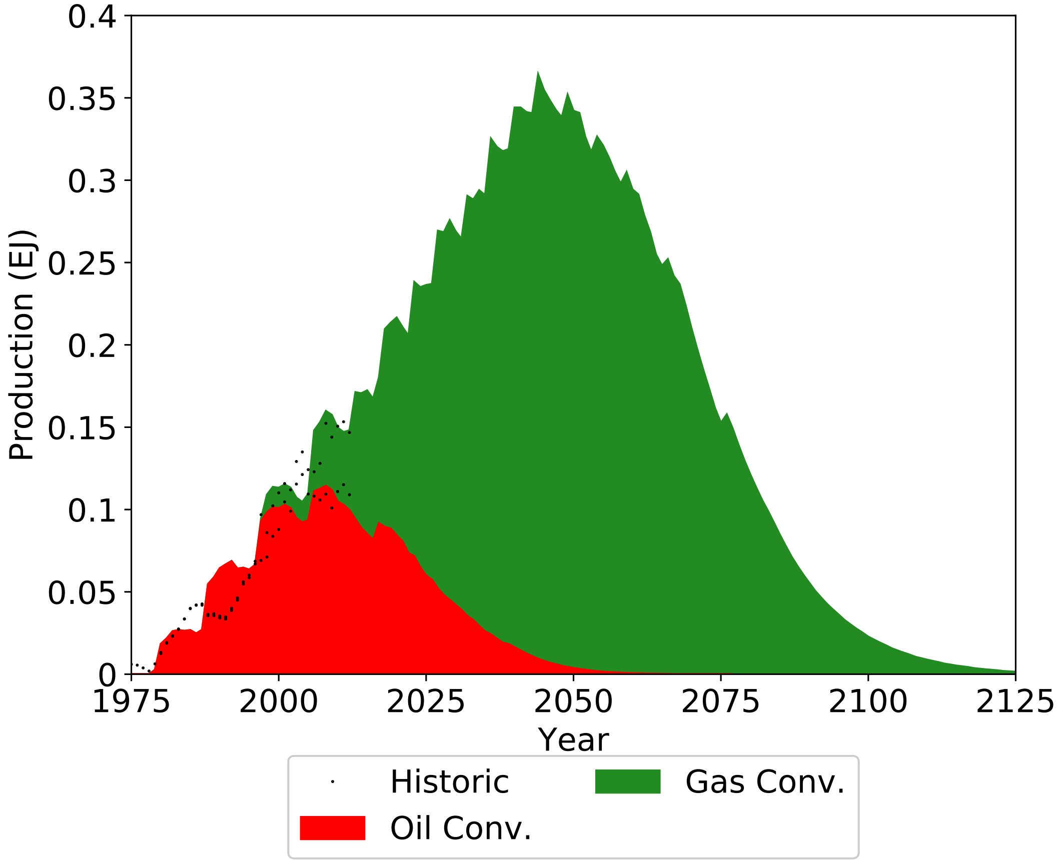

Figure 7.16: Cuba projection by mineral type

Table 7.16: Peak years - Minerals

Name	URR	Peak Year	Peak Rate
Oil Conv.	4.07	2008	0.11
Gas Conv.	17.83	2044	0.35
**Total**	**21.9**	**2044**	**0.36**

7.9 Ecuador

7.9.1 All Projections

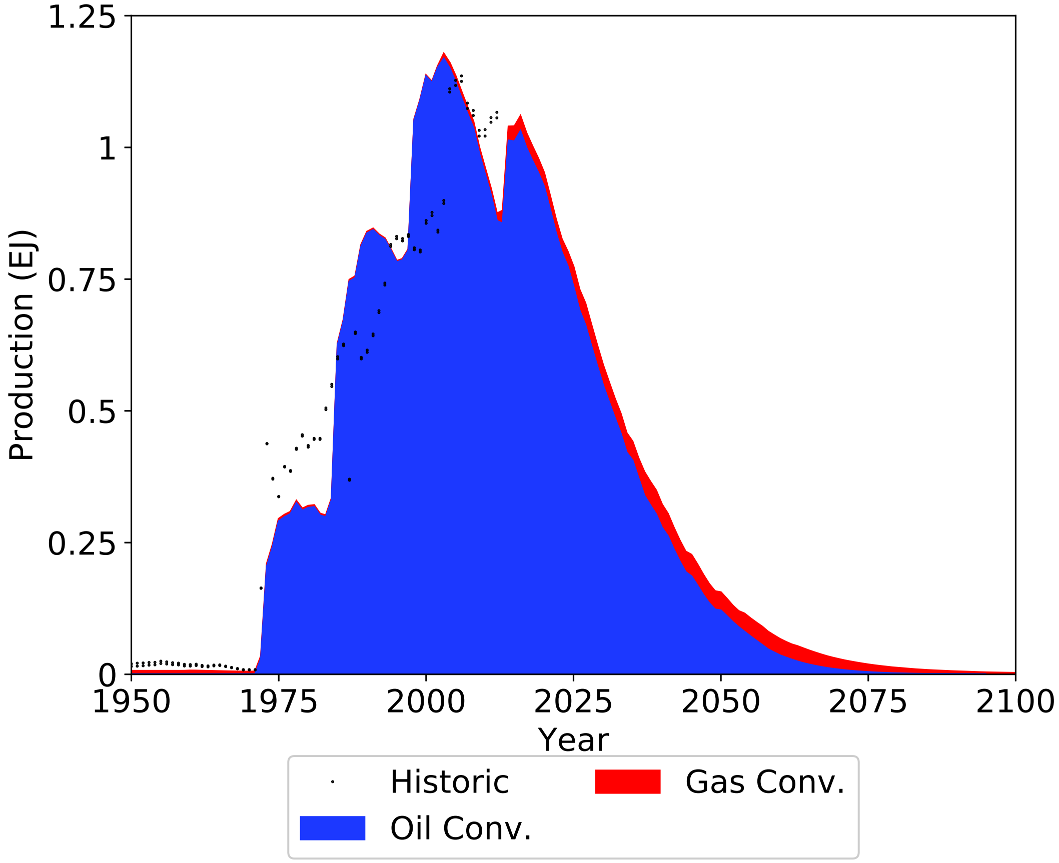

Figure 7.17: Ecuador projections capped at 16

Table 7.17: Peak years - All

Name	URR	Peak Year	Peak Rate
Oil Conv.	51.11	2003	1.17
Gas Conv.	2.6	2039	0.05
**Total**	**53.71**	**2003**	**1.18**

7.9.2 By Mineral

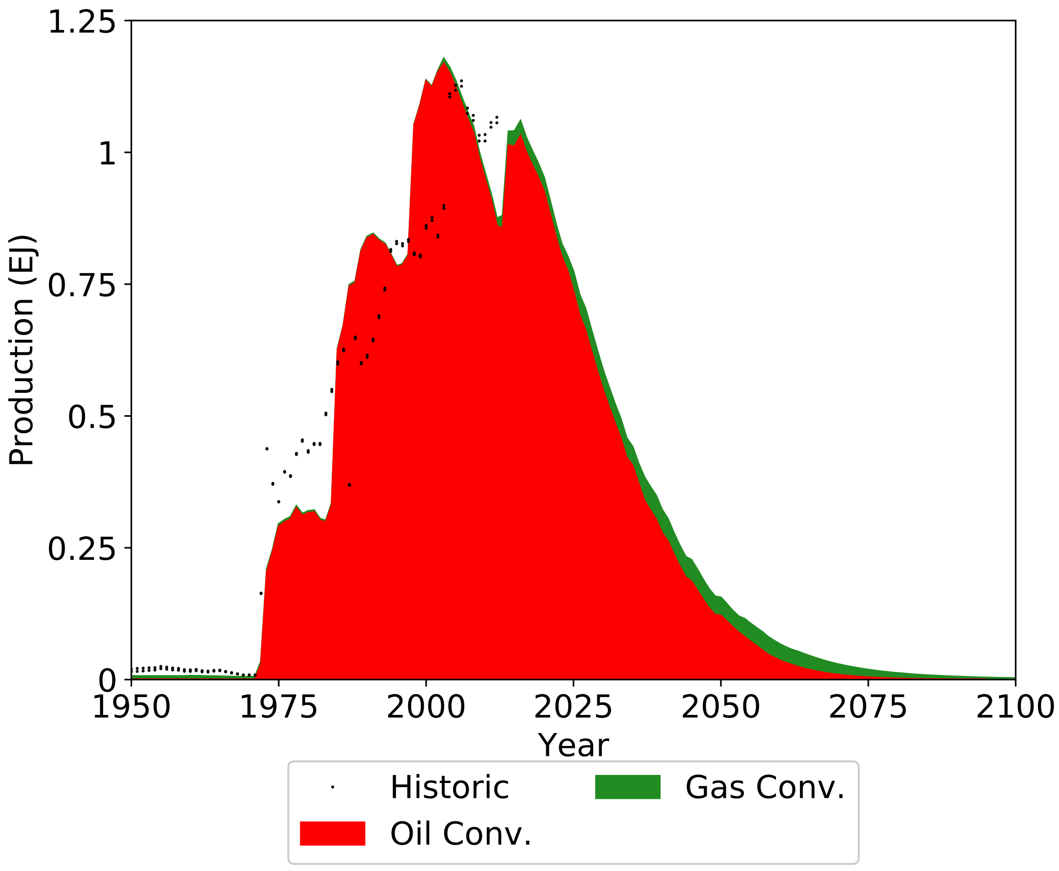

Figure 7.18: Ecuador projection by mineral type

Table 7.18: Peak years - Minerals

Name	URR	Peak Year	Peak Rate
Oil Conv.	51.11	2003	1.17
Gas Conv.	2.6	2039	0.05
**Total**	**53.71**	**2003**	**1.18**

7.10 Grenada

7.10.1 All Projections

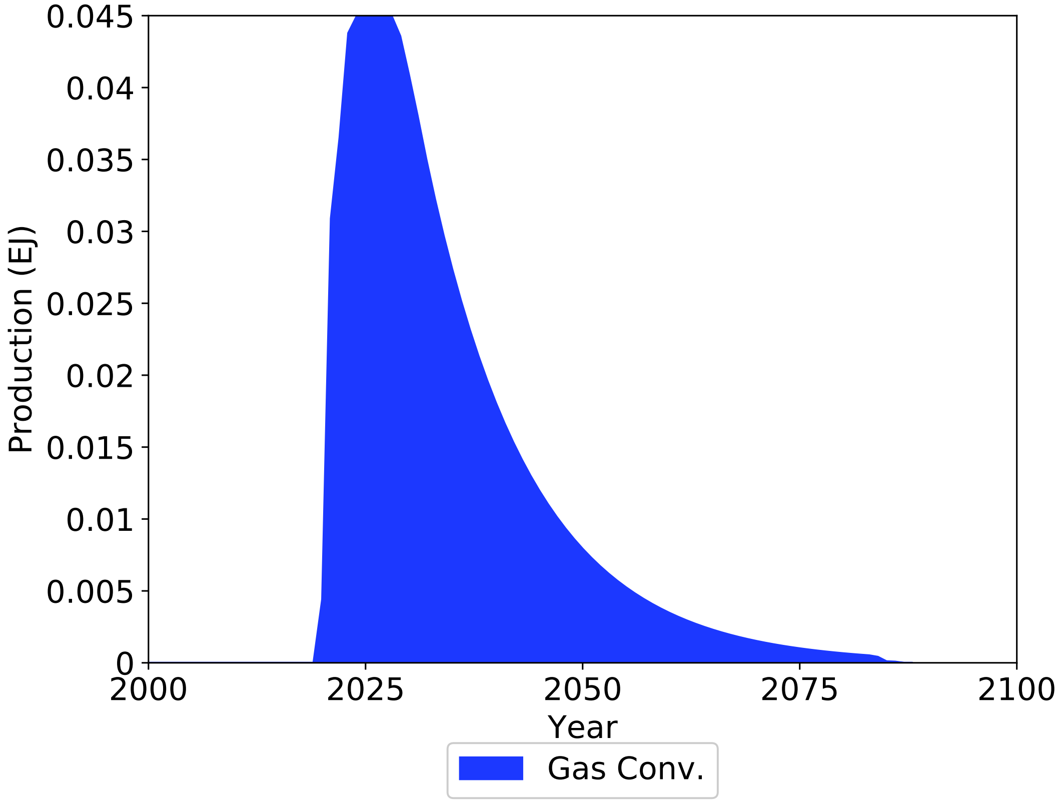

Figure 7.19: Grenada projections capped at 16

Table 7.19: Peak years - All

Name	URR	Peak Year	Peak Rate
Gas Conv.	0.9	2024	0.05
**Total**	**0.9**	**2024**	**0.05**

7.10.2 By Mineral

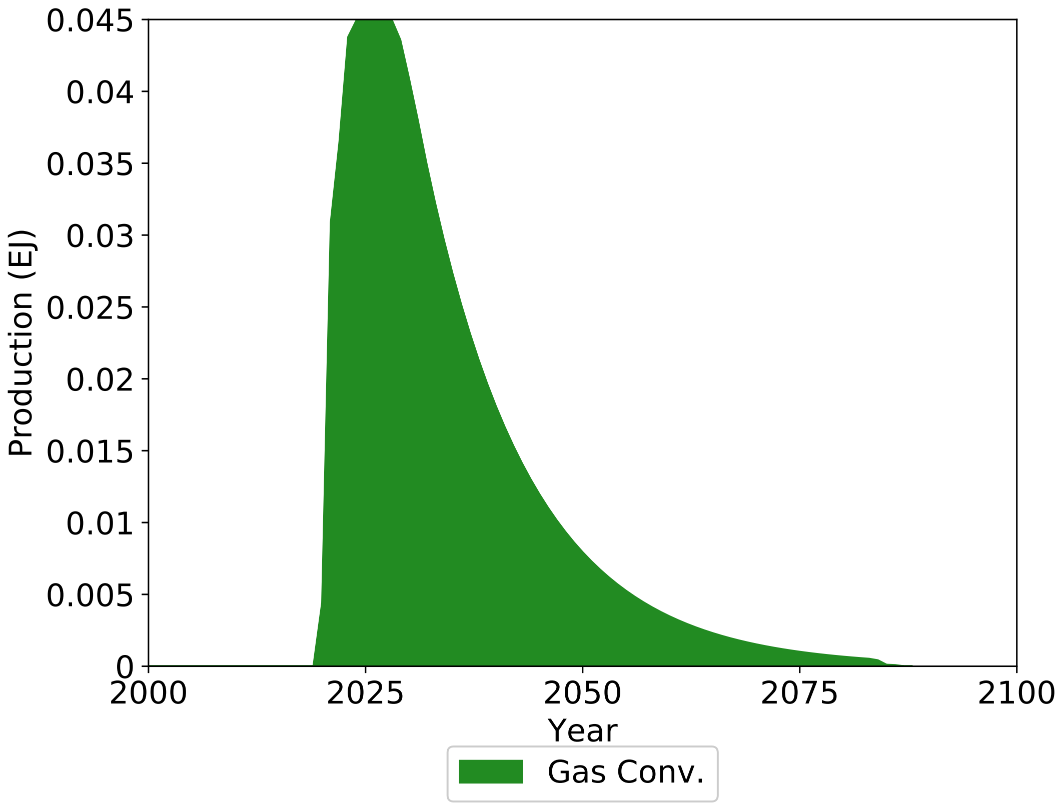

Figure 7.20: Grenada projection by mineral type

Table 7.20: Peak years - Minerals

Name	URR	Peak Year	Peak Rate
Gas Conv.	0.9	2024	0.05
**Total**	**0.9**	**2024**	**0.05**

7.11 Guatemala

7.11.1 All Projections

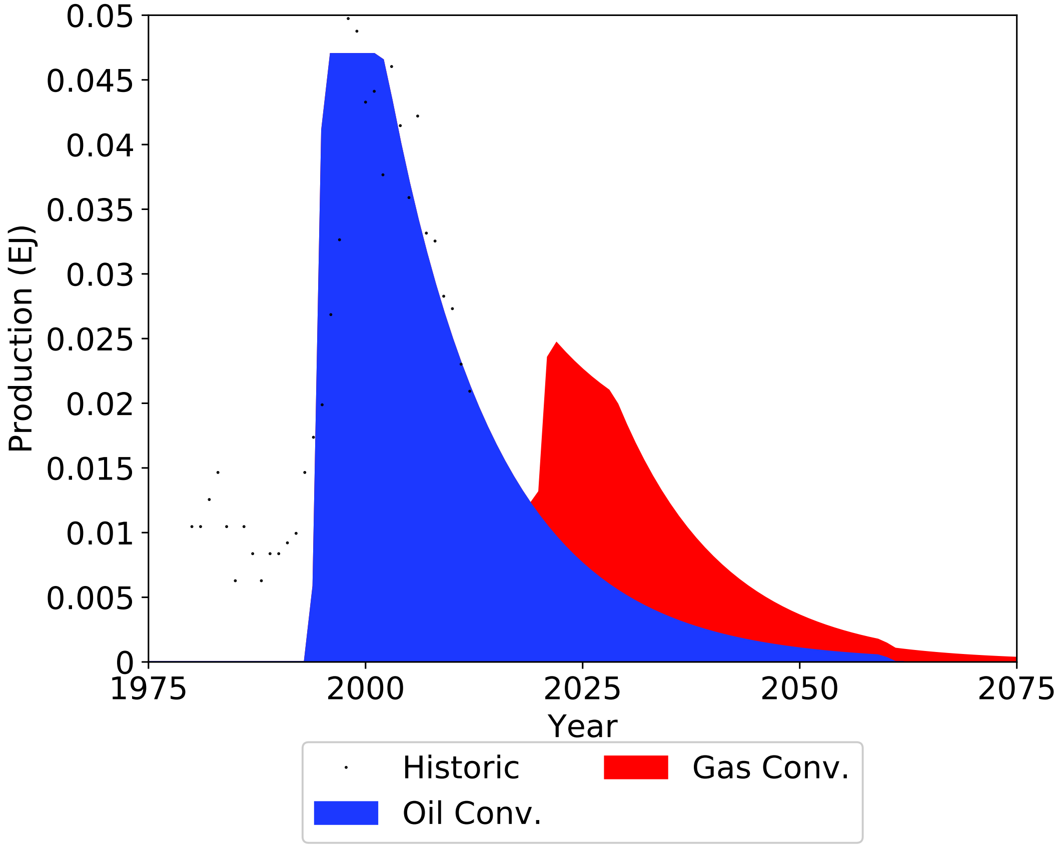

Figure 7.21: Guatemala projections capped at 16

Table 7.21: Peak years - All

Name	URR	Peak Year	Peak Rate
Oil Conv.	0.94	1996	0.05
Gas Conv.	0.3	2022	0.02
**Total**	**1.24**	**1996**	**0.05**

7.11.2 By Mineral

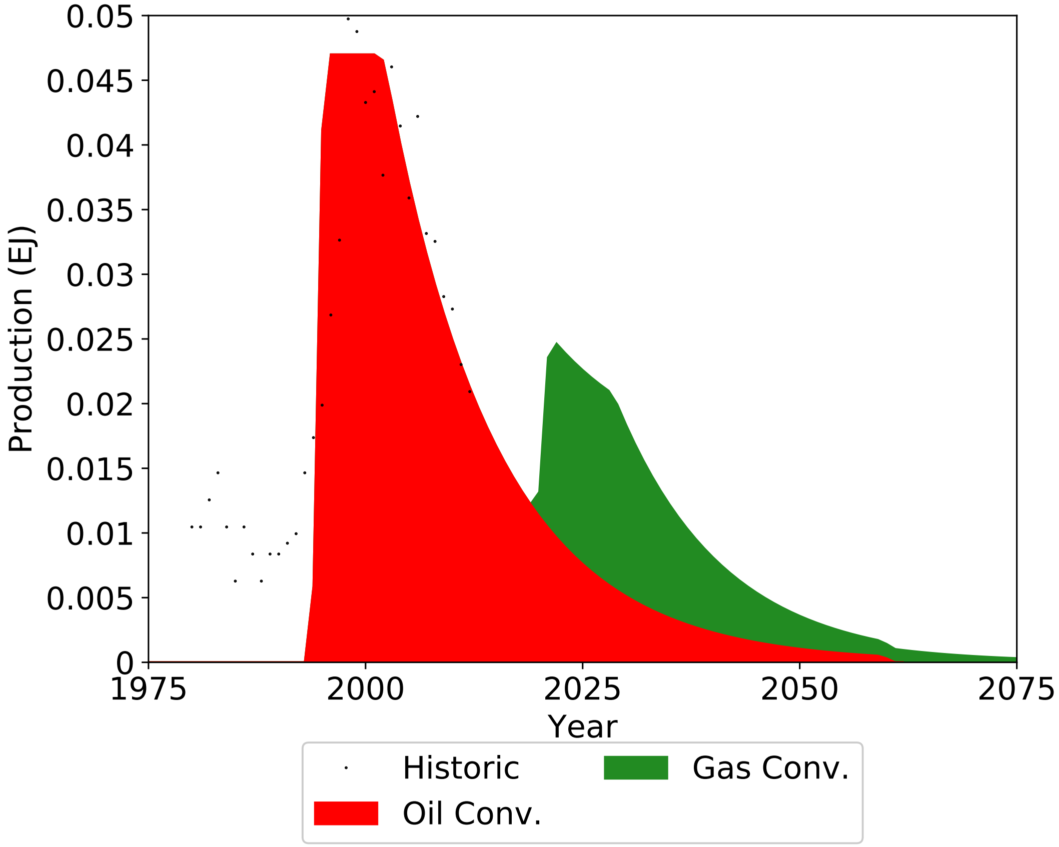

Figure 7.22: Guatemala projection by mineral type

Table 7.22: Peak years - Minerals

Name	URR	Peak Year	Peak Rate
Oil Conv.	0.94	1996	0.05
Gas Conv.	0.3	2022	0.02
**Total**	**1.24**	**1996**	**0.05**

7.12 Guyana

7.12.1 All Projections

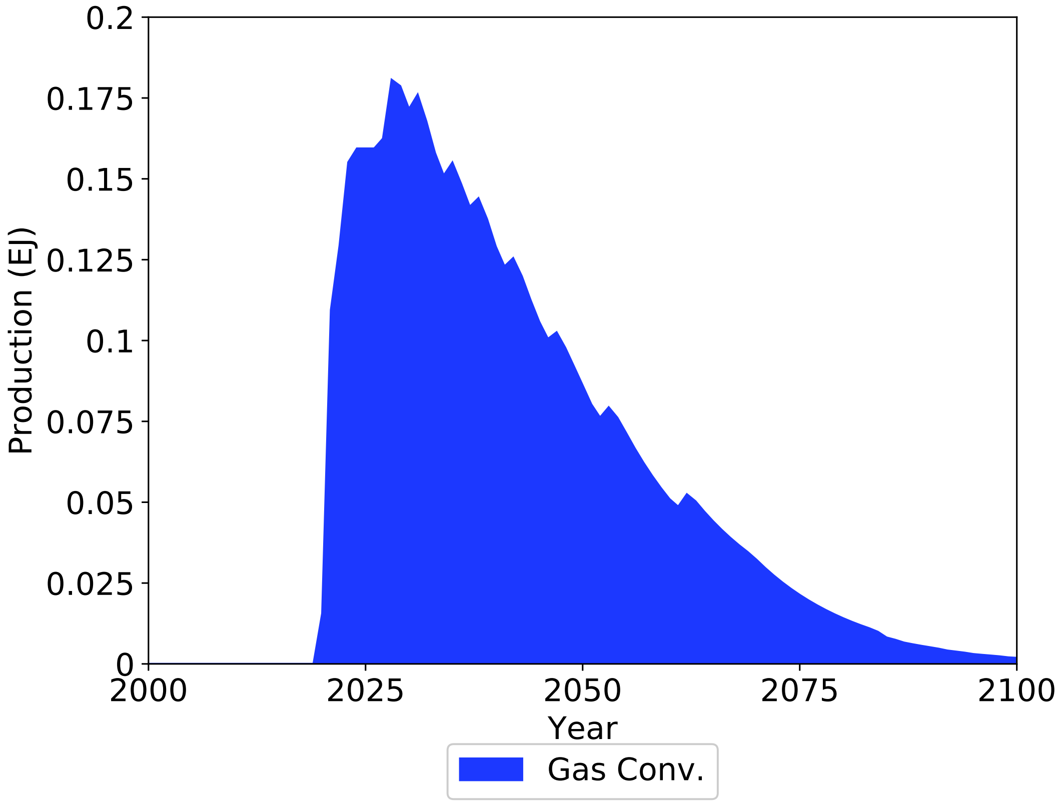

Figure 7.23: Guyana projections capped at 16

Table 7.23: Peak years - All

Name	URR	Peak Year	Peak Rate
Gas Conv.	5.6	2028	0.18
**Total**	**5.6**	**2028**	**0.18**

7.12.2 By Mineral

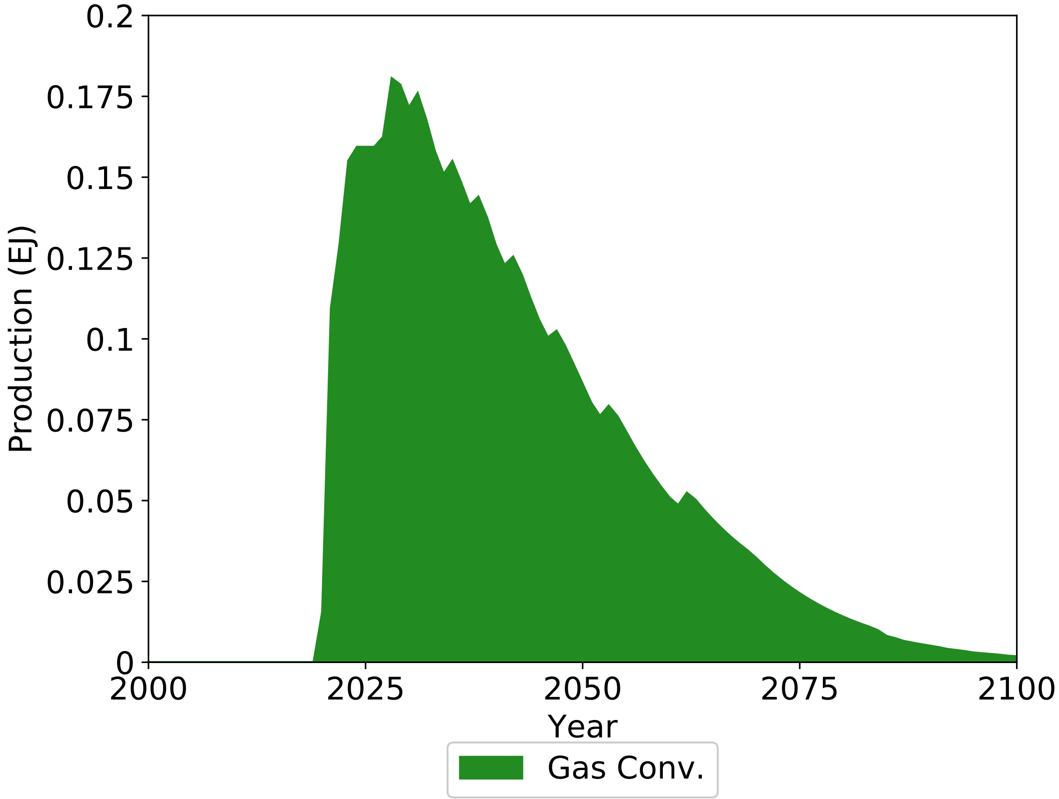

Figure 7.24: Guyana projection by mineral type

Table 7.24: Peak years - Minerals

**Name**	**URR**	**Peak Year**	**Peak Rate**
Gas Conv.	5.6	2028	0.18
**Total**	**5.6**	**2028**	**0.18**

7.13 Mexico

7.13.1 All Projections

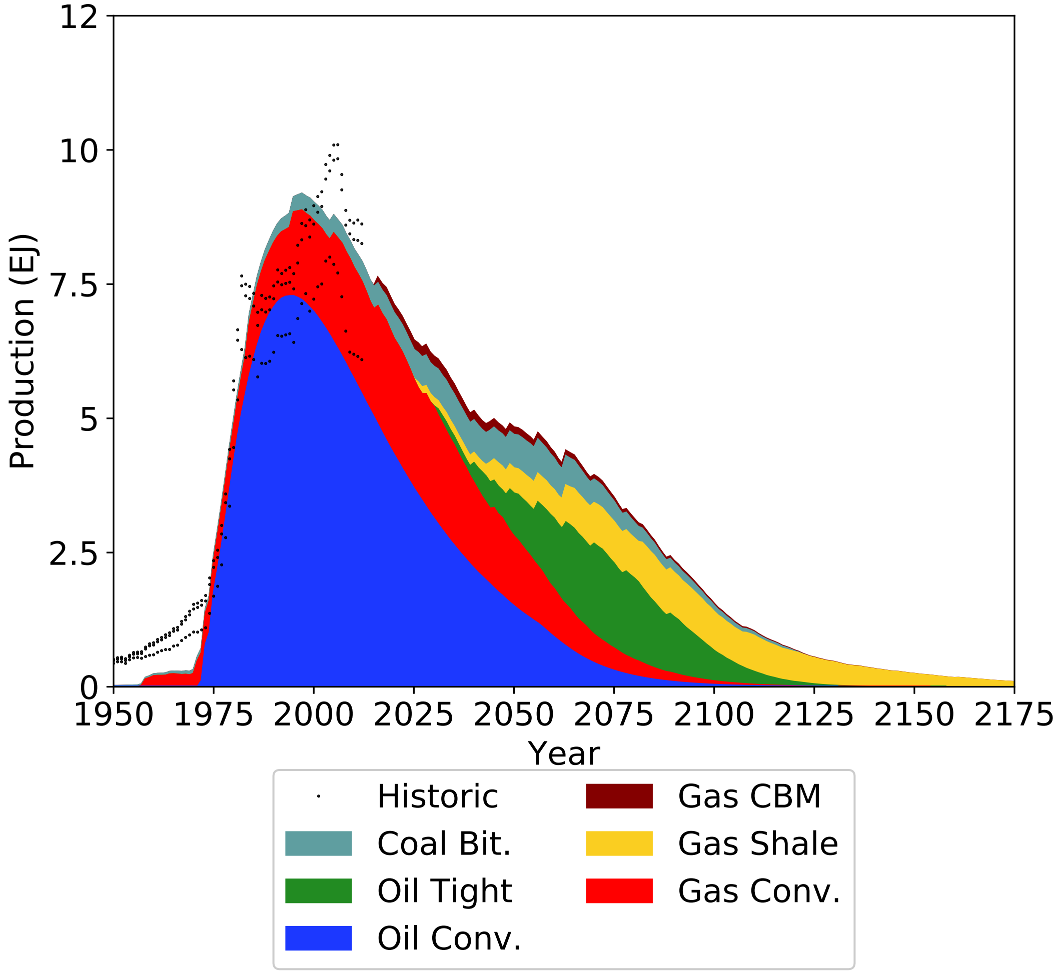

Figure 7.25: Mexico projections capped at 16

Table 7.25: Peak years - All

**Name**	**URR**	**Peak Year**	**Peak Rate**
Oil Conv.	368.91	1995	7.28
Gas Conv.	153.2	2018	2.24
Oil Tight	75.06	2072	1.7
Gas Shale	71.5	2085	0.87
Coal Bit.	51.4	2054	0.65
Gas CBM	10.5	2029	0.2
**Total**	**730.57**	**1997**	**9.19**

7.13.2 By Mineral

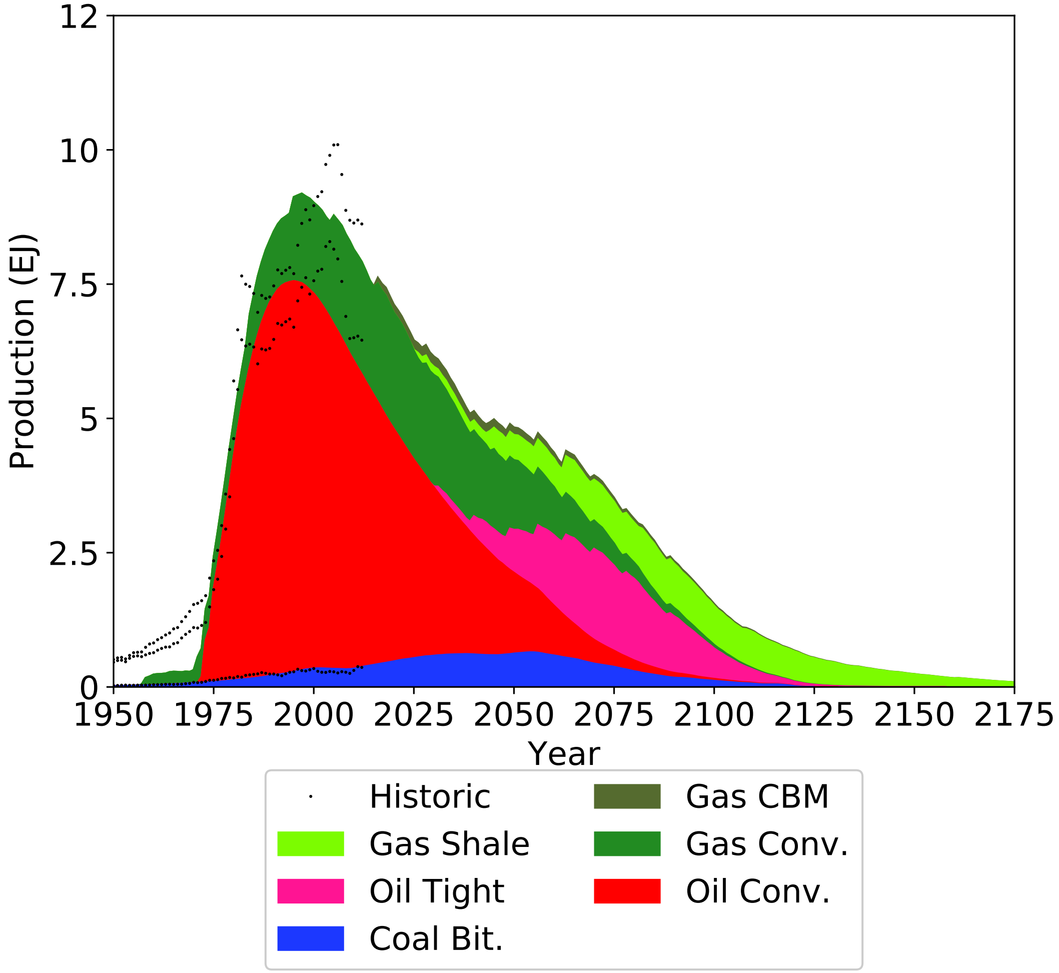

Figure 7.26: Mexico projection by mineral type

Table 7.26: Peak years - Minerals

**Name**	**URR**	**Peak Year**	**Peak Rate**
Coal Bit.	51.4	2054	0.65
Oil Conv.	368.91	1995	7.28
Oil Tight	75.06	2072	1.7
Gas Conv.	153.2	2018	2.24
Gas Shale	71.5	2085	0.87
Gas CBM	10.5	2029	0.2
**Total**	**730.57**	**1997**	**9.19**

7.14 Paraguay

7.14.1 All Projections

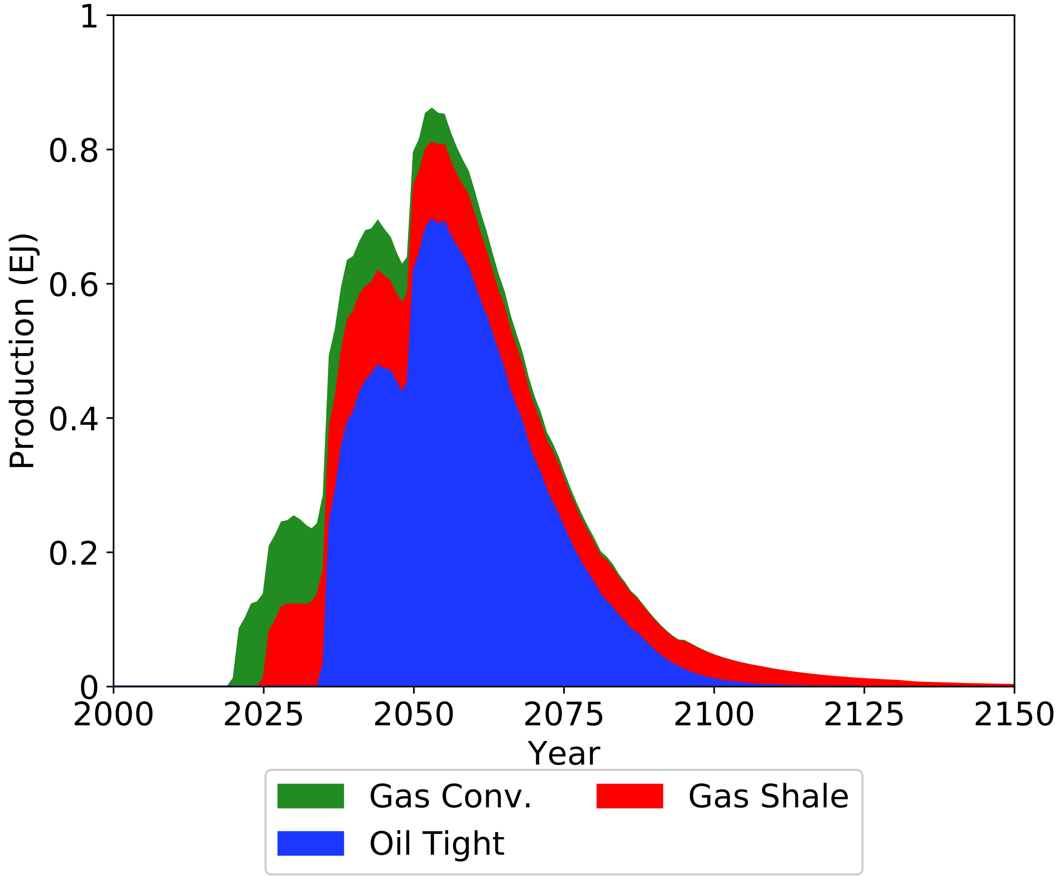

Figure 7.27: Paraguay projections capped at 16

Table 7.27: Peak years - All

Name	URR	Peak Year	Peak Rate
Oil Tight	21.2	2053	0.7
Gas Shale	7.62	2039	0.15
Gas Conv.	3.7	2030	0.13
**Total**	**32.52**	**2053**	**0.86**

7.14.2 By Mineral

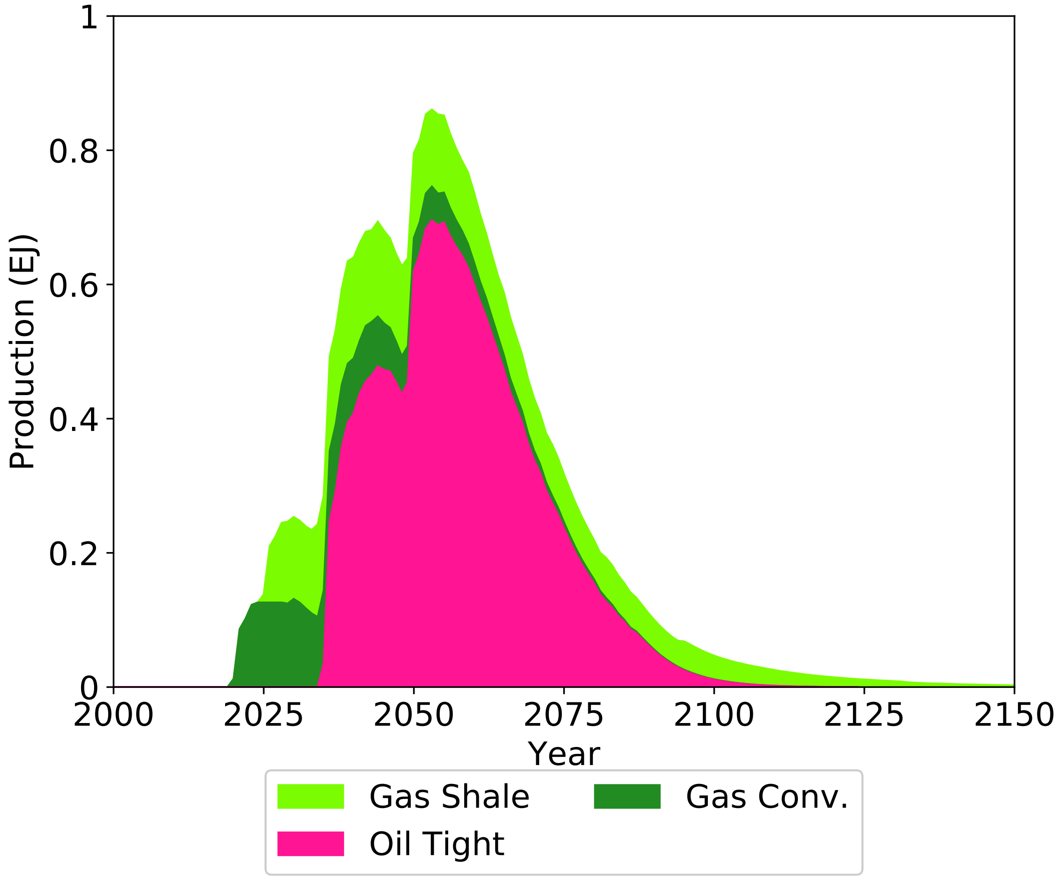

Figure 7.28: Paraguay projection by mineral type

Table 7.28: Peak years - Minerals

Name	URR	Peak Year	Peak Rate
Oil Tight	21.2	2053	0.7
Gas Conv.	3.7	2030	0.13
Gas Shale	7.62	2039	0.15
**Total**	**32.52**	**2053**	**0.86**

7.15 Peru

7.15.1 All Projections

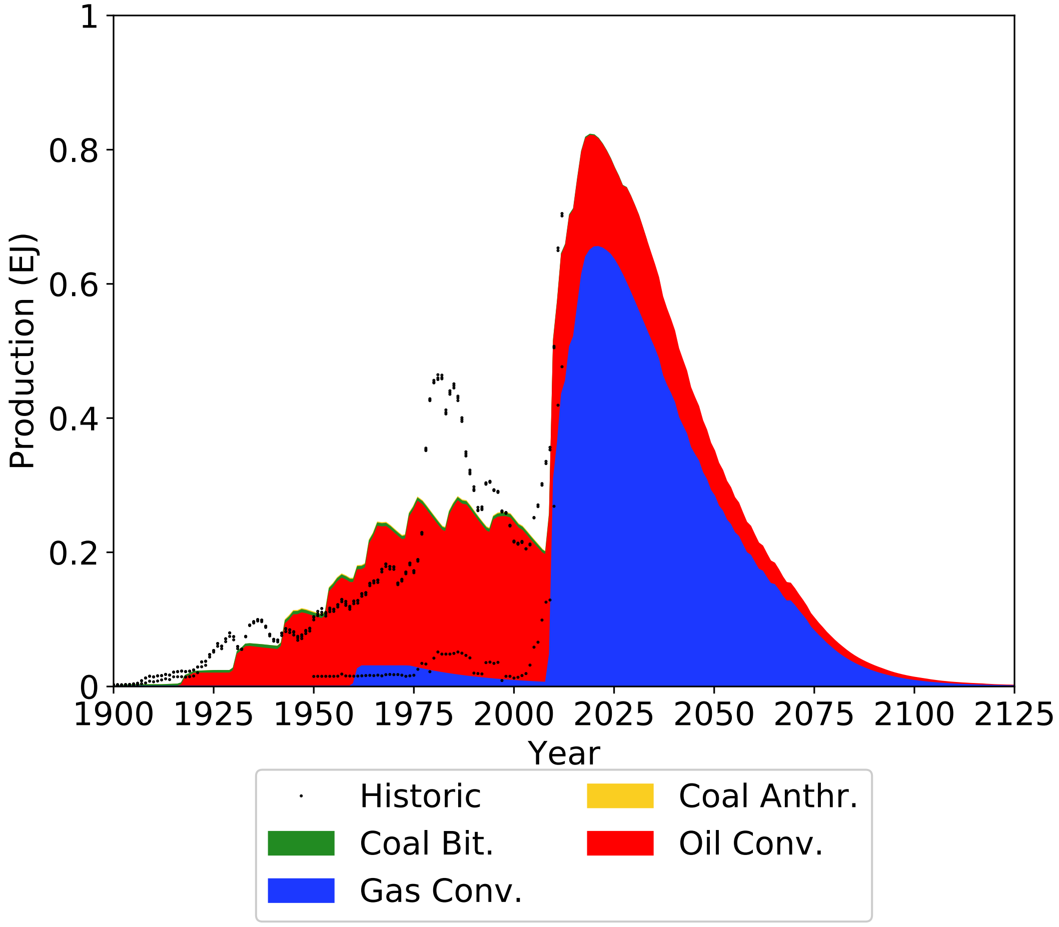

Figure 7.29: Peru projections capped at 16

Table 7.29: Peak years - All

**Name**	**URR**	**Peak Year**	**Peak Rate**
Gas Conv.	26.3	2021	0.65
Oil Conv.	20.64	1986	0.26
Coal Bit.	0.48	1938	–
Coal Anthr.	0.1	1944	–
**Total**	**47.52**	**2019**	**0.82**

7.15.2 By Mineral

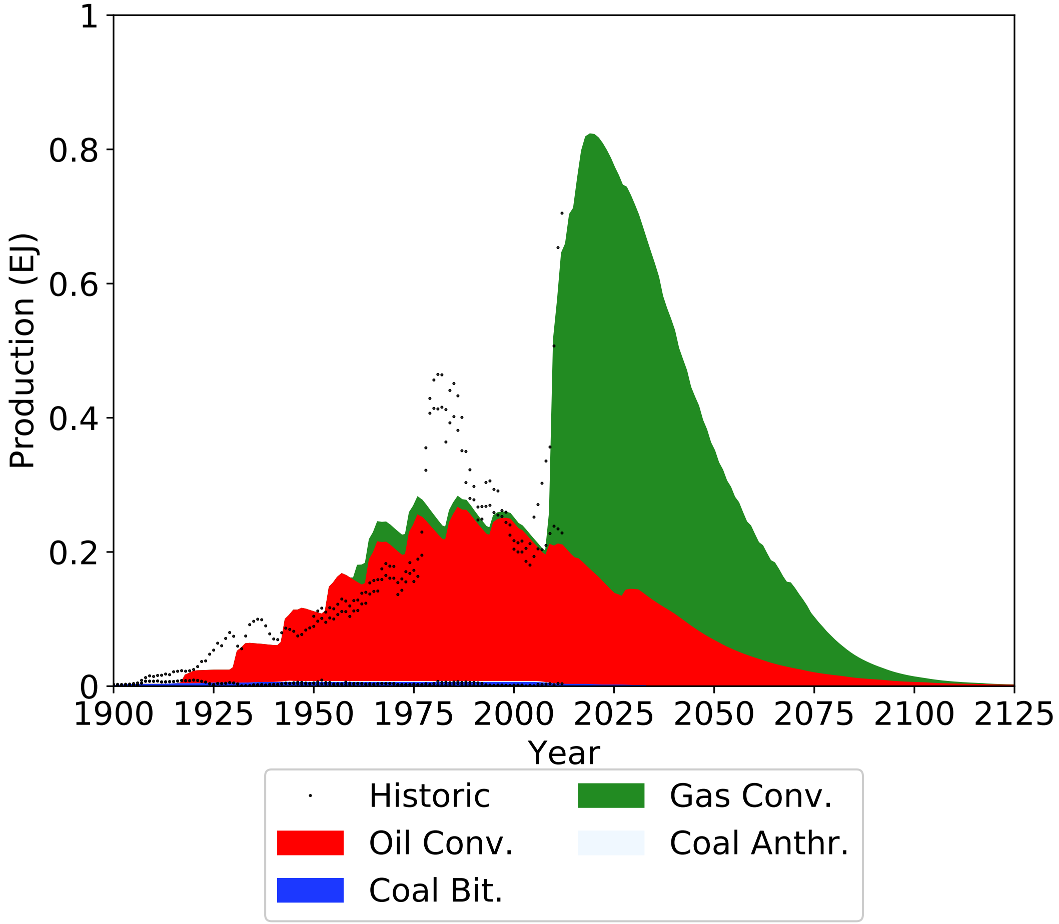

Figure 7.30: Peru projection by mineral type

Table 7.30: Peak years - Minerals

**Name**	**URR**	**Peak Year**	**Peak Rate**
Coal Bit.	0.48	1938	–
Coal Anthr.	0.1	1944	–
Oil Conv.	20.64	1986	0.26
Gas Conv.	26.3	2021	0.65
**Total**	**47.52**	**2019**	**0.82**

7.16 Suriname

7.16.1 All Projections

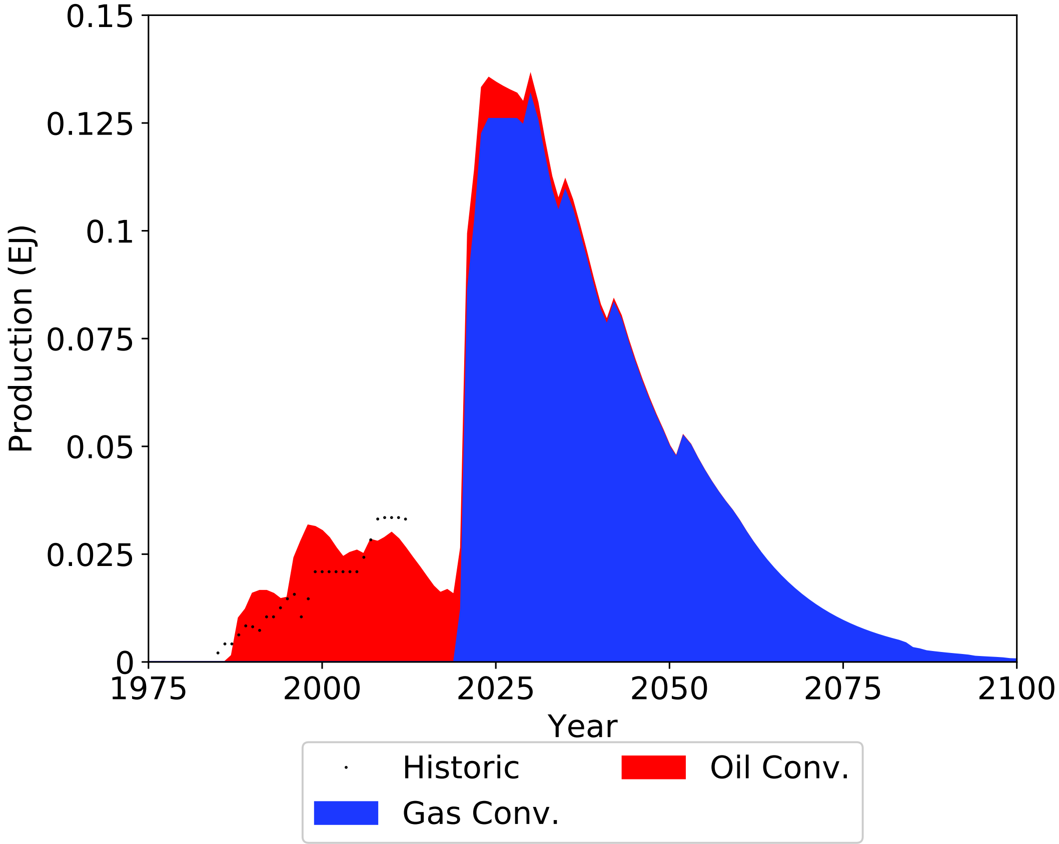

Figure 7.31: Suriname projections capped at 16

Table 7.31: Peak years - All

Name	URR	Peak Year	Peak Rate
Gas Conv.	3.7	2030	0.13
Oil Conv.	0.85	1998	0.03
**Total**	**4.55**	**2030**	**0.14**

7.16.2 By Mineral

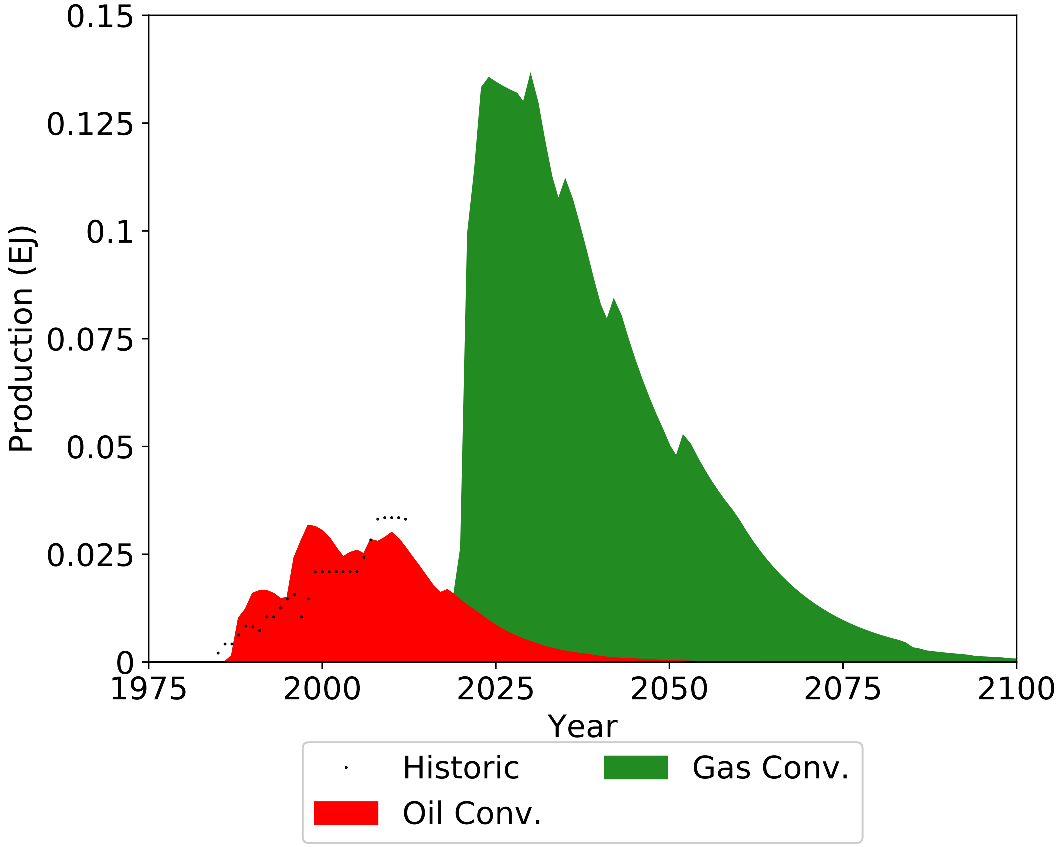

Figure 7.32: Suriname projection by mineral type

Table 7.32: Peak years - Minerals

Name	URR	Peak Year	Peak Rate
Oil Conv.	0.85	1998	0.03
Gas Conv.	3.7	2030	0.13
**Total**	**4.55**	**2030**	**0.14**

7.17 Trinidad and Tobago

7.17.1 All Projections

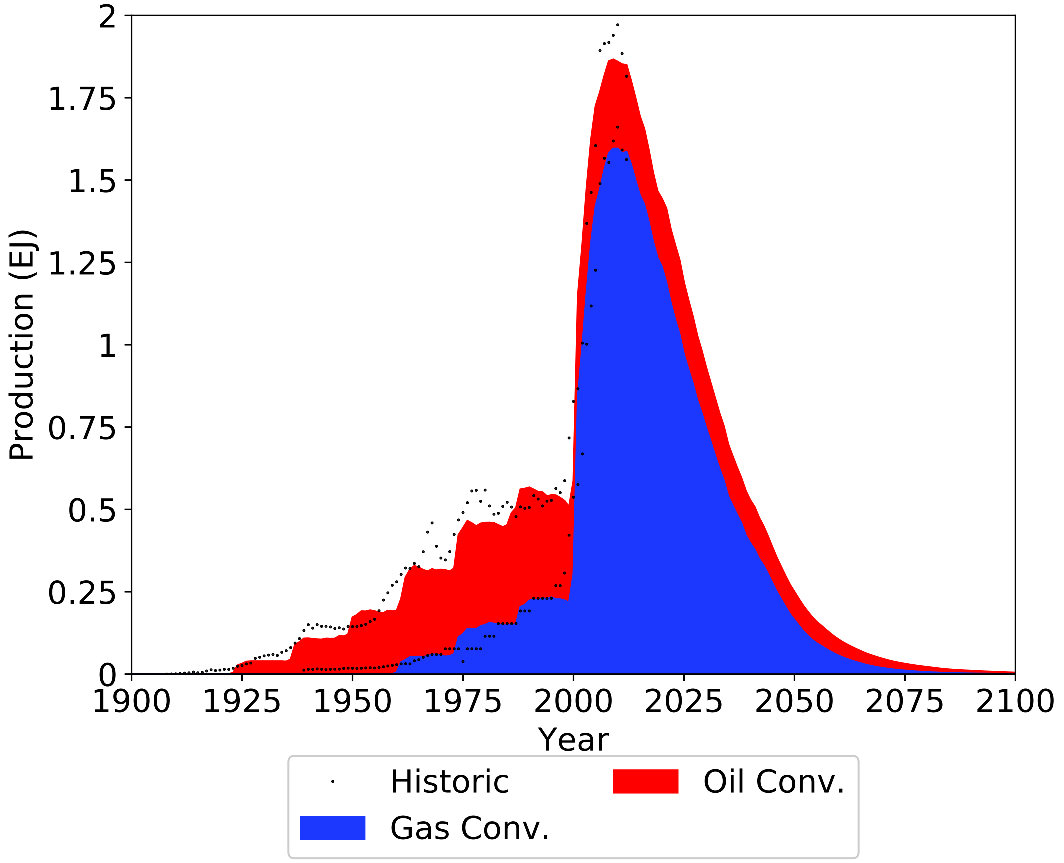

Figure 7.33: Trinidad and Tobago projections capped at 16

Table 7.33: Peak years - All

**Name**	**URR**	**Peak Year**	**Peak Rate**
Gas Conv.	52.5	2010	1.6
Oil Conv.	27.29	1988	0.36
**Total**	**79.79**	**2009**	**1.87**

7.17.2 By Mineral

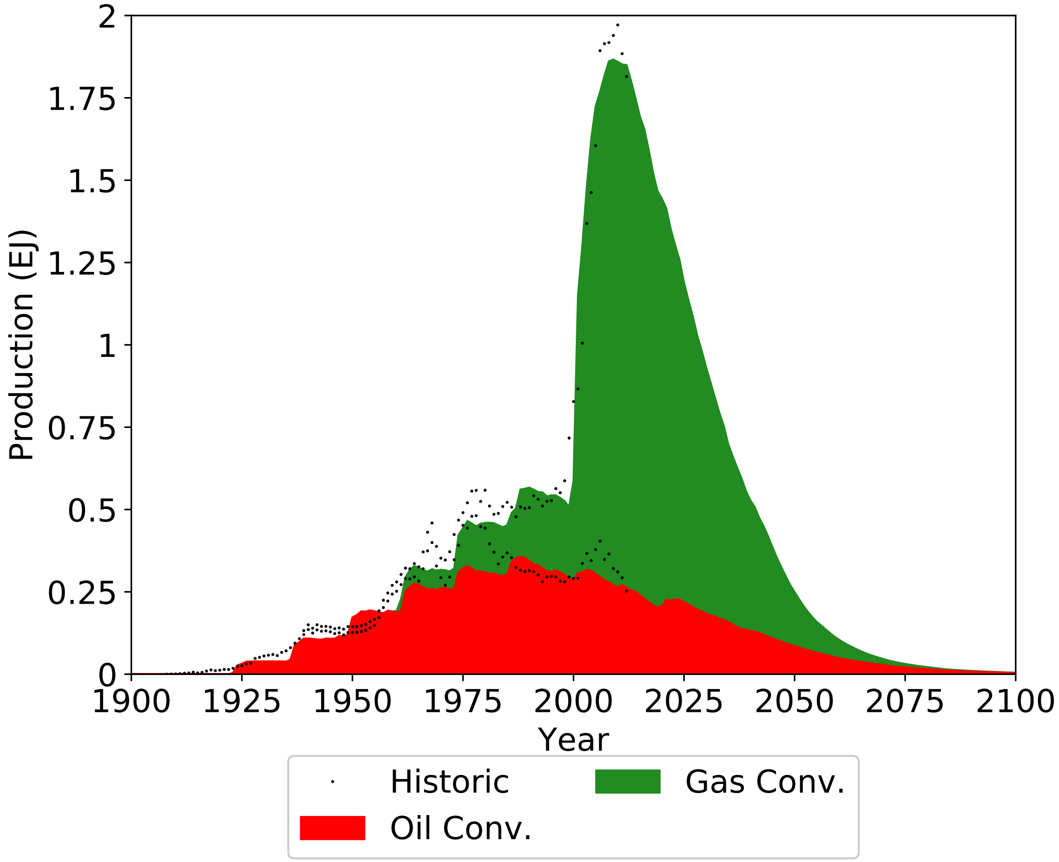

Figure 7.34: Trinidad and Tobago projection by mineral type

Table 7.34: Peak years - Minerals

**Name**	**URR**	**Peak Year**	**Peak Rate**
Oil Conv.	27.29	1988	0.36
Gas Conv.	52.5	2010	1.6
**Total**	**79.79**	**2009**	**1.87**

7.18 Uruguay

7.18.1 All Projections

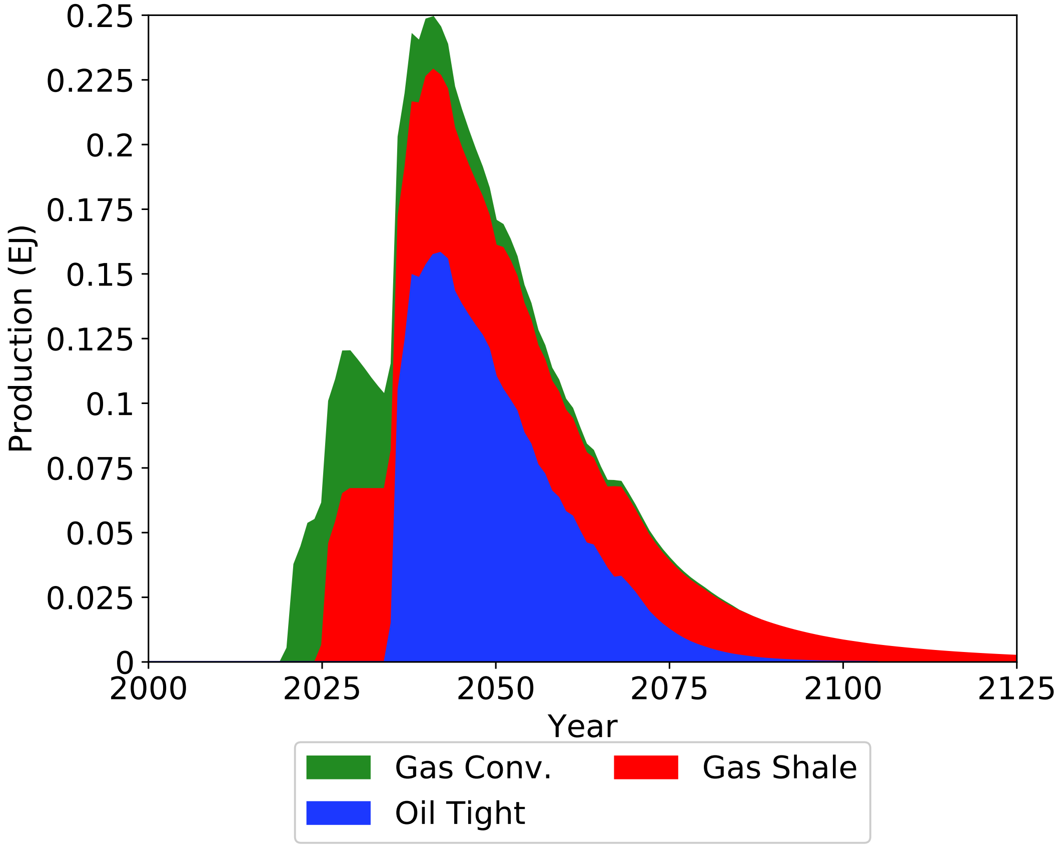

Figure 7.35: Uruguay projections capped at 16

Table 7.35: Peak years - All

Name	URR	Peak Year	Peak Rate
Oil Tight	3.44	2042	0.16
Gas Shale	3.07	2040	0.07
Gas Conv.	1.1	2024	0.06
**Total**	**7.61**	**2041**	**0.25**

7.18.2 By Mineral

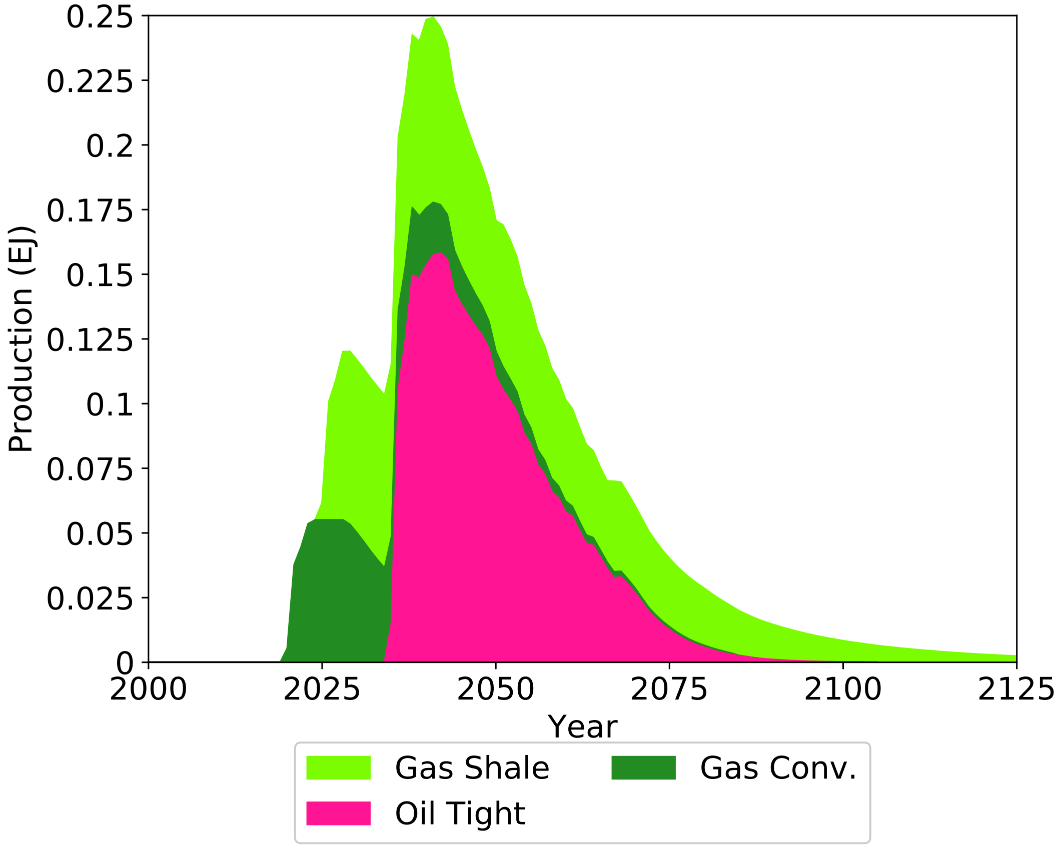

Figure 7.36: Uruguay projection by mineral type

Table 7.36: Peak years - Minerals

Name	URR	Peak Year	Peak Rate
Oil Tight	3.44	2042	0.16
Gas Conv.	1.1	2024	0.06
Gas Shale	3.07	2040	0.07
**Total**	**7.61**	**2041**	**0.25**

7.19 Venezuela

7.19.1 All Projections

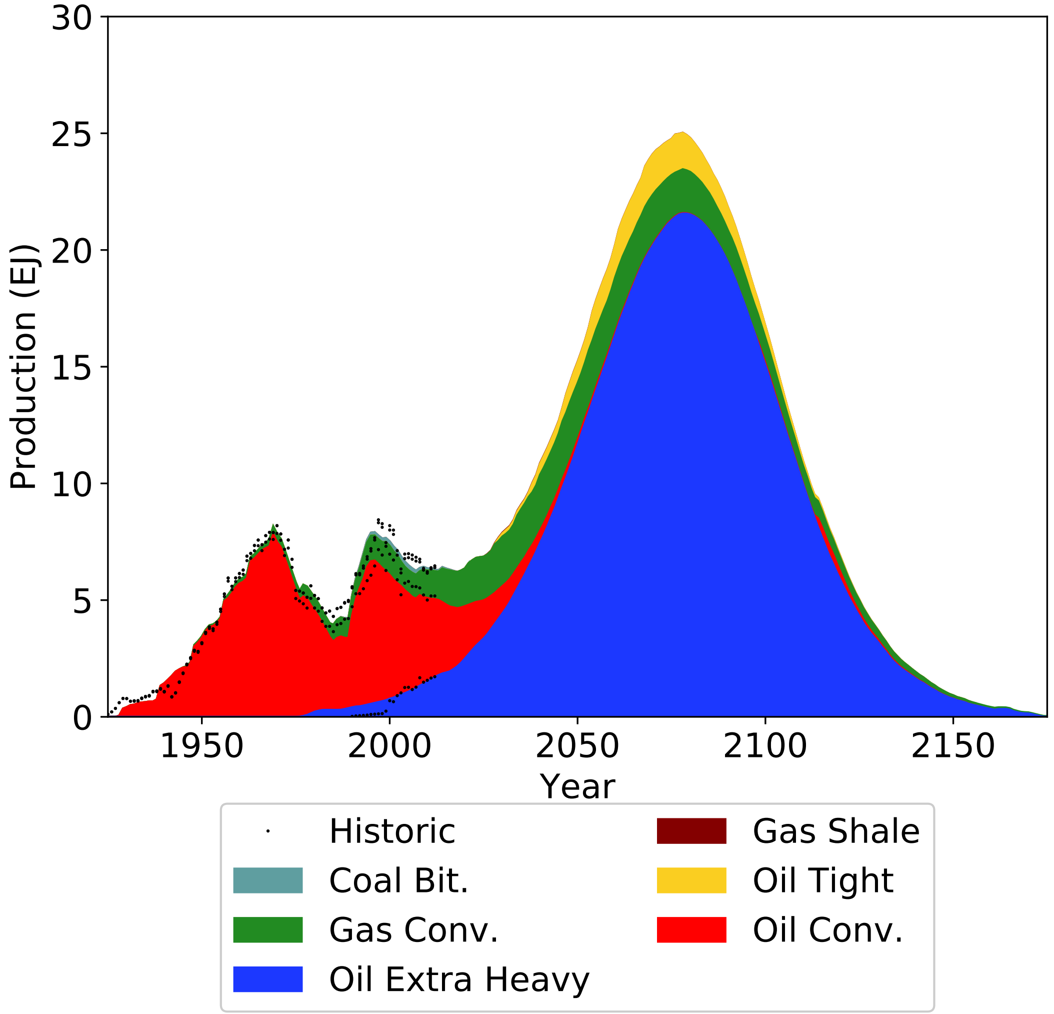

Figure 7.37: Venezuela projections capped at 16

Table 7.37: Peak years - All

**Name**	**URR**	**Peak Year**	**Peak Rate**
Oil Extra Heavy	1432.5	2078	21.56
Oil Conv.	399.42	1969	7.84
Gas Conv.	241.5	2048	2.49
Oil Tight	76.78	2070	1.73
Coal Bit.	3.7	2003	0.22
Gas Shale	1.16	2029	0.03
**Total**	**2155.06**	**2078**	**25.02**

7.19.2 By Mineral

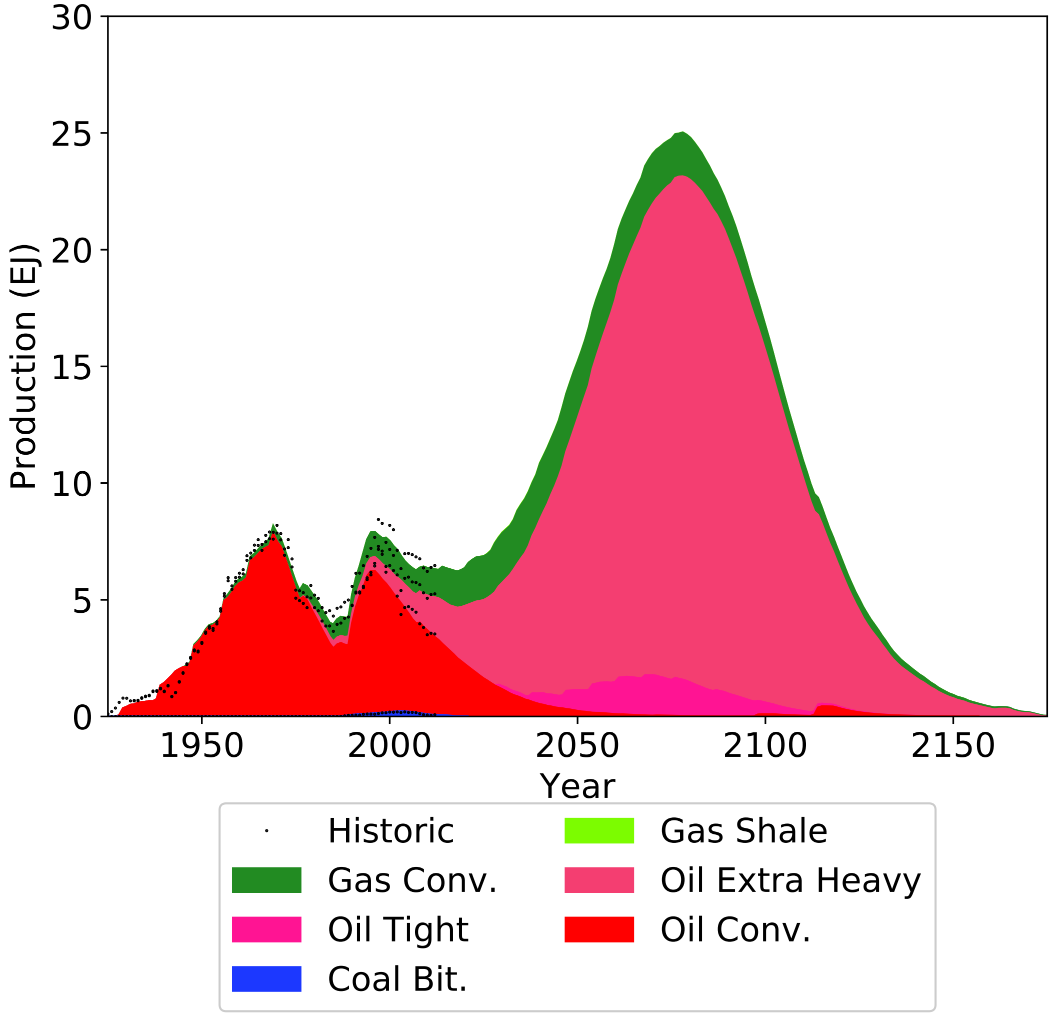

Figure 7.38: Venezuela projection by mineral type

Table 7.38: Peak years - Minerals

**Name**	**URR**	**Peak Year**	**Peak Rate**
Coal Bit.	3.7	2003	0.22
Oil Conv.	399.42	1969	7.84
Oil Tight	76.78	2070	1.73
Oil Extra Heavy	1432.5	2078	21.56
Gas Conv.	241.5	2048	2.49
Gas Shale	1.16	2029	0.03
**Total**	**2155.06**	**2078**	**25.02**

7.20 Total

7.20.1 By country

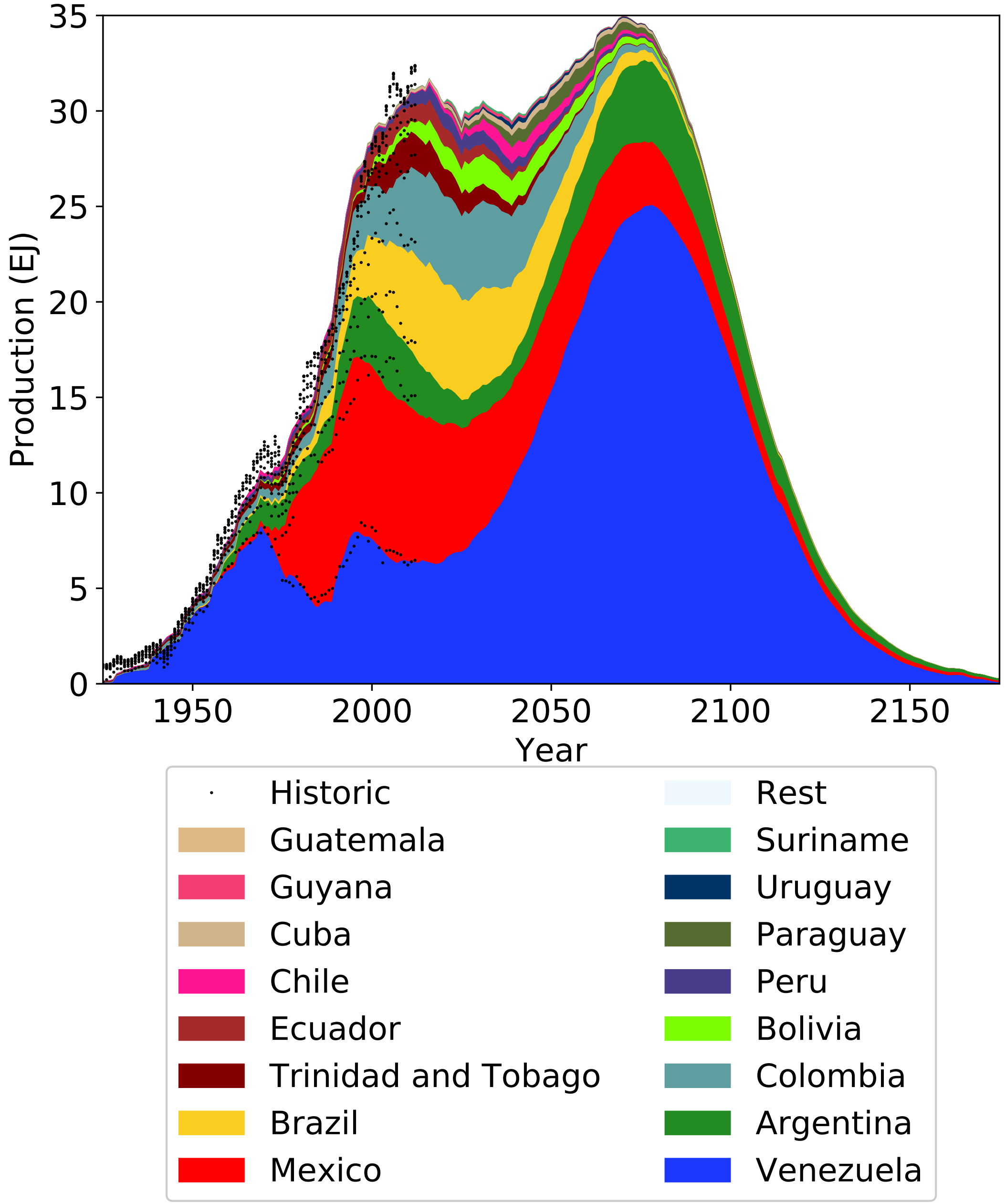

Figure 7.39: South America projections by country

Table 7.39: Peak years - All

Name	URR	Peak Year	Peak Rate
Venezuela	2155.06	2078	25.02
Mexico	730.57	1997	9.19
Argentina	402.22	2076	4.26
Brazil	316.19	2016	5.73
Colombia	277.63	2017	4.76
Trinidad and Tobago	79.79	2009	1.87
Bolivia	77.04	2028	1.57
Ecuador	53.71	2003	1.18
Peru	47.52	2019	0.82
Chile	38.49	2040	0.84
Paraguay	32.52	2053	0.86
Cuba	21.9	2044	0.36
Uruguay	7.61	2041	0.25
Guyana	5.6	2028	0.18
Suriname	4.55	2030	0.14
Guatemala	1.24	1996	0.05
Grenada	0.9	2024	0.05
Barbados	0.72	2032	0.02
Belize	0.11	2008	0.01
**Total**	**4253.37**	**2070**	**34.94**

7.20.2 By mineral

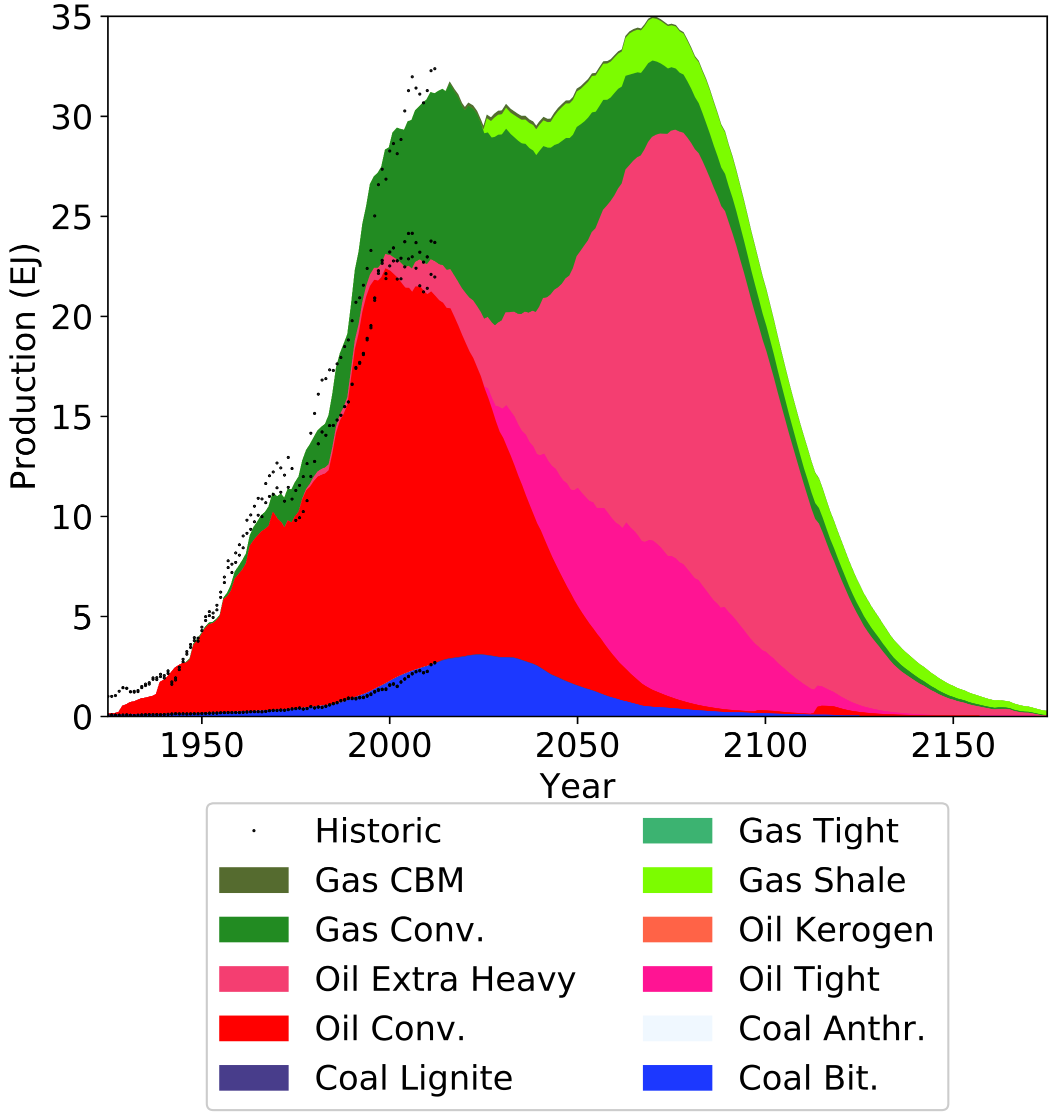

Figure 7.40: South America projection by mineral type

Table 7.40: Peak years - Minerals

**Name**	**URR**	**Peak Year**	**Peak Rate**
Coal Bit.	179.08	2024	3.03
Coal Lignite	2.85	2016	0.04
Coal Anthr.	0.1	1944	–
Oil Conv.	1299.35	1999	20.71
Oil Tight	417.14	2070	7.47
Oil Extra Heavy	1432.5	2078	21.56
Oil Kerogen	0.17	1993	0.01
Gas Conv.	710.97	2023	9.6
Gas Shale	200.68	2085	2.17
Gas CBM	10.5	2029	0.2
Gas Tight	0.04	2029	–
**Total**	**4253.37**	**2070**	**34.94**

Chapter 8

Total

8.1 By continent

Table 8.1: Peak years - All

Name	URR	Peak Year	Peak Rate
Asia	11056.18	2021	215.71
North America	9713.65	2013	76.21
Middle East	8517.97	2022	87.32
FSU	7067.67	2019	73.91
South America	4253.37	2070	34.94
Europe	3849.93	1996	39.05
Africa	3122.08	2026	39.36
**Total**	**47580.85**	**2021**	**539.59**

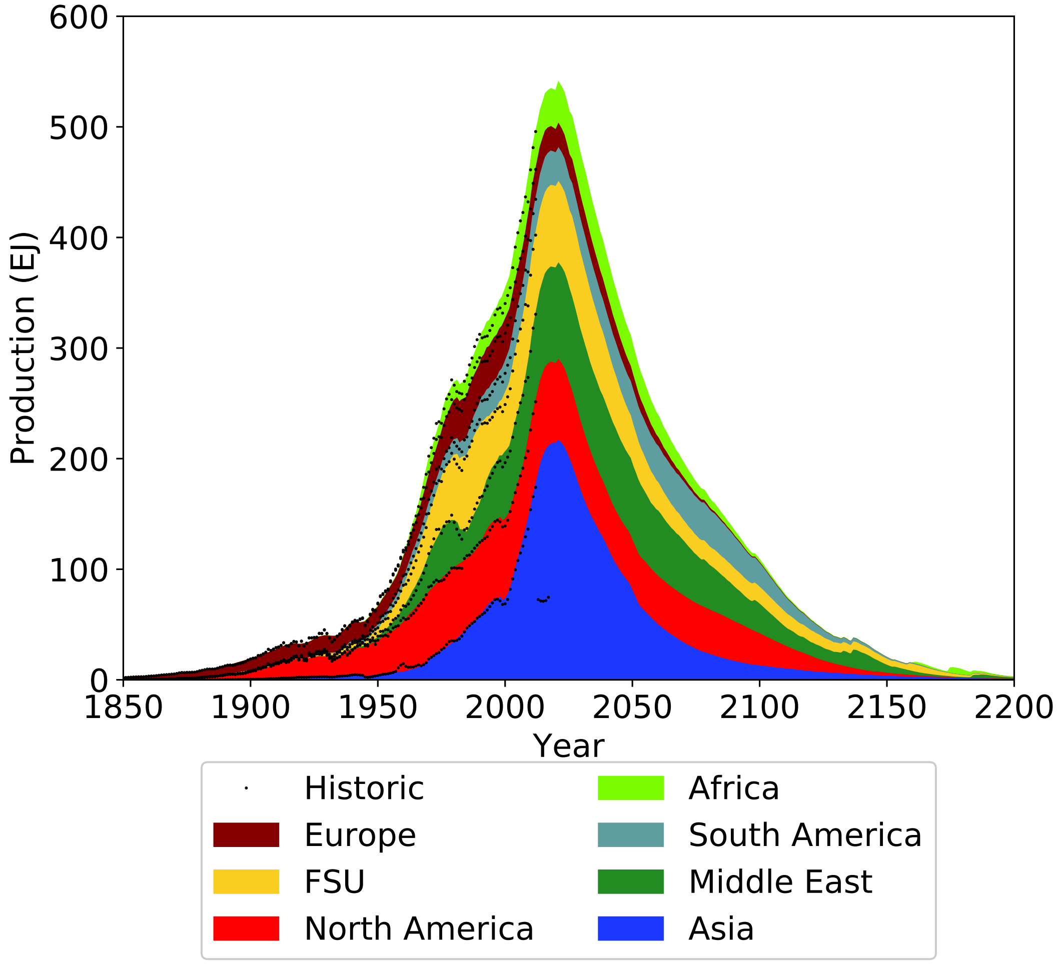

Figure 8.1: Total projections by continent

8.2 By mineral

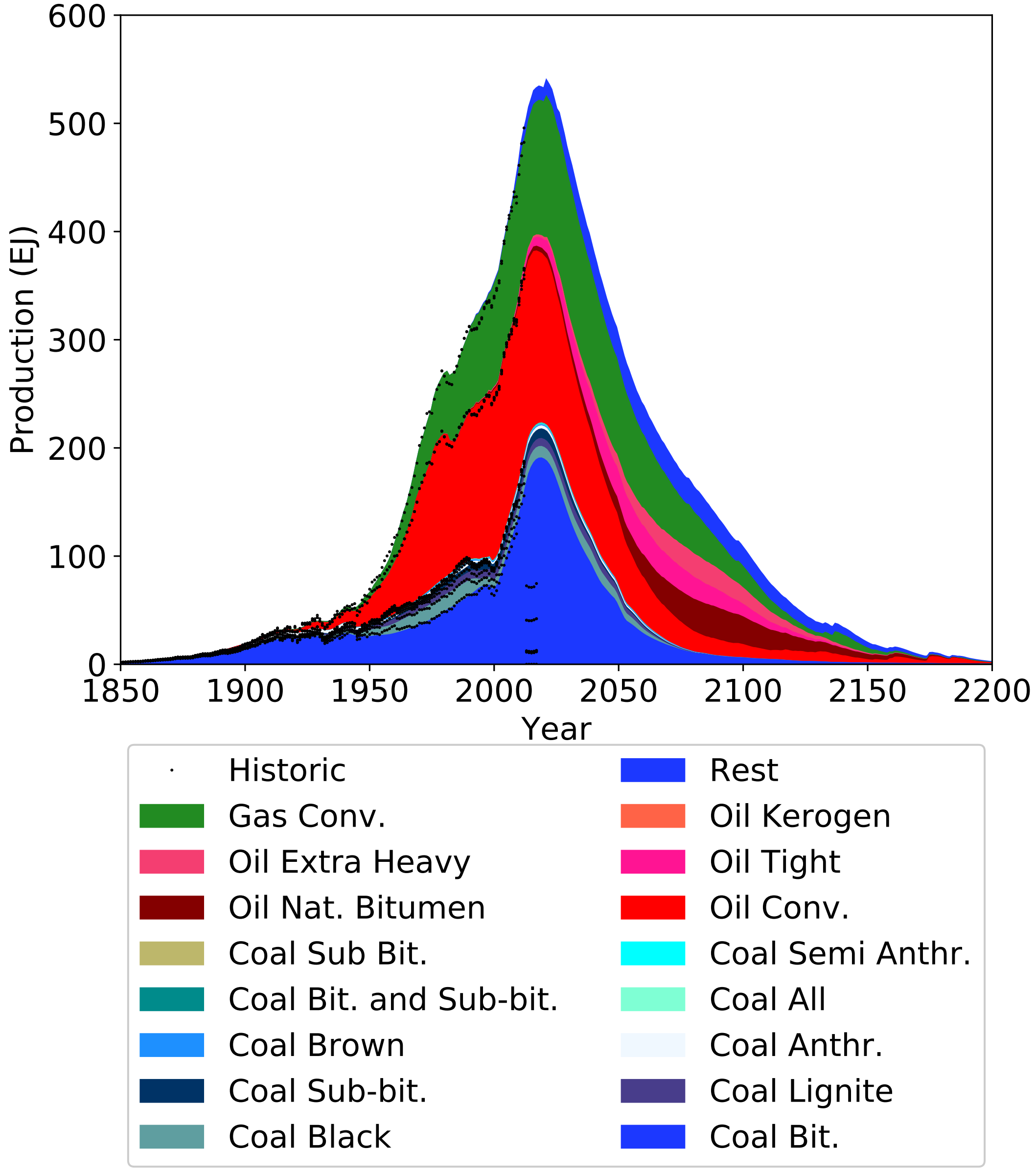

Figure 8.2: Total projection by mineral type

Table 8.2: Peak year by mineral

**Name**	**URR**	**Peak Year**	**Peak Rate**
Oil Conv.	13666.36	2011	167.17
Gas Conv.	11048.56	2023	132.56
Coal Bit.	10893.88	2019	190.31
Oil Nat. Bitumen	2373.84	2084	30.17
Oil Tight	2006.89	2051	28.6
Oil Extra Heavy	1432.54	2078	21.56
Gas Shale	1362.3	2050	13.59
Coal Black	1275.31	1985	14.99
Gas CBM	871.98	2039	9.01
Coal Lignite	798.69	2033	8.55
Gas Tight	579.36	2056	6.7
Coal Sub-bit.	567.65	2011	9.3
Coal Anthr.	430.39	1918	3.22
Coal Brown	165.77	1990	1.63
Coal All	43.63	2025	0.55
Coal Bit. and Sub-bit.	25.4	2019	0.31
Coal Semi Anthr.	22.82	2026	0.77
Oil Kerogen	13.11	2037	0.22
Coal Sub Bit.	2.37	1950	0.04
**Total**	**47580.85**	**2021**	**539.59**

8.3 By Country

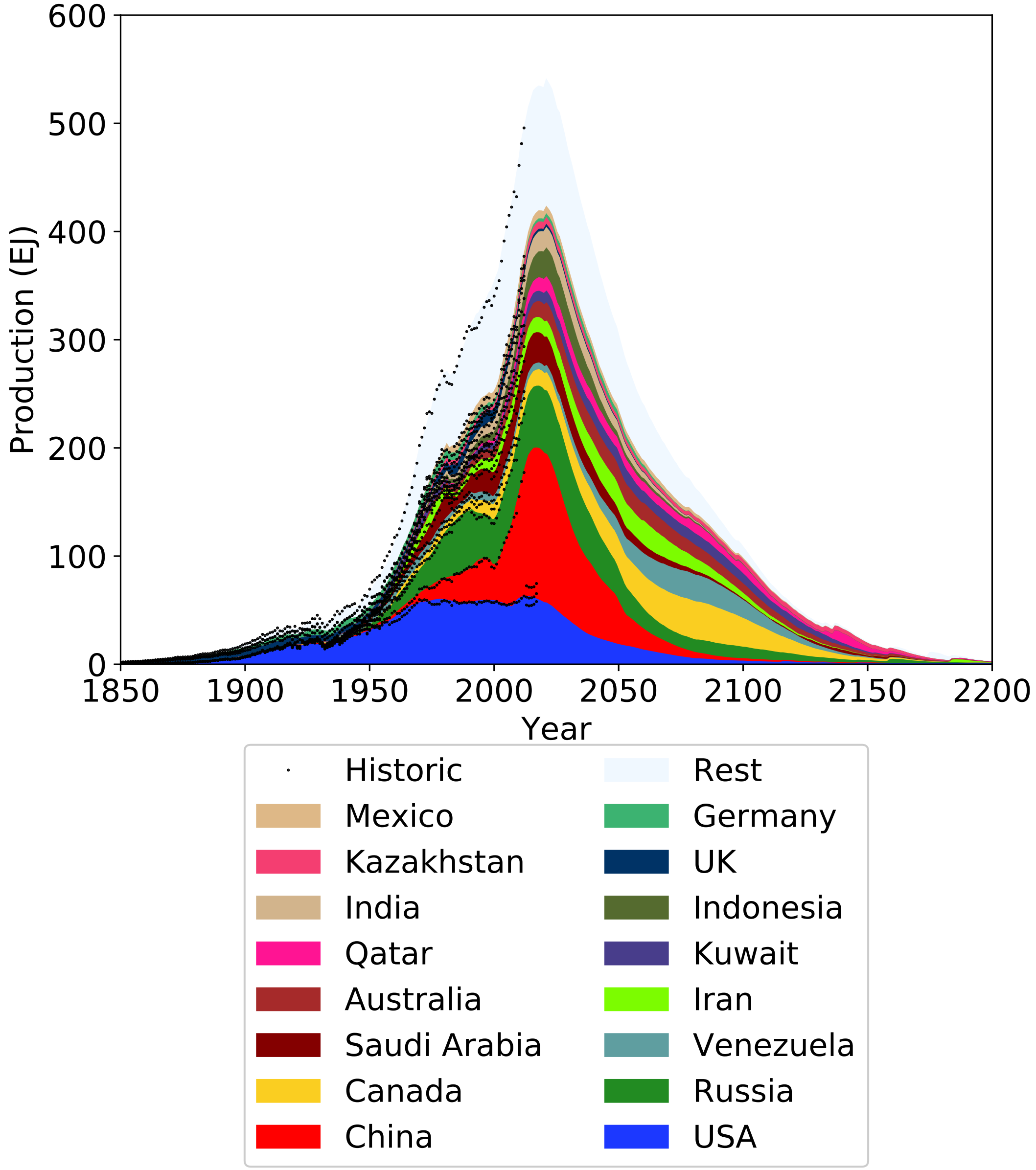

Figure 8.3: Total projection by country

Table 8.3: Peak year - Country

**Name**	**URR**	**Peak Year**	**Peak Rate**
USA	6205.04	2013	62.27
China	5833.28	2018	140.16
Russia	5079.49	2021	58.51
Canada	3508.61	2078	35.34
Venezuela	2155.06	2078	25.02
Saudi Arabia	2008.07	2016	28.67
Iran	1997.63	2047	22.97
Australia	1950.01	2034	19.47
Kuwait	1534.81	2051	13.85
Qatar	1334.55	2023	13.52
Indonesia	1113.61	2024	27.4
India	1101.66	2023	19.13
UK	995.13	1998	11.34
Kazakhstan	884.1	2012	7.66
Germany	823.0	1976	7.49
Mexico	730.57	1997	9.19
Iraq	704.3	2027	7.59
UAE	654.71	2019	10.5
Libya	653.66	1970	7.51
Nigeria	597.85	2018	7.23
Algeria	569.48	2026	8.25
South Africa	542.14	2023	6.47
Argentina	402.22	2076	4.26
Poland	388.3	1984	5.33
Norway	349.88	2005	9.81
Brazil	316.19	2016	5.73
Egypt	279.2	2035	4.46
Colombia	277.63	2017	4.76
Turkmenistan	235.93	2017	3.46
Netherlands	212.16	1978	3.8
Ukraine	211.51	1974	3.59
Azerbaijan	192.77	2013	2.61
Czech Republic	191.54	1977	2.34
Malaysia	186.52	2007	3.71
France	170.75	1927	1.26
Donetsk	169.01	1972	3.03
North Korea	146.97	2036	2.34
Angola	145.59	2008	3.71
Uzbekistan	144.04	2004	2.84
Romania	142.63	1982	2.28
Oman	141.43	2013	3.52
Pakistan	129.38	2012	1.69
Yugoslavia	123.24	1990	1.28
Luhansk	117.14	1971	2.12
Japan	82.91	1953	1.21
Vietnam	80.1	2010	2.28
Trinidad and Tobago	79.79	2009	1.87
Sudan	79.55	2029	2.24
Turkey	79.38	2052	1.01

Table 8.3: Peak year - Country – Continued

Name	URR	Peak Year	Peak Rate
Thailand	77.39	2010	2.34
Bolivia	77.04	2028	1.57
Belgium	67.12	1913	0.66
Bangladesh	59.03	2028	1.02
Burma	56.72	2033	0.88
Spain	55.23	2030	0.62
Mongolia	53.98	2022	2.3
Ecuador	53.71	2003	1.18
Syria	53.68	1998	1.4
Brunei	51.45	2002	0.89
Italy	50.65	1986	0.86
Hungary	48.29	1977	0.87
Bulgaria	48.2	2032	0.83
Peru	47.52	2019	0.82
Congo	42.64	2023	0.91
Chile	38.49	2040	0.84
Gabon	37.65	1994	0.77
Bahrain	37.2	2014	0.61
Yemen	35.24	1998	0.99
Paraguay	32.52	2053	0.86
Greece	29.11	2001	0.67
PNG	28.27	1993	0.62
Denmark	28.14	2001	0.99
Tunisia	26.24	2031	0.54
Afghanistan	24.68	2032	0.57
Equatorial Guinea	23.06	2006	0.89
New Zealand	23.04	2009	0.39
South Korea	22.7	1982	0.69
Cuba	21.9	2044	0.36
Austria	19.4	1960	0.22
Philippines	18.98	2022	0.5
Mozambique	16.3	2026	0.37
Cameroon	16.15	1986	0.36
Somalia	15.1	2038	0.39
Namibia	13.4	2030	0.37
Tanzania	9.26	2028	0.2
Israel	8.89	1970	0.22
Belarus	8.72	1976	0.29
Zimbabwe	8.28	1992	0.11
Albania	8.2	2019	0.19
Uruguay	7.61	2041	0.25
Ghana	7.0	2019	0.33
Taiwan	6.83	1968	0.17
Slovakia	6.44	2028	0.11
Niger	6.24	2017	0.26
Estonia	5.73	2048	0.08
Eritrea	5.6	2028	0.19
Guyana	5.6	2028	0.18
Tajikistan	5.2	2039	0.12

Table 8.3: Peak year - Country – Continued

**Name**	**URR**	**Peak Year**	**Peak Rate**
Sweden	5.0	2037	0.09
Zaire	5.0	2024	0.09
Jordan	4.68	2028	0.17
Ireland	4.58	2019	0.11
Suriname	4.55	2030	0.14
Kyrgyzstan	4.42	1972	0.09
Ivory Coast	4.38	2006	0.17
Lithuania	4.22	2033	0.21
Chad	4.17	2005	0.35
East Timor	4.11	2007	0.21
Georgia	3.66	1979	0.18
Rwanda	2.9	2030	0.11
Morocco	2.78	2033	0.09
Lebanon	2.78	2040	0.07
Portugal	2.39	2014	0.17
Mauritania	2.34	2024	0.08
Cambodia	1.9	2024	0.08
Crimea	1.71	2010	0.07
Ethiopia	1.71	2024	0.07
Laos	1.39	2023	0.06
Guatemala	1.24	1996	0.05
Sri Lanka	1.11	2032	0.03
Benin	0.97	2024	0.04
Botswana	0.93	1998	0.03
Cyprus	0.93	2029	0.03
Grenada	0.9	2024	0.05
Barbados	0.72	2032	0.02
Seychelles	0.7	2024	0.04
Zambia	0.44	1978	0.02
Togo	0.4	2022	0.02
Swaziland	0.3	1991	0.01
Madagascar	0.2	2022	0.01
Guinea-Bissau	0.2	2022	0.01
Malta	0.2	2024	0.01
Bhutan	0.15	2017	–
Belize	0.11	2008	0.01
Senegal	0.1	2010	–
Western Sahara	0.1	2022	0.01
Malawi	0.08	2020	–
Switzerland	0.03	1944	0.01
Greenland	0.02	1953	–
Moldova	0.02	1994	0.01
Nepal	0.01	2018	–
New Caledonia	–	1928	–
**Total**	**47580.85**	**2021**	**539.59**
